# COVID-19 engages clinical markers for the management of cancer and cancer-relevant regulators of cell proliferation, death, migration, and immune response

**DOI:** 10.1038/s41598-021-84780-y

**Published:** 2021-03-04

**Authors:** Serhiy Souchelnytskyi, Andriy Nera, Nazariy Souchelnytskyi

**Affiliations:** 1 College of Medicine, QU Health, Qatar University, Building H12, 2713 Al-Tarfa, Doha, Qatar; 2 Pustomyty Regional Hospital, Pustomyty, 81100 Ukraine; 3 Oranta CancerDiagnostics AB, 75263 Uppsala, Sweden; 4 Angstrom Laboratory, Polacksbacken, Uppsala University, 75237 Uppsala, Sweden

**Keywords:** Cancer therapy, Regulatory networks, SARS-CoV-2

## Abstract

Clinical reports show that the management of cancer patients infected with SARS-CoV-2 requires modifications. Understanding of cancer-relevant mechanisms engaged by the virus is essential for the evidence-based management of cancer. The network of SARS-CoV-2 regulatory mechanisms was used to study potential engagement of oncogenes, tumor suppressors, other regulators of tumorigenesis and clinical markers used in the management of cancer patients. Our network analysis confirms links between COVID-19 and tumorigenesis that were predicted in epidemiological reports. The COVID-19 network shows the involvement of tumorigenesis regulators and clinical markers. Regulators of cell proliferation, death, migration, and the immune system were retrieved. Examples are pathways initiated by EGF, VEGF, TGFβ and FGF. The SARS-CoV-2 network engages markers for diagnosis, prognosis and selection of treatment. Intersection with cancer diagnostic signatures supports a potential impact of the virus on tumorigenesis. Clinical observations show the diversity of symptoms correlating with biological processes and types of cells engaged by the virus, e.g. epithelial, endothelial, smooth muscle, glial and immune system cells. Our results describe an extensive engagement of cancer-relevant mechanisms and clinical markers by COVID-19. Engagement by the virus of clinical markers provides a rationale for clinical decisions based on these markers.

## Introduction

Cancer patients are under the risk of developing severe form of COVID-19^1–4^. Reports of the higher rate of SARS-CoV-2 infection by cancer patients led to recommendations of delaying treatments and changes in the management of such patients^4–9^. Clinical observations suggest that the SARS-CoV-2 infection may have an impact on the course of cancer progression and response to anti-cancer treatment^1–9^.

Clinical manifestation of COVID-19 have a broad range, and are related to the response of the patient. The response might be classified in 4 types, i.e. mild, moderate, severe and critical^10–15^. Fewer, fatigue, dry cough, anosmia and dysgeusia are among the initial symptoms of SARS-CoV-2 infection^10^. The respiratory system can be heavily affected, with the development of pneumonia and acute respiratory distress syndrome^10,11^. A strong impact of SARS-CoV-2 on the cardiovascular system has been reported, with a manifestation of arrhythmia, cardiac injury, myocarditis, heart failure, pulmonary embolism, and disseminated intravascular coagulation^10,12^. Dermatological symptoms were also observed, e.g. rush and skin discolorations^13^. The gastrointestinal system can be affected with a development of diarrhea, vomiting and nausea^14^. SARS-CoV-2 impact on the central nervous system was reported, with manifestations of seizures, neuropathy, headache and cerebrovascular events^15^. Some of the COVID-19 manifestations are also observed in cancer patients, e.g. of gastrointestinal, respiratory and cardiovascular symptoms. Therefore, it is crucial to discriminate and manage symptoms caused by COVID-19 and/or cancer in the treatment of cancer patients who contracted COVID-19.

An insight into a possible link between cancer and COVID-19 comes from the analysis of molecular mechanisms engaged by the virus. The RNA of SARS-CoV-2 codes for 14 open reading frames that can produce 29 proteins^16–19^. These proteins are divided into 3 groups: 16 non-structural, 4 structural and 9 accessory factors^16–19^. These proteins re-program cells to produce the virus by interacting with intracellular proteins^20,21^. Many of these intracellular proteins are involved in controlling cellular physiology, including processes of potential relevance to tumorigenesis. Regulation of the virus-controlled protein translation is one such example^20^. The host receptor of SARS-CoV-2 is angiotensin-converting enzyme 2 (ACE2), and the entry of the virus in cells requires the processing of the spike protein by a type 2 transmembrane serine protease (TMPRSS2)^22,23^. The binding of SARS-CoV-2 to ACE2 may lead to the downregulation of ACE2, as was reported for SARS-CoV-2 virus^24^. The surface concentration of ACE2 may fall to 10% of its normal level, as was shown for SARS-CoV-2 virus^24^. Decreased expression of ACE2 is associated with tumor growth and metastases^25–28^. Non-small cell lung cancer, gallbladder, pancreatic, breast, renal, thyroid, liver, and head and neck cancers were reported having low levels of ACE2 associated with aggressive development^25–35^. Therefore, the downregulation of ACE2 by the virus may contribute to the cancer-relevant effect of the virus.

The complexity of the virus-engaged mechanisms requires a systemic analysis. Reported studies of other viruses, e.g. influenza, showed large regulatory networks initiated by viruses^36–38^. Virus regulatory networks may contain thousands of nodes and edges. The networks provided insights into molecular mechanisms leading to clinical manifestations of the virus infection in patients^36–38^.

Currently, initiatives of systemic study of COVID-19 have been reported^39^. Knowledge of SARS-CoV-2 products allows a large-scale interactome screening. Previously, a yeast two-hybrid screening was used extensively^38–40^. Proteomics and transcriptomics studies were recently reported^40–42^. These studies described lists of molecules forming direct and indirect complexes with SARS-COV-2 proteins and lists of genes affected upon infection with SARS-CoV-2. These lists are a rich source for an exploration of SARS-CoV-2 regulatory networks for solving clinical questions.

Clinical observation of severe effects of the virus on lungs, gastrointestinal tract, metabolism and cardiovascular system prompted modifications in the treatment of cancer patients infected with the virus^8,9,43–45^. Precautions are recommended in the management of patients undergoing chemotherapy and/or radiotherapy, due to impairment of the patients’ immune system^5,9^. Cancer patients under treatment with immune checkpoint inhibitors (ICIs) may need a close monitoring due to a pneumological toxicity and cytokine-release syndrome^9^. ICIs may help to restore immunocompetence in cancer patients undergoing chemotherapy or radiotherapy and subsequently contribute to resistance to SARS-CoV-2 infection. ICIs may also exacerbate the cytokine storm if applied in the COVID-19 infectious phase^46–48^.

Management of cancer patients infected with SARS-CoV-2 requires knowledge of the impact of COVID-19 on cancer-relevant molecular mechanisms. Knowledge of SARS-CoV-2-targeted cancer markers and regulators would provide a rationale for decisions about treatment and prognosis. Here, we show that COVID-19 engages regulators of tumorigenesis and clinically used markers. Reporting virus-engaged cancer markers would help to assess the value of these markers in managing cancer patients infected with the virus.

## Results

### COVID-19 network

**Figure 1:**
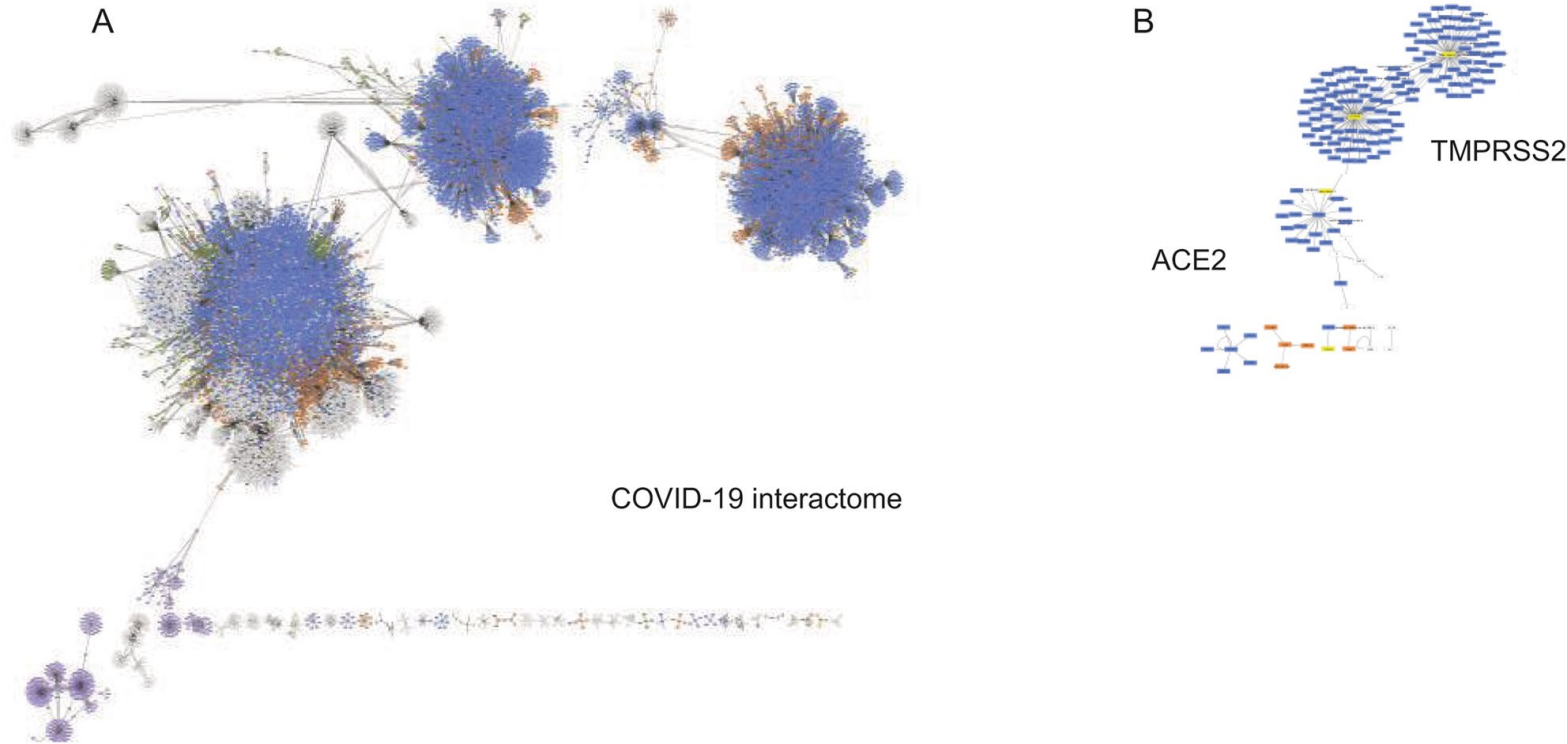
Networks formed by SARS-CoV-2 interactors. Cluster structures of the networks are shown. The merged network formed by SARS-CoV-2 interactors and built with data from databases mentioned in the text is shown (**A**). The network formed by ACE2 and TMPRSS2 is shown (**B**). Compare the structure of the COVID-19 networks built with data retrieved from each database separately (Supplementary Figure S1), for illustration of contributions of databases to the merged network. The panels A and B of this figure shows cluster structure of the network; to zoom-in on identifiers of nodes and edges, use the “Main merged Network” in the Cytoscape Session file (.cys) (Supplementary File S1 at https://figshare.com/articles/online_resource/Cancer_and_COVID-19_systems_biology_Suppl_File_S1/12793883).

To build a network of SARS-CoV-2-engaged proteins and genes, we used 2 input datasets. The first list contained 73 cancer-relevant SARS-CoV-2 interactors (Supplementary Table S1). This dataset was retrieved from the data published by Gordon et al.; Supplementary file 2 in the paper by Gordon et al. lists all detected 322 interactors with 27 proteins of SARS-CoV-2^19^. The second list contained ACE2 and TMPRSS2 only. The networks were built as described in the Material and Methods section. (Fig. 1A, B; Supplementary Figure S1). Figure 1 shows networks built for SARS-CoV-2 interactors (Fig. 1A) and for ACE2 and TMPRSS2 (Fig. 1B). Figure 1A shows a merged network combining all networks generated with each database listed in the Material and Methods section, for SARS-CoV-2 interactors. Figure 1 and Supplementary Figure S1 show structure of the networks to illustrate functional and interactome clusters. Cluster structure of the merged network (Fig. 1A) reflects diversity of data deposited in different databases. To read identifiers of nodes and edges, the Supplementary File S1 should be used. This file contains the Cytoscape Session file (.cys file) that can be used for such analysis, zooming in on nodes and edges, and for analysis of biological processes, if a reader would be interested in own analysis of the networks.

The networks generated with databases reflect the representation of COVID-19-relevant nodes and links in different databases. The numbers of nodes are 5,205 for BioGrid, 5,385 nodes for Mentha, 2,601 nodes for MINT, 465 nodes for UniProt, 7,021 nodes for IntAct, 107 nodes for HPIDb, 8 nodes for MPIDB, 291 nodes for InnateDB-ML, 28 nodes for MBinfo, 898 nodes MatrixDB, 3,790 nodes for ChEMBL, 8,405 nodes for IMEx, 1,343 nodes for Reactome-FIs, 716 nodes for EBI-GOA-nonIntAct, 37 nodes for EBI-GOA-miRNA, and 3,953 nodes for InnateDB (Supplementary Figure S1; online at FigShare.com as above). The sum of all the nodes is 40,253 but the merged network contains 24,656 nodes. This shows that up to 40% of the nodes overlap across the databases. Our results also demonstrate that merging data from different databases increases coverage of COVID-19-engaged biological processes by adding interactions described in selected databases only. The network formed by ACE2 and TMPRSS2 is less complex and contained three well-defined clusters. Two clusters are related to TMPRSS2 (124 nodes) and the third cluster to ACE2 (168 nodes), as shown in Fig. 1B. The high coverage of SARS-CoV-2 interactors (Fig. 1) leads to a comprehensive insight into COVID-19 regulatory mechanisms.

Further insight into the COVID-19 networks comes from the analysis of links/edges between nodes. The merged COVID-19 network showed 3 main and a number of smaller interconnected clusters. There were also small clusters not connected to the main interactome network (Fig. 1A). Clusters represent closely connected regulators. An analysis of biological processes of the 3 main clusters showed that all of them had similar profiles of biological processes but differ in involved nodes. This confirms that the observed clustering reflected the use of different databases that contributed to different aspects of interactions.

The network contains nodes and edges retrieved from different databases. Therefore, there was a possibility that the same nodes would be present in different clusters due to differences in edges retrieved from different databases. To estimate the number of unique nodes, we searched the network with unique identifiers of nodes. We observed that there were 2 or more nodes for unique identifiers in the merged network, e.g. 3 nodes of TP53, 2 of BRCA1, 2 of EGFR, 3 of VEGF. This is the consequence of using multiple databases that may have unique and non-overlapping clusters, e.g. unique edges between nodes and isoforms of nodes. On the other side, it confirms a broad and inclusive coverage of interactions. The interactome network contains 24,656 nodes. The observation of multiple locations of the same node in different clusters of the network suggests that the number of unique nodes is less than 10,000. This number is in the range observed for networks describing carcinogenesis and viruses^19,38,49–51^.

The databases provide information about physical and functional interactions. It is unlikely that all identified nodes directly interact with SARS-CoV-2 proteins. The nodes may be present in SARS-CoV-2-initiated complexes or functionally affected by SARS-CoV-2. Another important point is that the databases provide data for different types of cells. Analysis of the network for types of cells retrieved epithelial, endothelial, smooth muscle cells, fibroblasts, leukocytes (including T- and B-lymphocytes), neurons and glial cells. The edges of the network are not directional and leave open interpretation of a functional strength and directionality of the interactions. Despite the multiple representations of nodes, interactions not defined as direct vs indirect, and not defined directionality of edges, the network provides the most comprehensive sum of all interactions that could be initiated by SARS-CoV-2. The comprehensiveness of the network allows zooming in it for a specific type of cancer, type of cells or a type of medical condition, by selecting relevant nodes. The merged network can be used as a library of all possibilities, and a specific task can be completed by zooming on nodes and edges relevant to the task.

### COVID-19 network and focus on cancer

**Figure 2:**
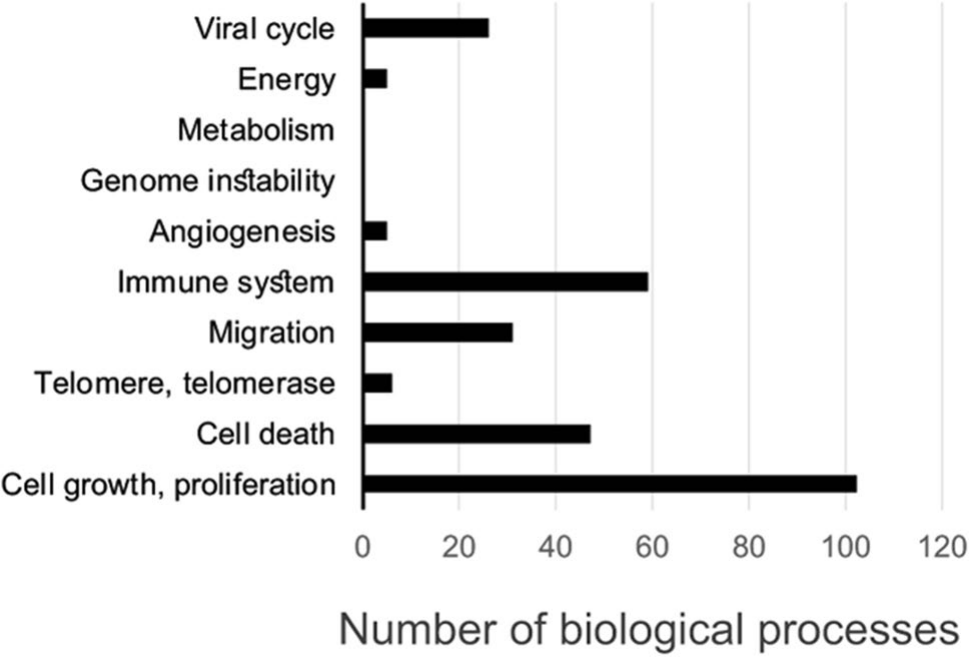
Biological processes affected by COVID-19. The graph shows numbers of biological processes identified in the COVID-19 network. Search of the network was performed for all cancer hallmarks, as described in the text. A complete list of the retrieved biological processes is presented in Supplementary Table S2. The BiNGO network can be explored by using the “Main merged Network BiNGO” in the Cytoscape Session file (Supplementary File S1 at https://figshare.com/articles/online_resource/Cancer_and_COVID-19_systems_biology_Suppl_File_S1/12793883).

We focused on the network nodes and edges of relevance to cancer. We analyzed the network for a representation of cancer hallmarks. BiNGO tool was used to retrieve biological processes of relevance to each cancer hallmark, e.g. search for biological processes controlling cell proliferation, growth, death, apoptosis, autophagy, migration, invasiveness, immune system, inflammation, immortalization, telomere, genome instability, metabolism, stroma, energy and angiogenesis. The search produced networks of biological processes (Supplementary Figure S2). We also searched the network for biological processes of relevance to the viral cycle and virus propagation. Figure 2 shows retrieved biological processes. Four categories of biological processes were predominant for SARS-CoV-2, ACE2 and TMPRSS2 interactors. They are regulation of cell proliferation, death, migration and control of immune system. All biological processes retrieved from the network in Fig. 1 and biological processes of relevance to cancer hallmarks are listed in the Supplementary Table S2.

As an example, the search of the network for the term “oncogene” retrieved 68 nodes. Among them were identified oncogenes, such as SRC, MYC, MET, LYN, KIT, JUN, FOS, FGFR1, EGFR, ERBB2, ERBB3, ERBB4, BRAF, CCND1, ABL2, ABL1 and SKI. Involvement of well-known oncogenes is a strong indication that the COVID-19 may interfere with tumorigenesis.

Recent OMICs studies confirmed an impact of SARS-CoV-2 infection of human cells on expression of c-Myc, Met, Jun, Fos, B-Raf, FGF, EGF and VEGF signaling^52–55^.

We observed an engagement of many polypeptide growth factors in the regulation of cell proliferation. These are EGF, FGF, VEGF, PDGF, TGFbeta and IGF1 (Table 1, Supplementary Table S2). These growth factors have a profound impact on normal cell physiology and cancer. An engagement of them by SARS-CoV-2 indicates a broad range of the virus-stimulated responses. Each of these growth factors initiates large signaling networks. When all these growth factors are engaged by the virus, it may contribute to the generation of a relatively large network of SARS-CoV-2 interactors (Fig. 1A).

**Table 1:** Biological processes of importance for carcinogenesis and relevant to the cancer hallmarks.

	Interactome SARS-CoV-2	ACE2-TMPRSS2
Growth, proliferation	**85** TGFb, activin, VEGF, JAK_STAT, growth hormone, PDGF, EGF, insulin-like growth factor receptor, T cell B cell proliferation, leukocyte, epithelial cells, fibroblasts, smooth muscle cells, mononuclear, glial	**17** Growth hormone, insulin-like growth factor, fibroblasts, smooth muscle cells
Death, apoptosis	**38** T cells, DNA damage P53, muscle, neurons, T cells, death domain receptors, chaperone-mediated autophagy	**9** Negative regulation of cell death, anti-apoptosis, general regulation
Telomerase, telomere	**4** telomere maintenance via telomerase	**2** Telomere maintenance
Migration	**27** Endothelial cells, epithelial cells, smooth muscle cells, glia, neuron, sprouting angiogenesis	**4** Endothelial cell migration
Immune system regulation	**37** Regulators, innate immune response, activation of immune response, adaptive immune response	**22** Multiple regulations of innate immune response
Angiogenesis	**5** Negative regulation of angiogenesis, sprouting angiogenesis	0
Energy regulation	**5** Energy derivation by oxidation of organic compounds	0
Virus, viral cycle regulation	**22** Virulence, defense, response to virus, virus-host interaction, viral reproduction, viral transcription, virus infection cycle, non-lytic virus release	**4** Initiation of virus infection, reproduction, viral cycle, reproductive process

The biological processes were retrieved from the networks formed by SARS-CoV-2 (COVID-19) interactome and ACE2 + TMPRSS2. BiNGO tool was used to search for the processes in *H. sapiens*, with p < 0.05 for significance of retrieved data. Hypergeometric statistical test was used, with Benjamini and Hochberg false discovery rate correction.

Numbers (in bold) and examples of retrieved biological processes are shown; cancer hallmarks searches that did not retrieve processes are not shown.

An analysis of retrieved cancer-relevant biological processes showed that the regulation of cell death involved predominantly apoptotic mechanisms. Biological processes relevant to the regulation of cell migration showed a representation of different types of cells. Regulation of migration of epithelial, endothelial, smooth muscle cells, fibroblasts, glial cells and neurons was retrieved. Published studies confirmed the capacity of SARS-CoV-2 to infect different cell types, e.g. epithelial cells and hepatocytes^52–63^. The migration of endothelial cells was associated to a regulation of angiogenesis (Table 1; Supplementary Table S2). Mechanisms affecting the immune system showed a high representation of regulators of innate immunity. This impact on the immune system could be linked to virus propagation but may also have an impact on tumorigenesis.

Representation of many potent regulators of tumorigenesis in the SARS-CoV-2 network provides a strong support of the impact of the viral infection on the course of cancer. To strengthen this conclusion, we analyzed the SARS-CoV-2 network for markers used in clinical practice.

### COVID-19 network engages clinical markers and components of diagnostic signatures

An engagement of clinical cancer markers by the COVID-19 network would further support prediction of the SARS-CoV-2 impact on tumorigenesis. Therefore, we analyzed the representation of cancer markers approved for clinical use, markers of companion diagnostic of anti-cancer drugs, and targets of drugs in trials for the treatment of COVID-19.

**Figure 3:**
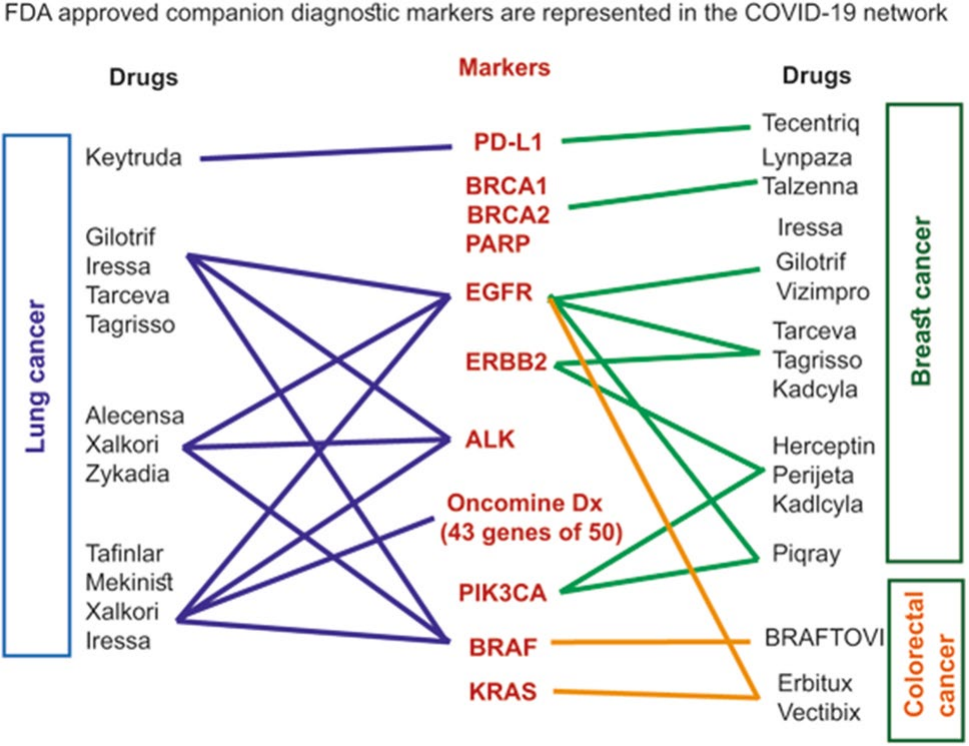
COVID-19 network engages companion diagnostic markers of anti-cancer drugs. Approved by FDA markers for companion diagnostic were retrieved from the COVID-19 network. Retrieved markers are shown in connection to the corresponding anti-cancer drugs used for treatment of lung, breast and colorectal cancers.

Firstly, we analyzed a representation in the COVID-19 network markers used for companion diagnostic of anti-cancer drugs that are approved for clinical use by the FDA (Supplementary Table S3A)^64^. The representation of these makers in the network indicates that the virus may affect a response of cancer patients to treatments. Drugs and their companion diagnostic markers for lung, breast and colorectal cancer are shown in Fig. 3. Note that all these companion diagnostic markers were retrieved from the COVID-19 network. These markers were also observed changing expression upon SARS-CoV-2 infection^52,54^. Therefore, COVID-19 may affect decisions about the use of anti-cancer drugs by engaging markers of companion diagnostic.

**Figure 4:**
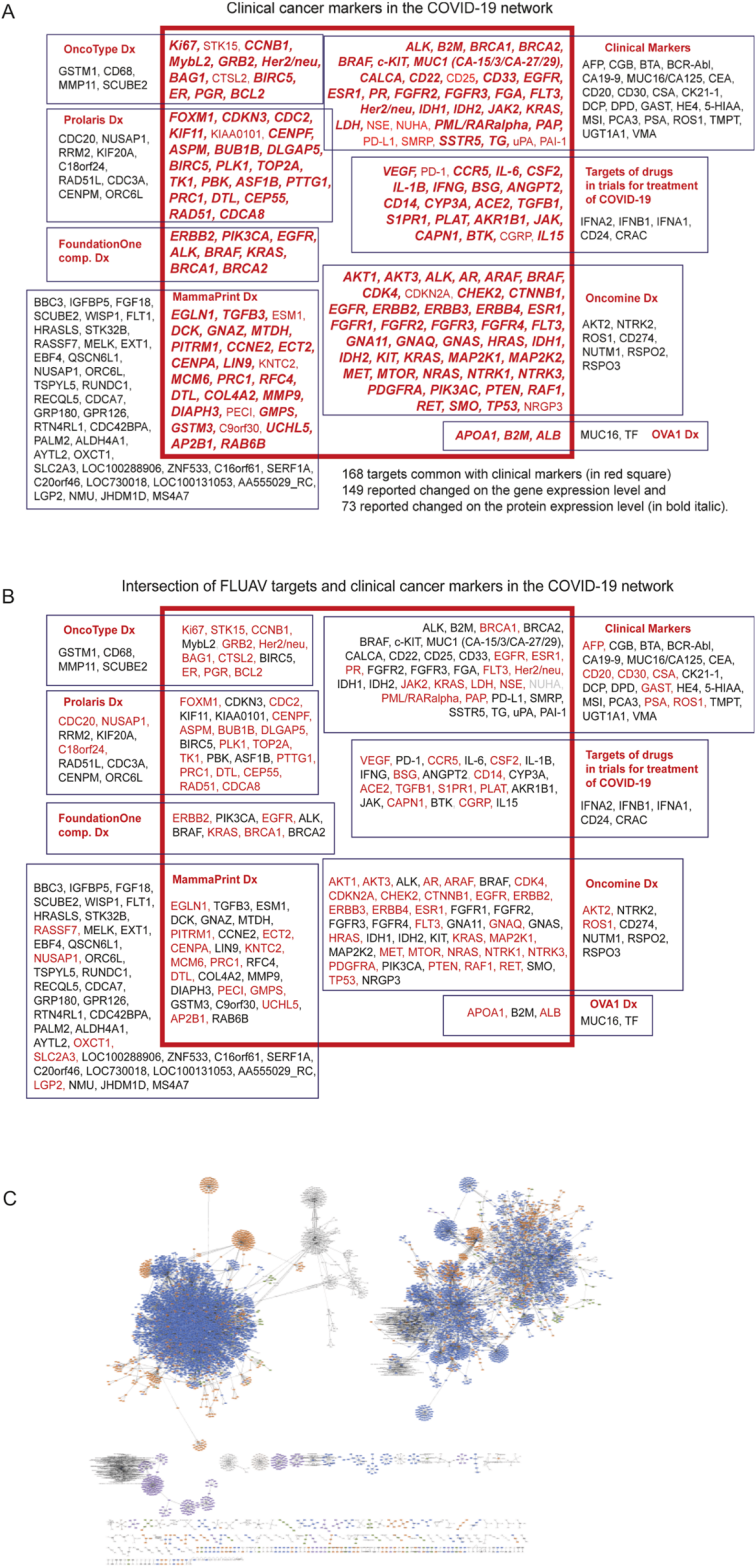
COVID-19 network engages clinically used markers and drug targets. The COVID-19 network was searched for single markers and diagnostic signatures used in clinical practice (**A**). Markers and signatures are annotated in respective boxes. The dark-red box encloses the nodes retrieved from the network. The network of influenza A virus contained a half of the cancer-relevant markers (**B**). The COVID-19-engaged nodes are in bold-red are indicated nodes confirmed by independent transcriptomics and proteomics studies. The structure of the network of influenza A virus is shown in panel C, to illustrate the cluster structure of the network in comparison with the structure of COVID-19 network (Fig. 1). To explore identifiers of the nodes and edges, zoom-in the “NetworkFLUAV01” network in the Supplementary File S1 online at FigShare.com (https://figshare.com/articles/online_resource/Cancer_and_COVID-19_systems_biology_Suppl_File_S1/12793883).

Secondly, we explored a representation of all markers recommended for clinical use for detection, diagnostic and prognosis of cancer^65^. The list of markers was retrieved from the NIH NCI depository (Supplementary Table S3B). Thirty-four individual cancer markers out of listed 57 were retrieved in the COVID-19 network (Fig. 4A). For 6 diagnostic signatures, all 8 companion markers in FoundationOne signature, 43 out of 50 markers in Oncomine Dx, 3 out of 5 markers in OVA1, 12 out of 16 markers in Oncotype Dx, 21 out of 31 markers of Prolaris and 26 out of 70 markers of Mammaprint Dx signatures were retrieved from the COVID-19 network. List of retrieved and all markers of each signature are presented in Fig. 4A and Supplementary Tables S3C-H.

Finally, we searched the network for targets of proposed anti-COVID-19 drugs^66^. Out of 26 drug targets, 21 were retrieved from the network (Fig. 4A; Supplementary Table S3I). Sixteen of the 26 targets were reported as involved in the regulation of tumorigenesis^66^.

The high representation of cancer markers and cancer regulators in the COVID-19 network concludes that the virus affects mechanisms of tumorigenesis, may influence the course of the disease and may modify the diagnostic and anti-cancer treatment protocols. Our data provide rationale for the reported clinical observations of higher risk of severe effects for cancer patients as compared to non-cancer infected individuals.

Recent proteomics and transcriptomics studies of SARS-CoV-2 infection confirmed predicted by us engagement of the cancer markers and regulators^52–54^. On the gene expression level, 149 out of 168 predicted targets were reported as changed expression upon SARS-CoV-2 infection^54^. On the protein expression level, more than a half of predicted targets were reported as regulated by SARS-CoV-2 (Fig. 4A)^52,53^. Dynamics of the gene and protein expression changes vary from unidirectional (e.g. inhibition or stimulation over a time period) to an oscillation of directionality between the first 1–2 h and 24 h infection with the virus^52,54^. For example, EGFR and TGFbeta showed the oscillation of inhibition and then stimulation of expression, while interferon-gamma showed stimulation and ACE2 showed inhibition^52,54^. Figure 4A shows in bold-red the predicted targets that were reported as changing expression upon SARS-CoV-2 infection.

To compare our data with a similar analysis of another virus, we performed a systemic network analysis of the interactome of the influenza A virus (FLUAV) (Fig. 4B, C)^38^. The list of interacting proteins was retrieved form a dataset reported by Shapira et al.^38^. The network was built with Cytoscape^67^. The size of the FLUAV-engaged interactome network was in the same range as that for COVID-19. FLUAV engaged 10,545 nodes and 15,489 edges (Fig. 4C). We searched the FLUAV interactome for the same clinical markers as we did for COVID-19. We observed a significantly lower representation of cancer markers in the FLUAV interactome as compared to COVID-19. Of 165 COVID-19-engaged cancer markers, only 92 were retrieved in the FLUAV interactome. The fact that the COVID-19 network contained almost twice as many clinical markers as the FLUAV network supports the stronger impact of COVID-19 on the course of cancer and on the management of patients, as compared to the influenza A virus.

Our analysis shows a significant representation of cancer regulators in the COVID-19 network. The virus engages markers for diagnosis, selection of treatment and prognosis. Our data may serve as the basis for a re-evaluation of the maker values for cancer patients infected with SARS-CoV-2. If COVID-19 affects cancer markers, the changes in marker values may influence decisions by a clinician.

## Discussion

Management of cancer patients infected with SARS-CoV-2 requires knowledge of the impact of the virus on tumorigenesis. Relevant questions include an exploration (i) of the virus impact on the mechanisms driving tumorigenesis, and (ii) the virus influence on a clinical monitoring of cancer patients, e.g. markers used for diagnostic and treatment. The systemic analysis of the COVID-19 network described here provides a systems biology-generated evidence of mechanisms engaged by the virus (Figs. 1 and 2: Supplementary Figures S1 and S2, and Table S2). Of clinical importance is an identification of clinical markers affected by the virus (Figs. 3 and 4A; Supplementary Table S3).

SARS-CoV-2 proteins, ACE2 and TMPRSS2 engage a large network of proteins and genes (Fig. 1, Table 1, Supplementary Figures S1 and S2 ad Tables S1, S2 and S3; for original files see links at FigShare.com as in figure legends and Supplementary Materials information). The number of SARS-CoV-2 interactors is comparable to reported numbers of interactors for influenza A virus^38^. Therefore, the complexity of the SARS-CoV-2 network does not differ significantly from the networks engaged by other viruses^36–38,40^.

Reported cancer networks show similar or higher complexity^49–51^. Profound re-arrangements of regulatory processes during carcinogenesis may engage tenth of thousands of nodes. Hundreds of biological processes are affected during the acquisition of carcinogenic properties by cells^49–51,56^. The high complexity of cancer regulatory mechanisms is expected due to the consideration that the network analysis combines thousands of studies and original data from different depositories and databases.

Tumorigenesis is controlled by mechanisms of cancer hallmarks^56^. We observed that out of 10 main cancer hallmarks, only 4 were engaged by COVID-19. The 4 hallmarks are regulation of cell proliferation, death, migration and immune system (Fig. 2). This rather limited engagement of the hallmark mechanisms suggests selectivity of COVID-19 actions on tumorigenesis.

The viral cycle and virus-relevant mechanisms are highly represented in the network (Fig. 2, Supplementary Table S2). Together with an impact on the immune system, the network-identified virus-relevant mechanisms may contribute to the development of the cytokine storm in some COVID-19 patients^47,48,57^. The identification of IL-1β, IL-6, interferon-γ and IL-16 (Fig. 4A) is an example of cytokines in the COVID-19 network.

Companion diagnostic (CDx) markers are used to guide the selection and application of anti-cancer drugs^64^. If COVID-19 affects CDx markers, it would mean that the treatment of cancer patients would also be affected. We observed an extensive representation of CDx markers in the COVID-19 network (Fig. 3; Supplementary Table S3A). Identification of these markers provides a basis for their re-evaluation in guiding drug selection for SARS-CoV-2-infected cancer patients.

The multiplicity of COVID-19 symptoms shows that the virus affects different types of cells, e.g. in lungs, gastrointestinal tract and the vasculature^58–63^. The COVID-19 network showed an impact of the virus on epithelial, endothelial, smooth muscle cells, glia and neurons (Table 1; Supplementary Table S2). The example of regulation of endothelial cells migration and cell–cell interaction may reflect the symptoms of damage to the vasculature^58,59^. The regulation of proliferation and death of epithelial cells may be engaged in tissue damage observed in lungs and gastrointestinal tract^60–62^. Damage in the central nervous system may be attributed to the effects on glial cells, neurons and endothelial cells^15,63^. That is in line with the reported multiple symptoms of COVID-19 infection^10–15,58–63^.

Recent studies of gene and protein expression in cells infected by SARS-CoV-2 confirmed changes of predicted here targets (Figs. 2, 3 and 4)^52–55^. Despite limitations in a research design, these studies are a significant step forward in understanding COVID-19 mechanisms. The reported datasets provided original data of SARS-CoV-2 impact on targets and markers described here (Figs. 2, 3, 4). Predicted by us regulatory mechanisms, companion diagnostic and clinical signature markers were reported changing expression upon SARS-CoV-2 infection^52–54^. This confirms the data of the network analysis. Reported here network analysis covers all nodes and edges available in databases. Reported proteomics and transcriptomics studies used selected types of cells, e.g. epithelial and hepatocellular^52–54^. This may contribute to the difference in representation of reported targets. Methodological limitations may also contribute to the difference, as proteomics studies were performed by a peptidomics LC–MS/MS technology that is far from delivering a full proteome coverage^52,55^.

Systemic analysis of COVID-19 impact on tumorigenesis is a step forward for better management of patients. However, it does have limitations to be considered when clinical applications are designed. These limitations are common to all network studies, and are related to the directionality of edges, the strength of functional impact between nodes, and incorporation of data from different models^49–51^. An introduction of a dynamic modeling of networks is under development. Despite these limitations, the current level of network analysis is a powerful tool to bring basic research to clinics. Reported data could be the basis for clinical trials of predictive markers for cancer management in SARS-CoV-2-infected patients. Presented here data show that COVID-19 has a strong impact on mechanisms of tumorigenesis. Moreover, COVID-19 may affect the responsiveness of cancer patients to anti-cancer drugs, by affecting diagnostic markers that are also regulators of tumorigenesis.

## Methods

### Retrieval of data for network analysis

Data for the network analysis were retrieved from published reports and from depositories. The workflow consisted of identification of SARS-CoV-2 protein–protein interactions and evaluation of cancer-relevance of interacting proteins was followed by retrieval of data relevant to companion diagnostic, clinical diagnostic signatures and FLUAV interactome (Supplementary Figure S3). The retrieved data were used for building and analysis of networks. Data collection was performed in 2 steps. The first step was a retrieval of SARS-CoV-2 targets. PubMed was searched for publications with search words “COVID-19” “SARS-CoV-2” and “Covid19”. Publications of SARS-CoV-2 protein-interacting proteins were manually evaluated, and the data were arranged in a list. 129 publications were retrieved, with 13 publications referring to proteomics and 38 publications referring to gene expression studies. The selection criterium was reporting SARS-CoV-2 interacting proteins and a comprehensiveness of a study. The publication by Gordon et al*.* provided a richest source of SARS-CoV-2 interactors^19^. Searches for ACE2 and TMPRSS2 interactors were also included.

The second step was to evaluate a cancer-relevance of the SARS-CoV-2 interacting proteins. Selected in the first step publications were searched for links of SARS-CoV-2 interactors and cancer. Search words “COVID-19 & cancer” retrieved 899 publications, as of July 2020. The publications were manually screened for reporting mechanisms and targets (genes and proteins) linking COVID-19 and cancer. A list of retrieved COVID-19-engaged regulators was revised after searches with these regulators of NCBI databases to confirm relevance to cancer, e.g. databases for Gene, Protein, ClinVar and OMIM. Criteria of a target inclusion were that original datasets were available for an evaluation of data quality, and cancer relevance was confirmed by at least two independent sources.

The list of clinically used markers was retrieved from NIH National Cancer Institute depository^65^. The list of commonly used cancer markers and diagnostic signatures was retrieved from the Food and Drug Administration depository^64^. The list of targets of drugs used for the treatment of COVID-19 was retrieved from the list updated at the Milken Institute^66^. The interactome data of the influenza A virus were retrieved from a publication by Shapira et al*.*^38^.

Therefore, we generated a list of cancer-relevant SARS-CoV-2 interactors (73 nodes), ACE2 and TMPRSS2 for generation of COVID-19 regulatory networks, i.e. merged and ACE2 + TMPRSS2 networks. Other lists used in this study were the list of cancer drug companion diagnostic targets, the list of commonly used cancer markers, the lists of diagnostic signatures, and the list of targets of drugs used for treatment of COVID-19. The list of the influenza A virus (FLUAV) interacting proteins was retrieved from the publication by Shapira et al*.*^38^.

### Network analysis

The list of SARS-CoV-2 products (RNAs and proteins), ACE2, TMPRSS2, and SARS-CoV-2-interacting proteins of relevance to cancer were used to build networks. Data for the network were retrieved from the following databases: BioGrid, Mentha, MINT, UniProt, IntAct, HPIDb, MPIDB, InnateDB-ML, MBinfo, MatrixDB, ChEMBL, IMEx, Reactome-FIs, EBI-GOA-nonIntAct, EBI-GOA-miRNA and InnateDB. Cytoscape version 3.7.2 was used for the generation of the network^67^. Statistical significance of network building (inclusion of nodes and confidence of edges) was set on p < 0.05. BiNGO tool was used to extract affected biological processes. For statistical significance of networks analysis, the level was set at p < 0.05, and the hypergeometric statistical test was used, with Benjamini and Hochberg false discovery rate correction.

The networks were searched for nodes that could be retrieved with one of the following key words: “cancer”, “oncogene”, “tumor”, proliferation”, “death”, “apoptosis”, “immortalization”, “telomerase” “migration”, “invasiveness”, “immune”, “inflammation”, “metabolism”, “energy”, “angiogenesis”, “vasculature”, “genome instability”, “stroma” and “metastasis”. These searches were to extract nodes of relevance to tumorigenesis.

The same protocol was used to build a network of influenza A virus interactome, e.g. the network was built with Cytoscape.

Extracted COVID-19 nodes were validated by independent searches for their links to tumorigenesis. In these searches were used NCBI databases, e.g. PubMed, Gene, Protein, ClinVar, OMIM (at https://www.ncbi.nlm.nih.gov/search/ ), and the Human Protein Atlas (www.proteinatlas.org). Nodes with confirmed impact on tumorigenesis were included, e.g. multiple independent publications and clinical observations supported links to tumorigenesis.

### Ethics approval and consent to participate

This work did/does not require ethical approval neither consent to participate.

## Supplementary Information

Supplementary Information 1.

Supplementary Information 2.

Supplementary Information 3.

Supplementary Information 4.

Supplementary Information 5.

## Data Availability

All data are freely available. The Cytoscape Session File (.cyv file) is available at https://figshare.com/articles/online_resource/Cancer_and_COVID-19_systems_biology_Suppl_File_S1/12793883. Supplementary Figures S1 and S2 can be retrieved online at https://figshare.com/articles/figure/Supplementary_Figures_S1_S2/12804881. Supplementary Figure S3 is available at figshare at https://doi.org/10.6084/m9.figshare.13633793. Supplementary Tables S1, S2 and S3 can be retrieved online at https://figshare.com/articles/dataset/Supplementary_Tables_S1_S2_and_S3/12804887.
