# Supplementary material for: COVID-19 engages clinical markers for the management of cancer and cancer-relevant regulators of cell proliferation, death, migration, and immune response
Source: Sci Rep. 2021 Mar 4;11:5228. doi: 10.1038/s41598-021-84780-y (PMC7933131; doi:10.1038/s41598-021-84780-y)
Supplement: Supplementary file 1 — Supplementary Information 1. [file 41598_2021_84780_MOESM1_ESM.pdf]

Supplementary materials for the paper:

COVID-19 engages clinical markers for the management of cancer and cancer-relevant regulators of cell proliferation, death, migration, and immune response.

Serhiy Souchelnytskyi[1]*, Andriy Nera[2], Nazariy Souchelnytskyi[3,4]

1. College of Medicine, QU Health, Qatar University, Doha 2713, Qatar

2. Pustomyty Regional Hospital, Pustomyty, 81100, Ukraine

3. Oranta CancerDiagnostics AB, Uppsala, 75263, Sweden

4. Angstrom Laboratory, Polacksbacken, Uppsala University, Uppsala, 75237, Sweden

*Correspondence to: Serhiy Souchelnytskyi, College of Medicine, building H12, QU Health, Qatar University, Al-Tarfa, 2713, Doha, Qatar. Email: serhiy@qu.edu.qa

Keywords: cancer, COVID-19, SARS-CoV-2, systems biology, markers

Running title: COVID-19 engages cancer clinical markers

LEGEND

Supplementary Figure S1, Networks retrieved with databases (A) BAR, (B) bhf-ucl , (C) BioGrid, (D) ChEMBL, (E) EBI-GOA-miRNA, (F) EBI-GOA-nonIntAct, (G) HPIDb, (H) IMEx, (I) InnateDB, (J) InnateDB-all, (K) IntAct, (L) MatrixDB, (M) MBinfo, (N) Mentha, (O) MINT, (P) MPIDB, (Q) Reactome-FIs, and (R) UniProt are shown. Numbers of nodes and edges are annotated.

Supplementary Figure S2, BiNGO networks for SARS-CoV-2 (A) and ACE2 and TMPRSS2 (B).

Supplementary Figure S3. Workflow of the project. Search for SARS-CoV-2 interacting proteins was followed by searches for cancer relevance of the interacting proteins. Identified interactors were validated by searches in NCBI cancer-relevant databases. Clinical markers, diagnostic signatures and data about COVID-19 treatment modalities were retrieved from NIH NCI, FDA and Milken Institute databases. The retrieved data were used for building networks. The network analysis was used for identification of SARS-CoV-2 targets of relevance for carcinogenesis and treatment of cancer. Supplementary Figure S3 is available at figshare at doi: 10.6084/m9.figshare.13633793

Supplementary Table S1. 73 SARS-CoV-2 targets of relevance to cancer.

Supplementary Table S2. List of all biological processes retrieved from the COVID-19 network with BiNGO (A). Biological processes of relevance to cancer are listed in (B).

Supplementary Table S3, Lists of CDx markers approved by FDA for anti-cancer drugs (40) (A), clinically used cancer markers (41) (B), clinically used diagnostic signatures (41) (C, D, E, F, G, H), and targets of COVID-19 drugs (42) (I) are shown.

Supplementary File S1 (at). The file of all networks is available in Supplementary Materials. This file can be used for an analysis of the COVID-19 network for multiple applications, e.g. search for various biological activities, nodes and edges.

Supplementary materials of this report are available online at figshare.com. The links are given in legends to the Supplementary Figures, Tables and .cys file.

Supplementary materials allow search of data, e.g. in Excel files or in .cys file.

*Supplementary Figures S1 and S2 can be retrieved online from:

https://figshare.com/articles/figure/Supplementary_Figures_S1_S2/12804881

* Supplementary Figure S3 is available at figshare at doi: 10.6084/m9.figshare.13633793

*Supplementary Tables S1, S2 and S3 can be retrieved online from:

https://figshare.com/articles/dataset/Supplementary_Tables_S1_S2_and_S3/12804887

*Supplementary File S1 can be retrieved online from:

https://figshare.com/articles/online_resource/Cancer_and_COVID-19_systems_biology_Suppl_File_S1/12793883

Supplementary Figure S1. Networks of COVID-19 interactors retrieved from different databases. Networks were generated with Cytoscape, with retrieval of nodes and edges from databases, as annotated. The network structure differs depending on the database. Merging the retrieved data, as we did in this publication, allowed combine and use information from these database

NOTE: The networks are shown to illustrate their structure. For zooming in on nodes and edges and for analysis of the networks, please use Cytoscape .cys file at https://figshare.com/articles/online_resource/Cancer_and_COVID-19_systems_biology_Suppl_File_S1/12793883 .

A. BAR (87 nodes, 104 edges). Reference: bar.utoronto.ca

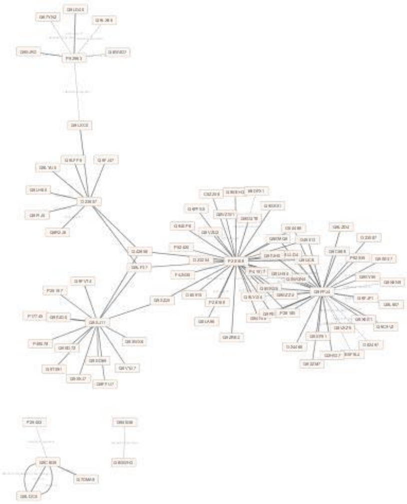

B. bhf-ucl (248 nodes, 271 edges). Reference: British Heart Foundation – University College London; http://www.ebi.ac.uk/Tools/webservices/psicquic/view/main.xhtml;jsessionid=D76DAB6186454EA73BCC448B7027EF9B?conversationContext=1

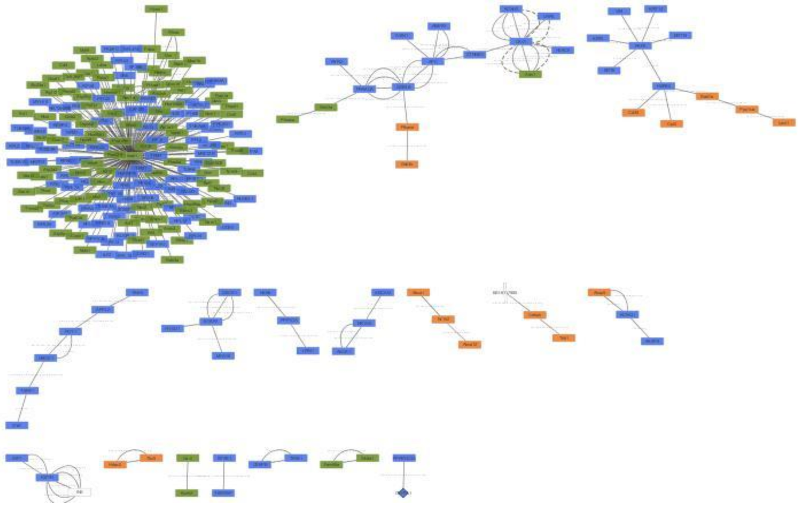

C. BioGrid (5,205 nodes, 10,204 edges). Reference: https://thebiogrid.org/

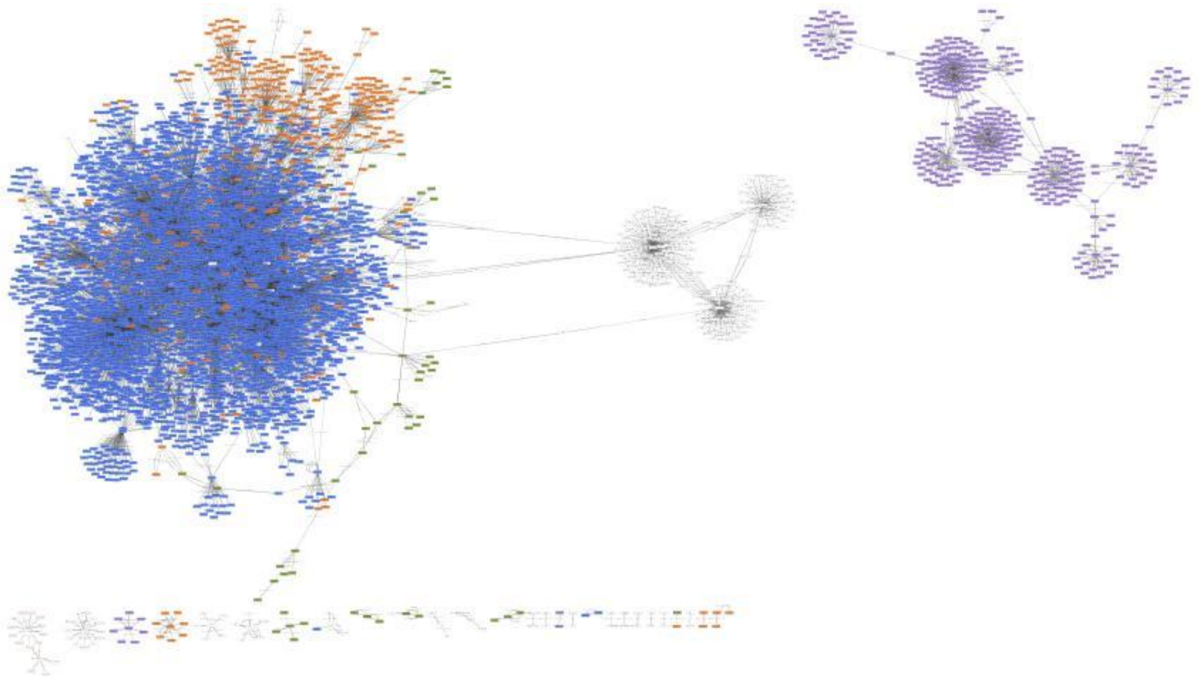

D. ChEMBL (3,790 nodes, 5,230 edges). Reference: https://www.ebi.ac.uk/chembl/

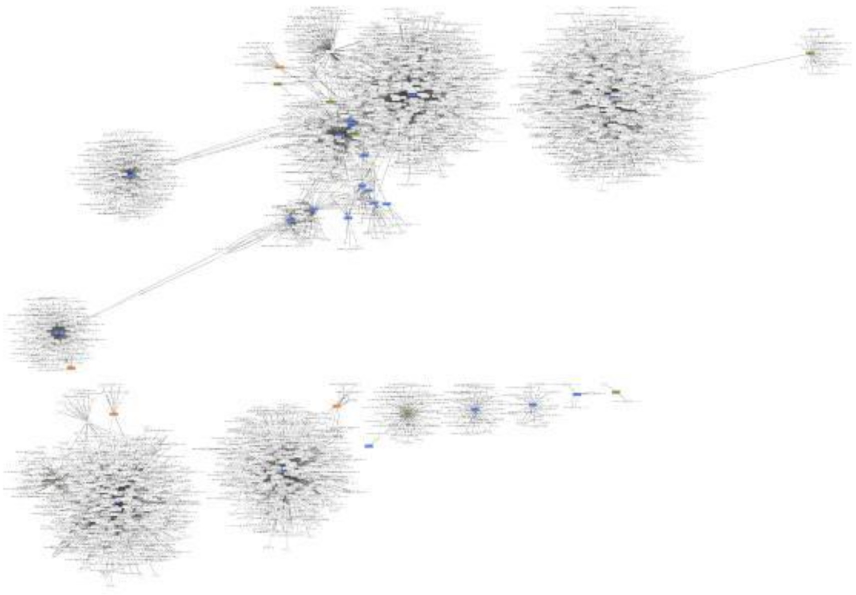

E. EBI-GOA-miRNA (37 nodes, 54 edges). Reference:
http://www.ebi.ac.uk/Tools/webservices/psicquic/view/main.xhtml?conversationContext=1

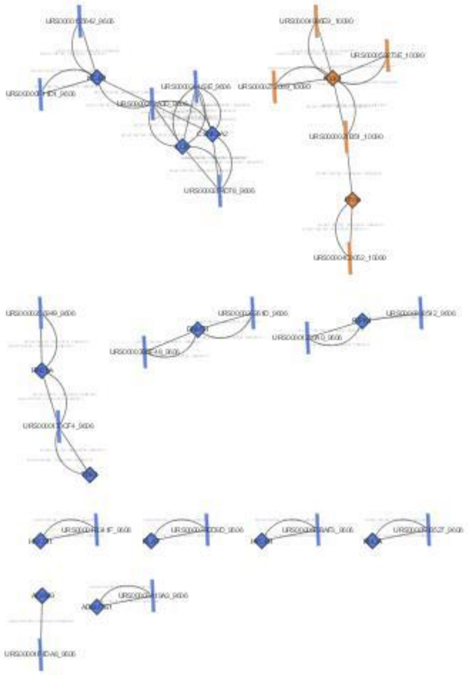

F. EBI-GOA-nonIntAct (716 nodes, 1,260 edges). Reference:
http://www.ebi.ac.uk/Tools/webservices/psicquic/view/main.xhtml?conversationContext=1

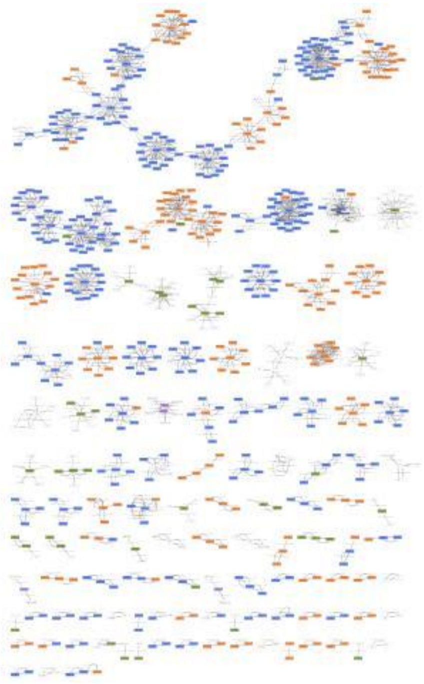

G. HPIDb (107 nodes, 146 edges). Reference: hpidb.igbb.msstate.edu

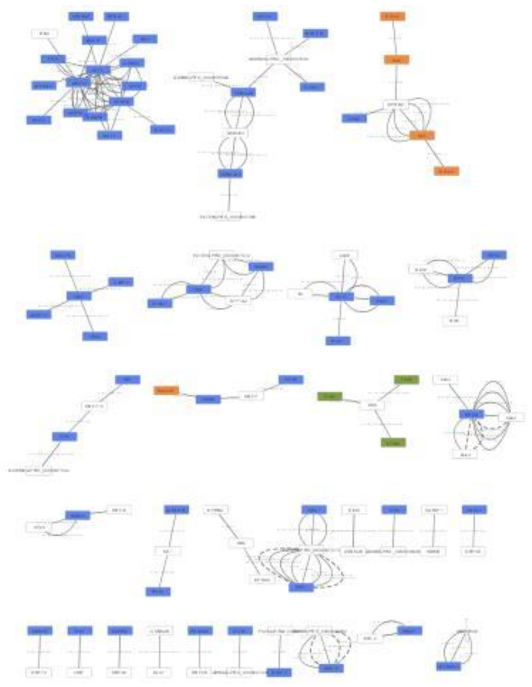

H. IMEx (8,405 nodes, 23,138 edges). Reference: www.imexconsortium.org

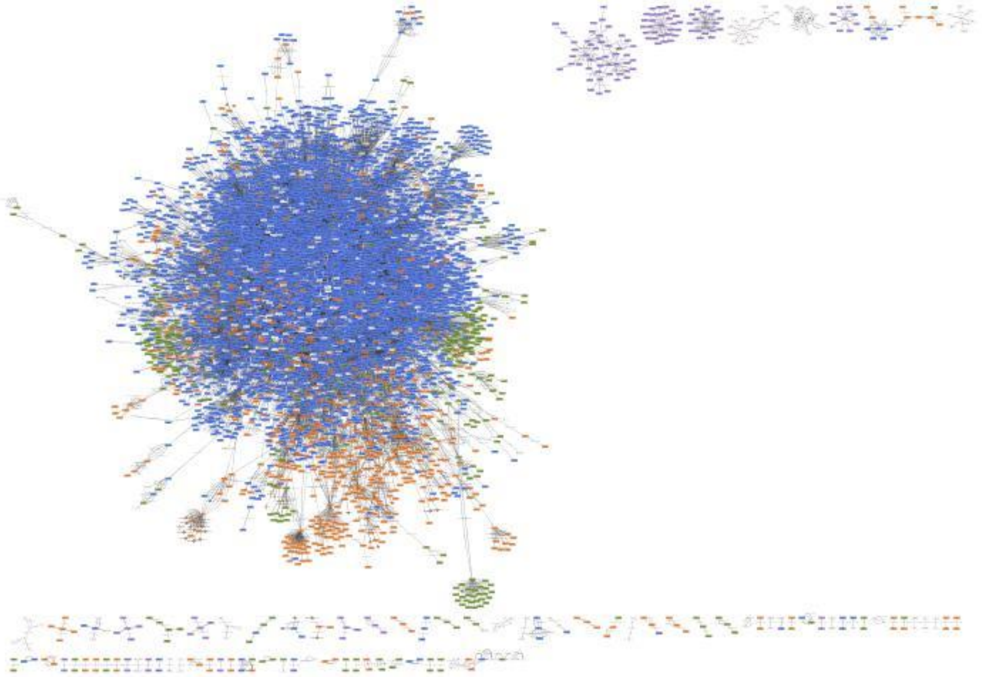

I. Innate-DB (291 nodes, 599 edges). Reference: www.innatedb.com

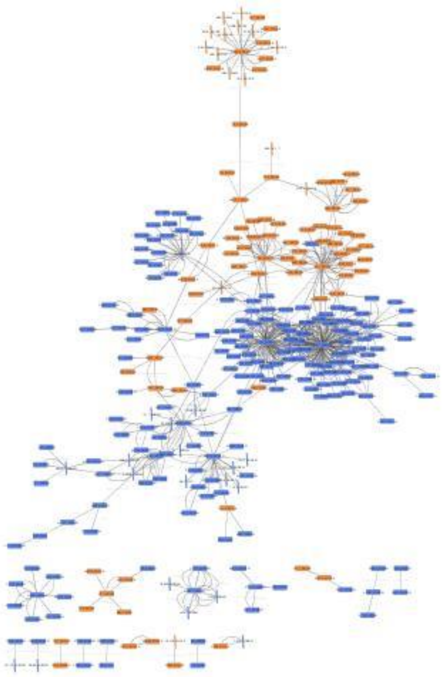

J. InnateDB-All (3,953 nodes, 10,819 edges). Reference: www.innatedb.com

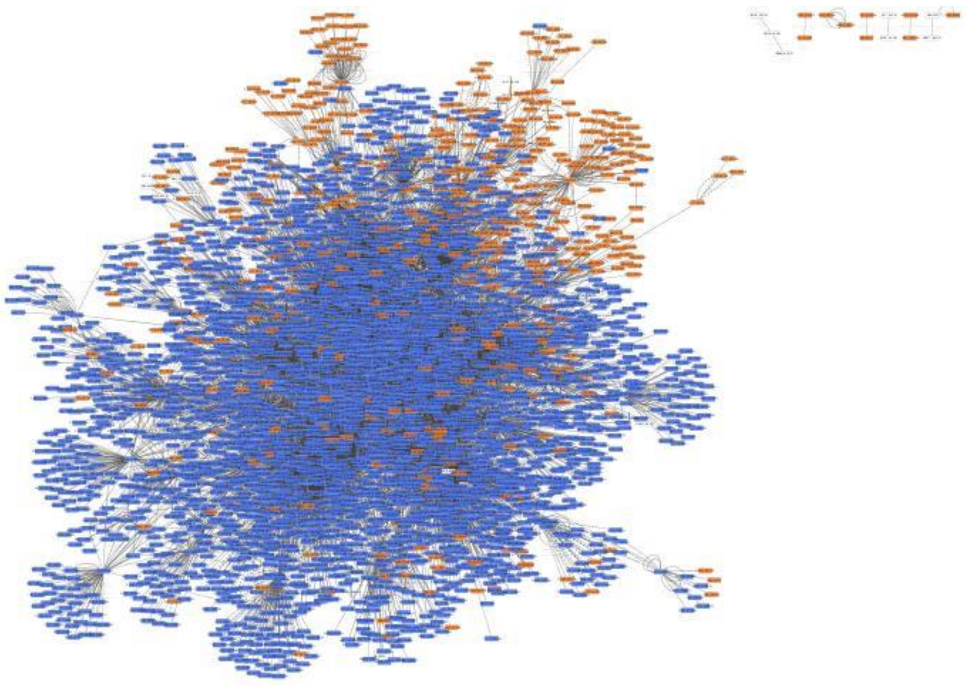

K. IntAct (7.021 nodes, 18,203 edges). Reference: www.ebi.ac.uk › intact

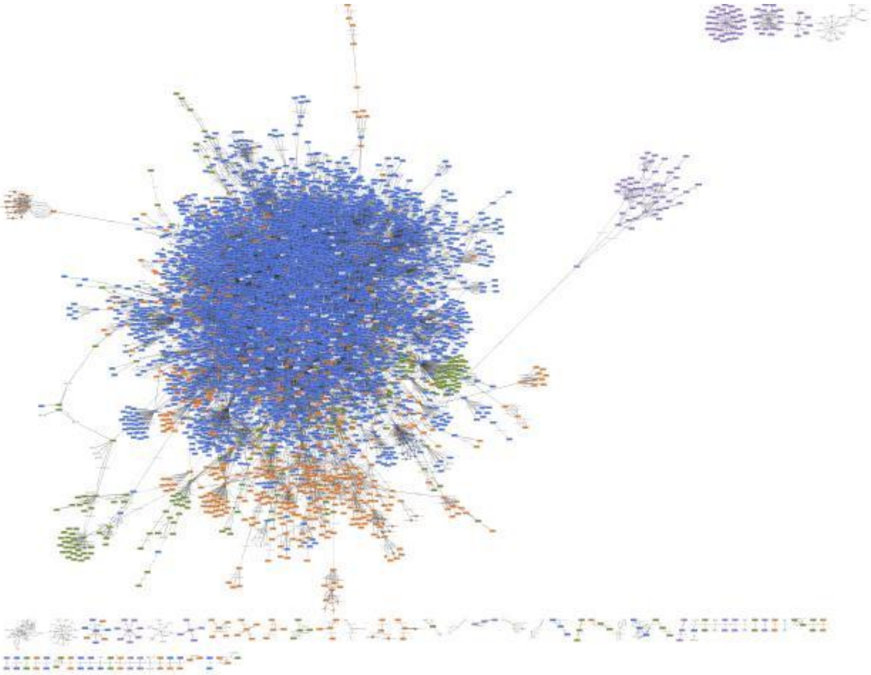

L. MatrixDB (898 nodes, 1,397 edges). Reference: matrixdb.univ-lyon1.fr

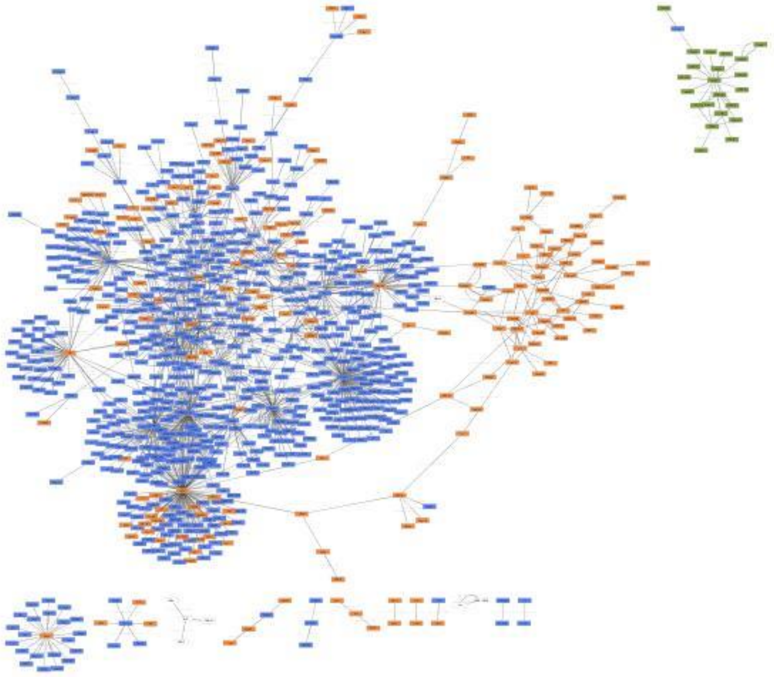

M. MBInfo (28 nodes, 44 edges). Reference: http://www.ebi.ac.uk/Tools/webservices/psicquic/view/main.xhtml?conversationContext=1

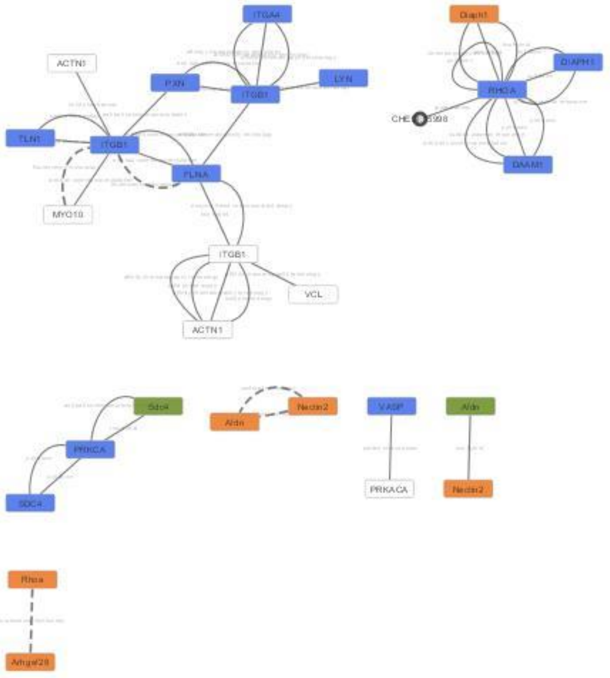

N. Mentha (5,385 nodes, 13,733 edges). Reference: mentha.uniroma2.it

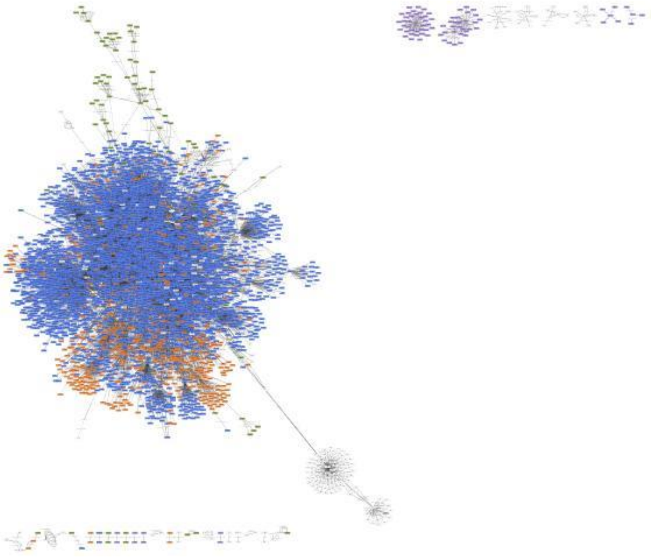

O. MINT (2,601 nodes, 4,823 edges). References: mint.bio.uniroma2.it
http://cbm.bio.uniroma2.it/mint/index.html

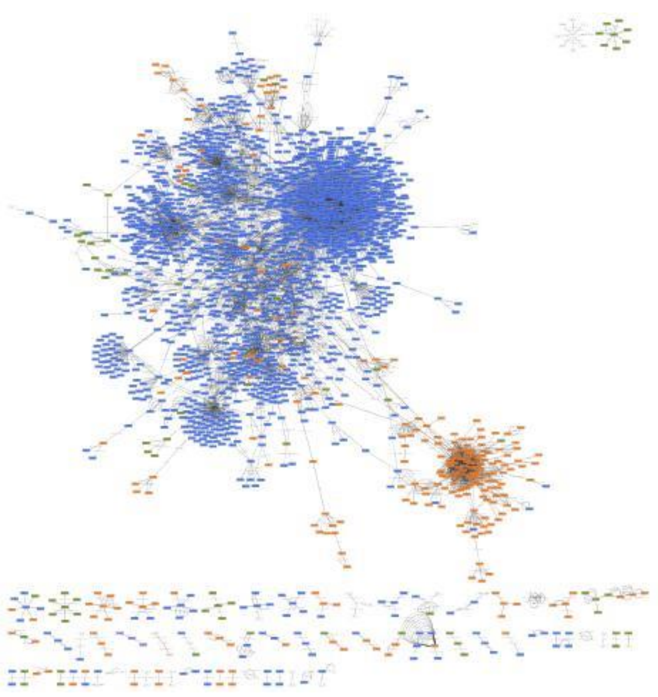

P. MPIDB (8 nodes, 9 edges). Reference: http://www.jcvi.org/mpidb/

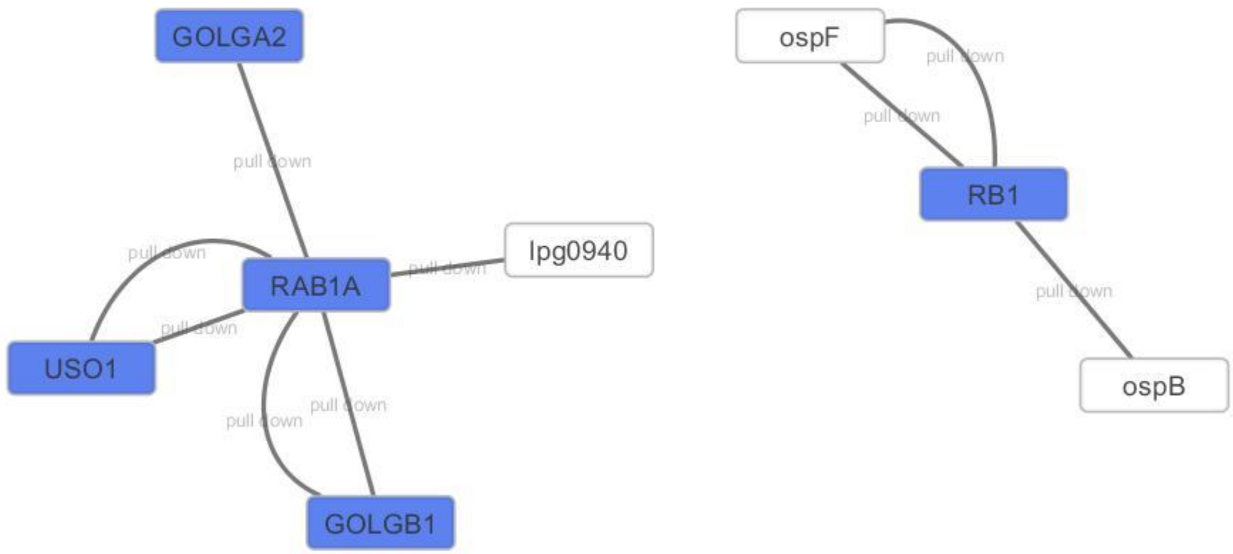

Q. Reactome-FIs (1,343 nodes, 2,296 edges). Reference: reactome.org

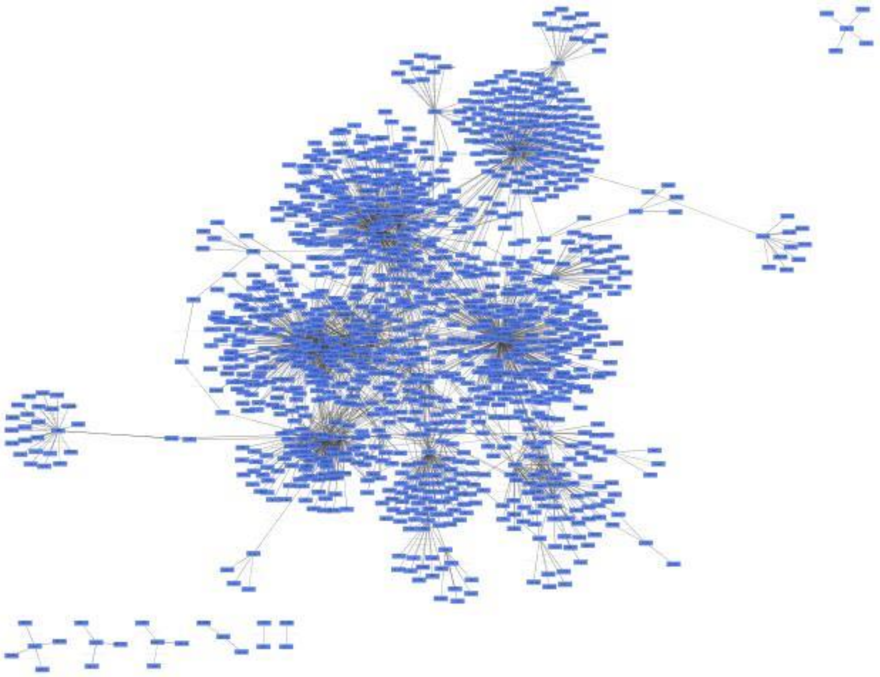

R. UniProt (465 nodes, 851 edges). Reference: www.uniprot.org

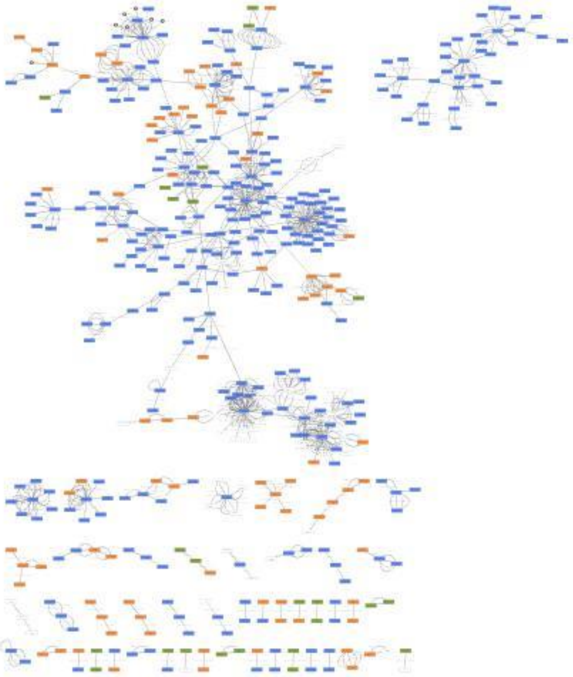

Supplementary Figure S2. BiNGO networks

A. BiNGO of the merged network of COVID-19 interactors (Figure 1A)

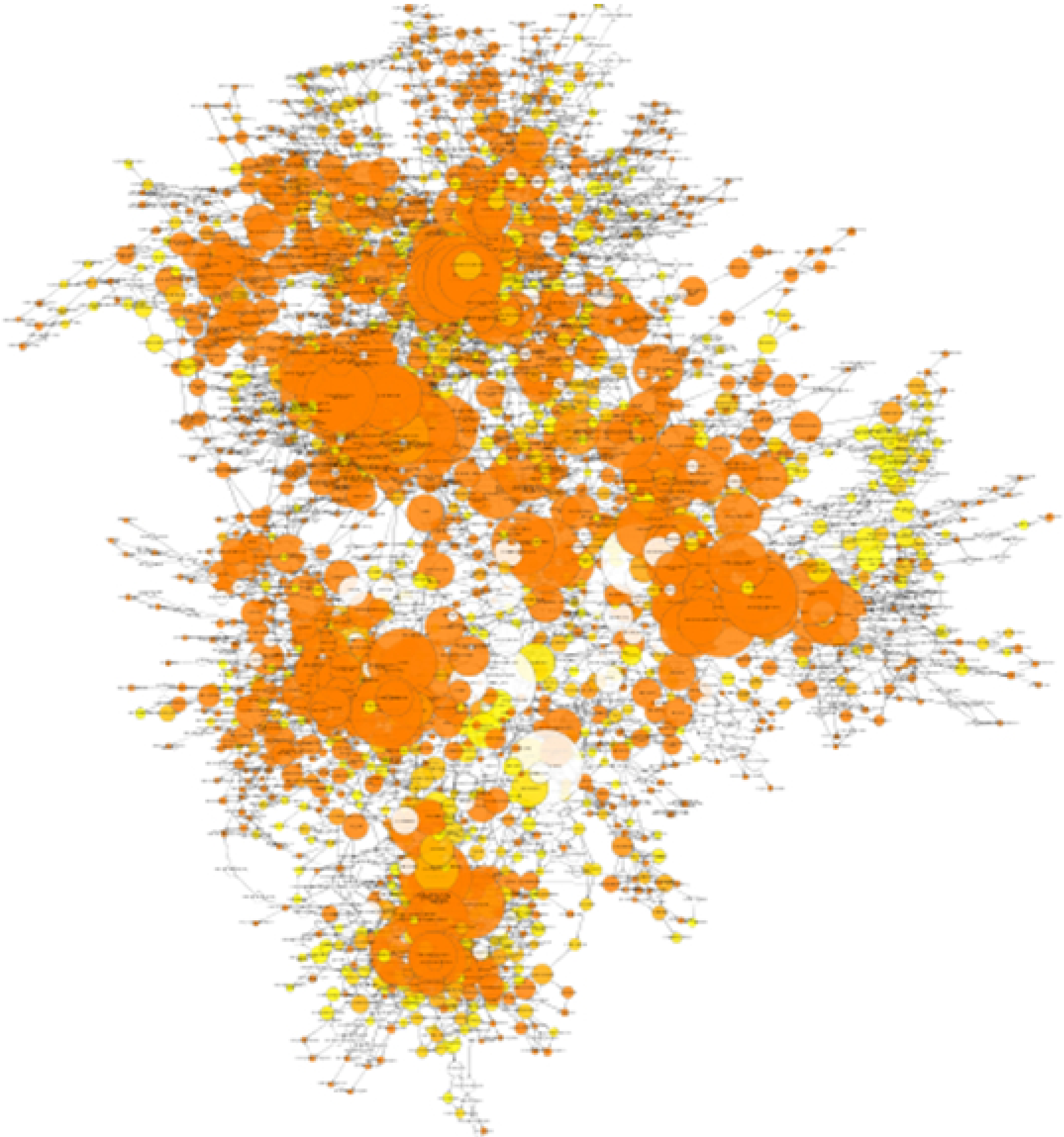

B. BiNGO of the network generated by ACE2 and TMPRSS2 (Figure 1B)

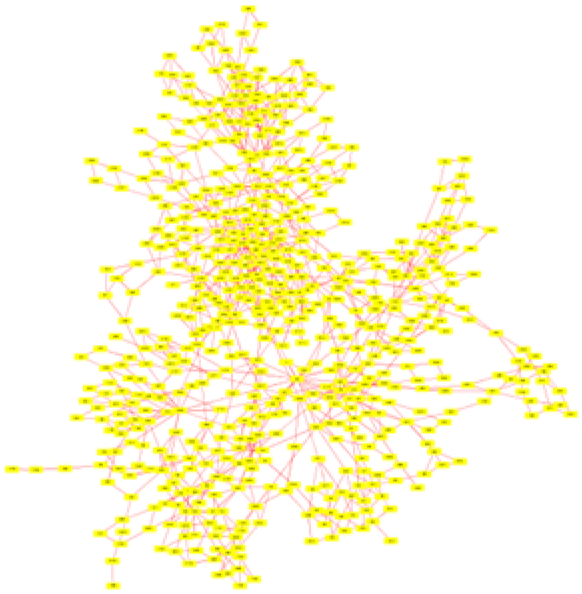

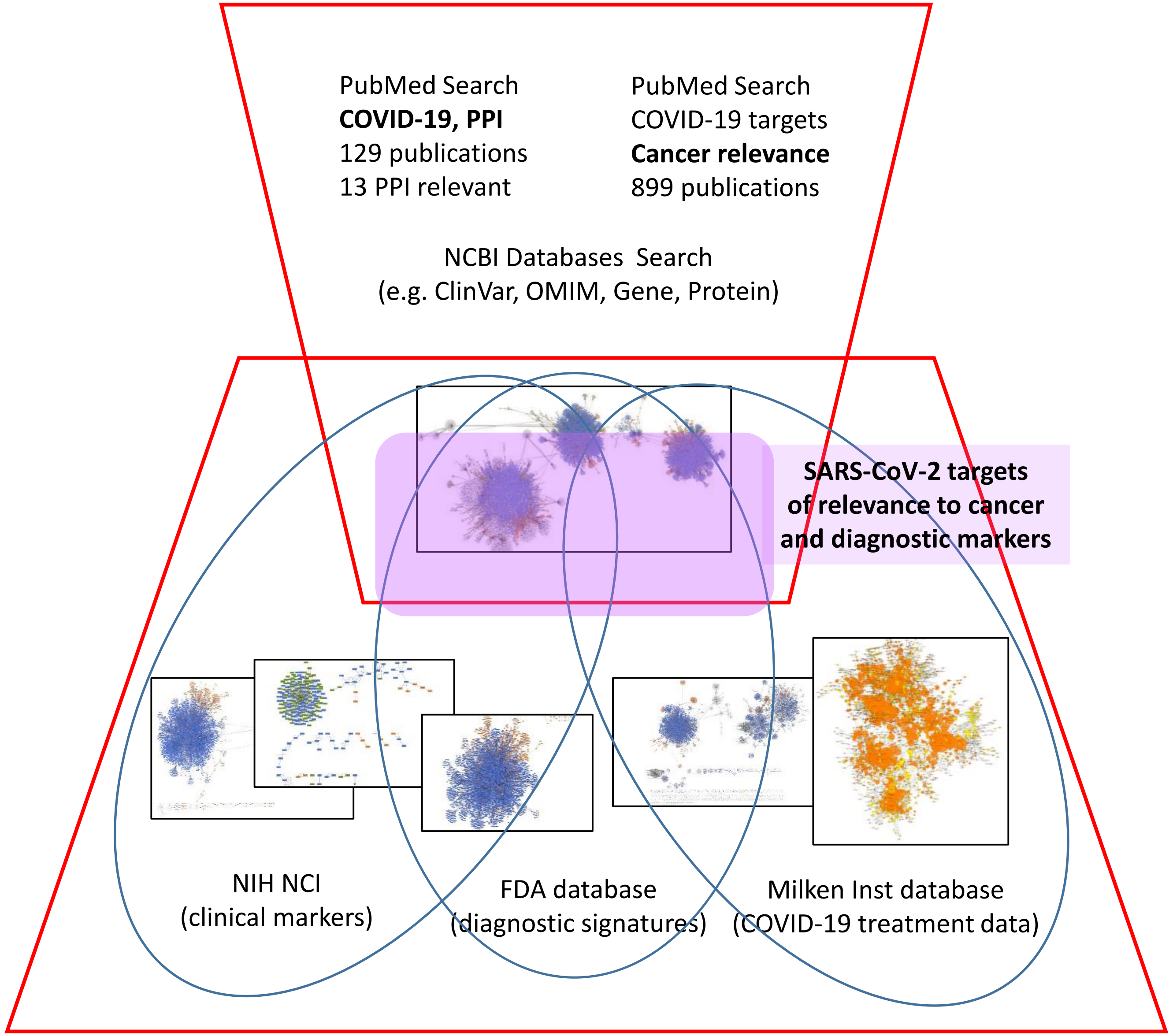

Supplementary Figure S3.

Workflow of the study.

Search for SARS-CoV-2 interacting proteins was followed by searches for cancer relevance of the interacting proteins. Identified interactors were validated by searches in NCBI cancer-relevant databases. Clinical markers, diagnostic signatures and data about COVID-19 treatment modalities were retrieved from NIH NCI, FDA and Milken Institute databases.
The retrieved data were used for building networks. The network analysis was used for identification of SARS-CoV-2 targets of relevance for carcinogenesis and treatment of cancer.
