# Supplementary material for: COVID-19 engages clinical markers for the management of cancer and cancer-relevant regulators of cell proliferation, death, migration, and immune response
Source: Sci Rep. 2021 Mar 4;11:5228. doi: 10.1038/s41598-021-84780-y (PMC7933131; doi:10.1038/s41598-021-84780-y)
Supplement: Supplementary file 2 — Supplementary Information 2. [file 41598_2021_84780_MOESM2_ESM.pdf]

Supplementary Table S1

73 COVID-19 targets of relevance to cancer.

*Supplementary Tables S1, S2 and S3 can be retrieved online from:

https://figshare.com/articles/dataset/Supplementary_Tables_S1_S2_and_S3/12804887

Name,	GO term Name
AP3B1	AP-3 complex subunit beta-1
CWC27	Spliceosome-associated protein CWC27
AKAP8L	A-kinase anchor protein 8-like
CSNK2A2	Casein kinase II subunit alpha
CSNK2B	Casein kinase II subunit beta
FAM98A	Protein FAM98A
SNIP1	Smad nuclear-interacting protein 1
MOV10	Helicase MOV-10
G3BP2	Ras GTPase-activating protein-binding protein 2 (G3BP-2)
ZDHHC5	Palmitoyltransferase ZDHHC5
COLGALT1	Procollagen galactosyltransferase 1
PKP2	Plakophilin-2
AKAP8	A-kinase anchor protein 8 (AKAP-8)
RIPK1	Receptor-interacting serine/threonine-protein kinase 1
CRTC3	CREB-regulated transcription coactivator 3
PPIL3	Peptidyl-prolyl cis-trans isomerase-like 3 (PPIase)
PLEKHA5	Pleckstrin homology domain-containing family A member 5 (
TBKBP1	TANK-binding kinase 1-binding protein 1 (TBK1-binding protein 1)
CIT	Citron Rho-interacting kinase (CRIK)
CEP43	FGFR1 oncogene partner
PRKAR2A	cAMP-dependent protein kinase type II-alpha regulatory subunit
PRKACA	cAMP-dependent protein kinase catalytic subunit alpha (PKA C-alpha)

PRKAR2B	cAMP-dependent protein kinase type II-beta regulatory subunit
TLE5	TLE family member 5
CNTRL	Centriolin (Centrosomal protein 1)
ERC1	ELKS/Rab6-interacting/CAST family member 1
GCC2	GRIP and coiled-coil domain-containing protein 2
JAKMIP1	Janus kinase and microtubule-interacting protein 1
CDK5RAP2	CDK5 regulatory subunit-associated protein 2
AKAP9	A-kinase anchor protein 9 (AKAP-9)
TBK1	Serine/threonine-protein kinase TBK1
IMPDH2	Inosine-5'-monophosphate dehydrogenase 2 (IMP dehydrogenase 2)
FKBP15	FK506-binding protein 15 (FKBP-15)
IDE	Insulin-degrading enzyme
HDAC2	Histone deacetylase 2 (HD2)
RALA	Ras-related protein Ral-A
RAB5C	Ras-related protein Rab-5C
RAB7A	Ras-related protein Rab-7a
RAB8A	Ras-related protein Rab-8A (Oncogene c-mel)
RAB2A	Ras-related protein Rab-2A
RAB10	Ras-related protein Rab-10
RAB14	Ras-related protein Rab-14
RHOA	Transforming protein RhoA
RAB1A	Ras-related protein Rab-1A (YPT1-related protein)
FAM162A	Protein FAM162A (E2-induced gene 5 protein)
RAB18	Ras-related protein Rab-18
NSD2	Histone-lysine N-methyltransferase NSD2
EXOSC5	Exosome complex component RRP46 (Chronic myelogenous leukemia tumor antigen 28)
MRPS27	28S ribosomal protein S27, mitochondrial (MRP-S27)
NEK9	Serine/threonine-protein kinase
HMOX1	Heme oxygenase 1 (HO-1)

TRIM59 Tripartite motif-containing protein 59 (RING finger protein 104) (Tumor suppressor TSBF-1)

MTCH1 Mitochondrial carrier homolog 1 (Presenilin-associated protein)

PLAT Tissue-type plasminogen activator (t-PA) (t-plasminogen activator) (tPA)

ITGB1 Integrin beta-1 (Fibronectin receptor subunit beta)

CISD3 CDGSH iron-sulfur domain-containing protein 3, mitochondrial

COL6A1 Collagen alpha-1(VI) chain

PVR Poliovirus receptor (Nectin-like protein 5) (NECL-5)

DNMT1 DNA (cytosine-5)-methyltransferase 1 (Dnmt1)

LOX Protein-lysine 6-oxidase

INHBE Inhibin beta E chain (Activin beta-E chain)

MFGE8 Lactadherin (Breast epithelial antigen BA46) (HMFG) (MFGM) (Milk fat globule-EGF factor 8)

OS9 Protein OS-9 (Amplified in osteosarcoma 9)

GDF15 Growth/differentiation factor 15 (GDF-15)

ADAMTS1 A disintegrin and metalloproteinase with thrombospondin motifs 1

HYOU1 Hypoxia up-regulated protein 1 (150 kDa oxygen-regulated protein)

ADAM9 Disintegrin and metalloproteinase domain-containing protein 9 (ADAM 9)

MARK2 Serine/threonine-protein kinase MARK2

MARK1 Serine/threonine-protein kinase MARK1

ABCC1 Multidrug resistance-associated protein 1

SCAP Sterol regulatory element-binding protein cleavage-activating protein (SCAP) (SREBP cleavage-activating protein)

TMEM97 Sigma intracellular receptor 2 (Sigma-2 receptor) (Sigma2 receptor) (Meningioma-associated protein 30)

GHITM Growth hormone-inducible transmembrane protein (Dermal papilla-derived protein 2)

Name, GO term	Name
AP3B1	AP-3 complex subunit beta-1
CWC27	Spliceosome-associated protein CWC27
AKAP8L	A-kinase anchor protein 8-like
CSNK2A2	Casein kinase II subunit alpha
CSNK2B	Casein kinase II subunit beta
FAM98A	Protein FAM98A
SNIP1	Smad nuclear-interacting protein 1
MOV10	Helicase MOV-10
G3BP2	Ras GTPase-activating protein-binding protein 2 (G3BP-2)
ZDHHC5	Palmitoyltransferase ZDHHC5
COLGALT1	Procollagen galactosyltransferase 1
PKP2	Plakophilin-2
AKAP8	A-kinase anchor protein 8 (AKAP-8)
RIPK1	Receptor-interacting serine/threonine-protein kinase 1
CRTC3	CREB-regulated transcription coactivator 3
PPIL3	Peptidyl-prolyl cis-trans isomerase-like 3 (PPIase)
PLEKHA5	Pleckstrin homology domain-containing family A member 5 (
TBKBP1	TANK-binding kinase 1-binding protein 1 (TBK1-binding protein 1)
CIT	Citron Rho-interacting kinase (CRIK)
CEP43	FGFR1 oncogene partner
PRKAR2A	cAMP-dependent protein kinase type II-alpha regulatory subunit
PRKACA	cAMP-dependent protein kinase catalytic subunit alpha (PKA C-alpha)
PRKAR2B	cAMP-dependent protein kinase type II-beta regulatory subunit
TLE5	TLE family member 5
CNTRL	Centriolin (Centrosomal protein 1)
ERC1	ELKS/Rab6-interacting/CAST family member 1
GCC2	GRIP and coiled-coil domain-containing protein 2
JAKMIP1	Janus kinase and microtubule-interacting protein 1
CDK5RAP2	CDK5 regulatory subunit-associated protein 2
AKAP9	A-kinase anchor protein 9 (AKAP-9)
TBK1	Serine/threonine-protein kinase TBK1
IMPDH2	Inosine-5'-monophosphate dehydrogenase 2 (IMP dehydrogenase 2)
FKBP15	FK506-binding protein 15 (FKBP-15)
IDE	Insulin-degrading enzyme
HDAC2	Histone deacetylase 2 (HD2)
RALA	Ras-related protein Ral-A
RAB5C	Ras-related protein Rab-5C
RAB7A	Ras-related protein Rab-7a
RAB8A	Ras-related protein Rab-8A (Oncogene c-mel)
RAB2A	Ras-related protein Rab-2A
RAB10	Ras-related protein Rab-10
RAB14	Ras-related protein Rab-14
RHOA	Transforming protein RhoA
RAB1A	Ras-related protein Rab-1A (YPT1-related protein)
FAM162A	Protein FAM162A (E2-induced gene 5 protein)
