# Supplementary material for: COVID-19 engages clinical markers for the management of cancer and cancer-relevant regulators of cell proliferation, death, migration, and immune response
Source: Sci Rep. 2021 Mar 4;11:5228. doi: 10.1038/s41598-021-84780-y (PMC7933131; doi:10.1038/s41598-021-84780-y)
Supplement: Supplementary file 3 — Supplementary Information 3. [file 41598_2021_84780_MOESM3_ESM.pdf]

Supplementary Table S2A
List of biological processes represented in the networks shown in Figure 1.

Biological processes were retrieved with BiNGO tool. Homo sapience and $p<0.05$ for significance of representation were selected. Hypergeometric statistical test was used, with Benjamini and Hochberg false discovery rate correction.

Note a large number of affected biological processes. Analysis of the network showed that there many biological processes that control the same process in a similar way. However, we show the whole list as it was retrieved from the network analysis. This would allow other researchers to perform their own analysis.

*Supplementary Tables S1, S2 and S3 can be retrieved online from:

https://figshare.com/articles/dataset/Supplementary_Tables_S1_S2_and_S3/12804887

DNA methylation on cytosine 1789 1788 Q9UBC3 Q9Y6K1

regulation of mitotic recombination 4361 P49959 641 P40692 P54132 Q92878

mitotic spindle elongation O43663 Q02241 9493

negative regulation of CREB transcription factor activity P35638 Q9BZS1 50943

ribosomal small subunit assembly P62263 P62851 P62753 6194

protein targeting to Golgi Q6VY07 10133 Q96CV9

protein import into nucleus, docking P52948 23165 Q92621

glomerular basement membrane development P53420 Q01955 P39210 1285 4358 P19544 7490 P14543

G1 phase of mitotic cell cycle 6872 9238 P30307 1021 P15923 Q01094 4193 990 1017 6929 Q9UJX2 Q00987 Q969Z0 Q99741 995 Q00534 P24941 8328 Q5VTD9 1869 P21675

G1/S transition of mitotic cell cycle 8453 8454 Q13554 8452 6198 P11802 2672 O75461 O00743 6194 25988 595 Q7L0Q8 Q99684 997 91 6502 5537 P62753 1978 P78536 P36896 26524 58480 8065 1029 1027 1104 1026 207 P00533 P42771 P11309 P24385 P30304 P10415 Q8N726 Q13618 Q13617 Q13616 P46527 Q9BQA5 1874 Q9NRM7 898 1956 P23443 Q16254 P06400 P54687 A4D1W7 P31749 Q04771 P38936 Q13309 Q93034 Q08209 P24864 2935 Q96FF9 P49427 5925

mitotic telophase 8379 Q9Y6D9

activation of Rho GTPase activity 7248 Q92949 Q86WV8 23513 Q14160 Q96N67 81565 Q6PID4 Q6ZW31 Q92574 Q9GZM8 Q14289 85440 85360

activation of Rac GTPase activity Q14160 Q96N67 Q6PID4 Q14289 85440 23513

activation of Cdc42 GTPase activity 81565 Q9GZM8

negative regulation of transcription from RNA polymerase III promoter Q9H063 84232

cytoplasmic transport Q8WXF0 10772 O75494 135295

negative regulation of stress-activated MAPK cascade 2305 Q12778 Q08050 3476 P78318 2308

regulation of DNA endoreduplication 4361 P49959 Q9UQE7 9126 Q969H0 Q8N3U4 55294 Q14683

negative regulation of DNA endoreduplication 4361 P49959 Q9UQE7 9126 Q969H0 Q8N3U4 55294 Q14683

regulation of transcription involved in G1 phase of mitotic cell cycle 8328 Q5VTD9

regulation of transcription involved in G2/M-phase of mitotic cell cycle 6872 P21675

establishment of mitotic spindle orientation Q9NXR1 O43264 54908 P49450 54820 6993 1058 P43034 P63172 O14777 Q96EA4

regulation of transforming growth factor-beta1 production P78423 P09958 7057 O75787 P07996 10159

regulation of transforming growth factor-beta2 production 3091 4088 4089 P84022 Q16665 Q13485

regulation of transforming growth factor-beta3 production 4088 P16220 P84022 1385 Q53X93

positive regulation of transforming growth factor-beta1 production P78423 7057 O75787 P07996 10159

positive regulation of transforming growth factor-beta3 production 4088 P16220 P84022 1385 Q53X93

regulation of activin receptor signaling pathway P27037 4221 A4D1W7 P36896 Q04771 O00255 Q13705 P12755 P19883 4092 2280 91 O15105 10468 P62942

positive regulation of activin receptor signaling pathway P27037 91 P36896 Q13705

regulation of superoxide anion generation P13686 Q9NY61 54

negative regulation of superoxide anion generation P13686 Q9NY61 54

chromatin silencing at rDNA O43463 6839 O43159 8467 P17480 11176 O60264 23411 23378 Q9UIF9 Q96EB6

histone deacetylation 5245 3066 3065 Q9UIF9 Q9UQL6 11107 83933 10933 Q96PK6 O15379 55869 10013 Q9UBU8 10014 10432 Q6IT96 P56524 Q92769 Q969S8 Q13547 8841 Q9BY41 23411 79885 P35232 11176 Q9UBN7 Q96EB6

histone demethylation	Q9UPP1 23210 23133 Q6NYC1 23135 O75164 Q9H3R0 23028 9682 Q8NB78 221656 Q9H6W3 23081 Q9Y4C1 O60341 Q9UGL1 10765

collagen biosynthetic process	P02452 P02461 871 1289 1277 P20908 P50454

nucleosome positioning	Q96T23 3008 3007 3006 51773 8467 P49711 O60264 P10412 P16403 P16402

meiotic spindle organization	26271 9700 P23258 Q14674 Q9UKT4 P35579 4627 7283

protein-DNA complex disassembly	Q969G3 3159 Q9UIG0 Q01105 P17096 11198 P09430 6605 Q9Y5B9 7141

assembly of spliceosomal tri-snRNP	11218 O95400 Q8WXF0 26121 Q8WWY3 10772 Q9UHI6 O75494 10421 O94906 135295

spliceosome assembly	Q15459 10291 6430 4154 6431 O75643 10907 O95391 P52756 51340 1653 P62316 7536 Q92499 10772 6426 P83876 Q13243 P62314 O94906 Q13242 135295 Q13247 Q16637 Q12874 10181 Q9UMS4 Q12872 8683 Q8WXF0 Q15428 O75494 27339 10569 Q07955 6733 6633 P78362 P62308 6637 Q9BZJ0 P62304

cytokinesis after mitosis4628 P35580 P25054 324 1616 Q9UER7

retrograde transport, vesicle recycling within Golgi	9950 Q6VY07 10133 Q96CV9 Q8TBA6

regulation of immature T cell proliferation	O43353 1029 Q8N726 P04626 8767 2064 P42771

regulation of immature T cell proliferation in the thymus	1029 Q8N726 P04626 2064 P42771

negative regulation of immature T cell proliferation	1029 Q8N726 P04626 2064 P42771

negative regulation of immature T cell proliferation in the thymus	1029 Q8N726 P04626 2064 P42771

protein deneddylation	10987 Q92905

negative regulation of RNA splicing	P62277 6231 Q8WXF0 10772 6207 O75494 4869 135295 P62854

serine phosphorylation of STAT3 protein	Q00535 A1XKG3 1020 Q9UBE8

RNA splicing, via transesterification reactions	O43395 O75940 P52272 10250 6631 P14678 O95391 O00148 Q96DI7 Q8WWY3 4686 10772 P09012 6625 P09651 Q9UK45 6627 Q13243 P62995 O94906 Q13242 135295 Q13247 P07910 Q16637 P54105 O75934 Q15428 Q15427 79084 O75494 P22626 27257 23451 O43290 6633 P09661 Q15393 O43172 10421 6637 P08579 P52298 Q9BRX9 9128 9129 O75643 10907 Q96IZ7 54496 10594 P09234 P83876 P23246 P31942 9410 Q9UMS4 84292 8683 6421 Q9H814 Q9Y333 Q9UHI6 11218 11338 29896 Q07955 57819 P62308 P26368 51319 P62304 Q15459 50628 Q13435 9785 10291 6430 6431 6434 P52756 1653 P62316 Q8IYB3 Q92499 P62318 6426 O14744 P62314 Q12874 10181 Q12872 O95400 Q8WXF0 Q09161 3178 27339 26121 Q6P2Q9 Q13573 3181 4670 4154 25929 22938 3189 Q8TEQ6 51340 Q5TAL4 Q96MU7 Q9NVM4 7536 91746 Q9BQA1 P08621 Q08170 10569 10286 6733 10285 Q92620 P78362 Q13595 Q9BZJ0 1207

negative regulation of steroid hormone receptor signaling pathway 9420 2100 29966 O75881 P51843 Q99623 387 3479 11331 Q92731 811 P61586 O75928 P27797 P01343 Q13033

RNA splicing, via transesterification reactions with bulged adenosine as nucleophile O43395 P52272 6631 P14678 O95391 O00148 Q8WWY3 4686 10772 P09012 6625 P09651 Q9UK45 6627 Q13243 P62995 O94906 Q13242 135295 Q13247 P07910 Q16637 P54105 Q15428 Q15427 79084 O75494 P22626 23451 O43290 6633 P09661 O43172 10421 6637 P08579 P52298 Q9BRX9 9128 9129 O75643 10907 Q96IZ7 54496 10594 P09234 P83876 P23246 P31942 Q9UMS4 84292 8683 6421 Q9H814 Q9Y333 Q9UHI6 11218 11338 29896 Q07955 57819 P62308 P26368 51319 P62304 Q15459 50628 Q13435 9785 10291 6430 6431 6434 P52756 1653 P62316 Q92499 P62318 6426 O14744 P62314 Q12874 10181 Q12872 O95400 Q8WXF0 Q09161 3178 27339 26121 Q6P2Q9 Q13573 3181 4670 4154 25929 22938 3189 Q8TEQ6 51340 Q5TAL4 Q96MU7 Q9NVM4 7536 91746 Q9BQA1 P08621 10569 6733 Q92620 P78362 Q13595 Q9BZJ0 1207

negative regulation of estrogen receptor signaling pathway Q99623 9420 11331 29966 O75881 Q13033

alternative nuclear mRNA splicing, via spliceosome 10569 4670 6421 P52272 3178 O95391 P09651 Q96IZ7 51319 P23246

regulation of intracellular protein transport 7046 Q15654 2033 O43557 P10599 O60603 Q04759 P84022 999 Q7Z6C1 6901 Q13485 P25963 Q9UBC1 4188 Q16635 7295 P50148 2316 9908 Q9HD26 Q99750 7205 P00533 Q96EB6 O00206 Q09472 7067 Q8N726 57120 Q06124 Q00535 P01137 Q9Y6A5 P01133 P01375 5716 8165 4088 4089 8720 Q9BZF9 Q9NR96 9495 387 P21359 O60674 P10071 5566 5569 Q9Y2B9 2737 Q92667 P24588 5460 7124 11142 1020 Q9UNN5 O00221 114548 Q93062 Q6PJW8 Q99728 P61586 7099 7098 Q6IR47 Q9NYA1 11030 10460 P14923 P36894 P10600 23411 P13796 1029 3728 1540 Q9NQB0 P42771 P49841 Q99835 P21333 P61925 4792 Q14703 P12830 A1XKG3 2010 Q96P20 11124 O15455 Q9UN86 1956 Q9NQC7 P10827 Q60FE5 O75832 Q06830 580 P50402 P17612 2932 Q9UMX1 54106 Q15653

nuclear mRNA 3'-splice site recognition 10569 Q15459 Q12874 10291 Q15428 7536 O95391

regulation of protein import into nucleus, translocation 5460 Q09472 7046 2033 2010 Q93062 P84022 Q7Z6C1 P01137 6901 Q13485 1956 P01133 Q6IR47 4088 11030 4089 Q16635 P36894 P10600 23411 P50402 P50148 O60674 P00533 Q96EB6

negative regulation of protein import into nucleus, translocationQ16635 2010 6901 P50402

positive regulation of protein import into nucleus, translocation 5460 Q09472 Q6IR47 7046 4088 11030 4089 2033 P36894 P10600 Q93062 O60674 P84022 Q7Z6C1 P00533 P01137 Q13485 1956 P01133

nuclear mRNA splicing, via spliceosome O43395 P52272 6631 P14678 O95391 O00148 Q8WWY3 4686 10772 P09012 6625 P09651 Q9UK45 6627 Q13243 P62995 O94906 Q13242 135295 Q13247 P07910 Q16637 P54105 Q15428 Q15427 79084 O75494 P22626 23451 O43290 6633 P09661 O43172 10421 6637 P08579 P52298 Q9BRX9 9128 9129 O75643 10907 Q96IZ7 54496 10594 P09234 P83876 P23246 P31942 Q9UMS4 84292 8683 6421 Q9H814 Q9Y333 Q9UHI6 11218 11338 29896 Q07955 57819 P62308 P26368 51319 P62304 Q15459 50628 Q13435 9785 10291 6430 6431 6434 P52756 1653 P62316 Q92499 P62318 6426 O14744 P62314 Q12874 10181 Q12872 O95400 Q8WXF0 Q09161 3178 27339

26121 Q6P2Q9 Q13573 3181 4670 4154 25929 22938 3189 Q8TEQ6 51340 Q5TAL4 Q96MU7 Q9NVM4 7536 91746 Q9BQA1 P08621 10569 6733 Q92620 P78362 Q13595 Q9BZJ0 1207

cell cycle cytokinesis 4628 Q9H0H5 P35580 P25054 29127 Q9NZ56 324 Q96RK4 1616 P51587 Q9UER7

leptin-mediated signaling pathway P48357 3953 P41159

maturation of 5.8S rRNA P78316 23481 55759 8602 O00541 Q9GZL7

maturation of LSU-rRNA from tricistronic rRNA transcript (SSU-rRNA, 5.8S rRNA, LSU-rRNA) Q14137 23481 55759 O00541 Q9GZL7

maturation of 5.8S rRNA from tricistronic rRNA transcript (SSU-rRNA, 5.8S rRNA, LSU-rRNA) P78316 23481 55759 8602 O00541 Q9GZL7

maturation of LSU-rRNAQ14137 23481 55759 O00541 Q9GZL7

DNA replication involved in S phase 7884 Q14493

response to vitamin B2 1050 P49715

protein sumoylation 7341 Q9UBT2 P55854 P63165 10055 6612 6613 P61956 O75928 Q9NS56

protein desumoylation Q9HC62 Q9P0U3 29843 59343

regulation of collagen binding P17301 Q9BZE4 23560 3673

negative regulation of collagen binding Q9BZE4 23560

positive regulation of collagen binding P17301 3673

protein amino acid nitrosylation128 P11766

fatty acid beta-oxidation using acyl-CoA dehydrogenase 34 P28330 Q16134 2110 P11310

DNA replication, Okazaki fragment processing 2237 1763 P39748

peptidyl-lysine hydroxylation 23210 Q6NYC1

transferrin transport 1213 1785 P50570 Q00610

mammary gland epithelial cell proliferation 595 P24385 1147 O15111 Q15648 8945 2099 Q02363 Q9Y297 P03372 O14788 P17676

negative regulation of dopamine secretion Q9UBS5 2550 P21554

cell adhesion mediated by integrin P08648 P04004 P05556 P78536 Q13443 8754 P23229 7448 P05362 3655 3688 3678

cell-cell adhesion mediated by integrin P08648 P05556 Q13443 8754 3688 3678

assembly of actomyosin apparatus involved in cell cycle cytokinesis Q9H0H5 29127

cytokinesis, actomyosin contractile ring assembly Q9H0H5 29127

septin ring assembly 54443 Q9NQW6

beta-amyloid metabolic process 3416 P49810 P56817 23621 2280 P62942 P49768

stress granule assembly Q99700 6311 1153 Q14011

regulation of homotypic cell-cell adhesion P09382 Q02750 O14788 5604 5270 3956

positive regulation of homotypic cell-cell adhesion P09382 O14788 3956

regulation of erythrocyte aggregation P09382 3956

positive regulation of erythrocyte aggregation P09382 3956

toll-like receptor 3 signaling pathway 81622 Q9H1C4

regulation of toll-like receptor 3 signaling pathway 57162 Q96FA3

positive regulation of toll-like receptor 3 signaling pathway 57162 Q96FA3

toll-like receptor 4 signaling pathway O43353 57402 4792 P51617 8767 P25963 Q9HCY8

toll-like receptor 7 signaling pathway 81622 Q9H1C4

toll-like receptor 9 signaling pathway 81622 Q9H1C4

activation of protein kinase A activity Q08462 B3KY43 P13861 P40145 O60503 P51828 O60266 5577 P22694 P22612 O95622 5573 5575 5576 O43306 P10644 P31321 P31323 Q9BUB1 P17612 Q8NFM4 Q8NFM5 5566 5567 5568

beta-amyloid formation 2280 P62942

macrophage fusion 960 P16070

regulation of macrophage fusion Q13443 8754

positive regulation of macrophage fusion Q13443 8754

positive regulation of homocysteine metabolic process P21964 1312

negative regulation of mRNA processing Q8WXF0 Q99728 10772 O75494 580 4869 135295

cell junction assembly Q13753 P25054 Q08043 Q15654 Q13751 Q16787 Q8TEW0 Q8WUI4 P12814 7082 Q07157 4092 P11047 Q4VCS5 2702 P16144 1499 P23229 P35221 P21926 Q9UMD9 51564 Q9Y4G6 Q02413 Q6NVY8 3678 5339 3911 P08648 3690 Q15149 O15530 P35609 Q96QB1 10580 5796 P14923 7094 P36382 P18206 3728 324 Q96S53 7205 10420 1828 928 P05106 P10415 59341 P13647 O43815 P48509 7414 977 3852 3655 Q9Y490 8443 Q9BX66 O15230 P35222 5170 P17302 56288 2697 3909 Q6PID4 O15105 P02533 Q14289 Q9UL54 87 O15228

cell junction organization Q13753 P25054 Q08043 8650 Q15654 Q13751 5584 Q16787 Q8TEW0 4771 Q8WUI4 P12814 P15924 7082 Q07157 4092 P11047 Q4VCS5 2702 P16144 1499 57662 P23229 P35221 P84022 P21926 Q9UMD9 51564 Q9Y4G6 Q02413 Q6NVY8 3678 5339 3911 P08648 3690 Q15149 O15530 P35609 Q96QB1 10580 5796 P14923 P10600 7094 P36382 P18206 3728 324 Q96S53 7205 10420 1828 Q6IQ23 3801 928 P05106 P10415 59341 P13647 O43815 P48509 1832 Q9BVG8

P35240 7414 977 3852 3655 P01137 50855 Q9Y490 8443 4088 Q9BX66 Q9Y6R0 O15230 P35222 9253 5170 P17302 56288 2697 3909 Q6PID4 O15105 P02533 Q14289 Q9UL54 P41743 Q9NPB6 87 O15228

adherens junction organization 1832 P18206 8650 1499 Q9Y6R0 P15924 O15105 7414 P35222 4092 9253

adherens junction assembly P18206 1499 O15105 7414 P35222 4092

regulation of transcription from RNA polymerase II promoter by nuclear hormone receptor Q16512 5585 1609 O60341 5579 23411 P52824 23028 P05771 Q96EB6

response to type I interferon 6672 P23497

negative regulation of peptidyl-tyrosine phosphorylation Q9UJM3 5580 4771 P30153 5590 P67775 Q05513 Q05655 P35240 54206 857 5515 O15524 5518

activated T cell proliferation 6304 P06241 P11215 Q01826

regulation of acyl-CoA biosynthetic process 6622 P37840

peptide stabilization 6892 O15533

peptide antigen stabilization 6892 O15533

tRNA 5'-leader removal Q99575 10940

N-terminal peptidyl-lysine acetylation Q09472 Q92793 1387 2033 Q7Z6C1 Q4LE28 Q92831

endodermal cell fate commitment 1499 P35222

white fat cell differentiation P37231 P56545 1488 9612 1487 Q9Y618 5468 23411 Q7Z2Q5 P15090 Q96EB6 Q13363

peptidyl-threonine phosphorylation P25098 Q7Z727 7046 6872 P10415 4133 5585 Q9H422 O95271 P42345 156 P27708 P17252 91 5599 5578 5579 P05771 Q6IR47 Q16512 O15530 P36896 1195 Q9UBE8 5170 P37173 2475 P45983 P21675

protein localization to kinetochore 9212 O43264 54908 O60566 Q96GD4 Q96EA4

peptidyl-cysteine S-nitrosylation 128 P11766

natural killer cell differentiation 6670 Q02447 Q13422 10320

peptidyl-lysine modification P56524 Q09472 Q9BU89 23210 Q6NYC1 2033 23411 1491 7052 Q92793 1387 Q04656 P32929 Q7Z6C1 P21980 10013 83475 Q4LE28 Q9UBN7 Q96EB6 Q92831

peptidyl-threonine modification P25098 Q7Z727 7046 6872 P10415 4133 5585 Q9H422 O95271 2590 P42345 156 2589 P27708 P17252 91 5599 5578 5579 P05771 Q10472 Q10471 Q6IR47 Q16512 O15530 P36896 1195 Q9UBE8 5170 P37173 2475 P45983 P21675

trophectodermal cellular morphogenesis 8452 Q96S42 Q13618

regulation of complement activation, lectin pathway 2 710 P05155 P01023

negative regulation of complement activation, lectin pathway 2 710 P05155 P01023

rRNA transport 7248 Q92574 Q86WV8 4869

protein-pyridoxal-5-phosphate linkage via peptidyl-N6-pyridoxal phosphate-L-lysine P32929 1491

dihydrolipoamide metabolic process 1738 P09622

positive regulation of receptor recycling Q6FGG2 Q14160 Q15836 6622 P37840 9341 23513

nuclear envelope disassembly 81565 P43034 Q9GZM8

PMA-inducible membrane protein ectodomain proteolysis P78536 Q13443 8754 O14672

negative regulation of helicase activity 7157 P04637 23411 Q96EB6

negative regulation of binding P23396 O95271 Q96RU8 P41134 Q96JM2 23560 P63165 P25963 2307 Q9NSA3 P56524 4188 4221 O75381 2316 51773 Q99750 408 P49407 409 Q96EB6 7341 P11309 P35638 7067 Q8N726 Q5TAX3 P32121 23028 Q9BYM8 P40424 P06400 5716 Q92949 Q9NR96 9093 O60674 Q05655 Q92786 Q96EY1 P23497 P43489 5580 O43593 6672 114548 4092 3162 P53567 O43638 154 Q02535 57669 Q9BZE4 10616 P07550 P04040 55806 P05412 5195 Q9Y2T7 Q92769 64127 Q08117 23411 1029 166 Q9HCM4 1540 Q9NQB0 P42771 P49841 847 11315 Q99835 P23510 P21333 7023 4792 P19474 Q02363 3066 P51843 Q96T23 Q96P20 Q01664 O60341 857 Q9GZT9 Q9NQC7 6188 P10827 Q60FE5 O75832 Q9BZS1 O00255 2932 54583 P51617 Q9HC29 Q99497 O15105 54106 P09601 50943 5925

histone H3-K4 demethylation O60341 Q9UGL1 23028 10765 Q8NB78 221656 Q9H6W3

negative regulation of cell-matrix adhesion 7057 Q8N726 4771 P41182 5728 1029 P37023 P20936 P60484 P35240 604 94 P42771 O43182 P07996

positive regulation of cell-matrix adhesion P35968 3791 4088 7248 7402 1021 Q13418 Q86WV8 P31431 5590 57669 P16671 Q05513 P46939 3611 P84022 Q00534 1012 Q9HCM4 Q92574 948 P55290

blood vessel maturation 960 P33151 4313 P08253 P16070

endochondral ossification P02452 Q15742 Q14814 O00628 P56178 P63092 Q13506 Q9NX62 1277 4664 Q5TAT6 4665 P02458 4209

protein-pyridoxal-5-phosphate linkage Q99259 P32929 2571 1491

regulation of NK T cell differentiation Q13422 8546 P37173 10320 O00203

positive regulation of NK T cell differentiation Q13422 8546 P37173 10320 O00203

negative regulation of striated muscle cell differentiation P56524 10014 Q9UQL6 P52952

peptidyl-lysine acetylation Q09472 Q92793 1387 2033 Q7Z6C1 Q4LE28 Q92831

peptidyl-lysine hydroxylation to 5-hydroxy-L-lysine 23210 Q6NYC1

positive regulation of sulfur metabolic process P21964 1312 1499 Q9NQB0 P35222

osteoblast fate commitment 4086 Q15797 4221 O00255

spindle assembly O95271 55142 26271 203068 54930 Q9H4B7 Q9H6D7 55125 O15379 O94927 P07437 93323 Q96CS2 Q7Z4H7 8841 Q68CZ6 23354 O75376 79441 80254 P40692 Q96MT8 Q99871 54801 55559 Q9UKT4

spindle elongation O43663 Q02241 9493

store-operated calcium entry 283229 84766 Q8N4Y2 Q9BSW2

aggressive behavior P01185 P37288 552 6869 P25103 P01178

establishment of spindle localization O43264 54908 P49450 6993 9700 P63172 P35579 Q9NXR1 54820 1058 Q9NZ56 P43034 O14777 Q14674 4627 Q96EA4

establishment of spindle orientation Q9NXR1 O43264 54908 P49450 54820 6993 1058 P43034 P63172 O14777 Q96EA4

establishment of meiotic spindle localization Q9NZ56 P35579 4627

centrosome organization O15182 Q9BV73 9662 Q14008 55142 P51587 P52732 1499 54930 Q9H6D7 Q6UVJ0 Q66GS9 55125 Q96RK4 9738 3832 Q9HC77 O94927 4869 Q9UBK9 93323 11190 Q96CS2 9793 Q7Z4H7 Q68CZ6 P51955 163786 23354 O43303 P35222 79441 Q9NXR1 54820 1069 Q99871 116840 Q15154 54801 55835 55559 P41208 5108 1647 P24522 Q8N137

centrosome duplication 9662 163786 O43303 P51587 Q9NXR1 54820 1069 Q6UVJ0 116840 Q66GS9 55125 9738 55835 P41208 Q9HC77 Q8N137

centrosome separation 116840 P51955 3832 Q8N137 P52732

desmosome assembly 3728 P14923

S phase 8099 O14519 P00519 84967 7884 Q969L4 P28340 5422 P09884 5424 Q14493

telophase 8379 Q9Y6D9

histone lysine methylation Q9C005 11091 4297 30827 Q9P0U4 Q96MX6 Q6UXN9 Q03164 11107 5929 Q15291 80335 84661 P61964 Q9UBL3

peptidyl-lysine deacetylation P56524 10013 23411 Q9UBN7 Q96EB6

positive regulation of defense response to virus by host 5371 O95786 60489 Q9HC16 Q7Z434 P29590 23586 57506

wound healing involved in inflammatory response 3091 960 P01137 Q16665 P16070 P09601 3162 P25116

connective tissue replacement involved in inflammatory response wound healing 3091 P01137 Q16665 P25116

negative regulation of Rho protein signal transduction 8452 Q8WZ19 253980 Q8WWN8 Q96QB1 7126 Q13618 P41182 Q8N9R8 604 P35348 Q13829

response to nitrosative stress P48507 2730 128 P11766 1848 Q16828

regulation of histone acetylation Q9BZS1 Q01105 6622 P49711 P01137 50943 P37840 408 P49407 Q9UBK2

positive regulation of histone acetylation Q9BZS1 P01137 50943 408 P49407 Q9UBK2

establishment of apical/basal cell polarity 163183 Q8N205 5584 Q14160 284217 P25391 Q92949 P41743 23513

embryonic arm morphogenesis 1499 Q6KC79 P35222 O15119 6926

arm morphogenesis 1499 Q6KC79 P35222 O15119 6926

negative regulation of NFAT protein import into nucleus 8165 Q92667

elastin metabolic process 3091 Q04656 Q16665

embryonic hemopoiesis P25791 4297 P15692 7422 P37173 Q15648 4005 10661 P40424 Q13351 Q03164

histone H3-K4 methylation Q9C005 11091 4297 30827 Q9P0U4 Q6UXN9 Q03164 5929 Q15291 80335 84661 P61964 Q9UBL3

regulation of histone H3-K9 methylation 1789 1786 Q9UBC3 3720 Q92833 P26358

positive regulation of histone H3-K4 methylation 1789 1786 Q9UBC3 P26358

positive regulation of histone H3-K9 methylation 3720 Q92833

gene silencing by miRNA Q9UKV8 Q15797 Q9UL18 P98179 Q5TAX3 27327 79753 P84022 Q8WYQ5 Q8TAD8 27161 Q9UPY3 5935 4086 4087 4088 4343 26523 Q8NDV7 Q9HCK5 Q9HCE1 51593 Q9H9G7 54487 192669 Q9BXP5 Q15796

negative regulation of neurotransmitter uptake 6622 P37840

complement receptor mediated signaling pathway Q16581 719

dopamine uptake Q99497 5071 O60260 11315

negative regulation of dopamine uptake 6622 P37840

regulation of serotonin uptake 6622 P37840

negative regulation of serotonin uptake 6622 P37840

regulation of norepinephrine uptake 6622 P37840

negative regulation of norepinephrine uptake 6622 P37840

centrosome localization O75051 Q8N4C6 5362 51199 81565 Q8IZT6 259266 Q9GZM8

nucleus localization Q9Y266 Q8NF91 10460 Q658W2 Q86VS8 Q03014 25777 Q05397 Q8IYN9 10726 Q15154 Q9UH99 84376 Q9Y6A5 5108 5747 Q8N960

spindle localization O43264 54908 P49450 6993 9700 P63172 P35579 Q9NXR1 54820 1058 Q9NZ56 P43034 O14777 Q14674 4627 Q96EA4

maintenance of centrosome location Q8IZT6 259266

negative regulation of translation involved in gene silencing by miRNA Q9HCK5 Q9UKV8 Q9H9G7 Q9UL18 192669 27327 26523 Q8NDV7 27161

regulation of protein amino acid dephosphorylation 8165 P31946 7531 Q96QB1 5524 P01137 Q15257 7529 P62258 P30542 Q92667

negative regulation of dephosphorylation 8165 P31946 7531 5524 Q15257 7529 P62258 Q92667

negative regulation of protein amino acid dephosphorylation 8165 P31946 7531 5524 Q15257 7529 P62258 Q92667

hippo signaling cascade 8463 Q9H8S9 O95835 Q16635 26524 P46937 Q9H4B6 Q9NRM7 Q15561 Q7L9L4 6789 Q13188 6901 10413 Q13043 Q15562

regulation of hippo signaling cascade 4771 23286 P35240

fatty-acyl-CoA metabolic process P11182 1629

protein localization to microtubule 22919 Q15691

actin crosslink formation 2316 Q60FE5 P21333

thioester biosynthetic process P11182 1629

positive regulation of nitric-oxide synthase biosynthetic process O00206 7099 P43490 Q9NR96 54106 O60603

negative regulation of cell division 641 P54132 Q8IZT6 259266

response to misfolded protein Q99942 Q9UNE7 10273 6048 10013 Q9UBN7 1191

histone-threonine phosphorylation Q7Z727 Q16512 5585 P17252 5578 5579 P05771

histone H3-T11 phosphorylation Q16512 5585

medium-chain fatty acid catabolic process 34 P11310

regulation of catenin protein nuclear translocation 5460 4088 P84022 Q16635 2010 P00533 6901 1956 P50402 P50148 P01133

regulation of hair follicle development Q15628 7124 P19883 P08138 10468 8717 P01375

positive regulation of catenin protein nuclear translocation 5460 4088 P84022 P00533 1956 P01133

positive regulation of hair follicle development Q15628 7124 P19883 10468 8717 P01375

negative regulation of catenin protein nuclear translocation Q16635 2010 6901 P50402

latent virus infection O43889 3054 10488 P51610

extracellular matrix-cell signaling Q9HB63 59277

reactivation of latent virus O43889 3054 10488 P51610

evasion of host defenses by virus 4088 P84022 P01137

avoidance of defenses of other organism involved in symbiotic interaction 4088 P84022 P01137

evasion or tolerance of defenses of other organism involved in symbiotic interaction 4088 P84022 P01137

viral assembly, maturation, egress, and release P09958 Q92824 90678 Q99816 Q6UWE0 7251

response to interferon-alpha 27252 Q9Y2M5

release of virus from host 90678 Q99816 Q6UWE0 7251

negative regulation of production of molecular mediator of immune response Q9BZS1 P41182 604 P09601 50943 3162

negative regulation of cytokine production involved in immune response Q9BZS1 P41182 604 P09601 50943 3162

regulation of T cell cytokine production 7186 7189 Q12933 Q9BZS1 Q9UDY8 6885 50943 Q9Y4K3 O43318

negative regulation of T cell cytokine production Q9BZS1 50943

regulation of focal adhesion assembly 7057 P35968 3791 4088 7248 Q86WV8 P31431 5728 P37023 57669 P84022 P60484 Q9HCM4 7249 94 Q92574 P49815 O43182 P07996

positive regulation of focal adhesion assembly P35968 57669 3791 4088 P84022 7248 Q9HCM4 Q92574 Q86WV8 P31431

MyD88-independent toll-like receptor signaling pathway Q8IUC6 148022

catecholamine uptake involved in synaptic transmission Q99497 5071 O60260 11315

negative regulation of catecholamine uptake involved in synaptic transmission 6622 P37840

lysophospholipid transport 8399 O15496

carnitine metabolic process, CoA-linked 34 P28330 P11310

glycine biosynthetic process from serine 6470 P34896

D-ribose biosynthetic process P06737 P11413 5836

pentose biosynthetic process P06737 P52209 5226 P11413 5836

glutamate deamidation P49448 P00367 2747

cardiac right ventricle morphogenesis 9421 O96004 P43694 2626

cardiac right ventricle formation 9421 O96004

atrial septum development P43694 2626 P52952

septum primum development P43694 2626

atrial septum primum morphogenesis P43694 2626

ribose phosphate metabolic process P06737 P11413 5836

pancreatic D cell differentiation Q8HWS3 222546

epithelial cilium movement 5991 O75665 P48380 8481

histone arginine demethylation 23210 Q6NYC1

histone lysine demethylation 23081 Q9Y4C1 Q9UPP1 23133 O60341 Q9H3R0 Q9UGL1 23028 10765 Q8NB78 221656 Q9H6W3

histone H4-R3 demethylation 23210 Q6NYC1

histone H3-R2 demethylation 23210 Q6NYC1

ubiquitin-dependent endocytosis 90678 Q6UWE0

mitochondrial outer membrane translocase complex assembly Q9Y512 Q9NS69 P07900 56993 3320 P34932 25813 3308

response to UV-A 595 P24385 P31749 207 P00533 1956

lymphocyte apoptosis 10758 O43521 P31749 O43734 207 P01589 Q96EY1 9093 P62753 6194

T cell apoptosis P31749 207 P01589 Q96EY1 9093 P62753 6194

necrotic cell death 8772 O60502 10724 Q13158 356 7124 P48023 P01375

chaperone cofactor-dependent protein refolding 573 7266 3337 10728 Q15185 P25685

nucleotide-binding oligomerization domain containing signaling pathway O43353 4792 5970 Q04206 Q9HC29 64127 8767 P25963

nucleotide-binding oligomerization domain containing 1 signaling pathway O43353 4792 8767 P25963

nucleotide-binding oligomerization domain containing 2 signaling pathway O43353 4792 5970 Q04206 Q9HC29 64127 8767 P25963

negative regulation of thrombin receptor signaling pathway 6622 P37840

regulation of thrombin receptor signaling pathway 6622 P37840

protein K63-linked deubiquitination Q5VVQ6 Q8TEY7 Q9UGI0 P40818 P21580 Q9Y2K6 O00487 55432 P46736 10868 Q9NWV8 54764 23032 1540 51720 7128 Q96DC9 Q96RL1 10213 29086 Q9NQC7

olfactory nerve development 1756 P11532 Q9NQC3 Q9NSC2

olfactory nerve morphogenesis 1756 P11532

olfactory nerve structural organization 1756 P11532

heterochromatin organization 3070 Q8TBE0 Q7Z7K6 22893 Q9NRZ9

cerebellar granular layer development O00755 136319 7476 P58546 O75385 8408

cerebellar granular layer morphogenesis O00755 136319 7476 P58546 O75385 8408

cerebellar granular layer formation O00755 136319 7476 P58546 O75385 8408

inclusion body assembly 7415 10013 P55072 5071 Q9UBN7 O60260

misfolded protein transport 10013 Q9UBN7

aggresome assembly 7415 10013 P55072 5071 Q9UBN7 O60260

polyubiquitinated misfolded protein transport 10013 Q9UBN7

polyubiquitinated protein transport 10013 Q9UBN7

Hsp90 deacetylation 10013 Q9UBN7

cerebellar granule cell differentiation O00755 136319 7476 P58546 O75385 8408

positive regulation of glycogen metabolic process Q9BX66 P01308 10580 8445 P31749 Q9Y4H2 P35368 207 Q92630 3667 P35568 P06213

regulation of ATP:ADP antiporter activity Q13546 8737

CRD-mediated mRNA stabilization 3192 P67809 10492 1660 10642 O60506 Q00839 Q08211

cerebral cortex radial glia guided migration Q14114 Q00535 O75553 A1XKG3 1020 P43034 7804

layer formation in the cerebral cortex Q14114 Q00535 A1XKG3 1020 P43034 7804

hindbrain radial glia guided cell migration P26232 O75553 O75385 8408

radial glia guided migration of granule cell O75385 8408

protein K48-linked deubiquitination Q5VVQ6 Q8TEY7 8078 P40818 Q96FW1 Q9Y2K6 55432 10868 55611 23032 Q92560 Q96DC9 P45974

regulation of cell cycle arrest 5371 Q6ZN33 7023 Q9NP71 Q8N726 Q08050 11186 P29590 P38936 Q9NYZ3 O43715 Q9NS23 4193 1029 2305 1026 Q00987 P14174 Q01664 P42771 51512

corpus callosum development Q00535 A1XKG3 1020 23322 P43034 Q68CZ1

cellular response to misfolded protein Q99942 Q9UNE7 10273 6048

cellular response to follicle-stimulating hormone stimulus P43694 2626

cellular response to fatty acid 891 P05091 5155 Q01094 P01127 P14635 P18146 1869

cellular response to lipoprotein stimulus1012 P55290

cellular response to low-density lipoprotein stimulus 1012 P55290

cellular response to organic cyclic substance 891 P41231 P09382 5029 P14635 3956

chloride ion homeostasis 773 356 P48023 1152 P12277

manganese ion homeostasis 27032 P98194

protein localization to chromosome, centromeric region P49450 1058

monovalent inorganic anion homeostasis 773 356 P48023 1152 P12277

lipoprotein mediated signaling 1012 P55290

low-density lipoprotein mediated signaling 1012 P55290

cellular response to sterol depletion 3638 51141 22937 Q9Y5U4 Q12770 O15503

glycogen biosynthetic process 178 P13807 Q16816 2632 P35573 Q04446 10728 Q9UGI9 Q15185 P15735 P46976

genetic imprinting Q9UBC3 346171 Q9NU63 O95661 9077 3481 29947 Q9Y6K1 Q8NB78 P51787 221656 1789 1788 54496 Q9NVM4 3784 P49711 P01344

dopaminergic neuron differentiation 2253 P55075

5-phosphoribose 1-diphosphate biosynthetic process P06737 5836

glucosamine catabolic process 10007 P46926

endothelial cell-cell adhesion 3728 P14923 P35443

acyl-CoA biosynthetic process P11182 1629

tricarboxylic acid cycle 5162 48 P40926 3420 P50213 O43837 6390 4191 Q9P2R7 3417 O75390 1431 P31040 8803 8801 3419 P07954 Q13423 P51553 P53597 P36957 O75874 6389 23530 P11177 P21912 1743 3421 P21399

regulation of transforming growth factor-beta production P78423 P09958 7057 3091 4088 4089 O75787 1385 P16220 P84022 Q53X93 Q16665 Q13485 P07996 10159

citrate metabolic process O75390 48 P53396 1431 P51692 6776 P42229 P21399

positive regulation of transforming growth factor-beta production P78423 7057 4088 P16220 P84022 O75787 1385 Q53X93 P07996 10159

succinate metabolic process P53597 P51692 6389 2572 2571 6390 Q9P2R7 P21912 Q05329 Q99259 P31040 8803 6776 P42229

cellular response to indole-3-methanol P38398 672 3728 1499 P35221 P14923 P12830 999 P35222

response to indole-3-methanol P38398 672 3728 1499 P35221 P14923 P12830 999 P35222

GTP catabolic process 2643 P17081 P30793

IMP biosynthetic process 5198 10606 P31939 P22102 P22234

'de novo' IMP biosynthetic process 5198 10606 P22102 P22234

membrane to membrane docking P15311 7430 7412 P19320 4478 P05362 P26038 Q13464 6093

DNA strand elongation involved in DNA replication 2237 1763 P40938 5422 5983 P39748 P09884 P35249

lagging strand elongation 2237 1763 5422 P39748 P09884

nucleosome disassembly Q969G3 3159 Q9UIG0 Q01105 P17096 11198 P09430 6605 Q9Y5B9 7141

chromatin silencing O43159 23409 3066 Q9UIF9 Q9UQL6 Q9Y6K1 7141 Q9Y6E7 O60264 P09430 51548 10014 51547 O43463 3070 Q9NRC8 O95983 Q92769 Q9UBC3 8467 23411 23378 Q9NRZ9 1789 1788 6839 P17480 11176 Q96EB6 53615

establishment of chromatin silencing 23411 Q96EB6

methylation-dependent chromatin silencing 1789 1788 3070 O95983 Q9UBC3 Q9NRZ9 Q9Y6K1 53615

regulation of gene expression by genetic imprinting Q9UBC3 346171 Q9NU63 O95661 9077 3481 29947 Q9Y6K1 Q8NB78 P51787 221656 1789 1788 54496 Q9NVM4 3784 P49711 P01344

rRNA processing 92345 9221 Q92979 O43159 11340 Q12788 P62081 O00541 Q06265 P18124 6194 10813 Q9H6Y2 Q9Y3A5 54512 Q9Y2X3 6229 Q14690 P62913 P62753 65083 1736 Q15024 Q9UQ80 P38919 5393 5394 Q14978 5036 6125 51096 51010 Q9Y3B2 51013 Q9NPD3 P46777 Q96EB6 P62249 Q8N726 O00567 Q8TED0 Q9NV06 Q5TAP6 Q8WVM0 P78316 P46087 P08708 9775 P18077 Q96B26 10528 P62263 55759 Q9UET6 134430 O95478 50628 Q9NYH9 P39019 23404 23481 Q8NI36 8602 P62312 Q8NEC7 Q14137 Q9Y2L1 51729 6201 Q13601 23411 23378 23016 Q96G21 Q9Y5J1 1029 6839 Q9NY12 11157 P42771 P62847 118460 P62841 Q8IY81 2091 9045 P22087 22894 10438 54853 Q5RKV6 P50914 88745 56915 P62857 Q9GZL7 P56182 O43463 Q9UNQ2 Q13901 Q13868 Q9H6R4 Q01780 Q969E8 Q9NQT4 Q9NQT5 Q96EU6 4839 Q9BVJ6 P61254 Q96HR8 23076 10607 Q9Y2W2 6217 Q14684

mRNA splice site selection Q15459 Q13247 Q12874 10291 6430 Q12872 8683 Q8WXF0 4154 6431 Q15428 O95391 O75494 10569 Q07955 7536 10772 6426 Q13243 Q13242 135295

rRNA export from nucleus 7248 Q92574 Q86WV8 4869

translational initiation P55884 8892 P23396 O75821 O75822 6193 O00303 P55010 P61247 Q14152 Q9UBQ5 8669 Q13144 Q14232 8662 P49770 O15371 Q13347 O15372 6188 8667 P08708 P46782 8665 Q9NR50 P41091 Q7L2H7 Q9Y262 1968 P60228

translational elongation 6231 P23396 6233 P62917 P62081 6193 P18124 6194 P62750 P47914 P62910 P46783 6229 P83731 P62913 P62753 P49411 P13639 6123 P46782 6125 P46781 P36578 25873 P62244 P84098 P63173 P68104 P62241 P62888 P61313 P46777 P46776 P27635 P60866 P46779 P46778 P62249 P26641 6133 P25398 P26640 6132 6134 6137 P18621 P63220 6128 P40429 P62899 1915 6141 P08708 P26373 P62829 P18077 P42677 P62266 P15880 Q02543 P62263 P62701 6138 P62945 P62269 P62424 Q9Y3U8 6156 6158 P62280 P39019 P62277 P30050 P39023 1937 1936 1933 Q07020 6164 6168 6201 6202 6160 P61353 P83881 P62847 P05386 P05387 P62841 P05388 Q9UNX3 P61513 P49207 P61927 6175 6210 9045 P32969 P42766 Q02878 P50914 P24534 P61247 6204 6207 P62857 P62979 P62851 P08865 P62854 6188 6187 Q92901 7311 P62906 P35268 P61254 P62988 6217 Q9HD40

alanyl-tRNA aminoacylation 80755 16 P49588 Q5JTZ9

arginyl-tRNA aminoacylation Q5T160 Q5D0E6 P54136 57038 55152

histidyl-tRNA aminoacylation 23438 P12081 3035 P49590

tyrosyl-tRNA aminoacylation P54577 51067 8565 Q9Y2Z4

regulation of translational initiation by iron 27102 7124 Q9BQI3 P01375

N-terminal protein amino acid acetylation Q09472 80155 79612 Q92793 1387 Q9BXJ9 2033 Q7Z6C1 Q4LE28 P41227 Q92831

protein amino acid deacetylation 23409 5245 3066 3065 Q9UIF9 Q9UQL6 11107 Q9Y6E7 83933 10933 Q96PK6 O15379 55869 10013 Q9UBU8 51548 10014 10432 51547 Q6IT96 P56524 Q9NRC8 Q92769 Q969S8 Q13547 8841 Q9BY41 23411 79885 P35232 11176 Q9UBN7 Q96EB6

protein amino acid demethylation Q9UPP1 23210 23133 Q6NYC1 23135 O75164 Q9H3R0 23028 9682 Q8NB78 221656 Q9H6W3 23081 Q9Y4C1 O60341 Q9UGL1 10765

misfolded or incompletely synthesized protein catabolic process Q99942 Q9UNE7 10273 6048 10013 Q9UBN7

glutamate catabolic process Q05329 Q99259 P00505 P17174 P49448 2572 P00367 2571 2805 2747

glutamate decarboxylation to succinate Q05329 Q99259 2572 2571

phosphagen metabolic process P50440 P17540 P51692 6776 1152 2593 P12277 P42229 P12532 Q14353

creatine metabolic process P50440 P17540 P51692 6776 1152 2593 P12277 P42229 P12532 Q14353

creatine biosynthetic process P50440 2593 Q14353

protein export from nucleus Q9BY84 Q8TD31 P62826 11097 Q9HCE7 Q9UKK6 57154 23214 P35658 8021 23039 P38646 Q96QU8 3313 Q7RTN6 Q9UIA9 811 54535 Q9C0K7 P01137 O15504 5901 P27797

3-keto-sphinganine metabolic process 2531 Q06136

regulation of metanephros development 4609 P01106 Q99966

glucocorticoid catabolic process 7533 Q04917

NADH metabolic process P51553 P36957 P40926 3420 P50213 O43837 4191 P43304 1743 2820 P05062 3421 3419

FAD biosynthetic process 80308 Q8NFF5

nicotinamide metabolic process P43490 93100 Q6XQN6

riboflavin metabolic process 55312 Q969G6

succinyl-CoA pathway 8803 Q9P2R7

tricarboxylic acid transport 6576 P53007

mitochondrial citrate transport 6576 P53007

retrograde vesicle-mediated transport, Golgi to ER P53621 6892 9463 Q9UBF2 Q92538 Q9NRW1 Q8WVM8 1315 375 1314 51560 O15533 O15258 Q96KG9 P83436 91949 P35606 9276 Q9Y678 P53618 23256 83548 57410 P84077 Q9H9E3 11079 O14579 Q9NRD5 Q8N6T3

activation-induced cell death of T cells P31749 207 P01589 Q96EY1 9093 P62753 6194

substrate-bound cell migration, cell extension 6386 23607 4983 O60890 O00560 Q9Y5K6

hypotonic response P17301 3673 59341

positive regulation of gene-specific transcription involved in unfolded protein response 22926 P18850

response to sterol depletion 3638 51141 22937 Q9Y5U4 Q12770 O15503

nuclear envelope organization O95476 4000 O75844 2010 23399 25777 P50402 81565 Q9UH99 P43034 P02545 Q9GZM8 P42574 10269

outer mitochondrial membrane organization Q9Y512 Q9NS69 P07900 56993 3320 P34932 25813 3308

microtubule nucleation Q9UGJ1 Q96CW5 Q6QNY1 10426 10844 Q9NXR1 Q96RT7 114791 54820 85378 55835 Q9BSJ2 10048 Q96S59 Q96RT8 Q9HC77

vacuolar protein catabolic process 1358 P48052

lysosomal lumen acidification 1203 O75503 P50897 Q9NWW5 Q13286

cell-substrate junction assemblyQ13753 Q08043 P05106 Q15654 Q13751 Q16787 P10415 P12814 P13647 P11047 P48509 P16144 P23229 Q9UMD9 977 3852 Q6NVY8 3655 3678 5339 3911 Q9Y490 P08648 3690 Q15149 Q9BX66 O15530 O15230 P35609 Q96QB1 10580 5796 7094 5170 Q96S53 3909 Q6PID4 7205 10420 P02533 Q14289 Q9UL54 87

spindle organization 6790 Q14204 9700 11065 Q14008 O95271 P35579 7283 1778 26271 O60566 203068 55722 Q9H6D7 55125 3796 3832 Q13561 Q96R06 P07437 Q96CS2 9793 10460 Q68CZ6 O75376 79441 3925 1104 Q9UQE7 Q99871 54801 5901 9126 O95613 Q02241 55142 O00762 P52732 57405 54930 Q9H4B7 O15379 5116 Q9Y6A5 Q14674 O94927 Q8N960 O14965 4627 93323 O00139 P62826 O43663 Q7Z4H7 8841 23354 25777 9493 7272 80254 Q9P209 P40692 Q96MT8 P23258 Q9UH99 55559 Q9HBM1 O14777 Q9UKT4 Q2M2Z5 Q14683

mitotic spindle organization O00139 Q14204 P62826 O43663 9126 Q02241 O95271 25777 P52732 9493 7272 1778 3925 1104 57405 Q9UQE7 Q9UH99 3796 Q9HBM1 3832 O14777 5901 Q13561 Q14683

positive regulation of transcription on exit from mitosis Q9NRC8 51547

nuclear migration 10726 Q9Y266 Q9UH99 25777

centrosome cycle O15182 Q9BV73 9662 P51587 P52732 1499 Q6UVJ0 Q66GS9 55125 9738 3832 Q9HC77 4869 11190 P51955 163786 O43303 P35222 Q9NXR1 54820 1069 116840 55835 P41208 1647 P24522 Q8N137

centriole replication 9662 1069 Q6UVJ0 116840 Q66GS9 163786 9738 55835 P41208 O43303 Q9HC77 Q8N137

mitotic centrosome separation 3832 P52732

cytokinesis, initiation of separation Q9H0H5 29127

meiotic metaphase I Q9NZ56 P35579 4627

cell-matrix adhesion Q08043 Q15654 Q9UQP3 P18084 P12814 P56199 P49023 Q9Y3I0 O95274 Q9BUG9 Q92574 P98161 3911 P04004 O15530 5310 O95965 5311 Q96QB1 5796 5829 Q96S53 7448 7205 10420 P16070 O14713 Q13136 Q13418 Q00535 P53708 P01019 3655 P25940 P26012 P05556 P02461 3672 P26010 3673 Q14118 P35222 5170 P14543 27076 P17301 387 Q9UKX5 54822 P26006 P49746 P08514 7248 1020 P18564 P16144 1499 P23229 Q9UMD9 3674 3675 P61586 Q13563 3679 Q13683 P31249 3690 Q13444 Q13443 3693 O43521 P35609 10580 O75578 9270 Q86WV8 Q9UBX5 P18206 960 Q9Y5J6 3685 3688 3689 64098 P05106 P05107 8754 P10415 A1XKG3 3611 Q5TAT6 7414 Q9BX66 O15230 Q9HBI0 Q96QT4 8516 Q6PID4 8515 Q14289 Q9UL54 1605 87

signal complex assembly P07766 4690 9463 P49023 A1XKG3 1020 P12931 O43639 O00468 Q05397 Q8IYN9 773 Q00535 O14640 Q60FE5 Q658W2 P16333 2316 5829 Q6PID4 375790 Q14289 6714 5747 Q9NRD5 P21333

common-partner SMAD protein phosphorylation 5371 4087 6711 P29590 Q01082 Q15796

SMAD protein complex assembly 4086 4087 Q15797 4088 O95405 4089 Q8N6I1 163126 2280 P84022 P01137 Q15796 Q13485 P62942

integrin-mediated signaling pathway Q13797 P08514 3680 P18084 O14672 P56199 Q08722 P18564 P35579 P15498 Q8IYN9 P16144 P23229 P38570 Q9BUG9 3674 10451 3675 3676 3678 3679 Q13683 3911 P08648 9510 3690 Q13443 3693 O95965 O75578 P11215 Q96RT1 961 3685 Q14185 3688 3689 5747 9564 P05106 P05107 8754 6850 Q07092 Q13418 Q05397 3611 P53708 1793 Q9UKW4 3655 4627 P26012 P05556 P02461 Q14511 3672 P26010 O15230 3673 Q9UHI8 Q03001 Q658W2 P13612 P20702 P17301 P43405 Q9UKX5 8516 P60201 8515 Q96HB1 P26006

establishment of mitotic spindle localization O43264 54908 P49450 6993 9700 P63172 Q9NXR1 54820 1058 P43034 O14777 Q14674 Q96EA4

I-kappaB phosphorylation O00206 7099 7098 O15111 6885 O95163 Q9NR96 3551 O60603 O43318 Q8IUD2 10549 1147 23085 Q13162 54106 O15455 O14920

establishment of nucleus localization Q8IYN9 10726 Q9Y266 Q8NF91 Q9UH99 Q658W2 25777 5747 Q05397

negative regulation of translation, ncRNA-mediated Q9HCK5 Q9UKV8 Q9H9G7 Q9UL18 192669 27327 26523 Q8NDV7 27161

Ras protein signal transduction O15085 P36507 Q9BST9 P04637 11186 Q9Y6W5 P12931 O75385 Q6PCE1 Q7L0Q8 P62993 3479 P01116 5898 P49137 P62873 10256 8408 P01112 P01111 P52565 6242 Q7L591 P27361 19 1072 P52566 P84095 9908 Q15392 6239 Q14344 10146 P98171 P01343 Q13017 P29353 Q16644 Q9UQ13 8315 Q8N726 Q9NR81 O15264 O00560 3265 6093 Q9Y2I1 Q7Z569 Q8TEU7 Q9NS23 P53667 890 P23528 P01138 P55290 Q8IV61 P06400 Q16539 6386 P31946 9138 P04629 Q07960 O00212 4914 27352 387 388 P21359 Q13393 7867 Q59EA4 5604 Q969H4 O95477 O14593 P28482 Q03113 391 392 9261 394 396 P46109 397 1017 398 25780 Q6KH11 1012 Q13283 P08123 8844 5337 P61586 P30556 Q52LW3 P04049 Q96HU1 Q8IVT5 O15211 58480 1029 P20248 1026 1147 O60496 5863 9826 P42771 7529 51735 6714 Q92888 Q92766 9046 Q15759 Q9P035 2247 2885 1399 1432 Q07889 50650 1718 10672 8625 Q9UN86 Q13464 O43182 P09038 O15111 6464 7157 5894 Q8NHX1 P62070 Q02750 P38936 O75791 P11234 P11233 P24941 P62745 Q99819 Q9H4E5 5925

foregut morphogenesis 4087 4088 Q13705 P35222 57669 1499 116113 P84022 Q9HCM4 P46531 4851 P43694 Q15796 2626

imaginal disc development O14641 1856

imaginal disc pattern formation O14641 1856

synaptic target recognition Q96FJ2 140735

establishment of blood-nerve barrier P01019 P21266 2947

regulation of establishment of protein localization in plasma membrane 5295 5584 Q9BX66 O15530 10580 P31749 Q9UKG1 5170 207 P17081 P27986 P41743 26060

establishment of protein localization in plasma membrane 54361 P56705

positive regulation of establishment of protein localization in plasma membrane 5295 5584 Q9BX66 O15530 10580 P31749 207 P27986 5170 P41743

retrograde axon cargo transportP07196 81565 P43034 Q9GZM8

regulation of steroid hormone biosynthetic process 476 27122 Q9UBP4 54361 P05023 P56705

negative regulation of steroid hormone biosynthetic process 476 27122 Q9UBP4 P05023

positive regulation of cellular chaperone-mediated protein complex assembly Q9UNE7 10273 10013 Q9UBN7

regulation of cellular chaperone-mediated protein complex assembly Q9UNE7 10273 10013 Q9UBN7

tubulin deacetylation 10013 Q9UBN7

negative regulation of transcription regulator activity O43593 6672 P23396 114548 4092 3162 P53567 O43638 Q02535 Q96RU8 P41134 10616 P63165 P04040 55806 P25963 2307 Q9NSA3 5195 P56524 Q92769 4221 64127 23411 1029 O75381 2316 1540 Q9NQB0 P42771 847 408 P49407 409 Q96EB6 P23510 P21333 7341 P11309 P35638 7067 4792 Q8N726 Q5TAX3 P32121 P19474 Q02363 3066 23028 P51843 Q9BYM8 Q96P20 O60341 P40424 Q9GZT9 P06400 5716 Q9NQC7 6188 P10827 Q60FE5 O75832 Q9BZS1 Q92949 Q9NR96 O00255 9093 54583 P51617 Q9HC29 Q92786 O15105 Q96EY1 54106 P09601 50943 P23497 P43489 5925

negative regulation of cell migration involved in sprouting angiogenesis 10014 Q9UQL6

regulation of metanephric cap mesenchymal cell proliferation 4609 P01106

positive regulation of metanephric cap mesenchymal cell proliferation 4609 P01106

pancreatic E cell differentiation Q8HWS3 222546

regulation of high-density lipoprotein particle assembly 7376 P55055

regulation of cell-substrate junction assembly 7057 P35968 3791 4088 7248 Q86WV8 P31431 5728 P37023 57669 P84022 P60484 Q9HCM4 7249 94 Q92574 P49815 O43182 P07996

positive regulation of high-density lipoprotein particle assembly 7376 P55055

protein amino acid dealkylation Q9UPP1 23210 23133 Q6NYC1 23135 O75164 Q9H3R0 23028 9682 Q8NB78 221656 Q9H6W3 23081 Q9Y4C1 O60341 Q9UGL1 10765

epithelial cell-cell adhesion P18206 3728 57662 Q9BVG8 P14923 7414 P35443 Q6IQ23 3801

establishment of protein localization in membrane 54361 P56705

establishment of epithelial cell polarity 163183 Q8N205 56288 Q8TEW0 284217 P25391

organelle membrane fusion 80124 Q12981 Q96JH7 Q13286

regulation of release of cytochrome c from mitochondria Q9BXM7 O75832 65018 5071 O60260 5716

negative regulation of release of cytochrome c from mitochondria O75832 5071 O60260 5716

insulin receptor signaling pathway 9564 O14492 64397 P08833 P29353 P49840 6599 3484 Q9H2Y7 3481 10458 2303 P62993 2885 Q92572 Q9UQB8 P08069 8503 Q13480 P27986 1978 2308 2549 5295 Q13322 Q12778 6464 Q9BX66 10580 P31749 1176 Q9Y4H2 Q9UKG1 P18433 P35568 Q6ZWJ1 2931 207 3667 P17081 Q92922 Q99958 P01344 Q92569 26060 P06213

intracellular mRNA localization 6780 22794 O15234 O95793

positive regulation of lipid kinase activity P60033 7410 975 Q9UKW4 10451 P52735 P01137 3667 P35568

positive regulation of canonical Wnt receptor signaling pathway O00755 3728 4088 P84022 7476 1453 P14923 P48730 857 54361 P56705

regulation of somatostatin secretion 3667 P35568

negative regulation of somatostatin secretion 3667 P35568

asymmetric cell division Q05516 56288 Q8TEW0 7704

negative regulation of intracellular protein transport P61925 7067 4792 11142 57120 A1XKG3 1020 P10599 2010 Q9UNN5 O00221 114548 Q96P20 Q00535 Q99728 11124 Q9UN86 Q9Y6A5 6901 P25963 5716 Q9NQC7 Q9UBC1 8165 P10827 O75832 4188 10460 Q16635 Q9BZF9 580 P50402 7295 9908 P21359 Q9HD26 Q99750 1540 Q9UMX1 5569 Q9Y2B9 Q15653 Q92667

regulation of superoxide metabolic process P13686 Q9NY61 54

ether lipid biosynthetic process 8443 O00628 O15228

activation of pro-apoptotic gene products 8772 8795 7186 Q13158 Q92934 8655 P48454 P10415 P50591 572 P63167 355 356 P84022 5533 5599 Q14790 637 O14763 P25445 P31946 8743 4088 Q15628 O43521 Q12933 P48023 Q13546 P31749 P55957 841 8737 207 P45983 7529 8717

lipopolysaccharide metabolic process Q8IYK4 Q9BT22 Q8NFW8 55907 Q8NBJ5 Q9Y223 56052 51148 23127 Q9NR45 Q5T4B2 79709

pentose-phosphate shunt, non-oxidative branch22934 P37837 P49247

lipopolysaccharide biosynthetic process Q8IYK4 Q9BT22 Q8NFW8 55907 Q8NBJ5 Q9Y223 56052 51148 23127 Q9NR45 Q5T4B2 79709

purine base biosynthetic process 5471 5631 Q06203 6470 P34896 353 P49915 10606 P60891 P22102 P22234 8833 P07741

riboflavin biosynthetic process 55312 Q969G6

glucan biosynthetic process 178 P13807 Q16816 2632 P35573 Q04446 10728 Q9UGI9 Q15185 P15735 P46976

protein refolding 7266 P08107 P07900 3320 2280 3329 573 3304 3337 10728 Q15185 P25685 P10809 P62942

interleukin-2 biosynthetic process 5074 Q14116 Q96IZ0

cellular response to glucose starvation 81788 P11021 7157 P04637 Q9H093 P08243 3309

interleukin-8 biosynthetic process Q9Y239 10392

mature ribosome assembly 2 3692 P56537

ribosomal subunit assembly P62263 P83731 P62851 P62753 6194

ribosomal large subunit biogenesis 9045 6125 P18077 O00541 55153 P18124 Q9NVU7 23481 P50914 P61254 55759 P83731 P62913 Q9GZL7 P46777 4869

ribosomal small subunit biogenesis Q92979 P78316 6201 P08708 P62081 Q96EU6 6194 P39019 88745 P62263 8602 6229 P62857 6217 P62847 P62851 P62841 P62753 4869 P62249

regulation of protein import into nucleus 5460 7046 Q15654 7124 11142 2033 O43557 Q9UNN5 O00221 114548 O60603 Q93062 Q04759 P84022 999 Q7Z6C1 P61586 6901 Q13485 P25963 7099 Q9UBC1 7098 Q6IR47 Q9NYA1 11030 4188 10460 Q16635 P14923 P36894 P10600 23411 P50148 2316 3728 9908 Q99750 1540 7205 P00533 Q96EB6 Q99835 P21333 O00206 Q09472 P61925 7067 4792 Q14703 P12830 2010 Q96P20 11124 P01137 O15455 Q9UN86 Q9Y6A5 1956 P01133 P01375 5716 Q9NQC7 8165 P10827 Q60FE5 4088 O75832 4089 8720 Q9BZF9 Q06830 Q9NR96 9495 P50402 387 P21359 O60674 P10071 Q9UMX1 54106 5569 Q9Y2B9 Q15653 2737 Q92667 P24588

negative regulation of protein import into nucleus P61925 7067 4792 11142 Q9UNN5 O00221 114548 Q96P20 11124 Q9UN86 Q9Y6A5 P25963 5716 Q9NQC7 Q9UBC1 8165 P10827 O75832 4188 10460 Q9BZF9 9908 P21359 Q99750 1540 Q9UMX1 5569 Q9Y2B9 Q15653 Q92667

negative regulation of NF-kappaB import into nucleus O75832 4792 Q9BZF9 Q9UNN5 114548 9908 Q96P20 1540 11124 Q9UN86 Q15653 P25963 5716 Q9NQC7

cristae formation O95202 Q16635 3954 6901

serine phosphorylation of STAT protein Q00535 P01579 A1XKG3 1020 Q9UBE8

negative regulation of tyrosine phosphorylation of Stat3 protein P67775 4771 P35240 P30153 5515 O15524 5518

negative regulation of tyrosine phosphorylation of Stat5 protein 4771 P35240 857

negative regulation of tyrosine phosphorylation of STAT protein P67775 4771 P35240 P30153 857 5515 O15524 5518

regulation of asymmetric cell division Q8IZT6 259266

regulation of abscisic acid mediated signaling pathway Q9NS86 55915

positive regulation of abscisic acid mediated signaling pathway Q9NS86 55915

regulation of mitotic cell cycle, embryonic 1877 Q66K89

regulation of hair cycle Q15628 7124 Q6KC79 P19883 P08138 10468 8717 P01375

auditory receptor cell fate specification 474 Q92858

sperm ejaculation P27037 P37288 552 P30559 6869 P25103 P01178

riboflavin and derivative metabolic process 55312 80308 Q8NFF5 Q969G6

riboflavin and derivative biosynthetic process 55312 80308 Q8NFF5 Q969G6

regulation of sulfur metabolic process P21964 1312 1499 Q9NQB0 P35222

nucleosome mobilization 8289 O14497

DNA damage response, detection of DNA damage P82912 64949 Q9BYN8 Q96T60 60488 P82673 64963 64965 P09874 142 P82933

mitochondrial ATP synthesis coupled proton transport P30049 O75947 O75964 P48047 P56134 P36542 498 P00846 P25705 521 P24539 522 P18859 513 P56385 514 515 539 P56381 10476 509 10632 P06576

protein ubiquitination involved in ubiquitin-dependent protein catabolic processQ8IUQ4 26994 O95376 4193 Q8TBB1 Q8WY64 Q9Y3C5 10956 Q16531 O60260 Q13049 Q7Z419 Q9HCE7 57154 Q96J02 83737 5071 84708 P46934 1642 Q00987 255488 4214 4734 Q13233

mRNA transcription from RNA polymerase II promoter P27540 P35638 3091 Q16665

response to carbon dioxide 2 P01023

response to iron(II) ion 48 6622 P37840 P21399

pyridoxal phosphate metabolic process 8566 O00764

pyridoxal phosphate biosynthetic process 8566 O00764

glucocorticoid receptor signaling pathway 8289 Q14686 7533 P46934 10728 Q96PK6 811 O14497 Q15185 4734 10432 P27797 Q04917

maintenance of DNA methylation 3070 1786 P49711 Q9NRZ9 P26358

positive regulation of amyloid precursor protein biosynthetic process 5074 Q96IZ0 P35610 6646

negative regulation of transcription factor import into nucleus 7067 4792 Q9UNN5 O00221 114548 Q96P20 11124 Q9UN86 Q9Y6A5 P25963 5716 Q9NQC7 Q9UBC1 8165 P10827 O75832 4188 10460 Q9BZF9 9908 P21359 Q99750 1540 Q9UMX1 Q15653 Q92667

Golgi to plasma membrane CFTR protein transport 3875 P05783

Golgi to plasma membrane protein transport Q9H2G9 3875 10133 8548 P05783 Q96CV9

DNA methylation involved in embryonic development 1788 9463 346171 Q9NU63 Q9NRD5 Q9Y6K1

positive regulation of Cdc42 GTPase activity Q96P48 81565 10928 Q9GZM8 Q15311

receptor clustering 2316 9463 773 Q60FE5 Q00535 O14640 A1XKG3 1020 375790 Q9NRD5 O00468 P21333

DNA replication, removal of RNA primer 2237 1763 P39748

stress fiber assembly 8452 Q8WZ19 253980 Q9BX66 10580 7126 Q6PID4 Q13618 408 Q14289 P49407 Q13829

DNA methylation on cytosine within a CG sequence 1789 1788 Q9UBC3 Q9Y6K1

response to leucine 6198 P23443

exit from mitosis Q9NRC8 Q7Z460 11065 23332 P83731 O00762 51547

apoptotic cell clearance 7057 4035 23210 Q6NYC1 P10276 7052 P16671 Q07954 3685 P21980 Q13133 948 P07996 5914

regulation of phosphatidylinositol biosynthetic process 5154 5155 P04085 P01127

negative regulation of phosphatidylinositol biosynthetic process 5154 5155 P04085 P01127

regulation of glycoprotein biosynthetic process 5074 Q09472 Q96IZ0 P10415 P35610 2033 P35222 Q9NY61 O60502 10724 1499 Q7Z6C1 Q9NQB0 6646 P06213

positive regulation of glycoprotein biosynthetic process 5074 Q09472 1499 Q96IZ0 P35610 2033 Q7Z6C1 Q9NQB0 6646 P35222 P06213

response to exogenous dsRNA O43353 7098 4792 6772 P28482 P27361 Q8NHX1 64127 Q9H257 148022 P42224 Q8IUC6 Q9HC29 8767 O15455 64170 P25963

response to dsRNA O43353 Q9UKV8 Q15797 4792 O75569 6772 P98179 P28482 Q5TAX3 148022 57162 P42224 79753 P84022 8767 Q8WYQ5 O15455 Q8TAD8 64170 27161 Q9UPY3 P25963 5935 Q96FA3 7098 4086 4087 4088 P27361 Q8NHX1 64127 Q9H257 51593 Q8IUC6 Q9HC29 54487 Q9BXP5 Q15796 Q13794

enucleate erythrocyte differentiation 6670 Q02447 6667 P08047 Q02363 P53567 P06400 5925

regulation of cardiac muscle adaptation O60502 10724

regulation of memory T cell differentiation P41182 604

negative regulation of cardiac muscle adaptation O60502 10724

negative regulation of DNA binding O43593 6672 P23396 O95271 114548 4092 3162 P53567 O43638 Q02535 Q96RU8 P41134 Q96JM2 10616 P63165 P04040 55806 P25963 2307 Q9NSA3 P05412 5195 P56524 Q92769 4188 4221 64127 23411 1029 O75381 2316 51773 Q99750 1540 Q9NQB0 P42771 847 408 P49407 409 Q96EB6 Q99835 P23510 P21333 7341 P11309 P35638 7023 7067 4792 Q8N726 Q5TAX3 P32121 P19474 Q02363 3066 23028 P51843 Q9BYM8 Q96T23 Q96P20 Q01664 O60341 P40424 Q9GZT9 P06400 5716 Q9NQC7 6188 P10827 Q60FE5 O75832 Q9BZS1 Q92949 Q9NR96 O00255 9093 54583 O60674 P51617 Q9HC29 Q92786 O15105 Q96EY1 54106 P09601 50943 P23497 P43489 5925

negative regulation of transcription factor activity O43593 6672 P23396 114548 4092 3162 P53567 O43638 Q02535 Q96RU8 P41134 10616 P63165 P04040 55806 P25963 2307 Q9NSA3 5195 P56524 Q92769 4221 64127 23411 1029 O75381 2316 1540 Q9NQB0 P42771 847 408 P49407 409 Q96EB6 P23510 P21333 7341 P11309 P35638 7067 4792 Q8N726 Q5TAX3 P32121 P19474 Q02363 3066 23028 P51843 Q9BYM8 Q96P20 O60341 P40424 Q9GZT9 P06400 5716 Q9NQC7 6188 P10827 Q60FE5 O75832 Q9BZS1 Q92949 Q9NR96 O00255 9093 54583 P51617 Q9HC29 Q92786 O15105 Q96EY1 54106 P09601 50943 P23497 P43489 5925

negative regulation of oxygen and reactive oxygen species metabolic process 10013 P13686 Q9UBN7 Q9NY61 54

positive regulation of cellular carbohydrate metabolic process P27540 3091 Q9NP71 Q9BX66 10580 8445 P31749 Q9Y4H2 P35368 P37840 P35568 3479 P01308 6622 P01579 207 Q92630 Q16665 3667 P01343 Q9UBK2 P06213 P04201

regulation of alkaline phosphatase activity P17301 4088 P84022 3673

positive regulation of alkaline phosphatase activity P17301 4088 P84022 3673

RNA stabilization Q13148 3192 Q9Y2T7 Q14103 7422 P39905 P67809 10492 1660 P11940 26986 1153 Q15717 O60506 23435 3184 Q08211 9261 1994 P15692 P49137 10642 Q00839 Q14011

protein kinase B signaling cascade Q09472 6199 6198 P31749 2033 P35568 Q9H244 Q9UBS0 207 7249 Q7Z6C1 3667 P49815 P23443 P42336

negative regulation of hydrogen peroxide metabolic process 10013 Q9UBN7

regulation of DNA damage response, signal transduction by p53 class mediator O75832 Q8N726 Q13315 Q13535 23411 4193 472 1029 960 Q00987 P14174 545 P42771 P16070 Q96EB6 P04233 4869 5716

negative regulation of DNA damage response, signal transduction by p53 class mediator 960 Q00987 O75832 P14174 23411 P16070 Q96EB6 P04233 4193 5716

regulation of lipid kinase activity 7410 P52735 P35568 P60033 Q08999 975 Q9UKW4 10451 P28749 P01137 3667 5933 P06400 5925

regulation of phosphoinositide 3-kinase activity 7410 Q9UKW4 10451 P52735 P01137 3667 P35568

positive regulation of phosphoinositide 3-kinase activity 7410 Q9UKW4 10451 P52735 P01137 3667 P35568

regulation of mRNA export from nucleus 23144 Q8IXZ2

regulation of peptidyl-threonine phosphorylation Q9UGJ0 O15105 P01137 O15169 4092

negative regulation of peptidyl-threonine phosphorylation O15105 4092

negative regulation of cell-substrate adhesion P02452 7057 Q8N726 4771 P41182 5728 1029 P37023 P00747 P20936 P60484 1277 P09382 P35240 P46531 4851 604 94 P42771 O43182 P07996 3956

skin morphogenesis Q9UJM3 P02452 P17301 1277 3673 P08123 54206

negative regulation of mitochondrion organization O75832 5071 O60260 5716

regulation of centrosome duplication P38398 10733 672 9475 91147 O75116 Q5HYA8 79959 O00444 Q8TAP6 4869

negative regulation of myotube differentiation P56524 10014 Q9UQL6 P52952

regulation of transcription from RNA polymerase II promoter in response to stress P46934 P27540 3091 2034 Q16665 Q99814 P09601 4734 3162

regulation of transcription from RNA polymerase II promoter in response to oxidative stress P27540 3091 2034 Q16665 Q99814 P09601 3162

regulation of transcription in response to stress P46934 P27540 P35638 3091 2034 Q16665 Q99814 P09601 4734 3162

cholesterol storage P35610 6646

notochord regression 2735 Q1PSW9 P10070 P08151 2736

notochord cell differentiation 1969 P29317

notochord cell development 1969 P29317

negative regulation of steroid biosynthetic process 4790 476 P19838 27122 Q99684 2672 Q92786 Q9UBP4 54361 P05023 P56705

regulation of UDP-glucose catabolic process 2931 P49840

negative regulation of UDP-glucose catabolic process 2931 P49840

regulation of heparan sulfate proteoglycan biosynthetic process 1499 Q9NQB0 P35222

positive regulation of heparan sulfate proteoglycan biosynthetic process 1499 Q9NQB0 P35222

uterus development 2100 Q92731 2099 Q6KC79 P03372

dedifferentiation Q00534 1021

cell dedifferentiation Q00534 1021

regulation of necrotic cell death 355 356 7124 P48023 Q13546 8737 2495 291 P12235 P02794 P25445 P01375

positive regulation of necrotic cell death 355 356 7124 P48023 Q13546 8737 P25445 P01375

regulation of phagocytosis, engulfment P17301 3673

positive regulation of phagocytosis, engulfment P17301 3673

thyroid-stimulating hormone-secreting cell differentiation 54361 P56705

embryonic process involved in female pregnancy 6670 Q02447 Q00613 P17275 O60488 Q96S42 6667 P08047 3297

regulation of syncytium formation by plasma membrane fusion Q13443 8754

positive regulation of syncytium formation by plasma membrane fusion Q13443 8754

regulation of eIF2 alpha phosphorylation by heme 27102 Q9BQI3

contact inhibition P46937 10413

cilium movement involved in determination of left/right asymmetry 5991 O75665 P48380 8481

transdifferentiation 4088 P84022

interferon-gamma-mediated signaling pathway 6672 P23497

regulation of interferon-gamma-mediated signaling pathway P02790 3326 11140 Q96EY1 Q16543 9093 3263 P08238

negative regulation of interferon-gamma-mediated signaling pathway Q96EY1 9093

type I interferon-mediated signaling pathway 6672 P23497

regulation of type I interferon-mediated signaling pathway 3326 11140 Q16543 P08238

bone trabecula formation P02452 1277 4313 P08253

histone H2A acetylation 55929 Q96L91 Q9Y265 O96019 57634 10856 10933 O95619 Q9NPF5 Q9Y230 Q9UBU8 86 8607

leucine import 6520 P08195

regulation of Fc receptor mediated stimulatory signaling pathway Q15762 10666

positive regulation of Fc receptor mediated stimulatory signaling pathway Q15762 10666

histone H4-R3 methylation 55352 Q9NQ92 O14744

regulation of ventricular cardiomyocyte membrane depolarization O15105 4092

regulation of mast cell differentiation 6776 P42229

positive regulation of mast cell differentiation 6776 P42229

JAK-STAT cascade involved in growth hormone signaling pathway O60674 P51692 6774 6776 P42229 P40763

cardiac septum morphogenesis 9421 8100 O96004 4092 P52952 54583 Q92786 O15105 P43694 Q9GZT9 Q96T37 Q13099 2626

ventricular septum morphogenesis 54583 Q92786 O15105 4092 Q9GZT9 Q96T37 P52952

atrial septum morphogenesis P43694 2626 P52952

regulation of DNA methylation Q8NB78 221656

secretory columnal luminar epithelial cell differentiation involved in prostate glandular acinus development 6256 3169 P46531 4851 P19793 79084 Q9BQA1

regulation of necroptosis 356 7124 P48023 Q13546 8737 P01375

positive regulation of necroptosis 356 7124 P48023 Q13546 8737 P01375

induction of necroptosis 356 7124 P48023 Q13546 8737 P01375

induction of necroptosis by extracellular signals 356 7124 P48023 Q13546 8737 P01375

neuroepithelial cell differentiation 2253 P55075

regulation of branching involved in salivary gland morphogenesis by extracellular matrix-epithelial cell signaling Q9HB63 59277

regulation of branching involved in salivary gland morphogenesis by epithelial-mesenchymal signaling 5154 P04085

cell differentiation involved in embryonic placenta development 9421 O95377 O96004 3880 4188 P08727 P31749 O00444 9021 10733 P00747 Q99750 207 O14543

trophoblast giant cell differentiation 9421 10733 O96004 P00747 4188 Q99750 O14543 O00444 9021

glycogen cell development involved in embryonic placenta development P31749 207

positive regulation of inositol phosphate biosynthetic process 6622 P37840 P04201

epithelial cell differentiation involved in prostate gland development Q92826 6256 2100 P19793 P35222 79084 P10275 1499 367 3169 P46531 4851 Q92731 6776 P42229 Q9BQA1

mammary gland branching involved in pregnancy	367 Q15648 2099 P03372 54361 P56705 P10275 P06401

tertiary branching involved in mammary gland duct morphogenesis	367 54361 P56705 P10275 P06401

negative regulation of androgen receptor signaling pathway	3479 2100 Q92731 O75928 P01343

avoidance of host defenses	4088 P84022 P01137

evasion or tolerance of host defenses	4088 P84022 P01137

establishment of monopolar cell polarity	163183 Q8N205 5584 Q14160 284217 P25391 Q92949 P41743 23513

depyrimidination	8930 Q13569 P22674 O95243 P78549 4913

transcytosis	11261 Q99653 P23945 O60763 8615 P01266

positive regulation of interleukin-12 biosynthetic process	O00206 7099 P10914 7189 Q04864 5970 Q04206 Q06643 P01579 5966 Q9Y4K3 3659

integrin biosynthetic process	1289 P20908

homologous chromosome segregation	P40692 9700 Q14674

establishment or maintenance of neuroblast polarity	Q96N67 85440

establishment of epithelial cell apical/basal polarity	163183 Q8N205 284217 P25391

establishment of neuroblast polarity	Q96N67 85440

zonula adherens maintenance	57662 Q9BVG8 Q6IQ23 3801

carnitine biosynthetic process	34 O75936 8424 P11310

positive regulation of B cell differentiation	572 P43405 Q92934 Q92835 6850 P51692 6776 P42229

regulation of gamma-delta T cell differentiation	P43405 6850 P51692 6776 P42229

positive regulation of gamma-delta T cell differentiation P43405 6850 P51692 6776 P42229

positive regulation of auditory receptor cell differentiation	474 Q92858

positive regulation of mechanoreceptor differentiation	474 Q92858

regulation of neutrophil differentiation	Q13422 Q92835 10320

positive regulation of neutrophil differentiation	Q13422 10320

positive regulation of glycogen biosynthetic process	Q9BX66 P01308 10580 8445 P31749 Q9Y4H2 207 Q92630 3667 P35568 P06213

negative regulation of cyclin-dependent protein kinase activity P25054 O94992 7023 O95835 4221 Q8N726 O00255 26524 P38936 124790 1029 10614 1026 Q96SZ6 324 Q01664 Q9NRM7 51654 P42771 P42574 Q96MH2

negative regulation of DNA repair 6188 P23396

positive regulation of cellular amino acid metabolic process P21964 1312

negative regulation of asymmetric cell division Q8IZT6 259266

negative regulation of gene expression, epigenetic O43159 23409 3066 Q9UIF9 Q9UQL6 Q9Y6K1 7141 Q9Y6E7 O60264 P09430 51548 10014 51547 O43463 3070 Q9NRC8 O95983 Q92769 Q9UBC3 8467 23411 23378 Q9NRZ9 1789 1788 6839 Q9NS37 P14373 58487 P17480 11176 5987 Q96EB6 53615

negative regulation of isotype switching Q9BT67 P41182 604

positive regulation of proteolysis 7341 Q09472 8078 8754 6872 7124 10273 2033 3066 4092 Q9NWW5 4193 O60502 Q96RU8 1454 1453 P63165 7415 P01579 Q7Z6C1 54998 P45974 P55072 P01375 5716 P09958 Q9UKS6 8100 Q13443 Q92769 O75832 P49674 P53350 P31749 O43847 Q9UNE7 10724 Q00987 Q9NWT8 O15105 207 5347 P48730 6613 4898 P61956 P21675 Q13099

positive regulation of survival gene product expression Q6IR47 7046 P51692 2100 2099 P14784 P52952 1012 Q92731 6776 10673 Q9Y275 P03372 P42229 P55290

positive regulation of carbohydrate metabolic process P27540 3091 Q9NP71 Q9BX66 10580 8445 P31749 Q9Y4H2 P35368 P37840 P35568 3479 P01308 6622 P01579 207 Q92630 Q16665 3667 P01343 Q9UBK2 P06213 P04201

negative regulation of catecholamine metabolic process P21964 1312

negative regulation of dopamine metabolic process P21964 1312

regulation of translation, ncRNA-mediated Q9HCK5 Q9UKV8 Q9H9G7 Q9UL18 192669 27327 26523 Q8NDV7 27161

negative regulation of translational initiation by iron 27102 Q9BQI3

IMP metabolic process 5198 10606 P31939 P22102 P22234

adenosine biosynthetic process 4907 P21589

aldehyde catabolic process P14550 128 P11766

formaldehyde metabolic process Q9Y4C1 128 P11766

formaldehyde catabolic process 128 P11766

amino sugar catabolic process 10007 P46926

ribose phosphate biosynthetic process P06737 P11413 5836

5-phosphoribose 1-diphosphate metabolic process P06737 5836

establishment of cell polarity O15085 54908 Q8N205 6993 5584 Q8TEW0 P25391 P63172 P35579 163183 60412 Q96N67 1058 284217 P23528 P08069 P43034 4627 Q96EA4 O43264 P49450 Q92949 1072 23513 Q9NXR1 54820 Q14160 56288 9826 O14777 85440 P41743

cellular manganese ion homeostasis 27032 P98194

lamellipodium assembly23647 4690 P53365 7410 Q9Y6W5 Q96M96 P52735 Q7L576 O43639 Q3V6T2 P16333 P18206 121512 1012 P63000 Q9UKW4 10451 55704 Q9NRF2 7414 5879 P55290

negative regulation of JAK-STAT cascade9146 P67775 4771 P35240 P30153 857 O14964 5515 O15524 5518

FAD metabolic process 80308 Q8NFF5

ether lipid metabolic process 8443 O00628 O15228

S-adenosylmethioninamine metabolic process 1789 1788 Q9UBC3 23463 Q9Y6K1 O60725

uroporphyrinogen III metabolic process 7389 8803 P06132 Q9P2R7

development of secondary male sexual characteristics P51692 6776 P42229

positive regulation of Wnt receptor signaling pathway O00755 5494 Q9UGI0 P35813 4088 7476 P14923 Q03014 Q9UBB5 54361 P56705 3728 27185 54764 P84022 1453 Q9NRI5 O14640 P48730 8932 857 Q9NSC2 9839

negative regulation of Ras protein signal transduction 23636 8452 Q8WZ19 O43597 Q8WWN8 Q96QB1 7126 O95140 Q13618 P41182 Q8N9R8 P35348 Q13829 P37198 O43609 253980 P62714 604 Q13574 5516 9927

positive regulation of calcium-dependent cell-cell adhesion Q92845 22920

regulation of centriole replication P38398 10733 672 79959 O00444 Q8TAP6

positive regulation of centriole replication 10733 O00444

platelet formation Q96AX1 65082 P35579 4627

regulation of centrosome cycle P38398 10733 672 Q9Y6G9 9475 91147 O75116 Q5HYA8 79959 O00444 Q8TAP6 4869

positive regulation of centrosome cycle 10733 O00444

monocyte differentiation 6670 P37231 3428 Q02447 Q16666 5468 P35579 4627

female sex determination P58012 668 54361 P56705

positive regulation of insulin receptor signaling pathway P01308 3667 P01344 3481 P35568

regulation of gamma-delta T cell activation P43405 6850 P51692 6776 P42229

positive regulation of gamma-delta T cell activation P43405 6850 P51692 6776 P42229

tetrahydrofolate biosynthetic process 2643 P30793

folic acid metabolic process 2348 P15328 6470 P34896

cellular monovalent inorganic anion homeostasis 773 356 P48023 1152 P12277

non-lytic viral release 90678 Q99816 Q6UWE0 7251

non-lytic virus budding 90678 Q99816 Q6UWE0 7251

glial cell proliferation 1499 4771 P35240 Q6PID4 P35222 Q14289

Schwann cell proliferation 1499 4771 P35240 P35222

membrane disassembly 81565 P43034 Q9GZM8

notochord formation P20827 1969 P29317

regulation of nucleocytoplasmic transport 7046 Q15654 2033 O43557 P10599 O60603 10657 Q04759 P84022 999 Q7Z6C1 6901 Q13485 P25963 Q9UBC1 4188 Q16635 7295 P50148 P46934 2316 9908 Q99750 7205 P00533 4734 Q96EB6 O00206 Q09472 P52298 7067 Q8N726 23144 Q06124 Q00535 P01137 Q9Y6A5 P01133 P01375 5716 8165 4088 4089 8720 Q9BZF9 Q9NR96 9495 387 P21359 O60674 P10071 5566 5569 Q9Y2B9 O14974 2737 Q8IXZ2 Q92667 P24588 5460 7124 11142 1020 Q9UNN5 O00221 114548 Q93062 Q99728 P61586 7099 7098 Q6IR47 Q9NYA1 11030 10460 Q07666 P14923 P36894 P10600 23411 1029 3728 1540 Q9NQB0 P42771 P49841 4659 Q99835 P21333 P61925 4792 Q14703 P12830 A1XKG3 2010 Q96P20 11124 O15455 Q9UN86 1956 Q9NQC7 P10827 Q60FE5 O75832 Q06830 580 P50402 P17612 2932 Q9UMX1 54106 Q15653

negative regulation of nucleocytoplasmic transport P61925 7067 4792 11142 A1XKG3 1020 P10599 Q9UNN5 O00221 114548 Q96P20 Q00535 Q99728 11124 Q9UN86 Q9Y6A5 P25963 5716 Q9NQC7 Q9UBC1 8165 P10827 O75832 4188 10460 Q9BZF9 580 7295 9908 P21359 Q99750 1540 Q9UMX1 5569 Q9Y2B9 Q15653 Q92667

regulation of protein export from nucleus Q8N726 A1XKG3 1020 P10599 2010 Q06124 580 P50402 7295 1029 P17612 2932 Q00535 5566 Q9NQB0 Q99728 P42771 P49841

negative regulation of protein export from nucleus Q00535 A1XKG3 1020 Q99728 P10599 580 7295

positive regulation of protein export from nucleus P17612 2932 5566 Q9NQB0 2010 P49841 P50402

regulation of RNA export from nucleus 10657 P52298 Q07666 23144 Q8IXZ2

inositol and derivative phosphorylation 64768 Q9H8X2

phosphoinositide dephosphorylation 5728 P60484 22908 Q9NTJ5 4952 Q01968

estrogen receptor signaling pathway O75528 8289 O43251 Q14686 2100 2099 P10276 Q96KS0 Q8TDD1 79039 Q96PK6 Q92731 O14497 P03372 10432 5914

embryonic genitalia morphogenesis Q01974 Q9NQB0 4920

fatty-acyl-CoA biosynthetic process P11182 1629

ubiquitin-dependent SMAD protein catabolic process Q9UNE7 64750 10273 Q9HCE7 57154 O43294 Q9HAU4

regulation of hemoglobin biosynthetic process 27102 Q9BQI3

negative regulation of hemoglobin biosynthetic process 27102 Q9BQI3

cellular chloride ion homeostasis 773 356 P48023 1152 P12277

regulation of oxygen and reactive oxygen species metabolic process Q86UR1 7057 10193 Q08050 Q9H4P4 Q13546 P13686 5071 Q9NY61 10811 2305 P15153 P39210 P63000 8737 10013 4358 Q9UBN7 O60260 5879 P07996 54 P06213

paranodal junction assembly 8443 P21926 O15228 928

establishment or maintenance of microtubule cytoskeleton polarity 9793 Q99661 Q14008 11004

ER-nuclear sterol response pathway 3638 51141 22937 Q9Y5U4 Q12770 O15503

retrograde protein transport, ER to cytosol 79139 7415 Q9GZP9 51009 P55072 Q9BUN8 P60468

regulation of centriole-centriole cohesion 11190 Q9BV73 1499 P35222

microtubule organizing center organization O15182 Q9BV73 9662 Q14008 55142 P51587 P52732 1499 54930 Q9H6D7 Q6UVJ0 Q66GS9 55125 Q96RK4 9738 P43034 3832 Q9HC77 O94927 4869 Q9UBK9 93323 11190 Q96CS2 9793 Q7Z4H7 Q68CZ6 P51955 163786 23354 O43303 P35222 79441 Q9NXR1 54820 1069 Q99871 116840 Q15154 54801 55835 55559 P41208 5108 1647 P24522 Q8N137

primary microRNA processing 4086 51593 4087 Q15797 4088 P84022 54487 Q9BXP5 Q8WYQ5 Q15796

chromatin remodeling at centromere 55355 3070 Q7Z7K6 Q8NCD3 Q9NRZ9

positive regulation of histone modification 5371 7334 4221 Q9BZS1 7157 P04637 Q9UBC3 O00255 P29590 Q6KC79 P14635 1789 891 1786 P61088 3720 P01137 50943 408 P49407 Q92833 P26358 Q9UBK2

positive regulation of histone methylation 1789 1786 4221 Q9UBC3 3720 O00255 Q92833 P26358

regulation of histone deacetylation 5371 Q9BZS1 7157 P04637 P29590 Q6KC79 P01137 50943

negative regulation of histone deacetylation Q9BZS1 50943

septin ring organization 54443 Q9NQW6

vesicle transport along microtubule 9001 P54257 P33176 Q02750 5590 Q9NXR1 54820 Q05513 P42858 81565 P43034 Q9GZM8 3799 5604

skeletal muscle atrophy 6198 O14793 P23443

negative regulation of muscle adaptation O60502 10724 P29474

SCF-dependent proteasomal ubiquitin-dependent protein catabolic process 8454 6500 Q969H0 Q13616 79791 Q9UKA1 Q9UKB1 55294 23291 26232 Q5XUX0 26234 P62877 P63208 Q9NRD1 9978 Q9UK22 26270

response to stimulus involved in regulation of muscle adaptation P56524 6198 P01019 P23443

response to electrical stimulus involved in regulation of muscle adaptation 6198 P23443

striated muscle atrophy 6198 O14793 P23443

positive regulation of smooth muscle cell migration P04004 6198 5155 P10415 3673 P17301 P41231 3479 Q15077 7448 P01127 5029 P01343 P23443

positive regulation of sulfur amino acid metabolic process P21964 1312

nuclear envelope reassembly 2010 P50402

chromatin disassembly Q969G3 3159 Q9UIG0 Q01105 P17096 11198 P09430 6605 Q9Y5B9 7141

heterochromatin formation 3070 Q8TBE0 Q7Z7K6 22893 Q9NRZ9

centromeric heterochromatin formation 3070 Q7Z7K6 Q9NRZ9

hemidesmosome assembly Q13753 Q15149 Q13751 Q16787 P13647 P11047 P48509 P16144 P23229 Q9UMD9 3909 977 3852 Q6NVY8 3655 P02533 5339

receptor internalization 8301 P50570 P32121 P42261 P37840 O75385 P46934 P20339 P62993 1213 1785 O14939 2885 Q00610 6622 Q13492 857 5338 4734 408 P49407 409 5868 8408

antigen processing and presentation, exogenous lipid antigen via MHC class Ib 8546 O00203

ephrin receptor signaling pathway 6760 P29323 Q13009 P20827 Q15768 P21709 1969 Q15532 P29317 7074

negative regulation of nuclear mRNA splicing, via spliceosome Q8WXF0 10772 O75494 4869 135295

focal adhesion assembly Q08043 Q15654 P10415 Q9BX66 O15530 O15230 P35609 Q96QB1 10580 P12814 5796 5170 Q96S53 Q6PID4 7205 10420 Q14289 Q9UL54 3911 87

regulation of cyclin-dependent protein kinase activity involved by G1/S P11309 P78536 P31749 207 P00533 1956

positive regulation of cyclin-dependent protein kinase activity involved in G1/S P11309 P78536 P31749 207 P00533 1956

lipopolysaccharide-mediated signaling pathway O43353 P29474 4792 6772 P28482 P27361 Q8NHX1 148022 P42224 P51617 Q8IUC6 8767 P25963

plasma membrane to endosome transport Q99523 P62491 8766

mRNA stabilization Q13148 3192 Q9Y2T7 Q14103 7422 P39905 P67809 10492 1660 P11940 26986 1153 Q15717 O60506 23435 3184 Q08211 9261 1994 P15692 P49137 10642 Q00839 Q14011

vesicle fusion with Golgi apparatus O60763 8615

regulation of isotype switching to IgE isotypes Q9BT67 P41182 604 P05112 P42226

negative regulation of isotype switching to IgE isotypes Q9BT67 P41182 604

negative regulation of synaptic plasticity Q00535 A1XKG3 1020

immunoglobulin secretion Q8IWV1 10758 O43734 10673 Q9Y275

TOR signaling cascade P42345 6198 Q8TAI7 2475 55704 P62753 1978 P23443 6194 Q3V6T2

axial mesoderm morphogenesis Q9HCS4 1969 P29317

axial mesoderm formation 1969 P29317

positive regulation of chromatin silencing 23411 Q96EB6

paraxial mesoderm morphogenesis 6927 P20823 4087 2303 4088 P42858 P84022 Q9UJU2 Q12948 P36894 Q15796 Q99958

corticosteroid receptor signaling pathway 8289 Q14686 7533 P46934 O60674 10728 Q96PK6 811 O14497 Q15185 4734 10432 P27797 Q04917

negative regulation of fatty acid beta-oxidation P31749 207 P21554

positive regulation of retinoic acid receptor signaling pathway 10626 2099 O95361 P03372

response to cobalt ion Q09472 P18846 466 841 Q14790 2033 Q7Z6C1 Q8N465

ferric iron transport 1213 1785 P50570 Q00610

negative regulation of NF-kappaB transcription factor activity P23396 4792 Q8N726 Q5TAX3 P32121 P19474 114548 Q9BYM8 Q96P20 10616 P04040 P25963 5716 Q9NQC7 6188 O75832 Q9BZS1 Q92949 64127 Q9NR96 9093 1029 P51617 Q9HC29 1540 Q96EY1 54106 P42771 847 50943 408 P49407 409

iodide transport 1080 P13569

positive regulation of protein binding P04004 3673 Q9BUZ4 P35612 148022 Q05682 2280 P35611 P17301 Q96RU7 57669 57761 Q8IUC6 P11171 Q9HCM4 118 7448 800 Q9NQB0 P62942

synaptic vesicle endocytosis Q15811 6453 8411 A1XKG3 1020 O00499 P37840 274 P20336 Q96MV8 Q00535 Q8TEH3 P61764 6622 Q15075 6812 57706

citrate transport 6576 P53007

regulation of pinocytosis 7376 O43707 P55055 Q13133 P50897

negative regulation of pinocytosis 7376 P55055 Q13133

septin cytoskeleton organization 54443 Q9NQW6

notochord morphogenesis 2735 Q1PSW9 P20827 P10070 P08151 1969 P29317 2736

branched-chain aliphatic amino acid transport 6520 P08195

leucine transport 6520 P08195

tryptophan transport 6520 P08195

negative regulation of actin filament bundle assembly Q96P48 Q96QB1 4214 Q13233 5071 O60260 O43182

embryonic foregut morphogenesis 4087 57669 1499 116113 4088 P84022 Q9HCM4 Q13705 P43694 P35222 Q15796 2626

regulation of nucleobase, nucleoside, nucleotide and nucleic acid transport 10657 P52298 Q07666 23144 P30542 Q8IXZ2

regulation of skeletal muscle tissue growth 154 6198 O14793 P07550 P23443

positive regulation of skeletal muscle tissue growth 154 6198 P07550 P23443

regulation of collateral sprouting 6696 P10451 O75385 8408

negative regulation of collateral sprouting 6696 P10451 O75385 8408

myelin assembly 8443 3611 P21926 P18074 Q13418 O15228 928

axon extension involved in regeneration 3913 P55268

sprouting of injured axon 3913 P55268

peroxisomal membrane transport 215 P33897

negative regulation of aldosterone metabolic process 27122 Q9UBP4

regulation of aldosterone biosynthetic process 27122 Q9UBP4 54361 P56705

negative regulation of aldosterone biosynthetic process 27122 Q9UBP4

negative regulation of hormone metabolic process 476 773 27122 Q9UBP4 P05023

negative regulation of hormone biosynthetic process 476 773 27122 Q9UBP4 P05023

oxygen homeostasis 54583 3091 Q9H6Q4 Q16665 64428 Q9GZT9 P04179

negative regulation of intracellular transport P61925 7067 4792 11142 57120 A1XKG3 1020 P10599 2010 Q9UNN5 O00221 114548 Q96P20 Q00535 Q99728 11124 Q9UN86 Q9Y6A5 6901 P25963 5716 Q9NQC7 Q9UBC1 8165 P10827 O75832 4188 10460 Q16635 Q9BZF9 580 P50402 7295 9908 P21359 Q9HD26 Q99750 1540 Q9UMX1 5569 Q9Y2B9 Q15653 Q92667

lysosome localization Q96AX1 10013 65082 Q9UBN7

positive regulation of proteasomal ubiquitin-dependent protein catabolic process 7341 8078 6872 10273 4092 4193 Q96RU8 1454 1453 P63165 7415 P45974 P55072 5716 O75832 P49674 P53350 P31749 Q9UNE7 Q00987 O15105 207 5347 P48730 6613 P61956 P21675

tRNA catabolic process Q99575 10940

regulation of cytokinesis 808 1069 Q7Z7K6 Q96RK4 9738 P08069 P41208 O43303 P62158 P51587

Golgi calcium ion homeostasis 27032 P98194

Golgi calcium ion transport 27032 P98194

Rap protein signal transduction 27352 Q96HU1

response to muramyl dipeptide 4792 5970 Q04206 Q9HC29 64127 Q9H257 64170 P25963

endosome transport via multivesicular body sorting pathway Q99698 Q99523 9525 1130 O75351

endosome to lysosome transport via multivesicular body sorting pathway Q99698 9525 1130 O75351

somite rostral/caudal axis specification 134701 57669 4089 92129 Q9HCM4 Q5TAB7 Q13485

synaptic vesicle to endosome fusion 8411 Q15075

chaperone-mediated autophagyQ13501 8878

negative regulation of interleukin-2 production 10524 Q9BZS1 Q8WUI4 51564 Q9HC29 64127 Q92993 50943 11326

endoplasmic reticulum membrane fusion 80124 Q12981 Q96JH7

calcium-dependent cell-matrix adhesionQ96QT4 Q14118 54822 1605

positive regulation of interferon-alpha production O00206 7099 3329 O95786 Q7Z434 23586 57506 P10809

positive regulation of interleukin-18 production Q9NR96 54106 O60603

regulation of mast cell cytokine production O95999 P41182 604 P09601 3162

negative regulation of mast cell cytokine production P41182 604 P09601 3162

cellular process P54852 P28562 259266 3638 2305 P40200 2304 81855 2303 55561 55568 284217 2300 O14543 2308 4967 4968 Q9H4L7 P30876 O14561 P52209 2317 2316 2 Q96QZ7 P42858 P39210 Q9UKV3 Q9UKV5 2319 Q9UKV8 3661 55584 2324 Q9UKW4 4983 3655 Q9Y6A5 Q9UKW6 3659 P54886 Q13702 3672 3673 Q13705 2335 Q9UKX5 3667 O14579 O14578 Q9UKX7 P42830 Q9UL15 Q8WXG6 3680 Q92538 Q9UL18 1022 1021 1020 Q06587 1018 1017 2348 79577 P54819 1012 3674 Q6Y7W6 3675 3676 Q92530 3678 3679 Q9UL26 3690 3692 Q8WXF0 3693 Q13724 Q9H4P4 Q9BUB7 1029 P42898 Q9BUB1 O14519 1027 1026 1025 Q9BUB5 Q9BUB4 3685 Q92522 3688 3689 Q07890 1040 79595 P40222 Q07889 Q9UL46 Q9UL45 Q13748 1050 1047 Q9UL51 Q92504 Q9UL54 Q9UKT4 Q9H4M9 Q9UKL0 Q9UKL4 Q9Y5U4 Q96A33 11108 11107 1058 Q8WWY3 85378 Q9BTU6 Q9Y676 Q9BU64 Q9Y678 Q9Y5V3 P57057 Q9UKM9 Q9NWZ3 P57059 115426 1072 Q9BU61 Q99996 1069 84034 Q9Y680 84033 11113 11116 1080 84061 Q9Y5W9 Q9UKN5 Q9UKN8 Q9H4B7 Q8WWW0 Q9Y5X1 55504 Q9Y5X2 55503 84062 Q99988 Q9Y5X9 Q9UL03 Q86X95 Q9Y697 Q9Y696 Q96A08 P17812 P17813 Q00403 P57087 9804 P57081 Q5VVQ6 9821 9820 Q9NX61 Q9NX62 Q9Y5Q8 Q9NWU5 P61981 Q96A72 9818 Q9Y5R4 8502 Q5JSP0 Q9NWV8 P60660 Q96QV6 9825 9826 207 Q9Y5S2 P30825 Q96A54 Q9NWW5 Q9Y5S9 P17844 211 Q96QU8 215 8503 9839 Q9H4F1 Q7Z4H7 Q9BU89 Q9UKK6 Q9UKK9 56882 P17858 Q96QT4 8516 8517 Q9H4E5 55559 8515 55558 9846 11060 8535 11065 7204 11064 23048 Q6NZI2 P35658 230 23043 P08047 11072 8546 P48960 8543 Q9ULG1 P58304 P09382 Q9NY59 9869 8539 7205 Q96RL7 8536 11079 9868 Q96RL1 P61956 Q9BUN8 7220 9883 P35638 P61968 11041 8555 23025 Q9Y6Q5 Q9ULH7 23028

Q9NY61 Q9Y6Q6 Q96RK4 P61962 8548 P61960 P22314 P61966 P61964 8563 Q9Y6R4 P36957 11051 8565 Q9Y6R0 8566 9897 Q86XR8 P36952 Q86Y37 P36956 23039 P36954 O75592 267 P61970 23031 23032 Q86Y39 P08034 P61978 O95907 8574 8576 11021 7248 23002 26994 Q9Y6K5 O75582 271 Q9Y6K1 O75581 P10398 Q9Y6K0 274 Q9ULB5 Q96RQ3 Q9Y6K9 8569 7252 Q86XX4 11030 Q9BV47 7257 P22392 O75578 11031 O75575 23014 23016 7251 285 Q9NY12 Q9Y6L7 11034 7249 P48995 11035 P61927 7266 Q86Y82 Q9BV36 O75569 7267 O95931 Q9Y6M4 291 292 Q8WY64 Q9Y6M9 Q9NY26 P08069 Q6I9Y2 11004 7277 O75558 26985 O75553 Q9Y6N6 26986 Q01780 Q86Y79 7272 Q9NXR1 114088 11014 O95947 11017 Q13753 Q13751 375519 P12931 Q7Z589 7283 Q05329 Q9Y6G9 Q92572 Q9BUG9 Q92574 Q13761 7297 Q13769 O95965 O75531 7291 O75530 7290 7295 P24928 Q9BUF5 Q9Y6H3 Q92560 Q9Y6H5 Q92569 O75528 Q5D0E6 Q8WXI4 O95977 Q06643 O75521 Q9Y6I3 92483 P40145 Q92551 P00966 Q92558 O95983 50618 Q8WXH2 O95989 91147 A4D1W7 63931 Q9Y6J0 3704 3705 63929 3708 Q07954 Q9Y6J8 Q07955 Q92542 Q13794 50628 O95995 Q13797 O75503 O95999 O14595 O95997 P35610 P35612 P35611 23081 Q9Y6C2 23085 Q9BUK6 O75508 Q9Y6C9 O75509 P35606 P35609 Q86Y13 Q9H596 23092 3728 Q9Y6D6 23095 Q9Y6D5 Q5QGT7 23097 Q9BUJ2 3720 P22303 Q9Y6D9 50649 11080 Q9NXH8 P12956 P12955 3737 Q9Y6E7 Q9Y6E0 50650 Q9Y6E2 Q96AE4 Q96RG2 11091 63971 11097 63976 23076 23077 P24941 11099 Q96RF0 Q5FWF5 Q7KZF4 285672 65003 O14672 399687 Q5VV41 P15374 79658 O14662 Q9UK45 P27348 P27361 65018 54361 1107 55696 1104 92609 1108 Q9UK53 Q9NWB1 P52333 P29992 3783 1121 P53667 1111 Q86WA8 Q9UJU2 P03372 P26012 P53675 3791 P26010 1130 P53677 1122 3784 Q6NXT2 P26006 Q9Y584 P11802 147746 1137 3796 3799 Q7KZI7 Q7Z419 Q9Y4X5 Q13601 1153 Q14938 P53618 1152 Q06455 P53611 1147 2475 P26045 P53621 O43175 Q13615 Q9Y4Y9 2495 Q13618 Q13617 Q13616 1161 284359 O14641 O14640 P26038 O43184 Q9Y4Z0 Q9UK22 65061 O43182 O43186 Q13620 Q13625 O43189 1176 1174 1173 Q13627 O14656 P40337 Q9UK32 O14657 O43159 Q9BST9 P04637 O43157 25862 1186 25861 P16615 1185 Q9NVW2 Q01650 1181 Q96PQ1 55605 11222 O43166 O43169 Q86W92 1198 25873 23210 Q99880 1196 23212 1195 Q9HDC5 23214 Q9BT40 1191 56940 Q99877 Q96Q15 9908 84159 Q99873 55611 Q99871 11231 11234 O43172 56946 Q99879 25847 9921 Q86VW1 P33176 9919 Q02952 55626 Q9Y572 Q7Z3C6 Q96Q05 O43143 Q9BT22 P04626 P04629 25853 P54920 Q96PN1 11218 O14617 301 302 P17936 9924 Q7Z3B4 Q96PN8 Q9Y580 11214 9928 9927 25828 Q3SY69 Q5U0I6 P15328 25822 Q96PU8 310 Q96PU4 79612 314 Q96PU5 Q02930 8604 8602 Q9H3F6 8607 Q9Y512 Q9Y4P1 9950 P16671 324 8615 328 329 84197 25804 9961 Q9NW64 25801 P15311 331 Q9NVU7 Q96PS8 333 55660 55666 56993 56994 P83436 Q9BT67 9972 P05997 7311 9973 Q96Q40 P17980 Q9NVV4 25813 P15313 Q9NVV9 Q17RW2 79622 9967 8650 8655 Q6DKI1 11186 7325 8654 Q9UKD1 351 353 355 356 23163 P09493 23165 P23786 9978 Q96QK1 P21127 P47755 P47756 8662 11190 7332 9997 7334 16 8667 Q9BTM1 7336 8665 11196 7335 19 Q9UKE5 Q9H3Z4 23170 367 369 11198 7329 7328 22 Q9Y600 7341 11160 8677 7347 29 6015 8676 23144 253260 P57103 P59768 Q9UKF6 P10451 Q9NWS0 Q9Y606 375 85437 P09471 11165 11168 8669 30 31 P23763 34 11171 Q9Y613 8683 7353 7358 Q8WWN8 Q9Y619 P0C0S5 Q9Y618 Q9UKG1 381 382 Q9H492 387 P59780 388 P0C0S8 23154 P08151 11177 11176 P60763 P23771 48 11140 23122 Q9Y5I4 390 391 25788 23127 394 396 Q99965 397 Q99962 Q99963 11146 52 Q99966 54 Q96R06 7373 6045 7376 7375 P60709 6048 23133 O75694 23135 Q9Y5J5 23136 Q86WV8 Q9UKA1 Q9Y5J1 7372 Q86WV6 Q9Y5J6 P33151 60 11157 85440 Q99958 Q99956 P46459 7385 7384 7386 7389 6059 O75683 Q9Y5K6 Q9Y5K5 6050 7381 Q9UKB1 Q99942 Q99943 11129 Q99941 Q01664 P08183 56915 P21145 56916 11124 11128 11127 P22492 O75676 O75674 23111 23112 Q9Y5L4 25776 P33121 25778 25777 Q9NX14 Q00325 P08195 84134 Q6R327 86 87 Q9NWH9 Q7KZN9 Q96QC0 Q13636 Q14966 O75665 P52272 Q05209 Q9Y5E8 Q86WK7 Q86WK6 91 Q8WWI1 94 Q9Y5E9 Q14974 Q13643

Q13642 Q14978 Q8WWH4 Q96QB1 Q86WJ1 Q9H3R0 Q7Z460 P52292 Q52LR7 3801 Q14980 O94763 P52298 O94768 O75643 P52294 Q6XUX3 Q9H422 Q7Z3S9 6093 6096 91272 P41597 Q9Y5G4 Q9Y5G3 Q14993 Q9BTC0 P11717 O94776 Q9H410 134430 Q9UJV9 O75626 Q6NYC1 Q9H488 P11766 P53567 B3KY43 3836 P22415 Q9UK80 P08123 Q92499 Q9UJW2 3832 Q13683 3835 P47712 Q07820 Q07817 Q9UJX2 Q9UJX0 Q9Y5B0 Q96QF0 3841 3843 P08138 P09467 2517 Q9Y5B9 Q9Y5B8 P09429 Q8WWK9 O75604 Q9NWF9 P10415 3860 387680 P10412 P35711 2521 23186 3852 P09430 3856 O75608 728642 P08107 P53597 374354 Q96QD9 23192 1203 2531 1207 65125 3880 25909 P54619 2550 O95793 P17676 54474 2547 1213 Q9HCJ0 Q9HCJ2 54476 79778 3875 2549 Q9H2H8 P28335 25913 Q9UIQ6 25915 P28330 P28331 2562 Q5VTR2 P55957 80755 54487 Q9H2G9 P67936 P30622 Q9H2G2 Q9BRX9 Q9H2G4 P28300 2572 2571 79791 Q96P70 54496 2569 3895 Q9UJ41 P31942 Q9UIS9 P31944 P31943 P31946 P31947 2582 P17661 P54646 P17655 P16333 80777 80776 388677 1244 P04350 Q9Y3U8 Q9Y468 P27037 Q9H2M9 Q9Y463 Q14807 2593 2590 Q8WVC6 P39019 P16389 P15056 2589 P39023 Q08999 Q9UIM3 Q9Y478 P30679 Q14814 P28370 Q07666 Q8WVB6 Q06330 Q9HCM9 Q9HCM4 Q9H2K2 P28340 1285 1284 Q9H2K0 169436 Q9HCL2 1277 Q9Y490 Q13501 50937 Q13505 Q9Y496 1297 Q13509 Q14839 1294 Q13506 1291 Q9HCK5 Q9HCK4 1289 50943 11340 Q86V81 23322 23325 23326 25988 23327 126328 55722 Q8WV60 10011 55726 10013 84289 55723 10015 10014 11345 10016 Q9UII4 Q9BS26 Q9NV70 23332 25998 23336 Q99755 400 402 Q99750 55737 406 Q9BS18 Q9BRP8 Q99759 408 409 Q99757 Q01518 P60468 Q9Y446 23307 23303 Q9NUW8 54413 79711 Q8WV41 Q99741 55743 8702 Q9Y450 84292 8721 23310 Q9NUX5 25975 Q99733 10007 11338 55750 55755 P60484 55759 55757 P48775 84299 8718 P23497 84296 8717 11336 79709 P31930 Q9Y3M2 8732 25942 25945 Q9NUQ2 54431 Q9NUQ3 Q8WUY8 Q96P48 433 435 A8K0Z3 55763 P31939 8726 Q9H2D6 79739 8724 140609 8743 8744 7410 7412 Q9HCE7 Q05086 80705 Q8WUX9 Q9HCE1 Q99797 55770 444 445 55775 54443 79723 8737 Q02809 P61758 7408 Q9H2C2 11315 284086 Q9BS57 8754 25929 7422 Q9BRT9 Q9HCD5 65108 Q9UIF9 Q9BRT3 25923 O75293 79753 55781 Q96P20 55787 55785 7415 P61764 7414 7417 7416 7419 8761 Q86UX7 7431 7430 7433 8766 Q5VTL8 Q9UIG0 6103 54460 Q9HCC0 Q9Y3P9 P17612 466 468 55796 7428 8772 8775 Q9Y4K4 O75386 P11441 O75385 Q9Y4K0 Q9H2Y7 Q86VQ3 Q9Y4K3 472 P48729 473 474 O75390 476 477 478 23286 Q99807 8767 Q8WW01 6123 6125 Q9Y4L5 Q86VP6 O75376 8780 481 Q9UJC3 Q86VP1 483 O75380 Q504Q3 O75381 23291 Q9NVP2 Q9NW13 488 6117 P22102 7448 6119 Q9BSJ2 6118 Q29RF7 8795 6133 6132 6134 P12757 7465 Q8WVM7 6137 7468 Q8WVM8 23265 O75367 P12755 O75365 490 O75362 O75360 495 498 P09110 23263 6128 Q5VUA4 Q8WVM0 Q9BSI4 6141 7476 Q01581 59277 O75351 O75352 Q9H2V7 Q9NVR5 P23416 Q96PF1 6138 11261 6156 6158 O75347 23244 P35453 O75340 P09172 P46109 84248 Q9Y4G6 P23467 11269 Q99848 P46108 Q86W54 6164 6168 P46100 11274 P36776 Q9Y4H2 23256 P35443 P35442 6160 7490 P11498 Q8WW38 11277 Q99835 6175 23229 Q86W42 O75326 Q02880 Q86VS8 25885 23225 Q9Y4I1 Q8WW22 Q02878 Q9NVM4 Q96PK6 11243 55704 Q9NVM6 55703 Q9NVM9 P23443 Q8WW24 55705 O75319 6185 6188 6187 11252 P10147 25896 6182 6184 23239 3909 Q99816 Q9NVN8 P48730 P23458 Q99814 P48736 Q8WW12 P78545 6196 P54577 P28288 6199 O75306 P28289 6198 O95714 Q13515 P78540 6193 P78549 6195 6194 3913 Q9Y4C1 P41252 P41250 65082 O95715 65083 3911 3912 Q13523 O95721 Q13522 P78536 Q13520 Q13526 Q9H2P9 P78537 Q9BSB4 3925 Q9HD26 Q9Y4D1 P41240 Q9NVH2 P42574 P42575 Q14865 P29597 Q9H305 Q13535 Q9H307 P29590 Q7Z2Q5 P41235 Q9Y4E8 3939 P41231 Q9HD15 P16284 Q9NVI1 50855 3932 Q9HCP0 Q13547 Q13546 3948 P41222 Q9Y4F9 P41220 Q9NVJ2 O95749 P41229 P41227 3945 Q13555 Q13554 O95757 Q13557 P78509 P78504 3958 3959 P00747 Q96PE2 Q8WVK2 3953 2629 Q13563 3954 Q13561 3956 2626 Q9NVC6 Q13569 Q9H2T7 P11413 P00738 2632 P00734 Q7Z2W7 P24723 Q13574 Q13573 Q5BJF6 P09104 Q13572 Q9HCU4 P55884

Q9NVD7 3980 100287932 P78527 P41279 2647 1315 1314 2644 1312 2643 P41273 Q9UJ83 3978 Q9Y4A8 133418 1326 Q63HQ2 121441 P67870 Q9HCS4 Q9HCS7 P00750 Q13596 Q9HD40 Q13595 Q9Y2W7 2672 Q06265 Q6QNY1 1337 Q9HBH0 Q9Y2X3 219931 Q9UI09 Q9Y399 79885 1345 P16473 2676 P16471 Q9UHP3 Q9UI12 P05787 65264 P54753 P53420 Q66PJ3 Q9Y2Y8 Q96NT1 P16435 P54756 1358 Q9Y2Z0 1355 Q9Y2Z2 Q9Y2Z4 Q9H1D0 P54764 P54762 Q9Y2Z9 P54760 1374 Q99698 Q8WTX9 Q9HBE1 2697 Q9UI32 Q9UI30 Q9UHR5 Q9UHR4 Q9H1C4 Q9Y342 P28482 1385 Q9UHJ9 Q9H1K0 P15173 Q9Y2T2 Q9Y2T1 Q9Y2T7 Q9UHK0 Q06210 Q5VST9 P30793 1388 1387 P54709 116840 P42771 Q06203 P42765 P42766 P42768 284119 P15153 1399 1398 P15151 Q9Y371 Q9H1H9 P27144 Q9Y375 P28472 P54727 P54725 Q9HBI0 Q9HBI1 Q9Y2W2 Q9Y385 Q6DHV7 Q9Y2W1 509 Q9Y305 Q8WTS6 Q8WTS1 54512 79810 Q99640 513 79811 514 8803 79813 515 10134 10133 8801 518 54517 220988 P04406 Q9UHG0 Q9Y2P8 Q8WTR2 521 522 523 23451 10142 526 8812 528 10147 Q99638 255626 8815 Q96NL6 Q86U44 23429 Q86U42 Q9Y320 Q9NU63 533 79834 535 55869 537 54536 54535 79837 539 Q8N8U2 8829 Q8N960 54539 23438 Q9Y336 23439 Q9Y2R5 Q9Y2R2 8841 Q9Y333 7511 Q9UHI6 23435 Q9Y2R9 10128 Q99611 545 546 10121 8836 Q8WTP8 8833 7508 23409 7520 8851 8852 Q9BQT9 Q9Y2K7 Q9Y2K6 Q9Y2K9 23400 Q9UHB6 Q9Y2K2 Q9UHB4 P17752 23404 54552 Q68EM7 54550 552 553 P42704 Q99683 Q8WTW3 Q99684 7514 8844 7518 P05771 7517 Q9Y2L1 6201 7532 7531 7534 6202 7533 23411 23414 10107 10109 563 Q99675 Q9H1A4 10100 P05783 7529 65220 6210 Q86U86 Q9UHD2 Q9UHD8 Q9Y2M5 572 573 Q99666 Q99661 54575 Q8N8Y2 7536 6204 6207 P04424 8880 Q9BQQ3 8881 8887 P16403 O75396 580 P16402 P16401 582 Q8WTT2 80854 Q99653 8879 79869 8878 6217 P60568 10090 Q8N9Q2 10093 Q13477 10092 6231 8892 10095 10094 6233 Q9Y3I0 593 595 Q9NUM3 Q9BRI3 Q8WUM4 6229 Q13480 Q13485 Q8WUM0 Q9HC98 6242 P35527 O75494 P35523 Q9UIA9 415117 6238 P22223 Q13492 6239 Q13490 6256 10075 Q9HBW0 O75489 Q9H269 23387 280636 23381 P23527 Q9NV06 P23528 P09234 P60510 Q8WUK0 Q9HC77 6262 344387 10081 O75478 163126 O75477 Q86UL3 23399 O75475 Q02790 P12883 23396 Q9NUP9 P24864 60386 10087 6259 P60520 Q8WUJ0 6275 O75469 10053 Q9Y3E0 23365 O75461 23368 P10275 P10276 P35579 O75460 P35580 23363 Q9Y3E5 10055 P23588 10057 Q8WV28 Q99728 Q99729 84365 10059 Q99726 Q8WV24 Q86UR1 Q5JQC9 P36896 P36894 23378 P35568 23370 P35573 Q02763 Q9Y3F4 Q01433 Q9BRL6 Q99715 Q99714 P61803 O95813 O75444 Q86V24 6294 P36888 P11586 Q99700 Q9H1Y0 Q99708 P22234 O95817 O95816 Q9BRK4 Q99707 O95819 55827 Q86UP0 O75438 O75436 23354 P36873 Q02750 O75431 P35548 Q86UP2 Q9H295 55832 55835 84376 10048 54509 Q5VTD9 P53367 O95831 Q14721 P53365 O95835 P12814 2702 Q9Y3A5 Q9HC16 P39060 O95837 Q6NVY8 P15090 Q8WUD6 Q6VN20 Q13405 Q13404 Q9H1N7 O75410 Q13409 Q9Y3B4 Q9Y3B7 2710 P39059 Q08945 Q9UHX1 Q9Y3B2 Q9UI95 P42695 O75400 Q13418 286826 Q9UHY1 Q9Y3C5 P00846 P42680 P42681 Q9UHY7 P42684 P42685 Q9NUG6 Q86UE8 Q13423 Q13422 O95861 P53396 Q13427 Q13426 O95865 P42677 2735 Q9Y3D6 1400 Q9Y3D3 2730 Q9Y3D5 Q9HBM1 1408 1407 1406 2737 2736 Q13435 Q9HC62 Q13432 P54652 Q8WUI4 Q86UK0 Q9H244 2744 2742 2741 Q9UI47 Q9HC52 2747 Q13444 Q14774 Q13443 Q08J23 P11532 Q9H1R2 O95881 P23515 P23511 1429 Q13451 Q14781 P23510 O14492 O14493 Q9H228 Q14789 O14495 P12830 59341 59343 Q9H223 163183 84305 1432 1431 2762 Q9HC35 Q14790 Q9HC36 Q13464 P54687 P53355 P53350 Q9H213 2773 O14498 Q7L9L4 P24821 O14497 Q9UHV9 Q9HC21 O75947 P25054 Q92979 O75940 P49023 1460 Q12824 Q5SQT9 Q5SR56 1459 Q9BYG3 1457 P13073 1454 O00391 1453 27089 2783 Q9BYG4 Q92973 Q00839 Q92974 O75935 O75936 O75934 P25067 Q12834 Q12837 1468 27090 P49006 O75937 P10809 Q96EB6 Q5S007 O75925 P27694 P27695 Q12846 P37023 1478 O75928 P26358 Q96EA4 Q12851 P26374 O75914 Q9H936 O75911 P26373 Q92949 Q12857 Q9BYD5 1491 27076 1488 1487 Q9BYD1 148789 P26368 64061 27072 P26367 Q5TCZ1 P25098 P08514

O75901 P49069 27043 1499 Q96EG1 O75909 O14986 Q12873 Q12874 Q12872 O00358 Q9H8X2 Q8TBC4 Q8NA29 604 Q12884 64098 Q12888 P53985 Q8TBB1 Q8NA19 P38398 610 Q92990 613 Q92995 Q5TCX8 Q92994 Q92993 P63302 Q8TBB6 P10826 P53992 P10827 P10828 P53990 221937 Q9H8V3 27032 P40692 27037 Q96ED9 Q92982 P63313 8916 Q8TBA6 Q15019 O96005 O96004 8930 O00308 10250 P56199 O96008 P14317 O00300 O00303 23569 O14949 23560 Q15022 8924 Q15021 55967 Q15020 637 79937 639 Q15025 Q15024 O96018 Q15029 O96017 O96019 23576 P14324 O96013 641 55973 196441 79923 8932 648 8939 Q15036 P27635 8936 Q16363 10269 Q15034 O00327 O96028 O00329 O96020 10239 10238 O14967 656 8945 P01019 8943 10232 O14964 Q9UP83 Q15047 Q15046 O14965 Q8TAT6 O14980 23552 23556 O14979 663 Q15050 53335 668 Q15054 Q15052 285193 Q15058 O14974 Q59EA4 O43474 P01042 Q92934 6311 P01040 8975 245972 P39656 23523 23524 672 673 53340 P15692 O14908 6304 6305 128308 10213 158135 23539 O43482 8985 O43488 23530 23536 10228 10229 688 Q15070 689 Q15075 Q92922 10226 10225 Q15078 23509 P56178 Q9H902 P56177 Q12802 Q12809 O14925 P13010 Q15084 A7KAX9 O14920 P56182 P56181 P56180 O43463 Q92903 6342 23517 6341 Q92901 O43464 Q92905 Q12816 23512 23513 79980 Q5TCQ9 10208 O14936 O14939 P40616 10200 P27658 10202 Q92900 10204 P56192 Q9H8M7 P19174 Q9BZ95 Q9UPN6 O43557 Q9UPN4 Q9UPN3 117584 P07196 84447 P46783 5017 P83731 5018 5034 6368 P46782 5036 P46781 5037 60489 P68104 60488 5029 Q9H9E3 Q16401 P46777 P46776 P46779 P46778 Q9BYV2 Q9BZ76 10193 P56134 Q9UPP1 P32121 10198 10197 Q05707 Q9BYU1 6383 6386 5054 55907 O43542 6389 Q9UPQ9 P68133 Q9UQ26 P04908 Q4AC94 Q5SRE5 10171 P32189 6390 23481 10179 5074 10181 55929 O43521 Q15109 O43524 O43529 5071 23492 Q15118 Q15116 10152 23463 A1XKG3 23466 Q9BYZ2 Q96EV8 23468 10155 2805 54617 2802 Q15126 5099 23476 5091 O43508 23478 5092 5093 Q96EU6 O43505 2810 Q9H9G7 79901 10166 54623 55959 Q6P1J9 P09769 Q96EL2 27005 27000 P22735 2821 2820 Q8NA72 1503 Q9BYN8 Q6P1K2 Q5JXB2 Q9BYM8 60412 Q8TBF4 201595 Q8TBE0 Q04446 P07101 P52597 Q96EP0 Q03113 Q9P015 Q96EP1 Q9H9B1 Q9H9B4 2869 1537 Q96F07 P46736 Q9H9A7 2875 2873 1540 P21453 124739 P46734 O75969 Q6P1L8 O75964 O75962 P34741 Q9P035 P63261 2885 O75953 P63279 P63272 P08473 23603 P10914 Q92858 23607 P10911 P51460 P52790 P52799 P52798 P13196 1576 P49137 P0CG13 P09936 Q9H832 P50148 Q92845 Q92838 Q92835 P50150 Q9BXC9 27185 125972 Q92830 P62136 Q92834 Q9H7P9 Q92833 Q92831 Q92826 91782 Q92824 P09917 Q9H814 P62140 705 Q92820 P07305 P34925 O00469 P07307 90480 P52756 O00468 27165 Q96DE5 O00471 P09972 27161 719 P08648 O00472 P37198 27173 P08651 Q92888 Q92887 Q9UNX3 137964 Q12768 O00483 Q12769 27148 O00487 Q96DC9 Q12770 Q92878 Q9UNY4 P08621 P51451 P09958 Q12778 Q05586 Q6KC79 O00499 P52788 P52789 740 P37173 P09960 7704 P02452 O00425 O00429 Q5VYS8 Q00796 P01116 Q16236 P01112 P01111 Q96CW9 P25100 10383 Q96CW5 118813 54764 P01106 768 Q96CW1 P01100 Q7Z7K6 P14416 P52735 O00444 Q96CV9 O00445 P52732 773 6402 6405 P01138 P01137 P01135 P01133 Q16254 P02462 P02461 P01130 Q9H7H0 6421 23673 780 10363 10362 P02458 P01127 6415 23647 6430 O43592 6432 6431 O43597 6434 7764 Q92817 64116 Q9UNN5 6427 10331 6426 P40763 23658 5111 23650 P37108 P83881 5104 5108 Q92800 P27797 7781 64130 7780 P01185 91754 6453 6456 Q8TA86 6455 Q9UNP9 O00400 23621 10318 10319 5116 Q9H7L9 91746 P25103 5119 P01178 23636 Q9UNQ2 7791 A2RTX5 O43581 Q5T1M5 6464 64145 O43586 P39748 O00411 146057 O00410 23633 P52701 Q5U4P2 6457 5127 10320 P26447 P25116 6472 5141 5142 Q9BY76 O43678 6470 P20273 5139 O43683 P20290 5154 O43687 5155 O43684 84552 5162 Q9BY50 6496 6495 6498 P32241 P32246 5160 5159 P83876 P21589 P32239 O43660 Q9BY44 O43663 Q9BY41 5170 P34896 P20264 5184 10291 2909 160287 Q6MZP7 O43639 P27708 2908 10296 2904 2902 5195 5198 Q09161 P15735 2917 2915 Q8TB22 10270 10273 O43613 O43612 P58876 Q9BXX0 O43615 Q53TN4 A8TX70 10276 2926

54739 Q15003 116150 54737 2923 Q15008 Q9BY84 Q9BXW4 Q15005 Q9BXW6 10283 116138 Q8TAP9 O43623 2932 2931 255488 10286 Q15011 10285 1605 Q15014 2935 P08559 Q12788 P62081 1613 Q96DI7 Q8TAF8 Q9BXM7 Q8TAF3 Q12792 P46821 1616 2947 P07237 Q12797 O43602 Q9H7Z7 Q05682 O43609 Q8TAE8 Q6IN84 29796 P08574 P08575 P08572 1629 2959 Q9BXL5 1627 P08579 2956 P07205 29767 Q03014 P34820 1634 Q9BXK5 27102 2961 84502 2960 1639 Q8TAD8 2969 P09874 Q6R6M4 Q03001 29777 1642 Q05655 2972 Q9BXJ9 P09884 1647 Q9BY32 1660 84525 2987 124626 1655 1653 1650 1659 P51398 P21580 1666 P20248 1665 P21579 Q9BXP5 Q9BY11 P19235 1676 P08581 1674 P08588 P33527 P62070 P62072 Q8TAG9 389840 P21554 134359 Q9H6Q4 P52434 O00148 P15498 Q9UMY4 Q92731 Q92730 O14786 O14788 P27469 10413 P27487 Q9NZG7 Q92729 O00154 P51114 P53779 P38117 O00159 10426 10427 P14174 800 10420 10421 64282 Q7Z727 23708 O00167 Q5T0N5 Q2VIQ3 P40429 811 P63104 Q5TAP6 P40424 P27448 Q676U5 Q5TAQ9 821 Q9NZJ4 P41743 829 P11926 Q13951 Q13956 Q5T160 27283 830 Q9BWG6 832 833 Q4G176 O14744 O14745 Q5TB80 Q92769 P09619 Q9NZC7 P10600 27297 841 Q9UN70 O14753 846 847 Q9UN75 7804 O14757 Q92766 P09622 Q13976 Q9NZD8 Q96TC7 7818 Q9BWE0 Q05469 Q92753 Q5TAT6 Q92752 857 O14763 Q9UN86 859 Q96CA5 P11908 Q92754 O00139 Q9UMW8 Q9H6R4 6500 P11912 Q4G0W2 861 862 863 O00141 865 867 Q9UMX1 Q6X784 Q9UMX0 P09601 O14777 10490 O43278 10492 P15408 Q09019 871 Q96T88 6502 7832 6504 6505 6520 Q7Z6J4 Q96T76 O14717 O43290 Q7Z6J9 7846 O43294 6517 O43293 O14713 O43252 Q6ZMR3 Q6ZN33 O43251 Q9UMR2 64215 23787 Q9BW92 23788 Q9BW91 890 Q00653 891 O14727 894 Q96T60 896 P53708 898 Q16134 10477 Q9UN42 10476 10478 O43264 Q9UMS4 5210 6541 10482 7874 5213 O14737 Q01974 Q96ST3 Q01970 Q96T51 10486 O14730 7867 10488 O14733 5207 10487 O43236 7884 23764 10459 10458 P60891 10451 10450 6548 7879 10452 10455 Q01968 O43242 10460 6566 Q9NYZ3 10469 Q01955 Q96SZ6 23770 5226 10466 10468 23746 5245 6576 Q9UMN6 10438 Q5TAL4 Q00613 Q00610 10432 5250 Q13901 Q96C86 P28749 P04792 10445 6595 6597 6599 6598 5268 P10599 6591 57062 O43318 P33240 P58546 5270 30813 P46531 54801 P60866 Q9UNL2 Q9UNL4 29803 P46527 O94804 O94805 54815 Q9BWQ8 5295 P35869 57092 O43306 P10586 P10588 57099 O43303 P10589 Q9NZZ3 54820 54822 O94817 Q9UNF0 P63092 Q3LXA3 O94826 O94827 Q96CS2 10390 10392 Q7Z7G2 Q9UNH7 54853 P21266 P47897 1718 P47895 1716 347733 Q9BWU1 Q9BX66 P21283 P21281 Q09028 O75791 Q9UNI6 O75792 Q9NZV6 Q9H7C4 Q00688 1729 1728 Q7Z7F7 P35813 P10515 27248 27242 Q9H6Y2 27246 1738 1737 1736 O75771 P10523 Q9H792 27252 1743 1742 27257 Q5TAX3 29889 84617 Q9H6W3 1756 29880 29886 Q92793 Q6ZNC8 57003 Q9NZQ3 Q7Z6Z7 Q92791 27236 Q9UNE2 112936 Q9UNE7 57017 Q9BWH6 653361 1763 29896 Q9NZR4 Q04206 29894 Q92786 Q92785 Q92784 Q9NZJ7 O75746 Q5GLZ8 1781 O00186 O00189 Q53SB5 1778 O00192 57026 P63000 60673 Q7Z7A1 P08243 P08246 30849 1789 1788 Q05516 1786 1785 Q05513 P63010 57038 339287 Q96CJ1 29843 57045 28513 1793 Q9NZM1 84661 Q9NZM3 Q9BWM7 O75716 30827 O75712 Q06830 Q9H6Z9 Q9H6Z4 P22528 Q9UNA1 P47804 900 Q9NZN5 902 P08237 P08238 Q9NZN9 Q7Z628 64397 O75821 Q9NYD6 O75822 O00268 51081 O00267 Q9BVA0 10535 O00273 Q9BVA1 Q9ULW3 P63208 Q9UM82 90678 Q6NZY4 Q9ULW0 Q92614 10534 10533 Q92611 P52564 P52565 51090 P52566 51094 51096 P63211 10549 51099 P63218 200081 928 Q9ULX9 Q05397 10514 P63220 O75807 26091 Q13823 Q13829 10524 10528 Q9ULZ3 10521 948 10523 Q5D1E8 O00220 O00221 58513 58517 Q9UM47 O00238 Q96SB4 Q96SB3 Q9NYA1 O00231 O00233 P10721 O00232 Q5JTZ9 Q96SB8 Q8WYH8 960 961 Q92643 967 7922 Q9UM54 Q9NYB0 Q9NYB9 O00244 6609 58533 975 6601 976 977 Q92630 6605 Q92636 Q9UM63 Q92633 161823 Q92626 Q13868 O00255 O00257 984 6612 Q92621 988 Q92620 989 6613 Q92624 Q9ULV4 26061 26060 Q9Y6W6 O43395 Q9Y6W5 5300 6631 P53804 Q9BV90 P53803 P15531 990 991 Q96RU8 O14827 Q96RU7 Q96RU2 O14829 994 O14828 51000 995 Q96RU3 997 998 6622 999 6625

6624 6627 51009 Q9UM07 Q9Y6X3 5310 Q7Z5H3 6643 5311 6642 Q96RT7 51010 Q96RT1 Q96S53 51013 6633 Q9Y6Y0 O14830 5305 Q96S59 Q96RT8 6637 O00206 Q9BV73 Q9BV79 5321 6653 O00203 51021 51023 Q00535 Q00534 Q96S42 10594 51026 6647 5315 6646 5316 10598 91949 5318 O00217 6660 4000 5331 5333 O00213 Q96B26 Q96AQ6 Q96RR4 Q96RR1 Q00526 6657 5327 Q9ULR0 O43390 O43353 6670 Q9H5K3 6672 Q96B97 Q8WYA0 6667 5335 5336 Q96AX1 10574 5337 5338 4008 5339 10577 P26232 Q7Z5L4 5351 Q9H5J4 5352 10580 5356 Q9ULK4 P68032 Q01831 78991 4015 5347 4017 Q9H5I1 5362 6696 5364 4035 51061 O14802 51067 P68036 10552 Q68J44 10554 Q9H5H4 Q01826 5371 P27540 5378 P15559 P16885 10569 51074 51075 O14818 6699 4038 Q01813 10564 10566 54908 O43432 57187 29948 29947 94121 Q9NYU1 94120 Q9NYU2 29941 Q96BM9 P22695 Q7Z6C1 83475 P22694 Q7Z6C3 O94905 O94906 5393 5394 4067 29959 4060 P47985 P60983 Q96SN8 54921 O94913 P60981 84790 4074 29928 P22681 29922 29927 P35998 29924 54930 115024 O94925 P22674 54931 54938 O94927 Q8WZ42 4086 4087 4088 P22692 4089 Q86YT9 29934 Q86YT6 Q96BK5 Q9NZ94 P21359 1808 P20020 53615 1801 4099 Q6ZN16 Q7Z6G3 Q9BW71 4092 Q4G0F5 4093 4094 Q5JUK2 Q00597 Q9NZ20 Q9H6D7 Q9UMD9 Q9NYQ6 P45379 Q96C24 P04843 Q86YZ3 P04844 O94955 29916 Q9BW60 1822 54961 1829 1828 1824 1823 O94966 Q96T21 1832 Q96T23 Q9NZ43 Q00577 1837 P60953 O94973 P20073 Q9BVS5 Q8WZ73 1843 Q9NZ53 1841 Q9NZ56 54984 O94979 1848 Q6VAB6 P21399 Q13873 P10636 26038 26037 Q86Z02 P63172 26039 1854 P63167 1852 P47914 1850 P23945 Q4KMG0 Q92692 P63165 Q92696 P09651 1859 P22612 Q13882 1856 Q13885 Q13887 P10644 O75891 84708 Q96BD5 P22626 P63173 P09661 P60900 1869 Q13895 P10619 O75886 P35916 27348 P11940 57120 26015 O75881 27347 O75880 26019 1877 1874 Q9BVG8 1870 Q5TA76 Q92673 P23921 Q9NZ08 P35908 O75874 1891 P10620 P35900 27352 84733 Q9NYP7 Q92667 P33316 Q9NYH9 58473 27327 57144 P51148 P51149 1896 58478 1892 Q8WYQ5 P35968 Q8WZ19 P51153 A4D0S4 26005 57154 26007 Q96BI3 58480 P51159 84749 27339 27330 58487 27332 57159 26002 Q8WYP5 Q9BVL2 Q8WYP3 P21333 P09669 O75843 O75844 377630 Q5RKV6 57169 P51178 O75832 27315 Q86YP4 Q06710 Q9BVJ6 P47929 10657 Q5QP82 O60443 Q96IK1 P21926 10651 Q8N3U4 10653 Q8N465 Q8NE71 Q8NDV7 Q8IWJ2 P19623 10667 P61158 10661 10666 136319 Q8NE63 P61163 P62495 P61160 P62491 10632 P21917 P21912 10645 O60437 10640 10642 10643 P51784 Q8IX01 10609 P50454 P51787 10613 P21964 10616 26191 P61106 6720 P33947 Q96J02 P51797 P51798 10626 10625 P20645 P20648 6711 P05023 P07686 6714 P05026 O95136 P19634 391627 57326 401505 P20618 6723 6726 Q8N488 O95147 6741 5411 O95140 Q38SD2 Q8IWL3 84932 10606 253980 57332 10607 6733 O95159 64432 5422 P50416 6748 6747 6749 P25445 6760 O95169 P49411 5430 112399 5432 O95163 O95166 51132 Q8IWA4 Q14186 5424 Q14185 P49407 Q14188 P02794 5440 5441 6772 Q08380 5442 6774 346171 P12111 Q66K89 P14780 51141 O95180 51144 5434 51148 Q14197 51147 6780 P50440 5451 Q8N3J5 A7MD48 O95182 O95183 Q53H96 124359 O95190 5445 6776 4116 P62424 6790 O00744 5460 6793 4131 O00746 6794 4133 10690 O95197 O00743 Q8IWF2 4125 6789 Q8N423 Q07020 5470 4140 O00755 P49450 5471 O00757 5476 O00754 Q9UBX5 Q9UBX3 P49448 5467 4137 P49447 5468 4139 4150 O00767 5481 4152 4154 4155 Q08345 O00762 P13489 O00764 51185 5478 5479 10673 4149 10672 5493 5494 5495 4168 5499 P50406 P50402 P13497 51199 P49427 4172 O15085 4173 Q14204 4174 P55268 4176 Q14209 Q16864 P55265 4171 Q6P5Z2 Q9NPC1 Q8NEH6 Q9NPC8 Q8IWZ8 4188 Q16877 Q9NPD3 Q15554 197259 1902 Q16881 Q96IZ0 P31273 4191 Q96IZ7 4193 Q6DD88 Q6UVJ0 Q15561 1915 Q93009 P02741 Q93008 Q14232 P55290 Q8IWX8 Q8N4C7 Q8N4C6 Q8N4C8 P31270 Q9NPF5 Q03518 Q8IX90 P43246 Q14249 P56556 Q93063 Q93062 Q04837 1937 1936 Q14254 1933 P12109 64403 P12110 Q93050 O15047 1948 1947 118460 1945 P02751 O15061 P02790 O15068 Q07092 O15066 Q14C86 51100 Q8NEJ0 51102 P02786 51107 1956 O15075 Q9NPA8 O15078 64423 64426

Q8N4H5 64428 1965 Q93034 Q93038 51116 1969 Q14289 1968 Q9NPB6 Q70CQ3 Q4VCS5 Q70CQ1 1975 Q6P5R6 1978 Q58WW2 P56524 Q02297 117178 117177 1982 P06276 O15020 9001 P06239 P55209 P56539 P56537 1994 26133 P06241 P21860 P56545 Q16816 340061 P55210 O15027 P07585 Q16825 Q16827 Q9NP61 Q9NP66 O43915 9021 Q16828 9020 O60487 O60488 P85037 P20594 84881 Q8NEC7 Q8IWV1 P31249 Q8NEC5 Q9NP71 Q15506 Q9NP72 Q8IWV7 O43921 O43920 P61081 Q96IW7 P61073 83548 Q6N021 26121 O60499 P61077 26123 O60493 Q6P5W5 9047 9045 Q9NP81 Q16849 Q8IWU5 P45880 Q8NEB9 Q02241 P33897 P02708 P61088 131474 Q9NP90 Q16854 Q15526 27436 Q9NP97 Q9NP98 9054 84893 Q15532 9061 Q9UBB6 Q9UBB5 9066 Q9P2R6 Q9P2R7 Q9UBB9 O60568 O60566 Q8IVH8 154091 10772 Q9UBC1 O95202 Q9UBC3 Q9GZZ9 Q9UBC2 9077 10783 10785 Q9UBD6 Q9GZY0 Q8TED0 57403 O60547 10758 6809 57405 Q8IVF5 O60543 P61289 P06401 P06400 Q9GZX9 P49591 P07737 Q9GZX7 Q8NCR0 9093 Q9UBE8 P56705 P49590 9099 10767 P20702 10768 57410 O60551 P61296 6810 6812 6811 P49588 6814 P07741 10762 10765 P05129 P50570 O95239 P50579 O95232 83706 Q8IVM8 6829 10733 Q9P2N5 10736 Q6FHQ0 P61221 6827 Q10570 O95249 P19784 O95243 56104 6839 10746 5500 P05141 5501 A6NMZ7 6834 6838 O95259 P07766 P05106 O95257 P05107 6850 P50591 O60508 O60506 Q9P2P5 O60502 O60503 10714 57448 P61244 Q8TEH3 5511 P61247 5514 5515 P05112 5516 5518 Q5QNW6 Q02078 56121 Q60I27 P18433 O60513 10725 10724 Q8IVI9 P61254 10726 10728 Q6FI13 P05121 P06454 Q8N2K1 O95278 6872 Q96HA8 6871 O95271 O95274 P13591 5533 4205 5536 6869 Q15389 5537 Q15388 Q9GZR7 4209 Q96HA1 Q9GZR5 6883 P48201 4221 6885 Q6P4A7 P13598 P50542 P12268 O76074 Q9P2K2 O76071 51251 Q15392 51253 Q9P2K8 4214 6878 4215 P01589 4216 Q15393 P01588 4218 Q9GZQ8 6890 6892 P50552 5562 5563 5565 P13569 P12236 O95292 O95298 P51884 O95299 P12235 O76061 51264 P61204 Q53X93 6886 Q9GZP9 5557 P61201 5558 4240 5573 5575 5576 Q08257 221613 5566 5567 5568 Q8TDZ2 P61218 5580 5581 5582 5583 5584 5585 4255 5586 22794 O76031 Q2M389 Q14093 P51828 5577 5578 5579 Q8N302 5591 Q6IR47 P24298 5596 5597 5598 P50502 O76024 P80370 O76021 Q5T686 5590 Q9GZU7 Q8N2M8 P12277 P51843 Q8NCN5 5599 Q96HC4 4285 4287 O76003 Q08211 221656 Q08209 Q96HB1 Q2M2Z5 Q96HZ4 4297 Q6ZT98 Q15418 Q8TEW0 P30085 P30086 Q04760 4291 Q03426 Q9UBS0 Q04759 Q9UBS5 Q9UBS4 Q15424 Q15428 Q15427 26205 Q8TF76 Q9UBT2 Q9UBT6 Q70SY1 P30084 Q15436 Q15434 Q14103 Q14108 Q15438 P29074 Q53GQ0 Q9UBU3 P51808 Q03405 P51805 P17096 Q16774 Q9UBU8 P43378 Q53H12 Q14116 Q8IW75 Q14114 Q16777 Q16778 Q14119 Q6PEY0 Q14118 P51812 P51813 P29084 Q96HW7 Q9UBV7 P51817 Q15459 P55347 O15160 Q14126 P55345 Q16787 O15164 Q14129 Q16795 Q16790 Q14134 Q15464 Q14139 Q14137 Q16799 163786 Q14141 O15169 51218 Q15475 Q6ZSZ5 Q8TEY7 O15182 P14859 Q6VY07 51222 Q9UBQ7 P01579 Q14152 Q9UBQ5 Q14155 O15197 O15198 P14868 P14866 Q8TEX9 Q14160 51231 51234 Q14164 Q14166 9100 O15123 Q9NNW5 P33992 P33993 Q8TF05 P33991 84958 26271 84967 Q1PSW9 P20671 O15119 P45974 P45973 26270 P82921 O15118 Q9UBK2 Q9UBK9 P82912 9114 P82914 O15131 Q6DT37 Q96I24 P31321 P55318 P31323 P55316 P43304 P43307 84950 P05062 O15126 P05067 Q9UBL3 9128 9129 9126 O15143 Q8TEM1 26258 Q96I15 9120 P55327 283989 Q8TEL6 P21980 Q9UBM7 9135 O15151 9133 O15155 O15156 9138 Q02156 26260 26262 57379 283970 Q9UBN7 P82932 Q9UBN6 P82933 P30049 9146 Q9UBF2 Q9UBF6 9149 56052 P30041 P30043 P30044 P30046 P30047 P30048 P05091 26232 P05093 P30050 Q03468 26234 Q6ZSS7 Q9UBG3 Q96HU1 Q8IVT5 9150 57396 Q16718 P17081 Q8TEQ6 83696 81037 Q6P4R8 Q5T6F0 9179 O15111 Q9P2Y4 Q04771 Q96HR8 Q8TF09 O15105 P45983 P45984 9182 22872 9181 P51587 9184 Q8TDD1 Q9P218 P37288 Q8N1Q1 Q8TDD5 6901 A1A4S6 Q6IQ55 P08729 P08727 124540 Q53EZ4 9197 P37268 O60216 P62244 Q8IUE6 57510 P62241 P49247 253725 57519 P62249 Q8N205 Q8NBQ5 6927 Q8IUD2 6929 P62256 57520 57521 6921 P26599 P62258 6926 P08708 57534 Q96GD0

P62266 Q8IUC6 P62263 5602 Q96GD4 P62269 5604 22839 Q8TDI0 P50213 22832 10855 P19404 10856 57544 Q8N264 5610 5613 6945 P07437 22846 P51553 5631 P80098 P52888 10868 10869 Q8N257 P49286 4311 P21709 Q8IUH5 Q71DI3 5636 Q8N1S5 Q10472 Q10471 P51572 P51575 Q5MAI5 22826 22827 22828 P51571 P51570 Q8NC51 Q9P202 10844 10845 Q9P209 Q8NBS9 192669 P08754 4318 P01241 6993 O00541 P14555 51362 Q8NBJ5 51363 P25205 135295 64682 4343 3010 Q6FGD7 P13236 P51513 3009 3008 3007 3006 Q8NBI2 51377 P01222 Q15276 Q09472 5682 5683 5684 5685 5686 3024 O00560 O00562 O00567 51380 3014 Q15286 P03891 Q15287 P51531 4361 5692 5693 4363 P40818 3035 Q8N1F7 3033 3032 P51532 3030 P14543 P14550 P15880 3028 Q15291 5687 5688 4358 P26583 O00505 Q6FGG2 O00506 Q641Q2 P14598 P38570 P55075 P55072 Q96GC5 P01275 P52803 3054 P37231 Q4LDE5 Q8NBM8 P82673 P82675 P55085 P55084 P55083 Q6IQ23 P01266 P49207 Q96GA9 P52815 22893 3066 22894 3065 22895 22897 3070 3074 P40855 O43791 53938 Q16629 Q16623 P18084 P18085 3084 Q96GX9 28973 P20396 Q8NCD3 Q16630 P43034 3093 Q16637 Q15303 3091 Q16633 P55036 Q16635 Q15306 64601 3099 28986 3098 Q05952 28981 Q16643 P43026 O00602 Q6YP21 Q16649 Q16644 Q16647 O43776 Q96H78 P31040 P56385 P82663 P82664 P56381 Q8NCB2 Q16650 Q16659 Q16656 P18074 P55055 Q16658 P18077 93166 P68371 144983 P68366 28960 Q9P2E3 Q16665 51317 Q5J8M3 Q9P2E9 51319 P43005 Q8TDY2 Q16666 O43752 Q14008 51324 P27824 Q14012 51329 Q14011 Q15349 Q8TDX7 Q7L2H7 Q9GZM8 Q9GZM5 9201 P98175 Q3ZCQ8 P98179 O43734 O43739 Q08188 51340 51341 P02549 Q15363 Q14032 Q16695 P55010 Q14031 P02545 Q16698 Q9GZL7 Q15365 Q8TE73 9212 Q15369 Q8IUX7 Q8IUX8 O43741 9217 Q08170 P55011 O43747 Q15370 P02538 P27815 P02533 P98194 Q15375 9223 9221 P98155 P10997 57461 O43711 O43715 P32302 10811 56137 10813 83752 56134 P62195 O60285 P08684 Q4LE39 9218 9219 P98161 P07355 P98160 O60282 9232 22803 9238 Q9P1Y6 Q8NBZ7 9230 83737 9231 Q8IUN9 Q3V6T2 P46934 P46937 83746 O60296 Q4LE28 P98170 P98172 P98171 O60293 P98174 P34947 P83916 Q4LE60 P46926 Q96GM5 O60266 O60264 O60260 P07339 9255 Q32P51 P19320 P34931 9252 83759 P34932 9253 O43707 P20309 P08670 Q5T5A8 9267 Q9P253 Q9P258 9261 9276 Q9P1U0 9270 P19387 P19388 93100 P62166 O60256 P46977 P46976 53916 Q8IUQ4 28998 P32320 P32322 O60229 P20339 P20336 Q04656 P20333 Q16611 P18031 Q9P286 P34972 Q9P287 P62191 Q8TDN6 P19367 9296 Q8TDM6 O60232 O60231 P20340 P21675 P08833 P48052 Q12948 22992 22994 P61020 Q8NB16 Q7L0Q8 57620 P48047 Q12952 Q12959 Q8NB12 5706 57634 5707 5708 5709 P21810 O60333 O60331 P21815 5700 P49368 5701 5702 5704 5705 P25398 Q12965 P25391 Q9BZG8 Q12968 22974 O60306 O60307 5717 150737 57646 83933 P12081 Q9UQB3 Q9UQB8 5713 5716 Q12972 Q12974 P48023 Q9BZF9 Q6P2C8 Q12979 22985 O60318 5728 253827 5725 Q6PCD5 Q12981 Q12982 Q12986 P51668 57661 P51665 Q9P0J0 Q9P0J1 P19525 5739 57669 57662 P07550 P62316 P62318 5733 56339 10972 Q9BZM4 P62312 10971 P08887 P62314 Q9BZM1 P51671 P51679 P19544 10987 57678 P18206 10988 56342 P62328 5747 Q8N137 22934 22937 22938 P51681 Q9H9Y6 Q9P0L0 P51686 Q9P0L2 22931 P62330 10953 57689 Q56NI9 10956 P62333 10959 Q9BZK7 22929 P61006 5757 P61009 P08865 10951 P51693 5770 P51692 P48061 Q9P0M2 Q03164 Q9P0M6 55035 Q9BZJ4 Q9P0M9 57695 57697 P48059 5768 Q9BZJ0 P61019 Q96FF9 P61018 P06213 Q15139 Q08043 5780 Q9UPV9 5783 P80188 P13349 P14678 P38646 P52952 Q66GS9 5775 5778 P27986 P02686 Q9UQ80 Q8TBX8 Q15149 Q9UQ88 5796 P51636 Q15154 P02675 P01344 P01343 Q96EZ8 P26641 P26640 O95059 3142 P26639 Q15165 5798 Q9H9L3 Q9UPY3 Q3ZAQ7 P01375 P01374 Q9UPY8 P40939 P40938 P40937 P51659 O95067 3151 P12004 O95070 3149 O95071 3148 3146 4478 P40933 Q96EY1 P62308 P25311 Q9UPZ3 P62304 O00623 O00628 Q9UPR5 Q9UQ35 Q9UPR3 O00629 Q12904 Q9H9S0 3162 O00622 Q12906 Q9BZE0 Q9BZE4 3159 Q9BZE1 Q15185 P49336 3178 Q5T442 Q9BZD4 3171 P12074 Q00987 3169 Q6PCB8 P49327 P24046 3181 P49321 Q9UPT5 3189 3188 Q12923

3187 Q9UPT9 Q12929 3185 3184 P12036 3182 P26678 3192 3191 3190 Q60FE5 Q12933 Q12931 P51610 P51617 P52948 57610 Q9UPV0 57617 Q9BZZ5 Q9UQP3 P15927 P15924 P15923 Q96FV9 Q8IU60 Q8TD31 P03950 O00716 Q16512 Q16513 Q16514 Q7LBC6 51412 Q8TD20 Q16526 P31153 P31152 O43897 Q2M1K9 Q5T4F4 Q16531 Q9BZW7 51426 51429 51428 Q16539 Q6PD62 Q15208 Q8TD19 64745 P52907 Q16543 64750 P40926 Q8TD84 P14635 P38606 64759 Q9UQL6 Q8NBF2 Q16555 Q15223 197322 9314 O43889 64768 P01308 120892 P01303 Q15233 9322 P02671 Q8TCU4 Q96G97 Q96FX7 Q8TCU6 P14618 Q5SSJ5 Q96FX2 P68431 51465 88745 9319 Q16576 9318 64780 64782 Q08050 P39905 Q96FW1 P14625 O43865 51474 Q2M1P5 Q16581 Q15256 9342 Q08AF3 22913 Q7L0Y3 10929 P18124 O43837 9341 10933 10935 10938 Q7RTN6 22908 Q9BZQ6 Q9P0W2 22926 22928 Q8TCJ0 10939 Q8TCJ2 8021 O43847 22920 9350 P57735 10947 56267 10949 57599 22919 10940 9368 10907 O43812 P57740 10908 O43815 9360 9361 10910 O43819 Q8N163 O60383 O60381 P21741 22901 Q8NAV1 O43823

cellular macromolecule metabolic process P28562 10657 2305 55561 55568 Q5QP82 2300 Q8N465 4968 P30876 Q8NE71 10667 2316 2 Q9UKV5 Q9UKV8 3661 Q8NE63 P62495 2324 Q9UKW6 3659 3672 Q13705 P21917 10645 2335 10642 O14578 10643 Q9UL15 Q8WXG6 P51784 Q9UL18 Q8IX01 1022 1021 1020 Q06587 10613 1018 1017 79577 10616 Q92530 26191 3692 Q8WXF0 Q13724 Q9H4P4 Q96J02 1029 10626 O14519 10625 1025 Q9BUB5 Q9BUB4 6711 P07686 6714 O95136 79595 391627 P20618 Q9UL46 O95147 6741 5411 Q38SD2 Q8IWL3 1050 253980 57332 1047 10607 6733 Q9UL54 64432 5422 11108 11107 Q8WWY3 6749 Q9Y676 P49411 5430 5432 Q9UKM9 O95163 Q9NWZ3 P57059 115426 1072 51132 Q9Y680 84033 5424 11113 P49407 84061 5440 5441 6772 5442 Q9UKN5 Q9UKN8 346171 Q66K89 5434 51148 Q14197 51147 Q9UL03 Q86X95 Q8N3J5 Q9Y697 A7MD48 Q00403 6776 4116 P62424 P57081 6790 Q5VVQ6 6793 6794 4133 10690 9820 Q9NX62 O00743 Q9Y5Q8 Q9NWU5 Q8IWF2 4125 6789 Q96A72 Q07020 5470 4140 Q9Y5R4 O00754 Q9NWV8 207 5468 4139 4150 5481 4152 4154 Q9Y5S2 Q08345 O00762 P13489 Q9NWW5 Q9Y5S9 P17844 51185 5478 5479 4149 Q9H4F1 5494 5495 Q9BU89 5499 Q96QT4 P49427 4172 4173 4174 11060 4176 Q14209 11065 7204 P55265 Q6NZI2 4171 Q6P5Z2 23043 11072 Q8IWZ8 4188 8543 Q9NPD3 8536 Q96RL1 P61956 Q15554 197259 Q9BUN8 P35638 P61968 11041 8555 23028 Q96IZ7 4193 P61962 1915 P61960 Q93009 P22314 Q93008 Q14232 P61964 8563 Q9Y6R4 11051 Q8IWX8 8565 Q86Y37 P36954 Q8N4C8 267 23031 23032 Q9NPF5 P61978 P43246 Q14249 8576 26994 O75582 Q9Y6K1 P10398 Q93063 Q93062 Q04837 8569 1937 1936 1933 11030 Q9BV47 O75575 23014 23016 7251 Q9NY12 11035 118460 P02751 P61927 7266 P02790 O75569 7267 Q9Y6M4 Q8WY64 Q8NEJ0 P08069 Q6I9Y2 51107 1956 O15075 Q9NPA8 26986 Q01780 Q86Y79 7272 1965 114088 Q93034 51116 1969 Q14289 1968 11017 P12931 Q7Z589 Q70CQ3 Q70CQ1 1975 Q6P5R6 Q13761 Q58WW2 7297 P56524 Q13769 O75531 P24928 1982 Q9Y6H3 Q92560 O75528 Q5D0E6 P06239 P55209 P56537 1994 26133 P06241 P21860 O95983 Q16816 91147 A4D1W7 63931 Q07954 Q9Y6J8 Q07955 P07585 Q92542 50628 Q16825 Q16827 O14595 O95997 Q16828 9020 23081 23085 P20594 84881 Q8NEC7 Q8IWV7 Q86Y13 P61081 Q9H596 P61073 83548 23097 26121 P61077 Q9BUJ2 P22303 11080 Q9NXH8 9045 Q9NP81 Q16849 P12956 Q8IWU5 Q9Y6E7 Q9Y6E0 P61088 Q96AE4 Q96RG2 11091 Q16854 9054 23076 P24941 11099 84893 Q5FWF5 285672 65003 O14672 399687 Q9UBB9 O60568 O60566 P15374 Q8IVH8 10772 Q9UK45 Q9UBC3 P27361 Q9GZZ9 65018 54361 55696 10783 10785 92609 Q9NWB1 P52333 P29992 1121 P53667 Q8TED0 O60543 1111 Q86WA8 P61289 P03372 P49591 3791 Q9GZX7 Q8NCR0 9093 Q9UBE8 P56705 P49590 9099 10767 57410 O60551 P49588 10765 P05129 P50570 P11802 P50579 O95232 6829 10733 147746 Q9P2N5 1137 Q6FHQ0 P61221 6827 Q10570 Q7KZI7 Q7Z419 Q9Y4X5 Q13601 1153 Q14938 P19784 O95243 P53611 6839 10746 1147 2475 5500 5501 P26045 P53621 O95257 6850 Q13615 Q9Y4Y9 Q13618 Q13617 O60508 Q13616 1161 O60506 Q9P2P5 O60502 10714 57448 P61244 O14641 5511 P61247 O14640

5514 Q9Y4Z0 5515 Q9UK22 65061 5516 5518 Q13620 Q13627 P18433 10725 10724 P61254 10728 O14656 P40337 P06454 Q9UK32 O14657 Q8N2K1 O95278 O43159 6872 Q96HA8 6871 P04637 25862 Q9NVW2 O95271 11222 5536 5537 Q9GZR7 6883 4221 6885 1198 25873 23210 1196 1195 56940 Q96Q15 Q15392 Q99873 55611 51253 Q9P2K8 4214 11231 6878 4215 4216 Q15393 P01588 O43172 56946 25847 5562 5563 51264 Q53X93 Q9GZP9 Q9Y572 5557 P61201 5558 O43143 Q9BT22 P04626 5575 P04629 25853 11218 301 P17936 9924 5566 Q96PN8 5567 5568 P61218 5580 5581 5582 5583 5584 5585 4255 5586 22794 25822 O76031 Q96PU8 Q96PU4 79612 Q96PU5 Q02930 5578 8602 5579 Q9H3F6 8607 Q8N302 5591 9950 Q6IR47 5596 5597 5598 P50502 O76024 O76021 5590 P16671 328 329 84197 25804 Q9GZU7 Q8N2M8 Q9NW64 333 55660 55666 5599 P83436 4285 4287 7311 Q96Q40 P17980 Q9NVV4 Q08211 221656 Q08209 79622 9967 4297 Q6DKI1 Q6ZT98 Q15418 7325 Q9UKD1 4291 351 Q9UBS0 Q04759 Q9UBS4 9978 P21127 8662 7332 7334 16 8667 Q15428 7336 8665 Q15427 7335 19 26205 Q8TF76 Q9UKE5 Q9H3Z4 Q9UBT2 23170 367 Q9UBT6 369 11198 7329 7328 7341 11160 Q15434 Q14103 7347 6015 P29074 23144 Q9UKF6 Q9Y606 Q9UBU3 Q03405 85437 P17096 11168 8669 Q9UBU8 P43378 11171 8683 7353 P51812 P51813 P29084 Q96HW7 Q9UBV7 P51817 11176 P23771 Q15459 O15160 P55345 O15164 25788 23127 52 Q14134 Q14139 6045 Q14137 7375 6048 23133 23135 Q9UKA1 Q9Y5J1 11157 Q99956 Q8TEY7 6059 Q9Y5K6 Q9Y5K5 6050 Q9UKB1 Q99942 11129 Q99941 51222 56915 P01579 Q14152 11124 Q9UBQ5 11128 O75676 O15197 O15198 O75674 P14868 P14866 25778 51231 Q14164 86 Q14166 9100 Q96QC0 Q14966 P52272 P33992 P33993 Q8TF05 P33991 Q05209 84967 91 Q8WWI1 94 P45974 26270 P82921 Q9UBK2 Q9UBK9 P82912 P82914 Q14978 Q8WWH4 Q86WJ1 Q6DT37 Q96I24 Q9H3R0 P31321 84950 P52292 Q52LR7 P05067 Q9UBL3 O94763 P52298 9128 O94768 9129 O75643 9126 Q6XUX3 Q9H422 6093 283989 Q8TEL6 P21980 Q9BTC0 Q02156 26260 26262 57379 134430 Q9UBN7 P82932 P82933 Q9UJV9 Q9UBF6 9149 56052 Q6NYC1 Q9H488 P11766 P53567 P22415 26232 Q9UK80 P30050 Q03468 26234 Q92499 3835 Q8IVT5 9150 57396 Q9UJX2 Q9Y5B0 P08138 Q9Y5B9 P09429 O75604 P10415 Q8TEQ6 P35711 2521 Q6P4R8 P09430 Q5T6F0 728642 P08107 O15111 Q9P2Y4 Q04771 Q96HR8 O15105 P45983 P45984 1207 65125 P54619 P51587 9184 P17676 Q8TDD1 2547 1213 Q6IQ55 Q9H2H8 25913 P08729 Q5VTR2 O60216 P62244 80755 54487 P62241 P62249 Q9H2G2 Q9BRX9 P28300 2571 79791 54496 Q8IUD2 6929 P62256 57520 6921 P26599 P31942 Q9UIS9 P31943 P08708 P54646 P62266 P62263 5602 Q96GD4 P62269 5604 Q9Y3U8 Q9Y463 Q14807 2590 P39019 10855 P15056 10856 2589 P39023 5610 5613 Q9UIM3 P30679 Q07666 Q8WVB6 Q06330 10868 10869 Q9H2K2 P28340 P21709 Q8IUH5 169436 Q10472 Q10471 Q13501 Q5MAI5 22826 22827 22828 Q8NC51 Q9HCK5 10845 192669 11340 Q86V81 O00541 23326 25988 23327 51362 Q8NBJ5 P25205 10013 84289 10014 135295 64682 Q9UII4 4343 Q9BS26 Q6FGD7 P51513 51377 Q99750 Q9BS18 Q9BRP8 Q99759 408 409 P60468 23307 Q09472 5682 5683 5684 5685 5686 O00567 Q9NUW8 Q99741 55743 3014 Q9Y450 Q15287 4361 84292 5692 5693 P40818 3035 Q9NUX5 P14543 11338 P15880 3028 P60484 Q15291 55759 5687 5688 55757 84296 79709 P26583 8732 O00506 25942 54431 Q8WUY8 433 Q9H2D6 79739 P55072 Q96GC5 140609 Q9HCE7 3054 Q05086 P37231 Q9HCE1 Q99797 444 55775 8737 Q02809 P82675 P61758 284086 P49207 8754 25929 7422 Q9BRT9 Q9HCD5 P52815 3066 22894 3065 Q9UIF9 22897 O75293 79753 55781 7415 8761 3070 Q5VTL8 Q9UIG0 54460 3074 P17612 55796 7428 O43791 53938 Q16629 Q9Y4K4 P11441 O75385 Q9Y4K0 Q86VQ3 Q9Y4K3 472 P48729 474 28973 Q16630 8767 Q8WW01 3093 Q16637 Q15303 3091 Q16633 P55036 6123 6125 Q15306 Q9Y4L5 Q86VP6 O75376 8780 Q504Q3 23291 Q9NW13 6117 6119 6118 Q16649 6133 6132 Q16644 6134 7465 6137 O43776 O75365 O75362 6128 P82663 P82664 Q8NCB2 Q8WVM0 Q9BSI4 Q16659 6141 P18074 P18077 O75352 144983 28960 Q96PF1 Q9P2E3 6138 Q16665 Q9P2E9 51319 6156 6158 O75347 P35453 P23467 P27824 Q14012 51329 Q14011 6164 Q15349

Q8TDX7 6168 P46100 11274 P36776 6160 7490 Q7L2H7 11277 6175 9201 Q86W42 P98175 Q3ZCQ8 P98179 Q02880 25885 Q08188 Q8WW22 Q02878 51340 Q9NVM4 Q96PK6 11243 P55010 Q9NVM6 55703 Q9GZL7 Q15365 P23443 O75319 9212 6185 6188 Q15369 6187 Q08170 25896 6182 6184 Q15370 Q99816 P48730 P23458 Q99814 P48736 Q8WW12 Q15375 P78545 6196 P54577 6199 9221 6198 O95714 57461 6193 P78549 6195 6194 10813 83752 Q9Y4C1 P41252 P62195 P41250 O60285 65083 Q13523 Q13522 P78536 9232 22803 Q13526 Q9P1Y6 Q9H2P9 83737 P46934 P41240 O60296 Q9NVH2 Q4LE28 P42574 O60293 P34947 P29597 Q13535 Q9H307 P29590 Q7Z2Q5 Q9Y4E8 Q9NVI1 O60264 3932 O60260 Q9HCP0 9255 Q32P51 Q13547 Q13546 P34931 9252 83759 P20309 P41227 Q13555 Q13554 O95757 Q13557 P78509 9261 P00747 Q8WVK2 2626 Q9NVC6 Q13569 P19387 P19388 2632 P00734 P24723 Q13573 P46977 P46976 P55884 3980 Q8IUQ4 28998 P78527 O60229 P41279 P20339 1314 Q04656 3978 Q9Y4A8 P18031 Q9P286 Q9P287 P62191 1326 Q63HQ2 O60231 Q9HCS7 P20340 P00750 Q9HD40 Q13595 P21675 P48052 Q06265 Q8NB16 Q9Y2X3 Q12952 Q9Y399 5706 57634 5707 79885 5708 5709 P21810 P16471 5700 P49368 5701 5702 Q9UHP3 5704 5705 65264 P54753 P25398 Q66PJ3 Q9BZG8 Q12968 P54756 O60306 O60307 5717 1358 57646 83933 P12081 Q9Y2Z2 5713 Q9Y2Z4 5716 Q12972 P54764 P54762 P54760 Q12974 Q6P2C8 O60318 5728 Q8WTX9 253827 Q9UI30 5725 Q6PCD5 P28482 Q12986 1385 Q9UHJ9 P51668 57661 P51665 Q9P0J1 P19525 P62316 P62318 5733 56339 P62312 P62314 P51671 Q9Y2T7 Q9UHK0 Q5VST9 P19544 10987 10988 56342 1388 1387 P42771 5747 22938 Q9H9Y6 Q9P0L2 P42766 Q56NI9 10956 P62333 284119 Q9BZK7 22929 5757 P61009 P08865 P51693 5770 P54727 P54725 Q9P0M2 Q03164 55035 Q9P0M9 57695 57697 Q9Y2W2 Q9Y385 5768 Q9BZJ0 Q9Y2W1 P06213 Q15139 5780 Q9UPV9 5783 P14678 P38646 54512 Q99640 5775 79813 5778 Q9UQ80 54517 220988 Q9UQ88 5796 Q8WTR2 23451 8812 10147 Q99638 8815 P01343 Q96EZ8 Q86U44 P26641 Q86U42 P26640 O95059 Q9NU63 P26639 79834 55869 5798 Q9UPY3 P01375 23438 Q9Y2R5 Q9Y2R2 P40938 P40937 8841 Q9Y333 Q9UHI6 23435 Q9Y2R9 P12004 3149 O95071 3148 545 546 3146 Q96EY1 P62308 P62304 7508 23409 Q9UQ35 7520 8851 Q9UPR3 Q12904 Q9Y2K6 Q9Y2K2 23404 3159 Q99683 Q9BZE1 8844 Q15185 P49336 7518 P05771 7517 Q9Y2L1 6201 6202 3178 23411 Q00987 Q9H1A4 3181 6210 P49321 3189 3188 Q9UHD2 Q12923 3187 Q9UPT9 Q9Y2M5 3185 3184 573 7536 6204 6207 3192 3191 8880 3190 8881 Q60FE5 Q12933 Q12931 P51610 580 P51617 P52948 79869 8878 6217 Q9UPV0 10090 Q8N9Q2 6231 8892 6233 P15927 P15924 P15923 Q96FV9 6229 Q8IU60 Q9HC98 P03950 O00716 Q16512 Q16513 Q16514 O75494 6238 Q13492 6239 Q13490 10075 Q9HBW0 Q16526 23387 P31152 23381 Q9NV06 P23528 P09234 Q16531 Q8WUK0 51426 51428 Q16539 344387 10081 Q6PD62 O75478 Q15208 O75477 23399 O75475 Q8TD19 64745 Q02790 10087 6259 Q8WUJ0 64750 P14635 Q9UQL6 P10275 P10276 P35579 O75460 Q9Y3E5 10055 P23588 Q99728 84365 Q8WV24 O43889 P36896 P36894 23378 P35573 Q02763 Q9Y3F4 120892 Q9BRL6 Q15233 Q99714 Q96FX7 P61803 Q96FX2 O75444 P36888 Q99700 Q9H1Y0 51465 88745 Q99708 O95817 9319 O95816 Q16576 Q99707 9318 O95819 55827 Q08050 P39905 Q96FW1 P14625 P36873 Q02750 55832 Q15256 22913 O95835 Q7L0Y3 10929 P18124 10933 Q9Y3A5 Q9HC16 O95837 Q9BZQ6 22926 Q13405 Q13404 Q8TCJ0 Q8TCJ2 Q9Y3B4 Q9Y3B7 10949 Q08945 Q9UHX1 Q9Y3B2 57599 10940 10907 O43812 O75400 Q13418 286826 9360 9361 Q9UHY1 Q9Y3C5 P42680 P42681 P42684 P42685 Q9NUG6 Q86UE8 Q13427 Q13426 Q8NAV1 Q8NB78 10921 P42677 P68400 Q9Y3D3 Q9Y3D5 1408 1407 Q13435 Q9HC62 P62280 P62277 Q9HC52 P43115 9397 Q13443 Q08J23 P11532 Q9H1R2 8065 Q96G21 O43809 Q8N0Z6 Q9UQE7 Q8N0Z8 P23511 Q6P2Q9 Q13451 8078 P19474 59343 84305 1432 Q14790 O60341 Q9HC36 Q13464 Q5T4B2 P53355 P53350 Q9P0U3 Q9P0U4 26469 Q7L9L4 5800 Q9UHV9 8099 Q92979 Q9H0E3 Q9Y265 O75940 Q12824 O95551 1459 Q9BYG3 1457 1454 O00391 1453 Q00839 P05455 Q9H0D6 O75934 Q12834 P16104 27090 Q9H0C8 Q9Y285 140801 O75937 P10809 Q96EB6 Q5S007 P27694

Q9UGN5 P27695 Q06124 A0AVT1 P37023 1478 57761 Q9Y295 55109 Q9Y297 O75928 Q9Y291 5836 P26358 Q12851 O75914 P26373 Q99583 Q12857 1491 55110 Q99575 1487 Q9BYD1 148789 55119 P26368 P25098 P49069 Q9Y223 Q96MX6 Q9NSU2 27043 57787 O60884 Q9Y230 O75909 Q9Y231 5859 Q12874 Q9UGI0 Q12872 5870 Q9H0H0 5871 Q9UGI9 Q08752 Q8TBC4 57794 Q96MV8 Q5C9Z4 5868 Q9UGJ0 Q12888 Q9Y243 5883 Q8TBB1 P38398 Q9BQ90 Q96MU7 55149 Q9Y250 613 Q92995 Q5TCX8 Q9Y251 Q92994 5875 Q92993 4548 P10827 P30414 P10828 5892 5894 P31749 55152 P40692 27037 55159 O60870 P04150 Q9Y262 5886 5887 5888 8916 Q9UGL1 Q8TBA6 O96004 8930 O00308 10250 P56199 O00303 P50897 23569 Q15022 P62877 8924 Q15020 Q15025 Q15024 P62875 Q15029 P23284 O96017 Q86SR1 O96019 P36578 P35244 O96013 P35249 Q03933 641 P35251 P62888 196441 8939 P27635 P60228 10269 Q15034 O00329 P35236 O96020 3263 10238 O14967 P11274 8945 P62899 Q15046 O14965 P23246 Q8TAT6 23552 3276 23556 P35227 O14979 P35232 Q9H093 668 Q15054 285193 O14974 6311 P01040 P62837 P06737 8975 Q99570 P39656 23524 672 673 P15692 6304 6305 128308 10213 158135 8985 3297 23536 Q6PJP8 Q99558 688 689 Q86T82 P62847 P62841 P61513 Q15078 Q9BQ15 23509 P13010 Q15084 Q99543 P62857 O14920 P62851 P56182 57727 P56180 P62854 O43463 Q9BQ04 23517 Q92901 Q92905 Q8NHX1 P35268 23512 Q7L7X3 O75031 10208 O14936 O14939 Q56UN5 Q92900 P56192 P36507 Q14686 Q9BZ95 Q9UPN6 Q86TM6 Q9H0U6 O75150 Q9H0U3 84447 P46783 P49810 P83731 Q14690 Q14694 Q13363 Q9HAV7 5034 P46782 5036 P46781 P11216 79084 P41091 Q9Y2H1 60489 P68104 P00533 Q16401 P46777 Q9HAU5 P46776 Q9HAU4 P46779 P24522 P46778 10193 Q9UPP1 P32121 10197 51548 51547 Q9BYU1 9410 55907 P62826 O43542 O75116 P62829 9421 P78347 26512 Q9Y2C2 23481 51567 10181 55929 Q9HAZ1 26524 26523 5071 P49841 P49848 Q15118 P78368 P49840 P11229 9448 10152 23463 A1XKG3 51585 P24534 P78371 10155 Q9BQI3 9439 Q15126 Q9HB90 23476 23478 P37840 5093 Q96EU6 O43505 Q9H173 51593 Q9H9G7 Q92187 P78362 54623 55959 9465 9463 Q6P1J9 P09769 P29375 P29374 Q96EL2 P29372 27005 79012 Q9H0M0 27000 P42345 P22735 P04083 P31689 9477 9474 9475 Q9H0L4 79005 Q6P1K2 P42338 P42336 P29353 Q5JXB2 P29350 Q9H0K1 Q9BYM8 79035 79039 Q8TBF4 Q9Y2A9 80020 P78316 55072 P78317 Q8N8D1 201595 Q04446 P53041 P52597 Q96EP0 Q13315 Q9P015 Q96EP1 Q9H9B1 2869 Q14653 55093 P46736 Q9H9A7 1540 124739 5901 P46734 P13807 Q6P1L8 O75962 P28065 26576 Q9P035 Q00059 Q9BQA5 2885 P43694 O15455 Q9BQA1 5914 Q13347 Q14676 O75953 P28074 P28072 P28070 5927 5928 P63279 P00519 5929 P63272 P43681 51514 P43686 Q14684 Q14683 P10914 O95677 Q92858 23607 P67809 Q9NRW4 5939 P16220 5931 P49137 5935 P0CG13 P09936 O95685 O15530 Q9NS91 Q9NRX2 Q8IZP0 Q9H832 P50148 P18887 P16234 55215 P67812 O95696 Q9NRY2 Q9NRY4 Q99471 P18858 125972 Q92830 P62136 4627 Q92831 P55854 Q92824 P46087 5970 Q9H814 Q99460 Q9NRZ9 Q99459 3304 P62140 55236 3301 3309 P04233 P34925 O00469 P07307 133584 5981 5983 3320 P52756 O00468 Q96DE5 3313 3312 5976 27161 O00472 Q9NS56 3329 3326 3324 P08651 4659 Q9UNX3 4670 4673 2011 27148 O00487 P30542 80222 3337 Q96DC9 Q92878 Q9UNY4 P08621 P51451 P09958 P30530 740 Q96LR5 P37173 Q99496 157570 4677 4678 O00425 P23396 Q5VYS8 2034 2033 P62993 4686 Q16236 P62995 Q96LI5 Q9C0K0 P25100 P46020 2043 Q9NRP7 54764 P46019 P01100 Q8N726 O00444 P11388 P11387 O60942 773 P01137 P01135 P01133 P02461 P01130 Q9H7H0 6421 2065 3396 2064 780 Q9NS37 Q8IZE3 P09086 P02458 57819 2070 Q5TKA1 6430 6432 6431 6434 7764 Q92817 Q9UNN5 O60925 Q8IZL8 O75190 Q8IZL9 6427 10331 6426 2081 23658 P46063 5111 23650 Q5HYA8 P18850 P83881 P27797 P50990 2091 P50991 P22087 91754 Q8TA86 Q9UNP9 2099 O60907 O75164 23621 10318 Q9NRM7 91746 P62979 Q9UNQ2 A2RTX5 Q5T1M5 6464 391356 P39748 O00411 146057 P52701 Q5U4P2 P58012 P62988 5127 Q8N752 P25116 Q13237 Q14566 64951 P62917 P13984 P62910 P09012 P62913 Q13243 Q13242 O43683 Q13247 P20290 O43687 64960 O43684 64963 64965

64969 80198 51650 51651 51654 51657 Q9BY50 P11309 Q6Y2X3 Q13257 P62937 P11308 64975 P49916 5159 P61604 P83876 P21589 Q13263 9531 O43660 9530 Q9BY44 9533 Q9BY41 5170 Q02543 P09001 9526 P62945 P49903 P62942 10291 9541 2909 121512 P27708 Q8NI36 2908 P48643 Q13287 Q9NSE4 Q13286 Q09161 P15735 Q8NI27 8237 10273 P49959 P50914 2926 P09038 Q15008 Q9BY84 Q15005 10283 P62906 9578 116138 8241 2932 2931 255488 10286 10285 Q15014 2935 P78424 Q12788 92105 Q15819 P62081 1613 80124 Q96DI7 Q9BXM7 79139 Q8TAF3 Q12792 1616 P07237 8266 Q12797 P55795 O95602 55173 P67775 Q6IN84 P08575 2959 P08579 2956 Q15831 Q96M96 1634 80145 27102 2961 2960 P04183 O95619 Q8TAD8 2969 P09874 Q15843 Q6R6M4 55192 29777 1642 Q05655 2972 Q9BXJ9 P09884 1647 Q15853 Q14527 1660 1655 1653 Q86SG6 1650 Q13200 1659 O95644 150094 Q13202 P21580 Q8N7H5 64919 80155 1665 Q9BXP5 51611 Q13216 93492 P55769 1676 P08581 Q8NHH1 389840 Q13233 Q14562 Q96L12 Q9NQU5 81605 P52434 O00148 Q8IYN9 P84022 Q70EL4 Q9C026 Q8NFZ0 Q92729 P53779 10420 4734 10421 64282 Q7Z727 23708 81629 O00167 P56817 3416 P40429 811 Q8N684 Q5TAP6 P27448 Q9C005 P99999 Q9NR96 Q8IYK4 Q8NFW8 Q5TAQ9 55352 O60674 P20827 P19838 821 Q9NZJ4 P41743 Q13951 Q13956 4772 Q5SGD2 O95352 2107 Q5T160 Q6PI48 80324 Q9BWG6 833 O14744 4780 Q92769 P09619 80308 O95361 P10600 O95363 Q7LGA3 27297 P61353 167227 841 4775 847 O14757 Q92766 4790 Q13976 2132 Q96L34 O95376 Q9NR45 P18545 Q9NR48 Q9BWE0 Q05469 2125 P60033 857 Q96CA5 Q9UMW8 Q9H6R4 6500 Q9NR50 O95382 Q96L21 Q9NQT4 Q9NR55 Q9NQT5 Q4G0W2 2139 O00141 80335 865 867 4799 10492 O95391 O60603 Q96T88 3479 2147 7832 Q96KG9 P25685 P23025 P51991 Q96T76 O14717 O43290 Q7Z6J9 P61313 P49643 O43294 O43293 Q6ZN33 O43251 P49642 64215 Q9BW92 891 29088 O14727 126789 Q8IYD1 Q96T60 Q9NR09 Q8IYD8 10477 29086 P61326 Q9UMS4 7874 Q658W2 114799 Q01974 Q96ST3 29093 O14730 7867 10488 O14733 7884 P50613 P51946 23764 10450 O43242 P51955 P51956 29079 Q96SZ6 23770 29074 23746 P24386 5245 10438 Q5TAL4 Q00613 Q00610 10432 Q13901 P07814 P49674 Q96C86 56647 10445 Q13115 6598 57062 O43318 Q01130 P33240 9616 Q9C0C2 P38919 P13639 8301 51773 Q9NRH2 24144 Q15796 P60866 Q13131 9641 Q15797 9646 9643 Q9UNL4 Q6UXN9 29803 Q01105 O94804 Q13144 54815 Q13148 P35869 57092 9656 P10586 Q13151 54822 Q49AN0 Q13153 P54136 O15287 P54132 Q13162 Q8IZD4 Q14494 Q14493 Q14498 Q13164 O15294 Q9NRC8 7013 7014 P13674 P11021 Q9NRD1 Q13177 Q9NRD5 9682 54853 P47897 1718 Q9UEW8 1716 Q13188 Q9BWU1 9695 Q09028 Q9UNI6 O75792 26747 Q9NZV6 Q00688 Q6PID4 Q7Z7F7 Q9NRF9 7046 P35813 Q96L91 27248 Q9H6Y2 27246 O15234 1736 P54105 375743 7057 O75771 Q9H792 55294 7052 27252 27257 Q9UER7 7068 7067 Q5TAX3 Q15714 O15264 Q15717 Q8IZ69 Q9H6W3 1756 80267 29880 Q92793 Q7Z6Z7 Q8IYT8 Q9UNE7 Q9BWH6 P43405 1763 29896 Q04206 29894 Q9UET6 Q5GLZ8 Q8N6G5 7083 4809 51720 4800 51726 7099 7098 51729 30849 Q15746 P30153 1789 1788 1786 1785 Q05513 57038 375790 Q15751 80273 51734 Q15759 29843 84661 4820 O15212 P05198 51747 O75716 30827 Q6XPS3 Q13107 P22528 4839 P47804 51752 902 Q13112 P08238 Q9NZN9 O95433 O75821 O75822 Q969L4 O00268 Q8IXM3 51081 O00267 3516 10535 Q8IXL7 O00273 Q9ULW3 P63208 4841 90678 Q6NZY4 Q92614 P07996 10533 Q92611 P52564 Q9NQ55 55454 51096 10549 Q9ULX9 29128 Q05397 P18621 P63220 55466 O60783 P32929 26091 Q6IT96 4882 3551 Q13829 152926 10524 10528 10521 948 10523 O95478 O95476 O95477 O60762 29102 P04040 P84103 58517 O00238 P04049 Q96SB4 Q96SB3 O00231 O00233 P10721 O00232 Q5JTZ9 Q96SB8 Q8WYH8 29110 2237 79444 Q92643 P05386 P05387 P05388 Q9NYB0 P30304 P30305 P32969 P30307 Q9NYB9 2247 975 Q6GMV3 Q92630 Q969M7 P29323 P29320 Q13868 O00255 81788 Q99259 984 6612 988 Q92620 6613 P29317 Q9Y6W6 O43395 P07900 5300 6631 P53804 Q9BV90 P53803 O60725 990 991 P62750 Q96RU7 Q96RU2 O14829 994 995 997 6622 6625 6627 51009 P62753 P07910 P49770 200916 Q9UM07 2287 2286 2280 Q93096 51010 Q96S53 51013 6633 Q9Y6Y0

O14830 6637 P49768 O00206 P49761 P49760 51021 51023 Q00535 Q8IXB1 Q00534 10594 P49756 P25789 P25788 6647 10598 91949 P25787 6660 O00213 Q96B26 Q96RR4 Q96RR1 Q00526 5327 Q9ULR0 O43390 O43353 Q9H5K3 P07947 P07949 Q969H0 P07948 10574 P62714 5338 4008 5351 5352 5356 Q9ULK4 Q01831 4015 5347 4017 Q96JH7 P50750 4035 O14802 Q8NER5 51067 P68036 9716 Q68J44 Q9H5H4 Q01826 P49792 5371 P27540 9733 5378 Q969E8 10569 54101 55432 O14818 Q9NPJ6 6699 54107 54106 O43432 57187 29947 Q9NYU1 Q9NYU2 29941 Q7Z6C1 83475 P22694 O94905 8408 O94906 5393 5394 4067 63875 29959 Q6UX04 128 P60983 54921 O94913 P22681 Q76FK4 P35998 Q9UDY4 P42285 8428 P22674 54931 54938 Q8WZ42 P00441 4086 4087 8446 4088 P22692 9775 8445 142 P62701 Q8IXZ2 53615 1801 8453 8454 9785 8452 Q6ZN16 8458 7124 P54252 P54253 7126 Q96KC8 8450 4092 4093 4094 Q00597 156 Q8IYB3 Q13042 8449 Q13043 P49736 Q13049 P04843 P04844 Q96KB5 8467 P11142 54961 7128 P49721 P49720 8473 O94966 P54277 P54274 7141 Q96T21 1832 Q96T23 178 Q00577 P49711 P49716 P49715 Q9NQC7 7153 7155 7158 Q9BVS5 7157 7150 1843 1848 Q6VAB6 92345 Q13873 Q96K76 Q86Z02 7161 1854 P42224 1852 P47914 1850 P63165 O15355 Q92696 P09651 1859 P22612 P42229 Q13882 Q969T4 1856 O15371 O15372 Q969S8 Q13887 Q01082 84708 P22626 P84098 P63173 P09661 P60900 Q969S2 7186 7189 P35916 27348 P11940 26015 27347 26019 7184 1877 4907 P17252 1870 O15379 P23921 Q9NZ08 Q6UWE0 4914 Q969Q1 4913 Q969Q0 P33316 Q9NYH9 57144 Q93100 Q8WYQ5 4920 Q8NFF5 Q8IY92 P35968 O95400 Q8WZ19 Q9NQ92 P42261 57154 Q96BI3 84749 27339 27330 58487 27332 Q15631 P21333 Q8IY81 Q8IY84 O75844 Q5W0Q7 377630 Q5RKV6 3611 Q5XUX0 57169 P01730 O15350 Q15643 O75832 O95427 Q14318 Q15648 27315 Q86YP4 Q9BVJ6 P30260 O15347 O95429 Q15653

cellular component organization P25054 Q9H0E3 Q9Y265 Q9Y266 P49023 1460 Q12824 8091 259266 Q9BYG3 2303 55561 Q96IK1 284217 P21926 10651 Q8N3U4 Q9H0D6 Q92974 O75935 Q9BPU9 Q9H4L7 P30876 P25067 Q12834 Q12837 Q8IWJ2 P16104 2317 2316 2 5829 Q96QZ7 P42858 P39210 Q9UKV3 10661 Q9UKV5 P10809 Q96EB6 5825 O75925 P27694 Q12846 Q06124 Q9UKW4 4983 5830 3655 Q9Y6A5 P26358 Q96EA4 3672 Q92949 1491 2335 3667 O14579 64061 O14578 Q92538 1022 Q96MX6 10609 1020 Q06587 114548 P50454 57787 1018 1017 2348 79577 P16144 1499 1012 55125 P61587 Q9Y230 P61586 O75909 3678 Q9UL26 Q12873 Q9H0H5 Q12874 3690 Q12872 Q9Y239 3692 Q8WXF0 Q15906 Q9BUB7 Q9NSV4 1029 Q9BUB1 10626 P20645 Q96MV8 1025 O60895 3685 Q92522 O60890 604 P07686 6714 5868 5869 P04179 Q15910 Q9UGJ1 266812 55142 79595 Q8TBB1 Q8NA19 401505 P84243 Q96N67 Q92994 Q92993 5877 5878 Q9H0F6 5879 P53992 Q9UL45 P31749 O95140 Q13748 1050 O60879 27032 253980 P40692 57332 Q96MT8 6733 P04150 Q96ED9 5888 P63313 Q9UGL1 Q9UL54 Q9UKT4 Q9H4M9 Q8TBA6 127829 A6NHR9 P04156 Q15019 Q9UKL0 P56199 O96008 P50416 O00300 P50897 23569 11107 Q99523 1058 Q8WWY3 Q15022 85378 Q15021 637 5898 P62875 P25445 4580 6760 P06702 Q9Y678 5430 5432 O96019 O95163 P36575 O95166 1072 Q9BU61 Q8NHV4 O75096 641 1069 P35251 Q8IWA4 Q14185 8932 11113 648 P47224 Q15036 P49407 8936 10269 P02794 11116 O96028 5440 5441 5442 3265 Q9UKN8 Q66K89 P14780 Q9H4B7 51144 P35240 P01019 5434 Q14197 51147 Q15047 84062 O14965 Q9Y5X9 Q8TAT6 Q9Y697 P35222 P14784 P35227 Q96A08 P35226 124359 Q15051 P17813 P35232 Q00403 Q15050 Q9H093 P23258 668 9804 P24588 6790 4131 6311 9821 4133 23523 673 Q68DK7 6304 6789 57706 P05412 O00755 P49450 5471 O43482 Q5JSP0 O60814 10228 Q6PJP8 P14373 Q9NWV8 10229 Q96QV6 688 Q15070 4137 9826 207 Q15075 4139 Q92922 Q15078 4150 Q86T65 4154 P56178 Q9Y5S2 Q9H902 Q12809 O00762 Q9NWW5 O14925 P13010 215 4149 P62851 Q99549 O43463 6342 6341 Q7Z4H7 O43464 P06729 23512 P50402 79980 O14939 56882 P17858 Q96QT4 8516 P40616 Q9H4E5 55559 55558 O15085 Q14204 P55268 Q14686 Q14209 8535 11065 Q9BZ95 O75146 Q9H0U9 23048 Q6NZI2 4171 P13861 230 O75150 P07196 23043

Q9H160 P83731 5018 P49815 Q9NPC8 Q9HAV7 8546 P62805 P62807 Q9ULG1 79084 9869 7205 Q9H9E3 P00533 9868 Q16401 Q96RL1 Q15554 P24522 9404 Q9UPP1 P32121 O75122 Q9Y6Q5 Q9Y2I1 23028 4193 Q6DD88 Q6UVJ0 51542 Q96RK4 8548 P02741 P61966 Q05707 Q93008 P55290 P61964 Q9BQE3 6383 6386 P62826 Q9Y6R0 O75116 Q86XR8 P36952 P68133 P04908 Q9Y2J2 P36954 O75592 Q4AC94 267 23032 Q6PKD3 Q9NPF5 P08034 Q8IX90 P43246 Q14249 P78347 9425 8576 7248 23002 O75582 Q9Y6K1 O75581 274 Q04837 P23229 P35221 51564 10181 8100 55929 Q15109 26524 5071 P12110 23492 11034 7249 O15047 1947 P49848 P02751 8110 O15061 10152 O95931 A1XKG3 291 23466 Q96EV8 23468 Q14C86 P48509 221150 222484 P08069 Q9NTI5 10155 9439 P02786 11004 1956 54617 O15075 Q9NPA8 7277 26509 O15078 O75558 64423 64426 Q8N4H5 P37840 5092 P11233 7272 Q9NXR1 2810 Q9NTJ3 P78362 54623 Q14289 Q9NPB6 Q13753 9463 Q96RE7 Q6P1J9 Q13751 P29375 P29374 27005 P12931 Q7Z589 7283 Q4VCS5 P42345 Q07507 P22735 Q9Y6G9 P04085 Q92574 P56524 9474 Q8IX30 9475 O15496 Q02297 O75530 7290 55054 P24928 Q9BUF5 117177 Q92560 Q6P1K2 P16070 O75528 Q9Y6I3 P55209 P56539 P56537 P21860 P04062 Q92558 93323 O95983 50618 91147 P55210 O95503 9495 Q9Y6J0 79023 9493 Q8TBE0 Q07954 Q07955 Q9UH99 P53041 P07585 Q13794 50628 O95996 O75503 O95999 O95997 P35610 Q9NP66 Q96EP1 P35612 P35611 23081 Q9Y6C2 Q9H9B1 O60488 Q9BUK6 P35606 Q8NEC5 Q9NP72 P35609 O43921 O43920 23092 P46736 3728 Q5QGT7 26121 3720 O60493 P22303 5901 Q27J81 Q9Y6D9 P12956 Q02241 P34741 P33897 3737 Q00059 P02708 2885 P61088 Q14674 Q9BQA1 5914 11091 Q14677 132320 Q15526 27436 Q9NP98 9054 79065 5927 5928 P63279 P00519 5929 P63272 23077 P24941 51510 Q15532 Q13351 51517 Q14683 5925 23603 O95677 Q92858 P06899 23607 Q9UBB6 Q9UBB5 9066 Q9P2R6 O60566 Q96M29 P16220 79658 10772 5931 5933 O95202 O15530 Q9UBC3 Q9UBC2 Q8IZP0 O95680 54361 O95684 1107 P16234 1104 10783 92609 Q92845 1108 O95696 3783 P53667 Q9BXC9 57403 6809 27185 57405 O60543 Q86WA8 O15533 Q92830 Q9UJU2 Q92833 4627 Q92831 P06400 P53675 P07737 Q9H814 1130 9093 Q9NRZ9 P56705 6810 6812 Q6NXT2 6814 10765 P04233 3308 P07305 P30566 P34925 P07307 133584 5981 P50570 O95239 3320 P52756 O00468 83706 6829 10736 Q96DE5 Q6FHQ0 3312 3796 P09972 3799 4646 6827 P08648 P30556 1153 P53618 Q8IZT6 3329 6839 2475 6834 P41208 5987 P53621 P07766 P05106 6850 4673 2495 Q13618 2010 P30542 Q9NS69 P61244 284359 Q8TEH3 O14640 Q92878 O43182 Q9UNY4 5518 P30536 O43189 Q5QNW6 1174 Q6KC79 Q60I27 O00499 Q8IVI9 10726 Q99496 10728 Q6FI13 4676 4678 P40337 P04275 P02452 4690 O43159 6872 O00429 6871 P04637 2034 2033 25861 O95271 P62993 4686 P01116 Q9C0K7 5536 Q15389 Q15388 P01112 P01111 O43166 Q9C0K0 Q96CW9 6883 4221 10383 Q96CW5 23210 Q99880 2043 23212 2040 Q9BT40 P50542 1191 Q99877 2039 Q15392 2037 Q99871 4214 6878 11234 Q15393 4218 56946 Q99879 25847 6892 P50552 Q8N726 5563 Q7Z7K6 P14416 2054 P52735 P33176 P51884 Q96CV9 P12235 P11388 P52732 773 Q53X93 P01138 P01137 Q7Z3C6 P48668 Q16254 P02461 4240 P01130 P04626 5576 2065 3396 2064 Q08257 221613 P54920 11218 Q9NRR8 2059 302 P10070 P10071 10363 10362 P02458 P01127 Q8TDZ2 P61218 9927 2070 23647 O43593 6430 Q8N307 5584 6431 5585 P15328 O75190 5578 5579 6426 8607 Q9Y512 5591 Q9Y4P1 9950 Q6IR47 64127 Q5HYA8 O75177 5590 Q9NRL2 P18846 P16671 324 8615 5104 329 5108 Q92800 P27797 91754 6453 6456 6455 O75164 25801 P15311 330 333 55666 56993 Q9NRM7 5116 Q9H7L9 5119 O43581 4285 P05997 Q5T1M5 O00411 25813 221656 P58012 6457 10320 9967 Q99418 Q2M2Z5 P26447 P25116 Q8N6T3 8650 8655 4297 Q8TEW0 Q9BY76 P13984 6470 351 355 356 P09493 23165 Q13243 P21127 P47755 Q13242 P47756 O43683 Q13247 11190 Q15424 5154 9997 7334 5155 Q9BTM1 Q15428 O43684 11196 19 Q8TF76 Q9UKE5 11198 7329 Q15436 Q13257 6498 9525 6015 8676 253260 Q53GQ0 Q9NWS0 Q9UBU3 P51808 375 P51805 P09471 P17096 Q9UBU8 P83876 Q13263 P25940 Q9Y613 8683 Q14114 Q9BY44 Q16777 O43663 Q16778 Q8WWN8 Q9BY41 Q14118

P0C0S5 P29084 382 5170 P34896 Q9H492 387 P0C0S8 Q9UBV7 Q7L7L0 23154 11177 11176 P60763 P62942 Q15459 10291 Q16787 O15164 23122 390 391 O43639 Q99965 397 Q99962 Q99963 121512 2908 Q02539 Q99966 Q96R06 Q13286 5195 7373 6045 Q09161 P60709 9555 23133 163786 23135 23136 Q86WV8 Q9Y5J6 60 8218 85440 Q99958 9564 Q6ZSZ5 Q8TEY7 O15182 10270 Q9Y5K6 P58876 O43615 P49959 Q01664 P21145 56916 Q14152 Q15003 54737 11127 8242 P22492 9578 P14868 Q8TAP9 Q9Y5L4 25776 25777 Q6R327 1605 Q15014 86 87 Q15811 Q7KZN9 O75665 1613 80124 26271 Q8TAF8 P41134 55165 Q86WK6 Q1PSW9 P20671 P46821 Q9Y5E9 P45973 O15118 Q9UBK2 Q9UBK9 Q13643 Q14978 Q96QB1 Q86WJ1 Q6DT37 O43602 Q9H3R0 P55318 Q05682 55172 P55316 Q7Z460 P05062 Q52LR7 P08572 P05067 2959 1627 3801 Q14980 Q9UBL3 P52298 P29474 Q15836 O75643 9126 O95613 26258 Q96M96 6093 91272 1634 2961 2960 O95619 P21980 Q14993 1639 Q9NSC2 2969 P09874 9135 O15151 P11717 8289 9133 Q14511 O94776 Q03001 Q8N7B1 Q9H410 2972 Q9UBN7 1647 Q14527 Q6NYC1 P41182 P41181 8290 P30047 P30048 1653 Q9UK80 Q03468 P08123 Q92499 O15554 3832 3835 51606 P51398 Q2NL82 Q68CZ6 Q8N7H5 Q68CZ1 Q07817 1666 P20248 64919 P21579 Q9UJX2 Q96QF0 P17081 P08138 Q9Y5B9 P09429 Q9BY11 P10415 Q8TEQ6 P10412 Q7Z494 1676 1674 23186 3852 P09430 3856 80184 728642 P62072 23192 1203 O15105 Q13233 1207 3880 25909 P54619 22872 9181 P51587 P52434 9184 9185 P15498 54474 4728 2547 Q8IYN9 1213 P84022 79778 Q92730 O14786 O14788 6901 A1A4S6 4725 25913 25915 P08729 P08727 Q5VTR2 Q53EZ4 P55957 10426 O60216 10427 Q8IUE6 P14174 30011 800 Q9H2G9 10420 4734 10421 64282 P30622 Q7Z727 Q9H2G4 P28300 O00167 Q9NQW6 Q5T0N5 54496 6927 3416 811 Q9UJ41 P63104 P35080 Q9C005 O95347 Q9NQX0 P31946 P31947 P99999 P17661 P54646 Q676U5 55355 P16333 P20823 81624 55352 O60674 80776 P62263 P04350 Q96GD4 5604 P41743 829 Q9Y468 P08758 4771 Q14807 Q8TDI0 22832 O95352 P16389 P15056 10856 114791 830 Q08999 832 O14744 O14745 Q5TB80 P07437 Q9Y478 P32856 Q92769 P28370 O95361 P10600 Q06330 10868 841 Q9HCM4 847 Q8N257 7804 4791 Q13976 1285 1284 Q9H2K0 Q96L34 Q9NR48 7818 Q71DI3 1277 Q96KQ7 Q92752 857 859 Q96CA5 Q9Y490 O00139 P51575 Q13505 Q9Y496 Q13509 Q14839 Q9P202 10844 Q9HCK4 2139 Q9P209 Q8NBS9 1289 80335 P09601 O14777 50943 P08754 4318 P06576 6993 10490 O43278 O95391 23322 P14555 3482 23326 P11047 871 203068 55722 Q8WV60 2146 55726 Q96KG7 10013 84289 55723 10014 6505 135295 64682 Q7Z6J4 23332 3010 Q6FGD7 23336 3009 3008 O60610 3007 400 3006 402 O43290 7846 Q9BS18 408 O43293 409 Q01518 Q15276 Q13099 23307 Q09472 3024 O00560 23787 890 Q00653 891 O14727 54413 Q99741 55743 P53708 3014 29086 P49639 O43264 Q9UMS4 P51531 4361 P40818 5213 23310 Q658W2 Q9NUX5 P51532 P14543 Q99733 3028 Q15291 Q96T51 10486 4358 10487 P26583 O00505 Q9Y3M2 Q6FGG2 P50613 P51946 10459 10458 Q8WUY8 Q96KK5 Q96P48 433 A8K0Z3 10451 8726 Q9H2D6 7879 10452 8724 P55072 7410 10460 P51955 P52803 P51956 Q05086 80705 29079 10469 P36382 Q99797 Q01955 P51959 54443 79723 7408 Q6IQ23 Q9BS57 25929 Q96GA9 5245 P52815 22893 3066 Q9UMN6 3065 Q9UIF9 22897 25923 Q5TAL4 Q96P20 Q00610 55785 7415 P61764 7414 10432 3070 Q86UX7 7430 P07814 Q9UIG0 3074 466 P28749 7428 P40855 8772 6595 6597 6599 8775 6598 5268 Q16625 24137 O75385 3084 P48729 473 474 Q99807 Q8NCD3 51763 P43034 Q15785 Q9C0C2 Q16637 Q15303 3091 8301 9632 Q16635 Q15306 O75376 Q9UJC3 O75381 Q9NVP2 51773 Q9NRH3 6117 P46531 7448 54801 Q9BSJ2 Q16643 Q13133 Q15796 Q29RF7 O00602 Q15797 P12757 9646 7465 Q8WVM7 Q8N6I1 9643 7468 P54198 O75367 Q9UNL4 Q6UXN9 P12755 51780 Q01105 Q9NRI5 O94805 Q9BSI4 Q16650 9657 7476 P18074 Q16658 P13611 O43303 93166 O75351 Q9NZZ3 P68371 Q9NVR5 54820 P23416 P68366 54822 9648 Q16665 51317 O94817 Q13153 P43005 Q13158 9662 Q8TDY2 O15287 O43752 O75347 P54132 Q14008 Q9UNF0 23244 Q9UEU0 Q9Y4G6 Q02413 O94826 8328 P46108 Q14011 O15294 Q96CS2 7013 8348 7014 P36776 7490 P13674 8338 Q9GZM8 Q9NRD5 Q99835

9201 9685 Q3ZCQ8 7023 10392 P12314 Q02880 P13647 Q86VS8 9682 Q08188 51340 Q9NVM4 P02549 Q96PK6 11243 55704 Q16695 Q13185 Q14031 1718 8349 P02545 Q16698 Q9NVM9 347733 Q8WW24 9212 7037 Q9BX66 11252 P10147 Q09028 6182 O43747 26747 Q15370 3909 Q6PID4 Q9NRF2 1729 Q9BWT7 Q99814 P02533 P98194 Q15375 P78545 9223 P28288 9221 7046 P28289 P98155 Q96L91 Q13515 8379 6194 3913 83752 Q9Y4C1 Q4LE39 9218 9219 P07355 O60282 3911 1736 3912 O95721 P54105 9232 7057 22803 Q8IYV9 P78537 Q8IUN9 55294 Q3V6T2 7052 27252 Q9BSB4 3925 81567 P46934 1742 83746 Q9HD26 Q9Y4D1 81565 Q4LE28 P42574 P98172 80230 P98171 P98174 8399 P83916 P19793 P29590 84617 Q9H6W3 1756 Q92793 Q96GM5 P16284 Q9NZQ3 Q7Z6Z7 Q92791 O15259 O60264 50855 O60260 P07339 Q13547 Q8IYT8 P34931 9252 P34932 9253 80254 P43405 O43707 1763 Q92786 P05164 Q92784 P41229 P41227 Q9NZJ7 Q9P253 P78509 O00186 7082 Q53SB5 Q9P258 1778 3958 P00747 P63000 60673 51720 Q96PE2 2629 3954 Q13561 P08246 9276 Q9NVC6 Q9NQZ2 P14923 P19387 P30153 P19388 7094 1789 1788 1786 1785 Q05513 Q7Z2W7 375790 80273 Q9HCU4 Q9NVD7 100287932 Q8IUQ4 339287 P32320 P78527 O60229 2647 1315 P20339 1314 P20336 2644 2643 1793 Q04656 Q9NZM1 84661 51742 Q9NZM3 O15212 O75716 Q16611 30827 O15230 Q15768 Q9UNA1 Q63HQ2 121441 P67870 O60232 Q9HCS4 4830 900 902 Q13596 P08237 Q13112 P21675 O15228 Q8NEY1 Q12948 22992 O00268 Q6QNY1 O00267 P61020 Q9BVA0 3516 Q7L0Q8 Q96JM7 O00273 Q9HBH0 Q9BVA1 90678 Q9ULW0 10534 P07996 10533 P27105 P04004 Q8NF91 Q12959 P52566 Q8NB12 Q08431 57634 79885 P16471 O60331 P21815 4851 Q9UI12 928 P05787 P54753 P53420 P25391 Q96NT1 22974 Q05397 150737 Q9Y2Z0 83933 29127 1355 P32929 Q9UQB8 4867 Q6IT96 4869 5716 P54764 O95466 P54762 P48023 1374 Q6P2C8 Q7L576 22985 Q13829 P17302 Q96JJ3 10524 Q99698 2697 948 Q9UHR4 Q12981 O95476 O95477 1385 Q9P0J0 58513 57669 57662 O60763 P07550 P62316 P04040 P62318 P62314 O00238 O95487 Q9UHK0 2243 O00233 P19544 P30793 79441 Q8WYH8 P18206 79444 960 1387 116840 P62328 4898 P42771 Q9UM54 5747 Q8N137 Q9NYB0 P30304 P30305 Q06203 Q9NYB9 Q9H9Y6 Q9P0L0 O60749 Q9P0L2 22931 P62330 10953 P42768 284119 P15153 1398 2244 Q9BZK7 P61006 6601 977 6605 10951 P51693 P29323 Q92626 Q9Y375 O00255 Q8NEZ4 O00257 Q03164 Q9P0M6 81788 440193 984 Q92621 989 Q9BZJ0 Q9ULV4 Q9Y2W1 Q96FF9 P06213 Q08043 P07900 Q9Y6W5 6631 P13349 2275 P14678 P53803 P52952 P15531 990 Q8WTS6 991 79810 994 P11171 Q99640 Q96RU3 Q66GS9 998 6622 79813 999 10133 6624 P62753 Q15149 Q9UM07 Q9Y6X3 6643 Q8N4N8 Q9UQ88 6642 5796 P51636 P07919 2280 Q96RT7 Q96RT1 Q96S53 Q15154 P48436 6633 8812 Q96S59 Q96RT8 P02675 6637 255626 Q96NL6 P49768 2290 Q9BV73 P48431 6653 O00203 Q00535 Q96S42 55869 6647 6646 Q8N8U2 8829 Q9UPY3 Q8N960 Q3ZAQ7 P01375 O00217 Q08495 Q9UPY8 4000 8841 Q9UHI6 O00213 O95067 3151 Q9P0I2 Q93079 3148 Q96RR1 Q93077 3146 P61457 Q96EY1 Q00526 6657 P62308 Q9UPZ3 P62304 O00623 P07942 O00628 9700 7520 8851 Q969H0 8852 Q96B97 Q9Y2K7 Q9Y2K6 Q9UHB6 Q07157 3162 O00622 Q68EM7 3159 Q8WTW3 Q15185 5338 5339 P05771 P26232 Q969G3 7534 10580 Q9ULK4 P68032 23411 Q9BZD4 3171 Q00987 P50748 3169 Q9NPH5 4015 Q9H1A4 5347 9702 7529 Q96JH7 Q9H5I1 P50750 Q86U86 4035 P49321 Q9UPT9 Q9UHD8 Q12929 Q9Y2M5 Q9NU22 P12036 3182 Q99661 7536 10552 Q01826 5371 Q9BQQ3 8881 Q60FE5 Q12933 P20908 P16403 P16402 P16401 582 10569 80854 P51617 O14817 P52948 Q9NPJ6 57610 4038 Q01813 Q9UPV0 Q8N4P2 57617 54908 10093 10092 Q15654 Q9UQP3 8411 P15924 Q9Y3I0 Q96BM9 118 9738 Q7Z6C1 Q7Z6C3 Q8NFJ9 Q13485 Q8WUM0 8408 Q9HC98 O94906 Q13489 P03950 P35527 O00716 Q16512 Q16514 P12429 O75494 Q7LBC6 4060 51412 Q13492 Q13490 P60981 84790 4074 6256 10075 29924 54930 P23527 Q9UDY8 P23528 P09234 Q8NFH4 P60510 Q9BZW7 Q8NFH8 Q9HC77 O94927 Q8WZ42 P00441 8443 4086 4087 Q6PD62 4088 4089 O75478 163126 23399 Q8TD19 Q96BK5 Q02790 Q9NZ94 142 P21359 P52907 23396 10087 6259 7106 Q15691 53615 9784 8452 6275

Q8N5M1 8458 7124 10053 Q8TD84 7126 P14635 Q9BW71 Q9UQL6 4092 P10276 P35579 154 Q00597 158 Q9H6D7 Q9UMD9 23363 Q13042 Q99729 84365 10059 Q15223 55806 P45379 Q13043 P49736 7132 9793 Q96KB5 8467 Q5JQC9 23378 161 P13796 P35568 P11142 23370 Q02763 1828 Q99715 Q99714 9322 P02671 Q8TCU4 8473 P00492 Q8TCU6 8479 Q5SSJ5 P54274 P36404 6294 7141 Q99700 1832 Q96T23 Q9H1Y0 P68431 P49711 Q9BRK4 Q16576 P49715 P60953 64780 O94973 7153 7155 P39905 7157 P14625 23354 Q02750 O75431 8481 138151 51474 Q9NZ56 54984 55835 O94979 84376 10048 54509 Q5VTD9 Q13077 P53365 9342 O95835 P10636 P12814 26038 26037 P63172 26039 7161 9341 2702 P63167 10933 10935 10938 Q92692 P39060 Q6NVY8 Q13885 Q6VN20 Q9P0W2 Q969S8 Q13887 10939 9351 O43847 84708 22920 P57735 Q96BD5 P84095 22919 Q9UI95 P42695 7186 9368 10907 57120 Q13418 O43815 O75880 7185 9361 7184 1877 10910 O43819 1874 P17252 Q9BVG8 1870 O15379 Q969R5 Q92673 P23921 O60383 Q86UE8 Q13422 O15392 Q8IY33 O43823 10919 Q6UWE0 P35900 Q8NB78 P19438 Q9Y3D6 P21757 56288 84733 Q9HBM1 2737 2736 9388 Q8WUI4 P51148 9382 P51149 4926 2742 Q9HC52 O95400 Q8WZ19 O95405 Q9NQ92 P42261 26005 P11532 58480 27339 Q9UQE7 Q8WYP5 Q8WYP3 1429 Q14781 P21333 O14492 P33778 O75844 Q6ZV73 Q14789 P12830 P19474 59341 Q9H223 3611 Q9HC35 Q14790 O60341 Q15642 Q13464 O15350 O75832 Q9BZS1 P53350 Q15648 Q9P0U4 Q96BF6 3622 P84077 2773 Q9HC29 P30260 P24821 O14497 Q71U36 Q15652 Q9BZR6

macromolecule metabolic process P28562 10657 2305 55561 55568 Q5QP82 2300 Q8N465 4968 Q9H4L4 P30876 Q8NE71 10667 2316 2 Q9UKV5 Q9UKV8 3661 Q8NE63 P62495 2324 P48147 Q9UKW6 3659 3672 Q13705 P21917 10645 2335 10642 O14578 10643 Q9UL15 Q8WXG6 P51784 Q9Y5Y6 Q9UL18 Q8IX01 1022 1021 1020 Q06587 P50454 10613 1018 1017 79577 10616 Q92530 26191 3692 Q8WXF0 Q13724 Q9H4P4 Q96J02 1029 10626 O14519 10625 1025 Q9BUB5 Q9BUB4 6711 P07686 6714 O95136 79595 391627 P20618 Q9UL46 O95147 6741 5411 Q38SD2 Q8IWL3 1050 253980 57332 1047 10607 6733 Q9UL54 64432 5422 11108 11107 Q8WWY3 6749 Q9Y676 P49411 5430 5432 Q9UKM9 O95163 Q9NWZ3 P57059 115426 1072 51132 Q9Y680 84033 5424 11113 P49407 84061 5440 5441 6772 5442 Q9UKN5 Q9UKN8 346171 Q66K89 P14780 5434 51148 Q14197 51147 Q9UL03 Q86X95 Q8N3J5 Q9Y697 A7MD48 Q9H4A9 Q00403 6776 4116 P62424 P57081 6790 Q5VVQ6 6793 6794 4133 10690 9820 Q9NX62 O00743 Q9Y5Q8 Q9NWU5 Q8IWF2 4125 6789 Q96A72 Q07020 5470 4140 Q9Y5R4 5476 O00754 Q9NWV8 207 5468 4139 4150 5481 4152 4154 Q9Y5S2 Q08345 O00762 P13489 Q9NWW5 Q9Y5S9 P17844 51185 5478 5479 4149 Q9H4F1 5494 5495 Q9BU89 5499 P13497 Q96QT4 P49427 4172 4173 4174 11060 4176 Q14209 11065 7204 P55265 4179 Q6NZI2 4171 Q9NXS2 Q6P5Z2 23043 11072 Q8IWZ8 4188 8543 Q9NPD3 8536 Q96RL1 P61956 Q15554 197259 Q9BUN8 P35638 P61968 11041 Q96IZ0 8555 23028 Q96IZ7 4193 Q8WXQ8 P61962 1915 P61960 Q93009 P02741 P22314 Q93008 Q14232 P61964 8563 Q9Y6R4 11051 Q8IWX8 8565 Q9Y6R0 Q86Y37 P36954 Q8N4C8 267 23031 23032 Q9NPF4 Q9NPF5 P61978 P43246 Q14249 8576 26994 O75582 Q9Y6K1 P10398 Q93063 Q93062 Q04837 8569 1937 1936 1933 11030 Q9BV47 O75575 23014 23016 7251 Q9NY12 Q9Y6L7 11035 118460 P02751 P61927 7266 P02790 O75569 7267 Q9Y6M4 Q8WY64 Q8NEJ0 P08069 P02786 Q6I9Y2 51107 1956 O15075 Q9NPA8 26986 Q01780 Q86Y79 7272 1965 114088 Q93034 51116 1969 Q14289 1968 11017 P12931 Q7Z589 Q70CQ3 Q70CQ1 1975 Q6P5R6 Q13761 Q58WW2 7297 P56524 Q13769 O75531 P24928 Q7Z4W2 1982 Q9Y6H3 Q92560 26168 O75528 Q5D0E6 P06239 Q06643 P55209 P56537 1994 26133 P06241 P21860 O95983 Q16816 P55212 91147 P55210 A4D1W7 63931 Q07954 Q9Y6J8 Q07955 P07585 Q92542 50628 Q16825 Q16827 O75503 O95999 O14595 O95997 Q16828 9020 23081 23085 P20594 84881 Q8NEC7 Q8IWV7 Q86Y13 P61081 Q9H596 P61073 83548 23097 26121 P61077 Q9BUJ2 P22303 11080 Q9NXH8 9045 Q9NP81 Q16849 P12956 P12955 Q8IWU5 Q9Y6E7 Q9Y6E0 P61088 Q96AE4 Q96RG2 11091 Q16854 9054 23076 P24941 11099 84893 Q5FWF5 285672 65003 O14672

399687 Q9UBB9 O60568 O60566 P15374 Q8IVH8 10772 Q9UK45 Q9UBC3 P27361 Q9GZZ9 65018 54361 55696 10783 10785 92609 Q9NWB1 P52333 P29992 1121 P53667 Q8TED0 O60543 1111 Q86WA8 P61289 P03372 P49591 3791 Q9GZX7 Q8NCR0 9093 Q9UBE8 P56705 P49590 9099 10767 57410 O60551 P49588 Q9H3G5 10765 P05129 P50570 P11802 P50579 O95232 6829 10733 147746 Q9P2N5 1137 Q6FHQ0 P61221 6827 Q10570 Q7KZI7 Q7Z419 Q9Y4X5 Q13601 1153 Q14938 P19784 O95243 P53611 6839 10746 1147 2475 5500 5501 P26045 P53621 O95257 6850 Q13615 Q9Y4Y9 Q13618 Q13617 O60508 Q13616 1161 O60506 Q9P2P5 O60502 10714 57448 P61244 O14641 5511 P61247 O14640 5514 O43184 Q9Y4Z0 5515 Q9UK22 P05112 65061 5516 5518 Q13620 Q13627 P18433 10725 10724 P61254 10728 O14656 P40337 P06454 Q9UK32 O14657 Q8N2K1 P04632 O95278 O43159 6872 Q96HA8 6871 P04637 25862 Q9NVW2 O95271 23203 11222 5536 Q15389 5537 Q9GZR7 6883 4221 6885 1198 25873 23210 1196 1195 1191 56940 Q96Q15 Q15392 Q99873 55611 51253 Q9P2K8 4214 11231 6878 4215 4216 Q15393 P01588 O43172 56946 25847 5562 5563 51264 Q53X93 Q9GZP9 Q9Y572 5557 P61201 5558 O43143 Q9BT22 P04626 5575 P04629 25853 11218 301 P17936 9924 5566 Q96PN8 5567 5568 P61218 5580 5581 5582 5583 5584 5585 4255 5586 22794 25822 O76031 Q96PU8 Q96PU4 79612 55644 Q96PU5 Q02930 5578 8602 5579 Q9H3F6 8607 Q8N302 5591 P07711 Q9Y4P1 9950 Q6IR47 5596 5597 5598 P50502 O76024 O76021 5590 P16671 327 328 329 84197 25804 Q9GZU7 Q8N2M8 Q9NW64 333 55660 55666 5599 P83436 4285 4287 25818 7311 Q96Q40 P17980 Q9NVV4 Q08211 221656 Q08209 79622 9967 4297 Q6DKI1 Q6ZT98 Q15418 7325 Q9UKD1 4291 351 Q9UBS0 Q04759 Q9UBS4 9978 P21127 8662 7332 7334 16 8667 Q15428 7336 8665 Q15427 7335 19 26205 Q8TF76 Q9UKE5 Q9H3Z4 Q9UBT2 23170 367 Q9UBT6 369 11198 7329 7328 7341 11160 Q15434 Q14103 7347 6015 P29074 23144 Q9UKF6 Q9Y606 Q9UBU3 Q03405 85437 P17096 11168 8669 Q9UBU8 P43378 31 Q14116 11171 8683 7353 Q14114 7358 P51812 P51813 P29084 Q96HW7 Q9UBV7 P51817 11176 P23771 Q15459 O15160 P55345 O15164 25788 23127 Q99965 52 Q14134 Q14139 6045 Q14137 7375 6048 23133 23135 Q9UKA1 Q9Y5J1 11157 O15169 Q99956 P46459 7385 7384 Q8TEY7 6059 Q9Y5K6 Q9Y5K5 6050 Q9UKB1 Q99942 11129 Q99941 51222 56915 P01579 Q14152 11124 Q9UBQ5 11128 O75676 O15197 O15198 O75674 P14868 P14866 25778 51231 Q14164 86 Q14166 9100 Q96QC0 Q14966 P52272 P33992 P33993 Q8TF05 P33991 Q05209 P0C0L4 84967 91 Q8WWI1 94 P45974 26270 P82921 Q9UBK2 Q9UBK9 P82912 P82914 Q14978 Q8WWH4 Q86WJ1 Q6DT37 Q96I24 Q9H3R0 P31321 84950 P52292 Q52LR7 P05067 Q9UBL3 O94763 P52298 9128 O94768 9129 O75643 9126 O94766 Q6XUX3 Q9H422 6093 283989 3817 Q8TEL6 P21980 Q9BTC0 Q5JRX3 Q02156 26260 26262 57379 134430 Q9UBN7 P82932 P82933 Q9UJV9 Q9UBF6 9149 56052 Q8TF47 Q6NYC1 Q9H488 P11766 P53567 P22415 26232 Q9UK80 P30050 Q03468 26234 Q92499 Q9UJW2 3835 Q8IVT5 9150 57396 Q9UJX2 Q9Y5B0 P08138 2517 Q9Y5B9 P09429 O75604 P10415 Q8TEQ6 P35711 2521 Q6P4R8 P09430 Q5T6F0 728642 P08107 O15111 Q9P2Y4 Q04771 23192 1203 Q96HR8 O15105 P45983 P45984 1207 Q7Z2K6 65125 P54619 P51587 9184 P17676 Q8TDD1 2547 1213 10893 Q6IQ55 Q9H2H8 25913 Q9UIQ6 P08729 Q5VTR2 O60216 P62244 80755 54487 P62241 P62249 Q9H2G2 Q9BRX9 P28300 2571 79791 54496 Q8IUD2 6929 P62256 57520 119180 6921 P26599 P31942 Q9UIS9 P31944 P31943 P08708 P54646 P17655 P62266 P62263 5602 Q96GD4 P62269 5604 Q9Y3U8 Q9Y463 Q14807 2590 P39019 10855 P15056 10856 2589 P39023 5610 5613 Q9UIM3 P30679 Q07666 Q8WVB6 P52888 Q06330 10868 10869 5624 Q9H2K2 P28340 4311 P21709 Q8IUH5 169436 1277 Q10472 Q10471 Q13501 Q5MAI5 22826 22827 22828 Q8NC51 Q9HCK5 10845 1289 192669 4313 4318 11340 Q86V81 O00541 23326 25988 23327 51362 Q8NBJ5 5654 P25205 10013 84289 10014 135295 64682 Q15269 Q9UII4 4343 Q9BS26 Q6FGD7 P51513 51377 Q99750 Q9BS18 Q9BRP8 Q99759 408 409 P60468 23307 Q09472 5682 5683 5684 5685 5686 O00567 Q9NUW8 Q99741 55743 3014 55748 Q9Y450 11326 Q15287 4361 84292

5692 5693 P40818 8720 3035 Q9NUX5 P14543 11338 P15880 3028 P60484 Q15291 55759 5687 5688 55757 84296 79709 P31930 P26583 8732 O00506 25942 54431 Q8WUY8 433 Q9H2D6 79739 P55072 Q96GC5 140609 Q9HCE7 3054 Q05086 P37231 Q9HCE1 Q99797 444 55775 8737 Q02809 P82675 P61758 8738 284086 P49207 8754 25929 7422 Q9BRT9 Q9HCD5 P52815 3066 22894 3065 Q9UIF9 22897 O75293 79753 55781 7415 8761 3070 Q5VTL8 Q9UIG0 54460 3075 3074 P17612 55796 7428 O43791 53938 Q16629 Q9Y4K4 P11441 O75385 Q9Y4K0 Q86VQ3 Q9Y4K3 472 P48729 474 28973 Q99807 Q16630 8767 Q8WW01 3093 Q16637 Q15303 3091 Q16633 P55036 6123 6125 Q15306 Q9Y4L5 Q86VP6 O75376 8780 Q504Q3 23291 Q9NW13 Q4J6C6 6117 6119 6118 Q16649 6133 6132 Q16644 6134 7465 6137 O43776 O75365 O75362 6128 P82663 P82664 Q8NCB2 Q8WVM0 Q9BSI4 Q16659 6141 P18074 P18077 O75352 144983 28960 Q96PF1 Q9P2E3 6138 Q16665 Q9P2E9 51319 6156 6158 O75347 P35453 P23467 P27824 Q14012 51329 Q14011 6164 Q15349 Q8TDX7 6168 P46100 11274 P36776 6160 7490 Q7L2H7 11277 6175 9201 Q86W42 P98175 Q3ZCQ8 P98179 Q02880 25885 Q08188 Q8WW22 Q02878 51340 Q9NVM4 Q96PK6 11243 P55010 Q9NVM6 55703 Q9GZL7 Q15365 P23443 O75319 9212 6185 6188 Q15369 Q8IUX7 6187 Q08170 25896 6182 6184 Q15370 Q99816 P48730 P23458 Q99814 P48736 Q8WW12 Q15375 P78545 6196 P54577 6199 9221 6198 O95714 Q9H324 57461 6193 P78549 6195 6194 10813 83752 Q9Y4C1 P41252 P62195 P41250 O60285 65083 Q13523 Q13522 P78536 9232 22803 Q13526 Q9P1Y6 Q9H2P9 83737 P46934 P41240 O60296 Q9NVH2 Q4LE28 P42574 P42575 O60293 P34947 P29597 Q13535 Q9H307 P29590 Q7Z2Q5 Q9Y4E8 Q9NVI1 O60264 3932 O60260 Q9HCP0 P07339 9255 Q32P51 Q13547 Q13546 P34931 9252 83759 9253 P20309 P78560 P41227 Q13555 Q13554 O95757 Q13557 P78509 9261 P00749 P00747 Q8WVK2 P00742 2626 Q9NVC6 Q13569 P19387 P19388 P00738 2632 P00734 P24723 Q13573 P46977 P46976 P55884 3980 Q8IUQ4 28998 P78527 O60229 P41279 Q58DX5 P20339 1314 Q04656 3978 Q9Y4A8 P18031 Q9P286 Q9P287 P62191 1326 Q63HQ2 2651 P07384 O60231 Q9HCS7 P20340 P00750 Q9HD40 Q13595 P21675 P48052 Q12948 Q06265 Q8NB16 Q9Y2X3 Q12952 Q9Y399 5706 57634 5707 79885 5708 5709 P21810 P16471 5700 P49368 5701 5702 Q9UHP3 5704 5705 65264 P54753 P25398 Q66PJ3 Q9BZG8 Q12968 P54756 O60306 O60307 P16444 5717 1358 57646 83933 P12081 Q9Y2Z2 5713 Q9Y2Z4 5716 Q12972 P54764 P54762 P54760 Q12974 Q6P2C8 O60318 5728 1368 Q8WTX9 253827 Q9UI30 5725 Q6PCD5 P28482 Q12986 1385 Q9UHJ9 P51668 57661 P51665 Q9P0J1 P19525 P62316 P62318 5733 56339 P62312 P62314 P51671 Q9Y2T7 Q9UHK0 Q5VST9 P19544 10987 10988 56342 1388 1387 P42771 5747 Q14703 22938 Q9H9Y6 Q9P0L2 P42766 Q56NI9 10956 P62333 284119 Q9BZK7 22929 5757 P61009 P08865 P51693 5770 P54727 P54725 Q9P0M2 Q03164 55035 Q9P0M9 57695 57697 Q9Y2W2 Q9Y385 5768 Q9BZJ0 Q9Y2W1 P06213 Q15139 5780 Q9UPV9 5783 P14678 P38646 54512 Q99640 5775 79813 5778 Q9UQ80 54517 220988 Q9UHG2 Q9UQ88 5796 Q8WTR2 23451 8812 10147 Q99638 8815 P01343 Q96EZ8 Q86U44 P26641 Q86U42 P26640 O95059 Q9NU63 P26639 79834 55869 5798 Q9UPY3 P01375 23438 Q9Y2R5 Q9Y337 Q9Y2R2 P40938 P40937 8841 Q9Y333 7511 Q9UHI8 Q9UHI6 23435 Q9Y2R9 P12004 3149 O95071 3148 545 546 3146 Q96EY1 P62308 8837 P62304 7508 23409 Q9UQ35 7520 8851 Q9UPR3 Q12904 Q9Y2K6 Q9Y2K2 23404 3159 Q99683 Q9BZE1 8844 Q15185 P49336 7518 P05771 7517 Q9Y2L1 6201 6202 3178 23411 Q00987 Q9H1A4 3181 6210 P49321 3189 3188 Q9UHD2 Q12923 3187 Q9UPT9 Q9Y2M5 3185 3184 573 7536 6204 6207 3192 3191 8880 3190 8881 Q60FE5 Q12933 Q12931 P51610 580 P51617 P52948 79869 8878 6217 Q9UPV0 10090 Q8N9Q2 6231 8892 6233 P15927 P15924 P15923 Q96FV9 6229 Q8IU60 Q9HC98 P03950 O00716 Q16512 Q16513 Q16514 O75494 P27918 6238 Q13492 6239 Q13490 10075 Q9HBW0 Q16526 23387 P31152 O43897 23381 Q9NV06 P23528 P09234 Q16531 Q8WUK0 51426 P09237 51428 Q16539 344387 10081 Q6PD62 O75478 Q15208 O75477 23399 O75475 Q8TD19 64745 Q02790 10087 6259 Q8WUJ0 64750 P14635 Q9UQL6 P10275 P10276 P35579 O75460

Q9Y3E5 10055 P23588 Q99728 84365 Q8WV24 O43889 P36896 P36894 23378 P35573 Q02763 Q9Y3F4 120892 Q9BRL6 Q15233 Q99714 Q96FX7 P61803 Q96FX2 O75444 P36888 Q99700 Q9H1Y0 51465 88745 Q99708 O95817 9319 O95816 Q16576 Q99707 9318 O95819 55827 O75439 Q08050 P39905 Q96FW1 P14625 P36873 Q02750 55832 54504 Q15256 22913 O95835 O95834 Q7L0Y3 10929 P18124 10933 Q9Y3A5 Q9HC16 O95837 Q9BZQ6 22926 Q13405 Q13404 Q8TCJ0 10939 Q8TCJ2 O43847 Q9Y3B4 Q9Y3B7 10949 Q08945 Q9UHX1 Q9Y3B2 57599 10940 10907 O43812 O75400 Q13418 286826 9360 9361 Q9UHY1 Q9Y3C5 P42680 P42681 P42684 P42685 Q9NUG6 Q86UE8 Q13427 Q13426 Q8NAV1 Q8NB78 10921 P42677 P68400 Q9Y3D3 Q9Y3D5 1408 1407 2737 Q13435 Q9HC62 Q9UI38 P62280 P62277 Q9HC52 P43115 Q13444 9397 Q13443 Q08J23 P11532 Q9H1R2 8065 Q96G21 O43809 Q8N0Z6 Q9UQE7 Q8N0Z8 P23511 Q6P2Q9 Q13451 8078 P12830 P19474 59343 84305 1432 Q14790 O60341 Q9HC36 Q13464 Q5T4B2 P53355 P53350 Q9P0U3 Q9P0U4 26469 Q7L9L4 5800 Q9UHV9 8099 Q92979 Q9H0E3 Q9Y265 O75940 Q12824 O95551 1459 Q9BYG3 1457 1454 O00391 1453 Q00839 P05455 Q9H0D6 O75934 Q12834 P16104 27090 Q9H0C8 Q9Y285 5822 140801 O75937 P10809 Q96EB6 Q5S007 P27694 Q9UGN5 P27695 Q06124 A0AVT1 P37023 P14384 1478 57761 Q9Y295 55109 Q9Y297 O75928 Q9Y291 5836 P26358 Q12851 O75914 P26373 Q99583 Q12857 1491 55110 Q99575 1487 Q9BYD1 148789 55119 P26368 P25098 P49069 Q9Y223 Q96MX6 Q9NSU2 27043 57787 O60884 Q9Y230 O75909 Q9Y231 5859 Q12874 Q9UGI0 Q12872 P29466 Q9Y239 5870 Q9H0H0 5871 Q9UGI9 Q08752 Q8TBC4 Q96EF6 57794 Q96MV8 Q5C9Z4 5868 Q12884 Q9UGJ0 Q12888 Q9Y243 5883 Q8TBB1 P38398 Q9BQ90 Q96MU7 55149 Q9Y250 613 Q92995 Q5TCX8 Q9Y251 Q92994 5875 254827 Q92993 4548 P10827 P30414 P10828 5892 5894 P31749 55152 P40692 27037 55159 O60870 P04150 Q9Y262 5886 5887 5888 8916 Q9UGL1 Q8TBA6 O96004 8930 O00308 O96009 10250 P56199 O00303 P50897 23569 Q15022 P62877 8924 Q15020 Q15025 Q15024 P62875 Q15029 P23284 O96017 Q86SR1 O96019 P36578 P35244 O96013 P35249 Q03933 641 P35251 P62888 196441 P23276 8939 P27635 P60228 10269 Q15034 O00329 P35236 O96020 3263 10238 O14967 P11274 8945 P62899 79956 Q15046 O14965 P23246 Q8TAT6 23552 3276 23556 P35227 O14979 P35232 Q9H093 668 Q15054 285193 O14974 6311 P01040 P62837 P06737 8975 P05408 Q99570 P39656 23524 672 673 P15692 6304 6305 128308 10213 158135 8985 3297 23536 Q6PJP8 Q99558 10229 688 689 Q86T82 P62847 P62841 P61513 Q15078 Q9BQ15 23509 P13010 Q15084 Q99543 P62857 O14920 P62851 P56182 57727 P56180 P62854 O43463 Q9BQ04 23517 Q92901 O43464 Q92905 Q8NHX1 P35268 23512 Q7L7X3 O75031 10208 O14936 O14939 Q56UN5 Q92900 P56192 P36507 Q14686 Q9BZ95 Q9UPN6 Q86TM6 Q9H0U6 Q8TC27 O75150 Q9H0U3 84447 P46783 P49810 P83731 Q14690 Q14694 Q13363 Q9HAV7 5034 P46782 5036 P46781 P11216 79084 P41091 Q9Y2H1 60489 P68104 P00533 Q16401 P46777 Q9HAU5 P46776 Q9HAU4 P46779 P24522 P46778 10193 Q9UPP1 P32121 60496 10197 51548 51547 Q9BYU1 9410 55907 P62826 P20151 O43542 O75116 P62829 Q4AC94 9421 P78347 P49862 26512 Q9Y2C2 23481 51567 5074 10181 8100 55929 Q9HAZ1 26524 26523 5071 P49841 P49848 Q15118 P78368 P49840 P11229 9448 10152 23463 A1XKG3 51585 P24534 P78371 10155 Q9BQI3 9439 10159 Q15126 Q9HB90 23476 23478 P37840 5093 Q96EU6 O43505 Q9H173 51593 Q9H9G7 Q92187 P78362 54623 55959 9465 9463 Q6P1J9 P09769 P29375 P29374 Q96EL2 P29372 27005 79012 Q9H0M0 27000 P42345 P22735 P04083 9476 P31689 9477 9474 9475 Q9H0L4 79005 Q6P1K2 P42338 P42336 P29353 Q5JXB2 P29350 Q9H0K1 1514 Q9BYM8 79035 79039 Q8TBF4 P04066 Q9Y2A9 80020 P78316 55072 P78317 Q8N8D1 201595 Q04446 P04070 P53041 P52597 Q96EP0 Q13315 Q86TI2 Q9P015 Q96EP1 Q9H9B1 2869 Q14653 55093 P46736 Q9H9A7 1540 124739 5901 P46734 P13807 Q6P1L8 O75962 P28065 26576 Q9P035 Q00059 Q9BQA5 2885 P43694 O15455 Q14674 Q9BQA1 5914 Q13347 Q14676 O75953 P28074 P28072 P28070 5927 5928 P63279 P00519 5929 P63272 P43681 P08473 Q9BZ11 51514 P43686 Q14684 Q14683 P10914 O95677

Q92858 23607 P67809 Q9NRW4 O15519 5939 P16220 Q92851 5931 P49137 5935 P0CG13 P09936 O95685 O15530 Q9NS91 Q9NRX2 Q8IZP0 O95680 Q9H832 P50148 P18887 P16234 55215 P67812 64174 O95696 Q9NRY2 Q9NRY4 Q99471 P18858 125972 Q92830 P62136 4627 Q92831 P55854 Q92824 P46087 5970 Q9H814 Q99460 Q9NRZ9 Q99459 3304 P62140 55236 3301 3309 P04233 P34925 O00469 P07307 133584 5981 5983 3320 P52756 O00468 Q96DE5 3313 710 3312 5976 27161 O00472 Q9NS56 3329 3326 720 3324 722 P08651 4659 Q9UNX3 4670 4673 2011 27148 O00487 P30542 80222 3337 Q96DC9 Q92878 P04264 Q9UNY4 P08621 P51451 P09958 P30530 740 Q96LR5 P37173 Q99496 P09960 157570 4677 4678 P02452 O00425 P23396 Q5VYS8 2034 2033 P09093 P62993 4686 Q16236 P62995 Q96LI5 Q9C0K0 P25100 P46020 2043 Q9NRP7 54764 P46019 P01100 Q8N726 O00444 P11388 P11387 O60942 773 P01138 P01137 P01135 P01133 P02461 P01130 Q9H7H0 6421 2065 3396 2064 780 Q9NS37 Q8IZE3 P09086 P10071 P02458 57819 2070 Q5TKA1 6430 6432 6431 6434 7764 Q92817 Q9UNN5 O60925 Q8IZL8 O75190 Q8IZL9 6427 10331 6426 P61626 2081 23658 P46063 5111 23650 Q5HYA8 P18850 P83881 P27797 P50990 2091 P50991 P22087 91754 Q8TA86 Q9UNP9 2099 O60907 O75164 23621 10318 Q9NRM7 91746 P62979 Q9UNQ2 A2RTX5 Q5T1M5 6464 391356 P39748 O00411 146057 P52701 O60911 Q5U4P2 Q9NRN7 P58012 P62988 5127 Q8N752 P25116 Q13237 Q14566 64951 P62917 P13984 84569 P62910 P09012 P62913 Q13243 Q13242 O43683 Q13247 P20290 9510 O43687 64960 O43684 9512 64963 64965 64969 80198 51650 51651 51654 51657 Q9BY50 P11309 Q6Y2X3 Q13257 P62937 P11308 64975 P49916 5159 P61604 P83876 P21589 Q13263 9531 O43660 9530 Q9BY44 9533 Q9BY41 5170 Q02543 P09001 9526 P62945 P49903 P62942 5184 10291 9541 2909 121512 P27708 Q8NI36 2908 P48643 Q13287 Q9NSE4 Q13286 Q09161 P15735 Q8NI27 Q96E52 8237 10273 P49959 P50914 2926 P09038 Q15008 Q9BY84 Q15005 10283 P62906 9578 116138 8241 2932 2931 255488 10286 10285 Q15014 2935 P78424 P55786 Q12788 92105 Q15819 P62081 1613 80124 Q96DI7 P41134 Q9BXM7 79139 Q8TAF3 Q12792 1616 P07237 8266 Q12797 P55795 O95602 55173 P67775 Q6IN84 P08575 2959 P08579 2956 Q15831 Q96M96 1634 80145 27102 2961 2960 P04183 O95619 Q8TAD8 2969 P09874 Q6V1X1 Q15843 Q6R6M4 55192 29777 1642 Q05655 2972 Q9BXJ9 P09884 1647 Q15853 Q14527 1660 1655 1653 Q86SG6 1650 Q13200 1659 O95644 150094 Q13202 P21580 Q8N7H5 64919 80155 1665 Q9BXP5 51611 Q13216 93492 P55769 1676 P08581 Q8NHH1 389840 Q13233 Q14562 Q96TA2 Q96L12 Q9NQU5 81605 P52434 O00148 Q8IYN9 P84022 Q70EL4 P27487 Q9C026 Q8NFZ0 Q92729 P53779 10420 4734 10421 64282 Q7Z727 23708 81629 O00167 P56817 3416 P40429 811 Q8N684 Q5TAP6 P27448 Q9C005 P07858 P99999 Q9NR96 Q8IYK4 Q8NFW8 Q5TAQ9 55352 O60674 P20827 P19838 821 823 826 Q9NZJ4 P41743 Q13951 Q13956 4772 Q5SGD2 O95352 2107 Q5T160 27283 Q6PI48 80324 Q9BWG6 833 O14744 4780 Q92769 P09619 80308 O95361 P10600 O95363 Q7LGA3 27297 P61353 167227 841 843 4775 847 7804 O14757 Q92766 P09622 4790 Q13976 2132 Q96L34 O95376 Q9NR45 P18545 Q9NR48 Q9BWE0 Q05469 2125 P60033 857 Q96CA5 Q9UMW8 Q9H6R4 6500 Q9NR50 O95382 Q96L21 Q9NQT4 Q9NR55 Q9NQT5 80332 Q4G0W2 2139 Q96KP4 O00141 80335 865 867 Q9UMX1 4799 Q92743 10492 O95391 O60603 871 Q96T88 3479 2147 Q13085 7832 Q96KG9 P25685 P23025 P51991 Q96T76 O14717 2159 O43290 Q7Z6J9 P61313 P49643 O43294 O43293 Q13099 Q6ZN33 O43251 P49642 64215 Q9BW92 891 29088 O14727 126789 Q8IYD1 Q96T60 Q9NR09 Q8IYD8 10477 29086 P61326 Q9UMS4 7874 Q658W2 114799 Q01974 Q96ST3 29093 O14730 7867 10488 O14733 7884 P50613 P51946 23764 10450 O43242 P51955 P51956 29079 Q96SZ6 23770 29074 23746 P24386 5245 10438 Q5TAL4 Q00613 Q00610 10432 Q13901 P07814 P49674 Q96C86 56647 10445 Q13115 6598 24139 57062 O43318 Q01130 P33240 9616 Q9C0C2 P38919 P13639 8301 51773 Q9NRH2 24144 Q15796 P60866 Q13131 9641 Q15797 9646 9643 Q9UNL4 Q6UXN9 29803 Q01105 O94804 Q13144 54815 Q13148 P35869 57092 9656 P10586 P26927 Q13151 54822 Q49AN0

Q13153 P54136 O15287 P54132 Q13162 Q8IZD4 Q14494 Q14493 Q14498 Q13164 O15294 Q9NRC8 7013 7014 P13674 P11021 Q9NRD1 Q13177 Q9NRD5 10392 9682 54853 Q9NRE1 P47897 1718 Q9UEW8 1716 Q13188 Q9BWU1 9695 7037 Q09028 Q9UNI6 O75792 26747 Q9NZV6 Q00688 Q6PID4 Q7Z7F7 Q9NRF9 7046 P35813 Q96L91 O75787 27248 Q9H6Y2 27246 O15234 1738 1736 P54105 375743 7057 P29144 O75771 Q9H792 55294 7052 27252 27257 Q9UER7 7068 7067 Q5TAX3 Q15714 O15264 Q15717 Q8IZ69 7064 Q9H6W3 1756 80267 29880 Q92793 Q7Z6Z7 P05155 Q8IYT8 Q9UNE7 Q9BWH6 P43405 1763 29896 Q04206 29894 Q9UET6 Q5GLZ8 Q8N6G5 7083 4809 51720 4800 P08246 51726 7099 7098 51729 30849 Q15746 P30153 1789 1788 1786 1785 Q05513 57038 375790 P08253 Q15751 80273 51734 Q15759 29843 84661 4820 O15212 P05198 51747 O75716 30827 Q6XPS3 Q13107 P22528 4839 P47804 51752 Q6ZVX7 902 Q13112 P08238 Q9NZN9 O95433 O75821 O75822 Q969L4 O00268 Q8IXM3 51081 O00267 3516 203102 10535 Q8IXL7 O00273 Q9ULW3 P63208 4841 90678 Q6NZY4 Q92614 10531 P07996 10533 Q92611 P04003 P52564 Q9NQ55 55454 51096 10549 Q9ULX9 29128 Q05397 P18621 29122 P63220 55466 O60783 P32929 26091 Q6IT96 4882 3551 Q13829 152926 10524 10528 Q9ULZ3 10521 948 10523 O95478 O95476 O95477 29108 O60762 29102 P04040 P84103 58517 O00238 P04049 Q96SB4 Q96SB3 O00231 O00233 P10721 O00232 Q5JTZ9 Q96SB8 Q8WYH8 29110 2237 79444 Q92643 4898 P05386 P05387 P05388 Q9NYB0 P30304 P30305 P32969 P30307 Q9NYB9 2247 975 Q6GMV3 Q92630 Q969M7 P29323 P29320 Q13868 O00255 81788 Q99259 984 6612 988 Q92620 6613 P29317 Q9Y6W6 O43395 P07900 5300 6631 P15529 P53804 Q9BV90 P53803 O60725 990 991 P62750 Q96RU7 Q96RU2 O14829 994 995 997 P28838 6622 999 6625 6627 51009 P62753 P07910 P49770 200916 Q9UM07 2287 2286 2280 Q93096 51010 Q96S53 51013 6633 Q9Y6Y0 O14830 6637 P49768 O00206 P49761 P49760 O60701 51021 51023 Q00535 Q8IXB1 Q00534 10594 P49756 P25789 P25788 6647 10598 91949 P25787 6660 Q10713 O00213 Q96B26 Q96RR4 Q96RR1 Q00526 5327 5328 Q9ULR0 O43390 O43353 Q9H5K3 P07947 9700 P07949 Q969H0 P07948 10574 P62714 5338 4008 5351 5352 5356 Q9ULK4 Q01831 51056 4015 5347 4017 Q96JH7 P50750 4035 O14802 Q8NER5 51067 P68036 9716 Q68J44 Q9H5H4 Q01826 P49792 5371 P27540 9733 5378 P20908 Q969E8 10569 54101 55432 O14818 Q9NPJ6 6699 54107 54106 O43432 57187 29947 Q9NYU1 Q9NYU2 29941 P22695 Q7Z6C1 83475 P22694 O94905 8408 O94906 5393 5394 4067 63875 29959 Q6UX04 128 P60983 54921 O94913 P22681 Q76FK4 P35998 Q9UDY4 Q9UDY8 P42285 8428 P22674 54931 54938 Q8WZ42 P00441 4086 4087 8446 4088 P22692 9775 8445 142 1800 P62701 Q8IXZ2 53615 1801 8453 8454 9785 8452 Q6ZN16 8458 7124 P54252 P54253 7126 Q96KC8 8450 4092 4093 4094 Q00597 156 Q8IYB3 Q13042 8449 Q13043 P49736 Q13049 P04843 P04844 Q96KB5 8467 P13798 P11142 54961 7128 P49721 P49720 8473 O94966 P54277 P54274 7141 Q96T21 1832 Q96T23 178 Q9NZ43 Q00577 P49711 P49716 P49715 Q9NQC7 7153 7155 7158 Q9BVS5 7157 7150 1843 Q6UWQ5 1848 Q6VAB6 92345 Q13873 Q96K76 Q86Z02 7161 8492 1854 P42224 1852 P47914 1850 P63165 O15355 Q92696 P09651 1859 P22612 P42229 Q13882 Q969T4 1856 O15371 O15372 Q969S8 Q13887 Q01082 84708 P22626 P84098 P63173 P09661 P60900 Q969S2 7186 7189 P10619 91039 P35916 27348 P11940 26015 27347 26019 7184 1877 4907 P17252 1870 O15379 P23921 Q9NZ08 Q6UWE0 4914 Q969Q1 4913 Q969Q0 P33316 Q9NYH9 57144 Q93100 Q8WYQ5 4920 Q8NFF5 Q8IY92 P35968 O95400 Q8WZ19 Q9NQ92 P42261 26005 57154 Q96BI3 84749 27339 27330 58487 27332 Q15631 P21333 Q8IY81 Q8IY84 O75844 Q5W0Q7 377630 Q5RKV6 3611 Q5XUX0 57169 P01730 Q969V3 O15350 Q15643 O75832 O95427 Q14318 Q15648 27315 Q86YP4 Q9BVJ6 P30260 O15347 O95429 Q15653

cellular metabolic process P28562 10657 2305 55561 55568 Q5QP82 2300 Q8N465 4967 4968 Q9H4L7 P30876 O14561 P52209 Q8NE71 P19623 10667 2316 2 P42858 P39210 Q9UKV5 136319 Q9UKV8 3661 Q8NE63 P62495 2324 Q9UKW6 3659 10632 P54886 3672 Q13705 P21917 P21912 10645

2335 10642 O14578 10643 Q9UL15 Q8WXG6 P51784 Q9UL18 Q8IX01 1022 1021 1020 Q06587 P51787 10613 1018 1017 2348 79577 P21964 10616 P54819 Q92530 26191 3692 Q8WXF0 Q13724 Q9H4P4 Q96J02 1029 P42898 10626 O14519 10625 1025 Q9BUB5 P20648 Q9BUB4 6711 P05023 P07686 6714 P05026 O95136 1040 79595 391627 P20618 6723 Q9UL46 O95147 6741 5411 Q38SD2 Q8IWL3 1050 10606 253980 57332 1047 10607 6733 Q9UL54 64432 5422 P50416 11108 11107 Q8WWY3 6749 Q9BTU6 Q9Y676 O95169 P49411 5430 5432 Q9UKM9 O95163 Q9NWZ3 P57059 115426 1072 51132 Q9Y680 84033 5424 11113 P49407 84061 5440 5441 6772 5442 Q9UKN5 Q9UKN8 346171 Q66K89 5434 51148 Q14197 51147 Q9Y5X9 P50440 Q9UL03 Q86X95 Q8N3J5 Q9Y697 A7MD48 O95182 Q53H96 P17812 Q00403 O95190 5445 6776 4116 P62424 P57081 6790 Q5VVQ6 6793 O00746 9821 6794 4133 10690 9820 Q9NX62 O00743 Q9Y5Q8 Q9NWU5 Q8IWF2 4125 6789 Q96A72 Q07020 5470 4140 5471 O00757 Q9Y5R4 O00754 Q9NWV8 Q9UBX3 P49448 5467 P49447 207 5468 4139 4150 O00767 5481 4152 4154 Q9Y5S2 Q96A54 Q08345 O00762 P13489 Q9NWW5 O00764 Q9Y5S9 P17844 211 51185 215 5478 5479 4149 Q9H4F1 5494 5495 Q9BU89 5499 Q9UKK9 P17858 Q96QT4 P49427 4172 4173 4174 11060 4176 Q14209 Q16864 11065 7204 P55265 Q6NZI2 4171 230 Q6P5Z2 23043 11072 Q8IWZ8 4188 8543 Q16877 Q9NY59 Q9NPD3 8536 Q96RL1 P61956 Q15554 197259 Q9BUN8 Q16881 P35638 P61968 11041 8555 4191 23028 Q96IZ7 4193 P61962 1915 P61960 Q93009 P22314 Q93008 Q14232 P61964 8563 Q9Y6R4 P36957 11051 Q8IWX8 8565 8566 Q86Y37 P36954 Q8N4C8 267 23031 23032 Q86Y39 Q9NPF5 P61978 P43246 8574 Q14249 P56556 8576 26994 Q9Y6K5 O75582 271 Q9Y6K1 P10398 Q9Y6K0 Q93063 Q93062 Q04837 Q96RQ3 8569 1937 1936 1933 11030 Q9BV47 P22392 O75575 23014 23016 7251 Q93050 Q9NY12 11035 118460 P02751 P61927 7266 P02790 O75569 7267 Q9Y6M4 291 Q8WY64 Q9Y6M9 Q8NEJ0 51102 P08069 Q6I9Y2 51107 1956 O15075 Q9NPA8 26986 Q01780 Q86Y79 64428 7272 1965 114088 Q93034 51116 1969 Q14289 1968 11017 P12931 Q7Z589 Q70CQ3 Q70CQ1 1975 Q05329 Q9BUG9 Q6P5R6 Q13761 Q58WW2 7297 P56524 Q13769 O75531 7295 P24928 1982 Q9Y6H3 Q92560 Q9Y6H5 P06276 O75528 Q5D0E6 P06239 Q8WXI4 O75521 92483 P55209 P56539 P56537 1994 Q92551 26133 P00966 P06241 P21860 O95983 Q16816 O95989 91147 A4D1W7 63931 3704 3705 Q07954 Q9Y6J8 Q07955 P07585 Q92542 50628 Q16825 Q16827 O14595 O95997 P35610 Q16828 9020 23081 23085 O60488 P20594 84881 Q8NEC7 Q8IWV7 O43920 Q86Y13 P61081 Q9H596 P61073 83548 23097 26121 P61077 Q9BUJ2 P22303 11080 Q9NXH8 9045 Q9NP81 Q16849 P12956 P12955 Q8IWU5 Q8NEB9 P33897 Q9Y6E7 Q9Y6E0 P61088 Q96AE4 Q96RG2 11091 Q16854 Q15526 9054 23076 P24941 11099 84893 Q5FWF5 285672 65003 O14672 399687 9061 Q9P2R7 Q9UBB9 O60568 O60566 P15374 Q8IVH8 10772 Q9UK45 Q9UBC3 P27361 Q9GZZ9 65018 54361 9077 55696 10783 10785 92609 Q9NWB1 P52333 P29992 1121 P53667 Q8TED0 O60547 O60543 1111 Q86WA8 P61289 P03372 P26012 P49591 3791 Q9GZX7 Q8NCR0 9093 Q9UBE8 P56705 P49590 9099 10767 10768 57410 O60551 3784 P49588 P07741 10765 P05129 P50570 P11802 P50579 O95232 6829 10733 147746 Q9P2N5 1137 Q6FHQ0 P61221 6827 Q10570 Q7KZI7 Q7Z419 Q9Y4X5 Q13601 1153 Q14938 1152 P19784 Q06455 O95243 P53611 6839 10746 1147 2475 5500 5501 6834 P26045 P53621 O43175 O95257 6850 Q13615 Q9Y4Y9 Q13618 Q13617 O60508 Q13616 1161 O60506 Q9P2P5 O60502 10714 57448 P61244 O14641 5511 P61247 O14640 5514 Q9Y4Z0 5515 Q9UK22 65061 5516 5518 Q13620 Q13627 P18433 O60513 10725 10724 P61254 10728 O14656 P40337 P06454 Q9UK32 O14657 Q8N2K1 O95278 O43159 6872 Q96HA8 6871 P04637 25862 P16615 Q9NVW2 O95271 Q01650 11222 5536 Q15389 5537 Q9GZR7 Q9GZR5 6883 O43169 P48201 4221 6885 1198 25873 23210 1196 1195 P50542 P12268 56940 O76074 Q96Q15 O76071 51251 Q15392 Q99873 55611 51253 Q9P2K8 4214 11231 6878 4215 4216 Q15393 P01588 O43172 56946 Q9GZQ8 25847 6892 5562 5563 5565 O95298 O95299 P12235 51264 Q53X93 55626 Q9GZP9 Q9Y572 5557 P61201 Q7Z3C6 5558 O43143 Q9BT22 P04626

5575 P04629 25853 Q08257 11218 301 P17936 9924 5566 Q96PN8 5567 5568 P61218 5580 5581 25828 5582 5583 5584 Q3SY69 5585 4255 5586 22794 P15328 25822 O76031 Q96PU8 Q96PU4 79612 314 Q96PU5 Q02930 8604 5577 5578 8602 5579 Q9H3F6 8607 Q8N302 5591 Q9Y4P1 9950 Q6IR47 P24298 5596 5597 5598 P50502 O76024 O76021 5590 P16671 328 329 84197 25804 Q9GZU7 Q8N2M8 P12277 Q9NW64 Q8NCN5 333 55660 55666 56994 5599 P83436 4285 4287 7311 9973 Q96Q40 P17980 Q9NVV4 P15313 Q08211 221656 Q08209 79622 9967 4297 Q6DKI1 Q6ZT98 Q15418 7325 8654 Q9UKD1 P30085 Q04760 4291 351 Q03426 353 Q9UBS0 Q04759 Q9UBS4 P23786 9978 P21127 8662 7332 7334 16 8667 Q15428 7336 8665 Q15427 7335 19 26205 Q8TF76 Q9UKE5 Q9H3Z4 Q9UBT2 23170 367 Q9UBT6 369 P30084 11198 7329 7328 Q9Y600 7341 11160 Q15434 Q14103 7347 6015 P29074 23144 Q9UKF6 Q9Y606 Q9UBU3 Q03405 85437 P17096 11165 11168 Q16774 8669 Q9UBU8 30 P43378 31 34 Q53H12 11171 8683 Q8IW75 7353 7358 P51812 Q9Y619 P51813 P29084 Q96HW7 Q9H492 Q9UBV7 P51817 11176 P23771 Q15459 O15160 P55345 48 O15164 25788 23127 Q16795 Q16790 52 Q14134 Q99966 54 Q14139 6045 7376 Q14137 7375 6048 23133 23135 Q9UKA1 Q9Y5J1 7372 11157 Q99956 7385 7384 Q8TEY7 7386 7389 6059 Q9Y5K6 Q9Y5K5 6050 7381 Q9UKB1 Q99942 Q99943 11129 Q99941 51222 56915 Q9UBQ7 56916 P01579 Q14152 11124 Q9UBQ5 11128 O75676 O15197 O15198 O75674 P14868 P33121 P14866 25778 Q9NX14 51231 Q00325 Q14164 86 Q14166 9100 Q7KZN9 Q96QC0 Q14966 P52272 P33992 P33993 Q8TF05 P33991 Q05209 84967 91 Q8WWI1 94 P45974 26270 P82921 O15118 Q9UBK2 Q9UBK9 P82912 9114 P82914 Q14978 Q8WWH4 Q86WJ1 Q6DT37 Q96I24 Q9H3R0 P31321 P31323 P43304 84950 P05062 P52292 Q52LR7 P05067 Q9UBL3 O94763 P52298 9128 O94768 9129 O75643 9126 Q6XUX3 Q9H422 Q96I15 6093 283989 Q8TEL6 P21980 Q9BTC0 Q02156 26260 26262 57379 283970 134430 Q9UBN7 P82932 P82933 P30049 Q9UJV9 Q9UBF6 9149 56052 P30041 Q6NYC1 Q9H488 P30043 P11766 P30046 P53567 P30047 P30048 B3KY43 P22415 26232 Q9UK80 P05093 P30050 Q03468 26234 Q92499 3835 P47712 Q8IVT5 9150 57396 Q16718 Q9UJX2 Q9Y5B0 P17081 P08138 P09467 2517 Q9Y5B9 Q9Y5B8 P09429 O75604 P10415 Q8TEQ6 P35711 2521 Q6P4R8 P09430 Q5T6F0 O75608 728642 P08107 P53597 O15111 Q9P2Y4 Q04771 23192 Q96HR8 2531 O15105 P45983 P45984 1207 65125 P54619 P51587 9184 P17676 Q8TDD1 2547 P37288 1213 Q8N1Q1 6901 Q6IQ55 Q9H2H8 P28335 25913 P28330 P08729 P28331 Q5VTR2 P37268 O60216 P62244 80755 54487 P62241 P49247 P62249 Q9H2G2 Q9BRX9 P28300 2572 2571 79791 Q8NBQ5 54496 6927 Q8IUD2 6929 P62256 57520 6921 P26599 P31942 Q9UIS9 P31943 P08708 2582 P54646 P62266 80777 P62263 5602 Q96GD4 P62269 5604 Q9Y3U8 Q9Y463 Q14807 2593 P50213 2590 Q8WVC6 P39019 10855 P19404 P15056 10856 2589 P39023 5610 5613 Q9UIM3 Q9Y478 P51553 P30679 5631 Q07666 Q8WVB6 P52888 Q06330 10868 10869 Q9H2K2 P28340 4311 P21709 Q8IUH5 169436 Q9HCL2 5636 Q10472 Q10471 Q13501 P51575 Q5MAI5 22826 22827 22828 P51570 Q8NC51 Q9HCK5 10845 192669 11340 Q86V81 O00541 P14555 23326 25988 23327 51362 126328 Q8NBJ5 51363 P25205 10013 84289 10014 11345 135295 64682 Q9UII4 4343 Q9BS26 Q6FGD7 P51513 Q99755 Q8NBI2 51377 Q99750 Q9BS18 Q9BRP8 Q99759 408 409 Q99757 P60468 23307 Q09472 5682 5683 5684 5685 5686 O00567 51380 Q9NUW8 Q99741 55743 3014 8702 Q9Y450 P03891 Q15287 4361 84292 5692 5693 P40818 3035 3033 3032 Q9NUX5 3030 P14543 10007 11338 55750 P14550 P15880 3028 P60484 Q15291 55759 5687 5688 4358 55757 P48775 84296 79709 P31930 P26583 8732 O00506 25942 P14598 Q9NUQ2 54431 Q8WUY8 433 435 P31939 Q9H2D6 79739 P55072 Q96GC5 140609 Q9HCE7 3054 Q05086 P37231 Q9HCE1 Q99797 Q8NBM8 444 445 55775 P82673 8737 Q02809 P82675 P55084 P61758 Q9H2C2 11315 284086 P49207 8754 25929 7422 Q9BRT9 Q9HCD5 P52815 3066 22894 3065 Q9UIF9 22897 O75293 79753 55781 7415 8761 3070 Q5VTL8 Q9UIG0 54460 Q9HCC0 3074 P17612 468 55796 7428 O43791 53938 Q16629 Q9Y4K4 P11441 O75385 Q9Y4K0 Q86VQ3 Q9Y4K3 472 Q96GX9 P48729 474 O75390 476 477 478 28973

Q99807 Q16630 8767 P43034 Q8WW01 3093 Q16637 Q15303 3091 Q16633 P55036 6123 Q16635 6125 Q15306 Q9Y4L5 Q86VP6 3099 O75376 3098 8780 481 483 O75380 Q504Q3 23291 Q9NW13 488 6117 P22102 6119 6118 Q6YP21 Q16649 6133 6132 Q16644 6134 7465 6137 Q16647 O43776 O75365 490 O75362 495 498 P09110 P31040 6128 P56385 P82663 P82664 P56381 Q8NCB2 Q8WVM0 Q9BSI4 Q16659 6141 Q16656 P18074 P55055 Q01581 P18077 O75351 O75352 144983 28960 Q96PF1 Q9P2E3 6138 Q16665 Q9P2E9 51319 Q8TDY2 6156 6158 O75347 P35453 P09172 P23467 P27824 Q14012 51329 Q14011 6164 Q15349 Q8TDX7 6168 P46100 11274 P36776 Q9Y4H2 6160 7490 P11498 Q7L2H7 11277 6175 9201 Q86W42 P98175 Q3ZCQ8 P98179 Q02880 25885 Q08188 Q8WW22 Q02878 51340 Q9NVM4 P02549 Q96PK6 11243 Q14032 P55010 Q9NVM6 55703 Q16698 Q9GZL7 Q15365 P23443 O75319 9212 6185 6188 Q15369 6187 O43741 Q08170 25896 6182 6184 Q15370 P27815 Q99816 P48730 P23458 Q99814 P48736 P98194 Q8WW12 Q15375 P78545 6196 P54577 6199 O75306 9221 6198 O95714 P78540 57461 6193 P78549 6195 6194 10811 10813 83752 Q9Y4C1 P41252 P62195 P41250 O60285 P08684 65083 Q13523 Q13522 P78536 9232 22803 Q13526 Q9P1Y6 Q9H2P9 Q8NBZ7 83737 Q9BSB4 P46934 P41240 O60296 Q9NVH2 Q4LE28 P42574 O60293 P34947 P29597 Q13535 Q9H307 P29590 Q7Z2Q5 P41235 P46926 Q9Y4E8 3939 Q9NVI1 O60264 3932 O60260 Q9HCP0 P07339 9255 Q32P51 Q13547 Q13546 P34931 9252 83759 3948 P41222 P20309 O95749 P41227 3945 Q13555 Q13554 O95757 Q13557 P78509 9261 P00747 Q8WVK2 3953 2629 2626 Q9NVC6 Q9P1U0 Q13569 P19387 P19388 P11413 93100 2632 O60256 P00734 P24723 Q13573 P09104 P46977 Q13572 P46976 P55884 3980 Q8IUQ4 28998 P32320 P78527 P32322 O60229 P41279 P20339 1314 2644 1312 2643 Q04656 Q9UJ83 3978 Q9Y4A8 P18031 Q9P286 Q9P287 P62191 P19367 9296 1326 Q63HQ2 O60231 Q9HCS7 P20340 P00750 Q9HD40 Q13595 P21675 P48052 Q06265 1337 Q8NB16 Q9Y2X3 P48047 Q9UI09 Q12952 Q9Y399 5706 57634 5707 79885 5708 5709 P21810 1345 P16471 O60331 5700 P49368 5701 5702 Q9UHP3 Q9UI12 5704 5705 65264 P54753 P25398 Q66PJ3 Q9Y2Y8 Q9BZG8 Q12968 P16435 P54756 O60306 O60307 5717 1358 57646 83933 1355 P12081 Q9Y2Z2 5713 Q9Y2Z4 5716 Q12972 P54764 P54762 Q9Y2Z9 P54760 Q12974 1374 Q6P2C8 O60318 5728 Q8WTX9 Q9UI32 253827 Q9UI30 5725 Q6PCD5 P28482 Q12986 1385 Q9UHJ9 P51668 57661 P51665 Q9P0J0 Q9P0J1 P19525 P62316 P62318 5733 56339 P62312 P62314 Q9BZM1 P51671 Q9Y2T7 Q9UHK0 Q06210 Q5VST9 P19544 P30793 10987 57678 10988 56342 1388 1387 P54709 P42771 5747 22934 22938 Q06203 Q9H9Y6 Q9P0L2 P42765 P42766 Q56NI9 10956 P62333 284119 Q9BZK7 22929 5757 P61009 P08865 P51693 P27144 5770 Q9Y375 P51692 P54727 P54725 Q9P0M2 Q03164 55035 Q9BZJ4 Q9P0M9 57695 57697 Q9Y2W2 Q9Y385 Q6DHV7 5768 Q9BZJ0 Q9Y2W1 509 P06213 Q15139 Q9Y305 5780 Q9UPV9 5783 P14678 P38646 Q8WTS1 54512 Q99640 513 514 5775 8803 79813 515 8801 5778 P27986 518 Q9UQ80 54517 220988 Q8TBX8 P04406 Q9UQ88 5796 Q8WTR2 521 522 523 23451 526 8812 528 10147 Q99638 P01344 8815 P01343 Q96EZ8 Q86U44 P26641 Q86U42 P26640 O95059 Q9NU63 P26639 533 79834 535 Q15165 55869 537 5798 79837 539 Q9UPY3 54539 P01375 23438 23439 Q9Y2R5 Q9Y2R2 P40939 P40938 P40937 8841 Q9Y333 7511 Q9UHI6 P51659 23435 Q9Y2R9 P12004 Q99611 3149 O95071 3148 545 546 3146 8836 8833 Q96EY1 P62308 P62304 7508 O00628 23409 Q9UQ35 7520 8851 Q9UPR3 Q12904 Q9Y2K6 23400 Q9Y2K2 3162 P17752 23404 54550 552 3159 Q99683 Q9BZE1 8844 Q15185 P49336 7518 P05771 7517 Q9Y2L1 6201 6202 7533 3178 23411 P12074 Q00987 P49327 Q9H1A4 65220 3181 6210 P49321 3189 3188 Q9UHD2 Q12923 3187 Q9UPT9 Q9Y2M5 3185 3184 572 573 54575 Q8N8Y2 7536 6204 6207 3192 3191 P04424 8880 3190 8881 Q60FE5 Q12933 Q12931 P51610 580 P51617 P52948 8879 79869 8878 6217 Q9UPV0 10090 Q8N9Q2 6231 8892 6233 P15927 P15924 P15923 Q96FV9 593 6229 Q8IU60 Q9HC98 P03950 O00716 Q16512 Q16513 Q16514 O75494 6238 Q13492 6239 Q13490 10075 Q9HBW0 O75489 Q16526 P31153 23387 P31152 23381 Q9NV06 P23528

P09234 Q16531 Q8WUK0 51426 51428 Q16539 344387 10081 Q6PD62 O75478 Q15208 O75477 Q86UL3 23399 O75475 Q8TD19 64745 Q02790 P12883 23396 10087 6259 P60520 Q8WUJ0 O75469 64750 P40926 P14635 P38606 Q9UQL6 P10275 P10276 P35579 O75460 Q9Y3E5 10055 P23588 Q99728 84365 Q16555 Q8WV24 197322 Q86UR1 O43889 P36896 64768 P36894 23378 P35573 Q02763 Q9Y3F4 P01308 Q01433 120892 Q9BRL6 Q15233 Q99714 Q8TCU4 Q96FX7 P61803 Q8TCU6 P14618 Q96FX2 O75444 Q86V24 P36888 P11586 Q99700 Q9H1Y0 51465 88745 Q99708 P22234 O95817 9319 O95816 Q16576 Q99707 9318 O95819 55827 O75438 Q08050 P39905 Q96FW1 P14625 O43865 P36873 Q02750 55832 Q15256 22913 O95835 Q7L0Y3 10929 P18124 O43837 10933 Q9Y3A5 Q9HC16 10935 22908 O95837 P15090 Q9BZQ6 Q8WUD6 22926 Q13405 Q13404 22928 Q8TCJ0 Q8TCJ2 Q9Y3B4 Q9Y3B7 2710 56267 10949 Q08945 Q9UHX1 Q9Y3B2 57599 10940 10907 O43812 O75400 10908 Q13418 286826 9360 9361 Q9UHY1 Q9Y3C5 P00846 P42680 P42681 Q9UHY7 P42684 P42685 Q9NUG6 Q86UE8 Q13423 22901 O95861 P53396 Q13427 Q13426 O95865 Q8NAV1 Q8NB78 10921 P42677 P19438 1400 P68400 Q9Y3D3 2730 Q9Y3D5 P06132 1408 1407 Q13435 9388 Q9HC62 9380 P62280 2744 P62277 Q9HC52 P43115 2747 9397 Q13443 Q08J23 9391 P11532 Q9H1R2 8065 Q96G21 O43809 Q8N0Z6 Q9UQE7 Q8N0Z8 P23511 Q6P2Q9 1429 Q13451 8078 O14495 P19474 59343 84305 1432 1431 2762 Q14790 O60341 Q9HC36 Q13464 Q5T4B2 P54687 P53355 P53350 Q9P0U3 Q9P0U4 26469 Q7L9L4 5800 Q9UHV9 Q9H0E2 O75947 8099 Q92979 Q9H0E3 Q9Y265 O75940 Q12824 O95551 1459 Q9BYG3 1457 P13073 1454 O00391 1453 27089 Q00839 P05455 Q9H0D6 O75936 O75934 Q12834 Q06136 P16104 1468 27090 Q9H0C8 Q9Y285 140801 O75937 P10809 Q96EB6 Q5S007 P27694 Q9UGN5 P27695 Q06124 P17405 A0AVT1 4507 P37023 1478 57761 5830 Q9Y295 55109 5832 Q9Y297 5833 O75928 Q9Y291 5836 P26358 Q12851 O75914 O75911 P26373 Q99583 Q12857 1491 55110 Q99575 1487 Q9BYD1 148789 55119 P26368 4514 P25098 P49069 Q9Y223 Q9UGH3 Q96MX6 Q9NSU2 27043 57787 Q96EG1 O60884 Q9Y230 O75909 Q9Y231 O14986 5859 Q12874 Q9UGI0 Q12872 5870 Q9H0H0 5871 Q9UGI9 Q15904 Q08752 Q9H8X2 Q8TBC4 57794 Q96MV8 Q5C9Z4 P16152 4535 5868 P04179 Q9UGJ0 Q12888 Q9Y243 5883 P16118 Q8TBB1 P38398 Q9BQ90 Q96MU7 55149 Q96N66 Q9Y250 613 Q92995 Q5TCX8 Q9Y251 Q92994 5875 Q92993 4548 P10827 P30414 P10828 5892 5894 P31749 55152 27032 P40692 27037 55159 O60870 P04150 Q9Y262 5886 5887 5888 8916 Q9UGL1 Q8TBA6 O96004 8930 O00308 10250 P56199 O00303 P50897 23569 O14949 Q15022 P62877 8924 55967 Q15020 Q15025 Q15024 P62875 Q15029 P23284 O96017 Q86SR1 O96019 P36578 23576 P35244 P14324 O96013 P35249 Q03933 641 P35251 P62888 196441 8939 P27635 P60228 10269 Q15034 O00329 P35236 O96020 3263 10238 O14967 P11274 8945 P01019 P62899 Q15046 O14965 P23246 Q9Y215 Q8TAT6 23552 P11279 3276 23556 P35227 O14979 P35232 Q9H093 4598 668 Q15054 285193 O14974 Q59EA4 P06733 Q92934 6311 P01040 P62837 P06737 8975 245972 Q99570 P39656 23524 672 673 P15692 6304 6305 128308 10213 158135 P06744 P05413 8985 O43488 23530 3297 23536 Q6PJP8 Q99558 10229 688 Q15070 689 Q86T82 P62847 P62841 P61513 Q15078 Q9BQ15 23509 P13010 Q15084 Q99543 P62857 O14920 P62851 P56182 P56181 57727 P56180 P62854 O43463 Q9BQ04 Q92903 23517 6341 Q92901 Q92905 Q8NHX1 P35268 23512 Q7L7X3 O75031 10208 O14936 O14939 Q56UN5 P60201 10202 Q92900 P56192 P36507 Q14686 P37837 P19174 Q9BZ95 Q9UPN6 Q86TM6 64834 Q9H0U6 P12532 O75150 Q9H0U3 84447 P46783 P49810 P83731 5018 Q14690 Q14694 Q13363 Q14693 Q9HAV7 5034 P46782 5036 P46781 P11216 79084 P41091 Q9Y2H1 60489 P68104 60488 P00533 Q13370 Q16401 P46777 Q9HAU5 P46776 Q9HAU4 P46779 P24522 P46778 10193 P56134 Q9UPP1 P32121 51540 10197 P00568 51548 51547 Q9BYU1 9410 55907 P62826 O43542 O75116 6389 P62829 P00558 Q13393 9421 P78347 9420 O75106 P32189 P36542 26512 6390 Q9Y2C2 23481 51567 Q9BQK8 10181 55929 Q9HAZ1 26524 26523 5071 P49841 P49848 Q15118 P78368 P49840 P11229 9448 10152 23463 A1XKG3 Q9BYZ2 P48507

P24539 51585 P24534 P78371 10155 Q9BQI3 9439 2805 Q15126 Q9HB90 P78356 23476 5091 23478 P37840 5092 5093 Q96EU6 O43505 Q9H173 51593 Q9H9G7 Q92187 P49821 79901 Q9NTJ5 P78362 10166 54623 55959 Q9HAW8 9465 P53004 9463 Q6P1J9 P09769 P29375 P29374 Q96EL2 P29372 27005 79012 Q9H0M0 27000 P42345 P22735 2821 2820 P04083 P31689 9477 9474 9475 O15496 Q9H0L4 55054 1503 Q9BYN8 79005 Q6P1K2 P42338 P42336 P29353 Q5JXB2 P29350 Q9H0K1 Q9BYM8 79035 55066 79039 Q8TBF4 P04062 P04066 Q9Y2A9 80020 P54368 P78316 55072 P78317 Q8N8D1 Q9Y2B5 201595 Q04446 P53041 P07101 P52597 Q96EP0 Q13315 Q9P015 Q96EP1 P00505 Q9H9B1 2869 1537 Q14653 55093 64801 Q9H0P0 P46736 Q9H9A7 2875 1540 124739 5901 P46734 P13807 Q6P1L8 O75964 P13804 O75962 P28065 26576 Q9P035 Q00059 Q9BQA5 2885 P43694 O15455 Q9BQA1 5914 O95544 Q13347 Q14676 O75953 P28074 P28072 79065 P28070 P42357 5927 5928 P63279 P00519 5929 P63272 Q9HB03 P43681 P08473 51514 P43686 Q14684 Q14683 P10914 O95677 Q92858 23607 P67809 P52790 Q9NRW4 P30520 P13196 5939 1576 P16220 5931 P49137 5935 P0CG13 P09936 O95685 O15530 Q9NS91 Q9NRX2 Q8IZP0 Q9H832 Q07343 P50148 P18887 P16234 55215 P30519 P67812 O95696 Q92835 Q9NRY2 Q9NRY4 Q99471 P18858 P18859 125972 O15533 Q92830 P62136 4627 Q92831 P55854 Q92824 P46087 P09917 5970 Q9H814 Q99460 Q9NRZ9 Q99459 3304 P17540 P62140 55236 3301 3309 P04233 Q92820 P30566 P34925 O00469 P07307 133584 5981 5983 3320 P52756 O00468 27165 Q96DE5 3313 3312 P09972 5976 27161 P30556 O00472 Q6PIW4 Q9NS56 3329 3326 3324 P08651 Q6XQN6 Q6ZWT7 4659 Q9UNX3 4670 P28223 137964 4673 O00483 2011 27148 O00487 P30542 P17568 80222 3337 Q96DC9 Q92878 Q9UNY4 P08621 Q15800 P51451 P09958 P30536 2023 P52788 P30530 P52789 740 Q96LR5 P37173 Q99496 Q99497 P09960 157570 4677 4678 O00425 P23396 4695 Q5VYS8 2034 2033 Q00796 P62993 2026 4686 Q16236 P62995 Q96LI5 Q9C0K0 P25100 P46020 2043 Q9NRP7 54764 4697 4698 768 P46019 P01100 Q8N726 P14416 O00444 P11388 P11387 O60942 773 P01137 P01135 P01133 P02461 P01130 Q9H7H0 6421 2065 3396 2064 780 Q9NRR6 Q9NS37 Q8IZE3 P09086 P02458 57819 2070 Q5TKA1 6430 6432 6431 6434 7764 Q92817 Q9UNN5 O60925 Q8IZL8 O75190 Q8IZL9 6427 10331 6426 2081 23658 P46063 5111 23650 Q5HYA8 P18850 Q99437 P18847 P18848 P83881 P27797 P50990 2091 P01185 P50991 P22087 91754 Q8TA86 Q9UNP9 2099 O60907 O75164 23621 P50993 10318 Q9NRM7 91746 P62979 Q9UNQ2 A2RTX5 Q5T1M5 6464 391356 P39748 O00411 146057 P52701 Q5U4P2 P58012 P62988 5127 Q8N752 P25116 6472 Q13237 5141 5142 Q14566 64951 P62917 O43678 P13984 6470 64949 P62910 P09012 5139 P62913 Q13243 Q13242 O43683 Q13247 P20290 O43687 64960 O43684 64963 64965 P13995 64969 80198 51650 51651 51654 51657 5162 Q9BY50 P11309 Q6Y2X3 Q13257 9525 P62937 P11308 64975 5160 P49916 P49915 5159 P61604 P83876 P49914 P21589 Q13263 9531 O43660 Q13268 9530 Q9BY44 9533 Q9BY41 5170 P34896 P11310 Q02543 P09001 9526 P62945 P49903 P62942 5184 10291 9541 P48651 2909 160287 P12694 121512 P27708 Q8NI36 2908 P48643 Q13287 Q9NSE4 Q13286 5198 Q09161 P15735 Q8NI27 Q8TB22 O75208 8237 10273 Q9HA82 Q53TN4 P49959 P50914 2926 P09038 Q15008 Q9BY84 Q9BXW4 Q15005 10283 P62906 9578 116138 8241 2932 2931 255488 221264 10286 10285 Q15014 2935 P78424 P08559 9588 Q12788 92105 Q15819 P62081 1613 80124 Q96DI7 Q9BXM7 79139 Q8TAF3 Q12792 1616 P07237 8266 Q12797 P55795 O95602 Q9H7Z7 55173 P67775 Q6IN84 29796 P08574 P08575 1629 2959 P08579 2956 P07205 Q15831 P29474 P29475 Q96M96 80146 1634 80145 27102 2961 2960 P04183 O95619 Q8TAD8 2969 P09874 Q15843 Q6R6M4 55192 64900 29777 79143 1642 Q05655 2972 Q9BXJ9 P09884 1647 Q15853 Q9BY32 Q14527 P13945 1660 Q86SG2 2987 1655 1653 Q86SG6 1650 Q13200 1659 O95644 150094 51606 Q13202 P21580 Q8N7H5 1666 64919 80155 1665 Q9BXP5 51611 Q13216 93492 P55769 4705 4707 1676 P08581 Q8NHH1 4701 4702 P41159 51629 Q14558 O95661 4718 4719 389840 4710 4713 Q13233 4714 Q14562 4715 Q9H6Q4 Q96L12 Q9NQU5

81605 P52434 O00148 4728 4729 Q8IYN9 P84022 Q70EL4 4720 4722 4723 4725 4726 Q9C026 Q8NFZ0 Q92729 O00154 P53779 P38117 P14174 4731 10420 4734 10421 64282 Q7Z727 23708 81629 3420 O00167 P56817 3417 3416 81631 P40429 811 Q8N684 Q5TAP6 P27448 3419 Q9C005 P07858 P99999 Q9NR96 Q676U5 Q8IYK4 P20823 Q8NFW8 Q5TAQ9 55352 O60674 P20827 P19838 821 3421 Q9NZJ4 P41743 P07864 P11926 Q13951 Q13956 4772 2110 Q5SGD2 55361 O95352 2108 2107 Q5T160 Q6PI48 80324 Q9BWG6 833 Q4G176 O14744 4780 Q92769 P09619 80308 O95361 P10600 O95363 Q7LGA3 27297 P61353 167227 841 4775 847 O14757 Q92766 P09622 4790 Q13976 2132 Q96L34 O95376 Q9NR45 P18545 Q9NR48 Q9BWE0 Q05469 2125 80347 P60033 857 859 Q96CA5 P11908 Q9UMW8 Q9H6R4 6500 Q9NR50 O95382 Q96L21 Q9NQT4 Q9NR55 Q9NQT5 P19878 Q4G0W2 2139 862 O00141 80335 865 867 4799 P09601 P06576 P00390 10492 O95391 3481 O60603 Q96T88 3479 2147 Q13085 7832 Q96KG9 P25685 P23025 Q6UWV6 P51991 Q96T76 O14717 O43290 Q7Z6J9 P61313 P49643 O43294 O43293 O43252 Q6ZMR3 Q6ZN33 O43251 P49642 64215 P13686 Q9BW92 Q9BW91 891 29088 O14727 126789 Q8IYD1 Q96T60 Q9NR09 Q16134 Q8IYD8 10477 Q9UN42 29086 10476 29085 P61326 Q9UMS4 5210 7874 5213 Q658W2 114799 Q01974 Q96ST3 29093 O14730 7867 10488 O14733 5207 7884 P50613 P51946 23764 2194 P60891 10450 10455 Q01968 O43242 P51955 P51956 29079 Q96SZ6 23770 5226 P48357 29074 Q7LFX5 23746 P24386 5245 10438 Q5TAL4 Q00613 Q00610 10432 5250 P51970 Q13901 P07814 P49674 Q96C86 55312 56647 10445 Q13115 6598 P10599 57062 O43318 Q01130 Q9C0C7 P33240 9616 Q9C0C2 P38919 Q13126 P13639 8301 P13637 P58546 51773 Q9NRH2 24144 Q15796 P60866 Q13131 9641 Q15797 9646 9643 Q9UNL4 Q6UXN9 29803 Q01105 O94804 Q13144 54815 Q13148 5295 P35869 57092 9656 P43490 P10586 Q13151 54822 Q49AN0 O94817 Q13153 P54136 O15287 P54132 P63092 Q3LXA3 Q13162 Q8IZD4 Q14494 Q14493 Q14498 Q13164 O15294 Q9NRC8 7013 7014 P13674 P11021 50487 Q9NRD1 Q13177 Q9NRD5 10390 9682 54853 P47897 1718 P00367 Q9UEW8 P47895 1716 Q13188 Q9BWU1 9695 P21283 P21281 Q09028 Q9UNI6 O75792 26747 Q9NZV6 Q00688 Q6PID4 1728 Q7Z7F7 P00352 Q9NRF9 7046 P35813 Q96L91 P10515 27248 Q9H6Y2 27246 O15234 1738 P05177 1737 1736 P54105 375743 7057 O75771 Q9H792 55294 7052 27252 1743 27257 P05181 Q9UER7 8399 8396 7068 7067 Q5TAX3 Q15714 O15264 Q15717 Q8IZ69 8394 7064 Q9H6W3 1756 80267 29880 Q92793 Q6ZNC8 Q7Z6Z7 O15270 Q8IYT8 Q9UNE7 57017 Q9BWH6 653361 P43405 1763 29896 Q04206 29894 O15269 Q9UET6 P05164 O75746 Q5GLZ8 Q8N6G5 7083 4809 60673 51720 P08243 4800 51726 7099 7098 51727 51729 P14927 30849 Q15746 P14920 P30153 1789 1788 1786 1785 Q05513 57038 80270 375790 Q15751 80273 51734 Q15759 29843 P17174 84661 4820 O15212 P05198 Q14432 51747 O75716 30827 Q6XPS3 Q13107 Q06830 P22528 4839 P47804 4830 51752 902 4833 P08237 Q13112 P08238 Q9NZN9 O15228 O95433 O75821 O75822 Q969L4 O00268 Q8IXM3 51081 O00267 3516 10535 Q8IXL7 O00273 P60174 Q9ULW3 P63208 4841 90678 Q6NZY4 Q92614 P07996 10533 Q92611 P52564 4860 Q9NQ55 51094 55454 51096 10549 51099 Q9ULX9 Q08426 29128 Q05397 P18621 P63220 55466 O60783 P32929 26091 Q6IT96 4882 3551 Q13829 152926 10524 10528 10521 948 10523 O95478 O95476 O95477 O95470 O60762 2224 O60760 29102 P04040 P84103 58517 O00238 P04049 Q96SB4 Q96SB3 Q9NYA1 O00231 O00233 P10721 O00232 Q5JTZ9 Q8IY17 Q96SB8 Q8WYH8 29110 2237 79444 Q92643 4899 P05386 P05387 P05388 Q9NYB0 P30304 P30305 P32969 P30307 Q9NYB9 6609 2247 975 Q6GMV3 Q92630 Q92636 Q969M7 161823 P29323 Q92626 P29320 Q13868 O00255 81788 Q99259 984 6612 988 Q92620 6613 P29317 26061 Q9Y6W6 O43395 P07900 5300 6631 P53804 Q9BV90 P53803 O60725 P15531 990 P11172 991 P62750 Q96RU7 Q96RU2 O14829 994 995 997 6622 6625 P61421 6627 51009 P62753 Q04917 P07910 P49770 200916 Q9UM07 2287 2286 P07919 P11177 2280 Q93096 51010 Q96S53 P11182 51013 6633 Q9Y6Y0 O14830 5305 6637 P49768 O00206 Q9BV79 5321 P49761 P49760 O60701 51021 51023 Q00535

Q8IXB1 Q00534 10594 P49756 P25789 P25788 6647 5315 6646 10598 91949 P48426 P25787 O00217 6660 5333 O00213 Q96B26 Q96RR4 Q96RR1 P61457 Q00526 5327 Q9ULR0 O43390 O43353 Q9H5K3 P07947 P07949 Q969H0 P07948 5335 5336 10574 P62714 5337 5338 4008 P07954 5351 Q9H5J4 5352 5356 Q9ULK4 Q969G6 Q01831 78991 Q9NPH5 4015 5347 4017 Q6UVY6 Q96JH7 Q08462 P50750 4035 O14802 Q8NER5 51067 P68036 9716 Q68J44 10554 Q9H5H4 Q01826 P49792 5371 P27540 9733 5378 P15559 Q969E8 P16885 10569 54101 55432 51074 O14818 Q9NPJ6 6699 54107 54106 Q01813 O43432 57187 29947 Q9NYU1 Q9NYU2 29941 P22695 Q7Z6C1 83475 P22694 O94905 8408 O94906 5393 5394 8424 4067 63875 29959 Q6UX04 128 P00414 P47985 P60983 54921 O94913 Q13011 P22681 Q76FK4 29922 P35998 Q9UDY4 P25705 115024 P42285 O94925 8428 P22674 54931 54938 Q8WZ42 Q14353 P00441 8443 4086 4087 8446 4088 P22692 9775 8445 142 1808 8439 P62701 P20020 Q8IXZ2 53615 1801 8453 8454 P54259 9785 8452 Q6ZN16 8458 7124 P54252 P54253 7126 Q7Z6G3 Q96KC8 8450 4092 4093 4094 Q00597 Q9NZ20 156 158 Q8IYB3 Q13042 8449 Q13043 P49736 Q13049 7132 P04843 P04844 Q96KB5 8467 Q9BW60 1822 P11142 Q8NFM5 54961 7128 P49721 P49720 Q13057 8473 P00491 P00492 O94966 P54277 P54274 7141 Q96T21 1832 Q96T23 178 Q00577 P49711 P49716 P49715 Q49A26 Q9NQC7 7153 7155 7158 Q9BVS5 7157 7150 1843 1841 1848 Q6VAB6 P21399 92345 Q13873 7167 Q96K76 Q86Z02 7161 1854 P42224 1852 P47914 1850 P63165 O15355 Q92696 P09651 Q969T7 1859 P22612 P42229 Q13882 Q969T4 1856 O15371 O15372 Q969S8 Q13887 Q01082 O75891 84708 P22626 P84098 P63173 P09661 P60900 Q969S2 7186 7189 P35916 27348 P11940 26015 O75881 27347 O75880 26019 7184 1877 4907 P17252 1870 O15379 P23921 Q9NZ08 O75874 1891 P10620 Q6UWE0 4914 Q9NYP7 Q969Q1 4913 Q969Q0 P33316 Q9NYH9 57144 Q93100 58478 1892 Q8WYQ5 4920 Q8NFF5 Q8IY92 P35968 O95400 Q8WZ19 Q9NQ92 P42261 57154 26007 Q96BI3 84749 27339 27330 58487 27332 26002 Q15631 P21333 Q8IY81 P09669 Q8IY84 O75844 Q5W0Q7 377630 P84090 4947 3615 3614 Q5RKV6 3611 Q5XUX0 57169 P00403 P01730 4946 O15350 P51178 Q15643 O75832 O95427 Q14318 Q15648 27315 Q86YP4 Q9BVJ6 P30260 O15347 4952 O95429 4953 Q15653

cellular protein metabolic process 8099 Q9H0E3 Q9Y265 P28562 1459 1457 55561 55568 Q5QP82 1454 O00391 1453 Q8N465 Q12834 Q8NE71 10667 2 27090 Q9H0C8 Q9Y285 140801 Q9UKV5 P10809 Q96EB6 Q5S007 Q9UGN5 Q9UKV8 Q8NE63 Q06124 A0AVT1 P62495 P37023 2324 57761 Q9Y297 O75928 Q9Y291 Q12851 O75914 3672 P26373 Q13705 P21917 1491 10645 2335 1487 Q9BYD1 148789 O14578 10643 Q9UL15 Q8WXG6 P25098 P51784 1022 Q96MX6 1021 1020 Q06587 10613 57787 1018 1017 79577 10616 O60884 Q9Y230 Q9Y231 Q92530 26191 5859 Q9UGI0 3692 5870 Q13724 Q9H4P4 5871 Q96J02 Q08752 10626 O14519 Q8TBC4 Q96MV8 1025 Q9BUB5 6711 P07686 6714 O95136 Q9Y243 79595 Q8TBB1 P38398 391627 P20618 613 Q92995 Q5TCX8 5875 Q92993 4548 Q9UL46 P30414 O95147 5894 P31749 Q38SD2 Q8IWL3 55152 253980 57332 1047 55159 6733 Q9Y262 5886 5887 8916 Q9UGL1 Q9UL54 Q8TBA6 64432 O00308 P56199 O00303 P50897 23569 11107 Q15022 P62877 8924 Q15025 Q9Y676 P23284 O96017 P49411 Q86SR1 O96019 P36578 O95163 Q9NWZ3 P57059 115426 1072 O96013 51132 Q9Y680 P62888 84033 11113 P27635 P49407 P60228 10269 Q15034 O00329 84061 6772 P35236 10238 O14967 P11274 8945 P62899 Q14197 51147 Q15046 O14965 Q8TAT6 Q8N3J5 Q9Y697 23552 3276 23556 P35227 P35232 Q9H093 6776 285193 O14974 P62424 6790 Q5VVQ6 6793 P01040 P62837 8975 6794 4133 10690 9820 Q99570 P39656 O00743 Q9NWU5 Q8IWF2 672 673 6304 4125 6305 6789 128308 10213 158135 Q07020 5470 4140 8985 3297 O00754 Q99558 Q9NWV8 Q86T82 207 4139 P62847 P62841 P61513 Q15078 5481 23509 Q9Y5S2 Q08345 O00762 51185 Q15084 Q99543 5478 5479 P62857 O14920 P62851 57727 P56180 P62854 Q9H4F1 5494 Q92901 5495 Q9BU89 Q92905 5499 Q8NHX1 P35268 23512 Q7L7X3 10208 O14936 Q96QT4 Q56UN5 P56192 P49427 P36507 11060 11065 7204 Q9BZ95 Q86TM6 Q9H0U6 O75150

Q9H0U3 Q6P5Z2 23043 84447 P46783 P49810 P83731 Q14694 Q13363 Q9HAV7 11072 5034 4188
P46782 P46781 P41091 Q9Y2H1 P68104 8536 P00533 Q16401 Q96RL1 P61956 P46777 197259 P46776
Q9HAU4 P46779 Q9BUN8 P46778 10193 Q9UPP1 8555 P32121 23028 Q96IZ7 4193 10197 P61962 1915
P61960 51548 Q93009 P22314 51547 Q93008 Q14232 P61964 Q9Y6R4 8565 O75116 P62829 Q86Y37
Q8N4C8 267 23031 23032 Q9NPF5 8576 26994 O75582 P10398 Q9Y2C2 8569 1937 1936 1933 55929
Q9BV47 Q9HAZ1 23014 26524 5071 7251 11035 P49841 P02751 P61927 Q15118 P78368 7266 P49840
P11229 O75569 9448 7267 10152 23463 Q9Y6M4 A1XKG3 Q8WY64 Q8NEJ0 P24534 P78371 P08069
10155 Q9BQI3 51107 1956 O15075 Q9NPA8 Q15126 23476 Q86Y79 23478 7272 1965 Q9H173 114088
Q93034 Q92187 P78362 54623 51116 1969 Q14289 1968 9463 Q6P1J9 P09769 Q96EL2 27005 P12931
Q70CQ3 79012 Q9H0M0 Q70CQ1 1975 27000 P42345 P22735 P04083 Q6P5R6 Q58WW2 7297 P31689
P56524 9474 9475 Q92560 P42338 P42336 O75528 Q5D0E6 P06239 P29353 Q5JXB2 P29350 Q9H0K1
P56537 Q9BYM8 26133 P06241 P21860 Q9Y2A9 80020 O95983 Q16816 91147 55072 63931 P78317
201595 Q9Y6J8 P53041 P07585 Q92542 Q16825 Q96EP0 Q16827 O14595 Q13315 Q9P015 Q16828
Q96EP1 9020 23081 23085 Q9H9B1 P20594 2869 Q8IWV7 55093 Q86Y13 P61081 Q9H596 P46736
P61073 83548 23097 P61077 1540 124739 P46734 11080 Q9NXH8 9045 Q9NP81 Q16849 Q6P1L8
O75962 P28065 26576 Q9P035 Q9Y6E7 Q9Y6E0 P61088 Q96RG2 O15455 5914 Q13347 11091 O75953
Q16854 P28074 P28072 9054 P28070 P63279 P00519 5929 P24941 11099 51514 P43686 23607 65003
O14672 Q9NRW4 O60568 O60566 P15374 Q8IVH8 P16220 P49137 P09936 O15530 Q9NRX2 P27361
Q9GZZ9 Q8IZP0 65018 Q9H832 54361 P50148 P16234 10783 92609 P67812 P52333 O95696 P29992
1121 Q99471 P53667 1111 125972 Q86WA8 Q92830 P61289 P62136 4627 Q92831 P55854 P49591
Q92824 3791 5970 Q8NCR0 9093 Q9UBE8 Q99460 P56705 P49590 9099 10767 3304 P62140 57410
55236 O60551 3301 P49588 10765 3309 P04233 P34925 O00469 P05129 133584 P11802 P50579 3320
10733 147746 Q96DE5 3313 3312 27161 Q7KZI7 Q7Z419 Q9Y4X5 P19784 P53611 Q9NS56 3329 10746
3326 1147 3324 2475 5500 4659 Q9UNX3 P26045 P53621 O95257 6850 Q13615 2011 Q13618 27148
Q13617 Q13616 1161 O00487 P30542 Q9P2P5 80222 57448 3337 P61247 Q96DC9 5515 Q9UK22 65061
5516 5518 P51451 P09958 Q13620 Q13627 P30530 P18433 740 Q96LR5 P61254 P37173 Q99496 10728
4677 O14656 P40337 Q9UK32 O14657 Q8N2K1 O00425 O95278 P23396 6872 Q96HA8 6871 25862
2033 Q9NVW2 O95271 11222 5536 5537 6883 6885 1198 25873 23210 1196 2043 1195 56940 Q96Q15
Q9NRP7 Q99873 54764 55611 51253 Q9P2K8 4214 11231 4215 4216 P46019 25847 5562 5563 O00444
O60942 51264 Q53X93 Q9GZP9 Q9Y572 P01137 P01135 P01133 Q9BT22 P02461 P01130 Q9H7H0
P04626 5575 P04629 2065 25853 3396 2064 780 301 P17936 Q8IZE3 9924 5566 Q96PN8 5567 5568
5580 5581 5582 5583 5584 5585 5586 Q92817 Q9UNN5 25822 O60925 O76031 Q96PU4 79612 O75190
Q8IZL9 Q96PU5 5578 5579 10331 Q9H3F6 8607 2081 5591 9950 Q6IR47 5596 5597 5598 P50502
Q5HYA8 O76024 P18850 O76021 5590 P83881 329 84197 P27797 P50990 Q9GZU7 P50991 91754
Q9UNP9 O60907 O75164 23621 10318 55666 Q9NRM7 5599 P62979 P83436 A2RTX5 4285 Q5T1M5
6464 7311 391356 Q96Q40 P17980 146057 Q5U4P2 221656 Q08209 P62988 5127 Q8N752 P25116
Q13237 4297 Q6DKI1 Q6ZT98 Q15418 64951 7325 P62917 351 Q9UBS0 Q04759 Q9UBS4 P62910
P62913 9978 P21127 O43683 8662 7332 64960 7334 16 8667 7336 O43684 8665 7335 19 64963 64965
Q8TF76 Q9UKE5 64969 Q9H3Z4 Q9UBT2 23170 51650 369 51651 7329 7328 51657 7341 Q9BY50
P11309 11160 Q6Y2X3 Q13257 P62937 P11308 7347 6015 P29074 64975 Q9UBU3 Q03405 5159 8669
Q9UBU8 P61604 P43378 Q13263 9531 9530 7353 Q9BY44 Q9BY41 P51812 P51813 5170 Q9UBV7
Q02543 P51817 11176 P09001 9526 P62945 P49903 P62942 P55345 O15164 121512 P27708 P48643
Q9NSE4 52 Q13286 Q14139 6045 7375 6048 23133 23135 P15735 Q9UKA1 Q99956 Q8TEY7 8237 10273
Q9Y5K6 Q9Y5K5 Q9UKB1 Q99942 P50914 P01579 Q14152 11124 Q9UBQ5 P09038 Q15008 Q9BY84

Q15005 O75676 O15197 10283 O15198 P62906 9578 O75674 P14868 25778 2932 2931 255488 51231 Q14164 86 2935 Q14166 9100 Q15819 P62081 Q8TF05 1613 Q05209 80124 Q9BXM7 79139 Q8TAF3 91 Q8WWI1 94 Q12792 P45974 1616 P07237 26270 P82921 8266 Q9UBK9 P82912 Q12797 P82914 Q6DT37 Q9H3R0 P31321 55173 P67775 P08575 P05067 Q9UBL3 O94763 Q15831 O94768 Q6XUX3 Q9H422 Q96M96 6093 1634 27102 Q8TEL6 O95619 P21980 P09874 Q15843 Q6R6M4 55192 Q02156 26260 26262 1642 Q05655 Q9BXJ9 Q9UBN7 P82932 P82933 Q9UBF6 9149 56052 Q6NYC1 Q9H488 P11766 26232 Q86SG6 Q9UK80 P30050 Q03468 26234 1650 Q13200 150094 Q13202 Q8IVT5 P21580 9150 57396 Q8N7H5 80155 Q9UJX2 Q9Y5B0 51611 P08138 O75604 P10415 Q13216 93492 P08581 Q8NHH1 Q5T6F0 728642 P08107 O15111 Q04771 389840 O15105 P45983 Q13233 P45984 65125 P54619 Q96L12 Q9NQU5 9184 Q8IYN9 Q70EL4 Q6IQ55 Q9H2H8 Q9C026 Q92729 Q5VTR2 P53779 P62244 80755 P62241 10420 4734 P62249 Q9H2G2 Q7Z727 P28300 23708 81629 O00167 P56817 2571 79791 54496 Q8IUD2 3416 P62256 57520 P40429 811 6921 P27448 Q9C005 P08708 Q9NR96 P54646 P62266 Q5TAQ9 55352 O60674 P20827 P19838 P62263 821 5602 Q96GD4 P62269 5604 Q9NZJ4 P41743 Q9Y3U8 Q13956 Q9Y463 Q5SGD2 2590 O95352 P39019 2107 Q5T160 P15056 10856 2589 Q6PI48 P39023 833 5610 5613 O14744 Q9UIM3 P30679 Q92769 P09619 80308 O95361 P10600 O95363 10868 10869 P61353 841 O14757 4790 Q9H2K2 Q13976 P21709 Q96L34 O95376 P18545 Q8IUH5 169436 Q05469 2125 P60033 Q96CA5 Q10472 Q10471 Q13501 Q9UMW8 Q5MAI5 6500 Q9NR50 O95382 Q96L21 Q4G0W2 2139 10845 O00141 80335 867 23326 O60603 23327 2147 7832 10013 84289 Q96KG9 10014 P25685 64682 Q9UII4 Q9BS26 Q6FGD7 51377 Q99750 P61313 Q9BS18 Q99759 O43294 408 O43293 409 P60468 23307 Q6ZN33 Q09472 5682 5683 5684 5685 5686 64215 Q9BW92 891 29088 Q8IYD1 55743 Q9NR09 Q9Y450 10477 29086 Q9UMS4 5692 5693 P40818 7874 3035 Q658W2 P14543 Q01974 P15880 P60484 Q15291 29093 5687 5688 55757 O14730 7867 O14733 8732 O00506 P50613 54431 10450 Q9H2D6 79739 P55072 Q96GC5 O43242 140609 Q9HCE7 P51955 P51956 Q05086 Q99797 444 23770 8737 Q02809 P82675 P61758 29074 284086 P49207 23746 P24386 8754 5245 Q9HCD5 P52815 3066 3065 Q9UIF9 O75293 55781 Q00613 7415 10432 8761 P07814 P49674 Q9UIG0 54460 3074 P17612 7428 10445 Q13115 53938 Q9Y4K4 P11441 O75385 Q9Y4K0 Q86VQ3 Q9Y4K3 O43318 472 P48729 28973 9616 8767 3093 Q15303 P13639 P55036 6123 6125 Q15306 Q9Y4L5 Q86VP6 8780 Q504Q3 23291 Q9NRH2 Q15796 P60866 Q13131 9641 6133 6132 Q15797 Q16644 6134 9646 7465 6137 Q9UNL4 Q6UXN9 O43776 O75365 O94804 6128 P82663 P82664 Q13144 Q8NCB2 Q16659 6141 57092 P10586 P18077 O75352 Q96PF1 Q9P2E3 6138 54822 Q49AN0 Q9P2E9 Q13153 51319 P54136 6156 6158 O75347 Q13162 P23467 P27824 Q13164 Q14012 6164 Q15349 O15294 Q9NRC8 Q8TDX7 6168 11274 P36776 6160 P13674 P11021 Q7L2H7 Q9NRD1 Q13177 Q9NRD5 6175 9201 Q3ZCQ8 9682 Q08188 Q8WW22 Q02878 Q9NVM4 Q96PK6 P55010 Q9NVM6 P47897 Q9UEW8 1716 Q13188 P23443 O75319 9212 6185 6188 Q15369 Q9BWU1 6187 9695 6182 Q9UNI6 6184 Q9NZV6 Q00688 Q6PID4 Q99816 P48730 P23458 P48736 Q7Z7F7 Q8WW12 Q15375 6196 P54577 6199 7046 6198 P35813 Q96L91 O95714 27248 6193 6195 6194 83752 Q9Y4C1 P41252 27246 P62195 P41250 O60285 Q13523 375743 P78536 7057 Q13526 Q9H2P9 83737 Q9H792 55294 7052 27252 P46934 P41240 O60296 Q4LE28 Q9UER7 P34947 P29597 Q13535 O15264 P29590 Q7Z2Q5 Q9H6W3 80267 29880 Q9Y4E8 Q92793 Q7Z6Z7 3932 O60260 Q9HCP0 9255 Q13547 Q13546 Q8IYT8 9252 Q9UNE7 P43405 P20309 Q04206 P41227 Q13555 Q13554 Q5GLZ8 O95757 Q13557 P78509 9261 P00747 51720 51726 7099 7098 30849 Q15746 P30153 Q05513 P00734 57038 P24723 Q15751 80273 51734 P46977 P55884 Q15759 Q8IUQ4 28998 29843 P78527 O60229 P41279 1314 Q04656 84661 4820 O15212 P05198 O75716 30827 Q6XPS3 Q13107 P18031 Q9P286 P62191 P22528 1326 Q63HQ2 P47804 51752 P20340 P00750 Q9HD40 P08238 P21675 Q9NZN9 P48052 O95433 O75821 O75822 Q8IXM3 51081 Q8IXL7 Q8NB16 P63208

90678 P07996 10533 Q92611 P52564 Q9Y399 5706 57634 5707 10549 79885 5708 5709 P21810 P16471 5700 P49368 5701 5702 Q9UHP3 5704 5705 65264 P54753 P25398 Q9BZG8 Q05397 P54756 P18621 O60307 5717 1358 57646 83933 P12081 P63220 55466 O60783 P32929 5713 26091 Q6IT96 Q9Y2Z4 5716 P54764 P54762 P54760 Q12974 4882 3551 Q13829 152926 5728 10524 Q8WTX9 253827 Q9UI30 Q6PCD5 O95476 O95477 P28482 1385 Q9UHJ9 P51668 P51665 Q9P0J1 P19525 O60762 P51671 O00238 P04049 Q96SB4 O00231 O00233 P10721 O00232 Q5JTZ9 Q5VST9 Q8WYH8 10987 29110 10988 79444 1387 Q92643 P05386 P05387 5747 P05388 P30304 P30305 P32969 P30307 Q9NYB9 Q9P0L2 P42766 10956 P62333 2247 Q9BZK7 975 22929 Q6GMV3 Q92630 P61009 P08865 Q969M7 P29323 5770 P29320 P54727 P54725 Q03164 81788 Q99259 Q9P0M9 57695 984 6612 Q9Y385 6613 P29317 5768 P06213 Q15139 Q9Y6W6 5780 P07900 Q9UPV9 5783 5300 P53804 P38646 O60725 991 P62750 Q96RU7 Q96RU2 O14829 994 995 Q99640 997 5775 79813 5778 51009 P62753 P49770 200916 Q9UM07 Q9UQ88 5796 2287 2286 2280 Q93096 Q8WTR2 Q96S53 O14830 P49768 O00206 Q96EZ8 P26641 P26640 P49761 P49760 51021 51023 Q00535 P26639 Q8IXB1 Q00534 79834 P25789 55869 P25788 5798 6647 10598 91949 P25787 P01375 23438 Q9Y2R5 Q9Y2R2 8841 O00213 Q9Y2R9 Q96RR4 O95071 545 Q96EY1 Q00526 5327 O43353 Q9H5K3 P07947 23409 8851 P07949 Q969H0 P07948 Q12904 Q9Y2K6 Q9Y2K2 Q99683 Q9BZE1 10574 8844 Q15185 P49336 P62714 4008 P05771 5351 5352 6201 6202 23411 Q00987 4015 Q9H1A4 5347 4017 Q96JH7 6210 P50750 Q9UHD2 Q12923 Q9UPT9 Q9Y2M5 573 Q8NER5 51067 P68036 6204 6207 Q68J44 Q01826 P49792 5371 8881 Q12933 Q12931 580 54101 55432 O14818 P51617 6699 54106 8878 6217 10090 6231 8892 6233 P15924 Q9NYU1 Q9NYU2 29941 Q7Z6C1 83475 6229 P22694 O94905 8408 Q9HC98 Q16512 4067 Q16513 Q16514 63875 29959 Q6UX04 128 6238 P60983 Q13490 P22681 10075 Q9HBW0 Q16526 23387 P31152 P35998 Q9UDY4 Q9NV06 P23528 8428 Q16531 Q8WUK0 54938 Q8WZ42 P00441 Q16539 4086 344387 4087 Q6PD62 8446 O75478 Q15208 8445 O75477 23399 Q8TD19 64745 Q02790 142 P62701 10087 6259 Q8WUJ0 53615 1801 8453 8454 8452 Q6ZN16 64750 7124 7126 Q96KC8 P14635 Q9UQL6 8450 4092 P10276 4093 P35579 O75460 156 Q9Y3E5 10055 P23588 Q13042 Q99728 Q13043 Q13049 P04843 P04844 Q96KB5 P36896 P36894 P11142 Q02763 54961 120892 7128 P49721 P49720 8473 P61803 O94966 Q96FX2 P36888 Q96T21 1832 Q9H1Y0 51465 O95817 O95816 Q99707 O95819 55827 Q9NQC7 Q96FW1 P14625 Q02750 1843 55832 1848 Q6VAB6 Q15256 Q13873 O95835 Q96K76 Q86Z02 P18124 P42224 1852 10933 P47914 1850 P63165 O95837 O15355 Q92696 1859 P22612 P42229 Q13882 Q9BZQ6 Q969T4 O15371 O15372 22926 Q13405 Q969S8 Q13404 Q8TCJ0 Q8TCJ2 Q01082 84708 Q9Y3B7 P84098 P63173 57599 P60900 7186 7189 P35916 27348 27347 Q13418 9360 9361 7184 Q9UHY1 Q9Y3C5 P17252 P42680 O15379 P42681 Q9NZ08 P42684 P42685 Q9NUG6 Q86UE8 Q13427 Q6UWE0 Q8NB78 4914 P42677 P68400 Q9Y3D3 Q9Y3D5 1408 1407 Q969Q1 Q969Q0 Q9HC62 P62280 57144 P62277 4920 Q8NFF5 Q9HC52 9397 P35968 Q13443 Q8WZ19 Q9NQ92 57154 Q96BI3 Q9H1R2 8065 84749 27339 27330 Q13451 8078 Q8IY84 O75844 Q5W0Q7 377630 P19474 59343 3611 1432 Q5XUX0 57169 Q14790 O60341 Q13464 P01730 P53355 O75832 O95427 Q14318 P53350 Q9P0U3 Q9P0U4 27315 26469 P30260 Q7L9L4 O95429 5800 Q9UHV9

organelle organization P25054 Q9H0E3 Q9Y265 3880 Q9Y266 25909 Q12824 8091 259266 9181 P51587 9184 54474 4728 2547 Q8IYN9 55561 1213 Q96IK1 P84022 Q92730 10651 Q8N3U4 6901 A1A4S6 4725 Q92974 O75935 25913 Q9H4L7 25915 P08729 Q12834 P08727 Q5VTR2 Q53EZ4 P55957 Q8IWJ2 P16104 10426 2317 O60216 2316 2 Q8IUE6 P42858 P39210 Q9UKV3 800 Q9H2G9 10420 10661 Q96EB6 5825 64282 P30622 Q7Z727 P27694 Q9H2G4 O00167 Q9NQW6 54496 6927 Q9UKW4 4983 811 5830 P63104 Q9Y6A5 P35080 P26358 Q96EA4 Q9C005 O95347 Q9NQX0 P31947 Q92949 P17661 Q676U5 55355 P16333 P20823 81624 55352 O60674 P62263 O14579 64061 Q96GD4 O14578 5604

P41743 829 Q9Y468 Q92538 4771 Q14807 Q96MX6 10609 1020 Q8TDI0 Q06587 22832 57787 1018 1017 79577 10856 114791 1499 830 55125 Q08999 832 P61587 Q9Y230 P61586 O75909 O14744 Q9UL26 Q12873 Q5TB80 P07437 Q9H0H5 Q92769 3692 P28370 Q15906 O95361 Q9BUB7 Q9NSV4 1029 10626 Q96MV8 Q92522 O60890 604 P07686 Q8N257 5869 P04179 Q15910 Q9UGJ1 266812 Q13976 Q96L34 55142 79595 Q8NA19 Q9NR48 7818 401505 P84243 Q96N67 Q71DI3 Q96KQ7 Q92993 857 5878 Q9H0F6 5879 Q9Y490 O00139 Q13505 Q9Y496 P31749 O95140 Q14839 1050 O60879 10844 27032 2139 Q9P209 253980 P40692 57332 80335 Q96MT8 P04150 Q96ED9 P63313 O14777 50943 P08754 Q9UGL1 Q9UL54 Q9UKT4 Q8TBA6 127829 A6NHR9 Q15019 Q9UKL0 6993 O96008 23326 P50897 23569 11107 Q99523 203068 55722 1058 Q8WV60 2146 Q15022 55726 85378 Q15021 10013 84289 637 5898 55723 10014 4580 6760 P06702 64682 Q9Y678 Q7Z6J4 O96019 23332 3010 O95166 1072 Q9BU61 23336 Q8NHV4 3009 3008 O60610 641 3007 400 3006 1069 P35251 Q8IWA4 11113 648 Q9BS18 408 O43293 P49407 8936 Q01518 10269 11116 O96028 Q09472 5442 3024 O00560 3265 890 Q66K89 891 Q9H4B7 Q99741 55743 P35240 3014 Q14197 51147 29086 Q15047 84062 O14965 O43264 P51531 Q8TAT6 4361 P40818 23310 Q658W2 P35222 Q9NUX5 P51532 P35227 Q96A08 P35226 Q99733 124359 P35232 Q15050 Q15291 Q9H093 P23258 9804 10486 4358 10487 P26583 6790 4131 9821 4133 23523 10459 Q96KK5 Q96P48 Q68DK7 A8K0Z3 10451 6304 8726 Q9H2D6 10452 P05412 P49450 O43482 10460 P51955 P51956 Q5JSP0 O60814 10228 10469 Q6PJP8 Q99797 Q9NWV8 10229 P51959 Q96QV6 54443 Q15070 79723 4137 9826 207 Q15075 4139 7408 Q92922 Q86T65 Q96GA9 Q9Y5S2 5245 P52815 22893 3066 Q9UMN6 3065 Q9UIF9 O00762 22897 25923 Q9NWW5 O14925 P13010 215 Q00610 55785 P61764 P62851 10432 Q99549 O43463 3070 6342 7430 Q9UIG0 Q7Z4H7 O43464 23512 3074 P50402 79980 O14939 Q96QT4 P28749 P40616 Q9H4E5 55559 P40855 O15085 Q14204 6595 6597 8535 6599 11065 6598 Q9BZ95 Q9H0U9 24137 4171 P48729 473 O75150 P07196 23043 Q9H160 Q99807 Q8NCD3 51763 P83731 5018 P43034 Q15785 Q9C0C2 Q9HAV7 P62805 8301 Q16635 Q15306 P62807 Q9ULG1 O75376 Q9UJC3 O75381 Q9NVP2 51773 6117 9869 54801 Q9BSJ2 Q9H9E3 9868 Q16643 Q96RL1 Q15554 P24522 Q29RF7 9646 7465 Q8WVM7 9643 7468 Q9UPP1 P54198 O75367 Q9UNL4 O75122 Q6UXN9 Q9Y2I1 23028 51780 Q6DD88 Q6UVJ0 51542 Q01105 Q96RK4 Q9NRI5 O94805 8548 Q93008 P61964 Q9BSI4 6386 P62826 O75116 Q16658 P68133 O43303 93166 O75351 P04908 Q9NZZ3 Q9Y2J2 54820 Q9NPF5 54822 9648 51317 Q8IX90 P43246 O94817 Q13153 9662 Q8TDY2 9425 O15287 O43752 P54132 Q14008 Q9UNF0 23002 23244 O75582 Q9Y6K1 Q04837 51564 Q9Y4G6 O94826 8328 P46108 Q96CS2 7013 55929 8348 7014 P36776 26524 23492 11034 8338 O15047 Q9GZM8 9685 8110 O15061 Q3ZCQ8 10152 O95931 A1XKG3 291 Q86VS8 23466 Q96EV8 23468 9682 221150 Q9NVM4 P02549 Q96PK6 11243 Q16695 Q13185 8349 Q9NTI5 P02545 Q9NVM9 347733 11004 54617 Q8WW24 9212 Q9NPA8 Q9BX66 64423 11252 P10147 Q09028 64426 Q8N4H5 6182 P37840 P11233 7272 Q9NXR1 2810 Q9NTJ3 Q6PID4 1729 54623 Q99814 P02533 P98194 Q14289 P28288 9221 P28289 Q6P1J9 Q96L91 P29375 Q13515 8379 P29374 27005 Q7Z589 6194 7283 Q4VCS5 Q9Y6G9 83752 Q9Y4C1 Q4LE39 9219 O60282 3911 1736 P56524 9232 9474 9475 O75530 7290 55054 P78537 55294 27252 Q9BSB4 3925 83746 Q9HD26 Q9Y4D1 Q92560 81565 Q4LE28 Q6P1K2 P42574 P98171 P98174 O75528 P83916 P29590 P55209 P56537 Q9H6W3 Q92793 Q96GM5 Q9NZQ3 Q7Z6Z7 Q92791 P04062 O15259 O60264 Q92558 93323 O95983 P07339 Q13547 P55210 O95503 P34931 9252 P34932 Q9Y6J0 79023 9493 80254 O43707 1763 Q8TBE0 Q92786 Q9UH99 P53041 Q92784 P41229 Q13794 O75503 O95997 Q9P253 Q9NP66 Q53SB5 Q9P258 Q96EP1 P35612 P35611 1778 23081 Q9H9B1 P63000 60673 51720 Q96PE2 2629 3954 Q9BUK6 Q13561 P35606 9276 Q9NQZ2 O43920 7094 1789 23092 1788 P46736 1786 Q05513 3720 80273 5901 Q27J81 Q9Y6D9 Q9NVD7 P12956 100287932 339287 Q02241 P78527 P33897 2647 Q00059 1315 1314 P20336 P61088 Q04656 84661 51742 Q14674 11091 Q14677

Q16611 30827 O15230 27436 Q9NP98 79065 5927 Q9UNA1 5928 P63279 P00519 5929 P63272 121441 O60232 P24941 Q9HCS4 900 51510 Q15532 Q13112 Q13351 51517 Q14683 5925 O95677 P06899 23607 Q8NEY1 22992 Q6QNY1 O00267 Q9P2R6 P61020 Q9BVA0 O60566 Q7L0Q8 Q96M29 Q96JM7 79658 Q9HBH0 Q9BVA1 5931 Q9ULW0 5933 10534 O95202 O15530 Q8NF91 Q9UBC3 Q8IZP0 Q12959 O95680 O95684 P52566 Q8NB12 1107 57634 79885 1104 10783 92609 1108 P05787 O95696 Q96NT1 22974 Q05397 P53667 Q9BXC9 57403 Q9Y2Z0 83933 27185 57405 29127 Q86WA8 Q92830 4867 Q92833 Q6IT96 4627 4869 Q92831 P06400 P53675 O95466 P07737 P48023 1130 9093 Q9NRZ9 22985 Q13829 10524 Q99698 6812 Q6NXT2 10765 3308 P07305 Q12981 O95476 O95477 5981 O95239 3320 6829 Q96DE5 57662 Q6FHQ0 O60763 3796 6827 P53618 Q8IZT6 79441 Q8WYH8 6839 1387 116840 P62328 P41208 P42771 5747 Q8N137 P53621 Q9NYB0 P30304 P30305 4673 Q13618 Q9NYB9 2010 Q9H9Y6 Q9P0L2 P62330 Q9NS69 10953 P42768 P15153 1398 Q9BZK7 P61006 6601 6605 Q92878 O43182 10951 P30536 O43189 Q5QNW6 O00255 Q8NEZ4 Q6KC79 Q60I27 O00257 Q03164 Q9P0M6 81788 10726 984 Q99496 10728 Q6FI13 4676 989 4678 Q96FF9 4690 P07900 O43159 O00429 6871 P04637 Q9Y6W5 2034 2033 2275 O95271 P52952 990 Q8WTS6 991 79810 994 P11171 Q99640 Q66GS9 998 6622 79813 P01116 10133 6624 5536 Q15388 P62753 P01112 P01111 O43166 Q9UM07 6883 4221 Q9Y6X3 Q8N4N8 Q96CW5 Q9UQ88 P51636 23210 Q99880 23212 Q9BT40 P50542 1191 Q99877 Q96RT7 2039 Q96RT1 2037 Q96S53 Q99871 Q15154 4214 11234 8812 Q96S59 Q96RT8 4218 56946 255626 Q99879 P49768 25847 P50552 Q9BV73 P48431 Q8N726 Q7Z7K6 Q96CV9 P12235 P11388 P52732 Q00535 55869 6647 Q7Z3C6 Q8N8U2 P48668 Q8N960 O00217 Q08495 Q9UPY8 4000 8841 3396 O00213 O95067 3151 221613 Q93079 2059 3148 Q96RR1 Q93077 10363 10362 P01127 Q96EY1 Q00526 6657 Q8TDZ2 Q9UPZ3 9927 2070 23647 O00623 O00628 9700 5584 7520 5585 Q969H0 Q9Y2K7 Q9UHB6 Q68EM7 O75190 3159 Q8WTW3 5578 Q15185 5579 5338 P05771 8607 Q9Y512 5591 Q9Y4P1 9950 Q969G3 7534 10580 O75177 P68032 23411 Q9BZD4 5590 3171 Q9NRL2 P50748 324 3169 8615 Q9H1A4 5347 5108 Q92800 P27797 Q96JH7 Q9H5I1 Q86U86 91754 P49321 Q9UPT9 Q12929 O75164 Q9Y2M5 P12036 P15311 55666 Q99661 56993 Q9NRM7 5116 Q9H7L9 10552 5119 Q01826 5371 Q9BQQ3 8881 4285 Q60FE5 O00411 P16403 P16402 25813 P16401 221656 80854 P52948 57610 10320 Q99418 Q2M2Z5 Q9UPV0 P25116 57617 Q8N6T3 54908 10093 10092 8655 4297 8411 356 Q96BM9 P09493 118 9738 Q7Z6C1 P21127 Q8WUM0 P47755 Q9HC98 P47756 O43683 11190 P03950 Q15424 P35527 7334 5155 Q16512 Q9BTM1 Q16514 O43684 11196 19 Q8TF76 Q7LBC6 Q9UKE5 51412 11198 Q13492 7329 P60981 Q13257 9525 10075 6015 253260 Q9UBU3 P51808 375 54930 P23527 P23528 P17096 Q8NFH4 P60510 Q9UBU8 P83876 Q9HC77 O94927 Q8WZ42 P00441 Q9Y613 Q6PD62 4088 Q9BY44 Q16777 O43663 O75478 Q16778 Q8WWN8 Q9BY41 Q14118 P0C0S5 23399 Q8TD19 Q96BK5 382 5170 142 Q9H492 387 P21359 P52907 P0C0S8 Q7L7L0 11177 10087 11176 P60763 Q15691 53615 8452 7126 23122 390 P14635 391 Q9BW71 O43639 Q9UQL6 P35579 397 121512 Q9H6D7 23363 2908 Q13042 10059 Q02539 P45379 P49736 Q96R06 Q13286 5195 6045 9793 Q96KB5 9555 8467 23133 163786 23135 23136 23378 P13796 23370 Q9Y5J6 8218 85440 9322 9564 Q6ZSZ5 O15182 Q8TCU6 8479 10270 Q5SSJ5 P54274 Q9Y5K6 P58876 6294 7141 O43615 Q96T23 P49959 Q9H1Y0 P68431 56916 P49711 Q9BRK4 Q16576 Q15003 P49715 P60953 54737 11127 64780 7153 8242 7157 P22492 9578 P14625 Q8TAP9 Q9Y5L4 23354 Q02750 O75431 25777 8481 51474 Q9NZ56 54984 55835 84376 Q6R327 10048 54509 Q5VTD9 1605 Q15014 86 P53365 O95835 O75665 P10636 26038 26037 P63172 26039 7161 1613 80124 P63167 26271 10933 55165 10935 P20671 P46821 P45973 Q9UBK2 Q9UBK9 Q13643 Q13885 Q6VN20 Q9P0W2 Q14978 Q969S8 Q96QB1 Q86WJ1 Q6DT37 Q9H3R0 P55318 Q05682 Q96BD5 P84095 Q7Z460 Q52LR7 22919 Q9UI95 P42695 1627 3801 Q14980 Q9UBL3 P29474 9126 O95613 10907 57120 Q96M96 6093 91272 9361 7184 1877 10910 P17252 Q9BVG8 O15379

O95619 Q969R5 1639 Q86UE8 P09874 8289 Q13422 9133 Q14511 O15392 O94776 O43823 Q03001 10919 P35900 Q8N7B1 Q8NB78 Q9H410 Q9Y3D6 84733 Q9HBM1 Q9UBN7 1647 Q14527 Q8WUI4 Q6NYC1 P41182 P41181 8290 P51148 9382 P30048 4926 Q9UK80 3832 Q9HC52 3835 P51398 Q8WZ19 Q2NL82 Q9NQ92 Q68CZ6 58480 Q8N7H5 Q07817 P20248 Q9UJX2 Q9UQE7 Q8WYP5 P17081 Q9Y5B9 Q14781 P21333 O14492 P33778 Q9BY11 P10415 O75844 Q6ZV73 Q14789 P10412 1674 23186 Q9HC35 O60341 P09430 Q15642 Q13464 3856 O15350 728642 Q9BZS1 P53350 Q9P0U4 P62072 23192 1203 3622 P84077 2773 P30260 O14497 Q13233 Q15652

regulation of cellular process Q9UKT9 P54852 P28562 Q8NDW4 259266 10657 2305 2304 2303 114991 284217 2300 O14544 Q8N3U4 O14543 2308 Q9UL63 2307 Q9H4L7 Q8WXB4 Q8NDV7 2317 2316 P42858 P39210 P15260 Q9UKV3 P61158 10661 Q9UKV5 10666 136319 Q9UKV8 4990 3660 3661 P62495 81876 84901 2324 P62491 55588 81873 Q9UKW4 4983 3655 Q9Y6A5 Q9UKW6 10633 3659 3672 Q5BKZ1 3673 Q13705 P21917 10645 2335 2332 3665 3667 10642 10641 10644 10643 P42830 Q9UL15 Q8WXG6 Q92538 Q9UL18 1022 1021 1020 Q06587 Q8IX07 Q5JT82 P51787 1017 10614 6709 79576 P21964 10616 1012 Q92530 3678 26191 3679 P61106 O14503 Q9UL26 Q9UL25 3690 Q8WXF0 Q9Y5Z7 Q8WXF1 Q9H4P4 6720 285527 Q96J02 P51797 1029 Q9BUB1 10626 1027 1026 1025 Q9BUB5 3685 6711 6710 3689 P05023 P07686 6714 Q9UL36 Q07890 O95136 1040 79595 P19634 57326 P20618 Q07889 6721 6726 Q8N488 Q9UKS7 Q9UKS6 Q9UL46 Q9UL45 5411 O95140 Q8NDX6 Q38SD2 1050 56005 84932 253980 57332 79589 10607 6733 Q8N3V7 Q9UL54 Q9UKT4 Q92502 O95159 Q9UKL0 P50416 11108 11107 Q8WWY6 1059 6749 P25445 6760 Q9Y5V3 5430 112399 O95163 Q9NWZ3 P57059 115426 1072 51131 Q99996 1069 P33076 51132 Q14186 84033 Q14185 P49407 85360 Q14188 P02794 11116 5440 5441 6772 Q08380 Q9UKN5 6774 Q99990 346171 Q66K89 P14780 Q2M3W8 51144 Q9H4B6 Q14192 Q8WWW0 51147 55504 Q9Y692 55503 Q99988 Q9Y5X9 Q86X95 5451 A7MD48 Q9Y696 Q8N3J9 Q5T6S3 P14784 P57082 P17813 Q00403 56849 6776 4116 6790 O00744 5460 4131 Q9Y5Q3 9821 6794 4133 P25490 9820 Q9NX61 Q9NX65 P61981 Q9UBW7 9815 6789 Q6UUV7 O00755 Q9Y5R5 5473 9831 Q9NX70 Q5JSP0 Q9UBX0 Q9NWV8 148327 5467 4137 9826 207 5468 54206 4139 4150 4152 4154 4155 Q9Y5S2 9841 P30825 Q96A54 O00762 Q9NWW5 Q9Y5S9 P17844 127435 10673 4149 10672 9839 5494 4168 P50406 P08908 P50402 Q96QT6 Q3KRB8 56882 8516 10681 8517 Q9H4E5 55558 9846 9849 P49427 326624 4172 O15085 4173 4174 P35659 4176 Q14209 8535 11065 7204 8533 Q6NZI2 4171 Q6P5Z2 23043 Q9NPC1 P08047 Q9NPC8 94234 8546 4188 8543 P58304 O15090 Q9UD71 23051 Q04864 P09382 9869 8539 7205 Q8WY36 8536 Q96RL1 P61956 Q15554 1902 Q16881 7220 8553 P35638 P61968 9882 Q8IWY8 Q96IZ0 8555 P31274 P31273 P31276 Q9ULH1 Q9ULH7 23028 Q9NY61 4193 Q9Y6Q6 257 Q6DD87 Q96RK4 Q15561 8548 Q96RK0 Q93009 P02741 Q14232 P55290 P61964 Q15562 Q9Y6R4 Q8IWX8 Q9Y6R0 P36952 Q86XR7 P36956 P35625 O75592 Q8N4C8 267 Q9NPF0 P31270 Q9NPF5 P61978 Q8IX90 P43246 Q14249 11021 7248 O75586 O75582 Q9Y6K1 O75581 P10398 274 Q93063 Q93062 Q9Y6K9 Q15583 8569 1936 11030 P22392 11031 O15055 7251 O75570 94274 285 7249 O15047 P48995 11035 1947 P02751 Q15596 P61925 P02790 O15062 O75569 166968 O15068 O95931 Q9Y6M4 291 Q9Y6M1 O15060 Q14C86 Q8WY64 51100 P08069 Q9NPA3 11004 51107 1956 286075 Q8NEJ9 Q9NPA8 O75553 26986 64426 64428 7272 1965 Q93034 Q93038 O95947 Q9NPB3 1969 P02775 Q14289 Q8IWS0 Q96RE7 P12931 Q7Z589 Q4VCS5 1975 Q9BUG6 Q9Y6G9 Q92574 Q13761 1978 Q58WW2 7297 P56524 O75534 Q02297 7291 O75530 7290 7295 P24928 1982 Q92560 Q9Y6H5 P06276 O75528 O15020 P06239 O95977 Q06643 P0C7X2 P55209 P56539 Q7Z569 Q8WXI9 P40145 1994 1993 Q96IQ9 P06241 P21860 P56545 O95983 50618 Q8WXH2 P55212 91147 A4D1W7 Q07960 3705 3708 Q07954 Q92542 Q13794 O95996 O95995 O14593 Q8IWW6 O95999 Q9NP61 P35610 Q9NP66 O43915 9021 Q16828 P35612 9020 P35611 23081 O43918 Q9Y6C2 23085 Q9UKY1 P85037 P31260 P20594

O75509 Q8IWV1 P31249 Q9NP71 Q15506 Q9NP72 P35609 23092 3728 P61073 23094 Q9Y6D6 Q9Y6D5 23098 O60496 Q9BUJ2 3720 P22303 Q9Y6D9 9047 50649 9046 Q16849 P12956 Q8NEB9 Q9NP86 Q9H4W6 P02708 Q9Y6E7 Q9H582 Q9Y6E0 P61088 50650 Q96AE4 147808 Q96RG2 11091 Q9NP90 A4D1S5 222068 63976 63978 9052 O43909 23077 P24941 Q92585 Q15532 Q14201 Q7KZF4 O14672 399687 Q5VV41 Q9UBB6 Q9UBB5 9066 A5YKK6 Q9P2R6 55684 O60566 Q8IVH8 79658 10772 P27348 Q9UBC1 Q9UBC3 P27361 65018 54361 9077 1107 1104 O60573 284323 10783 1108 Q9UK53 P52333 Q9UK58 Q7Z3K6 P29992 3783 1121 339122 Q6P4F7 Q7Z3K3 O60548 P53667 57402 57403 10758 Q8IVF5 O60543 1111 Q9UJU2 284312 P61289 P03372 P06401 P06400 Q9GZX9 3791 P07737 Q5JS13 1130 9093 Q9UBE8 P56705 9099 10767 57410 1122 P61296 3784 6812 6814 10765 P05129 P50570 Q9Y586 P11802 Q9P2N2 83706 O95231 O14627 6829 10733 10736 1137 Q6FHQ0 P61225 P61224 Q8N2W9 6827 Q7KZI7 65056 93986 1153 Q14938 P19784 Q06455 O95243 79685 6839 10746 1147 2475 5500 P26045 O95259 P07766 Q10586 P05106 O95257 P05107 6850 Q8ND82 P50591 Q6IA86 2495 Q13618 Q13617 Q13616 1161 O60506 Q7Z406 Q02086 O60502 O60503 57448 P61244 O14641 5511 P61247 O14640 5515 P05112 5516 O43182 26292 5518 O43186 Q13625 O43189 Q02078 10725 57459 10724 10728 O14654 P05120 5524 P05121 P40337 Q9UK32 5528 5529 P04632 O43159 6872 6871 Q9BST9 P04637 O43157 1185 Q9NVW2 O95271 11228 P13591 5533 4204 4205 Q15382 5536 6869 Q15389 4209 O43166 6883 Q9BT49 4221 6885 Q9GZR2 O43167 23210 Q9HDC5 P50548 Q9BT40 1192 23216 1191 56940 O76074 Q9P2K2 9908 O76071 Q15392 84159 O76070 Q9P2K8 4214 6878 4215 P01589 4216 P01588 11236 4218 56946 5562 Q86VW2 9921 5563 5565 P12235 Q02952 P61204 Q53X93 6886 Q9GZP9 Q9Y572 P61201 Q9UJM3 4240 5573 P04626 5575 Q9GZP0 5576 P04629 11218 Q99853 O14617 301 55633 302 P17936 5566 5567 307 5568 Q8TDZ2 5569 11214 O43150 11215 9927 5580 5581 25828 5582 5583 5584 5585 Q5U0I6 4255 5586 Q9UJF2 22794 25822 Q96PU8 310 Q96PU4 79612 Q96PU5 Q02930 P51828 5577 Q8TEB7 5578 Q9P2F6 5579 8607 Q8N302 Q495A1 5591 Q9GZV8 4261 Q6IR47 5597 Q3SY56 5598 O76024 P15336 Q9BSW2 5590 56987 51295 P16671 324 326 328 4259 Q8TEA7 329 55657 Q9GZU7 P51843 P15311 330 Q8NCN5 55662 331 Q8NCN2 333 56994 5599 10794 8625 Q9GZT9 Q96HC4 Q9BT67 9975 4287 25818 P62508 4289 Q5T5U3 P51858 O76003 P17980 Q08211 221656 Q9NVV9 Q08209 9967 Q96HB1 Q96HZ4 8650 8655 4297 Q15418 Q8TEW0 11186 8654 Q9UKD1 P30086 Q04760 4291 351 Q9UBS0 355 Q04759 356 Q9UBS5 85415 P09493 P23786 P21127 P47755 P47756 8662 11190 Q15424 7332 6003 9997 7334 8667 7336 7335 19 26205 Q8TF76 Q9UKE5 Q9H3Z4 85403 367 369 Q70SY1 Q9UBT3 11198 7329 20 Q8IW93 7341 Q14106 Q8IVW6 Q14103 29 Q99909 6015 Q15438 Q8N3C0 23144 Q86X27 253260 Q53GQ0 P10451 Q8TEU7 Q9Y606 84108 Q9UBU3 P51808 Q03405 375 P51805 P09471 P17096 6009 11168 Q9UBU8 Q8TF50 Q53H12 Q14116 Q14114 Q14119 Q8WWN8 Q14118 P51812 Q9Y618 Q9UKG1 P29084 381 Q86WP2 382 Q04725 Q04724 387 Q04727 Q9NWT6 388 Q04726 389 Q9NWT8 23152 Q9UBV7 Q66K14 23154 P08151 11177 11176 P60763 P23771 41 P55347 P55345 11140 Q16787 11142 O15164 23122 390 391 23126 392 25788 394 396 397 Q99962 398 Q99963 25780 Q6KH11 11146 Q15466 Q99966 Q15464 54 Q96R06 6045 Q9BU20 7376 Q14137 7375 Q16799 23133 23135 Q9Y5J5 Q86WV8 Q3KQV3 Q9UKA4 Q9UKA8 P33151 Q14140 Q9UBP4 O15169 51218 85440 Q99958 Q14145 Q15475 Q99956 P46459 Q8TEY5 Q6ZSZ5 P14859 Q9Y5K6 Q99943 Q99941 Q01664 51222 P21145 56916 P01579 11124 Q14151 Q9UBQ5 Q14155 P82979 O75676 O15198 23112 25776 P33121 23118 51230 Q14160 51231 Q14164 Q5VVH5 Q14161 Q6R327 Q14168 86 87 Q14964 Q9NWH9 Q13637 Q13636 O15123 Q9NNW5 P33992 P33993 Q8TF05 Q13639 P33991 26271 Q86WK6 Q1PSW9 91 P17030 94 O15119 P45974 Q6ZT07 P45973 26270 O15118 Q9UBK2 9112 Q96QB1 Q96I24 Q9H3R0 P31321 P55318 Q99081 P31323 P55316 Q7Z460 P43307 P05062 P52292 Q52LR7 P17040 P05067 Q9UBL3 O94763 P52298 O94768 P52294 9126 O15143 26258 Q9H422 6093

6096 P41597 56034 Q9BTC8 P21980 Q9UBM7 O15151 P11717 O15156 O94776 9138 Q7Z434 202559 Q02156 P17023 Q8N392 Q86WH2 P17020 Q8N393 Q9UBN7 Q9UBN6 9146 O75626 Q9UBF6 9149 Q8TF47 P30041 Q6NYC1 Q9H488 P30044 114822 Q58EX7 P53567 P30048 B3KY43 3836 P22415 Q9UK80 Q03468 P08123 Q92499 Q9UJW9 Q13683 O75629 P47712 Q52LW3 Q96HU1 Q8IVT5 Q9UBG7 Q07820 Q07817 Q9UJX2 Q9UJX0 P08134 P17081 P08138 Q9Y5B6 Q9Y5B9 P09429 O75604 P10415 Q9P2X3 Q9P2X7 23189 P35711 Q7Z494 23186 P09430 728642 P08107 O15111 374354 Q9P2Y4 Q04771 O15105 P45983 P45984 Q2M1Z3 Q6NX49 65125 25909 P54619 9182 22870 2550 9181 P51587 9184 P17676 Q8TDD1 54474 2547 P37288 57504 Q9HCJ0 Q9HCJ2 10890 3875 6901 57506 A1A4S6 P28335 25913 P28330 P08729 Q08AM6 2562 Q5VTR2 P55957 O60216 P62244 Q9H2G9 Q17R89 Q9H2G4 22850 Q8NC24 79791 54496 6927 Q8IUD2 6929 Q6AHZ1 57521 57522 6921 P62258 10875 6926 6925 Q6IQ32 Q17R98 22866 Q9UIS9 P31943 P31946 P31947 P54646 57534 79784 P16333 6938 Q8IUC4 Q8IUC6 P62263 P31949 5602 5604 6936 Q9Y468 P08758 P27037 Q9H2M9 Q9Y463 Q9Y466 Q8TDI0 Q9UIL8 Q9NUY8 P39019 P15056 10856 Q86VE0 P16383 Q6ZRI8 57544 Q08999 Q8N264 644943 5610 6945 P07437 Q9Y478 22846 P30679 P50222 Q14814 P28370 Q07666 P80098 124583 P52888 Q06330 10865 P19419 Q9HCM4 5624 P49286 Q9H2K2 1285 Q9H2K0 P21709 Q8IUH5 Q9HCL2 1277 83853 4302 Q13501 P51575 22827 Q13503 Q14839 Q8NC51 Q13506 Q9HCK5 Q9HCK4 Q8NBS9 192669 153090 50943 P08754 4318 Q9Y3Y2 Q86UW7 Q9BS34 P01241 6993 Q9BRR9 O00541 23322 P14555 Q9Y3Q8 Q9UIH9 23326 25988 23327 P15884 Q8WUU5 Q8WV60 10011 55726 5654 10010 P25205 10013 84289 P01236 55723 11346 10014 11345 10016 135295 64682 Q15269 Q9UII4 4343 Q9BS26 23332 25998 P13236 Q99755 400 402 Q99750 406 Q9BRP8 Q99759 408 84271 409 Q99757 Q01518 Q09472 Q8N1G0 23309 5682 5683 5684 4354 5685 4355 5686 116986 O00560 116987 O00562 23303 Q99743 54413 Q99741 55743 3014 Q15286 Q9Y450 11326 P51531 4361 5692 5693 8721 8720 Q8TCX5 Q9NUX5 P51532 P14543 P15882 55750 55755 P60484 Q15291 11331 5687 55758 5688 4358 10001 84299 8718 P23497 8717 P01243 P26583 Q9Y3M2 Q6FGG2 O00506 P46199 7402 Q9Y3M8 25942 54431 Q9NUQ3 Q8WUY8 Q8WUY9 Q96P48 79735 8726 Q9H2D6 P55075 P55072 P01275 8743 8744 7410 90993 7412 Q9HCE7 Q08117 3054 Q05086 Q9BRU2 P37231 Q9HCE1 444 54443 79723 8737 P55085 P55083 8738 Q6IQ22 P01266 8754 7422 Q96GA9 3068 Q9HCD5 P52815 22893 3066 65108 3065 Q9UIF9 Q9BRT3 O75293 O75290 79753 Q96P20 55787 55785 7415 P61764 7414 3070 Q86UX7 7430 7433 8766 Q9UIG0 P52824 3074 Q9Y3P9 80728 P17612 466 468 55796 7428 8772 Q16623 P18085 P10114 Q9Y4K4 O75386 O75385 Q86VQ3 Q9Y4K3 Q76L83 3084 472 P48729 473 474 476 23286 P20396 8767 P43034 P35408 Q15303 3091 Q16633 P55036 Q16635 Q15306 Q86VP6 O75376 3096 Q86VP1 O75381 Q9NVP2 Q9H2X0 6117 7448 P55042 P55040 Q16643 Q15311 P43026 Q9HCY8 O00602 8795 Q16649 Q16644 P12757 7468 Q8WVM8 P19397 P12755 O75362 Q8TDS5 O75360 498 23263 6128 7458 Q5VUA4 Q8WVM0 Q9BSI4 Q8IV61 Q16650 Q16659 P20393 7476 Q16656 Q15329 Q8NCA9 P18074 Q8WVL7 P55055 93166 Q86VN1 P68371 Q16665 51317 P55061 Q9P2E9 Q8TDY2 11261 Q16666 Q5VUG0 P35453 O75340 P09172 P46109 11269 Q16670 P46108 Q14012 Q14011 Q86W54 Q15349 P46100 Q9Y4H2 23256 P35443 Q5PSV4 7490 84232 Q8WW38 Q9GZM8 Q99835 23229 49854 P98179 O43734 Q86VS8 P15822 O43739 Q8WVQ1 Q02878 51341 Q9NVM4 P02549 Q96PK6 11243 55704 P55010 Q8NCF5 Q9NVM9 P48745 P23443 6188 Q15369 Q8IUX7 O43741 11252 Q9BSM1 P10147 O75312 6182 P11474 Q15370 P27816 P57682 3909 P02538 Q99819 P27815 Q99816 P48730 P23458 9208 Q99814 P48736 P98194 Q15375 P78545 6196 6199 6198 P98155 P10997 O95714 P78540 O43711 6193 O43715 6195 6194 10811 O43719 Q9Y4C1 P62195 Q86VI3 10817 Q4LE39 P46940 9218 O95715 9219 P98161 P07355 A8K8V0 3911 3912 Q13522 P78536 22803 9238 Q13526 22806 22807 57473 9230 83737 9231 Q3V6T2 3925 P46934 P46937 83746 P46939 Q9HD26 Q96GN5 P41240 O60296 Q32P28 Q4LE28 P98170 P42574

P98172 O60292 P42575 P98171 P98174 Q14865 P34947 P29597 Q9H305 Q13535 Q9H307 P29590 Q7Z2Q5 Q63HK5 P41235 Q9HCP6 P41231 Q96GM5 Q9HD15 P16284 Q9Y4E5 O60266 O60264 3932 O60260 9255 P78552 Q13547 Q13546 P19320 9252 9253 P41223 O43707 P41220 P20309 Q9NVJ2 P41229 P78560 O60271 9267 Q13554 O95751 Q8IV45 Q13557 P78509 P78504 9261 3959 P00749 Q63HR2 P00747 Q9BSG1 Q96PE2 P00742 3953 Q14894 Q13563 P62158 3956 2626 Q9NVC6 9270 P11413 P62166 Q9UIV1 P00734 Q9HD67 Q5BJF2 P24723 Q13574 3965 Q13573 Q13572 Q9HCU4 53916 P55884 93134 Q9H2S9 P32320 P78527 O60229 85509 P41279 P20338 P20339 P20336 121457 P20337 1312 P41273 Q86VK4 Q04656 Q16610 P20333 Q9Y4A8 Q13586 Q16611 P18031 Q9P286 P34972 Q9P287 P62191 Q96H20 O60238 1326 Q63HQ2 Q8TDM6 P67870 Q9HCS4 P07384 P20340 P00750 Q9HD40 P21675 P08833 Q9Y2W7 Q6P2D0 P48058 2672 Q12948 22992 Q6QNY1 P61020 79894 Q6PCE1 Q7L0Q8 Q9BZI1 2665 2664 57620 Q9HBH0 P61026 Q8N103 P08842 Q12950 Q12955 Q9Y2X7 Q12952 Q9Y2X9 Q12959 Q8NB12 Q8NAP3 Q9P107 5706 5707 79885 5708 5709 P16473 2676 P16471 P48039 5700 5701 5702 Q9Y2Y0 5704 Q008S8 5705 P54753 P50395 Q9Y2Y8 P25391 Q12968 22974 5717 57646 83933 Q9UQB3 Q9UQB8 5713 57649 5716 Q12972 Q9H1D0 P54764 P54762 Q0D2J5 Q9H9T3 P48023 1374 Q9BZF9 Q6P2C8 Q12979 22985 5728 Q99698 Q9HBE1 Q99697 2697 400961 Q9UHR5 Q9UHR4 Q6PCD5 Q12981 Q12982 P28482 Q9UHJ3 Q12986 1385 Q9UHJ9 P51668 P51665 P19532 Q9P0J0 Q9P0J1 P19525 5739 57669 57662 P15173 P07550 Q9Y2T1 10971 P08887 10973 P51671 Q9Y2T7 Q9UHK0 Q5VST9 P19544 10987 57678 P18206 1388 Q9P0K8 1387 1386 P62328 10981 P42771 Q9H1I8 5747 10985 Q14703 22937 22938 Q9UHL9 Q9P0L0 Q9P0L2 22931 P62330 57689 P62333 284119 P15153 1399 1398 Q9BZK7 P61006 Q9Y2V2 Q9Y371 P51693 5770 Q9Y376 P51692 P28472 Q9BQY4 P48061 Q9P0M2 Q03164 55035 440193 5764 Q5H9I0 5768 P61019 Q9Y2W1 P61018 P06213 Q15139 Q08043 Q9UPV9 P80188 P13349 115557 P38646 P52952 Q8WTS6 126208 Q8WTS1 79810 Q99640 79811 79813 10133 P27986 P02686 10138 Q9UQ80 Q9Y2P0 Q9UQ88 5796 P51636 Q8WTR2 8819 Q15154 10142 8812 Q99638 10146 P02675 P01344 P01343 23429 Q9Y320 3142 Q9NU63 Q99623 Q8NAF0 55869 5798 8829 Q9UPY3 8826 Q8N960 P01375 P01374 Q9UPY8 23439 Q9Y337 Q9Y2R2 8841 Q9UHI8 Q9UHI6 Q3KNV8 23435 3151 P12004 10128 Q15172 Q3KNW1 3148 Q15170 545 546 3146 Q8WTP8 P40933 Q96EY1 Q15173 Q8WU17 P25311 8837 7508 P24043 23409 7520 8851 Q9UPR3 8852 Q9H9S4 Q9Y2K7 Q12904 Q8WU90 Q9H9S0 Q9UHB6 3162 Q12905 O00622 O95076 Q12906 Q68EM7 Q9BZE0 552 553 Q9BZE4 P42704 3159 Q99683 Q8WTW4 Q99684 8844 Q15185 P49336 7518 P05771 Q96FA3 O00635 8864 7532 7531 7534 7533 P55198 Q9UHC3 23411 23414 3171 10109 563 Q00987 Q99675 3169 Q9NU19 Q9H1A4 P05783 7529 7528 6210 Q86U86 Q9UHD2 3187 Q9UPT9 Q12929 Q9Y2M5 3185 3184 571 3182 572 573 Q99661 P51608 7536 6207 8882 3192 8880 3190 8881 Q60FE5 Q9UPU9 Q12933 8887 7555 P51610 580 P16410 54583 80854 Q99653 P51617 7549 8878 P60568 Q9BZZ5 Q13478 Q13477 10092 6231 8892 10095 Q9UQP3 10094 6236 8896 P15923 Q96FV9 595 84324 Q9BRI3 Q13485 Q9HC98 Q13489 P03950 6242 O00716 Q16512 Q16513 Q16514 Q9UIA0 O75496 O75494 Q7LBC6 P10242 P10243 P10244 84312 6238 51412 Q13492 Q16520 6239 Q13490 Q9UQR1 6256 Q9HBW0 O75489 Q16526 Q2M1K9 280636 23380 23381 P23528 7579 Q16531 P09237 6262 Q16539 10081 Q8N9N2 O75478 Q15208 Q16537 163126 Q9H257 O75475 Q9Y3L3 Q9Y3L5 Q02790 P52907 P31150 P24864 10087 6259 84333 P60520 Q16543 6275 O75469 64750 Q9Y3E0 23365 P14635 O75461 64759 23368 Q9UQL6 P10275 P10276 P35579 O75460 P35580 Q8NBF2 P23588 Q99728 Q99729 Q16555 Q99726 Q15223 55806 Q8WV24 9311 Q86UR1 9314 Q5JQC9 O43889 P36896 P36894 23378 P15976 P35568 6282 23370 55810 Q02763 P01308 120892 Q99717 9306 P01303 Q15233 9322 P02671 Q8TCU4 P61803 Q8TCU6 O95813 9326 O75446 Q96FX2 O75444 Q86V24 6294 P36888 Q99700 Q9H1Y0 Q99708 O95817 Q9BRK4 Q16576 9318 O95819 55827 64780 64782 64784 O75437 Q08050 P39905 64786 P14625

Q8N9R8 Q02750 P35548 138151 55832 51474 84376 54509 Q5VTD9 Q15257 Q16581 P53367 O95831 P53365 O95835 P57729 O95833 P12814 10928 56252 57584 Q9H204 9341 56259 10933 Q9Y3A5 10935 Q7RTN6 P39060 83871 O95837 Q6NVY8 P15090 Q8WUD6 Q9P0W2 22926 22927 Q13404 57592 57594 Q14738 O43847 22920 57591 9350 P57735 P39059 Q08945 Q9UHX1 22919 Q9UI95 9367 9368 O43812 Q13418 O43815 O43819 Q86UE4 Q86UE3 Q9Y3C7 Q8N163 P42680 P42681 O60383 P42684 P42685 O60381 Q86UE8 P21741 Q13422 Q13426 O95865 O43823 B2RTY4 Q8NB78 2735 P19438 P21757 O43829 P68400 56288 2730 1408 1407 1406 2737 2736 9388 Q13432 Q96G30 Q8WUI4 8050 Q7RTR2 Q9H244 P23508 P62277 P43119 2741 Q9HC57 P09211 Q9HC52 Q5H9R7 Q14774 Q13443 9391 P11532 Q96G25 8065 O95881 P23515 Q9UQE7 P23511 Q8WUH2 Q14781 P23510 O14492 8078 Q9H228 O14495 Q9P0T4 P12830 P19474 P18146 Q9H223 84305 1432 O60343 Q14790 O60341 Q13464 P53355 Q9BZS1 P53350 Q9P0U3 Q9P0U4 P19484 Q9UHV2 O60356 2773 Q9HC29 Q7L9L4 P24821 O14497 Q9BZR8 Q9BZR6 Q9H0E2 P25054 Q9H0E3 Q9Y265 O75940 P49023 1460 Q12824 8091 124790 1459 1457 1454 O00391 1453 155061 Q9Y275 284695 Q00839 Q9H0D6 Q92974 Q12834 Q12837 P16104 5829 Q9H0C8 P49006 5822 P10809 Q96EB6 Q59EK9 Q5S007 O75925 P27694 P27695 Q9H8S9 Q92956 Q9BQ51 Q9BYE7 Q06124 P17405 P37023 P13056 57761 55109 Q9Y297 O75928 P26358 Q12851 P26374 Q6NUN9 Q86SX6 Q92949 Q99583 Q12857 1491 1488 1487 283337 Q99576 Q99574 64061 P26367 P25098 O75901 P49069 5861 5862 Q96N96 Q8NA42 114548 Q96MX3 57787 Q9HAJ7 1499 O60884 Q6PJW8 O14981 P61587 Q9Y230 P61586 O75909 Q12873 Q9H0H5 Q9UGI0 Q12872 P29466 Q9Y239 5870 5871 Q15907 Q15906 Q9UGI9 O00358 Q9BPY8 57798 3207 Q8TBC4 P17482 5863 O60890 P17480 Q9Y240 604 5865 Q9Y242 5867 5868 5869 P04179 Q12884 Q15910 Q9UGJ0 Q12888 Q9Y243 5883 Q9BPX5 Q9BQ95 O60869 P16118 Q8NA19 P38398 55145 3215 Q96N67 Q92990 Q5T2W1 4543 Q9Y250 613 Q5TCX8 Q92994 Q6NUQ1 Q92993 5877 P63302 5878 5879 Q92997 P10826 P10827 P10828 Q9UGK8 221937 5894 P31749 Q7L8A9 Q9H8V3 P53999 27032 79101 P40692 3226 3225 55159 Q96MT8 P04150 5888 P63313 Q9UGL1 127829 P04156 Q92985 O96005 O96004 8930 P56199 P14317 P14316 P14314 O00300 23568 P50897 23569 Q99523 23560 Q15022 8924 637 79937 5898 P62873 639 10256 P62875 O96017 P06702 Q15027 O96019 P16989 P36575 O96013 P35249 Q03933 641 P35251 Q6B0B8 79923 8932 648 P47224 8939 Q15036 P60228 Q16363 O00327 O96028 O00329 P48552 Q8TAU3 P48551 O96020 3265 3263 O60828 P15621 P11274 Q99500 P35240 8945 P01019 8943 79959 O14964 Q15047 O14965 P23246 O00330 P35225 P35222 P35227 P35226 P11277 O14979 O75084 O75081 663 Q15051 P35232 53335 3269 Q9H093 668 Q15052 Q96MH2 O14974 Q59EA4 Q15057 Q16385 P24588 Q15056 7629 O43474 P06733 P01042 Q92934 6311 P05408 3281 79971 672 673 53340 P15692 O14908 6304 57708 P62834 10213 Q92930 P05412 P05413 6320 3297 23532 Q99558 P14373 57713 688 Q15070 689 Q15077 Q8N895 Q92922 Q15078 Q9BQ15 23509 P56178 P56177 Q12802 Q8NHY6 Q12809 O75044 O75051 P13010 Q15084 Q0IIM8 Q99543 A7KAX9 O14920 57727 P56180 O14921 P62854 O43463 Q92903 O43464 Q92905 Q8NHX1 Q12816 P06729 23512 23513 57732 O14936 O14939 P40616 10202 Q92900 Q9BYX4 P36508 P36507 Q14686 P19174 Q9BZ95 O43559 O43557 Q9UPN3 Q9H165 Q9UPN7 P13861 Q9H161 Q9H9F9 Q9H0U4 P07196 Q9H160 P49810 5017 P83731 P78395 Q9BQG0 5018 P07199 P49815 Q9HB75 Q13363 Q14693 Q8TBP0 5034 P62805 6368 P46782 5036 P46781 5037 79084 Q96F45 Q9Y2H1 Q9H0T7 Q9Y2H8 5029 P00533 Q13370 Q16401 Q9HAU4 P24522 Q9BYV2 Q9BYV6 Q9BZ76 10193 Q9UQ13 9404 Q6PKC3 Q9UPP1 P32121 O75122 O75123 64857 Q9Y2I1 Q01344 Q00013 Q9H9D4 10197 51548 51547 Q9BYU1 Q9HAT0 6386 5054 5055 P62826 Q9UPQ3 9412 O75116 Q9UPQ9 58189 Q96F24 51552 Q00005 Q13393 Q6PKD3 Q13398 P62820 55915 9421 P78347 P78348 9420 9425 Q03989 51560 Q9Y2C9 Q71SY5 23481 P23229 51564 55922 5074 10181 P78337 8100 55929 Q9HAZ2 O43521 Q15109 O43524 26524 26523 5071 23492 P49841 P49848 Q96NH3 Q15118 P78368 Q9BYZ6

8110 Q3KNS6 P49840 P11229 5089 9448 23463 A1XKG3 P58753 23466 23468 P48507 221150 79913 51588 Q9NTI5 10155 Q9BQI3 9439 Q96NG5 10159 P78357 Q9HB90 10160 O43504 O43508 P37840 5092 P11234 P11233 2810 Q9H9G7 54625 P78362 54623 9465 9463 9464 P29375 9467 P29374 27005 Q9H0M0 27000 P22736 P42345 Q07507 Q96EK4 Q9UGU0 P04083 Q9UGU5 P04085 Q15942 9477 9474 9475 O15496 Q08830 9470 P42331 Q6P1K2 P16070 P42338 P42336 P29353 P29350 Q9H0K1 Q9BYM8 79035 55066 60412 79039 O15499 P78314 8165 O95503 55072 P78317 P78318 9495 Q8N8D1 Q8TBE0 Q9HAK2 P04070 Q9UH92 P53041 Q9Y2B9 P52597 Q96EP0 Q14643 Q13315 Q03113 Q86TI0 8174 Q96EP1 Q9H9B1 Q6P1N0 2869 Q13322 Q13323 Q14653 P46736 5906 Q9BQB4 5908 2873 1540 5901 5902 P46734 Q13330 Q6P1L6 O75962 Q9P031 P28065 Q9P035 2889 Q00059 Q9BQA5 Q86TG7 2885 Q68DY9 P43694 O15455 5911 Q14674 Q9BQA1 5914 Q14678 Q14676 O75953 P28074 P28072 P28070 Q9H0N0 5927 5928 P63279 P00519 5929 P63272 2896 P43681 51510 51512 51514 P43686 Q13351 51517 Q14683 P07148 Q13352 5925 23603 P10914 O95677 Q92858 23607 P10911 P67809 Q9NRW1 Q9NRW4 Q9NS86 Q8IZQ5 Q99490 O15519 4609 P16220 Q92851 55207 5931 P49137 5933 O15516 5935 P09936 O15530 Q9NS91 Q8IZP0 O95680 Q07343 O95684 115704 P50148 P16234 4613 Q92845 Q92844 O15524 Q92843 O15525 Q92838 Q92835 P50150 P05549 Q9NRY4 Q99471 Q9BXC9 4628 27185 Q92830 O15534 P49116 64170 Q9H7P9 Q92833 4627 Q92831 Q92826 P46087 5970 P46089 5971 Q99460 Q9NRZ9 P17535 P17544 Q99459 P17542 3304 P62140 400720 5966 3309 P04233 P34925 133584 5981 4651 3320 P52757 O00463 P52756 O00468 Q9UFF9 3315 3313 5976 P41218 4646 27161 5978 4649 719 P08648 P30556 Q5HYI8 P30559 5991 5993 O00472 Q8IZT6 P30550 55250 Q9NS56 P37198 3329 3326 3324 P08651 P41208 Q92882 5987 Q92888 4659 P28223 4673 P51449 2011 27148 2010 O00488 O00487 P30542 Q9NS68 Q9BXG8 4664 5997 Q12770 Q92878 Q9UNY4 P08621 Q12774 P51451 P09958 Q12772 Q12778 Q5TC82 Q05586 2023 Q6KC79 O00499 3350 P30530 P37173 Q99496 7704 P02452 O00425 4690 P23396 P38405 2034 2033 O00422 Q9UFB7 P62993 4686 P01116 Q9C0K7 Q16236 285282 P01112 P01111 Q9C0K0 Q9UNS2 P25100 10383 P35368 2043 2039 Q9NRP7 54764 54763 P01106 7727 Q9C0J9 P01100 Q7Z7K2 7741 Q8N726 Q7Z7K6 P14416 2054 P52735 P52736 O00444 Q96CV9 P11388 Q9NS23 773 6405 P01138 P01137 Q6UXV0 P01135 P01133 Q16254 O00459 P52740 7750 6421 2065 2064 P52747 2063 P35348 Q9NS37 Q9NRR8 2059 P10070 P09086 P10071 10363 Q9C0H5 10362 P02458 P01127 6415 2070 23647 O43593 6430 O43597 7764 23641 Q9UNN5 O75190 7756 10336 57826 P40763 P04201 2081 P46060 5111 64127 P37108 Q5HYA8 O75177 P18850 O75182 Q9NRL2 P18846 Q68CJ9 P18847 P18848 5104 5108 Q92800 P27797 7781 O43572 7780 P01185 64135 O00408 6453 6456 6455 Q9UNP9 2099 O60907 O75164 Q8IZJ4 Q9NRM6 Q9NRM7 91748 Q9H7L9 P25103 5119 P01178 23636 7791 O43581 6464 O43586 P39748 146050 P52701 P58012 6457 10320 P60321 Q99418 P26447 P25116 Q8N6T3 Q99417 Q13237 5141 O43670 5142 Q14566 Q9BY77 5144 Q9BY76 Q5VZM2 9500 P13984 5138 5139 P62913 Q13243 P25963 P09017 O43683 P20290 9510 5154 O43687 5155 O43684 51654 Q14586 5162 P11309 6496 6495 Q13257 6498 P11308 P32242 P32241 P32246 5160 116113 Q13261 5159 9518 Q14592 Q96MA1 P61604 Q13263 P32239 9531 5174 Q13268 9530 8204 Q9BY44 Q9BY41 125058 P12645 5170 222546 P20264 Q13275 P62942 Q9NSD7 9541 5187 10293 2909 Q6MZP7 O43639 O43638 201633 Q02535 Q01201 121512 Q9H082 Q13283 2908 10296 Q9UFW8 Q13287 Q6PJG2 2904 Q13285 2902 Q13286 5195 Q09161 Q13291 Q6PRX2 P09067 2917 Q8NHQ8 Q6PJH3 2915 9564 Q96ME7 10270 10273 Q9HA82 9567 O43612 Q8TAQ5 Q96DT7 Q9H063 P49959 10276 2925 P09038 2923 Q15008 Q9BY84 9575 8242 149041 10284 9578 O43623 Q8TAP6 2932 2931 Q15011 10285 1609 1605 10289 Q15014 2935 Q15811 P78423 P78424 P08559 9588 Q12788 Q15819 Q08722 1613 27121 Q8TAF7 P41134 Q9BXM7 27122 P46821 1616 Q9NSA1 P07237 Q9NSA3 P78412 Q12797 9590 Q9H7Z7 O43609 P67775 P08575 2959 Q9BXL5 1627 2956 Q15831

P29474 Q15836 P29475 92129 27107 Q96M96 Q460N5 29767 Q03014 Q9BXK5 27102 2961 84502 2960 O95619 Q8TAD8 Q9NSC2 2969 P09874 8289 Q15843 O95622 Q14511 O95625 Q6R6M4 Q03001 29777 P31785 55198 1642 Q05655 2972 79149 Q9BXJ9 Q8HWS3 P04198 1647 Q15853 Q14527 P13945 1660 Q96DN5 P41182 Q8TAK5 P41180 93474 79175 Q499Z4 79177 84525 1655 84528 1653 O15554 Q13200 O95644 150094 P51398 Q68CZ2 Q13202 P21580 Q8N7H5 Q68CZ1 P20248 64919 80155 P21579 Q8TAI7 P41162 Q9BY11 Q13216 P41161 P19235 1676 P08581 P08588 P41159 Q86SE9 P55773 Q13227 P55771 P62070 O95661 Q13233 P21554 Q92737 Q9H6Q4 Q5TB30 O14793 P15498 Q8IYN9 P84022 O00151 Q92731 Q92730 O14786 4722 O14788 10413 P28799 P27487 Q7Z6R9 64284 Q92729 Q8NFZ5 P51114 P53779 P14174 493856 4734 808 Q7Z727 Q9NR80 Q9NR81 O00167 Q9NQW6 Q7L5Y9 Q9C010 81628 3416 Q96KM6 Q8N680 811 P63104 P40425 P40424 P35080 Q9C005 333929 Q9NZI7 P48382 Q9NQX0 Q9C009 P07858 P99999 2100 Q9NR96 O00178 P48380 O95343 3428 P20823 55352 O60674 O00182 P20827 P19838 O60675 P18509 823 Q8N5V2 826 P41743 829 P11926 Q13956 Q8IZ40 4771 4772 Q5SGD2 O95352 2107 55364 2103 830 27287 80324 55367 832 O14744 O14745 4780 P32856 Q92769 P09619 Q9NZC7 O95361 80306 P10600 O95365 Q96KS0 Q9UEE9 2119 Q9BWF2 841 Q6PI57 843 4775 O14753 846 Q6ZW31 847 7804 O14757 Q92766 P09622 4790 O43295 4791 4792 Q13976 2132 Q9NQS1 Q96L34 2130 Q96L33 81669 P18545 Q9NR48 7818 3459 P60033 Q96KQ4 Q92753 Q92752 P05230 857 O14763 Q9UN86 859 Q96CA5 Q92754 6500 Q9NR50 O95382 P19883 Q9NR55 Q9NQT8 2139 861 2137 862 O95390 863 867 Q9UMX1 O14775 Q9UMX0 4799 P09601 O14777 Q92743 P06576 P00390 10492 P15408 Q96C74 3484 3482 2150 3481 P11047 O60603 Q96T88 203068 3479 2147 2146 3476 7832 6504 Q96KG9 10499 P23025 Q6UWV6 Q7Z6J4 Q96T76 2159 O43290 O43294 O43293 O14713 Q13098 Q13099 Q96C55 Q6ZN33 O43251 Q9UMR2 Q7Z6I6 64215 2173 P13686 23787 890 Q00653 P13693 891 O14727 894 896 Q9NR09 P53708 898 29080 Q9UN42 29086 P49639 P61326 O43264 Q9UMS4 64223 6541 7874 5213 148156 Q658W2 Q9NR11 O14737 Q01974 Q96ST3 10486 P60002 7867 10488 Q96T58 O14733 5207 10487 P28702 Q3MII6 O43236 7884 Q96BZ9 P50613 Q5R372 P51946 23764 Q8NFW5 10459 10458 P50616 10451 10450 6548 7879 10454 Q01968 Q8N5U6 O43242 10460 P51955 Q9NYZ3 29079 Q01955 Q96SZ6 P51959 P48357 10468 23746 P24385 P24386 9601 5245 9600 Q9UMN6 10437 10438 Q00613 10432 P24394 Q13901 9612 P49674 Q13905 P28749 Q9NQL9 P04792 56647 Q13115 6595 Q13114 6597 6599 Q14449 6598 5268 P10599 6591 O43318 Q6UY14 51762 9616 9617 51763 P21246 Q14451 P38919 Q13127 B2RXF5 5274 8301 P58546 P13631 Q96D03 24149 5270 Q02447 51773 30813 P46531 9628 Q13133 Q15796 Q13131 9641 Q13136 Q15797 9640 8315 Q8N6I1 9643 P54198 Q9UNL4 Q96CN4 P46527 51780 Q01101 P42167 Q01105 Q9NRI5 O94805 Q13144 Q13145 54815 Q9BWQ8 Q13148 5295 5296 P35869 9656 P43490 9657 O43306 P10586 P10588 57099 O43303 P10589 Q9NZZ3 Q8N6H7 9649 P43487 Q49AN0 Q13153 P43489 Q13158 9667 O15287 P54132 Q9UNF0 P63092 P13667 P0C1Z6 Q13162 Q14494 O94827 Q14493 8328 Q14498 Q13164 O15294 Q9NRC8 7013 7014 P13682 Q9NZT1 P11021 P63096 Q9C0F3 Q7Z7H5 Q9NRD1 Q13177 Q9NRD5 Q96LC9 7024 7023 10392 O94844 Q9C0F0 Q7Z7G2 P12314 9682 7020 Q9UNH7 54851 Q13185 1718 P00367 Q9BX69 P47895 Q13188 Q9BX66 Q09028 P38936 O75791 Q9UNI6 26747 54862 Q6PID4 1728 Q9BWT7 7046 P35813 P10515 8379 O75787 27242 O15234 1738 1737 7057 112950 55291 P10523 55294 7052 27252 81567 1742 1741 81565 Q9UER7 8399 7068 7067 Q5TAX3 Q15714 O15264 P19793 Q15717 8394 P30101 P31431 7064 Q01196 84619 Q9H6W3 1756 Q92793 Q9NZQ3 O15259 Q92796 Q9NRA1 P59817 27236 Q8IYT8 7073 Q9UNE2 7074 Q9UNE7 57018 80254 P43405 1763 Q9NZR4 Q04206 Q92786 Q92785 P05164 Q92784 P09525 7088 Q9NZJ7 Q15735 O00186 93594 Q01167 O00194 P63000 51720 P08243 4800 P08246 7099 7098 O15211 Q14416 7091 P14923 7090 Q9NQZ8 P30153 1789 1788 Q05516 1786 1785 Q05513 375790 51735 Q6SZW1 Q15750 Q15759 Q96CJ1 29843 57045 92283 P85298 1793 84661

51742 Q6ZNG0 51741 Q9NZM3 Q5T9L3 P05198 Q14432 Q15762 Q8IYX1 30827 O15230 Q13107 Q06830 Q9H6Z9 Q9UNA1 4839 P47804 4830 900 Q9NZN5 902 P08237 Q13112 P08238 Q9NZN8 P08235 Q9NZN9 Q92618 Q7Z628 Q9H609 Q92616 Q9NYD6 Q14CB8 O75820 Q92619 O00268 O00267 Q9BVA0 3516 Q96JM7 Q96JM2 Q5VWX1 283248 O00273 Q9ULW3 Q92610 P63208 4841 90678 Q9ULW0 Q8N587 Q92614 P07996 10533 P52564 P04004 O75815 P52565 Q7L591 4860 Q66LE6 P52566 Q08431 51094 P63211 10549 51099 P63218 4851 4854 Q96JL9 10542 Q92600 Q9NYF5 Q9ULX9 Q13813 Q9NYF8 P32927 29128 Q05397 10514 26097 29127 P20936 4862 P32929 O75807 4867 4868 Q6IT96 4869 Q96JK9 4882 3551 O00299 Q7L576 Q13829 P17302 10524 P17301 P20941 Q8NEU8 Q9ULZ3 948 Q96JJ6 10523 Q9ULR5 O95477 O00220 29108 O00221 O60760 P04040 58517 7913 O14867 Q96BA8 Q9UM47 O00238 P04049 Q96SB4 Q96SB3 Q9NYA1 O14880 O00231 2243 O00233 P10721 O00232 Q8WYH8 29110 2237 79444 960 961 29115 963 967 4898 Q9UM54 Q70UQ0 4899 Q9NYB0 P30304 P30305 P30307 O00241 2253 6609 2247 2246 Q9BVC4 58533 2244 975 6601 Q92630 6605 Q92636 57209 Q9UM63 Q92633 P29323 P29320 P31629 O00255 Q8NEZ4 Q9NQ33 O00257 81788 P17342 984 988 P05362 6613 P29317 Q9ULV4 26060 26959 Q9Y6W6 P07900 P48454 O43399 Q9Y6W5 5300 2274 P53803 O60725 P15531 990 991 Q96RU8 O14827 Q7L523 Q96RU7 Q96RU2 994 995 2267 51003 997 998 6622 999 6625 54998 51009 P62753 51008 Q04917 P49770 Q9UM07 5310 Q7Z5H3 Q8N4N8 5311 Q5T7W0 O60739 P11177 266747 2280 Q93096 Q04900 Q96RT1 2277 P48436 P49768 O00206 2290 Q9Y6Y9 P25791 Q9BV73 P48431 5321 Q96JC9 148022 O00203 Q00535 Q8IXB1 Q00534 Q96S42 P49756 P25789 P25788 7975 51026 6647 6646 5316 51028 P25787 P49758 Q08495 6660 64343 P49750 O00213 Q08499 O00212 Q96AQ6 O60716 Q96RR4 P61457 Q96JB5 6657 5327 5328 P49747 O43353 6670 P07942 6672 Q969H4 9700 P07949 Q969H0 P07948 Q8WYA6 P20916 Q9ULJ3 Q969H8 Q8IXI1 Q8WYA1 Q8IXI2 Q01844 6667 5335 Q96AX2 4005 5336 P62714 5337 5338 Q9H5J8 10577 P07951 P26232 Q969G3 Q7Z5L7 10580 Q9ULK4 Q01831 P50749 Q96S94 P50748 Q9NPH5 5347 Q96JH8 Q76NI1 Q08462 Q9H5I1 5361 64375 P50750 5362 6696 5364 4035 Q5VWQ8 Q9BUZ4 51061 Q8NER5 P68036 10552 10554 Q9H5H4 Q01826 5371 P27540 Q9ULM3 P15559 Q9ULM6 Q9BUY5 P16885 51075 O14818 Q9NPJ6 P62745 54106 10564 Q15654 9744 O43432 8412 9743 Q86YW9 29948 94121 29941 Q96BM9 116 118 Q8NFJ5 9738 Q7Z6C1 P22694 Q8WZ64 8408 O94906 P32019 Q13009 4067 P12429 Q15669 O94915 P60983 Q96SN8 Q14344 54925 Q7Z6B7 Q15672 Q13017 7101 P22681 Q9BVP2 Q76FK4 P35998 Q969Z0 P25705 Q9UDY8 8428 142678 Q8WZ42 P00441 9774 4086 7112 4087 4088 P22692 4089 9775 8445 Q86YT6 Q96BK5 9770 Q8N5F7 Q9NZ94 142 P21359 1808 8439 P62701 Q15691 Q15697 Q8IXZ2 53615 Q13033 8453 9784 8454 P54259 8452 4099 8458 7124 P54252 P54253 7126 Q96KC8 4092 4093 4094 154 156 Q13042 Q96KC2 Q13045 P45379 Q13043 P49736 7132 Q96C24 8463 7133 8467 P13796 Q96C28 1822 166 Q8NFM4 Q04941 Q8NFM5 338917 7128 Q9NQB0 P49721 P49720 Q96T37 8473 P00491 P00492 Q8N5K1 P54274 P36402 8471 P36404 7141 1831 Q96T23 Q9NZ45 Q00577 P49711 Q9NQC3 1837 P49716 P49715 P60953 Q9NQC7 Q96C00 7153 P20073 7158 7157 P19086 162239 7159 1843 Q9UDV6 54984 Q13075 1848 Q13077 7165 Q13873 7169 O94988 P10636 26038 26037 Q06787 Q86Z02 Q01094 P63172 26039 7161 P63167 P42224 1852 1850 P17275 P23945 Q92692 P63165 Q9BVI0 O94989 O15355 P22612 P42229 57109 1856 P42226 Q53QZ3 O15372 O94992 Q969S8 Q13887 Q01085 Q8IY57 P10644 Q01082 79363 Q96BD5 79365 P84095 P60900 1869 7187 7186 7189 7188 Q8WYK2 P35916 P11940 57120 P18583 27346 O75881 7181 7182 7185 7184 1877 1874 P17252 P68871 1870 P09630 O15379 Q969R5 O15391 O15392 O15397 Q6UWE0 P35900 4914 27352 84733 Q969Q1 Q92667 58473 27327 Q86YS6 Q02386 57144 P51148 P51149 1896 84766 P42262 4920 50514 P51157 P35968 P51151 Q8WZ19 P22670 Q15628 Q9NQ92 P51153 P42261 57154 Q96BI3 58480 P51159 27339 P30279 27330 84759 58487 P30281 57159 Q8WYP5 Q8WYP3 3609 3608 3607 P21333 Q9NYJ8 94104 Q6ZV73

377630 29966 Q02363 58491 57162 P84085 27300 3611 Q5XUX0 O15335 4943 Q15642 P01730 Q969V6 O15350 Q14314 O75832 O15353 Q15649 Q15648 27315 Q86Z14 Q86YP4 Q06710 Q96BF6 Q8N5A5 3622 P84077 3621 P30260 4952 O95429 4953 Q15653 Q15652

regulation of biological process Q9UKT9 P54852 P28562 Q8NDW4 259266 10657 2305 2304 2303 114991 284217 2300 O14544 Q8N3U4 O14543 2308 Q9UL63 2307 Q9H4L7 Q8WXB4 Q8NDV7 2317 2316 2 P42858 P39210 P15260 Q9UKV3 P61158 10661 Q9UKV5 10666 136319 Q9UKV8 4990 3660 3661 P62495 81876 84901 2324 P62491 55588 81873 Q9UKW4 4983 3655 Q9Y6A5 Q9UKW6 10633 3659 3672 Q5BKZ1 3673 Q13705 P21917 10645 2335 2332 3665 3667 10642 10641 10644 10643 P42830 Q9UL15 Q8WXG6 Q92538 Q9UL18 1022 1021 1020 Q06587 Q8IX07 Q5JT82 P51787 1017 10614 6709 79576 P21964 10616 1012 Q92530 3678 26191 3679 P61106 O14503 Q9UL26 Q9UL25 3690 Q8WXF0 Q9Y5Z7 Q8WXF1 Q9H4P4 6720 285527 Q96J02 P51797 1029 Q9BUB1 10626 1027 1026 1025 Q9BUB5 P20648 3685 6711 6710 3689 P05023 P07686 6714 Q9UL36 Q07890 O95136 1040 79595 P19634 57326 P20618 Q07889 6721 6726 Q8N488 Q9UKS7 Q9UKS6 Q9UL46 Q9UL45 5411 O95140 Q8NDX6 Q38SD2 1050 56005 84932 253980 57332 79589 10607 Q9UL51 6733 Q8N3V7 Q9UL54 Q9UKT4 Q9H4M9 Q92502 O95159 Q9UKL0 P50416 11108 11107 Q8WWY6 1059 6749 P25445 6760 Q9Y5V3 5430 112399 O95163 Q9NWZ3 P57059 115426 1072 51131 Q99996 1069 P33076 51132 Q14186 84033 Q14185 P49407 85360 Q14188 P02794 11116 1080 5440 5441 6772 Q08380 Q9UKN5 6774 Q99990 346171 Q66K89 P14780 O95180 Q2M3W8 51144 Q9H4B6 Q14192 Q8WWW0 51147 55504 Q9Y692 55503 84062 Q99988 Q9Y5X9 Q86X95 5451 A7MD48 Q9Y696 Q8N3J9 Q5T6S3 P14784 P57082 P17813 Q00403 56849 6776 4116 6790 O00744 5460 4131 Q9Y5Q3 9821 6794 4133 P25490 9820 Q9NX61 Q9NX65 P61981 Q9UBW7 9815 6789 Q6UUV7 O00755 Q9Y5R5 Q9Y5R4 5473 9831 Q9NX70 Q5JSP0 Q9UBX0 Q9NWV8 148327 5467 4137 9826 207 5468 54206 4139 4150 4152 4154 4155 Q9Y5S2 9841 P30825 Q96A54 O00762 P13489 Q9NWW5 Q9Y5S9 P17844 127435 5478 10673 4149 10672 9839 5494 4168 P50406 P08908 P50402 P13497 Q96QT6 Q3KRB8 56882 8516 10681 8517 Q9H4E5 55558 9846 9849 P49427 326624 4172 O15085 4173 4174 11060 P35659 4176 Q14209 8535 11065 7204 P55265 8533 4179 Q6NZI2 4171 Q6P5Z2 23043 Q9NPC1 P08047 Q9NPC8 94234 8546 4188 8543 P58304 O15090 Q9UD71 23051 Q04864 P09382 9869 8539 7205 Q8WY36 8536 Q96RL1 P61956 Q15554 1902 Q16881 7220 8553 P35638 P61968 9882 Q8IWY8 Q96IZ0 8555 P31274 P31273 P31276 Q9ULH1 Q9ULH7 23028 Q9NY61 4193 Q9Y6Q6 257 Q6DD87 Q96RK4 Q15561 8548 Q96RK0 Q93009 P02741 Q14232 P55290 P61964 Q15562 Q9Y6R4 Q8IWX8 Q9Y6R0 P36952 Q86XR7 P36956 P35625 O75592 Q8N4C8 267 Q9NPF0 P31270 23032 Q9NPF5 P61978 Q03518 Q8IX90 P43246 Q14249 11021 7248 O75586 O75582 Q9Y6K1 O75581 P10398 274 Q93063 Q93062 Q9Y6K9 Q15583 8569 1936 11030 P22392 11031 O15055 7251 O75570 94274 285 7249 O15047 P48995 11035 1947 P02751 Q15596 P61925 Q86Y82 P02790 O15062 O75569 166968 O15068 O95931 Q9Y6M4 291 Q9Y6M1 O15060 Q14C86 Q8WY64 51100 P08069 Q9NPA3 11004 51107 1956 286075 Q8NEJ9 Q9NPA8 O75553 26986 64426 64428 7272 1965 Q93034 Q93038 O95947 Q9NPB3 1969 P02775 Q14289 Q8IWS0 Q96RE7 P12931 Q7Z589 Q4VCS5 1975 Q9BUG6 Q9Y6G9 Q92574 Q13761 1978 Q58WW2 7297 P56524 O75534 Q02297 7291 O75530 7290 7295 P24928 Q96AB3 1982 Q92560 Q9Y6H5 P06276 O75528 O15020 P06239 O95977 Q06643 P0C7X2 P55209 P56539 Q7Z569 Q8WXI9 P40145 1994 1993 Q96IQ9 P06241 P21860 P56545 O95983 50618 Q8WXH2 P55212 91147 A4D1W7 Q07960 3705 3708 Q07954 Q92542 Q13794 O95996 O95995 O14593 Q8IWW6 O95999 Q9NP61 P35610 Q9NP66 O43915 9021 Q16828 P35612 9020 P35611 23081 O43918 Q9Y6C2 23085 Q9UKY1 P85037 P31260 P20594 O75509 Q8IWV1 P31249 Q9NP71 Q15506 Q9NP72 P35609 Q86XP3 P61081 23092 3728 P61073 23094 Q9Y6D6 83548 Q9Y6D5 P61077 23098 O60496 Q9BUJ2 3720 P22303 Q9Y6D9 9047 50649 9046 Q16849 P12956 Q8NEB9 Q9NP86 Q9H4W6 P02708

Q9Y6E7 Q9H582 Q9Y6E0 P61088 50650 Q96AE4 147808 Q96RG2 11091 Q9NP90 A4D1S5 222068 63976 63978 9052 O43909 23077 P24941 Q92585 Q15532 Q14201 Q7KZF4 O14672 399687 Q5VV41 Q9UBB6 Q9UBB5 9066 A5YKK6 Q9P2R6 55684 O60566 Q8IVH8 79658 10772 P27348 Q9UBC1 Q9UBC3 P27361 65018 54361 55692 9077 1107 1104 O60573 284323 10783 1108 Q9UK53 P52333 Q9UK58 Q7Z3K6 P29992 3783 1121 339122 Q6P4F7 Q7Z3K3 O60548 P53667 57402 57403 10758 Q8IVF5 O60543 1111 Q9UJU2 284312 P61289 P03372 P06401 P06400 P53675 Q9GZX9 3791 P07737 Q5JS13 1130 9093 Q9UBE8 P56705 9099 10767 57410 1122 P61296 3784 6812 6814 10765 P05129 P50570 Q9Y586 P11802 Q9P2N2 83706 O95231 O14627 6829 10733 10736 1137 Q6FHQ0 P61225 P61224 Q8N2W9 6827 Q7KZI7 65056 93986 1153 Q14938 P19784 Q06455 O95243 79685 6839 10746 1147 2475 5500 P26045 O95259 P07766 Q10586 P05106 O95257 P05107 6850 Q8ND82 P50591 Q6IA86 2495 Q13618 Q13617 Q13616 1161 O60506 Q7Z406 Q02086 O60502 O60503 57448 P61244 O14641 5511 P61247 O14640 5515 P05112 5516 O43182 26292 5518 O43186 Q13625 O43189 Q02078 1173 10725 57459 10724 10728 O14654 P05120 5524 P05121 P40337 Q9UK32 5528 5529 Q8N2K1 P04632 O43159 6872 6871 Q9BST9 P04637 O43157 P16615 1185 Q9NVW2 O95271 11228 P13591 5533 4204 4205 Q15382 5536 6869 Q15389 4209 O43166 6883 Q9BT49 4221 6885 Q9GZR2 O43167 23210 Q9HDC5 P50548 Q9BT40 P50542 1192 23216 1191 56940 O76074 Q9P2K2 9908 O76071 Q15392 84159 O76070 Q9P2K8 4214 6878 4215 P01589 4216 P01588 11236 4218 56946 6890 5562 Q86VW2 9921 5563 5565 P13569 O95292 P12235 Q02952 P61204 Q53X93 6886 Q9GZP9 Q9Y572 P61201 Q9UJM3 4240 5573 P04626 5575 Q9GZP0 5576 P04629 11218 Q99853 O14617 301 55633 302 P17936 5566 5567 307 5568 Q8TDZ2 5569 11214 309 O43150 11215 9927 5580 5581 25828 5582 5583 5584 5585 Q5U0I6 4255 5586 Q9UJF2 22794 25822 Q96PU8 310 Q96PU4 79612 Q96PU5 Q02930 P51828 5577 Q8TEB7 5578 Q9P2F6 5579 8607 Q8N302 Q495A1 5591 Q9GZV8 4261 Q9Y4P1 Q6IR47 5597 Q3SY56 5598 O76024 P15336 O76021 Q9BSW2 5590 56987 51295 P16671 324 326 328 4259 Q8TEA7 329 55657 Q9GZU7 P51843 P15311 330 Q8NCN5 55662 331 Q8NCN2 333 56994 5599 10794 8625 P83436 Q9GZT9 Q96HC4 Q9BT67 9975 4287 25818 P62508 4289 Q5T5U3 P51858 O76003 P17980 Q08211 221656 Q9NVV9 Q08209 9967 Q96HB1 Q96HZ4 8650 8655 4297 Q15418 Q8TEW0 11186 7325 8654 Q9UKD1 P30086 Q04760 4291 351 Q9UBS0 355 Q04759 356 Q9UBS5 85415 P09493 P23786 P21127 P47755 P47756 8662 11190 Q15424 7332 6003 9997 7334 8667 7336 7335 19 26205 Q8TF76 Q9UKE5 Q9H3Z4 85403 367 369 Q70SY1 Q9UBT3 11198 7329 7328 20 Q8IW93 7341 Q14106 Q8IVW6 Q14103 29 Q99909 6015 Q15438 Q8N3C0 23144 Q86X27 253260 Q53GQ0 P10451 Q8TEU7 Q9Y606 84108 Q9UBU3 P51808 Q03405 375 P51805 P09471 P17096 6009 11168 Q9UBU8 Q8TF50 Q53H12 Q14116 Q14114 Q14119 Q8WWN8 Q14118 P51812 Q9Y618 Q9UKG1 P29084 381 Q86WP2 382 Q04725 Q04724 387 Q04727 Q9NWT6 388 Q04726 389 Q9NWT8 23152 Q9UBV7 Q66K14 23154 P08151 11177 11176 P60763 P23771 41 P55347 P55345 11140 Q16787 11142 O15164 23122 390 391 23126 392 25788 394 396 397 Q99962 398 Q99963 25780 Q6KH11 11146 Q15466 Q99966 Q15464 54 Q96R06 6045 Q9BU20 7376 Q14137 7375 Q16799 23133 23135 Q9Y5J5 Q86WV8 Q3KQV3 Q9UKA4 Q9UKA8 P33151 Q14140 Q9UBP4 O15169 51218 85440 Q99958 Q14145 Q15475 Q99956 P46459 Q8TEY5 Q6ZSZ5 Q8TEY7 P14859 Q9Y5K6 6050 Q99943 Q99941 Q01664 51222 P21145 56916 P01579 11124 Q14151 Q9UBQ5 11128 Q14155 P82979 O75676 O15198 23112 25776 P33121 23118 51230 Q14160 51231 Q14164 Q5VVH5 Q14161 Q6R327 Q14168 86 87 Q14964 Q9NWH9 Q13637 Q13636 O15123 Q9NNW5 P33992 P33993 Q8TF05 Q13639 P33991 26271 P0C0L4 Q86WK6 Q1PSW9 91 P17030 94 O15119 P45974 Q6ZT07 P45973 26270 O15118 Q9UBK2 9112 Q8WWH4 Q96QB1 Q96I24 Q9H3R0 P31321 P55318 Q99081 P31323 P55316 Q7Z460 P43307 P05062 P52292 Q52LR7 P17040 P05067 Q9UBL3 O94763 P52298 O94768 P52294 9126 O15143 26258 Q9H422 6093 6096 P41597 56034 Q9BTC8 P21980 Q9UBM7 O15151 P11717 O15156 O94776 9138 Q7Z434 202559

Q02156 P17023 Q8N392 Q86WH2 P17020 Q8N393 Q9UBN7 Q9UBN6 9146 O75626 Q9UBF6 9149 Q8TF47 P30041 Q6NYC1 Q9H488 P30044 114822 Q58EX7 P53567 P30047 P30048 B3KY43 3836 P22415 Q9UK80 Q03468 P08123 Q92499 Q9UJW9 Q13683 O75629 P47712 Q52LW3 Q96HU1 Q8IVT5 Q9UBG7 Q07820 Q07817 Q9UJX2 Q9UJX0 P08134 P08133 P17081 P08138 Q9Y5B6 Q9Y5B9 P09429 O75604 P10415 Q9P2X3 Q9P2X7 23189 P35711 Q7Z494 23186 P09430 728642 P08107 O15111 374354 Q9P2Y4 Q04771 23192 O15105 P45983 P45984 Q2M1Z3 Q6NX49 65125 25909 P54619 9182 22870 2550 9181 P51587 9184 P17676 Q8TDD1 54474 2547 P37288 57504 Q9HCJ0 Q9HCJ2 10890 3875 6901 57506 A1A4S6 Q9Y3Z3 P28335 25913 P28330 P08729 Q08AM6 2562 Q5VTR2 P55957 O60216 P62244 79763 57510 54487 Q9H2G9 Q17R89 Q9H2G4 22850 Q8NC24 79791 54496 6927 Q8IUD2 6929 P62256 Q6AHZ1 57521 57522 6921 P62258 10875 6926 6925 Q6IQ32 Q17R98 22866 Q9UIS9 P31943 P31946 P31947 P17661 P54646 57534 79784 P16333 6938 Q8IUC4 Q8IUC6 P62263 P31949 5602 5604 6936 Q9Y468 P08758 P27037 Q9H2M9 Q9Y463 Q9Y466 Q8TDI0 Q9UIL8 Q9NUY8 P39019 P15056 10856 Q86VE0 P16383 Q6ZRI8 57544 Q08999 Q8N264 644943 5610 6945 P07437 Q9Y478 22846 P51553 P30679 P50222 Q14814 P28370 Q07666 P80098 124583 P52888 Q06330 10865 10868 P19419 Q9HCM4 5624 P49286 Q9H2K2 1285 1284 Q9H2K0 P21709 Q8IUH5 Q9HCL2 1277 83853 4302 Q13501 P51575 22827 Q13503 Q14839 Q8NC51 Q13506 Q9HCK5 Q9HCK4 Q8NBS9 1289 192669 153090 50943 P08754 4318 Q9Y3Y2 Q86UW7 Q9BS34 P01241 6993 Q9BRR9 O00541 23322 P14555 Q9Y3Q8 Q9UIH9 23326 25988 23327 P15884 Q8WUU5 Q8WV60 10011 55726 5654 10010 P25205 10013 84289 P01236 55723 11346 10014 11345 10016 135295 64682 Q15269 Q9UII4 4343 Q9BS26 23332 25998 P13236 Q99755 400 402 Q99750 406 Q9BRP8 Q99759 408 84271 409 Q99757 Q01518 Q09472 Q8N1G0 23309 5682 5683 5684 4354 5685 4355 5686 116986 O00560 116987 O00562 23303 Q99743 54413 Q99741 55743 3014 Q15286 Q9Y450 11326 11325 P51531 4361 5692 5693 8721 8720 Q8TCX5 Q9NUX5 P51532 P14543 P15882 55750 55755 P60484 Q15291 11331 5687 55758 5688 4358 10001 84299 8718 P23497 8717 P01243 P26583 Q9Y3M2 Q6FGG2 O00506 P46199 7402 Q9Y3M8 25942 54431 Q9NUQ3 Q8WUY8 Q8WUY9 Q96P48 433 79735 8726 Q9H2D6 P55075 P55072 P01275 8743 8744 7410 90993 7412 Q9HCE7 Q08117 3054 Q05086 Q9BRU2 P37231 Q9HCE1 444 54443 79723 8737 P55085 P55083 8738 Q6IQ22 11315 P01266 8754 7422 Q96GA9 3068 Q9HCD5 P52815 22893 3066 65108 3065 Q9UIF9 Q9BRT3 O75293 O75290 79753 Q96P20 55787 55785 7415 P61764 7414 3070 Q86UX7 7430 7433 8766 Q9UIG0 P52824 3075 3074 Q9Y3P9 80728 462 P17612 466 468 55796 7428 8772 Q16623 P18085 P10114 Q9Y4K4 O75386 O75385 Q86VQ3 Q9Y4K3 Q76L83 3084 472 P48729 473 474 476 477 23286 P20396 8767 P43034 3093 P35408 Q15303 3091 Q16633 P55036 Q16635 Q15306 Q86VP6 O75376 3096 Q86VP1 O75381 Q9NVP2 488 Q9H2X0 6117 7448 P55042 P55040 Q16643 Q15311 P43026 Q9HCY8 O00602 8795 Q16649 Q16644 P12757 7468 Q8WVM8 O75367 P19397 P12755 O75362 Q8TDS5 O75360 495 498 23263 6128 7458 Q5VUA4 Q8WVM0 Q9BSI4 Q8IV61 Q16650 Q16659 P20393 7476 Q16656 Q15329 Q8NCA9 P18074 Q8WVL7 P55055 59277 93166 Q86VN1 P68371 Q16665 51317 P55061 Q9P2E9 Q8TDY2 11261 Q16666 Q5VUG0 P35453 O75340 P09172 P46109 51324 11269 Q16670 P46108 Q14012 Q14011 Q86W54 Q15349 P46100 Q9Y4H2 23256 P35443 Q5PSV4 7490 84232 Q8WW38 Q9GZM8 Q99835 23229 49854 P98179 O43734 Q86VS8 P15822 O43739 Q8WVQ1 Q02878 51341 Q9NVM4 P02549 Q96PK6 11243 55704 P55010 Q8NCF5 55703 Q9NVM9 P48745 P23443 6188 Q15369 Q8IUX7 O43741 9217 11252 Q9BSM1 P10147 O75312 6182 P11474 Q15370 P27816 P57682 3909 P02538 Q99819 P27815 Q99816 P48730 P23458 9208 Q99814 P48736 P98194 Q15375 P78545 6196 6199 6198 P98155 P10997 O95714 P78540 O43711 6193 O43715 6195 6194 10811 O43719 Q9Y4C1 P62195 Q86VI3 10817 Q4LE39 P46940 9218 65082 O95715 9219 P98161 P07355 A8K8V0 3911 3912 Q13522 P78536 22803 9238 Q13526 22806 22807 57473 9230 83737 9231 Q3V6T2 3925 P46934

P46937 83746 P46939 Q9HD26 Q96GN5 P41240 O60296 Q32P28 Q4LE28 P98170 P42574 P98172 O60292 P42575 P98171 P98174 Q14865 P34947 P29597 Q9H305 Q13535 Q9H307 P29590 Q7Z2Q5 Q63HK5 P41235 Q9HCP6 P41231 Q96GM5 Q9HD15 P16284 Q9Y4E5 O60266 O60264 3932 O60260 9255 P78552 Q13547 Q13546 P19320 9252 9253 P41223 P41222 O43707 P41220 P20309 Q9NVJ2 P41229 P78560 O60271 9267 Q13554 O95751 Q8IV45 Q13557 P78509 P78504 9261 3959 P00749 Q63HR2 P00747 Q9BSG1 Q96PE2 P00742 3953 Q14894 Q13563 P62158 3956 2626 Q9NVC6 9270 P11413 P62166 Q9UIV1 P00734 Q9HD67 Q5BJF2 P24723 Q13574 3965 Q13573 Q13572 Q9HCU4 53916 P55884 93134 Q9H2S9 P32320 P78527 O60229 85509 P41279 P20338 P20339 P20336 121457 P20337 2644 1312 P41273 Q86VK4 Q04656 Q16610 P20333 Q9Y4A8 O95786 Q13586 Q16611 P18031 Q9P286 P34972 Q9P287 P62191 Q96H20 O60238 1326 Q63HQ2 Q8TDM6 P67870 Q9HCS4 P07384 P20340 P00750 Q9HD40 P21675 P08833 Q9Y2W7 Q6P2D0 P48058 2672 Q12948 22992 Q6QNY1 P61020 79894 Q6PCE1 Q7L0Q8 Q9BZI1 2665 2664 57620 Q9HBH0 P61026 Q8N103 P08842 Q12950 Q12955 Q9Y2X7 Q12952 Q9Y2X9 Q12959 Q8NB12 Q8NAP3 Q9P107 5706 5707 79885 5708 5709 P16473 2676 P16471 P48039 5700 5701 5702 Q9Y2Y0 Q9UI12 5704 Q008S8 5705 65264 P54753 P50395 Q9Y2Y8 P25391 Q12968 22974 5717 57646 83933 Q9UQB3 Q9UQB8 5713 57649 5716 Q12972 Q9H1D0 P54764 P54762 Q0D2J5 P54760 Q9H9T3 P48023 1374 Q9BZF9 Q6P2C8 Q12979 22985 5728 Q99698 Q9HBE1 Q99697 2697 400961 Q9UHR5 Q9UHR4 Q6PCD5 Q9H1C4 Q12981 Q12982 P28482 Q9UHJ3 Q12986 1385 Q9UHJ9 P51668 P51665 P19532 Q9P0J0 Q9P0J1 P19525 5739 57669 57662 P15173 P07550 5733 Q9Y2T1 10971 P08887 10973 P51671 Q9Y2T7 Q9UHK0 P51679 Q5VST9 P19544 10987 57678 P18206 1388 Q9P0K8 1387 1386 P62328 10981 P42771 Q9H1I8 5747 10985 Q14703 22937 22938 Q9UHL9 Q9P0L0 Q9P0L2 22931 P62330 57689 P62333 284119 P15153 1399 1398 P15151 Q9BZK7 P61006 Q9Y2V2 Q9Y371 P51693 5770 Q9Y376 P51692 P28472 Q9BQY4 P48061 Q9P0M2 Q03164 Q9P0M6 55035 440193 5764 Q5H9I0 Q9Y385 5768 P61019 Q9Y2W1 P61018 P06213 Q15139 Q08043 Q9UPV9 P80188 P13349 115557 P38646 P52952 Q8WTS6 126208 Q8WTS1 79810 Q99640 79811 79813 10133 P27986 P02686 10138 Q9UQ80 Q9Y2P0 Q9UHG2 Q9UQ88 5796 P51636 Q8WTR2 8819 Q15154 10142 8812 Q99638 10146 P02675 P01344 P01343 23429 Q9Y320 3142 Q9NU63 Q99623 Q8NAF0 55869 5798 8829 Q9UPY3 8826 Q8N960 P01375 P01374 Q9UPY8 23439 Q9Y337 Q9Y2R2 8841 Q9UHI8 Q9UHI6 Q3KNV8 23435 3151 P12004 10128 Q15172 Q3KNW1 3148 Q15170 545 546 3146 Q8WTP8 P40933 Q96EY1 Q15173 Q8WU17 P25311 8837 7508 P24043 23409 7520 8851 Q9UPR3 8852 Q9H9S4 Q9Y2K7 Q12904 Q9Y2K6 Q8WU90 Q9H9S0 Q9UHB6 3162 Q12905 O00622 O95076 Q12906 Q68EM7 Q9BZE0 552 553 Q9BZE4 P42704 3159 Q99683 Q8WTW4 Q99684 8844 Q15185 P49336 7518 P05771 Q96FA3 O00635 8864 7532 7531 7534 7533 P55198 Q9UHC3 23411 O00631 23414 3171 10109 563 Q00987 Q99675 3169 Q9NU19 Q9H1A4 P05783 7529 7528 6210 Q86U86 Q9UHD2 3187 Q9UPT9 Q12929 Q9Y2M5 3185 3184 Q9NU22 571 3182 572 573 Q99661 P51608 7536 6207 P26678 8882 3192 8880 3190 8881 Q60FE5 Q9UPU9 Q12933 8887 7555 P51610 580 P16410 54583 80854 Q99653 P51617 8879 7549 8878 P60568 Q9BZZ5 Q13478 Q13477 10092 6231 8892 10095 Q9UQP3 10094 6236 8896 P15923 Q96FV9 595 84324 Q9BRI3 Q13485 Q9HC98 Q13489 P03950 6242 O00716 Q16512 Q16513 Q16514 Q9UIA0 O75496 O75494 Q7LBC6 P10242 P10243 P10244 84312 P27918 6238 51412 Q13492 Q16520 6239 Q13490 Q9UQR1 6256 Q9HBW0 O75489 Q16526 Q2M1K9 280636 23380 23381 P23528 7579 Q16531 P09237 6262 Q16539 10081 Q8N9N2 O75478 Q15208 Q16537 163126 Q9H257 Q86UL3 O75475 Q9Y3L3 Q9Y3L5 Q02790 P12883 P52907 P31150 P24864 10087 6259 84333 P60520 Q16543 6275 O75469 64750 Q9Y3E0 23365 P14635 O75461 64759 23368 Q9UQL6 P10275 P10276 P35579 O75460 P35580 Q8NBF2 P23588 Q99728 Q99729 Q16555 Q99726 Q15223 55806 Q8WV24 9311 Q86UR1 9314 Q5JQC9 O43889 P36896 P36894 23378 P15976 P35568 6282 23370 55810 Q02763

P01308 120892 Q99717 9306 P01303 Q15233 9322 P02671 Q8TCU4 P61803 Q8TCU6 O95813 9326 O75446 Q96FX2 O75444 Q86V24 6294 P36888 Q99700 Q9H1Y0 51465 Q99708 O95817 Q9BRK4 Q16576 9318 O95819 55827 64780 64782 64784 O75437 Q08050 P39905 64786 P14625 Q8N9R8 Q02750 P35548 138151 55832 51474 84376 54509 Q5VTD9 Q15257 Q16581 P53367 O95831 P53365 O95835 P57729 O95833 P12814 10928 56252 57584 Q9H204 9341 56259 10933 Q9Y3A5 Q9HC16 10935 10938 Q7RTN6 P39060 83871 O95837 Q6NVY8 P15090 Q8WUD6 Q9P0W2 22926 22927 Q13404 57592 10939 57594 Q14738 O43847 22920 57591 9350 P57735 P39059 Q08945 Q9UHX1 22919 Q9UI95 9367 9368 O43812 Q13418 O43815 O43819 Q86UE4 Q86UE3 Q9Y3C7 Q8N163 P42680 P42681 O95859 O60383 P42684 P42685 O60381 Q86UE8 P21741 Q13422 Q13426 O95865 O43823 B2RTY4 Q8NB78 2735 P19438 P21757 O43829 P68400 56288 2730 1408 1407 1406 2737 2736 9388 Q9HC62 Q13432 Q96G30 Q8WUI4 8050 Q7RTR2 Q9H244 P23508 P62277 P43119 2741 Q9HC57 P09211 Q9HC52 P43115 Q5H9R7 Q14774 Q13443 9391 P11532 Q96G25 8065 O95881 P23515 Q9UQE7 P23511 Q8WUH2 P24844 Q14781 P23510 O14492 8078 Q9H228 O14495 Q9P0T4 P12830 P19474 59341 59343 P18146 Q9H223 84305 1432 O60343 Q14790 O60341 Q13464 P53355 Q9BZS1 P53350 Q9P0U3 Q9P0U4 P19484 Q9UHV2 O60356 2773 Q9HC29 Q7L9L4 P24821 O14497 Q9BZR8 Q9BZR6 Q9H0E2 P25054 Q9H0E3 Q9Y265 O75940 P49023 1460 Q12824 8091 124790 1459 5817 1457 1454 O00391 1453 155061 Q9Y275 284695 Q00839 Q9H0D6 Q92974 Q12834 Q12837 P16104 5829 Q9H0C8 P49006 5822 P10809 Q96EB6 Q59EK9 Q5S007 O75925 P27694 P27695 Q9H8S9 Q92956 Q9BQ51 Q9BYE7 Q06124 P17405 P37023 P13056 57761 5830 55109 Q9Y297 O75928 P26358 Q12851 P26374 Q6NUN9 Q86SX6 Q92949 Q99583 Q12857 1491 1488 1487 283337 Q99576 Q99574 64061 P26367 O75915 P25098 O75901 P49069 5861 5862 Q96N96 Q8NA42 114548 Q96MX3 57787 Q9HAJ7 1499 O60884 Q6PJW8 O14981 P61587 Q9Y230 P61586 O75909 Q12873 Q9H0H5 Q9UGI0 Q12872 P29466 Q9Y239 5870 5871 Q15907 Q15906 Q9UGI9 O00358 Q9BPY8 57798 3207 Q8TBC4 O60895 P17482 5863 O60890 P17480 Q9Y240 604 5865 Q9Y242 5867 5868 5869 P04179 Q12884 Q15910 Q9UGJ0 Q12888 Q9Y243 5883 Q9BPX5 Q9BQ95 Q15915 O60869 P16118 Q8NA19 P38398 55145 3215 Q96N67 610 Q92990 Q5T2W1 4543 Q9Y250 613 Q5TCX8 Q92994 Q6NUQ1 Q92993 5877 P63302 5878 5879 Q92997 P10826 P10827 P10828 Q9UGK8 221937 5894 P31749 Q7L8A9 Q9H8V3 P53999 27032 79101 P40692 3226 3225 55159 Q96MT8 P04150 5888 P63313 Q9UGL1 127829 A6NHR9 P04156 Q92985 Q15019 O96005 O96004 8930 O00308 P56199 P14317 P14316 P14314 O00300 23568 P50897 23569 Q99523 23560 Q15022 8924 637 79937 5898 P62873 639 10256 Q15025 P62875 O96018 O96017 P06702 Q15027 O96019 P16989 P36575 O96013 P35249 Q03933 641 P35251 Q6B0B8 79923 8932 648 P47224 8939 Q15036 P60228 Q16363 O00327 O96028 O00329 P48552 Q8TAU3 P48551 O96020 3265 3263 O60828 P15621 P11274 Q99500 P35240 8945 P01019 8943 79959 O14964 Q15047 O14965 P23246 O00330 P35225 P35222 P35227 P35226 P11277 O14979 O75084 O75081 663 Q15051 P35232 53335 3269 Q9H093 P01008 668 Q15052 Q96MH2 O14974 Q59EA4 Q15057 Q16385 P24588 Q15056 7629 O43474 P06733 P01042 Q92934 6311 P62837 P05408 3281 79971 672 673 53340 P15692 O14908 6304 57708 P62834 10213 Q92930 P05412 P05413 6320 3297 23532 Q99558 P14373 57713 688 Q15070 689 Q15077 Q8N895 Q92922 P06753 Q15078 P01023 Q9BQ15 23509 P48594 P56178 P56177 Q12802 Q8NHY6 Q12809 O75044 O75051 P13010 Q15084 Q0IIM8 Q99543 A7KAX9 O14920 57727 P56180 O14921 P62854 O43463 Q92903 O43464 Q92905 Q8NHX1 Q12816 P06729 23512 23513 57732 O14936 O14939 P40616 10202 Q92900 Q9BYX4 P36508 P36507 Q14686 P19174 Q9BZ95 O43559 O43557 Q9UPN3 Q9H165 Q9UPN7 P13861 Q9H161 Q9H9F9 Q9H0U4 P07196 Q9H160 P49810 5017 P83731 P78395 Q9BQG0 5018 P07199 P49815 Q9HB75 Q13363 Q14693 Q8TBP0 Q9HAV4 5034 P62805 6368 P46782 5036 P46781 5037 79084 Q96F45 Q9Y2H1 Q9H0T7 60489 Q9Y2H8 5029 P00533 Q13370 Q16401 Q9HAU4 P24522 Q9BYV2 Q9HB63

Q9BYV6 Q9BZ76 10193 Q9UQ13 9404 Q6PKC3 Q9UPP1 P32121 O75122 O75123 64857 Q9Y2I1 Q01344 Q00013 Q9H9D4 10197 51548 51547 Q9BYU1 Q9HAT0 6386 5054 5055 P62826 Q9UPQ3 9412 O75116 Q9UPQ9 58189 Q96F24 Q4AC94 51552 Q00005 Q13393 Q6PKD3 Q13398 P62820 55915 9421 P78347 P78348 9420 9425 Q03989 51560 Q9Y2C9 Q71SY5 23481 P23229 51564 55922 5074 10181 P78337 8100 55929 Q9HAZ2 O43521 Q15109 O43524 26524 26523 5071 23492 P49841 P49848 Q96NH3 Q15118 P78368 Q9BYZ6 8110 Q3KNS6 P49840 P11229 5089 9448 23463 A1XKG3 P58753 23466 Q96EV8 23468 P48507 221150 79913 51588 Q9NTI5 10155 Q9BQI3 9439 Q96NG5 10159 P78357 Q9HB90 10160 O43504 O43508 P37840 5092 P11234 P11233 2810 51593 Q9H9G7 54625 P78362 54623 9465 9463 9464 P29375 9467 P29374 27005 Q9H0M0 27000 P22736 P42345 Q07507 Q96EK4 Q9UGU0 P04083 Q9UGU5 P04085 Q15942 9477 9474 9475 O15496 Q08830 9470 P42331 Q6P1K2 P16070 P42338 P42336 P29353 Q5JXB2 P29350 Q9H0K1 Q9BYM8 79035 55066 60412 79039 O15499 P78314 8165 O95503 55072 P78317 P78318 9495 Q8N8D1 Q8TBE0 Q9HAK2 P04070 Q9UH92 P53041 P07101 Q9Y2B9 P52597 Q96EP0 Q14643 Q13315 Q03113 Q86TI0 8174 Q96EP1 Q9H9B1 Q6P1N0 2869 Q13322 Q13323 Q14653 P46736 5906 Q9BQB4 5908 2873 1540 5901 5902 P46734 Q13330 Q6P1L6 O75962 Q9P031 P28065 Q9P035 2889 Q00059 Q9BQA5 Q86TG7 2885 Q68DY9 P43694 O15455 5911 Q14674 Q9BQA1 5914 Q14678 Q14676 O75953 P28074 P28072 P28070 Q9H0N0 5927 5928 P63279 P00519 5929 P63272 2896 P43681 51510 51512 51514 P43686 Q13351 51517 Q14683 P07148 Q13352 5925 23603 P10914 O95677 Q92858 23607 P10911 P67809 Q9NRW1 Q9NRW4 Q9NS86 Q8IZQ5 Q99490 O15519 4609 P16220 Q92851 55207 5931 P49137 5933 O15516 5935 P09936 O15530 Q9NS91 Q8IZP0 O95680 Q9H832 Q07343 O95684 115704 P50148 P16234 4613 Q92845 Q92844 O15524 Q92843 O15525 Q92838 Q92835 P50150 P05549 Q9NRY4 Q99471 Q9BXC9 4628 27185 Q92830 O15534 P49116 64170 Q9H7P9 Q92833 4627 Q92831 Q92826 P46087 P09917 5970 P46089 5971 Q99460 Q9NRZ9 P17535 P17544 Q99459 P17542 3304 P62140 400720 5966 3309 P04233 P34925 P07307 133584 5981 4651 3320 P52757 O00463 P52756 O00468 Q9UFF9 3315 3313 710 4645 5976 P41218 4646 27161 5978 4649 719 P08648 P30556 Q5HYI8 P30559 5991 5993 O00472 Q8IZT6 P30550 55250 Q9NS56 P37198 3329 3326 720 3324 722 P08651 P41208 Q92882 5987 Q92888 4659 P28223 137964 4673 P51449 2011 27148 2010 O00488 O00487 P30542 Q9NS68 Q9BXG8 4664 4665 5997 Q12770 Q92878 P04264 Q9UNY4 P08621 Q12774 P51451 P09958 Q12772 P30536 Q12778 Q5TC82 Q05586 2023 Q6KC79 O00499 3350 P30530 Q96LR5 P37173 Q99496 Q99497 7704 P02452 O00425 4690 P23396 P38405 2034 2033 O00422 Q9UFB7 P62993 4686 P01116 Q9C0K7 Q16236 285282 P01112 P01111 Q9C0K0 Q9UNS2 P25100 10383 P35368 2043 2039 Q9NRP7 54764 54763 P01106 Q96CW1 7727 Q9C0J9 P01100 Q7Z7K2 7741 Q8N726 Q7Z7K6 P14416 2054 P52735 P52736 O00444 Q96CV9 P11388 Q9NS23 773 6405 P01138 P01137 Q6UXV0 P01135 P01133 Q16254 O00459 P52740 P02461 7750 6421 23673 2065 2064 P52747 2063 P35348 781 Q9NS37 Q9NRR8 2059 P10070 P09086 P10071 10363 Q9C0H5 10362 P02458 P01127 6415 2070 23647 O43593 6430 O43597 7764 23641 Q9UNN5 O75190 7756 10336 57826 P40763 P04201 2081 P46060 5111 64127 P37108 Q5HYA8 O75177 P18850 O75182 Q9NRL2 P18846 Q68CJ9 P18847 P18848 5104 P46059 5108 Q92800 P27797 7781 O43572 7780 P01185 64135 O00408 6453 6456 6455 Q9UNP9 2099 O60907 O75164 P50993 10318 Q8IZJ4 Q9NRM6 Q9NRM7 91748 Q9H7L9 P25103 5119 P01178 23636 7791 O43581 6464 O43586 P39748 146050 P52701 P58012 6457 10320 P60321 Q99418 P26447 P25116 Q8N6T3 Q99417 Q13237 5141 O43670 5142 Q14566 Q9BY77 5144 Q9BY76 Q5VZM2 9500 P13984 5138 5139 P62913 Q13243 P25963 P09017 O43683 P20290 9510 5154 O43687 5155 O43684 51654 Q14586 5162 P11309 6496 6495 Q13257 6498 P62937 P11308 P32242 P32241 P32246 5160 116113 Q13261 5159 9518 Q14592 Q96MA1 P61604 P21589 Q13263 P32239 9531 5174 Q13268 9530 8204 Q9BY44 Q9BY41 125058 P12645 5170 222546 P20264 Q13275 P62942

Q9NSD7 9541 5187 10293 9546 2909 Q6MZP7 O43639 O43638 201633 Q02535 Q01201 121512 Q9H082 Q13283 2908 10296 Q9UFW8 Q13287 Q6PJG2 2904 Q13285 2902 Q13286 5195 Q09161 9555 Q13291 Q6PRX2 P09067 2917 Q8NHQ8 8218 Q6PJH3 2915 9564 Q96ME7 10270 10273 Q9HA82 9567 O43612 23586 Q8TAQ5 Q96DT7 Q9H063 P49959 10276 2925 P09038 2923 Q15008 Q9BY84 9575 8242 149041 10284 9578 O43623 Q8TAP6 2932 2931 Q15011 10285 1609 1605 10289 Q15014 2935 Q15811 P78423 P78424 P08559 9588 Q12788 Q15819 Q08722 1613 27121 Q8TAF7 P41134 Q9BXM7 27122 P46821 1616 Q9NSA1 P07237 Q9NSA3 P78412 Q12797 9590 Q9H7Z7 O43609 P67775 P08575 P08572 2959 Q9BXL5 1627 2956 Q15831 P29474 Q15836 P29475 92129 27107 Q96M96 Q460N5 29767 Q03014 Q9BXK5 27102 2961 84502 2960 O95619 Q8TAD8 Q9NSC2 2969 P09874 8289 Q15843 O95622 Q14511 O95625 Q6R6M4 Q03001 29777 P31785 55198 1642 Q05655 2972 79149 Q9BXJ9 Q8HWS3 P04198 1647 Q15853 Q14527 P13945 1660 Q96DN5 P41182 Q8TAK5 P41181 P41180 93474 79175 Q499Z4 79177 84525 1655 84528 1653 O15554 Q13200 O95644 150094 51606 P51398 Q68CZ2 Q13202 P21580 Q8N7H5 Q68CZ1 P20248 64919 80155 P21579 Q8TAI7 P41162 Q9BXP5 Q9BY11 Q13216 P41161 P19235 P19237 1676 P08581 1674 P08588 P41159 Q86SE9 P55773 Q13227 P55771 P62070 O95661 Q13233 P21554 Q92737 Q9H6Q4 Q5TB30 O14793 P15498 Q8IYN9 P84022 O00151 Q92731 Q92730 O14786 4722 O14788 10413 P28799 P27487 Q7Z6R9 64284 Q92729 Q8NFZ5 P51114 P53779 P14174 493856 4734 808 Q7Z727 Q9NR80 Q9NR81 O00167 Q9NQW6 Q7L5Y9 Q9C010 81628 3416 Q96KM6 Q8N680 811 P63104 P40425 P40424 P35080 Q9C005 333929 Q9NZI7 P48382 Q9NQX0 Q9C009 P07858 P99999 2100 Q9NR96 O00178 P48380 O95343 3428 81622 P20823 55352 O60674 O00182 P20827 P19838 O60675 3421 P18509 823 Q8N5V2 826 Q9NZJ4 P41743 829 P11926 Q13956 Q8IZ40 4771 4772 Q5SGD2 O95352 2107 55364 2103 830 27287 80324 55367 832 O14744 O14745 4780 P32856 Q92769 P09619 Q9NZC7 O95361 80306 P10600 O95365 Q96KS0 Q9UEE9 2119 Q9BWF2 841 Q6PI57 843 4775 O14753 846 Q6ZW31 847 7804 O14757 Q92766 P09622 4790 O43295 4791 4792 Q13976 Q9NZD8 2132 Q9NQS1 Q96L34 2130 Q96L33 81669 P18545 Q9NR48 7818 3459 P60033 Q96KQ4 Q92753 Q92752 P05230 857 O14763 Q9UN86 859 Q96CA5 Q92754 6500 P11912 Q9NR50 O95382 P19883 Q9NR55 Q9NQT8 2139 861 2137 862 O95390 863 867 Q9UMX1 O14775 Q9UMX0 4799 P09601 O14777 Q92743 P06576 P00390 10492 P15408 Q96C74 3484 3482 2150 3481 P11047 O60603 Q96T88 203068 3479 2147 2146 3476 7832 6504 Q96KG9 10499 P23025 Q6UWV6 Q7Z6J4 Q96T76 O14717 2159 O43290 O43294 O43293 O14713 Q13098 Q13099 Q96C55 Q6ZN33 O43251 Q9UMR2 Q7Z6I6 64215 2173 P13686 23787 890 Q00653 P13693 891 O14727 894 896 Q9NR09 P53708 898 29080 10477 Q9UN42 29086 P49639 P61326 O43264 Q9UMS4 64223 6541 7874 5213 148156 Q658W2 Q9NR11 O14737 Q01974 Q96ST3 10486 P60002 7867 10488 Q96T58 O14733 5207 10487 P28702 Q3MII6 O43236 7884 Q96BZ9 P50613 Q5R372 P51946 23764 Q8NFW5 10459 10458 P50616 10451 10450 6548 7879 10454 Q01968 Q8N5U6 O43242 10460 P51955 Q9NYZ3 29079 Q01955 Q96SZ6 P51959 P48357 10468 23746 P24385 P24386 9601 5245 9600 Q9UMN6 10437 10438 Q00613 10432 P24394 Q13901 9612 P49674 Q13905 P28749 Q9NQL9 P04792 56647 Q13115 6595 Q13114 6597 6599 Q14449 6598 5268 P10599 6591 O43318 Q6UY14 51762 9616 9617 51763 P21246 Q14451 P38919 Q13127 B2RXF5 5274 8301 P58546 P13631 Q96D03 24149 5270 Q02447 51773 30813 P46531 9628 Q13133 Q15796 Q13131 9641 Q13136 Q15797 9640 8315 Q8N6I1 9643 P54198 Q9UNL4 Q96CN4 P46527 51780 Q01101 P42167 Q01105 Q9NRI5 O94805 Q13144 Q13145 54815 Q9BWQ8 Q13148 5295 5296 P35869 9656 P43490 9657 O43306 P10586 P10588 57099 O43303 P10589 Q9NZZ3 Q8N6H7 9649 P43487 Q49AN0 Q13153 P43489 Q13158 9667 O15287 P54132 Q9UNF0 P63092 P13667 P0C1Z6 Q02413 Q13162 Q14494 O94827 Q14493 8328 Q14498 Q13164 O15294 Q9NRC8 7013 7014 P13682 Q9NZT1 P11021 P63096 Q9C0F3 Q7Z7H5 Q9NRD1 Q13177 Q9NRD5 Q96LC9 7024 7023 10392 O94844 Q9C0F0 Q7Z7G2 P12314

9682 7020 Q9UNH7 54851 Q13185 1718 P00367 Q9BX69 Q9UEW8 P47895 Q13188 Q9BX66 Q09028 P38936 O75791 Q9UNI6 26747 54862 Q6PID4 1728 Q9BWT7 7046 P35813 P10515 8379 O75787 27242 O15234 1738 P05177 1737 7057 112950 55291 P10523 55294 7052 27252 81567 1742 1741 81565 Q9UER7 8399 7068 7067 Q5TAX3 Q15714 O15264 P19793 Q15717 8394 P30101 P31431 7064 Q01196 84619 Q9H6W3 1756 Q92793 Q9NZQ3 O15259 Q92796 P05155 Q9NRA1 P59817 27236 Q8IYT8 7073 Q9UNE2 7074 Q9UNE7 57018 80254 P43405 1763 Q9NZR4 Q04206 Q92786 Q92785 P05164 Q92784 P09525 7088 Q9NZJ7 Q15735 O00186 Q53SB5 93594 Q01167 O00194 P63000 51720 P08243 4800 P08246 7099 7098 Q15742 Q9NQZ2 O15211 Q14416 7091 P14923 7090 Q9NQZ8 P30153 1789 1788 Q05516 1786 1785 Q05513 P63010 375790 51735 Q6SZW1 Q15750 Q15759 Q96CJ1 29843 57045 92283 P85298 1793 84661 51742 Q6ZNG0 51741 Q9NZM3 Q5T9L3 P05198 Q14432 Q15762 Q8IYX1 30827 O15230 Q13107 Q06830 Q9H6Z9 Q9UNA1 4839 P47804 4830 900 51752 Q9NZN5 902 P08237 Q13112 P08238 Q9NZN8 P08235 Q9NZN9 Q92618 Q7Z628 Q9H609 Q92616 Q9NYD6 Q14CB8 O75820 Q92619 O00268 O00267 Q9BVA0 3516 Q96JM7 Q96JM2 Q5VWX1 283248 O00273 Q9ULW3 Q92610 P63208 4841 90678 Q9ULW0 Q8N587 Q92614 P07996 10533 P04003 P52564 P04004 O75815 P52565 Q7L591 4860 Q66LE6 P52566 Q08431 51094 P63211 10549 P20963 51099 P63218 4851 4854 Q96JL9 10542 Q92600 Q9NYF5 Q9ULX9 Q13813 Q9NYF8 P32927 29128 Q05397 10514 26097 29127 P20936 4862 P32929 O75807 4867 4868 Q6IT96 4869 Q96JK9 4882 3551 O00299 Q7L576 Q13829 P17302 10524 P17301 P20941 Q8NEU8 Q9ULZ3 948 Q96JJ6 10523 Q9ULR5 O95477 O00220 O95470 29108 O00221 O60760 29102 P04040 58517 7913 O14867 Q96BA8 Q9UM47 O00238 P04049 Q96SB4 Q96SB3 Q9NYA1 O14880 O00231 2243 O00233 P10721 O00232 Q8WYH8 29110 2237 79444 960 961 29115 963 967 4898 Q9UM54 Q70UQ0 4899 Q9NYB0 P30304 P30305 P30307 O00241 2253 6609 Q9NQ29 2247 2246 Q9BVC4 58533 2244 975 6601 Q92630 6605 Q92636 57209 Q9UM63 Q92633 Q969M7 P29323 P29320 P31629 O00255 Q8NEZ4 Q9NQ33 O00257 81788 P17342 984 988 Q9ULV0 P05362 6613 P29317 Q9ULV4 26060 26959 Q9Y6W6 P07900 P48454 O43399 Q9Y6W5 5300 P15529 2274 P53803 O60725 P15531 990 991 Q96RU8 O14827 Q7L523 Q96RU7 Q96RU2 994 995 2267 51003 997 998 6622 999 6625 54998 51009 P62753 51008 Q04917 P49770 Q9UM07 5310 Q7Z5H3 Q8N4N8 5311 Q5T7W0 O60739 P11177 266747 2280 Q93096 Q04900 Q96RT1 2277 P48436 P49768 O00206 2290 Q9Y6Y9 P25791 Q9BV73 P48431 5321 Q96JC9 148022 O00203 Q00535 Q8IXB1 Q00534 Q96S42 P49756 P25789 P25788 7975 51026 6647 6646 5316 51028 91949 P25787 P49758 Q08495 6660 64343 P49750 O00213 Q08499 O00212 Q96AQ6 O60716 Q96RR4 P61457 Q96JB5 6657 5327 5328 P49747 O43353 6670 P07942 6672 Q969H4 9700 P07949 Q969H0 P07948 Q8WYA6 P20916 Q9ULJ3 Q969H8 Q8IXI1 Q8WYA1 Q8IXI2 Q01844 6667 5335 Q96AX2 4005 5336 Q96AX1 P62714 5337 5338 Q9H5J8 10577 P07951 P26232 Q969G3 Q7Z5L7 10580 Q9ULK4 Q01831 P50749 Q96S94 P50748 Q9NPH5 5347 Q96JH8 Q76NI1 Q08462 Q9H5I1 5361 64375 P50750 5362 6696 5364 4035 Q5VWQ8 Q9BUZ4 51061 O14802 Q8NER5 P68036 10552 10554 Q9H5H4 Q01826 5371 P27540 Q9ULM3 P20908 P15559 Q9ULM6 Q9BUY5 P16885 51075 O14818 Q9NPJ6 P62745 54106 10564 Q15654 9744 O43432 8412 9743 Q86YW9 29948 29947 94121 Q8WZ60 29941 Q96BM9 116 118 Q8NFJ5 9738 Q7Z6C1 P22694 Q8WZ64 8408 O94906 P32019 Q13009 4067 P12429 Q15669 O94915 P60983 Q96SN8 Q14344 54925 Q7Z6B7 Q15672 Q13017 7101 P22681 Q9BVP2 Q76FK4 P35998 Q969Z0 P25705 Q9UDY8 8428 142678 Q8WZ42 P00441 9774 4086 7112 4087 4088 P22692 4089 9775 8445 Q86YT6 Q96BK5 9770 Q8N5F7 Q9NZ94 142 P21359 1808 8439 P62701 Q15691 Q15697 Q8IXZ2 53615 Q13033 8453 9784 8454 P54259 8452 4099 8458 7124 P54252 P54253 7126 Q96KC8 4092 4093 4094 154 156 Q13042 Q96KC2 Q13045 P45379 Q13043 P49736 7132 Q96C24 8463 7133 8467 161 P13796 Q96C28 1822 166 Q8NFM4 Q04941 Q8NFM5 338917 7128 Q9NQB0 1828 P49721 P49720 Q96T37 8473 P00491 P00492 Q8N5K1 P54274 P36402 8471 P36404 7141 1831 Q96T23

Q9NZ45 Q00577 P49711 Q9NQC3 1837 P49716 P49715 P60953 Q9NQC7 Q96C00 O94973 7153 P54289 P20073 7158 7157 P19086 162239 7159 1843 Q9UDV6 54984 Q13075 1848 Q13077 7165 Q13873 7169 O94988 P10636 26038 26037 Q06787 Q86Z02 Q01094 P63172 26039 7161 P63167 P42224 1852 1850 P17275 P23945 Q92692 P63165 Q9BVI0 O94989 O15355 P22612 P42229 57109 Q969T4 1856 P42226 Q53QZ3 O15372 O94992 Q969S8 Q13887 Q01085 Q8IY57 7170 P10644 Q01082 79363 Q96BD5 79365 P84095 P60900 1869 7187 7186 7189 7188 Q8WYK2 P35916 P11940 57120 P18583 27346 O75881 7181 27347 7182 7185 7184 1877 1874 4907 P17252 P68871 1870 P09630 O15379 Q969R5 Q9NZ08 O15391 O15392 O15397 Q6UWE0 P35900 4914 27352 84733 Q969Q1 Q92667 58473 27327 Q86YS6 Q02386 57144 P51148 P51149 1896 84766 P42262 Q8WYQ5 4920 50514 P51157 P35968 P51151 Q8WZ19 P22670 Q15628 Q9NQ92 P51153 P42261 26005 57154 Q96BI3 58480 P51159 27339 P30279 27330 84759 58487 P30281 57159 Q8WYP5 Q8WYP3 3609 3608 3607 P21333 Q9NYJ8 94104 Q6ZV73 377630 29966 Q02363 58491 57162 P84085 27300 3611 Q5XUX0 O15335 4943 Q15642 P01730 Q969V6 O15350 Q14314 O75832 O15353 Q15649 Q15648 27315 Q86Z14 Q86YP4 Q06710 Q96BF6 Q8N5A5 3622 P84077 3621 P30260 4952 O95429 4953 Q15653 Q15652

cellular component biogenesis P25054 Q92979 3880 25909 P49023 P54619 1460 P52434 9185 Q9BYG3 4728 Q8IYN9 P84022 P21926 O14788 6901 4725 25913 Q9H4L7 P30876 25915 P08727 P16104 2316 2 5829 Q8IUE6 Q96QZ7 P14174 800 10420 10421 Q9UKV5 P10809 Q96EB6 P62249 O75925 Q9H2G4 P28300 Q12846 Q9NQW6 54496 3416 Q9UKW4 4983 811 5830 3655 Q5TAP6 P31946 P08708 Q92949 1491 P54646 Q676U5 55355 P16333 P62263 P04350 3667 64061 5604 829 P08758 1022 10609 1020 114548 P39019 P16389 P15056 P16144 1499 830 1012 55125 3678 O14744 O14745 P07437 Q9H0H5 Q12874 Q9Y478 3690 Q12872 Q9Y239 P32856 3692 Q8WXF0 Q9BUB7 1029 Q9BUB1 1025 841 Q92522 O60890 847 Q8N257 6714 P04179 Q9UGJ1 266812 55142 Q8TBB1 P84243 Q71DI3 Q92994 857 5879 Q9Y490 P51575 Q9H6R4 Q13509 Q13748 Q9NQT4 55153 Q9NQT5 10844 253980 P40692 Q96MT8 10607 6733 5888 P09601 Q9UL54 Q9UKT4 P04156 11340 O00541 O95391 23322 P50416 P11047 203068 1058 23560 Q8WWY3 10013 55723 6505 Q15024 P62875 P25445 135295 5430 Q7Z6J4 5432 O95163 3010 Q6FGD7 Q9BU61 3009 3008 O60610 641 3007 3006 402 O43290 8932 7846 408 P49407 8936 Q09472 5440 5441 3024 O00560 3265 Q9UKN8 O00567 891 Q9H4B7 3014 5434 O43264 Q9UMS4 Q9Y697 5213 Q658W2 P35222 Q9NUX5 P14784 Q96A08 Q99733 3028 Q00403 Q15050 P23258 55759 P62424 P26583 O00505 Q9Y3M2 4131 Q6FGG2 6311 9821 P50613 P51946 10458 Q8WUY8 673 Q96KK5 10451 P55072 P49450 5471 7410 Q5JSP0 O60814 80705 29079 P36382 P14373 Q96QV6 688 54443 Q15070 P62847 P62841 4150 4154 25929 22893 22894 Q12809 O00762 25923 10438 Q5TAL4 Q96P20 55785 7415 7414 4149 P62857 P62851 P56182 O43463 3070 Q86UX7 6341 7430 Q13901 P07814 Q7Z4H7 466 P17858 55559 7428 P40855 8772 Q14686 Q14209 11065 Q16625 Q9H0U9 Q6NZI2 3084 4171 P13861 230 P07196 Q8NCD3 P83731 5018 P43034 Q14690 P49815 P38919 Q16637 P62805 8301 5036 Q16635 6125 P62807 O75376 79084 O75381 Q9NVP2 51773 Q9NRH3 54801 7205 Q9NPD3 Q9BSJ2 Q9H9E3 Q16401 P46777 Q15796 Q15797 P12757 9404 Q8N6I1 O75367 P12755 Q9NY61 4193 Q6DD88 Q01105 Q96RK4 P55290 Q8WVM0 Q9BQE3 Q9BSI4 6386 P18074 Q16658 Q86XR8 P68133 P18077 P04908 P68371 P36954 Q9NVR5 Q4AC94 267 P23416 P68366 Q6PKD3 P08034 O94817 P43005 P78347 Q13158 Q8TDY2 10171 8576 7248 O75347 P54132 23481 P23229 P35221 51564 Q9Y4G6 Q02413 Q99848 Q14011 O15294 Q96CS2 10181 7013 8348 P36776 23016 5071 Q9NY12 7249 8338 118460 P49848 Q9NRD5 6175 9685 7023 10392 P13647 A1XKG3 Q08188 P48509 51340 222484 54853 Q9NVM4 55704 Q16695 P08069 8349 9439 Q16698 Q9GZL7 347733 7277 Q9BX66 Q01780 5092 Q96EU6 26747 Q15370 3909 Q6PID4 Q9NRF2 Q9NVN8 1729 P78362 Q9BWT7 P02533 Q14289 9223 Q13753 9221 9463 Q96RE7 P28289 Q13751 P12931 6194 7283 Q4VCS5 10813 Q9H6Y2 P04085 Q92574

65083 3911 1736 P54105 9474 Q8IX30 Q02297 55054 Q3V6T2 7052 Q9BSB4 P24928 Q9BUF5 1742 117177 Q9HD26 Q4LE28 P98174 29889 P29590 P55209 84617 P56537 60412 Q92793 Q92791 O60264 Q92558 O60260 93323 P07339 P78316 91147 P34932 80254 P43405 O43707 Q8TBE0 Q07955 Q92786 P53041 Q9UET6 50628 O95996 O95999 P35610 O00186 7082 P35612 P35611 4809 P63000 60673 Q9BUK6 Q8NEC7 Q9NVC6 51729 P35609 P14923 O43920 P19387 P30153 P19388 7094 23092 3728 Q05513 26121 Q7Z2W7 375790 P22303 9045 Q9NVD7 P32320 3737 Q00059 2644 2643 2885 Q04656 O15212 Q9BQA1 O75716 Q14677 O15230 Q15526 Q9NP98 Q8TDN6 9054 79065 Q9UNA1 4839 P67870 23076 902 P08237 Q13112 Q14684 P21675 O15228 P06899 23607 Q9UBB5 O00268 Q06265 Q9BVA1 Q9Y2X3 10772 P27105 O15530 O95680 51096 P16471 Q92845 928 Q96NT1 Q05397 Q9BXC9 Q8TED0 29127 1355 P32929 O15533 Q9UQB8 4869 5716 P46087 Q13823 Q9H814 1374 Q6P2C8 Q9NRZ9 Q7L576 Q13829 P17302 10528 2697 6810 Q6NXT2 6814 705 Q9UHR4 P04233 3308 P07305 P30566 O95478 O95477 3320 P52756 O00468 83706 29102 P07550 P62316 P04040 P09972 P62318 4646 P62312 P62314 P08648 Q9UHK0 Q13601 2243 1153 O00233 P30793 79441 3329 6839 P18206 1387 6834 P42771 5987 Q9UM54 5747 6838 P05388 P07766 P05106 6850 4673 Q06203 Q13618 Q9H9Y6 Q9NS69 P42768 P61244 284119 P15153 2244 P61006 O14640 977 O43182 5518 Q13868 Q9Y375 Q5QNW6 Q03164 Q9P0M6 440193 P61254 Q92621 Q6FI13 4676 Q9Y2W2 989 P40337 P04275 Q9BZJ0 Q9Y2W1 P06213 4690 Q08043 P07900 O43159 6872 P04637 Q9Y6W5 6631 2033 P14678 P53803 O95271 P52952 P62993 54512 4686 999 6624 5536 Q15389 P62753 P01112 Q9UQ80 Q15149 6883 10383 5796 P51636 Q99880 23212 P07919 Q9Y2P8 2040 P50542 2280 Q99877 51010 2039 Q96S53 Q99871 51013 Q15154 6633 4214 6878 Q15393 Q96S59 P02675 6637 4218 255626 Q99879 P49768 6892 Q8N726 5563 Q7Z7K6 P14416 2054 P52735 773 Q00535 6646 P01137 Q7Z3C6 Q9H9L3 Q9UPY3 Q3ZAQ7 Q16254 O00217 Q08495 8841 5576 Q9UHI6 Q08257 Q96B26 221613 11218 Q93079 3148 Q96RR1 Q93077 P61457 P01127 P62308 P61218 P62304 23647 O43593 6430 Q8N307 6431 Q9UHB6 Q07157 3162 23404 54552 Q9BZE4 3159 8602 6426 5339 Q9Y512 Q9Y4P1 Q9Y2L1 6201 10580 64127 Q5HYA8 Q9ULK4 P68032 23411 5590 P18846 Q00987 P50748 324 4015 9702 329 7529 5108 P27797 2091 P50750 P22087 Q9UHD8 Q9NU22 P12036 P15311 330 Q9NVU7 56993 7536 5116 5371 Q9UNQ2 Q60FE5 Q12933 Q969E8 P16403 P16402 25813 P16401 582 10569 P51617 O14817 P52948 Q9NPJ6 6217 9967 Q01813 10093 Q15654 4297 Q8TEW0 Q9BY76 P13984 6470 351 355 P09493 23165 118 Q7Z6C1 6229 P62913 Q13243 Q8NFJ9 Q13485 P47755 O94906 Q13242 Q13247 5393 Q13489 P03950 5394 5154 O00716 9997 5155 Q9BTM1 Q15428 Q16514 19 O75494 Q13492 Q13490 84790 6498 Q9NWS0 54930 P23527 Q9UDY8 Q9NV06 P09234 P17096 Q9BZW7 P83876 Q8NFH8 Q9HC77 O94927 Q8WZ42 8443 4086 8683 4087 4088 Q9BY44 4089 Q16777 9775 Q16778 P0C0S5 163126 P29084 5170 P34896 Q02790 Q9H492 P52907 P0C0S8 Q7L7L0 7106 P60763 P62942 Q15459 10291 8452 Q8N5M1 Q16787 O15164 7126 P14635 O43639 4092 154 Q00597 121512 158 Q9H6D7 Q9UMD9 23363 Q8NI36 84365 Q02539 55806 P45379 P49736 7132 5195 Q14137 Q09161 9555 8467 23378 P13796 Q86WV8 Q9Y5J1 P35568 Q02763 1828 11157 Q99714 P02671 Q8TCU4 8473 P00492 Q8TCU6 Q5SSJ5 P54274 O75683 Q9Y5K6 P58876 P36404 7141 Q99700 Q96T23 P50914 Q9H1Y0 P68431 Q01664 88745 56915 56916 P49711 Q14152 7157 P22492 P14625 P14868 23354 25776 Q02750 8481 138151 51474 55835 10048 87 Q13077 92345 P53365 Q7KZN9 O75665 Q12788 P12814 P62081 P18124 9341 2702 26271 Q9Y3A5 P20671 Q6NVY8 P46821 Q9Y5E9 Q9UBK2 Q13885 Q14978 Q96QB1 Q13887 9351 84708 22920 Q05682 55172 P05062 Q9Y3B2 P05067 2959 7186 P52298 Q13895 Q15836 O75643 9368 O95613 10907 57120 Q96M96 Q13418 O43815 6093 O75880 7185 9361 7184 O43819 1874 2961 2960 1870 O15379 P21980 P23921 2969 O15151 Q14511 P19438 56288 2972 134430 Q9UBN7 Q9NYH9 Q8WUI4 8290 P30047 1653 2742 Q92499 O95400 Q8WZ19 Q2NL82 O95405 Q68CZ6 26005 Q68CZ1 Q96G21 27339

1666 P21579 Q96QF0 Q8WYP5 1429 P21333 Q8IY81 P33778 P10415 Q6ZV73 P12830 Q8TEQ6 P19474 59341 P10412 P55769 Q5RKV6 3611 Q14790 3852 P09430 Q13464 O75832 Q15648 23192 Q96BF6 Q9BVJ6 Q96HR8 Q9HC29 O15105 Q71U36 Q13233 1207

positive regulation of cellular process P25054 P28562 O75940 Q12824 2305 2304 2303 1454 1453 O14543 Q9Y275 2308 Q92974 Q9H4L7 Q12834 Q12837 P16104 2316 P42858 Q9UKV3 P61158 P49006 10666 P10809 Q96EB6 Q5S007 O75925 P27694 Q92956 Q9BQ51 Q06124 P17405 P37023 2324 57761 Q9UKW4 55109 3655 Q9Y297 O75928 3659 P26358 3672 3673 Q13705 10645 3667 P42830 P25098 1022 1021 1020 114548 57787 1017 79576 P21964 1499 10616 O60884 Q6PJW8 1012 P61586 Q92530 3678 3690 Q9UGI0 P29466 Q9Y239 Q9H4P4 6720 O00358 1029 3207 10626 1027 1026 P17482 P17480 Q9Y240 604 P04179 Q07890 O95136 Q9UGJ0 Q12888 5883 P38398 Q96N67 P20618 Q92990 Q5T2W1 Q07889 6721 Q92993 5879 Q92997 Q9UKS7 P10826 Q9UKS6 Q9UL46 P10827 Q9UL45 P31749 Q9H8V3 1050 56005 27032 P40692 3226 5888 Q8N3V7 Q9UL54 Q9UKT4 O96004 8930 P56199 P14317 P50416 P50897 Q99523 Q15022 637 5898 P25445 O96017 6760 Q9Y5V3 1072 641 P33076 84033 8932 648 P49407 11116 O00327 P48552 6772 6774 3265 3263 P14780 Q99500 51144 Q14192 P35240 8945 P01019 51147 55504 5451 P35225 P35222 P35226 O75084 P17813 3269 668 6776 Q15052 Q59EA4 P24588 O00744 5460 P01042 4131 Q9Y5Q3 Q92934 9821 Q9NX61 79971 672 673 P15692 O14908 57708 6789 10213 Q92930 P05412 O00755 6320 5473 23532 Q5JSP0 Q99558 Q9NWV8 688 148327 Q15077 5467 4137 9826 207 5468 Q92922 Q15078 P56178 Q12802 O00762 Q9NWW5 P17844 P13010 10673 9839 5494 O43464 4168 P06729 P08908 23512 23513 O14936 O14939 8516 8517 9846 O15085 Q14686 11065 7204 O43557 Q9H161 P07196 Q9H160 P49810 P08047 P78395 Q9NPC8 8546 P46781 5037 79084 P58304 Q04864 P09382 7205 8536 5029 P00533 Q16401 Q96RL1 P61956 Q15554 1902 7220 P35638 Q96IZ0 10193 Q9UQ13 8555 P32121 Q9ULH7 64857 Q9NY61 4193 Q9Y6Q6 Q96RK4 Q15561 10197 51547 P55290 Q15562 5054 P62826 Q9Y6R0 9412 O75116 Q86XR7 P36956 P31270 Q13393 55915 9421 Q14249 9420 7248 O75586 Q93062 Q9Y2C9 P23229 Q9Y6K9 51564 1936 5074 10181 8100 11030 Q9HAZ2 O43521 Q15109 P22392 O43524 5071 P48995 11035 1947 P02790 P11229 5089 O75569 O15068 23463 A1XKG3 51588 P08069 10155 51107 1956 Q9NPA8 O43508 P37840 5092 P11233 7272 2810 Q93034 Q93038 P02775 Q14289 Q96RE7 9464 P29375 27005 Q4VCS5 P22736 P42345 Q9Y6G9 P04083 P04085 Q92574 Q13761 1978 Q58WW2 P56524 9475 O15496 Q02297 P16070 O75528 P06239 P29353 Q06643 P55209 P56539 Q9BYM8 60412 P21860 P55212 A4D1W7 55072 P78317 9495 Q8N8D1 Q9HAK2 P53041 Q92542 Q13794 Q96EP0 O14593 O95999 Q13315 P35610 O43915 8174 9021 Q16828 9020 Q9Y6C2 Q6P1N0 P31249 Q13322 Q13323 Q9NP71 P46736 3728 3720 P22303 5901 P46734 50649 P12956 O75962 P28065 Q9H4W6 Q9BQA5 P61088 50650 P43694 O15455 Q14674 Q9BQA1 5914 P28074 222068 63976 P28072 P28070 5927 P00519 P63272 2896 O43909 P24941 Q92585 51512 Q15532 P43686 P07148 Q13352 5925 P10914 Q92858 O14672 P10911 Q5VV41 Q9UBB5 Q9NS86 O15519 4609 P16220 Q92851 O15516 5935 O15530 Q9UBC3 65018 54361 O95684 P16234 10783 Q92845 Q92835 3783 P53667 57402 10758 27185 Q8IVF5 O60543 Q9UJU2 P49116 P61289 P03372 64170 Q9H7P9 Q92833 P06401 P06400 Q9GZX9 3791 P46087 5970 1130 9093 Q99460 P56705 9099 P17535 P17542 P61296 5966 3309 P04233 P05129 133584 P50570 Q9Y586 P11802 3320 O00463 P52756 O00468 6829 10733 10736 Q8N2W9 4646 6827 719 P08648 P30556 P30559 5991 1153 Q14938 O95243 Q9NS56 P37198 3329 3326 1147 3324 2475 P08651 Q92888 P07766 P05106 P28223 6850 4673 P50591 Q13618 Q13617 Q13616 1161 O00487 P30542 Q9NS68 O60502 57448 O14641 P61247 O14640 Q12770 Q92878 5515 P05112 5518 P09958 O43186 Q12772 Q12778 Q13625 Q05586 Q6KC79 3350 10724 P37173 5524 P05121 P40337 7704 P04632 4690 P23396 6872 P04637 O43157 2034 2033 O95271 P13591 4686 5533 P01116 Q16236 5536 6869 Q15389 P01112 P01111 Q9C0K0

P25100 4221 10383 6885 P35368 1191 O76074 O76071 54764 4214 P01106 4215 P01589 P01588 P01100 Q8N726 9921 Q7Z7K6 P14416 P52735 O00444 P11388 Q9NS23 Q02952 Q53X93 6886 Q9GZP9 Q9Y572 P01138 P01137 P01135 P01133 4240 P04626 Q9GZP0 2065 2064 P35348 11218 301 302 P10070 P17936 P10071 P01127 11214 5581 5582 5584 Q9UNN5 79612 Q96PU5 Q02930 P51828 5578 5579 Q8N302 P40763 P04201 2081 5591 4261 Q6IR47 Q3SY56 64127 P18850 5590 P18846 P16671 P18847 324 P18848 329 P27797 P01185 6453 2099 O60907 5599 8625 P25103 P01178 Q9BT67 23636 6464 25818 P62508 P17980 P52701 P58012 10320 9967 P26447 P25116 8650 8655 4297 Q9BY77 11186 9500 8654 P30086 P13984 351 Q9UBS0 355 Q04759 356 Q9UBS5 P09493 P62913 P25963 5154 7334 5155 7335 19 367 Q70SY1 7341 P11309 6496 6495 6498 29 253260 P32241 Q53GQ0 P10451 Q9Y606 Q9UBU3 P17096 5159 Q13263 P32239 5174 Q14116 8204 Q9BY44 P12645 382 5170 222546 387 389 Q9NWT8 P08151 P20264 P62942 P55347 10293 391 O43639 394 396 Q02535 121512 11146 Q15466 2904 Q99966 Q13285 2902 7376 Q09161 Q9Y5J5 Q86WV8 Q13291 Q14140 O15169 85440 Q99958 Q15475 Q8TEY5 9564 Q6ZSZ5 10273 P14859 O43612 Q99943 P49959 Q01664 P21145 56916 P01579 11124 10276 P09038 Q14155 2923 Q15008 9575 O15198 23118 Q14160 Q14164 10285 Q6R327 Q15811 P78423 Q08722 Q13639 1613 26271 Q9BXM7 Q86WK6 Q1PSW9 91 94 P46821 O15119 P45974 1616 Q9NSA1 Q9UBK2 Q9NSA3 Q96QB1 9590 P55318 P55316 P67775 P43307 P08575 P05067 2956 Q9UBL3 O94768 Q15836 26258 Q96M96 29767 Q03014 6096 Q9BXK5 56034 O95619 P21980 Q9NSC2 O15151 P11717 8289 O15156 O94776 9138 Q7Z434 Q02156 Q9BXJ9 Q8HWS3 Q9UBN7 Q15853 P13945 Q9UBF6 9149 P41182 Q8TAK5 P53567 P30048 1655 P22415 Q9UK80 Q03468 O15554 Q13200 P47712 P51398 Q68CZ2 Q07817 P20248 64919 80155 Q9UJX2 P08134 P17081 P08138 P09429 O75604 P10415 Q13216 P35711 1676 P08588 P41159 O15111 P55771 P62070 Q04771 O15105 P45983 Q13233 P45984 P21554 O14793 2550 9181 P51587 P17676 P15498 2547 P37288 P84022 Q92731 O14786 4722 O14788 57506 P28799 P27487 25913 Q5VTR2 P55957 P62244 P14174 4734 Q7Z727 Q9NR80 Q9NR81 Q8NC24 6927 6929 57521 811 P40425 P40424 P62258 6926 6925 P31946 P31947 2100 Q9NR96 P48380 O95343 57534 3428 P16333 P20823 O60674 O00182 P19838 Q8IUC6 823 Q8N5V2 826 5604 P41743 P11926 P27037 Q13956 4771 Q9Y463 O95352 P39019 P15056 80324 P07437 4780 Q92769 P09619 P28370 Q9NZC7 O95361 124583 P10600 Q06330 Q9BWF2 P19419 841 Q9HCM4 843 4775 847 4790 4792 Q9H2K2 1285 Q96L34 P18545 Q8IUH5 7818 Q9HCL2 P60033 Q96KQ4 Q92753 P05230 857 O14763 859 Q13501 6500 Q13503 Q9HCK4 861 2137 867 P09601 50943 4318 Q86UW7 P01241 P15408 3484 P14555 3482 2150 3481 Q9UIH9 23326 P11047 O60603 25988 23327 P15884 203068 3479 2147 10011 10013 6504 84289 P01236 11346 10014 P23025 10016 64682 Q7Z6J4 Q96T76 2159 O43290 406 Q9BRP8 Q99759 O43294 408 O43293 84271 409 Q13099 Q6ZN33 Q09472 Q9UMR2 5682 5683 5684 5685 5686 23787 890 891 Q99743 894 896 Q9NR09 P53708 898 3014 29086 P49639 Q9UMS4 P51531 4361 5692 5693 64223 5213 Q9NUX5 P51532 P14543 O14737 Q01974 P60484 5687 5688 10001 10488 8718 P23497 8717 P26583 Q6FGG2 7402 P50613 P51946 Q96P48 10451 P55075 P55072 Q8N5U6 O43242 8743 7410 7412 Q9HCE7 3054 Q9NYZ3 Q05086 P37231 Q01955 8737 P55085 P48357 8738 P24385 8754 7422 P52815 3066 Q9UMN6 65108 3065 Q96P20 7415 10432 P24394 7433 P49674 Q9UIG0 466 468 7428 8772 6595 Q13114 6597 Q16623 6599 6598 Q9Y4K3 3084 O43318 472 474 Q6UY14 51762 23286 P20396 9616 8767 P21246 P38919 Q15303 3091 P55036 8301 Q15306 Q86VP6 P13631 5270 51773 Q9H2X0 6117 P46531 7448 Q13133 Q15796 P43026 Q9HCY8 9641 8795 Q15797 P12757 Q9UNL4 P12755 O75360 P46527 23263 Q9NRI5 Q13144 Q13145 Q9BSI4 Q16650 5295 P35869 P43490 7476 P18074 Q8WVL7 P55055 P10586 P68371 Q16665 P43489 Q13158 Q8TDY2 Q16666 P54132 P63092 P35453 O75340 P0C1Z6 O94827 11269 Q14012 Q14011 Q9NRC8 7013 7014 Q9Y4H2 P35443 7490 P11021 Q7Z7H5 Q8WW38 Q9GZM8 Q99835 23229 7023 10392 P98179 O43734 O43739

Q8WVQ1 54851 Q96PK6 P00367 P47895 Q13188 P23443 6188 Q9BX66 P38936 6182 P11474 26747 54862 P02538 Q6PID4 P48730 1728 Q99814 P98194 Q15375 6199 7046 6198 P35813 O43715 6194 Q9Y4C1 P62195 9218 9219 P07355 3912 P78536 7057 9238 Q13526 22807 7052 P46934 P46939 Q96GN5 81565 Q4LE28 P42574 P98172 P42575 P98171 P98174 Q9UER7 8399 7067 Q9H305 Q13535 P19793 P29590 Q63HK5 P30101 P31431 Q01196 P41235 P41231 Q92793 Q96GM5 Q9HD15 Q9NRA1 3932 O60260 P78552 Q13547 Q13546 P19320 7073 9253 7074 Q9UNE7 P43405 O43707 1763 Q04206 Q92786 Q92785 P78560 O60271 Q9NZJ7 P78509 P78504 P00747 P63000 51720 Q96PE2 P00742 P08243 3953 4800 P08246 3956 2626 7099 7098 Q9NVC6 P14923 P30153 1789 Q05516 P62166 1786 Q9UIV1 1785 Q05513 P00734 375790 3965 29843 57045 P78527 O60229 121457 1312 51741 Q5T9L3 Q16610 Q15762 Q16611 Q06830 P62191 O60238 Q63HQ2 4839 Q9HCS4 P07384 4830 Q9NZN5 902 P08237 P08238 P21675 Q7Z628 P08833 Q9NYD6 P48058 2672 Q12948 Q6QNY1 O00267 Q9BVA0 3516 Q96JM2 O00273 P63208 P08842 P07996 10533 P52564 P04004 P52565 4860 Q08431 Q8NAP3 5706 5707 5708 5709 P16473 51099 4851 5700 5701 5702 5704 5705 Q9NYF8 Q9Y2Y8 Q12968 5717 57646 4862 5713 Q6IT96 4869 5716 Q96JK9 P48023 1374 Q9BZF9 Q12979 22985 P17302 5728 10524 P17301 Q99698 2697 Q9ULZ3 Q9UHR5 948 Q12981 O95477 P28482 1385 O00220 P51668 29108 P51665 P19532 Q9P0J0 57669 P15173 P07550 P04040 P08887 P51671 O00238 Q9UHK0 Q9NYA1 O00231 O00233 P10721 O00232 Q5VST9 P19544 Q8WYH8 57678 29110 960 961 29115 Q9P0K8 1387 967 4898 P42771 Q9UM54 Q70UQ0 P30305 22937 2253 Q9P0L0 6609 P62330 P62333 2247 P15153 2246 Q9BVC4 Q9BZK7 975 6601 Q92630 Q9UM63 Q92633 P29323 P51692 O00255 P48061 Q03164 55035 5764 P05362 6613 Q9Y2W1 P06213 P07900 P48454 5300 P13349 2274 P52952 O60725 P15531 991 Q96RU8 O14827 Q7L523 Q96RU7 Q8WTS1 Q96RU2 994 998 6622 54998 P27986 51009 P62753 10138 Q04917 P51636 2280 Q93096 2277 P48436 Q99638 P01344 P01343 P49768 O00206 2290 P48431 5321 3142 148022 O00203 Q00535 Q00534 Q96S42 P25789 P25788 51026 6647 6646 5316 8829 P25787 P01375 P01374 Q9UPY8 6660 Q9Y337 Q9UHI6 O00213 Q96RR4 3148 545 3146 P61457 Q8WTP8 P40933 Q96EY1 6657 8837 O43353 P07942 6672 9700 7520 8851 P07948 Q8WYA6 3162 Q12905 O00622 Q969H8 Q12906 Q9BZE0 552 553 3159 Q99683 Q99684 6667 5337 5338 7518 P05771 Q96FA3 O00635 7531 10580 7533 23411 23414 3171 10109 Q00987 3169 Q9H1A4 5347 7529 6210 6696 5364 Q9UHD2 Q9UPT9 Q9BUZ4 3182 572 10554 5371 P27540 8881 Q60FE5 Q9UPU9 Q12933 P15559 P51610 580 O14818 P51617 54106 8878 P60568 Q13477 Q15654 P15923 595 Q7Z6C1 Q13485 Q9HC98 O94906 P03950 O00716 Q13009 4067 P12429 Q13492 Q13490 Q9UQR1 Q13017 6256 P22681 Q9HBW0 O75489 Q76FK4 P35998 Q2M1K9 Q969Z0 Q9UDY8 P23528 142678 P00441 6262 9774 4086 10081 4087 4088 4089 9775 8445 Q9H257 Q86YT6 Q8N5F7 P21359 1808 P24864 P62701 Q13033 8453 8454 8452 6275 O75469 7124 P54253 Q9Y3E0 23365 P14635 64759 23368 Q9UQL6 P10275 4092 P10276 4093 O75460 154 156 Q13042 Q99728 Q99729 Q16555 Q8WV24 Q13043 7132 8463 O43889 P36896 P36894 P35568 23370 55810 Q02763 P01308 120892 338917 Q9NQB0 P49721 P49720 Q96T37 P00491 P00492 Q8TCU6 P54274 P36888 Q96T23 Q00577 P49711 Q9BRK4 Q9NQC3 P49715 55827 P60953 7153 64782 7158 Q08050 P39905 7157 7159 Q02750 P35548 138151 1843 55832 1848 Q15257 Q16581 Q13077 Q13873 O95835 P10636 Q86Z02 Q01094 7161 9341 P63167 56259 P42224 10935 P17275 Q92692 P63165 P39060 Q6NVY8 P42229 P15090 1856 P42226 22926 Q13404 Q13887 Q01085 Q8IY57 O43847 22920 P57735 P84095 P60900 1869 7187 7186 7189 7188 P35916 O75881 Q13418 7182 7185 Q8N163 P17252 P68871 P21741 Q13422 O15392 Q13426 2735 P19438 P21757 1406 2737 2736 Q96G30 Q8WUI4 4920 P35968 Q14774 Q13443 Q15628 9391 57154 Q96BI3 P51159 8065 27339 P30279 P30281 P23511 3609 3608 P23510 P21333 Q9NYJ8 8078 29966 P19474 Q02363 P18146 57162 84305 3611 Q14790 P01730

O15350 P53355 O75832 Q9BZS1 P53350 Q9P0U3 Q15648 P19484 Q86Z14 Q06710 Q96BF6 Q9UHV2 3622 O60356 Q9HC29 P30260 O14497 4953

post-translational protein modification 8099 Q9H0E3 Q9Y265 65125 P28562 P54619 Q9NQU5 1459 1457 Q8IYN9 55561 Q5QP82 1454 O00391 1453 Q6IQ55 Q92729 Q5VTR2 P53779 Q9H0C8 10420 4734 Q9UKV5 Q96EB6 Q9H2G2 Q5S007 Q7Z727 Q9UGN5 81629 Q8NE63 O00167 2571 Q06124 A0AVT1 P62495 54496 P37023 Q8IUD2 P62256 2324 57761 O75928 P27448 Q12851 Q9C005 O75914 3672 Q9NR96 Q13705 P21917 1491 P54646 10645 Q5TAQ9 55352 2335 O60674 1487 P20827 5602 Q96GD4 O14578 5604 P41743 Q8WXG6 P25098 P51784 Q13956 Q9Y463 1022 Q96MX6 1021 1020 Q06587 Q5SGD2 57787 1018 2107 1017 P15056 79577 10856 10616 5610 Q9Y230 5613 O14744 26191 P30679 Q9UGI0 Q92769 5870 Q9H4P4 5871 P09619 O95361 P10600 Q96J02 10626 10868 O14519 Q8TBC4 1025 Q9BUB5 6711 6714 O14757 O95136 Q9Y243 Q9H2K2 Q13976 P21709 Q96L34 O95376 79595 P18545 Q8TBB1 P38398 169436 Q05469 2125 P60033 613 Q5TCX8 Q92993 Q96CA5 4548 O95147 Q5MAI5 6500 5894 P31749 O95382 Q38SD2 Q4G0W2 2139 253980 57332 O00141 55159 80335 6733 867 Q9UGL1 Q9UL54 Q8TBA6 O00308 P56199 23326 O60603 23327 11107 2147 Q15022 P62877 7832 10013 84289 Q96KG9 10014 O96017 64682 O96019 O95163 Q9NWZ3 P57059 115426 1072 O96013 51132 51377 Q99750 84033 11113 Q9BS18 Q99759 408 O43293 P49407 409 Q09472 O00329 6772 P35236 10238 P11274 55743 Q9NR09 51147 10477 29086 O14965 Q9UMS4 P40818 Q8N3J5 Q9Y697 7874 23552 3276 Q658W2 P35227 P14543 Q01974 P35232 P60484 Q15291 Q9H093 6776 O14730 7867 285193 O14733 O14974 6790 Q5VVQ6 6793 8732 P01040 P62837 6794 4133 O00506 P50613 Q99570 O00743 672 673 6304 Q9H2D6 6305 6789 10213 P55072 5470 4140 140609 Q9HCE7 P51955 3297 P51956 Q05086 Q99558 Q9NWV8 444 8737 207 4139 Q15078 284086 8754 Q9Y5S2 5245 3066 3065 Q9UIF9 Q08345 O00762 O75293 55781 51185 Q00613 7415 O14920 10432 P56180 5494 5495 P49674 Q9UIG0 Q92905 5499 Q8NHX1 23512 Q7L7X3 P17612 O14936 Q96QT4 Q56UN5 7428 P49427 Q13115 P36507 11060 11065 7204 Q9BZ95 Q9Y4K4 O75385 Q86VQ3 Q9Y4K3 O43318 472 P48729 O75150 Q6P5Z2 23043 9616 8767 Q14694 Q13363 3093 Q15303 11072 4188 Q15306 Q9Y4L5 Q86VP6 8780 Q9Y2H1 23291 Q9NRH2 8536 P00533 Q96RL1 P61956 Q15796 197259 Q13131 9641 Q15797 Q16644 10193 9646 7465 Q9UPP1 8555 P32121 Q9UNL4 Q6UXN9 O75365 23028 Q96IZ7 4193 O94804 P61962 P61960 51548 Q93009 51547 Q93008 Q8NCB2 P61964 Q16659 Q9Y6R4 57092 O75116 P10586 Q8N4C8 267 23031 23032 Q96PF1 Q9NPF5 54822 Q49AN0 Q13153 51319 8576 26994 O75582 P10398 Q9Y2C2 Q13162 P23467 8569 Q13164 Q14012 Q15349 Q9NRC8 Q8TDX7 55929 Q9BV47 Q9HAZ1 26524 5071 7251 11035 P49841 Q13177 Q9NRD5 P02751 Q15118 P78368 9201 Q3ZCQ8 P49840 O75569 9448 7267 10152 23463 Q9Y6M4 A1XKG3 9682 Q08188 Q8WY64 Q8NEJ0 Q9NVM4 Q96PK6 P08069 Q9UEW8 10155 Q9BQI3 1716 Q13188 1956 P23443 O75319 9212 O15075 Q9NPA8 Q9BWU1 Q15126 23476 Q9UNI6 7272 1965 Q6PID4 Q99816 P48730 P78362 P23458 54623 1969 P48736 Q14289 Q8WW12 Q15375 6196 6199 9463 7046 Q6P1J9 6198 P09769 P35813 Q96L91 27005 P12931 6195 79012 Q9H0M0 P42345 P22735 Q9Y4C1 27246 P04083 O60285 Q13523 Q58WW2 7297 P56524 9474 7057 9475 83737 Q9H792 55294 7052 27252 P46934 P41240 Q92560 Q4LE28 P42338 Q9UER7 P42336 O75528 P34947 P29597 P06239 P29353 Q5JXB2 Q13535 O15264 P29350 P29590 Q9H0K1 Q7Z2Q5 Q9H6W3 Q9BYM8 Q9Y4E8 Q92793 26133 Q7Z6Z7 P06241 P21860 3932 O60260 Q9HCP0 O95983 Q16816 Q13547 Q13546 55072 Q8IYT8 P78317 9252 Q9UNE7 P43405 Q9Y6J8 P53041 P07585 Q13555 Q16825 Q13554 Q96EP0 Q16827 O14595 Q13315 Q13557 P78509 Q16828 Q96EP1 9261 9020 23081 23085 Q9H9B1 P20594 51720 2869 7099 7098 30849 Q15746 P30153 Q86Y13 P61081 Q9H596 P46736 P61073 Q05513 23097 P61077 P00734 1540 P24723 P46734 Q16849 Q15759 O75962 Q8IUQ4 26576 Q9P035 29843 P78527 O60229 P41279 Q9Y6E7 Q9Y6E0 P61088 84661 Q96RG2 P05198 O15455 5914

O75716 11091 30827 Q16854 Q6XPS3 Q13107 P18031 Q9P286 9054 P22528 1326 Q63HQ2 P63279 P00519 P47804 5929 P24941 11099 P20340 51514 P21675 O14672 Q9NRW4 Q8NB16 O60566 Q8IVH8 P16220 P63208 90678 P49137 P07996 P52564 P09936 O15530 P27361 Q9GZZ9 Q8IZP0 65018 Q9H832 P50148 57634 P16234 10549 79885 P21810 P16471 10783 92609 P52333 65264 P54753 O95696 P29992 Q05397 P54756 P53667 O60307 57646 83933 1111 P32929 Q92830 P62136 Q6IT96 Q92831 P55854 P54764 P54762 3791 P54760 Q12974 4882 3551 Q9UBE8 9099 Q13829 152926 5728 10524 P62140 57410 55236 O60551 Q9UI30 10765 P04233 Q6PCD5 P34925 O95476 P05129 133584 P28482 P11802 1385 P51668 Q9P0J1 P19525 10733 147746 Q96DE5 Q7KZI7 P51671 O00238 Q7Z419 P04049 Q96SB4 P10721 P19784 Q5VST9 Q9NS56 Q8WYH8 10987 29110 10746 79444 1147 1387 2475 Q92643 5500 5747 4659 P26045 P30304 O95257 P30305 6850 Q13615 P30307 2011 Q13618 Q9NYB9 27148 Q13616 1161 O00487 Q9P0L2 P30542 10956 57448 2247 975 Q96DC9 Q6GMV3 Q92630 5515 Q9UK22 65061 5516 5518 Q969M7 P51451 P29323 5770 P29320 Q13627 P30530 P18433 Q03164 81788 Q99259 Q96LR5 P37173 984 Q99496 6612 Q9Y385 6613 P29317 P40337 5768 Q9UK32 P06213 Q8N2K1 Q15139 Q9Y6W6 O95278 5780 6872 6871 5783 P53804 2033 Q9NVW2 O95271 O60725 Q96RU7 Q96RU2 O14829 994 995 Q99640 997 5775 79813 5536 5778 5537 6883 6885 Q9UQ88 5796 1198 23210 1196 2043 1195 56940 Q93096 Q96Q15 Q8WTR2 Q9NRP7 Q99873 54764 55611 Q96S53 Q9P2K8 4214 O14830 4215 4216 P49768 O00206 25847 5562 5563 P49761 P49760 O00444 O60942 Q00535 Q00534 79834 Q53X93 55869 5798 6647 Q9Y572 P01137 P01135 P01133 P01375 P02461 Q9Y2R2 P04626 5575 8841 P04629 2065 25853 2064 O00213 780 Q96RR4 301 P17936 Q8IZE3 545 5566 Q96PN8 5567 5568 Q00526 O43353 Q9H5K3 5580 5581 5582 5583 P07947 23409 5584 5585 8851 P07949 Q969H0 5586 P07948 Q92817 Q9Y2K6 Q9Y2K2 Q96PU4 Q8IZL9 Q99683 Q96PU5 5578 8844 P49336 P62714 5579 4008 P05771 Q9H3F6 8607 2081 5591 9950 Q6IR47 5596 5597 5598 23411 5590 Q00987 Q9H1A4 5347 329 84197 Q96JH7 Q9GZU7 P50750 91754 Q9UHD2 Q12923 Q9UPT9 O75164 Q9Y2M5 Q8NER5 Q9NRM7 P68036 5599 Q68J44 Q01826 5371 8881 Q12933 6464 391356 Q96Q40 146057 580 221656 54101 55432 P51617 Q08209 6699 5127 54106 Q8N752 P25116 10090 Q13237 4297 Q15418 7325 P15924 351 Q9UBS0 Q04759 29941 Q7Z6C1 P22694 9978 P21127 8408 Q9HC98 O43683 7332 7334 Q16512 4067 Q16513 7336 Q16514 7335 Q8TF76 Q9UKE5 29959 Q9UBT2 369 128 P60983 7329 Q13490 7328 51657 7341 P11309 P22681 10075 Q9HBW0 P11308 6015 Q16526 P29074 23387 P31152 Q9UBU3 Q03405 Q9NV06 P23528 8428 5159 Q16531 Q9UBU8 Q8WUK0 P43378 Q8WZ42 Q13263 P00441 Q16539 4086 344387 4087 Q6PD62 8446 Q9BY44 O75478 Q15208 8445 Q9BY41 P51812 23399 P51813 Q8TD19 5170 142 P51817 10087 6259 11176 Q8WUJ0 53615 8454 8452 P55345 Q6ZN16 7124 O15164 7126 Q9UQL6 4092 P10276 4093 O75460 156 121512 P27708 10055 Q13042 Q99728 52 Q13043 Q13049 6045 7375 Q96KB5 6048 23133 P36896 23135 P15735 P36894 Q9UKA1 Q02763 54961 120892 7128 Q99956 Q8TEY7 8237 10273 Q9Y5K5 Q9UKB1 P36888 Q99942 1832 Q9H1Y0 51465 P01579 Q99707 P09038 O95819 55827 Q9NQC7 Q9BY84 O75676 O15197 O15198 9578 Q96FW1 Q02750 25778 1843 2932 2931 255488 55832 51231 Q14164 1848 Q6VAB6 Q15256 86 2935 Q13873 9100 O95835 Q15819 Q86Z02 Q8TF05 1613 Q05209 80124 P42224 1852 10933 Q9BXM7 1850 Q8TAF3 P63165 91 Q8WWI1 O95837 O15355 94 Q12792 1859 P22612 P42229 Q13882 P45974 1616 Q969T4 Q12797 Q969S8 Q13404 Q6DT37 Q8TCJ0 Q01082 Q9H3R0 P31321 84708 P67775 57599 P08575 P05067 Q9UBL3 7186 Q15831 7189 O94768 Q6XUX3 P35916 Q9H422 Q96M96 27347 Q13418 6093 Q9UHY1 1634 Q9Y3C5 27102 Q8TEL6 P17252 P42680 O15379 O95619 P42681 P21980 P42684 P42685 Q86UE8 P09874 Q15843 Q6R6M4 Q02156 Q6UWE0 Q8NB78 4914 26260 26262 1642 P68400 Q05655 1408 1407 Q9UBN7 Q9HC62 Q9UBF6 9149 Q6NYC1 P11766 57144 26232 Q86SG6 Q9UK80 Q03468 26234 4920 Q9HC52 150094 9397 P35968 Q13202 Q13443 Q8WZ19 Q9NQ92 Q8IVT5 P21580 9150 57154 57396 Q8N7H5

Q9H1R2 27339 27330 Q9UJX2 Q9Y5B0 8078 O75604 Q8IY84 P10415 Q13216 93492 377630 P19474 59343 P08581 3611 1432 O60341 Q5T6F0 Q13464 728642 P53355 O15111 P53350 Q9P0U3 Q9P0U4 Q04771 26469 389840 P30260 Q7L9L4 O15105 P45983 5800 Q13233 P45984

negative regulation of cellular process P54852 P25054 1460 259266 124790 10657 2305 2304 2303 O14544 Q8N3U4 O14543 Q9Y275 2308 Q92974 Q12834 Q12837 Q8NDV7 P42858 Q9H0C8 P10809 Q96EB6 O75925 Q9UKV8 Q92956 3660 Q9BQ51 Q9BYE7 Q06124 P37023 P13056 57761 O75928 P26358 Q92949 Q99583 P21917 1491 1488 1487 Q99576 2332 3665 3667 10642 64061 10644 10643 Q9UL15 P25098 Q9UL18 1021 1020 P51787 10614 6709 P21964 1499 1012 O14981 P61586 Q92530 26191 O14503 3690 Q8WXF0 Q15906 Q96J02 O00358 Q9BPY8 1029 1027 1026 3685 6711 6710 P17480 604 P05023 P04179 Q12884 Q15910 5883 P38398 57326 P20618 Q92990 Q92993 6726 Q8N488 5879 P10826 Q9UKS6 Q9UL46 P10827 Q9UGK8 5894 P31749 O95140 Q7L8A9 1050 253980 P40692 79589 P63313 Q9UGL1 Q9UKT4 P04156 Q92985 O96004 P14316 P50897 11108 Q99523 23560 Q15022 639 P25445 Q9Y5V3 P16989 P57059 1072 641 P33076 51132 79923 8932 P49407 P60228 P02794 P48552 Q9UKN5 6774 3265 P35240 P01019 51147 O14964 Q86X95 5451 Q9Y696 P35222 P14784 P35227 P11277 663 Q15051 P17813 P35232 Q9H093 668 6776 Q96MH2 O43474 P06733 O00744 5460 P01042 4131 Q9Y5Q3 9821 6794 4133 P25490 Q9NX61 3281 672 673 P15692 O14908 6304 6789 10213 P05412 O00755 P05413 3297 23532 P14373 5467 4137 207 5468 54206 4152 4155 P56177 Q96A54 O00762 P13010 Q99543 127435 10673 O14920 O14921 P62854 9839 O43463 Q12816 23512 23513 P50402 Q96QT6 57732 O14939 55558 10202 P49427 8535 11065 Q9UPN3 P07196 P49810 5017 P78395 P49815 Q13363 Q9NPC8 4188 P62805 6368 5036 5037 79084 P58304 23051 P09382 8539 P00533 Q13370 Q16401 Q96RL1 Q15554 Q9HAU4 P24522 Q9BYV2 8553 P35638 Q96IZ0 P32121 P31273 O75122 Q9Y2I1 23028 Q9NY61 4193 10197 51548 P02741 51547 Q14232 P55290 5054 5055 Q8IWX8 58189 P35625 Q6PKD3 Q9NPF5 P43246 55915 9421 9420 7248 Q9Y6K1 O75581 Q03989 51564 Q15583 55922 5074 10181 55929 Q9HAZ2 P22392 O15055 O43524 26524 26523 5071 7251 285 7249 P49841 P49848 P61925 P49840 O75569 A1XKG3 291 Q9Y6M1 23468 P48507 51100 51588 P08069 Q9NTI5 10155 Q9BQI3 Q9NPA3 1956 O43504 O75553 64426 P37840 7272 2810 Q9H9G7 Q93034 Q96RE7 P29374 Q9H0M0 27000 P42345 Q07507 P04083 Q9UGU5 P04085 Q92574 Q13761 1978 P56524 9474 Q02297 7291 O75530 7295 1982 Q92560 P06276 P16070 P42336 O15020 P56539 Q7Z569 P21860 8165 P56545 O95983 91147 A4D1W7 P78318 Q8TBE0 P04070 Q07954 Q9UH92 Q9Y2B9 O95995 O95999 Q13315 9021 Q16828 P35612 P35611 Q9UKY1 Q9H9B1 Q8IWV1 Q13322 Q9NP71 Q9BQB4 1540 3720 P22303 P28065 Q9BQA5 Q9Y6E7 Q86TG7 O15455 Q14674 Q9BQA1 5914 Q14678 O75953 P28074 63976 P28072 P28070 5928 P63279 P63272 Q14201 P43686 Q14683 5925 O14672 399687 Q9UBB5 Q9NS86 O15519 O60566 4609 10772 5931 P27348 5933 Q9NS91 Q9UBC3 Q8IZP0 54361 Q92845 O15524 Q92843 Q9UK53 Q92835 P50150 Q9NRY4 Q99471 P53667 O60543 1111 Q9UJU2 O15534 P61289 P03372 Q92833 P06401 Q92831 P06400 Q9GZX9 5970 9093 Q9UBE8 Q99460 Q9NRZ9 P56705 9099 3304 3784 6812 6814 10765 3309 P04233 P05129 P52756 Q9UFF9 O14627 6829 10736 3315 Q6FHQ0 3313 Q8N2W9 27161 5978 6827 5991 93986 5993 Q14938 Q8IZT6 P37198 3329 6839 3326 2475 P08651 5987 Q92888 P26045 P07766 P05106 P28223 2495 Q13618 2010 Q13617 Q13616 O00488 O00487 P30542 O60502 57448 5997 5515 P05112 5516 O43182 5518 P09958 Q12778 Q13625 Q5TC82 2023 Q6KC79 10724 Q99496 P05120 5524 P05121 P40337 7704 P02452 O00425 P23396 O43159 P04637 2033 Q9NVW2 4204 P01116 Q9C0K7 Q15389 P01112 P01111 Q9C0K0 Q9BT49 4221 6885 P35368 Q9BT40 1191 2039 Q15392 84159 Q9P2K8 4214 P01106 6878 P01589 7727 5562 Q8N726 9921 P14416 P12235 Q9NS23 773 6405 P01138 P01137 Q6UXV0 Q9UJM3 P04626 P04629 2065 2064 2063 P35348 11218 Q9NS37 301 P10070 P17936 P10071 5566 P02458 P01127 307 5569 9927 5580 O43593

5582 5584 O43597 23641 25822 O75190 Q8TEB7 5578 Q495A1 P40763 P04201 2081 4261 Q6IR47 64127 P37108 Q5HYA8 O76024 5590 O75182 324 5104 5108 P27797 P01185 6453 2099 O60907 P51843 330 331 333 Q9NRM7 5599 Q9H7L9 5119 Q9BT67 23636 P17980 P52701 P58012 10320 P25116 8655 4297 Q9BY76 11186 9500 P30086 Q04760 4291 355 Q9UBS5 P09493 5139 P25963 P47755 P47756 O43683 9510 5154 6003 5155 O43684 19 Q9H3Z4 367 Q9UBT3 51654 7329 7341 Q14106 P11309 6496 6495 Q13257 6498 P10451 84108 Q9UBU3 116113 P51805 P17096 Q13263 9531 Q13268 9530 8204 Q8WWN8 Q9BY41 Q14118 Q9Y618 382 Q04724 387 388 Q9NWT8 Q9UBV7 P20264 11176 Q13275 9541 5187 11142 O15164 2909 23122 O43639 201633 396 Q02535 397 398 10296 11146 Q15466 Q99966 54 Q13286 5195 6045 7376 7375 23135 Q9Y5J5 Q86WV8 P33151 2917 Q14140 Q9UBP4 O15169 Q99958 Q15475 P14859 O43612 Q9H063 P49959 Q01664 P01579 P09038 Q15008 149041 O43623 25776 2932 2931 Q14160 51231 1605 87 Q15811 P78423 O15123 Q9NNW5 27121 26271 P41134 Q1PSW9 27122 91 94 P46821 O15119 1616 P45973 O15118 Q9NSA3 Q12797 Q96QB1 P55318 P55316 O43609 P67775 Q7Z460 P08575 2956 Q15831 P29474 P29475 92129 9126 Q9H422 Q03014 6093 27102 Q9NSC2 O15151 O94776 9138 Q03001 Q05655 Q9UBN7 Q9UBN6 1647 9146 O75626 Q9UBF6 P41182 P30044 P30048 84525 Q9UJW9 Q13200 150094 Q96HU1 P21580 Q07820 Q68CZ1 Q07817 64919 Q9UJX2 Q9UJX0 P41162 P17081 P08138 P09429 O75604 Q9BY11 P10415 Q9P2X7 23189 P35711 Q7Z494 1676 P09430 P08107 P55773 Q13227 P55771 Q04771 O15105 P45983 Q13233 P21554 2550 9181 P51587 9184 P17676 P37288 Q8IYN9 P84022 Q92731 Q92730 O14786 3875 4722 6901 P27487 25913 P28330 Q92729 Q5VTR2 P51114 P14174 4734 Q7Z727 Q9H2G4 Q7L5Y9 6927 3416 811 P63104 P40424 P62258 6926 Q9UIS9 Q9NZI7 P48382 Q9NQX0 P31946 P07858 P31947 2100 Q9NR96 P48380 O95343 57534 P20823 O60674 P19838 P62263 P18509 P31949 5604 P41743 829 Q9Y468 P08758 Q8IZ40 4771 P15056 Q86VE0 830 832 5610 6945 22846 Q92769 Q9NZC7 Q07666 P10600 O95365 Q06330 Q9UEE9 10865 Q9HCM4 O14753 847 5624 O14757 4790 4792 1285 Q9NQS1 Q9HCL2 Q96KQ4 1277 Q92752 857 859 Q96CA5 Q13501 Q9NR50 P19883 Q9HCK5 Q9HCK4 Q8NBS9 861 O95390 192669 Q9UMX1 4799 P09601 50943 P08754 Q92743 P06576 6993 23322 P14555 25988 3479 2147 2146 3476 5654 7832 10013 84289 10014 Q6UWV6 135295 64682 23332 25998 Q99750 O43290 O43294 408 409 Q6ZN33 Q09472 O43251 23309 5682 5683 5684 5685 5686 64215 2173 P13686 P13693 891 Q99741 Q9NR09 11326 Q9UMS4 P51531 4361 5692 5693 Q658W2 Q9NUX5 P51532 Q01974 Q96ST3 P60484 11331 5687 5688 10488 Q96T58 P23497 P26583 Q9Y3M2 25942 Q8NFW5 10459 Q96P48 P50616 8726 Q9H2D6 Q8N5U6 O43242 Q9HCE7 Q08117 P37231 Q01955 Q96SZ6 P51959 444 10468 23746 P24385 7422 5245 22893 3066 3065 Q9UIF9 10437 10438 Q00613 P61764 7414 P24394 3070 Q13901 9612 P17612 55796 P28749 7428 P04792 6595 6597 Q14449 O75386 P10599 O75385 6591 Q9Y4K3 3084 O43318 472 476 P20396 9616 51763 8767 P38919 Q13127 P55036 8301 Q16635 O75376 P13631 Q96D03 3096 5270 Q86VP1 Q02447 O75381 51773 Q9H2X0 P46531 9628 P55042 Q13133 Q15796 Q13131 Q15797 8315 P12757 Q8N6I1 Q9UNL4 P12755 O75360 P46527 498 Q01105 Q13144 Q13145 54815 Q9BSI4 Q9BWQ8 Q13148 9657 7476 P18074 P55055 P10586 P10588 57099 P10589 93166 51317 P55061 P43489 Q8TDY2 P54132 Q9UNF0 8328 Q9NRC8 7013 7014 Q9Y4H2 7490 P11021 84232 Q8WW38 Q99835 7023 Q86VS8 P15822 Q9UNH7 51341 P02549 Q13185 1718 Q13188 P23443 6188 11252 Q09028 P38936 P27816 Q99819 Q99816 9208 P48736 7046 6198 P10997 O43711 O43715 6194 P62195 9219 P98161 P78536 7057 9238 Q13526 83737 9231 55294 27252 81567 P46934 1741 Q9HD26 P41240 Q32P28 Q4LE28 P98170 P42574 Q9UER7 Q14865 7067 Q5TAX3 Q13535 P19793 P29590 Q7Z2Q5 Q01196 84619 Q9H6W3 P41235 Q9HCP6 Q92793 Q92796 O60264 O60260 9255 Q13547 Q9UNE2 O43707 P41220 Q04206 Q92786 P05164 P09525 7088 O95751 Q15735 O00186 P78504 Q63HR2 P00747 P63000 51720 P08243 Q14894 Q13563 P08246 3956 7099 7098 P30153 P11413 1789 1788 Q05516 1786 Q05513

P00734 Q13574 Q13573 Q9H2S9 P32320 57045 1312 51741 Q14432 Q13107 P18031 Q9P286 P34972 P62191 O60238 Q8TDM6 P67870 Q9HCS4 4830 900 P00750 P08238 Q9NZN9 Q9Y2W7 O75820 2672 Q12948 O00267 Q9BVA0 3516 283248 O00273 90678 Q92614 P07996 P52564 P52565 Q9Y2X9 Q12959 P52566 Q8NB12 51094 5706 5707 5708 5709 P16471 4851 5700 4854 5701 5702 10542 5704 5705 Q13813 Q9NYF8 Q9Y2Y8 Q05397 5717 83933 P20936 P32929 O75807 5713 57649 Q6IT96 4869 5716 3551 Q13829 P17302 5728 10524 2697 400961 10523 Q9ULR5 Q12981 Q12982 O95477 P28482 Q12986 Q9UHJ9 P51668 P51665 Q9P0J0 P19525 57669 P07550 P04040 Q9Y2T1 10971 Q9UM47 Q9Y2T7 P04049 Q96SB3 Q9NYA1 O00231 O00233 P10721 O00232 P19544 Q8WYH8 57678 P18206 79444 960 1387 P62328 967 P42771 5747 Q9NYB0 22938 P62330 P62333 2247 58533 Q9BZK7 6601 Q9Y371 6605 Q9UM63 P51693 P29323 5770 P51692 O00255 O00257 Q03164 81788 P06213 26959 5300 P38646 P52952 P15531 990 991 Q96RU8 Q96RU7 997 6622 79813 54998 P02686 P62753 10138 Q9UQ80 Q04917 5310 5311 5796 P51636 Q04900 Q15154 P48436 Q99638 P01343 P49768 O00206 2290 P25791 23429 P48431 3142 Q99623 Q00535 Q00534 Q96S42 P25789 55869 P25788 6647 8829 Q9UPY3 P25787 P49758 P01375 P01374 Q08495 6660 Q9Y2R2 8841 Q9UHI8 Q9UHI6 O00213 23435 Q96AQ6 O60716 3148 Q15170 545 3146 P40933 Q96EY1 6657 5327 P25311 8837 P49747 O43353 6670 6672 23409 9700 7520 Q969H0 Q12904 P20916 Q9H9S0 Q9UHB6 3162 Q12906 Q9BZE0 552 Q9BZE4 3159 Q99684 4005 5336 P62714 5338 7518 Q969G3 8864 7531 Q7Z5L7 7534 7533 23411 23414 3171 563 Q00987 Q99675 3169 Q9NPH5 Q9H1A4 P05783 7529 7528 64375 6696 4035 Q9Y2M5 3182 573 Q8NER5 P51608 6207 Q01826 5371 8881 8887 580 P16885 P16410 O14818 P51617 P62745 54106 8878 P60568 Q9BZZ5 6231 6236 29948 94121 595 116 118 Q7Z6C1 Q13485 8408 Q13489 P03950 Q15669 O75496 O75494 Q13492 Q15672 Q9UQR1 6256 O75489 P35998 Q2M1K9 Q969Z0 P25705 Q9UDY8 P23528 P00441 9774 4086 4087 4088 4089 9775 163126 Q86YT6 Q96BK5 Q02790 P21359 P52907 Q15691 Q15697 53615 Q13033 8453 8454 P54259 8452 O75469 4099 64750 7124 P54253 7126 Q96KC8 P14635 O75461 23368 Q9UQL6 P10275 4092 P10276 O75460 154 156 Q13042 Q99728 Q99729 55806 Q13043 Q96C24 8467 9314 O43889 P36896 P36894 23378 P35568 6282 1822 166 P01308 338917 7128 Q9NQB0 9306 P49721 P49720 Q96T37 P61803 O95813 P54274 Q96FX2 Q86V24 P36888 7141 1831 Q96T23 Q9H1Y0 Q00577 O95817 P49711 Q16576 Q9NQC3 P49715 Q9NQC7 O75437 Q08050 P39905 7157 7159 P14625 Q8N9R8 Q02750 P35548 138151 51474 54984 Q13075 84376 1848 Q5VTD9 Q15257 Q13873 O95835 P10636 P12814 Q06787 Q01094 P63172 7161 P63167 Q9Y3A5 10935 P63165 P39060 O15355 P42229 O94992 Q969S8 Q8IY57 Q01082 22920 9350 Q96BD5 22919 Q9UI95 P60900 1869 7189 Q8WYK2 57120 P18583 O75881 7181 Q13418 O43815 7184 Q86UE4 P17252 O15379 O60383 P42685 O60381 Q13422 O15392 Q13426 O95865 Q6UWE0 4914 27352 O43829 2730 84733 2737 Q92667 2736 Q8WUI4 27327 57144 P23508 P62277 2741 Q9HC57 P09211 4920 50514 Q14774 Q8WZ19 57154 8065 27339 P23515 58487 Q9UQE7 57159 3609 Q14781 P23510 O14495 29966 P19474 Q02363 P18146 3611 O60341 Q13464 O15350 P53355 O75832 Q9BZS1 27315 Q86YP4 Q96BF6 Q8N5A5 3621 2773 Q9HC29 P30260 O95429 Q9BZR6

gene expression Q92979 O75940 81605 P52434 O00148 P17676 10657 Q8TDD1 Q9BYG3 2305 P84022 Q00839 P05455 Q9H0D6 Q9H2H8 O75934 P30876 Q8NE71 10667 2 P62244 80755 54487 P62241 Q9Y285 140801 10421 O75937 P10809 Q96EB6 P62249 Q9BRX9 Q9UKV8 P27695 3661 23708 54496 1478 P40429 811 Q9Y295 55109 Q8N684 P26599 Q5TAP6 Q9Y291 Q9UKW6 3659 P31942 Q9UIS9 P31943 P08708 P26373 Q99583 P17655 P62266 55110 Q99575 P19838 Q9BYD1 P62263 55119 P26368 P62269 10643 Q9Y3U8 Q13951 4772 Q8IX01 1022 P50454 P39019 27043 Q5T160 79577 Q6PI48 P39023 80324 Q9BWG6 833 5610 O75909 O14744 5859 Q12874 Q12872 P29466 4780 3692 Q8WXF0 Q9H0H0 Q07666 O95363 1029 27297 10625 57794 1025 P61353 Q9BUB4 4775 Q92766 4790 Q9NR48 Q96MU7

55149 Q9Y250 Q92994 P10827 P10828 22826 22827 Q9H6R4 22828 6741 5411 Q9NR50 Q96L21 Q9NQT4 Q9NR55 Q9NQT5 1050 55152 P40692 27037 O60870 10607 865 6733 P04150 Q9Y262 4799 O96004 64432 11340 Q86V81 10250 10492 O00541 O95391 O00303 11108 871 51362 Q8WWY3 Q15020 Q15025 Q15024 P62875 135295 P51991 Q15029 Q9Y676 P49411 5430 4343 5432 P36578 Q9UKM9 P51513 Q96T76 Q03933 O14717 O43290 P62888 Q7Z6J9 P61313 196441 P49643 O43294 408 8939 P27635 P49407 P60228 409 Q13099 O43251 5440 P49642 5441 6772 5442 Q9UKN5 Q9UKN8 Q9BW92 O00567 29088 126789 Q8IYD1 P62899 5434 Q14197 Q9Y450 Q15046 11326 P61326 Q15287 P23246 Q9UL03 Q86X95 Q9UMS4 84292 A7MD48 3035 O14979 11338 P15880 3028 Q00403 29093 55759 4116 10488 P62424 P57081 8732 7884 P05408 P50613 P51946 23764 Q9Y5Q8 23524 Q9NWU5 Q8WUY8 10450 Q96A72 128308 Q96GC5 Q07020 3054 23536 29079 Q9HCE1 Q99797 Q96SZ6 688 689 P82675 P62847 29074 P62841 P61513 P49207 4150 4152 4154 25929 Q9HCD5 P52815 22894 Q9Y5S9 P17844 79753 10438 Q5TAL4 4149 P62857 P62851 P56182 57727 P62854 O43463 8761 Q9BQ04 23517 Q92901 Q13901 Q5VTL8 P07814 Q92905 P35268 54460 3075 O75031 55796 P56192 O43791 53938 Q16629 Q14686 Q14209 P55265 4179 Q9UPN6 Q9H0U6 Q6NZI2 Q01130 474 P33240 28973 P46783 P49810 Q16630 P83731 Q14690 Q8WW01 P38919 Q16637 3091 Q8IWZ8 P13639 Q16633 6123 8543 P46782 5036 6125 P46781 O75376 79084 P41091 Q9NW13 60489 P68104 51773 24144 Q9NPD3 P00533 P46777 Q15796 P46776 P60866 P46779 P46778 Q16649 6133 P35638 P61968 6132 Q15797 6134 6137 P32121 O43776 O75362 Q96IZ7 6128 P82663 P82664 1915 Q13144 P02741 51547 Q14232 Q8WVM0 8563 Q13148 Q9BYU1 9410 6141 P35869 11051 Q8IWX8 8565 P18074 P62829 P18077 P36954 144983 Q4AC94 Q9P2E3 Q13151 6138 Q16665 P61978 Q9P2E9 51319 9421 P78347 P54136 6156 6158 P35453 26512 Q93062 23481 Q14494 Q14493 1937 1936 Q14498 1933 51329 6164 Q9NRC8 10181 8100 6168 11030 O75575 6160 23016 7490 Q9NY12 Q7L2H7 118460 P49848 P61927 6175 Q86W42 P98175 O75569 P98179 25885 Q02878 51340 54853 Q9NVM4 51585 P24534 11243 P55010 55703 P47897 1718 9439 Q9GZL7 Q6I9Y2 51107 1956 Q15365 10159 O75319 6188 6187 Q9HB90 Q08170 26986 Q01780 Q86Y79 25896 6182 23478 5093 Q96EU6 26747 51593 Q15370 P78362 51116 Q99814 1968 Q7Z7F7 11017 P78545 P54577 6199 9221 Q6P1J9 P29375 O75787 P29374 Q96EL2 57461 6193 6194 1975 10813 Q9H6Y2 83752 P41252 P62195 P41250 Q6P5R6 O15234 Q13761 65083 1736 Q13523 P54105 9477 9232 22803 Q13769 Q9H0L4 Q9P1Y6 P24928 79005 27257 Q9NVH2 Q6P1K2 P42575 O60293 Q5D0E6 7068 7067 Q5TAX3 Q15714 Q9H307 Q06643 Q8IZ69 P56537 79039 Q8TBF4 O60264 P05155 P78316 9255 Q32P51 63931 83759 Q8N8D1 Q9UNE7 Q9BWH6 29896 Q07955 29894 P53041 Q9UET6 Q92542 50628 P52597 O95997 Q9P015 4809 Q8WVK2 4800 2626 Q8NEC7 Q9NVC6 Q14653 51729 P19387 P30153 P19388 26121 1540 57038 Q9BUJ2 Q13573 9045 P55884 Q9NP81 Q6P1L8 28998 Q00059 Q96AE4 P43694 Q9BQA1 Q9Y4A8 51747 Q13347 5927 4839 P63272 23076 O60231 Q9HCS7 902 Q9HD40 Q13595 Q14684 P21675 Q14683 P10914 285672 Q92858 O75821 O75822 65003 Q969L4 P67809 O00268 Q8IXM3 Q06265 51081 O00267 Q9UBB9 5939 P16220 Q9ULW3 4841 Q9Y2X3 10772 Q6NZY4 Q9UK45 5935 P04003 Q9NRX2 Q9Y399 Q9NQ55 55696 51096 10785 P67812 5705 Q9NWB1 P25398 Q9ULX9 Q66PJ3 Q9NRY4 Q9BZG8 Q12968 O60306 P18621 Q8TED0 P12081 P63220 O60783 Q86WA8 Q92830 P03372 Q9Y2Z2 Q9Y2Z4 Q12972 P49591 Q92824 P46087 Q9H814 Q9GZX7 Q6P2C8 P49590 10767 Q99459 10528 P49588 10521 5725 10523 O95478 Q12986 1385 P50579 57661 O95232 P52756 6829 P19525 Q9P2N5 710 29102 P62316 P84103 P62318 5733 P62312 58517 27161 6827 P62314 Q10570 Q9Y2T7 Q96SB4 Q9UHK0 Q96SB3 Q13601 Q14938 O00472 Q5JTZ9 P19544 Q9NS56 10987 3329 6839 10988 56342 1388 720 722 P08651 P42771 P05386 P05387 P05388 Q9UNX3 4670 P32969 22938 Q9Y4Y9 O60508 Q9H9Y6 O60506 P42766 80222 P61244 284119 O14641 5511 P61247 O14640 Q6GMV3 5514 Q9Y4Z0 5515 5757 P04264 P61009 P08865 Q9UNY4 P08621 5518

P51693 P09958 Q13868 Q03164 10725 740 Q9P0M9 P61254 Q9Y2W2 988 Q92620 4677 P06454 Q9BZJ0 Q9Y2W1 O00425 O43395 P23396 O43159 6872 6871 P04637 Q5VYS8 6631 P15529 2034 Q9BV90 P14678 P53803 P62750 54512 4686 6625 11222 Q16236 5536 6627 P62995 P62753 Q9UQ80 54517 Q96LI5 P07910 P49770 220988 200916 6883 Q9UHG2 25873 23210 1191 2280 51010 Q15392 51253 23451 51013 6633 Q9Y6Y0 6878 8812 Q15393 10147 O43172 6637 P49768 P26641 Q86U42 P26640 O95059 Q8N726 O60942 51021 51023 P26639 51264 10594 Q53X93 P49756 P01138 5557 P61201 5558 Q9UPY3 23438 O43143 Q9Y2R5 Q9H7H0 6660 6421 Q9Y333 3396 Q9UHI6 23435 Q9Y2R9 Q96B26 11218 Q9NS37 Q96RR1 P09086 P10071 57819 P62308 P61218 Q9ULR0 O43390 P62304 6430 6432 Q9UQ35 6431 6434 7764 Q12904 22794 23404 Q96PU8 Q8IZL8 Q02930 Q9BZE1 8602 6427 6426 Q8N302 2081 23658 Q9Y2L1 6201 6202 5356 23650 3178 Q9ULK4 23411 O76024 O76021 P83881 328 P27797 25804 3181 2091 6210 P50750 P22087 Q8TA86 Q8N2M8 3189 Q9UNP9 2099 3188 3187 3185 3184 Q9NW64 O14802 10318 333 55660 51067 7536 6204 9716 6207 91746 P62979 Q9H5H4 3192 Q9UNQ2 3191 8880 A2RTX5 P27540 3190 4285 9733 7311 391356 Q969E8 P51610 O00411 Q9NVV4 Q08211 10569 79622 Q9NPJ6 79869 P62988 6217 9967 Q8N9Q2 6231 8892 4297 Q6DKI1 6233 64951 P62917 Q9UKD1 57187 P13984 4291 Q96FV9 351 Q9UBS0 P62910 P09012 6229 P62913 Q13243 O94906 Q13242 8662 Q13247 5393 P20290 P03950 5394 O00716 64960 16 8667 Q15428 Q16514 8665 Q15427 64963 63875 26205 64965 O75494 64969 367 51650 P27918 51651 6238 51654 11198 O94913 6239 Q9BY50 Q15434 Q14103 64975 23144 Q9UKF6 Q9Y606 85437 Q9NV06 P42285 P09234 8669 54931 P83876 54938 51428 O43660 4086 11171 10081 8683 4087 8446 4088 O75478 9533 9775 P29084 64745 142 Q96HW7 Q02543 P62701 P09001 P62945 P23771 Q8IXZ2 1801 P62942 Q15459 9785 10291 O15160 9541 8458 O15164 P54253 2909 P10275 O75460 4094 Q9Y3E5 Q8NI36 2908 Q8IYB3 P23588 8449 84365 Q13287 Q9NSE4 Q14134 Q13286 Q14137 Q09161 8467 O43889 23378 Q9Y5J1 Q9Y3F4 Q8NI27 Q9BRL6 11157 Q15233 Q99714 Q96FX7 10273 O75444 Q96T21 11129 Q96T23 Q99941 P50914 88745 51222 56915 9319 Q14152 Q9UBQ5 9318 2926 P49716 P49715 11128 Q9NQC7 Q15005 Q9BVS5 Q08050 7157 P62906 P14868 P14866 8241 10286 10285 2935 P78424 92345 Q13873 22913 Q96QC0 Q14966 Q7L0Y3 P52272 Q12788 92105 10929 P62081 P18124 8492 P42224 Q96DI7 P0C0L4 Q9Y3A5 84967 P47914 Q9HC16 P09651 P82921 1856 Q9UBK2 O15371 P82912 O15372 P82914 P55795 Q14978 Q13405 O95602 Q13887 Q96I24 55173 P22626 Q9Y3B4 Q9Y3B7 P84098 P67775 P63173 Q6IN84 84950 10949 Q9UHX1 Q9Y3B2 P09661 P05067 2959 P08579 10940 Q9UBL3 P52298 9128 9129 O75643 10907 O43812 P11940 O75400 26015 9360 80145 283989 2961 2960 1870 Q8TAD8 Q9BTC0 2969 P09874 Q13427 Q8NAV1 29777 10921 P42677 Q9Y3D3 2972 Q9Y3D5 57379 134430 2737 P82932 Q969Q0 P82933 Q13435 Q15853 Q9UJV9 Q9NYH9 Q14527 1660 Q6NYC1 P62280 1655 P62277 P22415 1653 P30050 Q03468 Q92499 Q8WYQ5 1659 P43115 O95644 O95400 Q08J23 26005 Q96BI3 Q96G21 27339 O43809 1665 58487 Q8N0Z8 27332 Q9BXP5 P23511 Q6P2Q9 Q9Y5B9 Q8IY81 Q8TEQ6 P55769 P35711 Q5RKV6 2521 57169 Q6P4R8 Q9HC36 Q15643 Q9P2Y4 Q15648 Q9BVJ6 Q96HR8 Q15653 Q14562 1207

primary metabolic process P28562 3638 10657 2305 55561 55568 Q5QP82 Q8N465 4967 4968 Q9H4L5 Q9H4L4 Q9H4L7 P30876 O14561 P52209 Q8NE71 P19623 10667 2 P42858 Q9UKV5 136319 Q9UKV8 3661 Q8NE63 P62495 2324 P48147 Q9UKW6 3659 10632 P54886 3672 Q13705 P21917 10645 2335 10642 O14578 10643 Q9UL15 Q8WXG6 P51784 Q9Y5Y6 Q9UL18 Q8IX01 1022 1021 1020 Q06587 P50454 P51787 10613 1018 1017 2348 79577 P21964 10616 P54819 Q92530 26191 3692 Q8WXF0 Q13724 Q9H4P4 6720 Q96J02 1029 P42898 10626 O14519 10625 1025 Q9BUB5 P20648 Q9BUB4 6711 P05023 P07686 6714 P05026 Q9UKR5 O95136 1040 79595 391627 P20618 6721 6723 Q9UL46 O95147 6741 5411 Q38SD2 Q8IWL3 1050 10606 253980 57332 1047 10607 6733 Q9UL54 64432 Q9Y5U4 5422

Q9UKL6 P50416 11108 11107 Q8WWY3 6749 Q9BTU6 Q9Y676 P49411 5430 5432 Q9UKM9 O95163 Q9NWZ3 P57059 115426 1072 51132 Q9Y680 84033 5424 11113 P49407 1080 84061 5440 5441 6772 5442 Q9UKN5 Q9UKN8 346171 Q66K89 P14780 51141 51144 5434 51148 Q14197 51147 Q9Y5X9 P50440 Q9UL03 Q86X95 Q8N3J5 Q9Y697 A7MD48 Q53H96 Q9H4A9 P17812 Q00403 O95190 6776 4116 P62424 P57081 6790 Q5VVQ6 6793 O00746 6794 4133 10690 9820 Q9NX62 O00743 Q9Y5Q8 Q9NWU5 Q8IWF2 4125 6789 Q96A72 Q07020 5470 4140 5471 O00757 Q9Y5R4 P30837 5476 O00754 Q9NWV8 Q9UBX3 P49448 5467 207 5468 4139 4150 O00767 5481 4152 4154 Q9Y5S2 Q96A54 Q08345 O00762 P13489 Q9NWW5 Q9Y5S9 P17844 51185 215 5478 5479 4149 Q9H4F1 5494 5495 Q9BU89 5499 P13497 Q9UKK9 P17858 Q96QT4 10682 P49427 4172 4173 4174 11060 4176 Q14209 Q16864 11065 7204 P55265 4179 Q6NZI2 4171 230 Q9NXS2 Q6P5Z2 23043 11072 Q8IWZ8 4188 8543 Q16877 Q9NY59 Q9NPD3 8536 Q96RL1 P61956 Q15554 197259 Q9BUN8 P35638 P61968 11041 8555 4191 23028 Q96IZ7 4193 Q8WXQ8 P61962 1915 P61960 Q93009 P02741 P22314 Q93008 Q14232 P61964 8563 Q9Y6R4 P36957 11051 Q8IWX8 8565 Q9Y6R0 Q86Y37 P36956 P36954 Q8N4C8 267 23031 23032 Q9NPF4 Q9NPF5 P61978 P43246 8574 Q14249 8576 26994 Q9Y6K5 O75582 271 Q9Y6K1 P10398 Q9Y6K0 Q93063 Q93062 Q04837 Q96RQ3 278 8569 1937 1936 1933 11030 Q9BV47 P22392 O75575 23014 23016 7251 Q93050 Q9NY12 Q9Y6L7 11035 118460 P02751 P61927 7266 P02790 O75569 7267 Q9Y6M4 Q8WY64 Q8NEJ0 51102 P08069 P02786 Q6I9Y2 51107 1956 O15075 Q9NPA8 26986 Q01780 Q86Y79 7272 1965 114088 Q93034 51116 1969 Q14289 1968 11017 P12931 Q7Z589 Q70CQ3 Q70CQ1 1975 Q05329 Q9BUG9 Q6P5R6 Q13761 Q58WW2 7297 P56524 Q13769 O75531 P24928 1982 Q9Y6H3 Q92560 26168 Q9Y6H5 P06276 O75528 Q5D0E6 P06239 Q8WXI4 O75521 92483 P55209 P56539 P56537 1994 Q92551 26133 P00966 P06241 P21860 O95983 Q16816 O95989 P55212 91147 P55210 A4D1W7 63931 3704 3705 Q07954 Q9Y6J8 Q07955 P07585 Q92542 50628 Q16825 Q16827 O75503 O14595 O95997 P35610 Q16828 9020 23081 23085 O60488 P20594 84881 Q8NEC7 Q8IWV7 Q86Y13 P61081 Q9H596 P61073 83548 23097 26121 P61077 Q9BUJ2 P22303 11080 Q9NXH8 9045 Q9NP81 Q16849 P12956 P12955 Q8NEB9 P33897 Q9Y6E7 Q9Y6E0 P61088 Q96AE4 Q96RG2 11091 Q16854 9054 23076 P24941 11099 84893 Q5FWF5 285672 65003 O14672 399687 9061 Q9UBB9 O60568 O60566 P15374 Q8IVH8 10772 Q9UK45 Q9UBC3 P27361 Q9GZZ9 65018 54361 9077 55696 10783 10785 92609 Q9NWB1 P52333 P29992 1121 O95210 P53667 Q8TED0 O60547 O60543 1111 Q86WA8 P61289 P03372 P26012 P49591 3791 Q9GZX7 Q8NCR0 9093 Q9UBE8 P56705 P49590 9099 10767 57410 O60551 3784 P49588 P07741 Q9H3G5 10765 P05129 P11802 P50579 O95232 6829 10733 147746 Q9P2N5 1137 Q6FHQ0 P61221 6827 Q10570 Q7KZI7 Q7Z419 Q9Y4X5 Q13601 1153 Q14938 1152 P19784 O95243 P53611 6839 10746 1147 2475 5500 5501 P26045 P53621 O43175 O95257 6850 Q13615 Q9Y4Y9 Q13618 Q13617 O60508 Q13616 1161 O60506 Q02083 Q9P2P5 O60502 10714 57448 P61244 O14641 5511 P61247 O14640 5514 O43184 Q9Y4Z0 5515 Q9UK22 P05112 65061 5516 5518 50814 Q13620 Q13627 P18433 O60513 10725 10724 O60512 P61254 10728 O14656 P40337 P06454 Q9UK32 O14657 Q8N2K1 P04632 O95278 O43159 6872 Q96HA8 6871 P04637 25862 P16615 Q9NVW2 O95271 Q01650 23203 11222 5536 Q15389 5537 Q9GZR7 Q9GZR5 6883 P48201 4221 6885 1198 25873 23210 1196 1195 P50542 P12268 1191 56940 O76074 Q96Q15 51251 Q15392 Q99873 55611 51253 Q9P2K8 4214 11231 6878 4215 4216 Q15393 P01588 O43172 56946 25847 5562 5563 5565 P13569 O95299 51264 Q53X93 Q9GZP9 Q9Y572 5557 P61201 5558 O43143 Q9BT22 P04626 5575 P04629 25853 11218 301 P17936 9924 5566 Q96PN8 5567 5568 P61218 5580 5581 5582 5583 5584 5585 4255 5586 22794 P15328 25822 O76031 Q96PU8 Q96PU4 79612 55644 314 Q96PU5 Q02930 5577 5578 8602 5579 Q9H3F6 8607 Q8N302 5591 P07711 Q9Y4P1 9950 Q6IR47 P24298 5596 5597 5598 P50502 O76024 O76021 5590 P16671 327 328 4259 329 84197 25804 Q9GZU7 Q8N2M8 P12277 P51843 Q9NW64

Q8NCN5 333 55660 55666 56994 5599 P83436 4285 4287 25818 7311 Q96Q40 P17980 Q9NVV4 P15313 Q08211 221656 Q08209 79622 9967 4297 Q6DKI1 Q6ZT98 Q15418 7325 8654 Q9UKD1 P30085 Q04760 4291 351 Q03426 353 Q9UBS0 Q04759 Q9UBS4 P23786 9978 P21127 8662 7332 7334 16 8667 Q15428 7336 8665 Q15427 7335 19 26205 Q8TF76 Q9UKE5 Q9H3Z4 Q9UBT2 23170 367 Q9UBT6 369 P30084 11198 7329 7328 20 7341 11160 Q15434 Q14103 11161 7347 6015 P29074 23144 Q53GQ0 Q9UKF6 Q9Y606 Q9UBU3 Q03405 85437 P17096 11165 11168 Q16774 8669 Q9UBU8 30 P43378 31 34 Q53H12 11171 8683 Q8IW75 7353 Q14114 39 7358 P51812 Q9Y619 P51813 P29084 Q96HW7 Q9UBV7 P51817 11176 P23771 Q15459 O15160 P55345 O15164 25788 23127 Q99965 Q15466 52 Q14134 Q14139 6045 7376 Q14137 7375 6048 23133 23135 Q9UKA1 Q9Y5J1 7372 11157 O15169 Q99956 P46459 7385 7384 Q8TEY7 6059 Q9Y5K6 Q9Y5K5 6050 Q9UKB1 Q99942 Q99943 11129 Q99941 51222 56915 56916 P01579 Q14152 11124 Q9UBQ5 11128 O75676 O15197 O15198 O75674 P14868 P33121 P14866 25778 51231 P08195 Q14164 86 Q14166 Q14165 9100 Q96QC0 Q14966 P52272 P33992 P33993 Q8TF05 P33991 Q05209 P0C0L4 84967 91 Q8WWI1 94 P45974 26270 P82921 O15118 Q9UBK2 Q9UBK9 P82912 9114 P82914 Q14978 Q8WWH4 Q86WJ1 Q6DT37 Q96I24 Q9H3R0 P31321 P31323 P43304 84950 P05062 P52292 Q52LR7 P05067 Q9UBL6 Q9UBL3 O94763 P52298 9128 O94768 9129 O75643 9126 O94766 Q6XUX3 Q9H422 Q96I15 6093 283989 3817 Q8TEL6 P21980 Q9BTC0 Q9UBM7 Q5JRX3 Q02156 26260 26262 57379 134430 Q9UBN7 P82932 P82933 P30049 Q9UJV9 Q9UBF6 9149 56052 Q8TF47 P30041 Q6NYC1 Q9H488 P11766 P53567 B3KY43 P05090 P22415 P05091 26232 Q9UK80 P05093 P30050 Q03468 26234 Q92499 Q9UJW2 3835 P47712 Q8IVT5 9150 57396 Q9UJX2 Q9Y5B0 P17081 P08138 P09467 2517 Q9Y5B9 Q9Y5B8 P09429 O75604 P10415 Q8TEQ6 P35711 Q7Z494 2521 Q6P4R8 P09430 Q5T6F0 O75608 728642 P08107 O15111 Q9P2Y4 Q04771 23192 23193 1203 Q96HR8 2531 O15105 P45983 P45984 1207 Q7Z2K6 65125 P54619 P51587 9184 P17676 Q8TDD1 2547 10893 6901 Q6IQ55 Q9H2H8 P28335 25913 Q9UIQ6 P28330 P08729 P28331 Q5VTR2 P37268 O60216 P62244 80755 54487 P62241 P49247 P62249 Q9H2G2 Q9BRX9 P28300 2572 2571 79791 Q8NBQ5 54496 6927 Q8IUD2 6929 P62256 57520 6921 P26599 P31942 Q9UIS9 P31944 P31943 P08708 2582 P54646 P17655 P62266 P62263 5602 Q96GD4 P62269 5604 Q9Y3U8 Q9Y463 Q14807 2593 P50213 2590 Q8WVC6 P39019 P15056 10856 2589 P39023 5610 5613 Q9UIM3 Q9Y478 P51553 P30679 5631 Q07666 Q8WVB6 P52888 Q06330 10868 10869 5624 Q9H2K2 P28340 4311 P21709 Q8IUH5 169436 Q9HCL2 5636 Q10472 Q10471 Q13501 P51575 Q5MAI5 22826 22827 22828 P51570 Q8NC51 Q9HCK5 10845 192669 4313 4318 11340 Q86V81 O00541 P14555 23326 25988 23327 51362 Q8NBJ5 51363 5654 P25205 10013 84289 10014 135295 64682 Q15269 Q9UII4 4343 Q9BS26 Q6FGD7 P51513 Q99755 51377 Q99750 Q9BS18 Q9BRP8 Q99759 408 409 P60468 23307 Q09472 5682 5683 5684 5685 5686 O00562 O00567 Q9NUW8 Q99741 55743 3014 8703 55748 8702 Q9Y450 11326 Q15287 4361 84292 5692 5693 P40818 8720 3035 3033 3032 Q9NUX5 3030 P14543 10007 11338 55750 P14550 P15880 3028 P60484 Q15291 55759 5687 5688 55757 P48775 84296 79709 P31930 P26583 8732 O00506 25942 Q9NUQ2 54431 Q8WUY8 435 P31939 Q9H2D6 79739 P55072 Q96GC5 140609 Q9HCE7 3054 Q05086 P37231 Q9HCE1 Q99797 Q8NBM8 444 445 55775 8737 Q02809 P82675 P55084 P61758 Q9H2C2 8738 284086 P49207 8754 25929 7422 Q9BRT9 Q9HCD5 P52815 3066 22894 3065 Q9UIF9 22897 O75293 79753 55781 7415 8761 3070 Q5VTL8 Q9UIG0 54460 3075 Q9HCC0 3074 P17612 468 55796 7428 O43791 53938 Q16629 Q9Y4K4 P11441 O75385 Q9Y4K0 Q86VQ3 Q9Y4K3 472 Q96GX9 P48729 474 O75390 476 477 478 28973 Q99807 Q16630 8767 P43034 Q8WW01 6120 3093 Q16637 Q15303 3091 Q16633 P55036 6123 Q16635 6125 Q15306 Q9Y4L5 Q86VP6 3099 O75376 3098 8780 481 483 Q504Q3 23291 Q9NW13 488 Q4J6C6 6117 P22102 6119 6118 Q6YP21 Q16649 6133 6132 Q16644 6134 7465 6137 Q16647 O43776 O75365 490 O75362 495 498 P09110 6128 P56385 P82663 P82664 P56381 Q8NCB2 Q8WVM0 Q9BSI4 Q16659

6141 P18074 P55055 Q01581 P18077 O75351 O75352 144983 28960 Q96PF1 Q9P2E3 6138 Q16665 Q9P2E9 51319 6156 6158 O75347 131601 P35453 P09172 P23467 P27824 Q14012 51329 Q14011 6164 Q15349 Q8TDX7 6168 P46100 11274 P36776 Q9Y4H2 6160 7490 P11498 Q7L2H7 11277 6175 9201 Q86W42 P98175 Q3ZCQ8 P98179 Q02880 25885 Q08188 Q8WW22 Q02878 51340 Q9NVM4 Q96PK6 11243 Q14032 P55010 Q9NVM6 55703 Q16698 Q9GZL7 Q15365 P23443 O75319 9212 6185 6188 Q15369 Q8IUX7 6187 O43741 Q08170 25896 6182 6184 Q86W33 P48739 Q15370 P27815 Q99816 P48730 P23458 Q99814 P48736 P98194 Q8WW12 Q15375 P78545 6196 P54577 6199 9221 6198 P98155 O95714 P78540 Q9H324 57461 6193 P78549 Q8IV08 6195 6194 10813 83752 Q9Y4C1 P41252 P62195 P41250 O60285 P08684 65083 Q13523 Q13522 P78536 9232 22803 Q13526 Q9P1Y6 Q9H2P9 83737 P46934 Q9Y4D2 P41240 O60296 Q9NVH2 Q4LE28 P42574 P42575 O60293 P34947 P29597 Q13535 Q9H307 P29590 Q7Z2Q5 P41235 P46926 Q9Y4E8 3939 Q9NVI1 O60264 3932 O60260 Q9HCP0 P07339 9255 Q32P51 Q13547 Q13546 P34931 9252 83759 9253 3948 P41222 P20309 O95749 P78560 P41227 3945 Q13555 Q13554 O95757 Q13557 P78509 9261 P00749 P00747 Q8WVK2 P00742 O60240 3953 2629 2626 Q9NVC6 Q9P1U0 Q13569 P19387 P19388 P11413 93100 P00738 Q9UIV1 2632 O60256 P00734 P24723 Q13573 P09104 P46977 Q13572 P46976 P55884 3980 Q8IUQ4 28998 P32320 P78527 P32322 O60229 P41279 Q58DX5 1314 1312 2643 Q04656 Q9UJ83 3978 Q9Y4A8 P18031 Q9P286 Q9P287 P62191 P19367 9296 1326 Q63HQ2 2651 P07384 O60231 Q9HCS7 P20340 P00750 Q9HD40 Q13595 P21675 P48052 Q12948 Q06265 Q8NB16 Q9Y2X3 P08842 P48047 Q9Y399 2681 5706 57634 5707 79885 5708 5709 P21810 P16471 O60331 5700 P49368 5701 5702 Q9UHP3 Q9UI12 5704 5705 65264 P54753 P25398 Q66PJ3 Q9Y2Y8 Q9BZG8 Q12968 P54756 O60306 O60307 P16444 5717 1358 57646 83933 P12081 Q9Y2Z2 5713 Q9Y2Z4 5716 Q12972 P54764 P54762 P54760 Q12974 1374 Q6P2C8 O60318 5728 1368 Q8WTX9 P15121 Q9BZF3 Q9UI32 253827 Q9UI30 5725 Q6PCD5 P28482 Q12986 1385 Q9UHJ9 P51668 57661 P51665 Q9P0J1 P19525 P62316 P62318 5733 56339 P62312 P62314 Q9BZM1 P51671 Q9Y2T7 Q9UHK0 Q06210 Q5VST9 P19544 P30793 10987 57678 10988 56342 1388 1387 P54709 P42771 5747 22934 Q14703 22937 22938 Q06203 Q9H9Y6 Q9P0L2 P42765 P42766 Q56NI9 10956 P62333 284119 Q9BZK7 22929 5757 P61009 P08865 P51693 P27144 5770 P51692 P54727 P54725 Q9P0M2 Q03164 55035 Q9P0M9 57695 57697 Q9Y2W2 Q9Y385 Q6DHV7 5768 Q9BZJ0 Q9Y2W1 509 P06213 Q15139 5780 Q9UPV9 5783 P14678 P38646 Q8WTS1 54512 Q99640 513 514 5775 79813 515 5778 P27986 518 Q9UQ80 54517 220988 Q8TBX8 Q9UHG2 P04406 Q9UQ88 5796 Q8WTR2 521 522 523 23451 526 8812 528 10147 Q99638 P01344 8815 P01343 Q96EZ8 Q86U44 P26641 Q86U42 P26640 O95059 Q9NU63 P26639 533 79834 535 55869 537 5798 79837 539 Q9UPY3 P01375 23438 23439 Q9Y2R5 Q9Y337 Q9Y2R2 P40939 P40938 P40937 8841 Q9Y333 7511 Q9UHI8 Q9UHI6 P51659 23435 Q9Y2R9 P12004 Q99611 3149 O95071 3148 545 546 3146 8836 8833 Q96EY1 P62308 P25311 8837 P62304 7508 Q9UPR0 O00628 23409 Q9UQ35 7520 8851 Q9UPR3 Q12904 Q9Y2K6 23400 Q9Y2K2 P17752 23404 Q99685 3159 Q99683 Q9BZE1 8844 Q15185 P49336 7518 P05771 7517 Q9Y2L1 6201 6202 7533 3178 23411 563 Q00987 P49327 Q9H1A4 65220 3181 6210 P49321 3189 3188 Q9UHD2 Q12923 3187 Q9UPT9 Q9Y2M5 3185 3184 572 573 54575 Q8N8Y2 7536 6204 6207 3192 3191 P04424 8880 3190 8881 Q12933 Q12931 7555 P51610 580 P51617 P52948 8879 79869 8878 6217 Q9UPV0 10090 Q8N9Q2 6231 8892 6233 P15927 P15924 P15923 Q96FV9 593 6229 Q8IU60 Q9HC98 P03950 O00716 Q16512 Q16513 Q16514 O75494 P27918 6238 6239 Q13490 6256 10075 Q9HBW0 Q16526 P31153 23387 P31152 O43897 Q01469 23381 Q9NV06 P23528 P09234 Q16531 Q8WUK0 51426 P09237 51428 Q16539 344387 10081 Q6PD62 O75478 Q15208 O75477 Q86UL3 23399 O75475 Q8TD19 64745 Q02790 P12883 23396 10087 6259 Q8WUJ0 O75469 64750 P40926 P14635 P38606 Q9UQL6 P10275 P10276 P35579 O75460 Q99720 Q9Y3E5 10055 P23588 Q99728 84365 Q16555 197322 O43889 P36896

64768 P36894 23378 P35573 Q02763 Q9Y3F4 P01308 Q01433 120892 Q9BRL6 Q15233 Q99714 Q8TCU4 Q96FX7 P61803 P14618 Q96FX2 O75444 Q86V24 P36888 P11586 Q99700 Q9H1Y0 51465 88745 Q99708 P22234 O95817 9319 O95816 Q16576 Q99707 9318 O95819 55827 O75439 Q08050 P39905 Q96FW1 P14625 P36873 Q02750 55832 54504 Q15256 22913 O95835 O95834 Q7L0Y3 10929 P18124 O43837 10933 Q9Y3A5 Q9HC16 22908 O95837 P15090 Q9BZQ6 Q8WUD6 22926 Q13405 Q13404 22928 Q8TCJ0 10939 Q8TCJ2 O43847 Q9Y3B4 Q9Y3B7 2710 56267 10949 Q08945 Q9UHX1 Q9Y3B2 57599 10940 10907 O43812 O75400 10908 Q13418 286826 9360 9361 Q9UHY1 Q9Y3C5 P00846 2720 P42680 P42681 Q9UHY7 P42684 P42685 Q9NUG6 Q86UE8 O95861 P53396 Q13427 Q13426 O95865 Q8NAV1 Q8NB78 10921 P42677 P19438 P68402 1400 P68400 Q9Y3D3 2730 Q9Y3D5 1408 1407 2737 Q13435 9388 Q9HC62 Q9UI38 P62280 2744 P62277 Q9HC52 P43115 2747 Q13444 9397 Q13443 Q08J23 Q9H1R2 8065 Q96G21 O43809 Q8N0Z6 Q9UQE7 Q8N0Z8 P23511 Q6P2Q9 Q13451 P23510 8078 O14495 P12830 P19474 59343 84305 1432 1431 2762 Q14790 O60341 Q9HC36 Q13464 Q5T4B2 P54687 P53355 P53350 Q9P0U3 Q9P0U4 26469 Q00169 Q7L9L4 5800 Q9UHV9 O75947 8099 Q92979 Q9H0E3 Q9Y265 O75940 Q12824 O95551 1459 Q9BYG3 1457 1454 O00391 1453 Q00839 P05455 Q9H0D6 O75936 O75934 Q12834 Q06136 P16104 1468 27090 Q9H0C8 Q9Y285 5822 140801 O75937 P10809 Q96EB6 Q5S007 P27694 Q9UGN5 P27695 Q06124 P17405 A0AVT1 4507 P37023 P14384 1478 57761 5830 Q9Y295 55109 5832 Q9Y297 5833 O75928 Q9Y291 5836 P26358 Q12851 O75914 O75911 P26373 Q99583 Q12857 1491 55110 Q99575 1487 Q9BYD1 148789 55119 P26368 P25098 Q9Y223 Q9UGH3 Q96MX6 Q9NSU2 27043 57787 O60884 Q9Y230 O75909 Q9Y231 O14986 5859 Q12874 Q9UGI0 Q12872 P29466 5870 Q9H0H0 5871 Q9UGI9 Q15904 Q08752 Q9H8X2 Q8TBC4 Q96EF6 57794 Q96MV8 Q5C9Z4 P04179 Q12884 Q9UGJ0 Q12888 Q9Y243 5883 P16118 Q8TBB1 P38398 Q9BQ90 Q96MU7 Q9BPX1 55149 Q96N66 Q9Y250 613 Q92995 Q5TCX8 Q92994 5875 254827 Q92993 4548 P10827 P30414 P10828 5892 5894 P31749 55152 27032 P40692 27037 55159 O60870 P04150 Q9Y262 5886 5887 5888 8916 Q9UGL1 P04155 Q8TBA6 O96004 8930 O00308 O96009 10250 P56199 O00303 P50897 23569 Q15022 P62877 8924 Q15020 Q15025 Q15024 P62875 Q15029 P23284 O96017 Q15027 Q86SR1 O96019 P36578 23576 P35244 P14324 O96013 P35249 Q03933 641 P35251 P62888 196441 P23276 8939 Q15036 P27635 P60228 10269 Q15034 O00329 P35236 O96020 3263 10238 O14967 P11274 8945 P62899 79956 Q15046 O14965 P23246 Q9Y215 Q8TAT6 23552 3276 23556 P35227 O14979 P35232 Q9H093 4598 668 Q15054 285193 O14974 Q59EA4 P06733 Q92934 6311 P01040 P62837 P06737 8975 P05408 245972 Q99570 P39656 23524 672 673 P15692 6304 6305 128308 10213 158135 P06744 P05413 3290 8985 O43488 3297 23536 Q6PJP8 Q99558 10229 688 689 Q86T82 P62847 P62841 P61513 Q15078 Q9BQ15 23509 P13010 Q15084 Q99543 8987 P62857 O14920 P62851 P56182 57727 P56180 P62854 O43463 Q9BQ04 Q92903 6342 23517 Q92901 O43464 Q92905 Q8NHX1 P35268 23512 Q7L7X3 O75031 10208 O14936 O14939 Q56UN5 P60201 10202 Q92900 P56192 P36507 Q14686 P37837 P19174 Q9BZ95 Q9UPN6 Q86TM6 64834 Q9H0U6 P12532 Q8TC27 O75150 Q9H0U3 84447 P46783 P49810 P83731 Q14690 Q14694 Q13363 Q14693 Q9HAV7 Q14697 5034 P46782 5036 P46781 P11216 79084 P41091 Q9Y2H1 60489 P68104 P00533 Q13370 Q16401 P46777 Q9HAU5 P46776 Q9HAU4 P46779 P24522 P46778 10193 P56134 Q9UPP1 P32121 51540 10197 P00568 51548 51547 Q9BYU1 9410 55907 P62826 P20151 O43542 O75116 P62829 Q4AC94 P00558 Q13393 5049 9421 P78347 9420 O75106 P49862 P32189 P36542 26512 Q9Y2C2 23481 51567 Q9BQK8 10181 8100 55929 Q9HAZ1 26524 26523 O43529 5071 P49841 P49848 Q15111 Q15118 P78368 P49840 P11229 9448 10152 23463 A1XKG3 Q9BYZ2 P48507 P24539 51585 P24534 P78371 10155 Q9BQI3 9439 Q15125 2805 10159 Q15126 Q9HB90 P78356 23476 5091 23478 P37840 5093 Q96EU6 O43505 Q9H173 51593 Q9H9G7 Q92187 Q9NTJ5 P78362 10166 54623 Q9HAW8 9465 9463 Q6P1J9 P09769 P29375 P29374 Q96EL2 P29372 27005 79012

Q9H0M0 27000 P42345 P22735 2821 2820 P04083 9476 P31689 9477 9474 9475 O15496 Q9H0L4 1503 79005 Q6P1K2 P42338 P42336 P29353 Q5JXB2 P29350 Q9H0K1 1514 Q9BYM8 79035 55066 79039 Q8TBF4 P04062 P04066 Q9Y2A9 80020 P54368 P78316 55072 P78317 Q8N8D1 Q9Y2B5 201595 Q04446 P04070 P53041 P07101 P52597 Q96EP0 Q13315 Q86TI2 Q9P015 Q96EP1 P00505 Q9H9B1 2869 Q14653 55093 64801 Q9H0P0 P46736 Q9H9A7 2875 1540 124739 5901 P46734 P13807 Q6P1L8 O75964 O75962 P28065 26576 Q9P035 Q00059 Q9BQA5 P43694 O15455 Q14674 Q9BQA1 5914 O95544 Q13347 Q14676 O75953 P28074 P28072 P28070 P42357 5927 5928 P63279 P00519 5929 P63272 Q9HB03 P43681 P08473 Q9BZ11 51514 P43686 Q14684 Q14683 P10914 O95677 Q92858 23607 P67809 P52790 Q9NRW4 P30520 O15519 5939 1576 P16220 Q92851 5931 P49137 5935 P0CG13 P09936 O95685 O15530 Q9NS91 Q9NRX2 Q8IZP0 Q9H832 Q07343 P50148 P18887 P16234 55215 P67812 64174 O95696 Q9NRY2 Q9NRY4 Q99471 P18858 P18859 125972 Q92830 P62136 4627 Q92831 P55854 Q92824 P46087 P09917 5970 Q9H814 Q99460 Q9NRZ9 Q99459 3304 P17540 P62140 55236 3301 3309 P04233 Q92820 P30566 P34925 O00469 133584 5981 5983 3320 P52756 27165 27163 Q96DE5 3313 710 3312 P09972 5976 27161 O00472 Q6PIW4 Q9NS56 3329 P16278 3326 720 3324 722 P08651 Q6XQN6 Q6ZWT7 4659 Q9UNX3 4670 P28223 137964 4673 2011 27148 O00487 P30542 80222 3337 Q96DC9 Q12770 Q92878 P04264 Q9UNY4 P08621 Q15800 P51451 P09958 Q12772 P30536 2023 P52788 P30530 P52789 740 Q96LR5 P37173 Q99496 P09960 157570 4677 747 4678 O15503 O00425 P23396 Q5VYS8 2034 2033 Q00796 P09093 2026 4686 Q16236 P62995 Q96LI5 Q9C0K0 P25100 P46020 2043 Q9NRP7 54764 P46019 P01100 Q8N726 P14416 O00444 P14410 P11388 P11387 O60942 773 P01138 P01137 P01135 P01133 P02461 P01130 Q9H7H0 6421 2065 3396 2064 780 Q9NRR6 Q9NS37 Q8IZE3 P09086 P10071 57819 2070 Q5TKA1 6430 6432 6431 6434 7764 Q92817 Q9UNN5 O60925 Q8IZL8 O75190 Q8IZL9 6427 10331 6426 2081 23658 P46063 5111 23650 Q5HYA8 P18850 Q99437 P18847 P18848 P83881 P27797 P50990 2091 P50991 P22087 91754 Q8TA86 Q9UNP9 2099 O60907 O75164 23621 P50993 10318 Q9NRM7 91746 P62979 Q9UNQ2 A2RTX5 Q5T1M5 6464 391356 P39748 O00411 146057 P52701 O60911 Q5U4P2 P58012 P62988 5127 Q8N752 P25116 6472 Q13237 5141 5142 Q14566 64951 P62917 P13984 6470 P62910 P09012 5139 P62913 Q13243 Q13242 O43683 Q13247 P20290 9510 O43687 64960 O43684 9512 64963 64965 64969 80198 51650 51651 51654 51657 5162 A2RUU4 Q9BY50 P11309 Q6Y2X3 Q13257 9524 9525 P62937 P11308 64975 5160 P49916 P49915 5159 P61604 P83876 P21589 Q13263 9531 O43660 Q13268 9530 Q9BY44 9533 Q9BY41 5170 P34896 P11310 Q02543 P09001 9526 P62945 P49903 P62942 5184 10291 9541 P48651 Q8TB40 2909 160287 P12694 121512 P27708 Q8NI36 2908 P48643 Q13287 Q9NSE4 Q13286 5198 Q09161 P15735 Q8NI27 Q8TB22 Q96E52 8237 10273 Q9HA82 P49959 P50914 2926 P09038 Q15008 Q9BY84 10280 Q15005 Q9BXW6 10283 P62906 9578 116138 8241 2932 2931 255488 221264 10286 10285 Q15014 2935 P78424 P55786 P08559 9588 Q12788 92105 Q15819 P62081 1613 80124 Q96DI7 Q9BXM7 79139 Q8TAF3 Q12792 1616 P07237 8266 Q12797 P55795 O95602 Q9H7Z7 55173 27132 P67775 Q6IN84 P08575 2959 P08579 2956 P07205 Q15831 P29474 P29475 Q96M96 1634 80145 27102 2961 2960 P04183 O95619 Q8TAD8 2969 P09874 Q6V1X1 Q15843 Q6R6M4 55192 64900 29777 79143 1642 Q05655 2972 Q9BXJ9 P09884 1647 Q15853 Q9BY32 Q14527 P13945 1660 Q86SG2 2987 1655 1653 Q86SG6 1650 Q13200 1659 O95644 150094 51606 Q13202 P21580 Q8N7H5 1666 64919 80155 1665 Q9BXP5 51611 Q13216 93492 P55769 4705 1676 P08581 Q8NHH1 P41159 Q14558 O95661 4719 389840 Q13233 Q14562 Q96TA2 Q96L12 Q9NQU5 81605 P52434 O00148 Q8IYN9 P84022 Q70EL4 P27487 Q9C026 Q8NFZ0 Q92729 O00154 P53779 P14174 10420 4734 10421 64282 Q7Z727 23708 81629 3420 O00167 P56817 3417 3416 P40429 811 Q8N684 Q5TAP6 P40424 P27448 3419 Q9C005 P07858 P99999 Q9NR96 Q8IYK4 P20823 Q8NFW8 Q5TAQ9 55352 O60674 P20827 P19838 821 3421 823 826 Q9NZJ4 P41743 P07864 P11926 Q13951

Q13956 4772 2110 Q5SGD2 55361 O95352 2107 Q5T160 27283 Q6PI48 80324 Q9BWG6 833 Q4G176
O14744 340204 4780 Q92769 P09619 Q9NZC7 80308 O95361 P10600 O95363 Q7LGA3 27297 P61353
167227 841 843 4775 847 7804 O14757 Q92766 P09622 4790 Q13976 2132 Q96L34 O95376 Q9NR45
P18545 Q9NR48 Q9BWE0 Q05469 2125 80347 P60033 857 859 Q96CA5 P11908 Q9UMW8 Q92748
Q9H6R4 6500 Q9NR50 O95382 Q96L21 Q9NQT4 Q9NR55 Q9NQT5 Q9BWD1 80332 Q4G0W2 2139
Q96KP4 O00141 80335 865 867 Q9UMX1 4799 Q92743 P06576 P00390 10492 O95391 3481 O60603
871 Q96T88 3479 2147 Q13085 7832 Q96KG9 P00387 P25685 P23025 Q6UWV6 P51991 6520 Q96T76
O14717 2159 O43290 Q7Z6J9 P61313 P49643 O43294 6517 O43293 Q13099 O43252 Q6ZMR3 Q6ZN33
O43251 P49642 64215 Q9BW92 Q9BW91 891 29088 O14727 126789 Q8IYD1 Q96T60 Q9NR09 Q16134
Q8IYD8 10477 Q9UN42 29086 10476 P61326 Q9UMS4 P04745 5210 P04746 7874 5213 Q658W2
114799 Q01974 Q96ST3 Q01970 29093 O14730 7867 10488 O14733 5207 7884 23760 P50613 P51946
23762 23764 2194 P60891 10450 10455 Q01968 O43242 P51955 P51956 29079 Q96SZ6 23770 5226
P48357 29074 Q7LFX5 23746 P24386 5245 9600 10438 Q5TAL4 Q00613 10432 Q13901 P07814 P49674
Q96C86 56647 10445 Q13115 6598 24139 57062 O43318 Q01130 P33240 9616 Q9C0C2 P38919 Q13126
P13639 P13637 P58546 51773 Q9NRH2 24144 Q15796 P60866 Q13131 9641 Q15797 9646 9643
Q9UNL4 Q6UXN9 29803 Q01105 O94804 Q13144 54815 Q13148 5295 P35869 57092 9656 P43490
P10586 P26927 Q13151 54822 Q49AN0 Q13153 P54136 Q9BX93 O15287 P54132 Q3LXA3 Q13162
Q8IZD4 Q14494 Q14493 Q14498 Q13164 O15294 Q9NRC8 7013 7014 P13674 P11021 50487 Q9NRD1
Q13177 Q9NRD5 10390 9682 54853 Q9NRE1 P47897 1718 P00367 Q9UEW8 P47895 1716 Q13188
Q9BWU1 9695 7037 P21283 P21281 Q09028 Q9UNI6 O75792 26747 Q9NZV6 Q00688 Q6PID4 1727
Q7Z7F7 Q9NRF9 7046 P35813 Q96L91 P10515 O75787 27248 Q9H6Y2 27246 O15234 1738 P05177 1737
1736 P54105 375743 7057 P29144 O75771 Q9H792 55294 7052 27252 1743 27257 P05181 Q9UER7
8399 8396 7068 7067 Q5TAX3 Q15714 O15264 P19793 Q15717 Q8IZ69 8394 7064 Q9H6W3 80267
29880 Q92793 Q6ZNC8 Q7Z6Z7 P05155 O15270 Q8IYT8 Q9UNE7 Q9BWH6 P43405 1763 29896 Q04206
29894 O15269 Q9UET6 Q5GLZ8 Q15738 Q8N6G5 7083 84647 4809 51720 P08243 4800 P08246 51726
7099 7098 51727 51729 30849 Q15746 P14920 P30153 1789 1788 1786 Q05513 57038 80270 P08253
Q15751 80273 51734 Q15759 29843 P17174 84661 51741 4820 O15212 P05198 Q14432 51747 O75716
30827 Q6XPS3 Q13107 P22528 4839 P47804 4830 51752 Q6ZVX7 902 4833 P08237 Q13112 P08238
Q9NZN9 O15228 O95433 O75821 O75822 Q969L4 O00268 Q8IXM3 51081 O00267 3516 203102 10535
Q8IXL7 O00273 P60174 Q9ULW3 P63208 4841 90678 Q6NZY4 Q92614 10531 P07996 10533 Q92611
P04003 P52564 4860 Q9NQ55 51094 55454 51096 10549 51099 Q9ULX9 Q08426 29128 Q05397 P18621
29122 P63220 55466 O60783 P32929 26091 Q6IT96 4882 3551 Q13829 152926 10524 10528 Q9ULZ3
10521 948 10523 O95478 O95476 O95477 O95470 29108 O60762 2224 O60760 29102 P04040 P84103
58517 O00238 P04049 Q96SB4 Q96SB3 Q9NYA1 O14880 O00231 O00233 P10721 O00232 Q5JTZ9
Q8IY17 Q96SB8 Q8WYH8 29110 2237 79444 Q92643 4898 P05386 P05387 P05388 Q9NYB0 P30304
P30305 P32969 P30307 P31639 Q9NYB9 6609 2247 975 Q6GMV3 Q92630 Q92636 Q969M7 161823
P29323 P29320 Q13868 O00255 81788 Q99259 984 6612 988 Q92620 6613 P29317 26061 Q9Y6W6
O43395 P07900 5300 6631 P15529 P53804 Q9BV90 P53803 Q96AT9 O60725 P15531 990 P11172 991
P62750 Q96RU7 Q96RU2 O14829 994 995 997 P28838 6622 999 6625 P61421 6627 51009 P62753
Q04917 P07910 P49770 200916 Q9UM07 2287 2286 P11177 2280 Q93096 51010 Q96S53 51013 6633
Q9Y6Y0 O14830 5305 5306 P28845 6637 P49768 O00206 Q9BV79 5321 P49761 6653 P49760 O60701
51021 51023 Q00535 Q8IXB1 Q00534 10594 P49756 P25789 P25788 6647 5315 6646 10598 91949
P48426 P25787 6660 5331 5333 Q10713 O00213 Q96B26 Q96RR4 Q96RR1 Q00526 5327 5328 Q9ULR0
O43390 O43353 Q9H5K3 P07947 9700 P07949 Q969H0 P07948 5335 5336 10574 P62714 5337 5338

4008 5351 Q9H5J4 5352 5356 Q9ULK4 Q01831 78991 51056 4015 5347 4017 Q6UVY6 Q96JH7 Q08462 P50750 4035 O14802 Q8NER5 51067 P68036 9716 Q68J44 10554 Q9H5H4 Q01826 P49792 5371 P27540 9733 5378 Q969E8 P16885 10569 54101 55432 51074 O14818 Q9NPJ6 6699 54107 54106 Q01813 9744 O43432 57187 29947 Q9NYU1 Q9NYU2 29941 P22695 Q7Z6C1 83475 P22694 O94905 8408 O94906 5393 5394 8424 4067 63874 63875 29959 Q6UX04 128 P60983 54921 O94913 Q13011 9761 P22681 Q76FK4 29922 P35998 Q9UDY4 P25705 115024 Q9UDY8 P42285 O94925 8428 P22674 54931 54938 Q8WZ42 Q14353 P00441 8443 4086 4087 8446 4088 P22692 9775 8445 142 1800 1808 8439 P62701 P20020 Q8IXZ2 53615 1801 8453 9784 8454 9785 8452 Q6ZN16 8458 7124 P54252 P54253 7126 Q96KC8 8450 4092 4093 4094 Q00597 Q9NZ20 156 158 Q8IYB3 Q13042 8449 Q13043 P49736 Q13049 7132 P04843 P04844 Q96KB5 8467 P13798 Q9BW60 P11142 Q8NFM5 54961 7128 P49721 P49720 Q13057 8473 P00491 P00492 O94966 P54277 P54274 7141 Q96T21 1832 Q96T23 178 Q9NZ43 Q00577 P49711 P49716 P49715 Q49A26 Q9NQC7 7153 7155 7158 Q9BVS5 7157 7150 1843 1841 1848 Q6VAB6 92345 Q13873 7167 Q96K76 Q86Z02 7161 8492 1854 P42224 1852 P47914 1850 P63165 O15355 Q92696 P09651 Q969T7 1859 P22612 P42229 Q13882 Q969T4 1856 O15371 O15372 Q969S8 Q13887 Q01082 84708 P22626 P84098 P63173 P09661 P60900 Q969S2 7186 7189 P10619 91039 P35916 27348 P11940 26015 O75881 27347 26019 7184 1877 4907 Q9NZ01 P17252 1870 O15379 Q92673 P23921 Q9NZ08 Q969R2 O75874 1891 P10620 Q6UWE0 4914 Q9NYP7 Q969Q1 4913 Q969Q0 P33316 Q9NYH9 57144 Q93100 58478 1892 Q8WYQ5 4920 Q8NFF5 Q8IY92 P35968 O95400 Q8WZ19 Q9NQ92 26005 57154 26007 Q96BI3 84749 27339 27330 58487 27332 26002 Q15631 Q8IY81 Q8IY84 O75844 Q5W0Q7 377630 P84090 4947 3615 3614 Q5RKV6 3611 Q5XUX0 57169 P01730 Q969V3 4946 O15350 P51178 Q15643 O75832 O95427 Q14318 Q15648 27315 Q86Z14 Q86YP4 Q9BVJ6 P30260 O15347 4952 O95429 4953 Q15653

positive regulation of biological process P25054 P28562 O75940 Q12824 2305 5817 2304 2303 1454 1453 O14543 Q9Y275 2308 2307 Q92974 Q9H4L7 Q12834 Q12837 P16104 2316 P42858 Q9UKV3 P61158 P49006 10666 P10809 Q96EB6 Q5S007 O75925 P27694 Q92956 Q9BQ51 Q06124 P17405 P37023 2324 57761 Q9UKW4 5830 55109 3655 Q9Y297 O75928 3659 P26358 3672 3673 Q92949 Q13705 P21917 10645 3667 P42830 P25098 1022 1021 1020 114548 57787 1017 79576 P21964 1499 10616 O60884 Q6PJW8 1012 P61586 Q92530 3678 3690 Q9UGI0 P29466 Q9Y239 Q9H4P4 6720 Q96J02 O00358 1029 3207 10626 1027 1026 P17482 P17480 Q9Y240 604 P05023 6714 P04179 Q07890 O95136 Q9UGJ0 Q12888 5883 P19634 P38398 Q96N67 P20618 610 Q92990 Q5T2W1 Q07889 6721 Q92993 5879 Q92997 Q9UKS7 P10826 Q9UKS6 Q9UL46 P10827 Q9UL45 P31749 Q9H8V3 1050 56005 27032 P40692 3226 Q9UL51 5888 Q8N3V7 Q9UL54 Q9UKT4 Q9H4M9 O96004 8930 P56199 P14317 P50416 P50897 Q99523 Q15022 637 5898 639 P25445 O96017 6760 Q9Y5V3 P16989 1072 641 P33076 84033 8932 648 P49407 11116 1080 O00327 P48552 6772 6774 3265 3263 P14780 Q99500 51144 Q14192 P35240 8945 P01019 51147 55504 Q9Y5X9 5451 P35225 P35222 P14784 P35226 O75084 P17813 3269 668 6776 Q15052 Q59EA4 P24588 O00744 5460 P01042 4131 Q9Y5Q3 Q92934 9821 Q9NX61 79971 672 673 P15692 O14908 57708 6789 10213 Q92930 P05412 O00755 6320 5473 3297 23532 Q5JSP0 Q99558 Q9NWV8 688 148327 Q15077 5467 4137 9826 207 5468 Q92922 Q15078 P56178 Q12802 O00762 Q9NWW5 P17844 P13010 10673 9839 5494 O43464 4168 P06729 P08908 23512 23513 P50402 P13497 O14936 O14939 8516 8517 9846 O15085 Q14686 11065 7204 4179 O43557 Q9H161 P07196 Q9H160 P49810 P08047 P78395 Q9NPC8 8546 P46781 5037 79084 P58304 Q04864 P09382 7205 8536 5029 P00533 Q16401 Q96RL1 P61956 Q15554 1902 7220 P35638 Q96IZ0 10193 Q9UQ13 8555 P32121 Q9ULH7 64857 Q9NY61 4193 Q9Y6Q6 Q96RK4 Q15561 10197 P02741 51547 P55290 Q15562 5054 P62826 Q9Y6R0 9412 O75116 Q86XR7 P36956 P31270 Q13393 55915 9421 Q14249 9420 7248 O75586

Q93062 Q9Y2C9 P23229 Q9Y6K9 51564 1936 5074 10181 8100 11030 Q9HAZ2 O43521 Q15109 P22392 O43524 5071 285 P48995 11035 P49841 1947 P02790 P11229 5089 O75569 O15068 23463 A1XKG3 Q8WY64 51588 P08069 10155 51107 1956 10159 Q9NPA8 O43508 P37840 5092 P11233 7272 2810 Q93034 Q93038 P02775 Q14289 Q96RE7 9464 P29375 27005 P12931 Q4VCS5 P22736 P42345 Q9Y6G9 P04083 P04085 Q92574 Q13761 1978 Q58WW2 P56524 9475 O15496 Q02297 P16070 O75528 P06239 P29353 Q06643 P55209 P56539 Q9BYM8 60412 P06241 P21860 P55212 A4D1W7 55072 P78317 9495 Q8N8D1 Q9HAK2 Q07954 P53041 Q92542 Q13794 Q96EP0 O14593 O95999 Q13315 P35610 O43915 8174 9021 Q16828 9020 Q9Y6C2 23085 Q6P1N0 Q8IWV1 P31249 Q13322 Q13323 Q9NP71 P46736 3728 3720 P22303 5901 P46734 50649 P12956 O75962 P28065 Q9H4W6 Q9BQA5 P61088 50650 P43694 O15455 Q14674 Q9BQA1 5914 P28074 222068 63976 P28072 P28070 5927 P00519 P63272 2896 O43909 P24941 Q92585 51512 Q15532 P43686 P07148 Q13352 5925 P10914 Q92858 O14672 P10911 Q5VV41 Q9UBB5 Q9NS86 O15519 4609 P16220 Q92851 O15516 5935 O15530 Q9UBC3 65018 54361 O95684 P16234 10783 Q92845 Q92835 P05549 3783 P53667 57402 10758 27185 Q8IVF5 O60543 Q9UJU2 P49116 P61289 P03372 64170 Q9H7P9 Q92833 P06401 P06400 P53675 Q9GZX9 3791 P46087 P09917 5970 5971 1130 9093 Q99460 P56705 9099 P17535 P17542 P61296 5966 3309 P04233 P05129 133584 P50570 Q9Y586 P11802 3320 O00463 P52756 O00468 6829 10733 10736 710 Q8N2W9 4646 6827 719 P08648 P30556 P30559 5991 1153 Q14938 O95243 Q9NS56 P37198 3329 3326 1147 720 3324 2475 722 P08651 Q92888 P07766 P05106 P28223 6850 4673 P50591 Q13618 2010 Q13617 Q13616 1161 O00487 P30542 Q9NS68 O60502 57448 O14641 P61247 O14640 Q12770 Q92878 5515 P04264 P05112 5518 P09958 O43186 Q12772 Q12778 Q13625 Q05586 Q6KC79 3350 10724 P37173 5524 P05121 P40337 7704 P04632 4690 P23396 6872 P04637 O43157 2034 2033 O95271 P13591 4686 5533 P01116 Q16236 5536 6869 Q15389 P01112 P01111 Q9C0K0 P25100 4221 10383 6885 P35368 P50542 1191 O76074 O76071 54764 4214 P01106 4215 P01589 P01588 P01100 5562 Q8N726 9921 Q7Z7K6 P14416 P13569 O95292 P52735 O00444 P11388 Q9NS23 Q02952 Q53X93 6886 Q9GZP9 Q9Y572 P01138 P01137 P01135 P01133 4240 P04626 Q9GZP0 2065 2064 P35348 11218 301 302 P10070 P17936 P10071 5566 P01127 11214 5581 5582 5584 Q9UNN5 79612 Q96PU5 Q02930 P51828 5578 5579 Q8N302 Q495A1 P40763 P04201 2081 5591 4261 Q9Y4P1 Q6IR47 Q3SY56 64127 O76024 P18850 Q9BSW2 5590 P18846 P16671 P18847 324 P18848 329 P27797 7780 P01185 6453 2099 O60907 5599 8625 P25103 P01178 Q9BT67 23636 6464 25818 P62508 P17980 P52701 P58012 10320 9967 P26447 P25116 8650 8655 4297 Q9BY77 Q9BY76 11186 9500 8654 P30086 P13984 351 Q9UBS0 355 Q04759 356 Q9UBS5 P09493 P62913 P25963 5154 7334 5155 7335 19 367 Q70SY1 7341 Q14106 P11309 6496 6495 6498 29 253260 P32241 Q53GQ0 P10451 Q9Y606 Q9UBU3 P17096 5159 Q13263 P32239 5174 Q14116 8204 Q9BY44 P12645 382 5170 222546 387 388 389 Q9NWT8 P08151 P20264 P62942 P55347 10293 391 O43639 O43638 201633 394 396 Q02535 Q01201 121512 11146 Q15466 2904 Q99966 Q13285 2902 7376 Q09161 Q9Y5J5 Q86WV8 Q13291 Q14140 8218 O15169 85440 Q99958 Q15475 Q8TEY5 9564 Q6ZSZ5 10273 P14859 O43612 23586 Q99943 P49959 Q01664 P21145 56916 P01579 11124 10276 P09038 11128 Q14155 2923 Q15008 9575 O15198 23118 2932 Q14160 Q14164 10285 Q6R327 Q15811 P78423 O15123 Q08722 Q13639 1613 26271 P0C0L4 Q9BXM7 Q86WK6 Q1PSW9 91 94 P46821 O15119 P45974 1616 Q9NSA1 Q9UBK2 Q9NSA3 Q96QB1 9590 P55318 P55316 O43609 P67775 P43307 P08575 P05067 2956 Q9UBL3 P52298 P29474 O94768 Q15836 P29475 26258 Q96M96 29767 Q03014 6096 Q9BXK5 56034 O95619 P21980 Q9NSC2 O15151 P11717 8289 O15156 O94776 9138 Q7Z434 Q02156 Q9BXJ9 Q8HWS3 Q9UBN7 Q15853 O75626 P13945 Q9UBF6 9149 P41182 Q8TAK5 P41181 P53567 P30048 1655 P22415 Q9UK80 Q03468 O15554 Q13200 P47712 P51398 Q68CZ2 Q96HU1 Q07817 P20248 64919 80155 Q9UJX2 P08134 P17081 P08138 P09429 O75604 P10415 Q13216

P35711 1676 P08588 P41159 O15111 P55771 P62070 Q04771 23192 O15105 P45983 Q13233 P45984 P21554 O14793 2550 9181 P51587 P17676 P15498 2547 P37288 P84022 Q92731 O14786 4722 O14788 57506 P28799 P27487 25913 Q5VTR2 P55957 P62244 P14174 4734 808 Q7Z727 Q9NR80 Q9NR81 Q8NC24 6927 Q8IUD2 6929 57521 811 P40425 P40424 P62258 6926 6925 P31946 P31947 2100 Q9NR96 P48380 O95343 57534 3428 P16333 81622 P20823 O60674 O00182 P19838 Q8IUC6 823 Q8N5V2 826 5604 P41743 P11926 P27037 Q13956 4771 Q9Y463 O95352 P39019 P15056 80324 P07437 4780 Q92769 P09619 P28370 Q9NZC7 O95361 124583 P10600 Q06330 Q96KS0 Q9BWF2 P19419 841 Q9HCM4 843 4775 847 4790 4792 Q9H2K2 Q9NZD8 1285 Q96L34 P18545 Q8IUH5 7818 Q9HCL2 P60033 Q96KQ4 Q92753 P05230 857 O14763 859 Q13501 P51575 6500 Q13503 P11912 P19883 Q9HCK4 861 2137 867 P09601 50943 4318 Q86UW7 P01241 P15408 3484 P14555 3482 2150 3481 Q9UIH9 23326 P11047 O60603 25988 23327 P15884 203068 3479 2147 10011 7832 10013 6504 84289 P01236 11346 10014 P23025 10016 64682 Q7Z6J4 Q96T76 2159 O43290 406 Q9BRP8 Q99759 O43294 408 O43293 84271 409 Q13099 Q6ZN33 Q09472 Q9UMR2 5682 5683 5684 5685 5686 23787 890 891 Q99743 894 896 Q9NR09 P53708 898 3014 29086 P49639 11326 Q9UMS4 P51531 4361 5692 5693 64223 5213 Q9NUX5 P51532 P14543 O14737 Q01974 P60484 5687 5688 10001 10488 8718 P23497 8717 P26583 Q6FGG2 7402 P50613 P51946 Q96P48 10451 P55075 6548 P55072 Q8N5U6 O43242 8743 7410 7412 Q9HCE7 3054 Q9NYZ3 Q05086 P37231 Q01955 8737 P55085 P48357 10468 8738 P24385 8754 7422 P52815 3066 Q9UMN6 65108 3065 Q00613 Q96P20 7415 10432 P24394 7433 P49674 Q9UIG0 3075 P17612 466 468 7428 8772 6595 Q13114 6597 Q16623 6599 6598 Q9Y4K3 3084 O43318 472 474 476 Q6UY14 51762 23286 P20396 9616 8767 P21246 P38919 Q15303 3091 P55036 8301 Q15306 Q86VP6 P13631 5270 51773 Q9H2X0 6117 P46531 7448 Q13133 Q15796 P43026 Q13131 Q9HCY8 9641 8795 Q15797 P12757 Q9UNL4 P12755 O75360 P46527 23263 Q9NRI5 Q13144 Q13145 Q9BSI4 Q16650 Q13148 5295 P35869 P43490 7476 P18074 Q8WVL7 P55055 P10586 P68371 Q16665 P43489 Q13158 Q8TDY2 Q16666 P54132 P63092 P35453 O75340 P0C1Z6 P09172 51324 O94827 11269 Q14012 Q14011 Q9NRC8 7013 7014 Q9Y4H2 P35443 7490 P11021 Q7Z7H5 Q8WW38 Q9GZM8 Q99835 23229 7023 10392 P98179 O43734 O43739 7020 Q8WVQ1 54851 Q96PK6 55703 P00367 Q9UEW8 P47895 Q13188 P23443 6188 Q9BX66 9217 P38936 6182 P11474 26747 54862 P02538 Q6PID4 P48730 1728 Q99814 P98194 Q15375 6199 7046 6198 P35813 O75787 O43715 6194 Q9Y4C1 P62195 9218 9219 P07355 3912 P78536 7057 9238 Q13526 22807 83737 7052 P46934 P46939 Q96GN5 81565 Q4LE28 P42574 P98172 P42575 P98171 P98174 Q9UER7 8399 7067 Q9H305 Q13535 P19793 P29590 Q63HK5 P30101 P31431 Q01196 P41235 P41231 Q92793 Q96GM5 Q9HD15 P05155 Q9NRA1 3932 O60260 P78552 Q13547 Q13546 P19320 7073 9253 7074 Q9UNE7 P43405 O43707 1763 P20309 Q04206 Q92786 Q92785 P78560 O60271 Q9NZJ7 P78509 P78504 P00747 P63000 51720 Q96PE2 P00742 P08243 3953 4800 P62158 P08246 3956 2626 7099 7098 Q9NVC6 P14923 P30153 1789 Q05516 P62166 1786 Q9UIV1 1785 Q05513 P00734 375790 3965 29843 57045 P78527 O60229 121457 1312 51741 Q5T9L3 Q16610 Q15762 O95786 Q13586 Q16611 Q06830 P62191 O60238 Q63HQ2 4839 Q9HCS4 P07384 4830 Q9NZN5 902 P08237 P08238 P21675 Q7Z628 P08833 Q9NYD6 P48058 2672 Q12948 Q6QNY1 O00267 Q9BVA0 3516 Q96JM2 O00273 57620 P63208 P08842 P07996 10533 P04003 P52564 P04004 P52565 4860 Q08431 Q8NAP3 5706 5707 P20963 5708 5709 P16473 51099 4851 5700 5701 5702 5704 5705 Q9NYF8 Q9Y2Y8 Q12968 5717 57646 P20936 4862 5713 Q6IT96 4869 5716 Q96JK9 P48023 1374 Q9BZF9 Q12979 22985 P17302 5728 10524 P17301 Q99698 2697 Q9ULZ3 Q9UHR5 948 Q9H1C4 Q12981 O95477 P28482 1385 O00220 P51668 29108 P51665 P19532 Q9P0J0 57669 P15173 P07550 P04040 5733 P08887 P51671 O00238 Q9UHK0 Q9NYA1 O00231 P51679 O00233 P10721 O00232 Q5VST9 P19544 Q8WYH8 57678 29110 960 961 29115 Q9P0K8 1387 967 4898 P42771 Q9UM54 Q70UQ0

P30305 22937 2253 Q9P0L0 6609 P62330 P62333 2247 P15153 2246 P15151 Q9BVC4 Q9BZK7 975 6601 Q92630 Q9UM63 Q92633 P29323 P51692 O00255 P48061 Q03164 55035 5764 P05362 6613 Q9Y2W1 P06213 P07900 P48454 5300 P15529 P13349 2274 P52952 O60725 P15531 991 Q96RU8 O14827 Q7L523 Q96RU7 Q8WTS1 Q96RU2 994 998 6622 999 54998 P27986 51009 P62753 10138 Q04917 P51636 2280 Q93096 2277 P48436 Q99638 P01344 P01343 P49768 O00206 2290 P48431 5321 3142 148022 O00203 Q00535 Q00534 Q96S42 P25789 P25788 51026 6647 6646 5316 8829 P25787 P01375 P01374 Q9UPY8 6660 Q9Y337 Q9UHI6 O00213 23435 Q96RR4 3148 545 3146 P61457 Q8WTP8 P40933 Q96EY1 6657 8837 O43353 P07942 6672 9700 7520 8851 P07948 Q8WYA6 3162 Q12905 O00622 Q969H8 Q12906 Q9BZE0 552 553 3159 Q99683 Q99684 6667 5336 5337 5338 7518 P05771 Q96FA3 O00635 7531 10580 7533 23411 23414 3171 10109 Q00987 3169 Q9H1A4 5347 7529 6210 6696 5364 4035 Q9UHD2 Q9UPT9 Q9BUZ4 3182 572 O14802 10554 5371 P27540 8881 Q60FE5 Q9UPU9 Q12933 P15559 P51610 580 P16885 O14818 P51617 P62745 54106 8878 P60568 Q13477 Q15654 P15923 595 Q8WZ60 Q9BRI3 Q7Z6C1 Q13485 Q9HC98 O94906 P03950 O00716 Q13009 4067 P12429 P27918 Q13492 Q13490 Q9UQR1 Q13017 6256 P22681 Q9HBW0 O75489 Q76FK4 P35998 Q2M1K9 Q969Z0 Q9UDY8 P23528 142678 P00441 6262 9774 4086 10081 4087 4088 4089 9775 8445 Q9H257 Q86YT6 Q8N5F7 P21359 1808 P24864 P62701 Q13033 8453 8454 8452 6275 O75469 7124 P54253 Q9Y3E0 23365 P14635 64759 23368 Q9UQL6 P10275 4092 P10276 4093 O75460 154 156 Q13042 Q99728 Q99729 Q16555 Q8WV24 Q13043 7132 8463 O43889 P36896 P36894 P35568 23370 55810 Q02763 P01308 120892 338917 Q9NQB0 P01303 P49721 P49720 Q96T37 P00491 P00492 Q8TCU6 P54274 Q86V24 P36888 Q96T23 Q00577 P49711 Q9BRK4 Q9NQC3 P49715 55827 P60953 7153 64782 7158 Q08050 P39905 7157 7159 Q02750 P35548 138151 1843 55832 1848 Q15257 Q16581 Q13077 Q13873 O95835 P10636 Q86Z02 Q01094 7161 9341 P63167 56259 P42224 10935 P17275 10938 Q92692 P63165 P39060 Q6NVY8 P42229 P15090 1856 P42226 22926 Q13404 Q13887 Q01085 Q8IY57 O43847 22920 P57735 P84095 P60900 1869 7187 7186 7189 7188 P35916 O75881 27347 Q13418 7182 7185 Q8N163 P17252 P68871 P21741 Q13422 O15392 Q13426 2735 27352 P19438 P21757 1406 2737 2736 9388 Q96G30 Q8WUI4 Q9H244 84766 4920 P43115 P35968 Q14774 Q13443 Q15628 9391 57154 Q96BI3 P51159 8065 27339 P30279 P30281 P23511 3609 3608 P23510 P21333 Q9NYJ8 O14492 8078 P12830 29966 P19474 Q02363 P18146 57162 84305 3611 Q14790 P01730 O15350 P53355 O75832 Q9BZS1 P53350 Q9P0U3 Q15648 P19484 Q86Z14 Q06710 Q96BF6 Q9UHV2 3622 O60356 Q9HC29 P30260 O14497 4953

biological regulation Q9UKT9 P54852 P28562 Q8NDW4 259266 10657 2305 2304 2303 114991 284217 P21926 2300 O14544 Q8N3U4 O14543 2308 Q9UL63 2307 Q9H4L7 Q8WXB4 Q8NDV7 2317 2316 2 P42858 P39210 P15260 Q9UKV3 P61158 10661 Q9UKV5 10666 136319 Q9UKV8 4990 3660 3661 P62495 81876 55584 84901 2324 P62491 55588 81873 Q9UKW4 4983 3655 Q9Y6A5 Q9UKW6 10633 3659 3672 Q5BKZ1 3673 Q13705 P21917 10645 2335 2332 3665 3667 10642 10641 10644 10643 P42830 Q9UL15 Q8WXG6 Q92538 Q9UL18 1022 1021 1020 Q06587 Q8IX07 Q5JT82 P51787 1017 10614 6709 79576 P21964 10616 1012 Q92530 3678 26191 3679 P61106 O14503 Q9UL26 Q9UL25 3690 Q8WXF0 Q9Y5Z7 Q8WXF1 Q9H4P4 6720 P33947 285527 Q96J02 P51797 1029 Q9BUB1 10626 1027 1026 1025 Q9BUB5 P20648 3685 6711 6710 3689 P05023 P07686 6714 Q9UL36 Q07890 O95136 1040 79595 P19634 57326 P20618 Q07889 6721 6726 Q8N488 Q9UKS7 Q9UKS6 Q9UL46 Q9UL45 5411 O95140 Q8NDX6 Q38SD2 1050 56005 84932 253980 57332 79589 10607 Q9UL51 6733 Q8N3V7 Q9UL54 Q9UKT4 Q9H4M9 Q92502 O95159 Q9UKL0 Q9UKL4 Q96A33 P50416 11108 11107 Q8WWY6 1059 6749 P25445 6760 Q9Y5V3 5430 112399 O95163 Q9NWZ3 P57059 115426 1072 51131 Q99996 1069 P33076 51132 Q14186 84033 Q14185 P49407 85360 Q14188 P02794 11116 1080 5440 5441 6772

Q08380 Q9UKN5 6774 Q99990 346171 Q66K89 P14780 O95180 Q2M3W8 51144 Q9H4B6 Q14192 Q8WWW0 51147 55504 Q9Y692 55503 84062 Q99988 Q9Y5X9 Q86X95 5451 A7MD48 Q9Y696 Q8N3J9 Q5T6S3 P14784 P57082 P17813 Q00403 56849 6776 4116 6790 O00744 5460 4131 Q9Y5Q3 9821 6794 4133 P25490 9820 Q9NX61 Q9NX65 P61981 Q9UBW7 9815 6789 Q6UUV7 O00755 Q9Y5R5 Q9Y5R4 5473 9831 Q9NX70 Q5JSP0 Q9UBX0 Q9NWV8 148327 5467 4137 9826 207 5468 54206 4139 4150 4152 4154 4155 Q9Y5S2 9841 P30825 Q96A54 O00762 P13489 Q9NWW5 Q9Y5S9 P17844 127435 5478 10673 4149 10672 9839 5494 4168 P50406 P08908 P50402 P13497 Q96QT6 Q3KRB8 56882 8516 10681 8517 Q9H4E5 55558 9846 9849 P49427 326624 4172 O15085 4173 4174 11060 P35659 P55268 4176 Q14209 8535 11065 7204 P55265 8533 4179 Q6NZI2 4171 Q6P5Z2 23043 Q9NPC1 P08047 Q9NPC8 94234 8546 4188 8543 P58304 O15090 Q9UD71 23051 Q04864 P09382 Q9NY59 9869 8539 7205 Q8WY36 8536 Q96RL1 P61956 Q15554 1902 Q9BUN8 Q16881 7220 8553 P35638 P61968 9882 Q8IWY8 Q96IZ0 8555 P31274 P31273 P31276 Q9ULH1 Q9ULH7 23028 Q9NY61 4193 Q9Y6Q6 257 Q6DD87 Q96RK4 Q15561 8548 Q96RK0 Q93009 P02741 Q14232 P55290 P61964 Q15562 Q9Y6R4 Q8IWX8 Q9Y6R0 P36952 Q86XR7 P36956 P35625 O75592 Q8N4C8 267 Q9NPF0 P31270 23032 Q9NPF5 P61978 Q03518 Q8IX90 P43246 Q14249 11021 7248 O75586 O75582 Q9Y6K1 O75581 P10398 274 Q93063 Q93062 Q04837 Q9Y6K9 Q15583 8569 1936 11030 P22392 11031 O15055 7251 O75570 94274 285 7249 O15047 P48995 11035 1947 P02751 Q15596 P61925 Q86Y82 P02790 O15062 O75569 166968 O15068 O95931 Q9Y6M4 291 Q9Y6M1 O15060 Q14C86 Q8WY64 51100 P08069 P02786 Q9NPA3 11004 51107 1956 286075 Q8NEJ9 O15075 Q9NPA8 O75553 26986 64426 64428 7272 1965 Q93034 Q93038 11014 O95947 Q9NPB3 1969 P02775 Q14289 Q8IWS0 Q96RE7 P12931 Q7Z589 Q4VCS5 1975 Q05329 Q9BUG6 Q9Y6G9 Q92574 Q13761 1978 Q58WW2 7297 P56524 O95967 O75534 Q02297 7291 O75530 7290 7295 P24928 Q96AB3 1982 Q92560 Q9Y6H5 P06276 O75528 O15020 P06239 O95977 Q06643 P0C7X2 P55209 P56539 Q7Z569 Q8WXI9 P40145 1994 1993 Q96IQ9 P06241 P21860 P56545 O95983 50618 Q8WXH2 P55212 91147 A4D1W7 Q07960 3705 3708 Q07954 Q92542 Q13794 O95996 O95995 O95994 O14593 Q8IWW6 O75503 O95999 Q9NP61 P35610 Q9NP66 O43915 9021 Q16828 P35612 9020 P35611 23081 O43918 Q9Y6C2 23085 Q9UKY1 P85037 P31260 P20594 O75508 O75509 Q8IWV1 P31249 Q9NP71 Q15506 Q9NP72 P35609 Q86XP3 P61081 23092 3728 P61073 23094 Q9Y6D6 83548 Q9Y6D5 P61077 23098 O60496 Q9BUJ2 3720 P22303 Q6P5W5 Q9Y6D9 9047 50649 9046 Q16849 P12956 Q8NEB9 Q9NP86 Q9H4W6 P02708 Q9Y6E7 Q9H582 Q9Y6E0 P61088 50650 Q96AE4 147808 Q96RG2 11091 Q9NP90 A4D1S5 222068 63976 63978 9052 O43909 23077 P24941 Q92585 Q15532 Q14201 Q7KZF4 O14672 399687 Q5VV41 Q9UBB6 Q9UBB5 9066 A5YKK6 Q9P2R6 55684 O60566 Q8IVH8 79658 10772 P27348 Q9UBC1 Q9UBC3 P27361 65018 54361 55692 9077 1107 1104 O60573 284323 10783 1108 Q9UK53 P52333 Q9UK58 Q7Z3K6 P29992 Q9UBD6 3783 1121 339122 Q6P4F7 Q7Z3K3 O60548 P53667 57402 57403 10758 Q8IVF5 O60543 1111 Q9UJU2 284312 P61289 P03372 P06401 P06400 P53675 Q9GZX9 3791 P07737 Q5JS13 1130 9093 Q9UBE8 P56705 9099 10767 57410 1122 P61296 3784 6810 6812 6814 10765 P05129 P50570 Q9Y586 P11802 Q9P2N2 83706 O95231 O14627 6829 10733 10736 1137 Q6FHQ0 P61225 P61224 Q8N2W9 6827 Q7KZI7 65056 93986 1153 Q14938 1152 P19784 Q06455 O95243 79685 6839 10746 1147 2475 5500 P26045 P53621 O95259 P07766 Q10586 P05106 O95257 P05107 6850 Q8ND82 P50591 Q6IA86 2495 Q13618 Q13617 Q13616 1161 O60506 Q7Z406 Q02086 O60502 O60503 57448 P61244 O14641 5511 P61247 O14640 5515 P05112 5516 O43182 26292 5518 O43186 Q13625 O43189 Q02078 1173 10725 57459 10724 10728 O14654 P05120 5524 P05121 P40337 Q9UK32 5528 5529 Q8N2K1 P04632 O43159 6872 6871 Q9BST9 P04637 O43157 25861 P16615 1185 Q9NVW2 O95271 11228 P13591 5533 4204 4205 Q15382 5536 6869 Q15389 4209 O43166 6883 Q9BT49 4221 6885 Q9GZR2 O43167 23210 Q9HDC5 P50548 Q9BT40

P50542 1192 23216 1191 56940 O76074 Q9P2K2 9908 O76071 Q15392 84159 O76070 Q9P2K8 4214 6878 4215 11234 P01589 4216 P01588 11236 4218 56946 6890 5562 Q86VW2 9921 5563 5565 P13569 O95292 P12235 Q02952 P61204 Q53X93 6886 Q9GZP9 Q9Y572 P61201 Q9UJM3 4240 5573 P04626 5575 Q9GZP0 5576 P04629 11218 Q99853 O14617 301 55633 302 P17936 5566 5567 307 5568 Q8TDZ2 5569 11214 309 O43150 11215 9927 5580 5581 25828 5582 5583 5584 5585 Q5U0I6 4255 5586 Q9UJF2 22794 25822 Q96PU8 310 Q96PU4 79612 Q96PU5 Q02930 P51828 5577 Q8TEB7 5578 Q9P2F6 5579 8607 Q8N302 Q495A1 5591 Q9GZV8 4261 Q9Y4P1 Q6IR47 5597 Q3SY56 5598 O76024 P15336 O76021 Q9BSW2 5590 56987 51295 P16671 324 326 328 4259 Q8TEA7 329 55657 Q9GZU7 P12277 P51843 P15311 330 Q8NCN5 55662 331 Q8NCN2 333 56994 5599 10794 8625 P83436 Q9GZT9 Q96HC4 Q9BT67 9975 4287 25818 P62508 9973 4289 Q5T5U3 P51858 O76003 P17980 P15313 Q08211 221656 Q9NVV9 Q08209 9967 Q96HB1 Q96HZ4 8650 8655 4297 Q15418 Q8TEW0 11186 7325 8654 Q9UKD1 P30086 Q04760 4291 351 Q9UBS0 355 Q04759 356 Q9UBS5 85415 P09493 P23786 P21127 P47755 P47756 8662 11190 Q15424 7332 6003 9997 7334 8667 7336 7335 19 26205 Q8TF76 Q9UKE5 Q9H3Z4 85403 367 369 Q70SY1 Q9UBT3 11198 7329 7328 20 Q8IW93 7341 Q14106 Q8IVW6 Q14103 29 Q99909 6015 Q15438 Q8N3C0 23144 Q86X27 253260 Q53GQ0 P10451 Q8TEU7 Q9Y606 84108 Q9UBU3 P51808 Q03405 375 P51805 P09471 P17096 6009 11168 Q9UBU8 31 Q8TF50 Q53H12 Q14116 Q14114 Q14119 Q8WWN8 Q14118 P51812 Q9Y618 Q9UKG1 P29084 381 Q86WP2 382 Q04725 Q04724 387 Q04727 Q9NWT6 388 Q04726 389 Q9NWT8 23152 Q9UBV7 Q66K14 23154 P08151 11177 11176 P60763 P23771 41 P55347 P55345 11140 Q16787 11142 O15164 23122 390 391 23126 392 25788 394 396 397 Q99962 398 Q99963 25780 Q6KH11 11146 Q15466 Q99966 Q15464 54 Q96R06 6045 Q9BU20 7376 Q14137 7375 Q16799 23133 23135 Q9Y5J5 Q86WV8 Q9UKA1 Q3KQV3 Q9UKA4 Q9UKA8 P33151 Q14140 Q9UBP4 O15169 51218 85440 Q99958 Q14145 Q15475 Q99956 P46459 Q8TEY5 Q6ZSZ5 Q8TEY7 P14859 Q9Y5K6 6050 Q99943 Q99941 Q01664 51222 P21145 56916 P01579 11124 Q14151 Q9UBQ5 11128 Q14155 P82979 O75676 O15198 23112 25776 P33121 25777 23118 51230 Q14160 51231 P08195 Q14164 Q5VVH5 Q14161 Q6R327 Q14168 86 87 Q14964 Q9NWH9 Q13637 Q13636 O15123 Q9NNW5 P33992 P33993 Q8TF05 Q13639 P33991 26271 P0C0L4 Q86WK6 Q1PSW9 91 P17030 94 O15119 P45974 Q6ZT07 P45973 26270 O15118 Q9UBK2 Q13642 9112 Q8WWH4 Q96QB1 Q96I24 Q9H3R0 P31321 P55318 Q99081 P31323 P55316 Q7Z460 P43307 P05062 P52292 Q52LR7 P17040 P05067 Q9UBL3 O94763 P52298 O94768 P52294 9126 O15143 26258 Q9H422 6093 6096 P41597 56034 Q9BTC8 P21980 Q9UBM7 O15151 P11717 9133 O15156 O94776 9138 Q7Z434 202559 Q02156 P17023 Q8N392 Q86WH2 P17020 Q8N393 Q9UBN7 Q9UBN6 9146 O75626 Q9UBF6 9149 Q8TF47 P30041 Q6NYC1 Q9H488 P11766 P30044 114822 Q58EX7 P53567 P30047 P30048 B3KY43 3836 P22415 Q9UK80 P05093 Q03468 26234 P08123 Q92499 Q9UJW9 Q13683 O75629 P47712 Q52LW3 Q96HU1 Q8IVT5 Q9UBG7 Q07820 Q07817 Q9UJX2 Q9UJX0 P08134 P08133 P17081 P08138 Q9Y5B6 Q9Y5B9 P09429 O75604 P10415 Q9P2X3 Q9P2X7 23189 P35711 Q7Z494 23186 P09430 728642 P08107 O15111 374354 Q9P2Y4 Q04771 23192 1203 O15105 P45983 P45984 1207 Q2M1Z3 Q6NX49 65125 25909 P54619 9182 22870 2550 9181 P51587 9184 P17676 Q8TDD1 54474 2547 P37288 57504 Q9HCJ0 Q9HCJ2 10890 3875 6901 57506 A1A4S6 Q9Y3Z3 P28335 25913 P28330 P08729 P28331 Q08AM6 2562 Q5VTR2 P55957 O60216 P62244 79763 57510 54487 Q9H2G9 Q17R89 Q9H2G4 2572 2571 22850 Q8NC24 79791 Q8NBQ5 54496 6927 Q8IUD2 6929 P62256 Q6AHZ1 57521 57522 6921 P62258 10875 6926 6925 Q6IQ32 Q17R98 22866 Q9UIS9 P31943 P31946 P08708 P31947 P17661 P54646 57534 79784 P16333 6938 Q8IUC4 Q8IUC6 P62263 P31949 5602 5604 6936 Q9Y468 P08758 P27037 Q9H2M9 Q9Y463 Q9Y466 Q8TDI0 Q9UIL8 Q9NUY8 P39019 P15056 10856 Q86VE0 P16383 Q6ZRI8 57544 Q08999 Q8N264 644943 5610 6945 P07437 Q9Y478 22846 P51553 P30679 P50222 Q14814 P28370 Q07666 P80098

124583 P52888 Q06330 10865 10868 P19419 Q9HCM4 5624 5627 P49286 Q9H2K2 1285 1284 Q9H2K0 P21709 Q8IUH5 Q9HCL2 1277 83853 4302 Q9Y490 Q13501 P51575 22827 Q13503 1297 Q14839 Q8NC51 Q13506 Q9P202 Q9HCK5 Q9HCK4 Q8NBS9 1289 192669 153090 50943 P08754 4318 Q9Y3Y2 Q86UW7 Q9BS34 P01241 6993 Q9BRR9 O00541 23322 P14555 Q9Y3Q8 Q9UIH9 23326 25988 23327 P15884 Q8WUU5 Q8WV60 10011 55726 5654 10010 P25205 10013 84289 P01236 55723 11346 10014 11345 10016 135295 64682 Q15269 Q9UII4 4343 Q9BS26 23332 25998 P13236 Q99755 400 402 Q99750 406 Q9BRP8 Q99759 408 84271 409 Q99757 Q01518 Q09472 Q8N1G0 23309 5682 5683 5684 4354 5685 4355 5686 116986 O00560 116987 O00562 23303 Q99743 54413 Q99741 55743 3014 Q15286 Q9Y450 11326 11325 P51531 4361 5692 5693 8721 8720 Q8TCX5 Q9NUX5 P51532 P14543 P15882 55750 55755 P60484 Q15291 11331 5687 55758 5688 4358 10001 84299 8718 P23497 8717 P01243 P26583 Q9Y3M2 Q6FGG2 O00506 P46199 7402 Q9Y3M8 25942 54431 Q9NUQ3 Q8WUY8 Q8WUY9 Q96P48 433 79735 8726 Q9H2D6 P55075 P55072 P01275 8743 8744 7410 90993 7412 Q9HCE7 Q08117 3054 Q05086 Q9BRU2 P37231 Q9HCE1 444 54443 79723 8737 P55085 P55083 8738 Q6IQ22 11315 P01266 8754 7422 Q96GA9 3068 Q9HCD5 P52815 22893 3066 65108 3065 Q9UIF9 Q9BRT3 O75293 O75290 79753 Q96P20 55787 55785 7415 P61764 7414 8761 3070 Q86UX7 7430 7433 8766 Q9UIG0 P52824 3075 3074 Q9Y3P9 80728 462 P17612 466 468 55796 7428 8772 Q16623 P18085 P10114 Q9Y4K4 O75386 O75385 Q86VQ3 Q9Y4K3 Q76L83 P35414 3084 472 P48729 473 474 476 477 23286 P20396 8767 P43034 3093 P35408 Q15303 3091 Q16633 P55036 Q16635 Q15306 Q86VP6 O75376 3096 Q86VP1 O75381 Q9NVP2 488 Q9H2X0 6117 7448 P55042 P55040 Q16643 Q15311 P43026 Q9HCY8 O00602 8795 Q16649 Q16644 P12757 7468 Q8WVM8 O75367 P19397 P12755 O75362 Q8TDS5 O75360 495 498 23263 6128 7458 Q5VUA4 Q8WVM0 Q9BSI4 Q8IV61 Q16650 Q16659 P20393 7476 Q16656 Q15329 Q8NCA9 P18074 Q8WVL7 P55055 59277 93166 Q86VN1 P68371 Q16665 51317 P55061 Q9P2E9 Q8TDY2 11261 Q16666 Q5VUG0 P35453 O75340 P09172 P46109 Q9Y4G6 51324 11269 Q16670 P46108 Q14012 Q14011 Q86W54 Q15349 P46100 Q9Y4H2 23256 P35443 Q5PSV4 7490 84232 Q8WW38 Q9GZM8 Q99835 23229 9201 49854 P98179 O43734 Q86VS8 P15822 O43739 Q8WVQ1 Q02878 51341 Q9NVM4 P02549 Q96PK6 11243 55704 P55010 Q8NCF5 55703 Q9NVM9 P48745 P23443 6188 Q15369 Q8IUX7 O43741 9217 11252 Q9BSM1 P55011 P10147 O75312 6182 P11474 Q15370 P27816 P57682 3909 P02538 Q99819 P27815 Q99816 P48730 P23458 9208 Q99814 P48736 P98194 Q15375 P78545 6196 6199 6198 P98155 P10997 O95714 P78540 O43711 6193 O43715 6195 6194 3913 10811 O43719 Q9Y4C1 P62195 Q86VI3 10817 P08684 Q4LE39 P46940 9218 65082 O95715 9219 P98161 P07355 A8K8V0 3911 3912 Q13522 P78536 22803 9238 Q13526 22806 22807 57473 9230 83737 9231 Q3V6T2 3925 P46934 P46937 83746 P46939 Q9HD26 Q96GN5 P41240 O60296 Q32P28 Q4LE28 P98170 P42574 P98172 O60292 P42575 P98171 P98174 Q14865 P34947 P29597 Q9H305 Q13535 Q9H307 P29590 Q7Z2Q5 Q63HK5 P41235 Q9HCP6 P41231 Q96GM5 Q9HD15 P16284 Q9Y4E5 O60266 O60264 3932 O60260 9255 P78552 Q13547 Q13546 P19320 P34931 9252 9253 P41223 P41222 O43707 P41220 P20309 Q9NVJ2 P41229 P78560 O60271 9267 Q13555 Q13554 O95751 Q8IV45 Q13557 P78509 P78504 9261 3959 P00749 Q63HR2 P00747 Q9BSG1 Q96PE2 P00742 3953 Q14894 Q13563 P62158 3956 2626 Q9NVC6 9270 P11413 P62166 P00738 Q9UIV1 P00734 Q7Z2W7 Q9HD67 Q5BJF2 P24723 Q13574 3965 Q13573 Q13572 Q9HCU4 53916 P55884 93134 Q9H2S9 P32320 P78527 O60229 85509 P41279 P20338 P20339 1314 P20336 121457 P20337 2644 1312 2643 P41273 Q86VK4 Q04656 Q16610 P20333 Q9Y4A8 O95786 Q13586 Q16611 P18031 Q9P286 P34972 Q9P287 P62191 Q96H20 O60238 1326 Q63HQ2 Q8TDM6 P67870 Q9HCS4 P07384 P20340 P00750 Q9HD40 P21675 P08833 Q9Y2W7 Q6P2D0 P48058 2672 Q12948 22992 Q6QNY1 P61020 79894 Q6PCE1 Q7L0Q8 Q9BZI1 2665 2664 57620 Q9HBH0 Q9Y2X3 P61026 Q8N103 P08842 Q12950 Q12955 Q9Y2X7 Q12952 Q9Y2X9 Q12959 Q8NB12 Q8NAP3 Q9P107

5706 5707 79885 5708 5709 P16473 2676 P16471 P48039 5700 5701 5702 Q9Y2Y0 Q9UI12 5704 Q008S8 5705 65264 P54753 P50395 Q9Y2Y8 P25391 Q12968 P16435 22974 5717 57646 83933 Q9UQB3 Q9UQB8 5713 57649 5716 Q12972 Q9H1D0 P54764 P54762 Q0D2J5 P54760 Q9H9T3 P48023 1374 Q9BZF9 Q6P2C8 Q12979 22985 5728 Q99698 Q9HBE1 Q99697 2697 400961 Q9UHR5 Q9UHR4 Q6PCD5 Q9H1C4 Q12981 Q12982 Q9Y342 P28482 Q9UHJ3 Q12986 1385 Q9UHJ9 P51668 P51665 P19532 Q9P0J0 Q9P0J1 P19525 5739 57669 57662 P15173 P07550 5733 Q9Y2T1 10971 P08887 10973 P51671 Q9Y2T7 Q9UHK0 P51679 Q6UB99 Q5VST9 P19544 P30793 10987 57678 P18206 1388 Q9P0K8 1387 1386 P62328 10981 P42771 Q9H1I8 5747 10985 Q14703 22937 22938 P51681 Q9UHL9 Q9P0L0 P51686 Q9P0L2 22931 P62330 57689 10956 P42768 P62333 284119 P15153 1399 1398 P15151 Q9BZK7 P61006 Q9Y2V2 Q9Y371 P51693 5770 Q9Y376 P51692 P28472 Q9BQY4 P48061 Q9P0M2 Q03164 Q9P0M6 55035 440193 5764 Q5H9I0 Q9Y385 5768 P61019 Q9Y2W1 P61018 P06213 Q15139 Q08043 Q9UPV9 P80188 P13349 Q9UHF4 115557 P38646 P52952 Q8WTS6 126208 Q8WTS1 79810 Q99640 79811 79813 10133 P27986 P02686 10138 Q9UQ80 Q9Y2P0 Q9UHG2 Q9UQ88 5796 P51636 Q8WTR2 8819 Q15154 10142 8812 Q99638 10146 P02675 P01344 P01343 23429 Q9Y320 3142 Q9NU63 Q99623 Q8NAF0 55869 5798 8829 Q9UPY3 8826 Q8N960 P01375 P01374 Q9UPY8 23439 Q9Y337 Q9Y2R2 8841 Q9UHI8 Q9UHI6 O95067 Q3KNV8 23435 3151 P12004 10128 Q15172 Q3KNW1 3148 Q15170 545 546 3146 Q8WTP8 P40933 Q96EY1 Q15173 Q8WU17 P25311 8837 Q9UPZ3 7508 P24043 23409 7520 8851 Q9UPR3 8852 Q9H9S4 Q9Y2K7 Q12904 Q9Y2K6 Q8WU90 Q9H9S0 Q9UHB6 3162 Q12905 O00622 O95076 Q12906 Q68EM7 Q9BZE0 552 553 Q9BZE4 P42704 3159 Q99683 Q8WTW4 Q99684 8844 Q15185 P49336 7518 P05771 Q96FA3 O00635 8864 7532 7531 7534 7533 P55198 Q9UHC3 23411 O00631 23414 3171 10107 10109 563 Q00987 Q99675 3169 Q9NU19 Q9H1A4 P05783 7529 7528 6210 Q86U86 Q9UHD2 3187 Q9UPT9 Q12929 Q9Y2M5 3185 3184 Q9NU22 571 3182 572 573 Q99661 P51608 7536 6207 P26678 8882 3192 8880 3190 8881 Q60FE5 Q9UPU9 Q12933 8887 7555 P51610 580 582 P16410 54583 80854 Q99653 P51617 8879 7549 8878 P60568 Q9BZZ5 Q13478 Q13477 10092 6231 8892 10095 Q9UQP3 10094 6236 8896 P15923 Q96FV9 595 84324 Q9BRI3 6229 Q13485 Q9HC98 Q13489 P03950 6242 O00716 Q16512 Q16513 Q16514 Q9UIA0 O75496 O75494 Q7LBC6 P10242 P10243 P10244 84312 P27918 6238 51412 Q13492 Q16520 6239 Q13490 Q9UQR1 6256 Q9HBW0 O75489 Q16526 Q2M1K9 280636 23380 23381 P23528 7579 Q16531 P09237 6262 Q16539 10081 Q8N9N2 O75478 Q15208 Q16537 163126 Q9H257 Q86UL3 O75475 Q9Y3L3 Q9Y3L5 Q02790 P12883 P52907 P31150 Q9NUP9 P24864 10087 6259 84333 P60520 Q16543 6275 O75469 64750 Q9Y3E0 23365 P14635 O75461 64759 23368 Q9UQL6 P10275 P10276 P35579 O75460 P35580 Q8NBF2 P23588 Q99728 Q99729 Q16555 Q99726 Q15223 55806 Q8WV24 9311 Q86UR1 9314 Q5JQC9 O43889 P36896 P36894 23378 P15976 P35568 6282 23370 55810 Q02763 P01308 120892 Q99717 9306 P01303 Q15233 9322 P02671 Q8TCU4 P61803 Q8TCU6 O95813 9326 O75446 Q96FX2 O75444 Q86V24 6294 P36888 Q99700 Q9H1Y0 51465 Q99708 O95817 Q9BRK4 Q16576 9318 O95819 55827 64780 64782 64784 O75437 Q08050 P39905 64786 P14625 Q8N9R8 Q02750 P35548 138151 55832 51474 84376 54509 Q5VTD9 Q15257 Q16581 P53367 O95831 P53365 O95835 P57729 O95833 P12814 10928 56252 57584 Q9H204 9341 56259 10933 Q9Y3A5 Q9HC16 10935 10938 Q7RTN6 P39060 83871 O95837 Q6NVY8 P15090 Q8WUD6 Q9P0W2 22926 22927 Q13404 57592 10939 57594 Q14738 O43847 22920 57591 9350 P57735 P39059 Q08945 Q9UHX1 22919 Q9UI95 9367 9368 O43812 Q13418 O43815 O43819 Q86UE4 Q86UE3 Q9Y3C7 Q8N163 P42680 P42681 O95859 O60383 P42684 P42685 O60381 Q86UE8 P21741 Q13422 Q13426 O95865 O43823 B2RTY4 Q8NB78 2735 P19438 P21757 O43829 P68400 56288 2730 1408 1407 1406 2737 2736 9388 Q9HC62 Q13432 Q96G30 Q8WUI4 8050 Q86UK0 Q7RTR2 Q9H244 P23508 P62277 P43119 2741 Q9HC57 P09211 Q9HC52 P43115 Q5H9R7 Q14774 Q13443 9391 P11532

Q96G25 8065 O95881 P23515 Q9UQE7 P23511 Q8WUH2 P24844 Q14781 P23510 O14492 8078 Q9H228 O14495 Q9P0T4 P12830 P19474 59341 59343 P18146 Q9H223 84305 1432 O60343 Q14790 O60341 Q13464 P53355 Q9BZS1 P53350 Q9P0U3 Q9P0U4 P19484 Q9UHV2 O60356 2773 Q9HC29 Q7L9L4 P24821 O14497 Q9BZR8 Q9BZR6 Q9H0E2 P25054 Q9H0E3 Q9Y265 O75940 P49023 1460 Q12824 8091 124790 1459 5817 1457 1454 O00391 1453 155061 Q9Y275 284695 Q9UGM1 Q00839 Q9H0D6 Q92974 Q12834 Q12837 P16104 5829 Q9H0C8 P49006 5822 P10809 Q96EB6 Q59EK9 Q5S007 O75925 P27694 P27695 Q9H8S9 Q92956 Q9BQ51 Q12846 Q9BYE7 Q06124 P17405 P37023 P13056 57761 5830 55109 Q9Y297 O75928 5836 P26358 Q12851 P26374 Q6NUN9 O75911 Q86SX6 Q92949 Q99583 Q12857 1491 1488 1487 283337 Q99576 Q99574 64061 P26367 O75915 P25098 O75901 P49069 5861 5862 Q96N96 Q8NA42 114548 Q96MX3 57787 Q9HAJ7 1499 O60884 Q6PJW8 O14981 P61587 Q9Y230 P61586 O75909 Q12873 Q9H0H5 Q9UGI0 Q12872 P29466 Q9Y239 5870 5871 Q15907 Q15906 Q9UGI9 O00358 Q9BPY8 57798 3207 Q8TBC4 O60895 P17482 5863 O60890 P17480 Q9Y240 604 5865 Q9Y242 5867 5868 5869 P04179 Q12884 Q15910 Q9UGJ0 Q12888 Q9Y243 5883 Q9BPX5 Q9BQ95 Q15915 O60869 P16118 Q8NA19 P38398 55145 3215 Q96N67 610 Q92990 Q5T2W1 4543 Q9Y250 613 Q5TCX8 Q92994 Q6NUQ1 Q92993 5877 P63302 5878 5879 Q92997 P10826 P10827 P10828 Q9UGK8 221937 5894 P31749 Q7L8A9 Q9H8V3 P53999 27032 79101 P40692 3226 3225 55159 Q96MT8 P04150 5888 P63313 Q9UGL1 P04155 127829 A6NHR9 P04156 Q92985 Q15019 O96005 O96004 8930 O00308 P56199 P14317 P14316 P14314 O00300 23568 P50897 23569 Q99523 23560 Q15022 8924 637 79937 5898 P62873 639 10256 Q15025 P62875 O96018 O96017 P06702 Q15027 O96019 P16989 P36575 O96013 P35249 Q03933 641 P35251 Q6B0B8 79923 8932 648 P47224 P23276 8939 Q15036 P60228 Q16363 O00327 O96028 O00329 P48552 Q8TAU3 P48551 O96020 3265 3263 O60828 P15621 P11274 Q99500 P35240 8945 P01019 8943 79959 O14964 Q15047 O14965 P23246 Q9Y215 O00330 P35225 P35222 P35227 P35226 P11277 O14979 O75084 O75081 663 Q15051 P35232 53335 3269 P01009 Q9H093 P01008 668 Q15052 Q96MH2 O14974 Q59EA4 Q15057 Q16385 P24588 Q15056 7629 O43474 P06733 P01042 Q92934 6311 P01040 P62837 P06737 P05408 3281 79971 672 673 53340 P15692 O14908 6304 57708 P62834 10213 Q92930 P05412 P06744 P05413 6320 3297 23532 Q99558 P14373 57713 688 Q15070 689 Q15077 P62847 Q8N895 Q92922 P06753 Q15078 P01023 Q9BQ15 23509 P48594 P56178 P56177 Q12802 Q8NHY6 Q12809 O75044 O75051 P13010 Q15084 Q0IIM8 Q99543 A7KAX9 O14920 57727 P56180 O14921 P62854 O43463 Q92903 6341 O43464 Q92905 Q8NHX1 Q12816 P06729 23512 23513 57732 O14936 O14939 P40616 P60201 10202 Q92900 Q9BYX4 P36508 P36507 Q14686 P19174 Q9BZ95 O43559 O43557 Q9UPN3 Q9H165 Q9UPN7 P13861 Q9H161 Q9H9F9 Q9H0U4 P07196 Q9H160 P49810 5017 P83731 P78395 Q9BQG0 5018 P07199 P49815 Q9HB75 Q13363 Q14693 Q8TBP0 Q9HAV4 5034 P62805 6368 P46782 5036 P46781 5037 79084 Q96F45 Q9Y2H1 Q9H0T7 60489 Q9Y2H8 5029 P00533 Q13370 Q16401 Q9HAU4 P24522 Q9BYV2 Q9HB63 Q9BYV6 Q9BZ76 10193 Q9UQ13 9404 Q6PKC3 Q9UPP1 P32121 O75122 O75123 64857 Q9Y2I1 Q01344 Q00013 Q9H9D4 51542 10197 51548 51547 Q9BYU1 Q9HAT0 6386 5054 5055 P62826 Q9UPQ3 9412 O75116 Q9UPQ9 P68133 58189 Q96F24 Q4AC94 51552 Q00005 Q13393 Q6PKD3 Q13398 P62820 55915 9421 P78347 P78348 9420 9425 Q03989 51560 Q9Y2C9 Q71SY5 23481 P23229 51564 55922 5074 10181 P78337 8100 55929 Q9HAZ2 O43521 Q15109 O43524 26524 26523 5071 23492 P49841 P49848 Q96NH3 Q15118 P78368 Q9BYZ6 8110 Q3KNS6 P49840 P11229 5089 9448 23463 A1XKG3 P58753 23466 Q96EV8 23468 P48507 221150 79913 51588 Q9NTI5 10155 Q9BQI3 9439 Q96NG5 2805 10159 P78357 Q9HB90 10160 O43504 23476 O43508 P37840 5092 P11234 P11233 2810 51593 Q9H9G7 54625 P78362 54623 9465 9463 9464 P29375 9467 P29374 27005 Q9H0M0 27000 P22736 P42345 Q07507 2821 Q96EK4 Q9UGU0 P04083 Q9UGU5 P04085 Q15942 9477 9474 9475 O15496 Q08830 9470 P42331 Q6P1K2 P16070 P42338

P42336 P29353 Q5JXB2 P29350 Q9H0K1 Q9BYM8 79035 55066 60412 79039 O15499 P78314 8165 O95503 55072 P78317 P78318 9495 Q8N8D1 Q8TBE0 Q9HAK2 P04070 Q9UH92 Q9UH99 P53041 P07101 Q9Y2B9 P52597 Q96EP0 Q14643 Q13315 Q03113 Q86TI0 8174 Q96EP1 Q9H9B1 Q9H9B4 Q6P1N0 2869 Q13322 Q13323 Q14653 P46736 5906 Q9BQB4 5908 2873 1540 5901 5902 P46734 Q13330 Q6P1L6 O75962 Q9P031 P28065 Q9P035 2889 Q00059 Q9BQA5 Q86TG7 2885 Q68DY9 Q9HAP6 P43694 O15455 5911 Q14674 Q9BQA1 5914 Q14678 Q14676 O75953 P28074 P28072 P28070 Q9H0N0 5927 5928 P63279 P00519 5929 P63272 2896 P43681 51510 51512 51514 P43686 Q13351 51517 Q14683 P07148 Q13352 5925 23603 P10914 O95677 Q92858 23607 P10911 P67809 Q9NRW1 Q9NRW4 Q9NS86 Q8IZQ5 Q99490 O15519 4609 1576 P16220 Q92851 55207 5931 P49137 5933 O15516 5935 P09936 O15530 Q9NS91 Q8IZP0 O95680 Q9H832 Q07343 O95684 115704 P50148 P16234 P38484 4613 Q92845 Q92844 O15524 Q92843 O15525 Q92838 Q92835 P50150 P05549 Q9NRY4 Q99471 Q9BXC9 4628 27185 Q92830 O15534 P49116 64170 Q9H7P9 Q92833 4627 Q92831 Q92826 Q92824 P46087 P09917 5970 P46089 5971 Q99460 Q9NRZ9 P17535 P17544 Q99459 P17542 3304 P62140 400720 5966 3309 P04233 P34925 P07307 133584 5981 4651 3320 P52757 O00463 P52756 O00468 Q9UFF9 3315 3313 710 4645 5976 P41218 4646 27161 5978 4649 719 P08648 P30556 Q5HYI8 P30559 5991 5993 O00472 Q8IZT6 P30550 55250 Q9NS56 P37198 3329 3326 720 3324 722 P08651 P41208 Q92882 5987 Q92888 4659 P28223 137964 4673 P51449 2011 27148 2010 O00488 O00487 P30542 Q9NS68 Q9BXG8 4664 4665 5997 Q12770 Q92878 P04264 Q9UNY4 P08621 Q12774 P51451 P09958 Q12772 P30536 Q12778 Q5TC82 Q05586 2023 Q6KC79 O00499 3350 P30530 Q96LR5 P37173 Q99496 Q99497 P04275 7704 P02452 O00425 4690 P23396 P38405 2034 2033 O00422 Q9UFB7 P62993 4686 P01116 Q9C0K7 Q16236 285282 P01112 P01111 Q9C0K0 Q9UNS2 P25100 10383 P35368 2043 2039 Q9NRP7 54764 54763 P01106 Q96CW1 7727 Q9C0J9 P01100 Q7Z7K2 7741 Q8N726 Q7Z7K6 P14416 2054 P52735 P52736 O00444 Q96CV9 P11388 Q9NS23 773 6405 P01138 P01137 Q6UXV0 P01135 P01133 Q16254 O00459 P52740 P02461 P01130 7750 6421 23673 2065 2064 P52747 2063 P35348 781 Q9NS37 Q9NRR8 2059 P10070 P09086 P10071 10363 Q9C0H5 10362 P02458 P01127 6415 2070 23647 O43593 6430 O43597 7764 Q9UNN8 23641 Q9UNN5 O75190 7756 10336 57826 P40763 P04201 2081 P46060 5111 64127 P37108 Q5HYA8 O75177 P18850 O75182 Q9NRL2 P18846 Q68CJ9 P18847 P18848 5104 P46059 5108 Q92800 P27797 7781 O43572 64130 7780 P01185 64135 O00408 6453 6456 6455 Q9UNP9 2099 O60907 O75164 P50993 10318 Q8IZJ4 Q9NRM6 Q9NRM7 91748 Q9H7L9 P25103 5119 P01178 23636 7791 O43581 6464 O43586 P39748 146050 P52701 P58012 6457 10320 P60321 Q99418 P26447 P25116 Q8N6T3 Q99417 Q13237 5141 O43670 5142 Q14566 Q9BY77 5144 Q9BY76 Q5VZM2 9500 P13987 P13984 5138 5139 P62913 Q13243 P25963 P09017 O43683 P20290 9510 5154 O43687 5155 O43684 51654 Q14586 5162 P11309 6496 6495 Q13257 6498 P62937 P11308 P32242 P32241 P32246 5160 116113 Q13261 5159 9518 Q14592 Q96MA1 P61604 P21589 Q13263 P32239 9531 5174 Q13268 9530 8204 Q9BY44 Q9BY41 125058 P12645 5170 222546 P20264 Q13275 P62942 Q9NSD7 9541 5187 10293 9546 2909 Q6MZP7 O43639 O43638 201633 Q02535 Q01201 121512 Q9H082 Q13283 2908 10296 Q9UFW8 Q13287 Q6PJG2 2904 Q13285 2902 Q13286 5195 Q09161 9555 Q13291 Q6PRX2 P09067 2917 Q8NHQ8 8218 Q6PJH3 2915 9564 Q96ME7 10270 10273 Q9HA82 9567 O43612 23586 Q8TAQ5 Q96DT7 Q9H063 P49959 10276 2925 P09038 2923 Q15008 Q9BY84 9575 8242 149041 10284 9578 O43623 Q8TAP6 2932 2931 Q15011 10285 1609 1605 10289 Q15014 2935 Q15811 P78423 P78424 P08559 9588 Q12788 Q15819 Q08722 1613 27121 Q8TAF7 P41134 Q9BXM7 79139 27122 P46821 1616 Q9NSA1 P07237 Q9NSA3 P78412 Q12797 O43602 9590 Q9H7Z7 Q05682 O43609 P67775 P08575 P08572 2959 Q9BXL5 1627 2956 Q15831 P29474 Q15836 P29475 92129 27107 Q96M96 Q460N5 29767 Q03014 Q9BXK5 27102 2961 84502 2960 O95619 Q8TAD8 Q9NSC2 2969 P09874 8289 Q15843 O95622 Q14511

O95625 Q6R6M4 Q03001 29777 P31785 55198 1642 Q05655 2972 79149 Q9BXJ9 Q8HWS3 P04198 1647 P07225 Q15853 Q14527 P13945 1660 Q96DN5 P41182 Q8TAK5 P41181 P41180 93474 79175 Q499Z4 79177 84525 1655 84528 1653 O15554 Q13200 O95644 150094 51606 P51398 Q68CZ2 Q13202 P21580 Q8N7H5 Q68CZ1 P20248 64919 80155 P21579 Q8TAI7 P41162 Q9BXP5 Q9BY11 Q13216 P41161 P19235 P19237 1676 P08581 1674 P08588 P41159 Q86SE9 P55773 Q13227 P55771 P62070 O95661 4719 Q13233 P21554 Q92737 Q9H6Q4 Q5TB30 O14793 P15498 Q8IYN9 P84022 O00151 Q92731 Q92730 O14786 4722 O14788 10413 P28799 P27487 Q7Z6R9 64284 Q92729 Q8NFZ5 P51114 P53779 P14174 P20849 800 493856 4734 808 Q7Z727 Q9NR80 Q9NR81 O00167 Q9NQW6 Q7L5Y9 Q9C010 81628 3416 Q96KM6 Q8N680 811 P63104 P40425 P40424 P35080 Q9C005 333929 Q9NZI7 P48382 Q9NQX0 Q9C009 P07858 P99999 2100 Q9NR96 O00178 P48380 O95343 3428 81622 P20823 55352 O60674 O00182 P20827 P19838 O60675 3421 P18509 823 Q8N5V2 826 Q9NZJ4 P41743 829 P11926 Q13956 Q8IZ40 4771 4772 Q5SGD2 O95352 2107 55364 2103 830 27287 80324 55367 832 O14744 O14745 4780 P32856 Q92769 P09619 Q9NZC7 O95361 80306 P10600 O95365 Q96KS0 Q9UEE9 2119 Q9BWF2 841 Q6PI57 843 4775 O14753 846 Q6ZW31 847 7804 O14757 Q92766 P09622 4790 O43295 4791 4792 Q13976 Q9NZD8 2132 Q9NQS1 Q96L34 2130 Q96L33 81669 O95376 P18545 Q9NR48 7818 3459 P60033 Q96KQ4 Q92753 Q92752 P05230 857 O14763 Q9UN86 859 Q96CA5 Q92754 6500 P11912 Q9NR50 O95382 P19883 Q9NR55 Q9NQT8 2139 861 2137 862 O95390 863 867 Q9UMX1 O14775 Q9UMX0 4799 P09601 O14777 Q92743 P06576 O95399 P00390 10492 P15408 Q96C74 3484 3482 2150 3481 P11047 O60603 Q96T88 203068 3479 2147 2146 3476 Q13085 7832 6504 Q96KG9 10499 P23025 Q6UWV6 6520 Q7Z6J4 Q96T76 O14717 2159 O43290 O43294 6517 O43293 O14713 Q13098 Q13099 Q96C55 Q6ZN33 O43251 Q9UMR2 Q7Z6I6 64215 2173 P13686 23787 890 Q00653 P13693 891 O14727 894 896 Q9NR09 P53708 898 29080 10477 Q9UN42 29086 P49639 P61326 O43264 Q9UMS4 64223 6541 7874 5213 148156 Q658W2 Q9NR11 O14737 Q01974 Q96ST3 10486 P60002 7867 10488 Q96T58 O14733 5207 10487 P28702 Q3MII6 O43236 7884 Q96BZ9 P50613 Q5R372 P51946 23764 Q8NFW5 10459 10458 P50616 10451 10450 6548 7879 10454 Q01968 Q8N5U6 O43242 10460 P51955 Q9NYZ3 29079 Q01955 Q96SZ6 P51959 P48357 10468 23746 P49682 P24385 P24386 9601 5245 9600 Q9UMN6 10437 10438 Q00613 10432 P24394 Q13901 9612 P49674 Q13905 P28749 Q9NQL9 P04792 56647 Q13115 6595 Q13114 6597 5265 6599 Q14449 6598 5268 P10599 6591 O43318 Q6UY14 51762 9616 9617 51763 Q9C0C2 P21246 Q14451 P38919 Q13127 B2RXF5 5274 8301 P58546 P13631 Q96D03 24149 5270 Q02447 51773 30813 P46531 9628 Q13133 Q15796 Q13131 9641 Q13136 Q15797 9640 8315 Q8N6I1 9643 P54198 Q9UNL4 Q96CN4 P46527 51780 Q01101 P42167 Q01105 Q9NRI5 O94805 Q13144 Q13145 54815 Q9BWQ8 Q13148 5295 5296 P35869 9656 P43490 9657 O43306 P10586 P10588 57099 O43303 P10589 Q9NZZ3 Q8N6H7 P26927 9649 P43487 Q49AN0 Q13153 P43489 Q13158 9667 O15287 P54132 Q9UNF0 P63092 P13667 P0C1Z6 Q02413 Q13162 Q14494 O94827 Q14493 8328 Q14498 Q13164 O15294 Q9NRC8 7013 7014 P13682 Q9NZT1 P11021 P63096 Q9C0F3 Q7Z7H5 Q9NRD1 Q13177 Q9NRD5 Q96LC9 7024 7023 10392 O94844 Q9C0F0 Q7Z7G2 P12314 9682 7020 Q9UNH7 54851 Q13185 1718 P00367 Q9BX69 Q9UEW8 P47895 Q13188 7037 Q9BX66 Q09028 P38936 O75791 Q9UNI6 26747 54862 Q6PID4 1728 Q9BWT7 7046 P35813 P10515 8379 O75787 27242 O15234 1738 P05177 1737 1736 P54105 7057 112950 55291 P10523 55294 7052 27252 81567 1742 1741 81565 Q9UER7 8399 7068 7067 Q5TAX3 Q15714 O15264 P19793 Q15717 8394 P30101 P31431 7064 Q01196 84619 Q9H6W3 1756 Q92793 57003 Q9NZQ3 O15259 Q92796 P05155 Q9NRA1 P59817 27236 Q8IYT8 7073 Q9UNE2 7074 Q9UNE7 57018 80254 P43405 1763 Q9NZR4 Q04206 Q92786 Q92785 P05164 Q92784 P09525 7088 Q9NZJ7 Q15735 O00186 Q53SB5 93594 Q01167 O00194 P63000 51720 P08243 4800 P08246 7099 7098 Q15742 Q9NQZ2 O15211 Q14416 7091 P14923 7090 Q9NQZ8 P30153 7094 1789 1788 Q05516

1786 1785 Q05513 P63010 375790 51735 Q6SZW1 Q15750 Q15759 Q96CJ1 29843 57045 92283 P85298
1793 P17174 84661 51742 Q6ZNG0 51741 Q9NZM3 Q5T9L3 P05198 Q14432 Q15762 Q8IYX1 30827
O15230 Q13107 Q06830 Q9H6Z9 Q9UNA1 4839 P47804 4830 900 51752 Q9NZN5 902 P08237 Q13112
P08238 Q9NZN8 P08235 Q9NZN9 O15228 Q92618 Q7Z628 Q9H609 Q92616 Q9NYD6 Q14CB8 O75820
Q92619 O00268 O00267 Q9BVA0 3516 Q96JM7 Q96JM2 Q5VWX1 283248 O00273 Q9ULW3 Q92610
P63208 4841 90678 Q9ULW0 Q8N587 Q92614 P07996 10533 P04003 P52564 P04004 O75815 P52565
Q7L591 Q8NF91 4860 Q66LE6 51090 P52566 Q08431 51094 P63211 10549 P20963 51099 P63218 4851
4854 Q96JL9 10542 928 Q92600 Q9NYF5 Q9ULX9 Q13813 Q9NYF8 P32927 29128 Q05397 10514 26097
29127 P20936 4862 P32929 O75807 4867 4868 Q6IT96 4869 Q96JK9 4882 3551 O00299 Q7L576
Q13829 P17302 10524 P17301 P20941 Q8NEU8 Q9ULZ3 948 Q96JJ6 10523 Q9ULR5 O95477 O00220
O95470 29108 O00221 O60760 29102 P04040 58517 7913 O14867 Q96BA8 Q9UM47 O00238 P04049
Q96SB4 Q96SB3 Q9NYA1 O14880 O00231 2243 O00233 P10721 O00232 Q8WYH8 29110 2237 79444
960 961 29115 963 967 4898 Q9UM54 Q70UQ0 4899 Q9NYB0 P30304 P30305 P30307 O00241 2253
O00244 6609 Q9NQ29 2247 2246 Q9BVC4 58533 2244 975 6601 Q92630 6605 Q92636 57209 Q9UM63
Q92633 Q969M7 P29323 P29320 P31629 O00255 Q8NEZ4 Q9NQ33 O00257 81788 Q99259 P17342 984
988 Q9ULV0 P05362 6613 P29317 Q9ULV4 26060 26959 Q9Y6W6 P07900 P48454 O43399 Q9Y6W5
5300 P15529 2274 P53803 O60725 P15531 990 991 Q96RU8 O14827 Q7L523 Q96RU7 Q96RU2 994 995
2267 P11171 51003 997 998 6622 999 6625 54998 51009 P62753 51008 Q04917 P49770 Q9UM07 5310
Q7Z5H3 Q8N4N8 5311 Q5T7W0 O60739 P11177 266747 2280 Q93096 Q04900 Q96RT1 2277 P48436
P49768 O00206 2290 Q9Y6Y9 P25791 Q9BV73 P48431 5321 Q96JC9 148022 O00203 Q00535 Q8IXB1
Q00534 Q96S42 P49756 P25789 P25788 7975 51026 6647 6646 5316 51028 91949 P25787 P49758
Q08495 6660 64343 P49750 O00213 Q08499 O00212 Q96AQ6 O60716 Q96RR4 P61457 Q96JB5 6657
5327 5328 P49747 O43353 6670 P07942 6672 Q969H4 P07947 9700 P07949 Q969H0 P07948 Q8WYA6
P20916 Q9ULJ3 Q969H8 Q8IXI1 Q8WYA1 Q8IXI2 Q01844 6667 5335 Q96AX2 4005 5336 Q96AX1 P62714
5337 5338 Q9H5J8 10577 P07951 P26232 P07954 Q969G3 Q7Z5L7 10580 Q9ULK4 Q01831 P50749
Q96S94 P50748 Q9NPH5 5347 Q96JH8 Q76NI1 Q08462 Q9H5I1 5361 64375 P50750 5362 6696 5364
4035 Q5VWQ8 Q9BUZ4 51061 O14802 Q8NER5 P68036 10552 P62736 10554 Q9H5H4 Q01826 5371
P27540 Q9ULM3 P20908 P15559 Q9ULM6 Q9BUY5 P16885 51075 O14818 Q9NPJ6 P62745 54106 10564
Q15654 9744 O43432 8412 9743 Q86YW9 29948 29947 94121 Q8WZ60 29941 Q96BM9 116 118
Q8NFJ5 9738 Q7Z6C1 P22694 Q7Z6C3 Q8NFJ9 Q8WZ64 8408 O94906 P32019 Q13009 4067 P12429
Q15669 128 O94915 P60983 Q96SN8 Q14344 54925 Q7Z6B7 Q15672 Q13017 7101 P22681 Q9BVP2
Q76FK4 P35998 Q969Z0 P25705 Q9UDY8 Q9UDY6 8428 142678 Q8WZ42 P00441 8443 9774 4086 7112
4087 4088 P22692 4089 9775 8445 Q86YT6 Q96BK5 9770 Q8N5F7 Q9NZ94 142 P21359 1808 8439
P62701 Q15691 Q15697 Q8IXZ2 53615 Q13033 8453 9784 8454 P54259 8452 4099 8458 7124 P54252
P54253 7126 Q96KC8 4092 4093 4094 154 Q00597 156 Q13042 Q96KC2 Q13045 P45379 Q13043
P49736 7132 Q96C24 8463 7133 8467 161 P13796 Q96C28 1822 166 Q8NFM4 Q04941 Q8NFM5 338917
7128 Q9NQB0 1828 P49721 P49720 Q96T37 8473 P00491 P00492 Q8N5K1 P54274 P36402 8471 P36404
7141 1831 Q96T23 Q9NZ45 Q00577 P49711 Q9NQC3 1837 P49716 P49715 P60953 Q9NQC7 Q96C00
O94973 7153 P54289 P20073 7158 7157 P19086 162239 7159 1843 Q9UDV6 54984 Q13075 1848
Q13077 7165 Q13873 7169 O94988 P10636 26038 26037 Q06787 Q86Z02 Q01094 P63172 26039 7161
P63167 P42224 1852 1850 P17275 P23945 Q92692 P63165 Q9BVI0 O94989 O15355 P22612 P42229
57109 Q969T4 1856 P42226 Q53QZ3 O15372 O94992 Q969S8 Q13887 Q01085 Q8IY57 7170 P10644
Q01082 79363 Q96BD5 79365 P84095 P60900 1869 7187 7186 7189 7188 Q8WYK2 P35916 P11940
57120 P18583 27346 O75881 7181 27347 O75880 7182 7185 7184 1877 1874 4907 P17252 P68871

1870 P09630 O15379 Q969R5 Q9NZ08 O15391 O15392 O15397 Q6UWE0 P35900 4914 27352 84733 Q969Q1 Q92667 58473 27327 Q86YS6 Q02386 57144 P51148 P51149 1896 84766 P42262 Q8WYQ5 4920 50514 P51157 P35968 P51151 Q8WZ19 P22670 Q15628 Q9NQ92 P51153 P42261 26005 57154 Q96BI3 58480 P51159 27339 P30279 27330 84759 58487 P30281 57159 Q8WYP5 Q8WYP3 3609 3608 3607 P21333 Q9NYJ8 94104 Q6ZV73 377630 29966 Q02363 58491 57162 P84085 27300 3611 Q5XUX0 O15335 4943 Q15642 P01730 Q969V6 O15350 Q14314 O75832 O15353 Q15649 Q15648 27315 Q86Z14 Q86YP4 Q06710 Q96BF6 Q8N5A5 3622 P84077 3621 P30260 4952 O95429 4953 Q15653 Q15652

negative regulation of biological process P54852 P25054 1460 259266 124790 10657 2305 2304 2303 O14544 Q8N3U4 O14543 Q9Y275 2308 Q92974 Q12834 Q12837 Q8NDV7 2316 2 P42858 Q9H0C8 P10809 Q96EB6 O75925 Q9UKV8 Q92956 3660 Q9BQ51 Q9BYE7 Q06124 P37023 P13056 57761 O75928 Q9Y6A5 P26358 Q92949 Q99583 P21917 1491 1488 1487 Q99576 2332 3665 3667 10642 64061 10644 O75915 10643 Q9UL15 P25098 Q9UL18 1021 1020 114548 P51787 10614 6709 P21964 1499 1012 O14981 P61586 Q92530 26191 O14503 3690 Q8WXF0 Q15906 Q96J02 O00358 Q9BPY8 1029 1027 1026 3685 6711 6710 P17480 604 P05023 P04179 Q12884 Q15910 5883 P38398 57326 P20618 Q92990 Q92993 6726 Q8N488 5879 P10826 Q9UKS6 Q9UL46 P10827 Q9UGK8 5894 P31749 O95140 Q7L8A9 1050 253980 P40692 79589 P63313 Q9UGL1 Q9UKT4 P04156 Q92985 O96004 O00308 P14316 O00300 P50897 11108 Q99523 23560 Q15022 639 Q15025 P25445 Q9Y5V3 P16989 P57059 1072 641 P33076 51132 79923 8932 P49407 P60228 P02794 P48552 Q9UKN5 6774 3265 P35240 P01019 51147 O14964 Q86X95 5451 Q9Y696 P35222 P14784 P35227 P11277 663 Q15051 P17813 P35232 Q9H093 P01008 668 6776 Q96MH2 P24588 O43474 P06733 O00744 5460 P01042 4131 Q9Y5Q3 6311 9821 6794 4133 P25490 Q9NX61 3281 672 673 P15692 O14908 6304 6789 10213 P05412 O00755 P05413 3297 23532 P14373 5467 4137 207 5468 54206 P01023 4152 4155 P56177 Q96A54 O00762 P13010 Q99543 127435 10673 O14920 O14921 P62854 9839 O43463 Q12816 23512 23513 P50402 Q96QT6 57732 O14939 55558 10202 P49427 11060 8535 11065 P55265 Q9UPN3 P07196 P49810 5017 P78395 P49815 Q13363 Q9NPC8 Q9HAV4 4188 P62805 6368 5036 5037 79084 P58304 23051 60489 P09382 8539 5029 P00533 Q13370 Q16401 Q96RL1 Q15554 Q9HAU4 P24522 Q9BYV2 8553 P35638 Q96IZ0 P32121 P31273 O75122 Q9Y2I1 23028 Q9NY61 4193 10197 51548 P02741 51547 Q14232 P55290 5054 5055 Q8IWX8 Q9UPQ9 58189 P35625 Q6PKD3 Q9NPF5 Q03518 P43246 55915 9421 9420 7248 Q9Y6K1 O75581 Q03989 51564 Q15583 55922 5074 10181 55929 Q9HAZ2 P22392 O15055 O43524 26524 26523 5071 7251 285 7249 P49841 P49848 P61925 P49840 O75569 A1XKG3 291 Q9Y6M1 23468 Q8WY64 P48507 51100 51588 P08069 Q9NTI5 10155 Q9BQI3 Q9NPA3 1956 O43504 O75553 64426 P37840 7272 2810 51593 Q9H9G7 Q93034 Q14289 Q96RE7 P29374 Q9H0M0 Q4VCS5 27000 P42345 Q07507 P04083 Q9UGU5 P04085 Q92574 Q13761 1978 P56524 9474 Q02297 7291 O75530 7295 1982 Q92560 P06276 P16070 P42336 O15020 P06239 P56539 Q7Z569 P21860 8165 P56545 O95983 91147 A4D1W7 P78318 9495 Q8TBE0 P04070 Q07954 Q9UH92 Q9Y2B9 O95995 O95999 Q13315 9021 Q16828 P35612 P35611 Q9UKY1 Q9H9B1 Q8IWV1 Q13322 Q9NP71 Q9BQB4 1540 3720 P22303 P28065 Q9BQA5 Q9Y6E7 Q86TG7 O15455 Q14674 Q9BQA1 5914 Q14678 O75953 P28074 63976 P28072 P28070 5928 P63279 P63272 Q14201 P43686 Q14683 5925 Q7KZF4 O14672 399687 Q9UBB5 Q9NS86 O15519 O60566 4609 10772 5931 P27348 5933 5935 Q9UBC1 Q9NS91 Q9UBC3 Q8IZP0 54361 55692 Q92845 O15524 Q92843 Q9UK53 Q92835 P50150 Q9NRY4 Q99471 P53667 Q9BXC9 O60543 1111 Q9UJU2 O15534 P61289 P03372 Q92833 P06401 Q92831 P06400 Q9GZX9 5970 9093 Q9UBE8 Q99460 Q9NRZ9 P56705 9099 3304 3784 6812 6814 10765 3309 P04233 P05129 P52756 Q9UFF9 O14627 6829 10736 3315 Q6FHQ0 3313 710 Q8N2W9 27161 5978 6827 5991 93986 5993 Q14938 Q8IZT6 P37198 3329 6839 3326 2475 P08651 5987 Q92888 P26045 P07766 P05106 P28223 2495 Q13618 2010 Q13617 Q13616 O00488

O00487 P30542 O60502 57448 5997 Q12770 5515 P04264 P05112 5516 O43182 5518 P09958 Q12778 Q13625 Q5TC82 2023 Q6KC79 10724 Q99496 P05120 5524 P05121 P40337 7704 P02452 O00425 P23396 O43159 P04637 2033 P16615 Q9NVW2 4686 4204 P01116 Q9C0K7 Q15389 P01112 P01111 Q9C0K0 Q9BT49 4221 6885 P35368 Q9BT40 1191 9908 2039 Q15392 84159 Q9P2K8 4214 P01106 6878 P01589 7727 6890 5562 Q8N726 9921 P14416 P12235 Q9NS23 773 6405 P01138 P01137 Q6UXV0 P01133 Q9UJM3 O00459 P02461 P04626 P04629 2065 2064 2063 P35348 11218 Q9NS37 301 302 P10070 P17936 P10071 5566 P02458 P01127 307 5569 9927 5580 O43593 5582 5584 O43597 23641 Q9UNN5 25822 O75190 Q8TEB7 5578 Q495A1 P40763 P04201 2081 4261 Q6IR47 64127 P37108 Q5HYA8 O76024 5590 O75182 324 5104 P46059 5108 P27797 P01185 6453 2099 O60907 P50993 P51843 330 331 10318 333 Q9NRM7 5599 Q9H7L9 5119 P01178 Q9BT67 23636 O76003 P17980 P52701 P58012 10320 P25116 8655 4297 Q9BY76 11186 9500 P30086 Q04760 4291 355 356 Q9UBS5 P09493 5139 P25963 P47755 P47756 O43683 9510 5154 6003 5155 O43684 19 Q9H3Z4 367 Q9UBT3 51654 7329 7341 Q14106 P11309 6496 6495 Q13257 6498 P10451 84108 Q9UBU3 116113 P51805 P09471 P17096 P21589 Q13263 9531 Q13268 9530 8204 Q8WWN8 Q9BY41 Q14118 Q9Y618 382 Q04724 387 388 Q9NWT8 Q9UBV7 P20264 11176 Q13275 9541 5187 11142 O15164 2909 23122 O43639 201633 396 Q02535 397 398 10296 11146 Q15466 Q99966 54 Q13286 5195 6045 7376 7375 Q09161 23135 Q9Y5J5 Q86WV8 P33151 2917 Q14140 Q9UBP4 O15169 Q99958 Q15475 P14859 O43612 Q9H063 P49959 Q01664 P01579 11124 P09038 Q15008 149041 23112 O43623 25776 2932 2931 Q14160 51231 1605 87 Q15811 P78423 O15123 Q9NNW5 27121 26271 P41134 Q1PSW9 27122 91 94 P46821 O15119 1616 P45973 O15118 Q9NSA3 Q12797 Q8WWH4 Q96QB1 P55318 P55316 O43609 P67775 Q7Z460 P08575 P08572 2956 Q15831 P52298 P29474 P29475 92129 9126 Q9H422 Q03014 6093 27102 Q8TAD8 Q9NSC2 O15151 O94776 9138 Q03001 Q05655 Q9UBN7 Q9UBN6 1647 9146 O75626 Q9UBF6 P41182 P30044 P30047 P30048 84525 P22415 Q9UJW9 Q13200 150094 Q96HU1 P21580 Q07820 Q68CZ1 Q07817 64919 Q9UJX2 Q9UJX0 P41162 Q9BXP5 P17081 P08138 P09429 O75604 Q9BY11 P10415 Q9P2X7 23189 P35711 Q7Z494 1676 P09430 P08588 P41159 P08107 P55773 Q13227 P55771 Q04771 O15105 P45983 Q13233 P21554 O14793 2550 9181 P51587 9184 P17676 P37288 Q8IYN9 Q9HCJ0 P84022 Q92731 Q92730 O14786 3875 4722 6901 P27487 P28335 25913 P28330 Q92729 Q5VTR2 P51114 P14174 57510 54487 4734 808 Q7Z727 Q9H2G4 Q7L5Y9 6927 3416 811 P63104 P40424 P62258 6926 Q9UIS9 Q9NZI7 P48382 Q9NQX0 P31946 P07858 P31947 2100 Q9NR96 P48380 O95343 57534 P20823 O60674 P19838 P62263 3421 P18509 P31949 5604 Q9NZJ4 P41743 829 Q9Y468 P08758 Q8IZ40 4771 P15056 Q86VE0 830 832 5610 6945 22846 P51553 Q92769 Q9NZC7 Q07666 P10600 O95365 Q06330 Q9UEE9 10865 Q9HCM4 O14753 847 5624 O14757 4790 4792 1285 Q9NQS1 1284 Q9HCL2 Q96KQ4 1277 Q92752 857 Q9UN86 859 Q96CA5 Q13501 Q9NR50 P19883 Q9HCK5 Q9HCK4 Q8NBS9 861 O95390 192669 Q9UMX1 4799 P09601 50943 P08754 Q92743 P06576 6993 23322 P14555 O60603 25988 3479 2147 2146 3476 5654 7832 10013 84289 10014 Q6UWV6 135295 64682 4343 23332 25998 Q99750 O43290 O43294 408 409 Q6ZN33 Q09472 O43251 23309 5682 5683 5684 5685 5686 64215 2173 P13686 P13693 891 Q99741 Q9NR09 11326 Q9UMS4 P51531 4361 5692 5693 Q658W2 Q9NUX5 P51532 Q01974 Q96ST3 P60484 11331 5687 5688 10488 Q96T58 P23497 P26583 Q9Y3M2 25942 Q8NFW5 10459 Q96P48 P50616 8726 Q9H2D6 Q8N5U6 O43242 10460 Q9HCE7 Q08117 P37231 Q9HCE1 Q01955 Q96SZ6 P51959 444 10468 23746 P24385 7422 5245 22893 3066 3065 Q9UIF9 10437 79753 10438 Q00613 Q96P20 P61764 7414 P24394 3070 Q13901 9612 462 P17612 55796 P28749 7428 P04792 6595 6597 Q14449 O75386 P10599 O75385 6591 Q9Y4K3 3084 O43318 472 476 477 P20396 9616 51763 8767 P38919 Q13127 P55036 8301 Q16635 O75376 P13631 Q96D03 3096 5270 Q86VP1 Q02447 O75381 488 51773 Q9H2X0 P46531 7448 9628 P55042 Q13133 Q15796 Q13131 Q15797 8315

P12757 Q8N6I1 Q9UNL4 P12755 O75360 P46527 498 Q01105 Q13144 Q13145 54815 Q9BSI4 Q9BWQ8 Q13148 5296 9657 7476 P18074 P55055 P10586 P10588 57099 P10589 93166 51317 P55061 P43489 Q8TDY2 P54132 Q9UNF0 8328 Q9NRC8 7013 7014 Q9Y4H2 P35443 7490 P11021 84232 Q8WW38 Q99835 7023 P98179 Q86VS8 P15822 Q9UNH7 51341 P02549 Q13185 1718 Q13188 P23443 6188 11252 Q09028 P38936 P27816 Q6PID4 Q99819 Q99816 9208 P48736 7046 6198 P10997 P78540 O43711 O43715 6194 P62195 9219 P98161 P07355 P78536 7057 9238 Q13526 83737 9231 55294 27252 81567 P46934 1741 Q9HD26 P41240 Q32P28 Q4LE28 P98170 P42574 Q9UER7 Q14865 7067 Q5TAX3 Q13535 P19793 P29590 Q7Z2Q5 Q01196 84619 Q9H6W3 P41235 Q9HCP6 P41231 Q92793 Q92796 O60264 P05155 3932 O60260 9255 Q13547 Q9UNE2 O43707 P41220 Q04206 Q92786 P05164 P09525 7088 O95751 Q15735 O00186 P78504 P00749 Q63HR2 P00747 P63000 51720 P08243 Q14894 Q13563 P62158 P08246 3956 7099 7098 Q9NQZ2 P30153 P11413 1789 1788 Q05516 P62166 1786 Q05513 P00734 Q13574 Q13573 Q9H2S9 P32320 57045 2644 1312 51741 Q16610 Q14432 P20333 Q13107 P18031 Q9P286 P34972 P62191 O60238 Q8TDM6 P67870 Q9HCS4 4830 900 P00750 P08238 Q9NZN9 Q9Y2W7 O75820 2672 Q12948 O00267 Q9BVA0 3516 283248 O00273 57620 90678 Q92614 P07996 P52564 P04004 P52565 Q9Y2X9 Q12959 P52566 Q8NB12 51094 5706 5707 5708 5709 51099 P16471 4851 5700 4854 5701 5702 10542 5704 5705 Q13813 Q9NYF8 Q9Y2Y8 Q05397 5717 83933 P20936 P32929 O75807 5713 57649 Q6IT96 4869 5716 P48023 Q9BZF9 3551 Q13829 P17302 5728 10524 2697 400961 10523 Q9ULR5 Q12981 Q12982 O95477 P28482 Q12986 Q9UHJ9 P51668 O00221 P51665 Q9P0J0 P19525 57669 29102 P07550 P04040 Q9Y2T1 10971 P08887 Q9UM47 Q9Y2T7 P04049 Q96SB3 Q9NYA1 O00231 O00233 P10721 O00232 P19544 Q8WYH8 57678 P18206 79444 960 1387 P62328 967 P42771 5747 Q9NYB0 22937 22938 P62330 Q9NQ29 P62333 2247 58533 Q9BZK7 6601 Q9Y371 6605 Q9UM63 P51693 P29323 5770 P51692 O00255 O00257 Q03164 81788 P06213 26959 5300 P38646 P52952 P15531 990 991 Q96RU8 Q96RU7 Q8WTS1 997 998 6622 79813 54998 P02686 P62753 10138 Q9UQ80 Q04917 5310 5311 5796 P51636 Q04900 Q15154 P48436 Q99638 P01343 P49768 O00206 2290 P25791 23429 P48431 3142 Q99623 Q00535 Q00534 Q96S42 P25789 55869 P25788 6647 8829 Q9UPY3 P25787 P49758 P01375 P01374 Q08495 6660 Q9Y2R2 8841 Q9UHI8 Q9UHI6 O00213 23435 Q96AQ6 O60716 3148 Q15170 545 3146 P40933 Q96EY1 6657 5327 5328 P25311 8837 P49747 O43353 6670 6672 23409 9700 7520 Q969H0 Q12904 P20916 Q9H9S0 Q9UHB6 3162 Q12906 Q9BZE0 552 Q9BZE4 3159 Q99684 4005 5336 P62714 5338 7518 P26232 Q969G3 8864 7531 Q7Z5L7 7534 7533 23411 23414 3171 563 Q00987 Q99675 3169 Q9NPH5 Q9H1A4 P05783 7529 7528 64375 6696 4035 Q9Y2M5 3182 573 Q8NER5 P51608 6207 P26678 Q01826 5371 8881 Q60FE5 8887 580 P16885 P16410 O14818 P51617 P62745 54106 8878 P60568 Q9BZZ5 6231 6236 29948 94121 595 116 118 Q7Z6C1 Q13485 8408 Q13489 P03950 Q15669 O75496 O75494 Q13492 Q15672 Q9UQR1 6256 O75489 P35998 Q2M1K9 Q969Z0 P25705 Q9UDY8 P23528 P00441 6262 9774 4086 4087 4088 4089 9775 163126 Q86YT6 Q96BK5 Q02790 P21359 P52907 Q15691 Q15697 53615 Q13033 8453 8454 P54259 8452 O75469 4099 64750 7124 P54253 7126 Q96KC8 P14635 O75461 23368 Q9UQL6 P10275 4092 P10276 O75460 154 156 Q13042 Q99728 Q99729 55806 Q13043 Q96C24 7133 8467 9314 O43889 P36896 P36894 23378 P35568 6282 1822 166 P01308 338917 7128 Q9NQB0 9306 P49721 P49720 Q96T37 P61803 O95813 P54274 Q96FX2 Q86V24 P36888 7141 Q99700 1831 Q96T23 Q9H1Y0 Q00577 O95817 P49711 Q16576 Q9NQC3 P49715 P60953 Q9NQC7 O75437 Q08050 P39905 7157 7159 P14625 Q8N9R8 Q02750 P35548 138151 51474 54984 Q13075 84376 1848 Q5VTD9 Q15257 Q13873 O95835 P10636 P12814 Q06787 Q01094 P63172 7161 P63167 Q9Y3A5 Q9HC16 10935 P63165 P39060 O15355 P42229 P42226 O94992 Q969S8 Q8IY57 Q01082 22920 9350 Q96BD5 22919 Q9UI95 P60900 1869 7189 Q8WYK2 57120 P18583 O75881 7181 Q13418 O43815 7184 Q86UE4 4907 P17252 O15379 O60383

P42685 O60381 Q13422 O15392 Q13426 O95865 Q6UWE0 4914 27352 O43829 2730 84733 2737 Q92667 2736 Q8WUI4 27327 57144 P23508 P62277 2741 Q9HC57 Q8WYQ5 P09211 4920 50514 Q14774 Q8WZ19 57154 8065 27339 P23515 58487 Q9UQE7 57159 3609 Q14781 P23510 P21333 O14495 29966 P19474 Q02363 P18146 3611 O60341 Q13464 O15350 P53355 O75832 Q9BZS1 27315 Q86YP4 Q96BF6 Q8N5A5 3621 2773 Q9HC29 P30260 O95429 Q15653 Q9BZR6

nucleic acid metabolic process 8099 Q92979 Q9Y265 O75940 Q12824 O95551 P51587 81605 P52434 O00148 P17676 10657 Q8TDD1 Q9BYG3 2305 2547 1454 P84022 1453 Q00839 P05455 4968 Q9H0D6 Q9H2H8 25913 O75934 P30876 P08729 Q8NFZ0 P16104 O60216 10667 80755 54487 Q9Y285 10421 O75937 P10809 Q96EB6 64282 P62249 Q9H2G2 Q9BRX9 P27694 Q9UGN5 Q9UKV8 P27695 3661 O00167 54496 1478 6929 Q9Y295 55109 Q8N684 P26599 Q5TAP6 Q9UKW6 3659 P26358 P31942 Q9UIS9 P31943 P08708 P99999 Q99583 Q12857 55110 Q99575 P19838 P62263 55119 P26368 10642 Q13951 Q9UL18 4772 Q8IX01 1022 Q14807 Q9NSU2 P39019 27043 Q5T160 1017 79577 10856 Q6PI48 80324 Q9BWG6 833 Q9Y230 O75909 O14744 5859 Q12874 Q12872 4780 Q8WXF0 Q9H0H0 Q07666 Q8WVB6 O95363 Q06330 1029 27297 O14519 10625 57794 1025 167227 Q5C9Z4 Q9BUB4 4775 O14757 Q92766 4790 Q12888 5883 P28340 Q9NR48 P38398 Q9BWE0 Q9BQ90 Q96MU7 55149 Q9Y250 Q92994 Q92993 Q96CA5 P10827 P10828 22826 5892 22827 Q9H6R4 22828 6741 5411 Q8NC51 Q9NQT4 Q9NR55 Q9NQT5 1050 55152 Q9HCK5 2139 253980 P40692 27037 55159 O60870 10607 192669 865 6733 P04150 5886 5887 4799 5888 O96004 8930 11340 Q86V81 10250 10492 5422 O00541 O95391 P50897 25988 11108 51362 Q96T88 3479 Q8WWY3 P62877 8924 P25205 7832 Q15020 84289 Q15024 P62875 P23025 6749 135295 P51991 Q15029 5430 4343 5432 Q9UKM9 P35244 P51513 P35249 Q96T76 Q03933 O14717 641 P35251 O43290 Q7Z6J9 196441 5424 Q9BRP8 P49643 O43294 408 8939 P49407 P60228 409 O43251 5440 P49642 5441 6772 5442 Q9UKN5 O96020 Q9UKN8 346171 Q9BW92 O00567 Q9NUW8 Q66K89 O14727 126789 Q99741 Q96T60 3014 5434 51147 Q8IYD8 29086 Q15046 P61326 Q15287 P23246 Q9UL03 Q86X95 Q9UMS4 4361 84292 A7MD48 3035 114799 Q9NUX5 O14979 Q96ST3 11338 P35232 3028 Q00403 55759 668 Q15054 4116 10488 84296 P57081 P26583 8732 6311 7884 P50613 P51946 23764 25942 Q9Y5Q8 23524 672 Q8WUY8 P15692 10450 Q96A72 P55072 Q9Y5R4 3054 23536 29079 Q6PJP8 Q9HCE1 Q9NWV8 Q96SZ6 55775 688 689 P62847 P62841 4150 Q9BQ15 4152 4154 25929 7422 Q9BRT9 5245 P52815 22894 Q9UIF9 P13489 22897 Q9Y5S9 P17844 79753 10438 Q5TAL4 P13010 Q99543 7415 5478 4149 P62857 10432 P56182 O43463 8761 Q9BQ04 3070 23517 Q13901 Q5VTL8 P07814 P49674 Q9UIG0 Q96C86 Q92905 O75031 55796 56647 Q92900 P56192 P49427 4172 4173 O43791 4174 53938 Q16629 Q14686 4176 Q14209 P55265 6598 Q9UPN6 Q6NZI2 57062 4171 Q01130 472 474 P33240 Q16630 Q14690 Q8WW01 Q9C0C2 Q14694 P38919 Q16637 3091 Q8IWZ8 Q16633 8543 5036 6125 O75376 79084 Q504Q3 Q9NW13 60489 51773 24144 6117 Q9NPD3 6119 6118 Q96RL1 P46777 Q15554 Q15796 Q9HAU5 P24522 Q16649 P35638 P61968 Q15797 9643 8555 P32121 Q9UNL4 O43776 O75362 Q96IZ7 29803 Q01105 51547 54815 Q8WVM0 Q9BSI4 8563 Q13148 Q9BYU1 9410 P35869 P62826 9656 11051 Q8IWX8 O43542 8565 P18074 P18077 P36954 144983 28960 Q9P2E3 Q13151 Q9NPF5 Q16665 P61978 Q49AN0 P43246 51319 9421 Q14249 P78347 P54136 O15287 P54132 P35453 26512 Q9Y6K1 Q93062 Q04837 23481 Q8IZD4 Q14494 Q14493 51567 Q14498 51329 Q14011 Q9NRC8 10181 7013 55929 11030 7014 P46100 O75575 P36776 26523 23016 7490 Q9NY12 Q9NRD1 11277 118460 P49848 Q9NRD5 Q86W42 P98175 O75569 P98179 Q02880 25885 51340 54853 Q9NVM4 51585 Q96PK6 11243 55703 P47897 9439 Q9GZL7 Q6I9Y2 Q15365 O75319 Q9HB90 Q08170 26986 Q01780 Q09028 25896 6182 O75792 5093 Q96EU6 26747 51593 Q15370 Q9H9G7 P48730 P78362 Q99814 11017 Q9NRF9 P78545 9465 P54577 9221 9463 Q6P1J9 P29375 O95714 P29374 P29372 57461 Q7Z589 P78549 6194 27000 10813 Q9H6Y2 P41252 P62195 P41250

O15234 Q13761 65083 1736 Q13523 P54105 9477 9232 22803 Q13769 Q9H0L4 Q9P1Y6 O75531
O75771 P24928 79005 1982 27257 Q9Y6H3 Q9NVH2 Q6P1K2 P42574 O60293 Q5D0E6 7068 7067
Q5TAX3 Q15714 Q13535 Q9H307 Q15717 Q8IZ69 P55209 79035 1994 79039 Q8TBF4 Q9NVI1 O60264
P78316 9255 Q32P51 P34931 83759 Q8N8D1 Q9BWH6 1763 29896 Q07955 29894 P53041 Q9UET6
50628 P52597 Q13315 O95997 7083 9261 4809 84881 51720 Q8WVK2 4800 51726 2626 Q8NEC7
Q9NVC6 Q14653 51729 Q13569 P19387 P30153 P19388 1789 1788 P46736 Q9H9A7 1786 26121
P61077 57038 Q9BUJ2 P22303 Q13573 5901 9045 Q9NP81 P12956 3980 P78527 Q00059 Q9BQA5
P61088 Q96AE4 P43694 Q9BQA1 3978 Q9Y4A8 51747 Q14676 Q9P287 5927 4839 5928 P00519 P63272
23076 P24941 P43681 O60231 Q9HCS7 84893 902 Q13112 Q13595 51514 Q14684 P21675 Q14683
Q5FWF5 P10914 O95677 285672 Q92858 Q969L4 P67809 399687 O00268 Q06265 O00267 Q9UBB9
3516 10535 5939 P16220 O00273 Q9ULW3 4841 Q9Y2X3 10772 Q6NZY4 5931 P49137 Q9UK45 Q92614
5935 P0CG13 Q9NS91 Q9UBC3 Q9NQ55 55696 51096 P18887 55215 10785 5705 Q9NWB1 Q9ULX9
Q66PJ3 Q9NRY2 Q9NRY4 Q12968 29128 O60306 Q8TED0 57646 P18858 P12081 O60543 1111 Q92830
P03372 Q9Y2Z2 Q9Y2Z4 Q12972 P49591 P46087 Q9H814 Q9GZX7 Q6P2C8 9093 Q9NRZ9 P49590
Q13829 O60318 10524 Q99459 3304 10528 P49588 10521 5725 10523 Q6PCD5 O95478 5981 Q12986
5983 1385 57661 O95232 P52756 6829 Q9P2N5 1137 Q6FHQ0 P61221 29102 P62316 P84103 P62318
5733 5976 56339 P62312 58517 27161 6827 P62314 Q10570 Q9Y2T7 Q96SB4 Q9UHK0 Q96SB3 Q13601
1153 Q14938 O00472 Q5JTZ9 O95243 P19544 Q96SB8 Q9NS56 Q8WYH8 10987 3329 6839 2237 79444
56342 1388 P08651 P42771 Q9NYB0 P30304 O95257 4670 P30307 22938 4673 Q9Y4Y9 O60508 Q9H9Y6
1161 O60506 Q56NI9 80222 10714 P61244 284119 O14641 5511 O14640 5514 Q9Y4Z0 Q92878 5515
5757 Q9UNY4 P08621 5518 P51693 Q13620 Q13868 O00255 P54727 P54725 Q9P0M2 Q03164 55035
10725 P61254 57697 10728 157570 Q9Y2W2 988 Q92620 4677 4678 P06454 Q9BZJ0 Q9Y2W1 O43395
O43159 6872 6871 P04637 Q5VYS8 6631 2034 Q9BV90 P14678 P53803 990 Q96RU2 54512 995 997
4686 6625 Q16236 5536 6627 P62995 P62753 Q9GZR7 Q9UQ80 54517 Q96LI5 Q9C0K0 P07910 220988
P25100 6883 4221 23210 Q96Q15 51010 23451 51013 6633 Q9Y6Y0 6878 8812 Q15393 10147 Q99638
O43172 6637 56946 8815 P01343 P01100 Q86U44 Q86U42 P26640 O95059 Q8N726 Q9NU63 P11388
P11387 O60942 P26639 10594 Q53X93 P49756 6647 5557 P61201 5558 Q9UPY3 P01133 23438 O43143
6660 6421 P40938 P40937 Q9Y333 Q9UHI6 23435 Q96B26 P12004 11218 Q9NS37 3149 3148 Q96RR1
545 P09086 546 3146 9924 57819 Q96EY1 P62308 P61218 Q9ULR0 O43390 P62304 7508 2070 Q5TKA1
6430 6432 Q9UQ35 6431 7520 6434 4255 Q9UPR3 7764 Q12904 22794 23404 Q96PU8 Q8IZL8 3159
Q02930 8602 6427 Q15185 6426 7518 7517 8607 Q8N302 2081 23658 5591 P46063 Q9Y2L1 6201 5111
5356 23650 3178 Q9ULK4 23411 O76021 Q01831 328 25804 3181 2091 P50750 P22087 Q8TA86 P49321
Q8N2M8 3189 Q9UNP9 2099 3188 3187 3185 3184 Q9NW64 O14802 333 55660 51067 7536 9716
91746 Q9H5H4 3192 Q9UNQ2 3191 8880 A2RTX5 P27540 3190 9733 4287 5378 P39748 Q969E8 P51610
O00411 P52701 580 Q9NVV4 Q08211 221656 10569 79622 P58012 P52948 Q9NPJ6 79869 54107 6217
9967 Q9UPV0 Q8N9Q2 Q14566 4297 P15927 O43432 Q9UKD1 P15923 57187 P13984 29947 4291
Q96FV9 351 Q9UBS4 P09012 6229 P62913 9978 Q13243 Q8IU60 O94906 Q13242 Q13247 5393 P20290
P03950 5394 O43687 O00716 7334 16 Q15428 7336 Q16514 Q15427 26205 O75494 80198 367 Q9UBT6
51654 11198 54921 O94913 6239 7341 Q15434 Q14103 P62937 Q76FK4 Q16526 23144 Q9UKF6
Q9Y606 P49916 23381 85437 Q9NV06 P42285 P09234 P22674 P17096 Q16531 11168 54931 Q9UBU8
P83876 54938 P21589 51426 P00441 51428 O43660 4086 11171 10081 8683 4087 8446 4088 P22692
O75478 9533 9775 O75475 P29084 142 Q96HW7 11176 P23771 Q8IXZ2 Q15459 9785 10291 O15160
9541 8458 P54252 O15164 P54253 2909 7126 8450 P10275 25788 O75460 4094 Q00597 Q8NI36 2908
Q8IYB3 Q99728 8449 84365 Q13287 Q9NSE4 Q14134 P49736 Q14137 Q09161 8467 O43889 23378

Q9Y5J1 Q9Y3F4 Q8NI27 Q9BRL6 11157 Q15233 Q99714 Q96FX7 P54277 P54274 6059 O75444 6050 7141 Q99700 11129 Q96T23 Q99941 P49959 P50914 Q00577 88745 51222 Q99708 56915 9319 P49711 Q16576 9318 2926 P49716 P49715 11128 7153 7155 7158 Q9BVS5 Q08050 P39905 7157 P62906 116138 P14868 P14866 7150 8241 10286 10285 Q15014 2935 P78424 92345 Q13873 9100 22913 Q96QC0 Q14966 Q7L0Y3 P52272 Q12788 92105 10929 Q15819 P33992 P62081 P33993 P33991 7161 P18124 1854 P42224 Q96DI7 10933 Q9Y3A5 84967 Q9HC16 P63165 P09651 26270 1856 Q9UBK2 P55795 Q14978 Q8WWH4 O95602 Q13887 Q86WJ1 Q96I24 P22626 Q9Y3B4 P67775 Q6IN84 84950 10949 Q08945 Q9UHX1 Q9Y3B2 P52292 Q52LR7 P09661 P05067 2959 P08579 10940 2956 Q969S2 Q9UBL3 P52298 9128 9129 O75643 9126 10907 O43812 P11940 O75400 26015 286826 26019 9360 9361 1877 80145 283989 4907 2961 2960 1870 P04183 P23921 Q8TAD8 Q9BTC0 2969 P09874 Q13427 Q13426 Q8NAV1 29777 Q8NB78 10921 1642 2972 57379 P09884 134430 1408 1407 1647 4913 Q13435 Q15853 P33316 Q9UJV9 Q9NYH9 Q14527 1660 Q6NYC1 P53567 1655 P22415 1653 Q03468 Q92499 Q8WYQ5 1659 P43115 3835 Q8IY92 O95644 O95400 Q8WZ19 Q08J23 Q96G21 27339 O43809 64919 1665 Q8N0Z6 58487 Q9UQE7 Q8N0Z8 27332 Q9BXP5 P23511 Q6P2Q9 Q15631 Q9Y5B9 P09429 Q8IY81 Q13216 Q8TEQ6 P55769 P35711 84305 1676 Q5RKV6 2521 57169 Q6P4R8 P09430 Q9HC36 O15350 P08107 Q15643 Q9P2Y4 Q15648 Q86YP4 Q9BVJ6 Q96HR8 O15347 Q15653 Q14562 1207

protein metabolic process 8099 Q9H0E3 Q9Y265 P28562 1459 1457 55561 55568 Q5QP82 1454 O00391 1453 Q8N465 Q9H4L4 Q12834 Q8NE71 10667 2 27090 Q9H0C8 Q9Y285 5822 140801 Q9UKV5 P10809 Q96EB6 Q5S007 Q9UGN5 Q9UKV8 Q8NE63 Q06124 A0AVT1 P62495 P37023 P14384 2324 57761 P48147 Q9Y297 O75928 Q9Y291 Q12851 O75914 3672 P26373 Q13705 P21917 1491 10645 2335 1487 Q9BYD1 148789 O14578 10643 Q9UL15 Q8WXG6 P25098 P51784 Q9Y5Y6 1022 Q96MX6 1021 1020 Q06587 P50454 10613 57787 1018 1017 79577 10616 O60884 Q9Y230 Q9Y231 Q92530 26191 5859 Q9UGI0 P29466 3692 5870 Q13724 Q9H4P4 5871 Q96J02 Q08752 10626 O14519 Q8TBC4 Q96EF6 Q96MV8 1025 Q9BUB5 6711 P07686 6714 Q12884 O95136 Q9Y243 79595 Q8TBB1 P38398 391627 P20618 613 Q92995 Q5TCX8 5875 254827 Q92993 4548 Q9UL46 P30414 O95147 5894 P31749 Q38SD2 Q8IWL3 55152 253980 57332 1047 55159 6733 Q9Y262 5886 5887 8916 Q9UGL1 Q9UL54 Q8TBA6 64432 O00308 O96009 P56199 O00303 P50897 23569 11107 Q15022 P62877 8924 Q15025 Q9Y676 P23284 O96017 P49411 Q86SR1 O96019 P36578 O95163 Q9NWZ3 P57059 115426 1072 O96013 51132 Q9Y680 P62888 84033 11113 P23276 P27635 P49407 P60228 10269 Q15034 O00329 84061 6772 P35236 3263 10238 O14967 P14780 P11274 8945 P62899 Q14197 79956 51147 Q15046 O14965 Q8TAT6 Q8N3J5 Q9Y697 23552 3276 23556 P35227 Q9H4A9 P35232 Q9H093 6776 285193 O14974 P62424 6790 Q5VVQ6 6793 P01040 P62837 8975 6794 4133 10690 P05408 9820 Q99570 P39656 O00743 Q9NWU5 Q8IWF2 672 673 6304 4125 6305 6789 128308 10213 158135 Q07020 5470 4140 8985 5476 3297 O00754 Q99558 Q9NWV8 10229 Q86T82 207 4139 P62847 P62841 P61513 Q15078 5481 23509 Q9Y5S2 Q08345 O00762 Q9NWW5 51185 Q15084 Q99543 5478 5479 P62857 O14920 P62851 57727 P56180 P62854 Q9H4F1 5494 Q92901 5495 O43464 Q9BU89 Q92905 5499 Q8NHX1 P35268 23512 Q7L7X3 P13497 10208 O14936 Q96QT4 Q56UN5 P56192 P49427 P36507 11060 11065 7204 4179 Q9BZ95 Q86TM6 Q9H0U6 Q8TC27 Q9NXS2 O75150 Q9H0U3 Q6P5Z2 23043 84447 P46783 P49810 P83731 Q14694 Q13363 Q9HAV7 11072 5034 4188 P46782 P46781 P41091 Q9Y2H1 P68104 8536 P00533 Q16401 Q96RL1 P61956 P46777 197259 P46776 Q9HAU4 P46779 Q9BUN8 P46778 10193 Q9UPP1 8555 P32121 23028 Q96IZ7 4193 Q8WXQ8 10197 P61962 1915 P61960 51548 Q93009 P02741 P22314 51547 Q93008 Q14232 P61964 Q9Y6R4 P20151 8565 Q9Y6R0 O75116 P62829 Q86Y37 Q4AC94 Q8N4C8 267 23031 23032 Q9NPF4 Q9NPF5 P49862 8576 26994 O75582 P10398 Q9Y2C2 8569 1937 1936 1933 8100 55929 Q9BV47 Q9HAZ1 23014 26524 5071 7251 Q9Y6L7 11035 P49841 P02751 P61927

Q15118 P78368 7266 P02790 P49840 P11229 O75569 9448 7267 10152 23463 Q9Y6M4 A1XKG3 Q8WY64 Q8NEJ0 P24534 P78371 P08069 10155 Q9BQI3 P02786 51107 1956 10159 O15075 Q9NPA8 Q15126 23476 Q86Y79 23478 7272 1965 Q9H173 114088 Q93034 Q92187 P78362 54623 51116 1969 Q14289 1968 9463 Q6P1J9 P09769 Q96EL2 27005 P12931 Q70CQ3 79012 Q9H0M0 Q70CQ1 1975 27000 P42345 P22735 P04083 Q6P5R6 Q58WW2 9476 7297 P31689 P56524 9474 9475 Q9Y6H3 Q92560 26168 P42338 P42336 O75528 Q5D0E6 P06239 P29353 Q5JXB2 P29350 Q9H0K1 P56537 1514 Q9BYM8 26133 P06241 P21860 Q9Y2A9 80020 O95983 Q16816 P55212 91147 P55210 A4D1W7 55072 63931 P78317 201595 P04070 Q9Y6J8 P53041 P07585 Q92542 Q16825 Q96EP0 Q16827 O75503 O14595 Q13315 Q86TI2 Q9P015 Q16828 Q96EP1 9020 23081 23085 Q9H9B1 P20594 2869 Q8IWV7 55093 Q86Y13 P61081 Q9H596 P46736 P61073 83548 23097 P61077 1540 124739 P46734 11080 Q9NXH8 9045 Q9NP81 Q16849 Q6P1L8 P12955 O75962 P28065 26576 Q9P035 Q9Y6E7 Q9Y6E0 P61088 Q96RG2 O15455 Q14674 5914 Q13347 11091 O75953 Q16854 P28074 P28072 9054 P28070 P63279 P00519 5929 P24941 11099 P08473 Q9BZ11 51514 P43686 23607 65003 O14672 Q9NRW4 O15519 O60568 O60566 P15374 Q8IVH8 P16220 Q92851 P49137 P09936 O15530 Q9NRX2 P27361 Q9GZZ9 Q8IZP0 65018 Q9H832 54361 P50148 P16234 10783 92609 P67812 P52333 64174 O95696 P29992 1121 Q99471 P53667 1111 125972 Q86WA8 Q92830 P61289 P62136 4627 Q92831 P55854 P49591 Q92824 3791 5970 Q8NCR0 9093 Q9UBE8 Q99460 P56705 P49590 9099 10767 3304 P62140 57410 55236 O60551 3301 P49588 Q9H3G5 10765 3309 P04233 P34925 O00469 P05129 133584 P11802 P50579 3320 10733 147746 Q96DE5 3313 710 3312 27161 Q7KZI7 Q7Z419 Q9Y4X5 P19784 P53611 Q9NS56 3329 10746 3326 1147 720 3324 2475 722 5500 4659 Q9UNX3 P26045 P53621 O95257 6850 Q13615 2011 Q13618 27148 Q13617 Q13616 1161 O00487 P30542 Q9P2P5 80222 57448 3337 P61247 Q96DC9 O43184 5515 P04264 Q9UK22 65061 5516 5518 P51451 P09958 Q13620 Q13627 P30530 P18433 740 Q96LR5 P61254 P37173 Q99496 10728 P09960 4677 O14656 P40337 Q9UK32 O14657 Q8N2K1 P04632 O00425 O95278 P23396 6872 Q96HA8 6871 25862 2033 Q9NVW2 O95271 23203 P09093 11222 5536 5537 6883 6885 1198 25873 23210 1196 2043 1195 1191 56940 Q96Q15 Q9NRP7 Q15392 Q99873 54764 55611 51253 Q9P2K8 4214 11231 4215 4216 P01588 P46019 25847 5562 5563 O00444 O60942 51264 Q53X93 Q9GZP9 Q9Y572 P01138 P01137 P01135 P01133 Q9BT22 P02461 P01130 Q9H7H0 P04626 5575 P04629 2065 25853 3396 2064 780 301 P17936 Q8IZE3 P10071 9924 5566 Q96PN8 5567 5568 5580 5581 5582 5583 5584 5585 5586 Q92817 Q9UNN5 25822 O60925 O76031 Q96PU4 79612 O75190 Q8IZL9 55644 Q96PU5 5578 5579 10331 Q9H3F6 8607 2081 5591 P07711 Q9Y4P1 9950 Q6IR47 5596 5597 5598 P50502 Q5HYA8 O76024 P18850 O76021 5590 P83881 327 329 84197 P27797 P50990 Q9GZU7 P50991 91754 Q9UNP9 O60907 O75164 23621 10318 55666 Q9NRM7 5599 P62979 P83436 A2RTX5 4285 Q5T1M5 6464 25818 7311 391356 Q96Q40 P17980 146057 O60911 Q5U4P2 221656 Q08209 P62988 5127 Q8N752 P25116 Q13237 4297 Q6DKI1 Q6ZT98 Q15418 64951 7325 P62917 351 Q9UBS0 Q04759 Q9UBS4 P62910 P62913 9978 P21127 O43683 8662 9510 7332 64960 7334 16 8667 7336 O43684 8665 9512 7335 19 64963 64965 Q8TF76 Q9UKE5 64969 Q9H3Z4 Q9UBT2 23170 51650 369 51651 7329 7328 51657 7341 Q9BY50 P11309 11160 Q6Y2X3 Q13257 P62937 P11308 7347 6015 P29074 64975 Q9UBU3 Q03405 5159 8669 Q9UBU8 P61604 P43378 31 Q13263 9531 9530 7353 Q14114 Q9BY44 Q9BY41 P51812 P51813 5170 Q9UBV7 Q02543 P51817 11176 P09001 9526 P62945 P49903 P62942 5184 P55345 O15164 Q99965 121512 P27708 P48643 Q9NSE4 52 Q13286 Q14139 6045 7375 6048 23133 23135 P15735 Q9UKA1 O15169 Q99956 P46459 7385 7384 Q96E52 Q8TEY7 8237 10273 Q9Y5K6 Q9Y5K5 Q9UKB1 Q99942 P50914 P01579 Q14152 11124 Q9UBQ5 P09038 Q15008 Q9BY84 Q15005 O75676 O15197 10283 O15198 P62906 9578 O75674 P14868 25778 2932 2931 255488 51231 Q14164 86 2935 Q14166 9100 P55786 Q15819 P62081 Q8TF05 1613 Q05209 80124 P0C0L4

Q9BXM7 79139 Q8TAF3 91 Q8WWI1 94 Q12792 P45974 1616 P07237 26270 P82921 8266 Q9UBK9 P82912 Q12797 P82914 Q6DT37 Q9H3R0 P31321 55173 P67775 P08575 P05067 Q9UBL3 O94763 Q15831 O94768 Q6XUX3 Q9H422 Q96M96 6093 1634 3817 27102 Q8TEL6 O95619 P21980 P09874 Q6V1X1 Q15843 Q5JRX3 Q6R6M4 55192 Q02156 26260 26262 1642 Q05655 Q9BXJ9 Q9UBN7 P82932 P82933 Q9UBF6 9149 56052 Q8TF47 Q6NYC1 Q9H488 P11766 26232 Q86SG6 Q9UK80 P30050 Q03468 26234 1650 Q9UJW2 Q13200 150094 Q13202 Q8IVT5 P21580 9150 57396 Q8N7H5 80155 Q9UJX2 Q9Y5B0 51611 P08138 O75604 P10415 Q13216 93492 P08581 Q8NHH1 Q5T6F0 728642 P08107 O15111 Q04771 23192 1203 389840 O15105 P45983 Q13233 P45984 Q7Z2K6 Q96TA2 65125 P54619 Q96L12 Q9NQU5 9184 Q8IYN9 Q70EL4 10893 Q6IQ55 Q9H2H8 P27487 Q9UIQ6 Q9C026 Q92729 Q5VTR2 P53779 P62244 80755 P62241 10420 4734 P62249 Q9H2G2 Q7Z727 P28300 23708 81629 O00167 P56817 2571 79791 54496 Q8IUD2 3416 P62256 57520 P40429 811 6921 P27448 Q9C005 P31944 P07858 P08708 Q9NR96 P54646 P17655 P62266 Q5TAQ9 55352 O60674 P20827 P19838 P62263 821 823 5602 826 Q96GD4 P62269 5604 Q9NZJ4 P41743 Q9Y3U8 Q13956 Q9Y463 Q5SGD2 2590 O95352 P39019 2107 Q5T160 27283 P15056 10856 2589 Q6PI48 P39023 833 5610 5613 O14744 Q9UIM3 P30679 Q92769 P09619 80308 O95361 P10600 P52888 O95363 10868 10869 P61353 841 843 847 5624 7804 O14757 P09622 4790 Q9H2K2 Q13976 4311 P21709 Q96L34 O95376 P18545 Q8IUH5 169436 Q05469 2125 P60033 Q96CA5 Q10472 Q10471 Q13501 Q9UMW8 Q5MAI5 6500 Q9NR50 O95382 Q96L21 80332 Q4G0W2 2139 10845 Q96KP4 O00141 80335 4313 867 Q9UMX1 4318 Q92743 23326 O60603 23327 871 2147 Q13085 5654 7832 10013 84289 Q96KG9 10014 P25685 64682 Q15269 Q9UII4 Q9BS26 Q6FGD7 2159 51377 Q99750 P61313 Q9BS18 Q99759 O43294 408 O43293 409 Q13099 P60468 23307 Q6ZN33 Q09472 5682 5683 5684 5685 5686 64215 Q9BW92 891 29088 Q8IYD1 55743 Q9NR09 55748 Q9Y450 10477 29086 11326 Q9UMS4 5692 5693 P40818 8720 7874 3035 Q658W2 P14543 Q01974 P15880 P60484 Q15291 29093 5687 5688 55757 O14730 7867 O14733 P31930 8732 O00506 P50613 54431 10450 Q9H2D6 79739 P55072 Q96GC5 O43242 140609 Q9HCE7 P51955 P51956 Q05086 Q99797 444 23770 8737 Q02809 P82675 P61758 29074 8738 284086 P49207 23746 P24386 8754 5245 Q9HCD5 P52815 3066 3065 Q9UIF9 O75293 55781 Q00613 7415 10432 8761 P07814 P49674 Q9UIG0 54460 3075 3074 P17612 7428 10445 Q13115 53938 Q9Y4K4 P11441 O75385 Q9Y4K0 24139 Q86VQ3 Q9Y4K3 O43318 472 P48729 28973 Q99807 9616 8767 3093 Q15303 P13639 P55036 6123 6125 Q15306 Q9Y4L5 Q86VP6 8780 Q504Q3 23291 Q4J6C6 Q9NRH2 Q15796 P60866 Q13131 9641 6133 6132 Q15797 Q16644 6134 9646 7465 6137 Q9UNL4 Q6UXN9 O43776 O75365 O94804 6128 P82663 P82664 Q13144 Q8NCB2 Q16659 6141 57092 P10586 P18077 O75352 P26927 Q96PF1 Q9P2E3 6138 54822 Q49AN0 Q9P2E9 Q13153 51319 P54136 6156 6158 O75347 Q13162 P23467 P27824 Q13164 Q14012 6164 Q15349 O15294 Q9NRC8 Q8TDX7 6168 11274 P36776 6160 P13674 P11021 Q7L2H7 Q9NRD1 Q13177 Q9NRD5 6175 9201 Q3ZCQ8 9682 Q08188 Q8WW22 Q02878 Q9NVM4 Q96PK6 P55010 Q9NRE1 Q9NVM6 P47897 1718 Q9UEW8 1716 Q13188 P23443 O75319 9212 6185 6188 Q15369 Q9BWU1 Q8IUX7 6187 9695 7037 6182 Q9UNI6 6184 Q9NZV6 Q00688 Q6PID4 Q99816 P48730 P23458 P48736 Q7Z7F7 Q8WW12 Q15375 6196 P54577 6199 7046 6198 P35813 Q96L91 O95714 O75787 Q9H324 27248 6193 6195 6194 83752 Q9Y4C1 P41252 27246 P62195 P41250 O60285 1738 Q13523 375743 P78536 7057 P29144 Q13526 Q9H2P9 83737 Q9H792 55294 7052 27252 P46934 P41240 O60296 Q4LE28 P42574 P42575 Q9UER7 P34947 P29597 Q13535 O15264 P29590 Q7Z2Q5 7064 Q9H6W3 80267 29880 Q9Y4E8 Q92793 Q7Z6Z7 P05155 3932 O60260 Q9HCP0 P07339 9255 Q13547 Q13546 Q8IYT8 9252 9253 Q9UNE7 P43405 P20309 Q04206 P78560 P41227 Q13555 Q13554 Q5GLZ8 O95757 Q13557 P78509 9261 P00749 P00747 51720 P00742 P08246 51726 7099 7098 30849 Q15746 P30153 P00738 Q05513 P00734 57038 P24723 P08253 Q15751 80273 51734 P46977 P55884 Q15759

Q8IUQ4 28998 29843 P78527 O60229 P41279 Q58DX5 1314 Q04656 84661 4820 O15212 P05198 O75716 30827 Q6XPS3 Q13107 P18031 Q9P286 P62191 P22528 1326 Q63HQ2 P47804 P07384 51752 Q6ZVX7 P20340 P00750 Q9HD40 P08238 P21675 Q9NZN9 P48052 O95433 O75821 O75822 Q8IXM3 51081 203102 Q8IXL7 Q8NB16 P63208 90678 10531 P07996 10533 Q92611 P04003 P52564 Q9Y399 5706 57634 5707 10549 79885 5708 5709 P21810 P16471 5700 P49368 5701 5702 Q9UHP3 5704 5705 65264 P54753 P25398 Q9BZG8 Q05397 P54756 P18621 O60307 P16444 5717 29122 1358 57646 83933 P12081 P63220 55466 O60783 P32929 5713 26091 Q6IT96 Q9Y2Z4 5716 P54764 P54762 P54760 Q12974 4882 3551 Q13829 152926 5728 10524 1368 Q8WTX9 253827 Q9UI30 Q9ULZ3 Q6PCD5 O95476 O95477 P28482 1385 Q9UHJ9 P51668 29108 P51665 Q9P0J1 P19525 O60762 P04040 P51671 O00238 P04049 Q96SB4 O00231 O00233 P10721 O00232 Q5JTZ9 Q5VST9 Q8WYH8 10987 29110 10988 79444 1387 Q92643 4898 P05386 P05387 5747 P05388 P30304 P30305 P32969 Q14703 P30307 Q9NYB9 Q9P0L2 P42766 10956 P62333 2247 Q9BZK7 975 22929 Q6GMV3 Q92630 P61009 P08865 Q969M7 P29323 5770 P29320 P54727 P54725 Q03164 81788 Q99259 Q9P0M9 57695 984 6612 Q9Y385 6613 P29317 5768 P06213 Q15139 Q9Y6W6 5780 P07900 Q9UPV9 5783 5300 P15529 P53804 P38646 O60725 991 P62750 Q96RU7 Q96RU2 O14829 994 995 Q99640 997 5775 P28838 79813 999 5778 51009 P62753 P49770 200916 Q9UM07 Q9UHG2 Q9UQ88 5796 2287 2286 2280 Q93096 Q8WTR2 Q96S53 O14830 P49768 O00206 Q96EZ8 P26641 P26640 P49761 P49760 51021 51023 Q00535 P26639 Q8IXB1 Q00534 79834 P25789 55869 P25788 5798 6647 10598 91949 P25787 P01375 23438 Q9Y2R5 Q9Y337 Q9Y2R2 8841 7511 Q10713 Q9UHI8 O00213 Q9Y2R9 Q96RR4 O95071 545 Q96EY1 Q00526 5327 5328 8837 O43353 Q9H5K3 P07947 23409 9700 8851 P07949 Q969H0 P07948 Q12904 Q9Y2K6 Q9Y2K2 Q99683 Q9BZE1 10574 8844 Q15185 P49336 P62714 4008 P05771 5351 5352 6201 6202 23411 Q00987 51056 4015 Q9H1A4 5347 4017 Q96JH7 6210 P50750 Q9UHD2 Q12923 Q9UPT9 Q9Y2M5 573 Q8NER5 51067 P68036 6204 6207 Q68J44 Q01826 P49792 5371 8881 Q12933 Q12931 580 54101 55432 O14818 P51617 6699 54106 8878 6217 10090 6231 8892 6233 P15924 Q9NYU1 Q9NYU2 29941 P22695 Q7Z6C1 83475 6229 P22694 O94905 8408 Q9HC98 Q16512 4067 Q16513 Q16514 63875 29959 P27918 Q6UX04 128 6238 P60983 Q13490 P22681 10075 Q9HBW0 Q16526 23387 P31152 O43897 P35998 Q9UDY4 Q9UDY8 Q9NV06 P23528 8428 Q16531 Q8WUK0 54938 Q8WZ42 P00441 P09237 Q16539 4086 344387 4087 Q6PD62 8446 O75478 Q15208 8445 O75477 23399 Q8TD19 64745 Q02790 142 1800 P62701 10087 6259 Q8WUJ0 53615 1801 8453 8454 8452 Q6ZN16 64750 7124 7126 Q96KC8 P14635 Q9UQL6 8450 4092 P10276 4093 P35579 O75460 156 Q9Y3E5 10055 P23588 Q13042 Q99728 Q13043 Q13049 P04843 P04844 Q96KB5 P36896 P13798 P36894 P11142 Q02763 54961 120892 7128 P49721 P49720 8473 P61803 O94966 Q96FX2 P36888 Q96T21 1832 Q9NZ43 Q9H1Y0 51465 O95817 O95816 Q99707 O95819 55827 Q9NQC7 O75439 Q96FW1 P14625 Q02750 1843 55832 54504 1848 Q6VAB6 Q15256 Q13873 O95835 O95834 Q96K76 Q86Z02 P18124 8492 P42224 1852 10933 P47914 1850 P63165 O95837 O15355 Q92696 1859 P22612 P42229 Q13882 Q9BZQ6 Q969T4 O15371 O15372 22926 Q13405 Q969S8 Q13404 Q8TCJ0 10939 Q8TCJ2 Q01082 O43847 84708 Q9Y3B7 P84098 P63173 57599 P60900 7186 7189 P10619 91039 P35916 27348 27347 Q13418 9360 9361 7184 Q9UHY1 Q9Y3C5 P17252 P42680 O15379 P42681 Q9NZ08 P42684 P42685 Q9NUG6 Q86UE8 Q13427 Q6UWE0 Q8NB78 4914 P42677 P68400 Q9Y3D3 Q9Y3D5 1408 1407 2737 Q969Q1 Q969Q0 Q9HC62 Q9UI38 P62280 57144 P62277 4920 Q8NFF5 Q9HC52 Q13444 9397 P35968 Q13443 Q8WZ19 Q9NQ92 26005 57154 Q96BI3 Q9H1R2 8065 84749 27339 27330 Q13451 8078 Q8IY84 O75844 Q5W0Q7 377630 P12830 P19474 59343 3611 1432 Q5XUX0 57169 Q14790 O60341 Q13464 P01730 Q969V3 P53355 O75832 O95427 Q14318 P53350 Q9P0U3 Q9P0U4 27315 26469 P30260 Q7L9L4 O95429 5800 Q9UHV9

negative regulation of cellular metabolic process P51587 124790 9184 10657 2304 P84022 Q8N3U4 6901 25913 P28330 Q12834 Q92729 Q12837 Q8NDV7 P51114 4734 Q96EB6 O75925 Q7Z727 Q9H2G4 Q9UKV8 3660 Q9BYE7 6927 P13056 3416 57761 811 P62258 6926 P26358 Q9UIS9 Q9NZI7 P48382 Q9NQX0 P31946 Q92949 P21917 P48380 O95343 P20823 1487 P19838 2332 P62263 P31949 3665 10642 64061 10644 10643 Q9UL15 Q9Y468 Q8IZ40 Q9UL18 4771 1020 10614 P21964 1499 Q86VE0 O14981 Q92530 6945 O14503 3690 Q92769 Q8WXF0 Q07666 Q15906 P10600 O95365 O00358 Q06330 1029 10865 1027 1026 3685 P17480 O14753 604 P05023 4790 5883 P38398 57326 P20618 Q92993 857 6726 Q8N488 P10826 Q9UL46 P10827 P31749 Q9NR50 P19883 1050 Q9HCK5 P40692 79589 192669 Q9UMX1 4799 50943 Q9UGL1 Q9UKT4 Q92985 O96004 P14316 25988 2147 23560 3476 Q15022 10013 639 10014 Q6UWV6 135295 64682 P16989 P57059 25998 641 P33076 51132 Q99750 8932 408 P49407 P60228 409 Q09472 O43251 23309 5682 P48552 5683 5684 5685 6774 5686 64215 P13686 891 Q99741 P35240 51147 Q86X95 P51531 4361 5692 5451 5693 P35222 Q9NUX5 P51532 P35227 Q96ST3 P17813 P35232 P60484 11331 668 5687 5688 Q96T58 Q96MH2 P23497 O43474 P06733 P26583 Q9Y3M2 P25490 25942 Q8NFW5 3281 672 O14908 6304 8726 10213 P05412 O43242 Q08117 P37231 P14373 5467 207 5468 54206 10468 4152 5245 P56177 22893 3066 3065 Q9UIF9 O00762 10438 P62854 9839 O43463 3070 Q13901 9612 23512 Q96QT6 P28749 7428 6595 6597 8535 11065 P10599 6591 Q9Y4K3 3084 476 P49810 5017 51763 Q13363 P38919 Q13127 P55036 4188 5036 Q16635 5037 O75376 P13631 3096 5270 Q02447 O75381 23051 51773 P46531 Q16401 Q96RL1 Q15554 Q15796 Q9HAU4 Q13131 8553 Q96IZ0 P12757 Q8N6I1 P32121 P31273 Q9UNL4 P12755 23028 Q9NY61 O75360 4193 P46527 Q01105 10197 Q13144 51548 51547 54815 Q14232 Q9BSI4 Q13148 P55055 P10588 P10589 93166 P35625 Q9NPF5 51317 P43246 55915 P43489 9421 7248 P54132 Q9Y6K1 Q03989 51564 Q15583 55922 8328 5074 Q9NRC8 7013 55929 Q9HAZ2 7014 O15055 O43524 26523 7490 5071 7251 84232 Q8WW38 P61925 7023 P49840 A1XKG3 P15822 Q9Y6M1 23468 Q9UNH7 51341 51588 Q13185 10155 Q9BQI3 6188 P38936 64426 P37840 Q9H9G7 Q99816 9208 Q96RE7 P29374 Q9H0M0 P42345 P62195 P04085 9219 Q92574 1978 P56524 9474 Q02297 7291 O75530 55294 7295 P46934 Q9UER7 Q14865 7067 Q13535 P19793 P29590 Q7Z2Q5 84619 Q9H6W3 Q9HCP6 O60264 O60260 8165 O95983 Q13547 A4D1W7 P78318 Q8TBE0 Q04206 Q9UH92 Q92786 Q9Y2B9 7088 Q15735 Q9UKY1 Q9H9B1 51720 Q14894 P08246 Q13322 Q9NP71 P30153 P11413 1789 1788 Q05516 1786 Q05513 P00734 3720 Q13573 Q9H2S9 P28065 P32320 Q9BQA5 Q9Y6E7 1312 5914 O75953 Q13107 P62191 P28074 63976 P28072 P28070 P63279 P63272 Q9HCS4 P00750 P08238 P43686 Q14683 5925 Q9Y2W7 O75820 2672 Q9UBB5 Q9NS86 O00267 3516 O60566 283248 10772 P27348 5933 Q9NS91 Q9UBC3 Q9Y2X9 54361 Q8NB12 5706 5707 5708 5709 4851 5700 5701 5702 O15524 5704 5705 Q92835 Q9NYF8 Q9Y2Y8 Q9NRY4 Q99471 P53667 5717 83933 Q9UJU2 O15534 P61289 5713 57649 Q92833 Q6IT96 P06401 4869 P06400 5716 5970 Q99460 Q9NRZ9 P56705 9099 5728 10524 400961 10765 Q9ULR5 P05129 Q12986 P51668 P51665 Q9P0J0 O14627 6829 Q8N2W9 10971 27161 5978 6827 Q9Y2T7 5991 93986 5993 O00231 Q14938 O00233 O00232 P19544 6839 3326 2475 P08651 P42771 5987 P26045 Q9NYB0 P05106 22938 O00488 O00487 O60502 P62333 2247 58533 Q9BZK7 6601 6605 5515 5518 P51693 P09958 2023 O00255 Q6KC79 O00257 10724 Q99496 5524 P40337 7704 P06213 O00425 P23396 O43159 P04637 2033 Q9NVW2 P52952 990 991 Q96RU7 6622 79813 4204 10138 Q9UQ80 Q9BT49 4221 5796 P35368 Q9BT40 84159 Q9P2K8 P48436 Q99638 7727 P49768 2290 23429 P48431 5562 Q8N726 Q99623 773 Q00535 Q96S42 P25789 55869 P25788 P01137 P25787 P01375 Q9UJM3 6660 8841 Q9UHI6 2063 23435 Q96AQ6 11218 Q9NS37 3148 P10070 Q15170 P17936 545 P10071 3146 P01127 6657 5327 5569 6670 5580 O43593 6672 5582 23409 Q969H0 25822 Q12906 Q9BZE0 Q9BZE4 O75190 Q99684 Q8TEB7 5578 P40763 P04201 4261 Q969G3 8864 7531 P37108 23411

23414 5590 O75182 Q00987 3169 5104 Q9H1A4 7529 7528 P27797 64375 O60907 P51843 3182 333 P51608 Q9H7L9 6207 5119 Q01826 Q9BT67 5371 8881 P17980 P52701 580 O14818 P58012 10320 P60568 6231 8655 P30086 Q7Z6C1 Q13485 P03950 5154 5155 O43684 O75496 O75494 7329 Q15672 Q9UQR1 7341 6496 Q13257 6256 6498 P35998 Q2M1K9 84108 Q9UBU3 116113 Q13263 9774 4087 8204 4088 4089 9775 Q9BY41 163126 Q9Y618 Q04724 P20264 11176 Q15697 53615 Q13033 P54259 9541 5187 O75469 64750 7124 11142 O15164 P54253 2909 Q96KC8 P14635 O75461 Q9UQL6 4092 P10276 Q02535 Q13042 Q99728 Q99729 Q15466 55806 Q99966 54 Q13286 5195 6045 7376 7375 8467 9314 23135 23378 Q86WV8 6282 1822 166 P01308 Q9NQB0 2917 P49721 Q9UBP4 P49720 Q96T37 P54274 P14859 O43612 7141 Q9H063 Q96T23 P49959 Q9H1Y0 Q01664 Q00577 P49711 P49715 P09038 Q15008 O75437 7157 O43623 25776 138151 2931 Q5VTD9 Q15257 Q06787 Q01094 P63167 26271 P41134 Q1PSW9 27122 P63165 O15119 1616 P45973 O15118 Q9NSA3 O94992 Q969S8 Q8IY57 P55316 Q96BD5 P67775 P60900 1869 2956 7189 92129 Q8WYK2 9126 7181 Q03014 Q86UE4 27102 P17252 O15379 Q9NSC2 O15151 Q13422 O94776 O43829 Q05655 84733 Q9UBN7 2737 Q92667 2736 O75626 Q8WUI4 27327 P41182 P62277 Q13200 150094 Q9UJX2 58487 Q9UQE7 P41162 3609 Q14781 P23510 P09429 O75604 O14495 29966 P19474 Q02363 P18146 P35711 O60341 P09430 O75832 Q9BZS1 Q13227 P55771 Q86YP4 Q96BF6 Q8N5A5 P30260 O15105 P21554

negative regulation of macromolecule metabolic process P51587 124790 9184 10657 2304 Q9HCJ0 P84022 Q8N3U4 6901 25913 Q12834 Q92729 Q12837 Q8NDV7 P51114 2316 2 P14174 57510 54487 4734 Q96EB6 O75925 Q7Z727 Q9H2G4 Q9UKV8 3660 Q9BYE7 6927 P13056 3416 811 P62258 6926 P26358 Q9UIS9 Q9NZI7 P48382 Q9NQX0 P31946 Q92949 P48380 O95343 P20823 1487 P19838 2332 P62263 P31949 3665 10642 64061 10644 10643 Q9UL15 Q9Y468 Q8IZ40 Q9UL18 4771 1020 P51787 10614 1499 Q86VE0 O14981 Q92530 6945 O14503 3690 Q92769 Q8WXF0 Q07666 Q15906 P10600 O95365 O00358 Q06330 1029 10865 1027 3685 P17480 O14753 604 4790 5883 P38398 57326 P20618 Q92993 857 6726 Q8N488 P10826 Q9UL46 P10827 Q9NR50 P19883 1050 Q9HCK5 P40692 79589 192669 Q9UMX1 4799 50943 Q9UGL1 Q9UKT4 Q92985 O96004 O00308 P14316 25988 2147 23560 3476 Q15022 10013 639 10014 Q6UWV6 135295 64682 4343 P16989 P57059 25998 641 P33076 51132 Q99750 8932 408 P49407 P60228 409 O43251 23309 5682 P48552 5683 5684 5685 6774 5686 64215 891 Q99741 P35240 51147 Q86X95 P51531 4361 5692 5451 5693 P35222 Q9NUX5 P51532 P35227 Q96ST3 P17813 P35232 P60484 11331 668 5687 5688 Q96T58 Q96MH2 P23497 O43474 P06733 P26583 Q9Y3M2 P25490 25942 Q8NFW5 3281 672 O14908 6304 8726 10213 P05412 O43242 Q08117 P37231 Q9HCE1 P14373 5467 5468 54206 10468 P01023 4152 5245 P56177 22893 3066 3065 Q9UIF9 O00762 79753 10438 P62854 9839 O43463 3070 Q13901 9612 23512 Q96QT6 P28749 7428 6595 11060 6597 8535 11065 P55265 P10599 6591 Q9Y4K3 3084 P49810 5017 51763 Q13363 P38919 Q13127 Q9HAV4 P55036 4188 5036 Q16635 5037 O75376 P13631 3096 5270 Q02447 O75381 23051 51773 P46531 Q16401 Q96RL1 Q15554 Q15796 Q9HAU4 8553 Q15797 Q96IZ0 P12757 Q8N6I1 P32121 P31273 Q9UNL4 P12755 23028 Q9NY61 O75360 4193 P46527 Q01105 10197 Q13144 51548 51547 54815 Q14232 Q9BSI4 Q13148 5054 P55055 Q9UPQ9 P10588 P10589 93166 P35625 Q9NPF5 51317 P43246 55915 P43489 9421 7248 P54132 Q9Y6K1 Q03989 51564 Q15583 55922 8328 5074 Q9NRC8 7013 55929 Q9HAZ2 7014 O15055 O43524 26523 7490 5071 7251 84232 Q8WW38 P61925 7023 P49840 O75569 P98179 A1XKG3 P15822 Q9Y6M1 23468 Q9UNH7 51341 51588 Q13185 10155 Q9BQI3 6188 64426 P37840 51593 Q9H9G7 Q99816 9208 Q96RE7 P29374 Q9H0M0 P62195 9219 Q92574 1978 P56524 9474 7057 Q02297 7291 O75530 55294 7295 P46934 Q9UER7 Q14865 7067 Q5TAX3 Q13535 P19793 P29590 Q7Z2Q5 84619 Q9H6W3 Q9HCP6 O60264 P05155 O60260 8165 O95983 Q13547 A4D1W7 P78318 Q8TBE0 Q04206 Q9UH92 Q92786 Q9Y2B9 7088 Q15735 Q9UKY1 Q9H9B1 51720

Q14894 P08246 Q13322 Q9NQZ2 Q9NP71 P30153 P11413 1789 1788 Q05516 1786 Q05513 P00734 3720 Q13573 Q9H2S9 P28065 Q9BQA5 Q9Y6E7 5914 O75953 Q13107 P62191 P28074 63976 P28072 P28070 O60238 P63279 P63272 Q9HCS4 P00750 P08238 P43686 Q14683 5925 Q7KZF4 Q9Y2W7 O75820 2672 Q9UBB5 Q9NS86 O00267 3516 O60566 283248 10772 P27348 5933 P07996 5935 Q9NS91 Q9UBC3 Q9Y2X9 54361 Q8NB12 5706 5707 5708 5709 4851 5700 5701 5702 O15524 5704 5705 Q92835 Q9NYF8 Q9Y2Y8 Q9NRY4 Q99471 P53667 5717 83933 Q9UJU2 O15534 P61289 5713 57649 Q92833 Q6IT96 P06401 4869 P06400 5716 5970 Q99460 Q9NRZ9 P56705 9099 5728 10524 400961 3784 10765 Q9ULR5 P05129 Q12986 P51668 P51665 Q9P0J0 O14627 6829 710 29102 Q8N2W9 10971 27161 P08887 5978 6827 Q9Y2T7 5991 93986 5993 O00231 Q14938 O00233 O00232 P19544 6839 3326 P08651 P42771 5987 P26045 Q9NYB0 P05106 22938 O00488 O00487 O60502 P62333 2247 58533 Q9BZK7 6601 6605 5515 5518 P09958 2023 O00255 Q6KC79 O00257 10724 Q99496 5524 P05121 P40337 7704 P06213 O00425 P23396 O43159 P04637 Q9NVW2 P52952 990 991 4686 6622 79813 4204 10138 Q9UQ80 Q9BT49 4221 5796 P35368 Q9BT40 84159 Q9P2K8 P48436 Q99638 7727 P49768 2290 23429 P48431 Q8N726 Q99623 Q00535 Q96S42 P25789 55869 P25788 P01137 Q9UPY3 P25787 P01375 Q9UJM3 6660 8841 Q9UHI6 O00213 2063 23435 Q96AQ6 11218 Q9NS37 3148 P10070 Q15170 P17936 545 P10071 3146 6657 5327 5569 6670 5580 O43593 6672 5582 23409 Q969H0 25822 Q12906 Q9BZE0 Q9BZE4 O75190 Q99684 Q8TEB7 5578 P40763 P04201 4261 Q969G3 8864 7531 P37108 23411 23414 5590 O75182 Q00987 3169 5104 Q9H1A4 7529 7528 P27797 64375 O60907 P51843 3182 P51608 Q9H7L9 6207 5119 Q01826 Q9BT67 5371 8881 Q60FE5 P17980 P52701 580 O14818 P58012 10320 P60568 6231 P30086 Q13485 P03950 O43684 O75496 O75494 7329 Q15672 Q9UQR1 7341 6496 Q13257 6256 6498 P35998 Q2M1K9 84108 Q9UBU3 116113 Q13263 9774 4086 4087 8204 4088 4089 9775 Q9BY41 163126 Q9Y618 Q04724 P20264 11176 Q15697 53615 Q13033 P54259 9541 5187 O75469 64750 7124 11142 O15164 P54253 2909 Q96KC8 P14635 O75461 Q9UQL6 4092 P10276 Q02535 Q13042 Q99728 Q99729 Q15466 55806 Q99966 Q13286 5195 6045 7376 7375 Q09161 8467 9314 23135 23378 Q86WV8 6282 1822 166 P01308 Q9NQB0 P49721 Q9UBP4 P49720 Q96T37 P54274 P14859 O43612 7141 Q9H063 Q96T23 P49959 Q9H1Y0 Q01664 Q00577 P49711 P49715 P09038 Q15008 O75437 7157 23112 O43623 25776 138151 2931 Q5VTD9 Q15257 Q06787 Q01094 26271 P41134 Q1PSW9 27122 P63165 O15119 1616 P45973 Q9NSA3 O94992 Q8WWH4 Q969S8 Q8IY57 P55316 Q96BD5 P67775 P60900 1869 2956 P52298 7189 92129 Q8WYK2 9126 7181 Q03014 Q86UE4 27102 P17252 O15379 Q8TAD8 Q9NSC2 O15151 Q13422 O94776 O43829 Q05655 84733 Q9UBN7 2737 Q92667 2736 O75626 Q8WUI4 27327 P41182 P62277 Q8WYQ5 Q13200 150094 Q07817 Q9UJX2 58487 Q9UQE7 P41162 Q9BXP5 3609 Q14781 P23510 P21333 P09429 O75604 O14495 29966 P19474 Q02363 P18146 P35711 O60341 P09430 O75832 Q9BZS1 Q13227 P55771 Q86YP4 Q96BF6 Q8N5A5 P30260 O15105

mitotic cell cycle P25054 8099 Q9Y265 23607 Q9Y266 Q969L4 2672 8091 259266 9181 9184 10657 Q9BVA0 O60566 Q7L0Q8 1213 Q96IK1 Q9BVA1 P63208 Q8N3U4 Q9ULW0 10534 Q92974 O75935 Q12834 Q53EZ4 O60216 5706 5707 5708 1104 5709 10783 5700 5701 5702 5704 64282 5705 P30622 Q9NQW6 22974 5717 Q9Y2Z0 6929 57405 P61289 Q9Y297 5713 Q96EA4 P06400 5716 P53675 O95347 Q99460 Q9NRZ9 Q96GD4 O14578 5604 P50570 P11802 Q14807 1021 P51668 P51665 22832 1018 1017 Q96DE5 3796 O75909 Q92530 Q5TB80 P07437 Q07666 O00231 O00233 Q8IZT6 O00232 79441 1029 O14519 Q8TBC4 1027 1026 P41208 P42771 P30304 P30305 P30307 P28340 Q13618 Q13617 Q13616 55142 O00487 P62333 P20618 O00139 Q9UL46 6500 P31749 Q6KC79 10726 984 Q96MT8 Q99496 989 O14777 Q9UKT4 127829 Q96FF9 Q15019 6872 6993 5422 O95271 25988 990 11107 991 203068 994 1058 995 Q99640 997 6502 55726 Q15021 5536 5537 P62753 64682 Q9Y6X3

Q8N4N8 Q9UQ88 23332 23212 Q8NHV4 641 1069 Q99871 5424 11113 8812 Q9BS18 25847 Q9BV73 5682 5683 Q8N726 5684 5685 Q7Z7K6 5686 P52732 890 Q66K89 891 Q99741 55743 Q00534 898 8945 P25789 P25788 P25787 O14965 Q16254 O43264 Q9UPY8 5692 5693 23310 O95067 Q96ST3 Q15050 5687 5688 Q00526 6790 9700 25942 O00743 10459 Q99684 10213 8607 O43242 P49450 O43482 P51955 P51956 Q9BZD4 Q9NYZ3 Q6PJP8 Q00987 P51959 P50748 324 54443 207 Q9H1A4 5347 Q96JH7 P24385 Q86U86 91754 Q99550 O00762 22897 Q99543 Q99661 Q9NRM7 Q00610 5119 3070 8881 Q7Z4H7 P17980 79980 O14818 Q08209 10200 55559 Q9UPV0 P49427 54908 Q14204 11065 P15923 351 P48729 595 Q96BM9 P83731 P43034 P21127 Q9HC98 O43683 11190 P55036 O43684 54801 7329 P00533 Q16401 P24522 Q29RF7 P11309 Q13257 7465 Q8WVM7 O75122 P35998 4193 P51808 P46527 54930 Q969Z0 10198 10197 Q8NFH4 P83876 51547 O94927 Q8WZ42 Q93008 P62826 O43663 Q8TD19 Q96BK5 54820 P24864 Q15691 Q8IX90 8453 8454 8452 P54132 Q14008 23122 23244 P14635 O75461 Q9H6D7 Q13042 8328 Q96R06 Q96CS2 Q9NRC8 6045 7013 9793 Q96KB5 P36896 26524 P49721 8218 P49720 O15182 10270 P54274 Q9Y5K6 221150 11243 Q9BRK4 Q9NTI5 Q9NVM9 347733 Q15003 11004 1956 P23443 Q15008 9212 Q8TAP9 23354 Q02750 P38936 25777 7272 Q9NXR1 Q93034 54984 Q9NTJ3 Q5VTD9 2935 9221 6198 O95835 8379 Q01094 P63172 6194 80124 27000 26271 Q9Y6G9 84967 55165 P62195 91 1978 Q13885 P78536 9232 9238 Q14978 3925 Q7Z460 P52292 22919 Q9UI95 Q6P1K2 P05067 P42695 P60900 1869 Q14980 9126 10907 91272 1877 10910 1874 O95619 1639 93323 9133 Q14511 O15392 A4D1W7 O43823 Q13309 Q8N7B1 79023 Q9H410 9493 80254 Q9UH99 P09884 P53041 Q9HBM1 1647 Q13554 O95997 Q9P258 Q96EP1 1778 4926 3832 Q13200 Q13561 3835 Q68CZ6 58480 8065 P20248 Q9UJX2 1785 Q9UQE7 5901 Q9Y6D9 P10415 P28065 Q02241 Q9BQA5 Q9HC35 Q14674 P54687 728642 O75832 P53350 27436 P62191 Q04771 P28074 P28072 P28070 P63279 P00519 121441 O60232 P30260 P24941 900 51512 P43686 P21675 Q14683 5925

cellular component assembly P25054 3880 25909 P49023 P54619 1460 P52434 9185 Q9BYG3 4728 Q8IYN9 P84022 P21926 O14788 6901 4725 25913 Q9H4L7 P30876 25915 P08727 P16104 2316 2 5829 Q8IUE6 Q96QZ7 P14174 800 10420 10421 Q9UKV5 P10809 O75925 Q9H2G4 P28300 Q12846 Q9NQW6 54496 3416 Q9UKW4 4983 811 5830 3655 P31946 Q92949 1491 P54646 Q676U5 55355 P16333 P62263 P04350 3667 64061 5604 829 P08758 1022 10609 1020 114548 P16389 P15056 P16144 1499 830 1012 55125 3678 O14744 O14745 P07437 Q9H0H5 Q12874 Q9Y478 3690 Q12872 Q9Y239 P32856 3692 Q8WXF0 Q9BUB7 Q9BUB1 1025 841 Q92522 O60890 847 Q8N257 6714 P04179 Q9UGJ1 266812 55142 Q8TBB1 P84243 Q71DI3 Q92994 857 5879 Q9Y490 P51575 Q13509 Q13748 10844 253980 P40692 Q96MT8 6733 5888 P09601 Q9UL54 Q9UKT4 P04156 O95391 23322 P50416 P11047 203068 1058 Q8WWY3 10013 55723 6505 P62875 P25445 135295 5430 Q7Z6J4 5432 O95163 3010 Q6FGD7 Q9BU61 3009 3008 O60610 641 3007 3006 402 O43290 8932 7846 408 P49407 8936 Q09472 5440 5441 3024 O00560 3265 Q9UKN8 891 Q9H4B7 3014 5434 O43264 Q9UMS4 Q9Y697 5213 Q658W2 P35222 Q9NUX5 P14784 Q96A08 Q99733 3028 Q00403 P23258 P26583 O00505 Q9Y3M2 4131 Q6FGG2 6311 9821 P50613 P51946 10458 Q8WUY8 673 Q96KK5 10451 P55072 P49450 5471 7410 Q5JSP0 O60814 80705 29079 P36382 P14373 Q96QV6 688 54443 Q15070 4150 4154 25929 22893 Q12809 O00762 25923 Q5TAL4 Q96P20 55785 7415 7414 4149 P62851 3070 Q86UX7 6341 7430 P07814 Q7Z4H7 466 P17858 55559 7428 8772 Q14686 Q14209 11065 Q16625 Q9H0U9 Q6NZI2 3084 4171 P13861 230 P07196 Q8NCD3 P83731 5018 P43034 P49815 Q16637 P62805 8301 Q16635 P62807 O75376 79084 O75381 Q9NVP2 51773 Q9NRH3 54801 7205 Q9BSJ2 Q9H9E3 Q16401 Q15796 Q15797 P12757 9404 Q8N6I1 O75367 P12755 4193 Q6DD88 Q01105 Q96RK4 P55290 Q9BQE3 Q9BSI4 6386 P18074 Q16658 Q86XR8 P68133 P04908 P68371 P36954 Q9NVR5 Q4AC94 267 P23416 P68366 Q6PKD3 P08034 O94817 P43005 P78347 Q13158 Q8TDY2 8576 7248 O75347 P54132 P23229 P35221 51564 Q9Y4G6 Q02413

Q14011 O15294 Q96CS2 10181 7013 8348 P36776 5071 7249 8338 P49848 Q9NRD5 9685 7023 10392 P13647 A1XKG3 Q08188 P48509 51340 222484 Q9NVM4 55704 Q16695 P08069 8349 9439 Q16698 347733 7277 Q9BX66 5092 26747 Q15370 3909 Q6PID4 Q9NRF2 1729 P78362 Q9BWT7 P02533 Q14289 9223 Q13753 9463 Q96RE7 P28289 Q13751 P12931 6194 7283 Q4VCS5 P04085 Q92574 3911 P54105 9474 Q8IX30 Q02297 55054 Q3V6T2 7052 Q9BSB4 P24928 Q9BUF5 1742 117177 Q9HD26 Q4LE28 P98174 P29590 P55209 84617 P56537 Q92793 Q92791 O60264 Q92558 O60260 93323 P07339 91147 P34932 80254 P43405 O43707 Q8TBE0 Q07955 Q92786 P53041 50628 O95996 O95999 P35610 O00186 7082 P35612 P35611 P63000 60673 Q9BUK6 Q9NVC6 P35609 P14923 O43920 P19387 P30153 P19388 7094 23092 3728 Q05513 26121 Q7Z2W7 375790 P22303 Q9NVD7 P32320 3737 Q00059 2644 2643 2885 Q04656 O15212 Q9BQA1 O75716 Q14677 O15230 Q15526 Q9NP98 9054 79065 Q9UNA1 P67870 902 P08237 Q13112 P21675 O15228 P06899 23607 Q9UBB5 O00268 Q9BVA1 10772 P27105 O15530 O95680 P16471 Q92845 928 Q96NT1 Q05397 Q9BXC9 29127 1355 P32929 O15533 Q9UQB8 4869 5716 Q9H814 1374 Q6P2C8 Q9NRZ9 Q7L576 Q13829 P17302 2697 6810 Q6NXT2 6814 Q9UHR4 P04233 3308 P07305 P30566 O95477 3320 P52756 O00468 83706 P07550 P62316 P04040 P09972 P62318 4646 P62314 P08648 Q9UHK0 2243 1153 O00233 P30793 79441 3329 P18206 1387 6834 5987 Q9UM54 5747 P07766 P05106 6850 4673 Q06203 Q13618 Q9NS69 P42768 P61244 284119 P15153 2244 P61006 O14640 977 O43182 5518 Q9Y375 Q5QNW6 Q03164 Q9P0M6 440193 Q92621 Q6FI13 4676 989 P40337 P04275 Q9BZJ0 Q9Y2W1 P06213 4690 Q08043 P07900 6872 P04637 Q9Y6W5 6631 2033 P14678 P53803 O95271 P52952 P62993 4686 999 6624 5536 Q15389 P62753 P01112 Q15149 6883 10383 5796 P51636 Q99880 P07919 2040 P50542 2280 Q99877 2039 Q96S53 Q99871 Q15154 6633 4214 6878 Q15393 Q96S59 P02675 6637 4218 255626 Q99879 P49768 6892 5563 Q7Z7K6 P14416 2054 P52735 773 Q00535 6646 P01137 Q7Z3C6 Q9UPY3 Q3ZAQ7 Q16254 O00217 Q08495 8841 5576 Q9UHI6 Q08257 221613 11218 Q93079 3148 Q96RR1 Q93077 P61457 P01127 P62308 P61218 P62304 23647 O43593 6430 Q8N307 6431 Q9UHB6 Q07157 3162 3159 6426 5339 Q9Y512 Q9Y4P1 10580 64127 Q5HYA8 Q9ULK4 P68032 5590 P18846 Q00987 P50748 324 4015 9702 329 7529 5108 P27797 P50750 Q9UHD8 Q9NU22 P12036 P15311 330 56993 7536 5116 5371 Q60FE5 Q12933 P16403 P16402 25813 P16401 582 10569 P51617 O14817 P52948 Q9NPJ6 9967 Q01813 10093 Q15654 4297 Q8TEW0 Q9BY76 P13984 6470 351 355 P09493 23165 118 Q7Z6C1 Q13243 Q8NFJ9 Q13485 P47755 O94906 Q13242 Q13247 Q13489 P03950 5154 O00716 9997 5155 Q9BTM1 Q15428 Q16514 19 O75494 Q13492 Q13490 84790 6498 Q9NWS0 54930 P23527 Q9UDY8 P09234 P17096 Q9BZW7 P83876 Q8NFH8 Q9HC77 O94927 Q8WZ42 8443 4086 8683 4087 4088 Q9BY44 4089 Q16777 Q16778 P0C0S5 163126 P29084 5170 P34896 Q02790 Q9H492 P52907 P0C0S8 Q7L7L0 7106 P60763 P62942 Q15459 10291 8452 Q8N5M1 Q16787 O15164 7126 P14635 O43639 4092 154 Q00597 121512 158 Q9H6D7 Q9UMD9 23363 84365 Q02539 55806 P45379 P49736 7132 5195 Q09161 9555 8467 P13796 Q86WV8 P35568 Q02763 1828 Q99714 P02671 Q8TCU4 8473 P00492 Q8TCU6 Q5SSJ5 P54274 Q9Y5K6 P58876 P36404 7141 Q99700 Q96T23 Q9H1Y0 P68431 Q01664 56916 P49711 Q14152 7157 P22492 P14625 P14868 23354 25776 Q02750 8481 138151 51474 55835 10048 87 Q13077 P53365 Q7KZN9 O75665 P12814 9341 2702 26271 P20671 Q6NVY8 P46821 Q9Y5E9 Q9UBK2 Q13885 Q96QB1 Q13887 9351 84708 22920 Q05682 55172 P05062 P05067 2959 7186 P52298 Q15836 O75643 9368 O95613 10907 57120 Q96M96 Q13418 O43815 6093 O75880 7185 9361 7184 O43819 1874 2961 2960 1870 O15379 P21980 P23921 2969 O15151 Q14511 P19438 56288 2972 Q9UBN7 Q8WUI4 8290 P30047 1653 2742 Q92499 O95400 Q8WZ19 Q2NL82 O95405 Q68CZ6 26005 Q68CZ1 27339 1666 P21579 Q96QF0 Q8WYP5 1429 P21333 P33778 P10415 Q6ZV73 P12830 Q8TEQ6 P19474 59341 P10412 3611 Q14790 3852 P09430 Q13464 O75832 Q15648 23192 Q96BF6 Q9HC29 O15105 Q71U36 Q13233 1207

nucleobase, nucleoside, nucleotide and nucleic acid metabolic process O75947 8099 Q92979 Q9Y265 O75940 Q12824 O95551 10657 Q9BYG3 2305 1454 1453 Q00839 P05455 4968 Q9H0D6 Q9H4L7 O75934 P30876 P52209 P16104 10667 Q9Y285 O75937 P10809 Q96EB6 P27694 Q9UGN5 Q9UKV8 P27695 3661 4507 1478 Q9Y295 55109 Q9UKW6 3659 P26358 10632 Q99583 Q12857 55110 Q99575 55119 P26368 10642 Q9UL18 Q9Y223 Q9UGH3 Q8IX01 1022 Q9NSU2 P51787 27043 1017 79577 P54819 Q9Y230 O75909 5859 Q12874 Q12872 Q8WXF0 Q9H0H0 Q15904 1029 O14519 10625 57794 1025 Q5C9Z4 P20648 Q9BUB4 P05023 P05026 Q9UGJ0 Q12888 5883 P38398 Q9BQ90 Q96MU7 55149 Q9Y250 Q92994 Q92993 P10827 P10828 5892 6741 5411 1050 55152 27032 10606 253980 P40692 27037 55159 O60870 10607 6733 P04150 5886 5887 5888 O96004 8930 10250 5422 P50897 11108 Q8WWY3 P62877 8924 Q15020 Q15024 P62875 6749 Q15029 5430 5432 Q9UKM9 P35244 P35249 Q03933 641 P35251 196441 5424 8939 P49407 P60228 5440 5441 6772 5442 Q9UKN5 O96020 Q9UKN8 346171 Q66K89 5434 51147 Q15046 P23246 Q9UL03 Q86X95 A7MD48 O14979 P17812 P35232 Q00403 668 Q15054 4116 P57081 O00746 6311 245972 Q9Y5Q8 23524 672 P15692 Q96A72 5471 Q9Y5R4 23536 Q6PJP8 Q9NWV8 688 689 P62847 P62841 4150 Q9BQ15 4152 4154 P13489 Q9Y5S9 P17844 P13010 Q99543 5478 4149 P62857 P56182 O43463 Q9BQ04 23517 Q92905 Q9UKK9 O75031 Q92900 P56192 P49427 4172 4173 4174 Q14686 4176 P37837 Q14209 Q16864 P55265 Q9UPN6 Q6NZI2 4171 Q14690 Q14694 Q8IWZ8 8543 5036 79084 60489 Q9NPD3 Q13370 Q96RL1 P46777 Q15554 Q9HAU5 P24522 P35638 P61968 P56134 8555 P32121 4191 Q96IZ7 P00568 51547 8563 Q9BYU1 9410 P36957 P62826 11051 Q8IWX8 O43542 8565 P36954 Q9NPF5 P61978 P43246 9421 Q14249 P78347 Q9Y6K5 P36542 26512 271 Q9Y6K1 Q93062 Q04837 23481 51567 10181 55929 11030 P22392 O75575 26523 23016 Q93050 Q9NY12 118460 P49848 O75569 23463 P24539 51585 9439 Q6I9Y2 Q9HB90 26986 Q01780 5093 Q96EU6 51593 Q9H9G7 P78362 11017 9465 9463 Q6P1J9 P29375 P29374 P29372 Q7Z589 27000 2820 Q13761 9477 Q13769 Q9H0L4 O75531 1503 P24928 79005 1982 Q9Y6H3 Q6P1K2 Q5D0E6 P55209 79035 1994 79039 Q8TBF4 P78316 O95989 Q8N8D1 3704 Q9Y2B5 Q07955 P53041 50628 P52597 Q13315 O95997 P20594 84881 Q8NEC7 Q14653 Q9H0P0 P46736 Q9H9A7 26121 P61077 Q9BUJ2 P22303 5901 9045 Q9NP81 P12956 O75964 Q00059 Q9BQA5 P61088 Q96AE4 P43694 Q9BQA1 O95544 Q14676 Q16854 5927 5928 P00519 P63272 23076 P24941 P43681 84893 51514 Q14684 Q14683 Q5FWF5 P10914 O95677 285672 Q92858 P67809 399687 9061 P30520 Q9UBB9 5939 P16220 10772 5931 P49137 Q9UK45 5935 P0CG13 Q9NS91 Q9UBC3 Q07343 9077 55696 P18887 55215 10785 Q9NWB1 Q9NRY2 Q9NRY4 Q8TED0 O60547 P18858 O60543 P18859 1111 Q92830 P03372 P49591 P46087 Q9H814 Q9GZX7 9093 Q9NRZ9 P49590 Q99459 3304 3784 P49588 P07741 P30566 5981 5983 O95232 P52756 6829 Q9P2N5 1137 Q6FHQ0 P61221 5976 27161 6827 Q10570 Q13601 1153 Q14938 O00472 O95243 Q6PIW4 Q9NS56 3329 6839 P08651 Q6XQN6 O95257 4670 4673 Q9Y4Y9 O60508 1161 O60506 O60502 80222 10714 P61244 O14641 5511 O14640 5514 Q9Y4Z0 Q92878 5515 Q9UNY4 P08621 5518 Q13620 10725 10724 P61254 10728 157570 4677 4678 P06454 O43159 6872 6871 P04637 Q5VYS8 2034 P16615 4686 Q16236 5536 P62995 Q9GZR7 Q96LI5 Q9C0K0 P25100 6883 P48201 4221 23210 P12268 O76074 Q96Q15 51251 6878 Q15393 O43172 56946 P01100 Q8N726 O95299 P11388 P11387 O60942 Q53X93 5557 P61201 P01137 5558 P01133 O43143 6421 11218 Q9NS37 P09086 9924 57819 P61218 2070 Q5TKA1 6430 6432 6431 6434 4255 7764 22794 Q96PU8 Q8IZL8 Q02930 8602 6427 6426 8607 Q8N302 2081 23658 5591 P46063 5111 23650 O76021 Q99437 328 25804 2091 P22087 Q8TA86 Q8N2M8 Q9UNP9 2099 P50993 Q9NW64 333 55660 91746 Q9UNQ2 A2RTX5 4287 P39748 O00411 P52701 Q9NVV4 P15313 Q08211 221656 79622 P58012 9967 5141 5142 Q14566 4297 8654 Q9UKD1 P30085 P13984 6470 4291 351 353 Q9UBS4 P09012 5139 P62913 9978 Q13243 Q13242 Q13247 P20290 O43687 7334 16 Q15428 7336 Q15427 26205 80198 367 Q9UBT6

51654 11198 7341 Q15434 Q14103 9525 P62937 23144 Q9UKF6 Q9Y606 P49916 P49915 85437 P17096 11165 11168 Q16774 Q9UBU8 P83876 P21589 O43660 11171 8683 9533 7358 P29084 P34896 Q96HW7 11176 P23771 Q15459 10291 O15160 9541 O15164 2909 25788 P27708 Q8NI36 2908 Q13287 Q9NSE4 Q14134 Q14137 5198 Q09161 Q9Y5J1 7372 Q8NI27 11157 6059 6050 11129 Q99941 P49959 P50914 51222 56915 56916 2926 11128 P62906 116138 P14868 P14866 8241 221264 10286 10285 Q15014 2935 P78424 9100 Q96QC0 Q14966 P52272 Q12788 92105 Q15819 P33992 P62081 P33993 P33991 Q96DI7 84967 26270 Q9UBK2 9114 P55795 Q14978 Q8WWH4 O95602 Q86WJ1 Q96I24 P43304 P67775 Q6IN84 84950 P05062 P52292 Q52LR7 P05067 2959 P08579 2956 Q9UBL3 P52298 9128 9129 O75643 9126 80145 283989 2961 2960 P04183 Q8TAD8 Q9BTC0 2969 P09874 29777 1642 2972 57379 P09884 134430 1647 P30049 Q15853 Q9UJV9 Q9BY32 Q14527 1660 Q6NYC1 P53567 2987 1655 P22415 1653 Q03468 Q92499 1659 3835 O95644 51606 64919 1665 Q9BXP5 P17081 Q9Y5B9 Q9Y5B8 P09429 Q13216 Q8TEQ6 P55769 P35711 4705 1676 2521 Q6P4R8 P09430 P08107 Q9P2Y4 Q14558 O95661 4719 Q96HR8 Q14562 1207 P51587 81605 P52434 O00148 P17676 Q8TDD1 2547 P84022 Q9H2H8 P28335 25913 P08729 P28331 Q8NFZ0 O60216 80755 54487 P49247 10421 64282 P62249 Q9H2G2 Q9BRX9 3420 O00167 3417 54496 6929 Q8N684 P26599 Q5TAP6 3419 P31942 Q9UIS9 P31943 P08708 P99999 P19838 P62263 3421 Q13951 4772 Q14807 P50213 Q8WVC6 P39019 Q5T160 10856 Q6PI48 80324 Q9BWG6 833 O14744 P51553 4780 5631 Q07666 80308 Q8WVB6 O95363 Q06330 27297 167227 4775 O14757 Q92766 4790 P28340 Q9NR48 Q9BWE0 80347 Q96CA5 P11908 5636 22826 22827 Q9H6R4 22828 Q8NC51 Q9NQT4 Q9NR55 Q9NQT5 Q9HCK5 2139 192669 865 4799 P06576 11340 Q86V81 10492 O00541 O95391 3481 25988 51362 Q96T88 3479 P25205 7832 84289 P23025 135295 P51991 4343 P51513 Q96T76 O14717 O43290 Q7Z6J9 Q9BRP8 P49643 O43294 408 409 O43252 O43251 P49642 Q9BW92 O00567 Q9BW91 Q9NUW8 O14727 126789 Q99741 Q96T60 3014 Q8IYD8 Q9UN42 29086 10476 P61326 Q15287 Q9UMS4 4361 84292 3035 114799 Q9NUX5 Q96ST3 11338 3028 55759 10488 84296 P26583 8732 7884 P50613 P51946 23764 25942 Q8WUY8 P60891 P31939 10450 P55072 3054 29079 Q9HCE1 Q96SZ6 55775 5226 25929 7422 Q9BRT9 5245 P52815 22894 Q9UIF9 22897 79753 10438 Q5TAL4 7415 10432 8761 3070 Q13901 Q5VTL8 P07814 P49674 Q9UIG0 Q96C86 55796 56647 O43791 53938 Q16629 6598 57062 Q01130 472 474 476 477 P33240 478 Q16630 Q8WW01 Q9C0C2 P38919 Q13126 Q16637 3091 Q16633 P13637 6125 O75376 481 483 Q504Q3 Q9NW13 488 51773 24144 6117 P22102 6119 6118 Q15796 Q16649 Q15797 9643 Q9UNL4 O43776 490 O75362 29803 495 498 Q01105 P56385 P56381 54815 Q8WVM0 Q9BSI4 Q13148 P35869 9656 P43490 P18074 P18077 O75351 144983 28960 Q9P2E3 Q13151 Q16665 Q49AN0 51319 P54136 O15287 P54132 P35453 Q8IZD4 Q14494 Q14493 Q14498 51329 Q14011 Q9NRC8 7013 7014 P46100 P36776 7490 Q9NRD1 11277 Q9NRD5 Q86W42 P98175 P98179 Q02880 25885 51340 54853 Q9NVM4 Q96PK6 11243 55703 P47897 Q9GZL7 1716 Q15365 O75319 P21283 Q08170 P21281 Q09028 25896 6182 O75792 26747 Q15370 P27815 P48730 Q99814 P98194 Q9NRF9 P78545 P54577 9221 O95714 57461 P78549 6194 10813 Q9H6Y2 P41252 P62195 P41250 O15234 65083 1736 Q13523 P54105 9232 22803 Q9P1Y6 O75771 1743 27257 Q9NVH2 P42574 O60293 7068 7067 Q5TAX3 Q15714 Q13535 Q9H307 Q15717 Q8IZ69 Q9NVI1 O60264 9255 Q32P51 P34931 83759 Q9BWH6 1763 29896 29894 Q9UET6 3945 7083 9261 4809 51720 Q8WVK2 4800 51726 2626 51727 Q9NVC6 51729 Q9P1U0 Q13569 P19387 P30153 P19388 P11413 93100 1789 1788 1786 O60256 57038 Q13573 3980 P32320 P78527 2643 Q04656 Q14432 3978 Q9Y4A8 51747 Q9P287 9296 4839 4830 O60231 Q9HCS7 902 4833 Q13112 Q13595 P21675 Q969L4 O00268 Q06265 O00267 3516 10535 O00273 P60174 Q9ULW3 4841 Q9Y2X3 Q6NZY4 Q92614 P48047 4860 Q9NQ55 51096 Q9UI12 5705 Q9ULX9 Q66PJ3 Q12968 29128 O60306 57646 P12081 Q9Y2Z2 Q9Y2Z4 Q12972 4882 Q6P2C8 Q13829 O60318 10524 10528 10521 5725 10523

Q6PCD5 O95478 P28482 Q12986 1385 57661 29102 P62316 P84103 P62318 5733 56339 P62312 58517 P62314 Q9Y2T7 Q96SB4 Q9UHK0 Q96SB3 Q5JTZ9 P19544 P30793 Q96SB8 Q8WYH8 10987 2237 79444 56342 1388 P54709 P42771 Q9NYB0 22934 P30304 P30307 22938 Q06203 Q9H9Y6 Q56NI9 284119 5757 161823 P51693 P27144 Q13868 O00255 P54727 P54725 Q9P0M2 Q03164 55035 57697 Q9Y2W2 988 Q92620 Q6DHV7 Q9BZJ0 Q9Y2W1 509 O43395 6631 Q9BV90 P14678 P53803 O60725 P15531 990 P11172 Q96RU2 54512 995 513 997 514 515 6625 P61421 6627 518 P62753 Q9UQ80 54517 P07910 220988 51010 521 522 523 23451 51013 6633 Q9Y6Y0 526 8812 528 10147 Q99638 6637 P01344 8815 P01343 Q86U44 Q86U42 P26640 O95059 Q9NU63 O60701 P26639 533 535 10594 P49756 537 6647 539 Q9UPY3 23438 23439 6660 P40938 P40937 Q9Y333 Q9UHI6 23435 Q96B26 P12004 3149 3148 Q96RR1 545 546 3146 8833 Q96EY1 P62308 Q9ULR0 O43390 P62304 7508 Q9UQ35 7520 Q9UPR3 Q12904 23400 23404 3159 Q15185 7518 7517 Q9Y2L1 6201 5356 3178 Q9ULK4 23411 Q01831 Q08462 65220 3181 P50750 P49321 3189 3188 3187 3185 3184 O14802 51067 Q8N8Y2 7536 9716 Q9H5H4 3192 3191 8880 P27540 3190 9733 5378 Q969E8 P51610 580 10569 P52948 Q9NPJ6 79869 54107 6217 Q9UPV0 Q8N9Q2 P15927 O43432 P15923 57187 29947 Q96FV9 6229 Q8IU60 O94906 5393 P03950 5394 O00716 Q16514 O75494 54921 O94913 6239 Q76FK4 Q16526 P31153 29922 23381 P25705 115024 Q9NV06 P42285 P09234 P22674 Q16531 54931 54938 51426 P00441 51428 4086 10081 4087 8446 4088 P22692 O75478 9775 O75475 P12883 142 1808 P20020 Q8IXZ2 9785 8458 P54252 P40926 P54253 7126 P38606 8450 P10275 O75460 4094 Q00597 158 Q8IYB3 Q99728 8449 84365 Q16555 P49736 8467 O43889 23378 Q9Y3F4 Q8NFM5 Q01433 Q9BRL6 Q13057 Q15233 Q99714 P00491 Q96FX7 P00492 P54277 P54274 O75444 7141 P11586 Q99700 Q96T23 Q00577 88745 Q99708 P22234 9319 P49711 Q16576 9318 P49716 P49715 Q49A26 7153 7155 7158 Q9BVS5 Q08050 P39905 7157 7150 1841 92345 Q13873 7167 22913 Q7L0Y3 10929 7161 P18124 O43837 1854 P42224 10933 Q9Y3A5 Q9HC16 P63165 P09651 Q969T7 1856 Q13887 P22626 Q9Y3B4 10949 Q08945 Q9UHX1 Q9Y3B2 P09661 10940 Q969S2 10907 O43812 P11940 O75400 26015 286826 26019 9360 9361 1877 P00846 4907 1870 P23921 O95861 P53396 Q13427 O75874 Q13426 Q8NAV1 Q8NB78 10921 1400 1408 1407 4913 Q13435 P33316 Q9NYH9 Q8WYQ5 Q8NFF5 P43115 Q8IY92 O95400 Q8WZ19 Q08J23 Q96G21 27339 O43809 Q8N0Z6 58487 Q9UQE7 Q8N0Z8 27332 P23511 Q6P2Q9 Q15631 Q8IY81 P84090 3615 84305 3614 Q5RKV6 2762 57169 Q9HC36 O15350 Q15643 Q15648 Q86YP4 Q9BVJ6 O15347 Q15653

cell cycle P25054 134359 8099 Q9Y265 P28562 Q9Y266 25909 Q12824 8091 259266 9181 P51587 9184 10657 2305 1213 Q96IK1 P84022 Q9BYG4 Q8N3U4 3875 P27469 Q92974 O75935 P08729 Q12834 Q53EZ4 P16104 O60216 64282 P30622 P27694 23708 Q9NQW6 79791 Q7L5Y9 6929 P62491 811 Q9Y297 Q9Y6A5 Q5TAP6 Q96EA4 O95347 55355 Q96GD4 O14578 5604 Q14807 1021 10609 22832 1018 1017 79577 1499 55125 Q08999 O75909 Q92530 Q5TB80 P07437 Q9H0H5 22846 Q07666 Q15907 Q8WVB6 1029 O14519 Q8TBC4 1027 1026 O14753 O14757 P28340 55142 P38398 55145 Q9BQ90 P20618 Q9Y250 Q6NUQ1 O00139 Q9UL46 P53990 5892 6500 P31749 Q7L8A9 Q9P209 P40692 Q96MT8 5888 O14777 Q9UKT4 127829 P04156 Q15019 6993 5422 Q09019 23326 25988 11107 Q96T88 203068 55722 1058 6502 55726 Q15021 P25205 5898 10015 O96017 64682 23332 P57059 Q8NHV4 641 1069 402 O43290 Q14186 5424 11113 Q9BS18 Q13098 Q14188 Q09472 5682 5683 5684 5685 5686 3265 890 Q66K89 891 Q8IYD1 Q9H4B7 Q99741 55743 898 3014 8945 51147 O14965 O43264 4361 5692 5693 P40818 23310 114799 P35222 25975 Q96ST3 Q15050 P23258 5687 5688 6790 O43236 9821 7884 6794 P51946 25942 O00743 10459 Q9NUQ3 672 Q96P48 P38570 10213 O43242 P49450 O43482 10460 P51955 P51956 Q9NYZ3 Q6PJP8 P51959 54443 79723 207 P24385 Q99550 O00762 22897 Q8NFT6 55787 Q99543 Q00610 Q99549 O43463 3070 8766 Q7Z4H7 5499 Q8NHX1 Q9Y3P9 79980 P28749 10200 55559 56647 Q92900 P49427 4172 Q14204 4176 11065 6598 11064 Q9UPN3 4171 472 P48729 Q8NCD3

5017 P83731 P43034 Q8NEH6 P55036 5036 O75376 24149 23291 Q9NY59 6117 54801 P00533 Q16401 Q15554 P24522 Q29RF7 P35638 P12757 7465 Q8WVM7 Q9UNL4 O75122 4193 P46527 Q6UVJ0 Q96RK4 10198 10197 Q5VUA4 51547 Q93008 Q16659 P35869 57092 P62826 9656 O43303 O75351 54820 Q8IX90 P43246 9662 Q8TDY2 P54132 Q14008 23244 Q14493 8328 10179 Q13164 Q96CS2 Q9NRC8 7013 7014 26524 7251 221150 11243 1718 Q9NTI5 Q9NVM9 347733 11004 1956 P23443 9212 Q8IUX8 Q09028 P38936 P11234 P11233 7272 Q9NXR1 Q93034 Q9NTJ3 Q99816 Q8WW12 Q9NPB6 9221 Q6P1J9 6198 8379 6194 7283 27000 Q9Y6G9 P62195 P04083 P98161 1978 P78536 9232 7057 9238 Q13526 Q8NA72 O75771 9230 55294 3925 1982 Q6P1K2 Q9UER7 Q13535 O15264 P29590 Q92791 O60266 Q9NVI1 50855 93323 A4D1W7 Q13309 79023 9493 80254 P41220 Q9NVJ2 Q9UH99 P53041 Q13554 Q13315 O95997 Q9P258 Q96EP1 1778 Q7Z7A1 Q13563 Q13561 Q6N021 1785 2873 1540 5901 Q9Y6D9 3980 Q8IUQ4 P28065 Q02241 P41279 Q9BQA5 Q14674 3978 Q14676 27436 Q9P287 P62191 P28074 P28072 P28070 1326 5928 P63279 P00519 121441 O60232 26586 P24941 900 902 51512 Q13112 P43686 P21675 Q14683 5925 Q5FWF5 23607 Q969L4 2672 Q8IXM3 O00267 Q9BVA0 O60566 Q7L0Q8 4609 Q9BVA1 P63208 55207 Q9ULW0 5933 10534 P07996 P52564 P0CG13 P27361 5706 5707 5708 1104 5709 55215 10783 5700 5701 5702 5704 Q9UK53 5705 29128 22974 4628 5717 Q9Y2Z0 57405 29127 P18858 1111 P61289 O75807 P62136 5713 4627 4869 Q92831 P06400 5716 P53675 Q99460 Q9NRZ9 9099 Q99459 Q8NEU8 P62140 P28482 P50570 P11802 P51668 P51665 90480 6829 Q96DE5 3796 5976 Q96SB3 O00231 O00233 Q8IZT6 O00232 79441 6839 116840 5500 5501 P41208 P42771 Q8N137 P30304 P30305 P30307 Q13618 Q13617 Q13616 O00487 Q56NI9 P62333 5997 Q92878 5515 Q9UM63 Q13620 O00255 Q6KC79 10726 984 Q99496 157570 988 989 4678 Q96FF9 26060 26959 6872 P04637 5300 2033 O95271 990 991 994 995 Q99640 Q66GS9 997 Q9C0K7 5536 5537 P62753 P01112 Q9UQ80 4221 Q9Y6X3 5310 Q8N4N8 5311 Q9UQ88 23212 Q93096 Q96RT1 Q15392 Q99871 P46013 P01106 8812 25847 Q9BV73 P48431 Q8N726 Q7Z7K6 Q9NS23 P52732 Q00534 P25789 P25788 P01137 Q8N960 P25787 Q16254 Q9UPY8 8841 O00213 O95067 301 545 10362 Q9Y580 Q00526 6657 Q5TKA1 9700 Q969H0 O60925 Q12906 Q99684 8607 2081 5596 5597 5598 Q9BZD4 Q5T686 Q00987 P50749 Q99675 P50748 324 Q9H1A4 5347 P05783 P27797 Q96JH7 Q9H5I1 Q86U86 91754 P49321 Q9UPT9 Q9UHD8 Q99661 Q9NRM7 5116 5119 5371 8881 P17980 P52701 580 Q9NVV9 O14818 Q08209 10320 Q2M2Z5 Q9UPV0 54908 Q14566 Q8TEW0 11186 P15923 4291 351 595 Q96BM9 9738 Q7Z6C1 Q8WUM4 P21127 Q9HC98 O43683 11190 O00716 O43684 Q8TF76 54921 7329 84552 P11309 Q13257 6498 9525 64975 P31152 P35998 P51808 P49916 54930 Q969Z0 Q8NFH4 P83876 Q9HC77 O94927 Q8WZ42 4088 O43663 Q9UKG1 Q8TD19 Q96BK5 9770 P24864 Q15691 8453 8454 8452 23122 P14635 O75461 Q6MZP7 8450 25788 P35579 O75460 P35580 Q9H6D7 10296 Q13042 Q99728 P49736 Q96R06 6045 9793 Q96KB5 9798 163786 P36896 Q14141 Q9NQB0 P49721 8218 P49720 O15182 10270 P54274 Q9Y5K6 Q9UKB1 P36404 P49959 9319 P01579 Q9BRK4 Q15003 54737 Q9NQC7 Q15008 Q08050 7157 116138 Q8TAP9 23354 P36873 Q02750 25777 1843 1841 Q9NZ56 54984 55835 Q5VTD9 2935 O95835 Q9NNW5 P33993 Q01094 P63172 7161 80124 26271 Q9Y3A5 84967 55165 Q7RTN6 91 O15355 1616 Q13885 Q9P0W2 Q14978 Q8WWH4 O75410 P67775 Q8TAE8 Q7Z460 P52292 22919 Q9UI95 P05067 P42695 P60900 1869 Q14980 2956 Q15831 9126 O95613 10907 Q03014 Q13418 286826 91272 1877 10910 1874 O15379 O95619 1639 O60381 Q86UE8 O15151 Q13422 9133 Q14511 O15392 Q6R6M4 O43823 Q03001 Q8N7B1 Q9H410 27352 55198 56288 P09884 Q9HBM1 1647 P54652 4926 3832 Q13200 3835 150094 Q96HU1 Q68CZ6 58480 8065 P20248 Q9UJX2 Q9UQE7 Q8WYP5 3609 Q8WWK9 O75604 P10415 377630 P84090 3611 Q5XUX0 Q9HC35 P54687 O15350 728642 O75832 P53350 Q13227 Q04771 3621 P30260

metabolic process P28562 3638 10657 2305 2304 55561 55568 Q5QP82 2300 Q8N465 4967 4968 Q9H4L5 Q9NXC2 Q9H4L4 Q9H4L7 P30876 Q7Z4N8 O14561 P52209 Q8NE71 P19623 10667 2316 2 55572 P42858 P39210 Q9UKV5 136319 Q9UKV8 3661 Q8NE63 P62495 2324 P48147 Q9UKW6 3659 10632 P54886 3672 Q13705 P21917 P21912 10645 2335 10642 O14578 10643 Q9UL15 Q8WXG6 P51784 Q9Y5Y6 Q9UL18 Q8IX01 1022 1021 1020 Q06587 P50454 P51787 10613 1018 1017 2348 79577 P21964 10616 P54819 Q92530 26191 3692 Q8WXF0 Q13724 Q9H4P4 6720 Q96J02 1029 P42898 10626 O14519 10625 1025 Q9BUB5 P20648 Q9BUB4 6711 P05023 P07686 6714 P05026 Q9UKR5 O95136 1040 79595 391627 P20618 6721 6723 Q9UL46 O95147 6741 5411 Q38SD2 Q8IWL3 1050 10606 253980 57332 1047 10607 6733 Q9UL54 64432 Q9Y5U4 5422 Q9UKL6 P50416 11108 11107 Q8WWY3 6749 Q9BTU6 Q9Y676 O95169 P49411 5430 112399 5432 Q9UKM9 O95163 Q9NWZ3 P57059 115426 1072 51132 Q9Y680 84033 5424 11113 P49407 P02794 1080 84061 5440 5441 6772 5442 Q9UKN5 Q9UKN8 346171 Q66K89 P14780 51141 51144 5434 51148 Q14197 51147 Q9Y5X9 P50440 Q9UL03 Q86X95 Q8N3J5 Q9Y697 A7MD48 O95182 Q53H96 Q9H4A9 P17812 Q00403 O95190 5445 6776 4116 P62424 P57081 6790 Q5VVQ6 6793 O00746 9821 6794 4133 10690 9820 Q9NX62 O00743 Q9Y5Q8 Q9NWU5 Q8IWF2 4125 6789 Q96A72 Q07020 5470 4140 5471 O00757 Q9Y5R4 P30837 5476 O00754 Q9NWV8 Q9UBX3 P49448 5467 P49447 207 5468 4139 4150 O00767 5481 4152 4154 Q9Y5S2 Q96A54 Q08345 O00762 P13489 Q9NWW5 O00764 Q9Y5S9 P17844 211 51185 215 5478 5479 4149 Q9H4F1 5494 5495 Q9BU89 5499 P13497 Q9UKK9 P17858 Q96QT4 10682 P49427 4172 4173 4174 11060 4176 Q14209 Q16864 11065 7204 P55265 4179 Q6NZI2 4171 230 Q9NXS2 Q6P5Z2 23043 11072 Q8IWZ8 4188 8543 Q16877 Q6N063 Q9NY59 Q9NPD3 8536 Q96RL1 P61956 Q15554 197259 Q9BUN8 Q16880 197257 Q16881 P35638 P61968 11041 Q96IZ0 8555 4191 23028 Q96IZ7 4193 Q8WXQ8 P61962 1915 P61960 Q93009 P02741 P22314 Q93008 Q14232 P61964 8563 Q9Y6R4 P36957 11051 Q8IWX8 8565 Q9Y6R0 8566 Q86Y37 P36956 P36954 Q8N4C8 267 23031 23032 Q86Y39 Q9NPF4 Q9NPF5 P61978 P43246 8574 Q14249 P56556 8576 26994 Q9Y6K5 O75582 271 Q9Y6K1 P10398 Q9Y6K0 Q93063 Q93062 Q04837 Q96RQ3 278 8569 1937 1936 1933 11030 Q9BV47 P22392 O75575 23014 23016 7251 Q93050 Q9NY12 Q9Y6L7 11035 118460 P02751 P61927 7266 P02790 O75569 7267 Q9Y6M4 291 Q8WY64 Q9Y6M9 Q8NEJ0 51102 P08069 P02786 Q6I9Y2 51107 1956 O15075 Q9NPA8 26986 Q01780 Q9Y6N5 Q86Y79 64428 7272 1965 114088 Q93034 51116 1969 Q14289 1968 11017 P12931 Q7Z589 Q70CQ3 Q70CQ1 1975 Q05329 Q9BUG9 Q6P5R6 Q13761 Q58WW2 7297 P56524 Q13769 O75531 7295 P24928 Q7Z4W2 1982 Q9Y6H3 Q92560 26168 Q9Y6H5 P06276 O75528 Q5D0E6 P06239 Q8WXI4 Q06643 O75521 92483 P55209 P56539 P56537 1994 Q92551 26133 P00966 P06241 P21860 P56545 O95983 Q16816 O95989 P55212 91147 P55210 A4D1W7 63931 3704 3705 Q07954 Q9Y6J8 Q07955 P07585 Q92542 50628 Q16825 Q16827 O75503 O95999 O14595 O95997 P35610 Q16828 9020 23081 23085 O60488 P20594 84881 Q8NEC7 Q8IWV7 O43920 Q86Y13 P61081 Q9H596 P61073 83548 Q6N021 23097 26121 P61077 Q9BUJ2 P22303 11080 Q9NXH8 9045 Q9NP81 Q16849 P12956 P12955 Q8IWU5 Q8NEB9 P33897 Q9Y6E7 Q9Y6E0 P61088 Q96AE4 Q96RG2 11091 Q16854 Q15526 9054 23076 P24941 11099 84893 Q5FWF5 285672 65003 O14672 399687 9061 Q9P2R7 Q9UBB9 O60568 O60566 P15374 Q8IVH8 10772 Q9UK45 Q9UBC3 P27361 Q9GZZ9 65018 54361 9077 55696 10783 10785 92609 Q9NWB1 P52333 P29992 1121 O95210 P53667 Q8TED0 Q8TED1 O60547 79676 O60543 1111 Q86WA8 P61289 P03372 P26012 P49591 3791 Q9GZX7 Q8NCR0 9093 Q9UBE8 P56705 P49590 9099 10767 10768 57410 O60551 3784 P49588 P07741 Q9H3G5 10765 P05129 P50570 P11802 P50579 O95232 6829 10733 147746 Q9P2N5 1137 Q6FHQ0 P61221 6827 Q10570 Q7KZI7 Q7Z419 Q9Y4X5 Q13601 1153 Q14938 1152 P19784 Q06455 O95243 P53611 Q8IVL6 6839 10746 1147 2475 5500 5501 6834 P26045 P53621 O43175 O95257 6850 Q13615 Q9Y4Y9 2495 Q13618 Q13617 O60508 Q13616 1161 O60506 Q02083

Q9P2P5 O60502 10714 57448 P61244 O14641 5511 P61247 O14640 5514 O43184 Q9Y4Z0 5515 Q9UK22 P05112 65061 5516 5518 50814 Q13620 Q13627 P18433 O60513 10725 10724 O60512 P61254 10728 O14656 P40337 P06454 Q9UK32 O14657 Q8N2K1 P04632 O95278 O43159 6872 Q96HA8 6871 P04637 25862 P16615 Q9NVW2 O95271 Q01650 23203 11222 5536 Q15389 5537 Q9GZR7 Q9GZR5 6883 O43169 P48201 4221 6885 Q9HDC9 1198 25873 23210 1196 1195 P50542 P12268 1191 56940 O76074 Q96Q15 O76071 51251 Q15392 84159 Q99873 55611 51253 Q9P2K8 4214 11231 6878 4215 4216 Q15393 P01588 O43172 56946 Q9GZQ8 25847 6892 5562 5563 5565 P13569 O95298 O95299 P12235 51264 Q53X93 55626 Q9GZP9 Q9Y572 5557 P61201 Q7Z3C6 5558 O43143 Q9BT22 P04626 5575 Q8N2H3 P04629 25853 Q08257 11218 O14617 301 P17936 9924 5566 Q96PN8 5567 5568 Q8TDZ2 O43151 P61218 5580 5581 25828 5582 5583 5584 Q3SY69 5585 4255 5586 22794 P15328 25822 O76031 Q96PU8 Q96PU4 79612 55644 314 Q96PU5 Q02930 8604 5577 5578 8602 5579 Q9H3F6 8607 Q8N302 5591 P07711 Q9Y4P1 9950 Q6IR47 P24298 5596 5597 5598 P50502 O76024 O76021 5590 P16671 327 328 4259 329 84197 25804 Q9GZU7 Q8N2M8 P12277 P51843 Q9NW64 Q8NCN5 55662 333 55660 55666 56994 5599 P83436 Q9GZT9 4285 4287 25818 7311 9973 Q96Q40 P17980 Q9NVV4 P15313 Q08211 221656 Q08209 79622 9967 4297 Q6DKI1 Q6ZT98 Q15418 7325 8654 Q9UKD1 P30085 Q04760 4291 351 Q03426 353 Q9UBS0 Q04759 Q9UBS4 P23786 9978 P21127 8662 7332 9997 7334 16 8667 Q15428 7336 8665 Q15427 7335 19 26205 Q8TF76 Q9UKE5 Q9H3Z4 Q9UBT2 23170 367 Q9UBT6 369 P30084 11198 7329 7328 20 Q9Y600 7341 11160 Q15434 Q14103 11161 7347 6015 P29074 23144 Q53GQ0 Q9UKF6 Q9Y606 Q9UBU3 Q03405 85437 P17096 11165 11168 Q16774 8669 Q9UBU8 30 P43378 31 34 Q53H12 Q14116 11171 8683 Q8IW75 7353 Q14114 39 7358 P51812 Q9Y619 P51813 P29084 Q96HW7 Q9H492 Q9NWT6 Q9UBV7 P51817 11176 P23771 Q15459 O15160 P55345 48 O15164 7368 25788 23127 Q99965 84129 Q16795 Q15466 Q16790 52 Q14134 Q99966 54 Q14139 6045 7376 Q14137 7375 6048 23133 23135 Q9UKA1 Q9Y5J1 7372 Q86X76 11157 O15169 Q99956 P46459 7385 7384 Q86WU2 Q8TEY7 7386 7389 6059 P14854 Q9Y5K6 Q9Y5K5 6050 7381 Q9UKB1 Q99942 Q99943 11129 Q99941 51222 56915 Q9UBQ7 56916 P01579 Q14152 11124 Q9UBQ5 11128 O75676 O15197 O15198 O75674 P14868 P33121 P14866 25778 Q9NX14 51231 Q00325 P08195 Q14164 86 Q14166 Q14165 9100 Q7KZN9 Q96QC0 Q14966 P52272 P33992 P33993 Q8TF05 P33991 Q05209 P0C0L4 84967 91 Q8WWI1 94 P45974 26270 P82921 O15118 Q9UBK2 Q9UBK9 P82912 9114 P82914 Q14978 Q8WWH4 Q86WJ1 Q6DT37 Q96I24 Q9H3R0 P31321 P31323 P43304 84950 P05062 P52292 Q52LR7 P05067 Q9UBL6 Q9UBL3 O94763 P52298 9128 O94768 9129 O75643 9126 O94766 Q6XUX3 Q9H422 Q96I15 6093 283989 3817 Q8TEL6 P21980 Q9BTC0 Q9UBM7 Q5JRX3 Q02156 26260 26262 57379 283970 134430 Q9UBN7 P82932 P82933 P30049 Q9UJV9 Q9UBF6 9149 56052 Q8TF47 P30041 Q6NYC1 Q9H488 P30043 P11766 P30044 P30046 P53567 P30047 P30048 B3KY43 P05090 P22415 P05091 26232 Q9UK80 P05093 P30050 Q03468 26234 Q92499 Q9UJW2 3835 P47712 Q8IVT5 9150 57396 Q16718 Q9UJX2 Q9Y5B0 P17081 P08138 P09467 2517 Q9Y5B9 Q9Y5B8 P09429 O75604 P10415 Q8TEQ6 P35711 Q7Z494 2521 Q6P4R8 P09430 Q5T6F0 O75608 728642 P08107 P53597 O15111 Q9P2Y4 Q04771 23192 23193 1203 Q96HR8 2531 O15105 P45983 P45984 1207 Q7Z2K6 65125 P54619 P51587 9184 P17676 Q8TDD1 2547 P37288 1213 10893 Q8N1Q1 6901 Q6IQ55 Q9H2H8 P28335 25913 Q9UIQ6 P28330 P08729 P28331 Q5VTR2 P37268 O60216 P62244 80755 54487 P62241 P49247 P62249 Q9H2G2 Q9BRX9 P28300 2572 2571 79791 Q8NBQ5 54496 6927 Q8IUD2 6929 P62256 57520 119180 6921 P26599 P31942 Q9UIS9 P31944 P31943 P08708 2582 P54646 P17655 P62266 80777 P62263 5602 Q96GD4 P62269 5604 Q9Y3U8 Q9Y463 Q14807 2593 P50213 2590 Q8WVC6 P39019 10855 P19404 P15056 10856 2589 P39023 5610 5613 Q9UIM3 Q9Y478 P51553 P30679 5631 Q07666 Q8WVB6 P52888 Q06330 10868 10869 5624 Q9H2K2 P28340 4311 P21709 Q8IUH5 169436 Q9HCL2 1277 5636 Q10472 Q10471

Q13501 P51575 Q5MAI5 22826 22827 22828 P51570 Q8NC51 Q9HCK5 10845 1289 192669 4313 4318 11340 Q86V81 O00541 P14555 23326 25988 23327 51362 126328 Q8NBJ5 51363 5654 P25205 10013 84289 10014 11345 135295 64682 Q15269 Q9UII4 4343 Q9BS26 Q6FGD7 P51513 Q99755 Q8NBI2 51377 Q99750 Q9BS18 Q9BRP8 Q99759 408 409 Q99757 P60468 23307 Q09472 5682 5683 5684 5685 5686 O00562 O00567 51380 Q9NUW8 Q99741 55743 3014 8703 55748 8702 Q9Y450 P03891 11326 Q15287 25979 4361 84292 5692 5693 P40818 8720 3035 3033 3032 Q9NUX5 3030 P14543 10007 11338 55750 P14550 P15880 3028 P60484 Q15291 55759 5687 5688 4358 55757 P48775 84296 79709 P31930 P26583 8732 O00506 25942 P14598 Q9NUQ2 54431 Q8WUY8 433 Q8NBN7 435 P31939 54438 Q9H2D6 79739 P55072 Q96GC5 140609 Q9HCE7 3054 Q05086 3052 P37231 Q9HCE1 Q99797 Q8NBM8 444 445 55775 P82673 8737 Q02809 P82675 P55084 P61758 Q9H2C2 8738 11315 P01266 284086 P49207 8754 25929 7422 Q9BRT9 Q9HCD5 P52815 3066 22894 3065 Q9UIF9 22897 O75293 79753 55781 7415 8761 3070 Q5VTL8 Q9UIG0 54460 3075 Q9HCC0 3074 P17612 468 55796 7428 O43791 53938 Q16629 Q9Y4K4 P11441 O75385 Q9Y4K0 Q86VQ3 Q9Y4K3 472 Q96GX9 P48729 474 O75390 476 477 478 28973 Q99807 Q16630 8767 P43034 Q8WW01 6120 3093 Q16637 Q15303 3091 Q16633 P55036 6123 Q16635 6125 Q15306 Q9Y4L5 Q86VP6 3099 O75376 3098 8780 481 483 Q709F0 O75380 Q504Q3 23291 Q9NW13 488 Q4J6C6 6117 P22102 6119 6118 Q6YP21 Q16649 6133 6132 Q16644 6134 7465 6137 Q16647 O43776 O75365 490 O75362 495 498 P09110 P31040 6128 P56385 P82663 P82664 P56381 Q8NCB2 Q8WVM0 Q9BSI4 Q16659 6141 Q16656 P18074 P55055 Q01581 P18077 O75351 O75352 144983 28960 Q96PF1 Q9P2E3 6138 Q16665 Q9P2E9 51319 Q8TDY2 6156 6158 O75347 131601 P35453 P09172 P23467 P27824 Q14012 51329 Q14011 6164 Q15349 Q8TDX7 6168 P46100 11274 P36776 Q9Y4H2 6160 7490 P11498 Q7L2H7 11277 6175 9201 Q86W42 P98175 Q3ZCQ8 P98179 Q02880 25885 Q08188 Q8WW22 Q02878 51340 Q9NVM4 P02549 Q96PK6 11243 Q14032 P55010 Q9NVM6 55703 Q16698 Q9GZL7 Q15365 84263 P23443 O75319 9212 6185 6188 Q15369 Q8IUX7 6187 O43741 Q08170 25896 6182 6184 Q86W33 P48739 Q15370 P27815 Q99816 P48730 P23458 Q99814 P48736 P98194 Q8WW12 Q15375 P78545 6196 P54577 6199 O75306 9221 6198 P98155 O95714 P78540 Q9H324 57461 6193 P78549 Q8IV08 6195 6194 10811 10813 83752 Q9Y4C1 P41252 P62195 P41250 O60285 P08684 65083 Q13523 Q13522 P78536 9232 22803 Q13526 Q9P1Y6 Q9H2P9 Q8NBZ7 83737 Q9BSB4 P46934 Q9Y4D2 P41240 O60296 Q32P28 Q9NVH2 Q4LE28 P42574 P42575 O60293 Q14865 P07327 P34947 P29597 Q13535 Q9H307 P29590 Q7Z2Q5 P41235 P46926 Q9Y4E8 3939 Q9NVI1 O60264 3932 O60260 Q9HCP0 P07339 9255 Q32P51 Q13547 Q13546 P34931 9252 83759 9253 O43709 3948 P41222 P20309 O95749 P41229 P78560 P41227 3945 Q13555 Q13554 O95757 Q13557 P78509 9261 P00749 P00747 Q8WVK2 P00742 O60240 3953 Q14894 2629 2626 Q9NVC6 Q9P1U0 Q13569 P19387 P19388 P11413 93100 P00738 Q9UIV1 2632 O60256 P00734 P24723 Q13573 P09104 P46977 Q13572 P46976 P55884 3980 Q8IUQ4 28998 P32320 P78527 P32322 O60229 P41279 Q58DX5 P20339 1314 2644 1312 2643 Q04656 Q9UJ83 3978 Q9Y4A8 P18031 Q9P286 Q9P287 P62191 P19367 9296 1326 Q63HQ2 2651 P21673 P07384 O60231 Q9HCS7 P20340 P00750 Q9HD40 Q13595 P21675 P48052 1340 Q12948 22992 Q06265 1337 Q8NB16 Q9Y2X3 P08842 P48047 Q9UI09 Q12952 Q9Y399 2681 5706 57634 5707 79885 5708 5709 P21810 1345 P16471 O60331 5700 P49368 5701 5702 Q9UHP3 Q9UI12 5704 5705 65264 P54753 P25398 Q66PJ3 Q12965 Q9Y2Y8 Q9BZG8 Q12968 P16435 P54756 O60306 O60307 P16444 5717 1358 57646 83933 1355 P12081 Q9Y2Z2 5713 Q9Y2Z4 5716 Q12972 P54764 P54762 Q9Y2Z9 P54760 Q12974 1374 Q6P2C8 O60318 5728 1368 Q8WTX9 P15121 Q9BZF3 Q9UI32 253827 Q9UI30 5725 Q6PCD5 P28482 Q12986 1385 Q9UHJ9 P51668 57661 P51665 Q9P0J0 Q9P0J1 P19525 P62316 P62318 5733 56339 P62312 P62314 Q9BZM1 P51671 Q9Y2T7 Q9UHK0 Q06210 Q5VST9 P19544 P30793 10987 57678 10988 56342 1388 1387 P54709 P42771 5747 22934 Q14703 22937 22938

Q06203 Q9H9Y6 Q9P0L2 P42765 P42766 Q56NI9 10956 P62333 284119 Q9BZK7 22929 5757 P61009 P08865 P51693 P27144 5770 Q9Y375 P51692 P54727 P54725 Q9P0M2 Q03164 55035 Q9BZJ4 Q9P0M9 57695 57697 Q9Y2W2 Q9Y385 Q6DHV7 5768 Q9BZJ0 Q9Y2W1 509 P06213 Q15139 Q9Y305 5780 Q9UPV9 5783 440138 P14678 P38646 Q8WTS1 54512 Q99640 513 514 5775 8803 79813 515 8801 5778 P27986 518 Q9UQ80 54517 220988 Q8TBX8 Q9UHG2 P04406 Q9UQ88 5796 Q8WTR2 521 522 523 23451 526 8812 528 10147 Q99638 P01344 8815 P01343 Q96EZ8 Q86U44 P26641 Q86U42 P26640 O95059 Q9NU63 P26639 533 79834 535 Q15165 55869 537 5798 79837 539 Q9UPY3 54539 P01375 23438 23439 Q9Y2R5 Q9Y337 Q9Y2R2 P40939 P40938 P40937 8841 Q9Y333 7511 Q9UHI8 Q9UHI6 P51659 23435 Q9Y2R9 P12004 Q99611 3149 Q3KNW1 O95071 3148 545 546 3146 8836 8833 Q96EY1 P62308 P25311 8837 P62304 7508 Q9UPR0 O00628 23409 Q9UQ35 7520 8851 Q9UPR3 Q9Y2K7 Q12904 Q9Y2K6 23400 Q9Y2K2 3162 Q9UHB4 P17752 23404 54550 552 Q99685 3159 Q99683 Q9BZE1 8844 Q15185 P49336 7518 P05771 7517 Q9Y2L1 6201 6202 7533 3178 23411 P12074 563 Q00987 3169 P49327 Q9H1A4 65220 3181 6210 P49321 3189 3188 Q9UHD2 Q12923 3187 Q9UPT9 Q9Y2M5 3185 3184 572 573 54575 Q8N8Y2 7536 6204 6207 3192 3191 P04424 8880 3190 8881 Q60FE5 Q12933 Q12931 7555 P51610 580 54583 P51617 P52948 8879 79869 8878 6217 Q9UPV0 10090 Q8N9Q2 6231 8892 6233 P15927 P15924 P15923 Q96FV9 593 6229 Q8IU60 Q9HC98 P03950 O00716 Q16512 Q16513 Q16514 O75494 Q7LBC6 P27918 6238 Q13492 6239 Q13490 6256 10075 Q9HBW0 O75489 Q16526 P31153 23387 P31152 O43897 Q01469 23381 Q9NV06 P23528 P09234 Q16531 Q8WUK0 51426 P09237 51428 Q16539 344387 10081 Q6PD62 O75478 Q15208 O75477 Q86UL3 23399 O75475 Q8TD19 64745 Q02790 P12883 23396 10087 6259 P60520 Q8WUJ0 O75469 64750 P40926 P14635 P38606 Q9UQL6 P10275 P10276 P35579 O75460 Q99720 Q9Y3E5 10055 P23588 Q99728 84365 Q16555 Q8WV24 197322 Q86UR1 O43889 P36896 64768 P36894 23378 P35573 Q02763 Q9Y3F4 P01308 Q01433 120892 Q9BRL6 Q15233 Q99714 Q8TCU4 Q96FX7 P61803 Q8TCU6 P14618 Q96FX2 O75444 Q86V24 P36888 P11586 Q99700 Q9H1Y0 51465 88745 Q99708 P22234 O95817 9319 O95816 Q16576 Q99707 9318 O95819 55827 64780 O75438 O75439 Q08050 P39905 Q96FW1 P14625 O43865 P36873 Q02750 55832 54504 Q15256 O95831 22913 O95835 O95834 Q7L0Y3 10929 P18124 O43837 10933 Q9Y3A5 Q9HC16 10935 22908 O95837 P15090 Q9BZQ6 Q8WUD6 22926 Q13405 Q13404 22928 Q8TCJ0 10939 Q8TCJ2 O43847 Q9Y3B4 Q9Y3B7 2710 56267 10949 Q08945 Q9UHX1 Q9Y3B2 57599 10940 10907 O43812 O75400 10908 Q13418 286826 9360 9361 Q9UHY1 O43819 Q9Y3C5 P00846 2720 P42680 P42681 Q9UHY7 P42684 P42685 Q9NUG6 Q86UE8 Q13423 22901 O95861 P53396 Q13427 Q13426 O95865 Q8NAV1 Q8NB78 10921 P42677 P19438 P68402 1400 P68400 Q9Y3D3 2730 Q9Y3D5 P06132 1408 1407 2737 Q13435 9388 Q9HC62 Q9UI38 9380 8050 P62280 2744 P62277 P09211 Q9HC52 P43115 2747 Q13444 9397 Q13443 Q08J23 9391 P11532 Q9H1R2 8065 O95881 Q96G21 O43809 Q8N0Z6 Q9UQE7 Q8N0Z8 P23511 Q6P2Q9 1429 Q13451 P23510 8078 O14495 Q7RTP6 P12830 P19474 59343 84305 1432 1431 2762 Q14790 O60341 Q9HC36 Q13464 Q5T4B2 P54687 P53355 P53350 Q9P0U3 Q9P0U4 26469 Q00169 Q7L9L4 5800 Q9UHV9 Q9H0E2 O75947 8099 Q92979 Q9H0E3 Q9Y265 O75940 Q12824 O95551 1459 Q9BYG3 1457 P13073 1454 O00391 1453 27089 Q00839 P05455 Q9H0D6 O75936 O75934 Q12834 Q06136 P16104 1468 27090 Q9H0C8 Q9Y285 5822 140801 O75937 P10809 Q96EB6 Q5S007 P27694 Q9UGN5 P27695 Q06124 P17405 A0AVT1 4507 P37023 P14384 1478 57761 5830 Q9Y295 55109 5832 Q9Y297 5833 O75928 Q9Y291 5836 P26358 Q12851 O75914 Q92947 O75911 P26373 Q99583 Q12857 1491 55110 1488 Q99575 1487 Q9BYD1 148789 55119 P26368 4514 P25098 P49069 Q9Y223 Q9UGH3 Q96MX6 Q9NSU2 27043 57787 Q96EG1 O60884 Q9Y230 O75909 Q9Y231 O14986 5859 Q12874 Q9UGI0 Q12872 P29466 Q9Y239 5870 Q9H0H0 5871 Q9UGI9 Q15904 Q08752 O00358 Q9H8X2 Q8TBC4 Q96EF6 57794 Q96MV8 Q5C9Z4 P16152 P61599 4535 5868 P04179 Q12884 Q9UGJ0

Q12888 Q9Y243 5883 P16118 Q8TBB1 P38398 Q9BQ90 Q96MU7 Q9BPX1 55149 Q96N66 Q9Y250 613 Q92995 Q5TCX8 Q9Y251 Q92994 5875 254827 Q92993 4548 P10827 P30414 P10828 5892 5894 P31749 55152 27032 P40692 27037 55159 O60870 P04150 Q9Y262 5886 5887 5888 8916 Q9UGL1 P04155 Q8TBA6 P04156 O96004 8930 O00308 O96009 10250 P56199 O00303 P50897 23569 O14949 Q15022 P62877 8924 55967 Q15020 Q15025 Q15024 P62875 Q15029 P23284 O96017 Q15027 Q86SR1 O96019 P36578 23576 P35244 P14324 O96013 P35249 Q03933 641 P35251 P62888 196441 P23276 8939 Q15036 P27635 P60228 10269 Q15034 O00329 P35236 O96020 3263 10238 O14967 P11274 8945 P01019 P62899 79956 8943 Q15046 O14965 P23246 Q9Y215 Q8TAT6 O00330 23552 P11279 3276 23556 P35227 O14979 P35232 Q9H093 4598 668 Q15054 285193 O14974 Q59EA4 P06733 Q9H0A0 Q92934 6311 P01040 P62837 P06737 8975 P05408 245972 Q99570 P39656 23524 672 673 P15692 6304 6305 128308 10213 158135 P06744 P05413 3290 8985 O43488 23530 3297 23536 Q6PJP8 Q99558 10229 688 Q15070 689 Q86T82 P62847 P62841 P61513 Q15078 Q9BQ15 23509 P13010 Q15084 Q99543 8987 P62857 O14920 P62851 P56182 P56181 57727 P56180 P62854 O43463 Q9BQ04 Q92903 6342 23517 6341 Q92901 O43464 Q92905 Q8NHX1 P35268 23512 Q7L7X3 O75031 10208 O14936 O14939 Q56UN5 P60201 10202 Q92900 P56192 P36507 Q14686 P37837 P19174 Q9BZ95 Q9UPN6 Q86TM6 64834 Q9H0U6 P12532 Q8TC27 O75150 Q9H0U3 84447 P46783 P49810 P83731 5018 Q14690 Q14694 Q13363 Q14693 Q9HAV7 Q14697 Q6YN16 5034 P46782 5036 P46781 P11216 79084 P41091 Q9Y2H1 60489 P68104 60488 P00533 Q13370 Q16401 P46777 Q9HAU5 P46776 Q9HAU4 P46779 P24522 P46778 10193 P56134 Q9UPP1 P32121 51540 60496 10197 P00568 51548 51547 Q9BYU1 9410 55907 P62826 P20151 O43542 O75116 6389 P62829 Q4AC94 P00558 Q13393 5049 9421 P78347 9420 O75106 P49862 P32189 P36542 26512 6390 P11245 Q9Y2C2 23481 51567 Q9BQK8 5074 10181 8100 55929 Q9HAZ1 26524 26523 O43529 5071 P49841 P49848 Q15111 Q15118 P78368 P49840 P11229 9448 10152 23463 A1XKG3 Q9BYZ2 P48507 P24539 51585 P24534 P78371 10155 Q9BQI3 9439 Q15125 2805 10159 Q15126 Q9HB90 P78356 23476 5091 23478 P37840 5092 5093 Q96EU6 O43505 Q9H173 51593 Q9H9G7 Q92187 P49821 79901 Q9NTJ5 P78362 10166 54623 55959 Q9HAW8 9465 112724 P53004 9463 Q6P1J9 P09769 P29375 P29374 Q96EL2 P29372 27005 79012 Q9H0M0 27000 P42345 P22735 2821 2820 P04083 9476 P31689 9477 9474 9475 O15496 Q9H0L4 55054 1503 Q9BYN8 79005 Q6P1K2 P42338 P42336 P29353 Q5JXB2 P29350 Q9H0K1 1514 Q9BYM8 79035 55066 79039 Q8TBF4 P04062 P04066 Q9Y2A9 80020 P54368 P78316 55072 P78317 Q8N8D1 Q9Y2B5 201595 Q04446 P04070 P53041 P07101 P52597 Q96EP0 Q13315 Q86TI2 Q9P015 Q96EP1 P00505 Q9H9B1 2869 1537 Q14653 55093 64801 Q9H0P0 P46736 Q9H9A7 2875 1540 124739 5901 P46734 P13807 Q6P1L8 O75964 P13804 O75962 P28065 26576 Q9P035 Q00059 Q9BQA5 2885 2882 P16083 P43694 O15455 Q14674 Q9BQA1 5914 O95544 Q13347 Q14676 O75953 P28074 P28072 79065 P28070 P42357 5927 5928 P63279 P00519 5929 P63272 Q9HB03 P43681 P08473 Q9BZ11 51514 P43686 Q14684 Q14683 P10914 O95677 Q92858 23607 P67809 P52790 Q9NRW4 P30520 O15519 P13196 5939 1576 P16220 Q92851 5931 P49137 5935 P0CG13 P09936 O95685 O15530 Q9NS91 Q9NRX2 Q8IZP0 O95680 Q9H832 Q07343 P50148 P18887 P16234 55215 P30519 P67812 64174 O95696 Q92835 Q9NRY2 Q9NRY4 Q99471 P18858 P18859 125972 O15533 Q92830 P62136 4627 Q92831 P55854 Q92824 P46087 P09917 5970 Q9H814 Q99460 Q9NRZ9 Q99459 3304 P17540 P62140 55236 3301 3309 P04233 Q92820 P30566 P34925 O00469 P07307 133584 5981 5983 3320 P52756 O00468 27165 27163 Q96DE5 3313 710 3312 P09972 4643 5976 27161 P30556 O00472 Q6PIW4 Q9NS56 3329 P16278 3326 720 3324 722 P08651 Q6XQN6 Q6ZWT7 4659 Q9UNX3 4670 P28223 137964 4673 O00483 2011 27148 O00487 P30542 P17568 80222 3337 Q96DC9 Q12770 Q92878 P04264 Q9UNY4 P08621 Q15800 P51451 P09958 Q12772 P30536 2023 P52788 P30530 P52789 740 Q96LR5 P37173 Q99496 Q99497 P09960 157570 4677 747 4678 O15503

P02452 O00425 P23396 4695 Q5VYS8 2034 2033 Q00796 P09093 P62993 2026 4686 Q16236 P62995 Q96LI5 Q9C0K0 P25100 P46020 2043 Q9NRP7 54764 4697 4698 768 P46019 P01100 Q8N726 Q7Z7K6 P14416 O00444 P14410 P11388 P11387 O60942 773 P01138 P01137 P01135 P01133 P02461 P01130 Q9H7H0 6421 2065 3396 2064 Q96CU9 780 Q9NRR6 Q9NS37 Q8IZE3 P09086 P10071 P02458 57819 2070 Q5TKA1 6430 6432 6431 6434 7764 Q92817 Q9UNN5 O60925 Q8IZL8 O75190 Q8IZL9 6427 10331 6426 P61626 2081 23658 P46063 5111 23650 Q5HYA8 P18850 Q99437 P18847 P18848 P83881 P27797 P50990 2091 P01185 P50991 P22087 91754 Q8TA86 Q9UNP9 2099 O60907 O75164 23621 P50993 10318 Q9NRM7 91746 P62979 Q9UNQ2 A2RTX5 Q5T1M5 6464 391356 P39748 O00411 146057 P52701 O60911 Q5U4P2 Q9NRN7 P58012 P62988 5127 Q8N752 P25116 6472 Q13237 5141 5142 Q14566 64951 P62917 O43678 P13984 6470 64949 84569 P62910 P09012 5139 P62913 Q13243 Q13242 O43683 Q13247 P20290 9510 O43687 64960 O43684 9512 64963 64965 P13995 64969 80198 51650 51651 51654 51657 5162 A2RUU4 Q9BY50 P11309 Q6Y2X3 Q13257 9524 9525 P62937 P11308 64975 5160 P49916 P49915 5159 P61604 P83876 P49914 P21589 Q13263 9531 O43660 Q13268 9530 Q9BY44 9533 Q9BY41 5170 P34896 P11310 Q02543 P09001 9526 P62945 P49903 P62942 5184 10291 9541 P48651 Q8TB40 2909 160287 P12694 121512 P27708 Q8NI36 2908 P48643 Q13287 Q9NSE4 Q13286 5198 Q09161 P15735 Q8NI27 Q8TB22 Q96E52 O75208 8237 10273 Q9HA82 Q53TN4 P49959 P50914 2926 P09038 Q15008 Q9BY84 Q9BXW4 10280 8242 Q15005 Q9BXW6 10283 P62906 9578 116138 8241 2932 2931 255488 221264 10286 10285 Q15014 2935 P78424 P55786 P08559 9588 Q12788 92105 Q15819 P62081 1613 80124 Q96DI7 P41134 Q9BXM7 79139 Q8TAF3 29785 Q12792 1616 2947 P07237 8266 Q12797 P55795 O95602 Q9H7Z7 55173 27132 P67775 Q6IN84 29796 P08574 P08575 1629 2959 P08579 2956 P07205 Q15831 P29474 P29475 Q96M96 80146 1634 80145 27102 2961 2960 P04183 O95619 Q8TAD8 2969 P09874 Q6V1X1 Q15843 Q6R6M4 55192 64900 29777 79143 1642 Q05655 2972 Q9BXJ9 P09884 1647 Q15853 Q9BY32 Q14527 P13945 1660 Q86SG2 2987 1655 1653 Q86SG6 1650 Q13200 1659 O95644 150094 51606 Q13202 P21580 Q8N7H5 1666 64919 80155 1665 Q9BXP5 51611 Q13216 93492 P55769 4705 4707 1676 P08581 Q8NHH1 4701 4702 P41159 51629 Q14558 O95661 4718 4719 389840 4710 4713 Q13233 4714 Q14562 4715 Q96TA2 Q9H6Q4 Q96L12 Q9NQU5 81605 P52434 O00148 4728 4729 Q8IYN9 P84022 Q70EL4 4720 4722 4723 4725 4726 P27487 Q9C026 Q8NFZ0 Q92729 O00154 P53779 P38117 P14174 4731 10420 4734 10421 64282 Q7Z727 23708 81629 3420 O00167 P56817 3417 3416 81631 P40429 811 Q8N684 Q5TAP6 P40424 P27448 3419 Q9C005 333929 P07858 P99999 Q9NR96 Q676U5 Q8IYK4 P20823 Q8NFW8 Q5TAQ9 55352 O60674 P20827 P19838 821 3421 823 826 Q9NZJ4 P41743 P07864 P11926 Q13951 Q13956 4772 2110 Q5SGD2 55361 O95352 2108 2107 Q5T160 27283 Q6PI48 80324 Q9BWG6 833 Q4G176 O14744 340204 4780 Q92769 P09619 Q9NZC7 80308 O95361 P10600 O95363 Q96KS0 Q7LGA3 27297 P61353 167227 841 843 4775 847 7804 O14757 Q92766 P09622 4790 Q13976 2132 Q96L34 O95376 Q9NR45 P18545 Q9NR48 Q9BWE0 Q05469 2125 80347 P60033 857 859 Q96CA5 P11908 Q9UMW8 Q92748 Q9H6R4 6500 Q9NR50 O95382 Q96L21 Q9NQT4 Q9NR55 Q9NQT5 P19878 Q9BWD1 80332 Q4G0W2 2139 862 Q96KP4 O00141 80335 865 867 Q9UMX1 4799 P09601 Q92743 P06576 P00390 10492 O95391 3481 O60603 871 Q96T88 3479 2147 Q13085 7832 Q96KG9 P00387 P25685 P23025 Q6UWV6 P51991 6520 Q96T76 O14717 2159 O43290 Q7Z6J9 P61313 P49643 O43294 6517 O43293 Q13099 O43252 Q6ZMR3 Q6ZN33 O43251 P49642 64215 P53701 P13686 Q9BW92 Q9BW91 891 29088 O14727 126789 Q8IYD1 Q96T60 Q9NR09 Q16134 Q8IYD8 10477 Q9UN42 29086 10476 29085 P61326 Q9UMS4 P04745 5210 P04746 7874 5213 Q658W2 114799 Q01974 Q96ST3 Q01970 29093 O14730 7867 10488 O14733 5207 7884 23760 P50613 P51946 23762 23764 2194 P60891 10450 10455 Q01968 O43242 P51955 P51956 29079 Q8NFU7 Q96SZ6 23770 5226 P48357 29074 Q7LFX5 23746 P24386 5245 9600 10437 10438 Q5TAL4 Q00613 Q00610 10432 5250

P51970 Q13901 P07814 P49674 Q96C86 55312 56647 10445 Q13115 6598 P10599 24139 57062 O43318 Q01130 Q9C0C7 P33240 9616 Q9C0C2 P38919 Q13126 P13639 8301 P13637 P58546 51773 Q9NRH2 24144 Q15796 P60866 Q13131 9641 Q15797 9646 9643 Q9UNL4 Q6UXN9 29803 51780 Q01105 O94804 Q13144 54815 Q13148 5295 P35869 57092 9656 P43490 P10586 P26927 Q13151 54822 Q49AN0 O94817 Q13153 P54136 Q9BX93 O15287 P54132 P63092 Q3LXA3 Q13162 Q8IZD4 Q14494 Q14493 Q14498 Q13164 O15294 Q9NRC8 7013 7014 P13674 P11021 50487 Q9NRD1 Q13177 Q9NRD5 10390 10392 9682 54853 P21266 Q9NRE1 P47897 1718 P00367 Q9UEW8 P47895 1716 Q13188 Q9BWU1 9695 7037 P21283 P21281 Q09028 Q9UNI6 O75792 26747 Q9NZV6 Q00688 Q6PID4 1728 1727 Q7Z7F7 P00352 Q9NRF9 7046 P35813 Q96L91 P10515 O75787 27248 Q9H6Y2 27246 O15234 1738 P05177 1737 1736 P54105 375743 7057 P29144 O75771 Q9H792 55294 7052 27252 1743 27257 P05181 Q9UER7 8399 8396 7068 7067 Q5TAX3 Q15714 O15264 P19793 Q15717 Q8IZ69 8394 7064 Q9H6W3 1756 80267 29880 Q92793 Q6ZNC8 Q7Z6Z7 P05155 O15270 Q8IYT8 Q9UNE7 57017 Q9BWH6 653361 P43405 1763 29896 Q04206 29894 O15269 Q9UET6 P05164 O75746 Q5GLZ8 Q15738 Q8N6G5 7083 Q8N6F8 84647 4809 60673 51720 P08243 4800 P08246 51726 7099 7098 51727 51729 P14927 30849 Q15746 P14920 P30153 1789 1788 4817 1786 1785 Q05513 57038 80270 375790 P08253 Q15751 80273 51734 Q15759 29843 P17174 84661 51741 4820 O15212 P05198 Q14432 51747 O75716 30827 Q6XPS3 Q13107 Q06830 Q9H6Z9 P22528 4839 P47804 4830 51752 Q6ZVX7 902 4833 P08237 Q13112 P08238 4835 Q9NZN9 O15228 O95433 O75821 O75822 Q969L4 O00268 Q8IXM3 51081 O00267 10536 3516 203102 10535 Q8IXL7 O00273 P60174 Q9ULW3 P63208 4841 90678 Q6NZY4 Q92614 10531 P07996 10533 Q92611 P04003 P52564 4860 Q9NQ55 Q7Z5P4 51094 55454 51096 10549 51099 Q9ULX9 Q08426 29128 Q05397 P18621 29122 P63220 55466 O60783 P32929 26091 Q6IT96 4882 3551 Q13829 152926 10524 10528 Q9ULZ3 10521 948 10523 O95478 O95476 O95477 O95470 29108 O60762 2224 O60760 29102 P04040 P84103 58517 O00238 P04049 Q96SB4 Q96SB3 Q9NYA1 O14880 O00231 O00233 P10721 O00232 Q5JTZ9 Q8IY17 Q96SB8 Q8WYH8 29110 2237 79444 Q92643 4898 4899 P05386 P05387 P05388 Q9NYB0 P30304 P30305 P32969 P30307 P31639 Q9NYB9 6609 2247 975 Q6GMV3 Q92630 Q92636 Q969M7 161823 P29323 Q92626 P29320 Q13868 O00255 81788 Q99259 984 6612 988 Q92620 6613 P29317 26061 Q9Y6W6 O43395 P07900 5300 6631 P15529 P53804 Q9BV90 P53803 Q96AT9 O60725 P15531 990 P11172 991 P62750 Q96RU7 Q96RU2 O14829 994 995 997 P28838 6622 999 6625 P61421 6627 51009 P62753 Q04917 P07910 P49770 200916 Q9UM07 2287 2286 P07919 P11177 2280 Q93096 51010 Q96S53 P11182 51013 6633 Q9Y6Y0 O14830 5305 5306 P28845 6637 P49768 O00206 P48431 Q9BV79 5321 P49761 6653 P49760 O60701 51021 51023 Q00535 Q8IXB1 Q00534 10594 P49756 P25789 P25788 6647 5315 6646 10598 91949 P48426 P25787 O00217 6660 5331 Q96JB6 5333 Q10713 O00213 Q96B26 Q96RR4 Q96RR1 P61457 Q00526 6657 5327 5328 Q9ULR0 O43390 O43353 Q9H5K3 P07947 9700 P07949 Q969H0 P07948 Q9NXW9 5335 5336 10574 P62714 5337 5338 4008 P07954 5351 Q9H5J4 5352 5356 Q9ULK4 Q969G6 Q01831 78991 51056 Q9NPH5 4015 5347 4017 Q6UVY6 Q96JH7 Q08462 P50750 4035 O14802 Q8NER5 51067 P68036 9716 Q68J44 10554 Q9H5H4 Q01826 P49792 5371 P27540 9733 5378 P20908 P15559 Q969E8 P16885 10569 54101 55432 51074 O14818 Q9NPJ6 6699 54107 54106 Q01813 9744 O43432 57187 29947 Q9NYU1 Q9NYU2 Q02338 29941 P22695 Q7Z6C1 83475 P22694 O94905 8408 O94906 5393 5394 8424 4067 63874 63875 29959 124 Q6UX04 128 P00414 P47985 P60983 54921 84795 O94913 Q13011 9761 P22681 Q76FK4 29922 P35998 Q9UDY4 P25705 115024 Q9UDY8 P42285 O94925 8428 P22674 54931 54938 Q8WZ42 Q14353 P00441 8443 4086 4087 8446 4088 P22692 9775 8445 142 1800 1808 Q96SL4 8439 P62701 P20020 Q8IXZ2 53615 1801 8453 9784 8454 P54259 9785 8452 Q6ZN16 8458 7124 P54252 P54253 7126 Q7Z6G3 Q96KC8 8450 4092 4093 4094 Q00597 Q9NZ20 156 158 Q8IYB3 Q13042 8449 Q13043

P49736 Q13049 7132 P04843 P04844 Q96KB5 8467 P13798 Q9BW60 1822 P11142 Q8NFM5 54961 7128 P49721 P49720 Q13057 8473 P00491 P00492 O94966 P54277 P54274 7141 Q96T21 1832 Q96T23 178 Q9NZ43 Q00577 P49711 P49716 P49715 Q49A26 Q9NQC7 7153 7155 7158 Q9BVS5 7157 7150 1843 Q6UWQ5 1841 1848 Q6VAB6 P21399 92345 Q13873 7167 Q96K76 Q86Z02 7161 8492 1854 P42224 1852 P47914 1850 P63165 O15355 Q92696 P09651 Q969T7 1859 P22612 P42229 Q13882 Q969T4 1856 O15371 O15372 Q969S8 Q13887 Q01082 O75891 84708 P22626 P84098 P63173 P09661 P60900 Q969S2 7186 7189 P10619 91039 P35916 27348 P11940 26015 O75881 27347 O75880 26019 7184 1877 4907 Q9NZ01 P17252 1870 O15379 Q92673 P23921 Q9NZ08 Q969R2 O75874 1891 P10620 Q6UWE0 4914 57136 Q9NYP7 Q969Q1 4913 Q969Q0 P33316 Q9NYH9 58472 57144 Q93100 58478 1892 Q8WYQ5 4920 Q8NFF5 Q8IY92 P35968 O95400 Q8WZ19 Q9NQ92 P42261 26005 57154 26007 Q96BI3 84749 27339 27330 58487 27332 26002 Q15631 P21333 Q8IY81 P09669 Q8IY84 O75844 Q5W0Q7 377630 P84090 4947 3615 3614 Q5RKV6 3611 Q5XUX0 57169 P00403 P01730 Q969V3 4946 O15350 P51178 Q15643 O75832 O95427 Q14318 Q15648 27315 Q86Z14 Q86YP4 Q9BVJ6 P30260 O15347 4952 O95429 4953 Q15653 Q15652

negative regulation of metabolic process P51587 124790 9184 10657 2304 Q9HCJ0 P84022 Q8N3U4 6901 25913 P28330 Q12834 Q92729 Q12837 Q8NDV7 P51114 2316 2 P14174 57510 54487 4734 Q96EB6 O75925 Q7Z727 Q9H2G4 Q9UKV8 3660 Q9BYE7 6927 P13056 3416 57761 811 P62258 6926 P26358 Q9UIS9 Q9NZI7 P48382 Q9NQX0 P31946 Q92949 P21917 P48380 O95343 P20823 1487 P19838 2332 P62263 P31949 3665 10642 64061 10644 10643 Q9UL15 Q9Y468 Q8IZ40 Q9UL18 4771 1020 P51787 10614 P21964 1499 Q86VE0 O14981 Q92530 6945 O14503 3690 Q92769 Q8WXF0 Q07666 Q15906 P10600 O95365 O00358 Q06330 1029 10865 1027 1026 3685 P17480 O14753 604 P05023 4790 5883 P38398 57326 P20618 Q92993 857 6726 Q8N488 P10826 Q9UL46 P10827 P31749 Q9NR50 P19883 1050 Q9HCK5 P40692 79589 192669 Q9UMX1 4799 50943 Q9UGL1 Q9UKT4 Q92985 O96004 O00308 P14316 25988 2147 23560 3476 Q15022 10013 639 10014 Q6UWV6 135295 64682 4343 P16989 P57059 25998 641 P33076 51132 Q99750 8932 408 P49407 P60228 409 Q09472 O43251 23309 5682 P48552 5683 5684 5685 6774 5686 64215 P13686 891 Q99741 P35240 51147 Q86X95 P51531 4361 5692 5451 5693 P35222 Q9NUX5 P51532 P35227 Q96ST3 P17813 P35232 P60484 11331 668 5687 5688 Q96T58 Q96MH2 P23497 O43474 P06733 P26583 Q9Y3M2 P25490 25942 Q8NFW5 3281 672 O14908 6304 8726 10213 P05412 O43242 Q08117 P37231 Q9HCE1 P14373 5467 207 5468 54206 10468 P01023 4152 5245 P56177 22893 3066 3065 Q9UIF9 O00762 79753 10438 P62854 9839 O43463 3070 Q13901 9612 23512 Q96QT6 P28749 7428 6595 11060 6597 8535 11065 P55265 P10599 6591 Q9Y4K3 3084 476 P49810 5017 51763 Q13363 P38919 Q13127 Q9HAV4 P55036 4188 5036 Q16635 5037 O75376 P13631 3096 5270 Q02447 O75381 23051 51773 P46531 Q13370 Q16401 Q96RL1 Q15554 Q15796 Q9HAU4 Q13131 8553 Q15797 Q96IZ0 P12757 Q8N6I1 P32121 P31273 Q9UNL4 P12755 23028 Q9NY61 O75360 4193 P46527 Q01105 10197 Q13144 51548 51547 54815 Q14232 Q9BSI4 Q13148 5054 P55055 Q9UPQ9 P10588 P10589 93166 P35625 Q9NPF5 51317 P43246 55915 P43489 9421 7248 P54132 Q9Y6K1 Q03989 51564 Q15583 55922 8328 5074 Q9NRC8 7013 55929 Q9HAZ2 7014 O15055 O43524 26523 7490 5071 7251 84232 Q8WW38 P61925 7023 P49840 O75569 P98179 A1XKG3 P15822 Q9Y6M1 23468 Q9UNH7 51341 51588 Q13185 10155 Q9BQI3 6188 P38936 64426 P37840 51593 Q9H9G7 Q99816 9208 Q96RE7 P29374 Q9H0M0 P42345 P62195 P04085 9219 Q92574 1978 P56524 9474 7057 Q02297 7291 O75530 55294 7295 P46934 Q9UER7 Q14865 7067 Q5TAX3 Q13535 P19793 P29590 Q7Z2Q5 84619 Q9H6W3 Q9HCP6 O60264 P05155 O60260 8165 O95983 Q13547 A4D1W7 P78318 Q8TBE0 Q04206 Q9UH92 Q92786 Q9Y2B9 7088 Q15735 Q9UKY1 Q9H9B1 51720 Q14894 P08246 Q13322 Q9NQZ2 Q9NP71 P30153 P11413 1789 1788 Q05516 1786 Q05513 P00734 3720 Q13573 Q9H2S9 P28065

P32320 Q9BQA5 Q9Y6E7 2644 1312 5914 O75953 Q13107 P62191 P28074 63976 P28072 P28070 O60238 P63279 P63272 Q9HCS4 P00750 P08238 P43686 Q14683 5925 Q7KZF4 Q9Y2W7 O75820 2672 Q9UBB5 Q9NS86 O00267 3516 O60566 283248 10772 P27348 5933 P07996 5935 Q9NS91 Q9UBC3 Q9Y2X9 54361 Q8NB12 5706 5707 5708 5709 4851 5700 5701 5702 O15524 5704 5705 Q92835 Q9NYF8 Q9Y2Y8 Q9NRY4 Q99471 P53667 5717 83933 O60543 Q9UJU2 O15534 P61289 5713 57649 Q92833 Q6IT96 P06401 4869 P06400 5716 5970 Q99460 Q9NRZ9 P56705 9099 5728 10524 400961 3784 10765 Q9ULR5 P05129 Q12986 P51668 P51665 Q9P0J0 O14627 6829 710 29102 Q8N2W9 10971 27161 P08887 5978 6827 Q9Y2T7 5991 93986 5993 O00231 Q14938 O00233 O00232 P19544 6839 3326 2475 P08651 P42771 5987 P26045 Q9NYB0 P05106 22937 22938 O00488 O00487 P30542 O60502 P62333 2247 58533 Q9BZK7 6601 Q12770 6605 5515 5518 P51693 P09958 2023 O00255 Q6KC79 O00257 10724 Q99496 5524 P05121 P40337 7704 P06213 O00425 P23396 O43159 P04637 2033 Q9NVW2 P52952 990 991 Q96RU7 4686 6622 79813 4204 10138 Q9UQ80 Q9BT49 4221 5796 P35368 Q9BT40 84159 Q9P2K8 P48436 Q99638 7727 P49768 2290 23429 P48431 5562 Q8N726 Q99623 773 Q00535 Q96S42 P25789 55869 P25788 6647 P01137 Q9UPY3 P25787 P01375 Q9UJM3 6660 8841 Q9UHI6 O00213 2063 23435 Q96AQ6 11218 Q9NS37 3148 P10070 Q15170 P17936 545 P10071 3146 P01127 6657 5327 5569 6670 5580 O43593 6672 5582 23409 Q969H0 25822 Q12906 Q9BZE0 Q9BZE4 O75190 Q99684 Q8TEB7 5578 P40763 P04201 4261 Q969G3 8864 7531 P37108 23411 23414 5590 O75182 Q00987 3169 5104 Q9H1A4 7529 7528 P27797 64375 O60907 P51843 3182 333 P51608 Q9H7L9 6207 5119 Q01826 Q9BT67 5371 8881 Q60FE5 P17980 P52701 580 O14818 P58012 10320 P60568 6231 8655 P30086 Q7Z6C1 Q13485 P03950 5154 5155 O43684 O75496 O75494 7329 Q15672 Q9UQR1 7341 6496 Q13257 6256 6498 P35998 Q2M1K9 84108 Q9UBU3 116113 Q13263 P00441 9774 4086 4087 8204 4088 4089 9775 Q9BY41 163126 Q9Y618 Q04724 P20264 11176 Q15697 53615 Q13033 P54259 9541 5187 O75469 64750 7124 11142 O15164 P54253 2909 Q96KC8 P14635 O75461 Q9UQL6 4092 P10276 Q02535 Q13042 Q99728 Q99729 Q15466 55806 Q99966 54 Q13286 5195 6045 7376 7375 Q09161 8467 9314 23135 23378 Q86WV8 6282 1822 166 P01308 Q9NQB0 2917 P49721 Q9UBP4 P49720 Q96T37 P54274 P14859 O43612 7141 Q9H063 Q96T23 P49959 Q9H1Y0 Q01664 Q00577 P49711 P49715 P09038 Q15008 O75437 7157 23112 O43623 25776 138151 2931 Q5VTD9 Q15257 Q06787 Q01094 P63167 26271 P41134 Q1PSW9 27122 P63165 O15119 1616 P45973 O15118 Q9NSA3 O94992 Q8WWH4 Q969S8 Q8IY57 P55316 Q96BD5 P67775 P60900 1869 2956 P52298 7189 92129 Q8WYK2 9126 7181 Q03014 Q86UE4 27102 P17252 O15379 Q8TAD8 Q9NSC2 O15151 Q13422 O94776 O43829 Q05655 84733 Q9UBN7 2737 Q92667 2736 O75626 Q8WUI4 27327 P41182 P30047 P62277 Q8WYQ5 Q13200 150094 Q07817 Q9UJX2 58487 Q9UQE7 P41162 Q9BXP5 3609 Q14781 P23510 P21333 P09429 O75604 O14495 29966 P19474 Q02363 P18146 P35711 O60341 P09430 P41159 O75832 Q9BZS1 Q13227 P55771 Q86YP4 Q96BF6 Q8N5A5 P30260 O15105 P21554

intracellular transport O75947 Q9NRW1 9182 22872 9181 Q9NRW7 Q5SQT9 Q9P2R6 O00148 Q9BVA0 1213 Q92973 O14662 Q9Y2X3 P60059 10651 10772 3875 P27348 Q92974 P48047 P55957 Q8IWJ2 55696 2316 1468 10427 O60333 P42858 Q9H2G9 O15400 4734 253725 Q12965 1121 Q12846 Q96P70 Q9GZY0 10514 4628 Q8IUD2 6809 55341 P62491 P18859 30000 Q86WA8 O15533 811 5830 P63104 Q92834 P62258 10632 4627 4869 P53675 Q9C005 P26374 91782 P55851 P31946 Q9H814 1130 56681 O95343 P53677 O60318 81622 Q99698 Q8NFW9 O60674 57410 1122 6810 10640 6811 O14579 27072 10762 5604 Q9UKX7 P41743 4637 P04233 Q9H1C4 O75901 O95477 Q92538 Q9H2M9 P28482 P50570 O95239 5862 3320 1020 Q9H1K0 Q9P0J0 Q9NRS6 10736 3313 O60763 O00471 3312 P07550 Q4VX76 4643 4644 Q9Y2T2 5976 3799 10972 4646 10971 6945 P61106 O95487 P32856 Q8WXF0 O95249 P33947 P53618 Q9NR31 P20645 O60895 6711 604 Q9UM54 P53621 4792 2495 Q12769 O60749

O60502 Q9NS69 10953 401505 P42768 2247 58533 P61006 5514 6726 Q13501 P51572 P53992 Q9UL45 P30536 Q13505 P51571 P31749 1176 O95140 1174 1173 Q9NQT8 27032 10724 Q8NBS9 Q92621 Q96ED9 O15504 Q9UL54 Q9H4M9 Q92624 P61019 Q8TBA6 509 P06576 P07900 6993 10490 Q9UPV9 P04637 Q9Y6W5 Q86V81 O96008 P16615 23325 P38646 O60725 Q99523 O14828 2147 513 4686 514 8924 Q15021 515 10013 10134 637 Q96KG9 10133 Q9C0K7 6748 51009 6747 Q15388 11345 135295 Q04917 4580 Q9Y678 6643 6642 O95166 23214 P50542 Q96Q15 400 521 522 55973 55737 11231 406 Q96CW1 408 Q15036 4218 P49407 P02794 P49768 P60468 O00327 6890 6892 Q86U42 Q9UMR2 O00560 2054 P33176 23303 Q96CV9 O00203 10239 79711 Q00535 Q02952 Q15286 Q9Y5X1 P49755 Q9GZP9 8943 P49754 54535 P01137 539 O14964 91949 Q9UP83 10476 84062 P01375 O43264 4000 5331 10482 O14980 23673 P12004 P54920 10128 O14617 Q01970 9804 P24588 9927 O00623 O00505 O43592 O00628 5584 Q9UPR3 O00629 P05408 Q641Q2 Q8IXI1 P14598 Q8IXI2 Q8IYJ3 P61981 Q2M389 P42704 Q8WTW3 O14908 A8K0Z3 8604 7514 10452 P55072 10577 P05412 9950 7532 5111 7531 7534 5476 7533 Q9HCE7 3178 P37108 P68032 5590 Q8WUX9 10228 10469 Q99797 Q9UBX3 P60660 8615 207 Q15075 9702 10466 P05783 7529 P62841 P27797 64130 P24386 6456 6576 22895 Q9NW64 Q96QU8 Q99666 O14925 56993 215 Q00610 7415 P83436 P49792 5371 4285 Q60FE5 8766 64145 9973 6103 Q9UKK6 O75396 O00410 23633 Q6ZWJ1 Q08209 P52948 P40616 P62745 8878 P40855 Q92900 10204 P25116 Q8N6T3 Q8TEW0 Q16623 8411 8775 57187 Q9UPN3 24137 P35658 94121 Q96FV9 94120 351 473 117584 23163 P07196 P09493 23165 P23786 Q9BQG0 P43034 P62913 Q96QK1 Q15785 P49815 Q8WUM0 P25963 Q9NPC8 Q9HAV7 Q8TD31 8546 8301 9632 11196 19 64601 79083 O75494 29959 Q9UJC3 O75381 488 Q9UIA9 415117 8417 Q13492 Q96RL7 8536 Q9H9E3 11079 9868 Q9HAU5 Q9BUN8 4074 Q15436 6496 Q9UNL2 8677 9525 P56134 9522 Q8WVM8 8676 23144 Q9H269 29927 Q9Y6Q5 Q96IZ7 375 23381 498 P25705 Q9UDY8 51542 Q01105 Q96RK4 P56385 8548 P56381 Q13023 P61966 51429 P61619 8563 6262 6386 Q9Y2J0 P62826 P62829 Q86XR8 Q9Y619 Q9UQ26 O75351 Q9NZZ3 23039 P12883 Q8N4C7 P59780 388 P61970 54820 51552 P60520 Q16543 9527 9648 Q03518 P43487 Q49AN0 Q8IXZ2 51319 9784 P54257 11140 O43752 7124 10053 11021 7248 P54253 P63092 P36542 Q9H089 Q4G0F5 P35579 154 P35580 Q9UEU0 Q99962 51560 84248 O94826 11269 P45379 Q14012 5195 9554 Q96C24 Q09161 O94955 11031 O75694 23256 161 Q86WV8 P11142 Q9Y5J6 Q8NI27 51693 7249 8218 Q9GZM8 Q9NRD5 9201 9685 Q86W42 Q86Y82 Q9BV36 O15066 23463 A1XKG3 Q86VS8 Q96EV8 Q9Y4I1 O43615 Q9UNH7 Q9NZ43 P24539 Q6VY07 P01579 9559 P02545 Q6I9Y2 P09038 2802 2923 55705 Q9BY84 O94973 O15075 O75436 O75558 7157 26985 O75674 Q9Y5L4 Q02750 O75431 Q8N4H5 Q8TEX9 O43747 Q8WZ73 Q9H173 Q9NXR1 Q13190 11014 10166 O94979 84376 P98194 P25874 P53367 8498 9463 O95714 Q96QC0 Q13636 P63172 84958 P53007 83752 79139 Q92572 10938 Q7RTN6 Q9UGU5 P09651 Q92574 O15118 Q9UBK9 Q14974 Q13769 O15131 9590 Q01082 9472 8021 P78537 81567 P46934 117177 P43307 10947 Q9HD26 81565 P52292 O15126 O60296 P05067 P52298 9001 P10619 P52294 O75886 26258 57120 P57740 P29590 Q03014 P30101 26019 Q4LE60 Q9UHY1 80145 P00846 60412 Q9NZQ3 O15258 Q9NSC5 O15155 O15397 27236 Q9UNE2 64901 9495 112936 9371 653361 56288 Q9UH92 1408 Q9UBN7 P30049 9146 O75746 Q9UBF2 Q9NP61 P41182 O00189 9382 3836 23085 P35606 9276 P56962 O95405 Q9H2T7 57154 Q15629 P51159 Q96IW7 Q07817 83548 23095 1785 Q05513 O60499 P63010 P00734 Q96QF0 3841 3843 80273 5901 5902 P21333 O75964 O75843 P10415 100287932 387680 P33897 2647 1315 1314 84661 Q9HAP6 9179 Q9NP90 Q14677 Q16611 11097 P62072 Q96QD9 Q9H6Z4 P84077 51510 Q13596 51517

intracellular signaling pathway Q9H0E2 Q2M1Z3 Q92737 65125 P51587 3638 Q8TDD1 P15498 P37288 55561 Q92731 10890 Q92730 O14544 O14543 6901 10413 P28335 64284 Q8NFZ5 P53779 P16104 2316 4734 Q9UKV5 Q59EK9 Q5S007 O75925 Q7Z727 Q9H8S9 Q9NR80 Q9NR81 3661 Q06124 79791 Q8IUD2

81876 P62491 Q9UKW4 811 O75928 P62258 10633 Q12851 P31946 3672 P31947 2100 Q9NR96 P21917 3428 10645 O60674 P20827 Q8IUC6 5602 3667 5604 P41743 Q8WXG6 Q13956 4772 5861 5862 1022 1020 Q9H765 Q5SGD2 1017 P15056 1499 1012 5610 P61587 P61586 P61106 Q9UL26 Q9H0H5 Q9UL25 P30679 Q9Y239 4780 5870 5871 Q15907 Q9UGI9 P10600 P52888 Q96KS0 1029 Q9BUB1 1026 Q9BUB5 5863 846 5865 5867 6714 5868 O14757 Q92766 5869 P49286 Q07890 O95136 Q9UGJ0 4792 5883 Q96L33 P16118 P19634 P18545 P38398 P60033 Q07889 4543 Q5TCX8 6721 P05230 Q6NUQ1 Q92993 857 O14763 5877 Q9UN86 Q96CA5 5878 5879 Q92997 Q13501 5894 P31749 O95382 Q38SD2 84932 P40692 55159 Q96MT8 10607 6733 P09601 O14777 Q9UL54 127829 P01241 8930 Q9Y5U4 Q96A33 P56199 P14317 P14314 O60603 25988 3479 2147 10010 5898 10499 P62873 10256 P23025 O96018 O96017 6760 Q15027 O95163 Q9NWZ3 P57059 1072 P35249 641 400 402 Q99750 Q14185 P47224 Q99759 O43294 O43293 O14713 Q13098 Q6ZN33 Q09472 O00329 O43251 P48552 6772 P48551 6774 116986 Q8WWX0 O00560 3265 116987 890 Q99500 51141 Q9H4B6 Q14192 898 3014 Q15286 P01019 51147 55504 29086 O14965 P35222 Q01974 Q01970 3269 6776 Q15052 7867 O14733 5207 P23497 Q59EA4 P26583 6790 Q5VVQ6 9821 P50613 672 673 Q8WUY9 10451 P62834 6548 7879 6789 P55072 10454 Q92930 7410 Q9NYZ3 Q05086 29079 Q9NWV8 P51959 23770 Q15077 8737 9826 207 4139 Q6IQ22 P24385 8754 Q9Y5S2 O00762 O75293 79753 7415 10672 O14920 10432 7433 8766 Q8NHX1 P50406 P52824 P08908 Q5TCQ9 P17612 O14939 P40616 8517 Q9H4E5 9846 326624 Q13115 O15085 P36507 Q14686 11065 P18085 P10114 Q9Y4K4 O43557 O75385 Q9Y4K3 O43318 P13861 472 Q9H0U4 23043 51762 P49815 Q14694 P35408 4188 P62805 Q16635 P13631 Q9Y2H1 Q9H0T7 488 7205 P55042 5029 P00533 P55040 Q96RL1 Q15796 Q15311 Q13131 Q9BUN8 9641 8795 P35638 Q15797 Q16644 Q9UQ13 8315 8555 Q9UNL4 Q9Y2I1 Q15561 P55290 Q15562 Q8IV61 5295 6386 Q9Y6R4 P62826 9656 Q9UPQ3 O75116 P55055 O43306 Q86XR7 Q8N4C8 267 51552 Q13393 9649 P43246 Q13153 P62820 Q8TDY2 11261 Q16666 11021 P54132 P63092 Q9Y4G2 O75582 P10398 P46109 51560 Q9Y2C9 Q9Y6K9 Q13162 8569 Q13164 Q15349 Q15109 11031 26524 P11021 P63096 7249 Q9NRD1 11035 P49841 Q15596 Q15111 Q15118 Q9BYZ6 7023 10392 O94844 P11229 Q7Z7G1 9448 O43734 A1XKG3 P58753 Q96PK6 55704 P08069 1718 P02545 Q13188 1956 P23443 Q9HB90 9217 O75553 P38936 O75791 P11234 P11233 2810 Q6PID4 Q99819 P78362 P23458 Q9BWT7 P48736 Q14289 9465 6196 6199 7046 6198 9467 P12931 O43715 6195 6194 P42345 Q9Y4C1 P98161 1978 7297 P31689 Q8WXJ9 7057 9475 9230 9231 Q3V6T2 7052 3925 P46934 P46937 P41240 P42338 P98171 Q9UER7 P42336 O75528 P34947 P29597 P29353 Q8WXI4 O95977 Q9H305 Q13535 O15264 P19793 P29590 Q9H0K1 Q7Z569 7064 P40145 P41231 57003 79039 O60266 P06241 P21860 Q13546 P78317 9252 Q07960 7074 80254 P43405 Q9Y6J8 Q9NVJ2 O14593 Q13315 Q03113 9021 Q16828 9261 O00194 23085 P63000 51720 Q13563 2869 Q8IWV1 7099 7098 Q9NVC6 Q14653 Q9NP71 O15211 Q9NP72 9270 P30153 P46736 P62166 P61073 5906 Q9Y6D5 Q05513 5908 2873 P00734 O60496 P24723 Q13574 51735 5901 Q15750 50649 53916 9046 Q15759 Q8NEB9 Q9P035 O60229 P20338 P20339 Q9BQA5 P20336 P20337 2885 1793 50650 O15455 5911 5914 Q9NP90 Q14676 A4D1S5 P34972 Q9H0N0 P00519 Q8TDM6 P24941 900 P20340 51512 Q15532 51514 Q14683 5925 Q9NRW1 Q92619 Q9NRW4 Q99490 P61020 55684 Q6PCE1 O60566 Q7L0Q8 Q8IVH8 2664 O00273 Q9HBH0 P61026 55207 P49137 P27348 P07996 P52564 O75815 P52565 Q7L591 O15530 P27361 65018 9077 P52566 P50148 Q9P107 10549 P16473 P16471 P48039 Q92844 O15524 P52333 P29992 339122 P53667 57403 10758 57646 29127 P20936 O60543 1111 P03372 O75807 4868 P06401 P06400 5716 P46089 Q92828 Q6PJ21 Q9BZF9 Q5JS13 3551 Q12979 9093 Q9UBE8 10524 3301 948 3309 P04233 Q6PCD5 O95477 P28482 O00220 Q9UHJ9 P19525 5739 P07550 P61225 P61224 4646 10971 Q7KZI7 P30556 Q5HYI8 P04049 Q96SB4 Q9NYA1 P30559 P10721 O95243 Q9NS56 29110 10746 2237 79444 1147 2475 10981 P42771 Q9UM54 Q92888 O95257 P28223 6850

22937 2011 Q9P0L2 P30542 22931 Q9NS68 P62330 O60503 2247 P15153 1399 2246 975 P61006 O14641 Q9Y2V2 Q92630 Q12770 5515 O43182 5518 P51451 P51693 Q12772 P51692 O00255 3350 Q9P0M2 P17342 10728 Q9UK32 P61019 Q9Y2W1 P61018 O15503 P06213 Q9Y6W6 Q9BST9 P04637 O43157 Q9Y6W5 P38405 2033 P16615 2274 Q96RU8 P62993 Q96RU2 998 P01116 Q15382 Q9C0K7 Q16236 6869 P27986 51009 285282 P62753 P01112 P01111 Q04917 P25100 4221 5310 6885 5311 P35368 266747 56940 Q8WTR2 9908 Q15392 Q9P2K8 4214 4215 4216 Q99638 10146 4218 P01343 P49768 O00206 Q9Y6Y9 5562 Q8N726 90864 P14416 O95292 P52735 P11388 Q9NS23 148022 Q00535 P61204 Q9GZP9 6647 Q9Y572 P01138 Q6UXV0 P01135 P01133 P01375 4000 5331 5573 64343 P04626 5333 5576 P04629 2065 2064 O00212 P12004 P35348 Q96RR4 O95071 3148 545 5566 5567 Q8WTP8 5568 Q96EY1 11215 7508 23647 5580 5581 6672 Q969H4 5583 5584 5585 Q5U0I6 P07949 Q9UNN5 3162 Q8IXI1 Q8IXI2 552 553 Q99683 P51828 55884 Q96AX2 5577 5336 5578 8844 Q15185 5337 5338 57826 P40763 P04201 2081 Q6IR47 5111 7531 7533 5598 64127 O76024 P18850 5590 Q01831 P16671 5347 7529 P27797 Q08462 5364 6453 2099 Q9UHD2 Q8IZJ4 333 Q9NRM7 5599 8625 P25103 5371 Q60FE5 Q12933 6464 P39748 P52701 P16885 55432 Q99653 P51617 Q9NPJ6 P62745 54106 8878 9967 10564 P25116 Q15654 9744 Q15418 6236 11186 8412 595 Q9UBS0 Q04759 Q96BM9 Q7Z6C1 P22694 8408 P25963 6242 O43687 Q13009 Q16512 Q15669 19 Q8TF76 Q9UKE5 367 369 6239 Q14344 51657 Q13017 6256 Q9HBW0 29 127247 Q86X27 P32241 P32246 Q8TEU7 Q9UBU3 375 Q9UDY8 P23528 6009 P00441 P32239 Q16539 4086 4087 8204 Q9BY44 Q15208 8445 P51812 P51813 381 382 5170 Q9Y3L5 Q02790 387 388 P21359 389 P31150 P24864 P60763 Q9NSD7 9546 7124 390 391 392 P10275 P10276 394 O75460 154 396 397 398 121512 25780 Q6KH11 Q8NI35 Q9H082 Q13283 Q96KC2 Q8WV28 Q15466 Q13287 Q13043 Q96R06 Q9BU20 7376 8463 Q9Y5J5 P35568 Q9UKA4 23370 Q8NFM4 P01308 Q8NFM5 Q9UKA8 120892 9306 P01303 2915 Q99956 Q8TCU4 Q6ZSZ5 O43612 P36404 Q01664 Q9NYS7 P01579 11124 140458 P09038 O95819 P60953 Q14155 Q9BY84 7153 64782 7157 O75676 P19086 9578 Q02750 2932 140461 Q9NZ56 Q15011 Q14164 1609 Q5VVH5 1848 54509 Q14964 Q15811 P53365 9100 Q13637 O95835 P57729 Q13636 Q12788 10928 Q86Z02 Q13639 7161 1613 P42224 1852 Q9BXM7 79139 1850 P23945 83871 P22612 P42229 1616 26270 1856 Q9UBK2 22926 Q6DT37 P10644 79363 P31323 P57735 P84095 P67775 Q96BD6 2956 9367 7186 7189 O94768 Q96M96 6093 P41597 P17252 P42680 P42681 P21980 Q8TAD8 Q86UE8 8289 O95622 9138 Q02156 4914 27352 Q05655 P13945 Q86YS6 P41180 P51148 Q7RTR2 P51149 B3KY43 Q9H244 P43119 Q03468 P08123 4920 O95644 150094 Q52LW3 P51157 Q13202 Q13443 Q96HU1 P51151 Q8IVT5 P51153 58480 P51159 27330 P20248 Q8TAI7 P08134 Q8WYP3 P17081 P21333 O14492 P84085 1676 1432 Q5XUX0 Q13464 P08588 O15350 P53355 P51178 O75832 O15111 Q14318 P53350 Q13227 Q15648 27315 P62070 O95661 P84077 Q9HC29 Q7L9L4 O14497 P45983 Q15653 Q13233 P45984 P21554

protein localization 25909 9182 22872 9181 Q96KP1 Q5SQT9 9184 1213 Q9UMY4 Q92973 10890 P60059 10651 3875 Q9Y275 Q92974 64284 Q9UGM3 Q92729 P55957 Q8IWJ2 O00159 2317 2316 10427 P42858 57510 Q9H2G9 O15400 4734 Q12846 Q96P70 6927 Q8IUD2 81876 55341 P62491 30000 811 5830 Q9UJ41 P63104 Q9Y6A5 P62258 Q96EA4 P26374 P31946 3673 56681 O95343 Q676U5 Q96N16 81622 P20823 Q8NFW9 O60674 821 10640 O14579 27072 Q96GD4 Q9UKX7 P41743 O75901 Q9H2M9 5861 5862 1020 114548 O95352 O60645 1499 P61106 Q9UL26 Q9UL25 P32856 5870 P33947 Q15907 Q9NR31 22841 Q96MV8 O60895 6711 604 5865 5868 5869 4792 Q9H2K2 Q8IUH5 401505 Q9HCL2 P60033 1277 Q6NUQ1 857 5877 Q9UN86 6726 859 5878 Q9Y490 Q13501 P51572 P53992 P10827 Q9UL45 Q13505 P51571 P31749 O95140 55153 Q9NQT8 84932 Q96ED9 Q9UMX1 P63313 O14777 Q9UL54 Q86UW7 Q9Y3Q3 6993 10490 O96008 P50897 25988 Q99523 1058 2147 8924 Q15021 10013 637 10015 6748 6747 11345 23339 O96018 4580 Q15027 Q9Y678 Q9NV70 O95166 55973 Q99750

55737 406 P47224 408 Q15036 P49407 409 Q15276 11116 P60468 O00327 Q9UMR2 Q9Y5W9 116986 O00560 116987 O00562 23303 10239 Q5VIR6 79711 Q15286 Q9Y5X1 8943 Q8NFP9 Q9Y5X2 O14964 Q9UP83 11325 O43264 Q9Y215 O14980 Q8N1F7 P35222 Q8N1F8 25978 Q96ST3 Q96T51 9804 P24588 11336 O00505 Q9Y3M2 P05408 Q9UID3 25942 P14598 Q8IYJ3 Q99567 P61981 O14908 55763 7879 10452 8724 9818 P55072 Q92930 P05412 P49450 10460 5476 Q9HCE7 10228 P37231 10469 Q99797 79720 55770 Q8IYI6 Q15070 207 5468 10466 Q6IQ22 P24386 9601 Q9H902 9600 Q8WV92 22895 Q96QU8 O14925 Q96P20 Q00610 7415 P61764 7414 10673 7430 8766 6103 Q9UKK6 P40855 10204 326624 Q16623 P18085 8775 P10114 Q9UPN3 P35658 O75385 473 Q9H0U4 117584 51762 P49810 5018 Q15785 P49815 Q9NPC8 Q9HAV7 Q9HAV4 8546 4188 9632 64601 79083 Q9UJC3 Q9H0T7 O75381 Q96RL7 Q9H9E3 P00533 9868 Q15554 Q9BUN8 9883 Q9UNL2 Q8WVM8 P32121 23265 Q9Y6Q5 4193 51542 Q96RK4 8548 P61966 Q9BSI4 6386 Q9Y2J0 129531 P62826 Q9UPQ3 P62829 Q86XR8 Q9UQ26 O75351 Q86VN1 Q9NZZ3 23039 Q8N6H7 Q8N4C7 Q709C8 P61970 51552 Q6PKD3 Q5SRE5 9648 Q03518 Q49AN0 Q9P2E9 P62820 149371 O43752 11021 P63092 P13667 Q9UEU0 51560 Q9Y4G6 O94826 54832 P27824 11269 7014 11031 23256 Q96D46 7251 152789 7249 Q9NRD5 Q86Y82 Q3ZCQ8 Q9BV36 23463 O43734 A1XKG3 Q86VS8 23225 Q9UNH7 Q15363 Q9C0E2 Q9H9H4 1718 P02545 1956 55705 9212 O15078 O75558 26985 Q8N4H5 O43747 Q9H173 Q13190 Q99816 11014 9465 9463 O95714 83752 Q92572 27243 55048 65082 O95721 9230 55054 9472 P46934 117177 Q9HD26 O60296 80230 7067 P29590 P56539 P30101 Q4LE60 29886 60412 Q9NZQ3 27236 Q9UNE2 9495 112936 79023 653361 O43707 O15027 Q9UH99 Q96NA2 Q9NP61 Q9P253 O00186 O00189 O00194 23085 Q8IWV1 P35606 9276 P56962 Q9NP72 Q9H2T7 Q86XP3 7094 Q96IW7 83547 Q05516 83548 1785 Q5QGT7 O60499 P63010 P00734 O60493 80273 5901 O75969 53916 100287932 1315 P20339 Q9BQA5 1314 P20336 P20337 Q9HAP6 5911 131474 Q9NP90 A4D1S5 63971 Q13107 11097 79065 Q9H6Z4 Q96H20 Q9H0N0 Q63HQ0 51510 Q96RF0 P20340 Q13596 51517 Q9NRW1 Q9NRW7 Q99490 Q9P2R6 P61020 Q9BVA0 O60566 2665 2664 O14662 Q9Y2X3 90678 P61026 P27348 10533 Q9UBC1 Q12955 Q8NF91 4860 54361 O95684 55696 P63211 92609 Q92845 P50395 1121 339122 Q9BXC9 57403 10758 6809 Q86WA8 Q92834 4627 4869 5716 P53675 Q92824 Q9H814 4641 1130 P56705 P53677 O60318 P17301 Q99698 1122 6810 6812 6811 6814 10762 948 P04233 Q9H1C4 O95477 P28482 P50570 O00221 Q9H1K0 Q9P0J0 Q9NRS6 10736 57662 3313 O60763 O00471 Q4VX76 Q9Y2T2 4645 10972 4646 10971 O95487 O95249 P53618 Q9NS56 P37198 57678 P18206 P62328 10981 Q9UM54 P53621 Q9NYB0 Q12769 O60749 22931 81555 P62330 80223 O60502 Q9NS69 10953 10956 2247 26056 58533 10959 975 P61006 O14640 5514 738 Q9H1H9 P30536 Q8NEZ2 Q969M3 1176 1174 1173 Q6KC79 Q60I27 Q9P0M2 10724 55275 Q9BXF6 Q92621 Q9ULV0 Q969M1 4678 O15504 10960 7704 Q92624 P61019 P61018 P02452 Q9UPV9 P04637 O95271 P38646 O60725 O14828 128866 10134 10133 Q9C0K7 Q15389 51009 Q15388 Q96HA1 Q04917 6643 6642 5796 Q6WKZ4 23214 P50542 9908 Q96RT1 Q15392 Q15154 11231 Q96CW1 4218 P49768 6890 Q9BV73 P14416 2054 23423 Q96CV9 O00203 9919 Q00535 Q02952 P61204 P49755 51026 Q9GZP9 54536 P49754 54535 P01137 Q7Z3C6 51028 91949 P01133 P01375 Q96JC1 4000 Q8N2H4 Q96EY5 23673 P12004 P54920 O14617 O95070 Q7Z3B4 9927 O00623 O43592 O00628 5584 Q5U0I6 8852 O00629 Q9Y2K9 Q9UNN5 Q12907 Q8WTW3 7514 Q96AX2 Q96AX1 5579 P05771 Q9Y4P1 7532 5111 7531 7534 7533 P37108 P16671 Q00987 8615 9702 P46059 P05783 7529 5108 P27797 O43572 64130 9961 P49321 Q9UPT5 P51843 Q9NW64 P15311 Q9NVU7 56993 51068 P83436 Q8N4Q1 5119 A5D8V6 23636 P49792 5371 9972 Q9BQQ3 P49790 4285 Q60FE5 64145 O75396 O00410 23633 Q969E2 Q6ZWJ1 Q08209 P52948 P62745 8878 P25116 57617 10566 Q8N6T3 54908 9744 Q8TEW0 94121 94120 Q7L804 23163 23165 Q8WUM4 P62913 Q96QK1 Q8WUM0 8408 P25963 Q8TD31 11190 O43687 O43684 11196 19 Q9H270 Q9UIA9 415117 8417 6238 Q15436 6496 Q6Y2X3

6495 8677 9525 9522 8676 Q9H269 29927 375 84342 Q8NFH4 9515 Q13023 51429 P61619 Q14118 29934 381 382 P59780 388 P31150 Q9NUP9 Q15691 P60520 Q16543 9527 9784 11140 9546 7124 10053 Q9Y3E0 O43633 Q9H089 Q4G0F5 P35579 Q9H082 Q13285 5195 9554 Q96C24 7375 Q5JQC9 O75694 29916 161 Q9Y5J6 Q9NWN3 8218 51699 Q15475 P46459 P00491 Q9UQN3 O43615 6293 Q9NZ43 Q6VY07 Q9UBQ0 P21145 P01579 11124 55823 P09038 2923 Q9BY84 O94973 7157 P14625 O75674 Q9Y5L4 25776 O75431 Q8TEX9 25777 Q8WZ73 O94979 84134 84376 1605 Q14964 P53367 8498 9342 Q13637 O95835 P57729 Q96QC0 Q13636 Q9H444 P63172 84958 P41134 79139 Q7RTN6 83871 Q14974 O15131 9590 Q01082 8021 22920 P57735 P43307 10947 P52292 O15126 22919 9367 P10619 P52294 O75886 Q8TEM1 26258 57120 Q9H1M0 P57740 Q03014 7184 Q9NSC5 9135 Q15843 O15155 O15397 Q6UWE0 64901 P21757 56288 1408 Q9UBN7 9146 Q9HC62 Q9UBF2 Q96G30 P41182 Q86YS6 P51148 9382 P51149 3836 4927 P51151 O95405 P51153 57154 Q15629 P51159 Q96QF0 Q8WYP5 Q9BVL2 3841 3843 P21333 O75843 59343 P84085 P01730 80184 9179 O75832 P62072 23192 Q8TAG9 P84077 Q15653

interspecies interaction between organisms Q7KZF4 3880 Q12824 O00268 P52799 O15519 5817 P16220 P84022 54476 Q92973 3875 5933 57506 Q9UGM3 P08729 Q08AM6 P08727 P27361 Q12959 Q08431 P20963 5701 4734 5702 Q9UI12 P10809 Q96EB6 P05787 Q92956 3661 79671 3416 Q92830 6921 Q9Y297 P62258 Q6IT96 4869 Q92831 P06400 P05556 O95347 3791 5970 3673 P17544 10524 P17301 1487 3665 708 P28482 Q12986 1385 90480 P18564 P19525 10616 P61221 2224 3312 5610 P61586 3678 P08648 3690 Q96SB4 P53618 Q96J02 3329 6839 29110 10625 1387 3685 P05141 3688 10980 6714 P05388 P05106 6850 4792 P30307 P51681 Q13617 Q13616 O60506 P20618 P60033 P15151 975 Q92993 857 O00499 P54725 1050 3107 O60870 5886 P05362 4799 Q92985 7706 P06213 Q9UKL0 6872 6993 O00308 P04637 Q86V81 10492 P15529 5422 2033 O60603 Q9BU70 23327 Q7L523 P62993 1058 995 6504 5898 10015 P27986 P14324 Q9Y6Y0 8815 O00206 6890 Q09472 6772 5684 5685 6774 P12236 O95292 3263 P12235 P11387 Q66K89 O60942 8945 Q53X93 P25789 Q9H6F5 5434 P25788 P01137 Q15046 4240 P01130 P23142 5692 5693 10482 O14980 7874 O00571 23435 P14784 Q86UT6 Q00403 5688 10488 Q96T58 P23497 8837 Q15056 6672 8732 P04439 Q96BZ9 P07948 O95197 3159 Q99683 Q96PU5 6304 6667 7514 5335 P40763 Q6UUV7 P05412 Q07021 P49450 7531 7412 O75179 3178 23411 Q08357 3054 Q05086 Q00987 23770 P05783 64135 6575 Q9UHD2 3065 O75164 5478 P25106 7416 Q01826 O43463 5371 9730 7791 7431 Q5T4S7 3190 Q8NHX1 P17980 P51610 O00410 Q5TCQ9 O14818 P52948 P28749 8517 8772 Q9BYX4 11060 O43432 P19174 6598 4179 57187 Q96FV9 P08047 Q7Z6C1 Q8WUM4 P25963 Q13363 4067 60489 7329 Q9BUN8 6256 P62937 P35998 4193 23264 P17096 7458 Q16531 11168 Q93009 8563 Q13148 5295 4088 P62826 P18074 O75475 Q9NXV2 387 P01889 P61978 Q03518 8453 8454 Q13158 274 Q9Y2C9 Q9Y6K9 Q15223 7132 Q14258 O43889 O43765 7251 Q7L5N1 P11142 Q13291 Q8NI27 Q14141 P49721 1948 P49720 Q13177 Q96T37 6175 Q86W42 P02790 7023 6059 291 292 9682 Q6VY07 Q01664 P02786 Q6I9Y2 P49715 Q15369 64784 7037 9217 7157 7150 P11233 Q93034 23352 Q99816 P63172 P12931 Q9H0M0 P42224 79139 Q9HC16 Q8TAF3 Q92692 P09651 1616 Q14974 Q15942 Q13769 O75531 83737 P46934 Q8TAE8 57599 P52292 Q4LE28 2959 Q9UER7 P06239 P52294 P19793 P29590 Q7Z2Q5 1877 P41597 Q9BYM8 80145 Q92793 2960 P06241 3932 Q13547 Q7Z434 P19320 P31785 P19438 P43405 1642 P08670 Q04206 P09884 Q13435 3836 1654 P08246 7099 7098 51606 P35968 Q14653 P19388 8065 P10321 P61073 Q9UGR2 3843 5901 Q9NWF9 O75843 P28065 1315 2885 23186 O15455 P01730 3856 Q14318 Q15768 P28074 P28072 P28070 O60238 P63279 P21675 P21796 5925

establishment of protein localization 25909 9182 22872 9181 Q96KP1 Q5SQT9 1213 Q9UMY4 Q92973 10890 P60059 10651 3875 Q9Y275 Q92974 64284 Q9UGM3 P55957 Q8IWJ2 O00159 2316 10427 P42858 57510 Q9H2G9 O15400 4734 Q12846 Q96P70 Q8IUD2 81876 55341 P62491 30000 811 5830 Q9UJ41 P63104 P62258 P26374 P31946 3673 56681 O95343 Q676U5 Q96N16 81622 Q8NFW9 O60674 821 10640 O14579 27072 Q9UKX7 P41743 O75901 Q9H2M9 5861 5862 1020 114548 O95352 O60645 P61106 Q9UL26 Q9UL25 P32856 5870 P33947 Q15907 Q9NR31 22841 Q96MV8 O60895 6711 604 5865 5868 5869 4792 Q8IUH5 401505 Q9HCL2 1277 Q6NUQ1 5877 6726 5878 P51572 P53992 Q9UL45 Q13505 P51571 P31749 O95140 55153 Q9NQT8 84932 Q96ED9 Q9UL54 Q86UW7 Q9Y3Q3 6993 10490 O96008 P50897 25988 Q99523 2147 8924 Q15021 10013 637 10015 6748 6747 11345 23339 O96018 4580 Q15027 Q9Y678 Q9NV70 O95166 55973 55737 406 P47224 408 Q15036 P49407 409 Q15276 P60468 O00327 Q9UMR2 Q9Y5W9 116986 O00560 116987 O00562 23303 10239 Q5VIR6 79711 Q15286 Q9Y5X1 8943 Q9Y5X2 O14964 Q9UP83 O43264 O14980 Q8N1F7 25978 Q96T51 9804 P24588 11336 O00505 P05408 Q9UID3 P14598 Q8IYJ3 Q99567 P61981 O14908 55763 7879 10452 8724 9818 P55072 Q92930 P05412 5476 Q9HCE7 10228 P37231 10469 Q99797 79720 55770 Q8IYI6 207 5468 10466 Q6IQ22 P24386 9601 9600 Q8WV92 22895 Q96QU8 O14925 Q96P20 Q00610 7415 P61764 10673 8766 6103 Q9UKK6 P40855 10204 326624 Q16623 P18085 8775 P10114 Q9UPN3 P35658 473 Q9H0U4 117584 51762 P49810 Q15785 P49815 Q9NPC8 Q9HAV7 Q9HAV4 8546 9632 64601 79083 Q9UJC3 Q9H0T7 O75381 Q96RL7 Q9H9E3 9868 Q9BUN8 9883 Q9UNL2 Q8WVM8 P32121 23265 Q9Y6Q5 4193 51542 8548 P61966 6386 Q9Y2J0 129531 P62826 Q9UPQ3 P62829 Q86XR8 Q9UQ26 O75351 Q86VN1 Q9NZZ3 23039 Q8N6H7 Q8N4C7 P61970 51552 Q5SRE5 9648 Q03518 Q49AN0 Q9P2E9 P62820 149371 O43752 11021 P63092 P13667 Q9UEU0 51560 O94826 P27824 11269 11031 23256 Q96D46 7251 152789 7249 Q9NRD5 Q86Y82 Q3ZCQ8 Q9BV36 23463 O43734 A1XKG3 Q86VS8 23225 Q9UNH7 Q15363 Q9C0E2 Q9H9H4 P02545 55705 O15078 O75558 26985 Q8N4H5 O43747 Q9H173 Q13190 Q99816 11014 9463 O95714 83752 Q92572 27243 55048 65082 O95721 9230 55054 9472 P46934 117177 Q9HD26 O60296 80230 P29590 P30101 Q4LE60 29886 60412 Q9NZQ3 27236 Q9UNE2 9495 112936 79023 653361 O43707 O15027 Q96NA2 Q9NP61 Q9P253 O00186 O00189 O00194 23085 Q8IWV1 P35606 9276 P56962 Q9NP72 Q9H2T7 Q96IW7 83547 83548 1785 O60499 P63010 P00734 O60493 80273 5901 53916 100287932 1315 P20339 Q9BQA5 1314 P20336 P20337 Q9HAP6 5911 131474 Q9NP90 A4D1S5 63971 11097 79065 Q9H6Z4 Q96H20 Q9H0N0 Q63HQ0 51510 Q96RF0 P20340 Q13596 51517 Q9NRW1 Q9NRW7 Q99490 Q9P2R6 P61020 Q9BVA0 2665 2664 O14662 Q9Y2X3 90678 P61026 P27348 10533 Q12955 4860 54361 55696 92609 P50395 1121 339122 57403 10758 6809 Q86WA8 Q92834 4627 4869 P53675 Q92824 Q9H814 4641 1130 P56705 P53677 O60318 P17301 Q99698 1122 6810 6812 6811 6814 10762 948 P04233 Q9H1C4 O95477 P28482 P50570 Q9H1K0 Q9P0J0 Q9NRS6 10736 3313 O60763 O00471 Q4VX76 Q9Y2T2 4645 10972 4646 10971 O95487 O95249 P53618 P37198 57678 10981 Q9UM54 P53621 Q12769 O60749 22931 81555 P62330 80223 O60502 Q9NS69 10953 2247 26056 58533 10959 P61006 5514 738 Q9H1H9 P30536 Q8NEZ2 Q969M3 1176 1174 1173 10724 55275 Q9BXF6 Q92621 Q9ULV0 Q969M1 4678 O15504 10960 Q92624 P61019 P61018 P02452 Q9UPV9 P04637 O95271 P38646 O60725 O14828 128866 10134 10133 Q9C0K7 51009 Q15388 Q96HA1 Q04917 6643 6642 Q6WKZ4 23214 P50542 11231 Q96CW1 4218 P49768 6890 2054 23423 Q96CV9 O00203 9919 Q00535 Q02952 P61204 P49755 51026 Q9GZP9 54536 P49754 54535 P01137 Q7Z3C6 51028 91949 P01375 Q96JC1 4000 Q8N2H4 Q96EY5 23673 P12004 P54920 O14617 O95070 Q7Z3B4 9927 O00623 O43592 O00628 5584 Q5U0I6 O00629 Q9Y2K9 Q12907 Q8WTW3 7514 Q96AX2 Q96AX1 5579 P05771 Q9Y4P1 7532 5111 7531 7534 7533 P37108 P16671 Q00987 8615 9702 P46059 P05783 7529 P27797 64130 9961 P49321 Q9UPT5 Q9NW64 Q9NVU7 56993 51068 P83436

Q8N4Q1 5119 A5D8V6 23636 P49792 5371 9972 Q9BQQ3 P49790 4285 Q60FE5 64145 O75396 O00410 23633 Q969E2 Q6ZWJ1 Q08209 P52948 P62745 P25116 57617 Q8N6T3 9744 Q8TEW0 94121 94120 Q7L804 23163 23165 Q8WUM4 P62913 Q96QK1 Q8WUM0 P25963 Q8TD31 11196 19 Q9H270 Q9UIA9 415117 8417 6238 Q15436 6496 Q6Y2X3 8677 9525 9522 8676 Q9H269 29927 375 84342 Q8NFH4 9515 Q13023 51429 P61619 29934 381 382 P59780 388 P31150 Q9NUP9 P60520 Q16543 9527 9784 11140 9546 7124 10053 Q9Y3E0 O43633 Q9H089 Q4G0F5 P35579 Q9H082 5195 9554 Q96C24 O75694 29916 161 Q9Y5J6 Q9NWN3 8218 51699 P46459 P00491 Q9UQN3 O43615 6293 Q9NZ43 Q6VY07 Q9UBQ0 P01579 55823 P09038 2923 Q9BY84 O94973 7157 P14625 O75674 Q9Y5L4 O75431 Q8TEX9 Q8WZ73 O94979 84134 84376 Q14964 P53367 8498 9342 Q13637 P57729 Q96QC0 Q13636 Q9H444 P63172 84958 79139 Q7RTN6 83871 Q14974 O15131 9590 Q01082 8021 P57735 P43307 10947 P52292 O15126 9367 P10619 P52294 O75886 Q8TEM1 26258 57120 Q9H1M0 P57740 7184 Q9NSC5 9135 O15155 O15397 Q6UWE0 64901 P21757 56288 1408 Q9UBN7 9146 Q9HC62 Q9UBF2 P41182 Q86YS6 P51148 9382 P51149 3836 4927 P51151 O95405 P51153 57154 Q15629 P51159 Q96QF0 Q8WYP5 Q9BVL2 3841 3843 P21333 O75843 59343 P84085 80184 9179 P62072 23192 Q8TAG9 P84077

protein modification process 8099 Q9H0E3 Q9Y265 65125 P28562 P54619 Q9NQU5 1459 1457 Q8IYN9 55561 55568 Q5QP82 1454 O00391 1453 Q6IQ55 Q92729 Q5VTR2 P53779 27090 Q9H0C8 10420 4734 Q9UKV5 Q96EB6 Q9H2G2 Q5S007 Q7Z727 Q9UGN5 P28300 81629 Q8NE63 O00167 2571 Q06124 A0AVT1 P62495 54496 P37023 Q8IUD2 P62256 2324 57761 57520 O75928 P27448 Q12851 Q9C005 O75914 3672 Q9NR96 Q13705 P21917 1491 P54646 10645 Q5TAQ9 55352 2335 O60674 1487 P20827 148789 5602 Q96GD4 O14578 5604 P41743 Q8WXG6 P25098 P51784 Q13956 Q9Y463 1022 Q96MX6 1021 1020 Q06587 Q5SGD2 2590 O95352 57787 1018 2107 1017 P15056 79577 10856 2589 10616 5610 Q9Y230 Q9Y231 5613 O14744 26191 P30679 Q9UGI0 Q92769 5870 Q13724 Q9H4P4 5871 P09619 O95361 P10600 Q96J02 10626 10868 O14519 Q8TBC4 Q96MV8 1025 Q9BUB5 6711 6714 O14757 O95136 Q9Y243 Q9H2K2 Q13976 P21709 Q96L34 O95376 79595 P18545 Q8TBB1 Q8IUH5 P38398 169436 Q05469 2125 P60033 613 Q5TCX8 5875 Q92993 Q96CA5 4548 Q10472 Q10471 O95147 Q5MAI5 6500 5894 P31749 O95382 Q38SD2 Q4G0W2 2139 253980 57332 O00141 55159 80335 6733 867 8916 Q9UGL1 Q9UL54 Q8TBA6 O00308 P56199 23326 P50897 O60603 23327 23569 11107 2147 Q15022 P62877 8924 7832 10013 84289 Q96KG9 10014 O96017 64682 Q9UII4 Q86SR1 O96019 O95163 Q9NWZ3 P57059 115426 1072 O96013 51132 51377 Q99750 84033 11113 Q9BS18 Q99759 408 O43293 P49407 409 Q15034 Q09472 O00329 84061 6772 P35236 10238 P11274 55743 Q9NR09 51147 10477 29086 O14965 Q9UMS4 P40818 Q8N3J5 Q9Y697 7874 23552 3276 Q658W2 23556 P35227 P14543 Q01974 P35232 P60484 Q15291 Q9H093 6776 55757 O14730 7867 285193 O14733 O14974 6790 Q5VVQ6 6793 8732 P01040 P62837 6794 4133 O00506 10690 P50613 Q99570 P39656 O00743 672 673 6304 4125 Q9H2D6 79739 6305 6789 10213 P55072 158135 5470 4140 140609 8985 Q9HCE7 P51955 3297 P51956 Q05086 O00754 Q99558 Q9NWV8 444 8737 207 Q02809 4139 Q15078 284086 23746 P24386 8754 23509 Q9Y5S2 5245 3066 3065 Q9UIF9 Q08345 O00762 O75293 55781 51185 Q00613 7415 O14920 10432 P56180 Q9H4F1 5494 5495 P49674 Q9UIG0 Q9BU89 Q92905 5499 Q8NHX1 23512 Q7L7X3 P17612 O14936 Q96QT4 Q56UN5 7428 10445 P49427 Q13115 P36507 11060 11065 7204 Q9BZ95 Q9Y4K4 P11441 O75385 Q9Y4K0 Q86VQ3 Q9Y4K3 O43318 472 P48729 O75150 Q9H0U3 Q6P5Z2 23043 9616 8767 Q14694 Q13363 3093 Q15303 11072 5034 4188 Q15306 Q9Y4L5 Q86VP6 8780 Q9Y2H1 23291 Q9NRH2 8536 P00533 Q96RL1 P61956 Q15796 197259 Q9HAU4 Q13131 9641 Q15797 Q16644 10193 9646 7465 Q9UPP1 8555 P32121 Q9UNL4 Q6UXN9 O75365 23028 Q96IZ7 4193 O94804 P61962 P61960 51548 Q93009 P22314 51547 Q93008 Q8NCB2 P61964 Q16659 Q9Y6R4 57092 O75116 P10586 O75352 Q8N4C8 267 23031 23032 Q96PF1 Q9NPF5 54822 Q49AN0 Q13153 51319 8576

26994 O75582 P10398 Q9Y2C2 Q13162 P23467 8569 Q13164 Q14012 Q15349 O15294 Q9NRC8 Q8TDX7 55929 Q9BV47 Q9HAZ1 26524 5071 7251 P13674 11035 P49841 Q13177 Q9NRD5 P02751 Q15118 P78368 9201 Q3ZCQ8 P49840 P11229 O75569 9448 7267 10152 23463 Q9Y6M4 A1XKG3 9682 Q08188 Q8WY64 Q8NEJ0 Q9NVM4 Q96PK6 P08069 Q9UEW8 10155 Q9BQI3 1716 Q13188 1956 P23443 O75319 9212 6185 O15075 Q9NPA8 Q9BWU1 Q15126 23476 Q9UNI6 6184 7272 1965 Q92187 Q6PID4 Q99816 P48730 P78362 P23458 54623 1969 P48736 Q14289 Q8WW12 Q15375 6196 6199 9463 7046 Q6P1J9 6198 P09769 P35813 Q96L91 O95714 27005 P12931 6195 79012 Q9H0M0 P42345 P22735 Q9Y4C1 27246 P04083 O60285 Q13523 Q58WW2 7297 P56524 375743 9474 7057 9475 Q9H2P9 83737 Q9H792 55294 7052 27252 P46934 P41240 Q92560 O60296 Q4LE28 P42338 Q9UER7 P42336 O75528 P34947 P29597 P06239 P29353 Q5JXB2 Q13535 O15264 P29350 P29590 Q9H0K1 Q7Z2Q5 Q9H6W3 Q9BYM8 29880 Q9Y4E8 Q92793 26133 Q7Z6Z7 P06241 P21860 3932 Q9Y2A9 O60260 Q9HCP0 O95983 Q16816 Q13547 Q13546 55072 Q8IYT8 P78317 9252 Q9UNE7 P43405 201595 P20309 Q9Y6J8 P53041 P07585 P41227 Q13555 Q16825 Q13554 Q96EP0 Q5GLZ8 Q16827 O14595 Q13315 Q13557 P78509 Q16828 Q96EP1 9261 9020 23081 23085 Q9H9B1 P20594 51720 2869 7099 7098 30849 Q15746 55093 P30153 Q86Y13 P61081 Q9H596 P46736 P61073 83548 Q05513 23097 P61077 P00734 1540 P24723 Q15751 P46977 P46734 Q16849 Q15759 O75962 Q8IUQ4 26576 Q9P035 29843 P78527 O60229 P41279 Q9Y6E7 Q9Y6E0 P61088 Q04656 84661 Q96RG2 P05198 O15455 5914 O75716 11091 30827 Q16854 Q6XPS3 Q13107 P18031 Q9P286 9054 P22528 1326 Q63HQ2 P63279 P00519 P47804 5929 P24941 11099 P20340 P00750 51514 P21675 Q9NZN9 O14672 Q9NRW4 O60568 Q8NB16 O60566 Q8IVH8 P16220 P63208 90678 P49137 P07996 10533 P52564 P09936 O15530 P27361 Q9GZZ9 Q8IZP0 65018 Q9H832 54361 P50148 57634 P16234 10549 79885 P21810 P16471 10783 92609 Q9UHP3 P52333 65264 P54753 O95696 P29992 1121 Q05397 P54756 P53667 O60307 57646 83933 1111 P32929 Q92830 P62136 26091 Q6IT96 Q92831 P55854 P54764 P54762 3791 P54760 Q12974 4882 Q8NCR0 3551 Q9UBE8 P56705 9099 Q13829 152926 5728 10524 Q8WTX9 P62140 57410 55236 O60551 Q9UI30 10765 P04233 Q6PCD5 P34925 O00469 O95476 P05129 O95477 133584 P28482 P11802 1385 Q9UHJ9 P51668 P50579 Q9P0J1 P19525 10733 147746 Q96DE5 O60762 Q7KZI7 P51671 O00238 Q7Z419 P04049 Q96SB4 P10721 P19784 Q5VST9 P53611 Q9NS56 Q8WYH8 10987 29110 10746 10988 79444 1147 1387 2475 Q92643 5500 5747 4659 P26045 P30304 O95257 P30305 6850 Q13615 P30307 2011 Q13618 Q9NYB9 27148 Q13616 1161 O00487 Q9P0L2 P30542 Q9P2P5 10956 57448 2247 975 22929 Q96DC9 Q6GMV3 Q92630 5515 Q9UK22 65061 5516 5518 Q969M7 P51451 P29323 5770 P29320 Q13627 P30530 P18433 Q03164 81788 Q99259 Q96LR5 P37173 984 Q99496 6612 Q9Y385 6613 P29317 P40337 5768 Q9UK32 P06213 Q8N2K1 Q15139 Q9Y6W6 O95278 5780 6872 Q96HA8 Q9UPV9 6871 5783 P53804 2033 Q9NVW2 O95271 O60725 Q96RU7 Q96RU2 O14829 994 995 Q99640 997 5775 79813 5536 5778 5537 Q9UM07 6883 6885 Q9UQ88 5796 1198 23210 1196 2043 1195 56940 Q93096 Q96Q15 Q8WTR2 Q9NRP7 Q99873 54764 55611 Q96S53 Q9P2K8 4214 O14830 4215 4216 P46019 P49768 O00206 Q96EZ8 25847 5562 5563 P49761 P49760 O00444 O60942 Q00535 Q00534 79834 Q53X93 55869 5798 6647 Q9Y572 P01137 91949 P01135 P01133 P01375 Q9BT22 P02461 P01130 Q9Y2R2 P04626 5575 8841 P04629 2065 25853 2064 O00213 780 Q96RR4 301 O95071 P17936 Q8IZE3 545 5566 Q96PN8 5567 5568 Q00526 5327 O43353 Q9H5K3 5580 5581 5582 5583 P07947 23409 5584 5585 8851 P07949 Q969H0 5586 P07948 Q92817 Q9Y2K6 Q9Y2K2 Q96PU4 79612 Q8IZL9 Q99683 Q96PU5 5578 8844 P49336 P62714 5579 10331 4008 P05771 Q9H3F6 8607 2081 5591 9950 Q6IR47 5351 5352 5596 5597 5598 23411 5590 Q00987 4015 Q9H1A4 5347 4017 329 84197 Q96JH7 Q9GZU7 P50750 91754 Q9UHD2 Q12923 Q9UPT9 O75164 Q9Y2M5 Q8NER5 Q9NRM7 P68036 5599 Q68J44 P83436 Q01826 5371 8881 Q12933 6464 7311 391356 Q96Q40 146057 580 Q5U4P2 221656 54101 55432 P51617 Q08209 6699 P62988 5127 54106

Q8N752 P25116 10090 Q13237 4297 Q6ZT98 Q15418 7325 P15924 Q9NYU1 351 Q9NYU2 Q9UBS0 Q04759 29941 Q7Z6C1 83475 P22694 9978 P21127 8408 Q9HC98 O43683 7332 7334 Q16512 4067 Q16513 7336 Q16514 7335 19 Q8TF76 Q9UKE5 29959 Q9UBT2 23170 369 128 P60983 7329 Q13490 7328 51657 7341 P11309 P22681 10075 Q9HBW0 P11308 6015 Q16526 P29074 23387 P31152 Q9UBU3 Q03405 Q9NV06 P23528 8428 5159 Q16531 Q9UBU8 Q8WUK0 P43378 Q8WZ42 Q13263 P00441 Q16539 4086 344387 4087 Q6PD62 8446 Q9BY44 O75478 Q15208 8445 Q9BY41 P51812 23399 P51813 Q8TD19 5170 142 Q9UBV7 P51817 10087 6259 11176 9526 P49903 Q8WUJ0 53615 8454 8452 P55345 Q6ZN16 64750 7124 O15164 7126 Q9UQL6 4092 P10276 4093 O75460 156 121512 P27708 10055 Q13042 Q99728 52 Q13043 Q13049 6045 P04843 7375 P04844 Q96KB5 6048 23133 P36896 23135 P15735 P36894 Q9UKA1 Q02763 54961 120892 7128 Q99956 8473 Q8TEY7 P61803 8237 10273 Q96FX2 Q9Y5K5 Q9UKB1 P36888 Q99942 1832 Q9H1Y0 51465 P01579 Q99707 P09038 O95819 55827 Q9NQC7 Q9BY84 O75676 O15197 O15198 9578 Q96FW1 Q02750 25778 1843 2932 2931 255488 55832 51231 Q14164 1848 Q6VAB6 Q15256 86 2935 Q14166 Q13873 9100 O95835 Q15819 Q86Z02 Q8TF05 1613 Q05209 80124 P42224 1852 10933 Q9BXM7 1850 Q8TAF3 P63165 91 Q8WWI1 O95837 O15355 Q92696 94 Q12792 1859 P22612 P42229 Q13882 P45974 1616 P07237 Q969T4 8266 Q12797 Q969S8 Q13404 Q6DT37 Q8TCJ0 Q8TCJ2 Q01082 Q9H3R0 P31321 84708 P67775 57599 P08575 P05067 Q9UBL3 7186 Q15831 7189 O94768 Q6XUX3 P35916 Q9H422 Q96M96 27347 Q13418 6093 Q9UHY1 1634 Q9Y3C5 27102 Q8TEL6 P17252 P42680 O15379 O95619 P42681 P21980 P42684 P42685 Q86UE8 P09874 Q15843 Q6R6M4 Q02156 Q6UWE0 Q8NB78 4914 26260 26262 1642 P68400 Q05655 Q9BXJ9 1408 1407 Q9UBN7 Q9HC62 Q9UBF6 9149 56052 Q6NYC1 Q9H488 P11766 57144 26232 Q86SG6 Q9UK80 Q03468 26234 1650 4920 Q9HC52 150094 9397 P35968 Q13202 Q13443 Q8WZ19 Q9NQ92 Q8IVT5 P21580 9150 57154 57396 Q8N7H5 Q9H1R2 27339 27330 80155 Q9UJX2 Q9Y5B0 51611 8078 O75604 Q8IY84 P10415 Q13216 93492 377630 P19474 59343 P08581 3611 1432 Q8NHH1 O60341 Q5T6F0 Q13464 P01730 728642 P53355 O95427 O15111 P53350 Q9P0U3 Q9P0U4 27315 Q04771 26469 389840 P30260 Q7L9L4 O15105 P45983 5800 Q13233 P45984

macromolecule modification 8099 Q9H0E3 Q9Y265 65125 P28562 P54619 Q9NQU5 81605 1459 1457 Q8IYN9 55561 55568 Q5QP82 1454 O00391 1453 Q6IQ55 P05455 4968 Q92729 Q5VTR2 P53779 27090 Q9H0C8 10420 4734 Q9UKV5 Q96EB6 Q9H2G2 Q5S007 Q7Z727 Q9UGN5 P28300 81629 Q8NE63 O00167 2571 Q06124 A0AVT1 P62495 54496 P37023 Q8IUD2 P62256 2324 57761 57520 O75928 P27448 P26358 Q12851 Q9C005 O75914 3672 Q9NR96 Q13705 P21917 1491 P54646 10645 Q5TAQ9 55352 2335 O60674 1487 P20827 148789 5602 Q96GD4 O14578 5604 P41743 Q8WXG6 P25098 P51784 Q13956 Q9Y463 1022 Q96MX6 1021 1020 Q06587 Q5SGD2 2590 O95352 57787 1018 2107 1017 P15056 79577 10856 2589 10616 80324 5610 Q9Y230 Q9Y231 5613 O14744 26191 P30679 Q9UGI0 Q92769 5870 Q13724 Q9H4P4 5871 P09619 O95361 P10600 Q96J02 10626 10868 O14519 Q8TBC4 Q96MV8 1025 Q9BUB5 6711 6714 O14757 O95136 Q9Y243 Q9H2K2 Q13976 P21709 Q96L34 O95376 79595 P18545 Q8TBB1 Q8IUH5 P38398 169436 Q05469 2125 P60033 613 Q5TCX8 5875 Q92993 Q96CA5 4548 Q10472 Q10471 O95147 Q5MAI5 6500 5894 6741 P31749 O95382 Q38SD2 Q4G0W2 2139 253980 57332 O00141 55159 80335 6733 867 8916 Q9UGL1 Q9UL54 Q8TBA6 8930 O00308 P56199 23326 P50897 O60603 23327 23569 11107 2147 Q15022 P62877 8924 7832 10013 84289 Q96KG9 10014 O96017 64682 Q9UII4 Q86SR1 O96019 O95163 Q9NWZ3 P57059 115426 1072 O96013 O14717 51132 51377 Q99750 84033 11113 Q9BS18 Q99759 408 O43293 P49407 409 Q15034 Q09472 O00329 84061 6772 P35236 346171 10238 P11274 126789 55743 Q9NR09 51147 10477 29086 O14965 Q9UMS4 P40818 Q8N3J5 Q9Y697 7874 23552 3276 Q658W2 23556 P35227 P14543 Q01974 P35232 P60484 Q15291 Q9H093 6776 55757 O14730 7867 285193 O14733 O14974 P57081 6790 Q5VVQ6 6793

8732 P01040 P62837 6794 4133 O00506 10690 P50613 Q99570 P39656 O00743 672 673 6304 4125 Q9H2D6 79739 6305 6789 10213 P55072 158135 5470 4140 140609 Q9Y5R4 8985 Q9HCE7 P51955 3297 P51956 Q05086 O00754 Q99558 Q9NWV8 Q96SZ6 444 8737 207 Q02809 4139 Q15078 284086 23746 P24386 8754 23509 Q9Y5S2 5245 3066 3065 Q9UIF9 Q08345 O00762 O75293 55781 51185 Q00613 7415 O14920 10432 P56180 3070 Q9H4F1 5494 5495 P49674 Q9UIG0 Q9BU89 Q92905 5499 Q8NHX1 23512 Q7L7X3 P17612 O14936 Q96QT4 Q56UN5 7428 10445 P49427 Q13115 P36507 11060 11065 7204 P55265 Q9BZ95 Q9Y4K4 P11441 O75385 Q9Y4K0 Q86VQ3 Q9Y4K3 O43318 472 P48729 O75150 Q9H0U3 Q6P5Z2 23043 9616 8767 Q14694 Q13363 3093 Q15303 11072 5034 4188 Q15306 Q9Y4L5 Q86VP6 8780 Q9Y2H1 23291 60489 Q9NRH2 8536 P00533 Q96RL1 P61956 Q15796 197259 Q9HAU4 Q13131 9641 Q15797 Q16644 10193 9646 7465 Q9UPP1 8555 P32121 Q9UNL4 Q6UXN9 O75365 23028 Q96IZ7 4193 O94804 P61962 P61960 51548 Q93009 P22314 51547 Q93008 54815 Q8NCB2 Q8WVM0 P61964 Q16659 Q9Y6R4 57092 O75116 P10586 O75352 Q8N4C8 267 23031 23032 Q96PF1 Q9NPF5 54822 Q49AN0 Q13153 51319 8576 26994 O75582 Q9Y6K1 P10398 Q9Y2C2 Q13162 P23467 8569 Q13164 Q14012 Q15349 O15294 Q9NRC8 Q8TDX7 55929 Q9BV47 Q9HAZ1 P46100 26524 5071 7251 P13674 11035 P49841 Q13177 Q9NRD5 P02751 Q15118 P78368 9201 Q3ZCQ8 P49840 P11229 O75569 9448 7267 10152 23463 Q9Y6M4 A1XKG3 9682 Q08188 Q8WY64 Q8NEJ0 Q9NVM4 Q96PK6 P08069 Q9UEW8 10155 Q9BQI3 1716 Q13188 1956 P23443 O75319 9212 6185 O15075 Q9NPA8 Q9BWU1 Q15126 23476 Q9UNI6 6184 7272 1965 Q92187 Q6PID4 Q99816 P48730 P78362 P23458 54623 1969 P48736 Q14289 Q8WW12 Q15375 6196 6199 9463 7046 Q6P1J9 6198 P09769 P35813 Q96L91 O95714 P29372 27005 P12931 P78549 6195 79012 Q9H0M0 P42345 P22735 Q9Y4C1 27246 P04083 O60285 1736 Q13523 Q58WW2 7297 P56524 375743 9474 7057 9475 Q9H2P9 83737 Q9H792 55294 7052 27252 P46934 P41240 Q92560 O60296 Q4LE28 P42338 Q9UER7 P42336 O75528 P34947 P29597 P06239 P29353 Q5JXB2 Q13535 O15264 P29350 P29590 Q9H0K1 Q7Z2Q5 Q9H6W3 Q9BYM8 29880 Q9Y4E8 Q92793 26133 Q7Z6Z7 P06241 P21860 3932 Q9Y2A9 O60260 Q9HCP0 O95983 Q16816 Q13547 Q13546 55072 Q8IYT8 P78317 9252 Q9UNE7 P43405 201595 P20309 Q9Y6J8 P53041 Q9UET6 P07585 P41227 Q13555 Q16825 Q13554 Q96EP0 Q5GLZ8 Q16827 O14595 Q13315 Q13557 P78509 Q16828 Q96EP1 9261 9020 23081 23085 Q9H9B1 P20594 84881 51720 2869 51726 7099 7098 30849 Q13569 Q15746 55093 P30153 Q86Y13 P61081 Q9H596 1789 1788 P46736 P61073 1786 83548 Q05513 23097 P61077 P00734 1540 P24723 Q15751 P46977 P46734 Q16849 Q15759 O75962 Q8IUQ4 26576 Q9P035 29843 P78527 O60229 P41279 Q9Y6E7 Q9Y6E0 P61088 Q04656 84661 Q96RG2 P05198 O15455 5914 O75716 11091 30827 Q16854 Q6XPS3 Q13107 P18031 Q9P286 9054 P22528 1326 Q63HQ2 P63279 P00519 P47804 5929 P24941 11099 P20340 P00750 51514 P21675 Q9NZN9 O14672 Q9NRW4 O60568 Q8NB16 O60566 Q8IVH8 P16220 P63208 90678 P49137 P07996 10533 P52564 P09936 O15530 Q9UBC3 P27361 Q9GZZ9 Q8IZP0 65018 Q9H832 54361 P50148 57634 P16234 10549 79885 P21810 P16471 10783 10785 92609 Q9UHP3 P52333 65264 P54753 O95696 P29992 1121 Q05397 P54756 P53667 O60307 57646 83933 1111 P32929 Q92830 P62136 26091 Q6IT96 Q92831 P55854 P54764 P54762 3791 P54760 Q12974 4882 Q8NCR0 3551 Q9UBE8 Q9NRZ9 P56705 9099 Q13829 152926 5728 10524 Q8WTX9 P62140 57410 55236 O60551 Q9UI30 10765 P04233 Q6PCD5 P34925 O00469 O95476 P05129 O95477 133584 P28482 P11802 1385 Q9UHJ9 P51668 P50579 Q9P0J1 P19525 10733 147746 Q96DE5 O60762 56339 Q7KZI7 P51671 O00238 Q7Z419 P04049 Q96SB4 P10721 P19784 O95243 Q5VST9 P53611 Q9NS56 Q8WYH8 10987 29110 10746 10988 79444 1147 1387 2475 Q92643 5500 5747 4659 P26045 P30304 O95257 P30305 6850 Q13615 P30307 2011 Q13618 Q9NYB9 27148 Q13616 1161 O00487 Q9P0L2 P30542 Q9P2P5 10956 57448 2247 975 22929 Q96DC9 Q6GMV3 Q92630 5515 Q9UK22 65061 5516 5518 Q969M7 P51451 P29323 5770 P29320 Q13627 P30530 P18433

Q03164 81788 Q99259 Q96LR5 P37173 984 Q99496 6612 Q9Y385 6613 P29317 P40337 5768 Q9UK32 P06213 Q8N2K1 Q15139 Q9Y6W6 O95278 5780 6872 Q96HA8 Q9UPV9 6871 5783 P53804 2033 Q9NVW2 O95271 O60725 Q96RU7 Q96RU2 O14829 994 995 Q99640 997 5775 79813 5536 5778 5537 54517 Q9UM07 6883 6885 Q9UQ88 5796 1198 23210 1196 2043 1195 56940 Q93096 Q96Q15 Q8WTR2 Q9NRP7 Q99873 54764 55611 Q96S53 Q9P2K8 4214 O14830 4215 4216 P46019 P01100 P49768 O00206 Q96EZ8 Q86U44 25847 5562 5563 P49761 P49760 Q9NU63 O00444 O60942 Q00535 Q00534 79834 Q53X93 55869 5798 6647 Q9Y572 P01137 91949 P01135 P01133 P01375 Q9BT22 P02461 P01130 Q9Y2R2 P04626 5575 8841 P04629 2065 25853 2064 O00213 780 Q96RR4 301 O95071 P17936 Q8IZE3 545 546 5566 Q96PN8 5567 5568 Q00526 5327 O43353 Q9H5K3 5580 5581 5582 5583 P07947 23409 5584 5585 8851 4255 P07949 Q969H0 5586 P07948 Q92817 Q9Y2K6 Q9Y2K2 Q96PU4 79612 Q8IZL9 Q99683 Q96PU5 5578 8844 P49336 P62714 5579 10331 4008 P05771 Q9H3F6 8607 2081 5591 9950 Q6IR47 5351 5352 5596 5597 5598 23411 5590 Q00987 4015 Q9H1A4 5347 4017 329 84197 Q96JH7 Q9GZU7 P50750 91754 Q9UHD2 Q12923 Q9UPT9 O75164 Q9Y2M5 Q8NER5 Q9NRM7 P68036 5599 Q68J44 P83436 Q01826 5371 Q9UNQ2 8881 Q12933 6464 7311 391356 Q96Q40 146057 580 Q5U4P2 221656 54101 55432 P51617 Q08209 6699 P62988 5127 54106 Q8N752 P25116 10090 Q13237 4297 Q6ZT98 Q15418 7325 P15924 29947 Q9NYU1 351 Q9NYU2 Q9UBS0 Q04759 29941 Q9UBS4 Q7Z6C1 83475 P22694 9978 P21127 8408 Q9HC98 O43683 7332 7334 Q16512 4067 Q16513 7336 Q16514 7335 19 Q8TF76 Q9UKE5 29959 Q9UBT2 23170 369 128 51654 P60983 7329 Q13490 7328 51657 7341 P11309 P22681 10075 Q9HBW0 P11308 6015 Q16526 P29074 23387 P31152 Q9Y606 Q9UBU3 Q03405 Q9NV06 P23528 8428 P22674 5159 Q16531 Q9UBU8 Q8WUK0 P43378 Q8WZ42 Q13263 P00441 Q16539 4086 344387 4087 Q6PD62 8446 Q9BY44 O75478 Q15208 8445 Q9BY41 P51812 23399 P51813 Q8TD19 5170 142 Q9UBV7 P51817 10087 6259 11176 9526 P49903 Q8WUJ0 53615 8454 8452 P55345 Q6ZN16 64750 7124 O15164 7126 Q9UQL6 4092 P10276 4093 O75460 156 121512 P27708 10055 Q13042 Q99728 52 Q13043 Q13049 6045 P04843 7375 P04844 Q96KB5 6048 23133 P36896 23135 P15735 P36894 Q9UKA1 Q02763 54961 120892 7128 Q99956 8473 Q8TEY7 Q96FX7 P61803 8237 10273 Q96FX2 Q9Y5K5 Q9UKB1 P36888 Q99942 1832 Q9H1Y0 51465 P01579 P49711 Q99707 P09038 O95819 55827 Q9NQC7 Q9BY84 Q9BVS5 O75676 O15197 O15198 9578 Q96FW1 Q02750 25778 1843 2932 2931 255488 55832 51231 Q14164 1848 Q6VAB6 Q15256 86 2935 Q14166 Q13873 9100 O95835 Q15819 Q86Z02 Q8TF05 1613 Q05209 80124 P42224 1852 10933 Q9BXM7 Q9HC16 1850 Q8TAF3 P63165 91 Q8WWI1 O95837 O15355 Q92696 94 Q12792 1859 P22612 P42229 Q13882 P45974 1616 P07237 Q969T4 8266 Q12797 Q8WWH4 Q969S8 Q13404 Q6DT37 Q8TCJ0 Q8TCJ2 Q01082 Q9H3R0 P31321 84708 P67775 57599 P08575 P05067 Q9UBL3 7186 Q15831 7189 O94768 Q6XUX3 P35916 Q9H422 Q96M96 27347 Q13418 6093 Q9UHY1 1634 Q9Y3C5 27102 Q8TEL6 P17252 P42680 O15379 O95619 P42681 P21980 P42684 P42685 Q86UE8 P09874 Q15843 Q6R6M4 Q02156 Q6UWE0 Q8NB78 4914 26260 26262 1642 P68400 Q05655 Q9BXJ9 1408 1407 Q9UBN7 4913 Q9HC62 Q9UBF6 9149 56052 Q6NYC1 Q9H488 P11766 57144 26232 Q86SG6 Q9UK80 Q03468 26234 1650 4920 Q9HC52 150094 9397 P35968 Q13202 Q13443 Q8WZ19 Q9NQ92 Q08J23 Q8IVT5 P21580 9150 57154 57396 Q8N7H5 Q9H1R2 27339 27330 80155 Q9UJX2 Q8N0Z8 Q9Y5B0 51611 Q8IY81 8078 O75604 Q8IY84 P10415 Q13216 93492 377630 P19474 59343 P08581 3611 1432 Q8NHH1 O60341 Q5T6F0 Q13464 P01730 728642 P53355 O95427 O15111 P53350 Q9P0U3 Q9P0U4 27315 Q86YP4 Q04771 26469 389840 P30260 Q7L9L4 O15105 P45983 5800 Q13233 P45984

RNA metabolic process Q92979 O75940 81605 P52434 O00148 P17676 10657 Q8TDD1 Q9BYG3 2305 P84022 Q00839 P05455 Q9H0D6 Q9H2H8 O75934 P30876 10667 80755 54487 Q9Y285 10421 O75937 Q96EB6 64282 P62249 Q9BRX9 Q9UKV8 P27695 3661 54496 1478 55109 Q8N684 P26599 Q5TAP6

Q9UKW6 3659 P31942 Q9UIS9 P31943 P08708 Q99583 55110 Q99575 P19838 P62263 55119 P26368 10642 Q13951 Q9UL18 4772 Q8IX01 1022 P39019 Q5T160 Q6PI48 80324 Q9BWG6 833 O75909 O14744 5859 Q12874 Q12872 4780 Q8WXF0 Q9H0H0 Q07666 O95363 1029 27297 10625 57794 1025 167227 Q5C9Z4 Q9BUB4 4775 Q92766 4790 Q9NR48 Q96MU7 55149 Q92994 P10827 P10828 22826 22827 Q9H6R4 22828 6741 5411 Q8NC51 Q9NQT4 Q9NR55 Q9NQT5 1050 55152 Q9HCK5 P40692 27037 O60870 10607 192669 865 6733 P04150 4799 O96004 11340 Q86V81 10250 10492 O00541 O95391 11108 51362 Q8WWY3 Q15020 Q15024 P62875 135295 P51991 Q15029 5430 4343 5432 Q9UKM9 P51513 Q03933 O14717 O43290 Q7Z6J9 196441 Q9BRP8 P49643 O43294 408 P49407 P60228 409 O43251 5440 P49642 5441 6772 5442 Q9UKN5 Q9UKN8 Q9BW92 O00567 126789 5434 Q15046 P61326 Q15287 P23246 Q9UL03 Q86X95 Q9UMS4 84292 A7MD48 3035 O14979 11338 3028 Q00403 55759 4116 P57081 8732 6311 7884 P50613 P51946 23764 Q9Y5Q8 23524 Q8WUY8 P15692 10450 Q96A72 3054 23536 29079 Q9HCE1 Q96SZ6 688 689 P62847 P62841 4150 4152 4154 25929 7422 P52815 22894 P13489 Q9Y5S9 P17844 79753 10438 Q5TAL4 4149 P62857 P56182 O43463 8761 Q9BQ04 23517 Q13901 Q5VTL8 P07814 Q96C86 Q92905 O75031 55796 Q92900 P56192 O43791 53938 Q16629 Q14686 Q14209 P55265 Q9UPN6 Q6NZI2 57062 Q01130 472 474 P33240 Q16630 Q14690 Q8WW01 P38919 Q16637 3091 Q8IWZ8 Q16633 8543 5036 6125 O75376 79084 Q504Q3 Q9NW13 60489 51773 24144 Q9NPD3 P46777 Q15796 Q9HAU5 Q16649 P35638 P61968 Q15797 P32121 O43776 O75362 Q96IZ7 51547 Q8WVM0 8563 Q13148 Q9BYU1 9410 P35869 11051 Q8IWX8 8565 P18074 P18077 P36954 144983 28960 Q9P2E3 Q13151 Q16665 P61978 51319 9421 P78347 P54136 P35453 26512 Q93062 23481 Q8IZD4 Q14494 Q14493 Q14498 51329 Q14011 Q9NRC8 10181 11030 O75575 26523 23016 7490 Q9NY12 118460 P49848 Q86W42 P98175 O75569 P98179 25885 51340 54853 Q9NVM4 51585 11243 P47897 9439 Q9GZL7 Q6I9Y2 Q15365 O75319 Q9HB90 Q08170 26986 Q01780 25896 6182 O75792 5093 Q96EU6 26747 51593 Q15370 Q9H9G7 P78362 Q99814 11017 P78545 9465 P54577 9221 P29375 P29374 57461 6194 10813 Q9H6Y2 P41252 P62195 P41250 O15234 Q13761 65083 1736 Q13523 P54105 9232 22803 Q13769 Q9H0L4 Q9P1Y6 P24928 79005 1982 27257 Q9NVH2 Q6P1K2 O60293 Q5D0E6 7068 7067 Q5TAX3 Q15714 Q9H307 Q15717 Q8IZ69 1994 79039 Q8TBF4 O60264 P78316 9255 Q32P51 83759 Q8N8D1 1763 29896 Q07955 29894 Q9UET6 50628 P52597 Q13315 O95997 9261 4809 84881 Q8WVK2 4800 51726 2626 Q8NEC7 Q9NVC6 Q14653 51729 P19387 P30153 P19388 26121 57038 Q9BUJ2 Q13573 9045 Q9NP81 Q00059 Q96AE4 P43694 Q9BQA1 Q9Y4A8 51747 5927 4839 P63272 23076 O60231 Q9HCS7 902 Q13595 Q14684 P21675 Q14683 P10914 285672 Q92858 Q969L4 P67809 O00268 Q06265 O00267 Q9UBB9 10535 5939 P16220 Q9ULW3 4841 Q9Y2X3 10772 Q6NZY4 P49137 Q9UK45 5935 Q9NQ55 55696 51096 10785 5705 Q9NWB1 Q9ULX9 Q66PJ3 Q12968 O60306 Q8TED0 P12081 Q92830 P03372 Q9Y2Z2 Q9Y2Z4 Q12972 P49591 P46087 Q9H814 Q9GZX7 Q6P2C8 P49590 Q99459 3304 10528 P49588 10521 5725 10523 O95478 Q12986 1385 57661 O95232 P52756 6829 Q9P2N5 P61221 29102 P62316 P84103 P62318 5733 5976 56339 P62312 58517 27161 6827 P62314 Q10570 Q9Y2T7 Q96SB4 Q9UHK0 Q96SB3 Q13601 1153 Q14938 O00472 Q5JTZ9 P19544 10987 6839 2237 56342 P08651 P42771 4670 22938 Q9Y4Y9 O60508 Q9H9Y6 O60506 80222 P61244 284119 O14641 5511 O14640 Q9Y4Z0 5515 Q9UNY4 P08621 5518 P51693 Q13868 Q9P0M2 Q03164 10725 P61254 Q9Y2W2 988 Q92620 4677 Q9BZJ0 Q9Y2W1 O43395 O43159 6872 6871 P04637 Q5VYS8 6631 2034 Q9BV90 P14678 P53803 54512 4686 6625 Q16236 6627 P62995 P62753 Q9GZR7 Q9UQ80 54517 Q96LI5 P07910 220988 6883 23210 Q96Q15 51010 23451 51013 6633 Q9Y6Y0 6878 8812 Q15393 10147 O43172 6637 Q86U44 Q86U42 P26640 O95059 Q8N726 O60942 P26639 10594 Q53X93 P49756 5557 P61201 5558 Q9UPY3 23438 O43143 6660 6421 Q9Y333 Q9UHI6 23435 Q96B26 11218 Q96RR1 P09086 9924 57819 P62308 P61218 Q9ULR0 O43390 P62304 6430 6432 Q9UQ35 6431

6434 Q9UPR3 7764 Q12904 22794 23404 Q96PU8 Q02930 8602 6427 6426 Q8N302 2081 23658 Q9Y2L1 6201 5356 23650 3178 Q9ULK4 23411 O76021 328 25804 3181 2091 P50750 P22087 Q8TA86 Q8N2M8 3189 Q9UNP9 2099 3188 3187 3185 3184 Q9NW64 O14802 333 55660 51067 7536 9716 91746 Q9H5H4 3192 Q9UNQ2 3191 8880 A2RTX5 P27540 3190 9733 P39748 Q969E8 P51610 O00411 Q9NVV4 Q08211 10569 79622 Q9NPJ6 79869 6217 9967 Q8N9Q2 4297 O43432 Q9UKD1 57187 P13984 Q96FV9 351 Q9UBS4 P09012 6229 P62913 Q13243 Q8IU60 O94906 Q13242 Q13247 5393 P20290 P03950 5394 O43687 O00716 16 Q15428 Q16514 Q15427 26205 O75494 367 51654 11198 O94913 6239 Q15434 Q14103 23144 Q9UKF6 Q9Y606 23381 85437 Q9NV06 P42285 P09234 54931 P83876 54938 51428 O43660 4086 11171 10081 8683 4087 8446 4088 O75478 9533 9775 P29084 142 Q96HW7 P23771 Q8IXZ2 Q15459 9785 10291 O15160 9541 8458 O15164 P54253 P10275 O75460 4094 Q8NI36 2908 Q8IYB3 8449 84365 Q13287 Q9NSE4 Q14134 Q14137 Q09161 8467 23378 Q9Y5J1 Q9Y3F4 Q8NI27 Q9BRL6 11157 Q15233 Q99714 Q96FX7 6059 O75444 6050 Q99700 11129 Q96T23 P50914 88745 51222 56915 9319 9318 2926 P49716 P49715 11128 Q9BVS5 Q08050 P39905 7157 P62906 P14868 P14866 8241 10286 10285 2935 P78424 92345 Q13873 22913 Q14966 Q7L0Y3 P52272 Q12788 92105 10929 P62081 P18124 P42224 Q96DI7 Q9Y3A5 84967 Q9HC16 P09651 1856 Q9UBK2 P55795 Q14978 Q8WWH4 O95602 Q13887 P22626 Q9Y3B4 P67775 Q6IN84 84950 10949 Q9UHX1 Q9Y3B2 P09661 P05067 2959 P08579 10940 Q9UBL3 P52298 9128 9129 O75643 10907 O43812 P11940 O75400 26019 9360 80145 283989 2961 2960 1870 Q8TAD8 2969 P09874 Q13427 Q8NAV1 29777 10921 2972 57379 134430 Q13435 Q15853 Q9UJV9 Q9NYH9 1660 Q6NYC1 P53567 1655 P22415 1653 Q03468 Q92499 Q8WYQ5 1659 P43115 O95644 O95400 Q08J23 Q96G21 27339 O43809 1665 Q8N0Z8 27332 Q9BXP5 P23511 Q6P2Q9 Q9Y5B9 Q8IY81 Q8TEQ6 P55769 P35711 84305 Q5RKV6 2521 57169 Q6P4R8 Q9HC36 P08107 Q15643 Q9P2Y4 Q15648 Q9BVJ6 Q96HR8 Q14562 1207

phosphorus metabolic process Q9H0E2 O75947 8099 65125 P28562 P54619 Q9NQU5 1459 4728 1457 4729 Q8IYN9 55561 1454 1453 4720 4722 4723 6901 Q6IQ55 4725 4726 P28331 O14561 Q92729 P53779 Q9H0C8 4731 10420 Q9H2G2 Q5S007 Q7Z727 81629 Q8NE63 O00167 Q06124 P37023 Q8IUD2 2324 57761 P27448 10632 Q12851 O75914 3672 Q9NR96 Q13705 P21917 P54646 10645 O60674 1487 P20827 5602 4514 Q96GD4 O14578 5604 P41743 Q8WXG6 P25098 Q13956 Q9Y463 1022 1021 1020 Q5SGD2 P19404 57787 1018 1017 P15056 5610 5613 O14986 26191 5871 P09619 P10600 Q15904 Q9H8X2 O14519 1025 Q9BUB5 6711 4535 6714 O14757 P09622 O95136 Q9Y243 Q13976 P21709 Q96L34 P16118 P18545 169436 Q05469 P60033 613 Q5TCX8 Q96CA5 O95147 Q5MAI5 5894 P51570 P31749 O95382 Q38SD2 Q4G0W2 2139 O00141 6733 Q9UL54 Q8TBA6 P06576 P56199 O60603 2147 Q96KG9 O96017 O95169 O95163 Q9NWZ3 P57059 1072 O96013 Q99755 Q99750 84033 11113 Q99759 O43293 O00329 6772 P35236 P13686 P11274 Q96T60 10476 29085 P03891 O14965 Q8N3J5 23552 Q658W2 O95182 Q01974 P60484 Q9H093 6776 O14730 7867 285193 O14733 5207 O14974 P31930 6790 6793 8732 6794 4133 O00506 P50613 245972 Q99570 O00743 673 6305 6789 Q01968 5470 4140 140609 P51955 3297 P51956 Q99558 10229 8737 207 4139 Q15078 284086 8754 Q9Y5S2 Q08345 O75293 55781 Q00613 O14920 P56181 P56180 5494 P51970 5495 P49674 Q9UIG0 5499 Q8NHX1 Q7L7X3 P17612 O14936 Q96QT4 Q56UN5 Q13115 P36507 Q16864 7204 Q9Y4K4 O75385 O43318 472 P48729 Q6P5Z2 23043 Q99807 8767 Q13363 Q15303 11072 4188 Q16635 3099 8780 Q9Y2H1 O75380 Q9NRH2 8536 P00533 Q15796 197259 Q13131 9641 Q15797 Q16644 7465 P56134 8555 O75365 Q96IZ7 498 O94804 P56385 P56381 Q8NCB2 Q16659 5295 Q9Y6R4 O75116 P10586 Q8N4C8 23031 P00558 54822 P43246 Q13153 51319 P56556 8576 P32189 P36542 O75582 P10398 Q13162 P23467 8569 Q13164 Q14012 Q15349 Q8TDX7 Q9BV47 Q9HAZ1 26524 Q93050 11035 P49841 Q13177 Q9NRD5 Q15118 P78368 9201 Q3ZCQ8 P49840 O75569 9448 10152 Q9Y6M4 A1XKG3 Q9Y6M9 P24539 Q8NEJ0

P08069 Q9UEW8 10155 Q9BQI3 1716 Q13188 1956 P23443 O75319 9212 O15075 Q9BWU1 Q15126 P21283 P21281 23476 P37840 Q9UNI6 7272 1965 Q6PID4 P49821 Q9NTJ5 P48730 P78362 P23458 1969 P48736 Q14289 Q15375 6196 6199 O75306 9463 7046 6198 P09769 P35813 P12931 6195 79012 P42345 2820 O60285 1738 Q13523 7297 7057 9475 Q9H792 P41240 P42338 Q9UER7 P42336 P34947 P29597 P06239 P29353 Q13535 O15264 P29350 P29590 Q9H0K1 Q7Z2Q5 8394 Q92551 P06241 P21860 3932 Q9HCP0 Q16816 Q13546 Q8IYT8 9252 Q9Y2B5 P43405 Q9Y6J8 P53041 Q13555 Q16825 Q13554 Q16827 O14595 Q13315 Q13557 P78509 Q16828 9261 9020 23085 P20594 2869 7099 7098 P14927 30849 Q15746 O43920 P30153 Q9H596 P61073 Q05513 23097 P00734 P24723 P46734 Q16849 O75964 Q15759 O75962 Q8NEB9 26576 Q9P035 P78527 O60229 P41279 Q9Y6E0 Q96RG2 P05198 O15455 5914 O95544 O75716 Q16854 Q6XPS3 P18031 Q9P286 9296 1326 P00519 P24941 11099 P21675 O14672 Q9NRW4 Q8NB16 O60566 Q8IVH8 P16220 P49137 P07996 P52564 P48047 O15530 P27361 Q8IZP0 65018 P16234 10549 P16471 10783 92609 Q9UI12 P52333 P54753 Q92835 Q05397 P54756 P53667 O60307 1355 P18859 1111 P62136 P54764 P54762 3791 P54760 Q12974 4882 3551 Q9UBE8 152926 5728 P62140 57410 P04233 P34925 O95476 P05129 P28482 P11802 1385 Q9P0J1 P19525 10733 147746 Q7KZI7 P51671 O00238 P04049 Q96SB4 P10721 P19784 Q5VST9 29110 10746 79444 1147 2475 5500 5747 4659 P26045 P30304 O95257 P30305 6850 Q13615 P30307 O00483 2011 Q9NYB9 27148 Q9P0L2 P30542 P17568 2247 975 Q6GMV3 Q92630 5515 65061 5516 5518 P51451 P29323 5770 P29320 Q9Y375 Q13627 P30530 P52789 P18433 81788 P37173 984 P29317 Q9UK32 509 P06213 Q15139 Q9Y6W6 O95278 5780 6872 5783 4695 O95271 Q96RU7 O14829 994 995 Q99640 513 514 5775 6622 515 P61421 5536 5778 P27986 518 5537 P48201 6885 Q9UQ88 5796 1198 1196 2043 1195 P07919 56940 Q93096 Q96Q15 Q8WTR2 Q9NRP7 521 522 Q96S53 523 Q9P2K8 4697 4214 4698 526 O14830 4215 4216 528 5305 P49768 O00206 5562 5563 P49761 P49760 O00444 O95298 O95299 P11387 O60942 Q00535 533 Q00534 79834 535 Q53X93 537 5798 6647 Q9Y572 P01137 539 P01135 P48426 P01133 P01375 O00217 Q9Y2R2 P04626 5575 P04629 2065 2064 780 Q96RR4 P17936 Q8IZE3 545 5566 Q96PN8 5567 5568 Q00526 O43353 Q9H5K3 5580 5581 5582 5583 P07947 5584 5585 8851 P07949 5586 P07948 Q9Y2K2 Q8IZL9 Q99683 5578 8844 P49336 P62714 5579 P05771 2081 5591 9950 Q6IR47 5596 5597 5598 5590 Q99437 5347 84197 65220 Q9GZU7 P50750 91754 Q9UHD2 Q12923 Q8NER5 Q9NRM7 Q8N8Y2 5599 Q68J44 5371 6464 391356 Q96Q40 146057 P15313 54101 P51617 Q08209 5127 54106 Q8N752 P25116 Q13237 Q15418 O43678 351 Q9UBS0 Q04759 29941 P22695 P22694 P21127 8408 Q9HC98 O43683 Q16512 4067 Q16513 Q8TF76 Q9UKE5 29959 369 P00414 P60983 51657 P11309 Q9HBW0 O75489 P11308 P29074 23387 P31152 Q9UBU3 P25705 P23528 8428 5159 Q8WUK0 P43378 Q8WZ42 Q13263 P00441 Q16539 4086 344387 4087 8446 Q9BY44 Q15208 8445 P51812 23399 P51813 Q8TD19 5170 P51817 10087 6259 Q8WUJ0 Q6ZN16 7124 O15164 P38606 4092 P10276 4093 O75460 156 121512 P27708 Q16795 52 Q13043 54 Q96KB5 P36896 64768 P15735 P36894 Q02763 54961 120892 Q99956 7385 7384 7381 P36888 P01579 P09038 O95819 Q9BY84 O75438 O75676 O15197 O15198 9578 Q02750 25778 7150 1843 2932 2931 51231 Q14164 1848 Q6VAB6 Q15256 Q13873 Q7KZN9 O95835 Q86Z02 Q8TF05 1613 Q05209 P42224 1852 Q9BXM7 1850 91 22908 O15355 94 Q12792 1859 P22612 P42229 Q13882 1616 9114 Q6DT37 Q01082 P31321 P43304 P67775 2710 29796 P08575 P05067 P07205 Q15831 O94768 Q6XUX3 P35916 Q9H422 Q96M96 27347 Q13418 6093 Q9UHY1 P00846 27102 P17252 P42680 P42681 P42684 P42685 Q86UE8 Q02156 4914 P68400 Q05655 P30049 9149 57144 Q86SG6 Q03468 4920 150094 51606 P35968 Q13202 Q13443 Q8IVT5 9150 57396 Q9H1R2 Q16718 27330 Q9Y5B0 Q8IY84 P10415 93492 4705 4707 P08581 3611 1432 P00403 4701 Q13464 4702 728642 P53355 O15111 P53350 Q04771 26469 4718 4719 389840 Q7L9L4 O15105 4710 4952 P45983 5800 4713 Q13233 P45984 4714 4715

phosphate metabolic process	Q9H0E2 O75947 8099 65125 P28562 P54619 Q9NQU5 1459 4728 1457 4729 Q8IYN9 55561 1454 1453 4720 4722 4723 6901 Q6IQ55 4725 4726 P28331 O14561 Q92729 P53779 Q9H0C8 4731 10420 Q9H2G2 Q5S007 Q7Z727 81629 Q8NE63 O00167 Q06124 P37023 Q8IUD2 2324 57761 P27448 10632 Q12851 O75914 3672 Q9NR96 Q13705 P21917 P54646 10645 O60674 1487 P20827 5602 4514 Q96GD4 O14578 5604 P41743 Q8WXG6 P25098 Q13956 Q9Y463 1022 1021 1020 Q5SGD2 P19404 57787 1018 1017 P15056 5610 5613 O14986 26191 5871 P09619 P10600 Q15904 Q9H8X2 O14519 1025 Q9BUB5 6711 4535 6714 O14757 P09622 O95136 Q9Y243 Q13976 P21709 Q96L34 P16118 P18545 169436 Q05469 P60033 613 Q5TCX8 Q96CA5 O95147 Q5MAI5 5894 P51570 P31749 O95382 Q38SD2 Q4G0W2 2139 O00141 6733 Q9UL54 Q8TBA6 P06576 P56199 O60603 2147 Q96KG9 O96017 O95169 O95163 Q9NWZ3 P57059 1072 O96013 Q99755 Q99750 84033 11113 Q99759 O43293 O00329 6772 P35236 P13686 P11274 Q96T60 10476 29085 P03891 O14965 Q8N3J5 23552 Q658W2 O95182 Q01974 P60484 Q9H093 6776 O14730 7867 285193 O14733 5207 O14974 P31930 6790 6793 8732 6794 4133 O00506 P50613 245972 Q99570 O00743 673 6305 6789 Q01968 5470 4140 140609 P51955 3297 P51956 Q99558 10229 8737 207 4139 Q15078 284086 8754 Q9Y5S2 Q08345 O75293 55781 Q00613 O14920 P56181 P56180 5494 P51970 5495 P49674 Q9UIG0 5499 Q8NHX1 Q7L7X3 P17612 O14936 Q96QT4 Q56UN5 Q13115 P36507 Q16864 7204 Q9Y4K4 O75385 O43318 472 P48729 Q6P5Z2 23043 Q99807 8767 Q13363 Q15303 11072 4188 Q16635 3099 8780 Q9Y2H1 O75380 Q9NRH2 8536 P00533 Q15796 197259 Q13131 9641 Q15797 Q16644 7465 P56134 8555 O75365 Q96IZ7 498 O94804 P56385 P56381 Q8NCB2 Q16659 5295 Q9Y6R4 O75116 P10586 Q8N4C8 23031 P00558 54822 P43246 Q13153 51319 P56556 8576 P32189 P36542 O75582 P10398 Q13162 P23467 8569 Q13164 Q14012 Q15349 Q8TDX7 Q9BV47 Q9HAZ1 26524 Q93050 11035 P49841 Q13177 Q9NRD5 Q15118 P78368 9201 Q3ZCQ8 P49840 O75569 9448 10152 Q9Y6M4 A1XKG3 Q9Y6M9 P24539 Q8NEJ0 P08069 Q9UEW8 10155 Q9BQI3 1716 Q13188 1956 P23443 O75319 9212 O15075 Q9BWU1 Q15126 P21283 P21281 23476 P37840 Q9UNI6 7272 1965 Q6PID4 P49821 Q9NTJ5 P48730 P78362 P23458 1969 P48736 Q14289 Q15375 6196 6199 O75306 9463 7046 6198 P09769 P35813 P12931 6195 79012 P42345 2820 O60285 1738 Q13523 7297 7057 9475 Q9H792 P41240 P42338 Q9UER7 P42336 P34947 P29597 P06239 P29353 Q13535 O15264 P29350 P29590 Q9H0K1 Q7Z2Q5 8394 Q92551 P06241 P21860 3932 Q9HCP0 Q16816 Q13546 Q8IYT8 9252 Q9Y2B5 P43405 Q9Y6J8 P53041 Q13555 Q16825 Q13554 Q16827 O14595 Q13315 Q13557 P78509 Q16828 9261 9020 23085 P20594 2869 7099 7098 P14927 30849 Q15746 O43920 P30153 Q9H596 P61073 Q05513 23097 P00734 P24723 P46734 Q16849 O75964 Q15759 O75962 Q8NEB9 26576 Q9P035 P78527 O60229 P41279 Q9Y6E0 Q96RG2 P05198 O15455 5914 O95544 O75716 Q16854 Q6XPS3 P18031 Q9P286 9296 1326 P00519 P24941 11099 P21675 O14672 Q9NRW4 Q8NB16 O60566 Q8IVH8 P16220 P49137 P07996 P52564 P48047 O15530 P27361 Q8IZP0 65018 P16234 10549 P16471 10783 92609 Q9UI12 P52333 P54753 Q92835 Q05397 P54756 P53667 O60307 1355 P18859 1111 P62136 P54764 P54762 3791 P54760 Q12974 4882 3551 Q9UBE8 152926 5728 P62140 57410 P04233 P34925 O95476 P05129 P28482 P11802 1385 Q9P0J1 P19525 10733 147746 Q7KZI7 P51671 O00238 P04049 Q96SB4 P10721 P19784 Q5VST9 29110 10746 79444 1147 2475 5500 5747 4659 P26045 P30304 O95257 P30305 6850 Q13615 P30307 O00483 2011 Q9NYB9 27148 Q9P0L2 P30542 P17568 2247 975 Q6GMV3 Q92630 5515 65061 5516 5518 P51451 P29323 5770 P29320 Q9Y375 Q13627 P30530 P52789 P18433 81788 P37173 984 P29317 Q9UK32 509 P06213 Q15139 Q9Y6W6 O95278 5780 6872 5783 4695 O95271 Q96RU7 O14829 994 995 Q99640 513 514 5775 6622 515 P61421 5536 5778 P27986 518 5537 P48201 6885 Q9UQ88 5796 1198 1196 2043 1195 P07919 56940 Q93096 Q96Q15 Q8WTR2 Q9NRP7 521 522 Q96S53 523 Q9P2K8 4697 4214 4698 526 O14830 4215 4216 528 5305 P49768 O00206 5562 5563 P49761 P49760 O00444 O95298 O95299

P11387 O60942 Q00535 533 Q00534 79834 535 Q53X93 537 5798 6647 Q9Y572 P01137 539 P01135 P48426 P01133 P01375 O00217 Q9Y2R2 P04626 5575 P04629 2065 2064 780 Q96RR4 P17936 Q8IZE3 545 5566 Q96PN8 5567 5568 Q00526 O43353 Q9H5K3 5580 5581 5582 5583 P07947 5584 5585 8851 P07949 5586 P07948 Q9Y2K2 Q8IZL9 Q99683 5578 8844 P49336 P62714 5579 P05771 2081 5591 9950 Q6IR47 5596 5597 5598 5590 Q99437 5347 84197 65220 Q9GZU7 P50750 91754 Q9UHD2 Q12923 Q8NER5 Q9NRM7 Q8N8Y2 5599 Q68J44 5371 6464 391356 Q96Q40 146057 P15313 54101 P51617 Q08209 5127 54106 Q8N752 P25116 Q13237 Q15418 O43678 351 Q9UBS0 Q04759 29941 P22695 P22694 P21127 8408 Q9HC98 O43683 Q16512 4067 Q16513 Q8TF76 Q9UKE5 29959 369 P00414 P60983 51657 P11309 Q9HBW0 O75489 P11308 P29074 23387 P31152 Q9UBU3 P25705 P23528 8428 5159 Q8WUK0 P43378 Q8WZ42 Q13263 P00441 Q16539 4086 344387 4087 8446 Q9BY44 Q15208 8445 P51812 23399 P51813 Q8TD19 5170 P51817 10087 6259 Q8WUJ0 Q6ZN16 7124 O15164 P38606 4092 P10276 4093 O75460 156 121512 P27708 Q16795 52 Q13043 54 Q96KB5 P36896 64768 P15735 P36894 Q02763 54961 120892 Q99956 7385 7384 7381 P36888 P01579 P09038 O95819 Q9BY84 O75438 O75676 O15197 O15198 9578 Q02750 25778 7150 1843 2932 2931 51231 Q14164 1848 Q6VAB6 Q15256 Q13873 Q7KZN9 O95835 Q86Z02 Q8TF05 1613 Q05209 P42224 1852 Q9BXM7 1850 91 22908 O15355 94 Q12792 1859 P22612 P42229 Q13882 1616 9114 Q6DT37 Q01082 P31321 P43304 P67775 2710 29796 P08575 P05067 P07205 Q15831 O94768 Q6XUX3 P35916 Q9H422 Q96M96 27347 Q13418 6093 Q9UHY1 P00846 27102 P17252 P42680 P42681 P42684 P42685 Q86UE8 Q02156 4914 P68400 Q05655 P30049 9149 57144 Q86SG6 Q03468 4920 150094 51606 P35968 Q13202 Q13443 Q8IVT5 9150 57396 Q9H1R2 Q16718 27330 Q9Y5B0 Q8IY84 P10415 93492 4705 4707 P08581 3611 1432 P00403 4701 Q13464 4702 728642 P53355 O15111 P53350 Q04771 26469 4718 4719 389840 Q7L9L4 O15105 4710 4952 P45983 5800 4713 Q13233 P45984 4714 4715

phosphorylation Q9H0E2 O75947 8099 65125 O14672 P54619 Q9NQU5 1459 4728 1457 4729 Q8NB16 Q8IYN9 O60566 55561 Q8IVH8 P16220 1454 1453 4720 4722 P49137 4723 6901 Q6IQ55 4725 P07996 4726 P52564 P28331 P48047 O15530 O14561 P27361 Q8IZP0 65018 P53779 P16234 10549 P16471 4731 10420 10783 Q9UI12 P52333 Q9H2G2 Q5S007 P54753 Q7Z727 81629 Q8NE63 Q06124 Q05397 P54756 P53667 O60307 P37023 Q8IUD2 2324 57761 1355 P18859 1111 P27448 10632 Q12851 O75914 P54764 P54762 3791 P54760 3672 4882 Q9NR96 3551 Q13705 P21917 Q9UBE8 P54646 10645 O60674 1487 P20827 57410 5602 4514 Q96GD4 O14578 5604 P41743 P04233 Q8WXG6 P34925 P25098 P05129 Q13956 Q9Y463 P28482 P11802 1022 1385 1021 1020 P19404 P19525 57787 1018 10733 147746 1017 P15056 5610 5613 O14986 Q7KZI7 P51671 O00238 P04049 Q96SB4 5871 P09619 P10600 P10721 P19784 Q15904 Q5VST9 Q9H8X2 29110 O14519 10746 79444 1147 1025 Q9BUB5 2475 6711 4535 6714 5747 O14757 P09622 O95136 O95257 6850 Q9Y243 Q13976 O00483 2011 Q9NYB9 27148 P21709 Q96L34 P16118 Q9P0L2 P30542 P17568 P18545 169436 Q05469 2247 P60033 975 613 Q5TCX8 Q92630 Q96CA5 65061 P51451 P29323 Q5MAI5 P29320 Q9Y375 5894 P51570 P31749 O95382 Q38SD2 Q13627 P30530 P52789 P18433 81788 P37173 984 O00141 6733 P29317 Q9UL54 Q9UK32 Q8TBA6 509 P06213 P06576 Q15139 6872 4695 P56199 O95271 O60603 Q96RU7 2147 Q99640 513 514 6622 515 Q96KG9 P61421 P27986 518 O96017 O95169 P48201 6885 Q9UQ88 1198 O95163 Q9NWZ3 1196 2043 P57059 1195 P07919 1072 O96013 Q99755 Q96Q15 Q9NRP7 521 522 Q96S53 523 Q99750 Q9P2K8 4697 4214 4698 84033 526 4215 11113 4216 528 5305 Q99759 O43293 P49768 O00206 O00329 5562 6772 5563 P49761 P49760 O00444 O95298 O95299 P11387 P11274 Q96T60 Q00535 533 Q00534 79834 535 Q53X93 537 6647 Q9Y572 P01137 539 10476 P01135 P03891 P48426 O14965 P01133 P01375 O00217 P04626 5575 P04629 23552 Q658W2 O95182 2065 2064 780 Q01974 Q96RR4 P17936 Q8IZE3 545 Q9H093 5566 Q96PN8 6776 5567 5568 Q00526 O14730 7867 O14733 5207 O43353 Q9H5K3 5580

P31930 6790 5581 6793 5582 5583 P07947 5584 6794 4133 O00506 5585 8851 P07949 5586 P07948 P50613 245972 Q99570 Q9Y2K2 673 Q8IZL9 Q99683 5578 8844 P49336 5579 6789 P05771 2081 5591 4140 9950 Q6IR47 140609 5596 5597 5598 P51955 3297 P51956 5590 Q99558 Q99437 10229 8737 207 5347 4139 84197 Q15078 284086 65220 P50750 8754 91754 Q9Y5S2 Q9UHD2 Q08345 O75293 Q8NER5 55781 Q00613 Q9NRM7 Q8N8Y2 5599 O14920 P56181 5371 P51970 6464 P49674 Q9UIG0 Q8NHX1 Q96Q40 146057 Q7L7X3 P15313 54101 P17612 O14936 P51617 Q96QT4 Q56UN5 5127 54106 Q8N752 P25116 Q13237 P36507 Q16864 Q15418 7204 Q9Y4K4 O43678 O75385 O43318 351 472 P48729 Q9UBS0 Q04759 Q6P5Z2 29941 23043 Q99807 P22695 8767 P22694 P21127 8408 Q9HC98 Q13363 O43683 Q15303 Q16512 4067 4188 Q16513 Q16635 3099 Q8TF76 Q9UKE5 8780 29959 Q9Y2H1 O75380 369 Q9NRH2 P00414 P60983 8536 P00533 Q15796 197259 Q13131 9641 P11309 Q15797 Q16644 7465 Q9HBW0 O75489 P56134 P11308 23387 P31152 Q96IZ7 Q9UBU3 498 P25705 P23528 O94804 8428 P56385 5159 P56381 Q8WZ42 Q13263 Q8NCB2 P00441 Q16659 5295 Q16539 4086 Q9Y6R4 344387 4087 Q9BY44 Q15208 O75116 8445 P51812 P51813 Q8TD19 5170 Q8N4C8 23031 P00558 P51817 10087 6259 54822 P43246 Q13153 51319 P56556 Q6ZN16 7124 8576 O15164 P38606 P36542 O75582 4092 P10276 4093 P10398 O75460 156 121512 P27708 Q13162 Q16795 8569 Q13164 Q13043 Q14012 Q15349 Q8TDX7 Q9HAZ1 Q96KB5 P36896 64768 P15735 26524 P36894 Q93050 Q02763 120892 11035 P49841 Q13177 Q9NRD5 Q15118 P78368 9201 7385 7384 P49840 O75569 9448 10152 Q9Y6M4 A1XKG3 7381 P36888 Q9Y6M9 P24539 P01579 P08069 Q9UEW8 10155 Q9BQI3 1716 Q13188 1956 P09038 O95819 P23443 9212 O15075 O75438 Q9BWU1 Q15126 P21283 O75676 O15197 O15198 9578 P21281 23476 Q02750 25778 P37840 7150 7272 1965 2932 2931 51231 Q6PID4 Q14164 P49821 P48730 P78362 P23458 1969 P48736 Q14289 Q6VAB6 Q15375 6196 6199 O75306 Q13873 9463 7046 6198 P09769 Q7KZN9 O95835 Q86Z02 Q8TF05 P12931 6195 79012 1613 P42345 P42224 Q9BXM7 91 O60285 94 Q12792 1738 1859 P22612 P42229 Q13882 1616 Q13523 7297 9114 7057 9475 Q6DT37 Q01082 Q9H792 P31321 P41240 29796 P05067 P42338 Q9UER7 P42336 P07205 P34947 Q15831 P29597 P06239 P29353 O94768 Q6XUX3 Q13535 O15264 P35916 Q9H422 Q96M96 P29590 27347 Q13418 Q9H0K1 Q7Z2Q5 8394 6093 Q9UHY1 P00846 27102 Q92551 P17252 P42680 P06241 P21860 P42681 P42684 3932 P42685 Q9HCP0 Q86UE8 Q16816 Q13546 Q8IYT8 Q02156 9252 Q9Y2B5 4914 P43405 P68400 Q05655 P30049 Q13555 Q13554 Q13315 Q13557 9149 P78509 57144 9261 9020 23085 Q86SG6 Q03468 P20594 4920 2869 7099 150094 7098 51606 P35968 Q13443 P14927 30849 Q8IVT5 Q15746 O43920 57396 Q16718 27330 P61073 Q05513 23097 P00734 P24723 P46734 Q8IY84 O75964 P10415 Q15759 O75962 Q8NEB9 26576 Q9P035 P78527 O60229 P41279 4705 4707 P08581 3611 1432 Q9Y6E0 P00403 Q96RG2 P05198 O15455 4701 Q13464 4702 5914 O95544 O75716 728642 P53355 O15111 Q16854 P53350 Q9P286 Q04771 9296 1326 4718 P00519 4719 389840 P24941 Q7L9L4 O15105 4710 P45983 4713 Q13233 P45984 4714 P21675 4715

protein transport 25909 Q9NRW1 9182 22872 9181 Q96KP1 Q9NRW7 Q99490 Q5SQT9 Q9P2R6 P61020 Q9BVA0 1213 2665 2664 Q9UMY4 Q92973 10890 O14662 Q9Y2X3 P60059 90678 10651 P61026 3875 Q9Y275 P27348 10533 Q92974 64284 Q9UGM3 4860 P55957 Q8IWJ2 O00159 55696 10427 P42858 57510 Q9H2G9 O15400 4734 92609 P50395 1121 339122 Q12846 Q96P70 57403 Q8IUD2 10758 81876 6809 55341 P62491 30000 Q86WA8 811 5830 Q9UJ41 P63104 Q92834 P62258 4627 4869 P53675 P26374 Q92824 P31946 Q9H814 4641 1130 56681 O95343 P53677 Q676U5 O60318 Q96N16 81622 Q99698 Q8NFW9 O60674 1122 821 6810 10640 6812 6811 O14579 27072 6814 10762 948 Q9UKX7 P41743 P04233 Q9H1C4 O75901 O95477 Q9H2M9 P28482 P50570 5861 5862 1020 Q9H1K0 114548 Q9P0J0 O95352 Q9NRS6 O60645 10736 3313 O60763 O00471 Q4VX76 Q9Y2T2 4645 10972 4646 10971 P61106 Q9UL26 Q9UL25 O95487 P32856 5870 O95249 P33947 Q15907 P53618 Q9NR31

22841 P37198 57678 O60895 6711 10981 604 5865 Q9UM54 5868 5869 P53621 4792 Q12769 O60749 22931 81555 Q8IUH5 P62330 80223 O60502 Q9NS69 10953 401505 Q9HCL2 2247 26056 1277 58533 10959 P61006 Q6NUQ1 5514 5877 6726 738 5878 Q9H1H9 P51572 P53992 Q9UL45 P30536 Q13505 Q8NEZ2 Q969M3 P51571 P31749 1176 O95140 1174 1173 55153 Q9NQT8 84932 10724 55275 Q9BXF6 Q92621 Q96ED9 Q9ULV0 Q969M1 4678 O15504 Q9UL54 10960 Q92624 P61019 P61018 P02452 Q86UW7 Q9Y3Q3 6993 10490 Q9UPV9 P04637 O96008 O95271 P50897 P38646 O60725 Q99523 O14828 2147 128866 8924 Q15021 10013 10134 637 10133 Q9C0K7 10015 6748 51009 6747 Q15388 11345 Q96HA1 Q04917 23339 O96018 4580 Q15027 Q9Y678 6643 6642 Q9NV70 Q6WKZ4 O95166 23214 P50542 55973 55737 11231 406 Q96CW1 P47224 408 Q15036 4218 P49407 409 Q15276 P49768 P60468 O00327 6890 Q9UMR2 Q9Y5W9 116986 O00560 2054 116987 O00562 23423 23303 Q96CV9 O00203 10239 Q5VIR6 9919 79711 Q00535 Q02952 P61204 Q15286 Q9Y5X1 P49755 51026 Q9GZP9 54536 8943 P49754 54535 Q9Y5X2 P01137 Q7Z3C6 51028 O14964 91949 Q9UP83 P01375 Q96JC1 O43264 4000 O14980 Q8N2H4 Q96EY5 23673 Q8N1F7 P12004 25978 P54920 O14617 O95070 Q96T51 Q7Z3B4 9804 P24588 11336 9927 O00623 O00505 O43592 O00628 5584 Q5U0I6 O00629 P05408 Q9UID3 Q9Y2K9 Q12907 P14598 Q8IYJ3 Q99567 P61981 Q8WTW3 O14908 55763 7514 Q96AX2 Q96AX1 7879 5579 10452 8724 9818 P55072 P05771 Q92930 P05412 Q9Y4P1 7532 5111 7531 7534 5476 7533 Q9HCE7 P37108 10228 P37231 10469 Q99797 79720 55770 P16671 Q8IYI6 8615 207 5468 9702 P46059 10466 P05783 7529 Q6IQ22 P27797 64130 9961 P24386 9601 P49321 Q9UPT5 9600 Q8WV92 22895 Q9NW64 Q9NVU7 Q96QU8 O14925 Q96P20 56993 Q00610 51068 7415 P61764 10673 P83436 Q8N4Q1 5119 A5D8V6 23636 P49792 5371 9972 Q9BQQ3 P49790 4285 8766 64145 6103 Q9UKK6 O75396 O00410 23633 Q969E2 Q6ZWJ1 Q08209 P52948 P62745 P40855 10204 P25116 57617 Q8N6T3 326624 9744 Q8TEW0 Q16623 P18085 8775 Q9UPN3 P35658 94121 94120 473 Q9H0U4 117584 Q7L804 23163 51762 23165 P49810 Q8WUM4 P62913 Q96QK1 Q15785 P49815 Q8WUM0 P25963 Q9NPC8 Q9HAV7 Q8TD31 Q9HAV4 8546 9632 11196 19 64601 79083 Q9UJC3 Q9H0T7 O75381 Q9H270 Q9UIA9 415117 8417 6238 Q96RL7 Q9H9E3 9868 Q9BUN8 9883 Q15436 6496 Q6Y2X3 Q9UNL2 8677 9525 9522 Q8WVM8 8676 P32121 23265 Q9H269 29927 Q9Y6Q5 375 51542 84342 Q8NFH4 9515 8548 Q13023 P61966 51429 P61619 6386 Q9Y2J0 129531 P62826 Q9UPQ3 P62829 Q86XR8 29934 Q9UQ26 O75351 381 Q86VN1 Q9NZZ3 23039 382 Q8N6H7 Q8N4C7 P59780 388 P61970 P31150 51552 Q9NUP9 Q5SRE5 P60520 Q16543 9527 9648 Q03518 Q49AN0 Q9P2E9 P62820 9784 149371 11140 9546 O43752 7124 10053 11021 P63092 Q9Y3E0 O43633 P13667 Q9H089 Q4G0F5 P35579 Q9UEU0 51560 Q9H082 O94826 P27824 11269 5195 9554 Q96C24 11031 O75694 23256 29916 161 Q96D46 7251 Q9Y5J6 152789 Q9NWN3 7249 8218 51699 Q9NRD5 P46459 P00491 Q86Y82 Q3ZCQ8 Q9BV36 23463 O43734 A1XKG3 Q9UQN3 Q86VS8 23225 O43615 6293 Q9UNH7 Q9NZ43 Q6VY07 Q9UBQ0 Q15363 Q9C0E2 Q9H9H4 P01579 55823 P02545 P09038 2923 55705 Q9BY84 O94973 O15078 O75558 7157 26985 P14625 O75674 Q9Y5L4 O75431 Q8N4H5 Q8TEX9 O43747 Q8WZ73 Q9H173 Q13190 Q99816 11014 O94979 84134 84376 Q14964 P53367 8498 9342 9463 Q13637 O95714 P57729 Q96QC0 Q13636 Q9H444 P63172 84958 83752 79139 Q92572 27243 Q7RTN6 55048 83871 65082 O95721 Q14974 O15131 9590 Q01082 9230 55054 9472 8021 P57735 P46934 117177 P43307 10947 Q9HD26 P52292 O15126 O60296 80230 9367 P10619 P52294 O75886 Q8TEM1 26258 57120 Q9H1M0 P57740 P29590 P30101 Q4LE60 7184 29886 60412 Q9NZQ3 Q9NSC5 9135 O15155 O15397 27236 Q6UWE0 Q9UNE2 64901 9495 112936 79023 P21757 653361 O43707 56288 O15027 1408 Q9UBN7 9146 Q9HC62 Q9UBF2 Q96NA2 Q9NP61 Q9P253 O00186 P41182 Q86YS6 O00189 P51148 9382 P51149 3836 4927 O00194 23085 Q8IWV1 P35606 9276 P56962 P51151 O95405 Q9NP72 P51153 Q9H2T7 57154 Q15629 P51159 Q96IW7 83547 83548 1785 O60499 P63010 P00734 Q96QF0 Q8WYP5 Q9BVL2 3841 O60493 3843

80273 5901 53916 O75843 100287932 59343 P84085 1315 P20339 1314 P20336 P20337 Q9HAP6 80184 9179 131474 Q9NP90 A4D1S5 63971 11097 P62072 79065 Q9H6Z4 Q96H20 Q9H0N0 Q63HQ0 23192 Q8TAG9 P84077 51510 Q96RF0 P20340 Q13596 51517

regulation of protein metabolic process P25054 Q92616 9184 O60566 Q9HCJ0 1454 P84022 1453 P63208 O14543 6901 P07996 5935 10533 P04004 P08729 Q12834 Q9UBC3 65018 Q9H832 Q8NDV7 P51114 5706 2316 5707 2 5708 5709 P14174 O60573 5700 5701 4734 5702 O15524 5704 5705 65264 Q5S007 O75925 Q7Z727 136319 Q9UKV8 Q92956 Q9Y2Y8 P53667 P62495 5717 3416 P62256 57761 811 P61289 Q9Y297 O75807 5713 P62258 Q92833 P26358 5716 P31946 5970 3673 Q9NR96 9093 Q99460 P17302 5728 P16333 P17301 O60674 P19838 2697 Q8IUC6 400961 2332 10642 10644 3309 P04233 10643 Q9UL15 Q9ULR5 P27037 P05129 Q9UL18 4771 P28482 P51668 1020 P51665 Q9P0J0 O95352 2107 55364 3315 710 Q92530 5976 3678 Q8N2W9 27161 P08887 P08648 P30556 Q9Y2T7 3690 Q9NYA1 Q92769 P09619 O00231 1153 O00233 P10600 O00232 Q96J02 P19544 Q96KS0 1029 Q9BUB1 10746 79444 960 3326 Q9BUB5 2475 3685 4898 P42771 3689 10985 P26045 P07766 P05106 4790 P05107 Q9UGJ0 6850 4792 Q9H2K0 Q13616 O00487 P30542 O60502 P38398 57448 P62333 2247 Q96N67 P20618 2246 P60033 975 O14641 Q5TCX8 P05230 857 6726 Q92878 Q96CA5 5515 P05112 Q92997 5518 Q969M7 P51693 P09958 Q9UKS6 Q9UL46 6500 P31749 Q9NR50 O95382 O00255 Q6KC79 Q03164 Q9HCK5 10724 440193 Q96LR5 192669 Q9Y385 5524 Q9UMX0 6613 P05121 50943 Q9UKT4 P06213 Q8N2K1 O00425 4690 P01241 6872 P04637 5300 P14317 2033 23327 991 Q96RU8 Q96RU7 3479 2147 23560 997 4686 6622 10013 54998 Q15389 Q9UQ80 P49770 64682 4221 6885 O60739 P36575 25998 Q9BT40 2280 Q99750 Q9P2K8 4214 406 648 4216 Q9BRP8 P01588 11236 408 84271 P49407 P60228 409 P01343 Q13099 P49768 O00327 O00206 Q09472 5682 5683 Q8N726 5684 5685 5686 64215 3263 Q9NS23 148022 891 894 Q00535 896 Q9NR09 P35240 8945 P01019 P25789 P25788 P01137 10477 O14964 P25787 P61326 P01133 P01375 Q9UJM3 4361 5692 5693 5573 5575 Q9GZP0 5576 P35226 P17813 P60484 P17936 5687 5688 4116 P01127 Q96EY1 5327 Q8WU17 P24588 Q15056 O43353 5580 5581 5582 6311 P62837 9821 5585 P46199 P07948 22794 Q9UNN5 Q96PU8 672 P15692 Q9BZE4 Q99683 O14908 Q96PU5 5577 5578 10213 P55072 Q96FA3 P04201 P05412 O43242 Q9Y4P1 Q6IR47 7531 Q9HCE7 64127 P37108 O76024 5590 Q00987 324 5104 8737 207 Q9H1A4 5347 54206 7529 P27797 P01023 P24385 8754 7422 P48594 3066 O00762 Q9NWW5 Q9Y5S9 333 P68036 7415 9839 Q9BT67 5371 P27540 8881 Q60FE5 Q9UPU9 Q12933 P49674 P17980 580 O14818 54106 P60321 P04792 Q92900 P60568 P49427 8892 4297 Q9BY77 O43432 11065 11186 7325 P30086 Q9Y4K4 Q9Y4K3 O43318 P13861 351 595 Q9UBS0 84324 P49810 9617 Q7Z6C1 51763 8767 Q13485 P25963 P38919 8662 3093 P03950 7332 5154 7334 5155 P55036 Q16512 8667 4067 4188 P46782 7336 5036 Q16635 O43684 7335 5037 P58546 5270 7448 7329 P00533 7328 Q16401 P61956 Q13133 7341 Q13257 8555 P32121 P35998 4193 Q9Y6Q6 Q03405 Q01105 5159 10197 7458 Q13144 Q14232 Q9BSI4 Q9Y6R4 5054 4088 Q9BY44 4089 9775 P55055 Q9UPQ9 P35625 Q4AC94 Q9NWT8 P62701 Q13153 P62942 8454 Q8TDY2 7124 7248 Q96KC8 P14635 O43639 4092 Q93062 Q9Y2C9 121512 P23588 Q13042 Q99728 8569 Q13286 Q14011 7376 8100 7375 11030 Q09161 P36896 P35443 P36894 26523 Q86WV8 7490 5071 7251 O75570 P11021 P01308 120892 P49721 O15169 P49720 85440 P02790 P98179 10273 A1XKG3 Q9Y6M1 Q99700 Q8WY64 P49959 Q9H1Y0 51465 55704 P55010 51588 P01579 P49711 11124 Q9UBQ5 Q9BQI3 1956 P09038 P23443 10159 Q8NEJ9 Q15008 P82979 7157 23112 P37840 7272 1965 Q9H9G7 Q6PID4 Q99816 P48730 Q15257 Q14289 10289 6199 Q13873 7046 6198 O95835 O75787 Q15819 Q06787 6193 7161 1975 P42345 26271 Q9BXM7 P62195 P63165 91 P04085 O15234 Q92574 P45974 1616 1978 Q969T4 1856 Q9UBK2 P56524 O15372 P78536 9474 7057 O15496 Q13526 Q13404 Q96QB1 P10644 83737 P31321 O43847 9470 Q3V6T2 P31323 P46934 P67775 1982 P16070 P05067 P60900

Q9UER7 8399 7186 P52298 7189 Q5JXB2 Q9H422 Q96M96 P29590 Q9HCP6 27102 P17252 P05155 O60260 O15151 8165 Q13546 Q02156 9495 Q9UNE7 27352 P43405 Q05655 Q04206 Q9UBN7 Q92667 9146 O95999 Q15735 27327 9021 B3KY43 1653 Q03468 Q92499 Q13200 7099 Q13443 Q96HU1 Q9NP71 26005 P30153 57154 P11413 P61081 P30279 1789 1786 Q9UJX2 Q05513 P61077 P00734 P30281 3720 P21333 P55884 8078 P10415 Q9P2X3 O14495 P28065 P19474 Q9P035 57162 84305 P61088 P05198 P01730 O15350 O75832 Q9BZS1 P53350 Q13107 P62191 P28074 P28072 P28070 23192 P63279 Q9HC29 P30260 O15105 P00750 Q9HD40 Q13233 P08238 P43686 P21675

RNA splicing Q8N9Q2 285672 53938 Q16629 O75940 Q969L4 P67809 Q9UPN6 57187 P13984 P52434 Q96FV9 O00148 Q01130 Q9UBB9 P33240 4841 10772 P09012 Q6NZY4 Q9UK45 Q13243 Q00839 O94906 Q13242 Q9H2H8 P38919 Q13247 Q16637 O75934 P30876 Q8IWZ8 Q15428 Q15427 79084 Q9NQ55 O75494 55696 Q9NW13 24144 O94913 10421 O75937 Q9NWB1 Q9BRX9 Q66PJ3 Q14103 Q9UKF6 Q96IZ7 O60306 54496 1478 85437 P42285 P09234 Q8N684 P26599 P83876 51428 P31942 Q12972 8563 Q13148 9410 O43660 11171 P31943 10081 8683 11051 9775 Q9H814 P36954 144983 Q99459 55110 Q13151 55119 P26368 P61978 5725 51319 Q15459 9785 10291 9541 8458 Q8IX01 O95232 P52756 Q9BWG6 P62316 Q8IYB3 P84103 P62318 8449 O14744 P62312 Q14498 58517 P62314 Q10570 51329 Q12874 10181 Q96SB4 Q12872 Q96SB3 Q8WXF0 Q09161 P19544 7490 10625 56342 57794 Q9Y3F4 Q8NI27 Q9BRL6 11157 Q15233 Q86W42 P98175 4670 22938 Q9Y4Y9 O60508 O60506 11129 51340 Q96MU7 Q9NVM4 51585 5511 Q9Y4Z0 5515 Q6I9Y2 Q15365 Q9UNY4 P08621 5518 22826 Q9HB90 22827 22828 Q08170 5411 26986 P14866 8241 5093 10286 6733 10285 Q9Y2W2 988 Q92620 P78362 Q9BZJ0 11017 O43395 22913 Q14966 P52272 Q86V81 10250 10492 6631 10929 Q9BV90 57461 P14678 O95391 P53803 Q96DI7 51362 84967 Q8WWY3 4686 6625 O15234 P09651 6627 P62995 P62875 135295 Q9UBK2 P51991 P07910 Q13523 220988 Q15029 P54105 Q13769 5430 P55795 5432 23210 Q9UKM9 P51513 P24928 P22626 Q9Y3B4 79005 P67775 27257 84950 10949 Q9UHX1 23451 O43290 Q7Z6J9 6633 Q9Y6Y0 P09661 Q15393 10147 O43172 6637 P08579 P52298 Q86U42 O43251 5440 9128 5441 9129 O75643 Q9H307 10907 P11940 O75400 9360 80145 283989 Q8TBF4 10594 P49756 5434 P61326 Q15287 P23246 O43143 Q86X95 Q9UMS4 84292 Q32P51 6421 Q13427 Q9Y333 Q8NAV1 Q9UHI6 83759 23435 Q8N8D1 10921 11218 11338 29896 Q07955 29894 4116 57819 P62308 P61218 Q9ULR0 O43390 P62304 50628 Q13435 P52597 6430 Q9UJV9 6432 Q9UQ35 6431 6434 1660 Q6NYC1 22794 23524 Q96PU8 1655 1653 4809 10450 Q92499 6427 Q8WVK2 6426 Q96A72 1659 23658 O95400 51729 5356 3178 P19387 P30153 P19388 27339 O43809 1665 26121 27332 Q9BUJ2 Q6P2Q9 Q13573 25804 3181 4154 25929 Q8TA86 Q8N2M8 3189 Q9UNP9 Q8TEQ6 3188 3187 3185 3184 P55769 Q9NW64 Q9Y5S9 P17844 Q5TAL4 55660 2521 7536 9716 91746 Q9BQA1 51747 3192 Q9BQ04 3191 23517 3190 Q5VTL8 Q08211 10569 79622 79869 O60231 Q9HCS7 Q13595 Q14683 Q14562 1207

signal transduction Q9H0E2 P25054 P49023 1460 10657 1457 1454 1453 O14544 O14543 Q9Y275 Q9UL63 P16104 2317 2316 5829 P15260 5822 Q9UKV5 10666 Q59EK9 Q5S007 O75925 Q9H8S9 3661 Q06124 P17405 P37023 81876 P62491 Q9UKW4 4983 Q9Y297 10633 Q12851 3672 Q13705 P21917 10645 3667 P42830 Q8WXG6 O75901 P49069 5861 5862 1020 114548 1017 1012 P61587 P61586 26191 P61106 Q9UL26 Q9H0H5 Q9UL25 P29466 Q9Y239 5870 5871 Q15907 Q9UGI9 P51797 1029 Q9BUB1 1026 Q9BUB5 5863 O60890 5865 5867 6714 5868 5869 Q07890 O95136 Q9UGJ0 Q9Y243 5883 1040 P16118 P19634 P38398 Q07889 4543 613 Q5TCX8 Q6NUQ1 5877 5878 5879 Q92997 P10826 Q9UGK8 5894 P31749 Q38SD2 1050 84932 55159 Q96MT8 10607 6733 P04150 Q9UL54 127829 Q92502 P56199 P14314 O00300 23568 11108 79937 5898 P62873 10256 P25445 O96017 6760 O96019 O95163 Q9NWZ3 P57059 P36575 1072 O96013 P35249 Q99996 641 Q14185 P47224 Q15036 P49407 85360

O00329 6772 Q08380 Q9UKN5 P48551 6774 3265 P11274 Q99500 Q9H4B6 Q8WWW0 8945 P01019 55504 O14964 Q99988 O14965 P35225 P14784 P35232 3269 6776 Q15052 Q59EA4 P24588 6790 Q92934 9821 672 673 53340 P62834 6789 Q92930 P05412 Q9NWV8 Q15077 9826 207 5468 4139 Q9Y5S2 O75044 O00762 O75051 A7KAX9 10673 10672 O14920 P56180 Q92903 Q8NHX1 P50406 P08908 O14939 Q3KRB8 56882 P40616 10681 8517 Q9H4E5 55558 9846 326624 O15085 P36507 P35659 P19174 11065 8533 O43559 O43557 P13861 Q9H0U4 Q6P5Z2 23043 Q9H160 P83731 P49815 Q9HB75 4188 P62805 6368 Q9Y2H1 Q9H0T7 Q9UD71 7205 8536 5029 P00533 Q13370 Q96RL1 Q16881 Q9BYV2 Q9BYV6 Q9BZ76 Q9UQ13 9404 8555 Q9Y2I1 Q9Y6Q6 Q01344 Q00013 Q15561 P55290 Q15562 Q9HAT0 6386 Q9Y6R4 P62826 Q9UPQ3 Q9Y6R0 Q86XR7 Q8N4C8 267 51552 Q00005 Q13393 P61978 P43246 P62820 P78347 P78348 11021 O75582 P10398 Q93063 51560 Q9Y2C9 Q9Y6K9 8569 Q15109 11031 26524 285 7249 11035 Q15118 P78368 Q9BYZ6 P11229 9448 Q9Y6M4 A1XKG3 P58753 Q14C86 P08069 1956 P78357 Q9HB90 O75553 O43508 P11234 P11233 7272 2810 Q93038 P78362 Q9NPB3 1969 Q14289 9467 P12931 Q9H0M0 P22736 P42345 P04083 1978 7297 Q15942 Q08830 7295 P42331 P42338 P42336 P29353 O95977 Q06643 P29350 Q9H0K1 Q7Z569 P40145 P06241 P21860 P78314 Q07960 P78318 9495 3705 3708 Q9Y2B9 O95996 Q14643 O14593 Q8IWW6 Q13315 Q03113 8174 9021 Q16828 Q96EP1 23085 P20594 2869 O75509 Q8IWV1 Q13322 Q14653 Q9NP71 Q15506 Q9NP72 23092 P46736 P61073 5906 5908 2873 23098 O60496 5901 5902 P46734 Q13330 Q9Y6D9 9047 9046 Q16849 Q8NEB9 Q9NP86 Q9P035 2889 Q9BQA5 P02708 2885 Q9Y6E0 50650 P43694 O15455 5911 5914 Q9NP90 Q14676 A4D1S5 9052 Q9H0N0 P00519 2896 P24941 P43681 51512 Q15532 51514 51517 Q13352 5925 23603 23607 Q9NRW1 Q9NRW4 Q99490 55684 O60566 Q8IVH8 P16220 79658 55207 P49137 P27348 O15516 P27361 O95680 65018 Q07343 9077 P50148 Q92845 Q92844 O15524 P52333 Q92838 Q92835 P29992 Q9NRY4 339122 Q6P4F7 P53667 57403 Q8IVF5 1111 P03372 P06401 P06400 5970 P46089 Q5JS13 9093 Q9UBE8 10767 P04233 P34925 4651 P50570 P11802 3320 P52757 O00463 O00468 Q9P2N2 1137 P61225 P61224 4649 Q7KZI7 P30556 Q5HYI8 P30559 10746 1147 2475 Q92882 Q92888 O95257 P28223 6850 P50591 2011 P30542 Q9NS68 O60503 O14640 5515 O43182 5518 P51451 Q13625 3350 P30530 10725 10728 O14654 Q9UK32 5528 5529 Q9BST9 P04637 O43157 P38405 2034 2033 1185 11228 P62993 P01116 Q15382 Q9C0K7 6869 Q15389 285282 P01112 P01111 Q9UNS2 P25100 4221 6885 P35368 2043 1192 56940 O76074 9908 Q15392 54763 4214 4215 4216 P01588 4218 P01100 5562 Q8N726 5563 5565 P14416 2054 P52735 Q96CV9 P11388 Q9NS23 Q02952 P61204 Q53X93 Q9Y572 P01138 P61201 P01137 Q6UXV0 P01135 P01133 O00459 5573 P04626 5575 5576 P04629 2065 2064 2063 P35348 Q9NRR8 301 2059 Q9C0H5 5566 5567 307 5568 Q8TDZ2 11215 23647 5580 5581 5583 5585 O43597 Q5U0I6 5586 Q9UJF2 Q9UNN5 P51828 5577 5578 Q9P2F6 5579 57826 P40763 P04201 P46060 Q6IR47 5111 5597 5598 P18850 5590 P16671 324 4259 O43572 P01185 O00408 6453 6456 6455 2099 Q8IZJ4 333 Q9NRM7 5599 8625 P25103 P01178 7791 6464 O43586 4289 P39748 Q5T5U3 P51858 6457 Q96HB1 P25116 Q13237 5141 5142 5144 Q15418 Q9BY76 Q5VZM2 11186 8654 Q9UBS0 355 356 85415 5138 5139 P25963 O43683 19 Q8TF76 Q9UKE5 367 369 Q13257 P11308 29 Q86X27 P32241 P32246 Q8TEU7 Q9UBU3 Q03405 375 P51805 Q13261 5159 9518 6009 P32239 Q14114 Q8WWN8 P51812 Q9UKG1 381 382 Q04725 Q04724 387 388 Q04726 389 P60763 41 Q9NSD7 P55345 10293 11142 2909 390 391 392 O43639 394 396 397 Q99962 398 Q99963 121512 25780 Q6KH11 Q9H082 Q13283 2908 Q13287 Q99966 Q15464 Q96R06 Q9BU20 Q16799 Q9UKA4 Q9UKA8 Q6PRX2 Q8NHQ8 Q6PJH3 2915 Q99956 Q6ZSZ5 10270 9567 O43612 Q9Y5K6 Q99941 P01579 11124 10276 P09038 Q14155 2923 Q9BY84 9575 O75676 9578 1609 Q5VVH5 Q14168 86 Q14964 Q15811 P78423 Q13637 Q13636 O15123 Q12788 Q8TF05 Q13639 1613 Q9BXM7 91 94 1616 Q9NSA1 26270 9112 Q96QB1 9590 P31321 P31323 P67775 P29474 O94768 9126 Q96M96 6093 P41597 P21980 Q8TAD8

2969 P11717 O95622 Q14511 Q6R6M4 9138 Q02156 P31785 55198 Q8N392 Q86WH2 Q05655 Q9UBN6 9146 P13945 P41180 114822 B3KY43 Q03468 P08123 150094 Q52LW3 Q13202 Q96HU1 Q8IVT5 Q9UBG7 P20248 Q8TAI7 P08134 P17081 P19235 P08581 P08588 P41159 O15111 P55773 Q13227 P62070 O95661 P45983 Q13233 P45984 P21554 Q2M1Z3 Q92737 65125 Q5TB30 P54619 9182 P37288 Q92731 10890 Q92730 O14786 O14788 6901 A1A4S6 10413 P28799 P28335 64284 Q08AM6 2562 Q8NFZ5 P53779 Q17R89 Q7Z727 Q9NR81 79791 6927 Q8IUD2 3416 57522 P63104 10875 P31946 P31947 2100 Q9NR96 O00178 P54646 P20823 Q8IUC4 O60674 P20827 P19838 Q8IUC6 P31949 5602 5604 P08758 Q13956 Q5SGD2 P15056 Q6ZRI8 55367 Q8N264 644943 Q9Y478 P30679 P32856 P09619 Q07666 P80098 P10600 P52888 Q9BWF2 Q6PI57 846 Q6ZW31 7804 O14757 Q92766 P49286 4790 O43295 4791 4792 Q13976 2132 P21709 Q96L33 P18545 3459 P60033 83853 Q92752 P05230 857 O14763 Q9UN86 Q96CA5 P51575 O95382 Q9NQT8 153090 Q9UMX1 O14775 P09601 O14777 P01241 Q9BRR9 Q96C74 3484 3482 O60603 25988 3479 2147 3476 10010 Q15269 P13236 Q99755 400 402 Q99750 Q99759 408 O43293 O14713 Q13098 Q01518 Q6ZN33 Q09472 4354 Q7Z6I6 4355 116986 O00560 116987 O00562 23303 890 Q00653 891 Q99741 55743 896 3014 Q15286 Q9Y450 29086 O43264 Q8TCX5 Q01974 P15882 10486 7867 8718 O14733 5207 10487 P23497 8717 P01243 P26583 O00506 Q9Y3M8 10459 10458 Q8WUY9 Q96P48 10451 6548 7879 10454 Q01968 P01275 8743 8744 7410 Q9NYZ3 P37231 P51959 8737 P48357 P55083 8738 Q6IQ22 P01266 23746 P24385 8754 Q96GA9 5245 9600 3068 O75293 79753 Q96P20 P24394 7433 8766 P49674 Q13905 P52824 80728 P17612 8772 Q13115 Q13114 P18085 Q14449 P10114 Q9Y4K4 P10599 O75385 Q9Y4K3 O43318 472 P48729 51762 P20396 8767 Q14451 P35408 Q15303 3091 Q16635 P55042 P55040 Q15311 Q13131 O00602 8795 Q13136 Q15797 Q16644 8315 P19397 P12755 Q8IV61 Q16659 5295 5296 9656 P43490 O43306 P10588 P10589 9649 Q16665 P43487 Q9P2E9 Q13158 Q8TDY2 11261 P54132 P63092 P46109 Q13162 P46108 Q13164 Q14012 Q15349 O15294 7013 Q9Y4H2 Q9NZT1 P63096 Q9NRD1 Q13177 Q99835 10392 O94844 P12314 55704 1718 Q13188 P23443 O43741 P10147 O75312 P38936 O75791 Q6PID4 Q99819 P27815 P48730 P23458 Q9BWT7 Q99814 P48736 6196 6199 7046 6198 P98155 P10997 8379 O43715 6195 6194 27242 Q86VI3 10817 P46940 O95715 P98161 Q13522 7057 P10523 9230 9231 Q3V6T2 7052 3925 P46937 1742 Q4LE28 P98171 P98174 Q9UER7 P34947 P29597 Q9H305 Q13535 O15264 P29590 8394 P30101 7064 P41231 Q92793 P16284 Q9NZQ3 O60266 O15259 9255 Q13546 Q8IYT8 9252 9253 7074 80254 P43405 P20309 Q04206 Q9NVJ2 P78560 P09525 7088 Q13554 Q8IV45 9261 3959 P00749 O00194 P63000 51720 3953 Q13563 2626 7099 7098 O15211 9270 7090 P30153 P62166 Q9UIV1 1785 Q05513 P00734 Q9HD67 P24723 375790 Q13574 51735 Q6SZW1 Q15750 Q13572 53916 Q15759 O60229 P41279 P20338 P20339 P20336 P20337 P85298 P41273 1793 Q14432 Q15762 P18031 P34972 1326 Q9UNA1 P47804 Q8TDM6 P67870 900 P20340 P08235 Q9NZN9 Q7Z628 P08833 Q9Y2W7 Q14CB8 Q92619 P61020 Q6PCE1 Q7L0Q8 2665 2664 Q9HBH0 P61026 Q8N103 P07996 P52564 O75815 P52565 Q12955 Q7L591 Q66LE6 Q12959 P52566 Q9P107 P63211 10549 P16473 2676 P16471 P63218 P48039 Q9Y2Y0 Q92600 P54753 P50395 Q9NYF5 P32927 57646 29127 P20936 Q9UQB3 Q9UQB8 4867 4868 4869 5716 P54764 P54762 4882 P48023 3551 O00299 Q12979 P20941 Q8NEU8 Q9ULZ3 948 Q9UHR4 Q6PCD5 O95477 P28482 1385 O00220 29108 5739 O60760 P07550 Q9Y2T1 10971 7913 P08887 P51671 P04049 Q96SB4 Q9NYA1 O14880 2243 P10721 29110 2237 79444 1388 1387 963 10981 P42771 O00241 Q9P0L2 22931 6609 P62330 2247 P15153 1399 2246 1398 2244 975 P61006 Q92636 P51693 5770 P29320 P51692 P28472 O00255 P48061 P17342 P29317 Q9ULV4 P61019 P61018 26060 P06213 Q15139 Q9Y6W6 P07900 Q9Y6W5 990 Q96RU8 Q96RU2 2267 998 10133 P27986 P62753 5310 Q7Z5H3 5311 5796 266747 Q04900 Q8WTR2 Q96RT1 10142 Q99638 10146 P02675 P01343 P49768 O00206 Q9Y6Y9 148022 Q00535 Q96S42 5798 6647 8829 8826 P01375 P01374 Q9Y2R2 64343 O00213 Q08499 O00212

P12004 Q96RR4 Q15172 3148 545 P40933 Q96EY1 Q15173 5328 7508 O43353 6672 Q969H4 P07949 8852 P07948 Q12904 Q8WU90 3162 Q8IXI1 Q8IXI2 Q68EM7 552 553 Q99683 5335 Q96AX2 5336 8844 Q15185 5337 5338 P05771 7534 Q9UHC3 Q01831 P50749 P50748 5347 Q96JH8 7529 Q08462 5361 5362 5364 Q5VWQ8 Q9UHD2 Q12929 Q9BUZ4 572 8882 5371 3190 Q60FE5 Q12933 P16885 Q99653 P51617 P62745 54106 Q13478 Q13477 Q15654 Q9UQP3 6236 8412 9743 Q96FV9 595 29941 Q96BM9 Q8NFJ5 Q7Z6C1 P22694 Q13485 Q8WZ64 8408 P32019 6242 Q13009 Q16512 4067 Q16513 Q15669 6238 P60983 6239 Q14344 Q7Z6B7 Q13017 Q9HBW0 23380 Q9UDY8 P23528 8428 P00441 Q16539 4086 P22692 4089 Q15208 Q16537 Q9Y3L3 9770 Q9Y3L5 P21359 P31150 1808 8439 10087 6259 9784 O75469 7124 P14635 P10275 P10276 154 Q96KC2 Q16555 Q13043 9311 7132 8463 Q5JQC9 P36896 P35568 6282 23370 Q8NFM4 Q04941 Q02763 P01308 Q8NFM5 120892 Q99717 9306 P01303 9322 P02671 Q8TCU4 8473 P54274 8471 P36404 9318 1837 P49715 O95819 P60953 64780 7153 P39905 7157 P19086 7159 Q02750 1848 54509 Q13077 P53365 O95835 P57729 O94988 O95833 10928 57584 P42224 1852 1850 P23945 83871 O95837 P22612 P42229 P42226 Q53QZ3 22926 Q14738 P10644 22920 79363 P57735 P84095 P39059 Q9UI95 7187 9367 7186 7189 7188 7185 P17252 P42680 P42681 P42684 P21741 O15397 O43823 B2RTY4 4914 27352 P19438 P68400 Q969Q1 Q13432 58473 Q86YS6 P51148 Q7RTR2 P51149 Q9H244 P23508 P43119 1896 P42262 4920 P51157 Q13443 P51151 Q15628 P51153 P42261 58480 P51159 27330 Q9UQE7 P30281 57159 Q8WUH2 Q8WYP3 P23510 P21333 O14492 377630 P84085 1432 Q5XUX0 Q15642 Q13464 P01730 Q14314 P53355 O75832 P53350 3622 P84077 Q7L9L4 P24821 4952 Q15653

cell cycle process P25054 8099 Q9Y265 23607 Q9Y266 Q969L4 2672 8091 259266 9181 P51587 9184 10657 Q9BVA0 O60566 Q7L0Q8 4609 1213 Q96IK1 P84022 Q9BVA1 P63208 Q8N3U4 Q9ULW0 10534 P07996 Q92974 O75935 P52564 P08729 Q12834 Q53EZ4 P16104 O60216 5706 5707 5708 1104 5709 10783 5700 5701 5702 5704 64282 5705 P30622 P27694 Q9NQW6 22974 4628 5717 Q9Y2Z0 6929 57405 29127 1111 811 P61289 Q9Y297 O75807 Q9Y6A5 5713 Q5TAP6 4627 4869 Q96EA4 Q92831 P06400 5716 P53675 O95347 Q99460 Q9NRZ9 Q96GD4 O14578 5604 P50570 P11802 Q14807 1021 10609 P51668 P51665 22832 1018 1017 Q96DE5 1499 55125 3796 O75909 Q92530 Q5TB80 P07437 Q9H0H5 22846 Q96SB3 Q07666 O00231 O00233 Q8IZT6 O00232 79441 1029 O14519 1027 1026 116840 O14753 P41208 P42771 O14757 Q8N137 P30304 P30305 P30307 P28340 Q13618 Q13617 Q13616 55142 O00487 P62333 Q9BQ90 P20618 Q92878 5515 Q9UM63 O00139 Q9UL46 5892 6500 P31749 Q7L8A9 O00255 Q6KC79 Q9P209 P40692 10726 984 Q96MT8 989 5888 O14777 Q9UKT4 127829 Q96FF9 P04156 Q15019 26959 6872 6993 P04637 5422 Q09019 O95271 25988 990 991 203068 55722 994 1058 995 Q99640 Q66GS9 997 6502 55726 Q15021 5536 5537 P62753 P01112 Q9UQ80 64682 4221 Q9Y6X3 5310 Q8N4N8 5311 Q9UQ88 23332 23212 Q8NHV4 641 Q15392 1069 Q99871 O43290 P46013 P01106 5424 11113 8812 Q9BS18 25847 Q9BV73 5682 P48431 5683 Q8N726 5684 5685 Q7Z7K6 5686 3265 Q9NS23 P52732 890 Q66K89 891 Q9H4B7 Q99741 55743 Q00534 898 3014 8945 P25789 P25788 51147 P01137 Q8N960 P25787 O14965 Q16254 O43264 Q9UPY8 4361 5692 5693 8841 23310 P35222 O00213 O95067 Q96ST3 Q15050 P23258 5687 Q9Y580 5688 Q00526 6657 6790 9700 7884 6794 Q969H0 25942 O00743 Q12906 10459 Q96P48 Q99684 10213 8607 2081 O43242 P49450 O43482 10460 P51955 P51956 Q9BZD4 Q9NYZ3 Q6PJP8 Q00987 P51959 Q99675 P50748 324 54443 79723 207 Q9H1A4 5347 P27797 Q96JH7 Q9H5I1 P24385 Q86U86 91754 Q99550 O00762 22897 Q99543 Q99661 Q9NRM7 Q00610 5116 5119 5371 3070 8881 Q7Z4H7 P17980 P52701 580 79980 O14818 Q08209 10200 55559 Q2M2Z5 Q9UPV0 P49427 54908 Q14204 11065 11186 P15923 Q9UPN3 4291 351 472 P48729 595 Q96BM9 9738 5017 P83731 P43034 P21127 Q8NEH6 Q9HC98 O43683 11190 P55036 5036 O43684 O75376 24149 6117 54801 7329 P00533 Q16401 P24522 Q29RF7 P11309 P35638

Q13257 6498 P12757 7465 Q8WVM7 Q9UNL4 O75122 P35998 4193 P51808 P46527 P49916 54930 Q969Z0 Q6UVJ0 Q96RK4 10198 10197 Q8NFH4 P83876 Q5VUA4 51547 Q9HC77 O94927 Q8WZ42 Q93008 4088 P62826 O43663 O43303 Q8TD19 Q96BK5 54820 P24864 Q15691 Q8IX90 P43246 8453 8454 8452 9662 P54132 Q14008 23122 23244 P14635 O75461 25788 P35579 O75460 P35580 Q9H6D7 Q13042 Q14493 Q99728 8328 10179 Q96R06 Q96CS2 Q9NRC8 7013 9793 Q96KB5 163786 P36896 26524 7251 Q9NQB0 P49721 8218 P49720 O15182 10270 P54274 Q9Y5K6 221150 P49959 11243 9319 P01579 1718 Q9BRK4 Q9NTI5 Q9NVM9 347733 Q15003 11004 1956 P23443 Q15008 9212 7157 116138 Q8TAP9 23354 Q02750 P38936 25777 7272 Q9NXR1 Q9NZ56 Q93034 54984 Q9NTJ3 55835 Q99816 Q5VTD9 2935 9221 6198 O95835 Q9NNW5 8379 Q01094 P63172 7161 6194 7283 80124 27000 26271 Q9Y6G9 Q9Y3A5 84967 55165 P62195 91 O15355 P98161 1616 1978 Q13885 P78536 9232 7057 9238 Q14978 Q8WWH4 O75771 55294 3925 P67775 Q7Z460 1982 P52292 22919 Q9UI95 Q6P1K2 P05067 P42695 P60900 1869 Q14980 Q9UER7 2956 Q15831 9126 O95613 10907 P29590 Q13418 91272 1877 10910 1874 Q92791 O60266 O15379 O95619 1639 O60381 O15151 93323 9133 Q14511 O15392 A4D1W7 O43823 Q03001 Q13309 Q8N7B1 79023 Q9H410 9493 27352 80254 Q9UH99 P09884 P53041 Q9HBM1 1647 Q13554 P54652 Q13315 O95997 Q9P258 Q96EP1 1778 4926 3832 Q13563 Q13200 Q13561 3835 Q96HU1 Q68CZ6 58480 8065 P20248 Q9UJX2 1785 Q9UQE7 3609 5901 Q9Y6D9 P10415 3980 P28065 Q02241 Q9BQA5 3611 Q9HC35 Q14674 P54687 O15350 728642 O75832 P53350 27436 P62191 Q04771 P28074 P28072 P28070 P63279 P00519 121441 O60232 P30260 P24941 900 51512 P43686 P21675 Q14683 5925

regulation of cellular protein metabolic process Q92616 9184 O60566 Q9HCJ0 1454 P84022 1453 P63208 O14543 6901 P07996 5935 10533 P04004 P08729 Q12834 Q9UBC3 65018 Q8NDV7 P51114 5706 5707 5708 5709 P14174 O60573 5700 5701 5702 O15524 5704 5705 Q5S007 O75925 Q7Z727 136319 Q9UKV8 Q92956 Q9Y2Y8 P53667 P62495 5717 3416 57761 811 P61289 Q9Y297 O75807 5713 P62258 Q92833 P26358 5716 P31946 3673 Q9NR96 9093 Q99460 5728 P16333 P17301 O60674 P19838 Q8IUC6 400961 2332 10642 10644 3309 P04233 10643 Q9UL15 Q9ULR5 P27037 P05129 Q9UL18 4771 P28482 P51668 1020 P51665 Q9P0J0 O95352 2107 55364 3315 Q92530 5976 3678 Q8N2W9 27161 P08887 P08648 P30556 Q9Y2T7 3690 Q9NYA1 Q92769 P09619 O00231 1153 O00233 P10600 O00232 P19544 1029 Q9BUB1 10746 79444 960 3326 Q9BUB5 2475 3685 4898 P42771 3689 10985 P26045 P07766 P05106 4790 P05107 Q9UGJ0 6850 4792 Q9H2K0 Q13616 O00487 P30542 O60502 P38398 P62333 2247 Q96N67 P20618 2246 P60033 975 O14641 Q5TCX8 P05230 857 6726 Q92878 Q96CA5 5515 P05112 Q92997 5518 P51693 P09958 Q9UKS6 Q9UL46 6500 P31749 Q9NR50 O95382 O00255 Q6KC79 Q03164 Q9HCK5 10724 440193 192669 5524 Q9UMX0 6613 50943 Q9UKT4 P06213 O00425 4690 P01241 6872 P04637 5300 P14317 2033 991 Q96RU8 Q96RU7 3479 2147 23560 4686 6622 10013 54998 Q15389 Q9UQ80 P49770 64682 4221 6885 O60739 P36575 25998 Q9BT40 2280 Q99750 Q9P2K8 4214 648 4216 Q9BRP8 P01588 11236 408 84271 P49407 P60228 409 P01343 Q13099 P49768 O00206 Q09472 5682 5683 Q8N726 5684 5685 5686 64215 3263 Q9NS23 148022 891 894 Q00535 896 P35240 8945 P01019 P25789 P25788 P01137 P25787 P61326 P01133 P01375 Q9UJM3 4361 5692 5693 5573 5575 Q9GZP0 5576 P35226 P17813 P60484 P17936 5687 5688 4116 P01127 Q96EY1 5327 Q8WU17 P24588 Q15056 O43353 5580 5581 5582 6311 9821 5585 P46199 P07948 22794 Q96PU8 672 P15692 Q9BZE4 Q99683 O14908 5577 5578 10213 P55072 Q96FA3 P04201 P05412 O43242 Q6IR47 7531 Q9HCE7 64127 P37108 5590 Q00987 5104 8737 207 Q9H1A4 5347 54206 7529 P27797 P24385 8754 7422 3066 O00762 Q9NWW5 Q9Y5S9 333 7415 9839 Q9BT67 5371 P27540 8881 Q9UPU9 Q12933 P49674 P17980 O14818 54106 P60321 P04792 Q92900 P60568 8892 4297 Q9BY77 O43432 11065 11186 P30086 Q9Y4K4 Q9Y4K3 O43318 P13861 351 595 Q9UBS0 84324 P49810 9617 Q7Z6C1 51763

8767 Q13485 P25963 P38919 8662 P03950 5154 7334 5155 P55036 Q16512 8667 4067 4188 P46782 5036 Q16635 O43684 5037 P58546 5270 7448 P00533 Q16401 P61956 Q13133 7341 Q13257 8555 P32121 P35998 4193 Q9Y6Q6 Q01105 5159 10197 7458 Q13144 Q14232 Q9BSI4 Q9Y6R4 4088 Q9BY44 4089 9775 P55055 Q9UPQ9 P35625 Q9NWT8 P62701 Q13153 P62942 8454 Q8TDY2 7124 7248 Q96KC8 P14635 O43639 4092 Q93062 Q9Y2C9 121512 P23588 Q13042 8569 Q13286 Q14011 7376 8100 7375 11030 Q09161 P36896 P35443 P36894 26523 Q86WV8 7490 5071 O75570 P11021 P01308 120892 P49721 O15169 P49720 85440 P02790 P98179 10273 A1XKG3 Q9Y6M1 Q99700 Q8WY64 P49959 Q9H1Y0 55704 P55010 51588 P01579 P49711 Q9UBQ5 Q9BQI3 1956 P09038 P23443 Q8NEJ9 Q15008 P82979 7157 23112 P37840 7272 1965 Q9H9G7 Q6PID4 P48730 Q15257 Q14289 10289 6199 Q13873 7046 6198 O95835 Q06787 6193 7161 1975 P42345 26271 Q9BXM7 P62195 P63165 91 P04085 O15234 Q92574 P45974 1616 1978 1856 Q9UBK2 P56524 O15372 P78536 9474 7057 O15496 Q13526 Q96QB1 P10644 P31321 O43847 9470 Q3V6T2 P31323 P67775 1982 P16070 P05067 P60900 Q9UER7 8399 7186 P52298 7189 Q9H422 Q96M96 P29590 Q9HCP6 27102 P17252 O60260 8165 Q13546 Q02156 9495 Q9UNE7 P43405 Q05655 Q9UBN7 Q92667 O95999 Q15735 27327 9021 B3KY43 1653 Q03468 Q92499 Q13200 7099 Q13443 Q9NP71 P30153 57154 P11413 P30279 1789 1786 Q9UJX2 Q05513 P00734 P30281 3720 P55884 8078 P10415 Q9P2X3 O14495 P28065 P19474 Q9P035 57162 84305 P61088 P05198 P01730 O15350 O75832 Q9BZS1 P53350 Q13107 P62191 P28074 P28072 P28070 Q9HC29 P30260 O15105 P00750 Q9HD40 Q13233 P08238 P43686 P21675

cellular nitrogen compound metabolic process O75947 8099 Q92979 Q9Y265 O75940 Q12824 O95551 10657 Q9BYG3 2305 1454 1453 Q00839 P05455 4968 Q9H0D6 O75936 Q9H4L7 O75934 P30876 P52209 P19623 P16104 10667 P42858 Q9Y285 O75937 P10809 Q96EB6 136319 P27694 Q9UGN5 Q9UKV8 P27695 3661 4507 1478 Q9Y295 55109 5832 Q9UKW6 3659 P26358 10632 P54886 Q99583 Q12857 P21917 1491 55110 Q99575 55119 P26368 10642 Q9UL18 Q9Y223 Q9UGH3 Q8IX01 1022 Q9NSU2 P51787 27043 1017 2348 79577 P21964 P54819 Q9Y230 O75909 5859 Q12874 Q12872 Q8WXF0 Q9H0H0 Q15904 1029 P42898 O14519 10625 57794 1025 Q5C9Z4 P20648 Q9BUB4 P05023 P05026 Q9UGJ0 Q12888 5883 P38398 Q9BQ90 Q96MU7 55149 Q9Y250 Q92994 Q92993 6723 4548 P10827 P10828 5892 6741 5411 P31749 1050 55152 27032 10606 253980 P40692 27037 55159 O60870 10607 6733 P04150 5886 5887 5888 O96004 8930 10250 5422 P50897 23569 11108 Q8WWY3 P62877 8924 Q15020 Q15024 P62875 6749 Q15029 5430 5432 Q9UKM9 23576 P35244 P35249 Q03933 641 P35251 196441 5424 8939 P49407 P60228 5440 5441 6772 5442 Q9UKN5 O96020 Q9UKN8 346171 Q66K89 5434 51147 Q15046 P23246 Q9UL03 Q9Y215 Q86X95 Q9Y697 A7MD48 Q53H96 O14979 P17812 P35232 Q00403 O95190 668 Q15054 6776 4116 P57081 O00746 6311 245972 Q9Y5Q8 23524 672 P15692 Q96A72 P05413 5471 Q9Y5R4 23536 Q6PJP8 Q9NWV8 688 689 P49448 207 P62847 P62841 4150 Q9BQ15 4152 4154 P13489 Q9Y5S9 P17844 211 P13010 Q99543 5478 4149 P62857 P56182 O43463 Q9BQ04 23517 Q92905 Q9UKK9 O75031 Q92900 P56192 P49427 4172 4173 4174 Q14686 4176 P37837 Q14209 Q16864 P55265 Q9UPN6 Q6NZI2 4171 Q14690 Q14694 Q8IWZ8 8543 5036 79084 60489 Q9NPD3 Q13370 Q96RL1 P46777 Q15554 Q9HAU5 P24522 P35638 P61968 P56134 8555 P32121 4191 Q96IZ7 51540 P00568 51547 8563 Q9BYU1 9410 P36957 P62826 11051 Q8IWX8 O43542 8565 P36954 Q9NPF5 P61978 P43246 9421 Q14249 P78347 O75106 Q9Y6K5 P36542 26512 271 Q9Y6K1 Q93062 Q04837 Q96RQ3 23481 51567 10181 55929 11030 P22392 O75575 26523 23016 5071 Q93050 Q9NY12 118460 P49848 O75569 23463 P48507 P24539 51585 9439 2805 Q6I9Y2 Q9HB90 26986 Q01780 P37840 5092 5093 Q96EU6 51593 Q9H9G7 P78362 10166 11017 9465 P53004 9463 Q6P1J9 P29375 P29374 P29372 Q7Z589 Q05329 27000 2820 Q13761 9477 Q13769 Q9H0L4 O75531 1503 P24928 79005 1982 Q9Y6H3 Q9Y6H5 P06276 Q6P1K2 Q5D0E6 P55209 79035 55066 1994 79039 P00966

Q8TBF4 P54368 P78316 O95989 Q8N8D1 3704 Q9Y2B5 Q07955 P53041 P07101 50628 P52597 Q13315 O95997 P00505 P20594 84881 Q8NEC7 Q14653 Q9H0P0 P46736 Q9H9A7 26121 P61077 Q9BUJ2 P22303 5901 9045 Q9NP81 P12956 O75964 P12955 Q00059 Q9BQA5 P61088 Q96AE4 P43694 Q9BQA1 O95544 Q14676 Q16854 9054 P42357 5927 5928 P00519 P63272 23076 P24941 P43681 84893 51514 Q14684 Q14683 Q5FWF5 P10914 O95677 285672 Q92858 P67809 399687 9061 P30520 Q9P2R7 Q9UBB9 P13196 5939 1576 P16220 10772 5931 P49137 Q9UK45 5935 P0CG13 Q9NS91 Q9UBC3 Q07343 9077 55696 P18887 55215 P30519 10785 Q9NWB1 Q9NRY2 Q9NRY4 Q8TED0 O60547 P18858 O60543 P18859 1111 Q92830 P03372 P49591 P46087 Q9H814 Q9GZX7 9093 Q9NRZ9 P49590 Q99459 3304 3784 P49588 P07741 Q92820 P30566 5981 5983 O95232 P52756 6829 27165 Q9P2N5 1137 Q6FHQ0 P61221 5976 27161 6827 Q10570 Q13601 1153 Q14938 O00472 O95243 Q6PIW4 Q9NS56 3329 6839 P08651 Q6XQN6 O43175 O95257 4670 137964 4673 Q9Y4Y9 O60508 1161 O60506 O60502 80222 10714 P61244 O14641 5511 O14640 5514 Q9Y4Z0 Q92878 5515 Q9UNY4 P08621 5518 Q13620 P30536 P52788 10725 10724 P61254 10728 157570 4677 4678 P06454 O43159 6872 6871 P04637 Q5VYS8 2034 P16615 Q01650 4686 Q16236 5536 P62995 Q9GZR7 Q96LI5 Q9C0K0 P25100 6883 P48201 4221 23210 P12268 O76074 Q96Q15 51251 6878 Q15393 O43172 56946 P01100 Q8N726 P14416 O95299 P11388 P11387 O60942 773 Q53X93 5557 P61201 P01137 5558 P01133 O43143 6421 11218 Q9NS37 P09086 9924 57819 P61218 2070 Q5TKA1 6430 6432 6431 Q3SY69 6434 4255 7764 22794 P15328 Q96PU8 Q8IZL8 314 Q02930 8602 6427 6426 8607 Q8N302 2081 23658 5591 P46063 5111 23650 O76021 Q99437 P18848 328 25804 2091 P22087 Q8TA86 Q8N2M8 Q9UNP9 2099 P50993 Q9NW64 Q8NCN5 333 55660 56994 91746 Q9UNQ2 A2RTX5 4287 P39748 O00411 P52701 Q9NVV4 P15313 Q08211 221656 79622 P58012 9967 6472 5141 5142 Q14566 4297 8654 Q9UKD1 P30085 P13984 6470 4291 351 353 Q9UBS4 P09012 5139 P62913 9978 Q13243 Q13242 Q13247 P20290 O43687 7334 16 Q15428 7336 Q15427 26205 80198 367 Q9UBT6 51654 11198 7341 Q15434 Q14103 9525 P62937 23144 Q9UKF6 Q9Y606 P49916 P49915 85437 P17096 11165 11168 Q16774 Q9UBU8 P83876 P21589 34 O43660 11171 8683 9533 7358 Q9Y619 P29084 P34896 P11310 Q96HW7 11176 P23771 Q15459 5184 10291 O15160 9541 O15164 2909 P12694 25788 P27708 Q8NI36 2908 Q13287 Q9NSE4 Q14134 Q13286 Q14137 5198 Q09161 Q9Y5J1 7372 Q8NI27 11157 7389 6059 6050 11129 Q99941 P49959 P50914 51222 56915 56916 2926 11128 P62906 116138 P14868 P14866 8241 221264 10286 10285 Q15014 2935 P78424 9100 Q7KZN9 Q96QC0 Q14966 P52272 Q12788 92105 Q15819 P33992 P62081 P33993 P33991 Q96DI7 84967 26270 Q9UBK2 9114 P55795 Q14978 Q8WWH4 O95602 Q86WJ1 Q96I24 P43304 P67775 Q6IN84 84950 P05062 P52292 Q52LR7 P05067 2959 P08579 2956 Q9UBL3 P52298 P29474 9128 P29475 9129 O75643 9126 Q96I15 80145 283989 2961 2960 P04183 Q8TAD8 Q9BTC0 2969 P09874 29777 1642 2972 57379 P09884 134430 1647 P30049 Q15853 Q9UJV9 Q9BY32 Q14527 1660 Q6NYC1 P30043 P53567 P30047 2987 1655 P22415 1653 Q03468 Q92499 1659 3835 P47712 O95644 51606 64919 1665 Q9BXP5 P17081 Q9Y5B9 Q9Y5B8 P09429 Q13216 Q8TEQ6 P55769 P35711 4705 1676 2521 Q6P4R8 P09430 51629 P08107 Q9P2Y4 Q14558 O95661 4719 Q96HR8 Q14562 1207 P51587 81605 P52434 O00148 P17676 Q8TDD1 2547 P84022 Q9H2H8 P28335 25913 P28330 P08729 P28331 Q8NFZ0 O60216 80755 54487 P49247 10421 64282 P62249 Q9H2G2 Q9BRX9 3420 2572 O00167 2571 3417 54496 6927 6929 Q8N684 P26599 Q5TAP6 3419 P31942 Q9UIS9 P31943 P08708 P99999 P20823 P19838 P62263 3421 P11926 Q13951 4772 Q14807 P50213 Q8WVC6 P39019 Q5T160 10856 Q6PI48 80324 Q9BWG6 833 O14744 P51553 4780 5631 Q07666 80308 Q8WVB6 O95363 Q06330 27297 167227 4775 O14757 Q92766 P09622 4790 P28340 Q9NR48 Q9BWE0 80347 Q96CA5 P11908 5636 22826 22827 Q9H6R4 22828 Q8NC51 Q9NQT4 Q9NR55 Q9NQT5 Q9HCK5 2139 192669 865 4799 P09601 P06576 11340 Q86V81 10492 O00541 O95391 3481 25988 51362 Q96T88 3479

P25205 7832 84289 P23025 135295 P51991 4343 P51513 Q96T76 O14717 O43290 Q7Z6J9 Q9BRP8 P49643 O43294 408 409 O43252 O43251 P49642 Q9BW92 O00567 Q9BW91 Q9NUW8 O14727 126789 Q99741 Q96T60 3014 Q8IYD8 Q9UN42 29086 10476 P61326 Q15287 Q9UMS4 4361 84292 3035 114799 Q9NUX5 Q96ST3 11338 3028 55759 P48775 10488 84296 P26583 8732 7884 P50613 P51946 23764 25942 Q8WUY8 P60891 435 P31939 10450 P55072 3054 29079 Q9HCE1 Q96SZ6 445 55775 5226 Q02809 25929 7422 Q9BRT9 5245 P52815 22894 Q9UIF9 22897 79753 10438 Q5TAL4 7415 10432 8761 3070 Q13901 Q5VTL8 P07814 P49674 Q9UIG0 Q96C86 Q9HCC0 468 55312 55796 56647 O43791 53938 Q16629 6598 57062 Q01130 472 Q96GX9 474 476 477 P33240 478 Q16630 P43034 Q8WW01 Q9C0C2 P38919 Q13126 Q16637 3091 Q16633 P13637 6125 O75376 P58546 481 483 Q504Q3 Q9NW13 488 51773 24144 6117 P22102 6119 6118 Q15796 Q6YP21 Q16649 Q15797 9643 Q9UNL4 O43776 490 O75362 29803 495 498 Q01105 P56385 P56381 54815 Q8WVM0 Q9BSI4 Q13148 P35869 9656 P43490 P18074 P18077 O75351 144983 28960 Q9P2E3 Q13151 Q16665 Q49AN0 51319 P54136 O15287 P54132 P35453 P09172 Q8IZD4 Q14494 Q14493 Q14498 51329 Q14011 Q9NRC8 7013 7014 P46100 P36776 7490 Q9NRD1 11277 Q9NRD5 Q86W42 P98175 P98179 Q02880 25885 51340 54853 Q9NVM4 P02549 Q96PK6 11243 Q14032 55703 P47897 P00367 Q9GZL7 1716 Q15365 O75319 P21283 Q08170 P21281 Q09028 25896 6182 O75792 26747 Q15370 P27815 P48730 1728 Q99814 P98194 Q9NRF9 P78545 P54577 9221 O95714 P78540 57461 P78549 6194 10813 Q9H6Y2 P41252 P62195 P41250 P08684 O15234 1738 65083 1736 Q13523 P54105 9232 22803 Q9P1Y6 O75771 1743 27257 Q9NVH2 P42574 O60293 7068 7067 Q5TAX3 Q15714 Q13535 Q9H307 Q15717 Q8IZ69 P41235 Q9NVI1 O60264 O60260 O15270 9255 Q32P51 P34931 83759 Q9BWH6 1763 29896 29894 Q9UET6 3945 7083 9261 4809 51720 Q8WVK2 P08243 4800 51726 2626 51727 Q9NVC6 51729 Q9P1U0 Q13569 P14920 P19387 P30153 P19388 P11413 93100 1789 1788 1786 O60256 57038 Q13573 3980 P32320 P78527 P32322 2644 1312 2643 P17174 Q04656 Q14432 3978 Q9Y4A8 51747 Q9P287 9296 4839 4830 O60231 Q9HCS7 902 4833 Q13112 Q13595 P21675 Q969L4 O00268 Q06265 O00267 3516 10535 O00273 P60174 Q9ULW3 4841 Q9Y2X3 Q6NZY4 Q92614 P48047 4860 Q9NQ55 51096 Q9UI12 5705 Q9ULX9 Q66PJ3 Q9Y2Y8 Q12968 29128 O60306 57646 1355 P12081 P32929 Q9Y2Z2 Q9Y2Z4 Q12972 4882 Q6P2C8 Q13829 O60318 10524 10528 Q9UI32 10521 5725 10523 Q6PCD5 O95478 P28482 Q12986 1385 57661 29102 P62316 P84103 P62318 5733 56339 P62312 58517 P62314 Q9Y2T7 Q96SB4 Q9UHK0 Q96SB3 Q5JTZ9 Q06210 P19544 P30793 Q8IY17 Q96SB8 Q8WYH8 10987 2237 79444 56342 1388 P54709 P42771 Q9NYB0 22934 P30304 P30307 22938 Q06203 Q9H9Y6 Q56NI9 284119 5757 161823 P51693 P27144 Q13868 P51692 O00255 P54727 P54725 Q9P0M2 Q03164 55035 Q9BZJ4 Q99259 57697 Q9Y2W2 988 Q92620 Q6DHV7 Q9BZJ0 Q9Y2W1 509 O43395 6631 Q9BV90 P14678 P53803 O60725 P15531 990 P11172 Q96RU2 54512 995 513 997 514 8803 6622 515 6625 P61421 6627 518 P62753 Q9UQ80 54517 P07910 220988 Q9UM07 51010 521 522 523 23451 51013 6633 Q9Y6Y0 526 8812 528 10147 Q99638 6637 P01344 8815 P01343 Q86U44 Q86U42 P26640 O95059 5321 Q9NU63 O60701 P26639 533 535 10594 P49756 537 6647 539 Q9UPY3 23438 23439 6660 P40938 P40937 Q9Y333 Q9UHI6 23435 Q96B26 P12004 Q99611 3149 3148 Q96RR1 545 546 3146 P61457 8836 8833 Q96EY1 P62308 Q9ULR0 O43390 P62304 7508 Q9UQ35 7520 Q9UPR3 Q12904 23400 3162 P17752 23404 3159 Q15185 7518 7517 5351 Q9Y2L1 6201 5356 3178 Q9ULK4 23411 Q969G6 Q01831 Q6UVY6 Q08462 65220 3181 P50750 P49321 3189 3188 3187 3185 3184 O14802 51067 Q8N8Y2 7536 9716 Q9H5H4 3192 3191 P04424 8880 P27540 3190 9733 5378 P15559 Q969E8 P51610 580 10569 51074 P52948 Q9NPJ6 79869 54107 6217 Q9UPV0 Q8N9Q2 P15927 O43432 P15923 57187 29947 Q96FV9 593 6229 Q8IU60 O94906 5393 P03950 5394 8424 O00716 Q16514 O75494 54921 O94913 6239 Q76FK4 Q16526 P31153 29922 23381 P25705 115024 Q9NV06 P42285 O94925 P09234 P22674 Q16531 54931 54938 51426 P00441 51428 4086

10081 4087 8446 4088 P22692 O75478 9775 Q86UL3 O75475 P12883 142 1808 P20020 Q8IXZ2 9785 8458 P54252 P40926 P54253 7126 P38606 8450 P10275 O75460 4094 Q00597 158 Q8IYB3 Q99728 8449 84365 Q16555 P49736 8467 O43889 23378 Q9Y3F4 Q8NFM5 Q01433 Q9BRL6 Q13057 Q15233 Q99714 P00491 Q96FX7 P00492 P54277 P54274 O75444 7141 P11586 Q99700 Q96T23 Q00577 88745 Q99708 P22234 9319 P49711 Q16576 Q99707 9318 P49716 P49715 Q49A26 7153 7155 7158 Q9BVS5 Q08050 P39905 7157 7150 1841 92345 Q13873 7167 22913 Q7L0Y3 10929 7161 P18124 O43837 1854 P42224 10933 Q9Y3A5 Q9HC16 P63165 P09651 Q969T7 P42229 1856 Q8WUD6 22928 Q13887 O75891 P22626 Q9Y3B4 56267 10949 Q08945 Q9UHX1 Q9Y3B2 P09661 10940 Q969S2 10907 O43812 P11940 O75400 26015 10908 286826 26019 9360 9361 1877 P00846 4907 1870 Q9UHY7 P23921 O95861 P53396 Q13427 O75874 Q13426 O95865 Q8NAV1 Q8NB78 10921 1400 2730 P06132 1408 1407 4913 Q13435 P33316 Q9NYH9 2744 58478 Q8WYQ5 Q8NFF5 P43115 2747 Q8IY92 O95400 Q8WZ19 Q08J23 Q96G21 27339 O43809 Q8N0Z6 58487 Q9UQE7 Q8N0Z8 27332 26002 P23511 Q6P2Q9 Q15631 Q8IY81 P84090 4947 3615 84305 3614 Q5RKV6 2762 57169 Q9HC36 4946 P54687 O15350 Q15643 Q15648 Q86YP4 Q9BVJ6 O15347 4953 Q15653

regulation of cell death P25054 P28562 O75940 9181 P51587 P17676 P15498 2303 P84022 Q92731 3875 O14543 Q9Y275 4722 2308 Q92974 P55957 P14174 P42858 P10809 Q96EB6 Q5S007 Q7Z727 Q9NR80 Q9NR81 P17405 Q7L5Y9 Q9UKW4 811 P63104 P62258 6926 P31946 P07858 3672 P31947 P99999 2100 Q99583 3428 O60674 P19838 Q99576 Q8IUC6 Q8N5V2 P41743 P08758 Q8WXG6 1020 114548 57787 P15056 1499 1012 P61586 P07437 P29466 Q9Y239 Q9H4P4 Q9NZC7 P10600 Q96J02 Q06330 Q9UEE9 1029 Q9BWF2 1027 1026 841 843 604 847 5624 P04179 Q07890 4790 4792 5883 1285 Q9NQS1 Q96L34 P38398 7818 Q9HCL2 Q96KQ4 Q07889 O14763 Q96CA5 5879 Q13501 P10826 P51575 P10827 P31749 Q9H8V3 Q8NBS9 P40692 P09601 4318 P04156 8930 P56199 3482 P50897 O60603 Q99523 203068 3479 2147 3476 7832 10013 637 P23025 10016 P25445 O96017 Q9Y5V3 Q7Z6J4 P16989 1072 O43290 84033 O43293 P02794 Q6ZN33 Q9UMR2 6772 3265 23787 P13693 P14780 O14727 Q9NR09 P01019 51147 55504 P35222 P14784 P35227 O14737 Q96ST3 663 P35232 P60484 Q9H093 668 6776 Q15052 8718 8717 O00744 P01042 Q92934 O43236 9821 Q9NX61 25942 672 673 P15692 10451 6789 P55072 P05412 8743 7410 23532 Q5JSP0 P37231 Q01955 P51959 8737 9826 207 5468 8738 Q15078 23746 7422 5245 P56177 Q12802 3065 P13010 Q96P20 7415 10673 O14920 3070 O43464 4168 P06729 23513 8517 7428 10202 P04792 8772 Q9BYX4 O15085 Q13114 8535 7204 O43557 Q9Y4K3 3084 O43318 472 Q9H161 Q6UY14 P07196 9616 P49810 8767 P78395 P13631 Q86VP1 P09382 P46531 8539 P00533 P43026 9641 8795 P35638 Q96IZ0 10193 P12757 Q9UNL4 P12755 64857 Q9NY61 O75360 P46527 257 23263 10197 P55290 Q9BWQ8 5054 5055 P18074 P10586 57099 P68371 P55061 P43246 P43489 Q14249 Q13158 Q8TDY2 Q16666 O75340 P0C1Z6 P09172 Q9Y6K9 O94827 11269 5074 10181 7013 O43521 P22392 Q9Y4H2 O43524 7490 5071 P11021 11035 P49841 Q99835 23229 Q96LC9 7023 10392 O75569 O15068 A1XKG3 291 P48507 51100 P08069 1718 Q9BX69 P47895 Q13188 51107 1956 P23443 6188 O43504 P38936 O43508 P37840 2810 Q93034 Q93038 1728 Q9BWT7 P48736 Q15375 7046 Q96RE7 6198 O43715 6194 P22736 P62195 P04083 Q13761 P78536 9474 7057 Q02297 83737 9231 7052 27252 81567 P98170 P16070 P42574 P42575 P98171 P98174 Q9UER7 P42336 7067 P06239 Q9H305 P19793 P29590 P30101 P21860 3932 O60260 P55212 Q13547 A4D1W7 Q13546 7073 P78318 Q8N8D1 7074 O43707 P04070 Q04206 Q92785 P05164 Q92542 P78560 Q13794 P09525 Q9NZJ7 O95999 Q13315 9021 Q16828 P00747 P63000 Q96PE2 P08243 3956 7099 Q13323 P35609 P30153 Q05516 1785 Q05513 P00734 50649 O75962 29843 P78527 O60229 121457 50650 Q04656 51741 Q14432 Q16611 Q9P286 Q06830 O60238 P00519 Q8TDM6 4830 900 Q9NZN5 Q9NZN9 Q13352 Q7Z628 Q9Y2W7 P10911 399687 Q12948 Q5VV41 Q6QNY1 Q9BVA0 O15519 3516 P16220 O00273

Q92851 Q92614 P07996 P52564 P52565 65018 P16471 4851 10542 Q92843 5705 Q92835 Q9NYF8 57646 Q8IVF5 P20936 O60543 Q92830 P61289 P03372 64170 Q9H7P9 Q6IT96 4869 5716 5970 P48023 Q9BZF9 3551 1130 Q12979 9093 Q9NRZ9 5728 Q99698 3304 Q9ULZ3 Q9UHR5 3309 P04233 Q12981 Q12982 P28482 P50570 1385 O00220 Q9UHJ9 29108 O00463 P52756 Q9P0J0 3315 3313 P07550 P04040 P08887 Q9NYA1 P10721 O95243 Q5VST9 P19544 Q9NS56 P37198 3329 57678 79444 960 29115 P42771 Q70UQ0 P07766 P50591 2495 Q13618 Q13617 Q13616 P30542 Q9NS68 6609 57448 2247 P61247 Q9Y371 Q92630 5515 P05112 5516 Q9UM63 5518 P09958 Q12778 Q13625 P51692 O00255 O00257 81788 P05120 5524 P05121 P40337 7704 Q08043 P23396 P48454 P04637 P80188 P38646 P52952 P15531 O14827 Q7L523 Q96RU2 5533 6622 999 P01116 Q9C0K7 Q15389 P62753 P01112 P01111 Q9C0K0 4221 10383 6885 1191 O76074 Q15392 P48436 4214 P01589 P01588 Q99638 P01343 P49768 O00206 P48431 Q8N726 5321 P52735 P12235 P11388 148022 773 Q00535 Q53X93 6647 Q9Y572 P01138 P01137 Q6UXV0 P01375 P01374 P04626 8841 P04629 2065 Q9UHI6 2064 O00213 11218 301 P17936 P10071 3146 Q8WTP8 P02458 307 Q96EY1 6657 11214 8837 P49747 O43353 5581 5584 7520 8851 4255 Q8WYA6 Q9UNN5 3162 O95076 O75190 Q99683 5336 5578 P62714 7518 2081 5591 Q6IR47 7531 7534 64127 23411 O76024 5590 324 3169 329 P05783 7529 P27797 64135 6453 2099 Q9Y2M5 Q9BUZ4 330 572 331 573 Q8NER5 5599 23636 5371 Q12933 8887 P15559 P52701 580 P16885 P51617 P58012 8878 P25116 P60568 Q9BZZ5 8655 Q9BY76 9500 8654 Q04760 351 355 356 5139 P62913 Q13485 P25963 Q13489 Q13009 Q9H3Z4 Q13490 P11309 6495 6256 6498 O75489 29 Q9UBU3 Q9UDY8 P23528 P61604 P00441 9531 6262 9774 Q14116 10081 Q13268 9530 4088 4089 8445 Q9H257 387 P21359 P20264 8453 8454 8452 10293 7124 23365 23368 O75460 154 396 Q02535 121512 10296 Q99728 Q8WV24 Q13043 Q13286 P36896 Q9Y5J5 23370 P01308 120892 7128 Q9NQB0 Q99958 Q15475 9564 Q6ZSZ5 P61803 Q8TCU6 P54274 1831 Q9H1Y0 Q01664 O95817 P21145 P01579 11124 Q9BRK4 10276 Q9NQC3 P09038 Q14155 2923 7153 64782 P39905 7157 7159 P14625 P35548 1843 2932 Q14160 Q15011 Q14164 10285 Q13075 1848 Q15257 2935 87 Q13077 Q15811 P78423 P12814 Q86Z02 7161 1613 P63167 56259 P42224 Q9BXM7 10935 91 P39060 P42229 O15119 1616 Q96QB1 Q01085 P67775 P05067 2956 7187 7186 7189 7188 P29474 O94768 Q9H422 Q96M96 P18583 Q13418 6093 7185 7184 Q9BXK5 Q8N163 P17252 O15379 P21980 P11717 O15392 Q13426 O95865 Q02156 4914 2730 Q9UBN7 2737 Q9UBN6 Q9UBF6 P41182 P30044 57144 P53567 P30048 Q03468 P09211 P47712 P51398 Q15628 P21580 Q07820 Q96BI3 P51159 8065 Q07817 64919 P08138 P09429 P10415 P12830 1676 3611 Q14790 Q13464 O15350 P08107 P53355 O75832 Q9P0U3 27315 Q04771 O60356 Q9HC29 O95429 P45983 Q13233 Q9BZR8 P45984 P21554

macromolecule localization 25909 9182 22872 9181 Q96KP1 O95793 Q5SQT9 9184 O00148 1213 Q9UMY4 Q92973 10890 P60059 10651 3875 Q9Y275 Q92974 Q9H4L5 64284 Q9UGM3 Q92729 P55957 Q8IWJ2 O00159 2317 2316 10427 P42858 57510 Q9H2G9 O15400 4734 5825 Q12846 Q96P70 6927 Q8IUD2 81876 55341 P62491 30000 811 5830 Q9UJ41 P63104 Q9Y6A5 P62258 Q96EA4 P26374 P31946 3673 56681 P21917 O95343 Q676U5 Q96N16 81622 P20823 55110 Q8NFW9 O60674 2332 821 10640 10642 O14579 27072 Q96GD4 Q9UKX7 P41743 O75901 Q9H2M9 5861 5862 1020 114548 O95352 O60645 1499 P61106 Q9UL26 Q9UL25 P32856 5870 P33947 Q15907 Q9NR31 22841 Q96MV8 O60895 6711 604 5865 P07686 5868 5869 4792 Q9H2K2 Q8IUH5 401505 Q9HCL2 P60033 1277 Q6NUQ1 857 5877 Q9UN86 6726 859 5878 Q9Y490 Q13501 P51572 P53992 P10827 Q9UL45 Q13505 P51571 P31749 O95140 55153 Q9NQT8 84932 Q96ED9 Q9UMX1 P63313 O14777 Q9UL54 Q86UW7 Q9Y3Q3 6993 10490 Q86V81 O96008 Q9UKL6 P50897 25988 Q99523 1058 2147 8924 Q15021 10013 637 10015 6748 6747 11345 23339 O96018 4580 Q15027 Q9Y678 Q9NV70 O95166 55973 Q99750 55737 406 P47224 408 Q15036 P49407 409 Q15276 11116 P60468 1080 O00327 Q9UMR2 Q9Y5W9 P48552 116986

O00560 116987 O00562 23303 10239 Q5VIR6 79711 Q15286 Q9Y5X1 8943 Q8NFP9 Q9Y5X2 O14964 Q9UP83 P61326 11325 6780 Q9Y5X9 O43264 Q9Y215 10482 O14980 Q8N1F7 P35222 Q8N1F8 25978 Q96ST3 Q96T51 6776 9804 4116 P24588 11336 O00505 Q9Y3M2 6311 7884 P05408 Q9UID3 23762 25942 P14598 Q8IYJ3 Q99567 P61981 O14908 55763 7879 10452 8724 Q96A72 9818 P55072 Q92930 P05412 P49450 10460 5476 Q9HCE7 10228 P37231 10469 Q99797 79720 55770 Q8IYI6 Q15070 5467 207 5468 10466 Q6IQ22 P24386 9601 Q9H902 9600 Q8WV92 22895 Q9Y5S9 Q96QU8 O14925 Q96P20 Q00610 7415 P61764 7414 10673 6342 7430 8766 6103 Q9UKK6 3074 P40855 Q92900 10204 326624 Q16623 P18085 8775 P10114 Q9UPN3 P35658 O75385 473 Q9H0U4 117584 51762 P49810 5018 Q15785 P49815 Q9NPC8 Q9HAV7 P38919 Q9HAV4 8546 4188 9632 64601 79083 Q9UJC3 Q9H0T7 O75381 Q96RL7 Q9H9E3 P00533 9868 Q15554 Q9HAU5 Q9BUN8 9883 Q9UNL2 Q8WVM8 P32121 23265 Q9Y6Q5 4193 51542 Q96RK4 8548 P61966 Q9BSI4 8563 6386 Q9Y2J0 129531 P62826 Q9UPQ3 P62829 Q86XR8 Q9UQ26 O75351 Q86VN1 Q9NZZ3 23039 Q9H2V7 Q8N6H7 Q8N4C7 144983 Q709C8 P61970 51552 Q6PKD3 Q5SRE5 9648 Q03518 Q49AN0 Q9P2E9 P62820 149371 O43752 11021 7248 Q14008 P63092 P13667 Q9UEU0 51560 84248 Q9Y4G6 O94826 Q14493 54832 P27824 11269 7014 11031 23256 Q96D46 7251 152789 7249 Q9NRD5 Q86W42 Q86Y82 Q3ZCQ8 Q9BV36 23463 O43734 A1XKG3 Q86VS8 23225 Q9UNH7 Q15363 Q9C0E2 Q9H9H4 1718 P02545 Q6I9Y2 1956 55705 9212 Q9NPA8 O15078 O75558 26985 Q8N4H5 O43747 Q9H173 Q13190 Q99816 11014 9465 P28288 9463 P98155 O95714 83752 Q92572 27243 55048 P04083 O15234 65082 Q92574 O95721 Q13769 O15496 9230 55054 9472 P46934 117177 Q9HD26 83985 O60296 80230 8399 7067 P29590 P56539 P30101 Q4LE60 29886 60412 Q9NZQ3 Q32P51 A4D1W7 27236 Q9UNE2 9495 112936 79023 653361 O43707 O15027 Q9UH99 Q96NA2 Q9NP61 Q9P253 P35610 O00186 O00189 P00505 O00194 23085 O60488 Q8IWV1 P35606 9276 P56962 Q9NP72 Q9H2T7 Q86XP3 7094 Q96IW7 83547 Q05516 83548 1785 Q5QGT7 O60499 P63010 P00734 O60493 80273 5901 O75969 53916 100287932 1315 P20339 Q9BQA5 1314 P20336 P20337 Q9HAP6 5911 131474 Q9NP90 A4D1S5 63971 Q13107 11097 79065 Q9H6Z4 Q96H20 Q9H0N0 Q63HQ0 51510 Q96RF0 P20340 Q13596 51517 Q9NRW1 Q9NRW7 Q99490 Q9P2R6 P61020 Q9BVA0 O60566 2665 2664 O14662 Q9Y2X3 90678 10772 P61026 P27348 10533 Q9UBC1 Q12955 Q8NF91 4860 54361 O95684 55696 P63211 92609 Q92845 Q9NWB1 P50395 3783 1121 339122 Q9BXC9 Q9GZY0 57403 10758 6809 O60543 Q86WA8 Q92834 4627 4869 5716 P53675 Q92824 Q9H814 4641 1130 P56705 P53677 O60318 P17301 Q99698 Q9BZF3 1122 6810 6812 6811 6814 10762 948 P04233 Q9H1C4 O95477 P28482 P50570 O00221 Q9H1K0 Q9P0J0 Q9NRS6 10736 57662 3313 O60763 O00471 Q4VX76 Q9Y2T2 4645 5976 10972 4646 10971 O95487 O95249 P53618 Q9NS56 P37198 57678 P18206 P62328 10981 Q9UM54 P53621 Q9NYB0 Q12769 O60749 22931 81555 P62330 80223 O60502 Q9NS69 10953 10956 2247 26056 58533 10959 975 P61006 O14640 5514 738 Q9H1H9 P30536 Q8NEZ2 Q969M3 P51692 1176 1174 1173 Q6KC79 Q60I27 Q9P0M2 10724 55275 Q9BXF6 Q92621 Q9ULV0 Q969M1 4678 O15504 10960 7704 Q92624 P61019 P61018 P02452 Q9UPV9 P04637 O95271 P38646 O60725 O14828 4686 128866 10134 10133 Q9C0K7 Q15389 51009 Q15388 Q96HA1 Q04917 6643 6642 5796 Q6WKZ4 23214 P50542 1191 Q96Q15 9908 Q96RT1 Q15392 Q15154 11231 Q96CW1 5306 4218 P49768 6890 Q86U42 Q9BV73 6653 P14416 P13569 2054 23423 Q96CV9 O00203 9919 Q00535 Q02952 P61204 P49755 51026 Q9GZP9 54536 P49754 54535 6646 P01137 Q7Z3C6 51028 91949 P01133 P01375 Q96JC1 P01130 4000 Q8N2H4 Q96EY5 23673 P12004 P54920 10128 O14617 O95070 301 Q7Z3B4 9927 O00623 O43592 O00628 5584 Q5U0I6 Q9UPR3 8852 O00629 22794 Q9Y2K9 Q9UNN5 Q12907 Q96PU8 P42704 Q8WTW3 7514 Q96AX2 Q96AX1 5579 P05771 10577 Q9Y4P1 7532 5111 7531 7534 7533 3178 P37108 P16671 Q00987 8615 9702 P46059 P05783 7529 5108 P27797 3181 O43572 64130 9961 P49321 Q9UPT5 P51843 Q9NW64 P15311 Q9NVU7 Q8NER5 56993 51068 P83436 Q8N4Q1 5119

A5D8V6 23636 P49792 5371 9972 Q9BQQ3 P49790 4285 Q60FE5 64145 O75396 O00410 23633 Q969E2 Q6ZWJ1 Q08209 P52948 P62745 8878 P25116 57617 10566 Q8N6T3 54908 9744 Q8TEW0 57187 94121 Q96FV9 94120 Q7L804 23163 23165 P23786 Q8WUM4 P62913 Q96QK1 Q8WUM0 8408 P25963 Q8TD31 11190 O43687 O43684 11196 19 O75494 Q9H270 Q9UIA9 415117 8417 6238 Q15436 6496 Q6Y2X3 6495 8677 9525 9522 8676 23144 Q9H269 29927 375 23381 84342 Q8NFH4 9515 Q13023 51429 P61619 8204 9775 Q14118 29934 381 382 P59780 388 P31150 Q9NUP9 10087 Q15691 P60520 Q16543 9527 Q8IXZ2 9784 11140 9546 7124 10053 Q9Y3E0 O43633 Q9H089 Q4G0F5 P35579 Q99720 Q9H082 Q13285 5195 9554 9793 Q96C24 7375 Q09161 Q5JQC9 O75694 29916 161 Q86WV8 Q9Y5J6 Q8NI27 Q9NWN3 8218 51699 Q15475 P46459 P00491 Q9UQN3 O43615 6293 Q99700 Q9NZ43 Q6VY07 Q9UBQ0 P21145 P01579 11124 55823 P09038 2923 Q9BY84 O94973 10280 7157 Q9BXW6 P14625 O75674 Q9Y5L4 25776 O75431 Q8TEX9 25777 Q8WZ73 O94979 84134 84376 1605 Q14964 P53367 8498 9342 Q13637 O95835 P57729 Q96QC0 Q13636 Q9H444 Q06787 P63172 84958 P41134 79139 Q7RTN6 83871 P09651 P42229 O15118 Q14974 O15131 9590 Q01082 8021 22920 P57735 P22626 P43307 10947 P52292 O15126 22919 9367 P52298 P10619 P52294 O75886 Q8TEM1 26258 57120 Q9H1M0 P57740 Q03014 26019 7184 80145 Q92673 Q969R2 Q9NSC5 9135 Q15843 O15155 O15397 Q6UWE0 64901 P21757 56288 1408 Q9UBN7 9146 9388 Q9HC62 Q9UBF2 Q96G30 P41182 Q86YS6 Q86UK0 P51148 9382 P51149 3836 4927 O15554 P51151 O95405 P51153 57154 Q15629 P51159 Q96QF0 Q8WYP5 Q9BVL2 3841 3843 P21333 O75843 59343 P84085 P01730 80184 9179 O75832 P62072 Q96QD9 Q00169 23192 Q8TAG9 P84077 Q15653

regulation of programmed cell death P25054 P28562 O75940 9181 P51587 P17676 P15498 2303 P84022 Q92731 3875 O14543 Q9Y275 4722 2308 Q92974 P55957 P14174 P42858 P10809 Q96EB6 Q5S007 Q7Z727 Q9NR80 Q9NR81 P17405 Q7L5Y9 Q9UKW4 811 P63104 P62258 6926 P31946 P07858 3672 P31947 P99999 2100 Q99583 3428 O60674 P19838 Q99576 Q8IUC6 Q8N5V2 P41743 P08758 Q8WXG6 1020 114548 57787 P15056 1499 1012 P61586 P07437 P29466 Q9Y239 Q9H4P4 Q9NZC7 P10600 Q96J02 Q06330 Q9UEE9 1029 Q9BWF2 1027 1026 841 843 604 847 5624 P04179 Q07890 4790 4792 5883 1285 Q9NQS1 Q96L34 P38398 7818 Q9HCL2 Q96KQ4 Q07889 O14763 Q96CA5 5879 Q13501 P10826 P51575 P10827 P31749 Q9H8V3 Q8NBS9 P40692 P09601 4318 P04156 8930 P56199 3482 P50897 O60603 Q99523 203068 3479 2147 3476 7832 10013 637 P23025 10016 P25445 O96017 Q9Y5V3 Q7Z6J4 P16989 1072 O43290 84033 O43293 Q6ZN33 Q9UMR2 6772 3265 23787 P13693 P14780 O14727 Q9NR09 P01019 51147 55504 P35222 P14784 P35227 O14737 Q96ST3 663 P35232 P60484 Q9H093 668 6776 Q15052 8718 8717 O00744 P01042 Q92934 O43236 9821 Q9NX61 25942 672 673 P15692 10451 6789 P55072 P05412 8743 7410 23532 Q5JSP0 P37231 Q01955 P51959 8737 9826 207 5468 8738 Q15078 23746 7422 5245 P56177 Q12802 3065 P13010 Q96P20 7415 10673 O14920 3070 O43464 4168 P06729 23513 8517 7428 10202 P04792 8772 Q9BYX4 O15085 Q13114 8535 7204 O43557 Q9Y4K3 3084 O43318 472 Q9H161 Q6UY14 P07196 9616 P49810 8767 P78395 P13631 Q86VP1 P09382 P46531 8539 P00533 P43026 9641 8795 P35638 Q96IZ0 10193 P12757 Q9UNL4 P12755 64857 Q9NY61 O75360 P46527 257 23263 10197 P55290 Q9BWQ8 5054 5055 P18074 P10586 57099 P68371 P55061 P43246 P43489 Q14249 Q13158 Q8TDY2 Q16666 O75340 P0C1Z6 P09172 Q9Y6K9 O94827 11269 5074 10181 7013 O43521 P22392 Q9Y4H2 O43524 7490 5071 P11021 11035 P49841 Q99835 23229 Q96LC9 7023 10392 O75569 O15068 A1XKG3 P48507 51100 P08069 1718 Q9BX69 P47895 Q13188 51107 1956 P23443 6188 O43504 P38936 O43508 P37840 2810 Q93034 Q93038 1728 Q9BWT7 P48736 Q15375 7046 Q96RE7 6198 O43715 6194 P22736 P62195 P04083 Q13761 P78536 9474 7057 Q02297 83737 9231 7052 27252 81567 P98170 P16070 P42574 P42575 P98171 P98174 Q9UER7 P42336 7067 P06239 Q9H305 P19793 P29590 P30101 P21860 3932 O60260 P55212 Q13547 A4D1W7 Q13546

7073 P78318 Q8N8D1 7074 O43707 P04070 Q04206 Q92785 P05164 Q92542 P78560 Q13794 P09525 Q9NZJ7 O95999 Q13315 9021 Q16828 P00747 P63000 Q96PE2 P08243 3956 7099 Q13323 P35609 P30153 Q05516 1785 Q05513 P00734 50649 O75962 29843 P78527 O60229 121457 50650 Q04656 51741 Q14432 Q16611 Q9P286 Q06830 O60238 P00519 Q8TDM6 4830 900 Q9NZN5 Q9NZN9 Q13352 Q7Z628 Q9Y2W7 P10911 399687 Q12948 Q5VV41 Q6QNY1 O15519 3516 P16220 O00273 Q92851 Q92614 P07996 P52564 P52565 65018 P16471 4851 10542 Q92843 5705 Q92835 Q9NYF8 57646 Q8IVF5 P20936 O60543 Q92830 P61289 P03372 64170 Q9H7P9 Q6IT96 4869 5716 5970 P48023 Q9BZF9 3551 1130 Q12979 9093 Q9NRZ9 5728 Q99698 3304 Q9ULZ3 Q9UHR5 3309 P04233 Q12981 Q12982 P28482 P50570 1385 O00220 Q9UHJ9 29108 O00463 P52756 Q9P0J0 3315 3313 P07550 P04040 P08887 Q9NYA1 P10721 O95243 Q5VST9 P19544 Q9NS56 P37198 3329 57678 79444 960 29115 P42771 Q70UQ0 P07766 P50591 Q13618 Q13617 Q13616 P30542 Q9NS68 6609 57448 2247 P61247 Q9Y371 Q92630 5515 P05112 5516 Q9UM63 5518 P09958 Q12778 Q13625 P51692 O00255 O00257 81788 P05120 5524 P05121 P40337 7704 Q08043 P23396 P48454 P04637 P80188 P38646 P52952 P15531 O14827 Q96RU2 5533 6622 999 P01116 Q9C0K7 Q15389 P62753 P01112 P01111 Q9C0K0 4221 10383 6885 1191 O76074 Q15392 P48436 4214 P01589 P01588 Q99638 P01343 P49768 O00206 P48431 Q8N726 5321 P52735 P11388 148022 773 Q00535 Q53X93 6647 Q9Y572 P01138 P01137 Q6UXV0 P01375 P01374 P04626 8841 P04629 2065 Q9UHI6 2064 O00213 11218 301 P17936 P10071 3146 Q8WTP8 P02458 307 Q96EY1 6657 11214 8837 P49747 O43353 5581 5584 7520 8851 4255 Q8WYA6 Q9UNN5 3162 O95076 O75190 Q99683 5336 5578 P62714 7518 2081 5591 Q6IR47 7531 7534 64127 23411 O76024 5590 324 3169 329 P05783 7529 P27797 64135 6453 2099 Q9Y2M5 Q9BUZ4 330 572 331 573 Q8NER5 5599 23636 5371 Q12933 8887 P15559 P52701 580 P16885 P51617 P58012 8878 P25116 P60568 Q9BZZ5 8655 Q9BY76 9500 8654 Q04760 351 355 356 5139 P62913 P25963 Q13489 Q13009 Q9H3Z4 Q13490 P11309 6495 6256 6498 O75489 29 Q9UBU3 Q9UDY8 P23528 P61604 P00441 9531 6262 9774 Q14116 10081 Q13268 9530 4088 8445 Q9H257 387 P21359 P20264 8453 8454 8452 10293 7124 23365 23368 O75460 154 396 Q02535 121512 10296 Q99728 Q8WV24 Q13043 Q13286 P36896 Q9Y5J5 23370 P01308 120892 7128 Q9NQB0 Q99958 Q15475 9564 Q6ZSZ5 P61803 Q8TCU6 P54274 1831 Q9H1Y0 Q01664 O95817 P21145 P01579 11124 10276 Q9NQC3 P09038 Q14155 2923 7153 64782 P39905 7157 7159 P14625 P35548 1843 2932 Q14160 Q15011 Q14164 10285 Q13075 1848 Q15257 2935 87 Q13077 Q15811 P78423 P12814 Q86Z02 7161 1613 P63167 56259 P42224 Q9BXM7 10935 91 P39060 P42229 O15119 1616 Q96QB1 Q01085 P67775 P05067 2956 7187 7186 7189 7188 P29474 O94768 Q9H422 Q96M96 P18583 Q13418 6093 7185 7184 Q9BXK5 Q8N163 P17252 O15379 P21980 P11717 O15392 Q13426 O95865 Q02156 4914 2730 Q9UBN7 2737 Q9UBN6 Q9UBF6 P41182 P30044 57144 P53567 P30048 Q03468 P09211 P47712 P51398 Q15628 P21580 Q07820 Q96BI3 P51159 8065 Q07817 64919 P08138 P09429 P10415 P12830 1676 3611 Q14790 Q13464 O15350 P08107 P53355 O75832 Q9P0U3 27315 Q04771 O60356 Q9HC29 O95429 P45983 Q13233 Q9BZR8 P45984 P21554

RNA processing 285672 Q92979 O75940 Q969L4 P67809 Q06265 81605 P52434 O00148 Q9UBB9 10657 Q8TDD1 5939 P84022 4841 Q9Y2X3 10772 Q6NZY4 Q9UK45 Q00839 5935 P05455 Q9H0D6 Q9H2H8 O75934 P30876 Q9NQ55 55696 51096 10667 54487 10785 10421 O75937 Q96EB6 P62249 Q9NWB1 Q9BRX9 Q9UKV8 Q66PJ3 O60306 Q8TED0 54496 1478 55109 Q8N684 P26599 Q9Y2Z2 Q5TAP6 P31942 Q12972 P31943 P49591 P46087 P08708 Q9H814 Q9GZX7 Q99459 55110 Q99575 10528 P62263 55119 P49588 P26368 10521 5725 10523 O95478 Q8IX01 57661 O95232 P52756 P39019 Q9P2N5 80324 29102 Q9BWG6 P62316 P84103 P62318 O14744 P62312 58517 27161 P62314 Q10570 Q12874 Q96SB4 Q9UHK0 Q12872 Q96SB3 Q8WXF0 Q9H0H0 Q13601 Q07666 P19544 O95363 1029 6839 10625 56342

57794 Q9BUB4 P42771 4670 22938 Q9Y4Y9 O60508 O60506 Q96MU7 55149 5511 Q9Y4Z0 5515 Q9UNY4 P08621 5518 P51693 22826 Q13868 22827 Q9H6R4 22828 6741 5411 Q9NQT4 Q9NQT5 P40692 P61254 27037 O60870 10607 6733 Q9Y2W2 988 Q92620 Q9BZJ0 O43395 O43159 11340 Q86V81 10250 10492 Q5VYS8 6631 Q9BV90 P14678 O00541 O95391 P53803 51362 54512 Q8WWY3 4686 Q15020 6625 6627 P62995 P62753 Q15024 P62875 Q9UQ80 54517 135295 Q96LI5 P51991 P07910 220988 Q15029 5430 4343 5432 23210 Q9UKM9 P51513 O14717 51010 23451 51013 O43290 Q7Z6J9 196441 6633 Q9Y6Y0 Q15393 10147 O43172 6637 Q86U42 O43251 O95059 5440 5441 Q8N726 O00567 O60942 126789 10594 P49756 5434 Q9UPY3 Q15046 P61326 Q15287 P23246 Q9UL03 O43143 Q86X95 Q9UMS4 84292 6421 A7MD48 Q9Y333 Q9UHI6 23435 Q96B26 O14979 11218 11338 3028 55759 4116 57819 P62308 P61218 Q9ULR0 O43390 P62304 P57081 6430 8732 6432 7884 Q9UQ35 6431 6434 22794 23524 23404 Q96PU8 10450 8602 6427 6426 Q96A72 Q8N302 2081 23658 Q9Y2L1 6201 5356 3178 23411 O76021 23536 Q9HCE1 Q96SZ6 P62847 P62841 25804 3181 2091 P22087 4154 25929 Q8TA86 Q8N2M8 3189 Q9UNP9 3188 3187 22894 3185 3184 Q9NW64 Q9Y5S9 P17844 79753 10438 Q5TAL4 333 55660 7536 9716 P62857 91746 P56182 O43463 8761 3192 Q9UNQ2 Q9BQ04 3191 23517 3190 Q13901 9733 Q5VTL8 Q969E8 Q9NVV4 Q08211 10569 79622 55796 79869 6217 Q8N9Q2 O43791 53938 Q16629 P55265 Q9UPN6 57187 P13984 Q96FV9 Q01130 351 P33240 Q16630 P09012 6229 Q14690 P62913 Q13243 Q8WW01 O94906 Q13242 P38919 Q13247 5393 Q16637 5394 Q8IWZ8 16 Q15428 5036 6125 Q15427 79084 O75494 Q9NW13 24144 51654 Q9NPD3 O94913 P46777 Q15796 Q15434 Q15797 Q14103 23144 Q9UKF6 Q96IZ7 Q9Y606 85437 Q9NV06 P42285 P09234 54931 P83876 Q8WVM0 51428 8563 Q13148 9410 O43660 4086 11171 10081 8683 4087 8446 4088 11051 Q8IWX8 9775 P18077 P36954 Q96HW7 144983 Q13151 P61978 Q8IXZ2 51319 Q15459 9785 10291 9541 8458 P54253 26512 O75460 Q93062 23481 Q8NI36 Q8IYB3 Q14493 8449 Q14498 51329 10181 Q14137 11030 Q09161 23378 23016 7490 Q9Y5J1 Q9NY12 Q9Y3F4 Q8NI27 Q9BRL6 11157 118460 Q15233 Q99714 Q86W42 P98175 Q96FX7 O75569 P98179 11129 51340 54853 P50914 Q9NVM4 88745 51585 56915 Q9GZL7 2926 Q6I9Y2 Q15365 O75319 Q9HB90 Q9BVS5 Q08170 P62906 26986 Q01780 P14866 25896 8241 5093 Q96EU6 26747 51593 10286 10285 P78362 11017 92345 9221 22913 Q14966 Q7L0Y3 P52272 Q12788 92105 10929 P62081 57461 P18124 6194 Q96DI7 10813 Q9H6Y2 Q9Y3A5 84967 O15234 P09651 65083 1736 Q9UBK2 Q13523 P54105 22803 Q13769 P55795 Q14978 Q9H0L4 Q9P1Y6 P24928 P22626 Q9Y3B4 79005 P67775 Q6IN84 27257 84950 10949 Q9UHX1 Q9Y3B2 Q9NVH2 P09661 P05067 P08579 O60293 10940 P52298 9128 9129 Q5TAX3 O75643 Q9H307 10907 P11940 O75400 Q8IZ69 9360 80145 283989 79039 Q8TBF4 Q8TAD8 P78316 Q32P51 Q13427 Q8NAV1 83759 Q8N8D1 10921 29896 Q07955 29894 57379 Q9UET6 134430 50628 Q13435 P52597 Q9UJV9 Q9NYH9 1660 Q6NYC1 1655 1653 4809 Q92499 Q8WVK2 Q8WYQ5 1659 Q8NEC7 O95400 51729 Q08J23 P19387 P30153 P19388 Q96G21 27339 O43809 1665 26121 Q8N0Z8 27332 Q9BUJ2 Q9BXP5 Q6P2Q9 Q13573 Q8IY81 9045 Q8TEQ6 P55769 Q5RKV6 2521 Q9HC36 Q9BQA1 51747 4839 Q9BVJ6 Q96HR8 23076 O60231 Q9HCS7 Q13595 Q14684 Q14683 Q14562 1207

regulation of apoptosis P25054 P28562 O75940 9181 P51587 P17676 P15498 2303 P84022 Q92731 3875 O14543 Q9Y275 4722 2308 Q92974 P55957 P14174 P42858 P10809 Q96EB6 Q7Z727 Q9NR80 Q9NR81 P17405 Q7L5Y9 Q9UKW4 811 P63104 P62258 6926 P31946 P07858 3672 P31947 P99999 2100 Q99583 3428 O60674 P19838 Q99576 Q8IUC6 Q8N5V2 P41743 P08758 Q8WXG6 1020 114548 P15056 1499 1012 P61586 P07437 P29466 Q9Y239 Q9H4P4 Q9NZC7 P10600 Q96J02 Q06330 Q9UEE9 1029 Q9BWF2 1027 1026 841 843 604 847 5624 P04179 Q07890 4790 4792 5883 1285 Q9NQS1 P38398 7818 Q9HCL2 Q96KQ4 Q07889 O14763 Q96CA5 5879 Q13501 P10826 P51575 P10827 P31749 Q9H8V3 Q8NBS9 P40692 P09601 4318 P04156 8930 P56199 3482 P50897 O60603 Q99523 203068 3479 2147

3476 7832 10013 637 P23025 10016 P25445 O96017 Q9Y5V3 Q7Z6J4 P16989 1072 O43290 84033 O43293 Q6ZN33 Q9UMR2 6772 3265 23787 P13693 P14780 O14727 Q9NR09 P01019 51147 55504 P35222 P14784 P35227 O14737 Q96ST3 663 P35232 P60484 Q9H093 668 6776 Q15052 8718 8717 O00744 P01042 Q92934 O43236 9821 Q9NX61 25942 672 673 P15692 10451 6789 P55072 P05412 8743 7410 23532 Q5JSP0 P37231 Q01955 P51959 8737 9826 207 5468 8738 Q15078 23746 7422 5245 P56177 Q12802 3065 P13010 Q96P20 7415 10673 O14920 3070 O43464 4168 P06729 23513 8517 7428 10202 P04792 8772 Q9BYX4 O15085 Q13114 8535 7204 O43557 Q9Y4K3 3084 O43318 472 Q9H161 Q6UY14 P07196 9616 P49810 8767 P78395 P13631 Q86VP1 P09382 P46531 8539 P00533 P43026 9641 8795 P35638 Q96IZ0 10193 P12757 Q9UNL4 P12755 64857 Q9NY61 O75360 P46527 257 23263 10197 P55290 Q9BWQ8 5054 5055 P18074 P10586 57099 P68371 P55061 P43246 P43489 Q14249 Q13158 Q8TDY2 Q16666 O75340 P0C1Z6 P09172 Q9Y6K9 O94827 11269 5074 10181 7013 O43521 P22392 Q9Y4H2 O43524 7490 5071 P11021 11035 P49841 Q99835 23229 Q96LC9 7023 10392 O75569 O15068 A1XKG3 P48507 51100 P08069 1718 Q9BX69 P47895 Q13188 51107 1956 P23443 6188 O43504 P38936 O43508 P37840 2810 Q93034 Q93038 1728 Q9BWT7 P48736 Q15375 7046 Q96RE7 6198 O43715 6194 P22736 P04083 Q13761 P78536 9474 7057 Q02297 83737 9231 7052 27252 81567 P98170 P16070 P42574 P42575 P98171 P98174 Q9UER7 P42336 7067 P06239 Q9H305 P19793 P29590 P30101 P21860 3932 O60260 P55212 Q13547 A4D1W7 Q13546 7073 P78318 Q8N8D1 7074 O43707 P04070 Q04206 Q92785 P05164 Q92542 P78560 Q13794 P09525 Q9NZJ7 O95999 Q13315 9021 Q16828 P00747 P63000 Q96PE2 P08243 3956 7099 Q13323 P35609 P30153 Q05516 1785 Q05513 P00734 50649 O75962 29843 P78527 O60229 121457 50650 Q04656 51741 Q14432 Q16611 Q9P286 Q06830 O60238 P00519 Q8TDM6 4830 900 Q9NZN5 Q9NZN9 Q13352 Q7Z628 Q9Y2W7 P10911 399687 Q12948 Q5VV41 Q6QNY1 O15519 3516 P16220 O00273 Q92851 Q92614 P07996 P52564 P52565 65018 P16471 4851 10542 Q92843 Q92835 Q9NYF8 57646 Q8IVF5 P20936 O60543 Q92830 P61289 P03372 64170 Q9H7P9 Q6IT96 4869 5716 5970 P48023 Q9BZF9 3551 1130 Q12979 9093 Q9NRZ9 5728 Q99698 3304 Q9ULZ3 Q9UHR5 3309 P04233 Q12981 Q12982 P28482 P50570 1385 O00220 Q9UHJ9 29108 O00463 P52756 Q9P0J0 3315 3313 P07550 P04040 P08887 Q9NYA1 O95243 Q5VST9 P19544 Q9NS56 P37198 3329 57678 79444 960 29115 P42771 Q70UQ0 P07766 P50591 Q13618 Q13617 Q13616 P30542 Q9NS68 6609 57448 P61247 Q9Y371 Q92630 5515 P05112 5516 Q9UM63 5518 P09958 Q12778 Q13625 P51692 O00255 O00257 81788 P05120 5524 P05121 P40337 7704 Q08043 P23396 P48454 P04637 P80188 P38646 P52952 P15531 O14827 Q96RU2 5533 6622 999 P01116 Q9C0K7 Q15389 P62753 P01112 P01111 Q9C0K0 4221 10383 6885 1191 O76074 Q15392 P48436 4214 P01589 P01588 Q99638 P01343 P49768 O00206 P48431 Q8N726 5321 P52735 P11388 148022 773 Q00535 Q53X93 6647 Q9Y572 P01138 P01137 Q6UXV0 P01375 P01374 P04626 8841 P04629 2065 Q9UHI6 2064 O00213 11218 301 P17936 P10071 3146 Q8WTP8 P02458 307 Q96EY1 6657 11214 8837 P49747 O43353 5581 5584 7520 8851 4255 Q8WYA6 Q9UNN5 3162 O95076 O75190 Q99683 5578 P62714 7518 2081 5591 Q6IR47 7531 7534 64127 23411 O76024 5590 324 3169 329 P05783 7529 P27797 64135 6453 2099 Q9Y2M5 Q9BUZ4 330 572 331 573 Q8NER5 5599 23636 5371 Q12933 8887 P15559 P52701 580 P51617 P58012 8878 P25116 P60568 Q9BZZ5 8655 Q9BY76 9500 8654 Q04760 351 355 356 5139 P62913 P25963 Q13489 Q13009 Q9H3Z4 Q13490 P11309 6495 6256 6498 O75489 29 Q9UBU3 Q9UDY8 P23528 P61604 P00441 9531 6262 9774 Q14116 10081 Q13268 9530 4088 8445 Q9H257 387 P21359 P20264 8453 8454 8452 10293 7124 23365 23368 O75460 154 396 Q02535 121512 10296 Q99728 Q8WV24 Q13043 Q13286 P36896 Q9Y5J5 23370 P01308 7128 Q9NQB0 Q99958 Q15475 9564 Q6ZSZ5 P61803 Q8TCU6 P54274 1831 Q9H1Y0 Q01664 O95817 P21145 P01579 11124 10276 Q9NQC3 Q14155 2923 7153 64782 P39905 7157 7159 P14625 P35548 1843 2932 Q14160 Q15011 Q14164 10285 Q13075 1848 Q15257 2935 87

Q13077 Q15811 P78423 P12814 Q86Z02 7161 1613 P63167 56259 P42224 Q9BXM7 10935 91 P39060 P42229 O15119 1616 Q96QB1 Q01085 P67775 P05067 2956 7187 7186 7189 7188 P29474 O94768 Q9H422 Q96M96 P18583 Q13418 6093 7185 7184 Q9BXK5 Q8N163 P17252 O15379 P21980 P11717 O15392 Q13426 O95865 Q02156 4914 2730 Q9UBN7 2737 Q9UBN6 Q9UBF6 P41182 P30044 57144 P53567 P30048 Q03468 P09211 P47712 P51398 Q15628 P21580 Q07820 Q96BI3 P51159 8065 Q07817 64919 P08138 P09429 P10415 P12830 1676 3611 Q14790 Q13464 O15350 P08107 P53355 O75832 Q9P0U3 27315 Q04771 O60356 Q9HC29 O95429 P45983 Q13233 Q9BZR8 P45984 P21554

cellular localization O75947 Q9Y266 9182 22872 259266 9181 Q96KP1 O95793 Q5SQT9 9184 O00148 Q8IYN9 1213 Q92973 P60059 10651 3875 Q9Y275 Q92974 Q92729 P55957 Q8IWJ2 2317 2316 1468 10427 P42858 Q9H2G9 O15400 4734 253725 Q12846 Q96P70 6927 Q8IUD2 55341 P62491 30000 811 5830 P63104 Q9Y6A5 P62258 6926 10632 Q96EA4 Q12851 Q9C005 P26374 P31946 56681 Q13705 O95343 81622 P20823 Q8NFW9 O60674 821 10640 O14579 27072 Q96GD4 5604 Q9UKX7 P41743 O75901 Q92538 Q9H2M9 5862 1020 114548 55361 O60645 1499 1012 6945 P61106 P32856 Q8WXF0 5871 P33947 Q9NR31 27297 P20645 O60895 6711 604 4792 Q9H2K2 P40222 401505 Q9HCL2 857 Q9UN86 6726 5879 Q9Y490 Q13501 P51572 P53992 P51575 P10827 Q9UL45 Q13505 P51571 P31749 O95140 Q9NQT8 27032 Q9P209 Q8NBS9 P40692 Q96ED9 Q9UMX1 O14777 Q9UL54 Q9H4M9 Q8TBA6 P06576 6993 10490 Q86V81 O96008 23325 Q99523 55722 1058 2147 8924 Q15021 10013 637 5898 Q96KG9 6748 6747 11345 135295 Q9BTU6 4580 Q9Y678 Q9NV70 O95166 400 55973 Q99750 55737 406 408 Q15036 P49407 P02794 11116 P60468 O00327 Q9UMR2 O00560 23303 10239 891 79711 Q15286 Q9Y5X1 8943 O14964 Q9UP83 10476 84062 6780 O43264 10482 O14980 Q658W2 P35222 Q96ST3 Q01970 Q15050 9804 P24588 11336 O00505 Q6FGG2 P05408 Q641Q2 25942 P14598 Q8IYJ3 P61981 O14908 A8K0Z3 55763 10452 P55072 P05412 O00755 P49450 10460 5476 Q9HCE7 Q8WUX9 10228 10469 Q99797 55770 Q9UBX3 Q8IYI6 P60660 Q15070 207 Q15075 10466 P62841 P24386 9601 Q9H902 6576 22895 O75051 Q96QU8 O14925 Q96P20 215 Q00610 7415 P61764 7414 10673 7430 8766 6103 P06729 Q9UKK6 51199 P40616 P40855 Q92900 10204 Q16623 8775 Q9UPN3 24137 P35658 473 117584 P07196 Q9BQG0 5018 P43034 Q15785 P49815 Q9NPC8 Q9HAV7 8546 4188 8301 9632 64601 79083 Q9UJC3 O75381 488 Q05952 Q9NY59 Q96RL7 8536 Q9H9E3 P00533 11079 9868 Q15554 Q15796 Q9HAU5 Q9BUN8 Q9UNL2 P56134 Q8WVM8 23265 23025 Q9Y6Q5 Q96IZ7 4193 498 51542 Q01105 Q96RK4 P56385 8548 P56381 P61966 P55290 Q9BSI4 8563 6386 Q9Y2J0 P62826 7476 P62829 Q86XR8 Q9UQ26 O75351 Q9NZZ3 23039 Q8N4C7 Q8N4C6 P61970 54820 51552 9648 Q03518 P43487 Q49AN0 51319 149371 11261 O43752 11021 7248 P63092 P36542 P13667 Q9UEU0 51560 84248 Q9Y4G6 O94826 P27824 11269 Q14012 7014 11031 O75575 23256 7249 Q9GZM8 Q9NRD5 9201 9685 Q86W42 Q86Y82 Q9BV36 O15066 Q7Z7G2 23463 O43734 A1XKG3 Q86VS8 Q96EV8 Q9Y4I1 Q9UNH7 P24539 P02545 Q6I9Y2 1956 2802 55705 9212 O15075 O75558 26985 P10147 Q8N4H5 O43747 P11233 Q9H173 Q9NXR1 Q13190 11014 10166 P98194 P25874 9463 O95714 P53007 83752 Q92572 P41250 Q9UGU5 O15234 65082 Q92574 O95721 Q13769 9472 P78537 81567 P46934 117177 Q9HD26 81565 O60296 O15020 9001 7067 P29590 P30101 Q4LE60 60412 Q9NZQ3 O15258 A4D1W7 27236 Q9UNE2 9495 112936 653361 P43405 Q9UH92 Q9UH99 Q13555 O75746 Q9NP61 O00186 O00189 23085 P63000 Q9BUK6 Q8IWV1 P35606 9276 P56962 Q14416 Q9H2T7 7094 Q96IW7 Q05516 Q9Y6D6 83548 23095 Q9Y6D5 1785 Q05513 Q5QGT7 O60499 P63010 P00734 80273 5901 5902 O75969 O75964 100287932 P33897 2647 1315 1314 P20336 84661 Q9HAP6 Q14674 Q9NP90 Q14677 Q16611 Q13107 11097 Q9H6Z4 51510 Q13596 51517 Q9NRW1 Q9NRW7 Q9P2R6 Q9BVA0 O60566 O14662 Q9Y2X3 10772 P27348 Q9UBC1 P48047 Q8NF91 4860 O95684 55696 O60333 200081 Q12965 1121 Q05397 Q9BXC9 Q9GZY0 10514 4628 10758 6809 P18859 Q86WA8 O15533 Q92834 4627 4869 5716 P53675 91782

Q92824 P55851 Q9H814 1130 P53677 O60318 Q99698 57410 1122 6810 6812 6811 6814 10762 4637 P04233 Q9H1C4 O95477 P28482 P50570 O95239 3320 O00221 Q9H1K0 Q9P0J0 Q9NRS6 10736 57662 3313 O60763 O00471 3312 P07550 Q4VX76 4643 4644 Q9Y2T2 5976 3799 10972 4646 10971 O95487 O95249 P53618 Q8IZT6 Q9NS56 57678 P18206 Q9UM54 5747 P53621 Q9NYB0 6850 2495 Q12769 O60749 O60502 Q9NS69 10953 10956 P42768 2247 58533 P61006 O14640 5514 P09958 P30536 1176 1174 1173 Q6KC79 10724 10726 Q92621 O15504 7704 Q92624 P61019 509 Q96FF9 P07900 Q9UPV9 P04637 Q9Y6W5 P16615 O95271 P38646 O60725 O14828 513 4686 514 515 10134 10133 Q9C0K7 Q15389 51009 Q15388 Q04917 6643 6642 5796 23212 23214 P50542 Q96Q15 9908 521 522 Q15154 11231 Q96CW1 4218 P49768 6890 6892 Q86U42 2054 P33176 Q96CV9 O00203 773 Q00535 Q02952 P49755 Q9GZP9 54536 P49754 54535 P01137 539 91949 Q8N960 P01133 P01375 4000 5331 23673 P12004 P54920 10128 O14617 9927 O00623 O43592 O00628 9700 5584 Q9UPR3 O00629 22794 Q9Y2K9 Q9UNN5 Q8IXI1 Q8IXI2 Q68EM7 Q2M389 P42704 Q8WTW3 8604 7514 Q96AX1 10577 9950 7532 5111 7531 7534 7533 3178 P37108 P68032 5590 Q00987 8615 9702 P05783 7529 5108 P27797 64130 5362 6456 Q9UPT5 Q9NW64 P15311 Q99666 56993 P83436 P49792 5371 4285 Q60FE5 64145 9973 O75396 O00410 23633 Q6ZWJ1 Q99653 Q08209 P52948 P62745 8878 10564 P25116 10566 Q8N6T3 54908 Q8TEW0 8411 57187 94121 Q96FV9 94120 351 23163 P09493 23165 P23786 P62913 Q96QK1 Q8WUM0 P25963 Q8TD31 O43684 P12429 11196 19 O75494 29959 Q9UIA9 415117 8417 Q13492 4074 Q15436 6496 6495 8677 9525 9522 8676 23144 Q9H269 29927 Q9UBU3 375 23381 P25705 Q9UDY8 9515 Q13023 51429 P61619 6262 4087 Q14118 Q9Y619 Q96BK5 P12883 P59780 388 Q9NUP9 Q15691 P60520 Q16543 9527 Q8IXZ2 9784 P54257 11140 7124 10053 P54253 P14635 Q9H089 4092 Q4G0F5 P35579 154 P35580 Q99962 P45379 Q13285 5195 9554 Q96C24 7375 Q09161 O94955 O75694 161 Q86WV8 P11142 Q9Y5J6 Q8NI27 51693 8218 Q15475 7142 P00491 O43615 Q9NZ43 Q6VY07 P21145 P01579 11124 9559 P09038 2923 Q9BY84 O94973 O75436 7157 O75674 Q9Y5L4 Q02750 O75431 Q8TEX9 25777 Q8WZ73 Q9NZ56 54984 O94979 84376 1605 P53367 8498 9342 O95835 Q96QC0 Q13636 P63172 84958 9341 P41134 79139 10938 Q7RTN6 P09651 O15119 O15118 Q9UBK9 Q14974 O15131 9590 Q01082 8021 P43307 10947 P52292 O15126 22919 P05067 P52298 P10619 Q15836 P52294 O75886 26258 57120 P57740 Q03014 26019 Q9UHY1 80145 P00846 Q9NSC5 O15392 O15155 O15397 64901 9371 56288 1408 Q9UBN7 P30049 9146 Q9UBF2 Q96G30 P41182 9382 3836 2741 O95405 57154 Q15629 P51159 Q07817 P21579 Q9UJX2 Q96QF0 3841 3843 P21333 O75843 P10415 59341 387680 P01730 9179 O75832 P62072 Q96QD9 Q8TAG9 P84077 O15105 Q15653

signaling pathway Q9H0E2 P25054 P49023 1460 O95551 3638 1459 10657 1457 2303 55561 284217 1453 2783 O14544 O14543 2308 P16104 2316 5829 Q96QZ7 P42858 Q9UKV5 P10809 Q59EK9 Q12841 Q5S007 O75925 Q9H8S9 Q92956 3661 Q06124 P37023 81876 2324 P62491 Q9UKW4 3655 Q9Y297 O75928 10633 Q12851 3672 3673 Q13705 P21917 10645 Q9UKX5 3667 Q8WXG6 P25098 P08514 3680 P49069 5861 5862 1022 1020 1017 79576 P16144 1499 10616 1012 3674 3675 3676 P61587 P61586 3678 3679 P61106 Q9UL26 Q9H0H5 Q9UL25 3690 Q9Y239 5870 3693 5871 Q15907 Q9UGI9 1029 Q9BUB1 1026 Q9BUB5 3685 6711 5863 5865 3688 3689 P40238 5867 6714 5868 5869 Q07890 O95136 Q9UGJ0 5883 Q9BQ95 P16118 P19634 P38398 Q07889 4543 Q5TCX8 6721 Q6NUQ1 Q92993 5877 5878 5879 Q92997 P10827 5894 P31749 Q38SD2 1050 84932 P40692 55159 Q96MT8 10607 6733 Q9UL54 127829 8930 Q9Y5U4 Q96A33 P56199 P14317 P14314 Q99523 P62879 5898 P62873 10256 O96018 O96017 6760 Q15027 10261 O95163 Q9NWZ3 P57059 23576 1072 P35249 641 Q14185 P47224 O00329 P48552 6772 P48551 6774 Q8WWX0 3265 Q99500 51141 Q9H4B6 Q14192 8945 P01019 51147 55504 Q99988 O14965 3276 P35222 P14784 O75084 P17813 3269 6776 Q15052 Q59EA4 6790 O00744 5460 Q5VVQ6 P56159 Q92934 9821 6794 P05408 23523 79971 672 673 P15692

O14908 P62834 6789 Q8N423 Q92930 P05412 O00755 682 Q9NWV8 Q15077 9826 207 4139 Q92922 Q15078 90780 23509 Q9Y5S2 Q96A54 Q08345 O00762 O75051 8503 10672 O14920 5494 Q8NHX1 P06729 P50406 P08908 Q5TCQ9 O14939 8516 P40616 10681 8517 Q9H4E5 P60201 8515 9846 326624 O15085 P36507 Q14686 11065 7204 O43559 O43557 Q9UPN3 P13861 Q9H0U4 23043 P49810 P49815 Q14694 P48960 4188 P62805 6368 Q9HAV0 P11215 Q9Y2H1 Q9H0T7 Q96F46 7205 5029 P00533 Q96RL1 1902 Q9HAU4 Q9BUN8 P35638 Q9UQ13 8555 P32121 Q9Y2I1 Q9Y6Q6 Q15561 Q93008 P55290 Q15562 6386 Q9Y6R4 P62826 Q9UPQ3 Q9Y6R0 O75116 Q5JTC6 Q86XR8 Q86XR7 Q8N4C8 267 51552 Q13393 P43246 P62820 11021 O75582 O75581 P10398 51560 Q9Y2C9 P23229 Q9Y6K9 8569 51567 7252 8100 Q15109 O75578 P78334 11031 26524 285 7249 11035 P49841 Q15596 Q15111 Q15118 P78368 Q9BYZ6 P49840 P11229 9448 Q07092 Q9Y6M4 A1XKG3 P58753 Q9Y2E6 51588 P08069 51107 1956 10161 Q9HB90 P78356 O75553 P11234 P11233 2810 Q93038 P78362 1969 Q14289 9465 9463 9467 P12931 P42345 Q92572 Q9BUG9 P04083 P04085 1978 7297 P31689 Q8WXJ9 9475 O95965 Q02297 P42338 Q92569 P42336 O75528 O95971 P29353 Q8WXI4 O95977 O95976 P29350 221188 Q9H0K1 Q7Z569 P40145 2844 Q9BYM8 P43657 79039 P06241 P21860 A4D1W7 55072 P78317 Q07960 Q9Y6J0 Q07954 Q9Y6J8 Q92542 O95996 Q13797 Q96EP0 O14593 O95999 Q13315 Q03113 O43915 9021 Q16828 P35613 23085 2861 P20594 2869 Q8IWV1 P31249 Q13322 Q14653 Q9NP71 Q9NP72 P46736 P61073 5906 Q9BQB4 Q9Y6D5 5908 2873 P61077 O60496 1540 P21453 5901 O75969 50649 9046 Q16849 O75962 Q8NEB9 Q9P035 2889 Q9BQA5 2885 P61088 50650 P43694 O15455 5911 5914 Q9NP90 Q14676 A4D1S5 Q9H0N0 P00519 P24941 Q92585 51512 Q15532 51514 Q14683 5925 O14672 Q9NRW1 Q9NRW4 Q99490 55684 O60566 Q8IVH8 P16220 55207 P49137 P27348 O15530 P27361 Q8IZP0 65018 54361 9077 P50148 P16234 P38484 Q92844 O15524 P52333 Q06481 P29992 P50150 339122 P53667 57402 57403 10758 O60543 1111 Q9UJU2 P03372 P06401 4627 P06400 P26012 P05556 3791 O15552 5970 P26010 P46089 Q92828 Q6PJ21 Q5JS13 9093 Q9UBE8 P56705 P20702 3301 3309 P04233 P26006 P34925 P07307 O00468 P61225 4643 P61224 Q8N2W9 4646 719 Q7KZI7 P08648 P30556 Q5HYI8 P30559 P19784 P30550 O95243 Q9NS56 P37198 3329 10746 1147 2475 Q92888 P07766 P05106 O95257 P05107 P28223 6850 2011 P30542 Q9NS68 O60503 O14641 O14640 Q12770 5515 O43182 5518 P51451 50814 Q12772 Q12778 1176 Q05586 3350 P30530 P18433 P37173 10728 Q9UK32 O15503 4690 Q9BST9 P04637 O43157 P38405 2033 P16615 P62993 P13591 P01116 Q15382 Q9C0K7 Q16236 6869 Q15389 285282 P01112 P01111 P25100 4221 6885 Q53G59 6400 23210 P35368 2043 56940 9908 Q15392 84159 Q99873 Q9P2K8 4214 4215 P01589 4216 4218 P01100 5562 Q8N726 90864 P14416 O95292 P52735 P11388 Q9NS23 O76061 773 Q8IZF4 Q02952 P61204 Q53X93 Q9GZP9 Q9Y572 P01138 P01137 Q6UXV0 P01135 P01133 P02461 5573 P04626 5575 5576 P04629 2065 2064 P35348 780 301 2059 P10070 P10071 5566 5567 P01127 5568 11215 23647 5580 5581 5583 5584 5585 Q5U0I6 64599 Q9UNN5 P51828 5577 5578 5579 57826 P40763 P04201 2081 Q6IR47 5111 5598 64127 O76024 P18850 5590 51295 P16671 324 329 P27797 6453 2099 O60907 330 Q8IZJ4 333 Q9NRM7 5599 8625 P25103 23636 6464 P39748 P52701 Q6ZWJ1 P18827 9967 Q8N752 Q96HB1 P25116 Q15418 Q8TEW0 11186 P13987 351 Q9UBS0 Q04759 Q9UBS5 P25963 9510 5154 O43687 7334 5155 19 Q8TF76 Q9UKE5 367 369 51657 Q13258 29 127247 Q86X27 P32241 P32246 P59768 Q8TEU7 Q9UBU3 375 P09471 5159 9518 6009 P32239 Q53H12 Q14114 8204 Q9BY44 P51812 Q9UKG1 P51813 381 382 5170 Q04725 387 Q9UBV2 Q04727 388 Q04726 389 P08151 P60763 P62942 Q9NSD7 9546 390 391 392 O43639 394 396 397 398 121512 25780 Q6KH11 Q8NI35 Q9H082 Q13283 Q15466 Q13287 2904 Q99966 2902 Q96R06 Q13286 Q9BU20 7376 Q9Y5J5 Q9UKA4 Q9UKA8 Q6PRX2 O15169 2915 Q99958 Q99956 9564 Q6ZSZ5 10273 O43613 O43612 Q9UKB1 Q01664 51222 P01579 11124 2925 P09038 Q14155 Q9BY84 O75676 O15197 O15198 9578 P14867 2932 2931 Q15011 Q14164 1609 Q5VVH5

Q14964 Q15811 P78423 9100 Q13637 Q13636 O15123 Q12788 Q08722 Q13639 1613 P41134 Q9BXM7 79139 Q1PSW9 91 94 1616 Q9NSA1 26270 Q9UBK2 Q6DT37 9590 P31321 P31323 P67775 P08575 P05067 2956 Q15831 P29474 O94768 Q96M96 Q7Z3S9 6093 P41597 56034 P21980 Q8TAD8 Q9NSC5 8289 O95622 Q14511 9138 Q03001 Q02156 Q05655 P13945 Q6NYC1 Q9H488 P41180 B3KY43 Q03468 P08123 Q13683 O95644 150094 Q52LW3 Q13202 Q96HU1 Q8IVT5 P20248 Q8TAI7 P08134 P17081 Q9UBH6 1676 P08581 1672 P08588 P41159 O15111 P55773 Q9P2Y4 Q13227 P62070 Q04771 O95661 O15105 P45983 Q13233 P45984 P21554 Q2M1Z3 Q92737 65125 O14793 2550 P51587 9185 Q8TDD1 P15498 P37288 Q8IYN9 P84022 Q92731 10890 Q92730 O14788 6901 10413 2549 P28335 64284 Q92729 Q8NFZ5 P53779 P14174 2554 4734 6915 808 Q7Z727 Q9NR80 Q9NR81 79791 6927 2569 Q8IUD2 2564 811 P62258 P31946 P31947 2100 Q9NR96 57534 3428 P16333 81622 P20823 O60674 P20827 388677 P18507 Q8IUC6 P18509 5602 5604 P41743 P27037 Q13956 4772 Q9H765 Q5SGD2 P18564 P15056 5610 O14745 P30679 4780 P09619 Q07666 P10600 P52888 Q06330 Q96KS0 P60022 Q6PI57 846 7804 P21731 O14757 Q92766 P49286 4792 Q9NZD8 1285 P21709 Q96L33 P18545 P60033 P05230 857 O14763 Q9UN86 Q96CA5 Q13501 50937 P11912 O95382 P19883 867 O14775 P09601 O14777 P08754 P01241 3484 2150 3481 O60603 25988 3479 2147 10010 P01236 10499 P23025 Q9BRQ0 400 402 Q99750 Q13094 Q99759 O43294 O43293 409 O14713 Q13098 P01222 Q13099 Q6ZN33 Q09472 O43251 116986 O00560 116987 890 P53708 898 3014 Q15286 29086 Q658W2 Q01974 55750 Q01970 P60484 55759 7867 Q96T58 8718 O14733 5207 P23497 8717 P26583 P50613 23765 10458 Q8WUY9 433 P38570 10451 P55075 6548 7879 P55072 10454 P01275 7410 Q9HCE7 Q08117 Q9NYZ3 Q05086 29079 Q01955 P51959 23770 8737 P55085 P48357 10468 Q6IQ22 P24385 8754 7422 O75293 79753 7415 10432 7433 8766 Q13905 P52824 P17612 Q13115 7442 P18084 6599 P18085 P10114 Q9Y4K4 O75386 O75385 Q9H2Y7 Q9Y4K3 P35414 3084 O43318 472 P48729 51762 P20396 8767 P21246 Q14451 P35408 Q15303 Q16635 P13631 23291 488 Q9H2X0 P46531 P55042 P55040 Q15796 Q15311 P43026 Q13131 Q9HCY8 9641 8795 Q15797 Q16644 8315 Q8N6I1 Q9UNL4 P12755 Q8TDS5 O75360 Q8IV61 5295 9656 7476 8323 P55055 O43306 P10586 P13612 P23416 9649 Q13153 Q8TDY2 11261 Q16666 P54132 P63092 Q9Y4G2 P46109 Q13162 51324 Q13164 Q15349 Q9Y4H2 P11021 P63096 Q9NRD1 51339 Q9NRD5 Q99835 7023 10392 O94844 Q7Z7G1 O43734 23220 Q96PK6 55704 1718 P02545 Q9GZL7 Q13188 P23443 9213 Q9BX66 9217 P55011 P10147 P38936 O75791 Q6PID4 Q99819 P48730 P23458 Q9BWT7 P48736 Q15375 9223 6196 6199 7046 6198 P35813 O43715 6195 6194 P32302 Q9Y4C1 10817 P98161 3911 P78536 7057 P10523 9230 9231 Q3V6T2 7052 3925 P46934 P46937 P41240 P98171 Q9UER7 Q14865 P34947 8396 P29597 7067 Q9H305 Q13535 O15264 P19793 P29590 7064 P41231 57003 O60266 Q9NRA1 Q9HCP0 P78552 Q13546 P49190 9252 9253 7074 Q9UNE7 80254 P43405 P20309 Q04206 Q9NVJ2 A8MTJ3 Q15738 P78504 9261 O00194 P63000 51720 3953 Q13563 P62158 2626 7099 7098 Q9NVC6 O15211 9270 7091 7090 P30153 P62166 Q05513 P00734 P24723 375790 Q13574 51735 Q15750 Q9HCU4 53916 Q15759 P32320 O60229 P20338 P20339 P20336 P20337 1793 Q5T9L3 P20333 O15230 Q15768 P34972 P47804 Q8TDM6 P67870 900 Q9NZN5 P20340 P00750 64397 P08833 P48058 Q92619 Q12948 P61020 3516 Q6PCE1 Q7L0Q8 2664 O00273 Q9HBH0 P61026 P07996 P52564 O75815 P52565 Q7L591 P52566 51094 Q9P107 P63211 10549 P20963 P16473 P16471 P63218 P48039 4851 4854 Q92600 P54753 Q12965 P25391 P50391 Q05397 P54756 57646 29127 P20936 Q9UQB8 O75807 4868 5716 Q96JK9 P54764 P54762 P54760 4882 Q9BZF9 3551 Q12979 5728 10524 P17301 P20941 Q9ULZ3 948 Q6PCD5 Q9H1C4 O95477 P28482 1385 O00220 O95470 Q9UHJ9 P51668 29108 P19525 5739 P07550 5733 10971 P08887 Q9UM47 O00238 P04049 Q96SB4 Q9NYA1 P10721 29110 2237 79444 961 10981 P42771 Q9UM54 5747 22937 P51681 O00241 2253 P51686 Q9P0L2 22931 P62330 2247 P15153 1399 2246 Q9BZK7 975 P61006 Q9Y2V2 976 Q92630

Q92633 P51693 P29323 P29320 P51692 O00255 P48061 Q9P0M2 440193 P17342 5764 P29317 P61019 Q9Y2W1 P61018 26060 P06213 26959 Q9Y6W6 Q9Y6W5 2274 Q96RU8 Q96RU2 998 P27986 51009 P62753 Q04917 Q9UHG2 5310 5311 5796 266747 2280 Q8WTR2 Q96RT1 2277 Q99638 10146 P01344 P01343 P49768 O00206 Q9Y6Y9 148022 Q00535 Q96S42 5798 6647 P01375 Q9Y336 4000 5331 64343 5333 Q9UHI8 O00212 O60716 P12004 Q96RR4 O95071 3148 545 Q8WTP8 Q96EY1 5327 7508 O43353 6672 Q969H4 8851 P07949 8852 Q8WU90 3162 Q8IXI1 Q8IXI2 552 553 Q99683 55884 Q96AX2 5336 8844 Q15185 5337 5338 P05771 7531 10580 7533 Q01831 Q99679 Q99677 5347 9702 P24046 7529 Q08462 5362 5364 4035 Q9UHD2 Q12929 572 573 5371 Q60FE5 Q12933 P16885 Q8NER1 55432 Q99653 P51617 Q9NPJ6 8879 P62745 54106 8878 10564 P60568 10566 Q15654 9744 6236 8412 595 Q8WZ60 Q96BM9 116 Q7Z6C1 P22694 Q13480 Q9HC97 Q13485 8408 Q13489 6242 O94910 Q13009 Q16512 Q15669 6239 Q13490 Q14344 Q13017 6256 P22681 Q9HBW0 Q9UDY8 P23528 142678 Q8NFH8 P00441 346562 Q16539 4086 4087 4088 4089 Q15208 8445 O43427 163126 Q86YT6 Q8N5F7 Q9Y3L5 Q02790 P21359 P31150 P24864 6259 64750 7124 23365 P10275 4092 P10276 4093 P35579 O75460 154 156 Q9NYQ6 Q96KC2 Q8WV28 Q13043 7132 8463 7133 Q5JQC9 P36896 P36894 P35568 166 23370 Q8NFM4 Q04941 Q02763 P01308 Q8NFM5 120892 Q9NQB0 Q99717 9306 P01303 Q96T37 Q8TCU4 O95813 P36402 Q86V24 P36404 P36888 Q9NYS7 140458 Q9BRK4 P49715 O95819 P60953 Q9NQC7 7153 64782 7157 P19086 Q02750 P35548 140461 Q9NZ56 1848 54509 Q16581 P53365 Q13873 O95835 P57729 10928 Q86Z02 O75420 7161 P42224 1852 1850 P23945 Q4KMG0 83871 O95837 P22612 P42229 1856 P42226 22926 P10644 Q01082 9350 79363 P57735 P84095 Q96BD6 9367 7186 7189 9368 P35916 Q13418 O43815 P17252 P42680 P42681 O95859 O60383 O60381 Q86UE8 O95865 4914 2735 27352 P19438 P68400 56288 2737 2736 Q86YS6 P51148 Q7RTR2 P51149 Q9H244 P23508 P43119 2742 2741 4920 P43115 P51157 P35968 Q13443 P51151 Q15628 O95405 P51153 P30273 57154 Q96BI3 58480 P51159 27330 Q8WUH2 Q8WYP3 P21333 O14492 O14495 59341 P84085 3611 1432 Q5XUX0 Q13464 P01730 O15350 P53355 P51178 O75832 O15354 Q14318 P53350 Q15648 27315 Q86Z14 P84077 2773 O60353 Q9HC29 Q7L9L4 O14497 Q15653

regulation of molecular function P25054 P54619 22870 2550 1460 Q12824 9181 124790 9184 P15498 P37288 P84022 O14788 6901 2307 Q92974 25913 Q12834 2316 800 P10809 Q96EB6 808 Q5S007 Q7Z727 Q9H2G4 P27695 Q06124 Q9C010 54496 Q8IUD2 57761 Q9UKW4 Q9Y297 P40424 P26374 P31946 P07858 3672 3673 P31947 Q92949 P99999 Q9NR96 Q13705 P21917 10645 O60674 P20827 P19838 Q8IUC6 P18509 3667 64061 10641 5604 Q9UL15 Q8WXG6 P27037 Q9H2M9 Q13956 4771 1022 1020 114548 Q9NUY8 10614 10616 Q08999 O75909 Q92530 O14745 Q9Y478 3690 P30679 Q9Y239 Q92769 P10600 1029 Q9BUB1 1027 1026 Q9HCM4 604 Q6ZW31 847 7804 O14757 P04179 4790 O95136 Q9UGJ0 4792 1285 Q9BQ95 O60869 P16118 P18545 Q96N67 P20618 P60033 Q5TCX8 857 O14763 859 Q96CA5 Q9UL46 P51575 P10827 6500 P31749 O95382 P09601 50943 P08754 Q9UL54 Q9UKT4 P01241 O00308 P56199 O60603 Q99523 2147 23560 3476 10013 P62873 10014 11345 10016 Q15027 64682 Q9UII4 Q7Z6J4 641 Q99750 648 408 P49407 409 85360 Q13098 Q01518 11116 Q09472 5682 5683 6772 5684 5685 5686 116986 O96020 116987 23787 891 Q99500 O14727 894 Q99741 896 P35240 8945 P01019 4361 5692 5693 8721 7874 Q9NUX5 P51532 P35226 55750 P17813 P60484 3269 668 5687 5688 10486 5207 Q96MH2 10487 P23497 Q15057 Q3MII6 P26583 Q92934 P01040 Q96BZ9 P50613 Q5R372 Q96P48 P61981 79735 10451 9815 10213 P55072 10454 P05412 O43242 7410 Q08117 Q5JSP0 P37231 Q01955 Q96SZ6 P51959 Q15070 Q15077 8737 207 5468 54206 P48357 11315 Q15078 P24385 P24386 8754 3066 O00762 O75293 Q96P20 Q0IIM8 55785 7415 10672 O14920 9839 Q8NHX1 P52824 P08908 Q9Y3P9 23513 57732 P17612 P28749 8517 56647 11060 6597 11065 6598 Q9Y4K4 Q9Y4K3 3084 Q9UPN7 O43318 P13861 23043 Q9NPC1 P49810 8767 5018 P43034 P49815 Q8TBP0 3091

P55036 4188 Q16635 Q15306 Q86VP6 O75381 51773 7448 5029 P00533 Q16401 Q13133 Q15796 1902 Q15311 Q13131 P24522 8795 P35638 9882 8555 P32121 Q9ULH1 P12755 23028 Q96CN4 Q9Y6Q6 P46527 10197 Q93009 Q8IV61 Q9Y6R4 5054 Q9UPQ3 P18074 P55055 O43306 Q8N6H7 Q16665 P43246 Q13153 P43489 7248 P54132 P63092 O75340 Q04837 Q9Y2C9 Q9Y6K9 7013 Q15109 Q9Y4H2 26524 P11021 P63096 7249 P49841 Q9GZM8 Q13177 P49848 Q9NRD5 Q96NH3 Q99835 P61925 7023 10392 P49840 P11229 A1XKG3 P48507 Q9UNH7 Q9NVM4 55704 1718 51107 1956 6188 O43504 O75553 23476 P38936 P37840 5092 Q9UNI6 2810 Q6PID4 1728 Q9BWT7 Q14289 9463 7046 P98155 P78540 9467 P22736 P42345 P62195 Q92574 P56524 P78536 7057 Q13526 55291 Q02297 Q3V6T2 7052 81565 P42574 P42338 P98174 Q9UER7 7067 P06239 P29353 O95977 Q5TAX3 Q13535 P29590 P56539 P31431 84619 P40145 Q9BYM8 P41231 O60266 3932 Q8WXH2 Q13546 55072 P78318 P43405 O43707 Q07954 Q04206 Q92786 Q92542 Q9Y2B9 Q13794 Q9NZJ7 Q96EP0 O95999 Q9NP61 P78509 Q86TI0 Q16828 93594 P35612 P35611 P00749 23085 3953 Q13563 P62158 P08246 Q8IWV1 7099 7098 Q14416 P14923 P30153 P62166 3728 P61073 Q05513 2873 P00734 1540 Q13574 51735 Q15750 P46734 P28065 Q9P035 29843 2644 2643 P61088 Q04656 O15455 Q16610 5914 Q8IYX1 Q13586 Q16611 P34972 Q9P287 P62191 P28074 P28072 P28070 P00519 P67870 4830 900 P43686 P07148 5925 2672 Q9NRW4 Q99490 O60566 2665 Q96JM2 2664 57620 P63208 Q9ULW0 5933 P07996 P52564 P04004 P09936 Q9Y2X7 O15530 P27361 O95684 9077 115704 P50148 Q9P107 5706 5707 5708 5709 P16473 51099 P16471 5700 5701 5702 10542 5704 5705 Q92838 P50395 P29992 1121 P16435 22974 P53667 5717 1111 P61289 5713 4869 P06400 5716 5970 P46089 P48023 Q9BZF9 3551 9093 Q99460 5728 P17301 1122 Q9ULZ3 Q96JJ6 3309 P04233 O00220 P51668 29108 O00463 P51665 57669 P07550 P04040 P51671 P30556 Q9Y2T7 Q9NYA1 O00231 O00233 P10721 O00232 P30793 P37198 3329 10746 79444 2475 4898 P42771 P05106 P30304 O95257 P28223 6850 P30307 27148 Q13616 O00487 P30542 O60503 P62333 2247 58533 975 O14640 Q92878 5515 P05112 O43182 Q92633 5518 P51693 P09958 O00255 3350 P17342 P37173 Q99497 P05362 5524 P05121 P06213 P23396 P04637 5300 P38405 2033 O95271 P52952 P15531 990 991 Q96RU8 Q96RU7 Q8WTS1 995 P11171 Q99640 6622 999 P01116 Q9C0K7 6869 Q15389 O43166 4221 6885 5311 Q9HDC5 23216 2280 56940 Q8WTR2 Q9NRP7 Q15392 4214 6878 8812 4216 P49768 O00206 P48431 5562 Q8N726 P14416 P52735 148022 Q00535 P25789 P25788 6647 P01137 P01135 P25787 P01133 P01375 Q9UJM3 5573 P04626 5575 5576 P04629 2064 P35348 Q9NS37 Q96RR4 55633 3148 545 3146 P61457 5566 5567 P01127 5568 Q96EY1 Q96JB5 6657 5569 5328 O43150 O43353 5580 O43593 6672 5585 O43597 8851 4255 3162 552 553 Q9BZE4 O75190 Q99683 Q8WTW4 Q99684 P51828 5577 5336 5578 7518 P07951 2081 Q6IR47 7532 64127 23411 Q9BSW2 5590 51295 324 3169 Q9NU19 328 Q9H1A4 5347 Q8TEA7 7529 Q08462 4035 P51843 Q9BUZ4 572 333 Q9NRM7 P25103 Q9GZT9 Q9BT67 23636 5371 8881 Q60FE5 Q12933 6464 9973 P15559 P17980 P52701 P16885 54583 O14818 P51617 P58012 54106 P25116 P60568 Q8N6T3 9744 Q8TEW0 Q9BY76 351 595 356 Q9UBS5 116 P09493 118 Q7Z6C1 P22694 Q13485 Q8WZ64 P25963 P03950 7334 5155 Q16512 O43684 P12429 7335 Q9UKE5 367 51654 Q14344 7341 P11309 Q13257 Q9HBW0 P35998 Q8TEU7 Q9UBU3 Q9UDY8 P09471 P61604 Q8WZ42 P00441 P32239 Q53H12 4087 Q14114 4088 4089 Q15208 Q8WWN8 125058 Q96BK5 5170 P21359 Q66K14 P31150 P60520 Q16543 P62942 Q9NSD7 8454 11140 7124 11142 P14635 O43639 Q9UQL6 P10275 4092 O43638 P10276 O75460 154 Q02535 121512 25780 Q13042 55806 P45379 Q13286 5195 7376 Q86WV8 P35568 166 Q8NFM4 P01308 Q8NFM5 120892 Q9NQB0 2917 P49721 P49720 2915 85440 Q99956 P54274 Q96FX2 O43612 Q96T23 P49959 Q01664 P01579 P49711 P09038 Q9NQC7 Q15008 Q9BY84 P39905 7157 P19086 64786 P14625 Q02750 23118 2932 2931 55832 Q14160 54984 51231 Q15011 1609 Q14161 1848 Q15257 2935 Q13077 O95835 7169 10928 26037 7161 P42224 26271 P41134 1852 1850 10935 P23945 Q7RTN6 P63165

P22612 P15090 1616 Q6ZT07 Q9UBK2 Q9NSA3 O94992 Q13404 Q96QB1 P10644 P31321 O43847 Q05682 P31323 O43609 P67775 P05062 P08575 P05067 P60900 2956 7186 7189 7188 P29474 9368 Q9H422 Q96M96 Q13418 7185 7184 P41597 Q86UE4 Q9BXK5 P17252 84502 P21980 P42684 O95622 O15392 Q13426 4914 27352 56288 Q05655 2730 Q9UBN7 1647 P13945 Q96DN5 P41182 P53567 P30047 Q7RTR2 P30048 B3KY43 1896 Q03468 84766 Q13200 Q5H9R7 Q13202 Q13443 Q96HU1 Q96BI3 P30279 Q9UJX2 58487 Q8TAI7 P30281 P23510 P21333 P09429 Q9NYJ8 P10415 Q6ZV73 O14495 P12830 P19474 Q02363 3611 O60343 O60341 4943 P01730 P08588 O15350 O75832 Q9BZS1 P53350 Q9P0U3 Q13227 O95661 Q8N5A5 Q9UHV2 2773 Q9HC29 P30260 O15105 Q13233 P21554

cellular macromolecule localization 9182 9181 Q9NRW7 O95793 Q5SQT9 Q9P2R6 Q9BVA0 O60566 1213 Q92973 O14662 Q9Y2X3 P60059 10651 3875 P27348 Q92974 Q8NF91 Q92729 P55957 Q8IWJ2 55696 2316 10427 P42858 Q9H2G9 O15400 4734 1121 Q12846 Q96P70 Q9BXC9 6809 P62491 30000 Q86WA8 811 5830 P63104 Q92834 P62258 4869 Q96EA4 P53675 P26374 P31946 56681 O95343 P53677 O60318 81622 Q8NFW9 O60674 1122 6810 6811 27072 Q96GD4 P41743 P04233 Q9H1C4 O75901 Q9H2M9 P28482 1020 Q9P0J0 Q9NRS6 10736 1499 3313 O60763 Q4VX76 Q9Y2T2 4646 10971 O95487 P32856 O95249 P33947 P53618 Q9NR31 Q9NS56 P18206 O60895 6711 604 Q9UM54 P53621 Q9NYB0 4792 Q9H2K2 O60749 O60502 Q9NS69 10953 401505 10956 2247 58533 O14640 5514 6726 P51572 P53992 P30536 Q13505 P51571 P31749 1176 O95140 1174 1173 Q6KC79 Q9NQT8 10724 Q92621 O15504 Q9UL54 7704 Q92624 6993 10490 Q9UPV9 P04637 O96008 O95271 P38646 O60725 Q99523 1058 2147 8924 Q15021 10013 10134 637 10133 Q9C0K7 6748 Q15389 51009 6747 Q15388 Q04917 4580 Q9Y678 6643 6642 5796 O95166 23214 P50542 55973 Q15154 11231 406 Q96CW1 Q15036 P60468 O00327 6890 O00560 2054 23303 Q96CV9 O00203 10239 79711 Q00535 Q02952 Q9Y5X1 Q9GZP9 8943 P49754 54535 P01137 O14964 91949 P01133 P01375 6780 O43264 4000 O14980 23673 P35222 P12004 P54920 Q96ST3 O14617 9804 P24588 9927 O00623 O00505 O43592 O00628 5584 O00629 P05408 22794 25942 P14598 Q8IYJ3 P61981 O14908 7514 10452 P55072 P05412 P49450 7532 5111 7531 7534 5476 7533 Q9HCE7 P37108 10228 10469 Q99797 Q00987 Q15070 8615 207 9702 P05783 7529 5108 P27797 64130 P24386 Q9H902 22895 Q9NW64 Q96QU8 O14925 56993 Q00610 7415 7414 P83436 P49792 5371 4285 Q60FE5 8766 6103 Q9UKK6 O00410 23633 Q6ZWJ1 Q08209 P52948 P40855 10204 P25116 54908 Q8TEW0 Q16623 8775 Q9UPN3 P35658 94121 94120 473 117584 23163 23165 5018 P62913 Q15785 P49815 P25963 Q9NPC8 Q9HAV7 Q8TD31 8546 9632 11196 64601 79083 O75381 Q9UIA9 415117 8417 P00533 9868 Q15554 Q9BUN8 Q15436 6496 6495 Q9UNL2 8677 8676 Q9H269 29927 Q9Y6Q5 4193 Q96RK4 8548 Q13023 P61966 51429 Q9BSI4 P61619 6386 Q9Y2J0 P62826 P62829 Q86XR8 Q9UQ26 23039 Q8N4C7 P59780 P61970 Q15691 Q16543 9527 9648 Q03518 Q49AN0 9784 11140 O43752 7124 10053 Q9UEU0 O94826 Q13285 5195 Q96C24 7375 7014 161 Q9Y5J6 7249 8218 Q9NRD5 Q15475 Q86Y82 Q9BV36 23463 A1XKG3 Q86VS8 O43615 Q9UNH7 Q6VY07 P01579 P02545 1956 P09038 2923 55705 Q9BY84 9212 O94973 O75558 7157 26985 O75674 Q9Y5L4 O75431 Q8N4H5 Q8TEX9 25777 O43747 Q8WZ73 Q9H173 Q13190 11014 84376 P53367 9463 O95714 Q96QC0 P63172 84958 P41134 83752 79139 Q92572 Q7RTN6 O15234 Q14974 O15131 9590 Q01082 9472 8021 P46934 117177 P43307 10947 P52292 O60296 22919 P10619 P52294 O75886 P29590 Q03014 P30101 Q4LE60 60412 Q9NZQ3 Q9NSC5 O15397 27236 Q9UNE2 64901 9495 653361 56288 Q9UH99 1408 Q9UBN7 9146 Q9UBF2 Q96G30 Q9NP61 P41182 O00189 3836 P35606 9276 P56962 O95405 Q9H2T7 57154 Q15629 P51159 Q05516 83548 Q5QGT7 O60499 P63010 P00734 Q96QF0 3841 3843 80273 5901 P21333 O75843 100287932 1315 1314 Q9HAP6 9179 Q13107 11097 P62072 Q13596 51517

negative regulation of macromolecule biosynthetic process Q9Y2W7 O75820 2672 Q9UBB5 Q9NS86 P51587 O00267 124790 10657 3516 2304 P84022 283248 Q8N3U4 P27348 5933 25913 Q9UBC3 Q9Y2X9 Q92729 Q12837 54361 Q8NDV7 P51114 Q8NB12 4851 4734 Q96EB6 5705 O75925 Q7Z727 Q9H2G4 Q9UKV8 Q92835 3660 Q9NYF8 Q9Y2Y8 Q9NRY4 Q9BYE7 Q99471 6927 P13056 83933 811 Q9UJU2 O15534 57649 Q92833 6926 Q6IT96 P06401 P26358 P06400 5716 Q9UIS9 Q9NZI7 P48382 Q9NQX0 P31946 5970 Q92949 Q9NRZ9 P56705 P48380 O95343 9099 10524 P20823 1487 P19838 400961 2332 P62263 P31949 3665 10642 64061 10644 10765 10643 Q9Y468 Q9ULR5 Q8IZ40 Q9UL18 4771 Q12986 Q9P0J0 O14627 6829 10614 1499 Q86VE0 O14981 Q8N2W9 10971 27161 6945 P08887 5978 6827 O14503 Q9Y2T7 3690 Q92769 5991 93986 5993 Q07666 Q14938 Q15906 P10600 P19544 O95365 O00358 Q06330 10865 6839 1027 3685 P08651 P17480 O14753 604 5987 P05106 4790 22938 5883 O00488 O60502 P38398 57326 2247 58533 Q9BZK7 6601 Q92993 857 6605 6726 Q8N488 P10826 P10827 Q9NR50 2023 O00255 Q6KC79 P19883 O00257 1050 Q9HCK5 10724 79589 Q99496 192669 Q9UMX1 4799 P40337 50943 Q9UGL1 7704 Q92985 O00425 O96004 O43159 P04637 Q9NVW2 P14316 25988 P52952 990 23560 3476 Q15022 79813 4204 639 10014 10138 Q6UWV6 Q9UQ80 Q9BT49 4221 5796 P16989 P57059 641 84159 P33076 51132 Q99750 Q9P2K8 P48436 8932 Q99638 7727 P60228 2290 23429 O43251 23309 P48552 P48431 6774 Q99623 Q99741 Q96S42 P35240 55869 51147 P01137 P01375 Q86X95 P51531 4361 6660 5451 8841 Q9UHI6 P35222 Q9NUX5 O00213 2063 P51532 23435 Q96AQ6 P35227 11218 Q96ST3 Q9NS37 P17813 P35232 3148 P10070 Q15170 545 P10071 3146 11331 668 6657 5569 Q96T58 Q96MH2 P23497 O43474 6670 P06733 P26583 O43593 6672 Q9Y3M2 23409 P25490 Q969H0 25822 25942 Q12906 Q8NFW5 3281 Q9BZE0 672 Q9BZE4 O75190 Q99684 6304 8726 Q8TEB7 5578 P40763 4261 Q969G3 8864 P37108 Q08117 23411 23414 O75182 P37231 P14373 Q00987 3169 5467 5468 7529 7528 10468 P27797 64375 4152 5245 P56177 O60907 22893 3066 3065 Q9UIF9 P51843 3182 10438 P51608 Q9H7L9 5119 Q01826 9839 O43463 5371 3070 Q13901 9612 23512 Q96QT6 P58012 P28749 7428 10320 6595 6597 8535 P10599 6591 Q9Y4K3 3084 5017 Q13485 Q13363 P38919 Q13127 P03950 4188 5036 O75496 O75376 P13631 3096 Q02447 O75381 23051 51773 P46531 7329 Q96RL1 Q15554 Q15796 Q9HAU4 Q15672 Q9UQR1 7341 8553 6496 6256 6498 Q96IZ0 P12757 Q8N6I1 P31273 Q9UNL4 P12755 23028 Q9NY61 O75360 4193 Q2M1K9 84108 Q9UBU3 P46527 116113 Q13144 51548 51547 54815 Q13263 Q14232 Q9BSI4 Q13148 9774 4087 8204 4088 4089 9775 Q9BY41 P55055 163126 Q9Y618 P10588 P10589 93166 Q04724 P20264 11176 Q9NPF5 Q15697 51317 55915 53615 P43489 Q13033 9421 P54259 9541 5187 O75469 64750 7124 11142 O15164 7248 P54132 P54253 2909 O75461 Q9UQL6 4092 P10276 Q9Y6K1 Q02535 Q03989 51564 Q15583 55922 Q99729 8328 Q15466 55806 Q99966 5074 5195 Q9NRC8 6045 7013 7376 55929 Q9HAZ2 7014 8467 O15055 9314 O43524 23135 23378 26523 Q86WV8 7490 7251 6282 1822 166 84232 Q9NQB0 Q8WW38 Q9UBP4 Q96T37 P61925 7023 P54274 P14859 O43612 P15822 Q9Y6M1 23468 7141 Q9H063 Q96T23 Q9UNH7 P49959 51341 Q01664 Q00577 51588 Q13185 P49711 10155 Q9BQI3 P49715 P09038 O75437 7157 O43623 25776 64426 138151 Q9H9G7 Q99816 9208 Q5VTD9 Q96RE7 P29374 Q06787 Q01094 Q9H0M0 P41134 Q1PSW9 P62195 27122 P63165 9219 Q92574 O15119 1616 P45973 1978 Q9NSA3 P56524 O94992 Q969S8 Q8IY57 Q02297 7291 O75530 55294 7295 P55316 Q96BD5 P46934 1869 Q9UER7 Q14865 7189 7067 92129 Q8WYK2 9126 Q13535 P19793 P29590 7181 Q03014 Q7Z2Q5 84619 Q9H6W3 Q86UE4 Q9HCP6 27102 P17252 O15379 O60264 Q9NSC2 O15151 O95983 Q13422 O94776 Q13547 A4D1W7 P78318 O43829 Q8TBE0 84733 Q04206 Q9UH92 Q92786 Q9Y2B9 2737 2736 7088 O75626 Q8WUI4 27327 P41182 Q9UKY1 Q9H9B1 51720 Q14894 P08246 150094 Q13322 Q9NP71 1789 1788 Q05516 1786 58487 Q9UQE7 P41162 3720 3609 Q13573 Q14781 P23510 P09429 O75604 Q9H2S9

29966 Q02363 P18146 P35711 Q9BQA5 Q9Y6E7 O60341 P09430 5914 O75953 O75832 Q9BZS1 Q13227 P55771 Q86YP4 63976 Q96BF6 P63279 Q8N5A5 P63272 Q9HCS4 O15105 Q14683 5925

negative regulation of nucleobase, nucleoside, nucleotide and nucleic acid metabolic process
Q9Y2W7 O75820 2672 Q9UBB5 Q9NS86 P51587 O00267 124790 10657 3516 2304 P84022 283248 10772 Q8N3U4 P27348 5933 25913 Q9NS91 Q9UBC3 Q9Y2X9 Q92729 Q12837 54361 Q8NB12 4851 4734 Q96EB6 5705 O75925 Q9H2G4 3660 Q9NYF8 Q9NRY4 Q9BYE7 Q99471 6927 P13056 83933 811 Q9UJU2 O15534 57649 Q92833 6926 Q6IT96 P06401 P26358 4869 P06400 5716 Q9UIS9 Q9NZI7 P48382 Q9NQX0 P31946 5970 Q92949 P21917 Q9NRZ9 P56705 P48380 O95343 9099 10524 P20823 1487 P19838 P62263 P31949 3665 64061 10765 Q9Y468 Q8IZ40 4771 Q12986 Q9P0J0 O14627 6829 10614 1499 Q86VE0 O14981 Q8N2W9 10971 6945 5978 6827 O14503 Q92769 Q8WXF0 5991 93986 5993 Q07666 Q14938 Q15906 P10600 P19544 O95365 O00358 Q06330 10865 6839 1027 P08651 P17480 O14753 604 5987 Q9NYB0 4790 22938 5883 O00488 P38398 57326 2247 58533 Q9BZK7 6601 Q92993 857 6605 Q8N488 P51693 P10826 P10827 2023 O00255 Q6KC79 P19883 O00257 1050 P40692 Q99496 Q9UMX1 4799 P40337 50943 Q9UGL1 7704 Q92985 P23396 O96004 O43159 P04637 Q9NVW2 P14316 25988 P52952 990 23560 3476 Q15022 79813 4204 639 10014 10138 Q6UWV6 Q9UQ80 135295 Q9BT49 4221 5796 P16989 P57059 641 84159 P33076 51132 Q99750 P48436 8932 Q99638 7727 2290 23429 O43251 23309 P48552 P48431 6774 Q99623 Q99741 Q96S42 P35240 55869 51147 P01137 P01375 Q86X95 P51531 4361 6660 5451 8841 Q9UHI6 P35222 Q9NUX5 2063 P51532 23435 Q96AQ6 P35227 11218 Q96ST3 Q9NS37 P17813 P35232 3148 P10070 Q15170 545 P10071 3146 11331 668 6657 5569 Q96T58 Q96MH2 P23497 O43474 6670 P06733 P26583 O43593 6672 Q9Y3M2 23409 P25490 Q969H0 25822 25942 Q12906 Q8NFW5 3281 Q9BZE0 672 Q9BZE4 O75190 Q99684 6304 8726 P40763 4261 Q969G3 8864 Q08117 23411 23414 O75182 P37231 P14373 Q00987 3169 5467 5468 7529 7528 10468 P27797 64375 4152 5245 P56177 O60907 22893 3066 3065 Q9UIF9 P51843 3182 10438 333 P51608 Q9H7L9 6207 5119 Q01826 P62854 9839 O43463 Q9BT67 5371 3070 Q13901 9612 23512 P52701 580 Q96QT6 P58012 P28749 7428 10320 6595 6231 6597 8535 P10599 6591 Q9Y4K3 3084 5017 Q13485 Q13363 Q13127 4188 5036 O75496 O75376 O75494 P13631 3096 Q02447 O75381 23051 51773 P46531 7329 Q96RL1 Q15554 Q15796 Q9HAU4 Q15672 Q9UQR1 7341 8553 6496 6256 6498 Q96IZ0 P12757 Q8N6I1 P31273 Q9UNL4 P12755 23028 O75360 4193 Q2M1K9 84108 P46527 116113 51548 51547 54815 Q13263 Q9BSI4 Q13148 9774 4087 8204 4088 4089 Q9BY41 P55055 163126 Q9Y618 P10588 P10589 93166 Q04724 P20264 11176 Q9NPF5 Q15697 51317 P43246 55915 53615 P43489 Q13033 9421 P54259 9541 5187 O75469 64750 7124 11142 O15164 P54132 P54253 2909 O75461 Q9UQL6 4092 P10276 Q9Y6K1 Q02535 Q03989 51564 Q15583 Q99728 55922 Q99729 8328 Q15466 55806 Q99966 5074 5195 Q9NRC8 6045 7013 7376 55929 Q9HAZ2 7014 8467 O15055 9314 O43524 23135 23378 7490 7251 6282 1822 166 84232 Q9NQB0 Q8WW38 2917 Q9UBP4 Q96T37 P61925 7023 P49840 P54274 P14859 O43612 P15822 23468 7141 Q9H063 Q96T23 Q9UNH7 P49959 51341 Q01664 Q00577 51588 Q13185 P49711 10155 P49715 P09038 6188 O75437 7157 O43623 25776 64426 138151 2931 Q99816 9208 Q5VTD9 Q96RE7 P29374 Q01094 Q9H0M0 P41134 Q1PSW9 P62195 27122 P63165 9219 O15119 1616 P45973 Q9NSA3 P56524 O94992 Q969S8 Q8IY57 Q02297 7291 O75530 55294 7295 P55316 Q96BD5 P46934 1869 Q9UER7 2956 Q14865 7189 7067 92129 Q8WYK2 9126 Q13535 P19793 P29590 7181 Q03014 Q7Z2Q5 84619 Q9H6W3 Q86UE4 O15379 O60264 Q9NSC2 O15151 O95983 Q13422 O94776 Q13547 P78318 O43829 Q8TBE0 84733 Q04206 Q9UH92 Q92786 Q9Y2B9 2737 2736 7088 O75626 Q8WUI4 P41182 P62277 Q9UKY1 Q9H9B1 51720 Q14894 150094 Q9NP71 1789 1788 Q05516 1786 58487 Q9UQE7 P41162 3720 3609 Q13573 Q14781 P23510 P09429 O75604 Q9H2S9 29966 Q02363 P32320 P18146 P35711 Q9BQA5 Q9Y6E7 O60341 P09430 5914 O75953

O75832 Q9BZS1 Q13227 P55771 Q86YP4 63976 Q96BF6 P63279 Q8N5A5 P63272 Q9HCS4 O15105 Q14683 5925

signaling Q9H0E2 P25054 P49023 1460 O95551 3638 1459 10657 1457 2303 55561 1454 284217 P21926 1453 2300 2783 O14544 O14543 Q9Y275 2308 Q9UL63 P16104 2317 2316 5829 Q96QZ7 P42858 P15260 5822 Q9UKV5 10666 P10809 Q59EK9 Q12841 Q5S007 O75925 Q9H8S9 Q92956 3661 Q12846 Q06124 P17405 P37023 81876 2324 P62491 Q9UKW4 4983 3655 Q9Y297 O75928 10633 Q12851 Q13702 3672 3673 Q13705 P21917 10645 Q9UKX5 3667 P42830 Q8WXG6 P25098 P08514 3680 O75901 P49069 5861 5862 1022 1020 114548 1017 79576 P21964 P16144 1499 10616 1012 3674 3675 3676 P61587 P61586 3678 26191 3679 P61106 Q9UL26 Q9H0H5 Q9UL25 3690 P29466 Q9Y239 5870 3693 5871 Q15907 Q9UGI9 P51797 1029 Q9BUB1 3207 1026 Q9BUB5 3685 6711 5863 O60890 5865 3688 3689 P07686 P40238 5867 6714 5868 5869 Q07890 O95136 Q9UGJ0 Q9Y243 5883 Q9BQ95 1040 P16118 P19634 P38398 610 Q07889 4543 613 Q5TCX8 6721 Q6NUQ1 Q92993 5877 5878 5879 Q92997 P10826 P10827 Q9UL45 Q9UGK8 5894 P31749 Q38SD2 1050 84932 P40692 55159 Q96MT8 10607 Q9UL51 6733 P04150 Q9UL54 127829 Q92502 8930 Q9UKL4 Q9Y5U4 Q96A33 P56199 P14317 P14314 O00300 23568 P50897 11108 Q99523 P62879 79937 5898 P62873 10256 P25445 O96018 O96017 6760 P06702 Q15027 10261 O96019 O95163 Q9NWZ3 P57059 P36575 23576 O95166 1072 O96013 P35249 Q99996 641 Q14185 P47224 Q15036 P49407 85360 O00329 P48552 6772 Q08380 Q9UKN5 P48551 6774 Q8WWX0 3265 P11274 Q99500 51141 Q9H4B6 Q14192 Q8WWW0 8945 P01019 51147 55504 O14964 Q99988 O14965 Q9Y215 5451 P35225 3276 P35222 P14784 O75084 P17813 P35232 3269 6776 Q15052 Q59EA4 P24588 6790 O00744 5460 Q5VVQ6 P56159 Q92934 9821 6794 P05408 23523 79971 672 673 53340 P15692 O14908 P62834 6789 Q8N423 Q92930 P05412 O00755 682 Q9NWV8 Q15077 5467 9826 207 5468 4139 Q92922 Q15078 90780 4155 23509 Q9Y5S2 Q96A54 Q08345 Q12809 O75044 O00762 O75051 A7KAX9 10673 8503 10672 O14920 P56180 Q92903 5494 Q8NHX1 P06729 P50406 P08908 Q5TCQ9 O14939 Q3KRB8 56882 8516 P40616 10681 8517 Q9H4E5 P60201 8515 55558 9846 326624 O15085 P36507 P35659 Q14686 P19174 11065 7204 8533 O43559 O43557 Q9UPN3 P13861 Q9H0U4 Q6P5Z2 23043 Q9H160 P49810 P83731 P49815 Q9HB75 Q14694 P48960 4188 P62805 6368 Q9HAV0 5037 P11215 Q9Y2H0 Q9Y2H1 Q9H0T7 Q96F46 Q9UD71 Q9NY59 7205 8536 5029 P00533 Q13370 Q96RL1 1902 Q9HAU4 Q9BUN8 Q16881 Q9BYV2 P35638 Q9HB63 Q9BYV6 Q9BZ76 Q9UQ13 9404 8555 P32121 Q9Y2I1 Q9Y6Q6 Q01344 Q00013 Q15561 Q93008 P55290 Q15562 Q9HAT0 6386 Q9Y6R4 P62826 Q9UPQ3 Q9Y6R0 O75116 Q5JTC6 Q86XR8 Q86XR7 Q8N4C8 267 P31270 51552 Q00005 Q13393 P08034 P61978 P43246 P62820 P78347 P78348 11021 7248 O75582 O75581 P10398 Q93063 51560 Q9Y2C9 P23229 Q9Y6K9 8569 51567 7252 8100 Q15109 O75578 P78334 11031 26524 5071 285 7249 1948 11035 P49841 1947 1945 Q15596 Q15111 Q15118 P78368 Q9BYZ6 P49840 P11229 9448 Q07092 Q9Y6M4 A1XKG3 P58753 291 Q14C86 Q9Y2E6 51588 P08069 51107 1956 P78357 10161 Q9HB90 P78356 O75553 O43508 P37840 P11234 P11233 7272 2810 Q93038 P78362 Q9NPB3 1969 Q14289 9465 9463 9467 P12931 Q9H0M0 Q05329 P22736 P42345 Q92572 Q9BUG9 P04083 P04085 Q92574 1978 7297 P31689 Q8WXJ9 Q15942 9475 O95965 Q02297 Q08830 7295 P42331 P42338 Q92569 P42336 O75528 O15020 9001 O95971 P29353 Q8WXI4 O95977 O95976 Q06643 P29350 221188 Q9H0K1 Q7Z569 P40145 2844 Q9BYM8 P43657 60412 79039 P06241 P21860 P78314 O95989 A4D1W7 55072 P78317 Q07960 P78318 9495 Q9Y6J0 3705 3708 Q07954 Q9Y6J8 P07101 Q92542 Q9Y2B9 O95996 Q13797 Q96EP0 Q14643 O14593 Q8IWW6 O95999 Q13315 Q03113 O43915 8174 9021 Q16828 P35613 Q96EP1 23085 2861 P20594 O75508 2869 O75509 Q8IWV1 P31249 Q13322 Q14653 Q9NP71 Q15506 Q9NP72 O43921 23092 P46736 P61073 5906 Q9BQB4 23095 Q9Y6D5 5908 2873 P61077 23098 O60496 1540 P21453 P22303 5901 5902 P46734 Q13330 Q9Y6D9 9047 O75969 50649

9046 Q16849 O75962 Q8NEB9 Q9NP86 Q9P035 2889 Q9BQA5 P02708 2885 Q9Y6E0 P61088 50650
Q9HAP6 P43694 O15455 5911 5914 Q9NP90 Q14676 A4D1S5 9052 Q9H0N0 P00519 2896 P24941
P43681 P08473 Q92585 51512 Q15532 51514 51517 Q14683 Q13352 5925 23603 23607 O14672
Q9NRW1 P51460 Q9NRW4 P52799 Q99490 P52798 55684 O60566 Q8IVH8 P16220 79658 55207
P49137 P27348 O15516 O15530 P27361 Q8IZP0 O95680 65018 Q07343 54361 9077 P50148 P16234
P38484 Q92845 Q92844 O15524 P52333 Q06481 Q92838 Q92835 P29992 P50150 Q9NRY4 339122
Q6P4F7 P53667 57402 57403 10758 Q8IVF5 O60543 1111 Q9UJU2 P03372 P06401 4627 P06400 P26012
P05556 Q92824 3791 O15552 5970 P26010 P46089 Q92828 Q6PJ21 Q5JS13 9093 Q9UBE8 P56705
10767 P20702 3301 6810 3309 P04233 P26006 P34925 P07307 4651 P50570 P11802 3320 P52757
O00463 O00468 Q9P2N2 1137 P61225 4643 P61224 Q8N2W9 4646 4649 719 Q7KZI7 P08648 P30556
Q5HYI8 P30559 P19784 P30550 O95243 Q9NS56 P37198 3329 10746 1147 2475 Q92882 Q92888
O95259 P07766 P05106 O95257 P05107 P28223 6850 P50591 2011 P30542 Q9NS68 O60503 O14641
4664 O14640 4665 Q12770 5515 O43182 5518 P51451 50814 Q12772 Q12778 Q13625 1176 Q05586
3350 P30530 P18433 10725 P37173 10728 Q99497 O14654 Q9UK32 5528 O15503 5529 4690 Q9BST9
P04637 O43157 P38405 2034 2033 25861 P16615 1185 11228 P62993 P13591 P01116 Q15382 Q9C0K7
Q16236 6869 Q15389 285282 P01112 P01111 Q9UNS2 P25100 4221 6885 Q53G59 6400 23210 P35368
2043 1192 56940 O76074 9908 Q15392 84159 Q99873 54763 Q9P2K8 4214 4215 P01589 4216 P01588
4218 P01100 5562 Q8N726 5563 90864 5565 P14416 O95292 2054 P52735 Q96CV9 P12235 P11388
Q9NS23 O00445 O76061 773 Q8IZF4 Q02952 P61204 Q53X93 Q9GZP9 Q9Y572 P01138 P61201 P01137
Q6UXV0 P01135 P01133 O00459 P02461 5573 P04626 5575 5576 P04629 2065 2064 2063 P35348 780
Q9NRR8 301 2059 P10070 P10071 Q9C0H5 5566 5567 P01127 307 5568 Q8TDZ2 11215 23647 5580
5581 5583 5584 5585 O43597 Q5U0I6 64599 5586 Q9UJF2 Q9UNN5 Q96PU8 P51828 5577 5578 Q9P2F6
5579 57826 P40763 P04201 2081 P46060 Q6IR47 5111 5597 5598 64127 O76024 P18850 5590 51295
P16671 324 4259 329 P27797 O43572 64130 P01185 O00408 6453 6456 6455 2099 O60907 P50993 330
Q8IZJ4 333 Q9NRM7 5599 8625 P25103 P01178 Q96HC4 23636 7791 6464 4287 O43586 4289 P39748
Q5T5U3 P51858 P52701 Q6ZWJ1 Q08209 P18827 6457 9967 Q8N752 Q96HB1 P25116 Q13237 5141
5142 5144 Q15418 Q8TEW0 Q9BY76 Q5VZM2 11186 8654 P13987 P30086 351 Q9UBS0 355 Q04759 356
Q9UBS5 85415 5138 5139 P25963 O43683 9510 5154 O43687 7334 5155 19 Q8TF76 Q9UKE5 367 369
51657 Q13258 6496 6495 Q13257 P11308 29 127247 Q86X27 P32241 P32246 P59768 Q8TEU7 Q9UBU3
Q03405 375 P51805 P09471 Q13261 5159 11165 9518 6009 P32239 Q53H12 Q14116 Q14114 8204
Q9BY44 Q8WWN8 P51812 Q9UKG1 P51813 P12645 381 382 5170 Q04725 Q04724 387 Q9UBV2
Q04727 388 Q04726 389 P08151 P60763 41 P62942 Q9NSD7 P55345 10293 9546 11142 2909 390 391
392 O43639 394 396 397 Q99962 398 Q99963 121512 25780 Q6KH11 Q8NI35 Q9H082 Q13283 2908
Q15466 Q13287 2904 Q99966 Q15464 Q13285 2902 Q96R06 Q13286 Q9BU20 7376 Q16799 Q9Y5J5
Q86WV8 Q9UKA4 Q9UKA8 Q6PRX2 2917 Q8NHQ8 O15169 Q6PJH3 2915 Q99958 Q15475 Q99956 9564
Q6ZSZ5 10270 10273 P14859 9567 O43613 O43612 Q9Y5K6 Q9UKB1 Q99941 Q01664 51222 P21145
P01579 11124 10276 2925 P09038 Q14155 2923 Q9BY84 9575 O75676 O15197 O15198 9578 P14867
O43623 2932 2931 Q15011 Q14164 1609 Q5VVH5 Q14168 86 Q14964 Q15811 P78423 9100 Q13637
Q13636 O15123 Q12788 Q15818 Q08722 Q8TF05 Q13639 1613 P41134 Q9BXM7 Q86WK6 79139
Q1PSW9 91 94 O15119 1616 Q9Y5E9 Q9NSA1 26270 Q9UBK2 9112 Q96QB1 Q6DT37 9590 P31321
P31323 O43609 P67775 P08575 P05067 2956 Q15831 P29474 O94768 P29475 9126 26258 Q96M96
29767 Q7Z3S9 6093 P41597 56034 P21980 Q8TAD8 Q9NSC2 2969 Q9NSC5 P11717 8289 O95622
Q14511 Q14517 Q6R6M4 9138 Q03001 Q02156 P31785 55198 Q8N392 Q86WH2 Q05655 Q9UBN6
9146 P13945 Q6NYC1 Q9H488 P41180 114822 P30047 B3KY43 Q03468 P08123 Q13683 O95644 150094

Q52LW3 Q13202 Q96HU1 Q8IVT5 Q9UBG7 P20248 P21579 Q8TAI7 P08134 P17081 Q9UBH6 P19235 1676 P08581 1672 P08588 P41159 O15111 P55773 Q9P2Y4 Q13227 P62070 Q04771 O95661 O15105 P45983 Q13233 P45984 P21554 Q2M1Z3 Q92737 65125 Q5TB30 O14793 P54619 9182 2550 P51587 9185 Q8TDD1 P15498 P37288 Q8IYN9 P84022 Q92731 10890 Q92730 O14786 O14788 6901 A1A4S6 10413 P28799 2549 P28335 64284 Q9UIQ6 Q08AM6 Q92729 2562 Q8NFZ5 P53779 P14174 2554 30011 Q17R89 4734 6915 808 Q7Z727 Q9NR80 Q9NR81 2572 2571 79791 6927 2569 Q8IUD2 3416 2564 57522 811 P63104 P62258 10875 6926 P31946 P31947 2100 Q9NR96 O00178 O95343 P54646 57534 3428 P16333 81622 P20823 Q8IUC4 O60674 P20827 P19838 388677 P18507 Q8IUC6 P18509 P31949 5602 5604 P41743 P08758 P27037 Q13956 22839 4772 Q9H765 Q5SGD2 P18564 P15056 Q6ZRI8 55367 Q8N264 644943 5610 O14745 Q9Y478 P30679 4780 P32856 P09619 Q07666 P80098 P10600 P52888 Q06330 Q96KS0 Q9BWF2 P60022 Q6PI57 846 Q6ZW31 7804 P21731 O14757 Q92766 P49286 4790 O43295 4791 4792 Q13976 4311 Q9NZD8 1285 2132 P21709 Q96L33 P18545 Q9NR48 3459 P60033 83853 Q92752 P05230 857 O14763 Q9UN86 Q96CA5 Q92754 Q13501 P51575 50937 P11912 O95382 P19883 Q13506 Q9P202 Q9NQT8 Q9HCK4 153090 867 Q9UMX1 O14775 P09601 O14777 P08754 P01241 O95399 Q9BRR9 Q96C74 3484 3482 2150 3481 O60603 25988 3479 2147 3476 10010 P01236 10499 6505 P23025 Q15269 Q9BRQ0 P13236 P51513 Q99755 Q96T76 400 402 Q99750 Q13094 Q99759 O43294 408 O43293 409 O14713 Q13098 Q01518 P01222 Q13099 Q6ZN33 Q09472 O43251 4354 Q7Z6I6 4355 116986 O00560 116987 O00562 23303 890 Q00653 891 54413 Q99741 55743 896 P53708 898 3014 Q15286 Q9Y450 29086 O43264 Q8TCX5 Q658W2 Q01974 P15882 55750 Q01970 P60484 55759 10486 7867 Q96T58 8718 O14733 5207 10487 P23497 8717 P01243 P26583 O00506 Q9Y3M8 P50613 2195 23765 10459 10458 Q8WUY9 Q96P48 433 P38570 10451 P55075 6548 7879 P55072 10454 Q01968 P01275 8743 8744 7410 Q9HCE7 P52803 Q08117 Q9NYZ3 Q05086 29079 P37231 P36382 Q01955 P51959 23770 8737 P55085 P48357 P55083 10468 8738 Q6IQ22 11315 P01266 23746 P24385 8754 7422 Q96GA9 5245 9600 3068 O75293 79753 Q96P20 7415 7416 10432 P24394 7433 8766 P49674 Q13905 P52824 3074 80728 P17612 8772 Q13115 7442 Q13114 Q16623 P18084 6599 P18085 Q14449 P10114 Q9Y4K4 O75386 P10599 O75385 Q9H2Y7 6591 Q9Y4K3 P35414 3084 O43318 472 P48729 477 51762 P20396 8767 P43034 Q13123 P21246 Q14451 P35408 Q15303 3091 Q16635 P13631 23291 488 Q9H2X0 P46531 P55042 P55040 Q15796 Q15311 P43026 Q13131 Q9HCY8 O00602 9641 8795 Q13136 Q15797 Q16644 8315 Q8N6I1 P19397 Q9UNL4 P12755 Q8TDS5 O75360 Q13144 Q8IV61 Q16659 5295 5296 9656 P43490 7476 8323 P18074 P55055 O43306 P10586 P10588 P10589 59277 P13612 P23416 9649 Q16665 P43487 Q9P2E9 Q13153 P43005 Q13158 Q8TDY2 11261 Q16666 P54132 P63092 Q9Y4G2 P09172 P46109 Q13162 51324 P46108 Q13164 Q14012 Q15349 O15294 7013 Q9Y4H2 Q9NZT1 P11021 P63096 Q9NRD1 Q13177 51339 Q9NRD5 Q99835 7023 10392 O94844 Q7Z7G1 P12314 O43734 23220 Q96PK6 55704 1718 P02545 Q9GZL7 Q13188 P23443 9213 O43741 Q9BX66 9217 P55011 P10147 O75312 P38936 O75791 Q6PID4 Q99819 P27815 P48730 P23458 1728 Q9BWT7 Q99814 P48736 Q15375 9223 6196 6199 7046 6198 P98155 P35813 P10997 8379 O43715 6195 6194 P32302 27242 Q9Y4C1 Q86VI3 10817 P46940 O95715 P98161 3911 Q13522 P78536 7057 P10523 9230 9231 Q3V6T2 7052 3925 P46934 P46937 1742 P41240 Q4LE28 P98172 P98171 P98174 Q9UER7 Q14865 P34947 8396 P29597 7067 Q9H305 Q13535 O15264 P19793 P29590 8394 P30101 7064 1756 P41231 Q92793 57003 P16284 Q9NZQ3 O60266 O15259 Q9NRA1 O60260 Q9HCP0 9255 P78552 Q13546 P49190 Q8IYT8 9252 9253 7074 Q9UNE7 80254 P43405 P20309 Q04206 Q9NVJ2 P78560 P09525 7088 Q13555 Q13554 A8MTJ3 Q15738 Q8IV45 P78504 9261 3959 P00749 O00194 P63000 51720 3953 Q13563 P62158 2626 7099 7098 Q9NVC6 Q15742 O15211 Q14416 9270 7091 7090 P30153 P62166 Q9UIV1 1785 Q05513 P00734 Q9HD67 P24723 375790 Q13574 51735 Q6SZW1 Q15750 Q13572 Q9HCU4 53916 Q15759

P32320 O60229 P41279 P20338 P20339 P20336 P20337 2644 1312 P85298 P41273 1793 Q5T9L3 Q14432 Q15762 P20333 O15230 Q15768 P18031 P34972 1326 Q9UNA1 P47804 Q8TDM6 P67870 900 Q9NZN5 P20340 P00750 P08235 Q9NZN9 O15228 Q7Z628 64397 P08833 Q9Y2W7 P48058 Q14CB8 Q92619 Q12948 P61020 3516 Q6PCE1 Q7L0Q8 2665 2664 O00273 Q9HBH0 P61026 Q8N103 P07996 P52564 O75815 P52565 Q12955 Q7L591 Q12952 Q66LE6 Q12959 51090 P52566 51094 Q9P107 P63211 10549 P20963 P16473 2676 O60333 P16471 P63218 P48039 4851 4854 Q9Y2Y0 928 Q92600 P54753 P50395 Q9NYF5 Q12965 P25391 P50391 P32927 Q05397 P54756 57646 29127 P20936 Q9UQB3 Q9UQB8 O75807 4867 4868 4869 5716 Q96JK9 P54764 P54762 P54760 4882 P48023 Q9BZF9 3551 O00299 3550 Q12979 P17302 5728 10524 P17301 P20941 Q8NEU8 2697 Q9ULZ3 948 Q9UHR4 Q6PCD5 Q9H1C4 O95477 Q9Y342 P28482 1385 O00220 O95470 Q9UHJ9 P51668 29108 P19525 5739 O60760 P07550 5733 Q9Y2T1 10971 7913 P08887 Q9UM47 P51671 O00238 P04049 Q96SB4 Q9NYA1 O14880 2243 P10721 29110 2237 79444 961 1388 1387 963 10981 P42771 Q9UM54 5747 22937 P51681 O00241 2253 P51686 Q9P0L2 22931 6609 P62330 2247 P15153 1399 2246 1398 2244 Q9BZK7 975 P61006 Q9Y2V2 976 Q92630 Q92636 Q92633 P51693 P29323 5770 P29320 P51692 P28472 O00255 P48061 Q9P0M2 Q99259 440193 P17342 5764 P29317 Q9ULV4 P61019 Q9Y2W1 P61018 26060 P06213 26959 Q15139 Q9Y6W6 P07900 Q9Y6W5 2274 990 Q96RU8 Q96RU2 2267 998 6622 10133 P27986 51009 P02686 P62753 Q04917 P49770 Q9UHG2 5310 Q7Z5H3 5311 5796 P51636 266747 2280 Q04900 Q8WTR2 Q96RT1 2277 10142 Q99638 10146 P02675 P01344 P01343 P49768 O00206 Q9Y6Y9 P48431 148022 Q00535 Q96S42 5798 6647 8829 8826 P01375 Q9Y336 P01374 4000 Q9Y2R2 5331 64343 5333 Q9UHI8 O00213 Q08499 O00212 O60716 P12004 Q96RR4 Q15172 O95071 3148 545 Q8WTP8 P40933 Q96EY1 6657 Q15173 5327 5328 7508 O43353 6672 Q969H4 8851 P07949 8852 P07948 Q96B97 Q12904 Q8WU90 3162 Q8IXI1 Q8IXI2 Q68EM7 552 553 Q99683 55884 5335 Q96AX2 5336 8844 Q15185 5337 5338 P05771 P26232 7531 7534 10580 7533 Q9UHC3 Q5T442 Q01831 Q99679 Q99677 P50749 P50748 3169 5347 Q96JH8 9702 P24046 7529 Q08462 5361 5362 5364 4035 Q5VWQ8 Q9UHD2 Q12929 Q9BUZ4 572 573 8882 5371 3190 Q60FE5 Q12933 P15559 P16885 Q8NER1 55432 Q99653 P51617 Q9NPJ6 8879 P62745 54106 8878 10564 P60568 10566 Q13478 Q13477 8892 Q15654 Q9UQP3 9744 6236 8412 9743 Q96FV9 595 Q8WZ60 29941 Q96BM9 116 Q8NFJ5 Q7Z6C1 P22694 Q13480 Q9HC97 Q13485 Q8WZ64 8408 P32019 Q13489 6242 O94910 Q13009 Q16512 4067 Q16513 Q15669 P35523 6238 P60983 6239 Q13490 Q14344 Q7Z6B7 Q13017 6256 P22681 Q9HBW0 23380 Q9UDY8 P23528 8428 142678 Q8NFH8 P00441 346562 8443 Q16539 4086 4087 4088 P22692 4089 Q15208 8445 Q16537 O43427 163126 Q86YT6 Q9Y3L3 9770 Q8N5F7 Q9Y3L5 Q02790 Q9NZ94 P21359 P31150 Q9NUP9 1808 8439 P24864 10087 6259 9784 P54257 O75469 64750 7124 P54252 P54253 23365 P14635 P10275 4092 P10276 4093 P35579 O75460 154 156 Q9NYQ6 Q96KC2 Q8WV28 Q16555 Q15223 Q13043 9311 7132 8463 7133 Q5JQC9 P36896 P36894 P35568 6282 166 23370 Q8NFM4 Q04941 Q02763 P01308 Q8NFM5 120892 Q9NQB0 Q99717 9306 P01303 Q96T37 9322 P02671 Q8TCU4 8473 O95813 P54274 P36402 8471 Q86V24 P36404 P36888 Q9NYS7 140458 Q9BRK4 9318 1837 P49715 O95819 P60953 Q9NQC7 64780 7153 64782 P39905 7157 P19086 7159 Q02750 P35548 140461 Q9NZ56 1848 54509 Q16581 Q13077 P53365 Q13873 O95835 P57729 O94988 O95833 10928 Q86Z02 O75420 57584 7161 2702 P42224 1852 1850 P23945 Q4KMG0 83871 O95837 P22612 P42229 1856 P42226 Q53QZ3 22926 10939 Q14738 P10644 Q01082 22920 9350 79363 P57735 P84095 P39059 Q96BD6 Q9UI95 7187 9367 7186 7189 7188 9368 P35916 Q13418 O43815 7185 P17252 P42680 P42681 O95859 O60383 P42684 O60381 Q86UE8 P21741 O15397 O95865 O43823 B2RTY4 4914 2735 27352 P19438 P68400 56288 2737 Q969Q1 2736 Q13432 58473 Q86YS6 P51148 Q7RTR2 P51149 Q9H244 P23508 P43119 1896 2742 2741 P42262 4920 P43115 P51157 P35968 Q13443 P51151 Q15628 O95405

P51153 P42261 P30273 P11532 57154 Q96BI3 58480 P51159 27330 Q9UQE7 P30281 57159 Q8WUH2 Q8WYP3 P23510 P21333 O14492 O14495 377630 59341 P84085 3611 1432 Q5XUX0 Q15642 Q13464 P01730 O15350 Q14314 P53355 P51178 O75832 O15354 Q14318 P53350 Q15648 27315 Q86Z14 3622 P84077 2773 O60353 Q9HC29 Q7L9L4 P24821 O14497 4952 Q15653 P21796

negative regulation of nitrogen compound metabolic process Q9Y2W7 O75820 2672 Q9UBB5 Q9NS86 P51587 O00267 124790 10657 3516 2304 P84022 283248 10772 Q8N3U4 P27348 5933 25913 Q9NS91 Q9UBC3 Q9Y2X9 Q92729 Q12837 54361 Q8NB12 4851 4734 Q96EB6 5705 O75925 Q9H2G4 3660 Q9NYF8 Q9NRY4 Q9BYE7 Q99471 6927 P13056 83933 811 Q9UJU2 O15534 57649 Q92833 6926 Q6IT96 P06401 P26358 4869 P06400 5716 Q9UIS9 Q9NZI7 P48382 Q9NQX0 P31946 5970 Q92949 P21917 Q9NRZ9 P56705 P48380 O95343 9099 10524 P20823 1487 P19838 P62263 P31949 3665 64061 10765 Q9Y468 Q8IZ40 4771 Q12986 Q9P0J0 O14627 6829 10614 P21964 1499 Q86VE0 O14981 Q8N2W9 10971 6945 5978 6827 O14503 Q92769 Q8WXF0 5991 93986 5993 Q07666 Q14938 Q15906 P10600 P19544 O95365 O00358 Q06330 10865 6839 1027 P08651 P17480 O14753 604 5987 Q9NYB0 4790 22938 5883 O00488 P38398 57326 2247 58533 Q9BZK7 6601 Q92993 857 6605 Q8N488 P51693 P10826 P10827 2023 O00255 Q6KC79 P19883 O00257 1050 P40692 Q99496 Q9UMX1 4799 P40337 50943 Q9UGL1 7704 Q92985 P23396 O96004 O43159 P04637 Q9NVW2 P14316 25988 P52952 990 23560 3476 Q15022 79813 4204 639 10014 10138 Q6UWV6 Q9UQ80 135295 Q9BT49 4221 5796 P16989 P57059 641 84159 P33076 51132 Q99750 P48436 8932 Q99638 7727 2290 23429 O43251 23309 P48552 P48431 6774 P13686 Q99623 Q99741 Q96S42 P35240 55869 51147 P01137 P01375 Q86X95 P51531 4361 6660 5451 8841 Q9UHI6 P35222 Q9NUX5 2063 P51532 23435 Q96AQ6 P35227 11218 Q96ST3 Q9NS37 P17813 P35232 3148 P10070 Q15170 545 P10071 3146 11331 668 6657 5569 Q96T58 Q96MH2 P23497 O43474 6670 P06733 P26583 O43593 6672 Q9Y3M2 23409 P25490 Q969H0 25822 25942 Q12906 Q8NFW5 3281 Q9BZE0 672 Q9BZE4 O75190 Q99684 6304 8726 P40763 4261 Q969G3 8864 Q08117 23411 23414 O75182 P37231 P14373 Q00987 3169 5467 5468 7529 7528 10468 P27797 64375 4152 5245 P56177 O60907 22893 3066 3065 Q9UIF9 P51843 3182 10438 333 P51608 Q9H7L9 6207 5119 Q01826 P62854 9839 O43463 Q9BT67 5371 3070 Q13901 9612 23512 P52701 580 Q96QT6 P58012 P28749 7428 10320 6595 6231 6597 8535 P10599 6591 Q9Y4K3 3084 5017 Q13485 Q13363 Q13127 4188 5036 O75496 O75376 O75494 P13631 3096 Q02447 O75381 23051 51773 P46531 7329 Q96RL1 Q15554 Q15796 Q9HAU4 Q15672 Q9UQR1 7341 8553 6496 6256 6498 Q96IZ0 P12757 Q8N6I1 P31273 Q9UNL4 P12755 23028 O75360 4193 Q2M1K9 84108 P46527 116113 51548 51547 54815 Q13263 Q9BSI4 Q13148 9774 4087 8204 4088 4089 Q9BY41 P55055 163126 Q9Y618 P10588 P10589 93166 Q04724 P20264 11176 Q9NPF5 Q15697 51317 P43246 55915 53615 P43489 Q13033 9421 P54259 9541 5187 O75469 64750 7124 11142 O15164 P54132 P54253 2909 O75461 Q9UQL6 4092 P10276 Q9Y6K1 Q02535 Q03989 51564 Q15583 Q99728 55922 Q99729 8328 Q15466 55806 Q99966 54 5074 5195 Q9NRC8 6045 7013 7376 55929 Q9HAZ2 7014 8467 O15055 9314 O43524 23135 23378 7490 7251 6282 1822 166 84232 Q9NQB0 Q8WW38 2917 Q9UBP4 Q96T37 P61925 7023 P49840 P54274 P14859 O43612 P15822 23468 7141 Q9H063 Q96T23 Q9UNH7 P49959 51341 Q01664 Q00577 51588 Q13185 P49711 10155 P49715 P09038 6188 O75437 7157 O43623 25776 64426 138151 2931 Q99816 9208 Q5VTD9 Q96RE7 P29374 Q01094 Q9H0M0 P41134 Q1PSW9 P62195 27122 P63165 9219 O15119 1616 P45973 Q9NSA3 P56524 O94992 Q969S8 Q8IY57 Q02297 7291 O75530 55294 7295 P55316 Q96BD5 P46934 1869 Q9UER7 2956 Q14865 7189 7067 92129 Q8WYK2 9126 Q13535 P19793 P29590 7181 Q03014 Q7Z2Q5 84619 Q9H6W3 Q86UE4 O15379 O60264 Q9NSC2 O15151 O95983 Q13422 O94776 Q13547 P78318 O43829 Q8TBE0 84733 Q04206 Q9UH92 Q92786 Q9Y2B9 2737 2736 7088 O75626 Q8WUI4 P41182 P62277 Q9UKY1 Q9H9B1 51720 Q14894 150094 Q9NP71 1789 1788

Q05516 1786 58487 Q9UQE7 P41162 3720 3609 Q13573 Q14781 P23510 P09429 O75604 Q9H2S9 29966 Q02363 P32320 P18146 P35711 Q9BQA5 Q9Y6E7 1312 O60341 P09430 5914 O75953 O75832 Q9BZS1 Q13227 P55771 Q86YP4 63976 Q96BF6 P63279 Q8N5A5 P63272 Q9HCS4 O15105 Q14683 5925

macromolecular complex subunit organization P25054 P06899 23607 25909 P49023 P54619 1460 Q9UBB5 O00268 P52434 9185 Q9BYG3 4728 Q8IYN9 P84022 Q9BVA1 10772 O14788 6901 4725 P27105 Q9H0D6 Q9H4L7 P30876 25915 Q8IZP0 P16104 2316 2 5829 Q8IUE6 Q96QZ7 P14174 P16471 Q92845 10421 Q9UKV5 P10809 O75925 Q9H2G4 Q96NT1 Q12846 Q9NQW6 Q05397 54496 3416 1355 P32929 O15533 811 5830 4869 5716 P31946 Q9H814 1374 Q6P2C8 Q9NRZ9 1491 P54646 Q676U5 P17302 55355 P16333 2697 P62263 6810 P04350 Q6NXT2 3667 64061 6814 5604 P04233 829 3308 P07305 P30566 P08758 O95477 1022 3320 1020 114548 P52756 O00468 83706 P16389 P15056 1499 830 P07550 P62316 P04040 P09972 P62318 O14744 P62314 O14745 P07437 Q12874 P30556 Q9Y478 Q9UHK0 Q12872 Q9Y239 3692 Q8WXF0 2243 1153 O00233 Q9BUB7 P30793 3329 Q9BUB1 1025 841 1387 Q92522 6834 847 5987 Q8N257 6714 5747 P04179 P07766 Q9UGJ1 266812 6850 4673 Q06203 Q9NYB9 Q9H2K0 Q8TBB1 Q9NS69 P42768 P61244 284119 P84243 Q71DI3 2244 O14640 Q92994 857 6605 5879 O43182 Q9UNY4 5518 P51575 Q9Y375 Q13509 Q5QNW6 Q13748 Q03164 10844 Q9P0M6 440193 P40692 6733 Q92621 Q6FI13 4676 989 4678 5888 P09601 P40337 P04275 Q9UKT4 Q9BZJ0 Q9Y2W1 P04156 P06213 4690 P07900 6872 P04637 6631 2033 P14678 O95391 P53803 P50416 P14555 P11047 P62993 203068 1058 Q8WWY3 4686 999 55723 5536 Q15389 6505 P62753 P01112 P62875 P25445 135295 6883 5430 10383 Q8N4N8 5432 P51636 Q99880 O95163 P07919 3010 Q6FGD7 2040 Q9BU61 P50542 2280 Q99877 3009 3008 O60610 641 3007 3006 402 O43290 6633 4214 8932 6878 7846 Q15393 Q96S59 P02675 6637 255626 8936 Q99879 Q09472 6892 5440 5441 5563 Q7Z7K6 3024 3265 Q9UKN8 891 773 Q9H4B7 Q00535 3014 P01019 5434 Q14197 6646 P01137 Q9UPY3 Q3ZAQ7 O00217 Q9Y5X9 O43264 Q9UMS4 Q9Y697 5213 5576 Q658W2 3396 Q9UHI6 P35222 Q08257 P14784 221613 Q96A08 Q99733 11218 Q93079 3028 Q00403 3148 Q96RR1 Q93077 P61457 P23258 P62308 P61218 P24588 P62304 P26583 O43593 6430 Q8N307 O00505 Q6FGG2 6311 6431 P50613 P51946 3162 Q8WUY8 673 Q96KK5 3159 6426 P55072 Q9Y512 P49450 5471 Q969G3 64127 Q9ULK4 O60814 5590 29079 P14373 P18846 Q00987 P50748 324 Q96QV6 54443 Q15070 9702 329 7529 P27797 4150 P50750 4154 25929 P49321 Q9UHD8 Q12809 O00762 25923 Q9NU22 330 Q5TAL4 Q96P20 Q99661 56993 7415 7536 4149 P62851 5371 3070 Q86UX7 6341 Q60FE5 Q12933 P07814 Q9UIG0 P16403 P16402 25813 P16401 10569 466 P51617 O14817 P17858 P52948 Q9NPJ6 7428 9967 Q01813 8772 10093 Q14686 4297 Q14209 Q8TEW0 Q9BY76 11065 Q16625 Q9H0U9 Q6NZI2 P13984 6470 3084 4171 P13861 230 355 P07196 23165 Q8NCD3 Q7Z6C1 P83731 5018 Q13243 P49815 Q13485 Q8WUM0 P47755 O94906 Q13242 Q13247 Q16637 Q13489 P03950 O00716 9997 Q9BTM1 P62805 8301 Q15428 Q16635 Q16514 P62807 19 79084 O75494 O75381 Q9NVP2 51773 Q9NRH3 11198 Q13492 Q9BSJ2 Q9H9E3 Q13490 Q16401 P60981 Q15796 84790 Q15797 6498 P12757 9404 Q8N6I1 O75367 P12755 Q9NWS0 4193 Q9UBU3 Q6DD88 P23527 Q9UDY8 Q01105 P09234 P17096 P83876 Q8NFH8 Q9HC77 Q8WZ42 Q9BQE3 4086 8683 4087 4088 Q9BY44 4089 Q16777 Q16778 P18074 Q86XR8 P0C0S5 163126 P29084 P04908 P68371 P36954 P34896 Q02790 Q9NVR5 267 P52907 P0C0S8 P68366 Q7L7L0 7106 P08034 P43005 P62942 Q15459 P78347 10291 Q13158 Q8N5M1 8458 8576 O15164 7248 O75347 P54132 P14635 O43639 154 Q00597 158 84365 Q02539 55806 P49736 Q14011 O15294 7132 5195 10181 7013 Q09161 8348 9555 8467 P36776 Q86WV8 P35568 Q02763 11034 7249 8338 P49848 Q9NRD5 Q99714 9685 P02671 8473 P00492 Q8TCU6 7023 10392 Q5SSJ5 10152 P54274 A1XKG3 Q9Y5K6 P58876 P36404 7141 Q08188 Q99700 Q96T23 51340 222484 P68431 Q01664 Q9NVM4 Q16695 56916 P49711 P08069 Q14152 8349 9439 Q16698 347733 11004 7277 7157 P22492 P14868 Q02750 5092 26747 138151

Q15370 Q6PID4 55835 1729 P78362 Q9BWT7 10048 Q14289 Q13077 9223 9463 Q96RE7 Q7KZN9 P12931 9341 6194 7283 26271 P20671 Q6NVY8 Q92574 Q9UBK2 P54105 Q13885 22803 Q8IX30 Q02297 9351 55054 84708 22920 55172 7052 3925 P24928 Q9BUF5 1742 Q9HD26 P05062 Q4LE28 2959 7186 P52298 Q15836 O75643 9368 10907 57120 P29590 Q13418 P55209 O75880 84617 7185 P56537 9361 O43819 Q92793 2961 2960 1870 P21980 O60264 P23921 2969 Q92558 O15151 8289 P34932 9495 P19438 P43405 56288 2972 Q07955 P53041 P05164 50628 O95996 9388 O95999 P35610 O00186 P41181 8290 P30047 1653 P63000 Q92499 Q9BUK6 Q9NVC6 O95400 Q2NL82 O95405 P35609 P14923 O43920 P19387 P30153 P19388 27339 3728 1666 P21579 Q05513 26121 Q7Z2W7 Q8WYP5 375790 1429 Q9Y5B9 P21333 P33778 Q9NVD7 P12830 Q8TEQ6 P19474 P10412 P32320 3737 Q00059 2644 2643 2885 3611 Q14790 P09430 O15212 Q9BQA1 O75716 Q14677 O75832 Q15648 Q15526 9054 Q96BF6 P67870 Q9HC29 O14497 Q71U36 902 P08237 Q13233 Q13112 P21675 1207

chromatin organization O95677 Q9H0E3 P06899 Q9Y265 Q12824 22992 8091 O00267 Q9P2R6 Q96JM7 5931 5933 Q9H4L7 Q9UBC3 Q5VTR2 Q8NB12 P16104 1107 57634 79885 Q8IUE6 10661 Q96EB6 1108 Q7Z727 O95696 Q9H2G4 Q96NT1 O00167 54496 6927 83933 Q92830 Q92833 Q6IT96 P26358 4869 Q92831 P06400 Q9C005 Q9NQX0 Q9NRZ9 55355 10524 P20823 55352 O60674 Q6NXT2 64061 10765 P07305 Q9Y468 Q96MX6 Q8TDI0 Q06587 6829 79577 10856 Q6FHQ0 Q08999 Q9Y230 O14744 6827 Q12873 Q92769 P28370 Q15906 O95361 Q8WYH8 6839 10626 1387 Q92522 Q8N257 Q15910 266812 4673 79595 Q8NA19 Q9NR48 P84243 Q71DI3 Q96KQ7 Q9BZK7 6601 Q92993 6605 10951 O43189 Q14839 Q5QNW6 O00255 Q8NEZ4 O00257 Q03164 Q9P0M6 2139 57332 80335 Q99496 Q6FI13 P04150 4676 4678 50943 Q9UGL1 Q9UKL0 O43159 6871 2033 23326 23569 Q8WTS6 11107 1058 2146 Q15022 79813 10013 84289 55723 10014 Q9UM07 6883 4221 O96019 23210 Q99880 3010 Q99877 3009 3008 3007 3006 648 408 O43293 P49407 56946 255626 Q99879 O96028 Q09472 P48431 Q7Z7K6 3024 3014 55869 51147 29086 Q8N8U2 Q15047 P51531 8841 O00213 P51532 3151 221613 P35227 Q96A08 P35226 Q99733 124359 Q93079 P35232 3148 Q93077 Q15291 10363 10362 6657 2070 P26583 5585 Q9Y2K7 23523 Q96KK5 3159 Q68DK7 6304 8726 5578 5579 P05771 8607 P49450 Q969G3 O75177 23411 O60814 3171 Q9NRL2 Q9NWV8 Q96QV6 3169 79723 Q92922 Q92800 Q9H5I1 Q86U86 P49321 5245 22893 3066 Q9UMN6 Q9UPT9 3065 Q9UIF9 O75164 Q9H7L9 10432 Q01826 Q99549 O43463 3070 Q9UIG0 P16403 23512 P16402 P16401 221656 80854 P28749 10320 6595 6597 4297 8535 6599 6598 Q9BZ95 Q9H0U9 4171 473 O75150 Q9H160 Q8NCD3 Q7Z6C1 Q15424 7334 Q16512 Q9BTM1 P62805 Q16514 Q15306 P62807 Q9ULG1 O75376 Q8TF76 Q7LBC6 Q9NVP2 51773 9869 51412 11198 Q96RL1 Q15554 9646 10075 9643 7468 Q9UPP1 6015 P54198 O75367 Q9UNL4 Q6UXN9 23028 51780 P23527 Q01105 P17096 O94805 Q9UBU8 P61964 Q6PD62 Q16777 O75478 Q16778 Q9BY41 P0C0S5 93166 P04908 P0C0S8 Q7L7L0 11177 11176 Q9NPF5 51317 53615 9425 O75582 Q9BW71 Q9UQL6 Q9Y6K1 51564 2908 8328 Q02539 P49736 6045 7013 55929 8348 7014 9555 8467 23133 23135 23378 23492 8338 O15047 8110 8479 Q5SSJ5 P54274 O95931 23466 P58876 23468 9682 6294 7141 Q96T23 P68431 Q9NVM4 Q96PK6 Q16695 56916 Q13185 P49711 8349 Q16576 54617 54737 Q9NPA8 8242 P22492 Q09028 64426 54623 Q5VTD9 Q15014 86 Q6P1J9 Q96L91 P29375 P29374 26038 27005 Q7Z589 26039 1613 10933 Q9Y4C1 P20671 Q4LE39 9219 P45973 P56524 Q9P0W2 Q969S8 Q86WJ1 O75530 7290 Q9H3R0 P55318 Q96BD5 83746 Q92560 Q52LR7 Q4LE28 Q9UBL3 O75528 P83916 P55209 Q9H6W3 Q92793 Q96GM5 P17252 Q7Z6Z7 O15379 O95619 O60264 Q969R5 Q86UE8 8289 O95983 Q13422 O94776 Q13547 O95503 10919 9252 Q9Y6J0 Q8NB78 Q8TBE0 84733 Q92784 P41229 Q9UBN7 Q14527 Q8WUI4 Q6NYC1 Q9NP66 8290 23081 Q9UK80 Q9H9B1 51720 Q9HC52 Q9NQZ2 Q9NQ92 Q8N7H5 1789 1788 P46736 1786 3720 Q9Y5B9 Q14781 P33778 339287 P10412 P61088 23186 84661 51742 O60341

P09430 11091 30827 Q9BZS1 Q9P0U4 5927 5928 5929 P63272 3622 Q9HCS4 O14497 Q13112 Q15652 Q13351 5925

nitrogen compound metabolic process O75947 8099 Q92979 Q9Y265 O75940 Q12824 O95551 10657 Q9BYG3 2305 2304 1454 1453 Q00839 P05455 4968 Q9H0D6 O75936 Q9H4L7 O75934 P30876 P52209 Q06136 P19623 P16104 10667 P42858 Q9Y285 O75937 P10809 Q96EB6 136319 P27694 Q9UGN5 Q9UKV8 P27695 3661 4507 1478 Q9Y295 55109 5832 Q9UKW6 3659 P26358 10632 P54886 Q99583 Q12857 P21917 1491 55110 Q99575 55119 P26368 10642 Q9UL18 Q9Y223 Q9UGH3 Q8IX01 1022 Q9NSU2 P51787 27043 1017 2348 79577 P21964 P54819 Q9Y230 O75909 5859 Q12874 Q12872 Q8WXF0 Q9H0H0 Q15904 O00358 1029 P42898 O14519 10625 57794 1025 Q5C9Z4 P20648 Q9BUB4 P05023 P07686 P05026 Q9UGJ0 Q12888 5883 P38398 Q9BQ90 Q96MU7 55149 Q9Y250 Q92994 Q92993 6723 4548 P10827 P10828 5892 6741 5411 P31749 1050 55152 27032 10606 253980 P40692 27037 55159 O60870 10607 6733 P04150 5886 5887 5888 O96004 8930 10250 5422 P50897 23569 11108 Q8WWY3 P62877 8924 Q15020 Q15024 P62875 6749 Q15029 5430 5432 Q9UKM9 23576 P35244 P35249 Q03933 641 P35251 196441 5424 8939 P49407 P60228 5440 5441 6772 5442 Q9UKN5 O96020 Q9UKN8 346171 3263 Q66K89 5434 51147 Q15046 P23246 P50440 Q9UL03 Q9Y215 Q86X95 Q9Y697 A7MD48 Q53H96 O14979 P17812 P35232 Q00403 O95190 668 Q15054 6776 4116 P57081 O00746 6311 245972 Q9NX62 Q9Y5Q8 23524 672 P15692 Q96A72 P05413 5471 Q9Y5R4 23536 Q6PJP8 Q9NWV8 688 689 P49448 207 P62847 P62841 4150 Q9BQ15 4152 4154 P13489 Q9NWW5 Q9Y5S9 P17844 211 P13010 Q99543 5478 4149 P62857 P56182 O43463 Q9BQ04 23517 Q92905 Q9UKK9 O75031 Q92900 P56192 P49427 4172 4173 4174 Q14686 4176 P37837 Q14209 Q16864 P55265 Q9UPN6 Q6NZI2 P12532 4171 Q14690 Q14694 Q8IWZ8 5034 8543 5036 79084 60489 Q9NPD3 Q13370 Q96RL1 P46777 Q15554 Q9HAU5 P24522 P35638 P61968 11041 P56134 8555 P32121 4191 Q96IZ7 51540 P00568 51547 8563 Q9BYU1 9410 P36957 P62826 11051 Q8IWX8 O43542 8565 P36954 Q9NPF5 P61978 P43246 9421 Q14249 P78347 O75106 Q9Y6K5 P36542 26512 271 Q9Y6K1 Q93063 Q93062 Q04837 Q96RQ3 23481 51567 10181 55929 11030 P22392 O75575 26523 23016 5071 Q93050 Q9NY12 118460 P49848 P02790 O75569 23463 P48507 P24539 51585 Q9BQI3 9439 2805 Q6I9Y2 Q9HB90 26986 Q01780 P37840 5092 5093 Q96EU6 O43505 51593 Q9H9G7 P78362 10166 11017 9465 P53004 9463 Q6P1J9 P29375 P29374 P29372 Q7Z589 Q05329 27000 2820 Q13761 9477 Q13769 Q9H0L4 O75531 1503 P24928 79005 1982 Q9Y6H3 Q9Y6H5 P06276 Q6P1K2 Q5D0E6 P55209 79035 55066 1994 79039 P00966 Q8TBF4 P04066 P54368 P78316 O95989 Q8N8D1 3704 Q9Y2B5 Q07955 P53041 P07101 P07585 50628 P52597 Q13315 O95997 P00505 P20594 84881 Q8NEC7 Q14653 Q9H0P0 P46736 Q9H9A7 2875 26121 P61077 Q9BUJ2 P22303 5901 9045 Q9NP81 P12956 O75964 P12955 Q00059 Q9BQA5 P61088 Q96AE4 P43694 Q9BQA1 O95544 Q14676 Q16854 9054 P42357 5927 5928 P00519 P63272 23076 P24941 P43681 84893 51514 Q14684 Q14683 Q5FWF5 P10914 O95677 285672 Q92858 P67809 399687 9061 P30520 Q9P2R7 Q9UBB9 P13196 5939 1576 P16220 10772 5931 P49137 Q9UK45 5935 P0CG13 Q9NS91 Q9UBC3 Q07343 9077 55696 P18887 55215 P30519 10785 Q9NWB1 Q9NRY2 Q9NRY4 Q8TED0 O60547 P18858 O60543 P18859 1111 Q92830 P03372 P49591 P46087 Q9H814 Q9GZX7 9093 Q9NRZ9 P49590 Q99459 3304 P17540 3784 P49588 P07741 Q92820 P30566 133584 5981 5983 O95232 P52756 6829 27165 Q9P2N5 1137 Q6FHQ0 P61221 4643 5976 27161 6827 Q10570 Q13601 1153 Q14938 O00472 1152 O95243 Q6PIW4 Q9NS56 3329 6839 P08651 Q6XQN6 O43175 O95257 4670 137964 4673 Q9Y4Y9 O60508 1161 O60506 O60502 80222 10714 P61244 O14641 5511 O14640 5514 Q9Y4Z0 Q92878 5515 Q9UNY4 P08621 5518 Q13620 P30536 P52788 10725 10724 P61254 10728 157570 4677 4678 P06454 O43159 6872 6871 P04637 Q5VYS8 2034 P16615 Q01650 4686 Q16236 5536 Q15389 P62995 Q9GZR7 Q96LI5 Q9C0K0 P25100 6883 P48201 4221 23210 P12268 O76074 Q96Q15 51251

84159 6878 Q15393 O43172 56946 P01100 Q8N726 P14416 O95299 P11388 P11387 O60942 773 Q53X93 5557 P61201 P01137 5558 P01133 O43143 6421 11218 Q9NS37 P09086 9924 57819 P61218 2070 Q5TKA1 6430 6432 6431 Q3SY69 6434 4255 7764 22794 P15328 Q96PU8 Q8IZL8 314 Q02930 8602 6427 6426 8607 Q8N302 2081 23658 5591 P46063 P24298 5111 23650 O76021 Q99437 P18848 328 25804 2091 P22087 Q8TA86 Q8N2M8 Q9UNP9 2099 P12277 P50993 Q9NW64 Q8NCN5 333 55660 56994 91746 Q9UNQ2 A2RTX5 4287 P39748 O00411 P52701 Q9NVV4 P15313 Q08211 221656 79622 P58012 9967 6472 5141 5142 Q14566 4297 8654 Q9UKD1 P30085 P13984 6470 4291 351 353 Q9UBS4 P09012 5139 P62913 9978 Q13243 Q13242 Q13247 P20290 O43687 7334 16 Q15428 7336 Q15427 26205 P13995 80198 367 Q9UBT6 51654 11198 7341 Q15434 Q14103 9525 P62937 23144 Q9UKF6 Q9Y606 P49916 P49915 85437 P17096 5159 11165 11168 Q16774 Q9UBU8 P83876 P49914 P21589 34 O43660 11171 8683 9533 7358 Q9Y619 P29084 P34896 P11310 Q96HW7 Q9UBV7 11176 P23771 Q15459 5184 10291 O15160 9541 O15164 2909 P12694 25788 P27708 Q8NI36 2908 Q13287 Q9NSE4 Q14134 Q13286 Q14137 5198 Q09161 Q9Y5J1 7372 Q86X76 Q8NI27 11157 7389 6059 6050 11129 Q99941 P49959 P50914 51222 56915 56916 2926 11128 P62906 116138 P14868 P14866 8241 221264 10286 10285 Q15014 2935 P78424 9100 Q7KZN9 Q96QC0 Q14966 P52272 Q12788 92105 Q15819 P33992 P62081 P33993 P33991 Q96DI7 84967 P07237 26270 Q9UBK2 9114 P55795 Q14978 Q8WWH4 O95602 Q86WJ1 Q96I24 P43304 P67775 Q6IN84 84950 P05062 P52292 Q52LR7 P05067 2959 P08579 2956 Q9UBL3 P52298 P29474 9128 P29475 9129 O75643 9126 O94766 Q96I15 1634 80145 283989 27102 2961 2960 P04183 Q8TAD8 Q9BTC0 2969 P09874 29777 1642 2972 57379 P09884 134430 1647 P30049 Q15853 Q9UJV9 Q9BY32 Q14527 1660 Q6NYC1 P30043 P53567 P30047 2987 1655 P22415 1653 Q03468 Q92499 1659 3835 P47712 O95644 51606 64919 1665 Q9BXP5 P17081 2517 Q9Y5B9 Q9Y5B8 P09429 Q13216 Q8TEQ6 P55769 P35711 4705 1676 2521 Q6P4R8 P09430 51629 P08107 Q9P2Y4 Q14558 O95661 4719 Q96HR8 2531 Q14562 1207 P51587 81605 P52434 O00148 P17676 Q8TDD1 2547 P84022 Q9H2H8 P28335 25913 P28330 P08729 P28331 Q8NFZ0 O60216 80755 54487 P49247 10421 64282 P62249 Q9H2G2 Q9BRX9 3420 2572 O00167 2571 3417 54496 6927 6929 Q8N684 P26599 Q5TAP6 3419 P31942 Q9UIS9 P31943 P08708 P99999 P20823 P19838 P62263 3421 P11926 Q13951 4772 Q14807 2593 P50213 Q8WVC6 P39019 Q5T160 10856 Q6PI48 80324 Q9BWG6 833 O14744 P51553 4780 P09619 5631 Q07666 80308 Q8WVB6 O95363 Q06330 Q7LGA3 27297 167227 4775 O14757 Q92766 P09622 4790 P28340 2132 Q9NR48 Q9BWE0 80347 Q96CA5 P11908 5636 22826 22827 Q9H6R4 22828 Q8NC51 Q9NQT4 Q9NR55 Q9NQT5 Q9HCK5 2139 192669 865 4799 P09601 P06576 11340 Q86V81 10492 O00541 O95391 3481 25988 51362 Q96T88 3479 P25205 7832 84289 P23025 135295 P51991 4343 P51513 Q96T76 O14717 O43290 Q7Z6J9 Q9BRP8 P49643 O43294 408 409 O43252 O43251 P49642 Q9BW92 O00567 Q9BW91 Q9NUW8 O14727 126789 Q99741 Q96T60 3014 Q8IYD8 Q9UN42 29086 10476 P61326 Q15287 Q9UMS4 4361 84292 3035 114799 Q9NUX5 Q96ST3 11338 3028 55759 P48775 10488 84296 P26583 8732 7884 P50613 P51946 23764 25942 Q8WUY8 P60891 435 P31939 10450 P55072 3054 29079 Q9HCE1 Q96SZ6 445 55775 5226 Q02809 P01266 25929 7422 Q9BRT9 5245 P52815 22894 Q9UIF9 22897 79753 10438 Q5TAL4 7415 10432 8761 3070 Q13901 Q5VTL8 P07814 P49674 Q9UIG0 Q96C86 Q9HCC0 3074 468 55312 55796 56647 O43791 53938 Q16629 6598 57062 Q01130 472 Q96GX9 474 476 477 P33240 478 Q16630 P43034 Q8WW01 Q9C0C2 P38919 Q13126 Q16637 3091 Q16633 P13637 6125 O75376 P58546 481 483 Q504Q3 Q9NW13 488 51773 24144 6117 P22102 6119 6118 Q15796 Q6YP21 Q16649 Q15797 9643 Q9UNL4 O43776 490 O75362 29803 495 498 Q01105 P56385 P56381 54815 Q8WVM0 Q9BSI4 Q13148 P35869 9656 P43490 P18074 P18077 O75351 144983 28960 Q9P2E3 Q13151 Q16665 Q49AN0 51319 P54136 O15287 P54132 P35453 P09172 Q8IZD4 Q14494 Q14493 Q14498 51329 Q14011 Q9NRC8 7013 7014 P46100 P36776 7490

P13674 Q9NRD1 11277 Q9NRD5 Q86W42 P98175 P98179 Q02880 25885 51340 54853 Q9NVM4 P02549 Q96PK6 11243 Q14032 55703 P47897 P00367 Q9GZL7 1716 Q15365 O75319 P21283 Q08170 P21281 Q09028 25896 6182 O75792 26747 Q15370 P27815 P48730 1728 Q99814 P98194 Q9NRF9 P78545 P54577 9221 O95714 P78540 57461 P78549 6194 10813 Q9H6Y2 P41252 P62195 P41250 P08684 O15234 1738 P05177 65083 1736 Q13523 P54105 9232 22803 Q9P1Y6 O75771 1743 27257 Q9NVH2 P42574 O60293 Q14865 7068 7067 Q5TAX3 Q15714 Q13535 Q9H307 Q15717 Q8IZ69 P41235 Q9NVI1 O60264 O60260 O15270 9255 Q32P51 P34931 83759 Q9BWH6 1763 29896 29894 Q9UET6 3945 7083 9261 4809 51720 Q8WVK2 P08243 Q14894 4800 51726 2626 51727 Q9NVC6 51729 Q9P1U0 Q13569 P14920 P19387 P30153 P19388 P11413 93100 1789 1788 4817 1786 O60256 57038 Q13573 3980 P32320 P78527 P32322 2644 1312 2643 P17174 Q04656 Q14432 3978 Q9Y4A8 51747 Q9P287 9296 Q63HQ2 4839 2651 4830 O60231 Q9HCS7 902 4833 Q13112 Q13595 P21675 Q969L4 Q12948 O00268 Q06265 O00267 3516 10535 O00273 P60174 Q9ULW3 4841 Q9Y2X3 Q6NZY4 Q92614 P48047 4860 Q9NQ55 51096 P21810 Q9UI12 5705 Q9ULX9 Q66PJ3 Q12965 Q9Y2Y8 Q12968 29128 O60306 57646 1355 P12081 P32929 Q9Y2Z2 Q9Y2Z4 Q12972 4882 Q6P2C8 Q13829 O60318 10524 10528 Q9UI32 10521 5725 10523 Q6PCD5 O95478 P28482 Q12986 1385 O95470 57661 29102 P62316 P84103 P62318 5733 56339 P62312 58517 P62314 Q9Y2T7 Q96SB4 Q9UHK0 Q96SB3 Q9NYA1 Q5JTZ9 Q06210 P19544 P30793 Q8IY17 Q96SB8 Q8WYH8 10987 2237 79444 56342 1388 P54709 P42771 Q9NYB0 22934 P30304 P30307 22938 Q06203 Q9H9Y6 Q56NI9 284119 5757 161823 P51693 P27144 Q13868 P51692 O00255 P54727 P54725 Q9P0M2 Q03164 55035 Q9BZJ4 Q99259 57697 Q9Y2W2 988 Q92620 Q6DHV7 Q9BZJ0 Q9Y2W1 509 O43395 6631 Q9BV90 P14678 P53803 O60725 P15531 990 P11172 Q96RU2 54512 995 513 997 514 8803 6622 515 6625 P61421 6627 518 P62753 Q9UQ80 54517 P07910 220988 Q9UM07 51010 521 522 523 23451 51013 6633 Q9Y6Y0 526 8812 528 10147 Q99638 6637 P01344 8815 P01343 Q86U44 Q86U42 P26640 O95059 5321 Q9NU63 O60701 P26639 533 535 10594 P49756 537 6647 539 Q9UPY3 23438 23439 6660 P40938 P40937 Q9Y333 Q9UHI6 23435 Q96B26 P12004 Q99611 3149 3148 Q96RR1 545 546 3146 P61457 8836 8833 Q96EY1 P62308 Q9ULR0 O43390 P62304 7508 Q9UQ35 7520 Q9UPR3 Q12904 23400 3162 P17752 23404 3159 Q15185 7518 7517 5351 Q9Y2L1 6201 5356 3178 Q9ULK4 23411 Q969G6 Q01831 Q6UVY6 Q08462 65220 3181 P50750 P49321 3189 3188 3187 3185 3184 O14802 51067 Q8N8Y2 7536 9716 Q9H5H4 3192 3191 P04424 8880 P27540 3190 9733 5378 P15559 Q969E8 P51610 580 10569 51074 P52948 Q9NPJ6 8879 79869 54107 6217 Q9UPV0 Q8N9Q2 P15927 O43432 P15923 57187 29947 Q96FV9 593 6229 Q8IU60 O94906 5393 P03950 5394 8424 O00716 Q16514 O75494 54921 O94913 6239 Q76FK4 Q16526 P31153 29922 23381 P25705 115024 Q9NV06 P42285 O94925 P09234 P22674 Q16531 54931 54938 51426 Q14353 P00441 51428 4086 10081 4087 8446 4088 P22692 O75478 9775 Q86UL3 O75475 P12883 142 1808 P20020 Q8IXZ2 9785 8458 P54252 P40926 P54253 7126 P38606 8450 P10275 O75460 4094 Q00597 158 Q8IYB3 Q99728 8449 84365 Q16555 P49736 8467 O43889 23378 Q9Y3F4 Q8NFM5 Q01433 Q9BRL6 Q13057 Q15233 Q99714 P00491 Q96FX7 P00492 P54277 P54274 O75444 7141 P11586 Q99700 Q96T23 Q00577 88745 Q99708 P22234 9319 P49711 Q16576 Q99707 9318 P49716 P49715 Q49A26 7153 7155 7158 Q9BVS5 Q08050 P39905 7157 7150 1841 92345 Q13873 7167 22913 Q7L0Y3 10929 7161 P18124 O43837 1854 P42224 10933 Q9Y3A5 Q9HC16 P63165 P09651 Q969T7 P42229 1856 Q8WUD6 22928 Q13887 O75891 P22626 Q9Y3B4 56267 10949 Q08945 Q9UHX1 Q9Y3B2 P09661 10940 Q969S2 10907 O43812 P11940 O75400 26015 10908 286826 26019 9360 9361 1877 P00846 4907 1870 Q9UHY7 P23921 O95861 P53396 Q13427 O75874 Q13426 O95865 Q8NAV1 Q8NB78 10921 1400 2730 P06132 1408 1407 4913 Q13435 P33316 Q9NYH9 2744 58478 Q8WYQ5 Q8NFF5 P43115 2747 Q8IY92 O95400 Q8WZ19 Q08J23 Q96G21 27339 O43809 Q8N0Z6 58487 Q9UQE7 Q8N0Z8 27332 26002 P23511 Q6P2Q9

Q15631 Q8IY81 P84090 4947 3615 84305 3614 Q5RKV6 2762 57169 Q9HC36 4946 P54687 O15350 Q15643 Q15648 Q86YP4 Q9BVJ6 O15347 4953 Q15653

protein amino acid phosphorylation 8099 65125 O14672 P54619 Q9NQU5 1459 1457 Q8NB16 Q8IYN9 O60566 55561 Q8IVH8 P16220 1454 1453 P49137 Q6IQ55 P07996 P52564 O15530 P27361 Q8IZP0 65018 P53779 P16234 10549 P16471 10420 10783 P52333 Q9H2G2 Q5S007 P54753 Q7Z727 81629 Q8NE63 Q06124 Q05397 P54756 P53667 O60307 P37023 Q8IUD2 2324 57761 1111 P27448 Q12851 O75914 P54764 P54762 3791 P54760 3672 4882 Q9NR96 3551 Q13705 P21917 Q9UBE8 P54646 10645 O60674 1487 P20827 57410 5602 Q96GD4 O14578 5604 P41743 P04233 Q8WXG6 P34925 P25098 P05129 Q13956 Q9Y463 P28482 P11802 1022 1385 1021 1020 P19525 57787 1018 10733 147746 1017 P15056 5610 5613 Q7KZI7 P51671 O00238 P04049 Q96SB4 5871 P09619 P10600 P10721 P19784 Q5VST9 29110 O14519 10746 79444 1147 1025 Q9BUB5 2475 6711 6714 5747 O14757 O95136 O95257 6850 Q9Y243 Q13976 2011 Q9NYB9 27148 P21709 Q96L34 Q9P0L2 P30542 P18545 169436 Q05469 2247 P60033 975 613 Q5TCX8 Q92630 Q96CA5 65061 P51451 P29323 Q5MAI5 P29320 5894 P31749 O95382 Q38SD2 Q13627 P30530 P18433 81788 P37173 984 O00141 6733 P29317 Q9UL54 Q9UK32 Q8TBA6 P06213 Q15139 6872 P56199 O95271 O60603 Q96RU7 2147 Q99640 Q96KG9 O96017 6885 Q9UQ88 1198 O95163 Q9NWZ3 1196 2043 P57059 1195 1072 O96013 Q96Q15 Q9NRP7 Q96S53 Q99750 Q9P2K8 4214 84033 4215 11113 4216 Q99759 O43293 P49768 O00206 O00329 5562 6772 5563 P49761 P49760 O00444 P11274 Q00535 Q00534 79834 Q53X93 6647 Q9Y572 P01137 P01135 O14965 P01133 P01375 P04626 5575 P04629 23552 Q658W2 2065 2064 780 Q01974 Q96RR4 P17936 Q8IZE3 545 Q9H093 5566 Q96PN8 6776 5567 5568 Q00526 O14730 7867 O14733 O43353 Q9H5K3 5580 6790 5581 6793 5582 5583 P07947 5584 6794 4133 O00506 5585 8851 P07949 5586 P07948 P50613 Q99570 Q9Y2K2 673 Q8IZL9 Q99683 5578 8844 P49336 5579 6789 P05771 2081 5591 4140 9950 Q6IR47 140609 5596 5597 5598 P51955 3297 P51956 5590 Q99558 8737 207 5347 4139 84197 Q15078 284086 P50750 8754 91754 Q9Y5S2 Q9UHD2 Q08345 O75293 Q8NER5 55781 Q00613 Q9NRM7 5599 O14920 5371 6464 P49674 Q9UIG0 Q8NHX1 Q96Q40 146057 Q7L7X3 54101 P17612 O14936 P51617 Q96QT4 Q56UN5 5127 54106 Q8N752 P25116 Q13237 P36507 Q15418 7204 Q9Y4K4 O75385 O43318 351 472 P48729 Q9UBS0 Q04759 Q6P5Z2 29941 23043 8767 P22694 P21127 8408 Q9HC98 Q13363 O43683 Q15303 Q16512 4067 4188 Q16513 Q8TF76 Q9UKE5 8780 29959 Q9Y2H1 369 Q9NRH2 P60983 8536 P00533 Q15796 197259 Q13131 9641 P11309 Q15797 Q16644 7465 Q9HBW0 P11308 23387 P31152 Q96IZ7 Q9UBU3 P23528 O94804 8428 5159 Q8WZ42 Q13263 Q8NCB2 P00441 Q16659 Q16539 4086 Q9Y6R4 344387 4087 Q9BY44 Q15208 O75116 8445 P51812 P51813 Q8TD19 5170 Q8N4C8 23031 P51817 10087 6259 54822 Q13153 51319 Q6ZN16 7124 8576 O15164 O75582 4092 P10276 4093 P10398 O75460 156 121512 P27708 Q13162 8569 Q13164 Q13043 Q14012 Q15349 Q8TDX7 Q9HAZ1 Q96KB5 P36896 P15735 26524 P36894 Q02763 120892 11035 P49841 Q13177 Q9NRD5 Q15118 P78368 9201 P49840 O75569 9448 10152 Q9Y6M4 A1XKG3 P36888 P01579 P08069 Q9UEW8 10155 Q9BQI3 1716 Q13188 1956 P09038 O95819 P23443 9212 O15075 Q9BWU1 Q15126 O75676 O15197 O15198 9578 23476 Q02750 25778 7272 1965 2932 2931 51231 Q6PID4 Q14164 P48730 P78362 P23458 1969 P48736 Q14289 Q6VAB6 Q15375 6196 6199 Q13873 9463 7046 6198 P09769 O95835 Q86Z02 Q8TF05 P12931 6195 79012 1613 P42345 P42224 Q9BXM7 91 O60285 94 Q12792 1859 P22612 P42229 Q13882 1616 Q13523 7297 7057 9475 Q6DT37 Q01082 Q9H792 P31321 P41240 P05067 P42338 Q9UER7 P42336 P34947 Q15831 P29597 P06239 P29353 O94768 Q6XUX3 Q13535 O15264 P35916 Q9H422 Q96M96 P29590 27347 Q13418 Q9H0K1 Q7Z2Q5 6093 Q9UHY1 27102 P17252 P42680 P06241 P21860 P42681 P42684 3932 P42685 Q9HCP0 Q86UE8 Q16816 Q13546 Q8IYT8 Q02156 9252 4914 P43405 P68400 Q05655 Q13555 Q13554 Q13315 Q13557 9149 P78509 57144 9261

9020 23085 Q86SG6 Q03468 P20594 4920 2869 7099 150094 7098 P35968 Q13443 30849 Q8IVT5 Q15746 57396 27330 P61073 Q05513 23097 P00734 P24723 P46734 Q8IY84 P10415 Q15759 O75962 26576 Q9P035 P78527 O60229 P41279 P08581 3611 1432 Q9Y6E0 Q96RG2 P05198 O15455 Q13464 5914 O75716 728642 P53355 O15111 Q16854 P53350 Q9P286 Q04771 1326 P00519 389840 P24941 Q7L9L4 O15105 P45983 Q13233 P45984 P21675

cellular protein localization 9182 9181 Q9NRW7 Q5SQT9 Q9P2R6 Q9BVA0 O60566 1213 Q92973 O14662 Q9Y2X3 P60059 10651 3875 P27348 Q92974 Q8NF91 Q92729 P55957 Q8IWJ2 55696 2316 10427 P42858 Q9H2G9 O15400 4734 1121 Q12846 Q96P70 Q9BXC9 6809 P62491 30000 Q86WA8 811 5830 P63104 Q92834 P62258 4869 Q96EA4 P53675 P26374 P31946 56681 O95343 P53677 O60318 81622 Q8NFW9 O60674 1122 6810 6811 27072 Q96GD4 P41743 P04233 Q9H1C4 O75901 Q9H2M9 P28482 1020 Q9P0J0 Q9NRS6 10736 1499 3313 O60763 Q4VX76 Q9Y2T2 4646 10971 O95487 P32856 O95249 P33947 P53618 Q9NR31 Q9NS56 P18206 O60895 6711 604 Q9UM54 P53621 Q9NYB0 4792 Q9H2K2 O60749 O60502 Q9NS69 10953 401505 10956 2247 58533 O14640 5514 6726 P51572 P53992 P30536 Q13505 P51571 P31749 1176 O95140 1174 1173 Q6KC79 Q9NQT8 10724 Q92621 O15504 Q9UL54 7704 Q92624 6993 10490 Q9UPV9 P04637 O96008 O95271 P38646 O60725 Q99523 1058 2147 8924 Q15021 10013 10134 637 10133 Q9C0K7 6748 Q15389 51009 6747 Q15388 Q04917 4580 Q9Y678 6643 6642 5796 O95166 23214 P50542 55973 Q15154 11231 406 Q96CW1 Q15036 P60468 O00327 6890 O00560 2054 23303 Q96CV9 O00203 10239 79711 Q00535 Q02952 Q9Y5X1 Q9GZP9 8943 P49754 54535 P01137 O14964 91949 P01133 P01375 O43264 4000 O14980 23673 P35222 P12004 P54920 Q96ST3 O14617 9804 P24588 9927 O00623 O00505 O43592 O00628 5584 O00629 P05408 25942 P14598 Q8IYJ3 P61981 O14908 7514 10452 P55072 P05412 P49450 7532 5111 7531 7534 5476 7533 Q9HCE7 P37108 10228 10469 Q99797 Q00987 Q15070 8615 207 9702 P05783 7529 5108 P27797 64130 P24386 Q9H902 22895 Q9NW64 Q96QU8 O14925 56993 Q00610 7415 7414 P83436 P49792 5371 4285 Q60FE5 8766 6103 Q9UKK6 O00410 23633 Q6ZWJ1 Q08209 P52948 P40855 10204 P25116 54908 Q8TEW0 Q16623 8775 Q9UPN3 P35658 94121 94120 473 117584 23163 23165 5018 P62913 Q15785 P49815 P25963 Q9NPC8 Q9HAV7 Q8TD31 8546 9632 11196 64601 79083 O75381 Q9UIA9 415117 8417 P00533 9868 Q15554 Q9BUN8 Q15436 6496 6495 Q9UNL2 8677 8676 Q9H269 29927 Q9Y6Q5 4193 Q96RK4 8548 Q13023 P61966 51429 Q9BSI4 P61619 6386 Q9Y2J0 P62826 P62829 Q86XR8 Q9UQ26 23039 Q8N4C7 P59780 P61970 Q15691 Q16543 9527 9648 Q03518 Q49AN0 9784 11140 O43752 7124 10053 Q9UEU0 O94826 Q13285 5195 Q96C24 7375 7014 161 Q9Y5J6 7249 8218 Q9NRD5 Q15475 Q86Y82 Q9BV36 23463 A1XKG3 Q86VS8 O43615 Q9UNH7 Q6VY07 P01579 P02545 1956 P09038 2923 55705 Q9BY84 9212 O94973 O75558 7157 26985 O75674 Q9Y5L4 O75431 Q8N4H5 Q8TEX9 25777 O43747 Q8WZ73 Q9H173 Q13190 11014 84376 P53367 9463 O95714 Q96QC0 P63172 84958 P41134 83752 79139 Q92572 Q7RTN6 Q14974 O15131 9590 Q01082 9472 8021 P46934 117177 P43307 10947 P52292 O60296 22919 P10619 P52294 O75886 P29590 Q03014 P30101 Q4LE60 60412 Q9NZQ3 Q9NSC5 O15397 27236 Q9UNE2 64901 9495 653361 56288 Q9UH99 1408 Q9UBN7 9146 Q9UBF2 Q96G30 Q9NP61 P41182 O00189 3836 P35606 9276 P56962 O95405 Q9H2T7 57154 Q15629 P51159 Q05516 83548 Q5QGT7 O60499 P63010 P00734 Q96QF0 3841 3843 80273 5901 P21333 O75843 100287932 1315 1314 Q9HAP6 9179 Q13107 11097 P62072 Q13596 51517

macromolecular complex assembly P25054 P06899 23607 25909 P49023 P54619 1460 Q9UBB5 O00268 P52434 9185 Q9BYG3 4728 Q8IYN9 P84022 Q9BVA1 10772 O14788 6901 4725 P27105 Q9H4L7 P30876 25915 P16104 2316 2 5829 Q8IUE6 Q96QZ7 P14174 P16471 Q92845 10421 Q9UKV5 P10809 O75925 Q9H2G4 Q96NT1 Q12846 Q9NQW6 Q05397 54496 3416 1355 P32929 O15533 811 5830 4869

5716 P31946 Q9H814 1374 Q6P2C8 Q9NRZ9 1491 P54646 Q676U5 P17302 55355 P16333 2697 P62263 6810 P04350 Q6NXT2 3667 64061 6814 5604 P04233 829 3308 P07305 P30566 P08758 O95477 1022 3320 1020 114548 P52756 O00468 83706 P16389 P15056 1499 830 P07550 P62316 P04040 P09972 P62318 O14744 P62314 O14745 P07437 Q12874 Q9Y478 Q9UHK0 Q12872 Q9Y239 3692 Q8WXF0 2243 1153 O00233 Q9BUB7 P30793 3329 Q9BUB1 1025 841 1387 Q92522 6834 847 5987 Q8N257 6714 5747 P04179 P07766 Q9UGJ1 266812 6850 4673 Q06203 Q8TBB1 Q9NS69 P42768 P61244 284119 P84243 Q71DI3 2244 O14640 Q92994 857 5879 O43182 5518 P51575 Q9Y375 Q13509 Q5QNW6 Q13748 Q03164 10844 Q9P0M6 440193 P40692 6733 Q92621 Q6FI13 4676 989 5888 P09601 P40337 P04275 Q9UKT4 Q9BZJ0 Q9Y2W1 P04156 P06213 4690 P07900 6872 P04637 6631 2033 P14678 O95391 P53803 P50416 P11047 P62993 203068 1058 Q8WWY3 4686 999 55723 5536 Q15389 6505 P62753 P01112 P62875 P25445 135295 6883 5430 10383 5432 P51636 Q99880 O95163 P07919 3010 Q6FGD7 2040 Q9BU61 P50542 2280 Q99877 3009 3008 O60610 641 3007 3006 402 O43290 6633 4214 8932 6878 7846 Q15393 Q96S59 P02675 6637 255626 8936 Q99879 Q09472 6892 5440 5441 5563 Q7Z7K6 3024 3265 Q9UKN8 891 773 Q9H4B7 Q00535 3014 5434 6646 P01137 Q9UPY3 Q3ZAQ7 O00217 O43264 Q9UMS4 Q9Y697 5213 5576 Q658W2 Q9UHI6 P35222 Q08257 P14784 221613 Q96A08 Q99733 11218 Q93079 3028 Q00403 3148 Q96RR1 Q93077 P61457 P23258 P62308 P61218 P62304 P26583 O43593 6430 Q8N307 O00505 Q6FGG2 6311 6431 P50613 P51946 3162 Q8WUY8 673 Q96KK5 3159 6426 P55072 Q9Y512 P49450 5471 64127 Q9ULK4 O60814 5590 29079 P14373 P18846 Q00987 P50748 324 Q96QV6 54443 Q15070 9702 329 7529 P27797 4150 P50750 4154 25929 Q9UHD8 Q12809 O00762 25923 Q9NU22 330 Q5TAL4 Q96P20 56993 7415 7536 4149 P62851 5371 3070 Q86UX7 6341 Q60FE5 Q12933 P07814 P16403 P16402 25813 P16401 10569 466 P51617 O14817 P17858 P52948 Q9NPJ6 7428 9967 Q01813 8772 10093 Q14686 4297 Q14209 Q8TEW0 Q9BY76 11065 Q16625 Q9H0U9 Q6NZI2 P13984 6470 3084 4171 P13861 230 355 P07196 23165 Q8NCD3 Q7Z6C1 P83731 5018 Q13243 P49815 Q13485 P47755 O94906 Q13242 Q13247 Q16637 Q13489 P03950 O00716 9997 Q9BTM1 P62805 8301 Q15428 Q16635 Q16514 P62807 19 79084 O75494 O75381 Q9NVP2 51773 Q9NRH3 Q13492 Q9BSJ2 Q9H9E3 Q13490 Q16401 Q15796 84790 Q15797 6498 P12757 9404 Q8N6I1 O75367 P12755 Q9NWS0 4193 Q6DD88 P23527 Q9UDY8 Q01105 P09234 P17096 P83876 Q8NFH8 Q9HC77 Q8WZ42 Q9BQE3 4086 8683 4087 4088 Q9BY44 4089 Q16777 Q16778 P18074 Q86XR8 P0C0S5 163126 P29084 P04908 P68371 P36954 P34896 Q02790 Q9NVR5 267 P52907 P0C0S8 P68366 Q7L7L0 7106 P08034 P43005 P62942 Q15459 P78347 10291 Q13158 Q8N5M1 8576 O15164 7248 O75347 P54132 P14635 O43639 154 Q00597 158 84365 Q02539 55806 P49736 Q14011 O15294 7132 5195 10181 7013 Q09161 8348 9555 8467 P36776 Q86WV8 P35568 Q02763 7249 8338 P49848 Q9NRD5 Q99714 9685 P02671 8473 P00492 Q8TCU6 7023 10392 Q5SSJ5 P54274 A1XKG3 Q9Y5K6 P58876 P36404 Q08188 Q99700 Q96T23 51340 222484 P68431 Q01664 Q9NVM4 Q16695 56916 P08069 Q14152 8349 9439 Q16698 347733 7277 7157 P22492 P14868 Q02750 5092 26747 138151 Q15370 Q6PID4 55835 1729 P78362 Q9BWT7 10048 Q14289 Q13077 9223 9463 Q96RE7 Q7KZN9 P12931 9341 6194 7283 26271 P20671 Q6NVY8 Q92574 Q9UBK2 P54105 Q13885 Q8IX30 Q02297 9351 55054 84708 22920 55172 7052 P24928 Q9BUF5 1742 Q9HD26 P05062 Q4LE28 2959 7186 P52298 Q15836 O75643 9368 10907 57120 P29590 Q13418 P55209 O75880 84617 7185 P56537 9361 O43819 Q92793 2961 2960 1870 P21980 O60264 P23921 2969 Q92558 O15151 P34932 P19438 P43405 56288 2972 Q07955 P53041 50628 O95996 O95999 P35610 O00186 8290 P30047 1653 P63000 Q92499 Q9BUK6 Q9NVC6 O95400 Q2NL82 O95405 P35609 P14923 O43920 P19387 P30153 P19388 27339 3728 1666 P21579 Q05513 26121 Q7Z2W7 Q8WYP5 375790 1429 P21333 P33778 Q9NVD7 P12830 Q8TEQ6 P19474 P10412 P32320 3737 Q00059 2644 2643

2885 3611 Q14790 O15212 Q9BQA1 O75716 Q14677 O75832 Q15648 Q15526 9054 Q96BF6 P67870 Q9HC29 Q71U36 902 P08237 Q13233 Q13112 P21675 1207

positive regulation of macromolecule metabolic process P25054 O14793 Q12824 P17676 2547 2304 2303 1454 P84022 1453 Q92731 Q9Y275 2308 25913 Q9H4L7 Q12834 Q12837 Q5VTR2 P16104 P14174 4734 Q5S007 O75925 Q7Z727 Q92956 6927 P37023 6929 57761 811 Q9Y297 O75928 P40425 P40424 6926 3659 P26358 6925 3673 2100 Q9NR96 P48380 O95343 10645 P20823 O60674 P19838 Q8IUC6 3667 5604 P27037 Q9Y463 1022 O95352 1017 1499 80324 1012 Q92530 3678 3690 4780 Q92769 6720 P28370 O95361 P10600 Q96J02 O00358 Q06330 Q96KS0 10626 P19419 P17482 P17480 4775 4790 Q9UGJ0 Q12888 4792 Q9H2K2 P38398 Q96N67 P20618 P60033 Q92990 Q92753 6721 P05230 Q92993 857 Q92997 Q13501 Q9UKS7 P10826 Q9UKS6 Q9UL46 P10827 6500 Q13503 P31749 1050 861 5888 P09601 50943 Q9UKT4 P01241 O96004 P14317 Q9UIH9 23326 O60603 25988 P15884 3479 2147 10011 10013 84289 639 10014 6760 64682 Q96T76 641 P33076 406 648 Q9BRP8 O43294 408 84271 P49407 Q13099 O00327 Q09472 5682 P48552 5683 5684 5685 6774 5686 3265 3263 890 891 Q99743 894 896 Q14192 898 3014 8945 P01019 29086 P49639 P51531 4361 5692 5451 5693 P35222 Q9NUX5 P51532 P14784 P35226 P17813 668 5687 6776 5688 10001 P23497 P24588 P26583 O00744 5460 Q9Y5Q3 Q6FGG2 9821 P50613 P51946 Q9NX61 672 P15692 10213 P55072 Q8N5U6 P05412 O43242 O00755 Q9HCE7 Q05086 P37231 Q9NWV8 688 148327 9826 207 5468 Q92922 P24385 8754 7422 P52815 3066 Q9UMN6 3065 O00762 Q9NWW5 P17844 7415 10673 10432 5494 P49674 Q9UIG0 23513 O14936 466 468 7428 O15085 6595 Q14686 6597 6599 11065 6598 Q9Y4K3 Q9H161 474 P08047 8767 Q9NPC8 P38919 3091 P55036 8301 Q15306 Q86VP6 P58304 P13631 Q04864 51773 P46531 7448 Q16401 Q96RL1 P61956 Q15554 Q13133 Q15796 P35638 Q15797 Q96IZ0 8555 P12755 Q9ULH7 Q9NY61 O75360 4193 Q15561 10197 Q13144 51547 P55290 Q15562 Q9BSI4 Q16650 P35869 P62826 P43490 7476 9412 P18074 Q8WVL7 P55055 P36956 Q16665 9421 Q8TDY2 P54132 O75586 P35453 Q93062 Q9Y2C9 Q14011 5074 Q9NRC8 8100 11030 Q9HAZ2 7014 Q9Y4H2 O43524 P35443 7490 P11021 Q8WW38 Q99835 P02790 7023 5089 P98179 Q8WY64 54851 Q96PK6 51588 P08069 10155 P23443 Q9NPA8 Q9BX66 6182 P37840 P11474 5092 7272 26747 Q6PID4 P48730 Q99814 Q14289 6199 7046 6198 9464 P35813 P29375 27005 P22736 P42345 Q9Y4C1 P62195 P04085 9219 Q58WW2 P56524 P78536 7057 O15496 Q13526 22807 83737 P46934 Q4LE28 P16070 O75528 8399 7067 P29353 Q06643 P19793 P29590 Q01196 P41235 Q92793 Q96GM5 Q9HD15 Q9NRA1 Q13547 A4D1W7 P78317 9495 Q9UNE7 P43405 1763 Q9HAK2 Q04206 Q92786 O14593 O95999 P35610 51720 4800 P08246 2626 7099 P31249 7098 Q9NVC6 Q9NP71 1789 P46736 1786 Q9UIV1 P00734 3720 375790 5901 P46734 P12956 P28065 P78527 Q9H4W6 Q9BQA5 P61088 P43694 O15455 5914 P62191 P28074 63976 P28072 P28070 5927 P63272 P24941 Q9HCS4 Q92585 902 Q15532 P43686 P21675 5925 P10914 Q92858 Q12948 Q6QNY1 O00267 3516 4609 P16220 Q96JM2 P63208 O15516 P07996 5935 10533 P04004 Q9UBC3 54361 Q8NAP3 5706 5707 P16234 5708 5709 4851 5700 5701 5702 5704 5705 Q9Y2Y8 Q12968 5717 4862 Q9UJU2 P49116 P61289 P03372 5713 Q92833 Q6IT96 P06401 P06400 5716 Q96JK9 5970 5971 9093 Q99460 P56705 P17302 10524 P17301 2697 P61296 5966 3309 P04233 P05129 P28482 1385 P51668 P51665 P19532 O00468 6829 10736 P15173 P07550 Q8N2W9 4646 P08887 6827 P08648 P30556 Q9UHK0 Q9NYA1 5991 O00231 1153 Q14938 O00233 P10721 O00232 P19544 Q9NS56 Q8WYH8 P37198 29110 960 Q9P0K8 1387 2475 P08651 4898 Q9UM54 P07766 P05106 6850 22937 Q13616 1161 O00487 P30542 O60502 P62333 2247 2246 Q9BZK7 975 O14641 6601 Q92630 Q12770 Q92878 P05112 Q9UM63 P09958 O43186 Q12772 Q12778 P51692 Q05586 O00255 Q6KC79 Q03164 10724 5524 6613 P40337 Q9Y2W1 P06213 6872 P04637 5300 2034 P13349 2033 2274 O95271 P52952 991 Q96RU8 Q96RU7 4686 6622 54998 Q16236 Q15389 10138 P01112 Q04917 Q9C0K0 4221 P35368

2280 P48436 4214 P01106 P01588 P01343 P01100 P49768 O00206 P48431 9921 Q9NS23 148022 Q96S42 Q53X93 P25789 P25788 6646 P01137 5316 P25787 P01375 Q9UPY8 6660 Q96RR4 3148 P10070 P10071 3146 P61457 P01127 Q96EY1 6657 O43353 6672 5582 P07948 3162 Q12905 Q12906 Q9BZE0 79612 3159 Q02930 6667 5578 P40763 Q96FA3 P04201 5591 4261 Q9Y4P1 Q6IR47 10580 7533 64127 O76024 P18850 23414 3171 P18846 Q00987 324 P18848 3169 Q9H1A4 5347 P27797 P01185 2099 O60907 Q9UHD2 Q9UPT9 3182 8625 Q9BT67 23636 5371 P27540 8881 Q9UPU9 6464 P62508 P17980 580 O14818 P51617 P58012 10320 54106 8878 9967 P25116 P60568 4297 Q9BY77 11186 P15923 P13984 351 595 Q9UBS0 Q04759 Q7Z6C1 Q13485 P25963 O94906 5154 O00716 7334 5155 4067 P12429 367 Q70SY1 Q13492 Q9UQR1 7341 6496 6495 6256 P35998 Q9Y606 Q2M1K9 P17096 Q13263 4086 4087 8204 4088 4089 9775 8445 P12645 222546 Q9NWT8 P08151 P24864 P20264 P62701 Q13033 P62942 8454 P55347 O75469 7124 P54253 P14635 391 Q9UQL6 P10275 4092 P10276 4093 154 Q01201 Q13042 Q99728 11146 Q99729 Q99966 Q13285 2902 7132 7376 8463 Q09161 P36896 P36894 P35568 55810 P01308 120892 338917 Q9NQB0 P49721 Q14140 O15169 P49720 85440 Q99958 Q15475 Q96T37 Q8TEY5 10273 P14859 Q96T23 P49959 Q01664 56916 P01579 P49711 P49715 P09038 55827 Q15008 9575 7158 P39905 7157 O15198 Q02750 55832 Q14160 Q15257 Q13873 O95835 Q01094 7161 9341 26271 Q1PSW9 P17275 P63165 91 94 P42229 O15119 P45974 1856 P42226 Q9UBK2 22926 Q96QB1 Q13887 Q8IY57 P55318 O43847 P84095 P05067 P60900 1869 7189 Q15836 Q03014 7182 6096 56034 P17252 O95619 Q9NSC2 8289 Q13422 O15156 O94776 2735 27352 P19438 Q9BXJ9 Q8HWS3 Q9UBN7 1406 2737 2736 Q15853 O75626 9149 Q8TAK5 P53567 1655 P22415 Q9UK80 Q03468 Q13200 Q13443 Q96HU1 57154 P30279 P20248 64919 80155 Q9UJX2 P30281 P23511 3609 P17081 3608 P09429 8078 P10415 Q13216 29966 P18146 P35711 57162 84305 P01730 O15350 O75832 Q9BZS1 P53350 Q15648 P55771 P19484 Q06710 Q04771 23192 Q9HC29 P30260 O14497 O15105 Q13233 P45984

negative regulation of biosynthetic process Q9Y2W7 O75820 2672 Q9UBB5 Q9NS86 P51587 O00267 124790 10657 3516 2304 P84022 283248 Q8N3U4 P27348 5933 25913 P28330 Q9UBC3 Q9Y2X9 Q92729 Q12837 54361 Q8NDV7 P51114 Q8NB12 4851 4734 Q96EB6 5705 O75925 Q7Z727 Q9H2G4 Q9UKV8 Q92835 3660 Q9NYF8 Q9Y2Y8 Q9NRY4 Q9BYE7 Q99471 6927 P13056 83933 57761 811 Q9UJU2 O15534 57649 Q92833 6926 Q6IT96 P06401 P26358 P06400 5716 Q9UIS9 Q9NZI7 P48382 Q9NQX0 P31946 5970 Q92949 P21917 Q9NRZ9 P56705 P48380 O95343 9099 10524 P20823 1487 P19838 400961 2332 P62263 P31949 3665 10642 64061 10644 10765 10643 Q9Y468 Q9ULR5 Q8IZ40 Q9UL18 4771 Q12986 Q9P0J0 O14627 6829 10614 1499 Q86VE0 O14981 Q8N2W9 10971 27161 6945 P08887 5978 6827 O14503 Q9Y2T7 3690 Q92769 5991 93986 5993 Q07666 Q14938 Q15906 P10600 P19544 O95365 O00358 Q06330 10865 6839 1027 3685 P08651 P17480 O14753 604 5987 P05023 P05106 4790 22938 5883 O00488 O60502 P38398 57326 2247 58533 Q9BZK7 6601 Q92993 857 6605 6726 Q8N488 P51693 P10826 P10827 Q9NR50 2023 O00255 Q6KC79 P19883 O00257 1050 Q9HCK5 10724 79589 Q99496 192669 Q9UMX1 4799 P40337 50943 Q9UGL1 7704 Q92985 O00425 O96004 O43159 P04637 Q9NVW2 P14316 25988 P52952 990 Q96RU7 23560 3476 Q15022 79813 4204 639 10014 10138 Q6UWV6 Q9UQ80 Q9BT49 4221 5796 P16989 P57059 641 84159 P33076 51132 Q99750 Q9P2K8 P48436 8932 Q99638 7727 P60228 2290 23429 O43251 23309 P48552 P48431 5562 6774 P13686 Q99623 773 Q99741 Q96S42 P35240 55869 51147 P01137 P01375 Q86X95 P51531 4361 6660 5451 8841 Q9UHI6 P35222 Q9NUX5 O00213 2063 P51532 23435 Q96AQ6 P35227 11218 Q96ST3 Q9NS37 P17813 P35232 3148 P10070 Q15170 545 P10071 3146 11331 668 P01127 6657 5569 Q96T58 Q96MH2 P23497 O43474 6670 P06733 P26583 O43593 6672 Q9Y3M2 23409 P25490 Q969H0 25822 25942 Q12906 Q8NFW5 3281 Q9BZE0 672 Q9BZE4 O75190 Q99684 6304 8726 Q8TEB7 5578 P40763

4261 Q969G3 8864 P37108 Q08117 23411 23414 O75182 P37231 P14373 Q00987 3169 5467 5468 7529 7528 10468 P27797 64375 4152 5245 P56177 O60907 22893 3066 3065 Q9UIF9 P51843 3182 10438 333 P51608 Q9H7L9 5119 Q01826 9839 O43463 5371 3070 Q13901 9612 23512 Q96QT6 P58012 P28749 7428 10320 6595 6597 8535 P10599 6591 Q9Y4K3 3084 476 5017 Q13485 Q13363 P38919 Q13127 P03950 5154 5155 4188 5036 O75496 O75376 P13631 3096 Q02447 O75381 23051 51773 P46531 7329 Q96RL1 Q15554 Q15796 Q9HAU4 Q13131 Q15672 Q9UQR1 7341 8553 6496 6256 6498 Q96IZ0 P12757 Q8N6I1 P31273 Q9UNL4 P12755 23028 Q9NY61 O75360 4193 Q2M1K9 84108 Q9UBU3 P46527 116113 Q13144 51548 51547 54815 Q13263 Q14232 Q9BSI4 Q13148 9774 4087 8204 4088 4089 9775 Q9BY41 P55055 163126 Q9Y618 P10588 P10589 93166 Q04724 P20264 11176 Q9NPF5 Q15697 51317 55915 53615 P43489 Q13033 9421 P54259 9541 5187 O75469 64750 7124 11142 O15164 7248 P54132 P54253 2909 O75461 Q9UQL6 4092 P10276 Q9Y6K1 Q02535 Q03989 51564 Q15583 55922 Q99729 8328 Q15466 55806 Q99966 54 5074 5195 Q9NRC8 6045 7013 7376 55929 Q9HAZ2 7014 8467 O15055 9314 O43524 23135 23378 26523 Q86WV8 7490 7251 6282 1822 166 P01308 84232 Q9NQB0 Q8WW38 2917 Q9UBP4 Q96T37 P61925 7023 P54274 P14859 O43612 P15822 Q9Y6M1 23468 7141 Q9H063 Q96T23 Q9UNH7 P49959 51341 Q01664 Q00577 51588 Q13185 P49711 10155 Q9BQI3 P49715 P09038 O75437 7157 O43623 25776 64426 138151 Q9H9G7 Q99816 9208 Q5VTD9 Q96RE7 P29374 Q06787 Q01094 Q9H0M0 P41134 Q1PSW9 P62195 27122 P63165 P04085 9219 Q92574 O15119 1616 P45973 1978 Q9NSA3 P56524 O94992 Q969S8 Q8IY57 Q02297 7291 O75530 55294 7295 P55316 Q96BD5 P46934 1869 Q9UER7 Q14865 7189 7067 92129 Q8WYK2 9126 Q13535 P19793 P29590 7181 Q03014 Q7Z2Q5 84619 Q9H6W3 Q86UE4 Q9HCP6 27102 P17252 O15379 O60264 Q9NSC2 O15151 O95983 Q13422 O94776 Q13547 A4D1W7 P78318 O43829 Q8TBE0 84733 Q04206 Q9UH92 Q92786 Q9Y2B9 2737 2736 7088 O75626 Q8WUI4 27327 P41182 P30047 Q9UKY1 Q9H9B1 51720 Q14894 P08246 150094 Q13322 Q9NP71 1789 1788 Q05516 1786 58487 Q9UQE7 P41162 3720 3609 Q13573 Q14781 P23510 P09429 O75604 Q9H2S9 29966 Q02363 P18146 P35711 Q9BQA5 Q9Y6E7 2644 O60341 P09430 5914 O75953 O75832 Q9BZS1 Q13227 P55771 Q86YP4 63976 Q96BF6 P63279 Q8N5A5 P63272 Q9HCS4 O15105 Q14683 5925

establishment of localization in cell O75947 Q9Y266 9182 22872 9181 Q96KP1 Q5SQT9 O00148 Q8IYN9 1213 Q92973 P60059 10651 3875 Q9Y275 Q92974 P55957 Q8IWJ2 2316 1468 10427 P42858 Q9H2G9 O15400 4734 253725 Q12846 Q96P70 6927 Q8IUD2 55341 P62491 30000 811 5830 P63104 P62258 6926 10632 Q96EA4 Q12851 Q9C005 P26374 P31946 56681 Q13705 O95343 81622 P20823 Q8NFW9 O60674 821 10640 O14579 27072 5604 Q9UKX7 P41743 O75901 Q92538 Q9H2M9 5862 1020 114548 55361 O60645 6945 P61106 P32856 Q8WXF0 5871 P33947 Q9NR31 27297 P20645 O60895 6711 604 4792 P40222 401505 Q9HCL2 6726 Q13501 P51572 P53992 P51575 Q9UL45 Q13505 P51571 P31749 O95140 Q9NQT8 27032 Q8NBS9 P40692 Q96ED9 O14777 Q9UL54 Q9H4M9 Q8TBA6 P06576 6993 10490 Q86V81 O96008 23325 Q99523 1058 2147 8924 Q15021 10013 637 Q96KG9 6748 6747 11345 135295 Q9BTU6 4580 Q9Y678 Q9NV70 O95166 400 55973 55737 406 408 Q15036 P49407 P02794 P60468 O00327 Q9UMR2 O00560 23303 10239 891 79711 Q15286 Q9Y5X1 8943 O14964 Q9UP83 10476 84062 O43264 10482 O14980 Q658W2 Q01970 Q15050 9804 P24588 11336 O00505 Q6FGG2 P05408 Q641Q2 P14598 Q8IYJ3 P61981 O14908 A8K0Z3 55763 10452 P55072 P05412 O00755 P49450 5476 Q9HCE7 Q8WUX9 10228 10469 Q99797 55770 Q9UBX3 Q8IYI6 P60660 207 Q15075 10466 P62841 P24386 9601 6576 22895 Q96QU8 O14925 Q96P20 215 Q00610 7415 P61764 10673 8766 6103 Q9UKK6 P40616 P40855 Q92900 10204 Q16623 8775 Q9UPN3 24137 P35658 473 117584 P07196 Q9BQG0 P43034 Q15785 P49815 Q9NPC8 Q9HAV7 8546 8301 9632 64601 79083 Q9UJC3 O75381 488 Q05952 Q9NY59 Q96RL7 8536 Q9H9E3 11079 9868 Q15796 Q9HAU5 Q9BUN8 Q9UNL2 P56134

Q8WVM8 23265 23025 Q9Y6Q5 Q96IZ7 498 51542 Q01105 Q96RK4 P56385 8548 P56381 P61966 8563 6386 Q9Y2J0 P62826 7476 P62829 Q86XR8 Q9UQ26 O75351 Q9NZZ3 23039 Q8N4C7 P61970 54820 51552 9648 Q03518 P43487 Q49AN0 51319 149371 11261 O43752 11021 7248 P63092 P36542 P13667 Q9UEU0 51560 84248 O94826 P27824 11269 Q14012 11031 O75575 23256 7249 Q9GZM8 Q9NRD5 9201 9685 Q86W42 Q86Y82 Q9BV36 O15066 Q7Z7G2 23463 O43734 A1XKG3 Q86VS8 Q96EV8 Q9Y4I1 Q9UNH7 P24539 P02545 Q6I9Y2 2802 55705 O15075 O75558 26985 P10147 Q8N4H5 O43747 Q9H173 Q9NXR1 Q13190 11014 10166 P98194 P25874 9463 O95714 P53007 83752 Q92572 P41250 Q9UGU5 65082 Q92574 O95721 Q13769 9472 P78537 81567 P46934 117177 Q9HD26 81565 O60296 O15020 9001 P29590 P30101 Q4LE60 60412 Q9NZQ3 O15258 A4D1W7 27236 Q9UNE2 9495 112936 653361 P43405 Q9UH92 Q9UH99 Q13555 O75746 Q9NP61 O00186 O00189 23085 Q8IWV1 P35606 9276 P56962 Q14416 Q9H2T7 Q96IW7 Q9Y6D6 83548 23095 Q9Y6D5 1785 Q05513 O60499 P63010 P00734 80273 5901 5902 O75969 O75964 100287932 P33897 2647 1315 1314 P20336 84661 Q9HAP6 Q14674 Q9NP90 Q14677 Q16611 11097 Q9H6Z4 51510 Q13596 51517 Q9NRW1 Q9NRW7 Q9P2R6 Q9BVA0 O14662 Q9Y2X3 10772 P27348 P48047 Q8NF91 4860 55696 O60333 200081 Q12965 1121 Q05397 Q9BXC9 Q9GZY0 10514 4628 10758 6809 P18859 Q86WA8 O15533 Q92834 4627 4869 P53675 91782 Q92824 P55851 Q9H814 1130 P53677 O60318 Q99698 57410 1122 6810 6812 6811 6814 10762 4637 P04233 Q9H1C4 O95477 P28482 P50570 O95239 3320 Q9H1K0 Q9P0J0 Q9NRS6 10736 3313 O60763 O00471 3312 P07550 Q4VX76 4643 4644 Q9Y2T2 5976 3799 10972 4646 10971 O95487 O95249 P53618 57678 Q9UM54 5747 P53621 6850 2495 Q12769 O60749 O60502 Q9NS69 10953 P42768 2247 58533 P61006 O14640 5514 P09958 P30536 1176 1174 1173 10724 10726 Q92621 O15504 Q92624 P61019 509 Q96FF9 P07900 Q9UPV9 P04637 Q9Y6W5 P16615 P38646 O60725 O14828 513 4686 514 515 10134 10133 Q9C0K7 51009 Q15388 Q04917 6643 6642 23212 23214 P50542 Q96Q15 521 522 11231 Q96CW1 4218 P49768 6890 6892 Q86U42 2054 P33176 Q96CV9 O00203 773 Q00535 Q02952 P49755 Q9GZP9 54536 P49754 54535 P01137 539 91949 P01375 4000 5331 23673 P12004 P54920 10128 O14617 9927 O00623 O43592 O00628 9700 5584 Q9UPR3 O00629 Q9Y2K9 Q8IXI1 Q8IXI2 Q68EM7 Q2M389 P42704 Q8WTW3 8604 7514 Q96AX1 10577 9950 7532 5111 7531 7534 7533 3178 P37108 P68032 5590 8615 9702 P05783 7529 P27797 64130 6456 Q9UPT5 Q9NW64 Q99666 56993 P83436 P49792 5371 4285 Q60FE5 64145 9973 O75396 O00410 23633 Q6ZWJ1 Q99653 Q08209 P52948 P62745 8878 10564 P25116 10566 Q8N6T3 54908 Q8TEW0 8411 57187 94121 Q96FV9 94120 351 23163 P09493 23165 P23786 P62913 Q96QK1 Q8WUM0 P25963 Q8TD31 P12429 11196 19 O75494 29959 Q9UIA9 415117 8417 Q13492 4074 Q15436 6496 8677 9525 9522 8676 23144 Q9H269 29927 Q9UBU3 375 23381 P25705 Q9UDY8 9515 Q13023 51429 P61619 6262 4087 Q9Y619 Q96BK5 P12883 P59780 388 Q9NUP9 P60520 Q16543 9527 Q8IXZ2 9784 P54257 11140 7124 10053 P54253 P14635 Q9H089 Q4G0F5 P35579 154 P35580 Q99962 P45379 5195 9554 Q96C24 Q09161 O94955 O75694 161 Q86WV8 P11142 Q9Y5J6 Q8NI27 51693 8218 7142 P00491 O43615 Q9NZ43 Q6VY07 P01579 9559 P09038 2923 Q9BY84 O94973 O75436 7157 O75674 Q9Y5L4 Q02750 O75431 Q8TEX9 25777 Q8WZ73 Q9NZ56 54984 O94979 84376 P53367 8498 9342 Q96QC0 Q13636 P63172 84958 9341 79139 10938 Q7RTN6 P09651 O15119 O15118 Q9UBK9 Q14974 O15131 9590 Q01082 8021 P43307 10947 P52292 O15126 P05067 P52298 P10619 Q15836 P52294 O75886 26258 57120 P57740 Q03014 26019 Q9UHY1 80145 P00846 Q9NSC5 O15392 O15155 O15397 64901 9371 56288 1408 Q9UBN7 P30049 9146 Q9UBF2 P41182 9382 3836 2741 O95405 57154 Q15629 P51159 Q07817 P21579 Q9UJX2 Q96QF0 3841 3843 P21333 O75843 P10415 59341 387680 9179 P62072 Q96QD9 Q8TAG9 P84077

negative regulation of cellular biosynthetic process Q9Y2W7 O75820 2672 Q9UBB5 Q9NS86 P51587 O00267 124790 10657 3516 2304 P84022 283248 Q8N3U4 P27348 5933 25913 P28330 Q9UBC3

Q9Y2X9 Q92729 Q12837 54361 Q8NDV7 P51114 Q8NB12 4851 4734 Q96EB6 5705 O75925 Q7Z727 Q9H2G4 Q9UKV8 Q92835 3660 Q9NYF8 Q9Y2Y8 Q9NRY4 Q9BYE7 Q99471 6927 P13056 83933 57761 811 Q9UJU2 O15534 57649 Q92833 6926 Q6IT96 P06401 P26358 P06400 5716 Q9UIS9 Q9NZI7 P48382 Q9NQX0 P31946 5970 Q92949 P21917 Q9NRZ9 P56705 P48380 O95343 9099 10524 P20823 1487 P19838 400961 2332 P62263 P31949 3665 10642 64061 10644 10765 10643 Q9Y468 Q9ULR5 Q8IZ40 Q9UL18 4771 Q12986 Q9P0J0 O14627 6829 10614 1499 Q86VE0 O14981 Q8N2W9 10971 27161 6945 5978 6827 O14503 Q9Y2T7 Q92769 5991 93986 5993 Q07666 Q14938 Q15906 P10600 P19544 O95365 O00358 Q06330 10865 6839 1027 P08651 P17480 O14753 604 5987 P05023 4790 22938 5883 O00488 O60502 P38398 57326 2247 58533 Q9BZK7 6601 Q92993 857 6605 6726 Q8N488 P51693 P10826 P10827 Q9NR50 2023 O00255 Q6KC79 P19883 O00257 1050 Q9HCK5 10724 79589 Q99496 192669 Q9UMX1 4799 P40337 50943 Q9UGL1 7704 Q92985 O00425 O96004 O43159 P04637 Q9NVW2 P14316 25988 P52952 990 Q96RU7 23560 3476 Q15022 79813 4204 639 10014 10138 Q6UWV6 Q9UQ80 Q9BT49 4221 5796 P16989 P57059 641 84159 P33076 51132 Q99750 Q9P2K8 P48436 8932 Q99638 7727 P60228 2290 23429 O43251 23309 P48552 P48431 5562 6774 P13686 Q99623 773 Q99741 Q96S42 P35240 55869 51147 P01137 P01375 Q86X95 P51531 4361 6660 5451 8841 Q9UHI6 P35222 Q9NUX5 2063 P51532 23435 Q96AQ6 P35227 11218 Q96ST3 Q9NS37 P17813 P35232 3148 P10070 Q15170 545 P10071 3146 11331 668 P01127 6657 5569 Q96T58 Q96MH2 P23497 O43474 6670 P06733 P26583 O43593 6672 Q9Y3M2 23409 P25490 Q969H0 25822 25942 Q12906 Q8NFW5 3281 Q9BZE0 672 Q9BZE4 O75190 Q99684 6304 8726 Q8TEB7 5578 P40763 4261 Q969G3 8864 P37108 Q08117 23411 23414 O75182 P37231 P14373 Q00987 3169 5467 5468 7529 7528 10468 P27797 64375 4152 5245 P56177 O60907 22893 3066 3065 Q9UIF9 P51843 3182 10438 333 P51608 Q9H7L9 5119 Q01826 9839 O43463 5371 3070 Q13901 9612 23512 Q96QT6 P58012 P28749 7428 10320 6595 6597 8535 P10599 6591 Q9Y4K3 3084 476 5017 Q13485 Q13363 P38919 Q13127 P03950 5154 5155 4188 5036 O75496 O75376 P13631 3096 Q02447 O75381 23051 51773 P46531 7329 Q96RL1 Q15554 Q15796 Q9HAU4 Q13131 Q15672 Q9UQR1 7341 8553 6496 6256 6498 Q96IZ0 P12757 Q8N6I1 P31273 Q9UNL4 P12755 23028 Q9NY61 O75360 4193 Q2M1K9 84108 Q9UBU3 P46527 116113 Q13144 51548 51547 54815 Q13263 Q14232 Q9BSI4 Q13148 9774 4087 8204 4088 4089 9775 Q9BY41 P55055 163126 Q9Y618 P10588 P10589 93166 Q04724 P20264 11176 Q9NPF5 Q15697 51317 55915 53615 P43489 Q13033 9421 P54259 9541 5187 O75469 64750 7124 11142 O15164 7248 P54132 P54253 2909 O75461 Q9UQL6 4092 P10276 Q9Y6K1 Q02535 Q03989 51564 Q15583 55922 Q99729 8328 Q15466 55806 Q99966 54 5074 5195 Q9NRC8 6045 7013 7376 55929 Q9HAZ2 7014 8467 O15055 9314 O43524 23135 23378 26523 Q86WV8 7490 7251 6282 1822 166 P01308 84232 Q9NQB0 Q8WW38 2917 Q9UBP4 Q96T37 P61925 7023 P54274 P14859 O43612 P15822 Q9Y6M1 23468 7141 Q9H063 Q96T23 Q9UNH7 P49959 51341 Q01664 Q00577 51588 Q13185 P49711 10155 Q9BQI3 P49715 P09038 O75437 7157 O43623 25776 64426 138151 Q9H9G7 Q99816 9208 Q5VTD9 Q96RE7 P29374 Q06787 Q01094 Q9H0M0 P41134 Q1PSW9 P62195 27122 P63165 P04085 9219 Q92574 O15119 1616 P45973 1978 Q9NSA3 P56524 O94992 Q969S8 Q8IY57 Q02297 7291 O75530 55294 7295 P55316 Q96BD5 P46934 1869 Q9UER7 Q14865 7189 7067 92129 Q8WYK2 9126 Q13535 P19793 P29590 7181 Q03014 Q7Z2Q5 84619 Q9H6W3 Q86UE4 27102 P17252 O15379 O60264 Q9NSC2 O15151 O95983 Q13422 O94776 Q13547 A4D1W7 P78318 O43829 Q8TBE0 84733 Q04206 Q9UH92 Q92786 Q9Y2B9 2737 2736 7088 O75626 Q8WUI4 27327 P41182 Q9UKY1 Q9H9B1 51720 Q14894 P08246 150094 Q13322 Q9NP71 1789 1788 Q05516 1786 58487 Q9UQE7 P41162 3720 3609 Q13573 Q14781 P23510 P09429 O75604 Q9H2S9 29966 Q02363 P18146 P35711 Q9BQA5 Q9Y6E7 O60341 P09430 5914 O75953 O75832 Q9BZS1 Q13227 P55771 Q86YP4 63976 Q96BF6 P63279 Q8N5A5 P63272 Q9HCS4 O15105 Q14683 5925

regulation of binding 6597 6598 1460 Q12824 9181 Q9Y4K3 3084 Q96JM2 P84022 118 Q7Z6C1 8767 O14788 Q13485 P25963 2307 Q92974 P04004 7334 4188 P12429 Q15306 P27361 7335 O75381 2316 51773 7448 800 Q15796 Q96EB6 7341 Q92838 P11309 P35638 P27695 P32121 P12755 23028 Q9Y6Q6 54496 Q8IUD2 57761 Q9UDY8 Q93009 P40424 4869 P06400 5716 4087 4088 4089 5970 P18074 3673 Q92949 Q9NR96 3551 9093 5728 P17301 O60674 Q8IUC6 Q9ULZ3 P43489 P62942 7124 P54132 29108 O00463 Q9UQL6 114548 4092 O43638 P10276 154 Q02535 57669 10616 Q9Y6K9 P07550 P04040 55806 5195 Q9Y2T7 Q9NYA1 Q9Y239 Q92769 Q15109 1029 166 P01308 Q9HCM4 Q9NQB0 P42771 P49841 847 P49848 Q99835 7023 10392 4792 Q96FX2 27148 O60869 Q96T23 Q01664 Q9NVM4 857 P05112 Q9NQC7 6188 P10827 O00255 23476 5092 2932 Q99497 P05362 P09601 50943 Q13077 P23396 2033 O95271 O60603 P15531 Q96RU8 P41134 Q96RU7 10935 23560 P11171 P63165 P01116 10014 Q9UBK2 Q9NSA3 P56524 4221 Q13404 Q02297 Q05682 2280 641 Q9NRP7 Q99750 6878 408 P49407 409 P49768 O00206 7186 Q09472 7189 7067 7188 Q8N726 Q5TAX3 Q13535 7185 148022 Q9BYM8 Q86UE4 P01019 P01137 P01375 8721 Q13546 7874 55072 P51532 Q9NS37 Q05655 P60484 3148 545 Q04206 3146 P61457 Q92786 Q96EY1 P23497 O43353 5580 P26583 O43593 6672 Q96EP0 O95999 3162 P53567 Q7RTR2 P35612 P30048 P35611 1896 23085 Q9BZE4 P05412 7099 7098 P14923 64127 Q08117 23411 P37231 3728 58487 Q8TAI7 3169 1540 8737 328 5468 11315 P23510 P21333 P09429 P10415 O14495 P19474 Q02363 3066 P51843 Q9BUZ4 Q96P20 P61088 O60341 O15455 O14920 Q9GZT9 5914 Q60FE5 O75832 Q16611 Q12933 Q9BZS1 Q8NHX1 54583 P51617 P67870 Q9HC29 8517 4830 O15105 54106 5925

intracellular signal transduction Q2M1Z3 Q92737 65125 P37288 10890 Q92730 O14544 O14543 6901 10413 P28335 64284 Q8NFZ5 P53779 2316 Q59EK9 Q5S007 O75925 Q7Z727 Q9H8S9 Q9NR81 3661 Q06124 Q8IUD2 81876 P62491 Q9UKW4 10633 Q12851 P31946 3672 Q9NR96 P21917 10645 O60674 P20827 Q8IUC6 5602 3667 5604 Q8WXG6 Q13956 5861 5862 1020 Q5SGD2 1017 P15056 1012 P61587 P61586 P61106 Q9UL26 Q9UL25 P30679 5870 5871 Q15907 Q9UGI9 P10600 P52888 1029 1026 Q9BUB5 5863 846 5865 5867 6714 5868 Q92766 5869 P49286 Q07890 O95136 Q9UGJ0 4792 Q96L33 P16118 P19634 P18545 P60033 Q07889 4543 Q5TCX8 P05230 857 O14763 5877 Q9UN86 Q96CA5 5878 5879 5894 P31749 O95382 Q38SD2 84932 10607 6733 P09601 O14777 Q9UL54 127829 P01241 P56199 P14314 O60603 3479 2147 10010 5898 P62873 10256 6760 O95163 Q9NWZ3 P57059 1072 P35249 400 402 Q99750 Q14185 P47224 Q99759 O43293 O14713 Q13098 Q09472 O00329 6772 P48551 6774 116986 O00560 3265 116987 890 Q99500 Q9H4B6 Q15286 P01019 55504 O14965 Q01974 3269 6776 Q15052 7867 O14733 5207 Q59EA4 P26583 6790 9821 673 10451 P62834 6548 7879 6789 10454 Q92930 7410 Q15077 8737 9826 207 4139 Q6IQ22 8754 O00762 O75293 79753 10672 O14920 7433 8766 Q8NHX1 P50406 P52824 P08908 P17612 O14939 P40616 8517 Q9H4E5 9846 326624 Q13115 O15085 P36507 11065 P18085 P10114 Q9Y4K4 O43557 O75385 Q9Y4K3 O43318 Q9H0U4 23043 51762 P49815 P35408 4188 P62805 Q16635 Q9Y2H1 Q9H0T7 7205 P55042 5029 P00533 P55040 Q15311 Q13131 8795 Q15797 Q16644 Q9UQ13 8315 Q9Y2I1 Q15561 P55290 Q15562 Q8IV61 5295 6386 Q9Y6R4 Q9UPQ3 O43306 Q86XR7 Q8N4C8 51552 Q13393 9649 P62820 Q8TDY2 11261 11021 P63092 O75582 P46109 51560 Q9Y2C9 Q9Y6K9 Q13162 8569 Q13164 Q15349 Q15109 11031 26524 P63096 7249 11035 Q15118 Q9BYZ6 O94844 P11229 9448 A1XKG3 P58753 55704 P08069 1718 Q13188 1956 P23443 Q9HB90 O75553 P38936 O75791 P11234 P11233 Q6PID4 Q99819 P78362 P23458 Q9BWT7 P48736 Q14289 6196 6199 7046 6198 P12931 6195 6194 P42345 P98161 1978 7297 7057 9230 Q3V6T2 7052 P46937 P42338 P98171 Q9UER7 P42336 P34947 P29597 P29353 O95977 O15264 Q9H0K1 Q7Z569 7064 P40145 P41231 O60266 P06241 P21860 Q13546 9252 Q07960 7074 P43405 Q9NVJ2 O14593 Q03113 9021 Q16828 9261 O00194 23085 P63000 Q13563 2869 Q8IWV1 7099 7098 Q14653 Q9NP71

O15211 Q9NP72 9270 P30153 P62166 P61073 5906 5908 2873 P00734 O60496 51735 Q15750 53916 9046 Q15759 Q8NEB9 Q9P035 P20338 P20339 P20336 P20337 2885 1793 50650 O15455 5911 Q9NP90 A4D1S5 P34972 Q9H0N0 P24941 P20340 Q15532 5925 Q9NRW1 Q9NRW4 Q99490 P61020 55684 Q6PCE1 O60566 Q7L0Q8 Q8IVH8 2664 Q9HBH0 P61026 55207 P49137 P27348 P07996 P52564 O75815 P52565 Q7L591 P27361 65018 9077 P52566 P50148 10549 P16473 P16471 P48039 Q92844 O15524 P52333 P29992 339122 P53667 57403 4868 P06400 5716 P46089 Q5JS13 3551 Q12979 9093 Q9UBE8 948 P04233 O95477 P28482 O00220 5739 P07550 P61225 P61224 10971 Q7KZI7 P30556 Q5HYI8 P04049 Q96SB4 Q9NYA1 P30559 P10721 29110 10746 2237 79444 1147 2475 10981 P42771 Q92888 O95257 P28223 6850 2011 Q9P0L2 P30542 22931 Q9NS68 P62330 O60503 2247 P15153 1399 2246 975 P61006 5515 O43182 5518 P51451 P51693 P51692 O00255 3350 P17342 Q9UK32 P61019 P61018 P06213 Q9Y6W6 Q9BST9 P04637 Q9Y6W5 P38405 2033 Q96RU8 P62993 998 P01116 Q15382 Q9C0K7 6869 P27986 285282 P62753 P01112 P01111 P25100 4221 5310 6885 5311 P35368 266747 56940 Q8WTR2 9908 Q15392 4214 4215 4216 10146 4218 P01343 P49768 O00206 Q9Y6Y9 5562 Q8N726 P14416 P52735 P11388 Q9NS23 148022 Q00535 P61204 6647 Q9Y572 P01138 Q6UXV0 P01135 P01133 P01375 64343 P04626 P04629 2065 2064 O00212 P12004 P35348 Q96RR4 3148 5566 5567 5568 Q96EY1 11215 23647 Q969H4 5585 Q5U0I6 P07949 Q9UNN5 3162 Q8IXI1 Q8IXI2 552 553 Q99683 P51828 Q96AX2 5578 8844 5337 5338 57826 P40763 P04201 Q6IR47 5111 5598 P16671 7529 Q08462 6453 Q9UHD2 Q8IZJ4 333 Q9NRM7 5599 8625 P25103 Q60FE5 Q12933 6464 P39748 Q99653 P51617 P62745 54106 P25116 Q15654 Q15418 6236 11186 8412 Q9UBS0 Q96BM9 Q7Z6C1 P22694 8408 P25963 6242 Q13009 Q16512 Q15669 19 Q8TF76 Q9UKE5 6239 Q14344 Q13017 Q9HBW0 29 Q86X27 P32241 P32246 Q8TEU7 Q9UBU3 375 Q9UDY8 P23528 6009 P00441 P32239 Q16539 4086 Q15208 P51812 381 382 Q9Y3L5 387 388 P21359 389 P31150 P60763 Q9NSD7 7124 390 391 392 394 154 396 397 398 121512 25780 Q6KH11 Q9H082 Q13283 Q96KC2 Q13287 Q13043 Q96R06 Q9BU20 8463 P35568 Q9UKA4 23370 Q8NFM4 P01308 Q8NFM5 Q9UKA8 120892 9306 P01303 2915 Q99956 Q8TCU4 Q6ZSZ5 O43612 P36404 P01579 11124 P09038 O95819 P60953 Q14155 Q9BY84 7153 7157 O75676 P19086 Q02750 1609 Q5VVH5 1848 54509 Q14964 Q15811 P53365 Q13637 O95835 P57729 Q13636 Q12788 10928 Q13639 1613 P42224 1852 Q9BXM7 1850 P23945 83871 P22612 P42229 1616 79363 P57735 P84095 P67775 9367 7186 7189 O94768 Q96M96 6093 P41597 P17252 P42680 P42681 P21980 Q8TAD8 O95622 9138 4914 27352 P13945 Q86YS6 P41180 P51148 Q7RTR2 P51149 Q9H244 P43119 Q03468 P08123 4920 150094 Q52LW3 P51157 Q13202 Q13443 Q96HU1 P51151 Q8IVT5 P51153 58480 P51159 27330 P20248 Q8TAI7 P08134 Q8WYP3 P17081 P21333 P84085 1432 Q13464 P08588 P53355 O75832 O15111 Q13227 P62070 O95661 P84077 Q7L9L4 P45983 Q15653 Q13233 P45984 P21554

ribonucleoprotein complex biogenesis Q92979 Q06265 Q9Y2X3 10772 6229 P83731 Q14690 P62913 Q13243 O94906 Q13242 P38919 Q13247 5393 Q16637 5394 Q15428 5036 6125 79084 O75494 51096 2 Q9NPD3 P46777 10421 Q96EB6 P62249 Q9NY61 Q9NWS0 Q8TED0 54496 Q9NV06 P09234 P83876 Q5TAP6 Q8WVM0 4869 8683 P46087 Q9BY44 Q13823 P08708 9775 Q9H814 P18077 10528 P62263 705 Q15459 O95478 10291 10171 7248 P52756 P39019 23481 29102 Q8NI36 P62316 P62318 O14744 P62312 Q99848 P62314 Q14011 Q12874 10181 Q14137 Q9UHK0 Q12872 3692 Q8WXF0 Q09161 Q13601 1153 23378 23016 Q86WV8 Q9Y5J1 1029 6839 Q9NY12 11157 P42771 118460 6838 P05388 6175 O75683 Q99700 51340 54853 P50914 Q9NVM4 88745 56915 Q14152 Q9GZL7 Q13868 Q9H6R4 Q01780 Q9NQT4 55153 Q9NQT5 Q96EU6 26747 P61254 10607 6733 Q9Y2W2 Q9NVN8 P78362 Q9BZJ0 92345 9221 O43159 11340 Q12788 6631 P62081 P14678 O00541 O95391 P18124 6194 10813 Q9H6Y2 Q9Y3A5 54512 23560 Q8WWY3 4686 Q92574 P62753 65083 1736 Q15024 Q9UQ80 135295 P54105 Q14978 23212 Q9Y2P8 51010 Q9Y3B2 51013 O43290 6633 6637 P52298 Q13895 Q8N726 O75643

10907 29889 O00567 P56537 Q9H9L3 Q9UPY3 Q9UMS4 P78316 Q9UHI6 Q96B26 11218 Q15050 Q07955 55759 Q9UET6 P62308 134430 P62424 P62304 50628 6430 Q9NYH9 6311 6431 23404 54552 1653 Q9BZE4 4809 Q92499 8602 6426 Q8NEC7 O95400 Q9Y2L1 51729 Q2NL82 6201 23411 Q96G21 27339 26121 P62847 P62841 Q8IY81 2091 9045 P22087 4154 25929 Q8TEQ6 22894 P55769 Q9NVU7 10438 Q5TAL4 Q5RKV6 7536 P62857 P62851 P56182 Q9BQA1 O43463 Q9UNQ2 Q13901 Q8TDN6 Q969E8 10569 4839 Q9BVJ6 Q96HR8 23076 6217 Q14684 1207

regulation of cellular metabolic process Q9UKT9 P25054 Q9H0E3 Q9Y265 Q8NDW4 Q12824 8091 124790 10657 2305 2304 2303 1454 114991 1453 2300 Q8N3U4 O14543 155061 284695 Q00839 2308 2307 Q9H0D6 Q92974 Q9H4L7 Q12834 Q12837 Q8WXB4 Q8NDV7 P16104 2316 P39210 10661 Q96EB6 Q5S007 O75925 136319 Q9UKV8 Q92956 4990 3660 3661 Q9BYE7 Q06124 P62495 P37023 P13056 84901 57761 55588 Q9UKW4 Q9Y297 O75928 Q9UKW6 3659 P26358 P26374 Q6NUN9 3672 Q5BKZ1 3673 Q92949 Q99583 Q12857 Q13705 P21917 10645 1488 1487 283337 Q99576 2332 3665 3667 10642 64061 P26367 10641 10644 10643 Q9UL15 Q8WXG6 Q9UL18 1022 1020 Q8NA42 Q06587 114548 Q8IX07 Q5JT82 Q96MX3 1017 10614 79576 P21964 Q9HAJ7 1499 10616 O14981 Q9Y230 P61586 O75909 Q92530 3678 O14503 Q12873 3690 Q12872 Q9Y239 Q8WXF0 Q9Y5Z7 Q8WXF1 Q9H4P4 6720 Q15906 Q9UGI9 285527 O00358 Q9BPY8 57798 1029 Q9BUB1 3207 10626 1027 1026 1025 Q9BUB5 P17482 3685 P17480 604 Q9Y242 3689 P05023 P07686 P04179 Q15910 Q9UL36 O95136 Q9UGJ0 Q12888 5883 Q9BQ95 O60869 P16118 79595 Q8NA19 P38398 57326 55145 3215 Q96N67 P20618 Q92990 Q9Y250 Q5TCX8 6721 Q92994 Q92993 6726 Q8N488 5879 Q92997 Q9UKS7 P10826 Q9UKS6 Q9UL46 P10827 P10828 221937 5411 P31749 Q8NDX6 1050 P53999 79101 P40692 57332 3226 79589 3225 P04150 5888 Q9UGL1 Q9UL54 Q9UKT4 Q92985 O95159 Q9UKL0 O96004 P56199 P14317 P14316 P50416 23569 11108 11107 Q8WWY6 1059 23560 Q15022 639 P62875 6749 6760 Q15027 Q9Y5V3 5430 O96019 O95163 P16989 P57059 P36575 Q03933 51131 641 P33076 51132 P35251 Q14186 Q6B0B8 79923 8932 648 8939 P49407 P60228 85360 Q14188 11116 O00327 O96028 5440 P48552 5441 6772 Q9UKN5 Q8TAU3 P48551 6774 Q99990 O96020 3265 346171 3263 O60828 P15621 Q66K89 Q99500 Q2M3W8 Q14192 P35240 8945 P01019 51147 Q9Y692 Q15047 P23246 Q9Y5X9 Q86X95 5451 A7MD48 O00330 Q8N3J9 P35222 Q5T6S3 P35227 P35226 O14979 P57082 O75081 P17813 P35232 Q00403 53335 3269 56849 668 6776 4116 Q96MH2 Q15057 Q16385 P24588 Q15056 7629 O43474 P06733 O00744 5460 Q9Y5Q3 6311 9821 6794 P25490 Q9NX61 3281 Q9NX65 672 P61981 P15692 O14908 Q9UBW7 6304 57708 9815 10213 Q6UUV7 P05412 O00755 Q9Y5R5 9831 3297 Q9NX70 23532 Q5JSP0 Q9UBX0 P14373 Q9NWV8 57713 688 148327 Q15070 689 5467 9826 207 5468 54206 Q8N895 Q92922 Q15078 4150 4152 4154 23509 P56178 9841 P56177 Q8NHY6 Q12809 O00762 Q9NWW5 Q9Y5S9 P17844 Q0IIM8 4149 O14920 57727 P62854 9839 O43463 5494 Q8NHX1 P08908 23512 23513 Q96QT6 57732 O14936 8517 Q92900 9849 4172 O15085 P36508 4173 4174 P35659 Q14686 4176 Q14209 8535 11065 Q9BZ95 Q6NZI2 Q9H165 Q9UPN7 4171 P13861 Q9H161 Q9H9F9 23043 Q9H160 Q9NPC1 P49810 P08047 5017 P78395 Q9BQG0 5018 P07199 P49815 Q13363 Q9NPC8 Q14693 Q8TBP0 94234 4188 8543 P46782 5036 5037 79084 P58304 Q96F45 O15090 23051 Q04864 Q9Y2H8 9869 7205 Q8WY36 P00533 Q16401 Q96RL1 P61956 Q15554 Q9HAU4 P24522 8553 P35638 P61968 9882 Q8IWY8 Q96IZ0 10193 Q9UPP1 8555 P32121 P31274 P31273 P31276 Q9ULH1 O75123 Q9ULH7 23028 Q9NY61 4193 Q9Y6Q6 257 Q9H9D4 Q6DD87 Q15561 10197 8548 Q96RK0 51548 Q93009 51547 Q14232 P61964 Q15562 Q9BYU1 Q9Y6R4 P62826 Q9UPQ3 9412 Q9UPQ9 P36956 P35625 O75592 Q96F24 P31270 Q9NPF5 Q13398 P43246 55915 9421 P78347 9425 7248 O75586 O75582 Q9Y6K1 Q93062 Q03989 Q9Y2C9 Q71SY5 Q9Y6K9 51564 Q15583 8569 55922 5074 10181 P78337 8100 55929 11030 Q9HAZ2 Q15109 P22392 O15055 O43524 26524 26523 5071 7251 O75570 94274 23492 7249 O15047 P49848

Q15596 Q96NH3 Q15118 8110 P61925 Q3KNS6 P02790 O15062 P49840 P11229 5089 166968 O95931 A1XKG3 23466 Q9Y6M1 23468 O15060 Q8WY64 79913 51588 P08069 10155 Q9BQI3 9439 Q96NG5 1956 286075 Q8NEJ9 Q9NPA8 O75553 26986 64426 P37840 5092 64428 7272 1965 2810 Q9H9G7 54625 O95947 54623 Q14289 Q8IWS0 9463 Q96RE7 9464 P29375 9467 P29374 27005 Q7Z589 Q9H0M0 1975 P22736 P42345 Q9BUG6 Q96EK4 Q9UGU0 P04085 Q92574 Q13761 1978 Q58WW2 P56524 9477 9474 O15496 O75534 Q02297 7291 O75530 7290 9470 7295 P24928 1982 Q6P1K2 P16070 P42338 O75528 P29353 O95977 Q06643 P0C7X2 P56539 Q8WXI9 P40145 Q9BYM8 55066 1994 1993 79039 Q96IQ9 O15499 8165 P56545 O95983 A4D1W7 O95503 55072 P78317 P78318 9495 Q8TBE0 Q9HAK2 Q07954 Q9UH92 Q9Y2B9 P52597 Q96EP0 O14593 O95999 Q9NP61 P35610 Q9NP66 Q86TI0 9021 Q16828 23081 O43918 23085 Q9UKY1 Q9H9B1 P85037 P31260 Q6P1N0 Q8IWV1 P31249 Q13322 Q14653 Q9NP71 Q9NP72 P46736 3728 P61073 2873 1540 Q9BUJ2 3720 5901 P46734 Q13330 P12956 Q6P1L6 Q9P031 P28065 Q9P035 Q9H4W6 Q00059 Q9BQA5 Q9Y6E7 Q9H582 P61088 Q96AE4 147808 Q96RG2 Q68DY9 P43694 O15455 Q9BQA1 5914 11091 O75953 P28074 63976 P28072 63978 P28070 5927 5928 P63279 P00519 5929 P63272 23077 P24941 51510 Q92585 Q15532 P43686 Q13351 Q14683 P07148 Q13352 5925 Q7KZF4 P10914 O95677 Q92858 P67809 Q9NRW4 Q9UBB5 Q9NS86 Q99490 A5YKK6 Q9P2R6 O60566 4609 P16220 10772 5931 P49137 P27348 5933 O15516 5935 P09936 O15530 Q9NS91 Q9UBC3 P27361 65018 54361 O95684 9077 115704 P50148 1107 P16234 O60573 284323 4613 O15524 1108 O15525 Q92838 Q9UK58 Q7Z3K6 Q92835 P05549 Q9NRY4 1121 Q99471 Q7Z3K3 O60548 P53667 1111 Q92830 Q9UJU2 284312 O15534 P49116 P61289 P03372 64170 Q92833 P06401 Q92831 P06400 Q92826 P07737 5970 P46089 5971 9093 Q9UBE8 Q99460 Q9NRZ9 P56705 9099 P17535 P17544 Q99459 P17542 P62140 57410 400720 1122 P61296 5966 10765 3309 P04233 P05129 5981 P50570 3320 O00463 P52756 O00468 O95231 Q9UFF9 O14627 6829 10736 3315 Q6FHQ0 5976 P41218 Q8N2W9 4646 27161 5978 6827 P08648 P30556 65056 5991 93986 5993 1153 Q14938 O00472 Q06455 55250 Q9NS56 P37198 79685 6839 10746 3326 3324 2475 P08651 5500 5987 P26045 O95259 P07766 Q10586 P05106 O95257 P05107 P28223 6850 Q8ND82 P51449 Q6IA86 27148 Q13616 1161 O00488 O60506 O00487 P30542 Q02086 O60502 O60503 P61244 Q9BXG8 O14641 4664 5511 O14640 Q12770 Q92878 5515 P05112 O43182 26292 Q9UNY4 P08621 5518 P09958 O43186 Q12772 Q12778 O43189 Q05586 2023 Q02078 Q6KC79 3350 10725 57459 10724 P37173 Q99496 5524 P40337 7704 O00425 4690 P23396 O43159 6872 6871 P04637 P38405 2034 2033 Q9NVW2 O95271 O00422 Q9UFB7 4686 4204 P01116 4205 Q9C0K7 Q16236 Q15389 P01112 4209 O43166 Q9C0K0 6883 Q9BT49 4221 6885 Q9GZR2 O43167 23210 P35368 P50548 Q9BT40 23216 56940 O76071 Q9NRP7 84159 Q9P2K8 4214 P01106 6878 4216 P01588 11236 7727 56946 Q9C0J9 P01100 Q7Z7K2 7741 5562 Q8N726 9921 5563 5565 P14416 P52735 P52736 Q9NS23 773 Q02952 Q53X93 6886 P01137 P01135 P01133 Q16254 Q9UJM3 P52740 7750 6421 5573 P04626 5575 Q9GZP0 5576 P04629 2064 P52747 2063 11218 Q9NS37 Q99853 55633 P10070 P17936 P09086 P10071 10363 5566 10362 5567 P01127 5568 5569 O43150 2070 5580 5581 O43593 5582 5585 O43597 7764 22794 25822 Q96PU8 79612 O75190 Q02930 P51828 7756 5577 Q8TEB7 5578 5579 10336 8607 P40763 P04201 2081 5591 Q9GZV8 4261 Q6IR47 5111 Q3SY56 64127 P37108 O75177 P18850 P15336 5590 O75182 Q9NRL2 56987 51295 P18846 Q68CJ9 P18847 324 P18848 326 5104 Q8TEA7 55657 Q92800 P27797 Q9GZU7 P01185 Q9UNP9 2099 O60907 O75164 P51843 Q8NCN5 55662 Q8NCN2 333 Q9NRM7 91748 Q9H7L9 10794 8625 5119 Q9GZT9 Q9BT67 23636 9975 6464 4287 P62508 P51858 P17980 146050 P52701 Q08211 221656 Q9NVV9 P58012 10320 P60321 9967 P25116 Q8N6T3 Q99417 Q96HZ4 O43670 Q14566 8655 4297 Q9BY77 Q8TEW0 11186 9500 Q9UKD1 P30086 P13984 351 Q9UBS0 Q04759 Q9UBS5 P09493 P23786 P21127 P25963 P09017 8662 P20290 Q15424 7332 5154 7334 5155 8667 7336 O43684 7335 19 26205 Q9UKE5

85403 367 Q70SY1 51654 11198 7329 Q14586 20 7341 5162 P11309 6496 6495 Q13257 Q8IVW6 Q14103 6498 P11308 Q99909 6015 P32242 Q8N3C0 Q8TEU7 Q9Y606 5160 84108 Q9UBU3 116113 P09471 P17096 5159 Q14592 11168 Q96MA1 Q9UBU8 Q13263 Q8TF50 Q53H12 Q14114 8204 Q9BY44 Q14119 Q8WWN8 Q9BY41 Q9Y618 125058 P12645 P29084 Q86WP2 5170 222546 Q04725 Q04724 387 Q04727 Q9NWT6 Q04726 Q9NWT8 23152 Q66K14 P08151 P20264 11177 11176 P23771 P62942 P55347 9541 5187 11140 11142 O15164 2909 391 Q6MZP7 23126 O43639 25788 O43638 Q02535 Q01201 121512 25780 2908 Q9UFW8 11146 Q15466 Q6PJG2 Q99966 Q13285 2902 54 Q13286 5195 6045 7376 7375 Q09161 23133 23135 Q86WV8 Q3KQV3 Q6PRX2 P09067 2917 Q14140 Q9UBP4 O15169 85440 Q99958 Q15475 Q99956 Q8TEY5 Q96ME7 10273 Q9HA82 P14859 O43612 Q8TAQ5 Q96DT7 Q9H063 Q99941 P49959 Q01664 51222 56916 P01579 Q14151 Q9UBQ5 P09038 Q15008 Q9BY84 9575 P82979 8242 10284 O75676 O15198 23112 O43623 25776 P33121 23118 2931 51230 Q14160 51231 1609 Q5VVH5 Q14161 10289 Q15014 86 P78424 Q9NWH9 P08559 Q15819 P33992 P33993 P33991 Q8TAF7 26271 P41134 Q9BXM7 Q1PSW9 27122 91 P17030 94 O15119 P45974 1616 Q6ZT07 P45973 O15118 Q9UBK2 Q9NSA3 P78412 9112 Q96QB1 Q96I24 9590 Q9H7Z7 Q9H3R0 P31321 P55318 Q99081 P31323 P55316 O43609 P67775 P05062 P52292 Q52LR7 P17040 P08575 P05067 2959 2956 Q9UBL3 O94763 Q15831 P52298 P29474 Q15836 92129 P52294 9126 27107 Q9H422 Q96M96 Q460N5 Q03014 6096 P41597 56034 27102 Q9BTC8 2961 2960 O95619 Q8TAD8 Q9NSC2 2969 P09874 O15151 8289 Q15843 O95622 O95625 O15156 O94776 29777 202559 Q02156 P17023 Q05655 2972 79149 Q9BXJ9 P17020 Q8N393 Q8HWS3 P04198 Q9UBN7 1647 Q15853 O75626 Q14527 P13945 9149 1660 Q8TF47 Q6NYC1 Q96DN5 Q9H488 P41182 Q8TAK5 93474 79175 Q499Z4 P53567 79177 P30048 B3KY43 3836 84525 1655 84528 P22415 1653 Q9UK80 Q03468 Q92499 Q13200 O75629 P47712 O95644 150094 Q13202 Q96HU1 Q9UBG7 Q8N7H5 P20248 64919 80155 Q9UJX2 Q8TAI7 P41162 P17081 Q9Y5B6 Q9Y5B9 P09429 O75604 P10415 Q9P2X3 Q13216 P41161 P35711 23186 P09430 P08588 P41159 Q86SE9 728642 Q9P2Y4 Q13227 P55771 Q04771 O95661 O15105 Q13233 P21554 Q6NX49 Q9H6Q4 O14793 25909 P54619 22870 2550 9181 P51587 9184 P17676 Q8TDD1 P15498 2547 P37288 57504 Q9HCJ0 P84022 O00151 Q92731 O14788 6901 10413 Q7Z6R9 25913 P28330 P08729 Q92729 Q5VTR2 P51114 O60216 P14174 Q9H2G9 493856 4734 Q7Z727 Q9H2G4 O00167 22850 Q9C010 81628 54496 6927 Q8IUD2 3416 6929 Q6AHZ1 Q96KM6 Q8N680 811 6921 P40425 P40424 P62258 6926 6925 Q6IQ32 Q17R98 Q9C005 333929 Q9UIS9 Q9NZI7 P31943 P48382 Q9NQX0 P31946 Q9C009 P31947 2100 Q9NR96 P48380 O95343 P54646 3428 P16333 6938 P20823 55352 O60674 P20827 P19838 O60675 Q8IUC6 P62263 P18509 P31949 5604 6936 Q9Y468 P27037 Q9H2M9 Q13956 Q8IZ40 4771 Q9Y463 4772 Q9Y466 Q8TDI0 Q9UIL8 Q9NUY8 O95352 2107 55364 10856 Q86VE0 2103 P16383 27287 80324 Q08999 O14744 6945 Q9Y478 P50222 4780 Q92769 Q14814 P09619 P28370 Q07666 O95361 80306 P10600 O95365 Q06330 2119 10865 P19419 Q6PI57 4775 O14753 Q6ZW31 847 7804 O14757 Q92766 P09622 4790 4791 4792 Q9H2K2 Q9H2K0 2130 81669 P18545 Q9NR48 Q9HCL2 P60033 Q92753 4302 P05230 857 O14763 859 Q96CA5 Q92754 Q13501 22827 6500 Q13503 Q9NR50 O95382 Q14839 Q8NC51 P19883 Q13506 Q9NR55 Q9HCK5 2139 861 862 863 192669 Q9UMX1 Q9UMX0 4799 P09601 50943 P08754 Q9Y3Y2 Q9BS34 P01241 10492 P15408 Q9Y3Q8 3481 Q9UIH9 23326 O60603 25988 P15884 Q8WUU5 Q96T88 3479 2147 Q8WV60 2146 3476 10011 P25205 7832 10013 84289 Q96KG9 55723 10499 10014 11345 Q6UWV6 135295 64682 Q9UII4 Q7Z6J4 4343 25998 Q96T76 Q99750 406 Q9BRP8 O43294 408 84271 409 Q13098 Q01518 Q13099 Q96C55 Q09472 Q8N1G0 O43251 23309 5682 5683 5684 5685 5686 116986 64215 116987 P13686 890 Q00653 891 Q99743 894 Q99741 896 898 3014 29080 Q9UN42 29086 P49639 P61326 P51531 4361 5692 5693 8721 7874 148156 Q9NUX5 Q9NR11 P51532 Q96ST3 55750 P60484 Q15291 11331 5687 55758 5688 10486 4358 10001 P60002 10488

Q96T58 5207 10487 P28702 P23497 Q3MII6 P26583 Q9Y3M2 Q6FGG2 P46199 Q96BZ9 P50613 Q5R372 P51946 23764 25942 Q8NFW5 Q9NUQ3 Q8WUY8 Q96P48 79735 10451 10450 8726 P55072 10454 Q8N5U6 O43242 7410 90993 Q9HCE7 Q08117 3054 Q05086 Q9BRU2 29079 P37231 Q9HCE1 Q96SZ6 P51959 79723 8737 10468 23746 P24385 P24386 8754 7422 Q96GA9 5245 3068 Q9HCD5 P52815 22893 3066 Q9UMN6 3065 Q9UIF9 O75293 O75290 79753 10438 Q00613 Q96P20 55787 55785 7415 10432 3070 Q13901 9612 P49674 Q9UIG0 P52824 3074 Q9Y3P9 P17612 466 468 55796 P28749 Q9NQL9 7428 P04792 56647 6595 6597 6599 6598 Q9Y4K4 O75386 P10599 O75385 6591 Q9Y4K3 Q76L83 3084 O43318 473 474 476 23286 9617 51763 8767 P43034 P38919 Q13127 Q15303 B2RXF5 3091 Q16633 P55036 8301 Q16635 Q15306 Q86VP6 O75376 P58546 P13631 24149 3096 5270 Q02447 O75381 Q9NVP2 51773 30813 P46531 7448 Q13133 Q15796 Q15311 Q13131 8795 Q16649 Q15797 9640 P12757 Q8N6I1 9643 7468 P54198 Q9UNL4 P12755 O75362 Q8TDS5 O75360 Q96CN4 P46527 51780 Q01101 P42167 Q01105 6128 O94805 7458 Q13144 Q5VUA4 54815 Q8WVM0 Q9BSI4 Q8IV61 Q16650 Q13148 P35869 P20393 7476 Q16656 Q15329 Q8NCA9 P18074 Q8WVL7 P55055 O43306 P10588 P10589 93166 Q86VN1 Q9NZZ3 Q8N6H7 Q16665 51317 Q49AN0 Q13153 P43489 Q8TDY2 9667 Q16666 Q5VUG0 P54132 P63092 P35453 Q14494 8328 Q14498 Q16670 P46108 Q14011 Q86W54 Q9NRC8 7013 7014 P46100 Q9Y4H2 P35443 Q5PSV4 7490 P13682 P11021 P63096 Q9C0F3 84232 Q8WW38 Q9GZM8 Q13177 Q9NRD5 Q99835 7024 7023 10392 Q9C0F0 49854 P98179 P15822 9682 7020 Q02878 Q9UNH7 51341 54851 Q9NVM4 Q96PK6 11243 55704 P55010 Q13185 Q8NCF5 P23443 6188 Q15369 Q8IUX7 O43741 Q9BX66 Q9BSM1 Q09028 P38936 6182 P11474 Q9UNI6 26747 Q15370 54862 P57682 Q6PID4 Q99816 P48730 Q9BWT7 9208 Q99814 P78545 6199 7046 6198 P98155 P35813 P78540 P10515 O43711 6193 10811 O43719 Q9Y4C1 P62195 Q4LE39 O15234 9219 1738 1737 A8K8V0 P78536 7057 22803 Q13526 112950 22806 22807 55291 57473 55294 Q3V6T2 P46934 P46937 83746 Q96GN5 81565 O60296 Q4LE28 P42574 P98174 Q9UER7 Q14865 8399 7068 7067 Q5TAX3 Q15714 Q13535 Q9H307 P19793 P29590 Q15717 Q7Z2Q5 Q63HK5 P31431 Q01196 84619 Q9H6W3 P41235 1756 Q9HCP6 Q92793 Q96GM5 Q9HD15 Q9Y4E5 O60266 O60264 Q9NRA1 O60260 P59817 Q13547 Q13546 9252 7073 Q9UNE7 P41223 57018 P43405 1763 Q9NZR4 Q04206 Q92786 Q92785 Q92784 P41229 7088 Q15735 P78509 93594 9261 Q01167 P63000 Q9BSG1 51720 Q14894 4800 Q13563 P08246 2626 7099 7098 Q9NVC6 Q14416 7091 P14923 7090 Q9NQZ8 P30153 P11413 1789 1788 Q05516 1786 Q9UIV1 1785 Q05513 P00734 375790 Q13574 51735 Q13573 Q15750 Q9HCU4 P55884 93134 Q9H2S9 Q96CJ1 P32320 P78527 92283 85509 1312 Q86VK4 Q04656 84661 51742 Q6ZNG0 P05198 P20333 Q9Y4A8 Q8IYX1 30827 Q13107 Q9P287 P62191 Q96H20 Q9HCS4 900 902 P00750 Q9HD40 Q13112 P08238 Q9NZN8 P08235 P21675 Q9NZN9 Q92618 Q9H609 Q92616 Q9Y2W7 Q9NYD6 Q6P2D0 O75820 2672 Q12948 22992 O00268 Q6QNY1 O00267 79894 3516 Q9BZI1 Q96JM7 2665 Q96JM2 Q5VWX1 2664 283248 Q9ULW3 Q92610 P63208 4841 Q9ULW0 Q8N587 P07996 10533 Q12950 P52564 P04004 Q9Y2X7 Q12952 Q9Y2X9 Q8NB12 Q8NAP3 Q9P107 5706 5707 79885 5708 5709 P16473 51099 P16471 4851 5700 4854 5701 5702 Q96JL9 5704 Q92600 5705 P50395 Q9ULX9 Q9NYF8 Q9Y2Y8 Q12968 29128 22974 10514 5717 83933 26097 P20936 Q9UQB3 4862 O75807 5713 57649 Q6IT96 4869 5716 Q96JK9 Q12972 Q0D2J5 Q9H9T3 1374 3551 Q6P2C8 5728 10524 P17301 Q9HBE1 Q99697 400961 Q9ULZ3 Q9UHR5 Q9ULR5 O95477 P28482 Q9UHJ3 Q12986 1385 O00220 P51668 29108 P51665 P19532 Q9P0J0 Q9P0J1 P15173 P07550 P04040 58517 10971 7913 P08887 O14867 Q96BA8 10973 Q9UM47 P51671 Q9Y2T7 Q96SB4 Q9UHK0 Q9NYA1 O00231 O00233 P10721 O00232 P19544 Q8WYH8 57678 29110 79444 960 1388 29115 Q9P0K8 1387 1386 4898 P42771 Q9UM54 4899 Q9H1I8 10985 Q9NYB0 P30304 22937 P30307 22938 Q9UHL9 22931 P62333 2247 284119 P15153 2246 1398 58533 Q9BZK7 975 6601 Q9Y2V2 Q92630 6605 57209 Q9UM63 P51693 Q9Y376 P51692 P31629 Q9BQY4 O00255 Q8NEZ4

Q9NQ33 O00257 Q03164 440193 P17342 984 Q5H9I0 988 P05362 6613 Q9Y2W1 P06213 P07900 Q9UPV9 5300 P13349 2274 P53803 P52952 990 Q8WTS6 991 126208 Q96RU8 Q96RU7 Q8WTS1 79810 995 51003 Q99640 79811 6622 79813 6625 54998 51008 10138 Q9UQ80 Q04917 P49770 Q9UM07 Q9Y2P0 Q7Z5H3 5311 Q9UQ88 5796 Q5T7W0 O60739 P11177 2280 Q8WTR2 8819 P48436 8812 Q99638 P01344 P01343 P49768 O00206 2290 23429 P48431 5321 Q96JC9 3142 Q9NU63 148022 Q99623 Q00535 Q96S42 Q8NAF0 P49756 P25789 55869 P25788 7975 6647 6646 5316 51028 P25787 P01375 Q9UPY8 23439 6660 P49750 8841 Q9UHI6 O00213 Q3KNV8 23435 Q96AQ6 3151 O60716 P12004 10128 Q96RR4 Q3KNW1 3148 Q15170 545 546 3146 P61457 Q96EY1 Q96JB5 6657 5327 Q8WU17 O43353 6670 6672 23409 8851 Q969H0 Q9UPR3 P07948 Q9H9S4 Q9Y2K7 Q9ULJ3 Q9H9S0 3162 Q12905 O95076 Q8WYA1 Q12906 Q9BZE0 Q01844 552 Q9BZE4 P42704 3159 Q99683 Q8WTW4 Q99684 6667 P49336 P05771 Q9H5J8 10577 P07951 Q96FA3 Q969G3 8864 7532 7531 10580 7533 P55198 Q9ULK4 23411 23414 3171 Q00987 Q96S94 3169 Q9NU19 Q9H1A4 5347 7529 7528 Q08462 Q9H5I1 64375 P50750 Q86U86 4035 Q9UHD2 3187 Q9UPT9 3185 3184 Q9BUZ4 571 3182 P51608 P68036 7536 6207 Q9H5H4 Q01826 5371 3192 8880 P27540 8881 Q60FE5 Q9UPU9 Q12933 Q9ULM3 7555 Q9ULM6 P51610 Q9BUY5 580 54583 O14818 80854 P51617 Q9NPJ6 7549 54106 8878 P60568 6231 8892 Q15654 9744 O43432 8896 P15923 Q86YW9 Q96FV9 595 84324 116 Q7Z6C1 P22694 Q13485 Q8WZ64 8408 O94906 P03950 O00716 Q16512 4067 Q16514 P12429 Q15669 O75496 O75494 Q7LBC6 P10242 P10243 P10244 84312 51412 O94915 Q13492 Q16520 6239 54925 Q15672 Q9UQR1 7101 6256 Q9HBW0 Q16526 P35998 Q2M1K9 23381 Q9UDY8 7579 Q8WZ42 P00441 9774 4086 7112 4087 4088 Q8N9N2 4089 O75478 Q15208 9775 8445 163126 Q9H257 O75475 Q8N5F7 142 P21359 P31150 P24864 P62701 84333 P60520 Q16543 Q15697 53615 Q13033 8454 P54259 O75469 8458 64750 7124 P54252 P54253 Q96KC8 P14635 O75461 Q9UQL6 P10275 4092 P10276 4093 O75460 4094 154 P23588 Q13042 Q99728 Q99729 Q13045 55806 P45379 P49736 7132 Q86UR1 8463 7133 8467 9314 O43889 P36896 P36894 23378 P15976 P35568 Q96C28 6282 1822 166 Q8NFM4 55810 P01308 Q8NFM5 120892 338917 Q9NQB0 Q99717 P49721 P49720 Q15233 Q96T37 P00492 9326 Q8N5K1 P54274 O75446 P36402 O75444 6294 7141 Q99700 1831 Q96T23 Q9NZ45 Q9H1Y0 Q00577 Q99708 P49711 Q16576 P49716 P49715 55827 Q9NQC7 Q96C00 64784 7158 O75437 Q08050 P39905 7157 P19086 64786 162239 P14625 Q8N9R8 Q02750 P35548 138151 55832 Q9UDV6 1848 Q5VTD9 Q15257 Q13077 Q13873 O95835 7169 26038 10928 26037 Q06787 Q86Z02 56252 Q01094 Q9H204 26039 7161 9341 P63167 P42224 1852 10933 1850 10935 P17275 P23945 Q7RTN6 P63165 Q9BVI0 P22612 P42229 P15090 57109 1856 P42226 O15372 O94992 Q9P0W2 22926 Q969S8 22927 Q13404 Q13887 Q01085 57592 Q8IY57 57594 P10644 O43847 57591 Q96BD5 79365 P84095 Q08945 Q9UHX1 P60900 1869 7186 7189 7188 Q8WYK2 O43812 P11940 7181 Q13418 7182 7185 7184 1877 Q86UE4 Q86UE3 1874 Q9Y3C7 P17252 P68871 1870 P09630 O15379 Q969R5 Q13422 O15391 Q8NB78 4914 2735 27352 P19438 O43829 56288 84733 1408 1407 1406 2737 Q92667 2736 9388 Q96G30 Q8WUI4 8050 27327 Q02386 Q7RTR2 P62277 1896 Q9HC52 Q5H9R7 Q14774 Q13443 P22670 Q9NQ92 9391 P11532 Q96G25 57154 P30279 84759 58487 Q9UQE7 P30281 Q8WYP5 P23511 Q8WUH2 3609 3608 3607 Q14781 P23510 P21333 Q9NYJ8 94104 8078 Q6ZV73 O14495 Q9P0T4 29966 P19474 Q02363 58491 P18146 57162 84305 27300 3611 O60343 O60341 4943 P01730 Q969V6 O15350 O75832 O15353 Q15649 Q9BZS1 P53350 Q15648 Q9P0U4 P19484 Q86YP4 Q06710 Q96BF6 Q8N5A5 Q9UHV2 3622 2773 Q9HC29 P30260 O14497 Q15652

regulation of metabolic process Q9UKT9 P25054 Q9H0E3 Q9Y265 Q8NDW4 Q12824 8091 124790 10657 2305 2304 2303 1454 114991 1453 2300 Q8N3U4 O14543 155061 Q9Y275 284695 Q00839 2308 2307 Q9H0D6 Q92974 Q9H4L7 Q12834 Q12837 Q8WXB4 Q8NDV7 P16104 2316 2 P39210 10661 P10809

Q96EB6 Q5S007 O75925 136319 Q9UKV8 Q92956 4990 3660 3661 Q9BYE7 Q06124 P62495 P37023 P13056 84901 57761 55588 Q9UKW4 Q9Y297 O75928 Q9UKW6 3659 P26358 P26374 Q6NUN9 3672 Q5BKZ1 3673 Q92949 Q99583 Q12857 Q13705 P21917 10645 1488 1487 283337 Q99576 2332 3665 3667 10642 64061 P26367 10641 10644 10643 Q9UL15 Q8WXG6 Q9UL18 1022 1021 1020 Q8NA42 Q06587 114548 Q8IX07 Q5JT82 P51787 Q96MX3 1017 10614 79576 P21964 Q9HAJ7 1499 10616 1012 O14981 Q9Y230 P61586 O75909 Q92530 3678 O14503 Q12873 3690 Q12872 Q9Y239 Q8WXF0 Q9Y5Z7 Q8WXF1 Q9H4P4 6720 Q15906 Q9UGI9 285527 Q96J02 O00358 Q9BPY8 57798 1029 Q9BUB1 3207 10626 1027 1026 1025 Q9BUB5 P17482 3685 P17480 604 Q9Y242 3689 P05023 P07686 P04179 Q15910 Q9UL36 O95136 Q9UGJ0 Q12888 5883 Q9BQ95 O60869 P16118 79595 Q8NA19 P38398 57326 55145 3215 Q96N67 P20618 Q92990 Q9Y250 Q5TCX8 6721 Q92994 Q92993 6726 Q8N488 5879 Q92997 Q9UKS7 P10826 Q9UKS6 Q9UL46 P10827 P10828 221937 5411 P31749 Q8NDX6 1050 P53999 79101 P40692 57332 3226 79589 3225 P04150 5888 Q9UGL1 Q9UL54 Q9UKT4 A6NHR9 Q92985 O95159 Q9UKL0 O96004 O00308 P56199 P14317 P14316 P50416 23569 11108 11107 Q8WWY6 Q99523 1059 23560 Q15022 639 P62875 6749 O96018 6760 P06702 Q15027 Q9Y5V3 5430 O96019 O95163 P16989 P57059 P36575 Q03933 51131 641 P33076 51132 P35251 Q14186 Q6B0B8 79923 8932 648 8939 P49407 P60228 85360 Q14188 11116 O00327 O96028 5440 P48552 5441 6772 Q9UKN5 Q8TAU3 P48551 6774 Q99990 O96020 3265 346171 3263 O60828 P15621 Q66K89 Q99500 Q2M3W8 Q14192 P35240 8945 P01019 51147 Q9Y692 O14964 Q15047 O14965 P23246 Q9Y5X9 Q86X95 5451 A7MD48 O00330 Q8N3J9 P35222 Q5T6S3 P14784 P35227 P35226 O14979 P57082 O75081 P17813 P35232 Q00403 53335 3269 56849 668 6776 4116 Q96MH2 Q15057 Q16385 P24588 Q15056 7629 O43474 P06733 6790 O00744 5460 Q9Y5Q3 6311 P62837 9821 6794 P25490 Q9NX61 3281 Q9NX65 672 P61981 P15692 O14908 Q9UBW7 6304 57708 9815 10213 Q6UUV7 P05412 O00755 Q9Y5R5 Q9Y5R4 9831 3297 Q9NX70 23532 Q5JSP0 Q9UBX0 P14373 Q9NWV8 57713 688 148327 Q15070 689 5467 9826 207 5468 54206 Q8N895 Q92922 Q15078 P01023 4150 4152 4154 23509 P48594 P56178 9841 P56177 Q8NHY6 Q12809 O00762 Q9NWW5 Q9Y5S9 P17844 Q0IIM8 10673 4149 O14920 57727 P62854 9839 O43463 5494 Q8NHX1 P08908 23512 23513 Q96QT6 57732 O14936 8517 Q92900 9849 P49427 4172 O15085 P36508 4173 4174 11060 P35659 Q14686 4176 Q14209 8535 11065 P55265 Q9BZ95 Q6NZI2 Q9H165 Q9UPN7 4171 P13861 Q9H161 Q9H9F9 23043 Q9H160 Q9NPC1 P49810 P08047 5017 P78395 Q9BQG0 5018 P07199 P49815 Q13363 Q9NPC8 Q14693 Q8TBP0 Q9HAV4 94234 4188 8543 P46782 5036 5037 79084 P58304 Q96F45 O15090 23051 Q04864 Q9Y2H8 9869 7205 Q8WY36 P00533 Q13370 Q16401 Q96RL1 P61956 Q15554 Q9HAU4 P24522 8553 P35638 P61968 9882 Q8IWY8 Q96IZ0 10193 Q9UPP1 8555 P32121 P31274 P31273 P31276 Q9ULH1 O75123 Q9ULH7 23028 Q9NY61 4193 Q9Y6Q6 257 Q9H9D4 Q6DD87 Q96RK4 Q15561 10197 8548 Q96RK0 51548 Q93009 51547 Q14232 P55290 P61964 Q15562 Q9BYU1 Q9Y6R4 5054 P62826 Q9UPQ3 9412 Q9UPQ9 P36956 P35625 O75592 Q96F24 Q4AC94 P31270 Q9NPF5 Q13398 P43246 55915 9421 P78347 9425 7248 O75586 O75582 Q9Y6K1 Q93062 Q03989 Q9Y2C9 Q71SY5 Q9Y6K9 51564 Q15583 8569 55922 5074 10181 P78337 8100 55929 11030 Q9HAZ2 Q15109 P22392 O15055 O43524 26524 26523 5071 7251 O75570 94274 23492 7249 O15047 P49848 Q15596 Q96NH3 Q15118 8110 P61925 Q3KNS6 Q86Y82 P02790 O15062 P49840 P11229 5089 O75569 166968 O95931 A1XKG3 23466 Q9Y6M1 23468 O15060 Q8WY64 79913 51588 P08069 10155 Q9BQI3 9439 Q96NG5 1956 286075 10159 Q8NEJ9 Q9NPA8 O75553 26986 64426 P37840 5092 64428 7272 1965 2810 51593 Q9H9G7 54625 O95947 54623 Q14289 Q8IWS0 9463 Q96RE7 9464 P29375 9467 P29374 27005 Q7Z589 Q9H0M0 1975 P22736 P42345 Q9BUG6 Q96EK4 Q9UGU0 P04085 Q92574 Q13761 1978 Q58WW2 P56524 9477 9474 O15496 O75534 Q02297 7291 O75530 7290 9470 7295 P24928 Q96AB3 1982 Q6P1K2 P16070 P42338 O75528 P29353 Q5JXB2 O95977 Q06643 P0C7X2 P56539

Q8WXI9 P40145 Q9BYM8 55066 1994 1993 79039 Q96IQ9 O15499 8165 P56545 O95983 A4D1W7 O95503 55072 P78317 P78318 9495 Q8TBE0 Q9HAK2 Q07954 Q9UH92 Q9Y2B9 P52597 Q96EP0 O14593 O95999 Q9NP61 P35610 Q9NP66 Q86TI0 9021 Q16828 23081 O43918 23085 Q9UKY1 Q9H9B1 P85037 P31260 Q6P1N0 Q8IWV1 P31249 Q13322 Q14653 Q9NP71 Q9NP72 P61081 P46736 3728 P61073 83548 2873 P61077 1540 Q9BUJ2 3720 5901 P46734 Q13330 P12956 Q6P1L6 Q9P031 P28065 Q9P035 Q9H4W6 Q00059 Q9BQA5 Q9Y6E7 Q9H582 P61088 Q96AE4 147808 Q96RG2 Q68DY9 P43694 O15455 Q9BQA1 5914 11091 O75953 P28074 63976 P28072 63978 P28070 5927 5928 P63279 P00519 5929 P63272 23077 P24941 51510 Q92585 Q15532 P43686 Q13351 Q14683 P07148 Q13352 5925 Q7KZF4 P10914 O95677 Q92858 P67809 Q9NRW4 Q9UBB5 Q9NS86 Q99490 A5YKK6 Q9P2R6 O60566 4609 P16220 10772 5931 P49137 P27348 5933 O15516 5935 P09936 O15530 Q9NS91 Q9UBC3 P27361 65018 Q9H832 54361 O95684 9077 115704 P50148 1107 P16234 O60573 284323 4613 O15524 1108 O15525 Q92838 Q9UK58 Q7Z3K6 Q92835 P05549 Q9NRY4 1121 Q99471 Q7Z3K3 O60548 P53667 O60543 1111 Q92830 Q9UJU2 284312 O15534 P49116 P61289 P03372 64170 Q92833 P06401 Q92831 P06400 Q92826 P07737 5970 P46089 5971 9093 Q9UBE8 Q99460 Q9NRZ9 P56705 9099 P17535 P17544 Q99459 P17542 P62140 57410 400720 1122 P61296 3784 5966 10765 3309 P04233 P07307 P05129 5981 P50570 P11802 3320 O00463 P52756 O00468 O95231 Q9UFF9 O14627 6829 10736 3315 Q6FHQ0 710 5976 P41218 Q8N2W9 4646 27161 5978 6827 P08648 P30556 65056 5991 93986 5993 1153 Q14938 O00472 Q06455 55250 Q9NS56 P37198 79685 3329 6839 10746 3326 3324 2475 P08651 5500 5987 P26045 O95259 P07766 Q10586 P05106 O95257 P05107 P28223 6850 Q8ND82 P51449 Q6IA86 27148 Q13616 1161 O00488 O60506 O00487 P30542 Q02086 O60502 O60503 57448 P61244 Q9BXG8 O14641 4664 5511 O14640 Q12770 Q92878 5515 P05112 5516 O43182 26292 Q9UNY4 P08621 5518 P09958 O43186 Q12772 Q12778 O43189 Q05586 2023 Q02078 Q6KC79 3350 10725 57459 10724 Q96LR5 P37173 Q99496 5524 P05121 P40337 7704 Q8N2K1 O00425 4690 P23396 O43159 6872 6871 P04637 P38405 2034 2033 Q9NVW2 O95271 O00422 Q9UFB7 4686 4204 P01116 4205 Q9C0K7 Q16236 Q15389 P01112 4209 O43166 Q9C0K0 6883 Q9BT49 4221 6885 Q9GZR2 O43167 23210 P35368 P50548 Q9BT40 23216 56940 O76071 Q9NRP7 84159 Q9P2K8 4214 P01106 6878 4216 P01588 11236 7727 56946 Q9C0J9 P01100 Q7Z7K2 7741 5562 Q8N726 9921 5563 5565 P14416 P52735 P52736 Q9NS23 773 Q02952 Q53X93 6886 P01137 P01135 P01133 Q16254 Q9UJM3 P52740 7750 6421 5573 P04626 5575 Q9GZP0 5576 P04629 23673 2064 P52747 2063 11218 Q9NS37 Q99853 55633 P10070 P17936 P09086 P10071 10363 5566 10362 5567 P02458 P01127 5568 5569 O43150 2070 5580 5581 O43593 5582 5585 O43597 7764 22794 Q9UNN5 25822 Q96PU8 79612 O75190 Q96PU5 Q02930 P51828 7756 5577 Q8TEB7 5578 5579 10336 8607 P40763 P04201 2081 5591 Q9GZV8 4261 Q9Y4P1 Q6IR47 5111 Q3SY56 64127 P37108 O75177 O76024 P18850 P15336 5590 O75182 Q9NRL2 56987 51295 P18846 Q68CJ9 P18847 324 P18848 326 5104 Q8TEA7 55657 Q92800 P27797 Q9GZU7 P01185 Q9UNP9 2099 O60907 O75164 P51843 Q8NCN5 55662 Q8NCN2 333 Q9NRM7 91748 Q9H7L9 10794 8625 P83436 5119 Q9GZT9 Q9BT67 23636 9975 6464 4287 P62508 P51858 P17980 146050 P52701 Q08211 221656 Q9NVV9 P58012 10320 P60321 9967 P25116 Q8N6T3 Q99417 Q96HZ4 O43670 Q14566 8655 4297 Q9BY77 Q8TEW0 11186 9500 7325 Q9UKD1 P30086 P13984 351 Q9UBS0 Q04759 Q9UBS5 P09493 P23786 P21127 P25963 P09017 8662 P20290 Q15424 7332 5154 7334 5155 8667 7336 O43684 7335 19 26205 Q9UKE5 85403 367 Q70SY1 51654 11198 7329 7328 Q14586 20 7341 5162 Q14106 P11309 6496 6495 Q13257 Q8IVW6 Q14103 6498 P11308 Q99909 6015 P32242 Q8N3C0 Q8TEU7 Q9Y606 5160 84108 Q9UBU3 Q03405 116113 P09471 P17096 5159 Q14592 11168 Q96MA1 Q9UBU8 Q13263 Q8TF50 Q53H12 Q14114 8204 Q9BY44 Q14119 Q8WWN8 Q9BY41 Q9Y618 125058 P12645 P29084 Q86WP2 5170 222546 Q04725 Q04724 387 Q04727 Q9NWT6 Q04726 Q9NWT8 23152 Q66K14 P08151 P20264

11177 11176 P23771 P62942 P55347 9541 5187 11140 9546 11142 O15164 2909 391 Q6MZP7 23126 O43639 25788 O43638 Q02535 Q01201 121512 25780 2908 Q9UFW8 11146 Q15466 Q6PJG2 Q99966 Q13285 2902 54 Q13286 5195 6045 7376 7375 Q09161 9555 23133 23135 Q86WV8 Q3KQV3 Q6PRX2 P09067 2917 Q14140 Q9UBP4 O15169 85440 Q99958 Q15475 Q99956 Q8TEY5 Q96ME7 10273 Q9HA82 P14859 O43612 Q8TAQ5 Q96DT7 Q9H063 Q99941 P49959 Q01664 51222 56916 P01579 11124 Q14151 Q9UBQ5 P09038 Q15008 Q9BY84 9575 P82979 8242 10284 O75676 O15198 23112 O43623 25776 P33121 23118 2931 51230 Q14160 51231 1609 Q5VVH5 Q14161 10289 Q15014 86 P78424 Q9NWH9 P08559 Q15819 P33992 P33993 P33991 Q8TAF7 26271 P41134 Q9BXM7 Q1PSW9 27122 91 P17030 94 O15119 P45974 1616 Q6ZT07 P45973 O15118 Q9UBK2 Q9NSA3 P78412 9112 Q8WWH4 Q96QB1 Q96I24 9590 Q9H7Z7 Q9H3R0 P31321 P55318 Q99081 P31323 P55316 O43609 P67775 P05062 P52292 Q52LR7 P17040 P08575 P05067 2959 2956 Q9UBL3 O94763 Q15831 P52298 P29474 Q15836 92129 P52294 9126 27107 Q9H422 Q96M96 Q460N5 Q03014 6096 P41597 56034 27102 Q9BTC8 2961 2960 O95619 Q8TAD8 Q9UBM7 Q9NSC2 2969 P09874 O15151 8289 Q15843 O95622 O95625 O15156 O94776 29777 202559 Q02156 P17023 Q05655 2972 79149 Q9BXJ9 P17020 Q8N393 Q8HWS3 P04198 Q9UBN7 1647 9146 Q15853 O75626 Q14527 P13945 9149 1660 Q8TF47 Q6NYC1 Q96DN5 Q9H488 P41182 Q8TAK5 93474 79175 Q499Z4 P53567 P30047 79177 P30048 B3KY43 3836 84525 1655 84528 P22415 1653 Q9UK80 Q03468 Q92499 Q13200 O75629 P47712 O95644 150094 Q13202 Q96HU1 Q9UBG7 Q8N7H5 Q07817 P20248 64919 80155 Q9UJX2 Q8TAI7 P41162 Q9BXP5 P17081 P08138 Q9Y5B6 Q9Y5B9 P09429 O75604 P10415 Q9P2X3 Q13216 P41161 P35711 23186 P09430 P08588 P41159 Q86SE9 728642 Q9P2Y4 Q13227 P55771 Q04771 O95661 23192 O15105 Q13233 P45984 P21554 Q6NX49 Q9H6Q4 O14793 25909 P54619 22870 2550 9181 P51587 9184 P17676 Q8TDD1 P15498 2547 P37288 57504 Q9HCJ0 P84022 O00151 Q92731 O14788 6901 10413 Q7Z6R9 25913 P28330 P08729 Q92729 Q5VTR2 P51114 O60216 79763 P14174 57510 54487 Q9H2G9 493856 4734 Q7Z727 Q9H2G4 O00167 22850 Q9C010 81628 54496 6927 Q8IUD2 3416 6929 P62256 Q6AHZ1 Q96KM6 Q8N680 811 6921 P40425 P40424 P62258 6926 6925 Q6IQ32 Q17R98 Q9C005 333929 Q9UIS9 Q9NZI7 P31943 P48382 Q9NQX0 P31946 Q9C009 P31947 2100 Q9NR96 P48380 O95343 P54646 3428 P16333 6938 P20823 55352 O60674 P20827 P19838 O60675 Q8IUC6 P62263 P18509 P31949 5604 6936 Q9Y468 P27037 Q9H2M9 Q13956 Q8IZ40 4771 Q9Y463 4772 Q9Y466 Q8TDI0 Q9UIL8 Q9NUY8 O95352 2107 55364 10856 Q86VE0 2103 P16383 27287 80324 Q08999 O14744 6945 Q9Y478 P50222 4780 Q92769 Q14814 P09619 P28370 Q07666 O95361 80306 P10600 O95365 Q06330 Q96KS0 2119 10865 P19419 Q6PI57 Q9HCM4 4775 O14753 Q6ZW31 847 7804 O14757 Q92766 P09622 4790 4791 4792 Q9H2K2 Q9H2K0 2130 81669 P18545 Q9NR48 Q9HCL2 P60033 Q92753 4302 P05230 857 O14763 859 Q96CA5 Q92754 Q13501 22827 6500 Q13503 Q9NR50 O95382 Q14839 Q8NC51 P19883 Q13506 Q9NR55 Q9HCK5 2139 861 862 863 192669 Q9UMX1 Q9UMX0 4799 P09601 50943 P08754 Q9Y3Y2 Q9BS34 P01241 10492 P15408 Q9Y3Q8 3481 Q9UIH9 23326 O60603 25988 23327 P15884 Q8WUU5 Q96T88 3479 2147 Q8WV60 2146 3476 10011 P25205 7832 10013 84289 Q96KG9 55723 10499 10014 11345 Q6UWV6 135295 64682 Q9UII4 Q7Z6J4 4343 25998 Q96T76 O14717 Q99750 406 Q9BRP8 O43294 408 84271 409 Q13098 Q01518 Q13099 Q96C55 Q09472 Q8N1G0 O43251 23309 5682 5683 5684 5685 5686 116986 64215 116987 P13686 890 Q00653 891 Q99743 894 Q99741 896 Q9NR09 898 3014 29080 10477 Q9UN42 29086 P49639 P61326 P51531 4361 5692 5693 8721 7874 148156 Q9NUX5 Q9NR11 P51532 Q96ST3 55750 P60484 Q15291 11331 5687 55758 5688 10486 4358 10001 P60002 10488 Q96T58 5207 10487 P28702 P23497 Q3MII6 P26583 Q9Y3M2 Q6FGG2 P46199 Q96BZ9 P50613 Q5R372 P51946 23764 25942 Q8NFW5 Q9NUQ3 Q8WUY8 Q96P48 433 79735 10451 10450 8726 P55072 10454 Q8N5U6 O43242 7410 90993 Q9HCE7 Q08117 3054 Q05086 Q9BRU2 29079 P37231

Q9HCE1 Q96SZ6 P51959 79723 8737 10468 23746 P24385 P24386 8754 7422 Q96GA9 5245 3068 Q9HCD5 P52815 22893 3066 Q9UMN6 3065 Q9UIF9 10437 O75293 O75290 79753 10438 Q00613 Q96P20 55787 55785 7415 10432 3070 Q13901 9612 P49674 Q9UIG0 P52824 3074 Q9Y3P9 P17612 466 468 55796 P28749 Q9NQL9 7428 P04792 56647 6595 6597 6599 6598 Q9Y4K4 O75386 P10599 O75385 6591 Q9Y4K3 Q76L83 3084 O43318 473 474 476 23286 9617 51763 8767 P43034 P38919 3093 Q13127 Q15303 B2RXF5 3091 Q16633 P55036 8301 Q16635 Q15306 Q86VP6 O75376 P58546 P13631 24149 3096 5270 Q02447 O75381 Q9NVP2 51773 30813 P46531 7448 Q13133 Q15796 Q15311 Q13131 8795 Q16649 Q15797 9640 P12757 Q8N6I1 9643 7468 P54198 O75367 Q9UNL4 P12755 O75362 Q8TDS5 O75360 Q96CN4 P46527 51780 Q01101 P42167 Q01105 6128 O94805 7458 Q13144 Q5VUA4 54815 Q8WVM0 Q9BSI4 Q8IV61 Q16650 Q13148 P35869 P43490 P20393 7476 Q16656 Q15329 Q8NCA9 P18074 Q8WVL7 P55055 O43306 P10588 P10589 93166 Q86VN1 Q9NZZ3 Q8N6H7 Q16665 51317 Q49AN0 Q13153 P43489 Q8TDY2 9667 Q16666 Q5VUG0 P54132 P63092 P35453 Q02413 Q14494 8328 Q14498 Q16670 P46108 Q14011 Q86W54 Q9NRC8 7013 7014 P46100 Q9Y4H2 P35443 Q5PSV4 7490 P13682 P11021 P63096 Q9C0F3 84232 Q8WW38 Q9GZM8 Q13177 Q9NRD5 Q99835 7024 7023 10392 Q9C0F0 49854 P98179 P15822 9682 7020 Q02878 Q9UNH7 51341 54851 Q9NVM4 Q96PK6 11243 55704 P55010 Q13185 Q8NCF5 P23443 6188 Q15369 Q8IUX7 O43741 Q9BX66 Q9BSM1 Q09028 P38936 6182 P11474 Q9UNI6 26747 Q15370 54862 P57682 Q6PID4 Q99816 P48730 Q9BWT7 9208 Q99814 P78545 6199 7046 6198 P98155 P35813 P78540 P10515 O75787 O43711 6193 10811 O43719 Q9Y4C1 P62195 Q4LE39 O15234 9219 1738 P05177 1737 A8K8V0 P78536 7057 22803 Q13526 112950 22806 22807 55291 57473 83737 55294 Q3V6T2 P46934 P46937 83746 Q96GN5 81565 O60296 Q4LE28 P42574 P98174 Q9UER7 Q14865 8399 7068 7067 Q5TAX3 Q15714 Q13535 Q9H307 P19793 P29590 Q15717 Q7Z2Q5 Q63HK5 P31431 Q01196 84619 Q9H6W3 P41235 1756 Q9HCP6 Q92793 Q96GM5 Q9HD15 Q9Y4E5 O60266 O60264 P05155 Q9NRA1 O60260 P59817 Q13547 Q13546 9252 7073 Q9UNE7 P41223 57018 P43405 1763 Q9NZR4 Q04206 Q92786 Q92785 Q92784 P41229 7088 Q15735 P78509 93594 9261 Q01167 P63000 Q9BSG1 51720 Q14894 4800 Q13563 P08246 2626 7099 7098 Q9NVC6 Q9NQZ2 Q14416 7091 P14923 7090 Q9NQZ8 P30153 P11413 1789 1788 Q05516 1786 Q9UIV1 1785 Q05513 P00734 375790 Q13574 51735 Q13573 Q15750 Q9HCU4 P55884 93134 Q9H2S9 Q96CJ1 P32320 P78527 92283 85509 2644 1312 Q86VK4 Q04656 84661 51742 Q6ZNG0 P05198 P20333 Q9Y4A8 Q8IYX1 30827 Q13107 Q9P287 P62191 Q96H20 O60238 Q9HCS4 900 902 P00750 Q9HD40 Q13112 P08238 Q9NZN8 P08235 P21675 Q9NZN9 Q92618 Q9H609 Q92616 Q9Y2W7 Q9NYD6 Q6P2D0 O75820 2672 Q12948 22992 O00268 Q6QNY1 O00267 79894 3516 Q9BZI1 Q96JM7 2665 Q96JM2 Q5VWX1 2664 283248 Q9ULW3 Q92610 P63208 4841 Q9ULW0 Q8N587 P07996 10533 Q12950 P52564 P04004 Q9Y2X7 Q12952 Q9Y2X9 Q8NB12 Q8NAP3 Q9P107 5706 5707 79885 5708 5709 P16473 51099 P16471 4851 5700 4854 5701 5702 Q96JL9 5704 Q92600 5705 65264 P50395 Q9ULX9 Q9NYF8 Q9Y2Y8 Q12968 29128 22974 10514 5717 83933 26097 P20936 Q9UQB3 4862 O75807 5713 57649 Q6IT96 4869 5716 Q96JK9 Q12972 Q0D2J5 Q9H9T3 1374 3551 Q6P2C8 P17302 5728 10524 P17301 Q9HBE1 Q99697 2697 400961 Q9ULZ3 Q9UHR5 Q9ULR5 O95477 P28482 Q9UHJ3 Q12986 1385 O00220 P51668 29108 P51665 P19532 Q9P0J0 Q9P0J1 57669 P15173 29102 P07550 P04040 58517 10971 7913 P08887 O14867 Q96BA8 10973 Q9UM47 P51671 Q9Y2T7 Q96SB4 Q9UHK0 Q9NYA1 O00231 O00233 P10721 O00232 P19544 Q8WYH8 57678 29110 79444 960 1388 29115 Q9P0K8 1387 1386 4898 P42771 Q9UM54 4899 Q9H1I8 10985 Q9NYB0 P30304 22937 P30307 22938 Q9UHL9 22931 P62333 2247 284119 P15153 2246 1398 58533 Q9BZK7 975 6601 Q9Y2V2 Q92630 6605 57209 Q9UM63 Q969M7 P51693 Q9Y376 P51692 P31629 Q9BQY4 O00255 Q8NEZ4 Q9NQ33 O00257 Q03164 Q9P0M6 440193 P17342 984 Q5H9I0 988 P05362 Q9Y385 6613 Q9Y2W1 P06213 P07900 Q9UPV9 5300 P13349 2274 P53803 P52952 990 Q8WTS6

991 126208 Q96RU8 Q96RU7 Q8WTS1 79810 995 51003 Q99640 79811 997 6622 79813 6625 54998 51008 10138 Q9UQ80 Q04917 P49770 Q9UM07 Q9Y2P0 Q7Z5H3 5311 Q9UQ88 5796 Q5T7W0 O60739 P11177 2280 Q8WTR2 8819 P48436 8812 Q99638 P01344 P01343 P49768 O00206 2290 23429 P48431 5321 Q96JC9 3142 Q9NU63 148022 Q99623 Q00535 Q00534 Q96S42 Q8NAF0 P49756 P25789 55869 P25788 7975 6647 6646 5316 51028 91949 Q9UPY3 P25787 P01375 Q9UPY8 23439 6660 P49750 8841 Q9UHI6 O00213 Q3KNV8 23435 Q96AQ6 3151 O60716 P12004 10128 Q96RR4 Q3KNW1 3148 Q15170 545 546 3146 P61457 Q96EY1 Q96JB5 6657 5327 Q8WU17 O43353 6670 6672 23409 8851 Q969H0 Q9UPR3 P07948 Q9H9S4 Q9Y2K7 Q9ULJ3 Q9H9S0 3162 Q12905 O95076 Q8WYA1 Q12906 Q9BZE0 Q01844 552 Q9BZE4 P42704 3159 Q99683 Q8WTW4 Q99684 6667 5336 P49336 P62714 P05771 Q9H5J8 10577 P07951 Q96FA3 Q969G3 8864 7532 7531 10580 7533 P55198 Q9ULK4 23411 23414 3171 Q00987 Q96S94 3169 Q9NU19 Q9H1A4 5347 7529 7528 Q08462 Q9H5I1 64375 P50750 Q86U86 4035 Q9UHD2 3187 Q9UPT9 3185 3184 Q9BUZ4 571 3182 P51608 P68036 7536 6207 Q9H5H4 Q01826 5371 3192 8880 P27540 8881 Q60FE5 Q9UPU9 Q12933 Q9ULM3 7555 Q9ULM6 P51610 Q9BUY5 580 P16885 54583 O14818 80854 P51617 Q9NPJ6 7549 54106 8878 P60568 6231 8892 Q15654 9744 O43432 8896 P15923 Q86YW9 29947 Q96FV9 595 84324 116 Q7Z6C1 P22694 Q13485 Q8WZ64 8408 O94906 P03950 O00716 Q16512 4067 Q16514 P12429 Q15669 O75496 O75494 Q7LBC6 P10242 P10243 P10244 84312 51412 O94915 Q13492 Q16520 6239 54925 Q15672 Q9UQR1 7101 6256 Q9HBW0 Q16526 P35998 Q2M1K9 23381 Q9UDY8 7579 Q8WZ42 P00441 9774 4086 7112 4087 4088 Q8N9N2 4089 O75478 Q15208 9775 8445 163126 Q9H257 O75475 Q8N5F7 142 P21359 P31150 P24864 P62701 84333 P60520 Q16543 Q15697 53615 Q13033 8454 P54259 O75469 8458 64750 7124 P54252 P54253 Q96KC8 P14635 O75461 Q9UQL6 P10275 4092 P10276 4093 O75460 4094 154 P23588 Q13042 Q99728 Q99729 Q13045 55806 P45379 P49736 7132 Q86UR1 8463 7133 8467 9314 O43889 P36896 P36894 23378 P15976 P35568 Q96C28 6282 1822 166 Q8NFM4 55810 P01308 Q8NFM5 120892 338917 Q9NQB0 Q99717 1828 P49721 P49720 Q15233 Q96T37 P00492 9326 Q8N5K1 P54274 O75446 P36402 O75444 6294 7141 Q99700 1831 Q96T23 Q9NZ45 Q9H1Y0 51465 Q00577 Q99708 P49711 Q16576 P49716 P49715 55827 Q9NQC7 Q96C00 64784 7158 O75437 Q08050 P39905 7157 P19086 64786 162239 P14625 Q8N9R8 Q02750 P35548 138151 55832 Q9UDV6 1848 Q5VTD9 Q15257 Q13077 Q13873 O95835 7169 26038 10928 26037 Q06787 Q86Z02 56252 Q01094 Q9H204 26039 7161 9341 P63167 P42224 1852 10933 1850 10935 P17275 P23945 Q7RTN6 P63165 Q9BVI0 P22612 P42229 P15090 57109 Q969T4 1856 P42226 O15372 O94992 Q9P0W2 22926 Q969S8 22927 Q13404 Q13887 Q01085 57592 Q8IY57 57594 P10644 O43847 57591 Q96BD5 79365 P84095 Q08945 Q9UHX1 P60900 1869 7186 7189 7188 Q8WYK2 O43812 P11940 7181 Q13418 7182 7185 7184 1877 Q86UE4 Q86UE3 1874 Q9Y3C7 P17252 P68871 1870 P09630 O15379 Q969R5 Q13422 O15391 Q8NB78 4914 2735 27352 P19438 O43829 56288 84733 1408 1407 1406 2737 Q92667 2736 9388 Q96G30 Q8WUI4 8050 27327 Q02386 Q7RTR2 P62277 1896 Q8WYQ5 Q9HC52 Q5H9R7 Q14774 Q13443 P22670 Q9NQ92 9391 26005 P11532 Q96G25 57154 P30279 84759 58487 Q9UQE7 P30281 Q8WYP5 P23511 Q8WUH2 3609 3608 3607 Q14781 P23510 P21333 Q9NYJ8 94104 O14492 8078 Q6ZV73 O14495 Q9P0T4 29966 P19474 Q02363 58491 P18146 57162 84305 27300 3611 O60343 O60341 4943 P01730 Q969V6 O15350 O75832 O15353 Q15649 Q9BZS1 P53350 Q15648 Q9P0U4 P19484 Q86YP4 Q06710 Q96BF6 Q8N5A5 Q9UHV2 3622 2773 Q9HC29 P30260 O14497 Q15652

intracellular protein transport 9182 9181 Q9NRW7 Q5SQT9 Q9P2R6 Q9BVA0 1213 Q92973 O14662 Q9Y2X3 P60059 10651 3875 P27348 Q92974 P55957 Q8IWJ2 55696 10427 P42858 Q9H2G9 O15400 4734 1121 Q12846 Q96P70 6809 P62491 30000 Q86WA8 811 5830 P63104 Q92834 P62258 4869 P53675 P26374 P31946 56681 O95343 P53677 O60318 81622 Q8NFW9 O60674 1122 6810 6811 27072

P41743 P04233 Q9H1C4 O75901 Q9H2M9 P28482 1020 Q9P0J0 Q9NRS6 10736 3313 O60763 Q4VX76 Q9Y2T2 4646 10971 O95487 P32856 O95249 P33947 P53618 Q9NR31 O60895 6711 604 Q9UM54 P53621 4792 O60749 O60502 Q9NS69 10953 401505 2247 58533 5514 6726 P51572 P53992 P30536 Q13505 P51571 P31749 1176 O95140 1174 1173 Q9NQT8 10724 Q92621 O15504 Q9UL54 Q92624 6993 10490 Q9UPV9 P04637 O96008 P38646 O60725 Q99523 2147 8924 Q15021 10013 10134 637 10133 Q9C0K7 6748 51009 6747 Q15388 Q04917 4580 Q9Y678 6643 6642 O95166 23214 P50542 55973 11231 406 Q96CW1 Q15036 P60468 O00327 6890 O00560 2054 23303 Q96CV9 O00203 10239 79711 Q00535 Q02952 Q9Y5X1 Q9GZP9 8943 P49754 54535 P01137 O14964 91949 P01375 4000 O14980 23673 P12004 P54920 O14617 9804 P24588 9927 O00623 O00505 O43592 O00628 5584 O00629 P05408 P14598 Q8IYJ3 P61981 O14908 7514 10452 P55072 P05412 7532 5111 7531 7534 5476 7533 Q9HCE7 P37108 10228 10469 Q99797 8615 207 9702 P05783 7529 P27797 64130 P24386 22895 Q9NW64 Q96QU8 O14925 56993 Q00610 7415 P83436 P49792 5371 4285 8766 6103 Q9UKK6 O00410 23633 Q6ZWJ1 Q08209 P52948 P40855 10204 P25116 Q8TEW0 Q16623 8775 Q9UPN3 P35658 94121 94120 473 117584 23163 23165 P62913 Q15785 P49815 P25963 Q9NPC8 Q9HAV7 Q8TD31 8546 9632 11196 64601 79083 O75381 Q9UIA9 415117 8417 9868 Q9BUN8 Q15436 6496 Q9UNL2 8677 8676 Q9H269 29927 Q9Y6Q5 8548 Q13023 P61966 51429 P61619 6386 Q9Y2J0 P62826 P62829 Q86XR8 Q9UQ26 23039 Q8N4C7 P59780 P61970 Q16543 9527 9648 Q03518 Q49AN0 9784 11140 O43752 7124 10053 Q9UEU0 O94826 5195 Q96C24 161 Q9Y5J6 7249 8218 Q9NRD5 Q86Y82 Q9BV36 23463 A1XKG3 O43615 Q9UNH7 Q6VY07 P01579 P02545 P09038 2923 55705 Q9BY84 O94973 O75558 7157 26985 O75674 Q9Y5L4 O75431 Q8N4H5 Q8TEX9 O43747 Q8WZ73 Q9H173 Q13190 11014 P53367 9463 O95714 Q96QC0 P63172 84958 83752 79139 Q92572 Q7RTN6 Q14974 O15131 9590 Q01082 9472 8021 P46934 P43307 10947 P52292 O60296 P10619 P52294 O75886 P29590 P30101 Q4LE60 60412 Q9NZQ3 Q9NSC5 O15397 27236 Q9UNE2 64901 9495 653361 56288 1408 Q9UBN7 9146 Q9UBF2 Q9NP61 P41182 O00189 3836 P35606 9276 P56962 O95405 Q9H2T7 57154 Q15629 P51159 83548 O60499 P63010 P00734 3841 3843 80273 5901 O75843 100287932 1315 1314 Q9HAP6 9179 11097 P62072 Q13596 51517

positive regulation of cell death P25054 Q7Z628 P28562 O75940 P10911 Q5VV41 9181 P51587 P17676 P15498 Q9BVA0 O15519 P84022 O00273 Q92731 Q92851 4722 P07996 Q92974 P52564 P55957 P42858 4851 P10809 Q5S007 Q7Z727 Q92835 Q9NR80 Q9NYF8 Q9NR81 P17405 57646 Q8IVF5 O60543 Q9UKW4 P62258 Q9H7P9 P31946 3672 P31947 P48023 2100 Q9BZF9 1130 Q12979 9093 5728 3428 Q99698 O60674 Q8IUC6 Q9ULZ3 Q8N5V2 Q9UHR5 Q12981 P28482 P50570 O00220 29108 1020 114548 P52756 Q9P0J0 57787 1499 P07550 P07437 P29466 Q9Y239 Q9H4P4 Q9NZC7 P10600 O95243 Q5VST9 P19544 Q9NS56 3329 1029 Q9BWF2 1027 960 1026 29115 841 843 604 P42771 Q70UQ0 Q07890 P07766 5883 P50591 1285 Q13618 Q13617 Q96L34 Q13616 P30542 Q9NS68 6609 P38398 7818 Q96KQ4 Q07889 P61247 Q92630 O14763 5515 5879 Q9UM63 5518 Q13501 P10826 Q13625 P31749 Q9H8V3 O00255 P40692 5524 P09601 7704 4318 P23396 8930 P48454 P04637 P56199 3482 O60603 O14827 Q7L523 Q99523 203068 Q96RU2 5533 10013 637 P62753 P23025 10016 P25445 O96017 Q9Y5V3 4221 Q7Z6J4 10383 O76074 O43290 4214 84033 P01589 Q99638 O43293 P49768 O00206 Q6ZN33 Q9UMR2 6772 Q8N726 5321 P52735 23787 P11388 148022 P14780 Q00535 P01019 51147 6647 Q9Y572 P01138 55504 P01137 P01375 P01374 Q9UHI6 P35222 O00213 O14737 11218 P60484 P17936 3146 668 Q8WTP8 Q96EY1 Q15052 8718 11214 8837 8717 O43353 O00744 5581 P01042 Q92934 8851 Q8WYA6 Q9UNN5 3162 672 Q99683 10451 5578 6789 P05412 2081 5591 Q6IR47 8743 7410 7531 Q5JSP0 P37231 Q01955 324 3169 8737 9826 207 5468 7529 8738 Q15078 6453 Q12802 572 Q96P20 5599 5371 Q12933 O43464 4168 P06729 P15559 P52701 580 23513 P58012 8517 8878 8772

O15085 Q13114 8655 9500 7204 8654 O43557 Q9Y4K3 351 472 355 356 Q6UY14 9616 P49810 8767 P62913 Q13009 P13631 P46531 9641 8795 P35638 6256 Q96IZ0 10193 O75489 29 Q9UNL4 64857 Q9NY61 P46527 23263 P00441 6262 9774 Q14116 10081 4088 P18074 8445 P10586 P68371 P21359 8453 Q14249 8454 8452 Q13158 10293 Q16666 7124 23365 O75340 23368 P0C1Z6 O75460 154 Q02535 121512 Q9Y6K9 O94827 Q99728 11269 Q8WV24 Q13043 5074 10181 7013 O43521 P36896 O43524 Q9Y5J5 7490 23370 120892 11035 23229 Q6ZSZ5 Q8TCU6 7023 10392 O75569 O15068 P54274 A1XKG3 Q01664 P21145 P01579 11124 Q9BRK4 10276 P47895 Q13188 51107 Q14155 2923 7153 6188 64782 7157 7159 P38936 O43508 P35548 1843 2810 Q14160 Q93034 Q14164 10285 Q93038 1728 1848 Q15257 Q15375 Q15811 7046 Q96RE7 Q86Z02 7161 6194 1613 P63167 56259 P22736 P42224 91 P39060 Q13761 1616 7057 Q96QB1 Q01085 7052 P67775 P16070 P05067 P42574 P42575 P98171 P98174 Q9UER7 2956 7187 7186 7189 P06239 O94768 Q9H305 P19793 Q96M96 P29590 P30101 Q9BXK5 Q8N163 P17252 P21980 3932 P11717 P55212 A4D1W7 Q13546 Q02156 7073 Q8N8D1 7074 Q92785 Q92542 P78560 Q13794 Q9UBN7 Q9NZJ7 O95999 Q13315 Q9UBF6 P41182 Q16828 P53567 P00747 Q03468 P63000 Q96PE2 P47712 7099 P51398 Q13323 Q15628 P30153 Q96BI3 P51159 8065 Q07817 Q05516 1785 P08138 P09429 50649 P10415 O75962 29843 P78527 O60229 1676 121457 50650 Q14790 51741 O15350 P53355 Q16611 Q9P0U3 Q06830 O60238 P00519 O60356 P45983 Q9NZN5 Q13233 P45984 P21554 Q13352

positive regulation of programmed cell death P25054 Q7Z628 P28562 O75940 P10911 Q5VV41 9181 P51587 P17676 P15498 O15519 P84022 O00273 Q92731 Q92851 4722 P07996 Q92974 P52564 P55957 P42858 4851 P10809 Q5S007 Q7Z727 Q92835 Q9NR80 Q9NYF8 Q9NR81 P17405 57646 Q8IVF5 O60543 Q9UKW4 P62258 Q9H7P9 P31946 3672 P31947 P48023 2100 Q9BZF9 1130 Q12979 9093 5728 3428 Q99698 O60674 Q8IUC6 Q9ULZ3 Q8N5V2 Q9UHR5 Q12981 P28482 P50570 O00220 29108 1020 114548 P52756 Q9P0J0 57787 1499 P07550 P07437 P29466 Q9Y239 Q9H4P4 Q9NZC7 P10600 O95243 Q5VST9 P19544 Q9NS56 3329 1029 Q9BWF2 1027 960 1026 29115 841 843 604 P42771 Q70UQ0 Q07890 P07766 5883 P50591 1285 Q13618 Q13617 Q96L34 Q13616 P30542 Q9NS68 6609 P38398 7818 Q96KQ4 Q07889 P61247 Q92630 O14763 5515 5879 Q9UM63 5518 Q13501 P10826 Q13625 P31749 Q9H8V3 O00255 P40692 5524 P09601 7704 4318 P23396 8930 P48454 P04637 P56199 3482 O60603 O14827 Q99523 203068 Q96RU2 5533 10013 637 P62753 P23025 10016 P25445 O96017 Q9Y5V3 4221 Q7Z6J4 10383 O76074 O43290 4214 84033 P01589 Q99638 O43293 P49768 O00206 Q6ZN33 Q9UMR2 6772 Q8N726 5321 P52735 23787 P11388 148022 P14780 Q00535 P01019 51147 6647 Q9Y572 P01138 55504 P01137 P01375 P01374 Q9UHI6 P35222 O00213 O14737 11218 P60484 P17936 3146 668 Q8WTP8 Q96EY1 Q15052 8718 11214 8837 8717 O43353 O00744 5581 P01042 Q92934 8851 Q8WYA6 Q9UNN5 3162 672 Q99683 10451 5578 6789 P05412 2081 5591 Q6IR47 8743 7410 7531 Q5JSP0 P37231 Q01955 324 3169 8737 9826 207 5468 7529 8738 Q15078 6453 Q12802 572 Q96P20 5599 5371 Q12933 O43464 4168 P06729 P15559 P52701 580 23513 P58012 8517 8878 8772 O15085 Q13114 8655 9500 7204 8654 O43557 Q9Y4K3 351 472 355 356 Q6UY14 9616 P49810 8767 P62913 Q13009 P13631 P46531 9641 8795 P35638 6256 Q96IZ0 10193 O75489 29 Q9UNL4 64857 Q9NY61 P46527 23263 P00441 6262 9774 Q14116 10081 4088 P18074 8445 P10586 P68371 P21359 8453 Q14249 8454 8452 Q13158 10293 Q16666 7124 23365 O75340 23368 P0C1Z6 O75460 154 Q02535 121512 Q9Y6K9 O94827 Q99728 11269 Q8WV24 Q13043 5074 10181 7013 O43521 P36896 O43524 Q9Y5J5 7490 23370 120892 11035 23229 Q6ZSZ5 Q8TCU6 7023 10392 O75569 O15068 P54274 A1XKG3 Q01664 P21145 P01579 11124 10276 P47895 Q13188 51107 Q14155 2923 7153 6188 64782 7157 7159 P38936 O43508 P35548 1843 2810 Q14160 Q93034 Q14164 10285 Q93038 1728 1848 Q15257 Q15375 Q15811 7046 Q96RE7 Q86Z02 7161 6194 1613 P63167 56259 P22736 P42224 91 P39060 Q13761 1616 7057 Q96QB1

Q01085 7052 P67775 P16070 P05067 P42574 P42575 P98171 P98174 Q9UER7 2956 7187 7186 7189 P06239 O94768 Q9H305 P19793 Q96M96 P29590 P30101 Q9BXK5 Q8N163 P17252 P21980 3932 P11717 P55212 A4D1W7 Q13546 Q02156 7073 Q8N8D1 7074 Q92785 Q92542 P78560 Q13794 Q9UBN7 Q9NZJ7 O95999 Q13315 Q9UBF6 P41182 Q16828 P53567 P00747 Q03468 P63000 Q96PE2 P47712 7099 P51398 Q13323 Q15628 P30153 Q96BI3 P51159 8065 Q07817 Q05516 1785 P08138 P09429 50649 P10415 O75962 29843 P78527 O60229 1676 121457 50650 Q14790 51741 O15350 P53355 Q16611 Q9P0U3 Q06830 O60238 P00519 O60356 P45983 Q9NZN5 Q13233 P45984 P21554 Q13352

positive regulation of apoptosis P25054 Q7Z628 P28562 O75940 P10911 Q5VV41 9181 P51587 P17676 P15498 O15519 P84022 O00273 Q92731 Q92851 4722 P07996 Q92974 P52564 P55957 P42858 4851 P10809 Q7Z727 Q92835 Q9NR80 Q9NYF8 Q9NR81 P17405 57646 Q8IVF5 O60543 Q9UKW4 P62258 Q9H7P9 P31946 3672 P31947 P48023 2100 Q9BZF9 1130 Q12979 9093 5728 3428 Q99698 O60674 Q8IUC6 Q9ULZ3 Q8N5V2 Q9UHR5 Q12981 P28482 P50570 O00220 29108 1020 114548 P52756 Q9P0J0 1499 P07550 P07437 P29466 Q9Y239 Q9H4P4 Q9NZC7 P10600 O95243 Q5VST9 P19544 Q9NS56 3329 1029 Q9BWF2 1027 960 1026 29115 841 843 604 P42771 Q70UQ0 Q07890 P07766 5883 P50591 1285 Q13618 Q13617 Q13616 P30542 Q9NS68 6609 P38398 7818 Q96KQ4 Q07889 P61247 Q92630 O14763 5515 5879 Q9UM63 5518 Q13501 P10826 Q13625 P31749 Q9H8V3 O00255 P40692 5524 P09601 7704 4318 P23396 8930 P48454 P04637 P56199 3482 O60603 O14827 Q99523 203068 Q96RU2 5533 10013 637 P62753 P23025 10016 P25445 O96017 Q9Y5V3 4221 Q7Z6J4 10383 O76074 O43290 4214 84033 P01589 Q99638 O43293 P49768 O00206 Q6ZN33 Q9UMR2 6772 Q8N726 5321 P52735 23787 P11388 148022 P14780 Q00535 P01019 51147 6647 Q9Y572 P01138 55504 P01137 P01375 P01374 Q9UHI6 P35222 O00213 O14737 11218 P60484 P17936 3146 668 Q8WTP8 Q96EY1 Q15052 8718 11214 8837 8717 O43353 O00744 5581 P01042 Q92934 8851 Q8WYA6 Q9UNN5 3162 672 Q99683 10451 5578 6789 P05412 2081 5591 Q6IR47 8743 7410 7531 Q5JSP0 P37231 Q01955 324 3169 8737 9826 207 5468 7529 8738 Q15078 6453 Q12802 572 Q96P20 5599 5371 Q12933 O43464 4168 P06729 P15559 P52701 580 23513 P58012 8517 8878 8772 O15085 Q13114 8655 9500 7204 8654 O43557 Q9Y4K3 351 472 355 356 Q6UY14 9616 P49810 8767 P62913 Q13009 P13631 P46531 9641 8795 P35638 6256 Q96IZ0 10193 O75489 29 Q9UNL4 64857 Q9NY61 P46527 23263 P00441 6262 9774 Q14116 10081 4088 P18074 8445 P10586 P68371 P21359 8453 Q14249 8454 8452 Q13158 10293 Q16666 7124 23365 O75340 23368 P0C1Z6 O75460 154 Q02535 121512 Q9Y6K9 O94827 Q99728 11269 Q8WV24 Q13043 5074 10181 7013 O43521 P36896 O43524 Q9Y5J5 7490 23370 11035 23229 Q6ZSZ5 Q8TCU6 7023 10392 O75569 O15068 P54274 A1XKG3 Q01664 P21145 P01579 11124 10276 P47895 Q13188 51107 Q14155 2923 7153 6188 64782 7157 7159 P38936 O43508 P35548 1843 2810 Q14160 Q93034 Q14164 10285 Q93038 1728 1848 Q15257 Q15375 Q15811 7046 Q96RE7 Q86Z02 7161 6194 1613 P63167 56259 P22736 P42224 91 P39060 Q13761 1616 7057 Q96QB1 Q01085 7052 P67775 P16070 P05067 P42574 P42575 P98171 P98174 Q9UER7 2956 7187 7186 7189 P06239 O94768 Q9H305 P19793 Q96M96 P29590 P30101 Q9BXK5 Q8N163 P17252 P21980 3932 P11717 P55212 A4D1W7 Q13546 Q02156 7073 Q8N8D1 7074 Q92785 Q92542 P78560 Q13794 Q9UBN7 Q9NZJ7 O95999 Q13315 Q9UBF6 P41182 Q16828 P53567 P00747 Q03468 P63000 Q96PE2 P47712 7099 P51398 Q13323 Q15628 P30153 Q96BI3 P51159 8065 Q07817 Q05516 1785 P08138 P09429 50649 P10415 O75962 29843 P78527 O60229 1676 121457 50650 Q14790 51741 O15350 P53355 Q16611 Q9P0U3 Q06830 O60238 P00519 O60356 P45983 Q9NZN5 Q13233 P45984 P21554 Q13352

regulation of macromolecule metabolic process Q9UKT9 P25054 Q9H0E3 Q9Y265 Q8NDW4 Q12824 8091 124790 10657 2305 2304 2303 1454 114991 1453 2300 Q8N3U4 O14543 155061 Q9Y275 284695

Q00839 2308 2307 Q9H0D6 Q92974 Q9H4L7 Q12834 Q12837 Q8WXB4 Q8NDV7 P16104 2316 2 10661 P10809 Q96EB6 Q5S007 O75925 136319 Q9UKV8 Q92956 4990 3660 3661 Q9BYE7 P62495 P37023 P13056 84901 57761 55588 Q9Y297 O75928 Q9UKW6 3659 P26358 Q6NUN9 Q5BKZ1 3673 Q92949 Q99583 Q12857 Q13705 10645 1488 1487 283337 Q99576 2332 3665 3667 10642 64061 P26367 10644 10643 Q9UL15 Q9UL18 1022 1021 1020 Q8NA42 Q06587 114548 Q8IX07 Q5JT82 P51787 Q96MX3 1017 10614 79576 Q9HAJ7 1499 10616 1012 O14981 Q9Y230 P61586 O75909 Q92530 3678 O14503 Q12873 3690 Q12872 Q9Y239 Q8WXF0 Q9Y5Z7 Q8WXF1 6720 Q15906 285527 Q96J02 O00358 Q9BPY8 57798 1029 Q9BUB1 3207 10626 1027 1025 Q9BUB5 P17482 3685 P17480 604 Q9Y242 3689 P04179 Q15910 Q9UL36 Q9UGJ0 Q12888 5883 Q9BQ95 O60869 79595 Q8NA19 P38398 57326 55145 3215 Q96N67 P20618 Q92990 Q9Y250 Q5TCX8 6721 Q92994 Q92993 6726 Q8N488 Q92997 Q9UKS7 P10826 Q9UKS6 Q9UL46 P10827 P10828 221937 5411 P31749 Q8NDX6 1050 P53999 79101 P40692 57332 3226 79589 3225 P04150 5888 Q9UGL1 Q9UKT4 A6NHR9 Q92985 O95159 Q9UKL0 O96004 O00308 P14317 P14316 23569 11108 11107 Q8WWY6 Q99523 1059 23560 Q15022 639 P62875 6749 O96018 6760 P06702 Q9Y5V3 5430 O96019 O95163 P16989 P57059 P36575 Q03933 51131 641 P33076 51132 P35251 Q14186 Q6B0B8 79923 8932 648 8939 P49407 P60228 Q14188 O00327 O96028 5440 P48552 5441 6772 Q9UKN5 Q8TAU3 P48551 6774 Q99990 3265 346171 3263 O60828 P15621 Q66K89 Q2M3W8 Q14192 P35240 8945 P01019 51147 Q9Y692 O14964 Q15047 O14965 P23246 Q9Y5X9 Q86X95 5451 A7MD48 Q8N3J9 P35222 Q5T6S3 P14784 P35227 P35226 O14979 P57082 O75081 P17813 P35232 Q00403 53335 56849 668 6776 4116 Q96MH2 Q16385 P24588 Q15056 7629 O43474 P06733 6790 O00744 5460 Q9Y5Q3 6311 P62837 9821 P25490 Q9NX61 3281 Q9NX65 672 P15692 O14908 Q9UBW7 6304 57708 10213 Q6UUV7 P05412 O00755 Q9Y5R5 Q9Y5R4 9831 3297 Q9NX70 23532 Q9UBX0 P14373 Q9NWV8 57713 688 148327 689 5467 9826 207 5468 54206 Q8N895 Q92922 P01023 4150 4152 4154 23509 P48594 P56178 9841 P56177 Q8NHY6 Q12809 O00762 Q9NWW5 Q9Y5S9 P17844 10673 4149 O14920 57727 P62854 9839 O43463 5494 Q8NHX1 23512 23513 Q96QT6 O14936 8517 Q92900 9849 P49427 4172 O15085 P36508 4173 4174 11060 P35659 Q14686 4176 Q14209 8535 11065 P55265 Q9BZ95 Q6NZI2 Q9H165 4171 P13861 Q9H161 Q9H9F9 Q9H160 P49810 P08047 5017 P78395 Q9BQG0 P07199 Q13363 Q9NPC8 Q14693 Q9HAV4 94234 4188 8543 P46782 5036 5037 79084 P58304 Q96F45 O15090 23051 Q04864 Q9Y2H8 9869 7205 Q8WY36 P00533 Q16401 Q96RL1 P61956 Q15554 Q9HAU4 8553 P35638 P61968 Q8IWY8 Q96IZ0 Q9UPP1 8555 P32121 P31274 P31273 P31276 O75123 Q9ULH7 23028 Q9NY61 4193 Q9Y6Q6 257 Q9H9D4 Q6DD87 Q15561 10197 8548 Q96RK0 51548 Q93009 51547 Q14232 P55290 P61964 Q15562 Q9BYU1 Q9Y6R4 5054 P62826 9412 Q9UPQ9 P36956 P35625 O75592 Q96F24 Q4AC94 P31270 Q9NPF5 Q13398 P43246 55915 9421 P78347 9425 7248 O75586 O75582 Q9Y6K1 Q93062 Q03989 Q9Y2C9 Q71SY5 Q9Y6K9 51564 Q15583 8569 55922 5074 10181 P78337 8100 55929 11030 Q9HAZ2 Q15109 P22392 O15055 O43524 26523 5071 7251 O75570 23492 O15047 P49848 Q15596 8110 P61925 Q3KNS6 Q86Y82 P02790 O15062 P49840 5089 O75569 166968 O95931 A1XKG3 23466 Q9Y6M1 23468 O15060 Q8WY64 79913 51588 P08069 10155 Q9BQI3 9439 Q96NG5 1956 286075 10159 Q8NEJ9 Q9NPA8 26986 64426 P37840 5092 64428 7272 1965 51593 Q9H9G7 54625 O95947 54623 Q14289 Q8IWS0 9463 Q96RE7 9464 P29375 P29374 27005 Q7Z589 Q9H0M0 1975 P22736 P42345 Q9BUG6 Q96EK4 Q9UGU0 P04085 Q92574 Q13761 1978 Q58WW2 P56524 9477 9474 O15496 O75534 Q02297 7291 O75530 7290 9470 7295 P24928 Q96AB3 1982 Q6P1K2 P16070 O75528 P29353 Q5JXB2 Q06643 P0C7X2 Q8WXI9 Q9BYM8 1994 1993 79039 Q96IQ9 O15499 8165 P56545 O95983 A4D1W7 O95503 55072 P78317 P78318 9495 Q8TBE0 Q9HAK2 Q9UH92 Q9Y2B9 P52597 Q96EP0 O14593 O95999 P35610 Q9NP66 9021 23081 O43918 23085 Q9UKY1 Q9H9B1 P85037 P31260 Q6P1N0 P31249 Q13322 Q14653 Q9NP71 Q9NP72 P61081 P46736 3728 83548 P61077

1540 Q9BUJ2 3720 5901 P46734 Q13330 P12956 Q6P1L6 Q9P031 P28065 Q9P035 Q9H4W6 Q00059 Q9BQA5 Q9Y6E7 Q9H582 P61088 Q96AE4 147808 Q96RG2 Q68DY9 P43694 O15455 Q9BQA1 5914 11091 O75953 P28074 63976 P28072 63978 P28070 5927 5928 P63279 P00519 5929 P63272 23077 P24941 51510 Q92585 Q15532 P43686 Q13351 Q14683 Q13352 5925 Q7KZF4 P10914 O95677 Q92858 P67809 Q9UBB5 Q9NS86 A5YKK6 Q9P2R6 O60566 4609 P16220 10772 5931 P49137 P27348 5933 O15516 5935 Q9NS91 Q9UBC3 P27361 65018 Q9H832 54361 9077 P50148 1107 P16234 O60573 284323 4613 O15524 1108 O15525 Q92838 Q9UK58 Q7Z3K6 Q92835 P05549 Q9NRY4 Q99471 Q7Z3K3 O60548 P53667 Q92830 Q9UJU2 284312 O15534 P49116 P61289 P03372 64170 Q92833 P06401 Q92831 P06400 Q92826 P07737 5970 5971 9093 Q9UBE8 Q99460 Q9NRZ9 P56705 9099 P17535 P17544 Q99459 P17542 P62140 57410 400720 P61296 3784 5966 10765 3309 P04233 P07307 P05129 5981 P50570 P11802 O00463 P52756 O00468 O95231 Q9UFF9 O14627 6829 10736 3315 Q6FHQ0 710 5976 P41218 Q8N2W9 4646 27161 5978 6827 P08648 P30556 65056 5991 93986 5993 1153 Q14938 O00472 Q06455 55250 Q9NS56 P37198 79685 3329 6839 10746 3326 2475 P08651 5500 5987 P26045 O95259 P07766 Q10586 P05106 P05107 6850 Q8ND82 P51449 Q6IA86 27148 Q13616 1161 O00488 O60506 O00487 P30542 Q02086 O60502 57448 P61244 Q9BXG8 O14641 4664 5511 Q12770 Q92878 5515 P05112 5516 26292 Q9UNY4 P08621 5518 P09958 O43186 Q12772 Q12778 O43189 Q05586 2023 Q02078 Q6KC79 10725 57459 10724 Q96LR5 Q99496 5524 P05121 P40337 7704 Q8N2K1 O00425 4690 P23396 O43159 6872 6871 P04637 2034 2033 Q9NVW2 O95271 O00422 Q9UFB7 4686 4204 P01116 4205 Q16236 Q15389 P01112 4209 Q9C0K0 6883 Q9BT49 4221 6885 Q9GZR2 O43167 23210 P35368 P50548 Q9BT40 O76071 Q9NRP7 84159 Q9P2K8 4214 P01106 6878 4216 P01588 11236 7727 56946 Q9C0J9 P01100 Q7Z7K2 7741 5562 Q8N726 9921 P14416 P52736 Q9NS23 Q53X93 6886 P01137 P01133 Q16254 Q9UJM3 P52740 7750 6421 5573 P04626 5575 Q9GZP0 5576 23673 2064 P52747 2063 11218 Q9NS37 Q99853 P10070 P17936 P09086 P10071 10363 10362 P02458 P01127 5569 2070 5580 5581 O43593 5582 5585 7764 22794 Q9UNN5 25822 Q96PU8 79612 O75190 Q96PU5 Q02930 7756 5577 Q8TEB7 5578 5579 10336 8607 P40763 P04201 2081 5591 Q9GZV8 4261 Q9Y4P1 Q6IR47 5111 Q3SY56 64127 P37108 O75177 O76024 P18850 P15336 5590 O75182 Q9NRL2 56987 51295 P18846 Q68CJ9 P18847 324 P18848 326 5104 55657 Q92800 P27797 Q9GZU7 P01185 Q9UNP9 2099 O60907 O75164 P51843 55662 Q8NCN2 333 91748 Q9H7L9 10794 8625 P83436 5119 Q9GZT9 Q9BT67 23636 9975 6464 4287 P62508 P51858 P17980 146050 P52701 Q08211 221656 Q9NVV9 P58012 10320 P60321 9967 P25116 Q99417 Q96HZ4 O43670 Q14566 8655 4297 Q9BY77 11186 9500 7325 Q9UKD1 P30086 P13984 351 Q9UBS0 Q04759 P21127 P25963 P09017 8662 P20290 Q15424 7332 5154 7334 5155 8667 7336 O43684 7335 26205 85403 367 Q70SY1 11198 7329 7328 Q14586 20 7341 Q14106 P11309 6496 6495 Q13257 Q8IVW6 Q14103 6498 P11308 Q99909 6015 P32242 Q8N3C0 Q9Y606 84108 Q9UBU3 Q03405 116113 P17096 5159 Q14592 11168 Q96MA1 Q9UBU8 Q13263 Q8TF50 8204 Q9BY44 Q14119 Q9BY41 Q9Y618 P12645 P29084 Q86WP2 222546 Q04725 Q04724 387 Q04727 Q9NWT6 Q04726 Q9NWT8 23152 P08151 P20264 11177 11176 P23771 P62942 P55347 9541 5187 9546 11142 O15164 2909 391 Q6MZP7 23126 O43639 25788 O43638 Q02535 Q01201 121512 2908 Q9UFW8 11146 Q15466 Q6PJG2 Q99966 Q13285 2902 Q13286 5195 6045 7376 7375 Q09161 9555 23133 23135 Q86WV8 Q3KQV3 Q6PRX2 P09067 Q14140 Q9UBP4 O15169 85440 Q99958 Q15475 Q8TEY5 Q96ME7 10273 Q9HA82 P14859 O43612 Q8TAQ5 Q96DT7 Q9H063 Q99941 P49959 Q01664 51222 56916 P01579 11124 Q14151 Q9UBQ5 P09038 Q15008 9575 P82979 8242 10284 O75676 O15198 23112 O43623 25776 2931 51230 Q14160 1609 Q5VVH5 10289 Q15014 86 P78424 Q9NWH9 Q15819 P33992 P33993 P33991 Q8TAF7 26271 P41134 Q9BXM7 Q1PSW9 27122 91 P17030 94 O15119 P45974 1616 P45973 Q9UBK2 Q9NSA3 P78412 9112 Q8WWH4 Q96QB1 Q96I24 Q9H7Z7 Q9H3R0 P31321 P55318 Q99081 P31323 P55316

P67775 P52292 Q52LR7 P17040 P05067 2959 2956 Q9UBL3 O94763 P52298 Q15836 92129 P52294 9126 27107 Q9H422 Q96M96 Q460N5 Q03014 6096 56034 27102 Q9BTC8 2961 2960 O95619 Q8TAD8 Q9NSC2 2969 P09874 O15151 8289 Q15843 O95625 O15156 O94776 29777 202559 Q02156 P17023 Q05655 2972 79149 Q9BXJ9 P17020 Q8N393 Q8HWS3 P04198 Q9UBN7 9146 Q15853 O75626 Q14527 9149 1660 Q8TF47 Q6NYC1 Q9H488 P41182 Q8TAK5 93474 79175 Q499Z4 P53567 79177 P30048 B3KY43 3836 84525 1655 84528 P22415 1653 Q9UK80 Q03468 Q92499 Q13200 O75629 O95644 150094 Q96HU1 Q9UBG7 Q8N7H5 Q07817 P20248 64919 80155 Q9UJX2 Q8TAI7 P41162 Q9BXP5 P17081 P08138 Q9Y5B6 Q9Y5B9 P09429 O75604 P10415 Q9P2X3 Q13216 P41161 P35711 23186 P09430 Q86SE9 728642 Q9P2Y4 Q13227 P55771 Q04771 O95661 23192 O15105 Q13233 P45984 Q6NX49 Q9H6Q4 O14793 25909 P54619 9181 P51587 9184 P17676 Q8TDD1 2547 57504 Q9HCJ0 P84022 O00151 Q92731 O14788 6901 10413 Q7Z6R9 25913 P08729 Q92729 Q5VTR2 P51114 O60216 79763 P14174 57510 54487 Q9H2G9 4734 Q7Z727 Q9H2G4 O00167 22850 81628 54496 6927 Q8IUD2 3416 6929 P62256 Q6AHZ1 Q96KM6 Q8N680 811 6921 P40425 P40424 P62258 6926 6925 Q6IQ32 Q17R98 Q9C005 333929 Q9UIS9 Q9NZI7 P31943 P48382 Q9NQX0 P31946 Q9C009 2100 Q9NR96 P48380 O95343 3428 P16333 6938 P20823 55352 O60674 P19838 O60675 Q8IUC6 P62263 P31949 5604 6936 Q9Y468 P27037 Q8IZ40 4771 Q9Y463 4772 Q9Y466 Q8TDI0 Q9UIL8 O95352 2107 55364 10856 Q86VE0 2103 P16383 27287 80324 Q08999 O14744 6945 P50222 4780 Q92769 Q14814 P09619 P28370 Q07666 O95361 80306 P10600 O95365 Q06330 Q96KS0 2119 10865 P19419 Q6PI57 Q9HCM4 4775 O14753 847 Q92766 4790 4791 4792 Q9H2K2 Q9H2K0 2130 81669 Q9NR48 P60033 Q92753 4302 P05230 857 Q96CA5 Q92754 Q13501 22827 6500 Q13503 Q9NR50 O95382 Q14839 Q8NC51 P19883 Q13506 Q9NR55 Q9HCK5 2139 861 862 863 192669 Q9UMX1 Q9UMX0 4799 P09601 50943 Q9Y3Y2 Q9BS34 P01241 10492 P15408 Q9Y3Q8 3481 Q9UIH9 23326 O60603 25988 23327 P15884 Q8WUU5 Q96T88 3479 2147 Q8WV60 2146 3476 10011 P25205 7832 10013 84289 Q96KG9 55723 10499 10014 Q6UWV6 135295 64682 4343 25998 Q96T76 O14717 Q99750 406 Q9BRP8 O43294 408 84271 409 Q13099 Q96C55 Q09472 Q8N1G0 O43251 23309 5682 5683 5684 5685 5686 64215 890 Q00653 891 Q99743 894 Q99741 896 Q9NR09 898 3014 29080 10477 Q9UN42 29086 P49639 P61326 P51531 4361 5692 5693 8721 7874 148156 Q9NUX5 Q9NR11 P51532 Q96ST3 P60484 Q15291 11331 5687 55758 5688 10001 P60002 10488 Q96T58 P28702 P23497 P26583 Q9Y3M2 Q6FGG2 P46199 P50613 P51946 23764 25942 Q8NFW5 Q9NUQ3 Q8WUY8 433 10450 8726 P55072 Q8N5U6 O43242 90993 Q9HCE7 Q08117 3054 Q05086 Q9BRU2 29079 P37231 Q9HCE1 79723 8737 10468 P24385 8754 7422 Q96GA9 5245 3068 Q9HCD5 P52815 22893 3066 Q9UMN6 3065 Q9UIF9 10437 O75290 79753 10438 Q00613 Q96P20 55787 7415 10432 3070 Q13901 9612 P49674 Q9UIG0 P52824 466 468 55796 P28749 Q9NQL9 7428 P04792 6595 6597 6599 6598 Q9Y4K4 O75386 P10599 6591 Q9Y4K3 Q76L83 3084 O43318 473 474 23286 9617 51763 8767 P38919 3093 Q13127 Q15303 B2RXF5 3091 Q16633 P55036 8301 Q16635 Q15306 Q86VP6 O75376 P58546 P13631 24149 3096 5270 Q02447 O75381 Q9NVP2 51773 30813 P46531 7448 Q13133 Q15796 Q13131 Q16649 Q15797 9640 P12757 Q8N6I1 9643 7468 P54198 O75367 Q9UNL4 P12755 O75362 O75360 P46527 51780 Q01101 P42167 Q01105 6128 O94805 7458 Q13144 Q5VUA4 54815 Q8WVM0 Q9BSI4 Q16650 Q13148 P35869 P43490 P20393 7476 Q16656 Q15329 Q8NCA9 P18074 Q8WVL7 P55055 P10588 P10589 93166 Q86VN1 Q9NZZ3 Q16665 51317 Q49AN0 Q13153 P43489 Q8TDY2 9667 Q16666 Q5VUG0 P54132 P35453 Q02413 Q14494 8328 Q14498 Q16670 P46108 Q14011 Q86W54 Q9NRC8 7013 7014 P46100 Q9Y4H2 P35443 Q5PSV4 7490 P13682 P11021 Q9C0F3 84232 Q8WW38 Q9NRD5 Q99835 7024 7023 10392 Q9C0F0 49854 P98179 P15822 9682 7020 Q02878 Q9UNH7 51341 54851 Q9NVM4 Q96PK6 11243 55704 P55010 Q13185 Q8NCF5 P23443 6188 Q15369 Q8IUX7 Q9BX66 Q9BSM1 Q09028 6182 P11474 26747 Q15370 54862 P57682 Q6PID4 Q99816

P48730 9208 Q99814 P78545 6199 7046 6198 P35813 O75787 O43711 6193 O43719 Q9Y4C1 P62195 Q4LE39 O15234 9219 P05177 A8K8V0 P78536 7057 22803 Q13526 112950 22806 22807 57473 83737 55294 Q3V6T2 P46934 P46937 83746 Q96GN5 O60296 Q4LE28 Q9UER7 Q14865 8399 7068 7067 Q5TAX3 Q15714 Q13535 Q9H307 P19793 P29590 Q15717 Q7Z2Q5 Q63HK5 Q01196 84619 Q9H6W3 P41235 1756 Q9HCP6 Q92793 Q96GM5 Q9HD15 Q9Y4E5 O60264 P05155 Q9NRA1 O60260 P59817 Q13547 Q13546 9252 7073 Q9UNE7 P41223 57018 P43405 1763 Q9NZR4 Q04206 Q92786 Q92785 Q92784 P41229 7088 Q15735 9261 Q01167 Q9BSG1 51720 Q14894 4800 P08246 2626 7099 7098 Q9NVC6 Q9NQZ2 7091 P14923 7090 Q9NQZ8 P30153 P11413 1789 1788 Q05516 1786 Q9UIV1 1785 Q05513 P00734 375790 Q13573 Q9HCU4 P55884 93134 Q9H2S9 Q96CJ1 P78527 92283 85509 Q86VK4 84661 51742 Q6ZNG0 P05198 P20333 Q9Y4A8 30827 Q13107 P62191 Q96H20 O60238 Q9HCS4 902 P00750 Q9HD40 Q13112 P08238 Q9NZN8 P08235 P21675 Q92618 Q9H609 Q92616 Q9Y2W7 Q9NYD6 Q6P2D0 O75820 2672 Q12948 22992 O00268 Q6QNY1 O00267 79894 3516 Q9BZI1 Q96JM7 Q96JM2 Q5VWX1 283248 Q9ULW3 Q92610 P63208 4841 Q8N587 P07996 10533 Q12950 P04004 Q12952 Q9Y2X9 Q8NB12 Q8NAP3 5706 5707 79885 5708 5709 4851 5700 4854 5701 5702 Q96JL9 5704 Q92600 5705 65264 Q9ULX9 Q9NYF8 Q9Y2Y8 Q12968 29128 10514 5717 83933 26097 P20936 Q9UQB3 4862 O75807 5713 57649 Q6IT96 4869 5716 Q96JK9 Q12972 Q0D2J5 Q9H9T3 3551 Q6P2C8 P17302 5728 10524 P17301 Q9HBE1 Q99697 2697 400961 Q9ULZ3 Q9UHR5 Q9ULR5 P28482 Q9UHJ3 Q12986 1385 P51668 29108 P51665 P19532 Q9P0J0 57669 P15173 29102 P07550 P04040 58517 10971 7913 P08887 O14867 Q96BA8 10973 Q9UM47 Q9Y2T7 Q96SB4 Q9UHK0 Q9NYA1 O00231 O00233 P10721 O00232 P19544 Q8WYH8 29110 79444 960 1388 29115 Q9P0K8 1387 1386 4898 P42771 Q9UM54 4899 Q9H1I8 10985 Q9NYB0 22937 22938 Q9UHL9 22931 P62333 2247 284119 2246 1398 58533 Q9BZK7 975 6601 Q9Y2V2 Q92630 6605 57209 Q9UM63 Q969M7 P51693 P51692 P31629 Q9BQY4 O00255 Q8NEZ4 Q9NQ33 O00257 Q03164 Q9P0M6 440193 984 Q5H9I0 988 P05362 Q9Y385 6613 Q9Y2W1 P06213 Q9UPV9 5300 P13349 2274 P53803 P52952 990 Q8WTS6 991 126208 Q96RU8 Q96RU7 79810 51003 79811 997 6622 79813 6625 54998 51008 10138 Q9UQ80 Q04917 P49770 Q9UM07 Q9Y2P0 Q7Z5H3 Q9UQ88 5796 Q5T7W0 O60739 2280 8819 P48436 8812 Q99638 P01344 P01343 P49768 O00206 2290 23429 P48431 Q96JC9 3142 Q9NU63 148022 Q99623 Q00535 Q00534 Q96S42 Q8NAF0 P49756 P25789 55869 P25788 7975 6646 5316 51028 91949 Q9UPY3 P25787 P01375 Q9UPY8 23439 6660 P49750 8841 Q9UHI6 O00213 Q3KNV8 23435 Q96AQ6 3151 O60716 P12004 10128 Q96RR4 Q3KNW1 3148 Q15170 545 546 3146 P61457 Q96EY1 6657 5327 Q8WU17 O43353 6670 6672 23409 Q969H0 P07948 Q9Y2K7 Q9ULJ3 Q9H9S0 3162 Q12905 O95076 Q8WYA1 Q12906 Q9BZE0 Q01844 Q9BZE4 P42704 3159 Q99683 Q99684 6667 5336 P49336 P62714 P05771 Q9H5J8 Q96FA3 Q969G3 8864 7531 10580 7533 P55198 Q9ULK4 23411 23414 3171 Q00987 Q96S94 3169 Q9H1A4 5347 7529 7528 Q9H5I1 64375 P50750 Q86U86 Q9UHD2 3187 Q9UPT9 3185 3184 571 3182 P51608 P68036 7536 6207 Q9H5H4 Q01826 5371 3192 8880 P27540 8881 Q60FE5 Q9UPU9 Q12933 Q9ULM3 7555 Q9ULM6 P51610 Q9BUY5 580 P16885 54583 O14818 80854 P51617 Q9NPJ6 7549 54106 8878 P60568 6231 8892 Q15654 O43432 8896 P15923 Q86YW9 29947 Q96FV9 595 84324 Q7Z6C1 Q13485 O94906 P03950 O00716 Q16512 4067 Q16514 P12429 Q15669 O75496 O75494 Q7LBC6 P10242 P10243 P10244 84312 51412 O94915 Q13492 Q16520 6239 54925 Q15672 Q9UQR1 7101 6256 Q16526 P35998 Q2M1K9 Q9UDY8 7579 9774 4086 7112 4087 4088 Q8N9N2 4089 O75478 9775 8445 163126 Q9H257 O75475 Q8N5F7 142 P24864 P62701 84333 Q15697 53615 Q13033 8454 P54259 O75469 8458 64750 7124 P54252 P54253 Q96KC8 P14635 O75461 Q9UQL6 P10275 4092 P10276 4093 O75460 4094 154 P23588 Q13042 Q99728 Q99729 Q13045 55806 P49736 7132 8463 7133 8467 9314 O43889 P36896 P36894 23378 P15976 P35568 Q96C28 6282 1822 166 55810 P01308 120892 338917 Q9NQB0 Q99717 1828 P49721 P49720 Q15233 Q96T37 9326

P54274 O75446 P36402 O75444 6294 7141 Q99700 1831 Q96T23 Q9H1Y0 51465 Q00577 Q99708 P49711 Q16576 P49716 P49715 55827 Q9NQC7 Q96C00 64784 7158 O75437 Q08050 P39905 7157 162239 Q8N9R8 Q02750 P35548 138151 55832 Q9UDV6 Q5VTD9 Q15257 Q13077 Q13873 O95835 26038 Q06787 Q86Z02 56252 Q01094 Q9H204 26039 7161 9341 P63167 P42224 10933 10935 P17275 P63165 Q9BVI0 P42229 57109 Q969T4 1856 P42226 O15372 O94992 Q9P0W2 22926 Q969S8 22927 Q13404 Q13887 Q01085 57592 Q8IY57 57594 P10644 O43847 57591 Q96BD5 79365 P84095 Q08945 Q9UHX1 P60900 1869 7186 7189 7188 Q8WYK2 O43812 P11940 7181 7182 7185 1877 Q86UE4 Q86UE3 1874 Q9Y3C7 P17252 1870 P09630 O15379 Q969R5 Q13422 O15391 Q8NB78 2735 27352 P19438 O43829 84733 1408 1407 1406 2737 Q92667 2736 9388 Q8WUI4 27327 Q02386 Q7RTR2 P62277 1896 Q8WYQ5 Q9HC52 Q14774 Q13443 P22670 Q9NQ92 9391 26005 P11532 Q96G25 57154 P30279 84759 58487 Q9UQE7 P30281 Q8WYP5 P23511 Q8WUH2 3609 3608 3607 Q14781 P23510 P21333 94104 8078 O14495 Q9P0T4 29966 P19474 Q02363 58491 P18146 57162 84305 27300 O60341 P01730 Q969V6 O15350 O75832 O15353 Q15649 Q9BZS1 P53350 Q15648 Q9P0U4 P19484 Q86YP4 Q06710 Q96BF6 Q8N5A5 3622 Q9HC29 P30260 O14497 Q15652

negative regulation of gene expression Q7KZF4 Q9Y2W7 O75820 2672 Q9UBB5 Q9NS86 O00267 124790 10657 3516 2304 Q9HCJ0 P84022 283248 P27348 5933 5935 Q9UBC3 Q9Y2X9 Q92729 Q12837 54361 Q8NDV7 Q8NB12 P14174 57510 54487 4851 4734 Q96EB6 5705 O75925 Q9UKV8 3660 Q9NYF8 Q9NRY4 Q9BYE7 Q99471 6927 P13056 83933 811 Q9UJU2 O15534 57649 Q92833 6926 Q6IT96 P06401 P26358 P06400 5716 Q9UIS9 Q9NZI7 P48382 Q9NQX0 P31946 5970 Q92949 Q9NRZ9 P56705 P48380 O95343 9099 10524 P20823 1487 P19838 P62263 3784 3665 10765 Q9Y468 Q8IZ40 Q9UL18 Q12986 Q9P0J0 P51787 O14627 6829 10614 1499 Q86VE0 29102 O14981 Q8N2W9 10971 27161 6945 5978 6827 O14503 Q92769 5991 93986 5993 Q07666 Q14938 Q15906 P19544 O95365 O00358 Q06330 10865 6839 1027 P08651 P17480 O14753 604 5987 4790 22938 O00488 P38398 57326 2247 58533 Q9BZK7 6601 Q92993 857 6605 Q8N488 P10826 P10827 2023 O00255 Q6KC79 P19883 O00257 1050 Q9HCK5 Q99496 192669 Q9UMX1 4799 P40337 50943 Q9UGL1 7704 Q92985 P06213 O96004 O43159 O00308 P04637 Q9NVW2 P14316 25988 P52952 3476 Q15022 4686 79813 4204 639 10014 10138 Q9UQ80 Q9BT49 4221 4343 5796 P16989 P57059 84159 P33076 51132 Q99750 P48436 8932 7727 2290 23429 O43251 23309 P48552 P48431 6774 Q99623 891 Q96S42 55869 51147 P01137 Q9UPY3 P01375 Q86X95 P51531 6660 5451 8841 Q9UHI6 P35222 2063 P51532 23435 Q96AQ6 P35227 11218 Q96ST3 Q9NS37 P17813 P35232 3148 P10070 Q15170 P10071 3146 11331 668 6657 5569 Q96T58 Q96MH2 P23497 O43474 6670 P06733 P26583 O43593 6672 Q9Y3M2 23409 P25490 25822 25942 Q12906 Q8NFW5 3281 Q9BZE0 672 O75190 Q99684 6304 8726 P40763 4261 Q969G3 8864 Q08117 23411 23414 O75182 P37231 Q9HCE1 P14373 Q00987 3169 5467 5468 7529 7528 10468 P27797 64375 4152 5245 P56177 O60907 22893 3066 3065 Q9UIF9 P51843 3182 79753 10438 P51608 Q9H7L9 5119 Q01826 9839 O43463 Q9BT67 5371 3070 Q13901 9612 23512 Q96QT6 P58012 P28749 7428 10320 6595 11060 6597 8535 P55265 P10599 6591 Q9Y4K3 3084 5017 Q13485 Q13363 Q13127 Q9HAV4 4188 5036 O75496 O75376 P13631 3096 Q02447 O75381 23051 51773 P46531 7329 Q96RL1 Q15796 Q9HAU4 Q15672 Q9UQR1 7341 8553 6496 Q15797 6256 6498 Q96IZ0 P12757 Q8N6I1 P31273 Q9UNL4 P12755 23028 O75360 4193 Q2M1K9 84108 P46527 116113 51548 51547 54815 Q13263 Q13148 9774 4086 4087 8204 4088 4089 Q9BY41 P55055 Q9UPQ9 163126 Q9Y618 P10588 P10589 93166 Q04724 P20264 11176 Q9NPF5 Q15697 51317 55915 53615 P43489 Q13033 9421 P54259 9541 5187 O75469 64750 7124 11142 O15164 P54253 2909 P14635 O75461 Q9UQL6 4092 P10276 Q9Y6K1 Q02535 Q03989 51564 Q15583 55922 Q99729 8328 Q15466 55806 Q99966 5074 5195 Q9NRC8 6045 7376 55929 Q9HAZ2 Q09161 8467 O15055 9314 O43524 23135 23378 26523 7490 7251 1822 166 84232

Q9NQB0 Q8WW38 Q9UBP4 Q96T37 P61925 7023 O75569 P98179 P14859 P15822 23468 7141 Q9H063 Q96T23 Q9UNH7 51341 Q01664 Q00577 51588 Q13185 P49711 10155 P49715 P09038 O75437 7157 23112 O43623 25776 64426 138151 51593 Q9H9G7 Q99816 9208 Q5VTD9 Q96RE7 P29374 Q01094 Q9H0M0 P41134 Q1PSW9 P62195 27122 P63165 9219 O15119 1616 P45973 Q9NSA3 P56524 O94992 Q8WWH4 Q969S8 Q8IY57 Q02297 7291 O75530 7295 P55316 Q96BD5 P46934 1869 Q9UER7 Q14865 P52298 7189 7067 92129 Q5TAX3 Q8WYK2 P19793 P29590 7181 Q03014 Q7Z2Q5 84619 Q9H6W3 Q86UE4 O15379 O60264 Q8TAD8 Q9NSC2 O15151 O95983 Q13422 O94776 Q13547 P78318 O43829 Q8TBE0 84733 Q04206 Q9UH92 Q92786 Q9Y2B9 2737 2736 7088 O75626 Q8WUI4 27327 P41182 Q9UKY1 Q9H9B1 51720 Q8WYQ5 Q14894 150094 Q9NQZ2 Q9NP71 1789 Q07817 1788 Q05516 1786 58487 P41162 Q9BXP5 3720 3609 Q13573 Q14781 P23510 P09429 O75604 Q9H2S9 29966 Q02363 P18146 P35711 Q9BQA5 Q9Y6E7 O60341 P09430 5914 O75953 O75832 Q9BZS1 Q13227 P55771 Q86YP4 63976 O60238 Q96BF6 P63279 Q8N5A5 P63272 Q9HCS4 O15105 5925

signaling process Q9H0E2 P25054 P49023 1460 10657 1457 1454 P21926 1453 O14544 O14543 Q9Y275 Q9UL63 P16104 2317 2316 5829 P42858 P15260 5822 Q9UKV5 10666 Q59EK9 Q5S007 O75925 Q9H8S9 3661 Q12846 Q06124 P17405 P37023 81876 P62491 Q9UKW4 4983 Q9Y297 10633 Q12851 Q13702 3672 Q13705 P21917 10645 3667 P42830 Q8WXG6 O75901 P49069 5861 5862 1020 114548 1017 P21964 1499 1012 P61587 P61586 26191 P61106 Q9UL26 Q9H0H5 Q9UL25 P29466 Q9Y239 5870 5871 Q15907 Q9UGI9 P51797 1029 Q9BUB1 1026 Q9BUB5 5863 O60890 5865 P07686 5867 6714 5868 5869 Q07890 O95136 Q9UGJ0 Q9Y243 5883 1040 P16118 P19634 P38398 Q07889 4543 613 Q5TCX8 Q6NUQ1 5877 5878 5879 Q92997 P10826 Q9UL45 Q9UGK8 5894 P31749 Q38SD2 1050 84932 55159 Q96MT8 10607 6733 P04150 Q9UL54 127829 Q92502 Q9UKL4 P56199 P14314 O00300 23568 P50897 11108 79937 5898 P62873 10256 P25445 O96018 O96017 6760 O96019 O95163 Q9NWZ3 P57059 P36575 O95166 1072 O96013 P35249 Q99996 641 Q14185 P47224 Q15036 P49407 85360 O00329 6772 Q08380 Q9UKN5 P48551 6774 3265 P11274 Q99500 Q9H4B6 Q8WWW0 8945 P01019 55504 O14964 Q99988 O14965 Q9Y215 P35225 P35222 P14784 P35232 3269 6776 Q15052 Q59EA4 P24588 6790 Q92934 9821 672 673 53340 O14908 P62834 6789 Q92930 P05412 O00755 Q9NWV8 Q15077 5467 9826 207 5468 4139 4155 Q9Y5S2 Q12809 O75044 O00762 O75051 A7KAX9 10673 10672 O14920 P56180 Q92903 Q8NHX1 P50406 P08908 O14939 Q3KRB8 56882 P40616 10681 8517 Q9H4E5 P60201 55558 9846 326624 O15085 P36507 P35659 P19174 11065 8533 O43559 O43557 P13861 Q9H0U4 Q6P5Z2 23043 Q9H160 P83731 P49815 Q9HB75 4188 P62805 6368 5037 Q9Y2H1 Q9H0T7 Q9UD71 Q9NY59 7205 8536 5029 P00533 Q13370 Q96RL1 Q16881 Q9BYV2 Q9BYV6 Q9BZ76 Q9UQ13 9404 8555 Q9Y2I1 Q9Y6Q6 Q01344 Q00013 Q15561 P55290 Q15562 Q9HAT0 6386 Q9Y6R4 P62826 Q9UPQ3 Q9Y6R0 Q86XR7 Q8N4C8 267 51552 Q00005 Q13393 P61978 P43246 P62820 P78347 P78348 11021 7248 O75582 P10398 Q93063 51560 Q9Y2C9 Q9Y6K9 8569 Q15109 11031 26524 5071 285 7249 11035 Q15118 P78368 Q9BYZ6 P11229 9448 Q9Y6M4 A1XKG3 P58753 291 Q14C86 P08069 1956 P78357 Q9HB90 O75553 O43508 P37840 P11234 P11233 7272 2810 Q93038 P78362 Q9NPB3 1969 Q14289 9467 P12931 Q9H0M0 Q05329 P22736 P42345 P04083 Q92574 1978 7297 Q15942 Q08830 7295 P42331 P42338 P42336 O15020 9001 P29353 O95977 Q06643 P29350 Q9H0K1 Q7Z569 P40145 60412 P06241 P21860 P78314 A4D1W7 Q07960 P78318 9495 3705 3708 P07101 Q9Y2B9 O95996 Q14643 O14593 Q8IWW6 Q13315 Q03113 8174 9021 Q16828 Q96EP1 23085 P20594 O75508 2869 O75509 Q8IWV1 Q13322 Q14653 Q9NP71 Q15506 Q9NP72 23092 P46736 P61073 5906 23095 5908 2873 23098 O60496 P22303 5901 5902 P46734 Q13330 Q9Y6D9 9047 9046 Q16849 Q8NEB9 Q9NP86 Q9P035 2889 Q9BQA5 P02708 2885 Q9Y6E0 50650 Q9HAP6 P43694 O15455 5911 5914 Q9NP90 Q14676 A4D1S5 9052 Q9H0N0 P00519 2896 P24941 P43681 51512 Q15532 51514 51517 Q13352 5925 23603 23607

Q9NRW1 Q9NRW4 Q99490 55684 O60566 Q8IVH8 P16220 79658 55207 P49137 P27348 O15516 P27361 O95680 65018 Q07343 9077 P50148 Q92845 Q92844 O15524 P52333 Q92838 Q92835 P29992 Q9NRY4 339122 Q6P4F7 P53667 57403 Q8IVF5 1111 P03372 P06401 P06400 5970 P46089 Q5JS13 9093 Q9UBE8 10767 6810 P04233 P34925 4651 P50570 P11802 3320 P52757 O00463 O00468 Q9P2N2 1137 P61225 P61224 4646 4649 Q7KZI7 P30556 Q5HYI8 P30559 10746 1147 2475 Q92882 Q92888 O95259 O95257 P28223 6850 P50591 2011 P30542 Q9NS68 O60503 4664 O14640 4665 5515 O43182 5518 P51451 Q13625 Q05586 3350 P30530 10725 10728 Q99497 O14654 Q9UK32 5528 5529 Q9BST9 P04637 O43157 P38405 2034 2033 25861 1185 11228 P62993 P01116 Q15382 Q9C0K7 6869 Q15389 285282 P01112 P01111 Q9UNS2 P25100 4221 6885 P35368 2043 1192 56940 O76074 9908 Q15392 54763 4214 4215 4216 P01588 4218 P01100 5562 Q8N726 5563 5565 P14416 2054 P52735 Q96CV9 P12235 P11388 Q9NS23 O00445 773 Q02952 P61204 Q53X93 Q9Y572 P01138 P61201 P01137 Q6UXV0 P01135 P01133 O00459 5573 P04626 5575 5576 P04629 2065 2064 2063 P35348 Q9NRR8 301 2059 Q9C0H5 5566 5567 307 5568 Q8TDZ2 11215 23647 5580 5581 5583 5585 O43597 Q5U0I6 5586 Q9UJF2 Q9UNN5 Q96PU8 P51828 5577 5578 Q9P2F6 5579 57826 P40763 P04201 P46060 Q6IR47 5111 5597 5598 P18850 5590 P16671 324 4259 O43572 64130 P01185 O00408 6453 6456 6455 2099 P50993 Q8IZJ4 333 Q9NRM7 5599 8625 P25103 P01178 Q96HC4 7791 6464 4287 O43586 4289 P39748 Q5T5U3 P51858 Q08209 6457 Q96HB1 P25116 Q13237 5141 5142 5144 Q15418 Q9BY76 Q5VZM2 11186 8654 P30086 351 Q9UBS0 355 356 85415 5138 5139 P25963 O43683 19 Q8TF76 Q9UKE5 367 369 Q13257 P11308 29 Q86X27 P32241 P32246 Q8TEU7 Q9UBU3 Q03405 375 P51805 Q13261 5159 9518 6009 P32239 Q14114 Q8WWN8 P51812 Q9UKG1 381 382 Q04725 Q04724 387 388 Q04726 389 P60763 41 Q9NSD7 P55345 10293 9546 11142 2909 390 391 392 O43639 394 396 397 Q99962 398 Q99963 121512 25780 Q6KH11 Q9H082 Q13283 2908 Q13287 2904 Q99966 Q15464 2902 Q96R06 Q13286 Q9BU20 Q16799 Q86WV8 Q9UKA4 Q9UKA8 Q6PRX2 2917 Q8NHQ8 Q6PJH3 2915 Q99956 Q6ZSZ5 10270 9567 O43613 O43612 Q9Y5K6 Q99941 P21145 P01579 11124 10276 P09038 Q14155 2923 Q9BY84 9575 O75676 9578 1609 Q5VVH5 Q14168 86 Q14964 Q15811 P78423 Q13637 Q13636 O15123 Q12788 Q15818 Q8TF05 Q13639 1613 Q9BXM7 Q86WK6 91 94 O15119 1616 Q9Y5E9 Q9NSA1 26270 9112 Q96QB1 9590 P31321 P31323 P67775 P05067 P29474 O94768 P29475 9126 26258 Q96M96 29767 6093 P41597 P21980 Q8TAD8 2969 P11717 O95622 Q14511 Q6R6M4 9138 Q02156 P31785 55198 Q8N392 Q86WH2 Q05655 Q9UBN6 9146 P13945 P41180 114822 P30047 B3KY43 Q03468 P08123 150094 Q52LW3 Q13202 Q96HU1 Q8IVT5 Q9UBG7 P20248 P21579 Q8TAI7 P08134 P17081 P19235 P08581 P08588 P41159 O15111 P55773 Q13227 P62070 O95661 P45983 Q13233 P45984 P21554 Q2M1Z3 Q92737 65125 Q5TB30 P54619 9182 P37288 Q8IYN9 Q92731 10890 Q92730 O14786 O14788 6901 A1A4S6 10413 P28799 P28335 64284 Q08AM6 2562 Q8NFZ5 P53779 Q17R89 Q7Z727 Q9NR81 2572 2571 79791 6927 2569 Q8IUD2 3416 57522 P63104 10875 6926 P31946 P31947 2100 Q9NR96 O00178 P54646 P20823 Q8IUC4 O60674 P20827 P19838 Q8IUC6 P31949 5602 5604 P08758 Q13956 Q5SGD2 P15056 Q6ZRI8 55367 Q8N264 644943 Q9Y478 P30679 P32856 P09619 Q07666 P80098 P10600 P52888 Q9BWF2 Q6PI57 846 Q6ZW31 7804 O14757 Q92766 P49286 4790 O43295 4791 4792 Q13976 2132 P21709 Q96L33 P18545 3459 P60033 83853 Q92752 P05230 857 O14763 Q9UN86 Q96CA5 P51575 O95382 Q13506 Q9P202 Q9NQT8 Q9HCK4 153090 Q9UMX1 O14775 P09601 O14777 P01241 O95399 Q9BRR9 Q96C74 3484 3482 O60603 25988 3479 2147 3476 10010 6505 Q15269 P13236 P51513 Q99755 Q96T76 400 402 Q99750 Q99759 408 O43293 O14713 Q13098 Q01518 Q6ZN33 Q09472 4354 Q7Z6I6 4355 116986 O00560 116987 O00562 23303 890 Q00653 891 54413 Q99741 55743 896 3014 Q15286 Q9Y450 29086 O43264 Q8TCX5 Q658W2 Q01974 P15882 10486 7867 8718 O14733 5207 10487 P23497 8717 P01243 P26583 O00506 Q9Y3M8 10459 10458 Q8WUY9 Q96P48

10451 6548 7879 10454 Q01968 P01275 8743 8744 7410 Q9NYZ3 P37231 P51959 8737 P48357 P55083 8738 Q6IQ22 11315 P01266 23746 P24385 8754 Q96GA9 5245 9600 3068 O75293 79753 Q96P20 7416 P24394 7433 8766 P49674 Q13905 P52824 3074 80728 P17612 8772 Q13115 Q13114 Q16623 P18085 Q14449 P10114 Q9Y4K4 P10599 O75385 Q9Y4K3 O43318 472 P48729 477 51762 P20396 8767 P43034 Q14451 P35408 Q15303 3091 Q16635 P55042 P55040 Q15796 Q15311 Q13131 O00602 8795 Q13136 Q15797 Q16644 8315 P19397 P12755 Q13144 Q8IV61 Q16659 5295 5296 9656 P43490 7476 P18074 O43306 P10586 P10588 P10589 9649 Q16665 P43487 Q9P2E9 P43005 Q13158 Q8TDY2 11261 P54132 P63092 P09172 P46109 Q13162 P46108 Q13164 Q14012 Q15349 O15294 7013 Q9Y4H2 Q9NZT1 P63096 Q9NRD1 Q13177 Q99835 10392 O94844 P12314 55704 1718 Q13188 P23443 O43741 P10147 O75312 P38936 O75791 Q6PID4 Q99819 P27815 P48730 P23458 1728 Q9BWT7 Q99814 P48736 6196 6199 7046 6198 P98155 P10997 8379 O43715 6195 6194 27242 Q86VI3 10817 P46940 O95715 P98161 Q13522 7057 P10523 9230 9231 Q3V6T2 7052 3925 P46937 1742 Q4LE28 P98171 P98174 Q9UER7 P34947 P29597 Q9H305 Q13535 O15264 P29590 8394 P30101 7064 1756 P41231 Q92793 P16284 Q9NZQ3 O60266 O15259 O60260 9255 Q13546 Q8IYT8 9252 9253 7074 80254 P43405 P20309 Q04206 Q9NVJ2 P78560 P09525 7088 Q13555 Q13554 Q8IV45 9261 3959 P00749 O00194 P63000 51720 3953 Q13563 2626 7099 7098 Q15742 O15211 Q14416 9270 7090 P30153 P62166 Q9UIV1 1785 Q05513 P00734 Q9HD67 P24723 375790 Q13574 51735 Q6SZW1 Q15750 Q13572 53916 Q15759 O60229 P41279 P20338 P20339 P20336 P20337 2644 1312 P85298 P41273 1793 Q14432 Q15762 P18031 P34972 1326 Q9UNA1 P47804 Q8TDM6 P67870 900 P20340 P00750 P08235 Q9NZN9 O15228 Q7Z628 P08833 Q9Y2W7 Q14CB8 Q92619 P61020 Q6PCE1 Q7L0Q8 2665 2664 Q9HBH0 P61026 Q8N103 P07996 P52564 O75815 P52565 Q12955 Q7L591 Q66LE6 Q12959 51090 P52566 Q9P107 P63211 10549 P16473 2676 O60333 P16471 P63218 P48039 Q9Y2Y0 928 Q92600 P54753 P50395 Q9NYF5 P32927 Q05397 57646 29127 P20936 Q9UQB3 Q9UQB8 4867 4868 4869 5716 P54764 P54762 4882 P48023 3551 O00299 Q12979 P20941 Q8NEU8 Q9ULZ3 948 Q9UHR4 Q6PCD5 O95477 Q9Y342 P28482 1385 O00220 29108 5739 O60760 P07550 Q9Y2T1 10971 7913 P08887 P51671 P04049 Q96SB4 Q9NYA1 O14880 2243 P10721 29110 2237 79444 1388 1387 963 10981 P42771 Q9UM54 5747 O00241 Q9P0L2 22931 6609 P62330 2247 P15153 1399 2246 1398 2244 975 P61006 Q92636 P51693 5770 P29320 P51692 P28472 O00255 P48061 Q99259 P17342 P29317 Q9ULV4 P61019 P61018 26060 P06213 Q15139 Q9Y6W6 P07900 Q9Y6W5 990 Q96RU8 Q96RU2 2267 998 6622 10133 P27986 P02686 P62753 P49770 5310 Q7Z5H3 5311 5796 P51636 266747 Q04900 Q8WTR2 Q96RT1 10142 Q99638 10146 P02675 P01343 P49768 O00206 Q9Y6Y9 148022 Q00535 Q96S42 5798 6647 8829 8826 P01375 P01374 Q9Y2R2 64343 O00213 Q08499 O00212 P12004 Q96RR4 Q15172 3148 545 P40933 Q96EY1 Q15173 5327 5328 7508 O43353 6672 Q969H4 P07949 8852 P07948 Q12904 Q8WU90 3162 Q8IXI1 Q8IXI2 Q68EM7 552 553 Q99683 5335 Q96AX2 5336 8844 Q15185 5337 5338 P05771 P26232 7534 Q9UHC3 Q01831 P50749 P50748 5347 Q96JH8 P24046 7529 Q08462 5361 5362 5364 Q5VWQ8 Q9UHD2 Q12929 Q9BUZ4 572 8882 5371 3190 Q60FE5 Q12933 P15559 P16885 Q99653 P51617 P62745 54106 Q13478 Q13477 8892 Q15654 Q9UQP3 6236 8412 9743 Q96FV9 595 29941 Q96BM9 Q8NFJ5 Q7Z6C1 P22694 Q13485 Q8WZ64 8408 P32019 6242 Q13009 Q16512 4067 Q16513 Q15669 P35523 6238 P60983 6239 Q14344 Q7Z6B7 Q13017 Q9HBW0 23380 Q9UDY8 P23528 8428 P00441 8443 Q16539 4086 4087 P22692 4089 Q15208 Q16537 Q9Y3L3 9770 Q9Y3L5 Q9NZ94 P21359 P31150 Q9NUP9 1808 8439 10087 6259 9784 P54257 O75469 7124 P54252 P54253 P14635 P10275 P10276 154 Q96KC2 Q16555 Q15223 Q13043 9311 7132 8463 Q5JQC9 P36896 P35568 6282 23370 Q8NFM4 Q04941 Q02763 P01308 Q8NFM5 120892 Q99717 9306 P01303 9322 P02671 Q8TCU4 8473 P54274 8471 P36404 9318 1837 P49715 O95819 P60953 64780 7153 P39905 7157 P19086 7159 Q02750 1848 54509 Q13077 P53365 O95835

P57729 O94988 O95833 10928 57584 P42224 1852 1850 P23945 83871 O95837 P22612 P42229 P42226 Q53QZ3 22926 10939 Q14738 P10644 22920 79363 P57735 P84095 P39059 Q9UI95 7187 9367 7186 7189 7188 Q13418 7185 P17252 P42680 P42681 P42684 P21741 O15397 O43823 B2RTY4 4914 27352 P19438 P68400 Q969Q1 Q13432 58473 Q86YS6 P51148 Q7RTR2 P51149 Q9H244 P23508 P43119 1896 2741 P42262 4920 P51157 Q13443 P51151 Q15628 P51153 P42261 P11532 58480 P51159 27330 Q9UQE7 P30281 57159 Q8WUH2 Q8WYP3 P23510 P21333 O14492 377630 59341 P84085 3611 1432 Q5XUX0 Q15642 Q13464 P01730 Q14314 P53355 O75832 P53350 3622 P84077 Q7L9L4 P24821 4952 Q15653 P21796

signal transmission Q9H0E2 P25054 P49023 1460 10657 1457 1454 P21926 1453 O14544 O14543 Q9Y275 Q9UL63 P16104 2317 2316 5829 P42858 P15260 5822 Q9UKV5 10666 Q59EK9 Q5S007 O75925 Q9H8S9 3661 Q12846 Q06124 P17405 P37023 81876 P62491 Q9UKW4 4983 Q9Y297 10633 Q12851 Q13702 3672 Q13705 P21917 10645 3667 P42830 Q8WXG6 O75901 P49069 5861 5862 1020 114548 1017 P21964 1499 1012 P61587 P61586 26191 P61106 Q9UL26 Q9H0H5 Q9UL25 P29466 Q9Y239 5870 5871 Q15907 Q9UGI9 P51797 1029 Q9BUB1 1026 Q9BUB5 5863 O60890 5865 P07686 5867 6714 5868 5869 Q07890 O95136 Q9UGJ0 Q9Y243 5883 1040 P16118 P19634 P38398 Q07889 4543 613 Q5TCX8 Q6NUQ1 5877 5878 5879 Q92997 P10826 Q9UL45 Q9UGK8 5894 P31749 Q38SD2 1050 84932 55159 Q96MT8 10607 6733 P04150 Q9UL54 127829 Q92502 Q9UKL4 P56199 P14314 O00300 23568 P50897 11108 79937 5898 P62873 10256 P25445 O96018 O96017 6760 O96019 O95163 Q9NWZ3 P57059 P36575 O95166 1072 O96013 P35249 Q99996 641 Q14185 P47224 Q15036 P49407 85360 O00329 6772 Q08380 Q9UKN5 P48551 6774 3265 P11274 Q99500 Q9H4B6 Q8WWW0 8945 P01019 55504 O14964 Q99988 O14965 Q9Y215 P35225 P35222 P14784 P35232 3269 6776 Q15052 Q59EA4 P24588 6790 Q92934 9821 672 673 53340 O14908 P62834 6789 Q92930 P05412 O00755 Q9NWV8 Q15077 5467 9826 207 5468 4139 4155 Q9Y5S2 Q12809 O75044 O00762 O75051 A7KAX9 10673 10672 O14920 P56180 Q92903 Q8NHX1 P50406 P08908 O14939 Q3KRB8 56882 P40616 10681 8517 Q9H4E5 P60201 55558 9846 326624 O15085 P36507 P35659 P19174 11065 8533 O43559 O43557 P13861 Q9H0U4 Q6P5Z2 23043 Q9H160 P83731 P49815 Q9HB75 4188 P62805 6368 5037 Q9Y2H1 Q9H0T7 Q9UD71 Q9NY59 7205 8536 5029 P00533 Q13370 Q96RL1 Q16881 Q9BYV2 Q9BYV6 Q9BZ76 Q9UQ13 9404 8555 Q9Y2I1 Q9Y6Q6 Q01344 Q00013 Q15561 P55290 Q15562 Q9HAT0 6386 Q9Y6R4 P62826 Q9UPQ3 Q9Y6R0 Q86XR7 Q8N4C8 267 51552 Q00005 Q13393 P61978 P43246 P62820 P78347 P78348 11021 7248 O75582 P10398 Q93063 51560 Q9Y2C9 Q9Y6K9 8569 Q15109 11031 26524 5071 285 7249 11035 Q15118 P78368 Q9BYZ6 P11229 9448 Q9Y6M4 A1XKG3 P58753 291 Q14C86 P08069 1956 P78357 Q9HB90 O75553 O43508 P37840 P11234 P11233 7272 2810 Q93038 P78362 Q9NPB3 1969 Q14289 9467 P12931 Q9H0M0 Q05329 P22736 P42345 P04083 Q92574 1978 7297 Q15942 Q08830 7295 P42331 P42338 P42336 O15020 9001 P29353 O95977 Q06643 P29350 Q9H0K1 Q7Z569 P40145 60412 P06241 P21860 P78314 A4D1W7 Q07960 P78318 9495 3705 3708 P07101 Q9Y2B9 O95996 Q14643 O14593 Q8IWW6 Q13315 Q03113 8174 9021 Q16828 Q96EP1 23085 P20594 O75508 2869 O75509 Q8IWV1 Q13322 Q14653 Q9NP71 Q15506 Q9NP72 23092 P46736 P61073 5906 23095 5908 2873 23098 O60496 P22303 5901 5902 P46734 Q13330 Q9Y6D9 9047 9046 Q16849 Q8NEB9 Q9NP86 Q9P035 2889 Q9BQA5 P02708 2885 Q9Y6E0 50650 Q9HAP6 P43694 O15455 5911 5914 Q9NP90 Q14676 A4D1S5 9052 Q9H0N0 P00519 2896 P24941 P43681 51512 Q15532 51514 51517 Q13352 5925 23603 23607 Q9NRW1 Q9NRW4 Q99490 55684 O60566 Q8IVH8 P16220 79658 55207 P49137 P27348 O15516 P27361 O95680 65018 Q07343 9077 P50148 Q92845 Q92844 O15524 P52333 Q92838 Q92835 P29992 Q9NRY4 339122 Q6P4F7 P53667 57403 Q8IVF5 1111 P03372 P06401 P06400 5970 P46089 Q5JS13 9093 Q9UBE8 10767 6810 P04233 P34925 4651 P50570 P11802 3320 P52757 O00463 O00468 Q9P2N2 1137

P61225 P61224 4646 4649 Q7KZI7 P30556 Q5HYI8 P30559 10746 1147 2475 Q92882 Q92888 O95259 O95257 P28223 6850 P50591 2011 P30542 Q9NS68 O60503 4664 O14640 4665 5515 O43182 5518 P51451 Q13625 Q05586 3350 P30530 10725 10728 Q99497 O14654 Q9UK32 5528 5529 Q9BST9 P04637 O43157 P38405 2034 2033 25861 1185 11228 P62993 P01116 Q15382 Q9C0K7 6869 Q15389 285282 P01112 P01111 Q9UNS2 P25100 4221 6885 P35368 2043 1192 56940 O76074 9908 Q15392 54763 4214 4215 4216 P01588 4218 P01100 5562 Q8N726 5563 5565 P14416 2054 P52735 Q96CV9 P12235 P11388 Q9NS23 O00445 773 Q02952 P61204 Q53X93 Q9Y572 P01138 P61201 P01137 Q6UXV0 P01135 P01133 O00459 5573 P04626 5575 5576 P04629 2065 2064 2063 P35348 Q9NRR8 301 2059 Q9C0H5 5566 5567 307 5568 Q8TDZ2 11215 23647 5580 5581 5583 5585 O43597 Q5U0I6 5586 Q9UJF2 Q9UNN5 Q96PU8 P51828 5577 5578 Q9P2F6 5579 57826 P40763 P04201 P46060 Q6IR47 5111 5597 5598 P18850 5590 P16671 324 4259 O43572 64130 P01185 O00408 6453 6456 6455 2099 P50993 Q8IZJ4 333 Q9NRM7 5599 8625 P25103 P01178 Q96HC4 7791 6464 4287 O43586 4289 P39748 Q5T5U3 P51858 Q08209 6457 Q96HB1 P25116 Q13237 5141 5142 5144 Q15418 Q9BY76 Q5VZM2 11186 8654 P30086 351 Q9UBS0 355 356 85415 5138 5139 P25963 O43683 19 Q8TF76 Q9UKE5 367 369 Q13257 P11308 29 Q86X27 P32241 P32246 Q8TEU7 Q9UBU3 Q03405 375 P51805 Q13261 5159 9518 6009 P32239 Q14114 Q8WWN8 P51812 Q9UKG1 381 382 Q04725 Q04724 387 388 Q04726 389 P60763 41 Q9NSD7 P55345 10293 9546 11142 2909 390 391 392 O43639 394 396 397 Q99962 398 Q99963 121512 25780 Q6KH11 Q9H082 Q13283 2908 Q13287 2904 Q99966 Q15464 2902 Q96R06 Q13286 Q9BU20 Q16799 Q86WV8 Q9UKA4 Q9UKA8 Q6PRX2 2917 Q8NHQ8 Q6PJH3 2915 Q99956 Q6ZSZ5 10270 9567 O43613 O43612 Q9Y5K6 Q99941 P21145 P01579 11124 10276 P09038 Q14155 2923 Q9BY84 9575 O75676 9578 1609 Q5VVH5 Q14168 86 Q14964 Q15811 P78423 Q13637 Q13636 O15123 Q12788 Q15818 Q8TF05 Q13639 1613 Q9BXM7 Q86WK6 91 94 O15119 1616 Q9Y5E9 Q9NSA1 26270 9112 Q96QB1 9590 P31321 P31323 P67775 P05067 P29474 O94768 P29475 9126 26258 Q96M96 29767 6093 P41597 P21980 Q8TAD8 2969 P11717 O95622 Q14511 Q6R6M4 9138 Q02156 P31785 55198 Q8N392 Q86WH2 Q05655 Q9UBN6 9146 P13945 P41180 114822 P30047 B3KY43 Q03468 P08123 150094 Q52LW3 Q13202 Q96HU1 Q8IVT5 Q9UBG7 P20248 P21579 Q8TAI7 P08134 P17081 P19235 P08581 P08588 P41159 O15111 P55773 Q13227 P62070 O95661 P45983 Q13233 P45984 P21554 Q2M1Z3 Q92737 65125 Q5TB30 P54619 9182 P37288 Q8IYN9 Q92731 10890 Q92730 O14786 O14788 6901 A1A4S6 10413 P28799 P28335 64284 Q08AM6 2562 Q8NFZ5 P53779 Q17R89 Q7Z727 Q9NR81 2572 2571 79791 6927 2569 Q8IUD2 3416 57522 P63104 10875 6926 P31946 P31947 2100 Q9NR96 O00178 P54646 P20823 Q8IUC4 O60674 P20827 P19838 Q8IUC6 P31949 5602 5604 P08758 Q13956 Q5SGD2 P15056 Q6ZRI8 55367 Q8N264 644943 Q9Y478 P30679 P32856 P09619 Q07666 P80098 P10600 P52888 Q9BWF2 Q6PI57 846 Q6ZW31 7804 O14757 Q92766 P49286 4790 O43295 4791 4792 Q13976 2132 P21709 Q96L33 P18545 3459 P60033 83853 Q92752 P05230 857 O14763 Q9UN86 Q96CA5 P51575 O95382 Q13506 Q9P202 Q9NQT8 Q9HCK4 153090 Q9UMX1 O14775 P09601 O14777 P01241 O95399 Q9BRR9 Q96C74 3484 3482 O60603 25988 3479 2147 3476 10010 6505 Q15269 P13236 P51513 Q99755 Q96T76 400 402 Q99750 Q99759 408 O43293 O14713 Q13098 Q01518 Q6ZN33 Q09472 4354 Q7Z6I6 4355 116986 O00560 116987 O00562 23303 890 Q00653 891 54413 Q99741 55743 896 3014 Q15286 Q9Y450 29086 O43264 Q8TCX5 Q658W2 Q01974 P15882 10486 7867 8718 O14733 5207 10487 P23497 8717 P01243 P26583 O00506 Q9Y3M8 10459 10458 Q8WUY9 Q96P48 10451 6548 7879 10454 Q01968 P01275 8743 8744 7410 Q9NYZ3 P37231 P51959 8737 P48357 P55083 8738 Q6IQ22 11315 P01266 23746 P24385 8754 Q96GA9 5245 9600 3068 O75293 79753 Q96P20 7416 P24394 7433 8766 P49674 Q13905 P52824 3074 80728 P17612 8772 Q13115 Q13114 Q16623 P18085 Q14449 P10114 Q9Y4K4 P10599 O75385 Q9Y4K3 O43318 472 P48729 477 51762 P20396 8767 P43034

Q14451 P35408 Q15303 3091 Q16635 P55042 P55040 Q15796 Q15311 Q13131 O00602 8795 Q13136 Q15797 Q16644 8315 P19397 P12755 Q13144 Q8IV61 Q16659 5295 5296 9656 P43490 7476 P18074 O43306 P10586 P10588 P10589 9649 Q16665 P43487 Q9P2E9 P43005 Q13158 Q8TDY2 11261 P54132 P63092 P09172 P46109 Q13162 P46108 Q13164 Q14012 Q15349 O15294 7013 Q9Y4H2 Q9NZT1 P63096 Q9NRD1 Q13177 Q99835 10392 O94844 P12314 55704 1718 Q13188 P23443 O43741 P10147 O75312 P38936 O75791 Q6PID4 Q99819 P27815 P48730 P23458 1728 Q9BWT7 Q99814 P48736 6196 6199 7046 6198 P98155 P10997 8379 O43715 6195 6194 27242 Q86VI3 10817 P46940 O95715 P98161 Q13522 7057 P10523 9230 9231 Q3V6T2 7052 3925 P46937 1742 Q4LE28 P98171 P98174 Q9UER7 P34947 P29597 Q9H305 Q13535 O15264 P29590 8394 P30101 7064 1756 P41231 Q92793 P16284 Q9NZQ3 O60266 O15259 O60260 9255 Q13546 Q8IYT8 9252 9253 7074 80254 P43405 P20309 Q04206 Q9NVJ2 P78560 P09525 7088 Q13555 Q13554 Q8IV45 9261 3959 P00749 O00194 P63000 51720 3953 Q13563 2626 7099 7098 Q15742 O15211 Q14416 9270 7090 P30153 P62166 Q9UIV1 1785 Q05513 P00734 Q9HD67 P24723 375790 Q13574 51735 Q6SZW1 Q15750 Q13572 53916 Q15759 O60229 P41279 P20338 P20339 P20336 P20337 2644 1312 P85298 P41273 1793 Q14432 Q15762 P18031 P34972 1326 Q9UNA1 P47804 Q8TDM6 P67870 900 P20340 P00750 P08235 Q9NZN9 O15228 Q7Z628 P08833 Q9Y2W7 Q14CB8 Q92619 P61020 Q6PCE1 Q7L0Q8 2665 2664 Q9HBH0 P61026 Q8N103 P07996 P52564 O75815 P52565 Q12955 Q7L591 Q66LE6 Q12959 51090 P52566 Q9P107 P63211 10549 P16473 2676 O60333 P16471 P63218 P48039 Q9Y2Y0 928 Q92600 P54753 P50395 Q9NYF5 P32927 Q05397 57646 29127 P20936 Q9UQB3 Q9UQB8 4867 4868 4869 5716 P54764 P54762 4882 P48023 3551 O00299 Q12979 P20941 Q8NEU8 Q9ULZ3 948 Q9UHR4 Q6PCD5 O95477 Q9Y342 P28482 1385 O00220 29108 5739 O60760 P07550 Q9Y2T1 10971 7913 P08887 P51671 P04049 Q96SB4 Q9NYA1 O14880 2243 P10721 29110 2237 79444 1388 1387 963 10981 P42771 Q9UM54 5747 O00241 Q9P0L2 22931 6609 P62330 2247 P15153 1399 2246 1398 2244 975 P61006 Q92636 P51693 5770 P29320 P51692 P28472 O00255 P48061 Q99259 P17342 P29317 Q9ULV4 P61019 P61018 26060 P06213 Q15139 Q9Y6W6 P07900 Q9Y6W5 990 Q96RU8 Q96RU2 2267 998 6622 10133 P27986 P02686 P62753 P49770 5310 Q7Z5H3 5311 5796 P51636 266747 Q04900 Q8WTR2 Q96RT1 10142 Q99638 10146 P02675 P01343 P49768 O00206 Q9Y6Y9 148022 Q00535 Q96S42 5798 6647 8829 8826 P01375 P01374 Q9Y2R2 64343 O00213 Q08499 O00212 P12004 Q96RR4 Q15172 3148 545 P40933 Q96EY1 Q15173 5327 5328 7508 O43353 6672 Q969H4 P07949 8852 P07948 Q12904 Q8WU90 3162 Q8IXI1 Q8IXI2 Q68EM7 552 553 Q99683 5335 Q96AX2 5336 8844 Q15185 5337 5338 P05771 P26232 7534 Q9UHC3 Q01831 P50749 P50748 5347 Q96JH8 P24046 7529 Q08462 5361 5362 5364 Q5VWQ8 Q9UHD2 Q12929 Q9BUZ4 572 8882 5371 3190 Q60FE5 Q12933 P15559 P16885 Q99653 P51617 P62745 54106 Q13478 Q13477 8892 Q15654 Q9UQP3 6236 8412 9743 Q96FV9 595 29941 Q96BM9 Q8NFJ5 Q7Z6C1 P22694 Q13485 Q8WZ64 8408 P32019 6242 Q13009 Q16512 4067 Q16513 Q15669 P35523 6238 P60983 6239 Q14344 Q7Z6B7 Q13017 Q9HBW0 23380 Q9UDY8 P23528 8428 P00441 8443 Q16539 4086 4087 P22692 4089 Q15208 Q16537 Q9Y3L3 9770 Q9Y3L5 Q9NZ94 P21359 P31150 Q9NUP9 1808 8439 10087 6259 9784 P54257 O75469 7124 P54252 P54253 P14635 P10275 P10276 154 Q96KC2 Q16555 Q15223 Q13043 9311 7132 8463 Q5JQC9 P36896 P35568 6282 23370 Q8NFM4 Q04941 Q02763 P01308 Q8NFM5 120892 Q99717 9306 P01303 9322 P02671 Q8TCU4 8473 P54274 8471 P36404 9318 1837 P49715 O95819 P60953 64780 7153 P39905 7157 P19086 7159 Q02750 1848 54509 Q13077 P53365 O95835 P57729 O94988 O95833 10928 57584 P42224 1852 1850 P23945 83871 O95837 P22612 P42229 P42226 Q53QZ3 22926 10939 Q14738 P10644 22920 79363 P57735 P84095 P39059 Q9UI95 7187 9367 7186 7189 7188 Q13418 7185 P17252 P42680 P42681 P42684 P21741 O15397 O43823 B2RTY4 4914 27352 P19438 P68400 Q969Q1 Q13432 58473 Q86YS6 P51148 Q7RTR2 P51149 Q9H244 P23508 P43119 1896

2741 P42262 4920 P51157 Q13443 P51151 Q15628 P51153 P42261 P11532 58480 P51159 27330 Q9UQE7 P30281 57159 Q8WUH2 Q8WYP3 P23510 P21333 O14492 377630 59341 P84085 3611 1432 Q5XUX0 Q15642 Q13464 P01730 Q14314 P53355 O75832 P53350 3622 P84077 Q7L9L4 P24821 4952 Q15653 P21796

negative regulation of transcription Q9Y2W7 O75820 2672 Q9UBB5 Q9NS86 O00267 124790 10657 3516 2304 P84022 283248 P27348 5933 Q9UBC3 Q9Y2X9 Q92729 Q12837 54361 Q8NB12 4851 4734 Q96EB6 5705 O75925 3660 Q9NYF8 Q9NRY4 Q9BYE7 Q99471 6927 P13056 83933 811 Q9UJU2 O15534 57649 Q92833 6926 Q6IT96 P06401 P26358 P06400 5716 Q9UIS9 Q9NZI7 P48382 Q9NQX0 P31946 5970 Q92949 Q9NRZ9 P56705 P48380 O95343 9099 10524 P20823 1487 P19838 P62263 3665 10765 Q9Y468 Q8IZ40 Q12986 Q9P0J0 O14627 6829 10614 1499 Q86VE0 O14981 Q8N2W9 10971 6945 5978 6827 O14503 Q92769 5991 93986 5993 Q07666 Q14938 Q15906 P19544 O95365 O00358 Q06330 10865 6839 1027 P08651 P17480 O14753 604 5987 4790 22938 O00488 P38398 57326 2247 58533 Q9BZK7 6601 Q92993 857 6605 Q8N488 P10826 P10827 2023 O00255 Q6KC79 P19883 O00257 1050 Q99496 Q9UMX1 4799 P40337 50943 Q9UGL1 7704 Q92985 O96004 O43159 P04637 Q9NVW2 P14316 25988 P52952 3476 Q15022 79813 4204 639 10014 10138 Q9UQ80 Q9BT49 4221 5796 P16989 P57059 84159 P33076 51132 Q99750 P48436 8932 7727 2290 23429 O43251 23309 P48552 P48431 6774 Q99623 Q96S42 55869 51147 P01137 P01375 Q86X95 P51531 6660 5451 8841 Q9UHI6 P35222 2063 P51532 23435 Q96AQ6 P35227 11218 Q96ST3 Q9NS37 P17813 P35232 3148 P10070 Q15170 P10071 3146 11331 668 6657 5569 Q96T58 Q96MH2 P23497 O43474 6670 P06733 P26583 O43593 6672 Q9Y3M2 23409 P25490 25822 25942 Q12906 Q8NFW5 3281 Q9BZE0 672 O75190 Q99684 6304 8726 P40763 4261 Q969G3 8864 Q08117 23411 23414 O75182 P37231 P14373 Q00987 3169 5467 5468 7529 7528 10468 P27797 64375 4152 5245 P56177 O60907 22893 3066 3065 Q9UIF9 P51843 3182 10438 P51608 Q9H7L9 5119 Q01826 9839 O43463 5371 3070 Q13901 9612 23512 Q96QT6 P58012 P28749 7428 10320 6595 6597 8535 P10599 6591 Q9Y4K3 3084 5017 Q13485 Q13363 Q13127 4188 5036 O75496 O75376 P13631 3096 Q02447 O75381 23051 51773 P46531 7329 Q96RL1 Q15796 Q9HAU4 Q15672 Q9UQR1 7341 8553 6496 6256 6498 Q96IZ0 P12757 Q8N6I1 P31273 Q9UNL4 P12755 23028 O75360 4193 Q2M1K9 84108 P46527 116113 51548 51547 54815 Q13263 Q13148 9774 4087 8204 4088 4089 Q9BY41 P55055 163126 Q9Y618 P10588 P10589 93166 Q04724 P20264 11176 Q9NPF5 Q15697 51317 55915 53615 P43489 Q13033 9421 P54259 9541 5187 O75469 64750 7124 11142 O15164 P54253 2909 O75461 Q9UQL6 4092 P10276 Q9Y6K1 Q02535 Q03989 51564 Q15583 55922 Q99729 8328 Q15466 55806 Q99966 5074 5195 Q9NRC8 6045 7376 55929 Q9HAZ2 8467 O15055 9314 O43524 23135 23378 7490 7251 1822 166 84232 Q9NQB0 Q8WW38 Q9UBP4 Q96T37 P61925 7023 P14859 P15822 23468 7141 Q9H063 Q96T23 Q9UNH7 51341 Q01664 Q00577 51588 Q13185 P49711 10155 P49715 P09038 O75437 7157 O43623 25776 64426 138151 Q99816 9208 Q5VTD9 Q96RE7 P29374 Q01094 Q9H0M0 P41134 Q1PSW9 P62195 27122 P63165 9219 O15119 1616 P45973 Q9NSA3 P56524 O94992 Q969S8 Q8IY57 Q02297 7291 O75530 7295 P55316 Q96BD5 P46934 1869 Q9UER7 Q14865 7189 7067 92129 Q8WYK2 P19793 P29590 7181 Q03014 Q7Z2Q5 84619 Q9H6W3 Q86UE4 O15379 O60264 Q9NSC2 O15151 O95983 Q13422 O94776 Q13547 P78318 O43829 Q8TBE0 84733 Q04206 Q9UH92 Q92786 Q9Y2B9 2737 2736 7088 O75626 Q8WUI4 P41182 Q9UKY1 Q9H9B1 51720 Q14894 150094 Q9NP71 1789 1788 Q05516 1786 58487 P41162 3720 3609 Q13573 Q14781 P23510 P09429 O75604 Q9H2S9 29966 Q02363 P18146 P35711 Q9BQA5 Q9Y6E7 O60341 P09430 5914 O75953 O75832 Q9BZS1 Q13227 P55771 Q86YP4 63976 Q96BF6 P63279 Q8N5A5 P63272 Q9HCS4 O15105 5925

positive regulation of cellular metabolic process O14793 Q12824 P17676 2547 2304 P37288 2303 1454 P84022 1453 Q92731 2308 25913 Q9H4L7 Q12834 Q12837 Q5VTR2 P16104 P14174 Q5S007 O75925 Q7Z727 Q92956 6927 P37023 6929 57761 811 Q9Y297 O75928 P40425 P40424 6926 3659 P26358 6925 3673 2100 Q9NR96 P48380 O95343 10645 P20823 O60674 P19838 Q8IUC6 3667 5604 P27037 Q9Y463 1022 O95352 1017 P21964 1499 80324 Q92530 3678 3690 4780 Q92769 6720 P28370 O95361 P10600 O00358 Q06330 10626 P19419 P17482 P17480 4775 P04179 4790 Q9UGJ0 Q12888 4792 Q9H2K2 P38398 Q9HCL2 Q96N67 P20618 P60033 Q92990 Q92753 6721 P05230 Q92993 857 Q92997 Q13501 Q9UKS7 P10826 Q9UKS6 Q9UL46 P10827 6500 Q13503 P31749 1050 861 5888 P09601 50943 Q9UKT4 P01241 O96004 P14317 P50416 Q9UIH9 23326 O60603 25988 P15884 3479 2147 10011 10013 84289 10014 6760 64682 Q96T76 641 P33076 406 648 Q9BRP8 O43294 408 84271 P49407 Q13099 O00327 Q09472 5682 P48552 5683 5684 5685 6774 5686 3265 3263 890 891 Q99743 894 896 Q14192 898 3014 8945 P01019 29086 P49639 P51531 4361 5692 5451 5693 P35222 Q9NUX5 P51532 P35226 P17813 3269 668 5687 6776 5688 10001 P23497 P24588 P26583 O00744 5460 Q9Y5Q3 Q6FGG2 9821 P50613 P51946 Q9NX61 672 P15692 10213 P55072 Q8N5U6 P05412 O43242 O00755 Q9HCE7 Q05086 P37231 Q9NWV8 688 148327 9826 207 5468 Q92922 P24385 8754 7422 P52815 3066 Q9UMN6 3065 O00762 Q9NWW5 P17844 7415 10432 5494 P49674 Q9UIG0 23513 O14936 466 468 7428 O15085 6595 Q14686 6597 6599 11065 6598 Q9Y4K3 Q9H161 474 P08047 8767 Q9NPC8 P38919 3091 P55036 8301 Q15306 5037 Q86VP6 P58304 P13631 Q04864 51773 P46531 7448 P00533 Q16401 Q96RL1 P61956 Q15554 Q13133 Q15796 P35638 Q15797 Q96IZ0 8555 P12755 Q9ULH7 Q9NY61 O75360 4193 Q15561 10197 Q13144 51547 Q15562 Q9BSI4 Q16650 P35869 P62826 7476 9412 P18074 Q8WVL7 P55055 P36956 Q16665 9421 Q8TDY2 P54132 O75586 P35453 Q93062 Q9Y2C9 Q14011 5074 Q9NRC8 8100 11030 Q9HAZ2 7014 P22392 Q9Y4H2 O43524 P35443 7490 P11021 Q8WW38 Q99835 P02790 7023 5089 P98179 54851 Q96PK6 51588 P08069 10155 1956 P23443 Q9NPA8 Q9BX66 6182 P37840 P11474 5092 7272 26747 Q6PID4 P48730 Q99814 Q14289 6199 7046 6198 9464 P35813 P29375 27005 P22736 P42345 Q9Y4C1 P62195 P04085 9219 Q58WW2 P56524 P78536 7057 O15496 Q13526 22807 Q4LE28 P16070 O75528 8399 7067 P29353 Q06643 P19793 P29590 Q01196 P41235 Q92793 Q96GM5 Q9HD15 Q9NRA1 Q13547 A4D1W7 P78317 9495 Q9UNE7 P43405 1763 Q9HAK2 Q04206 Q92786 O14593 O95999 P35610 51720 4800 P08246 2626 7099 7098 Q13322 Q9NVC6 Q9NP71 1789 P46736 1786 Q9UIV1 P00734 3720 375790 5901 P46734 P12956 P28065 P78527 Q9H4W6 Q9BQA5 1312 P61088 P43694 O15455 5914 P62191 P28074 63976 P28072 P28070 5927 P63272 P24941 Q9HCS4 Q92585 902 Q15532 P08238 P43686 P21675 P07148 5925 P10914 Q92858 Q12948 Q6QNY1 O00267 3516 4609 P16220 Q96JM2 P63208 O15516 P07996 5935 10533 P04004 Q9UBC3 54361 Q8NAP3 5706 5707 P16234 5708 5709 51099 4851 5700 5701 5702 5704 5705 Q9Y2Y8 Q12968 5717 4862 Q9UJU2 P49116 P61289 5713 Q92833 Q6IT96 P06401 4869 P06400 5716 Q96JK9 5970 1374 9093 Q99460 P56705 10524 P17301 P61296 5966 3309 P04233 P05129 O95477 P28482 1385 P51668 3320 P51665 P19532 O00468 6829 10736 P15173 P07550 Q8N2W9 4646 P08887 6827 P08648 P30556 Q9UHK0 Q9NYA1 5991 O00231 1153 Q14938 O00233 O00232 P19544 Q9NS56 Q8WYH8 P37198 57678 29110 960 3326 Q9P0K8 3324 1387 2475 P08651 4898 Q9UM54 P07766 P05106 6850 22937 Q13616 1161 O00487 P30542 O60502 P62333 2247 2246 Q9BZK7 975 O14641 6601 Q92630 Q12770 Q92878 P05112 Q9UM63 P09958 O43186 Q12772 Q12778 P51692 Q05586 O00255 Q6KC79 Q03164 10724 P05362 5524 6613 P40337 Q9Y2W1 P06213 P07900 6872 P04637 5300 2034 P13349 2033 2274 O95271 P52952 991 Q96RU8 Q96RU7 Q8WTS1 4686 6622 54998 Q16236 Q15389 10138 P01112 Q04917 Q9C0K0 4221 P35368 2280 P48436 4214 P01106 P01588 P01343 P01100 P49768 O00206 P48431 9921 5321 Q9NS23 148022 Q96S42 Q02952 Q53X93 P25789 P25788 6646 P01137 5316 P25787 P01133 P01375 Q9UPY8

6660 Q96RR4 3148 P10070 P10071 3146 P61457 P01127 Q96EY1 6657 O43353 6672 5582 P07948 3162 Q12905 Q12906 Q9BZE0 552 79612 3159 Q02930 P51828 6667 5578 P40763 Q96FA3 P04201 5591 4261 Q6IR47 10580 7533 64127 P18850 23414 3171 P18846 Q00987 P18848 3169 Q9H1A4 5347 P27797 P01185 O60907 Q9UHD2 Q9UPT9 3182 8625 Q9BT67 23636 5371 P27540 8881 Q9UPU9 6464 P62508 P17980 O14818 P51617 P58012 10320 54106 8878 9967 P25116 P60568 4297 Q9BY77 11186 P30086 P15923 P13984 351 595 Q9UBS0 Q04759 Q7Z6C1 Q13485 P25963 O94906 P03950 5154 O00716 7334 5155 4067 P12429 19 367 Q70SY1 Q13492 Q9UQR1 7341 6496 6495 6256 P35998 Q9Y606 Q2M1K9 P17096 Q13263 4086 4087 8204 4088 4089 9775 8445 P12645 222546 Q9NWT8 P08151 P24864 P20264 P62701 Q13033 P62942 8454 P55347 O75469 7124 P54253 P14635 391 Q9UQL6 P10275 4092 P10276 4093 154 Q13042 11146 Q99729 Q99966 Q13285 2902 7132 7376 8463 Q09161 P36896 P36894 P35568 55810 P01308 120892 338917 Q9NQB0 P49721 Q14140 O15169 P49720 85440 Q99958 Q15475 Q96T37 Q8TEY5 P00492 10273 P14859 Q96T23 P49959 Q01664 56916 P01579 P49711 P49715 P09038 55827 Q15008 9575 7158 P39905 7157 O15198 Q02750 55832 Q14160 Q15257 Q13873 O95835 Q01094 7161 9341 26271 Q1PSW9 P17275 P63165 91 94 P42229 O15119 P45974 1856 P42226 Q9UBK2 22926 Q96QB1 Q13887 9590 Q8IY57 P55318 O43847 P84095 P05067 P60900 1869 7189 Q15836 Q03014 7182 6096 56034 P17252 P68871 O95619 Q9NSC2 8289 Q13422 O15156 O94776 2735 P19438 Q9BXJ9 Q8HWS3 Q9UBN7 1406 2737 2736 Q15853 Q96G30 9149 Q8TAK5 P53567 1655 P22415 Q9UK80 Q03468 Q13200 P47712 Q13443 57154 P30279 P20248 64919 80155 Q9UJX2 P30281 P23511 3609 P17081 3608 P09429 8078 P10415 Q13216 29966 P18146 P35711 57162 84305 P01730 P08588 O15350 O75832 Q9BZS1 P53350 Q15648 P55771 P19484 Q06710 Q04771 Q9HC29 P30260 O14497 O15105 Q13233

small GTPase mediated signal transduction 326624 O15085 P36507 Q2M1Z3 Q92737 6236 11186 P18085 Q9NRW1 8412 P10114 Q99490 O75385 P61020 55684 Q6PCE1 Q9H0U4 Q7L0Q8 2664 Q96BM9 51762 Q9HBH0 10890 Q92730 P61026 55207 P49137 P27348 8408 64284 O75815 P52565 6242 Q13009 Q7L591 Q15669 P27361 19 9077 P52566 Q9H0T7 P55042 6239 P55040 Q14344 Q15311 Q59EK9 Q5S007 Q13017 Q16644 Q9UQ13 8315 Q9NR81 29 339122 Q86X27 Q9Y2I1 Q8TEU7 P53667 57403 375 81876 P62491 P23528 Q9UKW4 6009 10633 P55290 Q8IV61 P06400 Q16539 6386 P31946 Q9UPQ3 Q5JS13 Q12979 9093 381 382 Q9Y3L5 387 388 P21359 389 P31150 51552 Q13393 9649 P60763 5604 P62820 O95477 11261 P28482 11021 5861 5862 390 391 392 394 396 P46109 397 1017 P15056 398 51560 25780 Q6KH11 1012 Q9H082 Q13283 P61225 Q96KC2 P61224 P61587 P61586 10971 P61106 Q9UL26 Q9UL25 P30556 Q5HYI8 P04049 Q9BU20 5870 11031 Q15907 1029 23370 1026 1147 120892 5863 10981 5865 P42771 5867 6714 Q92888 5868 Q92766 5869 Q15118 Q9BYZ6 Q07890 Q6ZSZ5 O94844 Q96L33 P36404 22931 P62330 2247 P15153 1399 Q07889 P61006 1718 5877 Q9UN86 5878 5879 O43182 P09038 P60953 Q14155 Q9HB90 7157 5894 O75553 Q38SD2 Q02750 P38936 O75791 P11234 P11233 84932 Q99819 54509 P09601 P61019 127829 P61018 Q14964 Q15811 P53365 Q13637 P57729 Q9BST9 Q13636 P04637 Q9Y6W5 10928 P12931 P62993 3479 998 83871 P01116 5898 Q15382 P62873 10256 285282 P01112 P01111 9230 266747 1072 79363 P57735 P84095 9908 Q15392 400 402 Q14185 P47224 10146 4218 P98171 P01343 9367 P29353 Q8N726 O15264 116986 O00560 3265 P52735 116987 6093 Q7Z569 Q9NS23 890 P61204 Q15286 P01138 9138 P04629 Q07960 O00212 7074 4914 27352 Q9NVJ2 Q96EY1 7867 Q59EA4 23647 Q969H4 O14593 Q5U0I6 Q86YS6 Q03113 P51148 3162 9261 Q8IXI1 P51149 Q8IXI2 673 O00194 P63000 10451 P08123 Q96AX2 P62834 8844 7879 5337 5338 57826 Q92930 Q52LW3 P51157 Q96HU1 P51151 Q8IVT5 O15211 7410 Q9NP72 P51153 58480 P51159 P20248 5906 5908 Q8TAI7 O60496 P08134 9826 Q8WYP3 P17081 7529 51735 Q6IQ22 53916 9046 6453 Q15759 Q9P035 P20338 P84085 P20339 P20336 Q8IZJ4 P20337 2885 1432 1793 50650

10672 8625 Q13464 5911 Q9NP90 O15111 A4D1S5 8766 6464 Q8NHX1 P62070 O95661 Q9H0N0 O14939 Q99653 P84077 P24941 P40616 P62745 Q9H4E5 P20340 5925

positive regulation of metabolic process P25054 O14793 Q12824 P17676 2547 2304 P37288 2303 1454 P84022 1453 Q92731 Q9Y275 2308 25913 Q9H4L7 Q12834 Q12837 Q5VTR2 P16104 P14174 4734 Q5S007 O75925 Q7Z727 Q92956 6927 P37023 6929 57761 Q9UKW4 811 Q9Y297 O75928 P40425 P40424 6926 3659 P26358 6925 3673 2100 Q9NR96 P48380 O95343 10645 P20823 O60674 P19838 Q8IUC6 3667 5604 P27037 Q9Y463 1022 O95352 1017 P21964 1499 80324 1012 Q92530 3678 3690 4780 Q92769 6720 P28370 O95361 P10600 Q96J02 O00358 Q06330 Q96KS0 10626 P19419 P17482 P17480 4775 P04179 4790 Q9UGJ0 Q12888 4792 Q9H2K2 P38398 Q9HCL2 Q96N67 P20618 P60033 Q92990 Q92753 6721 P05230 Q92993 857 Q92997 Q13501 Q9UKS7 P10826 Q9UKS6 Q9UL46 P10827 6500 Q13503 P31749 1050 861 5888 P09601 50943 Q9UKT4 P01241 O96004 P14317 P50416 Q9UIH9 23326 O60603 25988 P15884 3479 2147 10011 10013 84289 639 10014 6760 64682 Q96T76 641 P33076 406 648 Q9BRP8 O43294 408 84271 P49407 Q13099 O00327 Q09472 5682 P48552 5683 5684 5685 6774 5686 3265 3263 890 891 Q99743 894 896 Q14192 898 3014 8945 P01019 29086 P49639 P51531 4361 5692 5451 5693 P35222 Q9NUX5 P51532 P14784 P35226 P17813 3269 668 5687 6776 5688 10001 P23497 P24588 P26583 O00744 5460 Q9Y5Q3 Q6FGG2 9821 P50613 P51946 Q9NX61 672 P15692 10451 10213 P55072 Q8N5U6 P05412 O43242 O00755 7410 Q9HCE7 Q05086 P37231 Q9NWV8 688 148327 9826 207 5468 Q92922 P24385 8754 7422 P52815 3066 Q9UMN6 3065 O00762 Q9NWW5 P17844 7415 10673 10432 5494 P49674 Q9UIG0 23513 O14936 466 468 7428 O15085 6595 Q14686 6597 6599 11065 6598 Q9Y4K3 Q9H161 474 P08047 8767 Q9NPC8 P38919 3091 P55036 8301 Q15306 5037 Q86VP6 P58304 P13631 Q04864 51773 P46531 7448 P00533 Q16401 Q96RL1 P61956 Q15554 Q13133 Q15796 Q13131 P35638 Q15797 Q96IZ0 8555 P12755 Q9ULH7 Q9NY61 O75360 4193 Q15561 10197 Q13144 51547 P55290 Q15562 Q9BSI4 Q16650 P35869 P62826 P43490 7476 9412 P18074 Q8WVL7 P55055 P36956 Q16665 9421 Q8TDY2 P54132 O75586 P35453 Q93062 Q9Y2C9 Q14011 5074 Q9NRC8 8100 11030 Q9HAZ2 7014 P22392 Q9Y4H2 O43524 P35443 7490 P11021 Q8WW38 Q99835 P02790 7023 5089 P98179 Q8WY64 54851 Q96PK6 51588 P08069 10155 1956 P23443 Q9NPA8 Q9BX66 6182 P37840 P11474 5092 7272 26747 Q6PID4 P48730 Q99814 Q14289 6199 7046 6198 9464 P35813 P29375 27005 P22736 P42345 Q9Y4C1 P62195 P04085 9219 Q58WW2 P56524 P78536 7057 O15496 Q13526 22807 83737 P46934 Q4LE28 P16070 O75528 8399 7067 P29353 Q06643 P19793 P29590 Q01196 P41235 Q92793 Q96GM5 Q9HD15 Q9NRA1 Q13547 A4D1W7 P78317 9495 Q9UNE7 P43405 1763 Q9HAK2 Q04206 Q92786 O14593 O95999 P35610 51720 4800 P08246 2626 7099 P31249 7098 Q13322 Q9NVC6 Q9NP71 1789 P46736 1786 Q9UIV1 P00734 3720 375790 5901 P46734 P12956 P28065 P78527 Q9H4W6 Q9BQA5 1312 P61088 P43694 O15455 5914 P62191 P28074 63976 P28072 P28070 5927 P63272 P24941 Q9HCS4 Q92585 902 Q15532 P08238 P43686 P21675 P07148 5925 P10914 Q92858 Q12948 Q6QNY1 O00267 3516 4609 P16220 Q96JM2 P63208 O15516 P07996 5935 10533 P04004 Q9UBC3 54361 Q8NAP3 5706 5707 P16234 5708 5709 51099 4851 5700 5701 5702 5704 5705 Q9Y2Y8 Q12968 5717 4862 Q9UJU2 P49116 P61289 P03372 5713 Q92833 Q6IT96 P06401 4869 P06400 5716 Q96JK9 5970 5971 1374 9093 Q99460 P56705 P17302 10524 P17301 2697 P61296 5966 3309 P04233 P05129 O95477 P28482 1385 P51668 3320 P51665 P19532 O00468 6829 10736 P15173 P07550 Q8N2W9 4646 P08887 6827 P08648 P30556 Q9UHK0 Q9NYA1 5991 O00231 1153 Q14938 O00233 P10721 O00232 P19544 Q9NS56 Q8WYH8 P37198 57678 29110 960 3326 Q9P0K8 3324 1387 2475 P08651 4898 Q9UM54 P07766 P05106 6850 22937 Q13616 1161 O00487 P30542 O60502 P62333 2247 2246 Q9BZK7 975 O14641 6601 Q92630 Q12770 Q92878 P05112 Q9UM63 P09958 O43186 Q12772 Q12778 P51692 Q05586 O00255 Q6KC79 Q03164 10724 P05362 5524 6613 P40337 Q9Y2W1 P06213

P07900 6872 P04637 5300 2034 P13349 2033 2274 O95271 P52952 991 Q96RU8 Q96RU7 Q8WTS1 4686 6622 54998 Q16236 Q15389 10138 P01112 Q04917 Q9C0K0 4221 P35368 2280 P48436 4214 P01106 P01588 P01343 P01100 P49768 O00206 P48431 5562 9921 5321 P52735 Q9NS23 148022 Q96S42 Q02952 Q53X93 P25789 P25788 6646 P01137 5316 P25787 P01133 P01375 Q9UPY8 6660 Q96RR4 3148 P10070 P10071 3146 P61457 P01127 Q96EY1 6657 O43353 6672 5582 P07948 3162 Q12905 Q12906 Q9BZE0 552 79612 3159 Q02930 P51828 6667 5578 P40763 Q96FA3 P04201 5591 4261 Q9Y4P1 Q6IR47 10580 7533 64127 O76024 P18850 23414 3171 P18846 Q00987 324 P18848 3169 Q9H1A4 5347 P27797 P01185 2099 O60907 Q9UHD2 Q9UPT9 3182 8625 Q9BT67 23636 5371 P27540 8881 Q9UPU9 6464 P62508 P17980 580 O14818 P51617 P58012 10320 54106 8878 9967 P25116 P60568 4297 Q9BY77 11186 P30086 P15923 P13984 351 595 Q9UBS0 Q04759 Q7Z6C1 Q13485 P25963 O94906 P03950 5154 O00716 7334 5155 4067 P12429 19 367 Q70SY1 Q13492 Q9UQR1 7341 6496 6495 6256 P35998 Q9Y606 Q2M1K9 P17096 Q13263 4086 4087 8204 4088 4089 9775 8445 P12645 222546 Q9NWT8 P08151 P24864 P20264 P62701 Q13033 P62942 8454 P55347 O75469 7124 P54253 P14635 391 Q9UQL6 P10275 4092 P10276 4093 154 Q01201 Q13042 Q99728 11146 Q99729 Q99966 Q13285 2902 7132 7376 8463 Q09161 P36896 P36894 P35568 55810 P01308 120892 338917 Q9NQB0 P49721 Q14140 O15169 P49720 85440 Q99958 Q15475 Q96T37 Q8TEY5 P00492 10273 P14859 Q96T23 P49959 Q01664 56916 P01579 P49711 P49715 P09038 55827 Q15008 9575 7158 P39905 7157 O15198 Q02750 55832 Q14160 Q15257 Q13873 O95835 Q01094 7161 9341 26271 Q1PSW9 P17275 P63165 91 94 P42229 O15119 P45974 1856 P42226 Q9UBK2 22926 Q96QB1 Q13887 9590 Q8IY57 P55318 O43847 P84095 P05067 P60900 1869 7189 Q15836 Q03014 7182 6096 56034 P17252 P68871 O95619 Q9NSC2 8289 Q13422 O15156 O94776 2735 27352 P19438 Q9BXJ9 Q8HWS3 Q9UBN7 1406 2737 2736 Q15853 O75626 Q96G30 9149 Q8TAK5 P53567 1655 P22415 Q9UK80 Q03468 Q13200 P47712 Q13443 Q96HU1 57154 P30279 P20248 64919 80155 Q9UJX2 P30281 P23511 3609 P17081 3608 P09429 8078 P10415 Q13216 29966 P18146 P35711 57162 84305 P01730 P08588 O15350 O75832 Q9BZS1 P53350 Q15648 P55771 P19484 Q06710 Q04771 23192 Q9HC29 P30260 O14497 O15105 Q13233 P45984

cell death Q7Z628 Q92858 Q9Y2W7 O75940 P10911 Q9NRW4 Q5VV41 9181 Q8IXM3 P15498 54474 O60566 P84022 O00273 54476 Q9ULW0 Q6IQ55 P07996 Q92974 P28331 Q8NF91 65018 Q9H832 P55957 9197 P51114 O60216 P42858 30011 Q9UKV3 10783 Q8N695 92609 P10809 Q9H2G2 65264 Q5S007 Q9NR80 Q9NR81 O00167 P17405 22974 10758 Q8IVF5 57761 O60543 Q9UKW4 O75807 P62258 Q9H7P9 P31944 P31946 P31947 P48023 P99999 Q12979 9093 Q6IC98 22985 Q13829 P17302 Q96JJ3 P19838 2697 Q8IUC6 P61296 Q9ULZ3 Q8N5V2 Q9UHR5 Q9UL15 Q12981 Q12982 P05129 Q5D1E8 O00220 O95470 Q96MX6 29108 Q9NSU2 O95232 114548 Q9P0J0 P19525 2348 3315 55367 Q6Y7W6 5610 P09972 5733 Q9Y2T1 58517 Q7Z419 P04049 P29466 Q9Y239 Q14814 Q9H4P4 Q9NZC7 P10721 Q15904 Q5VST9 Q8IY17 Q9NS56 P37198 3329 1029 Q9HCM9 1027 79444 29115 841 P42771 3689 P04179 Q07890 4790 O95257 P05107 P28223 4792 Q12768 P50591 Q9NZD8 1285 Q9NQS1 Q13616 Q96TC7 Q9NS68 6609 P62330 O60502 P38398 391627 7818 57448 P61244 2247 Q96KQ4 Q07889 Q9Y371 Q92630 O14763 Q8N488 Q96CA5 Q9H0F6 5879 Q13501 P51572 P51693 P51575 P30536 22827 Q13625 5894 P31749 Q9H8V3 Q02078 Q03164 2139 10724 984 O00141 Q99497 Q9UMX0 P09601 P29317 Q9UL54 7704 P23396 P48454 O00429 Q9BST9 P04637 P15408 2033 O00300 P50897 P52952 O14827 Q7L523 Q96RU7 2147 79811 10011 5533 6622 10134 637 10133 4205 10015 P62753 P01112 10016 4209 P25445 Q9Y5V3 Q7Z6J4 112399 Q9UQ88 118813 23210 1191 56940 Q15392 55973 4214 84033 Q14185 P01589 P01588 O43293 Q15276 P49768 Q09472 23429 6772 Q8N726 P12236 3265 O95292 P52735 23787 Q96CV9 P11388 148022 P11387 Q9NUW8 O14727 773 Q9NR09 Q8WWW0 P49756 537 51147 6647 Q9Y572 P01138 55504 5315 P01137 P01375 2065 23435

P35348 O14737 663 Q96RR1 P10070 668 Q8WTP8 Q96EY1 Q15052 8718 11214 P49747 8717 O43353 5582 Q92934 O43236 6311 9700 Q96B97 Q12904 P15328 Q9UNN5 3162 Q9UHB4 O95197 Q8IXI1 Q8IXI2 672 Q99683 10451 5579 P61626 P55072 P05771 P05412 2081 5591 8743 8744 7410 7531 5598 Q5T442 P68032 Q5JSP0 P37231 Q01955 55775 23770 5467 8737 9826 207 5468 7529 11315 6453 Q9H902 Q12802 O00400 Q9NWW5 P12036 572 O75293 331 573 10438 333 Q96P20 7415 5599 4149 7416 23636 5371 Q13901 Q12933 8887 4287 O43464 4168 146057 23513 O60911 51074 Q5TCQ9 P58012 8879 P62745 P60201 8878 P04792 P25116 Q9H8M7 8772 O15085 Q9BZZ5 8655 4297 Q14209 9500 7204 Q9Y4K3 Q96FV9 230 351 472 Q96GX9 474 117584 355 356 P49810 Q7Z6C1 Q8WUM4 8767 P21127 Q9HB75 P25963 Q9HC98 O43683 Q16637 P03950 6242 Q13009 Q13007 28986 Q86VP1 367 P09382 51651 8539 P24522 Q15672 8795 Q96IZ0 10193 29 64975 Q6GPH4 Q9UNL4 64857 Q9Y2I1 Q9NY61 P46527 Q969Z0 23263 Q5T4F4 P61604 P22314 P00441 Q9BWQ8 51428 P32239 Q13148 P35869 10081 9530 4088 8565 8445 9897 57099 382 388 Q00005 Q5J8M3 Q13153 Q14249 8454 P54259 Q13158 9420 7124 P54252 P54253 7126 23365 O75340 23368 P10275 O75460 121512 Q9Y3E5 51324 O94827 10059 Q8WV24 Q15464 Q13164 Q13286 7132 5074 7013 7133 O43521 O43524 Q9Y5J5 Q86WV6 1822 23370 120892 7128 11035 11277 O15169 Q13177 23229 Q8TCU4 Q96LC9 Q6ZSZ5 Q15116 Q96G97 P61803 Q8TCU6 Q3ZCQ8 10392 P14618 Q96KA5 O15068 P54274 O43734 Q99700 51100 O95816 P01579 1718 11124 10276 Q9NQC3 54739 51107 P09038 Q14155 7153 6188 64782 Q9HB90 9217 7157 7159 23111 O43508 P37840 7150 P11234 Q14CZ7 23239 Q8WZ73 2810 255488 Q14160 51234 Q6PID4 10285 Q93038 Q13075 1969 Q14289 2935 Q15811 P54577 Q9NWH9 9464 P10997 Q01094 7161 O43715 6194 1613 P63167 27242 P42224 P41134 Q9BXM7 Q1PSW9 P41250 1616 7057 9238 Q96QB1 Q01085 7291 55177 1982 Q9UHX1 Q9Y6H5 P98170 P05067 P42574 1869 P42575 P98171 P98174 Q9UER7 O15020 7186 7189 P06239 O94768 Q9H305 P29350 Q9H422 Q96M96 O75881 P29590 10908 6093 Q9BXK5 Q9HD15 Q8N163 1870 P21860 P04062 1639 Q9BTC0 3932 9135 O15151 P07339 9255 O15392 340061 P55210 Q6R6M4 Q13546 7073 P35900 Q8N8D1 7074 P19438 Q9Y3D6 80013 3708 Q92785 Q92542 Q13794 Q9UBN6 1647 2736 Q9NZJ7 Q14643 Q9UJV9 O75503 O95999 Q13315 Q6NYC1 P41181 P00747 P63000 Q96PE2 2629 P43115 3956 O75509 P51398 Q15628 P21580 Q07820 Q96F07 Q96BI3 Q07817 Q05516 P61073 P61077 P00734 26123 P08138 50649 Q8WWK9 Q9NWF9 P10415 O75962 Q8IUQ4 377630 Q96CJ1 29843 P78527 O60229 O60346 Q86TG7 1676 2521 P41273 1793 81037 50650 Q14790 51741 Q13464 Q14674 P20333 51747 O15350 728642 Q16611 Q14318 Q9P0U3 Q9H6Z9 Q9H213 1203 4719 26586 P47929 Q8N0X7 O95429 P45983 Q9NZN5 Q13233 Q9BZR8 P21796 Q13352

regulation of primary metabolic process Q9UKT9 P25054 Q9H0E3 Q9Y265 Q8NDW4 Q12824 8091 124790 10657 2305 2304 2303 1454 114991 1453 2300 Q8N3U4 O14543 155061 284695 Q00839 2308 2307 Q9H0D6 Q92974 Q9H4L7 Q12834 Q12837 Q8WXB4 Q8NDV7 P16104 2316 2 10661 Q96EB6 Q5S007 O75925 136319 Q9UKV8 Q92956 4990 3660 3661 Q9BYE7 P62495 P37023 P13056 84901 57761 55588 Q9UKW4 Q9Y297 O75928 Q9UKW6 3659 P26358 P26374 Q6NUN9 Q5BKZ1 3673 Q92949 Q99583 Q12857 Q13705 P21917 10645 1488 1487 283337 Q99576 2332 3665 3667 10642 64061 P26367 10644 10643 Q9UL15 Q9UL18 1022 1020 Q8NA42 Q06587 114548 Q8IX07 Q5JT82 Q96MX3 1017 10614 79576 P21964 Q9HAJ7 1499 10616 O14981 Q9Y230 P61586 O75909 Q92530 3678 O14503 Q12873 3690 Q12872 Q9Y239 Q8WXF0 Q9Y5Z7 Q8WXF1 6720 Q15906 Q9UGI9 285527 Q96J02 O00358 Q9BPY8 57798 1029 Q9BUB1 3207 10626 1027 1025 Q9BUB5 P17482 3685 P17480 604 Q9Y242 3689 P05023 P04179 Q15910 Q9UL36 Q9UGJ0 Q12888 5883 Q9BQ95 O60869 P16118 79595 Q8NA19 P38398 57326 55145 3215 Q96N67 P20618 Q9Y250 Q5TCX8 6721 Q92994 Q92993 6726 Q8N488 Q92997 Q9UKS7 P10826 Q9UKS6 Q9UL46 P10827 P10828 221937 5411 P31749 Q8NDX6 1050 P53999 79101 P40692

57332 3226 3225 P04150 5888 Q9UGL1 Q9UKT4 Q92985 O95159 Q9UKL0 O96004 P14317 P14316
P50416 23569 11108 11107 Q8WWY6 1059 23560 Q15022 639 P62875 6749 6760 Q15027 Q9Y5V3
5430 O96019 O95163 P16989 P57059 P36575 Q03933 51131 641 P33076 51132 P35251 Q14186
Q6B0B8 79923 8932 648 8939 P49407 P60228 85360 Q14188 O00327 O96028 5440 P48552 5441 6772
Q9UKN5 Q8TAU3 P48551 6774 Q99990 3265 346171 3263 O60828 P15621 Q66K89 Q99500 Q2M3W8
Q14192 P35240 8945 P01019 51147 Q9Y692 O14964 Q15047 P23246 Q86X95 5451 A7MD48 Q8N3J9
P35222 Q5T6S3 P35227 P35226 O14979 P57082 O75081 P17813 P35232 Q00403 53335 56849 668
6776 4116 Q96MH2 Q15057 Q16385 P24588 Q15056 7629 O43474 P06733 O00744 5460 Q9Y5Q3 6311
P62837 9821 6794 P25490 Q9NX61 3281 Q9NX65 672 P15692 O14908 Q9UBW7 6304 57708 9815
10213 Q6UUV7 P05412 O00755 Q9Y5R5 9831 3297 Q9NX70 23532 Q5JSP0 Q9UBX0 P14373 Q9NWV8
57713 688 148327 Q15070 689 5467 9826 207 5468 54206 Q8N895 Q92922 P01023 4150 4152 4154
23509 P48594 P56178 9841 P56177 Q8NHY6 Q12809 O00762 Q9NWW5 Q9Y5S9 P17844 Q0IIM8 4149
O14920 57727 P62854 9839 O43463 5494 Q8NHX1 P08908 23512 23513 Q96QT6 O14936 8517 Q92900
9849 P49427 4172 O15085 P36508 4173 4174 P35659 Q14686 4176 Q14209 8535 11065 Q9BZ95
Q6NZI2 Q9H165 4171 P13861 Q9H161 Q9H9F9 Q9H160 Q9NPC1 P49810 P08047 5017 P78395 Q9BQG0
5018 P07199 P49815 Q13363 Q9NPC8 Q14693 Q8TBP0 94234 4188 8543 P46782 5036 5037 79084
P58304 Q96F45 O15090 23051 Q04864 Q9Y2H8 9869 7205 Q8WY36 P00533 Q13370 Q16401 Q96RL1
P61956 Q15554 Q9HAU4 8553 P35638 P61968 9882 Q8IWY8 Q96IZ0 Q9UPP1 8555 P32121 P31274
P31273 P31276 Q9ULH1 O75123 Q9ULH7 23028 Q9NY61 4193 Q9Y6Q6 257 Q9H9D4 Q6DD87 Q96RK4
Q15561 10197 8548 Q96RK0 51548 Q93009 51547 Q14232 P61964 Q15562 Q9BYU1 Q9Y6R4 5054
P62826 Q9UPQ3 9412 Q9UPQ9 P36956 P35625 O75592 Q96F24 Q4AC94 P31270 Q9NPF5 Q13398
P43246 55915 9421 P78347 9425 7248 O75586 O75582 Q9Y6K1 Q93062 Q03989 Q9Y2C9 Q71SY5
Q9Y6K9 51564 Q15583 8569 55922 5074 10181 P78337 8100 55929 11030 Q9HAZ2 Q15109 P22392
O15055 O43524 26523 5071 7251 O75570 23492 7249 O15047 P49848 Q15596 Q96NH3 8110 P61925
Q3KNS6 P02790 O15062 P49840 5089 166968 O95931 A1XKG3 23466 Q9Y6M1 23468 O15060 Q8WY64
79913 51588 P08069 10155 Q9BQI3 9439 Q96NG5 1956 286075 10159 Q8NEJ9 Q9NPA8 26986 64426
P37840 5092 64428 7272 1965 Q9H9G7 54625 O95947 54623 Q14289 Q8IWS0 Q96RE7 9464 P29375
P29374 27005 Q7Z589 Q9H0M0 1975 P22736 P42345 Q9BUG6 Q96EK4 Q9UGU0 P04085 Q92574
Q13761 1978 Q58WW2 P56524 9477 9474 O15496 O75534 Q02297 7291 O75530 7290 9470 7295
P24928 1982 Q6P1K2 P16070 O75528 P29353 Q5JXB2 O95977 P0C7X2 Q8WXI9 P40145 Q9BYM8 1994
1993 79039 Q96IQ9 O15499 8165 P56545 O95983 A4D1W7 O95503 55072 P78317 P78318 9495
Q8TBE0 Q9HAK2 Q9UH92 Q9Y2B9 P52597 Q96EP0 O14593 O95999 Q9NP61 Q9NP66 Q86TI0 9021
23081 O43918 23085 Q9UKY1 Q9H9B1 P85037 P31260 Q6P1N0 P31249 Q13322 Q14653 Q9NP71
Q9NP72 P61081 P46736 3728 P61077 1540 Q9BUJ2 3720 5901 P46734 Q13330 P12956 Q6P1L6 Q9P031
P28065 Q9P035 Q9H4W6 Q00059 Q9BQA5 Q9Y6E7 Q9H582 P61088 Q96AE4 147808 Q96RG2 Q68DY9
P43694 O15455 Q9BQA1 5914 11091 O75953 P28074 63976 P28072 63978 P28070 5927 5928 P63279
P00519 5929 P63272 23077 P24941 Q92585 Q15532 P43686 Q13351 Q14683 P07148 Q13352 5925
Q7KZF4 P10914 O95677 Q92858 P67809 Q9UBB5 Q9NS86 Q99490 A5YKK6 Q9P2R6 O60566 4609
P16220 10772 5931 P49137 P27348 5933 O15516 5935 Q9NS91 Q9UBC3 P27361 65018 Q9H832 54361
115704 P50148 1107 P16234 O60573 284323 4613 O15524 1108 O15525 Q92838 Q9UK58 Q7Z3K6
P05549 Q9NRY4 1121 Q99471 Q7Z3K3 O60548 P53667 O60543 Q92830 Q9UJU2 284312 O15534
P49116 P61289 P03372 Q92833 P06401 Q92831 P06400 Q92826 P07737 5970 P46089 5971 9093
Q9UBE8 Q99460 Q9NRZ9 P56705 9099 P17535 P17544 Q99459 P17542 P62140 57410 400720 1122
P61296 5966 10765 3309 P04233 P05129 5981 P50570 O00463 P52756 O00468 O95231 Q9UFF9

O14627 6829 10736 3315 Q6FHQ0 710 5976 P41218 Q8N2W9 4646 27161 5978 6827 P08648 P30556 65056 5991 93986 5993 1153 Q14938 O00472 Q06455 55250 Q9NS56 P37198 79685 6839 10746 3326 2475 P08651 5500 5987 P26045 O95259 P07766 Q10586 P05106 P05107 6850 Q8ND82 P51449 Q6IA86 27148 Q13616 1161 O00488 O60506 O00487 P30542 Q02086 O60502 O60503 57448 P61244 Q9BXG8 O14641 4664 5511 Q12770 Q92878 5515 P05112 O43182 26292 Q9UNY4 P08621 5518 P09958 O43186 Q12772 Q12778 O43189 Q05586 2023 Q02078 Q6KC79 3350 10725 57459 10724 Q96LR5 Q99496 5524 P05121 P40337 7704 Q8N2K1 O00425 4690 P23396 O43159 6872 6871 P04637 P38405 2034 2033 Q9NVW2 O95271 O00422 Q9UFB7 4686 4204 P01116 4205 Q9C0K7 Q16236 Q15389 P01112 4209 O43166 Q9C0K0 6883 Q9BT49 4221 6885 Q9GZR2 O43167 23210 P35368 P50548 Q9BT40 23216 O76071 Q9NRP7 84159 Q9P2K8 4214 P01106 6878 4216 P01588 11236 7727 56946 Q9C0J9 P01100 Q7Z7K2 7741 5562 Q8N726 9921 5563 5565 P14416 P52735 P52736 Q9NS23 Q02952 Q53X93 6886 P01137 P01133 Q16254 Q9UJM3 P52740 7750 6421 5573 P04626 5575 Q9GZP0 5576 P04629 2064 P52747 2063 11218 Q9NS37 Q99853 55633 P10070 P17936 P09086 P10071 10363 10362 P01127 5569 O43150 2070 5580 5581 O43593 5582 5585 O43597 7764 22794 Q9UNN5 25822 Q96PU8 79612 O75190 Q96PU5 Q02930 P51828 7756 5577 5578 5579 10336 8607 P40763 P04201 2081 5591 Q9GZV8 4261 Q9Y4P1 Q6IR47 5111 Q3SY56 64127 P37108 O75177 O76024 P18850 P15336 5590 O75182 Q9NRL2 56987 51295 P18846 Q68CJ9 P18847 324 P18848 326 5104 Q8TEA7 55657 Q92800 P27797 Q9GZU7 P01185 Q9UNP9 2099 O60907 O75164 P51843 55662 Q8NCN2 333 91748 Q9H7L9 10794 8625 5119 Q9GZT9 Q9BT67 23636 9975 6464 4287 P62508 P51858 P17980 146050 P52701 Q08211 221656 Q9NVV9 P58012 10320 P60321 9967 P25116 Q8N6T3 Q99417 Q96HZ4 O43670 Q14566 8655 4297 Q9BY77 11186 9500 7325 Q9UKD1 P30086 P13984 351 Q9UBS0 Q9UBS5 P09493 P23786 P21127 P25963 P09017 8662 P20290 Q15424 7332 5154 7334 5155 8667 7336 O43684 7335 19 26205 85403 367 Q70SY1 11198 7329 7328 Q14586 20 7341 P11309 6496 6495 Q13257 Q8IVW6 Q14103 6498 P11308 Q99909 6015 P32242 Q8N3C0 Q8TEU7 Q9Y606 84108 Q03405 116113 P09471 P17096 5159 Q14592 11168 Q96MA1 Q9UBU8 Q13263 Q8TF50 8204 Q9BY44 Q14119 Q8WWN8 Q9BY41 Q9Y618 125058 P12645 P29084 Q86WP2 222546 Q04725 Q04724 387 Q04727 Q9NWT6 Q04726 Q9NWT8 23152 Q66K14 P08151 P20264 11177 11176 P23771 P62942 P55347 9541 5187 11142 O15164 2909 391 Q6MZP7 23126 O43639 25788 O43638 Q02535 Q01201 121512 25780 2908 Q9UFW8 Q15466 Q6PJG2 Q99966 Q13285 2902 Q13286 5195 6045 7376 7375 Q09161 23133 23135 Q86WV8 Q3KQV3 Q6PRX2 P09067 2917 Q14140 Q9UBP4 O15169 85440 Q99958 Q15475 Q8TEY5 Q96ME7 10273 Q9HA82 P14859 O43612 Q8TAQ5 Q96DT7 Q9H063 Q99941 P49959 Q01664 51222 56916 P01579 11124 Q14151 Q9UBQ5 P09038 Q15008 9575 P82979 8242 10284 O75676 O15198 23112 O43623 25776 P33121 2931 51230 Q14160 1609 Q5VVH5 Q14161 10289 Q15014 86 P78424 Q9NWH9 Q15819 P33992 P33993 P33991 Q8TAF7 26271 P41134 Q9BXM7 Q1PSW9 27122 91 P17030 94 O15119 P45974 1616 Q6ZT07 P45973 Q9UBK2 Q9NSA3 P78412 9112 Q96QB1 Q96I24 9590 Q9H7Z7 Q9H3R0 P31321 P55318 Q99081 P31323 P55316 O43609 P67775 P05062 P52292 Q52LR7 P17040 P05067 2959 2956 Q9UBL3 O94763 Q15831 P52298 P29474 92129 P52294 9126 27107 Q9H422 Q96M96 Q460N5 Q03014 6096 P41597 56034 27102 Q9BTC8 2961 2960 O95619 Q8TAD8 Q9UBM7 Q9NSC2 2969 P09874 O15151 8289 Q15843 O95622 O95625 O15156 O94776 29777 202559 Q02156 P17023 Q05655 2972 79149 Q9BXJ9 P17020 Q8N393 Q8HWS3 P04198 Q9UBN7 9146 Q15853 O75626 Q14527 P13945 9149 1660 Q8TF47 Q6NYC1 Q96DN5 Q9H488 P41182 Q8TAK5 93474 79175 Q499Z4 P53567 79177 P30048 B3KY43 3836 84525 1655 84528 P22415 1653 Q9UK80 Q03468 Q92499 Q13200 O75629 P47712 O95644 150094 Q96HU1 Q9UBG7 Q8N7H5 P20248 64919 80155 Q9UJX2 Q8TAI7 P41162 P17081 Q9Y5B6 Q9Y5B9 P09429 O75604 P10415 Q9P2X3 Q13216 P41161 P35711 23186 P09430 P08588 P41159 Q86SE9 728642

Q9P2Y4 Q13227 P55771 Q04771 23192 O15105 Q13233 P21554 Q6NX49 Q9H6Q4 O14793 25909 P54619 2550 9181 P51587 9184 P17676 Q8TDD1 P15498 2547 P37288 57504 Q9HCJ0 P84022 O00151 Q92731 O14788 6901 10413 Q7Z6R9 25913 P28330 P08729 Q92729 Q5VTR2 P51114 O60216 P14174 Q9H2G9 4734 Q7Z727 Q9H2G4 O00167 22850 81628 54496 6927 Q8IUD2 3416 6929 P62256 Q6AHZ1 Q96KM6 Q8N680 811 6921 P40425 P40424 P62258 6926 6925 Q6IQ32 Q17R98 Q9C005 333929 Q9UIS9 Q9NZI7 P31943 P48382 Q9NQX0 P31946 Q9C009 2100 Q9NR96 P48380 O95343 P54646 3428 P16333 6938 P20823 55352 O60674 P19838 O60675 Q8IUC6 P62263 P18509 P31949 5604 6936 Q9Y468 P27037 Q9H2M9 Q8IZ40 4771 Q9Y463 4772 Q9Y466 Q8TDI0 Q9UIL8 Q9NUY8 O95352 2107 55364 10856 Q86VE0 2103 P16383 27287 80324 Q08999 O14744 6945 Q9Y478 P50222 4780 Q92769 Q14814 P09619 P28370 Q07666 O95361 80306 P10600 O95365 Q06330 Q96KS0 2119 10865 P19419 Q6PI57 4775 O14753 Q6ZW31 847 Q92766 4790 4791 4792 Q9H2K2 Q9H2K0 2130 81669 Q9NR48 Q9HCL2 P60033 Q92753 4302 P05230 857 Q96CA5 Q92754 Q13501 22827 6500 Q13503 Q9NR50 O95382 Q14839 Q8NC51 P19883 Q13506 Q9NR55 Q9HCK5 2139 861 862 863 192669 Q9UMX1 Q9UMX0 4799 P09601 50943 P08754 Q9Y3Y2 Q9BS34 P01241 10492 P15408 Q9Y3Q8 3481 Q9UIH9 23326 O60603 25988 23327 P15884 Q8WUU5 Q96T88 3479 2147 Q8WV60 2146 3476 10011 P25205 7832 10013 84289 Q96KG9 55723 10499 10014 11345 Q6UWV6 135295 64682 Q7Z6J4 4343 25998 Q96T76 Q99750 406 Q9BRP8 O43294 408 84271 409 Q01518 Q13099 Q96C55 Q09472 Q8N1G0 O43251 23309 5682 5683 5684 5685 5686 116986 64215 116987 890 Q00653 891 Q99743 894 Q99741 896 Q9NR09 898 3014 29080 10477 Q9UN42 29086 P49639 P61326 P51531 4361 5692 5693 8721 7874 148156 Q9NUX5 Q9NR11 P51532 Q96ST3 P60484 Q15291 11331 5687 55758 5688 10486 10001 P60002 10488 Q96T58 5207 10487 P28702 P23497 Q3MII6 P26583 Q9Y3M2 P46199 Q96BZ9 P50613 Q5R372 P51946 23764 25942 Q8NFW5 Q9NUQ3 Q8WUY8 Q96P48 79735 10451 10450 8726 P55072 Q8N5U6 O43242 7410 90993 Q9HCE7 Q08117 3054 Q05086 Q9BRU2 29079 P37231 Q9HCE1 79723 8737 10468 23746 P24385 P24386 8754 7422 Q96GA9 5245 3068 Q9HCD5 P52815 22893 3066 Q9UMN6 3065 Q9UIF9 O75290 79753 10438 Q00613 Q96P20 55787 55785 7415 10432 3070 Q13901 9612 P49674 Q9UIG0 P52824 Q9Y3P9 466 468 55796 P28749 Q9NQL9 7428 P04792 6595 6597 6599 6598 Q9Y4K4 O75386 P10599 6591 Q9Y4K3 Q76L83 3084 O43318 473 474 476 23286 9617 51763 8767 P43034 P38919 3093 Q13127 Q15303 B2RXF5 3091 Q16633 P55036 8301 Q16635 Q15306 Q86VP6 O75376 P58546 P13631 24149 3096 5270 Q02447 O75381 Q9NVP2 51773 30813 P46531 7448 Q13133 Q15796 Q15311 Q13131 Q16649 Q15797 9640 P12757 Q8N6I1 9643 7468 P54198 Q9UNL4 P12755 O75362 Q8TDS5 O75360 Q96CN4 P46527 51780 Q01101 P42167 Q01105 6128 O94805 7458 Q13144 Q5VUA4 54815 Q8WVM0 Q9BSI4 Q8IV61 Q16650 Q13148 P35869 P20393 7476 Q16656 Q15329 Q8NCA9 P18074 Q8WVL7 P55055 O43306 P10588 P10589 93166 Q86VN1 Q8N6H7 Q16665 51317 Q49AN0 Q13153 P43489 Q8TDY2 9667 Q16666 Q5VUG0 P54132 P63092 P35453 Q14494 8328 Q14498 Q16670 P46108 Q14011 Q86W54 Q9NRC8 7013 7014 P46100 Q9Y4H2 P35443 Q5PSV4 7490 P13682 P11021 P63096 Q9C0F3 84232 Q8WW38 Q9GZM8 Q99835 7024 7023 10392 Q9C0F0 49854 P98179 P15822 9682 7020 Q02878 Q9UNH7 51341 54851 Q9NVM4 Q96PK6 11243 55704 P55010 Q13185 Q8NCF5 P23443 6188 Q15369 Q8IUX7 O43741 Q9BX66 Q9BSM1 Q09028 6182 P11474 Q9UNI6 26747 Q15370 54862 P57682 Q6PID4 Q99816 P48730 9208 Q99814 P78545 6199 7046 6198 P35813 O75787 O43711 6193 O43719 Q9Y4C1 P62195 Q4LE39 O15234 9219 A8K8V0 P78536 7057 22803 Q13526 112950 22806 22807 57473 83737 55294 Q3V6T2 P46934 P46937 83746 Q96GN5 81565 O60296 Q4LE28 P98174 Q9UER7 Q14865 8399 7068 7067 Q5TAX3 Q15714 Q13535 Q9H307 P19793 P29590 Q15717 Q7Z2Q5 Q63HK5 Q01196 84619 Q9H6W3 P41235 1756 Q9HCP6 Q92793 Q96GM5 Q9HD15 Q9Y4E5 O60266 O60264 P05155 Q9NRA1 O60260 P59817 Q13547 Q13546 9252 7073 Q9UNE7 P41223 57018 P43405 1763 Q9NZR4 Q04206

Q92786 Q92785 Q92784 P41229 7088 Q15735 93594 9261 Q01167 Q9BSG1 51720 Q14894 4800 Q13563 2626 7099 7098 Q9NVC6 Q14416 7091 P14923 7090 Q9NQZ8 P30153 P11413 1789 1788 Q05516 1786 Q9UIV1 1785 Q05513 P00734 375790 51735 Q13573 Q9HCU4 P55884 93134 Q9H2S9 Q96CJ1 P32320 P78527 92283 85509 1312 Q86VK4 84661 51742 Q6ZNG0 P05198 P20333 Q9Y4A8 Q8IYX1 30827 Q13107 P62191 Q96H20 Q9HCS4 902 P00750 Q9HD40 Q13112 P08238 Q9NZN8 P08235 P21675 Q9NZN9 Q92618 Q9H609 Q92616 Q9Y2W7 Q9NYD6 Q6P2D0 O75820 2672 Q12948 22992 O00268 Q6QNY1 O00267 79894 3516 Q9BZI1 Q96JM7 2665 Q96JM2 Q5VWX1 2664 283248 Q9ULW3 Q92610 P63208 4841 Q8N587 P07996 10533 Q12950 P04004 Q9Y2X7 Q12952 Q9Y2X9 Q8NB12 Q8NAP3 Q9P107 5706 5707 79885 5708 5709 P16473 51099 4851 5700 4854 5701 5702 Q96JL9 5704 Q92600 5705 65264 P50395 Q9ULX9 Q9NYF8 Q9Y2Y8 Q12968 29128 10514 5717 83933 26097 P20936 Q9UQB3 4862 O75807 5713 57649 Q6IT96 4869 5716 Q96JK9 Q12972 Q0D2J5 Q9H9T3 1374 3551 Q6P2C8 P17302 5728 10524 P17301 Q9HBE1 Q99697 2697 400961 Q9ULZ3 Q9UHR5 Q9ULR5 O95477 P28482 Q9UHJ3 Q12986 1385 P51668 29108 P51665 P19532 Q9P0J0 P15173 P07550 P04040 58517 10971 7913 P08887 O14867 Q96BA8 10973 Q9UM47 P51671 Q9Y2T7 Q96SB4 Q9UHK0 Q9NYA1 O00231 O00233 O00232 P19544 Q8WYH8 57678 79444 960 1388 29115 Q9P0K8 1387 1386 4898 P42771 Q9UM54 4899 Q9H1I8 10985 Q9NYB0 22937 22938 Q9UHL9 22931 P62333 2247 284119 2246 1398 58533 Q9BZK7 975 6601 Q9Y2V2 Q92630 6605 57209 Q9UM63 Q969M7 P51693 Q9Y376 P51692 P31629 Q9BQY4 O00255 Q8NEZ4 Q9NQ33 O00257 Q03164 440193 P17342 984 Q5H9I0 988 P05362 Q9Y385 6613 Q9Y2W1 P06213 Q9UPV9 5300 P13349 2274 P53803 P52952 990 Q8WTS6 991 126208 Q96RU8 Q96RU7 Q8WTS1 79810 51003 79811 997 6622 79813 6625 54998 51008 10138 Q9UQ80 Q04917 P49770 Q9UM07 Q9Y2P0 Q7Z5H3 5311 Q9UQ88 5796 Q5T7W0 O60739 2280 8819 P48436 8812 Q99638 P01344 P01343 P49768 O00206 2290 23429 P48431 5321 Q96JC9 3142 Q9NU63 148022 Q99623 Q00535 Q96S42 Q8NAF0 P49756 P25789 55869 P25788 7975 6647 5316 51028 P25787 P01375 Q9UPY8 23439 6660 P49750 8841 Q9UHI6 O00213 Q3KNV8 23435 Q96AQ6 3151 O60716 P12004 10128 Q96RR4 Q3KNW1 3148 Q15170 545 546 3146 P61457 Q96EY1 6657 5327 Q8WU17 O43353 6670 6672 23409 Q969H0 P07948 Q9H9S4 Q9Y2K7 Q9ULJ3 Q9H9S0 3162 Q12905 O95076 Q8WYA1 Q12906 Q9BZE0 Q01844 552 Q9BZE4 P42704 3159 Q99683 Q99684 6667 P49336 P05771 Q9H5J8 10577 P07951 Q96FA3 Q969G3 8864 7531 10580 7533 P55198 Q9ULK4 23411 23414 3171 Q00987 Q96S94 3169 Q9NU19 Q9H1A4 5347 7529 7528 Q08462 Q9H5I1 64375 P50750 Q86U86 3187 Q9UPT9 3185 3184 571 3182 P51608 P68036 7536 6207 Q9H5H4 Q01826 5371 3192 8880 P27540 8881 Q60FE5 Q9UPU9 Q12933 Q9ULM3 7555 Q9ULM6 P51610 Q9BUY5 580 54583 O14818 80854 P51617 Q9NPJ6 7549 54106 8878 P60568 6231 8892 Q15654 9744 O43432 8896 P15923 Q86YW9 Q96FV9 595 84324 116 Q7Z6C1 Q13485 Q8WZ64 O94906 P03950 O00716 Q16512 4067 Q16514 P12429 Q15669 O75496 O75494 Q7LBC6 P10242 P10243 P10244 84312 51412 O94915 Q13492 Q16520 6239 54925 Q15672 Q9UQR1 7101 6256 Q16526 P35998 Q2M1K9 Q9UDY8 7579 P00441 9774 4086 7112 4087 4088 Q8N9N2 4089 O75478 9775 8445 163126 O75475 Q8N5F7 142 P21359 P31150 P24864 P62701 84333 P60520 Q15697 53615 Q13033 8454 P54259 O75469 8458 64750 7124 P54252 P54253 Q96KC8 P14635 O75461 Q9UQL6 P10275 4092 P10276 4093 O75460 4094 154 P23588 Q13042 Q99728 Q99729 Q13045 55806 P45379 P49736 7132 8463 7133 8467 9314 O43889 P36896 P36894 23378 P15976 P35568 Q96C28 6282 1822 166 Q8NFM4 55810 P01308 Q8NFM5 120892 338917 Q9NQB0 Q99717 P49721 P49720 Q15233 Q96T37 P00492 9326 P54274 O75446 P36402 O75444 6294 7141 Q99700 1831 Q96T23 Q9H1Y0 51465 Q00577 Q99708 P49711 Q16576 P49716 P49715 55827 Q9NQC7 Q96C00 64784 7158 O75437 Q08050 P39905 7157 P19086 64786 162239 Q8N9R8 Q02750 P35548 138151 55832 Q9UDV6 Q5VTD9 Q15257 Q13077 Q13873 O95835 7169 26038 10928 26037 Q06787 Q86Z02 56252 Q01094

Q9H204 26039 7161 P63167 P42224 10933 10935 P17275 P23945 Q7RTN6 P63165 Q9BVI0 P42229 57109 Q969T4 1856 P42226 O15372 O94992 Q9P0W2 22926 Q969S8 22927 Q13404 Q13887 Q01085 57592 Q8IY57 57594 P10644 O43847 57591 Q96BD5 79365 P84095 Q08945 Q9UHX1 P60900 1869 7186 7189 7188 Q8WYK2 O43812 P11940 7181 7182 7185 1877 Q86UE4 Q86UE3 1874 Q9Y3C7 P17252 1870 P09630 O15379 Q969R5 Q13422 O15391 Q8NB78 4914 2735 27352 P19438 O43829 84733 1408 1407 1406 2737 Q92667 2736 Q96G30 Q8WUI4 27327 Q02386 Q7RTR2 P62277 1896 Q9HC52 Q14774 Q13443 P22670 Q9NQ92 9391 26005 P11532 Q96G25 57154 P30279 84759 58487 Q9UQE7 P30281 Q8WYP5 P23511 Q8WUH2 3609 3608 3607 Q14781 P23510 P21333 94104 8078 Q6ZV73 O14495 Q9P0T4 29966 P19474 Q02363 58491 P18146 57162 84305 27300 O60343 O60341 4943 P01730 Q969V6 O15350 O75832 O15353 Q15649 Q9BZS1 P53350 Q15648 Q9P0U4 P19484 Q86YP4 Q06710 Q96BF6 Q8N5A5 3622 2773 Q9HC29 P30260 O14497 Q15652

regulation of protein modification process 9184 O60566 P84022 1453 P63208 O14543 6901 10533 P04004 Q12834 Q9UBC3 65018 5706 5707 5708 5709 P14174 5700 5701 5702 O15524 5704 5705 Q5S007 O75925 Q7Z727 Q92956 P53667 5717 57761 P61289 Q9Y297 5713 P62258 Q92833 P26358 5716 P31946 Q9NR96 9093 Q99460 5728 O60674 Q8IUC6 3309 P04233 Q9UL15 P27037 P05129 4771 P51668 1020 P51665 O95352 Q92530 3678 Q8N2W9 P08887 P08648 3690 Q9NYA1 P09619 O00231 O00233 P10600 O00232 1029 Q9BUB1 10746 79444 960 2475 P42771 3689 P07766 P05106 P05107 Q9UGJ0 6850 Q13616 O00487 P30542 O60502 P38398 P62333 2247 Q96N67 P20618 2246 P60033 975 O14641 Q5TCX8 P05230 857 Q92878 Q96CA5 5515 P05112 Q92997 5518 Q9UL46 6500 O95382 O00255 Q6KC79 Q03164 10724 440193 5524 Q9UMX0 50943 Q9UKT4 P06213 P01241 P04637 5300 P14317 2033 991 Q96RU7 3479 2147 23560 6622 Q15389 64682 4221 6885 P36575 25998 Q9BT40 2280 Q99750 4214 648 4216 P01588 11236 408 P49407 409 P01343 P49768 O00206 Q09472 5682 5683 Q8N726 5684 5685 5686 3263 Q9NS23 148022 891 894 Q00535 896 P35240 8945 P01019 P25789 P25788 P01137 P25787 P01133 P01375 Q9UJM3 4361 5692 5693 5573 5575 Q9GZP0 5576 P35226 P17813 P60484 P17936 5687 5688 P01127 Q96EY1 Q8WU17 P24588 O43353 5580 5581 5582 9821 5585 P07948 672 P15692 Q9BZE4 Q99683 5577 5578 10213 Q96FA3 P04201 P05412 O43242 Q6IR47 7531 Q9HCE7 64127 5590 8737 Q9H1A4 5347 54206 7529 P24385 7422 O00762 9839 Q9BT67 5371 P27540 8881 Q12933 P17980 O14818 54106 P60568 4297 11065 11186 P30086 Q9Y4K4 Q9Y4K3 O43318 P13861 595 P49810 Q7Z6C1 51763 8767 Q13485 5154 7334 5155 P55036 Q16512 4067 4188 Q16635 O43684 5037 7448 P00533 Q16401 Q13257 8555 P32121 P35998 Q9Y6Q6 Q01105 5159 10197 Q9BSI4 Q9Y6R4 4088 4089 Q13153 P62942 8454 Q8TDY2 7124 7248 P14635 4092 Q93062 Q9Y2C9 121512 Q13042 7375 11030 P36896 P35443 P36894 Q86WV8 5071 P11021 P01308 120892 P49721 O15169 P49720 85440 P02790 10273 A1XKG3 P49959 Q9H1Y0 55704 51588 P01579 P49711 Q9BQI3 1956 P09038 Q15008 7157 P37840 7272 Q6PID4 P48730 Q15257 Q14289 Q13873 7046 O95835 7161 P42345 26271 Q9BXM7 P62195 91 P04085 Q92574 1616 1856 Q9UBK2 P56524 P78536 9474 Q13526 Q96QB1 P10644 P31321 Q3V6T2 P31323 P67775 P16070 P60900 Q9UER7 7186 7189 Q9H422 Q96M96 P29590 Q9HCP6 27102 P17252 O60260 8165 Q13546 Q02156 9495 Q9UNE7 P43405 Q05655 Q92667 O95999 Q15735 9021 B3KY43 Q03468 Q13200 7099 Q9NP71 P30153 57154 P11413 P30279 1789 1786 Q9UJX2 Q05513 P00734 P30281 3720 P10415 O14495 P28065 P19474 Q9P035 57162 P61088 P01730 O15350 O75832 Q9BZS1 P53350 Q13107 P62191 P28074 P28072 P28070 Q9HC29 P30260 O15105 Q13233 P43686

death Q7Z628 Q92858 Q9Y2W7 O75940 P10911 Q9NRW4 Q5VV41 9181 Q8IXM3 P15498 54474 O60566 P84022 O00273 54476 Q9ULW0 Q6IQ55 P07996 Q92974 P28331 Q8NF91 65018 Q9H832

P55957 9197 P51114 O60216 P42858 30011 Q9UKV3 10783 Q8N695 92609 P10809 Q9H2G2 65264 Q5S007 Q9NR80 Q9NR81 O00167 P17405 22974 10758 Q8IVF5 57761 O60543 Q9UKW4 O75807 P62258 Q9H7P9 P31944 P31946 P31947 P48023 P99999 Q12979 9093 Q6IC98 22985 Q13829 P17302 Q96JJ3 P19838 2697 Q8IUC6 P61296 Q9ULZ3 Q8N5V2 Q9UHR5 Q9UL15 Q12981 Q12982 P05129 Q5D1E8 O00220 O95470 Q96MX6 29108 Q9NSU2 O95232 114548 Q9P0J0 P19525 2348 3315 55367 Q6Y7W6 5610 P09972 5733 Q9Y2T1 58517 Q7Z419 P04049 P29466 Q9Y239 Q14814 Q9H4P4 Q9NZC7 P10721 Q15904 Q5VST9 Q8IY17 Q9NS56 P37198 3329 1029 Q9HCM9 1027 79444 29115 841 P42771 3689 P04179 Q07890 4790 O95257 P05107 P28223 4792 Q12768 P50591 Q9NZD8 1285 Q9NQS1 Q13616 Q96TC7 Q9NS68 6609 P62330 O60502 P38398 391627 7818 57448 P61244 2247 Q96KQ4 Q07889 Q9Y371 Q92630 O14763 Q8N488 Q96CA5 Q9H0F6 5879 Q13501 P51572 P51693 P51575 P30536 22827 Q13625 5894 P31749 Q9H8V3 Q02078 Q03164 2139 10724 984 O00141 Q99497 Q9UMX0 P09601 P29317 Q9UL54 7704 P23396 P48454 O00429 Q9BST9 P04637 P15408 2033 O00300 P50897 P52952 O14827 Q7L523 Q96RU7 2147 79811 10011 5533 6622 10134 637 10133 4205 10015 P62753 P01112 10016 4209 P25445 Q9Y5V3 Q7Z6J4 112399 Q9UQ88 118813 23210 1191 56940 Q15392 55973 4214 84033 Q14185 P01589 P01588 O43293 Q15276 P49768 Q09472 23429 6772 Q8N726 P12236 3265 O95292 P52735 23787 Q96CV9 P11388 148022 P11387 Q9NUW8 O14727 773 Q9NR09 Q8WWW0 P49756 537 51147 6647 Q9Y572 P01138 55504 5315 P01137 P01375 2065 23435 P35348 O14737 663 Q96RR1 P10070 668 Q8WTP8 Q96EY1 Q15052 8718 11214 P49747 8717 O43353 5582 Q92934 O43236 6311 9700 Q96B97 Q12904 P15328 Q9UNN5 3162 Q9UHB4 O95197 Q8IXI1 Q8IXI2 672 Q99683 10451 5579 P61626 P55072 P05771 P05412 2081 5591 8743 8744 7410 7531 5598 Q5T442 P68032 Q5JSP0 P37231 Q01955 55775 23770 5467 8737 9826 207 5468 7529 11315 6453 Q9H902 Q12802 O00400 Q9NWW5 P12036 572 O75293 331 573 10438 333 Q96P20 7415 5599 4149 7416 23636 5371 Q13901 Q12933 8887 4287 O43464 4168 146057 23513 O60911 51074 Q5TCQ9 P58012 8879 P62745 P60201 8878 P04792 P25116 Q9H8M7 8772 O15085 Q9BZZ5 8655 4297 Q14209 9500 7204 Q9Y4K3 Q96FV9 230 351 472 Q96GX9 474 117584 355 356 P49810 Q7Z6C1 Q8WUM4 8767 P21127 Q9HB75 P25963 Q9HC98 O43683 Q16637 P03950 6242 Q13009 Q13007 28986 Q86VP1 367 P09382 51651 8539 P24522 Q15672 8795 Q96IZ0 10193 29 64975 Q6GPH4 Q9UNL4 64857 Q9Y2I1 Q9NY61 P46527 Q969Z0 23263 Q5T4F4 P61604 P22314 P00441 Q9BWQ8 51428 P32239 Q13148 P35869 10081 9530 4088 8565 8445 9897 57099 382 388 Q00005 Q5J8M3 Q13153 Q14249 8454 P54259 Q13158 9420 7124 P54252 P54253 7126 23365 O75340 23368 P10275 O75460 121512 Q9Y3E5 51324 O94827 10059 Q8WV24 Q15464 Q13164 Q13286 7132 5074 7013 7133 O43521 O43524 Q9Y5J5 Q86WV6 1822 23370 120892 7128 11035 11277 O15169 Q13177 23229 Q8TCU4 Q96LC9 Q6ZSZ5 Q15116 Q96G97 P61803 Q8TCU6 Q3ZCQ8 10392 P14618 Q96KA5 O15068 P54274 O43734 Q99700 51100 O95816 P01579 1718 11124 10276 Q9NQC3 54739 51107 P09038 Q14155 7153 6188 64782 Q9HB90 9217 7157 7159 23111 O43508 P37840 7150 P11234 Q14CZ7 23239 Q8WZ73 2810 255488 Q14160 51234 Q6PID4 10285 Q93038 Q13075 1969 Q14289 2935 Q15811 P54577 Q9NWH9 9464 P10997 Q01094 7161 O43715 6194 1613 P63167 27242 P42224 P41134 Q9BXM7 Q1PSW9 P41250 1616 7057 9238 Q96QB1 Q01085 10939 7291 55177 1982 Q9UHX1 Q9Y6H5 P98170 P05067 P42574 1869 P42575 P98171 P98174 Q9UER7 O15020 7186 7189 P06239 O94768 Q9H305 P29350 Q9H422 Q96M96 O75881 P29590 10908 6093 Q9BXK5 Q9HD15 Q8N163 1870 P21860 P04062 1639 Q9BTC0 3932 9135 O15151 P07339 9255 O15392 340061 P55210 Q6R6M4 Q13546 7073 P35900 Q8N8D1 7074 P19438 Q9Y3D6 80013 3708 Q92785 Q92542 Q13794 Q9UBN6 1647 2736 Q9NZJ7 Q14643 Q9UJV9 O75503 O95999 Q13315 Q6NYC1 P41181 P00747 P63000 Q96PE2 2629 P43115 3956 O75509 P51398 Q15628 P21580 Q07820 Q96F07 Q96BI3 Q07817 Q05516 P61073 P61077 P00734 26123 P08138 50649

Q8WWK9 Q9NWF9 P10415 O75962 Q8IUQ4 377630 Q96CJ1 29843 P78527 O60229 O60346 Q86TG7 1676 2521 P41273 1793 81037 50650 Q14790 51741 Q13464 Q14674 P20333 51747 O15350 728642 Q16611 Q14318 Q9P0U3 Q9H6Z9 Q9H213 1203 4719 26586 P47929 Q8N0X7 O95429 P45983 Q9NZN5 Q13233 Q9BZR8 P21796 Q13352

translation 6231 8892 O75821 Q6DKI1 6233 O75822 65003 64951 P62917 Q9H0U6 Q8IXM3 51081 Q9UBS0 28973 P62910 P46783 6229 P83731 P62913 8662 64960 P13639 16 8667 6123 P46782 Q9NRX2 8665 6125 P46781 Q9Y399 64963 63875 Q8NE71 64965 P41091 64969 10667 2 P62244 80755 51650 P68104 P62241 51651 6238 Q9Y285 P00533 P46777 140801 P46776 P60866 P46779 P46778 P62249 6133 P25398 Q9UKV8 6132 6134 6137 23708 64975 Q9BZG8 O43776 P18621 P12081 P63220 O60783 6128 P40429 P82663 P82664 1915 8669 54938 Q13144 Q9Y291 Q9Y2Z4 Q14232 6141 P49591 P08708 P26373 8565 P62829 P18077 64745 P49590 10767 P62266 Q02543 Q9BYD1 P62263 Q9P2E3 P62701 6138 P09001 P62945 P49588 P62269 Q9P2E9 1801 10643 Q9Y3U8 P54136 6156 6158 P39019 P19525 Q5T160 Q6PI48 P39023 Q9Y3E5 P23588 833 5610 1937 1936 Q9NSE4 27161 1933 5859 6164 3692 6168 Q5JTZ9 6160 O95363 10987 P61353 Q7L2H7 P05386 P05387 P05388 Q9UNX3 P61927 6175 P32969 Q96T21 P42766 80222 Q02878 P50914 P24534 P55010 P61247 P47897 Q14152 Q6GMV3 Q9UBQ5 P08865 1956 Q9NQC7 6188 6187 P62906 Q9NR50 Q96L21 P14868 Q86Y79 6182 55152 740 Q9P0M9 P61254 Q9Y262 4677 51116 1968 Q7Z7F7 2935 O00425 P54577 6199 P23396 64432 Q96EL2 P62081 6193 P18124 O00303 6194 1975 P62750 P47914 P41252 P41250 Q6P5R6 11222 Q15025 P62753 P82921 P49770 O15371 P82912 200916 Q9Y676 O15372 P82914 P49411 Q13405 P36578 25873 55173 Q9Y3B7 P84098 P63173 51253 P62888 P61313 P27635 P60228 P26641 Q5D0E6 P26640 Q9BW92 P56537 29088 51021 Q8IYD1 51023 P26639 51264 P62899 Q14197 Q9Y450 Q15046 23438 Q9Y2R5 Q9H7H0 9255 3035 3396 63931 Q9Y2R9 P42677 P15880 Q9Y3D3 Q9Y3D5 29093 P62424 P82932 Q969Q0 P82933 Q12904 P62280 Q9P015 Q9NWU5 P62277 P30050 Q9BZE1 128308 Q96GC5 Q07020 6201 6202 O76021 P83881 1540 57038 P82675 P62847 29074 P62841 P61513 P49207 6210 9045 P55884 Q9NP81 Q6P1L8 28998 Q9HCD5 P52815 10318 51067 57169 6204 6207 P62857 P62979 P62851 57727 P62854 8761 Q13347 A2RTX5 Q92901 P07814 7311 Q92905 391356 P35268 54460 P62988 6217 Q9HD40 P56192

programmed cell death Q7Z628 Q92858 Q9Y2W7 O75940 P10911 Q9NRW4 Q5VV41 9181 Q8IXM3 P15498 54474 O60566 P84022 O00273 54476 Q9ULW0 P07996 Q92974 P28331 Q9H832 P55957 P51114 O60216 P42858 30011 Q9UKV3 10783 Q8N695 92609 P10809 Q9H2G2 65264 Q9NR80 Q9NR81 O00167 22974 10758 Q8IVF5 57761 O60543 Q9UKW4 O75807 P62258 Q9H7P9 P31944 P31946 P31947 P48023 P99999 Q12979 9093 Q6IC98 22985 Q13829 P17302 Q96JJ3 P19838 2697 Q8IUC6 P61296 Q9ULZ3 Q8N5V2 Q9UHR5 Q9UL15 Q12981 Q12982 Q5D1E8 O00220 O95470 Q96MX6 29108 O95232 114548 Q9P0J0 P19525 55367 5610 P09972 58517 Q7Z419 P04049 P29466 Q9Y239 Q14814 Q9H4P4 Q9NZC7 P10721 Q5VST9 Q9NS56 3329 1029 Q9HCM9 1027 79444 29115 841 P42771 3689 P04179 Q07890 4790 O95257 P05107 4792 P50591 1285 Q9NQS1 Q13616 Q96TC7 Q9NS68 P62330 P38398 391627 7818 57448 P61244 2247 Q96KQ4 Q07889 Q9Y371 Q92630 O14763 Q8N488 Q96CA5 Q9H0F6 5879 Q13501 P51572 P51693 P51575 P30536 22827 Q13625 5894 P31749 Q9H8V3 Q02078 Q03164 2139 984 O00141 Q9UMX0 P29317 Q9UL54 7704 P23396 P48454 O00429 Q9BST9 P04637 2033 O00300 P50897 P52952 O14827 Q7L523 Q96RU7 2147 79811 10011 5533 10134 637 4205 10015 P62753 P01112 10016 4209 P25445 Q9Y5V3 Q7Z6J4 112399 Q9UQ88 23210 1191 56940 Q15392 55973 4214 84033 Q14185 P01589 P01588 O43293 Q15276 P49768 Q09472 23429 6772 Q8N726 P12236 3265 P52735 23787 P11388 148022 P11387 O14727 Q9NR09 Q8WWW0 P49756 51147 6647 Q9Y572 P01138

55504 5315 P01375 2065 P35348 O14737 663 P10070 668 Q8WTP8 Q96EY1 Q15052 8718 11214 P49747 8717 O43353 Q92934 O43236 9700 Q96B97 Q12904 O95197 Q8IXI1 Q8IXI2 672 Q99683 10451 5579 P55072 P05771 P05412 2081 5591 8743 8744 7410 7531 5598 P68032 Q5JSP0 P37231 Q01955 23770 5467 8737 9826 207 5468 7529 6453 Q12802 572 O75293 331 573 10438 333 Q96P20 7415 5599 4149 7416 5371 Q13901 Q12933 8887 4168 23513 O60911 51074 Q5TCQ9 P58012 8879 P62745 8878 P25116 Q9H8M7 8772 O15085 Q9BZZ5 8655 4297 Q14209 9500 7204 Q9Y4K3 Q96FV9 230 351 Q96GX9 474 117584 355 356 P49810 Q7Z6C1 Q8WUM4 8767 P21127 Q9HB75 P25963 Q9HC98 O43683 6242 Q13009 Q13007 28986 Q86VP1 P09382 51651 8539 P24522 Q15672 8795 Q96IZ0 10193 29 64975 Q6GPH4 Q9UNL4 64857 Q9Y2I1 Q9NY61 P46527 Q969Z0 23263 P61604 P00441 Q9BWQ8 51428 P32239 P35869 10081 9530 4088 8565 8445 57099 382 388 Q00005 Q5J8M3 Q13153 Q14249 8454 Q13158 7124 7126 23365 O75340 23368 O75460 121512 Q9Y3E5 O94827 10059 Q8WV24 Q15464 Q13164 7132 5074 7013 7133 O43521 O43524 Q9Y5J5 Q86WV6 23370 7128 11035 O15169 Q13177 23229 Q8TCU4 Q96LC9 Q6ZSZ5 Q15116 P61803 Q8TCU6 Q3ZCQ8 10392 P14618 Q96KA5 O15068 P54274 O43734 51100 O95816 P01579 1718 10276 Q9NQC3 54739 51107 P09038 Q14155 7153 6188 64782 Q9HB90 7157 7159 O43508 7150 P11234 Q14CZ7 23239 Q8WZ73 2810 255488 Q14160 51234 Q6PID4 10285 Q93038 Q13075 1969 Q14289 2935 Q15811 P54577 Q9NWH9 9464 P10997 Q01094 7161 O43715 6194 1613 P63167 27242 P42224 P41134 Q1PSW9 1616 7057 9238 Q96QB1 Q01085 7291 55177 Q9UHX1 P98170 P05067 P42574 1869 P42575 P98171 P98174 Q9UER7 7186 7189 P06239 O94768 Q9H305 P29350 Q9H422 Q96M96 P29590 6093 Q9BXK5 Q9HD15 Q8N163 1870 P21860 Q9BTC0 3932 9135 O15151 9255 O15392 340061 P55210 Q6R6M4 Q13546 7073 P35900 Q8N8D1 7074 P19438 Q9Y3D6 80013 Q92785 Q92542 Q13794 Q9UBN6 1647 2736 Q9NZJ7 Q9UJV9 O95999 Q6NYC1 P41181 P00747 P63000 Q96PE2 3956 O75509 P51398 Q15628 P21580 Q07820 Q96F07 Q96BI3 Q07817 Q05516 P61073 P61077 P00734 26123 P08138 50649 Q8WWK9 Q9NWF9 P10415 O75962 Q8IUQ4 377630 Q96CJ1 29843 P78527 O60229 O60346 Q86TG7 1676 P41273 1793 81037 50650 Q14790 51741 Q13464 Q14674 P20333 51747 O15350 728642 Q16611 Q14318 Q9P0U3 Q9H6Z9 Q9H213 4719 26586 P47929 O95429 P45983 Q9NZN5 Q13233 Q9BZR8 P21796 Q13352

cellular macromolecular complex subunit organization 10093 P06899 11065 25909 P49023 Q9H0U9 1460 Q9UBB5 Q6NZI2 3084 4171 4728 Q8IYN9 P84022 P07196 23165 Q9BVA1 Q8NCD3 Q7Z6C1 10772 P83731 5018 Q13243 6901 Q13485 Q8WUM0 4725 O94906 Q13242 Q9H0D6 Q13247 Q16637 P03950 25915 9997 Q9BTM1 P62805 8301 Q15428 Q16635 P62807 Q8IZP0 79084 O75494 P16104 2316 2 Q9NVP2 5829 Q8IUE6 P16471 51773 Q9NRH3 11198 Q13492 Q9H9E3 Q16401 P60981 Q15796 10421 P10809 84790 O75925 Q9H2G4 Q15797 Q8N6I1 Q96NT1 O75367 Q9NQW6 Q9NWS0 Q05397 Q9UBU3 54496 1355 P23527 Q01105 P09234 P17096 811 P83876 Q9HC77 Q8WZ42 Q9BQE3 4869 5716 4086 8683 4087 4088 Q9BY44 4089 Q16777 Q9H814 Q16778 P0C0S5 163126 Q9NRZ9 P04908 P68371 Q02790 55355 P16333 Q9NVR5 P0C0S8 P68366 Q7L7L0 P62263 P04350 Q6NXT2 64061 P62942 3308 P07305 Q15459 10291 Q8N5M1 8458 O75347 3320 1020 O43639 P52756 O00468 154 P07550 P62316 P62318 O14744 Q02539 P62314 P49736 Q14011 P07437 Q12874 10181 Q9UHK0 Q12872 3692 Q8WXF0 Q09161 8348 9555 8467 2243 1153 O00233 Q9BUB7 3329 11034 Q92522 8338 6834 Q8N257 6714 5747 Q9NRD5 9685 P02671 P07766 Q8TCU6 266812 Q5SSJ5 10152 4673 A1XKG3 Q9NYB9 Q9H2K0 P58876 P36404 7141 Q99700 Q9NS69 Q96T23 51340 P42768 284119 P84243 Q71DI3 P68431 Q9NVM4 2244 Q16695 O14640 P49711 Q14152 8349 6605 347733 11004 5879 O43182 Q9UNY4 7277 P22492 Q13509 Q5QNW6 Q13748 26747 Q9P0M6 P40692 6733 Q6PID4 Q92621 Q6FI13 4676 55835 1729 P78362 4678 Q14289 Q9UKT4 Q9BZJ0 4690 9463 P07900 Q7KZN9 6872 6631 2033 P14678 O95391 P12931 6194 7283 26271 203068 1058 Q8WWY3 P20671 4686 55723 Q15389 P62753 135295 P54105

Q13885 22803 10383 Q8N4N8 Q99880 Q02297 3010 Q6FGD7 Q9BU61 2280 55172 Q99877 3925 Q9BUF5 3009 3008 O60610 3007 3006 402 P05062 O43290 6633 8932 7846 P02675 6637 255626 8936 Q99879 Q09472 P52298 O75643 Q7Z7K6 10907 3024 P55209 O75880 84617 P56537 O43819 773 Q9H4B7 Q00535 3014 Q14197 O60264 P01137 Q92558 Q9UPY3 Q3ZAQ7 O00217 8289 Q9UMS4 Q658W2 3396 Q9UHI6 P34932 9495 221613 Q96A08 Q99733 11218 Q93079 3148 Q93077 Q07955 P23258 P62308 P24588 P62304 50628 P26583 6430 6311 6431 P41181 8290 Q96KK5 1653 3159 P63000 Q92499 6426 Q9BUK6 Q9Y512 P49450 Q969G3 O95400 Q2NL82 O95405 O43920 O60814 27339 P18846 26121 Q96QV6 54443 Q15070 Q8WYP5 375790 Q9Y5B9 P27797 P21333 4150 P33778 4154 25929 P49321 Q8TEQ6 P10412 O00762 Q00059 Q5TAL4 Q99661 56993 7536 P09430 O15212 P62851 Q9BQA1 3070 Q14677 6341 Q60FE5 O75832 Q9UIG0 Q15526 P16403 P16402 25813 P16401 10569 466 P67870 P52948 O14497 Q71U36 Q13112 P21675 1207

regulation of DNA binding 6597 6598 1460 Q12824 9181 Q9Y4K3 Q96JM2 Q7Z6C1 8767 O14788 P25963 2307 Q92974 7334 4188 P12429 Q15306 P27361 7335 O75381 2316 51773 Q96EB6 7341 Q92838 P11309 P35638 P27695 P32121 P12755 23028 Q9Y6Q6 Q8IUD2 Q9UDY8 Q93009 P40424 4869 P06400 5716 5970 P18074 3673 Q92949 Q9NR96 3551 9093 5728 P17301 O60674 Q8IUC6 Q9ULZ3 P43489 7124 29108 O00463 Q9UQL6 114548 4092 O43638 Q02535 10616 Q9Y6K9 P04040 55806 5195 Q9NYA1 Q9Y239 Q92769 Q15109 1029 P01308 Q9NQB0 P42771 847 P49848 Q99835 7023 10392 4792 27148 O60869 Q96T23 Q01664 P05112 Q9NQC7 6188 P10827 O00255 23476 P05362 P09601 50943 Q13077 P23396 2033 O95271 O60603 P15531 Q96RU8 P41134 10935 P63165 P01116 10014 Q9UBK2 Q9NSA3 P56524 4221 Q13404 Q9NRP7 Q99750 6878 408 P49407 409 O00206 7186 Q09472 7189 7067 7188 Q8N726 Q5TAX3 7185 148022 Q9BYM8 Q86UE4 P01019 P01137 P01375 8721 Q13546 7874 55072 P51532 Q9NS37 P60484 3148 Q04206 3146 Q92786 Q96EY1 P23497 O43353 P26583 O43593 6672 Q96EP0 O95999 3162 P53567 Q7RTR2 P30048 1896 23085 P05412 7099 7098 P14923 64127 23411 P37231 3728 58487 Q8TAI7 3169 1540 8737 328 5468 P23510 P21333 P09429 O14495 P19474 Q02363 3066 P51843 Q96P20 P61088 O60341 O15455 O14920 Q9GZT9 Q60FE5 O75832 Q12933 Q9BZS1 Q8NHX1 54583 P51617 P67870 Q9HC29 8517 4830 O15105 54106 5925

chromosome organization O95677 P25054 Q9H0E3 P06899 Q9Y265 Q12824 22992 8091 P51587 O00267 Q9P2R6 9184 2547 Q96JM7 Q8N3U4 5931 5933 25913 Q9H4L7 Q9UBC3 Q5VTR2 Q8NB12 P16104 1107 57634 79885 Q8IUE6 Q9UKV3 10661 Q96EB6 1108 Q7Z727 O95696 P27694 Q9H2G4 Q96NT1 O00167 54496 6927 83933 Q92830 Q92833 Q6IT96 P26358 4869 Q96EA4 Q92831 P06400 Q9C005 O95347 Q9NQX0 Q9NRZ9 22985 55355 10524 P20823 55352 O60674 Q6NXT2 64061 10765 P07305 Q9Y468 5981 Q96MX6 10609 Q8TDI0 Q06587 6829 79577 10856 Q6FHQ0 Q08999 Q9Y230 O14744 6827 Q12873 Q92769 P28370 Q15906 O95361 Q8WYH8 6839 10626 1387 Q92522 Q8N257 Q15910 Q9NYB0 266812 4673 79595 Q8NA19 Q9NR48 P84243 Q71DI3 Q96KQ7 Q9BZK7 6601 Q92993 6605 Q92878 10951 O43189 Q14839 Q5QNW6 O00255 Q8NEZ4 Q6KC79 O00257 Q03164 Q9P0M6 2139 57332 80335 Q99496 10728 Q6FI13 P04150 4676 4678 O14777 50943 Q9UGL1 Q96FF9 A6NHR9 Q9UKL0 O43159 6871 P04637 2033 23326 23569 Q8WTS6 11107 1058 2146 Q15022 Q15021 79813 10013 84289 55723 10014 Q9UM07 6883 4221 Q9Y6X3 O96019 23210 Q99880 23212 3010 Q99877 3009 3008 641 3007 3006 P35251 648 408 O43293 P49407 56946 255626 Q99879 O96028 Q09472 P48431 Q7Z7K6 3024 P11388 891 3014 55869 51147 29086 Q8N8U2 Q15047 O43264 P51531 4361 8841 23310 Q9NUX5 O00213 P51532 3151 221613 P35227 Q96A08 P35226 Q99733 124359 Q93079 P35232 Q15050 3148 Q93077 Q15291 10363 10362 6657 2070 P26583 9700 7520 5585 Q969H0 Q9Y2K7 23523 Q96KK5 3159 Q68DK7 6304 8726 5578 Q15185 5579 P05771 8607 5591 P49450 Q969G3

P51955 O75177 23411 O60814 3171 Q9NRL2 Q9NWV8 324 Q96QV6 3169 79723 Q92922 Q92800 Q9H5I1 Q86U86 P49321 5245 22893 3066 Q9UMN6 Q9UPT9 3065 Q9UIF9 O75164 P13010 Q9H7L9 5119 10432 Q01826 Q99549 O43463 3070 Q9UIG0 P16403 23512 P16402 P16401 221656 80854 P28749 10320 54908 6595 6597 4297 8535 6599 6598 Q9BZ95 Q9H0U9 4171 473 O75150 Q9H160 Q8NCD3 Q7Z6C1 Q9C0C2 Q15424 7334 Q16512 Q9BTM1 P62805 Q16514 O43684 Q15306 P62807 Q9ULG1 O75376 Q8TF76 Q7LBC6 Q9NVP2 51773 6117 9869 51412 11198 Q96RL1 Q15554 9646 10075 9643 7468 Q9UPP1 6015 P54198 O75367 Q9UNL4 Q6UXN9 23028 51780 P23527 Q01105 P17096 O94805 Q9UBU8 Q8WZ42 P61964 Q9BSI4 Q6PD62 Q16777 O75478 Q16778 Q9BY41 P0C0S5 Q96BK5 93166 P04908 142 P0C0S8 Q7L7L0 11177 11176 Q9NPF5 51317 P43246 53615 9425 P54132 P14635 O75582 Q9BW71 Q9UQL6 Q9Y6K1 51564 2908 8328 Q02539 P49736 6045 7013 55929 8348 7014 9555 8467 23133 23135 23378 23492 8338 O15047 8110 8479 10270 Q5SSJ5 P54274 O95931 23466 P58876 23468 9682 6294 7141 Q96T23 P49959 P68431 Q9NVM4 Q96PK6 Q16695 56916 Q13185 P49711 8349 Q16576 Q9NTI5 Q15003 54617 54737 7153 Q9NPA8 8242 7157 P22492 Q09028 64426 54984 Q9NTJ3 54623 Q5VTD9 Q15014 86 Q6P1J9 Q96L91 P29375 O95835 P29374 26038 27005 Q7Z589 26039 1613 10933 Q9Y4C1 P20671 Q4LE39 9219 P45973 1736 P56524 9232 Q9P0W2 Q969S8 Q86WJ1 O75530 7290 Q9H3R0 P55318 55294 Q96BD5 83746 Q92560 Q52LR7 Q4LE28 P42695 Q9UBL3 O75528 P83916 9126 P55209 Q9H6W3 Q92793 Q96GM5 P17252 Q7Z6Z7 Q92791 O15379 O95619 O60264 Q969R5 Q86UE8 P09874 8289 O95983 Q13422 O94776 Q13547 O95503 O43823 10919 P34931 9252 Q9Y6J0 Q8NB78 Q8TBE0 84733 Q92784 P41229 Q9UBN7 Q14527 Q8WUI4 O95997 Q6NYC1 Q9NP66 8290 23081 Q9UK80 Q9H9B1 51720 Q9HC52 Q9NQZ2 Q9NQ92 Q8N7H5 1789 1788 P46736 1786 Q9UJX2 Q9UQE7 3720 Q9Y5B9 Q14781 P33778 P12956 339287 P10412 P78527 P61088 23186 84661 51742 O60341 P09430 Q14674 11091 30827 Q9BZS1 Q9P0U4 5927 5928 5929 P63272 3622 Q9HCS4 O14497 Q13112 Q15652 Q13351 Q14683 5925

apoptosis Q7Z628 Q92858 Q9Y2W7 O75940 P10911 Q9NRW4 Q5VV41 9181 Q8IXM3 P15498 54474 O60566 P84022 O00273 54476 Q9ULW0 P07996 Q92974 P28331 Q9H832 P55957 P51114 O60216 P42858 30011 Q9UKV3 10783 Q8N695 92609 P10809 Q9H2G2 65264 Q9NR80 Q9NR81 O00167 22974 10758 Q8IVF5 57761 O60543 Q9UKW4 O75807 P62258 Q9H7P9 P31944 P31946 P31947 P48023 P99999 Q12979 9093 Q6IC98 22985 Q13829 P17302 Q96JJ3 P19838 2697 Q8IUC6 P61296 Q9ULZ3 Q8N5V2 Q9UHR5 Q9UL15 Q12981 Q12982 Q5D1E8 O00220 O95470 Q96MX6 29108 O95232 114548 Q9P0J0 P19525 55367 5610 P09972 58517 Q7Z419 P04049 P29466 Q9Y239 Q14814 Q9H4P4 Q9NZC7 Q5VST9 Q9NS56 3329 1029 Q9HCM9 79444 29115 841 P42771 3689 P04179 Q07890 4790 O95257 P05107 4792 P50591 1285 Q9NQS1 Q13616 Q96TC7 Q9NS68 P62330 P38398 391627 7818 57448 P61244 2247 Q96KQ4 Q07889 Q9Y371 Q92630 O14763 Q8N488 Q96CA5 Q9H0F6 5879 Q13501 P51572 P51693 P51575 P30536 22827 Q13625 5894 P31749 Q9H8V3 Q02078 Q03164 2139 984 O00141 Q9UMX0 P29317 Q9UL54 7704 P23396 P48454 O00429 Q9BST9 P04637 2033 O00300 P50897 P52952 O14827 Q7L523 Q96RU7 2147 79811 10011 5533 10134 637 4205 10015 P62753 P01112 10016 4209 P25445 Q9Y5V3 Q7Z6J4 112399 Q9UQ88 23210 1191 56940 Q15392 55973 4214 84033 Q14185 P01589 P01588 O43293 Q15276 P49768 Q09472 23429 6772 Q8N726 P12236 3265 P52735 23787 P11388 148022 O14727 Q9NR09 Q8WWW0 P49756 51147 6647 Q9Y572 P01138 55504 P01375 2065 P35348 O14737 663 P10070 668 Q8WTP8 Q96EY1 Q15052 8718 11214 P49747 8717 O43353 Q92934 O43236 9700 Q96B97 Q12904 O95197 Q8IXI1 Q8IXI2 672 Q99683 10451 5579 P55072 P05771 P05412 2081 8743 8744 7410 7531 5598 P68032 Q5JSP0 P37231 Q01955 23770 5467 8737 9826 207 5468 7529 6453 Q12802 572 O75293 331 573 10438 333 Q96P20 7415 5599 4149 7416 5371 Q13901 Q12933 8887 4168 23513 51074 Q5TCQ9 P58012 8879 P62745 8878 P25116 Q9H8M7 8772 O15085 Q9BZZ5 8655 4297

Q14209 9500 7204 Q9Y4K3 Q96FV9 230 351 Q96GX9 474 117584 355 356 P49810 Q7Z6C1 Q8WUM4 8767 P21127 Q9HB75 P25963 Q9HC98 O43683 6242 Q13009 Q13007 28986 Q86VP1 P09382 51651 8539 P24522 Q15672 8795 Q96IZ0 10193 29 64975 Q6GPH4 Q9UNL4 64857 Q9Y2I1 Q9NY61 Q969Z0 23263 P61604 P00441 Q9BWQ8 51428 P32239 P35869 10081 9530 4088 8565 8445 57099 382 388 Q00005 Q5J8M3 Q13153 Q14249 8454 Q13158 7124 7126 23365 O75340 23368 O75460 121512 Q9Y3E5 O94827 10059 Q8WV24 Q15464 Q13164 7132 5074 7013 7133 O43521 O43524 Q9Y5J5 Q86WV6 23370 7128 11035 O15169 Q13177 23229 Q8TCU4 Q96LC9 Q6ZSZ5 Q15116 P61803 Q8TCU6 Q3ZCQ8 10392 Q96KA5 O15068 P54274 O43734 51100 O95816 P01579 1718 10276 Q9NQC3 54739 51107 P09038 Q14155 7153 6188 64782 Q9HB90 7157 7159 O43508 P11234 Q14CZ7 23239 Q8WZ73 2810 255488 Q14160 51234 Q6PID4 10285 Q93038 Q13075 1969 Q14289 2935 Q15811 P54577 Q9NWH9 9464 P10997 Q01094 7161 O43715 6194 1613 P63167 27242 P42224 P41134 Q1PSW9 1616 7057 9238 Q96QB1 Q01085 7291 55177 Q9UHX1 P98170 P05067 P42574 1869 P42575 P98171 P98174 Q9UER7 7186 7189 P06239 O94768 Q9H305 P29350 Q9H422 Q96M96 P29590 6093 Q9BXK5 Q9HD15 Q8N163 1870 P21860 Q9BTC0 3932 9135 O15151 9255 O15392 340061 P55210 Q6R6M4 Q13546 7073 P35900 Q8N8D1 7074 P19438 Q9Y3D6 80013 Q92785 Q92542 Q13794 Q9UBN6 1647 2736 Q9NZJ7 Q9UJV9 O95999 Q6NYC1 P41181 P00747 P63000 Q96PE2 3956 O75509 P51398 Q15628 P21580 Q07820 Q96F07 Q96BI3 Q07817 Q05516 P61073 P61077 P00734 26123 P08138 50649 Q8WWK9 Q9NWF9 P10415 O75962 Q8IUQ4 377630 Q96CJ1 29843 O60229 O60346 Q86TG7 1676 P41273 1793 81037 50650 Q14790 51741 Q13464 Q14674 P20333 51747 O15350 728642 Q16611 Q14318 Q9P0U3 Q9H6Z9 Q9H213 4719 26586 P47929 O95429 P45983 Q9NZN5 Q13233 Q9BZR8 P21796 Q13352

mRNA metabolic process 285672 O75940 Q969L4 P67809 O00148 Q9UBB9 10657 4841 10772 Q6NZY4 P49137 Q9UK45 Q00839 P05455 Q9H0D6 Q9H2H8 O75934 55696 10421 64282 Q9NWB1 Q9BRX9 Q9UKV8 O60306 54496 1478 Q8N684 P26599 P31942 Q12972 P31943 Q9H814 Q9GZX7 Q99459 55110 3304 55119 P26368 10642 5725 Q9UL18 Q8IX01 57661 O95232 P52756 Q9P2N5 Q9BWG6 P62316 P84103 P62318 5976 O14744 P62312 58517 27161 P62314 Q10570 Q12874 Q9Y2T7 Q12872 Q8WXF0 Q07666 1153 57794 167227 4670 22938 Q9Y4Y9 O60508 O60506 Q96MU7 55149 5511 Q92994 Q9Y4Z0 Q9UNY4 P08621 P51693 22827 22828 6741 5411 Q8NC51 Q9HCK5 P40692 O60870 192669 6733 Q9Y2W2 988 Q92620 Q9BZJ0 O43395 Q86V81 10250 10492 6631 Q9BV90 P14678 O95391 51362 54512 Q8WWY3 4686 6625 6627 P62995 135295 Q96LI5 P51991 P07910 220988 Q15029 4343 23210 Q9UKM9 Q96Q15 23451 O43290 Q7Z6J9 6633 Q15393 Q9BRP8 10147 O43172 6637 P60228 Q86U42 O43251 O60942 10594 P49756 P61326 Q15287 P23246 O43143 Q86X95 Q9UMS4 84292 6421 A7MD48 Q9Y333 Q9UHI6 23435 11218 11338 9924 4116 57819 P62308 Q9ULR0 O43390 P62304 6430 8732 6432 7884 Q9UQ35 6431 6434 Q9UPR3 22794 23524 Q96PU8 P15692 10450 6427 6426 Q96A72 2081 23658 5356 3178 Q9HCE1 25804 3181 4154 25929 7422 Q8N2M8 3189 Q9UNP9 3187 3185 P13489 3184 Q9NW64 Q9Y5S9 P17844 Q5TAL4 333 55660 7536 9716 91746 3192 Q9BQ04 3191 23517 3190 Q5VTL8 Q96C86 Q9NVV4 Q08211 10569 79622 55796 79869 Q92900 Q8N9Q2 O43791 53938 Q16629 P55265 Q9UPN6 57187 Q96FV9 Q01130 351 472 P33240 Q9UBS4 Q16630 P09012 Q14690 Q13243 Q8IU60 Q8WW01 O94906 Q13242 P38919 Q13247 Q16637 5394 Q8IWZ8 Q15428 Q15427 79084 O75494 Q504Q3 Q9NW13 24144 Q9NPD3 O94913 Q9HAU5 Q14103 23144 Q9UKF6 Q96IZ7 23381 85437 P42285 P09234 P83876 51428 8563 Q13148 9410 O43660 11171 8683 11051 9775 144983 28960 Q13151 P61978 Q8IXZ2 51319 Q15459 9785 10291 9541 8458 O75460 Q8IZD4 Q8IYB3 Q14493 8449 Q14498 Q14011 10181 Q09161 26523 Q9Y3F4 Q8NI27 Q9BRL6 11157 Q15233 Q86W42 P98175 6050 11129 51340 Q9NVM4 51585 2926 Q6I9Y2 Q15365 P39905 Q08170 26986 Q01780 P14866 8241 5093 Q9H9G7 10286 10285 P78362 2935 11017 22913 P52272 10929

57461 Q96DI7 84967 O15234 P09651 Q9UBK2 Q13523 P54105 22803 Q13769 Q9H0L4 Q9P1Y6 P22626 Q9Y3B4 79005 27257 84950 10949 Q9UHX1 P09661 P05067 P08579 P52298 9128 9129 O75643 Q9H307 10907 P11940 O75400 Q15717 26019 80145 283989 1994 Q8TBF4 Q32P51 Q8NAV1 83759 10921 29896 2972 Q07955 29894 57379 50628 Q13435 P52597 Q9UJV9 Q13315 1660 Q6NYC1 P53567 9261 1655 1653 4809 Q92499 Q8WVK2 1659 51726 O95400 51729 27339 O43809 1665 26121 Q6P2Q9 Q13573 Q8TEQ6 P55769 84305 Q9BQA1 51747 P08107 O60231 Q9HCS7 Q13595 Q14562 1207

regulation of signaling pathway P25054 9181 P15498 2305 P84022 1453 2300 Q92731 O14543 O14788 6901 57506 2308 Q92974 2316 P14174 4734 10666 Q96EB6 Q7Z727 Q9NR80 Q9NR81 Q06124 6927 P37023 57761 57521 Q9UKW4 811 Q9Y297 O75928 3672 Q92949 2100 Q9NR96 Q13705 P21917 O95343 P20823 O60674 O00182 P20827 Q8IUC6 Q8N5V2 3667 5604 Q8WXG6 P27037 P25098 Q92538 Q13956 4771 Q96N96 Q9NUY8 P15056 1499 10616 1012 P61586 3678 26191 3690 Q9UGI0 P29466 Q9Y239 Q9NZC7 O95361 124583 P10600 1029 10626 10868 841 O60895 843 5863 604 Q6ZW31 847 Q15910 Q07890 O95136 4792 Q15915 P18545 Q8IUH5 Q96N67 P60033 Q07889 613 Q5TCX8 857 O14763 859 Q96CA5 5879 Q13501 P10827 P31749 O95140 O95382 Q9H8V3 P19883 27032 253980 153090 Q9UMX1 P09601 Q9UL54 Q92743 P01241 6993 P56199 P14317 23322 3481 O60603 3479 2146 3476 5654 10013 P01236 Q15027 Q7Z6J4 2159 Q99750 84033 8932 Q99759 O43294 408 P49407 409 85360 Q13098 116986 3265 116987 3263 P11274 P53708 P35240 8945 P01019 55504 O14964 84062 64223 6541 P35222 P17813 P60484 11331 Q15052 Q15057 P24588 8717 Q3MII6 5460 Q9Y3M2 6794 Q96BZ9 Q5R372 79971 673 Q96P48 P15692 O14908 79735 10451 57708 9815 6789 O00755 8743 7410 Q9HCE7 23532 Q5JSP0 Q05086 Q99558 5467 8737 9826 207 54206 P48357 10468 11315 P24385 7422 Q96GA9 P30825 Q12802 Q0IIM8 55785 O14920 O14921 9839 5494 4168 Q13905 Q9Y3P9 23513 P50402 57732 O14939 8516 8517 P49427 8772 O15085 7204 Q14449 Q9Y4K4 O75386 O75385 Q9Y4K3 O43318 472 Q9H160 23286 P49810 8767 P78395 P43034 P49815 Q8TBP0 3091 4188 Q16635 5037 O75376 Q04864 P09382 Q9H2X0 P46531 7448 9628 P00533 Q13370 Q15796 1902 Q15311 Q9HAU4 Q13131 9641 8795 9882 Q96IZ0 Q9UQ13 P12757 Q8N6I1 P32121 Q9ULH1 P12755 64857 Q96CN4 4193 Q9Y6Q6 23263 Q9NRI5 Q13145 P55290 Q8IV61 Q9Y6R4 7476 Q9UPQ3 P10586 Q86XR7 Q8N6H7 Q4AC94 23032 Q16665 Q13153 55915 Q13158 9420 7248 O75581 Q93062 Q9Y2C9 Q9Y6K9 O94827 1936 P46108 5074 11030 Q9HAZ2 26524 5071 P11021 7249 Q7Z7H5 Q9GZM8 Q96NH3 Q99835 23229 P02790 10392 P49840 O15068 23463 O43734 O43739 Q96EV8 Q14C86 Q8WVQ1 Q9UNH7 P08069 Q13188 1956 10159 10160 P37840 7272 54862 Q6PID4 P48730 Q9BWT7 P98194 Q14289 9465 7046 P35813 O75787 P42345 Q9Y6G9 Q86VI3 Q9UGU5 P46940 9218 Q92574 P78536 7057 Q13526 7052 P46934 81565 P16070 O60292 P42338 P98174 Q9UER7 P34947 7067 P06239 P29353 Q5TAX3 Q13535 Q9H0K1 P56539 84619 P41235 Q9BYM8 60412 P21860 3932 O60260 50618 A4D1W7 Q13546 55072 Q9UNE2 P78318 9495 7074 P43405 P41220 Q07954 Q04206 P53041 9267 7088 Q96EP0 O95999 Q9NP61 Q13315 P78509 Q86TI0 9021 Q16828 93594 P78504 9020 P63000 Q96PE2 P00742 3953 Q6P1N0 2869 P08246 3956 Q8IWV1 7099 7098 Q13322 O15211 P14923 P30153 3728 P61073 23094 Q9Y6D6 Q9BQB4 Q9Y6D5 Q05513 2873 1540 Q13574 51735 3965 50649 O75962 Q9P035 57045 O60229 2889 Q86TG7 2885 P61088 50650 51741 Q9NZM3 Q5T9L3 O15455 Q16610 Q15762 Q8IYX1 P18031 Q06830 222068 63976 Q9HCS4 Q9NZN5 P08238 Q7Z628 P10911 Q9NRW4 Q5VV41 Q9UBB5 Q9NS86 Q99490 O15519 Q6PCE1 Q92851 P07996 P52564 P04004 P09936 Q9Y2X7 Q12952 65018 54361 115704 Q9P107 4851 10783 O15524 Q008S8 P50150 Q99471 10758 27185 Q8IVF5 P20936 O60543 Q9UJU2 P03372 64170 Q9H7P9 4869 5716 Q9GZX9 5970 P48023 3551 Q12979 9093 Q9UBE8 P56705 Q13829 P17302 5728 2697 5966 3309 P04233 O00220 O00463 P07550 P04040 Q9Y2T1 P08887 P08648 P51671 Q9NYA1 P10721 Q5VST9 P37198 29110 10746 79444 960 3326 1147 2475 P42771 Q92888

P07766 P05106 P28223 6850 P50591 Q13618 2010 Q9P0L0 Q9NS68 P62330 2247 1398 Q9BVC4 58533 975 O14640 5997 5515 P05112 5516 Q92633 5518 Q12774 P09958 5770 Q12778 O00255 Q9P0M2 Q99497 P06213 P04637 5300 115557 P52952 O60725 Q96RU8 O14827 Q96RU7 P62993 P13591 997 6622 P01116 5536 Q15389 P01112 P01111 O43166 4221 6885 P51636 23216 2280 56940 Q8WTR2 54764 4214 4215 4216 P01588 P01344 P01343 P49768 O00206 P48431 5562 Q86VW2 Q8N726 P14416 P52735 148022 Q99623 Q02952 51026 6647 P01137 P01135 8826 P49758 P01133 P01375 Q9UJM3 Q9Y337 Q9Y2R2 P04626 8841 2065 2064 O60716 P35348 55633 P10070 545 P10071 P01127 Q96EY1 6657 11214 O43150 8837 9927 O43353 5580 5585 O43597 P07948 Q9Y2K6 Q9UJF2 3162 Q99683 5578 P62714 5579 5338 P05771 Q96FA3 O00635 Q6IR47 64127 23411 5590 Q00987 324 3169 Q9NU19 Q8TEA7 329 P27797 Q76NI1 6453 4035 Q5VWQ8 2099 Q9UHD2 P51843 Q9BUZ4 Q9NRM7 10554 Q9BT67 23636 P27540 Q60FE5 Q12933 6464 25818 P51617 54106 8878 Q99418 10564 P26447 P25116 P60568 Q8N6T3 9744 P30086 351 595 356 Q13485 Q8WZ64 8408 P25963 Q9HC98 O43687 6003 Q13009 7334 5155 Q16512 4067 Q15669 7335 Q9UIA0 367 Q9UBT3 Q13490 Q8IW93 6496 6498 Q9HBW0 29 Q15438 253260 Q8TEU7 Q9UBU3 Q9UDY8 142678 Q8WZ42 P00441 4087 4088 4089 Q15208 Q8WWN8 Q14118 163126 125058 Q9H257 382 Q04724 387 P21359 389 Q66K14 P08151 Q16543 Q13033 P62942 8452 6275 11140 64750 7124 O15164 P54253 7126 Q9Y3E0 23365 O43639 P10275 4092 154 156 121512 25780 Q6KH11 2904 Q99966 Q13043 7132 P36896 Q9Y5J5 P36894 Q86WV8 P35568 23370 Q02763 P01308 7128 Q9NQB0 Q9UBP4 O15169 85440 Q99956 Q6ZSZ5 Q8TEY7 Q8TCU6 O95813 Q99943 P01579 10276 P09038 Q14155 Q9NQC7 Q9BY84 Q08050 7157 64786 25776 Q8N9R8 Q02750 23118 2931 Q14160 51231 Q14164 Q14161 Q6R327 1848 1605 Q13077 Q15811 Q13873 O95835 10928 26037 P63172 7161 27121 P41134 1852 Q9BXM7 1850 Q1PSW9 27122 91 O94989 94 1616 Q6ZT07 Q9NSA1 Q9NSA3 Q13404 Q96QB1 9590 9350 O43609 P67775 P08575 P05067 7186 Q15831 7189 7188 Q9H422 Q96M96 29767 O75881 Q03014 Q13418 7185 P17252 O15379 P21980 Q9NSC2 9138 Q7Z434 2735 27352 P19438 Q05655 Q9UBN7 2737 2736 9146 Q9HC62 P13945 Q96DN5 P41182 Q58EX7 P23508 Q03468 Q13202 Q96HU1 Q8WZ19 Q15628 P21580 26005 57154 Q68CZ1 P08134 P08138 P21333 Q9NYJ8 O14492 Q6ZV73 O14495 29966 59343 57162 3611 O60343 Q14790 4943 P01730 P41159 O15350 O75832 O15111 Q13227 Q86Z14 Q04771 Q8N5A5 3622 Q9HC29 O15105 Q13233

cell cycle phase P25054 8099 Q9Y265 23607 Q9Y266 Q969L4 2672 8091 259266 9181 P51587 9184 10657 Q9BVA0 O60566 Q7L0Q8 1213 Q96IK1 Q9BVA1 Q8N3U4 Q9ULW0 10534 Q92974 O75935 P08729 Q12834 Q53EZ4 P16104 O60216 1104 10783 64282 P30622 P27694 Q9NQW6 22974 Q9Y2Z0 6929 57405 1111 Q9Y6A5 Q5TAP6 4627 Q96EA4 P06400 P53675 O95347 Q9NRZ9 Q96GD4 O14578 5604 P50570 P11802 Q14807 1021 10609 22832 1018 1017 Q96DE5 55125 3796 O75909 Q5TB80 P07437 Q07666 Q8IZT6 79441 1029 O14519 1027 1026 O14753 P41208 P42771 O14757 P30304 P30305 P30307 P28340 Q13618 Q13617 Q13616 55142 Q9BQ90 Q92878 5515 O00139 5892 P31749 Q6KC79 Q9P209 P40692 10726 984 Q96MT8 989 5888 O14777 Q9UKT4 127829 Q96FF9 Q15019 6872 6993 5422 Q09019 O95271 25988 990 991 203068 55722 994 995 Q99640 997 6502 55726 Q15021 5536 5537 P62753 64682 Q9Y6X3 Q8N4N8 Q9UQ88 23332 23212 Q8NHV4 641 1069 Q99871 P46013 5424 11113 8812 Q9BS18 25847 Q8N726 Q7Z7K6 P52732 890 Q66K89 891 Q9H4B7 Q99741 55743 Q00534 898 3014 Q8N960 O14965 Q16254 O43264 Q9UPY8 4361 8841 23310 O95067 Q96ST3 Q15050 P23258 Q9Y580 Q00526 6790 9700 7884 25942 O00743 Q12906 10459 Q99684 8607 O43482 10460 P51955 P51956 Q9BZD4 Q9NYZ3 Q6PJP8 Q00987 P51959 P50748 324 54443 79723 207 Q9H1A4 5347 Q96JH7 Q9H5I1 P24385 Q86U86 91754 Q99550 O00762 22897 Q99543 Q99661 Q9NRM7 Q00610 5116 5119 3070 8881 Q7Z4H7 P52701 79980 Q08209 10200 55559 Q2M2Z5 Q9UPV0 P49427 54908 Q14204 11065

P15923 351 472 P48729 595 Q96BM9 5017 P83731 P43034 P21127 Q8NEH6 Q9HC98 O43683 O43684 O75376 24149 6117 54801 7329 P00533 P24522 Q29RF7 P11309 Q13257 7465 Q8WVM7 O75122 4193 P51808 P46527 P49916 54930 Q969Z0 10198 Q8NFH4 P83876 Q5VUA4 51547 O94927 Q8WZ42 Q93008 P62826 O43663 Q8TD19 Q96BK5 54820 P24864 Q15691 Q8IX90 8453 8454 8452 P54132 Q14008 23122 23244 P14635 O75461 25788 P35579 Q9H6D7 Q13042 Q14493 8328 10179 Q96R06 Q96CS2 Q9NRC8 7013 9793 Q96KB5 P36896 26524 8218 O15182 10270 P54274 Q9Y5K6 221150 P49959 11243 9319 Q9BRK4 Q9NTI5 Q9NVM9 347733 Q15003 11004 1956 P23443 9212 116138 Q8TAP9 23354 Q02750 P38936 25777 7272 Q9NXR1 Q9NZ56 Q93034 54984 Q9NTJ3 Q5VTD9 2935 9221 6198 O95835 8379 Q01094 P63172 6194 7283 80124 27000 26271 Q9Y6G9 84967 55165 91 1978 Q13885 P78536 9232 9238 Q14978 Q8WWH4 O75771 3925 P67775 Q7Z460 P52292 22919 Q9UI95 Q6P1K2 P05067 P42695 1869 Q14980 2956 9126 O95613 10907 91272 1877 10910 1874 Q92791 O60266 O15379 O95619 1639 93323 9133 Q14511 O15392 A4D1W7 O43823 Q13309 Q8N7B1 79023 Q9H410 9493 80254 Q9UH99 P09884 P53041 Q9HBM1 1647 Q13554 P54652 Q13315 O95997 Q9P258 Q96EP1 1778 4926 3832 Q13561 3835 Q68CZ6 58480 8065 P20248 Q9UJX2 1785 Q9UQE7 3609 5901 Q9Y6D9 P10415 3980 Q02241 Q9BQA5 Q9HC35 Q14674 P54687 728642 P53350 27436 Q04771 P63279 P00519 121441 O60232 P30260 P24941 900 51512 P21675 Q14683 5925

chromatin modification O95677 6595 Q9H0E3 6597 4297 Q9Y265 8535 6599 6598 Q9BZ95 Q12824 22992 O00267 Q9P2R6 473 O75150 Q96JM7 Q9H160 Q8NCD3 Q7Z6C1 5931 5933 Q9H4L7 7334 Q16512 Q9UBC3 Q16514 Q15306 Q5VTR2 Q9ULG1 O75376 Q8TF76 Q7LBC6 Q8NB12 1107 57634 Q9NVP2 79885 51773 9869 51412 10661 Q96RL1 Q15554 Q96EB6 1108 Q7Z727 O95696 Q9H2G4 9646 10075 9643 7468 Q9UPP1 6015 P54198 O75367 Q9UNL4 O00167 Q6UXN9 23028 54496 6927 51780 83933 Q92830 O94805 Q9UBU8 Q92833 Q6IT96 P26358 P61964 Q92831 P06400 Q9C005 Q6PD62 Q9NQX0 O75478 Q9BY41 Q9NRZ9 93166 55355 10524 P20823 55352 O60674 11177 11176 Q9NPF5 64061 51317 10765 53615 Q9Y468 Q96MX6 Q8TDI0 Q06587 O75582 Q9UQL6 Q9Y6K1 6829 79577 10856 Q6FHQ0 51564 Q08999 2908 Q9Y230 8328 O14744 6827 Q12873 6045 7013 55929 Q92769 7014 9555 8467 P28370 23133 Q15906 O95361 23135 23378 Q8WYH8 6839 10626 23492 1387 O15047 Q15910 8110 P54274 O95931 23466 9682 7141 79595 Q8NA19 Q9NR48 Q96T23 Q96KQ7 Q9NVM4 Q9BZK7 Q96PK6 6601 56916 Q13185 P49711 Q92993 Q16576 6605 54617 Q9NPA8 8242 O43189 Q14839 O00255 Q8NEZ4 Q09028 64426 O00257 Q03164 Q9P0M6 2139 57332 80335 Q99496 P04150 54623 4678 Q5VTD9 50943 Q9UGL1 Q15014 86 Q9UKL0 Q6P1J9 Q96L91 O43159 P29375 6871 26038 2033 27005 Q7Z589 26039 23326 23569 1613 Q8WTS6 11107 10933 Q9Y4C1 2146 Q15022 79813 10013 84289 55723 10014 P56524 Q9P0W2 Q9UM07 6883 4221 Q969S8 O96019 Q86WJ1 23210 O75530 7290 Q9H3R0 P55318 Q96BD5 83746 Q92560 Q52LR7 Q4LE28 648 408 O43293 P49407 56946 Q9UBL3 O75528 O96028 Q09472 Q7Z7K6 Q9H6W3 Q92793 Q96GM5 P17252 Q7Z6Z7 O15379 55869 O95619 51147 O60264 Q969R5 29086 Q15047 Q86UE8 8289 P51531 O95983 Q13422 Q13547 8841 O95503 10919 O00213 9252 P51532 Q9Y6J0 Q8NB78 P35227 P35226 Q8TBE0 P35232 Q15291 84733 10363 10362 Q92784 P41229 Q9UBN7 2070 5585 Q14527 Q8WUI4 Q9Y2K7 Q6NYC1 Q9NP66 23523 23081 Q9UK80 Q9H9B1 Q68DK7 6304 51720 8726 5578 5579 Q9HC52 P05771 8607 Q969G3 Q9NQZ2 Q9NQ92 O75177 23411 Q8N7H5 3171 1789 1788 P46736 Q9NRL2 Q9NWV8 1786 3169 79723 3720 Q92922 Q92800 Q14781 Q9H5I1 Q86U86 P49321 5245 339287 22893 3066 Q9UMN6 Q9UPT9 3065 Q9UIF9 O75164 P61088 23186 84661 Q9H7L9 O60341 P09430 10432 Q01826 O43463 11091 3070 30827 Q9BZS1 Q9UIG0 Q9P0U4 23512 221656 5927 5928 5929 P63272 80854 3622 P28749 O14497 10320 Q15652 Q13351 5925

negative regulation of RNA metabolic process Q9Y2W7 O75820 2672 Q9UBB5 O00267 124790 3516 2304 P84022 10772 P27348 5933 Q9UBC3 Q9Y2X9 Q12837 54361 4851 4734 Q96EB6 O75925 3660 Q99471 P13056 83933 811 Q9UJU2 O15534 57649 Q92833 6926 Q6IT96 P06401 P26358 4869 P06400 5716 P48382 5970 Q92949 Q9NRZ9 P56705 O95343 9099 10524 1487 P19838 P62263 3665 10765 Q12986 Q9P0J0 O14627 6829 10614 1499 Q86VE0 Q8N2W9 10971 6945 5978 6827 O14503 Q92769 Q8WXF0 93986 5993 Q14938 Q15906 P19544 O95365 O00358 Q06330 10865 6839 1027 P08651 P17480 O14753 604 5987 4790 O00488 2247 Q9BZK7 6601 Q92993 857 6605 Q8N488 P10826 P10827 2023 O00255 Q6KC79 P19883 O00257 1050 Q99496 Q9UMX1 4799 P40337 50943 Q9UGL1 7704 Q92985 O96004 O43159 P04637 P14316 P52952 3476 Q15022 79813 4204 639 10014 Q9UQ80 135295 4221 P16989 P57059 84159 P33076 Q99750 P48436 8932 7727 2290 23429 23309 P48552 P48431 6774 Q96S42 55869 51147 P01137 P01375 Q86X95 P51531 6660 5451 8841 Q9UHI6 P35222 2063 P51532 P35227 11218 Q96ST3 P17813 P10070 Q15170 P10071 3146 6657 5569 Q96T58 Q96MH2 P23497 O43474 P06733 6672 Q9Y3M2 23409 P25490 25822 25942 Q12906 Q8NFW5 3281 O75190 Q99684 6304 P40763 4261 Q969G3 8864 Q08117 23411 23414 O75182 P37231 P14373 Q00987 3169 5467 5468 7528 10468 P27797 64375 P56177 O60907 22893 3066 3065 Q9UIF9 P51843 3182 P51608 6207 5119 Q01826 P62854 O43463 3070 9612 23512 580 Q96QT6 P28749 7428 10320 6595 6231 6597 8535 P10599 6591 Q9Y4K3 5017 Q13485 Q13363 Q13127 4188 5036 O75496 O75376 O75494 P13631 3096 O75381 23051 51773 P46531 7329 Q15796 Q15672 Q9UQR1 8553 6496 6256 6498 Q96IZ0 P12757 P31273 Q9UNL4 P12755 23028 O75360 4193 P46527 116113 51548 51547 54815 Q13263 4087 8204 4088 4089 Q9BY41 P55055 Q9Y618 P10588 P10589 P20264 11176 Q9NPF5 Q15697 51317 53615 P43489 Q13033 9421 P54259 9541 5187 7124 11142 O15164 O75461 Q9UQL6 4092 P10276 Q9Y6K1 Q02535 Q03989 51564 Q15583 Q99728 Q99729 8328 Q15466 5074 5195 Q9NRC8 6045 7376 55929 Q9HAZ2 8467 O15055 9314 O43524 23135 23378 7490 7251 1822 166 84232 Q9NQB0 Q8WW38 Q96T37 P61925 7023 P14859 P15822 23468 7141 Q9H063 Q96T23 51341 Q01664 Q00577 51588 P49711 10155 P49715 P09038 O75437 7157 O43623 25776 Q99816 Q5VTD9 P29374 Q01094 P41134 Q1PSW9 9219 O15119 1616 P45973 Q9NSA3 P56524 O94992 Q969S8 7291 7295 P55316 Q96BD5 P46934 1869 Q9UER7 Q14865 7189 7067 92129 Q8WYK2 P19793 7181 Q03014 Q7Z2Q5 84619 Q9H6W3 Q86UE4 O15379 O60264 Q9NSC2 O15151 O95983 Q13422 O94776 Q13547 P78318 O43829 Q8TBE0 84733 Q04206 Q9UH92 Q92786 Q9Y2B9 2737 2736 O75626 Q8WUI4 P41182 P62277 Q9UKY1 Q9H9B1 Q14894 150094 Q9NP71 1789 1788 Q05516 1786 P41162 3720 3609 Q14781 P23510 P09429 O75604 Q9H2S9 29966 Q02363 P18146 P35711 Q9Y6E7 O60341 P09430 5914 O75953 O75832 Q9BZS1 Q13227 P55771 Q86YP4 63976 P63279 Q8N5A5 P63272 Q9HCS4 O15105 5925

cellular macromolecular complex assembly 10093 P06899 11065 25909 P49023 Q9H0U9 1460 Q9UBB5 3084 4171 4728 Q8IYN9 P84022 P07196 23165 Q9BVA1 Q8NCD3 Q7Z6C1 10772 P83731 5018 Q13243 6901 Q13485 4725 O94906 Q13242 Q13247 Q16637 P03950 25915 9997 Q9BTM1 P62805 8301 Q15428 Q16635 P62807 79084 O75494 P16104 2316 2 Q9NVP2 5829 Q8IUE6 P16471 51773 Q9NRH3 Q13492 Q9H9E3 Q16401 Q15796 10421 P10809 84790 O75925 Q9H2G4 Q15797 Q8N6I1 Q96NT1 O75367 Q9NQW6 Q9NWS0 Q05397 54496 1355 P23527 Q01105 P09234 811 P83876 Q9HC77 Q8WZ42 Q9BQE3 4869 5716 4086 8683 4087 4088 Q9BY44 4089 Q16777 Q9H814 Q16778 P0C0S5 163126 Q9NRZ9 P04908 P68371 Q02790 55355 P16333 Q9NVR5 P0C0S8 P68366 Q7L7L0 P62263 P04350 Q6NXT2 64061 P62942 3308 P07305 Q15459 10291 Q8N5M1 O75347 3320 1020 O43639 P52756 O00468 154 P07550 P62316 P62318 O14744 Q02539 P62314 P49736 Q14011 P07437 Q12874 10181 Q9UHK0 Q12872 3692 Q8WXF0 Q09161 8348 9555 8467 2243 1153 O00233 Q9BUB7 3329 Q92522 8338 6834 Q8N257 6714 5747 Q9NRD5 9685 P02671 P07766 Q8TCU6 266812 Q5SSJ5 4673 A1XKG3

P58876 P36404 Q99700 Q9NS69 Q96T23 51340 P42768 P84243 Q71DI3 P68431 Q9NVM4 2244 Q16695 O14640 Q14152 8349 347733 5879 O43182 7277 P22492 Q13509 Q5QNW6 Q13748 26747 Q9P0M6 P40692 6733 Q6PID4 Q92621 Q6FI13 4676 55835 1729 P78362 Q14289 Q9UKT4 Q9BZJ0 4690 9463 P07900 Q7KZN9 6872 6631 2033 P14678 O95391 P12931 6194 7283 26271 203068 1058 Q8WWY3 P20671 4686 55723 Q15389 P62753 135295 P54105 Q13885 10383 Q99880 Q02297 3010 Q6FGD7 Q9BU61 2280 55172 Q99877 Q9BUF5 3009 3008 O60610 3007 3006 402 P05062 O43290 6633 8932 7846 P02675 6637 255626 8936 Q99879 Q09472 P52298 O75643 Q7Z7K6 10907 3024 P55209 O75880 84617 P56537 O43819 773 Q9H4B7 Q00535 3014 O60264 P01137 Q92558 Q9UPY3 Q3ZAQ7 O00217 Q9UMS4 Q658W2 Q9UHI6 P34932 221613 Q96A08 Q99733 11218 Q93079 3148 Q93077 Q07955 P23258 P62308 P62304 50628 P26583 6430 6311 6431 8290 Q96KK5 1653 P63000 Q92499 6426 Q9BUK6 Q9Y512 P49450 O95400 Q2NL82 O95405 O43920 O60814 27339 P18846 26121 Q96QV6 54443 Q15070 Q8WYP5 375790 P27797 P21333 P33778 4154 25929 Q8TEQ6 P10412 O00762 Q00059 Q5TAL4 56993 7536 O15212 P62851 Q9BQA1 3070 Q14677 6341 Q60FE5 O75832 Q15526 P16403 P16402 25813 P16401 10569 466 P67870 P52948 Q71U36 Q13112 P21675 1207

negative regulation of protein metabolic process 11065 P30086 9184 O60566 P84022 P49810 51763 6901 Q13485 P07996 P38919 P03950 P55036 Q12834 Q9UBC3 Q16635 O43684 5037 Q8NDV7 P51114 5270 5706 2316 5707 2 5708 5709 5700 Q16401 5701 5702 O15524 5704 5705 Q7Z727 Q9UKV8 Q13257 Q9Y2Y8 P32121 P35998 P53667 5717 3416 Q01105 811 10197 P61289 Q13144 5713 P62258 Q92833 Q14232 P26358 Q9BSI4 5716 5054 P31946 4088 4089 5970 9775 P55055 Q99460 P35625 5728 P19838 400961 2332 10642 10644 10643 Q9UL15 Q9ULR5 P05129 Q9UL18 4771 7248 Q96KC8 P51668 1020 P14635 P51665 4092 Q9P0J0 710 Q13042 Q92530 27161 Q13286 Q9Y2T7 3690 7376 7375 O00231 O00233 O00232 26523 Q86WV8 P19544 7490 5071 1029 3326 P01308 3685 P49721 P42771 P49720 P26045 P05106 4790 A1XKG3 Q9Y6M1 O00487 O60502 P62333 P20618 Q9H1Y0 857 Q9BQI3 6726 5515 5518 Q15008 P09958 Q9UL46 Q9NR50 O00255 P37840 Q9HCK5 10724 Q9H9G7 192669 5524 P05121 Q15257 50943 Q9UKT4 P06213 O00425 Q06787 991 26271 2147 P62195 23560 6622 10013 Q92574 1978 9474 7057 64682 4221 25998 Q9BT40 P67775 Q9P2K8 408 P60900 P49407 P60228 409 P49768 5682 5683 Q8N726 5684 5685 5686 64215 P29590 891 Q9HCP6 27102 Q00535 P17252 P35240 P25789 P25788 P01137 P05155 O60260 P25787 Q9UJM3 O15151 8165 5692 5693 P17813 Q05655 P60484 P17936 Q04206 5687 5688 5327 Q9UBN7 Q92667 5580 5582 Q15735 27327 Q9BZE4 O14908 5578 10213 Q13200 P04201 P05412 O43242 Q9NP71 7531 P37108 P30153 P11413 5590 1789 1786 Q9UJX2 Q05513 P00734 5104 Q9H1A4 3720 54206 7529 P27797 P01023 P21333 O14495 P28065 P19474 O00762 5371 8881 Q60FE5 O75832 Q9BZS1 Q13107 P62191 P28074 P17980 P28072 P28070 O14818 P30260 O15105 P00750 P08238 P43686 P60568

cellular macromolecule catabolic process P48052 23607 O14672 9184 10535 O60566 P15374 Q70EL4 O00273 P63208 4968 Q9H0D6 Q92611 P09936 Q9C026 Q12834 Q5VTR2 5706 5707 5708 5709 5700 5701 4734 5702 Q9UHP3 Q9UKV5 5704 64282 5705 P27694 P56817 79791 A0AVT1 5717 3416 1358 57646 P62256 O60543 P61289 6921 Q9Y297 5713 4627 5716 Q12972 5970 P99999 Q99460 9099 Q13829 3304 Q99575 P19838 55236 948 3309 P51784 Q9UL18 1022 P51668 P51665 10613 10616 P61221 29102 Q92530 5976 Q7Z419 Q9Y4X5 O00231 O00233 O00232 Q96J02 O95243 Q9NS56 1029 10868 2237 79444 10869 167227 841 P42771 4790 Q13618 Q13617 Q13616 1161 O00487 O95376 Q8TBB1 391627 10956 P62333 P20618 55149 Q9BZK7 Q92995 5511 Q96CA5 Q9UK22 5516 Q13501 Q9UL46 Q9UMW8 Q13620 6500 P54727 P54725 Q9HCK5 10845 253980 P40692 57695 192669 5886 5887 8930 O00308 P53804 25862 Q9NVW2 23326 P50897 991 Q96RU2 54512 997 4686 P62877 10013

51009 Q15024 P23025 64682 115426 P35244 Q96Q15 51132 51377 4214 Q9BRP8 O43294 408 P49407 P60228 409 10269 P49768 P60468 Q6ZN33 5682 5683 Q8N726 5684 5685 5686 891 O14727 Q96T60 55743 8945 P25789 P25788 Q9GZP9 6647 P25787 P61326 Q15287 Q8TAT6 P01130 5692 5693 P40818 7874 O95071 9924 668 5687 5688 4116 7508 Q5VVQ6 P62837 8975 Q969H0 Q9UPR3 9820 P50613 Q9Y2K6 22794 P51946 Q9UNN5 O76031 Q8IWF2 Q96PU4 P62714 10213 P55072 Q9H3F6 O43242 Q9HCE7 Q5HYA8 Q05086 Q01831 P16671 Q00987 Q86T82 Q9H1A4 329 8754 O60907 Q9UPT9 Q9Y2M5 23621 O00762 P13489 3184 Q9NWW5 Q9Y5S9 51185 55666 P68036 7415 8761 8881 Q96C86 P39748 P17980 Q9NVV4 55432 10208 O14818 P58012 8878 Q92900 P49427 11060 P15927 11065 Q86TM6 Q9Y4K3 472 O75150 Q04759 84447 P49810 9978 Q8IU60 O94905 Q14694 P38919 3093 5394 7332 7334 P55036 7336 O43684 Q504Q3 23291 6117 Q9NPD3 6119 7329 6118 Q13490 7328 Q16401 Q9HAU5 Q9HAU4 Q9BUN8 11160 Q13257 Q14103 7347 P32121 P35998 4193 23381 P22674 10197 Q16531 Q93009 Q93008 P00441 57092 7353 9775 P18074 Q86Y37 O75477 267 23032 28960 8453 Q14249 8454 8452 64750 7126 26994 P14635 8450 P35579 Q8IZD4 Q13042 Q13049 Q14139 7375 Q09161 11274 6048 P36776 23014 26523 23016 Q9UKA1 5071 7251 P11021 Q9NRD1 P49721 P49720 Q8TEY7 8237 O94966 7267 10273 6059 Q9Y5K6 Q9Y5K5 6050 Q9UKB1 Q99942 Q8WY64 11124 51107 Q9NQC7 Q15008 Q15369 9695 P14625 O75674 Q01780 O75792 114088 255488 Q9H9G7 Q93034 Q99816 Q8WW12 2935 9100 Q15819 P29372 Q96K76 27005 27248 P78549 Q70CQ3 Q70CQ1 79139 P62195 O15234 P45974 Q9BZQ6 26270 P78536 22803 83737 84708 55294 27252 P46934 27257 Q92560 P42574 P60900 10940 P52298 7189 Q5TAX3 26019 9361 7184 80267 Q9Y3C5 Q9BYM8 Q9Y4E8 26133 Q8TEL6 Q9NZ08 80020 O60260 Q15843 91147 Q6R6M4 P78317 Q9UNE7 10921 1642 1763 Q04206 Q92542 Q9UBN7 Q969Q1 4913 Q13315 Q96EP1 P00747 26232 Q9UK80 26234 Q13200 Q8IY92 Q13443 Q8WZ19 Q13569 Q8IWV7 57154 Q96BI3 8065 84749 Q9UJX2 P61077 1540 P08138 124739 8078 O75604 O75844 Q13216 Q8IUQ4 P28065 Q5W0Q7 377630 84305 1676 Q5XUX0 P61088 Q14790 P08107 O75832 Q13107 P62191 P28074 P28072 P28070 P63279 P30260 51752 902 Q13233 51514 P43686

cytoskeleton organization Q8NEY1 3880 9181 Q6QNY1 P51587 9184 54474 Q8IYN9 O60566 Q7L0Q8 55561 Q96M29 79658 Q9HBH0 Q92730 A1A4S6 Q92974 P08729 O15530 Q8NF91 P08727 Q8IZP0 Q12959 O95680 O95684 P52566 10426 2317 2316 1104 800 10420 P05787 Q9NQW6 Q05397 P53667 Q9BXC9 27185 57405 29127 4983 811 Q9Y6A5 4867 P35080 4627 4869 Q96EA4 O95466 P07737 Q92949 P17661 Q13829 P16333 81624 P41743 829 4771 1020 57787 114791 1499 57662 830 55125 832 3796 P61587 P61586 P07437 Q9H0H5 Q9NSV4 79441 116840 O60890 P62328 604 P41208 5747 Q8N137 Q9UGJ1 Q13976 Q13618 Q9NYB9 Q96L34 55142 Q9P0L2 P62330 P42768 P15153 Q96N67 1398 5879 O43182 Q9Y490 O00139 O60879 10844 27032 81788 Q9P209 253980 P40692 Q96MT8 P63313 O14777 Q9UL54 Q9UKT4 4690 6993 Q9Y6W5 2275 O95271 P52952 203068 55722 1058 P11171 Q66GS9 998 85378 P01116 5898 6624 P01112 P01111 O43166 6760 P06702 Q7Z6J4 Q8N4N8 Q96CW5 23332 O95166 1072 Q9BT40 23336 Q96RT7 O60610 2039 Q96RT1 1069 2037 Q96S53 Q99871 Q15154 Q96S59 408 Q96RT8 P49407 8936 Q01518 11116 P50552 Q9BV73 O00560 3265 P52732 Q9H4B7 Q00535 P35240 6647 84062 P48668 Q8N960 O14965 Q08495 O43264 8841 Q658W2 P35222 2059 Q9H093 P23258 10486 P01127 Q8TDZ2 10487 23647 6790 4131 9700 5584 4133 Q9UHB6 Q68EM7 Q96P48 O75190 A8K0Z3 Q9H2D6 5338 P49450 10460 10580 P51955 P68032 Q5JSP0 5590 54443 4137 9826 4139 7408 5108 P27797 Q86T65 Q96GA9 Q9Y5S2 Q12929 Q9Y2M5 O00762 P12036 P15311 Q99661 55785 5116 10552 7430 Q60FE5 Q7Z4H7 O14939 Q96QT4 57610 Q9H4E5 55559 Q99418 Q2M2Z5 O15085 54908 Q14204 10093 10092 8655 11065 P07196 23043 P09493 118 9738 51763 P43034 P47755 P47756 11190 P03950 P35527 5155 O43684 O75376 Q9UKE5 Q9UJC3 54801 Q9BSJ2

P60981 Q16643 P24522 253260 Q9Y2I1 Q9UBU3 54930 Q6UVJ0 P23528 51542 Q96RK4 Q9NRI5 P60510 Q9HC77 O94927 Q8WZ42 P00441 6386 Q9Y613 P62826 O43663 Q8WWN8 O75116 Q14118 Q16658 P68133 O43303 Q9Y2J2 382 5170 387 P21359 54820 P52907 P60763 54822 Q13153 8452 9662 7126 Q14008 Q9UNF0 23002 390 391 O43639 P35579 397 121512 Q9H6D7 Q9Y4G6 23363 P45379 P46108 Q96R06 Q96CS2 9793 163786 23136 P13796 23370 11034 Q9GZM8 85440 9322 9564 Q6ZSZ5 O15061 O15182 Q8TCU6 10152 A1XKG3 Q86VS8 Q96EV8 P02549 11004 P60953 Q8WW24 64780 Q9BX66 64423 11252 9578 P14625 P10147 23354 25777 8481 P11233 7272 Q9NXR1 51474 Q9NZ56 Q6PID4 55835 1729 84376 Q6R327 10048 P02533 54509 P98194 Q14289 1605 P53365 P28289 Q13515 O75665 P10636 26037 P63172 7283 Q4VCS5 P63167 26271 P46821 3911 Q9UBK9 Q13643 Q6VN20 9475 Q96QB1 Q6DT37 Q05682 27252 3925 P84095 Q7Z460 Q9Y4D1 81565 1627 P98171 P98174 9126 O95613 Q96M96 6093 7184 Q9NZQ3 O15379 O15259 Q92558 93323 Q14511 Q03001 P35900 9493 80254 O43707 Q92786 Q9UH99 Q9HBM1 1647 P41182 P41181 Q53SB5 P35612 P35611 1778 P63000 Q96PE2 3832 Q13561 Q8WZ19 Q68CZ6 58480 7094 23092 Q05513 Q9UQE7 P17081 5901 Q27J81 P21333 O14492 Q9NVD7 Q9BY11 P10415 Q6ZV73 Q02241 1674 Q15642 Q13464 Q14674 3856 O15230 Q9NP98 Q9UNA1 P00519 Q15532 51517 Q14683

cellular response to stimulus P25054 P28562 P49023 O95551 P51587 3638 54474 2547 P37288 2303 1454 P84022 1453 2783 O14788 2308 4968 2549 Q8NFZ0 Q8NDV7 P53779 P16104 O60216 5829 P39210 54487 4734 Q9UKV5 Q96EB6 Q9H2G2 Q7Z727 P27694 Q9UGN5 Q9UKV8 P27695 O00167 Q06124 79791 57761 57521 3655 Q12851 3672 3673 P31947 P54646 Q676U5 Q96GD0 3428 O60674 Q8IUC6 5602 3667 P41743 1022 Q14807 Q9NSU2 10856 1499 1012 5610 Q9Y230 4780 Q92769 5871 6720 P09619 2119 Q9BUB1 1026 Q9HCM4 604 847 3689 O14757 P04179 Q12888 4792 5883 P28340 P38398 Q9HCL2 1277 Q5TCX8 P05230 Q6NUQ1 Q92993 857 Q96CA5 Q13501 P10827 5892 P31749 Q9NR50 O95382 Q9HCK4 2139 P40692 55159 O60870 Q96MT8 5886 5887 5888 P09601 P01241 8930 Q9Y5U4 Q96A33 P56199 5422 3484 3481 O60603 25988 Q96T88 P62877 8924 7832 10013 P62879 P62873 10014 P23025 6749 O96017 P06702 P35244 P35249 Q96T76 641 Q99750 P35251 5424 408 P49407 409 Q99757 Q13098 1080 Q6ZN33 Q09472 6772 6774 O00562 890 Q9NUW8 891 894 51141 Q96T60 898 3014 51147 55504 Q8IYD8 29086 P23246 Q9UMS4 4361 Q9Y696 114799 P35222 Q01974 O75084 P17813 Q9H093 Q15054 6776 4358 Q15052 O14733 P23497 P24588 P26583 O00744 Q5VVQ6 4131 9821 P50613 P51946 Q9NX61 10458 672 P15692 P55072 P05412 P01275 O00755 5471 8985 Q9NYZ3 P37231 Q6PJP8 Q9NWV8 10229 P51959 55775 P82673 8737 207 5468 P48357 Q92922 23746 Q9BQ15 P24385 7422 9600 Q96A54 3066 22897 79753 P13010 7415 8503 4149 10432 P49674 Q9UIG0 Q8NHX1 P50402 P17612 468 8517 10202 56647 Q92900 P55268 Q14686 4176 6599 Q9Y4K4 Q9H2Y7 Q9Y4K3 P13861 472 P07196 23043 Q99807 P20396 8767 Q14694 3091 4188 Q9HAV0 P11215 60488 P09382 6117 6119 5029 6118 Q13370 Q96RL1 Q15554 Q15796 Q9BUN8 P24522 9641 P35638 Q15797 9643 8555 P32121 Q9UNL4 Q9NY61 Q9Y6Q6 Q13144 Q14232 P55290 5295 Q9Y6R4 5054 9656 7476 O43542 P18074 O43306 P13611 P36956 Q8N4C8 267 Q16665 Q49AN0 P43246 O94817 Q8TDY2 O15287 Q16666 P54132 P63092 O75581 P46109 Q9Y2C9 P23229 P35221 Q9Y6K9 51567 7014 P46100 P36776 Q9Y4H2 O43524 26524 P11021 Q9NRD1 11277 P49841 Q99835 7023 P49840 O75569 P98179 P13646 Q96PK6 P08069 2805 P23443 6188 26509 Q9BX66 9217 P38936 P37840 1965 2810 51593 P48730 1728 Q99814 P25874 6198 P98155 O95714 P29372 Q7Z589 P78549 O43715 3913 Q9Y4C1 Q92572 81570 P05177 1978 9232 9474 7057 O75771 P10523 55054 Q9BSB4 P46934 Q9BYN8 Q9Y6H3 P98170 P42574 Q92569 Q9UER7 7067 P29353 Q9H305 Q5TAX3 Q13535 P19793 P29590 P41235 P40145 79035 3939 P41231 57003 O60266 Q9NVI1 P21860 8165 P07339 Q13546 P34931 9495 Q9UNE7 80254 P43405 1763 P05164 Q13315 O95997 57026 60673 51720 P08243 3953 3956 2626 7099 Q13322 Q9NP71 Q13569

P14923 P11413 P46736 3728 2873 P61077 Q13574 P12956 3980 Q9P035 P78527 P32322 Q9BQA5 2885 P61088 P17174 Q04656 Q9NZM1 P43694 P05198 3978 5914 Q14676 Q06830 Q9P287 79065 P00519 P47804 P43681 900 Q9HCS7 84893 902 51512 Q13112 51514 P21675 Q14683 Q9NZN9 Q5FWF5 O95677 64397 P08833 O60568 Q8IVH8 4841 5931 P07996 5935 P52564 O15530 Q9NS91 P27361 54361 51094 P18887 P63211 55215 P63218 P50150 Q9NRY2 29128 6809 57646 P18858 O60543 1111 Q9UQB8 O75807 4868 Q92831 P55851 P48023 Q9BZF9 1130 P56705 10524 P17301 Q99698 P20941 3309 Q6PCD5 5981 P28482 5983 Q9UHJ9 P19525 1137 57669 Q6FHQ0 P04040 5976 4646 27161 P08887 P30556 Q96SB3 Q9NYA1 O95243 Q96SB8 Q9NS56 P37198 57678 10746 2237 79444 Q9UM54 Q9NYB0 O95257 P05107 6850 22937 Q06203 2010 1161 Q9NS68 Q56NI9 O60503 10714 10956 P61244 1399 2246 Q92630 Q12770 Q92878 Q13620 Q92626 Q12778 P51692 1176 O00255 P54727 Q6KC79 P54725 P30530 P18433 81788 57697 157570 P05121 O15503 26060 P06213 P02452 Q9Y6W6 P23396 6872 P04637 2034 2033 Q96RU8 Q96RU7 P62993 Q96RU2 6622 999 Q9C0K7 Q16236 P27986 51009 P49770 4221 5796 Q96Q15 Q8WTR2 Q9P2K8 4214 4216 Q99638 P01344 56946 P01100 P49768 O00206 5563 P13569 O95292 P11388 Q9NS23 148022 Q9GZP9 6647 P01137 Q7Z3C6 Q6UXV0 Q9UPY3 6421 5573 P40938 P40937 5575 5576 2065 O00213 Q08257 P12004 O95071 3148 545 546 3146 5566 5567 Q8WTP8 P01127 5568 7508 O43353 2070 6672 25828 5584 7520 5585 4255 P07948 3162 552 Q99683 P51828 5577 5578 7518 7517 P40763 P04201 2081 5591 Q9Y4P1 P46063 5111 10580 23411 O76024 P18850 3171 Q01831 324 P18848 328 5347 Q08462 331 Q9NRM7 P68036 5599 5119 23636 5371 P27540 6464 4287 Q12931 5378 P39748 P15559 P52701 580 Q08211 O60911 55432 Q6ZWJ1 P51617 8878 Q9UPV0 8892 P15927 11186 64949 595 356 P09493 Q7Z6C1 P22694 Q13480 9978 P25963 7332 7334 5155 Q16512 4067 7336 64963 64965 Q9UKE5 80198 Q9UBT6 Q70SY1 11198 7341 6256 Q16526 P59768 Q9UBU3 P49916 P22674 5159 Q16531 Q9UBU8 51426 P00441 4086 4087 Q13268 4088 8445 Q9UKG1 5170 142 Q9H492 P24864 10087 O75469 P54252 P14635 Q9UQL6 8450 25788 P10276 O75460 Q00597 121512 Q99728 Q13286 6048 Q9Y5J5 Q9H078 P35568 Q8NFM4 Q8NFM5 Q99958 Q15233 Q99956 9564 P54277 10273 Q86V24 7141 Q99942 P49959 Q9H1Y0 Q01664 Q99708 9319 P01579 Q16576 7153 64782 7158 7157 P14625 1843 2932 2931 Q15011 Q14164 Q15014 P78423 Q13873 9100 O95835 9588 Q15819 P33993 Q86Z02 Q01094 7161 P42224 1852 10933 Q9Y3A5 79139 10935 P17275 P63165 P46821 P22612 P42229 1616 26270 P82912 22926 Q96QB1 Q86WJ1 P10644 P31321 P55318 P31323 Q08945 P05062 Q52LR7 1869 2956 Q969S2 7189 P29474 9126 Q96M96 9361 7184 P17252 Q8TAD8 Q86UE8 P09874 O95622 Q13426 P35900 1642 P09884 1408 1407 Q9UBN7 Q92667 1647 4913 P82933 Q15853 Q13432 1660 58473 P30041 27327 P41182 P41181 P30044 P30048 B3KY43 P22415 P05091 Q03468 Q8WYQ5 4920 3835 Q8IY92 27339 P30279 P20248 Q8N0Z6 Q9UQE7 Q9BXP5 P17081 1429 P09467 Q9Y5B9 P09429 O14492 P10415 Q13216 3860 P41161 P12830 59341 Q02363 P18146 Q5XUX0 P09430 O15350 P53350 Q13227 27315 23192 P45983 Q13233 P45984

negative regulation of cellular protein metabolic process 11065 P30086 9184 O60566 P84022 P49810 51763 6901 P38919 P03950 P55036 Q12834 Q9UBC3 Q16635 O43684 5037 Q8NDV7 P51114 5270 5706 5707 5708 5709 5700 Q16401 5701 5702 O15524 5704 5705 Q7Z727 Q9UKV8 Q13257 Q9Y2Y8 P32121 P35998 P53667 5717 3416 Q01105 811 10197 P61289 Q13144 5713 P62258 Q92833 Q14232 P26358 Q9BSI4 5716 P31946 4088 9775 P55055 Q99460 P35625 5728 P19838 400961 2332 10642 10644 10643 Q9UL15 Q9ULR5 P05129 Q9UL18 4771 7248 Q96KC8 P51668 1020 P14635 P51665 4092 Q9P0J0 Q13042 Q92530 27161 Q13286 Q9Y2T7 3690 7376 7375 O00231 O00233 O00232 26523 Q86WV8 P19544 7490 5071 1029 3326 P01308 3685 P49721 P42771 P49720 P26045 P05106 4790 A1XKG3 Q9Y6M1 O00487 O60502 P62333 P20618 Q9H1Y0 857 Q9BQI3 6726 5515 5518 Q15008 P09958 Q9UL46 Q9NR50 O00255 P37840 Q9HCK5 10724 Q9H9G7 192669 5524 Q15257 50943 Q9UKT4

P06213 O00425 Q06787 991 26271 2147 P62195 23560 6622 10013 Q92574 1978 9474 64682 4221 25998 Q9BT40 P67775 Q9P2K8 408 P60900 P49407 P60228 409 P49768 5682 5683 Q8N726 5684 5685 5686 64215 P29590 891 Q9HCP6 27102 Q00535 P17252 P35240 P25789 P25788 P01137 O60260 P25787 Q9UJM3 8165 5692 5693 P17813 Q05655 P60484 P17936 5687 5688 5327 Q9UBN7 Q92667 5580 5582 Q15735 27327 Q9BZE4 O14908 5578 10213 Q13200 P04201 P05412 O43242 Q9NP71 7531 P37108 P30153 P11413 5590 1789 1786 Q9UJX2 Q05513 P00734 5104 Q9H1A4 3720 54206 7529 P27797 O14495 P28065 P19474 O00762 5371 8881 O75832 Q9BZS1 Q13107 P62191 P28074 P17980 P28072 P28070 O14818 P30260 O15105 P00750 P08238 P43686 P60568

mRNA processing Q8N9Q2 285672 O43791 53938 Q16629 O75940 Q969L4 P55265 P67809 Q9UPN6 57187 Q96FV9 O00148 Q01130 351 Q9UBB9 10657 P33240 4841 Q16630 10772 P09012 Q6NZY4 Q14690 Q9UK45 Q13243 Q8WW01 Q00839 O94906 Q13242 Q9H0D6 Q9H2H8 P38919 Q13247 Q16637 O75934 Q8IWZ8 Q15428 Q15427 79084 O75494 55696 Q9NW13 24144 O94913 10421 Q9NWB1 Q9BRX9 Q9UKV8 23144 Q9UKF6 Q96IZ7 O60306 54496 1478 85437 P42285 P09234 Q8N684 P26599 P83876 51428 P31942 Q12972 8563 Q13148 9410 O43660 11171 P31943 8683 11051 9775 Q9H814 Q9GZX7 144983 Q99459 55110 Q13151 55119 P26368 P61978 5725 Q8IXZ2 51319 Q15459 9785 10291 9541 8458 Q8IX01 57661 O95232 P52756 O75460 Q9P2N5 Q9BWG6 P62316 Q8IYB3 P84103 Q14493 P62318 8449 O14744 P62312 Q14498 58517 27161 P62314 Q10570 Q12874 10181 Q12872 Q8WXF0 Q09161 Q07666 57794 Q9Y3F4 Q8NI27 Q9BRL6 11157 Q15233 Q86W42 P98175 4670 22938 Q9Y4Y9 O60508 O60506 11129 51340 Q96MU7 55149 Q9NVM4 51585 5511 Q9Y4Z0 2926 Q6I9Y2 Q9UNY4 P08621 P51693 22827 22828 Q08170 5411 26986 P14866 8241 P40692 O60870 10286 6733 10285 Q9Y2W2 988 Q92620 P78362 Q9BZJ0 11017 O43395 22913 P52272 Q86V81 10250 10492 6631 10929 Q9BV90 57461 P14678 O95391 Q96DI7 51362 84967 Q8WWY3 4686 6625 O15234 P09651 6627 P62995 135295 Q9UBK2 Q96LI5 P51991 P07910 Q13523 220988 Q15029 P54105 22803 Q13769 Q9H0L4 4343 Q9P1Y6 23210 Q9UKM9 P22626 Q9Y3B4 79005 27257 84950 10949 Q9UHX1 23451 O43290 Q7Z6J9 6633 P09661 Q15393 10147 P05067 O43172 6637 P08579 P52298 Q86U42 O43251 9128 9129 O75643 Q9H307 10907 P11940 O75400 O60942 80145 283989 Q8TBF4 10594 P49756 P61326 Q15287 P23246 O43143 Q86X95 Q9UMS4 84292 Q32P51 6421 A7MD48 Q9Y333 Q8NAV1 Q9UHI6 83759 23435 10921 11218 11338 29896 Q07955 29894 57379 4116 57819 P62308 Q9ULR0 O43390 P62304 50628 Q13435 P52597 6430 Q9UJV9 8732 6432 7884 Q9UQ35 6431 6434 Q6NYC1 22794 23524 Q96PU8 1655 1653 4809 10450 Q92499 6427 Q8WVK2 6426 Q96A72 1659 2081 23658 O95400 51729 5356 3178 27339 O43809 Q9HCE1 1665 26121 Q6P2Q9 Q13573 25804 3181 4154 25929 Q8N2M8 3189 Q9UNP9 Q8TEQ6 3187 3185 P55769 Q9NW64 Q9Y5S9 P17844 Q5TAL4 333 55660 7536 9716 91746 Q9BQA1 51747 3192 Q9BQ04 3191 23517 3190 Q5VTL8 Q9NVV4 10569 79622 55796 79869 O60231 Q9HCS7 Q13595 Q14562 1207

negative regulation of transcription, DNA-dependent Q9Y2W7 O75820 2672 Q9UBB5 O00267 124790 3516 2304 P84022 P27348 5933 Q9UBC3 Q9Y2X9 Q12837 54361 4851 4734 Q96EB6 O75925 3660 Q99471 P13056 83933 811 Q9UJU2 O15534 57649 Q92833 6926 Q6IT96 P06401 P26358 P06400 5716 P48382 5970 Q92949 Q9NRZ9 P56705 O95343 9099 10524 1487 P19838 P62263 3665 10765 Q12986 Q9P0J0 O14627 6829 10614 1499 Q86VE0 Q8N2W9 10971 6945 5978 6827 O14503 Q92769 93986 5993 Q14938 Q15906 P19544 O95365 O00358 Q06330 10865 6839 1027 P08651 P17480 O14753 604 5987 4790 O00488 2247 Q9BZK7 6601 Q92993 857 6605 Q8N488 P10826 P10827 2023 O00255 Q6KC79 P19883 O00257 1050 Q99496 Q9UMX1 4799 P40337 50943 Q9UGL1 7704 Q92985 O96004 O43159 P04637 P14316 P52952 3476 Q15022 79813 4204 639 10014 Q9UQ80 4221 P16989 P57059 84159

P33076 Q99750 P48436 8932 7727 2290 23429 23309 P48552 P48431 6774 Q96S42 55869 51147 P01137 P01375 Q86X95 P51531 6660 5451 8841 Q9UHI6 P35222 2063 P51532 P35227 11218 Q96ST3 P17813 P10070 Q15170 P10071 3146 6657 5569 Q96T58 Q96MH2 P23497 O43474 P06733 6672 Q9Y3M2 23409 P25490 25822 25942 Q12906 Q8NFW5 3281 O75190 Q99684 6304 P40763 4261 Q969G3 8864 Q08117 23411 23414 O75182 P37231 P14373 Q00987 3169 5467 5468 7528 10468 P27797 64375 P56177 O60907 22893 3066 3065 Q9UIF9 P51843 3182 P51608 5119 Q01826 O43463 3070 9612 23512 Q96QT6 P28749 7428 10320 6595 6597 8535 P10599 6591 Q9Y4K3 5017 Q13485 Q13363 Q13127 4188 5036 O75496 O75376 P13631 3096 O75381 23051 51773 P46531 7329 Q15796 Q15672 Q9UQR1 8553 6496 6256 6498 Q96IZ0 P12757 P31273 Q9UNL4 P12755 23028 O75360 4193 P46527 116113 51548 51547 54815 Q13263 4087 8204 4088 4089 Q9BY41 P55055 Q9Y618 P10588 P10589 P20264 11176 Q9NPF5 Q15697 51317 53615 P43489 Q13033 9421 P54259 9541 5187 7124 11142 O15164 O75461 Q9UQL6 4092 P10276 Q9Y6K1 Q02535 Q03989 51564 Q15583 Q99729 8328 Q15466 5074 5195 Q9NRC8 6045 7376 55929 Q9HAZ2 8467 O15055 9314 O43524 23135 23378 7490 7251 1822 166 84232 Q9NQB0 Q8WW38 Q96T37 P61925 7023 P14859 P15822 23468 7141 Q9H063 Q96T23 51341 Q01664 Q00577 51588 P49711 10155 P49715 P09038 O75437 7157 O43623 25776 Q99816 Q5VTD9 P29374 Q01094 P41134 Q1PSW9 9219 O15119 1616 P45973 Q9NSA3 P56524 O94992 Q969S8 7291 7295 P55316 Q96BD5 P46934 1869 Q9UER7 Q14865 7189 7067 92129 Q8WYK2 P19793 7181 Q03014 Q7Z2Q5 84619 Q9H6W3 Q86UE4 O15379 O60264 Q9NSC2 O15151 O95983 Q13422 O94776 Q13547 P78318 O43829 Q8TBE0 84733 Q04206 Q9UH92 Q92786 Q9Y2B9 2737 2736 O75626 Q8WUI4 P41182 Q9UKY1 Q9H9B1 Q14894 150094 Q9NP71 1789 1788 Q05516 1786 P41162 3720 3609 Q14781 P23510 P09429 O75604 Q9H2S9 29966 Q02363 P18146 P35711 Q9Y6E7 O60341 P09430 5914 O75953 O75832 Q9BZS1 Q13227 P55771 Q86YP4 63976 P63279 Q8N5A5 P63272 Q9HCS4 O15105 5925

M phase of mitotic cell cycle 54908 P25054 Q9Y265 23607 Q9Y266 11065 8091 259266 9181 9184 P48729 Q9BVA0 O60566 1213 Q96IK1 Q96BM9 Q9BVA1 P83731 Q8N3U4 P43034 Q9ULW0 P21127 10534 Q9HC98 Q92974 O43683 O75935 Q12834 O43684 Q53EZ4 O60216 54801 10783 7329 64282 Q29RF7 P30622 Q13257 7465 Q8WVM7 O75122 Q9NQW6 22974 P51808 Q9Y2Z0 57405 54930 10198 Q8NFH4 P83876 51547 O94927 Q8WZ42 Q93008 Q96EA4 P53675 O95347 P62826 Q8TD19 Q96BK5 Q9NRZ9 54820 Q15691 Q96GD4 O14578 Q8IX90 5604 8452 Q14008 Q14807 23122 23244 P14635 22832 1018 1017 Q96DE5 Q9H6D7 3796 Q13042 O75909 Q96R06 Q5TB80 P07437 Q96CS2 Q9NRC8 7013 9793 Q96KB5 Q8IZT6 26524 79441 P41208 8218 P30304 O15182 P30305 10270 P30307 P54274 Q13618 Q9Y5K6 55142 221150 11243 Q9BRK4 Q9NTI5 Q9NVM9 347733 Q15003 11004 9212 O00139 Q8TAP9 23354 Q02750 Q6KC79 Q9NXR1 10726 54984 984 Q96MT8 Q9NTJ3 989 O14777 Q9UKT4 127829 Q96FF9 Q15019 9221 6993 O95835 8379 P63172 6194 80124 990 991 26271 Q9Y6G9 55165 203068 994 995 Q99640 55726 Q15021 5536 P62753 Q13885 9232 64682 Q14978 Q9Y6X3 Q8N4N8 Q9UQ88 23332 23212 Q8NHV4 Q7Z460 1069 Q99871 22919 11113 Q9UI95 Q6P1K2 8812 Q9BS18 P42695 Q14980 25847 9126 Q7Z7K6 10907 91272 P52732 890 1877 10910 Q66K89 891 Q99741 55743 O95619 1639 O14965 O43264 Q9UPY8 93323 9133 Q14511 O15392 O43823 23310 O95067 Q8N7B1 79023 Q9H410 9493 80254 Q15050 P53041 Q9HBM1 Q00526 6790 9700 O95997 Q9P258 Q96EP1 10459 4926 3832 Q13561 3835 8607 O43482 Q68CZ6 P51955 P51956 Q9BZD4 Q6PJP8 P20248 Q9UJX2 P51959 Q9UQE7 P50748 324 54443 Q9H1A4 5347 5901 Q96JH7 Q9Y6D9 Q86U86 91754 Q02241 Q99550 O00762 22897 Q99661 Q9NRM7 Q00610 Q9HC35 5119 Q14674 3070 728642 8881 P53350 Q7Z4H7 27436 79980 P63279 121441 O60232 P30260 P24941 900 10200 55559 Q9UPV0 Q14683

response to organic substance P28562 3880 Q9Y266 O14793 2550 P51587 P37288 2303 P84022 2783 Q92731 O14543 2308 2549 P08727 Q92729 2 54487 P61158 Q9UKV5 P10809 Q7Z727 Q9UKV8 P28300 Q06124 P17405 6927 57761 P07858 3673 2100 Q9NR96 Q13705 P21917 P54646 Q96GD0 P20823 O60674 Q8IUC6 3667 5604 P41743 P08758 P25098 1020 P50454 P15056 P21964 1499 1012 5610 5611 22846 P29466 4780 Q92769 O95361 P10600 Q9BUB1 10626 1026 841 Q9HCM4 4535 6714 4792 1285 P16118 P19634 P38398 Q9HCL2 610 1277 Q92753 6721 857 P51575 P10827 P31749 Q9NR50 Q7L8A9 1050 Q9HCK4 Q9UL51 P04150 P09601 P04155 P01241 P14317 3484 P50416 O00300 3481 O60603 871 Q99523 3476 Q13085 7832 10013 P62879 P62873 10014 P25685 Q9BS26 1072 Q96T76 O14717 P33076 6517 Q99757 P01222 1080 Q09472 6772 6774 P13686 890 891 894 Q14192 55741 898 O14965 P50440 P35222 P60484 Q15291 P01009 10241 6776 5207 P23497 Q59EA4 P31930 P26583 6790 Q5VVQ6 4131 P39656 10458 672 673 6548 P55072 P05412 P01275 O00755 P05413 5471 8985 90993 Q08117 3297 P37231 682 Q01955 P51959 445 148327 5467 8737 207 5468 P48357 Q92922 P01266 P01023 P24385 8754 Q96A54 3066 79753 Q00613 7415 8503 4149 10432 Q8NHX1 P52824 P50402 P17612 O14939 466 P04792 Q13115 P35659 Q14686 5265 6599 Q9H2Y7 Q9Y4K3 P13861 230 P07196 P20396 51763 8767 Q15303 Q9HAV0 5037 P09382 P22102 5029 P00533 Q13370 Q13133 Q15796 Q9BUN8 Q9HCY8 Q13137 P35638 Q15797 P12757 Q9Y6Q6 Q13144 Q14232 P55290 6383 5295 5054 9414 7476 O43306 Q01581 P68133 58189 O75351 P35625 267 Q13393 P43246 Q14249 9420 7248 P63092 O75581 P09172 Q9Y2C9 P35221 Q96CS3 7252 Q14258 Q9Y4H2 26524 285 P12110 P11021 Q9NRD1 P49840 O75569 P98179 A1XKG3 Q96PK6 P21266 P08069 P02786 2805 1956 P23443 9695 7037 Q9BX66 9217 P38936 P37840 51593 Q6PID4 Q14289 P25874 7046 6198 P98155 P12931 P42345 83752 Q9Y4C1 Q92572 P04085 Q92575 Q92574 1978 P31689 P56524 P78536 7057 27252 P05181 P06276 P16070 P42574 Q92569 7067 P29353 Q5TAX3 P19793 P29590 80267 P40145 P41231 P00966 O60266 P06241 P21860 8165 P55212 Q13546 P34931 P34932 P78318 Q8N8D1 Q9UNE7 Q04206 P53041 P07101 P07585 Q13794 A8MTJ3 O95999 O95757 9021 Q16828 P35613 P00505 O60488 57026 P08243 3953 P08246 3956 2626 7099 7098 Q13322 Q9NP71 Q14416 P14923 P30153 3728 P09104 11080 Q16849 P34741 P78527 1312 2643 2885 P17174 Q92598 P43694 O15455 Q14432 5914 O75953 P34972 5929 P43681 4830 900 P00750 P08238 O15228 64397 P08833 O14672 O00267 O60568 P16220 P08842 P07996 5935 Q92611 O15530 P27361 54361 Q08431 51094 Q16082 P18887 P16234 P63218 O15524 P50150 57402 Q86WA8 Q9UJU2 Q9UQB8 P03372 O75807 64170 Q92831 P55851 5970 P48023 1374 9093 P56705 P17302 5728 P17301 3304 2697 3301 3309 Q92820 3308 P28482 1385 3320 6829 P19525 3316 1137 57669 3315 3312 P09972 27161 7913 P08887 Q96BA8 Q9NYA1 P30559 P51679 P30793 P37198 3329 57678 960 3326 1147 1388 2475 22937 Q06203 2010 6609 O60502 O60503 3337 P61244 Q12770 5515 5518 Q12772 P27144 Q12778 P29320 P51692 1176 Q05586 P18433 10724 10726 P37173 P09960 P05121 O14656 O14657 7706 26060 P06213 P02452 Q8N2K1 P07900 2033 2274 Q9BV94 P52952 P15531 Q96RU8 Q96RU7 P62993 2026 6622 999 P01116 Q16236 5536 6869 Q15389 P27986 518 51009 P49770 P48201 P35368 Q9BT40 1191 O76074 Q9P2K8 768 P01588 P01344 P01100 O00206 Q9Y6Y9 P48431 5321 5563 P14416 P13569 O95292 P14410 148022 Q00535 Q53X93 6402 Q9GZP9 5798 6647 P01138 P01137 Q9UPY3 P01375 P01374 P02461 4240 P01130 5573 P04626 5575 5576 2065 2064 P12004 P35348 2059 3148 5566 8836 5567 P01127 5568 Q96EY1 6657 5327 O43353 6672 25828 5584 P07948 25822 3162 O00622 552 O75190 P51828 5577 5336 5578 5337 5338 P40763 Q96FA3 P04201 2081 5591 4261 Q6IR47 5111 10580 64127 P68032 P18850 P18846 Q68CJ9 3169 4015 329 Q08462 P01185 6696 2099 Q12929 Q9Y2M5 P51843 Q8NER5 Q9NRM7 P68036 P25103 P01178 23636 5371 6464 O60911 P16885 55432 Q6ZWJ1 P51617 Q08209 P18827 54106 P25116 8892 8654 P30086 595 Q04759 356 Q9UBS5 Q7Z6C1 P22694 Q13480 5139 P25963 P03950 7332 5154 5155 4067 P12429 367 Q70SY1 128 P47985

Q13490 20 6256 6498 9525 P31153 P59768 P10451 Q9UBU3 Q9UDY4 Q9UDY8 P23528 P09471 P61604 31 P00441 P32239 346562 8443 6262 4086 10081 4087 4088 Q14118 Q9H257 Q9UKG1 5170 142 1808 P24864 P23771 Q13033 7124 P14635 Q9UQL6 P10275 P10276 O75460 Q02535 156 2908 10057 Q16555 Q15466 2904 Q16790 Q99966 2902 54 7132 6048 Q86WV8 P35568 166 P11142 Q8NFM4 Q8NFM5 Q9NQB0 Q99958 Q8TEY5 9564 7384 7386 10273 Q86V24 Q99942 Q99941 P01579 P49715 P14625 Q02750 P35548 1843 2931 Q15011 1609 1848 1605 O95835 O15123 Q01094 P42224 P41134 79139 10935 P17275 P46821 P22612 P42229 P15090 2947 Q9BZQ6 26270 P42226 22926 Q96QB1 P10644 P31321 P31323 P67775 P05062 1869 Q9UBL3 7189 P29474 27348 O75881 Q03014 6096 7184 1634 P17252 Q8TAD8 P09874 Q15843 O95622 P10620 P19438 Q9UBN7 Q92667 P54652 P11766 P41181 P30048 B3KY43 P22415 P05091 1650 Q9HC57 Q8WYQ5 P47712 Q13443 Q07820 P30279 Q07817 P20248 Q9BXP5 P17081 P09467 O14492 P10415 Q13217 P12830 29966 Q02363 P18146 57162 Q14790 P01730 P41159 P08107 O15111 P17066 23197 Q9HC29 P21554

regulation of transcription factor activity P23396 6597 6598 2033 Q12824 9181 Q9Y4K3 O60603 Q96RU8 P41134 10935 P63165 Q7Z6C1 8767 P01116 O14788 10014 P25963 2307 Q92974 Q9UBK2 Q9NSA3 P56524 7334 4221 Q13404 P12429 P27361 7335 O75381 2316 Q9NRP7 6878 408 P49407 409 Q96EB6 O00206 7341 Q92838 P11309 7186 Q09472 7189 P35638 7067 7188 Q8N726 Q5TAX3 P32121 23028 7185 148022 Q9Y6Q6 Q9BYM8 Q8IUD2 Q86UE4 Q9UDY8 P01019 P01137 Q93009 P40424 4869 P01375 P06400 5716 5970 Q13546 Q92949 7874 55072 Q9NR96 3551 9093 P51532 5728 Q9NS37 O60674 P60484 Q8IUC6 Q04206 Q92786 Q96EY1 Q9ULZ3 P23497 P43489 O43353 O43593 6672 Q96EP0 O95999 7124 29108 O00463 Q9UQL6 114548 4092 3162 P53567 O43638 Q7RTR2 P30048 Q02535 1896 23085 10616 Q9Y6K9 P04040 55806 7099 5195 7098 Q9NYA1 Q9Y239 Q92769 Q15109 P14923 64127 23411 P37231 1029 3728 58487 P01308 Q8TAI7 3169 1540 8737 5468 Q9NQB0 P42771 847 P49848 P23510 P21333 10392 4792 O14495 P19474 Q02363 27148 3066 P51843 Q96P20 P61088 O60341 O15455 O14920 P05112 Q9GZT9 Q9NQC7 6188 P10827 Q60FE5 O75832 Q12933 Q9BZS1 Q8NHX1 O00255 54583 P51617 Q9HC29 8517 O15105 P05362 54106 P09601 50943 Q13077 5925

regulation of transcription regulator activity P23396 6597 6598 2033 Q12824 9181 Q9Y4K3 O60603 Q96RU8 P41134 10935 P63165 Q7Z6C1 8767 P01116 O14788 10014 P25963 2307 Q92974 Q9UBK2 Q9NSA3 P56524 7334 4221 Q13404 P12429 P27361 7335 O75381 2316 Q9NRP7 6878 408 P49407 409 Q96EB6 O00206 7341 Q92838 P11309 7186 Q09472 7189 P35638 7067 7188 Q8N726 Q5TAX3 P32121 23028 7185 148022 Q9Y6Q6 Q9BYM8 Q8IUD2 Q86UE4 Q9UDY8 P01019 P01137 Q93009 P40424 4869 P01375 P06400 5716 5970 Q13546 Q92949 7874 55072 Q9NR96 3551 9093 P51532 5728 Q9NS37 O60674 P60484 Q8IUC6 Q04206 Q92786 Q96EY1 Q9ULZ3 P23497 P43489 O43353 O43593 6672 Q96EP0 O95999 7124 29108 O00463 Q9UQL6 114548 4092 3162 P53567 O43638 Q7RTR2 P30048 Q02535 1896 23085 10616 Q9Y6K9 P04040 55806 7099 5195 7098 Q9NYA1 Q9Y239 Q92769 Q15109 P14923 64127 23411 P37231 1029 3728 58487 P01308 Q8TAI7 3169 1540 8737 5468 Q9NQB0 P42771 847 P49848 P23510 P21333 10392 4792 O14495 P19474 Q02363 27148 3066 P51843 Q96P20 P61088 O60341 O15455 O14920 P05112 Q9GZT9 Q9NQC7 6188 P10827 Q60FE5 O75832 Q12933 Q9BZS1 Q8NHX1 O00255 54583 P51617 Q9HC29 8517 O15105 P05362 54106 P09601 50943 Q13077 5925

nuclear division 54908 P25054 Q9Y265 23607 Q9Y266 11065 8091 259266 9181 9184 P48729 Q9BVA0 O60566 1213 Q96IK1 Q96BM9 Q9BVA1 Q8N3U4 P43034 Q9ULW0 P21127 10534 Q9HC98 Q92974 O43683 O75935 Q12834 O43684 Q53EZ4 O60216 54801 10783 7329 64282 Q29RF7 P30622 Q13257 7465 Q8WVM7 O75122 Q9NQW6 22974 P51808 Q9Y2Z0 57405 54930 Q8NFH4 P83876 O94927 Q8WZ42 Q93008 Q96EA4 P53675 O95347 P62826 Q8TD19 Q96BK5 Q9NRZ9 54820 Q15691 Q96GD4

O14578 Q8IX90 5604 8452 Q14008 Q14807 23122 23244 P14635 22832 1018 1017 Q96DE5 Q9H6D7 3796 Q13042 O75909 Q96R06 Q5TB80 P07437 Q96CS2 7013 9793 Q96KB5 Q8IZT6 26524 79441 P41208 8218 P30304 O15182 P30305 10270 P54274 Q13618 Q9Y5K6 55142 221150 11243 Q9BRK4 Q9NTI5 Q9NVM9 347733 Q15003 11004 9212 O00139 Q8TAP9 23354 Q02750 Q6KC79 Q9NXR1 10726 54984 984 Q96MT8 Q9NTJ3 989 O14777 Q9UKT4 127829 Q96FF9 Q15019 9221 6993 O95835 8379 P63172 6194 80124 990 991 26271 Q9Y6G9 55165 203068 994 Q99640 55726 Q15021 5536 P62753 Q13885 9232 64682 Q14978 Q9Y6X3 Q8N4N8 Q9UQ88 23332 23212 Q8NHV4 Q7Z460 1069 Q99871 22919 11113 Q9UI95 Q6P1K2 8812 Q9BS18 P42695 Q14980 25847 9126 Q7Z7K6 10907 91272 P52732 890 1877 10910 Q66K89 891 Q99741 55743 O95619 1639 O14965 O43264 Q9UPY8 93323 9133 Q14511 O15392 O43823 23310 O95067 Q8N7B1 79023 Q9H410 9493 80254 Q15050 P53041 Q9HBM1 Q00526 6790 9700 O95997 Q9P258 Q96EP1 10459 4926 3832 Q13561 3835 8607 O43482 Q68CZ6 P51955 P51956 Q9BZD4 Q6PJP8 P20248 Q9UJX2 P51959 Q9UQE7 P50748 324 54443 Q9H1A4 5347 5901 Q96JH7 Q9Y6D9 Q86U86 91754 Q02241 O00762 22897 Q99661 Q9NRM7 Q00610 Q9HC35 5119 Q14674 3070 728642 8881 P53350 Q7Z4H7 27436 79980 P63279 121441 O60232 P30260 P24941 900 55559 Q9UPV0 Q14683

mitosis 54908 P25054 Q9Y265 23607 Q9Y266 11065 8091 259266 9181 9184 P48729 Q9BVA0 O60566 1213 Q96IK1 Q96BM9 Q9BVA1 Q8N3U4 P43034 Q9ULW0 P21127 10534 Q9HC98 Q92974 O43683 O75935 Q12834 O43684 Q53EZ4 O60216 54801 10783 7329 64282 Q29RF7 P30622 Q13257 7465 Q8WVM7 O75122 Q9NQW6 22974 P51808 Q9Y2Z0 57405 54930 Q8NFH4 P83876 O94927 Q8WZ42 Q93008 Q96EA4 P53675 O95347 P62826 Q8TD19 Q96BK5 Q9NRZ9 54820 Q15691 Q96GD4 O14578 Q8IX90 5604 8452 Q14008 Q14807 23122 23244 P14635 22832 1018 1017 Q96DE5 Q9H6D7 3796 Q13042 O75909 Q96R06 Q5TB80 P07437 Q96CS2 7013 9793 Q96KB5 Q8IZT6 26524 79441 P41208 8218 P30304 O15182 P30305 10270 P54274 Q13618 Q9Y5K6 55142 221150 11243 Q9BRK4 Q9NTI5 Q9NVM9 347733 Q15003 11004 9212 O00139 Q8TAP9 23354 Q02750 Q6KC79 Q9NXR1 10726 54984 984 Q96MT8 Q9NTJ3 989 O14777 Q9UKT4 127829 Q96FF9 Q15019 9221 6993 O95835 8379 P63172 6194 80124 990 991 26271 Q9Y6G9 55165 203068 994 Q99640 55726 Q15021 5536 P62753 Q13885 9232 64682 Q14978 Q9Y6X3 Q8N4N8 Q9UQ88 23332 23212 Q8NHV4 Q7Z460 1069 Q99871 22919 11113 Q9UI95 Q6P1K2 8812 Q9BS18 P42695 Q14980 25847 9126 Q7Z7K6 10907 91272 P52732 890 1877 10910 Q66K89 891 Q99741 55743 O95619 1639 O14965 O43264 Q9UPY8 93323 9133 Q14511 O15392 O43823 23310 O95067 Q8N7B1 79023 Q9H410 9493 80254 Q15050 P53041 Q9HBM1 Q00526 6790 9700 O95997 Q9P258 Q96EP1 10459 4926 3832 Q13561 3835 8607 O43482 Q68CZ6 P51955 P51956 Q9BZD4 Q6PJP8 P20248 Q9UJX2 P51959 Q9UQE7 P50748 324 54443 Q9H1A4 5347 5901 Q96JH7 Q9Y6D9 Q86U86 91754 Q02241 O00762 22897 Q99661 Q9NRM7 Q00610 Q9HC35 5119 Q14674 3070 728642 8881 P53350 Q7Z4H7 27436 79980 P63279 121441 O60232 P30260 P24941 900 55559 Q9UPV0 Q14683

protein catabolic process Q96TA2 P48052 11060 23607 O14672 11065 Q86TM6 Q9Y4K3 9184 O75150 O60566 P15374 Q04759 Q70EL4 84447 P63208 P49810 90678 9978 O94905 Q14694 Q92611 3093 P09936 Q9UIQ6 7332 Q9C026 7334 P55036 Q12834 O43684 Q5VTR2 Q504Q3 5706 23291 5707 5708 5709 7329 5700 Q13490 7328 Q16401 5701 4734 5702 Q9UHP3 Q9HAU4 Q9UKV5 Q9BUN8 5704 5705 11160 Q13257 7347 P32121 P56817 79791 P35998 A0AVT1 4193 5717 3416 1358 57646 P62256 10197 Q16531 P61289 6921 Q9Y297 5713 Q93009 Q93008 4627 5716 57092 7353 5970 Q86Y37 O75477 Q99460 9099 Q13829 267 P19838 23032 55236 3309 8453 8454 P51784 8452 64750 7126 P51668 26994 P14635 P51665 8450 P35579 10613 10616 Q13042 Q92530 Q13286 Q13049 Q14139

Q7Z419 7375 Q9Y4X5 11274 6048 P36776 O00231 O00233 23014 O00232 Q96J02 Q9UKA1 5071 7251 Q9NS56 10868 P11021 Q96EF6 10869 841 2475 Q9NRD1 P49721 O15169 P49720 Q8TEY7 4790 8237 O94966 7267 10273 Q13618 Q13617 Q9Y5K6 Q13616 Q9Y5K5 1161 O00487 O95376 Q9UKB1 Q99942 Q8TBB1 Q8WY64 391627 10956 Q9NZ43 P62333 P20618 Q9BZK7 Q92995 11124 Q9UK22 51107 5516 Q9NQC7 Q15008 Q13501 Q9UL46 Q9UMW8 Q15369 Q13620 9695 6500 P31749 P14625 O75674 P54727 P54725 10845 114088 255488 253980 57695 Q93034 5886 Q99816 5887 P40337 Q8WW12 9100 O00308 P53804 25862 Q96K76 27005 Q9NVW2 27248 Q70CQ3 23326 P50897 Q70CQ1 P42345 991 Q96RU2 79139 P62195 997 P62877 10013 51009 P45974 Q9BZQ6 26270 P78536 64682 10939 115426 83737 84708 55294 27252 P46934 51132 Q92560 51377 4214 O43294 408 P60900 P49407 409 10269 P49768 P60468 Q6ZN33 7189 5682 5683 5684 5685 5686 9361 7184 80267 891 Q9Y3C5 Q9BYM8 Q9Y4E8 26133 55743 Q8TEL6 8945 P25789 P25788 Q9GZP9 Q9NZ08 80020 O60260 P25787 Q15843 Q8TAT6 5692 5693 P40818 91147 Q6R6M4 7874 Q6UWE0 P78317 Q9UNE7 1642 O95071 Q04206 9924 5687 5688 Q92542 Q9UBN7 Q969Q1 Q5VVQ6 P62837 O75503 8975 Q969H0 9820 Q9Y2K6 Q9UNN5 Q96EP1 O76031 Q8IWF2 Q96PU4 P00747 26232 Q9UK80 26234 P62714 10213 P55072 Q13200 Q9H3F6 P08246 O43242 Q13443 Q8WZ19 Q8IWV7 Q9HCE7 Q5HYA8 57154 Q96BI3 8065 Q05086 84749 Q9UJX2 Q00987 P61077 1540 Q86T82 207 Q9H1A4 329 P08138 124739 8078 O75604 8754 O75844 Q13216 Q8IUQ4 P28065 Q5W0Q7 377630 O60907 Q9UPT9 Q9Y2M5 23621 O00762 Q9NWW5 51185 Q5XUX0 55666 P61088 P68036 7415 Q14790 8881 O75832 Q13107 P62191 P28074 P17980 P28072 P28070 55432 10208 1203 P63279 O14818 P30260 51752 7428 Q6ZVX7 8878 Q13233 51514 P43686 P49427

regulation of cell cycle P25054 Q12948 P51587 124790 9184 1459 10657 2305 O60566 4609 P84022 Q8N3U4 Q9ULW0 P07996 P52564 Q12959 9077 P16104 P62244 1104 P14174 Q9H0C8 10783 Q9H2G4 Q06124 Q9NQW6 79791 Q7L5Y9 22974 57646 6929 1111 811 Q9Y297 O75807 Q9Y6A5 6926 Q6IT96 4869 Q92831 P06400 5716 P46089 P31947 Q99583 9099 5728 P18509 64061 5604 Q6PCD5 Q9Y468 Q8WXG6 P11802 1022 1021 1020 10733 1017 10614 O75909 O14744 22846 Q96SB3 Q9NYA1 Q92769 Q07666 P19784 10987 1029 Q8TBC4 1027 1026 1387 604 P42771 O14757 P26045 P30304 O95257 P30307 5883 Q13618 Q13617 Q13616 P38398 55145 Q96KQ4 613 Q6NUQ1 Q9UM63 Q13625 P51692 P31749 Q7L8A9 O00255 55159 Q96MT8 Q9UKT4 P04156 P06213 26959 P04637 5300 O00541 3481 23326 25988 11108 990 Q96RU2 3479 995 23560 Q99640 55726 8924 54998 P62753 P01112 Q9UQ80 O96017 Q9UII4 4221 5310 5311 5796 P51636 P57059 115426 641 Q15392 O43290 P01106 6878 8812 Q99638 P01344 P01343 2290 Q6ZN33 P48431 Q8N726 Q9UKN5 O96020 3265 O00444 Q9NS23 890 Q66K89 891 P11274 894 Q99741 Q00535 55743 896 Q00534 3014 8945 51147 P01138 79959 P01137 29086 P01135 P01133 P01375 O43264 4361 8841 O00213 P51532 P60484 545 5566 6776 Q96JB5 6657 5569 10488 Q96MH2 7508 6430 9700 7884 6794 8851 Q969H0 9820 P50613 10459 672 Q96PU4 Q9BZE4 P05412 2081 10460 P51955 Q5HYA8 3054 Q9NYZ3 Q01831 Q9NWV8 Q96SZ6 Q00987 P51959 Q99675 P50748 324 3169 54443 207 5347 P27797 Q15078 Q9BQ15 P24385 6210 3066 Q9UPT9 3065 O00762 O75293 Q9NRM7 P68036 5119 5371 8881 Q92905 P51610 580 23513 P17612 Q9NVV9 P62745 56647 6597 Q14209 11065 11186 P30086 P15923 Q9UPN3 4291 351 472 595 Q04759 116 P83731 5139 Q13243 P49815 Q9HC98 O43683 7332 5036 O43684 5037 O75496 51654 P00533 Q96RL1 P24522 P11309 P35638 Q13257 6498 P12757 8555 Q9UNL4 Q9NY61 4193 P51808 P46527 Q969Z0 Q16531 4088 9656 P18074 O75116 O75592 388 Q9NWT8 Q16543 P43246 8453 8454 8452 11140 O15287 7124 P54132 P14635 23368 P10276 O75460 Q02535 23481 10296 Q13042 Q14493 Q99728 7013 Q14137 O43889 26524 7251 P01308 7249 Q9NRD1 Q9NQB0 P49848 P61925 7023 P54274 A1XKG3 P49959 Q01664 Q99708 P01579 P49711 1718 Q9NVM9 1956 P23443 Q9NQC7 Q08050 7157 7159 Q02750 P38936 Q8TAP6 7272 2810 Q14160 Q93034 Q99816 6198 O95714 O95835 Q9NNW5 8379 Q01094 7161

O43715 6194 26271 Q9Y6G9 Q9Y3A5 P17275 O15355 P42229 Q13761 O15119 P98161 1978 26270 O94992 P78536 7057 9475 9238 Q13526 55294 P55316 1982 Q92560 P08575 Q4LE28 Q9UI95 P05067 P42574 1869 Q15831 9126 Q13535 P29590 Q13418 1877 79035 Q92793 1870 O15379 O60381 O15392 91147 Q13547 A4D1W7 Q6R6M4 Q03001 P78317 27352 80254 1642 Q92786 1647 Q13315 P41182 Q96EP1 51720 P08243 Q13563 150094 Q96HU1 Q9NP71 8065 P30279 P46736 P20248 Q9UJX2 Q9UQE7 P30281 1540 Q13574 P08138 Q9Y6D9 O75604 P10415 377630 P19474 Q02363 Q9BQA5 3611 Q5XUX0 Q14674 Q14432 5914 O15350 Q14676 O75832 P53350 Q9P287 O95661 Q9UHV2 23077 P24941 900 51512 Q14201 51514 Q14683 5925

proteolysis involved in cellular protein catabolic process 11060 23607 O14672 11065 Q86TM6 Q9Y4K3 9184 O75150 O60566 P15374 Q04759 Q70EL4 84447 P63208 P49810 9978 O94905 Q14694 Q92611 3093 P09936 7332 Q9C026 7334 P55036 Q12834 O43684 Q5VTR2 Q504Q3 5706 23291 5707 5708 5709 7329 5700 Q13490 7328 Q16401 5701 4734 5702 Q9UHP3 Q9HAU4 Q9UKV5 Q9BUN8 5704 5705 11160 Q13257 7347 P32121 P56817 79791 P35998 A0AVT1 4193 5717 3416 57646 P62256 10197 Q16531 P61289 6921 Q9Y297 5713 Q93009 Q93008 4627 5716 57092 7353 5970 Q86Y37 O75477 Q99460 9099 Q13829 267 P19838 23032 55236 3309 8453 8454 P51784 8452 64750 7126 P51668 26994 P14635 P51665 8450 P35579 10613 10616 Q13042 Q92530 Q13049 Q14139 Q7Z419 7375 Q9Y4X5 11274 6048 P36776 O00231 O00233 23014 O00232 Q96J02 Q9UKA1 5071 7251 Q9NS56 10868 P11021 10869 841 Q9NRD1 P49721 P49720 Q8TEY7 4790 8237 O94966 7267 10273 Q13618 Q13617 Q9Y5K6 Q13616 Q9Y5K5 1161 O00487 O95376 Q9UKB1 Q99942 Q8TBB1 Q8WY64 391627 10956 P62333 P20618 Q9BZK7 Q92995 11124 Q9UK22 51107 5516 Q9NQC7 Q15008 Q13501 Q9UL46 Q9UMW8 Q15369 Q13620 9695 6500 P14625 O75674 P54727 P54725 10845 114088 255488 253980 57695 Q93034 5886 Q99816 5887 Q8WW12 9100 O00308 P53804 25862 Q96K76 27005 Q9NVW2 27248 Q70CQ3 23326 Q70CQ1 991 Q96RU2 79139 P62195 997 P62877 10013 51009 P45974 Q9BZQ6 26270 P78536 64682 115426 83737 84708 55294 27252 P46934 51132 Q92560 51377 4214 O43294 408 P60900 P49407 409 10269 P49768 P60468 Q6ZN33 7189 5682 5683 5684 5685 5686 9361 7184 80267 891 Q9Y3C5 Q9BYM8 Q9Y4E8 26133 55743 Q8TEL6 8945 P25789 P25788 Q9GZP9 Q9NZ08 80020 O60260 P25787 Q15843 Q8TAT6 5692 5693 P40818 91147 Q6R6M4 7874 P78317 Q9UNE7 1642 O95071 Q04206 9924 5687 5688 Q92542 Q9UBN7 Q969Q1 Q5VVQ6 P62837 8975 Q969H0 9820 Q9Y2K6 Q9UNN5 Q96EP1 O76031 Q8IWF2 Q96PU4 P00747 26232 Q9UK80 26234 P62714 10213 P55072 Q13200 Q9H3F6 O43242 Q13443 Q8WZ19 Q8IWV7 Q9HCE7 Q5HYA8 57154 Q96BI3 8065 Q05086 84749 Q9UJX2 Q00987 P61077 1540 Q86T82 Q9H1A4 329 P08138 124739 8078 O75604 8754 O75844 Q13216 Q8IUQ4 P28065 Q5W0Q7 377630 O60907 Q9UPT9 Q9Y2M5 23621 O00762 51185 Q5XUX0 55666 P61088 P68036 7415 Q14790 8881 O75832 Q13107 P62191 P28074 P17980 P28072 P28070 55432 10208 P63279 O14818 P30260 51752 8878 Q13233 51514 P43686 P49427

macromolecule catabolic process Q96TA2 P48052 23607 O14672 9184 10535 O60566 P15374 Q70EL4 O00273 P63208 90678 4968 Q9H0D6 Q92611 P09936 Q9UIQ6 Q9C026 Q12834 Q5VTR2 5706 5707 5708 5709 5700 5701 4734 5702 Q9UHP3 Q9UKV5 5704 64282 5705 P27694 P56817 79791 A0AVT1 5717 3416 1358 57646 P62256 O60543 P61289 6921 Q9Y297 5713 5836 4627 5716 Q12972 5970 P99999 Q99460 9099 Q13829 3304 Q99575 P19838 55236 948 3309 P51784 Q9UL18 1022 P51668 1020 P51665 10613 10616 P61221 29102 Q92530 5976 Q7Z419 Q9Y4X5 O00231 O00233 O00232 Q96J02 O95243 Q9NS56 1029 10868 Q96EF6 2237 79444 10869 167227 841 2475 P42771 4790 Q13618 Q13617 Q13616 1161 O00487 O95376 Q8TBB1 O60502 391627 10956 P62333 P20618 55149 Q9BZK7 Q92995 5511 Q96CA5 Q9UK22 5516 Q13501 Q9UL46 Q9UMW8 Q13620 6500 P31749 P54727 P54725

Q9HCK5 10724 10845 253980 P40692 57695 192669 5886 5887 P40337 8930 O00308 P53804 25862 Q9NVW2 23326 P50897 991 Q96RU2 54512 997 4686 P62877 10013 51009 Q15024 P23025 64682 115426 P35244 2280 Q96Q15 Q15392 51132 51377 4214 Q9BRP8 O43294 408 P49407 P60228 409 10269 P49768 P60468 Q6ZN33 5682 5683 Q8N726 5684 5685 5686 891 O14727 Q96T60 Q00535 55743 8945 P25789 P25788 Q9GZP9 6647 P01137 P25787 P61326 Q15287 Q8TAT6 P01130 5692 5693 P40818 7874 O95071 9924 668 5687 5688 4116 7508 Q5VVQ6 P62837 P06737 8975 Q969H0 Q9UPR3 9820 P50613 Q9Y2K6 22794 P51946 Q9UNN5 O76031 Q8IWF2 Q96PU4 4125 P62714 10213 P55072 Q9H3F6 O43242 Q9HCE7 Q5HYA8 Q05086 O00754 Q01831 P16671 Q00987 Q86T82 207 Q9H1A4 329 8754 O60907 Q9UPT9 Q9Y2M5 23621 O00762 P13489 3184 Q9NWW5 Q9Y5S9 51185 55666 P68036 7415 8761 8881 Q96C86 P39748 P17980 Q9NVV4 55432 10208 O14818 P58012 7428 8878 Q92900 P49427 11060 P15927 11065 Q86TM6 Q9Y4K3 472 O75150 Q04759 84447 P49810 9978 Q8IU60 O94905 Q14694 P38919 3093 5394 7332 7334 P55036 7336 O43684 P11216 Q504Q3 23291 6117 Q9NPD3 6119 7329 6118 Q13490 7328 Q16401 Q9HAU5 Q9HAU4 Q9BUN8 11160 Q13257 Q14103 7347 P32121 P35998 4193 23381 P22674 10197 Q16531 Q93009 Q93008 P00441 57092 7353 9775 P18074 Q86Y37 O75477 267 23032 28960 P62942 8453 Q14249 8454 8452 64750 7126 26994 P14635 8450 P35579 Q8IZD4 Q13042 Q13286 Q13049 Q14139 7375 Q09161 11274 6048 P36776 23014 26523 23016 Q9UKA1 5071 7251 P11021 Q9NRD1 P49721 O15169 P49720 Q8TEY7 8237 O94966 7267 10273 6059 A1XKG3 Q9Y5K6 Q9Y5K5 6050 Q9UKB1 Q99942 Q8WY64 Q9NZ43 1718 11124 51107 Q9NQC7 Q15008 Q15369 9695 P14625 O75674 Q01780 O75792 114088 255488 Q9H9G7 Q93034 Q99816 Q8WW12 2935 9100 Q15819 P29372 Q96K76 27005 27248 P78549 Q70CQ3 Q70CQ1 P42345 79139 P62195 O15234 P45974 Q9BZQ6 26270 P78536 22803 10939 83737 84708 55294 27252 P46934 27257 Q92560 P42574 P60900 10940 P52298 7189 Q5TAX3 26019 9361 7184 80267 Q9Y3C5 Q9BYM8 Q9Y4E8 26133 Q8TEL6 Q9NZ08 P04066 80020 O60260 Q15843 91147 Q6R6M4 Q6UWE0 P78317 Q9UNE7 10921 1642 1763 Q04206 Q92542 Q9UBN7 Q969Q1 4913 O75503 Q13315 Q96EP1 P00747 26232 Q9UK80 26234 Q13200 P08246 Q8IY92 Q13443 Q8WZ19 Q13569 Q8IWV7 57154 Q96BI3 8065 84749 Q9UJX2 P61077 1540 P08138 124739 2517 8078 O75604 O75844 Q13216 Q8IUQ4 P28065 Q5W0Q7 377630 84305 1676 Q5XUX0 P61088 Q14790 P08107 O75832 Q13107 P62191 P28074 P28072 P28070 1203 P63279 P30260 51752 Q6ZVX7 902 Q13233 51514 P43686

cellular protein catabolic process P48052 11060 23607 O14672 11065 Q86TM6 Q9Y4K3 9184 O75150 O60566 P15374 Q04759 Q70EL4 84447 P63208 P49810 9978 O94905 Q14694 Q92611 3093 P09936 7332 Q9C026 7334 P55036 Q12834 O43684 Q5VTR2 Q504Q3 5706 23291 5707 5708 5709 7329 5700 Q13490 7328 Q16401 5701 4734 5702 Q9UHP3 Q9HAU4 Q9UKV5 Q9BUN8 5704 5705 11160 Q13257 7347 P32121 P56817 79791 P35998 A0AVT1 4193 5717 3416 1358 57646 P62256 10197 Q16531 P61289 6921 Q9Y297 5713 Q93009 Q93008 4627 5716 57092 7353 5970 Q86Y37 O75477 Q99460 9099 Q13829 267 P19838 23032 55236 3309 8453 8454 P51784 8452 64750 7126 P51668 26994 P14635 P51665 8450 P35579 10613 10616 Q13042 Q92530 Q13049 Q14139 Q7Z419 7375 Q9Y4X5 11274 6048 P36776 O00231 O00233 23014 O00232 Q96J02 Q9UKA1 5071 7251 Q9NS56 10868 P11021 10869 841 Q9NRD1 P49721 P49720 Q8TEY7 4790 8237 O94966 7267 10273 Q13618 Q13617 Q9Y5K6 Q13616 Q9Y5K5 1161 O00487 O95376 Q9UKB1 Q99942 Q8TBB1 Q8WY64 391627 10956 P62333 P20618 Q9BZK7 Q92995 11124 Q9UK22 51107 5516 Q9NQC7 Q15008 Q13501 Q9UL46 Q9UMW8 Q15369 Q13620 9695 6500 P14625 O75674 P54727 P54725 10845 114088 255488 253980 57695 Q93034 5886 Q99816 5887 Q8WW12 9100 O00308 P53804 25862 Q96K76 27005 Q9NVW2 27248 Q70CQ3 23326 Q70CQ1 991 Q96RU2 79139 P62195 997 P62877 10013 51009 P45974 Q9BZQ6 26270 P78536 64682 115426 83737 84708 55294 27252 P46934 51132 Q92560 51377 4214 O43294 408

P60900 P49407 409 10269 P49768 P60468 Q6ZN33 7189 5682 5683 5684 5685 5686 9361 7184 80267 891 Q9Y3C5 Q9BYM8 Q9Y4E8 26133 55743 Q8TEL6 8945 P25789 P25788 Q9GZP9 Q9NZ08 80020 O60260 P25787 Q15843 Q8TAT6 5692 5693 P40818 91147 Q6R6M4 7874 P78317 Q9UNE7 1642 O95071 Q04206 9924 5687 5688 Q92542 Q9UBN7 Q969Q1 Q5VVQ6 P62837 8975 Q969H0 9820 Q9Y2K6 Q9UNN5 Q96EP1 O76031 Q8IWF2 Q96PU4 P00747 26232 Q9UK80 26234 P62714 10213 P55072 Q13200 Q9H3F6 O43242 Q13443 Q8WZ19 Q8IWV7 Q9HCE7 Q5HYA8 57154 Q96BI3 8065 Q05086 84749 Q9UJX2 Q00987 P61077 1540 Q86T82 Q9H1A4 329 P08138 124739 8078 O75604 8754 O75844 Q13216 Q8IUQ4 P28065 Q5W0Q7 377630 O60907 Q9UPT9 Q9Y2M5 23621 O00762 51185 Q5XUX0 55666 P61088 P68036 7415 Q14790 8881 O75832 Q13107 P62191 P28074 P17980 P28072 P28070 55432 10208 P63279 O14818 P30260 51752 8878 Q13233 51514 P43686 P49427

macromolecule biosynthetic process 8099 P51587 P52434 P17676 Q9BYG3 2305 55568 2300 Q9H0D6 25913 P30876 P08729 Q8NE71 10667 2 P62244 80755 P62241 27090 Q9Y285 140801 Q96EB6 64282 P62249 P27694 Q9UKV8 P27695 3661 23708 P40429 Q9Y295 Q9Y291 Q9UKW6 3659 Q9UIS9 P08708 P26373 Q99583 Q12857 Q8IYK4 P62266 Q8NFW8 P19838 Q9BYD1 P62263 148789 P62269 10643 Q9Y3U8 Q13951 Q9Y223 4772 1022 Q9NSU2 2590 P50454 O95352 P39019 27043 Q5T160 1017 79577 2589 Q6PI48 P39023 833 5610 O75909 Q9Y231 5859 4780 3692 Q13724 Q9UGI9 Q8WVB6 O95363 Q7LGA3 27297 O14519 10625 Q96MV8 1025 P61353 4775 O14757 Q92766 4790 5883 P28340 2132 Q9NR45 Q8IUH5 Q9NR48 Q9BWE0 55149 1277 Q9Y250 Q92994 5875 Q10472 Q10471 P10827 P10828 22828 Q9NR50 Q96L21 Q9NR55 1050 55152 253980 1289 O60870 865 P04150 Q9Y262 4799 5888 O96004 64432 5422 O00303 11108 871 Q8NBJ5 3479 P25205 84289 Q15025 P62875 6749 Q9Y676 P49411 5430 Q86SR1 5432 P36578 P35244 P35249 Q96T76 Q03933 641 P35251 P62888 P61313 5424 P49643 O43294 408 8939 P27635 P49407 P60228 409 84061 5440 P49642 5441 6772 5442 Q9UKN5 O96020 Q9UKN8 Q9BW92 Q66K89 29088 Q8IYD1 Q99741 Q96T60 P62899 5434 51148 Q14197 51147 Q9Y450 Q15046 Q15287 4361 3035 Q9NUX5 23556 Q96ST3 P15880 P35232 Q00403 29093 Q15054 55757 10488 84296 P62424 79709 7884 10690 P50613 P51946 P39656 23764 25942 Q9Y5Q8 Q9NWU5 Q8WUY8 128308 Q96GC5 Q07020 3054 29079 P37231 688 689 5468 P82675 P62847 29074 P62841 P61513 P49207 4150 23746 4152 P24386 23509 Q9BRT9 5245 Q9HCD5 P52815 Q99543 4149 P62857 P62851 10432 57727 P62854 8761 Q9H4F1 Q92901 P07814 Q92905 P35268 54460 O75031 Q92900 P56192 P49427 4172 4173 4174 Q14686 4176 Q14209 Q9UPN6 Q9H0U6 Q6NZI2 4171 474 Q9H0U3 28973 P46783 P83731 Q9C0C2 3091 P13639 Q16633 6123 8543 P46782 6125 P46781 O75376 P41091 60489 P68104 51773 6117 6119 P00533 6118 P46777 Q15554 P46776 P60866 P46779 P46778 Q16649 6133 P35638 P61968 6132 11041 6134 6137 P32121 Q9UNL4 O43776 O75362 29803 Q01105 6128 60496 P82663 P82664 1915 Q13144 51547 Q14232 Q13148 Q9BYU1 6141 P35869 55907 8565 P18074 P62829 P18077 O75352 P36954 Q9P2E3 6138 Q16665 Q9P2E9 9421 P78347 P54136 6156 6158 P54132 P35453 Q93063 Q04837 Q14494 Q14493 1937 1936 1933 6164 O15294 Q9NRC8 7013 6168 7014 O75575 6160 Q7L2H7 11277 P49848 P61927 6175 25885 Q02878 Q96PK6 P24534 11243 P55010 55703 P47897 9439 1956 6185 6188 6187 Q9HB90 Q86Y79 Q09028 6182 O75792 6184 O43505 Q15370 Q92187 51116 Q99814 1968 Q7Z7F7 Q9NRF9 P78545 P54577 6199 Q6P1J9 P29375 P29374 Q96EL2 6193 6194 1975 27000 P41252 P62195 P41250 Q6P5R6 Q13761 1736 9477 375743 9232 22803 Q9P1Y6 P24928 O60296 Q6P1K2 Q5D0E6 7068 7067 Q15714 Q13535 P55209 P56537 29880 O60264 Q9Y2A9 9255 Q16816 A4D1W7 63931 Q9BWH6 201595 1763 Q04446 P53041 O95997 Q9P015 Q8N6G5 7083 4800 2626 Q9NVC6 Q14653 P19387 P19388 Q9H9A7 83548 2632 1540 57038 P22303 P46977 P46976 9045 P55884 P13807 Q9NP81 Q6P1L8 3980 28998 Q00059 Q96AE4 Q04656 P43694 3978 Q9Y4A8 Q13347 5927 5928 P63272 2651 P24941 Q9HCS7 902 Q9HD40 Q13112 51514 P21675 Q9NZN9 P10914

Q92858 O75821 O75822 65003 P67809 O00268 Q8IXM3 51081 O00267 10535 P16220 Q9ULW3 5931 10533 P0CG13 Q12952 Q9NRX2 Q9Y399 54361 55454 5705 P25398 Q9ULX9 Q9NRY4 1121 Q9BZG8 Q12968 P18621 P18858 P12081 1111 P63220 O60783 Q92830 P03372 Q9Y2Z4 P49591 Q8NCR0 Q6P2C8 9093 P56705 P49590 Q13829 O60318 10767 Q8WTX9 O60551 P49588 O95477 5981 Q12986 5983 1385 Q9UHJ9 57661 6829 P19525 Q6FHQ0 O60762 5733 5976 27161 6827 Q9Y2T7 Q14938 O00472 Q5JTZ9 Q9NS56 Q8WYH8 10987 2237 1388 P08651 Q92643 P05386 P05387 P05388 Q9UNX3 Q9NYB0 P30304 P32969 P30307 4673 Q9H9Y6 P42766 80222 10714 P61244 284119 O14641 P61247 O14640 Q6GMV3 5514 Q92878 5757 P08865 Q9UNY4 Q03164 55035 10725 740 Q9P0M9 P61254 10728 4677 4678 P06454 Q9Y2W1 P02452 O00425 P23396 6872 Q9UPV9 6871 P04637 2034 P53803 990 P62750 995 997 11222 Q16236 5536 Q15389 P62753 P49770 200916 6883 25873 51253 Q9Y6Y0 6878 8812 P01588 Q99638 P01343 P49768 P26641 P26640 P11388 P11387 O60701 51021 51023 P26639 51264 Q53X93 5557 P61201 5558 91949 P01133 P01375 23438 Q9BT22 Q9Y2R5 P02461 P01130 Q9H7H0 6660 P40938 P40937 3396 23435 Q9Y2R9 P12004 Q9NS37 Q96RR1 545 P09086 Q96EY1 P61218 Q5TKA1 7764 Q12904 Q8IZL8 3159 Q02930 Q9BZE1 Q15185 10331 P46063 6201 5111 6202 23650 Q9ULK4 23411 O76021 P83881 328 6210 P50750 P49321 2099 O14802 10318 51067 6204 6207 P62979 P83436 Q9H5H4 8880 A2RTX5 P27540 7311 391356 P39748 P20908 P51610 O00411 Q9NVV4 Q9NRN7 P52948 Q9NPJ6 P62988 54107 6217 9967 Q14566 6231 8892 4297 Q6DKI1 6233 P15927 64951 P62917 Q9UKD1 P13984 4291 Q9NYU1 Q9NYU2 Q9UBS0 P62910 6229 P62913 8662 P20290 P03950 O00716 64960 16 8667 Q16514 8665 19 64963 63875 26205 64965 64969 367 Q9UBT6 51650 51651 6238 11198 54921 6239 Q76FK4 64975 Q03405 P49916 P17096 8669 54938 51426 O75478 9533 7358 P29084 64745 142 Q9UBV7 Q02543 P62701 P09001 9526 P62945 P23771 1801 O15160 8458 7124 O15164 2909 7126 P10275 23127 4094 Q9Y3E5 2908 P23588 84365 Q13287 Q9NSE4 Q14134 Q8WV24 P49736 P04843 P04844 8467 O43889 P15735 P35573 8473 P61803 P54274 O75444 Q96T21 Q96T23 178 Q99941 P49959 P50914 Q00577 51222 9319 Q14152 Q9UBQ5 Q16576 9318 P49716 P49715 11128 Q9NQC7 7153 Q08050 7157 P62906 P14868 7150 2935 P78424 Q13873 Q96QC0 P33992 P62081 P33993 P33991 P18124 1854 P42224 P47914 Q9HC16 Q92696 P82921 1856 Q9UBK2 O15371 P82912 O15372 P82914 Q13405 O95602 Q13887 Q96I24 Q8TCJ2 55173 Q9Y3B7 P84098 P63173 Q08945 P08575 2959 Q9UBL3 O94766 O43812 26015 286826 1877 2961 2960 1870 P04183 P23921 Q9BTC0 2969 P09874 29777 10921 P42677 Q9Y3D3 2972 Q9Y3D5 P09884 P82932 Q969Q0 P82933 Q15853 P33316 Q14527 56052 Q9H488 P62280 P62277 P22415 P30050 Q03468 1650 P43115 O95644 9397 Q8WZ19 58487 P23511 Q9Y5B9 P35711 57169 Q6P4R8 P01730 Q5T4B2 Q15643 O95427 Q9P2Y4 Q15648 27315 Q15653

induction of apoptosis 8772 O15085 Q7Z628 Q13114 O75940 P10911 9500 7204 O43557 Q5VV41 9181 P51587 Q9Y4K3 P17676 351 472 P15498 O15519 355 356 P84022 O00273 9616 P49810 Q92851 4722 P62913 P07996 Q92974 Q13009 P55957 P42858 9641 8795 Q7Z727 Q9NR80 10193 Q9NYF8 Q9NR81 O75489 29 64857 Q9NY61 P46527 57646 Q8IVF5 O60543 23263 Q9UKW4 P62258 Q9H7P9 6262 9774 10081 4088 P18074 P31947 P48023 8445 Q9BZF9 1130 Q12979 P68371 5728 3428 Q99698 O60674 Q8IUC6 Q9ULZ3 Q8N5V2 Q9UHR5 8453 8454 Q12981 8452 Q13158 10293 Q16666 7124 P28482 O00220 29108 23365 O75340 23368 114548 P0C1Z6 Q9P0J0 O75460 121512 Q9Y6K9 O94827 11269 Q8WV24 P07437 7013 P29466 Q9Y239 O43521 Q9H4P4 Q9NZC7 P36896 O43524 Q9Y5J5 P10600 O95243 Q5VST9 P19544 7490 Q9NS56 1029 Q9BWF2 1027 23370 1026 29115 841 843 11035 P42771 Q70UQ0 23229 Q07890 P07766 Q6ZSZ5 Q8TCU6 10392 O75569 O15068 P54274 P50591 1285 Q13618 Q13617 Q13616 P30542 Q9NS68 P38398 7818 Q96KQ4 Q07889 P21145 P61247 Q92630 10276 O14763 5515 51107 5879 Q9UM63 Q14155 5518 Q13501 6188 64782 Q13625 7157 7159 Q9H8V3 P38936 O43508 2810 P40692 Q93034 Q14164 10285 Q93038 P09601 Q15811 7046 Q96RE7 P23396 8930

P04637 Q86Z02 7161 O60603 1613 P22736 P42224 O14827 Q99523 203068 Q96RU2 91 637 Q13761 1616 P23025 10016 P25445 O96017 7057 Q9Y5V3 Q7Z6J4 10383 Q96QB1 Q01085 7052 P67775 O43290 84033 P05067 P42574 O43293 P42575 P98171 P98174 Q9UER7 2956 P49768 7187 Q6ZN33 7189 Q9UMR2 P06239 O94768 6772 Q8N726 Q9H305 Q96M96 P29590 P52735 148022 Q9BXK5 P17252 P21980 Q9Y572 P01138 55504 P01137 3932 P01375 P01374 P55212 A4D1W7 Q13546 Q9UHI6 Q02156 7073 Q8N8D1 7074 O14737 11218 P60484 668 Q92785 Q8WTP8 Q92542 Q15052 P78560 8718 Q13794 11214 8837 8717 5581 Q92934 O95999 Q13315 Q9UBF6 3162 P53567 672 P00747 Q99683 Q03468 P63000 10451 5578 Q96PE2 2081 Q6IR47 P51398 Q13323 8743 Q15628 7410 7531 P30153 Q5JSP0 Q96BI3 P51159 8065 P37231 Q01955 8737 9826 5468 P08138 8738 50649 6453 O75962 Q12802 29843 O60229 572 1676 121457 Q96P20 50650 5599 Q14790 51741 5371 O15350 P53355 Q16611 Q9P0U3 O43464 4168 Q06830 P06729 P52701 O60238 P00519 O60356 P58012 8517 P45983 Q9NZN5 8878 P45984 Q13352

induction of programmed cell death 8772 O15085 Q7Z628 Q13114 O75940 P10911 9500 7204 O43557 Q5VV41 9181 P51587 Q9Y4K3 P17676 351 472 P15498 O15519 355 356 P84022 O00273 9616 P49810 Q92851 4722 P62913 P07996 Q92974 Q13009 P55957 P42858 9641 8795 Q7Z727 Q9NR80 10193 Q9NYF8 Q9NR81 O75489 29 64857 Q9NY61 P46527 57646 Q8IVF5 O60543 23263 Q9UKW4 P62258 Q9H7P9 6262 9774 10081 4088 P18074 P31947 P48023 8445 Q9BZF9 1130 Q12979 P68371 5728 3428 Q99698 O60674 Q8IUC6 Q9ULZ3 Q8N5V2 Q9UHR5 8453 8454 Q12981 8452 Q13158 10293 Q16666 7124 P28482 O00220 29108 23365 O75340 23368 114548 P0C1Z6 Q9P0J0 O75460 121512 Q9Y6K9 O94827 11269 Q8WV24 P07437 7013 P29466 Q9Y239 O43521 Q9H4P4 Q9NZC7 P36896 O43524 Q9Y5J5 P10600 O95243 Q5VST9 P19544 7490 Q9NS56 1029 Q9BWF2 1027 23370 1026 29115 841 843 11035 P42771 Q70UQ0 23229 Q07890 P07766 Q6ZSZ5 Q8TCU6 10392 O75569 O15068 P54274 P50591 1285 Q13618 Q13617 Q13616 P30542 Q9NS68 P38398 7818 Q96KQ4 Q07889 P21145 P61247 Q92630 10276 O14763 5515 51107 5879 Q9UM63 Q14155 5518 Q13501 6188 64782 Q13625 7157 7159 Q9H8V3 P38936 O43508 2810 P40692 Q93034 Q14164 10285 Q93038 P09601 Q15811 7046 Q96RE7 P23396 8930 P04637 Q86Z02 7161 O60603 1613 P22736 P42224 O14827 Q99523 203068 Q96RU2 91 637 Q13761 1616 P23025 10016 P25445 O96017 7057 Q9Y5V3 Q7Z6J4 10383 Q96QB1 Q01085 7052 P67775 O43290 84033 P05067 P42574 O43293 P42575 P98171 P98174 Q9UER7 2956 P49768 7187 Q6ZN33 7189 Q9UMR2 P06239 O94768 6772 Q8N726 Q9H305 Q96M96 P29590 P52735 148022 Q9BXK5 P17252 P21980 Q9Y572 P01138 55504 P01137 3932 P01375 P01374 P55212 A4D1W7 Q13546 Q9UHI6 Q02156 7073 Q8N8D1 7074 O14737 11218 P60484 668 Q92785 Q8WTP8 Q92542 Q15052 P78560 8718 Q13794 11214 8837 8717 5581 Q92934 O95999 Q13315 Q9UBF6 3162 P53567 672 P00747 Q99683 Q03468 P63000 10451 5578 Q96PE2 2081 Q6IR47 P51398 Q13323 8743 Q15628 7410 7531 P30153 Q5JSP0 Q96BI3 P51159 8065 P37231 Q01955 8737 9826 5468 P08138 8738 50649 6453 O75962 Q12802 29843 O60229 572 1676 121457 Q96P20 50650 5599 Q14790 51741 5371 O15350 P53355 Q16611 Q9P0U3 O43464 4168 Q06830 P06729 P52701 O60238 P00519 O60356 P58012 8517 P45983 Q9NZN5 8878 P45984 Q13352

regulation of transcription from RNA polymerase II promoter Q9UKT9 P10914 Q92858 Q9Y265 Q9Y2W7 O75820 2672 Q12948 Q12824 Q9UBB5 O00268 Q6QNY1 O00267 124790 P17676 2547 2304 4609 2303 P16220 Q96JM2 P84022 Q92731 5933 O15516 2308 Q9Y2X9 Q12837 Q8NAP3 1107 O60216 4851 Q9H2G9 4613 4734 Q96EB6 1108 O75925 3660 P05549 Q12968 29128 6927 P13056 83933 6929 4862 Q92830 811 Q9UJU2 O15534 P49116 6921 O75928 P40425 P40424 Q92833 6926 Q6IT96 3659 P26358 P06400 5716 Q96JK9 Q9NZI7 P48382 P07737 Q9H9T3 5970 Q92949 2100 Q9NR96 Q6P2C8

O95343 9099 P17535 10524 6938 P20823 P17542 1487 P19838 P61296 P62263 3665 5604 Q12986 1022 1385 P19532 O00468 Q5JT82 O14627 6829 10736 10614 1499 Q86VE0 P15173 80324 P07550 P61586 Q8N2W9 4646 7913 6827 Q12873 Q9UHK0 Q92769 Q9Y5Z7 6720 5993 Q14938 Q15906 P10600 P19544 O95365 O00358 55250 P19419 1387 P17482 P08651 4775 O14753 604 Q9Y242 5987 Q9UM54 4899 P04179 Q10586 4790 4792 22937 22938 Q6IA86 Q9BQ95 O00488 Q02086 P38398 2247 1398 Q9BZK7 6601 6721 Q92993 857 Q12770 6605 Q8N488 P05112 Q9UM63 Q92754 Q13501 Q9UKS7 P10826 O43186 Q12772 P10827 Q12778 P51692 Q13503 Q05586 2023 Q14839 O00255 Q6KC79 P19883 Q9NQ33 O00257 1050 P53999 Q03164 861 Q99496 Q9UMX1 4799 P09601 P40337 50943 Q9Y2W1 Q92985 O96004 6872 Q9UPV9 P04637 P15408 2034 P13349 2033 2274 P14316 O00422 O60603 25988 P52952 Q96T88 3479 3476 10011 Q15022 79813 4204 639 10014 Q9C0K0 6760 4221 O95163 P16989 P57059 P50548 O76071 P33076 Q99750 Q14186 P48436 4214 P01106 8932 406 P01588 408 7727 P49407 P01343 P01100 O00327 O00206 Q09472 23429 P48552 P48431 9921 P48551 6774 Q99743 Q14192 Q96S42 Q53X93 55869 6886 P01137 5316 P49639 P01375 Q16254 P51531 6660 5451 5573 8841 Q9UHI6 P52747 P35222 2063 P51532 P35227 11218 Q96ST3 P17813 3148 P10070 Q15170 P10071 3146 668 6776 10001 6657 5569 Q96T58 Q96MH2 P23497 7629 P06733 P26583 O00744 5460 6672 Q9Y5Q3 P25490 5585 P50613 P51946 25822 25942 3162 Q8WYA1 Q8NFW5 3281 672 P15692 Q99684 6304 6667 5579 P05771 Q8N5U6 8607 P40763 P05412 5591 4261 Q969G3 8864 Q08117 Q9ULK4 23411 P18850 Q05086 23414 29079 P37231 P14373 51295 P18846 Q00987 P18848 3169 148327 5467 5468 Q92922 7528 10468 P27797 Q9GZU7 7422 Q96GA9 P56177 O60907 3066 3065 P51843 P51608 8625 10432 Q01826 P27540 9612 P52824 23512 O14936 466 P58012 468 Q9NPJ6 P28749 7428 10320 54106 8878 9967 P60568 P36508 Q96HZ4 6595 P35659 Q14686 6597 4297 8535 6599 8896 6598 P15923 P10599 Q86YW9 P13984 6591 Q9Y4K3 351 Q9H161 474 P08047 5017 Q7Z6C1 Q13485 P25963 O94906 Q13363 Q9NPC8 3091 Q16512 4188 Q15306 Q86VP6 O75376 79084 P58304 P13631 3096 367 P46531 Q13133 Q15796 20 Q15672 Q9UQR1 6496 6495 Q15797 6256 6498 Q96IZ0 P12757 P54198 P32242 P31273 P12755 Q9ULH7 23028 Q9NY61 O75360 Q9Y606 4193 116113 Q15561 8548 Q13263 Q15562 Q16650 4086 P35869 4087 8204 4088 4089 Q16656 9412 Q14119 P18074 Q9BY41 P55055 Q9Y618 P10588 P12645 P10589 P36956 387 P08151 P20264 Q16665 Q15697 51317 53615 Q13033 9421 P54259 P55347 5187 7124 11142 P54253 O75586 P35453 O75461 Q9UQL6 P10275 4092 P10276 Q9Y6K1 154 Q02535 51564 Q15583 8328 Q15466 Q99966 P46108 Q13285 2902 7132 5074 6045 7376 8463 Q9HAZ2 8467 O15055 P36896 O43524 23135 P15976 7490 1822 166 338917 Q9NQB0 Q8WW38 Q99958 Q15475 Q99835 Q96T37 Q8TEY5 P61925 7024 7023 5089 P14859 P36402 P15822 7020 51341 Q01664 Q96PK6 Q99708 51588 10155 9439 P49715 P09038 55827 9575 Q15369 O75437 P39905 7157 10284 O43623 Q02750 P11474 26747 55832 1609 9208 Q99814 Q5VTD9 9464 Q01094 P22736 O43719 P41134 Q1PSW9 P17275 91 9219 P42229 O15119 P42226 Q9UBK2 Q9NSA3 Q58WW2 P56524 9477 O94992 22926 Q969S8 22806 22807 Q01085 7291 P10644 7290 7295 Q99081 Q96BD5 P46934 O60296 Q4LE28 P05067 1869 O94763 O75528 7189 7067 Q8WYK2 P19793 7181 Q03014 Q7Z2Q5 7182 Q01196 P41235 Q86UE4 1874 Q92793 Q96GM5 Q9HD15 P09630 O15379 O60264 O15499 Q9NSC2 O15151 Q15843 O95983 Q13422 O15156 O94776 Q13547 A4D1W7 P78317 7073 P78318 2735 P41223 P19438 O43829 84733 Q04206 Q92786 Q9Y2B9 P04198 1406 2737 2736 Q15853 O75626 O14593 Q14527 Q8WUI4 P41182 Q8TAK5 P53567 P22415 Q01167 Q9H9B1 Q14894 4800 O75629 2626 7099 150094 7098 Q9NVC6 Q9NP71 9391 1788 64919 1786 Q9UIV1 P41162 P23511 3720 375790 P17081 Q13573 3607 Q14781 P09429 O75604 P12956 29966 Q02363 P78527 P18146 P35711 Q9BQA5 O60341 P43694 O15455 Q9BQA1 5914 O75953 O75832 O15353 Q9BZS1 Q13227

Q15648 P55771 P19484 Q06710 63976 Q96H20 P00519 P63272 Q9HCS4 O15105 Q92585 902 Q15532 Q13233 Q9NZN8 P21675 5925

ribosome biogenesis 92345 9221 Q92979 O43159 11340 Q12788 P62081 O00541 Q06265 P18124 6194 10813 Q9H6Y2 Q9Y3A5 54512 23560 Q9Y2X3 6229 P83731 Q14690 P62913 Q92574 P62753 65083 1736 Q15024 Q9UQ80 P38919 5393 5394 Q14978 5036 6125 23212 Q9Y2P8 51096 2 51010 Q9Y3B2 51013 Q9NPD3 P46777 Q96EB6 P62249 Q13895 Q8N726 29889 Q9NY61 O00567 P56537 Q8TED0 Q9NV06 Q5TAP6 Q9H9L3 Q8WVM0 4869 P78316 P46087 Q9BY44 Q13823 P08708 9775 P18077 Q96B26 10528 Q15050 P62263 55759 Q9UET6 134430 705 P62424 O95478 50628 Q9NYH9 10171 7248 P39019 23404 54552 23481 Q9BZE4 4809 29102 Q8NI36 8602 P62312 Q99848 Q8NEC7 Q14137 Q9Y2L1 51729 3692 Q2NL82 6201 Q13601 23411 23378 23016 Q86WV8 Q96G21 Q9Y5J1 1029 6839 Q9NY12 11157 P42771 P62847 118460 P62841 6838 P05388 6175 Q8IY81 2091 9045 P22087 O75683 22894 P55769 Q9NVU7 10438 54853 Q5RKV6 P50914 88745 56915 P62857 Q9GZL7 P62851 P56182 O43463 Q9UNQ2 Q13901 Q13868 Q9H6R4 Q01780 Q8TDN6 Q969E8 Q9NQT4 55153 Q9NQT5 Q96EU6 4839 Q9BVJ6 P61254 Q96HR8 23076 10607 Q9Y2W2 Q9NVN8 6217 Q14684

cellular response to stress Q5FWF5 O95677 P25054 P49023 O95551 P51587 3638 54474 2547 P37288 Q8IVH8 1454 1453 4841 5931 P07996 4968 P52564 Q9NS91 P27361 Q8NFZ0 Q8NDV7 P53779 P16104 O60216 P18887 5829 55215 P39210 4734 Q9UKV5 Q96EB6 Q9H2G2 P27694 Q9UGN5 P27695 Q9NRY2 O00167 Q06124 29128 79791 6809 57646 P18858 O60543 1111 O75807 4868 Q12851 P31947 P48023 Q9BZF9 Q676U5 3428 10524 O60674 5602 3309 Q6PCD5 5981 P28482 5983 1022 Q14807 Q9UHJ9 Q9NSU2 P19525 1137 10856 Q6FHQ0 5610 P04040 Q9Y230 5976 4646 Q9NYA1 4780 Q92769 5871 6720 O95243 Q96SB8 Q9NS56 2119 10746 2237 79444 1026 604 847 Q9UM54 O14757 P04179 Q9NYB0 O95257 Q12888 6850 22937 5883 P28340 1161 Q9NS68 P38398 Q56NI9 10714 10956 P61244 1399 2246 Q5TCX8 P05230 Q6NUQ1 Q92993 857 Q92630 Q12770 Q92878 Q96CA5 Q13501 Q13620 Q92626 5892 Q9NR50 O95382 O00255 P54727 Q6KC79 P54725 81788 2139 P40692 57697 55159 O60870 Q96MT8 157570 5886 5887 5888 P09601 O15503 Q9Y6W6 P23396 8930 6872 P04637 Q9Y5U4 Q96A33 5422 2034 2033 25988 Q96RU8 Q96T88 Q96RU2 P62877 8924 6622 7832 10013 Q9C0K7 Q16236 51009 P23025 6749 O96017 4221 5796 P35244 P35249 Q96T76 Q96Q15 Q8WTR2 641 Q99750 Q9P2K8 P35251 4214 5424 4216 Q99638 56946 Q13098 P01100 P49768 Q6ZN33 Q09472 O95292 P11388 Q9NS23 890 Q9NUW8 51141 Q96T60 3014 51147 Q9GZP9 6647 55504 Q8IYD8 Q7Z3C6 Q6UXV0 29086 P23246 Q9UMS4 4361 6421 P40938 P40937 114799 O00213 P12004 Q01974 O95071 3148 545 Q9H093 546 3146 Q15054 Q8WTP8 4358 Q15052 O14733 P23497 7508 2070 P26583 Q5VVQ6 6672 4131 9821 7520 5585 4255 P07948 P50613 P51946 Q9NX61 3162 672 552 Q99683 7518 P55072 7517 P05412 2081 5591 Q9Y4P1 P46063 5111 23411 O76024 Q9NYZ3 P18850 3171 Q01831 Q6PJP8 Q9NWV8 10229 P51959 324 P18848 55775 P82673 8737 328 5347 Q9BQ15 P24385 3066 22897 331 P13010 7415 5599 4149 10432 5371 P27540 4287 P49674 Q9UIG0 Q12931 5378 P39748 Q8NHX1 P52701 580 Q08211 O60911 55432 468 8517 8878 10202 56647 Q92900 Q9UPV0 P55268 Q14686 4176 P15927 11186 Q9Y4K4 Q9Y4K3 472 64949 595 356 P07196 23043 P09493 Q99807 Q7Z6C1 9978 Q14694 3091 7334 Q16512 4067 4188 7336 64963 64965 Q9UKE5 80198 Q9UBT6 60488 Q70SY1 6117 11198 6119 6118 Q96RL1 Q15554 Q9BUN8 P24522 9641 7341 P35638 6256 9643 8555 Q16526 Q9UNL4 Q9NY61 P49916 P22674 Q16531 Q9UBU8 Q13144 51426 Q14232 P00441 Q9Y6R4 Q13268 9656 O43542 P18074 8445 P13611 P36956 142 Q9H492 Q8N4C8 267 10087 Q16665 Q49AN0 P43246 O94817 Q8TDY2 O15287 Q16666 P54252 P54132 8450 25788 O75460 Q00597 P46109 121512 Q9Y6K9 Q99728 51567 Q13286 7014 P46100 6048 P36776 O43524 Q9Y5J5 Q9H078 P11021 Q9NRD1 11277 P49841 Q15233

Q99956 Q99835 7023 P54277 10273 7141 Q99942 P49959 Q9H1Y0 Q01664 Q96PK6 Q99708 9319 P01579 Q16576 7153 6188 64782 26509 7158 9217 7157 P38936 P37840 1965 2932 2810 Q15011 Q14164 P48730 Q99814 Q15014 Q13873 9100 O95714 9588 Q15819 P29372 P33993 Q86Z02 Q7Z589 P78549 7161 O43715 3913 1852 10933 79139 10935 P63165 81570 P46821 1616 26270 P82912 9232 9474 7057 22926 Q86WJ1 O75771 55054 P55318 Q9BSB4 P46934 Q9BYN8 Q9Y6H3 Q08945 Q52LR7 P98170 P42574 Q9UER7 2956 Q969S2 7189 Q9H305 9126 Q13535 P19793 Q96M96 P29590 9361 79035 57003 Q9NVI1 Q86UE8 P09874 P07339 Q13426 Q13546 P34931 P35900 Q9UNE7 80254 P43405 1642 1763 P09884 P05164 1408 1407 Q9UBN7 1647 4913 P82933 Q13315 O95997 1660 P30041 27327 P41182 P41181 P30044 P30048 Q03468 60673 51720 4920 P08243 3835 Q8IY92 Q13569 P11413 27339 P46736 P20248 Q8N0Z6 Q9UQE7 2873 P61077 Q13574 Q9Y5B9 P09429 P12956 P10415 3980 Q13216 P41161 59341 Q9P035 P78527 P32322 Q9BQA5 Q5XUX0 P61088 Q04656 Q9NZM1 P09430 P05198 3978 O15350 Q14676 P53350 Q13227 27315 Q06830 Q9P287 79065 23192 P00519 P43681 900 Q9HCS7 P45983 84893 902 51512 Q13233 Q13112 P45984 51514 P21675 Q14683

regulation of cell communication P25054 2550 9181 P15498 2305 P37288 P84022 1453 Q92731 O14544 O14543 O14788 6901 57506 2308 Q92974 2316 P14174 P42858 4734 10666 Q96EB6 Q7Z727 Q9H2G4 Q9NR80 Q9NR81 Q06124 6927 P37023 P62491 57761 57521 Q9UKW4 811 Q9Y297 O75928 22866 3672 3673 Q92949 2100 Q9NR96 Q13705 P21917 P48380 O95343 P20823 O60674 O00182 P20827 Q8IUC6 Q8N5V2 3667 64061 5604 Q8WXG6 P27037 P25098 Q92538 Q13956 4771 Q96N96 1020 Q9NUY8 P51787 P15056 1499 10616 1012 P61586 3678 26191 O14503 3690 Q9UGI0 P29466 Q9Y239 Q9NZC7 O95361 124583 P10600 1029 10626 841 843 5863 604 Q6ZW31 847 5868 Q15910 Q07890 O95136 4792 P18545 Q8IUH5 Q96N67 P60033 Q07889 Q9Y250 613 Q5TCX8 857 O14763 859 Q96CA5 5879 Q13501 P31749 O95140 O95382 Q9H8V3 P19883 27032 253980 153090 Q9UMX1 P09601 P08754 Q9UL54 Q92743 P01241 P56199 P14317 23322 P50416 3481 O60603 3479 2146 3476 5654 10013 P01236 Q15027 Q7Z6J4 2159 Q99750 84033 8932 Q99759 O43294 408 P49407 409 85360 Q13098 116986 3265 116987 3263 23787 P11274 54413 P53708 P35240 8945 P01019 55504 O14964 64223 6541 5213 P35222 P17813 P60484 11331 Q15052 Q15057 P24588 8717 Q3MII6 5460 Q9Y3M2 4131 6794 Q96BZ9 P05408 Q5R372 79971 673 Q96P48 P61981 P15692 O14908 79735 10451 57708 9815 6789 Q8N5U6 Q92930 O00755 8743 7410 Q9HCE7 23532 Q5JSP0 Q05086 Q99558 5467 8737 9826 207 54206 P48357 10468 P24385 7422 Q96GA9 P30825 Q12802 Q0IIM8 55785 P61764 O14920 O14921 9839 5494 8766 4168 Q13905 Q9Y3P9 23513 57732 P17612 O14939 8516 8517 P49427 8772 O15085 Q16623 7204 Q14449 Q9Y4K4 O75386 Q9Y4K3 O43318 472 Q9H160 51762 23286 P20396 P49810 8767 P78395 P43034 P49815 Q8TBP0 Q15303 3091 4188 Q16635 5037 O75376 Q96D03 Q04864 P09382 Q9H2X0 P46531 7448 9628 P00533 Q13370 Q16643 1902 Q15311 Q13131 9641 8553 8795 9882 Q96IZ0 Q9UQ13 8315 P12757 Q8N6I1 P32121 Q9ULH1 P12755 64857 Q96CN4 4193 Q9Y6Q6 23263 Q9NRI5 Q13145 P55290 Q8IV61 Q9Y6R4 5054 7476 Q9UPQ3 P10586 Q86XR7 Q8N6H7 Q16665 Q13153 55915 Q13158 9420 7248 O75581 Q93062 Q9Y2C9 Q9Y6K9 O94827 1936 P46108 5074 11030 Q9HAZ2 Q9Y4H2 26524 5071 P11021 7249 Q7Z7H5 Q9GZM8 Q96NH3 Q99835 23229 P02790 10392 P49840 O15068 Q7Z7G2 23463 O43734 A1XKG3 O43739 Q14C86 Q8WVQ1 Q9UNH7 P08069 P00367 Q13188 1956 10159 10160 P37840 7272 54862 Q6PID4 P48730 Q9BWT7 P98194 Q14289 9465 7046 P35813 O75787 P42345 Q9Y6G9 Q86VI3 Q9UGU5 P46940 9218 Q92574 P78536 7057 Q13526 7052 P46934 81565 Q9Y6H5 P06276 P16070 O60292 P42338 P98174 Q9UER7 P06239 P29353 Q13535 P56539 Q7Z569 84619 Q9BYM8 60412 P21860 3932 O60260 50618 A4D1W7 Q13546 55072 Q9UNE2 P78318 9495 7074 P43405 P41220 Q07954 Q04206 P53041 9267 7088 Q9NZJ7 Q96EP0 O95999 Q9NP61 Q13315 P78509 Q86TI0 9021 Q16828 93594 P78504 9020 P00749 P63000 Q96PE2 P00742 3953 Q6P1N0 P08246 3956

Q8IWV1 7099 7098 Q13322 O15211 P14923 P30153 3728 P61073 23094 Q9Y6D6 Q9BQB4 Q9Y6D5 Q05513 2873 1540 Q13574 51735 3965 P22303 50649 O75962 Q9P035 57045 O60229 2889 P20339 P20336 Q9Y6E7 Q86TG7 2885 P61088 50650 51741 Q9NZM3 Q5T9L3 O15455 Q16610 Q15762 Q8IYX1 P18031 Q06830 P34972 222068 63976 P43681 Q9NZN5 P00750 P08237 P08238 Q7Z628 P48058 P10911 Q9NRW4 Q5VV41 Q9UBB6 Q9UBB5 Q9NS86 Q99490 9066 O15519 Q6PCE1 P16220 Q92851 P07996 P52564 P04004 P09936 Q9Y2X7 65018 54361 115704 Q9P107 4851 10783 O15524 Q008S8 Q92835 Q99471 10758 27185 Q8IVF5 P20936 O60543 Q9UQB3 P03372 64170 Q9H7P9 5716 Q9GZX9 5970 P48023 1374 3551 Q12979 9093 Q9UBE8 P56705 Q13829 P17302 5728 P17301 2697 3784 6812 5966 3309 P04233 1385 O00220 O00463 1137 P07550 P04040 Q9Y2T1 4646 P08887 P08648 P51671 Q9NYA1 P30559 5991 O00233 P10721 Q5VST9 P37198 29110 10746 79444 960 3326 1147 2475 P42771 Q9UM54 Q92888 P07766 P05106 P28223 6850 P50591 Q13618 Q9P0L0 P30542 Q9NS68 P62330 O60502 2247 1398 Q9BVC4 58533 975 O14640 5997 5515 P05112 5516 Q92633 5518 Q12774 P09958 P29323 5770 Q12778 O00255 Q9P0M2 10724 P05121 P06213 P04637 5300 115557 P52952 O60725 Q96RU8 O14827 Q96RU7 P62993 P13591 997 6622 P01116 5536 6869 Q15389 P01112 P01111 Q04917 O43166 4221 6885 P51636 23216 2280 56940 Q8WTR2 O76070 54764 4214 4215 4216 P01588 P01344 P01343 P49768 O00206 P48431 5562 Q86VW2 Q8N726 9921 P14416 P52735 148022 Q99623 773 Q00535 Q02952 Q53X93 51026 6647 P01138 P01137 P01135 8826 P49758 P01133 P01375 Q9UJM3 Q9Y337 Q9Y2R2 P04626 8841 2065 2064 O60716 P35348 55633 P17936 545 P10071 5566 P01127 Q96EY1 6657 5327 11214 5328 O43150 8837 9927 O43353 5580 23409 5585 O43597 P07948 Q9UJF2 3162 552 553 Q99683 5578 P62714 5579 5338 P05771 Q96FA3 O00635 Q6IR47 7532 7533 64127 23411 5590 Q00987 324 3169 Q9NU19 Q8TEA7 329 P27797 Q76NI1 P01185 6453 4035 Q5VWQ8 2099 Q9UHD2 P51843 Q9BUZ4 Q8NER5 Q9NRM7 10554 P25103 P01178 Q9BT67 23636 P27540 O43581 Q60FE5 Q12933 6464 25818 P51617 Q08209 54106 8878 Q99418 10564 P26447 P25116 P60568 Q8N6T3 9744 P30086 94121 351 595 356 Q9UBS5 Q13485 Q8WZ64 P25963 Q9HC98 O43687 6003 Q13009 7334 5155 Q16512 4067 Q15669 7335 Q9UIA0 367 Q9UBT3 Q13490 Q8IW93 6496 6498 Q9HBW0 29 Q15438 253260 Q8TEU7 Q9UBU3 Q9UDY8 142678 Q8WZ42 P00441 P32239 4088 Q9BY44 4089 Q15208 Q8WWN8 Q14118 163126 125058 Q9H257 382 Q9NZ94 222546 Q04724 387 P21359 389 Q66K14 23154 1808 Q16543 Q13033 P62942 8452 6275 11140 7124 O15164 P54253 7126 Q9Y3E0 23365 O43639 P10275 4092 154 156 121512 25780 Q6KH11 Q16555 Q15466 2904 Q99966 Q13043 Q13286 7132 Q96C24 P36896 Q9Y5J5 P36894 Q86WV8 P35568 23370 Q02763 P01308 7128 Q9NQB0 2917 9306 Q9UBP4 O15169 85440 Q99956 Q6ZSZ5 Q8TCU6 O95813 O43612 Q99943 P01579 10276 Q9NQC3 P09038 Q14155 Q9NQC7 Q9BY84 Q08050 P39905 7157 64786 25776 Q8N9R8 Q02750 23118 2931 Q14160 51231 Q14164 Q14161 Q6R327 1848 1605 Q13077 Q15811 Q13873 O95835 10928 26037 7161 27121 P41134 1852 Q9BXM7 1850 27122 91 O94989 94 P46821 1616 Q6ZT07 Q9NSA1 O15118 Q9NSA3 Q13404 Q96QB1 9590 9350 O43609 P67775 P08575 P05067 1627 7186 Q15831 7189 7188 Q9H422 Q96M96 29767 O75881 Q03014 Q13418 7185 P17252 O15379 P21980 Q9NSC2 9138 Q7Z434 27352 P19438 Q05655 Q8HWS3 Q9UBN7 2737 9146 P13945 Q96DN5 P41182 Q58EX7 P23508 2741 Q03468 Q13202 Q96HU1 Q8WZ19 Q15628 P21580 57154 Q68CZ1 P08134 P08138 P21333 Q9NYJ8 O14492 Q6ZV73 29966 P18146 57162 3611 O60343 Q14790 4943 P01730 P41159 O15350 O75832 O15111 Q13227 Q86Z14 Q04771 Q8N5A5 3622 2773 Q9HC29 O15105 Q13233 P21554

cellular macromolecule biosynthetic process 8099 P51587 P52434 P17676 Q9BYG3 2305 55568 2300 Q9H0D6 25913 P30876 P08729 Q8NE71 10667 2 P62244 80755 P62241 27090 Q9Y285 140801 Q96EB6 64282 P62249 P27694 Q9UKV8 P27695 3661 23708 P40429 Q9Y295 Q9Y291 Q9UKW6 3659 Q9UIS9 P08708 P26373 Q99583 Q12857 Q8IYK4 P62266 Q8NFW8 P19838 Q9BYD1 P62263 148789 P62269

10643 Q9Y3U8 Q13951 Q9Y223 4772 1022 Q9NSU2 2590 O95352 P39019 27043 Q5T160 1017 79577 2589 Q6PI48 P39023 833 5610 O75909 Q9Y231 5859 4780 3692 Q13724 Q9UGI9 Q8WVB6 O95363 Q7LGA3 27297 O14519 10625 Q96MV8 1025 P61353 4775 O14757 Q92766 4790 5883 P28340 2132 Q9NR45 Q8IUH5 Q9NR48 Q9BWE0 55149 Q9Y250 Q92994 5875 Q10472 Q10471 P10827 P10828 22828 Q9NR50 Q96L21 Q9NR55 1050 55152 253980 O60870 865 P04150 Q9Y262 4799 5888 O96004 64432 5422 O00303 11108 Q8NBJ5 3479 P25205 84289 Q15025 P62875 6749 Q9Y676 P49411 5430 Q86SR1 5432 P36578 P35244 P35249 Q96T76 Q03933 641 P35251 P62888 P61313 5424 P49643 O43294 408 8939 P27635 P49407 P60228 409 84061 5440 P49642 5441 6772 5442 Q9UKN5 O96020 Q9UKN8 Q9BW92 Q66K89 29088 Q8IYD1 Q99741 Q96T60 P62899 5434 51148 Q14197 51147 Q9Y450 Q15046 Q15287 4361 3035 Q9NUX5 23556 Q96ST3 P15880 P35232 Q00403 29093 Q15054 55757 10488 84296 P62424 79709 7884 10690 P50613 P51946 P39656 23764 25942 Q9Y5Q8 Q9NWU5 Q8WUY8 128308 Q96GC5 Q07020 3054 29079 688 689 P82675 P62847 29074 P62841 P61513 P49207 4150 23746 4152 P24386 23509 Q9BRT9 5245 Q9HCD5 P52815 Q99543 4149 P62857 P62851 10432 57727 P62854 8761 Q9H4F1 Q92901 P07814 Q92905 P35268 54460 O75031 Q92900 P56192 P49427 4172 4173 4174 Q14686 4176 Q14209 Q9UPN6 Q9H0U6 Q6NZI2 4171 474 Q9H0U3 28973 P46783 P83731 Q9C0C2 3091 P13639 Q16633 6123 8543 P46782 6125 P46781 O75376 P41091 60489 P68104 51773 6117 6119 P00533 6118 P46777 Q15554 P46776 P60866 P46779 P46778 Q16649 6133 P35638 P61968 6132 11041 6134 6137 P32121 Q9UNL4 O43776 O75362 29803 Q01105 6128 P82663 P82664 1915 Q13144 51547 Q14232 Q13148 Q9BYU1 6141 P35869 55907 8565 P18074 P62829 P18077 O75352 P36954 Q9P2E3 6138 Q16665 Q9P2E9 9421 P78347 P54136 6156 6158 P54132 P35453 Q93063 Q04837 Q14494 Q14493 1937 1936 1933 6164 O15294 Q9NRC8 7013 6168 7014 O75575 6160 Q7L2H7 11277 P49848 P61927 6175 25885 Q02878 Q96PK6 P24534 11243 P55010 55703 P47897 9439 1956 6185 6188 6187 Q9HB90 Q86Y79 Q09028 6182 O75792 6184 O43505 Q15370 Q92187 51116 Q99814 1968 Q7Z7F7 Q9NRF9 P78545 P54577 6199 Q6P1J9 P29375 P29374 Q96EL2 6193 6194 1975 27000 P41252 P62195 P41250 Q6P5R6 Q13761 1736 9477 375743 9232 22803 Q9P1Y6 P24928 O60296 Q6P1K2 Q5D0E6 7068 7067 Q15714 Q13535 P55209 P56537 29880 O60264 Q9Y2A9 9255 Q16816 A4D1W7 63931 Q9BWH6 201595 1763 Q04446 P53041 O95997 Q9P015 Q8N6G5 7083 4800 2626 Q9NVC6 Q14653 P19387 P19388 Q9H9A7 83548 2632 1540 57038 P22303 P46977 P46976 9045 P55884 P13807 Q9NP81 Q6P1L8 3980 28998 Q00059 Q96AE4 Q04656 P43694 3978 Q9Y4A8 Q13347 5927 5928 P63272 P24941 Q9HCS7 902 Q9HD40 Q13112 51514 P21675 Q9NZN9 P10914 Q92858 O75821 O75822 65003 P67809 O00268 Q8IXM3 51081 O00267 10535 P16220 Q9ULW3 5931 10533 P0CG13 Q12952 Q9NRX2 Q9Y399 54361 55454 5705 P25398 Q9ULX9 Q9NRY4 1121 Q9BZG8 Q12968 P18621 P18858 P12081 1111 P63220 O60783 Q92830 P03372 Q9Y2Z4 P49591 Q8NCR0 Q6P2C8 9093 P56705 P49590 Q13829 O60318 10767 Q8WTX9 O60551 P49588 O95477 5981 Q12986 5983 1385 Q9UHJ9 57661 6829 P19525 Q6FHQ0 O60762 5733 5976 27161 6827 Q9Y2T7 Q14938 O00472 Q5JTZ9 Q9NS56 Q8WYH8 10987 2237 1388 P08651 Q92643 P05386 P05387 P05388 Q9UNX3 Q9NYB0 P30304 P32969 P30307 4673 Q9H9Y6 P42766 80222 10714 P61244 284119 O14641 P61247 O14640 Q6GMV3 5514 Q92878 5757 P08865 Q9UNY4 Q03164 55035 10725 740 Q9P0M9 P61254 10728 4677 4678 P06454 Q9Y2W1 O00425 P23396 6872 Q9UPV9 6871 P04637 2034 P53803 990 P62750 995 997 11222 Q16236 5536 P62753 P49770 200916 6883 25873 51253 Q9Y6Y0 6878 8812 P01588 Q99638 P01343 P49768 P26641 P26640 P11388 P11387 51021 51023 P26639 51264 Q53X93 5557 P61201 5558 91949 P01133 23438 Q9BT22 Q9Y2R5 P01130 Q9H7H0 6660 P40938 P40937 3396 23435 Q9Y2R9 P12004 Q9NS37 Q96RR1 545 P09086 Q96EY1 P61218 Q5TKA1 7764 Q12904 Q8IZL8 3159 Q02930 Q9BZE1 Q15185 10331 P46063 6201 5111 6202 23650 Q9ULK4 23411 O76021 P83881 328 6210 P50750 P49321 2099 O14802 10318 51067 6204 6207

P62979 P83436 Q9H5H4 8880 A2RTX5 P27540 7311 391356 P39748 P51610 O00411 Q9NVV4 P52948 Q9NPJ6 P62988 54107 6217 9967 Q14566 6231 8892 4297 Q6DKI1 6233 P15927 64951 P62917 Q9UKD1 P13984 4291 Q9NYU1 Q9NYU2 Q9UBS0 P62910 6229 P62913 8662 P20290 P03950 O00716 64960 16 8667 Q16514 8665 19 64963 63875 26205 64965 64969 367 Q9UBT6 51650 51651 6238 11198 54921 6239 Q76FK4 64975 Q03405 P49916 P17096 8669 54938 51426 O75478 9533 P29084 64745 142 Q02543 P62701 P09001 9526 P62945 P23771 1801 O15160 8458 O15164 2909 7126 P10275 23127 4094 Q9Y3E5 2908 P23588 84365 Q13287 Q9NSE4 Q14134 Q8WV24 P49736 P04843 P04844 8467 O43889 P15735 P35573 8473 P61803 P54274 O75444 Q96T21 Q96T23 178 Q99941 P49959 P50914 Q00577 51222 9319 Q14152 Q9UBQ5 Q16576 9318 P49716 P49715 11128 Q9NQC7 7153 Q08050 7157 P62906 P14868 7150 2935 P78424 Q13873 Q96QC0 P33992 P62081 P33993 P33991 P18124 1854 P42224 P47914 Q9HC16 Q92696 P82921 1856 Q9UBK2 O15371 P82912 O15372 P82914 Q13405 O95602 Q13887 Q96I24 Q8TCJ2 55173 Q9Y3B7 P84098 P63173 Q08945 2959 Q9UBL3 O43812 26015 286826 1877 2961 2960 1870 P04183 P23921 Q9BTC0 2969 P09874 29777 10921 P42677 Q9Y3D3 2972 Q9Y3D5 P09884 P82932 Q969Q0 P82933 Q15853 P33316 Q14527 56052 Q9H488 P62280 P62277 P22415 P30050 Q03468 1650 P43115 O95644 9397 Q8WZ19 58487 P23511 Q9Y5B9 P35711 57169 Q6P4R8 P01730 Q5T4B2 Q15643 O95427 Q9P2Y4 Q15648 27315 Q15653

regulation of cellular component organization P25054 23607 9181 P61020 Q9BVA0 Q8IYN9 Q7L0Q8 4609 P84022 Q9HCJ2 90678 O14786 Q9ULW0 P07996 Q92974 P27487 P04004 25913 P52565 Q12834 O15530 Q9UBC3 65018 Q08431 1104 P61158 10783 Q7Z727 Q13813 Q9NRY4 22974 Q05397 P53667 4628 P62495 P37023 27185 P20936 81873 811 P35080 Q92833 P26358 4627 4869 5716 3791 3673 Q7L576 57534 79784 5728 P16333 P17301 3304 2335 P20827 6812 Q9NZJ4 P41743 829 Q9UL15 Q9Y468 4771 O00468 10733 2107 6709 57669 1499 57662 830 1012 832 P61586 5976 O14744 3679 P51671 Q9NYA1 Q92769 P30559 P10600 P10721 Q9UEE9 Q9HCM4 2475 6711 6710 P62328 967 3689 5867 6714 Q92888 5747 5869 Q12884 Q9NYB0 P05107 Q9H2K2 P30307 Q9BPX5 Q7Z406 P62330 O60502 P38398 57689 Q9BVC4 Q9Y250 857 859 5878 5879 O43182 Q9UKS6 P10827 P29323 P31749 O00255 Q6KC79 O00499 P48061 Q03164 Q9HCK4 10724 1289 867 P05121 P63313 Q8N3V7 50943 Q9UL54 26060 P06213 4690 6872 P04637 O43157 5300 2033 O95271 3481 P50897 23327 P52952 991 3479 995 Q99640 998 8924 6622 10013 54998 5898 6869 Q15389 11346 P27986 P02686 Q04917 O43166 P06702 4221 Q7Z6J4 P51636 23332 1072 1191 2039 4214 P01106 O43294 408 Q15036 P49407 P01344 P01343 P49768 Q09472 Q9BV73 5321 Q7Z7K6 O00444 891 773 P35240 6405 P01138 79959 P01137 8829 P01135 P01133 P01375 Q08495 4240 64223 Q9Y696 Q658W2 P35222 Q9NUX5 P11277 Q9NRR8 301 302 P60484 3146 P24588 5460 4131 9700 5584 4133 9820 P20916 Q9UNN5 Q9UHB6 10459 672 310 Q96P48 P15692 Q96PU5 Q9H2D6 5578 5338 P26232 O00755 Q6IR47 10580 7533 P51955 64127 Q5HYA8 Q5JSP0 3054 5590 10109 P18846 324 4137 207 P27797 8754 6696 7422 5364 4155 3066 Q9NU22 P15311 55785 P61764 10552 10672 P25103 P01178 Q96HC4 5371 8881 7430 P20908 P51610 O14939 466 56882 Q9H4E5 55558 Q92900 10092 8650 10095 4297 10094 P30086 O75385 472 Q04759 P07196 P09493 118 9617 Q7Z6C1 P49815 Q13485 8408 P47755 Q9HC98 P47756 O43683 11190 5154 Q13009 7334 8301 5037 Q86VP6 Q9Y2H1 P09382 7448 Q13492 5029 Q16643 Q15554 Q13133 Q15796 Q14344 Q9BYV2 7341 Q15797 6498 P22681 P12757 253260 O75122 P10451 Q9UBU3 P51805 P23528 Q01105 Q96RK4 Q9NRI5 Q13145 P55290 Q9BSI4 5295 4086 5054 P35869 4087 4088 4089 7476 Q9Y6R0 Q8WWN8 O75116 P55055 P10586 Q9UKG1 Q86YT6 382 5170 Q02790 O75592 387 Q9NWT8 P52907 Q6PKD3 Q15691 Q8IX90 Q13275 9784 4099 7124 7248 2909 Q9UNF0 23122 P14635 O43639 4092 P35579 274 396 P35580 121512 Q13042 Q15223 Q99966 7013 7376 7014 Q86WV8 5071 O75570 23370 P01308 7249 P49841 Q9GZM8 P02751 Q6ZSZ5 10273 P54274 Q9Y5K6

221150 P02549 55704 P49711 11124 Q9NQC3 Q9NPA3 P60953 Q9NQC7 P20073 Q9BX66 7157 11252 P37840 Q8TAP6 P11234 P11233 7272 2932 55832 51474 P27816 Q6R327 7046 O95714 O95835 P10636 26037 P12931 Q4VCS5 P42345 Q9Y6G9 Q9Y3A5 Q9BXM7 Q86WK6 P63165 P04083 P04085 94 Q92574 P46821 P07355 1978 Q9UBK2 Q53QZ3 7057 9475 Q13526 Q96QB1 Q01082 Q3V6T2 Q7Z460 81565 Q9Y6H5 22919 Q9UI95 1627 P98174 O15020 7067 O15143 Q96M96 P29590 Q13418 P56539 P31431 P41231 P17252 P06241 O60260 Q86UE8 91147 9138 Q6UWE0 P78317 9253 9495 7074 Q9UNE7 O43707 Q92786 Q9UBN7 Q13315 Q15735 Q03113 P51148 P35612 P35611 P63000 Q13683 3956 P47712 P35968 Q13443 58480 1789 P62166 1786 Q9UJX2 Q05513 P23515 1540 57159 3720 375790 P17081 P08138 P09429 Q9BY11 Q6ZV73 23189 Q9H223 O60229 P20338 P20336 3611 P61088 Q14674 Q14678 P08107 O75832 Q9BZS1 Q9HC29 23077 O15105 Q13233 P21554 P21675 Q9BZR6

organelle fission 54908 P25054 Q9Y265 23607 Q9Y266 11065 8091 259266 9181 9184 P48729 Q9BVA0 O60566 1213 Q96IK1 Q96BM9 Q9BVA1 Q8N3U4 P43034 Q9ULW0 P21127 10534 Q9HC98 Q92974 O43683 O75935 Q12834 O43684 Q53EZ4 O60216 54801 10783 7329 64282 Q29RF7 P30622 Q13257 7465 Q8WVM7 O75122 Q9NQW6 22974 P51808 Q9Y2Z0 57405 54930 Q8NFH4 P83876 O94927 Q8WZ42 Q93008 Q96EA4 P53675 O95347 P62826 Q8TD19 Q96BK5 Q9NRZ9 54820 Q15691 Q96GD4 O14578 Q8IX90 5604 8452 Q14008 Q14807 23122 23244 P14635 22832 1018 1017 Q96DE5 Q9H6D7 3796 Q13042 O75909 10059 Q96R06 Q5TB80 P07437 Q96CS2 7013 9793 Q96KB5 Q8IZT6 26524 79441 P41208 8218 P30304 O15182 P30305 10270 P54274 Q13618 Q9Y5K6 55142 221150 11243 Q9BRK4 Q9NTI5 Q9NVM9 347733 Q15003 11004 9212 O00139 Q8TAP9 23354 Q02750 Q6KC79 Q9NXR1 10726 54984 984 Q96MT8 Q9NTJ3 989 O14777 Q9UKT4 127829 Q96FF9 Q15019 9221 6993 O95835 O00429 8379 P63172 6194 80124 990 991 26271 Q9Y6G9 55165 203068 994 Q99640 55726 Q15021 5536 P62753 Q13885 9232 64682 Q14978 Q9Y6X3 Q8N4N8 Q9UQ88 23332 23212 Q8NHV4 Q7Z460 1069 Q99871 22919 11113 Q9UI95 Q6P1K2 8812 Q9BS18 P42695 Q14980 25847 9126 Q7Z7K6 10907 91272 P52732 890 1877 10910 Q66K89 891 Q99741 55743 O95619 1639 O14965 O43264 Q9UPY8 93323 9133 Q14511 O15392 O43823 23310 O95067 Q8N7B1 79023 Q9H410 9493 Q9Y3D6 80254 Q15050 P53041 Q9HBM1 Q00526 6790 9700 O95997 Q9P258 Q96EP1 10459 4926 3832 Q13561 3835 8607 O43482 Q68CZ6 P51955 P51956 Q9BZD4 Q6PJP8 P20248 Q9UJX2 P51959 Q9UQE7 P50748 324 54443 Q9H1A4 5347 5901 Q96JH7 Q9Y6D9 Q86U86 91754 Q02241 O00762 22897 Q99661 Q9NRM7 Q00610 Q9HC35 5119 Q14674 3070 728642 8881 P53350 Q7Z4H7 27436 79980 P63279 121441 O60232 P30260 P24941 900 55559 Q9UPV0 Q14683

negative regulation of molecular function P25054 11060 Q9BY76 11065 2672 2550 Q9NRW4 124790 9184 O60566 Q9UBS5 Q96JM2 57620 Q9NPC1 5018 P49815 6901 P07996 P25963 2307 P04004 P09936 25913 P55036 Q12834 4188 O15530 Q16635 O43684 Q86VP6 O95684 P50148 Q9P107 5706 O75381 2316 5707 5708 5709 51773 7448 51654 5700 Q16401 5701 5702 10542 Q96EB6 5704 P24522 5705 7341 808 P11309 Q7Z727 P35638 Q13257 P32121 23028 P35998 Q9C010 P53667 5717 P46527 57761 10197 P61289 5713 P40424 P06400 5716 5054 Q15208 P31947 Q92949 O43306 Q9NR96 9093 P21917 Q99460 5170 P21359 O60674 P19838 10641 3309 P43489 P62942 Q9UL15 4771 11142 P51668 P51665 114548 4092 O43638 154 Q02535 10614 57669 10616 P07550 P04040 Q13042 Q92530 55806 P45379 Q13286 5195 Q9Y2T7 7013 Q92769 O00231 Q9Y4H2 O00233 26524 O00232 P37198 1029 166 P11021 1027 1026 P63096 Q8NFM4 P01308 Q8NFM5 Q9HCM4 7249 Q9NQB0 2917 P49721 P42771 P49841 P49720 847 Q13177 Q99956 Q99835 P61925 4790 O95257 7023 Q9UGJ0 4792 P49840 P54274 O00487 P30542 O60503 Q96T23 Q9UNH7 P62333 P20618 Q01664 58533 O14640 1718 857 859 5515 5518 Q9NQC7 Q15008 Q9BY84 P51693 Q9UL46 6188 P10827 7157 P19086 P31749 O43504 O00255

P38936 P37840 3350 2932 2810 2931 P17342 55832 Q99497 Q15011 5524 1728 P05121 1848 P09601 Q15257 50943 P08754 Q9UKT4 P06213 P23396 O95835 O00308 P78540 P04637 9467 P38405 O95271 7161 P22736 991 26271 Q96RU8 P41134 Q96RU7 1852 Q99523 1850 10935 P62195 23560 P63165 3476 6622 10013 P15090 Q9NSA3 P56524 O94992 7057 64682 4221 5311 2280 56940 Q8WTR2 O43609 P67775 Q15392 Q99750 P08575 P42574 408 P60900 P49407 409 Q13098 11116 P49768 7067 5682 P29474 5683 Q8N726 5684 Q5TAX3 5685 5686 P14416 Q9H422 Q13418 P56539 84619 P41597 P40145 Q9BYM8 Q99500 P17252 P35240 O60266 P25789 P25788 P25787 Q9UJM3 O95622 5692 O15392 5693 Q9NUX5 P78318 P17813 Q05655 Q92786 5687 5688 Q96EY1 5569 Q9Y2B9 Q96MH2 P23497 Q9UBN7 1647 5580 O43593 6672 P01040 O43597 Q16828 3162 P53567 P30047 P30048 P61981 Q9BZE4 O75190 Q8WTW4 Q99684 P51828 5578 3953 10213 Q13563 P62158 Q13200 P05412 Q8IWV1 O43242 Q13202 7532 Q14416 64127 Q08117 P30153 23411 5590 P37231 P62166 Q9UJX2 Q96SZ6 Q05513 2873 324 1540 Q15070 207 Q9H1A4 5468 54206 P48357 11315 P23510 P21333 Q08462 P28065 P19474 Q02363 3066 O00762 P51843 O75293 2644 333 3611 Q96P20 Q9NRM7 Q04656 O60341 Q16610 Q9GZT9 Q9BT67 23636 O15350 8881 Q60FE5 O75832 Q9BZS1 Q13227 P34972 P62191 P28074 P15559 P17980 P08908 P28072 P28070 57732 54583 Q8N5A5 O14818 P51617 2773 Q9HC29 P30260 O15105 54106 P43686 P21554 5925

microtubule cytoskeleton organization 54908 Q14204 6993 Q8NEY1 O75665 P10636 11065 P63172 O95271 Q6QNY1 P51587 9184 7283 26271 Q8IYN9 O60566 Q96M29 203068 55722 1058 P07196 Q66GS9 85378 9738 P43034 P46821 Q9UBK9 11190 Q6VN20 6760 Q8N4N8 Q96CW5 O43684 23332 O75376 O95166 O95684 Q9UJC3 10426 3925 Q96RT7 1104 Q7Z460 1069 Q99871 81565 Q15154 54801 Q9BSJ2 Q96S59 Q96RT8 P24522 11116 Q9BV73 9126 O95613 Q05397 Q9BXC9 P52732 27185 57405 54930 Q9H4B7 Q6UVJ0 Q96RK4 O15379 Q9NRI5 P60510 Q9Y6A5 Q9HC77 O94927 Q8N960 O14965 4627 4869 Q96EA4 O43264 93323 P62826 O43663 8841 Q14118 Q658W2 O43303 P35222 9493 80254 54820 P23258 Q9UH99 Q9HBM1 1647 6790 4131 9662 9700 4133 Q14008 P35579 1778 57787 114791 1499 57662 Q9H6D7 55125 3796 3832 Q13561 Q96R06 P07437 P49450 Q96CS2 9793 10460 Q68CZ6 P51955 163786 79441 5590 Q05513 Q9UQE7 116840 4137 P41208 4139 Q9GZM8 5108 85440 5747 5901 Q8N137 O15182 Q9UGJ1 Q02241 Q96L34 Q86VS8 55142 O00762 Q9P0L2 P12036 Q96N67 Q99661 5116 Q14674 11004 Q8WW24 O00139 Q7Z4H7 23354 25777 8481 7272 10844 Q9NXR1 Q9P209 P40692 Q9NZ56 Q96MT8 57610 55835 55559 84376 10048 O14777 Q15532 Q9UKT4 1605 Q2M2Z5 Q14683

regulation of catalytic activity P25054 P54619 22870 2550 124790 9184 P15498 P37288 P84022 6901 25913 Q12834 2316 P10809 Q96EB6 Q5S007 Q7Z727 Q9H2G4 Q06124 Q9C010 57761 Q9UKW4 Q9Y297 P26374 P31946 P07858 3672 3673 P31947 Q92949 P99999 Q9NR96 Q13705 P21917 10645 O60674 P20827 P19838 P18509 3667 64061 10641 5604 Q9UL15 Q8WXG6 P27037 Q9H2M9 Q13956 4771 1022 1020 114548 Q9NUY8 10614 Q08999 O75909 Q92530 Q9Y478 3690 P30679 Q9Y239 P10600 1029 Q9BUB1 1027 1026 604 Q6ZW31 7804 O14757 P04179 4790 O95136 Q9UGJ0 1285 Q9BQ95 P16118 P18545 Q96N67 P20618 P60033 Q5TCX8 857 O14763 859 Q96CA5 Q9UL46 P51575 6500 P31749 O95382 P08754 Q9UL54 Q9UKT4 P01241 P56199 Q99523 2147 23560 3476 10013 P62873 11345 10016 Q15027 64682 Q9UII4 Q7Z6J4 641 Q99750 648 85360 Q13098 Q01518 11116 5682 5683 6772 5684 5685 5686 116986 O96020 116987 23787 891 Q99500 O14727 894 Q99741 896 P35240 8945 P01019 4361 5692 5693 Q9NUX5 P35226 55750 P17813 P60484 3269 668 5687 5688 10486 5207 Q96MH2 10487 Q15057 Q3MII6 P26583 Q92934 P01040 Q96BZ9 P50613 Q5R372 Q96P48 P61981 79735 10451 9815 10213 P55072 10454 O43242 7410 Q5JSP0 P37231 Q01955 Q96SZ6 P51959 Q15070 Q15077 8737

207 5468 54206 P48357 Q15078 P24385 P24386 8754 O00762 O75293 Q96P20 Q0IIM8 55785 7415 10672 9839 P52824 P08908 Q9Y3P9 23513 57732 P17612 P28749 8517 56647 11065 Q9Y4K4 Q9Y4K3 3084 Q9UPN7 O43318 P13861 23043 Q9NPC1 P49810 5018 P43034 P49815 Q8TBP0 3091 P55036 4188 Q16635 Q86VP6 7448 5029 P00533 Q16401 Q13133 1902 Q15311 Q13131 P24522 8795 9882 8555 Q9ULH1 Q96CN4 Q9Y6Q6 P46527 10197 Q8IV61 Q9Y6R4 5054 Q9UPQ3 P55055 O43306 Q8N6H7 Q16665 P43246 Q13153 P43489 7248 P54132 P63092 O75340 Q04837 Q9Y2C9 Q9Y6K9 7013 Q9Y4H2 26524 P11021 P63096 7249 Q9GZM8 Q13177 Q9NRD5 Q96NH3 P61925 7023 10392 P49840 P11229 A1XKG3 P48507 Q9UNH7 55704 1718 51107 1956 6188 O43504 O75553 P38936 P37840 Q9UNI6 2810 Q6PID4 1728 Q9BWT7 Q14289 9463 7046 P98155 P78540 9467 P22736 P42345 P62195 Q92574 P78536 7057 Q13526 55291 Q02297 Q3V6T2 7052 81565 P42574 P42338 P98174 Q9UER7 P06239 P29353 O95977 P29590 P56539 P31431 84619 P40145 P41231 O60266 3932 Q13546 P78318 P43405 Q07954 Q92786 Q92542 Q9Y2B9 Q13794 Q9NZJ7 Q9NP61 P78509 Q86TI0 Q16828 93594 3953 P08246 Q8IWV1 7099 7098 Q14416 P30153 P61073 Q05513 2873 P00734 Q13574 51735 Q15750 P46734 P28065 Q9P035 29843 2644 2643 P61088 Q04656 O15455 Q16610 Q8IYX1 P34972 Q9P287 P62191 P28074 P28072 P28070 P00519 900 P43686 P07148 5925 2672 Q9NRW4 Q99490 O60566 2665 2664 P63208 Q9ULW0 5933 P07996 P52564 P04004 P09936 Q9Y2X7 O15530 O95684 9077 115704 P50148 Q9P107 5706 5707 5708 5709 P16473 51099 P16471 5700 5701 5702 10542 5704 5705 P50395 P29992 1121 P16435 22974 P53667 5717 1111 P61289 5713 4869 P06400 5716 P46089 P48023 Q9BZF9 9093 Q99460 5728 P17301 1122 Q9ULZ3 3309 P04233 O00220 P51668 29108 P51665 P07550 P51671 P30556 Q9NYA1 O00231 O00233 P10721 O00232 P30793 P37198 3329 10746 79444 2475 4898 P42771 P05106 P30304 O95257 P28223 6850 P30307 Q13616 O00487 P30542 O60503 P62333 2247 58533 975 O14640 Q92878 5515 O43182 Q92633 5518 P51693 P09958 O00255 3350 P17342 P37173 5524 P05121 P06213 P23396 P04637 5300 P38405 990 991 Q96RU8 Q96RU7 Q8WTS1 995 Q99640 6622 999 P01116 Q9C0K7 6869 Q15389 O43166 4221 6885 23216 2280 56940 Q8WTR2 Q15392 4214 8812 4216 P49768 O00206 P48431 5562 Q8N726 P14416 P52735 Q00535 P25789 P25788 6647 P01137 P01135 P25787 P01133 P01375 Q9UJM3 5573 P04626 5575 5576 P04629 2064 P35348 Q96RR4 55633 3148 3146 5566 5567 P01127 5568 Q96EY1 Q96JB5 6657 5569 O43150 5585 O43597 8851 4255 552 553 Q9BZE4 O75190 Q99683 Q8WTW4 Q99684 P51828 5577 5578 7518 P07951 2081 Q6IR47 7532 23411 5590 51295 324 Q9NU19 Q9H1A4 5347 Q8TEA7 7529 Q08462 4035 Q9BUZ4 572 333 Q9NRM7 P25103 23636 5371 8881 Q60FE5 Q12933 6464 9973 P15559 P17980 P52701 O14818 P51617 P58012 54106 P25116 P60568 Q8N6T3 9744 Q8TEW0 Q9BY76 351 595 356 Q9UBS5 116 P09493 P22694 Q8WZ64 P03950 7334 5155 Q16512 O43684 Q9UKE5 367 51654 Q14344 P11309 Q13257 Q9HBW0 P35998 Q8TEU7 Q9UBU3 Q9UDY8 P09471 P61604 Q8WZ42 P00441 P32239 Q53H12 Q14114 4088 Q15208 Q8WWN8 125058 Q96BK5 5170 P21359 Q66K14 P31150 P60520 Q16543 P62942 Q9NSD7 8454 11140 7124 11142 P14635 O43639 P10275 4092 O75460 154 121512 25780 Q13042 P45379 Q13286 7376 Q86WV8 P35568 Q8NFM4 P01308 Q8NFM5 120892 2917 P49721 P49720 2915 85440 Q99956 P54274 O43612 P49959 Q01664 P01579 P09038 Q15008 Q9BY84 P39905 7157 P19086 64786 P14625 Q02750 23118 2931 55832 Q14160 54984 51231 Q15011 1609 Q14161 1848 Q15257 2935 O95835 7169 10928 26037 7161 P42224 26271 1852 1850 10935 P23945 Q7RTN6 P22612 P15090 1616 Q6ZT07 O94992 Q96QB1 P10644 P31321 O43847 P31323 O43609 P67775 P05062 P08575 P05067 P60900 2956 7186 7189 P29474 Q9H422 Q96M96 Q13418 7184 P41597 Q9BXK5 P17252 P21980 P42684 O95622 O15392 Q13426 4914 27352 56288 2730 Q9UBN7 1647 P13945 Q96DN5 P41182 P30047 P30048 B3KY43 Q03468 Q13200 Q5H9R7 Q13202 Q13443 Q96HU1 Q96BI3 P30279 Q9UJX2 P30281 P21333 P09429 Q9NYJ8 P10415

Q6ZV73 P12830 3611 O60343 4943 P01730 P08588 O15350 O75832 P53350 Q9P0U3 Q13227 O95661 Q8N5A5 Q9UHV2 2773 P30260 O15105 Q13233 P21554

ubiquitin-dependent protein catabolic process 11060 23607 11065 Q86TM6 9184 O75150 O60566 P15374 Q70EL4 84447 P63208 9978 O94905 Q14694 Q92611 3093 P09936 7332 Q9C026 7334 P55036 Q12834 O43684 Q5VTR2 Q504Q3 5706 23291 5707 5708 5709 7329 5700 Q13490 7328 Q16401 5701 4734 5702 Q9UHP3 Q9HAU4 Q9UKV5 Q9BUN8 5704 5705 11160 Q13257 7347 P32121 79791 P35998 A0AVT1 4193 5717 57646 P62256 10197 Q16531 P61289 6921 Q9Y297 5713 Q93009 Q93008 5716 57092 7353 Q86Y37 O75477 Q99460 9099 Q13829 267 23032 55236 3309 8453 8454 P51784 8452 64750 7126 P51668 26994 P14635 P51665 8450 10613 10616 Q13042 Q92530 Q13049 Q14139 Q7Z419 7375 Q9Y4X5 11274 6048 O00231 O00233 23014 O00232 Q96J02 Q9UKA1 5071 7251 Q9NS56 10868 P11021 10869 Q9NRD1 P49721 P49720 Q8TEY7 8237 O94966 7267 10273 Q13618 Q13617 Q9Y5K6 Q13616 Q9Y5K5 1161 O00487 O95376 Q9UKB1 Q99942 Q8TBB1 Q8WY64 391627 10956 P62333 P20618 Q9BZK7 Q92995 11124 Q9UK22 5516 Q9NQC7 Q15008 Q13501 Q9UL46 Q9UMW8 Q15369 Q13620 9695 6500 P14625 O75674 P54727 P54725 114088 255488 253980 57695 Q93034 5886 Q99816 5887 Q8WW12 9100 O00308 P53804 25862 Q96K76 27005 Q9NVW2 27248 Q70CQ3 23326 Q70CQ1 991 Q96RU2 79139 P62195 997 P62877 51009 P45974 Q9BZQ6 26270 64682 115426 83737 84708 55294 27252 P46934 51132 Q92560 51377 4214 O43294 408 P60900 P49407 409 P60468 Q6ZN33 5682 5683 5684 5685 5686 7184 80267 891 Q9Y3C5 Q9BYM8 Q9Y4E8 26133 55743 Q8TEL6 8945 P25789 P25788 Q9GZP9 80020 O60260 P25787 Q15843 Q8TAT6 5692 5693 P40818 91147 Q6R6M4 7874 P78317 Q9UNE7 1642 O95071 9924 5687 5688 Q969Q1 Q5VVQ6 P62837 8975 Q969H0 9820 Q9Y2K6 Q9UNN5 Q96EP1 Q8IWF2 Q96PU4 26232 Q9UK80 26234 P62714 10213 P55072 Q13200 Q9H3F6 O43242 Q8WZ19 Q8IWV7 Q9HCE7 Q5HYA8 57154 8065 Q05086 84749 Q9UJX2 Q00987 P61077 1540 Q86T82 Q9H1A4 329 124739 8078 O75604 Q13216 Q8IUQ4 P28065 Q5W0Q7 377630 O60907 Q9UPT9 Q9Y2M5 O00762 51185 Q5XUX0 55666 P61088 P68036 7415 8881 O75832 Q13107 P62191 P28074 P17980 P28072 P28070 55432 10208 P63279 O14818 P30260 8878 Q13233 51514 P43686 P49427

modification-dependent protein catabolic process 11060 23607 11065 Q86TM6 9184 O75150 O60566 P15374 Q70EL4 84447 P63208 9978 O94905 Q14694 Q92611 3093 P09936 7332 Q9C026 7334 P55036 Q12834 O43684 Q5VTR2 Q504Q3 5706 23291 5707 5708 5709 7329 5700 Q13490 7328 Q16401 5701 4734 5702 Q9UHP3 Q9HAU4 Q9UKV5 Q9BUN8 5704 5705 11160 Q13257 7347 P32121 79791 P35998 A0AVT1 4193 5717 57646 P62256 10197 Q16531 P61289 6921 Q9Y297 5713 Q93009 Q93008 5716 57092 7353 Q86Y37 O75477 Q99460 9099 Q13829 267 23032 55236 3309 8453 8454 P51784 8452 64750 7126 P51668 26994 P14635 P51665 8450 10613 10616 Q13042 Q92530 Q13049 Q14139 Q7Z419 7375 Q9Y4X5 11274 6048 P36776 O00231 O00233 23014 O00232 Q96J02 Q9UKA1 5071 7251 Q9NS56 10868 P11021 10869 Q9NRD1 P49721 P49720 Q8TEY7 8237 O94966 7267 10273 Q13618 Q13617 Q9Y5K6 Q13616 Q9Y5K5 1161 O00487 O95376 Q9UKB1 Q99942 Q8TBB1 Q8WY64 391627 10956 P62333 P20618 Q9BZK7 Q92995 11124 Q9UK22 5516 Q9NQC7 Q15008 Q13501 Q9UL46 Q9UMW8 Q15369 Q13620 9695 6500 P14625 O75674 P54727 P54725 114088 255488 253980 57695 Q93034 5886 Q99816 5887 Q8WW12 9100 O00308 P53804 25862 Q96K76 27005 Q9NVW2 27248 Q70CQ3 23326 Q70CQ1 991 Q96RU2 79139 P62195 997 P62877 51009 P45974 Q9BZQ6 26270 64682 115426 83737 84708 55294 27252 P46934 51132 Q92560 51377 4214 O43294 408 P60900 P49407 409 10269 P60468 Q6ZN33 5682 5683 5684 5685 5686 9361 7184 80267 891 Q9Y3C5 Q9BYM8 Q9Y4E8 26133 55743 Q8TEL6 8945 P25789 P25788 Q9GZP9 80020 O60260 P25787 Q15843 Q8TAT6 5692 5693

P40818 91147 Q6R6M4 7874 P78317 Q9UNE7 1642 O95071 9924 5687 5688 Q969Q1 Q5VVQ6 P62837 8975 Q969H0 9820 Q9Y2K6 Q9UNN5 Q96EP1 Q8IWF2 Q96PU4 26232 Q9UK80 26234 P62714 10213 P55072 Q13200 Q9H3F6 O43242 Q8WZ19 Q8IWV7 Q9HCE7 Q5HYA8 57154 8065 Q05086 84749 Q9UJX2 Q00987 P61077 1540 Q86T82 Q9H1A4 329 124739 8078 O75604 O75844 Q13216 Q8IUQ4 P28065 Q5W0Q7 377630 O60907 Q9UPT9 Q9Y2M5 O00762 51185 Q5XUX0 55666 P61088 P68036 7415 8881 O75832 Q13107 P62191 P28074 P17980 P28072 P28070 55432 10208 P63279 O14818 P30260 8878 Q13233 51514 P43686 P49427

modification-dependent macromolecule catabolic process 11060 23607 11065 Q86TM6 9184 O75150 O60566 P15374 Q70EL4 84447 P63208 9978 O94905 Q14694 Q92611 3093 P09936 7332 Q9C026 7334 P55036 Q12834 O43684 Q5VTR2 Q504Q3 5706 23291 5707 5708 5709 7329 5700 Q13490 7328 Q16401 5701 4734 5702 Q9UHP3 Q9HAU4 Q9UKV5 Q9BUN8 5704 5705 11160 Q13257 7347 P32121 79791 P35998 A0AVT1 4193 5717 57646 P62256 10197 Q16531 P61289 6921 Q9Y297 5713 Q93009 Q93008 5716 57092 7353 Q86Y37 O75477 Q99460 9099 Q13829 267 23032 55236 3309 8453 8454 P51784 8452 64750 7126 P51668 26994 P14635 P51665 8450 10613 10616 Q13042 Q92530 Q13049 Q14139 Q7Z419 7375 Q9Y4X5 11274 6048 P36776 O00231 O00233 23014 O00232 Q96J02 Q9UKA1 5071 7251 Q9NS56 10868 P11021 10869 Q9NRD1 P49721 P49720 Q8TEY7 8237 O94966 7267 10273 Q13618 Q13617 Q9Y5K6 Q13616 Q9Y5K5 1161 O00487 O95376 Q9UKB1 Q99942 Q8TBB1 Q8WY64 391627 10956 P62333 P20618 Q9BZK7 Q92995 11124 Q9UK22 5516 Q9NQC7 Q15008 Q13501 Q9UL46 Q9UMW8 Q15369 Q13620 9695 6500 P14625 O75674 P54727 P54725 114088 255488 253980 57695 Q93034 5886 Q99816 5887 Q8WW12 9100 O00308 P53804 25862 Q96K76 27005 Q9NVW2 27248 Q70CQ3 23326 Q70CQ1 991 Q96RU2 79139 P62195 997 P62877 51009 P45974 Q9BZQ6 26270 64682 115426 83737 84708 55294 27252 P46934 51132 Q92560 51377 4214 O43294 408 P60900 P49407 409 10269 P60468 Q6ZN33 5682 5683 5684 5685 5686 9361 7184 80267 891 Q9Y3C5 Q9BYM8 Q9Y4E8 26133 55743 Q8TEL6 8945 P25789 P25788 Q9GZP9 80020 O60260 P25787 Q15843 Q8TAT6 5692 5693 P40818 91147 Q6R6M4 7874 P78317 Q9UNE7 1642 O95071 9924 5687 5688 Q969Q1 Q5VVQ6 P62837 8975 Q969H0 9820 Q9Y2K6 Q9UNN5 Q96EP1 Q8IWF2 Q96PU4 26232 Q9UK80 26234 P62714 10213 P55072 Q13200 Q9H3F6 O43242 Q8WZ19 Q8IWV7 Q9HCE7 Q5HYA8 57154 8065 Q05086 84749 Q9UJX2 Q00987 P61077 1540 Q86T82 Q9H1A4 329 124739 8078 O75604 O75844 Q13216 Q8IUQ4 P28065 Q5W0Q7 377630 O60907 Q9UPT9 Q9Y2M5 O00762 51185 Q5XUX0 55666 P61088 P68036 7415 8881 O75832 Q13107 P62191 P28074 P17980 P28072 P28070 55432 10208 P63279 O14818 P30260 8878 Q13233 51514 P43686 P49427

positive regulation of protein metabolic process P25054 Q9BY77 11065 11186 595 Q9UBS0 1454 1453 P63208 Q7Z6C1 8767 Q13485 P07996 P25963 5935 10533 P38919 P04004 7334 P55036 Q12834 4067 Q9UBC3 5706 5707 5708 5709 P14174 7448 5700 Q16401 5701 P61956 4734 Q13133 5702 5704 5705 7341 Q5S007 O75925 Q7Z727 Q92956 8555 P35998 4193 5717 57761 10197 P61289 Q9Y297 Q13144 5713 Q92833 P26358 5716 4089 9775 3673 P55055 9093 Q99460 P17302 P17301 O60674 Q9NWT8 2697 Q8IUC6 P62701 3309 P04233 P62942 8454 P27037 Q8TDY2 7124 P28482 P51668 P14635 P51665 4092 O95352 Q93062 Q13042 Q99728 Q92530 3678 Q8N2W9 P08887 Q14011 P08648 P30556 3690 7376 Q9NYA1 8100 Q92769 11030 O00231 1153 P36896 O00233 P35443 P36894 P10600 O00232 Q96J02 Q96KS0 P11021 960 P01308 120892 2475 P49721 4898 O15169 P49720 85440 P07766 P05106 P02790 Q9UGJ0 6850 4792 P98179 10273 Q13616 O00487 P30542 O60502 Q8WY64 P38398 P62333 P49959 2247 Q96N67 P20618 2246 P60033 975 O14641 51588 P01579 P05230 857 Q92878 P05112 P09038 P23443 Q92997 Q15008 P09958 Q9UKS6 Q9UL46 7157 6500 P31749 O00255 Q6KC79 7272

10724 Q6PID4 P48730 5524 6613 Q15257 50943 Q14289 Q9UKT4 P06213 P01241 6199 Q13873 7046 6198 6872 O95835 P04637 5300 P14317 2033 P42345 991 26271 Q96RU8 Q96RU7 3479 2147 P62195 P63165 91 54998 Q15389 P45974 1856 Q9UBK2 P56524 P78536 7057 64682 O15496 4221 Q13526 Q96QB1 83737 O43847 2280 P46934 648 Q9BRP8 P01588 P16070 408 P60900 84271 P49407 P01343 Q13099 P49768 O00206 8399 Q09472 5682 5683 5684 5685 5686 P29590 3263 Q9NS23 148022 891 894 896 P17252 8945 P01019 P25789 P25788 P01137 P25787 P01375 4361 5692 5693 9495 P35226 Q9UNE7 27352 P43405 P17813 5687 5688 Q96EY1 P24588 O43353 9821 O95999 P07948 672 P15692 5578 10213 P55072 Q13200 Q96FA3 7099 O43242 Q9Y4P1 Q6IR47 Q13443 Q96HU1 Q9HCE7 64127 57154 O76024 P30279 1789 1786 Q9UJX2 Q00987 324 P00734 P30281 207 Q9H1A4 5347 3720 P24385 8078 8754 7422 P10415 P28065 3066 O00762 Q9NWW5 57162 84305 P61088 7415 P01730 Q9BT67 5371 P27540 8881 Q9UPU9 O75832 Q9BZS1 P49674 P53350 P62191 P28074 P17980 P28072 580 P28070 23192 O14818 Q9HC29 P30260 O15105 P43686 P60568 P21675

regulation of I-kappaB kinase/NF-kappaB cascade 8772 P35813 Q9Y4K3 O43318 O15519 Q9BXM7 356 Q92851 8767 9218 5536 O14788 57506 Q9HC98 7334 6885 Q13404 Q15669 7335 65018 2280 7052 2316 Q04864 P09382 10783 4215 Q13490 Q99759 1902 O00206 9641 8795 7189 7188 7185 148022 Q9Y6Q6 Q9BYM8 10758 Q9UDY8 8945 142678 51026 P21980 55504 Q9Y297 64170 O60260 P01375 5970 P48023 Q13546 55072 Q7Z434 Q86XR7 Q9H257 Q9NR96 3551 9093 P17302 P19438 387 389 O00182 2697 Q8IUC6 Q04206 P53041 Q96EY1 5966 8837 8717 P62942 O43353 Q96EP0 6275 Q13158 O95999 7124 Q9Y3E0 O00463 3162 9020 79971 Q9Y2C9 10616 O94827 57708 5579 P61586 1936 P05771 Q6P1N0 3956 Q96FA3 O00635 7099 7132 7098 P29466 Q9Y239 8743 Q15628 P21580 64127 124583 5071 P37198 Q99558 29110 1147 841 843 P08134 8737 7128 Q7Z7H5 329 3965 P21333 Q9NYJ8 10392 P50591 O43734 Q9UHD2 Q9P0L0 57162 Q9NS68 Q8WVQ1 Q8IUH5 P61088 Q14790 Q5T9L3 O14763 O15455 Q16610 O14920 Q92633 Q9BT67 Q13501 23636 5494 Q60FE5 O15111 Q9H8V3 222068 23118 27032 54862 Q9HC29 Q14164 54106 8878 P09601 P98194 P26447 P25116 Q13077

positive regulation of I-kappaB kinase/NF-kappaB cascade 8772 P35813 Q9Y4K3 O43318 O15519 Q9BXM7 356 Q92851 8767 9218 5536 O14788 57506 Q9HC98 7334 6885 Q13404 7335 65018 2280 7052 2316 Q04864 P09382 10783 4215 Q13490 Q99759 1902 O00206 9641 8795 7189 7188 7185 148022 Q9Y6Q6 Q9BYM8 10758 Q9UDY8 142678 51026 P21980 55504 64170 O60260 P01375 5970 P48023 Q13546 55072 Q7Z434 Q86XR7 Q9H257 Q9NR96 P17302 P19438 387 389 O00182 2697 Q8IUC6 Q04206 P53041 5966 8837 8717 P62942 O43353 Q96EP0 6275 Q13158 O95999 7124 Q9Y3E0 O00463 3162 9020 79971 Q9Y2C9 10616 O94827 57708 5579 P61586 1936 P05771 Q6P1N0 3956 Q96FA3 O00635 7099 7132 7098 P29466 Q9Y239 8743 Q15628 64127 124583 5071 P37198 Q99558 29110 1147 841 843 P08134 8737 Q7Z7H5 329 3965 P21333 Q9NYJ8 10392 P50591 O43734 Q9UHD2 Q9P0L0 57162 Q9NS68 Q8WVQ1 Q8IUH5 P61088 Q14790 Q5T9L3 O14763 O15455 Q16610 Q92633 Q9BT67 23636 5494 Q60FE5 O15111 Q9H8V3 222068 23118 27032 54862 Q9HC29 Q14164 54106 P09601 P98194 P26447 P25116 Q13077

proteasomal ubiquitin-dependent protein catabolic process 11060 23607 O00308 11065 Q86TM6 27248 9184 991 O60566 79139 P62195 84447 997 P62877 P63208 9978 51009 O94905 Q9BZQ6 26270 Q92611 64682 Q9C026 P55036 Q12834 O43684 55294 27252 5706 23291 5707 5708 5709 5700 Q13490 Q16401 5701 408 P60900 5702 P49407 409 Q9UKV5 Q9BUN8 5704 P60468 5705 Q6ZN33 11160 Q13257 5682 5683 5684 5685 5686 P32121 79791 P35998 7184 80267 891 5717 Q9BYM8 P25789 10197 P25788 Q9GZP9 Q16531 P61289 5713 80020 P25787 5716 Q8TAT6 5692 57092 5693 91147 O75477 Q99460 P78317 Q13829 Q9UNE7 267 1642 5687 5688 Q969Q1 3309 8454 Q5VVQ6 8452

Q969H0 7126 P51668 Q9UNN5 P14635 P51665 10613 Q8IWF2 26232 10616 26234 Q13042 P62714 Q92530 10213 P55072 Q13200 Q9H3F6 O43242 Q8WZ19 6048 Q9HCE7 O00231 Q5HYA8 O00233 57154 O00232 Q9UKA1 Q9NS56 P11021 Q9UJX2 10869 P61077 Q9H1A4 Q9NRD1 P49721 329 P49720 O94966 10273 Q13216 Q8IUQ4 P28065 Q13618 O60907 Q9Y5K6 Q13616 1161 Q9Y2M5 O00487 O00762 Q9UKB1 Q99942 10956 P62333 51185 Q5XUX0 P20618 55666 Q9BZK7 7415 11124 Q9UK22 5516 Q15008 Q9UL46 8881 9695 O75832 6500 P14625 P62191 P28074 P54727 P17980 P28072 P54725 P28070 55432 114088 O14818 253980 P30260 5886 5887 P43686 Q8WW12 P49427

proteasomal protein catabolic process 11060 23607 O00308 11065 Q86TM6 27248 9184 991 O60566 79139 P62195 84447 997 P62877 P63208 9978 51009 O94905 Q9BZQ6 26270 Q92611 64682 Q9C026 P55036 Q12834 O43684 55294 27252 5706 23291 5707 5708 5709 5700 Q13490 Q16401 5701 408 P60900 5702 P49407 409 Q9UKV5 Q9BUN8 5704 P60468 5705 Q6ZN33 11160 Q13257 5682 5683 5684 5685 5686 P32121 79791 P35998 7184 80267 891 5717 Q9BYM8 P25789 10197 P25788 Q9GZP9 Q16531 P61289 5713 80020 P25787 5716 Q8TAT6 5692 57092 5693 91147 O75477 Q99460 P78317 Q13829 Q9UNE7 267 1642 5687 5688 Q969Q1 3309 8454 Q5VVQ6 8452 Q969H0 7126 P51668 Q9UNN5 P14635 P51665 10613 Q8IWF2 26232 10616 26234 Q13042 P62714 Q92530 10213 P55072 Q13200 Q9H3F6 O43242 Q8WZ19 6048 Q9HCE7 O00231 Q5HYA8 O00233 57154 O00232 Q9UKA1 Q9NS56 P11021 Q9UJX2 10869 P61077 Q9H1A4 Q9NRD1 P49721 329 P49720 O94966 10273 Q13216 Q8IUQ4 P28065 Q13618 O60907 Q9Y5K6 Q13616 1161 Q9Y2M5 O00487 O00762 Q9UKB1 Q99942 10956 P62333 51185 Q5XUX0 P20618 55666 Q9BZK7 7415 11124 Q9UK22 5516 Q15008 Q9UL46 8881 9695 O75832 6500 P14625 P62191 P28074 P54727 P17980 P28072 P54725 P28070 55432 114088 O14818 253980 P30260 5886 5887 P43686 Q8WW12 P49427

positive regulation of cellular protein metabolic process Q9BY77 11065 11186 595 Q9UBS0 1454 1453 P63208 Q7Z6C1 8767 Q13485 P07996 P25963 5935 10533 P38919 P04004 7334 P55036 Q12834 4067 Q9UBC3 5706 5707 5708 5709 P14174 7448 5700 Q16401 5701 P61956 Q13133 5702 5704 5705 7341 Q5S007 O75925 Q7Z727 Q92956 8555 P35998 4193 5717 57761 10197 P61289 Q9Y297 Q13144 5713 Q92833 P26358 5716 4089 9775 3673 P55055 9093 Q99460 P17301 O60674 Q9NWT8 Q8IUC6 P62701 3309 P04233 P62942 8454 P27037 Q8TDY2 7124 P28482 P51668 P14635 P51665 4092 O95352 Q93062 Q13042 Q92530 3678 Q8N2W9 P08887 Q14011 P08648 P30556 3690 7376 Q9NYA1 8100 Q92769 11030 O00231 1153 P36896 O00233 P35443 P36894 P10600 O00232 P11021 960 P01308 120892 2475 P49721 4898 O15169 P49720 85440 P07766 P05106 P02790 Q9UGJ0 6850 4792 P98179 10273 Q13616 O00487 P30542 O60502 P38398 P62333 P49959 2247 Q96N67 P20618 2246 P60033 975 O14641 51588 P01579 P05230 857 Q92878 P05112 P09038 P23443 Q92997 Q15008 P09958 Q9UKS6 Q9UL46 7157 6500 P31749 O00255 Q6KC79 7272 10724 Q6PID4 P48730 5524 6613 Q15257 50943 Q14289 Q9UKT4 P06213 P01241 6199 Q13873 7046 6198 6872 O95835 P04637 5300 P14317 2033 P42345 991 26271 Q96RU8 Q96RU7 3479 2147 P62195 P63165 91 54998 Q15389 P45974 1856 Q9UBK2 P56524 P78536 7057 64682 O15496 4221 Q13526 Q96QB1 O43847 2280 648 Q9BRP8 P01588 P16070 408 P60900 84271 P49407 P01343 Q13099 P49768 O00206 8399 Q09472 5682 5683 5684 5685 5686 P29590 3263 Q9NS23 148022 891 894 896 P17252 8945 P01019 P25789 P25788 P01137 P25787 P01375 4361 5692 5693 9495 P35226 Q9UNE7 P43405 P17813 5687 5688 Q96EY1 P24588 O43353 9821 O95999 P07948 672 P15692 5578 10213 P55072 Q13200 Q96FA3 7099 O43242 Q6IR47 Q13443 Q9HCE7 64127 57154 P30279 1789 1786 Q9UJX2 Q00987 P00734 P30281 207 Q9H1A4 5347 3720 P24385 8078 8754 7422 P10415 P28065 3066 O00762 Q9NWW5 57162 84305 P61088 7415 P01730 Q9BT67 5371 P27540 8881

Q9UPU9 O75832 Q9BZS1 P49674 P53350 P62191 P28074 P17980 P28072 P28070 O14818 Q9HC29 P30260 O15105 P43686 P60568 P21675

protein complex biogenesis P25054 23607 25909 P49023 P54619 1460 Q9UBB5 O00268 P52434 9185 Q9BYG3 4728 Q8IYN9 P84022 Q9BVA1 O14788 6901 4725 P27105 Q9H4L7 P30876 25915 2316 5829 Q96QZ7 P14174 P16471 Q92845 Q9UKV5 P10809 Q12846 Q9NQW6 Q05397 3416 1355 P32929 O15533 811 5830 4869 5716 P31946 1374 Q6P2C8 1491 P54646 Q676U5 P17302 P16333 2697 6810 P04350 3667 6814 5604 P04233 829 3308 P30566 P08758 1022 3320 1020 114548 O00468 83706 P16389 P15056 1499 830 P07550 P04040 P09972 O14745 P07437 Q9Y478 Q9Y239 2243 O00233 Q9BUB7 P30793 3329 Q9BUB1 1025 841 1387 6834 847 5987 6714 5747 P04179 P07766 Q9UGJ1 6850 Q06203 Q8TBB1 Q9NS69 P42768 P61244 284119 2244 O14640 Q92994 857 5879 O43182 5518 P51575 Q9Y375 Q13509 Q13748 Q03164 10844 440193 P40692 Q92621 989 5888 P09601 P40337 P04275 Q9UKT4 Q9Y2W1 P04156 P06213 4690 P07900 6872 P04637 P53803 P50416 P11047 P62993 203068 1058 999 5536 Q15389 6505 P01112 P62875 P25445 6883 5430 10383 5432 P51636 O95163 P07919 Q6FGD7 2040 Q9BU61 P50542 2280 O60610 641 402 4214 8932 6878 7846 Q15393 Q96S59 P02675 8936 6892 5440 5441 5563 3265 Q9UKN8 891 773 Q9H4B7 Q00535 5434 P01137 Q3ZAQ7 O00217 O43264 Q9Y697 5213 5576 Q658W2 P35222 Q08257 P14784 3028 Q00403 Q96RR1 P61457 P23258 P61218 O43593 Q8N307 O00505 Q6FGG2 P50613 P51946 3162 Q8WUY8 673 3159 P55072 Q9Y512 P49450 5471 64127 Q9ULK4 5590 29079 P14373 P18846 Q00987 P50748 324 54443 Q15070 9702 329 7529 P27797 4150 P50750 25929 Q9UHD8 Q12809 O00762 25923 Q9NU22 330 Q96P20 56993 7415 4149 5371 Q86UX7 6341 Q60FE5 Q12933 P07814 25813 466 P51617 O14817 P17858 P52948 Q9NPJ6 7428 9967 Q01813 8772 10093 Q14686 4297 Q14209 Q8TEW0 Q9BY76 11065 Q16625 Q6NZI2 P13984 6470 3084 P13861 230 355 P07196 23165 5018 P49815 Q13485 P47755 Q13489 P03950 O00716 9997 8301 Q16635 Q16514 O75381 51773 Q9NRH3 Q13492 Q9BSJ2 Q9H9E3 Q13490 Q16401 Q15796 84790 Q15797 6498 P12757 9404 Q8N6I1 P12755 4193 Q6DD88 Q9UDY8 P17096 Q8NFH8 Q9HC77 Q8WZ42 Q9BQE3 4086 4087 4088 4089 P18074 Q86XR8 163126 P29084 P68371 P36954 P34896 Q02790 Q9NVR5 267 P52907 P68366 7106 P08034 P43005 P62942 P78347 Q13158 Q8N5M1 8576 O15164 7248 O75347 P54132 P14635 O43639 154 Q00597 158 84365 55806 O15294 7132 5195 7013 8467 P36776 Q86WV8 P35568 Q02763 7249 P49848 Q9NRD5 Q99714 9685 P02671 8473 P00492 Q8TCU6 7023 10392 P54274 A1XKG3 Q9Y5K6 P36404 Q08188 Q96T23 222484 Q01664 56916 P08069 9439 Q16698 347733 7277 7157 P14868 Q02750 5092 138151 Q15370 Q6PID4 55835 1729 Q9BWT7 10048 Q14289 Q13077 9223 9463 Q96RE7 Q7KZN9 P12931 9341 7283 26271 Q6NVY8 Q92574 Q9UBK2 Q13885 Q8IX30 Q02297 9351 55054 84708 22920 55172 7052 P24928 Q9BUF5 1742 Q9HD26 P05062 Q4LE28 2959 7186 Q15836 9368 57120 P29590 Q13418 O75880 84617 7185 9361 O43819 Q92793 2961 2960 1870 P21980 O60264 P23921 2969 Q92558 O15151 P34932 P19438 P43405 56288 2972 P53041 O95996 O95999 O00186 P30047 P63000 Q9BUK6 Q9NVC6 O95405 P35609 P14923 O43920 P19387 P30153 P19388 3728 1666 P21579 Q05513 Q7Z2W7 Q8WYP5 375790 1429 P21333 Q9NVD7 P12830 Q8TEQ6 P19474 P32320 3737 Q00059 2644 2643 2885 3611 Q14790 O15212 O75716 Q14677 O75832 Q15648 Q15526 9054 Q96BF6 P67870 Q9HC29 Q71U36 902 P08237 Q13233 Q13112 P21675

protein complex assembly P25054 23607 25909 P49023 P54619 1460 Q9UBB5 O00268 P52434 9185 Q9BYG3 4728 Q8IYN9 P84022 Q9BVA1 O14788 6901 4725 P27105 Q9H4L7 P30876 25915 2316 5829 Q96QZ7 P14174 P16471 Q92845 Q9UKV5 P10809 Q12846 Q9NQW6 Q05397 3416 1355 P32929 O15533 811 5830 4869 5716 P31946 1374 Q6P2C8 1491 P54646 Q676U5 P17302 P16333 2697 6810 P04350 3667 6814 5604 P04233 829 3308 P30566 P08758 1022 3320 1020 114548 O00468 83706

P16389 P15056 1499 830 P07550 P04040 P09972 O14745 P07437 Q9Y478 Q9Y239 2243 O00233 Q9BUB7 P30793 3329 Q9BUB1 1025 841 1387 6834 847 5987 6714 5747 P04179 P07766 Q9UGJ1 6850 Q06203 Q8TBB1 Q9NS69 P42768 P61244 284119 2244 O14640 Q92994 857 5879 O43182 5518 P51575 Q9Y375 Q13509 Q13748 Q03164 10844 440193 P40692 Q92621 989 5888 P09601 P40337 P04275 Q9UKT4 Q9Y2W1 P04156 P06213 4690 P07900 6872 P04637 P53803 P50416 P11047 P62993 203068 1058 999 5536 Q15389 6505 P01112 P62875 P25445 6883 5430 10383 5432 P51636 O95163 P07919 Q6FGD7 2040 Q9BU61 P50542 2280 O60610 641 402 4214 8932 6878 7846 Q15393 Q96S59 P02675 8936 6892 5440 5441 5563 3265 Q9UKN8 891 773 Q9H4B7 Q00535 5434 P01137 Q3ZAQ7 O00217 O43264 Q9Y697 5213 5576 Q658W2 P35222 Q08257 P14784 3028 Q00403 Q96RR1 P61457 P23258 P61218 O43593 Q8N307 O00505 Q6FGG2 P50613 P51946 3162 Q8WUY8 673 3159 P55072 Q9Y512 P49450 5471 64127 Q9ULK4 5590 29079 P14373 P18846 Q00987 P50748 324 54443 Q15070 9702 329 7529 P27797 4150 P50750 25929 Q9UHD8 Q12809 O00762 25923 Q9NU22 330 Q96P20 56993 7415 4149 5371 Q86UX7 6341 Q60FE5 Q12933 P07814 25813 466 P51617 O14817 P17858 P52948 Q9NPJ6 7428 9967 Q01813 8772 10093 Q14686 4297 Q14209 Q8TEW0 Q9BY76 11065 Q16625 Q6NZI2 P13984 6470 3084 P13861 230 355 P07196 23165 5018 P49815 Q13485 P47755 Q13489 P03950 O00716 9997 8301 Q16635 Q16514 O75381 51773 Q9NRH3 Q13492 Q9BSJ2 Q9H9E3 Q13490 Q16401 Q15796 84790 Q15797 6498 P12757 9404 Q8N6I1 P12755 4193 Q6DD88 Q9UDY8 P17096 Q8NFH8 Q9HC77 Q8WZ42 Q9BQE3 4086 4087 4088 4089 P18074 Q86XR8 163126 P29084 P68371 P36954 P34896 Q02790 Q9NVR5 267 P52907 P68366 7106 P08034 P43005 P62942 P78347 Q13158 Q8N5M1 8576 O15164 7248 O75347 P54132 P14635 O43639 154 Q00597 158 84365 55806 O15294 7132 5195 7013 8467 P36776 Q86WV8 P35568 Q02763 7249 P49848 Q9NRD5 Q99714 9685 P02671 8473 P00492 Q8TCU6 7023 10392 P54274 A1XKG3 Q9Y5K6 P36404 Q08188 Q96T23 222484 Q01664 56916 P08069 9439 Q16698 347733 7277 7157 P14868 Q02750 5092 138151 Q15370 Q6PID4 55835 1729 Q9BWT7 10048 Q14289 Q13077 9223 9463 Q96RE7 Q7KZN9 P12931 9341 7283 26271 Q6NVY8 Q92574 Q9UBK2 Q13885 Q8IX30 Q02297 9351 55054 84708 22920 55172 7052 P24928 Q9BUF5 1742 Q9HD26 P05062 Q4LE28 2959 7186 Q15836 9368 57120 P29590 Q13418 O75880 84617 7185 9361 O43819 Q92793 2961 2960 1870 P21980 O60264 P23921 2969 Q92558 O15151 P34932 P19438 P43405 56288 2972 P53041 O95996 O95999 O00186 P30047 P63000 Q9BUK6 Q9NVC6 O95405 P35609 P14923 O43920 P19387 P30153 P19388 3728 1666 P21579 Q05513 Q7Z2W7 Q8WYP5 375790 1429 P21333 Q9NVD7 P12830 Q8TEQ6 P19474 P32320 3737 Q00059 2644 2643 2885 3611 Q14790 O15212 O75716 Q14677 O75832 Q15648 Q15526 9054 Q96BF6 P67870 Q9HC29 Q71U36 902 P08237 Q13233 Q13112 P21675

cell division O15085 54908 P25054 8650 Q9Y265 23607 Q8TEW0 Q9Y266 11065 25909 11064 8091 259266 9181 P51587 9184 P48729 Q9BVA0 595 O60566 Q96IK1 Q96BM9 Q9BYG4 Q8WUM4 Q8N3U4 55207 P43034 Q9ULW0 10534 Q9HC98 Q92974 O43683 O75935 Q12834 O43684 Q53EZ4 O60216 1104 P46531 4851 54801 10783 7329 84552 64282 Q29RF7 Q13257 9525 7465 Q8WVM7 O75122 Q9NQW6 Q7L5Y9 Q9NY61 22974 4628 P51808 P49916 57405 29127 54930 P20936 P62491 P18858 P23528 Q96RK4 Q8NFH4 P83876 P62136 Q9HC77 O94927 Q93008 4627 Q96EA4 P06400 O95347 P62826 O43663 Q9Y6R0 P18074 O75116 Q8TD19 Q9NRZ9 O75351 54820 P62140 P24864 P20264 Q15691 Q96GD4 O14578 Q8IX90 8452 11021 P11802 Q14008 1022 23122 1021 23244 1020 P14635 P35579 22832 P35580 1018 1017 Q96DE5 Q9H6D7 3796 10296 Q13042 O75909 Q96R06 Q5TB80 Q9H0H5 Q96CS2 7013 9793 P32856 9798 Q8IZT6 26524 P10721 79441 7251 1029 116840 5500 5501 Q14141 P41208 P42771 Q8N137 9564 P30304 O15182 P30305 P30307 P54274 Q13618 A1XKG3 Q9Y5K6 55142 221150 11243 Q9BRK4 Q9NTI5 Q9NVM9 Q15003 11004 9212 O00139 P53990 Q8TAP9 23354 P36873 7150 P11234 P11233 O60879 Q9NXR1 Q9NZ56 10726 Q96MT8 Q9NTJ3 55835 Q99816 989 O14777

Q9UKT4 7704 127829 Q9NPB6 Q96FF9 Q15019 6993 O95835 8379 P63172 990 991 26271 Q9Y6G9 55165 994 995 55726 Q15021 5898 10015 1616 Q9BU64 9232 64682 9475 Q9Y6X3 Q8N4N8 O75410 23332 1072 Q8NHV4 Q7Z460 1069 Q99871 22919 11113 Q9UI95 Q6P1K2 8812 Q9BS18 P42695 P42338 Q9UER7 25847 Q8N726 9126 Q7Z7K6 10907 O96020 2054 6093 91272 P52732 P11387 890 1877 Q66K89 891 894 Q99741 Q00535 55743 896 Q00534 898 Q15286 50855 O14965 O43264 Q9UPY8 93323 9133 Q14511 O15392 23310 23552 O95067 9253 79023 Q9H410 9493 80254 56288 Q9NVJ2 Q9HBM1 Q00526 6790 O43236 9700 O95997 P50613 79172 Q9P258 Q96EP1 10459 Q8IZL9 Q7Z7A1 3832 8607 O43482 Q68CZ6 P51955 P51956 Q9BZD4 P30279 Q05516 Q6PJP8 P20248 Q9UJX2 P51959 Q9UQE7 P50748 324 P30281 54443 Q8WYP5 9826 Q9H1A4 5347 5901 Q9Y6D9 P24385 91754 3980 Q02241 Q9UHD8 O00762 22897 Q99661 Q9NRM7 Q13464 5119 Q14674 3978 3070 8881 8766 P53350 Q7Z4H7 5499 79980 P63279 121441 O60232 P24941 900 55559 Q9UPV0 Q14683 5925

regulation of phosphorus metabolic process P25054 P54619 22870 Q9NRW4 124790 P84022 1453 Q9ULW0 O14543 5933 6901 P07996 P52564 P04004 P09936 O15530 O95684 9077 P50148 P14174 P16471 O15524 Q5S007 Q7Z727 Q9H2G4 Q92956 Q06124 22974 Q9C010 57761 1111 Q9UKW4 P62258 4869 P06400 5716 P31946 3672 3673 P31947 Q9NR96 Q13705 9093 P21917 5728 10645 P17301 O60674 P20827 3667 64061 10641 5604 3309 P04233 Q8WXG6 P27037 Q13956 4771 1022 O00220 1020 10614 Q08999 P07550 O75909 3678 P08887 P08648 3690 Q9NYA1 P09619 P10600 P10721 P37198 1029 Q9BUB1 1027 10746 79444 960 1026 2475 P42771 3689 7804 O14757 P07766 P05106 P30304 O95136 O95257 P05107 Q9UGJ0 P28223 6850 P30307 P16118 P30542 P18545 O60503 2247 Q96N67 2246 P60033 Q92990 58533 975 O14641 Q5TCX8 O14640 P05230 857 O14763 Q92878 859 Q96CA5 5515 P05112 Q92997 5518 P31749 O95382 O00255 440193 P37173 5524 Q9UL54 P06213 P01241 5300 P56199 P14317 2033 990 Q96RU8 Q96RU7 3479 2147 995 23560 Q99640 P01116 Q9C0K7 Q15389 Q9UII4 4221 6885 P36575 25998 Q9BT40 2280 56940 Q8WTR2 641 Q99750 4214 8812 4216 P01588 Q13098 P01343 11116 P49768 O00206 Q09472 5562 Q8N726 O96020 P52735 3263 891 894 Q99741 Q00535 896 P35240 P01019 6647 P01137 P01135 P01133 P01375 Q9UJM3 4361 5573 P04626 5575 Q9GZP0 5576 2064 55750 Q96RR4 P17813 P60484 P17936 5566 5567 P01127 5568 Q96EY1 Q96JB5 5569 5207 Q96MH2 P24588 5580 5581 9821 5585 O43597 8851 Q9UPR3 P07948 P50613 P61981 P15692 Q9BZE4 Q99683 Q8WTW4 P51828 10451 5577 5578 10454 P04201 P05412 2081 Q6IR47 7532 7410 7531 64127 5590 Q96SZ6 P51959 324 8737 207 54206 7529 Q15078 Q08462 P24385 8754 7422 4035 Q9BUZ4 O75293 Q9NRM7 9839 23636 Q12933 6464 P52824 580 57732 P17612 P51617 P28749 8517 54106 56647 P25116 P60568 8655 4176 Q8TEW0 P30086 Q9Y4K4 Q9Y4K3 3084 Q9UPN7 O43318 P13861 351 595 23043 P49810 Q7Z6C1 51763 P22694 P49815 Q13485 P03950 5154 5155 Q16512 4067 4188 Q16635 5037 Q9UKE5 7448 51654 P00533 Q13131 P24522 8795 P11309 Q9HBW0 Q9Y6Q6 Q9UBU3 P46527 23381 Q9UDY8 5159 Q8WZ42 P00441 Q53H12 Q9Y6R4 Q14114 4088 4089 Q15208 O43306 5170 P21359 Q16543 Q13153 P43489 P62942 Q8TDY2 11140 7124 11142 7248 P54132 P54253 P14635 O43639 4092 O75460 154 Q93062 Q9Y2C9 121512 Q9Y6K9 Q99728 11146 11030 Q9Y4H2 P36896 P35443 26524 P36894 Q86WV8 5071 P35568 94274 P11021 Q8NFM4 P01308 Q8NFM5 120892 7249 O15169 Q13177 85440 Q9NRD5 Q99956 P61925 P02790 7023 P11229 A1XKG3 O43612 Q9UNH7 P49959 Q01664 55704 P01579 Q9BQI3 1956 P09038 Q9BY84 P14625 O75553 Q02750 P38936 Q9UNI6 23118 7272 2810 51231 Q6PID4 1609 P48730 Q9BWT7 1848 Q15257 Q14289 Q13873 9463 7046 P98155 O95835 9467 P33993 7161 P63167 P42345 1852 1850 10935 Q7RTN6 91 P04085 Q92574 P22612 P15090 1616 1856 O94992 P78536 7057 Q13526 Q96QB1 55291 Q02297 P10644 P31321 Q3V6T2 P31323 O43609 P67775 P08575 P16070 P05067 P42574 P42338 Q9UER7 7186 7189 P29353 Q9H422 Q96M96 Q13418 P56539 P31431 84619 7184 P40145 27102 P17252 O60266 O60260 O95622

8165 A4D1W7 Q13546 Q02156 9495 P43405 56288 Q05655 Q07954 Q92786 Q9Y2B9 Q92667 1647 O95999 Q15735 P78509 9021 Q16828 P30048 B3KY43 Q03468 P08246 Q5H9R7 Q8IWV1 7099 7098 Q13322 Q13202 Q13443 Q9NP71 P30153 P30279 P61073 Q05513 2873 P00734 P30281 Q13574 Q15750 P46734 Q9NYJ8 P10415 O14495 Q9P035 3611 Q04656 O15455 P01730 O15350 O75832 Q13227 Q9P287 O95661 Q8N5A5 Q9UHV2 Q9HC29 O15105 900 Q13233 5925

regulation of phosphate metabolic process P25054 P54619 22870 Q9NRW4 124790 P84022 1453 Q9ULW0 O14543 5933 6901 P07996 P52564 P04004 P09936 O15530 O95684 9077 P50148 P14174 P16471 O15524 Q5S007 Q7Z727 Q9H2G4 Q92956 Q06124 22974 Q9C010 57761 1111 Q9UKW4 P62258 4869 P06400 5716 P31946 3672 3673 P31947 Q9NR96 Q13705 9093 P21917 5728 10645 P17301 O60674 P20827 3667 64061 10641 5604 3309 P04233 Q8WXG6 P27037 Q13956 4771 1022 O00220 1020 10614 Q08999 P07550 O75909 3678 P08887 P08648 3690 Q9NYA1 P09619 P10600 P10721 P37198 1029 Q9BUB1 1027 10746 79444 960 1026 2475 P42771 3689 7804 O14757 P07766 P05106 P30304 O95136 O95257 P05107 Q9UGJ0 P28223 6850 P30307 P16118 P30542 P18545 O60503 2247 Q96N67 2246 P60033 Q92990 58533 975 O14641 Q5TCX8 O14640 P05230 857 O14763 Q92878 859 Q96CA5 5515 P05112 Q92997 5518 P31749 O95382 O00255 440193 P37173 5524 Q9UL54 P06213 P01241 5300 P56199 P14317 2033 990 Q96RU8 Q96RU7 3479 2147 995 23560 Q99640 P01116 Q9C0K7 Q15389 Q9UII4 4221 6885 P36575 25998 Q9BT40 2280 56940 Q8WTR2 641 Q99750 4214 8812 4216 P01588 Q13098 P01343 11116 P49768 O00206 Q09472 5562 Q8N726 O96020 P52735 3263 891 894 Q99741 Q00535 896 P35240 P01019 6647 P01137 P01135 P01133 P01375 Q9UJM3 4361 5573 P04626 5575 Q9GZP0 5576 2064 55750 Q96RR4 P17813 P60484 P17936 5566 5567 P01127 5568 Q96EY1 Q96JB5 5569 5207 Q96MH2 P24588 5580 5581 9821 5585 O43597 8851 Q9UPR3 P07948 P50613 P61981 P15692 Q9BZE4 Q99683 Q8WTW4 P51828 10451 5577 5578 10454 P04201 P05412 2081 Q6IR47 7532 7410 7531 64127 5590 Q96SZ6 P51959 324 8737 207 54206 7529 Q15078 Q08462 P24385 8754 7422 4035 Q9BUZ4 O75293 Q9NRM7 9839 23636 Q12933 6464 P52824 580 57732 P17612 P51617 P28749 8517 54106 56647 P25116 P60568 8655 4176 Q8TEW0 P30086 Q9Y4K4 Q9Y4K3 3084 Q9UPN7 O43318 P13861 351 595 23043 P49810 Q7Z6C1 51763 P22694 P49815 Q13485 P03950 5154 5155 Q16512 4067 4188 Q16635 5037 Q9UKE5 7448 51654 P00533 Q13131 P24522 8795 P11309 Q9HBW0 Q9Y6Q6 Q9UBU3 P46527 23381 Q9UDY8 5159 Q8WZ42 P00441 Q53H12 Q9Y6R4 Q14114 4088 4089 Q15208 O43306 5170 P21359 Q16543 Q13153 P43489 P62942 Q8TDY2 11140 7124 11142 7248 P54132 P54253 P14635 O43639 4092 O75460 154 Q93062 Q9Y2C9 121512 Q9Y6K9 Q99728 11146 11030 Q9Y4H2 P36896 P35443 26524 P36894 Q86WV8 5071 P35568 94274 P11021 Q8NFM4 P01308 Q8NFM5 120892 7249 O15169 Q13177 85440 Q9NRD5 Q99956 P61925 P02790 7023 P11229 A1XKG3 O43612 Q9UNH7 P49959 Q01664 55704 P01579 Q9BQI3 1956 P09038 Q9BY84 P14625 O75553 Q02750 P38936 Q9UNI6 23118 7272 2810 51231 Q6PID4 1609 P48730 Q9BWT7 1848 Q15257 Q14289 Q13873 9463 7046 P98155 O95835 9467 P33993 7161 P63167 P42345 1852 1850 10935 Q7RTN6 91 P04085 Q92574 P22612 P15090 1616 1856 O94992 P78536 7057 Q13526 Q96QB1 55291 Q02297 P10644 P31321 Q3V6T2 P31323 O43609 P67775 P08575 P16070 P05067 P42574 P42338 Q9UER7 7186 7189 P29353 Q9H422 Q96M96 Q13418 P56539 P31431 84619 7184 P40145 27102 P17252 O60266 O60260 O95622 8165 A4D1W7 Q13546 Q02156 9495 P43405 56288 Q05655 Q07954 Q92786 Q9Y2B9 Q92667 1647 O95999 Q15735 P78509 9021 Q16828 P30048 B3KY43 Q03468 P08246 Q5H9R7 Q8IWV1 7099 7098 Q13322 Q13202 Q13443 Q9NP71 P30153 P30279 P61073 Q05513 2873 P00734 P30281 Q13574 Q15750 P46734 Q9NYJ8 P10415 O14495 Q9P035 3611 Q04656 O15455 P01730 O15350 O75832 Q13227 Q9P287 O95661 Q8N5A5 Q9UHV2 Q9HC29 O15105 900 Q13233 5925

positive regulation of molecular function P54619 Q12824 9181 P37288 P84022 57620 P63208 Q9ULW0 O14788 P07996 Q92974 P52564 P04004 25913 Q12834 O15530 P50148 5706 5707 5708 5709 P16473 51099 P16471 800 5700 5701 5702 P10809 5704 5705 Q92838 808 Q5S007 P29992 Q06124 P16435 22974 5717 Q8IUD2 57761 Q9UKW4 P61289 Q9Y297 5713 4869 5716 P31946 5970 3672 P46089 3673 P48023 Q92949 P99999 Q9BZF9 Q9NR96 3551 Q13705 9093 P21917 Q99460 5728 P17301 O60674 P20827 Q8IUC6 P18509 Q9ULZ3 3667 5604 3309 P04233 Q8WXG6 Q13956 O00220 P51668 29108 1020 O00463 P51665 114548 57669 10616 P07550 P04040 Q92530 P51671 P30556 3690 P30679 Q9NYA1 Q9Y239 O00231 O00233 P10600 P10721 O00232 P30793 3329 1029 Q9BUB1 10746 79444 Q9HCM4 Q6ZW31 P42771 847 7804 P05106 O95136 O95257 Q9UGJ0 P28223 6850 1285 Q13616 O60869 P16118 O00487 P30542 P18545 O60503 P62333 2247 Q96N67 P20618 P60033 975 Q5TCX8 857 O14763 Q92878 Q96CA5 P05112 O43182 Q92633 Q9UL46 P51575 6500 P31749 O95382 O00255 P17342 P37173 P05362 5524 Q9UL54 Q9UKT4 P06213 P01241 P23396 P04637 5300 P56199 P38405 2033 O60603 P52952 P15531 991 Q96RU7 Q8WTS1 2147 P11171 P01116 Q9C0K7 6869 Q15389 P62873 10014 11345 10016 O43166 64682 4221 6885 2280 Q99750 4214 648 4216 85360 Q01518 P49768 O00206 Q09472 5682 5562 5683 6772 Q8N726 5684 5685 5686 P14416 P52735 23787 148022 891 O14727 894 Q00535 896 8945 P01019 P25789 P25788 6647 P01137 P01135 P25787 P01133 P01375 4361 5692 5693 8721 5573 P04626 5575 5576 P04629 2064 Q9NUX5 P51532 P35348 P35226 55750 P60484 3148 3269 3146 668 5566 5687 5567 5688 10486 P01127 5568 Q96EY1 5207 10487 P23497 O43353 P26583 6672 5585 552 Q96P48 553 Q99683 P51828 10451 5577 5336 10213 7518 P55072 10454 2081 O43242 Q6IR47 7410 64127 Q9BSW2 5590 P37231 Q01955 3169 Q15077 8737 207 Q9H1A4 5347 5468 7529 Q08462 P24385 8754 4035 O00762 Q9BUZ4 O75293 Q96P20 7415 10672 O14920 P25103 9839 5371 8881 Q12933 6464 9973 P17980 P52824 P52701 23513 P16885 P17612 O14818 P51617 P58012 8517 54106 P25116 P60568 6597 Q8TEW0 11065 6598 Q9Y4K4 Q9Y4K3 3084 O43318 P13861 595 356 23043 116 P09493 118 P49810 Q7Z6C1 8767 P22694 P49815 P03950 3091 7334 5155 P55036 Q16512 4188 P12429 Q15306 7335 Q9UKE5 7448 5029 P00533 Q16401 Q13133 Q14344 1902 Q15311 Q13131 8795 P11309 Q9HBW0 8555 P12755 P35998 Q9Y6Q6 Q9UBU3 Q9UDY8 P09471 10197 P61604 P00441 P32239 Q53H12 Q9Y6R4 Q14114 4088 P18074 P55055 O43306 5170 P21359 P60520 Q16665 P43246 Q13153 P62942 Q9NSD7 8454 7124 7248 P63092 P14635 O75340 Q9UQL6 P10276 O75460 154 Q04837 Q9Y2C9 121512 Q9Y6K9 Q13042 P45379 7013 7376 Q15109 Q86WV8 P35568 P11021 Q8NFM4 P01308 Q8NFM5 120892 7249 Q9NQB0 P49721 P49720 Q9GZM8 2915 85440 Q9NRD5 10392 P11229 P54274 Q96FX2 A1XKG3 O43612 P48507 P49959 55704 P01579 51107 1956 P09038 Q15008 6188 P39905 7157 O75553 23476 Q02750 Q9UNI6 23118 Q14160 51231 Q6PID4 1609 Q9BWT7 Q15257 Q14289 2935 Q13077 9463 7046 P98155 10928 26037 P42224 26271 10935 P62195 P23945 Q7RTN6 Q92574 P22612 1616 Q9UBK2 P56524 P78536 7057 Q13526 Q13404 Q96QB1 Q02297 P10644 P31321 Q05682 Q3V6T2 7052 P31323 81565 P05062 P08575 P60900 P42338 Q9UER7 2956 7186 7189 7188 P06239 P29353 P29474 O95977 Q96M96 P29590 Q13418 P31431 7185 P40145 Q9BYM8 Q86UE4 Q9BXK5 P41231 O60266 P21980 P42684 3932 O95622 Q13426 Q13546 55072 4914 P43405 O43707 56288 2730 Q07954 Q04206 Q92786 Q92542 Q13794 Q9NZJ7 Q96EP0 O95999 P13945 P78509 P53567 Q7RTR2 P35612 P30048 B3KY43 P35611 1896 23085 Q03468 84766 P62158 Q13200 P08246 7099 7098 Q13443 P14923 Q96BI3 P30279 3728 P61073 Q9UJX2 Q05513 P00734 Q8TAI7 P30281 Q13574 Q15750 P46734 P09429 Q9NYJ8 P10415 O14495 P28065 Q9P035 29843 2643 3611 P61088 Q04656 O15455 P01730 P08588 5914 Q13586 O75832 P53350 Q9P0U3 P62191 P28074 P28072 P28070 P00519 Q9HC29 P30260 4830 Q13233 P43686 P07148

signal transmission via phosphorylation event Q13115 P36507 Q15654 65125 Q15418 Q9Y4K4 Q9NRW4 O43557 Q9Y4K3 O43318 Q9UBS0 Q8IVH8 23043 Q7Z6C1 O14544 P22694 O14543 P49137 P49815 P07996 P25963 P52564 P28335 Q16512 4188 Q8NFZ5 65018 Q8TF76 Q9UKE5 P53779 Q9Y2H1 10549 P16471 7205 P00533 Q14344 Q92844 Q13131 O15524 P52333 8795 Q5S007 O75925 Q7Z727 Q15797 8315 3661 Q9HBW0 Q06124 Q9UBU3 Q8IUD2 Q9UDY8 4868 P00441 Q8IV61 5716 Q12851 P32239 Q16539 4086 Q9Y6R4 3672 Q15208 P51812 Q86XR7 Q9NR96 3551 P21917 Q9UBE8 10645 Q8N4C8 P21359 O60674 P20827 Q8IUC6 5602 3667 5604 P04233 Q8WXG6 Q8TDY2 Q13956 7124 P28482 O00220 1020 O75582 Q5SGD2 P46109 P15056 Q9Y2C9 121512 25780 Q9Y6K9 Q13162 8569 Q13287 Q13164 Q13043 Q15349 Q7KZI7 Q96SB4 P30559 5871 Q15109 Q9UGI9 26524 P10600 P10721 P52888 P35568 Q9UKA4 29110 10746 79444 1147 Q9BUB5 P01308 120892 7249 9306 11035 6714 Q99956 O95136 O95257 Q9UGJ0 P28223 6850 4792 9448 2011 A1XKG3 P58753 P16118 Q9P0L2 P30542 P19634 Q9NS68 P18545 2247 1399 2246 P60033 975 Q5TCX8 P01579 P05230 11124 857 O14763 Q9UN86 Q13188 Q96CA5 5515 1956 P09038 O95819 P23443 5518 Q9BY84 P51451 P51692 O75676 P31749 O95382 O00255 Q02750 6733 Q6PID4 1609 Q5VVH5 P78362 P23458 Q9BWT7 1848 P09601 Q14289 Q9UL54 Q9UK32 P06213 6196 Q9Y6W6 P01241 6199 7046 6198 P56199 2033 P12931 P14314 6195 O60603 1613 P42224 Q96RU8 1852 Q9BXM7 1850 2147 10010 Q9C0K7 P22612 P42229 P98161 1616 7297 6760 7057 4221 5310 6885 5311 P35368 O95163 Q9NWZ3 P57059 56940 Q8WTR2 9908 P67775 Q99750 4214 4215 4216 Q99759 O43293 P42338 O14713 Q13098 Q9UER7 P42336 P49768 O00206 Q9Y6Y9 7186 Q09472 7189 P29597 P29353 5562 O94768 6772 P48551 6774 O15264 P14416 Q96M96 Q9H0K1 Q7Z569 7064 148022 P41597 Q00535 P17252 P42680 P06241 P42681 6647 Q9Y572 55504 Q6UXV0 Q8TAD8 P01135 P01133 P01375 64343 Q13546 9252 P35348 Q01974 P43405 Q96RR4 5566 6776 5567 5568 Q15052 O14733 5207 11215 9821 5585 P07949 Q9UNN5 Q03113 9021 Q16828 3162 9261 Q7RTR2 Q9H244 673 23085 Q99683 Q03468 5578 6548 6789 4920 Q13563 10454 P40763 P04201 Q8IWV1 7099 150094 7098 Q6IR47 Q13202 Q13443 Q14653 Q9NP71 5598 9270 P30153 27330 P61073 2873 P00734 8737 207 4139 Q15750 8754 Q15759 Q9UHD2 Q9P035 O75293 79753 1432 Q9NRM7 5599 10672 O15455 O14920 P53355 O75832 O15111 Q12933 6464 Q13227 P52824 P17612 P51617 8517 P45983 54106 Q15532 Q15653 Q13233 P45984 P25116

intracellular protein kinase cascade Q13115 P36507 Q15654 65125 Q15418 Q9Y4K4 Q9NRW4 O43557 Q9Y4K3 O43318 Q9UBS0 Q8IVH8 23043 Q7Z6C1 O14544 P22694 O14543 P49137 P49815 P07996 P25963 P52564 P28335 Q16512 4188 Q8NFZ5 65018 Q8TF76 Q9UKE5 P53779 Q9Y2H1 10549 P16471 7205 P00533 Q14344 Q92844 Q13131 O15524 P52333 8795 Q5S007 O75925 Q7Z727 Q15797 8315 3661 Q9HBW0 Q06124 Q9UBU3 Q8IUD2 Q9UDY8 4868 P00441 Q8IV61 5716 Q12851 P32239 Q16539 4086 Q9Y6R4 3672 Q15208 P51812 Q86XR7 Q9NR96 3551 P21917 Q9UBE8 10645 Q8N4C8 P21359 O60674 P20827 Q8IUC6 5602 3667 5604 P04233 Q8WXG6 Q8TDY2 Q13956 7124 P28482 O00220 1020 O75582 Q5SGD2 P46109 P15056 Q9Y2C9 121512 25780 Q9Y6K9 Q13162 8569 Q13287 Q13164 Q13043 Q15349 Q7KZI7 Q96SB4 P30559 5871 Q15109 Q9UGI9 26524 P10600 P10721 P52888 P35568 Q9UKA4 29110 10746 79444 1147 Q9BUB5 P01308 120892 7249 9306 11035 6714 Q99956 O95136 O95257 Q9UGJ0 P28223 6850 4792 9448 2011 A1XKG3 P58753 P16118 Q9P0L2 P30542 P19634 Q9NS68 P18545 2247 1399 2246 P60033 975 Q5TCX8 P01579 P05230 11124 857 O14763 Q9UN86 Q13188 Q96CA5 5515 1956 P09038 O95819 P23443 5518 Q9BY84 P51451 P51692 O75676 P31749 O95382 O00255 Q02750 6733 Q6PID4 1609 Q5VVH5 P78362 P23458 Q9BWT7 1848 P09601 Q14289 Q9UL54 Q9UK32 P06213 6196 Q9Y6W6 P01241 6199 7046 6198 P56199 2033 P12931 P14314 6195 O60603 1613 P42224 Q96RU8 1852 Q9BXM7 1850 2147 10010 Q9C0K7 P22612 P42229 P98161 1616 7297 6760 7057 4221 5310 6885 5311 P35368 O95163 Q9NWZ3 P57059 56940 Q8WTR2 9908 P67775

Q99750 4214 4215 4216 Q99759 O43293 P42338 O14713 Q13098 Q9UER7 P42336 P49768 O00206 Q9Y6Y9 7186 Q09472 7189 P29597 P29353 5562 O94768 6772 P48551 6774 O15264 P14416 Q96M96 Q9H0K1 Q7Z569 7064 148022 P41597 Q00535 P17252 P42680 P06241 P42681 6647 Q9Y572 55504 Q6UXV0 Q8TAD8 P01135 P01133 P01375 64343 Q13546 9252 P35348 Q01974 P43405 Q96RR4 5566 6776 5567 5568 Q15052 O14733 5207 11215 9821 5585 P07949 Q9UNN5 Q03113 9021 Q16828 3162 9261 Q7RTR2 Q9H244 673 23085 Q99683 Q03468 5578 6548 6789 4920 Q13563 10454 P40763 P04201 Q8IWV1 7099 150094 7098 Q6IR47 Q13202 Q13443 Q14653 Q9NP71 5598 9270 P30153 27330 P61073 2873 P00734 8737 207 4139 Q15750 8754 Q15759 Q9UHD2 Q9P035 O75293 79753 1432 Q9NRM7 5599 10672 O15455 O14920 P53355 O75832 O15111 Q12933 6464 Q13227 P52824 P17612 P51617 8517 P45983 54106 Q15532 Q15653 Q13233 P45984 P25116

regulation of phosphorylation P25054 P54619 Q9NRW4 124790 P84022 1453 Q9ULW0 O14543 5933 6901 P07996 P52564 P04004 P09936 O15530 O95684 9077 P50148 P14174 P16471 O15524 Q5S007 Q7Z727 Q9H2G4 Q92956 Q06124 22974 Q9C010 57761 1111 Q9UKW4 4869 P06400 5716 3672 P31947 Q9NR96 Q13705 9093 P21917 5728 10645 O60674 P20827 3667 64061 10641 5604 3309 P04233 Q8WXG6 P27037 Q13956 4771 1022 O00220 1020 10614 Q08999 P07550 O75909 3678 P08887 P08648 3690 Q9NYA1 P09619 P10600 P10721 P37198 1029 Q9BUB1 1027 10746 79444 960 1026 2475 P42771 3689 7804 O14757 P07766 P05106 P30304 O95136 O95257 P05107 Q9UGJ0 P28223 6850 P30307 P16118 P30542 P18545 O60503 2247 Q96N67 2246 P60033 Q92990 58533 975 O14641 Q5TCX8 O14640 P05230 857 O14763 Q92878 859 Q96CA5 5515 P05112 Q92997 5518 P31749 O95382 O00255 440193 P37173 Q9UL54 P06213 P01241 5300 P56199 P14317 2033 990 Q96RU8 Q96RU7 3479 2147 995 23560 Q99640 P01116 Q9C0K7 Q15389 Q9UII4 4221 6885 P36575 25998 Q9BT40 56940 Q8WTR2 641 Q99750 4214 8812 4216 P01588 Q13098 P01343 11116 P49768 O00206 Q09472 5562 Q8N726 O96020 P52735 3263 891 894 Q99741 Q00535 896 P35240 P01019 6647 P01137 P01135 P01133 P01375 Q9UJM3 4361 5573 P04626 5575 Q9GZP0 5576 2064 55750 Q96RR4 P17813 P60484 P17936 5566 5567 P01127 5568 Q96EY1 Q96JB5 5569 5207 Q96MH2 P24588 5580 5581 9821 5585 O43597 8851 P07948 P50613 P61981 P15692 Q9BZE4 Q99683 Q8WTW4 P51828 10451 5577 5578 10454 P04201 P05412 2081 Q6IR47 7532 7410 64127 5590 Q96SZ6 P51959 324 8737 207 54206 Q15078 Q08462 P24385 8754 7422 4035 Q9BUZ4 O75293 Q9NRM7 9839 23636 Q12933 6464 P52824 580 57732 P17612 P51617 P28749 8517 54106 56647 P25116 P60568 8655 4176 Q8TEW0 P30086 Q9Y4K4 Q9Y4K3 3084 O43318 P13861 351 595 23043 P49810 Q7Z6C1 51763 P22694 P49815 Q13485 P03950 5154 5155 Q16512 4067 4188 Q16635 5037 Q9UKE5 7448 51654 P00533 Q13131 P24522 8795 P11309 Q9HBW0 Q9Y6Q6 Q9UBU3 P46527 Q9UDY8 5159 Q8WZ42 P00441 Q53H12 Q9Y6R4 Q14114 4088 4089 Q15208 O43306 5170 P21359 Q16543 Q13153 P43489 Q8TDY2 11140 7124 11142 7248 P54132 P54253 P14635 O43639 4092 O75460 154 Q93062 Q9Y2C9 121512 Q9Y6K9 Q99728 11146 11030 Q9Y4H2 P36896 P35443 26524 P36894 Q86WV8 5071 P35568 94274 P11021 Q8NFM4 P01308 Q8NFM5 120892 7249 O15169 Q13177 85440 Q9NRD5 Q99956 P61925 P02790 7023 P11229 A1XKG3 O43612 Q9UNH7 P49959 Q01664 55704 P01579 Q9BQI3 1956 P09038 Q9BY84 O75553 Q02750 P38936 Q9UNI6 23118 7272 2810 Q6PID4 1609 P48730 Q9BWT7 1848 Q14289 Q13873 9463 7046 P98155 O95835 9467 P33993 7161 P63167 P42345 1852 1850 10935 Q7RTN6 91 P04085 Q92574 P22612 P15090 1616 1856 O94992 P78536 7057 Q13526 Q02297 P10644 P31321 Q3V6T2 P31323 O43609 P67775 P08575 P16070 P05067 P42574 P42338 Q9UER7 7186 7189 P29353 Q9H422 Q96M96 Q13418 P56539 P31431 84619 P40145 27102 P17252 O60266 O60260 O95622 A4D1W7 Q13546 Q02156 9495 P43405 56288 Q05655 Q07954 Q92786 Q9Y2B9 1647 O95999 Q15735 P78509 9021 Q16828 P30048 B3KY43 Q03468 P08246 Q8IWV1 7099 7098 Q13322 Q13202 Q13443 Q9NP71 P30153 P30279 P61073 Q05513 2873 P00734

P30281 Q13574 Q15750 P46734 Q9NYJ8 P10415 O14495 Q9P035 3611 Q04656 O15455 P01730 O15350 O75832 Q13227 Q9P287 O95661 Q8N5A5 Q9UHV2 Q9HC29 O15105 900 Q13233 5925

cellular response to organic substance 64397 P08833 P28562 6599 Q9H2Y7 P13861 O60568 595 2303 P84022 2783 P20396 P22694 Q13480 2308 5935 2549 7332 5155 O15530 Q9HAV0 54361 51094 P09382 54487 P63218 5029 Q13370 Q15796 Q9UKV5 Q9BUN8 P35638 Q9UKV8 Q15797 6256 P50150 Q06124 P59768 Q9UBU3 57761 Q9UQB8 O75807 P55290 Q92831 5295 4086 4087 P55851 4088 7476 3673 O43306 Q9UKG1 P56705 5170 P54646 142 Q96GD0 P17301 267 O60674 3667 P41743 3309 P63092 P14635 Q9UQL6 P10276 O75581 O75460 P19525 57669 1499 P35221 1012 5610 27161 4780 6048 Q9Y4H2 26524 P35568 P37198 Q9BUB1 57678 P11021 Q8NFM4 Q8NFM5 Q9HCM4 Q99958 9564 P49840 O75569 P98179 10273 Q06203 2010 Q86V24 Q99942 P38398 O60503 Q9HCL2 P61244 1277 P01579 P08069 2805 P23443 P10827 Q12778 Q9BX66 9217 P51692 P31749 1176 P14625 P18433 Q9HCK4 1843 2931 51593 Q15011 P25874 26060 P06213 P02452 P01241 6198 P98155 O95835 Q01094 3484 3481 P42224 Q96RU7 P62993 Q9Y4C1 79139 P17275 Q92572 999 P62879 Q16236 P22612 P42229 P27986 P62873 51009 10014 1978 22926 Q96QB1 P10644 P31321 P31323 Q9P2K8 P05062 1869 P01344 Q92569 P01100 1080 7067 P29353 6772 5563 Q5TAX3 6774 P13569 P19793 O95292 7184 891 P40145 894 P41231 O60266 P21860 Q9GZP9 P01137 Q8TAD8 Q9UPY3 P09874 O95622 8165 5573 5575 5576 2065 P35222 Q9UNE7 5566 6776 5567 P01127 5568 Q92667 Q5VVQ6 5584 B3KY43 10458 672 P22415 P05091 57026 P51828 5577 Q8WYQ5 P08243 P55072 3956 2626 P40763 P04201 2081 P01275 5591 O00755 5471 Q13322 8985 Q9NP71 10580 P14923 P18850 P30279 P37231 3728 Q9BXP5 207 5468 P17081 Q92922 P09467 Q08462 P24385 O14492 Q96A54 P12830 P78527 P18146 79753 2885 Q9NRM7 P68036 P17174 7415 8503 4149 P43694 5914 23636 6464 P50402 55432 P17612 Q6ZWJ1

regulation of intracellular protein kinase cascade 8772 P25054 P30086 Q9Y4K4 Q9Y4K3 O43318 O15519 2305 356 23286 Q92851 8767 O14788 P49815 57506 P07996 2308 Q9HC98 O43687 7334 Q16512 4067 4188 Q15669 7335 5037 65018 O75376 2316 367 Q04864 P14174 P09382 10783 P00533 Q13490 1902 O15524 9641 8795 Q9HBW0 P32121 253260 Q9Y6Q6 10758 Q9UDY8 142678 Q9Y297 64170 5716 Q9Y6R4 5970 P48023 Q14118 Q86XR7 Q9H257 Q9NR96 3551 9093 P17302 5728 387 P21359 389 O60674 O00182 2697 Q8IUC6 5966 Q13153 P04233 P62942 6275 Q13158 4771 7124 Q9Y3E0 O00463 P10275 154 P15056 Q9Y2C9 121512 1499 10616 P07550 O94827 P61586 1936 2904 P08887 7132 P29466 Q9Y239 Q9Y5J5 124583 5071 P37198 29110 10746 79444 960 1147 Q02763 P01308 841 2475 843 7128 7249 Q7Z7H5 Q9NQB0 O15169 P02790 10392 6850 P50591 O43734 Q9P0L0 Q9NS68 Q8WVQ1 Q8IUH5 2247 Q5TCX8 P01579 P08069 857 O14763 859 Q96CA5 5515 P05112 1956 P09038 10159 Q92633 5518 Q13501 Q12778 Q08050 P31749 O95382 Q9H8V3 O00255 Q9P0M2 23118 27032 51231 54862 Q6PID4 Q14164 Q6R327 1848 P09601 P98194 Q14289 Q9UL54 1605 Q13077 P06213 Q15811 9465 P01241 7046 P35813 O75787 P14317 7161 3481 P42345 P41134 Q9BXM7 P62993 3479 3476 9218 P01236 5536 Q15389 1616 Q9NSA1 P01112 7057 4221 6885 Q13404 9590 2280 7052 O43609 P67775 2159 Q99750 4214 4215 4216 P01588 P16070 Q99759 408 P49407 P01344 409 P01343 Q9UER7 O00206 7186 Q15831 7189 7188 P48431 P14416 Q9H422 3265 Q96M96 Q13418 3263 P56539 7185 148022 Q9BYM8 Q02952 P35240 8945 P01019 O15379 51026 P21980 55504 P01137 O14964 O60260 P01375 P04626 8841 Q13546 55072 Q7Z434 2064 P35222 P78318 9495 P35348 P19438 P43405 P60484 Q04206 P53041 Q96EY1 6657 8837 P24588 8717 O43353 9146 Q96EP0 6794 O95999 5585 O43597 P13945 P07948 Q16828 3162 9020 79971 673 Q99683 Q03468 57708 5579 P00742 3953 P05771 Q6P1N0 3956 Q96FA3 O00635 7099 7098 Q6IR47 8743 Q15628 P21580 64127 P30153 Q99558 324 P08134 8737 207 329 P48357 3965 P21333 Q9NYJ8 6453 Q9UHD2 Q9P035 Q9BUZ4 57162 2885

3611 P61088 Q14790 Q5T9L3 O15455 Q16610 O14920 9839 P41159 Q9BT67 23636 O15350 5494 Q60FE5 O75832 O15111 Q12933 Q13227 Q06830 Q86Z14 222068 Q9HC29 54106 8878 Q13233 P26447 P25116 P60568

negative regulation of protein modification process 11065 P30086 9184 991 26271 O60566 P62195 P84022 23560 6622 P49810 51763 Q92574 6901 9474 64682 P55036 Q12834 4221 Q9UBC3 Q16635 O43684 5037 25998 Q9BT40 5706 5707 5708 P67775 5709 5700 Q16401 5701 408 P60900 5702 P49407 409 O15524 5704 P49768 5705 Q7Z727 Q13257 5682 5683 Q8N726 5684 5685 5686 P32121 P35998 P53667 891 5717 Q9HCP6 Q00535 P17252 Q01105 P35240 P25789 10197 P25788 P61289 P01137 5713 P62258 Q92833 O60260 P25787 P26358 Q9BSI4 5716 Q9UJM3 8165 5692 5693 P31946 4088 Q99460 5728 P17813 Q05655 P60484 P17936 5687 5688 Q92667 Q9UL15 5580 5582 P05129 4771 7248 Q15735 P51668 1020 P14635 P51665 4092 Q9BZE4 Q13042 5578 Q92530 10213 Q13200 P04201 P05412 O43242 7375 Q9NP71 7531 O00231 O00233 P30153 O00232 P11413 Q86WV8 5071 5590 1789 1029 1786 Q9UJX2 Q05513 Q9H1A4 3720 54206 P49721 P42771 P49720 7529 O14495 P28065 P19474 A1XKG3 O00487 O00762 O60502 P62333 P20618 Q9H1Y0 857 5515 5518 Q15008 Q9UL46 8881 O75832 Q9BZS1 Q13107 P62191 P28074 O00255 P17980 P28072 P37840 P28070 10724 O14818 P30260 O15105 5524 Q15257 50943 Q9UKT4 P43686 P60568 P06213

M phase 54908 P25054 Q14204 Q9Y265 23607 Q9Y266 11065 8091 259266 9181 P51587 9184 472 P48729 Q9BVA0 O60566 1213 Q96IK1 Q96BM9 Q9BVA1 5017 P83731 Q8N3U4 P43034 Q9ULW0 P21127 10534 Q8NEH6 Q9HC98 Q92974 O43683 O75935 Q12834 O43684 Q53EZ4 O75376 24149 P16104 O60216 1104 6117 54801 10783 7329 64282 Q29RF7 P30622 P27694 Q13257 7465 Q8WVM7 O75122 Q9NQW6 22974 P51808 Q9Y2Z0 P49916 57405 54930 1111 10198 Q8NFH4 P83876 Q9Y6A5 Q5TAP6 Q5VUA4 51547 O94927 Q8WZ42 Q93008 4627 Q96EA4 P06400 P53675 O95347 P62826 O43663 Q8TD19 Q96BK5 Q9NRZ9 54820 Q15691 Q96GD4 O14578 Q8IX90 5604 8452 Q14008 Q14807 23122 10609 23244 P14635 25788 P35579 22832 1018 1017 Q96DE5 Q9H6D7 55125 3796 Q13042 O75909 10179 Q96R06 Q5TB80 P07437 Q96CS2 Q9NRC8 7013 9793 Q96KB5 Q8IZT6 26524 79441 O14753 P41208 8218 O14757 P30304 O15182 P30305 10270 P30307 P54274 Q13618 Q9Y5K6 55142 221150 P49959 Q9BQ90 11243 9319 Q9BRK4 Q9NTI5 Q9NVM9 347733 Q92878 5515 Q15003 11004 9212 O00139 5892 116138 Q8TAP9 23354 Q02750 Q6KC79 25777 7272 Q9NXR1 Q9P209 P40692 Q9NZ56 10726 54984 984 Q96MT8 Q9NTJ3 989 5888 O14777 Q9UKT4 127829 Q96FF9 Q15019 9221 6993 O95835 8379 Q09019 P63172 O95271 6194 7283 80124 990 991 26271 Q9Y6G9 55165 203068 55722 994 995 Q99640 55726 Q15021 5536 P62753 Q13885 9232 64682 Q14978 Q8WWH4 Q9Y6X3 Q8N4N8 Q9UQ88 23332 O75771 23212 Q8NHV4 3925 P67775 Q7Z460 1069 Q99871 P52292 22919 P46013 11113 Q9UI95 Q6P1K2 8812 Q9BS18 P42695 Q14980 2956 25847 9126 O95613 Q7Z7K6 10907 91272 P52732 890 1877 10910 Q66K89 891 Q9H4B7 Q99741 55743 Q92791 3014 O60266 O15379 O95619 1639 Q8N960 O14965 O43264 Q9UPY8 93323 4361 9133 Q14511 O15392 8841 O43823 23310 O95067 Q8N7B1 79023 Q9H410 9493 80254 Q15050 P23258 Q9UH99 Q9Y580 P53041 Q9HBM1 Q00526 6790 P54652 9700 Q13315 O95997 Q9P258 Q96EP1 Q12906 10459 1778 4926 3832 Q13561 3835 8607 O43482 10460 Q68CZ6 P51955 P51956 Q9BZD4 Q6PJP8 P20248 Q9UJX2 P51959 Q9UQE7 P50748 324 54443 79723 Q9H1A4 5347 3609 5901 Q96JH7 Q9Y6D9 Q9H5I1 Q86U86 91754 3980 Q02241 Q99550 O00762 22897 Q99661 Q9NRM7 Q00610 Q9HC35 5116 5119 Q14674 3070 728642 8881 P53350 Q7Z4H7 27436 P52701 79980 P63279 121441 O60232 P30260 P24941 900 10200 55559 Q2M2Z5 Q9UPV0 Q14683 5925

anatomical structure morphogenesis	P25054 3880 O14793 9181 2304 Q8IYN9 2303 284217 P84022 P21926 2300 Q92731 O14786 3875 O14543 O14788 Q9UGM1 Q92974 P25067 Q9NZG7 P08727 Q92729 Q12837 Q96KN7 P20849 P42858 3886 Q8N205 Q7Z727 136319 4990 O00167 Q06124 6927 P37023 P14384 55584 Q9UKW4 4983 55109 3655 Q9Y297 P40425 P40424 6926 Q9NZI7 Q9C009 3672 3673 Q92949 2100 Q13705 O95343 P17655 57534 P20823 2335 O60674 P20827 P26367 5604 P41743 10644 10643 P27037 4771 Q9Y463 1020 Q06587 55366 27283 P15056 1499 1012 Q8N264 3676 P61586 3678 3679 3690 P50222 P29466 P32856 Q14814 P09619 Q9NZC7 P10600 O00358 Q9BPY8 3207 1026 841 Q6PI57 Q9HCM4 Q92764 O60890 604 846 7804 A6NI15 P09622 P04179 O95377 Q9BQ95 2132 Q15915 Q9NQS3 P16118 3215 Q96N67 Q92990 1277 Q92753 Q5TAT6 Q92752 P05230 857 5879 P10826 P10827 50937 1297 O95140 Q13509 P19883 Q13506 1050 Q9HCK4 2139 3226 1289 O95390 4313 Q9UMX1 Q92982 P09601 P06576 O96004 O43278 P56199 23322 3484 3482 P11047 3479 Q8WV60 6760 P16989 1072 Q99750 Q8IWA4 O43294 P23276 Q01518 P01222 Q13099 O96028 Q09472 6774 3265 P53701 P13686 890 891 Q99500 O14727 O95180 P53708 P35240 898 8945 P01019 P49639 84062 P50440 5451 Q658W2 P35222 P35227 Q01974 P57082 P17813 P60484 51156 11331 668 6776 5207 10487 O43474 5460 Q9Y3M2 P01042 4131 Q9Y5Q3 Q6FGG2 P25490 9820 2195 Q9NX62 25945 10458 673 P15692 P31939 10451 P55075 6789 10454 P05412 P06744 O00755 5471 7410 7412 P52803 Q08117 3052 Q9UBX0 P37231 682 P36382 10229 P51959 444 688 5468 54206 P48357 7408 Q92922 10468 Q15078 P24385 4154 7422 23509 P56178 P56177 O75051 P61764 7414 10672 9839 9612 Q9UIG0 Q8NHX1 23513 P13497 P17612 8516 P27658 7428 55558 P55268 6599 5268 O75386 Q9UPN3 O75385 Q9Y4K3 O43318 230 472 Q9H161 474 P07196 Q99807 P49810 P08047 P83731 P43034 P49815 Q9NPC8 Q15303 3091 94234 4188 8543 P58304 P58546 P13631 Q02447 Q9H2X0 30813 P46531 P00533 Q15796 P43026 Q16881 P61968 Q15797 P12757 7468 P54198 P31273 P31276 P12755 Q9ULH7 O75360 257 Q96RK4 Q15561 P55290 Q16650 6383 134701 5054 7476 8323 Q9Y6R0 P18074 P36952 P68133 P13611 P13612 P35625 O75592 Q4AC94 P31270 23032 Q16665 9421 9420 7248 P63092 P35453 O75581 Q93063 P09172 P46109 Q04837 P23229 51564 P23467 Q14494 7252 P78337 8100 O43521 7490 285 P11021 7249 Q8WW38 1948 Q9GZM8 1947 P02751 Q99835 9201 5089 O75569 P13646 Q02880 A1XKG3 Q9Y6M1 Q96EV8 7020 Q08188 P48507 P08069 10155 P47895 1956 O15075 O15078 O75553 P38936 Q6PID4 O95947 Q99814 1969 Q14289 Q15375 P60842 P78545 7046 P28289 9464 6194 Q4VCS5 3913 P22735 2821 P04085 65082 Q92574 1738 P98161 P07355 P98160 O60282 3911 3912 9474 7057 Q13520 O15496 7291 7290 7052 P19338 117177 81565 P16070 P98172 P98174 Q14865 8399 7067 P19793 P29590 Q63HK5 1756 P21860 O15499 9255 91147 A4D1W7 P19320 Q8IYT8 9253 P43405 Q9NZR4 Q04206 Q92786 P07101 P07585 7088 O95999 Q13315 P78509 7082 Q03113 O43915 9021 P78504 P35613 P00749 P00747 O60487 P63000 P31260 3953 Q13563 2626 P31249 Q15742 Q9NP71 O43921 7090 Q05516 P08253 Q15750 Q9HCU4 9047 Q8IUQ4 P34741 57045 P78527 O60229 P20336 2885 Q04656 51741 P43694 3978 O15230 Q15768 Q9NP98 23077 Q9HCS4 900 51752 P00750 Q15532 P07148 O15228 O95677 Q92858 P08833 Q9NYD6 2672 Q12948 P52799 4609 P16220 P07996 Q12952 P27361 54361 Q08431 P50148 P63211 P16234 P21810 O60331 4851 4854 Q9UK55 O15524 928 P54753 Q12965 P05549 P25391 Q9NRY4 Q05397 Q9BXC9 P20936 P18858 Q9UQB3 Q92830 Q9UJU2 Q9UQB8 P03372 P06401 4627 P53675 P54764 Q92826 Q9GZX9 P54762 Q92824 3791 P07737 P54760 5970 P48023 P56705 Q7L576 P17302 5728 P20702 P17301 1368 Q99697 2697 Q9BXB1 P61296 6812 3309 P34925 P28482 Q9Y586 Q5D1E8 1385 O95470 10736 57669 P07550 P09972 4643 Q9Y2T1 4646 Q9UM47 P08648 P51671 O00238 P30556 Q6UB99 Q06210 P19544 Q8IY17 2239 P18206 960 1147 Q9UM54 5747 O95259 P05106 6850 Q06203 2253 Q13618 Q13616 2006 2247 1399 2246 P61006 O14641 4664 O14640 4665 O43184 P51693 O43186 P29323 O00255 Q6KC79 P30530 P37173 Q99496

P05121 P29317 P40337 6615 7704 P06213 P02452 O00425 4691 O00429 P04637 Q9Y6W5 2034 P13349 2033 P52952 P62993 79810 999 Q9C0K7 Q15389 P62753 P01112 4209 Q9C0K0 Q96CW9 4221 5310 Q7Z5H3 6885 5311 P35368 2043 1191 2280 O76074 84159 2277 Q15154 P48436 P01106 768 4218 Q9C0J9 P01343 P49768 2290 P50552 P48431 P14416 2054 P52735 Q99623 O60701 773 Q00535 Q96S42 Q53X93 6647 P01137 5316 8829 Q9UPY3 P01133 P01375 Q16254 Q9UJM3 P02462 4240 5573 P04626 8841 Q9UHI8 2065 2064 O00213 O60716 302 P10070 P10071 5566 P02458 P01127 6657 5327 5328 P49747 9927 6670 2070 P07942 O00628 5584 O43597 8851 P07949 Q12904 Q07157 3162 O00622 O95076 Q96PU8 O75190 Q99684 6667 Q96AX1 5578 Q8N302 P40763 P04201 P26232 5591 Q6IR47 Q5HYA8 P68032 23414 51295 324 3169 Q9NPH5 P05783 5108 7528 Q92800 5361 5362 2099 3182 333 5116 P62736 Q9GZT9 5371 P05997 P20908 582 54583 P58012 P18827 8879 P26447 P25116 8650 Q9UQP3 8655 Q8TEW0 8654 Q9Y3I0 351 595 Q04759 356 P09493 Q7Z6C1 Q7Z6C3 Q8NFJ9 Q13485 8408 P09017 P03950 9510 5154 5155 P12429 O75496 Q5TAB7 367 Q14344 Q15672 6496 6495 6256 6498 6015 O75487 P32242 Q03405 116113 P51805 P23528 5159 Q8WZ42 Q13263 P00441 8443 Q14116 4086 4087 Q14114 4088 4089 7358 Q14119 Q14118 Q9Y618 Q86YT6 P12883 Q04725 Q04724 387 P21359 Q04726 23396 P08151 P24864 10087 6259 P23771 P62942 8454 P55347 8452 6275 7124 8214 Q8TD84 2909 Q14129 P14635 P10275 4092 4093 P35579 154 Q02535 23363 Q9NYQ6 11146 Q99729 10059 Q15466 Q16790 Q15223 P45379 Q99966 Q15464 Q13043 54 6045 P60709 9314 Q9Y5J5 P36894 Q86WV8 166 Q9UKA8 Q6PRX2 338917 Q9NQB0 60 P09067 Q9UBP4 8218 85440 Q99958 Q15475 Q96T37 Q8TCU4 Q8TEY7 O95813 P14859 Q9H1Y0 Q9NQC3 2926 P49715 P09038 116150 7155 P39905 7157 O15198 P62906 25776 Q02750 P35548 8481 Q14160 1605 P78423 Q13873 O15123 O75665 7161 9341 7163 2702 P63167 Q8TAF8 P41134 Q86WK6 Q1PSW9 P17275 Q4KMG0 27122 P39060 Q6NVY8 94 P46821 P42229 O15119 1856 Q12797 Q9NNX1 Q13642 Q96QB1 Q13887 O43602 10939 Q15828 P10644 9350 P55316 79365 P39059 P05067 7189 P29474 Q15836 P29475 92129 O95613 O75881 10908 Q03014 Q13418 6096 P55327 1634 1874 P17252 P68871 O15379 P21980 O95859 O60383 Q9NZ08 Q9NSC2 P11717 Q15843 O95863 Q14517 2735 Q9Y3D6 56288 2730 Q9BXJ9 1406 2737 2736 Q8WUI4 9149 Q9H488 P41182 P41180 84525 P08123 Q9UJW2 4920 Q13683 Q13444 P35968 26005 P11532 Q68CZ1 P20248 64919 80155 Q96QF0 P08138 P10415 3860 O14495 P12830 163183 3611 Q14790 80184 P08588 O15350 O15111 O15353 Q15648 Q06710 Q04771 O60353 P24821 O15105 Q9BZR6

regulation of kinase activity P25054 Q8TEW0 P54619 Q9Y4K4 Q9NRW4 124790 Q9Y4K3 3084 O43318 P13861 351 595 23043 P49810 P22694 Q9ULW0 P49815 5933 6901 P07996 P52564 P09936 P03950 5155 Q16512 4188 O15530 Q16635 Q9UKE5 O95684 9077 P50148 P16471 51654 P00533 Q13131 P24522 8795 Q5S007 P11309 Q7Z727 Q9H2G4 Q9HBW0 Q06124 22974 Q9C010 Q9Y6Q6 Q9UBU3 P46527 57761 Q9UDY8 1111 Q9UKW4 Q8WZ42 P00441 4869 P06400 5716 Q53H12 Q9Y6R4 Q14114 3672 Q15208 P31947 O43306 Q9NR96 Q13705 9093 P21917 5170 5728 10645 P21359 O60674 P20827 Q16543 3667 64061 10641 5604 Q13153 3309 P04233 P43489 Q8WXG6 11140 Q13956 4771 7124 11142 7248 P54132 1022 O00220 1020 O43639 O75460 154 10614 Q9Y2C9 121512 Q9Y6K9 Q08999 P07550 O75909 3690 Q9NYA1 Q9Y4H2 26524 P10600 P10721 Q86WV8 P35568 P37198 1029 Q9BUB1 P11021 1027 10746 79444 1026 Q8NFM4 P01308 Q8NFM5 120892 2475 7249 P42771 Q13177 7804 Q9NRD5 O14757 Q99956 P61925 P05106 P30304 O95136 O95257 7023 Q9UGJ0 P28223 6850 P11229 P30307 A1XKG3 O43612 P16118 P30542 P18545 O60503 Q9UNH7 P49959 2247 P60033 Q01664 58533 975 55704 Q5TCX8 O14640 857 O14763 Q92878 859 Q96CA5 5515 1956 P09038 5518 Q9BY84 P31749 O75553 O95382 O00255 Q02750 P38936 Q9UNI6 23118 2810 P37173 Q6PID4 1609 Q9BWT7 1848 Q14289 Q9UL54 P06213 P01241 9463 7046 P98155 O95835 9467 P56199 7161 990

P42345 Q96RU8 Q96RU7 1852 1850 10935 995 23560 Q7RTN6 Q99640 P01116 Q9C0K7 Q92574 P22612 Q15389 P15090 1616 O94992 P78536 7057 Q9UII4 4221 6885 Q02297 P10644 P31321 Q3V6T2 P31323 56940 Q8WTR2 O43609 P67775 641 Q99750 4214 P08575 8812 4216 P05067 P42574 P42338 Q13098 Q9UER7 11116 P49768 O00206 7186 7189 P29353 5562 Q8N726 Q9H422 O96020 Q96M96 P52735 Q13418 P56539 P31431 84619 P40145 894 Q99741 Q00535 896 P17252 P35240 O60266 6647 P01137 P01135 P01133 P01375 Q9UJM3 O95622 4361 5573 P04626 5575 Q13546 5576 2064 55750 P43405 Q96RR4 56288 P60484 Q07954 Q92786 5566 5567 P01127 5568 Q96EY1 Q96JB5 5569 Q9Y2B9 5207 Q96MH2 1647 5585 O43597 8851 P78509 P50613 Q16828 P30048 B3KY43 P61981 Q9BZE4 Q99683 Q03468 Q8WTW4 P51828 10451 5577 5578 10454 P08246 2081 Q8IWV1 7099 7098 Q6IR47 Q13202 Q13443 7532 7410 P30153 P30279 5590 P61073 Q96SZ6 P51959 Q05513 2873 324 P30281 8737 207 54206 Q13574 Q15750 Q15078 P46734 Q08462 Q9NYJ8 P24385 8754 4035 Q9P035 Q9BUZ4 O75293 3611 Q9NRM7 O15455 P01730 9839 23636 O15350 O75832 Q12933 6464 Q13227 Q9P287 P52824 O95661 57732 P17612 Q8N5A5 Q9UHV2 P51617 P28749 8517 900 54106 56647 Q13233 P25116 P60568 5925

regulation of gene expression Q9UKT9 Q9H0E3 Q9Y265 Q8NDW4 Q12824 8091 124790 10657 2305 2304 2303 114991 1453 2300 155061 Q9Y275 284695 Q00839 2308 2307 Q9H0D6 Q92974 Q9H4L7 Q12837 Q8WXB4 Q8NDV7 2316 2 10661 P10809 Q96EB6 O75925 136319 Q9UKV8 4990 3660 3661 Q9BYE7 P62495 P37023 P13056 84901 57761 55588 O75928 Q9UKW6 3659 P26358 Q6NUN9 Q5BKZ1 3673 Q92949 Q99583 Q12857 Q13705 10645 1488 1487 283337 Q99576 2332 3665 10642 64061 P26367 10644 10643 Q9UL18 1022 1021 Q8NA42 Q06587 114548 Q8IX07 Q5JT82 P51787 Q96MX3 1017 10614 79576 Q9HAJ7 1499 10616 1012 O14981 Q9Y230 P61586 O75909 O14503 Q12873 Q12872 Q9Y239 Q8WXF0 Q9Y5Z7 Q8WXF1 6720 Q15906 285527 O00358 Q9BPY8 57798 1029 3207 10626 1027 1025 Q9BUB5 P17482 P17480 604 Q9Y242 P04179 Q15910 Q9UL36 Q12888 Q9BQ95 O60869 79595 Q8NA19 P38398 57326 55145 3215 Q92990 Q9Y250 6721 Q92994 Q92993 6726 Q8N488 Q92997 Q9UKS7 P10826 P10827 P10828 221937 5411 P31749 Q8NDX6 1050 P53999 79101 57332 3226 3225 P04150 Q9UGL1 A6NHR9 Q92985 O95159 Q9UKL0 O96004 O00308 P14317 P14316 23569 11108 11107 Q8WWY6 Q99523 1059 23560 Q15022 639 P62875 6749 O96018 6760 Q9Y5V3 5430 O96019 O95163 P16989 P57059 Q03933 51131 641 P33076 51132 P35251 Q14186 Q6B0B8 79923 8932 648 8939 P49407 P60228 Q14188 O00327 O96028 5440 P48552 5441 6772 Q9UKN5 Q8TAU3 P48551 6774 Q99990 346171 O60828 P15621 Q66K89 Q2M3W8 Q14192 P01019 51147 Q9Y692 Q15047 O14965 P23246 Q86X95 5451 A7MD48 Q8N3J9 P35222 Q5T6S3 P14784 P35227 P35226 O14979 P57082 O75081 P17813 P35232 Q00403 53335 56849 668 6776 4116 Q96MH2 Q16385 Q15056 7629 O43474 P06733 6790 O00744 5460 Q9Y5Q3 6311 9821 P25490 3281 Q9NX65 672 P15692 O14908 Q9UBW7 6304 57708 Q6UUV7 P05412 O00755 Q9Y5R5 Q9Y5R4 9831 3297 Q9NX70 23532 Q9UBX0 P14373 57713 688 148327 689 5467 9826 207 5468 Q8N895 Q92922 P01023 4150 4152 4154 23509 P56178 9841 P56177 Q8NHY6 Q12809 Q9Y5S9 P17844 10673 4149 O14920 57727 P62854 9839 O43463 5494 Q8NHX1 23512 Q96QT6 O14936 8517 Q92900 9849 4172 O15085 P36508 4173 4174 11060 P35659 Q14686 4176 Q14209 8535 P55265 Q9BZ95 Q6NZI2 Q9H165 4171 Q9H161 Q9H9F9 Q9H160 P08047 5017 P78395 Q9BQG0 P07199 Q13363 Q9NPC8 Q14693 Q9HAV4 94234 4188 8543 P46782 5036 79084 P58304 Q96F45 O15090 23051 Q04864 Q9Y2H8 9869 7205 Q8WY36 Q96RL1 Q15554 Q9HAU4 8553 P35638 P61968 Q8IWY8 Q96IZ0 Q9UPP1 P32121 P31274 P31273 P31276 O75123 Q9ULH7 23028 Q9NY61 4193 Q9Y6Q6 257 Q9H9D4 Q6DD87 Q15561 8548 Q96RK0 51548 Q93009 51547 Q14232 P55290 P61964 Q15562 Q9BYU1 5054 P62826 9412 Q9UPQ9 P36956 O75592 Q96F24 P31270 Q9NPF5 Q13398 55915 9421 P78347 9425 7248 O75586 O75582 Q9Y6K1 Q93062 Q03989 Q71SY5 Q9Y6K9 51564 Q15583 8569

55922 5074 10181 P78337 55929 11030 Q9HAZ2 Q15109 P22392 O15055 O43524 26523 7251 O75570 23492 O15047 P49848 Q15596 8110 P61925 Q3KNS6 Q86Y82 O15062 5089 O75569 166968 O95931 23466 Q9Y6M1 23468 O15060 Q8WY64 79913 51588 10155 Q9BQI3 9439 Q96NG5 286075 Q8NEJ9 Q9NPA8 26986 64426 5092 64428 1965 51593 Q9H9G7 54625 O95947 54623 Q14289 Q8IWS0 9463 Q96RE7 9464 P29375 P29374 27005 Q7Z589 Q9H0M0 1975 P22736 P42345 Q9BUG6 Q96EK4 Q9UGU0 Q92574 Q13761 1978 Q58WW2 P56524 9477 O75534 Q02297 7291 O75530 7290 9470 7295 P24928 Q96AB3 1982 Q6P1K2 O75528 P0C7X2 Q8WXI9 Q9BYM8 1994 1993 79039 Q96IQ9 O15499 P56545 O95983 A4D1W7 O95503 55072 P78317 P78318 Q8TBE0 Q9HAK2 Q9UH92 Q9Y2B9 P52597 Q96EP0 O14593 O95999 Q9NP66 23081 O43918 23085 Q9UKY1 Q9H9B1 P85037 P31260 Q6P1N0 P31249 Q14653 Q9NP71 Q9NP72 3728 83548 1540 Q9BUJ2 3720 5901 P46734 Q13330 P12956 Q6P1L6 Q9P031 Q9H4W6 Q00059 Q9BQA5 Q9Y6E7 Q9H582 P61088 Q96AE4 147808 Q96RG2 Q68DY9 P43694 O15455 Q9BQA1 5914 11091 O75953 63976 63978 5927 5928 P63279 P00519 5929 P63272 23077 P24941 Q92585 Q15532 Q13351 Q13352 5925 Q7KZF4 P10914 O95677 Q92858 P67809 Q9UBB5 Q9NS86 A5YKK6 Q9P2R6 4609 P16220 10772 5931 P49137 P27348 5933 O15516 5935 Q9UBC3 P27361 54361 9077 P50148 1107 O60573 284323 4613 1108 O15525 Q92838 Q9UK58 Q7Z3K6 P05549 Q9NRY4 Q99471 Q7Z3K3 O60548 Q92830 Q9UJU2 284312 O15534 P49116 P03372 Q92833 P06401 Q92831 P06400 Q92826 P07737 5970 5971 9093 Q9UBE8 Q9NRZ9 P56705 9099 P17535 P17544 Q99459 P17542 57410 400720 P61296 3784 5966 10765 P07307 5981 P50570 P11802 O00463 P52756 O00468 O95231 Q9UFF9 O14627 6829 10736 3315 Q6FHQ0 710 5976 P41218 Q8N2W9 4646 27161 5978 6827 65056 5991 93986 5993 1153 Q14938 O00472 Q06455 55250 Q9NS56 P37198 79685 3329 6839 2475 P08651 5987 O95259 Q10586 Q8ND82 P51449 Q6IA86 27148 O00488 O60506 Q02086 P61244 Q9BXG8 4664 5511 Q12770 5515 P05112 5516 26292 Q9UNY4 P08621 5518 O43186 Q12772 Q12778 O43189 Q05586 2023 Q02078 Q6KC79 10725 57459 Q99496 P05121 P40337 7704 O00425 4690 P23396 O43159 6872 6871 P04637 2034 2033 Q9NVW2 O00422 Q9UFB7 4686 4204 P01116 4205 Q16236 4209 Q9C0K0 6883 Q9BT49 4221 Q9GZR2 O43167 23210 P50548 O76071 Q9NRP7 84159 Q9P2K8 4214 P01106 6878 P01588 7727 56946 Q9C0J9 P01100 Q7Z7K2 7741 Q8N726 9921 P14416 P52736 Q9NS23 Q53X93 6886 P01137 Q16254 P52740 7750 6421 5573 P04626 23673 2064 P52747 2063 11218 Q9NS37 Q99853 P10070 P09086 P10071 10363 10362 P02458 5569 2070 5580 O43593 5585 7764 22794 25822 Q96PU8 79612 O75190 Q02930 7756 5578 5579 10336 8607 P40763 2081 5591 Q9GZV8 4261 Q6IR47 Q3SY56 64127 P37108 O75177 P18850 P15336 O75182 Q9NRL2 56987 51295 P18846 Q68CJ9 P18847 P18848 326 55657 Q92800 P27797 Q9GZU7 P01185 Q9UNP9 2099 O60907 O75164 P51843 55662 Q8NCN2 333 91748 Q9H7L9 10794 8625 P83436 5119 Q9GZT9 Q9BT67 23636 9975 4287 P62508 P51858 146050 Q08211 221656 Q9NVV9 P58012 10320 P60321 9967 P25116 Q99417 Q96HZ4 O43670 Q14566 8655 4297 Q9BY77 11186 9500 Q9UKD1 P13984 351 Q9UBS0 P21127 P25963 P09017 8662 P20290 Q15424 7332 7334 8667 7335 26205 85403 367 Q70SY1 11198 7329 Q14586 20 7341 Q14106 P11309 6496 6495 Q8IVW6 Q14103 6498 P11308 Q99909 6015 P32242 Q8N3C0 Q9Y606 84108 116113 P17096 Q14592 11168 Q96MA1 Q9UBU8 Q13263 Q8TF50 8204 Q9BY44 Q14119 Q9BY41 Q9Y618 P12645 P29084 Q86WP2 222546 Q04725 Q04724 387 Q04727 Q9NWT6 Q04726 23152 P08151 P20264 11177 11176 P23771 P55347 9541 5187 9546 11142 O15164 2909 391 Q6MZP7 23126 O43639 25788 O43638 Q02535 Q01201 2908 Q9UFW8 11146 Q15466 Q6PJG2 Q99966 Q13285 2902 5195 6045 7376 7375 Q09161 9555 23133 23135 Q86WV8 Q3KQV3 Q6PRX2 P09067 Q14140 Q9UBP4 Q99958 Q15475 Q8TEY5 Q96ME7 Q9HA82 P14859 Q8TAQ5 Q96DT7 Q9H063 Q99941 Q01664 51222 56916 P01579 Q14151 Q9UBQ5 P09038 9575 P82979 8242 10284 O75676 O15198 23112 O43623 25776 51230 1609 Q5VVH5 10289 Q15014 86 P78424 Q9NWH9 P33992 P33993 P33991 Q8TAF7 P41134 Q1PSW9 27122

91 P17030 94 O15119 1616 P45973 Q9UBK2 Q9NSA3 P78412 9112 Q8WWH4 Q96I24 Q9H7Z7 Q9H3R0 P55318 Q99081 P55316 P67775 Q52LR7 P17040 P05067 2959 Q9UBL3 O94763 P52298 92129 27107 Q9H422 Q460N5 Q03014 6096 27102 Q9BTC8 2961 2960 O95619 Q8TAD8 Q9NSC2 2969 P09874 O15151 8289 Q15843 O95625 O15156 O94776 29777 202559 P17023 Q05655 2972 79149 Q9BXJ9 P17020 Q8N393 Q8HWS3 P04198 Q9UBN7 Q15853 O75626 Q14527 9149 1660 Q8TF47 Q6NYC1 Q9H488 P41182 Q8TAK5 93474 79175 Q499Z4 P53567 79177 P30048 84525 1655 84528 P22415 1653 Q9UK80 Q03468 Q92499 O75629 O95644 150094 Q9UBG7 Q8N7H5 Q07817 P20248 64919 80155 Q8TAI7 P41162 Q9BXP5 P17081 P08138 Q9Y5B6 Q9Y5B9 P09429 O75604 P10415 Q9P2X3 P41161 P35711 23186 P09430 Q86SE9 728642 Q9P2Y4 Q13227 P55771 Q04771 O95661 O15105 Q13233 P45984 Q6NX49 Q9H6Q4 O14793 25909 9181 P51587 P17676 Q8TDD1 2547 57504 Q9HCJ0 P84022 O00151 Q92731 O14788 10413 Q7Z6R9 P08729 Q92729 Q5VTR2 P51114 O60216 79763 P14174 57510 54487 Q9H2G9 4734 Q7Z727 Q9H2G4 O00167 22850 81628 54496 6927 Q8IUD2 6929 Q6AHZ1 Q96KM6 Q8N680 811 6921 P40425 P40424 6926 6925 Q6IQ32 Q17R98 Q9C005 333929 Q9UIS9 Q9NZI7 P31943 P48382 Q9NQX0 P31946 Q9C009 2100 Q9NR96 P48380 O95343 3428 P16333 6938 P20823 55352 O60674 P19838 O60675 Q8IUC6 P62263 5604 6936 Q9Y468 Q8IZ40 Q9Y463 4772 Q9Y466 Q8TDI0 Q9UIL8 2107 55364 10856 Q86VE0 2103 P16383 27287 80324 Q08999 O14744 6945 P50222 4780 Q92769 Q14814 P28370 Q07666 O95361 80306 P10600 O95365 Q06330 2119 10865 P19419 Q6PI57 Q9HCM4 4775 O14753 847 Q92766 4790 4791 4792 Q9H2K0 2130 81669 Q9NR48 Q92753 4302 857 Q92754 Q13501 22827 Q13503 Q9NR50 Q14839 Q8NC51 P19883 Q13506 Q9NR55 Q9HCK5 2139 861 862 863 192669 Q9UMX1 4799 P09601 50943 Q9Y3Y2 Q9BS34 10492 P15408 Q9Y3Q8 3481 Q9UIH9 23326 O60603 25988 P15884 Q8WUU5 Q96T88 3479 Q8WV60 2146 3476 10011 P25205 7832 10013 84289 Q96KG9 55723 10499 10014 135295 4343 Q96T76 O14717 Q99750 406 Q9BRP8 O43294 408 84271 409 Q96C55 Q09472 Q8N1G0 O43251 23309 64215 890 Q00653 891 Q99743 898 29080 Q9UN42 P49639 P61326 P51531 8721 7874 148156 Q9NR11 P51532 Q96ST3 P60484 Q15291 11331 55758 10001 P60002 10488 Q96T58 P28702 P23497 P26583 Q9Y3M2 P46199 P50613 P51946 23764 25942 Q8NFW5 Q9NUQ3 Q8WUY8 433 10450 8726 Q8N5U6 90993 Q08117 3054 Q05086 Q9BRU2 29079 P37231 Q9HCE1 79723 8737 10468 7422 Q96GA9 5245 3068 Q9HCD5 P52815 22893 3066 Q9UMN6 3065 Q9UIF9 10437 O75290 79753 10438 Q00613 Q96P20 55787 10432 3070 Q13901 9612 Q9UIG0 P52824 466 468 55796 P28749 Q9NQL9 7428 P04792 6595 6597 6599 6598 O75386 P10599 6591 Q9Y4K3 Q76L83 3084 473 474 23286 9617 8767 P38919 Q13127 Q15303 B2RXF5 3091 Q16633 8301 Q15306 Q86VP6 O75376 P58546 P13631 24149 3096 5270 Q02447 O75381 Q9NVP2 51773 30813 P46531 Q13133 Q15796 Q16649 Q15797 9640 P12757 Q8N6I1 9643 7468 P54198 O75367 Q9UNL4 P12755 O75362 O75360 P46527 51780 Q01101 P42167 6128 O94805 7458 Q13144 Q5VUA4 54815 Q8WVM0 Q16650 Q13148 P35869 P20393 7476 Q16656 Q15329 Q8NCA9 P18074 Q8WVL7 P55055 P10588 P10589 93166 Q86VN1 Q16665 51317 Q49AN0 P43489 Q8TDY2 9667 Q16666 Q5VUG0 P54132 P35453 Q02413 Q14494 8328 Q14498 Q16670 P46108 Q14011 Q86W54 Q9NRC8 7013 7014 P46100 Q5PSV4 7490 P13682 Q9C0F3 84232 Q8WW38 Q9NRD5 Q99835 7024 7023 10392 Q9C0F0 49854 P98179 P15822 9682 7020 Q02878 Q9UNH7 51341 54851 Q9NVM4 Q96PK6 11243 P55010 Q13185 Q8NCF5 P23443 6188 Q15369 Q8IUX7 Q9BSM1 Q09028 6182 P11474 26747 Q15370 54862 P57682 Q6PID4 Q99816 P48730 9208 Q99814 P78545 6199 7046 6198 P35813 O43711 6193 O43719 Q9Y4C1 P62195 Q4LE39 O15234 9219 P05177 A8K8V0 7057 22803 112950 22806 22807 57473 P46934 P46937 83746 Q96GN5 O60296 Q4LE28 Q9UER7 Q14865 7068 7067 Q5TAX3 Q15714 Q9H307 P19793 P29590 Q15717 Q7Z2Q5 Q63HK5 Q01196 84619 Q9H6W3 P41235 1756 Q92793 Q96GM5 Q9HD15 Q9Y4E5 O60264 P05155 P59817 Q13547 Q13546 9252 7073 P41223 57018 Q9NZR4 Q04206 Q92786 Q92785

Q92784 P41229 7088 9261 Q01167 Q9BSG1 51720 Q14894 4800 2626 7099 7098 Q9NVC6 Q9NQZ2 7091 P14923 7090 Q9NQZ8 P30153 1789 1788 Q05516 1786 Q9UIV1 1785 375790 Q13573 Q9HCU4 P55884 93134 Q9H2S9 Q96CJ1 P78527 92283 85509 Q86VK4 84661 51742 Q6ZNG0 P05198 P20333 Q9Y4A8 30827 Q13107 Q96H20 O60238 Q9HCS4 902 Q9HD40 Q13112 Q9NZN8 P08235 P21675 Q92618 Q9H609 Q92616 Q9Y2W7 Q9NYD6 Q6P2D0 O75820 2672 Q12948 22992 O00268 Q6QNY1 O00267 79894 3516 Q9BZI1 Q96JM7 Q96JM2 Q5VWX1 283248 Q9ULW3 Q92610 4841 Q8N587 P07996 Q12950 Q12952 Q9Y2X9 Q8NB12 Q8NAP3 79885 4851 4854 Q96JL9 Q92600 5705 Q9ULX9 Q9NYF8 Q9Y2Y8 Q12968 29128 10514 83933 26097 Q9UQB3 4862 O75807 57649 Q6IT96 4869 5716 Q96JK9 Q12972 Q0D2J5 Q9H9T3 3551 Q6P2C8 5728 10524 P17301 Q9HBE1 Q99697 400961 Q9ULZ3 Q9UHR5 Q9ULR5 P28482 Q9UHJ3 Q12986 1385 29108 P19532 Q9P0J0 57669 P15173 29102 P07550 P04040 58517 10971 7913 O14867 Q96BA8 10973 Q9UM47 Q9Y2T7 Q96SB4 Q9UHK0 Q9NYA1 O00233 P10721 P19544 Q8WYH8 1388 29115 Q9P0K8 1387 1386 P42771 Q9UM54 4899 Q9H1I8 10985 Q9NYB0 22937 22938 Q9UHL9 22931 2247 284119 1398 58533 Q9BZK7 6601 Q9Y2V2 6605 57209 Q9UM63 P51693 P51692 P31629 Q9BQY4 O00255 Q8NEZ4 Q9NQ33 O00257 Q03164 Q9P0M6 440193 984 Q5H9I0 988 P05362 Q9Y2W1 P06213 Q9UPV9 P13349 2274 P53803 P52952 Q8WTS6 126208 Q96RU8 Q96RU7 79810 51003 79811 79813 6625 51008 10138 Q9UQ80 Q04917 P49770 Q9UM07 Q9Y2P0 Q7Z5H3 Q9UQ88 5796 Q5T7W0 O60739 8819 P48436 8812 P01344 P01343 O00206 2290 23429 P48431 Q96JC9 3142 Q9NU63 148022 Q99623 Q00534 Q96S42 Q8NAF0 P49756 55869 7975 5316 51028 91949 Q9UPY3 P01375 Q9UPY8 23439 6660 P49750 8841 Q9UHI6 O00213 Q3KNV8 23435 Q96AQ6 3151 O60716 10128 Q96RR4 Q3KNW1 3148 Q15170 546 3146 P61457 Q96EY1 6657 O43353 6670 6672 23409 Q9Y2K7 Q9ULJ3 Q9H9S0 3162 Q12905 O95076 Q8WYA1 Q12906 Q9BZE0 Q01844 Q9BZE4 P42704 3159 Q99684 6667 5336 P49336 P62714 P05771 Q9H5J8 Q969G3 8864 7533 P55198 Q9ULK4 23411 23414 3171 Q00987 Q96S94 3169 7529 7528 Q9H5I1 64375 P50750 Q86U86 3187 Q9UPT9 3185 3184 571 3182 P51608 P68036 7536 6207 Q9H5H4 Q01826 5371 3192 8880 P27540 Q60FE5 Q9UPU9 Q12933 Q9ULM3 7555 Q9ULM6 P51610 Q9BUY5 580 P16885 54583 80854 P51617 Q9NPJ6 7549 54106 8878 P60568 6231 8892 Q15654 O43432 8896 P15923 Q86YW9 29947 Q96FV9 84324 Q7Z6C1 Q13485 O94906 P03950 O00716 Q16512 Q16514 P12429 Q15669 O75496 O75494 Q7LBC6 P10242 P10243 P10244 84312 51412 O94915 Q13492 Q16520 6239 54925 Q15672 Q9UQR1 7101 6256 Q16526 Q2M1K9 Q9UDY8 7579 9774 4086 7112 4087 4088 Q8N9N2 4089 O75478 9775 163126 O75475 Q8N5F7 142 P24864 P62701 84333 Q15697 53615 Q13033 P54259 O75469 8458 64750 7124 P54252 P54253 Q96KC8 P14635 O75461 Q9UQL6 P10275 4092 P10276 4093 O75460 4094 154 P23588 Q99728 Q99729 Q13045 55806 P49736 7132 8463 7133 8467 9314 O43889 P36896 23378 P15976 Q96C28 1822 166 55810 P01308 338917 Q9NQB0 Q99717 1828 Q15233 Q96T37 9326 P54274 O75446 P36402 O75444 6294 7141 Q99700 1831 Q96T23 Q00577 Q99708 P49711 Q16576 P49716 P49715 55827 Q9NQC7 Q96C00 64784 7158 O75437 Q08050 P39905 7157 162239 Q8N9R8 Q02750 P35548 138151 55832 Q9UDV6 Q5VTD9 Q13077 26038 Q06787 Q86Z02 56252 Q01094 Q9H204 26039 7161 P63167 P42224 10933 10935 P17275 P63165 Q9BVI0 P42229 57109 P42226 O15372 O94992 Q9P0W2 22926 Q969S8 22927 Q13404 Q13887 Q01085 57592 Q8IY57 57594 P10644 57591 Q96BD5 79365 P84095 Q08945 Q9UHX1 1869 7186 7189 7188 Q8WYK2 O43812 P11940 7181 7182 7185 1877 Q86UE4 Q86UE3 1874 Q9Y3C7 P17252 1870 P09630 O15379 Q969R5 Q13422 O15391 Q8NB78 2735 P19438 O43829 84733 1408 1407 1406 2737 2736 Q8WUI4 27327 Q02386 Q7RTR2 P62277 1896 Q8WYQ5 Q9HC52 Q14774 P22670 Q9NQ92 9391 P11532 Q96G25 84759 58487 Q8WYP5 P23511 Q8WUH2 3609 3608 3607 Q14781 P23510 P21333 94104 O14495 Q9P0T4 29966 P19474 Q02363 58491 P18146 84305 27300

O60341 Q969V6 O15350 O75832 O15353 Q15649 Q9BZS1 Q15648 Q9P0U4 P19484 Q86YP4 Q06710 Q96BF6 Q8N5A5 3622 Q9HC29 O14497 Q15652

rRNA metabolic process 92345 9221 Q92979 O43159 11340 Q12788 P62081 O00541 Q06265 P18124 6194 Q9BYG3 10813 Q9H6Y2 Q9Y3A5 54512 Q9Y2X3 6229 Q14690 P62913 P62753 65083 1736 Q15024 Q9UQ80 P38919 5393 5394 Q14978 5036 6125 51096 51010 Q9Y3B2 51013 Q9NPD3 P46777 Q96EB6 P62249 Q8N726 O00567 Q8TED0 Q9NV06 Q5TAP6 Q8WVM0 P78316 P46087 P08708 9775 P18077 Q96B26 10528 P62263 55759 Q9UET6 134430 O95478 50628 Q9NYH9 P39019 23404 23481 29102 Q8NI36 8602 84365 P62312 Q8NEC7 Q14137 Q9Y2L1 51729 6201 Q13601 23411 23378 23016 Q96G21 Q9Y5J1 1029 6839 Q9NY12 11157 P42771 P62847 118460 P62841 Q8IY81 2091 9045 P22087 22894 10438 54853 Q5RKV6 P50914 88745 56915 P62857 Q9GZL7 P56182 O43463 Q9UNQ2 Q13901 Q13868 Q9H6R4 Q01780 Q969E8 Q9NQT4 Q9NQT5 Q96EU6 4839 Q9BVJ6 P61254 Q96HR8 23076 10607 Q9Y2W2 6217 Q14684

regulation of transferase activity P25054 P54619 Q9NRW4 124790 Q9ULW0 5933 6901 P07996 P52564 P09936 25913 O15530 O95684 9077 P50148 P16471 Q5S007 Q7Z727 Q9H2G4 Q06124 22974 Q9C010 57761 1111 Q9UKW4 4869 P06400 5716 3672 P31947 Q9NR96 Q13705 9093 P21917 5728 10645 O60674 P20827 3667 64061 10641 5604 3309 P04233 Q8WXG6 Q13956 4771 1022 O00220 1020 10614 Q08999 P07550 O75909 3690 Q9NYA1 P10600 P10721 P37198 1029 Q9BUB1 1027 10746 79444 1026 2475 P42771 7804 O14757 P05106 P30304 O95136 O95257 Q9UGJ0 P28223 6850 P30307 P16118 P30542 P18545 O60503 2247 P60033 58533 975 Q5TCX8 O14640 857 O14763 Q92878 859 Q96CA5 5515 5518 P31749 O95382 O00255 P37173 Q9UL54 P06213 P01241 P56199 990 Q96RU8 Q96RU7 995 23560 Q99640 P01116 Q9C0K7 Q15389 Q9UII4 4221 6885 56940 Q8WTR2 641 Q99750 4214 8812 4216 Q13098 11116 P49768 O00206 5562 Q8N726 O96020 P52735 894 Q99741 Q00535 896 P35240 6647 P01137 P01135 P01133 P01375 Q9UJM3 4361 5573 P04626 5575 5576 2064 Q9NUX5 55750 Q96RR4 P60484 5566 5567 P01127 5568 Q96EY1 Q96JB5 5569 5207 Q96MH2 5585 O43597 8851 P50613 P61981 Q9BZE4 Q99683 Q8WTW4 P51828 10451 5577 5578 10454 2081 Q6IR47 7532 7410 5590 P37231 Q96SZ6 P51959 324 8737 207 5468 54206 Q15078 Q08462 P24385 8754 4035 Q9BUZ4 O75293 Q9NRM7 9839 23636 Q12933 6464 P52824 57732 P17612 P51617 P28749 8517 54106 56647 P25116 P60568 Q8TEW0 Q9Y4K4 Q9Y4K3 3084 O43318 P13861 351 595 23043 P49810 P22694 P49815 P03950 5155 Q16512 4188 Q16635 Q9UKE5 51654 P00533 Q13131 P24522 8795 P11309 Q9HBW0 Q9Y6Q6 Q9UBU3 P46527 Q9UDY8 Q8WZ42 P00441 Q53H12 Q9Y6R4 Q14114 Q15208 O43306 Q96BK5 5170 P21359 Q16543 Q13153 P43489 11140 7124 11142 7248 P54132 O43639 O75460 154 Q9Y2C9 121512 Q9Y6K9 7013 Q9Y4H2 26524 Q86WV8 P35568 P11021 Q8NFM4 P01308 Q8NFM5 120892 7249 Q13177 Q9NRD5 Q99956 P61925 7023 P49840 P11229 P54274 A1XKG3 O43612 Q9UNH7 P49959 Q01664 55704 1956 P09038 Q9BY84 O75553 Q02750 P38936 Q9UNI6 23118 2810 2931 54984 Q6PID4 1609 Q9BWT7 1848 Q14289 9463 7046 P98155 O95835 9467 7161 P42345 1852 1850 10935 Q7RTN6 Q92574 P22612 P15090 1616 O94992 P78536 7057 Q02297 P10644 P31321 Q3V6T2 P31323 O43609 P67775 P08575 P05067 P42574 P42338 Q9UER7 7186 7189 P29353 Q9H422 Q96M96 Q13418 P56539 P31431 84619 P40145 P17252 O60266 O95622 Q13546 P43405 56288 Q07954 Q92786 Q9Y2B9 1647 P78509 Q16828 P30048 B3KY43 Q03468 P08246 Q8IWV1 7099 7098 Q13202 Q13443 P30153 P30279 P61073 Q05513 2873 P30281 Q13574 Q15750 P46734 Q9NYJ8 Q9P035 3611 O15455 P01730 O15350 O75832 Q13227 Q9P287 O95661 Q8N5A5 Q9UHV2 900 Q13233 5925

regulation of localization P25054 23607 O14672 2550 9066 10657 P61020 3516 54474 P37288 P16220 284217 P84022 57620 90678 O14786 O14788 6901 P07996 Q9UBC1 P04004 P28335 P52565

O15530 Q92729 Q5VTR2 65018 O95684 Q08431 P50148 2316 P16234 P14174 51099 4734 Q96EB6 808 Q7Z727 P25391 3783 Q06124 Q9BXC9 57402 6927 P37023 P62491 57761 O60543 811 Q9Y6A5 5716 P53675 Q9H1D0 3791 P31946 3673 1374 Q9BZF9 Q9NR96 P21917 P48380 P17302 57534 5728 P20823 P17301 O60674 P20827 P19838 2697 3784 6812 Q9ULZ3 3667 6814 948 5604 Q96JJ6 P41743 O75915 P04233 P25098 O95477 4771 P28482 1385 29108 1020 O00221 114548 P51787 P39019 1137 57669 1499 Q6PJW8 1012 P07550 P61586 4645 4646 P08887 P61106 O14745 719 P51671 P30556 3690 22846 Q9NYA1 P29466 P30559 5991 P09619 Q07666 O95361 O00233 P10600 P10721 Q06330 1029 57678 P18206 10626 P20648 Q9HCM4 3685 604 967 P42771 Q9UM54 5867 4659 5869 P05106 4790 Q9UGJ0 P28223 6850 4792 Q14703 2010 P30542 P62330 O60502 Q9HCL2 2247 Q92990 Q5T2W1 857 Q9UN86 859 5878 5879 P09958 Q9UKS6 P51575 P10827 P30536 Q9UGK8 P51692 P31749 Q7L8A9 P19883 O00499 P48061 10724 867 Q9UMX1 Q9ULV0 P05362 P05121 P09601 P29317 50943 Q9H4M9 4318 26060 P06213 Q15019 Q86UW7 O00308 2033 P50416 2150 P50897 O60603 23327 P52952 Q96RU8 Q96RU7 Q8WTS1 3479 2147 23560 6622 999 10013 5898 6869 Q15389 P27986 10014 5311 5796 Q9HDC5 25998 2280 Q93096 9908 2159 O76070 Q99750 2277 4214 Q15036 P01343 Q16363 11116 O00206 Q09472 Q8N726 5321 4354 P14416 64215 891 P14780 773 Q00535 P35240 Q53X93 P01019 8943 6405 P01138 P01137 55503 8829 Q8N960 P01133 P01375 Q9Y5X9 4240 8720 Q9Y696 5213 P35225 2065 P35222 Q01974 781 O14617 301 302 P60484 P17936 P10071 5566 P01127 5569 10488 5328 O14974 P23497 Q59EA4 P24588 P24043 P07942 5460 6672 23409 5584 P05408 P07948 Q5R372 Q9UNN5 3162 552 Q96P48 553 P15692 Q9BZE4 Q96PU5 5336 5578 5337 5338 Q92930 Q6IR47 Q7Z5L7 10460 10580 64127 23411 O76024 O00631 O76021 Q9BSW2 5590 P37231 P16671 324 Q15077 8737 207 5468 P55085 P46059 7529 10468 P27797 7781 7780 P01185 8754 7422 4035 Q8NER5 Q96P20 127435 P61764 7414 10672 P25103 P26678 P01178 Q9BT67 O43581 Q60FE5 8766 580 P50402 P16885 P17612 O14939 54106 55558 9846 P25116 P60568 Q13477 Q15654 11060 Q16623 O43557 P10599 Q9Y4K3 94121 3084 Q04759 Q9UBS5 51762 P09493 P20396 Q9BRI3 Q7Z6C1 P49815 Q13485 P25963 P03950 Q15303 3091 5154 5155 4067 4188 8301 Q16635 P12429 19 5270 Q9H2X0 7448 Q13492 7205 5029 P00533 Q13370 Q13133 Q14344 20 Q9HCY8 7220 7341 Q13017 P22681 Q8WVM8 23144 Q9Y2I1 Q9Y6Q6 495 Q9UBU3 Q00013 P51805 P09471 Q96RK4 5159 P02741 P55290 6262 5174 5295 5054 4088 4089 Q8WWN8 Q14118 P55055 Q9UKG1 Q86YT6 382 5170 222546 O75592 387 P21359 51552 1808 Q13393 Q16665 Q8IXZ2 P43489 Q13275 P62942 9784 Q16787 7124 11142 Q96KC8 Q9UNF0 P14635 Q9UQL6 4092 394 274 154 396 Q93062 156 51564 Q99728 11146 Q16555 Q99726 Q15466 2904 7376 Q96C24 11030 O43889 Q9Y4H2 23256 P35443 P36894 P13796 5071 P35568 285 P01308 7249 Q9NQB0 2917 8218 P48995 P49841 Q99835 P46459 9564 P61925 P49840 Q7Z7G2 Q96FX2 A1XKG3 O43612 Q9Y5K6 Q86V24 Q14C86 P01579 P08069 P00367 11124 Q9UEW8 Q9NQC3 1956 P09038 P23443 Q9NQC7 P54289 Q9BX66 P39905 11252 Q8N9R8 Q02750 P37840 P11234 P11233 2932 2931 3909 Q6PID4 1969 Q14289 Q16581 1605 87 P53367 Q13873 7046 6198 O95835 O15123 P12814 Q4VCS5 Q9BXM7 10938 P63165 P04083 P39060 P04085 94 P07355 Q9NSA1 3911 3912 P78536 7057 O15496 Q96QB1 Q02297 7295 P46934 Q9HD26 Q9Y6H5 P08575 8399 P52298 7189 7067 P06239 P29474 P29475 9368 57120 27347 Q13418 P56539 P41231 60412 P17252 84502 P21860 3932 O60260 8165 Q8WXH2 A4D1W7 Q13546 27236 Q6UWE0 P35900 Q9UNE2 9495 P21757 P43405 O43707 Q07954 Q92786 Q8HWS3 Q9Y2B9 Q9UBN7 O60271 2737 Q92667 9388 Q8WUI4 O00186 P41182 P41181 P51148 O43915 8174 P78504 Q9H244 P23508 P00749 O00194 84766 P63000 P00742 4920 O15554 Q13563 P62158 P47712 7099 7098 Q13322 P35968 Q13443 P14923 P62166 3728 P21579 Q05513 P00734 1540 P17081 P22303 P23510 P21333 Q9BY11 P10415 P12830 Q9H223 P20338 P20336 Q9Y6E7

3611 O15455 P41159 Q13586 O75832 Q9BZS1 O15230 Q06830 P62070 Q9HC29 23077 P43681 O15105 Q15653 P08237 Q13233 P21554

positive regulation of macromolecule biosynthetic process P10914 Q92858 O14793 Q12948 Q12824 Q6QNY1 O00267 P17676 3516 2547 2304 4609 2303 P16220 Q96JM2 P84022 Q92731 O15516 P07996 2308 5935 25913 Q9H4L7 Q12837 Q5VTR2 54361 Q8NAP3 P16234 4851 5705 O75925 Q9Y2Y8 Q12968 6927 P37023 6929 4862 811 Q9UJU2 P49116 O75928 P40425 P40424 6926 Q6IT96 P06401 3659 6925 P06400 Q96JK9 5970 3673 2100 Q9NR96 P56705 P48380 O95343 10524 10645 P20823 P17301 P19838 Q8IUC6 P61296 3667 5966 Q9Y463 P28482 1022 1385 P19532 O00468 6829 1017 10736 1499 P15173 80324 P07550 4646 6827 Q9UHK0 4780 Q92769 5991 6720 P28370 1153 Q14938 O95361 O00233 P10600 P19544 O00358 Q9NS56 Q8WYH8 Q06330 P37198 10626 29110 P19419 Q9P0K8 1387 P17482 2475 P08651 P17480 4775 Q9UM54 P07766 4790 Q12888 6850 4792 Q9H2K2 22937 P38398 2247 Q92990 Q9BZK7 6601 Q92753 6721 Q92993 Q92630 Q12770 P05112 Q9UM63 Q92997 Q13501 Q9UKS7 P10826 O43186 Q12772 P10827 Q12778 P51692 Q13503 P31749 Q05586 O00255 1050 Q03164 861 P09601 P40337 50943 Q9Y2W1 P06213 O96004 6872 P04637 2034 P13349 2033 2274 O95271 Q9UIH9 23326 O60603 25988 P15884 P52952 3479 2147 10011 10013 84289 Q16236 10014 10138 P01112 Q04917 Q9C0K0 6760 4221 Q96T76 641 P33076 P48436 4214 P01106 406 Q9BRP8 P01588 O43294 408 84271 P49407 P01343 P01100 O00327 O00206 Q09472 P48552 P48431 9921 6774 3265 148022 890 Q99743 Q14192 Q96S42 898 Q53X93 6646 P01137 5316 P49639 P01375 Q9UPY8 P51531 6660 5451 P35222 Q9NUX5 P51532 Q96RR4 P17813 3148 P10070 P10071 3146 P61457 668 6776 P01127 10001 6657 P23497 P26583 O00744 5460 6672 Q9Y5Q3 P50613 P51946 3162 Q12905 Q12906 Q9BZE0 672 P15692 79612 3159 Q02930 6667 Q8N5U6 P40763 P04201 P05412 5591 O00755 4261 Q6IR47 10580 7533 P18850 Q05086 23414 3171 P37231 P18846 P18848 688 3169 148327 9826 207 5468 Q92922 P27797 7422 P52815 O60907 Q9UHD2 3066 Q9UMN6 Q9UPT9 3065 3182 P17844 8625 10432 23636 P27540 5494 Q9UPU9 6464 P62508 Q9UIG0 O14936 466 P51617 P58012 468 7428 10320 54106 8878 9967 P25116 P60568 O15085 6595 Q14686 6597 4297 Q9BY77 6599 6598 P15923 P13984 Q9Y4K3 351 Q9H161 474 Q9UBS0 Q04759 P08047 Q7Z6C1 Q13485 P25963 O94906 Q9NPC8 P38919 3091 5154 O00716 5155 8301 Q15306 Q86VP6 P58304 P13631 367 Q04864 51773 Q70SY1 P46531 Q13492 Q13133 Q15796 Q9UQR1 6496 P35638 6495 Q15797 6256 Q96IZ0 P12755 Q9ULH7 Q9NY61 O75360 Q9Y606 Q2M1K9 Q15561 P17096 Q13144 51547 Q13263 Q15562 Q16650 4086 P35869 4087 8204 4088 P62826 P43490 4089 7476 9412 9775 P18074 Q8WVL7 8445 P55055 P12645 P36956 222546 P08151 P24864 P20264 P62701 Q16665 Q13033 9421 P55347 O75469 7124 P54132 P54253 O75586 391 P35453 Q9UQL6 P10275 P10276 4093 154 Q9Y2C9 11146 Q99729 Q99966 Q13285 2902 Q14011 7132 5074 Q9NRC8 7376 8463 Q9HAZ2 Q9Y4H2 P36896 O43524 7490 P35568 55810 P01308 338917 Q9NQB0 Q8WW38 Q14140 Q99958 Q15475 Q99835 Q96T37 Q8TEY5 7023 5089 P98179 P14859 Q96T23 54851 Q01664 Q96PK6 56916 P01579 P49711 P08069 10155 P49715 P09038 P23443 55827 9575 Q9NPA8 7158 Q9BX66 P39905 7157 O15198 6182 P11474 5092 26747 55832 Q6PID4 Q99814 Q14289 6199 7046 6198 9464 P35813 P29375 27005 Q01094 7161 P22736 P42345 Q9Y4C1 Q1PSW9 P17275 P62195 91 P04085 94 9219 P42229 O15119 P42226 Q9UBK2 Q58WW2 P56524 7057 22926 22807 Q13887 Q8IY57 P55318 P84095 Q4LE28 P05067 1869 O75528 7189 7067 P29353 Q06643 P19793 Q03014 7182 6096 Q01196 P41235 56034 Q92793 Q96GM5 Q9HD15 O95619 Q9NSC2 Q9NRA1 8289 Q13422 O15156 O94776 Q13547 A4D1W7 P78317 2735 P19438 P43405 1763 Q9HAK2 Q9BXJ9 Q04206 Q92786 Q8HWS3 Q9UBN7 1406 2737 2736 Q15853 O14593 O95999 9149 P35610 Q8TAK5 P53567 1655 P22415 Q9UK80 4800 P08246 2626 7099 7098 Q9NVC6 Q9NP71 P20248 64919 80155 Q9UIV1 P00734 P23511 375790 3609 P17081 3608 5901 P46734 P09429 P12956 29966

P78527 P18146 Q9H4W6 P35711 Q9BQA5 84305 P43694 O15455 P01730 5914 O15350 Q9BZS1 Q15648 P55771 P19484 Q06710 Q04771 63976 5927 P63272 P24941 Q9HCS4 O14497 Q92585 902 Q15532 Q13233 P21675 5925

multi-organism process Q7KZF4 P06899 3880 Q12824 O00268 P52799 O15519 5817 P37288 P16220 P84022 54476 Q92973 3875 O14543 P08842 5933 57506 Q9Y3Z3 P28799 Q9UIQ6 Q9UGM3 P08729 Q08AM6 P08727 P27361 Q12959 54361 Q08431 P20963 P62244 P42858 P16471 P38484 P15260 P48039 5701 4734 5702 10542 Q9UI12 P10809 O15524 Q96EB6 P05787 Q9ULX9 Q92956 3661 79671 57402 3416 Q92830 6921 Q9Y297 P03372 64170 P62258 Q6IT96 P06401 4869 Q92831 P06400 P05556 O95347 Q92824 3791 5970 3673 P48023 Q9NR96 1130 P21917 P56705 P17544 3428 10524 81622 P17301 Q99698 1487 Q8IUC6 P18509 3665 705 Q9H1C4 708 P11926 P27037 P28482 Q12986 1385 3442 1021 90480 P18564 114548 P19525 P21964 3315 10616 P61221 2224 3312 5610 5611 P61586 3678 P08887 P08648 P51671 3690 Q96SB4 Q9NYA1 P29466 Q9Y239 P30559 P53618 P51679 3452 P10600 P30550 Q96J02 P30793 3329 27297 6839 57678 29110 3448 10625 1147 P60022 841 3445 1387 3443 3685 P05141 3688 10980 P07686 6714 P05388 P05106 6850 4792 P30307 P51681 Q06203 Q13617 Q13616 Q9H9Y6 O60506 3459 Q9HCL2 Q05469 P20618 P60033 P15151 975 4543 Q92993 857 P51575 P10827 P29320 P51692 2023 1174 O00255 O00499 P54725 1050 P48061 3107 O60870 5886 P05362 P05121 4799 Q92985 7706 P06213 Q9UKL0 6872 6993 O00308 P04637 Q86V81 10492 P15529 5422 2033 P14555 O60603 245934 Q9BU70 23327 Q01523 Q01524 Q96RU8 Q7L523 P62993 1058 995 6622 6504 P01116 5898 P01236 10015 6869 P27986 P01112 Q9UII4 4221 5553 P14324 1072 P13236 1191 O76074 Q9Y6Y0 56829 P01588 8815 P01100 O00206 6890 Q9Y6Y9 Q09472 P26641 5683 6772 5321 5684 5685 P48551 6774 P12236 3265 O95292 3263 P13686 P12235 148022 P11387 Q66K89 P13693 O60942 54413 Q00534 Q96S42 8945 Q53X93 P25789 Q9H6F5 5434 P25788 6647 P01138 P01137 Q15046 P25787 P01375 P01374 4240 P01130 P23142 5692 5693 10482 O14980 7874 O00571 23435 P14784 Q86UT6 Q9NS37 Q00403 3148 P01009 6776 5688 10488 Q96T58 5328 P23497 Q59EA4 8837 P24588 Q15056 P01243 O43353 6670 P06733 P26583 6672 8732 P04439 Q96BZ9 P07948 P59665 23764 O95197 552 3159 Q99683 Q96PU5 P51828 6304 6667 7514 5335 5336 5337 P61626 10577 P40763 Q6UUV7 Q96FA3 P05412 Q07021 P49450 5471 Q6IR47 5473 7531 7412 O75179 3178 64127 3297 23411 Q08357 3054 O60814 Q05086 682 Q00987 445 23770 5467 P48357 P05783 P01266 6210 P01185 64135 6575 6696 2099 Q9UHD2 3065 O75164 O14802 Q00613 Q96P20 5478 P25106 P62736 7416 P25103 P01178 Q01826 O43463 5371 9730 7791 7431 Q5T4S7 3190 Q8NHX1 Q12816 P17980 P51610 O00410 3074 O60911 P16885 Q5TCQ9 O14818 P51617 P52948 P28749 8517 54106 P04792 P25116 8772 Q9BYX4 11060 5265 O43432 P19174 8654 6598 4179 57187 Q96FV9 351 356 116 P08047 Q7Z6C1 Q8WUM4 8767 P25963 Q13363 Q9BYW3 4067 P12429 P62807 Q02447 60489 367 P27918 128 7329 Q9BUN8 Q9HCY8 P80217 6256 P13727 P62937 P10451 P35998 4193 Q9Y6Q6 Q9UBU3 Q9UDY8 P23528 23264 Q15561 P17096 7458 Q16531 11168 Q93009 P61966 P00441 8563 Q13148 5295 5054 4088 P62826 Q16778 P18074 Q9H257 O75475 Q9NXV2 Q02790 Q9NZ94 387 P01889 Q13393 P61978 Q03518 8453 8454 Q13158 Q16666 7124 P63092 P10275 274 Q9Y2C9 Q9Y6K9 10057 1937 P48643 2904 Q15223 Q99966 54 7132 Q14258 O43889 O75575 O43765 7251 285 Q7L5N1 P11142 Q13291 Q8NI27 Q14141 P49721 1948 P49720 Q13177 Q96T37 6175 9201 Q86W42 P02790 7023 10392 O75569 Q07092 6059 23586 291 292 9682 Q86V24 P11464 Q6VY07 Q01664 P01579 55703 8349 P02786 Q6I9Y2 2925 P49715 11128 P23443 O15075 9213 Q15369 P01570 64784 7037 9217 7157 O43504 P37840 7150 P11233 Q93034 23352 P01569 P01568 Q99816 P02775 7046 6198 P09769 P98155 O15123 P63172 P12931 Q9H0M0 P42224 P47914 79139 Q9HC16 10935 Q8TAF3 P17275 Q92692 P09651 P42229 1616 Q14974 Q15942 P78536 Q13769 O75531 83737 3925 P46934 Q8TAE8 57599 P52292 P08575 Q4LE28 P05067

2959 Q9UER7 O94763 Q13895 7067 P06239 P29474 P52294 9126 P19793 P29590 P32881 Q7Z2Q5 1877 P41597 1634 Q9BYM8 80145 Q92793 2960 P00966 P06241 Q9NZ08 3932 Q13547 Q7Z434 P10620 P19320 9495 P31785 P19438 P43405 1642 P08670 Q04206 P09884 P07101 P07585 P81605 Q5J5C9 Q13435 O95999 P11766 P41181 9021 P35613 P30048 3836 P00749 1654 P05091 O60488 3953 P08246 P47712 7099 7098 51606 P35968 Q14653 P19388 8065 P10321 1667 P61073 Q7Z2W4 58487 Q9UQE7 Q9BUJ2 Q9UGR2 3843 5901 Q9UBH6 Q9NWF9 O75843 P10415 Q13217 P28065 P19235 57162 1315 1312 2643 2885 23186 1672 Q14790 O15455 P01730 3856 O95786 O15111 Q14318 Q15648 Q15768 P34972 P28074 P28072 P28070 O60238 P63279 2896 Q9HC29 51752 4953 P21554 P21675 P21796 5925

induction of apoptosis by extracellular signals 8772 Q15811 O15085 Q7Z628 P10911 9500 7204 Q5VV41 9181 Q9Y4K3 P15498 O15519 O14827 355 Q99523 356 P49810 Q92851 637 1616 10016 P25445 Q92974 Q13009 Q9Y5V3 Q7Z6J4 P55957 84033 P42574 P42575 P98171 P98174 Q9UER7 P49768 8795 Q7Z727 7189 Q9NR80 Q9NR81 29 Q96M96 P52735 64857 Q9NY61 148022 Q8IVF5 P17252 23263 Q9UKW4 Q9Y572 P01138 P62258 Q9H7P9 P01375 6262 P48023 Q12979 7074 Q8IUC6 Q92785 Q92542 Q15052 Q8N5V2 P78560 8718 11214 8837 Q13158 Q92934 7124 O00220 23365 O75340 Q9P0J0 121512 Q99683 P63000 10451 O94827 5578 Q96PE2 P51398 O43521 7410 7531 Q5JSP0 Q96BI3 Q5VST9 23370 841 843 9826 11035 P08138 8738 23229 Q07890 P07766 50649 Q6ZSZ5 Q8TCU6 6453 O15068 O75962 Q12802 29843 P30542 O60229 572 7818 Q07889 50650 5599 Q14790 10276 O14763 51107 5879 Q14155 Q13501 P53355 Q9P0U3 4168 Q9H8V3 Q93038 P45983 Q9NZN5 8878 P45984 Q13352

regulation of cell proliferation P54852 P25054 Q9NYD6 O14672 2550 1460 9181 Q9UBB5 Q6QNY1 P51587 3516 2305 P37288 4609 P84022 Q92731 O14786 Q9Y275 P08842 P07996 2308 P28799 Q92974 P27487 Q12834 4860 Q92729 Q8IZP0 O95684 Q08431 P16234 P62244 P16473 P14174 4851 P49006 Q92845 Q96EB6 Q9UK53 O15525 Q7Z727 P27694 Q92956 Q92835 Q9BQ51 P37023 27185 6929 2324 1111 P32929 811 55109 P03372 P40424 Q92833 6926 Q6IT96 P06401 P26358 6925 4869 Q92831 P06400 3791 P46087 5970 3673 P31947 P48023 Q92949 2100 Q99583 9093 1491 P17302 5728 P16333 P17301 3304 1488 O60674 1487 2697 Q8IUC6 P31949 823 3667 826 5604 10523 P04233 P42830 P11926 4771 P28482 Q9Y586 P11802 1021 P52756 Q9UFF9 P19525 1017 P21964 1499 O60884 1012 P07550 5610 Q9Y2T1 P08887 P51671 P30556 P04049 3690 22846 Q96SB3 Q9NYA1 Q92769 P09619 P10600 P10721 P30550 Q96J02 P19544 Q9NS56 Q8WYH8 Q06330 Q9UEE9 P37198 1029 57678 1027 1026 961 2475 Q9Y240 604 P42771 O14757 P04179 P07766 Q10586 P05106 O95136 P30305 P28223 6850 4792 4673 1285 2253 2495 Q13618 2010 Q13617 Q13616 P30542 P38398 57448 Q9HCL2 2247 P15153 2246 P60033 Q92990 975 P05230 857 P05112 P10826 Q12778 P51692 5894 O95140 Q7L8A9 O00255 3350 1050 Q03164 56005 P17342 3226 O95390 P37173 5764 P05121 P09601 P40337 50943 7704 P06213 P04632 4690 P04637 O43399 5300 P15408 P14317 P14555 3481 P11047 P15884 P52952 O60725 P15531 990 991 Q96RU8 994 3479 23560 Q15022 7832 6504 84289 P01116 6869 Q15389 51009 P01112 Q6UWV6 P01111 Q9C0K0 P25100 Q9Y5V3 4221 112399 5796 P51636 1191 Q04900 O76071 641 Q15392 2277 P48436 P01106 8932 6878 P01589 648 P01588 O43294 P01344 P01343 P02794 11116 O00206 2290 Q6ZN33 O43251 P48431 6772 Q8N726 9921 5321 P14416 3265 3142 2173 148022 890 Q99623 891 Q99500 894 Q99741 Q9NR09 Q00534 Q96S42 P35240 P01019 51147 Q9GZP9 P01138 P01137 O14964 8829 P01135 11326 P01133 P01375 Q16254 P01374 P51531 4240 P04626 P35225 Q9UHI8 2065 2064 P35222 P35348 P35226 Q01974 O75084 P17813 P35232 301 P60484 3148 P10070 P17936 P10071 11331 6776 P01127 P40933 Q96EY1 6657 10488 5328 P25311 9927 O43474 O43353 P26583 P07942 6794 O43597 P07948 Q12904 23641 3162 Q969H8 672 552 P15692 Q9BZE4

P50616 5578 P55075 7518 Q8N5U6 Q8N302 Q96FA3 P04201 P05412 O00755 P05413 6320 Q6IR47 8744 Q7Z5L7 Q3SY56 7412 3297 23411 23532 5590 3171 P37231 563 Q01955 Q00987 444 P18847 Q99675 324 688 Q9NPH5 5467 5468 P27797 P24385 6210 P01185 7422 P56178 5245 2099 3066 65108 3065 10437 Q00613 127435 10673 P25103 Q9BT67 23636 5371 P27540 7433 6464 P08908 23512 P50402 P16410 7428 9846 10202 P25116 P60568 6595 4297 9500 5268 P15923 Q9Y4K3 3084 595 Q04759 356 Q9UBS5 8767 P78395 P49815 Q13485 P25963 P21246 Q13363 P03950 9510 Q15303 3091 5154 O00716 5155 4067 6368 P46781 79084 P58304 P13631 367 6117 P46531 P00533 Q15796 Q14106 Q13017 Q15797 6256 Q96IZ0 Q9BVP2 Q8N6I1 P32241 Q9UNL4 P12755 4193 Q9Y6Q6 Q9UBU3 P46527 498 Q969Z0 P25705 5159 Q9NRI5 P55290 P09237 Q9BSI4 P32239 Q14116 4086 5054 4087 Q13268 4088 P43490 4089 Q8IWX8 7476 P36952 P10586 163126 58189 Q96BK5 P21359 Q9UBV7 P08151 P20264 P62701 Q16665 P43489 8453 8454 8452 9420 7124 O15164 7248 P54132 P14635 391 P35453 64759 O43639 P10275 P10276 394 154 P09172 11146 5074 10181 Q15109 P22392 9314 O43889 Q9Y4H2 P35443 P36894 Q86WV8 7490 7251 P35568 6282 Q02763 Q13291 P01308 338917 P33151 7249 1947 P49848 Q99835 P00491 7023 O95813 P11229 O75569 23463 P36888 Q01664 Q00577 55704 P01579 P08069 1718 Q9NTI5 Q9BQI3 2925 P49715 1956 P09038 P23443 Q08050 7157 Q02750 Q09028 P38936 O43508 P11474 P35548 7272 138151 2810 Q93034 54984 P02538 Q6PID4 Q99816 Q14289 7165 Q13873 7046 Q96RE7 6198 Q9NNW5 Q08722 Q86Z02 Q13639 P42345 P42224 Q07507 Q1PSW9 10935 P04083 P39060 P04085 Q6NVY8 94 Q92574 P42229 Q13761 P15090 O15119 Q9NSA1 3911 3912 P42226 Q9NSA3 Q12797 P56524 P78536 7057 9238 Q13526 Q96QB1 Q13887 Q02297 83737 9231 P55318 22920 Q3V6T2 9350 7052 P55316 P57735 P84095 O43609 P43307 1741 Q96GN5 P41240 Q92560 Q32P28 P08575 P42574 P98172 Q9UBL3 Q15831 7189 P29353 P29474 P35916 P19793 O75881 P29590 Q03014 Q13418 Q7Z2Q5 P55209 O43815 P41235 1874 56034 27102 P17252 P21860 Q92796 P21980 Q9UBM7 Q9NRA1 P42685 O15151 P56545 9255 P78552 Q13547 Q13426 A4D1W7 P19320 2735 P43405 Q04206 Q92786 2737 2736 9146 O95995 O95751 P41182 O43915 P78504 P30048 P23508 P00749 Q63HR2 P00747 Q9HC57 4920 50514 P08246 2626 P47712 7099 P35968 Q14774 Q68CZ2 Q9NP71 9391 8065 P30279 Q07817 Q05516 P20248 64919 1786 Q05513 3720 P08138 P23510 P10415 Q9P2X7 Q02363 57162 1312 3611 P41273 P43694 Q16610 Q9BQA1 5914 P08107 P55773 Q9BZS1 Q15648 O15230 Q9H6Z9 Q86Z14 Q96BF6 4839 5928 Q9UHV2 Q8TDM6 2896 3621 P67870 P24941 P07384 4830 4953 Q14201 P07148 5925

regulation of macromolecule biosynthetic process Q9UKT9 Q9H0E3 Q9Y265 Q8NDW4 Q12824 8091 124790 10657 2305 2304 2303 114991 2300 Q8N3U4 155061 284695 2308 2307 Q9H0D6 Q92974 Q9H4L7 Q12837 Q8WXB4 Q8NDV7 2316 10661 Q96EB6 O75925 136319 Q9UKV8 4990 3660 3661 Q9BYE7 P62495 P37023 P13056 84901 57761 55588 O75928 Q9UKW6 3659 P26358 Q6NUN9 Q5BKZ1 3673 Q92949 Q99583 Q12857 Q13705 10645 1488 1487 283337 Q99576 2332 3665 3667 10642 64061 P26367 10644 10643 Q9UL18 1022 Q8NA42 Q06587 114548 Q8IX07 Q5JT82 Q96MX3 1017 10614 79576 Q9HAJ7 1499 10616 O14981 Q9Y230 P61586 O75909 O14503 Q12873 3690 Q12872 Q9Y239 Q9Y5Z7 Q8WXF1 6720 Q15906 285527 O00358 Q9BPY8 57798 1029 3207 10626 1027 1025 Q9BUB5 P17482 3685 P17480 604 Q9Y242 P04179 Q15910 Q9UL36 Q12888 5883 Q9BQ95 O60869 79595 Q8NA19 P38398 57326 55145 3215 Q92990 Q9Y250 6721 Q92994 Q92993 6726 Q8N488 Q92997 Q9UKS7 P10826 P10827 P10828 221937 5411 P31749 Q8NDX6 1050 P53999 79101 57332 3226 79589 3225 P04150 Q9UGL1 Q92985 O95159 Q9UKL0 O96004 P14317 P14316 23569 11108 11107 Q8WWY6 1059 23560 Q15022 639 P62875 6749 6760 P06702 Q9Y5V3 5430 O96019 O95163 P16989 P57059 Q03933 51131 641 P33076 51132 P35251 Q14186 Q6B0B8 79923 8932 648 8939 P49407 P60228 Q14188 O00327 O96028 5440 P48552 5441 6772 Q9UKN5 Q8TAU3 P48551 6774 Q99990 3265 346171

O60828 P15621 Q66K89 Q2M3W8 Q14192 P35240 P01019 51147 Q9Y692 Q15047 P23246 Q86X95 5451 Q8N3J9 P35222 Q5T6S3 P35227 P35226 O14979 P57082 O75081 P17813 P35232 Q00403 53335 56849 668 6776 4116 Q96MH2 Q16385 Q15056 7629 O43474 P06733 O00744 5460 Q9Y5Q3 6311 9821 P25490 3281 Q9NX65 672 P15692 Q9UBW7 6304 57708 Q6UUV7 P05412 O00755 Q9Y5R5 9831 3297 Q9NX70 23532 Q9UBX0 P14373 57713 688 148327 689 5467 9826 207 5468 Q8N895 Q92922 4150 4152 23509 P56178 9841 P56177 Q8NHY6 Q12809 Q9Y5S9 P17844 4149 O14920 57727 9839 O43463 5494 Q8NHX1 23512 Q96QT6 O14936 8517 Q92900 9849 4172 O15085 P36508 4173 4174 P35659 Q14686 4176 Q14209 8535 Q9BZ95 Q6NZI2 Q9H165 4171 Q9H161 Q9H9F9 Q9H160 P08047 5017 P78395 Q9BQG0 P07199 Q13363 Q9NPC8 Q14693 94234 4188 8543 P46782 5036 79084 P58304 Q96F45 O15090 23051 Q04864 Q9Y2H8 9869 7205 Q8WY36 Q96RL1 Q15554 Q9HAU4 8553 P35638 P61968 Q8IWY8 Q96IZ0 Q9UPP1 P32121 P31274 P31273 P31276 O75123 Q9ULH7 23028 Q9NY61 4193 Q9Y6Q6 257 Q9H9D4 Q6DD87 Q15561 8548 Q96RK0 51548 Q93009 51547 Q14232 P61964 Q15562 Q9BYU1 P62826 9412 Q9UPQ9 P36956 O75592 Q96F24 P31270 Q9NPF5 Q13398 55915 9421 P78347 9425 7248 O75586 O75582 Q9Y6K1 Q93062 Q03989 Q9Y2C9 Q71SY5 Q9Y6K9 51564 Q15583 8569 55922 5074 P78337 55929 11030 Q9HAZ2 Q15109 P22392 O15055 O43524 26523 7251 O75570 23492 O15047 P49848 Q15596 8110 P61925 Q3KNS6 O15062 5089 166968 O95931 23466 Q9Y6M1 23468 O15060 79913 51588 P08069 10155 Q9BQI3 9439 Q96NG5 286075 Q8NEJ9 Q9NPA8 64426 5092 64428 1965 Q9H9G7 54625 O95947 54623 Q14289 Q8IWS0 Q96RE7 9464 P29375 P29374 27005 Q7Z589 Q9H0M0 1975 P22736 P42345 Q9BUG6 Q96EK4 Q9UGU0 P04085 Q92574 Q13761 1978 Q58WW2 P56524 9477 O75534 Q02297 7291 O75530 7290 9470 7295 P24928 1982 Q6P1K2 O75528 P29353 Q06643 P0C7X2 Q8WXI9 Q9BYM8 1993 79039 Q96IQ9 O15499 P56545 O95983 A4D1W7 O95503 55072 P78317 P78318 Q8TBE0 Q9HAK2 Q9UH92 Q9Y2B9 Q96EP0 O14593 O95999 P35610 Q9NP66 23081 O43918 23085 Q9UKY1 Q9H9B1 P85037 P31260 Q6P1N0 P31249 Q13322 Q14653 Q9NP71 Q9NP72 3728 1540 Q9BUJ2 3720 5901 P46734 Q13330 P12956 Q6P1L6 Q9P031 Q9H4W6 Q00059 Q9BQA5 Q9Y6E7 Q9H582 P61088 Q96AE4 147808 Q96RG2 Q68DY9 P43694 O15455 Q9BQA1 5914 11091 O75953 63976 63978 5927 5928 P63279 P00519 5929 P63272 23077 P24941 Q92585 Q15532 Q13351 Q14683 Q13352 5925 Q7KZF4 P10914 O95677 Q92858 P67809 Q9UBB5 Q9NS86 A5YKK6 Q9P2R6 4609 P16220 10772 5931 P27348 5933 O15516 5935 Q9UBC3 P27361 54361 1107 P16234 O60573 284323 4613 1108 O15525 Q92838 Q9UK58 Q7Z3K6 Q92835 P05549 Q9NRY4 Q99471 Q7Z3K3 O60548 Q92830 Q9UJU2 284312 O15534 P49116 P03372 64170 Q92833 P06401 Q92831 P06400 Q92826 P07737 5970 5971 9093 Q9UBE8 Q9NRZ9 P56705 9099 P17535 P17544 Q99459 P17542 P62140 57410 400720 P61296 5966 10765 5981 P50570 O00463 O00468 O95231 Q9UFF9 O14627 6829 10736 3315 Q6FHQ0 5976 P41218 Q8N2W9 4646 27161 5978 6827 65056 5991 93986 5993 1153 Q14938 O00472 Q06455 55250 Q9NS56 P37198 79685 6839 2475 P08651 5500 5987 O95259 P07766 Q10586 P05106 6850 Q8ND82 P51449 Q6IA86 27148 O00488 Q02086 O60502 P61244 Q9BXG8 4664 5511 Q12770 5515 P05112 26292 Q9UNY4 5518 O43186 Q12772 Q12778 O43189 Q05586 2023 Q02078 Q6KC79 10725 57459 10724 Q99496 P40337 7704 O00425 4690 P23396 O43159 6872 6871 P04637 2034 2033 Q9NVW2 O95271 O00422 Q9UFB7 4686 4204 P01116 4205 Q16236 P01112 4209 Q9C0K0 6883 Q9BT49 4221 Q9GZR2 O43167 23210 P50548 O76071 Q9NRP7 84159 Q9P2K8 4214 P01106 6878 P01588 7727 56946 Q9C0J9 P01100 Q7Z7K2 7741 5562 Q8N726 9921 P52736 Q53X93 6886 P01137 Q16254 P52740 7750 6421 5573 P04626 2064 P52747 2063 11218 Q9NS37 Q99853 P10070 P09086 P10071 10363 10362 P01127 5569 2070 O43593 5585 7764 22794 25822 Q96PU8 79612 O75190 Q02930 7756 Q8TEB7 5578 5579 10336 8607 P40763 P04201 2081 5591 Q9GZV8 4261 Q6IR47 5111 Q3SY56 64127 P37108 O75177 P18850 P15336 O75182 Q9NRL2 56987 51295 P18846 Q68CJ9 P18847 P18848 326

55657 Q92800 P27797 Q9GZU7 Q9UNP9 2099 O60907 O75164 P51843 55662 Q8NCN2 333 91748 Q9H7L9 10794 8625 5119 Q9GZT9 23636 9975 6464 4287 P62508 P51858 146050 221656 Q9NVV9 P58012 10320 P60321 9967 P25116 Q99417 Q96HZ4 O43670 Q14566 8655 4297 Q9BY77 9500 Q9UKD1 P13984 351 Q9UBS0 Q04759 P21127 P25963 P09017 8662 P20290 Q15424 7332 5154 7334 5155 8667 7335 26205 85403 367 Q70SY1 11198 7329 Q14586 20 7341 P11309 6496 6495 Q8IVW6 Q14103 6498 P11308 Q99909 6015 P32242 Q8N3C0 Q9Y606 84108 Q9UBU3 116113 P17096 Q14592 11168 Q96MA1 Q9UBU8 Q13263 Q8TF50 8204 Q9BY44 Q14119 Q9BY41 Q9Y618 P12645 P29084 Q86WP2 222546 Q04725 Q04724 387 Q04727 Q9NWT6 Q04726 23152 P08151 P20264 11177 11176 P23771 P55347 9541 5187 11142 O15164 2909 391 Q6MZP7 23126 O43639 25788 O43638 Q02535 Q01201 2908 Q9UFW8 11146 Q15466 Q6PJG2 Q99966 Q13285 2902 5195 6045 7376 Q09161 23133 23135 Q86WV8 Q3KQV3 Q6PRX2 P09067 Q14140 Q9UBP4 Q99958 Q15475 Q8TEY5 Q96ME7 Q9HA82 P14859 O43612 Q8TAQ5 Q96DT7 Q9H063 Q99941 P49959 Q01664 51222 56916 P01579 Q14151 Q9UBQ5 P09038 9575 P82979 8242 10284 O75676 O15198 23112 O43623 25776 51230 1609 Q5VVH5 10289 Q15014 86 P78424 Q9NWH9 P33992 P33993 P33991 Q8TAF7 P41134 Q1PSW9 27122 91 P17030 94 O15119 1616 P45973 Q9UBK2 Q9NSA3 P78412 9112 Q96I24 Q9H7Z7 Q9H3R0 P55318 Q99081 P55316 P67775 Q52LR7 P17040 P05067 2959 Q9UBL3 O94763 P52298 92129 9126 27107 Q9H422 Q460N5 Q03014 6096 56034 27102 Q9BTC8 2961 2960 O95619 Q8TAD8 Q9NSC2 2969 P09874 O15151 8289 Q15843 O95625 O15156 O94776 29777 202559 P17023 2972 79149 Q9BXJ9 P17020 Q8N393 Q8HWS3 P04198 Q9UBN7 Q15853 O75626 Q14527 9149 Q8TF47 Q6NYC1 Q9H488 P41182 Q8TAK5 93474 79175 Q499Z4 P53567 79177 P30048 84525 1655 84528 P22415 1653 Q9UK80 Q03468 Q92499 O75629 O95644 150094 Q9UBG7 Q8N7H5 P20248 64919 80155 Q8TAI7 P41162 P17081 Q9Y5B6 Q9Y5B9 P09429 O75604 P10415 Q9P2X3 P41161 P35711 23186 P09430 Q86SE9 728642 Q9P2Y4 Q13227 P55771 Q04771 O15105 Q13233 Q6NX49 Q9H6Q4 O14793 25909 9181 P51587 P17676 Q8TDD1 2547 57504 Q9HCJ0 P84022 O00151 Q92731 O14788 10413 Q7Z6R9 25913 P08729 Q92729 Q5VTR2 P51114 O60216 Q9H2G9 4734 Q7Z727 Q9H2G4 O00167 22850 81628 54496 6927 Q8IUD2 6929 Q6AHZ1 Q96KM6 Q8N680 811 6921 P40425 P40424 6926 6925 Q6IQ32 Q17R98 Q9C005 333929 Q9UIS9 Q9NZI7 P48382 Q9NQX0 P31946 Q9C009 2100 Q9NR96 P48380 O95343 3428 P16333 6938 P20823 55352 O60674 P19838 O60675 Q8IUC6 P62263 P31949 5604 6936 Q9Y468 Q8IZ40 4771 Q9Y463 4772 Q9Y466 Q8TDI0 Q9UIL8 2107 55364 10856 Q86VE0 2103 P16383 27287 80324 Q08999 O14744 6945 P50222 4780 Q92769 Q14814 P28370 Q07666 O95361 80306 P10600 O95365 Q06330 2119 10865 P19419 Q6PI57 4775 O14753 847 Q92766 4790 4791 4792 Q9H2K2 Q9H2K0 2130 81669 Q9NR48 Q92753 4302 857 Q92754 Q13501 22827 Q13503 Q9NR50 Q14839 P19883 Q13506 Q9NR55 Q9HCK5 2139 861 862 863 192669 Q9UMX1 4799 P09601 50943 Q9Y3Y2 Q9BS34 P15408 Q9Y3Q8 3481 Q9UIH9 23326 O60603 25988 P15884 Q8WUU5 Q96T88 3479 2147 2146 3476 10011 P25205 7832 10013 84289 Q96KG9 55723 10499 10014 Q6UWV6 4343 Q96T76 Q99750 406 Q9BRP8 O43294 408 84271 409 Q96C55 Q09472 Q8N1G0 O43251 23309 64215 890 Q00653 Q99743 Q99741 898 29080 Q9UN42 P49639 P61326 P51531 4361 8721 7874 148156 Q9NUX5 Q9NR11 P51532 Q96ST3 P60484 Q15291 11331 55758 10001 P60002 10488 Q96T58 P28702 P23497 P26583 Q9Y3M2 P46199 P50613 P51946 23764 25942 Q8NFW5 Q9NUQ3 Q8WUY8 10450 8726 Q8N5U6 90993 Q08117 3054 Q05086 Q9BRU2 29079 P37231 Q9HCE1 79723 8737 10468 7422 Q96GA9 5245 3068 Q9HCD5 P52815 22893 3066 Q9UMN6 3065 Q9UIF9 O75290 79753 10438 Q00613 Q96P20 55787 10432 3070 Q13901 9612 Q9UIG0 P52824 466 468 P28749 Q9NQL9 7428 P04792 6595 6597 6599 6598 O75386 P10599 6591 Q9Y4K3 Q76L83 3084 473 474 23286 9617 8767 P38919 Q13127 Q15303 B2RXF5 3091 Q16633 8301 Q15306 Q86VP6 O75376 P58546 P13631 24149 3096 Q02447 O75381 Q9NVP2 51773 30813 P46531 Q13133 Q15796 Q13131 Q16649

Q15797 9640 P12757 Q8N6I1 9643 7468 P54198 Q9UNL4 P12755 O75362 O75360 P46527 51780 Q01101 P42167 6128 O94805 7458 Q13144 Q5VUA4 54815 Q8WVM0 Q9BSI4 Q16650 Q13148 P35869 P43490 P20393 7476 Q16656 Q15329 Q8NCA9 P18074 Q8WVL7 P55055 P10588 P10589 93166 Q86VN1 Q16665 51317 Q49AN0 P43489 Q8TDY2 9667 Q16666 Q5VUG0 P54132 P35453 Q14494 8328 Q14498 Q16670 P46108 Q14011 Q86W54 Q9NRC8 7013 7014 P46100 Q9Y4H2 Q5PSV4 7490 P13682 Q9C0F3 84232 Q8WW38 Q99835 7024 7023 10392 Q9C0F0 49854 P98179 P15822 9682 7020 Q02878 Q9UNH7 51341 54851 Q9NVM4 Q96PK6 11243 55704 P55010 Q13185 Q8NCF5 P23443 6188 Q15369 Q8IUX7 Q9BX66 Q9BSM1 Q09028 6182 P11474 26747 Q15370 54862 P57682 Q6PID4 Q99816 9208 Q99814 P78545 6199 7046 6198 P35813 O43711 6193 O43719 Q9Y4C1 P62195 Q4LE39 O15234 9219 A8K8V0 7057 22803 112950 22806 22807 57473 55294 Q3V6T2 P46934 P46937 83746 Q96GN5 O60296 Q4LE28 Q9UER7 Q14865 7068 7067 Q5TAX3 Q15714 Q13535 Q9H307 P19793 P29590 Q7Z2Q5 Q63HK5 Q01196 84619 Q9H6W3 P41235 1756 Q9HCP6 Q92793 Q96GM5 Q9HD15 Q9Y4E5 O60264 Q9NRA1 P59817 Q13547 Q13546 9252 7073 P41223 57018 P43405 1763 Q9NZR4 Q04206 Q92786 Q92785 Q92784 P41229 7088 Q01167 Q9BSG1 51720 Q14894 4800 P08246 2626 7099 7098 Q9NVC6 7091 P14923 7090 Q9NQZ8 P30153 1789 1788 Q05516 1786 Q9UIV1 1785 P00734 375790 Q13573 Q9HCU4 P55884 93134 Q9H2S9 Q96CJ1 P78527 92283 85509 Q86VK4 84661 51742 Q6ZNG0 P05198 Q9Y4A8 30827 Q96H20 Q9HCS4 902 Q9HD40 Q13112 Q9NZN8 P08235 P21675 Q92618 Q9H609 Q92616 Q9Y2W7 Q9NYD6 Q6P2D0 O75820 2672 Q12948 22992 O00268 Q6QNY1 O00267 79894 3516 Q9BZI1 Q96JM7 Q96JM2 Q5VWX1 283248 Q9ULW3 Q92610 4841 Q8N587 P07996 Q12950 Q12952 Q9Y2X9 Q8NB12 Q8NAP3 79885 4851 4854 Q96JL9 Q92600 5705 Q9ULX9 Q9NYF8 Q9Y2Y8 Q12968 29128 10514 83933 26097 Q9UQB3 4862 O75807 57649 Q6IT96 4869 5716 Q96JK9 Q12972 Q0D2J5 Q9H9T3 3551 Q6P2C8 5728 10524 P17301 Q9HBE1 Q99697 400961 Q9ULZ3 Q9UHR5 Q9ULR5 P28482 Q9UHJ3 Q12986 1385 29108 P19532 Q9P0J0 P15173 P07550 P04040 10971 7913 P08887 O14867 Q96BA8 10973 Q9UM47 Q9Y2T7 Q9UHK0 Q9NYA1 O00233 P19544 Q8WYH8 29110 1388 29115 Q9P0K8 1387 1386 P42771 Q9UM54 4899 Q9H1I8 10985 Q9NYB0 22937 22938 Q9UHL9 22931 2247 284119 1398 58533 Q9BZK7 6601 Q9Y2V2 Q92630 6605 57209 Q9UM63 P51693 P51692 P31629 Q9BQY4 O00255 Q8NEZ4 Q9NQ33 O00257 Q03164 984 Q5H9I0 988 P05362 Q9Y2W1 P06213 Q9UPV9 P13349 2274 P53803 P52952 990 Q8WTS6 126208 Q96RU8 Q96RU7 51003 79811 79813 51008 10138 Q9UQ80 Q04917 P49770 Q9UM07 Q9Y2P0 Q7Z5H3 Q9UQ88 5796 Q5T7W0 O60739 8819 P48436 8812 Q99638 P01344 P01343 O00206 2290 23429 P48431 Q96JC9 3142 Q9NU63 148022 Q99623 Q96S42 Q8NAF0 55869 7975 6646 5316 51028 P01375 Q9UPY8 23439 6660 P49750 8841 Q9UHI6 O00213 Q3KNV8 23435 Q96AQ6 3151 O60716 P12004 10128 Q96RR4 Q3KNW1 3148 Q15170 545 546 3146 P61457 Q96EY1 6657 O43353 6670 6672 23409 Q969H0 Q9Y2K7 Q9ULJ3 Q9H9S0 3162 Q12905 O95076 Q8WYA1 Q12906 Q9BZE0 Q01844 Q9BZE4 P42704 3159 Q99684 6667 P49336 P05771 Q9H5J8 Q969G3 8864 10580 7533 P55198 Q9ULK4 23411 23414 3171 Q00987 Q96S94 3169 7529 7528 Q9H5I1 64375 P50750 Q86U86 Q9UHD2 Q9UPT9 3184 571 3182 P51608 P68036 7536 Q9H5H4 Q01826 5371 8880 P27540 Q60FE5 Q9UPU9 Q12933 Q9ULM3 7555 Q9ULM6 P51610 Q9BUY5 54583 80854 P51617 Q9NPJ6 7549 54106 8878 P60568 8892 Q15654 O43432 8896 P15923 Q86YW9 Q96FV9 84324 Q7Z6C1 Q13485 O94906 P03950 O00716 Q16512 Q16514 P12429 Q15669 O75496 O75494 Q7LBC6 P10242 P10243 P10244 84312 51412 O94915 Q13492 Q16520 6239 54925 Q15672 Q9UQR1 7101 6256 Q16526 Q2M1K9 Q9UDY8 7579 9774 4086 7112 4087 4088 Q8N9N2 4089 O75478 9775 8445 163126 Q9H257 O75475 Q8N5F7 142 P24864 P62701 84333 Q15697 53615 Q13033 P54259 O75469 8458 64750 7124 P54252 P54253 Q96KC8 O75461 Q9UQL6 P10275 4092 P10276 4093 O75460 4094 154 P23588 Q99729 Q13045 55806 P49736 7132 8463 8467 9314 O43889 P36896 23378 P15976 P35568 Q96C28 6282 1822 166 55810 P01308 338917

Q9NQB0 Q99717 Q15233 Q96T37 9326 P54274 O75446 P36402 O75444 6294 7141 Q99700 1831 Q96T23 Q00577 Q99708 P49711 Q16576 P49716 P49715 55827 Q9NQC7 Q96C00 64784 7158 O75437 Q08050 P39905 7157 162239 Q8N9R8 Q02750 P35548 138151 55832 Q9UDV6 Q5VTD9 Q13077 26038 Q06787 Q86Z02 56252 Q01094 Q9H204 26039 7161 P63167 P42224 10933 10935 P17275 P63165 Q9BVI0 P42229 57109 P42226 O15372 O94992 Q9P0W2 22926 Q969S8 22927 Q13404 Q13887 Q01085 57592 Q8IY57 57594 P10644 57591 Q96BD5 79365 P84095 Q08945 Q9UHX1 1869 7186 7189 7188 Q8WYK2 O43812 7181 7182 7185 1877 Q86UE4 Q86UE3 1874 Q9Y3C7 P17252 1870 P09630 O15379 Q969R5 Q13422 O15391 Q8NB78 2735 P19438 O43829 84733 1408 1407 1406 2737 2736 Q8WUI4 27327 Q02386 Q7RTR2 1896 Q9HC52 Q14774 P22670 Q9NQ92 9391 P11532 Q96G25 84759 58487 Q9UQE7 Q8WYP5 P23511 Q8WUH2 3609 3608 3607 Q14781 P23510 P21333 94104 O14495 Q9P0T4 29966 P19474 Q02363 58491 P18146 84305 27300 O60341 P01730 Q969V6 O15350 O75832 O15353 Q15649 Q9BZS1 Q15648 Q9P0U4 P19484 Q86YP4 Q06710 Q96BF6 Q8N5A5 3622 Q9HC29 O14497 Q15652

regulation of protein ubiquitination 11065 11186 5300 9184 991 26271 O60566 Q96RU7 Q9BXM7 P62195 23560 P63208 8767 Q92574 Q15389 1616 9474 64682 7334 P55036 Q12834 Q13526 O43684 65018 2280 5706 5707 5708 5709 648 5700 Q16401 5701 11236 408 P60900 5702 P49407 409 5704 Q9UER7 5705 Q5S007 Q13257 5682 5683 Q8N726 5684 5685 8555 5686 P32121 P35998 Q9NS23 P53667 148022 891 5717 57761 Q00535 8945 P25789 10197 P25788 P61289 Q9Y297 5713 P25787 5716 5692 5693 9093 Q99460 P35226 Q9UNE7 Q8IUC6 5687 5688 Q96EY1 Q8WU17 3309 P62942 Q9UL15 O43353 8454 5582 P05129 O95999 7248 P51668 1020 P14635 P51665 4092 672 Q9BZE4 Q13042 Q92530 10213 Q13200 Q96FA3 O43242 Q9NYA1 7375 Q9HCE7 O00231 64127 O00233 57154 O00232 Q86WV8 1029 P11021 Q9UJX2 120892 Q9H1A4 5347 P49721 P42771 O15169 P49720 10273 P28065 A1XKG3 Q13616 O00487 O00762 57162 P38398 P62333 P20618 Q9H1Y0 P61088 Q15008 Q9BT67 Q9UL46 8881 O75832 P53350 6500 Q13107 P62191 P28074 P17980 P28072 P28070 O14818 Q9HC29 P30260 O15105 Q9UMX0 Q9UKT4 P43686

regulation of signaling process P25054 Q7Z628 P10911 Q9NRW4 Q5VV41 9181 Q99490 P15498 O15519 2305 Q6PCE1 Q92851 O14544 O14543 O14788 57506 P07996 2308 Q92974 P52564 P09936 Q9Y2X7 65018 115704 Q9P107 2316 P14174 10783 4734 O15524 Q008S8 Q7Z727 Q9H2G4 Q92835 Q9NR80 Q9NR81 Q06124 10758 Q8IVF5 P20936 57761 57521 Q9UKW4 Q9Y297 64170 Q9H7P9 5716 22866 5970 3672 P48023 Q92949 Q9NR96 3551 Q12979 9093 P21917 Q13829 P17302 5728 O60674 O00182 P20827 2697 Q8IUC6 Q8N5V2 64061 5966 5604 P04233 Q8WXG6 Q92538 Q13956 4771 O00220 Q96N96 O00463 Q9NUY8 P15056 1499 10616 1012 P07550 P04040 P61586 P08887 P51671 Q9NYA1 P29466 Q9Y239 124583 P10600 P10721 Q5VST9 P37198 29110 10746 79444 960 3326 1147 841 2475 843 5863 604 Q6ZW31 847 Q92888 Q07890 P07766 O95136 P28223 6850 P50591 Q13618 Q9P0L0 Q9NS68 P18545 Q8IUH5 P62330 2247 Q96N67 P60033 1398 Q9BVC4 58533 Q07889 975 613 Q5TCX8 857 5997 O14763 859 Q96CA5 5515 P05112 5516 5879 Q92633 5518 Q12774 Q13501 Q12778 P31749 O95140 O95382 Q9H8V3 O00255 Q9P0M2 27032 253980 153090 P05121 P09601 Q9UL54 P06213 P01241 P56199 P14317 115557 3481 O60725 Q96RU8 O14827 Q96RU7 P62993 3479 P13591 997 3476 6622 10013 P01116 P01236 5536 Q15389 P01112 P01111 O43166 Q15027 4221 Q7Z6J4 6885 23216 2280 56940 Q8WTR2 2159 Q99750 4214 84033 4215 4216 P01588 Q99759 408 P49407 P01344 409 85360 Q13098 P01343 P49768 O00206 P48431 5562 Q86VW2 116986 P14416 3265 P52735 116987 3263 23787 148022 P11274 Q02952 P35240 8945 P01019 51026 6647 55504 P01137 O14964 P01135 8826 P49758 P01133 P01375 Q9UJM3 64223 6541 P04626 8841 2065 2064 P35222 P35348 55633

P60484 P17936 P01127 Q96EY1 Q15052 6657 11214 5328 O43150 8837 Q15057 P24588 8717 9927 O43353 Q3MII6 5580 Q6FGG2 6794 5585 O43597 Q96BZ9 P07948 Q5R372 Q9UJF2 3162 79971 673 Q96P48 P61981 P15692 Q99683 79735 10451 57708 9815 5578 P62714 5579 5338 P05771 Q96FA3 O00635 Q6IR47 8743 7532 7410 64127 Q5JSP0 Q05086 Q99558 324 Q9NU19 5467 8737 9826 207 54206 Q8TEA7 329 P48357 Q76NI1 7422 Q96GA9 6453 Q5VWQ8 P30825 Q9UHD2 Q12802 Q9BUZ4 Q0IIM8 55785 10554 O14920 O14921 9839 Q9BT67 23636 5494 Q60FE5 Q12933 6464 4168 Q13905 Q9Y3P9 23513 57732 O14939 P51617 8517 54106 8878 Q99418 10564 P26447 P25116 P60568 Q8N6T3 P49427 8772 O15085 9744 7204 P30086 Q9Y4K4 Q9Y4K3 O43318 351 356 23286 P49810 8767 P43034 P49815 Q8WZ64 Q9HC98 Q8TBP0 O43687 6003 Q13009 7334 5155 Q16512 4067 4188 Q15669 7335 5037 Q9UIA0 O75376 Q96D03 367 Q04864 P09382 9628 P00533 Q13370 Q13490 1902 Q15311 Q13131 Q8IW93 9641 8795 9882 Q9UQ13 8315 Q9HBW0 29 Q15438 P32121 253260 Q9ULH1 64857 Q8TEU7 Q96CN4 Q9Y6Q6 Q9UBU3 Q9UDY8 23263 142678 Q8WZ42 P55290 P00441 Q8IV61 Q9Y6R4 5054 Q9BY44 Q9UPQ3 Q15208 Q8WWN8 Q14118 P10586 Q86XR7 125058 Q9H257 Q9NZZ3 382 Q8N6H7 387 P21359 389 Q66K14 Q16543 Q13153 P62942 8452 6275 Q13158 11140 7124 7248 7126 Q9Y3E0 23365 O43639 P10275 154 Q9Y2C9 121512 25780 Q6KH11 Q9Y6K9 O94827 1936 2904 P46108 7132 Q9Y5J5 Q86WV8 5071 23370 Q02763 P01308 7128 7249 Q7Z7H5 Q9NQB0 9306 O15169 Q9GZM8 85440 Q96NH3 Q99956 23229 Q6ZSZ5 P02790 Q8TCU6 10392 O15068 23463 O43734 O43739 Q14C86 Q8WVQ1 Q99943 Q9UNH7 P01579 P08069 10276 1956 P09038 Q14155 10159 Q9BY84 10160 Q08050 64786 Q8N9R8 Q02750 P37840 23118 Q14160 51231 54862 Q6PID4 Q14164 Q9BWT7 Q14161 Q6R327 1848 P98194 Q14289 1605 Q13077 Q15811 9465 7046 P35813 O75787 10928 26037 7161 9341 P42345 Q9Y6G9 P41134 1852 Q9BXM7 1850 Q86VI3 O94989 P46940 9218 Q92574 1616 Q6ZT07 Q9NSA1 P78536 7057 Q13404 Q96QB1 9590 7052 P46934 O43609 P67775 81565 P08575 P16070 P05067 O60292 P42338 P98174 Q9UER7 7186 Q15831 7189 7188 P29353 Q15836 Q9H422 Q96M96 Q13418 P56539 Q7Z569 7185 84619 Q9BYM8 60412 P17252 O15379 P21860 P21980 O60260 50618 Q13546 9138 55072 Q7Z434 P78318 9495 7074 27352 P19438 P43405 P41220 Q05655 Q04206 P53041 Q9UBN7 9146 9267 Q9NZJ7 Q96EP0 O95999 Q9NP61 P13945 P78509 Q96DN5 P41182 Q86TI0 9021 Q16828 93594 Q58EX7 9020 P00749 Q03468 P63000 Q96PE2 P00742 3953 Q6P1N0 P08246 3956 Q8IWV1 7099 7098 Q13202 Q96HU1 Q8WZ19 Q15628 O15211 P21580 P30153 P61073 23094 Q9Y6D6 Q9Y6D5 2873 P08134 Q13574 51735 3965 P21333 Q9NYJ8 50649 O14492 Q6ZV73 O75962 Q9P035 O60229 57162 2889 2885 3611 P61088 50650 O60343 Q14790 Q9NZM3 Q5T9L3 O15455 Q16610 4943 P01730 P41159 Q8IYX1 O15350 O75832 O15111 Q13227 Q06830 Q86Z14 Q04771 222068 Q8N5A5 Q9HC29 51510 Q9NZN5 Q13233 P08238

positive regulation of cellular biosynthetic process P10914 Q92858 O14793 Q12948 Q12824 Q6QNY1 O00267 P17676 3516 2547 2304 P37288 4609 2303 P16220 Q96JM2 P84022 Q92731 O15516 P07996 2308 5935 25913 Q9H4L7 Q12837 Q5VTR2 54361 Q8NAP3 P16234 4851 5705 O75925 Q9Y2Y8 Q12968 6927 P37023 6929 4862 811 Q9UJU2 P49116 O75928 P40425 P40424 6926 Q6IT96 P06401 3659 6925 4869 P06400 Q96JK9 5970 3673 2100 Q9NR96 P56705 P48380 O95343 10524 10645 P20823 P17301 O60674 P19838 Q8IUC6 P61296 3667 5966 O95477 Q9Y463 P28482 1022 1385 3320 P19532 O00468 6829 1017 10736 1499 P15173 80324 P07550 4646 6827 Q9UHK0 4780 Q92769 5991 6720 P28370 1153 Q14938 O95361 O00233 P10600 P19544 O00358 Q9NS56 Q8WYH8 Q06330 P37198 57678 10626 29110 3326 P19419 Q9P0K8 3324 1387 P17482 2475 P08651 P17480 4775 Q9UM54 P04179 P07766 4790 Q12888 6850 4792 Q9H2K2 22937 P38398 Q9HCL2 2247 Q92990 Q9BZK7 6601 Q92753 6721 Q92993 Q92630 Q12770 P05112 Q9UM63 Q92997 Q13501 Q9UKS7 P10826 O43186 Q12772 P10827 Q12778 P51692 Q13503 P31749 Q05586 O00255 1050 Q03164 861 P05362 P09601 P40337

50943 Q9Y2W1 P06213 P07900 O96004 6872 P04637 2034 P13349 2033 2274 O95271 Q9UIH9 23326 O60603 25988 P15884 P52952 3479 10011 6622 84289 Q16236 10014 10138 P01112 Q04917 Q9C0K0 6760 4221 Q96T76 641 P33076 P48436 4214 P01106 406 Q9BRP8 P01588 O43294 408 84271 P49407 P01343 P01100 O00327 O00206 Q09472 P48552 P48431 9921 5321 6774 3265 148022 890 Q99743 Q14192 Q96S42 Q02952 898 Q53X93 P01019 6646 P01137 5316 P49639 P01375 Q9UPY8 P51531 6660 5451 P35222 Q9NUX5 P51532 Q96RR4 P17813 3148 3269 P10070 P10071 3146 P61457 668 6776 P01127 10001 6657 P23497 P24588 P26583 O00744 5460 6672 Q9Y5Q3 P50613 P51946 3162 Q12905 Q12906 Q9BZE0 672 552 P15692 79612 3159 Q02930 P51828 6667 Q8N5U6 P40763 P04201 P05412 5591 O00755 4261 Q6IR47 10580 7533 64127 P18850 Q05086 23414 3171 P37231 P18846 P18848 688 3169 148327 9826 207 5468 Q92922 P27797 P01185 7422 P52815 O60907 Q9UHD2 3066 Q9UMN6 Q9UPT9 3065 3182 P17844 8625 10432 23636 P27540 5494 Q9UPU9 6464 P62508 Q9UIG0 O14936 466 P51617 P58012 468 7428 10320 54106 8878 9967 P25116 P60568 O15085 6595 Q14686 6597 4297 Q9BY77 6599 6598 P15923 P13984 Q9Y4K3 351 Q9H161 474 Q9UBS0 Q04759 P08047 Q7Z6C1 Q13485 P25963 O94906 Q9NPC8 P38919 3091 5154 O00716 5155 8301 Q15306 19 Q86VP6 P58304 P13631 367 Q04864 51773 Q70SY1 P46531 Q13492 P00533 Q13133 Q15796 Q9UQR1 6496 P35638 6495 Q15797 6256 Q96IZ0 P12755 Q9ULH7 Q9NY61 O75360 Q9Y606 Q2M1K9 Q15561 P17096 Q13144 51547 Q13263 Q15562 Q16650 4086 P35869 4087 8204 4088 P62826 4089 7476 9412 9775 P18074 Q8WVL7 8445 P55055 P12645 P36956 222546 P08151 P24864 P20264 P62701 Q16665 Q13033 9421 P55347 O75469 7124 P54132 P54253 O75586 391 P35453 Q9UQL6 P10275 P10276 4093 154 Q9Y2C9 11146 Q99729 Q99966 Q13285 2902 Q14011 7132 5074 Q9NRC8 7376 8463 Q9HAZ2 P22392 Q9Y4H2 P36896 O43524 7490 P35568 55810 P01308 338917 Q9NQB0 Q8WW38 Q14140 Q99958 Q15475 Q99835 Q96T37 Q8TEY5 7023 5089 P98179 P14859 Q96T23 54851 Q01664 Q96PK6 56916 P01579 P49711 P08069 10155 P49715 1956 P09038 P23443 55827 9575 Q9NPA8 7158 Q9BX66 P39905 7157 O15198 6182 P37840 P11474 5092 26747 55832 Q6PID4 Q99814 Q14289 6199 7046 6198 9464 P35813 P29375 27005 Q01094 7161 P22736 P42345 Q9Y4C1 Q1PSW9 P17275 P62195 91 P04085 94 9219 P42229 O15119 P42226 Q9UBK2 Q58WW2 P56524 7057 22926 22807 Q13887 9590 Q8IY57 P55318 P84095 Q4LE28 P05067 1869 O75528 7189 7067 P29353 Q06643 P19793 Q03014 7182 6096 Q01196 P41235 56034 Q92793 Q96GM5 Q9HD15 P68871 O95619 Q9NSC2 Q9NRA1 8289 Q13422 O15156 O94776 Q13547 A4D1W7 P78317 9495 2735 P19438 P43405 1763 Q9HAK2 Q9BXJ9 Q04206 Q92786 Q8HWS3 1406 2737 2736 Q15853 Q96G30 O14593 O95999 9149 P35610 Q8TAK5 P53567 1655 P22415 Q9UK80 4800 P08246 2626 P47712 7099 7098 Q9NVC6 Q9NP71 P20248 64919 80155 Q9UIV1 P23511 375790 3609 P17081 3608 5901 P46734 P09429 P12956 29966 P78527 P18146 Q9H4W6 P35711 Q9BQA5 84305 P43694 O15455 P01730 P08588 5914 O15350 Q9BZS1 Q15648 P55771 P19484 Q06710 Q04771 63976 5927 P63272 Q9HC29 P24941 Q9HCS4 O14497 Q92585 902 Q15532 Q13233 P08238 P21675 5925

chromatin assembly or disassembly P06899 8535 6599 P29374 26038 Q9H0U9 4171 1058 P20671 Q8NCD3 Q4LE39 9219 55723 P45973 Q9BTM1 P62805 P62807 Q99880 3010 P16104 1107 Q99877 3009 Q9NVP2 3008 3007 Q8IUE6 3006 51773 11198 255626 1108 Q99879 Q9H2G4 P83916 Q7Z7K6 Q96NT1 3024 O75367 P55209 P23527 Q01105 3014 P17096 55869 O60264 Q8N8U2 4869 Q16777 O94776 Q16778 O95503 Q9BY41 P0C0S5 Q9NRZ9 P04908 221613 Q96A08 Q99733 55355 10524 124359 Q93079 Q8TBE0 P0C0S8 3148 Q93077 84733 Q7L7L0 Q6NXT2 64061 P07305 P26583 9425 Q8TDI0 8290 Q9BW71 Q96KK5 3159 Q02539 Q9HC52 P49736 Q12873 P49450 Q969G3 8348 9555 8467 O60814 6839 23492 Q96QV6 79723 Q92522 8338 Q8N257 Q92922 Q9Y5B9 Q14781 Q9H5I1 P33778 266812 8479 Q5SSJ5 4673 O95931 22893 P10412 23466 P58876 23468 7141 Q96T23 P84243 Q71DI3 P68431 6601

Q16695 51742 Q13185 P49711 8349 P09430 Q92993 6605 10951 54737 Q99549 O43463 3070 Q9UIG0 P22492 Q14839 Q5QNW6 O00257 P16403 P16402 P16401 Q9P0M6 57332 Q6FI13 4676 Q13112

positive regulation of nitrogen compound metabolic process P10914 Q92858 O14793 Q12948 Q12824 Q6QNY1 O00267 P17676 3516 2547 2304 4609 2303 P16220 Q96JM2 P84022 Q92731 O15516 2308 25913 Q9H4L7 Q12837 Q5VTR2 54361 Q8NAP3 P16104 P16234 4851 5705 O75925 Q12968 6927 P37023 6929 4862 811 Q9UJU2 P49116 O75928 P40425 P40424 6926 Q6IT96 P06401 3659 6925 P06400 Q96JK9 5970 2100 Q9NR96 P56705 P48380 O95343 10524 10645 P20823 O60674 P19838 Q8IUC6 P61296 5966 5604 P05129 O95477 Q9Y463 P28482 1022 1385 3320 P19532 O00468 6829 1017 10736 P21964 1499 P15173 80324 P07550 4646 6827 Q9UHK0 4780 Q92769 5991 6720 P28370 Q14938 O95361 O00233 P10600 P19544 O00358 Q9NS56 Q8WYH8 Q06330 P37198 10626 3326 P19419 Q9P0K8 3324 1387 P17482 P08651 P17480 4775 Q9UM54 P04179 4790 Q12888 4792 Q9H2K2 22937 1161 O60502 P38398 2247 Q9BZK7 6601 Q92753 6721 Q92993 Q12770 P05112 Q9UM63 Q92997 Q13501 Q9UKS7 P10826 O43186 Q12772 P10827 Q12778 P51692 Q13503 P31749 Q05586 O00255 1050 Q03164 10724 861 P05362 5888 P40337 50943 Q9Y2W1 P06213 P07900 O96004 6872 P04637 2034 P13349 2033 2274 O95271 Q9UIH9 23326 O60603 25988 P15884 P52952 3479 10011 4686 84289 Q16236 10014 10138 P01112 Q04917 Q9C0K0 6760 4221 Q96T76 641 P33076 P48436 4214 P01106 406 P01588 O43294 408 P49407 P01343 P01100 O00327 O00206 Q09472 P48552 P48431 9921 6774 3265 148022 890 891 Q99743 Q14192 Q96S42 Q02952 898 3014 Q53X93 P01137 5316 29086 P49639 P01375 Q9UPY8 P51531 6660 5451 P35222 Q9NUX5 P51532 Q96RR4 P17813 3148 3269 P10070 P10071 3146 P61457 668 6776 P01127 10001 6657 P23497 P24588 P26583 O00744 5460 6672 5582 Q9Y5Q3 P50613 P51946 Q9NX61 Q12905 Q12906 Q9BZE0 672 P15692 79612 3159 Q02930 P51828 6667 Q8N5U6 P40763 P04201 P05412 5591 O00755 4261 Q6IR47 7533 P18850 Q05086 23414 3171 P37231 Q9NWV8 P18846 P18848 688 3169 148327 9826 207 5468 Q92922 P27797 P01185 7422 P52815 O60907 3066 Q9UMN6 Q9UPT9 3065 3182 P17844 8625 10432 23636 P27540 5494 6464 P62508 Q9UIG0 O14936 466 P51617 P58012 468 7428 10320 54106 8878 9967 P25116 P60568 O15085 6595 Q14686 6597 4297 6599 6598 P30086 P15923 P13984 Q9Y4K3 351 Q9H161 474 P08047 Q7Z6C1 Q13485 P25963 O94906 Q9NPC8 3091 5154 O00716 7334 5155 8301 P12429 Q15306 5037 19 Q86VP6 P58304 P13631 367 Q04864 51773 Q70SY1 P46531 Q13492 P00533 Q96RL1 Q15554 Q13133 Q15796 Q9UQR1 6496 P35638 6495 Q15797 6256 P12755 Q9ULH7 Q9NY61 O75360 Q9Y606 Q2M1K9 Q15561 P17096 51547 Q13263 Q15562 Q9BSI4 Q16650 4086 P35869 4087 8204 4088 P62826 4089 7476 9412 P18074 Q8WVL7 P55055 P12645 P36956 222546 P08151 P24864 P20264 Q16665 Q13033 9421 P55347 O75469 7124 P54132 P54253 O75586 P14635 391 P35453 Q9UQL6 P10275 P10276 4093 154 Q99729 Q99966 Q13285 2902 7132 Q9NRC8 7376 8463 Q9HAZ2 Q09161 7014 P22392 P36896 O43524 7490 55810 P01308 338917 Q9NQB0 Q8WW38 Q14140 Q99958 Q15475 Q99835 Q96T37 Q8TEY5 P00492 7023 5089 P14859 Q96T23 54851 Q01664 Q96PK6 56916 P01579 P49711 P08069 10155 P49715 1956 P09038 55827 9575 Q9NPA8 7158 P39905 7157 O15198 Q02750 6182 P11474 5092 26747 55832 Q99814 7046 9464 P35813 P29375 27005 Q01094 7161 P22736 Q9Y4C1 Q1PSW9 P17275 P62195 91 P04085 94 9219 P42229 O15119 P42226 Q9UBK2 Q58WW2 P56524 22926 22807 Q13887 9590 Q8IY57 P55318 P84095 Q4LE28 P05067 1869 O75528 7189 7067 P29353 P19793 Q03014 7182 6096 Q01196 P41235 56034 Q92793 Q96GM5 Q9HD15 P68871 O95619 Q9NSC2 Q9NRA1 8289 Q13422 O15156 O94776 Q13547 A4D1W7 P78317 9495 2735 P19438 1763 Q9HAK2 Q9BXJ9 Q04206 Q92786 Q8HWS3 1406 2737 2736 Q15853 Q96G30 O14593 O95999 9149 Q8TAK5 P53567 1655 P22415 Q9UK80 Q03468 51720 4800 2626 7099 7098 Q9NVC6 Q9NP71 P46736 P20248 64919 80155 Q9UIV1 P23511 375790 3609 P17081 3608 5901 P46734 P09429 P12956 Q13216 29966 P78527 P18146 Q9H4W6 P35711 Q9BQA5 1312 P61088

P43694 O15455 P08588 5914 O15350 Q9BZS1 Q15648 P55771 P19484 Q06710 Q04771 63976 5927 P63272 P24941 Q9HCS4 O14497 Q92585 902 Q15532 Q13233 P08238 P21675 5925

microtubule-based process 54908 Q14204 8655 Q8NEY1 11065 24137 Q6QNY1 P51587 9184 351 Q9BVA0 Q8IYN9 O60566 Q96M29 P07196 Q9BVA1 9738 P43034 O75935 11190 O43684 O75376 O95684 Q9UJC3 10426 1104 O60333 P42858 Q9NRH3 54801 Q9BSJ2 Q92845 146909 P24522 84790 57519 Q9BYV2 Q05397 Q9BXC9 27185 57405 54930 Q6UVJ0 Q2VIQ3 Q96RK4 Q9NRI5 3895 P60510 Q9Y6A5 Q9HC77 O94927 Q9BQE3 4627 4869 Q96EA4 P62826 O43663 Q14118 O43303 P48380 P68371 54820 P68366 P04350 5604 P54257 9662 O95239 Q14008 Q14807 P35579 57787 114791 1499 57662 Q9H6D7 55125 3796 3799 Q96R06 P07437 Q96CS2 9793 5991 163786 79441 116840 P41208 Q9GZM8 85440 5747 Q8N137 O15182 Q9UGJ1 O15066 Q96L34 Q86VS8 55142 Q9P0L2 Q96N67 347733 11004 Q8WW24 11127 Q9H1H9 Q8TE73 O00139 7277 Q9Y496 Q13509 23354 Q02750 Q13748 25777 Q86UP2 8481 7272 Q9NQT8 10844 Q9NXR1 Q9P209 P40692 Q9NZ56 Q2M1P5 Q96MT8 55835 84376 10048 O14777 Q9UKT4 1605 6993 O75665 P10636 P63172 O95271 7283 P63167 26271 203068 55722 1058 Q66GS9 55605 85378 P46821 O60282 Q9UBK9 Q13885 Q6VN20 6760 374654 10383 Q8N4N8 Q96CW5 23332 Q13409 O95166 22920 3925 Q9BUF5 Q96RT7 Q7Z460 1069 Q99871 81565 Q15154 P52292 7846 Q96S59 P05067 Q96RT8 3801 11116 9001 Q9BV73 9126 O95613 P33176 23303 84617 P52732 Q9H4B7 Q9BVG8 O15379 Q8N960 O14965 O43264 93323 8841 Q658W2 P35222 9371 9493 10128 80254 Q96FJ2 P23258 Q9UH99 Q9HBM1 9928 Q15058 Q96JB1 1647 6790 4131 9700 4133 1781 Q8IXI1 Q8IXI2 1778 P42704 3832 Q13561 3835 P49450 10460 Q68CZ6 P51955 Q9NYZ3 5590 23095 Q05513 Q9UQE7 57159 4137 4139 5108 5901 Q02241 O00762 P12036 Q99661 Q9HC35 5116 140735 Q14674 Q7Z4H7 63971 27436 Q9NP97 Q8TF09 57610 Q71U36 55559 51512 Q15532 Q2M2Z5 Q14683

regulation of cellular biosynthetic process Q9UKT9 Q9H0E3 Q9Y265 Q8NDW4 Q12824 8091 124790 10657 2305 2304 2303 114991 2300 Q8N3U4 155061 284695 2308 2307 Q9H0D6 Q92974 Q9H4L7 Q12837 Q8WXB4 Q8NDV7 2316 10661 Q96EB6 O75925 136319 Q9UKV8 4990 3660 3661 Q9BYE7 P62495 P37023 P13056 84901 57761 55588 O75928 Q9UKW6 3659 P26358 Q6NUN9 Q5BKZ1 3673 Q92949 Q99583 Q12857 Q13705 P21917 10645 1488 1487 283337 Q99576 2332 3665 3667 10642 64061 P26367 10644 10643 Q9UL18 1022 Q8NA42 Q06587 114548 Q8IX07 Q5JT82 Q96MX3 1017 10614 79576 Q9HAJ7 1499 10616 O14981 Q9Y230 P61586 O75909 O14503 Q12873 Q12872 Q9Y239 Q9Y5Z7 Q8WXF1 6720 Q15906 285527 O00358 Q9BPY8 57798 1029 3207 10626 1027 1025 Q9BUB5 P17482 P17480 604 Q9Y242 P05023 P04179 Q15910 Q9UL36 Q12888 5883 Q9BQ95 O60869 79595 Q8NA19 P38398 57326 55145 3215 Q92990 Q9Y250 6721 Q92994 Q92993 6726 Q8N488 Q92997 Q9UKS7 P10826 P10827 P10828 221937 5411 P31749 Q8NDX6 1050 P53999 79101 57332 3226 79589 3225 P04150 Q9UGL1 Q92985 O95159 Q9UKL0 O96004 P14317 P14316 23569 11108 11107 Q8WWY6 1059 23560 Q15022 639 P62875 6749 6760 Q9Y5V3 5430 O96019 O95163 P16989 P57059 Q03933 51131 641 P33076 51132 P35251 Q14186 Q6B0B8 79923 8932 648 8939 P49407 P60228 Q14188 O00327 O96028 5440 P48552 5441 6772 Q9UKN5 Q8TAU3 P48551 6774 Q99990 3265 346171 O60828 P15621 Q66K89 Q99500 Q2M3W8 Q14192 P35240 P01019 51147 Q9Y692 Q15047 P23246 Q86X95 5451 O00330 Q8N3J9 P35222 Q5T6S3 P35227 P35226 O14979 P57082 O75081 P17813 P35232 Q00403 53335 3269 56849 668 6776 4116 Q96MH2 Q16385 P24588 Q15056 7629 O43474 P06733 O00744 5460 Q9Y5Q3 6311 9821 P25490 3281 Q9NX65 672 P15692 Q9UBW7 6304 57708 Q6UUV7 P05412 O00755 Q9Y5R5 9831 3297 Q9NX70 23532 Q9UBX0 P14373 57713 688 148327 689 5467 9826 207 5468 Q8N895 Q92922 4150 4152 23509 P56178 9841 P56177 Q8NHY6 Q12809 Q9Y5S9 P17844 4149 O14920 57727 9839 O43463 5494 Q8NHX1 P08908 23512 Q96QT6 O14936 8517 Q92900 9849 4172 O15085

P36508 4173 4174 P35659 Q14686 4176 Q14209 8535 Q9BZ95 Q6NZI2 Q9H165 4171 Q9H161 Q9H9F9 Q9H160 Q9NPC1 P08047 5017 P78395 Q9BQG0 P07199 Q13363 Q9NPC8 Q14693 94234 4188 8543 P46782 5036 79084 P58304 Q96F45 O15090 23051 Q04864 Q9Y2H8 9869 7205 Q8WY36 P00533 Q96RL1 Q15554 Q9HAU4 8553 P35638 P61968 Q8IWY8 Q96IZ0 Q9UPP1 P32121 P31274 P31273 P31276 O75123 Q9ULH7 23028 Q9NY61 4193 Q9Y6Q6 257 Q9H9D4 Q6DD87 Q15561 8548 Q96RK0 51548 Q93009 51547 Q14232 P61964 Q15562 Q9BYU1 P62826 9412 Q9UPQ9 P36956 O75592 Q96F24 P31270 Q9NPF5 Q13398 55915 9421 P78347 9425 7248 O75586 O75582 Q9Y6K1 Q93062 Q03989 Q9Y2C9 Q71SY5 Q9Y6K9 51564 Q15583 8569 55922 5074 P78337 55929 11030 Q9HAZ2 Q15109 P22392 O15055 O43524 26523 7251 O75570 23492 O15047 P49848 Q15596 Q15118 8110 P61925 Q3KNS6 O15062 5089 166968 O95931 23466 Q9Y6M1 23468 O15060 79913 51588 P08069 10155 Q9BQI3 9439 Q96NG5 1956 286075 Q8NEJ9 Q9NPA8 64426 P37840 5092 64428 1965 Q9H9G7 54625 O95947 54623 Q14289 Q8IWS0 Q96RE7 9464 P29375 P29374 27005 Q7Z589 Q9H0M0 1975 P22736 P42345 Q9BUG6 Q96EK4 Q9UGU0 P04085 Q92574 Q13761 1978 Q58WW2 P56524 9477 O75534 Q02297 7291 O75530 7290 9470 7295 P24928 1982 Q6P1K2 O75528 P29353 O95977 Q06643 P0C7X2 Q8WXI9 P40145 Q9BYM8 55066 1993 79039 Q96IQ9 O15499 P56545 O95983 A4D1W7 O95503 55072 P78317 P78318 9495 Q8TBE0 Q9HAK2 Q9UH92 Q9Y2B9 Q96EP0 O14593 O95999 P35610 Q9NP66 23081 O43918 23085 Q9UKY1 Q9H9B1 P85037 P31260 Q6P1N0 P31249 Q13322 Q14653 Q9NP71 Q9NP72 3728 1540 Q9BUJ2 3720 5901 P46734 Q13330 P12956 Q6P1L6 Q9P031 Q9H4W6 Q00059 Q9BQA5 Q9Y6E7 Q9H582 P61088 Q96AE4 147808 Q96RG2 Q68DY9 P43694 O15455 Q9BQA1 5914 11091 O75953 63976 63978 5927 5928 P63279 P00519 5929 P63272 23077 P24941 Q92585 Q15532 Q13351 Q14683 Q13352 5925 Q7KZF4 P10914 O95677 Q92858 P67809 Q9UBB5 Q9NS86 A5YKK6 Q9P2R6 4609 P16220 10772 5931 P27348 5933 O15516 5935 Q9UBC3 P27361 54361 P50148 1107 P16234 O60573 284323 4613 1108 O15525 Q92838 Q9UK58 Q7Z3K6 Q92835 P05549 Q9NRY4 Q99471 Q7Z3K3 O60548 Q92830 Q9UJU2 284312 O15534 P49116 P03372 64170 Q92833 P06401 Q92831 P06400 Q92826 P07737 5970 P46089 5971 9093 Q9UBE8 Q9NRZ9 P56705 9099 P17535 P17544 Q99459 P17542 P62140 57410 400720 P61296 5966 10765 5981 P50570 3320 O00463 O00468 O95231 Q9UFF9 O14627 6829 10736 3315 Q6FHQ0 5976 P41218 Q8N2W9 4646 27161 5978 6827 65056 5991 93986 5993 1153 Q14938 O00472 Q06455 55250 Q9NS56 P37198 79685 6839 3326 3324 2475 P08651 5500 5987 O95259 P07766 Q10586 6850 Q8ND82 P51449 Q6IA86 27148 O00488 P30542 Q02086 O60502 O60503 P61244 Q9BXG8 4664 5511 Q12770 5515 P05112 26292 Q9UNY4 5518 O43186 Q12772 Q12778 O43189 Q05586 2023 Q02078 Q6KC79 3350 10725 57459 10724 Q99496 P40337 7704 O00425 4690 P23396 O43159 6872 6871 P04637 P38405 2034 2033 Q9NVW2 O95271 O00422 Q9UFB7 4686 4204 P01116 4205 Q16236 P01112 4209 Q9C0K0 6883 Q9BT49 4221 Q9GZR2 O43167 23210 P50548 O76071 Q9NRP7 84159 Q9P2K8 4214 P01106 6878 P01588 7727 56946 Q9C0J9 P01100 Q7Z7K2 7741 5562 Q8N726 9921 P14416 P52736 773 Q02952 Q53X93 6886 P01137 Q16254 P52740 7750 6421 5573 P04626 P04629 2064 P52747 2063 11218 Q9NS37 Q99853 P10070 P09086 P10071 10363 10362 P01127 5569 2070 O43593 5585 7764 22794 25822 Q96PU8 79612 O75190 Q02930 P51828 7756 Q8TEB7 5578 5579 10336 8607 P40763 P04201 2081 5591 Q9GZV8 4261 Q6IR47 5111 Q3SY56 64127 P37108 O75177 P18850 P15336 O75182 Q9NRL2 56987 51295 P18846 Q68CJ9 P18847 P18848 326 55657 Q92800 P27797 Q9GZU7 P01185 Q9UNP9 2099 O60907 O75164 P51843 Q8NCN5 55662 Q8NCN2 333 91748 Q9H7L9 10794 8625 5119 Q9GZT9 23636 9975 6464 4287 P62508 P51858 146050 221656 Q9NVV9 P58012 10320 P60321 9967 P25116 Q99417 Q96HZ4 O43670 Q14566 8655 4297 Q9BY77 9500 Q9UKD1 P13984 351 Q9UBS0 Q04759 Q9UBS5 P21127 P25963 P09017 8662 P20290 Q15424 7332 5154 7334 5155 8667 7335 19 26205 85403 367 Q70SY1 11198 7329 Q14586 20 7341 5162 P11309 6496 6495 Q8IVW6 Q14103 6498

P11308 Q99909 6015 P32242 Q8N3C0 Q9Y606 5160 84108 Q9UBU3 116113 P17096 Q14592 11168 Q96MA1 Q9UBU8 Q13263 Q8TF50 8204 Q9BY44 Q14119 Q9BY41 Q9Y618 P12645 P29084 Q86WP2 222546 Q04725 Q04724 387 Q04727 Q9NWT6 Q04726 23152 P08151 P20264 11177 11176 P23771 P55347 9541 5187 11142 O15164 2909 391 Q6MZP7 23126 O43639 25788 O43638 Q02535 Q01201 2908 Q9UFW8 11146 Q15466 Q6PJG2 Q99966 Q13285 2902 54 5195 6045 7376 Q09161 23133 23135 Q86WV8 Q3KQV3 Q6PRX2 P09067 2917 Q14140 Q9UBP4 Q99958 Q15475 Q8TEY5 Q96ME7 Q9HA82 P14859 O43612 Q8TAQ5 Q96DT7 Q9H063 Q99941 P49959 Q01664 51222 56916 P01579 Q14151 Q9UBQ5 P09038 9575 P82979 8242 10284 O75676 O15198 23112 O43623 25776 51230 1609 Q5VVH5 10289 Q15014 86 P78424 Q9NWH9 P08559 P33992 P33993 P33991 Q8TAF7 P41134 Q1PSW9 27122 91 P17030 94 O15119 1616 P45973 Q9UBK2 Q9NSA3 P78412 9112 Q96I24 9590 Q9H7Z7 Q9H3R0 P55318 Q99081 P55316 P67775 Q52LR7 P17040 P05067 2959 Q9UBL3 O94763 P52298 P29474 92129 9126 27107 Q9H422 Q460N5 Q03014 6096 P41597 56034 27102 Q9BTC8 2961 2960 O95619 Q8TAD8 Q9NSC2 2969 P09874 O15151 8289 Q15843 O95622 O95625 O15156 O94776 29777 202559 P17023 2972 79149 Q9BXJ9 P17020 Q8N393 Q8HWS3 P04198 Q9UBN7 Q15853 O75626 Q14527 P13945 9149 Q8TF47 Q6NYC1 Q9H488 P41182 Q8TAK5 93474 79175 Q499Z4 P53567 79177 P30048 84525 1655 84528 P22415 1653 Q9UK80 Q03468 Q92499 O75629 P47712 O95644 150094 Q9UBG7 Q8N7H5 P20248 64919 80155 Q8TAI7 P41162 P17081 Q9Y5B6 Q9Y5B9 P09429 O75604 P10415 Q9P2X3 P41161 P35711 23186 P09430 P08588 P41159 Q86SE9 728642 Q9P2Y4 Q13227 P55771 Q04771 O15105 Q13233 Q6NX49 Q9H6Q4 O14793 25909 2550 9181 P51587 P17676 Q8TDD1 2547 P37288 57504 Q9HCJ0 P84022 O00151 Q92731 O14788 10413 Q7Z6R9 25913 P28330 P08729 Q92729 Q5VTR2 P51114 O60216 Q9H2G9 4734 Q7Z727 Q9H2G4 O00167 22850 81628 54496 6927 Q8IUD2 6929 Q6AHZ1 Q96KM6 Q8N680 811 6921 P40425 P40424 6926 6925 Q6IQ32 Q17R98 Q9C005 333929 Q9UIS9 Q9NZI7 P48382 Q9NQX0 P31946 Q9C009 2100 Q9NR96 P48380 O95343 3428 P16333 6938 P20823 55352 O60674 P19838 O60675 Q8IUC6 P62263 P18509 P31949 5604 6936 Q9Y468 Q8IZ40 4771 Q9Y463 4772 Q9Y466 Q8TDI0 Q9UIL8 2107 55364 10856 Q86VE0 2103 P16383 27287 80324 Q08999 O14744 6945 P50222 4780 Q92769 Q14814 P28370 Q07666 O95361 80306 P10600 O95365 Q06330 2119 10865 P19419 Q6PI57 4775 O14753 847 Q92766 P09622 4790 4791 4792 Q9H2K2 Q9H2K0 2130 81669 Q9NR48 Q9HCL2 Q92753 4302 857 Q92754 Q13501 22827 Q13503 Q9NR50 Q14839 P19883 Q13506 Q9NR55 Q9HCK5 2139 861 862 863 192669 Q9UMX1 4799 P09601 50943 P08754 Q9Y3Y2 Q9BS34 P15408 Q9Y3Q8 3481 Q9UIH9 23326 O60603 25988 P15884 Q8WUU5 Q96T88 3479 2146 3476 10011 P25205 7832 10013 84289 Q96KG9 55723 10499 10014 Q6UWV6 4343 Q96T76 Q99750 406 Q9BRP8 O43294 408 84271 409 Q01518 Q96C55 Q09472 Q8N1G0 O43251 23309 64215 P13686 890 Q00653 Q99743 Q99741 898 29080 Q9UN42 P49639 P61326 P51531 4361 8721 7874 148156 Q9NUX5 Q9NR11 P51532 Q96ST3 P60484 Q15291 11331 55758 10486 10001 P60002 10488 Q96T58 10487 P28702 P23497 P26583 Q9Y3M2 P46199 P50613 P51946 23764 25942 Q8NFW5 Q9NUQ3 Q8WUY8 10450 8726 Q8N5U6 90993 Q08117 3054 Q05086 Q9BRU2 29079 P37231 Q9HCE1 79723 8737 10468 7422 Q96GA9 5245 3068 Q9HCD5 P52815 22893 3066 Q9UMN6 3065 Q9UIF9 O75290 79753 10438 Q00613 Q96P20 55787 10432 3070 Q13901 9612 Q9UIG0 P52824 466 468 P28749 Q9NQL9 7428 P04792 6595 6597 6599 6598 O75386 P10599 6591 Q9Y4K3 Q76L83 3084 473 474 476 23286 9617 8767 P38919 Q13127 Q15303 B2RXF5 3091 Q16633 8301 Q15306 Q86VP6 O75376 P58546 P13631 24149 3096 Q02447 O75381 Q9NVP2 51773 30813 P46531 Q13133 Q15796 Q13131 Q16649 Q15797 9640 P12757 Q8N6I1 9643 7468 P54198 Q9UNL4 P12755 O75362 Q8TDS5 O75360 P46527 51780 Q01101 P42167 6128 O94805 7458 Q13144 Q5VUA4 54815 Q8WVM0 Q9BSI4 Q16650 Q13148 P35869 P20393 7476 Q16656 Q15329 Q8NCA9 P18074 Q8WVL7 P55055 O43306 P10588 P10589 93166 Q86VN1 Q16665 51317

Q49AN0 P43489 Q8TDY2 9667 Q16666 Q5VUG0 P54132 P63092 P35453 Q14494 8328 Q14498 Q16670 P46108 Q14011 Q86W54 Q9NRC8 7013 7014 P46100 Q9Y4H2 Q5PSV4 7490 P13682 P63096 Q9C0F3 84232 Q8WW38 Q99835 7024 7023 10392 Q9C0F0 49854 P98179 P15822 9682 7020 Q02878 Q9UNH7 51341 54851 Q9NVM4 Q96PK6 11243 55704 P55010 Q13185 Q8NCF5 P23443 6188 Q15369 Q8IUX7 Q9BX66 Q9BSM1 Q09028 6182 P11474 26747 Q15370 54862 P57682 Q6PID4 Q99816 9208 Q99814 P78545 6199 7046 6198 P35813 P78540 P10515 O43711 6193 O43719 Q9Y4C1 P62195 Q4LE39 O15234 9219 1738 1737 A8K8V0 7057 22803 112950 22806 22807 57473 55294 Q3V6T2 P46934 P46937 83746 Q96GN5 O60296 Q4LE28 Q9UER7 Q14865 7068 7067 Q5TAX3 Q15714 Q13535 Q9H307 P19793 P29590 Q7Z2Q5 Q63HK5 Q01196 84619 Q9H6W3 P41235 1756 Q9HCP6 Q92793 Q96GM5 Q9HD15 Q9Y4E5 O60266 O60264 Q9NRA1 P59817 Q13547 Q13546 9252 7073 P41223 57018 P43405 1763 Q9NZR4 Q04206 Q92786 Q92785 Q92784 P41229 7088 Q01167 Q9BSG1 51720 Q14894 4800 P08246 2626 7099 7098 Q9NVC6 Q14416 7091 P14923 7090 Q9NQZ8 P30153 1789 1788 Q05516 1786 Q9UIV1 1785 375790 Q13573 Q9HCU4 P55884 93134 Q9H2S9 Q96CJ1 P78527 92283 85509 Q86VK4 84661 51742 Q6ZNG0 P05198 Q9Y4A8 30827 Q96H20 Q9HCS4 902 Q9HD40 Q13112 P08238 Q9NZN8 P08235 P21675 Q92618 Q9H609 Q92616 Q9Y2W7 Q9NYD6 Q6P2D0 O75820 2672 Q12948 22992 O00268 Q6QNY1 O00267 79894 3516 Q9BZI1 Q96JM7 Q96JM2 Q5VWX1 283248 Q9ULW3 Q92610 4841 Q8N587 P07996 Q12950 Q12952 Q9Y2X9 Q8NB12 Q8NAP3 79885 P16473 4851 4854 Q96JL9 Q92600 5705 Q9ULX9 Q9NYF8 Q9Y2Y8 Q12968 29128 10514 83933 26097 Q9UQB3 4862 O75807 57649 Q6IT96 4869 5716 Q96JK9 Q12972 Q0D2J5 Q9H9T3 3551 Q6P2C8 5728 10524 P17301 Q9HBE1 Q99697 400961 Q9ULZ3 Q9UHR5 Q9ULR5 O95477 P28482 Q9UHJ3 Q12986 1385 29108 P19532 Q9P0J0 Q9P0J1 P15173 P07550 P04040 10971 7913 O14867 Q96BA8 10973 Q9UM47 Q9Y2T7 Q9UHK0 Q9NYA1 O00233 P19544 Q8WYH8 57678 29110 1388 29115 Q9P0K8 1387 1386 P42771 Q9UM54 4899 Q9H1I8 10985 Q9NYB0 22937 22938 Q9UHL9 22931 2247 284119 1398 58533 Q9BZK7 6601 Q9Y2V2 Q92630 6605 57209 Q9UM63 P51693 P51692 P31629 Q9BQY4 O00255 Q8NEZ4 Q9NQ33 O00257 Q03164 P17342 984 Q5H9I0 988 P05362 Q9Y2W1 P06213 P07900 Q9UPV9 P13349 2274 P53803 P52952 990 Q8WTS6 126208 Q96RU8 Q96RU7 51003 79811 6622 79813 51008 10138 Q9UQ80 Q04917 P49770 Q9UM07 Q9Y2P0 Q7Z5H3 Q9UQ88 5796 Q5T7W0 O60739 P11177 8819 P48436 8812 Q99638 P01344 P01343 O00206 2290 23429 P48431 5321 Q96JC9 3142 Q9NU63 148022 Q99623 Q96S42 Q8NAF0 55869 7975 6646 5316 51028 P01375 Q9UPY8 23439 6660 P49750 8841 Q9UHI6 O00213 Q3KNV8 23435 Q96AQ6 3151 O60716 P12004 10128 Q96RR4 Q3KNW1 3148 Q15170 545 546 3146 P61457 Q96EY1 6657 O43353 6670 6672 23409 Q969H0 Q9Y2K7 Q9ULJ3 Q9H9S0 3162 Q12905 O95076 Q8WYA1 Q12906 Q9BZE0 Q01844 552 Q9BZE4 P42704 3159 Q99684 6667 P49336 P05771 Q9H5J8 Q969G3 8864 10580 7533 P55198 Q9ULK4 23411 23414 3171 Q00987 Q96S94 3169 7529 7528 Q08462 Q9H5I1 64375 P50750 Q86U86 Q9UHD2 Q9UPT9 3184 571 3182 P51608 P68036 7536 Q9H5H4 Q01826 5371 8880 P27540 Q60FE5 Q9UPU9 Q12933 Q9ULM3 7555 Q9ULM6 P51610 Q9BUY5 54583 80854 P51617 Q9NPJ6 7549 54106 8878 P60568 8892 Q15654 O43432 8896 P15923 Q86YW9 Q96FV9 84324 116 Q7Z6C1 Q13485 O94906 P03950 O00716 Q16512 Q16514 P12429 Q15669 O75496 O75494 Q7LBC6 P10242 P10243 P10244 84312 51412 O94915 Q13492 Q16520 6239 54925 Q15672 Q9UQR1 7101 6256 Q16526 Q2M1K9 Q9UDY8 7579 9774 4086 7112 4087 4088 Q8N9N2 4089 O75478 9775 8445 163126 Q9H257 O75475 Q8N5F7 142 P21359 P24864 P62701 84333 Q15697 53615 Q13033 P54259 O75469 8458 64750 7124 P54252 P54253 Q96KC8 O75461 Q9UQL6 P10275 4092 P10276 4093 O75460 4094 154 P23588 Q99729 Q13045 55806 P49736 7132 8463 8467 9314 O43889 P36896 23378 P15976 P35568 Q96C28 6282 1822 166 Q8NFM4 55810 P01308 Q8NFM5 338917 Q9NQB0 Q99717 Q15233 Q96T37 9326 P54274 O75446 P36402 O75444 6294 7141 Q99700 1831 Q96T23 Q00577 Q99708 P49711 Q16576

P49716 P49715 55827 Q9NQC7 Q96C00 64784 7158 O75437 Q08050 P39905 7157 P19086 162239 Q8N9R8 Q02750 P35548 138151 55832 Q9UDV6 Q5VTD9 Q13077 26038 Q06787 Q86Z02 56252 Q01094 Q9H204 26039 7161 P63167 P42224 10933 10935 P17275 P23945 P63165 Q9BVI0 P42229 57109 P42226 O15372 O94992 Q9P0W2 22926 Q969S8 22927 Q13404 Q13887 Q01085 57592 Q8IY57 57594 P10644 57591 Q96BD5 79365 P84095 Q08945 Q9UHX1 1869 7186 7189 7188 Q8WYK2 O43812 7181 7182 7185 1877 Q86UE4 Q86UE3 1874 Q9Y3C7 P17252 P68871 1870 P09630 O15379 Q969R5 Q13422 O15391 Q8NB78 4914 2735 P19438 O43829 84733 1408 1407 1406 2737 2736 Q96G30 Q8WUI4 8050 27327 Q02386 Q7RTR2 1896 Q9HC52 Q14774 P22670 Q9NQ92 9391 P11532 Q96G25 84759 58487 Q9UQE7 Q8WYP5 P23511 Q8WUH2 3609 3608 3607 Q14781 P23510 P21333 94104 O14495 Q9P0T4 29966 P19474 Q02363 58491 P18146 84305 27300 O60341 P01730 Q969V6 O15350 O75832 O15353 Q15649 Q9BZS1 Q15648 Q9P0U4 P19484 Q86YP4 Q06710 Q96BF6 Q8N5A5 3622 2773 Q9HC29 O14497 Q15652

vesicle-mediated transport 23603 Q9NRW1 9182 22872 Q96KP1 Q9NRW7 Q5SQT9 P15498 1213 79778 O14662 90678 3875 P07996 Q9UBC2 Q8IWJ2 Q08431 2316 10427 P42858 30011 Q9H2G9 200081 P49006 O15400 4734 Q9UI12 253725 Q9Y282 Q12846 Q5T0N5 P61163 57403 Q8IUD2 6809 O60664 Q9UKW4 4983 O15533 Q9UJ41 P63104 P53675 Q12851 Q9C005 91782 1130 56681 P53677 Q96JJ3 Q99698 57410 6810 10640 6812 6811 O14579 27072 6814 948 P41743 Q12981 P07307 O75901 O95477 Q92538 P50570 5861 5862 1020 Q9H1K0 55361 O60645 58513 2348 1499 O60763 O00471 3312 P07550 Q9Y2T2 10972 4646 P61106 Q9UL26 O95487 P32856 5870 5871 O95249 P33947 P53618 Q9NR31 27297 10868 P20645 Q96MV8 O60895 3685 O60890 Q9UM54 7804 5868 P53621 2495 O60749 P30542 22931 81555 P62330 P40222 P42768 10959 Q8TEH3 P61006 Q6NUQ1 857 Q13501 P51572 P51693 P53992 P51575 Q9UL45 Q969M3 1176 1174 1173 O00499 84932 Q8NBS9 Q8IVI9 Q96ED9 P08754 Q9H4M9 Q9ULV4 P61019 Q8TBA6 P61018 P06576 O95159 10490 Q9UPV9 Q9Y6W5 3482 23325 P50897 P15531 P62993 Q99523 O14828 Q96RU3 Q96KG7 Q15021 6622 10134 P01116 Q96KG9 10133 11345 P01112 P01111 Q9BTU6 Q9Y678 6643 6642 P51636 23210 Q9NV70 P36575 O75096 400 55973 55737 Q14185 Q96CW1 408 Q15036 4218 P49407 409 Q01518 P02794 Q15276 P49768 23307 6892 P48553 6653 3265 2054 Q96CV9 O00203 10239 9919 Q00535 P61204 Q15286 P49755 51026 54536 8943 P49754 O14964 91949 Q9UP83 84062 O43264 4240 P01130 5331 23673 O95183 P35222 P54920 Q9NRR8 O14617 O95070 Q01970 Q96T51 10121 10487 11336 Q6FGG2 5584 Q5U0I6 Q96B97 Q9Y2K6 Q641Q2 P15328 Q9Y2K9 O95197 Q68EM7 Q8IYJ3 Q2M389 433 Q8WTW3 A8K0Z3 55763 10451 Q96AX1 7879 8724 5338 P55072 57706 9950 7534 Q05086 Q8WUX9 10228 55770 P16671 Q8IYI6 8615 Q15075 10466 P05783 10226 P01266 64130 6453 6456 4035 Q9UPT5 6455 65108 333 Q00610 7415 P61764 P83436 5119 Q5T1M5 Q60FE5 64145 O75396 O14939 Q99653 56882 P40616 P62745 6457 4038 8878 Q99418 10564 10566 Q8N6T3 Q16623 8411 P18085 8775 O75146 23048 O75385 94121 351 23163 Q96QK1 P49815 8408 8546 8301 9632 P12429 19 29959 Q9UJC3 Q05952 415117 8417 Q13492 Q96RL7 Q9H9E3 11079 Q13133 O00602 4074 Q15436 8677 9525 9522 Q8WVM8 8676 Q15438 P32121 23265 23025 Q9Y6Q5 29924 375 51542 9515 8548 P02741 P61966 P23763 Q14114 Q8WWN8 O75351 381 Q9NZZ3 382 Q8N6H7 Q8N4C7 P59780 388 23032 51552 Q9NUP9 1808 P60520 9527 9648 P62820 9784 149371 11261 O43752 10053 11021 Q9Y3E0 P10276 O75581 274 154 Q9UEU0 Q99962 51560 Q99963 Q16555 Q13286 9554 Q96C24 O94955 11031 O75575 23256 161 P11142 51693 7249 8218 Q9NRD5 P46459 9201 9322 9685 7142 Q8TEY7 Q86Y82 Q7Z7G2 P12314 A1XKG3 Q86VS8 O43739 Q96EV8 Q14C86 Q9NZ43 Q6VY07 Q15363 9559 P02786 2802 O94973 O15075 O75436 7037 O75558 Q9BXW6 26985 P10147 P37840 O43747 Q13190 11014 O94979 84376 Q15811 9342 9463 P98155 Q13636 84958 9341 Q92572 P23945 10938 P41250 Q9UGU5 65082 O15118 O95721 7057 P78537 Q8IUN9 7052 81567

P46934 117177 10947 Q9HD26 O15126 P05067 80230 O15020 Q15836 26258 57120 Q9Y6I3 Q9UHY1 60412 P16284 O15258 P21980 Q92673 9135 P11717 50618 O15155 Q8IY33 Q6UWE0 Q9UNE2 P21757 Q07954 O15027 9146 9267 Q9UBF2 Q9NP61 Q6NYC1 O00186 O00189 9382 P51149 23085 2741 P08246 P35606 51606 9276 P56962 O95405 Q9NP72 P51153 P42261 P51159 Q96IW7 Q9Y6D6 83548 Q9Y6D5 1785 O60499 P63010 Q96QF0 Q8WYP3 O60493 P21333 O75969 53916 O75843 Q9BY11 387680 Q9H223 O60229 2647 P84085 1315 P20339 1314 P20336 2885 1793 84661 Q9NZM3 Q9HAP6 Q15642 5914 9179 Q9NP90 Q14677 Q8TAG9 P84077 2773 4830 51510 P20340 Q13596

regulation of signal transduction P25054 Q7Z628 P10911 Q9NRW4 Q5VV41 9181 Q99490 P15498 O15519 2305 Q6PCE1 Q92851 O14544 O14543 O14788 57506 P07996 2308 Q92974 P52564 P09936 Q9Y2X7 65018 115704 Q9P107 2316 P14174 10783 4734 O15524 Q008S8 Q7Z727 Q9H2G4 Q92835 Q9NR80 Q9NR81 Q06124 10758 Q8IVF5 P20936 57761 57521 Q9UKW4 Q9Y297 64170 Q9H7P9 5716 22866 5970 3672 P48023 Q92949 Q9NR96 3551 Q12979 9093 P21917 Q13829 P17302 5728 O60674 O00182 P20827 2697 Q8IUC6 Q8N5V2 64061 5966 5604 P04233 Q8WXG6 Q92538 Q13956 4771 O00220 Q96N96 O00463 Q9NUY8 P15056 1499 10616 1012 P07550 P04040 P61586 P08887 P51671 Q9NYA1 P29466 Q9Y239 124583 P10600 P10721 Q5VST9 P37198 29110 10746 79444 960 3326 1147 841 2475 843 5863 604 Q6ZW31 847 Q92888 Q07890 P07766 O95136 P28223 6850 P50591 Q13618 Q9P0L0 Q9NS68 P18545 Q8IUH5 P62330 2247 Q96N67 P60033 1398 Q9BVC4 58533 Q07889 975 613 Q5TCX8 857 5997 O14763 859 Q96CA5 5515 P05112 5516 5879 Q92633 5518 Q12774 Q13501 Q12778 P31749 O95140 O95382 Q9H8V3 O00255 Q9P0M2 27032 253980 153090 P05121 P09601 Q9UL54 P06213 P01241 P56199 P14317 115557 3481 O60725 Q96RU8 O14827 Q96RU7 P62993 3479 P13591 997 3476 10013 P01116 P01236 5536 Q15389 P01112 P01111 O43166 Q15027 4221 Q7Z6J4 6885 23216 2280 56940 Q8WTR2 2159 Q99750 4214 84033 4215 4216 P01588 Q99759 408 P49407 P01344 409 85360 Q13098 P01343 P49768 O00206 P48431 5562 Q86VW2 116986 P14416 3265 P52735 116987 3263 23787 148022 P11274 Q02952 P35240 8945 P01019 51026 6647 55504 P01137 O14964 P01135 8826 P49758 P01133 P01375 Q9UJM3 64223 6541 P04626 8841 2065 2064 P35222 P35348 55633 P60484 P17936 P01127 Q96EY1 Q15052 6657 11214 5328 O43150 8837 Q15057 P24588 8717 9927 O43353 Q3MII6 5580 6794 5585 O43597 Q96BZ9 P07948 Q5R372 Q9UJF2 3162 79971 673 Q96P48 P61981 P15692 Q99683 79735 10451 57708 9815 5578 P62714 5579 5338 P05771 Q96FA3 O00635 Q6IR47 8743 7532 7410 64127 Q5JSP0 Q05086 Q99558 324 Q9NU19 5467 8737 9826 207 54206 Q8TEA7 329 P48357 Q76NI1 7422 Q96GA9 6453 Q5VWQ8 P30825 Q9UHD2 Q12802 Q9BUZ4 Q0IIM8 55785 10554 O14920 O14921 9839 Q9BT67 23636 5494 Q60FE5 Q12933 6464 4168 Q13905 Q9Y3P9 23513 57732 O14939 P51617 8517 54106 8878 Q99418 10564 P26447 P25116 P60568 Q8N6T3 P49427 8772 O15085 9744 7204 P30086 Q9Y4K4 Q9Y4K3 O43318 351 356 23286 P49810 8767 P43034 P49815 Q8WZ64 Q9HC98 Q8TBP0 O43687 6003 Q13009 7334 5155 Q16512 4067 4188 Q15669 7335 5037 Q9UIA0 O75376 Q96D03 367 Q04864 P09382 9628 P00533 Q13370 Q13490 1902 Q15311 Q13131 Q8IW93 9641 8795 9882 Q9UQ13 8315 Q9HBW0 29 Q15438 P32121 253260 Q9ULH1 64857 Q8TEU7 Q96CN4 Q9Y6Q6 Q9UBU3 Q9UDY8 23263 142678 Q8WZ42 P55290 P00441 Q8IV61 Q9Y6R4 5054 Q9BY44 Q9UPQ3 Q15208 Q8WWN8 Q14118 P10586 Q86XR7 125058 Q9H257 382 Q8N6H7 387 P21359 389 Q66K14 Q16543 Q13153 P62942 8452 6275 Q13158 11140 7124 7248 7126 Q9Y3E0 23365 O43639 P10275 154 Q9Y2C9 121512 25780 Q6KH11 Q9Y6K9 O94827 1936 2904 P46108 7132 Q9Y5J5 Q86WV8 5071 23370 Q02763 P01308 7128 7249 Q7Z7H5 Q9NQB0 9306 O15169 Q9GZM8 85440 Q96NH3 Q99956 23229 Q6ZSZ5 P02790 Q8TCU6 10392 O15068 23463 O43734 O43739 Q14C86 Q8WVQ1 Q99943 Q9UNH7 P01579 P08069 10276 1956 P09038 Q14155 10159 Q9BY84 10160 Q08050 64786 Q8N9R8 Q02750 23118 Q14160 51231 54862 Q6PID4 Q14164 Q9BWT7 Q14161 Q6R327 1848 P98194

Q14289 1605 Q13077 Q15811 9465 7046 P35813 O75787 10928 26037 7161 P42345 Q9Y6G9 P41134 1852 Q9BXM7 1850 Q86VI3 O94989 P46940 9218 Q92574 1616 Q6ZT07 Q9NSA1 P78536 7057 Q13404 Q96QB1 9590 7052 P46934 O43609 P67775 81565 P08575 P16070 P05067 O60292 P42338 P98174 Q9UER7 7186 Q15831 7189 7188 P29353 Q9H422 Q96M96 Q13418 P56539 Q7Z569 7185 84619 Q9BYM8 60412 P17252 O15379 P21860 P21980 O60260 50618 Q13546 9138 55072 Q7Z434 P78318 9495 7074 27352 P19438 P43405 P41220 Q05655 Q04206 P53041 Q9UBN7 9146 9267 Q9NZJ7 Q96EP0 O95999 Q9NP61 P13945 P78509 Q96DN5 P41182 Q86TI0 9021 Q16828 93594 Q58EX7 9020 P00749 Q03468 P63000 Q96PE2 P00742 3953 Q6P1N0 P08246 3956 Q8IWV1 7099 7098 Q13202 Q96HU1 Q8WZ19 Q15628 O15211 P21580 P30153 P61073 23094 Q9Y6D6 Q9Y6D5 2873 P08134 Q13574 51735 3965 P21333 Q9NYJ8 50649 O14492 Q6ZV73 O75962 Q9P035 O60229 57162 2889 2885 3611 P61088 50650 O60343 Q14790 Q9NZM3 Q5T9L3 O15455 Q16610 4943 P01730 P41159 Q8IYX1 O15350 O75832 O15111 Q13227 Q06830 Q86Z14 Q04771 222068 Q8N5A5 Q9HC29 Q9NZN5 Q13233 P08238

positive regulation of signaling pathway 8772 P30086 Q9UBB5 Q9NS86 Q9Y4K3 O43318 472 O15519 356 P84022 1453 Q9H160 23286 Q92851 8767 O14788 Q13485 57506 P07996 Q9HC98 P04004 3091 7334 5155 4067 7335 5037 65018 54361 2316 367 Q04864 P14174 P09382 7448 10783 P00533 Q13490 4734 1902 10666 9641 8795 Q9UQ13 Q9HBW0 P32121 253260 Q9Y6Q6 Q9UBU3 P37023 10758 27185 Q9UDY8 57521 Q9NRI5 142678 P03372 64170 P55290 Q9GZX9 4088 4089 7476 5970 P48023 Q86XR7 Q9H257 Q9NR96 Q13705 P56705 P17302 387 389 O60674 O00182 2697 Q8IUC6 Q16665 3667 5966 55915 P04233 P62942 P27037 6275 Q13158 Q13956 7124 Q9Y3E0 O00463 P10275 154 Q93062 P15056 Q9Y2C9 1499 10616 1012 P07550 P04040 O94827 P61586 3678 1936 P08887 Q99966 7132 P08648 3690 Q9UGI0 P29466 Q9Y239 11030 P36896 O95361 124583 P36894 P10600 P10721 5071 P35568 P37198 1029 10626 29110 960 1147 Q02763 P01308 841 2475 843 Q7Z7H5 Q9NQB0 P42771 O15169 847 Q99835 P07766 P05106 P02790 10392 6850 P50591 O43734 Q9P0L0 Q9NS68 P18545 Q8WVQ1 Q8IUH5 Q99943 2247 Q9BVC4 O14640 P01579 P08069 857 O14763 P05112 5879 1956 P09038 Q92633 P09958 Q9H8V3 O00255 23118 7272 27032 54862 Q14164 P48730 Q6R327 P09601 P98194 Q9UL54 Q13077 P06213 Q15811 P01241 Q13873 7046 P35813 P14317 3481 O60603 P42345 Q9Y6G9 Q9BXM7 3479 P13591 91 P01116 9218 94 P01236 5536 Q15389 Q9NSA1 P01112 P01111 P78536 7057 4221 6885 Q13404 P51636 9590 2280 7052 P46934 2159 54764 P08575 8932 4215 P01588 P16070 Q99759 O43294 408 P49407 P01344 409 P01343 O00206 7189 7188 P06239 P48431 Q8N726 Q13535 3265 29767 Q03014 Q13418 3263 7185 148022 Q9BYM8 60412 Q02952 P53708 P01019 P21860 51026 P21980 55504 P01137 Q9NSC2 3932 P01135 O60260 P01133 P01375 Q9Y337 64223 P04626 Q13546 55072 Q7Z434 2065 2064 P35222 9495 P35348 P19438 P43405 P17813 545 Q04206 P53041 P01127 6657 8837 P24588 8717 O43353 5460 Q96EP0 O95999 Q13315 P13945 P07948 P78509 P78504 3162 9020 79971 673 P15692 O14908 P63000 57708 5579 P00742 5338 3953 P05771 Q6P1N0 3956 Q96FA3 O00635 7099 O00755 7098 Q6IR47 Q13322 8743 Q15628 P14923 64127 Q05086 Q99558 3728 3169 P08134 5467 8737 329 P48357 3965 P21333 Q9NYJ8 7422 6453 2099 Q9UHD2 57045 Q9BUZ4 57162 3611 P61088 Q14790 Q5T9L3 O15455 Q16610 10554 P01730 Q15762 9839 P41159 Q9BT67 23636 P27540 5494 Q60FE5 O15111 25818 Q86Z14 222068 O14939 3622 Q9HC29 8516 54106 P26447 P25116 P60568

cellular catabolic process P48052 23607 O14672 9184 Q9P2R7 10535 O60566 P15374 1576 Q70EL4 O00273 P63208 10533 4968 Q9H0D6 Q92611 P09936 Q9C026 P28330 Q12834 P48047 4860 O00154 Q5VTR2 Q07343 5706 5707 5708 5709 P30519 5700 5701 4734 5702 Q9UHP3 Q9UKV5 5704 64282 5705 P27694 Q08426 3420 2572 P56817 2571 P17405 79791 A0AVT1 5717 3417 3416 1358

57646 P62256 O60543 81631 5830 P61289 6921 Q9Y297 5713 3419 4627 5716 Q12972 P07858 5970 1374 P99999 Q99460 9099 Q13829 Q676U5 P21912 3304 Q99575 P19838 55236 3421 948 3309 P51784 Q9UL18 1022 O95470 P51668 2110 1020 P51665 P50213 O95352 10613 P21964 10616 P61221 29102 P04040 Q92530 5976 Q7Z419 P51553 Q9NYA1 Q9Y4X5 O00231 O00233 O00232 Q96J02 O95243 P30793 Q9NS56 1029 10868 2237 79444 10869 167227 841 P42771 847 P07686 4790 Q13618 Q13617 Q13616 1161 O00487 O95376 Q8TBB1 6609 391627 10956 P62333 Q05469 P20618 55149 Q9BZK7 Q92995 5511 Q96CA5 Q9UK22 5516 Q13501 Q9UL46 Q9UMW8 Q13620 Q92626 6500 P54727 P54725 Q9HCK5 10845 Q99259 253980 P40692 57695 192669 5886 5887 P09601 26061 8930 O00308 P53804 25862 Q9NVW2 P50416 23326 P50897 991 Q96RU2 54512 513 997 4686 P62877 8803 10013 8801 51009 11345 Q15024 P23025 64682 115426 23576 P35244 P11177 P50542 O76074 Q96Q15 51132 51377 4214 Q9BRP8 O43294 408 P49407 P60228 409 10269 P49768 P60468 Q9GZQ8 Q6ZN33 5682 5683 Q8N726 5321 5684 5685 5686 Q9BW91 891 O14727 Q96T60 Q00535 55743 8945 Q15165 P25789 Q16134 P25788 55626 Q9GZP9 6647 P01137 Q7Z3C6 539 P25787 P61326 Q15287 Q9Y5X9 Q9Y215 Q8TAT6 P01130 5692 5693 P40818 P40939 7511 7874 P11279 P51659 3032 Q08257 3030 P14550 O95071 9924 668 5445 5687 5688 4116 P48775 Q59EA4 7508 Q5VVQ6 O00628 P62837 9821 8975 Q3SY69 Q969H0 Q9UPR3 9820 P50613 Q9Y2K6 22794 P51946 Q9UNN5 3162 O76031 Q8IWF2 Q96PU4 435 5335 5336 P62714 5337 5338 10213 P55072 Q9H3F6 P07954 O43242 Q9Y4P1 Q9HCE7 23530 Q5HYA8 Q05086 Q01831 78991 P16671 Q00987 Q8NBM8 P49448 5467 Q86T82 Q9H1A4 329 Q6UVY6 P55084 8754 O60907 Q9UPT9 Q9Y2M5 23621 O00762 P13489 3184 Q9NWW5 Q9Y5S9 Q8NCN5 51185 55666 215 P68036 7415 8761 P04424 8881 Q96C86 P39748 P17980 Q9HCC0 3074 Q9NVV4 Q9UKK9 P16885 55432 10208 O14939 O14818 P58012 8879 8878 Q92900 P49427 5141 5142 11060 P15927 P19174 11065 8654 Q86TM6 6470 Q9Y4K3 472 593 O75150 O75390 Q9C0C7 Q04759 84447 P23786 P49810 5139 9978 Q8IU60 O94905 Q14694 Q14693 P38919 3093 5394 7332 7334 P55036 7336 O43684 Q504Q3 23291 Q9NY59 P30084 128 6117 Q9NPD3 6119 7329 6118 Q13370 Q13490 7328 Q16401 Q9HAU5 Q9HAU4 Q9BUN8 Q13011 5162 11160 Q13257 Q14103 9525 7347 P32121 4191 P35998 4193 23381 P09110 P31040 O94925 P22674 10197 11165 Q16531 P21589 30 Q93009 Q93008 P00441 34 P36957 57092 7353 9775 P18074 6389 Q86Y37 O75477 O75351 P34896 P12883 P11310 Q9H492 267 23032 28960 Q13393 P60520 O94817 8453 Q14249 8454 8452 48 O75469 Q8TDY2 64750 P40926 7126 26994 P14635 P12694 8450 6390 P35579 P09172 Q96RQ3 Q8IZD4 Q13042 Q13286 Q13049 Q14139 7375 Q09161 11274 6048 P36776 23014 26523 23016 Q9UKA1 5071 7251 P11021 Q9NRD1 P49721 P49720 P00491 Q8TEY7 8237 O94966 7267 10273 6059 A1XKG3 Q9Y5K6 Q9Y5K5 6050 Q9UKB1 P11586 Q99942 Q8WY64 Q9H1Y0 P00367 11124 Q16698 2805 51107 Q9NQC7 Q15008 Q9BXW4 Q15369 9695 P14625 O75674 Q01780 O75792 114088 255488 Q9H9G7 Q93034 P27815 Q99816 Q8WW12 2935 P21399 P53004 9100 9588 Q15819 P29372 Q96K76 27005 27248 P78549 O43837 Q70CQ3 Q70CQ1 Q05329 79139 10935 P62195 P08684 O15234 P05177 P45974 Q9BZQ6 26270 P78536 9474 22803 55054 83737 O75891 84708 55294 27252 Q9BSB4 P46934 1743 27257 Q92560 P42574 P60900 10940 P52298 7189 P29474 P29475 Q5TAX3 26019 9361 7184 80267 Q9Y3C5 Q9BYM8 Q9Y4E8 55066 4907 26133 Q8TEL6 Q9NZ08 80020 O60260 Q15843 Q13423 P07339 P53396 O95989 91147 O75874 Q6R6M4 O95865 1891 P78317 Q9UNE7 10921 1642 1763 Q04206 P05164 Q92542 Q9UBN7 Q969Q1 4913 P30049 9388 Q13315 P30041 P30043 P11766 Q96EP1 P30048 2744 P00505 P00747 26232 Q9UK80 26234 1892 60673 Q13200 2747 P47712 Q8IY92 Q13443 Q8WZ19 Q13569 Q8IWV7 57154 Q96BI3 8065 84749 1666 Q9UJX2 P61077 1540 26002 P17081 P08138 124739 1429 P22303 8078 O75604 O75844 Q13216 Q8IUQ4 P28065 Q5W0Q7 377630 P32320 P33897 84305 1676 1312 2643 Q5XUX0 1431 P61088

P17174 Q14790 Q9UJ83 Q14432 P41159 P54687 P08107 P53597 O75832 Q13107 Q06830 P62191 P28074 P28072 79065 P28070 P42357 23192 P63279 P30260 51752 902 Q13233 51514 P43686

protein targeting Q8TEW0 9182 Q9UPN3 P35658 Q9P2R6 Q9BVA0 473 23165 Q92973 Q9Y2X3 P60059 10651 P62913 Q15785 P27348 P49815 P25963 Q9NPC8 Q9HAV7 Q8TD31 8546 79083 P55957 Q8IWJ2 55696 O75381 P42858 Q9UIA9 9868 4734 6496 Q9UNL2 29927 Q9Y6Q5 Q96P70 Q86WA8 811 5830 P63104 Q13023 P62258 P61619 6386 P31946 P62826 P62829 Q86XR8 O95343 23039 O60318 P61970 O60674 Q16543 9648 Q49AN0 P41743 O75901 11140 7124 P28482 10053 Q9P0J0 10736 3313 O94826 Q9Y2T2 4646 10971 5195 Q9Y5J6 6711 7249 604 Q9UM54 Q9NRD5 4792 Q9BV36 23463 O43615 O60502 Q9NS69 10953 401505 2247 Q6VY07 P01579 P02545 5514 6726 P09038 2923 55705 Q9BY84 P30536 Q13505 7157 P31749 26985 O95140 Q9Y5L4 O75431 Q8N4H5 Q9NQT8 10724 Q92621 O15504 Q9UL54 9463 Q96QC0 Q9UPV9 P04637 O96008 P38646 O60725 83752 2147 Q7RTN6 637 10133 Q9C0K7 6747 Q15388 Q14974 4580 O15131 9590 Q01082 O95166 23214 9472 8021 P50542 P46934 P43307 P52292 O60296 11231 406 P60468 O00327 P52294 O00560 P29590 P30101 23303 Q96CV9 O00203 60412 Q9NZQ3 Q02952 54535 P01137 P01375 Q9NSC5 4000 64901 9495 653361 56288 9804 1408 P24588 9927 O00623 O00505 O00628 5584 O00629 P41182 P14598 3836 P61981 O14908 10452 P05412 O95405 7532 7531 7534 Q9HCE7 Q9H2T7 P37108 57154 Q15629 P51159 10469 Q99797 P00734 207 3841 9702 3843 7529 80273 5901 P27797 100287932 Q9NW64 Q96QU8 O14925 56993 P49792 5371 4285 11097 Q9UKK6 P62072 O00410 23633 Q6ZWJ1 Q08209 P52948 P40855 10204 P25116 51517

regulation of protein kinase activity P25054 Q8TEW0 P54619 Q9Y4K4 Q9NRW4 124790 Q9Y4K3 3084 O43318 P13861 351 595 23043 P49810 P22694 Q9ULW0 P49815 6901 P07996 P52564 P09936 P03950 5155 Q16512 4188 O15530 Q16635 Q9UKE5 O95684 9077 P50148 P16471 51654 P00533 Q13131 P24522 8795 Q5S007 P11309 Q7Z727 Q9H2G4 Q9HBW0 Q06124 22974 Q9C010 Q9Y6Q6 Q9UBU3 P46527 57761 Q9UDY8 1111 Q8WZ42 P00441 4869 P06400 5716 Q53H12 Q9Y6R4 Q14114 3672 Q15208 P31947 O43306 Q9NR96 Q13705 9093 P21917 5170 5728 10645 P21359 O60674 P20827 Q16543 64061 5604 Q13153 3309 P04233 P43489 Q8WXG6 11140 Q13956 4771 7124 11142 7248 P54132 1022 O00220 1020 O43639 O75460 154 10614 Q9Y2C9 121512 Q9Y6K9 P07550 O75909 3690 Q9NYA1 26524 P10600 P10721 Q86WV8 P37198 1029 Q9BUB1 P11021 1027 10746 79444 1026 Q8NFM4 P01308 Q8NFM5 120892 2475 7249 P42771 Q13177 7804 Q9NRD5 O14757 Q99956 P61925 P05106 P30304 O95136 O95257 7023 Q9UGJ0 P28223 6850 P11229 P30307 A1XKG3 O43612 P30542 P18545 O60503 Q9UNH7 2247 P60033 Q01664 58533 975 55704 Q5TCX8 O14640 857 O14763 859 Q96CA5 5515 1956 P09038 5518 Q9BY84 P31749 O75553 O95382 O00255 Q02750 P38936 23118 2810 P37173 Q6PID4 1609 Q9BWT7 1848 Q14289 Q9UL54 P06213 P01241 9463 7046 P98155 O95835 9467 P56199 7161 990 P42345 Q96RU8 Q96RU7 1852 1850 995 23560 Q7RTN6 Q99640 P01116 Q9C0K7 Q92574 P22612 Q15389 P15090 1616 O94992 P78536 7057 Q9UII4 4221 6885 Q02297 P10644 P31321 Q3V6T2 P31323 56940 Q8WTR2 O43609 P67775 641 Q99750 4214 P08575 8812 4216 P05067 P42574 P42338 Q13098 Q9UER7 11116 P49768 O00206 7186 7189 P29353 5562 Q8N726 Q9H422 O96020 Q96M96 Q13418 P56539 P31431 84619 P40145 894 Q99741 Q00535 896 P17252 P35240 O60266 6647 P01137 P01135 P01133 P01375 Q9UJM3 O95622 5573 P04626 5575 Q13546 5576 2064 55750 P43405 Q96RR4 56288 P60484 Q07954 Q92786 5566 5567 P01127 5568 Q96EY1 Q96JB5 5569 Q9Y2B9 Q96MH2 1647 5585 O43597 8851 P78509 P50613 Q16828 B3KY43 P61981 Q9BZE4 Q99683 Q03468 P51828 5577 5578 10454 P08246 2081 Q8IWV1 7099 7098 Q6IR47 Q13202 Q13443 7532 P30153 P30279 5590 P61073 Q96SZ6 P51959 Q05513 2873 324 P30281 8737 207 54206 Q13574 Q15750 Q15078 P46734

Q08462 Q9NYJ8 P24385 8754 4035 Q9P035 Q9BUZ4 O75293 3611 Q9NRM7 O15455 P01730 9839 23636 O15350 O75832 Q12933 6464 Q13227 Q9P287 P52824 O95661 57732 P17612 Q8N5A5 Q9UHV2 P51617 8517 900 54106 56647 Q13233 P25116 P60568 5925

positive regulation of biosynthetic process P10914 Q92858 O14793 Q12948 Q12824 Q6QNY1 O00267 P17676 3516 2547 2304 P37288 4609 2303 P16220 Q96JM2 P84022 Q92731 O15516 P07996 2308 5935 25913 Q9H4L7 Q12837 Q5VTR2 54361 Q8NAP3 P16234 4851 5705 O75925 Q9Y2Y8 Q12968 6927 P37023 6929 4862 811 Q9UJU2 P49116 O75928 P40425 P40424 6926 Q6IT96 P06401 3659 6925 4869 P06400 Q96JK9 5970 3673 2100 Q9NR96 P56705 P48380 O95343 10524 10645 P20823 P17301 O60674 P19838 Q8IUC6 P61296 3667 5966 O95477 Q9Y463 P28482 1022 1385 3320 P19532 O00468 6829 1017 10736 1499 P15173 80324 P07550 4646 6827 Q9UHK0 4780 Q92769 5991 6720 P28370 1153 Q14938 O95361 O00233 P10600 P19544 O00358 Q9NS56 Q8WYH8 Q06330 P37198 57678 10626 29110 3326 P19419 Q9P0K8 3324 1387 P17482 2475 P08651 P17480 4775 Q9UM54 P04179 P07766 4790 Q12888 6850 4792 Q9H2K2 22937 P38398 Q9HCL2 2247 Q92990 Q9BZK7 6601 Q92753 6721 Q92993 Q92630 Q12770 P05112 Q9UM63 Q92997 Q13501 Q9UKS7 P10826 O43186 Q12772 P10827 Q12778 P51692 Q13503 P31749 Q05586 O00255 1050 Q03164 861 P05362 P09601 P40337 50943 Q9Y2W1 P06213 P07900 O96004 6872 P04637 2034 P13349 2033 2274 O95271 Q9UIH9 23326 O60603 25988 P15884 P52952 3479 2147 10011 6622 10013 84289 Q16236 10014 10138 P01112 Q04917 Q9C0K0 6760 4221 Q96T76 641 P33076 P48436 4214 P01106 406 Q9BRP8 P01588 O43294 408 84271 P49407 P01343 P01100 O00327 O00206 Q09472 P48552 P48431 9921 5321 6774 3265 148022 890 Q99743 Q14192 Q96S42 Q02952 898 Q53X93 P01019 6646 P01137 5316 P49639 P01375 Q9UPY8 P51531 6660 5451 P35222 Q9NUX5 P51532 Q96RR4 P17813 3148 3269 P10070 P10071 3146 P61457 668 6776 P01127 10001 6657 P23497 P24588 P26583 O00744 5460 6672 Q9Y5Q3 P50613 P51946 3162 Q12905 Q12906 Q9BZE0 672 552 P15692 79612 3159 Q02930 P51828 6667 Q8N5U6 P40763 P04201 P05412 5591 O00755 4261 Q6IR47 10580 7533 64127 P18850 Q05086 23414 3171 P37231 P18846 P18848 688 3169 148327 9826 207 5468 Q92922 P27797 P01185 7422 P52815 O60907 Q9UHD2 3066 Q9UMN6 Q9UPT9 3065 3182 P17844 8625 10432 23636 P27540 5494 Q9UPU9 6464 P62508 Q9UIG0 O14936 466 P51617 P58012 468 7428 10320 54106 8878 9967 P25116 P60568 O15085 6595 Q14686 6597 4297 Q9BY77 6599 6598 P15923 P13984 Q9Y4K3 351 Q9H161 474 Q9UBS0 Q04759 P08047 Q7Z6C1 Q13485 P25963 O94906 Q9NPC8 P38919 3091 5154 O00716 5155 8301 Q15306 19 Q86VP6 P58304 P13631 367 Q04864 51773 Q70SY1 P46531 Q13492 P00533 Q13133 Q15796 Q9UQR1 6496 P35638 6495 Q15797 6256 Q96IZ0 P12755 Q9ULH7 Q9NY61 O75360 Q9Y606 Q2M1K9 Q15561 P17096 Q13144 51547 Q13263 Q15562 Q16650 4086 P35869 4087 8204 4088 P62826 P43490 4089 7476 9412 9775 P18074 Q8WVL7 8445 P55055 P12645 P36956 222546 P08151 P24864 P20264 P62701 Q16665 Q13033 9421 P55347 O75469 7124 P54132 P54253 O75586 391 P35453 Q9UQL6 P10275 P10276 4093 154 Q9Y2C9 11146 Q99729 Q99966 Q13285 2902 Q14011 7132 5074 Q9NRC8 7376 8463 Q9HAZ2 P22392 Q9Y4H2 P36896 O43524 7490 P35568 55810 P01308 338917 Q9NQB0 Q8WW38 Q14140 Q99958 Q15475 Q99835 Q96T37 Q8TEY5 7023 5089 P98179 P14859 Q96T23 54851 Q01664 Q96PK6 56916 P01579 P49711 P08069 10155 P49715 1956 P09038 P23443 55827 9575 Q9NPA8 7158 Q9BX66 P39905 7157 O15198 6182 P37840 P11474 5092 26747 55832 Q6PID4 Q99814 Q14289 6199 7046 6198 9464 P35813 P29375 27005 Q01094 7161 P22736 P42345 Q9Y4C1 Q1PSW9 P17275 P62195 91 P04085 94 9219 P42229 O15119 P42226 Q9UBK2 Q58WW2 P56524 7057 22926 22807 Q13887 9590 Q8IY57 P55318 P84095 Q4LE28 P05067 1869 O75528 7189 7067 P29353 Q06643 P19793 Q03014 7182 6096 Q01196 P41235 56034 Q92793 Q96GM5 Q9HD15 P68871 O95619 Q9NSC2 Q9NRA1 8289 Q13422 O15156 O94776 Q13547 A4D1W7 P78317 9495 2735 P19438 P43405 1763 Q9HAK2 Q9BXJ9 Q04206 Q92786

Q8HWS3 Q9UBN7 1406 2737 2736 Q15853 Q96G30 O14593 O95999 9149 P35610 Q8TAK5 P53567 1655 P22415 Q9UK80 4800 P08246 2626 P47712 7099 7098 Q9NVC6 Q9NP71 P20248 64919 80155 Q9UIV1 P00734 P23511 375790 3609 P17081 3608 5901 P46734 P09429 P12956 29966 P78527 P18146 Q9H4W6 P35711 Q9BQA5 84305 P43694 O15455 P01730 P08588 5914 O15350 Q9BZS1 Q15648 P55771 P19484 Q06710 Q04771 63976 5927 P63272 Q9HC29 P24941 Q9HCS4 O14497 Q92585 902 Q15532 Q13233 P08238 P21675 5925

regulation of biosynthetic process Q9UKT9 Q9H0E3 Q9Y265 Q8NDW4 Q12824 8091 124790 10657 2305 2304 2303 114991 2300 Q8N3U4 155061 284695 2308 2307 Q9H0D6 Q92974 Q9H4L7 Q12837 Q8WXB4 Q8NDV7 2316 10661 Q96EB6 O75925 136319 Q9UKV8 4990 3660 3661 Q9BYE7 P62495 P37023 P13056 84901 57761 55588 O75928 Q9UKW6 3659 P26358 Q6NUN9 Q5BKZ1 3673 Q92949 Q99583 Q12857 Q13705 P21917 10645 1488 1487 283337 Q99576 2332 3665 3667 10642 64061 P26367 10644 10643 Q9UL18 1022 Q8NA42 Q06587 114548 Q8IX07 Q5JT82 Q96MX3 1017 10614 79576 Q9HAJ7 1499 10616 O14981 Q9Y230 P61586 O75909 O14503 Q12873 3690 Q12872 Q9Y239 Q9Y5Z7 Q8WXF1 6720 Q15906 285527 O00358 Q9BPY8 57798 1029 3207 10626 1027 1025 Q9BUB5 P17482 3685 P17480 604 Q9Y242 P05023 P04179 Q15910 Q9UL36 Q12888 5883 Q9BQ95 O60869 79595 Q8NA19 P38398 57326 55145 3215 Q92990 Q9Y250 6721 Q92994 Q92993 6726 Q8N488 Q92997 Q9UKS7 P10826 P10827 P10828 221937 5411 P31749 Q8NDX6 1050 P53999 79101 57332 3226 79589 3225 P04150 Q9UGL1 Q92985 O95159 Q9UKL0 O96004 P14317 P14316 23569 11108 11107 Q8WWY6 1059 23560 Q15022 639 P62875 6749 6760 P06702 Q9Y5V3 5430 O96019 O95163 P16989 P57059 Q03933 51131 641 P33076 51132 P35251 Q14186 Q6B0B8 79923 8932 648 8939 P49407 P60228 Q14188 O00327 O96028 5440 P48552 5441 6772 Q9UKN5 Q8TAU3 P48551 6774 Q99990 3265 346171 O60828 P15621 Q66K89 Q99500 Q2M3W8 Q14192 P35240 P01019 51147 Q9Y692 Q15047 P23246 Q86X95 5451 O00330 Q8N3J9 P35222 Q5T6S3 P35227 P35226 O14979 P57082 O75081 P17813 P35232 Q00403 53335 3269 56849 668 6776 4116 Q96MH2 Q16385 P24588 Q15056 7629 O43474 P06733 O00744 5460 Q9Y5Q3 6311 9821 P25490 3281 Q9NX65 672 P15692 Q9UBW7 6304 57708 Q6UUV7 P05412 O00755 Q9Y5R5 9831 3297 Q9NX70 23532 Q9UBX0 P14373 57713 688 148327 689 5467 9826 207 5468 Q8N895 Q92922 4150 4152 23509 P56178 9841 P56177 Q8NHY6 Q12809 Q9Y5S9 P17844 4149 O14920 57727 9839 O43463 5494 Q8NHX1 P08908 23512 Q96QT6 O14936 8517 Q92900 9849 4172 O15085 P36508 4173 4174 P35659 Q14686 4176 Q14209 8535 Q9BZ95 Q6NZI2 Q9H165 4171 Q9H161 Q9H9F9 Q9H160 Q9NPC1 P08047 5017 P78395 Q9BQG0 P07199 Q13363 Q9NPC8 Q14693 94234 4188 8543 P46782 5036 79084 P58304 Q96F45 O15090 23051 Q04864 Q9Y2H8 9869 7205 Q8WY36 P00533 Q96RL1 Q15554 Q9HAU4 8553 P35638 P61968 Q8IWY8 Q96IZ0 Q9UPP1 P32121 P31274 P31273 P31276 O75123 Q9ULH7 23028 Q9NY61 4193 Q9Y6Q6 257 Q9H9D4 Q6DD87 Q15561 8548 Q96RK0 51548 Q93009 51547 Q14232 P61964 Q15562 Q9BYU1 P62826 9412 Q9UPQ9 P36956 O75592 Q96F24 P31270 Q9NPF5 Q13398 55915 9421 P78347 9425 7248 O75586 O75582 Q9Y6K1 Q93062 Q03989 Q9Y2C9 Q71SY5 Q9Y6K9 51564 Q15583 8569 55922 5074 P78337 55929 11030 Q9HAZ2 Q15109 P22392 O15055 O43524 26523 7251 O75570 23492 O15047 P49848 Q15596 Q15118 8110 P61925 Q3KNS6 O15062 5089 166968 O95931 23466 Q9Y6M1 23468 O15060 79913 51588 P08069 10155 Q9BQI3 9439 Q96NG5 1956 286075 Q8NEJ9 Q9NPA8 64426 P37840 5092 64428 1965 Q9H9G7 54625 O95947 54623 Q14289 Q8IWS0 Q96RE7 9464 P29375 P29374 27005 Q7Z589 Q9H0M0 1975 P22736 P42345 Q9BUG6 Q96EK4 Q9UGU0 P04085 Q92574 Q13761 1978 Q58WW2 P56524 9477 O75534 Q02297 7291 O75530 7290 9470 7295 P24928 1982 Q6P1K2 O75528 P29353 O95977 Q06643 P0C7X2 Q8WXI9 P40145 Q9BYM8 55066 1993 79039 Q96IQ9 O15499 P56545 O95983 A4D1W7 O95503 55072 P78317 P78318 9495 Q8TBE0 Q9HAK2 Q9UH92 Q9Y2B9 Q96EP0 O14593 O95999 P35610

Q9NP66 23081 O43918 23085 Q9UKY1 Q9H9B1 P85037 P31260 Q6P1N0 P31249 Q13322 Q14653 Q9NP71 Q9NP72 3728 1540 Q9BUJ2 3720 5901 P46734 Q13330 P12956 Q6P1L6 Q9P031 Q9H4W6 Q00059 Q9BQA5 Q9Y6E7 Q9H582 P61088 Q96AE4 147808 Q96RG2 Q68DY9 P43694 O15455 Q9BQA1 5914 11091 O75953 63976 63978 5927 5928 P63279 P00519 5929 P63272 23077 P24941 Q92585 Q15532 Q13351 Q14683 Q13352 5925 Q7KZF4 P10914 O95677 Q92858 P67809 Q9UBB5 Q9NS86 A5YKK6 Q9P2R6 4609 P16220 10772 5931 P27348 5933 O15516 5935 Q9UBC3 P27361 54361 P50148 1107 P16234 O60573 284323 4613 1108 O15525 Q92838 Q9UK58 Q7Z3K6 Q92835 P05549 Q9NRY4 Q99471 Q7Z3K3 O60548 Q92830 Q9UJU2 284312 O15534 P49116 P03372 64170 Q92833 P06401 Q92831 P06400 Q92826 P07737 5970 P46089 5971 9093 Q9UBE8 Q9NRZ9 P56705 9099 P17535 P17544 Q99459 P17542 P62140 57410 400720 P61296 5966 10765 5981 P50570 3320 O00463 O00468 O95231 Q9UFF9 O14627 6829 10736 3315 Q6FHQ0 5976 P41218 Q8N2W9 4646 27161 5978 6827 65056 5991 93986 5993 1153 Q14938 O00472 Q06455 55250 Q9NS56 P37198 79685 6839 3326 3324 2475 P08651 5500 5987 O95259 P07766 Q10586 P05106 6850 Q8ND82 P51449 Q6IA86 27148 O00488 P30542 Q02086 O60502 O60503 P61244 Q9BXG8 4664 5511 Q12770 5515 P05112 26292 Q9UNY4 5518 O43186 Q12772 Q12778 O43189 Q05586 2023 Q02078 Q6KC79 3350 10725 57459 10724 Q99496 P40337 7704 O00425 4690 P23396 O43159 6872 6871 P04637 P38405 2034 2033 Q9NVW2 O95271 O00422 Q9UFB7 4686 4204 P01116 4205 Q16236 P01112 4209 Q9C0K0 6883 Q9BT49 4221 Q9GZR2 O43167 23210 P50548 O76071 Q9NRP7 84159 Q9P2K8 4214 P01106 6878 P01588 7727 56946 Q9C0J9 P01100 Q7Z7K2 7741 5562 Q8N726 9921 P14416 P52736 773 Q02952 Q53X93 6886 P01137 Q16254 P52740 7750 6421 5573 P04626 P04629 2064 P52747 2063 11218 Q9NS37 Q99853 P10070 P09086 P10071 10363 10362 P01127 5569 2070 O43593 5585 7764 22794 25822 Q96PU8 79612 O75190 Q02930 P51828 7756 Q8TEB7 5578 5579 10336 8607 P40763 P04201 2081 5591 Q9GZV8 4261 Q6IR47 5111 Q3SY56 64127 P37108 O75177 P18850 P15336 O75182 Q9NRL2 56987 51295 P18846 Q68CJ9 P18847 P18848 326 55657 Q92800 P27797 Q9GZU7 P01185 Q9UNP9 2099 O60907 O75164 P51843 Q8NCN5 55662 Q8NCN2 333 91748 Q9H7L9 10794 8625 5119 Q9GZT9 23636 9975 6464 4287 P62508 P51858 146050 221656 Q9NVV9 P58012 10320 P60321 9967 P25116 Q99417 Q96HZ4 O43670 Q14566 8655 4297 Q9BY77 9500 Q9UKD1 P13984 351 Q9UBS0 Q04759 Q9UBS5 P21127 P25963 P09017 8662 P20290 Q15424 7332 5154 7334 5155 8667 7335 19 26205 85403 367 Q70SY1 11198 7329 Q14586 20 7341 5162 P11309 6496 6495 Q8IVW6 Q14103 6498 P11308 Q99909 6015 P32242 Q8N3C0 Q9Y606 5160 84108 Q9UBU3 116113 P17096 Q14592 11168 Q96MA1 Q9UBU8 Q13263 Q8TF50 8204 Q9BY44 Q14119 Q9BY41 Q9Y618 P12645 P29084 Q86WP2 222546 Q04725 Q04724 387 Q04727 Q9NWT6 Q04726 23152 P08151 P20264 11177 11176 P23771 P55347 9541 5187 11142 O15164 2909 391 Q6MZP7 23126 O43639 25788 O43638 Q02535 Q01201 2908 Q9UFW8 11146 Q15466 Q6PJG2 Q99966 Q13285 2902 54 5195 6045 7376 Q09161 23133 23135 Q86WV8 Q3KQV3 Q6PRX2 P09067 2917 Q14140 Q9UBP4 Q99958 Q15475 Q8TEY5 Q96ME7 Q9HA82 P14859 O43612 Q8TAQ5 Q96DT7 Q9H063 Q99941 P49959 Q01664 51222 56916 P01579 Q14151 Q9UBQ5 P09038 9575 P82979 8242 10284 O75676 O15198 23112 O43623 25776 51230 1609 Q5VVH5 10289 Q15014 86 P78424 Q9NWH9 P08559 P33992 P33993 P33991 Q8TAF7 P41134 Q1PSW9 27122 91 P17030 94 O15119 1616 P45973 Q9UBK2 Q9NSA3 P78412 9112 Q96I24 9590 Q9H7Z7 Q9H3R0 P55318 Q99081 P55316 P67775 Q52LR7 P17040 P05067 2959 Q9UBL3 O94763 P52298 P29474 92129 9126 27107 Q9H422 Q460N5 Q03014 6096 P41597 56034 27102 Q9BTC8 2961 2960 O95619 Q8TAD8 Q9UBM7 Q9NSC2 2969 P09874 O15151 8289 Q15843 O95622 O95625 O15156 O94776 29777 202559 P17023 2972 79149 Q9BXJ9 P17020 Q8N393 Q8HWS3 P04198 Q9UBN7 Q15853 O75626 Q14527 P13945 9149 Q8TF47 Q6NYC1 Q9H488 P41182 Q8TAK5 93474 79175 Q499Z4 P53567 P30047 79177 P30048 84525 1655 84528 P22415 1653 Q9UK80 Q03468 Q92499 O75629 P47712

O95644 150094 Q9UBG7 Q8N7H5 P20248 64919 80155 Q8TAI7 P41162 P17081 Q9Y5B6 Q9Y5B9 P09429 O75604 P10415 Q9P2X3 P41161 P35711 23186 P09430 P08588 P41159 Q86SE9 728642 Q9P2Y4 Q13227 P55771 Q04771 O15105 Q13233 Q6NX49 Q9H6Q4 O14793 25909 2550 9181 P51587 P17676 Q8TDD1 2547 P37288 57504 Q9HCJ0 P84022 O00151 Q92731 O14788 10413 Q7Z6R9 25913 P28330 P08729 Q92729 Q5VTR2 P51114 O60216 Q9H2G9 4734 Q7Z727 Q9H2G4 O00167 22850 81628 54496 6927 Q8IUD2 6929 Q6AHZ1 Q96KM6 Q8N680 811 6921 P40425 P40424 6926 6925 Q6IQ32 Q17R98 Q9C005 333929 Q9UIS9 Q9NZI7 P48382 Q9NQX0 P31946 Q9C009 2100 Q9NR96 P48380 O95343 3428 P16333 6938 P20823 55352 O60674 P19838 O60675 Q8IUC6 P62263 P18509 P31949 5604 6936 Q9Y468 Q8IZ40 4771 Q9Y463 4772 Q9Y466 Q8TDI0 Q9UIL8 2107 55364 10856 Q86VE0 2103 P16383 27287 80324 Q08999 O14744 6945 P50222 4780 Q92769 Q14814 P28370 Q07666 O95361 80306 P10600 O95365 Q06330 2119 10865 P19419 Q6PI57 4775 O14753 847 Q92766 P09622 4790 4791 4792 Q9H2K2 Q9H2K0 2130 81669 Q9NR48 Q9HCL2 Q92753 4302 857 Q92754 Q13501 22827 Q13503 Q9NR50 Q14839 P19883 Q13506 Q9NR55 Q9HCK5 2139 861 862 863 192669 Q9UMX1 4799 P09601 50943 P08754 Q9Y3Y2 Q9BS34 P15408 Q9Y3Q8 3481 Q9UIH9 23326 O60603 25988 P15884 Q8WUU5 Q96T88 3479 2147 2146 3476 10011 P25205 7832 10013 84289 Q96KG9 55723 10499 10014 Q6UWV6 4343 Q96T76 Q99750 406 Q9BRP8 O43294 408 84271 409 Q01518 Q96C55 Q09472 Q8N1G0 O43251 23309 64215 P13686 890 Q00653 Q99743 Q99741 898 29080 Q9UN42 P49639 P61326 P51531 4361 8721 7874 148156 Q9NUX5 Q9NR11 P51532 Q96ST3 P60484 Q15291 11331 55758 10486 10001 P60002 10488 Q96T58 10487 P28702 P23497 P26583 Q9Y3M2 P46199 P50613 P51946 23764 25942 Q8NFW5 Q9NUQ3 Q8WUY8 10450 8726 Q8N5U6 90993 Q08117 3054 Q05086 Q9BRU2 29079 P37231 Q9HCE1 79723 8737 10468 7422 Q96GA9 5245 3068 Q9HCD5 P52815 22893 3066 Q9UMN6 3065 Q9UIF9 O75290 79753 10438 Q00613 Q96P20 55787 10432 3070 Q13901 9612 Q9UIG0 P52824 466 468 P28749 Q9NQL9 7428 P04792 6595 6597 6599 6598 O75386 P10599 6591 Q9Y4K3 Q76L83 3084 473 474 476 23286 9617 8767 P38919 Q13127 Q15303 B2RXF5 3091 Q16633 8301 Q15306 Q86VP6 O75376 P58546 P13631 24149 3096 Q02447 O75381 Q9NVP2 51773 30813 P46531 Q13133 Q15796 Q13131 Q16649 Q15797 9640 P12757 Q8N6I1 9643 7468 P54198 Q9UNL4 P12755 O75362 Q8TDS5 O75360 P46527 51780 Q01101 P42167 6128 O94805 7458 Q13144 Q5VUA4 54815 Q8WVM0 Q9BSI4 Q16650 Q13148 P35869 P43490 P20393 7476 Q16656 Q15329 Q8NCA9 P18074 Q8WVL7 P55055 O43306 P10588 P10589 93166 Q86VN1 Q16665 51317 Q49AN0 P43489 Q8TDY2 9667 Q16666 Q5VUG0 P54132 P63092 P35453 Q14494 8328 Q14498 Q16670 P46108 Q14011 Q86W54 Q9NRC8 7013 7014 P46100 Q9Y4H2 Q5PSV4 7490 P13682 P63096 Q9C0F3 84232 Q8WW38 Q99835 7024 7023 10392 Q9C0F0 49854 P98179 P15822 9682 7020 Q02878 Q9UNH7 51341 54851 Q9NVM4 Q96PK6 11243 55704 P55010 Q13185 Q8NCF5 P23443 6188 Q15369 Q8IUX7 Q9BX66 Q9BSM1 Q09028 6182 P11474 26747 Q15370 54862 P57682 Q6PID4 Q99816 9208 Q99814 P78545 6199 7046 6198 P35813 P78540 P10515 O43711 6193 O43719 Q9Y4C1 P62195 Q4LE39 O15234 9219 1738 1737 A8K8V0 7057 22803 112950 22806 22807 57473 55294 Q3V6T2 P46934 P46937 83746 Q96GN5 O60296 Q4LE28 Q9UER7 Q14865 7068 7067 Q5TAX3 Q15714 Q13535 Q9H307 P19793 P29590 Q7Z2Q5 Q63HK5 Q01196 84619 Q9H6W3 P41235 1756 Q9HCP6 Q92793 Q96GM5 Q9HD15 Q9Y4E5 O60266 O60264 Q9NRA1 P59817 Q13547 Q13546 9252 7073 P41223 57018 P43405 1763 Q9NZR4 Q04206 Q92786 Q92785 Q92784 P41229 7088 Q01167 Q9BSG1 51720 Q14894 4800 P08246 2626 7099 7098 Q9NVC6 Q14416 7091 P14923 7090 Q9NQZ8 P30153 1789 1788 Q05516 1786 Q9UIV1 1785 P00734 375790 Q13573 Q9HCU4 P55884 93134 Q9H2S9 Q96CJ1 P78527 92283 85509 2644 Q86VK4 84661 51742 Q6ZNG0 P05198 Q9Y4A8 30827 Q96H20 Q9HCS4 902 Q9HD40 Q13112 P08238 Q9NZN8 P08235 P21675 Q92618 Q9H609 Q92616 Q9Y2W7 Q9NYD6 Q6P2D0 O75820 2672 Q12948 22992 O00268 Q6QNY1 O00267 79894 3516 Q9BZI1

Q96JM7 Q96JM2 Q5VWX1 283248 Q9ULW3 Q92610 4841 Q8N587 P07996 Q12950 Q12952 Q9Y2X9 Q8NB12 Q8NAP3 79885 P16473 4851 4854 Q96JL9 Q92600 5705 Q9ULX9 Q9NYF8 Q9Y2Y8 Q12968 29128 10514 83933 26097 Q9UQB3 4862 O75807 57649 Q6IT96 4869 5716 Q96JK9 Q12972 Q0D2J5 Q9H9T3 3551 Q6P2C8 5728 10524 P17301 Q9HBE1 Q99697 400961 Q9ULZ3 Q9UHR5 Q9ULR5 O95477 P28482 Q9UHJ3 Q12986 1385 29108 P19532 Q9P0J0 Q9P0J1 P15173 P07550 P04040 10971 7913 P08887 O14867 Q96BA8 10973 Q9UM47 Q9Y2T7 Q9UHK0 Q9NYA1 O00233 P19544 Q8WYH8 57678 29110 1388 29115 Q9P0K8 1387 1386 P42771 Q9UM54 4899 Q9H1I8 10985 Q9NYB0 22937 22938 Q9UHL9 22931 2247 284119 1398 58533 Q9BZK7 6601 Q9Y2V2 Q92630 6605 57209 Q9UM63 P51693 P51692 P31629 Q9BQY4 O00255 Q8NEZ4 Q9NQ33 O00257 Q03164 P17342 984 Q5H9I0 988 P05362 Q9Y2W1 P06213 P07900 Q9UPV9 P13349 2274 P53803 P52952 990 Q8WTS6 126208 Q96RU8 Q96RU7 51003 79811 6622 79813 51008 10138 Q9UQ80 Q04917 P49770 Q9UM07 Q9Y2P0 Q7Z5H3 Q9UQ88 5796 Q5T7W0 O60739 P11177 8819 P48436 8812 Q99638 P01344 P01343 O00206 2290 23429 P48431 5321 Q96JC9 3142 Q9NU63 148022 Q99623 Q96S42 Q8NAF0 55869 7975 6647 6646 5316 51028 P01375 Q9UPY8 23439 6660 P49750 8841 Q9UHI6 O00213 Q3KNV8 23435 Q96AQ6 3151 O60716 P12004 10128 Q96RR4 Q3KNW1 3148 Q15170 545 546 3146 P61457 Q96EY1 6657 O43353 6670 6672 23409 Q969H0 Q9Y2K7 Q9ULJ3 Q9H9S0 3162 Q12905 O95076 Q8WYA1 Q12906 Q9BZE0 Q01844 552 Q9BZE4 P42704 3159 Q99684 6667 P49336 P05771 Q9H5J8 Q969G3 8864 10580 7533 P55198 Q9ULK4 23411 23414 3171 Q00987 Q96S94 3169 7529 7528 Q08462 Q9H5I1 64375 P50750 Q86U86 Q9UHD2 Q9UPT9 3184 571 3182 P51608 P68036 7536 Q9H5H4 Q01826 5371 8880 P27540 Q60FE5 Q9UPU9 Q12933 Q9ULM3 7555 Q9ULM6 P51610 Q9BUY5 54583 80854 P51617 Q9NPJ6 7549 54106 8878 P60568 8892 Q15654 O43432 8896 P15923 Q86YW9 Q96FV9 84324 116 Q7Z6C1 Q13485 O94906 P03950 O00716 Q16512 Q16514 P12429 Q15669 O75496 O75494 Q7LBC6 P10242 P10243 P10244 84312 51412 O94915 Q13492 Q16520 6239 54925 Q15672 Q9UQR1 7101 6256 Q16526 Q2M1K9 Q9UDY8 7579 P00441 9774 4086 7112 4087 4088 Q8N9N2 4089 O75478 9775 8445 163126 Q9H257 O75475 Q8N5F7 142 P21359 P24864 P62701 84333 Q15697 53615 Q13033 P54259 O75469 8458 64750 7124 P54252 P54253 Q96KC8 O75461 Q9UQL6 P10275 4092 P10276 4093 O75460 4094 154 P23588 Q99729 Q13045 55806 P49736 7132 8463 8467 9314 O43889 P36896 23378 P15976 P35568 Q96C28 6282 1822 166 Q8NFM4 55810 P01308 Q8NFM5 338917 Q9NQB0 Q99717 Q15233 Q96T37 9326 P54274 O75446 P36402 O75444 6294 7141 Q99700 1831 Q96T23 Q00577 Q99708 P49711 Q16576 P49716 P49715 55827 Q9NQC7 Q96C00 64784 7158 O75437 Q08050 P39905 7157 P19086 162239 Q8N9R8 Q02750 P35548 138151 55832 Q9UDV6 Q5VTD9 Q13077 26038 Q06787 Q86Z02 56252 Q01094 Q9H204 26039 7161 P63167 P42224 10933 10935 P17275 P23945 P63165 Q9BVI0 P42229 57109 P42226 O15372 O94992 Q9P0W2 22926 Q969S8 22927 Q13404 Q13887 Q01085 57592 Q8IY57 57594 P10644 57591 Q96BD5 79365 P84095 Q08945 Q9UHX1 1869 7186 7189 7188 Q8WYK2 O43812 7181 7182 7185 1877 Q86UE4 Q86UE3 1874 Q9Y3C7 P17252 P68871 1870 P09630 O15379 Q969R5 Q13422 O15391 Q8NB78 4914 2735 P19438 O43829 84733 1408 1407 1406 2737 2736 Q96G30 Q8WUI4 8050 27327 Q02386 Q7RTR2 1896 Q9HC52 Q14774 P22670 Q9NQ92 9391 P11532 Q96G25 84759 58487 Q9UQE7 Q8WYP5 P23511 Q8WUH2 3609 3608 3607 Q14781 P23510 P21333 94104 O14495 Q9P0T4 29966 P19474 Q02363 58491 P18146 84305 27300 O60341 P01730 Q969V6 O15350 O75832 O15353 Q15649 Q9BZS1 Q15648 Q9P0U4 P19484 Q86YP4 Q06710 Q96BF6 Q8N5A5 3622 2773 Q9HC29 O14497 Q15652

cell-substrate adhesion Q08043 Q15654 Q9UQP3 P18084 P12814 P56199 P49023 Q9Y3I0 9341 O95274 P11047 Q9BUG9 Q6NVY8 Q92574 P98161 3911 3912 P04004 O15530 5310 O95965 5311 Q96QB1 5796 5829 Q96S53 7448 7205 10420 P16070 O14713 Q13136 Q15836 Q13418 Q00535 P53708 P01019 3655

P25940 P26012 P05556 P02461 3672 P26010 3673 Q14118 P35222 5170 P14543 27076 P17301 387 2335 Q9UKX5 54822 P26006 P49746 P07942 P08514 Q6FGG2 7248 1020 P18564 57669 P16144 1499 P23229 Q9UMD9 3674 3675 P61586 Q13563 3679 Q13683 P31249 3690 Q13444 Q13443 3693 O43521 P35609 10580 O75578 9270 Q86WV8 Q9UBX5 P18206 960 Q9Y5J6 Q9HCM4 3685 5467 3688 3689 P02751 64098 P05106 P05107 8754 P10415 A1XKG3 3611 Q5TAT6 7414 Q9BX66 O15230 Q9HBI0 Q96QT4 8516 Q6PID4 8515 Q14289 P04275 Q9UL54 1605 87

organ development Q9UKT9 Q7Z2K6 P25054 3880 3881 O14793 259266 P51587 P17676 4729 2304 P37288 Q8IYN9 2303 O60443 284217 P84022 2300 Q92731 O14786 O14543 O14788 Q8TDD5 6901 Q9UGM1 2308 2307 P25067 Q9UGM3 Q9NZG7 P08727 Q92729 Q12837 P51114 2317 Q96KN7 P20849 P42858 P39210 Q9UKV3 10661 2319 Q96EB6 Q8N205 Q7Z727 136319 P27694 P28300 4990 O00167 Q7L5Y9 6927 P37023 55584 6929 Q9UKW4 5830 55109 3655 Q9Y297 Q9Y6A5 P40424 P62258 Q9UKW6 6926 3659 P31944 Q9NZI7 P26371 Q9C009 P07858 Q86SX6 3673 P31947 2100 Q13705 Q9BYD5 P48380 O95343 57534 3428 6938 P20823 O60437 2335 O60674 P20827 P62263 Q9UKX5 3667 P26367 5604 P41743 Q9Y468 P11926 P27037 P25098 4771 1020 Q06587 Q8IX07 P39019 P19404 55366 P15056 10614 1499 1012 Q8N264 3676 P61586 3678 3679 3690 P50222 P32856 Q92769 Q14814 P09619 P28370 Q9NZC7 P10600 O00358 3207 1026 841 Q6PI57 Q9HCM4 P17482 3685 4775 O14753 604 3688 7804 P04179 O95377 4791 1285 Q9BQ95 2132 Q15915 Q9NQS3 O60869 P16118 O95376 3215 2125 610 Q92990 1277 613 Q92753 Q5TAT6 P05230 857 859 Q9H0F6 Q92997 Q92754 P10826 P10827 221937 P11912 P31749 1297 O95140 1294 P19883 Q13506 Q9P202 1050 27032 Q9HCK4 2139 861 1289 O95390 863 Q9UL51 4313 Q9UMX1 Q92982 P09601 4318 P06576 O96004 O43278 P14317 23322 3484 3482 P50897 P11047 3479 Q8WV60 P01236 639 10014 P16989 Q9BRQ0 1072 3009 641 Q99750 Q01995 648 O43294 Q16363 Q13099 1080 Q09472 P48552 6774 3265 O00562 P53701 P13686 P12111 890 Q00653 891 P14780 P11274 O14727 894 O95180 Q9NR09 P53708 P35240 656 898 8945 P01019 79956 55504 6525 P49639 P50440 5451 8721 Q9Y696 Q658W2 O95183 P35222 P35227 P14543 P35226 Q01974 P57082 O75081 Q15051 P17813 55755 3028 P60484 53335 51156 11331 668 6776 4358 5207 P24588 O43474 P26583 O00744 5460 Q9Y3M2 Q9Y5Q3 P01040 9821 P25490 7402 9820 Q9NX62 25945 Q8NFW5 673 P15692 P31939 10451 6304 P55075 10454 P05412 P06744 O00755 5471 3290 Q9Y5R5 7410 10460 7412 Q9HCE7 P52803 Q08117 3297 Q05086 3052 Q9UBX0 P37231 682 P36382 10229 Q01955 Q96SZ6 P51959 P60660 445 688 5467 207 5468 Q02809 54206 P48357 7408 Q92922 10468 P06753 Q15078 90780 P24385 P24386 8754 4154 7422 23509 P56178 P56177 9600 3066 22895 O75051 P13010 Q00613 7414 4149 10672 9839 5493 7430 9612 Q9UIG0 O43464 Q8NHX1 P35268 23513 P50402 P13497 P17612 10682 8516 P27658 7428 55558 9846 10202 Q14686 6599 P18085 8775 5268 O75386 Q9UPN3 O75385 Q9H165 6591 Q9Y4K3 3084 O43318 230 472 Q9H161 474 Q99807 P49810 P08047 5017 P83731 P43034 P49815 P21246 Q9NPC8 Q15303 3091 94234 4188 8543 Q16635 Q15306 5037 3099 79084 P58304 P58546 P13631 Q02447 488 Q9NRH2 Q9H2X0 24144 6117 P22102 30813 P46531 51535 P00533 Q13370 Q15796 Q16881 P35638 P61968 Q15797 P12757 Q8N6I1 P31273 P31276 P12755 Q9ULH7 O75360 Q9Y6Q6 Q01101 257 Q96RK4 Q15561 Q15323 Q13144 P55290 Q16650 5054 P35869 Q9Y2J0 9657 7476 8323 Q9Y6R0 P18074 P36952 P68133 P37802 P10589 P13612 P35625 Q4AC94 Q8N4C8 54820 P31270 Q16665 P55061 P43246 9421 9420 Q8TDY2 P49862 O15287 Q16666 7248 P54132 P63092 P35453 O75581 Q93063 P09172 P46109 P23229 51564 P23467 Q14254 P78337 8100 O43521 P46100 Q15109 Q9Y4H2 O43524 7490 7251 285 Q9NZT1 P11021 7249 Q8WW38 1948 1947 P02751 Q99835 9201 O75569 10152 23463 P13646 Q02880 P13647 A1XKG3 Q86VS8 P13645 7020 Q08188 P02549 P21266 P08069 1718 10155 P02545 P47895 Q15125 1956 O15075 Q8IUX7 O15078 O75553 P38936 P11474 Q9NXR1 2810 3909 P02538 Q6PID4 Q99816 O95947 Q99814 1969

P02533 P98194 Q14289 P60842 P78545 Q13753 7046 Q13751 9464 55283 6194 P32302 Q4VCS5 P22735 2821 Q9BUG9 P04083 P04085 65082 Q92574 P05177 P98161 P07355 P98160 3911 3912 P56524 P78536 9474 7057 Q13520 22806 O75534 Q02297 7291 7052 P19338 P46939 P16070 P42574 P98172 P98174 Q14865 9001 7067 P06239 Q06643 P19793 P29590 Q63HK5 P56539 Q01196 1756 P00966 P06241 P21860 O15259 3932 O95983 9255 P55212 P55210 A4D1W7 Q04695 P19320 9253 9495 P43405 Q9Y4F9 Q9NZR4 Q04206 Q5T5A8 Q92786 P07101 P07585 7088 Q15738 O75503 O95999 Q13315 P78509 O43915 8174 9021 P78504 P35613 P35612 P00749 P00747 P85037 Q9H9B4 P08243 3953 Q13563 2626 P31249 Q13323 Q15742 Q9NQZ2 O43921 P14923 7090 P11413 Q05516 3728 3720 375790 P08253 P22303 Q15750 9047 P13807 26576 57045 P78527 P02708 P20336 2885 Q04656 51741 P43694 Q16610 Q9BQA1 O75712 O15230 P22528 Q9HCS4 4830 900 51752 P00750 P08238 Q13351 P07148 O15228 5925 P10914 Q92858 P08833 2672 Q12948 O00268 P52799 Q8NEY8 Q9UBB9 4609 P16220 P08842 P07996 Q12952 P27361 54361 Q8NB12 Q08431 P50148 P63211 P16234 P21810 P16473 P16471 P21815 4851 4854 Q9UK55 O15524 Q92838 P53420 Q12965 P29992 Q9UBD6 P05549 P25391 Q9NRY4 1121 Q12968 Q05397 Q9BXC9 P20930 P20936 Q92830 Q9UJU2 P03372 4867 Q92833 P06401 4627 P06400 P26012 P05556 Q92826 Q9GZX9 P54762 Q92824 3791 P07737 P54760 5970 5971 P48023 Q9GZX7 9093 P56705 9099 22985 P17302 5728 81501 P20702 P17301 P17542 Q9HBE1 Q99697 2697 Q9BXB1 P61296 4637 3309 P04233 P34925 P28482 Q9Y586 Q5D1E8 1385 O95470 O00468 O14627 10733 10736 57669 P15173 P09972 4643 4646 P08887 Q9UM47 P08648 P51671 O00238 P30556 Q9NYA1 P30559 5991 93986 1152 Q8IZT6 P10721 Q6UB99 Q06210 P19544 Q8IY17 P18206 960 3326 1147 967 4898 Q9UM54 5747 P07766 O43175 P05106 P04259 137964 6850 Q06203 2253 Q9NYB9 2010 Q13616 Q9NS68 P62330 57448 2006 P42768 P61244 2247 1399 2246 O14641 4664 O14640 4665 5515 P04264 P05112 P51693 50814 O43186 P29323 Q12778 P51692 O00255 Q02078 Q6KC79 P30530 P52789 Q03164 P37173 10728 P09960 5764 P05121 P29317 P40337 P04275 6615 7704 P06213 P02452 4691 P04637 Q9Y6W5 2034 P13349 2033 2275 25861 P16615 1181 P52952 O60725 P15531 P62993 79810 Q14050 998 999 P61421 4205 Q15389 P62753 P01112 4209 Q9C0K0 P49770 Q9UHG2 4221 5310 Q7Z5H3 6885 5311 P51636 23210 P35368 Q9HDC5 P53814 P50542 1191 2280 Q04900 Q15392 84159 2277 Q15154 P48436 4214 P01106 768 P01588 P28845 Q9C0J9 P01343 P49768 2290 P25791 P50552 P48431 5321 P14416 P13569 2054 P52735 3142 O00444 Q99623 773 Q00535 Q96S42 Q53X93 6886 6405 6647 P01137 5316 8829 5318 Q9UPY3 Q8N960 P01133 P01375 Q16254 Q9UJM3 P01374 P02462 P02461 4240 6660 Q9Y337 4000 5573 P04626 8841 P04629 Q9UHI8 2065 2064 O95067 O60716 P12004 P54920 Q9P0I2 301 302 3148 P10070 P10071 546 5566 P02458 P01127 P40933 5568 Q96EY1 Q96JB5 6657 5327 5328 P49747 9927 6670 P24043 P07942 O00628 5584 7520 O43597 8851 P07949 P07948 Q92817 Q12904 3162 O00622 O95076 Q96PU8 552 O75190 Q99684 6667 4005 5336 Q96AX1 5578 Q15185 7518 Q8N302 P40763 P04201 P26232 5591 Q6IR47 5351 5111 7531 P68032 23411 O76024 23414 10107 51295 324 3169 Q9NPH5 4015 329 5108 7528 5361 5362 Q86U86 6696 2099 P12277 O76011 P51843 Q9BUZ4 3182 P15311 333 Q9GZT9 P26678 P01178 Q01826 5371 P27540 P05997 25818 P20908 O60911 P16401 P16885 54583 Q08209 P58012 P18827 8879 6699 10320 P26447 P25116 P60568 Q13477 8650 8892 4297 P15924 P30086 P15923 4291 351 595 Q04759 356 P09493 8407 Q7Z6C1 Q7Z6C3 Q13485 Q8WUM0 8408 P09017 P03950 9510 9750 P35527 5154 O94910 5155 4067 P12429 Q15669 O75496 367 Q70SY1 51654 Q96SN8 Q13490 Q14344 Q15672 6496 6495 6256 6498 6015 P32242 P10451 Q9UBU3 Q03405 Q01469 116113 P51805 Q9UDY8 P23528 P09471 Q9UDY6 5159 Q8WZ42 Q13263 P00441 P25940 8443 Q14116 4086 4087 Q13268 Q14114 8204 4088 4089 Q14119 Q14118 163126 Q9Y618 Q86UL3 Q86YT6 P51813 P12645 382 Q02790 222546 P12883 Q04725 Q04724 387 P21359 Q04726 P08151 1808 P24864 P20264 10087 6259 53615 Q13275 P62942 8454 P55347

6275 P54257 Q16787 7124 8214 Q8TD84 2909 Q14129 P14635 64759 Q9UQL6 P10275 4092 O43638 4093 P35579 Q02535 Q01201 156 Q9UMD9 23363 Q9NYQ6 10296 Q8WV28 11146 Q99729 Q16555 Q15466 2904 Q16790 Q15223 P45379 Q99966 Q15464 Q13285 54 Q86YZ3 9314 P36896 P36894 Q86WV8 P35568 166 Q6PRX2 338917 P33151 Q9NQB0 P09067 Q9UBP4 51218 Q99958 Q15475 Q99714 Q96T37 Q8TCU4 P14859 P35555 Q86V24 P36888 1832 Q9H1Y0 Q9NQC3 2926 P49715 P09038 P60953 116150 7155 P39905 7157 O15198 O43623 25776 Q02750 P35548 Q9H295 Q14160 Q16586 84376 1605 P78423 Q13873 O15123 Q01094 7161 7163 2702 Q8TAF8 P41134 Q9Y3A5 Q1PSW9 10935 P17275 P23945 27122 91 P39060 Q6NVY8 94 P22612 P42229 O15119 2947 1856 Q13643 9114 Q9NNX1 O94992 Q13642 Q96QB1 Q13887 O43602 10939 Q15828 7170 P10644 9351 O43847 Q99081 P55316 79365 O43609 P67775 P39059 P05062 P08575 P05067 1869 Q9UBL3 7189 P29474 O75881 10908 Q03014 Q13418 6096 P34820 P55327 1634 1874 P17252 O15379 P21980 Q5TA76 O95859 Q14993 Q9NZ08 Q9UBM7 Q9NSC2 P21741 P11717 O95863 Q13422 9133 P35908 O15156 Q13426 P10620 4914 2735 Q02388 Q9BXJ9 57379 Q8HWS3 1406 2737 2736 Q15853 O75626 Q8WUI4 Q6NYC1 P19012 Q9H488 P41182 P53567 P30048 1896 P05091 P08123 4920 Q13683 P47712 Q13444 P35968 Q14774 Q68CZ2 Q13443 26005 P11532 57154 Q68CZ1 P30279 Q07817 P20248 64919 80155 P08138 O75604 P10415 3860 O14495 P12830 Q02363 P19235 P18146 P35711 163183 Q7Z494 3611 Q14790 P00403 3852 P01730 80184 P41159 O15350 O15111 O15353 Q15648 P55771 P19484 Q06710 Q04771 1203 O60353 P24821 O15105 4953 3866 Q13233

posttranscriptional regulation of gene expression Q7KZF4 Q92616 8892 Q9BY77 O43432 11186 P67809 351 Q9UBS0 Q9HCJ0 84324 P84022 9617 Q7Z6C1 P49137 Q00839 P07996 5935 P38919 8662 P03950 P08729 8667 P46782 5036 P58546 Q8NDV7 P51114 P50148 2316 79763 O60573 54487 Q15796 P10809 Q7Z727 136319 Q9UKV8 Q15797 Q14103 Q9Y2Y8 4193 P62495 811 7458 O75807 Q13144 Q14232 Q13148 4086 4087 4088 Q9BY44 9775 3673 Q9UPQ9 9093 9099 5728 P16333 P17301 400961 2332 P62701 10642 10644 10643 Q9ULR5 P07307 Q9UL18 7124 P28482 7248 Q96KC8 O43639 4092 Q9P0J0 2107 55364 57669 3315 Q02413 P23588 8569 5976 27161 Q14011 Q9Y2T7 7133 7375 Q09161 1153 26523 Q86WV8 P19544 7490 O75570 3329 1029 Q9BUB5 Q9HCM4 2475 1828 P42771 10985 Q99835 Q86Y82 O75569 P98179 Q9H2K0 Q9Y6M1 O60506 Q99700 Q8WY64 P55010 Q92994 Q9UBQ5 Q9BQI3 6726 P23443 Q8NEJ9 P51693 P82979 P39905 P31749 Q9NR50 26986 23112 Q8NC51 Q9HCK5 1965 51593 440193 Q9H9G7 192669 Q6PID4 P40337 Q14289 10289 O00425 4690 6199 6198 10492 2033 Q06787 6193 1975 P42345 23560 4686 O15234 Q92574 1978 Q9UQ80 Q9UBK2 P49770 O15372 7057 4343 O60739 9470 Q96AB3 1982 Q9P2K8 Q9BRP8 P05067 84271 P60228 Q09472 P52298 Q8N726 Q5TAX3 P11940 64215 P29590 Q15717 Q9NS23 1994 27102 P17252 Q8TAD8 91949 Q9UPY3 P61326 O14965 P01375 O15151 23673 23435 Q05655 2972 P60484 4116 Q96EY1 Q15056 5580 6790 O00744 6311 P46199 1660 27327 22794 9261 Q96PU8 1653 P15692 Q9BZE4 433 O14908 Q92499 5578 Q8WYQ5 5591 P37108 Q9HCE1 83548 Q00987 Q9BXP5 207 P27797 P21333 P55884 O75604 7422 P10415 Q9P2X3 O14495 P19474 P78527 3184 Q9Y5S9 10437 84305 79753 333 P05198 P83436 P20333 5371 3192 Q60FE5 Q9UPU9 Q13107 Q08211 O15105 7428 P60321 P04792 Q92900 Q9HD40

regulation of nitrogen compound metabolic process Q9UKT9 P25054 Q9H0E3 Q9Y265 Q8NDW4 Q12824 8091 124790 10657 2305 2304 2303 114991 2300 Q8N3U4 155061 284695 Q00839 2308 2307 Q9H0D6 Q92974 Q9H4L7 Q12837 Q8WXB4 P16104 2316 10661 Q96EB6 O75925 Q9UKV8 4990 3660 3661 Q9BYE7 P37023 P13056 84901 57761 55588 Q9UKW4 O75928 Q9UKW6 3659 P26358 P26374 Q6NUN9 Q5BKZ1 Q92949 Q99583 Q12857 Q13705 P21917 10645 1488 1487 283337 Q99576 3665 10642 64061 P26367 Q9UL18 1022 Q8NA42 Q06587 114548 Q8IX07 Q5JT82 Q96MX3 1017 10614

79576 P21964 Q9HAJ7 1499 10616 O14981 Q9Y230 P61586 O75909 O14503 Q12873 Q12872 Q9Y239 Q8WXF0 Q9Y5Z7 Q8WXF1 6720 Q15906 285527 O00358 Q9BPY8 57798 1029 3207 10626 1027 1025 P17482 P17480 604 Q9Y242 P04179 Q15910 Q9UL36 Q12888 5883 Q9BQ95 O60869 79595 Q8NA19 P38398 57326 55145 3215 Q96N67 Q9Y250 6721 Q92994 Q92993 Q8N488 Q92997 Q9UKS7 P10826 P10827 P10828 221937 5411 P31749 Q8NDX6 1050 P53999 79101 P40692 57332 3226 3225 P04150 5888 Q9UGL1 Q92985 O95159 Q9UKL0 O96004 P14317 P14316 23569 11108 11107 Q8WWY6 1059 23560 Q15022 639 P62875 6749 6760 Q15027 Q9Y5V3 5430 O96019 O95163 P16989 P57059 Q03933 51131 641 P33076 51132 P35251 Q14186 Q6B0B8 79923 8932 648 8939 P49407 85360 Q14188 O00327 O96028 5440 P48552 5441 6772 Q9UKN5 Q8TAU3 P48551 6774 Q99990 3265 346171 O60828 P15621 Q66K89 Q99500 Q2M3W8 Q14192 P35240 P01019 51147 Q9Y692 Q15047 P23246 Q86X95 5451 A7MD48 Q8N3J9 P35222 Q5T6S3 P35227 P35226 O14979 P57082 O75081 P17813 P35232 Q00403 53335 3269 56849 668 6776 Q96MH2 Q15057 Q16385 P24588 7629 O43474 P06733 O00744 5460 Q9Y5Q3 9821 P25490 Q9NX61 3281 Q9NX65 672 P15692 Q9UBW7 6304 57708 9815 Q6UUV7 P05412 O00755 Q9Y5R5 9831 3297 Q9NX70 23532 Q5JSP0 Q9UBX0 P14373 Q9NWV8 57713 688 148327 Q15070 689 5467 9826 207 5468 Q8N895 Q92922 4150 4152 4154 23509 P56178 9841 P56177 Q8NHY6 Q12809 P17844 Q0IIM8 4149 O14920 57727 P62854 9839 O43463 5494 Q8NHX1 P08908 23512 23513 Q96QT6 O14936 8517 9849 4172 O15085 P36508 4173 4174 P35659 Q14686 4176 Q14209 8535 Q9BZ95 Q6NZI2 Q9H165 4171 Q9H161 Q9H9F9 Q9H160 Q9NPC1 P08047 5017 P78395 Q9BQG0 5018 P07199 P49815 Q13363 Q9NPC8 Q14693 Q8TBP0 94234 4188 8543 5036 5037 79084 P58304 Q96F45 O15090 23051 Q04864 Q9Y2H8 9869 7205 Q8WY36 P00533 Q96RL1 Q15554 Q9HAU4 8553 P35638 P61968 9882 Q8IWY8 Q96IZ0 Q9UPP1 P32121 P31274 P31273 P31276 Q9ULH1 O75123 Q9ULH7 23028 Q9NY61 4193 Q9Y6Q6 257 Q9H9D4 Q6DD87 Q15561 8548 Q96RK0 51548 Q93009 51547 P61964 Q15562 Q9BYU1 P62826 Q9UPQ3 9412 P36956 O75592 Q96F24 P31270 Q9NPF5 Q13398 P43246 55915 9421 P78347 9425 7248 O75586 O75582 Q9Y6K1 Q93062 Q03989 Q71SY5 Q9Y6K9 51564 Q15583 55922 5074 10181 P78337 55929 11030 Q9HAZ2 Q15109 P22392 O15055 O43524 26523 7251 23492 7249 O15047 P49848 Q15596 Q96NH3 8110 P61925 Q3KNS6 O15062 P49840 5089 166968 O95931 23466 23468 O15060 79913 51588 P08069 10155 9439 Q96NG5 1956 286075 Q9NPA8 26986 64426 5092 64428 54625 O95947 54623 Q14289 Q8IWS0 Q96RE7 9464 P29375 P29374 27005 Q7Z589 Q9H0M0 P22736 P42345 Q9BUG6 Q96EK4 Q9UGU0 P04085 Q92574 Q13761 Q58WW2 P56524 9477 O75534 Q02297 7291 O75530 7290 7295 P24928 Q6P1K2 O75528 P29353 O95977 P0C7X2 Q8WXI9 P40145 Q9BYM8 1994 1993 79039 Q96IQ9 O15499 P56545 O95983 A4D1W7 O95503 55072 P78317 P78318 9495 Q8TBE0 Q9HAK2 Q9UH92 Q9Y2B9 P52597 Q96EP0 O14593 O95999 Q9NP61 Q9NP66 Q86TI0 23081 O43918 23085 Q9UKY1 Q9H9B1 P85037 P31260 Q6P1N0 P31249 Q14653 Q9NP71 Q9NP72 P46736 3728 1540 Q9BUJ2 3720 5901 P46734 Q13330 P12956 Q6P1L6 Q9P031 Q9H4W6 Q00059 Q9BQA5 Q9Y6E7 Q9H582 P61088 Q96AE4 147808 Q96RG2 Q68DY9 P43694 O15455 Q9BQA1 5914 11091 O75953 63976 63978 5927 5928 P63279 P00519 5929 P63272 23077 P24941 Q92585 Q15532 Q13351 Q14683 Q13352 5925 Q7KZF4 P10914 O95677 Q92858 P67809 Q9UBB5 Q9NS86 Q99490 A5YKK6 Q9P2R6 4609 P16220 10772 5931 P49137 P27348 5933 O15516 Q9NS91 Q9UBC3 P27361 54361 115704 P50148 1107 P16234 284323 4613 1108 O15525 Q92838 Q9UK58 Q7Z3K6 P05549 Q9NRY4 1121 Q99471 Q7Z3K3 O60548 Q92830 Q9UJU2 284312 O15534 P49116 P03372 Q92833 P06401 Q92831 P06400 Q92826 P07737 5970 P46089 5971 9093 Q9UBE8 Q9NRZ9 P56705 9099 P17535 P17544 Q99459 P17542 57410 400720 1122 P61296 5966 10765 P05129 5981 P50570 3320 O00463 P52756 O00468 O95231 Q9UFF9 O14627 6829 10736 Q6FHQ0 P41218 Q8N2W9 4646 27161 5978 6827 65056 5991 93986 5993 1153 Q14938 O00472 Q06455 55250 Q9NS56 P37198 79685

6839 3326 3324 2475 P08651 5987 O95259 Q10586 Q8ND82 P51449 Q6IA86 27148 1161 O00488 O60506 P30542 Q02086 O60502 O60503 P61244 Q9BXG8 4664 5511 Q12770 Q92878 5515 P05112 O43182 26292 Q9UNY4 P08621 5518 O43186 Q12772 Q12778 O43189 Q05586 2023 Q02078 Q6KC79 3350 10725 57459 10724 Q99496 P40337 7704 P23396 O43159 6872 6871 P04637 P38405 2034 2033 Q9NVW2 O95271 O00422 Q9UFB7 4686 4204 P01116 4205 Q16236 P01112 4209 O43166 Q9C0K0 6883 Q9BT49 4221 Q9GZR2 O43167 23210 P50548 23216 O76071 Q9NRP7 84159 4214 P01106 6878 P01588 7727 56946 Q9C0J9 P01100 Q7Z7K2 7741 Q8N726 9921 P14416 P52736 Q02952 Q53X93 6886 P01137 Q16254 P52740 7750 6421 5573 P04626 P04629 2064 P52747 2063 11218 Q9NS37 Q99853 55633 P10070 P09086 P10071 10363 10362 P01127 5569 O43150 2070 O43593 5582 5585 O43597 7764 25822 79612 O75190 Q02930 P51828 7756 5579 10336 8607 P40763 P04201 2081 5591 Q9GZV8 4261 Q6IR47 5111 Q3SY56 64127 O75177 P18850 P15336 O75182 Q9NRL2 56987 51295 P18846 Q68CJ9 P18847 324 P18848 326 Q8TEA7 55657 Q92800 P27797 Q9GZU7 P01185 Q9UNP9 2099 O60907 O75164 P51843 55662 Q8NCN2 333 91748 Q9H7L9 10794 8625 5119 Q9GZT9 Q9BT67 23636 9975 6464 4287 P62508 P51858 146050 P52701 Q08211 221656 Q9NVV9 P58012 10320 9967 P25116 Q8N6T3 Q99417 Q96HZ4 O43670 Q14566 8655 4297 9500 Q9UKD1 P30086 P13984 351 Q9UBS5 P09493 P21127 P25963 P09017 P20290 Q15424 7332 5154 7334 5155 7336 7335 19 26205 85403 367 Q70SY1 11198 7329 Q14586 20 7341 P11309 6496 6495 Q8IVW6 Q14103 6498 P11308 Q99909 6015 P32242 Q8N3C0 Q8TEU7 Q9Y606 84108 116113 P09471 P17096 Q14592 11168 Q96MA1 Q9UBU8 Q13263 Q8TF50 8204 Q14119 Q8WWN8 Q9BY41 Q9Y618 125058 P12645 P29084 Q86WP2 222546 Q04725 Q04724 387 Q04727 Q9NWT6 Q04726 23152 Q66K14 P08151 P20264 11177 11176 P23771 P55347 9541 5187 11142 O15164 2909 391 Q6MZP7 23126 25788 O43638 Q02535 Q01201 121512 25780 2908 Q9UFW8 Q15466 Q6PJG2 Q99966 Q13285 2902 54 5195 6045 7376 Q09161 23133 23135 Q86WV8 Q3KQV3 Q6PRX2 P09067 2917 Q14140 Q9UBP4 85440 Q99958 Q15475 Q8TEY5 Q96ME7 Q9HA82 P14859 O43612 Q8TAQ5 Q96DT7 Q9H063 Q99941 P49959 Q01664 51222 56916 P01579 Q14151 P09038 9575 P82979 8242 10284 O75676 O15198 O43623 25776 2931 51230 Q14160 1609 Q5VVH5 Q14161 Q15014 86 P78424 Q9NWH9 Q15819 P33992 P33993 P33991 Q8TAF7 P41134 Q1PSW9 27122 91 P17030 94 O15119 1616 Q6ZT07 P45973 Q9UBK2 Q9NSA3 P78412 9112 Q96I24 9590 Q9H7Z7 Q9H3R0 P55318 Q99081 P55316 O43609 P67775 P05062 P52292 Q52LR7 P17040 P05067 2959 2956 Q9UBL3 O94763 P29474 92129 P52294 9126 27107 Q9H422 Q96M96 Q460N5 Q03014 6096 P41597 56034 Q9BTC8 2961 2960 O95619 Q8TAD8 Q9NSC2 2969 P09874 O15151 8289 Q15843 O95622 O95625 O15156 O94776 29777 202559 P17023 2972 79149 Q9BXJ9 P17020 Q8N393 Q8HWS3 P04198 Q9UBN7 Q15853 O75626 Q14527 P13945 9149 1660 Q8TF47 Q6NYC1 Q96DN5 Q9H488 P41182 Q8TAK5 93474 79175 Q499Z4 P53567 79177 P30048 3836 84525 1655 84528 P22415 Q9UK80 Q03468 O75629 O95644 150094 Q96HU1 Q9UBG7 Q8N7H5 P20248 64919 80155 Q8TAI7 P41162 P17081 Q9Y5B6 Q9Y5B9 P09429 O75604 Q13216 P41161 P35711 23186 P09430 P08588 Q86SE9 728642 Q9P2Y4 Q13227 P55771 Q04771 O15105 Q13233 Q6NX49 Q9H6Q4 O14793 25909 2550 9181 P51587 P17676 Q8TDD1 P15498 2547 57504 P84022 O00151 Q92731 O14788 10413 Q7Z6R9 25913 Q92729 Q5VTR2 O60216 Q9H2G9 4734 Q9H2G4 O00167 22850 81628 54496 6927 Q8IUD2 6929 Q6AHZ1 Q96KM6 Q8N680 811 6921 P40425 P40424 6926 6925 Q6IQ32 Q17R98 Q9C005 333929 Q9UIS9 Q9NZI7 P31943 P48382 Q9NQX0 P31946 Q9C009 2100 Q9NR96 P48380 O95343 3428 6938 P20823 55352 O60674 P19838 O60675 Q8IUC6 P62263 P18509 P31949 5604 6936 Q9Y468 Q9H2M9 Q8IZ40 4771 Q9Y463 4772 Q9Y466 Q8TDI0 Q9UIL8 Q9NUY8 10856 Q86VE0 2103 P16383 27287 80324 Q08999 O14744 6945 P50222 4780 Q92769 Q14814 P28370 Q07666 O95361 80306 P10600 O95365 Q06330 2119 10865 P19419 Q6PI57 4775 O14753 Q6ZW31 847 Q92766 4790 4791 4792 Q9H2K2 2130 81669 Q9NR48 Q92753 4302 857 Q92754

Q13501 22827 Q13503 Q14839 Q8NC51 P19883 Q13506 Q9NR55 2139 861 862 863 Q9UMX1 4799 P09601 50943 P08754 Q9Y3Y2 Q9BS34 10492 P15408 Q9Y3Q8 3481 Q9UIH9 23326 O60603 25988 P15884 Q8WUU5 Q96T88 3479 Q8WV60 2146 3476 10011 P25205 7832 10013 84289 Q96KG9 55723 10499 10014 11345 Q6UWV6 135295 Q7Z6J4 4343 Q96T76 Q99750 406 O43294 408 409 Q01518 Q96C55 Q09472 Q8N1G0 O43251 23309 116986 116987 P13686 890 Q00653 891 Q99743 Q99741 898 3014 29080 Q9UN42 29086 P49639 P51531 4361 8721 7874 148156 Q9NUX5 Q9NR11 P51532 Q96ST3 P60484 Q15291 11331 55758 10486 10001 P60002 10488 Q96T58 10487 P28702 P23497 Q3MII6 P26583 Q9Y3M2 Q96BZ9 P50613 Q5R372 P51946 23764 25942 Q8NFW5 Q9NUQ3 Q8WUY8 Q96P48 79735 10451 10450 8726 Q8N5U6 90993 Q08117 3054 Q05086 Q9BRU2 29079 P37231 Q9HCE1 79723 8737 10468 23746 P24386 7422 Q96GA9 5245 3068 Q9HCD5 P52815 22893 3066 Q9UMN6 3065 Q9UIF9 O75290 79753 10438 Q00613 Q96P20 55787 55785 10432 3070 Q13901 9612 Q9UIG0 P52824 Q9Y3P9 466 468 55796 P28749 Q9NQL9 7428 6595 6597 6599 6598 O75386 P10599 6591 Q9Y4K3 Q76L83 3084 473 474 23286 8767 P43034 Q13127 Q15303 B2RXF5 3091 Q16633 8301 Q15306 Q86VP6 O75376 P13631 24149 3096 Q02447 O75381 Q9NVP2 51773 30813 P46531 Q13133 Q15796 Q15311 Q16649 Q15797 9640 P12757 Q8N6I1 9643 7468 P54198 Q9UNL4 P12755 O75362 Q8TDS5 O75360 Q96CN4 P46527 51780 Q01101 P42167 6128 O94805 Q5VUA4 54815 Q8WVM0 Q9BSI4 Q8IV61 Q16650 Q13148 P35869 P20393 7476 Q16656 Q15329 Q8NCA9 P18074 Q8WVL7 P55055 O43306 P10588 P10589 93166 Q86VN1 Q8N6H7 Q16665 51317 Q49AN0 P43489 Q8TDY2 9667 Q16666 Q5VUG0 P54132 P63092 P35453 Q14494 8328 Q14498 Q16670 P46108 Q14011 Q86W54 Q9NRC8 7013 7014 P46100 Q5PSV4 7490 P13682 P63096 Q9C0F3 84232 Q8WW38 Q9GZM8 Q99835 7024 7023 10392 Q9C0F0 49854 P15822 9682 7020 Q02878 Q9UNH7 51341 54851 Q9NVM4 Q96PK6 11243 55704 Q13185 Q8NCF5 6188 Q15369 Q8IUX7 Q9BSM1 Q09028 6182 P11474 26747 Q15370 54862 P57682 Q6PID4 Q99816 9208 Q99814 P78545 7046 P35813 P78540 O43711 O43719 Q9Y4C1 P62195 Q4LE39 9219 A8K8V0 22803 112950 22806 22807 57473 55294 Q3V6T2 P46934 P46937 83746 Q96GN5 81565 O60296 Q4LE28 P98174 Q9UER7 Q14865 7068 7067 Q5TAX3 Q15714 Q13535 Q9H307 P19793 P29590 Q15717 Q7Z2Q5 Q63HK5 Q01196 84619 Q9H6W3 P41235 1756 Q92793 Q96GM5 Q9HD15 Q9Y4E5 O60266 O60264 Q9NRA1 P59817 Q13547 Q13546 9252 7073 P41223 57018 1763 Q9NZR4 Q04206 Q92786 Q92785 Q92784 P41229 7088 93594 9261 Q01167 Q9BSG1 51720 Q14894 4800 Q13563 2626 7099 7098 Q9NVC6 Q14416 7091 P14923 7090 Q9NQZ8 P30153 1789 1788 Q05516 1786 Q9UIV1 1785 375790 51735 Q13573 Q9HCU4 93134 Q9H2S9 Q96CJ1 P32320 P78527 92283 85509 1312 Q86VK4 84661 51742 Q6ZNG0 P20333 Q9Y4A8 Q8IYX1 30827 Q96H20 Q9HCS4 902 Q13112 P08238 Q9NZN8 P08235 P21675 Q9NZN9 Q92618 Q9H609 Q9Y2W7 Q9NYD6 Q6P2D0 O75820 2672 Q12948 22992 O00268 Q6QNY1 O00267 79894 3516 Q9BZI1 Q96JM7 2665 Q96JM2 Q5VWX1 2664 283248 Q9ULW3 Q92610 4841 Q8N587 Q12950 Q9Y2X7 Q12952 Q9Y2X9 Q8NB12 Q8NAP3 Q9P107 79885 P16473 4851 4854 Q96JL9 Q92600 5705 P50395 Q9ULX9 Q9NYF8 Q12968 29128 10514 83933 26097 P20936 Q9UQB3 4862 57649 Q6IT96 4869 5716 Q96JK9 Q12972 Q0D2J5 Q9H9T3 3551 Q6P2C8 5728 10524 Q9HBE1 Q99697 Q9ULZ3 Q9UHR5 O95477 P28482 Q9UHJ3 Q12986 1385 29108 P19532 Q9P0J0 P15173 P07550 P04040 58517 10971 7913 O14867 Q96BA8 10973 Q9UM47 P51671 Q9Y2T7 Q96SB4 Q9UHK0 Q9NYA1 O00233 P19544 Q8WYH8 1388 29115 Q9P0K8 1387 1386 P42771 Q9UM54 4899 Q9H1I8 Q9NYB0 22937 22938 Q9UHL9 22931 2247 284119 1398 58533 Q9BZK7 6601 Q9Y2V2 6605 57209 Q9UM63 P51693 P51692 P31629 Q9BQY4 O00255 Q8NEZ4 Q9NQ33 O00257 Q03164 P17342 984 Q5H9I0 988 P05362 Q9Y2W1 P06213 P07900 Q9UPV9 P13349 2274 P53803 P52952 990 Q8WTS6 126208 Q96RU8 Q96RU7 79810 51003 79811 79813 6625 51008 10138 Q9UQ80 Q04917 Q9UM07 Q9Y2P0 Q7Z5H3 5311 Q9UQ88 5796 Q5T7W0 8819 P48436 8812 Q99638 P01344 P01343 O00206 2290

23429 P48431 Q96JC9 3142 Q9NU63 148022 Q99623 Q96S42 Q8NAF0 P49756 55869 7975 5316 51028 P01375 Q9UPY8 23439 6660 P49750 8841 Q9UHI6 O00213 Q3KNV8 23435 Q96AQ6 3151 O60716 P12004 10128 Q96RR4 Q3KNW1 3148 Q15170 545 546 3146 P61457 Q96EY1 6657 O43353 6670 6672 23409 Q969H0 Q9Y2K7 Q9ULJ3 Q9H9S0 3162 Q12905 O95076 Q8WYA1 Q12906 Q9BZE0 Q01844 Q9BZE4 P42704 3159 Q99684 6667 P49336 P05771 Q9H5J8 P07951 Q969G3 8864 7533 P55198 Q9ULK4 23411 23414 3171 Q00987 Q96S94 3169 Q9NU19 7529 7528 Q08462 Q9H5I1 64375 P50750 Q86U86 3187 Q9UPT9 3185 3184 571 3182 P51608 P68036 7536 6207 Q9H5H4 Q01826 5371 3192 8880 P27540 Q60FE5 Q12933 Q9ULM3 7555 Q9ULM6 P51610 Q9BUY5 580 54583 80854 P51617 Q9NPJ6 7549 54106 8878 P60568 6231 Q15654 9744 8896 P15923 Q86YW9 Q96FV9 84324 116 Q7Z6C1 Q13485 Q8WZ64 O94906 O00716 Q16512 Q16514 P12429 Q15669 O75496 O75494 Q7LBC6 P10242 P10243 P10244 84312 51412 O94915 Q13492 Q16520 6239 54925 Q15672 Q9UQR1 7101 6256 Q16526 Q2M1K9 Q9UDY8 7579 9774 4086 7112 4087 4088 Q8N9N2 4089 O75478 163126 O75475 Q8N5F7 142 P21359 P31150 P24864 84333 P60520 Q15697 53615 Q13033 P54259 O75469 8458 64750 7124 P54252 P54253 P14635 O75461 Q9UQL6 P10275 4092 P10276 4093 O75460 4094 154 Q99728 Q99729 Q13045 55806 P45379 P49736 7132 8463 7133 8467 9314 O43889 P36896 23378 P15976 Q96C28 6282 1822 166 Q8NFM4 55810 P01308 Q8NFM5 338917 Q9NQB0 Q99717 Q15233 Q96T37 P00492 9326 P54274 O75446 P36402 O75444 6294 7141 1831 Q96T23 Q00577 Q99708 P49711 Q16576 P49716 P49715 55827 Q9NQC7 Q96C00 64784 7158 O75437 Q08050 P39905 7157 P19086 64786 162239 Q8N9R8 Q02750 P35548 138151 55832 Q9UDV6 Q5VTD9 Q13077 7169 26038 10928 26037 Q86Z02 56252 Q01094 Q9H204 26039 7161 P63167 P42224 10933 10935 P17275 P23945 P63165 Q9BVI0 P42229 57109 P42226 O94992 Q9P0W2 22926 Q969S8 22927 Q13404 Q13887 Q01085 57592 Q8IY57 57594 P10644 57591 Q96BD5 79365 P84095 Q08945 Q9UHX1 1869 7186 7189 7188 Q8WYK2 O43812 P11940 7181 7182 7185 1877 Q86UE4 Q86UE3 1874 Q9Y3C7 P68871 1870 P09630 O15379 Q969R5 Q13422 O15391 Q8NB78 4914 2735 27352 P19438 O43829 84733 1408 1407 1406 2737 2736 Q96G30 Q8WUI4 Q02386 Q7RTR2 P62277 1896 Q9HC52 Q14774 P22670 Q9NQ92 9391 P11532 Q96G25 84759 58487 Q9UQE7 Q8WYP5 P23511 Q8WUH2 3609 3608 3607 Q14781 P23510 P21333 94104 Q6ZV73 O14495 Q9P0T4 29966 P19474 Q02363 58491 P18146 27300 O60343 O60341 4943 Q969V6 O15350 O75832 O15353 Q15649 Q9BZS1 Q15648 Q9P0U4 P19484 Q86YP4 Q06710 Q96BF6 Q8N5A5 3622 2773 Q9HC29 O14497 Q15652

regulation of protein localization P25054 Q15654 11060 O43557 P10599 Q9Y4K3 54474 Q04759 P84022 Q7Z6C1 6901 Q13485 P25963 Q9UBC1 P52565 P03950 4188 O15530 8301 Q16635 P50148 2316 P14174 Q13492 7205 P00533 Q96EB6 7341 3783 Q06124 P62491 O60543 Q9Y6A5 5716 5295 P31946 4088 4089 Q9BZF9 Q9NR96 Q9UKG1 P21917 5170 O75592 387 P21359 O60674 51552 Q9ULZ3 P41743 P43489 7124 11142 Q96KC8 29108 1020 P14635 O00221 114548 396 Q93062 57669 Q6PJW8 Q99728 11146 P61586 4645 P61106 Q9NYA1 P29466 11030 O95361 P36894 P10600 P13796 1029 57678 10626 P01308 Q9HCM4 Q9NQB0 P42771 P49841 Q99835 P61925 4792 Q14703 Q96FX2 A1XKG3 2010 Q14C86 Q9HCL2 Q92990 P01579 11124 Q9UN86 1956 Q9NQC7 P10827 Q9UGK8 Q9BX66 P31749 2932 Q9UMX1 Q9ULV0 50943 26060 P53367 Q15019 7046 O95835 O00308 2033 O60603 P63165 999 P27986 7295 9908 Q9HD26 Q99750 O00206 Q09472 7189 7067 Q8N726 P14416 64215 57120 891 60412 Q00535 P01137 Q8N960 P01133 P01375 8165 8720 P35225 27236 P35900 9495 P10071 5566 5569 Q9Y2B9 2737 Q92667 P24588 5460 5584 Q5R372 Q9UNN5 O15554 7099 7098 Q6IR47 Q13443 10460 10580 P14923 64127 23411 O76021 5590 3728 Q05513 324 1540 207 P17081 7529 P22303 P23510 P21333 8754 P10415 P12830 Q96P20 O15455 Q9BT67 Q60FE5 O75832 8766 Q9BZS1 Q06830 580 P50402 P17612 Q9HC29 23077 54106 Q15653 P60568

positive regulation of nucleobase, nucleoside, nucleotide and nucleic acid metabolic process P10914 Q92858 O14793 Q12948 Q12824 Q6QNY1 O00267 P17676 3516 2547 2304 4609 2303 P16220 Q96JM2 P84022 Q92731 O15516 2308 25913 Q9H4L7 Q12837 Q5VTR2 54361 Q8NAP3 P16104 P16234 4851 5705 O75925 Q12968 6927 P37023 6929 4862 811 Q9UJU2 P49116 O75928 P40425 P40424 6926 Q6IT96 P06401 3659 6925 P06400 Q96JK9 5970 2100 Q9NR96 P56705 P48380 O95343 10524 10645 P20823 P19838 P61296 5966 5604 P05129 O95477 Q9Y463 P28482 1022 1385 P19532 O00468 6829 1017 10736 1499 P15173 80324 P07550 4646 6827 Q9UHK0 4780 Q92769 5991 6720 P28370 Q14938 O95361 O00233 P10600 P19544 O00358 Q9NS56 Q8WYH8 Q06330 P37198 10626 P19419 Q9P0K8 1387 P17482 P08651 P17480 4775 Q9UM54 4790 Q12888 4792 Q9H2K2 22937 1161 O60502 P38398 2247 Q9BZK7 6601 Q92753 6721 Q92993 Q12770 P05112 Q9UM63 Q92997 Q13501 Q9UKS7 P10826 O43186 Q12772 P10827 Q12778 P51692 Q13503 Q05586 O00255 1050 Q03164 10724 861 5888 P40337 50943 Q9Y2W1 P06213 O96004 6872 P04637 2034 P13349 2033 2274 O95271 Q9UIH9 23326 O60603 25988 P15884 P52952 3479 10011 4686 84289 Q16236 10014 10138 P01112 Q04917 Q9C0K0 6760 4221 Q96T76 641 P33076 P48436 4214 P01106 406 P01588 O43294 408 P49407 P01343 P01100 O00327 O00206 Q09472 P48552 P48431 9921 6774 3265 890 891 Q99743 Q14192 Q96S42 Q02952 898 3014 Q53X93 P01137 5316 29086 P49639 P01375 Q9UPY8 P51531 6660 5451 P35222 Q9NUX5 P51532 Q96RR4 P17813 3148 P10070 P10071 3146 P61457 668 6776 P01127 10001 6657 P23497 P24588 P26583 O00744 5460 6672 5582 Q9Y5Q3 P50613 P51946 Q9NX61 Q12905 Q12906 Q9BZE0 672 P15692 79612 3159 Q02930 P51828 6667 Q8N5U6 P40763 P04201 P05412 5591 O00755 4261 Q6IR47 7533 P18850 Q05086 23414 3171 P37231 Q9NWV8 P18846 P18848 688 3169 148327 9826 5468 Q92922 P27797 P01185 7422 P52815 O60907 3066 Q9UMN6 Q9UPT9 3065 3182 P17844 8625 10432 23636 P27540 5494 6464 P62508 Q9UIG0 O14936 466 P51617 P58012 468 7428 10320 54106 8878 9967 P25116 P60568 O15085 6595 Q14686 6597 4297 6599 6598 P15923 P13984 Q9Y4K3 351 Q9H161 474 P08047 Q7Z6C1 Q13485 P25963 O94906 Q9NPC8 3091 5154 O00716 7334 5155 8301 P12429 Q15306 19 Q86VP6 P58304 P13631 367 Q04864 51773 Q70SY1 P46531 Q13492 Q96RL1 Q15554 Q13133 Q15796 Q9UQR1 6496 P35638 6495 Q15797 6256 P12755 Q9ULH7 Q9NY61 O75360 Q9Y606 Q2M1K9 Q15561 P17096 51547 Q13263 Q15562 Q9BSI4 Q16650 4086 P35869 4087 8204 4088 P62826 4089 7476 9412 P18074 Q8WVL7 P55055 P12645 P36956 222546 P08151 P24864 P20264 Q16665 Q13033 9421 P55347 O75469 7124 P54132 P54253 O75586 P14635 391 P35453 Q9UQL6 P10275 P10276 4093 154 Q99729 Q99966 Q13285 2902 7132 Q9NRC8 7376 8463 Q9HAZ2 Q09161 7014 P22392 P36896 O43524 7490 55810 P01308 338917 Q9NQB0 Q8WW38 Q14140 Q99958 Q15475 Q99835 Q96T37 Q8TEY5 7023 5089 P14859 Q96T23 54851 Q01664 Q96PK6 56916 P01579 P49711 P08069 10155 P49715 P09038 55827 9575 Q9NPA8 7158 P39905 7157 O15198 Q02750 6182 P11474 5092 26747 55832 Q99814 7046 9464 P35813 P29375 27005 Q01094 7161 P22736 Q9Y4C1 Q1PSW9 P17275 P62195 91 P04085 94 9219 P42229 O15119 P42226 Q9UBK2 Q58WW2 P56524 22926 22807 Q13887 9590 Q8IY57 P55318 P84095 Q4LE28 P05067 1869 O75528 7189 7067 P29353 P19793 Q03014 7182 6096 Q01196 P41235 56034 Q92793 Q96GM5 Q9HD15 O95619 Q9NSC2 Q9NRA1 8289 Q13422 O15156 O94776 Q13547 A4D1W7 P78317 9495 2735 P19438 1763 Q9HAK2 Q9BXJ9 Q04206 Q92786 Q8HWS3 1406 2737 2736 Q15853 Q96G30 O14593 O95999 9149 Q8TAK5 P53567 1655 P22415 Q9UK80 Q03468 51720 4800 2626 7099 7098 Q9NVC6 Q9NP71 P46736 P20248 64919 80155 Q9UIV1 P23511 375790 3609 P17081 3608 5901 P46734 P09429 P12956 Q13216 29966 P78527 P18146 Q9H4W6 P35711 Q9BQA5 P61088 P43694 O15455 P08588 5914 O15350 Q9BZS1 Q15648 P55771 P19484 Q06710 Q04771 63976 5927 P63272 P24941 Q9HCS4 O14497 Q92585 902 Q15532 Q13233 P21675 5925

regulation of nucleobase, nucleoside, nucleotide and nucleic acid metabolic process Q9UKT9 Q9H0E3 Q9Y265 Q8NDW4 Q12824 8091 124790 10657 2305 2304 2303 114991 2300 Q8N3U4 155061 284695 Q00839 2308 2307 Q9H0D6 Q92974 Q9H4L7 Q12837 Q8WXB4 P16104 2316 10661 Q96EB6 O75925 Q9UKV8 4990 3660 3661 Q9BYE7 P37023 P13056 84901 57761 55588 Q9UKW4 O75928 Q9UKW6 3659 P26358 P26374 Q6NUN9 Q5BKZ1 Q92949 Q99583 Q12857 Q13705 P21917 10645 1488 1487 283337 Q99576 3665 10642 64061 P26367 Q9UL18 1022 Q8NA42 Q06587 114548 Q8IX07 Q5JT82 Q96MX3 1017 10614 79576 Q9HAJ7 1499 10616 O14981 Q9Y230 P61586 O75909 O14503 Q12873 Q12872 Q9Y239 Q8WXF0 Q9Y5Z7 Q8WXF1 6720 Q15906 285527 O00358 Q9BPY8 57798 1029 3207 10626 1027 1025 P17482 P17480 604 Q9Y242 P04179 Q15910 Q9UL36 Q12888 5883 Q9BQ95 O60869 79595 Q8NA19 P38398 57326 55145 3215 Q96N67 Q9Y250 6721 Q92994 Q92993 Q8N488 Q92997 Q9UKS7 P10826 P10827 P10828 221937 5411 Q8NDX6 1050 P53999 79101 P40692 57332 3226 3225 P04150 5888 Q9UGL1 Q92985 O95159 Q9UKL0 O96004 P14317 P14316 23569 11108 11107 Q8WWY6 1059 23560 Q15022 639 P62875 6749 6760 Q15027 Q9Y5V3 5430 O96019 O95163 P16989 P57059 Q03933 51131 641 P33076 51132 P35251 Q14186 Q6B0B8 79923 8932 648 8939 P49407 85360 Q14188 O00327 O96028 5440 P48552 5441 6772 Q9UKN5 Q8TAU3 P48551 6774 Q99990 3265 346171 O60828 P15621 Q66K89 Q99500 Q2M3W8 Q14192 P35240 P01019 51147 Q9Y692 Q15047 P23246 Q86X95 5451 A7MD48 Q8N3J9 P35222 Q5T6S3 P35227 P35226 O14979 P57082 O75081 P17813 P35232 Q00403 53335 56849 668 6776 Q96MH2 Q15057 Q16385 P24588 7629 O43474 P06733 O00744 5460 Q9Y5Q3 9821 P25490 Q9NX61 3281 Q9NX65 672 P15692 Q9UBW7 6304 57708 9815 Q6UUV7 P05412 O00755 Q9Y5R5 9831 3297 Q9NX70 23532 Q5JSP0 Q9UBX0 P14373 Q9NWV8 57713 688 148327 Q15070 689 5467 9826 5468 Q8N895 Q92922 4150 4152 4154 23509 P56178 9841 P56177 Q8NHY6 Q12809 P17844 Q0IIM8 4149 O14920 57727 P62854 9839 O43463 5494 Q8NHX1 P08908 23512 23513 Q96QT6 O14936 8517 9849 4172 O15085 P36508 4173 4174 P35659 Q14686 4176 Q14209 8535 Q9BZ95 Q6NZI2 Q9H165 4171 Q9H161 Q9H9F9 Q9H160 Q9NPC1 P08047 5017 P78395 Q9BQG0 5018 P07199 P49815 Q13363 Q9NPC8 Q14693 Q8TBP0 94234 4188 8543 5036 79084 P58304 Q96F45 O15090 23051 Q04864 Q9Y2H8 9869 7205 Q8WY36 Q96RL1 Q15554 Q9HAU4 8553 P35638 P61968 9882 Q8IWY8 Q96IZ0 Q9UPP1 P32121 P31274 P31273 P31276 Q9ULH1 O75123 Q9ULH7 23028 Q9NY61 4193 Q9Y6Q6 257 Q9H9D4 Q6DD87 Q15561 8548 Q96RK0 51548 Q93009 51547 P61964 Q15562 Q9BYU1 P62826 Q9UPQ3 9412 P36956 O75592 Q96F24 P31270 Q9NPF5 Q13398 P43246 55915 9421 P78347 9425 7248 O75586 O75582 Q9Y6K1 Q93062 Q03989 Q71SY5 Q9Y6K9 51564 Q15583 55922 5074 10181 P78337 55929 11030 Q9HAZ2 Q15109 P22392 O15055 O43524 26523 7251 23492 7249 O15047 P49848 Q15596 Q96NH3 8110 P61925 Q3KNS6 O15062 P49840 5089 166968 O95931 23466 23468 O15060 79913 51588 P08069 10155 9439 Q96NG5 286075 Q9NPA8 26986 64426 5092 64428 54625 O95947 54623 Q14289 Q8IWS0 Q96RE7 9464 P29375 P29374 27005 Q7Z589 Q9H0M0 P22736 P42345 Q9BUG6 Q96EK4 Q9UGU0 P04085 Q92574 Q13761 Q58WW2 P56524 9477 O75534 Q02297 7291 O75530 7290 7295 P24928 Q6P1K2 O75528 P29353 O95977 P0C7X2 Q8WXI9 P40145 Q9BYM8 1994 1993 79039 Q96IQ9 O15499 P56545 O95983 A4D1W7 O95503 55072 P78317 P78318 9495 Q8TBE0 Q9HAK2 Q9UH92 Q9Y2B9 P52597 Q96EP0 O14593 O95999 Q9NP61 Q9NP66 Q86TI0 23081 O43918 23085 Q9UKY1 Q9H9B1 P85037 P31260 Q6P1N0 P31249 Q14653 Q9NP71 Q9NP72 P46736 3728 1540 Q9BUJ2 3720 5901 P46734 Q13330 P12956 Q6P1L6 Q9P031 Q9H4W6 Q00059 Q9BQA5 Q9Y6E7 Q9H582 P61088 Q96AE4 147808 Q96RG2 Q68DY9 P43694 O15455 Q9BQA1 5914 11091 O75953 63976 63978 5927 5928 P63279 P00519 5929 P63272 23077 P24941 Q92585 Q15532 Q13351 Q14683 Q13352 5925 Q7KZF4 P10914 O95677 Q92858 P67809 Q9UBB5 Q9NS86 Q99490 A5YKK6 Q9P2R6 4609 P16220 10772 5931 P49137 P27348 5933 O15516 Q9NS91 Q9UBC3 P27361 54361 115704 P50148 1107 P16234 284323

4613 1108 O15525 Q92838 Q9UK58 Q7Z3K6 P05549 Q9NRY4 1121 Q99471 Q7Z3K3 O60548 Q92830 Q9UJU2 284312 O15534 P49116 P03372 Q92833 P06401 Q92831 P06400 Q92826 P07737 5970 P46089 5971 9093 Q9UBE8 Q9NRZ9 P56705 9099 P17535 P17544 Q99459 P17542 57410 400720 1122 P61296 5966 10765 P05129 5981 P50570 O00463 P52756 O00468 O95231 Q9UFF9 O14627 6829 10736 Q6FHQ0 P41218 Q8N2W9 4646 27161 5978 6827 65056 5991 93986 5993 1153 Q14938 O00472 Q06455 55250 Q9NS56 P37198 79685 6839 2475 P08651 5987 O95259 Q10586 Q8ND82 P51449 Q6IA86 27148 1161 O00488 O60506 P30542 Q02086 O60502 O60503 P61244 Q9BXG8 4664 5511 Q12770 Q92878 5515 P05112 O43182 26292 Q9UNY4 P08621 5518 O43186 Q12772 Q12778 O43189 Q05586 2023 Q02078 Q6KC79 3350 10725 57459 10724 Q99496 P40337 7704 P23396 O43159 6872 6871 P04637 P38405 2034 2033 Q9NVW2 O95271 O00422 Q9UFB7 4686 4204 P01116 4205 Q16236 P01112 4209 O43166 Q9C0K0 6883 Q9BT49 4221 Q9GZR2 O43167 23210 P50548 23216 O76071 Q9NRP7 84159 4214 P01106 6878 P01588 7727 56946 Q9C0J9 P01100 Q7Z7K2 7741 Q8N726 9921 P14416 P52736 Q02952 Q53X93 6886 P01137 Q16254 P52740 7750 6421 5573 P04626 P04629 2064 P52747 2063 11218 Q9NS37 Q99853 55633 P10070 P09086 P10071 10363 10362 P01127 5569 O43150 2070 O43593 5582 5585 O43597 7764 25822 79612 O75190 Q02930 P51828 7756 5579 10336 8607 P40763 P04201 2081 5591 Q9GZV8 4261 Q6IR47 5111 Q3SY56 64127 O75177 P18850 P15336 O75182 Q9NRL2 56987 51295 P18846 Q68CJ9 P18847 P18848 326 Q8TEA7 55657 Q92800 P27797 Q9GZU7 P01185 Q9UNP9 2099 O60907 O75164 P51843 55662 Q8NCN2 333 91748 Q9H7L9 10794 8625 5119 Q9GZT9 Q9BT67 23636 9975 6464 4287 P62508 P51858 146050 P52701 Q08211 221656 Q9NVV9 P58012 10320 9967 P25116 Q8N6T3 Q99417 Q96HZ4 O43670 Q14566 8655 4297 9500 Q9UKD1 P13984 351 Q9UBS5 P09493 P21127 P25963 P09017 P20290 Q15424 7332 5154 7334 5155 7336 7335 19 26205 85403 367 Q70SY1 11198 7329 Q14586 20 7341 P11309 6496 6495 Q8IVW6 Q14103 6498 P11308 Q99909 6015 P32242 Q8N3C0 Q8TEU7 Q9Y606 84108 116113 P09471 P17096 Q14592 11168 Q96MA1 Q9UBU8 Q13263 Q8TF50 8204 Q14119 Q8WWN8 Q9BY41 Q9Y618 125058 P12645 P29084 Q86WP2 222546 Q04725 Q04724 387 Q04727 Q9NWT6 Q04726 23152 Q66K14 P08151 P20264 11177 11176 P23771 P55347 9541 5187 11142 O15164 2909 391 Q6MZP7 23126 25788 O43638 Q02535 Q01201 121512 25780 2908 Q9UFW8 Q15466 Q6PJG2 Q99966 Q13285 2902 5195 6045 7376 Q09161 23133 23135 Q86WV8 Q3KQV3 Q6PRX2 P09067 2917 Q14140 Q9UBP4 85440 Q99958 Q15475 Q8TEY5 Q96ME7 Q9HA82 P14859 O43612 Q8TAQ5 Q96DT7 Q9H063 Q99941 P49959 Q01664 51222 56916 P01579 Q14151 P09038 9575 P82979 8242 10284 O75676 O15198 O43623 25776 2931 51230 Q14160 1609 Q5VVH5 Q14161 Q15014 86 P78424 Q9NWH9 Q15819 P33992 P33993 P33991 Q8TAF7 P41134 Q1PSW9 27122 91 P17030 94 O15119 1616 Q6ZT07 P45973 Q9UBK2 Q9NSA3 P78412 9112 Q96I24 9590 Q9H7Z7 Q9H3R0 P55318 Q99081 P55316 O43609 P67775 P05062 P52292 Q52LR7 P17040 P05067 2959 2956 Q9UBL3 O94763 P29474 92129 P52294 9126 27107 Q9H422 Q96M96 Q460N5 Q03014 6096 P41597 56034 Q9BTC8 2961 2960 O95619 Q8TAD8 Q9NSC2 2969 P09874 O15151 8289 Q15843 O95622 O95625 O15156 O94776 29777 202559 P17023 2972 79149 Q9BXJ9 P17020 Q8N393 Q8HWS3 P04198 Q9UBN7 Q15853 O75626 Q14527 P13945 9149 1660 Q8TF47 Q6NYC1 Q96DN5 Q9H488 P41182 Q8TAK5 93474 79175 Q499Z4 P53567 79177 P30048 3836 84525 1655 84528 P22415 Q9UK80 Q03468 O75629 O95644 150094 Q96HU1 Q9UBG7 Q8N7H5 P20248 64919 80155 Q8TAI7 P41162 P17081 Q9Y5B6 Q9Y5B9 P09429 O75604 Q13216 P41161 P35711 23186 P09430 P08588 Q86SE9 728642 Q9P2Y4 Q13227 P55771 Q04771 O15105 Q13233 Q6NX49 Q9H6Q4 O14793 25909 2550 9181 P51587 P17676 Q8TDD1 P15498 2547 57504 P84022 O00151 Q92731 O14788 10413 Q7Z6R9 25913 Q92729 Q5VTR2 O60216 Q9H2G9 4734 Q9H2G4 O00167 22850 81628 54496 6927 Q8IUD2 6929 Q6AHZ1 Q96KM6 Q8N680 811 6921 P40425 P40424 6926 6925 Q6IQ32 Q17R98 Q9C005 333929 Q9UIS9 Q9NZI7 P31943

P48382 Q9NQX0 P31946 Q9C009 2100 Q9NR96 P48380 O95343 3428 6938 P20823 55352 O60674 P19838 O60675 Q8IUC6 P62263 P18509 P31949 5604 6936 Q9Y468 Q9H2M9 Q8IZ40 4771 Q9Y463 4772 Q9Y466 Q8TDI0 Q9UIL8 Q9NUY8 10856 Q86VE0 2103 P16383 27287 80324 Q08999 O14744 6945 P50222 4780 Q92769 Q14814 P28370 Q07666 O95361 80306 P10600 O95365 Q06330 2119 10865 P19419 Q6PI57 4775 O14753 Q6ZW31 847 Q92766 4790 4791 4792 Q9H2K2 2130 81669 Q9NR48 Q92753 4302 857 Q92754 Q13501 22827 Q13503 Q14839 Q8NC51 P19883 Q13506 Q9NR55 2139 861 862 863 Q9UMX1 4799 P09601 50943 P08754 Q9Y3Y2 Q9BS34 10492 P15408 Q9Y3Q8 3481 Q9UIH9 23326 O60603 25988 P15884 Q8WUU5 Q96T88 3479 Q8WV60 2146 3476 10011 P25205 7832 10013 84289 Q96KG9 55723 10499 10014 11345 Q6UWV6 135295 Q7Z6J4 4343 Q96T76 Q99750 406 O43294 408 409 Q01518 Q96C55 Q09472 Q8N1G0 O43251 23309 116986 116987 890 Q00653 891 Q99743 Q99741 898 3014 29080 Q9UN42 29086 P49639 P51531 4361 8721 7874 148156 Q9NUX5 Q9NR11 P51532 Q96ST3 P60484 Q15291 11331 55758 10486 10001 P60002 10488 Q96T58 10487 P28702 P23497 Q3MII6 P26583 Q9Y3M2 Q96BZ9 P50613 Q5R372 P51946 23764 25942 Q8NFW5 Q9NUQ3 Q8WUY8 Q96P48 79735 10451 10450 8726 Q8N5U6 90993 Q08117 3054 Q05086 Q9BRU2 29079 P37231 Q9HCE1 79723 8737 10468 23746 P24386 7422 Q96GA9 5245 3068 Q9HCD5 P52815 22893 3066 Q9UMN6 3065 Q9UIF9 O75290 79753 10438 Q00613 Q96P20 55787 55785 10432 3070 Q13901 9612 Q9UIG0 P52824 Q9Y3P9 466 468 55796 P28749 Q9NQL9 7428 6595 6597 6599 6598 O75386 P10599 6591 Q9Y4K3 Q76L83 3084 473 474 23286 8767 P43034 Q13127 Q15303 B2RXF5 3091 Q16633 8301 Q15306 Q86VP6 O75376 P13631 24149 3096 Q02447 O75381 Q9NVP2 51773 30813 P46531 Q13133 Q15796 Q15311 Q16649 Q15797 9640 P12757 Q8N6I1 9643 7468 P54198 Q9UNL4 P12755 O75362 Q8TDS5 O75360 Q96CN4 P46527 51780 Q01101 P42167 6128 O94805 Q5VUA4 54815 Q8WVM0 Q9BSI4 Q8IV61 Q16650 Q13148 P35869 P20393 7476 Q16656 Q15329 Q8NCA9 P18074 Q8WVL7 P55055 O43306 P10588 P10589 93166 Q86VN1 Q8N6H7 Q16665 51317 Q49AN0 P43489 Q8TDY2 9667 Q16666 Q5VUG0 P54132 P63092 P35453 Q14494 8328 Q14498 Q16670 P46108 Q14011 Q86W54 Q9NRC8 7013 7014 P46100 Q5PSV4 7490 P13682 P63096 Q9C0F3 84232 Q8WW38 Q9GZM8 Q99835 7024 7023 10392 Q9C0F0 49854 P15822 9682 7020 Q02878 Q9UNH7 51341 54851 Q9NVM4 Q96PK6 11243 55704 Q13185 Q8NCF5 6188 Q15369 Q8IUX7 Q9BSM1 Q09028 6182 P11474 26747 Q15370 54862 P57682 Q6PID4 Q99816 9208 Q99814 P78545 7046 P35813 O43711 O43719 Q9Y4C1 P62195 Q4LE39 9219 A8K8V0 22803 112950 22806 22807 57473 55294 Q3V6T2 P46934 P46937 83746 Q96GN5 81565 O60296 Q4LE28 P98174 Q9UER7 Q14865 7068 7067 Q5TAX3 Q15714 Q13535 Q9H307 P19793 P29590 Q15717 Q7Z2Q5 Q63HK5 Q01196 84619 Q9H6W3 P41235 1756 Q92793 Q96GM5 Q9HD15 Q9Y4E5 O60266 O60264 Q9NRA1 P59817 Q13547 Q13546 9252 7073 P41223 57018 1763 Q9NZR4 Q04206 Q92786 Q92785 Q92784 P41229 7088 93594 9261 Q01167 Q9BSG1 51720 Q14894 4800 Q13563 2626 7099 7098 Q9NVC6 Q14416 7091 P14923 7090 Q9NQZ8 P30153 1789 1788 Q05516 1786 Q9UIV1 1785 375790 51735 Q13573 Q9HCU4 93134 Q9H2S9 Q96CJ1 P32320 P78527 92283 85509 Q86VK4 84661 51742 Q6ZNG0 P20333 Q9Y4A8 Q8IYX1 30827 Q96H20 Q9HCS4 902 Q13112 Q9NZN8 P08235 P21675 Q9NZN9 Q92618 Q9H609 Q9Y2W7 Q9NYD6 Q6P2D0 O75820 2672 Q12948 22992 O00268 Q6QNY1 O00267 79894 3516 Q9BZI1 Q96JM7 2665 Q96JM2 Q5VWX1 2664 283248 Q9ULW3 Q92610 4841 Q8N587 Q12950 Q9Y2X7 Q12952 Q9Y2X9 Q8NB12 Q8NAP3 Q9P107 79885 P16473 4851 4854 Q96JL9 Q92600 5705 P50395 Q9ULX9 Q9NYF8 Q12968 29128 10514 83933 26097 P20936 Q9UQB3 4862 57649 Q6IT96 4869 5716 Q96JK9 Q12972 Q0D2J5 Q9H9T3 3551 Q6P2C8 5728 10524 Q9HBE1 Q99697 Q9ULZ3 Q9UHR5 O95477 P28482 Q9UHJ3 Q12986 1385 29108 P19532 Q9P0J0 P15173 P07550 P04040 58517 10971 7913 O14867 Q96BA8 10973 Q9UM47 P51671 Q9Y2T7 Q96SB4 Q9UHK0 Q9NYA1 O00233 P19544 Q8WYH8 1388 29115 Q9P0K8 1387 1386 P42771 Q9UM54 4899 Q9H1I8

Q9NYB0 22937 22938 Q9UHL9 22931 2247 284119 1398 58533 Q9BZK7 6601 Q9Y2V2 6605 57209 Q9UM63 P51693 P51692 P31629 Q9BQY4 O00255 Q8NEZ4 Q9NQ33 O00257 Q03164 P17342 984 Q5H9I0 988 P05362 Q9Y2W1 P06213 Q9UPV9 P13349 2274 P53803 P52952 990 Q8WTS6 126208 Q96RU8 Q96RU7 79810 51003 79811 79813 6625 51008 10138 Q9UQ80 Q04917 Q9UM07 Q9Y2P0 Q7Z5H3 5311 Q9UQ88 5796 Q5T7W0 8819 P48436 8812 Q99638 P01344 P01343 O00206 2290 23429 P48431 Q96JC9 3142 Q9NU63 148022 Q99623 Q96S42 Q8NAF0 P49756 55869 7975 5316 51028 P01375 Q9UPY8 23439 6660 P49750 8841 Q9UHI6 O00213 Q3KNV8 23435 Q96AQ6 3151 O60716 P12004 10128 Q96RR4 Q3KNW1 3148 Q15170 545 546 3146 P61457 Q96EY1 6657 O43353 6670 6672 23409 Q969H0 Q9Y2K7 Q9ULJ3 Q9H9S0 3162 Q12905 O95076 Q8WYA1 Q12906 Q9BZE0 Q01844 Q9BZE4 P42704 3159 Q99684 6667 P49336 P05771 Q9H5J8 P07951 Q969G3 8864 7533 P55198 Q9ULK4 23411 23414 3171 Q00987 Q96S94 3169 Q9NU19 7529 7528 Q08462 Q9H5I1 64375 P50750 Q86U86 3187 Q9UPT9 3185 3184 571 3182 P51608 P68036 7536 6207 Q9H5H4 Q01826 5371 3192 8880 P27540 Q60FE5 Q12933 Q9ULM3 7555 Q9ULM6 P51610 Q9BUY5 580 54583 80854 P51617 Q9NPJ6 7549 54106 8878 P60568 6231 Q15654 9744 8896 P15923 Q86YW9 Q96FV9 84324 116 Q7Z6C1 Q13485 Q8WZ64 O94906 O00716 Q16512 Q16514 P12429 Q15669 O75496 O75494 Q7LBC6 P10242 P10243 P10244 84312 51412 O94915 Q13492 Q16520 6239 54925 Q15672 Q9UQR1 7101 6256 Q16526 Q2M1K9 Q9UDY8 7579 9774 4086 7112 4087 4088 Q8N9N2 4089 O75478 163126 O75475 Q8N5F7 142 P21359 P31150 P24864 84333 P60520 Q15697 53615 Q13033 P54259 O75469 8458 64750 7124 P54252 P54253 P14635 O75461 Q9UQL6 P10275 4092 P10276 4093 O75460 4094 154 Q99728 Q99729 Q13045 55806 P45379 P49736 7132 8463 7133 8467 9314 O43889 P36896 23378 P15976 Q96C28 6282 1822 166 Q8NFM4 55810 P01308 Q8NFM5 338917 Q9NQB0 Q99717 Q15233 Q96T37 9326 P54274 O75446 P36402 O75444 6294 7141 1831 Q96T23 Q00577 Q99708 P49711 Q16576 P49716 P49715 55827 Q9NQC7 Q96C00 64784 7158 O75437 Q08050 P39905 7157 P19086 64786 162239 Q8N9R8 Q02750 P35548 138151 55832 Q9UDV6 Q5VTD9 Q13077 7169 26038 10928 26037 Q86Z02 56252 Q01094 Q9H204 26039 7161 P63167 P42224 10933 10935 P17275 P23945 P63165 Q9BVI0 P42229 57109 P42226 O94992 Q9P0W2 22926 Q969S8 22927 Q13404 Q13887 Q01085 57592 Q8IY57 57594 P10644 57591 Q96BD5 79365 P84095 Q08945 Q9UHX1 1869 7186 7189 7188 Q8WYK2 O43812 P11940 7181 7182 7185 1877 Q86UE4 Q86UE3 1874 Q9Y3C7 1870 P09630 O15379 Q969R5 Q13422 O15391 Q8NB78 4914 2735 27352 P19438 O43829 84733 1408 1407 1406 2737 2736 Q96G30 Q8WUI4 Q02386 Q7RTR2 P62277 1896 Q9HC52 Q14774 P22670 Q9NQ92 9391 P11532 Q96G25 84759 58487 Q9UQE7 Q8WYP5 P23511 Q8WUH2 3609 3608 3607 Q14781 P23510 P21333 94104 Q6ZV73 O14495 Q9P0T4 29966 P19474 Q02363 58491 P18146 27300 O60343 O60341 4943 Q969V6 O15350 O75832 O15353 Q15649 Q9BZS1 Q15648 Q9P0U4 P19484 Q86YP4 Q06710 Q96BF6 Q8N5A5 3622 2773 Q9HC29 O14497 Q15652

positive regulation of transcription P10914 Q92858 O14793 Q12948 Q12824 Q6QNY1 O00267 P17676 3516 2547 2304 4609 2303 P16220 Q96JM2 P84022 Q92731 O15516 2308 Q9H4L7 Q12837 Q5VTR2 54361 Q8NAP3 4851 5705 O75925 Q12968 6927 P37023 6929 4862 Q9UJU2 P49116 O75928 P40425 P40424 6926 Q6IT96 P06401 3659 6925 P06400 Q96JK9 5970 2100 Q9NR96 P56705 P48380 O95343 10524 10645 P20823 P19838 P61296 5966 Q9Y463 P28482 1022 1385 P19532 O00468 6829 1017 10736 1499 P15173 80324 P07550 4646 6827 Q9UHK0 4780 Q92769 5991 6720 P28370 Q14938 O95361 O00233 P10600 P19544 O00358 Q9NS56 Q8WYH8 Q06330 P37198 10626 P19419 Q9P0K8 1387 P17482 P08651 P17480 4775 Q9UM54 4790 Q12888 4792 22937 P38398 2247 Q9BZK7 6601 Q92753 6721 Q92993 Q12770 P05112 Q9UM63 Q92997 Q13501 Q9UKS7 P10826 O43186 Q12772 P10827 Q12778 P51692 Q13503 Q05586 O00255 1050 Q03164 861 P40337 50943 Q9Y2W1 O96004 6872 P04637 2034 P13349 2033 2274 Q9UIH9 23326 O60603 25988 P15884 P52952 3479 10011 84289

Q16236 10014 10138 Q04917 Q9C0K0 6760 4221 Q96T76 641 P33076 P48436 4214 P01106 406 P01588 O43294 408 P49407 P01343 P01100 O00327 O00206 Q09472 P48552 P48431 9921 6774 890 Q99743 Q14192 Q96S42 898 Q53X93 P01137 5316 P49639 P01375 Q9UPY8 P51531 6660 5451 P35222 P51532 Q96RR4 P17813 3148 P10070 P10071 3146 P61457 668 6776 10001 6657 P23497 P26583 O00744 5460 6672 Q9Y5Q3 P50613 P51946 Q12905 Q12906 Q9BZE0 672 P15692 79612 3159 Q02930 6667 Q8N5U6 P40763 P05412 5591 O00755 4261 Q6IR47 7533 P18850 Q05086 23414 3171 P37231 P18846 P18848 688 3169 148327 9826 5468 Q92922 7422 P52815 O60907 3066 Q9UMN6 Q9UPT9 3065 3182 P17844 8625 10432 23636 P27540 5494 P62508 Q9UIG0 O14936 466 P51617 P58012 468 7428 10320 54106 8878 9967 P25116 P60568 O15085 6595 Q14686 6597 4297 6599 6598 P15923 P13984 Q9Y4K3 351 Q9H161 474 P08047 Q7Z6C1 Q13485 P25963 O94906 Q9NPC8 3091 O00716 8301 Q15306 Q86VP6 P58304 P13631 367 Q04864 51773 Q70SY1 P46531 Q13492 Q13133 Q15796 Q9UQR1 6496 P35638 6495 Q15797 6256 P12755 Q9ULH7 Q9NY61 O75360 Q9Y606 Q2M1K9 Q15561 P17096 51547 Q13263 Q15562 Q16650 4086 P35869 4087 8204 4088 P62826 4089 7476 9412 P18074 Q8WVL7 P55055 P12645 P36956 222546 P08151 P24864 P20264 Q16665 Q13033 9421 P55347 O75469 7124 P54132 P54253 O75586 391 P35453 Q9UQL6 P10275 P10276 4093 154 Q99729 Q99966 Q13285 2902 7132 Q9NRC8 7376 8463 Q9HAZ2 P36896 O43524 7490 55810 338917 Q9NQB0 Q8WW38 Q14140 Q99958 Q15475 Q99835 Q96T37 Q8TEY5 7023 5089 P14859 Q96T23 54851 Q01664 Q96PK6 56916 P01579 P49711 10155 P49715 P09038 55827 9575 Q9NPA8 7158 P39905 7157 O15198 6182 P11474 5092 26747 55832 Q99814 7046 9464 P35813 P29375 27005 Q01094 7161 P22736 Q9Y4C1 Q1PSW9 P17275 P62195 91 94 9219 P42229 O15119 P42226 Q9UBK2 Q58WW2 P56524 22926 22807 Q13887 Q8IY57 P55318 P84095 Q4LE28 P05067 1869 O75528 7189 7067 P19793 Q03014 7182 6096 Q01196 P41235 Q92793 Q96GM5 Q9HD15 O95619 Q9NSC2 8289 Q13422 O15156 O94776 Q13547 A4D1W7 P78317 2735 P19438 Q9HAK2 Q9BXJ9 Q04206 Q92786 Q8HWS3 1406 2737 2736 Q15853 O14593 O95999 9149 Q8TAK5 1655 P22415 Q9UK80 4800 2626 7099 7098 Q9NVC6 Q9NP71 P20248 64919 80155 Q9UIV1 P23511 375790 3609 P17081 3608 5901 P46734 P09429 P12956 29966 P78527 P18146 Q9H4W6 P35711 Q9BQA5 P43694 O15455 5914 O15350 Q9BZS1 Q15648 P55771 P19484 Q06710 Q04771 63976 5927 P63272 P24941 Q9HCS4 O14497 Q92585 902 Q15532 Q13233 P21675 5925

positive regulation of protein modification process 11065 11186 595 1453 P63208 Q7Z6C1 8767 Q13485 10533 P04004 7334 P55036 Q12834 4067 Q9UBC3 5706 5707 5708 5709 P14174 7448 5700 Q16401 5701 5702 5704 5705 Q5S007 O75925 Q7Z727 Q92956 8555 P35998 5717 57761 10197 P61289 Q9Y297 5713 Q92833 P26358 5716 4089 9093 Q99460 O60674 Q8IUC6 3309 P04233 P62942 8454 P27037 Q8TDY2 7124 P51668 P14635 P51665 4092 O95352 Q93062 Q13042 Q92530 3678 Q8N2W9 P08887 P08648 3690 Q9NYA1 11030 O00231 P36896 O00233 P35443 P36894 P10600 O00232 P11021 960 P01308 120892 2475 P49721 O15169 P49720 85440 P07766 P05106 P02790 Q9UGJ0 6850 10273 Q13616 O00487 P30542 P38398 P62333 P49959 2247 Q96N67 P20618 2246 P60033 975 O14641 51588 P01579 P05230 857 Q92878 P05112 P09038 Q92997 Q15008 Q9UL46 7157 6500 O00255 Q6KC79 7272 P48730 5524 Q15257 50943 Q9UKT4 P06213 P01241 Q13873 7046 O95835 P04637 5300 P14317 2033 P42345 991 26271 Q96RU7 3479 2147 P62195 91 Q15389 1856 Q9UBK2 P56524 P78536 64682 4221 Q13526 Q96QB1 2280 648 P01588 P16070 408 P60900 P49407 P01343 P49768 O00206 Q09472 5682 5683 5684 5685 5686 P29590 3263 Q9NS23 148022 891 894 896 P17252 8945 P01019 P25789 P25788 P01137 P25787 P01375 4361 5692 5693 9495 P35226 Q9UNE7 P43405 P17813 5687 5688 Q96EY1 P24588 O43353 9821 O95999 P07948 672 P15692 5578 10213 Q13200 Q96FA3 7099 O43242 Q6IR47 Q9HCE7 64127 57154 P30279 1789 1786 Q9UJX2 P00734 P30281 Q9H1A4 5347 3720 P24385 7422

P10415 P28065 O00762 57162 P61088 P01730 Q9BT67 5371 P27540 8881 O75832 Q9BZS1 P53350 P62191 P28074 P17980 P28072 P28070 O14818 Q9HC29 P30260 O15105 P43686 P60568

positive regulation of gene expression P10914 Q92858 O14793 Q12948 Q12824 Q6QNY1 O00267 P17676 3516 2547 2304 4609 2303 P16220 Q96JM2 P84022 Q92731 Q9Y275 O15516 2308 Q9H4L7 Q9UBC3 Q12837 Q5VTR2 54361 Q8NAP3 4851 5705 O75925 Q12968 6927 P37023 6929 4862 Q9UJU2 P49116 P03372 O75928 P40425 P40424 6926 Q6IT96 P06401 3659 P26358 6925 P06400 Q96JK9 5970 5971 2100 Q9NR96 P56705 P48380 O95343 10524 10645 P20823 P19838 P61296 5966 Q9Y463 P28482 1022 1385 P19532 O00468 6829 1017 10736 1499 P15173 80324 1012 P07550 4646 6827 Q9UHK0 4780 Q92769 5991 6720 P28370 Q14938 O95361 O00233 P10600 P10721 P19544 O00358 Q9NS56 Q8WYH8 Q06330 P37198 10626 P19419 Q9P0K8 1387 P17482 P08651 P17480 4775 Q9UM54 4790 Q12888 4792 22937 P38398 2247 Q9BZK7 6601 Q92753 6721 Q92993 Q12770 P05112 Q9UM63 Q92997 Q13501 Q9UKS7 P10826 O43186 Q12772 P10827 Q12778 P51692 Q13503 Q05586 O00255 1050 Q03164 861 P40337 50943 Q9Y2W1 O96004 6872 P04637 2034 P13349 2033 2274 Q9UIH9 23326 O60603 25988 P15884 P52952 3479 10011 84289 Q16236 639 10014 10138 Q04917 Q9C0K0 6760 4221 Q96T76 641 P33076 P48436 4214 P01106 406 P01588 O43294 408 P49407 P01343 P01100 O00327 O00206 Q09472 P48552 P48431 9921 6774 890 Q99743 Q14192 Q96S42 898 Q53X93 P01137 5316 P49639 P01375 Q9UPY8 P51531 6660 5451 P35222 P51532 P14784 Q96RR4 P17813 3148 P10070 P10071 3146 P61457 668 6776 10001 6657 P23497 P26583 O00744 5460 6672 Q9Y5Q3 P50613 P51946 Q12905 Q12906 Q9BZE0 672 P15692 79612 3159 Q02930 6667 Q8N5U6 P40763 P05412 5591 O00755 4261 Q6IR47 7533 P18850 Q05086 23414 3171 P37231 P18846 P18848 688 3169 148327 9826 5468 Q92922 P01185 7422 2099 P52815 O60907 3066 Q9UMN6 Q9UPT9 3065 3182 P17844 10673 8625 10432 23636 P27540 5494 P62508 Q9UIG0 O14936 466 P51617 P58012 468 7428 10320 54106 8878 9967 P25116 P60568 O15085 6595 Q14686 6597 4297 6599 6598 P15923 P13984 Q9Y4K3 351 Q9H161 474 P08047 Q7Z6C1 Q13485 P25963 O94906 Q9NPC8 3091 O00716 8301 Q15306 Q86VP6 P58304 P13631 367 Q04864 51773 Q70SY1 P46531 Q13492 Q13133 Q15796 Q9UQR1 6496 P35638 6495 Q15797 6256 P12755 Q9ULH7 Q9NY61 O75360 Q9Y606 Q2M1K9 Q15561 P17096 51547 Q13263 P55290 Q15562 Q16650 4086 P35869 4087 8204 4088 P62826 4089 7476 9412 P18074 Q8WVL7 P55055 P12645 P36956 222546 P08151 P24864 P20264 Q16665 Q13033 9421 P55347 O75469 7124 P54132 P54253 O75586 391 P35453 Q9UQL6 P10275 P10276 4093 154 Q01201 Q99729 Q99966 Q13285 2902 7132 Q9NRC8 7376 8463 Q9HAZ2 P36896 O43524 7490 55810 338917 Q9NQB0 Q8WW38 Q14140 Q99958 Q15475 Q99835 Q96T37 Q8TEY5 7023 5089 P14859 Q96T23 54851 Q01664 Q96PK6 56916 P01579 P49711 10155 P49715 P09038 55827 9575 Q9NPA8 7158 P39905 7157 O15198 6182 P11474 5092 26747 55832 Q99814 7046 9464 P35813 P29375 27005 Q01094 7161 P22736 Q9Y4C1 Q1PSW9 P17275 P62195 91 94 9219 P42229 O15119 P42226 Q9UBK2 Q58WW2 P56524 22926 22807 Q13887 Q8IY57 P55318 P84095 Q4LE28 P05067 1869 O75528 7189 7067 P19793 Q03014 7182 6096 Q01196 P41235 Q92793 Q96GM5 Q9HD15 O95619 Q9NSC2 8289 Q13422 O15156 O94776 Q13547 A4D1W7 P78317 2735 P19438 Q9HAK2 Q9BXJ9 Q04206 Q92786 Q8HWS3 1406 2737 2736 Q15853 O75626 O14593 O95999 9149 Q8TAK5 1655 P22415 Q9UK80 4800 2626 7099 P31249 7098 Q9NVC6 Q9NP71 1789 P20248 64919 80155 1786 Q9UIV1 P23511 375790 3609 P17081 3608 5901 P46734 P09429 P12956 29966 P78527 P18146 Q9H4W6 P35711 Q9BQA5 P43694 O15455 5914 O15350 Q9BZS1 Q15648 P55771 P19484 Q06710 Q04771 63976 5927 P63272 P24941 Q9HCS4 O14497 Q92585 902 Q15532 Q13233 P45984 P21675 5925

protein modification by small protein conjugation or removal 11060 11065 7325 Q9Y4K3 O75150 Q5QP82 9616 P63208 90678 9978 Q14694 3093 P09936 7332 7334 7336 7335 Q9GZZ9 Q5VTR2 Q9Y4L5 Q86VP6 Q9UBT2 23291 Q13490 7328 Q96RL1 P61956 4734 Q9UKV5 7341 P22681 10193 9646 10075 6015 P32121 A0AVT1 4193 57646 P62256 Q9NV06 Q92830 P61962 Q16531 P61960 O75928 Q93009 Q93008 P55854 Q6PD62 57092 9099 Q13829 Q5TAQ9 267 23032 55236 Q6PCD5 8454 P51784 8452 7126 P51668 26994 Q06587 79577 Q96DE5 10616 10055 Q13042 Q99728 Q13049 Q7Z419 6045 Q9UGI0 7375 Q9H4P4 6048 Q96J02 Q9UKA1 5071 7251 Q9NS56 10987 10868 Q8TBC4 7128 Q8TEY7 8237 7267 10273 Q13618 Q13616 Q9Y5K5 1161 O00487 O95376 Q9UKB1 Q99942 Q8TBB1 Q8WY64 P38398 10956 Q96DC9 Q9UK22 55827 Q9NQC7 Q969M7 Q9NPA8 6500 Q96FW1 P31749 255488 253980 57332 55832 Q96LR5 55159 Q99496 6612 867 Q99816 54623 6613 P40337 Q8WW12 2935 9100 Q6P1J9 O00308 P53804 Q15819 27005 Q9NVW2 23326 Q9H0M0 23327 80124 Q96RU2 Q8TAF3 27246 P63165 997 Q15022 P62877 Q8WWI1 P45974 Q969T4 Q58WW2 64682 Q13404 Q8TCJ0 115426 83737 84708 55294 27252 P46934 54764 55611 51132 Q92560 51377 57599 4214 Q9BS18 408 P49407 409 25847 7186 7189 10238 Q9Y3C5 Q9BYM8 Q9Y4E8 26133 55743 Q8TEL6 Q7Z6Z7 10477 29086 O60260 Q15843 Q9UMS4 P40818 Q6R6M4 7874 55072 25853 Q6UWE0 P78317 Q9UNE7 26260 26262 1642 Q9HC62 Q5VVQ6 Q96EP0 P62837 Q969H0 Q9UBF6 Q9Y2K6 Q96EP1 672 Q96PU4 26232 Q9UK80 26234 Q96PU5 51720 10213 4008 P55072 Q9HC52 Q9H3F6 Q8WZ19 P21580 Q9HCE7 57154 Q86Y13 Q8N7H5 P61081 Q05086 27339 P46736 Q9NWV8 Q9UJX2 Q00987 P61077 1540 207 Q9H1A4 329 Q96JH7 8078 O75604 P10415 Q13216 Q8IUQ4 377630 P19474 Q9UPT9 29843 59343 Q9Y2M5 O00762 51185 P61088 P68036 7415 Q5T6F0 8881 Q12933 Q9P0U3 Q13107 Q92905 23512 580 55432 P30260 7428 Q13233 51514 P49427

nucleocytoplasmic transport Q96QC0 P04637 Q86V81 57187 P35658 Q9P2R6 Q96FV9 P38646 O00148 473 2147 Q7RTN6 23165 4686 Q92973 Q9Y2X3 10772 Q9BQG0 P09651 Q9C0K7 Q92574 P49815 Q8WUM0 P25963 Q9NPC8 Q14974 Q8TD31 Q13769 O15131 Q01082 O75494 23214 8021 55696 Q96Q15 P42858 Q9UIA9 P52292 8536 406 Q9HAU5 P60468 O00327 6496 P52298 Q86U42 Q9UMR2 P52294 23144 P57740 P29590 Q03014 P30101 26019 Q96IZ7 Q96P70 Q9GZY0 10514 80145 23381 55341 Q00535 Q9UDY8 Q9NZQ3 Q01105 811 54535 P01137 4869 P01375 8563 4000 P62826 10482 Q9H814 P62829 Q86XR8 64901 O95343 23039 O60318 P61970 O60674 Q9UH92 1408 Q49AN0 Q8IXZ2 51319 O00505 O43592 7124 P28482 7248 Q9UPR3 O00629 P54253 P41182 1020 Q9H089 Q9P0J0 3836 10736 3313 84248 5976 11269 6945 Q14012 P05412 O95405 Q09161 Q9HCE7 Q9H2T7 3178 O75694 57154 Q86WV8 Q8NI27 P00734 6711 207 7249 3841 9702 604 3843 P62841 5901 P27797 Q86W42 4792 A1XKG3 Q12769 Q9NW64 Q96QU8 2247 P01579 P02545 5514 Q6I9Y2 P09038 2923 55705 Q9BY84 P49792 5371 7157 11097 P31749 Q9UKK6 Q96QD9 O00410 23633 Q08209 P52948 Q92621 O15504 Q92900 10204 P25116 51517

positive regulation of cell communication 8772 P48058 P30086 Q9UBB5 Q9NS86 Q9Y4K3 O43318 472 O15519 356 P84022 1453 Q9H160 23286 Q92851 8767 O14788 Q13485 57506 P07996 Q9HC98 P04004 Q15303 3091 7334 5155 4067 7335 5037 65018 54361 2316 367 Q04864 P14174 P09382 7448 10783 P00533 Q13490 4734 1902 10666 9641 8795 Q9UQ13 Q9HBW0 P32121 253260 Q06124 Q9Y6Q6 Q9UBU3 P37023 10758 27185 Q9UDY8 57521 Q9NRI5 142678 P03372 64170 P55290 P32239 Q9GZX9 4088 Q9BY44 4089 7476 5970 3673 P48023 Q86XR7 Q9H257 Q9NR96 Q13705 P56705 P17302 P17301 387 389 O60674 O00182 2697 Q8IUC6 Q16665 3667 5966 55915 P04233 P62942 P27037 6275 Q13158 Q13956 7124 Q9Y3E0 O00463 P10275 154 Q93062 P15056 Q9Y2C9 1499 10616 1012 P07550 P04040 O94827 P61586 3678 1936 P08887 Q99966 7132 P08648 3690 Q9UGI0 P29466 Q9Y239 P30559 11030

P36896 O95361 124583 P36894 P10600 P10721 5071 P35568 P37198 1029 10626 29110 960 1147 Q02763 P01308 841 2475 843 Q7Z7H5 Q9NQB0 P42771 O15169 847 Q99835 P07766 P05106 P02790 10392 6850 P50591 O43734 O43612 Q9P0L0 Q9NS68 P18545 Q8WVQ1 Q8IUH5 Q99943 2247 Q9BVC4 O14640 P01579 P08069 857 O14763 P05112 5879 1956 P09038 Q92633 P09958 Q9H8V3 O00255 P37840 23118 7272 27032 54862 Q14164 P48730 Q6R327 P09601 P98194 Q9UL54 Q13077 P06213 Q15811 P01241 Q13873 7046 P35813 P14317 3481 O60603 P42345 Q9Y6G9 Q9BXM7 3479 P13591 91 6622 10013 P01116 9218 94 P01236 5536 6869 Q15389 Q9NSA1 P01112 P01111 P78536 7057 4221 6885 Q13404 P51636 9590 2280 7052 P46934 2159 54764 P08575 8932 4215 P01588 P16070 Q99759 O43294 408 P49407 P01344 409 P01343 O00206 7189 7188 P06239 P48431 Q8N726 Q13535 3265 29767 Q03014 Q13418 3263 7185 148022 Q9BYM8 60412 Q02952 P53708 P01019 P21860 51026 P21980 55504 P01137 Q9NSC2 3932 P01135 O60260 P01133 P01375 Q9Y337 64223 P04626 Q13546 55072 Q7Z434 2065 2064 P35222 9495 P35348 P19438 P43405 P17813 545 Q04206 P53041 P01127 6657 Q9UBN7 8837 P24588 8717 O43353 5460 Q96EP0 O95999 Q13315 P13945 P07948 P78509 P78504 3162 9020 79971 673 P15692 O14908 P63000 57708 5579 P00742 5338 3953 P05771 Q6P1N0 3956 Q96FA3 O00635 7099 O00755 7098 Q6IR47 Q13322 8743 Q15628 P14923 64127 Q05086 5590 Q99558 3728 Q05513 3169 P08134 5467 8737 329 P48357 3965 P21333 Q9NYJ8 7422 6453 2099 Q9UHD2 57045 Q9BUZ4 57162 3611 P61088 Q14790 Q5T9L3 O15455 Q16610 10554 P25103 P01730 Q15762 P01178 9839 P41159 Q9BT67 23636 P27540 5494 Q60FE5 O15111 25818 Q86Z14 222068 O14939 3622 Q9HC29 8516 54106 P26447 P25116 P60568

response to chemical stimulus P25054 Q9H6Q4 P28562 3880 Q9Y266 O14793 P49023 2550 22872 P51587 Q5SR56 4728 P37288 2303 P13073 P84022 O00151 2783 Q92731 4720 O14543 O14788 Q8N465 2308 2549 P27487 P28335 P08727 Q92729 2 5829 P42858 P39210 54487 P61158 4734 P67936 Q9UKV5 P10809 808 Q5S007 Q7Z727 Q9UKV8 P28300 Q06124 P17405 6927 57761 2564 P63104 P07858 3672 3673 2100 Q9NR96 Q13705 P21917 P54646 P17655 Q96GD0 P20823 O60674 Q8IUC6 3667 5604 P41743 P42830 P08758 P25098 Q9UGH3 2110 1020 P50454 P51787 P15056 P21964 1499 1012 5610 5611 22846 P29466 4780 Q92769 P09619 P80098 O95361 P10600 Q96KS0 2119 Q9BUB1 10626 1026 P60022 841 P20648 Q9HCM4 846 4535 847 3689 P05023 6714 P05026 P04179 4792 1285 P16118 P19634 P38398 Q9HCL2 Q05469 610 1277 Q5T2W1 Q92753 6721 857 P51575 P10827 P31749 Q9NR50 Q7L8A9 1050 Q9HCK4 Q9UL51 P04150 4313 Q9UMX0 P09601 P04155 P04156 P01241 P56199 P14317 3484 P50416 3482 O00300 3481 O60603 23327 871 Q99523 3476 Q13085 55967 7832 10013 P62879 5898 P62873 10014 P25685 P23025 10016 6760 P06702 112399 Q9BS26 1072 P13236 Q96T76 O14717 P33076 6517 409 Q99757 P01222 1080 Q09472 Q9UMR2 6772 6774 3265 P13686 890 891 894 Q96T60 Q14192 55741 898 Q16134 O14965 Q9Y5X9 P50440 4363 Q9Y696 P35222 3030 O75084 P17812 P17813 P60484 Q15291 P01009 P01008 10241 6776 4358 10488 5207 P23497 Q59EA4 P24588 P31930 P26583 6790 O00744 Q5VVQ6 4131 O00506 P59665 P39656 Q9NX61 10458 672 673 P15692 6548 P55072 P05412 P01275 O00755 P05413 5471 5473 8985 90993 7412 Q08117 3297 P37231 682 10229 Q01955 P51959 445 148327 5467 8737 207 5468 Q02809 P48357 Q92922 11315 P01266 P01023 P49682 P24385 8754 7422 Q96A54 3066 79753 Q00613 7415 8503 4149 O14920 10432 Q8NHX1 P52824 23513 P50402 462 P17612 O14939 466 7428 55558 10202 P04792 Q13115 7442 P35659 Q14686 5265 6599 Q9H2Y7 Q9Y4K3 P13861 230 476 P07196 Q99807 P20396 9616 Q9NPC1 P49810 51763 8767 P49815 Q15303 3091 6368 Q9HAV0 5037 P11215 P13631 Q9Y4L1 481 P09382 P22102 5029 P00533 Q13370 Q13133 Q15796 Q15311 Q13131 Q9BUN8 Q9HCY8 Q13137 7220 P35638 Q15797 P12757 Q8WVM8 P32121 Q96IZ7 Q9Y6Q6 495 1917 Q13144 Q14232 P55290 6383 5295 5054 P35869 9414 7476 P18074 O43306 Q01581 P68133 58189 O75351 P35625 Q9NVR5 267 Q13393 Q16665

P43246 51319 Q14249 P56556 9420 7248 P63092 O75582 O75340 O75581 P09172 Q9Y2C9 P35221 11269 O15294 Q96CS3 7252 Q14258 P78334 P36776 Q9Y4H2 23256 26524 285 P12110 P11021 9429 7249 Q9NRD1 P48995 P49840 O75569 P98179 P13646 A1XKG3 P48507 140576 Q96PK6 P21266 P08069 1718 P47895 P02786 2805 1956 P23443 9695 7037 Q9BX66 9217 P10147 P38936 P37840 64428 P11233 51593 Q6PID4 P27815 79901 P23458 1728 Q99814 P02775 P02533 Q14289 P25874 O75306 7046 6198 P98155 Q9P296 P12931 Q4VCS5 P42345 83752 Q9Y4C1 Q92572 P08684 P04085 Q92575 O95715 Q92574 P05177 1978 P31689 P56524 P78536 9474 7057 1503 27252 P46934 P05181 Q4LE28 P06276 P16070 P42574 P42338 Q92569 P34947 7067 P06239 P29353 Q5TAX3 P19793 P29590 P41235 80267 P40145 P41231 Q92793 P00966 O60266 P06241 P21860 3932 8165 9255 P55212 Q13546 P19320 P34931 9252 P34932 P78318 9495 Q8N8D1 Q9UNE7 P43405 O43707 3708 Q04206 P53041 P07101 P07585 P05164 Q13794 O75746 Q14643 A8MTJ3 O95999 O95757 8170 9021 Q16828 P35613 P00749 P00505 27202 O60488 57026 P08243 3953 P62158 2869 P08246 3956 2626 7099 7098 Q13322 Q9NP71 Q14416 P14923 P30153 P11413 93100 1789 3728 P61073 P08253 P09104 11080 Q16849 P34741 P78527 P32322 1312 2643 2885 P17174 2882 Q04656 Q92598 P43694 O15455 Q14432 5914 Q13586 O75953 Q06830 Q9H6Z9 P34972 Q9H0N0 5929 P43681 4830 900 P00750 Q15532 P08238 O15228 64397 P08833 O14672 O00267 O60568 4609 1576 P16220 P08842 P07996 5935 Q92611 P52564 O75815 O15530 Q9UI09 4860 Q9UBC3 P27361 54361 Q08431 51094 Q16082 P18887 P16234 P63218 P30519 O15524 P50150 57402 Q8TED1 6809 Q86WA8 Q9UJU2 Q9UQB8 P03372 O75807 64170 Q92831 P55851 P09917 5970 P48023 1374 3551 1130 9093 P56705 10525 P17302 5728 P17301 Q99698 3304 2697 3301 3784 10763 3309 Q92820 3308 O00469 P28482 P11802 5983 1385 3320 6829 P19525 3316 1137 57669 3315 3312 P04040 P09972 27161 7913 P08887 Q96BA8 719 Q7KZI7 P51671 P30556 Q9NYA1 P30559 2243 P51679 P30793 P37198 3329 57678 960 3326 1147 1388 1387 2475 Q6XQN6 P05107 P28223 6850 22937 P05109 P51681 Q06203 2011 O00244 2010 1161 P51686 6609 O60502 O60503 3337 P61244 2247 P15153 2244 Q12770 5515 P04264 P05112 5516 5518 Q12772 P27144 Q92626 Q12778 P29320 P51692 1176 Q05586 P48061 P18433 10724 10726 P37173 Q99497 P09960 P05121 O14656 P40337 O14657 7706 26060 P06213 P02452 Q8N2K1 P07900 P04637 2034 2033 2274 Q9BV94 P52952 P15531 Q96RU8 Q96RU7 P62993 2026 513 6622 999 P01116 Q16236 5536 6869 Q15389 P27986 518 51009 P01112 P49770 P48681 P48201 5796 P35368 Q6AZY7 Q9BT40 1191 O76074 Q15392 Q9P2K8 P01106 768 P01588 P02675 P01344 P01100 P49768 O00206 Q9Y6Y9 P48431 5562 5321 5563 P14416 P13569 O95292 P14410 148022 P11387 O76061 Q00535 Q53X93 6402 Q9GZP9 5798 6647 P01138 P01137 Q9UPY3 P01375 O00217 P01374 P02461 4240 P01130 P40939 5573 P40938 P04626 5575 8841 5576 2065 2064 Q08257 P12004 P35348 2059 3148 3146 5566 8836 5567 P01127 5568 Q96EY1 6657 5327 5328 O43353 6672 25828 5584 P07948 Q12904 25822 3162 O00622 552 O75190 Q96PU5 P51828 8604 5577 5336 5578 P62714 5337 5338 P40763 Q96FA3 P04201 2081 5591 4261 Q6IR47 5351 5352 5111 7534 10580 64127 P37108 P68032 P18850 P18846 Q68CJ9 324 3169 4015 329 Q08462 9961 P01185 6696 2099 Q12929 Q9Y2M5 P51843 Q96PS8 Q8NER5 Q9NRM7 P68036 5599 P25103 5119 Q9GZT9 P01178 23636 5371 P27540 Q9UNQ0 6464 Q12931 P15559 O60911 P16885 Q8NER1 55432 Q6ZWJ1 54583 P51617 Q08209 P18827 54106 P25116 5141 8892 Q9BY76 8412 8654 P30086 595 Q04759 356 Q9UBS5 P09493 Q7Z6C1 P22694 Q13480 5139 Q13485 P25963 P03950 7332 5154 5155 4067 P12429 367 Q70SY1 128 P22223 P47985 Q13490 20 6256 6498 9525 P31153 P32246 P59768 P10451 Q9UBU3 Q03405 Q9UDY4 P51805 Q9UDY8 P23528 P09471 5159 P61604 31 Q8WZ42 P00441 P32239 346562 8443 6262 5174 Q14116 Q16539 4086 10081 4087 Q13268 7353 4088 4089 Q14118 Q9H257 Q9UKG1 5170 P12883 142 P21359 Q9UBV8 1808 Q96SL4 P24864 51435 P23771 Q13033 48 O75469 7124 P14635 Q9UQL6 P10275 25788 P10276 4093 O75460 Q02535 Q00597 156

2908 10057 Q16555 Q15466 2904 Q16790 P45379 Q99966 2902 54 7132 P60709 6048 O43889 Q86WV8 P35568 166 P11142 Q8NFM4 Q04941 Q8NFM5 120892 Q9NQB0 60 Q99958 Q8TEY5 9564 P02671 7384 8473 P00491 P61803 Q401N2 7386 10273 Q86V24 Q99942 Q53TN4 Q99941 Q9H1Y0 P08183 P01579 P49715 P09038 7157 O15198 P14625 Q02750 7150 P35548 1843 2931 Q14160 Q15011 1609 O94979 1848 Q16581 1605 P21399 P78423 O95835 O15123 9588 10928 Q01094 P54219 P42224 P41134 Q9Y3A5 79139 10935 P17275 P39060 P46821 P22612 P42229 P15090 2947 Q9BZQ6 26270 P42226 O15118 22926 Q96QB1 P10644 7171 P31321 55172 P31323 P67775 P05062 1869 Q9UBL3 7189 P29474 P29475 27348 O75881 Q03014 6096 9361 7184 P41597 1634 P00846 P17252 O15379 Q8TAD8 P09874 P11717 Q15843 O95622 P10620 Q15849 P19438 2730 Q9UBN7 Q92667 P30049 9388 Q15853 P54652 Q9UBF6 P30041 P11766 P41181 P30044 P41180 P30048 B3KY43 P22415 P05091 P05093 Q03468 1650 Q9HC57 Q8WYQ5 P47712 Q13443 Q07820 Q96FQ6 P30279 Q07817 1667 P20248 Q05639 P21579 Q9BXP5 P17081 1429 P09467 P09429 O14492 P10415 Q13216 3860 Q13217 P41161 P12830 29966 Q02363 P18146 57162 1432 1672 Q14790 P01730 P41159 P33527 P08107 O15111 P55773 P17066 23197 Q9HC29 4710 P45983 P45984 P21554

regulation of establishment of protein localization P53367 7046 Q15654 11060 O95835 O00308 2033 O43557 P10599 Q9Y4K3 O60603 54474 Q04759 P84022 999 Q7Z6C1 P27986 6901 Q13485 P25963 Q9UBC1 P03950 4188 O15530 Q16635 7295 P50148 2316 9908 Q9HD26 P14174 Q99750 7205 P00533 Q96EB6 O00206 Q09472 7189 7067 Q8N726 3783 P14416 64215 57120 Q06124 60412 P62491 Q00535 O60543 P01137 Q9Y6A5 P01133 P01375 5716 5295 8165 P31946 4088 4089 8720 Q9BZF9 P35225 Q9NR96 27236 Q9UKG1 P21917 P35900 9495 5170 387 P21359 O60674 P10071 5566 Q9ULZ3 5569 Q9Y2B9 P41743 2737 Q92667 P43489 P24588 5460 5584 7124 11142 Q96KC8 29108 1020 Q9UNN5 O00221 114548 Q93062 57669 Q6PJW8 Q99728 11146 P61586 O15554 7099 7098 Q6IR47 Q9NYA1 P29466 Q13443 11030 10460 10580 P14923 64127 O95361 P36894 P10600 23411 P13796 5590 1029 57678 3728 10626 Q05513 P01308 Q9HCM4 1540 207 Q9NQB0 P42771 P49841 P17081 7529 P22303 Q99835 P23510 P21333 P61925 8754 4792 Q14703 Q96FX2 P12830 A1XKG3 2010 Q14C86 Q9HCL2 Q96P20 Q92990 P01579 11124 O15455 Q9UN86 1956 Q9NQC7 Q9BT67 P10827 Q60FE5 O75832 Q9UGK8 8766 Q9BX66 Q9BZS1 P31749 Q06830 580 P50402 P17612 2932 Q9HC29 Q9UMX1 54106 Q15653 50943 P60568 26060

negative regulation of cell death Q9BZZ5 P25054 8535 Q9BY76 399687 Q12948 Q04760 Q9Y4K3 3084 O43318 P17676 472 O15519 3516 2303 355 P84022 P07196 O00273 9616 Q92731 P49810 8767 P78395 5139 3875 O14543 Q9Y275 Q92614 Q13485 P07996 2308 P25963 Q13489 P52565 Q86VP1 Q9H3Z4 P14174 P42858 P16471 P46531 4851 8539 P00533 10542 P10809 Q92843 5705 P11309 6495 Q7L5Y9 Q9NY61 O75360 Q9UBU3 P20936 O60543 Q9UDY8 P23528 P03372 P63104 6926 Q6IT96 P55290 P00441 4869 Q9BWQ8 5716 9531 5054 Q13268 9530 5055 4088 4089 P07858 5970 P18074 2100 3551 57099 9093 Q9NRZ9 5728 387 3304 P19838 Q99576 P20264 P55061 P43246 P41743 3309 P04233 P08758 Q12981 Q12982 Q8TDY2 7124 Q9UHJ9 396 P15056 3315 1499 3313 1012 P04040 10296 Q99728 P61586 Q13286 Q9NYA1 P22392 Q9Y4H2 P10600 P10721 Q96J02 P19544 7490 5071 Q06330 Q9UEE9 P37198 3329 57678 P11021 79444 960 1026 P01308 7128 Q9NQB0 604 P49841 847 5624 Q99958 Q15475 Q99835 P04179 4790 P61803 4792 2495 Q9NQS1 291 P30542 1831 P48507 57448 Q9HCL2 2247 51100 Q9H1Y0 O95817 Q9Y371 P08069 1718 Q96CA5 P05112 5516 1956 P09038 P23443 Q13501 P09958 Q12778 P39905 7157 P51692 P31749 O43504 P14625 P38936 O00257 P37840 P35548 2932 81788 Q8NBS9 Q13075 P05120 P05121 P48736 P09601 P40337 P04156 Q15811 P78423 7046 6198 P04637 7161 O43715 P50897 P38646 6194 P52952 Q99523 3479 10935 P62195 P04083 6622

7832 P01116 Q9C0K7 Q15389 P42229 O15119 P62753 P01112 P01111 P25445 Q9C0K0 P78536 9474 7057 6885 Q02297 P16989 1072 83737 1191 27252 81567 Q15392 P98170 P16070 P42574 P01343 P02794 P42336 P49768 7189 P29474 Q9H422 3265 P18583 Q13418 6093 P12235 7184 P13693 773 Q9NR09 P01019 O15379 P21860 6647 P01138 Q6UXV0 O60260 P01375 O15392 P04626 Q13547 Q13426 8841 O95865 P04629 2065 2064 P35222 P14784 P35227 4914 Q96ST3 663 301 2730 P60484 P04070 Q04206 Q9H093 6776 P02458 P05164 307 Q96EY1 P09525 8837 P49747 Q9UBN6 O43353 9821 5584 O95999 7520 Q13315 Q9UBF6 P41182 P30044 57144 9021 Q9NX61 25942 3162 P30048 673 P15692 5336 P62714 P09211 P08243 7518 Q6IR47 P21580 7534 Q07820 23532 O76024 5590 Q07817 64919 P51959 Q05513 324 3169 207 P05783 P08138 23746 7422 P10415 6453 P56177 2099 3065 Q9Y2M5 330 331 573 1676 P13010 3611 5599 10673 O14920 Q13464 Q14432 23636 O15350 3070 P08107 P53355 O75832 8887 27315 Q9P286 Q04771 580 P16885 O60238 P51617 900 O95429 7428 P45983 8878 10202 P04792 P25116 P60568 Q9NZN9

response to stress Q9H0E2 P25054 Q9H6Q4 P28562 O14793 P49023 O95551 P51587 P17676 3638 54474 4728 2547 P37288 1454 P84022 P21926 1453 O00151 Q92731 4720 O14544 O14786 O14543 O14788 57506 Q9Y3Z3 4968 P27487 Q9UGM3 Q9NZG7 Q8NFZ0 Q8NE71 Q8NDV7 P53779 P16104 O60216 2 5829 P14174 P39210 P15260 4734 P67936 Q9UKV5 P10809 Q96EB6 Q9H2G2 Q5S007 Q7Z727 136319 P27694 Q9UGN5 P28300 P27695 O00167 Q06124 79791 81628 6927 P37023 57761 P63104 Q9Y6A5 Q12851 P07858 P31948 3673 P31947 2100 Q9NR96 Q9NP55 P21917 P54646 Q676U5 P17655 3428 81622 P20823 2335 O60674 P19838 Q99576 Q8IUC6 5602 10642 P26367 5604 P08758 P25098 P49069 Q9UGH3 1022 Q14807 3442 2110 1020 Q9NSU2 P18564 114548 P50454 10856 P21964 O60884 5610 5611 Q9Y230 3678 P07437 3690 P29466 Q9Y239 4780 Q92769 5871 6720 P80098 3452 P10600 Q96J02 Q96KS0 2119 27297 3448 1026 Q9BUB5 P60022 841 3445 3443 4775 604 3688 4535 847 3689 O14757 5627 P05026 P04179 4790 Q12888 5883 P28340 Q9BQ95 P16118 P38398 3459 Q9HCL2 1277 Q5TCX8 P05230 Q6NUQ1 Q92993 857 Q96CA5 5879 Q13501 P51575 P10827 Q9UL45 5892 P31749 Q9NR50 O95382 1050 P19878 2139 P40692 1289 O00141 55159 O60870 Q96MT8 4313 79109 5886 Q92982 5887 Q86VB7 Q9UMX0 4799 5888 P09601 Q9UL54 P04155 P04156 8930 Q9Y5U4 Q96A33 5422 3484 P14555 Q9Y3Q8 O60603 245934 25988 Q01523 Q01524 871 Q96T88 203068 3479 2147 P62877 8924 55967 7832 10013 10014 Q15025 P25685 P23025 6749 O96017 P06702 Q9UII4 112399 Q9BS26 P16989 P35244 P13236 P35249 Q96T76 Q03933 641 2159 P33076 Q99750 P35251 5424 Q99757 Q13098 Q6ZN33 Q09472 6772 Q08380 P48551 6774 P13686 890 Q9NUW8 891 Q99500 O14727 51141 Q96T60 55741 3014 P01019 Q16134 51147 55504 Q8IYD8 29086 11326 P23246 P50440 Q9UMS4 4361 P35225 114799 Q86UT6 Q01974 O75084 P17813 3269 P01009 Q9H093 51156 P01008 10241 Q15054 4358 Q15052 7867 O14733 5207 P23497 Q59EA4 P26583 5460 Q5VVQ6 P01042 4131 9821 O00506 P50613 P59665 P51946 23764 Q9NX61 O95197 P14598 672 P15692 P55072 Q8N423 P05412 O00755 5473 90993 10460 7412 3297 Q9NYZ3 O60814 P37231 Q6PJP8 Q9NWV8 10229 P51959 445 55775 148327 5467 P82673 8737 207 5468 Q02809 54206 11315 P01023 Q9BQ15 P24385 8754 7422 Q9Y5S1 3066 22897 O75293 P13010 Q00613 Q96P20 7415 P61764 4149 10672 7416 10432 8761 Q86UX7 P49674 Q9UIG0 O43464 Q8NHX1 3075 462 O14939 468 8517 7428 10202 P04792 56647 Q92900 Q9BYX4 P55268 Q14686 4176 5265 P18085 4179 Q9Y4K4 Q86TM6 Q9Y4K3 3084 230 472 P07196 23043 84447 Q99807 P20396 P49810 8767 P49815 Q14694 Q9BYW3 Q15303 3091 8546 P48960 4188 6368 P62807 5037 P58546 Q9Y4L1 481 60489 60488 P09382 6117 P46531 6119 5029 P00533 6118 Q96RL1 Q15554 Q13131 Q9BUN8 Q9HCY8 P24522 Q13137 9641 P35638 Q15797 Q16644 9643 8555 Q9UNL4 Q96ER3 Q9NY61 Q13144 P02741 Q14232 6383 Q9Y6R4 5054 P35869 5055 9656 7476 O43542 P18074 Q86XR7 P13611 P36956 P35625 P68371 Q8N4C8 267 P26927 P01889 Q13393 Q16665 Q03518 Q49AN0

P43246 O94817 P43489 P56556 Q5VWK5 Q8TDY2 O15287 Q16666 P54132 O75582 P09172 P46109
Q9Y2C9 Q9Y6K9 Q14494 8569 51567 Q14011 Q96CS3 Q14258 7014 P46100 Q15109 O75575 P36776
O43524 285 P11021 7249 Q9NRD1 11277 P49841 P02751 Q99835 7023 10392 O75569 P98179 9448
P12314 A1XKG3 P58753 Q8WW22 P48507 Q96PK6 1718 8349 Q9UEW8 Q9BQI3 P02786 1956 P23443
6188 9695 26509 7037 9217 P55011 P10147 P38936 P37840 64428 1965 2810 Q93034 Q6PID4 P48730
1728 Q99814 P02775 Q14289 P78545 O75306 7046 6198 P09769 P98155 O95714 P29372 Q7Z589
P78549 O43715 3913 P04083 81570 P04085 O15234 Q92575 P31689 P56524 P78536 9232 9474 7057
O95967 Q02297 O75771 55054 83737 Q8IUN9 Q9BSB4 P46934 Q9BYN8 Q9Y6H3 Q4LE28 P98170
P16070 P42574 P42338 Q9UER7 O95971 7067 Q8WXI4 Q9H305 Q13535 O15264 P19793 P29590
P41235 80267 79035 P41231 Q92793 57003 P00966 Q9NVI1 P21860 P05155 P07339 9255 P55212
340061 A4D1W7 Q13546 P19320 P34931 9252 7073 P34932 Q9UNE7 80254 653361 P43405 O43707
3708 1763 Q04206 P07101 P07585 P05164 Q14643 O95999 O95757 Q13315 O95997 8170 P78509
O00186 Q03113 9021 Q16828 3959 P00749 P00747 P63000 60673 51720 P00742 P08243 P08246 3956
7099 7098 Q13569 P11413 26127 93100 P46736 P61073 P00738 2873 P61077 P00734 23098 Q7Z2W7
P08253 Q13574 P22303 Q6SZW1 11080 Q16849 P12956 Q15759 3980 P34741 Q9P035 P78527 P32322
Q9BQA5 1312 2643 P61088 2882 Q04656 Q9NZM1 Q92598 P05198 O15455 3978 51747 O95786
Q14676 O75953 Q06830 Q9H6Z9 Q9P287 79065 O60238 P00519 P43681 900 Q9HCS7 84893 902 51512
P00750 Q13112 Q15773 P08238 51514 P21675 Q14683 O15228 Q5FWF5 O95677 P08833 O95433
P06899 Q9Y2W7 9066 Q8IVH8 4841 5931 P07996 5935 Q92611 P04003 P52564 O15530 Q9NS91
Q9UI09 P27361 65018 51090 P50148 Q16082 P18887 P16234 55215 P38484 P30519 4851 4854
Q9UK55 928 Q9ULX9 Q9NRY2 3783 Q12968 29128 79671 57402 Q8TED1 6809 57646 P18858 O60543
1111 55466 O75807 64170 4868 4869 P05556 Q92826 P55851 P09917 5970 P48023 Q9BZF9 1130 9093
10525 P17302 10524 P17301 Q99698 P15121 3304 2697 3301 6812 6814 948 3309 P04233 Q6PCD5
Q9H1C4 3308 O00469 Q9Y342 5981 P28482 Q12986 5983 Q9UHJ9 3320 O95232 83706 P19525 3316
1137 3315 Q6FHQ0 710 3312 P07550 P04040 P09972 5976 P41218 4646 P43630 P08887 Q96BA8 719
Q9UM47 Q7KZI7 P08648 P51671 O00238 Q9NYA1 P30559 2243 1153 P51679 O95243 P30793 Q96SB8
Q9NS56 3329 57678 29110 10746 2237 79444 960 3326 1147 1388 720 3324 1387 722 Q9UM54
Q6XQN6 Q9NYB0 P05106 O95257 P05107 P28223 6850 22937 P05109 P51681 2011 O00244 1161
P51686 P30542 Q9NS68 Q56NI9 10714 10956 P42768 3337 P61244 2247 1399 2246 2244 976 Q92630
Q12770 Q92878 P04264 P05112 5516 Q13620 Q92626 P29320 P51692 O00255 P54727 Q6KC79 P54725
81788 57697 Q99497 P09960 157570 P05120 P05121 O14656 P40337 P04275 O14657 O15503 7706
P02452 Q8N2K1 Q9Y6W6 P07900 P23396 6872 P04637 P80188 P15529 2034 2033 Q9UHF4 Q9BV94
P11166 Q96RU8 Q96RU7 Q96RU2 6622 Q9C0K7 Q16236 6869 Q15389 51009 P49770 Q9UHG2 4221
10383 5553 Q9UHG0 5796 Q6AZY7 1191 O76074 Q96Q15 Q8WTR2 Q15392 Q9P2K8 4214 768 11234
P01589 4216 P01588 Q99638 P02675 56946 P01343 P01100 P49768 O00206 6890 Q9Y6Y9 P48431 5562
5321 5563 O95292 P14410 P11388 Q9NS23 148022 O00203 773 Q00535 Q9GZP9 5798 6647 P01138
P01137 Q7Z3C6 Q6UXV0 10598 8829 P01375 Q9UJM3 O00217 P02461 6421 P40938 P04626 P40937
2065 2064 O00213 P12004 P35348 301 O95071 3148 545 546 3146 Q8WTP8 P01127 P40933 Q96EY1
6657 5327 5328 Q9UPZ3 7508 O43353 2070 6672 25828 7520 5585 4255 P07948 Q9UNN8 Q12904
22794 25822 3162 310 552 553 Q99685 O75190 Q99683 5578 P62714 5337 5338 P61626 7518 7517
P40763 2081 5591 4261 Q9Y4P1 P46063 Q6IR47 5351 5352 5111 7534 64127 23411 O76024 P18850
3171 Q01831 51295 P16671 Q68CJ9 Q99675 324 P18848 Q9NPH5 4015 328 5347 329 P01185 64135
6696 Q9UHD2 P51843 O14802 331 10318 Q9NRM6 5599 P25103 Q9GZT9 P01178 5371 P27540 O43581
4287 Q12931 5378 P39748 P20908 P15559 P52701 580 Q08211 O60911 55432 54583 Q08209 P18827

54106 8878 Q96HB1 Q9UPV0 P25116 Q13478 8892 Q9BY76 P15927 11186 8654 P15924 P13987 P30086 64949 595 Q04759 356 P09493 Q7Z6C1 9978 Q13485 P03950 5154 9755 7334 5155 Q16512 4067 7336 P12429 64963 64965 Q9UKE5 80198 Q9UBT6 P27918 Q70SY1 128 P22223 11198 Q13490 Q14344 A7MCY6 Q9UQR1 7341 6256 P13727 Q16526 P32246 P10451 Q03405 Q9UDY4 P49916 Q9UDY8 P09471 P22674 Q16531 Q9UBU8 P61604 51426 P00441 P32239 8443 6262 Q14116 Q16539 4086 Q13268 4088 P22692 4089 Q16778 Q14119 8445 Q9H257 5170 P12883 142 Q9H492 P21359 Q96SL4 10087 51435 P23771 7124 P54252 P14635 Q9UQL6 8450 4092 25788 4093 O75460 154 Q02535 Q00597 156 121512 Q8WV28 Q99728 Q13287 2904 Q16790 Q99966 54 Q13286 7132 6048 23135 Q9Y5J5 Q9H078 Q86WV6 P11142 P01308 120892 9306 Q15233 Q99956 Q8TEY5 P02671 P54277 10273 23586 7141 Q99942 1832 1831 Q99941 P49959 Q9H1Y0 Q01664 Q99708 9319 P01579 Q16576 P49715 P09038 11128 O95819 7153 64782 P01570 9332 P20073 7158 7157 O15198 P14625 Q02750 1843 2932 Q15011 P01569 Q14164 P01568 1848 Q16581 Q15014 P78423 Q13873 9100 O15123 9588 Q15819 P33993 Q86Z02 Q01094 7161 P42224 P0C0L4 1852 10933 Q9BXM7 79139 Q9HC16 10935 P63165 P39060 94 P46821 1616 Q9BZQ6 26270 Q9UBK2 Q9NSA3 P82912 22926 Q86WJ1 Q01085 A8K1F4 7171 P55318 Q08945 P05062 Q52LR7 P08575 1869 2956 Q969S2 7189 P29474 P29475 9126 27348 26258 Q96M96 Q03014 27347 P32881 9361 7184 P41597 1634 P00846 27102 Q8TAD2 P17252 Q86UE8 P09874 P21741 Q13426 Q7Z434 Q15849 P35900 P19438 1642 2730 P09884 1408 1407 P81605 Q9UBN7 1647 P07225 4913 P82933 Q5J5C9 P54652 P13945 1660 P30041 27327 P41182 P11766 P41181 P30044 P53567 P30048 Q9H244 P22415 P05091 Q03468 4920 O15554 3835 P47712 Q8IY92 Q9UBG3 Q13443 P51159 8065 27339 Q07817 1667 P20248 Q8N0Z6 Q9UQE7 P08138 Q9Y5B9 P23510 8079 P09429 P10415 Q13216 Q13217 P41161 59341 1432 Q5XUX0 1672 Q14790 Q6P4R8 P00403 P09430 P08588 O15350 P08107 O15111 O15353 P55773 P53350 Q13227 27315 Q04771 23192 P17066 23197 Q9HC29 P24821 O15105 4710 P45983 Q13233 P45984 P21554 P21796

positive regulation of binding 6597 6598 2033 Q12824 9181 Q9Y4K3 O60603 P15531 Q96RU7 10935 P11171 118 Q7Z6C1 8767 P01116 O14788 10014 Q92974 Q9UBK2 P04004 P56524 7334 Q13404 P12429 Q15306 7335 Q05682 2280 7448 800 O00206 Q92838 7186 Q09472 7189 7188 P12755 7185 148022 Q9Y6Q6 Q9BYM8 Q8IUD2 Q86UE4 57761 Q9UDY8 P01019 P01137 4869 P01375 8721 5970 P18074 3673 Q13546 55072 Q9NR96 3551 P51532 5728 P17301 O60674 P60484 3148 Q8IUC6 Q04206 3146 Q9ULZ3 P23497 P62942 O43353 P26583 6672 Q96EP0 O95999 7124 29108 O00463 Q9UQL6 P53567 P10276 Q7RTR2 P35612 P30048 P35611 1896 57669 23085 10616 Q9Y6K9 P04040 7099 7098 Q9NYA1 Q9Y239 Q15109 P14923 64127 P37231 3728 P01308 Q8TAI7 3169 Q9HCM4 8737 5468 Q9NQB0 847 P09429 10392 O14495 Q96FX2 O60869 Q9BUZ4 P61088 O15455 O14920 P05112 5914 Q12933 23476 P51617 Q9HC29 8517 4830 P05362 54106 Q13077

nuclear transport Q96QC0 P04637 Q86V81 57187 P35658 Q9P2R6 Q96FV9 P38646 O00148 473 2147 Q7RTN6 23165 4686 Q92973 Q9Y2X3 10772 Q9BQG0 P09651 Q9C0K7 Q92574 P49815 Q8WUM0 P25963 Q9NPC8 Q14974 Q8TD31 Q13769 O15131 Q01082 O75494 23214 8021 55696 Q96Q15 P42858 Q9UIA9 P52292 8536 406 Q9HAU5 P60468 O00327 6496 P52298 Q86U42 Q9UMR2 P52294 23144 P57740 P29590 Q03014 P30101 26019 Q96IZ7 Q96P70 Q9GZY0 10514 80145 23381 55341 Q00535 Q9UDY8 Q9NZQ3 Q01105 811 54535 P01137 4869 P01375 8563 4000 P62826 10482 Q9H814 P62829 Q86XR8 64901 O95343 23039 O60318 P61970 O60674 Q9UH92 1408 Q49AN0 Q8IXZ2 51319 O00505 O43592 7124 P28482 7248 Q9UPR3 O00629 P54253 P41182 1020 Q9H089 Q9P0J0 3836 10736 3313 84248 5976 11269 6945 Q14012 P05412 O95405 Q09161 Q9HCE7 Q9H2T7 3178 O75694 57154 Q86WV8 Q8NI27 P00734 6711 207 7249 3841 9702 604 3843 P62841 5901 P27797 Q86W42 4792

A1XKG3 Q12769 Q9NW64 Q96QU8 2247 P01579 P02545 5514 Q6I9Y2 P09038 2923 55705 Q9BY84 P49792 5371 7157 11097 P31749 Q9UKK6 Q96QD9 O00410 23633 Q08209 P52948 Q92621 O15504 Q92900 10204 P25116 51517

regulation of intracellular transport 7046 Q15654 2033 O43557 P10599 O60603 10657 Q04759 P84022 999 Q7Z6C1 6901 Q13485 P25963 Q9UBC1 4188 Q16635 7295 P50148 P46934 2316 9908 Q9HD26 Q99750 7205 P00533 4734 20 Q96EB6 O00206 808 Q09472 P52298 7067 Q8N726 Q8WVM8 23144 57120 Q06124 Q00535 P01137 Q9Y6A5 P01133 P01375 5716 6262 8165 4088 4089 8720 Q9BZF9 Q9NR96 9495 387 P21359 O60674 P10071 5566 5569 Q9Y2B9 O14974 2737 Q8IXZ2 Q92667 P24588 5460 7124 11142 1020 Q9UNN5 O00221 114548 Q93062 Q6PJW8 Q99728 P61586 P62158 7099 7098 Q6IR47 Q9NYA1 11030 10460 Q07666 P14923 23256 P36894 P10600 23411 P13796 1029 3728 1540 Q9NQB0 P42771 P49841 4659 Q99835 P21333 P61925 4792 Q14703 P12830 A1XKG3 2010 Q96P20 11124 O15455 Q9UN86 1956 Q9NQC7 P10827 Q60FE5 O75832 Q06830 580 P50402 P17612 2932 Q9UMX1 54106 Q15653

membrane organization23603 8411 8775 O75146 22872 23048 O75385 9066 351 P15498 1213 P21926 79778 90678 5018 P43034 P49815 6901 8408 P07996 10533 O95202 8546 8301 9632 Q16635 P12429 Q9UBC2 19 P55957 54361 Q08431 10427 P42858 30011 Q13492 P00533 4734 Q13133 92609 Q9UI12 928 O00602 4074 Q15436 3783 8676 P32121 Q12846 Q9Y6Q5 Q5T0N5 29924 57403 375 6809 Q9UKW4 4983 5830 Q9UJ41 P02741 P61966 P00441 P53675 8443 Q14114 23399 P56705 Q96JJ3 23032 6810 6812 O14579 948 P41743 3308 9784 Q12981 P07307 O95476 O95477 Q92538 O43752 10053 P50570 3320 1020 P10276 O00468 O75581 O95352 274 154 Q9UEU0 Q99965 58513 Q99962 2348 Q99963 O60763 3312 P07550 10059 4646 Q13286 Q9UL26 O95487 P32856 P53618 161 10868 P11142 P20645 Q96MV8 Q9Y5J6 O60895 3685 O60890 7249 8218 Q9GZM8 Q9UM54 7804 5868 Q9NRD5 9322 9685 P53621 Q8TEY7 Q3ZCQ8 P12314 2495 A1XKG3 2010 Q9P0L0 Q96EV8 O60749 Q14C86 P30542 22931 Q9NS69 284359 Q8TEH3 P61006 P21145 55704 1718 857 P02545 5877 P02786 859 1956 O94973 P51693 P53992 Q9UL45 26509 7037 O75558 P39905 7157 O95140 Q9Y5L4 1174 O00499 P37840 25777 O43747 Q8NBS9 Q8IVI9 1289 Q96ED9 O94979 P08754 Q9H4M9 Q9ULV4 P06576 Q15811 9342 9463 P07900 P98155 10490 O00429 P04637 Q9Y6W5 3482 9341 P50897 P15531 80124 P62993 Q99523 10938 Q92692 Q96RU3 Q96KG7 6622 P01116 9218 637 P01112 P01111 O15118 O95721 7057 Q9Y678 6643 6642 P51636 23210 Q8IYV9 P36575 P78537 P50542 O75096 Q8IUN9 Q3V6T2 7052 81567 P46934 Q15392 81565 Q8IWA4 Q14185 P47224 P05067 P42574 80230 408 Q15036 4218 P49407 409 Q01518 Q96NL6 10269 P02794 Q15276 23307 Q15836 6653 26258 3265 2054 Q9Y6I3 P56539 23787 O00203 Q00535 P16284 P21980 6647 Q92673 84062 9135 P11717 Q8TAT6 4240 P01130 50618 4000 Q8IY33 Q6UWE0 P34932 P54920 P21757 Q9NRR8 Q07954 Q96T51 Q9UH99 10487 9927 Q9NZJ7 Q6FGG2 5584 Q96B97 Q6NYC1 Q9Y2K6 P15328 P51149 433 10451 7879 8724 O15554 5338 3954 P08246 57706 Q9Y512 P35606 51606 Q8NEC5 9276 O95405 Q9NP72 P42261 Q05086 10228 Q07817 P16671 1785 Q5QGT7 Q15070 8615 5104 Q8WYP3 Q15075 375790 O60493 Q96JH7 Q9BY11 P10415 6453 O75844 6456 4035 Q9H902 6455 Q9H223 25801 2647 1315 P20339 1314 P20336 333 2885 55666 56993 Q00610 1793 Q9NZM1 P61764 Q9NZM3 Q15642 5914 Q14677 132320 O43581 Q5T1M5 Q16611 P20908 P06729 P62072 25813 P50402 O14939 56882 P84077 2773 6457 4038 4830 Q13596 Q99418 Q8N6T3 O15228

cellular membrane organization 23603 8411 8775 O75146 22872 23048 O75385 9066 351 P15498 1213 P21926 79778 90678 5018 P43034 P49815 6901 8408 P07996 10533 O95202 8546 8301 9632 Q16635 P12429 Q9UBC2 19 P55957 54361 Q08431 10427 P42858 30011 Q13492 P00533 4734 Q13133 92609

Q9UI12 928 O00602 4074 Q15436 3783 8676 P32121 Q12846 Q9Y6Q5 Q5T0N5 29924 57403 375 6809 Q9UKW4 4983 5830 Q9UJ41 P02741 P61966 P00441 P53675 8443 Q14114 23399 P56705 Q96JJ3 23032 6810 6812 O14579 948 P41743 3308 9784 Q12981 P07307 O95476 O95477 Q92538 O43752 10053 P50570 3320 1020 P10276 O00468 O75581 O95352 274 154 Q9UEU0 Q99965 58513 Q99962 2348 Q99963 O60763 3312 P07550 10059 4646 Q13286 Q9UL26 O95487 P32856 P53618 161 10868 P11142 P20645 Q96MV8 Q9Y5J6 O60895 3685 O60890 7249 8218 Q9GZM8 Q9UM54 7804 5868 Q9NRD5 9322 9685 P53621 Q8TEY7 Q3ZCQ8 P12314 2495 A1XKG3 2010 Q9P0L0 Q96EV8 O60749 Q14C86 P30542 22931 Q9NS69 284359 Q8TEH3 P61006 P21145 55704 1718 857 P02545 5877 P02786 859 1956 O94973 P51693 P53992 Q9UL45 26509 7037 O75558 P39905 7157 O95140 Q9Y5L4 1174 O00499 P37840 25777 O43747 Q8NBS9 Q8IVI9 1289 Q96ED9 O94979 P08754 Q9H4M9 Q9ULV4 P06576 Q15811 9342 9463 P07900 P98155 10490 O00429 P04637 Q9Y6W5 3482 9341 P50897 P15531 80124 P62993 Q99523 10938 Q96RU3 Q96KG7 6622 P01116 9218 637 P01112 P01111 O15118 O95721 7057 Q9Y678 6643 6642 P51636 23210 Q8IYV9 P36575 P78537 P50542 O75096 Q8IUN9 Q3V6T2 7052 81567 P46934 Q15392 81565 Q8IWA4 Q14185 P47224 P05067 P42574 80230 408 Q15036 4218 P49407 409 Q01518 Q96NL6 10269 P02794 Q15276 23307 Q15836 6653 26258 3265 2054 Q9Y6I3 P56539 23787 O00203 Q00535 P16284 P21980 6647 Q92673 84062 9135 P11717 Q8TAT6 4240 P01130 50618 4000 Q8IY33 Q6UWE0 P34932 P54920 P21757 Q9NRR8 Q07954 Q96T51 Q9UH99 10487 9927 Q9NZJ7 Q6FGG2 5584 Q96B97 Q6NYC1 Q9Y2K6 P15328 P51149 433 10451 7879 8724 O15554 5338 3954 P08246 57706 Q9Y512 P35606 51606 Q8NEC5 9276 O95405 Q9NP72 P42261 Q05086 10228 Q07817 P16671 1785 Q5QGT7 Q15070 8615 5104 Q8WYP3 Q15075 375790 O60493 Q96JH7 Q9BY11 P10415 6453 O75844 6456 4035 Q9H902 6455 Q9H223 25801 2647 1315 P20339 1314 P20336 333 2885 55666 56993 Q00610 1793 Q9NZM1 P61764 Q9NZM3 Q15642 5914 Q14677 132320 O43581 Q5T1M5 Q16611 P20908 P06729 P62072 25813 P50402 O14939 56882 P84077 2773 6457 4038 4830 Q13596 Q99418 Q8N6T3 O15228

actin cytoskeleton organization O15085 10093 10092 8655 3880 9181 Q7L0Q8 55561 Q9HBH0 P09493 118 Q92730 51763 P43034 P47755 P47756 Q92974 P03950 5155 O15530 P08727 Q8IZP0 Q12959 O95680 P52566 2317 2316 800 10420 P60981 Q16643 253260 Q9Y2I1 P53667 Q9UBU3 29127 P23528 4983 811 4867 P35080 Q8WZ42 4627 6386 Q9Y613 O95466 P07737 O75116 Q92949 Q16658 P68133 Q9Y2J2 382 5170 Q13829 P16333 387 81624 P21359 P52907 P60763 54822 P41743 829 8452 4771 7126 Q9UNF0 23002 1020 390 391 O43639 P35579 397 121512 830 23363 832 P61587 P61586 P45379 P46108 Q9H0H5 23136 Q9NSV4 P13796 23370 11034 O60890 P62328 604 9322 9564 Q6ZSZ5 Q8TCU6 10152 Q13976 Q13618 A1XKG3 Q9NYB9 Q96EV8 P62330 P42768 P15153 1398 P02549 5879 O43182 P60953 Q9Y490 Q9BX66 64423 11252 9578 P14625 P11233 O60879 27032 81788 253980 51474 Q9NZ56 Q6PID4 1729 Q6R327 P63313 54509 P98194 Q14289 Q9UL54 4690 P53365 P28289 Q9Y6W5 2275 26037 P52952 Q4VCS5 P63167 P11171 998 P01116 5898 6624 P01112 P01111 O43166 Q13643 P06702 9475 Q7Z6J4 Q96QB1 Q6DT37 1072 Q9BT40 Q05682 P84095 O60610 2039 2037 Q9Y4D1 Q96S53 408 1627 P49407 8936 Q01518 P98174 P50552 O00560 3265 Q96M96 6093 7184 Q00535 P35240 O15259 84062 Q92558 Q08495 Q14511 O43707 2059 Q9H093 Q92786 P01127 10487 23647 5584 P41182 P41181 Q9UHB6 P35612 P35611 Q68EM7 Q96P48 P63000 A8K0Z3 Q9H2D6 Q96PE2 Q8WZ19 10580 P68032 Q5JSP0 58480 7094 5590 23092 Q05513 9826 P17081 7408 P27797 Q27J81 P21333 O14492 Q9NVD7 Q86T65 Q96GA9 P10415 Q6ZV73 Q9Y5S2 Q12929 P15311 55785 10552 Q15642 Q13464 7430 Q60FE5 Q9NP98 Q9UNA1 P00519 Q96QT4 Q9H4E5 Q99418

actin filament-based process O15085 10093 10092 8655 3880 9181 Q7L0Q8 55561 Q9HBH0 P09493 118 Q92730 51763 P43034 P47755 P47756 Q92974 P03950 5155 O15530 P08727 Q8IZP0 Q12959 O95680 P52566 2317 2316 800 10420 P60981 Q16643 Q12965 253260 Q9Y2I1 P53667 4628 Q9UBU3 29127 P23528 4983 811 4867 P35080 Q8WZ42 4627 6386 Q9Y613 O95466 P07737 O75116 Q92949 Q16658 P68133 Q9Y2J2 382 5170 P12883 Q13829 P16333 387 81624 P21359 P52907 P60763 54822 P41743 4637 829 8452 4771 7126 Q9UNF0 23002 1020 390 391 O43639 P35579 P35580 397 121512 830 23363 832 4643 P61587 4644 P61586 4646 P45379 P46108 Q9H0H5 23136 Q9NSV4 P13796 23370 11034 O60890 P62328 604 Q9UM54 9322 9564 Q6ZSZ5 Q8TCU6 10152 Q13976 Q13618 A1XKG3 Q9NYB9 Q96EV8 Q9Y4I1 P62330 P42768 P15153 1398 P02549 5879 O43182 P60953 Q9Y490 Q9BX66 64423 11252 9578 P14625 P11233 O60879 27032 81788 253980 51474 Q9NZ56 Q6PID4 1729 Q6R327 P63313 54509 P98194 Q14289 Q9UL54 4690 P53365 P28289 Q9Y6W5 2275 26037 P52952 Q4VCS5 P63167 P11171 998 P01116 5898 6624 P01112 P01111 O43166 Q13643 P06702 9475 Q7Z6J4 Q96QB1 Q6DT37 1072 Q9BT40 Q05682 P84095 O60610 2039 2037 Q9Y4D1 Q96S53 408 1627 P49407 8936 Q01518 P98174 P50552 O00560 3265 Q96M96 6093 7184 Q00535 P35240 O15259 84062 Q92558 Q08495 Q14511 O43707 2059 Q9H093 Q92786 P01127 10487 23647 5584 P41182 P41181 Q9UHB6 P35612 P35611 Q68EM7 Q96P48 P63000 A8K0Z3 Q9H2D6 Q96PE2 Q8WZ19 10580 P68032 Q5JSP0 58480 7094 5590 23092 Q05513 P60660 9826 P17081 7408 P27797 Q27J81 P21333 O14492 Q9NVD7 Q86T65 Q96GA9 P10415 Q6ZV73 Q9Y5S2 Q12929 P15311 55785 10552 Q15642 Q13464 7430 Q60FE5 Q9NP98 Q9UNA1 P00519 Q96QT4 Q9H4E5 Q99418

positive regulation of catalytic activity P54619 P37288 P84022 P63208 Q9ULW0 P07996 P52564 25913 Q12834 O15530 P50148 5706 5707 5708 5709 P16473 51099 P16471 5700 5701 5702 P10809 5704 5705 Q5S007 P29992 Q06124 P16435 22974 5717 57761 Q9UKW4 P61289 Q9Y297 5713 4869 5716 P31946 3672 P46089 3673 P48023 Q92949 P99999 Q9BZF9 Q9NR96 Q13705 9093 P21917 Q99460 P17301 O60674 P20827 P18509 Q9ULZ3 3667 5604 3309 P04233 Q8WXG6 Q13956 O00220 P51668 29108 1020 P51665 114548 P07550 Q92530 P51671 P30556 3690 P30679 Q9NYA1 Q9Y239 O00231 O00233 P10600 P10721 O00232 P30793 3329 1029 Q9BUB1 10746 79444 Q6ZW31 P42771 7804 P05106 O95136 O95257 Q9UGJ0 P28223 6850 1285 Q13616 P16118 O00487 P30542 P18545 O60503 P62333 2247 Q96N67 P20618 P60033 975 Q5TCX8 857 O14763 Q92878 Q96CA5 O43182 Q92633 Q9UL46 P51575 6500 P31749 O95382 O00255 P17342 P37173 5524 Q9UL54 Q9UKT4 P06213 P01241 P23396 P04637 5300 P56199 P38405 991 Q96RU7 Q8WTS1 2147 P01116 Q9C0K7 6869 Q15389 P62873 11345 10016 O43166 64682 4221 6885 Q99750 4214 648 4216 85360 Q01518 P49768 O00206 5682 5562 5683 6772 Q8N726 5684 5685 5686 P14416 P52735 23787 891 O14727 894 Q00535 896 8945 P01019 P25789 P25788 6647 P01137 P01135 P25787 P01133 P01375 4361 5692 5693 5573 P04626 5575 5576 P04629 2064 Q9NUX5 P35348 P35226 55750 3148 3269 3146 668 5566 5687 5567 5688 10486 P01127 5568 Q96EY1 5207 10487 P26583 5585 552 Q96P48 553 Q99683 P51828 10451 5577 10213 7518 P55072 10454 2081 O43242 Q6IR47 7410 5590 P37231 Q01955 Q15077 8737 207 Q9H1A4 5347 5468 7529 Q08462 P24385 8754 4035 O00762 Q9BUZ4 O75293 Q96P20 7415 10672 P25103 9839 5371 8881 Q12933 6464 9973 P17980 P52824 P52701 23513 P17612 O14818 P51617 P58012 8517 54106 P25116 P60568 Q8TEW0 11065 Q9Y4K4 Q9Y4K3 3084 O43318 P13861 595 356 23043 116 P09493 P49810 P22694 P49815 P03950 3091 7334 5155 P55036 Q16512 4188 Q9UKE5 5029 P00533 Q16401 Q13133 Q14344 1902 Q15311 Q13131 8795 P11309 Q9HBW0 8555 P35998 Q9Y6Q6 Q9UBU3 Q9UDY8 P09471 10197 P61604 P00441 P32239 Q53H12 Q9Y6R4 Q14114 4088 P55055 O43306 5170 P21359 P60520 Q16665 P43246 Q13153 Q9NSD7 8454 7124 7248 P63092 P14635 O75340 O75460 154 Q04837 Q9Y2C9 121512 Q9Y6K9 Q13042 P45379 7013 7376 Q86WV8 P35568 P11021 Q8NFM4 P01308

Q8NFM5 120892 7249 P49721 P49720 Q9GZM8 2915 85440 Q9NRD5 10392 P11229 P54274 A1XKG3 O43612 P48507 P49959 55704 P01579 51107 1956 P09038 Q15008 6188 P39905 7157 O75553 Q02750 Q9UNI6 23118 Q14160 51231 Q6PID4 1609 Q9BWT7 Q15257 Q14289 2935 9463 7046 P98155 10928 26037 P42224 26271 P62195 P23945 Q7RTN6 Q92574 P22612 1616 P78536 7057 Q13526 Q96QB1 Q02297 P10644 P31321 Q3V6T2 7052 P31323 81565 P05062 P08575 P60900 P42338 Q9UER7 2956 7186 7189 P06239 P29353 P29474 O95977 Q96M96 P29590 Q13418 P31431 P40145 Q9BXK5 P41231 O60266 P21980 P42684 3932 O95622 Q13426 Q13546 4914 P43405 56288 2730 Q07954 Q92786 Q92542 Q13794 Q9NZJ7 P13945 P78509 B3KY43 Q03468 Q13200 P08246 7099 7098 Q13443 Q96BI3 P30279 P61073 Q9UJX2 Q05513 P00734 P30281 Q13574 Q15750 P46734 P09429 Q9NYJ8 P10415 P28065 Q9P035 29843 2643 3611 P61088 Q04656 O15455 P01730 P08588 O75832 P53350 Q9P0U3 P62191 P28074 P28072 P28070 P00519 P30260 Q13233 P43686 P07148

negative regulation of programmed cell death Q9BZZ5 P25054 8535 Q9BY76 399687 Q12948 Q04760 Q9Y4K3 3084 O43318 P17676 472 O15519 3516 2303 355 P84022 P07196 O00273 9616 Q92731 P49810 8767 P78395 5139 3875 O14543 Q9Y275 Q92614 P07996 2308 P25963 Q13489 P52565 Q86VP1 Q9H3Z4 P14174 P42858 P16471 P46531 4851 8539 P00533 10542 P10809 Q92843 5705 P11309 6495 Q7L5Y9 Q9NY61 O75360 Q9UBU3 P20936 O60543 Q9UDY8 P23528 P03372 P63104 6926 Q6IT96 P55290 P00441 4869 Q9BWQ8 5716 9531 5054 Q13268 9530 5055 4088 5970 P18074 2100 3551 57099 9093 Q9NRZ9 5728 387 3304 P19838 Q99576 P20264 P55061 P43246 P41743 3309 P04233 P08758 Q12981 Q12982 Q8TDY2 7124 Q9UHJ9 396 P15056 3315 1499 3313 1012 P04040 10296 Q99728 P61586 Q13286 Q9NYA1 P22392 Q9Y4H2 P10600 P10721 Q96J02 P19544 7490 Q06330 Q9UEE9 P37198 3329 57678 P11021 79444 960 1026 P01308 7128 Q9NQB0 604 P49841 847 5624 Q99958 Q15475 Q99835 P04179 4790 P61803 4792 Q9NQS1 P30542 1831 P48507 57448 Q9HCL2 51100 Q9H1Y0 O95817 Q9Y371 P08069 1718 Q96CA5 P05112 5516 1956 P23443 Q13501 P09958 Q12778 P39905 7157 P51692 P31749 O43504 P14625 P38936 O00257 P37840 P35548 2932 81788 Q8NBS9 Q13075 P05120 P05121 P48736 P09601 P40337 P04156 Q15811 P78423 7046 6198 P04637 7161 O43715 P50897 P38646 6194 P52952 Q99523 3479 10935 P62195 P04083 6622 7832 P01116 Q9C0K7 Q15389 P42229 O15119 P62753 P01112 P01111 P25445 Q9C0K0 P78536 9474 7057 6885 Q02297 P16989 1072 83737 1191 27252 81567 Q15392 P98170 P16070 P42574 P01343 P42336 P49768 7189 P29474 Q9H422 3265 P18583 Q13418 6093 7184 P13693 773 Q9NR09 P01019 O15379 P21860 6647 P01138 Q6UXV0 P01375 O15392 P04626 Q13547 Q13426 8841 O95865 P04629 2065 2064 P35222 P14784 P35227 4914 Q96ST3 663 301 2730 P60484 P04070 Q04206 Q9H093 6776 P02458 P05164 307 Q96EY1 P09525 8837 P49747 Q9UBN6 O43353 9821 5584 O95999 7520 Q13315 Q9UBF6 P41182 P30044 57144 9021 Q9NX61 25942 3162 P30048 673 P15692 5336 P62714 P09211 P08243 7518 Q6IR47 P21580 7534 Q07820 23532 O76024 5590 Q07817 64919 P51959 Q05513 324 3169 207 P05783 P08138 23746 7422 P10415 6453 P56177 2099 3065 Q9Y2M5 330 331 573 1676 P13010 3611 5599 10673 O14920 Q13464 Q14432 23636 O15350 3070 P08107 P53355 O75832 8887 27315 Q9P286 Q04771 580 P16885 O60238 P51617 900 O95429 7428 P45983 8878 10202 P04792 P25116 P60568 Q9NZN9

regulation of gene-specific transcription 6595 6597 6598 2672 P15923 Q12824 2547 P16220 P84022 Q92731 P25963 7332 3091 Q16512 Q15306 Q5VTR2 O75496 O75376 O60216 O75381 367 P46531 4851 Q13133 Q15796 Q96EB6 5705 6256 6498 P12757 Q99471 23028 P46527 6927 6929 Q9UJU2 O15534 O75928 Q13263 Q6IT96 5716 4087 4088 7476 5970 Q92949 P55055 2100 Q9Y618 Q9NR96 10524 P20823 P17542 P19838 Q16665 P43489 9421 5187 7124 1385 P19532 Q9UQL6 P10275 4092 P10276 1499 Q99729 5195 7376 Q92769 93986 O15055 P28370 Q14938 O95361 O43524 P10600 P19544 7490

166 10626 1027 P01308 P08651 Q9NQB0 Q96T37 4790 7023 4792 22937 P38398 Q01664 Q9BZK7 Q92753 6721 Q92993 857 10155 Q12770 P05112 P49715 Q9UKS7 Q12772 P10827 P39905 7157 Q6KC79 1050 1609 Q9UMX1 50943 P06213 O96004 6872 P04637 7161 3481 O60603 25988 P52952 3479 P62195 3476 10014 Q9UBK2 P56524 22926 22807 P33076 4214 P01588 408 P49407 P01344 P01343 O00206 7067 P19793 Q03014 6096 P41235 Q53X93 O15379 6886 P01137 P01375 Q9UPY8 P51531 Q13422 Q13547 A4D1W7 8841 P35222 P78317 P51532 P78318 P17813 3148 Q04206 P10071 Q92786 2737 P26583 O00744 5460 Q15853 P25490 5585 Q8TAK5 672 P22415 Q99684 5579 P05771 2626 7099 5591 O00755 4261 7098 8864 Q9NP71 Q08117 23411 P18850 P37231 3169 5468 P17081 7528 Q9HCU4 P23510 P12956 P56177 O60907 3066 3065 P78527 3182 Q9BQA5 P68036 O60341 P43694 O15455 5914 O15350 P27540 O75832 9612 Q9BZS1 Q13227 P19484 P52824 O15105 10320 54106 Q13233 P21675

negative regulation of apoptosis Q9BZZ5 P25054 8535 Q9BY76 399687 Q12948 Q04760 Q9Y4K3 3084 O43318 P17676 472 O15519 3516 2303 355 P84022 P07196 O00273 9616 Q92731 P49810 8767 P78395 5139 3875 O14543 Q9Y275 Q92614 P07996 2308 P25963 Q13489 P52565 Q86VP1 Q9H3Z4 P14174 P42858 P16471 P46531 4851 8539 P00533 10542 P10809 Q92843 P11309 6495 Q7L5Y9 Q9NY61 O75360 Q9UBU3 P20936 O60543 Q9UDY8 P23528 P03372 P63104 6926 Q6IT96 P55290 P00441 4869 Q9BWQ8 5716 9531 5054 Q13268 9530 5055 4088 5970 P18074 2100 3551 57099 9093 Q9NRZ9 5728 387 3304 P19838 Q99576 P20264 P55061 P43246 P41743 3309 P04233 P08758 Q12981 Q12982 Q8TDY2 7124 Q9UHJ9 396 P15056 3315 1499 3313 1012 P04040 10296 Q99728 P61586 Q13286 Q9NYA1 P22392 Q9Y4H2 P10600 Q96J02 P19544 7490 Q06330 Q9UEE9 P37198 3329 57678 P11021 79444 960 1026 P01308 7128 Q9NQB0 604 P49841 847 5624 Q99958 Q15475 Q99835 P04179 4790 P61803 4792 Q9NQS1 P30542 1831 P48507 57448 Q9HCL2 51100 Q9H1Y0 O95817 Q9Y371 P08069 1718 Q96CA5 P05112 5516 1956 P23443 Q13501 P09958 Q12778 P39905 7157 P51692 P31749 O43504 P14625 P38936 O00257 P37840 P35548 2932 81788 Q8NBS9 Q13075 P05120 P05121 P48736 P09601 P40337 P04156 Q15811 P78423 7046 6198 P04637 7161 O43715 P50897 P38646 6194 P52952 Q99523 3479 10935 P04083 6622 7832 P01116 Q9C0K7 Q15389 P42229 O15119 P62753 P01112 P01111 P25445 Q9C0K0 P78536 9474 7057 6885 Q02297 P16989 1072 83737 1191 27252 81567 Q15392 P98170 P16070 P42574 P01343 P42336 P49768 7189 P29474 Q9H422 3265 P18583 Q13418 6093 7184 P13693 773 Q9NR09 P01019 O15379 P21860 6647 P01138 Q6UXV0 P01375 O15392 P04626 Q13547 Q13426 8841 O95865 P04629 2065 2064 P35222 P14784 P35227 4914 Q96ST3 663 301 2730 P60484 P04070 Q04206 Q9H093 6776 P02458 P05164 307 Q96EY1 P09525 8837 P49747 Q9UBN6 O43353 9821 5584 O95999 7520 Q13315 Q9UBF6 P41182 P30044 57144 9021 Q9NX61 25942 3162 P30048 673 P15692 P62714 P09211 P08243 7518 Q6IR47 P21580 7534 Q07820 23532 O76024 5590 Q07817 64919 P51959 Q05513 324 3169 207 P05783 P08138 23746 7422 P10415 6453 P56177 2099 3065 Q9Y2M5 330 331 573 1676 P13010 3611 5599 10673 O14920 Q13464 Q14432 23636 O15350 3070 P08107 P53355 O75832 8887 27315 Q9P286 Q04771 580 O60238 P51617 900 O95429 7428 P45983 8878 10202 P04792 P25116 P60568 Q9NZN9

response to endogenous stimulus 64397 P08833 P28562 3880 Q9Y266 O14793 P51587 O60568 P37288 2303 2783 Q92731 O14543 P08842 P07996 2308 2549 O15530 P08727 Q92729 54361 Q08431 51094 P16234 2 P63218 O15524 Q7Z727 P28300 P50150 Q06124 57761 Q9UQB8 P03372 Q92831 P55851 P07858 5970 3673 2100 P21917 P56705 P54646 P17302 5728 P17301 O60674 2697 3667 5604 P41743 Q92820 P28482 P15056 57669 1499 1012 P09972 P08887 Q9NYA1 P30559 O95361 P10600 P37198 Q9BUB1 57678 10626 1026 1147 841 Q9HCM4 2475 22937 Q06203 P16118 O60502 P38398

O60503 Q9HCL2 P61244 610 1277 Q92753 6721 857 Q12770 Q12772 P10827 P27144 Q12778 P51692 P31749 Q9NR50 1176 1050 P18433 Q9HCK4 10724 10726 Q9UL51 P09960 P05121 P09601 P04155 7706 26060 P06213 P02452 P01241 P14317 2033 2274 3484 O00300 3481 O60603 P52952 Q96RU7 P62993 Q99523 2026 7832 P01116 P62879 6869 Q15389 P27986 P62873 10014 P49770 P35368 1072 Q9BT40 Q96T76 O76074 O14717 768 P01588 P01344 Q99757 P01222 P01100 1080 O00206 Q09472 6772 5321 5563 6774 P14416 P13569 P14410 890 894 Q14192 898 5798 6647 P01138 P01137 O14965 P01375 P50440 4240 P01130 5573 P04626 5575 5576 2065 2064 P35222 P35348 P60484 3148 Q15291 P01009 5566 8836 6776 5567 P01127 5568 5327 5207 Q59EA4 P26583 6790 25828 4131 5584 P07948 3162 10458 672 552 673 P51828 5577 5578 5337 5338 P40763 P04201 P01275 5591 O00755 P05413 5471 Q6IR47 8985 10580 P37231 682 P51959 445 3169 4015 207 5468 329 P48357 Q92922 P01023 Q08462 P24385 8754 6696 Q96A54 2099 P51843 Q8NER5 Q9NRM7 P68036 8503 4149 P25103 10432 P01178 23636 6464 O60911 P17612 Q6ZWJ1 O14939 Q08209 P18827 8892 Q14686 5265 6599 8654 P30086 Q9H2Y7 P13861 230 595 Q04759 P07196 P20396 Q7Z6C1 51763 P22694 Q13480 P03950 Q15303 7332 5154 5155 4067 Q9HAV0 P12429 5037 367 P47985 Q13370 Q13490 Q13133 20 P35638 Q15797 6256 P31153 P59768 P10451 Q9UBU3 P23528 P09471 Q13144 Q14232 P55290 P00441 P32239 5295 4086 5054 10081 7476 Q14118 O43306 Q01581 P68133 58189 Q9UKG1 P35625 5170 142 1808 P24864 Q13393 P23771 P43246 Q13033 7124 7248 P63092 Q9UQL6 P10275 P10276 P09172 10057 Q16555 Q16790 Q99966 7252 Q14258 Q9Y4H2 26524 Q86WV8 P35568 Q8NFM4 Q8NFM5 Q99958 9564 7386 P49840 Q86V24 Q96PK6 P21266 P08069 2805 P49715 P23443 Q9BX66 Q02750 P38936 1843 2931 Q6PID4 Q14289 1605 P25874 7046 6198 P98155 O95835 P42345 P42224 Q9Y4C1 P17275 Q92572 P04085 Q92574 P46821 P22612 P42229 P15090 2947 1978 P78536 7057 Q96QB1 P10644 P31321 P31323 P05181 P05062 P06276 Q92569 Q9UBL3 7067 P29353 P29474 P19793 Q03014 6096 P40145 P17252 P00966 O60266 P21860 P09874 O95622 8165 P55212 P10620 Q8N8D1 Q04206 P07101 Q92667 P41181 9021 P35613 B3KY43 P22415 P05091 Q9HC57 P08243 3953 2626 P47712 7099 Q13322 Q13443 P30279 Q07817 P20248 P17081 P09467 P09104 O14492 Q16849 P10415 29966 P78527 P18146 2885 P17174 Q14790 P43694 5914 P41159 O15111 P34972 5929 900 P00750

cellular biosynthetic process O75947 8099 Q9H6Q4 P54619 P51587 P52434 P17676 Q9BYG3 2305 55568 2300 6901 Q9H0D6 P28335 O75936 25913 P30876 P08729 O14561 P52209 Q8NE71 Q06136 P37268 P19623 10667 2 1468 P62244 80755 P14174 P42858 P62241 27090 Q9Y285 140801 Q96EB6 64282 P62249 P27694 Q9UKV8 P27695 3661 23708 2572 2571 P17405 6927 P40429 Q9Y295 5832 5833 Q9Y291 Q9UKW6 5836 3659 10632 P54886 Q9UIS9 P08708 P26373 Q99583 Q12857 1491 P54646 Q8IYK4 P20823 P62266 Q8NFW8 P19838 Q9BYD1 P62263 148789 P62269 10643 Q9Y3U8 P11926 Q13951 Q9Y223 4772 1022 2593 Q9NSU2 2590 55361 Q8WVC6 O95352 P39019 27043 Q5T160 1017 79577 2589 Q6PI48 P39023 833 5610 O75909 Q9Y231 5859 Q9Y478 4780 3692 Q13724 5631 80308 Q9UGI9 Q15904 Q8WVB6 O95363 Q7LGA3 27297 P42898 O14519 10625 Q96MV8 1025 P61353 P20648 4775 P05023 P07686 O14757 Q92766 P05026 P04179 4790 Q9UGJ0 5883 P28340 2132 1040 P16118 Q9NR45 Q8IUH5 Q9NR48 Q9BWE0 Q9HCL2 55149 80347 P60033 Q96N66 Q9Y250 Q92994 5875 6723 P11908 5636 4548 Q10472 Q10471 P51575 P10827 P10828 22828 P31749 Q9NR50 Q96L21 Q9NR55 1050 55152 27032 10606 253980 O60870 865 P04150 Q9Y262 4799 5888 P06576 O96004 64432 5422 O00303 23569 11108 Q8NBJ5 3479 51363 Q13085 P25205 84289 Q15025 P62875 6749 Q9BTU6 Q9Y676 P49411 5430 Q86SR1 5432 P36578 P35244 P14324 P35249 Q99755 Q96T76 Q03933 641 P35251 P62888 P61313 5424 P49643 O43294 408 8939 P27635 P49407 P60228 409 O43252 84061 5440 P49642 5441 6772 5442 Q9UKN5 O96020 Q9UKN8 Q9BW92 Q66K89 29088 Q8IYD1 Q99741 Q96T60 P62899 5434 51148 Q14197 51147 Q9Y450 Q9UN42 10476 Q15046 Q15287 P50440 4361

Q9Y697 3035 Q9NUX5 Q53H96 23556 Q96ST3 55750 P14550 P17812 P15880 P35232 Q00403 29093 4598 Q15054 55757 10488 5207 Q59EA4 84296 P62424 79709 O00746 7884 P06737 10690 P50613 245972 P51946 P39656 23764 2194 25942 Q9Y5Q8 Q9NUQ2 Q9NWU5 Q8WUY8 P60891 435 P31939 128308 Q96GC5 Q07020 P06744 P05413 5471 O00757 3054 29079 10229 Q9UBX3 445 688 689 P49448 P82673 207 5226 Q02809 P82675 P62847 29074 P62841 Q7LFX5 P61513 P49207 4150 O00767 23746 4152 P24386 23509 Q9BRT9 5245 Q9HCD5 P52815 O00764 211 Q99543 4149 P62857 P62851 10432 57727 P62854 8761 Q92903 Q9H4F1 Q92901 P07814 Q92905 P35268 54460 3074 O75031 468 55312 P60201 Q92900 P56192 P49427 4172 4173 4174 Q14686 4176 Q14209 Q16864 Q9UPN6 64834 Q9H0U6 Q6NZI2 4171 Q96GX9 474 Q9H0U3 476 477 478 28973 Q99807 P46783 P83731 Q9C0C2 3091 P13639 Q16633 6123 8543 P46782 P13637 Q16635 6125 P46781 O75376 P41091 481 483 60489 488 P68104 60488 51773 6117 P22102 6119 P00533 6118 P46777 Q15554 P46776 P60866 P46779 Q13131 P46778 Q16649 6133 P35638 P61968 6132 11041 6134 6137 P56134 Q16647 P32121 Q9UNL4 O43776 490 O75362 29803 495 498 Q01105 6128 P56385 P82663 P82664 1915 P56381 Q13144 51547 Q14232 Q13148 Q9BYU1 6141 P35869 55907 P43490 8565 P18074 8566 P62829 Q01581 P18077 O75352 P36954 Q9P2E3 Q13393 6138 Q16665 Q9P2E9 9421 P78347 P54136 9420 6156 6158 P54132 P36542 P35453 271 Q9Y6K0 Q93063 P09172 Q04837 Q14494 Q14493 1937 1936 1933 6164 O15294 Q9NRC8 7013 6168 7014 P22392 O75575 6160 P11498 Q93050 Q7L2H7 11277 P49848 P61927 6175 10390 25885 Q02878 P48507 P24539 51102 P02549 Q96PK6 P24534 11243 P55010 55703 P47897 P00367 9439 P47895 2805 1956 6185 6188 6187 Q9HB90 O43741 P21283 P21281 Q86Y79 Q09028 5091 6182 P37840 5092 O75792 64428 6184 O43505 Q15370 Q92187 10166 1728 51116 Q99814 P98194 1968 Q7Z7F7 Q9NRF9 P78545 P54577 6199 Q6P1J9 P29375 P78540 P10515 P29374 Q96EL2 6193 6194 1975 Q05329 27000 2821 Q9Y4C1 P41252 P62195 P41250 Q6P5R6 P05177 Q13761 1737 1736 9477 375743 9232 22803 Q9P1Y6 Q9H2P9 1503 P24928 Q9BYN8 O60296 Q6P1K2 Q5D0E6 7068 7067 Q15714 Q13535 8394 P55209 P56537 1756 29880 Q6ZNC8 P00966 O60264 Q9Y2A9 P54368 O15270 9255 Q16816 A4D1W7 63931 Q9Y2B5 57017 Q9BWH6 P41222 P43405 201595 1763 Q04446 O15269 P53041 P07101 O95749 O95997 Q9P015 Q8N6G5 7083 P00505 P20594 P08243 4800 2626 51727 Q9NVC6 Q14653 P19387 P19388 P11413 93100 Q9H9A7 83548 2875 2632 O60256 1540 57038 80270 P22303 P09104 P46977 P46976 9045 P55884 P13807 Q9NP81 Q6P1L8 O75964 3980 28998 Q9P035 P32320 P32322 Q00059 2644 2643 P17174 Q96AE4 Q04656 P43694 3978 Q9Y4A8 Q13347 9054 9296 5927 5928 P63272 P24941 Q9HB03 4830 Q9HCS7 902 4833 Q9HD40 Q13112 51514 P21675 Q9NZN9 O15228 P10914 Q92858 O75821 O75822 65003 P67809 9061 O00268 Q8IXM3 51081 O00267 P30520 Q9P2R7 10535 P13196 P16220 P60174 Q9ULW3 5931 10533 P0CG13 P48047 Q12952 4860 Q9NRX2 Q9Y399 54361 55454 51099 Q9UI12 5705 P25398 Q9ULX9 Q9Y2Y8 Q9NRY4 1121 Q9BZG8 Q12968 P18621 O60547 P18858 1355 P12081 P18859 1111 P63220 O60783 P32929 Q92830 P03372 Q9Y2Z4 P49591 Q92824 P09917 Q9Y2Z9 4882 Q8NCR0 Q6P2C8 9093 P56705 P49590 Q13829 O60318 10767 Q8WTX9 O60551 P49588 P07741 P04233 P30566 O95477 5981 Q12986 5983 1385 Q9UHJ9 57661 6829 P19525 Q6FHQ0 O60762 2224 O60760 5733 5976 27161 6827 Q9Y2T7 Q9NYA1 Q14938 O00472 Q5JTZ9 P30793 Q9NS56 Q8WYH8 10987 57678 2237 1388 P54709 P08651 Q92643 Q6XQN6 P05386 P05387 Q6ZWT7 P05388 Q9UNX3 Q9NYB0 O43175 P30304 P28223 137964 6850 P32969 P30307 4673 Q06203 Q9H9Y6 6609 P42766 80222 10714 P61244 2247 284119 975 O14641 P61247 O14640 Q6GMV3 5514 Q92878 5757 P08865 Q9UNY4 Q15800 161823 P09958 P30536 P52788 Q03164 55035 Q9BZJ4 10725 740 Q99259 Q9P0M9 P61254 10728 P09960 4677 Q6DHV7 4678 P06454 Q9Y2W1 509 O00425 P23396 6872 Q9UPV9 6871 P04637 2034 P16615 P53803 Q00796 P15531 990 P11172 P62750 Q8WTS1 2026 995 513 997 514 8803 6622 515 P61421 11222 Q16236 5536 Q15389 518 P62753 P49770 200916 Q9GZR5

Q9UM07 6883 P48201 25873 P12268 O76071 521 522 51253 P11182 523 Q9Y6Y0 526 6878 8812 P01588 528 Q99638 P01343 P49768 P26641 P26640 5562 Q9BV79 5321 5563 5565 P11388 P11387 O60701 51021 51023 P26639 533 51264 535 Q53X93 537 6647 5557 P61201 P01137 539 5558 91949 P01133 23438 Q9BT22 23439 Q9Y2R5 P01130 Q9H7H0 6660 P40938 P40937 3396 23435 Q9Y2R9 P12004 Q9NS37 Q99611 Q96RR1 545 P09086 P61457 8833 Q96EY1 P61218 Q5TKA1 O00628 7764 Q12904 23400 P17752 Q96PU8 54550 Q8IZL8 3159 Q02930 Q9BZE1 5336 Q15185 5337 10331 P46063 5351 Q9H5J4 P24298 6201 5111 6202 23650 Q9ULK4 23411 O76021 Q969G6 Q99437 P18847 P18848 P83881 P49327 328 Q08462 6210 P50750 P49321 2099 P50993 O14802 10318 51067 56994 Q8N8Y2 6204 6207 P62979 10554 P83436 Q9H5H4 P04424 8880 A2RTX5 P27540 7311 391356 P39748 P15559 P51610 O00411 Q9NVV4 P15313 P16885 51074 P52948 Q9NPJ6 P62988 54107 6217 9967 Q14566 6231 8892 4297 Q6DKI1 6233 P15927 64951 P62917 Q9UKD1 P30085 P13984 6470 4291 Q9NYU1 Q9NYU2 Q03426 64949 353 Q9UBS0 P62910 6229 P62913 8662 P20290 P03950 8424 O00716 64960 16 8667 Q16514 8665 19 64963 63875 26205 64965 P13995 64969 367 Q9UBT6 51650 51651 6238 11198 54921 6239 Q76FK4 64975 P31153 29922 Q03405 P49916 P49915 P25705 P17096 8669 54938 P49914 P21589 51426 31 Q14353 P00441 34 Q53H12 8443 O75478 9533 7358 Q9Y619 Q86UL3 P29084 64745 P34896 P11310 142 Q02543 P62701 P09001 P20020 9526 P62945 P23771 1801 O15160 P48651 8458 O15164 2909 7126 Q7Z6G3 P38606 P10275 23127 4094 P27708 158 Q9Y3E5 2908 P23588 84365 Q13287 Q9NSE4 Q14134 Q99966 Q8WV24 P49736 P04843 5198 P04844 8467 O43889 P15735 Q9BW60 7372 P35573 Q8NFM5 Q01433 Q13057 8473 P00491 O75208 P00492 P61803 7389 P54274 Q9HA82 Q96FX2 O75444 P11586 Q96T21 Q99943 Q96T23 178 Q99941 P49959 P50914 Q00577 51222 P22234 9319 Q14152 Q9UBQ5 Q16576 Q99707 9318 P49716 P49715 P09038 11128 Q9NQC7 7153 Q08050 7157 P62906 P14868 7150 1841 2935 P78424 Q13873 7167 Q7KZN9 Q96QC0 P33992 P62081 P33993 P33991 P18124 1854 P42224 P47914 Q9HC16 Q92696 P82921 1856 Q9UBK2 O15371 P82912 Q8WUD6 9114 O15372 P82914 Q13405 22928 O95602 Q13887 Q96I24 Q9H7Z7 Q8TCJ2 55173 Q9Y3B7 P84098 P63173 Q08945 P05062 1629 2959 Q9UBL3 P29474 P29475 O43812 26015 O75881 286826 1877 P00846 4907 2961 2960 1870 P04183 Q9UHY7 P23921 Q9BTC0 2969 P09874 O95865 29777 79143 10921 P42677 Q9Y3D3 2730 2972 Q9Y3D5 P09884 Q9NYP7 P06132 P82932 Q969Q0 P82933 P30049 Q15853 P33316 Q14527 56052 Q9H488 P62280 P30046 P30047 P62277 P22415 P05093 P30050 Q03468 1650 58478 Q8NFF5 P43115 2747 P47712 O95644 51606 9397 Q8WZ19 9391 P11532 58487 P23511 51611 P09467 Q9Y5B9 Q9Y5B8 P35711 3615 3614 2762 57169 Q6P4R8 P01730 Q5T4B2 4946 51629 P54687 Q15643 O95427 Q9P2Y4 Q15648 Q14558 27315 2531 4953 Q15653

positive regulation of protein ubiquitination 11065 11186 5300 991 26271 Q96RU7 P62195 P63208 8767 Q15389 64682 7334 P55036 Q12834 Q13526 2280 5706 5707 5708 5709 648 5700 Q16401 5701 P60900 5702 5704 5705 Q5S007 5682 5683 5684 5685 8555 5686 P35998 Q9NS23 148022 891 5717 57761 8945 P25789 10197 P25788 P61289 Q9Y297 5713 P25787 5716 5692 5693 9093 Q99460 P35226 Q9UNE7 Q8IUC6 5687 5688 Q96EY1 3309 P62942 O43353 8454 O95999 P51668 P14635 P51665 4092 672 Q13042 Q92530 10213 Q13200 Q96FA3 O43242 Q9NYA1 Q9HCE7 O00231 64127 O00233 57154 O00232 P11021 Q9UJX2 120892 Q9H1A4 5347 P49721 O15169 P49720 10273 P28065 Q13616 O00487 O00762 57162 P38398 P62333 P20618 P61088 Q15008 Q9BT67 Q9UL46 8881 O75832 P53350 6500 P62191 P28074 P17980 P28072 P28070 O14818 Q9HC29 P30260 O15105 Q9UKT4 P43686

regulation of organelle organization P25054 10092 10095 4297 10094 P30086 9181 472 Q9BVA0 4609 Q04759 P84022 P09493 118 Q7Z6C1 Q9ULW0 P49815 P47755 Q9HC98 P47756 Q92974 O43683 11190 25913 5154 7334 Q9UBC3 5037 65018 1104 P61158 10783 Q15554 Q9BYV2 Q13813 253260

O75122 22974 P53667 P20936 P23528 81873 Q01105 P35080 Q92833 P26358 4869 Q9BSI4 5716 4088 O75116 382 Q02790 O75592 P16333 387 Q9NWT8 P52907 Q6PKD3 Q15691 Q8IX90 829 Q9Y468 4771 7124 7248 23122 P14635 O43639 10733 6709 1499 57662 830 832 Q13042 P61586 O14744 P51671 7013 7014 Q86WV8 5071 P01308 2475 6711 6710 P62328 7249 Q9NYB0 Q9H2K2 P30307 P54274 Q9BPX5 P62330 P38398 221150 Q9BVC4 P02549 55704 P49711 857 859 Q9NPA3 O43182 Q9NQC7 7157 O00255 Q6KC79 P37840 P48061 Q8TAP6 Q03164 7272 51474 P27816 Q6R327 P63313 Q8N3V7 50943 P06213 4690 O95714 O95835 P04637 P10636 5300 2033 O95271 3481 Q4VCS5 P42345 Q9Y6G9 Q9Y3A5 Q9BXM7 3479 995 Q99640 P04083 8924 6622 P04085 54998 Q92574 P46821 6869 11346 P07355 Q9UBK2 9475 4221 Q13526 Q96QB1 P51636 23332 Q01082 1072 Q3V6T2 2039 Q7Z460 22919 4214 P01106 Q9UI95 408 P49407 P01344 P01343 O15020 Q09472 Q9BV73 5321 O15143 Q7Z7K6 P29590 Q13418 O00444 P56539 P31431 891 P35240 79959 P01137 P01135 O60260 Q86UE8 P01133 P01375 Q08495 64223 91147 Q9Y696 P35222 Q9NUX5 P78317 9495 P11277 301 302 Q92786 P24588 5460 4131 9700 4133 Q13315 Q15735 9820 Q9UHB6 P35612 P35611 10459 672 Q96P48 Q9H2D6 P47712 P51955 Q5HYA8 1789 10109 1786 Q9UJX2 324 1540 57159 4137 3720 P17081 23189 P20336 3611 P61088 10552 P25103 Q14674 5371 Q14678 8881 O75832 Q9BZS1 23077 Q13233

regulation of developmental process P25054 Q92858 O14793 2672 259266 P51587 Q9BVA0 2547 Q8IYN9 Q7L0Q8 4609 284217 P84022 Q9HCJ2 O14786 O14543 O14788 P07996 P52565 Q12834 4860 Q9UBC3 Q12837 54361 55692 P50148 P16471 Q9UKV3 4851 P61158 4854 Q96EB6 O15525 O75925 Q7Z727 136319 Q9ULX9 Q92835 P29992 P05549 P25391 Q9NRY4 Q05397 P53667 4628 27185 P20936 57761 811 Q9UJU2 55109 P03372 O75928 P40424 6926 4627 P06400 Q9GZX9 Q9NQX0 3791 P54760 5970 P48023 Q92949 Q13705 P56705 Q7L576 22985 P17535 P17302 57534 79784 5728 P17542 2335 O60674 P20827 P19838 2697 948 5604 P04233 Q9Y468 P27037 O95477 P28482 1021 O00468 10736 57669 79576 1499 P07550 P61586 Q9Y2T1 3679 P08887 5978 719 Q9UM47 P51671 O00238 P30556 3690 22846 Q9NYA1 4780 Q92769 P30559 O95361 Q8IZT6 P10600 P10721 O95365 Q9BPY8 Q9UEE9 1029 3207 10626 1387 Q9HCM4 3685 604 P42771 3689 Q92888 5747 P04179 P05106 4790 P05107 6850 4792 1285 1284 Q7Z406 57689 Q9NQ29 2247 2246 Q9Y250 4664 4665 P05230 857 859 5515 P04264 P05112 5518 O43186 P10827 P29323 P51692 Q5TC82 P31749 Q7L8A9 O00255 Q6KC79 P19883 Q13506 1050 Q9HCK4 861 O95390 P37173 P05121 P09601 P29317 P40337 Q9UL54 7704 P06213 O96005 6993 P04637 O43157 2033 P14555 O00300 P52952 P15531 991 Q96RU7 3479 2147 Q15022 999 Q16236 10014 P02686 Q04917 O43166 4221 Q7Z6J4 P16989 P57059 1191 O43290 Q15154 P48436 P01106 79923 P01589 P01588 O43294 P01343 Q16363 Q13099 P49768 O00206 2290 P25791 Q09472 P48431 Q8N726 9921 5321 P14416 3142 O00203 Q99623 Q66K89 891 773 894 Q00534 Q96S42 898 P01019 6886 6405 6647 P01138 P01137 8829 Q9UPY3 P01133 P01375 Q9UJM3 Q9UMS4 6660 P04626 Q658W2 2064 P35222 Q9NRR8 Q15051 55755 P60484 3148 P10070 P17936 P10071 11331 6776 P40933 Q96JB5 6657 P24588 8717 O43353 P26583 P24043 O00744 5460 4131 Q9Y5Q3 Q92934 6794 7520 8851 P20916 Q9H9S0 23764 3162 310 Q96P48 P61981 P15692 Q99684 4005 Q96AX1 5578 7518 Q8N5U6 Q8N302 P05412 O00755 Q6IR47 7532 7533 5598 Q9HCE7 23411 23532 Q5JSP0 3171 P37231 Q01955 P18846 P16671 Q96SZ6 324 3169 5467 4137 207 5468 54206 5108 10468 P27797 Q15078 P24385 8754 6696 7422 5364 4155 P56178 P56177 2099 3066 P13489 P51843 P15311 572 P13010 55785 10672 P01178 Q96HC4 P24394 Q9BT67 5371 P27540 7430 9612 P06729 23512 P13497 P16410 466 56882 55796 P62745 Q9H4E5 7428 10320 55558 P60568 8650 Q9BY76 O75386 O75385 Q9Y4K3 3084 474 595 356 P07196 Q7Z6C1 8767 P78395 Q13485 8408 P25963 Q9NPC8 Q13127 P35408 3091 5154 Q13009 8546 P62805 P12429 Q15306 19 79084 P58546 5270 367 P09382 Q9H2X0 P46531 51654 Q96SN8 8536 5029 Q16643 Q13133 Q15796 Q14344 P43026 Q15672 Q14106 Q9HB63 6495 Q15797

6256 6498 P12757 P12755 Q9ULH7 P10451 Q9UBU3 P51805 Q9NRI5 P02741 Q13145 P00441 4086 5054 4087 4088 4089 9657 7476 Q9Y6R0 Q8WWN8 P55055 P10586 Q9Y618 Q86YT6 59277 93166 Q8N5F7 Q02790 387 388 P21359 P31270 P08151 1808 P24864 Q16665 Q13275 P54259 4099 Q16787 7124 2909 P63092 P14635 P35453 Q9UQL6 P10275 4092 P10276 4093 P35579 154 396 P35580 121512 51564 Q16555 Q15223 Q99966 Q13164 Q14012 7132 7376 8100 Q9HAZ2 O43521 P22392 P36896 O43524 P35443 P36894 285 1822 23370 P01308 Q9NQB0 Q9GZM8 Q14145 P02751 Q15475 Q99835 Q96T37 Q8TCU4 Q6ZSZ5 P00491 Q86VS8 6050 P36888 7020 51341 P02549 55704 P01579 Q9NQC3 P49715 P09038 P23443 149041 P20073 P39905 7157 O15198 Q02750 O43508 P11474 3909 Q6PID4 84376 1969 Q14289 Q16581 P78423 Q13873 7046 6198 P10997 O15123 P10636 26037 O43711 P63172 Q4VCS5 P41134 Q86WK6 Q1PSW9 P17275 91 P04085 65082 P46821 P42229 O15119 3911 P42226 Q9NSA3 Q53QZ3 P56524 7057 O15496 Q96QB1 Q02297 7291 P55318 Q3V6T2 P55316 O43609 P67775 81565 Q4LE28 P08572 Q9BXL5 1627 P98174 8399 Q15831 7189 7067 P29474 Q5TAX3 P19793 Q96M96 P29590 Q03014 Q13418 Q63HK5 P56539 Q01196 Q9H6W3 1877 P41231 Q92793 P17252 P06241 O95859 Q13422 P55212 Q13426 A4D1W7 9138 9253 9495 7074 2735 P19438 P21757 P43405 Q04206 Q92786 1406 2737 2736 Q8WUI4 Q15735 P41182 Q03113 9021 P78504 84525 Q13683 3956 2626 P47712 7099 P31249 150094 7098 P35968 Q14774 Q15742 Q13443 Q15628 P30153 57154 58480 27339 P30279 1789 Q05516 P62166 Q9BQB4 P23515 P00734 375790 P17081 P08138 P12956 P10415 Q9H228 Q6ZV73 P12830 Q02363 57045 P35711 O60229 Q7Z494 3611 P43694 O15455 Q16610 Q9BQA1 5914 P41159 Q15648 O15230 Q04771 63976 4830 O15105 P45984 P21554 Q9BZR6 5925

anatomical structure development Q9UKT9 P25054 Q12824 259266 2304 2303 O60443 284217 P21926 2300 O14543 P05452 Q9UGM1 2308 2307 Q92974 P25067 Q9UGM3 Q12834 Q12837 2317 P42858 P39210 Q9UKV3 P61158 10661 P10809 2319 Q96EB6 136319 P27694 4990 Q06124 P17405 P37023 P14384 55584 Q9UKW4 4983 5830 55109 3655 Q9Y297 Q9Y6A5 Q9UKW6 3659 P26371 Q86SX6 3672 3673 Q92949 Q13705 Q9BYD5 O60437 2335 Q99574 Q9UKX5 3667 P26367 O14578 10644 10643 P25098 1021 1020 Q06587 Q8IX07 57787 10614 1499 1012 3676 P61586 Q9Y231 3678 3679 Q9H0H5 3690 P29466 O00358 Q9BPY8 3207 1026 P17482 3685 O60890 604 3688 P07686 A6NI15 P04179 Q9BQ95 Q15915 O60869 P16118 3215 Q96N67 610 Q92990 Q9Y250 613 4548 Q9H0F6 5879 Q92997 P10826 P10827 221937 P31749 O95140 1050 27032 P40692 3226 Q9UL51 Q92982 Q9UKT4 Q15019 O96004 P56199 P14317 O00300 P50897 Q99523 639 6760 O96019 P16989 1072 Q8NHU6 641 Q8IWA4 79923 11113 648 P23276 Q16363 1080 O96028 P48552 6774 3265 P12111 P14780 P11274 Q99500 O95180 P35240 656 8945 P01019 79956 55504 Q15047 84062 P50440 5451 A7MD48 Q9Y696 O95183 P35222 P35227 P35226 P57082 O75084 O75081 Q15051 P17813 53335 51156 668 6776 P24588 O43474 O00744 5460 P01042 P56159 4131 Q9Y5Q3 P01040 9821 4133 P25490 10690 9820 Q9NX62 673 P61981 P15692 6304 6789 P05412 P06744 O00755 5471 3290 Q9Y5R5 3297 Q9UBX0 682 10229 P60660 688 5467 4137 207 5468 54206 Q92922 P06753 Q15078 90780 4154 4155 23509 P56178 P56177 O75051 P13010 4149 10672 9839 5493 O43464 Q8NHX1 P35268 Q09666 23513 P50402 P13497 O14939 10682 8516 P27658 P60201 55558 9846 10202 P55268 Q14686 Q9UPN3 Q9H165 230 Q9H161 P07196 P49810 P08047 5017 P83731 P49815 Q9NPC8 94234 4188 8543 5037 79084 P58304 P09382 9869 8536 5029 51535 P00533 Q13370 Q16880 Q16881 P35638 Q9HB63 P61968 P31273 P31276 Q9ULH7 Q9Y6Q6 257 Q96RK4 Q15561 Q14232 P55290 6383 5054 Q9Y2J0 Q8IWX8 Q9Y6R0 9412 6389 Q86XR8 P36952 P68133 P37802 P35625 O75592 Q4AC94 Q8N4C8 P31270 23032 P08034 P43246 9421 9420 P49862 7248 O75581 274 Q93063 Q04837 P23229 51564 Q14254 7252 P78337 8100 Q9HAZ2 O43521 Q15109 O43524 5071 7251 285 7249 1948 1947 P02751 8110 P11229 5089 O75569 10152 23463 A1XKG3 Q9Y6M1 Q96EV8 Q8WY64 P48507 P08069 10155 Q15125 1956 O15075 O15078 O75553

Q9NXR1 2810 Q92187 O95947 1969 Q14289 Q13753 9463 Q13751 9464 Q9H0M0 Q4VCS5 P42345 P22735 2821 Q9BUG9 P04083 P04085 Q92574 P56524 9474 O15496 O75534 Q02297 7291 7290 117177 P16070 9001 P06239 Q06643 P56539 60412 P00966 P06241 P21860 O15499 O95983 P55212 91147 P55210 A4D1W7 9495 P07101 P07585 O75503 O95999 Q13315 Q03113 O43915 8174 9021 P35613 P35612 O60487 O60488 P85037 Q9H9B4 P31260 P20594 O75508 P31249 Q13323 Q9NP71 O43921 23092 3728 3720 P22303 9047 P13807 P12956 P34741 26576 Q9BQA5 P02708 2885 Q9Y6E2 P43694 Q9BQA1 132320 Q9NP98 63976 2896 23077 Q15532 P43686 Q13351 P07148 5925 P10914 O95677 Q92858 9061 Q9UBB6 P52799 Q9UBB9 4609 P16220 Q9UBC3 P27361 54361 P50148 P16234 Q9UK55 O15524 Q92838 P29992 Q9UBD6 P05549 Q9NRY4 1121 P53667 Q9BXC9 6809 27185 P18858 Q92830 Q9UJU2 P49116 P03372 Q92833 P06401 4627 P06400 P26012 P53675 P05556 Q92826 Q9GZX9 Q92824 3791 P07737 5970 5971 Q9GZX7 9093 Q9NRZ9 P56705 9099 P17535 81501 P20702 P17542 Q9BXB1 P61296 6812 10763 4637 3309 P04233 P34925 Q9Y586 O00468 O14627 10733 10736 3316 3796 P09972 4643 4646 5978 P08648 P30556 P30559 5991 93986 Q8ND90 Q8IVL1 1152 Q8IZT6 3329 3326 1147 2475 Q92882 Q92888 O95259 P07766 O43175 P05106 P04259 137964 6850 Q13618 2010 Q13616 P30542 Q9NS68 57448 2006 P61244 O14641 4664 O14640 4665 O43184 5515 P04264 P05112 50814 O43186 Q12778 Q02078 Q6KC79 56121 Q13627 O00499 P30530 P52789 P37173 Q99496 10728 P09960 P05121 4678 P40337 P04275 7704 Q9UK32 5528 P02452 O00425 O95278 4691 O00429 P04637 O43157 2034 2033 25861 P16615 Q01650 1181 P62993 Q14050 P01116 4205 Q9C0K7 Q15389 P01112 4209 P01111 Q14055 O43166 Q9C0K0 P48681 Q96CW9 4221 6885 23210 P35368 2043 Q9HDC5 P50542 1191 O76074 Q15392 84159 4214 P01106 768 P01588 4218 Q9C0J9 P01100 P50552 9921 P14416 P13569 2054 P52735 O00444 Q5T5P2 773 Q53X93 6886 55626 6405 P01138 P01137 P01133 Q16254 Q9UJM3 P02462 P02461 4240 5573 P04626 P04629 2065 2064 2063 P54920 301 302 P10070 P10071 5566 P02458 P01127 5568 9927 2070 5584 O43597 Q92817 Q96PU8 O75190 5578 Q8N302 P40763 P04201 5591 Q6IR47 5111 Q5HYA8 O76024 P80370 51295 P18846 324 329 5108 Q92800 P27797 6456 6455 2099 P12277 O76011 P51843 P15311 333 5599 5116 140735 Q9GZT9 P01178 Q96HC4 P05997 4287 25818 P17980 P52701 P15313 O60911 Q08209 P58012 P18827 6457 10320 P26447 P25116 Q96HZ4 8650 8655 4297 Q6ZT98 Q8TEW0 8654 P30086 4291 351 Q04759 356 Q9UBS5 P09493 5139 P09017 9510 5154 5155 367 Q70SY1 51654 6496 6495 6498 6015 P32242 P10451 Q9UBU3 Q03405 116113 P51805 P09471 5159 Q13263 P25940 Q14116 Q13268 7353 Q14114 8204 7358 Q14119 Q14118 P51812 Q9Y618 P51813 P12645 382 222546 Q04725 Q04724 387 Q04726 23154 P08151 P20264 P60763 P23771 Q13275 P62942 P55347 Q16787 8214 2909 7368 Q14129 Q9Y5I4 O43638 396 Q02535 Q99962 Q01201 Q99963 10296 11146 Q15466 2904 Q16790 Q99966 Q15464 Q13285 54 6045 P60709 Q16799 Q9Y5J5 Q86WV8 Q9UKA8 Q6PRX2 P33151 60 P09067 Q9UBP4 8218 O15169 51218 85440 Q99958 Q15475 Q8TEY7 P14859 P21145 P01579 2926 P09038 Q14155 116150 10280 O15198 P62906 O43623 25776 Q14160 1605 86 P78423 P78424 O15123 O75665 Q15818 1613 26271 Q8TAF8 Q9Y5E8 P41134 Q86WK6 Q1PSW9 27122 91 94 P46821 O15119 Q9Y5E9 2947 Q12797 Q13643 9114 Q9NNX1 Q13642 Q96QB1 O43602 Q15828 Q99081 P55316 O43609 P67775 P05062 P08575 P05067 1627 2956 Q9UBL3 P29474 Q15836 P29475 92129 O95613 29767 Q03014 6096 P34820 P55327 1634 56034 P21980 Q14993 1639 Q9UBM7 Q9NSC2 P11717 8289 Q15843 9133 Q14517 O15156 9138 29777 Q9BXJ9 57379 Q8HWS3 Q15853 O75626 9149 Q6NYC1 Q9H488 P41182 P41180 P53567 P30048 84525 P05091 P08123 Q9UJW2 Q13683 P47712 Q68CZ2 Q68CZ1 Q07817 P20248 64919 80155 Q96QF0 P08138 P09429 O75604 P10415 3860 P19235 P35711 Q7Z494 3852 P09430 80184 P08588 P41159 O15111 P55771 Q04771 1203 O15105 P45983 3866 Q13233 P21554 Q7Z2K6 65125 3880 3881 O14793 2550 9181 P51587 P17676 2547 4729 P37288 Q8IYN9 P84022 Q9HCJ2 Q92731 Q92730 O14786 3875

O14788 Q8TDD5 6901 P28799 Q9NZG7 P08727 Q92729 124540 P51114 Q96KN7 P20849 3886 4734
Q8N205 Q7Z727 P28300 O00167 Q7L5Y9 6927 55342 6929 811 P40425 P40424 P62258 6926 P31944
Q9NZI7 Q9NQX0 Q9C009 P07858 P31947 2100 P48380 O95343 P17655 57534 3428 6938 P20823
O60674 P20827 O60675 P62263 Q8N5V2 5604 P41743 Q9Y468 P11926 P27037 4771 Q9Y463 Q9Y466
P39019 P19404 55366 6949 27283 P15056 Q8N264 P07437 P50222 P32856 Q92769 Q14814 P09619
5631 P28370 Q9NZC7 P10600 Q06330 841 Q6PI57 Q9HCM4 Q92764 4775 O14753 846 7804 P09622
O95377 4791 1285 2132 Q96L34 Q9NQS3 O95376 2125 1277 Q92753 Q5TAT6 Q92752 P05230 857 859
Q92754 O00139 50937 Q13503 P11912 Q9NR50 1297 Q13509 1294 P19883 Q13506 Q9P202 O60636
Q9HCK4 2139 861 56243 1289 O95390 863 4313 Q9UMX1 P09601 4318 P06576 6993 O43278 23322
3484 P14555 3482 3481 P11047 25988 203068 3479 2147 Q8WV60 7832 P01236 10014 Q9BRQ0 3009
Q99750 Q01995 O43294 O43293 Q01518 P01222 Q13099 O43252 Q09472 O00562 P53701 2173
P13686 23787 890 Q00653 891 Q99743 O14727 894 Q9NR09 P53708 898 6525 P49639 Q9UMS4
P51531 8721 Q658W2 P51532 P14543 Q01974 55755 3028 P60484 11331 4358 5207 10487 P26583
Q9Y3M2 Q6FGG2 7402 2195 25945 Q8NFW5 10458 P60891 P31939 10451 P55075 79739 10454
Q8N5U6 7410 10460 7412 Q9HCE7 P52803 Q08117 Q05086 3052 P37231 P36382 Q01955 Q96SZ6
P51959 444 445 Q02809 P48357 7408 10468 P24385 P24386 8754 7422 9600 3066 22895 Q00613
P61764 7414 3070 7430 9612 Q9UIG0 3074 P17612 466 7428 6595 6597 6599 P18085 8775 6598 5268
O75386 O75385 6591 Q9Y4K3 3084 O43318 472 474 Q9C0C7 Q99807 P43034 P21246 Q13127 Q15303
5274 3091 Q16635 Q15306 3099 P58546 P13631 Q9UJC3 5270 Q02447 488 Q9NRH2 Q9H2X0 24144
6117 P22102 30813 P46531 Q16643 Q15796 P43026 Q15797 P12757 Q8N6I1 7468 P54198 P12755
O75360 Q01101 P31040 Q9NRI5 O94805 Q15323 Q13144 Q9BWQ8 Q16650 134701 P35869 9657 7476
8323 P18074 P10586 P10588 P13611 P10589 59277 P13612 93166 54820 P23416 Q16665 P55061
Q8TDY2 O15287 Q16666 P54132 P63092 P35453 P09172 P46109 P23467 Q14494 Q14012 P46100
Q9Y4H2 7490 Q9NZT1 P11021 Q8WW38 Q9GZM8 Q9NRD5 Q99835 9201 O75326 P13646 Q02880
P13647 Q86VS8 P13645 7020 Q08188 P02549 55704 P21266 1718 P02545 P47895 347733 P23443
Q8IUX7 P38936 P11474 3909 P02538 Q6PID4 Q99816 Q99814 P02533 P98194 Q15375 P60842 P78545
7046 P28289 6198 P98155 P78540 O43711 55283 6194 P32302 3913 56134 65082 1738 P05177 P98161
P07355 P98160 O60282 3911 3912 P78536 7057 Q13520 22806 Q3V6T2 7052 P19338 3925 P46934
1742 P46939 Q9HD26 81565 Q4LE28 P42574 P98172 P98174 Q14865 8399 9244 7067 Q5TAX3 P19793
P29590 Q63HK5 P31431 Q01196 1756 P41231 Q92793 Q96GM5 O15259 Q9NRA1 3932 O60260 9255
Q04695 P19320 Q8IYT8 9253 7074 P43405 Q9Y4F9 P20309 Q9NZR4 Q04206 Q5T5A8 Q92786 Q92784
7088 Q9NZJ7 Q15738 Q15735 P78509 7082 P78504 3958 P00749 P00747 P63000 P08243 3953 Q13563
3956 2626 Q15742 Q9NQZ2 P14923 7090 P11413 1789 Q05516 P62166 P00734 375790 P08253 Q15750
Q9HCU4 Q8IUQ4 57045 P78527 O60229 P20336 Q04656 51741 Q16610 Q14432 3978 O15232 O75712
O15230 Q15768 Q06830 P22528 Q9UNA1 Q9HCS4 4830 900 51752 Q9HCS7 P00750 P08238 O15228
P08833 Q8NEY1 Q9NYD6 2672 Q12948 O00268 Q8NEY8 Q9BVA0 3516 Q9BVA1 Q9ULW3 P08842
P07996 P52565 Q8NF91 Q12952 51090 Q8NB12 Q08431 Q16082 P63211 79885 P21810 P16473 2676
P16471 O60331 P21815 4851 4854 5702 5704 928 P54753 P53420 Q12965 P25391 Q12968 Q05397
P20930 29127 P20936 Q9UQB3 4862 Q9UQB8 4867 P54764 P54762 P54760 4882 P48023 Q7L576
22985 P17302 5728 P17301 1368 Q9HBE1 Q99697 2697 10523 Q9Y342 P28482 Q5D1E8 1385 O95470
57669 P15173 P07550 Q9Y2T1 P08887 Q9UM47 P51671 O00238 Q9Y2T7 Q96SB3 Q9NYA1 P51679
P10721 Q6UB99 Q06210 P19544 Q8IY17 2239 P18206 960 1387 967 4898 Q9UM54 5747 Q06203 2253
Q9NYB9 6609 P62330 57689 P42768 2247 1399 2246 P61006 6601 6605 P51693 P29323 P51692
O00255 Q03164 P17342 5764 P29317 6615 P06213 Q9Y6W5 P13349 2275 P52952 O60725 P15531 991

79810 998 999 P61421 P02686 P62753 Q04917 P49770 Q9UHG2 5310 Q7Z5H3 10141 5311 Q9UHG0 P51636 P53814 2280 Q04900 2277 Q15154 P48436 P28845 P01344 P01343 Q96NL6 P49768 2290 P25791 P48431 5321 3142 Q99623 O60701 Q00535 Q00534 Q96S42 7975 6647 5316 8829 5318 Q9UPY3 Q8N960 P01375 P01374 6660 Q9Y337 4000 8841 Q9UHI8 O00213 O95067 O60716 P12004 Q9P0I2 3148 546 3146 P40933 Q96EY1 Q96JB5 6657 5327 5328 P49747 6670 P24043 P07942 O00628 7520 8851 P07949 P07948 Q8WYA6 Q12904 P20916 Q9H9S0 Q07157 3162 O00622 O95076 Q9BZE0 552 Q99684 6667 4005 5336 Q96AX1 Q15185 5338 7518 P26232 Q969G3 5351 7532 7531 7533 P68032 23411 23414 10107 3169 Q9NPH5 4015 9702 10100 P05783 7528 5361 5362 Q86U86 6696 5364 P49321 Q9BUZ4 P12036 3182 P62736 P26678 Q01826 5371 P27540 P20908 P16401 P16885 582 54583 8879 6699 P60568 Q13477 8892 Q9UQP3 P15924 P15923 Q9Y3I0 595 8407 Q7Z6C1 Q7Z6C3 Q8NFJ9 Q13485 Q8WUM0 8408 P03950 9750 P35527 O94910 Q13009 4067 P12429 Q15669 O75496 Q5TAB7 51412 P60983 Q96SN8 Q13490 Q14344 Q15672 7101 6256 O75487 O43897 Q2M1K9 Q01469 Q9UDY8 P23528 Q9UDY6 Q8WZ42 P00441 8443 4086 4087 4088 P22692 4089 163126 Q86UL3 Q86YT6 Q02790 P12883 P21359 23396 1808 P24864 10087 6259 53615 8454 P54259 7123 8452 6275 P54257 4099 7124 P54252 Q8TD84 P14635 64759 Q9UQL6 P10275 O75462 4092 4093 P35579 154 Q00597 Q99720 156 Q9UMD9 23363 Q9NYQ6 Q8WV28 Q99729 Q16555 10059 Q15223 P45379 Q13043 Q86YZ3 9314 P36896 P36894 P35568 1822 166 338917 Q9NQB0 Q99715 Q99714 Q96T37 Q8TCU4 P61803 O95813 P35555 Q86V24 P36888 7141 1832 Q9H1Y0 Q00577 9319 Q99707 Q9NQC3 P49715 P60953 7155 P39905 7157 Q02750 P35548 8481 Q9H295 Q13075 Q16586 84376 Q15256 O75427 Q13873 P10636 26037 Q01094 P63172 7161 9341 7163 2702 P63167 56259 Q9Y3A5 10935 P17275 P23945 Q4KMG0 Q92692 P39060 Q6NVY8 1859 P22612 P42229 1856 Q13885 O94992 Q13887 10939 7170 Q14738 P10644 9351 O43847 9350 79365 P39059 1869 7189 57120 O75881 10908 Q13418 O43815 7182 1874 P17252 P68871 P09630 O15379 Q5TA76 O95859 O60383 Q9NZ08 P21741 O95863 Q13422 O95861 Q13428 P35908 Q13426 P10620 4914 2735 Q9Y3D6 1400 Q02388 56288 2730 Q96FJ2 1406 2737 2736 P54652 Q8WUI4 P19012 1896 2742 P09211 4920 Q13444 P35968 Q14774 Q13443 26005 P11532 57154 27339 P30279 27330 P23515 O14492 P56851 Q9H228 O14495 P12830 Q02363 P18146 Q03692 163183 3611 Q14790 P00403 P01730 O15350 O15353 Q15648 P19484 Q06710 O60353 P24821 O14497 4953 Q9BZR6

response to hormone stimulus 64397 P08833 P28562 3880 Q9Y266 O14793 P51587 O60568 P37288 2303 2783 Q92731 O14543 P08842 P07996 2308 2549 O15530 P08727 Q92729 Q08431 51094 P16234 2 P63218 O15524 Q7Z727 P28300 P50150 Q06124 57761 Q9UQB8 P03372 Q92831 P55851 P07858 5970 3673 2100 P17302 5728 P17301 O60674 2697 3667 5604 P41743 Q92820 P28482 P15056 1499 P08887 Q9NYA1 P30559 O95361 P10600 P37198 Q9BUB1 57678 10626 1026 841 2475 22937 Q06203 P16118 O60502 P38398 O60503 Q9HCL2 P61244 610 1277 Q92753 6721 857 Q12770 Q12772 P10827 Q12778 P51692 P31749 Q9NR50 1176 1050 P18433 Q9HCK4 10724 10726 Q9UL51 P09960 P05121 P09601 P04155 7706 26060 P06213 P02452 P01241 P14317 2033 2274 3484 O00300 3481 O60603 P52952 Q96RU7 P62993 Q99523 2026 7832 P01116 P62879 6869 Q15389 P27986 P62873 10014 P49770 Q9BT40 Q96T76 O76074 768 P01588 P01344 Q99757 P01222 P01100 1080 O00206 Q09472 6772 5321 6774 P13569 P14410 890 894 Q14192 898 5798 P01138 P01137 O14965 P01375 P50440 4240 P01130 5573 P04626 5575 5576 2065 2064 P35222 P35348 P60484 3148 Q15291 P01009 5566 8836 6776 5567 P01127 5568 5327 5207 Q59EA4 P26583 6790 25828 4131 5584 P07948 3162 10458 672 552 673 P51828 5577 5578 5337 5338 P40763 P04201 P01275 5591 P05413 5471 Q6IR47 8985 10580 P37231 682 445 3169 4015 207 5468 P48357 Q92922 P01023 Q08462 P24385 8754 6696 Q96A54 2099 P51843 Q8NER5 Q9NRM7 P68036 8503 4149 P25103 10432 P01178 23636 6464 O60911 P17612 Q6ZWJ1

O14939 P18827 8892 Q14686 5265 6599 8654 P30086 Q9H2Y7 P13861 595 Q04759 P07196 P20396 Q7Z6C1 51763 P22694 Q13480 P03950 Q15303 7332 5154 5155 4067 Q9HAV0 P12429 5037 367 P47985 Q13370 Q13133 20 6256 P31153 P59768 P10451 Q9UBU3 Q13144 Q14232 P32239 5295 5054 10081 Q14118 O43306 P68133 58189 Q9UKG1 P35625 5170 142 P24864 Q13393 P23771 Q13033 7124 7248 P63092 Q9UQL6 P10275 P10276 10057 Q16790 Q99966 7252 Q14258 Q9Y4H2 26524 Q86WV8 P35568 Q8NFM4 Q8NFM5 Q99958 9564 7386 P49840 Q86V24 Q96PK6 P21266 P08069 2805 P49715 P23443 Q9BX66 Q02750 P38936 1843 2931 Q6PID4 Q14289 1605 P25874 7046 6198 P98155 O95835 P42345 P42224 Q9Y4C1 P17275 Q92572 P04085 Q92574 P46821 P22612 P42229 P15090 2947 1978 7057 Q96QB1 P10644 P31321 P31323 P05062 P06276 Q92569 Q9UBL3 7067 P29353 P29474 P19793 Q03014 6096 P40145 P17252 P00966 O60266 P21860 P09874 O95622 8165 P55212 Q8N8D1 Q04206 P07101 Q92667 P41181 9021 P35613 B3KY43 P22415 P05091 Q9HC57 P08243 3953 2626 P47712 7099 Q13322 Q13443 P30279 Q07817 P20248 P17081 P09467 P09104 O14492 Q16849 P10415 29966 P78527 P18146 2885 P17174 Q14790 P43694 5914 P41159 5929 P00750

cellular response to chemical stimulus 64397 P08833 P28562 6599 P49023 Q9H2Y7 P13861 O60568 595 P37288 2303 P84022 P09493 2783 Q99807 P20396 Q7Z6C1 P22694 Q13480 O14788 2308 5935 2549 7332 3091 5155 O15530 Q9HAV0 P11215 54361 51094 5829 P09382 P39210 54487 P63218 5029 Q13370 Q15796 Q9UKV5 Q9BUN8 Q7Z727 P35638 Q9UKV8 Q15797 6256 P50150 P32121 Q06124 P59768 Q9Y6Q6 Q9UBU3 6809 57761 5159 Q9UQB8 O75807 P55290 P00441 Q92831 5295 4086 5054 4087 Q13268 P55851 4088 7476 3672 3673 O43306 1130 Q9UKG1 P56705 5170 P54646 142 Q96GD0 P17301 Q99698 267 O60674 P24864 Q16665 3667 P41743 3309 O75469 P63092 P14635 Q9UQL6 P10276 O75581 O75460 P19525 Q00597 57669 1499 P35221 1012 5610 P04040 27161 P08887 P30556 Q9NYA1 4780 6048 P09619 P36776 Q9Y4H2 26524 P35568 2119 P37198 Q9BUB1 57678 P11021 Q8NFM4 Q8NFM5 Q9HCM4 847 3689 Q99958 P04179 9564 P05107 6850 P49840 O75569 P98179 10273 P13646 Q06203 2010 Q86V24 Q99942 P38398 O60503 Q9HCL2 P61244 1277 P01579 P08069 2805 P23443 P10827 Q92626 Q12778 Q9BX66 9217 P51692 P31749 1176 P14625 P37840 P18433 Q9HCK4 1843 2931 51593 Q15011 1728 P05121 Q99814 P09601 P25874 26060 P06213 P02452 P78423 P01241 6198 P98155 O95835 9588 P56199 2034 2033 Q01094 3484 3481 P42224 Q96RU7 Q9Y3A5 P62993 Q9Y4C1 79139 10935 P17275 Q92572 6622 999 10013 P62879 Q16236 P22612 P05177 P42229 P27986 P62873 51009 10014 1978 P06702 22926 Q96QB1 5796 P10644 P31321 P31323 Q9P2K8 P05062 1869 P01344 Q92569 409 P01100 1080 Q09472 7067 P29353 6772 5563 Q5TAX3 6774 P13569 P19793 O95292 P29590 9361 7184 P41235 891 P40145 894 P41231 P17252 898 O60266 P21860 Q9GZP9 6647 P01137 Q8TAD8 Q9UPY3 P09874 O95622 8165 5573 5575 Q9Y696 5576 2065 P35222 Q08257 9495 Q9UNE7 O75084 P43405 P17813 3148 3146 5566 6776 5567 4358 P05164 P01127 5568 Q9UBN7 Q92667 P24588 P26583 O00744 Q5VVQ6 Q15853 5584 P30041 P41181 P30044 Q9NX61 3162 P30048 B3KY43 10458 672 552 P22415 P05091 P15692 57026 P51828 5577 5578 Q8WYQ5 P08243 P55072 3956 2626 P40763 P04201 2081 P01275 5591 O00755 5471 Q13322 8985 Q9NP71 10580 P14923 P11413 P18850 P30279 P37231 3728 10229 Q9BXP5 207 5468 P17081 Q92922 1429 P09467 P09429 Q08462 P24385 O14492 7422 3860 Q96A54 P41161 P12830 Q02363 P78527 P18146 P32322 79753 2885 Q9NRM7 P68036 P17174 Q04656 7415 8503 4149 P43694 5119 5914 23636 5371 P27540 6464 Q12931 Q06830 P15559 P50402 55432 P17612 Q6ZWJ1 10202

in utero embryonic development Q14686 3880 O14672 8533 Q12948 O75386 P51587 29947 P17676 P84022 Q99807 P08047 51763 O14543 Q13485 P28799 P20290 4188 P08727 Q02447 P16234 367 6117 P46531 4851 9869 10661 P00533 Q15554 Q15796 5702 Q14344 5704 O15525 P27694

Q9ULX9 Q12965 6498 P12757 Q9ULH7 6927 P37023 Q15561 Q92830 5159 Q9UJU2 6926 4627 4087 4088 4089 9412 P18074 Q9Y618 Q86YT6 P13612 P17302 P17655 57534 P20823 Q4AC94 2697 P61296 10087 P43246 5604 53615 9421 Q14249 8452 9546 P28482 P10275 P35579 O14627 10733 57669 1499 4643 3676 2904 Q99966 8100 O43521 7014 P09619 P36896 P36894 P10600 Q6UB99 Q9BPY8 Q9HCM4 Q8WW38 Q9GZM8 Q14145 Q99835 O95377 P61803 23463 Q13618 Q9NPQ8 57448 P49715 1956 50814 7157 Q13503 P31749 O95140 Q02750 1050 4678 Q99814 Q15256 7046 9464 O96004 P04637 O43278 2034 23322 25988 60626 Q4VCS5 O60725 Q9Y3A5 P62993 Q1PSW9 P17275 91 999 94 O15119 P98161 639 O96018 Q9UNS2 5310 5311 P16989 Q9BRQ0 Q9BT40 Q99873 Q99750 81565 Q13099 O00444 Q63HK5 Q9NR09 Q96S42 Q15047 Q9UMS4 O95983 9133 6660 Q13426 P19320 3276 P35222 O95067 P35227 Q96ST3 P10070 P10071 2737 2736 9927 6670 O75626 Q15738 7082 Q03113 23764 9021 Q07157 25942 O00622 84525 P00747 P15692 O75190 6667 Q13563 7518 10454 2626 Q6IR47 7412 26005 3297 Q68CZ1 27339 23414 Q07817 10229 689 207 Q15750 90780 4154 7422 P49321 P12830 P35711 Q9BQA5 Q00613 2885 P43694 10672 Q9GZT9 P27540 9612 O75712 Q15648 P19484 Q04771 P17980 54583 2896 Q9HCS7 P43686 Q13351

interphase of mitotic cell cycle 8099 6198 6872 O95835 Q969L4 5422 2672 P15923 Q01094 6194 25988 351 10657 990 27000 595 Q7L0Q8 84967 995 997 91 6502 5537 P62753 1978 P78536 9238 1104 641 P52292 5424 P00533 P05067 1869 P24522 P11309 Q8N726 4193 891 P46527 1874 6929 Q969Z0 Q99741 Q00534 898 Q16254 P06400 O15392 A4D1W7 Q13309 Q96ST3 P24864 P09884 1647 8453 8454 Q13554 8452 P50570 P54132 P11802 1021 P14635 O75461 25942 O00743 1017 Q99684 8328 7013 Q07666 P36896 26524 Q9NYZ3 58480 8065 1029 O14519 1027 1026 Q9UJX2 1785 Q00987 207 P42771 P24385 P30304 P10415 P30307 P54274 P28340 Q13618 Q13617 Q13616 Q9BQA5 Q99543 Q9NRM7 1956 P23443 P54687 P31749 Q04771 P38936 P00519 Q93034 Q08209 P24941 51512 Q5VTD9 2935 P21675 Q96FF9 P49427 5925

covalent chromatin modification Q6P1J9 Q9H0E3 Q96L91 4297 Q9Y265 6871 Q9BZ95 2033 27005 23326 O75150 11107 10933 Q9Y4C1 Q15022 79813 Q7Z6C1 10013 84289 10014 P56524 6883 7334 Q16512 Q969S8 Q9UBC3 Q16514 Q15306 O96019 23210 Q5VTR2 Q9H3R0 57634 79885 Q92560 Q4LE28 Q96RL1 408 P49407 Q96EB6 Q9UBL3 O75528 Q7Z727 Q09472 O95696 9646 10075 Q9UPP1 6015 Q9UNL4 O00167 Q6UXN9 23028 Q9H6W3 54496 83933 Q92793 P17252 Q7Z6Z7 O15379 55869 Q92830 O95619 51147 Q9UBU8 Q6IT96 P61964 Q92831 Q9C005 O95983 Q6PD62 O75478 Q13547 8841 Q9BY41 Q9NRZ9 O00213 9252 Q8NB78 P35227 10524 55352 P35232 O60674 Q15291 11176 Q9NPF5 Q9UBN7 10765 53615 5585 Q6NYC1 Q96MX6 Q06587 O75582 Q9UQL6 Q9Y6K1 23081 79577 10856 Q9UK80 Q9H9B1 6304 51720 5578 Q9Y230 5579 O14744 Q9HC52 P05771 8607 6045 55929 Q92769 Q9NQ92 23133 O95361 23135 23411 Q8N7H5 Q8WYH8 1789 1788 P46736 10626 1387 5245 3066 Q9UPT9 3065 Q9UIF9 O75164 9682 79595 P61088 Q9NVM4 Q96PK6 84661 O60341 Q92993 10432 Q01826 11091 Q9NPA8 3070 30827 Q9UIG0 Q9P0U4 23512 Q03164 221656 2139 5929 57332 80335 Q99496 54623 Q9UGL1 86

interphase 8099 6198 6872 O95835 Q969L4 5422 2672 P15923 Q01094 6194 25988 351 10657 990 27000 595 Q7L0Q8 84967 995 997 91 6502 5537 P62753 1978 P78536 9238 P08729 1104 641 P52292 5424 P00533 P05067 1869 P24522 P11309 Q8N726 4193 891 P46527 1874 6929 Q969Z0 Q99741 Q00534 898 Q16254 P06400 O15392 A4D1W7 Q13309 Q96ST3 P24864 P09884 1647 8453 8454 Q13554 8452 7884 P50570 P54132 P11802 1021 P14635 O75461 25942 O00743 1017 Q99684 Q14493 8328 7013 Q07666 P36896 26524 Q9NYZ3 58480 8065 1029 O14519 1027 1026 Q9UJX2 1785 Q00987 207 P42771 P24385 P30304 P10415 P30307 P54274 P28340 Q13618 Q13617 Q13616 Q9BQA5 Q99543

Q9NRM7 1956 P23443 P54687 P31749 Q04771 P38936 P00519 Q93034 Q08209 P24941 51512 Q5VTD9 2935 P21675 Q96FF9 P49427 5925

cellular response to endogenous stimulus P01241 64397 P08833 6198 P98155 O95835 P28562 6599 3484 Q9H2Y7 3481 P13861 O60568 P42224 Q96RU7 2303 P62993 Q9Y4C1 P17275 Q92572 2783 P20396 P22694 Q13480 P62879 P22612 P42229 P27986 P62873 10014 1978 2308 2549 7332 O15530 Q9HAV0 Q96QB1 P10644 54361 P31321 51094 P31323 P05062 P63218 Q13370 P01344 Q92569 P01100 1080 7067 6256 P29353 P50150 6772 5563 6774 P13569 P19793 Q06124 P59768 Q9UBU3 P40145 894 57761 O60266 P21860 Q9UQB8 P01137 P09874 P55290 Q92831 O95622 5295 8165 P55851 5573 7476 5575 3673 5576 O43306 2065 Q9UKG1 P56705 5170 P54646 142 P17301 O60674 5566 6776 5567 5568 3667 P41743 Q92667 5584 P63092 Q9UQL6 P10276 B3KY43 10458 P22415 P05091 57669 1012 P51828 5577 P08243 2626 P40763 P04201 P01275 5591 O00755 5471 Q13322 8985 10580 Q9Y4H2 26524 P35568 P30279 P37198 P37231 Q9BUB1 57678 Q8NFM4 Q8NFM5 Q9HCM4 207 5468 P17081 Q92922 Q99958 P09467 9564 Q08462 O14492 P49840 Q96A54 Q06203 P78527 Q86V24 O60503 Q9HCL2 P61244 2885 Q9NRM7 P68036 P17174 P08069 8503 4149 P43694 2805 P23443 5914 23636 P10827 Q12778 6464 Q9BX66 P51692 P31749 1176 P18433 Q9HCK4 1843 P17612 Q6ZWJ1 2931 P25874 26060 P06213

DNA metabolic process Q5FWF5 O95677 8099 Q9Y265 399687 Q12824 O95551 P51587 3516 10535 2547 1454 1453 O00273 4841 5931 Q92614 4968 Q9H0D6 P0CG13 25913 P08729 Q9NS91 Q9UBC3 Q8NFZ0 P16104 O60216 P18887 55215 P10809 Q96EB6 64282 Q9H2G2 P27694 Q9UGN5 P27695 Q9NRY2 O00167 29128 54496 57646 6929 P18858 O60543 1111 P26358 Q9GZX7 P99999 Q12857 9093 Q9NRZ9 Q13829 O60318 10524 Q6PCD5 5981 5983 1022 Q14807 Q9NSU2 1017 1137 10856 Q6FHQ0 Q9Y230 5976 Q14938 Q8WVB6 O95243 Q96SB8 Q8WYH8 Q06330 3329 1029 O14519 2237 79444 P08651 P42771 O14757 Q9NYB0 P30304 O95257 Q12888 P30307 4673 5883 P28340 1161 P38398 Q56NI9 10714 Q9BWE0 Q9BQ90 Q92993 Q92878 Q96CA5 Q13620 5892 O00255 P54727 P54725 Q03164 55035 2139 253980 P40692 57697 55159 O60870 10728 157570 5886 5887 4678 5888 8930 P04637 5422 P50897 25988 990 Q96T88 Q96RU2 3479 995 997 P62877 8924 P25205 7832 84289 P23025 6749 Q9C0K0 P25100 4221 P35244 P35249 Q96T76 Q96Q15 O14717 641 P35251 5424 P49643 Q99638 56946 8815 P01343 P01100 P49642 Q8N726 O96020 346171 Q9NU63 P11388 P11387 Q9NUW8 Q66K89 O14727 Q99741 Q96T60 3014 51147 6647 5557 Q8IYD8 5558 29086 P01133 P23246 Q9UMS4 4361 6421 P40938 P40937 114799 Q9NUX5 P12004 Q96ST3 P35232 3149 3148 Q96RR1 545 546 3146 668 Q15054 Q96EY1 84296 7508 2070 P26583 Q5TKA1 7884 7520 4255 P50613 P51946 25942 672 3159 Q15185 7518 P55072 7517 8607 5591 P46063 Q9Y5R4 5111 23411 Q01831 Q6PJP8 Q9NWV8 55775 328 Q9BQ15 Q9BRT9 P49321 5245 Q9UIF9 22897 P13010 Q99543 7415 5478 10432 3070 4287 P49674 Q9UIG0 5378 P39748 P52701 580 221656 P58012 P52948 54107 56647 Q92900 Q9UPV0 P49427 4172 4173 4174 Q14566 Q14686 4176 4297 P15927 6598 P15923 29947 4171 472 9978 Q9C0C2 Q14694 7334 7336 80198 Q9UBT6 6117 11198 6119 54921 6118 Q96RL1 Q15554 P24522 7341 P62937 9643 Q76FK4 8555 Q16526 Q9UNL4 29803 P49916 Q01105 P22674 P17096 Q16531 11168 Q9UBU8 P21589 51426 54815 P00441 Q9BSI4 P22692 P62826 9656 O43542 P18074 O75475 142 11176 Q9NPF5 Q49AN0 P43246 Q14249 O15287 P54252 P54132 7126 8450 25788 Q9Y6K1 Q00597 Q04837 Q14493 Q99728 51567 P49736 7013 55929 7014 P46100 P36776 Q9NRD1 11277 Q9NRD5 Q15233 P54277 P54274 Q02880 7141 P49959 Q9NVM4 Q00577 Q96PK6 Q99708 9319 P49711 Q16576 7153 7155 7158 7157 116138 Q09028 7150 O75792 P48730 Q15014 Q9NRF9 9100 9463 O95714 Q15819 P33992 P29372 P33993 Q7Z589 P33991 P78549 7161 1854 27000 10933 P63165 1736 26270 9232 22803

Q8WWH4 Q86WJ1 O75531 O75771 Q9Y6H3 Q08945 P52292 Q52LR7 P42574 2956 Q969S2 9126 Q13535 286826 P55209 9361 1877 79035 4907 Q9NVI1 P04183 P23921 P09874 Q13426 P34931 Q8NB78 1642 1763 57379 P09884 1408 1407 1647 4913 P33316 Q13315 O95997 7083 Q03468 51720 3835 Q8IY92 Q8WZ19 Q13569 27339 1789 1788 P46736 64919 Q9H9A7 1786 Q8N0Z6 Q9UQE7 P61077 Q15631 P22303 5901 Q9Y5B9 P09429 P12956 3980 Q13216 P78527 Q00059 Q9BQA5 1676 P61088 P09430 3978 O15350 Q14676 Q15648 Q86YP4 Q9P287 5928 P00519 P24941 P43681 O15347 Q9HCS7 84893 902 Q13112 51514 Q14683

regulation of cellular component biogenesis 4690 P25054 7046 10092 10095 6872 O95835 10094 P10636 2033 P12931 P52952 Q4VCS5 P42345 Q8IYN9 Q9BXM7 Q04759 P84022 P63165 P09493 118 998 Q7Z6C1 10013 5898 94 Q92574 P46821 6869 11346 P49815 1978 P07996 P47755 P47756 7057 Q96QB1 65018 Q86VP6 Q01082 1191 2039 Q9Y6H5 22919 4214 P61158 Q13131 7341 O15020 Q7Z727 Q09472 7067 Q13813 5562 O15143 P56539 P31431 Q05397 P53667 Q9UBU3 P37023 P20936 P17252 81873 P35240 Q96RK4 O60260 Q08495 P35869 3791 4088 7476 P55055 Q658W2 P78317 382 P11277 Q9UNE7 5728 P16333 387 3304 P52907 P60484 3146 Q92786 Q6PKD3 6812 Q15691 Q9UBN7 Q9NZJ4 829 Q9UL15 4131 4771 7248 Q15735 Q9UNN5 O43639 O00468 P35612 P35611 Q96P48 6709 57669 830 P63000 832 Q9H2D6 5578 P61586 Q15223 P51671 O00755 Q6IR47 7013 7376 P35968 P30559 P10600 P10721 3054 Q86WV8 5071 5590 10109 Q05513 324 Q9HCM4 2475 6711 6710 4137 P62328 7249 375790 P49841 P17081 6714 5747 P09429 10273 P54274 Q9BPX5 23189 Q9NU22 P62330 P02549 P61764 10552 11124 857 P25103 859 5879 O43182 P01178 P60953 Q96HC4 Q14678 P10827 P08107 P51610 P11234 P11233 Q9HCK4 2932 55832 P63313 Q8N3V7 Q13233 P21675

positive regulation of signaling process 8772 P30086 Q9Y4K3 O43318 O15519 356 23286 Q92851 8767 O14788 57506 P07996 Q9HC98 7334 4067 7335 5037 65018 2316 367 Q04864 P14174 P09382 10783 P00533 Q13490 4734 1902 9641 8795 Q9UQ13 Q9HBW0 P32121 253260 Q06124 Q9Y6Q6 10758 Q9UDY8 57521 142678 64170 P55290 Q9BY44 5970 P48023 Q86XR7 Q9H257 Q9NR96 P17302 387 389 O60674 O00182 2697 Q8IUC6 5966 P04233 P62942 6275 Q13158 7124 Q9Y3E0 O00463 P10275 154 P15056 Q9Y2C9 1499 10616 1012 P07550 P04040 O94827 P61586 1936 P08887 7132 P29466 Q9Y239 124583 5071 P37198 29110 960 1147 Q02763 P01308 841 2475 843 Q7Z7H5 Q9NQB0 O15169 847 P07766 P02790 10392 6850 P50591 O43734 Q9P0L0 Q9NS68 Q8WVQ1 Q8IUH5 2247 Q9BVC4 P01579 P08069 857 O14763 P05112 5879 1956 P09038 Q92633 Q9H8V3 P37840 23118 27032 Q14160 54862 Q14164 Q6R327 P09601 P98194 Q9UL54 Q13077 P06213 Q15811 P01241 7046 P35813 P14317 9341 3481 P42345 Q9Y6G9 Q9BXM7 3479 P13591 6622 10013 P01116 9218 P01236 5536 Q15389 Q9NSA1 P01112 P01111 7057 6885 Q13404 9590 2280 7052 P46934 2159 4215 P01588 P16070 Q99759 408 P49407 P01344 409 P01343 O00206 7189 7188 P48431 Q15836 3265 Q13418 3263 7185 148022 Q9BYM8 60412 Q02952 P01019 P21860 51026 P21980 55504 P01137 O60260 P01375 64223 P04626 Q13546 55072 Q7Z434 2065 2064 P35222 9495 P35348 P19438 P43405 Q04206 P53041 6657 Q9UBN7 8837 P24588 8717 O43353 Q96EP0 Q6FGG2 O95999 P13945 P07948 P78509 3162 9020 79971 673 P15692 P63000 57708 5579 P00742 5338 3953 P05771 Q6P1N0 3956 Q96FA3 O00635 7099 7098 Q6IR47 8743 Q15628 64127 Q05086 Q99558 P08134 5467 8737 329 P48357 3965 P21333 Q9NYJ8 7422 6453 Q9UHD2 Q9BUZ4 57162 3611 P61088 Q14790 Q5T9L3 O15455 Q16610 P01730 P41159 Q9BT67 23636 5494 Q60FE5 O15111 Q86Z14 222068 23513 O14939 Q9HC29 54106 P26447 P25116 P60568

positive regulation of RNA metabolic process P10914 Q92858 Q12948 Q12824 Q6QNY1 O00267 P17676 2547 2304 2303 P16220 Q96JM2 P84022 Q92731 O15516 2308 Q12837 Q8NAP3 4851 5705 O75925 Q12968 6927 6929 4862 Q9UJU2 P49116 O75928 P40425 P40424 6926 Q6IT96 P06401 3659

6925 P06400 Q96JK9 5970 2100 Q9NR96 O95343 10524 P20823 P61296 5966 5604 Q9Y463 1022 1385 P19532 O00468 6829 10736 1499 P15173 80324 P07550 4646 6827 Q9UHK0 4780 Q92769 6720 P28370 Q14938 O95361 P10600 P19544 O00358 10626 P19419 Q9P0K8 1387 P17482 P08651 P17480 4775 Q9UM54 Q12888 4792 22937 P38398 2247 Q9BZK7 6601 Q92753 6721 Q92993 Q12770 P05112 Q9UM63 Q92997 Q13501 Q9UKS7 P10826 O43186 Q12772 P10827 Q12778 P51692 Q13503 Q05586 O00255 1050 Q03164 861 50943 Q9Y2W1 O96004 6872 P04637 2034 P13349 2033 2274 Q9UIH9 O60603 25988 P15884 P52952 3479 10011 4686 Q16236 10014 Q9C0K0 6760 4221 Q96T76 P33076 P48436 4214 406 P01588 O43294 408 P49407 P01343 P01100 O00327 O00206 Q09472 P48552 P48431 9921 6774 891 Q99743 Q14192 Q96S42 898 Q53X93 P01137 5316 P49639 P01375 Q9UPY8 P51531 6660 5451 P35222 P51532 P17813 3148 P10070 P10071 3146 668 6776 10001 6657 P23497 P26583 O00744 5460 6672 Q9Y5Q3 P50613 P51946 Q12905 Q12906 672 P15692 79612 3159 Q02930 6667 Q8N5U6 P40763 P05412 5591 O00755 4261 P18850 Q05086 23414 3171 P37231 P18846 P18848 3169 148327 9826 5468 Q92922 7422 P52815 O60907 3066 Q9UMN6 3065 3182 8625 10432 P27540 5494 P62508 Q9UIG0 O14936 466 P58012 468 10320 54106 8878 9967 P25116 P60568 O15085 6595 Q14686 6597 4297 6599 6598 P15923 P13984 Q9Y4K3 351 Q9H161 474 P08047 Q7Z6C1 Q13485 P25963 O94906 Q9NPC8 3091 O00716 Q15306 Q86VP6 P58304 P13631 367 Q04864 P46531 Q13133 Q15796 Q9UQR1 6496 6495 Q15797 6256 P12755 Q9ULH7 Q9NY61 O75360 Q9Y606 Q15561 P17096 Q13263 Q15562 Q16650 4086 P35869 4087 8204 4088 P62826 4089 7476 9412 P18074 Q8WVL7 P55055 P12645 P36956 P08151 P24864 P20264 Q16665 Q13033 9421 P55347 7124 P54253 O75586 P14635 P35453 Q9UQL6 P10275 P10276 154 Q99729 Q13285 2902 7132 7376 8463 Q9HAZ2 Q09161 P36896 O43524 7490 55810 338917 Q9NQB0 Q8WW38 Q14140 Q99958 Q15475 Q99835 Q96T37 Q8TEY5 7023 5089 P14859 54851 Q01664 Q96PK6 P01579 P49711 10155 P49715 P09038 55827 9575 Q9NPA8 7158 P39905 7157 Q02750 6182 P11474 26747 55832 Q99814 9464 P35813 27005 Q01094 7161 P22736 Q1PSW9 P17275 P62195 91 9219 P42229 O15119 P42226 Q9UBK2 Q58WW2 P56524 22926 22807 P55318 Q4LE28 P05067 1869 7189 7067 P19793 Q03014 7182 6096 Q01196 P41235 Q92793 Q96GM5 Q9HD15 O95619 Q9NSC2 8289 Q13422 O15156 O94776 Q13547 A4D1W7 P78317 2735 P19438 Q9BXJ9 Q04206 Q92786 1406 2737 2736 Q15853 O14593 O95999 9149 Q8TAK5 P22415 Q9UK80 Q03468 4800 2626 7099 7098 Q9NVC6 Q9NP71 64919 80155 Q9UIV1 P23511 375790 3609 P17081 3608 5901 P46734 P09429 P12956 29966 P78527 P18146 P35711 Q9BQA5 P43694 O15455 5914 O15350 Q9BZS1 Q15648 P55771 P19484 Q06710 63976 P63272 Q9HCS4 O14497 Q92585 902 Q15532 Q13233 P21675 5925

organ morphogenesis Q92858 2672 Q12948 P52799 2304 4609 2303 284217 P84022 2300 Q92731 O14786 O14543 O14788 Q9UGM1 P25067 Q12952 P27361 Q92729 54361 P63211 P16234 Q96KN7 P20849 P42858 4851 O15524 Q8N205 4990 P05549 P25391 Q9NRY4 O00167 Q9BXC9 6927 55584 Q92830 Q9UJU2 3655 Q9Y297 P03372 P40424 6926 P06401 Q92826 Q9GZX9 P54762 Q9C009 P07737 P54760 5970 3673 P48023 2100 Q13705 P56705 O95343 P17302 57534 P20702 P20823 P17301 Q99697 P20827 2697 P61296 P26367 P41743 P34925 4771 P28482 O95470 Q06587 10736 P15056 57669 1499 P09972 4646 P51671 O00238 P32856 Q14814 P09619 Q9NZC7 P10600 Q6UB99 Q06210 P19544 Q8IY17 O00358 P18206 3207 960 1026 1147 841 Q6PI57 Q9HCM4 Q9UM54 6850 Q06203 Q9BQ95 2132 2253 Q15915 Q13616 Q9NQS3 P16118 2006 2247 3215 1399 1277 O14641 4664 Q92753 Q5TAT6 4665 P51693 P10826 O43186 P10827 P29323 1297 O95140 O00255 Q6KC79 P19883 Q13506 1050 P30530 2139 1289 O95390 P37173 4313 Q9UMX1 P29317 6615 P06213 P02452 O96004 O43278 P13349 2033 3482 P52952 P62993 79810 3479 Q8WV60 Q15389 P01112 4209 4221 5310 6885 5311 P16989 1072 2280 84159 Q99750 P48436 P01106 768 O43294 Q9C0J9 P01343 Q13099 P49768 2290 Q09472 P50552 P48431 6774 3265 2054 P53701 P13686 890 Q99623 O14727 Q96S42 P53708 P35240 898 8945 P01019

6647 P01137 P49639 8829 P01375 Q16254 Q9UJM3 5451 5573 Q9UHI8 2065 P35222 O60716 P35227 Q01974 P57082 P17813 P10070 P10071 11331 668 5566 6776 P02458 P01127 6657 5207 P49747 9927 O43474 6670 P07942 Q9Y5Q3 O00628 5584 P25490 O43597 P07949 Q9NX62 O95076 25945 673 P15692 Q99684 P31939 6667 P55075 10454 P40763 P26232 5471 Q6IR47 Q08117 P68032 3052 Q9UBX0 P37231 682 10229 51295 3169 5468 54206 7408 Q92922 7528 10468 5361 P24385 7422 P56178 P56177 2099 333 7414 10672 Q9GZT9 9839 5371 P05997 9612 Q9UIG0 Q8NHX1 P20908 23513 P13497 P17612 54583 P58012 P18827 8516 8879 6599 5268 O75386 Q9UPN3 Q9Y4K3 O43318 230 Q9H161 474 595 356 P09493 Q99807 P49810 P08047 Q7Z6C1 P43034 Q7Z6C3 P49815 Q13485 P09017 Q9NPC8 9510 Q15303 94234 5154 5155 4188 8543 P12429 O75496 P58304 P13631 Q02447 367 Q9H2X0 30813 P46531 P00533 Q15796 Q14344 Q15672 Q16881 6496 P61968 6495 Q15797 6256 6015 P32242 P31273 P31276 P12755 Q9ULH7 O75360 257 P23528 Q96RK4 5159 Q8WZ42 P00441 8443 4086 4087 4088 4089 8323 Q14118 P36952 Q9Y618 Q86YT6 P12883 Q04725 Q04724 Q4AC94 P21359 Q04726 P31270 P08151 P24864 10087 6259 P62942 9421 8454 9420 7124 7248 8214 Q8TD84 2909 Q14129 P63092 P35453 P10275 4092 O75581 Q02535 Q93063 P46109 P23229 Q9NYQ6 Q15466 Q16790 Q15223 P45379 Q99966 54 8100 O43521 9314 P36894 Q86WV8 7490 285 166 Q6PRX2 338917 7249 Q9NQB0 P09067 1948 Q99958 Q15475 Q99835 Q96T37 Q8TCU4 O75569 P14859 P13646 7020 Q08188 P08069 P47895 2926 P49715 1956 P09038 O15078 P39905 P38936 P35548 Q14160 O95947 1969 1605 P60842 Q13873 7046 9464 O15123 P22735 Q8TAF8 P41134 Q1PSW9 P39060 P04085 Q92574 P42229 O15119 P98161 3911 3912 1856 Q9NNX1 Q13642 Q13520 Q96QB1 7291 P10644 7052 P55316 79365 P16070 P98174 Q14865 7189 7067 P19793 O75881 P29590 10908 Q03014 Q13418 Q63HK5 6096 1634 1874 P21860 P21980 O95859 Q9NSC2 P11717 O95863 A4D1W7 2735 P43405 Q9NZR4 Q04206 Q92786 P07101 P07585 1406 2737 2736 7088 O95999 9021 P78504 P35613 P08123 4920 Q13563 2626 P31249 Q15742 26005 7090 P20248 P08253 P08138 Q15750 P10415 3860 57045 163183 2885 3611 Q04656 Q14790 51741 P43694 80184 O15111 O15353 Q15648 O15230 Q04771 O60353 Q9HCS4 P24821 O15105 P07148 O15228

positive regulation of intracellular protein kinase cascade 8772 Q9Y4K3 O43318 O15519 356 23286 Q92851 8767 O14788 57506 P07996 Q9HC98 7334 4067 7335 65018 2316 367 Q04864 P14174 P09382 10783 P00533 Q13490 1902 9641 8795 Q9HBW0 P32121 Q9Y6Q6 10758 Q9UDY8 142678 64170 5970 P48023 Q86XR7 Q9H257 Q9NR96 P17302 387 389 O60674 O00182 2697 Q8IUC6 5966 P04233 P62942 6275 Q13158 7124 Q9Y3E0 O00463 P10275 154 P15056 Q9Y2C9 1499 10616 P07550 O94827 P61586 1936 P08887 7132 P29466 Q9Y239 124583 5071 P37198 29110 960 1147 Q02763 P01308 841 2475 843 Q7Z7H5 Q9NQB0 O15169 P02790 10392 P50591 O43734 Q9P0L0 Q9NS68 Q8WVQ1 Q8IUH5 2247 P01579 P08069 O14763 P05112 1956 P09038 Q92633 Q9H8V3 23118 27032 54862 Q14164 P09601 P98194 Q9UL54 Q13077 P06213 Q15811 P01241 7046 P35813 P14317 3481 P42345 Q9BXM7 3479 9218 P01236 5536 Q15389 Q9NSA1 P01112 7057 6885 Q13404 9590 2280 7052 2159 4215 P01588 P16070 Q99759 408 P49407 P01344 409 P01343 O00206 7189 7188 P48431 3265 Q13418 3263 7185 148022 Q9BYM8 Q02952 P01019 51026 P21980 55504 P01137 O60260 P01375 P04626 Q13546 55072 Q7Z434 2064 P35222 9495 P35348 P19438 Q04206 P53041 6657 8837 P24588 8717 O43353 Q96EP0 O95999 P13945 P07948 3162 9020 79971 673 57708 5579 P00742 3953 P05771 Q6P1N0 3956 Q96FA3 O00635 7099 7098 Q6IR47 8743 Q15628 64127 Q99558 P08134 8737 329 P48357 3965 P21333 Q9NYJ8 6453 Q9UHD2 Q9BUZ4 57162 3611 P61088 Q14790 Q5T9L3 O15455 Q16610 P41159 Q9BT67 23636 5494 Q60FE5 O15111 Q86Z14 222068 Q9HC29 54106 P26447 P25116 P60568

negative regulation of transcription from RNA polymerase II promoter 6595 6597 Q9Y2W7 8535 O75820 2672 P10599 Q9UBB5 O00267 124790 6591 Q9Y4K3 2304 P84022 5017 5933 Q13363 4188 Q9Y2X9 Q12837 O75376 P13631 3096 4734 Q15672 Q9UQR1 O75925 6256 6498 Q96IZ0 3660 P12757 P31273 P12755 23028 O75360 4193 P13056 83933 116113 811 Q9UJU2 O15534 Q92833 Q13263 Q6IT96 P26358 P06400 5716 P48382 8204 4088 Q9BY41 Q92949 P55055 Q9Y618 P10588 P10589 9099 10524 1487 P19838 P62263 P20264 3665 Q15697 51317 53615 Q13033 9421 P54259 5187 7124 11142 Q12986 O75461 Q9UQL6 4092 Q9Y6K1 O14627 6829 Q02535 10614 1499 Q86VE0 51564 Q15583 8328 Q8N2W9 Q15466 6827 5074 6045 7376 Q92769 Q9HAZ2 5993 O15055 Q14938 Q15906 O43524 23135 P19544 O95365 7490 O00358 1822 166 P08651 Q9NQB0 Q8WW38 O14753 604 5987 Q96T37 P61925 4790 P15822 O00488 51341 Q9BZK7 51588 Q92993 857 10155 Q8N488 P49715 P10826 P10827 O75437 7157 2023 O43623 O00255 Q6KC79 P19883 O00257 1050 Q99496 Q9UMX1 4799 Q5VTD9 P40337 Q92985 O96004 P04637 Q01094 P14316 P52952 P41134 Q1PSW9 3476 Q15022 79813 4204 9219 639 10014 Q9NSA3 P56524 O94992 4221 Q969S8 P16989 P57059 7291 7295 Q96BD5 P46934 P33076 Q99750 8932 7727 1869 23429 7189 7067 P48552 P48431 Q8WYK2 6774 P19793 7181 Q03014 Q7Z2Q5 Q86UE4 Q96S42 O15379 55869 P01137 Q9NSC2 P01375 O15151 P51531 O95983 Q13422 O94776 Q13547 8841 Q9UHI6 P35222 2063 P51532 P78318 P35227 11218 Q96ST3 O43829 P17813 P10070 Q15170 84733 P10071 3146 Q92786 6657 5569 Q9Y2B9 Q96MH2 P23497 2737 2736 P06733 6672 O75626 P25490 Q8WUI4 P41182 25822 25942 Q8NFW5 3281 Q9H9B1 Q99684 6304 Q14894 P40763 4261 150094 8864 Q9NP71 Q08117 23414 P37231 1788 P14373 1786 Q00987 3169 P41162 5467 5468 3720 7528 10468 P27797 Q14781 P09429 O75604 P56177 29966 O60907 Q02363 3066 3065 P18146 P51843 P51608 O60341 Q01826 O75953 O75832 9612 Q13227 63976 23512 P63272 P28749 O15105 7428 10320 5925

biosynthetic process O75947 8099 Q9H6Q4 P54619 P51587 P52434 P17676 Q9BYG3 2305 55568 2300 6901 Q9H0D6 P28335 O75936 25913 P30876 P08729 O14561 P52209 Q8NE71 Q06136 P37268 P19623 10667 2 1468 P62244 80755 P14174 P42858 P62241 27090 Q9Y285 140801 Q96EB6 64282 P62249 P27694 Q9UKV8 P27695 3661 23708 2572 2571 P17405 Q8NBQ5 6927 P40429 Q9Y295 5832 5833 Q9Y291 P40424 Q9UKW6 5836 3659 10632 P54886 Q9UIS9 P08708 P26373 Q99583 Q12857 1491 P54646 Q8IYK4 P20823 P62266 Q8NFW8 P19838 Q9BYD1 P62263 148789 P62269 10643 Q9Y3U8 P11926 Q13951 Q9Y223 4772 1022 2593 Q9NSU2 2590 P50454 55361 Q8WVC6 O95352 P39019 27043 Q5T160 1017 79577 2589 Q6PI48 P39023 833 5610 O75909 Q9Y231 5859 Q9Y478 4780 3692 Q13724 5631 80308 Q9UGI9 Q15904 Q8WVB6 O95363 Q7LGA3 27297 P42898 O14519 10625 Q96MV8 1025 P61353 P20648 4775 P05023 P07686 O14757 Q92766 P05026 P04179 Q9UKR5 4790 Q9UGJ0 5883 P28340 2132 1040 P16118 Q9NR45 Q8IUH5 Q9NR48 Q9BWE0 Q9HCL2 55149 80347 P60033 Q96N66 1277 Q9Y250 Q92994 5875 6723 P11908 5636 4548 Q10472 Q10471 P51575 P10827 P10828 22828 P31749 Q9NR50 Q96L21 Q9NR55 1050 55152 27032 10606 253980 1289 O60870 865 P04150 Q9Y262 4799 5888 P06576 O96004 64432 5422 O00303 23569 11108 871 Q8NBJ5 3479 51363 Q13085 P25205 84289 P00387 Q15025 P62875 6749 Q9BTU6 Q9Y676 P49411 5430 Q86SR1 5432 P36578 P35244 P14324 P35249 Q99755 Q96T76 Q03933 641 P35251 P62888 P61313 5424 P49643 O43294 408 8939 P27635 P49407 P60228 409 1080 O43252 84061 5440 P49642 5441 6772 5442 Q9UKN5 O96020 Q9UKN8 Q9BW92 Q66K89 29088 Q8IYD1 51144 Q99741 Q96T60 P62899 5434 51148 Q14197 51147 8943 Q9Y450 Q9UN42 10476 Q15046 Q15287 P50440 Q9UMS4 4361 Q9Y697 3035 Q9NUX5 Q53H96 23556 Q96ST3 55750 P14550 P17812 P15880 P35232 Q00403 29093 4598 Q15054 55757 10488 5207 Q59EA4 84296 P62424 79709 O00746 7884 P06737 10690 P50613 245972 P51946 P39656 23764 2194 25942 Q9Y5Q8 Q9NUQ2 Q9NWU5 Q8WUY8 P60891 435 P31939 128308 Q96GC5 Q07020 P06744

P05413 5471 3290 O00757 3054 29079 P37231 10229 Q9UBX3 445 688 689 P49448 P82673 207 5226 5468 Q02809 P82675 P62847 29074 P62841 P01266 Q7LFX5 P61513 P49207 4150 O00767 23746 4152 P24386 23509 Q9BRT9 5245 Q9HCD5 P52815 O00764 211 Q99543 4149 P62857 P62851 10432 57727 P62854 8761 Q92903 6342 Q9H4F1 Q92901 P07814 Q92905 P35268 54460 3074 O75031 468 55312 10682 P60201 Q92900 P56192 P49427 4172 4173 4174 Q14686 4176 Q14209 Q16864 Q9UPN6 64834 Q9H0U6 Q6NZI2 4171 Q96GX9 474 Q9H0U3 476 477 478 28973 Q99807 P46783 P83731 Q9C0C2 3091 P13639 Q16633 6123 8543 P46782 P13637 Q16635 6125 P46781 O75376 P41091 481 483 60489 488 P68104 60488 51773 6117 P22102 6119 P00533 6118 P46777 Q15554 P46776 P60866 P46779 Q13131 P46778 Q6YP21 Q16649 6133 P35638 P61968 6132 11041 6134 6137 P56134 Q16647 P32121 Q9UNL4 O43776 490 O75362 29803 495 498 Q01105 6128 P56385 60496 P82663 P82664 1915 P56381 Q13144 51547 Q14232 Q13148 Q9BYU1 6141 P35869 55907 P43490 8565 P18074 8566 P62829 Q01581 P18077 O75352 P36954 Q9P2E3 Q13393 6138 Q16665 Q9P2E9 9421 P78347 P54136 9420 6156 6158 P54132 P36542 P35453 271 Q9Y6K0 Q93063 P09172 Q04837 Q9Y2C2 Q14494 Q14493 1937 1936 1933 6164 O15294 Q9NRC8 7013 6168 7014 P22392 O75575 6160 O43529 P11498 Q93050 Q7L2H7 11277 P49848 P61927 6175 10390 25885 Q02878 P48507 P24539 51102 P02549 Q96PK6 P24534 11243 P55010 55703 P47897 1718 P00367 9439 P47895 Q15125 2805 1956 6185 6188 6187 Q15126 Q9HB90 O43741 P21283 P21281 Q86Y79 Q09028 5091 6182 P37840 5092 O75792 64428 6184 O43505 Q15370 Q92187 10166 1728 51116 Q99814 1727 P98194 1968 Q7Z7F7 Q9NRF9 P78545 P54577 6199 Q6P1J9 P29375 P78540 P10515 P29374 Q96EL2 6193 6194 1975 Q05329 27000 2821 Q9Y4C1 P41252 P62195 P41250 Q6P5R6 P05177 Q13761 1737 1736 9477 375743 9232 22803 Q9P1Y6 Q9H2P9 1503 P24928 Q9BYN8 O60296 Q6P1K2 Q5D0E6 7068 7067 Q15714 Q13535 8394 P55209 P56537 1756 29880 Q6ZNC8 P00966 O60264 Q9Y2A9 P54368 O15270 9255 Q16816 A4D1W7 63931 Q9Y2B5 57017 Q9BWH6 P41222 P43405 201595 1763 Q04446 O15269 P53041 P07101 O95749 Q15738 O95997 Q9P015 Q8N6G5 7083 P00505 P20594 P08243 4800 2626 51727 Q9NVC6 Q14653 P19387 P19388 P11413 93100 Q9H9A7 83548 2875 2632 O60256 1540 57038 80270 P22303 P09104 P46977 P46976 9045 P55884 P13807 Q9NP81 Q6P1L8 O75964 3980 28998 Q9P035 P32320 P32322 Q00059 2644 2643 P17174 Q96AE4 Q04656 P43694 3978 Q9Y4A8 Q13347 9054 9296 P42357 5927 5928 P63272 2651 P24941 Q9HB03 4830 Q9HCS7 902 4833 Q9HD40 Q13112 51514 P21675 Q9NZN9 O15228 P10914 Q92858 O75821 O75822 65003 P67809 9061 O00268 Q8IXM3 51081 O00267 P30520 Q9P2R7 10535 P13196 P16220 P60174 Q9ULW3 5931 10533 P0CG13 P48047 Q12952 4860 Q9NRX2 Q9Y399 54361 55454 51099 P16471 Q9UI12 5705 P25398 Q9ULX9 Q9Y2Y8 Q9NRY4 1121 Q9BZG8 Q12968 P18621 O60547 P18858 1355 P12081 P18859 1111 P63220 O60783 P32929 Q92830 P03372 Q9Y2Z4 P49591 Q92824 P09917 Q9Y2Z9 4882 Q8NCR0 Q6P2C8 9093 P56705 P49590 Q13829 O60318 10767 Q8WTX9 O60551 P49588 P07741 P04233 P30566 O95477 5981 Q12986 5983 1385 Q9UHJ9 57661 6829 P19525 Q6FHQ0 O60762 2224 O60760 5733 5976 27161 6827 Q9Y2T7 Q9NYA1 Q14938 O00472 Q5JTZ9 Q06210 P30793 Q9NS56 Q8WYH8 10987 57678 2237 1388 P54709 P08651 Q92643 Q6XQN6 P05386 P05387 Q6ZWT7 P05388 Q9UNX3 Q9NYB0 O43175 P30304 P28223 137964 6850 P32969 P30307 4673 Q06203 Q9H9Y6 6609 P42765 P42766 80222 10714 P61244 2247 284119 975 O14641 P61247 O14640 Q6GMV3 5514 Q92878 5757 P08865 Q9UNY4 Q15800 161823 P09958 50814 P30536 P52788 Q03164 55035 Q9BZJ4 10725 740 Q99259 Q9P0M9 P61254 10728 P09960 4677 Q6DHV7 4678 P06454 Q9Y2W1 509 P02452 O00425 P23396 6872 Q9UPV9 6871 P04637 2034 440138 P16615 P53803 Q00796 P15531 990 P11172 P62750 Q8WTS1 2026 995 513 997 514 8803 6622 515 P61421 11222 Q16236 5536 Q15389 518 P62753 P49770 200916 Q9GZR5 Q9UM07 6883 P48201 Q9HDC9 25873 P12268 O76071 Q15392 521 522 51253 P11182 523 Q9Y6Y0 526 6878 8812 P01588 528 Q99638 P28845 P01343 P49768 P26641 P26640 P48431 5562 Q9BV79 5321

5563 5565 P13569 P11388 P11387 O60701 51021 51023 P26639 533 51264 535 Q53X93 537 6647 5557 P61201 P01137 539 5558 91949 P01133 P01375 23438 Q9BT22 23439 Q9Y2R5 P02461 P01130 Q9H7H0 6660 P40938 P40937 3396 23435 Q9Y2R9 P12004 Q9NS37 Q99611 O14617 Q96RR1 545 P09086 P61457 8833 Q96EY1 6657 P61218 Q5TKA1 O00628 Q3SY69 7764 Q12904 23400 P17752 Q96PU8 54550 Q8IZL8 3159 Q02930 Q9BZE1 5336 Q15185 5337 10331 P46063 5351 Q9H5J4 P24298 6201 5111 6202 23650 Q9ULK4 23411 O76021 Q969G6 Q99437 P18847 P18848 P83881 P49327 328 Q08462 6210 P50750 P49321 2099 P50993 P51843 O14802 10318 51067 56994 Q8N8Y2 6204 6207 P62979 10554 P83436 Q9H5H4 P04424 8880 A2RTX5 P27540 7311 7555 391356 P39748 P20908 P15559 P51610 O00411 Q9NVV4 P15313 P16885 51074 Q9NRN7 P52948 Q9NPJ6 P62988 54107 6217 9967 10090 Q14566 6231 8892 4297 Q6DKI1 6233 P15927 64951 P62917 Q9UKD1 P30085 P13984 6470 4291 Q9NYU1 Q9NYU2 Q03426 64949 353 Q9UBS0 P62910 6229 P62913 8662 P20290 P03950 8424 O00716 64960 16 8667 Q16514 8665 19 64963 63875 26205 64965 P13995 64969 367 Q9UBT6 51650 51651 6238 11198 54921 6239 9524 11161 Q76FK4 64975 P31153 29922 Q53GQ0 Q03405 P49916 P49915 P25705 P17096 8669 54938 P49914 P21589 51426 31 Q14353 P00441 34 Q53H12 8443 O75478 9533 7358 Q9Y619 Q86UL3 P29084 64745 P34896 P11310 142 Q9UBV7 Q02543 P62701 P09001 P20020 9526 P62945 P23771 1801 O15160 P48651 8458 7124 O15164 2909 7126 Q7Z6G3 P38606 P10275 23127 4094 Q99720 P27708 158 Q9Y3E5 2908 P23588 84365 Q13287 Q9NSE4 Q14134 Q99966 Q8WV24 P49736 P04843 5198 P04844 8467 O43889 P15735 Q9BW60 7372 P35573 Q8NFM5 Q01433 Q13057 8473 P00491 O75208 P00492 P61803 7389 P54274 Q9HA82 Q96FX2 O75444 P11586 Q96T21 Q99943 Q96T23 178 Q99941 P49959 P50914 Q00577 51222 P22234 9319 Q14152 Q9UBQ5 Q16576 Q99707 9318 P49716 P49715 P09038 11128 Q9NQC7 7153 10280 Q08050 7157 P62906 P14868 7150 1841 2935 P78424 Q13873 7167 Q7KZN9 Q96QC0 P33992 P62081 P33993 P33991 P18124 1854 P42224 P47914 Q9HC16 Q92696 P82921 1856 Q9UBK2 O15371 P82912 Q8WUD6 9114 O15372 P82914 Q13405 22928 O95602 Q13887 Q96I24 Q9H7Z7 Q8TCJ2 O75891 55173 Q9Y3B7 P84098 P63173 56267 Q08945 P05062 P08575 1629 2959 Q9UBL3 P29474 P29475 O94766 O43812 26015 O75881 286826 1877 P00846 4907 Q9NZ01 2961 2960 1870 P04183 Q9UHY7 P23921 Q9BTC0 Q9UBM7 2969 P09874 P53396 O95865 29777 79143 10921 P42677 Q9Y3D3 2730 2972 Q9Y3D5 57136 P09884 Q9NYP7 P06132 P82932 Q969Q0 P82933 P30049 Q15853 P33316 Q14527 56052 Q9H488 P62280 P30046 P30047 P62277 P22415 P05093 P30050 Q03468 1650 58478 Q8NFF5 P43115 2747 P47712 O95644 51606 9397 Q8WZ19 9391 P11532 27339 58487 P23511 51611 P09467 Q9Y5B9 Q9Y5B8 P35711 3615 3614 2762 57169 Q6P4R8 P01730 Q5T4B2 4946 51629 P54687 Q15643 O95427 Q9P2Y4 Q15648 Q14558 27315 2531 4953 Q15653

negative regulation of catalytic activity P25054 Q9BY76 11065 2672 2550 Q9NRW4 124790 9184 O60566 Q9UBS5 Q9NPC1 5018 P49815 6901 P07996 P04004 P09936 25913 P55036 Q12834 O15530 Q16635 O43684 Q86VP6 O95684 P50148 Q9P107 5706 2316 5707 5708 5709 7448 51654 5700 Q16401 5701 5702 10542 Q96EB6 5704 P24522 5705 Q7Z727 Q13257 P35998 Q9C010 P53667 5717 P46527 57761 10197 P61289 5713 P06400 5716 5054 Q15208 P31947 O43306 9093 P21917 Q99460 5170 P21359 P19838 10641 3309 P62942 Q9UL15 4771 11142 P51668 P51665 4092 10614 Q13042 Q92530 P45379 Q13286 7013 O00231 Q9Y4H2 O00233 26524 O00232 P37198 1029 P11021 1027 1026 P63096 Q8NFM4 P01308 Q8NFM5 7249 2917 P49721 P42771 P49720 Q13177 Q99956 P61925 4790 O95257 7023 Q9UGJ0 P49840 P54274 O00487 P30542 O60503 Q9UNH7 P62333 P20618 Q01664 58533 O14640 1718 857 859 5515 5518 Q15008 Q9BY84 P51693 Q9UL46 7157 P19086 P31749 O43504 O00255 P38936 P37840 3350 2810 2931 P17342 55832 Q15011 5524 1728 P05121 1848 Q15257 P08754 Q9UKT4 O95835 P78540 P04637 9467 P38405 7161 P22736 991 26271 Q96RU8 Q96RU7 1852 Q99523 1850 10935 P62195 3476 6622 10013 P15090 O94992 7057 64682 4221 2280 56940 Q8WTR2 O43609

P67775 Q15392 P08575 P42574 P60900 Q13098 11116 P49768 5682 P29474 5683 Q8N726 5684 5685 5686 P14416 Q9H422 Q13418 P56539 84619 P41597 P40145 Q99500 P17252 P35240 O60266 P25789 P25788 P25787 Q9UJM3 O95622 5692 O15392 5693 Q9NUX5 P78318 P17813 5687 5688 Q96EY1 5569 Q9Y2B9 Q96MH2 Q9UBN7 1647 P01040 O43597 Q16828 P30047 P30048 P61981 O75190 Q8WTW4 Q99684 P51828 5578 3953 10213 Q13200 Q8IWV1 O43242 Q13202 7532 Q14416 P30153 23411 5590 P37231 Q9UJX2 Q96SZ6 Q05513 2873 324 Q15070 207 Q9H1A4 5468 54206 P48357 P21333 Q08462 P28065 O00762 O75293 2644 333 3611 Q9NRM7 Q04656 Q16610 23636 O15350 8881 Q60FE5 O75832 Q13227 P34972 P62191 P28074 P15559 P17980 P08908 P28072 P28070 57732 Q8N5A5 O14818 2773 P30260 O15105 P43686 P21554 5925

positive regulation of DNA binding 6597 6598 2033 Q12824 9181 Q9Y4K3 O60603 P15531 10935 Q7Z6C1 8767 P01116 O14788 10014 Q92974 Q9UBK2 P56524 7334 Q13404 P12429 Q15306 7335 O00206 Q92838 7186 Q09472 7189 7188 P12755 7185 148022 Q9Y6Q6 Q9BYM8 Q8IUD2 Q86UE4 Q9UDY8 P01019 P01137 4869 P01375 8721 5970 P18074 3673 Q13546 55072 Q9NR96 3551 P51532 5728 P17301 O60674 P60484 3148 Q8IUC6 Q04206 3146 Q9ULZ3 P23497 O43353 P26583 6672 Q96EP0 O95999 7124 29108 O00463 Q9UQL6 P53567 Q7RTR2 P30048 1896 23085 10616 Q9Y6K9 P04040 7099 7098 Q9NYA1 Q9Y239 Q15109 P14923 64127 P37231 3728 P01308 Q8TAI7 3169 8737 5468 847 P09429 10392 O14495 O60869 P61088 O15455 O14920 P05112 Q12933 23476 P51617 Q9HC29 8517 4830 P05362 54106 Q13077

regulation of transcription Q9UKT9 Q9H0E3 Q9Y265 Q8NDW4 Q12824 8091 124790 10657 2305 2304 2303 114991 2300 155061 284695 2308 2307 Q9H0D6 Q92974 Q9H4L7 Q12837 Q8WXB4 2316 10661 Q96EB6 O75925 Q9UKV8 4990 3660 3661 Q9BYE7 P37023 P13056 84901 57761 55588 O75928 Q9UKW6 3659 P26358 Q6NUN9 Q5BKZ1 Q92949 Q99583 Q12857 Q13705 10645 1488 1487 283337 Q99576 3665 64061 P26367 Q9UL18 1022 Q8NA42 Q06587 114548 Q8IX07 Q5JT82 Q96MX3 1017 10614 79576 Q9HAJ7 1499 10616 O14981 Q9Y230 P61586 O75909 O14503 Q12873 Q12872 Q9Y239 Q9Y5Z7 Q8WXF1 6720 Q15906 285527 O00358 Q9BPY8 57798 1029 3207 10626 1027 1025 P17482 P17480 604 Q9Y242 P04179 Q15910 Q9UL36 Q12888 Q9BQ95 O60869 79595 Q8NA19 P38398 57326 55145 3215 Q9Y250 6721 Q92994 Q92993 Q8N488 Q92997 Q9UKS7 P10826 P10827 P10828 221937 5411 Q8NDX6 1050 P53999 79101 57332 3226 3225 P04150 Q9UGL1 Q92985 O95159 Q9UKL0 O96004 P14317 P14316 23569 11108 11107 Q8WWY6 1059 Q15022 639 P62875 6749 6760 Q9Y5V3 5430 O96019 O95163 P16989 P57059 Q03933 51131 641 P33076 51132 P35251 Q14186 Q6B0B8 79923 8932 648 8939 P49407 Q14188 O00327 O96028 5440 P48552 5441 6772 Q9UKN5 Q8TAU3 P48551 6774 Q99990 346171 O60828 P15621 Q66K89 Q2M3W8 Q14192 P01019 51147 Q9Y692 Q15047 P23246 Q86X95 5451 Q8N3J9 P35222 Q5T6S3 P35227 P35226 O14979 P57082 O75081 P17813 P35232 Q00403 53335 56849 668 6776 Q96MH2 Q16385 7629 O43474 P06733 O00744 5460 Q9Y5Q3 9821 P25490 3281 Q9NX65 672 P15692 Q9UBW7 6304 57708 Q6UUV7 P05412 O00755 Q9Y5R5 9831 3297 Q9NX70 23532 Q9UBX0 P14373 57713 688 148327 689 5467 9826 5468 Q8N895 Q92922 4150 4152 23509 P56178 9841 P56177 Q8NHY6 Q12809 P17844 4149 O14920 57727 9839 O43463 5494 Q8NHX1 23512 Q96QT6 O14936 8517 9849 4172 O15085 P36508 4173 4174 P35659 Q14686 4176 Q14209 8535 Q9BZ95 Q6NZI2 Q9H165 4171 Q9H161 Q9H9F9 Q9H160 P08047 5017 P78395 Q9BQG0 P07199 Q13363 Q9NPC8 Q14693 94234 4188 8543 5036 79084 P58304 Q96F45 O15090 23051 Q04864 Q9Y2H8 9869 7205 Q8WY36 Q96RL1 Q15554 Q9HAU4 8553 P35638 P61968 Q8IWY8 Q96IZ0 Q9UPP1 P32121 P31274 P31273 P31276 O75123 Q9ULH7 23028 Q9NY61 4193 Q9Y6Q6 257 Q9H9D4 Q6DD87 Q15561 8548 Q96RK0 51548 Q93009 51547 P61964 Q15562 Q9BYU1 P62826 9412 P36956 O75592 Q96F24 P31270

Q9NPF5 Q13398 55915 9421 P78347 9425 O75586 O75582 Q9Y6K1 Q93062 Q03989 Q71SY5 Q9Y6K9 51564 Q15583 55922 5074 P78337 55929 11030 Q9HAZ2 Q15109 P22392 O15055 O43524 26523 7251 23492 O15047 P49848 Q15596 8110 P61925 Q3KNS6 O15062 5089 166968 O95931 23466 23468 O15060 79913 51588 10155 9439 Q96NG5 286075 Q9NPA8 64426 5092 64428 54625 O95947 54623 Q8IWS0 Q96RE7 9464 P29375 P29374 27005 Q7Z589 Q9H0M0 P22736 Q9BUG6 Q96EK4 Q9UGU0 Q13761 Q58WW2 P56524 9477 O75534 Q02297 7291 O75530 7290 7295 P24928 Q6P1K2 O75528 P0C7X2 Q8WXI9 Q9BYM8 1993 79039 Q96IQ9 O15499 P56545 O95983 A4D1W7 O95503 55072 P78317 P78318 Q8TBE0 Q9HAK2 Q9UH92 Q9Y2B9 Q96EP0 O14593 O95999 Q9NP66 23081 O43918 23085 Q9UKY1 Q9H9B1 P85037 P31260 Q6P1N0 P31249 Q14653 Q9NP71 Q9NP72 3728 1540 Q9BUJ2 3720 5901 P46734 Q13330 P12956 Q6P1L6 Q9P031 Q9H4W6 Q00059 Q9BQA5 Q9Y6E7 Q9H582 P61088 Q96AE4 147808 Q96RG2 Q68DY9 P43694 O15455 Q9BQA1 5914 11091 O75953 63976 63978 5927 5928 P63279 P00519 5929 P63272 23077 P24941 Q92585 Q15532 Q13351 Q13352 5925 Q7KZF4 P10914 O95677 Q92858 P67809 Q9UBB5 Q9NS86 A5YKK6 Q9P2R6 4609 P16220 10772 5931 P27348 5933 O15516 Q9UBC3 P27361 54361 1107 284323 4613 1108 O15525 Q92838 Q9UK58 Q7Z3K6 P05549 Q9NRY4 Q99471 Q7Z3K3 O60548 Q92830 Q9UJU2 284312 O15534 P49116 P03372 Q92833 P06401 Q92831 P06400 Q92826 P07737 5970 5971 9093 Q9UBE8 Q9NRZ9 P56705 9099 P17535 P17544 Q99459 P17542 57410 400720 P61296 5966 10765 5981 P50570 O00463 O00468 O95231 Q9UFF9 O14627 6829 10736 Q6FHQ0 P41218 Q8N2W9 4646 27161 5978 6827 65056 5991 93986 5993 Q14938 O00472 Q06455 55250 Q9NS56 P37198 79685 6839 P08651 5987 O95259 Q10586 Q8ND82 P51449 Q6IA86 27148 O00488 Q02086 P61244 Q9BXG8 4664 5511 Q12770 5515 P05112 26292 Q9UNY4 5518 O43186 Q12772 Q12778 O43189 Q05586 2023 Q02078 Q6KC79 10725 57459 Q99496 P40337 7704 P23396 O43159 6872 6871 P04637 2034 2033 Q9NVW2 O00422 Q9UFB7 4204 P01116 4205 Q16236 4209 Q9C0K0 6883 Q9BT49 4221 Q9GZR2 O43167 23210 P50548 O76071 Q9NRP7 84159 4214 P01106 6878 P01588 7727 56946 Q9C0J9 P01100 Q7Z7K2 7741 Q8N726 9921 P52736 Q53X93 6886 P01137 Q16254 P52740 7750 6421 5573 P04626 2064 P52747 2063 11218 Q9NS37 Q99853 P10070 P09086 P10071 10363 10362 5569 2070 O43593 5585 7764 25822 79612 O75190 Q02930 7756 5579 10336 8607 P40763 2081 5591 Q9GZV8 4261 Q6IR47 Q3SY56 64127 O75177 P18850 P15336 O75182 Q9NRL2 56987 51295 P18846 Q68CJ9 P18847 P18848 326 55657 Q92800 P27797 Q9GZU7 Q9UNP9 2099 O60907 O75164 P51843 55662 Q8NCN2 91748 Q9H7L9 10794 8625 5119 Q9GZT9 23636 9975 4287 P62508 P51858 146050 221656 Q9NVV9 P58012 10320 9967 P25116 Q99417 Q96HZ4 O43670 Q14566 8655 4297 9500 Q9UKD1 P13984 351 P21127 P25963 P09017 P20290 Q15424 7332 7334 7335 26205 85403 367 Q70SY1 11198 7329 Q14586 20 7341 P11309 6496 6495 Q8IVW6 Q14103 6498 P11308 Q99909 6015 P32242 Q8N3C0 Q9Y606 84108 116113 P17096 Q14592 11168 Q96MA1 Q9UBU8 Q13263 Q8TF50 8204 Q14119 Q9BY41 Q9Y618 P12645 P29084 Q86WP2 222546 Q04725 Q04724 387 Q04727 Q9NWT6 Q04726 23152 P08151 P20264 11177 11176 P23771 P55347 9541 5187 11142 O15164 2909 391 Q6MZP7 23126 25788 O43638 Q02535 Q01201 2908 Q9UFW8 Q15466 Q6PJG2 Q99966 Q13285 2902 5195 6045 7376 23133 23135 Q3KQV3 Q6PRX2 P09067 Q14140 Q9UBP4 Q99958 Q15475 Q8TEY5 Q96ME7 Q9HA82 P14859 Q8TAQ5 Q96DT7 Q9H063 Q99941 Q01664 51222 56916 P01579 Q14151 P09038 9575 P82979 8242 10284 O75676 O15198 O43623 25776 51230 1609 Q5VVH5 Q15014 86 P78424 Q9NWH9 P33992 P33993 P33991 Q8TAF7 P41134 Q1PSW9 27122 91 P17030 94 O15119 1616 P45973 Q9UBK2 Q9NSA3 P78412 9112 Q96I24 Q9H7Z7 Q9H3R0 P55318 Q99081 P55316 P67775 Q52LR7 P17040 P05067 2959 Q9UBL3 O94763 92129 27107 Q9H422 Q460N5 Q03014 6096 Q9BTC8 2961 2960 O95619 Q8TAD8 Q9NSC2 2969 P09874 O15151 8289 Q15843 O95625 O15156 O94776 29777 202559 P17023 2972 79149 Q9BXJ9 P17020 Q8N393 Q8HWS3 P04198 Q9UBN7 Q15853 O75626

Q14527 9149 Q8TF47 Q6NYC1 Q9H488 P41182 Q8TAK5 93474 79175 Q499Z4 P53567 79177 P30048 84525 1655 84528 P22415 Q9UK80 Q03468 O75629 O95644 150094 Q9UBG7 Q8N7H5 P20248 64919 80155 Q8TAI7 P41162 P17081 Q9Y5B6 Q9Y5B9 P09429 O75604 P41161 P35711 23186 P09430 Q86SE9 728642 Q9P2Y4 Q13227 P55771 Q04771 O15105 Q13233 Q6NX49 Q9H6Q4 O14793 25909 9181 P51587 P17676 Q8TDD1 2547 57504 P84022 O00151 Q92731 O14788 10413 Q7Z6R9 Q92729 Q5VTR2 O60216 Q9H2G9 4734 Q9H2G4 O00167 22850 81628 54496 6927 Q8IUD2 6929 Q6AHZ1 Q96KM6 Q8N680 811 6921 P40425 P40424 6926 6925 Q6IQ32 Q17R98 Q9C005 333929 Q9UIS9 Q9NZI7 P48382 Q9NQX0 P31946 Q9C009 2100 Q9NR96 P48380 O95343 3428 6938 P20823 55352 O60674 P19838 O60675 Q8IUC6 P62263 5604 6936 Q9Y468 Q8IZ40 Q9Y463 4772 Q9Y466 Q8TDI0 Q9UIL8 10856 Q86VE0 2103 P16383 27287 80324 Q08999 O14744 6945 P50222 4780 Q92769 Q14814 P28370 Q07666 O95361 80306 P10600 O95365 Q06330 2119 10865 P19419 Q6PI57 4775 O14753 847 Q92766 4790 4791 4792 2130 81669 Q9NR48 Q92753 4302 857 Q92754 Q13501 22827 Q13503 Q14839 P19883 Q13506 Q9NR55 2139 861 862 863 Q9UMX1 4799 P09601 50943 Q9Y3Y2 Q9BS34 P15408 Q9Y3Q8 3481 Q9UIH9 23326 O60603 25988 P15884 Q8WUU5 Q96T88 3479 2146 3476 10011 P25205 7832 10013 84289 Q96KG9 55723 10499 10014 4343 Q96T76 Q99750 406 O43294 408 409 Q96C55 Q09472 Q8N1G0 O43251 23309 890 Q00653 Q99743 898 29080 Q9UN42 P49639 P51531 8721 7874 148156 Q9NR11 P51532 Q96ST3 P60484 Q15291 11331 55758 10001 P60002 10488 Q96T58 P28702 P23497 P26583 Q9Y3M2 P50613 P51946 23764 25942 Q8NFW5 Q9NUQ3 Q8WUY8 10450 8726 Q8N5U6 90993 Q08117 3054 Q05086 Q9BRU2 29079 P37231 Q9HCE1 79723 8737 10468 7422 Q96GA9 5245 3068 Q9HCD5 P52815 22893 3066 Q9UMN6 3065 Q9UIF9 O75290 79753 10438 Q00613 Q96P20 55787 10432 3070 Q13901 9612 Q9UIG0 P52824 466 468 P28749 Q9NQL9 7428 6595 6597 6599 6598 O75386 P10599 6591 Q9Y4K3 Q76L83 3084 473 474 23286 8767 Q13127 Q15303 B2RXF5 3091 Q16633 8301 Q15306 Q86VP6 O75376 P13631 24149 3096 Q02447 O75381 Q9NVP2 51773 30813 P46531 Q13133 Q15796 Q16649 Q15797 9640 P12757 Q8N6I1 9643 7468 P54198 Q9UNL4 P12755 O75362 O75360 P46527 51780 Q01101 P42167 6128 O94805 Q5VUA4 54815 Q8WVM0 Q16650 Q13148 P35869 P20393 7476 Q16656 Q15329 Q8NCA9 P18074 Q8WVL7 P55055 P10588 P10589 93166 Q86VN1 Q16665 51317 Q49AN0 P43489 Q8TDY2 9667 Q16666 Q5VUG0 P54132 P35453 Q14494 8328 Q14498 Q16670 P46108 Q86W54 Q9NRC8 7013 7014 P46100 Q5PSV4 7490 P13682 Q9C0F3 84232 Q8WW38 Q99835 7024 7023 10392 Q9C0F0 49854 P15822 9682 7020 Q02878 Q9UNH7 51341 54851 Q9NVM4 Q96PK6 11243 Q13185 Q8NCF5 6188 Q15369 Q8IUX7 Q9BSM1 Q09028 6182 P11474 26747 Q15370 54862 P57682 Q99816 9208 Q99814 P78545 7046 P35813 O43711 O43719 Q9Y4C1 P62195 Q4LE39 9219 A8K8V0 22803 112950 22806 22807 57473 P46934 P46937 83746 Q96GN5 O60296 Q4LE28 Q9UER7 Q14865 7068 7067 Q5TAX3 Q15714 Q9H307 P19793 P29590 Q7Z2Q5 Q63HK5 Q01196 84619 Q9H6W3 P41235 1756 Q92793 Q96GM5 Q9HD15 Q9Y4E5 O60264 P59817 Q13547 Q13546 9252 7073 P41223 57018 Q9NZR4 Q04206 Q92786 Q92785 Q92784 P41229 7088 Q01167 Q9BSG1 51720 Q14894 4800 2626 7099 7098 Q9NVC6 7091 P14923 7090 Q9NQZ8 P30153 1789 1788 Q05516 1786 Q9UIV1 1785 375790 Q13573 Q9HCU4 93134 Q9H2S9 Q96CJ1 P78527 92283 85509 Q86VK4 84661 51742 Q6ZNG0 Q9Y4A8 30827 Q96H20 Q9HCS4 902 Q13112 Q9NZN8 P08235 P21675 Q92618 Q9H609 Q9Y2W7 Q9NYD6 Q6P2D0 O75820 2672 Q12948 22992 O00268 Q6QNY1 O00267 79894 3516 Q9BZI1 Q96JM7 Q96JM2 Q5VWX1 283248 Q9ULW3 Q92610 4841 Q8N587 Q12950 Q12952 Q9Y2X9 Q8NB12 Q8NAP3 79885 4851 4854 Q96JL9 Q92600 5705 Q9ULX9 Q9NYF8 Q12968 29128 10514 83933 26097 Q9UQB3 4862 57649 Q6IT96 4869 5716 Q96JK9 Q12972 Q0D2J5 Q9H9T3 3551 Q6P2C8 5728 10524 Q9HBE1 Q99697 Q9ULZ3 Q9UHR5 P28482 Q9UHJ3 Q12986 1385 29108 P19532 Q9P0J0 P15173 P07550 P04040 10971 7913 O14867 Q96BA8 10973 Q9UM47 Q9Y2T7 Q9UHK0 Q9NYA1 O00233 P19544 Q8WYH8 1388 29115

Q9P0K8 1387 1386 P42771 Q9UM54 4899 Q9H1I8 Q9NYB0 22937 22938 Q9UHL9 22931 2247 284119 1398 58533 Q9BZK7 6601 Q9Y2V2 6605 57209 Q9UM63 P51692 P31629 Q9BQY4 O00255 Q8NEZ4 Q9NQ33 O00257 Q03164 984 Q5H9I0 988 P05362 Q9Y2W1 P06213 Q9UPV9 P13349 2274 P53803 P52952 Q8WTS6 126208 Q96RU8 Q96RU7 51003 79811 79813 51008 10138 Q9UQ80 Q04917 Q9UM07 Q9Y2P0 Q7Z5H3 Q9UQ88 5796 Q5T7W0 8819 P48436 8812 P01344 P01343 O00206 2290 23429 P48431 Q96JC9 3142 Q9NU63 148022 Q99623 Q96S42 Q8NAF0 55869 7975 5316 51028 P01375 Q9UPY8 23439 6660 P49750 8841 Q9UHI6 O00213 Q3KNV8 23435 Q96AQ6 3151 O60716 10128 Q96RR4 Q3KNW1 3148 Q15170 546 3146 P61457 Q96EY1 6657 O43353 6670 6672 23409 Q9Y2K7 Q9ULJ3 Q9H9S0 3162 Q12905 O95076 Q8WYA1 Q12906 Q9BZE0 Q01844 P42704 3159 Q99684 6667 P49336 P05771 Q9H5J8 Q969G3 8864 7533 P55198 Q9ULK4 23411 23414 3171 Q00987 Q96S94 3169 7529 7528 Q9H5I1 64375 P50750 Q86U86 Q9UPT9 3184 571 3182 P51608 P68036 7536 Q9H5H4 Q01826 5371 8880 P27540 Q60FE5 Q12933 Q9ULM3 7555 Q9ULM6 P51610 Q9BUY5 54583 80854 P51617 Q9NPJ6 7549 54106 8878 P60568 Q15654 8896 P15923 Q86YW9 Q96FV9 84324 Q7Z6C1 Q13485 O94906 O00716 Q16512 Q16514 P12429 Q15669 O75496 O75494 Q7LBC6 P10242 P10243 P10244 84312 51412 O94915 Q13492 Q16520 6239 54925 Q15672 Q9UQR1 7101 6256 Q16526 Q2M1K9 Q9UDY8 7579 9774 4086 7112 4087 4088 Q8N9N2 4089 O75478 163126 O75475 Q8N5F7 142 P24864 84333 Q15697 53615 Q13033 P54259 O75469 8458 64750 7124 P54252 P54253 O75461 Q9UQL6 P10275 4092 P10276 4093 O75460 4094 154 Q99729 Q13045 55806 P49736 7132 8463 8467 9314 O43889 P36896 23378 P15976 Q96C28 1822 166 55810 P01308 338917 Q9NQB0 Q99717 Q15233 Q96T37 9326 P54274 O75446 P36402 O75444 6294 7141 1831 Q96T23 Q00577 Q99708 P49711 Q16576 P49716 P49715 55827 Q9NQC7 Q96C00 64784 7158 O75437 Q08050 P39905 7157 162239 Q8N9R8 Q02750 P35548 138151 55832 Q9UDV6 Q5VTD9 Q13077 26038 Q86Z02 56252 Q01094 Q9H204 26039 7161 P63167 P42224 10933 10935 P17275 P63165 Q9BVI0 P42229 57109 P42226 O94992 Q9P0W2 22926 Q969S8 22927 Q13404 Q13887 Q01085 57592 Q8IY57 57594 P10644 57591 Q96BD5 79365 P84095 Q08945 Q9UHX1 1869 7186 7189 7188 Q8WYK2 O43812 7181 7182 7185 1877 Q86UE4 Q86UE3 1874 Q9Y3C7 1870 P09630 O15379 Q969R5 Q13422 O15391 Q8NB78 2735 P19438 O43829 84733 1408 1407 1406 2737 2736 Q8WUI4 Q02386 Q7RTR2 1896 Q9HC52 Q14774 P22670 Q9NQ92 9391 P11532 Q96G25 84759 58487 Q8WYP5 P23511 Q8WUH2 3609 3608 3607 Q14781 P23510 P21333 94104 O14495 Q9P0T4 29966 P19474 Q02363 58491 P18146 27300 O60341 Q969V6 O15350 O75832 O15353 Q15649 Q9BZS1 Q15648 Q9P0U4 P19484 Q86YP4 Q06710 Q96BF6 Q8N5A5 3622 Q9HC29 O14497 Q15652

positive regulation of transcription, DNA-dependent P10914 Q92858 Q12948 Q12824 Q6QNY1 O00267 P17676 2547 2304 2303 P16220 Q96JM2 P84022 Q92731 O15516 2308 Q12837 Q8NAP3 4851 5705 O75925 Q12968 6927 6929 4862 Q9UJU2 P49116 O75928 P40425 P40424 6926 Q6IT96 P06401 3659 6925 P06400 Q96JK9 5970 2100 Q9NR96 O95343 10524 P20823 P61296 5966 Q9Y463 1022 1385 P19532 O00468 6829 10736 1499 P15173 80324 P07550 4646 6827 Q9UHK0 4780 Q92769 6720 P28370 Q14938 O95361 P10600 P19544 O00358 10626 P19419 Q9P0K8 1387 P17482 P08651 P17480 4775 Q9UM54 Q12888 4792 22937 P38398 2247 Q9BZK7 6601 Q92753 6721 Q92993 Q12770 P05112 Q9UM63 Q92997 Q13501 Q9UKS7 P10826 O43186 Q12772 P10827 Q12778 P51692 Q13503 Q05586 O00255 1050 Q03164 861 50943 Q9Y2W1 O96004 6872 P04637 2034 P13349 2033 2274 Q9UIH9 O60603 25988 P15884 P52952 3479 10011 Q16236 10014 Q9C0K0 6760 4221 Q96T76 P33076 P48436 4214 406 P01588 O43294 408 P49407 P01343 P01100 O00327 O00206 Q09472 P48552 P48431 9921 6774 Q99743 Q14192 Q96S42 898 Q53X93 P01137 5316 P49639 P01375 Q9UPY8 P51531 6660 5451 P35222 P51532 P17813 3148 P10070 P10071 3146 668 6776 10001 6657 P23497 P26583 O00744 5460 6672 Q9Y5Q3 P50613 P51946 Q12905 Q12906 672 P15692 79612 3159 Q02930 6667 Q8N5U6 P40763

P05412 5591 O00755 4261 P18850 Q05086 23414 3171 P37231 P18846 P18848 3169 148327 9826 5468 Q92922 7422 P52815 O60907 3066 Q9UMN6 3065 3182 8625 10432 P27540 5494 P62508 Q9UIG0 O14936 466 P58012 468 10320 54106 8878 9967 P25116 P60568 O15085 6595 Q14686 6597 4297 6599 6598 P15923 P13984 Q9Y4K3 351 Q9H161 474 P08047 Q7Z6C1 Q13485 P25963 O94906 Q9NPC8 3091 O00716 Q15306 Q86VP6 P58304 P13631 367 Q04864 P46531 Q13133 Q15796 Q9UQR1 6496 6495 Q15797 6256 P12755 Q9ULH7 Q9NY61 O75360 Q9Y606 Q15561 P17096 Q13263 Q15562 Q16650 4086 P35869 4087 8204 4088 P62826 4089 7476 9412 P18074 Q8WVL7 P55055 P12645 P36956 P08151 P24864 P20264 Q16665 Q13033 9421 P55347 7124 P54253 O75586 P35453 Q9UQL6 P10275 P10276 154 Q99729 Q13285 2902 7132 7376 8463 Q9HAZ2 P36896 O43524 7490 55810 338917 Q9NQB0 Q8WW38 Q14140 Q99958 Q15475 Q99835 Q96T37 Q8TEY5 7023 5089 P14859 54851 Q01664 Q96PK6 P01579 P49711 10155 P49715 P09038 55827 9575 Q9NPA8 7158 P39905 7157 6182 P11474 26747 55832 Q99814 9464 P35813 27005 Q01094 7161 P22736 Q1PSW9 P17275 P62195 91 9219 P42229 O15119 P42226 Q9UBK2 Q58WW2 P56524 22926 22807 P55318 Q4LE28 P05067 1869 7189 7067 P19793 Q03014 7182 6096 Q01196 P41235 Q92793 Q96GM5 Q9HD15 O95619 Q9NSC2 8289 Q13422 O15156 O94776 Q13547 A4D1W7 P78317 2735 P19438 Q9BXJ9 Q04206 Q92786 1406 2737 2736 Q15853 O14593 O95999 9149 Q8TAK5 P22415 Q9UK80 4800 2626 7099 7098 Q9NVC6 Q9NP71 64919 80155 Q9UIV1 P23511 375790 3609 P17081 3608 5901 P46734 P09429 P12956 29966 P78527 P18146 P35711 Q9BQA5 P43694 O15455 5914 O15350 Q9BZS1 Q15648 P55771 P19484 Q06710 63976 P63272 Q9HCS4 O14497 Q92585 902 Q15532 Q13233 P21675 5925

regulation of protein transport P53367 7046 Q15654 11060 O95835 O00308 2033 O43557 P10599 Q9Y4K3 O60603 54474 Q04759 P84022 999 Q7Z6C1 6901 Q13485 P25963 Q9UBC1 P03950 4188 Q16635 7295 P50148 2316 9908 Q9HD26 P14174 Q99750 7205 P00533 Q96EB6 O00206 Q09472 7189 7067 Q8N726 3783 P14416 64215 57120 Q06124 60412 P62491 Q00535 O60543 P01137 Q9Y6A5 P01133 P01375 5716 8165 P31946 4088 4089 8720 Q9BZF9 P35225 Q9NR96 27236 P21917 P35900 9495 387 P21359 O60674 P10071 5566 Q9ULZ3 5569 Q9Y2B9 2737 Q92667 P43489 P24588 5460 7124 11142 Q96KC8 29108 1020 Q9UNN5 O00221 114548 Q93062 Q6PJW8 Q99728 11146 P61586 O15554 7099 7098 Q6IR47 Q9NYA1 P29466 Q13443 11030 10460 P14923 64127 O95361 P36894 P10600 23411 P13796 5590 1029 57678 3728 10626 Q05513 P01308 1540 Q9NQB0 P42771 P49841 7529 P22303 Q99835 P23510 P21333 P61925 8754 4792 Q14703 Q96FX2 P12830 A1XKG3 2010 Q14C86 Q9HCL2 Q96P20 Q92990 P01579 11124 O15455 Q9UN86 1956 Q9NQC7 Q9BT67 P10827 Q60FE5 O75832 Q9UGK8 8766 Q9BZS1 Q06830 580 P50402 P17612 2932 Q9HC29 Q9UMX1 54106 Q15653 50943 P60568

enzyme linked receptor protein signaling pathway 64397 P08833 O14793 6599 7204 P18085 P49023 O43559 Q12948 Q9H2Y7 3084 O43318 9185 Q8IYN9 2303 P16220 P84022 116 Q13480 P49815 Q13485 2308 P21246 2549 Q14451 Q15303 5154 Q13009 5155 Q92729 Q8IZP0 2316 P16234 5829 P16471 Q9H2X0 P00533 Q15796 P43026 Q9HAU4 Q12841 P54753 Q15797 Q12965 P22681 Q9UQ13 Q8N6I1 P32121 Q06124 P12755 Q05397 P54756 6927 P37023 2324 5159 Q9UJU2 Q9UQB8 9518 Q8NFH8 Q93008 P54764 5295 4086 4087 P54762 3791 4088 4089 P54760 4882 Q86XR8 O43427 P10586 163126 Q13705 Q9UKG1 Q9UBE8 5728 P16333 P20823 O60674 P20827 P18509 3667 P62942 P27037 64750 P49069 1385 O95470 P51668 1020 O75582 Q5SGD2 O43639 4092 O00468 4093 154 P07550 4643 Q99966 O00238 P09619 Q5JQC9 Q9Y4H2 P36896 P36894 P10600 P10721 P35568 285 Q02763 6711 7249 Q99717 Q99958 6714 5747 Q9NRD5 9564 P07766 6850 O95813 P49840 Q7Z7G1 10273 Q9BQ95 2253 A1XKG3 P21709 P36888 2247 2246 Q07889 O14640 P08069 P05230 1956 P09038

P29323 Q12778 P29320 Q9BX66 P51692 O15197 O15198 P31749 1176 P19883 P35548 P30530 P18433 2931 P37173 Q6PID4 5764 867 P23458 1969 P29317 Q14289 Q15375 26060 P06213 4690 P01241 Q13873 9463 7046 O15123 3484 P12931 O75420 3481 P41134 P62993 Q99523 Q92572 10817 91 P04085 94 Q15389 P42229 P27986 10256 Q9NSA1 1978 6760 P78536 6885 5796 Q02297 2043 Q01082 2280 9350 Q96RT1 84159 2277 4214 Q13094 O43294 P01344 Q92569 409 P01100 Q14865 P29353 6774 P35916 P29590 773 56034 Q00535 Q96S42 Q53X93 P21860 P01138 P01137 O60383 Q9NRA1 Q99988 P01133 P02461 P04626 P04629 Q658W2 2065 2064 9252 7074 780 Q9UNE7 4914 Q01974 P43405 P17813 2059 P60484 6776 P01127 5327 5460 Q969H4 64599 P07949 8852 O43915 10458 P15692 P20594 P08123 P55075 4920 10454 2626 P40763 P05412 Q6IR47 Q13322 P35968 Q13443 O95405 10580 Q9HCE7 57154 51295 P61077 O60496 Q8WUH2 207 375790 9702 P17081 Q92922 10468 Q15750 P21333 O75969 O14492 9046 8754 7422 O75962 Q12929 Q08345 2889 2885 8503 P43694 P01730 5371 Q60FE5 6464 Q15768 Q13905 Q86Z14 Q04771 Q6ZWJ1 P51617 8879 O15105 P00750 Q15532 Q13233 10566

positive regulation of cellular component organization P25054 8650 P30086 Q9BVA0 Q04759 P84022 P07196 P09493 Q7Z6C1 Q13485 P04004 25913 P52565 Q13009 7334 O15530 Q9UBC3 5037 65018 Q86VP6 Q08431 7448 P61158 5029 Q15554 Q15796 7341 Q7Z727 Q15797 6498 P22681 P12757 P53667 Q9UBU3 P23528 Q96RK4 811 Q13145 Q92833 P26358 4869 Q9BSI4 5295 4086 5054 P35869 4087 3791 4088 4089 7476 Q9Y6R0 3673 O75116 P55055 P10586 Q86YT6 382 5170 57534 P16333 P17301 387 P41743 4771 7124 7248 P14635 O43639 396 10733 57669 P61586 P51671 7013 7376 Q9NYA1 P30559 7014 P10600 P10721 Q86WV8 P01308 Q9HCM4 2475 967 P49841 Q9GZM8 6714 Q92888 Q9H2K2 10273 P54274 P62330 O60502 11124 857 859 5879 P60953 Q9BX66 7157 P31749 O00255 Q6KC79 P37840 P11233 Q9HCK4 2932 10724 55832 867 P05121 Q8N3V7 50943 P06213 4690 7046 6872 P04637 O43157 P10636 2033 P12931 O95271 3481 P50897 23327 P52952 Q4VCS5 P42345 Q9BXM7 Q86WK6 3479 P63165 P04083 998 6622 10013 5898 Q92574 P46821 6869 Q15389 11346 P27986 P07355 Q9UBK2 9475 4221 1072 81565 4214 O43294 408 P49407 P01344 P01343 Q09472 5321 P29590 Q13418 O00444 P56539 P31431 891 P41231 P17252 P35240 P01138 P01137 P01135 P01133 P01375 4240 9138 Q9NUX5 9253 9495 7074 Q9UNE7 O43707 301 302 Q92786 Q9UBN7 P24588 4131 9700 5584 Q9UNN5 Q96P48 Q96PU5 P63000 5578 5338 P47712 O00755 Q6IR47 P35968 Q13443 10580 64127 1789 10109 1786 P18846 324 4137 207 3720 P17081 P08138 P27797 8754 5364 3611 P61088 P25103 Q14674 P01178 5371 Q9BZS1 O14939 466 Q9HC29 Q13233 P21554 P21675

RNA localization 25909 Q86V81 Q06787 57187 O95271 P35658 O95793 Q96FV9 O00148 23165 4686 10772 O15234 P09651 Q92574 Q8WUM0 Q96HA1 P38919 Q13769 O75494 8021 O00159 P22626 Q96Q15 9908 Q9UIA9 Q9HAU5 Q9NWB1 9883 P52298 Q86U42 Q9UMR2 Q8TEM1 23144 P57740 Q03014 26019 Q9GZY0 80145 23381 Q8NFH4 P61326 4869 8563 6780 Q32P51 P62826 10482 O14980 9775 Q9H814 4641 Q8N1F7 64901 23039 79023 10128 144983 55110 2332 Q7Z3B4 Q5SRE5 4116 10642 10762 Q9UKX7 Q8IXZ2 Q9HC62 O43592 6311 7884 7248 Q9UPR3 Q14008 22794 Q96PU8 4927 Q99567 P42704 84248 7514 Q14493 Q96A72 5976 11269 9818 9793 Q09161 Q9H2T7 3178 O75694 Q86WV8 P37198 Q8NI27 Q8WYP5 Q9BVL2 5901 3181 Q86W42 9961 Q12769 59343 23225 Q9Y5S9 Q99700 Q9UN86 Q6I9Y2 23636 P49792 9972 Q9NPA8 P49790 11097 Q9UKK6 Q96QD9 P52948 Q92621 O15504 Q92900

cellular response to hormone stimulus P01241 64397 P08833 6198 P98155 O95835 P28562 6599 3484 Q9H2Y7 3481 P13861 O60568 P42224 Q96RU7 2303 P62993 Q9Y4C1 P17275 Q92572 2783 P20396 P22694 Q13480 P62879 P22612 P42229 P27986 P62873 10014 1978 2308 2549 7332 O15530 Q9HAV0

Q96QB1 P10644 P31321 51094 P31323 P05062 P63218 Q13370 P01344 Q92569 P01100 1080 7067 6256 P29353 P50150 6772 6774 P13569 P19793 Q06124 P59768 Q9UBU3 P40145 894 57761 O60266 P21860 Q9UQB8 P01137 P09874 Q92831 O95622 5295 8165 P55851 5573 5575 3673 5576 O43306 2065 Q9UKG1 5170 142 P17301 O60674 5566 6776 5567 5568 3667 P41743 Q92667 5584 P63092 Q9UQL6 P10276 B3KY43 10458 P22415 P05091 P51828 5577 P08243 2626 P40763 P04201 P01275 5591 5471 Q13322 8985 10580 Q9Y4H2 26524 P35568 P30279 P37198 P37231 Q9BUB1 57678 Q8NFM4 Q8NFM5 207 5468 P17081 Q92922 Q99958 P09467 9564 Q08462 O14492 P49840 Q96A54 Q06203 P78527 Q86V24 O60503 Q9HCL2 P61244 2885 Q9NRM7 P68036 P17174 P08069 8503 4149 P43694 2805 P23443 5914 23636 P10827 Q12778 6464 Q9BX66 P51692 P31749 1176 P18433 Q9HCK4 1843 P17612 Q6ZWJ1 2931 P25874 26060 P06213

chromatin remodeling 6595 6597 6599 6598 Q12824 Q9UQL6 O00267 Q9P2R6 6829 473 Q8NCD3 10014 6827 P56524 Q92769 4221 8467 O96019 Q86WJ1 P28370 Q9ULG1 23411 Q8NB12 Q9NRL2 3169 51773 79723 10661 Q92922 Q96EB6 Q9H5I1 Q86U86 P49321 Q7Z7K6 22893 3066 Q9UMN6 3065 Q9UIF9 7141 6927 Q96T23 Q96GM5 6601 Q92830 Q13185 O60264 P09430 54617 Q6IT96 Q92831 P06400 8289 3070 P51531 Q9BZS1 Q9UIG0 Q13547 O00255 Q09028 Q9NRZ9 P51532 55355 P20823 5928 P63272 Q8TBE0 11177 O14497 11176 4678 50943 86 Q13351 5925

positive regulation of signal transduction 8772 P30086 Q9Y4K3 O43318 O15519 356 23286 Q92851 8767 O14788 57506 P07996 Q9HC98 7334 4067 7335 5037 65018 2316 367 Q04864 P14174 P09382 10783 P00533 Q13490 4734 1902 9641 8795 Q9UQ13 Q9HBW0 P32121 253260 Q06124 Q9Y6Q6 10758 Q9UDY8 57521 142678 64170 P55290 Q9BY44 5970 P48023 Q86XR7 Q9H257 Q9NR96 P17302 387 389 O60674 O00182 2697 Q8IUC6 5966 P04233 P62942 6275 Q13158 7124 Q9Y3E0 O00463 P10275 154 P15056 Q9Y2C9 1499 10616 1012 P07550 P04040 O94827 P61586 1936 P08887 7132 P29466 Q9Y239 124583 5071 P37198 29110 960 1147 Q02763 P01308 841 2475 843 Q7Z7H5 Q9NQB0 O15169 847 P07766 P02790 10392 6850 P50591 O43734 Q9P0L0 Q9NS68 Q8WVQ1 Q8IUH5 2247 Q9BVC4 P01579 P08069 857 O14763 P05112 5879 1956 P09038 Q92633 Q9H8V3 23118 27032 54862 Q14164 Q6R327 P09601 P98194 Q9UL54 Q13077 P06213 Q15811 P01241 7046 P35813 P14317 3481 P42345 Q9Y6G9 Q9BXM7 3479 P13591 10013 P01116 9218 P01236 5536 Q15389 Q9NSA1 P01112 P01111 7057 6885 Q13404 9590 2280 7052 P46934 2159 4215 P01588 P16070 Q99759 408 P49407 P01344 409 P01343 O00206 7189 7188 P48431 3265 Q13418 3263 7185 148022 Q9BYM8 60412 Q02952 P01019 P21860 51026 P21980 55504 P01137 O60260 P01375 64223 P04626 Q13546 55072 Q7Z434 2065 2064 P35222 9495 P35348 P19438 P43405 Q04206 P53041 6657 Q9UBN7 8837 P24588 8717 O43353 Q96EP0 O95999 P13945 P07948 P78509 3162 9020 79971 673 P15692 P63000 57708 5579 P00742 5338 3953 P05771 Q6P1N0 3956 Q96FA3 O00635 7099 7098 Q6IR47 8743 Q15628 64127 Q05086 Q99558 P08134 5467 8737 329 P48357 3965 P21333 Q9NYJ8 7422 6453 Q9UHD2 Q9BUZ4 57162 3611 P61088 Q14790 Q5T9L3 O15455 Q16610 P01730 P41159 Q9BT67 23636 5494 Q60FE5 O15111 Q86Z14 222068 O14939 Q9HC29 54106 P26447 P25116 P60568

anatomical structure formation involved in morphogenesis 3880 Q12948 O75386 Q9UPN3 O75385 Q9Y4K3 O43318 472 Q8IYN9 2303 P84022 P21926 P09493 Q99807 P49810 P08047 Q7Z6C1 O14786 P43034 P49815 Q13485 8408 P07996 P03950 9510 P25067 5154 8543 P08727 Q5TAB7 54361 P58546 Q08431 Q02447 367 P42858 Q9H2X0 P46531 4851 Q15796 Q14344 Q15672 928 Q16881 136319 P61968 Q15797 6498 P12757 P05549 Q9NRY4 O00167 P12755 Q05397 Q9BXC9 6927 P37023 P23528 Q9UKW4 Q96RK4 Q15561 Q92830 Q9UJU2 Q8WZ42 6926 P55290 4627 8443 Q92826 Q9GZX9 Q14116 Q9NZI7 4086 134701 4087 Q14114 3791 4088 4089 P07737 7476 8323 Q14119 P18074 P68133 Q86YT6

P56705 P17655 57534 5728 P20823 2335 P20827 P61296 P62942 9421 P55347 8452 4771 Q9Y463 7248 2909 Q5D1E8 1020 P10275 P35579 Q93063 57669 1499 1012 23363 Q9NYQ6 Q8N264 P23467 Q9Y2T1 3678 P45379 Q15464 P08648 3690 P50222 P29466 O43521 9314 P36894 Q86WV8 Q8IY17 Q9BPY8 960 841 Q9HCM4 7249 7804 A6NI15 Q99958 5747 P02751 Q96T37 O95259 P05106 P14859 Q9BQ95 2132 Q13618 A1XKG3 7020 2247 2246 1277 O14641 P05230 P47895 Q9NQC3 O43184 P09038 116150 50937 O15078 P39905 7157 O95140 2139 P37173 Q6PID4 4313 Q9UMX1 O95947 Q99814 1969 P09601 P29317 P40337 Q14289 P06576 P02452 P78423 4691 Q13873 P28289 9464 O96004 P04637 Q9Y6W5 2034 P13349 2033 P52952 2821 P41134 P62993 Q1PSW9 P17275 Q4KMG0 P39060 999 P04085 65082 94 Q92574 Q15389 O15119 P07355 P98160 3911 1856 7057 Q7Z5H3 6885 Q96QB1 Q13887 7291 P10644 1072 2280 7052 P19338 P39059 2277 P16070 P49768 Q09472 P50552 7189 P48431 P29475 92129 P52735 10908 Q03014 Q13418 O14727 773 O95180 Q00535 Q96S42 P35240 O15379 P21980 O95859 5316 Q9NZ08 P49639 Q9NSC2 8829 Q9UPY3 P01133 P02462 4240 9255 5451 5573 8841 Q9UHI8 Q658W2 P35222 Q01974 P17813 302 P60484 P10070 Q9BXJ9 P10071 Q92786 5566 6657 5328 2737 2736 9927 O43474 6670 5460 O95999 Q13315 P07949 9149 Q12904 Q9H488 7082 O43915 Q07157 P78504 3162 O00622 84525 P00749 P15692 10451 6667 Q96AX1 4920 3953 P05412 5591 P06744 O00755 Q13444 P35968 7410 P68032 Q9UBX0 80155 10229 51295 P51959 688 P08253 P48357 7408 9047 5362 7422 23509 P56178 P12830 57045 P78527 O75051 2885 3611 Q04656 Q14790 10672 Q9GZT9 80184 9839 O15230 Q9NP98 Q04771 P17612 54583 O60353 900 51752 P27658 7428 O15228

regulation of transport 23607 2550 9066 10657 P61020 54474 P37288 P16220 P84022 57620 90678 O14788 6901 P07996 Q9UBC1 P04004 65018 Q08431 P50148 2316 P14174 4734 Q96EB6 808 Q7Z727 3783 Q06124 6927 P62491 57761 O60543 811 Q9Y6A5 5716 P53675 Q9H1D0 P31946 3673 1374 Q9BZF9 Q9NR96 P21917 P48380 P17302 57534 5728 P20823 P17301 O60674 P19838 2697 3784 6812 Q9ULZ3 3667 6814 Q96JJ6 P41743 O75915 P04233 P25098 O95477 1385 29108 1020 O00221 114548 P51787 1137 1499 Q6PJW8 1012 P07550 P61586 4646 O14745 P30556 3690 Q9NYA1 P29466 P30559 5991 Q07666 O95361 O00233 P10600 1029 57678 10626 P20648 3685 967 P42771 Q9UM54 5867 4659 5869 P05106 4790 Q9UGJ0 P28223 6850 4792 Q14703 2010 P30542 P62330 O60502 Q9HCL2 2247 Q92990 Q5T2W1 857 Q9UN86 859 5878 5879 Q9UKS6 P51575 P10827 P30536 Q9UGK8 P31749 P19883 O00499 10724 867 Q9UMX1 P05121 P09601 50943 26060 P06213 Q15019 Q86UW7 O00308 2033 P50416 P50897 O60603 23327 P52952 Q96RU7 2147 6622 999 5898 6869 Q15389 P27986 5311 Q9HDC5 2280 9908 O76070 Q99750 Q15036 O00206 Q09472 Q8N726 5321 P14416 64215 773 Q00535 Q53X93 P01019 P01138 P01137 55503 P01133 P01375 Q9Y5X9 4240 8720 5213 P35225 2065 P35222 781 301 302 P60484 P10071 5566 5569 10488 O14974 P24588 5460 23409 5584 P05408 Q9UNN5 3162 552 553 Q96PU5 5336 5578 5338 Q92930 Q6IR47 10460 10580 64127 23411 O76024 O00631 Q9BSW2 5590 P37231 8737 207 5468 P46059 7529 10468 P27797 P01185 8754 4035 Q8NER5 Q96P20 P61764 P25103 P26678 P01178 Q9BT67 O43581 Q6OFE5 8766 580 P50402 P16885 P17612 O14939 54106 9846 P25116 P60568 Q15654 11060 Q16623 O43557 P10599 Q9Y4K3 94121 3084 Q04759 Q9UBS5 51762 P20396 Q7Z6C1 P49815 Q13485 P25963 P03950 Q15303 4188 8301 Q16635 19 7448 Q13492 7205 5029 P00533 Q13370 Q13133 20 7220 P22681 Q8WVM8 23144 Q9Y6Q6 495 Q9UBU3 P09471 P55290 6262 5174 5295 5054 4088 4089 P55055 Q9UKG1 Q86YT6 382 222546 387 P21359 1808 Q8IXZ2 P43489 P62942 9784 7124 11142 Q96KC8 Q9UNF0 274 154 Q93062 156 Q99728 11146 Q16555 Q15466 2904 7376 Q96C24 11030 O43889 Q9Y4H2 23256 P36894 P13796 5071 P35568 P01308 7249 Q9NQB0 2917 8218 P48995 P49841 Q99835 P46459 P61925 P49840 Q7Z7G2 Q96FX2 A1XKG3 O43612 Q9Y5K6 Q86V24 Q14C86 P01579 P00367 11124 Q9UEW8 Q9NQC3 1956 P09038 Q9NQC7 P54289 Q9BX66 P39905 11252 P37840 P11234 P11233 2932 2931 P53367 7046 O95835 Q9BXM7 P04083 P07355

Q9NSA1 7057 O15496 Q02297 7295 P46934 Q9HD26 Q9Y6H5 8399 P52298 7189 7067 P29474 P29475 9368 57120 27347 P56539 P41231 60412 P17252 84502 P21860 O60260 8165 Q8WXH2 A4D1W7 Q13546 27236 Q6UWE0 P35900 Q9UNE2 9495 P43405 O43707 Q07954 Q8HWS3 Q9Y2B9 2737 Q92667 9388 O00186 P41181 P51148 Q9H244 O00194 84766 P63000 O15554 Q13563 P62158 P47712 7099 7098 Q13322 Q13443 P14923 P62166 3728 P21579 Q05513 P00734 1540 P17081 P22303 P23510 P21333 Q9BY11 P10415 P12830 Q9H223 P20338 P20336 Q9Y6E7 O15455 P41159 Q13586 O75832 Q9BZS1 Q06830 Q9HC29 P43681 Q15653 P08237 P21554

system development Q9UKT9 P25054 Q12824 259266 2304 2303 O60443 284217 P21926 2300 O14543 P05452 Q9UGM1 2308 2307 P25067 Q9UGM3 Q12834 Q12837 2317 P42858 P39210 Q9UKV3 P61158 10661 P10809 2319 Q96EB6 136319 P27694 4990 Q06124 P17405 P37023 55584 Q9UKW4 4983 5830 55109 3655 Q9Y297 Q9Y6A5 Q9UKW6 3659 P26371 Q86SX6 3672 3673 Q92949 Q13705 Q9BYD5 O60437 2335 Q99574 Q9UKX5 3667 P26367 O14578 P25098 1021 1020 Q06587 Q8IX07 57787 10614 1499 1012 3676 P61586 Q9Y231 3678 3679 Q9H0H5 3690 O00358 3207 1026 P17482 3685 O60890 604 3688 P07686 P04179 Q9BQ95 Q15915 O60869 P16118 3215 Q96N67 610 Q92990 Q9Y250 613 4548 Q9H0F6 Q92997 P10826 P10827 221937 P31749 O95140 1050 27032 P40692 3226 Q9UL51 Q92982 Q15019 O96004 P56199 P14317 O00300 P50897 Q99523 639 O96019 P16989 1072 641 11113 648 Q16363 1080 P48552 6774 3265 P12111 P14780 P11274 O95180 P35240 656 8945 P01019 79956 55504 84062 P50440 5451 A7MD48 Q9Y696 O95183 P35222 P35227 P35226 P57082 O75084 O75081 Q15051 P17813 53335 51156 668 6776 P24588 O43474 O00744 5460 P56159 4131 Q9Y5Q3 P01040 9821 4133 P25490 10690 9820 Q9NX62 673 P61981 P15692 6304 P05412 P06744 O00755 5471 3290 Q9Y5R5 3297 Q9UBX0 682 10229 P60660 688 5467 4137 207 5468 54206 Q92922 P06753 Q15078 90780 4154 4155 23509 P56178 P56177 O75051 P13010 4149 10672 9839 5493 O43464 Q8NHX1 P35268 Q09666 23513 P50402 P13497 O14939 10682 8516 P27658 P60201 55558 9846 10202 P55268 Q14686 Q9UPN3 Q9H165 230 Q9H161 P07196 P49810 P08047 5017 P83731 P49815 Q9NPC8 94234 4188 8543 5037 79084 P58304 P09382 8536 5029 51535 P00533 Q13370 Q16880 Q16881 P35638 Q9HB63 P61968 P31273 P31276 Q9ULH7 Q9Y6Q6 257 Q96RK4 Q15561 Q14232 P55290 6383 5054 Q9Y2J0 Q8IWX8 Q9Y6R0 6389 P36952 P68133 P37802 P35625 O75592 Q4AC94 Q8N4C8 P31270 23032 P08034 P43246 9421 9420 P49862 7248 O75581 Q93063 P23229 51564 Q14254 P78337 8100 Q9HAZ2 O43521 Q15109 O43524 5071 7251 285 7249 1948 1947 P02751 8110 P11229 O75569 10152 23463 A1XKG3 Q96EV8 Q8WY64 P08069 10155 Q15125 1956 O15075 O15078 O75553 Q9NXR1 2810 Q92187 O95947 1969 Q14289 Q13753 9463 Q13751 9464 Q9H0M0 Q4VCS5 P22735 2821 Q9BUG9 P04083 P04085 Q92574 P56524 9474 O15496 O75534 Q02297 7291 P16070 9001 P06239 Q06643 P56539 60412 P00966 P06241 P21860 O95983 P55212 P55210 A4D1W7 9495 P07101 P07585 O75503 O95999 Q13315 O43915 8174 9021 P35613 P35612 O60488 P85037 Q9H9B4 P31260 P20594 O75508 P31249 Q13323 O43921 23092 3728 3720 P22303 9047 P13807 P12956 P34741 26576 P02708 2885 Q9Y6E2 P43694 Q9BQA1 132320 63976 23077 Q13351 P07148 5925 P10914 Q92858 9061 Q9UBB6 P52799 Q9UBB9 4609 P16220 Q9UBC3 P27361 54361 P50148 P16234 Q9UK55 O15524 Q92838 P29992 Q9UBD6 P05549 Q9NRY4 1121 P53667 Q9BXC9 6809 27185 Q92830 Q9UJU2 P49116 P03372 Q92833 P06401 4627 P06400 P26012 P05556 Q92826 Q9GZX9 Q92824 3791 P07737 5970 5971 Q9GZX7 9093 Q9NRZ9 P56705 9099 P17535 81501 P20702 P17542 Q9BXB1 P61296 6812 10763 4637 3309 P04233 P34925 Q9Y586 O00468 O14627 10733 10736 3796 P09972 4643 4646 5978 P08648 P30556 P30559 5991 93986 Q8ND90 Q8IVL1 1152 Q8IZT6 3329 3326 1147 Q92882 Q92888 P07766 O43175 P05106 P04259 137964 6850 2010 Q13616 P30542 Q9NS68 57448 2006 P61244 O14641 4664 O14640 4665 5515 P04264 P05112 50814 O43186 Q12778 Q02078 Q6KC79 56121 Q13627 P30530 P52789 P37173

10728 P09960 P05121 P40337 P04275 7704 Q9UK32 5528 P02452 O95278 4691 P04637 O43157 2034 2033 25861 P16615 Q01650 1181 P62993 Q14050 4205 Q15389 P01112 4209 Q14055 O43166 Q9C0K0 P48681 Q96CW9 4221 6885 23210 P35368 2043 Q9HDC5 P50542 1191 O76074 Q15392 84159 4214 P01106 768 P01588 Q9C0J9 P01100 P50552 9921 P14416 P13569 2054 P52735 O00444 Q5T5P2 773 Q53X93 6886 55626 6405 P01138 P01137 P01133 Q16254 Q9UJM3 P02462 P02461 4240 5573 P04626 P04629 2065 2064 2063 P54920 301 302 P10070 P10071 5566 P02458 P01127 5568 9927 5584 O43597 Q92817 Q96PU8 O75190 5578 Q8N302 P40763 P04201 5591 Q6IR47 5111 O76024 P80370 51295 P18846 324 329 5108 P27797 6456 6455 2099 P12277 O76011 P51843 P15311 333 5599 140735 Q9GZT9 P01178 Q96HC4 P05997 4287 25818 P52701 P15313 O60911 Q08209 P58012 P18827 6457 10320 P26447 P25116 Q96HZ4 8650 4297 Q6ZT98 Q8TEW0 8654 P30086 4291 351 Q04759 356 Q9UBS5 P09493 P09017 9510 5154 5155 367 Q70SY1 51654 6496 6495 6498 6015 P32242 P10451 Q9UBU3 Q03405 116113 P51805 P09471 5159 Q13263 P25940 Q14116 Q13268 7353 Q14114 8204 Q14119 Q14118 P51812 Q9Y618 P51813 P12645 382 222546 Q04725 Q04724 387 Q04726 23154 P08151 P20264 P60763 Q13275 P62942 P55347 Q16787 8214 2909 7368 Q14129 Q9Y5I4 O43638 396 Q02535 Q99962 Q01201 Q99963 10296 11146 Q15466 2904 Q16790 Q99966 Q15464 Q13285 54 P60709 Q16799 Q86WV8 Q6PRX2 P33151 60 P09067 Q9UBP4 51218 85440 Q99958 Q15475 Q8TEY7 P14859 P21145 P01579 2926 P09038 Q14155 116150 10280 O15198 O43623 25776 Q14160 1605 86 P78423 P78424 O15123 Q15818 1613 Q8TAF8 Q9Y5E8 P41134 Q86WK6 Q1PSW9 27122 91 94 P46821 O15119 Q9Y5E9 2947 Q13643 9114 Q9NNX1 Q13642 Q96QB1 O43602 Q15828 Q99081 P55316 O43609 P67775 P05062 P08575 P05067 1627 2956 Q9UBL3 P29474 29767 Q03014 6096 P34820 P55327 1634 56034 P21980 Q14993 1639 Q9UBM7 Q9NSC2 P11717 8289 9133 O15156 9138 29777 Q9BXJ9 57379 Q8HWS3 Q15853 O75626 Q6NYC1 Q9H488 P41182 P41180 P53567 P30048 P05091 P08123 Q9UJW2 Q13683 P47712 Q68CZ2 Q68CZ1 Q07817 P20248 64919 80155 P08138 P09429 O75604 P10415 3860 P19235 P35711 Q7Z494 3852 80184 P41159 O15111 P55771 Q04771 1203 O15105 P45983 3866 Q13233 P21554 Q7Z2K6 65125 3880 3881 O14793 2550 P51587 P17676 2547 4729 P37288 Q8IYN9 P84022 Q9HCJ2 Q92731 Q92730 O14786 O14788 Q8TDD5 6901 Q9NZG7 P08727 Q92729 P51114 Q96KN7 P20849 4734 Q8N205 Q7Z727 P28300 O00167 Q7L5Y9 6927 6929 811 P40424 P62258 6926 P31944 Q9NZI7 Q9NQX0 Q9C009 P07858 P31947 2100 P48380 O95343 57534 3428 6938 P20823 O60674 P20827 O60675 P62263 Q8N5V2 5604 P41743 Q9Y468 P11926 P27037 4771 Q9Y466 P39019 P19404 55366 6949 27283 P15056 Q8N264 P07437 P50222 P32856 Q92769 Q14814 P09619 5631 P28370 Q9NZC7 P10600 Q06330 841 Q6PI57 Q9HCM4 4775 O14753 846 7804 O95377 4791 1285 2132 Q96L34 Q9NQS3 O95376 2125 1277 Q92753 Q5TAT6 Q92752 P05230 857 859 Q92754 O00139 P11912 Q9NR50 1297 Q13509 1294 P19883 Q13506 Q9P202 O60636 Q9HCK4 2139 861 56243 1289 O95390 863 4313 Q9UMX1 P09601 4318 P06576 6993 O43278 23322 3484 3482 3481 P11047 203068 3479 2147 Q8WV60 7832 P01236 10014 Q9BRQ0 3009 Q99750 Q01995 O43294 O43293 Q13099 O43252 Q09472 O00562 P53701 2173 P13686 23787 890 Q00653 891 Q99743 O14727 894 Q9NR09 P53708 898 6525 P49639 Q9UMS4 P51531 8721 Q658W2 P51532 P14543 Q01974 55755 3028 P60484 11331 4358 5207 P26583 Q9Y3M2 7402 25945 Q8NFW5 10458 P60891 P31939 10451 P55075 79739 10454 Q8N5U6 7410 10460 7412 Q9HCE7 P52803 Q08117 Q05086 3052 P37231 P36382 Q01955 Q96SZ6 P51959 445 Q02809 P48357 7408 10468 P24385 P24386 8754 7422 9600 3066 22895 Q00613 P61764 7414 3070 7430 9612 Q9UIG0 3074 P17612 466 7428 6595 6597 6599 P18085 8775 6598 5268 O75386 O75385 6591 Q9Y4K3 3084 O43318 472 474 Q9C0C7 Q99807 P43034 P21246 Q13127 Q15303 5274 3091 Q16635 Q15306 3099 P58546 P13631 5270 Q02447 488 Q9NRH2 Q9H2X0 24144 6117 P22102 30813 P46531 Q16643 Q15796 Q15797 P12757 Q8N6I1 P12755 O75360 Q01101 P31040 Q9NRI5 O94805

Q15323 Q13144 Q9BWQ8 Q16650 134701 P35869 9657 7476 8323 P18074 P10586 P10588 P13611 P10589 59277 P13612 93166 54820 P23416 Q16665 P55061 Q8TDY2 O15287 Q16666 P54132 P63092 P35453 P09172 P46109 P23467 Q14012 P46100 Q9Y4H2 7490 Q9NZT1 P11021 Q8WW38 Q9GZM8 Q9NRD5 Q99835 9201 O75326 P13646 Q02880 P13647 Q86VS8 P13645 7020 Q08188 P02549 55704 P21266 1718 P02545 P47895 347733 Q8IUX7 P38936 P11474 3909 P02538 Q6PID4 Q99816 Q99814 P02533 P98194 Q15375 P60842 P78545 7046 P98155 O43711 55283 6194 P32302 3913 56134 65082 P05177 P98161 P07355 P98160 O60282 3911 3912 P78536 7057 Q13520 22806 Q3V6T2 7052 P19338 3925 P46934 1742 P46939 81565 P42574 P98172 P98174 Q14865 8399 7067 P19793 P29590 Q63HK5 Q01196 1756 P41231 Q96GM5 O15259 Q9NRA1 3932 O60260 9255 Q04695 P19320 Q8IYT8 9253 7074 P43405 Q9Y4F9 P20309 Q9NZR4 Q04206 Q5T5A8 Q92786 Q92784 7088 Q9NZJ7 Q15738 Q15735 P78509 P78504 3958 P00749 P00747 P08243 3953 Q13563 3956 2626 Q15742 Q9NQZ2 P14923 7090 P11413 1789 Q05516 P62166 P00734 375790 P08253 Q15750 Q9HCU4 Q8IUQ4 57045 P78527 O60229 P20336 Q04656 51741 Q16610 O15232 O75712 O15230 Q15768 Q06830 P22528 Q9UNA1 Q9HCS4 4830 900 51752 P00750 P08238 O15228 P08833 Q8NEY1 Q9NYD6 2672 Q12948 O00268 Q8NEY8 Q9BVA0 3516 Q9BVA1 Q9ULW3 P08842 P07996 P52565 Q12952 51090 Q8NB12 Q08431 P63211 79885 P21810 P16473 2676 P16471 O60331 P21815 4851 4854 928 P54753 P53420 Q12965 P25391 Q12968 Q05397 P20930 29127 P20936 4862 Q9UQB8 4867 P54764 P54762 P54760 4882 P48023 Q7L576 22985 P17302 5728 P17301 Q9HBE1 Q99697 2697 10523 Q9Y342 P28482 Q5D1E8 1385 O95470 57669 P15173 Q9Y2T1 P08887 Q9UM47 P51671 O00238 Q96SB3 Q9NYA1 P51679 P10721 Q6UB99 Q06210 P19544 Q8IY17 P18206 960 967 4898 Q9UM54 5747 Q06203 2253 Q9NYB9 6609 P62330 57689 P42768 2247 1399 2246 6601 6605 P51693 P29323 P51692 O00255 Q03164 P17342 5764 P29317 6615 P06213 Q9Y6W5 P13349 2275 P52952 O60725 P15531 991 79810 998 999 P61421 P02686 P62753 Q04917 P49770 Q9UHG2 5310 Q7Z5H3 10141 5311 Q9UHG0 P51636 P53814 2280 Q04900 2277 Q15154 P48436 P28845 P01344 P01343 Q96NL6 P49768 2290 P25791 P48431 5321 3142 Q99623 Q00535 Q00534 Q96S42 7975 6647 5316 8829 5318 Q9UPY3 Q8N960 P01375 P01374 6660 Q9Y337 4000 8841 Q9UHI8 O00213 O95067 O60716 P12004 Q9P0I2 3148 546 3146 P40933 Q96EY1 Q96JB5 6657 5327 5328 P49747 6670 P24043 P07942 O00628 7520 8851 P07949 P07948 Q8WYA6 Q12904 P20916 3162 O00622 O95076 Q9BZE0 552 Q99684 6667 4005 5336 Q96AX1 Q15185 5338 7518 P26232 Q969G3 5351 7532 7531 7533 P68032 23411 23414 10107 3169 Q9NPH5 4015 10100 7528 5361 5362 Q86U86 6696 5364 Q9BUZ4 P12036 3182 P26678 Q01826 5371 P27540 P20908 P16401 P16885 54583 8879 6699 P60568 Q13477 8892 Q9UQP3 P15924 P15923 595 8407 Q7Z6C1 Q7Z6C3 Q13485 Q8WUM0 8408 P03950 9750 P35527 O94910 Q13009 4067 P12429 Q15669 O75496 Q5TAB7 51412 P60983 Q96SN8 Q13490 Q14344 Q15672 7101 6256 O43897 Q2M1K9 Q01469 Q9UDY8 P23528 Q9UDY6 Q8WZ42 P00441 8443 4086 4087 4088 P22692 4089 163126 Q86UL3 Q86YT6 Q02790 P12883 P21359 23396 1808 P24864 10087 6259 53615 8454 P54259 7123 6275 P54257 4099 7124 P54252 Q8TD84 P14635 64759 Q9UQL6 P10275 4092 4093 P35579 Q99720 156 Q9UMD9 23363 Q9NYQ6 Q8WV28 Q99729 Q16555 Q15223 P45379 Q86YZ3 9314 P36896 P36894 P35568 1822 166 338917 Q9NQB0 Q99715 Q99714 Q96T37 Q8TCU4 P35555 Q86V24 P36888 1832 Q9H1Y0 Q00577 Q99707 Q9NQC3 P49715 P60953 7155 P39905 7157 Q02750 P35548 Q9H295 Q13075 Q16586 84376 Q15256 O75427 Q13873 P10636 26037 Q01094 P63172 7161 7163 2702 56259 Q9Y3A5 10935 P17275 P23945 P39060 Q6NVY8 1859 P22612 P42229 1856 Q13885 O94992 Q13887 10939 7170 Q14738 P10644 9351 O43847 79365 P39059 1869 7189 O75881 10908 Q13418 O43815 7182 1874 P17252 P09630 O15379 Q5TA76 O95859 Q9NZ08 P21741 O95863 Q13422 O95861 Q13428 P35908 Q13426 P10620 4914 2735 1400 Q02388 56288 Q96FJ2 1406 2737 2736 Q8WUI4 P19012 1896 2742 P09211 4920 Q13444 P35968 Q14774

Q13443 26005 P11532 57154 27339 P30279 27330 P23515 O14492 Q9H228 O14495 P12830 Q02363 P18146 Q03692 163183 3611 Q14790 P00403 P01730 O15350 O15353 Q15648 P19484 Q06710 O60353 P24821 O14497 4953 Q9BZR6

regulation of protein amino acid phosphorylation P30086 Q9Y4K4 Q9Y4K3 O43318 P13861 595 P84022 1453 P49810 Q7Z6C1 51763 O14543 6901 Q13485 P04004 5154 5155 Q16512 4067 4188 Q16635 5037 P14174 7448 P00533 O15524 Q7Z727 Q92956 Q9Y6Q6 5159 Q9Y6R4 4088 4089 Q9NR96 5728 O60674 Q13153 P04233 P27037 Q8TDY2 4771 7124 P14635 4092 Q93062 Q9Y2C9 121512 3678 P08887 P08648 3690 Q9NYA1 11030 P09619 P36896 P35443 P36894 P10600 5071 Q9BUB1 10746 79444 960 P01308 2475 O15169 3689 85440 P07766 P05106 P02790 P05107 Q9UGJ0 6850 P49959 2247 Q96N67 2246 P60033 975 O14641 55704 Q5TCX8 P01579 P05230 857 Q9BQI3 Q92878 Q96CA5 5515 P05112 1956 P09038 Q92997 5518 O95382 O00255 7272 440193 Q6PID4 P48730 Q14289 P06213 P01241 Q13873 7046 O95835 5300 P14317 2033 7161 P42345 3479 2147 91 P04085 Q15389 1616 1856 P78536 4221 Q13526 6885 P36575 P10644 25998 Q9BT40 P31321 Q3V6T2 P31323 P67775 Q99750 4214 4216 P01588 P16070 P01343 Q9UER7 P49768 O00206 7186 Q09472 7189 Q9H422 Q96M96 3263 891 894 27102 896 P17252 P35240 P01019 P01137 O60260 P01133 P01375 Q9UJM3 4361 5573 5575 Q9GZP0 Q13546 5576 Q02156 9495 P43405 P17813 Q05655 P60484 P17936 P01127 P24588 5580 5581 9821 5585 Q15735 P07948 9021 B3KY43 P15692 Q99683 Q03468 5577 5578 P04201 P05412 7099 Q6IR47 Q9NP71 64127 P30153 P30279 5590 Q05513 P00734 P30281 8737 54206 P24385 7422 P10415 O14495 Q9P035 P01730 9839 O15350 Q12933 Q9HC29 O15105 54106 Q13233 P60568

nucleic acid transport 25909 Q86V81 Q06787 57187 O95271 P35658 Q96FV9 O00148 23165 4686 10772 O15234 P09651 Q92574 Q8WUM0 Q96HA1 P38919 Q13769 O75494 8021 O00159 P22626 Q96Q15 9908 Q9UIA9 Q9HAU5 Q9NWB1 9883 P52298 Q86U42 Q9UMR2 Q8TEM1 23144 P57740 Q03014 26019 Q9GZY0 80145 23381 Q8NFH4 P61326 4869 8563 Q32P51 P62826 10482 O14980 9775 Q9H814 4641 Q8N1F7 64901 23039 79023 10128 144983 55110 2332 Q7Z3B4 Q5SRE5 4116 10762 Q9UKX7 Q8IXZ2 Q9HC62 O43592 6311 7884 7248 Q9UPR3 Q14008 22794 Q96PU8 4927 Q99567 P42704 84248 7514 Q14493 Q96A72 5976 11269 9818 9793 Q09161 Q9H2T7 3178 O75694 Q86WV8 P37198 Q8NI27 Q8WYP5 Q9BVL2 5901 3181 Q86W42 9961 Q12769 59343 23225 Q9Y5S9 Q99700 Q9UN86 Q6I9Y2 23636 P49792 9972 Q9NPA8 P49790 11097 Q9UKK6 Q96QD9 P52948 Q92621 O15504 Q92900

RNA transport 25909 Q86V81 Q06787 57187 O95271 P35658 Q96FV9 O00148 23165 4686 10772 O15234 P09651 Q92574 Q8WUM0 Q96HA1 P38919 Q13769 O75494 8021 O00159 P22626 Q96Q15 9908 Q9UIA9 Q9HAU5 Q9NWB1 9883 P52298 Q86U42 Q9UMR2 Q8TEM1 23144 P57740 Q03014 26019 Q9GZY0 80145 23381 Q8NFH4 P61326 4869 8563 Q32P51 P62826 10482 O14980 9775 Q9H814 4641 Q8N1F7 64901 23039 79023 10128 144983 55110 2332 Q7Z3B4 Q5SRE5 4116 10762 Q9UKX7 Q8IXZ2 Q9HC62 O43592 6311 7884 7248 Q9UPR3 Q14008 22794 Q96PU8 4927 Q99567 P42704 84248 7514 Q14493 Q96A72 5976 11269 9818 9793 Q09161 Q9H2T7 3178 O75694 Q86WV8 P37198 Q8NI27 Q8WYP5 Q9BVL2 5901 3181 Q86W42 9961 Q12769 59343 23225 Q9Y5S9 Q99700 Q9UN86 Q6I9Y2 23636 P49792 9972 Q9NPA8 P49790 11097 Q9UKK6 Q96QD9 P52948 Q92621 O15504 Q92900

establishment of RNA localization 25909 Q86V81 Q06787 57187 O95271 P35658 Q96FV9 O00148 23165 4686 10772 O15234 P09651 Q92574 Q8WUM0 Q96HA1 P38919 Q13769 O75494 8021 O00159 P22626 Q96Q15 9908 Q9UIA9 Q9HAU5 Q9NWB1 9883 P52298 Q86U42 Q9UMR2 Q8TEM1 23144 P57740 Q03014 26019 Q9GZY0 80145 23381 Q8NFH4 P61326 4869 8563 Q32P51 P62826 10482

O14980 9775 Q9H814 4641 Q8N1F7 64901 23039 79023 10128 144983 55110 2332 Q7Z3B4 Q5SRE5 4116 10762 Q9UKX7 Q8IXZ2 Q9HC62 O43592 6311 7884 7248 Q9UPR3 Q14008 22794 Q96PU8 4927 Q99567 P42704 84248 7514 Q14493 Q96A72 5976 11269 9818 9793 Q09161 Q9H2T7 3178 O75694 Q86WV8 P37198 Q8NI27 Q8WYP5 Q9BVL2 5901 3181 Q86W42 9961 Q12769 59343 23225 Q9Y5S9 Q99700 Q9UN86 Q6I9Y2 23636 P49792 9972 Q9NPA8 P49790 11097 Q9UKK6 Q96QD9 P52948 Q92621 O15504 Q92900

regulation of cytoskeleton organization 4690 P25054 10092 10095 O95835 10094 P10636 2033 9181 Q4VCS5 Q9BVA0 P42345 Q9Y6G9 Q9Y3A5 Q04759 P84022 P09493 118 Q7Z6C1 P04085 Q9ULW0 Q92574 P46821 6869 11346 P49815 P47755 P47756 Q92974 11190 9475 5154 Q96QB1 23332 Q01082 1072 Q3V6T2 2039 Q7Z460 22919 4214 P61158 Q9BYV2 O15020 Q09472 Q9BV73 Q13813 O15143 253260 O75122 Q13418 O00444 P56539 P31431 22974 P53667 891 P20936 P23528 81873 P35240 79959 P35080 O60260 4869 Q08495 64223 4088 91147 O75116 Q9Y696 P35222 P78317 9495 382 Q02790 O75592 P11277 P16333 387 P52907 Q92786 Q6PKD3 Q15691 Q8IX90 P24588 829 4131 4133 4771 7248 Q15735 23122 P14635 Q9UHB6 O43639 P35612 P35611 10733 672 Q96P48 6709 1499 57662 830 832 Q9H2D6 P61586 P51671 7013 Q5HYA8 Q86WV8 5071 10109 324 1540 2475 57159 6711 6710 4137 P62328 7249 P17081 P54274 Q9BPX5 23189 P62330 P38398 221150 3611 Q9BVC4 P02549 55704 10552 857 P25103 859 Q9NPA3 O43182 Q9NQC7 Q14678 P48061 Q8TAP6 51474 P27816 23077 Q6R327 P63313 Q8N3V7 Q13233

regulation of gene expression, epigenetic Q7KZF4 9463 O43159 4297 Q9NVW2 3481 29947 P84022 10014 5935 Q8WWH4 4343 Q9UBC3 Q8NDV7 9077 O14717 51132 54487 Q15796 P01344 Q96EB6 P01100 Q9UKV8 Q15797 Q5TAX3 O75367 346171 Q9NU63 54496 Q96GM5 P17096 O60264 Q8TAD8 51548 51547 Q9UPY3 54815 P26358 8289 4086 O95983 4087 4088 Q9NRZ9 Q8NB78 Q9NS37 546 3784 11176 Q9NPF5 53615 23409 Q9UL18 27327 Q9UQL6 Q9Y6K1 P51787 3159 11146 Q8WYQ5 27161 Q9NRC8 Q9Y5R4 55929 Q92769 P46100 9555 8467 23411 23378 26523 1789 1788 6839 Q9HCE1 P14373 1786 58487 Q9BXP5 P17480 5987 Q9NRD5 O75569 P98179 3066 Q9UMN6 Q9UIF9 7141 Q9Y6E7 79753 Q92990 Q9NVM4 P49711 P09430 O43463 3070 Q9UIG0 Q86YP4 O95661 Q03164 221656 Q9HCK5 Q9P0M6 51593 Q9H9G7 192669 O14497 A6NHR9

embryonic development Q92858 3880 Q9NYD6 O14672 2672 Q12948 P51587 P17676 2304 2303 P84022 P60174 O14543 Q9UGM1 P28799 P08727 54361 P50148 P16234 P42858 4851 10661 5702 5704 O15525 P27694 Q9ULX9 Q12965 P05549 Q9NRY4 O00167 6927 P37023 55584 29127 P20936 Q92830 Q9UJU2 P40425 P40424 6926 4627 Q9GZX9 Q92824 P07737 Q13705 9093 P56705 Q13829 P17302 P17655 57534 P20823 P20827 2697 P61296 5604 P27037 4771 P28482 Q9Y586 1020 O14627 10733 10736 27283 57669 1499 4643 3676 Q9Y2T1 4646 Q9H0H5 P50222 P09619 P10600 Q6UB99 Q06210 O00358 Q9BPY8 3207 841 Q9HCM4 P17482 Q9UM54 A6NI15 P09622 O95377 Q9BQ95 2132 2253 Q13618 Q15915 Q9H9Y6 Q9NPQ8 57448 2247 3215 1277 O14641 O14640 P10826 50814 P10827 50937 Q13503 P31749 O95140 O00255 Q6KC79 1050 Q03164 2139 56243 3226 P37173 Q99496 Q9UMX1 4678 P29317 7704 P02452 O96004 P04637 O43278 Q96A33 2034 P13349 2033 23322 23326 25988 P52952 O60725 P62993 999 639 P62753 O96018 Q9UNS2 4221 5310 6885 5311 P16989 Q9BRQ0 1072 Q9BT40 Q99873 Q99750 79923 O43294 Q13099 P49768 2290 P25791 Q09472 P50552 P48431 346171 Q9NU63 O00444 Q5T5P2 P11387 Q66K89 O60701 O14727 Q00535 Q9NR09 Q96S42 P53708 P35240 6647 P01137 P49639 Q15047 Q9UPY3 P50440 Q9UMS4 6660 5451 5573 3276 P35222 O95067 P35227 Q01974 Q96ST3 P17813 P10070 P10071 668 5566 P02458 Q96EY1 6657 9927 O43474 6670 5460 4131 Q9Y5Q3 O43597 8851 P07949 Q9H9S0 23764 Q07157 25942 Q9NX62 O00622 O95076 P15692 O75190

Q99684 6667 4005 P55075 7518 10454 5591 O00755 Q6IR47 7412 3297 P80370 23414 Q9UBX0 P36382 10229 51295 689 207 7408 Q15078 90780 5362 4154 7422 23509 P56178 P49321 P56177 Q9UPT9 O75051 Q00613 10672 Q9GZT9 9839 P27540 9612 P17980 P17612 54583 P58012 8516 Q13477 Q14686 4297 8533 O75386 Q9UPN3 29947 Q9Y4K3 3084 O43318 472 Q9H161 474 Q99807 P49810 P08047 Q7Z6C1 51763 P62913 P49815 Q13485 P09017 Q9NPC8 O43683 P20290 5154 4188 8543 Q5TAB7 P13631 Q02447 367 Q9H2X0 6117 P46531 9869 5029 P00533 Q15554 Q15796 Q14344 P43026 Q15672 Q16881 P35638 P61968 6495 Q15797 6498 P12757 P54198 P32242 253260 P12755 Q9ULH7 Q9NY61 498 116113 P25705 257 P23528 Q96RK4 Q15561 5159 P00441 4086 134701 4087 4088 4089 7476 8323 9412 7358 P18074 Q9Y618 Q86YT6 P13612 Q4AC94 P31270 P08151 10087 P43246 53615 9421 Q14249 8452 9546 7248 Q8TD84 2909 7126 P63092 P35453 P10275 Q9Y6K1 P35579 O75581 Q02535 Q93063 Q9NYQ6 2904 Q99966 6045 P78337 8100 O43521 7014 8467 9314 P36896 P36894 Q86WV8 7249 Q9NQB0 Q8WW38 Q99717 P09067 Q9GZM8 1947 Q99958 Q14145 Q9NRD5 Q15475 Q99835 P61803 O95813 5089 O75569 P14859 23463 A1XKG3 7020 P47895 2926 P49715 1956 P09038 O15078 P39905 7157 Q02750 7150 P35548 O95947 Q6R327 Q99814 1969 Q15256 P78545 Q13873 9463 7046 7167 9464 6194 60626 Q4VCS5 2702 Q8TAF8 Q9Y3A5 Q1PSW9 P17275 Q4KMG0 91 P04085 94 Q92574 1738 P46821 O15119 P98161 3911 1856 Q96QB1 Q02297 7291 P10644 7290 9350 P55316 81565 P98172 P42338 7189 7067 92129 Q03014 Q63HK5 1877 P41231 57003 P09630 O60264 Q9NSC2 O95983 9133 Q13426 P19320 2735 Q92786 P07101 2737 2736 O75626 Q15738 O95999 Q13315 Q9H488 7082 Q03113 8174 9021 84525 P00747 O60488 P31260 Q9UJW2 4920 Q13563 2626 P31249 26005 Q68CZ1 27339 Q07817 1788 Q05516 Q15750 O14495 P12830 57045 P19235 P78527 P35711 Q9BQA5 2885 Q14790 P43694 80184 O75712 Q15648 O15230 P19484 Q04771 2896 O60353 Q9HCS4 Q9HCS7 P43686 Q13351

response to protein stimulus Q8N2K1 P07900 P35659 O15123 2033 Q9BV94 595 871 P41134 79139 Q7Z6C1 10013 Q92575 Q16236 P46821 51009 P25685 Q9BZQ6 26270 Q92611 P31689 22926 Q9BS26 1191 Q16082 Q70SY1 Q9P2K8 P05062 P61158 Q9UKV5 P10809 Q9BUN8 P01100 Q09472 P35638 27348 O95292 80267 891 Q9UDY4 55741 Q9GZP9 P01137 P61604 O75807 P07858 3673 P34931 P34932 Q9UNE7 P17301 267 3304 3301 Q9UBN7 3309 3308 Q5VVQ6 4131 P54652 O95757 3320 P14635 25822 P50454 O00622 O75460 P19525 Q02535 3316 3315 O75190 3312 2908 5610 5611 P55072 7913 Q96BA8 2081 Q96CS3 22846 Q14258 Q9NYA1 4780 90993 6048 P51679 P18850 285 Q07817 3329 P11021 P11142 3326 Q68CJ9 1388 148327 Q9NRD1 Q8TEY5 11080 P24385 P10415 10273 Q13217 Q02363 P18146 Q99942 Q99941 3337 7415 P01579 Q92598 P01730 P01178 P08107 9695 O75953 9217 Q7L8A9 P35548 O60911 55432 P17066 23197 Q15011 P04150 P04792 O14656 P08238 O14657 7706 P06213

ribonucleoprotein complex assembly Q15459 50628 10291 6430 6311 6431 6631 P14678 O95391 P52756 6194 1653 Q8WWY3 4686 P62316 Q92499 10772 P83731 P62318 6426 O14744 Q13243 P62753 P62314 O94906 Q13242 135295 Q14011 Q13247 Q16637 Q12874 P54105 10181 Q9UHK0 Q12872 O95400 3692 Q8WXF0 Q2NL82 Q09161 Q15428 1153 79084 O75494 27339 2 26121 O43290 6633 10421 6637 P52298 4154 25929 O75643 10907 Q8TEQ6 Q9NWS0 P56537 Q99700 54496 51340 Q5TAL4 Q9NVM4 P09234 7536 Q14152 P83876 P62851 Q9BQA1 Q9UPY3 4869 Q9UMS4 8683 Q9BY44 Q9H814 Q9UHI6 26747 10569 11218 Q07955 P62263 6733 P78362 P62308 Q9BZJ0 1207 P62304

developmental process Q9UKT9 P25054 Q9Y266 Q12824 8091 259266 A0MZ66 2305 2304 2303 O60443 284217 P21926 2300 O14543 P05452 Q9UGM1 2308 2307 Q92974 P25067 Q9UGM3 Q12834 Q12837 P16104 2317 P42858 Q99598 P39210 Q9UKV3 P61158 10661 P10809 2319 Q96EB6 Q5S007

136319 P27694 4990 Q06124 P17405 P37023 P14384 55584 Q9UKW4 4983 5830 Q9Y295 55109 3655 Q9Y297 Q9Y6A5 Q9Y6A4 Q9UKW6 3659 O75914 P26371 Q86SX6 3672 3673 Q92949 Q99583 Q13705 Q9BYD5 O60437 1488 2335 1487 Q99574 Q9UKX5 3667 P26367 O14578 10644 10643 P25098 1021 1020 Q06587 Q8IX07 57787 10614 1499 1012 3676 P61586 Q9Y231 3678 3679 Q9H0H5 3690 P29466 Q15904 O00358 Q9BPY8 1029 3207 1026 Q96MV8 P17482 3685 O60890 Q92520 604 3688 P07686 A6NI15 P04179 Q9BQ95 Q15915 O60869 P16118 P19634 57326 3215 Q96N67 610 Q92990 Q9Y250 613 6721 Q8N488 4548 Q9H0F6 5879 Q92997 P10826 P10827 Q9UL45 221937 P31749 O95140 Q8NDX5 1050 O60879 27032 P40692 3226 3225 Q9UL51 6733 Q92982 Q9UKT4 Q15019 O96005 O96004 Q96A33 P56199 P14317 O00300 P50897 Q99523 639 O96018 O96017 6760 O96019 P16989 P57059 115426 1072 Q8NHU6 641 Q8IWA4 84033 79923 11113 648 P23276 Q16363 1080 O96028 P48552 6774 3265 346171 P12111 Q66K89 10238 P14780 P11274 Q99500 O95180 P35240 656 8945 P01019 79956 8943 55504 Q15047 84062 P50440 5451 A7MD48 Q9Y696 23552 3276 O95183 P35222 P35227 P35226 P57082 O75084 O75081 Q15051 P17813 53335 51156 668 6776 P24588 O43474 O00744 5460 P01042 P56159 4131 Q9Y5Q3 P01040 9821 4133 P25490 10690 9820 Q9NX62 79971 673 P61981 P15692 6304 6789 P05412 P06744 O00755 5471 3290 Q9Y5R5 3297 Q9UBX0 682 10229 P60660 688 689 9825 5467 4137 207 5468 54206 Q92922 P06753 Q15078 90780 4154 4155 23509 P56178 P56177 O75051 P13010 4149 10672 9839 O43463 5493 O43464 Q8NHX1 Q12816 P35268 Q09666 23513 P50402 P13497 O14939 10682 8516 P27658 P60201 55558 9846 10202 P55268 Q14686 8533 Q9BZ95 Q9UPN4 Q9UPN3 Q9H165 230 Q9H161 P07196 P49810 P08047 5017 P83731 P49815 Q13363 Q9NPC8 94234 4188 8543 5037 79083 79084 P58304 P09382 Q9NY59 9869 8536 5029 51535 P00533 Q13370 Q15554 Q16880 Q16881 Q9BYV2 P35638 Q9HB63 P61968 P31274 P31273 P31276 Q9ULH7 23028 Q9NY61 Q9Y6Q6 257 Q96RK4 Q15561 P61962 1912 Q14232 P55290 1911 8563 6383 5054 Q9Y2J0 Q8IWX8 Q9Y6R0 9412 6389 Q86XR8 P36952 P68133 P37802 P35625 O75592 Q4AC94 Q8N4C8 P31270 23032 P08034 P43246 9421 Q14249 9420 P49862 7248 8577 Q9Y6K1 O75581 274 Q93063 Q04837 P23229 51564 Q15583 Q14254 7252 P78337 8100 7257 Q9HAZ2 O43521 Q15109 O43524 5071 7251 285 Q9Y6L7 7249 1948 1947 P02751 Q96NH3 8110 Q15116 P78364 Q9BV36 P11229 5089 O75569 10152 O15066 23463 A1XKG3 Q9Y6M1 Q96EV8 Q8WY64 P48507 P08069 10155 P02786 Q15125 1956 O15075 O15078 O75553 O43508 Q9NXR1 2810 Q92187 P78362 O95947 1969 Q14289 P25874 Q13753 9463 Q13751 9464 P29375 Q9H0M0 Q4VCS5 P42345 P22735 2821 Q9BUG9 P04083 P04085 Q92574 P56524 9474 Q13769 O15496 O75534 Q02297 7291 7290 117177 P16070 P42338 9001 P06239 Q06643 P56539 1994 60412 P00966 P06241 P21860 O15499 P56545 O95983 P55212 91147 P55210 A4D1W7 9495 3708 Q9HAK2 Q07954 P07101 P07585 Q14643 O75503 O95999 Q13315 P35610 Q03113 O43915 8174 9021 Q16828 P35613 P35612 23085 O60487 O60488 P85037 Q9H9B4 P31260 P20594 P25800 O75508 P31249 Q8NEC5 Q13323 Q9NP71 O43921 23092 3728 3720 P22303 9047 P13807 P12956 P34741 26576 Q9H4W6 Q9BQA5 P02708 Q86TG7 2885 Q9Y6E2 P43694 Q9BQA1 132320 Q9NP98 63976 5927 2896 23077 Q15532 P43686 Q13351 P07148 5925 P10914 O95677 Q92858 O14672 9061 Q9NRW4 Q9UBB6 P52799 Q9P2R6 Q9UBB9 4609 P16220 5931 Q9UBC3 P27361 54361 P50148 P16234 Q9UK55 O15524 O15525 Q92838 Q92835 P29992 Q9UBD6 P05549 Q9NRY4 1121 Q7Z3K3 P53667 Q9BXC9 Q9GZY0 6809 27185 P18858 1111 Q92830 Q9UJU2 P49116 P03372 Q92833 P06401 4627 P06400 P26012 P53675 P05556 Q92826 Q9GZX9 Q92824 3791 P07737 5970 5971 Q9GZX7 9093 Q9NRZ9 P56705 9099 P17535 81501 P20702 P17542 Q9BXB1 P61296 6812 10763 4637 3309 P04233 P34925 Q9Y586 O00468 O95231 O14627 10733 10736 3316 Q6FHQ0 3796 P09972 4643 4646 5978 Q7KZI7 P08648 P30556 P30559 5991 93986 Q8ND90 Q8IVL1 1152 Q8IZT6 P37198 3329 6839 3326 1147 2475 Q92882 Q92888 O95259 P07766 O43175 P05106 P04259 O95257 P28223 137964 6850 2011 Q13618 27148 2010 Q13616

P30542 Q9NS68 O60502 57448 2006 P61244 O14641 4664 P61247 O14640 4665 5997 O43184 Q12770 5515 P04264 P05112 P09958 50814 O43186 Q12772 Q12778 Q02078 Q6KC79 56121 Q13627 O00499 P30530 P52789 10724 10726 P37173 Q99496 10728 P09960 P05121 4678 P40337 P04275 7704 Q9UK32 5528 P02452 O00425 O95278 4691 O00429 P04637 O43157 2034 2033 25861 P16615 Q01650 1181 P62993 Q14050 P01116 4205 Q9C0K7 Q15389 P01112 4209 P01111 Q14055 O43166 Q9C0K0 P48681 Q96CW9 Q9UNS2 P25100 P48201 4221 6885 23210 P35368 2043 Q9HDC5 Q9BT40 P50542 1191 56940 O76074 Q9NRP7 Q15392 84159 Q99873 4214 P01106 768 P01588 4218 Q9C0J9 P01100 P50552 Q8N726 9921 Q86VW1 P14416 P13569 2054 P52735 O00444 Q5T5P2 P14410 P11387 773 Q53X93 6886 55626 6405 54778 P01138 P01137 Q96Q05 P01133 Q16254 Q9UJM3 P02462 P02461 4240 5573 P04626 P04629 2065 2064 2063 P35348 P54920 O14617 301 302 P10070 P10071 5566 Q96PN8 P02458 P01127 5568 9927 2070 5580 5584 O43597 Q92817 Q96PU8 Q96PU4 O75190 Q8IZL9 Q14093 5578 Q8N302 P40763 P04201 5591 Q6IR47 5111 5598 Q5HYA8 O76024 P80370 51295 P18846 324 79600 329 5108 Q92800 P27797 Q05195 Q8N6U8 6456 6455 2099 P12277 O76011 P51843 P15311 333 5599 5116 140735 Q9GZT9 P01178 Q96HC4 23636 P05997 4287 25818 P17980 P52701 P15313 O60911 221656 Q08209 P58012 P18827 6457 10320 P60321 P26447 P25116 Q96HZ4 8650 8655 4297 Q6ZT98 Q8TEW0 Q9BY76 8654 P30086 4291 351 Q04759 356 Q9UBS5 P09493 5139 P62913 P09017 O43683 P20290 9510 5154 5155 7335 367 Q70SY1 Q9UBT3 51654 Q14586 Q13258 P11309 6496 6495 6498 P11308 6015 P32242 253260 P10451 6491 Q9UBU3 Q03405 116113 P51805 51663 P09471 5159 Q96MA1 Q13263 P25940 Q14116 Q13268 7353 Q14114 8204 7358 Q14119 Q14118 P51812 Q9Y618 P51813 P12645 382 222546 Q04725 Q04724 387 388 Q04726 23154 P08151 P20264 P60763 Q5VZL5 P23771 Q13275 P62942 P55347 9546 Q16787 8214 2909 7368 Q14129 Q9Y5I4 23126 O43638 396 Q2MV58 Q02535 397 Q99962 Q01201 Q99963 10296 11146 Q15466 2904 Q16790 Q99966 Q15464 Q13285 54 6045 Q15468 P60709 Q16799 Q9Y5J5 Q86WV8 Q9UKA8 Q6PRX2 P33151 60 P09067 Q9UBP4 8218 O15169 51218 85440 Q99958 Q14145 Q15475 Q8TB22 Q8TEY7 P14859 P21145 P01579 2926 P09038 Q14155 116150 10280 P22492 O15198 P62906 O43623 25776 Q14160 Q6R327 1605 86 P78423 P78424 O15123 O75665 Q15818 1613 27121 26271 Q8TAF8 Q9Y5E8 P41134 Q86WK6 Q1PSW9 27122 91 94 P46821 O15119 Q9Y5E9 2947 Q9UBK2 Q9NSA3 Q12797 Q13643 9114 Q9NNX1 Q13642 Q8WWH4 Q96QB1 O43602 Q15828 P55318 Q99081 P55316 O43609 55177 P67775 P05062 P08575 P05067 Q9BXL5 1627 2956 Q9UBL3 P29474 Q15836 P29475 92129 O95613 26258 29767 Q03014 Q7Z3S9 6096 P34820 P55327 1634 56034 P21980 Q14993 1639 Q9UBM7 Q9NSC2 P11717 8289 Q15843 9133 Q14517 O15156 9138 29777 Q05655 Q9BXJ9 57379 Q8HWS3 Q15853 Q9UJV9 O75626 P13945 9149 Q6NYC1 Q9H488 P41182 P41181 P41180 P53567 P30048 84525 P05091 1653 P05093 P08123 Q92499 Q9UJW2 Q13683 O75629 P47712 150094 Q68CZ2 Q07820 Q68CZ1 Q07817 P20248 64919 80155 Q9UJX0 Q96QF0 P08138 P09429 O75604 P10415 3860 P19235 P35711 23181 Q7Z494 P08581 83696 3852 P09430 80184 P08588 P41159 O15111 P55771 Q04771 1203 O15105 P45983 3866 Q13233 P21554 Q7Z2K6 65125 3880 3881 O14793 2550 9181 P51587 P17676 2547 4729 P37288 Q8IYN9 P84022 Q9HCJ2 Q92731 Q92730 O14786 3875 O14788 Q8TDD5 6901 P28799 Q9NZG7 P08727 Q92729 124540 P51114 Q96KN7 P20849 3886 493856 4734 Q8N205 Q7Z727 P28300 81629 O00167 Q7L5Y9 54496 6927 Q8IUD2 3416 55342 6929 811 P40425 Q5TAP6 P40424 P62258 6926 P31944 Q9NZI7 Q9NQX0 Q9C009 P07858 P31947 2100 P48380 O95343 P17655 57534 3428 6938 P20823 O60674 P20827 388677 O60675 P62263 821 Q8N5V2 5604 P41743 Q9Y468 P11926 P27037 4771 Q9Y463 Q9Y466 P39019 P19404 55366 6949 27283 P15056 27287 Q6ZNA4 Q8N264 P07437 P50222 P32856 Q92769 Q14814 P09619 5631 P28370 Q9NZC7 P10600 Q8IYR6 O95365 Q06330 Q9UEE9 841 Q6PI57 Q9HCM4 Q92764 4775 O14753 846 7804 O14757 Q92766 P09622 O95377 4791 1285 2132 Q96KR1 Q96L34 Q9NQS3 O95376 Q96TC7 2125 1277 Q92753 Q5TAT6 Q92752

P05230 857 859 Q92754 Q13501 O00139 50937 Q13503 P11912 Q9NR50 1297 Q13509 1294 P19883 Q13506 Q9P202 O60636 Q9HCK4 2139 861 56243 1289 O95390 863 4313 Q9UMX1 P09601 4318 P06576 6993 O43278 23322 3484 P14555 3482 3481 23326 P11047 25988 203068 3479 2147 Q8WV60 10011 7832 P01236 55723 10014 3010 Q9BRQ0 3009 Q99750 Q01995 O43294 6517 O43293 Q01518 P01222 Q13099 O43252 Q09472 O00562 P53701 2173 P13686 23787 890 Q00653 891 Q99743 O14727 894 Q9NR09 P53708 898 3014 6525 P49639 Q9UMS4 P51531 8721 Q658W2 P51532 25975 P14543 Q01974 Q96ST3 55755 3028 P60484 11331 4358 5207 10487 P26583 Q9Y3M2 Q6FGG2 7402 2195 23764 25942 25945 Q8NFW5 10458 P60891 P31939 10451 P55075 6548 79739 10454 Q8N5U6 7410 10460 7412 Q9HCE7 P52803 Q08117 Q05086 3052 P37231 P36382 Q01955 Q96SZ6 P51959 444 445 79723 Q02809 P48357 7408 10468 P24385 P24386 8754 7422 9600 3066 22895 O75293 Q00613 P61764 7414 3070 7430 9612 Q9UIG0 3074 10447 P17612 466 55796 Q9NQL9 7428 6595 6597 6599 P18085 8775 6598 5268 O75386 O75385 Q9Y4K0 Q86VQ3 6591 Q9Y4K3 3084 O43318 472 473 474 Q9C0C7 Q99807 51763 P43034 P21246 Q13127 Q15303 5274 3091 Q16635 Q15306 Q86VP6 3099 P58546 P13631 Q9UJC3 5270 Q02447 Q9NVP2 488 Q05952 28981 Q9NRH2 Q9H2X0 24144 6117 P22102 30813 P46531 Q16643 Q15796 P43026 Q15797 P12757 Q8N6I1 7468 P54198 P12755 O75360 Q01101 498 P31040 Q9NRI5 O94805 Q15323 Q13144 Q9BWQ8 Q16650 134701 P35869 9657 7476 8323 P18074 P10586 P10588 P13611 P10589 59277 P13612 93166 200894 54820 P23416 Q16665 P55061 Q8TDY2 O15287 Q16666 P54132 P63092 131601 P35453 P09172 P46109 P23467 Q14494 8328 P27824 Q13164 Q14012 Q86W54 7013 7014 P46100 Q9Y4H2 7490 Q9NZT1 P11021 Q8WW38 Q9GZM8 51339 Q9NRD5 Q9GZM5 Q99835 9201 9202 O75326 9203 P13646 Q02880 P13647 Q86VS8 P13645 7020 Q08188 51341 Q9NVM4 P02549 55704 P21266 1718 P02545 P47895 347733 P23443 Q8IUX7 Q8IUX8 7037 P38936 P11474 Q86W33 P57682 3909 P02538 Q6PID4 Q99816 Q99814 P02533 P98194 Q15375 P60842 P78545 7046 P28289 6198 P98155 P78540 O43711 55283 6194 P32302 60626 3913 Q9Y4C1 56134 65082 1738 P05177 P98161 P07355 P98160 O60282 3911 3912 Q2Q1W2 P78536 7057 Q13520 22806 Q3V6T2 7052 P19338 3925 P46934 1742 P46939 Q9HD26 81565 Q4LE28 P42574 P98172 P98174 Q14865 8399 9244 7067 Q5TAX3 Q13535 P19793 P29590 Q15717 Q7Z2Q5 Q63HK5 P31431 Q01196 1756 29880 P41231 Q92793 Q96GM5 Q9HD15 57003 Q7Z6Z7 O15259 O60264 Q9NRA1 3932 O60260 9255 Q04695 P19320 Q8IYT8 9253 7074 P43405 Q9Y4F9 P41220 P20309 Q9NZR4 Q04206 Q5T5A8 Q92786 Q92784 7088 Q9NZJ7 Q5GLZ8 Q15738 Q15735 P78509 7082 P78504 3958 P00749 O00192 P00747 P63000 P08243 3953 Q13563 3956 2626 Q15742 Q9NQZ2 P14923 7090 P11413 1789 1788 Q05516 P62166 P00734 375790 P08253 Q5BJF6 Q15750 Q9HCU4 Q8IUQ4 57045 P78527 O60229 P20336 Q04656 51741 Q5T9L3 Q16610 Q14432 3978 O15232 O75712 O15230 Q15768 Q06830 P22528 Q9UNA1 2651 Q9HCS4 4830 900 51752 Q9HCS7 P00750 P08238 O15228 P08833 Q8NEY1 Q9NYD6 2672 Q12948 22994 O00268 Q8NEY8 Q9BVA0 3516 Q9BVA1 P60174 Q9ULW3 Q9UM82 P08842 P07996 Q12950 P52565 Q8NF91 Q12952 51090 P52566 Q8NB12 Q08431 Q16082 P63211 79885 P21810 P16473 2676 51099 P16471 O60331 P21815 4851 4854 5702 5704 928 Q92600 P54753 P53420 Q9ULX9 Q12965 P25391 Q12968 Q8IXK0 Q05397 P20930 29127 P20936 Q9UQB3 4862 Q9UQB8 4867 26091 4869 P54764 P54762 P54760 4882 P48023 Q7L576 22985 Q13829 P17302 5728 P17301 1368 Q9HBE1 Q99697 2697 10523 Q9Y342 P28482 Q5D1E8 1385 O95470 57669 P15173 P07550 Q9Y2T1 P08887 Q9UM47 P51671 O00238 Q9Y2T7 Q96SB4 Q96SB3 Q9NYA1 P51679 P10721 Q6UB99 Q06210 Q5VST9 P19544 Q8IY17 2239 P18206 960 1387 967 4898 P42771 Q9UM54 5747 22937 Q06203 2253 Q9NYB9 Q9UHL9 Q9H9Y6 6609 P62330 57689 Q9NPQ8 P42768 2247 1399 2246 P61006 6601 6605 P51693 P29323 P51692 O00255 Q03164 P17342 5764 P48059 P29317 6615 P06213 Q9Y6W5 P13349 2275 P52952 O60725 P15531 991 Q8WTS1 79810 998 999 P61421 518 P02686 P62753 Q04917 P49770

Q9UHG2 5310 Q7Z5H3 10141 5311 Q9UHG0 P51636 P53814 2280 Q93096 Q04900 2277 23451 Q15154 P48436 P28845 P01344 P01343 Q96NL6 P49768 2290 P25791 23429 P48431 5321 3142 Q9NU63 Q99623 O60701 Q00535 Q00534 Q96S42 537 7975 6647 54535 6646 5316 8829 5318 Q9UPY3 Q8N960 P01375 P01374 6660 Q9Y337 4000 8841 Q9UHI8 23432 O00213 O95067 Q96AQ6 O60716 P12004 Q9P0I2 3149 3148 545 546 3146 P40933 Q96EY1 Q96JB5 6657 5327 5328 P49747 6670 P24043 P07942 O00628 7520 8851 P07949 P07948 Q8WYA6 Q12904 P20916 Q9H9S0 Q07157 3162 Q8WYA0 O00622 O95076 Q9BZE0 552 Q99684 4004 6667 4005 5336 Q96AX1 Q15185 5338 7518 P26232 Q7Z5L4 Q969G3 5351 7532 7531 7533 P68032 23411 23414 3171 10107 3169 Q9NPH5 4015 Q96JH8 4017 9702 10100 P05783 7528 Q9H5I1 5361 5362 Q86U86 6696 5364 4035 P49321 Q9UPT9 Q9BUZ4 P12036 3182 Q8NER5 P62736 P26678 Q01826 5371 P27540 P20908 P16401 P16885 582 54583 Q8WTT2 8879 P62745 6699 8878 P60568 Q13477 8892 Q9UQP3 P15924 P15923 Q9Y3I0 29948 29947 595 8407 Q7Z6C1 Q7Z6C3 Q8NFJ9 Q13485 Q8WUM0 8408 Q8TD31 P03950 9750 P35527 O94910 Q13009 4067 P12429 Q15669 O75496 Q5TAB7 51412 P60983 Q96SN8 6239 Q13490 Q14344 Q15672 7101 6256 10075 O75487 O43897 Q2M1K9 Q01469 P25705 Q9UDY8 P23528 Q9UDY6 Q8WZ42 P00441 51428 4084 8443 4086 4087 4088 P22692 4089 163126 Q86UL3 Q86YT6 Q02790 P12883 P21359 23396 1808 P24864 P62701 10087 6259 53615 8454 P54259 7123 8452 6275 P54257 4099 7124 P54252 Q8TD84 7126 P14635 64759 Q9UQL6 P10275 O75462 4092 4093 P35579 154 Q5JUK2 Q00597 Q99720 156 Q9UMD9 23363 Q9NYQ6 Q8WV28 Q99729 Q16555 10059 Q13045 Q15223 P45379 Q13043 Q13049 Q86YZ3 8467 9314 P36896 P36894 P15976 P35568 1822 166 120892 338917 Q9NQB0 Q99717 Q99715 Q99714 Q96T37 Q8TCU4 7142 P61803 O95813 Q8N5K1 P54274 P35555 Q86V24 P36888 7141 1832 Q9H1Y0 Q00577 9319 Q16576 Q99707 Q9NQC3 P49715 P60953 7155 Q08050 P39905 7157 Q02750 7150 P35548 8481 Q9H295 55832 Q9NZ56 Q13075 Q16586 84376 1848 Q5VTD9 Q15256 O75427 Q13873 7167 Q08AF3 P10636 26037 Q86Z02 Q01094 P63172 7161 9341 7163 2702 P63167 56259 Q9Y3A5 P47914 10935 P17275 P23945 Q4KMG0 Q92692 P39060 Q6NVY8 1859 P22612 P42229 P15090 1856 Q13885 O94992 Q13404 Q13887 10939 7170 Q14738 P10644 9351 O43847 9350 79365 P39059 1869 7189 57120 O75881 10908 Q13418 O43815 7182 1877 1874 P17252 P68871 P09630 O15379 Q5TA76 O95859 O60383 Q9NZ08 P42685 P21741 O95863 Q13422 O95861 Q13428 P35908 Q13426 P10620 9371 Q8NB78 4914 2735 Q9Y3D6 1400 Q02388 56288 2730 Q96FJ2 1406 2737 2736 P54652 Q8WUI4 P19012 Q02386 1896 2742 P09211 4920 Q13444 P35968 Q14774 Q13443 26005 P11532 57154 P51159 27339 P30279 27330 P23515 57159 O14492 P56851 Q9H228 O14495 P12830 Q02363 P18146 Q03692 163183 3611 Q14790 P00403 O60341 P01730 O15350 O15353 Q15648 P19484 Q06710 O60353 P24821 O14497 O15347 4953 4957 Q9BZR6

protein modification by small protein conjugation 11060 11065 7325 Q9Y4K3 O75150 Q5QP82 9616 P63208 90678 9978 3093 7332 7334 7336 7335 Q9GZZ9 Q5VTR2 Q9Y4L5 Q86VP6 Q9UBT2 23291 Q13490 7328 P61956 4734 Q9UKV5 7341 P22681 10193 9646 10075 6015 P32121 A0AVT1 4193 P62256 Q9NV06 P61962 Q16531 P61960 O75928 P55854 Q6PD62 57092 Q13829 Q5TAQ9 267 55236 Q6PCD5 8454 8452 7126 P51668 26994 Q06587 79577 Q96DE5 10616 10055 Q13042 Q99728 Q13049 Q7Z419 6045 Q9H4P4 6048 Q96J02 Q9UKA1 5071 7251 Q9NS56 Q8TBC4 7128 7267 10273 Q13618 Q13616 1161 O95376 Q9UKB1 Q99942 Q8TBB1 Q8WY64 P38398 10956 Q9UK22 55827 Q969M7 6500 P31749 255488 253980 57332 55832 Q96LR5 55159 Q99496 6612 867 Q99816 54623 6613 P40337 Q8WW12 2935 Q6P1J9 O00308 P53804 Q15819 Q9NVW2 23326 Q9H0M0 23327 80124 27246 P63165 997 Q15022 P62877 Q8WWI1 Q969T4 Q58WW2 64682 Q13404 Q8TCJ0 115426 83737 84708 55294 27252 P46934 51132 4214 Q9BS18 408 P49407 409 25847 7186 7189 10238 Q9Y3C5 Q9BYM8 26133 55743 Q8TEL6 Q7Z6Z7 10477 O60260 Q15843 Q9UMS4 55072 25853 Q6UWE0 P78317 Q9UNE7 26260 26262

1642 Q96EP0 P62837 Q969H0 Q9UBF6 Q96EP1 672 Q96PU4 26232 26234 Q96PU5 4008 P55072 Q9HC52 Q9H3F6 Q8WZ19 P21580 Q9HCE7 57154 Q86Y13 Q8N7H5 P61081 Q05086 27339 Q9UJX2 Q00987 P61077 207 Q9H1A4 329 Q96JH7 P10415 Q13216 Q8IUQ4 P19474 Q9UPT9 Q9Y2M5 O00762 51185 P61088 P68036 7415 Q5T6F0 8881 Q12933 23512 580 P30260 7428 Q13233 51514 P49427

negative regulation of signaling pathway P25054 Q14449 P30086 O75386 2305 595 Q92731 P78395 O14543 P49815 6901 P07996 2308 P25963 6003 4188 Q16635 Q15669 5037 O75376 54361 P14174 Q9H2X0 Q9UBT3 P46531 4851 Q13370 4734 O15524 Q96EB6 Q7Z727 6496 6498 Q96IZ0 P12757 Q8N6I1 P32121 P12755 Q99471 4193 O60543 811 O75928 Q13145 5716 Q9GZX9 5970 Q8WWN8 Q14118 P10586 2100 163126 Q9NR96 9093 Q9UBE8 P56705 O95343 Q13829 5728 Q04724 387 P21359 3667 3309 P04233 Q13033 P25098 8452 9420 4771 7248 P54253 7126 4092 O75581 154 156 P07550 P61586 Q9Y2T1 26191 Q99966 Q13043 5074 Q9HAZ2 Q9NZC7 Q9Y5J5 26524 P10600 Q86WV8 P35568 P37198 P11021 960 7128 7249 604 Q9UBP4 O15169 Q15910 P07766 4792 O95813 P49840 Q13618 Q9UNH7 58533 P08069 857 Q13188 859 5515 5516 5518 Q9NQC7 5770 Q12778 Q08050 7157 P31749 O95140 O00255 25776 Q8N9R8 P19883 P37840 2931 253980 51231 Q9UMX1 1848 1605 Q92743 O95835 P04637 5300 23322 P52952 27121 Q96RU8 3479 27122 2146 997 3476 5654 6622 Q9UGU5 Q92574 Q9NSA3 7057 4221 Q13526 Q96QB1 9350 P46934 O43609 P67775 Q99750 P08575 P16070 O43294 408 P49407 409 P01343 O00206 P48431 P14416 O75881 Q03014 P56539 84619 Q99623 P17252 P35240 P01019 O15379 O14964 Q9UJM3 Q9Y2R2 8841 Q9UNE2 P78318 O60716 P35348 P17813 Q05655 P60484 Q07954 Q04206 P10071 11331 Q96EY1 6657 2737 9927 9146 7088 5580 Q9Y3M2 O43597 P41182 9021 Q16828 P23508 5578 P62714 6789 5338 7099 Q13322 Q8WZ19 P21580 Q9HCE7 64127 P30153 57154 23411 23532 Q68CZ1 5590 Q9BQB4 Q00987 Q05513 324 1540 207 54206 Q13574 P08138 10468 P27797 P24385 4035 29966 57045 P51843 Q86TG7 Q9NRM7 51741 O14921 23636 O75832 Q13227 P18031 Q04771 63976 57732 Q8N5A5 O14939 Q9HC29 O15105 54106 P49427

response to peptide hormone stimulus P02452 P01241 64397 P08833 6198 8892 P98155 Q9Y266 6599 3484 Q9H2Y7 3481 O60603 P42345 P42224 Q96RU7 2303 P62993 Q04759 Q99523 P17275 Q92572 P07196 7832 51763 Q13480 O14543 Q92574 P46821 P42229 P27986 P08842 10014 1978 2308 2549 P49770 5155 O15530 Q96QB1 Q9BT40 367 P05062 Q13370 P01344 Q92569 O15524 1080 O00206 Q7Z727 6256 P29353 P29474 6772 6774 P13569 P19793 Q03014 P14410 890 894 57761 P17252 P00966 P21860 Q9UQB8 5798 P01138 Q13144 P09874 Q14232 Q92831 P32239 P50440 5295 8165 P55851 P07858 5970 Q14118 2065 Q9UKG1 5170 142 P17302 O60674 2697 Q04206 Q13393 8836 6776 P01127 5327 3667 5207 Q59EA4 P41743 Q92667 Q92820 4131 5584 7248 P41181 9021 Q9UQL6 P10275 P35613 10458 P15056 673 P22415 5578 10057 5337 5338 P08887 Q99966 P40763 P04201 7099 5591 P05413 5471 Q13322 P30559 10580 Q9Y4H2 O95361 Q86WV8 P35568 P30279 Q07817 P37231 682 57678 10626 P20248 445 2475 207 5468 P17081 Q92922 Q99958 P09467 9564 O14492 Q16849 P49840 22937 P10415 Q06203 P78527 P18146 P16118 P51843 Q8NER5 Q9HCL2 P61244 2885 1277 P17174 P08069 8503 4149 Q12770 2805 P01178 P23443 P41159 Q12778 6464 Q9BX66 P51692 P31749 Q9NR50 1176 P18433 Q6ZWJ1 2931 O14939 10726 P09960 P00750 1605 P04155 26060 P06213

histone modification Q6P1J9 Q9H0E3 Q96L91 4297 Q9Y265 6871 Q9BZ95 2033 27005 23326 O75150 11107 10933 Q9Y4C1 Q15022 79813 Q7Z6C1 10013 84289 10014 P56524 6883 7334 Q16512 Q969S8 Q16514 Q15306 O96019 23210 Q5VTR2 Q9H3R0 57634 79885 Q92560 Q4LE28 Q96RL1 408 P49407 Q96EB6 Q9UBL3 O75528 Q7Z727 Q09472 O95696 9646 10075 Q9UPP1 6015 Q9UNL4 O00167 Q6UXN9 23028 Q9H6W3 54496 83933 Q92793 P17252 Q7Z6Z7 O15379 55869 Q92830 O95619 51147 Q9UBU8

Q6IT96 P61964 Q92831 Q9C005 O95983 Q6PD62 O75478 Q13547 8841 Q9BY41 O00213 9252 Q8NB78 P35227 10524 55352 P35232 O60674 Q15291 11176 Q9NPF5 Q9UBN7 10765 53615 5585 Q6NYC1 Q96MX6 Q06587 O75582 Q9UQL6 23081 79577 10856 Q9UK80 Q9H9B1 6304 51720 5578 Q9Y230 5579 O14744 Q9HC52 P05771 8607 6045 55929 Q92769 Q9NQ92 23133 O95361 23135 23411 Q8N7H5 Q8WYH8 P46736 10626 1387 5245 3066 Q9UPT9 3065 Q9UIF9 O75164 9682 79595 P61088 Q9NVM4 Q96PK6 84661 O60341 Q92993 10432 Q01826 11091 Q9NPA8 30827 Q9UIG0 Q9P0U4 23512 Q03164 221656 2139 5929 57332 80335 Q99496 54623 Q9UGL1 86

negative regulation of protein ubiquitination 11065 9184 991 26271 O60566 P62195 23560 Q92574 9474 64682 P55036 Q12834 O43684 5706 5707 5708 5709 5700 Q16401 5701 408 P60900 5702 P49407 409 5704 5705 Q13257 5682 5683 Q8N726 5684 5685 5686 P32121 P35998 P53667 5717 Q00535 P25789 10197 P25788 P61289 5713 P25787 5716 5692 5693 Q99460 5687 5688 Q9UL15 5582 P05129 7248 P51668 1020 P51665 4092 Q9BZE4 Q13042 Q92530 10213 Q13200 O43242 7375 O00231 O00233 O00232 Q86WV8 1029 Q9UJX2 Q9H1A4 P49721 P42771 P49720 P28065 A1XKG3 O00487 O00762 P62333 P20618 Q9H1Y0 Q15008 Q9UL46 8881 O75832 Q13107 P62191 P28074 P17980 P28072 P28070 O14818 P30260 O15105 Q9UKT4 P43686

response to DNA damage stimulus Q5FWF5 O95677 P25054 Q14686 4176 P15927 11186 O95551 P51587 472 64949 2547 595 1454 1453 4841 9978 Q14694 4968 P52564 7334 4067 Q9NS91 7336 P27361 Q8NFZ0 64963 64965 P16104 O60216 P18887 80198 Q9UBT6 60488 55215 6117 11198 6119 6118 Q96RL1 Q15554 Q96EB6 P24522 9641 7341 Q9H2G2 P27694 P35638 Q9UGN5 P27695 Q9NRY2 9643 8555 Q16526 Q9UNL4 O00167 Q06124 29128 79791 Q9NY61 57646 P49916 P18858 O60543 1111 P22674 Q16531 Q9UBU8 O75807 51426 P00441 9656 O43542 P18074 P31947 8445 Q9BZF9 142 3428 10524 Q49AN0 P43246 Q6PCD5 O15287 Q16666 5981 P28482 P54252 P54132 5983 1022 Q14807 Q9UHJ9 Q9NSU2 8450 25788 Q00597 1137 10856 Q9Y6K9 Q99728 Q9Y230 51567 5976 4646 7014 P46100 O43524 Q9Y5J5 O95243 Q96SB8 Q9NS56 2237 1026 Q9NRD1 604 11277 Q9UM54 O14757 Q15233 Q9NYB0 O95257 7023 Q12888 P54277 5883 P28340 1161 7141 P38398 Q56NI9 10714 P49959 Q01664 Q96PK6 Q99708 9319 Q6NUQ1 Q92993 Q92630 Q92878 7153 6188 64782 Q13620 7158 5892 7157 O00255 P54727 Q6KC79 P38936 P54725 2139 2810 P40692 57697 55159 O60870 Q96MT8 Q14164 157570 5886 P48730 5887 5888 P09601 Q15014 9100 P23396 8930 6872 O95714 P04637 5422 Q15819 P29372 P33993 Q86Z02 Q7Z589 P78549 7161 O43715 25988 Q96T88 10933 Q96RU2 P63165 P62877 8924 7832 P23025 26270 6749 P82912 O96017 9232 4221 Q86WJ1 O75771 P35244 P35249 Q96T76 Q96Q15 Q9BYN8 641 Q9Y6H3 Q08945 P35251 Q52LR7 5424 P98170 Q99638 P42574 56946 2956 P49768 Q969S2 Q6ZN33 Q9H305 9126 Q13535 P29590 P11388 Q9NS23 890 Q9NUW8 79035 Q96T60 3014 Q9NVI1 51147 6647 Q8IYD8 29086 Q86UE8 P09874 P23246 Q9UMS4 4361 6421 P40938 Q13426 P40937 114799 O00213 P34931 P12004 80254 1642 1763 O95071 3148 545 546 3146 Q15054 P09884 Q8WTP8 1408 1407 P23497 1647 4913 7508 P82933 2070 P26583 6672 7520 4255 Q13315 P07948 O95997 P50613 P41182 P51946 3162 672 Q03468 51720 7518 P55072 7517 3835 Q8IY92 5591 P46063 5111 Q13569 23411 Q9NYZ3 27339 Q01831 P46736 Q6PJP8 P20248 Q9NWV8 P51959 Q8N0Z6 Q9UQE7 324 P61077 55775 P82673 328 5347 Q13574 Q9Y5B9 P09429 Q9BQ15 P24385 P12956 3980 Q13216 P78527 22897 Q9BQA5 331 P13010 Q5XUX0 P61088 7415 P09430 10432 3978 5371 O15350 Q14676 4287 P49674 Q9UIG0 P53350 5378 27315 P39748 Q8NHX1 Q9P287 P52701 580 P00519 P43681 8517 900 Q9HCS7 84893 902 51512 56647 Q92900 Q13112 51514 Q9UPV0 P21675 Q14683

generation of precursor metabolites and energy O75947 Q16864 P52790 O43678 P10599 Q9P2R7 230 1337 4728 4729 P37288 O75390 P13073 27089 P60174 Q99807 P22695 4720 5018 4722 4723 6901

4725 4967 4726 O95685 P28331 P48047 Q9UI09 Q16635 O14561 P11216 3099 3098 P38117 O75380 1345 P00414 4731 P47985 Q9UI12 Q13011 Q16881 5162 O75489 P56134 3420 Q12968 4191 5160 3417 498 P25705 1355 P09110 P18859 P31040 P56385 P56381 P62136 30 5836 3419 10632 P36957 Q16656 6389 P99999 P21912 80777 P62140 P00558 Q86Y39 3421 4514 P43246 P07864 P56556 48 P40926 P63092 2110 P38606 P36542 160287 P50213 6390 Q9P0J0 P19404 2108 P04040 P09972 Q16795 P51553 Q9UGI9 P15735 Q15904 Q06455 Q06210 Q93050 P35573 5500 4775 5501 6834 4535 P49841 847 4899 P09622 P04179 7385 7384 7386 Q9UGJ0 P14618 O00483 291 Q9BYZ2 7381 P16118 P17568 Q53TN4 178 Q9Y6M9 P24539 2805 P49715 O75438 Q9Y375 P21283 P21281 2023 P36873 P37840 1050 P52789 2932 862 Q9NX14 Q00325 10728 P49821 79901 509 P21399 P06576 O75306 O95278 7167 Q7KZN9 P08559 P10515 4695 O43837 2821 126328 O14949 2026 513 514 8803 6622 515 55967 P61421 8801 1738 P05177 518 1737 Q9UBK2 Q13522 9114 O95169 O43169 P48201 P46020 P04406 P07919 P11177 7295 1743 521 522 29796 Q8NBI2 523 P05062 4697 P08574 4698 526 528 Q99757 P46019 P07205 Q6ZMR3 O95298 92483 O75880 O95299 P12235 P46926 3939 P00846 533 535 Q16134 537 5315 539 10476 P03891 54539 O00217 Q13423 Q16816 O75874 5213 1891 O95182 Q9Y2B5 10007 3948 Q04446 5207 3945 P30049 P06733 P31930 O75746 25828 P06737 P13945 245972 552 Q93100 8604 Q15185 3953 1537 P07954 P06744 51606 P14927 23530 O43920 Q16718 P12074 Q99437 10229 2632 Q15070 5467 P49447 P48357 P09104 P46976 P09669 P01185 P13807 O75964 P13804 4705 4707 1431 P17174 Q8N8Y2 P00403 4701 4702 P56181 P41159 5250 P53597 P51970 6341 Q15526 5499 P19367 9296 P15313 4718 4719 P17858 4710 Q01813 P08237 4713 4714 4715

cell proliferation 8650 4176 Q9Y266 Q12948 Q9NRW4 O43557 P10599 259266 P51587 3084 P17676 3638 10657 595 O60566 4609 2303 8767 Q13480 P43034 5931 Q9ULW0 Q9Y275 O14788 P21127 10413 2549 Q15303 7332 P25067 5155 7336 5036 Q12959 P11215 367 P14174 9869 Q9H2G9 200081 P00533 P10809 Q16881 P11309 6256 3660 P11308 O75487 29128 P59768 22974 6491 Q01344 57646 29127 Q9UDY8 Q13261 Q92830 8548 Q9Y297 P03372 Q9Y6A5 Q9UKW6 P55290 Q12972 P32239 4084 5174 P22692 P54760 7476 Q9Y6R0 P18074 3673 P31947 8566 Q16658 Q9Y618 Q9UKG1 Q9NRZ9 5728 3428 P17301 P17542 Q8NEU8 54820 Q9BXB1 P20264 10087 Q15691 P04233 P43489 P27037 10293 Q16666 4771 7124 1022 1020 P10275 274 55366 Q9UEU0 1018 1499 23481 Q6FHQ0 1012 Q13042 P61224 8328 O14744 Q99966 Q15464 Q9H0H5 P04049 Q15468 Q14137 Q07666 Q8IZT6 P10721 P30550 3329 2239 Q9BWF2 1147 1025 1387 Q9NQB0 P01303 Q9Y242 4898 Q9GZM8 5987 P40238 Q99958 Q92888 9564 Q8TCU4 P30304 6850 P11229 P30307 1285 2495 A1XKG3 Q86VS8 O95376 P40222 55145 2006 P60033 Q5T2W1 975 5511 4665 Q16576 Q9NTI5 Q9GZL7 11004 2925 1956 P09958 P30536 P20073 7157 5894 P11912 O75312 P19883 O00499 Q9NXR1 2810 51593 1841 P17342 Q14160 Q93034 10726 863 984 10728 Q6PID4 4678 84376 Q5VTD9 P09601 Q14289 O15503 26060 Q15139 10490 P04637 5422 Q15819 P33993 O00541 Q01094 P14316 6194 11108 Q96T88 Q9Y3A5 Q96RU2 3479 Q1PSW9 P17275 995 10011 6502 6624 P01236 Q13761 P62873 P62753 3911 1736 Q9UQ80 P25100 P35368 Q02297 1195 115426 P50548 P12268 O43847 7295 56940 79365 P46937 81565 Q15154 Q14186 22919 P46013 P48436 P01106 79923 P08575 Q4LE28 P01589 1869 Q9C0J9 P01343 P02794 P49768 O95971 Q9UKN5 P48551 P19793 Q03014 Q13418 1877 Q66K89 Q92793 Q9HD15 Q00535 896 P35240 8945 P01019 P06241 6886 P01137 Q15047 P01135 Q8N960 P01375 Q9Y5X9 O15151 Q9UMS4 P40818 P35908 P04626 9138 Q13309 2064 P35222 9253 P12004 P42677 O75081 P43405 55198 P20309 Q05655 O95071 2059 P60484 P10070 Q07954 55759 P09884 P01127 P40933 Q00526 2736 O43353 9388 5580 O00744 Q92934 7520 8851 P50613 Q9H9S0 3162 O00622 310 Q96PU4 6304 4004 P25800 Q15185 O75629 Q13561 P04201 P01275 O00755 Q15742 5111 8744 10460 8065 27339 P14373 Q01955 5908 Q9UKZ1 P30281 Q9BXP5 5467 5347 5108 P22303 10468 Q15078

P23510 Q9UQV4 P24385 Q05195 P50750 P10415 P56178 4035 P49321 3068 2099 Q02363 Q12929 O00764 572 3615 3614 P13010 P08581 3611 P41273 Q99661 P68036 10673 Q01826 9839 8882 P54687 3070 8881 O15111 9612 O15353 P53350 Q15648 O15230 Q06830 P51858 23513 Q9NVV9 P30260

catabolic process Q96TA2 9184 Q70EL4 4967 4968 Q9H0D6 Q9UIQ6 Q9C026 P28330 Q12834 O00154 P52209 Q5VTR2 P49247 4734 Q9UKV5 64282 P27694 3420 2572 P56817 2571 P17405 79791 Q8NBQ5 A0AVT1 3417 3416 P62256 81631 5830 119180 6921 Q9Y297 5836 3419 P07858 P99999 2582 Q676U5 P21912 Q99575 P19838 3421 P07864 P51784 Q9UL18 1022 2110 1020 P50213 O95352 10613 P21964 10616 Q9Y231 Q92530 340204 P51553 Q96J02 1029 10868 Q96EF6 10869 167227 841 847 P07686 4790 P16118 O95376 Q8TBB1 391627 Q05469 P20618 Q9BPX1 55149 Q92995 Q96CA5 Q13501 Q9UL46 Q9UMW8 6500 P31749 Q9HCK5 10845 253980 P40692 192669 5886 5887 P09601 8930 O00308 P50416 P14555 23326 P50897 P62877 10013 11345 Q15024 P23025 64682 115426 23576 P35244 51132 51377 Q9BRP8 O43294 408 Q15036 P49407 P60228 409 10269 P60468 Q6ZMR3 Q6ZN33 5682 5683 5684 5685 5686 Q9BW91 891 O14727 Q96T60 55743 8945 Q16134 P61326 Q15287 Q9Y5X9 Q9Y215 Q8TAT6 5692 5693 P40818 P04746 7874 5213 P11279 3032 3030 10007 P14550 Q01970 668 5445 5687 5688 4116 P48775 5207 Q59EA4 P06733 Q5VVQ6 Q92934 P62837 9821 P06737 8975 10690 9820 P50613 P51946 Q8IWF2 435 4125 10213 P55072 P06744 O43242 Q9HCE7 23530 Q05086 O00754 Q8NBM8 P49448 5467 Q86T82 207 5226 P55084 8754 O00762 P13489 Q9NWW5 Q9Y5S9 51185 215 7415 8761 Q96C86 Q9HCC0 3074 Q9UKK9 10208 O14939 P17858 7428 Q92900 P49427 11060 P37837 P19174 11065 Q86TM6 Q9Y4K3 230 472 O75150 O75390 Q9C0C7 84447 P49810 P43034 Q14694 Q14693 P38919 3093 P55036 P11216 3099 3098 Q504Q3 23291 Q9NY59 6117 Q9NPD3 6119 6118 Q13370 Q16401 Q9HAU5 Q9HAU4 Q9BUN8 P32121 4191 4193 P09110 P31040 10197 Q93009 Q93008 P36957 57092 P18074 6389 Q86Y37 O75351 267 P00558 23032 28960 Q13393 5049 O94817 Q14249 Q9BX93 Q8TDY2 26994 6390 P09172 Q96RQ3 Q8IZD4 11274 P36776 23014 26523 23016 5071 7251 P11021 50487 Q9NRD1 7267 A1XKG3 Q9BYZ2 Q8WY64 1718 P00367 Q16698 2805 51107 Q15369 9695 Q01780 O75792 114088 Q9H9G7 Q93034 P27815 Q99816 Q8WW12 P53004 P10515 P29372 27005 27248 P78549 Q8IV08 Q70CQ3 Q70CQ1 Q05329 P42345 2821 P62195 P08684 O15234 P05177 1737 P78536 9474 22803 O15496 55054 83737 55294 27252 Q9BSB4 P46934 1743 Q7Z4W2 Q9Y4D2 27257 Q92560 P42574 8399 Q5TAX3 92483 80267 Q9BYM8 P46926 Q9Y4E8 55066 3939 26133 P04066 80020 O60260 P07339 O95989 91147 P78317 Q9UNE7 3948 1763 Q04206 P05164 Q92542 3945 O75503 Q13315 Q96EP1 84647 P00505 P00747 60673 O60240 P08246 Q13569 Q8IWV7 P11413 P61077 1540 124739 P22303 P09104 Q8IUQ4 P28065 P32320 P33897 1312 2643 P61088 P17174 Q9UJ83 Q14432 Q13107 Q06830 P62191 P28074 P28072 P19367 79065 P28070 P42357 P63279 51752 Q6ZVX7 902 P08237 51514 P43686 P48052 23607 O14672 P52790 Q9P2R7 10535 O60566 P15374 1576 O00273 P60174 P63208 90678 P08842 10533 Q92611 P09936 P48047 4860 Q07343 5706 5707 5708 5709 P30519 5700 5701 5702 Q9UHP3 5704 5705 Q08426 5717 1358 57646 O60543 P61289 5713 4627 5716 Q12972 5970 1374 Q99460 9099 Q13829 3304 55236 948 3309 O95470 P51668 P51665 P61221 29102 P04040 P09972 5976 Q9BZM1 Q7Z419 Q9NYA1 Q9Y4X5 O00231 O00233 O00232 O95243 P30793 Q8IY17 Q9NS56 2237 79444 2475 P42771 22934 Q13618 Q13617 Q13616 1161 O00487 6609 O60502 10956 P62333 Q9BZK7 5511 Q9UK22 5516 Q13620 Q92626 2023 P54727 P54725 P52789 10724 Q99259 57695 747 P40337 26061 P53804 25862 Q9NVW2 Q00796 991 Q96RU2 54512 2026 513 997 4686 8803 8801 51009 Q04917 P04406 P11177 P50542 2280 O76074 Q96Q15 Q15392 4214 P49768 Q9GZQ8 Q8N726 5321 Q00535 Q15165 P25789 P25788 55626 Q9GZP9 6647 5315 P01137 Q7Z3C6 539 P25787 P01130 P40939 5331 5333 7511 P51659 Q08257 O95071 9924 P25311 7508 O00628 Q3SY69 Q969H0 Q9UPR3 Q9Y2K6 22794 Q9UNN5 3162 O76031 Q96PU4 5335 5336 P62714 5337 5338 P61626 Q9H3F6

P07954 Q9Y4P1 7533 Q5HYA8 Q01831 78991 563 P16671 Q00987 Q9H1A4 329 Q6UVY6 O60907 Q9UPT9 Q9Y2M5 23621 3184 572 Q8NCN5 55666 P68036 P04424 8881 P39748 P17980 Q9NVV4 P16885 55432 O14818 P58012 8879 8878 Q01813 5141 5142 P15927 8654 6470 593 84569 Q04759 P23786 5139 9978 Q8IU60 O94905 5394 7332 7334 7336 O43684 63874 P30084 128 7329 Q13490 7328 Q13011 5162 A2RUU4 11160 Q13257 Q14103 9525 7347 P35998 5160 23381 O94925 P22674 11165 Q16531 P21589 30 P00441 34 7353 9775 O75477 P34896 P12883 P11310 Q9H492 P60520 P62942 8453 9784 8454 8452 48 O75469 Q8TB40 64750 P40926 7126 P14635 160287 P12694 8450 P35579 Q9NZ20 Q13042 Q13286 Q13049 Q14139 7375 Q09161 6048 Q9UKA1 P49721 O15169 P49720 P00491 Q8TEY7 P14618 8237 O94966 10273 6059 Q9Y5K6 Q9Y5K5 6050 Q9UKB1 P11586 Q99942 Q9NZ43 Q9H1Y0 11124 Q49A26 Q9NQC7 Q15008 Q9BXW4 P14625 O75674 Q6UWQ5 255488 2935 P21399 9100 7167 P08559 9588 Q15819 Q96K76 O43837 79139 10935 P45974 Q9BZQ6 26270 10939 O75891 84708 P05062 P60900 10940 P07205 P52298 7189 P29474 P29475 10908 26019 9361 7184 Q9Y3C5 4907 Q8TEL6 Q9NZ08 Q15843 Q13423 P53396 O75874 Q6R6M4 O95865 1891 Q6UWE0 10921 P68402 1642 Q9UBN7 Q969Q1 4913 P30049 9388 P30041 P30043 P11766 P30048 2744 26232 Q9UK80 26234 1892 Q13200 2747 P47712 Q8IY92 Q13443 Q8WZ19 57154 Q96BI3 8065 84749 1666 Q9UJX2 26002 P17081 P08138 1429 2517 8078 O75604 O75844 Q13216 Q5W0Q7 377630 84305 1676 Q5XUX0 1431 Q14790 P41159 P54687 P08107 P53597 P51178 O75832 23192 1203 P30260 Q13233

positive regulation of NF-kappaB transcription factor activity O43353 Q96EP0 O95999 7124 29108 O00463 9181 Q9Y4K3 Q7RTR2 O60603 P30048 1896 23085 10616 10935 Q9Y6K9 P04040 8767 P01116 O14788 Q92974 7099 7098 Q9NYA1 Q9Y239 7334 Q15109 Q13404 7335 64127 P01308 Q8TAI7 8737 847 O00206 Q92838 7186 7189 7188 10392 7185 148022 Q9Y6Q6 Q9BYM8 Q8IUD2 Q86UE4 Q9UDY8 P61088 P01019 P01137 O15455 O14920 4869 P01375 Q12933 5970 Q13546 55072 Q9NR96 3551 P51617 Q8IUC6 Q04206 Q9HC29 8517 P05362 Q9ULZ3 54106 Q13077

response to unfolded protein Q8N2K1 Q5VVQ6 P07900 P54652 O95757 3320 25822 Q9BV94 P50454 O75460 P19525 595 871 3316 3315 79139 O75190 3312 5610 5611 Q92575 Q16236 51009 P55072 P25685 Q9BZQ6 Q96BA8 26270 Q92611 2081 Q96CS3 P31689 4780 22926 90993 Q9BS26 P18850 Q16082 3329 P11021 P11142 3326 Q68CJ9 1388 148327 Q70SY1 Q9P2K8 Q9NRD1 Q9UKV5 P10809 Q9BUN8 Q8TEY5 11080 P24385 P35638 Q13217 27348 O95292 80267 Q9UDY4 Q99941 3337 55741 7415 P01579 Q92598 Q9GZP9 P61604 O75807 P08107 9695 O75953 9217 P34931 P34932 55432 267 3304 P17066 23197 3301 Q15011 P04792 O14656 P08238 3309 O14657 3308

cellular component movement Q92858 23607 Q8NEY1 Q12948 5817 2304 Q8IYN9 P21926 Q9BVA1 Q92731 O14786 10653 O14788 P07996 P27487 P52565 Q12837 Q8IZP0 P52566 2676 800 P61158 P67936 Q9UKV5 928 P54753 Q7Z727 Q05397 P37023 55342 P61160 27185 Q9UKW4 4983 5830 3655 P62258 4627 P05556 P54764 P54762 3672 3673 Q92949 2100 1130 P48380 Q13829 27076 P17302 5728 P16333 P17301 Q99698 2335 O60674 2697 3301 Q9UKX5 5604 829 O95470 1020 57669 3315 830 1012 832 3676 3678 P08887 P07437 P08648 O00238 P30556 3690 5991 P09619 P51679 P10721 O00358 P18206 960 Q9HCM4 O60890 3688 4898 3689 7804 5747 Q12884 P05106 P05107 6850 Q13618 Q9NYB9 P62330 2247 P15151 976 Q92752 P26038 5879 Q9Y490 P29323 P31749 Q13509 Q9HCK4 253980 1289 P05362 P40337 Q9UL54 P06576 4690 O43157 Q9Y6W5 P56199 O95274 P11047 Q00796 203068 3479 P06702 10383 Q9UHG0 5796 2043 3010 1072 P50542 P13236 O96013 Q99755 84159 409 8936 O14713 Q01518 P01343 P49768 2290 P50552 Q86VW1 6774 P14416 O00560 P52735 Q00535 Q96S42 51148 6405 P01137 P49639 8829 P01375 P04626 P35225 Q658W2 2064 P17813 301 P60484 3148 P10070 Q8IZE3 P10071 3146 4478 P01127 5327 10487 Q96JB1 23647 P26583 O00628 8851

P07949 8852 Q12904 P15692 O14908 10451 5578 P40763 P26232 5591 Q6IR47 7410 7531 7412 P52803 10109 5467 9826 207 7408 P06753 Q15078 P49682 5362 7422 5364 O75051 7414 10672 9839 7431 P20908 23513 8879 7428 55558 P04792 Q96HB1 10566 O15085 10092 10095 Q9UQP3 10094 5268 P10599 Q9UPN3 O75385 351 474 P09493 23286 Q9NPC1 P83731 P43034 8408 P47755 P47756 P03950 5155 P48960 P11215 Q05952 P00533 Q14344 Q15672 6495 P32121 Q9Y6Q6 Q03405 P51805 P23528 5159 Q9NRI5 Q13145 P55290 Q16650 Q16539 6386 Q14114 P36952 P13611 P10589 P13612 P68371 382 O75592 Q9NVR5 267 54820 P52907 23032 P20264 Q13275 8452 7124 7126 64759 O43639 P35579 O75581 396 P09172 397 P23229 Q15223 P60709 Q5JQC9 P35443 60 P01303 Q9GZM8 1947 P02751 Q15475 Q99835 9201 9564 7142 Q8TEY7 O95813 10152 Q02880 A1XKG3 Q9Y5K6 7141 Q8WY64 55704 P01579 347733 1956 P09038 P23443 Q14155 Q8TE73 O15075 7155 P39905 P22492 O75553 P10147 Q02750 O43508 8481 Q9NXR1 Q14160 Q6PID4 Q9NRF2 Q14289 Q15375 P78423 P53365 7046 6198 O75665 O43711 P32302 Q4VCS5 P41134 Q9Y3A5 Q1PSW9 P04083 Q6NVY8 94 O60282 3911 P31689 P78536 7057 O15496 O43602 7170 7291 7171 O43847 7295 Q05682 Q3V6T2 9350 55172 P55316 81565 P16070 P05067 P98172 Q14865 8399 P29474 O15143 8394 6093 P16284 P17252 P06241 Q92558 9255 P35908 P19320 P43405 P08670 2737 2736 O95995 P78509 P63000 P08246 Q8NEC5 Q68CZ2 Q8WZ19 9270 O43921 P14923 7094 3728 Q13574 P08138 P09429 O75969 Q8IUQ4 O14495 P78527 P63261 1432 P09430 Q13464 Q5T4B2 O15230 Q15768 Q04771 23077 P00750

positive regulation of transcription from RNA polymerase II promoter P10914 6595 Q92858 Q14686 6597 4297 6599 6598 Q12948 P15923 Q12824 Q6QNY1 O00267 P13984 Q9Y4K3 P17676 351 2547 Q9H161 474 2303 P16220 Q96JM2 P84022 Q92731 P08047 Q7Z6C1 Q13485 O15516 2308 P25963 O94906 Q9NPC8 3091 Q15306 Q12837 Q86VP6 P58304 P13631 Q8NAP3 367 P46531 4851 Q13133 Q15796 Q9UQR1 6496 6495 Q15797 6256 Q12968 P12755 Q9ULH7 Q9NY61 O75360 Q9Y606 6927 6929 Q15561 4862 Q9UJU2 P49116 O75928 P40425 P40424 Q6IT96 3659 Q15562 P06400 Q16650 Q96JK9 4086 P35869 4087 8204 4088 4089 5970 9412 P18074 P55055 2100 Q9NR96 P12645 P36956 O95343 P20823 P61296 P08151 P20264 Q16665 Q13033 9421 P55347 7124 P54253 1022 1385 O75586 P35453 P19532 Q9UQL6 P10275 P10276 O00468 6829 154 10736 1499 P15173 80324 P07550 4646 6827 Q13285 2902 7132 7376 Q9UHK0 8463 Q92769 6720 Q14938 P36896 O43524 P10600 P19544 7490 P19419 1387 P17482 338917 P08651 4775 Q9NQB0 Q8WW38 Q9UM54 Q99958 Q15475 Q99835 Q96T37 Q8TEY5 7023 4792 5089 22937 P14859 P38398 2247 Q01664 Q9BZK7 Q96PK6 6721 Q12770 P05112 P49715 Q9UM63 P09038 55827 Q13501 9575 Q9UKS7 P10826 O43186 Q12772 P10827 Q12778 P39905 7157 P51692 Q13503 Q05586 O00255 P11474 1050 Q03164 26747 861 55832 Q99814 50943 Q9Y2W1 9464 O96004 6872 P04637 2034 P13349 2033 Q01094 O60603 P52952 P22736 3479 Q1PSW9 P17275 91 10011 9219 P42229 10014 P42226 Q9UBK2 Q9C0K0 Q58WW2 P56524 6760 22926 4221 22807 P33076 P48436 4214 Q4LE28 406 P05067 408 1869 P49407 P01343 P01100 O00327 O00206 Q09472 7189 7067 P48552 P48431 9921 6774 P19793 Q03014 7182 Q01196 P41235 Q99743 Q92793 Q9HD15 Q96S42 Q53X93 P01137 5316 P49639 Q9NSC2 P01375 P51531 Q13422 6660 5451 O15156 O94776 Q13547 A4D1W7 P35222 P78317 P51532 2735 P19438 P17813 3148 P10070 Q04206 P10071 3146 668 6776 10001 6657 1406 2737 2736 P26583 O00744 5460 Q15853 Q9Y5Q3 O14593 P50613 P51946 Q8TAK5 672 P22415 P15692 6667 4800 Q8N5U6 2626 P40763 P05412 7099 5591 4261 7098 Q9NVC6 Q9NP71 P18850 Q05086 23414 P37231 64919 P18846 Q9UIV1 P18848 3169 148327 P23511 5468 375790 P17081 Q92922 P09429 P12956 7422 29966 O60907 3066 3065 P78527 P18146 P35711 P43694 8625 O15455 10432 5914 P27540 Q9BZS1 Q15648 P55771 P19484 Q06710 O14936 P63272 466 P58012 468 Q9HCS4 10320 54106 8878 Q92585 9967 902 Q15532 Q13233 P60568 P21675 5925

regulation of cell adhesion P02452 P78423 P25054 O14672 P13349 Q08722 3084 P15498 284217 P84022 23560 P09493 Q92730 94 Q92574 O14788 P42229 P49815 3911 P07996 P27487 P04004 P52565 7057 5274 Q02297 P52566 5270 22920 7052 Q04900 P67775 P46939 P16471 P09382 Q9H2X0 P46531 4851 7448 Q13370 Q92845 P42338 Q16363 Q8N726 P25391 Q15438 Q13418 Q53GQ0 Q8NC24 P10451 P31431 P37023 P20936 51144 Q00534 P35240 Q9UKW4 P21860 P21980 3655 P01137 P55290 P01375 Q14116 5054 3791 4088 P04626 3673 2065 2064 P14543 5728 P17301 P21359 P17813 O60674 P60484 Q99574 6776 5328 948 5604 9267 P24043 P01042 133584 Q16787 4771 7124 7248 7402 1021 P41182 Q9UNN5 4092 O00622 83706 396 Q9Y6C2 397 P00749 398 57669 P00747 Q9BZE4 P23229 1012 10451 5338 3956 P35968 Q13443 P30153 Q86WV8 5590 1029 P16671 Q05513 961 324 Q9HCM4 7249 604 967 P42771 Q9HCU4 8754 6696 P10415 O43739 3611 2246 1277 P01579 Q92752 P05230 11124 5515 O43182 5518 Q86UX7 P51692 O15230 O75553 Q02750 Q63HQ2 O14939 3909 Q99819 O15105 P05362 P05121 P06576

viral reproduction Q15853 11060 P35659 6993 O00308 7520 Q86V81 6598 2672 Q12824 57187 P63172 O00267 Q9H0M0 Q96FV9 6829 2547 O43719 Q7L523 P22415 79139 P84022 3159 Q92692 Q99684 90678 7518 Q15223 7913 Q13363 Q9Y5Z7 Q13769 O43889 O75531 Q96J02 3054 83737 P13236 7251 P46934 P61073 Q8NI27 4734 10542 8815 Q9BUN8 6890 Q13137 Q86W42 P12956 P62937 P51681 O95292 Q7Z2Q5 80145 P13010 P60033 975 P17096 5478 11168 P01137 50855 P01730 Q6I9Y2 8563 P09958 Q9NZI7 P56545 Q92824 4088 9217 Q13426 O43504 Q12857 O75475 Q6UWE0 P51610 1488 1487 10241 Q99816 P05362 10488 Q03518 Q9NPB6 P06213

negative regulation of cellular component organization P25054 P10636 9181 O75385 472 Q9BVA0 Q8IYN9 Q9Y3A5 118 998 6622 90678 10013 O14786 54998 94 P46821 P02686 1978 8408 P07996 P47755 P47756 Q92974 Q04917 O43683 P27487 25913 P52565 7057 8301 Q9UBC3 Q96QB1 23332 Q01082 1191 2039 Q7Z460 P09382 22919 4214 Q13492 Q9UI95 Q15554 Q13133 P49768 Q9BYV2 O15020 7067 Q13813 O75122 P10451 P56539 Q05397 891 P37023 P51805 Q01105 6405 P01137 8829 Q92833 O60260 P26358 4869 Q9BSI4 5716 Q08495 91147 9138 P55055 P10586 Q658W2 Q6UWE0 Q9NUX5 382 Q02790 P11277 5728 387 3304 Q9NWT8 P52907 P60484 3146 Q6PKD3 Q15691 Q9UBN7 Q9NZJ4 Q13275 829 4131 4099 9700 4133 Q13315 Q15735 Q9UNF0 23122 P20916 P14635 Q9UHB6 4092 P35612 P35611 10459 396 672 Q96P48 6709 830 P63000 832 Q9H2D6 P61586 Q99966 3956 7013 7376 Q92769 7014 7533 Q5HYA8 5071 5590 1789 1786 Q05513 P23515 324 57159 6711 6710 4137 P62328 3720 P17081 P08138 Q92888 5747 Q12884 P09429 Q9NYB0 6696 Q9BY11 4155 P54274 23189 3066 P62330 P38398 P02549 Q9NQC3 859 Q9NPA3 Q14674 5879 O43182 P60953 Q14678 Q9UKS6 P10827 P08107 P29323 O75832 Q9BZS1 11252 P37840 7272 Q9HCK4 51474 P27816 O15105 55558 P63313 50943 Q13233 Q9BZR6

cellular response to peptide hormone stimulus P01241 64397 P08833 P98155 5584 6599 3484 Q9UQL6 Q9H2Y7 3481 10458 P42224 Q96RU7 P22415 2303 P62993 Q92572 Q13480 P42229 P27986 10014 1978 2308 2549 P40763 P04201 5591 5471 Q13322 O15530 Q96QB1 10580 Q9Y4H2 P35568 P30279 P37231 57678 P05062 207 5468 Q13370 P17081 Q92922 Q99958 P01344 Q92569 P09467 9564 O14492 6256 P29353 P49840 6772 6774 Q06203 P19793 P78527 894 Q9HCL2 P61244 2885 57761 P17174 P21860 Q9UQB8 P08069 8503 4149 2805 P09874 Q92831 5295 8165 P55851 Q12778 6464 Q9BX66 P51692 P31749 1176 2065 Q9UKG1 5170 P18433 142 Q6ZWJ1 2931 O60674 6776 3667 P41743 Q92667 26060 P06213

Golgi vesicle transport 9463 Q13636 Q9NRW1 8775 22872 Q5SQT9 Q99523 1213 O14828 O14662 Q96KG9 3875 10133 11345 Q9Y678 9632 29959 P78537 Q8IWJ2 81567 10427 117177 400 Q9HD26 P42858 8417 O15126 Q9H2G9 Q96RL7 Q9H9E3 11079 O15400 408 4218 P49407 P02794 Q15436 6892 8677 9522 Q8WVM8 26258 57120 Q12846 Q9Y6Q5 Q96CV9 Q9UHY1 375 60412 O15533 O15258 P49755 P49754 8548 91949 Q9UP83 P61966 84062 O43264 O15155 5331 56681 P54920 Q01970 57410 51552 6810 10640 P60520 9527 9648 O14579 27072 P41743 Q9UBF2 Q92538 5584 O43752 10053 P50570 5862 9382 Q99962 51560 O60763 Q8WTW3 O00471 3312 10972 P55072 P61106 9554 P35606 9950 O95487 9276 O95249 11031 P53618 23256 Q9NR31 Q96IW7 10228 P11142 83548 1785 O60499 51693 Q96QF0 8615 10466 P05783 Q9NRD5 9685 P53621 6456 2495 Q96EV8 2647 1315 1314 Q6VY07 Q00610 P61006 7415 P83436 2802 Q9NP90 Q14677 P53992 Q9UL45 1174 O75396 O43747 Q8NBS9 P84077 P40616 O94979 P61019 Q8TBA6 Q8N6T3

viral reproductive process Q15853 11060 P35659 6993 O00308 7520 Q86V81 6598 Q12824 57187 P63172 O00267 Q9H0M0 Q96FV9 6829 2547 O43719 Q7L523 P22415 79139 P84022 3159 Q92692 90678 7518 Q15223 7913 Q13363 Q13769 O43889 O75531 Q96J02 3054 83737 P13236 7251 P46934 P61073 Q8NI27 4734 10542 8815 Q9BUN8 6890 Q86W42 P12956 P62937 P51681 O95292 Q7Z2Q5 80145 P13010 P60033 975 P17096 5478 11168 P01137 P01730 Q6I9Y2 8563 P09958 Q9NZI7 P56545 Q92824 4088 9217 Q13426 O43504 Q12857 O75475 Q6UWE0 P51610 1488 1487 Q99816 P05362 10488 Q03518 P06213

positive regulation of transcription factor activity O43353 6672 Q96EP0 6597 O95999 7124 6598 29108 2033 Q12824 O00463 9181 Q9UQL6 P53567 Q9Y4K3 Q7RTR2 O60603 P30048 1896 23085 10616 10935 Q9Y6K9 P04040 Q7Z6C1 8767 P01116 O14788 10014 Q92974 Q9UBK2 7099 P56524 7098 Q9NYA1 Q9Y239 7334 Q15109 Q13404 P12429 7335 P14923 64127 P37231 3728 P01308 Q8TAI7 3169 8737 5468 847 O00206 Q92838 7186 Q09472 7189 7188 10392 O14495 7185 148022 Q9Y6Q6 Q9BYM8 Q8IUD2 Q86UE4 Q9UDY8 P61088 P01019 P01137 O15455 O14920 P05112 4869 P01375 Q12933 5970 Q13546 55072 Q9NR96 3551 P51532 5728 O60674 P51617 P60484 Q8IUC6 Q04206 Q9HC29 8517 P05362 Q9ULZ3 54106 P23497 Q13077

positive regulation of transcription regulator activity O43353 6672 Q96EP0 6597 O95999 7124 6598 29108 2033 Q12824 O00463 9181 Q9UQL6 P53567 Q9Y4K3 Q7RTR2 O60603 P30048 1896 23085 10616 10935 Q9Y6K9 P04040 Q7Z6C1 8767 P01116 O14788 10014 Q92974 Q9UBK2 7099 P56524 7098 Q9NYA1 Q9Y239 7334 Q15109 Q13404 P12429 7335 P14923 64127 P37231 3728 P01308 Q8TAI7 3169 8737 5468 847 O00206 Q92838 7186 Q09472 7189 7188 10392 O14495 7185 148022 Q9Y6Q6 Q9BYM8 Q8IUD2 Q86UE4 Q9UDY8 P61088 P01019 P01137 O15455 O14920 P05112 4869 P01375 Q12933 5970 Q13546 55072 Q9NR96 3551 P51532 5728 O60674 P51617 P60484 Q8IUC6 Q04206 Q9HC29 8517 P05362 Q9ULZ3 54106 P23497 Q13077

regulation of Rho protein signal transduction Q15811 O15085 Q7Z628 P10911 7204 10928 Q5VV41 115557 9181 P15498 P42345 O14827 O94989 P43034 Q92574 Q92974 Q13009 Q7Z6J4 Q96QB1 Q9P107 81565 84033 408 Q15311 P49407 85360 P98174 Q008S8 Q8IW93 Q9NR80 Q86VW2 Q9NR81 Q9HBW0 29 Q96M96 P52735 64857 P11274 Q8IVF5 23263 Q9UKW4 Q9H7P9 Q8WZ42 50618 Q8WWN8 9138 Q92949 Q12979 P35348 Q13829 7074 Q15052 Q8N5V2 11214 8452 7248 7126 Q96N96 23365 P41182 Q58EX7 Q96P48 121512 P63000 10451 O94827 Q96PE2 P51671 Q8WZ19 7410 Q5JSP0 Q86WV8 Q5VST9 23370 2475 9826 604 Q6ZW31 Q9GZM8 85440 Q92888 23229 Q07890 50649 Q6ZSZ5 Q8TCU6 6453 O15068 Q6ZV73 O75962 Q13618 Q12802 O60229 Q96N67 Q07889 50650 55785 613 Q9NZM3

10276 5879 Q14155 Q12774 10160 4168 Q9H8V3 Q8N9R8 23513 253980 Q14160 Q6PID4 Q9NZN5 Q14289

protein ubiquitination 11060 11065 7325 Q9Y4K3 O75150 Q5QP82 P63208 90678 9978 3093 7332 7334 7336 7335 Q5VTR2 Q9Y4L5 Q86VP6 23291 Q13490 7328 4734 Q9UKV5 P22681 10193 9646 10075 6015 P32121 A0AVT1 4193 P62256 Q9NV06 P61962 Q16531 Q6PD62 57092 Q13829 Q5TAQ9 267 55236 Q6PCD5 8454 8452 7126 P51668 26994 Q06587 79577 Q96DE5 10616 10055 Q13042 Q99728 Q13049 Q7Z419 6045 Q9H4P4 6048 Q96J02 Q9UKA1 5071 7251 7128 7267 10273 Q13618 Q13616 1161 O95376 Q9UKB1 Q99942 Q8TBB1 Q8WY64 P38398 10956 Q9UK22 55827 6500 P31749 255488 253980 57332 55832 Q96LR5 55159 Q99496 867 Q99816 54623 P40337 Q8WW12 2935 Q6P1J9 O00308 P53804 Q15819 Q9NVW2 23326 Q9H0M0 23327 80124 27246 997 Q15022 P62877 Q8WWI1 Q969T4 Q58WW2 64682 Q13404 Q8TCJ0 115426 83737 84708 55294 27252 P46934 51132 4214 Q9BS18 408 P49407 409 25847 7186 7189 10238 Q9Y3C5 Q9BYM8 26133 55743 Q8TEL6 Q7Z6Z7 10477 O60260 Q9UMS4 55072 25853 Q6UWE0 P78317 Q9UNE7 26260 26262 1642 Q96EP0 P62837 Q969H0 Q96EP1 672 Q96PU4 26232 26234 Q96PU5 4008 P55072 Q9HC52 Q9H3F6 Q8WZ19 P21580 Q9HCE7 57154 Q86Y13 Q8N7H5 Q05086 27339 Q9UJX2 Q00987 P61077 207 Q9H1A4 329 Q96JH7 P10415 Q13216 Q8IUQ4 P19474 Q9UPT9 Q9Y2M5 O00762 51185 P61088 P68036 7415 Q5T6F0 8881 Q12933 23512 580 P30260 7428 Q13233 51514 P49427

peptidyl-amino acid modification 7046 6872 2033 O95271 23569 P42345 P42224 Q9BXM7 Q9H0U3 2147 91 Q7Z6C1 10013 83475 1859 P42229 P07237 7297 Q12797 P56524 Q15303 Q9UM07 5034 Q16512 4067 O15530 Q9H2P9 1198 23210 Q8IZP0 65018 Q8TCJ2 1195 7052 P16234 Q96Q15 P21810 P16471 128 4214 Q4LE28 Q96EB6 P52333 Q5S007 Q7Z727 Q09472 84061 P29597 6772 P49761 Q9H422 Q13418 1634 54496 Q92793 Q00535 P17252 P32929 5159 P06241 P21980 P42684 Q92831 Q9UBE8 1491 5170 201595 O60674 Q9BXJ9 O60551 5566 6776 P07585 P41227 Q9UBN7 P25098 133584 4133 5585 P07948 P78509 Q6NYC1 P50579 1020 P11766 P39656 2590 156 2589 P27708 79612 1650 5578 5579 6789 O14744 P05771 Q13043 5591 Q6IR47 9397 P04843 P04844 5870 Q9HAZ1 P09619 P36896 57396 P10721 23411 P13674 5590 80155 10988 Q05513 444 1387 P00734 120892 2475 207 P49841 51611 P46977 Q15118 P61803 P10415 10152 Q96FX2 A1XKG3 Q9NYB9 P78527 3611 Q9NVM4 Q04656 5599 P01579 Q10472 Q10471 6185 P29323 Q9BU89 P31749 Q13627 6184 Q5U4P2 P17612 2932 Q63HQ2 P00519 P37173 Q6PID4 P23458 P45983 P20340 Q14289 Q13233 P25116 P21675 P06213

cellular protein complex assembly 4690 10093 9463 P07900 Q7KZN9 11065 25909 P49023 1460 P12931 Q9UBB5 3084 7283 4728 26271 Q8IYN9 203068 1058 P84022 P07196 23165 Q9BVA1 5018 Q15389 6901 Q13485 4725 Q13885 P03950 25915 9997 10383 8301 Q16635 Q02297 Q6FGD7 Q9BU61 2280 55172 2316 Q9BUF5 5829 O60610 P16471 402 P05062 Q9NRH3 Q13492 8932 Q9H9E3 7846 Q16401 Q15796 P02675 P10809 8936 84790 Q15797 Q8N6I1 Q9NQW6 O75880 84617 Q05397 O43819 773 Q9H4B7 1355 Q00535 811 P01137 Q92558 Q9HC77 Q8WZ42 Q3ZAQ7 Q9BQE3 5716 O00217 4086 4087 4088 4089 163126 Q658W2 P68371 P34932 Q02790 P16333 Q9NVR5 P68366 P23258 P04350 P62942 3308 Q8N5M1 O75347 3320 1020 O43639 O00468 154 P63000 P07550 Q9BUK6 P07437 Q9Y512 P49450 O95405 2243 O43920 O00233 Q9BUB7 3329 P18846 54443 Q15070 Q8WYP5 6834 375790 6714 5747 Q9NRD5 P27797 P21333 9685 P02671 P07766 Q8TCU6 A1XKG3 O00762 P36404 Q00059 Q9NS69 P42768 56993 2244 O14640 O15212 347733 5879 O43182 Q14677 7277 6341 Q60FE5 O75832 Q15526 Q13509 Q13748 25813 P40692 466 P67870 P52948 Q6PID4 Q92621 55835 1729 Q71U36 Q14289 Q9UKT4

regulation of ligase activity 11065 5300 9184 991 26271 O60566 Q96RU7 P62195 P63208 64682 7334 P55036 Q12834 Q13526 O43684 5706 5707 5708 5709 648 5700 Q16401 5701 P60900 5702 5704 5705 Q13257 5682 5683 Q8N726 5684 5685 8555 5686 P35998 P53667 891 5717 57761 8945 P25789 10197 P25788 P61289 Q9Y297 5713 P25787 5716 5692 5693 Q13426 Q99460 P35226 2730 5687 5688 Q9UL15 8454 P51668 P14635 P51665 4092 Q13042 Q92530 10213 7518 Q13200 O43242 O00231 O00233 O00232 1029 Q9UJX2 Q9H1A4 5347 P49721 P42771 P49720 P28065 Q13616 O00487 O00762 P48507 P62333 P20618 P61088 Q15008 Q9UL46 8881 O75832 P53350 6500 P62191 P28074 P17980 P28072 P28070 O14818 P30260 O15105 Q9UKT4 P43686

regulation of cell differentiation P25054 Q92858 2672 259266 Q9BVA0 2547 Q8IYN9 P84022 Q9HCJ2 O14786 O14543 O14788 P52565 Q12834 4860 Q9UBC3 Q12837 54361 P50148 P16471 Q9UKV3 4851 P61158 4854 Q96EB6 O15525 O75925 Q9ULX9 Q92835 P29992 Q05397 P53667 27185 57761 811 O75928 P40424 6926 P06400 Q9GZX9 Q9NQX0 5970 Q92949 Q13705 P56705 22985 P17535 57534 5728 P17542 O60674 P20827 P19838 948 5604 P04233 Q9Y468 P27037 O95477 P28482 1021 O00468 10736 57669 79576 1499 P61586 Q9Y2T1 P08887 5978 Q9UM47 O00238 P30556 3690 Q9NYA1 Q92769 O95361 Q8IZT6 P10600 P10721 O95365 Q9BPY8 1029 3207 10626 1387 Q9HCM4 3685 604 P42771 Q92888 5747 P04179 P05106 4790 6850 4792 57689 2247 Q9Y250 857 859 5515 P05112 5518 O43186 P10827 P29323 P51692 Q5TC82 P31749 O00255 P19883 1050 Q9HCK4 861 O95390 P37173 P40337 7704 O96005 6993 P04637 O43157 2033 P14555 P52952 P15531 991 Q96RU7 3479 2147 Q15022 10014 P02686 Q04917 O43166 4221 P57059 1191 O43290 Q15154 P48436 79923 P01589 P01588 O43294 P01343 P49768 O00206 2290 P25791 Q09472 P48431 Q8N726 9921 P14416 3142 O00203 773 894 Q00534 Q96S42 898 P01019 6886 6405 6647 P01138 P01137 8829 Q9UPY3 P01375 Q9UJM3 Q9UMS4 6660 P04626 Q658W2 2064 P35222 Q15051 55755 P60484 3148 P10070 P17936 P10071 6776 P40933 Q96JB5 6657 P24588 O43353 P26583 O00744 5460 4131 Q9Y5Q3 Q92934 7520 8851 P20916 Q9H9S0 23764 P61981 Q99684 4005 7518 Q8N5U6 P05412 O00755 Q6IR47 7532 7533 23411 23532 3171 P37231 P18846 P16671 Q96SZ6 324 3169 5467 4137 207 5468 54206 5108 10468 P27797 Q15078 P24385 6696 5364 4155 P56178 P56177 3066 P51843 572 P13010 Q96HC4 P24394 Q9BT67 P27540 9612 P06729 23512 P16410 466 55796 7428 10320 55558 P60568 8650 O75385 Q9Y4K3 3084 474 595 P07196 Q7Z6C1 8767 P78395 Q13485 8408 P25963 Q9NPC8 Q13127 3091 Q13009 8546 P62805 Q15306 19 5270 367 P09382 Q9H2X0 P46531 51654 Q96SN8 8536 5029 Q16643 Q13133 Q15796 P43026 Q15672 Q14106 6495 Q15797 6498 P12757 P12755 P10451 P51805 Q9NRI5 P02741 Q13145 P00441 4086 4087 4088 4089 9657 7476 Q9Y6R0 P55055 P10586 Q9Y618 Q86YT6 93166 Q8N5F7 Q02790 387 P21359 P31270 1808 P24864 Q16665 Q13275 P54259 4099 7124 P63092 Q9UQL6 P10275 4092 P10276 4093 396 51564 Q16555 Q99966 Q14012 7376 Q9HAZ2 P22392 P36896 O43524 P36894 1822 P01308 Q9NQB0 Q9GZM8 Q14145 Q15475 Q99835 Q96T37 Q8TCU4 P00491 Q86VS8 P36888 51341 55704 P01579 Q9NQC3 P49715 P09038 149041 7157 O15198 Q02750 P11474 84376 Q13873 7046 P10997 P10636 26037 O43711 P63172 Q86WK6 Q1PSW9 P17275 91 P46821 P42229 O15119 Q9NSA3 P56524 O15496 Q02297 7291 P55318 Q3V6T2 P55316 P67775 81565 Q4LE28 Q9BXL5 1627 8399 7189 7067 Q5TAX3 Q13418 Q63HK5 P56539 Q01196 Q9H6W3 P41231 Q92793 Q13422 P55212 Q13426 A4D1W7 9138 9253 9495 7074 P21757 P43405 Q04206 Q92786 1406 2737 2736 Q8WUI4 Q15735 P41182 9021 P78504 84525 3956 2626 7099 P31249 150094 7098 Q14774 P30153 27339 P30279 1789 Q05516 P62166 P23515 P00734 375790 P08138 P12956 P10415 Q9H228 Q02363 57045 P35711 O60229 Q7Z494 3611 P43694 O15455 5914 P41159 Q04771 63976 4830 O15105 P45984 P21554 Q9BZR6 5925

immune system development P10914 P25054 Q14686 4297 P15923 P51587 Q9H165 4291 Q9Y4K3 472 2547 P84022 P49810 P08047 O14788 6901 4067 Q16635 Q15306 Q15669 Q02447 P16473 Q9NRH2 Q9UKV3 6117 10661 P10809 P27694 6495 Q12965 Q7L5Y9 Q9Y6Q6 6929 Q9UDY8 Q9UDY6 Q9Y6A5 P40424 Q92833 3659 P00441 4627 P06400 P05556 Q9GZX9 Q13268 3791 4088 Q86SX6 P18074 5971 Q92949 Q9GZX7 9093 P13612 22985 3428 81501 P17542 Q9HBE1 Q8N4C8 O60674 P62263 P43246 P04233 Q9Y468 P55347 Q16666 P54132 O95470 Q9UQL6 P35579 P39019 P46109 Q01201 1499 10296 4643 3676 Q8WV28 O43521 P10721 3329 1147 841 604 3688 51218 Q15475 Q96T37 P04179 P07766 4791 6850 O95376 P36888 1399 P02549 Q15125 P05112 P49715 P60953 P10827 7157 P51692 P11912 O00255 P19883 1050 Q03164 Q9H295 861 P40692 P37173 863 Q99814 7704 4318 7046 P04637 P14317 2034 7163 6194 P32302 P52952 56259 Q9Y3A5 10935 P17275 998 65082 P42229 10014 P62753 Q9C0K0 P56524 P78536 4221 23210 Q04900 641 P08575 648 P01588 2956 P49768 Q9UBL3 P25791 7189 7067 P06239 Q06643 P29590 Q03014 Q01196 P55327 Q00653 P14780 6886 6647 P01137 5316 3932 P01374 Q13422 9133 Q13426 A4D1W7 P04629 P19320 P35222 O95067 P35226 4914 O75081 P43405 53335 3148 57379 3146 6776 P40933 Q96EY1 6670 P26583 O00744 7520 Q13315 P07948 Q8WYA6 Q6NYC1 P41182 P78504 P53567 P35612 P30048 P15692 Q9H9B4 6304 6667 4005 5336 Q96AX1 7518 5591 Q6IR47 P35968 10460 7412 P11413 10107 P37231 Q05516 64919 324 5468 3720 10468 P09429 P12956 7422 P10415 Q02363 57045 P78527 P18146 P13010 Q04656 Q14790 P01730 Q01826 5371 O15111 Q15648 P35268 P52701 P16885 10682 8879 10320 9846 10202 Q13351 P60568 5925

regulation of ubiquitin-protein ligase activity 11065 5300 9184 991 26271 O60566 Q96RU7 P62195 P63208 64682 7334 P55036 Q12834 Q13526 O43684 5706 5707 5708 5709 648 5700 Q16401 5701 P60900 5702 5704 5705 Q13257 5682 5683 Q8N726 5684 5685 8555 5686 P35998 P53667 891 5717 57761 8945 P25789 10197 P25788 P61289 Q9Y297 5713 P25787 5716 5692 5693 Q99460 P35226 5687 5688 Q9UL15 8454 P51668 P14635 P51665 4092 Q13042 Q92530 10213 Q13200 O43242 O00231 O00233 O00232 1029 Q9UJX2 Q9H1A4 5347 P49721 P42771 P49720 P28065 Q13616 O00487 O00762 P62333 P20618 P61088 Q15008 Q9UL46 8881 O75832 P53350 6500 P62191 P28074 P17980 P28072 P28070 O14818 P30260 O15105 Q9UKT4 P43686

chordate embryonic development Q14686 3880 O14672 8533 Q12948 O75386 P51587 29947 Q9Y4K3 O43318 P17676 472 Q9H161 2303 P84022 Q99807 P49810 P08047 Q7Z6C1 51763 O14543 P49815 Q13485 P28799 Q9NPC8 P20290 4188 8543 P08727 Q5TAB7 Q02447 P16234 367 P42858 Q9H2X0 6117 P46531 4851 9869 10661 P00533 Q15554 Q15796 5702 Q14344 5704 Q15672 O15525 P27694 Q9ULX9 P61968 6495 Q12965 6498 P12757 P05549 Q9NRY4 P12755 Q9ULH7 6927 P37023 257 P23528 Q96RK4 Q15561 Q92830 5159 Q9UJU2 P40424 6926 4627 134701 4087 Q92824 4088 4089 P07737 8323 9412 P18074 Q9Y618 Q86YT6 P13612 P17302 P17655 57534 P20823 Q4AC94 2697 P61296 10087 P43246 5604 53615 9421 Q14249 P27037 8452 9546 P28482 7248 Q8TD84 2909 P63092 P10275 P35579 O14627 10733 10736 57669 1499 Q9NYQ6 4643 3676 Q9Y2T1 2904 Q99966 P50222 8100 O43521 7014 P09619 P36896 P36894 P10600 Q6UB99 Q06210 Q86WV8 Q9BPY8 841 Q9HCM4 P17482 7249 Q9NQB0 Q8WW38 P09067 Q9GZM8 A6NI15 Q99958 Q14145 Q15475 Q99835 O95377 P61803 23463 Q13618 7020 Q9NPQ8 57448 3215 1277 O14641 O14640 P49715 1956 50814 7157 Q13503 P31749 O95140 O00255 Q02750 Q6KC79 1050 56243 P37173 Q9UMX1 4678 Q99814 1969 P29317 Q15256 P02452 7046 9464 O96004 P04637 O43278 2034 P13349 2033 23322 25988 60626 P52952 Q4VCS5 O60725 Q9Y3A5 P62993 Q1PSW9 P17275 91 999 94 Q92574 O15119 P98161 639 1856 O96018 Q9UNS2 4221 5310 6885 5311 Q96QB1 P16989 7291 Q9BRQ0 1072 Q9BT40 Q99873 Q99750

81565 Q13099 P49768 Q09472 P50552 7189 92129 O00444 Q63HK5 Q5T5P2 O14727 Q9NR09 Q96S42 P09630 Q15047 Q9UMS4 O95983 9133 6660 Q13426 P19320 3276 P35222 O95067 P35227 Q01974 Q96ST3 P10070 P10071 Q92786 P02458 2737 2736 9927 6670 O75626 Q15738 O95999 Q13315 Q9H488 7082 Q03113 23764 9021 Q07157 25942 O00622 O95076 84525 P00747 P15692 O75190 6667 4920 Q13563 7518 10454 2626 P31249 5591 Q6IR47 7412 26005 3297 P80370 Q68CZ1 27339 23414 Q07817 10229 689 207 7408 Q15750 90780 5362 4154 7422 23509 P49321 P56177 P12830 P78527 P35711 O75051 Q9BQA5 Q00613 2885 Q14790 P43694 10672 Q9GZT9 9839 P27540 9612 O75712 Q15648 P19484 Q04771 P17980 54583 2896 O60353 Q9HCS7 P43686 Q13351

nucleosome organization P06899 Q9H0U9 4171 1058 P20671 Q8NCD3 55723 Q9BTM1 P62805 P62807 Q99880 3010 P16104 Q99877 3009 Q9NVP2 3008 3007 Q8IUE6 3006 51773 11198 255626 Q99879 Q9H2G4 Q96NT1 3024 O75367 P55209 P23527 Q01105 3014 P17096 O60264 4869 8289 Q16777 Q16778 P0C0S5 P04908 221613 Q96A08 Q99733 55355 Q93079 P0C0S8 3148 Q93077 Q7L7L0 Q6NXT2 64061 P07305 P26583 8290 Q96KK5 3159 Q02539 P49736 P49450 Q969G3 8348 9555 8467 O60814 Q96QV6 Q92522 8338 Q8N257 Q9Y5B9 P33778 266812 Q5SSJ5 4673 P49321 P10412 P58876 7141 Q96T23 P84243 Q71DI3 P68431 Q16695 P49711 8349 P09430 6605 Q9UIG0 P22492 Q5QNW6 P16403 P16402 P16401 Q9P0M6 Q6FI13 O14497 4676 4678 Q13112

hemopoiesis P10914 P25054 Q14686 4297 P15923 P51587 Q9H165 4291 Q9Y4K3 472 P49810 P08047 O14788 6901 4067 Q16635 Q15306 Q15669 Q02447 P16473 Q9NRH2 Q9UKV3 6117 10661 P27694 Q12965 Q7L5Y9 Q9Y6Q6 6929 Q9UDY8 Q9UDY6 Q9Y6A5 P40424 3659 4627 P06400 P05556 Q9GZX9 Q13268 3791 Q86SX6 P18074 5971 Q9GZX7 9093 P13612 22985 3428 81501 P17542 Q9HBE1 Q8N4C8 O60674 P62263 P43246 P04233 Q9Y468 P55347 Q16666 P54132 O95470 Q9UQL6 P35579 P39019 Q01201 1499 10296 4643 3676 Q8WV28 P10721 1147 841 604 3688 51218 P04179 P07766 6850 O95376 P36888 P02549 Q15125 P05112 P49715 P60953 P10827 7157 P51692 P11912 O00255 P19883 1050 Q03164 Q9H295 861 P37173 863 Q99814 7704 4318 P04637 P14317 2034 7163 6194 P52952 10935 P17275 998 65082 P42229 10014 P62753 Q9C0K0 P56524 P78536 4221 23210 Q04900 641 P08575 648 P01588 P49768 Q9UBL3 P25791 7189 7067 P06239 P29590 Q03014 Q01196 P55327 P14780 6886 P01137 5316 3932 Q13422 Q13426 A4D1W7 P04629 P19320 P35222 P35226 4914 O75081 P43405 53335 57379 6776 P40933 Q96EY1 6670 O00744 7520 Q13315 P07948 Q6NYC1 P41182 P78504 P53567 P35612 P30048 P15692 Q9H9B4 6304 6667 4005 5336 Q96AX1 7518 5591 P35968 10460 7412 P11413 10107 P37231 Q05516 64919 324 5468 10468 7422 P10415 Q02363 57045 P78527 P18146 P13010 Q04656 Q14790 P01730 Q01826 5371 O15111 Q15648 P35268 P16885 10682 8879 10320 9846 10202 Q13351 P60568 5925

regulation of gene-specific transcription from RNA polymerase II promoter 6595 O96004 6597 6872 P04637 6598 2672 P15923 Q12824 O60603 P52952 2547 P16220 3479 P84022 3476 10014 P25963 P56524 Q16512 22807 Q15306 O75376 O60216 367 P33076 P46531 4851 4214 Q13133 408 P49407 P01343 Q96EB6 O00206 7067 6256 6498 P12757 P19793 Q03014 23028 P41235 6927 6929 Q53X93 O15379 Q9UJU2 6886 O15534 P01137 O75928 Q6IT96 5716 P51531 Q13422 4088 Q13547 A4D1W7 8841 Q92949 P55055 Q9Y618 Q9NR96 P35222 P78317 P51532 P78318 10524 P20823 P17542 P17813 P19838 3148 P10071 2737 9421 P26583 O00744 5460 Q15853 5187 P25490 5585 1385 Q8TAK5 P19532 Q9UQL6 P10275 4092 672 P22415 1499 Q99684 5579 P05771 7099 5591 4261 7098 7376 Q92769 8864 Q9NP71 O15055 Q14938 Q08117 O43524 23411 P37231 166 3169 P08651 5468 Q9NQB0 P17081 7528 Q96T37 4790 P12956 4792 22937 P56177 O60907 3066 3065 P78527 P38398 Q9BZK7 6721 O60341 Q92993 857 O15455 Q12770 P49715 Q9UKS7 Q12772 P10827 O75832 9612 P39905

Q9BZS1 7157 Q13227 P19484 Q6KC79 P52824 1050 1609 O15105 Q9UMX1 10320 54106 50943 Q13233 P21675

regulation of cellular component movement P25054 Q13477 Q15654 O14672 3516 284217 P84022 P09493 O14786 P07996 P04004 Q15303 3091 5154 5155 4067 P12429 Q92729 Q5VTR2 O95684 5270 P16234 Q9H2X0 7448 7205 5029 P00533 Q14344 Q9HCY8 Q13017 P25391 Q9Y2I1 Q9BXC9 57402 P37023 Q00013 P51805 Q96RK4 5159 P55290 5054 3791 4088 3673 Q8WWN8 Q14118 5728 P17301 P21359 O60674 P20827 Q13393 Q16665 3667 5604 Q13275 Q16787 4771 P28482 1020 Q9UQL6 4092 394 P39019 57669 51564 1012 P08887 719 P51671 3690 22846 Q9NYA1 P09619 O43889 Q9Y4H2 P35443 P10721 P35568 285 Q06330 P18206 P01308 Q9HCM4 604 967 9564 P05106 A1XKG3 P30542 2247 P08069 Q9NQC3 1956 P09038 P23443 P09958 P51692 P31749 Q7L8A9 Q8N9R8 Q02750 P48061 3909 Q6PID4 P05362 P05121 1969 P09601 P29317 Q14289 Q16581 1605 4318 87 P06213 Q13873 7046 6198 O15123 P12814 2150 O60603 Q4VCS5 Q96RU8 3479 23560 P39060 P04085 10013 94 6869 Q15389 10014 3911 3912 P78536 7057 Q96QB1 5796 Q93096 2159 2277 4214 P01343 Q16363 11116 4354 P14416 Q13418 P14780 P41231 Q00535 P35240 P01019 6405 P01137 8829 Q9Y696 Q01974 O43707 P60484 P17936 Q92786 P01127 10488 5328 P23497 Q59EA4 Q9UBN7 O60271 P24043 P07942 6672 Q8WUI4 P07948 P41182 O43915 8174 P78504 3162 P23508 P00749 Q96P48 P15692 Q9BZE4 5337 P00742 4920 5338 Q6IR47 P35968 Q13443 Q7Z5L7 64127 324 Q15077 207 P55085 8754 7422 P10415 3611 127435 7414 10672 P25103 O15230 P62070 O14939 Q9HC29 O15105 55558 Q13233 P25116

regulation of translation O00425 4690 6199 Q92616 6198 8892 Q9BY77 O43432 2033 Q06787 6193 351 1975 P42345 Q9UBS0 Q9HCJ0 84324 4686 9617 Q7Z6C1 O15234 Q92574 1978 P07996 5935 Q9UQ80 P38919 8662 P49770 P03950 O15372 7057 P08729 8667 P46782 5036 O60739 P58546 Q8NDV7 P51114 9470 1982 O60573 Q9P2K8 Q9BRP8 P05067 84271 P60228 Q7Z727 136319 Q09472 P52298 Q9UKV8 Q9Y2Y8 64215 P29590 P62495 27102 P17252 811 7458 O75807 Q13144 Q14232 P61326 P01375 Q9BY44 9775 3673 Q9UPQ9 P16333 P17301 400961 2332 P62701 4116 10642 10644 10643 Q15056 Q9ULR5 6311 Q9UL18 7124 P28482 P46199 7248 Q96KC8 27327 22794 O43639 Q9P0J0 Q96PU8 2107 55364 3315 1653 P23588 Q92499 5578 8569 5976 27161 Q14011 Q9Y2T7 Q09161 1153 P37108 26523 Q86WV8 P19544 7490 O75570 Q9BUB5 2475 207 10985 P27797 P55884 Q9P2X3 P98179 Q9H2K0 Q9Y6M1 Q9Y5S9 Q99700 84305 333 P55010 Q9UBQ5 Q9BQI3 P05198 6726 P23443 Q8NEJ9 5371 P51693 P82979 Q9UPU9 P31749 Q9NR50 23112 Q9HCK5 1965 Q9H9G7 192669 Q6PID4 P60321 P04792 Q92900 Q14289 Q9HD40 10289

nuclear export O43592 7248 Q86V81 Q9UPR3 P54253 57187 Q9H089 P35658 Q96FV9 P38646 O00148 3313 Q7RTN6 84248 4686 10772 P09651 Q9C0K7 Q92574 5976 11269 Q8WUM0 Q8TD31 Q13769 Q09161 Q9HCE7 3178 57154 O75494 23214 Q86WV8 8021 Q96Q15 Q8NI27 Q9UIA9 Q9HAU5 P62841 5901 P27797 Q86W42 P52298 Q86U42 Q9UMR2 23144 P57740 Q12769 Q03014 26019 Q9GZY0 80145 Q96QU8 23381 55341 Q9UDY8 811 54535 P01137 Q6I9Y2 4869 8563 Q9BY84 P62826 10482 Q9H814 11097 Q9UKK6 Q96QD9 23039 O15504 Q92900 Q8IXZ2

hemopoietic or lymphoid organ development P10914 P25054 Q14686 4297 P15923 P51587 Q9H165 4291 Q9Y4K3 472 P49810 P08047 O14788 6901 4067 Q16635 Q15306 Q15669 Q02447 P16473 Q9NRH2 Q9UKV3 6117 10661 P27694 6495 Q12965 Q7L5Y9 Q9Y6Q6 6929 Q9UDY8 Q9UDY6 Q9Y6A5 P40424 Q92833 3659 P00441 4627 P06400 P05556 Q9GZX9 Q13268 3791 Q86SX6 P18074 5971 Q9GZX7 9093 P13612 22985 3428 81501 P17542 Q9HBE1 Q8N4C8 O60674 P62263 P43246 P04233 Q9Y468 P55347 Q16666 P54132 O95470 Q9UQL6 P35579 P39019 P46109 Q01201 1499 10296 4643 3676 Q8WV28

O43521 P10721 1147 841 604 3688 51218 Q15475 Q96T37 P04179 P07766 4791 6850 O95376 P36888 1399 P02549 Q15125 P05112 P49715 P60953 P10827 7157 P51692 P11912 O00255 P19883 1050 Q03164 Q9H295 861 P37173 863 Q99814 7704 4318 7046 P04637 P14317 2034 7163 6194 P32302 P52952 Q9Y3A5 10935 P17275 998 65082 P42229 10014 P62753 Q9C0K0 P56524 P78536 4221 23210 Q04900 641 P08575 648 P01588 P49768 Q9UBL3 P25791 7189 7067 P06239 Q06643 P29590 Q03014 Q01196 P55327 Q00653 P14780 6886 6647 P01137 5316 3932 P01374 Q13422 9133 Q13426 A4D1W7 P04629 P19320 P35222 O95067 P35226 4914 O75081 P43405 53335 57379 6776 P40933 Q96EY1 6670 O00744 7520 Q13315 P07948 Q6NYC1 P41182 P78504 P53567 P35612 P30048 P15692 Q9H9B4 6304 6667 4005 5336 Q96AX1 7518 5591 Q6IR47 P35968 10460 7412 P11413 10107 P37231 Q05516 64919 324 5468 3720 10468 7422 P10415 Q02363 57045 P78527 P18146 P13010 Q04656 Q14790 P01730 Q01826 5371 O15111 Q15648 P35268 P16885 10682 8879 10320 9846 10202 Q13351 P60568 5925

nucleobase, nucleoside, nucleotide and nucleic acid transport 25909 Q86V81 Q06787 57187 O95271 P35658 Q96FV9 O00148 23165 4686 10772 O15234 P09651 Q92574 Q8WUM0 Q96HA1 P38919 Q13769 Q9Y277 O75494 8021 O00159 P22626 Q96Q15 9908 Q9UIA9 Q9HAU5 Q9NWB1 9883 P52298 Q86U42 Q9UMR2 Q8TEM1 23144 P57740 Q03014 26019 P12235 Q9GZY0 80145 23381 Q8NFH4 P61326 4869 8563 Q32P51 P62826 10482 O14980 9775 Q9H814 4641 Q8N1F7 64901 23039 79023 P17302 10128 144983 55110 2697 2332 60386 Q7Z3B4 Q5SRE5 4116 P08034 10762 Q9UKX7 Q8IXZ2 Q9HC62 O43592 6311 7884 7248 Q9UGH3 Q9UPR3 Q14008 22794 Q96PU8 4927 Q99567 P42704 84248 7514 Q14493 Q96A72 5976 11269 9818 9793 Q09161 Q9H2T7 3178 O75694 Q86WV8 P37198 Q8NI27 Q8WYP5 Q9BVL2 5901 3181 Q86W42 9961 Q12769 291 59343 23225 Q9Y5S9 Q99700 Q9UN86 7419 Q6I9Y2 23636 P49792 9972 Q9NPA8 P49790 11097 Q9UKK6 Q96QD9 P52948 Q92621 O15504 Q92900 Q9HC21

regulation of cyclin-dependent protein kinase activity P25054 11140 O95835 8851 P54132 1022 P50613 124790 990 595 10614 Q9BZE4 995 23560 Q99640 O75909 O94992 P78536 Q9UII4 4221 26524 9077 P30279 1029 1027 641 1026 Q96SZ6 P51959 324 P30281 51654 207 P00533 8812 P42771 P42574 O14757 P24522 Q15078 P11309 P24385 P30304 7023 Q8N726 P30307 O96020 P46527 894 Q99741 896 Q01664 Q9NRM7 1111 1956 5716 O75832 P31947 P31749 Q9P287 O00255 P38936 O95661 5728 2810 Q9UHV2 P60484 Q92786 900 Q16543 Q96JB5 56647 Q96MH2 1647

embryonic development ending in birth or egg hatching Q14686 3880 O14672 8533 Q12948 O75386 P51587 29947 Q9Y4K3 O43318 P17676 472 Q9H161 2303 P84022 Q99807 P49810 P08047 Q7Z6C1 51763 O14543 P49815 Q13485 P28799 Q9NPC8 P20290 4188 8543 P08727 Q5TAB7 Q02447 P16234 367 P42858 Q9H2X0 6117 P46531 4851 9869 10661 P00533 Q15554 Q15796 5702 Q14344 5704 Q15672 O15525 P27694 Q9ULX9 P61968 6495 Q12965 6498 P12757 P05549 Q9NRY4 P12755 Q9ULH7 6927 P37023 257 P23528 Q96RK4 Q15561 Q92830 5159 Q9UJU2 P40424 6926 4627 134701 4087 Q92824 4088 4089 P07737 8323 9412 P18074 Q9Y618 Q86YT6 P13612 P17302 P17655 57534 P20823 Q4AC94 2697 P61296 10087 P43246 5604 53615 9421 Q14249 P27037 8452 9546 P28482 7248 Q8TD84 2909 P63092 P10275 P35579 O14627 10733 10736 57669 1499 Q9NYQ6 4643 3676 Q9Y2T1 2904 Q99966 P50222 8100 O43521 7014 P09619 P36896 P36894 P10600 Q6UB99 Q06210 Q86WV8 Q9BPY8 841 Q9HCM4 P17482 7249 Q9NQB0 Q8WW38 P09067 Q9GZM8 A6NI15 Q99958 Q14145 Q15475 Q99835 O95377 P61803 23463 Q13618 7020 Q9NPQ8 57448 3215 1277 O14641 O14640 P49715 1956 50814 7157 Q13503 P31749 O95140 O00255 Q02750 Q6KC79 1050 56243 P37173 Q9UMX1 4678 Q99814 1969 P29317 Q15256 P02452 7046 9464 O96004 P04637 O43278 2034 P13349 2033 23322 25988 60626 P52952 Q4VCS5 O60725 Q9Y3A5 P62993 Q1PSW9 P17275 91 999 94 Q92574 O15119 P98161 639 1856 O96018 Q9UNS2 4221 5310 6885 5311 Q96QB1 P16989 7291 Q9BRQ0 1072 Q9BT40

Q99873 Q99750 81565 Q13099 P49768 Q09472 P50552 7189 92129 O00444 Q63HK5 Q5T5P2 O14727 Q9NR09 Q96S42 P09630 Q15047 Q9UMS4 O95983 9133 6660 Q13426 P19320 3276 P35222 O95067 P35227 Q01974 Q96ST3 P10070 P10071 Q92786 P02458 2737 2736 9927 6670 O75626 Q15738 O95999 Q13315 Q9H488 7082 Q03113 23764 9021 Q07157 25942 O00622 O95076 84525 P00747 P15692 O75190 O60488 6667 4920 Q13563 7518 10454 2626 P31249 5591 Q6IR47 7412 26005 3297 P80370 Q68CZ1 27339 23414 Q07817 10229 689 207 7408 Q15750 90780 5362 4154 7422 23509 P49321 P56177 P12830 P78527 P35711 O75051 Q9BQA5 Q00613 2885 Q14790 P43694 10672 Q9GZT9 9839 P27540 9612 O75712 Q15648 P19484 Q04771 P17980 54583 2896 O60353 Q9HCS7 P43686 Q13351

transcription P10914 Q92858 Q14686 4297 Q14209 P67809 Q9UKD1 Q9UPN6 O00268 Q6NZI2 O00267 P13984 4291 P52434 P17676 Q9BYG3 2305 474 P16220 Q9ULW3 Q9H0D6 P20290 P03950 3091 P30876 O00716 Q16633 8543 Q16514 26205 O75376 60489 367 51773 11198 6239 5705 Q16649 P35638 Q9ULX9 P61968 P27695 3661 Q9NRY4 P32121 Q12968 O75362 Q92830 Q9Y295 P03372 51547 Q9UKW6 3659 Q13148 Q9BYU1 Q9UIS9 P35869 O75478 9533 P18074 Q99583 Q6P2C8 P29084 P36954 142 P19838 Q16665 P23771 9421 P78347 Q13951 O15160 8458 4772 O15164 Q12986 2909 1022 1385 57661 P35453 P10275 4094 27043 6829 79577 2908 Q14494 5733 O75909 84365 Q13287 Q14134 6827 Q9Y2T7 Q9NRC8 4780 8467 O43889 O75575 Q14938 O00472 Q9NS56 10987 27297 10625 1025 1388 P08651 4775 P49848 Q92766 4790 O75444 25885 Q9H9Y6 Q9NR48 Q96T23 Q99941 P61244 284119 55149 51222 O14641 Q9Y250 11243 9319 O14640 55703 Q92994 9318 9439 5514 P49716 5757 P49715 11128 Q9UNY4 P10827 P10828 Q9HB90 Q08050 7157 22828 6182 Q9NR55 1050 Q03164 10725 Q15370 865 P04150 4799 Q99814 P06454 Q9Y2W1 P78545 P78424 Q13873 Q6P1J9 O96004 6872 P29375 Q96QC0 6871 P04637 P29374 2034 P53803 11108 P42224 Q9HC16 P62195 Q16236 5536 Q13761 P62875 1856 Q9UBK2 9477 9232 22803 6883 5430 Q9P1Y6 5432 O95602 Q13887 Q96I24 Q96T76 P24928 Q03933 Q9Y6Y0 6878 Q6P1K2 8812 P49643 O43294 2959 408 8939 P49407 409 Q9UBL3 7068 7067 5440 P49642 5441 6772 5442 Q9UKN5 Q15714 O43812 26015 Q9UKN8 2961 2960 1870 Q53X93 5434 O60264 5557 P61201 5558 Q9BTC0 2969 P09874 Q15287 6660 29777 23435 10921 Q9BWH6 Q9NS37 Q00403 2972 Q96RR1 P09086 P53041 10488 P61218 Q15853 Q14527 O95997 7764 P50613 P51946 23764 Q9Y5Q8 Q8WUY8 P22415 Q8IZL8 Q03468 Q02930 4800 P43115 2626 O95644 Q9NVC6 Q14653 23650 P19387 Q9ULK4 P19388 3054 29079 58487 688 689 P23511 328 Q9Y5B9 4150 4152 P50750 2099 P52815 P35711 Q00059 O14802 Q96AE4 Q6P4R8 4149 P43694 Q9H5H4 Q9Y4A8 8880 P27540 Q15643 Q9P2Y4 Q15648 Q92905 P51610 O00411 Q9NVV4 O75031 5927 P63272 Q9NPJ6 Q9HCS7 9967 902 Q15653 P21675

positive regulation of ligase activity 8454 11065 5300 P51668 P14635 P51665 991 26271 Q96RU7 P62195 P63208 Q13042 Q92530 10213 7518 Q13200 O43242 64682 7334 P55036 Q12834 Q13526 O00231 O00233 O00232 5706 5707 5708 5709 Q9UJX2 Q9H1A4 5347 P49721 648 5700 P49720 Q16401 5701 P60900 5702 5704 5705 5682 5683 5684 5685 8555 5686 P28065 Q13616 O00487 O00762 P35998 891 5717 P48507 P62333 57761 P20618 P61088 8945 P25789 10197 P25788 P61289 Q9Y297 5713 P25787 5716 Q15008 Q9UL46 5692 8881 5693 O75832 P53350 6500 Q13426 P62191 P28074 P17980 P28072 Q99460 P28070 P35226 O14818 2730 P30260 5687 5688 Q9UKT4 P43686

positive regulation of kinase activity Q8TEW0 P54619 Q9Y4K4 Q9Y4K3 3084 O43318 P13861 595 23043 P22694 Q9ULW0 P07996 P52564 P03950 5155 Q16512 4188 O15530 Q9UKE5 P16471 P00533 Q13131 8795 Q5S007 P11309 Q9HBW0 Q06124 22974 Q9Y6Q6 Q9UBU3 Q9UDY8 Q9UKW4 P00441 4869 5716 Q53H12 Q9Y6R4 Q14114 3672 O43306 Q9NR96 Q13705 P21917 5170 O60674 P20827 3667 5604 Q13153 3309 P04233 Q8WXG6 Q13956 7124 O00220 1020 O75460 154 Q9Y2C9 121512 Q9Y6K9

P07550 3690 Q9NYA1 P10600 P10721 P35568 Q9BUB1 P11021 10746 79444 Q8NFM4 P01308 Q8NFM5 120892 7804 Q9NRD5 P05106 O95136 O95257 Q9UGJ0 P28223 6850 P11229 A1XKG3 O43612 P16118 P30542 P18545 O60503 P49959 2247 P60033 975 55704 Q5TCX8 O14763 Q92878 Q96CA5 1956 P09038 P31749 O75553 O95382 Q02750 Q9UNI6 23118 P37173 Q6PID4 1609 Q9BWT7 Q14289 Q9UL54 P06213 P01241 9463 7046 P98155 P56199 Q7RTN6 P01116 Q9C0K7 P22612 Q15389 1616 P78536 7057 6885 Q02297 P10644 P31321 Q3V6T2 P31323 Q99750 4214 P08575 4216 P42338 Q9UER7 P49768 O00206 7186 7189 P29353 5562 Q96M96 P52735 Q13418 P31431 P40145 894 Q00535 896 O60266 6647 P01137 P01135 P01133 P01375 O95622 4361 5573 P04626 5575 Q13546 5576 2064 55750 P43405 56288 Q07954 Q92786 5566 5567 P01127 5568 5207 5585 P78509 B3KY43 Q99683 Q03468 P51828 10451 5577 10454 P08246 2081 7099 7098 Q6IR47 Q13443 7410 P30279 5590 P61073 Q05513 P30281 8737 207 Q13574 Q15750 P46734 Q08462 Q9NYJ8 P24385 8754 4035 Q9P035 Q9BUZ4 O75293 3611 O15455 P01730 9839 O75832 Q12933 6464 P52824 P17612 P51617 8517 54106 Q13233 P25116 P60568

negative regulation of protein transport 11060 O95835 O00308 7124 11142 1020 P10599 Q9UNN5 O00221 114548 Q99728 6901 P25963 Q9UBC1 4188 10460 Q16635 7295 2316 9908 Q9HD26 P01308 Q99750 1540 7529 P23510 P21333 P61925 7067 4792 Q96FX2 P14416 57120 A1XKG3 2010 Q96P20 Q00535 O60543 11124 Q9UN86 Q9Y6A5 P01375 5716 Q9NQC7 Q9BT67 8165 P10827 Q60FE5 P31946 O75832 Q9UGK8 Q9BZS1 Q9BZF9 P21917 580 P50402 P21359 Q9UMX1 5569 Q9Y2B9 Q15653 50943 Q92667 P43489

positive regulation of ubiquitin-protein ligase activity 8454 11065 5300 P51668 P14635 P51665 991 26271 Q96RU7 P62195 P63208 Q13042 Q92530 10213 Q13200 O43242 64682 7334 P55036 Q12834 Q13526 O00231 O00233 O00232 5706 5707 5708 5709 Q9UJX2 Q9H1A4 5347 P49721 648 5700 P49720 Q16401 5701 P60900 5702 5704 5705 5682 5683 5684 5685 8555 5686 P28065 Q13616 O00487 O00762 P35998 891 5717 P62333 57761 P20618 P61088 8945 P25789 10197 P25788 P61289 Q9Y297 5713 P25787 5716 Q15008 Q9UL46 5692 8881 5693 O75832 P53350 6500 P62191 P28074 P17980 P28072 Q99460 P28070 P35226 O14818 P30260 5687 5688 Q9UKT4 P43686

protein localization in organelle Q96QC0 P04637 O96008 Q9UPN3 Q9P2R6 473 2147 23165 Q92973 Q9Y2X3 P60059 10651 637 10133 Q15785 P49815 Q15388 P25963 Q9NPC8 Q9HAV7 Q14974 4580 Q8NF91 O15131 Q01082 P55957 Q8IWJ2 55696 117177 P42858 Q15154 P52292 406 9868 P60468 O00327 6496 6495 P52294 P29590 Q03014 29927 P30101 Q96CV9 Q96P70 4193 Q9BXC9 Q9NZQ3 Q96RK4 P01137 P63104 91949 P01375 P61619 4000 P62829 Q86XR8 64901 O95343 O60318 P61970 O60674 Q9UH99 9804 9648 1408 Q49AN0 9927 O00505 7124 P28482 O00629 P41182 Q9P0J0 3836 10736 O94826 10452 Q13285 P05412 O95405 7534 P33947 Q9H2T7 P37108 Q9NS56 10469 Q05516 Q99797 83548 Q00987 Q9Y5J6 P00734 Q96QF0 6711 207 7249 3841 9702 604 3843 5108 80273 Q15475 4792 100287932 Q86VS8 Q9NW64 O43615 Q9NS69 10953 401505 10956 O14925 2247 56993 Q6VY07 O14640 P01579 P02545 5514 P83436 6726 P09038 2923 55705 P49792 5371 4285 P30536 Q13505 7157 P31749 O95140 Q9Y5L4 O75431 Q8N4H5 P62072 O00410 25777 23633 Q08209 P52948 Q92621 11014 84376 7704 10204 P25116 51517

regulation of microtubule-based process P25054 4131 4133 P10636 Q15735 23122 P14635 9181 Q9BVA0 10733 672 Q9Y6G9 Q9Y3A5 1499 57662 3476 10013 Q9ULW0 P46821 Q92974 11190 7013 9475 10460 23332 Q5HYA8 Q7Z460 324 1540 57159 22919 4137 Q9BYV2 Q9BV73 P54274 O75122

O00444 P56539 22974 Q9BXC9 891 P38398 221150 Q96RK4 79959 857 Q9Y6A5 859 Q9NPA3 Q8N960 4869 Q9NQC7 91147 O75116 P35222 P78317 P78318 Q8TAP6 Q02790 P27816 Q15691 Q8IX90 Q9UBN7

vasculature development Q14686 Q12948 P52799 O43318 Q8IYN9 2303 O14786 O14543 P07996 2308 2307 P03950 P25067 5154 Q08431 P21810 P46531 4851 Q14344 P28300 Q12965 1121 Q9ULH7 O75360 Q05397 P37023 P20936 Q9UKW4 55109 6926 P55290 4627 P26012 Q92826 Q14116 Q9NZI7 3791 Q14119 Q86YT6 P13612 P17302 57534 5728 P21359 2335 2697 P61296 5604 9421 P55347 P28482 Q5D1E8 O95470 4092 O43638 P35579 O14627 P09172 P46109 1499 51564 1012 Q8N264 P23467 4643 3676 11146 3678 3679 Q99966 Q15464 P08648 3690 P50222 Q9NYA1 8100 P19544 Q8IY17 7490 960 841 P33151 3685 Q9NQB0 Q8WW38 1948 Q99958 5747 P02751 Q99835 Q96T37 P05106 2247 1399 2246 Q9H1Y0 Q92990 1277 P05230 857 Q9NQC3 P09038 116150 50814 Q12778 P31749 Q02750 1289 P37173 Q6PID4 4313 Q99814 1969 P09601 P29317 P40337 Q14289 P06576 P02452 P78423 4691 7046 9464 O96004 O43278 Q9Y6W5 2034 P52952 Q4VCS5 2702 2821 P41134 3479 P17275 Q9BUG9 P39060 P04085 94 Q15389 O15119 P98161 P07355 P98160 3911 9474 7057 5310 Q7Z5H3 6885 Q13887 23210 P35368 7052 P19338 P39059 2277 P16070 P01343 Q16363 Q13099 P49768 P25791 P29474 P52735 10908 Q03014 Q96S42 P01019 O15379 P21980 5316 Q9NZ08 Q9UBM7 8829 Q9UPY3 P01133 Q9UJM3 P02462 P02461 4240 9255 8841 Q658W2 P35222 P17813 302 P60484 Q9BXJ9 P10071 Q92786 5327 5328 2737 Q15738 Q8WUI4 9820 Q6NYC1 Q12904 Q9H488 O43915 9021 P78504 3162 O00622 Q96PU8 P00749 P00747 P15692 10451 P08123 4005 3953 Q13683 Q8N302 P05412 P06744 Q6IR47 Q13444 P35968 7410 23414 P36382 80155 688 4015 207 54206 P08253 P48357 9047 P24386 7422 23509 O14495 Q04656 Q14790 10672 O15230 P20908 Q04771 8879 O15105 51752 P27658 7428 P00750

tissue morphogenesis 5268 Q12948 O75386 Q9UPN3 Q9Y4K3 O43318 2304 4609 2303 284217 P84022 P09493 Q99807 Q92731 O14786 O14543 P49815 Q13485 Q9NPC8 94234 5155 8543 54361 P13631 367 P42858 Q9H2X0 P46531 4851 P00533 Q15796 Q14344 Q15672 Q16881 Q8N205 P61968 6495 Q15797 6256 P05549 P25391 Q9NRY4 O00167 P12755 6927 P23528 Q96RK4 Q92830 Q9UJU2 Q9Y297 P03372 P40424 Q8WZ42 6926 P06401 Q92826 Q9GZX9 4086 4087 4088 Q9C009 4089 P07737 8323 3673 Q14118 P36952 2100 Q86YT6 P56705 P12883 P17302 57534 P20823 P17301 Q4AC94 P31270 2697 P61296 P62942 9421 9420 4771 7248 2909 P35453 P10275 4092 Q93063 10736 57669 1499 Q9NYQ6 Q16790 P45379 Q99966 P51671 9314 P36894 Q86WV8 P19544 7490 O00358 P18206 3207 960 1147 841 Q9HCM4 7249 Q99958 Q15475 Q96T37 Q9BQ95 2132 7020 Q08188 2247 1277 O14641 P08069 P47895 2926 1956 P09038 P39905 P19883 2139 Q14160 P37173 Q9UMX1 O95947 1969 P29317 1605 6615 P02452 Q13873 9464 O96004 O43278 P52952 P62993 79810 3479 Q1PSW9 Q8WV60 Q92574 P42229 O15119 3911 1856 6885 Q96QB1 7291 P10644 1072 2280 7052 P48436 P01106 768 P16070 O43294 P01343 P49768 P50552 7189 P48431 P19793 O75881 P29590 Q03014 Q13418 Q99623 O14727 Q96S42 P35240 8945 P01019 P21980 P01137 Q9NSC2 8829 Q9UJM3 O95863 5573 P35222 O60716 P17813 P10070 P10071 11331 Q92786 5566 6776 P01127 6657 2737 2736 O43474 O95999 P07949 9021 P78504 P15692 P08123 2626 26005 P68032 10229 51295 3169 54206 P08138 7408 10468 5361 7422 P10415 2099 57045 163183 2885 3611 Q04656 Q14790 7414 P43694 10672 Q9GZT9 9839 5371 O15111 Q15648 O15230 Q04771 23513 P17612 54583 O60353 Q9HCS4 P24821 O15105

positive regulation of intracellular protein transport 5460 7046 Q15654 7124 2033 O43557 O60603 Q93062 Q04759 P84022 Q6PJW8 999 Q7Z6C1 P61586 Q13485 7099 7098 Q6IR47 Q9NYA1 11030 P14923 P36894 P10600 2316 3728 7205 Q9NQB0 P00533 P49841 Q99835 P21333 O00206 Q09472

P12830 2010 P01137 O15455 1956 P01133 P01375 Q60FE5 4088 4089 Q9BZF9 Q9NR96 9495 P50402 P17612 2932 387 O60674 P10071 5566 54106 2737 P24588

cellular developmental process P25054 65125 3880 2550 259266 9181 P51587 P17676 2547 P37288 Q8IYN9 2303 O60443 284217 P84022 P21926 Q9HCJ2 Q92731 Q92730 O14786 O14543 O14788 Q8TDD5 Q92974 Q9UGM3 Q12834 P08727 Q92729 Q12837 124540 P51114 2317 Q96KN7 P20849 P42858 Q99598 Q9UKV3 P61158 10661 4734 Q96EB6 Q8N205 Q7Z727 136319 81629 O00167 Q06124 Q7L5Y9 54496 55342 6929 4983 811 5830 55109 3655 Q9Y6A5 Q5TAP6 P40424 P62258 Q9UKW6 6926 3659 P31944 Q9NQX0 3672 3673 P31947 Q92949 2100 Q9BYD5 P48380 O95343 P17655 57534 3428 O60437 1488 2335 O60674 1487 P20827 388677 P62263 Q8N5V2 P26367 O14578 5604 P41743 4771 Q9Y463 1021 1020 P39019 1499 Q8N264 3676 P61586 P07437 Q9H0H5 P29466 P32856 Q92769 Q14814 P28370 Q9NZC7 O95365 Q9BPY8 Q06330 3207 841 Q9HCM4 O60890 604 3688 A6NI15 P09622 P04179 O95377 4791 2132 Q15915 O60869 O95376 Q96TC7 P19634 57326 3215 Q96N67 2125 Q92990 1277 Q9Y250 Q92753 Q5TAT6 Q92752 P05230 857 859 Q9H0F6 Q13501 P10826 O00139 P10827 Q9UL45 50937 221937 P11912 P31749 Q9NR50 1297 O95140 Q13509 P19883 Q13506 Q9P202 1050 O60636 O60879 Q9HCK4 2139 861 3226 O95390 863 6733 Q9UKT4 4318 Q15019 O96005 O96004 6993 P56199 P14317 23322 P14555 P50897 P11047 25988 Q99523 203068 3479 2147 Q8WV60 10011 7832 55723 639 10014 6760 115426 3010 1072 Q8NHU6 641 Q99750 Q8IWA4 84033 79923 11113 O43294 6517 O43293 Q01518 Q16363 Q13099 Q09472 6774 3265 23787 Q00653 891 P14780 894 O95180 P53708 P35240 656 P01019 P49639 84062 Q9UMS4 5451 8721 A7MD48 Q9Y696 Q658W2 O95183 P35222 25975 Q01974 O75084 O75081 P17813 55755 3028 P60484 53335 668 6776 10487 P24588 O43474 P26583 O00744 5460 Q9Y3M2 4131 Q6FGG2 P01040 4133 9820 Q9NX62 10458 P61981 P15692 6304 P55075 6548 79739 6789 Q8N5U6 P05412 O00755 10460 7412 Q9HCE7 P52803 P37231 10229 Q96SZ6 79723 9825 5467 4137 207 5468 7408 10468 Q15078 P24385 8754 4154 7422 4155 P56178 P56177 3066 O75293 P13010 P61764 10672 9839 O43463 5493 7430 9612 O43464 P35268 23513 P13497 O14939 466 8516 Q9NQL9 P60201 7428 55558 9846 10202 P55268 Q14686 8775 Q9BZ95 Q9UPN4 O75386 O75385 Q9H165 Q86VQ3 Q9Y4K3 3084 472 474 Q9C0C7 P07196 Q99807 P49810 P08047 P83731 P43034 Q13363 Q9NPC8 Q13127 3091 4188 Q15306 5037 79083 Q86VP6 79084 P58304 P58546 Q9UJC3 5270 Q02447 Q9NVP2 Q05952 P09382 28981 Q9NRH2 30813 P46531 8536 5029 51535 P00533 Q16643 Q15796 Q9BYV2 Q9HB63 Q15797 P12757 Q8N6I1 P31273 P12755 Q9ULH7 O75360 Q9Y6Q6 Q01101 Q96RK4 Q15561 Q9NRI5 Q13144 Q14232 Q16650 8563 6383 7476 Q9Y6R0 P18074 Q86XR8 P10586 P68133 P10588 P13611 P10589 59277 P13612 93166 O75592 Q4AC94 Q8N4C8 54820 P31270 23032 Q16665 P43246 9421 O15287 Q16666 7248 P54132 274 Q93063 Q04837 P23229 51564 Q13164 Q14012 Q86W54 P78337 8100 7257 Q9HAZ2 Q15109 O43524 7490 7251 285 Q9Y6L7 Q8WW38 1948 Q9GZM8 1947 Q9NRD5 P02751 Q9GZM5 Q99835 9201 O75326 Q9BV36 10152 Q02880 A1XKG3 Q86VS8 Q96EV8 Q08188 51341 Q9NVM4 55704 P21266 P08069 10155 P02545 347733 1956 P23443 O15075 Q8IUX8 O15078 O75553 O43508 Q9NXR1 2810 P02538 Q6PID4 Q99816 P78362 O95947 Q99814 1969 P02533 Q14289 Q15375 P25874 P78545 9463 7046 P28289 6198 Q13751 9464 O43711 55283 6194 Q4VCS5 3913 P42345 P22735 Q9Y4C1 P04083 65082 Q92574 1738 O60282 3911 3912 P56524 P78536 9474 Q13769 O15496 Q02297 7291 Q3V6T2 3925 P46934 117177 Q9HD26 81565 Q4LE28 P16070 P42574 P98172 8399 7067 P06239 Q5TAX3 P19793 P29590 Q7Z2Q5 P56539 Q01196 1756 P41231 60412 Q92793 Q9HD15 Q7Z6Z7 P06241 P21860 O15259 3932 P56545 91147 A4D1W7 P19320 Q8IYT8 9253 9495 7074 P43405 Q9Y4F9 P41220 Q9NZR4 Q04206 Q5T5A8 Q92786 P07101 Q9NZJ7 Q5GLZ8 O75503 Q13315 Q15735 P78509 P35610 Q03113 O43915 8174 9021 Q16828 P78504 3958 P00747 O60488 P85037 Q9H9B4 3956 2626 P31249 Q8NEC5 Q15742 O43921 P11413 1789

Q05516 P62166 P00734 3720 375790 P22303 Q5BJF6 Q9HCU4 9047 P12956 Q8IUQ4 P34741 26576 57045 P78527 O60229 Q9BQA5 P02708 P20336 Q86TG7 2885 Q9Y6E2 Q04656 51741 P43694 Q14432 Q9BQA1 132320 O15230 Q15768 Q9NP98 63976 P22528 23077 Q9HCS4 4830 51752 Q15532 Q13351 O15228 5925 P10914 Q92858 Q8NEY1 Q9NYD6 2672 Q12948 Q9UBB6 22994 P52799 Q8NEY8 Q9BVA0 3516 P16220 Q9BVA1 Q9ULW3 Q9UM82 P52565 Q8NF91 Q9UBC3 54361 P50148 P63211 P16234 79885 P16473 2676 51099 P16471 O60331 4851 4854 O15524 928 Q92838 P54753 Q12965 Q9UBD6 P25391 Q05397 P53667 Q9BXC9 6809 P20930 27185 29127 P20936 Q9UJU2 Q9UQB8 P03372 4867 Q92833 26091 P06401 4627 P06400 P05556 P54764 Q92826 Q9GZX9 P54762 3791 5970 5971 P48023 Q9GZX7 9093 P56705 Q7L576 22985 P17535 P17302 5728 81501 P17301 P17542 Q9HBE1 2697 P61296 6812 P04233 P34925 Q5D1E8 1385 O95470 O00468 10733 10736 57669 P15173 P07550 3796 4643 4646 5978 Q9UM47 Q7KZI7 O00238 Q9Y2T7 Q96SB4 Q96SB3 Q9NYA1 5991 P51679 Q8IZT6 P10721 Q06210 Q5VST9 P19544 6839 960 1147 1387 2475 967 4898 Q9UM54 Q92888 5747 O95259 P07766 O95257 137964 6850 2011 2253 Q13618 Q9NYB9 57689 2247 2246 P61006 4664 P61247 O14640 4665 5997 O43184 P05112 O43186 P29323 P51692 O00255 Q02078 Q6KC79 O00499 P37173 P09960 P29317 P40337 7704 P06213 P02452 O00429 P04637 O43157 2034 P13349 2033 25861 Q01650 1181 P52952 P15531 991 P62993 Q8WTS1 79810 998 999 P01116 4205 Q9C0K7 Q15389 P02686 P62753 P01112 4209 P01111 Q04917 O43166 Q9C0K0 P49770 Q96CW9 4221 Q7Z5H3 Q9UHG0 P51636 23210 2043 P50542 1191 2277 Q15154 P48436 P01588 4218 Q9C0J9 P01343 Q96NL6 P49768 2290 P50552 P48431 9921 Q86VW1 P14416 2054 3142 O00444 773 Q00535 Q00534 Q96S42 Q53X93 6886 55626 6405 6647 P01138 54535 6646 P01137 5316 Q96Q05 8829 Q9UPY3 Q8N960 Q16254 P02462 6660 4000 P04626 P04629 2065 2064 O00213 2063 Q96AQ6 O60716 P54920 301 3148 P10070 P10071 3146 Q96PN8 P02458 P40933 Q96EY1 Q96JB5 6657 9927 6670 P07942 O00628 5584 7520 O43597 8851 P07949 P07948 Q92817 P20916 Q9H9S0 Q8WYA0 Q96PU8 Q9BZE0 552 Q96PU4 Q14093 Q99684 6667 5336 Q96AX1 5578 5338 7518 Q8N302 P40763 P26232 Q7Z5L4 5591 Q6IR47 7532 7531 7533 5598 Q5HYA8 P68032 23411 P80370 23414 3171 10107 P18846 324 3169 Q9NPH5 9702 10100 5108 P27797 Q9H5I1 6696 5364 2099 P51843 3182 P15311 Q8NER5 5116 140735 Q01826 Q96HC4 5371 P27540 O60911 P16885 582 Q8WTT2 Q08209 P58012 P18827 8879 P62745 6699 10320 8878 P60321 P26447 P25116 P60568 Q96HZ4 Q13477 8650 8892 Q9UQP3 Q6ZT98 Q8TEW0 Q9BY76 P15924 P30086 P15923 Q9Y3I0 29948 4291 351 595 Q04759 356 Q9UBS5 P09493 Q7Z6C1 5139 Q7Z6C3 Q8NFJ9 Q13485 8408 Q8TD31 P03950 9750 Q13009 4067 Q15669 7335 367 Q70SY1 51654 Q96SN8 Q14344 Q15672 6496 6495 6256 6498 10075 O43897 P10451 Q2M1K9 Q9UBU3 P51805 Q9UDY8 P23528 P09471 Q9UDY6 Q8WZ42 Q13263 P00441 8443 4086 4087 Q13268 4088 4089 Q14119 163126 Q9Y618 Q86UL3 Q86YT6 P12645 Q02790 222546 387 388 P21359 23154 23396 P08151 1808 P20264 10087 6259 P60763 Q13275 P54259 P55347 8452 6275 4099 Q8TD84 P14635 Q9UQL6 P10275 P35579 154 396 Q5JUK2 Q02535 Q00597 Q01201 23363 10296 Q8WV28 11146 Q99729 Q16555 10059 Q15223 P45379 Q99966 Q15464 Q13285 Q13043 Q13049 Q86YZ3 P60709 Q16799 9314 P36894 Q86WV8 1822 338917 Q9NQB0 60 P09067 O15169 85440 Q99958 Q15475 Q99714 Q8TB22 Q8TCU4 7142 Q8TEY7 P14859 P36888 7141 1832 Q9H1Y0 9319 P21145 P01579 Q9NQC3 P49715 P09038 P60953 Q14155 116150 7155 P39905 7157 P22492 25776 Q02750 P35548 8481 Q9H295 55832 Q14160 84376 1848 Q15256 P78424 Q08AF3 O15123 O75665 P10636 26037 P63172 7161 9341 7163 1613 26271 Q8TAF8 P41134 Q86WK6 Q1PSW9 10935 P17275 Q4KMG0 Q92692 P39060 Q6NVY8 P46821 P42229 P15090 O15119 2947 Q9UBK2 Q13885 Q13642 Q8WWH4 Q13404 O43602 10939 P55318 O43847 P55316 79365 O43609 55177 P39059 P08575 P05067 Q9BXL5 1627 7189 Q15836 P29475 O95613 26258 57120 Q03014 Q13418 Q7Z3S9 O43815 6096 P34820 P55327 1874 P17252 Q5TA76 O60383 Q14993 Q9NZ08 Q9UBM7 Q9NSC2

P42685 P21741 Q13422 P35908 O15156 Q13426 9138 P10620 29777 4914 2735 Q9Y3D6 56288 Q96FJ2 Q9BXJ9 57379 Q8HWS3 1406 2737 2736 O75626 P54652 Q8WUI4 9149 Q6NYC1 P41182 P53567 P30048 84525 1896 4920 P35968 Q14774 Q13443 Q07820 26005 P11532 57154 P51159 Q68CZ1 27339 P30279 Q07817 64919 80155 P23515 Q9UJX0 57159 Q96QF0 P08138 P09429 O14492 P56851 P10415 Q9H228 P12830 Q02363 P18146 P35711 163183 3611 83696 Q14790 P09430 P01730 80184 P08588 P41159 O15350 O15111 O15353 Q15648 Q06710 Q04771 1203 P24821 P21554 4957 Q9BZR6

blood vessel development Q14686 Q12948 O43318 Q8IYN9 2303 O14786 O14543 P07996 2308 2307 P03950 P25067 5154 Q08431 P21810 P46531 4851 Q14344 P28300 Q12965 1121 Q9ULH7 O75360 Q05397 P37023 P20936 Q9UKW4 55109 6926 P55290 4627 P26012 Q92826 Q14116 Q9NZI7 3791 Q14119 Q86YT6 P13612 P17302 57534 5728 P21359 2335 2697 P61296 5604 9421 P55347 P28482 Q5D1E8 O95470 4092 O43638 P35579 O14627 P09172 P46109 1499 51564 1012 Q8N264 P23467 4643 3676 11146 3678 3679 Q99966 Q15464 P08648 3690 P50222 Q9NYA1 P19544 Q8IY17 7490 960 841 P33151 3685 Q9NQB0 Q8WW38 Q99958 5747 P02751 Q99835 Q96T37 P05106 2247 1399 2246 Q9H1Y0 Q92990 1277 P05230 857 Q9NQC3 P09038 116150 50814 Q12778 P31749 Q02750 1289 P37173 Q6PID4 4313 Q99814 1969 P09601 P29317 P40337 Q14289 P06576 P02452 P78423 4691 7046 9464 O96004 O43278 Q9Y6W5 2034 P52952 Q4VCS5 2702 2821 P41134 3479 P17275 Q9BUG9 P39060 P04085 94 Q15389 O15119 P98161 P07355 P98160 3911 9474 7057 5310 Q7Z5H3 6885 Q13887 23210 P35368 7052 P19338 P39059 2277 P16070 P01343 Q16363 P49768 P25791 P29474 P52735 10908 Q03014 P01019 O15379 P21980 5316 Q9NZ08 Q9UBM7 8829 Q9UPY3 P01133 P02462 P02461 4240 9255 8841 Q658W2 P35222 P17813 302 P60484 Q9BXJ9 P10071 Q92786 5327 5328 2737 Q15738 Q8WUI4 9820 Q6NYC1 Q12904 Q9H488 O43915 9021 P78504 3162 O00622 Q96PU8 P00749 P00747 P15692 10451 P08123 4005 3953 Q13683 Q8N302 P05412 P06744 Q6IR47 Q13444 P35968 7410 23414 P36382 80155 688 4015 207 P08253 P48357 9047 P24386 7422 23509 O14495 Q04656 Q14790 10672 O15230 P20908 Q04771 8879 O15105 51752 P27658 7428 P00750

mRNA transport 25909 Q86V81 Q06787 57187 O95271 P35658 Q96FV9 O00148 23165 4686 10772 O15234 P09651 Q8WUM0 Q96HA1 P38919 Q13769 O75494 8021 O00159 Q96Q15 9908 Q9UIA9 Q9HAU5 9883 P52298 Q86U42 Q9UMR2 Q8TEM1 23144 P57740 Q03014 26019 Q9GZY0 80145 23381 Q8NFH4 P61326 8563 Q32P51 10482 O14980 9775 4641 Q8N1F7 64901 23039 79023 10128 144983 55110 2332 Q7Z3B4 Q5SRE5 4116 10762 Q9UKX7 Q8IXZ2 Q9HC62 7884 Q9UPR3 22794 Q96PU8 4927 Q99567 P42704 84248 7514 Q14493 Q96A72 5976 11269 9818 Q09161 Q9H2T7 3178 O75694 P37198 Q8NI27 Q8WYP5 Q9BVL2 Q86W42 9961 Q12769 59343 23225 Q9Y5S9 Q9UN86 Q6I9Y2 23636 P49792 9972 Q9NPA8 P49790 11097 Q96QD9 P52948 Q92621 O15504 Q92900

cellular respiration O75306 Q7KZN9 4695 O43678 O43837 Q9P2R7 4728 4729 O75390 Q99807 8803 6622 P22695 4720 5018 8801 4722 1738 4723 P05177 6901 4725 4726 Q9UBK2 O95169 P28331 Q16635 O14561 P07919 P11177 O75380 1743 29796 4697 P00414 4698 4731 5162 O75489 3420 Q12968 O95298 4191 O95299 3417 1355 P31040 P03891 3419 O00217 Q13423 P36957 O75874 6389 P99999 O95182 P21912 3421 4514 P31930 O75746 P56556 48 P40926 P50213 6390 P19404 8604 P04040 Q16795 P07954 P51553 P14927 23530 O43920 Q16718 10229 Q15070 4775 6834 4535 847 P09622 P04179 7385 7384 O00483 7381 P17568 4705 4707 Q9Y6M9 1431 P00403 4701 4702 P56181 O75438 P53597 P51970 Q9Y375 Q15526 P37840 4718 4719 P49821 4710 4713 4714 4715 P21399

cellular response to insulin stimulus 64397 P08833 P98155 5584 6599 3484 Q9UQL6 Q9H2Y7 3481 10458 P42224 Q96RU7 P22415 2303 P62993 Q92572 Q13480 P27986 10014 1978 2308 2549 5591 5471

Q13322 O15530 Q96QB1 10580 Q9Y4H2 P35568 P30279 P37231 57678 P05062 207 5468 Q13370 P17081 Q92922 Q99958 P01344 Q92569 P09467 9564 O14492 6256 P29353 P49840 6772 Q06203 P19793 P78527 894 Q9HCL2 2885 57761 P17174 P21860 Q9UQB8 P08069 8503 2805 P09874 Q92831 5295 8165 P55851 Q12778 6464 Q9BX66 P31749 1176 2065 Q9UKG1 5170 P18433 142 Q6ZWJ1 2931 3667 P41743 Q92667 26060 P06213

negative regulation of cell proliferation P54852 P25054 6595 4297 9500 2550 1460 P51587 Q9UBS5 P84022 Q92731 P49815 Q13485 P07996 Q13363 P03950 9510 6368 Q92729 Q8IZP0 79084 P58304 P13631 367 Q15796 Q92845 Q9UK53 Q14106 Q7Z727 Q15797 Q92956 Q92835 6256 Q96IZ0 Q9BQ51 Q9UNL4 P12755 Q9UBU3 P46527 P37023 498 P25705 1111 P32929 Q92833 P55290 4869 Q9BSI4 Q92831 P06400 4086 4087 Q13268 4088 4089 Q8IWX8 P31947 Q92949 P10586 2100 Q99583 58189 9093 Q96BK5 1491 P17302 5728 P21359 3304 1488 O60674 1487 2697 Q9UBV7 P31949 5604 10523 8453 8454 4771 7124 O15164 7248 1021 O43639 P10275 P52756 Q9UFF9 P19525 P21964 1499 1012 5610 11146 Q9Y2T1 5074 P04049 10181 22846 9314 P10600 Q96J02 Q86WV8 P19544 7490 7251 Q8WYH8 6282 P37198 1029 1027 1026 338917 P33151 7249 604 P42771 P49848 O14757 P04179 7023 O95813 O75569 1285 2495 2010 Q13617 Q13616 P36888 P30542 2247 Q92990 Q01664 P01579 1718 857 Q9NTI5 Q9BQI3 P49715 P09038 P10826 7157 5894 O95140 Q7L8A9 O00255 Q02750 Q09028 P38936 P35548 1050 Q03164 2810 Q93034 54984 O95390 Q99816 P09601 P40337 50943 7704 P04637 Q9NNW5 P14555 P15531 990 Q96RU8 Q07507 3479 23560 P39060 7832 84289 94 Q92574 Q13761 P01112 Q6UWV6 Q9NSA3 Q9C0K0 Q12797 P56524 7057 Q9Y5V3 4221 Q96QB1 5796 P51636 83737 9231 P55318 22920 9350 Q04900 O43609 1741 Q15392 P41240 Q92560 Q32P28 6878 P01589 O43294 P42574 P01343 P02794 Q6ZN33 Q15831 P48431 P29474 Q8N726 9921 P14416 P19793 3265 P29590 Q13418 Q7Z2Q5 2173 O43815 P41235 Q99623 27102 Q99741 Q00534 P17252 P35240 P01019 Q92796 51147 P01137 O14964 P42685 11326 P01375 O15151 P01374 P51531 P56545 9255 P04626 A4D1W7 Q9UHI8 2064 P35222 P35348 Q01974 P17813 P35232 P60484 P17936 P10071 11331 Q92786 P40933 Q96EY1 6657 P25311 2737 9927 O43474 9146 O95995 O95751 6794 O43597 Q12904 P41182 23641 3162 P23508 Q63HR2 P00747 Q9BZE4 P50616 Q9HC57 5578 4920 50514 Q8N5U6 P05412 P05413 Q7Z5L7 3297 8065 3171 P37231 Q05516 64919 563 Q01955 444 Q99675 324 Q9NPH5 5467 5468 3720 P10415 5245 Q9P2X7 10437 1312 Q00613 3611 127435 Q9BQA1 Q9BT67 23636 5371 P08107 P55773 Q9BZS1 P50402 P16410 5928 Q8TDM6 3621 P67870 4830 7428 10202 Q14201 P25116 P60568 5925

cell migration Q92858 Q9UQP3 23607 Q8NEY1 Q12948 O75385 474 5817 2304 Q8IYN9 23286 Q9BVA1 Q92731 Q9NPC1 O14786 P43034 O14788 8408 P07996 P27487 P03950 5155 P11215 2676 P00533 Q15672 Q7Z727 6495 P32121 Q05397 Q9Y6Q6 P37023 27185 P23528 Q9UKW4 4983 5159 5830 Q9NRI5 3655 Q13145 P62258 P55290 4627 P05556 6386 Q14114 3672 Q92949 2100 1130 P13611 P10589 P13612 Q13829 P17302 5728 P16333 Q99698 54820 2335 2697 23032 Q9UKX5 P20264 Q13275 8452 7124 7126 O95470 1020 64759 O43639 P35579 O75581 P09172 57669 P23229 1012 3676 3678 P08887 P08648 P30556 3690 P09619 P51679 P35443 P10721 O00358 960 Q9HCM4 O60890 3688 4898 Q9GZM8 3689 1947 7804 5747 P02751 Q15475 Q99835 Q12884 9201 9564 P05106 Q8TEY7 P05107 6850 O95813 10152 Q02880 Q13618 A1XKG3 Q9NYB9 Q9Y5K6 2247 P15151 55704 P01579 P26038 347733 1956 P09038 P23443 Q14155 O15075 7155 P39905 P31749 O75553 O43508 Q9NXR1 253980 Q14160 1289 Q6PID4 P05362 P40337 Q14289 Q9UL54 P06576 P78423 4690 7046 6198 O43157 Q9Y6W5 P56199 O43711 P11047 Q4VCS5 P41134 Q9Y3A5 Q6NVY8 94 3911 P78536 7057 P06702 Q9UHG0 5796 O43602 7291 1072 P50542 O43847 Q3V6T2 9350 Q99755 84159 81565 P16070 P98172 409 O14713 Q01518 P49768 Q14865 P29474 P14416 O00560 P52735 8394 6093 Q00535 P16284

Q96S42 P17252 P06241 6405 P01137 8829 P01375 9255 P35908 P19320 Q658W2 P43405 P17813 P60484 3148 Q8IZE3 3146 4478 P01127 5327 10487 P26583 O00628 8851 P07949 P78509 Q12904 P15692 O14908 10451 5578 P08246 P26232 5591 Q6IR47 Q68CZ2 Q8WZ19 7410 7531 7412 9270 P14923 3728 5467 207 Q13574 Q15078 P09429 5362 7422 5364 O14495 P78527 O75051 Q13464 9839 O15230 P20908 Q04771 23513 8879 7428 P00750

chromatin assembly P07305 P26583 P06899 Q9H0U9 8290 4171 Q96KK5 1058 P20671 Q8NCD3 55723 Q02539 P49736 P49450 8348 Q9BTM1 P62805 9555 8467 P62807 Q99880 3010 O60814 P16104 Q99877 3009 Q9NVP2 3008 3007 Q8IUE6 3006 Q96QV6 51773 Q92522 8338 Q8N257 255626 Q99879 Q9H2G4 P33778 266812 Q5SSJ5 4673 Q7Z7K6 Q96NT1 3024 O75367 22893 P10412 P58876 P55209 Q96T23 P84243 Q71DI3 P23527 P68431 Q01105 3014 Q16695 P49711 O60264 8349 4869 3070 Q16777 P22492 Q16778 P0C0S5 Q5QNW6 Q9NRZ9 P16403 P04908 P16402 221613 Q96A08 P16401 Q99733 55355 Q9P0M6 Q93079 Q8TBE0 P0C0S8 3148 Q93077 Q7L7L0 Q6FI13 4676 Q6NXT2 64061 Q13112

negative regulation of ligase activity Q9UL15 11065 P51668 P51665 4092 9184 991 26271 O60566 P62195 Q13042 Q92530 10213 Q13200 O43242 64682 P55036 Q12834 O43684 O00231 O00233 O00232 5706 1029 5707 5708 5709 Q9UJX2 Q9H1A4 P49721 5700 P42771 P49720 Q16401 5701 P60900 5702 5704 5705 Q13257 5682 5683 Q8N726 5684 5685 5686 P28065 O00487 O00762 P35998 P53667 5717 P62333 P20618 P25789 10197 P25788 P61289 5713 P25787 5716 Q15008 Q9UL46 5692 8881 5693 O75832 P62191 P28074 P17980 P28072 Q99460 P28070 O14818 P30260 5687 O15105 5688 Q9UKT4 P43686

negative regulation of ubiquitin-protein ligase activity Q9UL15 11065 P51668 P51665 4092 9184 991 26271 O60566 P62195 Q13042 Q92530 10213 Q13200 O43242 64682 P55036 Q12834 O43684 O00231 O00233 O00232 5706 1029 5707 5708 5709 Q9UJX2 Q9H1A4 P49721 5700 P42771 P49720 Q16401 5701 P60900 5702 5704 5705 Q13257 5682 5683 Q8N726 5684 5685 5686 P28065 O00487 O00762 P35998 P53667 5717 P62333 P20618 P25789 10197 P25788 P61289 5713 P25787 5716 Q15008 Q9UL46 5692 8881 5693 O75832 P62191 P28074 P17980 P28072 Q99460 P28070 O14818 P30260 5687 O15105 5688 Q9UKT4 P43686

transmembrane receptor protein tyrosine kinase signaling pathway 64397 P08833 6599 P18085 P49023 O43559 Q12948 Q9H2Y7 3084 9185 Q8IYN9 2303 116 Q13480 P49815 2308 2549 Q14451 Q15303 5154 Q13009 5155 Q8IZP0 2316 P16234 5829 P16471 P00533 P54753 Q12965 P22681 Q9UQ13 Q06124 Q05397 P54756 2324 5159 Q9UQB8 Q8NFH8 P54764 5295 P54762 3791 P54760 Q86XR8 O43427 Q9UKG1 5728 P16333 O60674 P20827 P18509 3667 P49069 O95470 1020 O75582 O43639 O00468 154 P07550 4643 P09619 Q9Y4H2 P10721 P35568 285 Q02763 7249 Q99958 6714 5747 Q9NRD5 9564 P07766 P49840 Q7Z7G1 2253 A1XKG3 P21709 P36888 2247 2246 Q07889 O14640 P08069 P05230 1956 P09038 P29323 Q12778 P29320 Q9BX66 P51692 O15197 P31749 1176 P30530 P18433 2931 Q6PID4 867 1969 P29317 Q14289 Q15375 26060 P06213 4690 P01241 Q13873 9463 O15123 3484 P12931 O75420 3481 P62993 Q99523 Q92572 10817 P04085 Q15389 P42229 P27986 10256 Q9NSA1 1978 6760 P78536 Q02297 2043 Q96RT1 84159 2277 Q13094 P01344 Q92569 Q14865 P29353 6774 P35916 773 56034 Q00535 P21860 P01138 P01137 Q9NRA1 P01133 P04626 P04629 Q658W2 2065 2064 9252 7074 780 4914 Q01974 2059 P60484 6776 P01127 5327 Q969H4 64599 P07949 O43915 10458 P15692 P55075 4920 P40763 Q13322 P35968 10580 O60496 207 375790 9702 P17081 Q92922 P21333 O14492 9046 7422 Q12929 Q08345 2889 2885 8503 P01730 Q60FE5 6464 Q15768 Q13905 Q86Z14 Q6ZWJ1 8879 P00750 Q15532

regulation of ubiquitin-protein ligase activity involved in mitotic cell cycle 8454 11065 P51668 P14635 P51665 9184 991 26271 O60566 P62195 P63208 Q13042 Q92530 10213 Q13200 O43242 64682 P55036 Q12834 O43684 O00231 O00233 O00232 5706 5707 5708 5709 Q9UJX2 Q9H1A4 5347 P49721 5700 P49720 Q16401 5701 P60900 5702 5704 5705 Q13257 5682 5683 5684 5685 5686 P28065 Q13616 O00487 O00762 P35998 891 5717 P62333 P20618 8945 P25789 10197 P25788 P61289 Q9Y297 5713 P25787 5716 Q15008 Q9UL46 5692 8881 5693 O75832 P53350 6500 P62191 P28074 P17980 P28072 Q99460 P28070 O14818 P30260 5687 5688 Q9UKT4 P43686

regulation of cellular localization P25054 Q15654 Q16623 2550 O43557 P10599 9066 Q9Y4K3 94121 10657 54474 P37288 Q04759 Q9UBS5 P16220 P84022 51762 P20396 Q7Z6C1 O14788 6901 Q13485 P25963 Q9UBC1 P03950 4188 Q16635 65018 P50148 2316 P14174 7205 P00533 Q13370 4734 20 Q96EB6 808 Q7Z727 3783 Q8WVM8 23144 Q06124 Q9UBU3 6927 O60543 Q9Y6A5 5716 Q9H1D0 6262 4088 4089 1374 Q9BZF9 Q9NR96 P21917 P48380 222546 P20823 387 P21359 O60674 3784 1808 6812 Q9ULZ3 3667 6814 Q8IXZ2 P43489 P25098 7124 11142 Q96KC8 1385 29108 1020 P14635 O00221 114548 P51787 Q93062 156 1137 Q6PJW8 Q99728 11146 P61586 Q16555 Q15466 2904 Q96C24 Q9NYA1 P29466 P30559 11030 5991 Q07666 Q9Y4H2 O95361 O00233 23256 P36894 P10600 P13796 5071 P35568 1029 57678 10626 P01308 Q9NQB0 2917 P42771 P49841 4659 Q99835 P46459 P61925 P28223 6850 4792 Q14703 Q7Z7G2 Q96FX2 A1XKG3 O43612 2010 P30542 O60502 Q9HCL2 2247 Q92990 P01579 P00367 11124 Q9NQC3 Q9UN86 1956 P09038 Q9NQC7 P10827 Q9UGK8 P39905 P19883 P37840 P11234 P11233 2932 10724 Q9UMX1 P09601 50943 P53367 Q86UW7 7046 2033 P50416 O60603 Q9BXM7 6622 999 5898 7295 P46934 9908 O76070 Q9HD26 Q99750 Q9Y6H5 O00206 Q09472 P52298 7189 7067 Q8N726 P14416 64215 57120 891 773 Q00535 P17252 Q53X93 P01019 P01138 P01137 55503 O60260 P01133 P01375 8165 A4D1W7 8720 5213 P35225 27236 P35900 Q9UNE2 9495 P43405 P10071 5566 Q8HWS3 5569 Q9Y2B9 O14974 2737 Q92667 P24588 5460 23409 P05408 O00186 Q9UNN5 3162 552 553 O00194 5578 O15554 5338 P62158 Q92930 7099 7098 Q6IR47 Q13443 10460 P14923 64127 23411 P62166 3728 P21579 324 1540 P22303 10468 P23510 P21333 P01185 8754 P12830 P20336 Q9Y6E7 Q8NER5 Q96P20 P61764 O15455 P01178 P41159 O43581 Q60FE5 O75832 Q9BZS1 Q06830 580 P50402 P17612 O14939 Q9HC29 P43681 54106 9846 Q15653 P08237 P60568 P21554

RNA biosynthetic process P10914 Q92858 Q14686 4297 Q14209 P67809 Q9UKD1 Q9UPN6 O00268 Q6NZI2 O00267 P13984 P52434 P17676 Q9BYG3 2305 474 P16220 Q9ULW3 Q9H0D6 P20290 P03950 3091 P30876 O00716 Q16633 8543 Q16514 26205 O75376 367 51773 11198 6239 5705 Q16649 P35638 Q9ULX9 P61968 P27695 3661 P32121 Q12968 O75362 Q92830 P03372 51547 Q9UKW6 3659 Q13148 Q9BYU1 Q9UIS9 P35869 O75478 9533 P18074 Q99583 Q6P2C8 P29084 P36954 142 P19838 Q16665 P23771 9421 P78347 Q13951 O15160 8458 4772 O15164 Q12986 1022 1385 57661 P35453 P10275 4094 6829 2908 Q14494 5733 O75909 84365 Q13287 Q14134 6827 Q9Y2T7 Q9NRC8 4780 8467 O75575 Q14938 O00472 10987 27297 10625 1025 P08651 4775 P49848 Q92766 4790 O75444 25885 Q9H9Y6 Q9NR48 Q96T23 P61244 284119 51222 O14641 11243 9319 O14640 Q92994 9318 9439 P49716 P49715 11128 Q9UNY4 P10827 P10828 Q08050 7157 22828 6182 Q9NR55 1050 Q03164 10725 Q15370 865 P04150 4799 Q99814 Q9Y2W1 P78545 P78424 Q13873 O96004 6872 P29375 6871 P04637 P29374 2034 P53803 11108 P42224 P62195 Q16236 Q13761 P62875 1856 Q9UBK2 9232 22803 6883 5430 Q9P1Y6 5432 O95602 Q13887 P24928 Q03933 Q9Y6Y0 6878 Q6P1K2 8812 P49643 O43294 2959 408 P49407 409 Q9UBL3 7068 7067 5440 P49642 5441 6772 5442 Q9UKN5 Q15714 O43812 Q9UKN8 2961 2960 1870 Q53X93 5434 O60264 5557 P61201 5558 2969 P09874 6660 29777 23435 Q00403 2972

Q96RR1 P09086 P61218 Q15853 O95997 7764 P50613 P51946 23764 Q9Y5Q8 Q8WUY8 P22415 Q03468 Q02930 4800 P43115 2626 O95644 Q9NVC6 Q14653 23650 P19387 Q9ULK4 P19388 3054 29079 688 689 P23511 328 Q9Y5B9 4150 4152 P50750 2099 P52815 P35711 Q00059 O14802 Q96AE4 Q6P4R8 4149 P43694 Q9H5H4 Q9Y4A8 8880 P27540 Q15643 Q9P2Y4 Q15648 Q92905 P51610 O00411 O75031 5927 P63272 Q9NPJ6 9967 902 P21675

tissue development Q9UKT9 P25054 P08833 3881 O14793 Q12948 P17676 Q8NEY8 Q9UBB9 4729 2304 4609 2303 284217 P84022 Q92731 O14786 O14543 O14788 P08842 6901 Q9UGM3 Q9NZG7 P27361 54361 P20849 P42858 P16471 P21815 4851 4854 Q9UK55 2319 Q92838 Q8N205 Q7Z727 136319 Q9UBD6 P05549 P25391 Q9NRY4 O00167 6927 P20930 Q92830 Q9UJU2 Q9Y297 P03372 P40424 Q9UKW6 6926 P06401 Q92826 Q9GZX9 P31944 3791 P26371 Q9C009 P07737 P07858 5970 3673 P31947 2100 Q13705 Q9BYD5 9093 P56705 P17302 57534 5728 P20823 P17301 O60437 O60674 2697 P61296 5604 4637 P27037 4771 1020 O00468 P19404 10736 57669 1499 P15173 P61586 Q9UM47 P51671 O00238 P50222 P32856 Q14814 P09619 Q06210 P19544 O00358 P18206 3207 960 1147 841 Q9HCM4 O14753 967 4898 O95377 P04259 137964 Q9BQ95 2132 O60869 Q9NS68 P42768 2247 3215 2125 1277 O14641 O14640 857 5515 P04264 Q9H0F6 P10826 50814 P10827 P31749 1297 1294 P19883 27032 2139 1289 O95390 P37173 10728 P09960 5764 Q9UMX1 Q92982 P05121 P29317 6615 P02452 O96004 O43278 P13349 2033 P16615 23322 3484 P11047 P52952 P62993 79810 3479 Q8WV60 4209 5310 6885 P51636 1072 2280 Q15392 P48436 P01106 768 O43294 P01343 P49768 Q09472 P50552 P48431 5321 2054 3142 Q99623 891 O14727 Q00535 Q96S42 P35240 656 8945 P01019 6405 55504 P01137 8829 Q9UPY3 Q16254 Q9UJM3 P50440 P02462 P02461 6660 Q9Y337 8721 4000 5573 P04626 Q9Y696 2065 O95183 2064 P35222 O60716 Q01974 P17813 301 P60484 P10070 P10071 51156 11331 668 5566 6776 P02458 P01127 Q96EY1 6657 5328 O43474 O00744 Q9Y3M2 P01040 P07949 Q92817 Q9NX62 P15692 5578 Q15185 P05412 5351 Q9HCE7 P68032 23414 P37231 682 10229 51295 P60660 324 3169 5467 207 5468 Q02809 54206 7408 10468 5361 P24385 8754 6696 4154 7422 P56178 2099 O76011 3182 P15311 7414 10672 Q9GZT9 P26678 9839 5371 5493 P05997 7430 25818 Q8NHX1 P20908 23513 P13497 O60911 P17612 54583 Q08209 P58012 6699 10320 P26447 P25116 Q13477 5268 P15924 O75386 Q9UPN3 6591 Q9Y4K3 3084 O43318 351 595 Q04759 P09493 Q99807 P49810 5017 Q7Z6C1 Q7Z6C3 P49815 Q13485 Q8WUM0 P21246 P09017 Q9NPC8 3091 94234 P35527 5154 5155 8543 Q16635 79084 P58546 P13631 367 488 Q70SY1 Q9H2X0 24144 P46531 51535 P00533 Q15796 Q14344 Q15672 Q16881 P61968 6495 Q15797 6256 6498 P12757 P31276 P12755 Q9ULH7 P10451 Q9UBU3 Q03405 Q01469 P23528 Q96RK4 5159 Q15323 Q8WZ42 Q13263 P25940 4086 5054 4087 4088 4089 8323 Q14119 P18074 Q14118 P36952 P68133 Q86UL3 Q86YT6 P51813 P12645 P35625 P12883 387 Q4AC94 P21359 P31270 P08151 P20264 Q16665 53615 Q13275 P62942 9421 6275 9420 P49862 Q16787 7248 2909 P63092 P14635 P35453 P10275 4092 Q02535 Q93063 Q9UMD9 23363 Q9NYQ6 Q99729 Q16790 P45379 Q99966 Q13285 Q14254 P78337 Q86YZ3 9314 P36896 P36894 Q86WV8 7490 7251 Q9NZT1 7249 Q8WW38 P09067 1947 Q99958 Q15475 Q99835 Q96T37 P13646 P13647 A1XKG3 P13645 7020 Q08188 1832 Q9H1Y0 P21266 P08069 1718 10155 P02545 P47895 2926 1956 P09038 P39905 O43623 25776 Q02750 P11474 2810 Q14160 3909 P02538 Q99816 O95947 1969 P02533 P98194 1605 P78545 Q13753 Q13873 Q13751 9464 P22735 P41134 Q9Y3A5 Q1PSW9 P17275 P04083 91 P39060 P04085 Q6NVY8 Q92574 P42229 O15119 P98161 2947 3911 1856 Q9NNX1 9474 22806 Q96QB1 10939 Q02297 Q15828 7291 P10644 O43847 7052 P67775 P16070 P05067 P42574 P98172 7189 7067 Q06643 P19793 O75881 P29590 Q03014 Q13418 Q63HK5 P34820 1756 1634 1874 P17252 P21860 P21980 Q5TA76 Q14993 Q9NSC2 O95863 O95983 Q13422 P35908 O15156 A4D1W7 Q04695 2735 Q02388 Q04206 Q5T5A8 Q92786 P07585 2737 2736 Q15738 O95999 P19012 8174 9021 P78504

P35613 P00749 1896 P00747 P08123 4920 2626 P47712 P31249 P35968 Q14774 Q13443 26005 P14923 P11532 57154 Q68CZ1 3728 375790 P08138 P10415 3860 Q02363 57045 P19235 P35711 163183 P02708 2885 3611 Q04656 Q14790 3852 P43694 Q16610 Q9BQA1 O15111 O15353 O75712 Q15648 O15230 P55771 Q04771 P22528 O60353 Q9HCS4 P24821 O15105 3866

transcription, DNA-dependent P10914 Q92858 Q14686 4297 Q14209 P67809 Q9UKD1 Q9UPN6 O00268 Q6NZI2 O00267 P13984 P52434 P17676 Q9BYG3 2305 474 P16220 Q9ULW3 Q9H0D6 P20290 P03950 3091 P30876 O00716 Q16633 8543 Q16514 26205 O75376 367 51773 11198 6239 5705 Q16649 P35638 Q9ULX9 P61968 P27695 3661 P32121 Q12968 O75362 Q92830 P03372 51547 Q9UKW6 3659 Q13148 Q9BYU1 Q9UIS9 P35869 O75478 9533 P18074 Q99583 Q6P2C8 P29084 P36954 142 P19838 Q16665 P23771 9421 P78347 Q13951 O15160 8458 4772 O15164 Q12986 1022 1385 57661 P35453 P10275 4094 6829 2908 Q14494 5733 O75909 84365 Q13287 Q14134 6827 Q9Y2T7 Q9NRC8 4780 8467 O75575 Q14938 O00472 10987 27297 10625 1025 P08651 4775 P49848 Q92766 4790 O75444 25885 Q9H9Y6 Q9NR48 Q96T23 P61244 284119 51222 O14641 11243 9319 O14640 Q92994 9318 9439 P49716 P49715 11128 Q9UNY4 P10827 P10828 Q08050 7157 22828 6182 Q9NR55 1050 Q03164 10725 Q15370 865 P04150 4799 Q99814 Q9Y2W1 P78545 P78424 Q13873 O96004 6872 P29375 6871 P04637 P29374 2034 P53803 11108 P42224 P62195 Q16236 Q13761 P62875 1856 Q9UBK2 9232 22803 6883 5430 Q9P1Y6 5432 O95602 Q13887 P24928 Q03933 Q9Y6Y0 6878 Q6P1K2 8812 O43294 2959 408 P49407 409 Q9UBL3 7068 7067 5440 5441 6772 5442 Q9UKN5 Q15714 O43812 Q9UKN8 2961 2960 1870 Q53X93 5434 O60264 P61201 2969 P09874 6660 29777 23435 Q00403 2972 Q96RR1 P09086 P61218 Q15853 O95997 7764 P50613 P51946 23764 Q9Y5Q8 Q8WUY8 P22415 Q03468 Q02930 4800 P43115 2626 O95644 Q9NVC6 Q14653 23650 P19387 Q9ULK4 P19388 3054 29079 688 689 P23511 328 Q9Y5B9 4150 4152 P50750 2099 P52815 P35711 Q00059 O14802 Q96AE4 Q6P4R8 4149 P43694 Q9H5H4 Q9Y4A8 8880 P27540 Q15643 Q9P2Y4 Q15648 Q92905 P51610 O00411 O75031 5927 P63272 Q9NPJ6 9967 902 P21675

positive regulation of intracellular transport 5460 7046 Q15654 7124 2033 O43557 O60603 Q93062 Q04759 P84022 Q6PJW8 999 Q7Z6C1 P61586 Q13485 7099 7098 Q6IR47 Q9NYA1 11030 P14923 P36894 P10600 P46934 2316 3728 7205 Q9NQB0 P00533 P49841 4734 Q99835 P21333 O00206 Q09472 P52298 P12830 2010 P01137 O15455 1956 P01133 P01375 Q60FE5 4088 4089 Q9BZF9 Q9NR96 9495 P50402 P17612 2932 387 O60674 P10071 5566 54106 2737 P24588

positive regulation of ubiquitin-protein ligase activity involved in mitotic cell cycle 8454 11065 P51668 P14635 P51665 991 26271 P62195 P63208 Q13042 Q92530 10213 Q13200 O43242 64682 P55036 Q12834 O00231 O00233 O00232 5706 5707 5708 5709 Q9UJX2 Q9H1A4 5347 P49721 5700 P49720 Q16401 5701 P60900 5702 5704 5705 5682 5683 5684 5685 5686 P28065 Q13616 O00487 O00762 P35998 891 5717 P62333 P20618 8945 P25789 10197 P25788 P61289 Q9Y297 5713 P25787 5716 Q15008 Q9UL46 5692 8881 5693 O75832 P53350 6500 P62191 P28074 P17980 P28072 Q99460 P28070 O14818 P30260 5687 5688 Q9UKT4 P43686

positive regulation of gene-specific transcription6595 O96004 6597 6872 P04637 6598 P15923 Q12824 7161 O60603 25988 P52952 2547 P16220 3479 P62195 P84022 Q92731 10014 P25963 Q9UBK2 P56524 3091 22926 22807 Q15306 367 P46531 4851 4214 P01588 Q13133 Q15796 408 P49407 P01343 5705 O00206 6256 P19793 Q03014 6096 P41235 6929 Q53X93 Q9UJU2 P01137 O75928 Q13263 Q6IT96 P01375 Q9UPY8 P51531 4087 4088 7476 Q13547 P55055 2100 Q9NR96 P35222 P78317 P51532 P17813 3148 P10071 Q92786 Q16665 2737 9421 P26583 O00744 5460 Q15853 7124 1385 Q8TAK5 P19532

Q9UQL6 P10275 P10276 672 P22415 1499 Q99729 2626 7099 5591 O00755 7098 7376 Q92769 Q9NP71 P28370 Q14938 O95361 P10600 P18850 P19544 7490 P37231 10626 3169 P08651 5468 Q9NQB0 P17081 Q96T37 P12956 4792 22937 3066 3065 P78527 3182 P38398 Q9BQA5 Q92753 6721 P43694 10155 O15455 Q12770 P05112 P49715 5914 Q9UKS7 O15350 Q12772 P27540 P39905 Q9BZS1 7157 P19484 1050 54106 50943 Q13233 P21675

energy derivation by oxidation of organic compounds O75306 O95278 Q7KZN9 4695 O43678 O43837 Q9P2R7 4728 4729 O75390 Q99807 8803 6622 P22695 4720 5018 8801 4722 1738 4723 P05177 6901 4725 4726 Q9UBK2 Q13522 O95685 O95169 P28331 P46020 Q16635 O14561 P11216 P07919 P11177 O75380 1743 29796 4697 P00414 4698 4731 P46019 5162 O75489 3420 Q12968 O95298 4191 O95299 3417 1355 P31040 P62136 P03891 5836 3419 O00217 Q13423 P36957 Q16816 O75874 6389 P99999 O95182 P21912 Q04446 P62140 3421 4514 P31930 O75746 P56556 48 P06737 P40926 P13945 P63092 P50213 6390 P19404 Q93100 8604 P04040 Q16795 Q15185 3953 P07954 P51553 P14927 23530 Q9UGI9 O43920 P15735 Q06210 Q16718 10229 2632 P35573 Q15070 5500 4775 5501 6834 P48357 4535 P49841 847 P09622 P46976 P04179 7385 7384 P13807 Q9UGJ0 O00483 7381 P17568 4705 4707 178 Q9Y6M9 1431 P17174 P00403 4701 2805 4702 P56181 P41159 O75438 P53597 P51970 Q9Y375 Q15526 5499 P36873 P37840 2932 4718 4719 10728 P49821 4710 4713 4714 4715 P21399

cell-cell junction organization P25054 8650 5584 Q8TEW0 4771 Q8WUI4 P15924 7082 Q07157 4092 Q4VCS5 2702 1499 57662 P35221 P84022 P21926 51564 Q9Y4G6 Q02413 P14923 P10600 7094 P36382 P18206 3728 324 1828 Q6IQ23 3801 928 59341 O43815 1832 Q9BVG8 P35240 7414 P01137 50855 Q9Y490 8443 4088 Q9Y6R0 P35222 9253 P17302 56288 2697 O15105 P41743 Q9NPB6 O15228

multicellular organismal development Q9UKT9 P25054 Q9Y266 Q12824 8091 259266 A0MZ66 2304 2303 O60443 284217 P21926 2300 O14543 P05452 Q9UGM1 2308 2307 P25067 Q9UGM3 Q12834 Q12837 2317 P42858 Q99598 P39210 Q9UKV3 P61158 10661 P10809 2319 Q96EB6 Q5S007 136319 P27694 4990 Q06124 P17405 P37023 55584 Q9UKW4 4983 5830 Q9Y295 55109 3655 Q9Y297 Q9Y6A5 Q9Y6A4 Q9UKW6 3659 O75914 P26371 Q86SX6 3672 3673 Q92949 Q99583 Q13705 Q9BYD5 O60437 2335 Q99574 Q9UKX5 3667 P26367 O14578 P25098 1021 1020 Q06587 Q8IX07 57787 10614 1499 1012 3676 P61586 Q9Y231 3678 3679 Q9H0H5 3690 O00358 Q9BPY8 3207 1026 P17482 3685 O60890 Q92520 604 3688 P07686 A6NI15 P04179 Q9BQ95 Q15915 O60869 P16118 57326 3215 Q96N67 610 Q92990 Q9Y250 613 Q8N488 4548 Q9H0F6 Q92997 P10826 P10827 221937 P31749 O95140 Q8NDX5 1050 O60879 27032 P40692 3226 3225 Q9UL51 Q92982 Q15019 O96005 O96004 Q96A33 P56199 P14317 O00300 P50897 Q99523 639 O96018 O96019 P16989 P57059 1072 641 84033 79923 11113 648 P23276 Q16363 1080 P48552 6774 3265 346171 P12111 Q66K89 10238 P14780 P11274 O95180 P35240 656 8945 P01019 79956 55504 Q15047 84062 P50440 5451 A7MD48 Q9Y696 23552 3276 O95183 P35222 P35227 P35226 P57082 O75084 O75081 Q15051 P17813 53335 51156 668 6776 P24588 O43474 O00744 5460 P01042 P56159 4131 Q9Y5Q3 P01040 9821 4133 P25490 10690 9820 Q9NX62 79971 673 P61981 P15692 6304 P05412 P06744 O00755 5471 3290 Q9Y5R5 3297 Q9UBX0 682 10229 P60660 688 689 9825 5467 4137 207 5468 54206 Q92922 P06753 Q15078 90780 4154 4155 23509 P56178 P56177 O75051 P13010 4149 10672 9839 5493 O43464 Q8NHX1 Q12816 P35268 Q09666 23513 P50402 P13497 O14939 10682 8516 P27658 P60201 55558 9846 10202 P55268 Q14686 8533 Q9UPN4 Q9UPN3 Q9H165 230 Q9H161 P07196 P49810 P08047 5017 P83731 P49815 Q9NPC8 94234 4188 8543 5037 79084 P58304 P09382 Q9NY59 9869 8536 5029 51535 P00533 Q13370 Q15554 Q16880 Q16881 Q9BYV2 P35638 Q9HB63 P61968 P31274 P31273 P31276 Q9ULH7 23028 Q9NY61 Q9Y6Q6 257 Q96RK4 Q15561 P61962 1912 Q14232 P55290 1911 6383 5054 Q9Y2J0 Q8IWX8 Q9Y6R0 9412 6389

P36952 P68133 P37802 P35625 O75592 Q4AC94 Q8N4C8 P31270 23032 P08034 P43246 9421 Q14249 9420 P49862 7248 8577 Q9Y6K1 O75581 274 Q93063 P23229 51564 Q15583 Q14254 P78337 8100 7257 Q9HAZ2 O43521 Q15109 O43524 5071 7251 285 Q9Y6L7 7249 1948 1947 P02751 Q96NH3 8110 Q15116 P78364 P11229 5089 O75569 10152 O15066 23463 A1XKG3 Q96EV8 Q8WY64 P48507 P08069 10155 Q15125 1956 O15075 O15078 O75553 O43508 Q9NXR1 2810 Q92187 O95947 1969 Q14289 Q13753 9463 Q13751 9464 P29375 Q9H0M0 Q4VCS5 P22735 2821 Q9BUG9 P04083 P04085 Q92574 P56524 9474 O15496 O75534 Q02297 7291 7290 P16070 P42338 9001 P06239 Q06643 P56539 1994 60412 P00966 P06241 P21860 O95983 P55212 91147 P55210 A4D1W7 9495 3708 Q9HAK2 Q07954 P07101 P07585 Q14643 O75503 O95999 Q13315 Q03113 O43915 8174 9021 P35613 P35612 23085 O60488 P85037 Q9H9B4 P31260 P20594 P25800 O75508 P31249 Q8NEC5 Q13323 O43921 23092 3728 3720 P22303 9047 P13807 P12956 P34741 26576 Q9H4W6 Q9BQA5 P02708 2885 Q9Y6E2 P43694 Q9BQA1 132320 63976 5927 2896 23077 P43686 Q13351 P07148 5925 P10914 O95677 Q92858 O14672 9061 Q9NRW4 Q9UBB6 P52799 Q9P2R6 Q9UBB9 4609 P16220 5931 Q9UBC3 P27361 54361 P50148 P16234 Q9UK55 O15524 O15525 Q92838 Q92835 P29992 Q9UBD6 P05549 Q9NRY4 1121 Q7Z3K3 P53667 Q9BXC9 Q9GZY0 6809 27185 Q92830 Q9UJU2 P49116 P03372 Q92833 P06401 4627 P06400 P26012 P05556 Q92826 Q9GZX9 Q92824 3791 P07737 5970 5971 Q9GZX7 9093 Q9NRZ9 P56705 9099 P17535 81501 P20702 P17542 Q9BXB1 P61296 6812 10763 4637 3309 P04233 P34925 Q9Y586 O00468 O95231 O14627 10733 10736 Q6FHQ0 3796 P09972 4643 4646 5978 Q7KZI7 P08648 P30556 P30559 5991 93986 Q8ND90 Q8IVL1 1152 Q8IZT6 3329 3326 1147 Q92882 Q92888 P07766 O43175 P05106 P04259 O95257 137964 6850 2011 Q13618 27148 2010 Q13616 P30542 Q9NS68 57448 2006 P61244 O14641 4664 O14640 4665 5515 P04264 P05112 50814 O43186 Q12778 Q02078 Q6KC79 56121 Q13627 O00499 P30530 P52789 10726 P37173 Q99496 10728 P09960 P05121 4678 P40337 P04275 7704 Q9UK32 5528 P02452 O95278 4691 O00429 P04637 O43157 2034 2033 25861 P16615 Q01650 1181 P62993 Q14050 4205 Q15389 P01112 4209 Q14055 O43166 Q9C0K0 P48681 Q96CW9 Q9UNS2 P25100 4221 6885 23210 P35368 2043 Q9HDC5 Q9BT40 P50542 1191 56940 O76074 Q9NRP7 Q15392 84159 Q99873 4214 P01106 768 P01588 Q9C0J9 P01100 P50552 9921 Q86VW1 P14416 P13569 2054 P52735 O00444 Q5T5P2 P11387 773 Q53X93 6886 55626 6405 54778 P01138 P01137 P01133 Q16254 Q9UJM3 P02462 P02461 4240 5573 P04626 P04629 2065 2064 2063 P54920 301 302 P10070 P10071 5566 Q96PN8 P02458 P01127 5568 9927 2070 5584 O43597 Q92817 Q96PU8 O75190 Q8IZL9 Q14093 5578 Q8N302 P40763 P04201 5591 Q6IR47 5111 Q5HYA8 O76024 P80370 51295 P18846 324 79600 329 5108 P27797 Q05195 Q8N6U8 6456 6455 2099 P12277 O76011 P51843 P15311 333 5599 140735 Q9GZT9 P01178 Q96HC4 P05997 4287 25818 P17980 P52701 P15313 O60911 221656 Q08209 P58012 P18827 6457 10320 P60321 P26447 P25116 Q96HZ4 8650 4297 Q6ZT98 Q8TEW0 Q9BY76 8654 P30086 4291 351 Q04759 356 Q9UBS5 P09493 P62913 P09017 O43683 P20290 9510 5154 5155 367 Q70SY1 Q9UBT3 51654 Q14586 Q13258 P11309 6496 6495 6498 P11308 6015 P32242 253260 P10451 6491 Q9UBU3 Q03405 116113 P51805 51663 P09471 5159 Q13263 P25940 Q14116 Q13268 7353 Q14114 8204 7358 Q14119 Q14118 P51812 Q9Y618 P51813 P12645 382 222546 Q04725 Q04724 387 388 Q04726 23154 P08151 P20264 P60763 Q5VZL5 Q13275 P62942 P55347 9546 Q16787 8214 2909 7368 Q14129 Q9Y5I4 23126 O43638 396 Q2MV58 Q02535 397 Q99962 Q01201 Q99963 10296 11146 Q15466 2904 Q16790 Q99966 Q15464 Q13285 54 6045 Q15468 P60709 Q16799 Q86WV8 Q6PRX2 P33151 60 P09067 Q9UBP4 O15169 51218 85440 Q99958 Q14145 Q15475 Q8TB22 Q8TEY7 P14859 P21145 P01579 2926 P09038 Q14155 116150 10280 P22492 O15198 O43623 25776 Q14160 Q6R327 1605 86 P78423 P78424 O15123 O75665 Q15818 1613 27121 Q8TAF8 Q9Y5E8 P41134 Q86WK6 Q1PSW9 27122 91 94 P46821 O15119 Q9Y5E9 2947 Q9NSA3 Q12797 Q13643 9114 Q9NNX1 Q13642

Q8WWH4 Q96QB1 O43602 Q15828 P55318 Q99081 P55316 O43609 P67775 P05062 P08575 P05067 Q9BXL5 1627 2956 Q9UBL3 P29474 92129 29767 Q03014 Q7Z3S9 6096 P34820 P55327 1634 56034 P21980 Q14993 1639 Q9UBM7 Q9NSC2 P11717 8289 9133 O15156 9138 29777 Q9BXJ9 57379 Q8HWS3 Q15853 Q9UJV9 O75626 Q6NYC1 Q9H488 P41182 P41180 P53567 P30048 84525 P05091 1653 P08123 Q92499 Q9UJW2 Q13683 O75629 P47712 150094 Q68CZ2 Q07820 Q68CZ1 Q07817 P20248 64919 80155 Q9UJX0 P08138 P09429 O75604 P10415 3860 P19235 P35711 23181 Q7Z494 P08581 3852 P09430 80184 P08588 P41159 O15111 P55771 Q04771 1203 O15105 P45983 3866 Q13233 P21554 Q7Z2K6 65125 3880 3881 O14793 2550 P51587 P17676 2547 4729 P37288 Q8IYN9 P84022 Q9HCJ2 Q92731 Q92730 O14786 O14788 Q8TDD5 6901 P28799 Q9NZG7 P08727 Q92729 P51114 Q96KN7 P20849 493856 4734 Q8N205 Q7Z727 P28300 81629 O00167 Q7L5Y9 6927 Q8IUD2 55342 6929 811 P40425 Q5TAP6 P40424 P62258 6926 P31944 Q9NZI7 Q9NQX0 Q9C009 P07858 P31947 2100 P48380 O95343 P17655 57534 3428 6938 P20823 O60674 P20827 388677 O60675 P62263 Q8N5V2 5604 P41743 Q9Y468 P11926 P27037 4771 Q9Y466 P39019 P19404 55366 6949 27283 P15056 27287 Q6ZNA4 Q8N264 P07437 P50222 P32856 Q92769 Q14814 P09619 5631 P28370 Q9NZC7 P10600 Q8IYR6 O95365 Q06330 Q9UEE9 841 Q6PI57 Q9HCM4 4775 O14753 846 7804 Q92766 P09622 O95377 4791 1285 2132 Q96KR1 Q96L34 Q9NQS3 O95376 2125 1277 Q92753 Q5TAT6 Q92752 P05230 857 859 Q92754 O00139 50937 Q13503 P11912 Q9NR50 1297 Q13509 1294 P19883 Q13506 Q9P202 O60636 Q9HCK4 2139 861 56243 1289 O95390 863 4313 Q9UMX1 P09601 4318 P06576 6993 O43278 23322 3484 3482 3481 23326 P11047 25988 203068 3479 2147 Q8WV60 7832 P01236 55723 10014 3010 Q9BRQ0 3009 Q99750 Q01995 O43294 O43293 Q13099 O43252 Q09472 O00562 P53701 2173 P13686 23787 890 Q00653 891 Q99743 O14727 894 Q9NR09 P53708 898 6525 P49639 Q9UMS4 P51531 8721 Q658W2 P51532 25975 P14543 Q01974 Q96ST3 55755 3028 P60484 11331 4358 5207 P26583 Q9Y3M2 7402 23764 25942 25945 Q8NFW5 10458 P60891 P31939 10451 P55075 79739 10454 Q8N5U6 7410 10460 7412 Q9HCE7 P52803 Q08117 Q05086 3052 P37231 P36382 Q01955 Q96SZ6 P51959 444 445 Q02809 P48357 7408 10468 P24385 P24386 8754 7422 9600 3066 22895 O75293 Q00613 P61764 7414 3070 7430 9612 Q9UIG0 3074 10447 P17612 466 55796 Q9NQL9 7428 6595 6597 6599 P18085 8775 6598 5268 O75386 O75385 Q86VQ3 6591 Q9Y4K3 3084 O43318 472 473 474 Q9C0C7 Q99807 51763 P43034 P21246 Q13127 Q15303 5274 3091 Q16635 Q15306 3099 P58546 P13631 Q9UJC3 5270 Q02447 Q9NVP2 488 Q05952 28981 Q9NRH2 Q9H2X0 24144 6117 P22102 30813 P46531 Q16643 Q15796 P43026 Q15797 P12757 Q8N6I1 P54198 P12755 O75360 Q01101 498 P31040 Q9NRI5 O94805 Q15323 Q13144 Q9BWQ8 Q16650 134701 P35869 9657 7476 8323 P18074 P10586 P10588 P13611 P10589 59277 P13612 93166 200894 54820 P23416 Q16665 P55061 Q8TDY2 O15287 Q16666 P54132 P63092 P35453 P09172 P46109 P23467 8328 Q14012 Q86W54 7014 P46100 Q9Y4H2 7490 Q9NZT1 P11021 Q8WW38 Q9GZM8 51339 Q9NRD5 Q99835 9201 9202 O75326 9203 P13646 Q02880 P13647 Q86VS8 P13645 7020 Q08188 51341 P02549 55704 P21266 1718 P02545 P47895 347733 Q8IUX7 Q8IUX8 P38936 P11474 P57682 3909 P02538 Q6PID4 Q99816 Q99814 P02533 P98194 Q15375 P60842 P78545 7046 P98155 P78540 O43711 55283 6194 P32302 60626 3913 56134 65082 1738 P05177 P98161 P07355 P98160 O60282 3911 3912 Q2Q1W2 P78536 7057 Q13520 22806 Q3V6T2 7052 P19338 3925 P46934 1742 P46939 81565 P42574 P98172 P98174 Q14865 8399 9244 7067 Q13535 P19793 P29590 Q15717 Q63HK5 P31431 Q01196 1756 29880 P41231 Q96GM5 57003 O15259 O60264 Q9NRA1 3932 O60260 9255 Q04695 P19320 Q8IYT8 9253 7074 P43405 Q9Y4F9 P20309 Q9NZR4 Q04206 Q5T5A8 Q92786 Q92784 7088 Q9NZJ7 Q15738 Q15735 P78509 7082 P78504 3958 P00749 O00192 P00747 P08243 3953 Q13563 3956 2626 Q15742 Q9NQZ2 P14923 7090 P11413 1789 1788 Q05516 P62166 P00734 375790 P08253 Q5BJF6 Q15750 Q9HCU4 Q8IUQ4 57045 P78527 O60229 P20336 Q04656 51741

Q5T9L3 Q16610 O15232 O75712 O15230 Q15768 Q06830 P22528 Q9UNA1 2651 Q9HCS4 4830 900 51752 Q9HCS7 P00750 P08238 O15228 P08833 Q8NEY1 Q9NYD6 2672 Q12948 22994 O00268 Q8NEY8 Q9BVA0 3516 Q9BVA1 P60174 Q9ULW3 Q9UM82 P08842 P07996 Q12950 P52565 Q12952 51090 P52566 Q8NB12 Q08431 P63211 79885 P21810 P16473 2676 P16471 O60331 P21815 4851 4854 5702 5704 928 P54753 P53420 Q9ULX9 Q12965 P25391 Q12968 Q8IXK0 Q05397 P20930 29127 P20936 Q9UQB3 4862 Q9UQB8 4867 P54764 P54762 P54760 4882 P48023 Q7L576 22985 Q13829 P17302 5728 P17301 Q9HBE1 Q99697 2697 10523 Q9Y342 P28482 Q5D1E8 1385 O95470 57669 P15173 P07550 Q9Y2T1 P08887 Q9UM47 P51671 O00238 Q96SB3 Q9NYA1 P51679 P10721 Q6UB99 Q06210 Q5VST9 P19544 Q8IY17 P18206 960 967 4898 Q9UM54 5747 Q06203 2253 Q9NYB9 Q9UHL9 Q9H9Y6 6609 P62330 57689 Q9NPQ8 P42768 2247 1399 2246 6601 6605 P51693 P29323 P51692 O00255 Q03164 P17342 5764 P29317 6615 P06213 Q9Y6W5 P13349 2275 P52952 O60725 P15531 991 79810 998 999 P61421 P02686 P62753 Q04917 P49770 Q9UHG2 5310 Q7Z5H3 10141 5311 Q9UHG0 P51636 P53814 2280 Q93096 Q04900 2277 23451 Q15154 P48436 P28845 P01344 P01343 Q96NL6 P49768 2290 P25791 23429 P48431 5321 3142 Q9NU63 Q99623 O60701 Q00535 Q00534 Q96S42 7975 6647 54535 5316 8829 5318 Q9UPY3 Q8N960 P01375 P01374 6660 Q9Y337 4000 8841 Q9UHI8 23432 O00213 O95067 Q96AQ6 O60716 P12004 Q9P0I2 3149 3148 545 546 3146 P40933 Q96EY1 Q96JB5 6657 5327 5328 P49747 6670 P24043 P07942 O00628 7520 8851 P07949 P07948 Q8WYA6 Q12904 P20916 Q9H9S0 Q07157 3162 Q8WYA0 O00622 O95076 Q9BZE0 552 Q99684 4004 6667 4005 5336 Q96AX1 Q15185 5338 7518 P26232 Q7Z5L4 Q969G3 5351 7532 7531 7533 P68032 23411 23414 3171 10107 3169 Q9NPH5 4015 Q96JH8 10100 7528 5361 5362 Q86U86 6696 5364 4035 P49321 Q9UPT9 Q9BUZ4 P12036 3182 P62736 P26678 Q01826 5371 P27540 P20908 P16401 P16885 54583 8879 P62745 6699 P60568 Q13477 8892 Q9UQP3 P15924 P15923 29948 29947 595 8407 Q7Z6C1 Q7Z6C3 Q13485 Q8WUM0 8408 Q8TD31 P03950 9750 P35527 O94910 Q13009 4067 P12429 Q15669 O75496 Q5TAB7 51412 P60983 Q96SN8 6239 Q13490 Q14344 Q15672 7101 6256 O43897 Q2M1K9 Q01469 P25705 Q9UDY8 P23528 Q9UDY6 Q8WZ42 P00441 51428 4084 8443 4086 4087 4088 P22692 4089 163126 Q86UL3 Q86YT6 Q02790 P12883 P21359 23396 1808 P24864 P62701 10087 6259 53615 8454 P54259 7123 8452 6275 P54257 4099 7124 P54252 Q8TD84 7126 P14635 64759 Q9UQL6 P10275 O75462 4092 4093 P35579 154 Q5JUK2 Q99720 156 Q9UMD9 23363 Q9NYQ6 Q8WV28 Q99729 Q16555 10059 Q13045 Q15223 P45379 Q86YZ3 8467 9314 P36896 P36894 P15976 P35568 1822 166 120892 338917 Q9NQB0 Q99717 Q99715 Q99714 Q96T37 Q8TCU4 7142 P61803 O95813 Q8N5K1 P35555 Q86V24 P36888 7141 1832 Q9H1Y0 Q00577 Q16576 Q99707 Q9NQC3 P49715 P60953 7155 P39905 7157 Q02750 7150 P35548 8481 Q9H295 Q9NZ56 Q13075 Q16586 84376 Q5VTD9 Q15256 O75427 Q13873 7167 P10636 26037 Q86Z02 Q01094 P63172 7161 7163 2702 56259 Q9Y3A5 P47914 10935 P17275 P23945 Q4KMG0 P39060 Q6NVY8 1859 P22612 P42229 1856 Q13885 O94992 Q13887 10939 7170 Q14738 P10644 9351 O43847 9350 79365 P39059 1869 7189 O75881 10908 Q13418 O43815 7182 1877 1874 P17252 P68871 P09630 O15379 Q5TA76 O95859 Q9NZ08 P21741 O95863 Q13422 O95861 Q13428 P35908 Q13426 P10620 9371 Q8NB78 4914 2735 1400 Q02388 56288 2730 Q96FJ2 1406 2737 2736 Q8WUI4 P19012 Q02386 1896 2742 P09211 4920 Q13444 P35968 Q14774 Q13443 26005 P11532 57154 27339 P30279 27330 P23515 57159 O14492 Q9H228 O14495 P12830 Q02363 P18146 Q03692 163183 3611 Q14790 P00403 O60341 P01730 O15350 O15353 Q15648 P19484 Q06710 O60353 P24821 O14497 O15347 4953 4957 Q9BZR6

positive regulation of cell proliferation Q9NYD6 O14672 P15923 Q6QNY1 Q9Y4K3 3084 3516 2305 595 P37288 4609 Q04759 356 8767 O14786 P78395 Q9Y275 P08842 P21246 P28799 P27487 P03950 Q15303 3091 5154 O00716 5155 Q12834 4067 4860 P46781 79084 P58304 O95684 Q08431 P16234

P62244 P16473 P14174 6117 P46531 4851 P49006 P00533 Q13017 Q7Z727 P27694 P32241 Q9BQ51 4193 Q9Y6Q6 27185 6929 2324 Q969Z0 5159 811 Q9NRI5 55109 P03372 P40424 6926 Q6IT96 P55290 4869 P32239 Q14116 3791 P46087 P43490 7476 5970 3673 P48023 9093 P16333 P17301 O60674 Q8IUC6 P08151 P20264 P62701 823 Q16665 3667 826 P04233 P43489 P42830 P11926 8452 9420 7124 P28482 P54132 Q9Y586 P11802 1021 P14635 391 64759 O43639 P10276 394 154 1017 1499 O60884 1012 P07550 P08887 P51671 3690 Q9NYA1 Q92769 Q15109 P22392 P09619 Q9Y4H2 P35443 P36894 P10721 P35568 Q06330 57678 1026 961 Q13291 P01308 338917 2475 Q9Y240 604 1947 Q99835 P07766 P00491 P05106 O95136 P30305 P28223 6850 P11229 4673 23463 2253 Q13618 P36888 57448 Q9HCL2 2247 P15153 2246 P60033 Q00577 975 P01579 P08069 P05230 P05112 1956 P09038 P23443 Q08050 P51692 P38936 O43508 3350 7272 56005 138151 3226 P37173 P02538 Q6PID4 5764 P09601 Q14289 P06213 P04632 4690 Q13873 7046 Q96RE7 6198 P15408 P14317 Q08722 Q86Z02 Q13639 3481 P11047 P52952 O60725 P15531 P42345 P42224 991 994 3479 Q1PSW9 10935 Q15022 P39060 P04085 6504 P01116 Q6NVY8 6869 P42229 P15090 O15119 51009 Q9NSA1 P01112 3912 P01111 P56524 P78536 P25100 9238 Q13887 Q02297 1191 7052 P55316 P57735 P84095 O76071 641 P43307 Q96GN5 2277 P01106 P08575 P01589 648 P01588 P98172 P01344 P01343 11116 Q9UBL3 O00206 2290 7189 P48431 P29353 6772 5321 P35916 P14416 3265 O75881 Q13418 3142 P55209 148022 890 891 Q99500 894 56034 Q9NR09 Q00534 Q96S42 P17252 P01019 Q9GZP9 P21980 P01138 P01137 Q9NRA1 8829 P01135 P01133 P01375 O15151 4240 P78552 P04626 Q13547 Q13426 P35225 P19320 2064 P35222 P35226 2735 O75084 P43405 3148 P10070 Q04206 Q92786 6776 P01127 P40933 Q96EY1 6657 2736 O43353 P26583 P07942 P07948 P41182 O43915 3162 Q969H8 P30048 552 P15692 5578 P55075 7518 P08246 Q8N302 P47712 Q96FA3 P04201 P05412 7099 O00755 6320 Q6IR47 P35968 Q14774 Q68CZ2 Q9NP71 Q3SY56 7412 9391 23532 P30279 5590 Q07817 P20248 Q00987 Q05513 P18847 688 5467 P08138 P27797 P23510 P24385 6210 P01185 7422 P10415 P56178 2099 Q02363 3066 65108 3065 57162 3611 10673 Q16610 P25103 Q9BQA1 5914 P27540 7433 6464 Q15648 Q86Z14 P08908 23512 Q96BF6 4839 Q9UHV2 2896 P24941 P07384 4830 4953 9846 P25116 P60568 P07148

localization O75947 Q9Y266 259266 Q5SQT9 Q5SR56 5817 2304 81855 27089 Q92973 10651 Q9Y275 Q9UGM1 Q92974 Q9H4L5 Q9UGM3 Q9Y277 O14561 Q8IWJ2 2317 2316 1468 P42858 27091 P49006 O15400 5825 Q9Y282 Q8WXA8 Q12846 P61163 P37023 81876 55584 P62491 27067 Q9UKW4 4983 5830 3655 Q9Y6A5 10632 Q96EA4 Q12851 P26374 Q9H936 O15431 3672 3673 Q92949 Q13705 P21917 P21912 Q96N16 55110 2335 2332 Q9UKX5 10640 10642 O14579 27072 O15427 Q9UKX7 O75915 O75901 Q92538 5861 Q9UGH3 5862 1020 114548 P51787 P37088 1017 2348 1499 1012 3676 3678 P61106 Q9UL26 Q9H0H5 Q9UL25 3690 Q8WXF0 5870 5871 P33947 Q15907 Q9UGI9 Q15904 P51797 O00358 P51798 Q8TBC3 P20645 Q8NA29 Q96MV8 O60895 P20648 3685 6711 O60890 604 5865 3688 4535 3689 P05023 P07686 Q6NUT3 5868 5869 P05026 Q12884 P53985 P19634 P40222 401505 610 Q5T2W1 Q6NUQ1 5877 6726 5878 Q8TBB6 5879 P53992 P10827 Q9UL45 P31749 O95140 55153 27032 84932 253980 P40692 Q9UL51 Q96ED9 P63313 Q92504 Q9UL54 Q9H4M9 Q8TBA6 O95159 P56199 O96008 Q9UKL6 P50416 Q8NE01 P50897 Q99523 1058 O14949 8924 Q15021 55967 637 5898 6748 79939 6747 Q9BTU6 O96018 4580 P06702 Q15027 O95169 Q9Y678 P57057 P36575 O95166 1072 O75096 Q99996 51133 55973 Q14185 P47224 Q15036 P49407 P02794 11116 1080 O00327 Q9Y5W9 P48552 P48553 3265 3263 10239 Q5VIR6 114571 O95180 Q9Y5X1 8943 P48544 Q9Y5X2 55503 O14964 Q9UP83 84062 6780 Q9Y5X9 Q9Y215 Q15049 O14980 Q9Y696 O95182 O95183 P35222 P17813 Q15050 6776 9804 4116 P24588 P01042 6311 P05408 245972 O95197 Q99567 P61981 P15692 O14908 Q96A72 9818 57706 Q92930 P05412 O00755 P49450 23539 5473 5476 23530 Q08357 10228 Q9UBX3 P60660 Q15070 Q15077 5467 P49447 207 5468 Q15075 P62841 10226 Q15078 Q9H902

Q9Y5S1 P30825 Q12809 Q9Y5S9 Q8NHX9 O75051 Q96QU8 O14925 215 10673 P56181 P56180 9839
6342 6341 P06729 Q9UKK6 23513 O14939 51199 56882 Q96QT4 6337 P40616 Q92900 10204 326624
Q14204 Q16864 O75146 Q9UPN3 23048 P35658 Q9H0U4 117584 P07196 Q9NPC1 P49810 Q9BQG0
5018 P49815 Q9NPC8 Q9HAV7 Q9HAV4 P04920 8546 4188 79083 93380 P11215 Q9H0T7 Q9NY59
Q96RL7 8536 Q9H9E3 P00533 11079 9868 Q15554 Q9HAU5 Q9BUN8 Q16881 7220 9883 P56134
P32121 23025 Q9Y6Q5 Q96IZ7 4193 Q9Y6Q6 51542 Q96RK4 8548 P02741 P61966 P55290 8563 6386
Q9Y2J0 129531 P62826 Q9UPQ3 6389 P62829 Q86XR8 Q9UQ26 23039 Q9NXV2 Q9NY72 Q8N4C7
Q8N4C6 P61970 23032 51552 Q6PKD3 Q5SRE5 P08034 Q03518 O95907 P62820 P56556 P78348 11021
7248 P36542 6390 O75581 274 51560 P23229 P02766 P78334 11031 P05814 O75575 5071 7251
Q93050 152789 9429 7249 P48995 1947 P02751 Q86Y82 P02790 Q9BV36 10152 O15066 23463 A1XKG3
291 292 Q96EV8 Q14C86 Q9Y6M9 P24539 Q9NY26 Q9H9H4 P02786 Q6I9Y2 1956 2802 O15075 Q9NPA8
O15078 O75558 26985 O75553 Q8N4H5 O43508 P37840 P11233 Q9H173 Q9NXR1 P49821 79901 11014
10166 P02775 Q14289 P02774 P25874 9465 9463 Q4VCS5 P53007 Q9Y6G9 Q92572 55048 P04083
Q9UGU5 Q92574 P31689 Q13769 O15496 7291 55054 9472 7295 117177 83985 P16070 O15020 9001
P06239 Q9Y6I3 P56539 60412 P06241 50618 Q8WXH2 A4D1W7 9495 79023 Q9Y2B5 3708 Q07954
Q9UH92 O15027 Q9UH99 O95995 O95994 Q14643 Q96NA2 Q9NP61 8170 P35610 P35612 P00505
23085 O60488 Q9H9B4 Q9BUK6 Q9Y6C9 1537 Q8IWV1 P35606 Q8NEC5 P56962 Q9NP72 O43920
Q86XP3 Q96IW7 83547 3728 Q9Y6D6 83548 23095 Q9Y6D5 Q5QGT7 O60499 O60493 5901 Q6P5W5
5902 O75969 O75964 P13804 P45880 P33897 3737 Q9BQA5 P02708 2885 Q9HAP6 5911 Q14674 5914
131474 Q9NP90 Q14677 A4D1S5 63971 11097 Q9NP97 222068 79065 Q9H0N0 P24941 P43681 51510
Q96RF0 51517 23603 Q92858 23607 Q9NRW1 Q9NRW7 Q99490 Q9P2R6 O60566 154091 O14662
10772 P27348 Q9UBC1 Q9UBC3 Q9UBC2 Q6PIS1 54361 O95684 55696 92609 Q92845 Q9NWB1
Q9UBD6 3783 1121 339122 Q9BXC9 Q9GZY0 4628 57403 10758 6809 27185 O60543 P18859 Q86WA8
O15533 Q92834 4627 P53675 P05556 91782 Q92824 P55851 Q9H814 4641 1130 P56705 P53677 57410
3301 1122 3784 6810 6812 6811 6814 10762 4637 P04233 P07307 Q9Y584 P50570 O95239 O00461
3320 Q8IVM8 Q9NRS6 10736 1137 3313 O00471 3312 Q4VX76 4643 4644 4645 5976 3799 4646 P08648
P30556 P30559 5991 O95249 Q9Y597 P53618 Q8IZT6 Q9NS56 P37198 27173 P05141 Q92887 P53621
O95259 P05106 P05107 P28223 6850 O00483 2495 Q13618 Q12769 P30542 P17568 81555 80223
O60502 Q9NS69 Q8TEH3 O14640 5514 P26038 738 P09958 P30536 1176 Q05586 1174 1173 Q6KC79
Q60I27 O00499 10725 10724 55275 Q8IVI9 10726 Q9BXF6 Q99497 4678 P40337 O15504 7704 P02452
4690 O95279 P04637 O43157 4695 1186 P16615 1185 O95271 Q01650 1181 Q00796 P62993 4686
P01116 Q9C0K7 6869 Q15389 Q15388 P01112 P01111 Q96HA1 Q9C0K1 O43169 P48201 23210 Q6P4A7
23212 Q9HDC5 23214 P50542 1192 1191 Q96Q15 9908 Q15392 84159 4697 4698 11231 768 Q96CW1
4218 6890 6892 Q86VW1 P14416 P13569 P12236 2054 P52735 P33176 O95298 O95299 Q96CV9 3382
P12235 9919 773 Q02952 P61204 Q9GZP9 6405 P01137 Q7Z3C6 P01133 4240 P01130 Q8N2H4 23673
P35348 P54920 781 Q9NRR8 O14617 301 302 Q8IZE3 Q7Z3B4 P01127 309 9927 25828 O43592 5584
Q5U0I6 64116 22794 P15328 Q9UNN5 Q96PU8 Q2M389 Q96PU5 8604 5578 5579 Q9H3F6 5591
Q9Y4P1 9950 Q6IR47 5111 P37108 Q9BSW2 5590 Q99437 P16671 8615 P46059 5108 P27797 7781
O43572 64130 9961 7780 P01185 6453 6456 6455 O00400 P50993 P51843 Q9NW64 P15311 Q9NVU7
Q96PS8 333 56993 P83436 P25103 140735 5119 A5D8V6 P01178 23636 9972 Q9UNQ0 4285 Q5T1M5
64145 9973 O00410 23633 P15313 Q6ZWJ1 Q08209 6457 Q99418 P25116 Q8N6T3 8655 Q8TEW0
O43678 351 Q7L804 23163 P09493 23165 P23786 P62913 Q96QK1 P25963 11190 O43687 9997 5155
O43684 11196 19 367 20 22 Q15436 6496 Q6Y2X3 6495 8677 9525 9522 8676 Q15438 23144 P57103
Q9UBU3 P51808 375 5159 9515 P23763 P61619 P32239 5174 Q14114 8204 Q8WWN8 Q14118 Q9Y619

381 382 P59780 388 P20264 9527 41 Q13275 11140 9546 O43633 Q9H089 O43639 Q99962 Q99963 Q9H082 Q13283 Q16795 2904 Q16790 Q13285 2902 Q13286 5195 9554 7375 Q09161 O75694 Q86WV8 9550 Q9Y5J6 Q8NI27 51693 Q9NWN3 8218 51699 Q6PJH3 Q15475 P46459 9564 7385 7384 Q8TEY7 7386 Q9Y5K6 7381 O43615 Q53TN4 54732 Q6VY07 P08183 Q9UBQ0 P21145 Q9UBQ7 P01579 11124 9559 P09038 Q14155 2923 Q9BY84 10280 P22492 Q9BXW6 P14867 O75674 Q9Y5L4 25776 Q8TEX9 25777 Q14160 Q9NX14 Q00325 P08195 84134 1605 Q14964 Q15811 P78423 Q13637 Q96QC0 Q13636 Q15818 Q9H444 84958 P41134 79139 94 O15119 O15118 Q9UBK9 Q14974 9114 O15131 O43602 9590 Q9H7Z7 55172 P43307 29796 P52292 O15126 P08574 P08575 P05067 P52298 P29474 Q15836 P52294 Q8TEM1 26258 Q03014 9120 6093 P55327 80145 Q9UG63 84502 P21980 Q9NSC5 9135 P11717 Q15843 O15155 Q15849 64901 Q9UBN7 P30049 9146 Q9UBF2 Q6NYC1 P41182 P41181 3836 Q6ZSS7 O15554 51606 Q68CZ2 Q16718 Q07817 P21579 Q9UJX2 Q96QF0 3841 P08133 3843 P09429 Q9BY11 P10415 93492 387680 4705 4707 P09430 4701 4702 80184 9179 P33527 51629 Q96QD8 Q04771 P62072 Q96QD9 23192 Q8TAG9 4718 4719 Q8TF09 O15105 4710 4713 4714 1207 4715 65125 92745 25909 9182 22872 9181 Q96KP1 O95793 9184 O00148 P15498 4728 4729 Q8IYN9 1213 P84022 79778 Q9UMY4 Q92731 10890 4720 P60059 O14786 3875 4722 O14788 Q8TDD5 4723 4725 4726 P27487 P28335 64284 P28331 Q92729 2562 P55957 9197 P38117 O00159 10427 2554 30011 57510 55334 4731 Q9H2G9 Q8N695 4734 253725 Q7Z727 Q5T0N5 Q96P70 6927 2569 Q8IUD2 55341 O60664 30000 2564 811 Q9UJ41 P63104 P62258 6926 Q9C005 P31946 P99999 2100 56681 P48380 O95343 Q676U5 P16333 81622 P20823 Q8NFW9 284427 80777 O60674 P18507 1244 821 Q96GD4 5604 P41743 91452 Q9H2M9 2110 Q8NBW4 55361 O95352 P39019 P16389 P19404 2108 O60645 6945 P32856 P09619 Q9NR31 22841 27297 10868 Q9HCM4 7804 4792 Q9H2K2 Q8IUH5 Q9HCL2 P60033 1277 124565 857 Q9UN86 859 Q8N1S5 Q9Y490 Q13501 P51572 P51575 Q13505 92799 P51571 Q9NQT8 Q9P209 Q8NBS9 1289 O00141 Q9UMX1 P09601 O14777 P08754 P06576 Q86UW7 Q9Y3Q3 P01241 6993 10490 Q86V81 6510 3482 23325 Q9UIH9 P11047 O60603 25988 23327 55722 2147 Q96KG7 10013 Q96KG9 10015 6505 11345 135295 23339 6520 6522 Q9NV70 3010 25998 Q99755 400 Q8NBI2 402 Q99750 55737 406 6517 408 409 Q99757 O14713 Q01518 54407 Q15276 P60468 23307 Q9UMR2 116986 O00560 116987 O00562 23303 23787 23788 P13693 891 79711 Q15286 Q16134 Q8NFP9 Q9UN42 10476 P03891 10478 P61326 11325 O43264 4363 6541 10482 Q658W2 Q8N1F7 Q8N1F8 25978 Q96ST3 Q01970 P60484 Q96T51 10487 11336 P31930 P26583 O00505 Q9Y3M2 Q6FGG2 7884 Q9UID3 23760 Q641Q2 23762 O43237 25942 P14598 Q8IYJ3 433 A8K0Z3 55763 10451 6548 7879 10452 8724 P55072 Q05084 7410 10460 6566 7412 Q9HCE7 Q05086 Q8WUX9 P37231 10469 P36382 Q99797 79720 55770 Q8IYI6 10466 Q6IQ22 11315 P01266 P24386 6575 9601 7422 6576 9600 Q8WUX1 Q8WV92 65108 22895 Q96P20 Q8WV83 Q00610 7415 P61764 7414 7417 7416 7419 Q8N5S1 5250 P51970 7430 8766 6103 3074 7428 P40855 7442 Q16623 P18085 8775 P10114 P10599 24137 O75385 473 474 476 477 478 51762 23286 Q99805 P43034 Q15785 P38919 8301 9632 P13637 64601 23299 481 Q9UJC3 483 O75380 O75381 488 Q05952 Q13133 Q15796 Q15311 O00602 Q9UNL2 Q8WVM8 23265 490 Q96H78 495 498 Q01105 P31040 P56385 Q9NRI5 P56381 Q13145 Q9BSI4 7476 P13611 P10589 P13612 O75351 Q86VN1 Q9NZZ3 Q9H2V7 Q8N6H7 144983 Q709C8 Q9NVR5 54820 P23416 54822 9648 P43487 Q49AN0 Q9P2E9 51319 P43005 149371 O75348 11261 O43752 Q14008 P63092 119559 P13667 Q9UEU0 P09172 84248 Q9Y4G6 O94826 Q14493 54832 P27824 11269 Q14012 9673 7014 23256 P35443 Q96D46 Q7Z7H5 Q9BX79 Q9GZM8 Q9NRD5 Q99835 9201 9685 Q86W42 Q3ZCQ8 Q7Z7G2 P12314 Q02880 O43734 Q86VS8 O43739 23225 Q9Y4I1 Q9UNH7 Q15363 Q9C0E2 55704 1718 P02545 347733 P23443 55705 Q8TE73 9212 7037 Q9BX66 P21283 P21281 P55011 P10147 O43747 P48739 Q13190 Q6PID4 Q99816 P98194 P28288 O75306 7046 6198 P98155 Q14847 O95714 O43711 55283 83752 27243 P41250 O15234 65082

P98161 P07355 3911 O95721 P54105 P78536 7057 Q13520 9230 P78537 Q8IUN9 Q3V6T2 7052 81567 P46934 3927 Q9HD26 81565 O60296 P98172 80230 Q14865 8399 7067 P29590 8394 P30101 Q4LE60 29886 P16284 Q9NZQ3 O15258 O15259 3932 O60260 9255 Q32P51 P19320 27236 Q9UNE2 112936 P41222 653361 P43405 O43707 9267 Q9NZJ7 Q13555 O75746 Q13554 1781 Q9P253 P78509 O00186 O00189 1778 O00194 P63000 Q14894 Q13563 P08246 9276 P14927 Q14416 9270 Q9H2T7 P14923 7094 1789 Q05516 1785 Q05513 P63010 P00734 Q7Z2W7 Q13574 Q15751 80273 53916 100287932 P78527 O60229 2647 1315 P20339 1314 P20336 P20337 1793 Q04656 84661 Q9UF02 Q9NZM3 Q9BWM7 1317 Q13586 Q16611 O15230 Q6XPS3 Q13107 113829 9296 Q9H6Z4 Q96H20 Q63HQ0 4830 P20340 P00750 Q13596 P48051 Q9Y2W7 Q8NEY1 P48058 Q12948 P61020 Q9BVA0 2665 2664 57620 Q9BVA1 Q9Y2X3 90678 P61026 P07996 10533 219931 P48047 Q12955 Q8NF91 Q9UI09 4860 51090 Q08431 P63211 2676 O60333 Q8N4V2 Q8N4V1 200081 Q9UI12 P50395 Q12965 Q05397 10514 283229 29127 4867 4868 4869 5716 Q9H1D0 1374 O00299 Q13829 O60318 P17302 5728 Q96JJ3 P17301 Q99698 2697 Q9BZF3 948 Q96JJ6 Q9H1C4 Q12981 O95477 Q9Y342 P28482 O95470 O00221 Q9H1K0 Q9P0J0 58513 57669 57662 O60763 P07550 Q9Y2T2 10972 10971 P08887 Q5T8D3 O95487 P51679 P10721 57678 P18206 960 P54709 P62328 10981 4898 7922 Q9UM54 5747 Q9NYB0 P31639 Q9NYB9 O00244 O60749 22931 P62330 10953 10956 P42768 2247 26056 P15151 58533 10959 Q8N4Y2 975 P61006 Q9H1H9 P51693 Q8NEZ2 Q969M3 P51692 P28472 Q9P0M2 Q9BZJ4 P17342 Q92621 Q9ULV0 P05362 Q969M1 10960 Q92624 Q9ULV4 P61019 509 Q96FF9 P61018 P07900 Q9UPV9 Q9Y6W5 P80188 P11166 P38646 O60725 P15531 O14828 51000 Q96RU3 513 514 128866 6622 515 10134 10133 P61421 518 51009 Q04917 5310 6643 5311 Q9UHG0 6642 5796 P51636 Q6WKZ4 P07919 Q96RT1 521 522 523 Q15154 10142 526 528 5306 10146 P49768 Q86U42 Q9BV73 6653 Q3KNW5 23423 O00203 Q00535 533 Q96S42 535 P49755 537 51026 54536 P49754 54535 6646 539 51028 91949 8829 Q8N960 54539 P01375 Q96JC1 O00217 23439 Q9Y2R5 4000 5331 Q96EY5 P12004 10128 O95070 3148 3146 10121 4478 5327 Q96JB1 O00623 O00628 P07947 9700 Q9UPR5 8851 P07949 Q9UPR3 8852 O00629 Q96B97 Q12904 Q9Y2K6 Q9Y2K9 23400 Q12907 3162 Q8IXI1 Q8IXI2 Q68EM7 P42704 Q8WTW3 7514 Q96AX2 Q96AX1 5338 P05771 10577 P26232 7532 7531 7534 10580 7533 Q9UHC3 3178 P68032 O00631 Q00987 9702 P24046 P05783 7529 3181 5362 5364 4035 P49321 Q9UPT5 Q99666 Q8NER5 51068 Q8N8Y2 Q8N4Q1 P49792 5371 Q9BQQ3 P49790 Q60FE5 8402 P20908 O75396 Q969E2 Q8NER1 Q99653 P52948 8879 P62745 4038 8878 10564 57617 10566 54908 Q9UQP3 9744 8411 O75027 57187 94121 Q96FV9 Q96G79 94120 Q9NUM3 P22695 Q9BRI3 Q8WUM4 Q8WUM0 8408 Q8TD31 P03950 P12429 O75494 29959 P35523 Q9H270 Q9UIA9 415117 8417 6238 Q13492 P47985 Q15672 4074 Q8TD20 29928 O75489 Q9H269 29927 29924 23381 P25705 Q9UDY8 P23528 84342 Q8NFH4 Q13023 51429 6262 4087 4088 9775 29934 Q8TD16 Q96BK5 Q02790 P12883 P31150 Q9NUP9 1808 60386 10087 P20020 Q15691 P60520 Q16543 Q8IXZ2 9784 8452 P54257 O75469 7124 10053 P54253 7126 Q9Y3E0 P14635 P38606 64759 P10275 4092 Q4G0F5 P10276 P35579 154 P35580 Q99720 10057 55800 Q16555 Q99726 P45379 9311 9793 Q96C24 10061 O94955 Q5JQC9 29916 161 P11142 Q04941 P01308 P01303 9322 7142 P00491 Q401N2 O95813 Q9UQN3 P36404 7141 6293 Q99700 Q9NZ43 55823 O94973 O75438 P54289 7155 O75436 P39905 7157 P14625 Q02750 O75431 Q8WZ73 Q9NZ56 54984 Q9NUL3 O94979 84376 P53367 Q14721 8498 9342 O95835 P57729 O95833 10928 Q06787 P63172 7161 P54219 9341 7163 2702 P63167 Q9Y3A5 P23945 10938 Q7RTN6 83871 Q6NVY8 P09651 P42229 Q9H1N7 Q13409 Q01082 8021 O43847 22920 9350 P57735 P22626 10947 22919 Q969S0 9367 P10619 O75886 57120 Q9H1M0 P57740 O75880 26019 7184 Q9UHY1 O43819 P00846 P17252 P68871 Q92673 Q969R2 Q13423 O15392 O15397 P35908 Q8IY33 Q6UWE0 9371 P21757 Q96K37 56288 Q96FJ2 1408 9388 Q9HC62 Q96G30 9380 Q86YS6 Q86UK0 P51148 9382 P51149 4927 2742 2741 84766 P42262 Q6UWJ1

P51151 Q8WZ19 O95405 P51153 P42261 57154 Q15629 P51159 O43808 27333 Q8WYP5 Q9BVL2 Q8WYP3 P21333 O75843 O14495 59341 59343 Q9H223 P84085 P00403 Q15642 Q13464 Q9BVK6 P01730 Q9HBR0 O15350 O75832 Q00169 1447 P84077 2773 Q15653 P21796 Q9HC21

protein amino acid autophosphorylation Q13554 7046 6872 P07947 Q13315 O15164 1020 O75385 O75460 472 P19525 P42345 Q8IYN9 P27708 23043 91 5610 6789 1859 8408 Q13164 Q13043 2081 Q6IR47 Q9HAZ1 P09619 5598 1198 P51955 P36896 57396 1195 P10721 Q9UKE5 P16234 Q96Q15 120892 2475 8737 4214 207 4215 P00533 Q99759 Q13177 5747 P78368 Q5S007 P11309 6850 P49761 A1XKG3 P36888 Q05397 27102 Q00535 5159 P06241 Q5TCX8 P08069 10155 Q9BQI3 P05198 1956 Q13263 P04626 Q13546 P31749 Q658W2 2064 Q13627 Q9UBE8 1965 10645 P17612 P43405 Q96RR4 O60674 P51617 Q96QT4 Q7L9L4 5566 54822 Q13233 Q13153 P21675 P06213

placenta development 6670 9421 O75626 O96004 Q14686 Q15738 3880 P28482 O43278 2034 9021 P35613 O00622 6194 P30048 P17676 O14627 10733 P62993 P00747 10935 O75190 P17275 Q9BUG9 6667 P08047 3676 O14543 Q13563 639 P62753 Q99966 P47712 P03950 4188 5311 P08727 7412 3297 P37231 682 Q02447 3326 Q99750 5467 207 5468 329 P00533 Q13490 Q96T37 O95377 Q86U86 6256 6696 5321 P19793 O00444 P19235 P10451 Q9UBU3 1634 6927 57448 Q00613 2885 Q9NR09 Q96S42 Q9UJU2 6647 P49715 Q9GZT9 1956 P00441 P26012 50814 P27540 O75712 Q15648 P31749 P19484 P19320 Q02750 P13612 1050 O60911 P20823 54583 P07585 Q99814 P04275 P08238 5604

negative regulation of cell cycle 26959 P25054 6597 O95835 P04637 11186 Q9NNW5 Q12948 Q9UPN3 7161 124790 4291 472 10657 11108 4609 P84022 116 54998 O15355 Q13761 P98161 P07996 P01112 Q9UQ80 O43683 P52564 O94992 7057 9238 4221 5310 5036 5311 5796 Q12959 O75496 Q15392 1982 O43290 Q9H0C8 51654 P01106 6878 Q9UI95 P00533 P42574 P24522 Q15831 P35638 Q9H2G4 P48431 6498 P12757 Q8N726 Q9UKN5 3265 Q9UNL4 P29590 Q13418 Q9NS23 P46527 Q969Z0 Q00535 Q00534 O15379 811 51147 P01138 P01137 O75807 O60381 Q6IT96 Q92831 P06400 4088 Q13547 A4D1W7 8841 Q03001 O00213 P51532 27352 388 Q9NWT8 P18509 5566 6657 10488 64061 Q96MH2 P43246 5604 1647 8453 8454 8452 6794 Q13315 1021 P41182 1020 23368 O75460 10459 10614 Q99728 Q13563 2081 22846 7013 Q96SB3 Q92769 Q96HU1 O43889 Q07666 26524 8065 7251 1029 1027 1026 Q96SZ6 Q99675 324 Q9NQB0 604 P42771 Q13574 P49848 P08138 P27797 P26045 7023 P10415 P54274 Q13618 A1XKG3 Q13617 3066 Q13616 3065 3611 Q96KQ4 Q01664 Q9NRM7 P01579 1718 5119 Q9UM63 1956 5371 O15350 Q13625 7157 7159 Q7L8A9 O00255 Q02750 P38936 580 23513 7272 P17612 Q14160 Q93034 P62745 Q99816 Q14201 P04156 5925

I-kappaB kinase/NF-kappaB cascade Q15654 O00220 O43557 Q9UNN5 Q9Y4K3 Q7RTR2 O60603 O43318 P42224 Q9Y2C9 23085 Q9Y6K9 Q13162 10010 P25963 7099 7098 Q14653 6885 Q8NFZ5 O95163 Q9NWZ3 10549 29110 9908 1147 8737 4214 7205 11035 Q92844 O00206 Q9Y6Y9 8795 7186 7189 4792 6772 3661 Q9UHD2 P58753 Q9P035 148022 Q8IUD2 79753 Q9UDY8 Q9Y572 11124 O14763 O15455 Q9UN86 O14920 Q8TAD8 5716 O75832 O15111 Q12933 64343 Q13546 Q86XR7 Q9NR96 3551 P51617 Q8IUC6 8517 Q5VVH5 54106 Q9BWT7 Q15653 Q13233

anti-apoptosis Q9BZZ5 7046 8535 399687 Q12948 Q04760 O43715 Q9Y4K3 3084 P38646 P17676 P52952 O15519 2303 355 3479 P04083 9616 Q92731 6622 8767 O14543 Q9Y275 Q9C0K7 Q15389 P42229 O15119 Q92614 P07996 2308 P25963 P25445 Q13489 P52565 P78536 7057 Q02297 1072 1191 Q86VP1 81567 Q15392 P42858 P16471 8539 P98170 10542 P01343 Q92843 P42336 P49768 7189 P29474 Q9H422 P18583 Q9NY61 7184 P13693 Q9UBU3 Q9NR09 Q9UDY8 P23528 O15379 6647 P01138 P03372 Q6UXV0 P63104 6926 Q6IT96 P55290 P00441 4869 Q9BWQ8 P01375 9531 9530 O15392 5055

5970 Q13547 8841 O95865 2100 3551 57099 Q9NRZ9 P14784 663 3304 301 P19838 Q99576 Q04206 6776 P05164 307 P09525 P41743 8837 P49747 Q9UBN6 3309 O43353 P08758 Q12981 Q12982 5584 7124 Q9UBF6 Q9UHJ9 57144 9021 3162 396 P15056 673 3315 P15692 3313 1012 P09211 Q6IR47 Q9NYA1 P21580 7534 Q07820 5590 Q9UEE9 Q07817 P11021 79444 Q05513 3169 7128 207 Q9NQB0 P08138 Q99958 P04179 4790 P61803 4792 7422 P10415 2099 Q9NQS1 3065 330 331 573 1831 57448 51100 O95817 Q9Y371 P08069 1718 10673 O14920 Q96CA5 Q13501 3070 P08107 P53355 Q12778 8887 P39905 P51692 P31749 O43504 P14625 27315 Q9P286 O00257 P37840 O60238 Q8NBS9 P51617 Q13075 P05120 O95429 7428 8878 P04792 P09601 P40337 P60568 P04156

regulation of cell migration P25054 Q13477 Q15654 O14672 3516 284217 P84022 P09493 P07996 P04004 Q15303 3091 5154 5155 P12429 Q92729 Q5VTR2 O95684 5270 P16234 Q9H2X0 7448 7205 5029 P00533 Q14344 Q9HCY8 Q13017 P25391 Q9Y2I1 57402 P37023 Q00013 5159 P55290 5054 3791 4088 3673 Q8WWN8 Q14118 5728 P17301 P21359 O60674 P20827 Q13393 Q16665 3667 5604 Q16787 4771 P28482 1020 Q9UQL6 4092 394 57669 51564 1012 P08887 719 P51671 3690 22846 Q9NYA1 P09619 O43889 Q9Y4H2 P35443 P10721 P35568 285 Q06330 P18206 P01308 Q9HCM4 967 9564 P05106 A1XKG3 P30542 2247 P08069 Q9NQC3 1956 P09038 P23443 P09958 P31749 Q7L8A9 Q8N9R8 Q02750 P48061 3909 Q6PID4 P05362 P05121 1969 P09601 P29317 Q14289 Q16581 1605 4318 P06213 Q13873 6198 O15123 2150 O60603 Q4VCS5 Q96RU8 3479 23560 P39060 P04085 10013 94 6869 Q15389 10014 3911 3912 P78536 7057 Q96QB1 5796 Q93096 2159 2277 4214 P01343 Q16363 11116 4354 P14416 Q13418 P14780 P41231 Q00535 P35240 P01019 P01137 Q9Y696 Q01974 P60484 P17936 Q92786 P01127 10488 5328 Q59EA4 Q9UBN7 O60271 P24043 P07942 Q8WUI4 O43915 8174 P78504 3162 P23508 P00749 P15692 Q9BZE4 5337 P00742 4920 5338 P35968 Q13443 Q7Z5L7 64127 324 Q15077 207 P55085 8754 7422 P10415 3611 127435 7414 10672 P25103 O15230 P62070 O14939 Q9HC29 O15105 Q13233 P25116

regulation of DNA metabolic process P23396 P04637 Q15819 O95271 P51587 990 4609 3479 Q1PSW9 23560 P04085 Q8N3U4 P01112 Q6UWV6 P42226 25913 Q9H4L7 5154 7334 5155 Q9NS91 Q13404 7336 P12429 7335 O75496 55294 Q3V6T2 P16104 P16234 P67775 641 P52292 P01106 P01588 Q96RL1 Q99638 Q15554 P01343 2956 Q9H2G4 P29353 P52294 9126 Q13535 3265 56034 Q99741 P35240 3014 811 P01137 29086 Q9NRA1 Q9BSI4 4361 Q9NUX5 P12004 Q8NB78 2735 1763 P10070 545 P08151 P31949 P01127 64061 P43246 2736 5582 P05129 4771 Q969H0 P54132 P41182 Q9NX61 P53567 3836 Q02535 672 1017 Q9BZE4 51720 P04201 P05412 7013 5111 7014 P30153 P10600 6282 P46736 Q9NWV8 P18846 Q9UQE7 P01308 604 P27797 Q9NYB0 Q9H2K2 Q13216 P54274 5883 O43612 1161 O60502 P38398 P49959 P61088 55704 56916 P01579 P08069 Q92878 5515 P05112 5518 Q9BT67 6188 6464 7157 P52701 221656 10724 P40692 466 P24941 5888 P60568 Q14683 P06213

positive regulation of transferase activity Q8TEW0 P54619 Q9Y4K4 Q9Y4K3 3084 O43318 P13861 595 23043 P22694 Q9ULW0 P07996 P52564 25913 P03950 5155 Q16512 4188 O15530 Q9UKE5 P16471 P00533 Q13131 8795 Q5S007 P11309 Q9HBW0 Q06124 22974 Q9Y6Q6 Q9UBU3 Q9UDY8 Q9UKW4 P00441 4869 5716 Q53H12 Q9Y6R4 Q14114 3672 O43306 Q9NR96 Q13705 P21917 5170 O60674 P20827 3667 5604 Q13153 3309 P04233 Q8WXG6 Q13956 7124 O00220 1020 O75460 154 Q9Y2C9 121512 Q9Y6K9 P07550 3690 Q9NYA1 P10600 P10721 P35568 Q9BUB1 P11021 10746 79444 Q8NFM4 P01308 Q8NFM5 120892 7804 Q9NRD5 P05106 O95136 O95257 Q9UGJ0 P28223 6850 P11229 A1XKG3 O43612 P16118 P30542 P18545 O60503 P49959 2247 P60033 975 55704 Q5TCX8 O14763 Q92878 Q96CA5 1956 P09038 P31749 O75553 O95382 Q02750 Q9UNI6 23118 P37173 Q6PID4 1609 Q9BWT7 Q14289 Q9UL54 P06213 P01241 9463 7046 P98155 P56199 Q7RTN6 P01116 Q9C0K7 P22612 Q15389

1616 P78536 7057 6885 Q02297 P10644 P31321 Q3V6T2 P31323 Q99750 4214 P08575 4216 P42338 Q9UER7 P49768 O00206 7186 7189 P29353 5562 Q96M96 P52735 Q13418 P31431 P40145 894 Q00535 896 O60266 6647 P01137 P01135 P01133 P01375 O95622 4361 5573 P04626 5575 Q13546 5576 2064 Q9NUX5 55750 P43405 56288 Q07954 Q92786 5566 5567 P01127 5568 5207 5585 P78509 B3KY43 Q99683 Q03468 P51828 10451 5577 10454 P08246 2081 7099 7098 Q6IR47 Q13443 7410 P30279 5590 P61073 Q05513 P30281 8737 207 Q13574 Q15750 P46734 Q08462 Q9NYJ8 P24385 8754 4035 Q9P035 Q9BUZ4 O75293 3611 O15455 P01730 9839 O75832 Q12933 6464 P52824 P17612 P51617 8517 54106 Q13233 P25116 P60568

positive regulation of protein kinase activity Q8TEW0 P54619 Q9Y4K4 Q9Y4K3 3084 O43318 P13861 595 23043 P22694 Q9ULW0 P07996 P52564 P03950 5155 Q16512 4188 O15530 Q9UKE5 P16471 P00533 Q13131 8795 Q5S007 P11309 Q9HBW0 Q06124 22974 Q9Y6Q6 Q9UBU3 Q9UDY8 P00441 4869 5716 Q53H12 Q9Y6R4 Q14114 3672 O43306 Q9NR96 Q13705 P21917 5170 O60674 P20827 5604 Q13153 3309 P04233 Q8WXG6 Q13956 7124 O00220 1020 O75460 154 Q9Y2C9 121512 Q9Y6K9 P07550 3690 Q9NYA1 P10600 P10721 Q9BUB1 P11021 10746 79444 Q8NFM4 P01308 Q8NFM5 120892 7804 Q9NRD5 P05106 O95136 O95257 Q9UGJ0 P28223 6850 P11229 A1XKG3 O43612 P30542 P18545 O60503 2247 P60033 975 55704 Q5TCX8 O14763 Q96CA5 1956 P09038 P31749 O75553 O95382 Q02750 23118 P37173 Q6PID4 1609 Q9BWT7 Q14289 Q9UL54 P06213 P01241 9463 7046 P98155 P56199 Q7RTN6 P01116 Q9C0K7 P22612 Q15389 1616 P78536 7057 6885 Q02297 P10644 P31321 Q3V6T2 P31323 Q99750 4214 P08575 4216 P42338 Q9UER7 P49768 O00206 7186 7189 P29353 5562 Q96M96 Q13418 P31431 P40145 894 Q00535 896 O60266 6647 P01137 P01135 P01133 P01375 O95622 5573 P04626 5575 Q13546 5576 2064 55750 P43405 56288 Q07954 Q92786 5566 5567 P01127 5568 5585 P78509 B3KY43 Q99683 Q03468 P51828 5577 10454 P08246 2081 7099 7098 Q6IR47 Q13443 P30279 5590 P61073 Q05513 P30281 8737 207 Q13574 Q15750 P46734 Q08462 Q9NYJ8 P24385 8754 4035 Q9P035 Q9BUZ4 O75293 3611 O15455 P01730 9839 O75832 Q12933 6464 P52824 P17612 P51617 8517 54106 Q13233 P25116 P60568

ER-nucleus signaling pathway Q5VVQ6 P04637 Q9Y5U4 Q96A33 P16615 O75460 3638 P19525 595 79139 5610 Q16236 51009 P55072 2081 4780 22926 O76024 P18850 P11021 488 Q9P2K8 P49841 Q9UKV5 Q9BUN8 P24385 P35638 22937 O95292 51141 57003 7415 6721 P01579 Q9GZP9 P02545 Q12770 O75807 Q12772 4000 Q9BY44 9217 7157 55432 2932 267 Q15011 Q13153 3309 O15503

protein-DNA complex assembly P07305 P26583 P06899 6872 2033 Q9H0U9 8290 4171 Q96KK5 1058 P20671 Q8NCD3 Q7Z6C1 55723 Q02539 P49736 P49450 8348 Q9BTM1 P62805 9555 8467 P62807 Q99880 3010 O60814 P16104 Q99877 3009 Q9NVP2 3008 3007 Q8IUE6 3006 Q96QV6 51773 Q92522 8338 Q8N257 255626 Q99879 O75925 Q09472 Q9H2G4 P33778 266812 Q5SSJ5 4673 Q7Z7K6 Q96NT1 3024 O75367 P10412 P58876 P55209 Q96T23 P84243 Q71DI3 P23527 P68431 Q01105 3014 Q16695 O60264 8349 4869 3070 Q16777 P22492 Q16778 P0C0S5 Q5QNW6 Q9NRZ9 P16403 P04908 P16402 221613 Q96A08 P16401 Q99733 55355 Q9P0M6 Q93079 P0C0S8 3148 Q93077 Q7L7L0 Q6FI13 4676 Q6NXT2 64061 Q13112 P21675

cell differentiation P25054 65125 3880 2550 259266 P51587 P17676 2547 P37288 Q8IYN9 2303 O60443 284217 P84022 Q9HCJ2 Q92731 Q92730 O14786 O14543 O14788 Q8TDD5 Q9UGM3 Q12834 P08727 Q92729 Q12837 124540 P51114 2317 Q96KN7 P20849 P42858 Q99598 Q9UKV3 P61158 10661 4734 Q96EB6 Q8N205 Q7Z727 136319 81629 O00167 Q06124 Q7L5Y9 54496 55342 6929 4983 811 5830 55109 3655 Q9Y6A5 Q5TAP6 P40424 P62258 Q9UKW6 6926 3659 P31944 Q9NQX0 3672 3673

P31947 2100 Q9BYD5 P48380 O95343 P17655 57534 3428 O60437 1488 2335 O60674 1487 P20827 388677 P62263 Q8N5V2 P26367 O14578 5604 P41743 4771 Q9Y463 1021 1020 P39019 1499 Q8N264 3676 P61586 P07437 Q9H0H5 P29466 P32856 Q92769 Q14814 P28370 Q9NZC7 O95365 Q9BPY8 Q06330 3207 841 Q9HCM4 O60890 604 3688 A6NI15 P09622 P04179 O95377 4791 2132 Q15915 O60869 O95376 Q96TC7 P19634 57326 3215 Q96N67 2125 Q92990 1277 Q9Y250 Q92753 Q5TAT6 Q92752 P05230 857 859 Q9H0F6 Q13501 P10826 O00139 P10827 Q9UL45 50937 221937 P11912 P31749 Q9NR50 1297 Q13509 P19883 Q13506 Q9P202 1050 O60636 O60879 Q9HCK4 2139 861 3226 O95390 863 6733 Q9UKT4 4318 Q15019 O96005 O96004 6993 P56199 P14317 P14555 P50897 P11047 25988 Q99523 203068 3479 2147 Q8WV60 10011 7832 55723 639 10014 115426 3010 1072 Q8NHU6 641 Q99750 84033 79923 11113 O43294 6517 O43293 Q16363 Q09472 6774 3265 23787 Q00653 891 P14780 894 O95180 P53708 P35240 656 P01019 P49639 84062 Q9UMS4 5451 8721 A7MD48 Q9Y696 Q658W2 O95183 P35222 25975 Q01974 O75084 O75081 P17813 55755 3028 P60484 53335 668 6776 P24588 O43474 P26583 O00744 5460 Q9Y3M2 4131 Q6FGG2 P01040 4133 9820 Q9NX62 10458 P61981 P15692 6304 P55075 6548 79739 Q8N5U6 P05412 O00755 10460 7412 Q9HCE7 P52803 P37231 10229 Q96SZ6 79723 9825 5467 4137 207 5468 7408 10468 Q15078 P24385 8754 4154 7422 4155 P56178 P56177 3066 O75293 P13010 P61764 10672 9839 O43463 5493 7430 9612 O43464 P35268 23513 P13497 O14939 466 8516 Q9NQL9 P60201 55558 9846 10202 P55268 Q14686 8775 Q9BZ95 Q9UPN4 O75386 O75385 Q9H165 Q86VQ3 Q9Y4K3 3084 472 474 Q9C0C7 P07196 Q99807 P49810 P08047 P83731 P43034 Q13363 Q9NPC8 Q13127 3091 4188 Q15306 5037 79083 Q86VP6 79084 P58304 P58546 Q9UJC3 5270 Q02447 Q9NVP2 Q05952 P09382 28981 Q9NRH2 30813 P46531 8536 5029 51535 P00533 Q16643 Q15796 Q9BYV2 Q9HB63 Q15797 P12757 Q8N6I1 P31273 P12755 Q9ULH7 O75360 Q9Y6Q6 Q01101 Q96RK4 Q15561 Q9NRI5 Q13144 Q14232 Q16650 8563 6383 7476 Q9Y6R0 P18074 Q86XR8 P10586 P68133 P10588 P13611 P10589 59277 P13612 93166 O75592 Q8N4C8 54820 P31270 23032 Q16665 P43246 9421 O15287 Q16666 7248 P54132 274 Q93063 P23229 51564 Q13164 Q14012 Q86W54 P78337 7257 Q9HAZ2 Q15109 O43524 7490 7251 285 Q9Y6L7 Q8WW38 1948 Q9GZM8 1947 Q9NRD5 P02751 Q9GZM5 Q99835 9201 O75326 Q9BV36 10152 Q02880 A1XKG3 Q86VS8 Q96EV8 Q08188 51341 Q9NVM4 55704 P21266 P08069 10155 P02545 347733 1956 P23443 O15075 Q8IUX8 O15078 O75553 O43508 Q9NXR1 2810 P02538 Q6PID4 Q99816 P78362 O95947 Q99814 1969 P02533 Q14289 Q15375 P25874 P78545 9463 7046 P28289 6198 Q13751 9464 O43711 55283 6194 Q4VCS5 3913 P42345 P22735 Q9Y4C1 P04083 65082 Q92574 1738 O60282 3911 3912 P56524 P78536 9474 Q13769 O15496 Q02297 7291 Q3V6T2 3925 P46934 Q9HD26 81565 Q4LE28 P16070 P42574 P98172 8399 7067 P06239 Q5TAX3 P19793 P29590 Q7Z2Q5 P56539 Q01196 1756 P41231 60412 Q92793 Q9HD15 Q7Z6Z7 P06241 P21860 O15259 3932 P56545 A4D1W7 P19320 Q8IYT8 9253 9495 7074 P43405 Q9Y4F9 P41220 Q9NZR4 Q04206 Q5T5A8 Q92786 P07101 Q9NZJ7 Q5GLZ8 O75503 Q13315 Q15735 P78509 P35610 Q03113 O43915 8174 9021 Q16828 P78504 3958 P00747 O60488 P85037 Q9H9B4 3956 2626 P31249 Q8NEC5 Q15742 O43921 P11413 1789 Q05516 P62166 P00734 3720 375790 P22303 Q5BJF6 Q9HCU4 9047 P12956 Q8IUQ4 P34741 26576 57045 P78527 O60229 Q9BQA5 P02708 P20336 Q86TG7 2885 Q9Y6E2 Q04656 51741 P43694 Q14432 Q9BQA1 132320 O15230 Q15768 Q9NP98 63976 P22528 23077 Q9HCS4 4830 51752 Q13351 5925 P10914 Q92858 Q8NEY1 Q9NYD6 2672 Q12948 Q9UBB6 22994 P52799 Q8NEY8 Q9BVA0 3516 P16220 Q9BVA1 Q9ULW3 Q9UM82 P52565 Q8NF91 Q9UBC3 54361 P50148 P63211 P16234 79885 P16473 2676 51099 P16471 O60331 4851 4854 O15524 Q92838 P54753 Q12965 Q9UBD6 P25391 Q05397 P53667 Q9BXC9 6809 P20930 27185 29127 P20936 Q9UJU2 Q9UQB8 P03372 4867 Q92833 26091 P06401 4627 P06400 P05556 P54764 Q92826 Q9GZX9 P54762 3791 5970 5971 P48023 Q9GZX7 9093 P56705 Q7L576 22985 P17535 P17302 5728

81501 P17301 P17542 Q9HBE1 2697 P61296 6812 P04233 P34925 Q5D1E8 1385 O95470 O00468 10733 10736 57669 P15173 P07550 3796 4643 4646 5978 Q9UM47 Q7KZI7 O00238 Q9Y2T7 Q96SB4 Q96SB3 Q9NYA1 5991 P51679 Q8IZT6 P10721 Q06210 Q5VST9 P19544 6839 960 1147 1387 2475 967 4898 Q9UM54 Q92888 5747 O95259 P07766 O95257 137964 6850 2011 2253 Q13618 Q9NYB9 57689 2247 2246 4664 P61247 O14640 4665 5997 O43184 P05112 O43186 P29323 P51692 O00255 Q02078 Q6KC79 O00499 P37173 P09960 P29317 7704 P06213 P02452 P04637 O43157 2034 P13349 2033 25861 Q01650 1181 P52952 P15531 991 P62993 Q8WTS1 79810 998 999 P01116 4205 Q15389 P02686 P62753 P01112 4209 P01111 Q04917 O43166 Q9C0K0 P49770 Q96CW9 4221 Q7Z5H3 Q9UHG0 P51636 23210 2043 P50542 1191 2277 Q15154 P48436 P01588 Q9C0J9 P01343 Q96NL6 P49768 2290 P50552 P48431 9921 Q86VW1 P14416 2054 3142 O00444 773 Q00535 Q00534 Q96S42 Q53X93 6886 55626 6405 6647 P01138 54535 6646 P01137 5316 Q96Q05 8829 Q9UPY3 Q8N960 Q16254 P02462 6660 4000 P04626 P04629 2065 2064 O00213 2063 Q96AQ6 O60716 P54920 301 3148 P10070 P10071 3146 Q96PN8 P02458 P40933 Q96EY1 Q96JB5 6657 6670 P07942 O00628 5584 7520 O43597 8851 P07949 P07948 Q92817 P20916 Q9H9S0 Q8WYA0 Q96PU8 Q9BZE0 552 Q96PU4 Q14093 Q99684 6667 5336 Q96AX1 5578 5338 7518 Q8N302 P40763 P26232 Q7Z5L4 5591 Q6IR47 7532 7531 7533 5598 P68032 23411 P80370 23414 3171 10107 P18846 324 3169 Q9NPH5 9702 10100 5108 P27797 Q9H5I1 6696 5364 2099 P51843 3182 P15311 Q8NER5 140735 Q01826 Q96HC4 5371 P27540 O60911 P16885 Q8WTT2 Q08209 P58012 P18827 8879 P62745 6699 10320 8878 P60321 P26447 P25116 P60568 Q96HZ4 Q13477 8650 8892 Q9UQP3 Q6ZT98 Q8TEW0 Q9BY76 P15924 P30086 P15923 Q9Y3I0 29948 4291 351 595 Q04759 356 Q9UBS5 P09493 Q7Z6C1 5139 Q7Z6C3 Q13485 8408 Q8TD31 P03950 9750 Q13009 4067 Q15669 7335 367 Q70SY1 51654 Q96SN8 Q14344 Q15672 6496 6495 6256 6498 10075 O43897 P10451 Q2M1K9 Q9UBU3 P51805 Q9UDY8 P23528 P09471 Q9UDY6 Q8WZ42 Q13263 P00441 4086 4087 Q13268 4088 4089 Q14119 163126 Q9Y618 Q86UL3 Q86YT6 P12645 Q02790 222546 387 388 P21359 23154 23396 P08151 1808 P20264 6259 P60763 Q13275 P54259 P55347 8452 6275 4099 Q8TD84 P14635 Q9UQL6 P10275 P35579 154 396 Q5JUK2 Q02535 Q00597 Q01201 23363 10296 Q8WV28 11146 Q99729 Q16555 Q15223 P45379 Q99966 Q15464 Q13285 Q13049 Q86YZ3 P60709 Q16799 9314 P36894 Q86WV8 1822 338917 Q9NQB0 60 P09067 O15169 85440 Q99958 Q15475 Q99714 Q8TB22 Q8TCU4 7142 Q8TEY7 P14859 P36888 7141 1832 Q9H1Y0 9319 P21145 P01579 Q9NQC3 P49715 P09038 P60953 Q14155 116150 7155 P39905 7157 P22492 25776 Q02750 P35548 Q9H295 55832 Q14160 84376 1848 Q15256 P78424 Q08AF3 O15123 P10636 26037 P63172 7161 9341 7163 1613 26271 Q8TAF8 P41134 Q86WK6 Q1PSW9 10935 P17275 Q4KMG0 Q92692 P39060 Q6NVY8 P46821 P42229 P15090 O15119 2947 Q9UBK2 Q13885 Q13642 Q8WWH4 Q13404 O43602 10939 P55318 O43847 P55316 79365 O43609 55177 P39059 P08575 P05067 Q9BXL5 1627 7189 Q15836 P29475 26258 57120 Q03014 Q13418 Q7Z3S9 O43815 6096 P34820 P55327 1874 P17252 Q5TA76 O60383 Q14993 Q9NZ08 Q9UBM7 Q9NSC2 P42685 P21741 Q13422 P35908 O15156 Q13426 9138 P10620 29777 4914 2735 56288 Q96FJ2 Q9BXJ9 57379 Q8HWS3 1406 2737 2736 O75626 P54652 Q8WUI4 9149 Q6NYC1 P41182 P53567 P30048 84525 1896 4920 P35968 Q14774 Q13443 Q07820 P11532 57154 P51159 27339 P30279 Q07817 64919 80155 P23515 Q9UJX0 57159 P08138 P09429 O14492 P56851 P10415 Q9H228 P12830 Q02363 P18146 P35711 163183 3611 83696 Q14790 P09430 P01730 80184 P08588 P41159 O15350 O15111 O15353 Q15648 Q06710 Q04771 1203 P24821 P21554 4957 Q9BZR6

negative regulation of transferase activity P25054 O95835 9467 Q9NRW4 7161 124790 Q96RU8 Q96RU7 1852 1850 10935 P15090 P49815 6901 P09936 25913 O94992 4221 O15530 Q16635 O95684 P50148 56940 Q8WTR2 O43609 P67775 51654 P08575 P42574 Q13098 P24522 11116 P49768 Q7Z727 Q8N726 Q9H422 Q13418 P56539 Q9C010 84619 P46527 57761 P17252 P35240 P06400 Q9UJM3

Q15208 P31947 9093 Q9NUX5 5170 P21359 Q96EY1 5569 Q9Y2B9 Q96MH2 10641 1647 4771 O43597 11142 Q16828 P30048 10614 P61981 Q8WTW4 5578 Q8IWV1 7013 Q13202 7532 Q9Y4H2 P30153 26524 P37198 P37231 1029 1027 1026 Q96SZ6 2873 324 207 7249 5468 54206 P42771 Q13177 Q99956 P61925 O95257 7023 Q9UGJ0 P49840 P54274 O75293 Q9UNH7 3611 Q01664 Q9NRM7 58533 O14640 857 859 5515 5518 23636 Q9BY84 O15350 Q13227 P31749 O00255 P38936 57732 2810 2931 Q8N5A5 1848 5925

oxidative phosphorylation O75947 O75306 Q7KZN9 Q16864 4695 O43678 4728 4729 513 Q99807 514 6622 P22695 4720 515 P61421 4722 1738 4723 518 6901 4725 4726 9114 O95169 P28331 P48201 P48047 Q16635 O14561 P07919 O75380 521 522 29796 523 4697 P00414 4698 4731 526 528 Q9UI12 O75489 P56134 O95298 O95299 498 P00846 P25705 1355 533 P18859 535 P56385 537 539 P56381 10476 P03891 10632 O00217 O95182 Q9Y2B5 4514 P43246 P30049 P31930 P56556 245972 P38606 P36542 P19404 Q16795 51606 P14927 O43920 Q15904 Q16718 Q99437 10229 Q93050 4535 P09622 7385 7384 O75964 O00483 7381 P17568 4705 4707 Q9Y6M9 P24539 Q8N8Y2 P00403 4701 4702 P56181 O75438 P51970 Q9Y375 P21283 P21281 9296 P37840 P15313 4718 4719 P49821 4710 4713 4714 509 4715 P06576

regulation of microtubule cytoskeleton organization P25054 4131 4133 P10636 Q15735 23122 P14635 9181 Q9BVA0 10733 672 Q9Y6G9 Q9Y3A5 1499 57662 Q9ULW0 P46821 Q92974 11190 7013 9475 23332 Q5HYA8 Q7Z460 324 1540 57159 22919 4137 Q9BYV2 Q9BV73 P54274 O75122 O00444 P56539 22974 891 P38398 221150 79959 857 859 Q9NPA3 4869 Q9NQC7 91147 O75116 P35222 P78317 Q8TAP6 Q02790 P27816 Q15691 Q8IX90

regulation of transcription factor import into nucleus Q15654 7124 O43557 Q9UNN5 O00221 114548 O60603 Q04759 P84022 999 P61586 P25963 7099 Q9UBC1 7098 Q9NYA1 4188 10460 2316 9908 Q99750 1540 7205 P21333 O00206 7067 4792 Q14703 P12830 Q96P20 11124 O15455 Q9UN86 Q9Y6A5 P01375 5716 Q9NQC7 8165 P10827 Q60FE5 4088 O75832 8720 Q9BZF9 Q06830 Q9NR96 9495 387 P21359 Q9UMX1 54106 Q15653 Q92667 P24588

regulation of Wnt receptor signaling pathway 7088 P25054 5460 Q9HC62 Q9Y3M2 P35813 O95835 6794 Q9UBB5 O75581 P52952 P23508 27121 595 P84022 1453 27122 2300 Q9UGU5 6789 Q9Y2T1 P49815 6901 Q99966 Q13043 Q9NSA3 O00755 Q13322 Q9UGI0 4188 Q12952 Q16635 Q9NZC7 P14923 P30153 26524 54361 3728 P67775 54764 324 Q99750 1540 Q9UBT3 7249 8932 Q9UBP4 O15169 P24385 6496 Q15831 P48431 4035 O14495 2010 Q03014 59343 Q99471 6927 27185 Q9NRM7 Q9NRI5 Q9UJU2 O14640 51741 857 Q13188 5515 Q9NSC2 9839 5518 Q9NQC7 5494 4088 7476 25776 Q9UBE8 P56705 O95343 O60716 P50402 P20823 Q04724 Q07954 P10071 Q9HCS4 P48730 6657 2737

negative regulation of cell communication P25054 Q14449 P30086 2550 O75386 2305 595 P37288 Q9UBS5 Q92731 O14544 P78395 O14543 P49815 6901 P07996 2308 P25963 6003 4188 Q16635 Q15669 5037 O75376 54361 Q96D03 P14174 Q9H2X0 Q9UBT3 P46531 4851 9628 Q13370 4734 O15524 Q96EB6 Q7Z727 6496 Q92835 6498 Q96IZ0 8315 P12757 Q8N6I1 P32121 P12755 Q99471 4193 O60543 811 O75928 Q13145 5716 Q9GZX9 5970 Q8WWN8 Q14118 P10586 2100 163126 Q9NR96 9093 Q9UBE8 P56705 O95343 Q13829 5728 Q04724 387 P21359 6812 3667 3309 P04233 Q13033 P25098 8452 9420 4771 7248 P54253 7126 4092 O75581 154 156 P07550 P61586 Q9Y2T1 26191 Q99966 Q13043 Q13286 5074 Q9HAZ2 Q9NZC7 Q9Y5J5 26524 P10600 Q86WV8 P35568 P37198 P11021 960 2475 7128 7249 9306 604 Q9UBP4 O15169 Q15910 P07766 P28223 4792 O95813 P49840 Q13618 O43612 P30542 Q9UNH7 58533 P08069 857 5997 Q13188 859 5515 5516 5518 Q9NQC7 5770 Q12778 Q08050 7157

P31749 O95140 O00255 25776 Q8N9R8 P19883 P37840 2931 253980 51231 Q9UMX1 1848 P08754 1605 Q92743 O95835 P04637 5300 23322 P52952 27121 P42345 Q96RU8 3479 27122 2146 997 3476 5654 6622 Q9UGU5 Q92574 O15118 Q9NSA3 7057 4221 Q13526 Q96QB1 9350 P46934 O43609 P67775 Q99750 P08575 P06276 P16070 O43294 408 P49407 409 P01343 O00206 P48431 P14416 O75881 Q03014 P56539 Q7Z569 84619 Q99623 P17252 P35240 P01019 O15379 P21860 O14964 P49758 Q9UJM3 Q9Y2R2 8841 2065 Q9UNE2 P78318 O60716 P35348 P41220 P17813 Q05655 P60484 Q07954 P17936 Q04206 P10071 11331 Q96EY1 6657 2737 9927 9146 7088 5580 Q9Y3M2 O43597 P41182 9021 Q16828 P23508 552 2741 5578 P62714 6789 5338 7099 Q13322 Q8WZ19 P21580 Q9HCE7 64127 P30153 57154 23411 23532 Q68CZ1 5590 Q9BQB4 Q00987 Q05513 324 1540 207 54206 Q13574 P08138 P22303 10468 P27797 P24385 P01185 4035 29966 57045 P51843 Q86TG7 Q9NRM7 P61764 51741 O14921 23636 O75832 Q13227 P18031 P34972 Q04771 63976 57732 Q8N5A5 O14939 2773 Q9HC29 O15105 54106 P49427

steroid hormone receptor signaling pathway Q14686 1022 P50613 2274 P10275 P10276 Q8TDD1 672 1499 Q9Y4C1 Q92731 Q15185 P61586 10499 Q15466 1616 Q9UBK2 Q04917 P31689 Q9NVC6 7533 Q05086 Q96KS0 29079 P46934 367 O43294 4734 5901 Q15596 P27797 Q9UER7 O75528 O75925 O43251 P48552 2099 P38398 79039 Q14192 898 Q96PK6 811 Q92993 P03372 O75928 10432 P06401 P06400 5914 8289 8204 P62826 Q15648 2100 P35222 P78317 Q02790 10524 387 O60674 O95071 3301 Q9NPJ6 10728 P24864 O14497 9967 Q9Y2W1 5925

localization of cell Q92858 Q9UQP3 23607 Q8NEY1 Q12948 O75385 474 5817 2304 Q8IYN9 23286 Q9BVA1 Q92731 Q9NPC1 O14786 P43034 O14788 8408 P07996 P27487 P03950 5155 P11215 2676 Q05952 P00533 Q15672 Q7Z727 6495 P32121 Q05397 Q9Y6Q6 P37023 27185 P23528 Q9UKW4 4983 5159 5830 Q9NRI5 3655 Q13145 P62258 P55290 4627 P05556 6386 Q14114 3672 Q92949 2100 1130 P13611 P10589 P13612 P48380 Q13829 P17302 5728 P16333 Q99698 Q9NVR5 54820 2335 2697 23032 3301 Q9UKX5 P20264 5604 Q13275 8452 7124 7126 O95470 1020 64759 O43639 P35579 O75581 P09172 57669 P23229 1012 3676 3678 P08887 P08648 P30556 3690 5991 P09619 Q5JQC9 P51679 P35443 P10721 O00358 960 Q9HCM4 O60890 3688 4898 Q9GZM8 3689 1947 7804 5747 P02751 Q15475 Q99835 Q12884 9201 9564 7142 P05106 Q8TEY7 P05107 6850 O95813 10152 Q02880 Q13618 A1XKG3 Q9NYB9 Q9Y5K6 7141 2247 P15151 55704 P01579 P26038 347733 1956 P09038 P23443 Q14155 Q8TE73 O15075 7155 P39905 P22492 P31749 O75553 Q02750 O43508 Q9NXR1 253980 Q14160 1289 Q6PID4 P05362 P40337 Q14289 Q9UL54 P06576 P78423 4690 7046 6198 O43157 Q9Y6W5 P56199 O43711 P11047 Q4VCS5 Q00796 P41134 Q9Y3A5 Q6NVY8 94 3911 P31689 P78536 7057 P06702 Q9UHG0 5796 O43602 7291 3010 1072 P50542 O43847 Q3V6T2 9350 55172 Q99755 84159 81565 P16070 P98172 409 O14713 Q01518 P49768 Q14865 P29474 Q86VW1 P14416 O00560 P52735 8394 6093 Q00535 P16284 Q96S42 P17252 P06241 6405 P01137 8829 P01375 9255 P35908 P19320 Q658W2 P43405 P17813 P60484 3148 Q8IZE3 3146 4478 P01127 5327 10487 Q96JB1 P26583 O95995 O00628 8851 P07949 8852 P78509 Q12904 P15692 O14908 10451 5578 P08246 P26232 5591 Q6IR47 Q8NEC5 Q68CZ2 Q8WZ19 7410 7531 7412 9270 P14923 3728 5467 207 Q13574 Q15078 P09429 5362 7422 5364 O14495 P78527 O75051 P09430 Q13464 9839 O15230 P20908 Q04771 23513 8879 7428 P00750

cell motility Q92858 Q9UQP3 23607 Q8NEY1 Q12948 O75385 474 5817 2304 Q8IYN9 23286 Q9BVA1 Q92731 Q9NPC1 O14786 P43034 O14788 8408 P07996 P27487 P03950 5155 P11215 2676 Q05952 P00533 Q15672 Q7Z727 6495 P32121 Q05397 Q9Y6Q6 P37023 27185 P23528 Q9UKW4 4983 5159 5830 Q9NRI5 3655 Q13145 P62258 P55290 4627 P05556 6386 Q14114 3672 Q92949 2100 1130

P13611 P10589 P13612 P48380 Q13829 P17302 5728 P16333 Q99698 Q9NVR5 54820 2335 2697 23032 3301 Q9UKX5 P20264 5604 Q13275 8452 7124 7126 O95470 1020 64759 O43639 P35579 O75581 P09172 57669 P23229 1012 3676 3678 P08887 P08648 P30556 3690 5991 P09619 Q5JQC9 P51679 P35443 P10721 O00358 960 Q9HCM4 O60890 3688 4898 Q9GZM8 3689 1947 7804 5747 P02751 Q15475 Q99835 Q12884 9201 9564 7142 P05106 Q8TEY7 P05107 6850 O95813 10152 Q02880 Q13618 A1XKG3 Q9NYB9 Q9Y5K6 7141 2247 P15151 55704 P01579 P26038 347733 1956 P09038 P23443 Q14155 Q8TE73 O15075 7155 P39905 P22492 P31749 O75553 Q02750 O43508 Q9NXR1 253980 Q14160 1289 Q6PID4 P05362 P40337 Q14289 Q9UL54 P06576 P78423 4690 7046 6198 O43157 Q9Y6W5 P56199 O43711 P11047 Q4VCS5 Q00796 P41134 Q9Y3A5 Q6NVY8 94 3911 P31689 P78536 7057 P06702 Q9UHG0 5796 O43602 7291 3010 1072 P50542 O43847 Q3V6T2 9350 55172 Q99755 84159 81565 P16070 P98172 409 O14713 Q01518 P49768 Q14865 P29474 Q86VW1 P14416 O00560 P52735 8394 6093 Q00535 P16284 Q96S42 P17252 P06241 6405 P01137 8829 P01375 9255 P35908 P19320 Q658W2 P43405 P17813 P60484 3148 Q8IZE3 3146 4478 P01127 5327 10487 Q96JB1 P26583 O95995 O00628 8851 P07949 8852 P78509 Q12904 P15692 O14908 10451 5578 P08246 P26232 5591 Q6IR47 Q8NEC5 Q68CZ2 Q8WZ19 7410 7531 7412 9270 P14923 3728 5467 207 Q13574 Q15078 P09429 5362 7422 5364 O14495 P78527 O75051 P09430 Q13464 9839 O15230 P20908 Q04771 23513 8879 7428 P00750

response to insulin stimulus 64397 P08833 6198 P98155 6599 3484 Q9H2Y7 3481 O60603 P42345 P42224 Q96RU7 2303 P62993 Q04759 Q99523 Q92572 51763 Q13480 O14543 Q92574 P27986 10014 1978 2308 2549 5155 O15530 Q96QB1 Q9BT40 367 P05062 Q13370 P01344 Q92569 O00206 6256 P29353 6772 P19793 P14410 894 57761 P21860 Q9UQB8 5798 P09874 Q92831 P32239 5295 8165 P55851 5970 2065 Q9UKG1 5170 142 Q04206 8836 P01127 3667 5207 P41743 Q92667 Q92820 5584 7248 9021 Q9UQL6 P10275 10458 P22415 10057 Q99966 7099 5591 P05413 5471 Q13322 10580 Q9Y4H2 Q86WV8 P35568 P30279 P37231 57678 2475 207 5468 P17081 Q92922 Q99958 P09467 9564 O14492 Q16849 P49840 22937 P10415 Q06203 P78527 P18146 P16118 Q8NER5 Q9HCL2 P61244 2885 P17174 P08069 8503 4149 Q12770 2805 P23443 P41159 Q12778 6464 Q9BX66 P31749 1176 P18433 Q6ZWJ1 2931 26060 P06213

regulation of locomotion P25054 Q13477 Q15654 O14672 3516 284217 P84022 P09493 P07996 P04004 Q15303 3091 5154 5155 P12429 Q92729 Q5VTR2 O95684 5270 P16234 P16473 Q9H2X0 7448 7205 5029 P00533 Q14344 Q9HCY8 Q5S007 Q13017 Q7Z727 P25391 Q9Y2I1 Q9BXC9 57402 Q9UBU3 P37023 Q00013 Q96RK4 5159 P55290 5054 3791 4088 3673 Q8WWN8 Q14118 5728 P17301 P21359 O60674 P20827 Q13393 Q16665 3667 5604 Q16787 4771 P28482 1020 Q9UQL6 4092 394 57669 51564 1012 P08887 719 P51671 3690 22846 Q9NYA1 Q15109 P09619 O43889 Q9Y4H2 P51679 P35443 P10721 P35568 285 Q06330 P18206 P01308 120892 Q9HCM4 967 9564 P05106 P11229 A1XKG3 P30542 2247 P08069 Q9NQC3 1956 P09038 P23443 P09958 P31749 Q7L8A9 Q8N9R8 Q02750 P37840 P48061 Q9HCK4 3909 Q6PID4 P05362 P05121 1969 P09601 P29317 Q14289 Q16581 1605 4318 P06213 Q13873 6198 O15123 2150 O60603 Q4VCS5 Q96RU8 3479 23560 6622 P39060 P04085 10013 94 6869 Q15389 10014 3911 3912 P78536 7057 Q96QB1 5796 Q93096 2159 2277 4214 P01343 Q16363 11116 4354 P14416 Q13418 P14780 P41231 Q00535 P17252 P35240 P01019 P01137 Q9Y696 Q01974 P60484 P17936 Q92786 P01127 10488 5328 Q59EA4 Q9UBN7 O60271 P24043 P07942 Q8WUI4 O43915 8174 P78504 3162 P23508 P00749 P15692 Q9BZE4 5578 5337 P00742 4920 5338 P08246 P35968 Q13443 Q7Z5L7 64127 P61073 324 Q15077 207 P55085 8754 7422 P10415 3611 127435 7414 10672 P25103 O15230 P62070 O14939 Q9HC29 O15105 Q13233 P25116

transmembrane receptor protein serine/threonine kinase signaling pathway Q13873 7046 O14793 O43318 P41134 P16220 P84022 91 P04085 94 Q13485 5154 5155 6885 5796 Q01082 2280 9350 Q9H2X0 4214 O43294 Q15796 409 P43026 Q9HAU4 P01100 Q12841 Q15797 Q8N6I1 P32121 P29590 P12755 6927 P37023 Q96S42 Q53X93 Q9UJU2 9518 P01137 O60383 Q99988 Q93008 P02461 4086 4087 4088 4089 163126 Q13705 Q9UBE8 Q9UNE7 P20823 P17813 P01127 P62942 P27037 5460 64750 8852 1385 P51668 Q5SGD2 4092 4093 P08123 10454 Q99966 2626 P05412 O00238 Q6IR47 Q13443 O95405 Q5JQC9 Q9HCE7 P36896 57154 P36894 P10600 51295 P61077 6711 Q8WUH2 Q99717 10468 Q15750 O75969 8754 O95813 10273 Q9BQ95 P43694 5371 O15198 Q04771 P19883 P35548 P51617 P37173 O15105 Q13233 10566

gene silencing Q7KZF4 O43159 23409 Q9UL18 P55265 27327 Q9UQL6 Q9Y6K1 P51787 Q9HCJ0 P84022 29102 4686 Q8WYQ5 10014 27161 5935 Q9NRC8 Q9HAV4 Q92769 Q9NQZ2 Q8WWH4 Q09161 4343 Q9UBC3 8467 23411 23378 26523 Q8NDV7 1789 1788 6839 Q9HCE1 1786 57510 54487 Q9BXP5 P17480 Q15796 Q96EB6 P52298 Q9UKV8 Q15797 O75569 P98179 Q5TAX3 3066 Q9UIF9 7141 Q9Y6E7 79753 O60264 P09430 Q8TAD8 5119 51548 51547 Q9UPY3 P26358 O43463 3070 4086 O95983 4087 4088 Q9UPQ9 23112 Q9NRZ9 Q9HCK5 51593 Q9H9G7 192669 3784 11176 53615

protein oligomerization 8772 Q96RE7 Q9BY76 P04637 P54619 P50416 6470 P13861 230 P62993 999 Q92574 5536 O14788 Q15389 P49815 6505 P01112 P27105 Q13489 Q9H4L7 Q8IX30 P51636 P07919 55054 2040 P50542 84708 7052 O75381 641 Q9HD26 P14174 4214 Q13490 Q9UKV5 7186 6498 P12757 5563 57120 3265 P12755 Q13418 9361 3416 Q6DD88 Q9UDY8 P32929 5830 P21980 P23921 4869 P31946 5213 5576 Q86XR8 1374 P35222 1491 Q08257 P34896 P54646 Q676U5 P17302 P19438 267 3028 2697 Q96RR1 P61457 P53041 P08034 3667 6814 5604 P43005 P30566 P08758 O43593 Q8N307 Q13158 O95999 7248 P54132 O00186 114548 3162 P30047 P16389 P15056 673 1499 158 P04040 P09972 P55072 55806 O15294 7132 5195 Q9Y478 5471 7013 Q9Y239 P35609 P36776 P14923 64127 Q86WV8 P30793 P35568 5590 Q9BUB1 3728 P14373 1666 P21579 Q05513 Q02763 841 Q7Z2W7 7249 9702 329 847 5987 7529 1429 Q99714 P04179 8473 Q9NVD7 P00492 10392 P54274 Q06203 P12830 P19474 P32320 Q9UHD8 Q12809 25923 Q08188 3737 Q8TBB1 330 2644 222484 2643 2885 3611 Q96P20 7415 Q14790 56916 P08069 857 Q16698 P51575 Q12933 7157 Q02750 5092 138151 Q96BF6 440193 P51617 P17858 Q9HC29 7428 989 5888 P09601 P40337 Q01813 P08237 P04275 Q13233 P04156 P06213

aging Q13477 7046 6198 P04637 P30086 P51587 Q9Y4K0 230 472 2305 P62993 Q04759 Q99807 P01116 O14543 1738 O15119 518 P01112 P01111 O96017 P48201 5037 P16104 P42858 5029 P01588 493856 Q15554 Q96EB6 P01100 2956 Q5S007 Q7Z727 P35638 Q92835 6256 P29474 Q8N726 5321 Q13535 P19793 3265 P29590 Q13418 P14410 1634 P41231 P17252 1111 P09471 3014 811 537 6647 P01137 6926 P00441 4869 5054 5970 P55210 P18074 P19320 Q99583 9093 P35625 P35348 5728 P17813 3028 Q05655 P60484 Q07954 545 Q04206 821 P07585 P40933 Q96EY1 P43246 5604 5580 P25098 Q13315 P13945 P41181 131601 8174 9021 156 P09972 5578 P27824 P47712 P05412 Q6IR47 7013 7014 Q15109 7412 P10600 Q15904 23411 P37198 1029 10229 1026 120892 Q9NPH5 4017 P42771 O14757 P27797 Q99714 P09622 P04179 P28223 22937 P10415 Q8N5K1 4035 P54274 O60502 2885 3611 610 6721 Q9NQC3 Q12770 P02786 P23443 23636 5371 P09958 Q12772 7037 Q08050 7157 Q02750 P38936 P52701 O60911 Q86W33 10724 Q9UL51 P48059 P05121 P21554

DNA damage response, signal transduction 9100 8930 P04637 Q86Z02 7161 P51587 O43715 25988 472 595 Q96RU2 P23025 26270 Q14694 O96017 P16104 641 Q96RL1 Q99638 2956 9641 Q6ZN33

Q9H305 8555 Q13535 Q9UNL4 P29590 Q06124 79791 890 57646 O60543 1111 3014 51147 29086 9656 P31947 8445 Q9BZF9 3428 10524 80254 545 Q8WTP8 P23497 P43246 Q6PCD5 7508 6672 Q16666 Q13315 P54132 Q9UHJ9 3162 672 Q03468 51720 4646 Q9Y5J5 Q9NYZ3 O95243 Q9NS56 Q01831 P46736 P20248 Q9NWV8 1026 P51959 5347 Q9NRD1 Q9UM54 Q13574 O14757 P24385 7023 5883 P38398 Q9BQA5 Q5XUX0 Q01664 Q6NUQ1 Q92993 Q92630 5371 O15350 64782 Q14676 7157 P53350 27315 P38936 P52701 2810 P00519 P40692 55159 Q96MT8 Q14164 900 51512 P09601 51514 Q14683

leukocyte differentiation P10914 P25054 P04637 P15923 Q9H165 Q9Y4K3 7163 6194 472 P17275 998 P49810 O14788 P42229 10014 P62753 Q9C0K0 P56524 P78536 Q15306 Q15669 23210 Q02447 641 P16473 P08575 P49768 7189 P06239 Q03014 Q9Y6Q6 P55327 P14780 6929 Q9UDY8 P01137 5316 3932 3659 4627 P05556 Q13422 Q13268 Q13426 5971 Q9GZX7 P04629 P19320 9093 P35222 P13612 4914 3428 81501 O75081 P43405 Q9HBE1 Q8N4C8 53335 57379 6776 P40933 Q96EY1 P43246 P04233 6670 P55347 Q16666 Q13315 P54132 Q6NYC1 P41182 Q9UQL6 P53567 P35579 Q01201 1499 6304 5336 3676 Q8WV28 7518 5591 7412 P10721 P37231 64919 1147 324 841 5468 604 3688 P07766 6850 P10415 P78527 P18146 P36888 Q04656 Q14790 P01730 P05112 P49715 Q01826 P60953 O15111 7157 P51692 P11912 P35268 1050 P16885 Q9H295 P37173 863 10320 9846 10202 4318 P60568

negative regulation of translation Q9ULR5 O00425 Q7Z727 Q9UKV8 Q9UL18 Q9Y2Y8 7248 27327 Q06787 P29590 Q9Y6M1 Q9P0J0 27102 P17252 811 5578 Q9BQI3 Q92574 6726 Q13144 27161 1978 Q14232 P38919 5371 Q9Y2T7 P03950 9775 Q9NR50 P37108 26523 Q8NDV7 Q86WV8 P51114 P19544 7490 Q9HCK5 Q9H9G7 400961 Q9P2K8 192669 2332 10642 P60228 10644 P27797 10643

cellular response to biotic stimulus O43353 P26583 Q5VVQ6 P04637 P28482 Q96A33 O60603 O75460 P19525 P42224 595 Q9Y2C9 79139 5610 8767 Q16236 51009 P55072 P25963 2081 7099 4780 22926 6048 P27361 O76024 P18850 P11021 Q9P2K8 P49841 Q9UKV5 Q9BUN8 O00206 P24385 P35638 P29474 4792 6772 10273 O95292 148022 Q99942 57003 7415 P01579 Q9GZP9 O75807 5054 9217 7157 Q8NHX1 Q9UNE7 55432 2932 267 P51617 3148 Q8IUC6 Q15011 P05121 3309

protein import Q96QC0 P04637 O96008 Q9P2R6 473 2147 23165 Q92973 Q9Y2X3 10651 637 Q15785 P49815 Q15388 P25963 Q9NPC8 Q9HAV7 Q14974 4580 O15131 Q01082 P55957 P50542 55696 O75381 P42858 P52292 406 9868 P60468 O00327 6496 P52294 P29590 P30101 Q96P70 Q9NZQ3 5830 P01137 P63104 P01375 4000 P62829 Q86XR8 64901 O95343 O60318 P61970 O60674 9804 1408 Q49AN0 9927 O00623 O00505 O00628 7124 P28482 O00629 P41182 Q9P0J0 3836 10736 O94826 10452 P05412 5195 O95405 7534 Q9H2T7 10469 Q99797 Q9Y5J6 P00734 6711 207 7249 3841 9702 604 3843 80273 4792 100287932 Q9NW64 O43615 Q9NS69 10953 401505 O14925 2247 56993 P01579 P02545 5514 P09038 2923 55705 P49792 5371 4285 P30536 Q13505 7157 P31749 O95140 Q9Y5L4 O75431 Q8N4H5 P62072 O00410 23633 Q08209 P52948 Q92621 10204 P25116 51517

regulation of mitotic cell cycle P25054 6198 6597 O95714 P04637 11065 5300 8379 Q12948 P30086 P51587 3481 O43715 23326 6194 351 472 990 595 26271 O60566 Q9Y3A5 Q04759 3479 P84022 995 Q99640 55726 8924 P83731 54998 Q9ULW0 P42229 P62753 1978 P01112 Q9HC98 O43683 7332 Q13526 5037 P51636 Q12959 P57059 P55316 1104 Q9H0C8 10783 Q9UI95 P00533 P05067 P01344 P01343 2290 Q6ZN33 Q13257 Q8N726 3265 P29590 Q9NQW6 79791 Q7L5Y9 Q9NY61 22974 4193 890 1877 Q66K89 891 P51808 Q99741 55743 O15379 P01137 P01135 P01133 P01375 P06400 O43264 O15392 4088 Q6R6M4 8841 O00213 P51532 9099 O75592 Q9NWT8 Q92786 6776 5569 Q6PCD5 Q9Y468 9700 7124 Q13315 9820 P41182 P14635 Q96EP1 10459 1017 10296 Q13042 P08243 O14744 150094 7013 Q96SB3 Q9NYA1 P51955 Q9NYZ3 1029 P20248 1026 Q9UJX2 Q00987 P51959 P01308

P50748 324 3169 1540 54443 5347 604 P42771 Q13574 Q9Y6D9 P26045 P24385 P61925 O75604 7023 P10415 P30307 P54274 377630 Q9UPT9 O00762 55145 Q5XUX0 Q01664 P68036 Q9NVM9 5119 Q14674 1956 P23443 Q9NQC7 5371 8881 7157 P53350 P51692 P38936 23513 7272 Q9NVV9 Q14160 55159 23077 P24941 900 51512 Q14201 Q9UKT4 P06213 5925

positive regulation of organelle organization 4690 P25054 P04637 P10636 P30086 2033 O95271 3481 Q4VCS5 Q9BVA0 P42345 Q9BXM7 Q04759 3479 P84022 P09493 P04083 Q7Z6C1 P46821 6869 11346 P07355 Q9UBK2 25913 9475 7334 4221 Q9UBC3 5037 65018 1072 4214 P61158 Q15554 408 P49407 P01344 P01343 Q09472 5321 P29590 O00444 P56539 P31431 P53667 891 P23528 P35240 P01137 P01135 Q92833 P26358 4869 Q9BSI4 P01133 P01375 4088 O75116 Q9NUX5 382 P16333 387 301 302 Q92786 4131 9700 4771 7124 P14635 O43639 10733 P61586 P47712 P51671 7013 7014 1789 10109 1786 P01308 324 2475 4137 3720 Q9H2K2 P54274 P62330 P61088 857 P25103 859 Q14674 5371 Q9BZS1 7157 O00255 Q6KC79 Q8N3V7 50943 Q13233 P06213

ncRNA processing 92345 9221 Q92979 O43159 Q7L0Y3 11340 Q12788 92105 P62081 O00541 Q06265 P18124 81605 6194 10813 Q9H6Y2 Q9Y3A5 54512 P84022 Q9Y2X3 6229 Q14690 P62913 P62753 65083 Q8WW01 1736 Q15024 Q9UQ80 54517 P05455 P38919 5393 5394 16 Q14978 5036 6125 51096 10667 O14717 51010 54487 Q9Y3B2 51013 Q7Z6J9 51654 Q9NPD3 Q9NVH2 10785 P46777 Q15796 Q96EB6 10940 P62249 Q9UKV8 O95059 Q15797 Q8N726 Q5TAX3 O00567 Q9Y606 Q8TED0 283989 126789 Q9NV06 54931 Q9Y2Z2 Q5TAP6 Q9UPY3 Q15046 Q8WVM0 Q9UL03 4086 4087 P78316 P49591 P46087 4088 P08708 9775 P18077 Q96B26 Q96HW7 3028 Q99575 10528 P62263 55759 Q9UET6 P49588 134430 P57081 O95478 50628 Q9NYH9 26512 P39019 23404 23481 80324 Q8NI36 8602 Q8WYQ5 P62312 27161 Q8NEC7 Q14137 Q9Y2L1 51729 6201 Q08J23 Q9H0H0 Q13601 23411 23378 23016 Q96G21 Q9Y5J1 O95363 23536 1029 6839 Q9NY12 Q96SZ6 Q8N0Z8 Q9BUB4 Q9BXP5 11157 P42771 P62847 118460 P62841 Q99714 Q8IY81 2091 9045 Q96FX7 P22087 22894 10438 54853 Q5RKV6 P50914 88745 56915 P62857 Q9GZL7 P56182 O43463 Q9UNQ2 Q13901 Q13868 Q9BVS5 Q9H6R4 6741 Q01780 25896 Q969E8 Q9NQT4 Q9NQT5 Q96EU6 4839 51593 Q9BVJ6 P61254 Q96HR8 23076 10607 Q9Y2W2 6217 Q14684

regulation of cellular component size P54852 P08833 10092 P55268 10095 Q9UQP3 10094 O14672 6236 Q9BZ95 O75385 29948 3084 351 P37288 P16220 P84022 P07196 118 Q92731 Q9Y2X3 Q7Z6C1 O14786 5931 4722 P49815 Q13485 8408 P47755 Q9H0D6 P47756 5155 Q12837 P58546 P13631 O95684 51094 367 P55042 P61158 Q9UK53 Q13017 136319 Q9H2G4 P50150 Q13813 O75489 Q76FK4 P10451 P53667 P46527 P37023 P51805 P20936 57521 P23528 81873 P32929 P35080 4869 P06400 5716 4088 4089 7476 P31947 2100 P68133 58189 1491 Q7L576 382 P16333 3304 P52907 Q6PKD3 64061 Q13275 829 Q8TDY2 7248 1385 O43639 P10275 Q9P0J0 394 6709 Q6FHQ0 830 832 P51671 Q96SB3 Q9NYA1 P36896 P10600 Q86WV8 P19544 7490 1029 1027 1026 P01308 2475 6711 6710 P62328 7249 604 Q14140 P42771 9201 Q9BPX5 O95376 Q86V24 P19634 P62330 2247 P02549 Q16576 Q9NQC3 859 5515 P09038 5518 O15075 Q9HB90 7157 P31749 2023 P38936 P48061 55035 2810 2137 51474 P08195 Q6PID4 P63313 Q14289 4690 Q13873 7046 O95835 P04637 P10636 2033 3484 7161 P50897 Q4VCS5 3913 11108 27000 P42345 91 94 Q92574 P46821 51009 Q13642 P78536 22803 6520 P35368 O43602 Q02297 Q01082 1072 P67775 2039 Q96RT1 4214 P05067 11116 O15020 Q09472 Q8N726 Q9UKN5 O15143 P29590 Q13418 P56539 P41235 1874 773 Q53X93 P01019 Q9GZP9 6405 P01138 P01137 O60383 8829 Q16254 Q08495 A4D1W7 O00213 P11277 P01127 P06733 4131 9821 P41182 Q9UHB6 P35612 P35611 1655 552 Q9HC57 Q9H2D6 6548 Q9UJW9 O00755 Q6IR47 P30153 P37231 10109 P51959 Q99675 Q9UJX0 4137 207 5468 P01185 6696 P10415 Q96A54 23189 P32320 P17844 3611

Q99543 10552 5371 Q14678 O15350 P08107 O75832 Q12816 O60356 3621 O43909 900 55558 Q13233 P60568 5925

regulation of protein catabolic process 9146 P25054 5582 P05129 6872 7124 Q9UNN5 4092 23327 Q96RU8 1454 P84022 1453 O14908 Q96PU5 P63165 Q99728 P45974 P55072 Q13200 Q13485 O43242 Q9Y4P1 Q13443 Q96HU1 Q96J02 83737 O43847 Q96KS0 P46934 2316 5707 5708 5709 3326 Q00987 P01308 324 207 5347 406 4898 P61956 4734 P21333 P26045 O00327 7341 8078 8754 10273 4193 Q8WY64 7415 P01579 11124 O14964 P01375 5716 Q9BT67 O15151 P09958 Q9UKS6 Q60FE5 4088 O75832 4089 P49674 5970 P53350 P31749 Q99460 P35625 580 Q9UNE7 P17302 27352 23192 2697 Q04206 O15105 P48730 6613 P08238 P21675

tube development 8654 Q12948 O75386 Q9Y4K3 O43318 4609 2303 284217 P84022 Q99807 P49810 P08047 Q7Z6C1 O14786 P49815 Q13485 5154 8543 54361 Q02447 P16234 367 P46531 4851 Q15796 Q14344 Q15672 P28300 P61968 6495 Q15797 P05549 P25391 Q9NRY4 P12755 P23528 Q96RK4 Q92830 Q9Y297 P03372 P40424 6926 P06401 P00441 4086 4087 Q92824 3791 4088 4089 P07737 3672 8323 Q14118 Q13705 Q86YT6 P56705 P17302 57534 Q4AC94 P31270 2697 Q9BXB1 P61296 P08151 P20264 P55061 9421 7248 2909 64759 P10275 O75462 4092 4093 55366 154 27283 1499 Q9NYQ6 P07550 Q99966 P51671 P30556 3690 8100 O43521 P36894 P10600 Q86WV8 P19544 7490 3207 960 841 7249 Q8WW38 Q99958 Q15475 Q96T37 P04179 P05106 7020 P48507 2247 2246 Q9H1Y0 O14641 P05230 P49715 P09038 P10826 P10827 P39905 O15198 1050 Q9HCK4 Q14160 O95390 P37173 10728 P09960 Q9UMX1 Q99814 1605 Q13873 7046 9464 O96004 P78540 P56199 2034 2033 1181 P52952 2702 P41134 3479 Q1PSW9 999 P04085 Q92574 P05177 O15119 3911 1856 9474 6885 Q96QB1 23210 P35368 7291 1072 7052 O76074 O43609 P01106 P16070 P23276 P28845 P01343 Q13099 P49768 1080 Q09472 P50552 7189 9244 7067 P48431 P29474 P13569 P29590 Q03014 Q13418 Q63HK5 P31431 Q99623 O14727 Q96S42 P68871 8945 P01019 P21980 6647 P01137 Q9UBM7 Q9NSC2 8829 Q9UPY3 P01133 Q9UJM3 91147 P35222 2735 P17813 2730 P10070 P10071 11331 Q92786 6657 2737 2736 6670 P01042 O95999 O43597 P07949 Q6NYC1 P15692 6667 Q15185 Q9UJW2 10454 2626 3290 Q6IR47 P35968 Q68CZ2 26005 Q5HYA8 23414 P36382 10229 3169 4015 54206 7408 Q15750 7422 P10415 P12830 2099 Q9BUZ4 P20336 3611 Q04656 Q14790 P62736 P43694 10672 9839 P08588 5371 Q15648 O15230 Q04771 23513 O60353 P18827 O15105

negative regulation of kinase activity P25054 O95835 9467 Q9NRW4 7161 124790 Q96RU8 Q96RU7 1852 1850 10935 P15090 P49815 6901 P09936 O94992 4221 O15530 Q16635 O95684 P50148 56940 Q8WTR2 O43609 P67775 51654 P08575 P42574 Q13098 P24522 11116 P49768 Q7Z727 Q8N726 Q9H422 Q13418 P56539 Q9C010 84619 P46527 57761 P17252 P35240 P06400 Q9UJM3 Q15208 P31947 9093 5170 P21359 Q96EY1 5569 Q9Y2B9 Q96MH2 10641 1647 4771 O43597 11142 Q16828 P30048 10614 P61981 Q8WTW4 5578 Q8IWV1 Q13202 7532 Q9Y4H2 P30153 26524 P37198 1029 1027 1026 Q96SZ6 2873 324 207 7249 54206 P42771 Q13177 Q99956 P61925 O95257 7023 Q9UGJ0 O75293 Q9UNH7 3611 Q01664 Q9NRM7 58533 O14640 857 859 5515 5518 23636 Q9BY84 O15350 Q13227 P31749 O00255 P38936 57732 2810 Q8N5A5 1848 5925

negative regulation of transport 11060 O95835 O00308 2550 P10599 94121 3084 Q9UBS5 57620 P20396 6622 90678 6901 P07996 P25963 Q9UBC1 7057 4188 8301 5311 Q16635 Q02297 7295 P46934 2316 9908 Q9HD26 Q99750 Q13492 Q13370 4734 Q13133 808 Q7Z727 7067 P29474 P29475 P14416 57120 Q06124 Q9UBU3 Q00535 O60543 P17252 P09471 P21860 P01137 Q9Y6A5 P01133 P01375 5716 8165 P31946 A4D1W7 P55055 Q9BZF9 2065 P21917 Q6UWE0 9495 382 5728 P21359 P19838 P60484

3784 5569 3667 6814 Q9Y2B9 O75915 Q92667 P04233 P43489 P24588 23409 7124 11142 Q9UNF0 O00186 1020 Q9UNN5 O00221 114548 3162 P51787 154 P63000 P07550 5578 Q99728 Q13563 P62158 3690 Q13322 7376 Q96C24 10460 Q9Y4H2 O00233 P35568 P62166 P01308 1540 3685 207 2917 P46059 7529 10468 P23510 P21333 P61925 P05106 4790 P28223 4792 Q9BY11 P49840 P10415 Q96FX2 A1XKG3 O43612 2010 P30542 P62330 Q9Y6E7 Q8NER5 Q96P20 11124 Q9UN86 5879 P01178 Q9NQC7 Q9BT67 Q9UKS6 P10827 Q60FE5 O75832 Q9UGK8 Q9BZS1 11252 P31749 P19883 P37840 580 P50402 2931 Q9UMX1 P09601 Q15653 50943 P21554 P06213

protein polyubiquitination Q96EP0 Q15819 P51668 Q96EP1 Q9Y4K3 10616 997 90678 7332 Q9H4P4 Q13404 7336 7335 Q9HCE7 Q5VTR2 57154 Q86Y13 Q96J02 83737 5071 27339 23291 P61077 4214 329 Q13490 Q9UKV5 7189 10193 P10415 10075 10273 Q13216 P19474 1161 O95376 Q9UKB1 Q9BYM8 55743 Q7Z6Z7 P68036 O60260 Q9UMS4 55072 Q6UWE0 Q9UNE7 267 Q13233 51514 P49427

DNA conformation change 4173 Q14566 P06899 4176 Q9H0U9 P33993 P33991 4171 1058 P20671 Q15021 Q8NCD3 55723 25913 Q9BTM1 P62805 P62807 Q99880 3010 P16104 Q99877 3009 Q9NVP2 3008 3007 Q8IUE6 3006 51773 Q9UKV3 P42695 255626 Q99879 Q9H2G4 Q7Z7K6 Q96NT1 3024 O75367 Q03014 P55209 P11388 P11387 P23527 Q01105 3014 P17096 O60264 Q8WZ42 4869 O95347 4361 Q16777 Q16778 O43823 P0C0S5 23310 Q9NRZ9 Q9NUX5 P04908 22985 221613 Q96A08 Q99733 55355 Q93079 Q8TBE0 P0C0S8 3148 Q96RR1 Q93077 Q7L7L0 3146 Q6NXT2 64061 P41227 P07305 P26583 8290 Q96KK5 3159 Q02539 P49736 P49450 8348 9555 8467 O60814 Q96QV6 Q92522 8338 Q8N257 P09429 P33778 266812 10270 Q5SSJ5 4673 Q02880 22893 P10412 P58876 Q9NR48 Q96T23 P49959 P84243 Q71DI3 P68431 Q00577 Q16695 P49711 8349 Q92878 5119 Q15003 7153 3070 7155 P22492 Q5QNW6 7150 P16403 P16402 P16401 Q9P0M6 Q9NTJ3 Q6FI13 4676 5888 Q13112 Q96FF9

nucleosome assembly P07305 P26583 P06899 Q9H0U9 8290 4171 Q96KK5 1058 P20671 Q8NCD3 55723 Q02539 P49736 P49450 8348 Q9BTM1 P62805 9555 8467 P62807 Q99880 3010 O60814 P16104 Q99877 3009 Q9NVP2 3008 3007 Q8IUE6 3006 Q96QV6 51773 Q92522 8338 Q8N257 255626 Q99879 Q9H2G4 P33778 266812 Q5SSJ5 4673 Q96NT1 3024 O75367 P10412 P58876 P55209 Q96T23 P84243 Q71DI3 P23527 P68431 Q01105 3014 Q16695 O60264 8349 4869 Q16777 P22492 Q16778 P0C0S5 Q5QNW6 P16403 P04908 P16402 221613 Q96A08 P16401 Q99733 55355 Q9P0M6 Q93079 P0C0S8 3148 Q93077 Q7L7L0 Q6FI13 4676 Q6NXT2 64061 Q13112

negative regulation of cell differentiation P25054 2672 259266 O75385 595 Q8IYN9 P84022 O14786 P78395 8408 P25963 Q9NPC8 Q13127 P52565 P62805 19 54361 Q9H2X0 P46531 4851 4854 Q13133 Q96EB6 Q15672 Q14106 Q92835 6498 P12757 P12755 P10451 Q05397 P51805 57761 811 P02741 P40424 6926 Q9GZX9 Q9NQX0 4088 9657 7476 Q92949 P55055 Q9Y618 Q86YT6 93166 P56705 57534 387 P21359 P04233 Q13275 O95477 4099 7124 P28482 1021 Q9UQL6 4092 P10276 396 10736 1499 51564 P61586 Q9Y2T1 5978 Q99966 Q9UM47 3690 7376 Q9HAZ2 P22392 Q8IZT6 P36894 P10721 Q9BPY8 1387 3685 Q9NQB0 604 Q92888 5747 Q96T37 P04179 P05106 4792 Q86VS8 P36888 857 Q9NQC3 P05112 P29323 149041 7157 P51692 Q5TC82 O00255 P19883 861 O95390 84376 7704 7046 6993 P10997 P04637 O43711 P63172 P14555 P52952 P15531 Q96RU7 3479 Q1PSW9 2147 Q15022 P42229 O15119 10014 P02686 Q04917 P56524 4221 7291 P55316 Q15154 P48436 79923 Q4LE28 O43294 P01343 P49768 O00206 2290 P25791 P48431 9921 Q5TAX3 3142 Q01196 Q9H6W3 Q92793 Q00534 Q96S42 6405 P01137 8829 Q9UPY3 P01375 Q9UMS4 A4D1W7 9138 Q658W2 P35222 Q15051 P10070 P10071 6776 6657 2737 2736 O00744 5460 Q9Y5Q3 Q8WUI4 P41182 P20916 Q9H9S0 P78504 84525 Q99684 4005 Q8N5U6 7099 O00755 7098 Q6IR47 Q14774 7533 23411 23532 27339 P37231

Q05516 P23515 324 P00734 5468 P08138 5108 10468 P27797 P24385 6696 4155 P56177 57045 P51843 Q7Z494 O15455 5914 P24394 9612 63976 23512 P16410 55796 4830 O15105 55558 Q9BZR6

response to drug P25054 5141 8412 P30086 Q5SR56 595 4609 476 1576 P16220 Q99807 Q7Z6C1 5139 O14543 P07996 P52564 P28335 O75815 Q15303 5154 5155 4860 Q9UBC3 5037 P18887 P09382 P22223 P47985 20 O15524 P35638 P28300 Q15797 P31153 P17405 495 2564 P09471 P63104 64170 31 P00441 8443 6262 5174 4086 5970 3673 1374 Q01581 Q9H257 3551 58189 5728 P17301 1808 P24864 10763 P43246 Q92820 9420 O75469 7124 P11802 1385 P63092 P14635 25788 P21964 1499 Q16555 Q16790 P29466 Q92769 P30559 P78334 1026 1147 P20648 9429 4535 P05023 P04179 P00491 P61803 7386 P28223 P19634 6609 P48507 Q05469 Q9H1Y0 610 P08183 Q5T2W1 6721 P01579 857 P47895 P23443 Q12772 P10827 7157 P38936 P37840 7150 P37173 Q99497 Q9UL51 Q6PID4 P27815 1848 Q14289 6198 P04637 2033 P50416 O00300 P54219 O60603 P15531 P42224 2026 P17275 Q13085 P08684 6622 P39060 999 P04085 P46821 P05177 P15090 P48681 P56524 6760 P78536 9474 7057 1503 P05062 P01106 768 P06276 Q99757 P01100 1080 P34947 Q09472 7067 P06239 P29474 6772 6774 P14416 P13569 O75881 P11387 891 P00966 898 Q53X93 O15379 P21860 6647 P01138 P01137 3932 P01375 4363 P40939 P04626 8841 P10620 2065 2064 P35222 3030 P35348 P17812 2730 P60484 Q04206 8836 P01127 25828 4131 9021 Q16828 Q9HC57 6548 2869 3956 P40763 P04201 P05412 P05413 7534 P37108 P68032 1789 P37231 10229 324 445 4015 5468 P09104 11315 9961 P24385 P10415 P12830 3066 1312 O14920 Q14432 P33527 Q9UNQ0 O15111 Q9H0N0 4830 Q15532 O15228

response to organic cyclic substance 7046 6198 5265 O15123 P30086 2033 2550 P50416 P51587 230 P42224 595 Q04759 83752 Q9UBS5 2026 P17275 P20396 Q13085 7832 Q7Z6C1 O14543 5536 6869 P08842 5155 5037 P35368 P09382 P05062 5029 Q13490 P16070 Q99757 P01100 Q7Z727 Q09472 6256 P29474 6772 5563 6774 P14416 P19793 P17405 891 894 P41231 Q00535 P17252 P09471 898 Q86WA8 Q9UJU2 P01138 P01137 31 346562 6262 6383 Q15843 P07858 5970 3673 1374 P35222 P21917 P35625 P54646 5728 P17301 P60484 Q04206 P01009 1808 P24864 P53041 P01127 P43246 P25098 25828 A8MTJ3 1020 P14635 9021 Q16828 3162 156 1137 P05091 P21964 1499 P09972 5578 6548 P08243 5338 Q16555 3953 3956 P40763 P05412 Q6IR47 Q9NYA1 P29466 Q92769 P30559 Q14416 P30279 285 Q07817 P37231 960 P18846 1026 5468 329 P48357 4535 P09104 P24385 P01185 P10415 P34741 A1XKG3 3066 P19634 6609 1312 P25103 P01178 P23443 P27144 P38936 O60911 O14939 466 Q6PID4 P43681 1848 P09601 Q14289 P21554

hepaticobiliary system development 6670 Q8TDY2 9821 2033 23322 3482 P53567 O60725 595 P05091 1499 79810 P84022 Q8WV60 6667 P08047 Q7Z6C1 P08243 Q14774 54361 Q68CZ1 P30279 Q02447 445 P05062 P46531 4851 3720 10661 P04179 Q09472 P24385 7067 6256 23463 P19793 Q03014 3142 Q9ULH7 P62330 6927 894 Q96S42 P00966 898 O15379 P49715 Q92833 P11717 P10827 4088 5970 P04626 P51692 Q15648 3673 8841 Q6KC79 2064 P35222 P56705 1050 382 P20823 P17301 P21359 Q04206 P24864 Q92786 P04275 Q13351

regulation of anatomical structure size P54852 P08833 10092 P55268 10095 Q9UQP3 10094 O14672 6236 8654 Q9BZ95 Q12948 O75385 29948 3084 351 P37288 2303 P16220 P84022 P07196 118 Q92731 Q9Y2X3 Q7Z6C1 O14786 5931 4722 P49815 Q13485 8408 P47755 Q9H0D6 P47756 5155 Q12837 P58546 P13631 O95684 51094 367 P55042 P61158 Q9UK53 Q13017 136319 Q9H2G4 P50150 Q13813 O75489 Q76FK4 P10451 P53667 P46527 P37023 P51805 P20936 57521 P23528 81873 P32929 P35080 P00441 4869 P06400 5716 4088 4089 7476 3672 P31947 2100 P68133 58189 1491 Q7L576 382 P16333 3304 P52907 Q6PKD3 64061 Q13275 829 Q8TDY2 7248 1385 O43639 P10275 Q9P0J0 394 154 6709

Q6FHQ0 830 832 P07550 P51671 P30556 Q96SB3 Q9NYA1 P36896 P10600 Q86WV8 P19544 7490 1029 1027 1026 P01308 2475 6711 6710 P62328 7249 604 Q14140 P42771 Q99958 P04179 9201 Q9BPX5 O95376 Q86V24 P19634 P62330 P48507 2247 Q9H1Y0 P02549 Q16576 Q9NQC3 859 5515 P09038 5518 O15075 Q9HB90 7157 P31749 2023 P38936 P48061 55035 2810 2137 51474 P08195 Q6PID4 P63313 Q14289 4690 Q13873 7046 O95835 P04637 P10636 P56199 2033 3484 7161 P50897 Q4VCS5 3913 11108 27000 P42345 91 94 Q92574 P46821 51009 Q13642 P78536 9474 22803 6520 P35368 O43602 Q02297 Q01082 1072 O76074 P67775 2039 Q96RT1 4214 P05067 P23276 11116 O15020 Q09472 P29474 Q8N726 Q9UKN5 O15143 P29590 Q13418 P56539 P41235 1874 773 P68871 Q53X93 P01019 Q9GZP9 6405 6647 P01138 P01137 O60383 8829 Q16254 Q08495 A4D1W7 O00213 P11277 2730 P01127 P06733 P01042 4131 9821 P41182 Q9UHB6 P35612 P35611 1655 552 Q9HC57 Q9H2D6 6548 Q9UJW9 O00755 Q6IR47 P30153 P37231 10109 P51959 Q99675 Q9UJX0 4137 207 5468 P01185 6696 P10415 Q96A54 23189 P32320 P17844 3611 Q99543 10552 P62736 P08588 5371 Q14678 O15350 P08107 O75832 Q12816 O60356 3621 O43909 900 55558 Q13233 P60568 5925

regulation of cell-matrix adhesion P10415 Q8N726 4771 7248 7402 P13349 1021 P41182 Q13418 P31431 P37023 57669 P20936 3611 P84022 Q00534 1012 P35240 94 Q92574 P49815 O43182 P07996 P55290 7057 P35968 3791 4088 Q86WV8 5590 5728 1029 P21359 P16671 Q05513 P46939 P60484 Q9HCM4 7249 604 P42771 P42338 948

transcription from RNA polymerase II promoter P10914 Q92858 Q14686 4297 Q14209 P67809 Q9UKD1 Q9UPN6 O00268 O00267 P13984 P52434 P17676 2305 474 Q9ULW3 P20290 3091 P30876 O00716 Q16633 8543 Q16514 26205 O75376 11198 6239 5705 Q16649 P35638 Q9ULX9 P61968 P27695 3661 P32121 Q12968 Q92830 Q9UKW6 3659 Q13148 Q9BYU1 Q9UIS9 P35869 O75478 P18074 Q99583 Q6P2C8 P29084 P36954 142 P19838 Q16665 P23771 9421 P78347 Q13951 4772 O15164 Q12986 1022 57661 P35453 4094 6829 2908 Q14494 O75909 Q13287 Q14134 6827 Q9Y2T7 4780 Q14938 O00472 10987 1025 P08651 4775 P49848 Q92766 4790 O75444 Q9NR48 P61244 O14641 11243 9319 O14640 9318 9439 P49716 P49715 P10827 Q08050 22828 Q9NR55 1050 Q03164 10725 Q15370 865 P04150 4799 Q99814 Q9Y2W1 P78545 P78424 Q13873 O96004 6872 P29375 6871 P29374 2034 P53803 11108 P42224 P62195 Q16236 Q13761 P62875 1856 Q9UBK2 9232 6883 5430 Q9P1Y6 5432 Q13887 P24928 Q03933 6878 Q6P1K2 8812 O43294 2959 408 P49407 409 Q9UBL3 7067 5440 5441 6772 Q9UKN5 Q15714 O43812 2961 2960 1870 5434 P61201 2969 P09874 6660 29777 23435 Q00403 P09086 P61218 O95997 P50613 P51946 23764 P22415 Q03468 Q02930 4800 2626 O95644 Q9NVC6 Q14653 23650 P19387 Q9ULK4 P19388 3054 29079 688 689 P23511 328 Q9Y5B9 4150 4152 P50750 P35711 Q96AE4 Q6P4R8 4149 P43694 Q9H5H4 Q9Y4A8 8880 P27540 Q15643 Q15648 Q92905 P51610 O75031 5927 P63272 Q9NPJ6 9967 902 P21675

liver development 6670 Q8TDY2 9821 2033 23322 3482 P53567 O60725 595 P05091 1499 79810 P84022 Q8WV60 6667 P08047 Q7Z6C1 P08243 Q14774 54361 Q68CZ1 P30279 Q02447 445 P05062 P46531 4851 3720 10661 P04179 Q09472 P24385 7067 6256 23463 P19793 Q03014 3142 Q9ULH7 P62330 6927 894 Q96S42 P00966 898 O15379 P49715 Q92833 P11717 P10827 4088 5970 P04626 P51692 Q15648 3673 8841 2064 P35222 P56705 1050 382 P20823 P17301 P21359 Q04206 P24864 Q92786 P04275 Q13351

ncRNA metabolic process Q92979 Q06265 81605 Q9BYG3 Q9Y2X3 6229 Q14690 P62913 Q8WW01 P05455 P38919 5393 5394 16 5036 6125 51096 10667 80755 51654 Q9NPD3 Q9Y285 10785 P46777 Q96EB6 P62249 O43776 Q9Y606 Q8TED0 P12081 Q9NV06 54931 54938 Q9Y2Z2 Q5TAP6

Q9Y2Z4 Q8WVM0 P49591 P46087 P08708 8565 9775 P18077 P49590 Q96HW7 Q99575 10528 P62263 Q9P2E3 P49588 O95478 P54136 26512 P39019 Q5T160 Q6PI48 23481 80324 29102 Q8NI36 833 84365 P62312 Q9NSE4 5859 Q14137 Q9H0H0 Q13601 23378 Q5JTZ9 23016 Q9Y5J1 O95363 1029 6839 Q9NY12 Q9BUB4 11157 P42771 118460 Q99714 Q96FX7 80222 54853 P50914 88745 56915 P47897 Q9GZL7 Q13868 Q9BVS5 Q9H6R4 6741 P14868 Q01780 25896 Q9NQT4 Q9NQT5 55152 Q96EU6 P61254 10607 Q9Y2W2 4677 P54577 92345 9221 O43159 Q7L0Y3 11340 Q12788 92105 P62081 O00541 P18124 6194 10813 Q9H6Y2 Q9Y3A5 54512 P41252 P41250 P62753 65083 1736 Q15024 Q9UQ80 54517 Q14978 Q8WWH4 O14717 51010 Q9Y3B2 51013 Q7Z6J9 Q9NVH2 10940 Q5D0E6 P26640 O95059 Q8N726 Q5TAX3 Q9BW92 O00567 283989 126789 P26639 Q15046 23438 Q9UL03 P78316 9255 3035 Q96B26 3028 55759 Q9UET6 134430 P57081 50628 Q9NYH9 Q12904 23404 8602 Q8NEC7 Q9Y2L1 51729 6201 Q08J23 23411 Q96G21 23536 Q96SZ6 Q8N0Z8 57038 P62847 P62841 Q8IY81 2091 9045 Q9NP81 P22087 22894 10438 Q5RKV6 51067 57169 P62857 P56182 O43463 Q9UNQ2 A2RTX5 Q13901 P07814 Q969E8 4839 Q9BVJ6 Q96HR8 23076 6217 Q14684 P56192

negative regulation of ubiquitin-protein ligase activity involved in mitotic cell cycle 11065 P51668 P51665 9184 991 26271 O60566 P62195 Q13042 Q92530 10213 Q13200 O43242 64682 P55036 Q12834 O43684 O00231 O00233 O00232 5706 5707 5708 5709 Q9UJX2 Q9H1A4 P49721 5700 P49720 Q16401 5701 P60900 5702 5704 5705 Q13257 5682 5683 5684 5685 5686 P28065 O00487 O00762 P35998 5717 P62333 P20618 P25789 10197 P25788 P61289 5713 P25787 5716 Q15008 Q9UL46 5692 8881 5693 O75832 P62191 P28074 P17980 P28072 Q99460 P28070 O14818 P30260 5687 5688 Q9UKT4 P43686

mitochondrion organization P07900 O00429 P04637 O96008 2034 7161 4728 79810 10935 P84022 Q8WV60 Q99807 6622 10651 637 5018 Q15785 Q15388 6901 4725 Q9UBK2 Q9HAV7 4580 O95202 25915 Q16635 P51636 P55957 Q9BU61 P50542 1191 P42858 P39210 Q8IWA4 4214 9868 P42574 92609 P29474 Q8N726 5442 P12235 9361 Q14197 5830 P63104 O00217 4088 P55210 P31947 3396 9093 P34932 Q9Y3D6 1763 Q96RR1 10087 9804 4358 Q96EY1 Q13794 9927 3308 O15287 3320 P30048 Q04837 O94826 10452 10059 3954 Q9BUK6 P05412 Q9Y512 P51398 7534 P36776 O43920 Q9BUB7 Q07817 1029 10469 Q99797 10229 Q9Y5J6 Q15070 207 P42771 80273 P04179 Q3ZCQ8 P10415 100287932 P52815 291 O43615 Q00059 Q9NS69 10953 7818 401505 P20336 O14925 56993 Q04656 Q9H0F6 P49715 O15350 4285 P30536 Q13505 Q16611 7157 O43464 P31749 O95140 Q9Y5L4 O75431 Q8N4H5 P62072 6182 O00411 P37840 1050 25813 2810 Q99814 Q13233

regulation of cellular response to stress P23396 Q15819 Q9Y4K4 7161 Q9Y4K3 O43318 472 P42345 2305 3476 8767 1616 2308 O15118 7334 Q16512 4067 4188 4221 6885 Q13404 7336 7335 O75376 P16104 P14174 Q99750 81565 4214 4216 P16070 Q96RL1 Q96EB6 Q9UER7 O00206 7186 7189 Q8N726 Q13535 Q9H422 Q96M96 P10451 4193 Q9Y6Q6 27102 3014 O15379 29086 64170 4869 P01375 5716 Q9Y6R4 8841 Q13546 Q9H257 Q9NR96 P78318 P43405 545 Q13153 P04233 O43353 5582 P05129 7124 5585 Q13315 P07948 Q9NX61 P53567 672 Q9Y2C9 121512 Q99683 Q03468 51720 Q13286 7099 7098 Q9Y239 64127 23411 1029 P46736 Q9NWV8 10746 79444 960 Q00987 2475 8737 207 P42771 O15169 Q9GZM8 10392 6696 6850 Q13216 Q9P035 1161 Q9BUZ4 P38398 P61088 Q5TCX8 Q9BQI3 O15455 Q9NQC3 Q96CA5 9839 O15350 6188 Q12778 O75832 Q12933 Q08050 Q13227 P31749 O95382 Q06830 O00255 Q9HC29 Q6PID4 54106 Q14289 Q9UL54 Q13233

negative regulation of gene-specific transcription 9421 6595 O96004 5187 6597 P25490 7124 2672 Q9UQL6 4092 P10276 P52952 P84022 Q99684 3476 10014 5195 P56524 4261 7376 Q92769 8864 93986 O15055 Q14938 Q08117 O43524 O75496 O75376 P19544 7490 P37231 O75381 166 1027

P33076 P46531 4851 P08651 5468 Q9NQB0 7528 Q96T37 P23510 4790 7023 6256 6498 P12757 P19793 O60907 3066 Q03014 3065 Q99471 23028 P46527 Q01664 Q9BZK7 O15379 O15534 O60341 Q92993 857 Q6IT96 P01375 5716 5914 P51531 Q13422 4088 O75832 9612 5970 Q13227 Q13547 8841 Q92949 P55055 Q9Y618 Q6KC79 P51532 P78318 10524 P17813 P19838 Q04206 Q92786 O15105 Q9UMX1 10320 P43489

regulation of biological quality P54852 P25054 65125 Q9H6Q4 259266 9184 2547 2304 P37288 Q8IYN9 2303 O00391 P84022 P21926 Q92731 O14786 Q9Y275 4722 Q9UGM1 Q9H0D6 P28335 25913 P28330 P28331 Q12837 P55957 2317 2316 79763 P20849 P42858 P39210 P15260 Q9UKV3 P61158 10661 P10809 Q7Z727 136319 P27694 Q9H2G4 P27695 2572 Q12846 2571 Q06124 Q7L5Y9 Q8NBQ5 6927 P37023 3416 55584 P62491 57521 81873 811 P63104 Q9Y6A5 P35080 6926 5836 O75911 P31946 P08708 Q86SX6 3672 3673 P31947 2100 Q9NR96 Q13705 P21917 1491 79784 P16333 P20823 2335 O60674 P62263 3667 64061 829 P08758 P25098 1020 P51787 P39019 6709 P21964 1499 830 57544 832 3679 P61106 O14503 O14745 3690 P30679 P33947 P09619 P80098 P10600 P51797 O00358 Q96KS0 Q9UEE9 1029 1027 1026 P20648 6711 6710 604 846 3689 P05023 P07686 6714 5868 5627 P09622 P04179 4792 Q9BPX5 O95376 P19634 3459 Q9HCL2 610 Q9Y250 857 P63302 Q9UN86 859 Q9Y490 P51575 P10827 Q9UL45 P31749 1297 Q13506 Q9P202 27032 Q9HCK4 Q8NBS9 2137 Q9UL51 Q9UMX1 P63313 P09601 O14777 50943 Q9UL54 Q9H4M9 P04155 P04156 P06576 O95399 P00390 6993 Q9UKL4 Q96A33 P56199 P14317 3484 2150 P50897 O60603 23327 11108 3479 2147 23560 Q13085 637 P62873 6520 Q7Z6J4 Q9BS26 25998 1072 641 2159 Q99750 P35251 6517 P23276 Q99757 P02794 11116 Q09472 Q9UKN5 P48551 6774 3265 3263 P13686 P13693 Q99500 54413 P01019 O14965 Q9Y5X9 Q9Y215 4361 5213 Q658W2 P35222 Q9NUX5 P11277 P17813 P60484 P01009 P01008 6776 4358 84299 P06733 6790 O00744 P01042 4131 Q92934 9821 P06737 6794 P05408 54431 Q96P48 P61981 P15692 433 O14908 Q9H2D6 6548 P05412 P06744 O00755 10460 Q5JSP0 P37231 P51959 5467 4137 207 5468 P55085 P62847 11315 P01266 23746 P49682 9601 7422 4155 Q96A54 3065 Q9BRT3 Q12809 Q9NWW5 10437 P17844 P13010 Q15084 Q99543 55785 P61764 10673 10672 O14920 8761 Q86UX7 6341 7430 9612 8766 Q12816 3074 462 56882 8517 Q9H4E5 P60201 7428 55558 10202 P55268 5265 Q16623 Q9BZ95 O43557 P10599 O75385 Q86VQ3 Q9Y4K3 P35414 3084 476 477 P07196 P49810 P08047 51763 P49815 Q9C0C2 Q15303 3091 5034 8546 4188 6368 P46782 5037 P58546 P13631 Q02447 488 Q9NY59 6117 P55042 5029 Q13370 Q16643 Q15554 Q15796 1902 Q9BUN8 Q9HCY8 Q16881 7220 8553 P35638 Q15797 P12757 Q6PKC3 P12755 4193 Q9Y6Q6 495 P46527 51542 Q96RK4 Q13144 Q13145 Q9BSI4 Q13148 5054 Q8IWX8 7476 P18074 P55055 P10586 P68133 58189 P26927 51552 Q6PKD3 Q16665 Q03518 Q8TDY2 7248 P54132 P13667 P09172 Q9Y2C9 Q9Y6K9 Q9Y4G6 Q02413 Q13162 7013 O43521 7014 Q9Y4H2 O43524 7490 5071 7249 P48995 P02751 Q99835 9201 Q86Y82 P02790 7023 A1XKG3 291 Q86VS8 Q8WY64 P48507 P02549 P47895 P02786 2805 10159 O15075 7037 Q9HB90 P55011 P10147 P38936 P37840 64428 2810 Q93034 Q6PID4 11014 Q99814 P98194 Q14289 7046 O75787 P12931 6193 6194 Q4VCS5 3913 Q05329 27000 P42345 2821 P08684 Q92574 1738 P07355 1736 P54105 P78536 9474 22803 O95967 Q02297 7295 7052 81567 Q96AB3 Q9HD26 Q4LE28 P42574 P42338 P98174 O15020 7067 P06239 O95977 P29590 P56539 P30101 P41235 1756 P41231 Q92793 57003 P06241 P05155 3932 O60260 Q8WXH2 Q13547 A4D1W7 P34931 Q9UNE2 3708 Q9UH99 P07101 Q13555 O95994 Q14643 O75503 P35610 O00186 Q03113 P35612 P35611 P00749 P00747 Q9H9B4 P20594 P00742 Q14894 Q13563 O75508 P08246 7099 7098 Q15742 Q9NP71 Q14416 P30153 P11413 7094 P61073 P00738 83548 Q05513 P00734 Q7Z2W7 Q5BJF2 375790 P22303 Q6P5W5 P12956 P32320 P78527 P20339 1314 P02708 P20336 2644 1312 2643 P17174 Q04656 Q9HAP6 O15455 Q14678 Q16611 Q13107 Q06830 P34972 O43909 P43681 900 51752 P00750 P08237

Q13351 Q9NZN9 O15228 5925 P08833 O14672 Q12948 Q9UBB6 Q8IZQ5 Q7L0Q8 4609 1576 P16220 57620 Q9Y2X3 5931 Q9UBC1 Q8NF91 51090 54361 O95684 51094 P50148 10549 P38484 Q9UI12 Q9UK53 O15525 928 P29992 P50150 Q13813 Q9UBD6 3783 Q9NRY4 Q05397 P53667 Q9BXC9 4628 57402 P20936 O60543 Q9UQB3 P32929 P03372 Q6IT96 4627 4869 P06400 5716 Q92824 3791 4882 P48023 3551 9093 P56705 Q7L576 9099 22985 5728 P17301 P17542 3304 3784 6810 6812 6814 948 Q96JJ6 10523 P07307 O95477 Q9Y342 5981 1385 O00221 O00468 Q9P0J0 83706 1137 57662 Q6FHQ0 710 P07550 4646 719 P51671 P30556 Q96SB3 Q9NYA1 P30559 2243 P51679 1152 Q8IZT6 P10721 Q6UB99 P19544 P30793 Q9NS56 3329 57678 1387 2475 P62328 P42771 Q9UM54 5747 P53621 Q9NYB0 P05106 P05107 P28223 P51681 2495 O00244 P51686 P30542 Q7Z406 P62330 10956 P42768 2247 P15153 2244 4664 O14640 4665 Q92878 5515 Q92633 5518 P09958 P29323 P29320 P51692 Q05586 2023 O00255 P48061 55035 Q99259 440193 P17342 10728 Q99497 P05121 P40337 5768 P04275 P06213 4690 P04637 P80188 2034 2033 25861 P16615 1185 Q9UHF4 6622 P01116 6869 Q15389 51009 P02686 P62753 P01112 P01111 Q04917 P49770 Q9UHG2 4221 5311 23210 P35368 Q9HDC5 Q9BT40 O76074 Q9P2K2 9908 2039 Q96RT1 Q15154 4214 P01106 11234 P01589 P01588 P02675 P01343 P49768 O00206 6890 Q8N726 Q9Y320 5321 P14416 P12235 Q9NS23 O00203 773 Q00535 Q8IXB1 Q53X93 6886 Q9GZP9 6405 6647 P01138 6646 P01137 5316 91949 8829 P01375 Q16254 Q08495 P02461 P01130 P04626 23673 2064 O00213 O95067 23435 P35348 Q9NRR8 302 P02458 P01127 Q96EY1 6415 5327 5328 Q9UPZ3 6670 5580 25828 P07947 7520 P07948 Q9UNN8 Q9UNN5 Q9UHB6 3162 Q8IXI1 Q8IXI2 Q96PU8 310 552 553 Q9BZE4 Q96PU5 6667 5336 5578 Q15185 10577 P40763 P26232 P07954 5591 Q6IR47 7532 7534 7533 O76024 5590 3171 10107 10109 P16671 Q00987 Q99675 324 3169 Q9NPH5 328 7529 5108 P27797 64130 P01185 6696 2099 P12277 P50993 51061 P15311 572 10552 P62736 P83436 P25103 Q9GZT9 P26678 P01178 Q96HC4 Q9BT67 5371 Q60FE5 O76003 580 P15313 P16885 582 54583 51075 Q08209 54106 P25116 P60568 10092 8892 10095 Q9UQP3 10094 Q9BY76 6236 11186 8654 P13987 P30086 29948 351 356 P09493 118 Q7Z6C1 6229 Q7Z6C3 Q8NFJ9 Q13485 8408 P25963 P47755 P47756 P03950 9997 5155 4067 O43684 19 367 128 Q14344 20 Q13017 6498 Q9HBW0 O75489 Q76FK4 P32246 P10451 Q9UBU3 280636 Q03405 P51805 P23528 Q9UDY6 5159 31 P00441 P32239 8443 6262 4086 4087 Q13268 4088 4089 Q8WWN8 Q14118 Q9Y618 382 Q9NZ94 222546 P12883 142 P21359 P52907 23154 Q9NUP9 10087 Q13275 Q9NSD7 P55347 7124 O15164 P54253 2909 O43639 P10275 4092 394 P35579 154 P35580 Q00597 156 121512 Q8NBF2 10296 Q99728 2904 Q15223 Q13285 2902 54 Q13286 7376 7375 P36896 Q86WV8 Q9UKA1 P35568 23370 P01308 Q9NQB0 1828 P01303 Q14140 51218 Q99958 P02671 Q8TCU4 Q6ZSZ5 P54274 O43612 Q86V24 P49959 Q9H1Y0 Q01664 P21145 P01579 11124 Q16576 Q9NQC3 P09038 2923 P20073 7157 P14625 25777 51474 P08195 Q15011 84376 Q16581 1605 O95831 Q13873 O95835 P10636 9588 P63172 7161 Q86WK6 79139 10935 10938 91 94 P46821 P42229 P15090 O15119 P07237 O15118 Q9UBK2 Q9NSA3 Q53QZ3 Q13642 Q96QB1 O43602 10939 Q9H7Z7 Q01082 P55318 P67775 P08575 P05067 1627 Q15831 7189 P29474 P29475 9368 O15143 26258 57120 Q96M96 27346 Q13418 O75880 7184 P41597 O43819 1874 P17252 P68871 84502 P21980 O60383 Q9NZ08 P09874 O15151 9133 Q05655 2730 Q8HWS3 P07225 9388 Q15853 P30041 Q6NYC1 P41182 P11766 P41181 P30044 P41180 Q86UK0 P53567 P30047 P30048 Q9H244 1655 P22415 P05093 2741 26234 Q9HC57 P08123 O15554 Q9UJW9 Q13683 P47712 51606 P35968 Q07820 P11532 58480 P51159 8065 O95881 Q07817 P21579 Q9UJX0 P17081 P21333 O14492 O75604 P10415 Q6ZV73 O14495 P19474 23189 59341 Q02363 P19235 P18146 3611 P01730 P08588 P41159 O15350 P08107 O75832 374354 P55773 Q9BZS1 Q15648 1203 4719 O60356 3621 O15105 Q15653 Q13233 P21554 1207

regulation of actin cytoskeleton organization 4690 10092 10095 O95835 10094 4771 7248 2033 Q9UHB6 O43639 P35612 P35611 Q4VCS5 P42345 Q96P48 6709 Q04759 P84022 830 P09493 832 118 Q9H2D6 Q7Z6C1 P04085 P61586 Q92574 6869 11346 P49815 P47755 P47756 P51671 5154 Q96QB1 Q01082 1072 Q86WV8 5071 Q3V6T2 10109 2039 2475 6711 6710 4214 P62328 7249 P61158 P17081 O15020 Q09472 Q13813 O15143 253260 Q9BPX5 23189 Q13418 P56539 P31431 P53667 P62330 P20936 3611 Q9BVC4 P23528 P02549 81873 P35240 55704 10552 P25103 859 P35080 O60260 O43182 Q08495 Q14678 64223 4088 P48061 382 P11277 P16333 387 51474 P52907 Q92786 Q6PKD3 Q6R327 P63313 Q8N3V7 Q13233 829

regulation of multicellular organismal process P25054 O14793 2550 259266 P51587 P17676 2547 P37288 Q8IYN9 284217 P84022 Q9HCJ2 O14786 O14788 57506 2307 Q12834 P14174 P42858 Q9UKV3 P61158 P10809 Q7Z727 136319 Q06124 P62491 811 5830 55109 O75928 P40424 6926 3659 3673 Q92949 Q9NR96 Q13705 P17661 P21917 57534 O60674 P20827 Q8IUC6 10642 5604 10644 10643 Q9Y468 P08758 P27037 P25098 1021 1020 114548 P51787 79576 1499 P61586 O14503 3690 22846 P29466 Q9Y239 4780 Q92769 O95361 P10600 O95365 1029 3207 10626 Q9HCM4 604 5624 P05023 6714 5868 4792 Q9H2K2 1285 1284 Q9HCL2 610 Q92990 Q9Y250 P05230 Q92993 857 859 P51575 P10827 P31749 Q7L8A9 P19883 Q13506 Q9HCK4 861 O95390 79589 Q9UL51 P09601 50943 P08754 P01241 O96005 6993 O00300 2150 O60603 3479 2147 10013 P01236 10014 P16989 P33076 O43290 O43294 408 P49407 Q16363 Q13099 1080 Q09472 6774 3265 P13686 Q66K89 891 Q99500 894 54413 O95180 P01019 11326 Q9Y5X9 Q9UMS4 Q658W2 P35222 55755 P60484 11331 6776 P24588 8717 P26583 5460 P01042 4131 Q9Y5Q3 Q92934 6311 6794 23764 P61981 P15692 O14908 Q8N5U6 P05412 O00755 Q9HCE7 3297 P37231 Q01955 Q96SZ6 4137 207 5468 54206 P55085 10468 P06753 Q15078 P24385 7422 4155 P56178 P56177 3066 Q12809 P13489 P13010 Q00613 Q96P20 P61764 P24394 8766 O43464 P06729 P13497 P17612 466 55796 55558 Q16623 O75386 O75385 Q9Y4K3 3084 O43318 474 476 477 P07196 P49810 8767 Q13127 P35408 Q15303 3091 8546 P62805 Q15306 5037 79084 P58546 5270 488 Q04864 P09382 Q9H2X0 P46531 7448 5029 P00533 Q16643 Q15796 P43026 8553 Q9HB63 Q15797 P12757 P12755 Q9ULH7 Q9Y6Q6 Q9NRI5 Q13145 5054 7476 Q9Y6R0 P10586 59277 P31270 Q16665 P43489 P63092 P35453 P09172 Q9Y2C9 51564 Q13164 8100 Q9HAZ2 P22392 O43524 P35443 5071 285 Q9GZM8 Q99835 10392 Q7Z7G2 A1XKG3 Q86VS8 Q9Y6M1 7020 Q8WY64 51341 55704 55703 1956 P23443 10159 O43508 P37840 P11474 3909 Q6PID4 Q99814 1969 Q14289 7046 6198 P10997 P78540 O75787 O43711 P12931 Q4VCS5 P04085 P07355 3911 P56524 P78536 7057 Q02297 7291 Q3V6T2 81565 Q9Y6H5 P06276 P42336 7067 Q06643 P19793 P29590 P56539 P31431 Q01196 Q9H6W3 P41231 O60260 8165 P55212 A4D1W7 9253 9495 7074 P41222 P43405 P20309 P04070 Q04206 Q92786 P07101 O95999 Q15735 Q53SB5 P78504 P00749 P00747 P08246 3956 2626 7099 P31249 7098 Q15742 1789 Q05516 P62166 Q9BQB4 Q05513 P00734 375790 P22303 P12956 57045 O60229 P20339 P20336 P43694 O15455 Q16610 Q9BQA1 5914 O95786 O15230 P34972 63976 4830 P00750 5925 P10914 Q92858 P48058 Q9UBB6 Q9BVA0 4609 P16220 P07996 P04004 P52565 4860 Q9UBC3 65018 54361 55692 P16473 P16471 4851 4854 O15525 Q9ULX9 Q92835 P05549 Q9Y2Y8 P25391 Q05397 P53667 Q9BXC9 27185 O60543 Q9UQB3 Q9UJU2 P03372 64170 P06400 Q9GZX9 P09917 P54760 5970 P48023 P56705 22985 P17535 P17302 5728 10524 P17301 P17542 2697 3784 6812 Q9ULZ3 5966 P04233 1385 O95470 29108 O00468 57669 P07550 5733 Q9Y2T1 4646 P08887 5978 719 Q9UM47 P51671 O00238 P30556 Q9NYA1 P30559 Q8IZT6 P10721 3329 57678 29110 967 P42771 Q9UM54 Q92888 5747 P07766 P05106 P28223 137964 6850 P30542 O60502 57689 Q9NQ29 2247 2246 4664 4665 P04264 P05112 P09958 O43186 P29323 P51692 Q5TC82 O00255 Q6KC79 10724 P37173 P05121 P29317 7704 P06213 O00425 P04637 O43157 2034 2033 P16615 P52952 P15531 991 6622 999 P01116

Q16236 6869 P02686 P01112 P01111 Q04917 O43166 Q9UHG2 4221 6885 P35368 P50542 O76074 O76070 Q15154 P48436 P01106 P01589 P01588 P01343 P49768 O00206 2290 P25791 P48431 Q8N726 9921 5321 P14416 P13569 3142 148022 O00203 Q99623 773 Q00535 Q00534 Q96S42 Q53X93 6886 6405 6647 P01138 P01137 8829 P01133 P01375 Q9UJM3 6660 P04626 8841 2064 P35348 302 3148 P10070 P17936 P10071 5566 P01127 P40933 Q96JB5 6657 5327 309 5328 O43353 P24043 7520 8851 P20916 3162 552 553 4005 Q8TEB7 5578 7518 Q8N302 Q495A1 P40763 Q96FA3 4261 Q6IR47 7532 7533 5598 64127 5590 3171 P18846 324 3169 5108 P27797 P01185 6696 5364 2099 Q9UHD2 P50993 572 O14802 Q8NER5 10554 P25103 P26678 P01178 Q96HC4 Q9BT67 5371 P27540 Q12933 O76003 P16410 Q08209 8879 P62745 10320 54106 P25116 P60568 8650 Q9BY76 8654 P30086 351 595 Q04759 356 Q9UBS5 P09493 Q7Z6C1 Q13485 8408 P25963 5154 Q13009 5155 P12429 367 51654 Q96SN8 Q15672 Q14106 6495 6256 6498 P10451 Q9UBU3 P51805 Q9UDY8 P09471 P00441 P32239 6262 Q14116 4086 4087 4088 4089 Q9H257 Q86UL3 Q86YT6 Q8N5F7 Q02790 Q9NZ94 P12883 387 388 P21359 23154 P08151 1808 Q13275 P54259 4099 Q16787 7124 P14635 Q9UQL6 P10275 4092 O43638 P10276 201633 154 396 156 11146 Q16555 2904 Q15223 P45379 Q99966 54 7132 P36896 P36894 1822 P01308 Q14145 Q15475 Q96T37 P00491 O43612 23586 6050 P36888 Q99700 Q99943 P01579 Q9NQC3 P09038 11128 149041 P39905 7157 Q02750 84376 Q16581 P78423 Q13873 O15123 P10636 26037 P63172 P41134 Q9BXM7 Q86WK6 Q1PSW9 91 P46821 P42229 O15119 P42226 Q9NSA3 10939 7170 P55318 P55316 O43609 P08572 P05067 Q9BXL5 1627 7186 Q15831 7189 P29474 P29475 Q03014 Q13418 1877 P17252 O15379 O95859 Q13422 Q13426 9138 Q7Z434 2735 P19438 Q9UBN7 1406 2737 Q92667 2736 9388 Q8WUI4 P41182 P53567 P23508 P22415 2741 P43115 P47712 Q14774 Q15628 57154 27339 P30279 P23515 P24844 P08133 P08138 P23510 P10415 Q9H228 P12830 Q02363 P18146 P19237 P35711 57162 3611 1674 P01730 P08588 P41159 Q9BZS1 Q15648 Q04771 2773 Q9HC29 O15105 P21554 Q9BZR6

regulation of proteolysis 6872 7124 Q96KC8 2033 4092 Q96RU8 1454 2147 1453 O14908 P63165 Q7Z6C1 10013 54998 P45974 P55072 Q13286 7376 8100 Q13443 Q92769 26005 O43847 5270 3326 Q00987 P01308 P00734 5104 207 5347 4898 P61956 Q13099 P26045 7341 Q09472 8078 8754 10273 P48594 64215 3066 Q9NWW5 4193 O60502 Q03405 3416 7415 P01579 P01375 5716 P09958 Q9UKS6 O75832 P49674 P53350 P55055 P31749 P35625 Q9UNE7 10724 Q4AC94 Q9NWT8 O15105 P48730 6613 5327 P00750 P08238 Q9UBN7 P21675

urogenital system development P25054 7046 5268 Q12948 23322 2303 79810 3479 Q1PSW9 Q8WV60 Q92731 Q92574 P42229 Q13485 Q9NPC8 9510 5311 23210 79084 54361 Q9BRQ0 P13631 O43609 84159 367 P16471 P39210 P46531 4851 P48436 P16070 P01343 Q14865 P53420 6495 Q15797 7067 Q12965 6256 P19793 O75881 Q63HK5 1634 P53708 P00966 P01019 5159 Q9UJU2 P03372 Q9NSC2 P40424 Q92826 4086 P35869 Q92824 4089 P36952 2100 Q9UHI8 Q13705 P35222 Q9NRZ9 P56705 Q02790 P14543 5728 P21359 P31270 P60484 P10070 P10071 P20264 Q92786 6776 P07585 4358 2737 2736 P11926 9420 7248 O95470 Q6NYC1 P35453 P10275 Q02535 10736 1499 P15692 4643 P55075 Q13563 Q99966 P30556 Q6IR47 O43521 P09619 O76024 Q68CZ1 Q86WV8 Q05086 P19544 7490 Q05516 3207 Q01955 960 324 445 3169 Q99958 Q15475 90780 7422 P10415 P35555 1285 2099 2253 P08069 P47895 Q9BQA1 80184 3070 P10827 O15078 P39905 Q06710 Q04771 Q6KC79 Q9HCK4 O95390 8516 8879 P24821 4953 7704

DNA repair Q5FWF5 O95677 Q14686 P15927 O95551 P51587 472 2547 1454 1453 4841 9978 Q14694 4968 7334 Q9NS91 7336 Q8NFZ0 P16104 O60216 P18887 80198 Q9UBT6 55215 6117 11198 6119 6118 Q96RL1 Q96EB6 P24522 7341 Q9H2G2 P27694 Q9UGN5 P27695 Q9NRY2 9643 8555 Q16526

O00167 29128 57646 P49916 P18858 1111 P22674 Q16531 Q9UBU8 51426 P00441 9656 O43542 P18074 142 10524 Q49AN0 P43246 Q6PCD5 O15287 5981 P54252 P54132 5983 1022 Q14807 Q9NSU2 8450 25788 Q00597 1137 10856 Q99728 Q9Y230 51567 5976 P46100 O95243 Q96SB8 2237 Q9NRD1 11277 O14757 Q15233 O95257 Q12888 P54277 5883 P28340 1161 7141 P38398 Q56NI9 10714 P49959 Q96PK6 Q99708 9319 Q92993 Q92878 7153 Q13620 7158 5892 7157 O00255 P54727 P54725 2139 P40692 57697 55159 O60870 157570 5886 P48730 5887 5888 Q15014 9100 8930 O95714 P04637 5422 Q15819 P29372 Q7Z589 P78549 7161 25988 Q96T88 10933 Q96RU2 P63165 P62877 8924 7832 P23025 26270 6749 9232 4221 Q86WJ1 O75771 P35244 P35249 Q96T76 Q96Q15 641 Q9Y6H3 Q08945 P35251 Q52LR7 5424 Q99638 56946 2956 Q969S2 9126 Q13535 P11388 Q9NUW8 79035 Q96T60 3014 Q9NVI1 6647 Q8IYD8 29086 P09874 P23246 Q9UMS4 4361 6421 P40938 Q13426 P40937 114799 P34931 P12004 1642 1763 3148 545 546 3146 Q15054 P09884 1408 1407 1647 4913 7508 2070 P26583 7520 4255 Q13315 O95997 P50613 P51946 672 Q03468 51720 7518 P55072 7517 3835 Q8IY92 5591 P46063 5111 Q13569 23411 27339 Q01831 P46736 Q6PJP8 Q9NWV8 Q8N0Z6 Q9UQE7 P61077 55775 328 Q9Y5B9 P09429 Q9BQ15 P12956 3980 Q13216 P78527 22897 Q9BQA5 P13010 P61088 7415 P09430 10432 3978 O15350 Q14676 4287 P49674 Q9UIG0 5378 P39748 Q9P287 P52701 580 P00519 P43681 Q9HCS7 84893 902 56647 Q92900 Q13112 51514 Q9UPV0 Q14683

positive regulation of nucleocytoplasmic transport O00206 P52298 Q15654 7124 P12830 O43557 2010 O60603 Q04759 P84022 999 P61586 O15455 P01375 7099 7098 Q9NYA1 Q60FE5 4088 Q9BZF9 P14923 Q9NR96 9495 P50402 P46934 2316 P17612 2932 3728 387 P10071 5566 7205 Q9NQB0 54106 P49841 4734 2737 Q99835 P24588 P21333

protein heterooligomerization 8772 O43593 Q13158 O95999 7248 P54619 O00186 P30047 P13861 230 P15056 673 P62993 1499 P09972 Q92574 5536 P49815 55806 P01112 7132 Q9Y478 Q13489 Q8IX30 P14923 P07919 Q86WV8 P35568 5590 Q9BUB1 3728 Q05513 841 7249 329 Q13490 7529 Q9NVD7 6498 P12757 5563 3265 P12755 Q13418 Q9UHD8 Q12809 330 3416 2644 2885 3611 Q14790 P08069 P23921 P51575 P31946 5576 Q02750 P35222 5092 P54646 P19438 P61457 P53041 7428 989 3667 6814 P40337 5604 P06213

positive regulation of cellular component movement P25054 Q13477 Q13873 7046 6198 Q15654 O14672 2150 O60603 Q4VCS5 3479 P84022 P39060 P04085 10013 6869 Q15389 P07996 3912 P04004 Q15303 P78536 7057 3091 5154 5155 4067 P12429 O95684 Q93096 P16234 2159 2277 7448 7205 5029 P00533 P01343 Q9HCY8 11116 Q13017 Q13418 57402 P14780 P41231 P01019 5159 P01137 P55290 5054 3791 4088 3673 Q01974 P17301 O43707 O60674 Q13393 Q92786 P01127 Q16665 3667 10488 Q59EA4 Q9UBN7 5604 O60271 P07942 P28482 Q8WUI4 P07948 P41182 O43915 8174 394 P39019 57669 P15692 51564 1012 5337 P00742 4920 5338 P08887 719 P51671 3690 Q6IR47 P35968 Q9NYA1 Q13443 P09619 O43889 Q9Y4H2 P35443 P10721 P35568 P01308 324 Q9HCM4 Q15077 604 P55085 9564 P05106 8754 7422 P10415 2247 3611 P08069 P25103 1956 P09038 P23443 P09958 P51692 P62070 Q02750 P48061 O14939 Q6PID4 P05362 P05121 Q14289 Q16581 4318 P25116 P06213

DNA replication 4172 4173 8099 4174 Q14566 Q14686 4176 P15927 5422 P33992 P33993 P33991 P51587 4171 990 10535 1854 27000 3479 995 997 P25205 84289 5931 1736 Q9C0C2 6749 P0CG13 25913 P08729 P35244 P35249 641 Q9UBT6 Q08945 P35251 6117 11198 5424 6119 54921 6118 P49643 Q99638 Q15554 P01343 Q96EB6 64282 P27694 P49642 Q76FK4 Q13535 O96020 Q9UNL4 286826 P55209 P11388 29803 P11387 1877 Q66K89 P49916 P18858 Q99741 Q96T60 1111 Q01105 P17096 P04183 51147 5557 P23921 5558 51426 P01133 4361 P40938 P40937 Q12857 9093 Q9NUX5 P12004

Q13829 O60318 Q96ST3 1763 P35232 Q96RR1 545 Q15054 P09884 Q96EY1 84296 Q5TKA1 P33316 7884 5981 P54132 7126 5983 Q9NSU2 25942 7083 1017 Q04837 Q6FHQ0 3159 Q14493 5976 P49736 P46063 7013 Q8WZ19 5111 7014 Q14938 23411 Q8WVB6 Q8WYH8 O14519 Q9H9A7 2237 P08651 11277 P22303 O14757 Q9Y5B9 Q9NYB0 P30304 P30307 3980 4673 P54274 Q9BRT9 P49321 5245 5883 P28340 Q00059 10714 Q9BWE0 P49959 Q99543 Q00577 Q96PK6 Q16576 Q92878 10432 3978 7153 Q15648 P39748 Q09028 7150 O75792 55035 5928 253980 P52948 O60870 P24941 54107 4678 5888 Q92900 Q13112 51514 P49427 Q9NRF9

regulation of protein binding 5580 Q9UQL6 P35612 3084 P35611 154 Q96RU7 57669 Q9BZE4 23560 P11171 P07550 118 10014 Q9NSA3 P04004 P56524 Q08117 Q02297 Q05682 2280 166 Q9HCM4 7448 800 Q9NQB0 P49841 409 11315 P49768 P10415 Q13535 P32121 Q9BUZ4 148022 54496 57761 Q9NVM4 857 Q16611 3673 5092 P17301 2932 Q05655 Q8IUC6 545 Q99497 P61457 P62942

regulation of protein complex assembly 4690 P25054 10092 10095 6872 O95835 10094 P10636 P12931 P52952 P42345 Q9BXM7 P63165 118 998 10013 P46821 1978 P47755 P47756 65018 Q86VP6 Q01082 1191 2039 22919 4214 P61158 7341 O15020 7067 Q13813 O15143 P56539 P20936 81873 Q08495 P35869 P78317 382 P11277 Q9UNE7 P16333 P52907 3146 Q6PKD3 6812 Q15691 Q9UBN7 829 4131 Q15735 Q9UNN5 O43639 P35612 P35611 6709 830 832 Q9H2D6 P51671 7013 3054 5590 10109 Q05513 324 2475 6711 6710 4137 P62328 P49841 6714 P09429 10273 P54274 Q9BPX5 23189 Q9NU22 P62330 P02549 P61764 10552 11124 857 859 P60953 Q14678 P10827 P51610 P11234 2932 55832 P63313 Q13233 P21675

anaphase-promoting complex-dependent proteasomal ubiquitin-dependent protein catabolic process 11065 P51668 P14635 P51665 9184 991 O60566 P62195 Q13042 Q92530 10213 Q13200 O43242 64682 P55036 Q12834 O43684 O00231 O00233 O00232 5706 5707 5708 5709 Q9UJX2 Q9H1A4 P49721 5700 P49720 Q16401 5701 P60900 5702 5704 5705 Q13257 5682 5683 5684 5685 5686 P28065 O00487 O00762 P35998 891 5717 P62333 P20618 P25789 10197 P25788 P61289 5713 P25787 5716 Q15008 Q9UL46 5692 8881 5693 O75832 P62191 P28074 P17980 P28072 Q99460 P28070 O14818 P30260 5687 5688 P43686

negative regulation of phosphorus metabolic process 5580 8655 4771 Q15735 P54253 P30086 P14635 4092 P63167 P84022 P49810 5578 51763 6901 P04201 P05412 Q9NP71 4221 7531 Q16635 5037 P30153 25998 Q9BT40 5071 5590 1029 P67775 1027 1026 Q05513 54206 P42771 7529 O15524 P49768 Q7Z727 Q8N726 O14495 891 P46527 P17252 P35240 857 P01137 5515 P62258 O60260 5518 Q9UJM3 8165 P31946 4088 A4D1W7 O00255 P38936 5728 P17813 Q05655 P60484 P17936 O15105 5524 Q15257 P60568 Q92667 P06213

negative regulation of phosphate metabolic process 5580 8655 4771 Q15735 P54253 P30086 P14635 4092 P63167 P84022 P49810 5578 51763 6901 P04201 P05412 Q9NP71 4221 7531 Q16635 5037 P30153 25998 Q9BT40 5071 5590 1029 P67775 1027 1026 Q05513 54206 P42771 7529 O15524 P49768 Q7Z727 Q8N726 O14495 891 P46527 P17252 P35240 857 P01137 5515 P62258 O60260 5518 Q9UJM3 8165 P31946 4088 A4D1W7 O00255 P38936 5728 P17813 Q05655 P60484 P17936 O15105 5524 Q15257 P60568 Q92667 P06213

regulation of DNA replication 4771 P04637 Q969H0 P54132 O95271 P51587 990 Q02535 1017 Q9BZE4 3479 Q1PSW9 23560 P04085 Q8N3U4 P01112 Q6UWV6 P04201 P05412 25913 7013 5154 5111 5155 7014 O75496 P30153 P10600 55294 6282 Q3V6T2 P16234 P67775 641 P18846 Q9UQE7 P01308

P01588 Q99638 Q15554 P27797 P01343 Q9H2G4 P29353 Q9H2K2 P54274 9126 5883 Q13535 3265 O43612 P49959 56034 Q99741 P35240 55704 811 P08069 P01137 5515 Q9NRA1 Q9BSI4 5518 4361 6464 7157 Q9NUX5 P12004 2735 1763 466 P10070 545 P08151 P24941 P31949 P01127 64061 Q14683 2736 P06213

protein import into nucleus O00505 Q96QC0 7124 P04637 P28482 O00629 P41182 Q9P2R6 Q9P0J0 3836 473 10736 2147 23165 Q92973 Q9Y2X3 P49815 P25963 Q9NPC8 P05412 Q14974 O95405 O15131 Q9H2T7 Q01082 55696 P42858 P00734 6711 P52292 207 7249 3841 9702 406 604 3843 P60468 O00327 6496 4792 P52294 P29590 P30101 Q96P70 Q9NW64 2247 Q9NZQ3 P01579 P02545 P01137 5514 P09038 2923 P01375 55705 P49792 5371 4000 7157 P62829 P31749 Q86XR8 O00410 64901 O95343 23633 O60318 P61970 O60674 Q08209 P52948 Q92621 1408 Q49AN0 10204 P25116 51517

cell projection organization P25054 Q92858 8650 P55268 Q9UQP3 23607 Q8TEW0 Q9UBB6 O75385 351 3516 474 Q8IYN9 P16220 284217 P07196 P09493 P83731 O14786 P43034 P49815 Q8NFJ9 8408 Q9BPU9 5154 5155 Q12837 O60331 P46531 4851 P00533 4734 P54753 Q7Z727 6256 P25391 Q06124 Q05397 Q9BXC9 Q9UBU3 150737 6809 P51805 P09471 Q9UKW4 Q96RK4 4983 Q9UQB8 3655 Q9BZW7 4867 P55290 P00441 4627 Q16650 6383 P54764 6386 P54762 3672 Q9Y6R0 Q92949 P13611 Q7L576 382 O75592 P17302 P16333 Q4AC94 O60674 2697 80776 23032 23154 23396 6259 Q6PKD3 P60763 6812 5604 Q9UHR4 P34925 7248 Q8TD84 1385 1020 O43639 P35579 121512 P16144 P23229 1012 4646 Q15223 O00238 8100 P32856 P60709 Q15109 Q5JQC9 Q86WV8 Q06330 P18206 960 2475 O60890 7249 60 Q9GZM8 Q9UM54 1947 85440 5747 Q99835 9201 Q8TCU4 Q8TEY7 10152 Q02880 A1XKG3 Q9NYB9 Q9Y5K6 Q96EV8 P62330 P15153 Q96N67 P61006 55704 O14640 P08069 Q92752 5879 1956 Q8WW24 O15075 P29323 7155 O15078 P39905 P31749 Q13509 25776 Q02750 Q9P202 8481 Q9HCK4 51474 Q6PID4 Q9NRF2 Q14289 Q15375 Q15019 4690 P53365 O75665 Q9Y6W5 P56199 25861 23322 P11047 3913 P42345 Q8TAF8 Q86WK6 Q1PSW9 999 P04085 Q6NVY8 Q92574 P46821 O60282 3911 3912 Q9C0K0 Q96CW9 O15496 Q7Z6J4 Q13887 O43602 10939 2043 1191 Q3V6T2 P55316 P57735 P46934 117177 81565 Q15154 P16070 P05067 P98172 4218 P98174 Q13099 2290 8399 P50552 O95613 P14416 O00560 P19793 Q96M96 2054 P52735 Q13418 O43815 6093 1756 1874 773 Q00535 P17252 P53708 Q53X93 P21860 O15259 6647 P01138 P49639 8829 84062 Q16254 P04626 91147 Q658W2 2065 2064 Q8IYT8 O00213 9253 56288 P10070 P10071 3146 P01127 2737 2736 23647 P07942 Q9Y3M2 4131 4133 8851 8852 P78509 Q9UHB6 10458 O60488 P63000 10451 5578 5338 P26232 7410 P35609 P52803 26005 O43921 P11532 Q5HYA8 Q5JSP0 Q68CZ1 80705 23092 64919 324 688 Q96QF0 207 P08138 7408 5108 Q9HCU4 Q15078 P09429 P10415 Q6ZV73 P56178 Q8IUQ4 P12830 P34741 O60229 P20336 3611 55785 Q04656 P61764 7414 5116 Q13464 80184 O15230 Q15768 582 Q9UNA1 O14939 23077 8516 55558 Q8N4P2 Q9BZR6

positive regulation of protein catabolic process P25054 6872 7124 4092 Q96RU8 1454 1453 P63165 Q99728 P45974 P55072 Q9Y4P1 Q13443 Q96HU1 Q96J02 83737 O43847 Q96KS0 P46934 Q00987 324 207 5347 4898 P61956 4734 7341 8078 8754 10273 4193 Q8WY64 7415 P01579 P01375 5716 Q9BT67 P09958 Q9UKS6 O75832 P49674 P53350 P31749 580 Q9UNE7 P17302 27352 23192 2697 O15105 P48730 6613 P21675

induction of apoptosis by intracellular signals 8453 8454 8452 8930 Q16666 P04637 Q13315 Q9UBF6 Q86Z02 7161 P51587 3162 472 672 Q96RU2 Q03468 9616 5578 P23025 O96017 Q9Y5J5 O95243 8065 Q9NS56 1026 O43290 P42574 2956 9641 Q7Z727 Q6ZN33 Q9H305 Q13618 Q13617 P29590 Q13616

P38398 57646 O60543 P17252 Q92630 5371 O15350 64782 7157 P31947 O43464 8445 Q9BZF9 P38936 P52701 3428 2810 P00519 P40692 O60674 Q93034 Q14164 Q8WTP8 P09601

negative regulation of developmental process P25054 O14793 2672 259266 O75385 595 Q8IYN9 356 P84022 O14786 P78395 8408 P07996 P25963 Q9NPC8 Q13127 P52565 P62805 19 54361 55692 Q9H2X0 P46531 4851 5029 4854 Q13133 Q96EB6 Q15672 Q14106 Q92835 6498 P12757 P12755 P10451 Q05397 Q9UBU3 P51805 57761 811 Q9UJU2 P02741 P40424 6926 Q9GZX9 5054 Q9NQX0 4088 9657 7476 P48023 Q92949 P55055 Q9Y618 Q86YT6 93166 P56705 57534 387 P21359 P04233 Q13275 O95477 4099 7124 P28482 1021 Q9UQL6 4092 P10276 154 396 10736 1499 51564 P07550 P61586 Q9Y2T1 5978 Q99966 Q9UM47 3690 22846 7376 Q9HAZ2 P22392 Q8IZT6 P35443 P36894 P10721 Q9BPY8 285 1387 3685 Q9NQB0 604 Q92888 5747 Q96T37 P04179 P05106 4792 1285 1284 Q86VS8 P36888 Q9NQ29 857 Q9NQC3 P05112 P29323 149041 7157 P51692 Q5TC82 Q7L8A9 O00255 P19883 Q9HCK4 861 O95390 Q6PID4 P05121 84376 Q14289 7704 7046 6993 P10997 O15123 P04637 O43711 P63172 P14555 O00300 P52952 Q4VCS5 P15531 Q96RU7 3479 Q1PSW9 2147 Q15022 P42229 O15119 10014 P02686 Q04917 P56524 7057 4221 P16989 7291 P55316 Q15154 P48436 79923 Q4LE28 P08572 O43294 P01343 P49768 O00206 2290 P25791 P48431 9921 Q5TAX3 P29590 Q03014 3142 Q01196 Q9H6W3 P41231 Q92793 Q00534 Q96S42 6405 P01137 8829 Q9UPY3 P01375 Q9UMS4 A4D1W7 9138 Q658W2 P35222 Q15051 P10070 P10071 6776 6657 2737 2736 O00744 5460 Q9Y5Q3 Q8WUI4 P41182 P20916 Q9H9S0 P78504 84525 Q99684 4005 Q8N5U6 7099 O00755 7098 Q6IR47 Q14774 7533 Q9HCE7 57154 23411 23532 27339 P37231 Q05516 Q9BQB4 Q01955 P23515 324 P00734 5468 P08138 5108 10468 P27797 P24385 6696 P10415 4155 P56177 57045 P51843 Q7Z494 O15455 Q16610 5914 P24394 5371 9612 63976 23512 P16410 55796 4830 O15105 55558 Q9BZR6

cell projection assembly23647 4690 P53365 Q9Y3M2 23607 O75665 Q9Y6W5 23322 O43639 10458 121512 P16144 P23229 1012 P63000 10451 P04085 Q8NFJ9 3911 P32856 5154 5155 7410 Q7Z6J4 P35609 Q13887 26005 Q5HYA8 Q5JSP0 Q68CZ1 Q3V6T2 80705 23092 P18206 117177 688 Q96QF0 Q15154 O60890 5108 4218 P98174 Q8TCU4 Q6ZV73 O95613 O00560 Q96M96 2054 Q9Y5K6 P52735 6093 Q9BXC9 1874 P15153 55785 Q9UKW4 Q96RK4 P61006 4983 55704 Q9UQB8 7414 5116 3655 Q9BZW7 Q13464 5879 P55290 Q16254 6386 91147 O15230 Q92949 25776 Q7L576 8481 582 P16333 Q9UNA1 Q4AC94 Q9NRF2 Q6PKD3 P60763 P01127 Q9UHR4

viral infectious cycle Q15853 11060 P35659 O00308 7520 6598 Q12824 O00267 Q9H0M0 6829 2547 O43719 P22415 3159 Q92692 90678 7518 Q15223 7913 Q13363 O75531 Q96J02 83737 P13236 7251 P61073 10542 8815 P12956 P62937 P51681 Q7Z2Q5 P13010 P60033 975 P17096 5478 11168 P01730 P09958 Q9NZI7 P56545 Q92824 Q13426 O43504 Q12857 O75475 Q6UWE0 1488 1487 Q99816 P05362

regulation of Ras protein signal transduction O15085 Q7Z628 9744 P10911 7204 Q5VV41 9181 Q99490 P15498 2305 P43034 Q8WZ64 Q92974 Q8TBP0 Q13009 Q9Y2X7 Q9UIA0 115704 Q9P107 Q15311 Q008S8 Q8IW93 9882 Q9NR80 Q9UQ13 Q9NR81 Q9HBW0 29 Q15438 Q9ULH1 64857 Q96CN4 Q8IVF5 23263 Q9UKW4 Q9H7P9 Q8WZ42 Q9UPQ3 Q8WWN8 Q92949 125058 Q12979 382 Q8N6H7 Q13829 P21359 Q66K14 Q8N5V2 5604 8452 Q92538 7248 7126 Q96N96 23365 Q9NUY8 121512 O94827 P46108 P51671 Q86WV8 Q5VST9 P37198 23370 2475 604 Q6ZW31 Q9GZM8 85440 Q92888 Q96NH3 23229 Q07890 Q6ZSZ5 Q8TCU6 O15068 23463 Q13618 O43739 P62330 Q96N67 1398 Q07889 613 10276 5516 5879 Q14155 Q12774 10160 Q08050 64786 O95140 Q9H8V3 Q8N9R8 Q02750 253980 Q14160 Q6PID4 Q14161 Q14289 Q15811 10928 26037 115557 O60725 P42345 O14827 3479 O94989 P01116 Q92574 Q6ZT07 P01112 P01111 O43166 Q15027 Q7Z6J4 Q96QB1 23216 O43609 81565 84033

P01588 408 P49407 85360 P98174 P01343 Q86VW2 116986 3265 Q96M96 P52735 116987 P11274 50618 P04626 9138 2064 P35348 7074 27352 55633 Q15052 11214 O43150 Q15057 9927 9267 Q3MII6 Q9NP61 O43597 Q96BZ9 Q96DN5 P41182 Q5R372 Q86TI0 93594 Q58EX7 Q96P48 79735 P63000 10451 9815 Q96PE2 P62714 Q96HU1 Q8WZ19 7410 Q5JSP0 Q9Y6D6 Q9Y6D5 Q9NU19 9826 Q8TEA7 Q13574 50649 O14492 Q96GA9 6453 Q6ZV73 O75962 Q12802 O60229 Q0IIM8 50650 55785 O60343 Q9NZM3 4943 23636 Q8IYX1 4168 Q9Y3P9 23513 Q9NZN5 Q99418 10564 Q8N6T3

negative regulation of protein kinase activity P25054 O95835 9467 Q9NRW4 7161 124790 Q96RU8 Q96RU7 1852 1850 P15090 P49815 6901 P09936 O94992 4221 O15530 Q16635 O95684 P50148 56940 Q8WTR2 O43609 P67775 51654 P08575 P42574 Q13098 P24522 11116 P49768 Q7Z727 Q8N726 Q9H422 Q13418 P56539 Q9C010 84619 57761 P17252 P35240 P06400 Q9UJM3 Q15208 P31947 9093 5170 P21359 Q96EY1 5569 Q9Y2B9 Q96MH2 1647 4771 O43597 11142 Q16828 10614 P61981 5578 Q8IWV1 Q13202 7532 P30153 26524 P37198 1029 1026 Q96SZ6 2873 324 207 7249 54206 P42771 Q13177 Q99956 P61925 O95257 7023 Q9UGJ0 O75293 Q9UNH7 3611 Q01664 Q9NRM7 58533 O14640 857 859 5515 5518 23636 Q9BY84 O15350 Q13227 P31749 O00255 P38936 57732 2810 Q8N5A5 1848 5925

Rho protein signal transduction O15085 Q13017 Q969H4 O95477 Q9BST9 Q9NR81 Q9P035 Q03113 391 392 6093 394 P53667 396 397 398 P23528 1012 50650 P08123 10672 P61586 Q13464 10256 O43182 P55290 P30556 P52565 Q52LW3 6242 O15111 9138 19 1072 Q07960 O00212 P52566 P84095 387 388 1147 P62745 Q99819 9826 Q9H4E5 Q14344 P98171 Q92888

regulation of cell-substrate adhesion P02452 133584 4771 7248 7402 P13349 1021 P41182 O00622 Q9Y6C2 57669 P00747 P84022 1012 94 Q92574 P49815 P07996 3956 P04004 7057 P35968 Q86WV8 5590 1029 P16671 Q05513 P46939 P09382 Q9HCM4 P46531 4851 7448 7249 604 P42771 P42338 6696 P10415 Q8N726 Q13418 Q53GQ0 Q8NC24 P10451 P31431 P37023 P20936 51144 3611 1277 Q00534 P35240 O43182 P55290 3791 4088 P14543 5728 Q63HQ2 P21359 P60484 948

protein localization in nucleus O00505 Q96QC0 7124 P04637 P28482 O00629 P41182 Q9P2R6 Q9P0J0 3836 473 10736 2147 23165 Q92973 Q9Y2X3 P49815 Q13285 P25963 Q9NPC8 P05412 Q14974 O95405 Q8NF91 O15131 Q9H2T7 Q01082 Q9NS56 55696 Q05516 Q00987 P42858 P00734 6711 P52292 207 7249 3841 9702 406 604 3843 Q15475 P60468 O00327 6496 6495 4792 P52294 P29590 Q03014 P30101 Q96P70 Q9NW64 4193 2247 Q9NZQ3 Q96RK4 O14640 P01579 P02545 P01137 5514 P09038 2923 P01375 55705 P49792 5371 4000 7157 P62829 P31749 Q86XR8 O00410 64901 O95343 25777 23633 O60318 P61970 O60674 Q08209 P52948 Q92621 Q9UH99 1408 Q49AN0 7704 10204 P25116 51517

gland development P78545 7046 5268 Q12948 P51587 1181 3084 P17676 P52952 P15531 595 2304 P16220 3479 Q1PSW9 284217 P84022 27122 Q92731 999 O14786 P01236 O14788 P42229 O15119 3911 Q15303 5155 Q02297 3099 79084 54361 P13631 7052 367 P16471 P46531 4851 P48436 P00533 P16070 P01343 6495 7067 P48431 6256 P25391 P14416 P19793 O75881 P29590 Q03014 O75360 Q9Y6Q6 Q99623 8945 Q53X93 P21860 Q9UJU2 P21980 Q9Y297 P03372 P01137 Q9NSC2 8829 P40424 Q9UKW6 6926 P06401 P21741 P01133 P01375 Q92826 Q9GZX9 Q13422 P35869 4088 P04626 3673 Q14118 P36952 2100 Q86UL3 2065 2064 P35222 P56705 O60716 Q02790 2735 5728 P17301 P21359 P60484 P10070 P10071 P08151 11331 6776 P01127 6657 3667 2737 2736 Q15853 9420 7124 1385 P35453 P10275 P46109 1499 P15692 Q13285 P31249 P51671 5471 Q6IR47 P78337 P30559 O43521 Q9Y4H2 P36894 P10600 Q05086 P19544 7490 O00358 P35568 960 1147 3169 P17482 Q9UBP4 Q15475 5361 P24385 137964 7422 P10415 Q06203 P12830 2099 Q02363 57045 P51843 2247 1399 P00403

P08069 857 P47895 Q9BQA1 1956 P09038 5371 P10827 O15111 P51692 Q15648 O15230 Q06710 P52789 23513 Q14160 P24821 4830 10320 1605

regulation of cell morphogenesis 8650 O43157 P10636 2033 26037 O75385 Q8IYN9 Q7L0Q8 Q86WK6 P84022 Q9HCJ2 P07196 Q7Z6C1 O14786 P46821 P02686 Q13485 8408 Q04917 O43166 Q53QZ3 P52565 Q13009 Q7Z6J4 Q96QB1 81565 P61158 O43294 Q15796 Q14344 P98174 P49768 Q09472 Q15797 6498 P12757 Q9NRY4 Q96M96 Q13418 P10451 Q05397 P53667 4628 773 P51805 P20936 P06241 6405 P01138 P01137 8829 Q13145 4627 4086 4087 3791 4088 4089 7476 Q9Y6R0 Q8WWN8 9138 P10586 Q658W2 Q7L576 9253 9495 7074 79784 387 Q9NRR8 2335 P20827 P24588 Q13275 4131 4099 2909 P20916 Q03113 4092 P35579 396 P35580 310 Q96P48 121512 57669 P15692 P61586 3679 Q13683 Q99966 O00755 P35968 7533 P10600 Q5JSP0 58480 Q9UEE9 23370 P23515 Q9HCM4 4137 Q9GZM8 3689 P17081 P08138 Q92888 5747 P02751 Q6ZSZ5 P05107 6696 7422 5364 4155 Q6ZV73 Q7Z406 P15311 57689 3611 P02549 55785 Q9Y250 10672 Q9NQC3 Q96HC4 P29323 7430 P20073 Q9HCK4 56882 O15105 Q9H4E5 55558 Q9UL54 Q9BZR6

negative regulation of gene-specific transcription from RNA polymerase II promoter 9421 6595 O96004 5187 6597 P25490 2672 Q9UQL6 4092 P52952 P84022 Q99684 3476 10014 P56524 4261 7376 Q92769 8864 O15055 Q14938 Q08117 O43524 O75376 P37231 166 P33076 P08651 5468 Q9NQB0 7528 Q96T37 4790 6256 6498 P12757 P19793 O60907 3066 Q03014 3065 23028 Q9BZK7 O15379 O15534 O60341 Q92993 857 Q6IT96 5716 P51531 Q13422 4088 O75832 9612 Q13227 Q13547 8841 Q92949 P55055 Q9Y618 Q6KC79 P51532 P78318 10524 P17813 P19838 O15105 Q9UMX1 10320

endocytosis 23603 8411 O75146 23048 O75385 351 P15498 1213 79778 90678 P49815 8408 P07996 8546 8301 P12429 Q9UBC2 19 Q08431 30011 Q13492 4734 Q13133 Q9UI12 O00602 4074 P32121 Q5T0N5 29924 57403 4983 Q9UJ41 P02741 P61966 P53675 Q14114 Q96JJ3 23032 6812 948 9784 P07307 O95477 P50570 1020 P10276 O75581 274 154 58513 Q99962 2348 Q99963 P07550 4646 Q13286 Q9UL26 161 10868 P20645 Q96MV8 O60895 3685 O60890 7249 8218 Q9UM54 7804 5868 9322 9685 Q8TEY7 P12314 A1XKG3 O60749 Q14C86 P30542 22931 Q8TEH3 857 P02786 O94973 P51693 7037 1174 O00499 P37840 O43747 Q8IVI9 Q96ED9 Q9H4M9 Q9ULV4 P06576 Q15811 P98155 Q9Y6W5 3482 P50897 P15531 P62993 Q99523 10938 Q96RU3 Q96KG7 6622 P01116 P01112 P01111 O15118 7057 6643 6642 23210 P36575 O75096 Q8IUN9 7052 P46934 Q14185 P05067 80230 408 Q15036 P49407 409 Q01518 Q15276 23307 6653 3265 Q9Y6I3 O00203 Q00535 P16284 P21980 Q92673 9135 P11717 4240 P01130 50618 Q8IY33 Q6UWE0 P21757 Q9NRR8 Q07954 Q96T51 10487 Q96B97 Q6NYC1 Q9Y2K6 P15328 P51149 433 7879 8724 5338 P08246 57706 51606 O95405 Q9NP72 P42261 Q05086 P16671 1785 Q8WYP3 Q15075 O60493 Q9BY11 6453 6456 4035 6455 Q9H223 P20339 P20336 333 2885 Q00610 1793 P61764 Q9NZM3 Q15642 5914 Q14677 Q5T1M5 O14939 56882 6457 4038 4830 Q13596 Q99418

membrane invagination 23603 8411 O75146 23048 O75385 351 P15498 1213 79778 90678 P49815 8408 P07996 8546 8301 P12429 Q9UBC2 19 Q08431 30011 Q13492 4734 Q13133 Q9UI12 O00602 4074 P32121 Q5T0N5 29924 57403 4983 Q9UJ41 P02741 P61966 P53675 Q14114 Q96JJ3 23032 6812 948 9784 P07307 O95477 P50570 1020 P10276 O75581 274 154 58513 Q99962 2348 Q99963 P07550 4646 Q13286 Q9UL26 161 10868 P20645 Q96MV8 O60895 3685 O60890 7249 8218 Q9UM54 7804 5868 9322 9685 Q8TEY7 P12314 A1XKG3 O60749 Q14C86 P30542 22931 Q8TEH3 857 P02786 O94973 P51693 7037 1174 O00499 P37840 O43747 Q8IVI9 Q96ED9 Q9H4M9 Q9ULV4 P06576 Q15811 P98155 Q9Y6W5 3482 P50897 P15531 P62993 Q99523 10938 Q96RU3 Q96KG7 6622 P01116 P01112 P01111

O15118 7057 6643 6642 23210 P36575 O75096 Q8IUN9 7052 P46934 Q14185 P05067 80230 408 Q15036 P49407 409 Q01518 Q15276 23307 6653 3265 Q9Y6I3 O00203 Q00535 P16284 P21980 Q92673 9135 P11717 4240 P01130 50618 Q8IY33 Q6UWE0 P21757 Q9NRR8 Q07954 Q96T51 10487 Q96B97 Q6NYC1 Q9Y2K6 P15328 P51149 433 7879 8724 5338 P08246 57706 51606 O95405 Q9NP72 P42261 Q05086 P16671 1785 Q8WYP3 Q15075 O60493 Q9BY11 6453 6456 4035 6455 Q9H223 P20339 P20336 333 2885 Q00610 1793 P61764 Q9NZM3 Q15642 5914 Q14677 Q5T1M5 O14939 56882 6457 4038 4830 Q13596 Q99418

regulation of growth O15085 Q7Z628 P08833 Q9Y265 O14672 6236 O14793 Q12948 8091 O75385 29948 3084 351 2305 P37288 Q8IYN9 2303 Q04759 P16220 P84022 Q9H160 Q92731 Q7Z6C1 O14544 O14786 P78395 5931 O14543 4722 P21127 Q13485 8408 2307 O95684 51094 P16473 367 84312 Q9H2G9 P55042 P43026 O15524 Q9UK53 Q9H2G4 P50150 O75489 9643 Q76FK4 Q9UNL4 Q06124 P10451 Q05397 P53667 Q9BXC9 Q9UBU3 P46527 P37023 P51805 P32929 5830 8548 Q9UBU8 P55290 P00441 P06400 5716 Q92826 4088 P22692 4089 P31947 2100 Q86UL3 58189 1491 142 10524 3304 Q9NPF0 3421 Q9NPF5 64061 Q13275 1385 O95470 P14635 P10275 O43638 O00468 Q9P0J0 154 10856 Q6FHQ0 1012 P07550 Q9Y230 P30556 P51553 Q96SB3 Q9NYA1 55929 O43521 P36896 Q96J02 Q5PSV4 P19544 7490 7251 Q8WYH8 Q96KS0 1029 57678 1027 960 1026 P01308 9306 604 Q14140 P42771 Q13177 Q99958 5747 9564 137964 Q9H2K2 Q86V24 Q99700 Q9HCL2 2247 P01579 Q92993 Q16576 10276 Q9NQC3 5515 P09038 P48745 P23443 5518 Q08050 7157 P51692 P31749 2023 O00255 Q6KC79 P38936 55035 2810 2137 984 Q6PID4 Q99816 Q14289 Q9UL54 Q15014 86 Q92743 P06213 P01241 Q13873 7046 6198 P04637 P10636 2033 3484 7161 P50897 O60603 11108 27000 10933 3479 91 5654 84289 94 P01236 P46821 P42229 51009 Q969T4 Q8WUD6 P78536 Q9UHG2 4221 Q9UQ88 O96019 10939 Q02297 P16989 83737 P50542 P67775 Q92560 P16070 P05067 P01343 P42336 11116 Q09472 P29353 Q8N726 Q9UKN5 6774 27346 P29590 Q13418 3142 P41235 1877 Q66K89 891 Q53X93 P01019 O15379 O95619 51147 Q9GZP9 6405 6647 P01138 P01137 O60383 10477 8829 P09874 Q13422 Q14511 A4D1W7 8841 Q658W2 O00213 P17936 Q92786 6776 P06733 4131 6311 Q13557 P41182 9021 O00622 552 Q9HC57 Q9UJW9 O75629 P08246 8607 2626 P40763 Q6IR47 Q14774 64127 P30153 3297 23532 P37231 Q99675 Q9UJX0 4137 9826 207 5468 Q5BJF2 375790 P01185 6696 P10415 Q96A54 P32320 Q8NER5 Q00613 3611 Q99543 Q9NRM6 56994 O15335 P43694 P08588 P41159 5371 O15350 728642 P08107 O75832 6464 O43464 Q12816 3622 3621 Q9HC29 O43909 8879 10320 55558 P60568 5925

organelle localization 54908 9342 Q92538 6993 9700 Q9Y266 10053 P63172 P14635 259266 Q8IXI1 P35579 Q8IXI2 Q8IYN9 1058 P42704 Q96AX1 10013 65082 P43034 10972 Q9BUK6 O95721 Q9UBK9 P49450 P35606 9276 Q9Y678 Q8NF91 5871 10460 79083 P53618 23212 Q8IZT6 P51159 Q9UJX2 81565 Q15154 Q9GZM8 5108 P62841 5747 P49768 P53621 5362 Q9BV36 Q86VS8 Q03014 Q9Y6Q5 Q05397 Q9BXC9 891 1315 O75051 375 1314 Q96RK4 P49755 Q9Y6A5 Q14674 Q8N960 4627 4869 Q96EA4 Q12851 O43264 O15392 O95140 Q658W2 Q02750 Q96BK5 25777 Q9NXR1 10128 Q8N4C6 54820 P40692 51199 Q9NZ56 10726 Q15050 54984 P84077 Q13190 Q9UH99 6811 84376 O14579 O14777 Q9UBN7 5604 Q96FF9 Q8N6T3 9927

regulation of MAPKKK cascade P25054 O75787 P30086 Q9Y4K4 7161 3481 Q9Y4K3 O43318 2305 P41134 P62993 3479 23286 3476 8767 O14788 Q15389 1616 Q9NSA1 2308 P01112 Q16512 4188 4221 6885 5037 O75376 O43609 367 P14174 Q99750 4214 4216 P16070 408 P49407 P01344 409 P01343 Q9UER7 O00206 7186 7189 P48431 Q9HBW0 P32121 Q9H422 3265 Q96M96 Q13418 P56539 Q9Y6Q6 P35240 P01019 O15379 64170 P01375 5716 Q9Y6R4 P04626 8841 Q13546 Q14118 Q9H257 Q9NR96

2064 P35222 P78318 P35348 P43405 P21359 6657 Q13153 P04233 O43353 4771 7124 5585 O43597 P13945 Q16828 P10275 154 P15056 673 Q9Y2C9 121512 1499 Q99683 Q03468 P07550 3953 2904 P08887 7099 7098 Q9Y239 64127 10746 79444 960 Q02763 P01308 324 8737 207 P48357 O15169 10392 6850 Q9P035 Q9BUZ4 2247 2885 3611 Q5TCX8 P08069 857 O15455 859 Q96CA5 P09038 10159 9839 P41159 O15350 Q12778 O75832 Q12933 Q08050 Q13227 P31749 O95382 Q06830 Q86Z14 O00255 51231 Q9HC29 Q6PID4 54106 1848 Q14289 Q9UL54 1605 Q13233 P25116 P06213

positive regulation of transport Q15654 Q16623 2550 O43557 P37288 Q04759 Q9UBS5 P16220 P84022 57620 51762 P20396 Q7Z6C1 O14788 Q13485 P25963 P04004 P03950 Q15303 19 65018 Q08431 2316 P14174 7448 7205 5029 P00533 4734 Q13133 7220 808 Q7Z727 P22681 3783 Q06124 Q9Y6Q6 Q9UBU3 811 P53675 5174 5295 5054 4088 4089 3673 P55055 Q9BZF9 Q9NR96 Q86YT6 P21917 57534 P17301 387 O60674 1808 Q9ULZ3 3667 P41743 P43489 P25098 O95477 7124 1385 29108 1020 114548 154 Q93062 156 Q6PJW8 P07550 11146 P61586 Q16555 Q15466 2904 7376 Q9NYA1 P29466 P30559 11030 O43889 Q9Y4H2 O95361 O00233 P36894 P10600 P35568 10626 P01308 Q9NQB0 967 8218 P48995 P49841 Q99835 6850 4792 A1XKG3 O43612 2010 Q86V24 P30542 O60502 Q92990 Q5T2W1 P01579 P00367 857 Q9UEW8 Q9NQC3 1956 P51575 Q9BX66 P39905 P31749 P37840 2932 10724 867 P05121 P06213 Q86UW7 7046 2033 P50897 O60603 23327 P52952 Q9BXM7 2147 P04083 6622 999 6869 Q15389 P27986 P07355 Q9NSA1 O15496 P46934 O00206 8399 Q09472 P52298 5321 P14416 27347 P41231 Q00535 P17252 Q53X93 P21860 P01137 P01133 P01375 Q9Y5X9 4240 A4D1W7 5213 P35225 2065 9495 P43405 O43707 301 302 Q07954 P10071 5566 10488 2737 P24588 9388 5460 5584 P41181 Q9H244 552 553 84766 Q96PU5 5336 5578 O15554 5338 P62158 P47712 Q92930 7099 7098 Q6IR47 Q13443 10580 P14923 64127 O76024 Q9BSW2 5590 P62166 3728 Q05513 P00734 207 P17081 P22303 P27797 P23510 P21333 P01185 8754 4035 P12830 Q96P20 O15455 P25103 P01178 Q13586 Q60FE5 P50402 P16885 P17612 O14939 Q9HC29 54106 9846 P08237 P25116 P60568

nuclear import O00505 Q96QC0 7124 P04637 P28482 O00629 P41182 Q9P2R6 Q9P0J0 3836 473 10736 2147 23165 Q92973 Q9Y2X3 P09651 P49815 P25963 Q9NPC8 P05412 Q14974 O95405 O15131 Q9H2T7 3178 Q01082 55696 P42858 P00734 6711 P52292 207 7249 3841 9702 406 604 3843 P60468 O00327 6496 4792 P52294 P29590 P30101 Q96P70 Q9NW64 2247 Q9NZQ3 P01579 P02545 P01137 5514 P09038 2923 P01375 55705 P49792 5371 4000 7157 P62829 P31749 Q86XR8 O00410 64901 O95343 23633 O60318 P61970 O60674 Q08209 P52948 Q92621 1408 Q49AN0 10204 P25116 51517

regulation of cell development Q92858 8650 259266 O75385 Q9BVA0 2547 474 Q8IYN9 P84022 Q9HCJ2 P07196 Q7Z6C1 O14786 Q13485 8408 Q13127 P52565 Q13009 Q12834 Q9UBC3 5270 P09382 P46531 4851 51654 Q96SN8 P61158 5029 4854 Q16643 Q15796 6495 Q15797 6498 P12757 P12755 P10451 Q05397 P53667 27185 P51805 811 Q9NRI5 Q13145 P40424 6926 4086 4087 4088 4089 7476 5970 Q9Y6R0 P10586 Q86YT6 Q02790 57534 5728 387 P21359 P31270 P20827 1808 Q13275 P54259 4099 Q9UQL6 4092 O00468 396 57669 P61586 Q16555 5978 Q99966 Q9UM47 Q9NYA1 Q92769 Q8IZT6 P36894 P10600 P10721 1822 3207 P01308 Q9HCM4 Q9GZM8 Q92888 5747 Q15475 Q99835 Q86VS8 57689 2247 Q9Y250 55704 P01579 Q9NQC3 859 P09038 O43186 P10827 P29323 7157 P31749 Q9HCK4 O95390 84376 6993 P04637 O43157 P10636 2033 26037 O43711 P63172 P52952 991 Q86WK6 Q1PSW9 2147 P46821 O15119 10014 P02686 Q04917 O43166 P56524 Q3V6T2 P55316 81565 Q15154 P48436 P01588 O43294 1627 P49768 2290 Q09472 7067 P48431 9921 P14416 Q13418 P56539 773 894 P41231 6405 P01138 P01137 8829 Q9UMS4 6660 P55212 Q13426 9138 Q658W2 9253 9495 7074 55755 P60484 P10070 P17936 Q04206 Q92786 Q96JB5 6657 1406 P24588 2736 4131 7520 8851 Q15735 P20916 P61981 7518 Q8N5U6 3956 P31249 O00755 7532 7533 27339 P30279 1789 P37231 P62166 P18846

Q96SZ6 P23515 P00734 3169 4137 207 5468 375790 P08138 5108 P27797 Q15078 P12956 6696 5364 P10415 4155 Q9H228 P56177 3066 P35711 O60229 P13010 3611 Q96HC4 466 55796 O15105 55558 P21554 Q9BZR6

myeloid cell differentiation 6670 P55347 Q14686 Q16666 P07948 P14317 2034 Q6NYC1 P41182 P53567 Q9Y4K3 P35579 6194 P30048 P39019 Q01201 P15692 10935 P17275 Q9H9B4 6667 998 10296 P49810 P08047 Q96AX1 65082 O14788 P62753 4067 Q15306 23210 P10721 P11413 10107 P37231 Q02447 Q05516 1147 841 Q9NRH2 Q9UKV3 5468 604 10661 P01588 P49768 7189 7067 7422 Q02363 P29590 Q03014 Q7L5Y9 Q01196 Q9Y6Q6 P14780 Q9UDY6 Q14790 6886 P01137 5316 P49715 P60953 4627 P06400 5371 P10827 Q13268 O15111 P18074 5971 A4D1W7 1050 22985 3428 81501 O75081 P17542 Q9H295 861 O60674 P37173 863 P62263 9846 Q99814 10202 7704 4318 Q13351 5925

receptor metabolic process 9463 P49069 7124 P50570 P32121 A1XKG3 1020 O75385 O00468 1756 P20339 P62993 773 1213 2885 Q00535 Q00610 O14640 6622 857 P01137 5338 8408 P01375 Q60FE5 8301 Q9HCE7 P42261 P11532 57154 P37840 P37231 P46934 2316 1785 O14939 Q13492 5468 375790 4734 408 P49407 409 5868 Q9NRD5 P21333

macromolecule methylation 9463 P55345 4297 Q9BZ95 Q96MX6 29947 Q9Y6K1 O60725 2107 11107 Q9H9B1 6304 79813 7832 56339 O14744 Q9Y5R4 55929 5870 Q9NQ92 Q8WWH4 Q08J23 P46100 Q9UBC3 1789 1788 O14717 1786 Q99873 Q9NRD5 P01100 Q9UBL3 Q8IY81 Q86U44 Q96FX7 23463 Q6UXN9 346171 Q9UIF9 Q9NU63 P62495 54496 Q9NVM4 84661 P49711 Q99707 4548 54815 Q01826 P26358 P61964 Q9C005 11091 3070 30827 Q9BVS5 Q9P0U4 Q86YP4 3276 Q9NRZ9 Q8NB78 Q03164 221656 55352 5929 Q15291 80335 546 11176 Q9NPF5 Q9UET6 Q9UI30 P20340 2935

methylation 9463 P55345 4297 Q9BZ95 Q96MX6 29947 Q9Y6K1 O60725 2107 11107 Q9H9B1 6304 79813 7832 56339 O14744 Q9Y5R4 55929 5870 Q9NQ92 Q8WWH4 Q08J23 P46100 Q9UBC3 1789 1788 O14717 1786 Q99873 Q9NRD5 P01100 Q9UBL3 Q8IY81 Q86U44 Q96FX7 23463 Q6UXN9 346171 Q9UIF9 Q9NU63 P62495 54496 Q9NVM4 84661 P49711 Q99707 4548 54815 Q01826 P26358 P61964 Q9C005 11091 3070 30827 Q9BVS5 Q9P0U4 Q86YP4 3276 Q9NRZ9 Q8NB78 Q03164 221656 55352 5929 Q15291 80335 546 11176 Q9NPF5 Q9UET6 Q9UI30 P20340 2935

positive regulation of cell migration P25054 Q13477 Q13873 6198 Q15654 O14672 2150 O60603 Q4VCS5 3479 P84022 P39060 P04085 10013 6869 Q15389 P07996 3912 P04004 Q15303 P78536 7057 3091 5154 5155 P12429 O95684 Q93096 P16234 2159 2277 7448 7205 5029 P00533 P01343 Q9HCY8 11116 Q13017 Q13418 57402 P14780 P41231 P01019 5159 P01137 P55290 5054 3791 4088 3673 Q01974 P17301 O60674 Q13393 Q92786 P01127 Q16665 3667 10488 Q59EA4 Q9UBN7 5604 O60271 P07942 P28482 Q8WUI4 O43915 8174 394 57669 P15692 51564 1012 5337 P00742 4920 5338 P08887 719 P51671 3690 P35968 Q9NYA1 Q13443 P09619 O43889 Q9Y4H2 P35443 P10721 P35568 P01308 324 Q9HCM4 Q15077 P55085 9564 P05106 8754 7422 P10415 2247 3611 P08069 P25103 1956 P09038 P23443 P09958 P62070 Q02750 P48061 O14939 Q6PID4 P05362 P05121 Q14289 Q16581 4318 P25116 P06213

intracellular receptor mediated signaling pathway 6672 Q14686 1022 P50613 2274 P10275 P10276 Q8TDD1 672 1499 Q9Y4C1 Q92731 Q15185 P61586 10499 Q15466 1616 Q9UBK2 Q04917 P31689 7376 Q9NVC6 7533 P13631 Q05086 Q96KS0 29079 P46934 367 O43294 4734 5901 Q15596 P27797 Q9UER7 O75528 O75925 O43251 P48552 6256 2099 P19793 P29590 P38398 79039 Q14192 898 Q96PK6 811 Q92993 P03372 O75928 10432 P06401 P06400 5914 5371 8289 8204 P62826 Q15648

P55055 2100 P35222 P78317 Q02790 10524 387 O60674 O95071 3301 Q9NPJ6 10728 P24864 O14497 9967 P23497 Q9Y2W1 5925

establishment or maintenance of cell polarity O15085 54908 P25054 6993 5584 Q8TEW0 23122 Q9UPN3 P63172 P35579 1058 284217 998 P43034 Q7KZI7 P49450 Q12959 23332 1072 P13236 Q7Z460 Q96RT1 324 9826 85440 Q01518 Q8N205 P25391 Q9Y5S2 2011 O75122 Q13418 163183 P15311 60412 Q96N67 3611 P23528 P08069 P60953 4627 Q96EA4 O43264 7430 Q9Y696 9578 Q92949 23513 Q9NXR1 54820 Q14160 56288 10486 O14777 10487 P41743

DNA packaging P06899 Q9H0U9 4171 1058 P20671 Q15021 Q8NCD3 55723 Q9BTM1 P62805 P62807 Q99880 3010 P16104 Q99877 3009 Q9NVP2 3008 3007 Q8IUE6 3006 51773 Q9UKV3 P42695 255626 Q99879 Q9H2G4 Q7Z7K6 Q96NT1 3024 O75367 P55209 P11388 P23527 Q01105 3014 O60264 Q8WZ42 4869 O95347 Q16777 Q16778 O43823 P0C0S5 23310 Q9NRZ9 P04908 22985 221613 Q96A08 Q99733 55355 Q93079 Q8TBE0 P0C0S8 3148 Q93077 Q7L7L0 Q6NXT2 64061 P41227 P07305 P26583 8290 Q96KK5 Q02539 P49736 P49450 8348 9555 8467 O60814 Q96QV6 Q92522 8338 Q8N257 P33778 266812 10270 Q5SSJ5 4673 22893 P10412 P58876 Q9NR48 Q96T23 P84243 Q71DI3 P68431 Q16695 P49711 8349 5119 Q15003 7153 3070 P22492 Q5QNW6 P16403 P16402 P16401 Q9P0M6 Q9NTJ3 Q6FI13 4676 Q13112 Q96FF9

peptidyl-serine phosphorylationQ5S007 P25098 Q7Z727 7046 6872 Q9H422 A1XKG3 1020 O95271 Q13418 P78527 P42345 156 Q9BXM7 3611 Q00535 P17252 P01579 5578 6789 Q13043 5591 Q6IR47 P31749 65018 1195 Q9UBE8 5590 P17612 2932 Q96Q15 Q05513 P37173 120892 2475 5566 4214 207 P49841 Q13233 P21675

regulation of cell size P54852 P08833 P55268 Q9UQP3 O14672 6236 Q9BZ95 O75385 29948 3084 351 P37288 P16220 P84022 Q92731 Q9Y2X3 Q7Z6C1 O14786 5931 4722 P49815 Q13485 8408 Q9H0D6 5155 Q12837 P58546 P13631 O95684 51094 367 P55042 Q9UK53 Q13017 136319 Q9H2G4 P50150 O75489 Q76FK4 P10451 P53667 P46527 P37023 P51805 57521 P32929 4869 P06400 5716 4088 4089 P31947 2100 P68133 58189 1491 Q7L576 3304 64061 Q13275 Q8TDY2 7248 1385 P10275 Q9P0J0 394 Q6FHQ0 Q96SB3 Q9NYA1 P36896 P10600 Q86WV8 P19544 7490 1029 1027 1026 P01308 2475 7249 604 Q14140 P42771 9201 O95376 Q86V24 P19634 2247 Q16576 Q9NQC3 859 5515 P09038 5518 O15075 Q9HB90 7157 P31749 2023 P38936 55035 2810 2137 P08195 Q6PID4 Q14289 Q13873 7046 P04637 P10636 2033 3484 7161 P50897 Q4VCS5 3913 11108 27000 P42345 91 94 Q92574 P46821 51009 Q13642 P78536 22803 6520 P35368 O43602 Q02297 P67775 Q96RT1 P05067 11116 Q09472 Q8N726 Q9UKN5 P29590 Q13418 P56539 P41235 1874 773 Q53X93 P01019 Q9GZP9 6405 P01138 P01137 O60383 8829 Q16254 A4D1W7 O00213 P01127 P06733 4131 9821 P41182 1655 552 Q9HC57 6548 Q9UJW9 Q6IR47 P30153 P37231 P51959 Q99675 Q9UJX0 4137 207 5468 P01185 6696 P10415 Q96A54 P32320 P17844 3611 Q99543 5371 O15350 P08107 O75832 Q12816 O60356 3621 O43909 900 55558 P60568 5925

transforming growth factor beta receptor signaling pathway 7046 O14793 1385 4093 O43318 P41134 P16220 P84022 P08123 P04085 94 10454 Q13485 Q99966 P05412 Q6IR47 Q13443 5154 O95405 5155 6885 5796 P36894 P10600 Q8WUH2 4214 Q99717 Q15796 409 P43026 Q15750 P01100 Q15797 8754 Q8N6I1 P32121 P29590 P37023 Q53X93 9518 P01137 O60383 Q99988 Q93008 5371 P02461 4086 4087 4088 4089 O15198 163126 Q04771 Q9UBE8 P17813 P37173 P01127 Q13233

negative regulation of protein amino acid phosphorylation 5580 Q7Z727 4771 O14495 Q15735 P30086 P14635 4092 891 P84022 P17252 P35240 P49810 5578 51763 857 P01137 5515 6901 O60260 5518 P04201 Q9UJM3 P05412 4088 Q9NP71 4221 Q16635 5037 O00255 P30153 25998 Q9BT40 5071 5590 5728 P67775 Q05513 P17813 Q05655 P60484 P17936 O15105 54206 O15524 P60568 P49768 P06213

regulation of actin filament-based process 4690 10092 10095 O95835 10094 4771 7248 2033 Q9UHB6 O43639 P35612 P35611 Q4VCS5 P42345 Q96P48 6709 Q04759 P84022 830 P09493 832 118 Q9H2D6 Q7Z6C1 P04085 P61586 Q92574 6869 11346 P49815 P47755 P47756 P51671 5154 Q96QB1 Q01082 1072 Q86WV8 5071 Q3V6T2 10109 2039 2475 6711 6710 4214 P62328 7249 P61158 P17081 O15020 Q09472 Q13813 O15143 253260 Q9BPX5 23189 Q13418 P56539 P31431 P53667 P62330 P20936 3611 Q9BVC4 P23528 P02549 81873 P35240 55704 10552 P25103 859 P35080 O60260 O43182 Q08495 Q14678 64223 4088 P48061 382 P11277 P16333 387 51474 P52907 Q92786 Q6PKD3 Q6R327 P63313 Q8N3V7 Q13233 829

positive regulation of developmental process P25054 Q92858 8650 Q9BY76 2672 Q9Y4K3 3084 2547 474 4609 P84022 P07196 Q7Z6C1 8767 O14543 O14788 Q13485 P07996 P52565 3091 Q13009 8546 4860 Q9UBC3 P12429 Q12837 54361 5270 Q9UKV3 P46531 4851 P61158 8536 Q15796 P43026 Q14106 O75925 Q7Z727 6495 Q15797 Q92835 6498 P12757 P05549 Q9ULH7 P53667 Q9UBU3 27185 Q9NRI5 55109 Q13145 P06400 4086 5054 4087 4088 4089 7476 5970 Q9Y6R0 P10586 Q13705 P56705 22985 P17535 Q8N5F7 P17302 P17542 387 388 P31270 O60674 P19838 2697 P24864 Q16665 948 5604 P04233 P27037 7124 P63092 P14635 P10276 4093 154 396 57669 79576 1499 51564 P07550 P61586 P08887 Q14012 719 7132 P51671 O00238 P30556 Q9NYA1 P30559 P22392 P36896 O95361 O43524 P36894 P10600 P10721 285 3207 10626 P01308 Q9HCM4 Q9GZM8 Q92888 Q15475 Q99835 P00491 4790 6850 7020 2247 2246 P01579 P05230 Q9NQC3 P49715 P09038 P23443 O43186 P10827 P29323 P39905 P51692 O15198 P31749 O00255 Q02750 Q6KC79 P19883 O43508 1050 Q9HCK4 861 P37173 P05121 P40337 Q16581 P06213 P78423 Q13873 6198 O15123 O43157 P10636 2033 P14555 P52952 Q4VCS5 Q86WK6 Q1PSW9 P17275 91 P46821 P42229 Q9NSA3 7057 O15496 4221 Q02297 P55318 1191 P55316 O43609 81565 O43290 P01106 P01589 P01588 O43294 2290 8399 Q09472 7189 7067 P48431 P29474 5321 P14416 Q13418 3142 Q63HK5 O00203 Q01196 891 P17252 898 P01019 6886 P01138 P01137 P01375 Q9UMS4 Q13422 P55212 Q13426 A4D1W7 9138 P35222 9253 9495 7074 P19438 P21757 P43405 3148 P10070 P17936 Q04206 P10071 Q92786 6776 6657 1406 2737 P24588 8717 2736 O43353 P26583 5460 4131 Q92934 7520 Q8WUI4 9021 P78504 P15692 Q99684 5578 7518 Q8N302 2626 P47712 P05412 P31249 O00755 Q14774 Q13443 Q15628 27339 3171 1789 P37231 P16671 324 3169 5467 4137 207 5468 P08138 10468 P12956 8754 7422 5364 P10415 P56178 Q02363 572 P13010 3611 P43694 Q16610 P01178 5914 P41159 P24394 P27540 Q04771 P13497 P62745 7428 10320 P45984 P60568 5925

endosome transport 9146 9784 O75901 O95477 Q9UPV9 8411 11021 9182 Q641Q2 Q9H1K0 23325 154 Q99523 Q2M389 23085 10938 A8K0Z3 P07550 O14662 Q9UGU5 Q96QK1 P61106 O94955 8301 O95249 6642 19 Q8IWJ2 Q9UJC3 Q8WUX9 2316 P20645 P42858 55737 Q13492 Q96RL7 Q15075 Q15036 253725 P21333 9201 4074 9525 387680 Q86VS8 Q8IUD2 P42768 51542 Q15286 84661 9559 O14964 Q9C005 Q13501 O15075 Q9NP90 91782 Q60FE5 O75436 64145 1130 O75351 Q9NZZ3 Q99698 388 Q13190 51552 P62745 Q96ED9 51510 8878 6811 84376 9527 9648 Q13596 Q9H4M9

regulation of nervous system development Q92858 8650 O75386 259266 O75385 Q9BVA0 2547 474 Q8IYN9 Q9HCJ2 P07196 Q7Z6C1 O14786 8408 Q13127 P52565 Q13009 Q12834 Q9UBC3 5270 P09382 Q9H2X0 P46531 4851 51654 Q96SN8 P61158 5029 4854 Q16643 Q7Z727 6495 Q15797 6498 P12757 P12755 P10451 Q05397 P53667 Q9UBU3 27185 P51805 811 Q9NRI5 P40424 4086 7476 5970 Q9Y6R0 P10586 Q86YT6 Q02790 57534 5728 387 P21359 P20827 P08151 1808 Q13275 P54259 4099 O00468 396 P61586 Q16555 Q15223 5978 Q9UM47 Q9NYA1 Q92769 P30559 Q8IZT6 P36894 P10721 1822 Q9GZM8 Q92888 5747 Q15475 Q99835 Q86VS8 57689 2247 Q9Y250 55704 P01579 Q9NQC3 P09038 O43186 P29323 7157 P31749 Q9HCK4 O95390 84376 6993 P04637 O43157 P10636 2033 26037 O43711 P63172 P52952 991 Q86WK6 3479 Q1PSW9 2147 P46821 P02686 Q04917 O43166 Q3V6T2 P55316 81565 Q15154 P01588 1627 P01343 P49768 2290 Q09472 P48431 9921 P14416 Q13418 773 894 P41231 P17252 6405 P01138 P01137 8829 P01133 Q9UMS4 6660 Q13426 9138 Q658W2 9253 9495 7074 2735 55755 P60484 P10070 Q04206 Q96JB5 6657 1406 P24588 2736 4131 7520 8851 Q15735 P20916 P61981 5578 7518 Q8N5U6 3956 P31249 O00755 7532 7533 27339 P30279 1789 P37231 P62166 P18846 Q96SZ6 P23515 P00734 3169 4137 207 5468 375790 P08138 5108 P27797 Q15078 P12956 6696 5364 P10415 4155 Q9H228 P56177 3066 P35711 O60229 P13010 3611 P01178 Q96HC4 466 55558 P21554 Q9BZR6

respiratory electron transport chain P31930 O75306 O75746 P56556 Q7KZN9 4695 O43678 6390 P19404 4728 4729 Q99807 8604 6622 4720 Q16795 4722 1738 4723 6901 4725 4726 Q9UBK2 O95169 P28331 P14927 Q16635 O14561 O43920 P07919 Q16718 O75380 10229 29796 4697 P00414 4698 4731 4535 P09622 P04179 7384 O75489 O00483 O95298 7381 O95299 P17568 4705 4707 Q9Y6M9 1355 P31040 P00403 4701 4702 P03891 P56181 O00217 O75438 P51970 Q9Y375 6389 O95182 P37840 P21912 4718 4719 P49821 4710 4514 4713 4714 4715

regulation of cell cycle process P25054 6597 O95714 11065 11186 5300 P30086 P51587 3481 O43715 472 2305 26271 Q9Y6G9 Q9Y3A5 Q04759 3479 995 Q99640 8924 54998 Q8N3U4 5139 Q9ULW0 Q9HC98 O43683 7332 9475 Q13526 5037 P51636 55294 1104 P14174 Q9H0C8 P08575 10783 Q4LE28 Q9UI95 P01344 P01343 Q6ZN33 Q8N726 9126 P29590 Q9NQW6 O00444 22974 Q9NS23 4193 891 Q92793 811 79959 P01137 P01135 4869 P01133 P01375 P06400 O43264 4361 O15392 P46089 91147 O75116 O00213 P78317 P51532 O75592 Q9NWT8 Q92786 5569 Q9Y468 9700 7884 7124 Q13315 Q969H0 9820 P41182 P14635 10459 10733 672 Q13042 Q14493 O14744 7013 Q96SB3 Q9NP71 P51955 Q5HYA8 Q9NYZ3 1029 1026 Q9UJX2 Q00987 Q9UQE7 P01308 P50748 324 1387 54443 604 P42771 P27797 P61925 7023 P30307 P54274 O00762 P38398 P49959 Q01664 P68036 P49711 5119 Q14674 Q14432 5371 8881 Q08050 P38936 Q8TAP6 7272 23077 51512 Q9UKT4 Q14683 P06213 5925

organelle assembly Q8TDY2 9821 O95271 6194 26271 203068 Q9H6D7 1058 60673 55125 P83731 P62753 P07437 Q9Y4P1 P49450 Q96CS2 9474 3692 Q2NL82 Q68CZ6 O75376 55054 79441 Q9BSB4 2 Q99871 54801 P49768 55142 7141 P56537 54930 Q9H4B7 Q9H1Y0 O15379 P09430 Q7Z3C6 P62851 O94927 4869 93323 P07339 Q9BY44 Q7Z4H7 8841 23354 79065 Q676U5 Q9H492 23192 80254 P40692 Q96MT8 P62263 55559 Q9UKT4 O94817

response to abiotic stimulus P48051 8892 P28562 Q16623 O14793 8533 2672 P30086 P51587 6591 351 472 2547 595 P37288 Q04759 477 P20396 5931 O14543 O14788 P08842 O15516 Q9UGM1 P07996 5935 Q16512 O15530 Q9NS91 Q9UBC3 5037 P16104 Q16082 P63211 P42858 P00533 Q96RL1 4734 8553 Q7Z727 Q9NRY2 P31153 81628 Q9UDY4 57646 55342 55584 55466 O15534 Q13144 4867 Q14232 P00441 Q14116 P07858 5970 P18074 3673 Q9BZF9 P68133 P10588 9093 P35625 5170 Q9NZ94 P17302

P17301 P20941 P21359 2697 Q99576 3301 51435 10763 41 P43246 Q6PCD5 3308 P78348 5187 O15287 7124 P54132 P54253 1020 P14635 25788 154 Q99965 P09172 3316 3315 Q6FHQ0 O60884 P07550 P04040 2904 P08887 2902 O14503 Q14011 Q9UIM3 P51671 Q92769 1153 P51679 P10721 O95243 Q9H078 285 2237 1026 841 847 Q70UQ0 P04179 P98179 P28340 P13646 A1XKG3 1161 P19634 Q8WW22 P38398 Q9NPQ8 1831 2246 1277 P05230 857 Q16576 1956 P23443 9575 P10827 64782 26509 P20073 7157 P55011 P31749 Q9NR50 Q05586 O43623 O00255 Q6KC79 P38936 1843 Q93034 55159 Q6PID4 P02533 Q14289 P02452 7046 6198 8930 O15123 P04637 Q9Y3Q8 P11166 60626 P42224 Q96RU2 P17275 81570 P39060 7832 P01116 P46821 6869 P98161 P01112 P23025 P01111 P49770 P31689 P48681 7057 Q9UNS2 Q9UHG2 4221 5310 5311 5796 P35368 P16989 P10523 Q6AZY7 P46934 641 4214 P08575 5424 P01588 P05067 P42574 408 P49407 P01100 2956 7067 P48431 P29474 Q8WXI4 6772 P29475 5321 Q13535 P14416 3265 P29590 O00562 891 1634 79035 54413 Q96T60 Q00535 P17252 3014 P01019 P06241 O15259 6647 P01138 P01137 29086 P01375 P02461 P55212 Q13426 P55210 P19320 Q15849 P34931 2063 P34932 9495 545 Q04206 Q8WTP8 P07585 Q96EY1 6657 P24588 7508 4131 Q13432 5585 Q13315 P13945 1660 58473 8170 P41181 9021 Q9NX61 672 310 552 P22415 553 P15692 Q03468 Q99684 51720 5578 6548 P08243 Q13563 7518 P08246 P47712 P05412 5591 7098 Q6IR47 Q9UBG3 7412 P14923 3297 8065 1789 Q07817 Q01831 P37231 P46736 3728 Q9NWV8 Q7Z2W7 207 5468 P08138 P01266 23746 Q9BQ15 11080 P24385 P01185 P12956 7422 P10415 Q13216 3860 Q9Y5S1 9600 59341 3066 P78527 P50993 P20336 121457 Q00613 Q9NZM1 5599 Q14790 P00403 O15455 P25103 P01178 P08588 5371 P39748 Q9NP97 P52701 Q08211 P47804 P45983 P04792 Q13233 51514 Q14683 Q9NZN9

regulation of catabolic process P25054 9744 P54619 Q99490 O75385 P15498 2665 1454 2664 P84022 1453 P09493 5018 P43034 P49815 Q13485 Q8WZ64 8408 Q8TBP0 3091 Q9Y2X7 115704 Q9P107 2316 5707 5708 5709 51099 Q13370 493856 P61956 4734 Q15311 7341 P50395 9882 1121 Q9ULH1 Q8TEU7 Q96CN4 4193 O60543 P09471 Q9UKW4 Q8IV61 5716 P26374 4088 4089 5970 Q9UPQ3 Q8WWN8 Q92949 1374 125058 Q99460 P35625 Q8N6H7 P17302 P21359 2697 P62140 Q66K14 1122 P31150 P60520 Q16665 3667 5604 P05129 Q9H2M9 7124 7248 4092 Q9NUY8 121512 25780 Q99728 P45379 Q13286 P51671 Q9Y4H2 Q96J02 Q86WV8 P35568 Q96KS0 3326 P01308 2475 5500 7249 604 Q6ZW31 4898 Q9GZM8 85440 Q96NH3 P26045 Q9UGJ0 P49840 Q8N5K1 10273 P30542 Q8WY64 Q96N67 P01579 11124 O43182 P09958 Q9UKS6 P10827 64786 P31749 Q02750 2931 Q14160 Q6PID4 P48730 6613 Q14161 Q14289 P06213 6872 7169 10928 26037 P50416 23327 P42345 Q96RU8 Q8WTS1 3479 P63165 Q92574 P45974 Q6ZT07 11345 O15118 O43166 P56524 Q15027 Q7Z6J4 P35368 83737 O43847 23216 P46934 O43609 81565 P05062 406 85360 P98174 P01343 O00327 7067 116986 Q96M96 116987 O14964 P01375 O15151 Q9UNE7 27352 55633 Q04206 O43150 Q15057 9146 Q3MII6 5582 Q9NP61 O43597 Q96BZ9 Q96DN5 P41182 Q5R372 Q9UNN5 Q86TI0 93594 Q96P48 O14908 79735 Q96PU5 10451 9815 P55072 Q13200 P07951 O43242 Q9Y4P1 Q13443 Q96HU1 Q9NP71 Q5JSP0 Q00987 324 Q9NU19 Q15070 207 5347 Q8TEA7 51735 P21333 8078 P24386 8754 Q6ZV73 Q0IIM8 55785 O60343 7415 4943 Q9BT67 Q8IYX1 P27540 Q60FE5 O75832 P49674 P53350 580 Q9Y3P9 23513 23192 O15105 P08238 P21554 P21675 P07148 Q8N6T3

negative regulation of organelle organization P25054 P10636 9181 472 Q9BVA0 Q9Y3A5 118 6622 54998 P46821 P47755 P47756 Q92974 O43683 25913 Q9UBC3 Q96QB1 23332 Q01082 2039 Q7Z460 22919 4214 Q9UI95 Q15554 Q9BYV2 O15020 Q13813 O75122 P56539 891 Q01105 Q92833 O60260 P26358 4869 Q9BSI4 5716 Q08495 91147 Q9NUX5 Q02790 P11277 Q9NWT8 P52907 Q6PKD3 Q15691 829 4131 9700 4133 Q13315 Q15735 23122 P14635 Q9UHB6 P35612 P35611 10459 672 Q96P48 6709

830 832 Q9H2D6 7013 7014 Q5HYA8 5071 1789 1786 324 57159 6711 6710 4137 P62328 3720 Q9NYB0 P54274 23189 P38398 P02549 859 Q9NPA3 Q14674 O43182 Q14678 O75832 Q9BZS1 P37840 7272 51474 P27816 P63313 50943 Q13233

cytokinesis O15085 P25054 8452 O43236 9700 11021 25909 P51587 P35579 P35580 5898 1616 O75935 Q9H0H5 P32856 9475 1072 324 116840 54443 Q8WYP5 9826 Q14141 Q9UER7 Q8N137 Q13618 2054 Q9NQW6 6093 4628 29127 P20936 P62491 P23528 Q96RK4 Q15286 Q13464 Q14674 4627 9212 O15392 8766 O43663 O75116 P11234 P11233 O60879 Q9NZ56 989 Q96GD4

regulation of small GTPase mediated signal transductionO15085 Q7Z628 9744 P10911 7204 Q5VV41 9181 Q99490 P15498 2305 Q6PCE1 P43034 P49815 Q8WZ64 Q92974 Q8TBP0 Q13009 Q9Y2X7 Q9UIA0 115704 Q9P107 Q15311 Q008S8 Q8IW93 9882 Q9NR80 Q9UQ13 Q9NR81 Q9HBW0 29 Q15438 Q9ULH1 64857 Q8TEU7 Q96CN4 Q8IVF5 P20936 23263 Q9UKW4 Q9H7P9 Q8WZ42 Q8IV61 Q9UPQ3 Q8WWN8 Q92949 125058 Q12979 382 Q8N6H7 Q13829 P21359 Q66K14 Q8N5V2 5604 8452 Q92538 7248 7126 Q96N96 23365 Q9NUY8 121512 25780 Q6KH11 O94827 P46108 P51671 Q86WV8 Q5VST9 P37198 23370 2475 5863 7249 604 Q6ZW31 Q9GZM8 85440 Q92888 Q96NH3 23229 Q07890 Q6ZSZ5 Q8TCU6 O15068 23463 Q13618 O43739 Q14C86 P62330 Q96N67 1398 Q07889 613 10276 5516 5879 Q14155 Q12774 10160 Q08050 64786 O95140 Q9H8V3 Q8N9R8 Q02750 253980 Q14160 153090 Q6PID4 Q14161 Q14289 Q15811 10928 26037 115557 O60725 P42345 O14827 3479 Q86VI3 O94989 P46940 P01116 Q92574 Q6ZT07 P01112 P01111 O43166 Q15027 Q7Z6J4 Q96QB1 23216 O43609 81565 84033 P01588 408 O60292 P49407 85360 P98174 P01343 Q86VW2 116986 3265 Q96M96 P52735 116987 P11274 8826 50618 P04626 9138 2064 P35348 7074 27352 55633 Q15052 11214 O43150 Q15057 9927 9267 Q3MII6 Q9NP61 O43597 Q96BZ9 P78509 Q96DN5 P41182 Q5R372 Q9UJF2 Q86TI0 93594 Q58EX7 Q96P48 79735 P63000 10451 9815 Q96PE2 P62714 Q96HU1 Q8WZ19 O15211 7410 Q5JSP0 23094 Q9Y6D6 Q9Y6D5 Q9NU19 9826 Q8TEA7 Q13574 51735 Q76NI1 50649 O14492 Q96GA9 6453 Q6ZV73 O75962 Q5VWQ8 Q12802 O60229 2889 Q0IIM8 50650 55785 O60343 Q9NZM3 4943 23636 Q8IYX1 4168 Q13905 Q9Y3P9 23513 Q9NZN5 Q99418 10564 Q8N6T3

morphogenesis of an epithelium 9464 O96004 O43278 5268 O75386 Q9Y4K3 O43318 P52952 4609 2303 P62993 3479 Q1PSW9 284217 P84022 Q99807 Q92731 O14786 O14543 Q92574 P42229 O15119 P49815 Q13485 3911 1856 Q9NPC8 5155 8543 6885 Q96QB1 7291 54361 P13631 1072 7052 367 P42858 P46531 4851 P48436 P01106 768 P00533 P16070 O43294 Q14344 P01343 Q15672 P49768 Q8N205 P50552 7189 P61968 6495 P48431 P05549 P25391 Q9NRY4 O75881 P29590 P12755 Q03014 Q13418 Q99623 O14727 Q96S42 P23528 Q96RK4 8945 P01019 Q92830 P21980 Q9Y297 P03372 P01137 Q9NSC2 8829 P40424 6926 P06401 Q92826 4088 4089 P07737 8323 Q14118 P36952 2100 Q86YT6 P35222 P56705 O60716 P17302 57534 Q4AC94 P17813 P31270 2697 P10070 P61296 P10071 11331 6776 P01127 6657 2737 2736 9421 9420 O95999 P07949 7248 2909 P35453 9021 P10275 P78504 10736 57669 1499 P15692 Q9NYQ6 Q16790 Q99966 2626 P51671 26005 Q86WV8 P19544 7490 P18206 3207 10229 960 1147 841 3169 Q9HCM4 7249 7408 Q99958 Q15475 Q96T37 5361 7422 P10415 2099 7020 163183 2247 2885 3611 O14641 Q14790 P08069 7414 P43694 10672 P47895 2926 1956 P09038 9839 5371 O15111 P39905 Q15648 O15230 Q04771 23513 Q14160 P37173 O60353 P24821 Q9UMX1 1605

protein modification by small protein removal Q9HC62 P51784 Q5VVQ6 9100 Q9Y2K6 27005 23326 Q96RU2 Q9UK80 Q8TAF3 51720 10213 P45974 Q14694 P09936 Q9UGI0 7375 P21580 10987 P46736 10868 Q9NWV8 54764 55611 Q92560 51377 1540 57599 7128 Q96RL1 Q8TEY7 8078 O75604 8237

377630 Q9UPT9 29843 Q9Y5K5 59343 O00487 57646 Q9Y4E8 Q92830 Q96DC9 29086 Q93009 Q93008 Q9NQC7 Q9NPA8 P40818 Q9P0U3 Q6R6M4 Q13107 Q92905 7874 Q96FW1 9099 55432 23032

positive regulation of cell differentiation P25054 Q92858 8650 2672 Q9Y4K3 3084 2547 474 P84022 P07196 Q7Z6C1 8767 O14543 O14788 Q13485 P52565 3091 Q13009 8546 4860 Q9UBC3 Q12837 54361 5270 Q9UKV3 P46531 4851 P61158 8536 Q15796 P43026 O75925 Q15797 Q92835 6498 P12757 P53667 27185 Q9NRI5 Q13145 P06400 4086 4087 4088 4089 5970 Q9Y6R0 P10586 Q13705 P56705 22985 P17535 Q8N5F7 P17542 387 P31270 O60674 P19838 P24864 Q16665 948 5604 P04233 P27037 P63092 P10276 4093 396 57669 79576 1499 P61586 P08887 Q14012 O00238 P30556 P22392 P36896 O95361 O43524 P36894 P10600 P10721 3207 10626 P01308 Q9HCM4 Q9GZM8 Q92888 Q99835 P00491 4790 6850 2247 P01579 Q9NQC3 P49715 P09038 O43186 P10827 P29323 P51692 O15198 P31749 O00255 Q02750 1050 Q9HCK4 861 P37173 P40337 Q13873 O43157 P10636 2033 P14555 P52952 Q86WK6 Q1PSW9 P17275 91 P46821 P42229 Q9NSA3 O15496 4221 Q02297 P55318 1191 P55316 81565 O43290 P01589 P01588 O43294 2290 8399 Q09472 7189 7067 P48431 P14416 Q13418 3142 Q63HK5 O00203 Q01196 898 P01019 6886 P01138 P01137 Q9UMS4 Q13422 Q13426 A4D1W7 9138 P35222 9253 9495 7074 P21757 P43405 3148 P10070 P17936 Q04206 P10071 6776 6657 1406 2737 P24588 2736 O43353 P26583 4131 Q92934 7520 9021 P78504 Q99684 7518 2626 P05412 P31249 Q14774 27339 3171 1789 P37231 P16671 324 3169 5467 4137 207 5468 P08138 P12956 5364 P10415 P56178 Q02363 572 P13010 3611 P43694 5914 P41159 P24394 P27540 Q04771 7428 10320 P45984 P60568 5925

regulation of response to stress 8654 Q9Y4K4 Q9Y4K3 O43318 472 2305 5817 8767 O14788 57506 P07996 2308 Q9Y3Z3 P25963 P04004 5154 7334 5155 Q16512 4067 4188 7336 7335 O75376 5270 P16104 2 60489 P14174 7448 Q96RL1 Q9UI12 10666 P10809 Q96EB6 Q9HCY8 Q7Z727 P32121 P10451 4193 Q9Y6Q6 57402 Q9UBU3 P21589 64170 4869 5716 Q9Y6R4 5054 5970 3673 Q9BZF9 Q9H257 Q9NR96 81622 P17301 O60674 Q8IUC6 Q03518 Q13153 P04233 Q9H1C4 P05129 7124 154 Q9Y2C9 121512 710 P07550 5733 54 Q13286 7132 P30556 Q9Y239 7133 Q15109 Q96J02 161 3329 1029 29110 10746 79444 960 P01308 2475 604 P42771 O15169 5624 Q9GZM8 P02790 10392 6850 4792 23586 1161 P30542 P38398 P15151 Q5TCX8 55703 857 Q9BQI3 Q9NQC3 Q96CA5 P04264 O94973 6188 Q12778 Q08050 P51692 P31749 O95382 O00255 1173 Q6PID4 P05121 Q14289 Q9UL54 P78423 P23396 Q15819 P14555 7161 2150 O60603 P42345 Q9HC16 2147 Q92692 3476 P04085 P42229 P15090 1616 P07355 O15118 7057 4221 6885 Q13404 83737 7052 O76074 Q99750 81565 4214 P01589 Q96CW1 4216 P16070 409 Q9UER7 O00206 6890 7186 7189 P29474 Q8N726 5321 Q13535 Q9H422 Q96M96 P29590 3263 P13686 148022 27102 4907 P17252 3014 P01019 O15379 P21980 Q9NZ08 29086 P05155 P01375 5692 8841 Q13546 Q7Z434 P78318 P19438 P43405 302 P04070 545 Q04206 P01008 6776 P01127 P40933 5328 O43353 P01042 5582 O95999 5585 Q13315 P07948 P41182 Q9NX61 P53567 672 P00749 P22415 P00747 Q99683 Q03468 51720 5578 P08246 P43115 P47712 7099 7098 51606 64127 23411 P37231 P46736 Q9NWV8 Q00987 P63010 P00734 8737 207 5468 P55085 P01023 6696 Q13216 59341 Q9UHD2 Q9P035 Q9BUZ4 P61088 O15455 Q15762 P20333 9839 Q9BT67 5371 O95786 O15350 O75832 Q12933 Q13227 Q06830 P34972 P28070 462 P51617 Q9HC29 51752 54106 Q13233 P25116 P60568 P21554

blastocyst development6670 9421 7046 8452 P61803 O96004 6498 P12757 P49321 P12830 Q13618 7082 P51587 Q07157 6927 84525 Q9Y3A5 P17275 Q96S42 6667 Q15561 P08047 999 Q15047 P28799 Q9UMS4 Q6IR47 Q13503 9412 O95140 P17980 27339 Q9BPY8 P17655 Q02447 P20823 2896 81565 9869 Q9HCS7 4678 Q9GZM8 5702 P43686 5704 9927

mesoderm morphogenesis O43474 9421 Q13873 Q15797 O96004 4771 Q12948 Q9BQ95 2132 O00167 Q9UPN3 57045 Q93063 6927 2303 57669 P84022 Q96S42 P35240 Q9UJU2 O15119 6926 Q9GZX9 4086 4087 4088 5573 9314 Q04771 P36894 P10644 P20823 P17612 2139 51295 P42858 Q9HCM4 Q9H2X0 5566 Q9HCS4 O95947 1969 Q15796 P29317 Q99958 Q16881

regulation of anatomical structure morphogenesis P25054 8650 Q9BY76 O75385 Q8IYN9 Q7L0Q8 4609 356 P84022 Q9HCJ2 P07196 Q7Z6C1 O14786 Q13485 8408 P07996 P52565 3091 5154 Q13009 P12429 367 P46531 4851 P61158 Q15796 Q14344 Q9HB63 6495 Q15797 6256 6498 P12757 P05549 Q9NRY4 P10451 Q05397 P53667 4628 Q9UBU3 P51805 P20936 55109 P03372 Q13145 4627 4086 5054 4087 3791 4088 4089 P54760 7476 Q9Y6R0 Q8WWN8 P48023 P10586 59277 Q7L576 79784 387 388 P21359 2335 P31270 P20827 Q16665 Q13275 4099 7124 2909 P35453 Q9UQL6 P10275 4092 P35579 396 P35580 121512 57669 1499 51564 P61586 3679 Q99966 Q13164 719 7132 P51671 22846 Q9NYA1 8100 P35443 P36894 P10600 285 Q9UEE9 3207 23370 Q9HCM4 Q9GZM8 3689 Q92888 5747 P02751 Q15475 Q6ZSZ5 P05107 1285 1284 6050 7020 Q7Z406 57689 2247 2246 P02549 Q9Y250 P05230 Q9NQC3 859 P04264 P09038 P29323 P20073 P39905 Q7L8A9 O43508 Q9HCK4 861 P05121 1969 P09601 P29317 Q16581 Q9UL54 P78423 O15123 O43157 P10636 2033 26037 O00300 Q4VCS5 P41134 Q86WK6 999 P04085 P46821 10014 P02686 Q04917 O43166 Q53QZ3 7057 Q7Z6J4 Q96QB1 O43609 81565 P01106 P08572 O43294 P98174 Q13099 P49768 Q09472 P29474 P19793 Q96M96 P29590 Q03014 Q13418 P56539 Q01196 Q99623 773 Q96S42 P06241 6405 O95859 P01138 P01137 8829 P01375 P55212 P04626 9138 Q658W2 2064 P35222 9253 9495 7074 P19438 Q9NRR8 11331 Q92786 P24588 5460 4131 Q8WUI4 P20916 Q03113 3162 310 Q96P48 P15692 Q13683 Q8N302 2626 O00755 P35968 Q13443 7533 5598 Q5JSP0 58480 Q01955 P23515 324 4137 P17081 P08138 8754 6696 7422 5364 P10415 4155 Q6ZV73 P12830 2099 P13489 P15311 3611 55785 P43694 10672 Q16610 Q96HC4 5371 7430 56882 P62745 O15105 Q9H4E5 55558 Q9BZR6

MAPKKK cascade Q13115 Q9Y6W6 P36507 7046 P56199 Q9Y4K4 Q9NRW4 Q9Y4K3 O43318 Q96RU8 1852 Q8IVH8 1850 23043 Q9C0K7 P49137 1616 P07996 P52564 P28335 7057 Q16512 4188 4221 6885 Q9UKE5 P53779 56940 Q8WTR2 P67775 Q99750 4214 4215 P00533 4216 Q99759 P42338 Q13098 Q13131 Q9UER7 P49768 O00206 Q5S007 Q7Z727 7189 Q15797 P29353 5562 8315 Q9HBW0 Q96M96 Q06124 Q7Z569 Q9UBU3 P17252 6647 55504 P01135 4868 P00441 P01133 Q8IV61 P01375 Q12851 P32239 4086 Q9Y6R4 3672 Q13546 P21917 Q01974 10645 P43405 Q96RR4 Q8N4C8 P21359 O60674 P20827 Q15052 5602 O14733 5604 P04233 Q8WXG6 Q8TDY2 Q13956 9821 7124 5585 P28482 P07949 Q5SGD2 Q16828 9261 P46109 P15056 673 121512 25780 Q99683 Q03468 5578 4920 10454 Q13164 Q8IWV1 7099 Q6IR47 Q13202 Q13443 P30559 5871 5598 P30153 P10600 P61073 10746 79444 2873 P01308 120892 8737 Q15750 Q99956 O95136 O95257 P28223 8754 6850 Q9P035 P30542 Q9NS68 P18545 O75293 2247 1399 P60033 975 5599 Q5TCX8 857 Q96CA5 5515 1956 P09038 5518 Q9BY84 6464 Q13227 O95382 O00255 Q02750 Q6PID4 P45983 1848 Q14289 Q9UL54 Q13233 P45984 P25116 P06213

mitochondrial transport P30049 O75947 O75746 P07900 P04637 O96008 3320 P36542 P53007 513 O94826 8604 P23786 514 515 10651 637 10452 Q15785 Q15388 Q9HAV7 4580 P48047 7534 P55957 Q07817 10469 Q99797 1468 Q9UBX3 521 P42858 Q9Y5J6 522 9868 80273 P49768 O75964 P10415 100287932 P56134 6576 O43615 Q9NS69 10953 401505 O14925 498 P00846 P25705 P24539 P18859 56993 P56385 539 P63104 P56381 10476 10632 4285 P30536 P55851 Q13505 Q16611 7157 O95140 Q9Y619 Q9Y5L4 O75431 Q8N4H5 P62072 9804 10166 509 P25874 9927 P06576

embryonic placenta development 6670 9421 O75626 O96004 Q14686 Q15738 3880 P28482 O43278 2034 9021 O00622 P17676 O14627 10733 P62993 P00747 O75190 P17275 6667 P08047 3676 O14543 Q13563 639 Q99966 4188 5311 P08727 7412 3297 Q02447 Q99750 207 P00533 O95377 O00444 57448 Q00613 2885 Q9NR09 Q96S42 Q9UJU2 P49715 Q9GZT9 1956 50814 P27540 Q15648 P31749 P19484 P19320 Q02750 P13612 1050 54583 Q99814 5604

positive regulation of cell adhesion P78423 P25054 133584 7124 7248 7402 1021 Q08722 4092 O00622 3084 P15498 Q9Y6C2 57669 P23229 P84022 1012 P09493 10451 Q92574 5338 O14788 P07996 3956 P04004 7057 P35968 Q13443 Q02297 Q86WV8 22920 7052 5590 P16671 Q05513 P46939 961 324 P09382 Q9HCM4 Q9H2X0 7448 967 Q92845 8754 6696 Q13418 Q53GQ0 O43739 Q8NC24 P10451 P31431 51144 3611 2246 Q00534 Q9UKW4 P01579 P21980 P05230 3655 P55290 P01375 3791 4088 P04626 3673 2064 P14543 P17301 Q63HQ2 O14939 O15105 948

neurogenesis P25054 Q92858 Q8NEY1 65125 Q9NYD6 2672 Q9UBB6 259266 P17676 Q9BVA0 3516 2547 Q8IYN9 O60443 P16220 284217 Q9HCJ2 Q9BVA1 Q9ULW3 Q92731 Q92730 O14786 Q8TDD5 P52565 Q12834 Q9UBC3 Q12837 P50148 P63211 P16234 79885 Q96KN7 2676 P42858 O60331 4851 P61158 4854 4734 P54753 Q7Z727 136319 P25391 Q06124 Q05397 P53667 6809 27185 29127 4983 811 5830 Q9UQB8 Q9Y6A5 P40424 P62258 P54764 P54762 Q9NQX0 5970 3672 2100 Q7L576 P17302 57534 5728 O60674 P20827 2697 6812 P26367 O14578 5604 P41743 P34925 4771 1385 1021 1020 O00468 1499 P61586 4646 5978 Q9UM47 P07437 Q9H0H5 O00238 Q9NYA1 Q92769 P28370 P51679 Q8IZT6 P10721 Q06330 960 O60890 Q9UM54 Q92888 5747 P04179 2253 Q9NYB9 57689 2247 Q96N67 Q9Y250 4664 Q92753 O14640 4665 Q92752 P10826 O43186 P29323 P31749 Q9NR50 Q13509 Q13506 Q9P202 O60636 Q9HCK4 3226 O95390 P29317 Q15019 6993 P04637 O43157 P56199 2033 25861 P50897 P11047 P52952 991 203068 3479 2147 7832 999 P02686 Q04917 O43166 Q9C0K0 P49770 Q96CW9 Q9UHG0 2043 P50542 1191 Q15154 P48436 11113 P01588 O43293 P01343 Q96NL6 P49768 2290 Q09472 P50552 P48431 9921 6774 P14416 23787 773 894 Q00535 Q00534 P35240 Q53X93 P01019 6405 6647 P01138 P01137 P49639 8829 84062 Q8N960 Q9UMS4 6660 P04626 P04629 Q658W2 2065 2064 P35222 O00213 2063 P54920 O75084 55755 P60484 P10070 P10071 3146 Q96JB5 6657 P24588 O00744 P07942 4131 O00628 5584 4133 7520 8851 P07949 P20916 10458 P61981 P15692 Q99684 5578 P55075 5338 7518 Q8N5U6 P40763 P26232 O00755 Q6IR47 7532 7531 10460 7533 P52803 P37231 10229 P18846 Q96SZ6 324 3169 4137 207 5468 10100 7408 5108 P27797 Q15078 6696 7422 5364 4155 P56178 P56177 3066 P13010 P61764 140735 Q96HC4 O43464 O14939 466 P60201 55558 8650 8892 P55268 Q9UQP3 Q8TEW0 8775 O75386 O75385 351 474 Q04759 P07196 Q99807 Q7Z6C1 P83731 P43034 Q13485 8408 Q13127 Q13009 P58304 P58546 5270 P09382 30813 P46531 51654 Q96SN8 5029 P00533 Q16643 Q15672 Q9HB63 6495 Q15797 6256 6498 P12757 P31273 P12755 P10451 Q9UBU3 P51805 P09471 Q96RK4 Q9NRI5 Q13144 Q14232 P00441 Q16650 6383 4086 4089 7476 Q9Y6R0 P18074 P10586 P10588 P13611 Q86YT6 P10589 59277 93166 Q02790 O75592 387 P21359 54820 23032 23154 23396 1808 6259 P60763 Q13275 P54259 4099 Q8TD84 396 Q02535 Q16555 Q15223 Q9HAZ2 P60709 Q16799 Q15109 P36894 1822 338917 60 Q9GZM8 1947 85440 Q9NRD5 Q15475 Q99835 9201 Q8TCU4 Q8TEY7 10152 Q02880 A1XKG3 Q86VS8 Q96EV8 55704 P01579 P08069 Q9NQC3 347733 1956 P09038 Q14155 O15075 7155 O15078 P39905 7157 O75553 Q02750 Q9NXR1 Q6PID4 84376 1969 Q14289 Q15256 Q15375 9463 7046 P10636 26037 O43711 P63172 7161 55283 3913 1613 Q8TAF8 Q86WK6 Q1PSW9 Q6NVY8 P46821 O60282 3912 Q13885 O15496 O43602 10939 7291 Q3V6T2 P55316 P46934 81565 P16070 P05067 P98172 1627 8399 P19793 Q13418 O43815 6096 1756 P41231 60412 P17252 P06241 P21860 Q13426 9138 29777 Q8IYT8 9253 9495 7074 4914

56288 Q96FJ2 Q9NZR4 Q04206 P07101 1406 2737 2736 Q9NZJ7 O75503 Q15735 P78509 P78504 O60488 3956 P31249 Q15742 O43921 P11532 27339 P30279 1789 P62166 64919 P23515 P00734 375790 P08138 Q9HCU4 P09429 P12956 P10415 Q9H228 Q8IUQ4 P12830 P34741 P35711 O60229 P20336 3611 Q04656 80184 P41159 O15350 132320 Q15768 63976 1203 23077 P21554 Q9BZR6

regulation of neuron differentiation Q92858 8650 O43157 P10636 2033 26037 O43711 259266 O75385 P52952 Q9BVA0 474 991 Q8IYN9 Q86WK6 Q1PSW9 Q9HCJ2 P07196 Q7Z6C1 O14786 P46821 P02686 8408 Q04917 O43166 Q13127 P52565 Q13009 Q12834 Q9UBC3 Q3V6T2 P55316 P09382 81565 P46531 4851 51654 Q96SN8 P61158 5029 4854 P01588 Q16643 1627 P49768 2290 Q09472 6495 Q15797 P48431 6498 P12757 Q13418 P10451 Q05397 P53667 27185 773 894 P41231 P51805 811 Q9NRI5 6405 P01138 8829 P40424 Q9UMS4 4086 6660 7476 Q9Y6R0 9138 P10586 Q658W2 Q86YT6 9253 9495 Q02790 7074 57534 5728 387 55755 P20827 P60484 P10070 1808 Q96JB5 6657 1406 P24588 Q13275 2736 P54259 4131 4099 8851 Q15735 P20916 396 P61981 P61586 Q16555 5978 3956 Q9UM47 P31249 O00755 Q9NYA1 Q92769 7532 7533 Q8IZT6 27339 P30279 1789 1822 P62166 P18846 Q96SZ6 P23515 3169 4137 207 Q9GZM8 P08138 Q92888 5747 Q15475 P27797 Q15078 6696 5364 P10415 4155 Q9H228 3066 P35711 O60229 57689 3611 Q9Y250 55704 P01579 Q9NQC3 Q96HC4 O43186 P29323 P31749 Q9HCK4 466 O95390 55558 P21554 Q9BZR6

lymphocyte differentiation 6670 P10914 P25054 P55347 P04637 Q13315 P54132 Q6NYC1 P15923 P41182 Q9UQL6 Q9H165 P53567 7163 6194 472 Q01201 1499 6304 5336 3676 Q8WV28 P42229 7518 10014 P62753 Q9C0K0 5591 P56524 P78536 Q15669 7412 23210 Q02447 64919 641 P16473 324 P08575 604 3688 P07766 P06239 6850 P10415 Q03014 P78527 P18146 P36888 P55327 6929 Q9UDY8 Q04656 P01137 5316 P01730 3932 P05112 Q01826 3659 P05556 Q13422 7157 P51692 Q13426 5971 P11912 Q9GZX7 P04629 P19320 P35268 9093 P35222 P13612 P16885 4914 P43405 Q9HBE1 Q8N4C8 53335 57379 6776 P40933 Q96EY1 10320 P43246 P60568 P04233

actin filament organization 4690 10093 8452 5584 7126 P41181 9181 Q9UHB6 O43639 P35612 P35611 Q68EM7 Q96P48 Q9HBH0 P63000 A8K0Z3 118 Q92730 6624 Q92974 P03950 Q8WZ19 10580 Q8IZP0 Q12959 P68032 P13796 1072 Q05682 2316 O60610 2039 11034 800 P60981 Q16643 408 1627 P49407 8936 P21333 9564 Q8TCU6 P10415 10152 Q13618 Q9NYB9 7184 P15311 Q9UBU3 P42768 P23528 P02549 Q92558 5879 O43182 Q8WZ42 Q08495 Q14511 7430 Q60FE5 Q9BX66 Q16658 P14625 P68133 Q13829 P16333 O43707 253980 51474 Q6PID4 Q92786 1729 54509 Q14289 P41743

positive regulation of locomotion P25054 Q13477 Q13873 6198 Q15654 O14672 2150 O60603 Q4VCS5 3479 P84022 P39060 P04085 10013 6869 Q15389 P07996 3912 P04004 Q15303 P78536 7057 3091 5154 5155 P12429 O95684 Q93096 P16234 2159 2277 7448 7205 5029 P00533 P01343 Q9HCY8 11116 Q13017 Q7Z727 Q13418 57402 P14780 P41231 P17252 P01019 5159 P01137 P55290 5054 3791 4088 3673 Q01974 P17301 O60674 Q13393 Q92786 P01127 Q16665 3667 10488 Q59EA4 Q9UBN7 5604 O60271 P07942 P28482 Q8WUI4 O43915 8174 394 57669 P15692 51564 1012 5578 5337 P00742 4920 5338 P08887 719 P51671 3690 P35968 Q9NYA1 Q13443 Q15109 P09619 O43889 Q9Y4H2 P51679 P35443 P10721 P35568 P01308 324 Q9HCM4 Q15077 P55085 9564 P05106 8754 7422 P10415 2247 3611 P08069 P25103 1956 P09038 P23443 P09958 P62070 Q02750 P48061 O14939 Q6PID4 P05362 P05121 Q14289 Q16581 4318 P25116 P06213

response to endoplasmic reticulum stress P24385 Q5VVQ6 P35638 P04637 Q96A33 O95292 O75460 P19525 595 10956 79139 57003 7415 5610 P01579 Q9GZP9 Q16236 O75807 51009 P55072

2081 4780 22926 9217 7157 O76024 P18850 55432 2932 P11021 267 P18848 468 Q70SY1 Q9P2K8 Q15011 10087 P49841 Q9UKV5 Q9BUN8 3309

DNA damage response, signal transduction resulting in induction of apoptosis 9641 Q6ZN33 8930 Q16666 Q9H305 P04637 Q13315 Q86Z02 P29590 7161 P51587 3162 472 P38398 672 57646 Q96RU2 O60543 Q03468 Q92630 P23025 5371 O15350 O96017 64782 7157 P31947 8445 Q9BZF9 Q9Y5J5 O95243 P52701 Q9NS56 3428 2810 P00519 P40692 Q14164 Q8WTP8 P09601 2956

regulation of chromosome organization Q9NYB0 5460 4297 9700 Q9H2K2 P04637 P54274 Q7Z7K6 P29590 P14635 O95271 891 4609 P61088 Q01105 6622 P49711 P01137 Q14674 Q92833 Q86UE8 P26358 Q9BSI4 Q9UBK2 O43683 5371 25913 7013 7334 4221 Q9BZS1 7014 7157 Q9UBC3 O00255 Q6KC79 Q9NUX5 P78317 P37840 Q03164 1789 1786 P01106 3720 Q15554 50943 408 P49407

locomotion Q92858 11060 Q9UQP3 23607 Q8NEY1 Q12948 O75385 474 5817 2304 Q8IYN9 477 P07196 23286 Q9BVA1 Q92731 Q9NPC1 P49810 O14786 P43034 O14788 P49815 8408 P07996 P27487 P03950 5154 5155 6368 P11215 2676 Q05952 P00533 4734 Q15311 Q15672 Q7Z727 6495 P32121 P32246 Q05397 Q9Y6Q6 Q03405 P37023 27185 P51805 P23528 Q9UKW4 4983 5159 5830 Q9NRI5 3655 Q13145 P62258 P55290 4627 P05556 Q16539 6386 Q14114 3672 Q92949 2100 1130 P13611 P10589 P13612 P48380 Q13829 P17302 5728 P16333 Q99698 Q9NVR5 54820 2335 2697 23032 3301 Q9UKX5 P20264 Q13393 5604 Q13275 P42830 8452 7124 P28482 7126 O95470 1020 64759 O43639 P35579 O75581 P09172 57669 P23229 1012 3676 3678 Q15223 P08887 719 P08648 P51671 P30556 3690 5991 P09619 Q5JQC9 O43889 P80098 P51679 P35443 P10721 Q96J02 O00358 960 Q04941 P60022 Q9HCM4 O60890 7249 3688 4898 Q9GZM8 3689 1947 7804 5747 P02751 Q15475 Q99835 Q12884 9201 9564 7142 P05106 Q8TEY7 P05107 6850 O95813 P05109 10152 P51681 Q02880 Q13618 A1XKG3 Q9NYB9 Q9Y5K6 P51686 7141 2247 P15153 P60033 P15151 975 55704 P01579 P26038 347733 P05112 1956 P09038 P23443 Q14155 Q8TE73 O15075 7155 P39905 P22492 P31749 O75553 P10147 Q02750 O43508 P48061 P11233 Q9NXR1 Q9HCK4 253980 Q14160 1289 Q6PID4 P05362 P02775 P40337 Q14289 Q16581 Q9UL54 P06576 P78423 4690 7046 6198 O00308 O43157 Q9Y6W5 P56199 Q9P296 10928 O43711 Q9H0M0 P11047 Q4VCS5 Q00796 P41134 Q9Y3A5 Q92692 P04085 Q6NVY8 5898 94 O95715 Q15389 3911 P01112 P31689 P78536 7057 P06702 Q9UHG0 5796 O43602 7291 3010 1072 83737 P50542 P13236 O43847 Q3V6T2 9350 55172 Q99755 P46934 84159 81565 P16070 P98172 P42338 409 O14713 Q01518 P49768 Q14865 P29474 Q86VW1 P14416 O00560 3265 P52735 8394 6093 P41597 Q00535 P16284 Q96S42 P17252 P06241 6405 P01137 8829 P01375 9255 Q8WXH2 P35908 P19320 Q658W2 P43405 P17813 P60484 3148 Q8IZE3 3146 4478 P01127 5327 10488 5328 10487 Q59EA4 Q96JB1 P26583 O95995 O00628 8851 P07949 8852 P78509 P59665 Q12904 O00622 P00749 P15692 27202 O14908 10451 5578 5337 P08246 P26232 5591 Q6IR47 Q8NEC5 Q68CZ2 5473 Q8WZ19 7410 7531 7412 9270 P14923 1667 3728 5467 207 Q13574 Q15078 P09429 P49682 5362 7422 5364 O14495 P78527 P50993 Q9NWW5 O75051 1432 1672 P09430 Q13464 9839 P55773 O15230 P20908 Q04771 23513 8879 7428 55558 P00750

epithelial tube morphogenesis 9464 O96004 O75386 Q9Y4K3 O43318 P52952 4609 Q1PSW9 284217 P84022 Q99807 Q92574 O15119 P49815 Q13485 3911 1856 8543 6885 Q96QB1 7291 54361 1072 367 P01106 P16070 Q15672 P49768 P50552 7189 P61968 6495 P48431 P05549 P25391 Q9NRY4 P29590 P12755 Q03014 Q13418 Q99623 O14727 Q96S42 P23528 Q96RK4 8945 P01019 Q92830 Q9Y297 P03372 P01137 Q9NSC2 P40424 6926 P06401 4088 4089 P07737 8323 Q14118 Q86YT6 P35222 P56705 P17302 57534 Q4AC94 P17813 P31270 2697 P10070 P61296 P10071 11331 6657 2737 2736 9421

O95999 P07949 7248 2909 P10275 1499 Q9NYQ6 Q99966 2626 P51671 26005 Q86WV8 P19544 7490 3207 10229 960 841 7249 7408 Q15475 P10415 2099 7020 2247 3611 O14641 Q14790 P43694 P09038 9839 5371 P39905 Q15648 O15230 23513 Q14160 O60353 Q9UMX1 1605

negative regulation of phosphorylation 5580 8655 4771 Q15735 P54253 P30086 P14635 4092 P63167 P84022 P49810 5578 51763 6901 P04201 P05412 Q9NP71 4221 Q16635 5037 P30153 25998 Q9BT40 5071 5590 1029 P67775 1027 1026 Q05513 54206 P42771 O15524 P49768 Q7Z727 Q8N726 O14495 891 P46527 P17252 P35240 857 P01137 5515 O60260 5518 Q9UJM3 4088 A4D1W7 O00255 P38936 5728 P17813 Q05655 P60484 P17936 O15105 P60568 P06213

peptidyl-serine modification P25098 7046 6872 133584 1020 O95271 2590 P42345 156 2589 Q9BXM7 5578 6789 Q13043 5591 Q6IR47 65018 1195 5590 Q96Q15 P21810 Q05513 120892 2475 4214 207 P49841 Q5S007 Q7Z727 Q9H422 A1XKG3 Q13418 P78527 1634 3611 Q00535 P17252 P01579 Q10472 Q10471 P31749 Q9UBE8 P17612 2932 Q63HQ2 P37173 5566 P07585 Q13233 P21675

regulation of smooth muscle cell proliferation 6198 7124 3162 Q9Y4K3 P15884 3516 P42224 Q96RU8 P21964 1499 P15692 3479 1012 5578 P08887 P08246 P05412 P03950 5155 Q15109 7052 Q06330 P00533 P01343 Q7Z727 7189 P29474 7422 6772 Q13418 1312 3611 P17252 P01579 P21980 1956 P23443 P55290 6925 P01375 P51692 3673 O95140 P35222 P17301 P17936 P01127 P40933 P09601 9927

positive regulation of gene-specific transcription from RNA polymerase II promoter 6595 O96004 6597 6872 P04637 6598 P15923 Q12824 O60603 P52952 2547 P16220 3479 P84022 10014 P25963 P56524 22807 Q15306 367 P46531 4851 4214 Q13133 408 P49407 P01343 O00206 6256 P19793 Q03014 P41235 6929 Q53X93 Q9UJU2 P01137 O75928 Q6IT96 P51531 4088 Q13547 P55055 Q9NR96 P35222 P78317 P51532 P17813 3148 P10071 2737 9421 P26583 O00744 5460 Q15853 1385 Q8TAK5 P19532 Q9UQL6 P10275 672 P22415 1499 7099 5591 7098 7376 Q92769 Q9NP71 Q14938 P37231 3169 P08651 5468 Q9NQB0 P17081 Q96T37 P12956 4792 22937 3066 3065 P78527 P38398 6721 O15455 Q12770 P49715 Q9UKS7 Q12772 P39905 Q9BZS1 7157 P19484 1050 54106 50943 Q13233 P21675

regulation of neurogenesis Q92858 8650 6993 P04637 O43157 P10636 2033 26037 O43711 P63172 259266 O75385 P52952 Q9BVA0 2547 474 991 Q8IYN9 Q86WK6 Q1PSW9 2147 Q9HCJ2 P07196 Q7Z6C1 O14786 P46821 P02686 8408 Q04917 O43166 Q13127 P52565 Q13009 Q12834 Q9UBC3 5270 Q3V6T2 P55316 P09382 81565 P46531 Q15154 4851 51654 Q96SN8 P61158 5029 4854 P01588 Q16643 1627 P49768 2290 Q09472 6495 Q15797 P48431 6498 P12757 9921 P14416 P12755 Q13418 P10451 Q05397 P53667 27185 773 894 P41231 P51805 811 Q9NRI5 6405 P01138 P01137 8829 P40424 Q9UMS4 4086 6660 7476 5970 Q9Y6R0 Q13426 9138 P10586 Q658W2 Q86YT6 9253 9495 Q02790 7074 57534 5728 387 P21359 55755 P20827 P60484 P10070 Q04206 1808 Q96JB5 6657 1406 P24588 Q13275 2736 P54259 4131 4099 7520 8851 Q15735 P20916 396 P61981 P61586 Q16555 7518 5978 Q8N5U6 3956 Q9UM47 P31249 O00755 Q9NYA1 Q92769 7532 7533 Q8IZT6 P36894 P10721 27339 P30279 1789 1822 P37231 P62166 P18846 Q96SZ6 P23515 P00734 3169 4137 207 5468 Q9GZM8 P08138 5108 Q92888 5747 Q15475 P27797 Q99835 Q15078 P12956 6696 5364 P10415 4155 Q9H228 P56177 3066 Q86VS8 P35711 O60229 57689 P13010 3611 Q9Y250 55704 P01579 Q9NQC3 Q96HC4 O43186 P29323 7157 P31749 Q9HCK4 466 O95390 84376 55558 P21554 Q9BZR6

protein amino acid dephosphorylation Q13115 Q9Y6W6 O95278 5780 P35813 P28562 5783 Q9NRW4 Q05209 1852 O14829 994 1850 995 5775 O15355 5536 5778 5537 11072 Q92729 5796 56940 Q93096

Q8WTR2 P67775 Q9H0C8 P08575 O14830 92609 51657 8555 P29074 P35236 P29350 O00167 Q06124 O75365 O60942 5798 Q8WUK0 P62136 P43378 8446 Q9Y2R2 Q12974 Q8N3J5 P10586 23399 152926 5728 P62140 P60484 Q9Y6J8 P53041 285193 Q8WUJ0 O14974 Q16825 O95476 Q16827 8732 O14595 Q5SGD2 Q16828 O00743 Q9P0J1 P23467 P62714 6305 26191 52 5470 Q13202 Q9BV47 9150 P30153 Q9H1R2 Q9H596 54961 Q9Y5B0 5500 4659 Q99956 P26045 Q9GZU7 P30304 P30305 Q3ZCQ8 Q16849 Q13615 P10415 P30307 93492 Q12923 Q8NEJ0 Q6GMV3 Q68J44 5515 5516 P56180 5518 O75319 Q9BY84 5494 O95147 5495 5770 Q6XPS3 5499 P18031 391356 26469 Q9UNI6 P18433 1843 Q4G0W2 2139 Q08209 11099 5800 1848 Q15256

regulation of neuron apoptosis Q15811 Q9Y2W7 7124 P04637 8851 Q13315 P56199 1020 7161 P50897 P30048 472 P15056 673 356 10935 P07196 Q92731 6622 P01116 P61586 7518 P01112 Q13286 P01111 P05412 P10600 O76024 Q07817 Q9H3Z4 Q15392 P42858 P01588 Q15475 Q15078 P49768 P04179 6495 P10415 6453 P56177 2099 A1XKG3 3265 Q13418 6093 P48507 773 P20936 3611 Q00535 Q04656 P01019 P21860 1718 6647 P01138 P03372 Q13464 P00441 P01375 P09958 O15350 Q14116 P55212 P39905 7157 3672 Q13426 P48023 P10586 2100 P04629 2065 P15559 P37840 4914 387 P21359 O60674 2730 1728 P43246 P25116 Q15375

ER-associated protein catabolic process Q5VVQ6 11160 Q86TM6 27248 7184 Q99942 10613 Q8IWF2 10956 79139 55666 84447 7415 Q9GZP9 51009 P55072 80020 O94905 26270 Q92611 Q8TAT6 9695 91147 6048 P14625 O75477 Q5HYA8 55432 P11021 267 Q9NRD1 Q9UKV5 Q9BUN8 3309 P60468

muscle cell differentiation P28289 3880 3084 25988 P52952 351 P37288 Q99523 79810 3479 Q8WV60 Q4KMG0 P09493 P01116 Q92574 O15119 P01112 P01111 9474 Q8NF91 P08727 P51636 10939 Q02297 54361 P58546 O43847 P09382 P05067 P01343 136319 6256 P29475 P19793 3265 P12755 Q9ULH7 P56539 891 O95180 P01019 Q8WZ42 Q9UPY3 6926 4627 P06400 4000 P04626 P68133 2064 9093 P35222 P56705 P17655 Q92786 Q96EY1 Q9Y3M2 Q9Y463 7248 9149 P14635 P78504 O00468 P35579 274 Q96PU8 552 1499 P00747 P15692 P15173 23363 11146 P45379 3956 P78337 P29466 P68032 Q86WV8 1147 Q9NQB0 375790 4898 O95259 4154 7422 Q9BQA5 P02708 Q9H1Y0 Q92990 O14640 P02545 O43184 859 P10826 50937 O15111 Q9NP98 Q04771 25776 O00499 Q08209 P18827 P24821 P25116 5925

ectoderm development P02452 P78545 Q13753 P25054 Q13477 Q13751 3881 P15924 P16615 Q8NEY8 P22735 2304 Q1PSW9 P04083 91 P49810 5017 P04085 P08842 3911 94234 P35527 5154 Q15828 Q15392 488 P46531 4851 P48436 51535 P00533 P42574 2319 P49768 Q92838 P05549 Q06643 2054 P31276 Q01469 P20930 P35240 Q9UJU2 Q5TA76 55504 Q15323 Q9UKW6 P25940 Q9UJM3 O95863 Q92826 P31944 P02461 Q9Y337 P26371 Q9C009 P35908 O15156 5970 P18074 3673 A4D1W7 P31947 Q9Y696 Q04695 Q9BYD5 P35222 2735 P17301 O60437 301 Q02388 P10070 Q04206 Q5T5A8 P08151 5604 2736 Q15738 P01040 P49862 Q16787 4771 Q92817 P19012 8174 P78504 1896 57669 1499 Q9UMD9 P08123 Q15185 Q14254 5351 P32856 Q13443 Q86YZ3 Q9HCE7 P14923 P36896 57154 P36894 O00358 7251 Q9NZT1 3728 324 Q9HCM4 5467 207 Q02809 54206 O14753 P08138 10468 O95377 P04259 8754 P10415 3860 P13646 P13647 P13645 O76011 7020 Q08188 Q9NS68 1832 P42768 2125 1277 Q04656 3852 1718 P04264 Q9H0F6 1956 50814 5493 P05997 O15353 O75712 25818 O15230 P31749 P20908 1294 Q02750 P19883 27032 P22528 2810 1289 3909 10728 P02538 6699 Q99816 Q9UMX1 P02533 P98194 3866 6615

chromosome segregation 54908 O95835 9700 Q969H0 O95997 P14635 79172 9184 672 Q96BM9 Q15021 Q8NCD3 Q8N3U4 55207 Q9HC98 Q96SB4 Q9BU64 9232 Q9Y6X3 O43684 P51955 P30153 23212

Q9BZD4 8780 55294 O60216 Q9UJX2 Q9UQE7 81565 10783 7329 Q6P1K2 Q9GZM8 P42695 10270 Q8WVM7 9126 P11388 891 P38398 221150 57405 P49959 11243 Q8NFH4 P49711 Q9NTI5 5119 Q14674 Q15003 Q8WZ42 Q93008 Q96EA4 5518 O43264 O95347 7153 4361 O15392 O43823 23310 Q6KC79 Q96BK5 79023 Q9H410 79980 55355 P63279 P40692 Q9NZ56 Q15050 54984 Q9NTJ3 Q9NVJ2 Q9HBM1 O14730 O14777 Q8IX90 127829 Q96FF9 Q14683

establishment of organelle localization 54908 9342 Q92538 6993 9700 Q9Y266 10053 P63172 P14635 Q8IXI1 P35579 Q8IXI2 Q8IYN9 1058 P42704 P43034 10972 O95721 Q9UBK9 P49450 P35606 9276 Q9Y678 Q8NF91 5871 P53618 23212 P51159 Q9UJX2 P62841 5747 P49768 P53621 Q9Y6Q5 Q05397 Q9BXC9 891 1315 375 1314 Q96RK4 P49755 Q14674 4627 4869 Q96EA4 Q12851 O43264 O15392 Q658W2 Q02750 Q96BK5 25777 Q9NXR1 10128 54820 P40692 Q9NZ56 10726 Q15050 54984 P84077 Q13190 Q9UH99 6811 O14579 O14777 5604 Q96FF9 Q8N6T3

aerobic respiration 7385 5162 P31930 7384 48 P40926 3420 P50213 7381 O43837 6390 4191 Q9P2R7 3417 O75390 1431 P31040 P04040 8803 P22695 5018 8801 3419 P07954 Q13423 P51553 P53597 P36957 P14927 O75874 6389 Q15526 23530 P07919 P11177 P21912 1743 Q15070 3421 6834 847 P21399

heart development 7046 9464 O96004 Q14686 Q12948 2033 23322 3084 P52952 472 2702 4729 P41134 2303 79810 Q1PSW9 P84022 Q8WV60 P09493 Q7Z6C1 O14786 Q92574 O15119 P98161 P49815 6901 1856 9510 O94992 9474 5310 5311 Q16635 Q96QB1 23210 P35368 Q02297 Q8NB12 2280 P50148 P46531 4851 P48436 Q15796 P42574 Q13099 P49768 Q09472 6256 P29992 P19793 Q12968 Q03014 Q9ULH7 891 116113 Q96S42 Q96RK4 P21860 Q9NSC2 8829 5318 Q8WZ42 Q9UPY3 6926 P02461 4087 Q92824 4000 4088 P04626 P55210 Q9UHI8 P19320 Q9Y618 2065 Q13705 Q86YT6 2064 P35222 P13612 P12004 P12883 P17302 57534 5728 Q4AC94 P21359 P17813 2697 P60484 P10070 P61296 P10071 Q92786 10087 P02458 P07101 2737 2736 P62942 9421 P25098 5460 Q9Y3M2 Q8TDY2 9821 Q13315 7248 Q6NYC1 Q9H488 P14635 4092 Q8IX07 P19404 Q02535 P46109 156 10614 1499 P15692 23363 3676 Q13563 10454 P45379 2626 5591 Q6IR47 8100 P30559 5111 7412 26005 P36894 P68032 Q68CZ1 Q86WV8 P19544 7490 23414 P36382 841 Q9NPH5 4775 7249 Q8WW38 Q99958 Q15750 Q99835 Q96T37 P04179 Q86U86 P13807 7422 23509 P35555 Q02363 P19235 P78527 Q86V24 1399 Q9H1Y0 O14641 Q14790 O14640 P43694 P02545 Q9GZT9 P26678 P01178 Q92997 P10826 9612 Q9UIG0 Q15648 P20908 Q04771 25776 Q6KC79 P35548 54583 1289 P37173 O15105 Q9UMX1 P06213

membrane fusion Q12981 9342 Q6FGG2 10490 O43752 8411 8775 9341 O95352 80124 Q9UEU0 Q99965 P21926 O60763 Q92692 10451 9218 10533 Q13286 O95721 Q8NEC5 P32856 P51636 Q8IYV9 10228 8615 Q8IWA4 5104 Q15075 P47224 4218 Q96JH7 Q15276 928 Q15836 8676 26258 Q12846 2054 Q9P0L0 25801 6809 55666 284359 Q9UKW4 P61006 5877 9135 Q8TAT6 Q9UL45 O75558 O95140 P54920 2773 6810 P08754 9927

regulation of response to stimulus O14672 Q9NS86 2305 5817 P84022 Q92731 O14543 Q9Y275 O14788 57506 P07996 2308 Q9Y3Z3 P04003 P27487 P04004 P16104 2 P20963 P14174 Q9UI12 10666 P10809 O15524 Q96EB6 Q7Z727 Q92835 57402 64170 4869 5716 3791 5970 3673 Q92949 2100 Q9BZF9 Q9NR96 81622 P17301 O60674 Q8IUC6 3667 P04233 Q9H1C4 P05129 P28482 10616 710 1012 P07550 5733 P08887 719 P30556 P29466 Q9Y239 P51679 Q96J02 3329 1029 29110 10746 79444 960 720 2475 722 604 P42771 5624 P07766 6850 4792 Q9NZD8 1161 P30542 P38398 P15151 Q5TCX8 857 Q96CA5 P04264 P05112 P10827 5770 Q12778 P51692 Q5TC82 P11912 P31749 O95382 O00255 1173 3350 P48061 Q9HCK4 P05362 P05121 P09601 50943 Q9UL54 P23396 P15529 P14555 2150 3481

O60603 Q96RU8 2147 3476 4221 6885 1191 2280 O76074 Q99750 2277 4214 P01589 Q96CW1 4216 P01344 409 P49768 O00206 6890 Q8N726 5321 4354 P14416 3142 3263 P13686 148022 3014 P01019 P01137 29086 11326 P01375 P02461 5692 8841 P35225 302 545 P01008 6776 P01127 P40933 10488 5328 O43353 5580 P01042 5582 5585 P07948 Q9NX61 3162 672 P15692 Q99683 5336 5578 5579 5338 P05771 P26232 64127 Q08117 23411 5590 P37231 Q9NWV8 Q00987 8737 207 5468 P55085 P01023 6696 7422 Q9UHD2 Q9BUZ4 10673 9839 P24394 Q9BT67 5371 Q12933 P08908 3075 P16885 462 P16410 O14939 P51617 8517 54106 9846 P25116 P60568 Q14449 8654 4179 Q9Y4K4 Q9Y4K3 O43318 472 Q8WZ60 P49810 8767 P49815 P25963 5154 7334 5155 Q16512 4067 4188 7336 Q15306 7335 O75376 5270 60489 P27918 7448 Q96RL1 Q9HCY8 P32121 P10451 4193 Q9Y6Q6 Q9UBU3 Q00013 Q9UDY8 P21589 P02741 P55290 Q9Y6R4 5054 4088 P10586 Q9H257 Q03518 Q13153 55915 P43489 P62942 7124 O15164 P10276 154 Q9Y2C9 121512 Q9Y6K9 51324 54 Q13286 7132 7133 Q15109 O43889 P35443 161 P35568 285 166 P01308 7249 P01303 O15169 Q9GZM8 9564 P02790 10392 P49840 23586 P01579 55703 Q9BQI3 Q9NQC3 O94973 6188 149041 Q08050 2931 Q6PID4 Q14289 Q16581 P78423 O15123 Q15819 7161 P42345 P0C0L4 Q9HC16 Q92692 P04085 P42229 P15090 1616 P07355 P42226 O15118 P78536 7057 Q13404 83737 7052 81565 P08575 P16070 Q9UER7 7186 7189 7067 P29474 Q5TAX3 Q13535 Q9H422 Q96M96 P29590 Q9H0K1 P41235 Q9BYM8 27102 4907 P17252 O15379 P06241 P21980 Q9NZ08 P05155 P78552 Q13546 55072 Q7Z434 P78318 P19438 P41222 P43405 Q05655 P04070 Q04206 Q96EP0 O95999 Q13315 P41182 O43915 9021 P53567 P00749 P22415 P00747 Q03468 51720 P08246 P43115 P47712 Q8IWV1 7099 7098 51606 Q13322 P35968 Q14774 P46736 P61073 Q05513 P63010 P00734 P23510 O14492 Q13216 59341 Q9P035 P61088 O15455 Q15762 P20333 5914 P41159 O95786 O15350 O75832 Q9BZS1 Q13227 P18031 Q06830 P34972 P28070 Q9HC29 51752 Q13233 P21554

homeostasis of number of cells 6670 P55347 P07948 P14317 2034 Q6NYC1 P41182 O43557 3162 P53567 6194 P39019 Q00597 P15692 Q9Y6K9 Q9H9B4 6667 10296 P08047 6229 Q9Y275 P42229 P62753 P07954 O43521 4067 4221 23210 P11413 10107 Q02447 Q9UKV3 6117 207 P01589 604 10661 P01588 P62847 P42574 O14492 P27694 7067 6498 7422 P10415 P12757 Q02363 Q7L5Y9 51542 Q9UDY6 6886 P01579 10673 6647 P01137 5316 O14920 P00441 P06400 P10827 9133 Q9BZS1 P08708 P51692 P18074 A4D1W7 P31749 Q06830 3551 O00255 9093 O95067 22985 P17542 O60674 P62263 8517 6776 Q96EY1 Q99814 P09601 50943 Q13351 P25116 5925

androgen receptor signaling pathway O75925 P48552 1022 P50613 2274 P10275 P38398 672 1499 Q9Y4C1 Q14192 898 Q92993 P61586 10499 O75928 Q15466 1616 P06400 Q9UBK2 P31689 8289 Q9NVC6 8204 P62826 Q15648 P35222 P78317 Q05086 Q02790 29079 10524 387 367 3301 Q9NPJ6 P24864 O14497 9967 O43294 5901 Q15596 Q9Y2W1 Q9UER7 5925

dephosphorylation Q13115 Q9Y6W6 O95278 5780 P35813 P28562 5783 Q9NRW4 Q05209 1852 O14829 994 1850 995 5775 22908 O15355 5536 5778 5537 11072 Q92729 5796 56940 Q93096 Q8WTR2 P67775 Q9H0C8 P08575 O14830 92609 51657 8555 P29074 P35236 P29350 O00167 Q06124 O75365 P13686 O60942 Q96T60 5798 Q8WUK0 P62136 29085 P43378 8446 Q9Y2R2 Q12974 Q8N3J5 P10586 23399 152926 5728 P62140 P60484 Q9Y6J8 P53041 285193 Q8WUJ0 O14974 Q16825 O95476 Q16827 8732 O14595 Q5SGD2 Q16828 O00743 Q9P0J1 P23467 P62714 6305 26191 52 Q01968 54 5470 Q13202 Q9BV47 9150 P30153 Q9H1R2 Q9H596 54961 Q9Y5B0 5500 4659 Q99956 P26045 Q9GZU7 P30304 P30305 Q3ZCQ8 Q16849 Q13615 P10415 P30307 93492 Q12923 Q8NEJ0 Q6GMV3 Q68J44 5515 5516 P56180 5518 O75319 Q9BY84 5494 O95147 5495 5770 Q6XPS3 5499 P18031 391356 26469 Q9UNI6 P18433 1843 Q4G0W2 2139 Q08209 11099 Q9NTJ5 4952 5800 1848 Q15256

muscle structure development 3880 O14793 Q12948 3084 351 Q9H161 4729 P37288 2303 P09493 8407 Q7Z6C1 6901 9750 Q8NF91 P08727 Q16635 54361 P58546 P51114 2317 Q16082 P09382 Q96EB6 Q15672 136319 6495 6256 6498 P12757 Q8N6I1 P12755 Q9ULH7 Q15561 5159 Q8WZ42 6926 4627 P06400 P07858 163126 P68133 P37802 9093 P56705 9099 P12883 P17655 5728 6938 387 P21359 Q9UKX5 4637 P62942 9421 Q9Y463 7248 1020 P14635 4092 O00468 P35579 274 P19404 3316 1499 P15173 23363 11146 P61586 3679 P45379 P78337 P50222 P29466 Q14814 P09619 Q86WV8 1147 Q9NQB0 Q8WW38 4898 Q99958 Q15475 O95259 A1XKG3 2010 Q9H1Y0 Q92990 O14640 857 P02545 O43184 859 P10826 Q8IUX7 50937 221937 25776 Q02078 O00499 Q16586 P28289 O96004 P13349 2033 2275 25988 P52952 Q99523 79810 3479 Q8WV60 Q4KMG0 P01116 4205 Q92574 O15119 3911 P01112 4209 P01111 Q13643 Q13642 9474 P51636 10939 Q02297 7291 Q9HDC5 P53814 O43847 Q99081 2280 3009 Q04900 P46939 84159 Q01995 P05067 P01343 Q14865 Q09472 P29475 P19793 3265 3142 P12111 Q63HK5 P56539 1756 891 1634 O95180 Q00535 P01019 P21860 6525 Q14993 Q9UPY3 4000 P04626 2065 O95183 2064 P35222 Q9Y4F9 P17813 P60484 668 Q92786 P07585 Q96EY1 P24043 Q9Y3M2 7402 9149 P78504 Q96PU8 552 P00747 P15692 P85037 Q13683 3956 Q14774 P11532 P68032 23411 23414 P60660 375790 P22303 O75604 4154 7422 26576 Q9BQA5 P02708 Q9GZT9 P26678 O15111 O15230 Q9NP98 Q04771 P50402 P16401 54583 Q08209 P58012 P18827 P24821 O15105 P25116 5925

spliceosomal snRNP assembly 50628 P52298 25929 6631 Q8TEQ6 P14678 54496 Q5TAL4 Q8WWY3 Q9NVM4 P09234 4686 P62316 10772 P62318 O14744 Q9BQA1 P62314 O94906 135295 Q16637 P54105 O95400 Q8WXF0 Q09161 Q9H814 Q9UHI6 79084 O75494 11218 26121 O43290 6633 P62308 10421 6637 1207 P62304

posttranscriptional gene silencing Q7KZF4 Q9UKV8 Q15797 Q9UL18 O75569 P98179 Q5TAX3 27327 79753 P84022 Q8WYQ5 Q8TAD8 27161 Q9UPY3 5935 4086 4087 4088 4343 26523 Q8NDV7 Q9HCK5 Q9HCE1 51593 Q9H9G7 54487 192669 Q9BXP5 Q15796

cellular response to unfolded protein P24385 Q5VVQ6 P35638 O95292 O75460 P19525 595 79139 7415 5610 P01579 Q9GZP9 Q16236 O75807 51009 P55072 2081 4780 22926 9217 P18850 55432 P11021 267 Q9P2K8 Q15011 Q9UKV5 Q9BUN8 3309

posttranscriptional gene silencing by RNA Q7KZF4 Q9UKV8 Q15797 Q9UL18 O75569 P98179 Q5TAX3 27327 79753 P84022 Q8WYQ5 Q8TAD8 27161 Q9UPY3 5935 4086 4087 4088 4343 26523 Q8NDV7 Q9HCK5 Q9HCE1 51593 Q9H9G7 54487 192669 Q9BXP5 Q15796

acetyl-CoA catabolic process 5162 48 P40926 3420 P50213 O43837 6390 4191 Q9P2R7 3417 O75390 1431 P31040 8803 8801 3419 P07954 Q13423 P51553 P53597 P36957 O75874 6389 23530 P11177 P21912 1743 3421 P21399

endoplasmic reticulum unfolded protein response P24385 Q5VVQ6 P35638 O95292 O75460 P19525 595 79139 7415 5610 P01579 Q9GZP9 Q16236 O75807 51009 P55072 2081 4780 22926 9217 P18850 55432 P11021 267 Q9P2K8 Q15011 Q9UKV5 Q9BUN8 3309

regulation of histone modification 4297 P04637 P29590 P14635 891 P61088 Q01105 6622 P49711 P01137 Q92833 P26358 Q9UBK2 5371 7334 4221 Q9BZS1 7157 Q9UBC3 O00255 Q6KC79 P37840 Q03164 1789 1786 3720 50943 408 P49407

regulation of actin filament bundle assembly 4771 7248 P31431 P53667 Q4VCS5 P42345 Q96P48 Q04759 P84022 P09493 P35240 P61586 Q92574 6869 11346 P25103 P49815 O60260 O43182 4088 Q96QB1 Q86WV8 5071 387 2475 4214 7249 Q8N3V7 Q13233

tube morphogenesis 9464 O96004 8654 P56199 Q12948 O75386 Q9Y4K3 O43318 P52952 4609 2303 3479 Q1PSW9 284217 P84022 Q99807 999 O14786 Q92574 O15119 P49815 Q13485 3911 1856 9474 8543 6885 Q96QB1 P35368 7291 54361 1072 7052 O76074 367 P46531 4851 P01106 P16070 P23276 Q14344 P01343 Q15672 P49768 P50552 7189 P61968 6495 P48431 P29474 P05549 P25391 Q9NRY4 P29590 P12755 Q03014 Q13418 Q99623 O14727 Q96S42 P68871 P23528 Q96RK4 8945 P01019 Q92830 P21980 6647 Q9Y297 P03372 P01137 Q9NSC2 8829 P40424 Q9UPY3 6926 P06401 P00441 P01133 3791 4088 4089 P07737 3672 8323 91147 Q14118 Q86YT6 P35222 P56705 P17302 57534 Q4AC94 P17813 P31270 2697 2730 P10070 Q9BXB1 P61296 P10071 11331 6657 2737 2736 9421 P01042 O95999 O43597 P07949 7248 2909 P10275 55366 154 27283 1499 P15692 Q9NYQ6 P07550 Q9UJW2 Q99966 2626 P51671 P30556 P35968 O43521 26005 Q5HYA8 Q86WV8 P19544 7490 3207 10229 960 841 3169 7249 7408 Q99958 Q15475 Q96T37 P04179 7422 P10415 P12830 2099 7020 P48507 2247 3611 Q9H1Y0 O14641 Q14790 P62736 P43694 10672 P09038 9839 P08588 5371 P39905 Q15648 O15230 Q04771 23513 Q14160 P37173 O60353 Q9UMX1 1605

cellular membrane fusion Q12981 9342 Q6FGG2 10490 O43752 8411 8775 9341 O95352 80124 Q9UEU0 Q99965 P21926 O60763 10451 9218 10533 Q13286 O95721 Q8NEC5 P32856 P51636 Q8IYV9 10228 8615 Q8IWA4 5104 Q15075 P47224 4218 Q96JH7 Q15276 928 Q15836 8676 26258 Q12846 2054 Q9P0L0 25801 6809 55666 284359 Q9UKW4 P61006 5877 9135 Q8TAT6 Q9UL45 O75558 O95140 P54920 2773 6810 P08754 9927

regulation of protein complex disassembly P25054 4131 4133 P10636 23122 P14635 9181 Q9UHB6 P35612 P35611 Q9BVA0 2107 6709 Q9Y3A5 830 832 118 9617 Q9H2D6 10013 5976 P46821 P47755 P47756 Q92974 23332 Q01082 1072 O75570 2039 Q7Z460 324 57159 6711 6710 4137 Q9BYV2 O15020 Q13813 O75122 P62495 891 O60502 P23528 P02549 Q9NPA3 Q08495 P11277 10724 51474 P52907 P27816 Q6PKD3 Q92900 Q9UBN7 829

negative regulation of cell size Q13873 P04637 6236 7161 O75385 29948 P50897 11108 27000 P42345 P84022 91 Q92731 O14786 5931 94 4722 Q92574 P49815 Q13485 8408 51094 P67775 P55042 Q9UK53 Q9H2G4 P50150 Q8N726 Q9UKN5 O75489 P29590 P10451 P56539 P41235 P46527 P37023 P51805 P32929 P01019 6405 P01137 O60383 8829 P06400 4088 4089 A4D1W7 2100 58189 1491 O00213 3304 64061 Q13275 P06733 7248 P41182 Q9P0J0 Q6FHQ0 Q9HC57 Q9UJW9 Q96SB3 P36896 P30153 Q86WV8 P19544 7490 P37231 1029 1027 1026 Q99675 Q9UJX0 2475 207 7249 5468 604 Q14140 P42771 6696 P10415 Q96A54 P32320 Q86V24 2247 Q99543 Q16576 Q9NQC3 859 5515 P09038 5518 5371 O15350 P08107 7157 P31749 2023 Q12816 P38936 3621 55558 5925

regulation of cell projection organization P25054 7046 8650 O43157 P10636 2033 26037 O75385 Q9BVA0 P42345 991 Q8IYN9 Q04759 Q86WK6 Q9HCJ2 P07196 998 Q7Z6C1 O14786 5898 P46821 P02686 8408 Q04917 O43166 P52565 Q13009 Q12834 Q3V6T2 P09382 81565 P61158 5029 Q16643 1627 P49768 Q09472 Q15797 6498 P12757 Q13418 P10451 Q05397 P53667 27185 773 P41231 P51805 Q96RK4 Q9NRI5 6405 P01138 8829 4086 7476 Q9Y6R0 3673 9138 P10586 Q658W2 9253 9495 Q02790 7074 5728 P17301 387 P20827 P60484 P24588 Q13275 4131 4099 Q15735 P20916 396 Q96P48 P63000 P61586 3956 O00755 Q6IR47 Q9NYA1 Q92769 7533 P10600 P10721 P62166 P18846 P23515 324 2475

4137 207 Q9GZM8 P17081 P08138 Q92888 5747 6696 5364 4155 3066 O60229 57689 3611 Q9Y250 55704 Q9NQC3 5879 P60953 Q96HC4 P29323 P31749 P11233 Q9HCK4 466 55558 P21554 Q9BZR6

ATP synthesis coupled electron transport P31930 O75306 P56556 Q7KZN9 4695 O43678 P19404 4728 4729 Q99807 6622 4720 Q16795 4722 1738 4723 6901 4725 4726 O95169 P28331 P14927 Q16635 O14561 O43920 P07919 Q16718 O75380 10229 29796 4697 P00414 4698 4731 4535 P09622 7384 O75489 O00483 O95298 7381 O95299 P17568 4705 4707 Q9Y6M9 1355 P00403 4701 4702 P03891 P56181 O00217 O75438 P51970 Q9Y375 O95182 P37840 4718 4719 P49821 4710 4514 4713 4714 4715

mitochondrial ATP synthesis coupled electron transport P31930 O75306 P56556 Q7KZN9 4695 O43678 P19404 4728 4729 Q99807 6622 4720 Q16795 4722 1738 4723 6901 4725 4726 O95169 P28331 P14927 Q16635 O14561 O43920 P07919 Q16718 O75380 10229 29796 4697 P00414 4698 4731 4535 P09622 7384 O75489 O00483 O95298 7381 O95299 P17568 4705 4707 Q9Y6M9 1355 P00403 4701 4702 P03891 P56181 O00217 O75438 P51970 Q9Y375 O95182 P37840 4718 4719 P49821 4710 4514 4713 4714 4715

response to oxygen levels 7046 P98155 Q9H6Q4 5265 Q9BY76 O15123 8654 2034 2033 Q01094 O60603 230 Q04759 P84022 O00151 P20396 P49810 Q7Z6C1 P04085 O14543 Q15389 Q13485 P07996 P27487 P03950 P78536 7057 3091 5154 5155 112399 Q9Y4L1 481 O76074 P18887 P16234 P30519 768 Q4LE28 Q13490 P01588 1869 Q99757 Q13131 O00206 Q09472 P29474 5562 P29475 P29590 9361 7184 891 P00846 Q92793 P01137 6262 6383 Q14116 P55851 4088 4089 3673 P19320 10525 P17655 P17301 O43707 P21359 3708 P17813 P01009 P07101 P01127 5327 Q16665 5328 O00469 25828 Q14643 P14635 9021 3162 4093 P00749 1137 P22415 P05091 P15692 P09972 5338 Q16790 7099 Q6IR47 5351 P29466 5352 P30559 7412 P36776 P10600 285 Q96KS0 Q07817 P61073 1026 1387 Q02809 329 P08253 P05026 P04179 7422 22937 P10415 P34741 857 Q12770 P02786 Q9GZT9 5371 P27540 7037 P51692 O15198 P14625 Q9H6Z9 P38936 64428 54583 O14939 Q6PID4 P43681 4313 7428 Q9UMX0 Q99814 P00750 P09601 P40337 Q14289

regulation of translational initiation Q9ULR5 6199 P52298 Q9UKV8 P55884 6198 8892 7124 O43432 P46199 Q06787 Q9H2K0 1975 Q9UBS0 3315 1653 27102 4686 P23588 P55010 Q92499 7458 Q9UBQ5 Q9BQI3 P05198 Q13144 27161 1978 P23443 Q14232 P01375 8662 P49770 O15372 Q09161 8667 Q9NR50 O60739 1965 1982 400961 Q9P2K8 2332 P04792 10289 P60228 Q15056

response to hypoxia 7046 P98155 Q9H6Q4 5265 Q9BY76 O15123 8654 2034 2033 Q01094 O60603 230 Q04759 P84022 O00151 P20396 P49810 Q7Z6C1 P04085 O14543 Q15389 Q13485 P07996 P27487 P03950 P78536 7057 3091 5154 5155 112399 Q9Y4L1 481 O76074 P18887 P30519 768 Q4LE28 Q13490 P01588 1869 Q99757 Q13131 O00206 Q09472 P29474 5562 P29475 P29590 9361 7184 891 Q92793 P01137 6262 6383 Q14116 P55851 4088 4089 3673 P19320 10525 P17655 P17301 O43707 P21359 3708 P17813 P01009 P07101 P01127 5327 Q16665 5328 O00469 25828 Q14643 P14635 9021 3162 4093 P00749 1137 P22415 P15692 P09972 5338 Q16790 7099 Q6IR47 5351 P29466 5352 7412 P36776 P10600 285 Q96KS0 Q07817 P61073 1387 Q02809 329 P08253 P05026 P04179 7422 22937 P10415 P34741 857 Q12770 P02786 Q9GZT9 5371 P27540 7037 P51692 O15198 P14625 Q9H6Z9 64428 54583 O14939 Q6PID4 P43681 4313 7428 Q9UMX0 Q99814 P00750 P09601 P40337 Q14289

negative regulation of cytoskeleton organization P25054 4131 4133 P10636 Q15735 23122 P14635 9181 Q9UHB6 P35612 P35611 Q9BVA0 672 Q96P48 6709 Q9Y3A5 830 832 118 Q9H2D6 P46821

P47755 P47756 Q92974 Q96QB1 23332 Q01082 5071 2039 Q7Z460 324 57159 6711 22919 6710 4137 4214 P62328 Q9BYV2 O15020 Q13813 23189 O75122 P56539 891 P38398 P02549 859 Q9NPA3 O60260 O43182 Q08495 Q14678 Q02790 P11277 51474 P52907 P27816 Q6PKD3 Q15691 P63313 Q13233 829

homeostatic process P25054 Q9H6Q4 Q9UBB6 Q8IZQ5 2547 P37288 4609 O00391 P21926 57620 Q9Y275 Q9UGM1 P28335 25913 P28330 P28331 51090 P50148 10549 P20849 P42858 P39210 Q9UKV3 10661 Q9UI12 O15525 928 Q7Z727 P27694 P27695 P29992 Q9UBD6 3783 Q06124 Q7L5Y9 57402 6927 55584 O60543 811 5836 4869 P06400 3791 P08708 Q86SX6 3673 P48023 Q9NR96 3551 9093 P21917 22985 P20823 P17301 P17542 O60674 P62263 3784 3667 Q96JJ6 10523 P07307 O95477 Q9Y342 5981 1020 P51787 P39019 1137 1499 57544 P07550 O14745 719 P51671 P30556 P30679 P30559 P09619 P80098 P51679 1152 Q6UB99 P51797 Q96KS0 57678 1387 P20648 604 846 P07686 P09622 P04179 Q9NYB0 P28223 P51681 2495 O00244 P51686 P30542 P19634 Q9HCL2 P15153 610 4664 4665 857 P63302 Q92878 859 Q92633 P51575 P10827 P29320 P51692 P31749 1297 Q05586 O00255 Q13506 Q9P202 P48061 27032 Q8NBS9 P17342 10728 Q99497 Q9UL51 P05121 P09601 50943 5768 Q9H4M9 P04155 P04156 P06213 P06576 P00390 6993 Q9UKL4 Q96A33 P80188 P14317 2034 25861 P16615 1185 2150 P50897 23327 3479 2147 Q13085 6622 6869 P62873 P02686 P62753 P49770 4221 Q9BS26 5311 23210 P35368 Q9HDC5 25998 Q9BT40 Q9P2K2 641 P35251 P01106 P01589 P01588 6517 Q99757 P01343 P02794 P49768 O00206 6890 Q9Y320 5321 6774 P14416 3263 P13686 P13693 Q99500 773 54413 Q00535 Q8IXB1 P01019 6886 6647 6646 P01137 5316 Q16254 Q9Y5X9 4361 P01130 P04626 5213 2064 P35222 Q9NUX5 O95067 P35348 6776 P02458 4358 Q96EY1 6415 84299 6670 25828 P01042 Q92934 P06737 6794 7520 P07948 3162 Q8IXI1 Q8IXI2 Q96PU8 54431 310 552 553 P15692 433 Q96PU5 6667 5336 5578 6548 Q15185 10577 P40763 P05412 P07954 5591 O76024 5590 3171 10107 P37231 324 3169 Q9NPH5 5467 207 328 5468 P55085 P62847 11315 P27797 23746 P49682 P01185 9601 7422 4155 P12277 Q9BRT3 Q12809 P50993 51061 Q9NWW5 572 P13010 Q15084 10673 10672 O14920 P25103 Q9GZT9 P26678 P01178 Q9BT67 6341 9612 O76003 580 3074 P15313 P16885 582 54583 51075 Q08209 8517 P60201 54106 P25116 P60568 8892 Q9BY76 O43557 P10599 Q86VQ3 Q9Y4K3 351 356 477 P08047 51763 6229 Q7Z6C3 Q8NFJ9 Q9C0C2 P03950 Q15303 3091 9997 5034 4067 6368 19 Q02447 488 6117 5029 Q13370 Q15554 Q14344 1902 20 Q9BUN8 Q9HCY8 Q16881 7220 P35638 Q15797 6498 P12757 Q9HBW0 Q6PKC3 P12755 P32246 Q9Y6Q6 495 Q9UBU3 280636 51542 Q9UDY6 Q96RK4 5159 Q13144 31 P00441 Q9BSI4 P32239 8443 6262 4086 5054 Q8IWX8 P18074 P55055 Q9Y618 Q9NZ94 222546 142 P21359 23154 10087 Q16665 Q03518 Q9NSD7 P55347 O15164 7248 P54132 P54253 P13667 154 Q00597 P09172 Q8NBF2 Q9Y6K9 Q13162 10296 Q99728 2904 2902 54 Q13286 7013 7376 O43521 7014 Q9Y4H2 O43524 Q86WV8 Q9UKA1 P35568 P01308 Q9NQB0 P48995 51218 Q8TCU4 P02790 P54274 A1XKG3 O43612 Q8WY64 P49959 P21145 P01579 P02786 2805 2923 P20073 7037 P55011 P14625 P10147 P37840 64428 Q93034 Q15011 Q6PID4 Q99814 P98194 Q14289 Q16581 O95831 9588 P63172 6194 Q86WK6 79139 10935 10938 Q92574 1738 P42229 P15090 1736 P07237 O15118 Q9UBK2 P54105 10939 Q9H7Z7 P55318 7295 7052 81567 P08575 Q4LE28 P05067 P42574 P42338 Q15831 7189 7067 P06239 O95977 9368 27346 Q13418 O75880 P56539 P30101 7184 P41235 1756 P41597 O43819 1874 P41231 Q92793 57003 P17252 84502 P21980 3932 P09874 9133 Q8WXH2 A4D1W7 P34931 Q9UNE2 3708 Q8HWS3 9388 Q15853 Q14643 O75503 P30041 P35610 Q6NYC1 P41182 P41181 P30044 P41180 Q86UK0 P53567 P30048 P22415 P00747 2741 Q9H9B4 26234 O15554 Q13563 O75508 P08246 P47712 7099 51606 P35968 Q15742 Q9NP71 Q07820 P11532 P11413 8065 O95881 Q07817 P61073 P00738 Q05513 P00734 Q7Z2W7 Q5BJF2 Q6P5W5 O14492 P12956 P10415 59341 Q02363 P19235 P78527 P02708 3611 P17174 Q04656 P08588 374354 Q16611 P55773 Q9BZS1 Q06830 P34972 1203 4719 P43681 P08237 Q13351 P21554 1207 Q9NZN9 O15228 5925

positive regulation of catabolic process P25054 6872 7124 P50416 4092 Q96RU8 Q8WTS1 3479 1454 1453 P63165 Q99728 P45974 P55072 Q9Y4P1 3091 Q13443 Q96HU1 Q9NP71 Q9Y4H2 P35368 Q96J02 83737 O43847 P35568 Q96KS0 P46934 Q00987 P01308 324 51099 207 5347 4898 P61956 4734 P01343 7341 8078 8754 10273 4193 Q8WY64 7415 P01579 P01375 5716 Q9BT67 P09958 Q9UKS6 P27540 O75832 P49674 P53350 P31749 1374 580 Q9UNE7 P17302 27352 23192 2697 O15105 P48730 6613 Q16665 3667 P21675 P07148 P06213

actin filament bundle assembly 8452 7126 Q13618 Q9UHB6 P35612 P35611 P15311 118 6624 Q08495 Q14511 7430 Q8WZ19 Q9BX66 10580 Q16658 P13796 Q05682 Q13829 O43707 2039 253980 51474 Q6PID4 800 408 Q14289 P49407

lymphocyte homeostasis O14492 6498 P10415 P12757 O43557 6194 Q9Y6K9 10673 Q9Y275 P01137 O14920 P42229 P62753 9133 O43521 Q9BZS1 P51692 P31749 3551 9093 O95067 8517 6776 207 P01589 Q96EY1 50943 P42574

erythrocyte homeostasis 6670 P55347 P07948 P14317 2034 Q6NYC1 P41182 3162 P53567 6194 P39019 P15692 Q9H9B4 6667 10296 P08047 6229 P62753 4067 23210 P11413 10107 Q02447 Q9UKV3 604 10661 P01588 P62847 7067 7422 Q02363 Q7L5Y9 Q9UDY6 6886 5316 P06400 P10827 P08708 P18074 A4D1W7 Q06830 22985 P17542 O60674 P62263 Q99814 P09601 Q13351 5925

regulation of endopeptidase activity Q9NZJ7 P23396 Q92934 7124 P04637 4255 O00220 29108 O75340 114548 P22736 P42224 356 O75190 2147 P84022 3476 6622 999 P55072 P07996 10016 P04004 7013 7057 Q9Y239 4221 Q96QB1 O43847 P37231 3329 1029 P11021 Q01955 Q15392 P00734 7448 5468 4898 P42771 10542 P10809 P09429 8795 P06239 10392 P48431 6772 Q8N726 P54274 1285 P12830 P29590 29843 23787 572 O14727 Q9BXK5 Q96P20 7415 1718 O14763 P61604 3932 P01375 5371 P09958 P51575 6188 5054 O15392 4088 7157 Q9P0U3 P31947 P48023 O43504 P99999 Q9BZF9 O00255 9093 P37840 P78318 2810 P58012 3146 Q15011 668 Q96EY1 Q9ULZ3 6657 P05121 Q13794 P25116 2935 3309

mesoderm formation O43474 9421 Q13873 Q15797 O96004 4771 Q12948 Q9BQ95 2132 O00167 Q9UPN3 57045 Q93063 6927 2303 57669 P84022 Q96S42 P35240 Q9UJU2 Q9GZX9 4086 4087 4088 5573 9314 Q04771 P36894 P10644 P20823 P17612 2139 51295 P42858 Q9HCM4 Q9H2X0 5566 O95947 1969 Q15796 P29317 Q99958 Q16881

RNA export from nucleus Q86W42 P52298 Q86U42 O43592 Q9UMR2 7248 Q86V81 Q9UPR3 23144 P57740 Q12769 57187 Q03014 26019 Q96FV9 O00148 Q9GZY0 80145 23381 84248 4686 10772 P09651 Q92574 5976 11269 Q6I9Y2 Q8WUM0 4869 8563 Q13769 P62826 Q09161 10482 Q9H814 3178 Q9UKK6 O75494 Q86WV8 Q96QD9 Q96Q15 Q8NI27 Q92900 Q9HAU5 5901 Q8IXZ2

cell cycle checkpoint P25054 P04637 8379 O43715 9184 6194 25988 472 990 595 O60566 Q96RU2 P83731 P62753 P01112 26270 O43683 O96017 O43684 Q12959 P16104 641 Q9UI95 Q96RL1 Q99638 Q6ZN33 Q13257 Q8N726 8555 Q13535 O96020 3265 P29590 Q06124 79791 890 891 79035 57646 Q99741 55743 1111 3014 Q16531 P01137 29086 P06400 O43264 O15392 9656 P18074 Q6R6M4 80254 1642 545 P43246 Q6PCD5 7508 O15287 Q13315 P54132 P14635 Q96EP1 10459 672 51720 7013 Q9NYZ3 Q01831 1029 P46736 P20248 Q9NWV8 1026 P51959 P50748 324 5347 Q9NRD1 P42771 Q13574 O14757 Q9Y6D9 Q9BQ15 P24385 P54274 5883 377630 P38398 Q9BQA5 Q5XUX0 Q99708 Q6NUQ1 5371 Q14676 7157 P53350 P38936 7272 55159 Q96MT8 900 51512 51514 Q14683 5925

DNA damage response, signal transduction by p53 class mediator Q6ZN33 6672 9100 7023 Q16666 Q9H305 P04637 Q9UNL4 Q86Z02 P29590 P51587 O43715 P38398 672 57646 Q96RU2 Q01664 51147 Q92993 Q92630 4646 Q14694 5371 64782 7157 8445 Q9Y5J5 P38936 Q9NYZ3 3428 10524 1026 Q8WTP8 51512 Q9UM54 P23497

DNA alkylation 9463 4297 346171 Q9UIF9 Q9NU63 29947 Q9Y6K1 54496 Q9NVM4 P49711 54815 P26358 3070 Q9Y5R4 55929 Q8WWH4 P46100 Q9UBC3 Q86YP4 Q9NRZ9 Q8NB78 Q03164 221656 1789 1788 O14717 1786 546 11176 Q9NPF5 Q9NRD5 P01100

DNA methylation 9463 4297 346171 Q9UIF9 Q9NU63 29947 Q9Y6K1 54496 Q9NVM4 P49711 54815 P26358 3070 Q9Y5R4 55929 Q8WWH4 P46100 Q9UBC3 Q86YP4 Q9NRZ9 Q8NB78 Q03164 221656 1789 1788 O14717 1786 546 11176 Q9NPF5 Q9NRD5 P01100

regulation of hydrolase activity 9744 Q9BY76 22870 Q99490 Q9UPN7 P15498 P37288 2665 356 2664 P84022 P09493 5018 P43034 P49815 Q8WZ64 P07996 P04004 Q8TBP0 25913 P03950 Q9Y2X7 115704 P50148 Q9P107 51099 7448 5029 P00533 Q13133 Q14344 10542 1902 Q15311 P10809 Q96EB6 8795 P50395 9882 P29992 Q9HBW0 1121 Q9ULH1 Q8TEU7 Q96CN4 P09471 Q9UKW4 P61604 Q8IV61 P32239 P26374 5054 4088 Q9UPQ3 3673 Q8WWN8 P31947 P48023 Q92949 P55055 P99999 Q9BZF9 125058 9093 Q8N6H7 P17301 P21359 O60674 Q66K14 1122 P31150 P60520 Q9ULZ3 P43246 5604 3309 P62942 Q9NSD7 Q9H2M9 7124 7248 O00220 29108 O75340 114548 Q9NUY8 O75460 Q04837 121512 25780 P45379 P51671 P30556 P30679 7013 7376 Q9Y239 Q86WV8 3329 1029 P11021 2475 7249 604 Q6ZW31 4898 P42771 Q9GZM8 2915 85440 Q96NH3 P28223 10392 P11229 P54274 1285 O43612 Q96N67 P01579 1718 857 O14763 O43182 1956 Q92633 P09958 P51575 6188 7157 64786 O43504 P14625 O00255 Q02750 P37840 2810 P17342 Q14160 51231 Q15011 Q6PID4 5524 P05121 Q14161 Q15257 Q14289 2935 P23396 7169 P04637 10928 26037 P22736 P42345 P42224 Q99523 Q8WTS1 2147 3476 6622 999 Q92574 6869 P62873 Q6ZT07 11345 10016 O43166 7057 Q15027 4221 Q7Z6J4 Q96QB1 55291 O43847 23216 2280 7052 O43609 Q15392 81565 P05062 85360 P98174 2956 P06239 P48431 P29474 6772 Q8N726 O95977 116986 P14416 Q96M96 P29590 116987 23787 7184 O14727 Q9BXK5 P41231 P01019 P21980 3932 P01375 O15392 Q9NUX5 P78318 P35348 27352 55633 3148 3269 Q07954 3146 668 Q96EY1 6657 Q13794 O43150 Q15057 Q3MII6 Q9NZJ7 P26583 Q92934 P01040 Q9NP61 O43597 4255 Q96BZ9 Q96DN5 P41182 Q5R372 Q86TI0 93594 P30047 552 Q96P48 553 O75190 79735 10451 9815 3953 P55072 P07951 Q5H9R7 2081 Q96HU1 23411 Q5JSP0 5590 P37231 Q01955 Q05513 P00734 Q9NU19 Q15070 Q15077 5468 Q8TEA7 P48357 51735 P09429 P24386 Q6ZV73 4035 P12830 29843 572 2644 Q96P20 Q0IIM8 55785 O60343 7415 10672 Q16610 4943 P25103 Q8IYX1 5371 Q9P0U3 P52701 Q9Y3P9 23513 P58012 P25116 P07148 Q8N6T3

negative regulation of cell adhesion P02452 P01042 4771 O14672 P41182 396 397 398 57669 P00747 Q9BZE4 23560 1012 Q92730 94 P07996 3956 P52565 7057 P52566 5270 1029 Q04900 P09382 Q9HCM4 P46531 4851 604 Q13370 P42771 Q8N726 P37023 P20936 1277 P35240 P21860 Q92752 P01137 O43182 P55290 5054 O75553 2065 Q02750 5728 O60674 P60484 Q99819 P05121 5604 P06576

blood vessel morphogenesis P78423 4691 7046 9464 O96004 Q9Y6W5 2034 Q12948 O43318 P52952 Q4VCS5 2821 Q8IYN9 P41134 2303 3479 P17275 P39060 P04085 O14786 94 Q15389 P07355 P98160 3911 P07996 P03950 9474 7057 P25067 5154 Q7Z5H3 6885 Q13887 P35368 Q08431 7052 P19338 P39059 P21810 2277 P46531 4851 Q14344 P01343 Q12965 P29474 P52735 10908 Q03014 Q9ULH7 Q05397 P37023 P20936 Q9UKW4 P01019 O15379 55109 P21980 5316 Q9NZ08 8829 Q9UPY3 P55290 4627 P01133 P02462 Q92826 Q14116 Q9NZI7 4240 9255 3791 Q14119 8841 Q658W2 P35222 P13612

P17302 5728 P21359 2335 P17813 2697 302 P60484 Q9BXJ9 P61296 Q92786 5327 5328 9421 P55347 Q8WUI4 Q5D1E8 9820 O95470 Q12904 Q9H488 O43915 4092 P78504 3162 O00622 P35579 Q96PU8 P09172 P00749 1499 P15692 51564 1012 10451 Q8N264 P23467 4643 3676 11146 3678 3953 3679 Q13683 Q99966 Q15464 Q8N302 P05412 P08648 P06744 3690 Q6IR47 Q13444 P50222 P35968 7410 P19544 Q8IY17 7490 23414 80155 841 688 Q8WW38 P08253 P48357 Q99958 5747 P02751 Q99835 Q96T37 9047 P05106 7422 23509 2247 2246 Q9H1Y0 Q92990 Q04656 Q14790 P05230 857 10672 Q9NQC3 P09038 116150 O15230 Q04771 P37173 8879 Q6PID4 4313 O15105 51752 P27658 7428 Q99814 1969 P00750 P09601 P29317 P40337 Q14289 P06576

regulation of peptidase activity P23396 P04637 P22736 P42224 356 2147 P84022 3476 6622 999 P07996 10016 P04004 7057 4221 Q96QB1 O43847 Q15392 7448 10542 P10809 8795 P06239 P48431 6772 Q8N726 P29590 23787 O14727 Q9BXK5 P61604 3932 P01375 5054 O15392 4088 P31947 P48023 P99999 Q9BZF9 9093 P78318 3146 668 Q96EY1 Q9ULZ3 6657 Q13794 3309 Q9NZJ7 Q92934 P01040 7124 4255 O00220 29108 O75340 114548 O75190 P55072 7013 Q9Y239 P37231 3329 1029 P11021 Q01955 P00734 5468 4898 P42771 P09429 10392 P54274 1285 P12830 29843 572 Q96P20 7415 1718 857 O14763 Q16610 5371 P09958 P51575 6188 7157 Q9P0U3 O43504 O00255 P37840 2810 P58012 Q15011 P05121 P25116 2935

striated muscle cell differentiation Q9Y3M2 P28289 3880 Q9Y463 7248 9149 P14635 O00468 P35579 3084 P52952 351 P37288 552 Q99523 P15692 79810 3479 Q8WV60 Q4KMG0 P15173 P09493 23363 P01116 Q92574 O15119 P45379 P01112 P01111 9474 P29466 P08727 P51636 10939 Q02297 P68032 P58546 Q86WV8 O43847 1147 375790 4898 P05067 P01343 O95259 136319 6256 7422 P29475 P19793 3265 P12755 891 P02708 O95180 Q9H1Y0 O14640 P02545 O43184 Q8WZ42 Q9UPY3 6926 4627 P06400 P10826 50937 4000 O15111 P04626 P68133 Q9NP98 25776 2064 9093 P17655 Q08209 P18827 Q92786 P24821 Q96EY1 P25116 5925

positive regulation of protein transport 5460 7046 Q15654 7124 29108 2033 O43557 114548 O60603 Q93062 Q04759 P84022 Q6PJW8 999 Q7Z6C1 11146 P61586 O15554 Q13485 7099 P03950 7098 Q6IR47 Q9NYA1 P29466 Q13443 11030 P14923 64127 O95361 P36894 P10600 5590 2316 3728 10626 Q05513 P14174 P01308 7205 Q9NQB0 P00533 P49841 P22303 Q99835 P23510 P21333 O00206 Q09472 8754 3783 P12830 2010 Q96P20 Q92990 P01579 P01137 O15455 1956 P01133 P01375 Q60FE5 4088 4089 Q9BZF9 P35225 Q9NR96 9495 P50402 P17612 2932 387 O60674 Q9HC29 P10071 5566 Q9ULZ3 54106 2737 P60568 P43489 P24588

gland morphogenesis P78545 9420 7124 5268 P35453 P10275 3479 Q1PSW9 284217 Q92731 999 O14786 P42229 O15119 3911 P51671 5155 P10600 54361 P13631 7052 960 367 3169 P46531 4851 P48436 P00533 P16070 P01343 5361 P10415 P25391 P12830 2099 O75881 P29590 57045 O75360 Q99623 8945 P21860 P08069 P21980 857 Q9Y297 P03372 P01137 8829 1956 6926 P06401 P01375 5371 Q92826 Q9GZX9 P04626 Q15648 O15230 Q14118 P36952 2100 2065 2064 P56705 O60716 23513 Q14160 P10070 11331 P24821 6776 P01127 1605 2736

cellular response to protein stimulus P24385 Q5VVQ6 P35638 10273 O95292 P14635 O75460 Q99942 P19525 891 595 79139 7415 5610 P01579 Q9GZP9 Q16236 O75807 51009 P55072 2081 4780 22926 9217 6048 P18850 Q9UNE7 55432 P11021 267 Q9P2K8 Q15011 Q9UKV5 Q9BUN8 3309

skin development P02452 P25054 P15924 Q06643 1832 1277 Q04656 P08123 Q9UJU2 P04085 1718 Q15185 P08842 P25940 Q9UJM3 P02461 P05997 P35527 5154 O75712 P18074 3673 P31947 P14923 P20908 P17301 3728 2810 Q15392 1289 324 10728 Q9UMX1 54206 P08138

kidney development P11926 P25054 7046 7248 O95470 Q6NYC1 Q12948 23322 Q02535 10736 2303 P15692 79810 Q1PSW9 Q8WV60 4643 P55075 Q92574 Q13563 Q13485 Q99966 Q9NPC8 P30556 9510 Q6IR47 O43521 5311 P09619 23210 O76024 54361 Q68CZ1 Q9BRQ0 Q86WV8 P19544 7490 Q05516 3207 O43609 Q01955 84159 324 445 P39210 Q99958 Q15475 Q14865 90780 P53420 6495 Q15797 7067 Q12965 7422 P10415 P35555 1285 2253 Q63HK5 1634 P53708 P00966 P01019 5159 Q9UJU2 P47895 Q9NSC2 80184 P10827 4086 Q92824 O15078 4089 P39905 Q9UHI8 Q06710 Q13705 Q6KC79 P56705 P14543 Q9HCK4 P21359 P31270 O95390 P10070 P10071 8516 8879 P20264 Q92786 P07585 4358 4953 7704 2737 2736

positive regulation of protein import into nucleus O00206 Q15654 7124 P12830 O43557 O60603 Q04759 P84022 999 P61586 O15455 P01375 7099 7098 Q9NYA1 Q60FE5 4088 Q9BZF9 P14923 Q9NR96 9495 2316 3728 387 P10071 7205 54106 2737 Q99835 P24588 P21333

negative regulation of DNA metabolic process P23396 4771 P04637 Q969H0 P54132 P41182 P51587 990 Q9BZE4 23560 Q8N3U4 Q6UWV6 25913 7013 7014 Q9NS91 O75496 P10600 55294 6282 641 Q9UQE7 604 Q99638 Q15554 2956 Q9NYB0 Q9H2G4 P54274 9126 5883 Q13535 O43612 P49959 Q99741 P35240 P01137 Q9BSI4 Q9BT67 6188 4361 7157 Q9NUX5 P52701 P40692 545 P31949 64061 P43246 Q14683

post-Golgi vesicle-mediated transport Q92538 10053 P50570 8775 Q99962 Q99523 1213 O14828 O00471 3312 3875 10133 P61106 23256 P78537 81567 P11142 117177 1785 Q9HD26 Q96QF0 8417 O15126 Q9H2G9 Q96RL7 O15400 P05783 408 P49407 P02794 9685 6456 9522 Q8WVM8 26258 2495 57120 Q12846 Q9Y6Q5 Q96EV8 Q96CV9 2647 375 Q00610 8548 P61966 84062 Q9NP90 Q14677 Q9UL45 5331 1174 O43747 P54920 Q8NBS9 Q01970 P84077 51552 6810 10640

gastrulation O43474 9421 P27037 5460 Q13873 8452 O96004 4771 P04637 Q12948 Q9UPN3 O75581 6194 Q4VCS5 Q93063 2303 57669 1499 P84022 P55075 1738 Q13485 P62753 2626 6045 9314 P36894 P10644 7290 9350 51295 P42858 Q9HCM4 Q9H2X0 Q15796 Q99958 P09622 Q16881 Q15797 O95813 O14495 P54198 Q9BQ95 2132 2253 Q13618 O00167 57045 6927 O60701 Q96S42 P35240 Q96RK4 Q9UJU2 P43694 Q9GZX9 4086 4087 4088 5573 4089 7157 7358 Q04771 Q13705 P35222 P20823 P17612 2139 Q99496 5566 O95947 1969 P29317

positive regulation of phosphorus metabolic process P01241 Q13873 7046 O95835 P14317 2033 P42345 595 3479 2147 1453 91 Q7Z6C1 Q15389 Q13485 P07996 1856 P04004 P03950 P78536 7057 4067 Q96QB1 P14174 7448 P00533 P01588 P16070 P01343 P49768 O00206 Q7Z727 Q09472 Q92956 3263 891 894 896 P17252 P01019 P01137 P01133 P01375 4361 4089 9495 P43405 P17813 O60674 3667 P04233 P24588 P27037 Q8TDY2 9821 O95999 7124 P07948 P14635 Q93062 P15692 5578 11146 3678 P08887 7099 P08648 3690 Q6IR47 Q13322 Q9NYA1 11030 P36896 64127 P35443 P36894 P10600 P35568 P30279 960 P01308 P00734 P30281 2475 O15169 85440 P07766 P24385 P05106 P02790 Q9UGJ0 6850 7422 P10415 P30542 P49959 2247 Q96N67 2246 P60033 Q92990 975 O14641 P01579 P05230 857 P01730 Q92878 P05112 1956 P09038 Q92997 7272 Q9HC29 P48730 5524 Q15257 P60568 P06213

positive regulation of phosphate metabolic process P01241 Q13873 7046 O95835 P14317 2033 P42345 595 3479 2147 1453 91 Q7Z6C1 Q15389 Q13485 P07996 1856 P04004 P03950 P78536 7057 4067 Q96QB1 P14174 7448 P00533 P01588 P16070 P01343 P49768 O00206 Q7Z727 Q09472 Q92956 3263 891 894 896 P17252 P01019 P01137 P01133 P01375 4361 4089 9495 P43405 P17813 O60674 3667 P04233 P24588 P27037 Q8TDY2 9821 O95999 7124 P07948 P14635 Q93062 P15692 5578 11146 3678 P08887 7099 P08648 3690 Q6IR47 Q13322 Q9NYA1 11030 P36896 64127 P35443 P36894 P10600 P35568 P30279 960 P01308 P00734 P30281 2475 O15169 85440 P07766 P24385 P05106 P02790 Q9UGJ0 6850 7422 P10415 P30542 P49959 2247 Q96N67 2246 P60033 Q92990 975 O14641 P01579 P05230 857 P01730 Q92878 P05112 1956 P09038 Q92997 7272 Q9HC29 P48730 5524 Q15257 P60568 P06213

cytoplasmic sequestering of protein 7067 4792 O95835 57120 Q9UNN5 O00221 11124 Q9UN86 Q9Y6A5 P25963 5716 Q9UBC1 P10827 Q60FE5 P31946 O75832 4188 10460 2316 9908 Q9HD26 Q99750 Q9UMX1 Q15653 7529 P21333

senescence 5580 Q8N726 P04637 Q13315 Q13535 3265 472 2305 1111 811 P01112 O96017 Q08050 7014 7157 Q02750 P38936 1029 1026 Q05655 545 P42771 Q15554 5604 O14757 P27797

epithelium development Q13477 5268 P15924 O75386 Q9Y4K3 O43318 P17676 Q8NEY8 595 4609 2303 284217 P84022 Q99807 Q92731 O14786 O14543 O14788 P49815 Q13485 Q9NPC8 Q9UGM3 5155 8543 79084 54361 P13631 367 P42858 P16471 P46531 4851 51535 P00533 Q15796 Q14344 Q15672 Q8N205 P61968 6495 6256 Q9UBD6 P05549 P25391 Q9NRY4 P12755 P20930 P23528 Q96RK4 Q92830 Q9Y297 P03372 P40424 Q9UKW6 6926 P06401 Q92826 4087 3791 4088 4089 P07737 8323 Q14119 P31947 Q14118 P36952 2100 Q86UL3 Q86YT6 Q9BYD5 P56705 P17302 57534 O60437 Q4AC94 P31270 2697 P61296 P20264 5604 9421 9420 7248 2909 P35453 P10275 Q02535 10736 57669 1499 Q9NYQ6 Q16790 Q99966 P51671 P32856 Q86YZ3 Q86WV8 P19544 7490 7251 P18206 3207 960 1147 841 Q9HCM4 7249 P09067 967 Q99958 Q15475 Q96T37 137964 O60869 7020 Q08188 1832 2247 3215 2125 O14641 P21266 P08069 P47895 2926 Q9H0F6 1956 P09038 P39905 Q02750 2810 Q14160 P37173 P09960 Q99816 Q9UMX1 P02533 1605 P78545 9464 O96004 O43278 23322 P52952 P22735 P41134 P62993 3479 Q1PSW9 P04083 P39060 Q92574 P42229 O15119 2947 3911 1856 6885 Q96QB1 7291 1072 7052 P48436 P01106 768 P16070 O43294 P42574 P01343 P49768 P50552 7189 P48431 P19793 O75881 2054 P29590 Q03014 Q13418 Q99623 1874 O14727 Q96S42 8945 P01019 P21980 Q5TA76 P01137 Q9NSC2 8829 Q16254 Q9UJM3 P02462 8721 P35908 Q9Y696 P35222 O60716 P17813 301 P10070 Q5T5A8 P10071 11331 Q92786 6776 P01127 6657 2737 2736 P01040 O95999 P07949 Q92817 8174 9021 P78504 P15692 2626 P05412 P35968 Q13443 26005 Q68CZ1 P37231 10229 3169 5468 54206 7408 5361 P24385 8754 7422 P10415 P56178 2099 Q02363 163183 P15311 2885 3611 Q14790 7414 P43694 10672 Q9BQA1 9839 5371 5493 7430 O15111 O15353 Q15648 O15230 Q04771 23513 P22528 O60353 P24821 6699

immune response-activating signal transduction O43353 Q96EP0 O95999 P28482 Q9Y4K3 O60603 O43318 Q8WZ60 Q9Y2C9 10616 Q9Y6K9 51324 P49810 5336 8767 5579 P05771 P25963 719 Q8IWV1 7099 7334 6885 64127 3329 P20963 P08575 P10809 Q9HCY8 P49768 O00206 9564 P07766 O14492 7189 6850 4792 Q9NZD8 148022 57402 Q9BYM8 Q9UDY8 P61088 P06241 5970 P11912 55072 P16885 81622 P43405 P51617 Q8IUC6 Q04206 Q9HC29 8517 Q16581 Q9H1C4

JNK cascade Q9Y6W6 Q8TDY2 9821 5585 Q9Y4K4 Q9Y4K3 P46109 Q96RU8 1852 121512 Q8IVH8 Q99683 Q03468 23043 Q9C0K7 4920 1616 Q16512 4188 5871 Q9UKE5 P53779 Q8WTR2 10746 79444

2873 Q99750 8737 4214 4216 Q13098 Q9UER7 Q99956 7189 6850 Q96M96 Q9P035 Q9NS68 1399 5599 Q5TCX8 55504 Q96CA5 4868 Q12851 Q9Y6R4 Q13227 Q13546 O95382 Q01974 P43405 Q8N4C8 P45983 Q15052 5602 Q13233 P45984

acetyl-CoA metabolic process 5162 48 P10515 P40926 3420 P50213 O43837 6390 4191 Q9P2R7 3417 O75390 1431 P31040 Q13085 8803 8801 1737 31 3419 P07954 Q13423 P51553 P53597 P36957 O75874 6389 23530 P11177 P21912 1743 3421 P48775 P21399

negative regulation of growth Q13873 P04637 6236 O14793 7161 O75385 29948 P50897 O60603 11108 27000 Q8IYN9 P84022 91 Q92731 O14786 84289 5931 94 4722 Q13485 8408 4221 51094 P67775 P55042 Q9UK53 Q9H2G4 P50150 Q8N726 Q9UKN5 O75489 Q9UNL4 P29590 P10451 Q05397 Q9BXC9 P41235 P46527 P37023 P51805 P32929 P01019 51147 6405 P01137 O60383 8829 P06400 4088 4089 A4D1W7 2100 Q658W2 58189 1491 O00213 3304 3421 64061 Q13275 P06733 6311 P41182 Q9P0J0 154 Q6FHQ0 Q9HC57 P07550 Q9UJW9 P08246 P51553 Q96SB3 P36896 64127 P30153 P19544 7490 Q8WYH8 P37231 1029 1027 1026 Q99675 Q9UJX0 5468 604 Q14140 P42771 5747 6696 P10415 Q96A54 P32320 Q86V24 Q99700 2247 Q99543 Q16576 Q9NQC3 5515 P09038 P08588 5518 5371 O15350 P08107 7157 2023 Q12816 O00255 P38936 3621 Q9HC29 55558 5925

cellular polysaccharide biosynthetic process P13807 11041 Q9Y223 56052 2132 Q8N6G5 23127 Q9NR45 Q93063 178 Q8NBJ5 51148 Q15185 Q5T4B2 Q9BT22 55907 Q16816 Q9UGI9 P15735 O43505 Q8IYK4 Q7LGA3 55454 Q8NFW8 2632 P35573 Q04446 10728 P46976 79709

lymphocyte activation P10914 4690 P25054 P04637 P15923 O43557 Q9H165 7163 6194 P32302 472 P15498 P84022 3476 P49810 8767 6504 P42229 10014 P62753 Q9C0K0 P27487 P56524 P78536 Q15306 Q15669 23210 P11215 P12268 2280 Q02447 641 P16473 P16471 P08575 P10809 2956 P49768 P06239 Q03014 23303 P55327 6929 896 Q9UDY8 P06241 P01137 5316 3932 3659 4627 P05556 Q13422 4088 Q13426 5971 Q9GZX7 P04629 P19320 9093 P35222 P13612 Q9NRZ9 P78318 4914 P16333 P43405 Q9HBE1 Q8N4C8 Q05655 53335 57379 6776 P40933 Q96EY1 P43246 P04233 P43489 P62942 O43353 6670 5580 P55347 Q13315 P54132 Q6NYC1 P41182 P39656 O43639 Q9UQL6 P53567 Q7RTR2 P35579 Q01201 1137 1499 1650 6304 5336 3676 Q8WV28 5579 Q9BZM4 7518 P05771 Q15464 Q8IWV1 5591 7412 P51159 P13796 3329 64919 Q13291 P01308 324 P30281 604 3688 P23510 P07766 6850 P10415 P78527 P18146 P36888 3615 3614 P42768 Q04656 P01730 P05112 Q01826 3070 Q9BZS1 7157 P51692 P11912 P35268 P06729 P52701 P16885 Q9NQT8 79465 P40692 P43681 P05362 10320 50943 Q96HB1 P60568

regulation of cell morphogenesis involved in differentiation 8650 4131 4099 O43157 P10636 2033 26037 P20916 O75385 4092 396 Q8IYN9 57669 Q86WK6 P84022 Q9HCJ2 P07196 Q7Z6C1 O14786 P61586 P46821 P02686 Q13485 Q99966 8408 Q04917 O43166 O00755 P52565 Q13009 7533 P10600 P23515 Q9HCM4 81565 4137 P61158 Q9GZM8 O43294 Q15796 P08138 Q92888 5747 P49768 Q09472 Q15797 6498 6696 5364 P12757 4155 Q13418 P10451 Q05397 P53667 57689 773 P51805 3611 Q9Y250 6405 P01138 P01137 Q9NQC3 8829 Q13145 Q96HC4 4086 4087 P29323 4088 4089 7476 Q9Y6R0 9138 P10586 Q658W2 9253 9495 7074 Q9HCK4 387 P20827 O15105 55558 P24588 Q13275 Q9BZR6

negative regulation of protein complex disassembly Q9BYV2 P25054 O15020 4131 Q13813 4133 P10636 23122 O75122 P14635 9181 Q9UHB6 P35612 P35611 Q9BVA0 891 6709 Q9Y3A5 830 P02549 832 118 Q9H2D6 10013 P46821 Q9NPA3 P47755 P47756 Q92974 Q08495 23332 Q01082 P11277 2039 Q7Z460 51474 P52907 324 P27816 57159 6711 6710 4137 Q6PKD3 Q9UBN7 829

learning or memory 6198 P98155 8654 60626 351 Q9Y3A5 477 Q92731 P49810 P01116 P43034 6869 P08842 P01112 P21246 P01111 P35368 P31321 P31323 O76074 Q96AB6 1742 P42858 P06276 P05067 P01100 P49768 Q7Z727 7067 P14416 3265 29767 O75881 P40145 54413 Q00535 Q9UQB3 P17252 P53708 P06241 P01138 O60260 5575 2100 Q9NZ94 5728 P21359 P60484 P07101 5582 P05129 9420 P54253 1020 B3KY43 Q99965 P09172 P21964 O60488 4125 5577 5578 3678 2904 2902 Q13286 P05412 P08648 P29466 P30559 P42261 P10721 P30550 O43529 5071 O00754 5590 Q05513 Q8NFM5 123803 P28223 10152 A1XKG3 Q9NYB9 P18146 P50993 Q9NPQ8 1312 7416 P25103 2925 P01178 P23443 P10827 P29323 Q05586 8516 5764 P21554 P21796

regulation of neuron projection development 8650 O43157 P10636 2033 26037 O75385 Q9BVA0 991 Q8IYN9 Q86WK6 Q9HCJ2 P07196 Q7Z6C1 O14786 P46821 P02686 8408 Q04917 O43166 P52565 Q13009 Q12834 Q3V6T2 P09382 81565 P61158 5029 Q16643 1627 P49768 Q09472 Q15797 6498 P12757 Q13418 P10451 Q05397 P53667 27185 773 P41231 P51805 Q9NRI5 6405 P01138 8829 4086 7476 Q9Y6R0 9138 P10586 Q658W2 9253 9495 Q02790 7074 5728 387 P20827 P60484 P24588 Q13275 4131 4099 Q15735 P20916 396 P61586 3956 O00755 Q9NYA1 Q92769 7533 P62166 P18846 P23515 4137 207 Q9GZM8 P08138 Q92888 5747 6696 5364 4155 3066 O60229 57689 3611 Q9Y250 55704 Q9NQC3 Q96HC4 P29323 P31749 Q9HCK4 466 55558 P21554 Q9BZR6

erythrocyte differentiation 6670 P55347 7067 7422 P07948 P14317 2034 Q6NYC1 P41182 Q02363 Q7L5Y9 P53567 6194 P39019 P15692 Q9H9B4 Q9UDY6 6667 10296 6886 P08047 5316 P62753 P06400 P10827 4067 P18074 A4D1W7 23210 P11413 22985 10107 Q02447 P17542 O60674 P62263 Q9UKV3 604 10661 P01588 Q99814 Q13351 5925

negative regulation of microtubule polymerization or depolymerization Q9BYV2 P25054 4131 4133 P10636 Q15735 23122 O75122 P14635 9181 Q9BVA0 891 Q9Y3A5 P46821 Q9NPA3 Q92974 23332 Q02790 Q7Z460 324 P27816 57159 22919 4137 Q15691

negative regulation of intracellular protein kinase cascade 9146 P25054 4771 O43597 P30086 Q16828 2305 3479 3476 P49815 2308 7099 4221 P21580 Q15669 5037 P30153 Q9Y5J5 O75376 O43609 P67775 324 7128 207 7249 P01343 O15524 O00206 P14416 P56539 P35240 O15379 P08069 857 O14964 859 5515 5518 5716 Q12778 O75832 Q08050 Q13227 8841 Q14118 P31749 O00255 9093 P78318 5728 P21359 P60484 51231 Q96EY1 1848 1605

cell surface receptor linked signaling pathway P25054 O14793 P49023 2550 1460 O95551 9185 1459 10657 P15498 1457 P37288 Q8IYN9 2303 284217 P84022 1453 2783 O14788 2308 2549 P28335 Q92729 2316 5829 Q96QZ7 P14174 P42858 2554 6915 808 Q12841 Q92956 Q06124 6927 2569 P37023 2324 2564 Q9UKW4 3655 Q9Y297 3672 3673 Q13705 P21917 57534 P16333 P20823 O60674 P20827 388677 P18507 Q8IUC6 P18509 Q9UKX5 3667 Q8WXG6 P27037 P25098 P08514 3680 P49069 1020 Q5SGD2 P18564 79576 P16144 1499 10616 3674 3675 3676 3678 3679 O14745 3690 P30679 3693 P09619 Q07666 P10600 Q06330 P60022 Q6PI57 3685 6711 846 3688 3689 P40238 7804 6714 P21731 P49286 O95136 4792 Q9NZD8 1285 Q9BQ95 P21709 Q07889 4543 P05230 O14763 Q92997 50937 P11912 P31749 P19883 1050 10607 867 O14775 P08754 P01241 P56199 3484 2150 3481 O60603 Q99523 2147 P62879 P01236 P62873 10256 6760 10261 Q9NWZ3 Q9BRQ0 Q14185 Q13094 O43294 409 P01222 Q13099 6772 P48551 6774 3265 Q99500 P53708 8945 P01019 Q99988 3276 Q658W2 P35222 P14784 Q01974 O75084 55750 P17813 Q01970 P60484 3269 55759 6776 Q96T58 8718 P23497 8717 O00744 5460 P56159 Q92934 6794 P05408 23523 23765 79971 10458 P15692 433 P38570 O14908 10451 P55075 10454 Q8N423 P05412 P01275 O00755 Q9HCE7 Q08117 682 Q01955 Q15077

8737 9826 207 P55085 P48357 Q92922 10468 Q15078 90780 P24385 8754 7422 23509 Q08345 O75051 8503 10672 5494 7433 Q13905 Q8NHX1 P06729 P50406 P52824 P08908 8516 10681 8517 P60201 8515 O15085 7442 P18084 6599 7204 P18085 O43559 O75386 Q9UPN3 Q9H2Y7 Q9Y4K3 P35414 3084 O43318 P48729 23043 P49810 8767 P49815 P21246 Q14451 P35408 Q15303 P48960 6368 P11215 P13631 Q96F46 23291 Q9H2X0 P46531 5029 P00533 P55040 Q15796 1902 P43026 Q9HAU4 8795 Q15797 Q9UQ13 Q8N6I1 P32121 P12755 Q8TDS5 O75360 Q9Y6Q6 Q93008 5295 7476 8323 Q9Y6R0 Q5JTC6 Q86XR8 O43306 P10586 P13612 P23416 P63092 O75582 O75581 P23229 Q9Y6K9 51324 51567 7252 8100 Q15109 O75578 P78334 Q9Y4H2 285 P63096 7249 P49841 51339 Q9NRD5 Q99835 P78368 P49840 P11229 Q7Z7G1 Q07092 Q9Y6M4 A1XKG3 Q9Y2E6 23220 51588 P08069 Q9GZL7 51107 1956 9213 10161 Q9BX66 P78356 P55011 P10147 Q6PID4 Q93038 P48730 P23458 1969 P48736 Q14289 Q15375 9223 9463 7046 P35813 P12931 P32302 Q92572 10817 Q9BUG9 P04083 P04085 P98161 3911 1978 P78536 O95965 Q02297 P10523 7052 P42338 Q92569 Q14865 P34947 8396 O95971 P29353 O95977 Q9H305 O95976 P29350 221188 P29590 P40145 2844 Q9BYM8 P43657 P41231 O60266 P06241 P21860 Q9NRA1 Q9HCP0 P78552 A4D1W7 Q13546 P49190 55072 9252 9253 Q9Y6J0 7074 Q9UNE7 P43405 P20309 Q07954 Q04206 Q92542 O95996 Q13797 Q96EP0 A8MTJ3 Q15738 O95999 Q03113 O43915 P78504 P35613 2861 P20594 3953 P62158 2869 2626 Q8IWV1 P31249 Q13322 7091 7090 P61073 Q9BQB4 Q05513 P61077 P00734 O60496 1540 P21453 375790 Q13574 Q15750 Q9HCU4 O75969 9046 Q16849 O75962 P32320 2889 2885 P61088 1793 P43694 Q5T9L3 P20333 O15230 Q15768 P34972 P47804 P67870 Q9NZN5 Q92585 P00750 Q15532 64397 P08833 P48058 O14672 Q12948 3516 P16220 P27361 Q8IZP0 54361 P50148 P63211 P16234 P20963 P16473 P16471 P38484 P63218 P48039 4851 4854 O15524 Q92600 Q06481 P54753 Q12965 P29992 P25391 P50391 Q05397 P54756 Q9UJU2 Q9UQB8 4627 Q96JK9 P26012 P05556 P54764 P54762 3791 P54760 O15552 5970 P26010 P46089 4882 Q9UBE8 P56705 5728 P20702 P17301 P20941 Q9ULZ3 P26006 P34925 P07307 O95477 P28482 1385 O95470 P51668 29108 O00468 5739 P07550 4643 5733 Q8N2W9 P08887 719 Q9UM47 P08648 O00238 P30556 Q9NYA1 P30559 P10721 P19784 P30550 P37198 961 5747 P07766 P05106 P05107 P28223 6850 P51681 O00241 2253 P51686 P30542 O60503 2247 2246 Q9BZK7 O14641 976 O14640 Q92633 P51693 50814 P29323 Q12778 P29320 P51692 1176 Q05586 3350 P48061 P30530 P18433 440193 P17342 P37173 5764 P29317 26060 P06213 26959 4690 Q9Y6W5 P38405 P62993 P13591 P01116 6869 Q15389 P27986 P01112 P25100 Q9UHG2 5310 6885 Q53G59 6400 5796 23210 P35368 2043 2280 Q96RT1 84159 Q99873 2277 4214 P01589 P01344 P01100 P49768 Q9Y6Y9 P14416 148022 O76061 773 Q00535 Q96S42 Q8IZF4 Q02952 Q53X93 5798 P01138 P01137 P01133 P01375 Q9Y336 P02461 5331 P04626 P04629 Q9UHI8 2065 2064 O60716 P35348 780 301 2059 P10070 P10071 5567 P01127 5327 O43353 6672 Q969H4 8851 64599 P07949 8852 Q8WU90 552 553 P51828 5336 5579 P05771 P40763 P04201 Q6IR47 10580 5590 Q99679 Q99677 51295 324 9702 329 P24046 Q08462 5362 4035 O60907 Q12929 330 572 573 333 P25103 23636 5371 Q60FE5 Q12933 6464 P16885 Q8NER1 Q6ZWJ1 P51617 P18827 8879 Q8N752 Q96HB1 P25116 P60568 10566 Q8TEW0 P13987 351 595 Q8WZ60 Q9UBS5 116 P22694 Q13480 Q9HC97 Q13485 P25963 Q13489 9510 5154 O94910 Q13009 7334 5155 19 Q9UKE5 Q13490 Q14344 Q13258 P22681 Q9HBW0 P32241 P32246 P59768 Q9UBU3 Q9UDY8 P09471 5159 142678 9518 Q8NFH8 P32239 346562 Q53H12 Q16539 4086 4087 Q14114 4088 4089 O43427 163126 Q9UKG1 Q86YT6 Q8N5F7 Q04725 Q9UBV2 Q04727 Q04726 P08151 6259 P62942 Q9NSD7 64750 7124 23365 O43639 4092 4093 P35579 154 156 Q9NYQ6 2904 Q99966 2902 Q13286 7132 7133 Q5JQC9 P36896 P36894 P35568 166 Q8NFM4 Q04941 Q02763 P01308 Q8NFM5 Q6PRX2 Q9NQB0 Q99717 P01303 O15169 2915 Q99958 Q96T37 9564 O95813 10273 P36402 O43613 O43612 Q9UKB1 P36888 51222 P01579 Q9BRK4 2925 P49715 P09038 Q9NQC7 P19086 O15197 O15198 P14867 P35548 2932 2931

1609 Q16581 P78423 Q13873 O15123 Q12788 Q08722 Q86Z02 Q13639 O75420 P42224 P41134 Q1PSW9 P23945 Q4KMG0 91 O95837 94 P42229 Q9NSA1 1856 P42226 9590 Q01082 9350 P08575 P05067 7186 Q15831 7189 P29474 9368 P35916 Q13418 Q7Z3S9 O43815 P41597 56034 P21980 O95859 O60383 O60381 Q9NSC5 O95622 Q14511 Q03001 4914 2735 P19438 P68400 56288 2737 2736 P13945 Q6NYC1 Q9H488 P41180 Q9H244 P23508 P43119 2742 2741 P08123 4920 Q13683 P43115 P35968 Q13443 Q15628 O95405 P30273 57154 Q96BI3 Q8WUH2 P17081 P21333 Q9UBH6 O14492 O14495 P08581 3611 1432 1672 P01730 P08588 P41159 P55773 Q9P2Y4 O15354 Q86Z14 Q04771 2773 O60353 O15105 Q13233 P21554

regulation of caspase activity Q9NZJ7 P23396 Q92934 7124 P04637 4255 O00220 29108 O75340 114548 P22736 P42224 356 O75190 2147 P84022 3476 6622 999 P55072 P07996 10016 7013 7057 Q9Y239 4221 Q96QB1 P37231 3329 1029 P11021 Q01955 Q15392 P00734 5468 P42771 10542 P10809 P09429 8795 P06239 10392 P48431 6772 Q8N726 P54274 1285 P12830 P29590 29843 23787 572 O14727 Q9BXK5 Q96P20 7415 1718 O14763 P61604 3932 P01375 5371 P51575 6188 O15392 4088 7157 Q9P0U3 P31947 P48023 O43504 P99999 Q9BZF9 O00255 9093 P37840 P78318 2810 P58012 3146 Q15011 668 Q96EY1 Q9ULZ3 6657 Q13794 P25116 2935 3309

embryonic morphogenesis Q92858 Q9NYD6 2672 Q12948 O75386 Q9UPN3 Q9Y4K3 O43318 Q9H161 474 2304 2303 P84022 Q99807 P49810 P08047 O14543 P49815 Q13485 Q9UGM1 P09017 Q9NPC8 4188 8543 54361 P13631 P50148 Q02447 P42858 Q9H2X0 P46531 4851 Q15796 P43026 Q15672 Q16881 P61968 6495 Q15797 P05549 P54198 Q9NRY4 P32242 O00167 P12755 6927 55584 116113 257 P23528 Q96RK4 Q92830 Q9UJU2 P40425 P40424 6926 P00441 Q9GZX9 4086 4087 4088 4089 P07737 7476 8323 7358 Q13705 Q86YT6 P13612 P56705 P17302 57534 P20823 Q4AC94 P31270 P20827 2697 P61296 P08151 9421 P27037 8452 4771 7248 Q8TD84 Q9Y586 2909 P63092 P35453 O75581 Q93063 10736 27283 57669 1499 Q9NYQ6 3676 4646 6045 P78337 8100 9314 P36894 P10600 Q06210 Q86WV8 O00358 3207 841 Q9HCM4 7249 Q9NQB0 P09067 Q9UM54 Q99958 Q15475 P09622 O95377 O95813 5089 O75569 P14859 Q9BQ95 2132 2253 Q13618 Q15915 7020 3215 O14641 P47895 2926 P10826 50937 O15078 P39905 7157 O00255 Q6KC79 P35548 2139 3226 P37173 Q99496 Q9UMX1 O95947 1969 P29317 7704 Q13873 7046 9464 O96004 P04637 O43278 P13349 23322 6194 P52952 Q4VCS5 2702 Q8TAF8 P62993 Q1PSW9 Q4KMG0 Q92574 1738 O15119 P62753 3911 1856 4221 6885 Q96QB1 7291 P10644 7290 1072 9350 P55316 Q99750 O43294 Q13099 P49768 2290 P50552 7189 P48431 Q03014 O60701 O14727 Q96S42 P53708 P35240 6647 P49639 Q9NSC2 Q9UPY3 5451 5573 P19320 P35222 P35227 2735 Q01974 P17813 P10070 P10071 668 Q92786 5566 P02458 P07101 6657 2737 2736 O43474 6670 5460 Q9Y5Q3 O95999 O43597 P07949 Q03113 9021 Q9NX62 O00622 O95076 O75190 Q99684 P31260 6667 P55075 Q9UJW2 4920 2626 P31249 O00755 Q6IR47 7412 26005 Q68CZ1 Q9UBX0 P36382 Q05516 10229 51295 7408 4154 P56178 O14495 57045 2885 Q14790 P43694 80184 9839 Q15648 O15230 Q04771 P17612 P58012 O60353 8516

extracellular matrix organization P02452 P78545 7046 O43278 5268 P13349 Q12948 O00300 P11047 351 Q07507 Q8IYN9 871 2303 P39060 Q6NVY8 Q7Z6C3 P07355 P04004 P25067 O95680 4060 2280 P16234 P39210 P21815 7448 P08572 P05067 P53420 P28300 Q13418 Q53GQ0 P51884 Q05397 Q00653 1634 P14780 51144 P53708 P01019 Q14993 Q05707 P01375 P25940 P02461 P18074 P36952 Q658W2 P14543 P21359 P17813 302 Q9UBV7 P02458 P07585 4358 P62942 133584 7124 P50454 O00622 3958 Q9Y6C2 P00747 P08123 7373 Q6IR47 P19544 7490 P13674 P12110 Q01955 4015 Q99715 Q99958 5747 Q9BS57 4791 1285 1284 333 3611 1277 Q04656 Q92752 Q14031 P51693 P05997 Q92626 P20908 Q63HQ2 1289 8516 7428 P40337 4318

regulation of peptidyl-tyrosine phosphorylation 5580 P01241 5581 4771 P07948 P14317 P42345 P15692 3479 5578 P04085 3678 Q15389 P08887 7099 P08648 P04004 3690 5154 5155 4067 P09619 64127 P30153 P35443 5590 P67775 960 Q05513 P14174 P01308 2475 7448 54206 P00533 P01588 P16070 3689 P01343 O15524 O00206 P07766 Q7Z727 P05106 P02790 P05107 Q92956 6850 7422 3263 P60033 P17252 P35240 975 P01019 5159 P01579 857 P01730 5515 P05112 1956 P01133 5518 Q9UJM3 Q9GZP0 Q02156 P43405 O60674 Q05655 Q9HC29 P01127 P60568 P04233

protein deubiquitination P51784 Q5VVQ6 9100 Q9Y2K6 27005 23326 Q96RU2 Q9UK80 Q8TAF3 51720 10213 P45974 Q14694 P09936 Q9UGI0 7375 P21580 P46736 10868 Q9NWV8 54764 55611 Q92560 51377 1540 57599 7128 Q96RL1 Q8TEY7 8078 O75604 8237 377630 Q9UPT9 Q9Y5K5 O00487 57646 Q9Y4E8 Q92830 Q96DC9 29086 Q93009 Q93008 Q9NQC7 Q9NPA8 P40818 Q6R6M4 Q13107 7874 Q96FW1 9099 55432 23032

cell-cell junction assembly P25054 Q8TEW0 Q8WUI4 59341 7082 O43815 Q07157 Q4VCS5 2702 P35221 P21926 51564 Q9Y4G6 Q02413 7414 Q9Y490 8443 P14923 7094 P17302 P36382 P18206 3728 56288 2697 324 1828 O15228 928

regulation of glucose import Q7Z727 Q9UGJ0 P49840 5584 7124 Q86V24 O60502 P17252 P21860 5578 P27986 Q9NSA1 P01375 P53675 5295 Q15303 Q13322 Q9BX66 10580 P31749 Q9Y4H2 2065 Q9UKG1 P35568 5590 2931 10724 Q05513 P01308 207 8218 3667 P17081 P41743 26060 P06213

regulation of JUN kinase activity 7186 7189 6850 5585 Q9Y4K4 Q9H422 Q96M96 Q9P035 7161 Q9Y4K3 O43318 Q9Y6Q6 Q9Y2C9 121512 Q99683 Q03468 Q5TCX8 Q96CA5 1616 9839 O15350 Q9Y6R4 Q16512 Q12933 4188 6885 Q13546 O95382 Q9NR96 P43405 10746 79444 Q99750 Q6PID4 8737 4214 54106 4216 Q14289 Q13233 Q13153 Q9UER7

regulation of anti-apoptosis Q15797 5562 P28562 Q9BST9 4035 4092 3162 P19634 Q8IUD2 1499 P20936 23085 P08069 7832 6548 857 Q9NQC3 Q15389 P08887 11325 O00459 4086 Q15303 5296 6242 Q13546 Q8NC51 Q86XP3 P38936 23411 P35222 Q07817 1843 1026 Q07954 Q6PID4 8737 O15105 P09601 Q14289 Q13131 Q96EB6

cellular component disassembly Q14249 8458 P41181 Q6NZI2 Q9P0J0 P50897 P11047 3084 P00747 3159 O00273 Q6NVY8 P43034 Q9H0D6 Q969G3 22803 Q8N4N8 Q02297 1029 3925 79444 81565 Q9UKV3 11198 P42771 Q9GZM8 P42574 Q9Y5B9 4150 Q8N726 Q9H2K0 P11388 7141 1676 O14727 284119 O60543 Q99661 Q01105 P17096 Q14197 P09430 6647 6605 Q96CA5 11004 Q9H0F6 Q9UNY4 P00441 7153 Q9UIG0 P99999 3396 9495 22985 P17813 P58012 668 Q13794 P24588

generation of neurons P25054 Q92858 Q8NEY1 65125 Q9NYD6 2672 Q9UBB6 259266 P17676 Q9BVA0 3516 2547 Q8IYN9 O60443 P16220 284217 Q9HCJ2 Q9BVA1 Q9ULW3 Q92731 Q92730 O14786 Q8TDD5 P52565 Q12834 Q9UBC3 Q12837 P50148 P63211 Q96KN7 2676 P42858 O60331 4851 P61158 4854 4734 P54753 Q7Z727 136319 P25391 Q06124 Q05397 P53667 6809 27185 29127 4983 811 5830 Q9UQB8 P40424 P62258 P54764 P54762 5970 3672 2100 Q7L576 P17302 57534 5728 O60674 P20827 2697 6812 P26367 O14578 5604 P41743 P34925 1385 1020 O00468 P61586 4646 5978 Q9UM47 P07437 Q9H0H5 O00238 Q9NYA1 Q92769 P28370 P51679 Q8IZT6 P10721 Q06330 960 O60890 Q9UM54 Q92888 5747 P04179 2253 Q9NYB9 57689 Q96N67 Q9Y250 Q92753 O14640 Q92752 O43186 P29323 P31749 Q13509 Q9P202 Q9HCK4 3226 O95390 P29317 Q15019 6993 P04637 O43157 P56199 2033 25861 P50897 P11047 P52952 991 203068 2147 7832 999 P02686 Q04917 O43166 Q9C0K0

Q96CW9 Q9UHG0 2043 P50542 1191 Q15154 11113 P01588 O43293 Q96NL6 P49768 2290 Q09472 P50552 P48431 9921 6774 P14416 23787 773 894 Q00535 Q53X93 6405 6647 P01138 P01137 P49639 8829 84062 Q9UMS4 6660 P04626 P04629 Q658W2 2065 2064 O00213 2063 P54920 O75084 55755 P60484 P10070 P10071 3146 Q96JB5 6657 P24588 O00744 P07942 4131 O00628 5584 4133 7520 8851 P07949 P20916 10458 P61981 P15692 Q99684 5578 P55075 5338 7518 Q8N5U6 P40763 P26232 O00755 Q6IR47 7532 7531 7533 P52803 P37231 P18846 Q96SZ6 324 3169 4137 207 5468 7408 5108 P27797 Q15078 6696 7422 5364 4155 P56178 P56177 3066 P13010 P61764 140735 Q96HC4 O43464 O14939 466 55558 8650 P55268 Q9UQP3 Q8TEW0 8775 O75386 O75385 351 474 Q04759 P07196 Q7Z6C1 P83731 P43034 Q13485 8408 Q13127 Q13009 P58304 P58546 5270 P09382 30813 P46531 51654 Q96SN8 5029 P00533 Q16643 Q15672 Q9HB63 6495 Q15797 6256 6498 P12757 P31273 P12755 P10451 Q9UBU3 P51805 P09471 Q96RK4 Q9NRI5 P00441 Q16650 6383 4086 4089 7476 Q9Y6R0 P10586 P10588 P13611 Q86YT6 P10589 59277 Q02790 O75592 387 P21359 54820 23032 23154 23396 1808 6259 P60763 Q13275 P54259 4099 Q8TD84 396 Q02535 Q16555 Q15223 P60709 Q16799 Q15109 P36894 1822 338917 60 Q9GZM8 1947 85440 Q9NRD5 Q15475 Q99835 9201 Q8TCU4 Q8TEY7 10152 Q02880 A1XKG3 Q86VS8 Q96EV8 55704 P01579 P08069 Q9NQC3 347733 1956 O15075 7155 O15078 P39905 7157 O75553 Q02750 Q9NXR1 Q6PID4 84376 1969 Q14289 Q15256 Q15375 9463 7046 P10636 26037 O43711 P63172 7161 55283 3913 1613 Q8TAF8 Q86WK6 Q1PSW9 Q6NVY8 P46821 O60282 3912 Q13885 O15496 O43602 10939 7291 Q3V6T2 P55316 P46934 81565 P16070 P05067 P98172 1627 8399 P19793 Q13418 O43815 6096 1756 P41231 P17252 P06241 P21860 Q13426 9138 29777 Q8IYT8 9253 9495 7074 4914 56288 Q96FJ2 Q9NZR4 Q04206 P07101 1406 2737 2736 Q9NZJ7 O75503 Q15735 P78509 P78504 O60488 3956 P31249 O43921 P11532 27339 P30279 1789 P62166 64919 P23515 P00734 375790 P08138 Q9HCU4 P09429 P12956 P10415 Q9H228 Q8IUQ4 P12830 P34741 P35711 O60229 P20336 3611 Q04656 80184 P41159 O15350 132320 Q15768 1203 23077 P21554 Q9BZR6

peptidyl-tyrosine phosphorylation P52333 P29597 6772 10152 P49761 P07948 P78509 Q9NYB9 P42224 2147 5159 P06241 P01579 1859 P42229 P42684 7297 Q15303 P29323 4067 Q9HAZ1 P09619 1198 Q8IZP0 57396 1195 P10721 Q13627 P16234 P00519 O60674 P16471 P00734 Q6PID4 6776 P23458 Q14289 P25116 P06213

positive regulation of JUN kinase activity 7186 7189 6850 5585 Q9Y4K4 Q96M96 Q9P035 Q9Y4K3 O43318 Q9Y6Q6 Q9Y2C9 121512 Q99683 Q03468 Q5TCX8 Q96CA5 1616 9839 Q9Y6R4 Q16512 Q12933 4188 6885 Q13546 O95382 Q9NR96 P43405 10746 79444 Q99750 Q6PID4 8737 4214 54106 4216 Q14289 Q13233 Q13153 Q9UER7

positive regulation of leukocyte activation 4690 P15923 Q08722 O43557 Q9Y4K3 O43318 Q04759 Q92692 8767 Q9Y275 O14788 6869 P42229 P07996 P42226 P27487 7057 8546 6885 4860 641 P14174 O43290 P08575 P01589 P98172 10666 P10809 O00206 7186 7189 Q92835 P06239 26258 Q9BQ51 3142 148022 O00203 6929 Q9UDY8 P01137 3932 Q14116 Q13422 P78552 P35225 P19320 9093 Q8N5F7 P16333 P43405 Q8IUC6 6776 P40933 Q96EY1 P04233 P43489 O43353 Q92934 O95999 P54132 P41182 O43639 P10276 79576 5338 Q96FA3 7099 Q14774 7412 Q9Y4H2 3329 57678 1026 961 604 1947 P07766 P00491 6850 57162 572 Q9HCL2 P01579 10673 857 P25103 P01730 P05112 Q15762 5914 P24394 Q9UL45 Q12933 P51692 P06729 P38936 O14939 P37173 10320 9846 P60568

cell cycle arrest 26959 P25054 P04637 11186 Q9NNW5 Q9UPN3 7161 4291 472 10657 4609 P84022 O15355 P98161 P07996 P01112 Q9UQ80 P52564 7057 9238 4221 5310 5036 5311 Q15392 1982 O43290 P01106 P24522 Q15831 P35638 P48431 6498 P12757 Q8N726 3265 Q9UNL4 P29590 Q13418

Q9NS23 P46527 Q969Z0 811 51147 P01137 O75807 O60381 Q92831 P06400 4088 A4D1W7 Q03001 O00213 27352 6657 P43246 5604 1647 8453 8454 8452 6794 Q13315 O75460 Q99728 Q13563 2081 22846 Q96SB3 Q96HU1 Q07666 8065 7251 1029 1027 1026 Q99675 324 Q9NQB0 P42771 P27797 Q13618 Q13617 Q13616 3611 P01579 1718 Q9UM63 5371 O15350 7157 Q7L8A9 O00255 Q02750 P38936 580 Q93034 Q99816 P04156 5925

regulation of MAP kinase activity P01241 P56199 Q9Y4K4 Q9NRW4 7161 Q9Y4K3 O43318 Q96RU8 Q96RU7 1852 1850 P01116 P49815 1616 P07996 P52564 P09936 7057 5155 Q16512 4188 6885 56940 Q8WTR2 O43609 P67775 Q99750 4214 P00533 4216 P42338 Q13098 Q13131 Q9UER7 P49768 O00206 7186 Q7Z727 7189 P29353 5562 Q9HBW0 Q9H422 Q96M96 Q06124 Q13418 P56539 Q9Y6Q6 Q9UBU3 57761 P17252 6647 P01137 P01135 P00441 P01133 P01375 Q9Y6R4 3672 P04626 Q15208 Q13546 Q9NR96 2064 P21917 P43405 P21359 P20827 P01127 5604 Q13153 P04233 Q8WXG6 Q13956 7124 5585 O43597 Q16828 Q9Y2C9 121512 Q99683 Q03468 5578 P08246 Q8IWV1 7099 Q13202 P30153 P10600 P10721 P37198 P61073 10746 79444 2873 8737 7249 Q99956 O95136 P28223 6850 Q9P035 P18545 2247 3611 P60033 975 Q5TCX8 857 859 Q96CA5 5515 1956 P09038 9839 5518 23636 Q9BY84 O15350 Q12933 6464 Q13227 O95382 Q02750 Q6PID4 54106 1848 Q14289 Q13233 P06213

intra-Golgi vesicle-mediated transport P35606 9950 9276 Q9UBF2 O95249 8775 P53618 Q96CV9 9382 P54920 1315 83548 Q8WTW3 Q6VY07 O14662 P60520 10133 9527 10466 O14579 Q9UP83 11345 Q8TBA6 2802

response to oxidative stress P02452 O75306 P28562 9588 P49023 2034 P30086 2033 4728 P42224 10935 P09493 O00151 Q99807 6622 4720 55967 Q7Z6C1 10013 P23025 Q15303 3091 Q9UI09 5037 5796 Q6AZY7 7171 1191 5829 Q15392 P39210 P30519 P00533 P67936 Q99757 P01100 P49768 O00206 Q5S007 Q7Z727 Q09472 P35638 6772 5321 P29590 9361 Q8TED1 6927 6809 Q96T60 P17252 P09471 Q16134 5798 6647 P00441 O00217 P50440 5054 Q13268 P55851 P55212 5970 P18074 P12883 P20823 O60674 2730 Q04206 Q96SL4 4358 P05164 Q16665 51435 Q9UBN7 5604 P25098 P56556 25828 O00506 Q9UGH3 P30041 2110 P30044 Q9NX61 3162 P30048 Q00597 156 1137 Q03468 P04040 5578 P62714 5338 P47712 P05412 Q7KZI7 7099 Q9NYA1 Q13443 P36776 P11413 93100 Q07817 2119 10229 1147 120892 4535 847 Q6XQN6 11315 P04179 Q16849 8754 P10415 Q13216 P41161 2011 O00244 1161 P32322 P48507 1277 2882 Q04656 1718 P04264 5516 1956 5371 P27540 Q92626 O15111 Q12931 Q06830 P15559 Q02750 P37840 1843 O14939 P18827 Q99497 Q6PID4 P43681 4710 1728 P05121 Q99814 10202 O14656 P09601 Q14289 P04156

positive regulation of phosphorylation P01241 Q13873 7046 O95835 P14317 2033 P42345 595 3479 2147 1453 91 Q7Z6C1 Q15389 Q13485 P07996 1856 P04004 P03950 P78536 7057 4067 P14174 7448 P00533 P01588 P16070 P01343 P49768 O00206 Q7Z727 Q09472 Q92956 3263 891 894 896 P17252 P01019 P01137 P01133 P01375 4361 4089 9495 P43405 P17813 O60674 3667 P04233 P24588 P27037 Q8TDY2 9821 O95999 7124 P07948 P14635 Q93062 P15692 5578 11146 3678 P08887 7099 P08648 3690 Q6IR47 Q13322 Q9NYA1 11030 P36896 64127 P35443 P36894 P10600 P35568 P30279 960 P01308 P00734 P30281 2475 O15169 85440 P07766 P24385 P05106 P02790 Q9UGJ0 6850 7422 P10415 P49959 2247 Q96N67 2246 P60033 Q92990 975 O14641 P01579 P05230 857 P01730 Q92878 P05112 1956 P09038 Q92997 7272 Q9HC29 P48730 P60568 P06213

tube formation O95999 P07949 7248 2909 O75386 Q9Y4K3 O43318 Q99807 Q9NYQ6 999 Q92574 P49815 1856 O43521 8543 6885 Q96QB1 7291 54361 1072 Q86WV8 7052 10229 841 7249 7408

Q15672 P50552 7189 P61968 P05549 Q9NRY4 P12830 P12755 7020 O14727 Q96S42 P23528 Q96RK4 O14641 Q92830 Q14790 P21980 9839 P07737 P39905 8323 Q86YT6 P56705 57534 O60353 Q9UMX1

negative regulation of neuron apoptosis Q15811 7161 P50897 P30048 P15056 673 10935 P07196 6622 P01116 P61586 7518 P01112 Q13286 P01111 P10600 O76024 Q07817 Q9H3Z4 P42858 Q15475 P49768 P04179 6495 P10415 6453 P56177 3265 Q13418 6093 P48507 773 P20936 3611 P01019 P21860 6647 P01138 Q13464 P00441 P09958 O15350 P39905 Q13426 P04629 2065 P37840 4914 387 2730 P43246 P25116

regulation of RNA metabolic process Q9UKT9 Q6NX49 Q9Y265 Q8NDW4 Q12824 8091 P51587 124790 P17676 2305 2547 2304 2303 57504 P84022 2300 Q92731 155061 Q00839 2308 2307 Q7Z6R9 Q12837 Q5VTR2 Q8WXB4 O60216 Q9H2G9 4734 Q96EB6 O75925 4990 3660 3661 22850 81628 6927 P13056 Q8IUD2 6929 84901 811 6921 O75928 P40425 P40424 Q9UKW6 6926 3659 P26358 6925 Q6IQ32 333929 Q6NUN9 Q9NZI7 P31943 P48382 Q9C009 Q92949 2100 Q99583 Q9NR96 Q12857 P48380 O95343 3428 6938 P20823 1487 P19838 Q99576 O60675 P62263 3665 10642 P26367 5604 6936 Q9Y468 Q9Y463 4772 Q9Y466 1022 Q8NA42 Q5JT82 10614 1499 Q86VE0 2103 P16383 27287 80324 P61586 6945 O14503 Q12873 P50222 4780 Q92769 Q8WXF0 Q14814 Q9Y5Z7 6720 P28370 Q15906 O95361 P10600 O95365 O00358 Q9BPY8 Q06330 2119 57798 10865 3207 10626 1027 P19419 Q6PI57 P17482 P17480 4775 O14753 604 Q9Y242 Q92766 P04179 Q15910 Q9UL36 4790 4791 Q12888 4792 Q9BQ95 O60869 Q8NA19 P38398 55145 3215 Q9Y250 Q92753 6721 4302 Q92994 Q92993 857 Q8N488 Q92997 Q92754 Q13501 Q9UKS7 P10826 P10827 P10828 221937 Q13503 Q14839 Q8NC51 P19883 Q13506 Q9NR55 1050 P53999 861 862 3226 863 3225 P04150 Q9UMX1 4799 P09601 50943 Q9UGL1 Q92985 Q9BS34 O96004 10492 P15408 P14317 P14316 Q9Y3Q8 3481 Q9UIH9 O60603 25988 P15884 Q8WUU5 Q96T88 3479 Q8WV60 2146 3476 10011 Q15022 10499 639 10014 P62875 135295 6760 Q9Y5V3 5430 O95163 P16989 P57059 Q96T76 Q03933 P33076 Q99750 Q14186 79923 8932 406 O43294 408 P49407 Q14188 O00327 Q09472 23309 5440 P48552 5441 6772 Q8TAU3 P48551 6774 Q99990 346171 O60828 P15621 Q00653 891 Q99743 Q2M3W8 Q14192 898 51147 P49639 Q86X95 P51531 5451 8721 A7MD48 148156 P35222 Q9NR11 P51532 P35227 P57082 Q96ST3 O75081 P17813 Q00403 668 6776 10001 10488 Q96T58 Q96MH2 P28702 P23497 Q16385 7629 O43474 P06733 P26583 O00744 5460 Q9Y3M2 Q9Y5Q3 P25490 P50613 P51946 23764 25942 Q8NFW5 3281 672 P15692 6304 Q8N5U6 P05412 O00755 Q9Y5R5 90993 Q08117 3297 Q05086 Q9UBX0 29079 P37231 P14373 688 148327 5467 9826 5468 Q92922 10468 4154 7422 Q96GA9 P56178 P56177 P52815 22893 3066 Q9UMN6 3065 Q9UIF9 Q8NHY6 Q12809 O75290 79753 Q00613 4149 10432 P62854 9839 O43463 3070 5494 9612 Q9UIG0 P52824 23512 Q96QT6 O14936 466 468 55796 P28749 Q9NQL9 7428 O15085 P36508 6595 P35659 Q14686 6597 Q14209 8535 6599 6598 O75386 P10599 6591 Q9Y4K3 473 Q9H161 474 Q9H160 P08047 5017 Q9BQG0 Q13363 Q9NPC8 Q13127 3091 94234 4188 5036 Q15306 Q86VP6 O75376 79084 P58304 P13631 3096 Q02447 O75381 23051 Q04864 Q9Y2H8 51773 30813 P46531 Q13133 Q15796 8553 Q16649 P35638 Q15797 Q8IWY8 Q96IZ0 P12757 P54198 P31274 P31273 Q9UNL4 P31276 P12755 Q9ULH7 23028 Q9NY61 O75360 4193 P46527 257 6128 Q15561 8548 51548 51547 54815 Q15562 Q16650 Q13148 Q9BYU1 P35869 P62826 P20393 7476 Q16656 Q15329 9412 P18074 Q8WVL7 P55055 P10588 P10589 P36956 P31270 Q9NPF5 Q16665 51317 Q13398 P43489 9421 Q16666 O75586 P35453 O75582 Q9Y6K1 Q03989 51564 Q15583 Q14494 8328 Q16670 P46108 Q14011 5074 Q9NRC8 10181 P78337 55929 Q9HAZ2 P46100 P22392 O15055 O43524 7490 7251 P13682 Q9C0F3 84232 Q8WW38 Q15596 Q99835 P61925 7024 Q3KNS6 7023 5089 P15822 23468 7020 Q02878 51341 54851 Q96PK6 51588 Q8NCF5 10155 9439 Q96NG5 286075 Q9NPA8 Q15369 Q8IUX7 26986 6182

P11474 26747 Q99816 O95947 9208 Q99814 P78545 9464 P35813 P29374 O43711 27005 P22736 Q9BUG6 O43719 P62195 Q9UGU0 9219 Q13761 A8K8V0 Q58WW2 P56524 9477 22806 22807 O75534 7291 7290 7295 P24928 P46934 O60296 Q4LE28 Q9UER7 Q14865 O75528 7068 7067 Q15714 P19793 P0C7X2 Q15717 Q7Z2Q5 Q63HK5 Q8WXI9 Q01196 84619 Q9H6W3 P41235 1994 Q92793 Q96GM5 Q9HD15 1993 O60264 O15499 O95983 Q13547 A4D1W7 P78317 9252 7073 P78318 P41223 Q8TBE0 Q9HAK2 Q9NZR4 Q04206 Q9UH92 Q92786 Q9Y2B9 P52597 O14593 O95999 Q9NP66 9261 O43918 Q01167 23085 Q9UKY1 Q9H9B1 P85037 P31260 Q9BSG1 Q14894 4800 2626 7099 P31249 7098 Q9NVC6 Q14653 Q9NP71 Q9NP72 7090 Q9NQZ8 1789 1788 Q05516 1786 Q9UIV1 3720 375790 Q13573 5901 Q9HCU4 P46734 Q13330 93134 P12956 Q9H2S9 Q6P1L6 P78527 92283 Q9H4W6 Q00059 Q9BQA5 Q9Y6E7 Q6ZNG0 Q96RG2 Q68DY9 P43694 O15455 Q9BQA1 P20333 Q9Y4A8 5914 O75953 30827 63976 Q96H20 P63279 P00519 P63272 Q9HCS4 Q92585 902 Q15532 Q9NZN8 P08235 P21675 5925 P10914 Q92858 Q9Y2W7 Q9NYD6 Q6P2D0 P67809 O75820 2672 Q12948 Q9UBB5 O00268 Q6QNY1 O00267 Q9P2R6 3516 4609 Q9BZI1 P16220 Q96JM2 10772 P49137 Q8N587 P27348 5933 O15516 Q12950 Q12952 Q9UBC3 Q9Y2X9 54361 Q8NAP3 1107 4851 284323 4854 4613 Q96JL9 1108 O15525 5705 Q9ULX9 P05549 Q12968 29128 Q99471 O60548 10514 83933 P20936 4862 Q92830 Q9UJU2 284312 O15534 P49116 P03372 57649 Q92833 Q6IT96 P06401 4869 Q92831 P06400 5716 Q96JK9 Q92826 Q0D2J5 P07737 Q9H9T3 5970 5971 Q6P2C8 Q9NRZ9 P56705 9099 P17535 P17544 10524 Q99459 P17542 Q9HBE1 Q99697 400720 P61296 5966 10765 Q12986 1385 P19532 P52756 O00468 Q9P0J0 O95231 Q9UFF9 O14627 6829 10736 P15173 P07550 Q8N2W9 4646 58517 10971 7913 5978 O14867 6827 Q96BA8 Q9UM47 Q9Y2T7 Q96SB4 Q9UHK0 5991 93986 5993 1153 Q14938 Q06455 P19544 55250 6839 1388 Q9P0K8 1387 1386 P08651 5987 Q9UM54 4899 O95259 Q10586 22937 22938 P51449 Q6IA86 Q9UHL9 O00488 O60506 22931 Q02086 P61244 2247 1398 Q9BZK7 6601 4664 Q9Y2V2 Q12770 6605 P05112 57209 Q9UM63 P08621 O43186 Q12772 Q12778 P51692 P31629 Q05586 2023 Q9BQY4 O00255 Q8NEZ4 Q02078 Q6KC79 Q9NQ33 O00257 Q03164 10725 57459 984 Q99496 Q5H9I0 988 P40337 7704 Q9Y2W1 P06213 O43159 6872 Q9UPV9 P04637 2034 P13349 2033 2274 P53803 O00422 P52952 Q8WTS6 79810 4686 79813 6625 4204 4205 Q16236 Q9UQ80 4209 Q9C0K0 4221 Q9GZR2 Q9UQ88 23210 P50548 O76071 84159 P48436 4214 P01106 P01588 7727 P01344 Q9C0J9 P01343 P01100 O00206 2290 23429 P48431 7741 9921 P52736 3142 Q9NU63 Q96S42 Q53X93 P49756 55869 6886 7975 P01137 5316 P01375 Q16254 P52740 Q9UPY8 6660 5573 8841 Q9UHI6 P52747 2063 23435 3151 11218 Q9NS37 Q99853 Q3KNW1 3148 P10070 Q15170 P09086 P10071 546 3146 10363 10362 6657 5569 6670 O43593 6672 23409 5585 Q9H9S0 25822 3162 Q12905 O95076 Q8WYA1 Q12906 79612 O75190 3159 Q02930 Q99684 6667 7756 5579 P05771 8607 P40763 5591 4261 Q969G3 8864 P55198 Q9ULK4 23411 P18850 P15336 23414 3171 O75182 Q9NRL2 51295 P18846 Q00987 Q68CJ9 P18847 P18848 3169 326 7528 P27797 Q9GZU7 64375 2099 O60907 3187 3185 3184 P51843 571 3182 P51608 P68036 10794 6207 8625 5119 Q01826 3192 P27540 9975 P62508 Q9ULM3 7555 146050 Q9BUY5 580 Q08211 Q9NVV9 80854 P58012 Q9NPJ6 7549 10320 54106 8878 9967 P25116 P60568 Q96HZ4 O43670 6231 4297 9500 8896 P15923 Q86YW9 P13984 351 84324 Q7Z6C1 P21127 Q13485 P25963 O94906 P09017 7332 O00716 Q16512 7335 O75496 O75494 P10242 P10243 367 Q70SY1 Q16520 7329 6239 Q14586 20 Q15672 Q9UQR1 6496 7101 6495 Q14103 6256 6498 P11308 Q99909 P32242 Q9Y606 116113 P17096 7579 Q14592 Q96MA1 Q13263 4086 4087 8204 4088 4089 Q14119 Q9BY41 Q9Y618 P12645 222546 387 Q04726 P08151 P24864 P20264 11177 11176 Q15697 P23771 53615 Q13033 P54259 P55347 9541 5187 O75469 7124 11142 O15164 P54253 P14635 O75461 Q9UQL6 P10275 4092 O43638 P10276 4093 4094 154 Q02535 Q01201 2908 Q99728 Q99729 Q15466 55806 Q99966 Q13285 2902 7132 5195 6045 7376 8463 7133 Q09161 8467 9314 O43889 P36896 23135

23378 P15976 Q96C28 Q3KQV3 1822 166 55810 P01308 Q6PRX2 338917 Q9NQB0 Q99717 P09067 Q14140 Q99958 Q15475 Q96T37 Q8TEY5 9326 Q9HA82 P14859 P36402 O75444 Q8TAQ5 7141 Q9H063 1831 Q96T23 Q99941 Q01664 Q00577 Q99708 P01579 P49711 P49716 P49715 P09038 55827 9575 P82979 7158 O75437 Q08050 P39905 7157 10284 O75676 162239 O15198 O43623 25776 Q02750 P35548 55832 Q9UDV6 1609 Q5VTD9 P78424 Q01094 7161 Q8TAF7 P42224 P41134 Q1PSW9 P17275 91 P17030 P42229 57109 O15119 1616 P45973 P42226 Q9UBK2 Q9NSA3 P78412 O94992 Q9P0W2 9112 22926 Q969S8 Q13404 Q13887 Q01085 57594 P10644 P55318 Q99081 P55316 Q96BD5 79365 P17040 P05067 2959 1869 Q9UBL3 O94763 7189 92129 Q8WYK2 O43812 P11940 7181 Q03014 7182 6096 Q86UE4 Q86UE3 1874 Q9BTC8 1870 P09630 O15379 O95619 Q8TAD8 Q9NSC2 O15151 8289 Q15843 Q13422 O15156 O94776 2735 P19438 O43829 P17023 2972 79149 Q9BXJ9 84733 Q8N393 Q8HWS3 P04198 1406 2737 2736 Q15853 O75626 Q14527 Q8WUI4 9149 1660 Q8TF47 Q6NYC1 P41182 Q8TAK5 Q02386 93474 79175 P53567 84525 84528 P62277 P22415 Q9UK80 Q03468 O75629 O95644 150094 Q14774 P22670 Q9UBG7 9391 64919 80155 58487 P41162 P23511 3609 P17081 3608 Q9Y5B6 3607 Q14781 P23510 P09429 94104 O75604 P41161 29966 Q02363 58491 P18146 P35711 27300 O60341 P09430 O15350 728642 O75832 O15353 Q15649 Q9BZS1 Q13227 Q15648 P55771 Q9P0U4 P19484 Q86YP4 Q06710 Q8N5A5 3622 O14497 O15105 Q13233 Q15652

protein stabilization 5580 O00744 Q86Y82 O75604 Q8N726 11186 O14495 P29590 4092 Q9NS23 10437 Q9BZE4 P84022 23560 Q02413 811 P83436 91949 Q9UBK2 O15151 5371 Q60FE5 4088 23673 9093 9099 P50148 5728 3329 1029 2316 83548 Q05655 P60484 O15105 1828 7428 Q96EY1 P42771 P40337 P10809 P27797 Q99835 P21333

stress-activated protein kinase signaling cascade Q9Y6W6 Q8TDY2 9821 5585 Q9Y4K4 Q9Y4K3 P46109 Q96RU8 1852 121512 Q8IVH8 Q99683 Q03468 23043 Q9C0K7 4920 1616 Q16512 4188 5871 Q9UKE5 P53779 Q8WTR2 10746 79444 2873 Q99750 8737 4214 4216 Q13098 Q9UER7 Q99956 7189 6850 Q96M96 Q9P035 Q9NS68 1399 5599 Q5TCX8 55504 Q6UXV0 Q96CA5 4868 Q12851 Q9Y6R4 Q13227 Q13546 O95382 Q01974 P43405 Q8N4C8 P45983 Q15052 5602 O14733 Q13233 P45984

response to steroid hormone stimulus P02452 P01241 7046 6198 5265 P28562 3880 O14793 8654 P30086 2033 P51587 O00300 O60603 P52952 595 P37288 2026 P17275 P07196 P20396 Q92731 Q7Z6C1 P04085 P01116 O14543 P46821 Q15389 P15090 P08842 2947 P07996 Q15303 7057 7332 5154 5155 P08727 P12429 5037 Q92729 Q08431 O76074 P16234 2 P05062 768 P06276 P01588 Q13133 20 P01222 O15524 P01100 Q9UBL3 1080 O00206 Q7Z727 Q09472 P28300 6256 P29474 5321 6774 P13569 P19793 P10451 P14410 890 Q9UBU3 894 P17252 P00966 898 5798 P01138 P03372 P01137 O14965 P01375 4240 P01130 5054 10081 P55212 5970 P04626 2100 P68133 58189 2064 P35222 P35625 Q8N8D1 5728 P60484 3148 Q15291 Q04206 P01009 P24864 P07101 P01127 5327 P23771 5207 5604 Q13033 P26583 6790 4131 7124 P28482 9021 3162 P10276 672 552 P05091 1499 Q9HC57 5578 3953 Q16790 P08887 Q99966 2626 P40763 P47712 7099 7252 Q6IR47 Q14258 Q9NYA1 Q13443 P30559 P10600 P30279 P37231 P20248 1026 445 841 3169 4015 5468 P48357 P09104 P01023 P24385 Q16849 8754 6696 P10415 29966 2099 P16118 O60502 P38398 1277 P68036 P17174 P21266 Q14790 857 P43694 2805 P49715 P01178 P23443 5914 P51692 Q02750 P38936 1050 O60911 1843 10724 5929 P18827 P05121 P00750 P09601 P04155 P25874 7706 P06213

positive regulation of lymphocyte activation O43353 4690 Q92934 O95999 P54132 P15923 Q08722 P41182 O43557 O43639 P10276 Q9Y4K3 O43318 79576 Q04759 8767 Q9Y275 O14788 6869 P42229 P42226 Q96FA3 P27487 7099 Q14774 8546 6885 4860 7412 Q9Y4H2 3329 57678 641 1026 961 P14174

O43290 P08575 P01589 604 1947 P98172 P10809 O00206 P07766 7186 P00491 7189 Q92835 P06239 6850 26258 Q9BQ51 3142 148022 O00203 57162 572 6929 Q9HCL2 Q9UDY8 P01579 10673 857 P01137 P25103 P01730 3932 P05112 5914 P24394 Q14116 Q13422 Q9UL45 P78552 Q12933 P51692 P35225 P19320 P38936 9093 Q8N5F7 P16333 P43405 P37173 Q8IUC6 6776 P40933 Q96EY1 10320 P60568 P04233 P43489

establishment of localization O75947 Q9Y266 Q5SQT9 Q5SR56 81855 27089 Q92973 10651 Q9Y275 Q9UGM1 Q92974 Q9H4L5 Q9UGM3 Q9Y277 O14561 Q8IWJ2 2316 1468 P42858 27091 P49006 O15400 5825 Q9Y282 Q8WXA8 Q12846 P61163 81876 55584 P62491 27067 Q9UKW4 4983 5830 10632 Q96EA4 Q12851 P26374 Q9H936 O15431 3673 Q13705 P21917 P21912 Q96N16 55110 2332 10640 O14579 27072 O15427 Q9UKX7 O75915 O75901 Q92538 5861 Q9UGH3 5862 1020 114548 P51787 P37088 1017 2348 1499 P61106 Q9UL26 Q9H0H5 Q9UL25 Q8WXF0 5870 5871 P33947 Q15907 Q9UGI9 Q15904 P51797 P51798 Q8TBC3 P20645 Q8NA29 Q96MV8 O60895 P20648 3685 6711 O60890 604 5865 4535 P05023 Q6NUT3 5868 5869 P05026 P53985 P19634 P40222 401505 610 Q5T2W1 Q6NUQ1 5877 6726 5878 Q8TBB6 P53992 Q9UL45 P31749 O95140 55153 27032 84932 253980 P40692 Q9UL51 Q96ED9 Q92504 Q9UL54 Q9H4M9 Q8TBA6 O95159 O96008 Q9UKL6 P50416 Q8NE01 P50897 Q99523 1058 O14949 8924 Q15021 55967 637 6748 79939 6747 Q9BTU6 O96018 4580 Q15027 O95169 Q9Y678 P57057 P36575 O95166 O75096 Q99996 51133 55973 Q14185 P47224 Q15036 P49407 P02794 1080 O00327 Q9Y5W9 P48553 3265 3263 10239 Q5VIR6 114571 O95180 Q9Y5X1 8943 P48544 Q9Y5X2 55503 O14964 Q9UP83 84062 Q9Y5X9 Q15049 O14980 Q9Y696 O95182 O95183 P35222 Q15050 9804 4116 P24588 P01042 6311 P05408 245972 O95197 Q99567 P61981 O14908 Q96A72 9818 57706 Q92930 P05412 O00755 P49450 23539 5473 5476 23530 Q08357 10228 Q9UBX3 P60660 Q15077 5467 P49447 207 5468 Q15075 P62841 10226 Q9Y5S1 P30825 Q12809 Q9Y5S9 Q8NHX9 Q96QU8 O14925 215 10673 P56181 P56180 6342 6341 Q9UKK6 O14939 56882 Q96QT4 6337 P40616 Q92900 10204 326624 Q14204 Q16864 O75146 Q9UPN3 23048 P35658 Q9H0U4 117584 P07196 P49810 Q9BQG0 P49815 Q9NPC8 Q9HAV7 Q9HAV4 P04920 8546 79083 93380 Q9H0T7 Q9NY59 Q96RL7 8536 Q9H9E3 11079 9868 Q9HAU5 Q9BUN8 Q16881 7220 9883 P56134 P32121 23025 Q9Y6Q5 Q96IZ7 4193 51542 Q96RK4 8548 P02741 P61966 8563 6386 Q9Y2J0 129531 P62826 Q9UPQ3 6389 P62829 Q86XR8 Q9UQ26 23039 Q9NXV2 Q9NY72 Q8N4C7 P61970 23032 51552 Q5SRE5 P08034 Q03518 O95907 P62820 P56556 P78348 11021 7248 P36542 6390 O75581 274 51560 P02766 P78334 11031 P05814 O75575 5071 7251 Q93050 152789 9429 7249 P48995 Q86Y82 P02790 Q9BV36 O15066 23463 A1XKG3 291 292 Q96EV8 Q14C86 Q9Y6M9 P24539 Q9NY26 Q9H9H4 P02786 Q6I9Y2 2802 O15075 Q9NPA8 O15078 O75558 26985 Q8N4H5 P37840 Q9H173 Q9NXR1 P49821 79901 11014 10166 P02775 P02774 P25874 9465 9463 P53007 Q9Y6G9 Q92572 55048 P04083 Q9UGU5 Q92574 Q13769 O15496 55054 9472 7295 117177 83985 O15020 9001 P06239 Q9Y6I3 60412 P06241 50618 Q8WXH2 A4D1W7 9495 79023 Q9Y2B5 3708 Q07954 Q9UH92 O15027 Q9UH99 O95994 Q14643 Q96NA2 Q9NP61 8170 P35610 P35612 P00505 23085 O60488 Q9H9B4 Q9Y6C9 1537 Q8IWV1 P35606 Q8NEC5 P56962 Q9NP72 O43920 Q96IW7 83547 Q9Y6D6 83548 23095 Q9Y6D5 O60499 O60493 5901 Q6P5W5 5902 O75969 O75964 P13804 P45880 P33897 3737 Q9BQA5 P02708 2885 Q9HAP6 5911 Q14674 5914 131474 Q9NP90 Q14677 A4D1S5 63971 11097 Q9NP97 222068 79065 Q9H0N0 P24941 P43681 51510 Q96RF0 51517 23603 Q9NRW1 Q9NRW7 Q99490 Q9P2R6 154091 O14662 10772 P27348 Q9UBC2 Q6PIS1 54361 55696 92609 Q9NWB1 Q9UBD6 3783 1121 339122 Q9BXC9 Q9GZY0 4628 57403 10758 6809 P18859 Q86WA8 O15533 Q92834 4627 P53675 91782 Q92824 P55851 Q9H814 4641 1130 P56705 P53677 57410 1122 3784 6810 6812 6811 6814 10762 4637 P04233 P07307 Q9Y584 P50570 O95239 O00461 3320 Q8IVM8 Q9NRS6 10736 1137 3313 O00471 3312 Q4VX76 4643 4644 4645 5976 3799 4646 P30559

O95249 Q9Y597 P53618 P37198 27173 P05141 Q92887 P53621 O95259 P28223 6850 O00483 2495 Q12769 P30542 P17568 81555 80223 O60502 Q9NS69 Q8TEH3 O14640 5514 738 P09958 P30536 1176 Q05586 1174 1173 O00499 10725 10724 55275 Q8IVI9 10726 Q9BXF6 Q99497 4678 O15504 P02452 O95279 P04637 4695 1186 P16615 1185 O95271 Q01650 1181 P62993 4686 P01116 Q9C0K7 6869 Q15388 P01112 P01111 Q96HA1 Q9C0K1 O43169 P48201 23210 Q6P4A7 23212 Q9HDC5 23214 P50542 1192 1191 Q96Q15 9908 4697 4698 11231 768 Q96CW1 4218 6890 6892 Q86VW1 P14416 P13569 P12236 2054 P33176 O95298 O95299 Q96CV9 3382 P12235 9919 773 Q02952 P61204 Q9GZP9 P01137 Q7Z3C6 4240 P01130 Q8N2H4 23673 P35348 P54920 781 Q9NRR8 O14617 301 302 Q7Z3B4 309 9927 25828 O43592 5584 Q5U0I6 64116 22794 P15328 Q96PU8 Q2M389 Q96PU5 8604 5579 Q9H3F6 Q9Y4P1 9950 5111 P37108 Q9BSW2 5590 Q99437 P16671 8615 P46059 P27797 7781 64130 9961 7780 P01185 6453 6456 6455 O00400 P50993 Q9NW64 Q9NVU7 Q96PS8 333 56993 P83436 P25103 140735 5119 A5D8V6 P01178 23636 9972 Q9UNQ0 4285 Q5T1M5 64145 9973 O00410 23633 P15313 Q6ZWJ1 Q08209 6457 Q99418 P25116 Q8N6T3 8655 Q8TEW0 O43678 351 Q7L804 23163 P09493 23165 P23786 P62913 Q96QK1 P25963 O43687 9997 11196 19 367 20 22 Q15436 6496 Q6Y2X3 8677 9525 9522 8676 Q15438 23144 P57103 Q9UBU3 P51808 375 9515 P23763 P61619 P32239 5174 Q14114 Q8WWN8 Q9Y619 381 382 P59780 388 9527 41 11140 9546 O43633 Q9H089 Q99962 Q99963 Q9H082 Q13283 Q16795 2904 Q16790 2902 Q13286 5195 9554 Q09161 O75694 Q86WV8 9550 Q9Y5J6 Q8NI27 51693 Q9NWN3 8218 51699 Q6PJH3 P46459 7385 7384 Q8TEY7 7386 7381 O43615 Q53TN4 54732 Q6VY07 P08183 Q9UBQ0 Q9UBQ7 P01579 9559 P09038 2923 Q9BY84 10280 Q9BXW6 P14867 O75674 Q9Y5L4 Q8TEX9 25777 Q9NX14 Q00325 P08195 84134 Q14964 Q15811 Q13637 Q96QC0 Q13636 Q15818 Q9H444 84958 79139 O15119 O15118 Q9UBK9 Q14974 9114 O15131 9590 Q9H7Z7 P43307 29796 P52292 O15126 P08574 P08575 P05067 P52298 Q15836 P52294 Q8TEM1 26258 Q03014 9120 P55327 80145 Q9UG63 84502 P21980 Q9NSC5 9135 P11717 O15155 Q15849 64901 Q9UBN7 P30049 9146 Q9UBF2 Q6NYC1 P41182 P41181 3836 Q6ZSS7 O15554 51606 Q16718 Q07817 P21579 Q9UJX2 Q96QF0 3841 P08133 3843 Q9BY11 P10415 93492 387680 4705 4707 4701 4702 80184 9179 P33527 51629 Q96QD8 P62072 Q96QD9 23192 Q8TAG9 4718 4719 Q8TF09 4710 4713 4714 1207 4715 65125 92745 25909 9182 22872 9181 Q96KP1 O00148 P15498 4728 4729 Q8IYN9 1213 P84022 79778 Q9UMY4 10890 4720 P60059 3875 4722 Q8TDD5 4723 4725 4726 P27487 P28335 64284 P28331 2562 P55957 9197 P38117 O00159 10427 2554 30011 57510 55334 4731 Q9H2G9 Q8N695 4734 253725 Q5T0N5 Q96P70 6927 2569 Q8IUD2 55341 O60664 30000 2564 811 Q9UJ41 P63104 P62258 6926 Q9C005 P31946 P99999 56681 O95343 Q676U5 81622 P20823 Q8NFW9 284427 80777 O60674 P18507 1244 821 5604 P41743 91452 Q9H2M9 2110 Q8NBW4 55361 O95352 P39019 P16389 P19404 2108 O60645 6945 P32856 Q9NR31 22841 27297 10868 7804 4792 Q8IUH5 Q9HCL2 1277 124565 857 Q9UN86 Q8N1S5 Q13501 P51572 P51575 Q13505 92799 P51571 Q9NQT8 Q8NBS9 O00141 P09601 O14777 P08754 P06576 Q86UW7 Q9Y3Q3 P01241 6993 10490 Q86V81 6510 3482 23325 Q9UIH9 O60603 25988 23327 2147 Q96KG7 10013 Q96KG9 10015 6505 11345 135295 23339 6520 6522 Q9NV70 25998 400 Q8NBI2 402 55737 406 6517 408 409 Q99757 Q01518 54407 Q15276 P60468 23307 Q9UMR2 116986 O00560 116987 O00562 23303 23787 23788 P13693 891 79711 Q15286 Q16134 Q9UN42 10476 P03891 10478 P61326 O43264 4363 6541 10482 Q658W2 Q8N1F7 25978 Q01970 Q96T51 10487 11336 P31930 O00505 Q6FGG2 7884 Q9UID3 23760 Q641Q2 23762 O43237 P14598 Q8IYJ3 433 A8K0Z3 55763 10451 6548 7879 10452 8724 P55072 Q05084 6566 Q9HCE7 Q05086 Q8WUX9 P37231 10469 P36382 Q99797 79720 55770 Q8IYI6 10466 Q6IQ22 11315 P01266 P24386 6575 9601 6576 9600 Q8WUX1 Q8WV92 65108 22895 Q96P20 Q8WV83 Q00610 7415 P61764 7417 7416 7419 Q8N5S1 5250 P51970 8766 6103 P40855 7442 Q16623 P18085 8775 P10114 P10599 24137 O75385 473 476 477 478 51762

Q99805 P43034 Q15785 P38919 8301 9632 P13637 64601 23299 481 Q9UJC3 483 O75380 O75381 488 Q05952 Q13133 Q15796 Q15311 O00602 Q9UNL2 Q8WVM8 23265 490 Q96H78 495 498 Q01105 P31040 P56385 P56381 7476 O75351 Q86VN1 Q9NZZ3 Q9H2V7 Q8N6H7 144983 54820 P23416 54822 9648 P43487 Q49AN0 Q9P2E9 51319 P43005 149371 O75348 11261 O43752 Q14008 P63092 119559 P13667 Q9UEU0 84248 O94826 Q14493 P27824 11269 Q14012 9673 23256 Q96D46 Q7Z7H5 Q9BX79 Q9GZM8 Q9NRD5 9201 9685 Q86W42 Q3ZCQ8 Q7Z7G2 P12314 O43734 Q86VS8 O43739 23225 Q9Y4I1 Q9UNH7 Q15363 Q9C0E2 P02545 55705 7037 Q9BX66 P21283 P21281 P55011 P10147 O43747 P48739 Q13190 Q99816 P98194 P28288 O75306 P98155 Q14847 O95714 55283 83752 27243 P41250 O15234 65082 P98161 P07355 O95721 P54105 7057 Q13520 9230 P78537 Q8IUN9 7052 81567 P46934 3927 Q9HD26 81565 O60296 80230 8399 P29590 P30101 Q4LE60 29886 P16284 Q9NZQ3 O15258 O15259 3932 O60260 Q32P51 27236 Q9UNE2 112936 P41222 653361 P43405 O43707 9267 Q9NZJ7 Q13555 O75746 Q13554 1781 Q9P253 O00186 O00189 1778 O00194 Q14894 Q13563 P08246 9276 P14927 Q14416 Q9H2T7 1785 Q05513 P63010 P00734 Q7Z2W7 Q15751 80273 53916 100287932 O60229 2647 1315 P20339 1314 P20336 P20337 1793 Q04656 84661 Q9UF02 Q9NZM3 Q9BWM7 1317 Q13586 Q16611 Q6XPS3 113829 9296 Q9H6Z4 Q96H20 Q63HQ0 4830 P20340 Q13596 P48051 Q9Y2W7 P48058 P61020 Q9BVA0 2665 2664 57620 Q9Y2X3 90678 P61026 P07996 10533 219931 P48047 Q12955 Q8NF91 Q9UI09 4860 51090 Q08431 O60333 Q8N4V2 Q8N4V1 200081 Q9UI12 P50395 Q12965 Q05397 10514 283229 29127 4867 4868 4869 Q9H1D0 1374 O00299 Q13829 O60318 P17302 Q96JJ3 P17301 Q99698 2697 Q9BZF3 948 Q96JJ6 Q9H1C4 Q12981 O95477 Q9Y342 P28482 O00221 Q9H1K0 Q9P0J0 58513 O60763 P07550 Q9Y2T2 10972 10971 Q5T8D3 O95487 57678 P54709 10981 7922 Q9UM54 5747 P31639 O00244 O60749 22931 P62330 10953 P42768 2247 26056 58533 10959 Q8N4Y2 P61006 Q9H1H9 P51693 Q8NEZ2 Q969M3 P28472 Q9P0M2 Q9BZJ4 P17342 Q92621 Q9ULV0 Q969M1 10960 Q92624 Q9ULV4 P61019 509 Q96FF9 P61018 P07900 Q9UPV9 Q9Y6W5 P80188 P11166 P38646 O60725 P15531 O14828 51000 Q96RU3 513 514 128866 6622 515 10134 10133 P61421 518 51009 Q04917 5310 6643 5311 6642 P51636 Q6WKZ4 P07919 521 522 523 10142 526 528 5306 10146 P49768 Q86U42 6653 Q3KNW5 23423 O00203 Q00535 533 535 P49755 537 51026 54536 P49754 54535 6646 539 51028 91949 54539 P01375 Q96JC1 O00217 23439 Q9Y2R5 4000 5331 Q96EY5 P12004 10128 O95070 10121 O00623 O00628 P07947 9700 Q9UPR5 Q9UPR3 O00629 Q96B97 Q9Y2K6 Q9Y2K9 23400 Q12907 3162 Q8IXI1 Q8IXI2 Q68EM7 P42704 Q8WTW3 7514 Q96AX2 Q96AX1 5338 P05771 10577 7532 7531 7534 10580 7533 Q9UHC3 3178 P68032 O00631 Q00987 9702 P24046 P05783 7529 3181 4035 P49321 Q9UPT5 Q99666 51068 Q8N8Y2 Q8N4Q1 P49792 5371 Q9BQQ3 P49790 Q60FE5 8402 O75396 Q969E2 Q8NER1 Q99653 P52948 P62745 4038 8878 10564 57617 10566 54908 9744 8411 O75027 57187 94121 Q96FV9 Q96G79 94120 Q9NUM3 P22695 Q9BRI3 Q8WUM4 Q8WUM0 8408 Q8TD31 P12429 O75494 29959 P35523 Q9H270 Q9UIA9 415117 8417 6238 Q13492 P47985 4074 Q8TD20 29928 O75489 Q9H269 29927 29924 23381 P25705 Q9UDY8 84342 Q8NFH4 Q13023 51429 6262 4087 4088 9775 29934 Q8TD16 Q96BK5 Q02790 P12883 P31150 Q9NUP9 1808 60386 10087 P20020 P60520 Q16543 Q8IXZ2 9784 P54257 O75469 7124 10053 P54253 7126 Q9Y3E0 P14635 P38606 P10275 Q4G0F5 P10276 P35579 154 P35580 Q99720 10057 55800 Q16555 Q99726 P45379 9311 9793 Q96C24 10061 O94955 29916 161 P11142 Q04941 P01308 P01303 9322 7142 P00491 Q401N2 Q9UQN3 P36404 6293 Q99700 Q9NZ43 55823 O94973 O75438 P54289 O75436 7157 P14625 Q02750 O75431 Q8WZ73 Q9NZ56 54984 Q9NUL3 O94979 84376 P53367 Q14721 8498 9342 P57729 O95833 10928 Q06787 P63172 7161 P54219 9341 7163 2702 P63167 P23945 10938 Q7RTN6 83871 P09651 Q9H1N7 Q13409 Q01082 8021 P57735 P22626 10947 Q969S0 9367 P10619 O75886 57120 Q9H1M0 P57740 O75880 26019 7184 Q9UHY1 O43819 P00846 P68871 Q92673 Q969R2 Q13423 O15392 O15397 Q8IY33 Q6UWE0 9371 P21757

Q96K37 56288 Q96FJ2 1408 9388 Q9HC62 9380 Q86YS6 Q86UK0 P51148 9382 P51149 4927 2742 2741 84766 P42262 Q6UWJ1 P51151 Q8WZ19 O95405 P51153 P42261 57154 Q15629 P51159 O43808 27333 Q8WYP5 Q9BVL2 Q8WYP3 P21333 O75843 59341 59343 Q9H223 P84085 P00403 Q15642 Q9BVK6 Q9HBR0 O15350 Q00169 1447 P84077 2773 P21796 Q9HC21

negative regulation of MAPKKK cascade O00206 P25054 4771 O43597 P30086 Q16828 P56539 2305 3479 P35240 3476 O15379 P08069 857 859 2308 5716 7099 Q12778 O75832 4221 Q08050 Q13227 8841 5037 Q14118 P31749 O00255 O75376 P78318 O43609 P21359 324 51231 207 1848 1605 P01343

positive regulation of DNA metabolic process 5582 P05129 O95271 Q9NX61 P53567 672 3479 Q1PSW9 51720 P04085 P01112 P42226 P04201 P05412 25913 5154 7334 5155 7014 P12429 P10600 P16104 P46736 P16234 Q9NWV8 P18846 P01308 P01588 Q96RL1 Q15554 P27797 P01343 P29353 Q9H2K2 Q13216 3265 1161 O60502 P38398 56034 P61088 3014 811 P01579 P08069 P01137 29086 Q9NRA1 P05112 Q9BSI4 6464 Q9NUX5 2735 10724 1763 466 P10070 P08151 P01127 5888 P60568 2736 P06213

regulation of stress-activated protein kinase signaling cascade O43353 7124 5585 P07948 Q9Y4K4 7161 Q9Y4K3 O43318 2305 Q9Y2C9 121512 Q99683 Q03468 3476 8767 1616 2308 7099 7098 Q9Y239 Q16512 4067 4188 4221 6885 64127 O75376 10746 79444 Q99750 8737 4214 207 4216 O15169 Q9UER7 O00206 7186 7189 10392 6850 Q9H422 Q96M96 Q9P035 Q9BUZ4 Q9Y6Q6 O15379 Q5TCX8 O15455 Q96CA5 64170 P01375 9839 O15350 Q9Y6R4 Q12778 Q12933 Q08050 Q13227 8841 Q13546 P31749 O95382 Q06830 Q9H257 Q9NR96 O00255 P78318 P43405 Q9HC29 Q6PID4 54106 Q14289 Q9UL54 Q13233 Q13153

cell activation Q9H0E2 P10914 P25054 O14672 P15923 O43557 Q9H165 Q9Y4K3 472 P15498 P84022 P21926 P49810 8767 P27487 5154 P12429 Q15306 Q15669 P11215 Q02447 P16234 P16473 P16471 P00533 Q14344 P10809 928 Q9Y2Y8 6929 Q9UDY8 P63104 3659 4627 P05556 Q13268 4088 5971 Q9GZX7 9093 P13612 Q9NRZ9 P16333 Q9HBE1 Q8N4C8 Q8IUC6 6812 6814 P43246 P04233 P43489 P62942 P55347 7124 P54132 O43639 Q9UQL6 55361 P35579 83706 Q01201 1137 Q9Y2C9 1499 3676 Q8WV28 Q9BZM4 Q15464 P29466 2243 P13796 3329 Q13291 P01308 604 3688 P02671 P07766 Q8TCU6 4791 6850 P36888 P42768 2244 P05112 1956 P51575 7157 P51692 P11912 P37840 Q9NQT8 79465 P40692 P37173 P05362 50943 P04275 P78423 4690 P04637 7163 O60603 6194 P32302 2147 3476 6622 P04085 6504 P42229 10014 P62753 Q9C0K0 Q9BTU6 P56524 P78536 23210 P12268 2280 641 P08575 P02675 P42338 2956 P49768 O00206 7189 P06239 Q03014 23303 148022 P55327 Q00653 896 P01019 P06241 P01137 5316 3932 P01375 P02461 Q13422 P35908 Q13426 P04629 P19320 P35222 P78318 4914 P43405 Q05655 53335 57379 3146 6776 P40933 Q96EY1 O43353 6670 5580 Q13315 Q6NYC1 O00186 P41182 P39656 P53567 Q7RTR2 Q9H244 1650 6304 5336 5579 7518 P05771 Q8IWV1 7099 5591 7098 Q13443 7534 7412 P51159 64919 324 P00734 P30281 P23510 P09429 8754 P10415 P78527 P18146 3615 3614 Q04656 P61764 10672 O15455 P01730 Q01826 3070 Q86UX7 Q9BZS1 P35268 P06729 P52701 P16885 P43681 10320 10202 Q96HB1 P25116 P60568

cellular carbohydrate biosynthetic process 7167 P06737 Q9Y223 56052 Q8N6G5 23127 Q00796 Q93063 2821 Q8NBJ5 2026 51363 P60174 5336 Q15185 Q9UBK2 P06744 P24298 O00757 P52209 Q9UGI9 P15735 P11413 P11498 Q7LGA3 55454 1468 2875 Q9UBX3 2632 P35573 P18847 P18848 P05062 5226 P09467 P09104 Q7LFX5 P46976 P13807 11041 2132 P16118 Q9NR45 O60547 178 2762 P17174 51148 2805 Q5T4B2 5836 Q9BT22 55907 Q16816 5091 O43505 P16885 Q8IYK4 Q8NFW8 Q04446 468 10728 5207 79709

renal system development P11926 P25054 7046 7248 O95470 Q6NYC1 Q12948 23322 Q02535 10736 2303 P15692 79810 Q1PSW9 Q8WV60 4643 P55075 Q92574 Q13563 Q13485 Q99966 Q9NPC8 P30556 9510 Q6IR47 O43521 5311 P09619 23210 O76024 54361 Q68CZ1 Q9BRQ0 Q86WV8 P19544 7490 Q05516 3207 O43609 Q01955 84159 324 445 P39210 Q99958 Q15475 Q14865 90780 P53420 6495 Q15797 7067 Q12965 7422 P10415 P35555 1285 2253 Q63HK5 1634 P53708 P00966 P01019 5159 Q9UJU2 P47895 Q9NSC2 80184 P10827 4086 Q92824 O15078 4089 P39905 Q9UHI8 Q06710 Q13705 Q6KC79 P56705 P14543 Q9HCK4 P21359 P31270 O95390 P10070 P10071 8516 8879 P20264 Q92786 P07585 4358 4953 7704 2737 2736

coenzyme catabolic process 5162 48 Q3SY69 P40926 3420 P50213 O43837 6390 4191 Q9P2R7 3417 O75390 1431 P31040 8803 8801 3419 P07954 Q13423 P51553 P53597 P36957 O75874 6389 23530 P11177 O75891 P21912 1743 3421 P21399

regulation of NF-kappaB import into nucleus O00206 Q15654 4792 7124 O43557 Q9UNN5 114548 O60603 Q04759 Q96P20 11124 P61586 O15455 Q9UN86 P25963 P01375 5716 Q9NQC7 7099 7098 Q9NYA1 O75832 Q9BZF9 Q06830 Q9NR96 9908 387 1540 7205 54106 Q15653

activation of protein kinase activity 9463 7046 Q8TEW0 Q9Y4K3 3084 O43318 P13861 23043 Q7RTN6 P22694 Q9ULW0 Q9C0K7 P22612 Q15389 P03950 O15530 6885 Q02297 P10644 Q9UKE5 P31321 Q3V6T2 P31323 P16471 4214 P00533 4216 P49768 8795 Q5S007 7186 7189 22974 P40145 Q9UDY8 O60266 P01133 Q53H12 O95622 Q9Y6R4 5573 5575 5576 O43306 Q13705 5170 55750 O60674 56288 Q07954 5566 5567 5568 O00220 B3KY43 154 Q9Y2C9 Q03468 Q9Y6K9 P51828 P07550 5577 10454 7098 3690 Q6IR47 Q9NYA1 Q13443 5590 Q9BUB1 Q05513 Q8NFM4 P01308 Q8NFM5 120892 Q13574 Q9NRD5 Q15750 Q08462 P05106 O95257 8754 P11229 4035 O43612 P30542 O75293 O60503 2247 55704 O14763 O15455 1956 P09038 Q12933 P52824 P17612 P51617 P37173 Q6PID4 1609 8517 Q9BWT7 Q14289 Q9UL54 Q13233 P25116 P60568 P06213

glucose catabolic process P06733 7167 Q92934 P08559 P37837 P10515 P40926 P52790 160287 230 2821 2026 P60174 P09972 1737 4967 P06744 P04406 P52209 3099 P11413 3098 P11177 P05062 5226 P49247 P09104 5162 P07205 Q6ZMR3 22934 P14618 Q9BYZ2 92483 P16118 4191 5160 572 3939 5315 Q49A26 5213 2023 P19367 P52789 3948 P17858 P00558 Q01813 5207 P08237 P07864 3945

positive regulation of cellular catabolic process 6872 7124 P50416 4092 Q96RU8 Q8WTS1 3479 1454 1453 P63165 P45974 P55072 3091 Q13443 Q9NP71 Q9Y4H2 P35368 O43847 P35568 Q00987 P01308 51099 207 5347 4898 P61956 P01343 7341 8078 8754 10273 4193 7415 P01579 P01375 5716 P09958 Q9UKS6 P27540 O75832 P49674 P53350 P31749 1374 Q9UNE7 O15105 P48730 6613 Q16665 3667 P21675 P07148 P06213

leukocyte activation P78423 Q9H0E2 P10914 4690 P25054 P04637 O14672 P15923 O43557 Q9H165 Q9Y4K3 7163 O60603 6194 P32302 472 P15498 P84022 3476 6622 P49810 8767 6504 P42229 10014 P62753 Q9C0K0 Q9BTU6 P27487 P56524 P78536 P12429 Q15306 Q15669 23210 P11215 P12268 2280 Q02447 641 P16473 P16471 P08575 P10809 2956 P49768 O00206 7189 P06239 Q9Y2Y8 Q03014 23303 148022 P55327 6929 896 Q9UDY8 P06241 P01137 5316 P63104 3932 3659 4627 P05556 Q13422 Q13268 4088 Q13426 5971 Q9GZX7 P04629 P19320 9093 P35222 P13612 Q9NRZ9 P78318 4914 P16333 P43405 Q9HBE1 Q8N4C8 Q05655 53335 Q8IUC6 57379 3146 6776 P40933 Q96EY1 6814 P43246 P04233 P43489 P62942 O43353 6670 5580 P55347 Q13315 P54132 Q6NYC1 O00186 P41182 P39656 O43639 Q9UQL6 55361 P53567 Q7RTR2 P35579 Q01201 1137 Q9Y2C9 1499 1650 6304 5336 3676 Q8WV28

5579 Q9BZM4 7518 P05771 Q15464 Q8IWV1 7099 5591 7098 P29466 Q13443 7534 7412 P51159 P13796 3329 64919 Q13291 P01308 324 P30281 604 3688 P23510 P09429 P07766 Q8TCU6 8754 6850 P10415 P78527 P18146 P36888 3615 3614 P42768 Q04656 O15455 P01730 P05112 Q01826 3070 Q9BZS1 7157 P51692 P11912 P35268 P06729 P37840 P52701 P16885 Q9NQT8 79465 P40692 P37173 P43681 P05362 10320 10202 50943 Q96HB1 P60568

leukocyte homeostasis O14492 6498 P10415 P12757 O43557 6194 Q00597 Q9Y6K9 P01579 10673 6647 Q9Y275 P01137 O14920 P42229 P62753 P00441 9133 O43521 4221 Q9BZS1 P51692 P31749 3551 O00255 9093 O95067 8517 6776 207 P01589 Q96EY1 50943 P42574

protein folding 53938 O95433 P07900 5300 Q96L12 Q86VQ3 P38646 Q9NYU2 27000 O00391 Q9UBS4 P25685 Q9H2H8 Q9HAV7 Q9UBK9 P31689 P23284 22926 Q13526 Q9BS26 2287 2286 Q6FGD7 2280 Q9H3Z4 Q6UX04 Q9Y680 11231 P49368 P10809 O94763 23307 Q6Y2X3 P62937 27348 64215 Q99471 9360 7184 O14967 Q9UDY4 Q8IXB1 125972 55466 811 10598 P61604 Q9NUG6 9531 9530 Q13427 55192 9093 O75352 Q02790 3304 3301 821 9526 Q96EY1 Q9NZJ4 P04233 P62942 Q9UL15 O95757 O75347 Q96KC8 3320 25822 O60925 O76031 54431 10856 O75190 O60884 3313 3312 10450 10574 Q15185 Q9Y230 P48643 P27824 51726 Q13286 Q9UIM3 P50502 O76024 Q08752 P18850 3329 P11142 3326 3324 23770 Q92643 P61758 80273 Q13451 P27797 23746 P50990 5481 P53621 11080 Q9NXH8 7266 P50991 Q9UNP9 Q8WW22 573 1314 3337 Q15084 Q99543 P78371 O95817 5478 Q9NVM6 O95816 5479 4820 O15212 P49792 P30414 P08107 Q5T1M5 O75953 Q14318 10283 Q12931 P14625 Q8IWL3 Q9H173 10845 1047 Q00688 10728 O95429 O14656 5768 Q9UHV9 P08238 Q9NZN9 O14657

regulation of cell growth O15085 Q7Z628 Q13873 7046 P08833 P04637 O14672 6236 P10636 2033 3484 7161 O75385 29948 P50897 3084 11108 27000 2305 P37288 Q04759 P84022 91 Q92731 5654 Q7Z6C1 O14786 5931 94 4722 P46821 P21127 51009 Q13485 8408 Q8WUD6 P78536 Q9UQ88 Q02297 83737 O95684 51094 P67775 Q92560 Q9H2G9 P55042 P16070 Q9UK53 11116 Q09472 Q9H2G4 P50150 Q8N726 Q9UKN5 O75489 9643 Q76FK4 27346 P29590 Q13418 P10451 P53667 P41235 P46527 P37023 P51805 P32929 P01019 Q9GZP9 6405 P01138 8548 P01137 O60383 8829 P55290 P06400 5716 4088 P22692 4089 A4D1W7 P31947 2100 58189 1491 O00213 3304 Q9NPF0 P17936 64061 Q13275 P06733 4131 Q13557 P41182 Q9P0J0 O00622 552 Q6FHQ0 1012 Q9HC57 Q9UJW9 P30556 Q6IR47 Q96SB3 Q9NYA1 P36896 P30153 Q96J02 P19544 7490 7251 Q96KS0 P37231 1029 1027 960 1026 Q99675 P01308 Q9UJX0 4137 9826 207 5468 Q5BJF2 604 Q14140 P42771 9564 P01185 6696 P10415 Q96A54 P32320 Q86V24 2247 3611 Q99543 Q9NRM6 56994 O15335 Q16576 10276 Q9NQC3 5515 P09038 P48745 5518 5371 O15350 728642 P08107 O75832 Q08050 7157 P31749 2023 Q12816 P38936 55035 2810 2137 984 3621 O43909 Q6PID4 Q99816 55558 Q14289 Q9UL54 Q15014 P60568 Q92743 5925

histone H4 acetylation Q09472 Q96L91 4297 Q9Y265 2033 Q9UNL4 10856 10933 Q92830 O95619 Q7Z6C1 51147 Q9Y230 Q9UBU8 8607 55929 Q15306 O96019 O95361 O00213 Q03164 57634 10626 Q9NPF5 408 P49407 86

negative regulation of cell growth P06733 Q13873 P04637 6236 P41182 7161 O75385 29948 Q9P0J0 P50897 11108 27000 Q6FHQ0 P84022 91 Q9HC57 Q92731 O14786 5931 94 4722 Q9UJW9 Q13485 8408 Q96SB3 P36896 P30153 P19544 7490 51094 P37231 1029 P67775 1027 1026 Q99675 Q9UJX0 5468 P55042 604 Q14140 P42771 Q9UK53 Q9H2G4 6696 P50150 P10415 Q8N726 Q9UKN5 O75489 Q96A54 P29590 P32320 P10451 Q86V24 P41235 P46527 P37023 P51805 2247 Q99543 P32929 P01019 6405 Q16576 P01137 O60383 Q9NQC3 5515 8829 P09038 P06400 5518 5371 O15350 P08107

4088 4089 7157 A4D1W7 2023 2100 Q12816 58189 P38936 1491 O00213 3304 3621 55558 64061 Q13275 5925

response to biotic stimulus Q9BYX4 P06899 5265 8654 595 356 P84022 8767 O14543 57506 Q9Y3Z3 P25963 Q92611 Q9BYW3 Q9UGM3 P12429 P27361 P62807 Q16082 P62244 60489 P27918 P38484 Q70SY1 P15260 128 10542 Q9UKV5 P10809 Q9BUN8 O15524 Q9HCY8 P80217 P35638 P13727 3661 Q9Y6Q6 57402 Q9UDY4 Q9UDY8 P23528 P61604 O75807 P61966 64170 5054 4088 5970 Q16778 P48023 Q9H257 Q9NR96 1130 3428 81622 Q99698 267 3304 Q8IUC6 3301 Q13393 3665 3309 Q9H1C4 3308 P11926 Q16666 7124 P28482 3442 1021 3320 114548 P50454 O75460 P19525 3316 Q9Y2C9 P21964 3315 P61221 3312 5610 5611 10057 1937 P48643 P08887 Q99966 Q96BA8 54 7132 Q96CS3 P51671 Q14258 P29466 Q9Y239 4780 6048 O75575 P51679 3452 Q96J02 P30793 3329 27297 57678 29110 P11021 P11142 3448 10625 3326 1147 1388 P60022 841 3445 3443 Q9NRD1 P49841 Q8TEY5 9201 10392 4792 O75569 10273 6059 23586 Q99942 3459 Q99941 3337 Q9HCL2 P01579 55703 8349 11128 P23443 O15075 9213 P01570 9695 P29320 9217 7157 P51692 O43504 2023 1174 P37840 P48061 2932 Q15011 P01569 P01568 P05121 P02775 O14656 O14657 Q92985 7706 Q8N2K1 6198 P09769 P07900 P98155 P04637 Q96A33 P14555 Q9BV94 7161 O60603 245934 Q01523 P42224 Q01524 Q96RU8 871 79139 Q9HC16 10935 3476 6622 10013 Q92575 Q16236 51009 P25685 Q9BZQ6 26270 P31689 P78536 Q9UII4 22926 Q9BS26 5553 O75531 1072 83737 P13236 1191 O76074 3925 Q9P2K8 Q9Y6Y0 P08575 56829 P01588 8815 P01100 O94763 O00206 Q9Y6Y9 P26641 P29474 5683 6772 5321 P48551 27348 O95292 P32881 P13686 148022 P13693 80267 1634 57003 Q00534 P00966 55741 Q9GZP9 P01138 P01137 Q9NZ08 P25787 P01375 P01374 Q7Z434 P10620 P34931 P34932 P78318 Q9UNE7 P19438 Q9NS37 3148 Q04206 P01009 P07585 P81605 Q59EA4 Q9UBN7 O43353 Q5J5C9 P06733 P26583 Q5VVQ6 P54652 O95999 O95757 P59665 P11766 P41181 25822 9021 P30048 P05091 O75190 5336 5337 P61626 P55072 P08246 10577 P47712 Q96FA3 P05412 2081 7099 7098 5473 Q14653 90993 64127 3297 O76024 O60814 P18850 1667 P61073 Q7Z2W4 Q68CJ9 58487 445 148327 Q9BUJ2 P01266 Q9UBH6 11080 P24385 6210 64135 P10415 Q13217 Q9UHD2 57162 O14802 1312 Q00613 2643 Q96P20 1672 7415 Q14790 Q92598 P62736 O15455 O95786 O15350 P08107 O75953 O15111 Q8NHX1 P34972 P16885 55432 O60238 P51617 P17066 23197 Q9HC29 51752 54106 4953 P04792 P08238 P25116 P21554

cellular component morphogenesis P25054 Q92858 8650 P55268 Q9UQP3 3880 Q8TEW0 9181 Q9Y3I0 O75385 351 474 Q8IYN9 P16220 P21926 P07196 P09493 Q99807 P83731 O14786 P43034 Q8NFJ9 8408 Q92974 Q9NPC8 3091 P08727 Q12837 54361 O60331 P46531 4851 P00533 928 P54753 Q7Z727 6256 Q06124 Q05397 Q9BXC9 P51805 Q96RK4 4983 Q9UJU2 Q9UQB8 Q8WZ42 Q13263 P00441 4627 Q16650 8443 6383 P54764 P54762 3672 Q9Y6R0 P18074 Q92949 P68133 P13611 P56705 Q7L576 O75592 P17302 387 Q4AC94 2335 O60674 2697 23032 23396 10087 6259 6812 Q16665 5604 P34925 8452 6275 Q8TD84 1385 1020 P35579 10736 Q04837 57669 1499 23363 Q99729 P61586 10059 Q15223 P45379 Q99966 Q13043 O00238 8100 P60709 P19544 7490 Q9HCM4 O60890 60 604 Q9GZM8 1947 85440 5747 P02751 Q99835 9201 Q8TCU4 Q8TEY7 Q02880 Q13618 A1XKG3 Q96EV8 Q96N67 P61006 O14640 P08069 Q92752 10155 1956 O15075 P29323 7155 P39905 O95140 Q13509 25776 Q02750 8481 Q9HCK4 P40337 Q15375 P28289 O00429 O75665 P56199 23322 9341 P11047 P52952 3913 Q8TAF8 P41134 Q86WK6 Q1PSW9 P39060 Q6NVY8 Q9C0K7 P46821 O60282 3911 3912 Q9C0K0 6760 Q96CW9 O15496 O43602 10939 2043 1191 P55316 117177 81565 Q15154 Q8IWA4 P48436 P05067 P98172 4218 Q01518 Q13099 2290 8399 P50552 Q15836 O95613 P14416 P19793 Q13418 1756 1874 773 Q00535 Q96S42 P17252 Q53X93 P21860 6647 O60383 P49639 Q9NSC2 8829 84062 Q16254

P04626 91147 Q658W2 2065 2064 P35222 Q8IYT8 O00213 9253 Q9Y3D6 56288 P10070 P10071 Q92786 10487 2737 2736 9927 P07942 Q9Y3M2 4131 Q6FGG2 8851 P78509 P41182 10458 5578 6789 P26232 P52803 26005 O43921 P11532 Q5HYA8 P68032 Q68CZ1 64919 10229 324 Q9NPH5 Q96QF0 P08138 7408 5108 Q9HCU4 Q15078 P10415 P56178 Q8IUQ4 P34741 O60229 3182 P20336 3611 Q04656 P61764 5116 O15230 Q15768 Q9NP98 582 23077 7428 55558 Q15532 P26447 Q9BZR6 O15228

energy coupled proton transport, down electrochemical gradient P30049 O75947 O75964 Q16864 P56134 245972 P38606 P36542 498 P00846 P25705 P24539 533 P18859 513 535 P56385 Q8N8Y2 514 515 537 P61421 539 P56381 518 10476 10632 9114 51606 P48201 P48047 P21283 P21281 Q15904 9296 P15313 Q9Y2B5 Q99437 Q93050 521 522 523 526 528 Q9UI12 509 P06576

ATP synthesis coupled proton transport P30049 O75947 O75964 Q16864 P56134 245972 P38606 P36542 498 P00846 P25705 P24539 533 P18859 513 535 P56385 Q8N8Y2 514 515 537 P61421 539 P56381 518 10476 10632 9114 51606 P48201 P48047 P21283 P21281 Q15904 9296 P15313 Q9Y2B5 Q99437 Q93050 521 522 523 526 528 Q9UI12 509 P06576

negative regulation of response to stimulus P23396 O15123 Q14449 P42345 2305 Q96RU8 3476 Q92731 O14543 2308 P42226 O15118 4221 O75376 83737 2 P14174 P08575 P01589 P16070 409 O15524 Q96EB6 6890 Q7Z727 Q92835 P14416 P32121 P13686 P10451 4193 Q9UBU3 4907 P17252 O15379 P01137 P05155 P21589 5716 P02461 5054 5692 5970 8841 Q92949 P10586 2100 Q9BZF9 P78318 Q05655 Q04206 P01008 3667 Q03518 P04233 5580 P41182 9021 154 710 P07550 5578 P08246 54 Q13286 P26232 Q13322 7133 Q08117 23411 Q96J02 P35568 285 5590 P37231 166 960 Q00987 Q05513 P01308 2475 207 5468 604 P01023 6696 P49840 P30542 Q9NQC3 P20333 P41159 Q9BT67 6188 5770 Q12778 O75832 Q08050 Q9BZS1 Q13227 P18031 P31749 P34972 O00255 P28070 462 Q9HCK4 P16410 2931 P05121 50943 P60568

negative regulation of DNA replication Q9H2G4 4771 P04637 P54274 9126 Q969H0 5883 P54132 Q13535 O43612 P51587 990 P49959 Q9BZE4 Q99741 23560 P35240 Q8N3U4 P01137 Q6UWV6 Q9BSI4 25913 4361 7013 7014 7157 O75496 P10600 Q9NUX5 55294 6282 641 Q9UQE7 545 P31949 Q99638 64061 Q15554 Q14683

regulation of protein stability 5580 6790 O00744 P07307 11186 4092 Q9BZE4 433 P84022 O14908 23560 Q02413 Q9UBK2 5591 7375 P50148 3329 1029 2316 Q96AB3 83548 79763 Q00987 1828 P42771 P10809 P27797 Q99835 P21333 Q86Y82 O75604 P10415 Q8N726 O14495 P19474 P29590 P78527 Q9NS23 4193 10437 Q8WY64 811 P83436 91949 O14965 O15151 5371 Q60FE5 4088 Q13107 23673 9093 9099 5728 440193 Q05655 P60484 O15105 7428 Q96EY1 P40337

mammary gland development P78545 Q15853 1385 P51587 P10275 3084 P17676 P15531 595 P15692 P16220 3479 Q1PSW9 P01236 O14788 P42229 O15119 P51671 5471 Q15303 P30559 O43521 Q9Y4H2 Q02297 3099 P10600 54361 P35568 1147 367 P16471 P17482 P01343 P24385 137964 7422 Q06203 2099 Q02363 P29590 Q9Y6Q6 Q99623 2247 8945 Q53X93 P21860 Q9UJU2 P00403 P08069 857 Q9Y297 P03372 P01137 Q9UKW6 P09038 6926 P06401 P01133 5371 O15111 P04626 P51692 Q15648 3673 Q86UL3 2065 2064 P56705 P52789 23513 P17301 Q14160 P10070 P10071 11331 6776 4830 3667 2737 2736

protein import into nucleus, translocation O00327 4792 7124 P04637 P28482 P41182 P29590 Q9NW64 2247 2147 Q92973 P01579 P02545 P01137 P09038 P25963 P01375 P05412 5371 Q14974

4000 O95405 7157 P31749 Q86XR8 Q01082 55696 O60674 P00734 6711 207 9702 406 604 P25116 P60468

cofactor catabolic process 5162 P53004 48 Q3SY69 P40926 3420 P30043 P50213 O43837 6390 4191 3162 Q9P2R7 3417 O75390 1431 P31040 8803 8801 3419 P07954 Q13423 P51553 P53597 P36957 O75874 6389 23530 P11177 O75891 P21912 1743 3421 P30519 P09601 P21399

forebrain development 8650 Q01094 23322 259266 7161 351 P37288 P16220 Q1PSW9 P49810 999 P43034 Q92574 O15119 Q15303 Q96QB1 P12429 54361 P50542 P50148 P55316 P42858 Q9H2X0 P46531 Q15154 4851 P00533 4854 P05067 1869 Q13099 P49768 2290 6496 P48431 Q9NRY4 P14416 P32242 P12755 Q03014 O75360 Q9BXC9 O14727 P51805 Q00535 P09471 Q96RK4 Q53X93 Q92830 P06241 5830 Q9UJU2 Q9Y6A5 Q9NSC2 P62258 6926 Q8N960 Q9GZX9 Q13422 Q14114 7476 Q9Y6R0 Q9Y618 P10589 P35222 P56705 O95343 9253 9495 2735 P21359 54820 P10070 P10071 P08151 546 1808 P20264 6657 2737 P24588 2736 7248 2909 1385 P78509 1020 552 1499 Q16555 P04201 Q9UM47 O00755 P78337 8100 Q92769 P30559 7531 10460 93986 P46100 O43921 Q8IZT6 P36894 Q68CZ1 Q86WV8 Q9UBX0 5108 7804 9201 P56177 Q02880 P12830 A1XKG3 3066 Q86VS8 57045 P51843 333 Q04656 P47895 1956 9839 P51693 P10826 O15075 O15350 7155 9612 O43464 O75553 Q9NXR1 Q9HCK4 10320 84376 55558

cell morphogenesis P25054 Q92858 8650 P55268 Q9UQP3 Q8TEW0 9181 Q9Y3I0 O75385 351 474 Q8IYN9 P16220 P07196 P83731 O14786 P43034 Q8NFJ9 8408 Q92974 Q9NPC8 3091 Q12837 54361 O60331 P46531 4851 P00533 P54753 Q7Z727 6256 Q06124 Q05397 Q9BXC9 P51805 Q96RK4 4983 Q9UJU2 Q9UQB8 Q13263 P00441 4627 Q16650 6383 P54764 P54762 3672 Q9Y6R0 Q92949 P13611 P56705 Q7L576 O75592 P17302 387 Q4AC94 2335 O60674 2697 23032 23396 10087 6259 6812 Q16665 5604 P34925 8452 6275 Q8TD84 1385 1020 P35579 10736 57669 1499 Q99729 P61586 Q15223 Q99966 Q13043 O00238 8100 P60709 P19544 7490 Q9HCM4 O60890 60 604 Q9GZM8 1947 85440 5747 P02751 Q99835 9201 Q8TCU4 Q8TEY7 Q02880 Q13618 A1XKG3 Q96EV8 Q96N67 P61006 O14640 P08069 Q92752 10155 1956 O15075 P29323 7155 P39905 Q13509 25776 Q02750 8481 Q9HCK4 P40337 Q15375 O75665 P56199 23322 9341 P11047 3913 Q8TAF8 P41134 Q86WK6 Q1PSW9 P39060 Q6NVY8 Q9C0K7 P46821 O60282 3911 3912 Q9C0K0 6760 Q96CW9 O15496 O43602 10939 2043 1191 P55316 117177 81565 Q15154 P48436 P05067 P98172 4218 Q01518 Q13099 2290 8399 P50552 Q15836 O95613 P14416 P19793 Q13418 1756 1874 773 Q00535 Q96S42 P17252 Q53X93 P21860 6647 O60383 P49639 Q9NSC2 8829 84062 Q16254 P04626 91147 Q658W2 2065 2064 P35222 Q8IYT8 O00213 9253 56288 P10070 P10071 Q92786 10487 2737 2736 P07942 Q9Y3M2 4131 Q6FGG2 8851 P78509 P41182 10458 5578 6789 P26232 P52803 26005 O43921 P11532 Q5HYA8 Q68CZ1 64919 324 Q9NPH5 Q96QF0 P08138 7408 5108 Q9HCU4 Q15078 P10415 P56178 Q8IUQ4 P34741 O60229 3182 P20336 3611 Q04656 P61764 5116 O15230 Q15768 582 23077 7428 55558 Q15532 P26447 Q9BZR6

one-carbon metabolic process 6472 9463 4297 Q9BZ95 6470 29947 O60725 11107 1576 Q9Y4C1 P08684 79813 7832 Q8N1Q1 P05177 Q8WWH4 Q9UBC3 23210 Q9H3R0 O75891 P13995 O14717 Q99873 768 P01100 Q9UBL3 Q86U44 Q9UPP1 P31153 Q6UXN9 346171 Q9NU63 23028 Q9H6W3 P62495 54496 54815 P26358 P61964 Q9C005 3276 Q9NRZ9 P34896 Q8NB78 10768 55352 Q15291 546 11176 Q9NPF5 Q9UET6 Q9UI30 10765 P55345 Q3SY69 Q6NYC1 Q96MX6 Q9Y6K1 23081 2107 Q9H9B1 6304 56339 O14744 Q16790 Q9Y5R4 55929 5870 Q9NQ92 Q08J23 P46100 23133 23135 1789 1788 1786 Q9NRD5 Q8IY81 Q96FX7 23463 Q9UIF9 O75164 9682 P11586 Q9NVM4 84661 P49711 O60341

Q99707 4548 Q01826 11091 3070 30827 Q9BVS5 Q9P0U4 Q86YP4 O43865 Q03164 221656 5929 80335 P20340 Q9UGL1 2935

regulation of smooth muscle cell migration 6198 P10415 Q13418 3516 Q96RU8 P00749 P41231 3479 3611 P04085 P23443 P04004 5054 5154 5155 3673 Q06330 P17301 P17936 Q15077 7448 P01127 5029 P05121 5328 P01343

brain development Q92858 8650 Q14686 P18085 8775 Q12948 P30086 O75386 259266 P51587 O75385 351 472 474 P37288 P16220 Q92731 P49810 P43034 8408 Q15303 O94910 P12429 5037 54361 P58546 P50148 P42858 Q9H2X0 P46531 4851 51654 Q96SN8 P00533 4854 136319 6496 Q15797 Q9NRY4 P32242 P12755 O75360 Q9BXC9 P51805 P09471 Q96RK4 Q92830 5830 Q9UJU2 Q9Y6A5 P62258 6926 Q16650 8443 Q9GZX9 4086 Q9Y2J0 Q14114 7476 Q9Y6R0 2100 Q9Y618 P10589 P56705 O95343 Q4AC94 P21359 54820 P08151 1808 P20264 3309 P54257 7248 Q8TD84 2909 1385 1020 4093 1499 Q16555 Q99966 Q9UM47 P78337 Q9NYA1 8100 Q92769 P30559 93986 P46100 Q15109 P28370 Q9Y4H2 1152 Q8IZT6 P36894 Q86WV8 P11021 7804 Q99835 9201 O43175 Q02880 A1XKG3 Q86VS8 Q15915 610 613 Q92753 P08069 P47895 1956 P51693 P10826 O15075 P10827 7155 O15078 O15198 O75553 Q6KC79 Q9NXR1 Q9HCK4 P37173 Q9UL51 84376 Q01094 23322 7161 P50897 Q1PSW9 999 P61421 Q92574 O15119 9114 Q9UHG2 Q96QB1 O43602 7170 Q9BRQ0 P50542 P55316 Q15154 P05067 1869 Q13099 P49768 2290 9001 7067 P48431 P14416 Q03014 O00562 6096 P11274 O14727 773 Q00535 Q96S42 P53708 Q53X93 P06241 P49639 Q9NSC2 Q8N960 Q13422 P35222 9253 9495 P54920 2735 55755 P10070 P10071 546 Q92786 Q96JB5 6657 2737 P24588 2736 Q9Y5Q3 O75503 8851 Q13315 P78509 Q8NFW5 552 P04201 P26232 5591 O00755 Q9NQZ2 7531 10460 P52803 26005 O43921 Q68CZ1 Q05086 Q9UBX0 Q96SZ6 P51959 5108 P06753 Q15078 90780 5362 P10415 P56177 9600 P12830 3066 57045 P12277 22895 P19235 P78527 P51843 O75051 333 Q04656 80184 9839 O15350 9612 Q15648 O43464 1203 8516 900 10320 55558 O15228

glycolysis 5162 P06733 P07205 Q6ZMR3 7167 P08559 P14618 P10515 P40926 P52790 160287 Q9BYZ2 92483 P16118 4191 5160 230 2821 3939 2026 P60174 P09972 5315 1737 4967 P06744 P04406 5213 2023 3099 3098 P19367 P11177 P52789 3948 P17858 P00558 P05062 Q01813 5207 P08237 P09104 P07864 3945

cellular response to starvation Q13873 Q8TDY2 9821 P04637 27327 P61244 Q9H1Y0 60673 10013 4149 857 Q7Z3C6 P08243 Q13286 P05412 Q13501 Q9Y4P1 9474 Q9NYA1 P07339 7157 6720 23411 P36956 Q8NDV7 55054 79065 P55318 O60911 Q676U5 3171 Q9BSB4 Q9H492 23192 81788 P11021 Q9P2K8 Q9H093 8878 Q9UBN7 O94817 Q96EB6 3309 P49768

positive regulation of cytoskeleton organization 4690 P25054 4131 4771 P10636 2033 P14635 O43639 Q4VCS5 Q9BVA0 10733 P42345 Q04759 P84022 P09493 Q7Z6C1 P61586 P46821 6869 11346 P51671 7013 1072 10109 324 2475 4137 4214 P61158 Q09472 P54274 O00444 P56539 P31431 P53667 P62330 891 P23528 P35240 857 P25103 859 4088 382 P16333 387 Q92786 Q8N3V7 Q13233

respiratory system development Q13873 7046 2034 2033 O75386 23322 1181 P41134 3479 Q1PSW9 284217 P49810 P08047 Q7Z6C1 P04085 P05177 3911 5154 23210 P13631 Q02447 P16234 P46531 4851 Q15796 P28845 P01343 Q13099 1080 Q09472 P28300 7067 P48431 P29474 P25391 P13569 P12755 Q03014 Q63HK5 Q96S42 Q9UBM7 Q9UPY3 Q9UJM3 4087 5451 3791 Q14118 Q13705 P35222 2735 P10070 P10071 P08151 Q92786 6657 P55061 2737 2736 6670 O43597 Q6NYC1 64759 1499 P15692 6667 Q15185 10454 3290 Q6IR47 P35968 8100 Q68CZ2 P36894 P10600 Q68CZ1 23414

Q9UBX0 3169 4015 Q8WW38 54206 Q15750 7422 P14859 P20336 2247 2246 Q04656 P05230 P47895 P49715 P09038 P10827 O15230 1050 10728 P09960 Q99814 1605

DNA modification 9463 8930 4297 4255 P29372 346171 Q9UIF9 Q9NU63 P78549 29947 Q9Y6K1 54496 Q9NVM4 P22674 P49711 54815 P26358 4968 3070 Q9Y5R4 55929 Q8WWH4 P46100 Q13569 Q9UBC3 Q86YP4 Q9NRZ9 O95243 Q8NB78 Q03164 221656 1789 1788 O14717 1786 546 11176 Q9NPF5 Q9NRD5 4913 P01100

formation of primary germ layer O43474 9421 5460 Q13873 Q15797 O96004 4771 Q12948 Q9BQ95 2132 O00167 Q9UPN3 57045 Q93063 6927 2303 57669 1499 P84022 Q96S42 P35240 Q9UJU2 Q9GZX9 4086 4087 4088 5573 9314 Q04771 P36894 P10644 P35222 P20823 P17612 2139 51295 P42858 Q9HCM4 Q9H2X0 5566 O95947 1969 Q15796 P29317 Q99958 Q16881

regulation of glucose transport Q7Z727 Q9UGJ0 P49840 5584 7124 Q86V24 O60502 Q96RU7 57761 P17252 P21860 5578 P27986 Q9NSA1 P01375 P53675 5295 Q15303 Q13322 Q9BX66 10580 P31749 Q9Y4H2 2065 Q9UKG1 P35568 5590 2931 10724 Q05513 P01308 207 8218 3667 P17081 P41743 26060 P06213

positive regulation of protein complex assembly 7341 4690 P25054 4131 6872 10273 P10636 P54274 Q9UNN5 P12931 O43639 P56539 P52952 P62330 P42345 P63165 10013 11124 857 P46821 859 P51671 7013 382 Q9UNE7 P16333 2932 10109 324 2475 4137 4214 P61158 P49841 6714 Q13233 Q9UBN7 P21675

epidermis development P02452 P78545 Q13753 P25054 Q13477 Q13751 3881 P15924 P16615 Q8NEY8 P22735 2304 Q1PSW9 P04083 91 P49810 5017 P04085 P08842 3911 94234 P35527 5154 Q15828 Q15392 488 P46531 4851 P48436 51535 P00533 P42574 2319 P49768 Q06643 P31276 Q01469 P20930 Q9UJU2 Q5TA76 55504 Q15323 P25940 Q9UJM3 O95863 Q92826 P31944 P02461 Q9Y337 P26371 Q9C009 P35908 5970 P18074 3673 A4D1W7 P31947 Q9Y696 Q04695 Q9BYD5 2735 P17301 O60437 301 Q02388 P10070 Q04206 Q5T5A8 P08151 5604 2736 Q15738 P01040 P49862 Q16787 Q92817 P19012 8174 P78504 Q9UMD9 P08123 Q15185 Q14254 5351 Q13443 Q86YZ3 P14923 P36896 O00358 7251 Q9NZT1 3728 324 5467 207 Q02809 54206 O14753 P08138 10468 O95377 8754 P10415 3860 P13646 P13647 P13645 O76011 Q08188 Q9NS68 1832 P42768 2125 1277 Q04656 3852 1718 P04264 Q9H0F6 1956 50814 5493 P05997 O15353 O75712 25818 O15230 P31749 P20908 1294 Q02750 P19883 27032 P22528 2810 1289 3909 10728 6699 Q99816 Q9UMX1 P02533 P98194 3866 6615

T cell activation P10914 4690 P25054 P04637 O43557 Q9H165 6194 P15498 P84022 P49810 8767 P42229 P62753 Q9C0K0 P27487 P78536 Q15306 Q15669 23210 P11215 2280 Q02447 641 P16471 P08575 P10809 P49768 P06239 23303 896 Q9UDY8 P06241 P01137 5316 3932 3659 4627 Q13422 4088 Q13426 5971 9093 P35222 P16333 P43405 Q9HBE1 Q8N4C8 53335 6776 P40933 Q96EY1 P04233 P43489 P62942 O43353 6670 P55347 P54132 Q6NYC1 P39656 O43639 Q7RTR2 P35579 Q01201 1499 1650 6304 7518 5591 P51159 P13796 3329 64919 P01308 324 P30281 P23510 P07766 6850 P10415 P78527 P18146 P36888 P42768 Q04656 P01730 Q01826 Q9BZS1 7157 P51692 P35268 P06729 Q9NQT8 P05362 10320 50943 Q96HB1 P60568

angiogenesis P78423 4691 9464 O96004 Q9Y6W5 2034 O43318 2821 Q8IYN9 P41134 2303 P39060 P04085 O14786 94 Q15389 P07355 P98160 3911 P07996 P03950 7057 P25067 5154 Q7Z5H3 6885 Q13887 Q08431 P19338 P39059 2277 P46531 4851 Q14344 P52735 10908 Q05397 P37023 Q9UKW4

O15379 5316 Q9NZ08 8829 Q9UPY3 P55290 4627 P01133 P02462 Q92826 Q14116 Q9NZI7 4240 9255 3791 Q14119 8841 Q658W2 P35222 5728 2335 P17813 302 P60484 Q9BXJ9 P61296 5328 9421 P55347 Q5D1E8 Q12904 Q9H488 O43915 P78504 3162 O00622 P35579 P00749 1499 P15692 1012 10451 Q8N264 P23467 3678 3953 Q15464 P05412 P08648 P06744 3690 Q13444 P50222 P35968 7410 Q8IY17 80155 841 688 P08253 P48357 Q99958 5747 P02751 Q96T37 9047 P05106 7422 23509 2247 2246 Q14790 P05230 10672 Q9NQC3 P09038 116150 O15230 Q04771 P37173 Q6PID4 4313 51752 P27658 7428 Q99814 P09601 P40337 Q14289 P06576

negative regulation of small GTPase mediated signal transduction 23636 8452 Q8WZ19 O43597 Q8WWN8 Q96QB1 7126 O95140 Q13618 P41182 Q8N9R8 P35348 Q13829 P37198 O43609 253980 P62714 604 Q13574 5516 9927

regulation of erythrocyte differentiationP26583 P27037 P25791 P27540 3091 Q9Y5Q3 Q92835 P51692 A4D1W7 1021 P36896 O43524 P17542 3148 Q00534 91 4005 6776 6886 Q16665 P42229

apoptotic mitochondrial changes Q3ZCQ8 P10415 O00429 Q8N726 P04637 7161 7818 P84022 637 10059 P05412 O15350 P51398 4088 Q16611 7157 P55210 P31947 P31749 P55957 1191 Q07817 1029 2810 4214 207 P42771 Q13794 P42574 92609 Q13233 P04179

regulation of microtubule polymerization or depolymerization Q9BYV2 P25054 4131 4133 P10636 P54274 Q15735 23122 O75122 P14635 9181 P56539 Q9BVA0 891 221150 Q9Y3A5 857 P46821 859 Q9NPA3 Q92974 7013 23332 Q02790 Q7Z460 324 P27816 57159 22919 4137 Q15691 Q8IX90

regulation of cellular catabolic process 6872 9744 7169 P54619 10928 26037 P50416 Q99490 O75385 P15498 P42345 Q96RU8 Q8WTS1 2665 3479 1454 2664 1453 P63165 P09493 5018 P43034 Q92574 P49815 P45974 Q6ZT07 11345 Q8WZ64 8408 O15118 O43166 Q8TBP0 P56524 3091 Q15027 Q9Y2X7 Q7Z6J4 P35368 O43847 23216 115704 Q9P107 O43609 51099 81565 P05062 493856 P61956 Q15311 85360 P98174 P01343 7341 P50395 9882 1121 116986 Q96M96 Q9ULH1 116987 Q8TEU7 Q96CN4 4193 P09471 Q9UKW4 Q8IV61 P01375 5716 P26374 Q9UPQ3 Q8WWN8 Q92949 1374 125058 P35625 Q8N6H7 Q9UNE7 27352 P21359 P62140 55633 Q66K14 1122 P31150 P60520 Q16665 3667 O43150 5604 Q15057 Q3MII6 Q9H2M9 7124 Q9NP61 O43597 7248 Q96BZ9 Q96DN5 P41182 Q5R372 Q86TI0 93594 4092 Q9NUY8 Q96P48 121512 25780 O14908 79735 10451 9815 P55072 P45379 Q13286 P07951 P51671 Q13443 Q96HU1 Q9NP71 Q9Y4H2 Q5JSP0 Q86WV8 P35568 3326 Q00987 P01308 Q9NU19 Q15070 2475 5500 207 7249 5347 Q8TEA7 604 Q6ZW31 4898 Q9GZM8 51735 85440 Q96NH3 P26045 8078 P24386 Q9UGJ0 8754 P49840 Q8N5K1 10273 Q6ZV73 Q8WY64 Q96N67 Q0IIM8 55785 O60343 7415 P01579 4943 O43182 Q8IYX1 P09958 Q9UKS6 P27540 O75832 P49674 P53350 64786 P31749 Q02750 Q9Y3P9 23513 2931 Q14160 Q6PID4 O15105 P48730 6613 Q14161 Q14289 P08238 P21554 P21675 P07148 Q8N6T3 P06213

respiratory tube development 6670 Q13873 7046 O43597 2034 Q6NYC1 2033 64759 1181 P41134 1499 P15692 3479 Q1PSW9 284217 6667 P49810 P08047 Q7Z6C1 P04085 Q15185 P05177 10454 3911 3290 Q6IR47 P35968 8100 Q68CZ2 5154 23210 P36894 P10600 23414 Q02447 P16234 3169 P46531 4015 4851 Q8WW38 54206 Q15796 P28845 Q15750 P01343 Q13099 1080 Q09472 P28300 7067 P48431 P29474 7422 P25391 P13569 Q03014 Q63HK5 Q9BUZ4 P20336 2247 2246 Q96S42 Q04656 P05230 Q9UBM7 P49715 Q9UPY3 P09038 Q9UJM3 P10827 4087 Q92824 3791 O15230 Q14118 Q13705 P35222 1050 2735 P10070 10728 P10071 P08151 P09960 Q92786 6657 Q99814 P55061 1605 2737 2736

lung development 6670 Q13873 7046 O43597 2034 Q6NYC1 2033 64759 1181 P41134 1499 P15692 3479 Q1PSW9 284217 6667 P49810 P08047 Q7Z6C1 P04085 Q15185 P05177 10454 3911 3290 Q6IR47 P35968 8100 Q68CZ2 5154 23210 P36894 P10600 23414 Q02447 P16234 3169 P46531 4015 4851 Q8WW38 54206 Q15796 P28845 Q15750 P01343 Q13099 1080 Q09472 P28300 7067 P48431 P29474 7422 P25391 P13569 Q03014 Q63HK5 P20336 2247 2246 Q96S42 Q04656 P05230 Q9UBM7 P49715 Q9UPY3 P09038 Q9UJM3 P10827 4087 3791 O15230 Q14118 Q13705 P35222 1050 2735 P10070 10728 P10071 P08151 P09960 Q92786 6657 Q99814 P55061 1605 2737 2736

RNA catabolic process Q9UL18 Q13315 Q9UPR3 22794 472 10535 54512 P61221 29102 4686 Q8IZD4 O15234 5976 Q8IU60 Q15024 Q9H0D6 P38919 5394 22803 Q09161 26523 23016 Q504Q3 Q96Q15 2237 27257 167227 Q9NPD3 Q9BRP8 Q9HAU5 P60228 10940 64282 P52298 Q14103 Q5TAX3 6059 6050 P13489 26019 3184 Q9Y5S9 84305 23381 55149 5511 P61326 Q15287 Q12972 8761 P08107 9775 Q96C86 P39748 Q01780 O75792 Q9NVV4 Q9HCK5 10921 3304 1763 P40692 Q9H9G7 Q99575 192669 28960 9924 4116 Q92900 2935

T cell differentiation 6670 P10914 P25054 P55347 P04637 P54132 Q6NYC1 Q9H165 6194 Q01201 1499 6304 P42229 7518 P62753 Q9C0K0 5591 P78536 Q15669 23210 Q02447 64919 641 324 P08575 P07766 P06239 6850 P10415 P78527 P18146 P36888 Q04656 P01137 5316 P01730 3932 Q01826 3659 Q13422 7157 P51692 Q13426 5971 P35268 9093 P35222 P43405 Q9HBE1 Q8N4C8 53335 6776 P40933 Q96EY1 10320 P60568 P04233

positive regulation of transcription factor import into nucleus O00206 Q15654 7124 P12830 O43557 O60603 Q04759 P84022 999 P61586 O15455 P01375 7099 7098 Q9NYA1 Q60FE5 4088 Q9NR96 9495 2316 387 7205 54106 P24588 P21333

positive regulation of cell activation 4690 P15923 Q08722 O43557 Q9Y4K3 O43318 Q04759 Q92692 8767 Q9Y275 O14788 6869 P42229 P07996 P42226 P27487 7057 8546 6885 4860 641 P14174 O43290 P08575 P01589 P98172 10666 P10809 O00206 7186 7189 Q92835 P06239 26258 Q9BQ51 3142 148022 O00203 6929 Q9UDY8 P01137 3932 Q14116 Q13422 P78552 P35225 P19320 9093 Q8N5F7 P16333 P43405 O60674 Q8IUC6 6776 P40933 Q96EY1 P04233 P43489 O43353 Q92934 O95999 P54132 P41182 O43639 P10276 79576 5338 Q96FA3 7099 Q14774 7412 Q9Y4H2 3329 57678 1026 961 604 1947 P07766 P00491 6850 57162 572 Q9HCL2 P01579 10673 857 P25103 P01730 P05112 Q15762 5914 P24394 Q9UL45 Q12933 P51692 P06729 P38936 O14939 P37173 10320 9846 P60568

response to estrogen stimulus P01241 7046 5265 P28562 3880 O14793 2033 P51587 O00300 P52952 595 2026 Q7Z6C1 P04085 O14543 P46821 Q15389 P08842 2947 5154 5155 P08727 Q08431 P16234 P01588 P01222 O15524 Q9UBL3 1080 Q7Z727 Q09472 P29474 6774 P13569 890 Q9UBU3 894 P17252 898 5798 P03372 P01137 O14965 4240 P01130 P55212 58189 P35222 P35625 5728 P60484 Q15291 P01009 P24864 P01127 P23771 Q13033 6790 4131 P28482 9021 3162 P10276 672 1499 Q9HC57 5578 3953 Q99966 2626 P40763 7252 Q6IR47 Q14258 P30559 P10600 P30279 P37231 P20248 841 3169 5468 P48357 P09104 P24385 Q16849 P10415 29966 2099 P38398 P21266 Q14790 857 P43694 P01178 5914 P51692 1843 5929 P09601 P04155 7706 P06213

prostate gland development 9420 6256 5268 2099 P19793 O75881 P35453 P10275 1499 3479 Q1PSW9 Q92731 P08069 P03372 P42229 Q9BQA1 Q92826 P35869 P36952 2100 P35222 79084 P13631 Q05086 Q02790 5728 960 367 P60484 P16471 P10070 3169 P46531 4851 P48436 P24821 6776 P16070 P01343 2736

tRNA aminoacylation for protein translation P54577 Q5D0E6 P26640 P54136 Q9NP81 Q12904 O43776 Q9BW92 80222 Q5T160 Q6PI48 51067 P41252 P12081 P26639 57169 P41250 833 P47897 54938 Q9NSE4 Q15046 Q9Y2Z4 5859 23438 A2RTX5 P49591 9255 16 P07814 8565 3035 P14868 Q5JTZ9 P49590 55152 O95363 10667 80755 57038 Q9P2E3 Q9Y285 4677 P49588 P56192

amino acid activation P54577 Q5D0E6 P26640 P54136 Q9NP81 Q12904 O43776 Q9BW92 80222 Q5T160 Q6PI48 51067 P41252 P12081 P26639 57169 P41250 833 P47897 54938 Q9NSE4 Q15046 Q9Y2Z4 5859 23438 A2RTX5 P49591 9255 16 P07814 8565 3035 P14868 Q5JTZ9 P49590 55152 O95363 10667 80755 57038 Q9P2E3 Q9Y285 4677 P49588 P56192

tRNA aminoacylation P54577 Q5D0E6 P26640 P54136 Q9NP81 Q12904 O43776 Q9BW92 80222 Q5T160 Q6PI48 51067 P41252 P12081 P26639 57169 P41250 833 P47897 54938 Q9NSE4 Q15046 Q9Y2Z4 5859 23438 A2RTX5 P49591 9255 16 P07814 8565 3035 P14868 Q5JTZ9 P49590 55152 O95363 10667 80755 57038 Q9P2E3 Q9Y285 4677 P49588 P56192

cellular response to oxidative stress 9588 P49023 P30041 2034 2033 P30044 Q9NX61 3162 P30048 Q00597 10935 P09493 Q99807 P04040 6622 Q7Z6C1 10013 3091 Q9NYA1 5796 P36776 P11413 2119 5829 10229 P39210 847 P01100 P04179 Q09472 P41161 P29590 P32322 9361 6809 Q04656 6647 P00441 5371 P27540 Q13268 Q92626 Q12931 Q06830 P37840 4358 P05164 Q16665 Q99814 10202 P09601 Q9UBN7

electron transport chain O75306 Q7KZN9 4695 O43678 P10599 4728 4729 126328 O14949 27089 Q99807 6622 P22695 4720 55967 4722 1738 4723 6901 4725 4726 Q9UBK2 O95169 O43169 P28331 Q9UI09 Q16635 O14561 P07919 P38117 7295 O75380 29796 Q8NBI2 4697 P08574 P00414 4698 4731 P47985 Q99757 Q16881 O75489 O95298 O95299 1355 P31040 Q16134 P03891 54539 O00217 6389 P99999 O95182 P21912 80777 Q86Y39 4514 P31930 O75746 P56556 25828 2110 6390 Q9P0J0 P19404 2108 8604 Q16795 1537 P14927 O43920 Q16718 10229 P49447 4535 P09622 P04179 7385 7384 7386 P13804 O00483 7381 P17568 Q53TN4 4705 4707 Q9Y6M9 P00403 4701 4702 P56181 O75438 P51970 Q9Y375 P37840 4718 4719 Q9NX14 P49821 79901 4710 4713 4714 4715

regulation of JNK cascade O43353 7124 5585 Q9Y4K4 7161 Q9Y4K3 O43318 Q9Y2C9 121512 Q99683 Q03468 8767 1616 7099 7098 Q9Y239 Q16512 4188 4221 6885 64127 O75376 10746 79444 Q99750 8737 4214 207 4216 O15169 Q9UER7 O00206 7186 7189 10392 6850 Q9H422 Q96M96 Q9P035 Q9BUZ4 Q9Y6Q6 O15379 Q5TCX8 O15455 Q96CA5 64170 P01375 9839 O15350 Q9Y6R4 Q12933 Q13227 8841 Q13546 P31749 O95382 Q9H257 Q9NR96 O00255 P43405 Q9HC29 Q6PID4 54106 Q14289 Q9UL54 Q13233 Q13153

embryonic epithelial tube formation P50552 7189 P61968 P05549 O95999 P07949 7248 2909 Q9NRY4 O75386 P12755 Q9Y4K3 7020 O43318 O14727 Q96S42 P23528 Q96RK4 Q99807 Q9NYQ6 O14641 Q92830 Q14790 Q92574 P49815 1856 9839 P07737 P39905 8323 8543 6885 Q96QB1 7291 Q86YT6 54361 P56705 1072 Q86WV8 57534 10229 841 O60353 7249 Q9UMX1 7408 Q15672

telencephalon development 8650 7248 P78509 1020 23322 7161 P37288 552 P43034 Q92574 Q16555 P04201 Q15303 8100 Q92769 P30559 7531 10460 93986 P12429 O43921 Q68CZ1 Q86WV8 P50542 P42858 P00533 7804 Q13099 P49768 6496 P48431 P56177 P14416 A1XKG3 3066 P12755 Q9BXC9 P51805 Q00535 Q96RK4 Q92830 5830 Q9UJU2 P47895 Q9Y6A5 Q9NSC2 P62258 1956 Q8N960

9839 P10826 O15350 Q14114 9612 Q9Y6R0 O75553 Q9Y618 O95343 9253 9495 Q9NXR1 Q9HCK4 P21359 54820 P10071 1808 P20264 6657 55558 2737 P24588

positive regulation of B cell activation O00206 Q92934 Q92835 6850 P15923 P41182 148022 57162 572 6929 P01579 10673 Q9Y275 P01137 P42229 P05112 P42226 Q96FA3 7099 P78552 P51692 Q9Y4H2 P35225 P38936 P43405 1026 P14174 Q8IUC6 6776 P08575 604 P60568 P04233 P43489

positive regulation of anti-apoptosis Q15797 5562 P28562 4035 4092 3162 P19634 Q8IUD2 1499 P20936 23085 P08069 7832 6548 857 Q15389 P08887 4086 Q15303 Q13546 P38936 23411 P35222 Q07817 1843 1026 Q07954 Q6PID4 8737 O15105 P09601 Q14289 Q13131 Q96EB6

positive regulation of smooth muscle cell proliferation Q7Z727 7189 6198 7422 6772 7124 3162 Q9Y4K3 3516 P42224 P15692 3479 P17252 1012 5578 P21980 P08887 1956 P23443 P08246 P55290 P01375 P05412 5155 P51692 Q15109 3673 7052 Q06330 P17301 P01127 P00533 P09601 P01343

regulation of lymphocyte proliferation O43353 4690 P54132 P15923 P41182 O43639 Q9Y4K3 Q04759 1499 8767 11146 Q9Y275 6869 P42229 Q96FA3 7099 5074 4860 7412 Q9Y4H2 Q96J02 83737 1029 57678 641 1026 P14174 P08575 P01589 604 P42771 1947 P42574 P98172 O00206 P07766 P00491 7189 Q92956 Q92835 6850 Q96IZ0 Q8N726 Q9BQ51 148022 57162 6929 Q9HCL2 Q92990 P01579 10673 P01137 P25103 P05112 11326 Q9BT67 Q14116 P78552 Q9BZS1 P04626 P51692 Q92949 P35225 P19320 P38936 2064 9093 P35222 P16333 P16410 P43405 Q8IUC6 6776 P40933 Q96EY1 50943 P60568 P04233 P43489

response to virus Q9BYX4 P09769 P42224 Q9HC16 57506 Q9Y3Z3 Q9UGM3 Q9UII4 O75531 1072 83737 P13236 1191 3925 P62244 60489 P38484 P15260 Q9Y6Y0 P08575 56829 10542 8815 P80217 O94763 P26641 5683 6772 3661 P48551 P32881 148022 P13693 Q00534 P23528 P61966 64170 P25787 P01375 5970 Q7Z434 Q9H257 1130 3428 81622 Q9NS37 Q99698 Q8IUC6 Q04206 3665 Q9H1C4 P11926 P06733 Q16666 7124 P59665 3442 1021 114548 P19525 3315 P61221 5610 5611 1937 P48643 10577 P51671 7098 Q14258 Q14653 O75575 3452 Q96J02 1667 27297 57678 P61073 29110 Q7Z2W4 3448 10625 58487 3445 3443 Q9BUJ2 9201 Q9UBH6 6210 64135 O75569 P10415 6059 Q13217 Q9UHD2 23586 O14802 3459 Q9HCL2 Q96P20 P01579 55703 P62736 O15455 11128 O95786 O15075 9213 P01570 O43504 2023 1174 P48061 O60238 P01569 P01568 4953 P04792 Q92985 7706

regulation of Rho GTPase activity Q6ZV73 7248 10928 Q96M96 P41182 P42345 Q96P48 121512 Q96N67 55785 P43034 Q92574 P51671 Q7Z6J4 Q92949 Q5JSP0 Q86WV8 23513 Q9P107 Q14160 81565 2475 Q6PID4 604 Q6ZW31 Q9GZM8 Q14289 85440 Q15311 85360 P98174

peptidyl-tyrosine modification P52333 P29597 6772 10152 P49761 P07948 P78509 Q9NYB9 P42224 2147 5159 P06241 P01579 1859 P42229 P42684 7297 Q15303 P29323 4067 Q9HAZ1 P09619 1198 Q8IZP0 57396 1195 P10721 Q13627 P16234 P00519 O60674 P16471 P00734 Q6PID4 6776 P23458 Q14289 P25116 P06213

myeloid leukocyte differentiation 6670 7189 Q16666 Q03014 Q9Y4K3 P35579 Q9Y6Q6 P14780 Q01201 P17275 998 Q14790 P49810 P01137 O14788 P49715 P60953 4627 Q13268 O15111 5971 Q15306 P10721 1050 P37231 3428 Q02447 81501 O75081 Q9H295 1147 P37173 841 863 5468 9846 10202 4318 P49768

regulation of stress fiber assembly 4088 4771 7248 Q96QB1 Q86WV8 P31431 Q4VCS5 P42345 387 Q96P48 Q04759 P84022 P09493 2475 P35240 7249 P61586 Q92574 Q8N3V7 6869 11346 P25103 P49815 O43182

regulation of T cell activation 4690 O96005 Q08722 O43557 Q9Y4K3 O43318 Q04759 8767 Q9Y275 O14788 P42229 P27487 8546 6885 4860 Q15306 83737 641 O43290 P08575 P01589 P42574 P98172 P10809 7186 7189 Q92956 P06239 Q96IZ0 Q8N726 Q9BQ51 3142 O00203 Q9UDY8 6647 P01137 3932 11326 P00441 Q14116 Q13422 P04626 Q92949 P19320 2064 9093 P35222 Q8N5F7 P16333 P43405 6776 P40933 Q96EY1 P04233 O43353 Q92934 O95999 P54132 P41182 O43639 P10276 201633 79576 1499 11146 Q495A1 Q8IWV1 5074 Q14774 7412 Q96J02 3329 1029 57678 961 604 P42771 1947 P07766 P00491 6850 572 Q9HCL2 Q92990 P01579 10673 857 P01730 P05112 5914 P24394 Q9BT67 149041 Q12933 Q9BZS1 P51692 Q5TC82 P06729 P16410 P37173 10320 50943 P60568

negative regulation of Wnt receptor signaling pathway 7088 P25054 Q9Y3M2 O95835 O75581 P52952 P23508 27121 595 27122 Q9UGU5 6789 Q9Y2T1 P49815 6901 Q99966 Q13043 Q9NSA3 Q13322 4188 Q16635 Q9NZC7 26524 54361 324 Q99750 1540 Q9UBT3 7249 Q9UBP4 O15169 P24385 6496 P48431 4035 Q99471 Q9NRM7 51741 857 Q13188 Q9NQC7 25776 Q9UBE8 P56705 O95343 O60716 Q04724 Q07954 P10071 6657 2737

regulation of myeloid cell differentiation Q9Y468 P26583 P27037 P25054 Q9Y5Q3 7124 P63092 1021 P78504 P10276 Q9Y4K3 P15531 1499 91 4005 O14788 P42229 P25963 Q9NSA3 P05412 7099 7098 3091 Q9HAZ2 P62805 P22392 P36896 O43524 O95365 Q05516 324 Q9UKV3 Q96T37 O00206 Q14106 P25791 7189 Q92835 4792 Q02363 Q01196 51341 Q00534 6886 O15455 P05112 P01375 P06400 5914 P41159 Q9BT67 Q13422 P27540 P51692 A4D1W7 63976 P35222 P11474 22985 P17542 861 3148 6776 4830 10320 Q16665 7704 5925

positive regulation of cell cycle 6198 9700 7124 P30086 P15923 P14635 P51587 3481 P10276 23326 351 595 3479 P08243 P42229 O15119 1978 P78536 7013 Q9NYA1 5037 3054 P30279 P55316 P62244 Q00987 P01308 P30281 3169 207 P00533 P05067 P01344 P27797 P01343 2290 P11309 P24385 6210 O75604 P54274 P19474 Q02363 Q9UPT9 4193 891 6929 894 896 811 Q14674 P01135 1956 6926 P23443 P01133 P01375 5716 5914 O15392 O75832 P51692 P31749 P51610 9099 Q92786 6776 P06213

nervous system development P25054 65125 Q12824 259266 P51587 P17676 2547 4729 P37288 Q8IYN9 O60443 284217 P21926 Q9HCJ2 Q92731 Q92730 O14786 Q8TDD5 Q9NZG7 Q12834 Q12837 Q96KN7 P42858 P61158 4734 Q7Z727 136319 Q06124 P17405 4983 811 5830 Q9Y6A5 P40424 P62258 6926 Q9NQX0 3672 2100 O95343 57534 O60674 P20827 O60675 Q99574 Q8N5V2 P26367 O14578 5604 P41743 4771 Q9Y466 1021 1020 P19404 57787 1499 P61586 Q9Y231 P07437 Q9H0H5 Q92769 Q14814 5631 P28370 Q06330 841 O60890 P07686 7804 P04179 Q96L34 Q15915 Q96N67 610 Q9Y250 613 Q92753 Q92752 P05230 4548 Q92997 P10826 O00139 P10827 P31749 Q9NR50 Q13509 Q13506 Q9P202 O60636 Q9HCK4 3226 O95390 Q9UL51 Q9UMX1 Q92982 Q15019 6993 P56199 23322 P50897 P11047 203068 3479 2147 7832 O96019 Q9BRQ0 1072 11113 O43293 Q13099 Q09472 6774 O00562 2173 23787 P11274 Q99743 O14727 894 P53708 P35240 P01019 P49639 84062 Q9UMS4 P51531 A7MD48 Q658W2 P35222 P51532 O75084 P17813 55755 P60484 P24588 O00744 P56159 4131 Q9Y5Q3 4133 10690 Q8NFW5 10458 P61981 P15692 P60891 P55075 79739 Q8N5U6 O00755 10460 P52803 Q05086 Q9UBX0 P37231 10229 Q96SZ6 P51959 5467 4137 207 5468 7408 Q92922 P06753 Q15078 90780 4154 7422 4155 23509 P56178 P56177 9600 3066 22895 O75051 P13010 P61764 9839 9612 O43464 Q09666 3074 O14939 466 8516 P60201 55558 6595 P55268 Q14686 6597 6599 P18085 8775

6598 O75386 O75385 Q9Y4K3 3084 O43318 472 474 Q9C0C7 P07196 Q99807 P49810 P83731 P43034 P49815 P21246 Q13127 Q15303 5274 8543 5037 P58304 P58546 5270 P09382 Q9H2X0 30813 P46531 8536 5029 P00533 Q16643 Q16880 Q9HB63 P61968 Q15797 P12757 P31273 P12755 O75360 P31040 Q96RK4 Q9NRI5 O94805 Q13144 Q14232 Q9BWQ8 Q16650 6383 Q9Y2J0 Q8IWX8 7476 8323 Q9Y6R0 P18074 6389 P10586 P10588 P13611 P10589 59277 93166 P35625 O75592 Q4AC94 54820 P23416 23032 P08034 7248 Q14012 P78337 8100 Q9HAZ2 P46100 Q15109 Q9Y4H2 5071 P11021 7249 1948 Q9GZM8 1947 Q9NRD5 Q99835 9201 8110 O75326 P11229 10152 Q02880 A1XKG3 Q86VS8 Q96EV8 7020 Q8WY64 55704 P21266 P08069 P47895 347733 1956 O15075 O15078 O75553 Q9NXR1 Q92187 Q6PID4 1969 Q14289 Q15375 9463 7046 P98155 O43711 Q9H0M0 55283 3913 56134 Q92574 O60282 3912 P56524 O15496 Q02297 7291 Q3V6T2 3925 P46934 1742 81565 P16070 P98172 8399 9001 7067 P19793 1756 P41231 60412 Q96GM5 P06241 P21860 Q9NRA1 O60260 A4D1W7 Q8IYT8 9253 9495 7074 P20309 Q9NZR4 Q04206 Q92786 P07101 Q92784 Q9NZJ7 O75503 O95999 Q13315 Q15735 P78509 P78504 O60488 O75508 3956 P31249 Q15742 Q9NQZ2 O43921 1789 23092 Q05516 P62166 P00734 3720 375790 P22303 Q9HCU4 P12956 Q8IUQ4 P34741 57045 P78527 O60229 P20336 Q9Y6E2 Q04656 132320 Q15768 63976 Q9UNA1 23077 4830 900 O15228 Q92858 Q8NEY1 Q9NYD6 2672 Q12948 Q9UBB6 P52799 Q9BVA0 3516 P16220 Q9BVA1 Q9ULW3 P52565 Q9UBC3 51090 54361 P50148 P63211 P16234 79885 2676 O60331 4851 4854 928 P54753 P05549 P25391 Q9NRY4 Q05397 P53667 Q9BXC9 6809 27185 29127 4862 Q92830 Q9UJU2 Q9UQB8 P49116 Q92833 P54764 Q9GZX9 P54762 P07737 5970 P56705 Q7L576 P17302 5728 2697 6812 10763 10523 3309 P34925 Q9Y342 Q9Y586 1385 O00468 3796 4646 5978 Q9UM47 O00238 Q96SB3 Q9NYA1 P30559 93986 Q8ND90 P51679 Q8IVL1 1152 Q8IZT6 P10721 960 Q9UM54 Q92888 5747 O43175 2253 Q9NYB9 P30542 6609 57689 2247 2246 O14641 6601 4664 O14640 4665 6605 P51693 O43186 P29323 Q02078 Q6KC79 56121 Q13627 P37173 5764 P29317 7704 Q9UK32 5528 O95278 P04637 O43157 2033 25861 Q01650 P52952 P15531 991 999 P61421 4205 P02686 4209 Q04917 O43166 Q9C0K0 P49770 P48681 Q96CW9 Q9UHG2 10141 6885 Q9UHG0 2043 P50542 1191 O76074 Q15154 P48436 P01588 P01343 Q96NL6 P01100 P49768 2290 P50552 P48431 9921 P14416 3142 773 Q00535 Q00534 Q96S42 Q53X93 55626 7975 6405 6647 P01138 P01137 8829 Q8N960 6660 P04626 P04629 2065 2064 O00213 2063 P54920 P10070 P10071 546 3146 Q96JB5 6657 P07942 O00628 5584 7520 8851 P07949 P20916 Q96PU8 Q9BZE0 552 Q99684 5578 5338 7518 P40763 P04201 P26232 5591 Q6IR47 Q969G3 7532 7531 7533 P18846 324 3169 10100 5108 P27797 5362 6696 5364 6456 6455 P12277 P51843 P12036 333 140735 Q96HC4 4287 O60911 6457 10320 Q96HZ4 8650 8892 Q9UQP3 Q6ZT98 Q8TEW0 8654 P30086 351 Q04759 Q7Z6C1 Q13485 8408 O94910 Q13009 P12429 51412 51654 P60983 Q96SN8 Q15672 6496 7101 6495 6256 6498 P32242 P10451 Q2M1K9 Q9UBU3 P51805 P23528 P09471 P00441 8443 4086 Q14114 4089 P51812 Q9Y618 Q86YT6 Q02790 387 P21359 23154 23396 P08151 1808 P20264 6259 P60763 Q13275 P54259 P54257 4099 P54252 Q8TD84 2909 7368 Q9Y5I4 4093 396 Q02535 Q99962 Q99720 Q99963 Q9NYQ6 Q16555 Q15223 Q99966 P60709 Q16799 P36896 P36894 Q86WV8 1822 338917 Q9NQB0 60 85440 Q15475 Q8TCU4 Q8TEY7 Q00577 P21145 P01579 Q99707 Q9NQC3 P09038 Q14155 10280 7155 P39905 7157 O15198 Q02750 Q13075 84376 Q15256 86 P78424 O75427 P10636 Q15818 26037 Q01094 P63172 7161 1613 Q8TAF8 Q9Y5E8 Q86WK6 Q1PSW9 91 Q6NVY8 1859 P46821 O15119 Q9Y5E9 2947 1856 Q13885 9114 Q96QB1 O43602 10939 7170 Q14738 P55316 P05067 1627 1869 7189 29767 Q03014 Q13418 O43815 7182 6096 56034 P17252 1639 Q9NSC2 P21741 8289 Q13422 O95861 Q13426 9138 29777 4914 2735 1400 56288 Q96FJ2 1406 2737 2736 Q9H488 2742 P09211 Q14774 26005 P11532 Q68CZ1 27339 P30279 27330 64919 P23515 P08138 P09429 O14492 P10415 Q9H228 P12830 P19235 P35711 3611 Q14790 80184 P41159 O15350 Q15648 1203 O60353 O14497 P21554 Q9BZR6

regulation of protein kinase B signaling cascade Q15811 Q15831 7046 6453 6794 P14416 253260 Q13418 3481 P42345 3611 P08069 P00742 P01137 O14788 Q15389 P49815 1956 P07996 Q6IR47 7057 Q14118 Q9Y5J5 5728 2159 Q02763 P01308 P60484 2475 7249 Q9NQB0 P00533 Q6R327 1605 P01344 P06213

membrane docking Q6FGG2 10490 Q15836 Q16623 Q8WVM8 26258 O00186 Q12846 Q9NRW7 6093 9341 P15311 Q9UEU0 60412 O00471 P61006 P61764 Q96AX1 54536 65082 P26038 Q13464 Q9UL45 7430 7412 P51636 P19320 23256 Q8TAG9 6810 4478 10640 6812 P05362 6814 4218

cytoskeleton-dependent intracellular transport 9001 P54257 Q12965 Q9Y6W5 O95239 O15066 P33176 24137 Q9Y4I1 Q8IXI1 P35579 Q8IXI2 351 4628 P35580 P42768 P42704 P07196 P09493 4643 4644 P43034 3799 4646 P45379 4627 Q9UBK9 Q02750 P68032 9371 P12883 5590 Q9NXR1 10128 23095 54820 Q05513 O60333 P42858 P60660 81565 Q9GZM8 Q9UM54 P05067 5604 4637

DNA-dependent DNA replication 4172 4173 8099 4174 Q14566 4176 7884 P15927 5981 P54132 5422 5983 P33992 P33993 P33991 P51587 4171 3159 997 P25205 Q14493 P49736 5111 P35249 O14519 2237 641 P35251 6117 6118 P49643 P27694 P49642 O96020 P11387 Q00059 10714 Q96T60 Q00577 P17096 5557 5558 P40938 P39748 9093 7150 P12004 1763 Q96RR1 Q15054 P09884 Q96EY1 5888 51514 P49427

striated muscle tissue development O96004 P13349 Q12948 2033 3084 P52952 351 4729 2303 79810 Q8WV60 P09493 Q7Z6C1 Q92574 O15119 6901 9474 Q16635 P51636 10939 Q02297 O43847 2280 P05067 Q09472 6495 6256 6498 P12757 P19793 P12755 3142 Q9ULH7 1756 891 1634 Q00535 P21860 Q14993 Q8WZ42 Q9UPY3 6926 4000 P07858 P04626 P68133 2065 O95183 2064 9093 P12883 5728 387 P60484 668 Q92786 P07585 Q96EY1 4637 P62942 9421 Q9Y3M2 7248 1020 P14635 4092 O00468 P19404 P15692 P15173 23363 P61586 P45379 P78337 P50222 Q14774 P11532 P68032 Q86WV8 23414 P60660 Q8WW38 375790 4898 Q99958 Q15475 4154 7422 A1XKG3 P02708 Q9H1Y0 O14640 857 P02545 Q9GZT9 P26678 P10826 25776 54583 Q08209 P58012 P24821 O15105 P25116

mitochondrial membrane organization P07900 Q3ZCQ8 P10415 O00429 P04637 3320 Q9NS69 56993 5830 6622 637 10059 3954 6901 Q9Y512 O95202 Q16611 7157 Q16635 O95140 Q9Y5L4 P62072 P55957 P37840 P34932 P50542 25813 Q07817 P42858 Q9Y5J6 92609 9927 3308

activation of caspase activity Q9NZJ7 P23396 7124 P04637 O00220 29108 O75340 114548 P42224 356 2147 P84022 P55072 10016 7013 Q9Y239 Q96QB1 P37231 3329 1029 Q01955 P00734 5468 P42771 P10809 8795 P06239 10392 6772 Q8N726 P54274 1285 P29590 29843 23787 O14727 Q9BXK5 Q96P20 7415 O14763 P61604 3932 P01375 5371 P51575 6188 4088 7157 Q9P0U3 P48023 P99999 9093 Q96EY1 Q9ULZ3 Q13794 P25116 2935

negative regulation of axonogenesis 6696 4099 4155 P20916 P10451 O75385 Q05397 396 Q8IYN9 P51805 6405 O14786 P61586 Q9NQC3 P02686 8829 8408 P52565 P29323 9138 Q658W2 387 P23515 55558 P08138 Q92888 5747 Q13275 Q9BZR6 P49768

regulation of proteasomal protein catabolic process 7341 8078 6872 10273 4092 4193 Q96RU8 1454 1453 O14908 P63165 7415 P45974 P55072 5716 O75832 P49674 P53350 P31749 Q9UNE7 3326 Q00987 O15105 207 5347 P48730 6613 P61956 P08238 P21675

regulation of proteasomal ubiquitin-dependent protein catabolic process 7341 8078 6872 10273 4092 4193 Q96RU8 1454 1453 O14908 P63165 7415 P45974 P55072 5716 O75832 P49674 P53350 P31749 Q9UNE7 3326 Q00987 O15105 207 5347 P48730 6613 P61956 P08238 P21675

maintenance of protein location in cell O95835 Q9UNN5 O00221 9184 57662 Q9Y4G6 Q13285 P25963 Q9UBC1 4188 Q8NF91 10460 P33947 O43684 O95684 7094 Q9NS56 2317 2316 9908 Q9HD26 Q99750 Q15154 7529 5108 11116 P21333 7067 4792 57120 P29590 Q86VS8 P30101 P15311 10956 Q96RK4 11124 857 Q9UN86 Q9Y6A5 P01730 Q9Y490 2923 5716 5371 P10827 7430 Q60FE5 P31946 O75832 Q14118 25777 Q9UH99 Q9UMX1 11014 84376 O14777 Q15653 1605

B cell differentiation 6670 P10415 P04637 Q13315 P15923 P41182 Q03014 P78527 Q9UQL6 Q9H165 P53567 7163 472 P55327 6929 Q9UDY8 5336 3676 Q8WV28 10014 P05112 5591 P56524 P05556 P78536 Q13422 7157 7412 P11912 Q9GZX7 P04629 P19320 P13612 P16885 4914 Q02447 P16473 53335 57379 604 3688 10320 P43246

regulation of mononuclear cell proliferation O43353 4690 P54132 P15923 P41182 O43639 Q9Y4K3 Q04759 1499 8767 11146 Q9Y275 6869 P42229 Q96FA3 7099 5074 4860 7412 Q9Y4H2 Q96J02 83737 1029 57678 641 1026 P14174 P08575 P01589 604 P42771 1947 P42574 P98172 O00206 P07766 P00491 7189 Q92956 Q92835 6850 Q96IZ0 Q8N726 Q9BQ51 148022 57162 6929 Q9HCL2 Q92990 P01579 10673 P01137 P25103 P05112 11326 Q9BT67 Q14116 P78552 Q9BZS1 P04626 P51692 Q92949 P35225 P19320 P38936 2064 9093 P35222 P16333 P16410 P43405 Q8IUC6 6776 P40933 Q96EY1 50943 P60568 P04233 P43489

anatomical structure homeostasis P25054 6794 7520 5981 P54132 2034 25861 Q9UBB6 Q9Y4K3 154 2547 1499 P00747 P15692 P07550 Q13085 Q99728 Q15185 Q7Z6C3 Q8NFJ9 1736 Q9C0C2 54 P47712 P07954 7099 5591 25913 Q15303 7013 P35968 7014 P09619 P11532 Q6UB99 641 P20849 324 P35251 Q9NPH5 6117 Q15554 O00206 23746 Q9NYB0 Q15831 P27694 7189 P12956 7422 P10415 5321 P54274 P78527 P13686 P56539 1756 6927 P02708 P13010 P49959 P15153 51542 Q96RK4 5159 6647 Q92878 859 31 P09874 P00441 Q9BSI4 4361 3791 3673 5213 1297 Q9NR96 P35222 Q9NUX5 Q9P202 P34931 580 142 582 P20823 P17301 10728 23154 P02458 54106 Q99814 P08237 P04155 P25116 Q9NZN9

production of small RNA involved in gene silencing by RNA 4086 Q9UKV8 4087 Q15797 4088 O75569 P98179 Q5TAX3 79753 51593 P84022 54487 Q9BXP5 Q8WYQ5 Q8TAD8 Q15796 27161 Q9UPY3 5935

cellular response to dsRNA 4086 Q9UKV8 4087 Q15797 4088 O75569 P98179 Q5TAX3 79753 51593 P84022 54487 Q9BXP5 Q8WYQ5 Q8TAD8 Q15796 27161 Q9UPY3 5935

T cell homeostasis 9133 O43521 P10415 Q9BZS1 P51692 P31749 O43557 9093 O95067 6194 6776 207 P01589 Q96EY1 P01137 P42229 50943 P42574 P62753

positive regulation of glucose metabolic process P27540 3091 Q9NP71 Q9BX66 10580 8445 P31749 Q9Y4H2 P35368 P35568 3479 P01308 207 Q92630 Q16665 3667 P01343 Q9UBK2 P06213

dsRNA fragmentation 4086 Q9UKV8 4087 Q15797 4088 O75569 P98179 Q5TAX3 79753 51593 P84022 54487 Q9BXP5 Q8WYQ5 Q8TAD8 Q15796 27161 Q9UPY3 5935

regulation of homeostatic process P10997 11065 P12931 O95271 O00300 P37288 4609 2147 91 6622 Q9BRI3 O14788 6869 P42229 P28335 25913 P78536 3091 5311 25998 P01106 P08575 P01589 P16070 Q15554 7220 808 P25791 Q92835 P06239 P29475 9921 5321 P14416 P10451 Q9Y6Q6 Q00534 P01019 6886 8943 P01137 3932 Q9BSI4 P01375 6262 A4D1W7 Q9NUX5 9495 P17542 O14617 P21359 2730 3148 6776 Q16665 P24588 P26583 P27037 Q9Y5Q3 O95999 7124 1021 4092 552 553 P15692 4005 5733 Q99726 Q13563 P62158 Q8N5U6 P43115 P47712 P30556 3690 7013 7014 P36896 O43524 57678 960 P00734 P48995 6714 7781 Q9NYB0 7780 P05106 P01185 P28223 6696 Q9H2K2 7422 P10415 P54274 O00762 O60502 P48507 Q9HCL2 P01579 857 P25103 P01178 P27540 P51692 P37840 10724 O15105 P25116 P60568 P21554

G1 phase 6872 9238 P30307 1021 P15923 Q01094 4193 990 1017 6929 Q9UJX2 Q00987 Q969Z0 Q99741 995 Q00534 P24941 8328 Q5VTD9 1869 P21675 P06400 5925

positive regulation of hydrolase activity P23396 P04637 10928 26037 P42224 P37288 Q8WTS1 356 2147 P84022 P09493 Q92574 6869 P62873 P49815 11345 10016 O43166 25913 P03950 4221 Q96QB1 P50148 7052 51099 81565 P05062 5029 P00533 Q13133 Q14344 1902 Q15311 85360 P10809 2956 8795 P06239 P29992 6772 Q8N726 O95977 Q9HBW0 P14416 P29590 23787 O14727 Q9BXK5 P41231 P09471 P01019 P21980 P61604 3932 P01375 P32239 4088 3673 P48023 Q92949 P55055 P99999 Q9BZF9 9093 Q9NUX5 P35348 P17301 P21359 O60674 3148 3269 3146 668 P60520 Q96EY1 Q9ULZ3 Q13794 P43246 5604 3309 Q9NSD7 Q9NZJ7 P26583 7124 7248 O00220 29108 O75340 114548 O75460 552 Q04837 Q96P48 553 P55072 P45379 2081 P51671 P30556 P30679 7013 7376 Q9Y239 Q86WV8 5590 P37231 3329 1029 P11021 Q01955 Q05513 P00734 Q15077 7249 5468 Q6ZW31 P42771 Q9GZM8 2915 85440 P09429 P28223 10392 P11229 P54274 1285 O43612 29843 Q96N67 Q96P20 7415 P01579 10672 O14763 P25103 O43182 1956 Q92633 5371 P51575 6188 7157 Q9P0U3 O00255 Q02750 P52701 23513 P17342 Q14160 51231 P58012 Q6PID4 5524 Q15257 Q14289 P25116 2935 P07148

cell development P25054 Q92858 65125 3880 Q12948 Q9UBB6 P51587 3516 Q8IYN9 2303 P16220 284217 P84022 Q92731 Q92730 O14786 P08727 Q12837 124540 54361 P50148 P63211 79885 Q96KN7 2676 P42858 O60331 4851 10661 4734 P54753 Q7Z727 P25391 Q06124 Q7L5Y9 Q05397 Q9BXC9 6809 55342 4983 5830 Q9UJU2 Q9UQB8 P03372 P06401 4627 P54764 Q92826 P54762 3791 3672 P48023 2100 9093 P56705 Q7L576 P17535 P17302 2335 O60674 2697 P61296 6812 5604 P41743 P34925 1385 1020 O00468 10736 57669 1499 P15173 4646 O00238 Q9Y2T7 P10721 Q06210 P19544 Q06330 960 1387 Q9HCM4 2475 O60890 604 4898 Q9UM54 5747 P09622 P04179 137964 Q13618 Q9NYB9 O95376 Q96N67 Q92753 O14640 Q92752 859 P29323 P31749 Q9NR50 Q13509 O00255 Q9P202 1050 Q9HCK4 O95390 P29317 Q9UKT4 7704 Q15019 P56199 2034 25861 P14555 P50897 P11047 P52952 79810 3479 Q8WV60 999 P62753 Q9C0K0 P49770 Q96CW9 4221 P51636 23210 2043 1072 P50542 1191 Q8NHU6 P48436 79923 P01588 P01343 Q96NL6 P49768 2290 P50552 P48431 P14416 23787 891 773 Q00535 Q96S42 Q53X93 P01019 6405 6647 P01138 P49639 8829 84062 Q9UPY3 Q16254 4000 P04626 Q658W2 2065 2064 P35222 O00213 2063 P17813 3148 P10070 P10071 3146 Q96EY1 6657 P26583 P07942 5460 4131 Q6FGG2 5584 4133 8851 P07949 Q9H9S0 Q9NX62 10458 P15692 5578 5338 P26232 5591 O00755 P52803 P68032 P37231 324 3169 207 5468 9702 7408 Q15078 7422 P56178 2099 3182 P61764 140735 9839 O43464 23513 O14939 Q08209 P18827 P60201 55558 P26447 P25116 8650 8892 P55268 Q9UQP3 Q8TEW0 P30086 Q9Y3I0 O75385 Q9Y4K3 3084 351 474 356 P07196 P09493 P83731 5139 P43034 8408 Q9NPC8 P03950 3091 5037 Q9UJC3 30813 P46531 P00533 Q9HB63 6256 P12755 Q9UBU3 P51805 P23528 P09471 Q96RK4 Q13144 Q8WZ42 Q13263 Q14232 P00441 Q16650 6383 4088 7476 Q9Y6R0 Q14119 P18074 Q86XR8 P68133 Q86UL3 P10588 P13611

59277 O75592 P21359 23032 23154 23396 6259 P60763 Q16665 P43246 Q13275 8452 6275 O15287 Q8TD84 P14635 P35579 Q00597 23363 10296 Q99729 Q15223 P45379 Q99966 P60709 Q15109 O43524 P36894 7490 285 Q9NQB0 60 O15169 Q9GZM8 1947 85440 Q99958 Q9NRD5 P02751 Q99835 9201 Q8TCU4 Q8TEY7 10152 Q02880 A1XKG3 Q96EV8 7141 Q9H1Y0 9319 P21266 P08069 10155 P02545 P49715 1956 P23443 O15075 7155 O15078 P39905 Q02750 Q14160 Q6PID4 Q99814 1969 Q14289 Q15375 9463 P28289 6198 9464 O15123 7161 9341 6194 3913 P42345 26271 Q8TAF8 P41134 Q86WK6 Q1PSW9 Q92692 P39060 Q6NVY8 1738 P46821 2947 O60282 3911 3912 P56524 9474 O15496 O43602 10939 Q02297 O43847 P55316 P46934 Q9HD26 81565 Q4LE28 P16070 P05067 P98172 8399 7189 Q15836 Q5TAX3 P19793 57120 Q13418 O43815 P56539 6096 1756 1874 Q92793 P17252 P21860 O60383 Q9NSC2 Q8IYT8 9253 56288 Q96FJ2 Q9NZR4 Q92786 P07101 2737 2736 Q9NZJ7 P54652 O75503 P78509 Q6NYC1 P41182 O60488 P35968 O43921 P11532 P11413 Q07817 Q05516 64919 375790 P08138 P22303 Q9HCU4 P09429 P56851 P10415 Q8IUQ4 P12830 P34741 P78527 O60229 P02708 P20336 3611 Q04656 P09430 Q14432 80184 P41159 O15350 132320 O15230 Q15768 Q9NP98 Q04771 1203 23077 P24821 Q13351 Q9BZR6

gene silencing by RNA Q7KZF4 P52298 Q9UKV8 Q15797 Q9UL18 O75569 P98179 Q5TAX3 P55265 27327 79753 Q9HCJ0 P84022 29102 4686 Q8WYQ5 Q8TAD8 27161 Q9UPY3 5935 4086 Q9HAV4 4087 4088 Q8WWH4 Q09161 4343 Q9UPQ9 23112 26523 Q8NDV7 Q9HCK5 Q9HCE1 51593 Q9H9G7 57510 54487 192669 Q9BXP5 Q15796

regulation of cell activation 4690 O96005 P15923 Q08722 O43557 Q9Y4K3 O43318 Q04759 2147 Q92692 6622 P04085 8767 Q9Y275 O14788 6869 P42229 P07996 P42226 P27487 7057 5154 O15496 5155 8546 6885 4860 Q15306 83737 5270 641 P14174 O43290 P08575 P01589 P42574 P98172 10666 P10809 O00206 8399 7186 7189 Q92956 Q92835 P06239 P29474 Q96IZ0 Q8N726 26258 Q9BQ51 3142 148022 O00203 6929 Q9UDY8 6647 P01137 3932 11326 P00441 Q14116 Q13422 P78552 P04626 A4D1W7 Q92949 P35225 P19320 2064 9093 P35222 Q8N5F7 P16333 P43405 O60674 Q8IUC6 6776 P01127 P40933 Q96EY1 P04233 P43489 O43353 Q92934 O95999 P54132 P41182 O43639 3162 P10276 201633 79576 1499 11146 5338 Q495A1 Q96FA3 Q8IWV1 7099 5074 Q14774 7412 Q9Y4H2 Q96J02 3329 1029 57678 1026 961 P00734 604 P42771 1947 P07766 P00491 6850 P36888 57162 572 Q9HCL2 Q92990 P01579 10673 857 P25103 P01730 P05112 Q15762 5914 P24394 Q9BT67 Q9UL45 149041 Q12933 Q9BZS1 P51692 Q5TC82 P34972 P06729 P38936 P37840 P16410 O14939 P37173 10320 9846 P09601 50943 P60568 P21554

epithelial tube formation P50552 7189 P61968 P05549 O95999 P07949 7248 2909 Q9NRY4 O75386 P12755 Q9Y4K3 7020 O43318 O14727 Q96S42 P23528 Q96RK4 Q99807 Q9NYQ6 O14641 Q92830 Q14790 Q92574 P49815 1856 9839 P07737 P39905 8323 8543 6885 Q96QB1 7291 Q86YT6 54361 P56705 1072 Q86WV8 57534 10229 841 O60353 7249 Q9UMX1 7408 Q15672

regulation of leukocyte activation 4690 O96005 P15923 Q08722 O43557 Q9Y4K3 O43318 Q04759 Q92692 6622 8767 Q9Y275 O14788 6869 P42229 P07996 P42226 P27487 7057 O15496 8546 6885 4860 Q15306 83737 641 P14174 O43290 P08575 P01589 P42574 P98172 10666 P10809 O00206 8399 7186 7189 Q92956 Q92835 P06239 Q96IZ0 Q8N726 26258 Q9BQ51 3142 148022 O00203 6929 Q9UDY8 6647 P01137 3932 11326 P00441 Q14116 Q13422 P78552 P04626 A4D1W7 Q92949 P35225 P19320 2064 9093 P35222 Q8N5F7 P16333 P43405 Q8IUC6 6776 P40933 Q96EY1 P04233 P43489 O43353 Q92934 O95999 P54132 P41182 O43639 3162 P10276 201633 79576 1499 11146 5338 Q495A1 Q96FA3 Q8IWV1 7099 5074 Q14774 7412 Q9Y4H2 Q96J02 3329 1029 57678 1026 961 604 P42771 1947 P07766

P00491 6850 P36888 57162 572 Q9HCL2 Q92990 P01579 10673 857 P25103 P01730 P05112 Q15762 5914 P24394 Q9BT67 Q9UL45 149041 Q12933 Q9BZS1 P51692 Q5TC82 P34972 P06729 P38936 P37840 P16410 O14939 P37173 10320 9846 P09601 50943 P60568 P21554

transport O75947 Q5SQT9 Q5SR56 81855 27089 Q92973 10651 Q9Y275 Q9UGM1 Q92974 Q9H4L5 Q9UGM3 Q9Y277 O14561 Q8IWJ2 2316 1468 P42858 27091 P49006 O15400 5825 Q9Y282 Q8WXA8 Q12846 P61163 81876 55584 P62491 27067 Q9UKW4 4983 5830 10632 Q12851 P26374 Q9H936 O15431 Q13705 P21917 P21912 Q96N16 55110 2332 10640 O14579 27072 O15427 Q9UKX7 O75915 O75901 Q92538 5861 Q9UGH3 5862 1020 114548 P51787 P37088 1017 2348 1499 P61106 Q9UL26 Q9H0H5 Q9UL25 Q8WXF0 5870 5871 P33947 Q15907 Q9UGI9 Q15904 P51797 P51798 Q8TBC3 P20645 Q8NA29 Q96MV8 O60895 P20648 3685 6711 O60890 604 5865 4535 P05023 Q6NUT3 5868 5869 P05026 P53985 P19634 P40222 401505 610 Q5T2W1 Q6NUQ1 5877 6726 5878 Q8TBB6 P53992 Q9UL45 P31749 O95140 55153 27032 84932 253980 Q9UL51 Q96ED9 Q92504 Q9UL54 Q9H4M9 Q8TBA6 O95159 O96008 Q9UKL6 P50416 Q8NE01 P50897 Q99523 O14949 8924 Q15021 55967 637 6748 79939 6747 Q9BTU6 O96018 4580 Q15027 O95169 Q9Y678 P57057 P36575 O95166 O75096 Q99996 51133 55973 Q14185 P47224 Q15036 P49407 P02794 1080 O00327 Q9Y5W9 P48553 3265 3263 10239 Q5VIR6 114571 O95180 Q9Y5X1 8943 P48544 Q9Y5X2 55503 O14964 Q9UP83 84062 Q9Y5X9 Q15049 O14980 Q9Y696 O95182 O95183 P35222 9804 4116 P24588 P01042 6311 P05408 245972 O95197 Q99567 P61981 O14908 Q96A72 9818 57706 Q92930 P05412 O00755 23539 5473 5476 23530 Q08357 10228 Q9UBX3 P60660 Q15077 5467 P49447 207 5468 Q15075 P62841 10226 Q9Y5S1 P30825 Q12809 Q9Y5S9 Q8NHX9 Q96QU8 O14925 215 10673 P56181 P56180 6342 6341 Q9UKK6 O14939 56882 Q96QT4 6337 P40616 Q92900 10204 326624 Q14204 Q16864 O75146 Q9UPN3 23048 P35658 Q9H0U4 117584 P07196 P49810 Q9BQG0 P49815 Q9NPC8 Q9HAV7 Q9HAV4 P04920 8546 79083 93380 Q9H0T7 Q9NY59 Q96RL7 8536 Q9H9E3 11079 9868 Q9HAU5 Q9BUN8 Q16881 7220 9883 P56134 P32121 23025 Q9Y6Q5 Q96IZ7 51542 Q96RK4 8548 P02741 P61966 8563 6386 Q9Y2J0 129531 P62826 Q9UPQ3 6389 P62829 Q86XR8 Q9UQ26 23039 Q9NXV2 Q9NY72 Q8N4C7 P61970 23032 51552 Q5SRE5 P08034 Q03518 O95907 P62820 P56556 P78348 11021 7248 P36542 6390 O75581 274 51560 P02766 P78334 11031 P05814 O75575 5071 7251 Q93050 152789 9429 7249 P48995 Q86Y82 P02790 Q9BV36 O15066 23463 A1XKG3 291 292 Q96EV8 Q14C86 Q9Y6M9 P24539 Q9NY26 Q9H9H4 P02786 Q6I9Y2 2802 O15075 Q9NPA8 O15078 O75558 26985 Q8N4H5 P37840 Q9H173 Q9NXR1 P49821 79901 11014 10166 P02775 P02774 P25874 9465 9463 P53007 Q9Y6G9 Q92572 55048 P04083 Q9UGU5 Q92574 Q13769 O15496 55054 9472 7295 117177 83985 O15020 9001 P06239 Q9Y6I3 60412 P06241 50618 Q8WXH2 A4D1W7 9495 79023 Q9Y2B5 3708 Q07954 Q9UH92 O15027 O95994 Q14643 Q96NA2 Q9NP61 8170 P35610 P35612 P00505 23085 O60488 Q9H9B4 Q9Y6C9 1537 Q8IWV1 P35606 Q8NEC5 P56962 Q9NP72 O43920 Q96IW7 83547 Q9Y6D6 83548 23095 Q9Y6D5 O60499 O60493 5901 Q6P5W5 5902 O75969 O75964 P13804 P45880 P33897 3737 P02708 2885 Q9HAP6 5914 131474 Q9NP90 Q14677 A4D1S5 63971 11097 Q9NP97 222068 79065 Q9H0N0 P24941 P43681 51510 Q96RF0 51517 23603 Q9NRW1 Q9NRW7 Q99490 Q9P2R6 154091 O14662 10772 P27348 Q9UBC2 Q6PIS1 55696 92609 Q9NWB1 Q9UBD6 3783 1121 339122 Q9BXC9 Q9GZY0 4628 57403 10758 6809 P18859 Q86WA8 O15533 Q92834 4627 P53675 91782 Q92824 P55851 Q9H814 4641 1130 P53677 57410 1122 3784 6810 6812 6811 6814 10762 4637 P04233 P07307 Q9Y584 P50570 O95239 O00461 3320 Q8IVM8 Q9NRS6 10736 1137 3313 O00471 3312 Q4VX76 4643 4644 4645 5976 3799 4646 P30559 O95249 Q9Y597 P53618 P37198 27173 P05141 Q92887 P53621 O95259 P28223 6850 O00483 2495 Q12769 P30542 P17568 81555 80223 O60502 Q9NS69 Q8TEH3 O14640 5514 738 P09958 P30536 1176 Q05586 1174 1173 O00499 10725 10724 55275 Q8IVI9 Q9BXF6 Q99497 4678 O15504 P02452 O95279 P04637 4695

1186 P16615 1185 O95271 Q01650 1181 P62993 4686 P01116 Q9C0K7 6869 Q15388 P01112 P01111 Q96HA1 Q9C0K1 O43169 P48201 23210 Q6P4A7 Q9HDC5 23214 P50542 1192 1191 Q96Q15 9908 4697 4698 11231 768 Q96CW1 4218 6890 6892 Q86VW1 P14416 P13569 P12236 2054 P33176 O95298 O95299 Q96CV9 3382 P12235 9919 773 Q02952 P61204 Q9GZP9 P01137 Q7Z3C6 4240 P01130 Q8N2H4 23673 P35348 P54920 781 Q9NRR8 O14617 301 302 Q7Z3B4 309 9927 25828 O43592 5584 Q5U0I6 64116 22794 P15328 Q96PU8 Q2M389 Q96PU5 8604 5579 Q9H3F6 Q9Y4P1 9950 5111 P37108 Q9BSW2 5590 Q99437 P16671 8615 P46059 P27797 7781 64130 9961 7780 P01185 6453 6456 6455 O00400 P50993 Q9NW64 Q9NVU7 Q96PS8 333 56993 P83436 P25103 140735 5119 A5D8V6 P01178 23636 9972 Q9UNQ0 4285 Q5T1M5 64145 9973 O00410 23633 P15313 Q6ZWJ1 Q08209 6457 Q99418 P25116 Q8N6T3 8655 Q8TEW0 O43678 351 Q7L804 23163 P09493 23165 P23786 P62913 Q96QK1 P25963 O43687 9997 11196 19 367 20 22 Q15436 6496 Q6Y2X3 8677 9525 9522 8676 Q15438 23144 P57103 Q9UBU3 P51808 375 9515 P23763 P61619 P32239 5174 Q14114 Q8WWN8 Q9Y619 381 382 P59780 388 9527 41 11140 9546 O43633 Q9H089 Q99962 Q99963 Q9H082 Q13283 Q16795 2904 Q16790 2902 Q13286 5195 9554 Q09161 O75694 Q86WV8 9550 Q9Y5J6 Q8NI27 51693 Q9NWN3 8218 51699 Q6PJH3 P46459 7385 7384 Q8TEY7 7386 7381 O43615 Q53TN4 54732 Q6VY07 P08183 Q9UBQ0 Q9UBQ7 P01579 9559 P09038 2923 Q9BY84 10280 Q9BXW6 P14867 O75674 Q9Y5L4 Q8TEX9 Q9NX14 Q00325 P08195 84134 Q14964 Q15811 Q13637 Q96QC0 Q13636 Q15818 Q9H444 84958 79139 O15119 O15118 Q9UBK9 Q14974 9114 O15131 9590 Q9H7Z7 P43307 29796 P52292 O15126 P08574 P08575 P05067 P52298 Q15836 P52294 Q8TEM1 26258 Q03014 9120 P55327 80145 Q9UG63 84502 P21980 Q9NSC5 9135 P11717 O15155 Q15849 64901 Q9UBN7 P30049 9146 Q9UBF2 Q6NYC1 P41182 P41181 3836 Q6ZSS7 O15554 51606 Q16718 Q07817 P21579 Q96QF0 3841 P08133 3843 Q9BY11 P10415 93492 387680 4705 4707 4701 4702 80184 9179 P33527 51629 Q96QD8 P62072 Q96QD9 23192 Q8TAG9 4718 4719 Q8TF09 4710 4713 4714 1207 4715 65125 92745 25909 9182 22872 9181 Q96KP1 O00148 P15498 4728 4729 1213 P84022 79778 Q9UMY4 10890 4720 P60059 3875 4722 Q8TDD5 4723 4725 4726 P28335 64284 P28331 2562 P55957 9197 P38117 O00159 10427 2554 30011 57510 55334 4731 Q9H2G9 Q8N695 4734 253725 Q5T0N5 Q96P70 6927 2569 Q8IUD2 55341 O60664 30000 2564 811 Q9UJ41 P63104 P62258 6926 Q9C005 P31946 P99999 56681 O95343 Q676U5 81622 P20823 Q8NFW9 284427 80777 O60674 P18507 1244 821 5604 P41743 91452 Q9H2M9 2110 Q8NBW4 55361 O95352 P39019 P16389 P19404 2108 O60645 6945 P32856 Q9NR31 22841 27297 10868 7804 4792 Q8IUH5 Q9HCL2 1277 124565 857 Q9UN86 Q8N1S5 Q13501 P51572 P51575 Q13505 92799 P51571 Q9NQT8 Q8NBS9 O00141 P09601 P08754 P06576 Q86UW7 Q9Y3Q3 P01241 6993 10490 Q86V81 6510 3482 23325 Q9UIH9 O60603 23327 2147 Q96KG7 10013 Q96KG9 10015 6505 11345 135295 23339 6520 6522 Q9NV70 25998 400 Q8NBI2 402 55737 406 6517 408 409 Q99757 Q01518 54407 Q15276 P60468 23307 Q9UMR2 116986 O00560 116987 O00562 23303 23787 23788 P13693 79711 Q15286 Q16134 Q9UN42 10476 P03891 10478 P61326 O43264 4363 6541 10482 Q8N1F7 25978 Q01970 Q96T51 10487 11336 P31930 O00505 Q6FGG2 7884 Q9UID3 23760 Q641Q2 23762 O43237 P14598 Q8IYJ3 433 A8K0Z3 55763 10451 6548 7879 10452 8724 P55072 Q05084 6566 Q9HCE7 Q05086 Q8WUX9 P37231 10469 P36382 Q99797 79720 55770 Q8IYI6 10466 Q6IQ22 11315 P01266 P24386 6575 9601 6576 9600 Q8WUX1 Q8WV92 65108 22895 Q96P20 Q8WV83 Q00610 7415 P61764 7417 7416 7419 Q8N5S1 5250 P51970 8766 6103 P40855 7442 Q16623 P18085 8775 P10599 24137 O75385 473 476 477 478 51762 Q99805 P43034 Q15785 P38919 8301 9632 P13637 64601 23299 481 Q9UJC3 483 O75380 O75381 488 Q05952 Q13133 Q15796 Q15311 O00602 Q9UNL2 Q8WVM8 23265 490 Q96H78 495 498 Q01105 P31040 P56385 P56381 7476 O75351 Q86VN1 Q9NZZ3 Q9H2V7 Q8N6H7 144983 54820 P23416 54822 9648 P43487 Q49AN0 Q9P2E9 51319 P43005 149371 O75348 11261 O43752 Q14008 P63092 119559

P13667 Q9UEU0 84248 O94826 Q14493 P27824 11269 Q14012 9673 23256 Q96D46 Q7Z7H5 Q9BX79 Q9GZM8 Q9NRD5 9201 9685 Q86W42 Q3ZCQ8 Q7Z7G2 P12314 O43734 Q86VS8 O43739 23225 Q9Y4I1 Q9UNH7 Q15363 Q9C0E2 P02545 55705 7037 Q9BX66 P21283 P21281 P55011 P10147 O43747 P48739 Q13190 Q99816 P98194 P28288 O75306 P98155 Q14847 O95714 55283 83752 27243 P41250 O15234 65082 P98161 P07355 O95721 P54105 7057 Q13520 9230 P78537 Q8IUN9 7052 81567 P46934 3927 Q9HD26 81565 O60296 80230 8399 P29590 P30101 Q4LE60 29886 P16284 Q9NZQ3 O15258 O15259 3932 O60260 Q32P51 27236 Q9UNE2 112936 P41222 653361 P43405 O43707 9267 Q9NZJ7 Q13555 O75746 Q13554 1781 Q9P253 O00186 O00189 1778 O00194 Q14894 Q13563 P08246 9276 P14927 Q14416 Q9H2T7 1785 Q05513 P63010 P00734 Q7Z2W7 Q15751 80273 53916 100287932 O60229 2647 1315 P20339 1314 P20336 P20337 1793 Q04656 84661 Q9UF02 Q9NZM3 Q9BWM7 1317 Q13586 Q16611 Q6XPS3 113829 9296 Q9H6Z4 Q96H20 Q63HQ0 4830 P20340 Q13596 P48051 Q9Y2W7 P48058 P61020 Q9BVA0 2665 2664 57620 Q9Y2X3 90678 P61026 P07996 10533 219931 P48047 Q9UI09 4860 51090 Q08431 O60333 Q8N4V2 Q8N4V1 200081 Q9UI12 P50395 Q12965 10514 283229 29127 4867 4868 4869 Q9H1D0 1374 O00299 Q13829 O60318 P17302 Q96JJ3 Q99698 2697 Q9BZF3 948 Q96JJ6 Q9H1C4 Q12981 O95477 Q9Y342 P28482 O00221 Q9H1K0 Q9P0J0 58513 O60763 P07550 Q9Y2T2 10972 10971 Q5T8D3 O95487 57678 P54709 10981 7922 Q9UM54 P31639 O00244 O60749 22931 P62330 10953 P42768 2247 26056 58533 10959 Q8N4Y2 P61006 Q9H1H9 P51693 Q8NEZ2 Q969M3 P28472 Q9P0M2 Q9BZJ4 P17342 Q92621 Q9ULV0 Q969M1 10960 Q92624 Q9ULV4 P61019 509 P61018 P07900 Q9UPV9 Q9Y6W5 P80188 P11166 P38646 O60725 P15531 O14828 51000 Q96RU3 513 514 128866 6622 515 10134 10133 P61421 518 51009 Q04917 5310 6643 5311 6642 P51636 Q6WKZ4 P07919 521 522 523 10142 526 528 5306 10146 P49768 Q86U42 6653 Q3KNW5 23423 O00203 Q00535 533 535 P49755 537 51026 54536 P49754 54535 6646 539 51028 91949 54539 P01375 Q96JC1 O00217 23439 Q9Y2R5 4000 5331 Q96EY5 P12004 10128 O95070 10121 O00623 O00628 P07947 Q9UPR5 Q9UPR3 O00629 Q96B97 Q9Y2K6 Q9Y2K9 23400 Q12907 3162 Q8IXI1 Q8IXI2 Q68EM7 P42704 Q8WTW3 7514 Q96AX2 Q96AX1 5338 P05771 10577 7532 7531 7534 10580 7533 Q9UHC3 3178 P68032 O00631 9702 P24046 P05783 7529 3181 4035 P49321 Q9UPT5 Q99666 51068 Q8N8Y2 Q8N4Q1 P49792 5371 Q9BQQ3 P49790 Q60FE5 8402 O75396 Q969E2 Q8NER1 Q99653 P52948 P62745 4038 8878 10564 57617 10566 9744 8411 O75027 57187 94121 Q96FV9 Q96G79 94120 Q9NUM3 P22695 Q9BRI3 Q8WUM4 Q8WUM0 8408 Q8TD31 P12429 O75494 29959 P35523 Q9H270 Q9UIA9 415117 8417 6238 Q13492 P47985 4074 Q8TD20 29928 O75489 Q9H269 29927 29924 23381 P25705 Q9UDY8 84342 Q8NFH4 Q13023 51429 6262 4087 4088 9775 29934 Q8TD16 Q02790 P12883 P31150 Q9NUP9 1808 60386 10087 P20020 P60520 Q16543 Q8IXZ2 9784 P54257 O75469 7124 10053 P54253 7126 Q9Y3E0 P38606 P10275 Q4G0F5 P10276 P35579 154 P35580 Q99720 10057 55800 Q16555 Q99726 P45379 9311 9793 Q96C24 10061 O94955 29916 161 P11142 Q04941 P01308 P01303 9322 7142 P00491 Q401N2 Q9UQN3 P36404 6293 Q99700 Q9NZ43 55823 O94973 O75438 P54289 O75436 7157 P14625 Q02750 O75431 Q8WZ73 Q9NUL3 O94979 84376 P53367 Q14721 8498 9342 P57729 O95833 10928 Q06787 P63172 7161 P54219 9341 7163 2702 P63167 P23945 10938 Q7RTN6 83871 P09651 Q9H1N7 Q13409 Q01082 8021 P57735 P22626 10947 Q969S0 9367 P10619 O75886 57120 Q9H1M0 P57740 O75880 26019 7184 Q9UHY1 O43819 P00846 P68871 Q92673 Q969R2 Q13423 O15397 Q8IY33 Q6UWE0 9371 P21757 Q96K37 56288 Q96FJ2 1408 9388 Q9HC62 9380 Q86YS6 Q86UK0 P51148 9382 P51149 4927 2742 2741 84766 P42262 Q6UWJ1 P51151 Q8WZ19 O95405 P51153 P42261 57154 Q15629 P51159 O43808 27333 Q8WYP5 Q9BVL2 Q8WYP3 P21333 O75843 59341 59343 Q9H223 P84085 P00403 Q15642 Q9BVK6 Q9HBR0 O15350 Q00169 1447 P84077 2773 P21796 Q9HC21

leukocyte cell-cell adhesion P05107 6850 7124 6093 83706 P15311 O60547 2762 51148 3676 3678 P26038 Q13464 Q5T4B2 P01375 P08648 P05556 Q86UX7 7430 7412 P19320 P11215 P13612 P43405 4478 P05362 3688 3689 Q96HB1

regulation of RNA stability Q14103 7422 P67809 10492 1660 P11940 Q15717 O60506 3184 9261 1994 P15692 Q92994 P49137 Q00839 P20333 Q14011 Q13148 3192 Q9Y2T7 7133 P39905 26986 1153 Q8NC51 23435 Q08211 2972 10642

response to cytokine stimulus O43353 Q14249 6672 5265 5584 O14672 P12931 P39656 9021 Q9Y4K3 P42224 1499 P17275 O60488 1650 3476 6622 5578 8767 O14543 P08887 Q99966 54 P42226 P40763 P05412 P56524 4261 Q9NYA1 Q13443 P30559 Q07820 Q08117 P30793 Q07817 27252 166 P16234 P33076 841 P05062 8737 5029 P01588 P42574 6714 O15524 P01100 1080 Q13137 Q7Z727 7189 8754 P29474 P10415 6772 6774 P13569 P29590 Q9Y2M5 P13686 Q9Y6Q6 P41231 2643 P17252 P09471 898 Q14790 5371 P02461 5054 P29320 P55212 P07858 5970 P51692 Q13546 9093 P35222 P37840 P78318 P51617 Q04206 P01009 P24864 10241 Q96EY1 P05121 P23497 P41743 P06213

mitochondrial electron transport, NADH to ubiquinone O75306 P56556 4695 O43678 P19404 4728 4729 4720 Q16795 4722 1738 4723 4725 4726 O95169 P28331 O14561 O43920 Q16718 O75380 4697 4698 4731 4535 P09622 O75489 O00483 O95298 O95299 P17568 4705 4707 Q9Y6M9 4701 4702 P03891 P56181 O00217 O75438 P51970 Q9Y375 O95182 4718 4719 P49821 4710 4713 4714 4715

regulation of lymphocyte activation 4690 O96005 P15923 Q08722 O43557 Q9Y4K3 O43318 Q04759 8767 Q9Y275 O14788 6869 P42229 P42226 P27487 8546 6885 4860 Q15306 83737 641 P14174 O43290 P08575 P01589 P42574 P98172 P10809 O00206 7186 7189 Q92956 Q92835 P06239 Q96IZ0 Q8N726 26258 Q9BQ51 3142 148022 O00203 6929 Q9UDY8 6647 P01137 3932 11326 P00441 Q14116 Q13422 P78552 P04626 A4D1W7 Q92949 P35225 P19320 2064 9093 P35222 Q8N5F7 P16333 P43405 Q8IUC6 6776 P40933 Q96EY1 P04233 P43489 O43353 Q92934 O95999 P54132 P41182 O43639 P10276 201633 79576 1499 11146 Q495A1 Q96FA3 Q8IWV1 7099 5074 Q14774 7412 Q9Y4H2 Q96J02 3329 1029 57678 1026 961 604 P42771 1947 P07766 P00491 6850 P36888 57162 572 Q9HCL2 Q92990 P01579 10673 857 P25103 P01730 P05112 5914 P24394 Q9BT67 Q9UL45 149041 Q12933 Q9BZS1 P51692 Q5TC82 P06729 P38936 P16410 P37173 10320 50943 P60568

nucleotide-excision repair P15927 5981 P04637 P54252 5983 1022 P50613 P51946 P78549 P51587 Q00597 Q03468 P23025 4968 Q8IY92 5111 P35244 P35249 Q96T76 Q01831 Q6PJP8 P35251 6117 5424 6119 6118 Q969S2 Q9H2G2 P27694 Q13216 P28340 1161 10714 P18858 Q96T60 Q16531 3978 4287 P40938 7157 P40937 P18074 P54727 P54725 P12004 1642 Q15054 5886 Q9HCS7 5887 902 4913 7508

regulation of B cell activation O00206 Q92934 Q92835 6850 Q96IZ0 Q8N726 P15923 P41182 P36888 148022 57162 572 6929 P01579 10673 Q9Y275 P01137 P42229 P05112 P42226 Q96FA3 Q9BT67 7099 5074 P78552 P51692 A4D1W7 Q92949 Q9Y4H2 P35225 P38936 1029 P43405 1026 P14174 Q8IUC6 6776 P08575 604 P42771 P42574 P60568 P04233 P43489

positive regulation of protein amino acid phosphorylation P01241 Q13873 7046 O95835 P14317 2033 P42345 595 3479 2147 1453 91 Q7Z6C1 Q15389 Q13485 1856 P04004 P78536 4067 P14174 7448 P01588 P16070 P01343 P49768 O00206 Q7Z727 Q09472 Q92956 3263 891 894 896 P17252 P01019 P01137 P01375 4361 4089 9495 P43405 P17813 O60674 P04233 P24588 P27037 Q8TDY2 9821 7124

P07948 P14635 Q93062 P15692 5578 3678 P08887 7099 P08648 3690 Q6IR47 Q9NYA1 11030 P36896 64127 P35443 P36894 P10600 P30279 960 P01308 P00734 P30281 2475 O15169 85440 P07766 P24385 P05106 P02790 Q9UGJ0 6850 7422 P10415 P49959 2247 Q96N67 2246 P60033 975 O14641 P01579 P05230 857 P01730 Q92878 P05112 P09038 Q92997 7272 Q9HC29 P48730 P60568 P06213

regulation of leukocyte proliferation O43353 4690 P54132 P15923 P41182 O43639 Q9Y4K3 Q04759 1499 8767 11146 Q9Y275 6869 P42229 Q96FA3 7099 5074 4860 7412 Q9Y4H2 Q96J02 83737 1029 57678 641 1026 P14174 P08575 P01589 604 P42771 1947 P42574 P98172 O00206 P07766 P00491 7189 Q92956 Q92835 6850 Q96IZ0 Q8N726 Q9BQ51 148022 57162 6929 Q9HCL2 Q92990 P01579 10673 P01137 P25103 P05112 11326 Q9BT67 Q14116 P78552 Q9BZS1 P04626 P51692 Q92949 P35225 P19320 P38936 2064 9093 P35222 P16333 P16410 P43405 Q8IUC6 6776 P40933 Q96EY1 50943 P60568 P04233 P43489

positive regulation of cell development 8650 4131 7520 O43157 P10636 2033 396 2547 57669 Q86WK6 P84022 P07196 Q7Z6C1 P61586 P46821 7518 Q13485 P52565 Q13009 P10600 P10721 27339 5270 P55316 P37231 3207 Q9HCM4 81565 P46531 4851 4137 5468 P61158 Q9GZM8 O43294 Q15796 P08138 Q92888 Q99835 2290 Q09472 Q15797 P48431 P12956 6498 5364 P10415 P12757 P14416 Q13418 P53667 27185 P13010 3611 Q9NRI5 P01138 P01137 Q9NQC3 Q13145 Q9UMS4 4086 4087 P29323 4088 4089 5970 Q9Y6R0 Q13426 9138 P10586 9253 9495 7074 Q9HCK4 387 P31270 Q04206 6657 P24588

branching morphogenesis of a tube O43597 P10275 4609 2303 1499 P15692 3479 Q1PSW9 284217 O14786 O15119 Q13485 Q99966 3911 P51671 P35968 Q5HYA8 54361 P19544 7490 3207 960 367 3169 P46531 4851 P01106 P16070 Q14344 Q99958 Q15475 P01343 Q96T37 6495 P48431 7422 P10415 P25391 2099 P29590 Q03014 Q13418 Q99623 2247 3611 8945 P01019 10672 Q9Y297 P03372 P01137 Q9NSC2 8829 P40424 Q9UPY3 P09038 6926 P06401 P01133 5371 3791 4089 91147 Q15648 O15230 Q14118 Q04771 P35222 P56705 P17813 P31270 P37173 P10070 P10071 11331 6657 1605 2737 2736

production of miRNAs involved in gene silencing by miRNA 4086 Q9UKV8 4087 Q15797 4088 P98179 Q5TAX3 79753 51593 P84022 54487 Q9BXP5 Q8WYQ5 Q8TAD8 Q15796 27161 Q9UPY3 5935

branching involved in mammary gland duct morphogenesis 5371 P51671 Q15648 2099 P29590 54361 P56705 P10275 Q99623 367 8945 11331 Q9Y297 P03372 P01137 O15119 6926 P06401

negative regulation of protein binding 5580 P32121 Q08117 154 2932 166 57669 Q9BZE4 Q05655 23560 Q9HCM4 Q99497 P07550 857 P49841 409 11315 Q9NSA3

regulation of immune system process P25054 Q13477 O14672 4179 P15923 O43557 Q9Y4K3 O43318 5817 Q8WZ60 Q04759 P84022 P49810 8767 Q9Y275 O14788 57506 P07996 Q9Y3Z3 P25963 P04003 P27487 3091 7334 8546 P62805 4860 Q15306 2 P20963 60489 P14174 P27918 Q9UKV3 Q9UI12 10666 P10809 Q9HCY8 Q14106 Q92956 Q92835 Q96IZ0 P32121 Q9BQ51 57402 Q00013 6929 Q9UDY8 P02741 64170 P00441 P06400 Q14116 5054 4088 5970 3673 Q92949 Q9H257 Q9NR96 9093 22985 Q8N5F7 P16333 81622 P17301 P17542 Q8IUC6 Q16665 Q03518 P04233 P43489 P62942 Q9H1C4 Q9Y468 P27037 7124 P28482 P54132 P63092 1021 O43639 P10276 201633 Q9Y2C9 79576 1499 10616 Q9Y6K9 710 51324 11146 26191 P08887 719 5074 Q9HAZ2 P22392 Q9Y4H2 P36896 O43524 P35443 Q96J02 161 O95365 3329 1029 57678 29110 960 1026 961 P01308 720 722 604 P42771 1947 Q96T37 9564 P07766 P00491 P02790 6850 4792 Q9NZD8 23586 P36888 P30542 Q9HCL2 Q92990 51341 P15151 P01579

55703 857 P04264 P05112 O94973 Q9UL45 149041 P51692 Q5TC82 P11912 1173 P38936 P37840 P11474 P48061 861 P37173 P05362 P05121 P09601 50943 Q16581 7704 4690 O96005 P15529 Q08722 2150 O60603 P15531 P0C0L4 Q9HC16 Q92692 91 6622 6869 P42229 P42226 Q9NSA3 P78536 7057 O15496 6885 83737 1191 2280 641 2277 O43290 P08575 P01589 Q96CW1 P16070 P42574 P98172 409 P49768 O00206 6890 8399 P25791 7186 7189 P06239 Q8N726 4354 26258 P29590 3142 3263 148022 O00203 Q01196 Q9BYM8 Q00534 P06241 6886 6647 P01137 Q9NZ08 P05155 3932 11326 P01375 P02461 Q13422 5692 P78552 Q9Y2R2 P04626 A4D1W7 P35225 55072 Q7Z434 P19320 2064 P35222 P43405 3148 Q04206 6776 P40933 Q96EY1 O43353 P26583 Q96EP0 Q9Y5Q3 Q92934 O95999 P41182 O43915 8174 P78504 3162 P15692 4005 5336 5579 5338 P05771 P08246 Q495A1 Q96FA3 P05412 Q8IWV1 7099 7098 51606 Q14774 7412 64127 Q05516 324 P63010 P55085 P01023 P23510 O14492 7422 Q02363 Q9UHD2 57162 572 P61088 10673 O15455 P25103 P01730 Q15762 5914 P41159 P24394 Q9BT67 5371 O95786 P27540 Q12933 Q9BZS1 P34972 P06729 63976 3075 P28070 P16885 P16410 O14939 P51617 Q9HC29 8517 4830 51752 10320 54106 9846 P60568 P21554 5925

protein localization in mitochondrion 100287932 O96008 O43615 Q9NS69 10953 401505 O14925 56993 O94826 10651 637 10452 P63104 Q15785 Q15388 Q9HAV7 4580 4285 P30536 Q13505 7534 O95140 Q9Y5L4 O75431 Q8N4H5 P62072 P55957 10469 Q99797 Q9Y5J6 9804 9868 80273 9927

protein targeting to mitochondrion 100287932 O96008 O43615 Q9NS69 10953 401505 O14925 56993 O94826 10651 637 10452 P63104 Q15785 Q15388 Q9HAV7 4580 4285 P30536 Q13505 7534 O95140 Q9Y5L4 O75431 Q8N4H5 P62072 P55957 10469 Q99797 Q9Y5J6 9804 9868 80273 9927

immune response-regulating signaling pathway O43353 Q96EP0 O95999 P28482 Q9Y4K3 O60603 O43318 Q8WZ60 Q9Y2C9 10616 Q9Y6K9 51324 P49810 5336 8767 5579 P05771 P25963 719 Q8IWV1 7099 7334 6885 64127 3329 P20963 P08575 P10809 Q9HCY8 P49768 O00206 9564 P07766 O14492 7189 6850 4792 Q9NZD8 148022 57402 Q9BYM8 Q9UDY8 P61088 P06241 5970 P11912 55072 P16885 81622 P43405 P51617 Q8IUC6 Q04206 Q9HC29 8517 Q16581 Q9H1C4

regeneration 7046 P08833 4131 P55268 O15123 O14793 3484 P14635 9021 3482 230 3913 595 P00749 Q04759 P00747 3479 P07196 P31939 P09972 O14543 P46821 Q15389 Q15466 Q9UM47 5471 Q6IR47 Q9NZG7 P12429 Q92729 P16989 P58546 285 P37231 P20248 1026 P46531 4851 5468 4854 Q9UK55 P01343 O15524 Q99835 136319 P24385 6256 P10415 Q06203 P19793 P16118 890 891 Q03405 898 P01137 P49715 P50440 P11717 5054 P13611 P38936 P35625 1050 P30530 P17302 O60674 2697 51156 P24864 Q92982 P05121 5207 5328 P60842

protein amino acid methylation P55345 4297 Q9BZ95 23463 Q96MX6 Q6UXN9 O60725 P62495 54496 2107 11107 Q9H9B1 Q9NVM4 6304 84661 79813 7832 Q99707 O14744 4548 Q01826 P61964 Q9C005 11091 5870 Q9NQ92 30827 Q9P0U4 3276 Q03164 55352 5929 Q99873 Q15291 80335 Q9UI30 P20340 2935 Q9UBL3

protein amino acid alkylation P55345 4297 Q9BZ95 23463 Q96MX6 Q6UXN9 O60725 P62495 54496 2107 11107 Q9H9B1 Q9NVM4 6304 84661 79813 7832 Q99707 O14744 4548 Q01826 P61964 Q9C005 11091 5870 Q9NQ92 30827 Q9P0U4 3276 Q03164 55352 5929 Q99873 Q15291 80335 Q9UI30 P20340 2935 Q9UBL3

regulation of cellular carbohydrate metabolic process Q9UGJ0 P49840 P54619 P16118 O60502 P42345 3479 6622 P01579 Q92630 Q9UBK2 P04201 P41159 P56524 Q13322 P27540 3091 Q9NP71

Q9BX66 10580 8445 P31749 Q9Y4H2 P35368 P37840 Q9UNI6 P35568 2931 10724 P62140 P01308 2475 5500 207 Q16665 3667 5207 P01343 P06213

regulation of cellular ketone metabolic process P08559 6794 P10515 Q9H9S4 P54619 8050 P50416 Q9P0J1 P42345 672 P37288 552 Q96RU7 P21964 Q7RTN6 P23786 6622 Q9C0K7 1738 1737 P47712 Q9UBK2 Q9Y478 7376 P28330 Q9NP71 Q9Y4H2 Q9UGI9 P11177 P35568 P37231 P01308 2475 207 5468 Q13133 Q13131 P09622 Q15118 5162 Q15831 P01185 Q9UGJ0 5562 22937 5321 5563 5565 5160 P38398 Q8NCN5 55066 1312 57761 P01019 857 Q12770 P41159 Q9Y376 O43741 P55055 P31749 1374 O00330 P33121 P37840 P54646 Q92786 3667 P21554 P07148

positive regulation of cell projection organization P25054 7046 8650 4131 O43157 P10636 2033 Q9BVA0 396 P42345 Q96P48 Q04759 Q86WK6 P07196 P63000 998 Q7Z6C1 5898 P61586 P46821 P52565 Q6IR47 Q9NYA1 Q13009 P10600 P10721 P18846 324 81565 2475 4137 P61158 5029 Q9GZM8 P17081 P08138 Q92888 Q09472 Q15797 6498 5364 P12757 Q13418 P53667 P41231 3611 Q96RK4 P01138 5879 P60953 4086 Q9Y6R0 3673 9138 P10586 9253 9495 P11233 7074 Q9HCK4 P17301 387 466 P21554 P24588

protein homooligomerization P08758 Q8N307 Q96RE7 O95999 Q9BY76 P50416 3162 6470 230 P09972 999 O14788 Q15389 P49815 P55072 6505 P27105 O15294 5195 5471 7013 Q9H4L7 Q8IX30 P35609 P36776 55054 2040 P30793 84708 7052 O75381 1666 P21579 Q9HD26 P14174 Q7Z2W7 7249 9702 1429 Q99714 P04179 7186 8473 P00492 6498 P12757 P54274 Q06203 P12830 57120 P12755 P32320 25923 9361 Q8TBB1 3416 222484 2643 Q6DD88 P32929 7415 56916 P21980 857 Q16698 P51575 Q12933 5213 Q86XR8 1374 1491 Q08257 5092 P34896 Q676U5 138151 Q96BF6 440193 3028 Q96RR1 P61457 5888 P09601 Q01813 P08237 P04275 P43005 P04156

interaction with host O00206 6890 Q86W42 11060 6993 O00308 Q86V81 O95292 57187 P63172 Q9H0M0 Q96FV9 O60603 80145 Q7L523 Q9Y2C9 79139 P84022 P60033 Q92692 975 P01137 O15455 P01730 Q6I9Y2 Q15223 8563 7099 7098 4088 Q13769 9217 Q96J02 83737 P46934 Q8NI27 P05362 Q03518 4734 Q9BUN8 P06213

regulation of mRNA processing 4154 Q6NYC1 P14635 P52756 891 79810 Q8WV60 4686 P49756 Q99728 10772 P21127 58517 4869 135295 728642 10181 Q96SB4 Q8WXF0 Q09161 Q9UQ88 23210 O75494 580 984

Golgi organization Q14789 Q96CV9 9382 80124 55666 Q8WTW3 Q9BVG8 10133 8548 9950 Q8TAT6 Q9BQQ3 Q8NF91 11196 Q02750 Q8IWJ2 400 P40616 Q9H2G9 Q9H9E3 9648 5604 3801 Q8TBA6 Q96JH7

positive regulation of glucose import 5584 Q86V24 O60502 P21860 P27986 Q9NSA1 P53675 5295 Q15303 Q9BX66 10580 P31749 Q9Y4H2 2065 P35568 5590 10724 Q05513 P01308 207 8218 3667 P17081 P41743 P06213

mRNA catabolic processQ9UL18 Q13315 Q9UPR3 22794 472 54512 4686 Q8IZD4 O15234 5976 Q8IU60 P38919 5394 Q09161 26523 Q504Q3 Q96Q15 27257 167227 Q9NPD3 Q9BRP8 Q9HAU5 P60228 64282 P52298 6050 P13489 26019 Q9Y5S9 84305 23381 55149 P61326 Q15287 P08107 9775 Q96C86 Q01780 Q9NVV4 Q9HCK5 10921 3304 P40692 Q9H9G7 192669 28960 9924 4116 Q92900 2935

cell part morphogenesisP25054 Q92858 8650 P55268 Q9UQP3 O00429 Q8TEW0 O75665 P56199 23322 O75385 351 3913 474 Q8IYN9 Q86WK6 P16220 Q1PSW9 P07196 Q99807 P83731 O14786 P43034 P46821 Q8NFJ9 O60282 3911 8408 Q9C0K0 Q96CW9 O15496 Q12837 O43602 10939 2043 1191 P55316 117177 O60331 81565 P46531 Q15154 4851 Q8IWA4 P00533 P05067 P98172 4218 Q13099 2290 8399 P54753 Q7Z727 P50552 6256 O95613 P14416 P19793 Q06124 Q13418 Q05397 Q9BXC9 1756 1874 773 P51805 Q00535 P17252 Q96RK4 Q53X93 4983 P21860 Q9UQB8 P49639 8829 84062 Q16254 Q16650 6383 P54764 P54762 3672 P04626 91147 Q9Y6R0 Q92949 Q658W2 2065 P13611 2064 Q8IYT8 O00213 Q7L576 9253 O75592 P17302 Q9Y3D6 Q4AC94 O60674 56288 2697 P10070 23032 P10071 23396 10087 6259 6812 5604 2737 2736 9927 P34925 Q9Y3M2 4131 8851 Q8TD84 1385 P78509 1020 10458 Q04837 5578 10059 Q15223 P26232 O00238 8100 P60709 P52803 26005 O43921 P11532 Q5HYA8 Q68CZ1 64919 10229 324 Q96QF0 O60890 60 Q9GZM8 1947 P08138 7408 5108 85440 5747 Q99835 Q9HCU4 Q15078 9201 Q8TCU4 Q8TEY7 P10415 P56178 Q8IUQ4 Q02880 P34741 A1XKG3 Q96EV8 O60229 P20336 Q96N67 3611 Q04656 P61006 O14640 P61764 P08069 5116 Q92752 1956 O15075 P29323 7155 O15230 Q15768 O95140 Q13509 25776 Q02750 8481 582 Q9HCK4 23077 55558 Q15375 Q9BZR6

fatty acid beta-oxidation O00628 Q08426 2110 P50416 P33897 P09110 215 1892 P23786 Q16134 5830 Q16698 30 34 P28330 P40939 1374 1891 P51659 3032 P50542 3030 P11310 1666 P30084 5467 P55084 Q13011

microtubule-based transport 9001 P54257 O95239 O15066 P33176 24137 Q8IXI1 Q8IXI2 351 P42704 P07196 P43034 3799 Q9UBK9 Q02750 9371 5590 Q9NXR1 10128 23095 54820 Q05513 O60333 P42858 81565 Q9GZM8 P05067 5604

muscle tissue development O96004 P13349 Q12948 2033 3084 P52952 351 4729 2303 79810 Q8WV60 P09493 Q7Z6C1 Q92574 O15119 6901 9474 Q16635 P51636 10939 Q02297 O43847 2280 P05067 Q09472 6495 6256 6498 P12757 P19793 P12755 3142 Q9ULH7 Q63HK5 1756 891 1634 Q00535 5159 P21860 Q14993 Q8WZ42 Q9UPY3 6926 4000 P07858 P04626 P68133 2065 O95183 2064 9093 P12883 5728 387 P21359 P17813 P60484 668 Q92786 P07585 Q96EY1 4637 P62942 9421 Q9Y3M2 7248 1020 P14635 4092 O00468 P19404 P15692 P15173 23363 P61586 P45379 P78337 P50222 Q14774 P09619 P11532 P68032 Q86WV8 23414 P60660 Q8WW38 375790 4898 Q99958 Q15475 4154 7422 A1XKG3 P02708 Q9H1Y0 O14640 857 P02545 Q9GZT9 P26678 P10826 25776 54583 Q08209 P58012 P24821 O15105 P25116

antigen receptor-mediated signaling pathway O43353 9564 P07766 O14492 7189 Q96EP0 6850 O95999 P28482 Q9NZD8 Q9Y4K3 O43318 Q9BYM8 Q8WZ60 10616 Q9UDY8 P61088 Q9Y6K9 P06241 51324 P49810 5336 8767 5579 P05771 Q8IWV1 7334 6885 P11912 55072 P16885 P20963 P43405 8517 P08575 P49768

positive regulation of cell-substrate adhesion 6696 133584 7248 7402 1021 Q13418 Q53GQ0 Q8NC24 P10451 P31431 O00622 Q9Y6C2 57669 51144 3611 P84022 Q00534 1012 Q92574 P07996 P55290 P04004 7057 P35968 3791 4088 Q86WV8 P14543 5590 Q63HQ2 P16671 Q05513 P46939 Q9HCM4 7448 948

positive regulation of MAP kinase activity Q8WXG6 P01241 Q13956 7124 5585 P56199 Q9Y4K4 Q9Y4K3 O43318 Q9Y2C9 121512 Q99683 Q03468 P01116 1616 P07996 P08246 P52564 7099 7057 5155 Q16512 4188 6885 P10600 P10721 P61073 10746 79444 Q99750 8737 4214 P00533 4216 P42338 Q13131 Q9UER7 P49768 O00206 7186 7189 O95136 P28223 6850 P29353 5562 Q9HBW0 Q96M96

Q9P035 Q06124 Q13418 Q9Y6Q6 P18545 Q9UBU3 2247 3611 P60033 975 Q5TCX8 6647 P01137 Q96CA5 P01135 1956 P09038 P00441 P01133 P01375 9839 Q9Y6R4 Q12933 6464 3672 P04626 Q13546 O95382 Q9NR96 Q02750 2064 P21917 P43405 P20827 Q6PID4 P01127 54106 Q14289 Q13233 5604 Q13153 P04233 P06213

response to starvation Q13873 Q8TDY2 9821 P04637 27327 P41181 60673 10013 P08243 Q13286 Q9UBK2 P05412 Q9Y4P1 9474 Q9NYA1 6720 23411 Q8NDV7 55054 P55318 3171 Q9BSB4 P11021 Q9P2K8 P05062 Q96EB6 P49768 P16118 P14410 P61244 Q9H1Y0 4149 857 Q7Z3C6 Q13501 8443 P07339 7157 P36956 79065 O60911 Q676U5 Q9H492 23192 81788 Q9H093 8878 5207 Q9UBN7 O94817 3309 O15228

protein amino acid acylation Q9H0E3 Q96L91 4297 Q9Y265 6871 2033 10856 10933 79612 Q7Z6C1 Q9Y230 84289 8607 9397 55929 6883 Q16514 Q15306 O96019 O95361 54361 Q8WYH8 57634 10626 80155 Q96MV8 1387 Q4LE28 408 P49407 O75528 Q09472 O95696 Q9UNL4 79595 Q8IUH5 Q92793 Q92830 O95619 51147 Q92993 Q9UBU8 P01730 Q92831 O95983 O75478 O00213 P56705 P35227 Q03164 10524 Q8WTX9 Q9BXJ9 O60551 Q9NPF5 P41227 86 53615

regulation of protein polymerization 4690 P25054 10092 4131 10095 O95835 10094 P10636 Q15735 O43639 P35612 P35611 P42345 6709 830 832 118 Q9H2D6 P46821 P47755 P47756 P51671 7013 Q01082 10109 2039 324 2475 6711 22919 6710 4137 4214 P62328 P61158 O15020 Q13813 P54274 O15143 Q9BPX5 23189 P56539 P62330 P20936 P02549 81873 10552 857 859 Q08495 Q14678 382 P11277 P16333 P52907 Q6PKD3 Q15691 P63313 Q13233 829

positive regulation of caspase activity Q9NZJ7 P23396 7124 P04637 O00220 29108 O75340 114548 P42224 356 2147 P84022 P55072 10016 7013 Q9Y239 4221 Q96QB1 P37231 3329 1029 Q01955 P00734 5468 P42771 P10809 P09429 8795 P06239 10392 6772 Q8N726 P54274 1285 P29590 29843 23787 O14727 Q9BXK5 Q96P20 7415 O14763 P61604 3932 P01375 5371 P51575 6188 4088 7157 Q9P0U3 P48023 P99999 Q9BZF9 O00255 9093 P58012 3146 668 Q96EY1 Q9ULZ3 Q13794 P25116 2935

positive regulation of peptidase activity Q9NZJ7 P23396 7124 P04637 O00220 29108 O75340 114548 P42224 356 2147 P84022 P55072 10016 7013 Q9Y239 4221 Q96QB1 P37231 3329 1029 Q01955 P00734 5468 P42771 P10809 P09429 8795 P06239 10392 6772 Q8N726 P54274 1285 P29590 29843 23787 O14727 Q9BXK5 Q96P20 7415 O14763 P61604 3932 P01375 5371 P51575 6188 4088 7157 Q9P0U3 P48023 P99999 Q9BZF9 O00255 9093 P58012 3146 668 Q96EY1 Q9ULZ3 Q13794 P25116 2935

extracellular structure organization P02452 P78545 7046 O43278 5268 P13349 Q12948 O00300 P11047 351 Q07507 Q8IYN9 871 2303 P39060 999 Q6NVY8 Q92574 Q7Z6C3 P46821 P07355 Q9Y5E9 P04004 Q15303 P25067 10939 O95680 O43847 4060 2280 P16234 P39210 P21815 7448 P08572 P05067 P53420 P28300 P14416 Q13418 Q53GQ0 P51884 Q05397 Q00653 1634 P14780 773 54413 51144 P53708 P01019 Q14993 Q05707 P01375 P25940 8443 P02461 7476 P04626 P18074 P36952 Q658W2 2064 9093 P35222 Q9NZ94 P14543 P21359 P17813 P23416 302 Q9UBV7 P02458 P07585 4358 Q96EY1 P62942 4131 133584 7124 7248 P50454 O00468 O00622 3958 Q9Y6C2 1499 P00747 2742 P08123 4646 O00755 7373 Q6IR47 Q86WV8 P19544 7490 P13674 P12110 Q01955 4015 375790 4898 Q99715 Q9UM54 Q99958 P22303 5747 Q9BS57 4791 1285 P12830 1284 P02708 333 3611 1277 Q04656 O14640 Q92752 Q14031 P51693 P05997 Q92626 P20908 Q63HQ2 1289 8516 P24821 7428 P40337 4318 P25116 O15228

response to lipid O00206 Q09472 7046 5265 9525 2033 Q01094 P14635 P18146 O60603 O75581 891 P05091 6721 Q7Z6C1 P01137 1956 P01178 7099 8443 P05413 Q12772 Q6IR47 4087 P55851 5111 5155 O75351 P12004 P37231 P37173 P01009 5468 P01127 P00533 Q15796 1869 O15228

cell aging P10415 P04637 P54274 3265 P29590 Q13418 P51587 3611 3014 6647 P01116 O15119 6926 P01112 P00441 4869 P01111 23636 5371 7013 5054 7014 7157 Q99583 23411 9093 P16104 P37198 P17813 3028 P42858 Q9NPH5 P48059 Q96EY1 P05121 Q15554 Q96EB6 Q99714

regulation of fatty acid oxidation Q15831 Q9UGJ0 5562 6794 5563 5565 Q9H9S4 P54619 P50416 P42345 Q7RTN6 P23786 Q9C0K7 Q9UBK2 Q9Y478 P28330 Q9Y376 O43741 P31749 1374 Q9Y4H2 Q9UGI9 P33121 P54646 P35568 P37231 2475 207 5468 3667 Q13131 P21554 P07148

negative regulation of microtubule depolymerization Q9BYV2 P25054 4131 4133 P10636 23122 23332 O75122 P14635 9181 Q9BVA0 891 Q9Y3A5 Q7Z460 324 P27816 57159 4137 P46821 Q9NPA3 Q92974

regulation of microtubule depolymerization Q9BYV2 P25054 4131 4133 P10636 23122 23332 O75122 P14635 9181 Q9BVA0 891 Q9Y3A5 Q7Z460 324 P27816 57159 4137 P46821 Q9NPA3 Q92974

response to testosterone stimulus 6198 P55212 P28562 O14793 8654 2100 P30279 O76074 1843 P05091 894 Q92731 768 P01588 Q16790 P23443 P06213

cerebellar cortex morphogenesis O00755 136319 7476 A1XKG3 1020 P58546 O75385 2735 773 Q00535 Q1PSW9 P10070 P08151 Q04656 8408 Q99835 2736

activation of NF-kappaB-inducing kinase activity 8795 7098 7186 7189 Q12933 6885 O00220 Q9Y4K3 O43318 Q9Y2C9 P51617 Q9UDY8 Q9Y6K9 8517 O14763 Q9BWT7 O15455

negative regulation of lipid storage 3690 P05106 7376 O95477 4792 7124 P55055 19 P37231 Q8WTS1 51099 3685 5468 Q13133 P02741 P25963 P01375

mammary gland alveolus development P24385 O15111 7422 2099 Q02363 P10275 Q9Y6Q6 Q99623 595 1147 P15692 367 P16471 11331 P03372 O14788 P01133

positive regulation of tumor necrosis factor production O43353 O00206 7099 7098 Q9Y239 10392 64127 Q9H257 Q9NR96 O60603 148022 Q8IUC6 Q9HC29 8767 54106 O15455 64170

collagen fibril organization P02452 7046 P28300 Q12948 P51884 P50454 Q07507 871 2303 1277 Q04656 P08123 Q7Z6C3 Q05707 P07355 P25940 7373 P02461 Q6IR47 P05997 P20908 4060 P13674 P21359 1289 302 4015 P02458 Q99715 Q99958

regulation of B cell proliferation O00206 Q92835 Q96IZ0 Q8N726 P15923 P41182 148022 57162 6929 10673 Q9Y275 P05112 Q96FA3 7099 5074 P78552 Q9Y4H2 P35225 P38936 1029 1026 P14174 Q8IUC6 P08575 604 P42771 P42574 P60568 P04233 P43489

neuron projection development Q15019 P25054 Q92858 8650 P55268 Q9UQP3 Q8TEW0 P56199 Q9UBB6 O75385 P11047 351 3913 3516 474 Q8IYN9 Q86WK6 P16220 Q1PSW9 284217 P07196 999 P83731 O14786 Q6NVY8 P43034 P46821 O60282 8408 3912 Q9C0K0 Q96CW9 O15496 Q12837 O43602 10939 2043 1191 P55316 P46934 O60331 81565 P46531 4851 P00533 P16070 P05067 4734 P98172 2290 8399 P54753 Q7Z727 P50552 6256 P25391 P14416 P19793 Q06124 Q13418 O43815 Q05397 1756

Q9UBU3 6809 773 P51805 Q00535 P17252 P09471 Q96RK4 Q53X93 4983 P21860 Q9UQB8 P01138 P49639 8829 84062 Q16650 6383 P54764 P54762 3672 P04626 Q9Y6R0 Q658W2 2065 P13611 2064 Q8IYT8 O00213 Q7L576 9253 O75592 P17302 O60674 56288 2697 P10070 23032 P10071 23154 23396 3146 6259 P60763 6812 5604 2737 2736 P34925 P07942 4131 4133 8851 Q8TD84 1385 P78509 1020 10458 O60488 5578 5338 4646 Q15223 P26232 O00238 P60709 Q15109 P52803 O43921 P11532 Q06330 64919 960 324 O60890 60 Q9GZM8 Q9UM54 1947 P08138 7408 85440 5747 Q99835 Q9HCU4 Q15078 P09429 9201 Q8TEY7 P10415 10152 P56178 Q8IUQ4 Q02880 P12830 P34741 A1XKG3 Q9NYB9 Q96EV8 O60229 P20336 Q96N67 3611 Q04656 O14640 P61764 P08069 Q92752 1956 O15075 P29323 7155 P39905 Q15768 Q13509 Q02750 Q9HCK4 O14939 23077 Q6PID4 55558 Q14289 Q15375 Q9BZR6

response to inorganic substance P02452 5265 P28562 9588 P49023 P30086 2033 22872 O00300 23327 P42224 595 P37288 10935 P09493 513 6622 999 Q7Z6C1 P04085 10013 P46821 Q8N465 P07996 10016 O15118 Q15303 7057 5154 5037 5796 P46934 P16234 2 5829 P42858 P39210 P05062 P22102 P00533 4734 P02675 P01222 P01100 7220 808 Q7Z727 Q09472 P35638 Q9UMR2 6256 P29474 6772 5321 P19793 891 P17252 P00966 P09471 Q9UJU2 1917 5798 6647 Q8WZ42 P00441 P50440 6262 5054 P55851 P55212 5970 Q9Y696 P68133 P35222 P12004 P12883 5728 Q9UBV8 P60484 Q04206 P01009 8836 4358 P05164 Q9UBN7 Q92820 P30049 P25098 O75746 4131 48 P30041 P41181 P14635 P30044 P41180 O75340 P35613 3162 P30048 Q00597 156 552 1499 Q03468 Q96PU5 8604 P04040 5336 5578 P62714 5338 11269 P62158 P45379 P47712 P05412 7252 Q9NYA1 Q13443 5111 P60709 2243 Q96FQ6 Q07817 682 57678 Q05639 P21579 P18846 1026 445 841 Q9BXP5 60 846 P48995 847 11315 P01023 P04179 P02671 P24385 Q401N2 Q16849 8754 P10415 P12830 Q02363 Q53TN4 Q9HCL2 1277 2244 140576 Q04656 5599 Q14790 6721 857 P02786 5516 1956 Q12772 Q13586 Q92626 7037 Q06830 P38936 P37840 P16885 1843 51593 O14939 466 P18827 Q99497 P09960 Q6PID4 79901 P45983 O94979 P05121 P02533 P09601 Q14289 P45984 P04155 P04156 P21399 P06213

glucose metabolic process O95278 7167 P08559 P37837 P10515 P52790 P50416 3481 230 Q00796 2821 2026 P60174 1737 4967 Q9UBK2 Q13522 O95685 P46020 P04406 P52209 P11216 3099 3098 P11177 1468 P05062 P49247 P01344 P46019 P42336 5162 P07205 Q6ZMR3 92483 4191 5160 Q9UBU3 O60701 773 3939 5315 P62136 5836 P01375 Q8IW75 9255 Q16816 7358 5213 1374 3948 P14550 Q04446 P62140 P00558 5207 P07864 3945 P06733 Q92934 P06737 7124 P40926 Q12904 160287 P22415 Q93100 P09972 Q15185 P06744 P24298 O00757 Q9Y4H2 Q9UGI9 P15735 P11413 P11498 2875 Q9UBX3 2632 P35573 P18847 P01308 P18848 5467 5500 5226 5501 P49841 P09467 P09104 P46976 Q15118 22934 P13807 Q9UGJ0 P14618 Q9BYZ2 P16118 572 178 Q49A26 P41159 5499 2023 P36873 5091 P19367 P52789 2932 P17858 468 10728 Q01813 P08237

positive regulation of T cell activation O43353 4690 Q92934 O95999 P54132 Q08722 O43557 O43639 P10276 Q9Y4K3 O43318 79576 Q04759 8767 Q9Y275 O14788 P42229 P27487 Q14774 8546 6885 4860 7412 3329 57678 641 961 O43290 P08575 P01589 1947 P98172 P10809 P07766 7186 P00491 7189 P06239 6850 Q9BQ51 3142 O00203 572 Q9HCL2 Q9UDY8 P01579 10673 857 P01730 3932 P05112 5914 P24394 Q14116 Q13422 Q12933 P51692 P19320 9093 Q8N5F7 P16333 P43405 P37173 6776 P40933 Q96EY1 10320 P60568 P04233

developmental maturation Q9NZJ7 9463 4131 O75503 P07949 2034 P14635 P51587 O00468 351 26271 1499 P15692 Q92731 10296 P49810 Q92730 5139 1738 P46821 P03950 Q15303 P35968 O43524 P11413 P50148 P37231 960 Q96MV8 3169 P33151 30813 P46531 4851 5467 207 5468 375790 P08253 10661 P01588 O15169 P16070 P05067 Q9NRD5 Q96NL6 P09622 P49768 Q9HB63 137964 7422 Q7L5Y9

23787 891 P20336 773 9319 P61764 Q14432 P49715 P06401 Q92826 132320 3791 P18074 P31749 2100 Q86UL3 59277 P35222 1050 P17302 1203 O95390 2697 Q9NZR4 Q6PID4 4313 6812 P60201 Q99814 Q14289 Q9UKT4 Q13351

initiation of viral infection 11060 P12956 O00308 P62937 7520 P51681 Q9H0M0 2547 P13010 P60033 3159 Q92692 975 P17096 5478 11168 P01730 7518 Q15223 Q13426 O75531 O75475 Q96J02 83737 P61073 P05362 8815

epidermal growth factor receptor signaling pathway 9564 P22681 P29353 P49069 P18085 Q12929 O75582 O43639 9185 P62993 2885 Q13480 P01137 Q8NFH8 1956 2549 P01133 Q14451 P78536 6464 9252 Q96RT1 2059 Q6PID4 867 P00533 Q14289

regulation of epidermal growth factor receptor signaling pathway P29353 Q13956 O43639 84619 351 P18545 Q9UNH7 356 1012 58533 P01019 P49810 P01135 P55290 P01133 Q9UJM3 23636 P78536 6464 P48023 P10586 P37198 57732 Q8N5A5 54206 P05067 P49768

regulation of mRNA stability Q14103 7422 P67809 10492 1660 P11940 Q15717 O60506 3184 9261 1994 P15692 Q92994 P49137 Q00839 Q14011 Q13148 3192 Q9Y2T7 P39905 26986 1153 Q8NC51 23435 Q08211 2972 10642

regulation of insulin receptor signaling pathway 5580 Q7Z727 P49840 Q14449 Q9H0K1 9021 3481 P17252 5578 O14543 P49815 Q13322 5770 5970 P18031 P10586 P35568 5590 2931 Q05513 Q05655 P01308 Q04206 7249 3667 P01344 O15524

regulation of interleukin-12 production O00206 P10914 7189 Q06643 P13686 Q9Y4K3 201633 O60603 P01579 O15455 P07996 3659 54 Q495A1 7099 7098 7057 5970 64127 Q9NR96 3329 Q04864 Q04206 Q9HC29 54106 5966 P10809

maintenance of location in cell O95835 Q9UNN5 O00221 259266 9184 57662 Q9Y4G6 Q13285 P25963 Q9UBC1 4188 Q8NF91 10460 P33947 O43684 Q8IZT6 O95684 7094 Q9NS56 2317 2316 9908 Q9HD26 Q99750 Q15154 7529 5108 P02794 11116 P21333 7067 4792 2495 57120 P29590 Q86VS8 P30101 P15311 10956 Q96RK4 11124 857 Q9UN86 Q9Y6A5 P01730 Q9Y490 2923 5716 5371 P10827 7430 Q60FE5 P31946 O75832 Q14118 25777 Q9UH99 Q9UMX1 11014 84376 O14777 Q15653 1605

regulation of transcription, DNA-dependent Q9UKT9 Q6NX49 Q9Y265 Q8NDW4 Q12824 8091 P51587 124790 P17676 2305 2547 2304 2303 57504 P84022 2300 Q92731 155061 2308 2307 Q7Z6R9 Q12837 Q5VTR2 Q8WXB4 O60216 Q9H2G9 4734 Q96EB6 O75925 4990 3660 3661 22850 81628 6927 P13056 Q8IUD2 6929 84901 811 6921 O75928 P40425 P40424 Q9UKW6 6926 3659 P26358 6925 Q6IQ32 333929 Q6NUN9 Q9NZI7 P48382 Q9C009 Q92949 2100 Q99583 Q9NR96 Q12857 P48380 O95343 3428 6938 P20823 1487 P19838 Q99576 O60675 P62263 3665 P26367 5604 6936 Q9Y468 Q9Y463 4772 Q9Y466 1022 Q8NA42 Q5JT82 10614 1499 Q86VE0 2103 P16383 27287 80324 P61586 6945 O14503 Q12873 P50222 4780 Q92769 Q14814 Q9Y5Z7 6720 P28370 Q15906 O95361 P10600 O95365 O00358 Q9BPY8 Q06330 2119 57798 10865 3207 10626 1027 P19419 Q6PI57 P17482 P17480 4775 O14753 604 Q9Y242 Q92766 P04179 Q15910 Q9UL36 4790 4791 Q12888 4792 Q9BQ95 O60869 Q8NA19 P38398 55145 3215 Q9Y250 Q92753 6721 4302 Q92994 Q92993 857 Q8N488 Q92997 Q92754 Q13501 Q9UKS7 P10826 P10827 P10828 221937 Q13503 Q14839 P19883 Q13506 Q9NR55 1050 P53999 861 862 3226 863 3225 P04150 Q9UMX1 4799 P09601 50943 Q9UGL1 Q92985 Q9BS34 O96004 P15408 P14317 P14316 Q9Y3Q8 3481 Q9UIH9 O60603 25988 P15884 Q8WUU5 Q96T88 3479 2146

3476 10011 Q15022 10499 639 10014 P62875 6760 Q9Y5V3 5430 O95163 P16989 P57059 Q96T76 Q03933 P33076 Q99750 Q14186 79923 8932 406 O43294 408 P49407 Q14188 O00327 Q09472 23309 5440 P48552 5441 6772 Q8TAU3 P48551 6774 Q99990 346171 O60828 P15621 Q00653 Q99743 Q2M3W8 Q14192 898 51147 P49639 Q86X95 P51531 5451 8721 148156 P35222 Q9NR11 P51532 P35227 P57082 Q96ST3 O75081 P17813 Q00403 668 6776 10001 10488 Q96T58 Q96MH2 P28702 P23497 Q16385 7629 O43474 P06733 P26583 O00744 5460 Q9Y3M2 Q9Y5Q3 P25490 P50613 P51946 23764 25942 Q8NFW5 3281 672 P15692 6304 Q8N5U6 P05412 O00755 Q9Y5R5 90993 Q08117 3297 Q05086 Q9UBX0 29079 P37231 P14373 688 148327 5467 9826 5468 Q92922 10468 7422 Q96GA9 P56178 P56177 P52815 22893 3066 Q9UMN6 3065 Q9UIF9 Q8NHY6 Q12809 O75290 79753 Q00613 4149 10432 9839 O43463 3070 5494 9612 Q9UIG0 P52824 23512 Q96QT6 O14936 466 468 P28749 Q9NQL9 7428 O15085 P36508 6595 P35659 Q14686 6597 Q14209 8535 6599 6598 O75386 P10599 6591 Q9Y4K3 473 Q9H161 474 Q9H160 P08047 5017 Q9BQG0 Q13363 Q9NPC8 Q13127 3091 94234 4188 5036 Q15306 Q86VP6 O75376 79084 P58304 P13631 3096 Q02447 O75381 23051 Q04864 Q9Y2H8 51773 30813 P46531 Q13133 Q15796 8553 Q16649 P35638 Q15797 Q8IWY8 Q96IZ0 P12757 P54198 P31274 P31273 Q9UNL4 P31276 P12755 Q9ULH7 23028 Q9NY61 O75360 4193 P46527 257 6128 Q15561 8548 51548 51547 54815 Q15562 Q16650 Q9BYU1 P35869 P62826 P20393 7476 Q16656 Q15329 9412 P18074 Q8WVL7 P55055 P10588 P10589 P36956 P31270 Q9NPF5 Q16665 51317 Q13398 P43489 9421 Q16666 O75586 P35453 O75582 Q9Y6K1 Q03989 51564 Q15583 Q14494 8328 Q16670 P46108 5074 Q9NRC8 P78337 55929 Q9HAZ2 P46100 P22392 O15055 O43524 7490 7251 P13682 Q9C0F3 84232 Q8WW38 Q15596 Q99835 P61925 7024 Q3KNS6 7023 5089 P15822 23468 7020 Q02878 51341 54851 Q96PK6 51588 Q8NCF5 10155 9439 Q96NG5 286075 Q9NPA8 Q15369 Q8IUX7 6182 P11474 26747 Q99816 O95947 9208 Q99814 P78545 9464 P35813 P29374 O43711 27005 P22736 Q9BUG6 O43719 P62195 Q9UGU0 9219 Q13761 A8K8V0 Q58WW2 P56524 9477 22806 22807 O75534 7291 7290 7295 P24928 P46934 O60296 Q4LE28 Q9UER7 Q14865 O75528 7068 7067 Q15714 P19793 P0C7X2 Q7Z2Q5 Q63HK5 Q8WXI9 Q01196 84619 Q9H6W3 P41235 Q92793 Q96GM5 Q9HD15 1993 O60264 O15499 O95983 Q13547 A4D1W7 P78317 9252 7073 P78318 P41223 Q8TBE0 Q9HAK2 Q9NZR4 Q04206 Q9UH92 Q92786 Q9Y2B9 O14593 O95999 Q9NP66 O43918 Q01167 23085 Q9UKY1 Q9H9B1 P85037 P31260 Q9BSG1 Q14894 4800 2626 7099 P31249 7098 Q9NVC6 Q14653 Q9NP71 Q9NP72 7090 Q9NQZ8 1789 1788 Q05516 1786 Q9UIV1 3720 375790 Q13573 5901 Q9HCU4 P46734 Q13330 93134 P12956 Q9H2S9 Q6P1L6 P78527 92283 Q9H4W6 Q00059 Q9BQA5 Q9Y6E7 Q6ZNG0 Q96RG2 Q68DY9 P43694 O15455 Q9BQA1 Q9Y4A8 5914 O75953 30827 63976 Q96H20 P63279 P00519 P63272 Q9HCS4 Q92585 902 Q15532 Q9NZN8 P08235 P21675 5925 P10914 Q92858 Q9Y2W7 Q9NYD6 Q6P2D0 P67809 O75820 2672 Q12948 Q9UBB5 O00268 Q6QNY1 O00267 Q9P2R6 3516 4609 Q9BZI1 P16220 Q96JM2 Q8N587 P27348 5933 O15516 Q12950 Q12952 Q9UBC3 Q9Y2X9 54361 Q8NAP3 1107 4851 284323 4854 4613 Q96JL9 1108 O15525 5705 Q9ULX9 P05549 Q12968 29128 Q99471 O60548 10514 83933 4862 Q92830 Q9UJU2 284312 O15534 P49116 P03372 57649 Q92833 Q6IT96 P06401 Q92831 P06400 5716 Q96JK9 Q92826 Q0D2J5 P07737 Q9H9T3 5970 5971 Q6P2C8 Q9NRZ9 P56705 9099 P17535 P17544 10524 Q99459 P17542 Q9HBE1 Q99697 400720 P61296 5966 10765 Q12986 1385 P19532 O00468 Q9P0J0 O95231 Q9UFF9 O14627 6829 10736 P15173 P07550 Q8N2W9 4646 10971 7913 5978 O14867 6827 Q96BA8 Q9UM47 Q9Y2T7 Q9UHK0 5991 93986 5993 Q14938 Q06455 P19544 55250 6839 1388 Q9P0K8 1387 1386 P08651 5987 Q9UM54 4899 O95259 Q10586 22937 22938 P51449 Q6IA86 Q9UHL9 O00488 22931 Q02086 P61244 2247 1398 Q9BZK7 6601 4664 Q9Y2V2 Q12770 6605 P05112 57209 Q9UM63 O43186 Q12772 Q12778 P51692 P31629 Q05586 2023 Q9BQY4 O00255 Q8NEZ4 Q02078 Q6KC79 Q9NQ33 O00257 Q03164 10725 57459 984 Q99496 Q5H9I0 988 P40337 7704 Q9Y2W1 P06213

O43159 6872 Q9UPV9 P04637 2034 P13349 2033 2274 P53803 O00422 P52952 Q8WTS6 79813 4204 4205 Q16236 Q9UQ80 4209 Q9C0K0 4221 Q9GZR2 Q9UQ88 P50548 O76071 84159 P48436 4214 P01106 P01588 7727 P01344 Q9C0J9 P01343 P01100 O00206 2290 23429 P48431 7741 9921 P52736 3142 Q9NU63 Q96S42 Q53X93 55869 6886 7975 P01137 5316 P01375 Q16254 P52740 Q9UPY8 6660 5573 8841 Q9UHI6 P52747 2063 3151 11218 Q9NS37 Q99853 Q3KNW1 3148 P10070 Q15170 P09086 P10071 546 3146 10363 10362 6657 5569 6670 O43593 6672 23409 5585 Q9H9S0 25822 3162 Q12905 O95076 Q8WYA1 Q12906 79612 O75190 3159 Q02930 Q99684 6667 7756 5579 P05771 8607 P40763 5591 4261 Q969G3 8864 P55198 Q9ULK4 23411 P18850 P15336 23414 3171 O75182 Q9NRL2 51295 P18846 Q00987 Q68CJ9 P18847 P18848 3169 326 7528 P27797 Q9GZU7 64375 2099 O60907 3184 P51843 571 3182 P51608 P68036 10794 8625 5119 Q01826 P27540 9975 P62508 Q9ULM3 7555 146050 Q9BUY5 Q9NVV9 80854 P58012 Q9NPJ6 7549 10320 54106 8878 9967 P25116 P60568 Q96HZ4 O43670 4297 9500 8896 P15923 Q86YW9 P13984 351 84324 Q7Z6C1 P21127 Q13485 P25963 O94906 P09017 7332 O00716 Q16512 7335 O75496 P10242 P10243 367 Q70SY1 Q16520 7329 6239 Q14586 20 Q15672 Q9UQR1 6496 7101 6495 Q14103 6256 6498 P11308 Q99909 P32242 Q9Y606 116113 P17096 7579 Q14592 Q96MA1 Q13263 4086 4087 8204 4088 4089 Q14119 Q9BY41 Q9Y618 P12645 222546 387 Q04726 P08151 P24864 P20264 11177 11176 Q15697 P23771 53615 Q13033 P54259 P55347 9541 5187 O75469 7124 11142 O15164 P54253 O75461 Q9UQL6 P10275 4092 O43638 P10276 4093 4094 154 Q02535 Q01201 2908 Q99729 Q15466 55806 Q99966 Q13285 2902 7132 5195 6045 7376 8463 8467 9314 O43889 P36896 23135 23378 P15976 Q96C28 Q3KQV3 1822 166 55810 P01308 Q6PRX2 338917 Q9NQB0 Q99717 P09067 Q14140 Q99958 Q15475 Q96T37 Q8TEY5 9326 Q9HA82 P14859 P36402 O75444 Q8TAQ5 7141 Q9H063 1831 Q96T23 Q99941 Q01664 Q00577 Q99708 P01579 P49711 P49716 P49715 P09038 55827 9575 P82979 7158 O75437 Q08050 P39905 7157 10284 O75676 162239 O15198 O43623 25776 Q02750 P35548 55832 Q9UDV6 1609 Q5VTD9 P78424 Q01094 7161 Q8TAF7 P42224 P41134 Q1PSW9 P17275 91 P17030 P42229 57109 O15119 1616 P45973 P42226 Q9UBK2 Q9NSA3 P78412 O94992 Q9P0W2 9112 22926 Q969S8 Q13404 Q13887 Q01085 57594 P10644 P55318 Q99081 P55316 Q96BD5 79365 P17040 P05067 2959 1869 Q9UBL3 O94763 7189 92129 Q8WYK2 O43812 7181 Q03014 7182 6096 Q86UE4 Q86UE3 1874 Q9BTC8 1870 P09630 O15379 O95619 Q8TAD8 Q9NSC2 O15151 8289 Q15843 Q13422 O15156 O94776 2735 P19438 O43829 P17023 2972 79149 Q9BXJ9 84733 Q8N393 Q8HWS3 P04198 1406 2737 2736 Q15853 O75626 Q14527 Q8WUI4 9149 Q8TF47 P41182 Q8TAK5 Q02386 93474 79175 P53567 84525 84528 P22415 Q9UK80 Q03468 O75629 O95644 150094 Q14774 P22670 Q9UBG7 9391 64919 80155 58487 P41162 P23511 3609 P17081 3608 Q9Y5B6 3607 Q14781 P23510 P09429 94104 O75604 P41161 29966 Q02363 58491 P18146 P35711 27300 O60341 P09430 O15350 728642 O75832 O15353 Q15649 Q9BZS1 Q13227 Q15648 P55771 Q9P0U4 P19484 Q86YP4 Q06710 Q8N5A5 3622 O14497 O15105 Q13233 Q15652

monosaccharide catabolic process P06733 7167 Q92934 P08559 P37837 P10515 P40926 10690 P52790 160287 230 2821 2026 P60174 P09972 Q9Y231 1737 4967 P06744 P04406 P52209 3099 P11413 3098 P11177 P05062 5226 P49247 P09104 5162 P07205 Q6ZMR3 22934 P14618 Q9BYZ2 92483 P16118 4191 5160 572 P46926 3939 5315 Q49A26 5213 2023 2582 P19367 P52789 Q9UKK9 10007 3948 P17858 P00558 Q01813 5207 P08237 P07864 3945

negative regulation of signal transduction P25054 P30086 2305 3479 997 3476 O14544 O14543 Q92574 P49815 P07996 2308 7057 6003 4221 Q96QB1 Q15669 5037 O75376 Q96D03 O43609 P67775 9628 Q13370 408 P49407 409 P01343 O15524 O00206 Q92835 8315 P14416 P32121 P56539 Q7Z569 P35240 O15379 P21860 O14964 P49758 5716 Q8WWN8 8841 Q14118 2065 9093 P78318 P35348

Q13829 5728 P21359 P41220 P60484 P17936 Q96EY1 9927 9146 8452 4771 O43597 7248 7126 P41182 9021 Q16828 P62714 7099 Q8WZ19 P21580 P30153 Q9Y5J5 Q86WV8 P37198 324 7128 207 7249 9306 604 Q13574 Q13618 P08069 857 5997 859 5515 5516 O14921 5518 23636 Q12778 O75832 Q08050 Q13227 P31749 O95140 Q04771 O00255 Q8N9R8 253980 51231 1848 1605 P49427

central nervous system development Q92858 8650 8892 Q14686 Q9NYD6 P18085 8775 Q12948 P30086 O75386 259266 P51587 O75385 351 472 474 P37288 Q8IYN9 P16220 Q9ULW3 Q92731 P49810 P43034 8408 Q15303 5274 O94910 P12429 5037 54361 P58546 P50148 79885 P42858 Q9H2X0 P46531 4851 51654 Q96SN8 P00533 4854 Q16880 P54753 Q7Z727 136319 6496 Q15797 Q9NRY4 P32242 P12755 O75360 Q05397 Q9BXC9 P51805 P09471 Q96RK4 4862 Q92830 5830 Q9UJU2 Q13144 Q9Y6A5 P62258 Q92833 6926 Q14232 Q9BWQ8 Q16650 8443 Q9GZX9 4086 Q9Y2J0 P54762 Q14114 7476 Q9Y6R0 P18074 P51812 2100 Q9Y618 P10589 P56705 P35625 O95343 O75592 5728 Q4AC94 P21359 54820 P08151 Q99574 1808 P20264 10763 P26367 3309 P54259 P54257 7248 Q8TD84 2909 7368 1385 1020 4093 Q02535 Q99962 Q99963 1499 Q9NYQ6 Q16555 Q99966 Q9UM47 P78337 Q9NYA1 8100 Q92769 P30559 93986 P46100 Q15109 Q8ND90 P28370 Q9Y4H2 P36896 1152 Q8IZT6 P36894 Q86WV8 5071 1822 P11021 Q9GZM8 7804 5747 Q99835 9201 O43175 Q02880 A1XKG3 Q86VS8 Q15915 610 P21145 613 Q92753 P08069 P47895 Q9NQC3 1956 P51693 P10826 O15075 P10827 P29323 7155 O15078 7157 O15198 Q9NR50 O75553 Q6KC79 Q9NXR1 Q9HCK4 3226 O95390 P37173 Q9UL51 Q9UMX1 84376 7704 Q9UK32 P78424 P04637 Q15818 O43711 Q01094 23322 7161 P50897 Q9H0M0 Q1PSW9 91 999 P61421 Q92574 P46821 O15119 P02686 Q9C0K0 P49770 P48681 9114 Q9UHG2 Q96QB1 O43602 7170 Q9BRQ0 P50542 P55316 81565 Q15154 P48436 P05067 1869 Q13099 P49768 2290 9001 7067 P48431 P14416 Q03014 O00562 6096 P11274 Q99743 O14727 773 56034 60412 Q00535 Q96S42 P17252 P53708 Q53X93 P01019 P06241 P49639 Q9NSC2 Q9NRA1 O60260 Q8N960 Q13422 6660 Q13426 Q658W2 29777 P35222 9253 9495 P54920 2735 P17813 55755 P60484 P10070 P10071 546 Q92786 Q96JB5 6657 2737 P24588 2736 4131 Q9Y5Q3 O75503 8851 Q13315 P78509 Q8NFW5 552 5578 P09211 7518 P04201 P26232 5591 O00755 Q9NQZ2 7531 10460 P52803 26005 O43921 Q68CZ1 Q05086 Q9UBX0 Q05516 27330 64919 Q96SZ6 P51959 3720 P08138 5108 P06753 Q15078 90780 5362 P10415 4155 6456 P56177 6455 9600 P12830 3066 57045 P12277 22895 P19235 P78527 P51843 P35711 O75051 333 Q04656 80184 9839 P41159 O15350 9612 Q15648 O43464 1203 23077 8516 6457 900 P60201 10320 55558 O15228

regulation of generation of precursor metabolites and energy Q9UGJ0 P54619 P42345 3479 Q9NZ45 Q04656 Q92630 P56524 Q13322 P27540 3091 Q9NP71 Q9HAZ2 Q9BX66 10580 8445 P31749 Q9Y4H2 P35368 63976 P35568 P17612 P62140 P01308 2475 5500 5566 207 Q16665 3667 P01343 P06213

cell projection morphogenesis P25054 Q92858 8650 P55268 Q9UQP3 Q8TEW0 O75665 P56199 23322 O75385 351 3913 474 Q8IYN9 Q86WK6 P16220 Q1PSW9 P07196 P83731 O14786 P43034 P46821 Q8NFJ9 O60282 3911 8408 Q9C0K0 Q96CW9 O15496 Q12837 O43602 10939 2043 1191 P55316 117177 O60331 81565 P46531 Q15154 4851 P00533 P05067 P98172 4218 Q13099 2290 8399 P54753 Q7Z727 P50552 6256 O95613 P14416 P19793 Q06124 Q13418 Q05397 Q9BXC9 1756 1874 773 P51805 Q00535 P17252 Q96RK4 Q53X93 4983 P21860 Q9UQB8 P49639 8829 84062 Q16254 Q16650 6383 P54764 P54762 3672 P04626 91147 Q9Y6R0 Q92949 Q658W2 2065 P13611 2064 Q8IYT8 O00213 Q7L576 9253 O75592 P17302 Q4AC94 O60674 56288 2697 P10070 23032 P10071 23396 6259 6812 5604 2737 2736 P34925 Q9Y3M2 4131 8851 Q8TD84 1385 P78509 1020 10458 5578 Q15223 P26232 O00238 8100 P60709 P52803 26005 O43921 P11532 Q5HYA8 Q68CZ1 64919 324 Q96QF0 O60890 60 Q9GZM8 1947 P08138 7408 5108 85440 5747 Q99835 Q9HCU4 Q15078 9201 Q8TCU4 Q8TEY7 P10415 P56178 Q8IUQ4

Q02880 P34741 A1XKG3 Q96EV8 O60229 P20336 Q96N67 3611 Q04656 P61006 O14640 P61764 P08069 5116 Q92752 1956 O15075 P29323 7155 O15230 Q15768 Q13509 25776 Q02750 8481 582 Q9HCK4 23077 55558 Q15375 Q9BZR6

learning P54253 1020 B3KY43 60626 351 Q99965 P09172 Q9Y3A5 P21964 477 5577 P01116 6869 2904 P01112 2902 P21246 Q13286 P01111 P05412 P35368 P10721 O43529 5071 P31323 1742 P42858 P06276 P05067 P01100 P14416 A1XKG3 3265 P50993 Q9NPQ8 1312 54413 Q00535 Q9UQB3 P06241 7416 P25103 O60260 P29323 Q05586 Q9NZ94 P21359 5764 P07101 P21796

regulation of carbohydrate metabolic process Q9UGJ0 P49840 P54619 P16118 O60502 P42345 3479 6622 P01579 Q92630 Q9UBK2 P04201 P41159 P56524 Q13322 P27540 3091 Q9NP71 Q9BX66 10580 8445 P31749 Q9Y4H2 P35368 P37840 Q9UNI6 P35568 2931 10724 P62140 P01308 2475 5500 207 Q16665 3667 5207 P01343 P06213

negative regulation of cell projection organization 6696 4099 4155 Q15735 P20916 3066 P10451 O75385 Q05397 396 Q8IYN9 P51805 6405 O14786 P61586 Q9NQC3 P02686 8829 8408 3956 Q04917 P52565 P29323 Q92769 9138 7533 P10586 Q658W2 Q02790 387 P23515 P09382 55558 P08138 Q92888 5747 Q13275 Q9BZR6 P49768

mesoderm development Q9UKT9 O43474 9421 P27037 Q13873 O96004 4771 Q12948 Q9UPN3 6591 Q93063 2303 57669 P15692 P84022 5017 O15119 Q13485 Q8WUM0 22806 9314 P36894 P10644 P67775 51295 P42858 Q9HCM4 Q9H2X0 O14753 Q15796 Q99958 Q16881 Q15797 7422 Q9BQ95 2132 O00167 57045 6927 Q96S42 P35240 Q9UJU2 5515 6926 Q9GZX9 4086 Q13422 4087 4088 5573 4089 Q04771 O43623 Q13705 P51813 P20823 P17612 2139 O60674 O95390 5566 Q9HCS4 O95947 10320 1969 P29317

positive regulation of lymphocyte proliferation O43353 4690 P54132 P15923 P41182 O43639 Q9Y4K3 Q04759 8767 Q9Y275 6869 P42229 Q96FA3 7099 4860 7412 Q9Y4H2 57678 641 1026 P14174 P08575 P01589 604 1947 P98172 O00206 P07766 P00491 7189 6850 Q9BQ51 148022 57162 6929 Q9HCL2 P01579 10673 P25103 P05112 Q14116 P78552 P51692 P35225 P19320 P38936 9093 P16333 P43405 Q8IUC6 6776 P40933 Q96EY1 P60568 P04233 P43489

regulation of cell-cell adhesion P78423 4771 7124 Q08722 4092 83706 57669 Q9BZE4 P23229 23560 P35240 3655 P01137 O14788 3956 P01375 P27487 Q86UX7 Q02750 5270 22920 961 O60674 P09382 Q9HCM4 O15105 Q92845 5604 Q9HCU4

DNA damage checkpoint P04637 Q13315 P54132 O43715 25988 472 595 672 Q96RU2 51720 26270 O96017 Q9NYZ3 P16104 Q01831 P46736 P20248 Q9NWV8 641 1026 P51959 5347 Q9NRD1 Q96RL1 Q99638 Q13574 O14757 Q6ZN33 P24385 5883 8555 Q13535 P29590 Q06124 79791 890 P38398 Q9BQA5 57646 Q5XUX0 1111 3014 Q6NUQ1 29086 5371 Q14676 9656 7157 P53350 P38936 80254 55159 545 Q96MT8 900 51512 P43246 51514 Q6PCD5 7508

tissue homeostasis 6794 2034 25861 Q9UBB6 Q9Y4K3 154 1499 P15692 P07550 Q13085 Q99728 Q7Z6C3 Q8NFJ9 54 P47712 P07954 7099 Q15303 P35968 P09619 Q6UB99 P20849 Q9NPH5 6117 O00206 23746 Q15831 P27694 7189 7422 P10415 5321 P13686 6927 P15153 51542 Q96RK4 5159 6647 31 P00441 3791 3673 1297 Q9NR96 P35222 Q9P202 580 582 P20823 P17301 23154 P02458 54106 Q99814 P04155 P25116 Q9NZN9

regulation of T cell proliferation O43353 4690 P54132 O43639 Q9Y4K3 Q04759 1499 8767 11146 Q9Y275 P42229 5074 4860 7412 Q96J02 83737 1029 57678 641 P08575 P01589 P42771 1947 P42574 P98172 P07766 P00491 7189 Q92956 6850 Q96IZ0 Q8N726 Q9BQ51 Q9HCL2 Q92990 P01579 10673 P01137 P05112 11326 Q9BT67 Q14116 Q9BZS1 P04626 P51692 Q92949 P19320 2064 9093 P35222 P16333 P16410 P43405 6776 P40933 Q96EY1 50943 P60568

2-oxoglutarate metabolic process Q6YP21 P51553 P36957 P51692 O75874 3420 P50213 O43837 3417 1743 P00505 56267 P17174 3421 6776 1738 P42229 2805 3419 P09622

nucleotide-excision repair, DNA gap filling P27694 5111 P40938 P15927 5981 P40937 P28340 5983 P35244 P12004 P35249 10714 P18858 P35251 6117 Q15054 5424 6119 6118 3978

glycoprotein catabolic process Q96BI3 O00754 2280 80267 O60502 10724 Q15392 26232 4125 P49810 Q9NRD1 1718 Q92542 Q9UK22 51107 Q9BZQ6 26270 Q13286 P62942 P49768

negative regulation of osteoblast differentiation P56524 Q9GZX9 P48431 4088 4221 Q8WUI4 1021 O00255 P12755 57045 Q9H6W3 P84022 Q00534 51564 Q9H2X0 P46531 4851 6657 Q9Y2T1 Q99966

regulation of survival gene product expression Q6IR47 7046 P51692 P31749 2100 2099 P14784 P52952 Q07817 O60238 3169 1012 Q92731 6776 207 10673 Q9Y275 P03372 P42229 P55290

regulation of phosphoinositide 3-kinase cascade P01241 P04626 2065 2064 Q05086 P46934 O14939 O60674 3479 P01308 P01019 5467 P21860 P04040 7249 847 5338 4734 P49815 P01343

response to temperature stimulus 6198 8892 O14793 P13945 1660 P30086 9021 154 3316 Q04759 3315 P15692 Q6FHQ0 O60884 P20396 P07550 81570 5931 O14543 6869 P07996 5935 P47712 Q14011 P49770 P31689 7057 Q9UBG3 Q92769 Q9UHG2 5037 1153 P16989 3297 Q9H078 P37231 Q16082 841 Q7Z2W7 207 5468 P08138 P01100 11080 7067 P29474 7422 Q8WXI4 P10415 P29475 P98179 5321 Q9Y5S1 3066 Q8WW22 Q9UDY4 Q00613 2246 55466 Q9NZM1 P01019 Q14790 P00403 6647 P05230 Q16576 Q13144 P25103 P23443 Q14232 P00441 P08588 Q14116 P10827 26509 P55212 P31749 Q9NR50 P10588 9093 P34931 2063 P34932 Q08211 3301 Q96EY1 P04792 3308

mRNA export from nucleus Q86W42 Q86U42 Q9UMR2 Q86V81 Q9UPR3 23144 P57740 Q12769 57187 Q03014 26019 Q96FV9 O00148 Q9GZY0 80145 23381 84248 4686 10772 5976 11269 Q6I9Y2 Q8WUM0 8563 Q13769 Q09161 10482 O75494 Q96QD9 Q96Q15 Q8NI27 Q92900 Q9HAU5 Q8IXZ2

positive regulation of DNA replication P29353 Q9H2K2 3265 O95271 56034 3479 Q1PSW9 811 P08069 P04085 Q9NRA1 P01112 P04201 P05412 25913 5154 5155 6464 P10600 Q9NUX5 2735 P16234 P18846 1763 466 P01308 P10070 P08151 P01127 P01588 P27797 P01343 2736 P06213

regulation of cofactor metabolic process Q15118 5162 P08559 P10515 O00330 8050 P37840 P11177 5160 Q9P0J1 Q8NCN5 55066 6622 1738 1737 P09622

regulation of coenzyme metabolic process Q15118 5162 P08559 P10515 O00330 8050 P37840 P11177 5160 Q9P0J1 Q8NCN5 55066 6622 1738 1737 P09622

nucleobase biosynthetic process 5471 5631 Q06203 6470 P34896 7372 P11172 353 P49915 10606 P27708 P60891 P22102 P22234 8833 P07741

embryonic organ development 7046 Q92858 O96004 Q14686 4297 3880 O43278 2034 2672 P13349 O75386 P17676 Q9H161 474 Q8TAF8 2304 2303 P62993 Q1PSW9 P17275 P84022 P08047 O14543 639 Q9UGM1 P09017 Q9NPC8 4188 4221 5311 P08727 P13631 P55316 Q02447 Q99750 10661 P00533 Q15796 Q13099 2290 P25791 P35638 6495 7067 P48431 P05549 P32242 Q03014 O00444 Q9ULH7 55584 257 Q9NR09 Q96S42 P53708 Q9UJU2 6647 P49639 Q9NSC2 P40424 P00441 4087 Q92824 4088 8323 P19320 P13612 P35227 2735 Q01974 P20827 P10070 P10071 P08151 668 Q92786 P02458 P07101 6657 5604 2737 2736 6670 9421 O75626 Q9Y5Q3 Q15738 P28482 O43597 Q8TD84 P63092 9021 O00622 O95076 O75581 O14627 Q02535 10733 10736 P00747 P15692 O75190 Q99684 6667 4005 3676 4920 4646 Q13563 Q99966 P31249 Q6IR47 8100 7412 3297 P36894 P10600 Q06210 O00358 23414 Q9UBX0 207 Q9NQB0 Q8WW38 P09067 Q9UM54 Q99958 Q15475 O95377 7422 O75569 P56178 Q15915 7020 57448 Q00613 2885 3215 P47895 P49715 Q9GZT9 1956 80184 P10826 50814 P10827 P27540 O15078 Q15648 P31749 P19484 O00255 Q02750 Q6KC79 1050 Q03164 54583 P37173 P58012 O60353 8516 Q99814 1969 P29317 Q13351

T cell receptor signaling pathway O43353 9564 P07766 7189 Q96EP0 O95999 P28482 Q9Y4K3 O43318 Q9BYM8 10616 Q9UDY8 P61088 Q9Y6K9 P06241 P49810 5336 8767 7334 6885 55072 P16885 P20963 8517 P08575 P49768

nuclear-transcribed mRNA catabolic process P52298 Q9UL18 Q13315 Q9UPR3 22794 26019 Q9Y5S9 472 84305 23381 54512 55149 4686 Q8IZD4 O15234 5976 Q8IU60 P61326 Q15287 P38919 5394 Q09161 9775 Q96C86 Q01780 26523 Q9NVV4 10921 Q504Q3 Q96Q15 P40692 27257 167227 28960 9924 Q9NPD3 4116 Q9BRP8 Q92900 Q9HAU5 P60228 2935 64282

neural tube formation P50552 7189 P61968 P05549 O95999 7248 2909 Q9NRY4 O75386 P12755 Q9Y4K3 7020 O43318 O14727 Q96S42 P23528 Q96RK4 Q99807 Q9NYQ6 O14641 Q92830 Q14790 Q92574 P49815 1856 9839 P07737 8323 8543 6885 Q96QB1 7291 Q86YT6 1072 Q86WV8 57534 10229 841 O60353 7249 Q9UMX1 7408 Q15672

regulation of steroid hormone receptor signaling pathway 9420 2100 29966 O75881 P10275 P51843 Q99623 387 367 3479 3169 Q99497 11331 Q92731 811 10013 P61586 O75928 Q9UBN7 11315 P27797 P01343 Q13033

membrane protein proteolysis 4790 P78536 7189 Q13443 8754 5970 O14672 P56817 Q96BI3 23621 Q9Y4K3 P35579 Q04759 P19838 Q04206 P49810 51752 Q92542 Q9NZ08 P08138 51107 4627 P49768

positive regulation of neuron apoptosis P05412 Q14116 P55212 7124 7157 3672 P04637 8851 Q13315 P48023 P56199 P10586 A1XKG3 1020 P15559 472 P21359 356 Q00535 1728 Q15078 Q15375 P01375

negative regulation of mitotic cell cycle P26045 5371 6597 4088 P10415 Q12948 Q12959 P41182 P29590 O00213 P51532 23513 Q14160 P84022 604 P00533 P01137 Q13574 5119 Q14201 1956 P06400 5925

platelet-derived growth factor receptor signaling pathway Q14865 9564 5154 Q12965 7422 5155 P09619 O95470 5728 P16234 56034 84159 P15692 O60674 P60484 8879 5159 4643 P04085 P01127 5327 P00750 Q9NRA1

regulation of canonical Wnt receptor signaling pathway P25054 5460 Q9Y3M2 P48431 O95835 2010 Q99471 P52952 P23508 P84022 1453 Q9NRM7 857 6789 Q13188 6901 Q13043 Q9NQC7 O00755 4088

7476 Q16635 P14923 25776 26524 54361 P56705 O60716 P50402 3728 324 1540 P10071 P48730 6657 2737

response to radiation 8930 P28562 O15123 P04637 8533 P51587 6591 60626 351 472 2547 595 Q96RU2 477 P17275 P01116 O14543 O14788 O15516 P01112 P23025 P01111 P48681 Q9UNS2 4221 Q9NS91 Q9UBC3 5796 P35368 P10523 Q6AZY7 P16104 P63211 P46934 641 P42858 P08575 5424 P00533 Q96RL1 P05067 4734 P42574 408 P49407 P01100 2956 8553 Q9NRY2 Q13535 P14416 P31153 3265 P29590 O00562 79035 57646 54413 Q96T60 Q00535 3014 O15534 O15259 P01138 P01137 29086 4867 P02461 P55212 5970 Q13426 P55210 P18074 Q9BZF9 P19320 P10588 2063 Q9NZ94 P20941 P21359 545 Q04206 Q8WTP8 51435 10763 P43246 Q6PCD5 7508 Q13432 5187 O15287 Q13315 P54132 P54253 58473 1020 9021 Q9NX61 25788 Q99965 P09172 672 P22415 Q03468 51720 P04040 P08243 7518 P08887 2902 P08246 O14503 Q14011 5591 Q9UIM3 P51671 7412 1153 P51679 P10721 O95243 285 1789 Q07817 Q01831 P46736 Q9NWV8 2237 1026 207 847 Q70UQ0 P04179 23746 Q9BQ15 P24385 P12956 P10415 Q13216 3860 P28340 9600 P13646 A1XKG3 1161 P78527 P50993 P38398 Q9NPQ8 121457 5599 857 1956 9575 5371 64782 7157 P31749 Q05586 P39748 Q9NP97 O43623 O00255 Q6KC79 P38936 P52701 1843 P47804 55159 P45983 P02533 51514 Q14683 Q9NZN9

hormone-mediated signaling pathway O95835 P63092 B3KY43 P13861 Q9Y4C1 P51828 2783 P20396 5577 P22694 P62879 P22612 P62873 P01275 Q9HAV0 26524 P10644 P31321 51094 P31323 P37198 Q9BUB1 Q8NFM4 Q8NFM5 P63218 Q08462 7067 P50150 Q96A54 Q06124 P59768 Q86V24 Q9UBU3 P40145 O60503 Q9NRM7 O60266 23636 O95622 P10827 5573 5575 5576 O43306 P17612 O60674 5566 5567 5568

morphogenesis of embryonic epithelium O95999 P07949 7248 2909 O75386 Q9Y4K3 O43318 57669 1499 Q1PSW9 Q99807 Q9NYQ6 Q92574 P49815 3911 1856 8543 6885 Q96QB1 26005 7291 54361 1072 Q86WV8 10229 P42858 841 Q9HCM4 7249 O43294 7408 Q15672 P50552 7189 P61968 P05549 Q9NRY4 P12755 Q03014 7020 O14727 Q96S42 P23528 Q96RK4 O14641 Q92830 Q14790 P47895 2926 9839 P07737 P39905 8323 O15230 Q86YT6 P35222 P56705 57534 Q4AC94 P10070 O60353 Q9UMX1 2736

DNA catabolic process Q14249 8930 P15927 1022 Q15819 P50613 P29372 P51946 P78549 P50897 O00273 P23025 4968 Q9H0D6 Q8IY92 22803 7334 Q13569 7336 P35244 O95243 Q01831 1029 79444 6117 6119 6118 P42771 P42574 P27694 Q8N726 1676 O14727 Q96T60 O60543 P61088 P22674 Q16531 6647 Q96CA5 P00441 P18074 P99999 P54727 1642 P58012 668 5887 902 4913 7508

regulation of cell shape 2909 Q03113 P35579 P35580 310 Q96P48 Q7L0Q8 121512 P15692 3679 Q13683 Q53QZ3 P35968 Q7Z6J4 Q96QB1 Q5JSP0 58480 Q9UEE9 23370 3689 P17081 Q14344 P02751 P98174 Q6ZSZ5 P05107 7422 Q6ZV73 Q9NRY4 Q96M96 Q7Z406 4628 P15311 P20936 P02549 55785 P06241 10672 Q13145 4627 7430 3791 P20073 Q8WWN8 Q7L576 79784 Q9NRR8 2335 56882 Q9H4E5 Q9UL54

ribonucleotide metabolic process O75947 Q16864 P30085 P16615 P30520 P15531 P11172 476 477 478 513 514 515 P61421 518 9114 P28331 P48201 P48047 P13637 481 P12268 483 1503 488 521 522 523 P22102 526 528 Q9UI12 9525 P56134 29922 490 Q9BW91 495 P49915 498 P00846 4907 P25705 533 P18859 535 P56385 P00568 537 P01137 539 Q9UN42 P56381 P21589 10476 10632 23439 P53396 O75351 P12883 Q9Y2B5 P17812 8833 P20020 P30049 P30566 O00746 245972 P38606 P36542 23400 271 158 P60891 P31939 5471 51606 51727 5198 P22392 5631 Q15904 Q6PIW4 P30793 7372

Q99437 Q93050 P20648 P54709 Q01433 P05023 P17081 P05026 Q9Y5B8 65220 Q9UGJ0 O75964 Q06203 P50993 3615 3614 2643 P24539 Q04656 P22234 Q8N8Y2 P11908 O95544 161823 P21283 P21281 9296 P15313 Q9UKK9 27032 4719 10606 4830 Q6DHV7 4833 P98194 509 P06576

vacuolar transport 4074 Q9UPV9 Q86VS8 Q4G0F5 O00203 154 Q9NZ43 Q99523 P07550 Q9UGU5 Q9Y2T2 9559 O15118 91782 O75436 8546 6642 26985 Q9NZZ3 Q8IWJ2 112936 Q9UJC3 Q8WUX9 P46934 P20645 388 P62745 Q96ED9 51510 84376 9648 4734 Q13596

cerebellar cortex development O00755 136319 7476 A1XKG3 1020 P58546 O75385 2735 P11021 773 Q00535 Q1PSW9 P10070 P08151 Q04656 8408 3309 Q99835 2736

response to growth factor stimulus P02452 P48431 6498 P12757 2010 Q16828 57669 P41231 1499 356 1277 10013 O00755 7476 P48023 P12429 P35222 54361 P56705 P50402 Q9HCM4 5029 6657 1848 Q9UBN7

regulation of receptor activity 5580 P29353 O43639 84619 351 P00749 Q9UNH7 58533 P49810 10013 P01135 P01133 Q9UJM3 P78536 5054 6464 57732 Q8N5A5 Q05655 54206 P05121 P05067 5328 Q9UBN7 P49768

positive regulation of glucose transport 5584 Q86V24 O60502 P21860 P27986 Q9NSA1 P53675 5295 Q15303 Q9BX66 10580 P31749 Q9Y4H2 2065 P35568 5590 10724 Q05513 P01308 207 8218 3667 P17081 P41743 P06213

thymus development P25054 7046 6495 P10415 P46109 1499 1399 P49810 6647 P40424 Q92833 P00441 Q9C0K0 Q6IR47 Q13422 9133 O43521 P35222 O95067 64919 324 3720 10320 Q15475 P49768

histone acetylation O75528 Q09472 O95696 Q9H0E3 Q96L91 4297 Q9Y265 6871 2033 Q9UNL4 79595 10856 10933 Q92793 Q92830 O95619 Q7Z6C1 51147 Q9Y230 84289 Q92993 Q9UBU8 8607 Q92831 O95983 55929 6883 O75478 Q16514 Q15306 O96019 O95361 O00213 P35227 Q8WYH8 Q03164 10524 57634 10626 1387 Q9NPF5 Q4LE28 408 P49407 86 53615

primary neural tube formation P50552 7189 P61968 P05549 O95999 7248 2909 Q9NRY4 O75386 P12755 Q9Y4K3 7020 O14727 Q96S42 P23528 Q96RK4 Q9NYQ6 O14641 Q92830 Q92574 P49815 1856 9839 P07737 8323 8543 Q96QB1 7291 1072 Q86WV8 O60353 7249 Q9UMX1 7408 Q15672

negative regulation of signaling process P25054 P30086 2305 3479 997 3476 O14544 O14543 Q92574 P49815 P07996 2308 7057 6003 4221 Q96QB1 Q15669 5037 O75376 Q96D03 O43609 P67775 9628 Q13370 408 P49407 409 P01343 O15524 O00206 Q92835 8315 P14416 P32121 P56539 Q7Z569 P35240 O15379 P21860 O14964 P49758 5716 Q8WWN8 8841 Q14118 2065 9093 P78318 P35348 Q13829 5728 P21359 P41220 P60484 P17936 Q96EY1 9927 9146 8452 4771 O43597 7248 7126 P41182 9021 Q16828 P62714 7099 Q8WZ19 P21580 P30153 Q9Y5J5 Q86WV8 P37198 324 7128 207 7249 9306 604 Q13574 Q13618 P08069 857 5997 859 5515 5516 O14921 5518 23636 Q12778 O75832 Q08050 Q13227 P31749 O95140 Q04771 O00255 Q8N9R8 253980 51231 1848 1605 P49427

cytokine-mediated signaling pathway P78423 6672 Q92934 7124 29108 Q8WU90 1460 Q9Y4K3 P42224 P01116 3953 O14788 P42229 P08887 P42226 P40763 7132 Q15628 Q9NWZ3 P10721 Q04941 8737 P48357 7804 O15524 Q92600 7186 O14492 7189 Q16849 6772 Q9H305 6774 P32246 Q9Y6Q6 572 P41597 5798 P49715 P01375 P41159 Q14114 Q12933 5970 P51692 Q9Y6R0 Q13546 1050 9253 P14784 P19438 O60674 P51617 P67870 Q04206 6776 P23458 Q9ULZ3 P23497 8717

regulation of lipid metabolic process P54619 2672 P50416 P42345 P37288 Q96RU7 476 Q8WTS1 27122 Q7RTN6 P23786 6622 P04085 Q9C0K7 P42229 5933 Q9UBK2 5154 P28330 5155 54361 51099 Q13370 Q13133 Q13131 Q15831 7067 5562 5321 5563 5565 P52735 P41235 57761 O60543 Q9UKW4 Q96RK4 P01019 6647 P01137 Q9UBM7 P00441 P01375 P06400 8721 P55055 1374 P56705 P54646 Q9UNE7 P19838 Q92786 6776 P01127 3667 6794 7124 Q9H9S4 672 552 Q99684 Q08999 10451 Q13285 10577 P47712 P30556 Q9Y478 7376 Q9NP71 7410 10580 Q9Y4H2 Q9UGI9 P35568 P37231 57678 P01308 2475 207 5468 Q9UBP4 P05023 4790 P01185 Q9UGJ0 22937 10273 O60869 P30542 P38398 Q9HCL2 P60033 975 P01579 857 Q12770 P41159 P10827 Q9Y376 O43741 Q9BX66 P51692 P31749 P33121 P37840 P28749 P21554 P07148 5925

regulation of myeloid cell apoptosis P10827 P78536 7067 P10415 Q8N726 Q7L5Y9 5728 1029 960 P60484 10296 6776 P42771 P16070 P42229

NLS-bearing substrate import into nucleus Q14974 O00505 P52294 O15131 O00629 O00410 23633 Q9P2R6 3836 473 Q9NZQ3 P52292 3841 3843 51517

pattern recognition receptor signaling pathway O43353 O00206 7099 4792 O95999 5970 64127 O60603 148022 57402 3329 81622 Q9Y2C9 P51617 Q8IUC6 Q04206 Q9HC29 8767 P10809 P25963 Q9HCY8 Q9H1C4

cerebellum morphogenesis 8443 O00755 136319 7476 O75553 A1XKG3 1020 P58546 O75385 2735 P11021 773 Q00535 Q1PSW9 P10070 P08151 Q04656 8408 3309 Q99835 2736 O15228

protein amino acid acetylation Q9H0E3 Q96L91 4297 Q9Y265 6871 2033 10856 10933 79612 Q7Z6C1 Q9Y230 84289 8607 55929 6883 Q16514 Q15306 O96019 O95361 Q8WYH8 57634 10626 80155 1387 Q4LE28 408 P49407 O75528 Q09472 O95696 Q9UNL4 79595 Q92793 Q92830 O95619 51147 Q92993 Q9UBU8 Q92831 O95983 O75478 O00213 P35227 Q03164 10524 Q9BXJ9 Q9NPF5 P41227 86 53615

positive regulation of mononuclear cell proliferation O43353 4690 P54132 P15923 P41182 O43639 Q9Y4K3 Q04759 8767 Q9Y275 6869 P42229 Q96FA3 7099 4860 7412 Q9Y4H2 57678 641 1026 P14174 P08575 P01589 604 1947 P98172 O00206 P07766 P00491 7189 6850 Q9BQ51 148022 57162 6929 Q9HCL2 P01579 10673 P25103 P05112 Q14116 P78552 P51692 P35225 P19320 P38936 9093 P16333 P43405 Q8IUC6 6776 P40933 Q96EY1 P60568 P04233 P43489

hexose catabolic process P06733 7167 Q92934 P08559 P37837 P10515 P40926 10690 P52790 160287 230 2821 2026 P60174 P09972 Q9Y231 1737 4967 P06744 P04406 P52209 3099 P11413 3098 P11177 P05062 5226 P49247 P09104 5162 P07205 Q6ZMR3 22934 P14618 Q9BYZ2 92483 P16118 4191 5160 572 3939 5315 Q49A26 5213 2023 2582 P19367 P52789 3948 P17858 P00558 Q01813 5207 P08237 P07864 3945

negative regulation of cell development 4099 6993 P04637 P20916 P63172 O75385 4092 396 Q8IYN9 2147 O14786 P61586 P02686 Q99966 8408 Q8N5U6 Q04917 O00755 P52565 7533 P36894 P23515 P00734 Q15154 P08138 5108 Q92888 5747 P49768 6696 4155 9921 P56177 Q86VS8 P12755 P10451 Q05397 P51805 6405 P01137 Q9NQC3 8829 P29323 7157 7476 9138 Q658W2 387 P21359 O15105 84376 55558 Q13275 Q9BZR6

hair follicle development P25054 Q15738 P10415 P31276 Q08188 Q9NS68 2304 Q04656 91 P49810 P04085 55504 3911 1956 50814 O95863 94234 5154 Q9C009 5970 O15230 P18074 A4D1W7

P31749 P36896 P19883 O00358 324 Q04206 P46531 4851 5467 P48436 207 P00533 P08138 6615 10468 P49768

immune response-activating cell surface receptor signaling pathway O43353 9564 P07766 O14492 7189 Q96EP0 6850 O95999 P28482 Q9NZD8 Q9Y4K3 O60603 O43318 Q9BYM8 Q8WZ60 10616 Q9UDY8 P61088 Q9Y6K9 P06241 51324 P49810 5336 8767 5579 P05771 719 Q8IWV1 7334 6885 P11912 55072 P16885 P20963 P43405 8517 P08575 Q16581 P49768

molting cycle process P25054 Q15738 P10415 P31276 Q08188 Q9NS68 2304 Q04656 91 P49810 P04085 55504 3911 1956 50814 O95863 94234 5154 Q9C009 5970 O15230 P18074 A4D1W7 P31749 P36896 P19883 O00358 324 Q04206 P46531 4851 5467 P48436 207 P00533 P08138 6615 10468 P49768

hair cycle process P25054 Q15738 P10415 P31276 Q08188 Q9NS68 2304 Q04656 91 P49810 P04085 55504 3911 1956 50814 O95863 94234 5154 Q9C009 5970 O15230 P18074 A4D1W7 P31749 P36896 P19883 O00358 324 Q04206 P46531 4851 5467 P48436 207 P00533 P08138 6615 10468 P49768

protein polymerization P02671 10093 Q8TCU6 11065 O00762 84617 7283 26271 P42768 203068 Q9H4B7 P07196 P63000 2244 Q9BVA1 347733 Q9BUK6 Q92558 5879 O43182 Q9HC77 Q9BQE3 P07437 Q13885 P03950 7277 10383 2243 Q13509 Q13748 P68371 Q9BUF5 O60610 P68366 Q9NRH3 P23258 55835 P04350 1729 Q71U36 7846 Q9UKT4 P02675 8936 84790

cell morphogenesis involved in differentiation P25054 Q92858 8650 P55268 Q9UQP3 Q8TEW0 Q9Y3I0 O75385 9341 P11047 351 3913 474 Q8TAF8 Q8IYN9 P41134 Q86WK6 P16220 Q1PSW9 P07196 P39060 P83731 O14786 Q6NVY8 P43034 P46821 O60282 3911 8408 3912 Q9NPC8 Q9C0K0 Q96CW9 3091 O15496 Q12837 O43602 10939 2043 54361 P55316 O60331 81565 P46531 4851 P48436 P05067 P98172 2290 8399 P54753 Q7Z727 P50552 6256 Q15836 P14416 P19793 Q06124 Q13418 Q05397 773 P51805 Q00535 Q96S42 P17252 Q53X93 4983 P21860 Q9UJU2 Q9UQB8 6647 O60383 P49639 Q9NSC2 8829 Q13263 P00441 4627 Q16650 6383 P54764 P54762 P04626 Q9Y6R0 Q658W2 2065 P13611 2064 P35222 Q8IYT8 O00213 P56705 Q7L576 9253 O75592 2335 O60674 56288 P10070 23032 P10071 23396 Q92786 6259 6812 Q16665 2737 2736 P34925 P07942 8452 6275 4131 Q6FGG2 8851 Q8TD84 1385 P78509 1020 P35579 10458 10736 57669 1499 5578 Q99729 Q15223 Q99966 P26232 O00238 P60709 P52803 O43921 P19544 7490 64919 324 Q9HCM4 O60890 60 Q9GZM8 1947 P08138 7408 85440 5747 P02751 Q9HCU4 Q15078 9201 Q8TEY7 P10415 P56178 Q8IUQ4 Q02880 P34741 Q13618 A1XKG3 O60229 3182 P20336 Q96N67 3611 O14640 P61764 P08069 Q92752 10155 O15075 P29323 7155 P39905 O15230 Q15768 Q13509 Q9HCK4 23077 55558 P26447 Q15375 Q9BZR6

regulation of fatty acid metabolic process 6794 Q9H9S4 P54619 P50416 P42345 672 P37288 552 Q96RU7 Q7RTN6 P23786 6622 Q9C0K7 P47712 Q9UBK2 Q9Y478 7376 P28330 Q9NP71 Q9Y4H2 Q9UGI9 P35568 P37231 P01308 2475 207 5468 Q13133 Q13131 Q15831 P01185 Q9UGJ0 5562 22937 5321 5563 5565 P38398 57761 P01019 857 Q12770 Q9Y376 O43741 P55055 P31749 1374 P33121 P37840 P54646 3667 P21554 P07148

epithelial cell proliferation Q8TCU4 P24385 P25067 O15111 O15353 5155 7124 Q15648 2099 Q02363 P10721 P17676 55366 595 1147 Q9BXB1 8945 P48436 P01127 Q9Y297 P03372 O14788 P01375 P49768

positive regulation of mitotic cell cycle	7013 Q9NYA1 O75604 6198 O15392 P51692 P54274 Q9UPT9 P14635 P51587 9099 23326 4193 351 891 Q00987 3169 Q92786 6776 P08243 P05067 P42229 1978 P23443

purine nucleotide metabolic process	O75947 5141 5142 Q16864 8654 9061 P29372 P16615 P30520 P15531 476 477 478 513 514 515 5139 P61421 518 4968 P28335 9114 P28331 P48201 P48047 P13637 Q07343 481 P12268 483 O76074 488 521 522 523 P22102 526 Q13370 528 Q9UI12 O43252 9525 P56134 29922 490 Q9BW91 495 P49915 498 P00846 4907 P25705 533 P18859 535 P56385 P00568 537 Q16774 P01137 539 Q9UN42 P56381 P21589 10476 10632 23439 P53396 4882 O75351 P12883 Q9Y2B5 8833 P20020 P30049 P30566 O00746 245972 P38606 P36542 23400 271 2987 158 P60891 P31939 P20594 5471 51606 5198 P22392 5631 Q15904 Q6PIW4 P30793 Q99437 Q93050 Q8NFM5 P20648 P54709 Q01433 P05023 P17081 P05026 Q9Y5B8 Q08462 65220 P00492 Q9UGJ0 O75964 Q06203 P50993 P11586 O60502 3615 3614 2643 P24539 Q04656 P22234 Q8N8Y2 1716 Q14432 O95544 161823 P21283 Q16854 P21281 9296 P15313 27032 10724 4719 10606 4830 P27815 Q6DHV7 4833 P98194 509 P06576

toll-like receptor signaling pathway	O43353 O00206 7099 4792 O95999 O60603 148022 57402 3329 81622 Q9Y2C9 P51617 Q8IUC6 8767 P10809 P25963 Q9HCY8 Q9H1C4

cytoplasmic sequestering of transcription factor Q9UBC1 P10827 7067 O75832 4792 4188 10460 Q9UNN5 O00221 9908 Q99750 Q9UMX1 11124 Q9UN86 Q15653 Q9Y6A5 P25963 5716

positive regulation of phosphoinositide 3-kinase cascade	P01241 P04626 2065 2064 Q05086 P46934 O14939 O60674 3479 P01308 P01019 5467 P21860 P04040 847 5338 4734 P01343

positive regulation of Rho GTPase activity	P51671 7248 Q92949 10928 Q86WV8 23513 Q96P48 Q14160 Q96N67 81565 Q6PID4 Q6ZW31 Q92574 Q9GZM8 Q14289 85440 Q15311 85360

regulation of epithelial cell proliferation P07942 P25054 9420 6794 9500 5268 1021 P14555 P51587 P10275 P11047 P15531 P23508 1499 P15692 3479 P84022 Q9HC57 Q92731 Q6NVY8 6869 Q13761 P49815 3912 P28799 O00755 Q15303 P35968 Q9Y5V3 P22392 P36894 79084 P30279 367 324 P46531 4851 5467 7249 P00533 P01343 Q15831 P48431 7422 P56178 O75881 Q99623 894 2247 2246 Q00534 P05230 P01137 P25103 Q9BQA1 P01135 1956 P09038 P06401 Q9BSI4 3791 4088 7476 P04626 Q15648 P36952 2100 58189 2064 P35222 5728 O75084 2896 P60484 11331 4830 6657

purine ribonucleotide metabolic process	O75947 Q16864 P16615 P30520 P15531 476 477 478 513 514 515 P61421 518 9114 P28331 P48201 P48047 P13637 481 P12268 483 488 521 522 523 P22102 526 528 Q9UI12 9525 P56134 29922 490 Q9BW91 495 P49915 498 P00846 4907 P25705 533 P18859 535 P56385 P00568 537 P01137 539 Q9UN42 P56381 P21589 10476 10632 23439 P53396 O75351 P12883 Q9Y2B5 8833 P20020 P30049 P30566 O00746 245972 P38606 P36542 23400 271 158 P31939 5471 51606 5198 P22392 Q15904 Q6PIW4 P30793 Q99437 Q93050 P20648 P54709 Q01433 P05023 P17081 P05026 Q9Y5B8 65220 Q9UGJ0 O75964 Q06203 P50993 3615 3614 2643 P24539 Q04656 P22234 Q8N8Y2 O95544 161823 P21283 P21281 9296 P15313 27032 4719 10606 4830 Q6DHV7 4833 P98194 509 P06576

macromolecular complex disassembly	4150 8458 Q9H2K0 P41181 Q6NZI2 7141 3084 284119 3159 Q99661 Q01105 P17096 Q14197 P09430 6605 11004 Q9UNY4 Q9H0D6 Q969G3 22803 Q9UIG0 Q8N4N8 Q02297 3396 9495 3925 11198 Q9Y5B9 P24588

cellular macromolecular complex disassembly 4150 8458 Q9H2K0 P41181 Q6NZI2 7141 3084 284119 3159 Q99661 Q01105 P17096 Q14197 P09430 6605 11004 Q9UNY4 Q9H0D6 Q969G3 22803 Q9UIG0 Q8N4N8 Q02297 3396 9495 3925 11198 Q9Y5B9 P24588

central nervous system neuron axonogenesis 9201 P54753 O15075 Q7Z727 P29323 P54762 O43602 Q658W2 Q05397 O75592 Q8IYN9 Q1PSW9 P10070 P17252 81565 23077 5578 P43034 Q9GZM8 5747 2736

macroautophagy Q13501 Q9Y4P1 9474 P07339 Q8TDY2 9821 55054 79065 Q676U5 Q9BSB4 Q9H492 23192 Q9H1Y0 60673 10013 8878 Q7Z3C6 Q9UBN7 O94817 Q13286 P49768

regulation of vesicle-mediated transport Q86UW7 23607 Q16623 9066 P50897 23327 P61020 P04083 6622 90678 5898 Q15389 P49815 P07355 P04004 8301 Q08431 Q9HD26 7448 Q13492 Q13133 Q15036 Q7Z727 P22681 5321 Q8WVM8 57120 773 Q00535 P17252 811 P01137 55503 P55290 Q9H1D0 4240 5054 3673 P55055 Q86YT6 Q6UWE0 Q9UNE2 382 57534 5728 P17301 P43405 O43707 301 302 P60484 6812 6814 9784 Q9UNF0 O00186 1020 P51148 3162 274 O00194 Q6PJW8 1012 Q96PU5 P63000 5578 5338 P47712 7376 64127 23256 P62166 P21579 7249 967 5867 P27797 5869 P46459 6850 Q9BY11 A1XKG3 Q9Y5K6 Q9H223 P20338 P62330 P20336 2247 P61764 857 5878 5879 P09038 Q9UKS6 O43581 11252 O00499 P37840 P11234 P11233 O14939 Q9HC29 867 P05121 9846 P09601

DNA integrity checkpoint P04637 Q13315 P54132 O43715 25988 472 990 595 672 Q96RU2 51720 26270 O96017 Q9NYZ3 P16104 Q01831 P46736 P20248 Q9NWV8 641 1026 P51959 5347 Q9NRD1 Q96RL1 Q99638 Q13574 O14757 Q6ZN33 P24385 5883 8555 Q13535 P29590 Q06124 79791 890 P38398 Q9BQA5 57646 Q99741 Q5XUX0 1111 3014 Q6NUQ1 29086 5371 Q14676 9656 7157 P53350 P38936 80254 55159 545 Q96MT8 900 51512 P43246 51514 Q6PCD5 7508

syncytium formation by plasma membrane fusion O95259 P29466 50937 P29475 Q9Y463 9149 P35579 P17655 960 O95180 Q4KMG0 P16070 O43184 4627

substrate adhesion-dependent cell spreading P07942 Q6FGG2 Q15836 O15230 Q13418 Q9Y3I0 9341 P11047 2335 3611 Q6NVY8 3911 P02751 3912

histone mRNA catabolic process5394 Q13315 Q01780 Q9NVV4 472 54512 27257 167227 55149 Q9NPD3 5976 Q92900 Q8IU60 64282

DNA unwinding involved in replication 4173 Q14566 4176 P33993 P33991 7150 4171 P11387 3159 Q96RR1 Q00577 P17096 5888 P49736

mRNA polyadenylation P51693 23144 P11940 26986 Q9UKF6 351 1478 333 P33240 29894 P05067 2926 Q8IXZ2 Q10570

telomere maintenance via telomerase Q9NYB0 25913 4361 7013 7014 5981 P54274 Q9NUX5 P49959 P35251 Q15554 Q92878 1736 Q9C0C2

regulation of acetyl-CoA biosynthetic process from pyruvate Q15118 5162 P08559 P10515 O00330 8050 P11177 5160 Q9P0J1 Q8NCN5 55066 1738 1737 P09622

initiation of signal transduction P78423 6672 Q92934 7124 29108 Q8WU90 1460 Q9Y4K3 P42224 P01116 3953 O14788 P42229 P08887 P42226 P40763 7132 Q15628 Q9NWZ3 P10721 Q04941 8737 P48357 7804 O15524 Q92600 7186 O14492 7189 Q16849 6772 Q9H305 6774 P32246 Q9Y6Q6 572

P41597 5798 P49715 P01375 P41159 Q14114 Q12933 5970 P51692 Q9Y6R0 Q13546 1050 9253 P14784 P19438 O60674 P51617 P67870 Q04206 6776 P23458 Q9ULZ3 P23497 8717

signal initiation by diffusible mediator P78423 6672 Q92934 7124 29108 Q8WU90 1460 Q9Y4K3 P42224 P01116 3953 O14788 P42229 P08887 P42226 P40763 7132 Q15628 Q9NWZ3 P10721 Q04941 8737 P48357 7804 O15524 Q92600 7186 O14492 7189 Q16849 6772 Q9H305 6774 P32246 Q9Y6Q6 572 P41597 5798 P49715 P01375 P41159 Q14114 Q12933 5970 P51692 Q9Y6R0 Q13546 1050 9253 P14784 P19438 O60674 P51617 P67870 Q04206 6776 P23458 Q9ULZ3 P23497 8717

signal initiation by protein/peptide mediator P78423 6672 Q92934 7124 29108 Q8WU90 1460 Q9Y4K3 P42224 P01116 3953 O14788 P42229 P08887 P42226 P40763 7132 Q15628 Q9NWZ3 P10721 Q04941 8737 P48357 7804 O15524 Q92600 7186 O14492 7189 Q16849 6772 Q9H305 6774 P32246 Q9Y6Q6 572 P41597 5798 P49715 P01375 P41159 Q14114 Q12933 5970 P51692 Q9Y6R0 Q13546 1050 9253 P14784 P19438 O60674 P51617 P67870 Q04206 6776 P23458 Q9ULZ3 P23497 8717

response to heat 6198 8892 O14793 1660 P30086 9021 3316 Q04759 3315 Q6FHQ0 O60884 81570 5931 O14543 6869 P07996 P47712 P49770 P31689 7057 Q9UBG3 Q92769 5037 3297 Q9H078 Q16082 207 11080 P29474 P10415 P29475 5321 Q9Y5S1 3066 Q8WW22 Q9UDY4 Q00613 2246 55466 Q9NZM1 6647 P05230 Q16576 Q13144 P25103 P23443 Q14232 P00441 26509 P55212 P31749 Q9NR50 9093 P34931 P34932 Q08211 3301 Q96EY1 P04792 3308

regulation of oxidoreductase activity P27037 4790 P78540 7124 2672 Q9BQ95 P56539 P16435 2643 Q99684 Q04656 P01019 6622 P01579 10013 P01116 5018 P42684 859 1956 P01375 P30556 3091 P39905 9973 P31749 P34972 P37840 P30793 P00519 51295 P17813 P19838 P01308 Q15070 207 P00533 Q16665 Q9UBN7 P21554

positive regulation of leukocyte proliferation O43353 4690 P54132 P15923 P41182 O43639 Q9Y4K3 Q04759 8767 Q9Y275 6869 P42229 Q96FA3 7099 4860 7412 Q9Y4H2 57678 641 1026 P14174 P08575 P01589 604 1947 P98172 O00206 P07766 P00491 7189 6850 Q9BQ51 148022 57162 6929 Q9HCL2 P01579 10673 P25103 P05112 Q14116 P78552 P51692 P35225 P19320 P38936 9093 P16333 P43405 Q8IUC6 6776 P40933 Q96EY1 P60568 P04233 P43489

regulation of viral reproduction 6672 7023 P10415 P62937 7124 O95292 3065 P11388 10318 Q96T23 Q9HC16 Q01664 6667 5478 P08047 Q15025 Q6IT96 P01375 P05412 Q13148 7153 9217 Q13547 P10147 23435 60489 51773 Q92786 4214 Q13233 P23497

regulation of osteoblast differentiation P27037 P25054 Q13873 Q15797 P48431 Q8WUI4 P56178 P63092 1021 P12755 57045 Q9H6W3 1499 P84022 Q00534 51564 Q9Y2T1 O75928 P08887 Q99966 Q9NSA3 O00238 P56524 Q9GZX9 4086 4088 4221 Q04771 O00255 Q13705 P36894 P35222 54361 P56705 P11474 P17535 324 Q9H2X0 P46531 4851 6657 Q9BXL5

ion transmembrane transport P30049 O75947 Q16864 245972 P38606 P36542 477 513 514 515 P61421 518 9114 51606 P48201 P48047 Q15904 Q99437 Q93050 521 522 P20648 523 526 528 Q9UI12 O75964 P56134 P50993 495 498 P00846 P25705 P24539 533 P18859 535 Q04656 P56385 Q8N8Y2 537 539 P56381 10476 10632 P21283 P21281 9296 P15313 Q9Y2B5 509 P06576

mitotic cell cycle checkpoint P25054 P04637 Q13315 8379 P14635 Q96EP1 O43715 6194 472 10459 595 O60566 P83731 P62753 P01112 O43683 7013 Q12959 Q9NYZ3 P20248 1026 P51959 P50748 324

Q9UI95 Q13574 Q9Y6D9 Q6ZN33 P24385 Q13257 P54274 377630 3265 P29590 79791 890 891 Q5XUX0 55743 P01137 P06400 5371 O43264 7157 Q6R6M4 P38936 7272 55159 900 51512 Q6PCD5 5925

regulation of actin polymerization or depolymerization 4690 10092 10095 O95835 10094 Q9UHB6 O43639 P35612 P35611 P42345 6709 830 832 118 Q9H2D6 P47755 P47756 P51671 Q01082 1072 10109 2039 2475 6711 6710 4214 P62328 P61158 O15020 Q13813 O15143 Q9BPX5 23189 P62330 P20936 P23528 P02549 81873 10552 P35080 Q08495 Q14678 P48061 382 P11277 P16333 51474 P52907 Q6PKD3 P63313 Q13233 829

cardiac muscle tissue development 9421 Q9Y3M2 O96004 7248 Q12948 P14635 4092 3084 P52952 P19404 4729 2303 P15692 79810 Q8WV60 P09493 23363 Q92574 O15119 6901 P45379 9474 Q16635 Q02297 P68032 Q86WV8 23414 2280 Q8WW38 Q99958 6256 7422 P19793 Q9ULH7 891 Q9H1Y0 P02545 Q9GZT9 Q8WZ42 Q9UPY3 P26678 6926 P10826 4000 25776 P12883 5728 54583 P60484 Q92786 O15105 P62942

receptor-mediated endocytosis 9784 P50570 O75146 P15328 3482 O75385 P50897 154 2348 P62993 1213 P07550 6622 5338 8408 Q13286 8301 P42261 P46934 P20645 1785 O60895 Q13492 8218 4734 408 Q15036 P49407 409 5868 Q01518 4074 4035 6653 P32121 P20339 2885 Q00610 857 Q92673 P61966 P53675 P11717 1174 P37840 P21757 O14939 Q07954 10487 P06576

regulation of transforming growth factor beta receptor signaling pathway 6498 P12757 64750 P04637 Q8N6I1 5300 P12755 4092 Q86TG7 Q9UNH7 P84022 O60543 O14908 Q9H160 P53708 58533 5654 P01137 Q13145 Q13485 Q99966 P07996 P09958 P78536 7057 4087 4088 Q9HAZ2 4089 4221 Q13526 7157 Q9HCE7 163126 63976 O00255 57154 P10600 P11021 P17813 3622 8516 O15105 O43294 Q15796 Q9HAU4 3309 Q92743

cellular polysaccharide metabolic process O95278 P13807 Q9UGJ0 11041 P06737 Q9Y223 56052 2132 Q8N6G5 23127 Q9NR45 Q93063 178 Q8NBJ5 Q93100 51148 Q15185 P62136 Q5T4B2 5836 Q9BT22 Q13522 O95685 55907 Q16816 P46020 5499 Q9UGI9 P36873 P15735 P11216 O43505 Q8IYK4 Q7LGA3 55454 2932 Q8NFW8 2632 P35573 Q04446 P62140 10728 5500 5501 P49841 P46019 P46976 79709

prostate gland epithelium morphogenesis 9420 2099 O75881 P35453 P10275 3479 Q1PSW9 Q92731 P08069 P03372 P42229 Q92826 2100 P13631 960 367 P10070 3169 P46531 4851 P48436 P24821 6776 P16070 P01343 2736

regulation of glucose metabolic process Q9UGJ0 P49840 P54619 P16118 P42345 3479 Q92630 Q9UBK2 P41159 P56524 Q13322 P27540 3091 Q9NP71 Q9BX66 10580 8445 P31749 Q9Y4H2 P35368 Q9UNI6 P35568 2931 P62140 P01308 2475 5500 207 Q16665 3667 5207 P01343 P06213

ossification P02452 6670 O00628 P63092 P13349 Q12948 2550 P41180 3481 Q9NX62 Q9Y4K3 Q93063 2303 Q9Y3A5 Q99523 Q9UBS5 Q1PSW9 P17275 P84022 P15173 6667 P20594 P08047 Q9Y2T1 O14788 P21246 4209 P56524 Q9NNX1 Q15742 Q14814 4221 Q9NZC7 P10600 Q5TAB7 Q02447 P21815 846 P08253 P00533 Q92882 Q99958 P22303 P01344 Q99835 7189 Q15797 7067 P48431 6696 P56178 2099 2132 57045 P10451 P34820 Q9Y6Q6 1277 656 4664 5599 Q5TAT6 4665 51741 P03372 P01137 Q16610 1956 Q9GZX9 P10827 4086 134701 P05997 4088 4882 P18074 O00255 P12645 Q13506 P35548 P17535 P15313 P13497 2735 P21359 P10070 P08151 5764 4313 P02458 P45983 6657 2736

bone development P02452 P13349 Q12948 2550 3481 Q9Y4K3 2303 Q9Y3A5 Q99523 Q9UBS5 Q1PSW9 P17275 P84022 P08047 O14788 P21246 4209 P56524 Q9NNX1 4221 Q5TAB7 P13631 Q02447 P21815 P48436 P00533 P01344 7189 Q15797 7067 P48431 P12755 P13686 P10451 P34820 Q9Y6Q6 656 P03372 P01137 Q9GZX9 4086 134701 4088 4882 P18074 P12645 P17535 2735 P21359 P31270 P10070 P08151 P02458 6657 2736 6670 O00628 P63092 P41180 Q9NX62 Q93063 P15173 6667 P20594 P55075 Q9Y2T1 54 Q15742 Q14814 Q9NZC7 P10600 Q6UB99 3207 846 P08253 Q92882 Q99958 P22303 Q99835 6696 P56178 2099 2132 2253 57045 1277 4664 5599 Q5TAT6 4665 51741 Q16610 1956 P10827 P05997 O00255 Q13506 P35548 P15313 P13497 5764 4313 P45983

response to osmotic stress P01185 7124 5585 P04637 59341 P41181 Q9Y3Q8 P11166 81628 1831 310 553 P01019 O15455 1956 P01178 P01375 7098 P20073 Q16512 O15530 7157 3673 P55011 8065 5170 P17301 Q93034 Q99576 Q6PID4 4214 P00533 P01588 Q14289 Q13233

virus-host interaction 8563 6890 Q86W42 4088 6993 Q13769 9217 Q86V81 O95292 57187 P63172 Q96FV9 80145 Q7L523 79139 Q8NI27 P84022 P01137 Q03518 P01730 Q6I9Y2 Q9BUN8 P06213

positive regulation of B cell proliferationO00206 7099 P78552 Q9Y4H2 P35225 P15923 P41182 P38936 148022 57162 6929 1026 P14174 Q8IUC6 P08575 10673 604 Q9Y275 P05112 P60568 P04233 P43489 Q96FA3

regulation of monooxygenase activity P27037 4790 P78540 7124 2672 P56539 P16435 2643 Q99684 6622 P01579 P01116 859 1956 P01375 3091 P39905 P31749 P34972 P37840 P30793 P17813 P19838 P01308 207 P00533 Q16665 P21554

visual behavior P54253 P14416 A1XKG3 3265 1020 P50993 60626 351 Q99965 P09172 Q9NPQ8 54413 477 Q00535 O15259 P01116 4867 P01112 2902 P01111 Q05586 P35368 Q9NP97 P10721 Q9NZ94 P21359 P42858 P05067

prostate gland morphogenesis 9420 5268 2099 O75881 P35453 P10275 3479 Q1PSW9 Q92731 P08069 P03372 P42229 Q92826 P36952 2100 P13631 960 367 P10070 3169 P46531 4851 P48436 P24821 6776 P16070 P01343 2736

G1/S transition checkpoint Q6ZN33 P24385 P04637 377630 3265 P29590 79791 O43715 6194 595 Q5XUX0 P83731 P01137 P62753 P01112 P06400 5371 7157 Q6R6M4 Q12959 P38936 Q9NYZ3 1026 55159 51512 Q13574 Q6PCD5 5925

proton transport P30049 O75947 O75348 Q16864 245972 P38606 P36542 477 513 514 515 P61421 518 9114 51606 P48201 P48047 23530 Q15904 9550 Q99437 Q93050 521 522 P20648 523 526 528 Q9UI12 O75964 P56134 P50993 495 498 P00846 P25705 P24539 533 P18859 535 P56385 Q8N8Y2 537 539 P56381 10476 10632 Q13423 P55851 P21283 P21281 9296 P15313 Q9Y2B5 509 P25874 P06576

regulation of transmembrane receptor protein serine/threonine kinase signaling pathway P27037 5460 Q13873 7046 64750 P04637 5300 4092 Q93062 P84022 O14908 Q9H160 91 5654 94 Q13485 Q99966 P07996 Q6IR47 P78536 7057 11030 Q9HAZ2 4221 Q13526 Q9HCE7 P36896 57154 P36894 P10600 2280 9350 P11021 Q9BQB4 Q9H2X0 P46531 4851 O43294 Q15796 Q9HAU4 10468 6498 O95813 P12757 Q8N6I1 P12755 57045 P37023 Q86TG7 Q9UNH7 O60543 P53708 58533 857 P01137

Q13145 P09958 Q9GZX9 4087 4088 4089 7157 A4D1W7 163126 Q04771 63976 O00255 Q13705 P19883 7272 P17813 3622 8516 O15105 3309 P62942 Q92743

establishment or maintenance of apical/basal cell polarity Q7KZI7 Q8N205 7430 5584 P25391 Q9Y696 Q92949 2011 Q13418 23513 163183 P15311 Q96RT1 Q14160 3611 284217 P41743

DNA-dependent DNA replication initiation 4172 4173 4174 Q14566 4176 5422 P33992 O96020 P33993 P33991 4171 Q00577 997 P09884 P25205 P49736 P49427

microtubule-based movement Q14204 P54257 O75665 O95239 Q14807 1781 24137 Q8IXI1 Q8IXI2 351 1778 203068 P42704 P07196 Q9BVA1 55605 3796 P43034 3799 3832 O60282 3835 P07437 Q9UBK9 Q13885 374654 5991 10383 Q8N4N8 Q13409 5590 Q9BUF5 23095 Q05513 O60333 P42858 81565 7846 Q9GZM8 P05067 146909 3801 84790 57519 9001 O15066 Q02241 P33176 23303 84617 P52732 Q9H4B7 Q99661 Q9BVG8 Q2VIQ3 3895 347733 11004 Q9BQE3 11127 Q9H1H9 Q8TE73 O00139 7277 Q9Y496 63971 Q13509 Q9NP97 Q02750 Q13748 P48380 P68371 9371 Q86UP2 8481 9493 Q9NQT8 Q9NXR1 10128 54820 Q8TF09 Q2M1P5 P68366 P04350 Q71U36 9928 Q15058 5604 Q96JB1

symbiosis, encompassing mutualism through parasitism 11060 6993 O00308 Q86V81 57187 P63172 Q9H0M0 Q96FV9 O60603 Q7L523 Q9Y2C9 79139 P84022 Q92692 6667 P08047 Q15223 P08246 P05412 7099 7098 Q13769 Q96J02 83737 P46934 Q8NI27 4734 Q9BUN8 O00206 6890 Q86W42 7023 O95292 3065 80145 P60033 Q01664 975 P01137 O15455 P01730 Q6I9Y2 Q6IT96 8563 Q13148 O95347 4088 9217 Q13547 23435 P05362 Q03518 P06213

dicarboxylic acid metabolic process Q6YP21 P40926 3420 2572 2571 P50213 O43837 6390 4191 Q9P2R7 3417 Q05329 O75390 P00505 1431 P17174 P31040 8803 1738 P42229 2805 3419 P07954 P51553 P53597 P36957 P51692 O75874 6389 5091 P11498 P21912 1743 Q99259 P42858 56267 3421 6776 P09622

regulation of fibroblast proliferation P04637 P15408 P11802 1021 2099 2010 P12755 890 10437 1499 56034 3479 Q00534 P01019 P04085 P03372 7518 Q9NRA1 P01374 Q9NYA1 5154 5155 7157 Q13426 P38936 P35222 P50402 P35226 P16234 P20248 1026 P14174 Q9UBV7 P01127 648 P08138 P01343 P04233 P04179

developmental growth P08833 P55268 O14793 3484 P14635 P35453 P51587 O75385 P10275 Q8NFW5 351 3913 P00749 Q9Y3A5 Q04759 P00747 3479 Q1PSW9 P84022 P49810 Q13485 8408 Q9UM47 P51671 O00238 Q9NZG7 Q12837 O43602 O43524 P36894 P58546 Q9BRQ0 Q05086 27339 3207 367 P16471 81565 P46531 4851 9869 5467 P48436 207 4854 Q9GZM8 P05067 Q15796 Q9UK55 P01343 Q99835 P49768 9201 Q8TCU4 90780 136319 P10415 2099 O95376 891 Q03405 P02708 898 P03372 P01137 O60383 Q9NSC2 Q15047 P50440 O15075 Q9UMS4 5054 4087 4088 4089 Q15648 P31749 Q6KC79 Q7L576 P35625 P17302 5728 P31270 2697 P60484 P10070 P10071 51156 P24864 Q92982 P05121 5328 2737 2736

embryonic hindlimb morphogenesis P10826 O00755 P78337 7476 Q15648 P63092 23322 P35222 Q68CZ1 P13631 O95076 O75581 Q05516 Q9H161 1499 257 P46531 4851 7704 Q9UPY3

protein K63-linked ubiquitination 7186 7189 11060 7334 Q12933 O00308 7325 6048 Q96J02 P78317 83737 O95376 5071 Q9Y4K3 Q99942 Q96LR5 P61088 10477 O60260 Q969T4

intermediate filament-based process O15061 7431 P35527 Q03001 P35900 23336 P12036 54474 Q96RT1 O75190 Q9H7C4 P08670 P07196 51542 81565 81493 6647 Q9GZM8 P02533 P00441

response to organic nitrogen 6198 P30086 230 P09172 P42345 595 P07196 P09972 7832 P08243 Q16555 P47712 Q9NYA1 P30559 5037 P35368 1072 Q07817 O14717 1026 1147 P51959 P05181 P05062 2475 329 Q13490 P24385 P35638 Q15797 P29474 5321 5563 P14416 P61244 P23528 P09471 898 6647 4149 P01178 P23443 P00441 4086 P27144 O15111 P55212 P07858 5970 3673 Q01581 P10620 P34972 P38936 P21917 P35625 P54646 P17301 Q08209 Q04206 1808 P24864 Q6PID4 900 Q14289 P43246

negative regulation of neurogenesis 4099 6993 P04637 P20916 P63172 O75385 396 Q8IYN9 2147 O14786 P61586 P02686 8408 Q8N5U6 Q04917 O00755 P52565 7533 P36894 P23515 P00734 Q15154 P08138 5108 Q92888 5747 P49768 6696 4155 9921 P56177 Q86VS8 P12755 P10451 Q05397 P51805 6405 P01137 Q9NQC3 8829 P29323 7157 7476 9138 Q658W2 387 P21359 84376 55558 Q13275 Q9BZR6

ureteric bud development P78540 P07949 Q12948 O75462 4092 4093 4609 2303 P84022 Q13485 Q99966 3911 P19544 7490 3207 O43609 960 P01106 P16070 Q15796 Q99958 Q15475 6495 Q15797 9244 P10415 Q13418 Q63HK5 P31431 2247 3611 P01019 P01137 Q9NSC2 P40424 P09038 P10826 4086 4087 4088 4089 P39905 O15230 O15198 Q9HCK4 P31270 O95390 P18827 P10071 O15105 2737

regulation of muscle organ development 136319 6198 7067 P10415 O14793 P14635 Q13418 Q9ULH7 Q9UQL6 O00468 3084 P52952 154 891 Q9NQ29 2247 3611 P84022 P07550 Q9UJU2 P43694 P01137 O15119 10014 P09038 6926 P23443 2626 P56524 P10827 4088 Q02297 Q04771 P16989 7291 P58546 55692 P17302 2697 P17936 55796 P46531 4851 P48436 375790 P08138 Q15672

tissue remodeling P78545 7189 P29474 7422 2034 Q12948 Q9UBB6 P13686 Q9Y4K3 154 P09172 6927 2303 1499 P00747 P15153 P15692 3479 Q9H1Y0 Q04656 P07550 P01019 P21860 P21980 3676 857 P01137 54 9474 3091 P04626 P35368 2065 P10600 2064 P35222 P13612 7052 P20823 P21810 23154 Q9NPH5 Q16665 Q99814 Q99958 P25116 P01343

muscle organ development O96004 O14793 P13349 Q12948 2033 2275 3084 P52952 351 Q9H161 4729 2303 79810 3479 Q8WV60 P09493 8407 Q7Z6C1 4205 Q92574 O15119 6901 3911 4209 Q13643 Q13642 9474 9750 Q16635 P51636 10939 Q02297 7291 Q9HDC5 P53814 P51114 O43847 Q99081 2280 2317 3009 Q04900 P46939 84159 Q01995 P05067 P01343 Q96EB6 Q15672 Q14865 Q09472 6495 6256 6498 P12757 Q8N6I1 P19793 P12755 3142 Q9ULH7 P12111 Q63HK5 P56539 1756 891 1634 O95180 Q00535 Q15561 5159 P21860 6525 Q14993 Q8WZ42 Q9UPY3 6926 4000 P07858 P04626 163126 P68133 2065 P37802 O95183 2064 9093 9099 P12883 5728 6938 387 Q9Y4F9 P21359 P17813 P60484 Q9UKX5 668 Q92786 P07585 Q96EY1 4637 P62942 9421 P24043 Q9Y3M2 7248 7402 1020 P14635 4092 O00468 P19404 P15692 P85037 P15173 23363 P61586 3679 Q13683 P45379 P78337 P50222 Q14774 Q14814 P09619 P11532 P68032 23411 Q86WV8 23414 P60660 Q8WW38 375790 4898 Q99958 P22303 Q15475 O75604 4154 7422 A1XKG3 26576 2010 P02708 Q9H1Y0 O14640 857 P02545 859 Q9GZT9 P26678 P10826 Q8IUX7 221937 O15230 25776 Q02078 P50402 P16401 54583 Q08209 P58012 P24821 O15105 Q16586 P25116

negative regulation of cellular component movement 6672 4771 O15123 P12814 Q9UQL6 4092 3162 P23508 3516 Q96RU8 Q9BZE4 23560 P09493 94 10014 P07996 22846 7057 5155 Q7Z5L7 Q96QB1 Q92729 5796 Q5VTR2 285 Q06330 P18206 Q9H2X0 967 P10415 P14416 Q13418 Q9Y2I1 P30542 P37023

2247 3611 127435 P35240 7414 P01137 P09038 5054 Q8WWN8 Q9Y696 Q14118 Q7L8A9 Q8N9R8 5728 O43707 P21359 P60484 P17936 O15105 P01127 P05121 P09601 1605 P23497 87

histone methylation 4297 Q9BZ95 Q96MX6 Q6UXN9 54496 11107 Q9H9B1 Q9NVM4 6304 84661 79813 O14744 Q01826 P61964 Q9C005 11091 Q9NQ92 30827 Q9P0U4 Q03164 55352 5929 Q15291 80335 Q9UBL3

negative regulation of canonical Wnt receptor signaling pathway P25054 Q9Y3M2 P48431 O95835 Q99471 P52952 P23508 Q9NRM7 857 6789 Q13188 6901 Q13043 Q9NQC7 Q16635 25776 26524 54361 P56705 O60716 324 1540 P10071 6657 2737

actin cytoskeleton reorganization Q9Y5S2 253260 26037 Q12929 Q96EV8 P35579 Q96P48 55561 5898 84062 4627 O43166 P06702 Q60FE5 9578 Q6DT37 P11233 5590 27032 2316 Q05513 2059 Q6R327 P98194 P21333

peptide or protein amino-terminal blocking Q09472 9397 2033 80155 79612 Q92793 1387 Q9BXJ9 O60551 Q7Z6C1 Q4LE28 P41227 Q92831

DNA damage response, signal transduction by p53 class mediator resulting in transcription of p21 class mediator 6672 7023 7157 P04637 Q9UNL4 P51587 P38398 10524 672 Q01664 51147 Q92993 P23497

DNA damage response, signal transduction resulting in transcription 6672 7023 7157 P04637 Q9UNL4 P51587 P38398 10524 672 Q01664 51147 Q92993 P23497

face morphogenesis Q14865 P02452 Q12797 O95470 Q6KC79 P12755 P16234 84159 444 1277 8879 4313 P08253

positive regulation of erythrocyte differentiation P26583 P27037 P27540 3091 Q92835 A4D1W7 P36896 O43524 P17542 3148 91 6886 Q16665

Schwann cell differentiation Q15742 A1XKG3 1020 2065 P12755 Q13506 P21359 Q00535 4664 P21860 4665 6647 P00441

erythrocyte development P18074 23210 Q6NYC1 P41182 P11413 Q7L5Y9 6194 10296 604 10661 P01588 P62753 Q13351

nuclear-transcribed mRNA catabolic process, nonsense-mediated decay P52298 Q9UPR3 22794 26019 Q9Y5S9 84305 23381 4686 Q8IZD4 O15234 5976 Q8IU60 P61326 Q15287 P38919 5394 Q09161 9775 Q96C86 Q01780 10921 Q504Q3 Q96Q15 167227 28960 9924 4116 Q9BRP8 Q92900 Q9HAU5 P60228 2935

neural tube closure P50552 7189 P61968 P05549 O95999 7248 2909 Q9NRY4 O75386 P12755 Q9Y4K3 7020 O14727 Q96RK4 Q9NYQ6 O14641 Q92830 Q92574 P49815 1856 9839 P07737 8323 8543 Q96QB1 7291 Q86WV8 O60353 7249 Q9UMX1 7408 Q15672

tube closure P50552 7189 P61968 P05549 O95999 7248 2909 Q9NRY4 O75386 P12755 Q9Y4K3 7020 O14727 Q96RK4 Q9NYQ6 O14641 Q92830 Q92574 P49815 1856 9839 P07737 8323 8543 Q96QB1 7291 Q86WV8 O60353 7249 Q9UMX1 7408 Q15672

synaptic vesicle transport Q15811 O15020 6453 Q16623 8411 6456 P50570 26258 A1XKG3 1020 Q12846 274 Q99962 P20336 1499 Q00535 Q8TEH3 P61764 6622 Q16555 57706 Q9UL45 P35222 O00499 P37840 1785 Q96MV8 1808 6810 Q15075 6812 P49768

hydrogen transport P30049 O75947 O75348 Q16864 Q9UGH3 245972 P38606 P36542 477 513 514 515 P61421 518 9114 51606 P48201 P48047 23530 Q15904 9550 Q99437 Q93050 521 522 P20648 523 526 528 Q9UI12 O75964 P56134 P50993 495 498 P00846 P25705 P24539 533 P18859 535 P56385 Q8N8Y2 537 539 P56381 10476 10632 Q13423 P55851 P21283 P21281 9296 P15313 Q9Y2B5 509 P25874 P06576

JAK-STAT cascade O75925 P01241 6772 P48551 6774 A1XKG3 1020 9021 P41597 P42224 Q00535 2147 P01579 O14544 O14543 Q13287 P42229 Q13563 P98161 P40763 5310 P51692 Q15109 5311 Q9UBE8 O60674 P16471 P00734 Q6PID4 6776 9306 Q14289 P25116 O15524

response to calcium ion O75746 P28562 P30086 2033 P41181 22872 P41180 O75340 595 8604 Q7Z6C1 P62158 P45379 P07996 10016 P47712 7252 7057 Q13443 P60709 5037 2243 Q96FQ6 P46934 P21579 P42858 60 846 P00533 P48995 4734 P02675 P01222 7220 808 P02671 Q09472 P24385 8754 5321 2244 140576 857 Q8WZ42 1956 6262 Q13586 Q9Y696 1843 Q9UBV8 P18827 Q6PID4 O94979 Q14289

response to mechanical stimulus P02452 6198 4131 O15123 7124 2672 P14635 P42224 P17275 Q99684 7832 5578 P08243 P46821 6869 Q13563 2904 P98161 Q9UGM1 P05412 5310 5311 P14923 285 3728 P01100 Q7Z727 P48431 6772 59341 891 1634 55342 55584 1277 P17252 P06241 P01138 857 P25103 P23443 P01375 P07858 5970 3673 P55011 P68133 P35625 P17301 Q04206 Q6PID4 P07585 6657 Q14289

neural tube development O95999 7248 2909 O75386 23322 Q9Y4K3 O43318 Q1PSW9 Q99807 Q9NYQ6 P49810 Q92574 P49815 1856 8543 6885 Q96QB1 26005 7291 Q68CZ1 1072 Q86WV8 10229 841 Q9H2X0 P46531 4851 7249 Q9NQB0 7408 Q99835 Q15672 P49768 P50552 7189 5362 P61968 P05549 Q9NRY4 P12755 7020 O75051 O14727 Q96S42 P23528 Q96RK4 O14641 Q92830 Q14790 O14640 9839 P07737 8323 Q86YT6 57534 Q4AC94 P10070 O60353 P10071 Q92786 Q9UMX1 1969 P29317 2737 2736

alcohol catabolic process P06733 7167 Q92934 P08559 P37837 P10515 P40926 10690 P52790 160287 230 Q00796 P09172 2821 P21964 2026 P60174 P09972 Q9Y231 1737 4967 P06744 P04406 P52209 3099 P11413 3098 P11177 P05062 5226 P49247 P09104 5162 P07205 Q6ZMR3 22934 P14618 Q9BYZ2 92483 P16118 4191 5160 572 P46926 3939 1312 11165 5315 Q49A26 O95989 5213 2023 2582 P19367 P52789 Q9UKK9 10007 3948 P17858 P00558 Q01813 5207 P08237 P07864 3945

DNA recombination P26583 Q14686 Q9Y265 7520 Q13315 P54132 P15923 Q9NSU2 P51587 25788 472 3516 2547 672 10856 3159 4841 Q9Y230 7518 7517 8607 Q9C0K0 Q8IY92 5591 P46063 7334 P46100 O75531 O75771 Q96SB8 Q06330 P16104 O60216 3329 64919 80198 641 6117 11277 Q15631 8815 P10809 O14757 Q15233 2956 P09429 Q9BQ15 P27694 P12956 P62937 3980 P78527 P38398 79035 P49916 6929 P13010 P49959 Q9BQ90 P18858 P61088 1111 3014 Q96PK6 P17096 9319 5478 11168 Q92878 10432 3978 P23246 4361 6421 5892 O43542 Q13426 Q9GZX7 116138 O75475 P52701 P40692 3149 3148 O60870 57379 546 3146 O15347 5888 P43246

morphogenesis of a branching structure O43597 O43278 P35453 9021 P10275 4609 2303 P62993 1499 P15692 3479 Q1PSW9 284217 O14786 O14543 O15119 Q13485 Q99966 3911 P51671 P35968 5155

Q5HYA8 54361 P19544 7490 7052 3207 960 367 3169 P46531 4851 P48436 P01106 P16070 Q14344 Q99958 Q15475 P01343 Q96T37 5361 6495 P48431 7422 P10415 P25391 P14416 2099 P29590 Q03014 Q13418 Q99623 2247 2885 3611 Q9UQB3 8945 P01019 Q5TAT6 P21980 10672 Q9Y297 P03372 P01137 Q9NSC2 8829 P40424 Q9UPY3 P09038 6926 P06401 P01133 5371 Q92826 3791 4089 91147 Q15648 O15230 Q14118 Q04771 P35222 P56705 P17813 P31270 P37173 P10070 P10071 11331 P01127 6657 1605 2737 Q15375 2736

positive regulation of response to external stimulus P78423 7124 O14672 8654 O43915 P14555 2150 O60603 P15692 P84022 1012 5578 5733 O14788 P42229 P15090 P08887 P07996 P43115 P47712 719 7099 7132 P30556 7098 P78536 7057 P35968 5155 Q15109 O43889 P51679 P35443 7052 O76074 81565 2277 P01303 P55085 Q9GZM8 Q9HCY8 O00206 Q7Z727 7422 5321 Q9Y6Q6 57402 Q9UBU3 P17252 P01019 P21980 P01137 O15455 P55290 P01375 5054 3791 4088 P51692 3673 Q9NR96 P48061 P19438 P17301 O60674 6776 P01127 54106 P05121 10488 Q16581 P21554

regulation of lipid storage 8399 P05106 4790 O95477 4792 7124 Q8WTS1 O60543 10938 P02741 P25963 P01375 3690 7376 O15496 P55055 19 P37231 P21757 P16671 P19838 51099 3685 5468 Q13133 Q9H4M9 948

regulation of dendrite development O43166 4086 8650 Q15797 Q12834 Q9Y6R0 7533 P10586 26037 Q13418 9253 9495 O60229 991 3611 Q9Y250 P61158 Q16643 1627 Q96HC4 P24588 Q04917

negative regulation of lipid biosynthetic process 4790 5154 P28330 5155 5562 2672 54361 P56705 P38398 672 Q96RU7 476 57761 P19838 27122 Q99684 Q92786 P04085 P01127 Q9UBP4 P05023 Q13131

regulation of striated muscle tissue development 136319 6198 7067 P10415 O14793 P14635 Q13418 Q9ULH7 Q9UQL6 O00468 3084 P52952 154 891 Q9NQ29 2247 3611 P84022 P07550 Q9UJU2 P43694 P01137 O15119 10014 P09038 6926 P23443 2626 P56524 P10827 4088 Q02297 Q04771 P16989 7291 P58546 55692 P17302 2697 P17936 55796 P46531 4851 P48436 375790 Q15672

carbohydrate homeostasis Q8TCU4 Q92934 P06737 6774 Q06124 P56539 6194 572 P09172 6927 P22415 51763 859 P62753 5836 P40763 Q9UBK2 5054 9612 Q9NP71 5213 P35368 Q9Y618 O43524 O76024 Q9UNE2 Q9BT40 P55318 P35568 222546 3171 P37231 P20823 P01308 3169 Q8HWS3 5468 Q9NQB0 P05121 Q13370 3667 6517 P08237 P06213

glucose homeostasis Q8TCU4 Q92934 P06737 6774 Q06124 P56539 6194 572 P09172 6927 P22415 51763 859 P62753 5836 P40763 Q9UBK2 5054 9612 Q9NP71 5213 P35368 Q9Y618 O43524 O76024 Q9UNE2 Q9BT40 P55318 P35568 222546 3171 P37231 P20823 P01308 3169 Q8HWS3 5468 Q9NQB0 P05121 Q13370 3667 6517 P08237 P06213

regulation of T cell differentiation O43353 P00491 O96005 Q92934 6850 Q8N726 P41182 3142 P10276 O00203 572 79576 8767 6647 P42229 P00441 5914 P24394 Q13422 Q14774 149041 8546 P04626 P51692 4860 Q5TC82 Q15306 Q92949 P06729 2064 Q8N5F7 1029 P16410 P43405 P37173 O43290 6776 P01589 P40933 604 10320 P42771 P60568 P04233

multicellular organismal homeostasis 6794 2034 25861 Q9UBB6 Q9Y4K3 154 P09172 1499 P15692 P07550 Q13085 Q99728 Q7Z6C3 Q8NFJ9 54 P40763 P47712 Q9UBK2 P07954 7099 Q15303 P35968 P28330 P09619 Q6UB99 P20849 Q9NPH5 6117 O00206 23746 Q15831 P27694 7189 P28223 7422

P10415 5321 6774 P14416 P13686 P30542 Q9Y6Q6 6927 P15153 O60543 51542 Q96RK4 5159 6647 31 P00441 P08588 3791 P29320 3673 1297 Q9NR96 P35222 Q9P202 580 582 P20823 P17301 23154 P02458 54106 Q99814 P04155 P25116 Q9NZN9

morphogenesis of a branching epithelium O43278 P35453 9021 P10275 4609 2303 P62993 1499 P15692 3479 Q1PSW9 284217 O14786 O14543 O15119 Q13485 Q99966 3911 P51671 5155 54361 P19544 7490 7052 3207 960 367 P48436 P01106 P16070 Q14344 Q99958 Q15475 P01343 Q96T37 5361 6495 P48431 7422 P10415 P25391 2099 P29590 Q03014 Q13418 Q99623 2247 2885 3611 8945 P01019 P21980 10672 Q9Y297 P03372 P01137 Q9NSC2 8829 P40424 P09038 6926 P06401 5371 Q92826 4089 Q15648 O15230 Q14118 Q04771 P35222 P56705 P17813 P31270 P37173 P10070 P10071 11331 P01127 6657 1605 2737 2736

histone mRNA metabolic process 5394 7884 Q13315 6741 Q01780 Q9UKF6 Q9NVV4 472 54512 27257 167227 55149 Q9NPD3 Q14493 5976 Q92900 Q8IU60 64282 P05455

cellular amide metabolic process P04424 P43490 P78540 P51692 Q9Y619 93100 1050 Q96RQ3 P42858 445 435 P00966 6776 10166 1738 Q6XQN6 P42229 P49715 P09622

activation of Ras GTPase activity O43166 7248 Q92949 26037 Q86WV8 23513 Q14160 Q96N67 81565 Q6PID4 Q6ZW31 Q92574 Q9GZM8 Q14289 85440 85360

protein localization at cell surface 7375 Q60FE5 Q96G30 Q13107 Q92729 5796 Q9HCE7 57154 P35222 2316 P18206 1499 7414 Q15389 P01133 P21333

membrane protein ectodomain proteolysis P78536 Q13443 8754 O14672 P56817 Q96BI3 23621 P35579 Q04759 P49810 51752 Q92542 Q9NZ08 51107 4627 P49768

syncytium formation O95259 P29466 50937 P29475 Q9Y463 9149 P35579 P17655 960 P51959 O95180 Q4KMG0 900 P16070 O43184 4627

induction of apoptosis via death domain receptors 8772 8795 Q13158 7124 P48023 O00220 P55957 356 637 O14763 P78560 P42574 1616 8738 Q9UER7 P01375

double-strand break repair 7520 P04637 P54132 5422 Q15819 O95551 P51587 25788 2547 672 51720 51567 7518 P55072 Q8IY92 5591 P46063 7334 7336 P16104 O60216 P46736 Q9NWV8 2237 641 Q9Y6H3 55775 6117 Q96RL1 Q9BQ15 P27694 P12956 P78527 Q9NUW8 P38398 79035 P13010 P49959 P61088 3014 9319 7415 6647 Q92993 29086 Q92878 P00441 4361 7157 Q9UIG0 Q13426 P39748 10524 P40692 P09884 5888 P43246

regulation of secretion P53367 Q86UW7 Q16623 2033 2550 P50416 9066 Q9Y4K3 94121 3084 54474 P37288 Q9BXM7 Q04759 Q9UBS5 P16220 51762 P20396 6622 Q7Z6C1 5898 O14788 6869 P03950 O15496 65018 Q02297 O76070 P14174 Q9Y6H5 5029 Q13370 8399 Q7Z727 Q09472 7189 3783 P14416 64215 Q06124 Q9Y6Q6 Q9UBU3 6927 773 P41231 Q00535 O60543 P17252 Q53X93 P01019 P21860 P01138 P01137 55503 O60260 P01133 P01375 Q9H1D0 A4D1W7 5213 1374 P35225 27236 2065 P21917 P35900 Q9UNE2 P48380 222546 P20823 P43405 O60674 3784 1808 Q8HWS3 6812 Q9ULZ3 3667 6814 P04233 P43489 P25098 23409 7124 P05408 Q96KC8 1385 29108 O00186 1020 114548 3162 P51787 156 552 1137 553 O00194 5578 11146 O15554 5338 Q16555 Q15466 4646 2904 Q92930 P30556 Q96C24 Q9NYA1 P29466 Q13443 P30559 5991 Q9Y4H2 64127 O95361 O00233 P10600 5071 P35568 P62166 57678 10626 P21579 P01308 Q9NQB0 2917 Q9UM54 P22303 10468 P46459 P23510

P01185 P28223 8754 6850 Q7Z7G2 Q96FX2 A1XKG3 O43612 P30542 O60502 P20336 Q9Y6E7 Q8NER5 Q9HCL2 2247 Q96P20 Q92990 P61764 P01579 P00367 Q9NQC3 P25103 P09038 P01178 P41159 O43581 Q9UGK8 P39905 Q9BZS1 P19883 P37840 P11234 P11233 10724 O14939 Q9HC29 P43681 9846 P09601 50943 P08237 P60568 P21554

cellular response to nutrient levels P02452 O00744 Q15853 25828 Q13873 Q8TDY2 9821 P04637 27327 3162 P10276 P22415 60673 10013 P08243 3953 Q13286 P05412 Q9Y4P1 9474 Q9NYA1 6720 23411 Q8NDV7 55054 P55318 3171 Q9BSB4 P11021 Q9P2K8 P48357 Q99757 Q96EB6 P49768 3860 P13646 P61244 Q9H1Y0 1277 57521 898 4149 857 Q7Z3C6 5119 5914 Q13501 P07339 7157 P36956 79065 O60911 Q676U5 Q9H492 O75084 23192 81788 Q9H093 P24864 8878 P09601 Q9UBN7 O94817 3309

reproduction Q7Z2K6 Q9Y265 O14793 P51460 P54619 Q12948 Q12824 Q9UBB5 22994 O00268 P51587 O00267 2547 P37288 P16220 P84022 P21926 Q92731 Q9UM82 90678 P08842 P28799 Q9H0D6 Q9UIQ6 Q9NS91 54361 Q08431 P16104 10426 P16234 P42858 Q99598 P16471 P48039 4851 10420 4734 140801 10542 Q96EB6 Q92843 928 Q92600 O75925 Q9ULX9 81629 Q06124 Q9BXC9 54496 6927 3416 55342 29127 1111 P03372 Q5TAP6 4867 P40424 26091 6926 P06401 Q92826 Q9NZI7 Q92824 3791 Q92949 2100 Q12857 P56705 5728 P20823 Q9HBE1 1488 1487 Q9BXB1 3301 P18509 705 P27037 1385 Q5JT82 6829 55366 P21964 1499 7913 Q9H0H5 O00238 Q9Y2T7 Q9NYA1 P32856 P30559 Q8ND90 P10600 P10721 Q96J02 P19544 27297 960 1147 2475 O14753 604 P41208 5987 P07686 O14757 P09622 137964 P51681 Q06203 2253 Q9H9Y6 Q9NQS3 O00124 Q05469 P60033 284359 975 4543 6721 857 5997 26292 Q92754 P51572 P09958 Q12772 P51575 P10827 P51692 P31749 Q96L21 O00255 P54727 Q6KC79 P19883 P52789 O60879 Q9HCK4 P40692 1047 Q9UMX2 P05362 Q6X784 5887 Q9UKT4 7704 P06213 P00390 6993 O00308 Q86V81 P15529 3482 P15531 Q7L523 Q14050 3479 10134 P01236 55723 6869 Q15389 P62753 P49770 4221 Q96CW5 P16989 3010 P13236 Q8NHU6 Q03933 Q15392 1069 Q96S53 P48436 8932 P01588 8815 P01343 P01100 6890 P48552 P48431 5321 Q86VW1 6774 3024 O95292 2054 Q99623 891 O14967 894 Q96S42 898 3014 Q53X93 P01019 79956 6647 P01137 P25440 4240 4000 7993 P04626 Q9UHI8 2064 P35222 Q01974 10007 3028 P60484 3148 P10070 11331 668 Q96PN8 6776 5568 6657 10488 5328 P24588 P01243 6670 P26583 6674 7520 8852 23764 Q8WYA0 25945 552 53340 P15692 Q14093 3159 P51828 6667 P55075 7518 8607 P40763 P04201 Q7Z5L4 5591 O00755 5471 Q6IR47 Q9Y5R5 3297 23411 3054 Q05086 80705 3171 682 P14373 3169 148327 9825 5104 5467 207 9702 P48357 10468 P24385 P01185 6696 7422 2099 P51843 330 P13010 Q00613 5478 P25103 P01178 Q12816 P51610 580 3074 O60911 O75031 221656 P58012 P18827 Q9NQL9 P60321 10566 Q99417 8892 11060 P35659 8655 6598 4179 5268 P30086 Q9UPN4 57187 Q86VQ3 29947 Q96FV9 351 472 595 116 P08047 5017 5139 P43034 Q13363 Q13489 P03950 9510 P35527 5037 3099 79084 P13631 Q9UJC3 Q02447 Q9NVP2 367 Q05952 28981 128 P46531 P00533 Q9BUN8 Q9UQR1 Q13258 Q14106 Q15797 6256 P62937 P10451 Q9UBU3 P49916 Q96RK4 Q15561 P17096 11168 Q96MA1 Q9BZW7 Q9NPE6 Q13144 Q93008 P00441 8563 P32239 4086 P35869 8204 4088 7476 Q86XR8 P36952 Q86UL3 O75475 Q02790 P08151 P24864 5049 Q03518 P55061 P43246 9420 9425 O15287 P63092 P14635 P35453 P10275 4093 Q9Y6K1 Q5JUK2 Q99965 Q00597 P09172 Q99728 Q02539 Q15223 Q13285 Q86W54 7257 O43521 6046 Q5JQC9 O43889 O75575 P36896 O43524 P36894 7490 7251 285 P13682 Q8NI27 Q9NQB0 O15169 Q9NRD5 Q99714 Q8TEY5 Q86W42 Q8TB22 Q8TCU4 7142 Q07092 Q86V24 7141 P11464 Q9NVM4 9319 P08069 1718 P02545 Q6I9Y2 1956 P23443 9217 P22492 O15198 O43504 Q9NZ56 Q6PID4 Q99816 Q14289 9463 7046 6198 O15123 Q01094 P63172 Q9H0M0 6194 P63167 P42345 26271 O43719 P47914 Q9Y4C1 79139 Q1PSW9 P17275 P23945 Q92692 91 1738 P22612

P42229 O15119 P31689 9232 22803 Q13769 Q8WWH4 O75534 O75531 Q9P0W8 Q8IYV9 83737 P55318 P46934 Q9HD26 P16070 P05067 1869 Q13895 7067 P29474 P19793 57120 O75881 Q7Z2Q5 80145 P46926 O15259 O60383 Q9NSC2 P11717 P56545 Q13426 A4D1W7 P10620 Q6UWE0 9495 Q8NB78 9130 2735 P68402 P41220 P07101 O60271 2736 Q15853 Q5GLZ8 P54652 Q13315 O95997 P41182 P11766 P41181 P35613 124626 P00749 P22415 P05093 2741 O60488 P31260 4920 3953 O75508 2626 P47712 Q8NEC5 P35968 Q13323 Q15506 P30279 Q07817 1788 Q05516 P61073 Q5BJF6 Q9HCU4 O75969 P12956 P56851 P10415 3980 Q8IUQ4 O14495 P19235 P78527 1312 P00403 P09430 P43694 P01730 Q14432 Q9BQA1 P41159 O15111 Q15648 Q04771 2896 P24821 4830 P21554 Q14320 O75718 4957

activation of innate immune response O43353 O00206 7099 4792 O95999 5970 64127 Q9UHD2 O60603 148022 57402 3329 81622 29110 Q9Y2C9 P51617 Q8IUC6 Q04206 Q9HC29 8767 P10809 P25963 Q9HCY8 Q9H1C4

cardiac muscle cell differentiation P10826 Q9Y3M2 9474 6256 4000 7248 P19793 Q02297 25776 P14635 P68032 Q86WV8 3084 P52952 891 Q9H1Y0 23363 Q92786 P02545 Q92574 O15119 Q8WZ42 Q9UPY3 6926

proteolysis Q7Z2K6 Q96TA2 P48052 23607 O14672 9184 O15519 203102 O60566 P15374 Q70EL4 P63208 Q92851 10893 10531 Q92611 P27487 P09936 Q9H4L4 Q9UIQ6 Q9C026 Q12834 Q5VTR2 5706 5707 5708 5709 5700 5701 5822 4734 5702 Q9UHP3 Q9UKV5 5704 P67812 5705 64174 P56817 79791 A0AVT1 P16444 5717 29122 3416 1358 P14384 57646 P62256 Q86WA8 P48147 P61289 6921 Q9Y297 5713 4627 5716 P31944 Q92824 P07858 5970 Q99460 9099 Q13829 P17655 1368 P19838 55236 823 Q9ULZ3 Q9H3G5 826 3309 P51784 Q9Y5Y6 P51668 29108 P50579 P51665 10613 27283 10616 Q92530 Q7Z419 P29466 Q9Y4X5 O00231 O00233 O00232 Q96J02 P52888 Q9NS56 10868 Q8TBC4 10988 10869 841 843 Q92643 4898 5624 7804 P09622 Q12884 4790 Q14703 4311 Q13618 Q13617 Q13616 1161 O00487 O95376 Q8TBB1 391627 10956 P62333 P20618 Q9BZK7 Q92995 254827 O43184 Q9UK22 5516 Q13501 P09958 Q9UL46 Q9UMW8 Q13620 6500 P54727 P54725 80332 10845 253980 57695 Q96KP4 P09960 4313 5886 Q9UMX1 5887 P40337 4318 Q92743 P04632 O00308 O96009 P53804 25862 Q9NVW2 23203 23326 991 P09093 Q96RU2 2147 997 P62877 5654 P28838 10013 51009 64682 Q15269 115426 2159 51132 51377 4214 O43294 P23276 408 P49407 409 10269 P49768 P60468 Q6ZN33 5682 5683 5684 5685 5686 891 P14780 55743 8945 P25789 55748 P25788 79956 Q9GZP9 P25787 Q8TAT6 5692 Q9Y337 5693 P40818 8720 7511 7874 Q10713 Q9UHI8 Q9H4A9 O95071 9924 5687 5688 5327 5328 8837 P31930 Q5VVQ6 P62837 9700 8975 Q969H0 9820 Q9Y2K6 Q9UNN5 O76031 Q8IWF2 Q96PU4 55644 P62714 10213 P55072 Q9H3F6 O43242 P07711 Q9Y4P1 5476 Q9HCE7 Q5HYA8 Q05086 Q99797 Q00987 51056 327 Q86T82 Q9H1A4 329 8738 8754 O60907 Q9UPT9 Q9Y2M5 23621 O00762 51185 55666 P68036 7415 8881 4285 25818 O43464 P17980 P13497 O60911 55432 10208 O14818 7428 8878 P49427 11060 11065 Q86TM6 24139 Q9Y4K3 Q8TC27 Q9NXS2 O75150 Q04759 84447 P22695 P49810 9978 O94905 Q14694 3093 9510 7332 7334 P55036 O43684 9512 Q504Q3 23291 Q4J6C6 7329 Q13490 7328 Q16401 Q9HAU4 Q9BUN8 Q9BY50 11160 Q13257 7347 P32121 O43897 P35998 4193 Q9UDY8 Q8WXQ8 10197 Q16531 Q93009 Q93008 P09237 57092 7353 Q14114 P20151 Q86Y37 O75477 1800 267 23032 P26927 Q9NPF4 8453 5184 8454 8452 P49862 64750 7126 26994 P14635 O75582 8450 P35579 Q99965 Q13042 Q13049 Q14139 7375 11274 6048 P36776 23014 P13798 Q9UKA1 5071 7251 P11021 Q9Y6L7 Q9NRD1 P49721 P49720 P46459 7385 7384 Q96E52 Q8TEY7 8237 O94966 7267 10273 Q9Y5K6 Q9Y5K5 Q9UKB1 Q99942 Q8WY64 Q9NRE1 11124 P02786 51107 Q9NQC7 Q15008 Q15369 Q8IUX7 O75439 9695 7037 P14625 O75674 23478 114088 255488 Q93034 54504

Q99816 Q8WW12 9100 P55786 O95834 Q9H324 Q96K76 27005 27248 Q70CQ3 8492 Q70CQ1 83752 79139 P62195 1738 P45974 Q9BZQ6 26270 9476 P78536 P29144 10939 83737 O43847 84708 55294 27252 P46934 Q9Y6H3 Q92560 26168 P42574 P60900 P42575 7189 P10619 91039 7064 9361 7184 1514 80267 Q9Y3C5 Q9BYM8 3817 Q9Y4E8 26133 Q8TEL6 Q9NZ08 80020 O60260 Q6V1X1 Q15843 Q5JRX3 P07339 P55212 91147 P55210 Q6R6M4 P78317 9252 Q9UNE7 1642 P04070 Q04206 Q92542 P78560 Q9UBN7 Q969Q1 Q9HC62 Q9UI38 Q8TF47 Q86TI2 Q96EP1 P00749 P00747 26232 Q9UK80 26234 Q9UJW2 P00742 Q13200 P08246 Q13444 Q13443 Q8WZ19 Q8IWV7 57154 Q96BI3 8065 84749 P00738 Q9UJX2 P61077 P00734 1540 P08253 P08138 124739 8078 O75604 P12955 O75844 Q13216 Q8IUQ4 P28065 Q5W0Q7 377630 29843 59343 Q58DX5 Q5XUX0 P61088 Q14790 Q14674 Q969V3 O75832 Q9P0U3 Q13107 P62191 P28074 P28072 P28070 23192 P63279 P30260 P07384 51752 P08473 Q9BZ11 P00750 Q13233 51514 P43686

reproductive process Q7Z2K6 Q9Y265 O14793 P51460 P54619 Q12948 Q12824 Q9UBB5 22994 O00268 P51587 O00267 2547 P37288 P16220 P84022 P21926 Q92731 Q9UM82 90678 P08842 P28799 Q9H0D6 Q9UIQ6 Q9NS91 54361 Q08431 P16104 10426 P16234 P42858 Q99598 P16471 P48039 4851 10420 4734 140801 10542 Q96EB6 Q92843 928 Q92600 O75925 Q9ULX9 81629 Q06124 Q9BXC9 54496 6927 3416 55342 29127 1111 P03372 Q5TAP6 4867 P40424 26091 6926 P06401 Q92826 Q9NZI7 Q92824 3791 Q92949 2100 Q12857 P56705 5728 P20823 Q9HBE1 1488 1487 Q9BXB1 3301 P18509 705 P27037 1385 Q5JT82 6829 55366 P21964 1499 7913 Q9H0H5 O00238 Q9Y2T7 Q9NYA1 P32856 P30559 Q8ND90 P10600 P10721 Q96J02 P19544 27297 960 1147 2475 O14753 604 P41208 5987 P07686 O14757 P09622 137964 P51681 Q06203 2253 Q9H9Y6 Q9NQS3 O00124 Q05469 P60033 284359 975 4543 6721 857 5997 26292 Q92754 P51572 P09958 Q12772 P51575 P10827 P51692 P31749 Q96L21 O00255 P54727 Q6KC79 P19883 P52789 O60879 Q9HCK4 P40692 1047 Q9UMX2 P05362 Q6X784 5887 Q9UKT4 7704 P06213 P00390 6993 O00308 Q86V81 P15529 3482 P15531 Q7L523 Q14050 3479 10134 P01236 55723 6869 Q15389 P62753 P49770 4221 Q96CW5 P16989 3010 P13236 Q8NHU6 Q03933 Q15392 1069 Q96S53 P48436 8932 P01588 8815 P01343 P01100 6890 P48552 P48431 5321 Q86VW1 3024 O95292 2054 Q99623 891 O14967 894 Q96S42 898 3014 Q53X93 P01019 79956 6647 P01137 P25440 4240 4000 7993 P04626 Q9UHI8 2064 P35222 Q01974 10007 3028 P60484 3148 P10070 11331 668 Q96PN8 6776 5568 6657 10488 5328 P24588 P01243 6670 P26583 6674 7520 8852 23764 Q8WYA0 25945 552 53340 P15692 Q14093 3159 P51828 6667 P55075 7518 8607 P04201 Q7Z5L4 5591 O00755 5471 Q6IR47 Q9Y5R5 3297 23411 3054 Q05086 80705 3171 682 P14373 3169 148327 9825 5104 5467 207 9702 P48357 10468 P24385 P01185 6696 7422 2099 P51843 330 P13010 Q00613 5478 P25103 P01178 Q12816 P51610 580 3074 O60911 O75031 221656 P58012 P18827 Q9NQL9 P60321 10566 Q99417 8892 11060 P35659 8655 6598 4179 5268 P30086 Q9UPN4 57187 Q86VQ3 29947 Q96FV9 351 472 595 116 P08047 5017 5139 P43034 Q13363 Q13489 P03950 9510 P35527 5037 3099 79084 P13631 Q9UJC3 Q02447 Q9NVP2 367 Q05952 28981 128 P46531 P00533 Q9BUN8 Q9UQR1 Q13258 Q14106 Q15797 6256 P62937 P10451 Q9UBU3 P49916 Q96RK4 Q15561 P17096 11168 Q96MA1 Q9BZW7 Q9NPE6 Q13144 Q93008 P00441 8563 P32239 4086 P35869 8204 4088 7476 Q86XR8 P36952 Q86UL3 O75475 Q02790 P08151 P24864 5049 Q03518 P55061 P43246 9420 9425 O15287 P63092 P14635 P35453 P10275 4093 Q9Y6K1 Q5JUK2 Q99965 Q00597 P09172 Q99728 Q02539 Q15223 Q13285 Q86W54 7257 O43521 6046 Q5JQC9 O43889 O75575 P36896 O43524 P36894 7490 7251 285 P13682 Q8NI27 Q9NQB0 O15169 Q9NRD5 Q99714 Q8TEY5 Q86W42 Q8TB22 Q8TCU4 7142 Q07092 Q86V24 7141 P11464 Q9NVM4 9319 P08069 1718 P02545 Q6I9Y2 1956 P23443 9217 P22492 O15198 O43504 Q9NZ56 Q6PID4 Q99816 Q14289 9463 7046 6198 O15123 Q01094 P63172 Q9H0M0 6194 P63167 P42345 26271 O43719 P47914 Q9Y4C1 79139 Q1PSW9 P17275 P23945 Q92692 91 1738 P22612 P42229

O15119 P31689 9232 22803 Q13769 Q8WWH4 O75534 O75531 Q9P0W8 Q8IYV9 83737 P55318 P46934 Q9HD26 P16070 P05067 1869 Q13895 7067 P29474 P19793 57120 O75881 Q7Z2Q5 80145 P46926 O15259 O60383 Q9NSC2 P11717 P56545 Q13426 A4D1W7 P10620 Q6UWE0 9495 Q8NB78 9130 2735 P68402 P41220 P07101 O60271 2736 Q15853 Q5GLZ8 P54652 Q13315 O95997 P41182 P11766 P41181 P35613 124626 P00749 P22415 P05093 2741 O60488 P31260 4920 3953 O75508 2626 P47712 Q8NEC5 P35968 Q13323 Q15506 P30279 Q07817 1788 Q05516 P61073 Q5BJF6 Q9HCU4 O75969 P12956 P56851 P10415 3980 Q8IUQ4 O14495 P19235 P78527 1312 P00403 P09430 P43694 P01730 Q14432 Q9BQA1 P41159 O15111 Q15648 Q04771 2896 P24821 4830 P21554 Q14320 O75718 4957

neuron projection morphogenesis P25054 Q92858 8650 P55268 Q9UQP3 Q8TEW0 P56199 O75385 351 3913 474 Q8IYN9 Q86WK6 P16220 Q1PSW9 P07196 P83731 O14786 P43034 P46821 O60282 8408 Q9C0K0 Q96CW9 O15496 Q12837 O43602 10939 2043 1191 P55316 O60331 81565 P46531 4851 P00533 P05067 P98172 2290 8399 P54753 Q7Z727 P50552 6256 P14416 P19793 Q06124 Q13418 Q05397 1756 773 P51805 Q00535 P17252 Q53X93 4983 P21860 Q9UQB8 P49639 8829 84062 Q16650 6383 P54764 P54762 3672 P04626 Q9Y6R0 Q658W2 2065 P13611 2064 Q8IYT8 O00213 Q7L576 9253 O75592 P17302 O60674 56288 2697 P10070 23032 P10071 23396 6259 6812 5604 2737 2736 P34925 4131 8851 Q8TD84 1385 P78509 1020 10458 5578 Q15223 P26232 O00238 P60709 P52803 O43921 P11532 64919 324 O60890 60 Q9GZM8 1947 P08138 7408 85440 5747 Q99835 Q9HCU4 Q15078 9201 Q8TEY7 P10415 P56178 Q8IUQ4 Q02880 P34741 A1XKG3 Q96EV8 O60229 P20336 Q96N67 3611 Q04656 O14640 P61764 P08069 Q92752 1956 O15075 P29323 7155 Q15768 Q13509 Q02750 Q9HCK4 23077 55558 Q15375 Q9BZR6

regulation of cell division 808 P25054 7422 P54132 Q7Z7K6 2253 259266 O43915 P51587 3481 56034 2247 P15692 2246 Q96RK4 P04040 9738 P08069 P55075 P04085 P05230 P01137 P62158 Q9NRA1 P01135 P09038 P21246 P21741 5154 5473 5155 4221 Q9GZP0 O00255 Q8IZT6 O43303 P10600 641 1069 324 2277 5764 P01127 P41208 847 P02775 P01344

molting cycle P25054 Q15738 P10415 P31276 Q08188 Q9NS68 2304 Q04656 91 P49810 P04085 55504 3911 1956 50814 O95863 94234 5154 Q9C009 5970 O15230 P18074 A4D1W7 P31749 P36896 P19883 O00358 324 Q04206 P46531 4851 5467 P48436 207 P00533 P08138 6615 10468 P49768

hair cycle P25054 Q15738 P10415 P31276 Q08188 Q9NS68 2304 Q04656 91 P49810 P04085 55504 3911 1956 50814 O95863 94234 5154 Q9C009 5970 O15230 P18074 A4D1W7 P31749 P36896 P19883 O00358 324 Q04206 P46531 4851 5467 P48436 207 P00533 P08138 6615 10468 P49768

regulation of axonogenesis 4131 4099 O43157 P10636 2033 P20916 O75385 396 Q8IYN9 Q86WK6 Q9HCJ2 P07196 Q7Z6C1 O14786 P61586 P46821 P02686 8408 O00755 P52565 Q13009 P23515 81565 4137 Q9GZM8 P08138 Q92888 5747 P49768 Q09472 6498 6696 5364 P12757 4155 Q13418 P10451 Q05397 P53667 57689 773 P51805 3611 6405 P01138 Q9NQC3 8829 P29323 7476 9138 Q658W2 7074 Q9HCK4 387 P20827 55558 Q13275 Q9BZR6

nitric oxide biosynthetic process2644 P29474 2643 P29475 P78540 O95865 P31749 207 P15559 1728 P30793 P30047

regulation of Cdc42 GTPase activity Q96P48 121512 Q7Z6J4 Q6ZV73 81565 55785 10928 Q96M96 Q5JSP0 Q9GZM8 Q15311 P98174

positive regulation of epithelial to mesenchymal transition 4087 57669 4088 4089 P84022 Q9HCM4 P10600 P01137 O43294 Q15796 Q13145 Q13485

positive regulation of cell morphogenesis involved in differentiation 4087 57669 4088 4089 P84022 Q9HCM4 P10600 P01137 O43294 Q15796 Q13145 Q13485

regulation of interleukin-12 biosynthetic process O00206 7099 P10914 7189 Q04864 5970 Q04206 Q06643 P01579 5966 Q9Y4K3 3659

insulin-like growth factor receptor signaling pathway 5295 Q13322 64599 P31749 207 7249 P08069 O75420 3667 P27986 P49815 P35568

regulation of Cdc42 protein signal transduction Q96P48 121512 Q7Z6J4 Q6ZV73 81565 55785 10928 Q96M96 Q5JSP0 Q9GZM8 Q15311 P98174

hippocampus development 7248 P56177 A1XKG3 1020 3066 7161 Q9BXC9 P51805 Q00535 Q96RK4 Q9UJU2 P43034 Q92574 P62258 9839 P04201 O15350 Q92769 Q14114 7531 P12429 Q86WV8 9495 55558 7804 P24588

blastocyst formation 6670 9421 8452 O96004 6498 P12757 O95140 P12830 Q13618 7082 Q07157 Q9BPY8 Q02447 84525 P17275 Q96S42 6667 Q15561 P08047 999 9927

purine nucleoside monophosphate metabolic process P30566 161823 5471 5198 Q06203 271 P12268 P30520 3615 3614 P49915 10606 4907 158 Q01433 P31939 P22102 P22234 8833 Q6DHV7 P21589

purine ribonucleoside monophosphate metabolic process P30566 161823 5471 5198 Q06203 271 P12268 P30520 3615 3614 P49915 10606 4907 158 Q01433 P31939 P22102 P22234 8833 Q6DHV7 P21589

negative regulation of insulin receptor signaling pathway 5580 Q7Z727 Q13322 5770 P49840 5970 Q14449 P18031 P10586 9021 P35568 5590 2931 Q05513 Q05655 P17252 Q04206 5578 O14543 3667 O15524

regulation of multicellular organism growth P01241 6311 1385 O95470 O43638 351 154 P16220 3479 P07550 P01236 P42229 2307 P40763 Q9UHG2 10939 3297 P50542 57678 P16473 P05067 P43026 P01343 P42336 137964 Q9H2K2 P10415 6774 Q06124 Q9BXC9 Q99700 Q9UBU3 Q8NER5 Q9HCL2 Q00613 Q53X93 P01019 O15379 5830 6647 P00441 P08588 P41159 Q13422 P51692 O43464 8841 Q86UL3 Q6KC79 8879 6776 10320

regulation of actin filament length 4690 10092 10095 O95835 10094 Q9UHB6 O43639 P35612 P35611 P42345 6709 830 832 118 Q9H2D6 P47755 P47756 P51671 Q01082 1072 10109 2039 2475 6711 6710 4214 P62328 P61158 O15020 Q13813 O15143 Q9BPX5 23189 P62330 P20936 P23528 P02549 81873 10552 P35080 Q08495 Q14678 P48061 382 P11277 P16333 51474 P52907 Q6PKD3 P63313 Q13233 829

maintenance of protein locationO95835 Q9UNN5 O00221 9184 57662 Q9Y4G6 Q13285 P25963 Q9UBC1 4188 Q8NF91 10460 P33947 O43684 O95684 7094 Q9NS56 2317 2316 9908 Q9HD26 Q99750 Q15154 P62328 7529 5108 11116 P21333 7067 4792 57120 P29590 Q86VS8 P30101 P15311 10956 Q96RK4 11124 857 Q9UN86 Q9Y6A5 P01730 Q9Y490 2923 5716 5371 P10827 7430 Q60FE5 P31946 O75832 Q14118 25777 Q9UH99 Q9UMX1 11014 84376 P63313 O14777 Q15653 1605

nucleobase, nucleoside and nucleotide metabolic process O75947 5141 5142 P37837 Q16864 8654 P30085 9061 6470 P30520 353 476 477 478 P60174 5139 4968 Q13126 P28335 Q9H4L7 P28331 P48047 4860 P13637 Q9UBC3 P52209 Q07343 481 483 488 P22102 Q13370 P49247 Q9UI12 9525 P56134 P31153 3420 29922 490 4191 4507 495 3417 P49915 498 P25705 115024 P18859 P22674 P56385 P00568 11165 Q16774 P56381 P21589 3419 10632 P36957 P43490 4882 O75351 P34896 P12883 3421 P20020 P07741 P30566 P28482 P40926 P38606 P36542 P50213 271 Q9Y6K1 Q8WVC6 P27708 158 P51553 5198 P22392 5631 80308 Q15904 O95243 Q6PIW4 P30793 7372 Q93050 Q8NFM5 P20648 P54709 Q01433 Q6XQN6 P05023 Q13057 P05026 22934 P00491 P00492 Q9UGJ0 23463 Q06203 P11586 O60502 P24539 80347 P22234 56916 1716 P11908 5636 Q49A26 161823 P21283 P21281 27032 1841 10724 10606 P27815 Q6DHV7 P98194 509 P06576 7167 8930 P29372 P16615 P78549 O43837 O60725 P15531 1854 P11172 2820 P41250 513 514 515 P61421 Q969T7 518 9114 P48201 P12268 1503 O76074 P43304 1743 51251 521 522 523 P05062 526 528 O43252 Q9BW91 P00846 4907 Q96T60 533 535 537 P01137 539 P23921 Q9UN42 10476 23439 P53396 O95989 O75874 3704 Q9Y2B5 P17812 8833 3945 4913 P30049 P33316 O00746 Q9BY32 245972 23400 2987 P60891 P31939 P20594 Q8NFF5 5471 51606 51727 Q13569 P11413 93100 Q9H0P0 1789 1788 Q99437 O60256 5226 P17081 Q9Y5B8 Q08462 65220 O75964 P84090 P32320 P50993 3615 3614 2643 Q04656 Q8N8Y2 Q14432 O95544 Q16854 Q14558 9296 P15313 Q9UKK9 4719 4830 4833

response to alkaloid P31930 7384 P01185 A8MTJ3 P10415 5563 P14416 P34741 2550 A1XKG3 1020 3066 P17405 3162 6609 1137 P05091 Q9UBS5 Q00535 P09471 5536 Q16555 3953 6869 P25103 P01178 346562 6262 6383 Q92769 P30559 5970 Q14416 P35368 P21917 P54646 P37231 Q04206 1808 Q6PID4 P43681 5468 P53041 P06276 P48357 P09601 Q14289 P21554

vesicle organization 9685 P53621 O95477 Q92538 5584 O43752 P10415 8411 Q6QNY1 2647 1315 375 26271 1314 P20336 Q99523 O60763 Q9UKW4 10451 P61006 P61764 857 P35606 Q14677 9276 Q9Y678 8301 19 P51636 P53618 1130 P78537 10228 Q99698 Q96MV8 P84077 2773 8615 Q13492 Q15075 6812 O14579 P08754 Q9UKT4 4218 P41743 P25116 Q8N6T3

regulation of GTP catabolic process 9744 10928 26037 Q99490 P15498 P42345 2665 2664 P43034 Q92574 P49815 Q6ZT07 Q8WZ64 O43166 Q8TBP0 Q15027 Q9Y2X7 Q7Z6J4 23216 115704 Q9P107 O43609 81565 Q15311 85360 P98174 P50395 9882 1121 116986 Q96M96 Q9ULH1 116987 Q8TEU7 Q96CN4 P09471 Q9UKW4 Q8IV61 P26374 Q9UPQ3 Q8WWN8 Q92949 125058 Q8N6H7 27352 P21359 55633 Q66K14 1122 P31150 O43150 5604 Q15057 Q3MII6 Q9H2M9 Q9NP61 O43597 7248 Q96BZ9 Q96DN5 P41182 Q5R372 Q86TI0 93594 Q9NUY8 Q96P48 121512 25780 79735 10451 9815 P51671 Q96HU1 Q5JSP0 Q86WV8 Q9NU19 2475 7249 Q8TEA7 604 Q6ZW31 Q9GZM8 51735 85440 Q96NH3 P24386 Q6ZV73 Q96N67 Q0IIM8 55785 O60343 4943 O43182 Q8IYX1 64786 Q02750 Q9Y3P9 23513 Q14160 Q6PID4 Q14161 Q14289 Q8N6T3

regulation of GTPase activity 9744 10928 26037 Q99490 P15498 P42345 2665 2664 P43034 Q92574 P49815 Q6ZT07 Q8WZ64 O43166 Q8TBP0 Q15027 Q9Y2X7 Q7Z6J4 23216 115704 Q9P107 O43609 81565 Q15311 85360 P98174 P50395 9882 1121 116986 Q96M96 Q9ULH1 116987 Q8TEU7 Q96CN4 P09471 Q9UKW4 Q8IV61 P26374 Q9UPQ3 Q8WWN8 Q92949 125058 Q8N6H7 27352 P21359 55633 Q66K14 1122 P31150 O43150 5604 Q15057 Q3MII6 Q9H2M9 Q9NP61 O43597 7248 Q96BZ9 Q96DN5 P41182 Q5R372 Q86TI0 93594 Q9NUY8 Q96P48 121512 25780 79735 10451 9815 P51671 Q96HU1 Q5JSP0 Q86WV8 Q9NU19 2475 7249 Q8TEA7 604 Q6ZW31 Q9GZM8 51735 85440 Q96NH3 P24386

Q6ZV73 Q96N67 Q0IIM8 55785 O60343 4943 O43182 Q8IYX1 64786 Q02750 Q9Y3P9 23513 Q14160 Q6PID4 Q14161 Q14289 Q8N6T3

response to fatty acid O00206 7099 8443 P05413 Q09472 P55851 5155 2033 Q01094 P14635 P18146 O60603 891 P05091 Q7Z6C1 P01127 1869 O15228

positive regulation of type I interferon production O00206 7099 O95786 7098 Q7Z434 Q9NR96 23586 O60603 148022 3329 O14802 Q8IUC6 55703 54106 O15455 57506 P10809 11128

regulation of chemotaxis Q7Z727 7422 O15123 O14672 4354 O43915 2150 57402 Q00013 P15692 P84022 P17252 1012 5578 P01137 P08887 P07996 P08246 P55290 719 P78536 7057 5054 P35968 3791 4088 5155 Q15109 3673 O43889 P51679 64127 P35443 P48061 285 Q9HCK4 P17301 P61073 Q9HC29 2277 P01127 P55085 P05121 10488 Q16581 Q9HCY8

regulation of actin filament polymerization 4690 O15020 10092 10095 O95835 Q13813 10094 O15143 Q9BPX5 23189 O43639 P35612 P35611 P62330 P42345 6709 P20936 830 P02549 81873 832 118 Q9H2D6 10552 P47755 P47756 Q08495 Q14678 P51671 Q01082 382 P11277 P16333 10109 2039 P52907 2475 6711 6710 4214 Q6PKD3 P62328 P61158 P63313 Q13233 829

ovulation from ovarian follicle 9510 P48552 8204 P29474 5321 Q9UHI8 O43524 23411 P01019 P48357 3953 Q96EB6 P06401 P47712 P41159

trophectodermal cell differentiation 6670 9421 8452 O96004 P12830 Q13618 Q9BPY8 Q02447 84525 P17275 Q96S42 6667 Q15561 P08047 999

positive regulation of cyclin-dependent protein kinase activity P11309 P24385 P78536 O75832 P31749 P30279 595 894 896 P30281 Q92786 207 P00533 1956 5716

paraxial mesoderm development 4087 4088 Q12948 P36894 6927 P20823 2303 57669 P42858 P84022 Q9HCM4 Q9UJU2 Q15796 Q99958 Q8WUM0

sister chromatid segregation 54908 O95835 9700 10270 P14635 9184 891 Q15021 Q9NTI5 5119 Q14674 Q15003 Q8WZ42 Q96EA4 O43264 O95347 Q9Y6X3 O43684 O43823 P51955 23310 23212 Q6KC79 Q96BK5 Q9UJX2 Q15050 54984 Q9NTJ3 O14777 P42695 Q96FF9 Q14683

central nervous system neuron development 2290 9201 P54753 Q7Z727 P14416 A1XKG3 1020 Q05397 Q8IYN9 P51805 Q00535 Q1PSW9 P17252 Q04656 5578 P43034 P41159 O15075 P29323 P54762 O43602 Q658W2 O75592 P50148 P55316 P10070 81565 23077 55558 Q9GZM8 5747 2736

regulation of positive chemotaxis Q7Z727 P35968 3791 4088 7422 O15123 Q15109 3673 O43889 P51679 O43915 2150 285 P17301 P15692 P84022 P17252 1012 2277 5578 P55085 10488 P55290

vesicle targeting Q12851 O95721 P53621 P35606 9276 9342 Q9Y678 Q92538 5871 10053 P53618 Q9Y6Q5 1315 375 1314 P84077 Q13190 P49755 6811 10972 O14579 Q8N6T3 P49768

activation of JUN kinase activity 6850 Q16512 4188 5585 Q13546 O95382 Q9Y4K4 Q96M96 Q9P035 P43405 10746 121512 79444 Q99683 Q03468 Q99750 Q5TCX8 8737 4214 Q96CA5 1616 Q13233 Q9UER7

mammary gland duct morphogenesis 5371 P51671 Q15648 2099 P29590 54361 P56705 P10275 23513 Q99623 367 Q14160 Q1PSW9 P10070 8945 11331 Q9Y297 P03372 P01137 O15119 6926 P06401 2736

lipid oxidation O00628 Q96A54 Q08426 2110 P50416 P33897 Q86V24 P09110 215 1892 P23786 Q16134 5830 Q9UJ83 Q16698 30 Q9UBK2 34 P28330 P40939 1374 1891 P51659 3032 P50542 3030 51094 P11310 1666 P30084 5467 P55084 26061 Q13011

fatty acid oxidation O00628 Q96A54 Q08426 2110 P50416 P33897 Q86V24 P09110 215 1892 P23786 Q16134 5830 Q9UJ83 Q16698 30 Q9UBK2 34 P28330 P40939 1374 1891 P51659 3032 P50542 3030 51094 P11310 1666 P30084 5467 P55084 26061 Q13011

positive regulation of transmembrane receptor protein serine/threonine kinase signaling pathway P27037 5460 Q13873 7046 57045 P37023 Q93062 O14908 Q9H160 P53708 91 94 P01137 Q13485 Q99966 P07996 P09958 Q9GZX9 Q6IR47 P78536 7057 11030 4089 4221 P36896 O00255 Q13705 P36894 P10600 7272 P17813 3622 8516 O43294

mammary gland epithelium development P24385 2099 Q02363 P29590 P10275 P17676 Q99623 595 2247 Q1PSW9 8945 Q9Y297 P03372 P01137 O14788 O15119 Q9UKW6 P09038 6926 P06401 5371 P51671 O15111 Q15648 54361 P56705 23513 1147 367 Q14160 P16471 P10070 11331 2736

positive regulation of neurogenesis 8650 4131 7520 O43157 P10636 2033 396 2547 Q86WK6 P07196 Q7Z6C1 P61586 P46821 7518 P52565 Q13009 P10721 27339 5270 P55316 P37231 81565 P46531 4851 4137 5468 P61158 Q9GZM8 P08138 Q92888 Q99835 2290 Q09472 Q15797 P48431 P12956 6498 5364 P12757 P14416 Q13418 P53667 27185 P13010 3611 Q9NRI5 P01138 Q9NQC3 Q9UMS4 4086 P29323 5970 Q9Y6R0 Q13426 9138 P10586 9253 9495 7074 Q9HCK4 387 Q04206 6657 P24588

cell maturation Q9NZJ7 9463 O75503 P07949 2034 P14635 P51587 O00468 351 26271 1499 P15692 Q92731 10296 Q92730 5139 1738 P03950 P35968 O43524 P11413 P50148 P37231 3169 30813 5468 375790 10661 P01588 O15169 P05067 Q9NRD5 Q96NL6 P09622 Q9HB63 137964 7422 Q7L5Y9 23787 891 9319 Q14432 P49715 P06401 Q92826 132320 3791 P18074 2100 Q86UL3 59277 P35222 1050 P17302 1203 O95390 2697 Q9NZR4 Q6PID4 P60201 Q99814 Q14289 Q9UKT4 Q13351

negative regulation of cell cycle process P25054 7023 6597 9700 Q13315 P54274 11186 9126 Q969H0 P41182 Q9NS23 4193 472 10459 P38398 672 P49959 Q01664 54998 Q8N3U4 5119 Q14674 P06400 O43683 4361 7013 Q9NP71 O00213 P51532 55294 7272 Q00987 Q9NWT8 P14174 Q9UQE7 324 604 Q9UI95 Q14683 5925

organ regeneration P24385 7046 O15123 Q06203 9021 P16118 3482 230 890 595 898 P31939 P09972 O14543 P01137 Q15389 Q15466 P49715 P11717 5471 Q6IR47 P12429 Q92729 P16989 P38936 1050 P30530 285 P37231 P20248 1026 P24864 5468 5207 O15524 P60842

positive regulation of inflammatory response O00206 P78423 Q7Z727 7124 5321 8654 P14555 O60603 Q9Y6Q6 P17252 P01019 5578 P21980 5733 O15455 O14788 P42229 P15090 P43115 P01375 P47712 7099 7132 P30556 7098 5054 P51692 3673 Q9NR96 7052 O76074 P19438 P17301 O60674 6776 54106 P05121 P21554

neuron development P25054 Q92858 8650 P55268 Q9UQP3 65125 Q8TEW0 Q9UBB6 O75385 351 3516 474 Q8IYN9 P16220 284217 P07196 Q92730 P83731 O14786 P43034 8408 Q12837 P50148 P63211 Q96KN7 2676 P42858 O60331 30813 P46531 4851 P00533 4734 P54753 Q7Z727 Q9HB63 6256 P25391 Q06124 Q05397 Q9UBU3 6809 P51805 P09471 Q96RK4 4983 Q9UQB8 P00441 Q16650 6383 P54764 P54762 3672 Q9Y6R0 P10588 P13611 59277 Q7L576 O75592 P17302 O60674 2697 23032 23154 23396 6259 P60763 6812 5604 P41743 P34925 Q8TD84 1385 1020 O00468 4646 Q15223 O00238 P60709 Q15109 Q06330 960 O60890 60 Q9GZM8 Q9UM54 1947 85440 5747 Q9NRD5 Q99835 P04179 9201 Q8TCU4 Q8TEY7 10152 Q02880 A1XKG3 Q9NYB9 Q96EV8 Q96N67 Q92753 O14640 P08069 Q92752 1956 O15075 P29323 7155 O15078 P39905 Q13509 Q02750 Q9P202 Q9HCK4 Q6PID4 Q14289 Q15375 Q15019 9463 P56199 25861 7161 P50897 P11047 3913 Q8TAF8 Q86WK6 Q1PSW9 999 Q6NVY8 P46821 O60282 3912 Q9C0K0 Q96CW9 O15496 O43602 10939 2043 1191 P55316 P46934 81565 P16070 P05067 P98172 Q96NL6 P49768 2290 8399 P50552 P14416 P19793 Q13418 O43815 23787 6096 1756 773 Q00535 P17252 Q53X93 P21860 6647 P01138 P49639 8829 84062 P04626 Q658W2 2065 2064 Q8IYT8 O00213 2063 9253 56288 Q96FJ2 Q9NZR4 P10070 P10071 3146 P07101 2737 2736 Q9NZJ7 P07942 4131 5584 O75503 4133 8851 P07949 P78509 10458 P15692 O60488 5578 5338 P26232 P52803 O43921 P11532 64919 324 375790 P08138 7408 Q9HCU4 Q15078 P09429 7422 P10415 P56178 Q8IUQ4 P12830 P34741 O60229 P20336 3611 Q04656 P61764 140735 80184 P41159 O15350 132320 O43464 Q15768 1203 O14939 23077 55558 Q9BZR6

response to ionizing radiation P04637 Q13315 P54132 9021 P51587 25788 472 2547 595 672 Q96RU2 Q03468 51720 O14543 7518 P08887 5591 P48681 4221 Q9UBC3 7412 P16104 1789 P46736 Q9NWV8 641 P08575 Q96RL1 Q70UQ0 P04179 Q9BQ15 P24385 P12956 P10415 Q9NRY2 Q13216 Q13535 P29590 1161 P78527 P38398 79035 57646 121457 3014 857 29086 5371 64782 P55212 7157 Q13426 P19320 O00255 Q6KC79 55159 545 Q8WTP8 10763 P02533 P43246 Q6PCD5

eye morphogenesis 6670 5584 P25490 Q06587 25945 O75581 1499 P15692 356 6667 P08047 Q15223 P40763 P25067 P58304 P13631 Q02447 P63211 Q96KN7 338917 30813 7528 Q8TCU4 6496 P48431 7422 P14859 6015 6774 P12755 Q9NQS3 6096 Q96RK4 Q92753 O95859 P47895 80184 P10826 P29323 P05997 P54762 5451 O15078 P48023 O95140 P20908 Q6KC79 P35222 O95343 P21359 1289 O95390 Q9NZR4 P58012 P10071 668 Q92786 P07101 6657 P41743 2737 9927

response to metal ion 5265 P28562 P30086 2033 22872 O00300 23327 595 513 6622 999 Q7Z6C1 Q8N465 P07996 10016 O15118 7057 5037 P46934 P42858 P05062 P00533 4734 P02675 P01222 7220 808 Q09472 Q9UMR2 6256 P29474 5321 P19793 891 P00966 Q9UJU2 6647 Q8WZ42 P00441 P50440 6262 P55212 Q9Y696 P68133 P35222 P12004 5728 Q9UBV8 P60484 P01009 8836 Q92820 P30049 O75746 48 P41181 P14635 P41180 O75340 P35613 1499 Q96PU5 8604 5336 11269 P62158 P45379 P47712 7252 Q9NYA1 Q13443 5111 P60709 2243 Q96FQ6 Q07817 682 57678 P21579 P18846 445 841 60 846 P48995 P02671 P24385 Q401N2 8754 P10415 P12830 Q02363 Q53TN4 Q9HCL2 2244 140576 Q04656 5599 Q14790 6721 857 P02786 1956 Q12772 Q13586 7037 P37840 P16885 1843 466 P18827 P09960 Q6PID4 79901 P45983 O94979 P02533 Q14289 P45984 P04155 P04156 P21399 P06213

visual learning P54253 P14416 A1XKG3 3265 1020 P50993 60626 351 Q99965 P09172 Q9NPQ8 54413 477 Q00535 P01116 P01112 2902 P01111 Q05586 P35368 P10721 Q9NZ94 P21359 P42858 P05067

regulation of actin filament depolymerization O15020 Q13813 Q9UHB6 P35612 P35611 6709 830 P23528 P02549 832 118 Q9H2D6 P47755 P47756 Q08495 Q01082 1072 P11277 2039 51474 P52907 6711 6710 Q6PKD3 829

response to progesterone stimulus O00206 9021 O60603 P05091 P17275 898 857 O14543 P01137 P07996 P01178 7099 Q15303 7057 Q9NYA1 P30559 5970 P04626 P10600 2064 Q04206 P24864 Q13133 O15524 P01100

reproductive structure development Q7Z2K6 8892 5268 Q12948 O00268 P51587 595 Q14050 3479 Q1PSW9 P23945 Q92731 P22612 Q15389 P42229 P49770 P03950 9510 O75534 P16989 79084 54361 P13631 367 P16471 P46531 4851 P48436 P16070 P01343 Q96EB6 P48552 6256 P29474 5321 P19793 O75881 6927 894 898 P01019 79956 6647 P03372 Q13144 Q9NSC2 P06401 P00441 Q92826 P35869 3791 8204 A4D1W7 P36952 2100 Q9UHI8 P10620 P35222 P56705 Q02790 5728 P20823 Q9HBE1 3028 P60484 3148 P10070 P24864 668 6776 5568 P43246 2736 P26583 P27037 9420 O15287 P35453 P10275 1499 P15692 P55075 3953 Q13285 2626 P47712 P04201 O00238 Q9Y5R5 P35968 Q13323 O43521 O43524 P10721 23411 Q05086 P19544 7490 P30279 Q07817 960 3169 P48357 10468 Q99714 P24385 7422 P10415 2099 2253 P51843 P08069 P43694 Q9BQA1 Q92754 P41159 P51692 Q6KC79 P19883 O60911 Q9HCK4 P58012 P18827 P24821

organelle fusionQ12981 O43752 8411 P51636 O95140 10228 80124 O60763 2773 Q9UKW4 10451 P61006 8615 Q8IWA4 Q15075 P08754 4218 Q96JH7 Q13286 9927

activation of MAPKK activity Q5S007 7046 O95257 8754 P30542 O43318 O75293 2247 Q03468 23043 1956 P09038 P01133 Q6IR47 Q9Y6R4 Q13443 6885 Q9UKE5 O60674 120892 4214 P00533 4216 Q9UL54 Q13233 P25116 P49768

lysosomal transport 4074 Q9UPV9 Q86VS8 O00203 154 Q9NZ43 Q99523 P07550 Q9UGU5 Q9Y2T2 O15118 8546 6642 26985 Q9NZZ3 Q8IWJ2 Q9UJC3 P46934 P20645 388 P62745 Q96ED9 51510 84376 9648 4734 Q13596

positive regulation of T cell differentiation O43353 P00491 Q92934 6850 3142 P10276 O00203 572 79576 8767 P42229 5914 P24394 Q13422 Q14774 8546 P51692 4860 Q8N5F7 P43405 P37173 O43290 6776 P01589 10320 P60568 P04233

vesicle docking Q6FGG2 10490 Q15836 Q16623 Q8WVM8 26258 O00186 Q12846 Q9NRW7 9341 Q9UEU0 60412 O00471 P61006 P61764 Q96AX1 54536 65082 Q9UL45 P51636 23256 Q8TAG9 6810 10640 6812 6814 4218

pallium development P48431 7248 P56177 P78509 A1XKG3 1020 3066 7161 Q9BXC9 P51805 Q00535 Q96RK4 5830 Q9UJU2 P43034 Q92574 Q9Y6A5 P62258 1956 Q8N960 9839 P04201 O15350 Q92769 Q14114 7531 10460 93986 P12429 O75553 Q86WV8 P50542 9495 Q9NXR1 P21359 54820 P10071 P20264 P00533 6657 55558 7804 2737 P24588 P49768

growth P54852 P08833 P55268 Q9UQP3 P04637 O14793 Q9BZ95 Q12948 3484 P51587 O75385 351 O60725 3913 P42345 2303 Q9Y3A5 Q04759 3479 Q1PSW9 P84022 Q9Y2X3 P49810 Q13485 8408 Q9H0D6 Q13642 22803 Q9NZG7 6520 5155 Q12837 P35368 O43602 P58546 Q9BRQ0 Q96RT1 84159 367 P16471 81565 P46531 4851 9869 P48436 4854 P05067 Q15796 Q9UK55 P43026 P01343 P49768 Q14865 136319 6256 P19793 Q06124 P56539 P34820 891 Q03405 773 Q96S42 P58166 51542 656 898 Q92830 P03372 P01137 O60383 Q9UBM7 Q9NSC2 Q15047 4869 P50440 Q9UMS4 9133 5054 4087 4088 4089 P18074 A4D1W7 P68133 Q13705 P12645 O95067 Q7L576 P35625 P17302 5728 P31270 2697 P60484 P10070 P10071 51156 P24864 P01127 5328 2737 2736 O43597 P14635 P35453 P10275 Q8NFW5 1655 P00749 P00747 P15692 6548 Q9UM47 P51671 O00238 O43524 P36894 P10600 Q6UB99

Q05086 27339 Q07817 3207 P51959 2475 5467 207 Q9GZM8 Q99958 Q99835 9201 Q8TCU4 90780 7422 P10415 23463 2099 O95376 83729 P19634 P02708 P17844 859 O15075 Q9HB90 7157 Q15648 P31749 Q6KC79 O95390 O60356 P08195 900 Q92982 P05121

SREBP-mediated signaling pathway 3638 Q12772 51141 4000 Q9BY44 22937 Q9Y5U4 6721 P02545 Q12770 O15503

histone phosphorylation Q7Z727 Q16512 O60674 5585 Q9UIG0 P17252 5578 5579 O75582 9252 P05771

positive regulation of dendrite morphogenesis 4086 8650 Q15797 3611 Q9Y6R0 P10586 P61158 Q13418 9253 9495 P24588

positive regulation of cell cycle arrest 5371 1029 Q6ZN33 1026 Q8N726 P29590 P38936 P42771 51512 Q9NYZ3 O43715

ribosome assembly 2 3692 Q2NL82 Q9BY44 P62263 P83731 P62851 P62753 6194 P56537 4869

positive regulation of epithelial cell migration P17301 Q13873 3091 57669 3673 Q9HCM4 10013 Q16665 6869 P25103 Q9UBN7

trabecula formation P02452 9510 54583 1277 Q9UHI8 4313 P08253 Q9GZT9 2280 P52952 P62942

response to growth hormone stimulus P01241 5295 10626 O60674 P51692 6774 6776 O95361 P42229 P27986 P40763

establishment or maintenance of epithelial cell apical/basal polarity 163183 Q7KZI7 Q8N205 Q96RT1 5584 3611 284217 P25391 2011 Q13418 P41743

positive regulation of epidermis development 10626 Q15628 7124 P22392 P46531 4851 O95361 P19883 10468 8717 P01375

negative regulation of histone modification 1789 1786 Q9BZS1 Q9UBC3 Q01105 6622 3720 50943 P37840 Q92833 P26358

regulation of histone methylation 1789 1786 4297 4221 Q9UBC3 P49711 3720 O00255 Q92833 P26358 Q03164

positive regulation of sterol transport Q9Y5X9 9388 7376 O95477 4792 Q07954 4035 P55055 19 Q13133 P25963

positive regulation of cholesterol transport Q9Y5X9 9388 7376 O95477 4792 Q07954 4035 P55055 19 Q13133 P25963

response to molecule of bacterial origin 6198 P98155 5265 8654 O60603 P42224 Q96RU8 356 10935 6622 8767 O14543 P25963 P78536 P27361 O76074 128 P01588 O15524 Q9HCY8 P01100 O00206 Q9Y6Y9 P29474 6772 5321 P13686 148022 Q9Y6Q6 57402 1634 Q9UDY8 P00966 P01138 64170 P01374 5054 5970 P48023 P10620 Q9H257 Q9NR96 P19438 3148 Q8IUC6 Q04206 P01009 P07585 O43353 P26583 O95999 P28482 P11766 P41181 9021 P30048 P05091 Q9Y2C9 P21964 5336 10057 P08887 Q99966 P08246 54 P47712 Q96FA3 P05412 7099 7132 P29466 64127 3297 P30793 1147 445 841

P01266 4792 57162 1312 Q00613 2643 Q14790 P23443 P29320 O15111 P51692 Q8NHX1 P34972 P37840 P16885 P51617 Q9HC29 54106 P05121 P25116 P21554

mammary gland morphogenesis P78545 2099 P29590 P10275 Q99623 Q1PSW9 8945 P21860 857 Q9Y297 P03372 P01137 O15119 6926 P06401 5371 P51671 P04626 Q15648 2065 2064 54361 P56705 23513 367 Q14160 P10070 11331 2736

labyrinthine layer development O95377 Q14686 Q15738 P28482 O43278 9021 O00622 O14627 57448 P62993 P00747 2885 O75190 P17275 Q9NR09 Q9UJU2 3676 O14543 Q9GZT9 Q99966 50814 7412 P31749 P19320 Q02750 P13612 54583 207 5604

regulation of interleukin-2 production O43353 P07766 7186 7189 Q8WUI4 Q9Y4K3 O43318 Q04759 Q92990 Q9UDY8 51564 8767 11146 Q92993 P42229 P01730 64170 11326 Q12933 Q9BZS1 P51692 6885 Q15306 64127 Q9H257 10524 Q9HC29 6776 50943

mitotic sister chromatid segregation 54908 9700 10270 P14635 9184 891 Q15021 Q9NTI5 5119 Q14674 Q15003 Q8WZ42 Q96EA4 O43264 O95347 Q9Y6X3 O43684 O43823 P51955 23310 23212 Q6KC79 Q96BK5 Q9UJX2 Q15050 54984 Q9NTJ3 O14777 P42695 Q96FF9 Q14683

negative regulation of myeloid cell differentiation O00206 Q14106 P25791 Q9Y5Q3 Q92835 4792 P10276 Q01196 P15531 1499 4005 O15455 P42229 P05112 P25963 5914 7099 7098 Q9HAZ2 P62805 P51692 A4D1W7 P22392 63976 P35222 Q05516 861 6776 4830 7704 Q96T37

female sex differentiation Q7Z2K6 8892 O15287 Q12948 O00268 P51587 P15692 Q14050 P23945 91 Q92731 3953 Q15389 P42229 O15119 Q13285 P47712 P49770 O00238 P03950 9510 P35968 P36896 O43524 23411 54361 Q05086 P30279 Q07817 P48357 10468 Q96EB6 P48552 P29474 7422 P10415 5321 2099 Q99623 894 898 P01019 79956 6647 P03372 P01137 Q13144 6926 P06401 P00441 P41159 3791 8204 P51692 Q15648 A4D1W7 2100 Q9UHI8 P19883 P56705 Q9HCK4 P58012 P24864 11331 668 6776

response to lipopolysaccharide O43353 P26583 6198 P98155 5265 P28482 8654 P11766 P41181 9021 O60603 P30048 P42224 Q96RU8 P05091 P21964 356 10935 6622 5336 10057 8767 O14543 P08887 Q99966 P25963 P08246 54 P47712 Q96FA3 P05412 7099 7132 P78536 P29466 P27361 64127 3297 P30793 O76074 1147 445 841 128 P01588 P01266 O15524 Q9HCY8 P01100 O00206 Q9Y6Y9 P29474 4792 6772 5321 P13686 148022 Q9Y6Q6 57162 57402 1634 1312 Q00613 2643 P00966 Q14790 P01138 P23443 P01374 5054 P29320 O15111 5970 P51692 P48023 Q8NHX1 P10620 P34972 P37840 P16885 P19438 P51617 3148 Q8IUC6 Q04206 Q9HC29 P01009 P07585 P05121 P25116 P21554

regulation of polysaccharide metabolic process Q13322 Q9BX66 10580 8445 P31749 Q9Y4H2 P35368 P35568 P42345 P62140 P01308 2475 5500 207 Q92630 3667 P06213

regulation of collagen biosynthetic process 4261 Q09472 Q92769 3673 2033 3066 P10600 54361 P56705 P17301 2147 P33076 P00734 Q7Z6C1 P01137 P08887 P25116

negative regulation of oxidoreductase activity 4790 P78540 2672 P34972 P37840 P56539 P17813 P19838 P01308 Q99684 Q15070 6622 10013 5018 859 Q9UBN7 P21554

regulation of glycogen metabolic process Q13322 Q9BX66 10580 8445 P31749 Q9Y4H2 P35368 P35568 P42345 P62140 P01308 2475 5500 207 Q92630 3667 P06213

endochondral bone morphogenesis P02452 Q15742 Q14814 O00628 P56178 P63092 Q13506 Q9NX62 3207 P31270 1277 4664 Q5TAT6 P48436 4665 P02458 4209

positive regulation of multicellular organismal metabolic process Q09472 Q92769 3673 2033 3066 P10600 54361 P56705 154 P17301 2147 P00734 P07550 Q7Z6C1 P01137 P25116 P08588

G1/S DNA damage checkpoint 5371 Q6ZN33 P24385 7157 P04637 P29590 P38936 Q9NYZ3 79791 O43715 595 1026 Q5XUX0 55159 51512 Q13574 Q6PCD5

inactivation of MAPK activity Q9BY84 Q8IWV1 Q7Z727 Q13202 Q13227 Q9NRW4 P30153 Q16828 56940 Q8WTR2 P67775 1852 1850 2873 P17252 5578 857 1848 5515 Q13098 Q99956 5518

histone ubiquitination 6045 Q6P1J9 Q6PD62 7334 9646 10075 6015 Q5VTR2 Q9UPT9 Q06587 Q8N7H5 23512 23326 O75150 79577 57332 P61088 Q7Z6Z7 Q99496 Q15022 54623 Q9HC52

innate immune response-activating signal transduction O43353 O00206 7099 4792 O95999 5970 64127 O60603 148022 57402 3329 81622 Q9Y2C9 P51617 Q8IUC6 Q04206 Q9HC29 8767 P10809 P25963 Q9HCY8 Q9H1C4

regulation of epidermis development Q9UJM3 Q9ULX9 Q15742 Q15628 7124 P22392 O95361 P19883 Q13506 23764 10626 P46531 4664 4851 4665 54206 P08138 Q14145 10468 8717 P01375 O15525

cellular response to extracellular stimulus P02452 O00744 Q15853 25828 Q13873 Q8TDY2 9821 P04637 27327 P41181 3162 P10276 P37288 552 P22415 P23229 60673 10013 P08243 3953 Q13286 P05412 Q9Y4P1 9474 Q96SB3 Q9NYA1 6720 23411 Q8NDV7 55054 P55318 3171 Q9BSB4 P11021 1026 Q9P2K8 P05062 P48357 Q99757 Q96EB6 P01100 P49768 3860 P13646 3939 P61244 Q9H1Y0 1277 57521 898 4149 857 3655 Q7Z3C6 5119 5914 Q13501 P07339 7157 P38936 P36956 79065 P30530 O60911 Q676U5 Q9H492 O75084 23192 81788 Q9H093 P24864 8878 P09601 Q9UBN7 O94817 3309

regulation of lymphocyte differentiationO43353 P00491 O96005 Q92934 Q92835 6850 Q8N726 P41182 3142 P10276 P36888 O00203 572 79576 8767 6647 P42229 P00441 5914 P24394 Q9BT67 Q13422 Q14774 149041 8546 P04626 P51692 4860 A4D1W7 Q5TC82 Q15306 Q92949 P06729 2064 Q8N5F7 1029 P16410 P43405 P37173 O43290 6776 P01589 P40933 604 10320 P42771 P60568 P04233

positive regulation of collagen biosynthetic process Q09472 Q92769 3673 2033 3066 P10600 54361 P56705 P17301 2147 P00734 Q7Z6C1 P01137 P25116

provirus integration P12956 P62937 7520 Q13426 O75531 O75475 2547 P13010 3159 P17096 5478 11168 7518 8815

double-strand break repair via nonhomologous end joining 5591 4361 P12956 7520 Q13426 5422 P78527 2547 P13010 P49959 P40692 Q9Y6H3 P09884 7518

cell volume homeostasis P54105 P20073 3783 P55011 59341 1185 P41181 P51797 310 1874 O15554 1207 4869 Q16254

positive regulation of collagen metabolic process Q09472 Q92769 3673 2033 3066 P10600 54361 P56705 P17301 2147 P00734 Q7Z6C1 P01137 P25116

regulation of epithelial to mesenchymal transition 4087 4088 4089 P10600 4092 57669 P84022 Q9HCM4 O15105 P01137 O43294 Q15796 Q13145 Q13485

regulation of receptor biosynthetic process 3690 P05106 Q92769 22937 Q13547 3066 3065 P37231 3685 5468 10013 Q12770 Q9UBN7 Q6IT96

lysogeny P12956 P62937 7520 Q13426 O75531 O75475 2547 P13010 3159 P17096 5478 11168 7518 8815

regulation of microtubule polymerization P25054 7013 4131 P10636 P54274 Q15735 P56539 324 22919 4137 Q15691 857 P46821 859

Golgi vesicle budding P53621 P35606 9276 Q9Y678 Q92538 5584 P53618 1315 375 1314 P84077 O14579 P41743 Q8N6T3

vesicle targeting, to, from or within Golgi P53621 P35606 9276 Q9Y678 Q92538 P53618 1315 375 1314 P84077 P49755 10972 O14579 Q8N6T3

negative regulation of cell migration 4771 O15123 Q9UQL6 4092 3162 P23508 3516 Q96RU8 Q9BZE4 23560 P09493 94 10014 P07996 22846 7057 5155 Q7Z5L7 Q96QB1 Q92729 5796 Q5VTR2 285 Q06330 P18206 Q9H2X0 967 P10415 P14416 Q13418 Q9Y2I1 P30542 P37023 2247 3611 127435 P35240 7414 P01137 P09038 5054 Q8WWN8 Q9Y696 Q14118 Q7L8A9 Q8N9R8 5728 P21359 P60484 P17936 O15105 P01127 P05121 P09601 1605

B cell activation 6670 5580 P04637 Q13315 P15923 P41182 Q9UQL6 Q9H165 P53567 7163 P32302 472 1137 3476 5336 3676 Q8WV28 5579 7518 10014 P05771 Q15464 Q8IWV1 5591 P56524 P78536 7412 3329 Q02447 P16473 P08575 604 3688 P10809 2956 P10415 Q03014 P78527 P55327 6929 Q9UDY8 P05112 P05556 Q13422 7157 Q13426 P11912 Q9GZX7 P04629 P19320 P13612 P78318 P52701 P16885 4914 P40692 Q05655 53335 57379 P43681 10320 P43246

development of primary female sexual characteristics Q7Z2K6 8892 O15287 Q12948 O00268 P51587 P15692 Q14050 P23945 91 Q92731 3953 Q15389 P42229 O15119 Q13285 P47712 P49770 O00238 P03950 9510 P35968 P36896 O43524 23411 54361 Q05086 P30279 Q07817 P48357 10468 Q96EB6 P48552 P29474 7422 P10415 5321 2099 894 898 P01019 79956 6647 P03372 Q13144 6926 P06401 P00441 P41159 3791 8204 P51692 A4D1W7 2100 Q9UHI8 P19883 P56705 Q9HCK4 P58012 P24864 668 6776

T cell differentiation in the thymus 5591 P07766 P25054 P78536 P10415 7157 P04637 P51692 Q13426 23210 Q6NYC1 9093 P35222 P78527 6194 Q8N4C8 1499 324 6776 Q96EY1 P42229 7518 P62753 P04233

positive regulation of protein kinase B signaling cascade Q15811 Q6IR47 7046 7057 6453 Q13418 3481 P42345 2159 3611 Q02763 P01308 2475 P08069 Q9NQB0 P00533 P00742 P01137 O14788 Q15389 P01344 1956 P07996 P06213

salivary gland morphogenesis 5361 Q9GZX9 5155 P25391 7124 O15230 Q14118 P12830 P10600 57045 O60716 7052 284217 999 P21980 O14786 P01127 P00533 P01137 1605 8829 3911 1956 P01375

regulation of macrophage derived foam cell differentiation 8399 P30556 3690 P05106 4790 7376 O95477 4792 O15496 P55055 19 P14555 P37231 P21757 P16671 P19838 3685 P01019 5468 Q13133 P02741 948 P45984 P25963

positive regulation of nitric oxide biosynthetic process O00206 7099 P07900 7124 P31749 3320 O60603 148022 3326 O60674 P01308 3269 3324 P68871 Q8IUC6 P01579 207 P05362 P00533 P08238 1956 P01375 P06213 P04179

immune system processQ9H0E2 P10914 P25054 O14672 Q06265 P51587 P30520 P17676 P15498 P40200 2547 P84022 910 Q9Y275 O14788 6901 57506 P07996 Q9Y3Z3 P04003 P27487 P04004 Q9UGM3 4860 P52566 P20963 P16473 P14174 P16471 Q9UKV3 4851 10661 P10809 Q92838 Q7Z727 Q9NZH6 P27694 Q92956 Q92835 Q12965 Q9Y2Y8 Q9BQ51 Q7L5Y9 79671 57402 10758 6929 O15533 811 3655 P63104 Q9Y6A5 64170 P40424 Q92833 3659 4627 P06400 Q12851 P05556 Q9GZX9 3791 Q86SX6 5970 3672 5971 P48023 Q92949 Q9GZX7 Q9NR96 3551 1130 O00178 Q9NP55 3550 9093 Q9NRZ9 22985 Q13829 3428 P16333 81501 81622 6938 Q99698 P17542 Q9HBE1 O60674 Q8IUC6 P62263 6814 P04233 P42830 Q9H1C4 708 Q9Y468 P28482 O95470 P19532 114548 2590 55361 P39019 27283 1137 1499 10616 710 4643 3676 O14862 Q9BZM4 P08887 719 P07437 P51671 P29466 5871 P80098 P51679 P10721 Q96J02 P30793 3329 27297 57678 29110 1147 P60022 720 841 P43628 722 604 3688 3689 P04179 P07766 P05107 4791 6850 4792 P51681 P50591 Q9NZD8 Q9BQ95 2495 P51686 O95376 Q02086 P42768 Q9HCL2 1399 976 857 P26038 P04264 P05112 Q10471 P51572 P10827 Q92626 P51692 P11912 P31749 O00255 P19883 Q9NS75 1050 P48061 Q03164 Q9NQT8 79465 P19878 3107 861 P40692 P37173 863 P05362 50943 7704 4318 7706 4690 Q9Y6W8 P04637 P80188 P56199 P15529 P14317 2034 3122 O60603 P52952 203068 3115 3476 998 6622 10134 6504 10014 P02686 P62753 P25445 Q9C0K0 Q9BTU6 P06702 Q9UII4 4221 10383 6885 5553 10261 23210 O95163 P13236 P12268 1191 2280 Q04900 641 P33076 Q13094 P01589 648 P01588 P02794 P49768 O00206 6890 Q9Y6Y9 P25791 6892 P13686 23303 148022 O00203 Q00653 P14780 896 6886 6647 P01137 5316 11326 P01375 P01374 P35225 P04629 P35222 O95067 Q86UT6 P35226 O75081 53335 3148 P09086 3146 10241 6776 4478 P01127 P40933 Q96EY1 P25311 P23497 O43353 6670 5580 P26583 O00744 6672 7520 P04439 P07948 Q9UNN8 Q8WYA6 P59665 Q12904 P39656 Q12905 P14598 P15692 6304 6667 4005 5336 Q96AX1 5578 5579 P04440 7518 P05771 Q8N423 5591 P06744 Q07021 4261 Q6IR47 5473 8743 8744 10460 7534 7412 64127 10107 P37231 563 51295 324 326 207 5468 10468 P27797 10225 8754 64135 7422 4155 P48594 Q9UHD2 10437 O14802 P13010 Q96P20 10673 O14920 Q01826 P24394 5371 3070 7433 P35268 P06729 3075 P52701 P16885 P16410 P51617 10682 8879 8517 10320 54106 9846 10202 Q96HB1 P60568 Q9BYX4 Q13478 Q13477 Q14686 4297 P13747 4179 P15923 O43557 Q9H165 4291 Q9Y4K3 O43318 472 Q8WZ60 355 356 P49810 P08047 8767 Q13123 P25963 5393 P35408 9755 7334 5155 Q16633 8546 P48960 4067 6368 Q16635 P12429 Q15306 Q15669 P11215 Q02447 60489 P27918 Q9NRH2 6117 P46531 7448 P55040 Q9HCY8 A7MCY6 P80217 O00602 Q13137 9641 Q16649 6495 6498 P13727 P12757 P32241 P32246 Q9Y6Q6 Q9UDY8 Q9UDY6 P02741 P00441 Q14116 Q13268 4088 P18074 Q86XR7 Q9H257 P13612 P68371 Q8N4C8 P01889 10087 Q03518 P43246 P43489 P62942 P55347 Q5VWK5 Q16666 7124 P54132 7126 Q9Y6K5 O43639 Q9UQL6 P35579 Q00597 P09172 P46109 397 Q01201 Q9Y2C9 P23229 Q9Y6K9 51324 10296 Q8WV28 Q15223 Q99966 Q15464 54 Q14258 7133 O43521 O75575 P36894 P13796 Q86WV6 Q13291 P01308 51218 Q15475 Q96T37 9564 P00491 Q15116 Q8TCU6 O75326 Q7Z7G1 O75569 9567 P12314 P36402 O43734 P58753 23586 P36888 P02549 P01579 P08069 Q15125 P49715 11128 P60953 7157 P10147 O43508 P37840 Q9H295 Q14164 Q99814 P02775 Q16581 P27930 P29016 P78423 7046 7163 6194 P32302 56259 2821 P0C0L4 Q9Y3A5 Q9HC16 10935 P17275 65082 O95715 P42229 P56524 P78536 7057 83737 Q8IUN9 P01909 Q99081 P01903 P08575 P42574 2956 Q9UBL3 7189 7067 P06239 O95977 O95976 Q06643 P29590 Q03014 6093 Q01196 P55327 P41597 Q9BYM8 P16284 P17252 P06241 Q9NZ08 P05155 3932 Q6V1X1 Q13422 9133 9255 340061 Q13426 A4D1W7 55072 Q7Z434 P19320 P78318 P31785 4914 653361 P43405 Q05655

Q04206 57379 Q96EP0 O95999 Q13315 Q6NYC1 O00186 P41182 8174 P78504 P53567 Q7RTR2 P35612 P30048 O43918 1896 Q9H9B4 1650 Q9UJW2 P08246 Q8IWV1 7099 7098 P35968 Q13443 P22670 P11413 P51159 P10321 P32456 1667 Q05516 64919 2634 P30281 23098 3720 3608 Q6SZW1 P23510 P09429 O14492 P12956 P10415 P28065 Q02363 57045 P78527 P18146 3615 3614 2643 P41273 P61088 1672 Q04656 Q14790 O15455 Q13464 P01730 P20333 Q15646 O95786 O15111 P55773 Q9BZS1 Q15648 P19484 Q06830 P34972 O60238 Q9HC29 P43681 51752 Q13351 5925

response to UV P04637 Q9NX61 P51587 595 P22415 Q03468 P04040 P23025 P08246 Q14011 4221 Q9NS91 5796 1153 Q6AZY7 Q01831 P46934 2237 1026 207 5424 P00533 847 4734 P42574 2956 P24385 P10415 Q13216 P28340 Q13535 P29590 1161 5599 1956 5371 7157 5970 P55210 P18074 P31749 P39748 Q9BZF9 O00255 P38936 P52701 545 Q04206 P45983 51435 P43246 51514 7508

ribonucleotide biosynthetic process O75947 Q16864 P30085 P16615 P30520 P15531 P11172 476 477 478 513 514 515 P61421 518 9114 P48201 P48047 P13637 481 P12268 483 1503 488 521 522 523 P22102 526 528 Q9UI12 P56134 29922 490 495 P49915 498 P00846 P25705 533 P18859 535 P56385 537 P01137 539 Q9UN42 P56381 10476 10632 23439 Q9Y2B5 P17812 8833 P20020 P30049 P30566 O00746 245972 P38606 P36542 23400 271 158 P60891 P31939 5471 51606 51727 5198 P22392 5631 Q15904 7372 Q99437 Q93050 P20648 P54709 Q01433 P05023 P05026 Q9Y5B8 Q9UGJ0 O75964 Q06203 P50993 3615 3614 P24539 Q04656 P22234 Q8N8Y2 P11908 161823 P21283 P21281 9296 P15313 27032 10606 4830 Q6DHV7 4833 P98194 509 P06576

digestive tract morphogenesis 7161 27283 57669 1499 116113 Q1PSW9 P84022 Q96S42 P43694 Q9UJW2 2626 O15350 4087 4088 Q13705 Q6KC79 P35222 P10070 Q9HCM4 P10071 P46531 4851 Q9NQB0 Q15796 2737 2736

hexose metabolic process O95278 7167 P08559 P37837 P10515 P52790 P50416 3481 230 Q00796 2821 2026 51363 P60174 1737 4967 Q9UBK2 Q13522 O95685 P46020 P04406 Q16877 P52209 P11216 3099 3098 P11177 1468 P05062 P49247 P01344 P46019 P42336 5162 P07205 Q6ZMR3 92483 4191 5160 Q9UBU3 O60547 O60701 773 3939 5315 P62136 P04066 5836 P01375 Q8IW75 9255 Q16816 5210 7358 5213 1374 2582 3948 P14550 Q04446 P62140 P00558 5207 P07864 3945 P06733 Q92934 P06737 7124 P40926 10690 Q12904 Q9H488 160287 P22415 Q93100 4125 P09972 Q15185 Q9Y231 P06744 P24298 O00757 Q9Y4H2 Q9UGI9 P15735 P11413 Q06210 O00754 P11498 2875 Q9UBX3 2632 P35573 P18847 P01308 P18848 5467 5500 5226 5501 P49841 P09467 P09104 2517 Q7LFX5 P46976 Q15118 Q8TB22 22934 P13807 Q9UGJ0 P14618 23509 Q9BYZ2 P16118 572 178 2762 Q49A26 P41159 P51570 5499 2023 P36873 5091 P19367 P52789 2932 P17858 468 10728 Q01813 P08237

cellular carbohydrate catabolic process P06733 7167 Q92934 P08559 P37837 P06737 P10515 P40926 10690 P52790 160287 230 Q00796 2821 2026 P60174 P09972 Q9Y231 1737 4967 P06744 P04406 P52209 P11216 3099 P11413 3098 P11177 P05062 5226 P49247 P09104 5162 P07205 Q6ZMR3 22934 P14618 Q9BYZ2 92483 P16118 4191 5160 572 P46926 3939 11165 5315 5836 Q49A26 O95989 5213 2023 2582 P19367 P52789 Q9UKK9 10007 3948 P17858 P00558 Q01813 5207 P08237 P07864 3945

regulation of endocytosis 9784 23607 Q9UNF0 P51148 P50897 23327 274 P61020 1012 Q96PU5 P63000 6622 90678 5578 5338 Q15389 P49815 P04004 7376 8301 64127 Q08431 7448 Q13492 7249 967 Q13133 5867 Q15036 P27797 5869 Q7Z727 P22681 Q9BY11 Q9Y5K6 Q9H223 P20338 P62330 P17252 811 857 P01137 5878 5879 P55290 Q9UKS6 4240 5054 11252 3673 P55055 Q86YT6 Q6UWE0 O00499 P37840 382 57534 5728 P17301 O43707 O14939 P60484 Q9HC29 867 P05121

response to glucose stimulus 25828 6198 8892 O15123 2033 Q04759 P20396 Q7Z6C1 Q15466 P07996 3956 P49770 7057 Q9NP71 Q9Y4H2 P35568 285 57678 P12110 Q01955 P09382 5467 Q9NQB0 Q15796 Q99757 Q09472 Q16849 1285 P18146 6927 Q8NER5 Q9HCL2 5798 P01137 Q13144 P23443 Q14232 4087 P55851 P55212 P07858 Q9NR50 Q13705 O60911 5728 P20823 P60484 Q6PID4 3667 Q14289 P06213

negative regulation of locomotion 4771 O15123 Q9UQL6 4092 3162 P23508 3516 Q96RU8 Q9BZE4 23560 P09493 94 10014 P07996 P08246 22846 7057 5155 Q7Z5L7 Q96QB1 Q92729 5796 Q5VTR2 285 Q06330 P18206 Q9H2X0 967 P10415 P14416 Q13418 Q9Y2I1 P30542 Q9UBU3 P37023 2247 3611 127435 P35240 7414 P01137 P09038 5054 Q8WWN8 Q9Y696 Q14118 Q7L8A9 Q8N9R8 5728 Q9HCK4 P21359 P60484 P17936 O15105 P01127 P05121 P09601 1605

female gonad development Q7Z2K6 8892 O15287 Q12948 O00268 P51587 P15692 Q14050 P23945 Q92731 3953 Q15389 P42229 Q13285 P47712 P49770 O00238 P03950 9510 P35968 O43524 23411 54361 Q05086 P30279 Q07817 P48357 10468 Q96EB6 P48552 P29474 7422 P10415 5321 2099 894 898 P01019 79956 6647 P03372 Q13144 P06401 P00441 P41159 3791 8204 P51692 A4D1W7 2100 Q9UHI8 P19883 P56705 Q9HCK4 P58012 P24864 668 6776

positive regulation of immune system process 4690 Q13477 O14672 4179 P15529 P15923 Q08722 O43557 2150 Q9Y4K3 O60603 O43318 5817 Q8WZ60 P0C0L4 Q04759 Q92692 P49810 8767 Q9Y275 O14788 6869 P42229 P07996 P25963 P42226 P04003 P27487 P78536 7057 7334 8546 6885 4860 83737 1191 P20963 641 P14174 P27918 2277 O43290 P08575 P01589 P98172 10666 P10809 Q9HCY8 P49768 O00206 7186 7189 Q92835 P06239 26258 Q9BQ51 3142 3263 148022 O00203 57402 Q9BYM8 6929 Q9UDY8 P06241 P01137 P05155 3932 P02741 64170 11326 Q14116 Q13422 5054 P78552 5970 3673 Q92949 P35225 55072 P19320 Q9H257 Q9NR96 9093 Q8N5F7 P16333 81622 P17301 P43405 Q8IUC6 Q04206 6776 P40933 Q96EY1 P04233 P43489 Q9H1C4 O43353 Q96EP0 Q92934 O95999 P28482 P54132 P41182 O43915 8174 O43639 P10276 Q9Y2C9 79576 P15692 10616 Q9Y6K9 710 51324 5336 5579 5338 P05771 P08887 P08246 719 Q96FA3 Q8IWV1 7099 7098 Q14774 7412 Q9Y4H2 64127 P35443 Q96J02 3329 57678 29110 1026 961 720 722 604 P55085 1947 9564 P07766 P00491 O14492 P02790 6850 4792 7422 Q9NZD8 Q9UHD2 57162 572 Q9HCL2 P61088 P15151 P01579 55703 10673 857 O15455 P25103 P01730 P04264 P05112 Q15762 5914 P24394 Q9UL45 Q12933 P51692 P11912 P06729 P38936 3075 P48061 P16885 O14939 P51617 P37173 Q9HC29 8517 P05362 10320 54106 P05121 9846 Q16581 P60568 P21554

cellular response to lipopolysaccharide O43353 O00206 7099 P26583 5054 P29474 4792 6772 P28482 P27361 Q8NHX1 148022 P42224 P51617 3148 Q8IUC6 8767 P05121 P25963

purine nucleoside monophosphate biosynthetic process P30566 161823 5471 5198 Q06203 271 P12268 P30520 3615 3614 P49915 10606 158 Q01433 P31939 P22102 P22234 8833 Q6DHV7

purine ribonucleoside monophosphate biosynthetic process P30566 161823 5471 5198 Q06203 271 P12268 P30520 3615 3614 P49915 10606 158 Q01433 P31939 P22102 P22234 8833 Q6DHV7

DNA geometric change 4173 25913 4361 Q14566 4176 P33993 P33991 Q9NUX5 7150 4171 P11387 P49959 3159 Q96RR1 Q00577 P17096 5888 Q92878 P49736

DNA duplex unwinding 4173 25913 4361 Q14566 4176 P33993 P33991 Q9NUX5 7150 4171 P11387 P49959 3159 Q96RR1 Q00577 P17096 5888 Q92878 P49736

regulation of nitric oxide biosynthetic process O00206 P07900 P78540 7124 3320 P13686 O60603 148022 P68871 P01579 857 1956 54 P01375 7099 P31749 3326 O60674 P01308 3269 3324 Q8IUC6 207 P05362 P00533 P08238 P06213 P04179

negative regulation of protein complex assembly O15020 Q13813 Q15735 23189 P35612 P35611 6709 830 P02549 832 118 998 Q9H2D6 1978 P47755 P60953 P47756 Q08495 Q14678 Q01082 1191 P11277 5590 2039 Q05513 P52907 6711 22919 6710 Q6PKD3 P62328 Q15691 P63313 829

skeletal muscle tissue development P13349 2033 1020 O00468 351 P15173 Q7Z6C1 P61586 6901 P78337 P50222 Q14774 Q16635 P51636 10939 P11532 O43847 P60660 375790 4898 P05067 Q15475 Q09472 6495 6498 4154 P12757 A1XKG3 P12755 3142 1756 1634 P02708 Q00535 P21860 O14640 857 Q14993 P07858 P04626 P68133 2065 O95183 2064 9093 387 Q08209 P58012 668 P24821 P07585 Q96EY1 4637 P25116

skeletal system development Q9NYD6 9061 Q12948 2550 O75386 Q9Y4K3 Q9H161 2303 Q9UBS5 P84022 P08047 Q7Z6C3 O14788 P05452 P21246 P09017 Q9NPC8 4188 P27361 Q5TAB7 P13631 P50148 Q02447 P16234 P20849 P21815 Q70SY1 Q9H2X0 P00533 Q15796 Q15672 Q7Z727 6495 Q15797 P29992 P05549 P31273 O43897 P12755 P10451 Q9Y6Q6 Q9UBU3 257 Q15561 5159 P03372 P40424 6926 Q9GZX9 4086 134701 4087 7353 Q92824 4088 P22692 4882 P18074 P51812 Q13705 P12645 P17535 P21359 P31270 P08151 6259 P34925 P27037 7123 Q8TD84 P63092 O95470 P35453 Q93063 6949 10736 1499 P15173 Q9Y2T1 54 O00238 P78337 Q14814 P09619 Q9NZC7 P36894 P10600 Q6UB99 Q06210 3207 P17482 P09067 846 Q92882 Q99715 P07686 Q99958 Q15475 Q99835 O75569 P35555 2132 2253 7020 2247 3215 1277 4664 Q5TAT6 4665 Q15125 1956 P09038 P10827 Q8IUX7 1297 O00255 Q6KC79 Q13506 P11474 P35548 56243 P17342 3226 O95390 P37173 5764 4313 Q9UMX1 1969 P29317 7704 4318 P02452 7046 P13349 O00300 3481 2702 Q9Y3A5 Q99523 3479 Q1PSW9 P17275 O15119 P98161 P07355 4209 Q14055 P56524 Q9NNX1 4221 5310 7291 84159 Q99750 P48436 P01344 P01343 P49768 Q14865 O43252 7189 7067 P48431 P13686 Q5T5P2 P34820 P14780 Q96S42 P17252 656 P09630 P01137 Q14993 P02461 6660 Q13428 P35222 P35227 2735 Q01974 P57082 302 P10070 P10071 P02458 6657 2737 P49747 2736 6670 O00744 O00628 P41180 Q9NX62 O95076 3958 P31260 6667 P20594 P08123 5578 P55075 4920 P31249 Q6IR47 Q15742 P80370 P36382 Q05516 P08253 P22303 6696 P56178 P56177 2099 57045 P35711 Q03692 Q04656 5599 51741 Q16610 P05997 O15232 Q8NHX1 Q06830 3074 P15313 P13497 10682 8879 P45983

cellular response to external stimulus P02452 O00744 Q15853 25828 Q13873 Q8TDY2 9821 P04637 27327 P41181 3162 P10276 P37288 552 P22415 P23229 60673 10013 P08243 3953 Q13286 P05412 Q9Y4P1 9474 Q96SB3 Q9NYA1 6720 23411 Q8NDV7 55054 P55318 3171 Q9BSB4 P11021 1026 Q9P2K8 P05062 P48357 Q99757 Q96EB6 P01100 P49768 3860 P13646 3939 P61244 Q9H1Y0 1277 57521 898 4149 857 3655 Q7Z3C6 5119 5914 Q13501 P07339 7157 P38936 P36956 79065 P30530 O60911 Q676U5 Q9H492 O75084 23192 81788 Q9H093 P24864 8878 P09601 Q9UBN7 O94817 3309

reproductive developmental process Q7Z2K6 8892 5268 Q12948 P30086 O00268 P51587 595 Q92731 5139 P43034 P03950 9510 5037 79084 54361 P13631 Q9UJC3 P16234 367 P16471 P46531 4851 Q96EB6 Q92600 Q13258 6256 P10451 Q9BXC9 Q9UBU3 6927 3416 55342 Q96RK4 Q96MA1 P03372 Q13144 4867 P40424 6926 P06401 P00441 Q92826 P35869 3791 8204 7476 Q86XR8 P36952 2100 P56705 Q02790 5728 P20823 Q9HBE1 Q9BXB1 P24864 P43246 P27037 9420 O15287 P14635 P35453 P10275 4093 55366 Q00597 1499 Q13285 O00238 Q9Y2T7 O43521 P36896 O43524 P36894 P10600 P10721 P19544 7490 285 960 2475 Q9NQB0 O15169 Q99714 P09622 Q8TCU4 2253 7141 9319

P08069 1718 P23443 Q92754 P51692 O15198 P31749 Q6KC79 P19883 Q9HCK4 Q6PID4 Q14289 Q9UKT4 7704 P06213 6198 O15123 P42345 26271 Q14050 3479 Q1PSW9 P17275 P23945 Q92692 91 1738 P22612 Q15389 P42229 O15119 P49770 O75534 P16989 Q8NHU6 Q15392 Q9HD26 P48436 P16070 P01343 P48552 P48431 P29474 5321 P19793 57120 O75881 Q99623 891 894 898 P01019 O15259 79956 6647 P01137 O60383 Q9NSC2 A4D1W7 Q9UHI8 P10620 P35222 Q01974 3028 P60484 3148 P10070 11331 668 6776 5568 6657 2736 P26583 P54652 P35613 P15692 P05093 P55075 4920 3953 2626 P47712 P04201 5591 O00755 Q9Y5R5 P35968 Q13323 23411 Q05086 P30279 Q07817 682 Q05516 3169 5467 207 9702 P48357 10468 P24385 6696 P56851 7422 P10415 2099 P19235 P78527 P51843 P09430 P43694 Q14432 Q9BQA1 P41159 Q15648 Q04771 O60911 P58012 P18827 Q9NQL9 P24821

positive regulation of positive chemotaxis Q7Z727 P35968 3791 4088 7422 Q15109 3673 O43889 P51679 O43915 2150 P17301 P15692 P84022 P17252 1012 2277 5578 P55085 10488 P55290

release of cytochrome c from mitochondria P05412 O15350 Q3ZCQ8 4088 Q16611 P10415 7157 P04637 P55210 P31947 P55957 7161 1191 Q07817 2810 P84022 637 Q13794 P42574 92609 P04179

actin filament capping Q08495 O15020 Q13813 Q01082 P35612 P11277 P35611 6709 2039 P52907 830 P02549 832 6711 118 6710 Q6PKD3 Q9H2D6 P47755 P47756 829

cellular response to molecule of bacterial origin O43353 O00206 7099 P26583 5054 P29474 4792 6772 P28482 P27361 Q8NHX1 O60603 148022 P42224 Q9Y2C9 P51617 3148 Q8IUC6 8767 P05121 P25963

negative regulation of organic acid transport 7057 Q9UBS5 7124 P31749 Q9Y4H2 2550 207 P46059 P07996 P01375

morphogenesis of a polarized epithelium 163183 Q8N205 284217 P25391 8323 O15230 O60353 Q9NYQ6 O60716 3911

B cell homeostasis O14492 O43521 P10415 Q9Y6K9 8517 3551 10673 Q9Y275 O14920 P42574

regulation of helicase activity 25913 Q04837 7157 P04637 23411 Q9NUX5 P52701 P43246 Q96EB6 2956

synaptic transmission, dopaminergic Q00535 Q99497 6622 A1XKG3 1020 P07101 P37840 5071 O60260 11315

Golgi to plasma membrane transport 117177 Q9HD26 Q96QF0 57120 Q9H2G9 3875 10133 8548 P05783 Q96CV9

amyloid precursor protein catabolic process Q15392 P49810 1718 Q92542 Q96BI3 51107 Q13286 2280 P62942 P49768

ATP-dependent chromatin remodeling 55355 Q9UIG0 P49321 8467 P28370 Q8NCD3 O60264 P09430 4678 7141

regulation of ryanodine-sensitive calcium-release channel activity 808 Q8WXH2 84502 5311 Q9HDC5 Q13563 P62158 Q96JJ6 2280 P62942

positive regulation of SMAD protein nuclear translocation 5460 Q93062 Q6IR47 7046 11030 4089 P36894 P10600 P01137 Q13485

regulation of interferon-beta biosynthetic process O00206 7099 7098 29110 Q8IUC6 Q9NR96 Q9UHD2 54106 O15455 148022

positive regulation of interferon-beta biosynthetic process O00206 7099 7098 29110 Q8IUC6 Q9NR96 Q9UHD2 54106 O15455 148022

negative regulation of DNA recombination Q9BT67 641 P40692 Q9NS91 P54132 P41182 604 P52701 P43246 2956

regulation of hormone biosynthetic process P27540 3091 476 773 27122 Q9UBP4 Q16665 54361 P05023 P56705

regulation of polysaccharide biosynthetic process Q13322 Q9BX66 10580 8445 P31749 Q9Y4H2 P35568 P42345 P62140 P01308 2475 5500 207 Q92630 3667 P06213

regulation of glycogen biosynthetic process Q13322 Q9BX66 10580 8445 P31749 Q9Y4H2 P35568 P42345 P62140 P01308 2475 5500 207 Q92630 3667 P06213

fructose metabolic process O00757 P37837 5210 Q16877 5213 Q06210 P16118 230 Q00796 P17858 P05062 P09972 Q01813 5207 P08237 P09467

positive regulation of NF-kappaB import into nucleus O00206 7099 7098 Q9NYA1 Q15654 7124 Q9NR96 O43557 O60603 387 Q04759 7205 P61586 54106 O15455 P01375

regulation of glucan biosynthetic process Q13322 Q9BX66 10580 8445 P31749 Q9Y4H2 P35568 P42345 P62140 P01308 2475 5500 207 Q92630 3667 P06213

microspike assembly Q7Z6J4 Q6ZV73 P35609 Q96M96 Q5JSP0 23092 10458 Q9UNA1 121512 P16144 P23229 55785 Q9UQB8 3655 Q9UHR4 P98174

regulation of viral transcription P05412 Q13148 6672 7023 Q13547 3065 23435 Q96T23 Q01664 51773 6667 4214 P08047 Q13233 P23497 Q6IT96

cell redox homeostasis O95831 25828 P00390 9588 P30041 P10599 P30044 P13667 Q8IZQ5 Q86VQ3 P30048 54431 10935 O00391 Q8NBF2 57544 Q13162 1738 P07237 9997 5034 Q9BS26 Q9H7Z7 O95881 7295 Q96KS0 81567 Q9P2K2 10549 328 51218 Q99757 P09622 Q16881 P35638 P27695 9601 Q9Y320 Q6PKC3 Q9BRT3 P30101 51061 O43819 280636 Q15084 Q8IXB1 P63302 2923 374354 Q86SX6 Q06830 O76003 Q8NBS9 51075 6415 84299 5768

neuron migration Q92858 O00628 Q8NEY1 8851 P78509 1020 O43711 474 Q8IYN9 Q9BVA1 Q92731 P43034 7531 Q9UHG0 O43602 P51679 7291 P50542 Q3V6T2 2676 81565 207 Q9GZM8 5747 Q15078 Q15672 P49768 9201 Q02880 A1XKG3 Q05397 27185 Q00535 55704 P06241 5830 Q9NRI5 347733 P62258 O15075 7155 P31749 O75553 2100 Q658W2 P10589 P17302 Q9NXR1 54820 2697

hindbrain morphogenesis 8443 O00755 136319 7476 Q96QB1 O75553 A1XKG3 1020 P58546 O75385 2735 P11021 773 Q00535 Q1PSW9 P10070 P08151 Q04656 8408 3309 Q99835 2736 O15228

regulation of blood vessel endothelial cell migration 22846 7057 7422 5155 O15123 Q8WUI4 Q7L8A9 Q9UQL6 285 Q4VCS5 P21359 2247 P15692 P20827 51564 P01127 P01137 1969 Q15389 P29317 10014 P09038 P07996

bone morphogenesis P02452 Q15742 Q14814 O00628 P56178 P63092 P12755 P10600 Q13506 P13686 P13631 Q9NX62 3207 P31270 1277 4664 Q5TAT6 P48436 4665 P02458 P01137 54 4209

negative regulation of actin filament depolymerization Q08495 O15020 Q13813 Q01082 Q9UHB6 P35612 P11277 P35611 6709 2039 51474 P52907 830 P02549 832 6711 118 6710 Q6PKD3 Q9H2D6 P47755 P47756 829

positive regulation of Ras GTPase activity O43166 P51671 7248 Q92949 10928 26037 Q02750 Q86WV8 23513 Q96P48 P21359 Q14160 Q96N67 81565 Q6PID4 Q6ZW31 Q92574 Q9GZM8 Q14289 85440 Q15311 5604 85360

immune response-regulating cell surface receptor signaling pathway O43353 9564 P07766 O14492 7189 Q96EP0 6850 O95999 P28482 Q9NZD8 Q9Y4K3 O60603 O43318 Q9BYM8 Q8WZ60 10616 Q9UDY8 P61088 Q9Y6K9 P06241 51324 P49810 5336 8767 5579 P05771 719 Q8IWV1 7334 6885 P11912 55072 P16885 P20963 P43405 8517 P08575 Q16581 P49768

somite development Q09472 5362 23509 92129 P04637 Q13315 P13349 Q12948 2033 Q9H488 P78527 472 O75051 57669 P84022 Q92830 Q9UJU2 P49810 Q7Z6C1 4920 Q9Y2T1 Q13485 9839 5591 P50222 134701 4088 4089 7157 Q86YT6 P36894 Q5TAB7 54361 P56705 Q01974 57534 Q9HCM4 A6NI15 P49768

regulation of purine nucleotide catabolic process 9744 7169 10928 26037 Q99490 P15498 P42345 2665 2664 P09493 5018 P43034 Q92574 P49815 Q6ZT07 11345 Q8WZ64 O43166 Q8TBP0 Q15027 Q9Y2X7 Q7Z6J4 23216 115704 Q9P107 O43609 81565 P05062 Q15311 85360 P98174 P50395 9882 1121 116986 Q96M96 Q9ULH1 116987 Q8TEU7 Q96CN4 P09471 Q9UKW4 Q8IV61 P26374 Q9UPQ3 Q8WWN8 Q92949 125058 Q8N6H7 27352 P21359 55633 Q66K14 1122 P31150 P60520 O43150 5604 Q15057 Q3MII6 Q9H2M9 Q9NP61 O43597 7248 Q96BZ9 Q96DN5 P41182 Q5R372 Q86TI0 93594 Q9NUY8 Q96P48 121512 25780 79735 10451 9815 P45379 P07951 P51671 Q96HU1 Q5JSP0 Q86WV8 Q9NU19 Q15070 2475 7249 Q8TEA7 604 Q6ZW31 Q9GZM8 51735 85440 Q96NH3 P24386 Q6ZV73 Q96N67 Q0IIM8 55785 O60343 4943 O43182 Q8IYX1 64786 Q02750 Q9Y3P9 23513 Q14160 Q6PID4 Q14161 Q14289 Q8N6T3

regulation of nucleotide catabolic process 9744 7169 10928 26037 Q99490 P15498 P42345 2665 2664 P09493 5018 P43034 Q92574 P49815 Q6ZT07 11345 Q8WZ64 O43166 Q8TBP0 Q15027 Q9Y2X7 Q7Z6J4 23216 115704 Q9P107 O43609 81565 P05062 Q15311 85360 P98174 P50395 9882 1121 116986 Q96M96 Q9ULH1 116987 Q8TEU7 Q96CN4 P09471 Q9UKW4 Q8IV61 P26374 Q9UPQ3 Q8WWN8 Q92949 125058 Q8N6H7 27352 P21359 55633 Q66K14 1122 P31150 P60520 O43150 5604 Q15057 Q3MII6 Q9H2M9 Q9NP61 O43597 7248 Q96BZ9 Q96DN5 P41182 Q5R372 Q86TI0 93594 Q9NUY8 Q96P48 121512 25780 79735 10451 9815 P45379 P07951 P51671 Q96HU1 Q5JSP0 Q86WV8 Q9NU19 Q15070 2475 7249 Q8TEA7 604 Q6ZW31 Q9GZM8 51735 85440 Q96NH3 P24386 Q6ZV73 Q96N67 Q0IIM8 55785 O60343 4943 O43182 Q8IYX1 64786 Q02750 Q9Y3P9 23513 Q14160 Q6PID4 Q14161 Q14289 Q8N6T3

somitogenesis Q09472 5362 23509 92129 P04637 Q13315 P13349 Q12948 2033 Q9H488 P78527 472 O75051 57669 P84022 Q92830 Q9UJU2 P49810 Q7Z6C1 4920 Q9Y2T1 Q13485 9839 5591 P50222 134701 4088 4089 7157 Q86YT6 P36894 Q5TAB7 Q01974 57534 Q9HCM4 A6NI15 P49768

positive regulation of chemotaxis Q7Z727 7422 O14672 O43915 2150 57402 P15692 P84022 P17252 1012 5578 P01137 P08887 P07996 P55290 719 P78536 7057 5054 P35968 3791 4088 5155 Q15109 3673 O43889 P51679 P35443 P48061 P17301 2277 P01127 P55085 P05121 10488 Q16581 Q9HCY8

tumor necrosis factor-mediated signaling pathway 7186 Q15628 Q12933 7124 Q9H305 Q13546 29108 Q9Y6Q6 8737 Q9ULZ3 O14788 8717 P01375

regulation of chromosome segregation O43683 P25054 9700 Q8N4N8 O43684 P14635 9184 891 324 Q99661 P49711 Q14674 11004

ventricular cardiac muscle cell differentiation P10826 9474 6256 4000 P19793 Q02297 P14635 3084 P52952 891 Q9H1Y0 Q92786 P02545

vesicle fusion O43752 8411 P51636 10228 O60763 2773 Q9UKW4 10451 P61006 8615 Q15075 P08754 4218

regulation of epithelial cell migration Q13873 3091 3673 P23508 P17301 57669 Q9HCM4 10013 967 Q16665 6869 P25103 Q9UBN7

negative regulation of macrophage derived foam cell differentiation 3690 P05106 7376 O95477 4792 P55055 19 P37231 3685 5468 Q13133 P02741 P25963

prostate glandular acinus development Q92826 6256 2099 P19793 79084 P35453 3479 3169 P46531 4851 P03372 Q9BQA1 P01343

early endosome to late endosome transport Q60FE5 9525 8411 1130 Q86VS8 O75351 Q9UJC3 2316 Q99698 Q96ED9 Q15075 84376 P21333

regulation of dopamine secretion P28223 P39905 2550 65018 P37840 1137 Q9BXM7 O76070 Q9UBS5 P43681 6622 Q9NQC3 P21554

regulation of hormone metabolic process P27540 3091 10273 54361 P56705 Q9UNE7 476 773 27122 Q9NQB0 Q9UBP4 Q16665 P05023

protein K48-linked ubiquitination P62837 7267 11065 7325 P53804 P51668 O00762 O95376 Q99942 P62256 997 10477 Q969T4 3093 P21580 6048 Q96J02 P78317 83737 Q05086 Q96LR5 P61077 7128 7328 P49427

purine nucleotide catabolic process P30049 5141 5142 9525 8654 P29372 Q9BW91 4907 2643 513 5139 539 P21589 Q14432 4968 P53396 P48047 Q07343 O75351 P30793 P12883 O76074 P27815 Q13370 P17081

salivary gland development 5361 P25391 7124 P12830 57045 1181 284217 999 P21980 O14786 P01137 8829 3911 1956 P01375 Q9GZX9 5155 O15230 Q14118 P10600 O60716 7052 P01127 P00533 1605

regulation of skeletal muscle tissue development 6198 7067 P10415 O14793 Q13418 Q9UQL6 O00468 P52952 154 3611 P07550 O15119 10014 6926 P23443 P56524 P10827 Q04771 P16989 P17936 55796 P46531 4851 P48436 375790

regulation of defense response P78423 8654 P14555 O60603 5817 Q9HC16 Q92692 8767 O14788 P42229 P15090 57506 Q9Y3Z3 P25963 83737 7052 O76074 2 60489 P01589 Q96CW1 409 Q9UI12 10666 P10809 Q9HCY8 O00206 6890 Q7Z727 5321 P32121 P29590 3263 P13686 148022 Q9Y6Q6 57402 Q9UBU3 4907 P17252 P01019 P21980 Q9NZ08 P05155 P21589 64170 P01375 5054 5692 5970 3673 Q9BZF9 Q7Z434 Q9H257 Q9NR96 81622 P19438 P17301 O60674 Q8IUC6 Q04206 P01008 6776 P40933 Q03518 Q9H1C4 O43353 O95999 7124 P41182 154 Q9Y2C9 710 P07550 5578 5733 P08246 P43115 54 P47712 7099 7132 P30556 7098 51606 7133 Q15109 64127 Q96J02 161 P37231 3329 29110 P01308 P63010 5468 604 P01023 P02790 4792 Q9UHD2 23586 P30542 P15151 55703 O15455 Q15762 P20333 Q9BT67 5371 O94973 O95786 P51692 P34972 1173 P28070 462 P51617 Q9HC29 51752 54106 P05121 P60568 P21554

positive regulation of GTPase activity 7248 10928 26037 Q96P48 Q96N67 P09471 Q92574 P49815 O43182 O43166 P51671 Q92949 Q02750 Q86WV8 23513 P21359 Q14160 81565 Q6PID4 7249 Q6ZW31 Q9GZM8 Q14289 85440 Q15311 5604 85360

osteoblast differentiation P02452 Q15797 P48431 6696 P56178 2099 2550 P10451 Q9UBS5 Q1PSW9 P17275 P84022 1277 51741 P03372 4209 P56524 4086 Q14814 4088 4221 Q9NZC7 O00255 P35548 P17535 2735 P21359 P10070 P08151 6657 P22303 Q99835 2736

circadian rhythm O00327 8553 9420 5187 Q16526 P14416 P31153 O75881 6096 Q8WYA1 Q9Y6Q6 Q9UBU3 O60346 Q99743 1453 4543 4862 O15379 Q92753 P21860 O15534 Q8NFJ6 O15516 1956 O14503 P05412 9575 O43186 P29466 8864 P20393 8841 O15055 2065 23239 79365 P41222 P48039 P48730 406 P00533 1408 1407 Q49AN0 1406 Q9C0J9

response to ethanol 6198 O14793 P30086 2033 2550 P09172 595 P00505 Q9UBS5 P51828 P20396 Q7Z6C1 5578 6869 2904 518 P08887 2902 P40763 7099 7132 P48201 5037 P68032 P30279 P05181 841 329 Q13490 6517 O00206 Q7Z727 Q09472 P24385 P01185 P29474 P10415 6774 P14416 Q12929 894 P17252 898 P06241 Q14790 6647 P25103 P23443 P00441 P07858 P51692 Q05586 P21917 5728 P19438 2059 P60484 P24864 Q6PID4 8836 P07101 Q14289 P21554 Q92820 P06213

regulation of dephosphorylation 7248 Q9UPR3 22870 P30542 Q9UPN7 7184 23381 P84022 P01579 P01137 Q92574 P62258 Q5H9R7 8165 P31946 4088 7531 3673 Q96QB1 P14625 55291 Q86WV8 2280 P17301 O60674 51231 5524 Q15257 7529 Q92667 P62942

positive regulation of translation Q09472 6199 6198 Q9BY77 P98179 7124 P28482 2033 P42345 84305 Q9UBS0 Q7Z6C1 Q13144 P07996 P23443 5935 P01375 Q14011 P38919 7057 Q9UPU9 9775 3673 1153 P17301 2475 Q6PID4 P62701 Q9BRP8 Q14289 84271

monosaccharide biosynthetic process 7167 P06737 P16118 Q00796 O60547 2821 2026 51363 2762 P60174 5836 Q9UBK2 P06744 P24298 O00757 P52209 P11413 5091 P11498 1468 2875 Q9UBX3 P18847 P18848 468 P05062 5226 5207 P09467 P09104 Q7LFX5

response to monosaccharide stimulus 25828 6198 8892 O15123 2033 Q04759 P20396 Q7Z6C1 Q15466 P07996 3956 P49770 7057 Q9NP71 Q9Y4H2 P35568 285 57678 P12110 Q01955 P09382 P05062 5467 Q9NQB0 Q15796 Q99757 Q09472 Q16849 1285 P18146 P14410 6927 Q8NER5 Q9HCL2 5798 P01137 Q13144 P23443 Q14232 4087 P55851 P55212 P07858 Q9NR50 Q13705 O60911 5728 P20823 P60484 Q6PID4 3667 Q14289 P06213

response to hexose stimulus 25828 6198 8892 O15123 2033 Q04759 P20396 Q7Z6C1 Q15466 P07996 3956 P49770 7057 Q9NP71 Q9Y4H2 P35568 285 57678 P12110 Q01955 P09382 P05062 5467 Q9NQB0 Q15796 Q99757 Q09472 Q16849 1285 P18146 P14410 6927 Q8NER5 Q9HCL2 5798 P01137 Q13144 P23443 Q14232 4087 P55851 P55212 P07858 Q9NR50 Q13705 O60911 5728 P20823 P60484 Q6PID4 3667 Q14289 P06213

nucleoside phosphate metabolic process O75947 5141 5142 P37837 Q16864 8654 P30085 9061 P30520 476 477 478 P60174 5139 4968 P28335 Q9H4L7 P28331 P48047 4860 P13637 P52209 Q07343 481 483 488 P22102 Q13370 P49247 Q9UI12 9525 P56134 3420 29922 490 4191 495 3417 P49915 498 P25705 115024 P18859 P22674 P56385 P00568 Q16774 P56381 P21589 3419 10632 P36957 P43490 4882 O75351 P12883 3421 P20020 P30566 P40926 P38606 P36542 P50213 271 Q8WVC6 P27708 158 P51553 5198 P22392 5631 80308 Q15904 O95243 Q6PIW4 P30793 7372 Q93050 Q8NFM5 P20648 P54709 Q01433 Q6XQN6 P05023 Q13057 P05026 22934 P00491 P00492 Q9UGJ0 Q06203 P11586 O60502 P24539 80347 P22234 56916 1716 P11908 5636 Q49A26 161823 P21283 P21281 27032 1841 10724 10606 P27815 Q6DHV7 P98194 509 P06576 7167 8930 P29372 P16615 P78549 O43837 P15531 1854 P11172 2820 513 514 515 P61421 Q969T7 518 9114 P48201 P12268 1503 O76074 P43304 1743 51251 521 522 523 P05062 526 528 O43252 Q9BW91 P00846 4907 Q96T60 533 535 537 P01137 539 P23921 Q9UN42 10476 23439 P53396 O75874 3704 Q9Y2B5 P17812 8833 3945 4913 P30049 P33316 O00746 Q9BY32 245972 23400 2987 P60891 P31939 P20594 Q8NFF5 5471 51606 51727 Q13569 P11413 93100 Q9H0P0 Q99437 O60256 5226 P17081 Q9Y5B8 Q08462 65220 O75964 P50993 3615 3614 2643 Q04656 Q8N8Y2 Q14432 O95544 Q16854 Q14558 9296 P15313 Q9UKK9 4719 4830 4833

nucleotide metabolic process O75947 5141 5142 P37837 Q16864 8654 P30085 9061 P30520 476 477 478 P60174 5139 4968 P28335 Q9H4L7 P28331 P48047 4860 P13637 P52209 Q07343 481 483 488 P22102 Q13370 P49247 Q9UI12 9525 P56134 3420 29922 490 4191 495 3417 P49915 498 P25705 115024 P18859 P22674 P56385 P00568 Q16774 P56381 P21589 3419 10632 P36957 P43490 4882 O75351 P12883 3421 P20020 P30566 P40926 P38606 P36542 P50213 271 Q8WVC6 P27708 158 P51553 5198 P22392 5631 80308 Q15904 O95243 Q6PIW4 P30793 7372 Q93050 Q8NFM5 P20648 P54709 Q01433 Q6XQN6 P05023 Q13057 P05026 22934 P00491 P00492 Q9UGJ0 Q06203 P11586 O60502 P24539 80347 P22234 56916 1716 P11908 5636 Q49A26 161823 P21283 P21281 27032 1841 10724 10606 P27815 Q6DHV7 P98194 509 P06576 7167 8930 P29372 P16615 P78549 O43837 P15531 1854 P11172 2820 513 514 515 P61421 Q969T7 518 9114 P48201 P12268 1503 O76074 P43304 1743 51251 521 522 523 P05062 526 528 O43252 Q9BW91 P00846 4907 Q96T60 533 535 537 P01137 539 P23921 Q9UN42 10476 23439 P53396 O75874 3704 Q9Y2B5 P17812 8833 3945 4913 P30049 P33316 O00746 Q9BY32 245972 23400 2987 P60891 P31939 P20594 Q8NFF5 5471 51606 51727 Q13569 P11413 93100 Q9H0P0 Q99437 O60256 5226 P17081 Q9Y5B8 Q08462 65220 O75964 P50993 3615 3614 2643 Q04656 Q8N8Y2 Q14432 O95544 Q16854 Q14558 9296 P15313 Q9UKK9 4719 4830 4833

actin polymerization or depolymerization P03950 10093 Q8TCU6 10152 Q8IZP0 Q9NYB9 P41181 Q9UBU3 O60610 P42768 P63000 11034 1729 P60981 Q92558 5879 O43182 8936

negative regulation of endocytosis Q9UKS6 7376 Q9BY11 8301 11252 P55055 Q9UNF0 Q6UWE0 382 P62330 5728 P60484 P63000 Q13492 90678 P01137 Q13133 5879

lipid modification O00628 P35610 2110 P50416 1892 P23786 22908 P27986 Q01968 Q9UBK2 P28330 P50542 51094 Q96Q15 1666 P30084 5467 P55084 P42338 P42336 Q13011 O00329 Q8NEB9

Q96A54 Q08426 P33897 Q86V24 Q92551 P09110 215 Q16134 5830 Q9UJ83 6646 Q16698 30 34 5295 P40939 1374 1891 P51659 3032 3030 P11310 5728 P60484 Q9NTJ5 4952 P48736 26061

regulation of myeloid leukocyte differentiation O00206 Q14106 P25054 7189 Q92835 7124 P63092 Q02363 P10276 Q9Y4K3 Q01196 P15531 1499 51341 O15455 O14788 P42229 P05112 P01375 P06400 5914 Q9NSA3 P05412 Q9BT67 7099 7098 Q13422 Q9HAZ2 A4D1W7 P22392 63976 P35222 P11474 O95365 22985 861 324 Q9UKV3 6776 4830 10320 5925

positive regulation of epithelial cell proliferation P07942 9420 7422 P56178 O75881 P11047 P15531 894 1499 2247 P15692 3479 2246 P05230 Q6NVY8 P01137 6869 P25103 P01135 1956 P09038 3912 P28799 O00755 Q15303 P35968 3791 7476 P04626 Q15648 P22392 P36894 2064 P35222 P30279 O75084 2896 4830 P00533 P01343

positive regulation of response to stimulus O14672 8654 4179 Q9NS86 Q9Y4K3 O43318 472 5817 Q8WZ60 P84022 P49810 8767 Q9Y275 O14788 P07996 P25963 P04003 P04004 7334 5155 4067 P16104 P20963 P27918 7448 Q96RL1 10666 P10809 Q9HCY8 Q7Z727 Q9Y6Q6 57402 Q9UBU3 Q9UDY8 P02741 64170 P55290 5054 3791 4088 5970 3673 Q9H257 Q9NR96 81622 P17301 O60674 Q8IUC6 3667 55915 Q9H1C4 P05129 7124 P28482 Q9Y2C9 10616 Q9Y6K9 710 1012 51324 5733 P08887 719 7132 P30556 P29466 Q9Y239 Q15109 O43889 P51679 P35443 P35568 3329 1029 29110 P01308 720 722 P01303 P42771 O15169 Q9GZM8 9564 P07766 P02790 10392 6850 4792 Q9NZD8 1161 P38398 P15151 55703 P04264 P51692 P11912 P48061 P05121 Q16581 Q9UL54 P78423 P15529 P14555 2150 3481 O60603 P0C0L4 Q92692 P42229 P15090 P78536 7057 6885 1191 7052 O76074 81565 2277 P08575 P01344 P49768 O00206 7186 7189 Q8N726 5321 Q13535 3263 148022 Q9BYM8 P17252 3014 P01019 P06241 P21980 P01137 29086 P05155 11326 P01375 55072 P19438 P43405 545 Q04206 6776 P01127 P40933 10488 O43353 Q96EP0 5582 O95999 Q13315 P07948 O43915 Q9NX61 P53567 672 P15692 51720 5336 5578 5579 P05771 P08246 P43115 P47712 Q8IWV1 7099 7098 P35968 64127 P46736 Q9NWV8 P55085 O14492 7422 Q13216 Q9UHD2 Q9BUZ4 P61088 10673 O15455 Q15762 Q12933 3075 P16885 P51617 Q9HC29 8517 54106 P21554

regulation of cytokine production P78423 P10914 O00425 O75787 Q9Y4K3 O60603 O43318 P17676 Q04759 P16220 P84022 8767 P42229 Q13485 57506 P07996 P78536 7057 3091 6885 Q15306 Q04864 P14174 5029 408 P49407 P10809 O00206 7186 7189 Q92835 Q9Y2Y8 Q06643 P13686 148022 Q9UBU3 Q99500 P41231 O60543 Q9UDY8 Q53X93 P01019 6647 P01137 64170 11326 3659 P00441 P01375 Q14116 5054 4088 4089 5970 A4D1W7 Q92949 Q7Z434 Q9H257 Q9NR96 10524 P43405 O60674 Q8IUC6 Q04206 6776 Q9ULZ3 Q16665 10642 5966 10644 P43489 10643 O43353 O95999 7124 Q8WUI4 1385 29108 P41182 114548 3162 P53567 P10276 201633 Q9Y2C9 51564 Q8TEB7 11146 P08887 P08246 54 Q495A1 2626 719 Q96FA3 7099 7098 Q9NYA1 P29466 Q9Y239 64127 O95361 3297 P37231 3329 57678 10626 29110 P01308 5468 604 P23510 P07766 10392 6850 Q9UHD2 23586 Q9Y6M1 57162 Q99943 O14802 Q9HCL2 Q00613 Q96P20 Q92990 P01579 55703 Q92993 P43694 O15455 10554 P01730 11128 10159 5914 Q9BT67 P09958 O95786 P27540 Q12933 Q9BZS1 P51692 79589 Q9HC29 54106 P05121 P09601 50943 Q16581 P25116

endosome organization Q9UL26 P40818 O43752 9525 P48023 Q9P253 Q86VS8 Q60I27 P51148 O75351 Q9UJC3 10228 P61020 57403 356 Q96ED9 84376 5878 57617 5869

regulation of multicellular organismal metabolic process 4261 Q09472 Q92769 3673 2033 3066 P10600 54361 P56705 154 P17301 2147 P33076 P00734 P07550 Q7Z6C1 P01137 P08887 P25116 P08588

positive regulation of viral reproduction P05412 Q13148 7153 6672 7023 9217 Q13547 O95292 3065 23435 P11388 Q96T23 Q01664 51773 6667 4214 P08047 Q13233 P23497 Q6IT96

regulation of transporter activity 808 11060 O00308 9368 P14416 P56539 P52952 154 84502 84766 57620 P07550 6622 5336 Q13563 P62158 859 O14745 Q9BT67 Q13586 Q8WXH2 5311 Q13546 Q9HDC5 P21917 P37840 P16885 2280 Q9BSW2 P37231 P62166 O43707 P01308 8737 5468 Q96JJ6 P62942 P06213

digestive tract development Q09472 7046 7067 6256 P07949 2033 P19793 P14635 7161 891 Q9H161 27283 57669 1499 116113 Q1PSW9 P84022 Q96S42 2300 Q7Z6C1 P43694 Q9UJW2 P01137 Q9NSC2 2626 O15350 P02461 P10827 Q6IR47 4087 Q92824 4088 P30559 Q12952 Q13705 Q6KC79 P10600 P35222 P10070 Q9HCM4 P10071 P46531 4851 Q9NQB0 Q15796 2737 2736

BMP signaling pathway Q12841 P27037 5460 Q13873 Q15797 O95813 64750 P51668 Q9BQ95 P12755 4092 4093 P41134 Q9UJU2 Q13485 Q93008 O00238 4086 4089 O15198 Q9HCE7 Q04771 Q13705 57154 P36894 P19883 P35548 9350 51295 P17813 P61077 Q9H2X0 O15105 Q99717 Q9HAU4 10468

ribonucleoside triphosphate metabolic process P30049 O75947 O00746 Q16864 245972 P16615 P38606 P36542 23400 P15531 476 477 478 513 514 515 P61421 518 9114 51606 P28331 P48201 P48047 P13637 P22392 Q15904 Q6PIW4 P30793 481 483 1503 Q99437 Q93050 488 521 522 P20648 P54709 523 526 528 P05023 P17081 Q9UI12 P05026 Q9Y5B8 65220 Q9UGJ0 O75964 9525 P56134 29922 490 P50993 495 498 P00846 2643 P25705 P24539 533 P18859 535 Q04656 P56385 Q8N8Y2 P00568 537 P01137 539 Q9UN42 P56381 10476 10632 O95544 23439 P53396 P21283 P21281 O75351 9296 P15313 P12883 27032 Q9Y2B5 4719 P17812 4830 P20020 4833 P98194 509 P06576

positive regulation of multicellular organismal process P48058 8654 Q9Y4K3 3084 O43318 P37288 4609 476 P16220 P84022 P09493 P49810 Q7Z6C1 8767 O14788 57506 P07996 2307 Q15303 3091 65018 54361 P16473 5029 P00533 P10809 P05549 P10451 Q9Y6Q6 Q9UBU3 Q9UDY8 5830 64170 P00441 P32239 Q14116 5054 P09917 4088 3673 Q9H257 Q9NR96 Q13705 P56705 P17301 O60674 Q8IUC6 Q9ULZ3 Q16665 P27037 7124 1385 29108 P14635 114548 O43638 P10276 201633 154 P09172 P07550 5733 P08887 719 O00238 Q9NYA1 P29466 Q9Y239 Q92769 P30559 O95361 P36894 P10600 3329 57678 10626 P01308 P05023 P07766 P28223 10392 O43612 23586 7020 Q99943 Q9HCL2 2247 610 P01579 55703 857 1956 P09038 11128 10159 O43186 P39905 P51692 Q6KC79 P19883 P37840 Q9UL51 P05121 P09601 Q16581 P78423 P01241 Q13873 O75787 2033 O60603 P52952 Q9BXM7 2147 6622 6869 P42229 P78536 7057 6885 P35368 Q02297 P50542 P55318 O76074 P01106 P49768 1080 O00206 7186 Q09472 7189 P29474 P29475 9921 5321 P13569 148022 891 P41231 Q53X93 P01019 6647 P01137 P01375 Q9Y5X9 Q13422 A4D1W7 Q7Z434 P35348 P20309 6776 1406 8717 O43353 9388 O95999 3162 552 P22415 553 P00747 P15692 Q8N5U6 P43115 Q495A1 2626 P47712 Q96FA3 7099 7098 Q15628 64127 3297 5590 3171 Q05513 P00734 10468 P01185 6696 7422 P10415 3066 57162 O14802 Q00613 Q96P20 P43694 O15455 10554 P25103 P01178 P08588 5914 O95786 P27540 Q12933 Q04771 Q9HC29 10320 54106 P25116 P21554

wound healing P78423 P08833 5265 O14793 P15924 P13987 Q9UHF4 3484 9066 3084 Q04759 3479 2147 P84022 P21926 P09493 P04085 94 P78536 3091 5154 Q9NZG7 O95967 5155 8546 Q02297 P58546 P50148 P16234 2159 P38484 P15260 P22223 P46531 4851 4214 11234 5029 4854 P16070 Q14344 Q9UK55 P02675 P42338 P01343 928 136319 P28300 Q15797 P48551 26258 O00203 P41235 891 1634 Q03405 P37023 P41231 P21860 P01137 P05155 P50440 6383 P02461 4086 5054 5055 4088 7476

P04626 3673 2065 2064 P35625 P17302 O75084 P17301 P21359 P17813 2697 P26927 P01009 51156 P01008 P07585 6812 P01127 5327 Q16665 6814 5328 948 Q9UPZ3 P07225 P08758 Q9UNN8 O00186 P14635 Q03113 3162 83706 Q9H244 P00749 P00747 710 P00742 3678 Q9UM47 P08648 O00755 3690 2243 P10600 P51159 960 P16671 P01308 P00734 4015 5467 5627 P02671 P05106 P34741 1832 3459 P42768 2247 2244 Q9NZM1 P61764 10672 P09038 8761 Q86UX7 P51575 Q9UL45 O43581 26509 P20908 462 1289 P18827 P05120 Q92982 P05121 P00750 P09601 P04275 Q13233 P25116

positive regulation of lipid biosynthetic process P01185 7376 Q9NP71 Q9BX66 7124 5321 10580 P55055 P31749 54361 P56705 57678 P37288 552 Q9HCL2 P01308 P01019 P01579 207 Q13133 P01375 P47712

positive regulation of growth P01241 7046 P08833 6198 4131 O14672 P10636 1385 2033 3484 O43638 3084 154 P37288 552 P16220 P07550 Q7Z6C1 P46821 P42229 51009 2307 Q6IR47 P78536 Q14774 Q9NYA1 Q02297 P16989 3297 O95684 P50542 57678 P16473 P01308 4137 207 11116 Q09472 P01185 P10415 Q76FK4 Q13418 3142 P53667 Q9UBU3 Q9HCL2 Q00613 3611 Q53X93 P01019 5830 Q9GZP9 P01138 P23443 5716 Q13422 O75832 P51692 P31947 P31749 Q6KC79 55035 2810 2137 O43909 Q6PID4 Q92786 6776 10320 Q14289 P60568 P06213

response to electrical stimulus P48051 Q6IR47 7046 6198 O14793 5037 P30086 9495 P20336 7832 P01138 P01588 P01178 P23443 P24588

telomere maintenance via telomere lengthening Q9NYB0 25913 4361 7013 7014 5981 P54274 Q9NUX5 P49959 P35251 Q15554 Q92878 1736 Q9C0C2 Q9BSI4

negative regulation of steroid metabolic process4790 22937 2672 54361 P56705 476 P19838 27122 Q99684 Q92786 6647 Q9UBP4 Q12770 P05023 P00441

filopodium assembly Q7Z6J4 Q6ZV73 Q96M96 Q5JSP0 23092 10458 Q9UNA1 121512 P16144 P23229 55785 Q9UQB8 3655 Q9UHR4 P98174

N-terminal protein amino acid modification Q09472 9397 2033 P50579 80155 10988 79612 Q92793 1387 Q9BXJ9 O60551 Q7Z6C1 Q4LE28 P41227 Q92831

rhythmic process Q7Z2K6 8892 O14793 Q12948 O00268 P23945 1453 Q92731 Q8NFJ6 Q15389 P42229 O15516 P49770 P03950 9510 23291 79365 128 P48039 406 P00533 Q9C0J9 Q96EB6 O00327 8553 Q16649 P48552 P29474 5321 Q16526 P14416 P31153 O75881 6096 Q9Y6Q6 Q9UBU3 Q99743 894 898 4862 P01019 O15379 P21860 O15534 79956 6647 P03372 Q13144 P06401 P00441 P32239 3791 8204 P20393 P04626 A4D1W7 8841 2100 Q9UHI8 2065 2064 P41222 P24864 668 6776 1408 1407 Q49AN0 1406 9420 5187 O15287 P11766 Q8WYA1 P15692 3953 2904 Q13285 O14503 P47712 P05412 O00238 P35968 P29466 8864 P30559 O15055 O43524 P10600 23411 Q05086 402055 P30279 Q07817 P48357 Q9UH36 Q10586 7422 P10415 2099 Q9UKB1 O60346 4543 Q92753 1956 P41159 9575 O43186 P51692 23239 Q9HCK4 P58012 P48730

positive regulation of myeloid cell differentiation P26583 P27037 7189 Q92835 P63092 Q02363 P78504 Q9Y4K3 Q01196 91 6886 O14788 P42229 P06400 Q9NSA3 P41159 P05412 Q13422 P27540 3091 A4D1W7 P36896 O43524 22985 P17542 861 3148 Q9UKV3 6776 10320 Q16665 5925

epithelial cell differentiation P78545 Q13477 P15924 P17676 Q8NEY8 P22735 P41134 3479 284217 P04083 Q92731 P39060 P42229 2947 3911 Q9NPC8 Q9UGM3 79084 54361 367 P16471 P46531 4851

51535 O43294 P42574 P01343 Q8N205 6495 6256 Q9UBD6 P25391 P19793 2054 P20930 1874 P01019 Q5TA76 P03372 Q9NSC2 Q9UKW6 P06401 Q16254 P02462 Q92826 3791 8721 P35908 Q14119 P31947 Q9Y696 2100 Q86UL3 Q9BYD5 P35222 P56705 P17302 O60437 P17813 301 2697 Q5T5A8 Q92786 6776 5604 P01040 Q92817 8174 P10275 P78504 Q02535 10736 57669 1499 P15692 Q99966 2626 P05412 P35968 P32856 Q13443 Q86YZ3 P19544 7490 7251 P37231 3169 Q9HCM4 5468 P09067 967 Q15475 8754 137964 7422 P56178 2099 O60869 Q08188 163183 P15311 1832 2247 3215 2125 P21266 P43694 Q9BQA1 Q9H0F6 P09038 5493 7430 O15353 P39905 O15230 Q02750 P22528 2810 P09960 6699 Q99816 P02533

peptide cross-linking	P02461 7057 133584 P01040 Q92817 P15924 Q08188 7052 P22528 1634 1832 Q63HQ2 P22735 P21810 2335 301 2125 P04083 Q96PF1 6699 P21980 P07585 P02751 P07996

nucleus organization	P26583 O95476 O75844 25909 57120 2010 P29590 Q9H9Y6 7141 4926 P09430 P43034 P02545 Q8WUM0 5371 4000 Q8NF91 23399 25777 P50402 Q9HD26 3148 P52948 81565 Q8WYP5 Q9UH99 Q9GZM8 P42574 Q14980 10269

associative learning	P54253 P14416 A1XKG3 3265 1020 P50993 60626 351 Q99965 P09172 Q9NPQ8 54413 477 Q00535 P01116 6869 P25103 2904 P01112 2902 Q13286 P01111 Q05586 P35368 P10721 Q9NZ94 P21359 P42858 P05067 P01100

segmentation	Q9Y5Q3 P04637 Q13315 P13349 Q12948 2033 Q9H488 23322 472 57669 Q1PSW9 P84022 P49810 Q7Z6C1 4920 Q9Y2T1 O15119 Q13485 1856 5591 P50222 P36894 Q5TAB7 Q68CZ1 Q9HCM4 648 A6NI15 P49768 Q09472 5362 23509 92129 P78527 O75051 O14641 Q92830 Q9UJU2 6926 9839 134701 4088 4089 7157 Q86YT6 P35226 Q01974 57534 P10070 Q9HCS4 2736

immunoglobulin production	Q8WYA6 O43734 P15923 P78527 2590 56259 10758 6929 10673 Q9Y275 7518 Q10471 Q8IWV1 5591 Q13426 Q9GZX7 64127 P52701 3329 P40692 Q9HC29 57379 P08575 P43246 P10809 2956

hexose biosynthetic process	7167 P16118 Q00796 O60547 2821 2026 51363 2762 P60174 Q9UBK2 P06744 P24298 O00757 5091 P11498 1468 2875 Q9UBX3 P18847 P18848 468 P05062 5207 P09467 P09104 Q7LFX5

ribonucleoside monophosphate metabolic process	P30566 Q06203 271 P30520 3615 P11172 3614 P49915 4907 158 P60891 P31939 P22234 P21589 P11908 161823 5471 5198 5631 P12268 7372 10606 Q01433 P22102 8833 Q6DHV7

positive regulation of lymphocyte differentiation	O43353 P00491 Q92934 Q92835 6850 3142 P10276 O00203 572 79576 8767 P42229 5914 P24394 Q13422 Q14774 8546 P51692 4860 Q8N5F7 P43405 P37173 O43290 6776 P01589 10320 P60568 P04233

regulation of transcription involved in G1/S-phase of mitotic cell cycle	Q9BQA5 1874 Q99684 2672 O75461 25988 Q16254 P06400 5925

S phase of mitotic cell cycle	8099 O14519 P00519 84967 Q969L4 P28340 5422 P09884 5424

positive regulation of viral transcription	P05412 Q96T23 7023 Q01664 51773 6667 4214 P08047 Q13233

glandular epithelial cell differentiation	6256 137964 3169 P46531 4851 P19793 Q86UL3 79084 Q9BQA1

natural killer cell mediated immunity P07437 Q99698 203068 10383 Q06830 1130 P51159 P68371 P53567

cellular response to growth hormone stimulus P01241 5295 O60674 P51692 6774 6776 P42229 P27986 P40763

cellular response to lipid 891 P05091 5155 Q01094 P01127 P14635 P18146 1869 O75581

cytoplasmic sequestering of NF-kappaB 9908 O75832 4792 11124 Q9UNN5 Q9UN86 Q15653 P25963 5716

natural killer cell mediated cytotoxicity P07437 Q99698 203068 10383 Q06830 1130 P51159 P68371 P53567

positive regulation of receptor biosynthetic process Q92769 22937 Q13547 10013 3066 3065 Q12770 Q9UBN7 Q6IT96

positive regulation of cholesterol efflux 7376 O95477 4792 Q07954 4035 P55055 19 Q13133 P25963

negative regulation of protein maturation by peptide bond cleavage 2 7057 5054 710 P05121 P05155 P07996 5270 P01023

mesenchymal to epithelial transition 10736 P39905 54361 P56705 Q9NSC2 P19544 7490 Q99966 Q9NPC8

growth hormone receptor signaling pathway P01241 5295 O60674 P51692 6774 6776 P42229 P27986 P40763

regulation of androgen receptor signaling pathway 3479 Q99497 2100 Q92731 10013 O75928 Q9UBN7 11315 P01343

intronless viral mRNA export from host nucleus 8563 Q86W42 80145 Q13769 Q8NI27 Q86V81 57187 Q6I9Y2 Q96FV9

response to reactive oxygen species P02452 P25098 P28562 9588 P49023 P30041 2033 P30044 3162 P30048 Q00597 P42224 156 10935 Q03468 P09493 P04040 Q7Z6C1 5578 10013 P62714 5338 P47712 P05412 Q15303 Q9NYA1 Q13443 5796 Q07817 5829 P39210 847 11315 P01100 P04179 Q7Z727 Q09472 P35638 Q16849 8754 P10415 6772 5321 1277 P17252 P09471 Q04656 5798 6647 5516 P00441 5054 Q92626 P55851 P55212 5970 Q06830 P12883 1843 O14939 Q04206 P18827 Q99497 Q6PID4 4358 P05164 P05121 P09601 Q14289 Q9UBN7

purine ribonucleotide biosynthetic process O75947 Q16864 P16615 P30520 P15531 476 477 478 513 514 515 P61421 518 9114 P48201 P48047 P13637 481 P12268 483 488 521 522 523 P22102 526 528 Q9UI12 P56134 29922 490 495 P49915 498 P00846 P25705 533 P18859 535 P56385 537 P01137 539 Q9UN42 P56381 10476 10632 23439 Q9Y2B5 8833 P20020 P30049 P30566 O00746 245972 P38606 P36542 23400 271 158 P31939 5471 51606 5198 P22392 Q15904 Q99437 Q93050 P20648 P54709 Q01433 P05023 P05026 Q9Y5B8 Q9UGJ0 O75964 Q06203 P50993 3615 3614 P24539 Q04656 P22234 Q8N8Y2 161823 P21283 P21281 9296 P15313 27032 10606 4830 Q6DHV7 4833 P98194 509 P06576

reproductive behavior in a multicellular organism P09172 P10827 P01185 P37288 552 7067 P30559 8932 Q9UBB5 P07686 3074 P01178

peptidyl-asparagine modification 6185 84061 P04843 P61803 201595 Q9H0U3 P04844 1650 Q8TCJ2 P39656 6184 P46977

protein amino acid N-linked glycosylation via asparagine 6185 84061 P04843 P61803 201595 Q9H0U3 P04844 1650 Q8TCJ2 P39656 6184 P46977

cellular response to growth factor stimulus P02452 O00755 57669 1499 7476 1277 Q9HCM4 2010 P35222 54361 P56705 P50402

oxaloacetate metabolic process O75390 P00505 1431 P51692 P40926 P17174 6776 5091 P42229 2805 4191 P11498

myoblast fusion P17655 O95259 P29466 50937 O95180 P29475 Q9Y463 Q4KMG0 9149 O43184 P35579 4627

nitric oxide metabolic process 2644 P29474 2643 P29475 P78540 O95865 P31749 207 P15559 1728 P30793 P30047

positive regulation of vascular endothelial growth factor receptor signaling pathway P08648 P04004 3690 P05106 Q13322 P27540 3091 P15692 7422 7448 Q16665 3678

protein destabilization 5591 1029 Q8WY64 Q96AB3 79763 440193 Q00987 Q8N726 P19474 P42771 P78527 4193

Golgi transport vesicle coating P53621 1315 P35606 375 1314 9276 Q9Y678 Q92538 P84077 P53618 O14579 Q8N6T3

COPI coating of Golgi vesicle P53621 1315 P35606 375 1314 9276 Q9Y678 Q92538 P84077 P53618 O14579 Q8N6T3

regulation of interferon-alpha production O00206 7099 3329 O95786 7098 Q7Z434 Q9NR96 23586 54106 O15455 57506 P10809

ovulation cycle process Q7Z2K6 8892 O15287 O14793 Q12948 O00268 P15692 P23945 Q92731 3953 Q15389 P42229 Q13285 P47712 P49770 O00238 P03950 9510 P35968 P30559 O43524 P10600 23411 Q05086 P30279 Q07817 P48357 Q96EB6 P48552 P29474 7422 P10415 5321 2099 894 898 P01019 79956 6647 P03372 Q13144 P06401 P00441 P41159 P32239 3791 8204 P04626 P51692 A4D1W7 2100 Q9UHI8 2064 Q9HCK4 P58012 P24864 668 6776

purine nucleoside triphosphate metabolic process P30049 O75947 O00746 Q16864 245972 P16615 P38606 P36542 23400 P15531 476 477 478 513 514 515 P61421 518 9114 51606 P28331 P48201 P48047 P13637 P22392 Q15904 Q6PIW4 P30793 481 483 Q99437 Q93050 488 521 522 P20648 P54709 523 526 528 P05023 P17081 Q9UI12 P05026 Q9Y5B8 65220 Q9UGJ0 O75964 9525 P56134 29922 490 P50993 495 O60502 498 P00846 2643 P25705 P24539 533 P18859 535 Q04656 P56385 Q8N8Y2 P00568 537 P01137 539 Q9UN42 1716 P56381 10476 10632 O95544 23439 P53396 P21283 Q16854 P21281 O75351 9296 P15313 P12883 27032 Q9Y2B5 10724 4719 4830 P20020 4833 P98194 509 P06576

regulation of nitric-oxide synthase activity P27037 3091 P78540 P31749 P34972 P56539 P30793 P17813 2643 P01308 207 P01116 P00533 Q16665 859 1956 P21554

viral genome replication Q9NZI7 P56545 P35659 6598 O43504 Q12824 Q12857 Q7Z2Q5 O00267 P13236 6829 O43719 1488 1487 10542 7913 Q13363

cell proliferation in forebrain O00755 8650 9612 7476 10460 Q9Y6R0 Q9Y618 Q86VS8 Q03014 9253 P20264 Q15154 84376 Q9Y6A5 5108 Q8N960 9839

regulation of epidermal growth factor receptor activity Q9UJM3 P78536 P29353 6464 O43639 84619 351 57732 Q9UNH7 Q8N5A5 58533 P49810 54206 P05067 P01135 P01133 P49768

endosome to lysosome transport 4074 Q9UPV9 6642 Q86VS8 Q9NZZ3 Q9UJC3 154 P20645 388 Q99523 P07550 P62745 Q96ED9 Q9UGU5 51510 84376 Q13596

DNA damage response, signal transduction by p53 class mediator resulting in induction of apoptosis 5371 Q6ZN33 64782 Q16666 7157 Q9H305 P04637 8445 Q86Z02 Q9Y5J5 P29590 P51587 3428 57646 Q96RU2 Q8WTP8 Q92630

response to X-ray P24385 7157 P04637 Q13216 Q13426 P54132 Q6KC79 1161 P51587 P46736 595 121457 641 Q03468 Q70UQ0 7518 P43246

regulation of collagen metabolic process 4261 Q09472 Q92769 3673 2033 3066 P10600 54361 P56705 P17301 2147 P33076 P00734 Q7Z6C1 P01137 P08887 P25116

positive regulation of actin filament bundle assembly 4088 4771 P31431 P53667 Q4VCS5 P42345 387 Q04759 P84022 P09493 2475 P35240 P61586 Q8N3V7 6869 11346 P25103

positive regulation of chemokine production O43353 O00206 7099 7098 P78536 3091 7124 Q9NR96 O60603 P41231 8767 5029 54106 O15455 Q16665 P08887 P01375

response to nutrient levels P02452 Q13873 6198 P98155 P28562 P04637 2033 3482 P51587 O00300 P42345 P42224 595 P13073 Q7Z6C1 P04085 10013 O14543 P46821 Q15389 Q9UBK2 P48681 9474 5154 Q8NDV7 55054 P13631 P55318 55172 Q9BSB4 2 Q9P2K8 P05062 P06276 P01588 P16070 Q99757 P01222 Q96EB6 P49768 Q09472 P35638 7067 P48431 6256 6772 5321 P19793 P10451 P14410 Q9UBU3 O76061 57521 P00966 898 6647 P01137 Q7Z3C6 P00441 Q9Y5X9 P50440 P01374 P11717 8443 6262 P07339 P09917 5970 3673 P19320 P36956 P35625 Q676U5 Q9H492 5728 O75084 P17301 Q9NVR5 P60484 Q04206 Q9H093 P24864 P01008 6657 10763 5207 P23497 Q9UBN7 O94817 3309 9388 O00744 6672 Q15853 25828 4131 Q8TDY2 9821 27327 P41181 9021 Q9NX61 3162 P10276 P22415 O60488 60673 P08243 3953 Q13286 P47712 P05412 O15294 Q9Y4P1 7252 Q14258 Q9NYA1 6720 7412 O95361 23411 3171 P37231 57678 10626 P11021 960 445 2475 207 5468 P01303 P48357 P01023 P04179 P24385 8473 P61803 6696 P10415 3860 P13646 P16118 Q86V24 Q9HCL2 P61244 Q9H1Y0 1277 P08069 4149 857 P02786 5119 P49715 P01178 P23443 5914 P41159 Q13501 P10827 7037 7157 P31749 79065 1050 O60911 462 1843 23192 81788 8878 P09601 P21554 O15228 7706 P06213

regulation of response to external stimulus P78423 O15123 O14672 8654 P14555 2150 O60603 P42345 2147 P84022 P04085 O14788 P42229 P15090 P07355 P07996 O15118 P04004 P78536 7057 5154 5155 5270 7052 O76074 2 81565 2277 7448 P01589 Q9HCY8 O00206 Q7Z727 P29474 5321 4354 P14416 P13686 P10451 Q9Y6Q6 57402 Q9UBU3 Q00013 4907 P17252 P01019 P21980 P01137 P05155 P21589 P55290 P01375 5054 5692 3791 4088 3673 Q9BZF9 Q9NR96 P19438 P17301 O60674 302 P04070 P01008 6776 P01127 10488 5328 P01042 7124 O15164 P41182 O43915 154 P00749 P22415 P00747 P15692 710 1012 P07550 5578 5733 P08887 P08246 P43115 54 Q13286 P47712 719 P26232

7099 7132 P30556 7098 P35968 7133 Q15109 O43889 P51679 64127 P35443 285 P37231 P61073 P01308 P00734 2475 5468 P01303 604 P55085 5624 Q9GZM8 P01023 6696 7422 P30542 857 O15455 Q9NQC3 P04264 P20333 P41159 Q9BT67 P51692 P34972 P48061 P28070 462 Q9HCK4 Q9HC29 54106 P05121 Q16581 P25116 P60568 P21554

eye development P25054 Q12948 O75386 23322 2303 356 P84022 P08047 P83731 Q9C0K0 P25067 Q12837 23210 P58304 Q9BRQ0 P13631 Q02447 P63211 Q96KN7 30813 4214 6496 P48431 6498 P12757 6015 6774 Q9NRY4 P12755 6096 Q96RK4 O15259 O95859 P01137 5316 4867 Q9GZX9 Q13422 P54762 5451 4088 P55212 7476 A4D1W7 P48023 P35222 O95343 Q9P0I2 P21359 Q9NZR4 P10071 668 Q92786 P07101 6657 P26367 P41743 2737 9927 6670 P55347 5584 P25490 Q9Y586 2909 Q6NYC1 Q06587 25945 O75581 1499 P15692 6667 Q15223 P40763 O00238 O00755 Q68CZ1 P19544 7490 64919 324 338917 Q99958 7528 Q8TCU4 90780 7422 10152 P14859 Q9NYB9 57045 Q9NQS3 P61244 Q92753 4149 P47895 80184 P10826 P29323 P05997 O15078 Q15648 O95140 P20908 Q6KC79 1289 O95390 P58012 10320 Q13233

regulation of transmembrane transport 808 9368 P14416 P56539 P52952 154 1499 84502 84766 57620 Q5T2W1 P07550 5336 Q13563 P62158 859 O14745 P01133 5174 Q13586 Q8WXH2 5311 Q13546 Q9HDC5 P35222 P21917 P16885 2280 Q9BSW2 P62166 O43707 P01308 8737 Q96JJ6 P62942

energy reserve metabolic process O95278 P13807 Q9UGJ0 P06737 P13945 P63092 178 Q93100 Q15185 3953 P62136 5836 P41159 Q13522 O95685 Q16816 P46020 5499 Q9UGI9 P36873 P15735 P11216 Q06210 2932 2632 P35573 Q04446 P62140 10728 5500 5501 P48357 P49841 P46019 P46976

fatty acid catabolic process O00628 Q08426 2110 P50416 P33897 P09110 215 1892 P23786 Q16134 5830 Q9UJ83 Q16698 30 Q14693 P41159 34 P28330 P40939 1374 1891 O00154 P51659 3032 P50542 3030 P11310 1666 P30084 5467 P55084 26061 Q13011

negative regulation of hydrolase activityP01040 Q9BY76 P04637 O43597 P30047 P22736 Q99523 O75190 3476 6622 5018 3953 P45379 P07996 P04004 7057 23411 2280 5590 Q9P107 O43609 P11021 Q05513 Q15392 Q15070 7448 P48357 10542 Q96EB6 P29474 2644 1718 Q16610 5054 O15392 7157 P31947 O43504 9093 P37840 P78318 2810 Q15011 5524 Q96EY1 P05121 Q15257 3309 P62942

regulation of lipid biosynthetic process 7124 2672 672 P37288 552 Q96RU7 476 27122 Q99684 P04085 Q13285 P47712 7376 5154 P28330 5155 Q9NP71 10580 54361 57678 P01308 207 Q9UBP4 P05023 Q13133 Q13131 4790 P01185 5562 22937 5321 P38398 Q9HCL2 57761 P01019 P01579 6647 Q12770 Q9UBM7 P00441 P01375 P41159 Q9BX66 P55055 P31749 P56705 P19838 Q92786 P01127

regulation of organic acid transport Q15019 8399 7057 O15496 7124 P31749 Q9Y4H2 2550 Q9Y6Q6 P41231 Q9UBS5 P20396 207 5029 P46059 O14788 P07996 P01178 P01375

negative regulation of immune effector process 6890 Q9BZS1 Q92949 P32121 P41182 Q96J02 83737 3162 2 P01308 710 P08575 604 P09601 Q03518 P05155 50943 409 P01023

endothelium development P35968 3791 8721 O15230 Q14119 P35222 O60869 P78504 P41134 1499 P17813 3215 P01019 P21266 Q92786 P39060 P09067 2947 3911

retrograde transport, endosome to Golgi O75436 O94955 O95249 387680 Q641Q2 Q8IWJ2 Q8IUD2 23085 51542 Q13190 A8K0Z3 55737 O14662 9559 6811 9527 9648 Q96QK1 253725

skeletal muscle organ development P13349 2033 1020 O00468 351 P15173 Q7Z6C1 P61586 6901 P78337 P50222 Q14774 Q16635 P51636 10939 P11532 O43847 P60660 375790 4898 P05067 Q15475 Q09472 6495 6498 4154 P12757 A1XKG3 P12755 3142 1756 1634 P02708 Q00535 P21860 O14640 857 Q14993 P07858 P04626 P68133 2065 O95183 2064 9093 387 Q08209 P58012 668 P24821 P07585 Q96EY1 4637 P25116

anterior/posterior pattern formation P25054 Q13873 7046 4297 Q9NYD6 P04637 P13349 Q12948 2033 Q86Z02 472 Q9H161 P84022 P49810 7832 Q7Z6C1 O15119 Q13485 P09017 Q9NSA3 Q5TAB7 P13631 9350 P42858 23451 P46531 4851 Q15796 Q13099 P49768 Q09472 6496 92129 6015 P32242 P31273 P31276 P12755 Q03014 Q96S42 P09630 Q92830 Q9UJU2 P49639 P40424 6926 134701 4087 Q92824 4088 4089 Q13705 Q86YT6 P35222 O95343 P35227 Q01974 57534 P31270 P10071 2737 P27037 P25490 Q13315 Q9H488 Q06587 P35453 O75581 O14627 P46109 57669 1499 P31260 4920 Q9Y2T1 2626 P31249 5591 6045 Q6IR47 P50222 8100 P36894 Q05516 3207 324 Q9HCM4 P17482 P09067 A6NI15 7528 5362 O95813 23509 P78527 O75051 3215 1399 P43694 2926 9839 7157 Q03164 3226 O95390 Q99496 Q9HCS4 7704

limbic system development 7248 P56177 A1XKG3 1020 3066 7161 P51843 O75360 Q9BXC9 P51805 Q00535 Q96RK4 Q9UJU2 P43034 Q92574 P47895 O15119 P62258 6926 9839 P04201 O15350 Q92769 Q14114 7531 P12429 Q86WV8 9495 55558 7804 P24588

canonical Wnt receptor signaling pathway O00744 P25054 P24385 Q15831 6794 O14495 O60907 O75360 O75581 595 1499 Q9BZK7 Q9UJU2 O14640 Q92997 O00755 7476 Q92729 P35222 54361 P56705 P13631 5728 O75084 2932 324 P60484 P18827 Q9NQB0 O15169 P49841

blood vessel remodeling 9474 P29474 7422 2034 P35368 Q12948 P13612 7052 P09172 2303 P21810 P15692 3479 Q9H1Y0 Q04656 P01019 P21980 3676 Q99814 Q99958 P01343

gluconeogenesis P06744 7167 P24298 O00757 5091 P16118 P11498 1468 2821 2875 Q9UBX3 P18847 2026 P18848 468 P05062 P60174 5207 P09467 P09104 Q9UBK2

regulation of alpha-beta T cell activation O43353 P07766 P00491 Q92956 6850 P54132 P41182 3142 P10276 O00203 79576 8767 5914 P24394 Q13422 Q14774 149041 8546 4860 Q5TC82 Q15306 Q96J02 83737 Q8N5F7 P43405 641 P37173 604 10320

positive regulation of defense response O43353 P78423 O95999 7124 8654 P14555 O60603 5817 Q9Y2C9 Q92692 5578 8767 5733 O14788 P42229 P15090 P25963 P43115 P47712 7099 7132 P30556 7098 64127 7052 O76074 3329 29110 10666 P10809 Q9HCY8 O00206 Q7Z727 4792 5321 Q9UHD2 148022 Q9Y6Q6 57402 P17252 P15151 P01019 55703 P21980 O15455 64170 Q15762 P01375 5054 5970 P51692 3673 Q9H257 Q9NR96 81622 P19438 P17301 O60674 P51617 Q8IUC6 Q04206 Q9HC29 6776 54106 P05121 P21554 Q9H1C4

cell adhesion P25054 23607 P49023 Q9P218 P40200 5817 P21926 O14786 P07996 P27487 P04004 P25067 Q9NZG7 O15530 Q92729 Q12959 O95680 Q08554 Q08431 5829 Q96QZ7 P20849 P21815 10420 Q9Y286 10666 2319 928 Q7L5Y9 O60547 Q9UQB3 Q8N6Y1 Q9UJU2 55109 3655 4867 4868 4627 P26012 P05556 3672 P26010 3673 27076 P20702 P17301 2335 Q9UKX5 6812 6814 705 948 P26006 P08514 3680 Q9Y223 O95471 1020 P18564 83706 27283 57669 P16144 1499 57662 1012 3674 3675 3676 P61587 P61586 3678 3679 P08648 P51671 O00238 3690 3693 29119 O95484 Q9UEE9 56104 P18206 960 961 Q9HCM4 3685 Q9UN70 A6NMZ7 3688 3689 Q9UN75 64098 P05106 P05107 6850 1285
[truncated: 34,136 more chars]
