# Supplementary material for: COVID-19 engages clinical markers for the management of cancer and cancer-relevant regulators of cell proliferation, death, migration, and immune response
Source: Sci Rep. 2021 Mar 4;11:5228. doi: 10.1038/s41598-021-84780-y (PMC7933131; doi:10.1038/s41598-021-84780-y)
Supplement: Supplementary file 4 — Supplementary Information 4. [file 41598_2021_84780_MOESM4_ESM.pdf]

Supplementary Table S2B

List of biological processes relevant to carcinogenesis.

The biological processes were retrieved from the networks (Figure 1), by searching with key words of cancer hallmarks.

ID of the nodes in each biological process can be retrieved from the Supplementary Figure S1. Copy a biological process of interest and search for it in the Supplementary Figure S1 to retrieve IDs of involved nodes.

Biological processes that were retrieved are listed. Data for no-hits searches are not shown.

Searches were performed with keywords representing proliferation, growth, death, apoptosis, autophagy, migration, invasiveness, immune system, inflammation, immortalization, telomere, genome instability, metabolism, energy, angiogenesis. We searched the network also for biological processes of relevance to viral cycle and propagation of a virus. If a search showed no hits, it is not listed.

*Supplementary Tables S1, S2 and S3 can be retrieved online from:
https://figshare.com/articles/dataset/Supplementary_Tables_S1_S2_and_S3/12804887

Growth	positive regulation of growth
	positive regulation of transforming growth factor beta receptor signaling pathway
	vascular endothelial growth factor receptor signaling pathway
	blastocyst growth
	positive regulation of skeletal muscle tissue growth
	JAK-STAT cascade involved in growth hormone signaling pathway
	negative regulation of developmental growth
	cell growth
	positive regulation of transforming growth factor-beta production
	regulation of vascular endothelial growth factor receptor signaling pathway
	growth
	cellular response to growth hormone stimulus
	platelet-derived growth factor receptor signaling pathway
	regulation of transforming growth factor beta receptor signaling pathway
	developmental growth
	negative regulation of transforming growth factor beta receptor signaling pathway
	regulation of developmental growth
	regulation of skeletal muscle tissue growth
	regulation of transforming growth factor-beta2 production
	response to growth factor stimulus

negative regulation of growth
epidermal growth factor receptor signaling pathway
regulation of cell growth
growth hormone receptor signaling pathway
regulation of transforming growth factor-beta3 production
regulation of growth
cellular response to growth factor stimulus
transforming growth factor beta receptor signaling pathway
positive regulation of developmental growth
negative regulation of cell growth
positive regulation of vascular endothelial growth factor receptor signaling pathway
developmental cell growth
regulation of multicellular organism growth
regulation of epidermal growth factor receptor signaling pathway
regulation of transforming growth factor-beta production
positive regulation of transforming growth factor-beta3 production
insulin-like growth factor receptor signaling pathway
regulation of transforming growth factor-beta1 production
regulation of epidermal growth factor receptor activity
positive regulation of transforming growth factor-beta1 production

ACE2 and TMPRSS2 contribution:

positive regulation of multicellular organism growth
regulation of developmental growth
regulation of organ growth
positive regulation of growth hormone receptor signaling pathway
positive regulation of growth
regulation of growth
regulation of multicellular organism growth
regulation of insulin-like growth factor receptor signaling pathway

Proliferation

Schwann cell proliferation
positive regulation of mononuclear cell proliferation
regulation of metanephric cap mesenchymal cell proliferation
regulation of cell proliferation
regulation of T cell proliferation
regulation of immature T cell proliferation
cell proliferation in forebrain
regulation of B cell proliferation
positive regulation of metanephric cap mesenchymal cell proliferation
regulation of leukocyte proliferation
positive regulation of epithelial cell proliferation
epithelial cell proliferation
regulation of epithelial cell proliferation
negative regulation of T cell proliferation
activated T cell proliferation
negative regulation of lymphocyte proliferation

positive regulation of leukocyte proliferation
regulation of fibroblast proliferation
positive regulation of lymphocyte proliferation
positive regulation of B cell proliferation
regulation of mesenchymal cell proliferation
negative regulation of cell proliferation
leukocyte proliferation
regulation of smooth muscle cell proliferation
cell proliferation
negative regulation of immature T cell proliferation
positive regulation of fibroblast proliferation
positive regulation of smooth muscle cell proliferation
negative regulation of smooth muscle cell proliferation
positive regulation of cell proliferation
T cell proliferation
regulation of mononuclear cell proliferation
inner cell mass cell proliferation
negative regulation of mononuclear cell proliferation
lymphocyte proliferation
positive regulation of T cell proliferation
mammary gland epithelial cell proliferation
negative regulation of immature T cell proliferation in the thymus
glial cell proliferation
regulation of lymphocyte proliferation

ACE2 and TMPRSS2 contribution:

positive regulation of fibroblast proliferation
cell proliferation
positive regulation of cell proliferation
negative regulation of cell proliferation
regulation of cell proliferation
muscle cell proliferation
regulation of fibroblast proliferation
smooth muscle cell proliferation

Death

induction of programmed cell death
positive regulation of programmed cell death
necrotic cell death
cell death
activation-induced cell death of T cells
regulation of programmed cell death
positive regulation of cell death
death
regulation of cell death
negative regulation of cell death
induction of apoptosis via death domain receptors
programmed cell death
negative regulation of programmed cell death

regulation of necrotic cell death
positive regulation of necrotic cell death
ACE2 and TMPRSS2 contribution:
regulation of cell death
death
negative regulation of programmed cell death
negative regulation of cell death
cell death
regulation of programmed cell death

Apoptosis

regulation of muscle cell apoptosis
DNA damage response, signal transduction resulting in induction of apoptosis
DNA damage response, signal transduction by p53 class mediator resulting in induction of apoptosis
regulation of neuron apoptosis
negative regulation of neuron apoptosis
regulation of apoptosis
positive regulation of apoptosis
induction of apoptosis by extracellular signals
T cell apoptosis
induction of apoptosis
positive regulation of anti-apoptosis
induction of apoptosis by intracellular signals
anti-apoptosis
regulation of anti-apoptosis
positive regulation of neuron apoptosis
negative regulation of apoptosis
regulation of myeloid cell apoptosis
apoptosis
lymphocyte apoptosis
cellular component disassembly involved in apoptosis
induction of apoptosis via death domain receptors
ACE2 and TMPRSS2 contribution:
regulation of apoptosis
negative regulation of apoptosis
anti-apoptosis

Autophagy

chaperone-mediated autophagy
autophagy

Telomerase

telomere maintenance via telomerase

Migration

regulation of blood vessel endothelial cell migration
telencephalon cell migration
cell migration in hindbrain
positive regulation of epithelial cell migration

positive regulation of smooth muscle cell migration
nuclear migration
negative regulation of cell migration
radial glia guided migration of granule cell
hindbrain radial glia guided cell migration
cerebral cortex radially oriented cell migration
neuron migration
cell migration
substrate-bound cell migration, cell extension
regulation of epithelial cell migration
regulation of cell migration
substrate-bound cell migration
forebrain cell migration
cerebral cortex radial glia guided migration
negative regulation of blood vessel endothelial cell migration
regulation of smooth muscle cell migration
negative regulation of endothelial cell migration
positive regulation of cell migration
interkinetic nuclear migration
regulation of cell migration involved in sprouting angiogenesis
negative regulation of cell migration involved in sprouting angiogenesis
regulation of endothelial cell migration

ACE2 and TMPRSS2 contribution:

positive regulation of cell migration
positive regulation of endothelial cell migration
regulation of cell migration
regulation of endothelial cell migration

Immune system

regulation of production of molecular mediator of immune response
immune response-regulating signaling pathway
regulation of immunoglobulin mediated immune response
immune response-regulating cell surface receptor signaling pathway
negative regulation of immune response
positive regulation of immune response
somatic diversification of immune receptors via germline recombination within a single locus
regulation of innate immune response
production of molecular mediator of immune response
negative regulation of immune effector process
negative regulation of adaptive immune response
regulation of immune response
somatic diversification of immune receptors
regulation of humoral immune response
immune effector process
negative regulation of adaptive immune response based on somatic recombination of immune receptors built from immunoglobulin superfamily domains

activation of innate immune response
immune response-activating cell surface receptor signaling pathway
immune system process
negative regulation of immune system process
regulation of adaptive immune response based on somatic recombination of immune receptors built from immunoglobulin superfamily domains
regulation of immune effector process
negative regulation of production of molecular mediator of immune response
regulation of immune system process
negative regulation of cytokine production involved in immune response
innate immune response-activating signal transduction
regulation of cytokine production involved in immune response
immune response
positive regulation of innate immune response
positive regulation of immune system process
immune response-activating signal transduction
activation of immune response
innate immune response
negative regulation of humoral immune response
negative regulation of innate immune response
regulation of adaptive immune response
ACE2 and TMPRSS2 contribution:
positive regulation of immune system process
immune system development
immune response-activating cell surface receptor signaling pathway
immune response
immune response-activating signal transduction
activation of innate immune response
immune response-regulating cell surface receptor signaling pathway
positive regulation of immune response
somatic diversification of immune receptors via germline recombination within a single locus
somatic diversification of immune receptors
regulation of innate immune response
activation of immune response
innate immune response activating cell surface receptor signaling pathway
regulation of humoral immune response
regulation of immune response
positive regulation of innate immune response
immune system process
immune response-regulating signaling pathway
regulation of immune system process
regulation of production of molecular mediator of immune response

Angiogenesis	negative regulation of angiogenesis
	angiogenesis
	regulation of angiogenesis
	regulation of cell migration involved in sprouting angiogenesis
	negative regulation of cell migration involved in sprouting angiogenesis
Energy, regulation of energy	energy derivation by oxidation of organic compounds
	generation of precursor metabolites and energy
	regulation of generation of precursor metabolites and energy
	energy reserve metabolic process
	energy coupled proton transport, down electrochemical gradient
Virus, viral cycle	latent virus infection
	regulation of defense response to virus
	regulation of defense response to virus by host
	evasion of host defenses by virus
	reactivation of latent virus
	response to virus
	non-lytic virus budding
	positive regulation of defense response to virus by host
	virus-host interaction
	release of virus from host
	regulation of viral reproduction
	viral assembly, maturation, egress, and release
	initiation of viral infection
	viral reproduction
	positive regulation of viral transcription
	regulation of viral transcription
	viral genome replication
	intronless viral mRNA export from host nucleus
	viral infectious cycle
	viral reproductive process
	positive regulation of viral reproduction
	non-lytic viral release
	ACE2 and TMPRSS2 contribution:
	initiation of viral infection
	viral reproduction
	viral infectious cycle
	viral reproductive process
