# Supplementary material for: COVID-19 engages clinical markers for the management of cancer and cancer-relevant regulators of cell proliferation, death, migration, and immune response
Source: Sci Rep. 2021 Mar 4;11:5228. doi: 10.1038/s41598-021-84780-y (PMC7933131; doi:10.1038/s41598-021-84780-y)
Supplement: Supplementary file 5 — Supplementary Information 5. [file 41598_2021_84780_MOESM5_ESM.pdf]

Supplementary Table S3

**Supplementary Tables S1, S2 and S3 in Excel format suitable for searches can be retrieved online from: https://figshare.com/articles/dataset/Supplementary_Tables_S1_S2_and_S3/12804887*

Page 2 Supplementary Table S3A, Tumor Markers used in companion diagnostics, approved by FDA.

Page 4 Supplementary Table S3B, Tumor Markers in Common Use

https://www.cancer.gov/about-cancer/diagnosis-staging/diagnosis/tumor-markers-list

Listed are tumor markers that are in common use, mainly to determine treatment or for diagnostic of cancer.

Page 6 Supplementary Table S3C, List of genes in FoundationOne CDx signature for companion diagnostic, and the list of genes tested for substitutions, indels and copy-number alterations.

Page 6 Supplementary Table S3D, List of genes of 5-protein signature OVA1.

Page 7 Supplementary Table S3E, List of genes of 21-gene Oncotype Dx.

Page 7 Supplementary Table S3F, List of genes of Oncomine Dx signature.

Page 8 Supplementary Table S3G, List of genes of Prolaris Dx signature.

Page 9 Supplementary Table S3H, List of genes of Mammaprint Dx signature.

Page 11 Supplementary Table S3I, List of targets of drugs proposed for treatment of COVID-19

Supplementary Table S3A, Tumor Markers used in companion diagnostics, approved by FDA.

Dx target	Dx Name	Type of cancer	Name of Drug
PARP	BRCAnalysis CDx	Breast, ovarian, pancreatic	Lynparza
BRCA1			Talzennna
BRCA2			Rubraca
EGFR	theracreen EGFR RGQ PCR kit	Non-small cell lung cancer	Iressa, Gilotrif, Vizimpro
EGFR	cobas EGFR mutation test v2	Non-small cell lung cancer	Tarceva, Tagrisso, Iressa
PD-L1	PD-L1 IHC 22C3 pharmDx	Non-small cell lung cancer	Keytruda
IDH1	Abbott RealTime IDH1	Acute myeloid leukemia	Tibsovo
BCR-ABL	MRDx BCR-ABL test	Chronic myeloid leukemia	Tasigna
EGFR	FoundationOne CDx	Non-small cell lung cancer	Gilotrif, Iressa, Tarceva
ALK			Tagriso, Alecensa, Xalkori
BRAF			Zykadia, Tafinlar
ERBB2		Melanoma	Tafinlar, Zelboraf, Melkinist
PIK3CA		Breast cancer	Herceptin, Perjeta, Kadcyla
KRAS			Piqray
		Colorectal cancer	Erbitux, Vectibix
		Ovarian cancer	Rubraca, Lynparza
		Cholangiocarcinoma	Pemazyre
ALK	Ventana ALK (D5F3) CDx assay	Non-small cell lung cancer	Zykadia, Xalkori, Alecensa
IDH2	Abbott RealTime IDH2	Acute myeloid leukemia	Idhifa
RAS	Praxis Extended RAS panel	Colorectal cancer	Vectibix
50 genes signature	Oncomine Dx target test	Non-small cell lung cancer	Tafinlar, Melkinist, Xalkori, Iressa

FLT3	LeukoStrat CDx FLT3 mutation assay	Acute myelogenous leukemia	Rydapt, Xospata
BRCA	FoundationFocus CDx BRCA assay	Ovarian cancer	Rubraca
TP53, ATM	Vysis CLL FISH probe kit	B-cell chronic lymphocytic leukemia	Venclexta
KIT	KIT D816V mutation Dx	Aggressive systemic mastocytosis	Gleevec
PDFGRB	PDGFRB FISH for Gleevec (MDS/MPD)	Myelodysplastic syndrome	Gleevec
KRAS	cobas KRAS mutation test	Colorectal cancer	Erbitux, Vectibix
KRAS	therascreen KRAS RGQ PCR kit	Colorectal cancer	Erbitux, Vectibix
EGFR	Dako EGFR pharmDx kit	Colorectal cancer	Erbitux, Vectibix
c-KIT	Dako c-KIT pharmDx	Gastrointestinal stromal tumors	Gleevec, Glivec
Her2/neu	INFORM HER-2/neu	Breast cancer	Herceptin
	PathVysion Her-2 DNA probe kit		
	Pathway anti-Her2/neu Ab		Herceptin, Kadcyla
	InSite Her-2/neu Kit		Herceptin
	SPOT-LIGHT HER2 CISH Kit		Herceptin
	Bond Oracle HER-2 IHC system		Herceptin
	HER2 CISH pharmDx kit		Herceptin
	INFORM HER2 Dual ISH DNA probe		Herceptin
	HerceptTest	Breast cancer	Herceptin, Perjeta, Kadcyla
	HER2 FISH pharmDx Kit		Herceptin, Perjeta, Kadcyla
BRAF	THXID	Melanoma	Braftovi, Melkinist, Tafinlar
ALK	Vysis ALK Break Apart FISH probe kit	Non-small cell lung cancer	Xalkori
BRAF	cobas 4800 BRAF V600 mutation test	Melanoma	Zelboraf, Cotellic

PD-1L	Ventana PD-1L (SP142) assay	urothelial carcinoma, breast cancer	Tecentriq
FGFR	therascreen FGFR RGQ RT_PCR kit	Urothelial cancer	Balversa
PIK3CA	therascreen PIK3CA RGQ PCR kit	Breast cancer	PIQRAY
BRCA1, BRCA2	Myriad mychoice CDx	Ovarian cancer	Zejula
BRAF	QIAGEN	Colorectal cancer	BRAFTOVI

Supplementary Table S3B, Tumor Markers in Common Use

Name	Type of cancer
ALK	Non-small cell lung cancer and anaplastic large cell lymphoma
Alpha-fetoprotein (AFP); AFPD, FETA, HPAFP	Liver cancer and germ cell tumors
Beta-2-microglobulin (B2M)	Multiple myeloma, chronic lymphocytic leukemia, and some lymphomas
Beta-human chorionic gonadotropin (Beta-hCG) CGB, CGB5, CGB7, CGB8, hCGB	Choriocarcinoma and germ cell tumors
Bladder Tumor Antigen (BTA)	Bladder cancer and cancer of the kidney or ureter
BRCA1, BRCA2	Ovarian and breast cancers
BCR-ABL	Chronic myeloid leukemia, acute lymphoblastic leukemia, and acute myelogenous leukemia
BRAF V600	Cutaneous melanoma, Erdheim-Chester disease, colorectal cancer, and non-small cell lung cancer
c-Kit/CD117	Gastrointestinal stromal tumor, mucosal melanoma, acute myeloid leukemia, and mast cell disease
CA15-3/CA27.29 MUC1, EMA; MCD; PEM; PUM; KL-6; MAM6; MCKD; PEMT; CD227; H23AG; MCKD1; MUC-1; ADMCKD; ADMCKD1; CA 15-3; MUC-1/X; MUC1/ZD; MUC-1/SEC	Breast cancer
CA19-9, 5-Acetylneuraminyl-2-3-Galactosyl-1-3-(Fucopyranosyl-1-4)-N-Acetylglucosamine	Pancreatic, gallbladder, bile duct, and gastric cancers
CA-125, MUC16	Ovarian cancer
CA 27.29, MUC1	Breast cancer

CALCA	Medullary thyroid cancer
Carcinoembryonic antigen (CEA)	Colorectal cancer and some other cancers
CD20 B1, Bp35, CD20, CVID5, LEU-16, MS4A2, S7	Non-Hodgkin lymphoma
CD22, SIGLEC-2, SIGLEC2	Hairy cell leukemia and B-cell neoplasms
CD25, IL2ra	Non-Hodgkin (T-cell) lymphoma
CD30, TNFRSF8	Mycosis fungoides and peripheral T-cell lymphoma
CD33	Acute myeloid leukemia
Chromogranin A (CgA), CG-ALPHA, FSHA, GPA1, GPHA1, GPHa, HCG, LHA, TSHA	Neuroendocrine tumors
Cytokeratin fragment 21-1	Lung cancer
Chromosome 17p deletion	Chronic lymphocytic leukemia
Chromosomes 3, 7, 17, and 9p21	Bladder cancer
Cellsearch, CTCs test	Breast, prostate, colorectal cancers
Des-gamma-carboxy prothrombin (DCP)	Hepatocellular carcinoma
DPD gene mutation DPYD – dihydropyrimidine dehydrogenase, DHP, DHPDHASE, DPD	Breast, colorectal, gastric, and pancreatic cancers
EGFR gene mutation	Non-small cell lung cancer
Estrogen receptor (ER, ESR1)/progesterone receptor (PR) PGR	Breast cancer
FGFR2 and FGFR3 gene mutations	Bladder cancer
Fibrin/fibrinogen FGA FGG, FGB	Bladder cancer
FLT3 gene mutations	Acute myeloid leukemia
Gastrin, GAST	Gastrin-producing tumor (gastrinoma)
HE4, WFDC2, EDDM4, HE4, WAP5, dJ461P17.6	Ovarian cancer
HER2/neu	Breast, ovarian, bladder, pancreatic, and stomach cancers
5-HIAA, 5-hydroxyindolacetic acid	Carcinoid tumors
IDH1, IDH2	Acute myeloid leukemia
Immunoglobulins	Multiple myeloma and Waldenström macroglobulinemia
JAK2	Leukemia
KRAS	Colorectal cancer and non-small cell lung cancer
Lactate dehydrogenase, LDH	Germ cell tumors, lymphoma, leukemia, melanoma, and neuroblastoma
Microsatellite instability (MSI) and/or mismatch repair deficient (dMMR)	Colorectal cancer and other solid tumors
Neuron-specific enolase (NSE)	Small cell lung cancer and neuroblastoma
Nuclear matrix protein 22, NMP22, NUMA, NMP-22	Bladder cancer
PCA3	Prostate cancer
PML/RARα fusion gene	Acute promyelocytic leukemia (APL)
Prostatic Acid Phosphatase (PAP)	Metastatic prostate cancer
Programmed death ligand 1 (PD-L1)	Non-small cell lung cancer, liver cancer, stomach cancer, gastroesophageal junction cancer,

	classical Hodgkin lymphoma, and other aggressive lymphoma subtypes
Prostate-specific antigen (PSA), KLK3, APS, KLK2A1, PSA, hK3	Prostate cancer
ROS1	Non-small cell lung cancer
Soluble mesothelin-related peptides (SMRP) MSLN, MPF	Mesothelioma
Somatostatin receptor, SSTR1, 2, 3, 4, SSTR5	Neuroendocrine tumors affecting the pancreas or gastrointestinal tract (GEP-NETs)
T-cell receptor gene rearrangement	T-cell lymphoma
Thiopurine S-methyltransferase (TPMT) enzyme activity or TPMT genetic test, TPMTD	Acute lymphoblastic leukemia
Thyroglobulin, TG, AITD3N	Thyroid cancer
UGT1A1*28 variant homozygosity	Colorectal cancer
Urine catecholamines: VMA and HVA	Neuroblastoma
Urokinase plasminogen activator (uPA) and plasminogen activator inhibitor (PAI-1)	Breast cancer
FoundationOne® CDx (F1CDx) genomic test	Any solid tumor
5-Protein signature (OVA1®)	Ovarian cancer
17-Gene signature (Oncotype DX GPS test®),	Prostate cancer
21-Gene signature (Oncotype DX®)	Breast cancer
46-Gene signature (Prolaris®)	Prostate cancer
70-Gene signature (Mammaprint®)	Breast cancer

Supplementary Table S3C, List of genes in FoundationOne CDx signature for companion diagnostic, and the list of genes tested for substitutions, indels and copy-number alterations.

FoundationOne **CDx**
EGFR
ALK
BRAF
ERBB2
PIK3CA
KRAS
BRCA1, BRCA2

Supplementary Table S3D, List of genes of 5-protein signature OVA1.

Name of gene
CA125, MUC16
Apolipoprotein A1, APOA1
Beta2 microglobulin, B2M
Transferrin, TF
Pre-albumin, ALB

<u>Supplementary Table S3E</u>, List of genes of 21-gene Oncotype Dx.

Name of gene
Ki67
STK15
CCNB1
Mybl2
GRB2
Her2/neu
GSTM1
CD68
BIRC5
BAG1
MMP11
CTSL2, CTSL
ER
PGR
BCL2
SVUBE2

houskeeping references are not listed

<u>Supplementary Table S3F</u>, List of genes of Oncomine Dx signature.

Name of genes
AKT1
CDKN2A
FGFR1
HRAS
MTOR
RAF1
AKT2
CHEK2
FGFR2
IDH1
NRAS
RET
AKT3
CTNNB1
FGFR3
IDH2
NTRK1
ROS1

ALK
EGFR
FGFR4
KIT
NTRK2
SMO
AR
ERBB2
FLT3
KRAS
NTRK3
TP53
ARAF
ERBB3
GNA11
MAP2K1
PDGFRA
BRAF
ERBB4
GNAQ
MAP2K2
PIK3CA
CDK4
ESR1
GNAS
MET
PTEN
CD274
NRG1
NUTM1
RSPO2
RSPO3

Supplementary Table S3G, List of genes of Prolaris Dx signature.

Name of gene
FOXM1
ASPM
TK1
PRC1
CDC20
BUB1B
PBK
DTL

CDKN3
RRM2
ASF1B
CEP55
CDC2
DLGAP5
C18orf24
RAD51
KIF11
BIRC5
RAD54L
CENPM
KIAA0101
KIF20A
PTTG1
CDCA8
NUSAP1
PLK1
CDCA3
ORC6L
CENPF
TOP2A
MCM10

Supplementary Table S3H, List of genes of Mammaprint Dx signature.

Name of gene
BBC3
IGFBP5
FGF18
SCUBE2
WISP1
FLT1
HRASLS
STK32B
RASSF7
MELK
EXT1
EBF4
QSCN6L1
NUSAP1
ORC6L
TSPYL5
RUNDC1

RECQL5
CDCA7
GRP180
GPR126
RTN4RL1
CDC42BPA
PALM2
ALDH4A1
AYTL2
OXCT1
SLC2A3
LOC100288906
ZNF533
C16orf61
SERF1A
C20orf46
LOC730018
LOC100131053
AA555029_RC
LGP2
NMU
JHDM1D
MS4A7
EGLN1
TGFB3
ESM1
DCK
GNAZ
MTDH
PITRM1
CCNE2
ECT2
CENPA
LIN9
KNTC2
MCM6
PRC1
RFC4
DTL
COL4A2
MMP9
DIAPH3
PECI
GMPS

GSTM3
C9orf30
UCHL5
AP2B1
RAB6B

Supplementary Table S3I, List of targets of drugs proposed for treatment of COVID-19

Name
VEGF
PD-1
CCR5
IL-6
CSF2
IL-1B
IFNG
BSG
ANGPT2
CD14
CYP3A
ACE2
TGFB1
S1PR1
PLAT
AKR1B1
JAK
CAPN1
BTK
CGRP
IL15
IFNA2
IFNB1
IFNA1
CD24
CRAC
